# Mechanisms maintaining right ventricular contractility-to-pulmonary arterial elastance ratio in VA ECMO: a retrospective animal data analysis of RV–PA coupling

**DOI:** 10.1186/s40560-024-00730-6

**Published:** 2024-05-11

**Authors:** Kaspar F. Bachmann, Per Werner Moller, Lukas Hunziker, Marco Maggiorini, David Berger

**Affiliations:** 1 Department of Intensive Care Medicine, Inselspital, Bern University Hospital, University of Bern, Bern, Switzerland; 2 Department of Anesthesia, SV Hospital Group, Institute of Clinical Sciences at the Sahlgrenska Academy, University of Gothenburg, Gothenburg, Sweden; 3 Department of Cardiology, Inselspital, Bern University Hospital, University of Bern, Bern, Switzerland; 4 Medical Intensive Care Unit, University Hospital Zürich, University of Zürich, Zurich, Switzerland

**Keywords:** Extracorporeal membrane oxygenation, Right ventricular function, Ventriculo-arterial coupling, Homeometric adaption, Heterometric adaption

## Abstract

**Background:**

To optimize right ventricular–pulmonary coupling during veno-arterial (VA) ECMO weaning, inotropes, vasopressors and/or vasodilators are used to change right ventricular (RV) function (contractility) and pulmonary artery (PA) elastance (afterload). RV–PA coupling is the ratio between right ventricular contractility and pulmonary vascular elastance and as such, is a measure of optimized crosstalk between ventricle and vasculature. Little is known about the physiology of RV–PA coupling during VA ECMO. This study describes adaptive mechanisms for maintaining RV–PA coupling resulting from changing pre- and afterload conditions in VA ECMO.

**Methods:**

In 13 pigs, extracorporeal flow was reduced from 4 to 1 L/min at baseline and increased afterload (pulmonary embolism and hypoxic vasoconstriction). Pressure and flow signals estimated right ventricular end-systolic elastance and pulmonary arterial elastance. Linear mixed-effect models estimated the association between conditions and elastance.

**Results:**

At no extracorporeal flow, end-systolic elastance increased from 0.83 [0.66 to 1.00] mmHg/mL at baseline by 0.44 [0.29 to 0.59] mmHg/mL with pulmonary embolism and by 1.36 [1.21 to 1.51] mmHg/mL with hypoxic pulmonary vasoconstriction (*p* < 0.001). Pulmonary arterial elastance increased from 0.39 [0.30 to 0.49] mmHg/mL at baseline by 0.36 [0.27 to 0.44] mmHg/mL with pulmonary embolism and by 0.75 [0.67 to 0.84] mmHg/mL with hypoxic pulmonary vasoconstriction (*p* < 0.001). Coupling remained unchanged (2.1 [1.8 to 2.3] mmHg/mL at baseline; − 0.1 [− 0.3 to 0.1] mmHg/mL increase with pulmonary embolism; − 0.2 [− 0.4 to 0.0] mmHg/mL with hypoxic pulmonary vasoconstriction, *p* > 0.05). Extracorporeal flow did not change coupling (0.0 [− 0.0 to 0.1] per change of 1 L/min, *p* > 0.05). End-diastolic volume increased with decreasing extracorporeal flow (7.2 [6.6 to 7.8] ml change per 1 L/min, *p* < 0.001).

**Conclusions:**

The right ventricle dilates with increased preload and increases its contractility in response to afterload changes to maintain ventricular–arterial coupling during VA extracorporeal membrane oxygenation.

**Supplementary Information:**

The online version contains supplementary material available at 10.1186/s40560-024-00730-6.

## Introduction

Despite improved treatment options, including mechanical circulatory support, cardiogenic shock has a high mortality of up to 50%. The entry and exit strategies for mechanical support still show a substantial gap in knowledge and evidence [1, 2]. Randomized trials of extracorporeal membrane oxygenation (ECMO) in scenarios of cardiogenic shock provide negative results [3]. Still, veno-arterial (VA) ECMO remains a short-term salvage option for severe acute cardiac failure [1], including right heart failure. ECMO is complex and resource intense, accompanied by profound and often harmful alterations of circulatory physiology [2], such as increases in left ventricular afterload [4], extensive changes in gas exchange [5, 6], fibrosis of the lung [7], and activation of coagulation and inflammatory pathways [8]. To gain therapeutic benefit from such complex supportive treatment, a better understanding of the pathophysiology is needed [2].

**Fig. 1:**
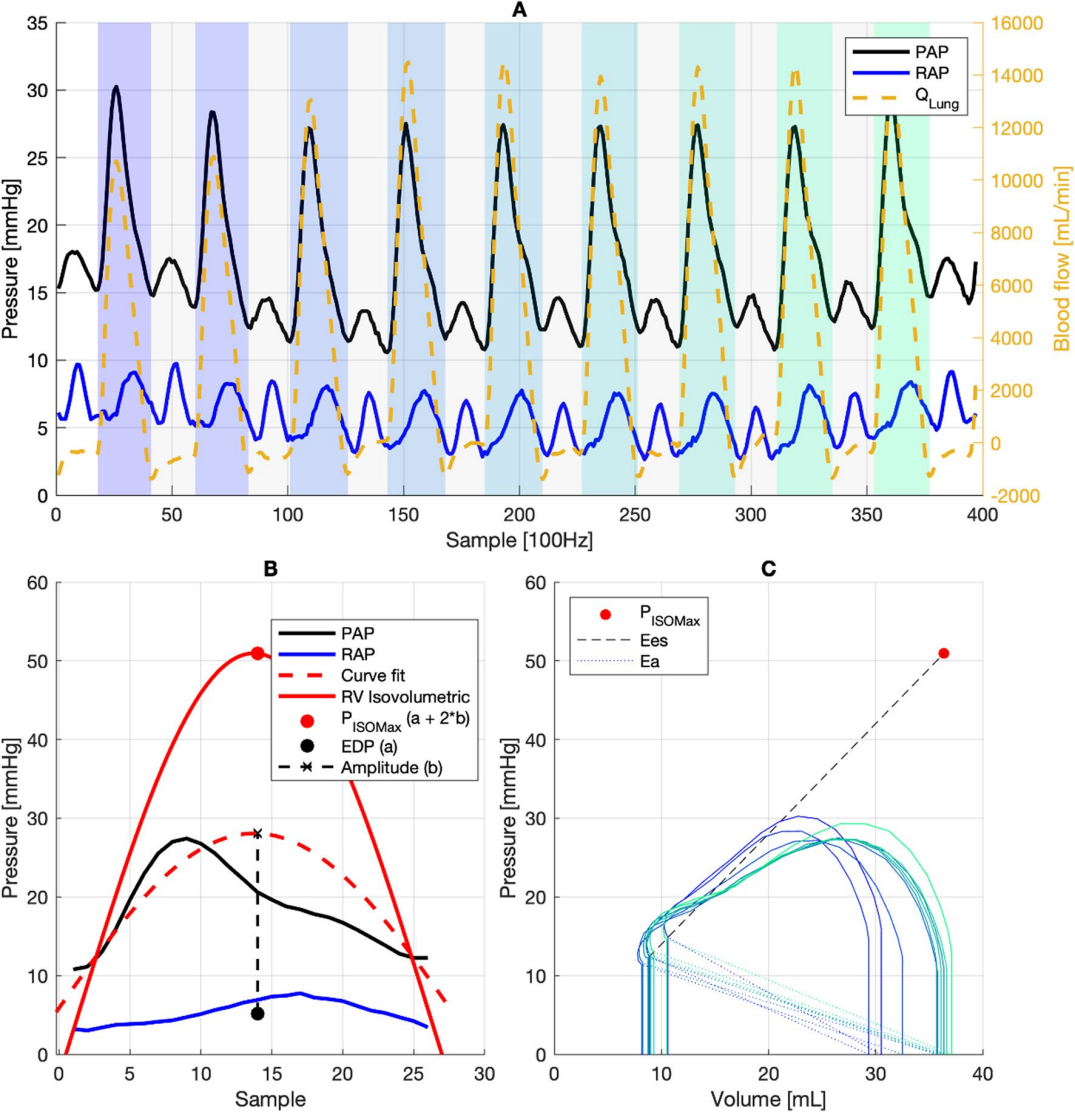
Exemplary analysis of a full respiratory cycle (baseline condition, animal 2, *Q*_ECMO_ 829 mL/min). **A** Systoles (blue to green shaded areas) were identified using peak width analysis of the derivative of each pulmonary flow tracing, where the beginning of each peak (positive d*Q*/d*t*_max_) defined the beginning of systoles. Beginnings of diastoles (uncolored areas) were identified through minimum pulmonary flow. Pressure and flow signals were aligned by determining the delay between d*p*/d*t*_max_ and d*Q*/d*t*_max_. **B** A sine wave function *f*(*x*) = *a* + *b* × sin(*c* × *x* + *d*) was fitted through the systolic data points before dp/dt_max_ of the pressure rise and the end-systolic pulmonary arterial pressure. Coefficient *a* of the fit was forced to equal right ventricular end-diastolic pressure (RV_EDP_), which was derived from the median right atrial pressure during the respective cardiac cycle. The curve fit was performed using a Levenberg–Marquardt algorithm [17]. *P*_isomax_ (maximum of isovolumetric right ventricular contraction) was then defined as *a* + 2 × *b* (**B**) [9, 17]. **C** Reconstruction of partial PV loops through calculation of end-diastolic volume, derived from RVEF = *E*_es_/(*E*_a_ + *E*_es_). *E*_es_ was extrapolated as the slope between end-systolic pressure and *P*_isomax_ from the cardiac cycle with the maximum stroke volume for a given respiratory cycle. As a load independent parameter, it was then assumed to be constant for all other cardiac cycles within a specific respiratory cycle [48, 49]. *E*_a_ was calculated as end-systolic PAP divided by stroke volume. *E*_es_: Ventricular elastance. *E*_a_: Arterial elastance

**Fig. 2:**
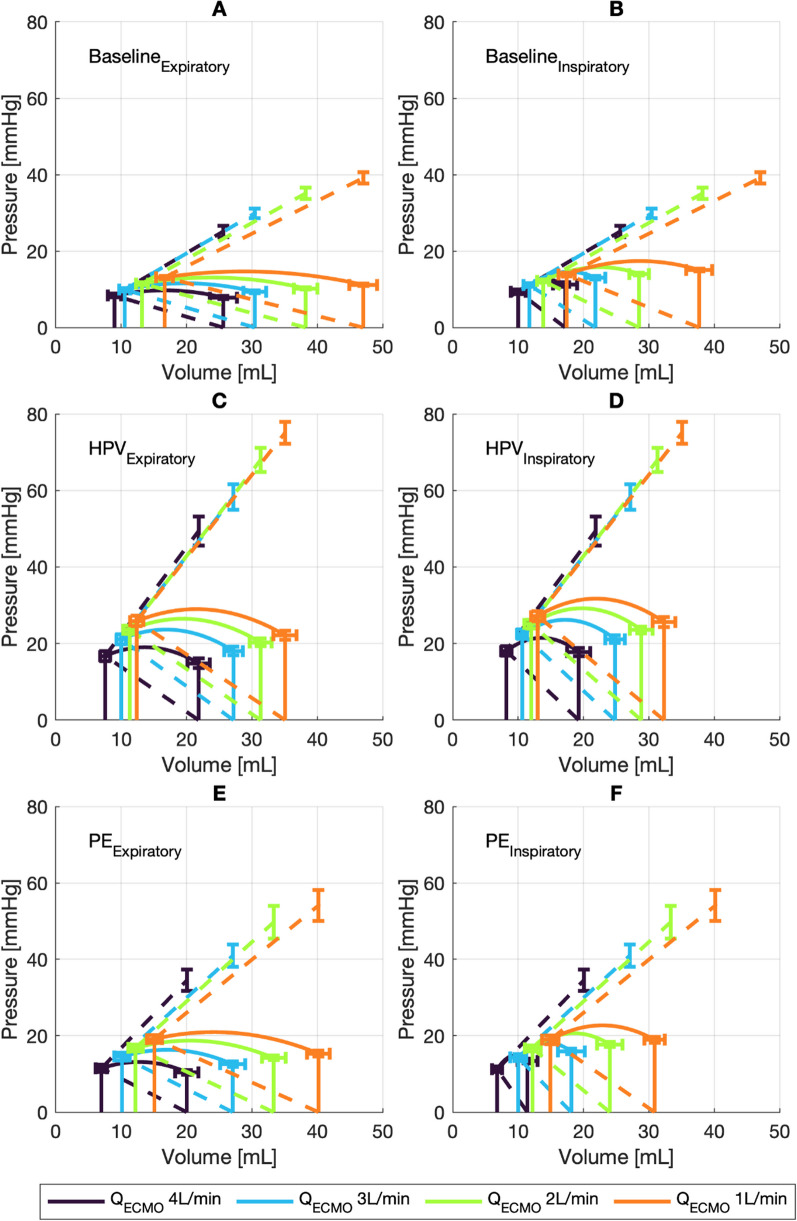
Averaged PV loops (full lines) for each experimental condition. The left column are expiratory loops, the right column inspiratory loops. The vertical and horizontal error bars indicate 95% CI intervals for the respective parameter (end-systolic and end-diastolic volume on the abscissa, end-systolic pressure, early systolic pressure and *P*_isomax_ on the ordinate). The dashed lines indicate ventricular and pulmonary arterial elastance, respectively. Panels **A** and **B** show the averaged expiratory and inspiratory loops for baseline condition. Panel **C** and **D** show the averaged expiratory and inspiratory loops for hypoxic pulmonary vasoconstriction condition. Panel **E** and **F** show the averaged expiratory and inspiratory loops for pulmonary embolism condition

Ventricular function is governed by contractility and afterload. Both parameters can be influenced by inotropes, vasopressors, or vasodilators. For a ventricle to work efficiently, contractility must match the afterload. This match is described by ventricular–arterial coupling—expressed as the ratio of ventricular elastance to arterial elastance in the pressure–volume loop of a cardiac beat. It is visually shown by the two lines intersecting at the end-systolic pressure (Figs. 1C and 2) [9]. Ventricular–arterial uncoupling occurs in various states of shock and heart failure and has prognostic value in left heart failure [10, 11], pulmonary hypertension and right heart failure [12, 13], including intensive care patients [14]. Little is known about ventricular–arterial coupling during veno-arterial ECMO. Because of its ability to provide a combined assessment of contractility and afterload and its importance for right ventricular energetics [12], right ventricular–pulmonary artery (RV–PA) coupling is an interesting therapeutic target for patients on ECMO and has prognostic relevance for successful weaning from VA EMCO [15]. Physiological data for RV–PA coupling under ECMO are lacking. This post hoc study [5, 16] describes RV–PA coupling during VA ECMO weaning trials and provides a physiological framework for mechanisms that determine RV–PA coupling during extracorporeal circulatory support.

## Methods

After approval by the animal welfare committee of Bern (BE 111/18) and in accordance with the Swiss Ordinance on the Protection of Animals (TSchV 2008 455.1), 16 pigs (3 pilot animals, *Sus scrofa*, 6 males, 45.5 ± 3.3 kg) were centrally cannulated for VA ECMO [5, 16]. After sedation with ketamine (15 mg kg^−1^), midazolam (0.5 mg kg^−1^) and methadone (0.2 mg kg^−1^) i.m., general anesthesia was induced with propofol to effect (1–4 mg kg^−1^ IV), and further maintained with continuous infusion of propofol (2–8 mg*kg^−1^*h^−1^) and fentanyl (5–10 μg*kg^−1^*h^−1^). More details on animal care, anesthesia, and experimental setup have been previously described [5, 16]. The report follows the applicable ARRIVE guidelines.

The protocol consisted of 1 L/min VA ECMO flow reductions (from 4 to 1 L/min) at baseline, followed by, in randomized order, experimental conditions simulating pulmonary embolization (PE) and hypoxic pulmonary vasoconstriction (HPV). HPV was achieved through left main bronchus intubation. PE was simulated by left pulmonary artery balloon inflation.

Tidal volume was 10 mL/kg for baseline and PE, and 6 mL/kg during HPV. Each condition was repeated with varying respiratory rates (10 and 15 for baseline and PE; 10 and 20 breaths per minute for HPV). Norepinephrine and epinephrine infusions were used to maintain adequate perfusion pressure.

Ultrasonic flow probes around the pulmonary artery and VA ECMO outlet measured pulmonary (*Q*_Lung_) and VA ECMO (*Q*_ECMO_) blood flows, respectively (Transonic, Ithaca, NY). Pressures were measured in the right and left atria, and pulmonary and left carotid arteries, with transducers leveled to the right atrium. Data were acquired using LabVIEW (National Instruments Corp., Austin, TX) with Soleasy (Alea Solutions, Zurich, Switzerland) and Hamilton Memory Box (Hamilton Medical, Bonaduz, Switzerland) at 100 Hz resolution.

We selected three respiratory cycles from each experimental condition (Fig. 1A). Datasets with missing data or artifacts were excluded after visual inspection. Stroke volumes (SV), pressure amplitudes, end-systolic pulmonary arterial and right atrial pressures were extracted from each cardiac cycle. Right ventricular maximum isovolumetric pressure (*P*_isomax_), pulmonary arterial elastance (*E*_a_), and end-systolic ventricular elastance (*E*_es_) were determined (Fig. 1B, C) [9, 17]. We determined the inspiratory and expiratory cardiac cycle at the highest and lowest SV of any given respiratory cycle, respectively. Ventricular coupling was defined as *E*_es_/*E*_a_ and ejection fraction (RVEF) as *E*_es_/(*E*_a_ + *E*_es_) [18]. End-diastolic volume was calculated from RVEF and SV, allowing for partial reconstruction of PV loops (Figs. 1C and 2). Stroke volume variation (SVV) was calculated as (amplitude_max_ − amplitude_min_) divided by (amplitude_max_/2 + amplitude_min_ /2).

Impedance describes the total opposition of a vascular bed to the pulsatile flow. It is a more comprehensive measure than resistance, as it considers not only the static component of blood flow but also the pulsatile component [19, 20]. Pulmonary vascular impedance was calculated as the ratio between pressure and flow moduli [19]. The input resistance was defined at 0 Hz (*Z*_0_), and *Z*_c_ represented the average of impedance moduli between 2 and 15 Hz [19]. Total hydraulic power (*W*_T_) was the integral of the instantaneous product of pressure times flow, and oscillatory hydraulic power (*W*_Osc_) was *W*_T_ minus the product of mean pressure × mean flow [20]. Data are presented as mean with standard deviation or boxplots. Linear mixed-effects models were used to assess the impact of *Q*_ECMO_ and experimental conditions on outcomes, with individual animals as random effects. Fixed effects (experimental condition and *Q*_ECMO_) were entered as individual and interaction variables. *r*^2^ represents goodness of fit. A *p*-value of < 0.05 was considered statistically significant. Data and statistical analysis were performed using MatLab (R2023a, MathWorks, Natick, MA, USA).

## Results

Data from 13 animals were available. We extracted 1074 respiratory cycles from 358 conditions, of which 928 (86%) were included after visual exclusion of artifacts. All extracted datasets are available in Additional file 1.

### Pressures and flows

*Q*_ECMO_ and ventilator settings followed the experimental protocol (Additional file 2: Table S1). At baseline, every 1 L/min reduction in *Q*_ECMO_ was associated with a 0.5 L/min increase in *Q*_Lung_ and approximately 0.5 mmHg increase in right and left atrial pressures (RAP and LAP, respectively). The increase in *Q*_Lung_ subsequently increased mean pulmonary artery pressure (mPAP) by approximately 1.8 mmHg for each 1 L/min decrease in *Q*_ECMO_. Mean PAP almost doubled under HPV conditions, while SV and the resulting *Q*_Lung_ decreased compared to baseline. Of note, the regression model estimated not only a fixed increase in mPAP (+ 14.7 mmHg), but also estimated a steeper change associated with variations in *Q*_ECMO_ (− 1.4 additional mmHg per increase of 1 L/min of *Q*_ECMO_). RAP remained stable and LAP increased only slightly during HPV condition (+ 1.4 mmHg). The hemodynamic consequences introduced by PE were similar, but less pronounced as compared to HPV (Table 1, Additional file 2: Fig. S1). Support with norepinephrine (0.04 [0.03 to 0.08] μg/kg/min) and epinephrine (0.02 [0.00 to 0.03] μg/kg/min) was low-throughout experimental conditions (Additional file 2: Fig. S2).

**Table 1:** Estimates from linear mixed-effect models predicting hemodynamic parameters

	RAP [mmHg]	LAP [mmHg]	Q_Lung_ [L/min]	mPAP [mmHg]
Intercept	6.1 [5.5 to 6.8]	6.4 [5.8 to 7.1]	3.1 [2.9 to 3.2]	17.8 [16.5 to 19.2]
HPV	− 0.1 [− 0.4 to 0.2]	1.4 [1.1 to 1.7]^‡^	− 0.4 [− 0.5 to − 0.2]^‡^	14.7 [13.8 to 15.7]^‡^
PE	0.9 [0.6 to 1.2]^‡^	0.8 [0.5 to 1.1]^‡^	− 0.2 [− 0.3 to − 0.1]^‡^	6.2 [5.3 to 7.2]^‡^
*Q*_ECMO_	− 0.5 [− 0.6 to − 0.4]^‡^	− 0.6 [− 0.7 to − 0.6]^‡^	− 0.5 [− 0.5 to − 0.5]^‡^	− 1.8 [− 2.0 to − 1.6]^‡^
*Q*_ECMO_ × HPV	− 0.4 [− 0.5 to − 0.2]^‡^	− 0.1 [− 0.2 to 0.1]	0.1 [0.1 to 0.2]^‡^	− 1.4 [− 1.7 to − 1.0]^‡^
*Q*_ECMO_ × PE	− 0.1 [− 0.2 to − 0.0]*	0.0 [− 0.1 to 0.1]	0.0 [− 0.0 to 0.1]	− 0.8 [− 1.2 to − 0.5]^‡^
Adjusted *r*^2^	0.76	0.75	0.76	0.84

Estimates from regression are shown with 95% CI. The intercept is the estimated value at 0 L/min *Q*_ECMO_ for baseline condition. HPV and PE estimates the change if the respective condition is present. *Q*_ECMO_ estimates the change per change of 1 L/min. *Q*_ECMO_ × HPV and *Q*_ECMO_ × PE estimates the additional change per change of 1 L/min of *Q*_ECMO_ during the respective condition. *Q*_ECMO_: ECMO blood flow [L/min]. HPV: hypoxic pulmonary vasoconstriction. PE: pulmonary embolism. RAP: right atrial pressure. LAP: left atrial pressure. *Q*_Lung_: pulmonary blood flow. mPAP: mean pulmonary arterial pressure. *p < 0.05. ^‡^*p* < 0.001

### Elastances

**Fig. 3:**
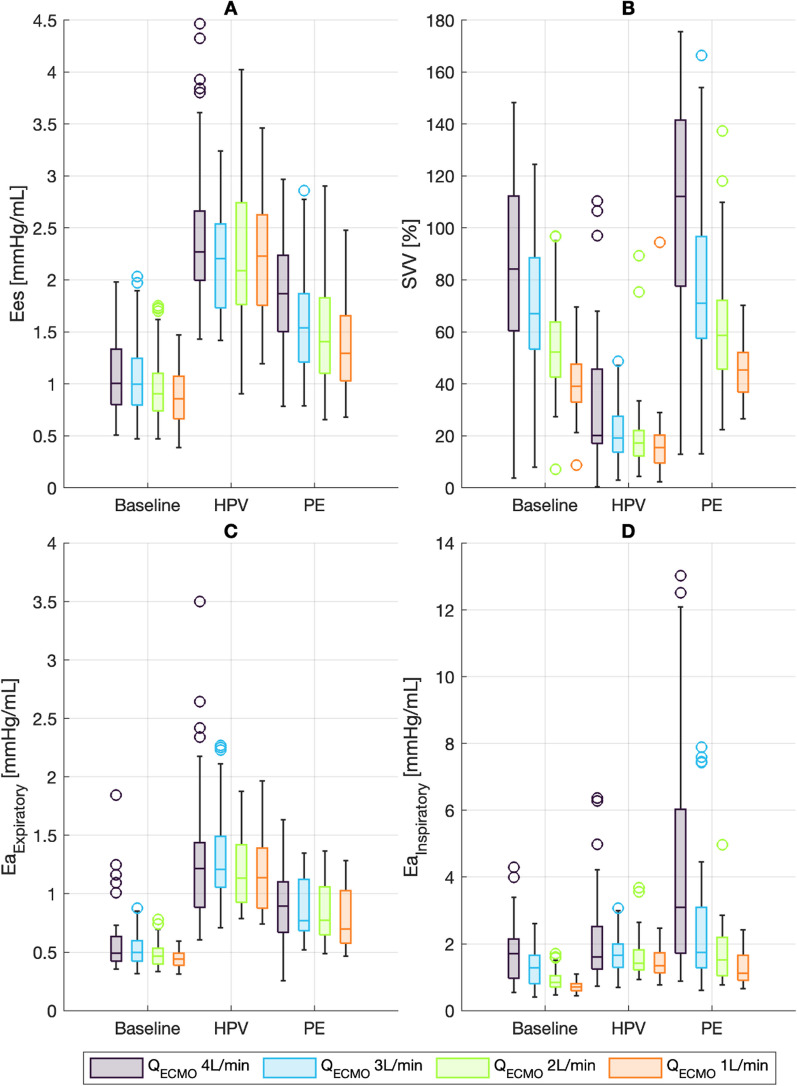
Boxplots of right ventricular elastance (*E*_es_) and pulmonary arterial elastance (*E*_a_), presented by experimental condition and Q_ECMO_ (4, 3, 2, 1 L/min) as well as stroke volume variation (SVV). HPV: hypoxic pulmonary vasoconstriction. PE: pulmonary embolism. **A** Right ventricular elastance, calculated from the cardiac cycle with maximum stroke volume. **B** Stroke volume variation (SVV). **C** Pulmonary arterial elastance during expiration (*E*_aExp_), calculated from the cardiac cycle with maximum stroke volume. **D** Pulmonary arterial elastance during inspiration (*E*_aInsp_), calculated from the cardiac cycle with minimal stroke volume

At baseline, end-systolic ventricular elastance (*E*_es_) was approximately 0.8 mmHg/mL at zero *Q*_ECMO_ and was only marginally increased by higher *Q*_ECMO_ (0.07 mmHg/mL per change of 1 L/min). Expiratory PA elastance was 0.4 mmHg/mL in baseline (Fig. 3) and increased only slightly with increasing *Q*_ECMO_ (change of 0.04 mmHg/mL per change of 1 L/min, Table 2). Inspiratory arterial elastance (*E*_aInsp_) remained unchanged from *E*_aExp_ at low *Q*_ECMO_ but increased markedly with increasing *Q*_ECMO_ (0.3 mmHg/mL, per change of 1 L/min) (Table 2, Figs. 2, 3).

**Table 2:** Estimates from linear mixed-effect models predicting right ventricular indices and ventricular–arterial coupling

	*E*_es_ [mmHg/mL]	*E*_aInsp_ [mmHg/mL]	*E*_aExp_ [mmHg/mL]
Intercept	0.83 [0.66 to 1.00]	0.37 [0.03 to 0.71]	0.39 [0.30 to 0.49]
HPV	1.36 [1.21 to 1.51]^‡^	0.89 [0.51 to 1.27]^‡^	0.75 [0.67 to 0.84]^‡^
PE	0.44 [0.29 to 0.59]^‡^	− 0.35 [− 0.73 to 0.02]	0.36 [0.27 to 0.44]^‡^
*Q*_ECMO_	0.07 [0.03 to 0.10]^‡^	0.30 [0.21 to 0.39]^‡^	0.04 [0.02 to 0.06]^‡^
*Q*_ECMO_ × HPV	− 0.03 [− 0.09 to 0.02]	− 0.12 [− 0.26 to 0.02]	0.00 [− 0.03 to 0.03]
*Q*_ECMO_ × PE	0.07 [0.01 to 0.12]*	0.65 [0.51 to 0.79]^‡^	0.01 [− 0.03 to 0.04]
Adjusted *r*^2^	0.69	0.45	0.69

Estimates from regression are shown with 95% CI. The intercept is the estimated value at 0 L/min *Q*_ECMO_ at baseline. HPV and PE estimates the change if the respective condition is present. *Q*_ECMO_ estimates the change per change of 1 L/min. Q_ECMO_ × HPV and *Q*_ECMO_ × PE estimates the additional change per change of 1 L/min of *Q*_ECMO_ during the respective condition. Inspiratory (Insp) and expiratory (exp) refer to the cardiac cycle with minimal and maximal stroke volumes, respectively. *Q*_ECMO_: ECMO blood flow [L/min]. HPV: hypoxic pulmonary vasoconstriction. PE: pulmonary embolism. *E*_es_: ventricular elastance. *E*_a_: arterial elastance. *p < 0.05. ^‡^*p* < 0.001

In HPV and PE conditions, *E*_es_ increased (by 1.4 and 0.4 mmHg/mL, respectively), but, as seen at baseline, remained almost constant with varying *Q*_ECMO_. In concordance with increasing *E*_es_, *E*_aExp_ increased in HPV and PE conditions (by 0.8 and 0.4 mmHg/mL, respectively), without changing further with changing Q_ECMO_. In contrast, *E*_aInsp_ showed substantial increases in HPV and PE conditions as compared to baseline. The increase was constant at HPV condition, without association to *Q*_ECMO_ (fixed increase of 0.9 mmHg/mL compared to baseline), while the increase at PE increased further with changing *Q*_ECMO_ (additional change of 0.7 mmHg/mL per increase of 1 L/min; Table 2, Figs. 2, 3).

### Impedances

**Fig. 4:**
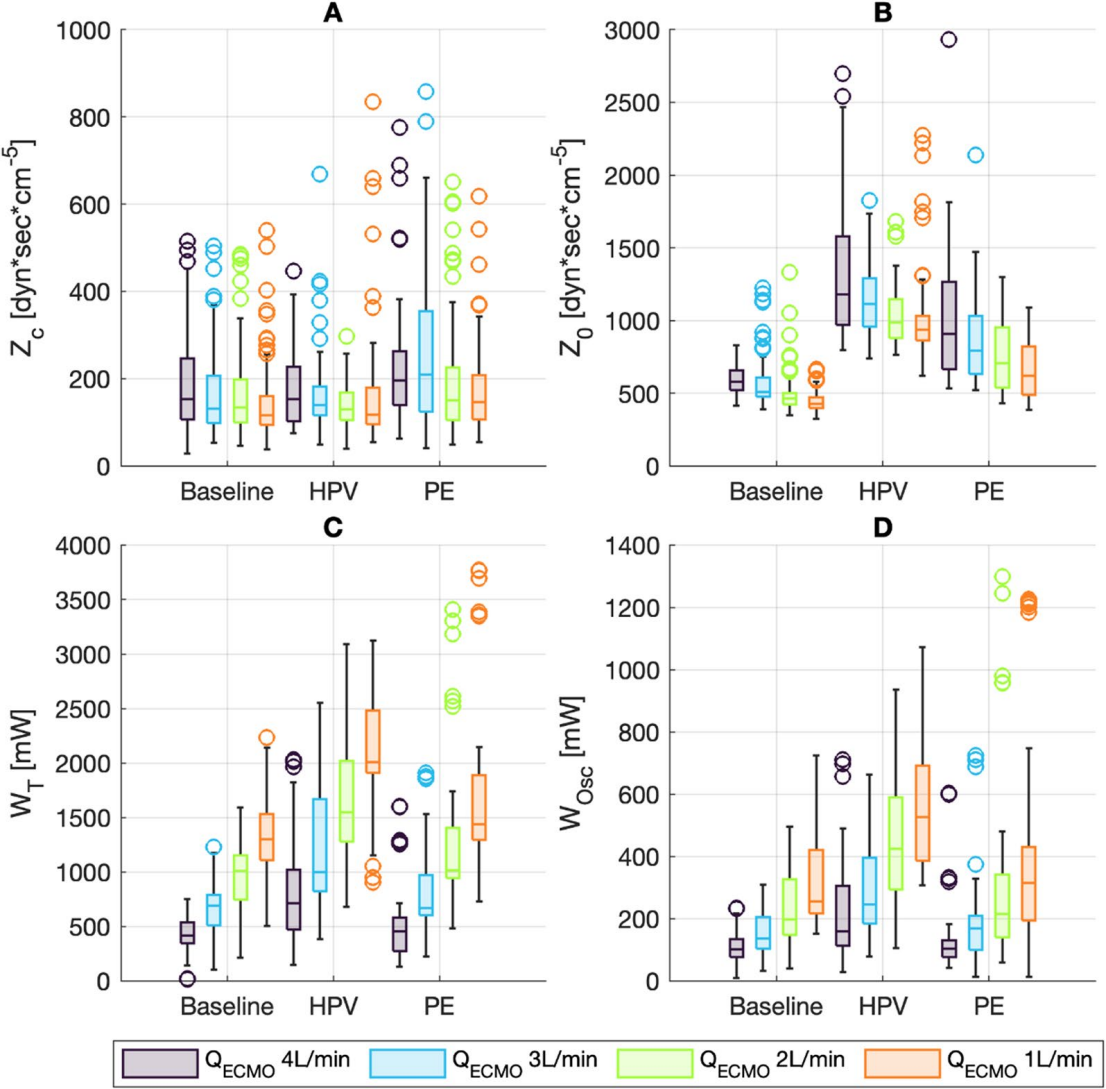
Boxplots of pulmonary artery impedance and hydraulic work, presented by experimental condition and *Q*_ECMO_ (4, 3, 2, 1 L/min). HPV: hypoxic pulmonary vasoconstriction. PE: pulmonary embolism. **A** Characteristic pulmonary vascular impedance (*Z*_c_), pressure and flow harmonics with amplitudes of < 1% of original signal excluded [19, 20]. **B** Input resistance (*Z*_0_), defined as the impedance at 0 Hz. **C** Total hydraulic work (*W*_T_). **D** Oscillatory hydraulic work (*W*_Osc_)

The pulmonary vascular impedance (*Z*_c_) was estimated at 133 dyn × sec × cm^−5^ for baseline condition at zero *Q*_ECMO_ and varied only minimally with *Q*_ECMO_ (13 dyn × sec × cm^−5^ per increase of 1 L/min). Vascular impedance was not significantly altered during PE and HPV conditions (Table 3, Fig. 4). In contrast, the input resistance Z_0_ increased significantly with increasing *Q*_ECMO_ (124 dyn × s × cm^−5^ per change of 1 L/min from baseline, with impedance at zero Q_ECMO_ estimated to 268 dyn × s × cm^−5^). HPV and PE conditions further increased *Z*_0_ (fixed increases of 610 and 251 dyn × s × cm^−5^, respectively), while the changes associated with *Q*_ECMO_ remained stable. The total hydraulic work (*W*_T_) increased with decreasing *Q*_ECMO_ (change of − 300 mW per increase of 1 L/min from an estimated 1609 mW at baseline and zero *Q*_ECMO_). *W*_T_ was highest during HPV condition (additional fixed change of 888 mW, with a further decrease of − 124 mW per change of 1 L/min). During PE condition, W_T_ increased significantly compared to baseline (increase of 251 mW, with further decrease of − 81 mW per change of 1 L/min *Q*_ECMO_,). Similarly, *W*_Osc_ was highest during HPV condition (fixed increase of 273 mW from an estimated baseline of 387 mW at zero Q_ECMO_,), clearly associated with changing *Q*_ECMO_ (− 72 mW per increase of 1 L/min *Q*_ECMO_, with an additional change of − 40 mW per increase of 1 L/min). PE condition increased *W*_Osc_ by only 82 mW without further change with Q_ECMO_ (Table 3 and Fig. 4).

**Table 3:** Estimates from linear mixed-effect models predicting pulmonary arterial impedance and total and oscillatory hydraulic power

	*Z*_c_ [dyn × sec × cm^−5^]	*Z*_0_ [dyn × sec × cm^−5^]	*W*_T_ [mW]	*W*_Osc_ [mW]
Intercept	132.8 [96.7 to 169.0]	267.7 [132.7 to 402.7]	1608.7 [1440.1 to 1777.3]	386.7 [320.4 to 453.0]
HPV	15.0 [− 34.1 to 64.1]	609.7 [460.8 to 758.5]^‡^	888.2 [763.6 to 1012.8]^‡^	273.4 [225.8 to 321.0]^‡^
PE	40.6 [− 8.2 to 89.4]	251.1 [103.2 to 399.1]^‡^	386.9 [263.1 to 510.7]^‡^	81.9 [34.6 to 129.2]^‡^
*Q*_ECMO_	13.4 [1.7 to 25.1]*	123.6 [88.1 to 159.1]^‡^	− 299.5 [− 329.2 to − 269.8]^‡^	− 71.8 [− 83.1 to − 60.4]^‡^
*Q*_ECMO_ × HPV	− 7.1 [− 25.3 to 11.1]	− 27.2 [− 82.4 to 28.0]	− 123.7 [− 169.9 to − 77.4]^‡^	− 40.0 [− 57.7 to − 22.4]^‡^
*Q*_ECMO_ × PE	9.1 [− 8.9 to 27.2]	0.7 [− 54.1 to 55.5]	− 80.8 [− 126.7 to − 35.0]^‡^	− 16.5 [− 34.0 to 1.1]
Adjusted *r*^2^	0.10	0.38	0.73	0.62

Estimates from regression are shown with 95% CI. The intercept is the estimated value at 0 L/min *Q*_ECMO_ for baseline condition. HPV and PE estimates the change if the respective condition is present. *Q*_ECMO_ estimates the change per change of 1 L/min. *Q*_ECMO_ × HPV and *Q*_ECMO_ × PE estimates the additional change per change of 1 L/min of *Q*_ECMO_ during the respective condition. *Q*_ECMO_: ECMO blood flow [L/min]. HPV: hypoxic pulmonary vasoconstriction. PE: pulmonary embolism. *Z*_c_: characteristic pulmonary vascular impedance averaged over frequencies 2–15 Hz. *Z*_0_: input resistance (impedance at 0 Hz). *W*_T_: total hydraulic power. *W*_Osc_: oscillatory hydraulic power. mW: milliwatt. *p < 0.05. ^‡^*p* < 0.001

### Coupling and stroke volume variations

**Fig. 5:**
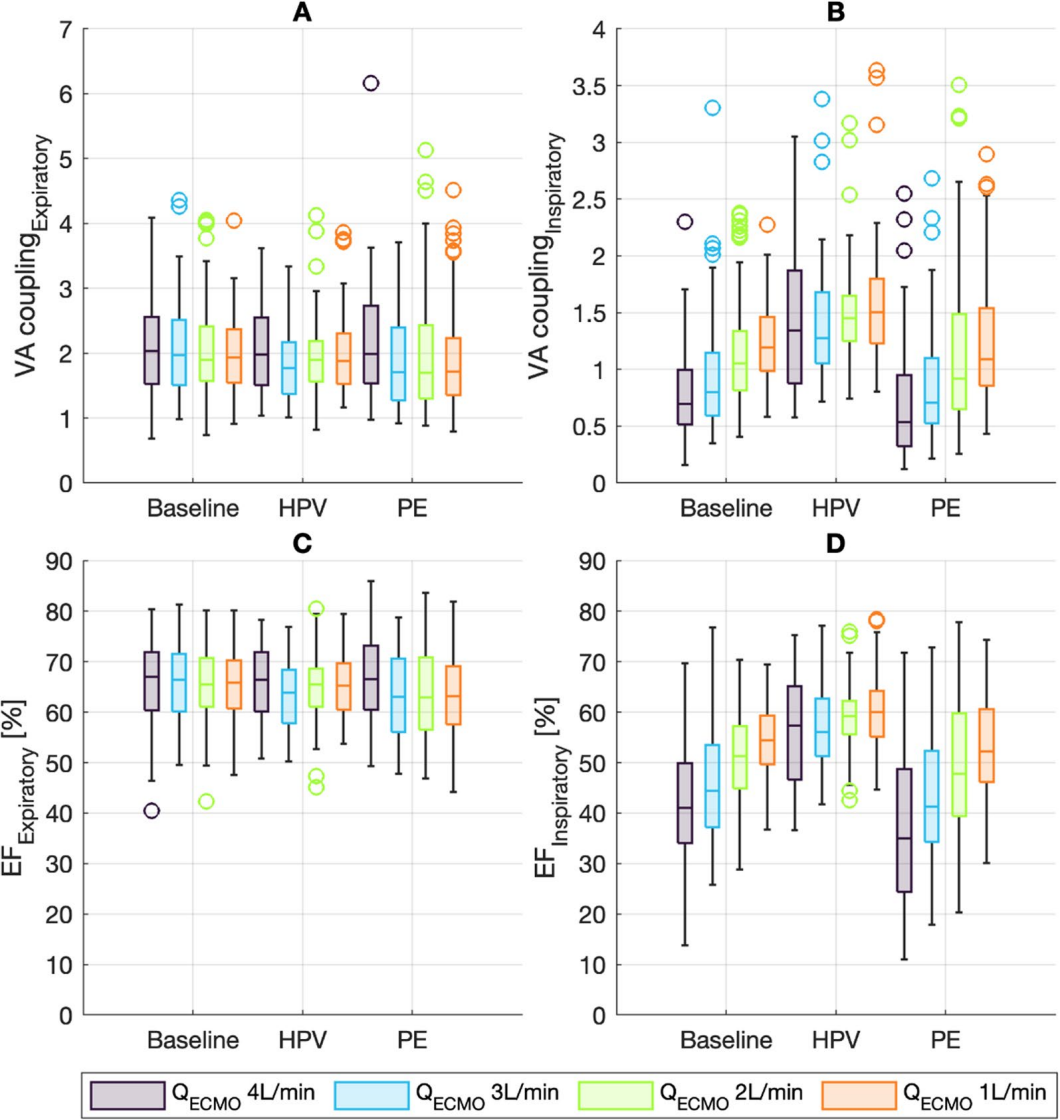
Boxplots of ventricular–arterial coupling and ejection fraction, presented by experimental condition and *Q*_ECMO_ (4, 3, 2, 1 L/min). HPV: hypoxic pulmonary vasoconstriction. PE: pulmonary embolism. **A** Ventricular–arterial coupling during inspiration (VA coupling_Insp_), calculated from the cardiac cycle with minimal stroke volume. **B** Ventricular–arterial coupling during expiration (VA coupling_Exp_), calculated from the cardiac cycle with maximum stroke volume. **C** Ejection fraction during expiration (EF_Exp_), calculated from the cardiac cycle with maximum stroke volume. **D** Ejection fraction during inspiration (EF_Insp_), calculated from the cardiac cycle with minimal stroke volume

The expiratory VA coupling was estimated to 2.1 at baseline and zero ECMO flow (Table 4, Figs. 2 and 5). The ratio remained stable at HPV and PE conditions, unaffected by changing *Q*_ECMO_. Inspiratory VA coupling followed the changes described for *E*_aInsp_, substantially decreasing with increasing *Q*_ECMO_ (− 0.15 per change of 1 L/min *Q*_ECMO_) at baseline and PE conditions. In contrast, HPV led to increases in inspiratory VA coupling (fixed increase of 0.2 and additional increase of 0.08 per increase of 1 L/min *Q*_ECMO_; Table 4, Figs. 2 and 5).

**Table 4:** Estimates from linear mixed-effect models predicting ventriculo-arterial coupling and ejection fraction

	VA coupling_insp_	VA coupling_Exp_	EF_Insp_	EF_Exp_
Intercept	1.4 [1.2 to 1.6]	2.1 [1.8 to 2.3]	59.4 [55.5 to 63.3]	66.0 [63.0 to 69.1]
HPV	0.2 [0.0 to 0.4]^†^	− 0.1 [− 0.3 to 0.1]	2.0 [− 1.1 to 5.1]	− 1.3 [− 3.4 to 0.8]
PE	0.0 [− 0.1 to 0.2]	− 0.2 [− 0.4 to 0.0]	− 0.3 [− 3.4 to 2.8]	− 3.7 [− 5.8 to − 1.6]^‡^
*Q*_ECMO_	− 0.1 [− 0.2 to − 0.1]^‡^	0.0 [− 0.0 to 0.1]	− 4.2 [− 4.9 to − 3.4]^‡^	0.0 [− 0.5 to 0.5]
*Q*_ECMO_ × HPV	0.1 [0.0 to 0.1]^†^	− 0.0 [− 0.1 to 0.1]	2.7 [1.6 to 3.9]^‡^	− 0.1 [− 0.9 to 0.6]
*Q*_ECMO_ × PE	− 0.0 [− 0.1 to 0.0]	0.0 [− 0.0 to 0.1]	− 1.2 [− 2.4 to − 0.1]*	0.8 [0.0 to 1.5]*
Adjusted *r*^2^	0.49	0.45	0.54	0.44

Estimates from regression are shown with 95% CI. The intercept is the estimated value at 0 L/min *Q*_ECMO_ for baseline condition. HPV and PE estimates the change if the respective condition is present. *Q*_ECMO_ estimates the change per change of 1 L/min. *Q*_ECMO_ × HPV and *Q*_ECMO_ × PE estimates the additional change per change of 1 L/min of *Q*_ECMO_ during the respective condition. *Q*_ECMO_: ECMO blood flow [L/min]. HPV: hypoxic pulmonary vasoconstriction. PE: pulmonary embolism. *p < 0.05. ^†^*p* < 0.01. ^‡^*p* < 0.001

The expiratory EF followed VA coupling_Exp_ and remained stable at approximately 66% of the ratio at baseline condition and zero *Q*_ECMO_, with only minor variations seen during PE condition (Table 4). Inspiratory EF was lower at 59% for baseline conditions with zero *Q*_ECMO_ and followed VA coupling_Insp_: EF_Insp_ was markedly reduced with increase in *Q*_ECMO_, most accentuated at baseline and PE conditions (− 4.2% per 1 L/min at baseline with an additional reduction of − 1.2% per 1 L/min during PE, Table 4, Fig. 5). EF_Insp_ was less affected during HPV (2.7% less change per 1 L/min compared to baseline condition, Table 4). This resulted in linear relationship between EDV_Exp_ and ESV_Exp_ without additional relevant impact of *Q*_ECMO_ (ESV_Exp_ = 0.40 × EDV_Exp_ + 0.009 × *Q*_ECMO_ × EDV_Exp_ − 2.3, *p* < 0.001, *r*^2^ = 0.85). EDV_Insp_ shared a linear relationship with ESV_Insp_, albeit with a higher coefficient which was additionally increased by Q_ECMO_ (ESV_Inspiratory_ = 0.43 × EDV_Inspiratory_ + 0.02 × Q_ECMO_ × EDV_Inspiratory_ + 0.2, *p* < 0.001, *r*^2^ = 0.82).

Stroke volume variation (SVV) was 28% at baseline and zero *Q*_ECMO_. SVV increased by 13% per increase of 1 L/min of *Q*_ECMO_ at baseline. These changes were amplified during PE condition, with an additional change of 7% per increase of 1 L/min of *Q*_ECMO_. During HPV, SVV was decreased compared to baseline ( − 18%, Table 4) and the variations associated with increasing *Q*_ECMO_ were also diminished (− 9% per change of 1 L/min of *Q*_ECMO,_ Table 5, Fig. 3B).

**Table 5:** Estimates from linear mixed-effect models predicting stroke volumes and end-diastolic volumes, including stroke volume variation (SVV)

	SV_Min_ [mL]	SV_Max_ [mL]	EDV_Insp_ [mL]	EDV_Exp_ [mL]	SVV [%]
Intercept	24 [22.3 to 25.6]	35 32.9 to 36.5]	43 [40.4 to 46.1]	53 [50.4 to 56.4]	28 [19.8 to 35.9]
HPV	− 2.0 [− 3.3 to − 0.7]^†^	− 9.5 [− 11.0 to − 8.0]^‡^	− 6.5 [− 8.75 to − 4.2]^‡^	− 14.1 [− 16.5 to − 11.6]^‡^	− 17.6 [− 24.7 to − 10.56]^‡^
PE	− 4.6 [− 5.9 to − 3.3]^‡^	− 5.9 [− 7.3 to − 4.4]^‡^	− 6.8 [− 9.1 to − 4.5]^‡^	− 7.3 [− 9.8 to − 4.9]^‡^	− 4.9 [− 11.9 to 2.1]
*Q*_ECMO_	− 4.3 [− 4.6 to − 4.0]^‡^	− 4.6 [− 5.0 to − 4.3]^‡^	− 6.8 [− 7.3 to − 6.2]^‡^	− 7.2 [− 7.8 to − 6.6]^‡^	13.3 [11.7 to 15]^‡^
*Q*_ECMO_ × HPV	1.7 [1.2 to 2.2]^‡^	2.0 [1.5 to 2.6]^‡^	2.6 [1.7 to 3.5]^‡^	3.1 [2.2 to 4.0]^‡^	− 8.7 [− 11.4 to − 6.1]^‡^
*Q*_ECMO_ × PE	0.6 [0.1 to 1.1]*	0.7 [0.1 to 1.2]*	0.54 [− 0.3 to 1.4]	0.7 [− 0.2 to 1.6]	6.7 [4.1 to 9.3]^‡^
Adjusted *r*^2^	0.70	0.66	0.65	0.64	0.70

Estimates from regression are shown with 95% CI. The intercept is the estimated value at 0 L/min *Q*_ECMO_ for baseline condition. HPV and PE estimates the change if the respective condition is present. *Q*_ECMO_ estimates the change per change of 1 L/min. *Q*_ECMO_ × HPV and *Q*_ECMO_ × PE estimates the additional change per change of 1 L/min of *Q*_ECMO_ during the respective condition. *Q*_ECMO_: ECMO blood flow [L/min]. HPV: hypoxic pulmonary vasoconstriction. PE: pulmonary embolism. SV: stroke volume. SVV: stroke volume variation. EDV_insp_: inspiratory end-diastolic volume. EDV_exp_: expiratory end-diastolic volume *p < 0.05. ^†^*p* < 0.01. ^‡^*p* < 0.001

## Discussion

### Main results

This post hoc study analyses the effects on the right ventricle and pulmonary vasculature of changing preload and afterload during VA ECMO weaning. Changing ECMO flow was used to model changing preload [21], such that increases in Q_ECMO_ reduced pulmonary blood flow, thereby unloading the right ventricle. These changes led to decreases in right and left atrial pressures, as blood volume was redistributed from the pulmonary to the systemic compartment [22, 23], and increased total blood flow (i.e., increased venous return) [21]. The decreasing left atrial pressures indicate that the left ventricle coped well with the afterload produced by the VA ECMO [2]. During *Q*_ECMO_ reductions, mean PA pressure increased as a consequence of increasing pulmonary blood flow [24], with a simultaneous decrease in input resistance (*Z*_0_) and characteristic impedance (*Z*_c_), attributable to improved distensibility and recruitment of the pulmonary vasculature [16, 25].

### Effects of afterload increase

HPV and PE conditions acutely increased RV afterload. These changes were accompanied by small increases in RAP, indicating that the ventricles did not fail [26]. Rather, they operated below their stressed volumes [9, 26]. Both HPV and PE caused major increases in *Z*_0._ *Z*_c_ remained unchanged compared to baseline as previously described [19, 20, 27].

During HPV and PE, the RV coped with acute afterload increases by increasing contractility (increased *E*_es_) to maintain *Q*_Lung_ when *Q*_ECMO_ was decreased. This is in line with homeometric adaption (RV Anrep effect) [12, 17, 28]. Increased contractility from homeometric adaptation manifests as increased ventricular elastance (*E*_es_), to match the increases in afterload (i.e., arterial elastance, *E*_aExp_). This mechanism restores RV–PA coupling to normal values (approximately 1.8 to 2.0; Figs. 2 and 5) [29] and establishes optimal energy transfer from the RV to the pulmonary vasculature [12]. Increases in ventricular elastance, in turn, lead to an increase in total (*W*_T_) and oscillatory hydraulic work (*W*_Osc_) with a constant *W*_T_/ *W*_Osc_ ratio.

### Effects of the respiratory cycle

When preload was reduced by increasing *Q*_ECMO_, the RV became more susceptible to afterload increases through the respiratory cycle, demonstrated by increases in *E*_aInsp_ during baseline and PE conditions. The resulting inspiratory RV–PA decoupling (Figs. 2 and 5) lead to reduced SV and enhanced SVV (Fig. 3B). This resulted from a combination of decreased filling and acute RV ejection inability. During states of normal preload (i.e., low *Q*_ECMO_), the main cause of SVV was decreased filling—as demonstrated by only minor changes in RV–PA coupling. The additional relative respiratory cycle change in SV during conditions of low preload (i.e., high *Q*_ECMO_) was a result of an acute RV inability to eject against the increased afterload (increased intrathoracic pressure). Previous studies explain inspiratory SV decrease predominantly as an effect of increased afterload [30, 31]. The time course of homeometric RV adaptation (Anrep effect) is unknown [12]. Respiratory cyclic changes of afterload occur too rapidly to allow for homeometric adaptation or “slow response” [32]. At low preload, the Starling mechanism (heterometric adaption), may not suffice to increase SV during inspiration, and does not restore ventricular–arterial coupling to preserve ejection fraction.

The increase in RV afterload was highest during HPV, as demonstrated by the highest input resistance (*Z*_0_), mPAP, and E_aExp_. Porcine pulmonary vasculature is highly reactive [20, 33]. Nevertheless, changes in inspiratory load at HPV condition were less pronounced compared to PE or baseline since only one lung was exposed to positive pressure ventilation.

### Right ventricular behavior

Our study group has demonstrated that during VA ECMO, cardiac output can be estimated using gas exchange or modified thermodilution and that RV ejection fraction can be assessed from the exponential decay of the thermodilution signal [5, 6, 16, 34, 35]. In line with the findings of the present analysis, we could demonstrate that the EDV/ESV relationship was linear [16, 36]. The slope of EDV/ESV represents 1 – RVEF. We can now extrapolate that the expiratory RVEF, remained constant during Q_ECMO_ variations. This suggests that the RV dilated to cope with increased preload [26], shifting the PV loop rightwards (Fig. 2). This dilation caused almost no increase in RAP. The RV end-diastolic PV relationship is flat at normal operating volumes [26]; increases in RAP by additional volume loading would therefore indicate RV failure [37]. During inspiration, the brisk combination of decreased preload and increased afterload prevented the ventricle from acute dilation and adaption, explaining the inspiratory decoupling and decrease in ejection fraction [30]. The PV loop is shifted left- and upwards (Fig. 2). While expiratory values remain constant and support the previous data on EDV/ESV relationship [16, 26, 36], we could demonstrate that acute inspiratory increases in afterload (e.g., *E*_aInsp_) inhibit adequate RV ejection. This was most pronounced during states of low preload. Vieillard-Baron and colleagues had similar findings in echocardiographic studies: the respiratory cycle induced decreases in RV stroke volume, which were not associated with decreases in filling parameters and therefore interpreted as an afterload phenomenon [30, 31, 38]. It appears that although the RV can adapt to increases in afterload by increasing its contractility [28], the time available during the respiratory cycle is insufficient to allow for this adaptation [39]. The large variation in SV (Additional file 2: Fig. S1) may reflect RV–PA uncoupling rather than a pure preload dependency and is a result of preload decrease and simultaneous afterload increase [30, 40].

### Clinical implications

Since the RV and PA are exposed to the same pressure at end-systole, ventricular–arterial coupling can be visualized as two intersecting lines at the end-systolic pressure (Fig. 2). This intersection divides the abscissa into end-systolic volume (ESV) on the side of *E*_es_, and SV on the side of *E*_a_. The RV and PA are exposed to the same pressure at end-systole. This end-systolic pressure provides a physiological and mathematical link between ventricular and arterial elastance, so that the ejection fraction can be determined by the *E*_es_/*E*_a_ ratio or VA coupling [18]:

$$\frac{1}{EF}=\frac{E_{\text{a}}}{E_{\text{es}}}+ 1 =\frac{1}{{\text{VAcoupling}}}+ 1.$$

This is illustrated in Fig. 5 when comparing plots A&B with C&D [18]. From this equation, optimal coupling (ratio 1.5 to 2) will result in a RVEF of 60 to 67%.

When assessing right ventricular performance in a VA ECMO weaning trial, the EF, commonly accepted as a surrogate for contractility, depends on loading conditions and is an expression of RV–PA coupling [41]. It, therefore, seems reasonable to hypothesize that therapeutic measures improving contractility (for example, inotropes) and afterload (vasopressors or vasodilators, depending on the clinical context) should aim to improve coupling. In return, the right ventricular ejection fraction will increase. Pathological conditions leading to RV–PA decoupling will, therefore, inevitably lead to reduced ejection fraction, and with a clear understanding of coupling mechanisms, therapeutic options may be tailored to the underlying pathology. It is common to interpret a reduced ejection fraction as a consequence of low contractility. While this may be true in many cases, our results demonstrate that the ejection fraction is determined by contractility in combination with afterload and preload conditions. Lowering pulmonary vascular resistance (i.e., lowering pulmonary vascular elastance, *E*_a_), optimizing volume status, or changing respiratory parameters and thereby decreasing right ventricular afterload may improve RV–PA coupling as efficiently as increasing contractility (*E*_es_). We demonstrate that positive pressure ventilation in states of high afterload impacts right ventricular performance significantly, and positive pressure ventilation, therefore, may contribute significantly to disturbances in RV–PA coupling. If high pulmonary vascular resistance (i.e., high pulmonary vascular elastance, *E*_a_) results from high left ventricular filling pressures, pharmacological or mechanical unloading of the left ventricular may optimize RV–PA coupling (and thereby RVEF) and interventions aimed directly at the right ventricle or pulmonary vasculature may not be necessary [2, 42]. It is therefore appealing to guide therapy and weaning from ECMO towards optimized coupling by directly influencing *E*_es_ (e.g., by increasing contractility) and *E*_a_ (by optimization of afterload and preload). Our analysis shows that right ventricular dilation is a physiological behavior of the right ventricle (i.e., heterometric adaption) to maintain RV–PA coupling. This should be considered in echocardiography guided weaning trials, where RV dilation is often seen as a weaning failure [43]. We demonstrate that dilation is a physiological adaptation if the RV operates below its stressed volume (i.e., at normal filling pressures). While we demonstrate the link between coupling and ejection fraction in the specific setting of VA ECMO, this physiological link, and the ensuing therapeutic targets (i.e., targeting *E*_a_ or *E*_es_) could be applied to any hemodynamic assessment.

### Limitations

We estimate higher RV ejection fractions in the current analysis than with our thermodilution approach. The reasons may be twofold: underestimation of the ejection fraction is common with thermodilution [44, 45]. We have shown that the exponential decay of thermodilution signals depends on the distance from the injection port [35]. Opposed to thermodilution studies where RVEF is estimated as a mean over multiple respiratory cycles, the elastance-based approach allows per beat calculation of EF during fractions of the respiratory cycle. Our findings are comparable to those of other groups [28].

We lack direct RV pressure signals. Instead, we rely on a modified single-beat method to estimate *P*_isomax_, suggested by Pinsky [9]. Our estimations of *E*_a_ and *E*_es_ match published data from porcine PV loops [28]. We, therefore, judge the method to describe RV behavior reliably but conductance catheters are the gold standard to assess end-diastolic and end-systolic pressure–volume relationships [46].

The creation of HPV through main-stem intubation instead of using low levels of oxygen is a consequence of the retrospective nature of our study. In the protocol, we investigated the effects of shunt on a modified Fick principle. Therefore, both lungs were not evenly pressurized during HPV. Nevertheless, the diminished effect of lower intrathoracic pressure and one-lung ventilation on the reduction of RVEF and stroke volume variation highlights the impact of optimized ventilatory strategy in the setting of high right ventricular afterload.

Last, we lack transmural pressures in this experiment, which precludes us from fully differentiating the changes in stroke volume as a change of pre- or afterload [38, 46]. Additionally, our data did not allow for reconstruction of the end-diastolic PV relationship. The method of single-beat estimation from left ventricular EDPVR is not validated for RV use [47].

## Conclusions

In conclusion, we have demonstrated that the RV, under support of VA ECMO, can increase its contractility in response to afterload changes to maintain adequate VA coupling. Abrupt additional increases in afterload by mechanical inspiration exhaust RV adaption ability. This effect was exacerbated in states of low preload. Assessment of RV function and RV–PA coupling using the here described approach is feasible with readily available bedside tools and provides an in-depth physiological in patients treated with VA ECMO. From this, future therapeutic concepts and weaning procedures may be built. We see RV–PA coupling as a key parameter for therapeutic optimization and guidance towards weaning success.

### Supplementary Information

**Additional file 1.** Visualization of full dataset.

**Additional file 2:** **Figure S1**. Boxplots of hemodynamic variables, presented by experimental condition and Q_ECMO_ (4, 3, 2, 1 L/min). HPV: Hypoxic pulmonary vasoconstriction. PE: Pulmonary embolism. **A**: Right atrial pressure. **B**: Left atrial pressure. **C**: pulmonary blood flow **D**: mean pulmonary arterial pressure. **E**: minimal stroke volume. **F**: maximal stroke volume. **G**: Heart rate. **H**: Mean systemic arterial blood pressure. **Figure S2**. Boxplots of peak vasopressor dose (Norepinephrine and epinephrine) for each condition. **A**: Peak norepinephrine dose. **B**: Peak epinephrine dose. **Table S1.** Protocol conditions.

## Data Availability

The dataset supporting the conclusions of this article is included within the article and its additional files.

## Abbreviations

VA ECMO: Veno-arterial extracorporeal membrane oxygenation
RV: Right ventricular
PA: Pulmonary artery
*E*_es_: End-systolic elastance
*E*_a_: Pulmonary arterial elastance
*Q*_Lung_: Pulmonary blood flow
*Q*_ECMO_: ECMO blood flow
RAP: Right atrial pressure
LAP: Left atrial pressure
mPAP: Mean pulmonary artery pressure
PE: Pulmonary embolism
HPV: Hypoxic pulmonary vasoconstriction
SV: Stroke volume
RVEF: Right ventricular ejection fraction
SVV: Stroke volume variation
*Z*_c_: Characteristic impedance
*Z*_0_: Input resistance
*W*_T_: Total hydraulic power
W_Osc_: Oscillatory hydraulic power
Ea_Exp_: Expiratory arterial elastance
Ea_Insp_: Inspiratory arterial elastance
ESV: End-systolic volume
EDV: End-diastolic volume
*P*_isomax_: Right ventricular maximum isovolumetric pressure
