# Supplementary material for: Mechanisms maintaining right ventricular contractility-to-pulmonary arterial elastance ratio in VA ECMO: a retrospective animal data analysis of RV–PA coupling
Source: J Intensive Care. 2024 May 11;12:19. doi: 10.1186/s40560-024-00730-6 (PMC11088130; doi:10.1186/s40560-024-00730-6)
Supplement: Supplementary file 2 — Additional file 2: Figure S1. Boxplots of hemodynamic variables, presented by experimental condition and QECMO (4, 3, 2, 1 L/min). HPV: Hypoxic pulmonary vasoconstriction. PE: Pulmonary embolism. A: Right atrial pressure. B: Left atrial pressure. C: pulmonary blood flow D: mean pulmonary arterial pressure. E: minimal stroke volume. F: maximal stroke volume. G: Heart rate. H: Mean systemic arterial blood pressure. Figure S2. Boxplots of peak vasopressor dose (Norepinephrine and epinephrine) for each condition. A: Peak norepinephrine dose. B: Peak epinephrine dose. Table S1. Protocol conditions. [file 40560_2024_730_MOESM2_ESM.docx]

**Figure S1**


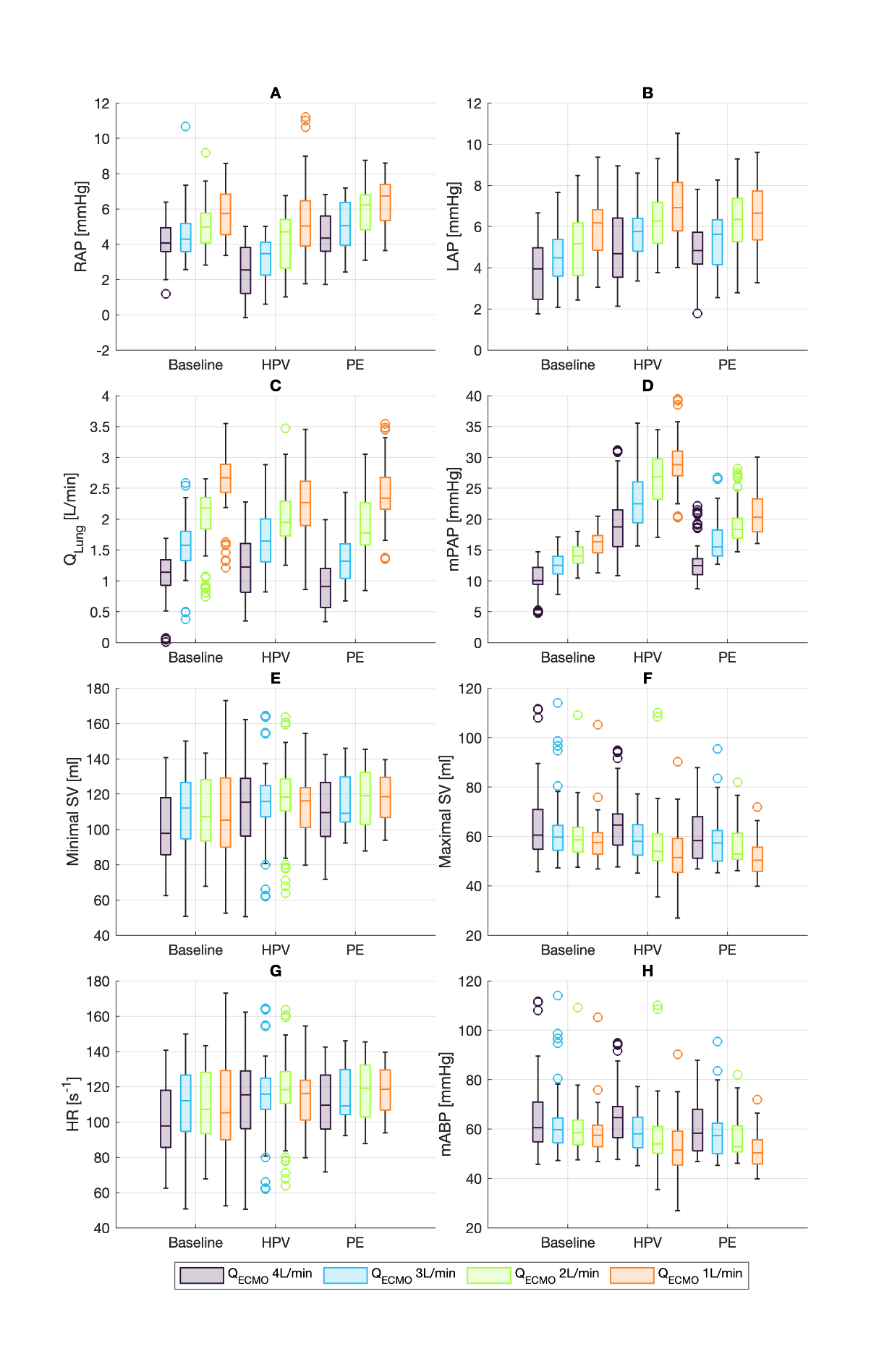


**Figure S1**. Boxplots of hemodynamic variables, presented by experimental condition and Q_ECMO_ (4, 3, 2, 1 L/min). HPV: Hypoxic pulmonary vasoconstriction. PE: Pulmonary embolism. **A**: Right atrial pressure. **B**: Left atrial pressue. **C**: pulmonary blood flow **D**: mean pulmonary arterial pressure. **E**: minimal stroke volume. **F**: maximal stroke volume. **G**: Heart rate. **H**: Mean systemic arterial blood pressure.

**Figure S2**


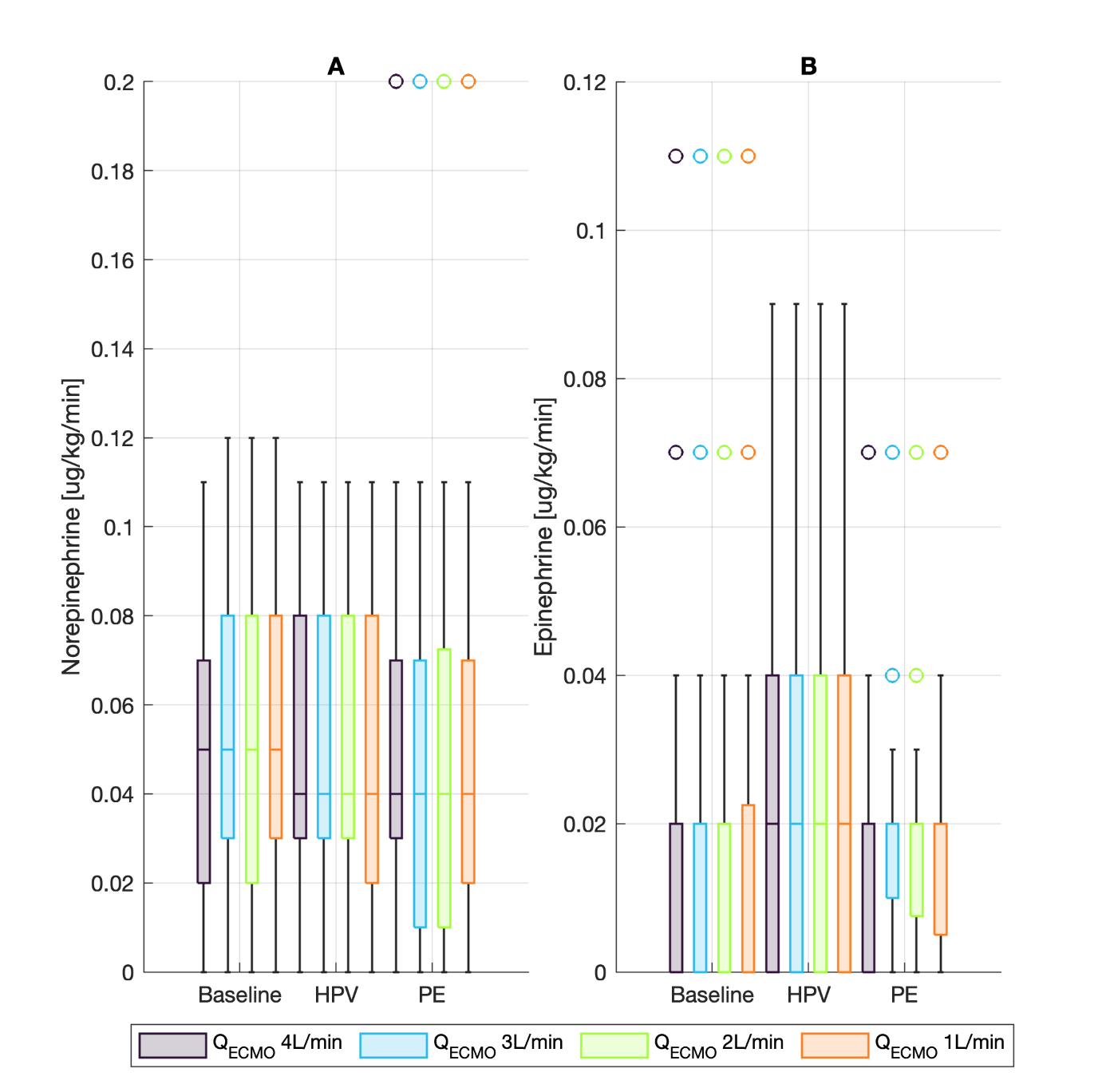


**Figure S2**. Boxplots of peak vasopressor dose (Norepinephrine and epinephrine) for each condition. **A**: Peak norepinephrine dose. **B**: Peak epinephrine dose.

**Table S1.** Supplementary table showing ECMO blood flow (Q_ECMO_) and respiratory parameters. PEEP: positive end-expiratory pressures, Vt: tidal volume, RR: respiratory rate.

| Condition | ECMO flow (per protocol) | QECMO [L/min] | Plateau pressure [cmH2O] | Peak pressure [cmH2O] | PEEP [cmH2O] | Vt [mL/kgBW] | RR [/min] |
| --- | --- | --- | --- | --- | --- | --- | --- |
| Baseline A | 4 L/min | 4.04 +/- 0.08 | 15.6 +/- 1.1 | 18.7 +/- 1.4 | 4.7 +/- 0.1 | 9.7 +/- 1.0 | 10.0 +/- 0.0 |
| Baseline A | 3 L/min | 3.01 +/- 0.08 | 15.4 +/- 1.3 | 18.4 +/- 1.5 | 4.7 +/- 0.1 | 9.9 +/- 1.1 | 10.0 +/- 0.0 |
| Baseline A | 2 L/min | 2.06 +/- 0.12 | 15.6 +/- 1.0 | 18.6 +/- 1.3 | 4.7 +/- 0.1 | 10.0 +/- 1.1 | 10.0 +/- 0.0 |
| Baseline A | 1 L/min | 0.95 +/- 0.08 | 15.4 +/- 1.3 | 18.5 +/- 1.4 | 4.7 +/- 0.1 | 9.9 +/- 1.1 | 10.0 +/- 0.0 |
| Baseline B | 4 L/min | 4.11 +/- 0.10 | 17.4 +/- 1.1 | 22.0 +/- 1.3 | 5.0 +/- 0.2 | 10.3 +/- 1.1 | 15.0 +/- 0.0 |
| Baseline B | 3 L/min | 3.07 +/- 0.08 | 17.0 +/- 1.3 | 21.8 +/- 1.6 | 4.9 +/- 0.1 | 10.1 +/- 1.2 | 15.0 +/- 0.0 |
| Baseline B | 2 L/min | 2.03 +/- 0.06 | 17.0 +/- 1.2 | 21.7 +/- 1.7 | 4.9 +/- 0.2 | 10.2 +/- 1.1 | 15.0 +/- 0.0 |
| Baseline B | 1 L/min | 0.91 +/- 0.09 | 16.8 +/- 1.3 | 21.6 +/- 1.6 | 4.9 +/- 0.2 | 10.2 +/- 1.1 | 15.0 +/- 0.0 |
| Baseline C | 4 L/min | 4.01 +/- 0.10 | 16.1 +/- 1.0 | 19.2 +/- 1.2 | 4.7 +/- 0.1 | 10.0 +/- 1.2 | 10.0 +/- 0.0 |
| Baseline C | 3 L/min | 3.05 +/- 0.07 | 16.0 +/- 0.9 | 19.1 +/- 1.1 | 4.7 +/- 0.1 | 9.9 +/- 1.2 | 10.0 +/- 0.0 |
| Baseline C | 2 L/min | 2.00 +/- 0.06 | 16.1 +/- 1.2 | 19.3 +/- 1.4 | 4.7 +/- 0.1 | 10.1 +/- 1.2 | 10.0 +/- 0.0 |
| Baseline C | 1 L/min | 0.92 +/- 0.10 | 15.9 +/- 1.3 | 19.0 +/- 1.6 | 4.6 +/- 0.1 | 10.0 +/- 1.1 | 10.0 +/- 0.0 |
| HPV A | 4 L/min | 4.08 +/- 0.10 | 20.3 +/- 2.8 | 23.4 +/- 3.3 | 4.7 +/- 0.1 | 5.9 +/- 0.6 | 10.0 +/- 0.0 |
| HPV A | 3 L/min | 2.97 +/- 0.06 | 20.3 +/- 2.5 | 23.5 +/- 3.0 | 4.7 +/- 0.1 | 6.0 +/- 0.6 | 10.0 +/- 0.0 |
| HPV A | 2 L/min | 2.00 +/- 0.11 | 20.6 +/- 2.6 | 23.7 +/- 3.2 | 4.3 +/- 1.3 | 6.0 +/- 0.6 | 10.0 +/- 0.0 |
| HPV A | 1 L/min | 0.96 +/- 0.07 | 20.8 +/- 2.8 | 24.0 +/- 3.4 | 4.6 +/- 0.1 | 5.9 +/- 0.6 | 10.0 +/- 0.0 |
| HPV B | 4 L/min | 4.05 +/- 0.14 | 23.6 +/- 2.5 | 27.0 +/- 2.8 | 4.6 +/- 0.1 | 6.0 +/- 0.7 | 20.0 +/- 0.0 |
| HPV B | 3 L/min | 3.01 +/- 0.08 | 23.0 +/- 3.2 | 26.5 +/- 3.4 | 4.6 +/- 0.1 | 6.1 +/- 0.8 | 20.0 +/- 0.0 |
| HPV B | 2 L/min | 1.99 +/- 0.06 | 24.1 +/- 5.2 | 28.0 +/- 6.8 | 4.6 +/- 0.2 | 6.1 +/- 0.8 | 20.0 +/- 0.0 |
| HPV B | 1 L/min | 0.98 +/- 0.16 | 23.8 +/- 3.4 | 28.9 +/- 6.2 | 4.6 +/- 0.3 | 6.2 +/- 0.9 | 20.0 +/- 0.0 |
| PE A | 4 L/min | 4.07 +/- 0.07 | 16.7 +/- 1.4 | 19.7 +/- 1.6 | 4.7 +/- 0.1 | 9.8 +/- 1.0 | 10.0 +/- 0.0 |
| PE A | 3 L/min | 3.01 +/- 0.06 | 16.7 +/- 1.4 | 19.8 +/- 1.7 | 4.7 +/- 0.1 | 9.9 +/- 1.2 | 10.0 +/- 0.0 |
| PE A | 2 L/min | 2.00 +/- 0.07 | 16.9 +/- 1.5 | 20.2 +/- 1.9 | 4.6 +/- 0.1 | 10.0 +/- 1.1 | 10.0 +/- 0.0 |
| PE A | 1 L/min | 0.99 +/- 0.12 | 17.1 +/- 1.6 | 20.4 +/- 2.0 | 4.6 +/- 0.1 | 10.0 +/- 1.1 | 10.0 +/- 0.0 |
| PE B | 4 L/min | 4.12 +/- 0.08 | 18.6 +/- 1.7 | 23.6 +/- 1.9 | 4.9 +/- 0.1 | 10.1 +/- 0.9 | 15.0 +/- 0.0 |
| PE B | 3 L/min | 3.01 +/- 0.09 | 18.8 +/- 1.8 | 23.8 +/- 2.2 | 4.8 +/- 0.1 | 10.1 +/- 1.0 | 15.0 +/- 0.0 |
| PE B | 2 L/min | 1.98 +/- 0.09 | 18.7 +/- 1.9 | 23.7 +/- 2.3 | 4.8 +/- 0.1 | 9.8 +/- 0.8 | 15.0 +/- 0.0 |
| PE B | 1 L/min | 0.95 +/- 0.12 | 18.7 +/- 1.9 | 23.7 +/- 2.3 | 4.7 +/- 0.2 | 9.9 +/- 1.0 | 15.0 +/- 0.0 |
