# Supplementary material for: Mechanisms maintaining right ventricular contractility-to-pulmonary arterial elastance ratio in VA ECMO: a retrospective animal data analysis of RV–PA coupling
Source: J Intensive Care. 2024 May 11;12:19. doi: 10.1186/s40560-024-00730-6 (PMC11088130; doi:10.1186/s40560-024-00730-6)
Supplement: Supplementary file 1 — Additional file 1. Visualization of full dataset. [file 40560_2024_730_MOESM1_ESM.pdf]

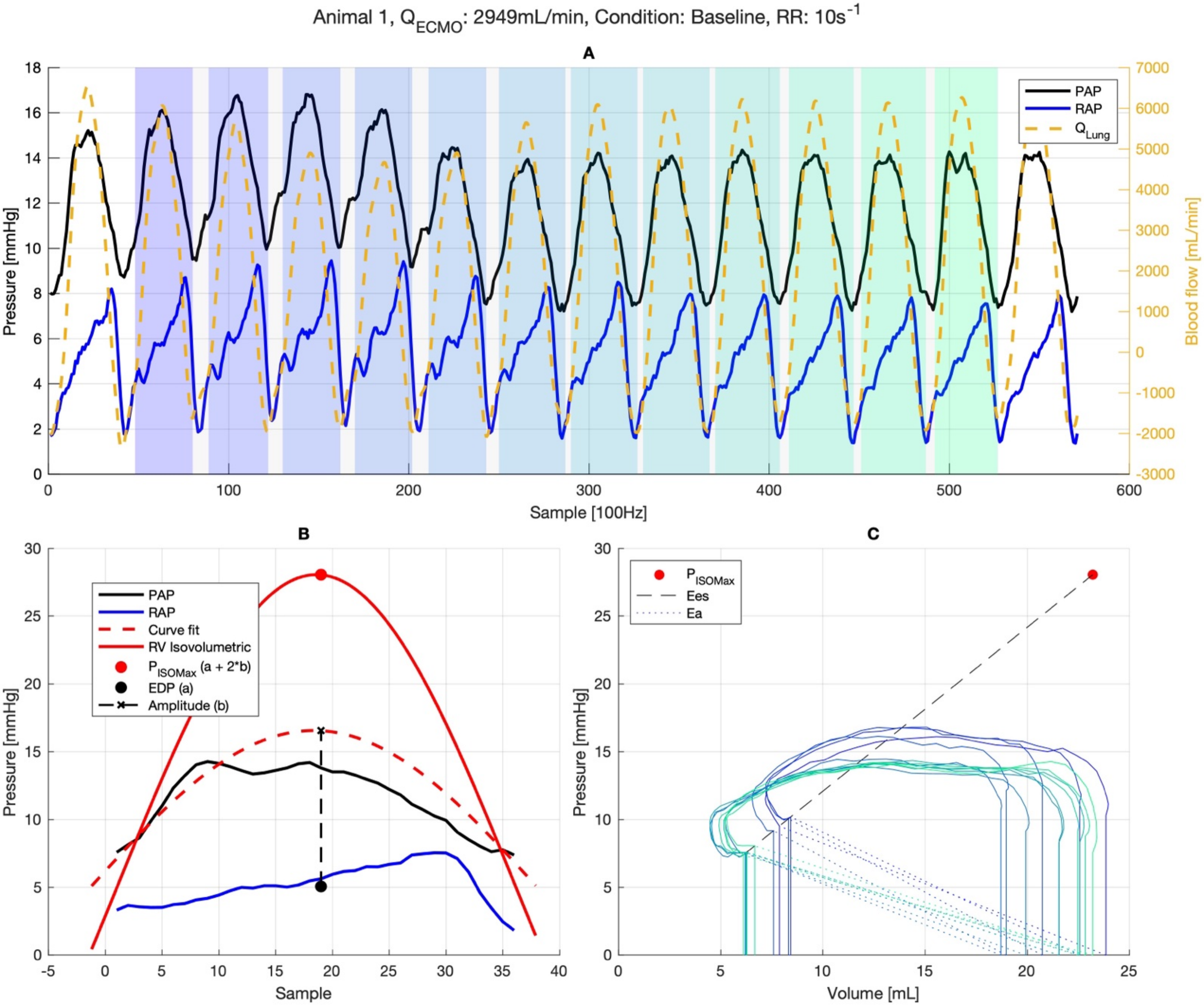

Animal 1, Q_{ECMO}: 2949mL/min, Condition: Baseline, RR: $10s^{-1}$
A
PAP
RAP
Q_{Lung}
Pressure [mmHg]
Blood flow [mL/min]
Sample [100Hz]
B
PAP
RAP
Curve fit
RV Isovolumetric
P_{ISOMax} (a + 2*b)
EDP (a)
Amplitude (b)
Pressure [mmHg]
Sample
C
P_{ISOMax}
Ees
Ea
Pressure [mmHg]
Volume [mL]

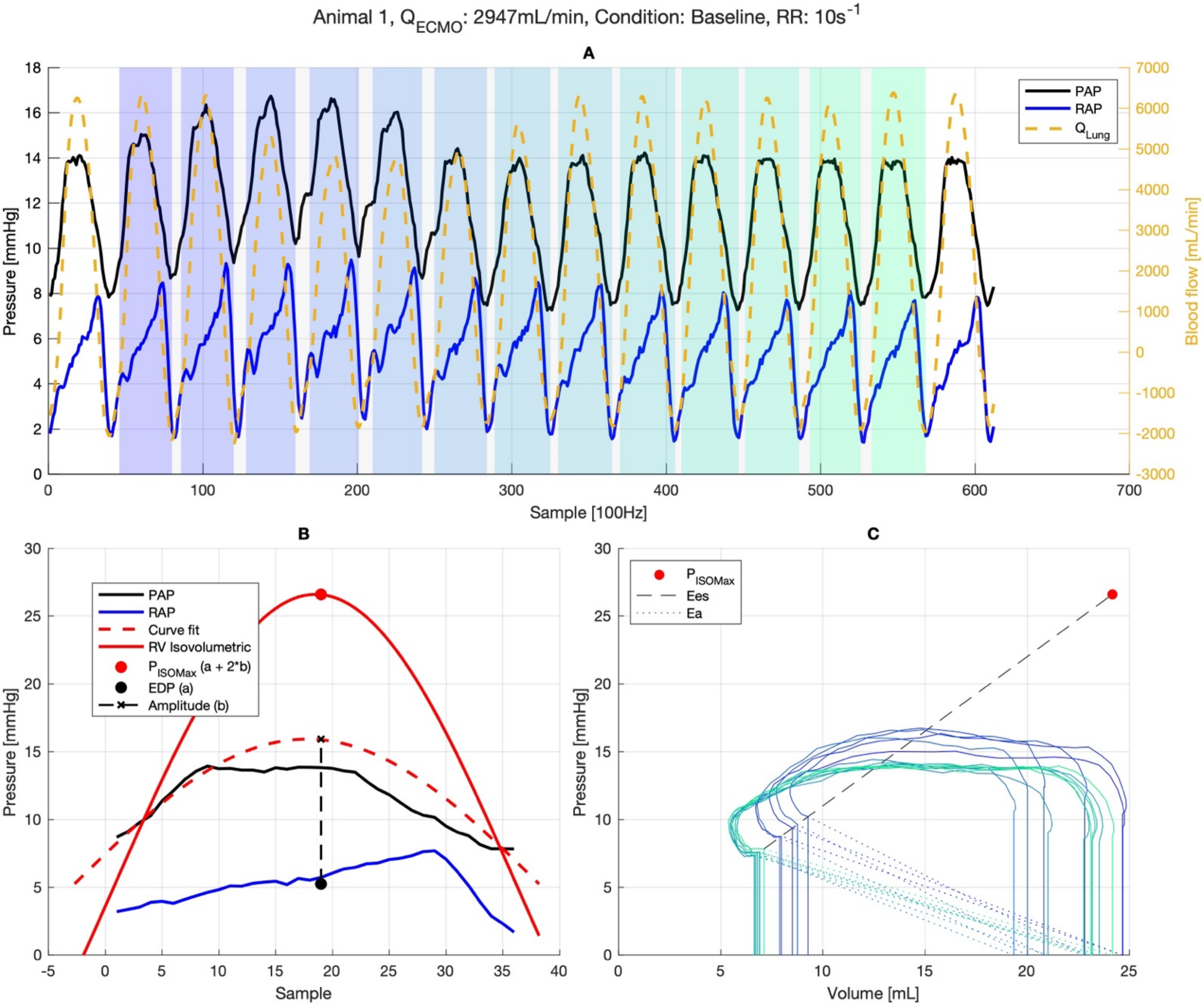

Animal 1, Q_{ECMO}: 2947mL/min, Condition: Baseline, RR: $10s^{-1}$
A
PAP
RAP
Q_{Lung}
Pressure [mmHg]
Blood flow [mL/min]
Sample [100Hz]
B
PAP
RAP
Curve fit
RV Isovolumetric
P_{ISOMax} (a + 2*b)
EDP (a)
Amplitude (b)
Pressure [mmHg]
Sample
C
P_{ISOMax}
Ees
Ea
Pressure [mmHg]
Volume [mL]

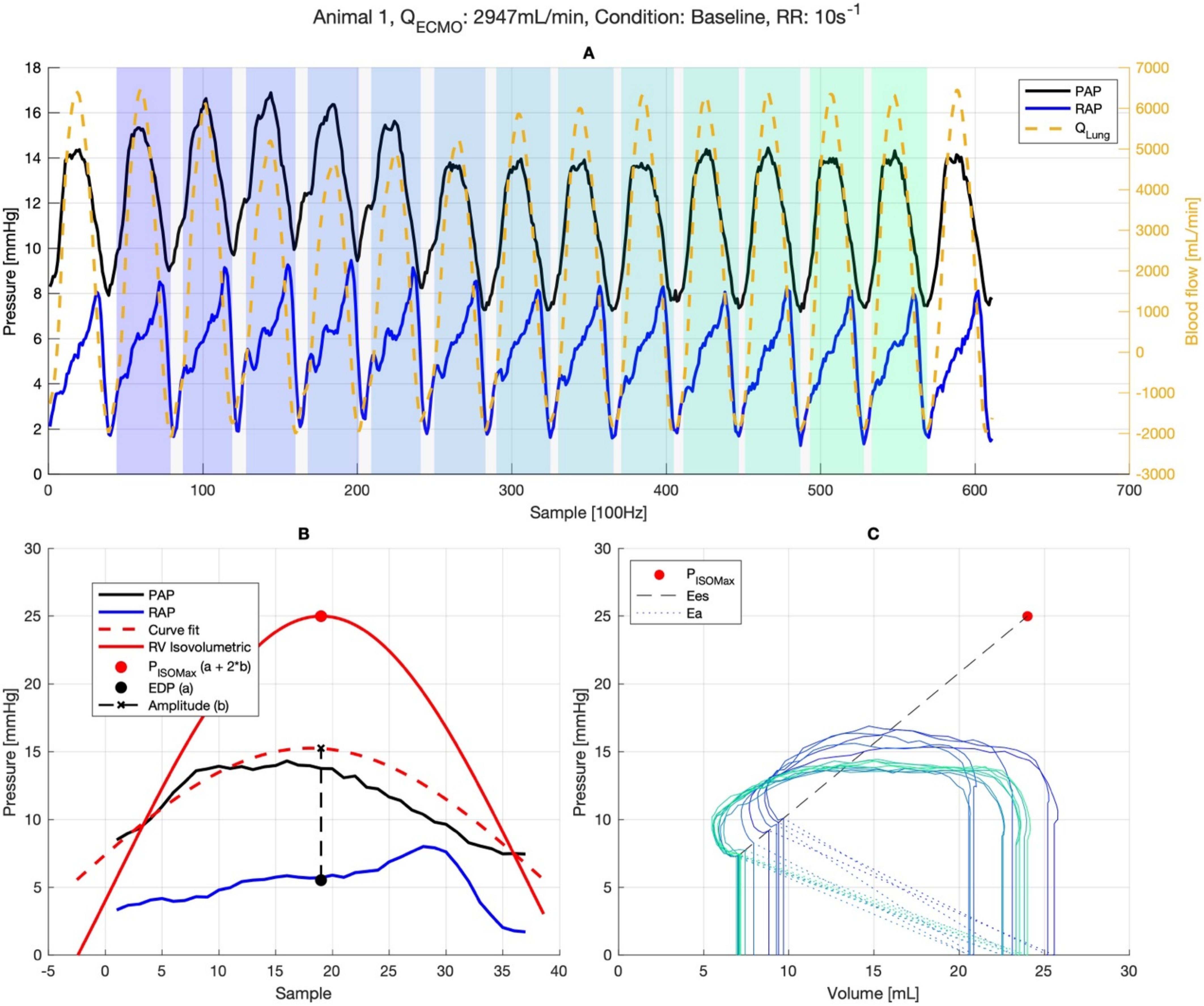

Animal 1, Q_ECMO: 2947mL/min, Condition: Baseline, RR: 10s^-1
A
PAP
RAP
Q_Lung
Pressure [mmHg]
Blood flow [mL/min]
Sample [100Hz]
B
PAP
RAP
Curve fit
RV Isovolumetric
P_ISOMax (a + 2*b)
EDP (a)
Amplitude (b)
Pressure [mmHg]
Sample
C
P_ISOMax
Ees
Ea
Pressure [mmHg]
Volume [mL]

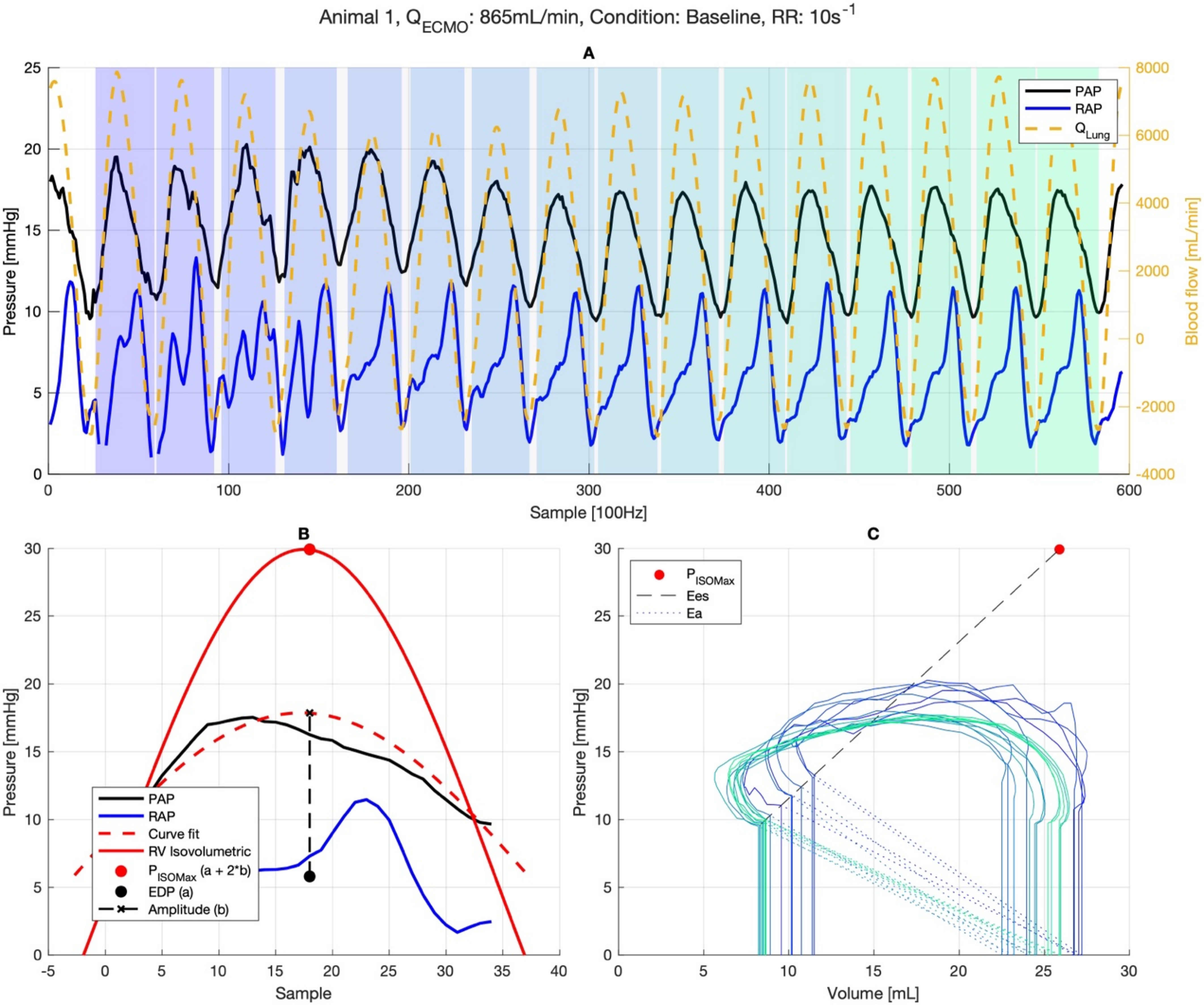

Animal 1, Q_ECMO: 865mL/min, Condition: Baseline, RR: 10s^-1
A
PAP
RAP
Q_Lung
Pressure [mmHg]
Blood flow [mL/min]
Sample [100Hz]
B
PAP
RAP
Curve fit
RV Isovolumetric
P_ISOMax (a + 2*b)
EDP (a)
Amplitude (b)
Pressure [mmHg]
Sample
C
P_ISOMax
Ees
Ea
Pressure [mmHg]
Volume [mL]

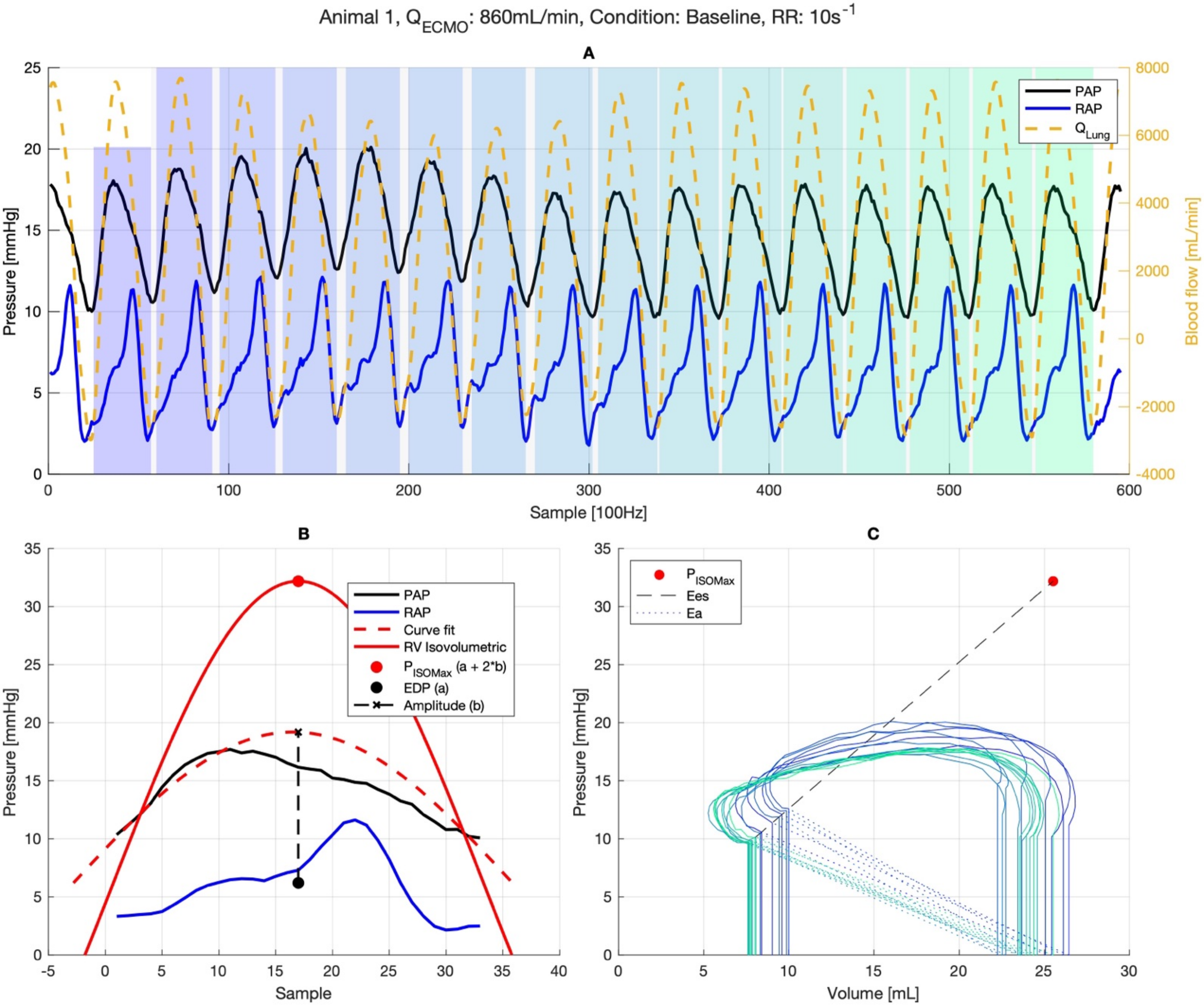

Animal 1, Q_ECMO: 860mL/min, Condition: Baseline, RR: 10s^-1
A
PAP
RAP
Q_Lung
Pressure [mmHg]
Blood flow [mL/min]
Sample [100Hz]
B
PAP
RAP
Curve fit
RV Isovolumetric
P_ISOMax (a + 2*b)
EDP (a)
Amplitude (b)
Pressure [mmHg]
Sample
C
P_ISOMax
Ees
Ea
Pressure [mmHg]
Volume [mL]

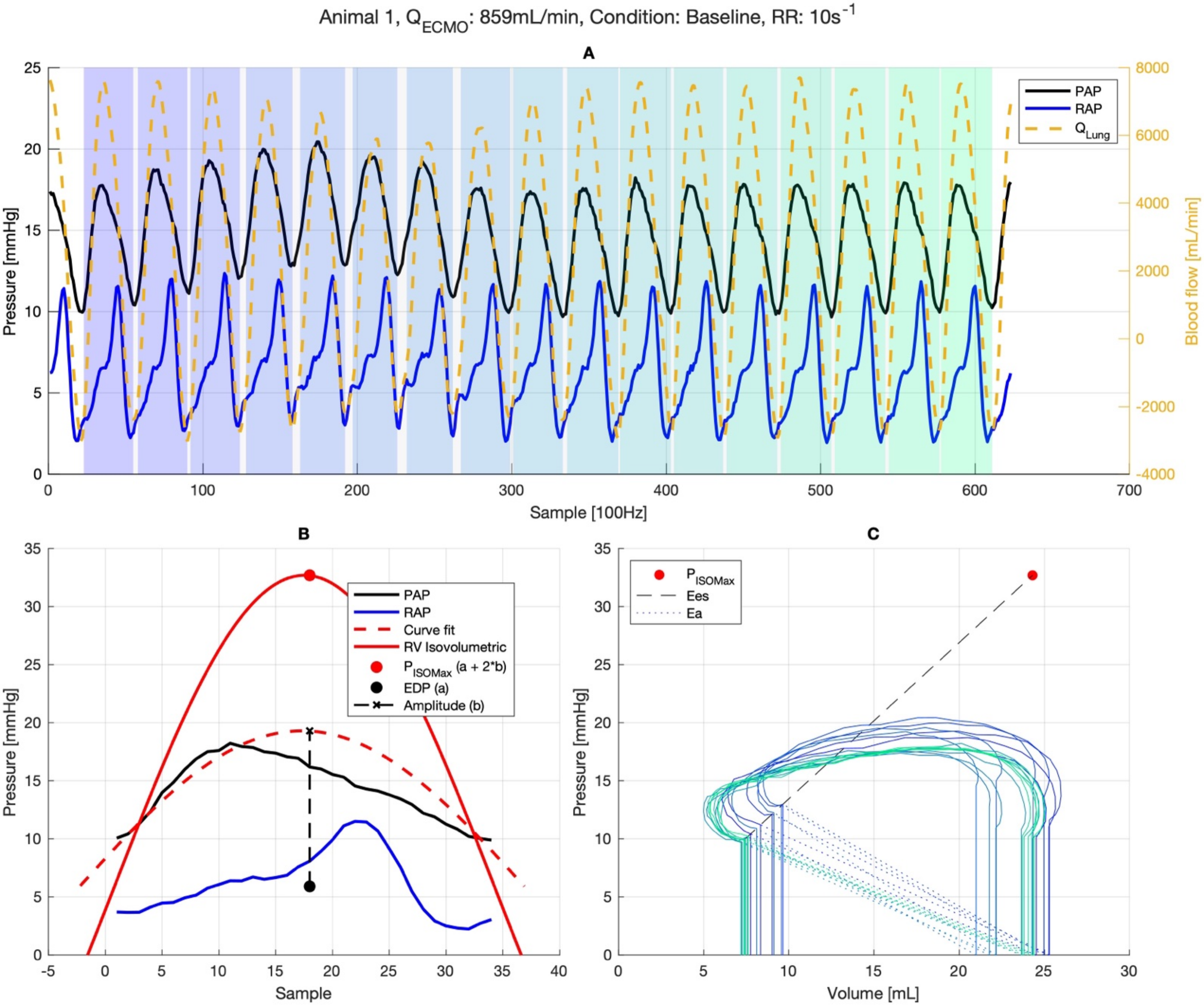

Animal 1, Q_ECMO: 859mL/min, Condition: Baseline, RR: 10s^-1
A
Pressure [mmHg]
Blood flow [mL/min]
Sample [100Hz]
PAP
RAP
Q_Lung
B
Pressure [mmHg]
Sample
PAP
RAP
Curve fit
RV Isovolumetric
P_ISOMax (a + 2*b)
EDP (a)
Amplitude (b)
C
Pressure [mmHg]
Volume [mL]
P_ISOMax
Ees
Ea

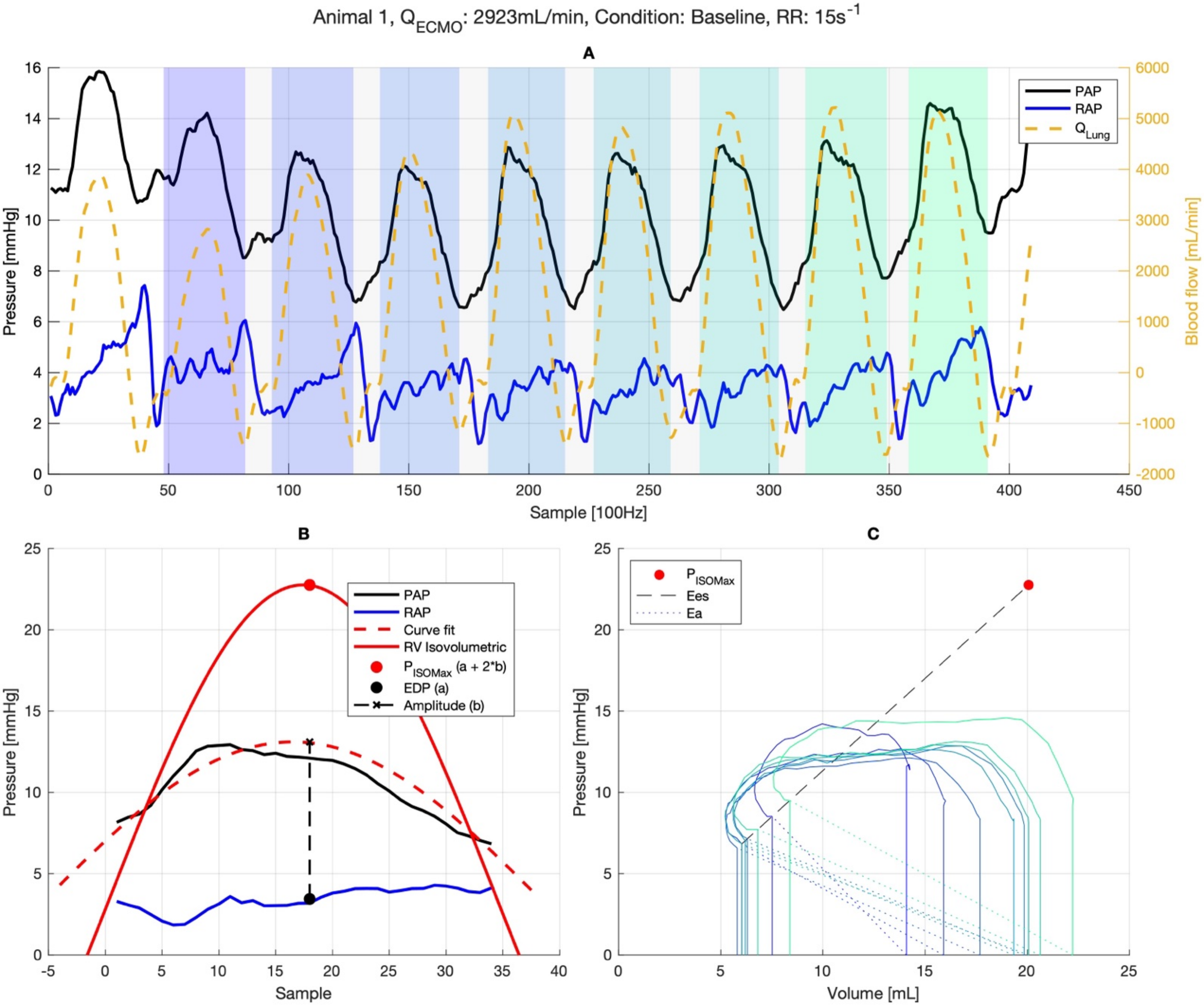

Animal 1, QECMO: 2923mL/min, Condition: Baseline, RR: 15s-1
A
PAP
RAP
QLung
Pressure [mmHg]
Blood flow [mL/min]
Sample [100Hz]
B
PAP
RAP
Curve fit
RV Isovolumetric
PISOMax (a + 2*b)
EDP (a)
Amplitude (b)
Pressure [mmHg]
Sample
C
PISOMax
Ees
Ea
Pressure [mmHg]
Volume [mL]

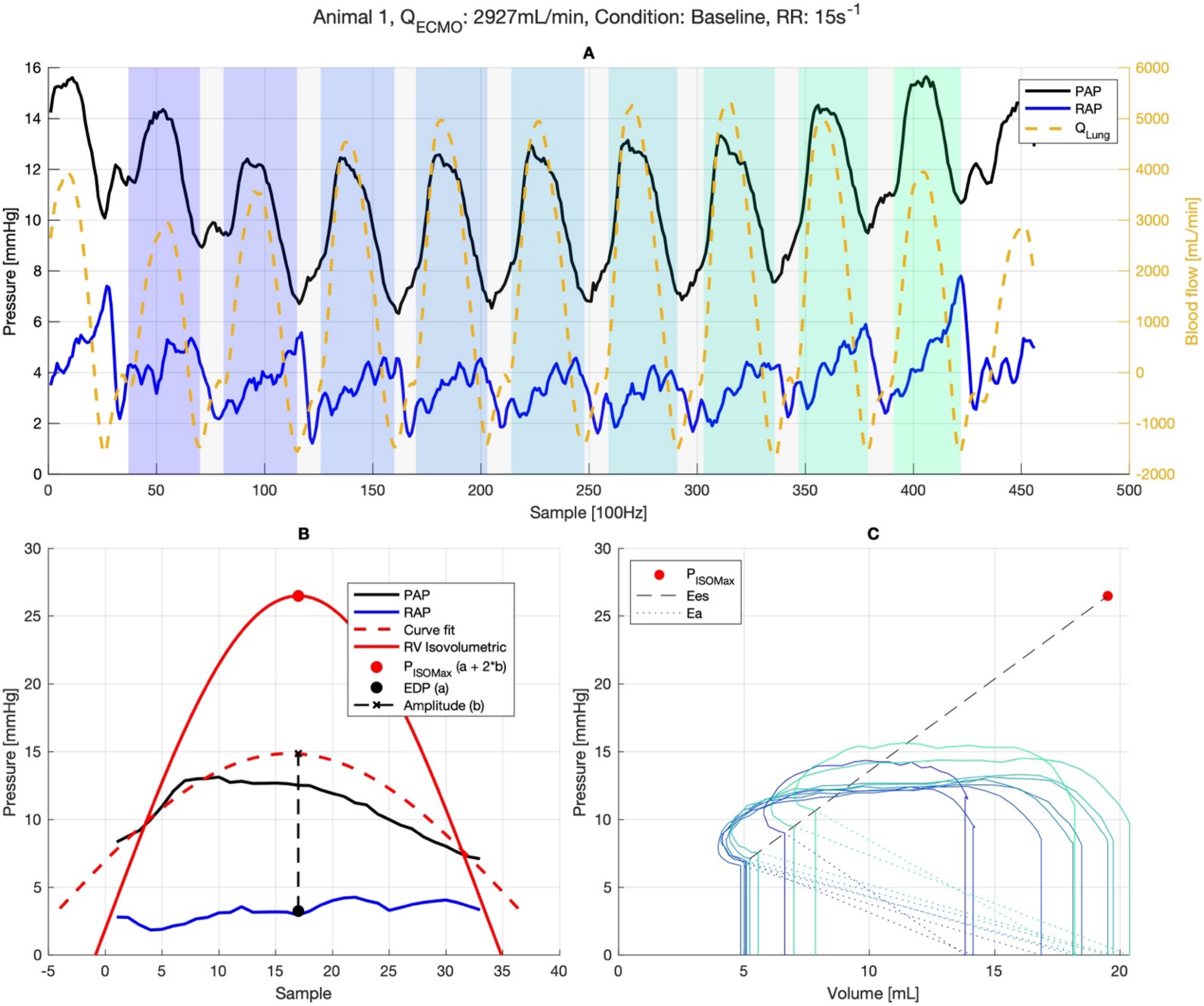

Animal 1, Q_ECMO: 2927mL/min, Condition: Baseline, RR: 15s^-1
A
Pressure [mmHg]
Blood flow [mL/min]
Sample [100Hz]
PAP
RAP
Q_Lung
B
Pressure [mmHg]
Sample
PAP
RAP
Curve fit
RV Isovolumetric
P_ISOMax (a + 2*b)
EDP (a)
Amplitude (b)
C
Pressure [mmHg]
Volume [mL]
P_ISOMax
Ees
Ea

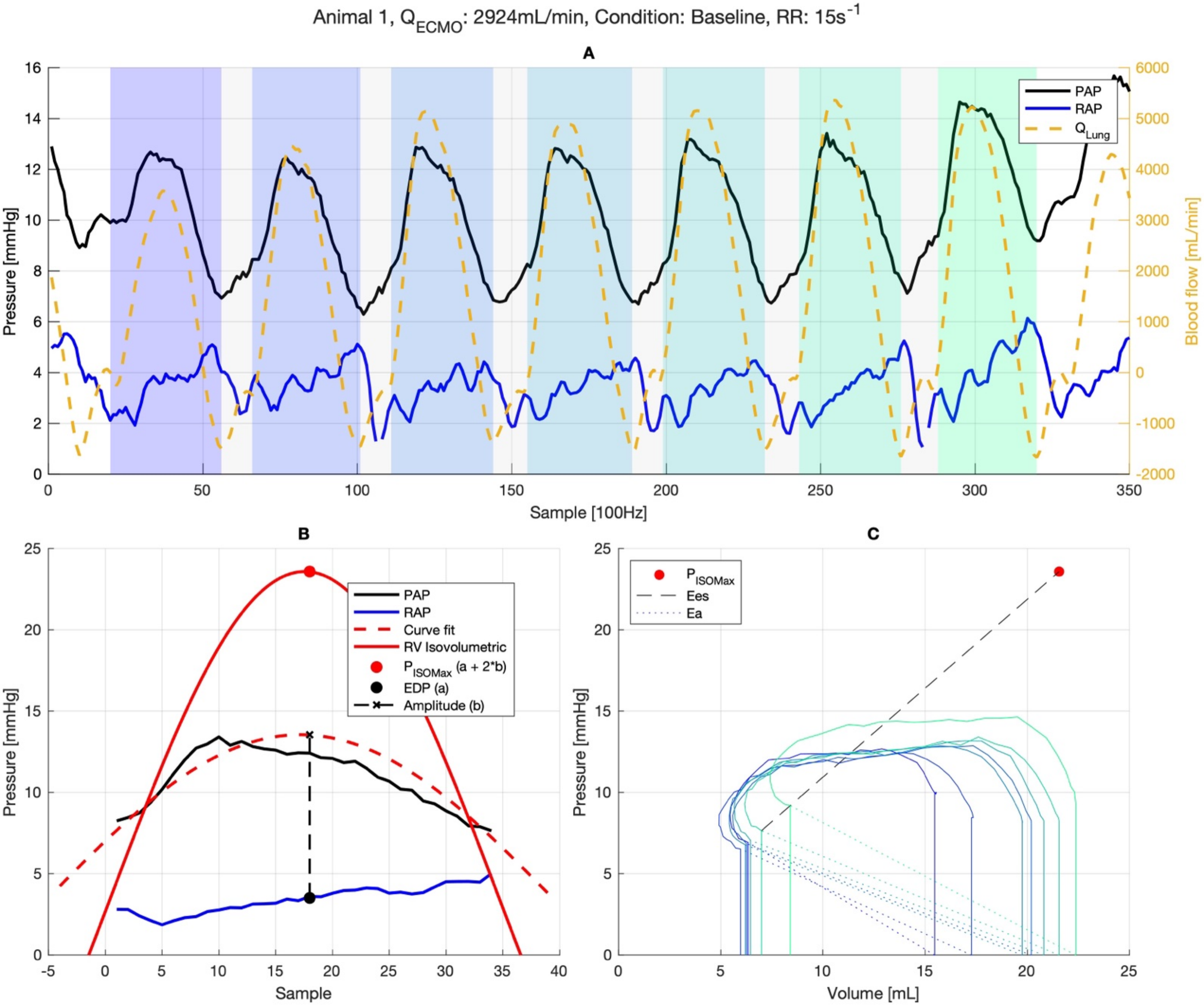
Animal 1, Q_ECMO: 2924mL/min, Condition: Baseline, RR: 15s^-1
A
Pressure [mmHg]
Blood flow [mL/min]
Sample [100Hz]
PAP
RAP
Q_Lung
B
Pressure [mmHg]
Sample
PAP
RAP
Curve fit
RV Isovolumetric
P_ISOMax (a + 2*b)
EDP (a)
Amplitude (b)
C
Pressure [mmHg]
Volume [mL]
P_ISOMax
Ees
Ea

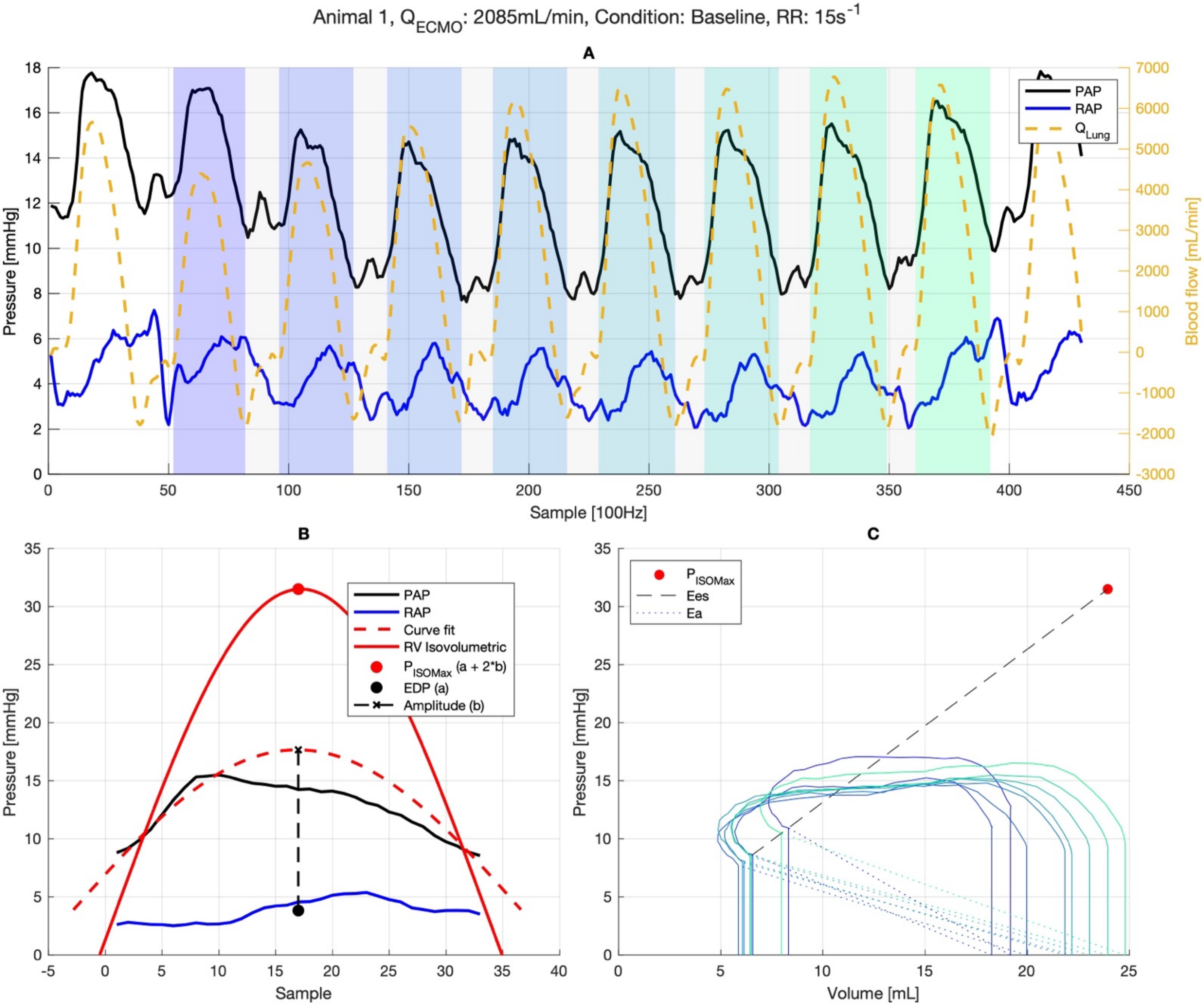

Animal 1, Q_{ECMO}: 2085mL/min, Condition: Baseline, RR: $15s^{-1}$
A
Pressure [mmHg]
Blood flow [mL/min]
Sample [100Hz]
PAP
RAP
Q_{Lung}
B
Pressure [mmHg]
Sample
PAP
RAP
Curve fit
RV Isovolumetric
P_{ISOMax} (a + 2*b)
EDP (a)
Amplitude (b)
C
Pressure [mmHg]
Volume [mL]
P_{ISOMax}
Ees
Ea

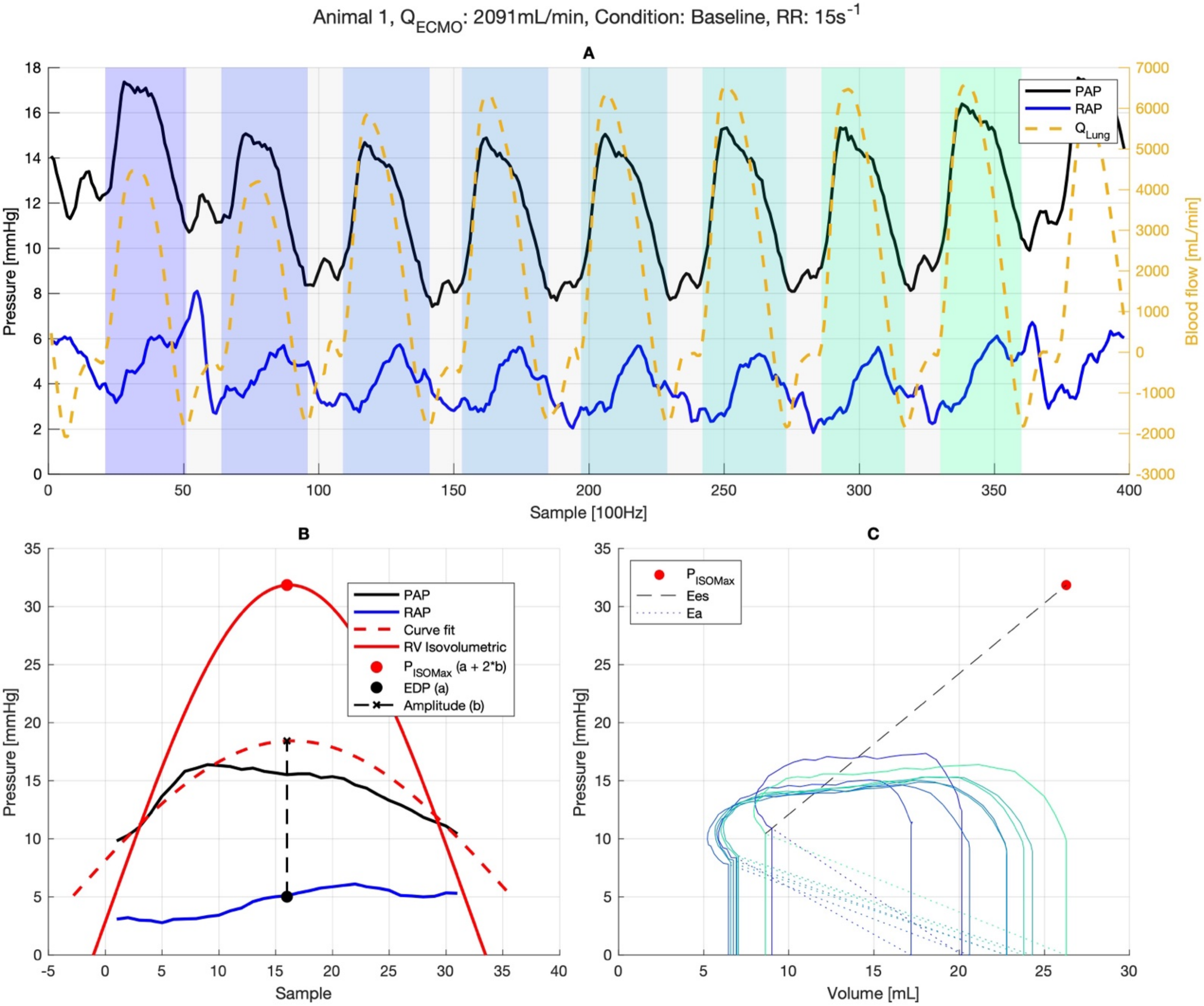

Animal 1, QECMO: 2091mL/min, Condition: Baseline, RR: 15s-1
A
PAP
RAP
QLung
Pressure [mmHg]
Blood flow [mL/min]
Sample [100Hz]
B
PAP
RAP
Curve fit
RV Isovolumetric
PISOMax (a + 2*b)
EDP (a)
Amplitude (b)
Pressure [mmHg]
Sample
C
PISOMax
Ees
Ea
Pressure [mmHg]
Volume [mL]

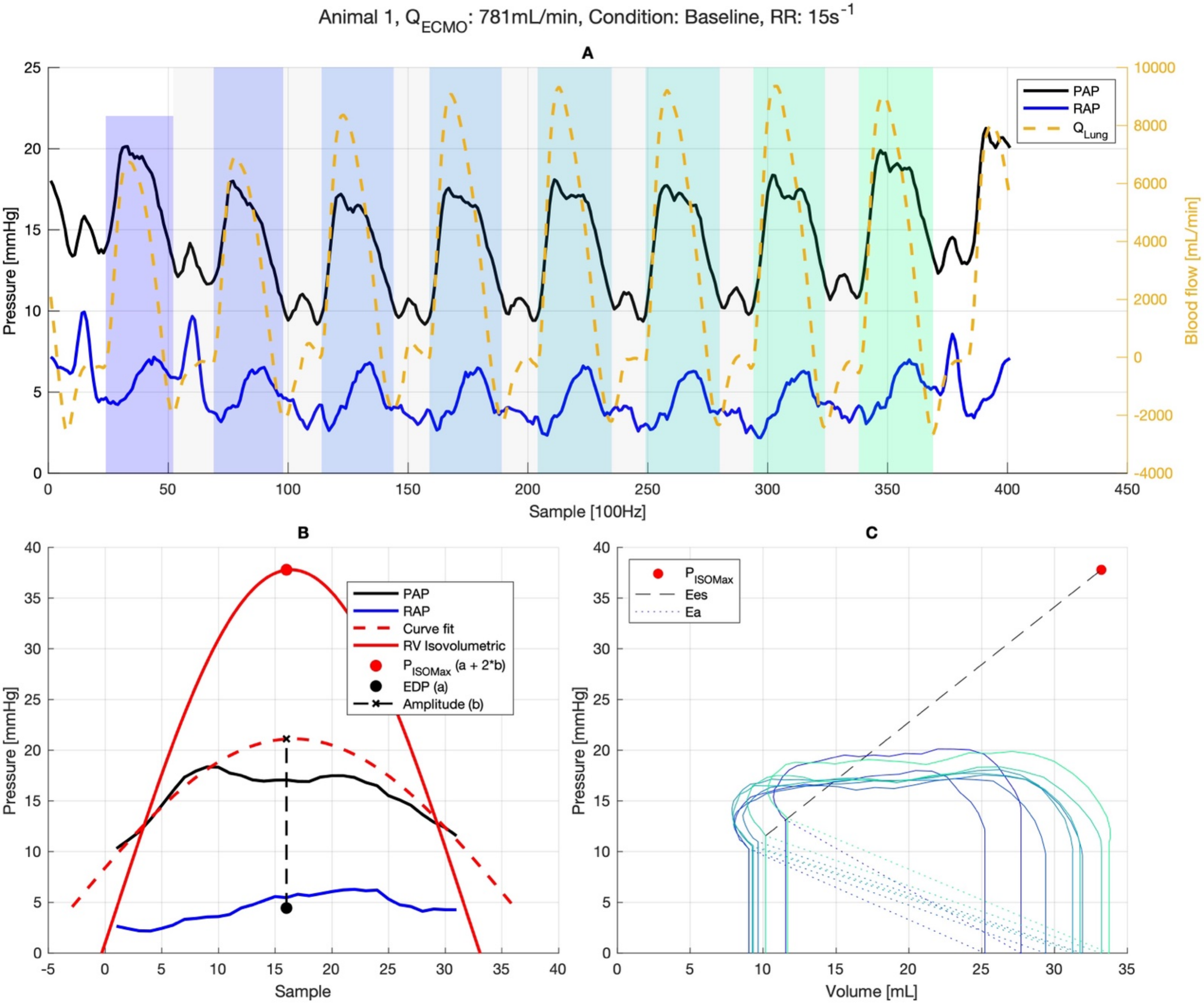

Animal 1, Q_{ECMO}: 781mL/min, Condition: Baseline, RR: $15s^{-1}$
A
Pressure [mmHg]
Blood flow [mL/min]
Sample [100Hz]
PAP
RAP
Q_{Lung}
B
Pressure [mmHg]
Sample
PAP
RAP
Curve fit
RV Isovolumetric
P_{ISOMax} (a + 2*b)
EDP (a)
Amplitude (b)
C
Pressure [mmHg]
Volume [mL]
P_{ISOMax}
Ees
Ea

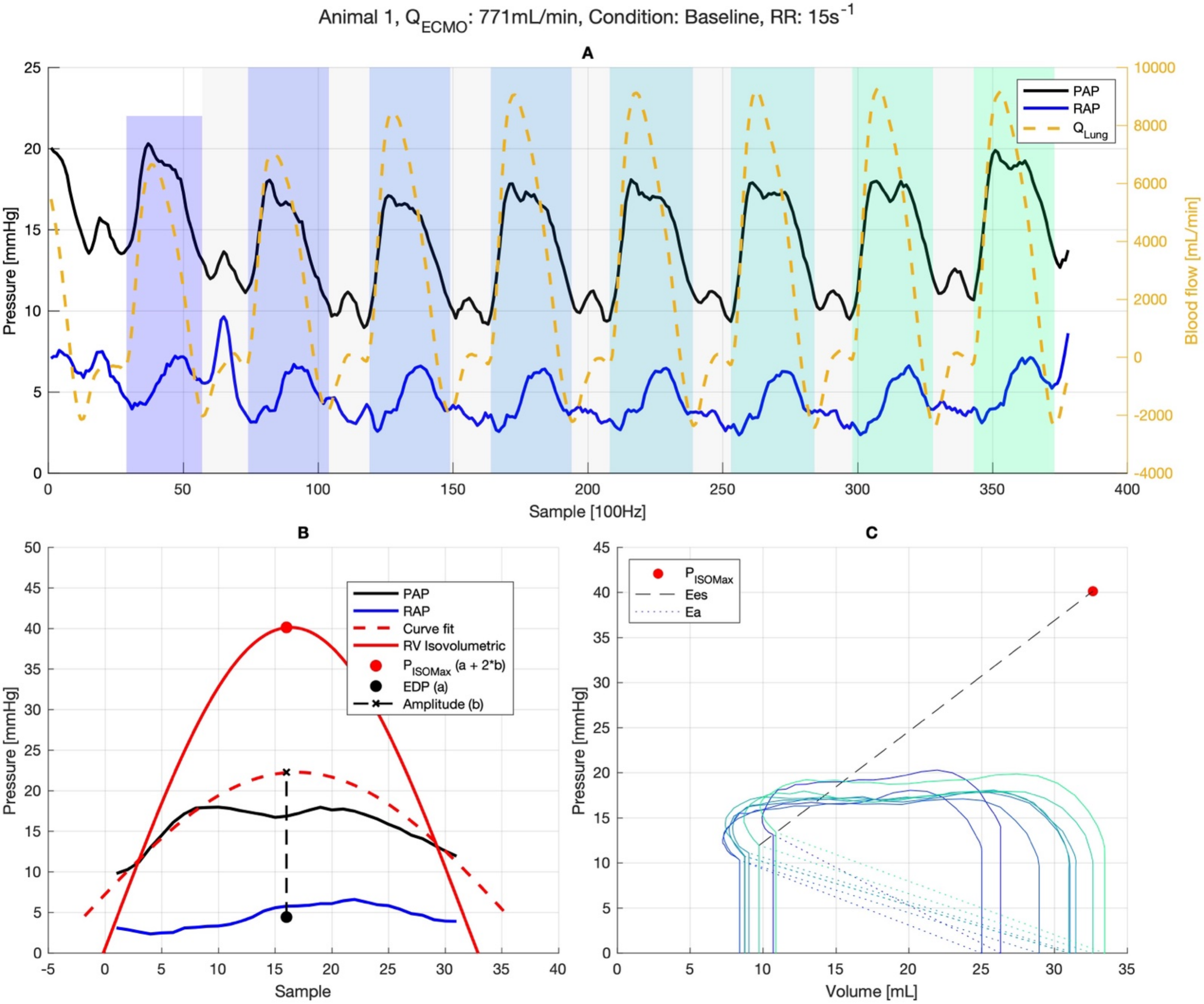

Animal 1, Q_ECMO: 771mL/min, Condition: Baseline, RR: 15s^-1
A
Pressure [mmHg]
Blood flow [mL/min]
Sample [100Hz]
PAP
RAP
Q_Lung
B
Pressure [mmHg]
Sample
PAP
RAP
Curve fit
RV Isovolumetric
P_ISOMax (a + 2*b)
EDP (a)
Amplitude (b)
C
Pressure [mmHg]
Volume [mL]
P_ISOMax
Ees
Ea

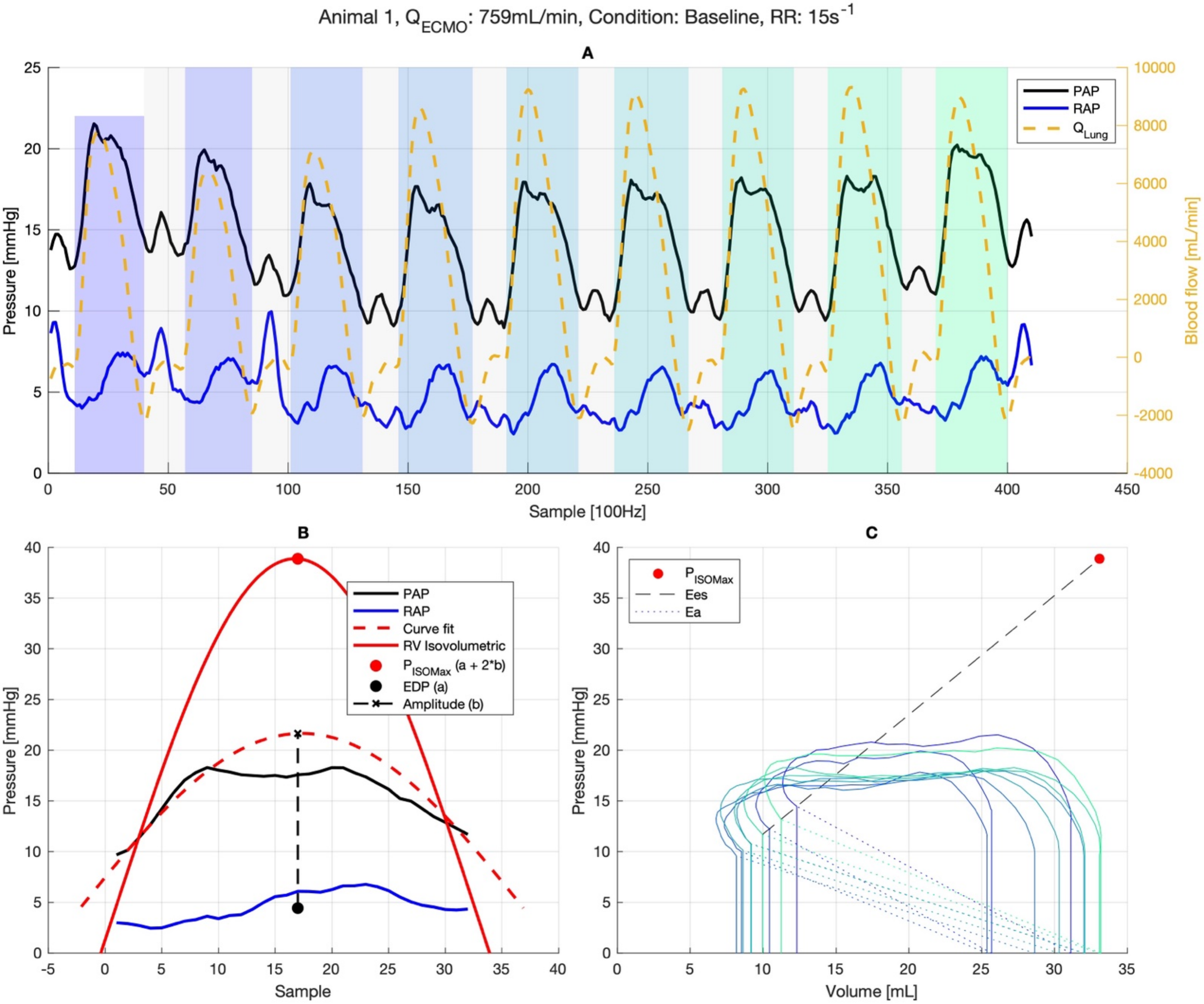

Animal 1, Q_ECMO: 759mL/min, Condition: Baseline, RR: 15s-1
A
Pressure [mmHg]
Blood flow [mL/min]
Sample [100Hz]
PAP
RAP
Q_Lung
B
Pressure [mmHg]
Sample
PAP
RAP
Curve fit
RV Isovolumetric
P_ISOMax (a + 2*b)
EDP (a)
Amplitude (b)
C
Pressure [mmHg]
Volume [mL]
P_ISOMax
Ees
Ea

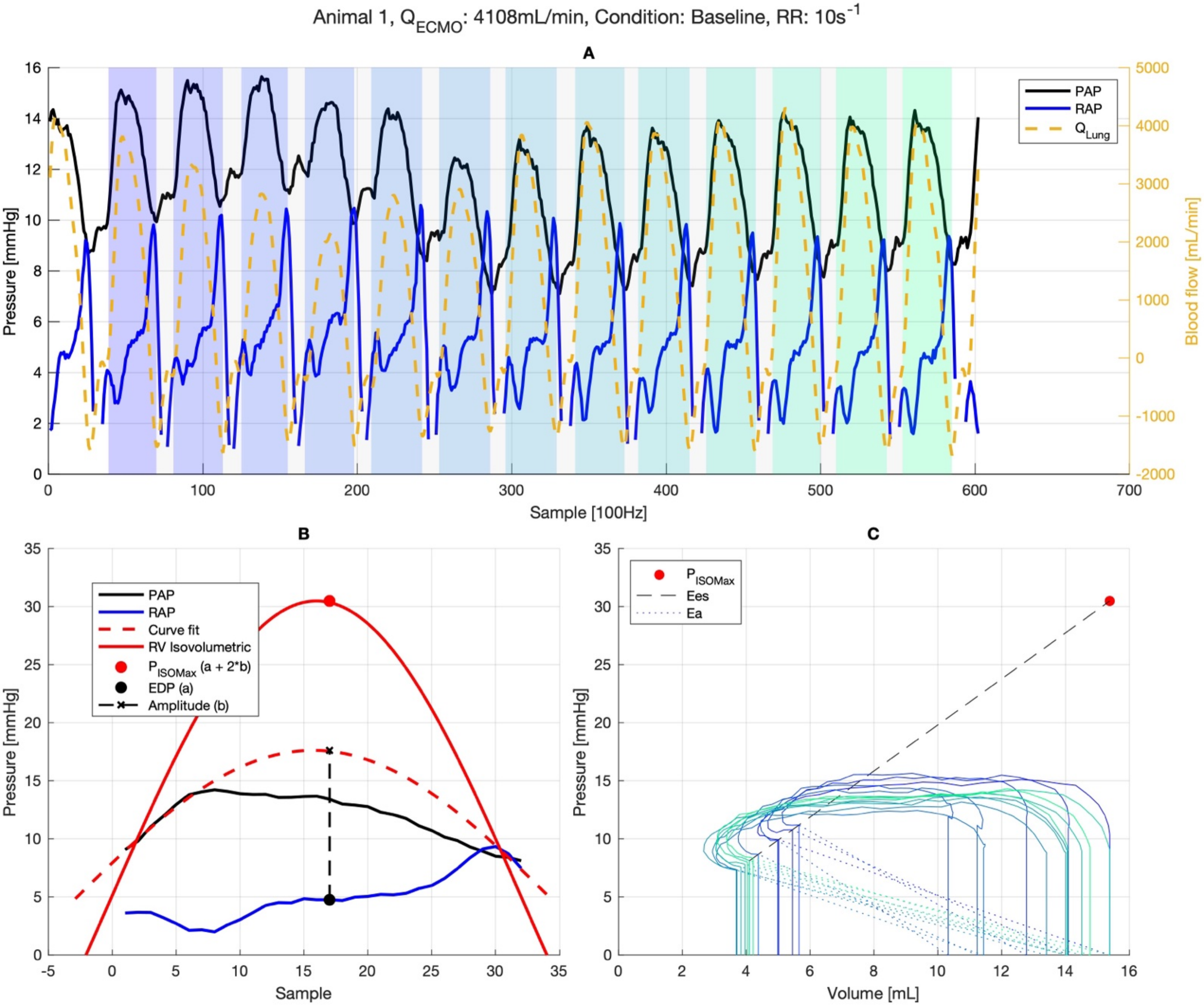

Animal 1, Q_{ECMO}: 4108mL/min, Condition: Baseline, RR: $10s^{-1}$
A
PAP
RAP
Q_{Lung}
Pressure [mmHg]
Blood flow [mL/min]
Sample [100Hz]
B
PAP
RAP
Curve fit
RV Isovolumetric
P_{ISOMax} (a + 2*b)
EDP (a)
Amplitude (b)
Pressure [mmHg]
Sample
C
P_{ISOMax}
Ees
Ea
Pressure [mmHg]
Volume [mL]

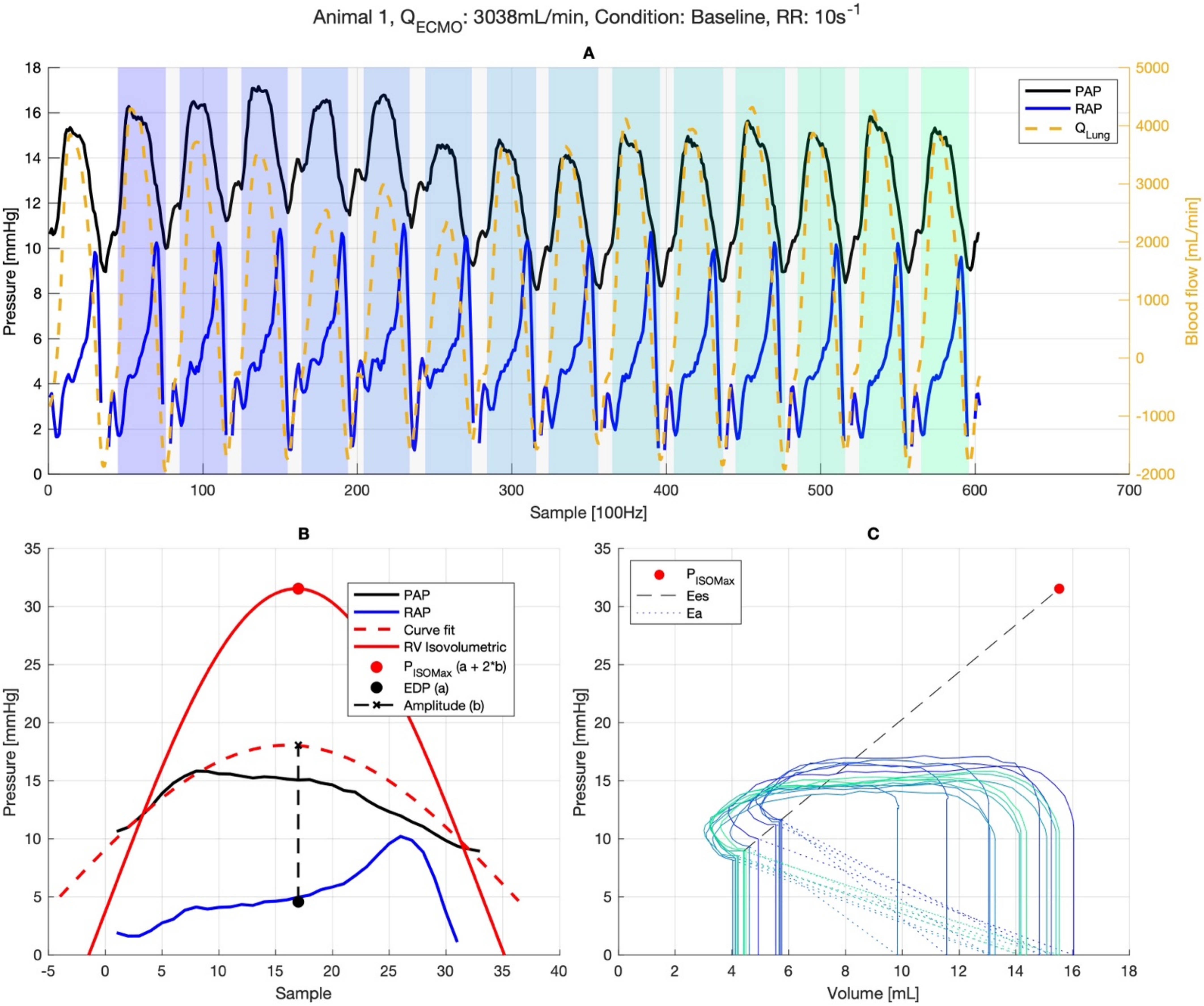

Animal 1, QECMO: 3038mL/min, Condition: Baseline, RR: 10s-1
A
PAP
RAP
QLung
Pressure [mmHg]
Blood flow [mL/min]
Sample [100Hz]
B
PAP
RAP
Curve fit
RV Isovolumetric
PISOMax (a + 2*b)
EDP (a)
Amplitude (b)
Pressure [mmHg]
Sample
C
PISOMax
Ees
Ea
Pressure [mmHg]
Volume [mL]

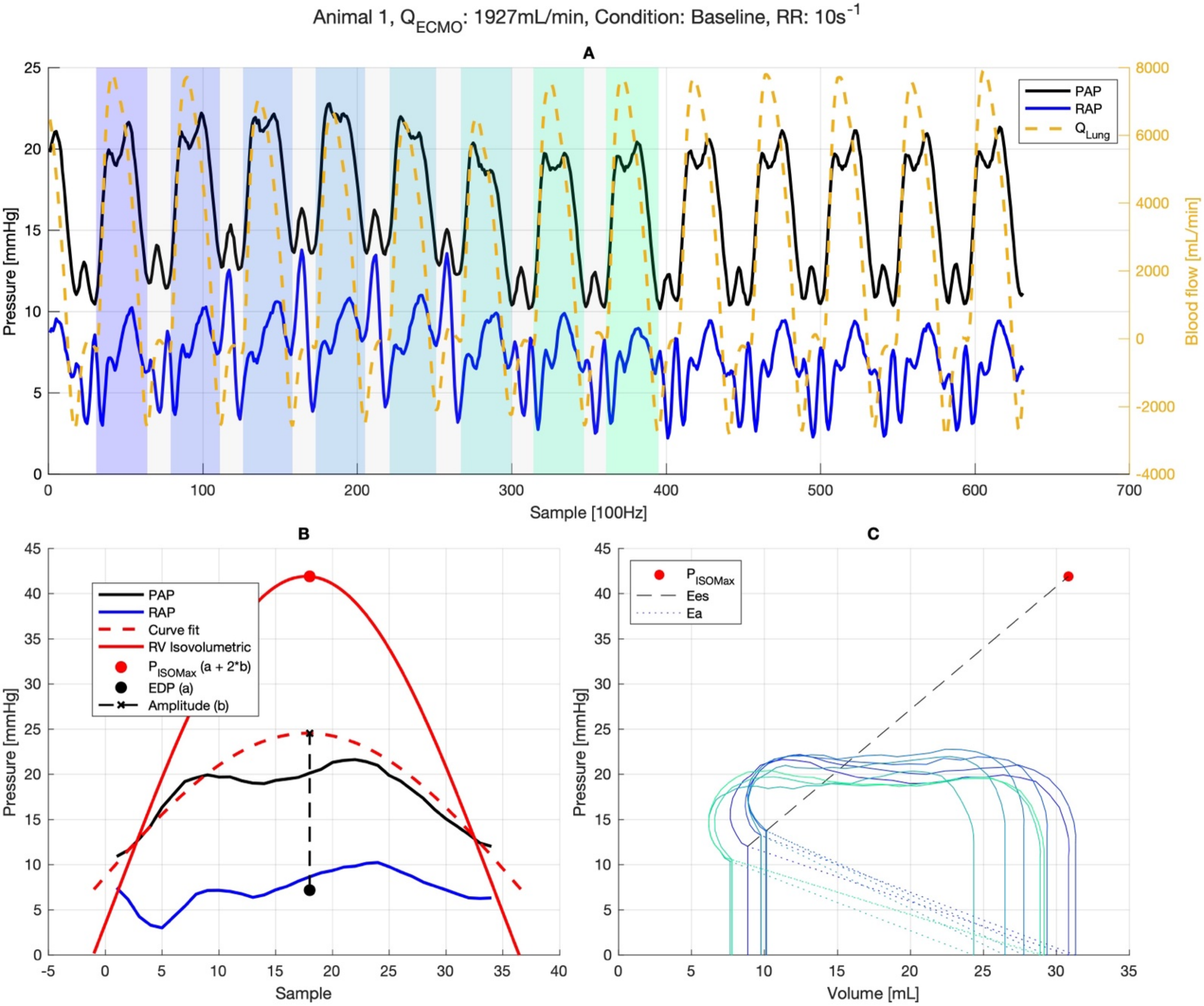

Animal 1, Q_{ECMO}: 1927mL/min, Condition: Baseline, RR: $10s^{-1}$
A
PAP
RAP
Q_{Lung}
Pressure [mmHg]
Blood flow [mL/min]
Sample [100Hz]
B
PAP
RAP
Curve fit
RV Isovolumetric
P_{ISOMax} (a + 2*b)
EDP (a)
Amplitude (b)
Pressure [mmHg]
Sample
C
P_{ISOMax}
Ees
Ea
Pressure [mmHg]
Volume [mL]

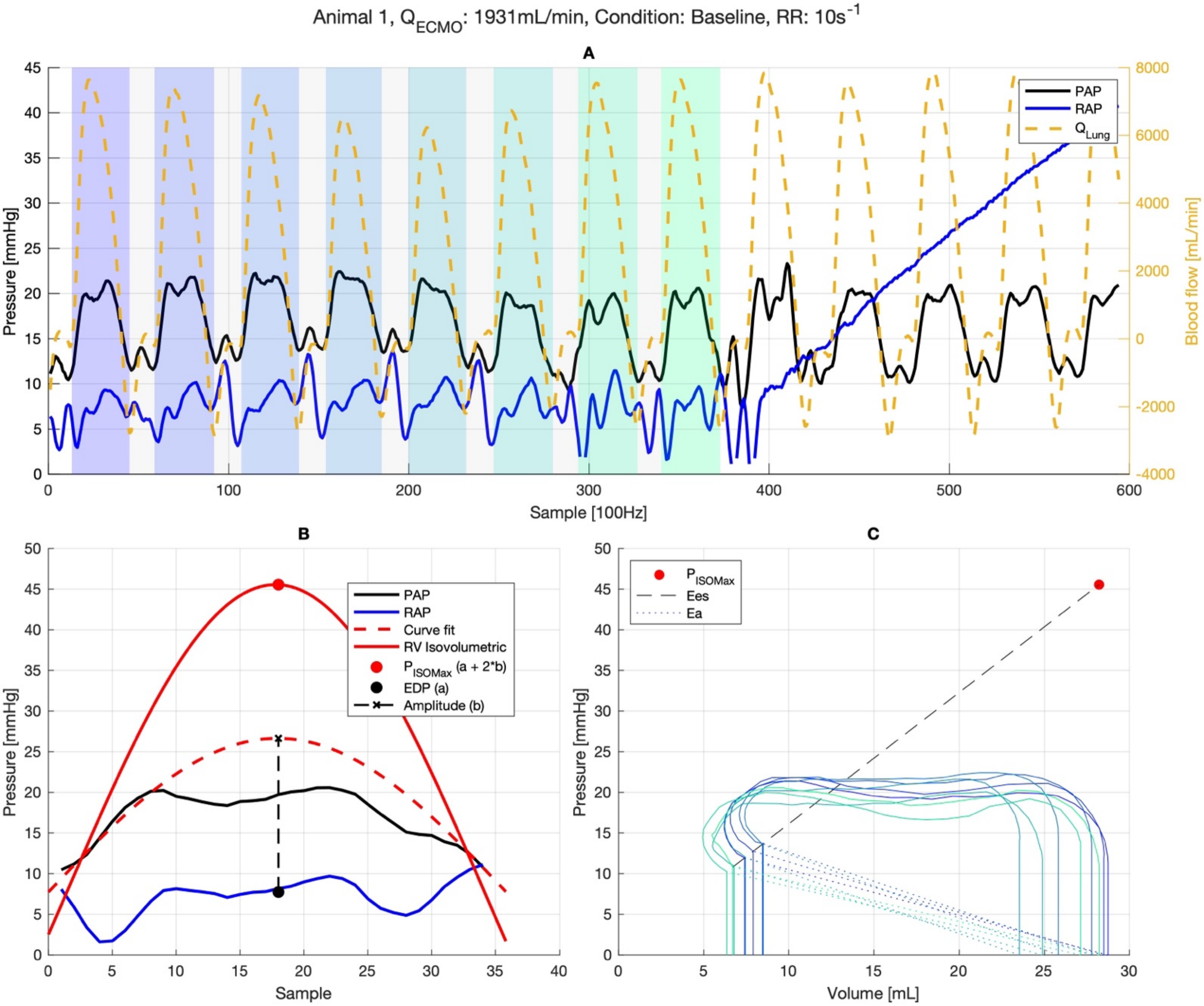

Animal 1, Q_{ECMO}: 1931mL/min, Condition: Baseline, RR: $10s^{-1}$
A
PAP
RAP
Q_{Lung}
Pressure [mmHg]
Blood flow [mL/min]
Sample [100Hz]
B
PAP
RAP
Curve fit
RV Isovolumetric
P_{ISOMax} (a + 2*b)
EDP (a)
Amplitude (b)
Pressure [mmHg]
Sample
C
P_{ISOMax}
Ees
Ea
Pressure [mmHg]
Volume [mL]

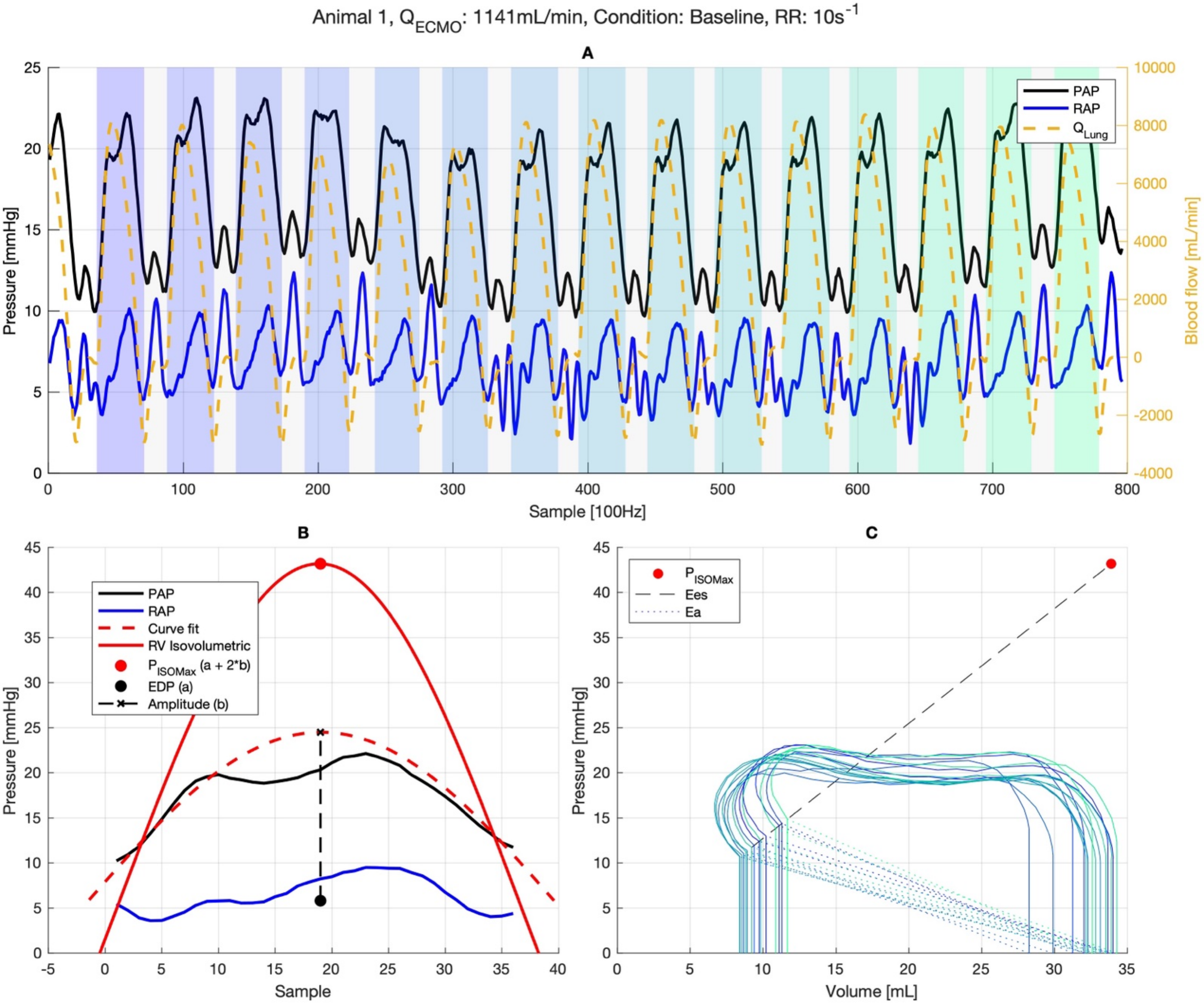

Animal 1, Q_{ECMO}: 1141mL/min, Condition: Baseline, RR: $10s^{-1}$
A
Pressure [mmHg]
Blood flow [mL/min]
Sample [100Hz]
PAP
RAP
Q_{Lung}
B
PAP
RAP
Curve fit
RV Isovolumetric
P_{ISOMax} (a + 2*b)
EDP (a)
Amplitude (b)
Pressure [mmHg]
Sample
C
P_{ISOMax}
Ees
Ea
Pressure [mmHg]
Volume [mL]

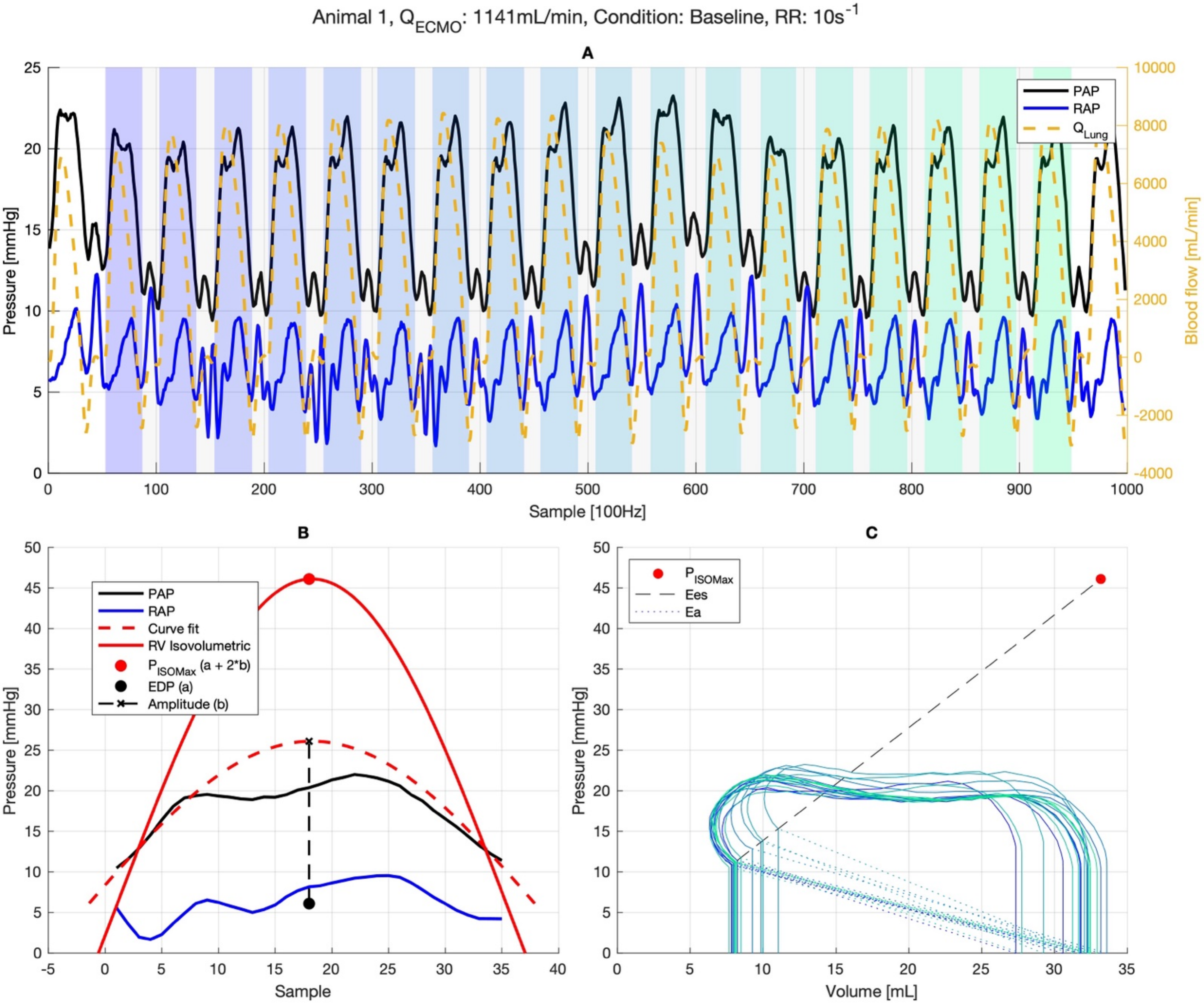

Animal 1, Q_ECMO: 1141mL/min, Condition: Baseline, RR: 10s^-1
A
PAP
RAP
Q_Lung
Pressure [mmHg]
Blood flow [mL/min]
Sample [100Hz]
B
PAP
RAP
Curve fit
RV Isovolumetric
P_ISOMax (a + 2*b)
EDP (a)
Amplitude (b)
Pressure [mmHg]
Sample
C
P_ISOMax
Ees
Ea
Pressure [mmHg]
Volume [mL]

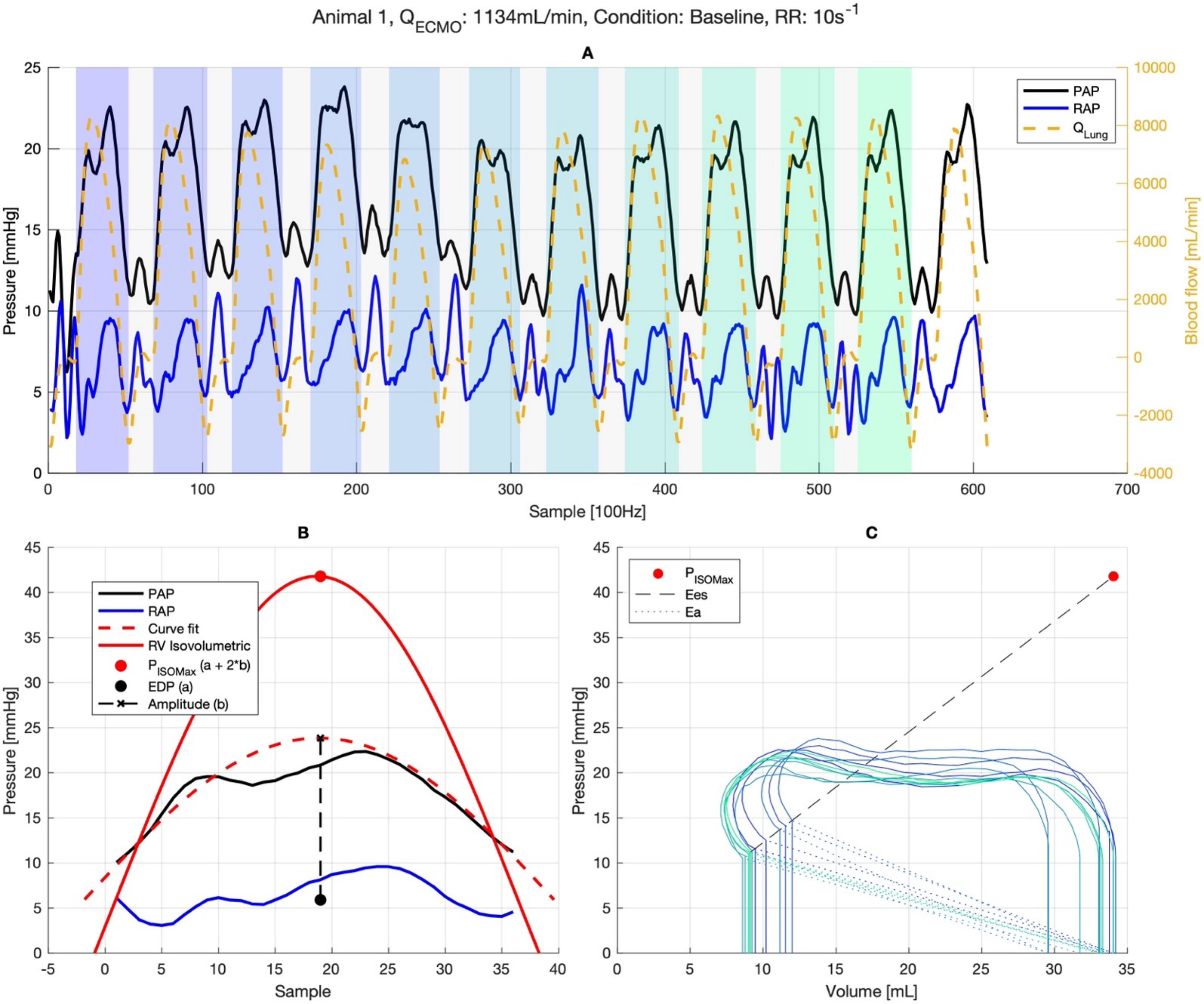

Animal 1, Q_ECMO: 1134mL/min, Condition: Baseline, RR: 10s^-1
A
Pressure [mmHg]
Blood flow [mL/min]
Sample [100Hz]
PAP
RAP
Q_Lung
B
Pressure [mmHg]
Sample
PAP
RAP
Curve fit
RV Isovolumetric
P_ISOMax (a + 2*b)
EDP (a)
Amplitude (b)
C
Pressure [mmHg]
Volume [mL]
P_ISOMax
Ees
Ea

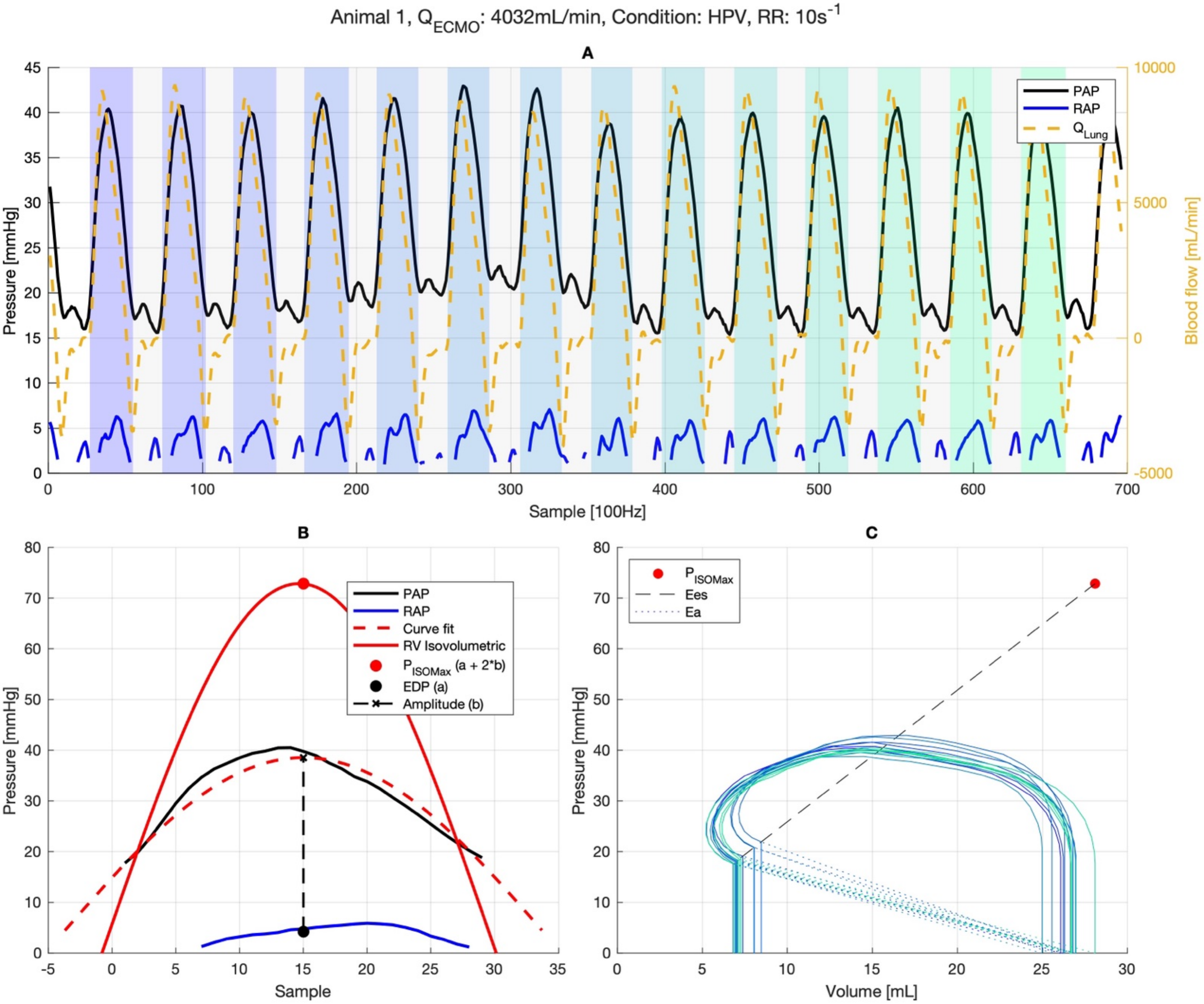

Animal 1, Q_ECMO: 4032mL/min, Condition: HPV, RR: 10s^-1
A
PAP
RAP
Q_Lung
Pressure [mmHg]
Blood flow [mL/min]
Sample [100Hz]
B
PAP
RAP
Curve fit
RV Isovolumetric
P_ISOMax (a + 2*b)
EDP (a)
Amplitude (b)
Pressure [mmHg]
Sample
C
P_ISOMax
Ees
Ea
Pressure [mmHg]
Volume [mL]

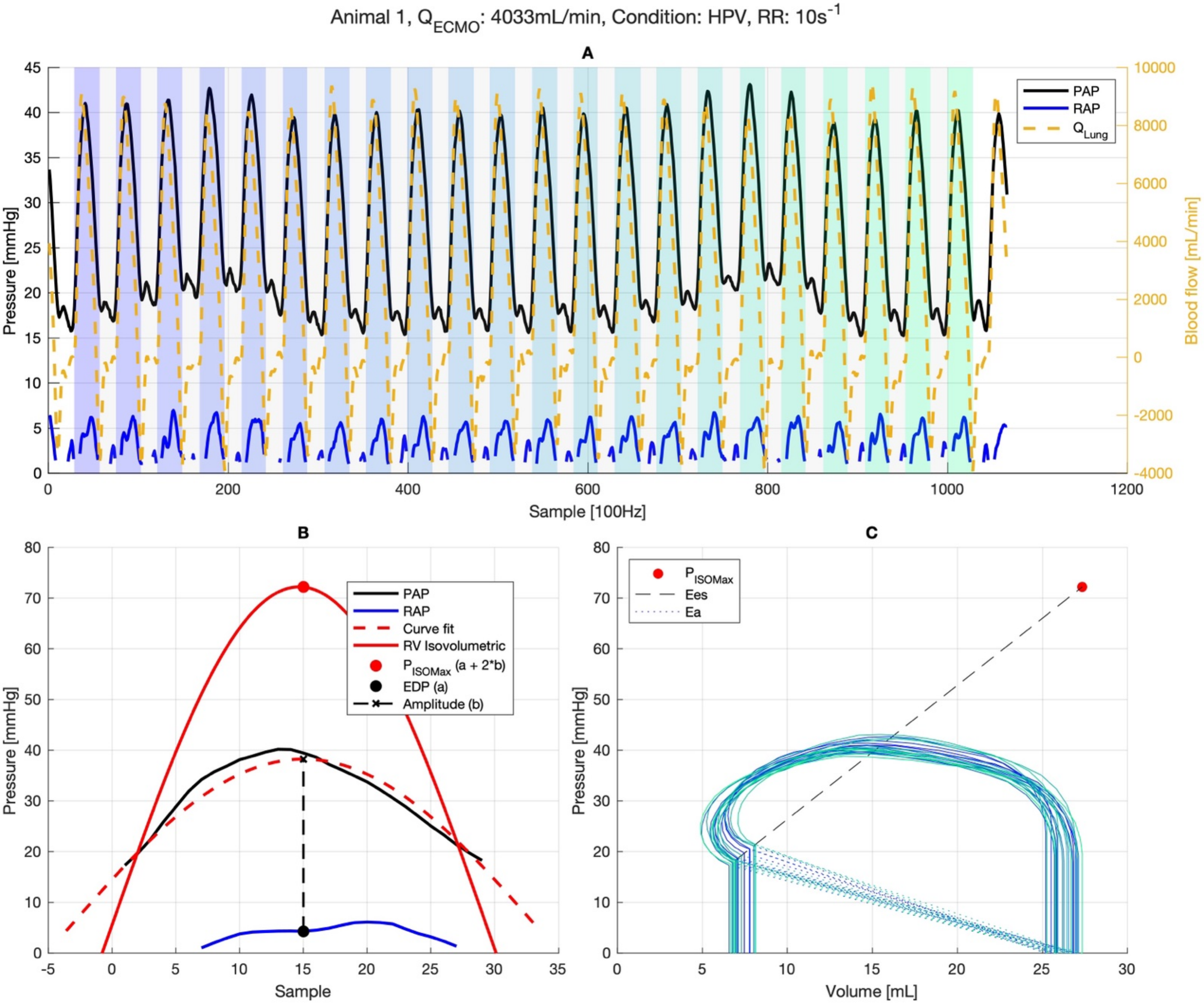

Animal 1, Q_{ECMO}: 4033mL/min, Condition: HPV, RR: $10s^{-1}$
A
PAP
RAP
Q_{Lung}
Pressure [mmHg]
Blood flow [mL/min]
Sample [100Hz]
B
PAP
RAP
Curve fit
RV Isovolumetric
P_{ISOMax} (a + 2*b)
EDP (a)
Amplitude (b)
Pressure [mmHg]
Sample
C
P_{ISOMax}
Ees
Ea
Pressure [mmHg]
Volume [mL]

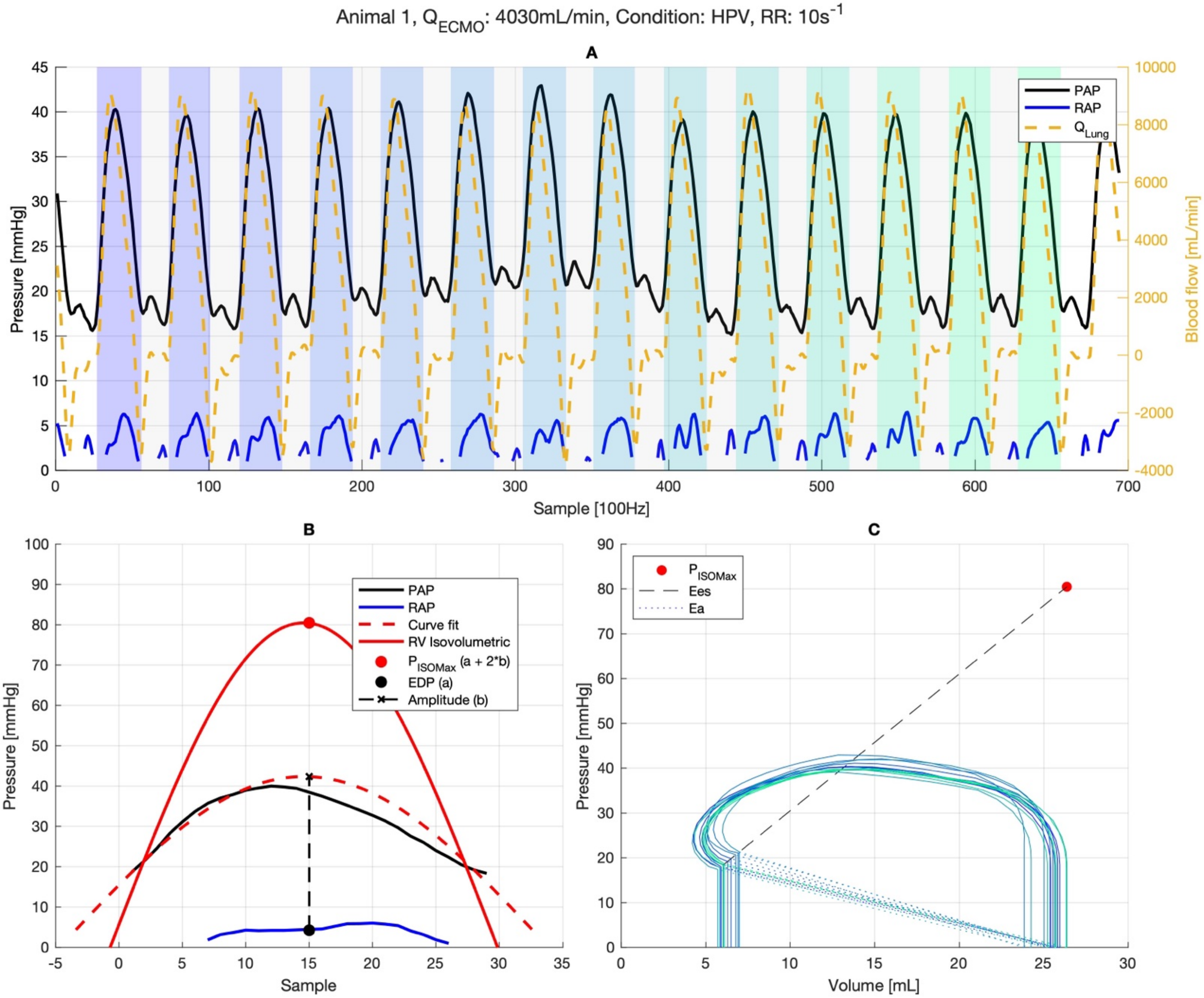

Animal 1, Q_{ECMO}: 4030mL/min, Condition: HPV, RR: $10s^{-1}$
A
Pressure [mmHg]
Blood flow [mL/min]
Sample [100Hz]
PAP
RAP
Q_{Lung}
B
PAP
RAP
Curve fit
RV Isovolumetric
P_{ISOMax} (a + 2*b)
EDP (a)
Amplitude (b)
Pressure [mmHg]
Sample
C
P_{ISOMax}
Ees
Ea
Pressure [mmHg]
Volume [mL]

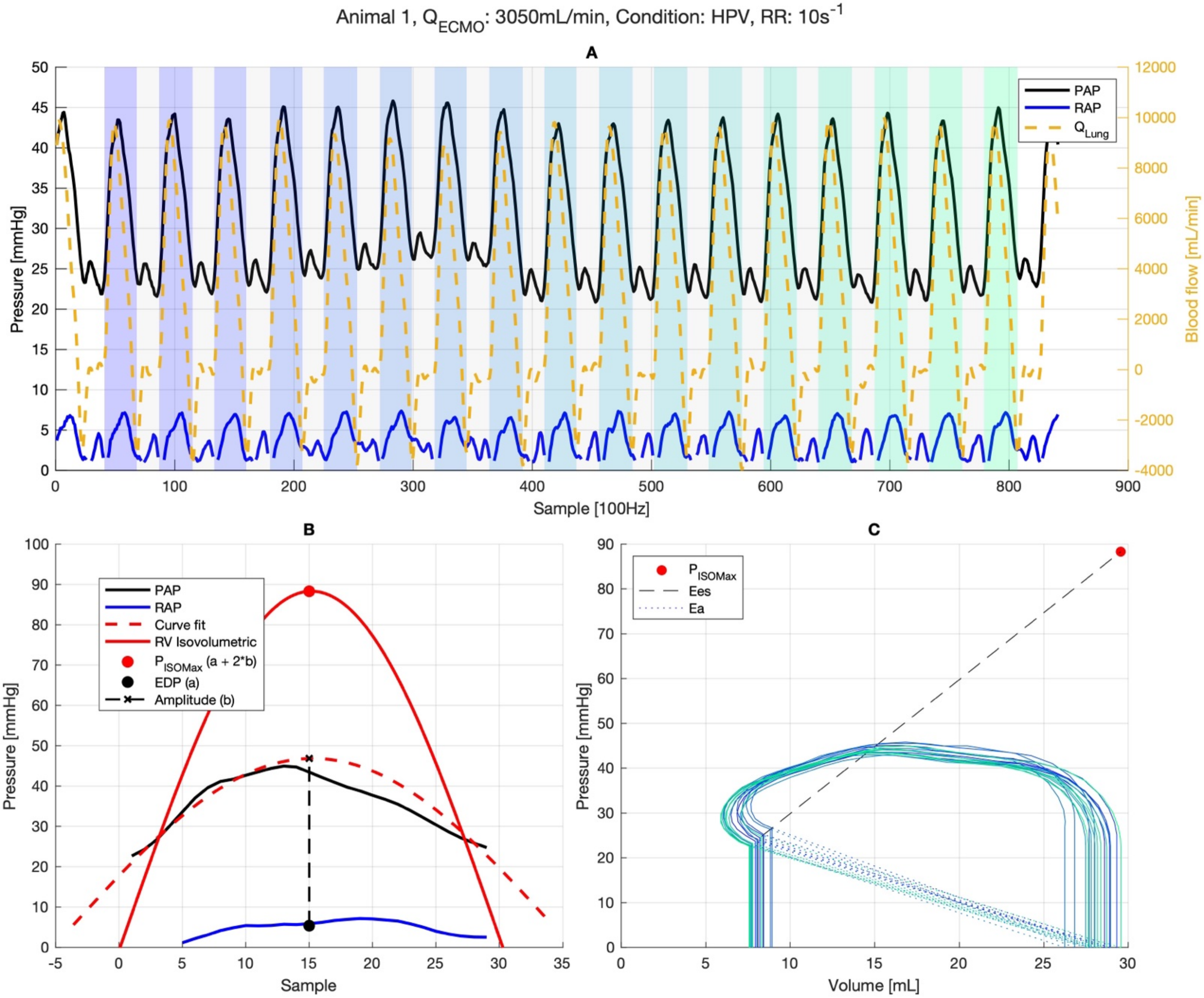

Animal 1, Q_{ECMO}: 3050mL/min, Condition: HPV, RR: $10s^{-1}$
A
PAP
RAP
Q_{Lung}
Pressure [mmHg]
Blood flow [mL/min]
Sample [100Hz]
B
PAP
RAP
Curve fit
RV Isovolumetric
P_{ISOMax} (a + 2*b)
EDP (a)
Amplitude (b)
Pressure [mmHg]
Sample
C
P_{ISOMax}
Ees
Ea
Pressure [mmHg]
Volume [mL]

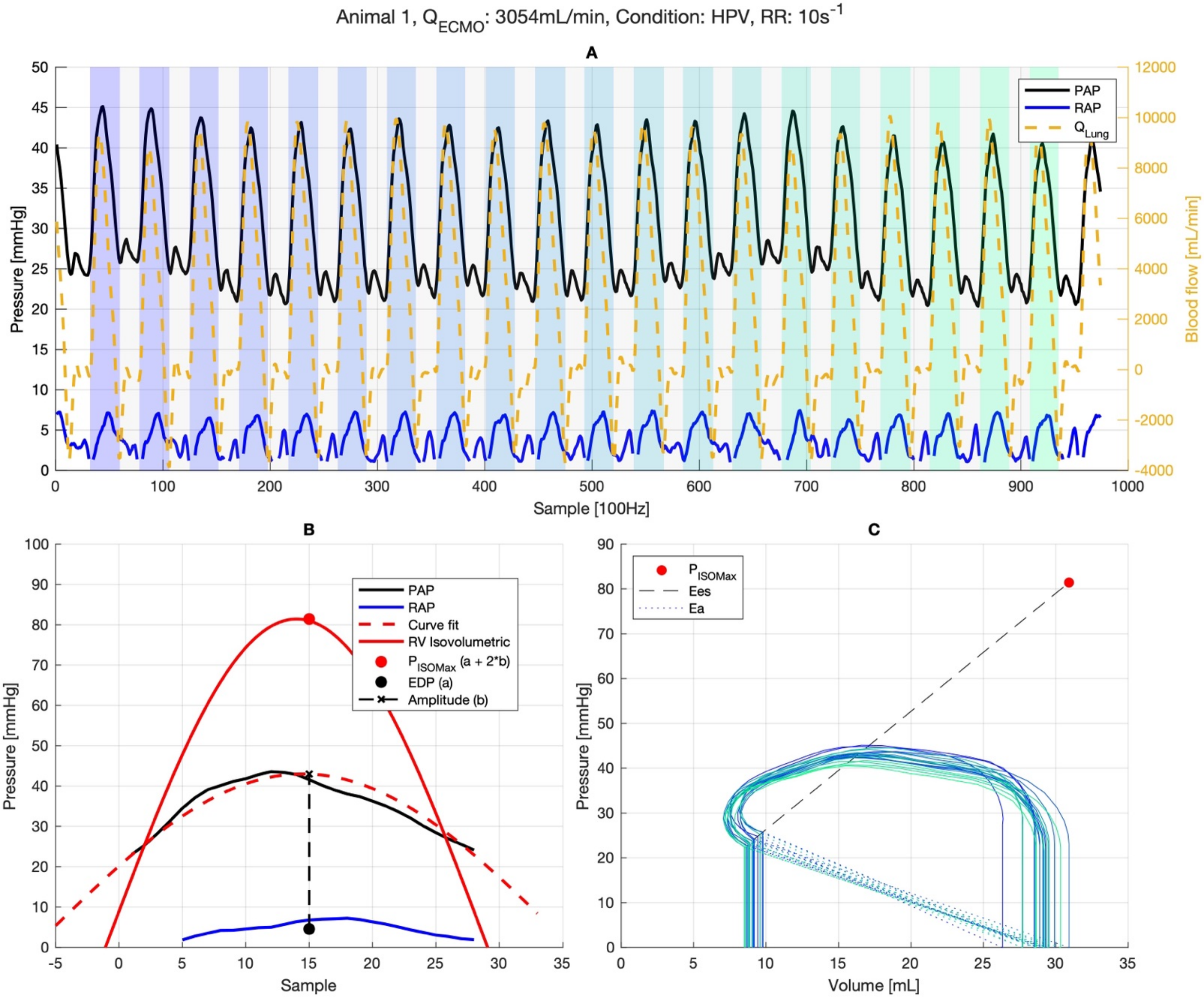

Animal 1, Q_ECMO: 3054mL/min, Condition: HPV, RR: 10s^-1
A
PAP
RAP
Q_Lung
Pressure [mmHg]
Blood flow [mL/min]
Sample [100Hz]
B
PAP
RAP
Curve fit
RV Isovolumetric
P_ISOMax (a + 2*b)
EDP (a)
Amplitude (b)
Pressure [mmHg]
Sample
C
P_ISOMax
Ees
Ea
Pressure [mmHg]
Volume [mL]

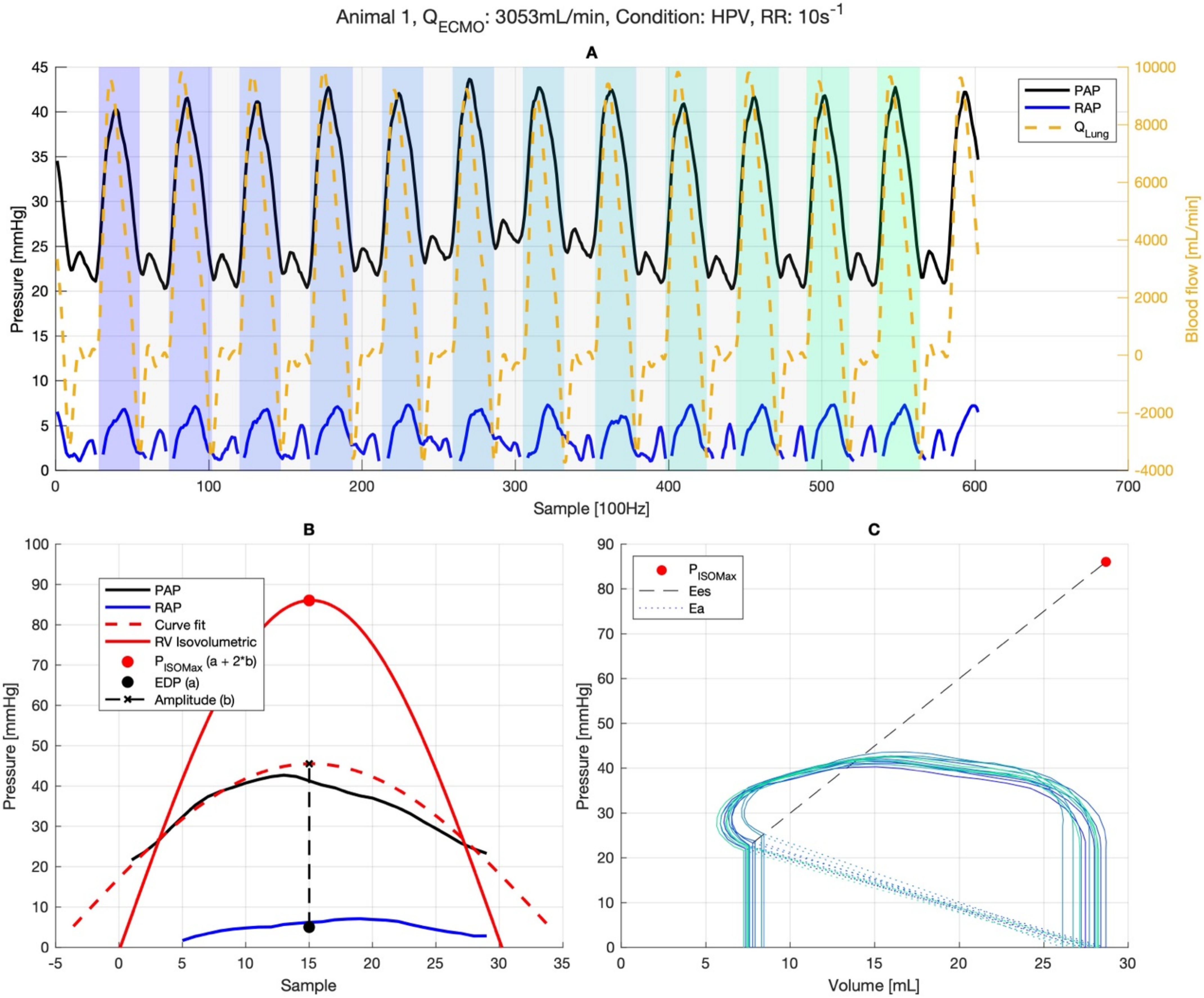

Animal 1, Q_ECMO: 3053mL/min, Condition: HPV, RR: 10s^-1
A
Pressure [mmHg]
Blood flow [mL/min]
Sample [100Hz]
PAP
RAP
Q_Lung
B
PAP
RAP
Curve fit
RV Isovolumetric
P_ISOMax (a + 2*b)
EDP (a)
Amplitude (b)
Pressure [mmHg]
Sample
C
P_ISOMax
Ees
Ea
Pressure [mmHg]
Volume [mL]

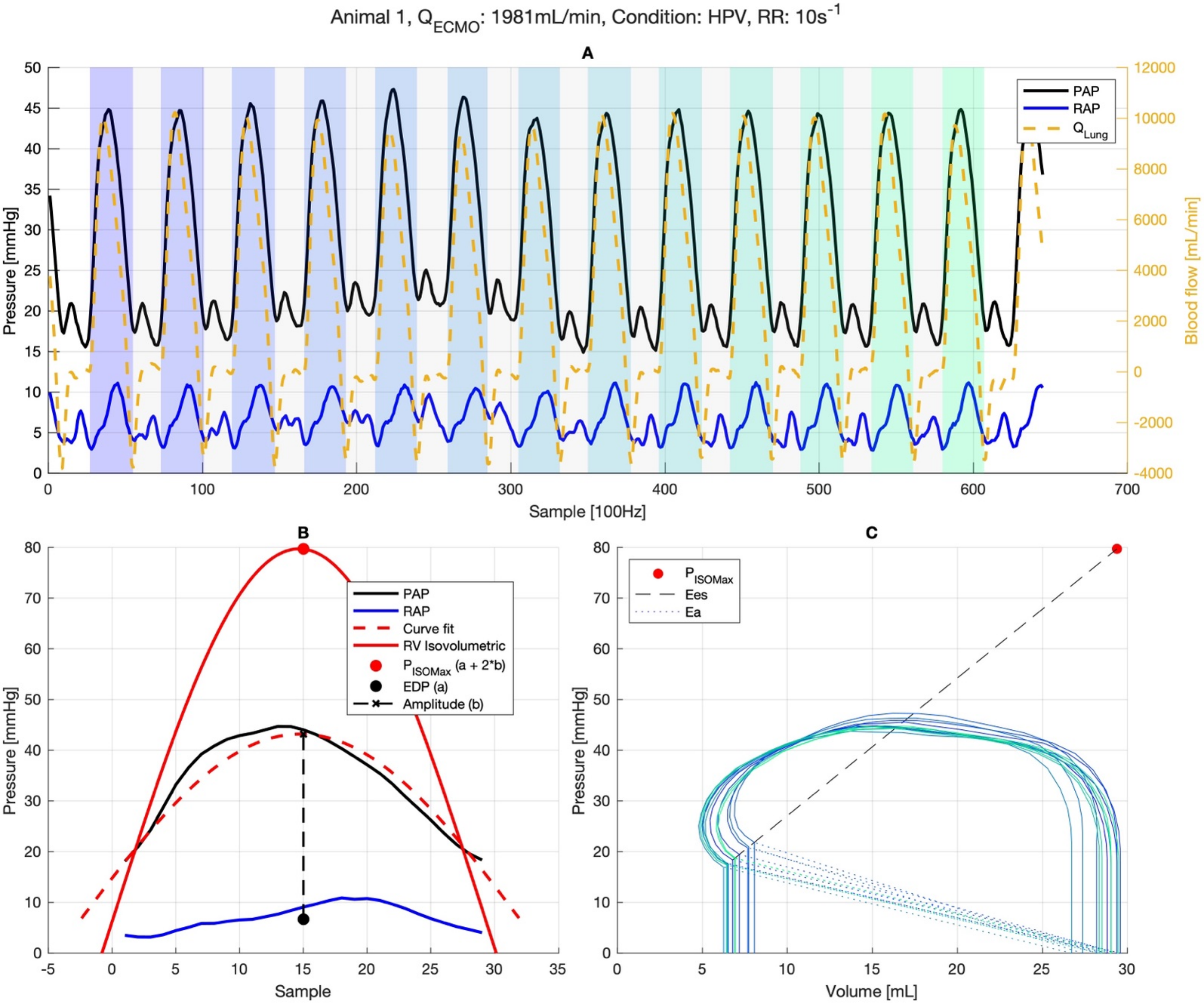

Animal 1, Q_ECMO: 1981mL/min, Condition: HPV, RR: 10s^-1
A
Pressure [mmHg]
Blood flow [mL/min]
Sample [100Hz]
PAP
RAP
Q_Lung
B
Pressure [mmHg]
Sample
PAP
RAP
Curve fit
RV Isovolumetric
P_ISOMax (a + 2*b)
EDP (a)
Amplitude (b)
C
Pressure [mmHg]
Volume [mL]
P_ISOMax
Ees
Ea

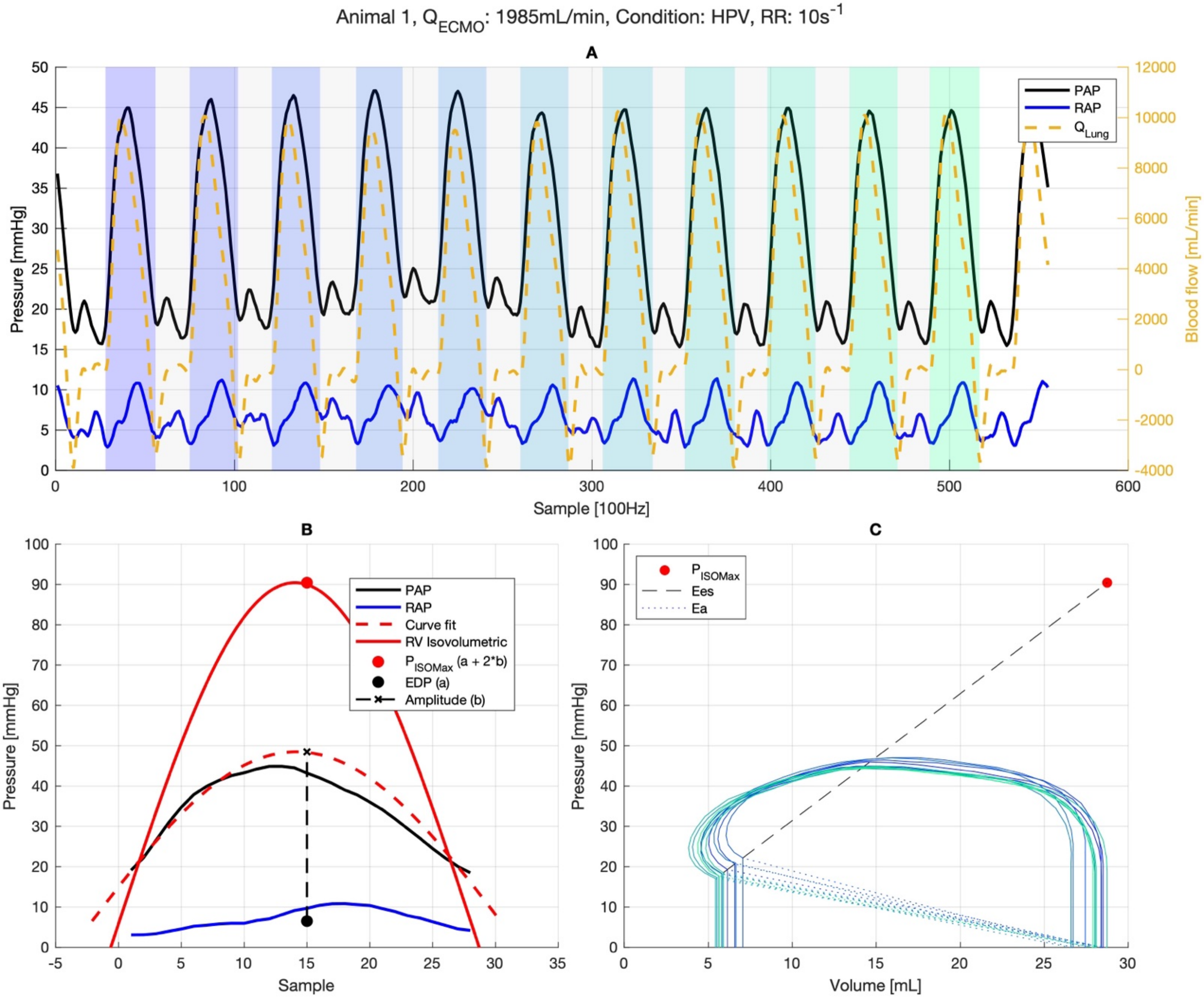

Animal 1, Q_{ECMO}: 1985mL/min, Condition: HPV, RR: $10s^{-1}$
A
Pressure [mmHg]
Blood flow [mL/min]
Sample [100Hz]
PAP
RAP
Q_{Lung}
B
Pressure [mmHg]
Sample
PAP
RAP
Curve fit
RV Isovolumetric
P_{ISOMax} (a + 2*b)
EDP (a)
Amplitude (b)
C
Pressure [mmHg]
Volume [mL]
P_{ISOMax}
Ees
Ea

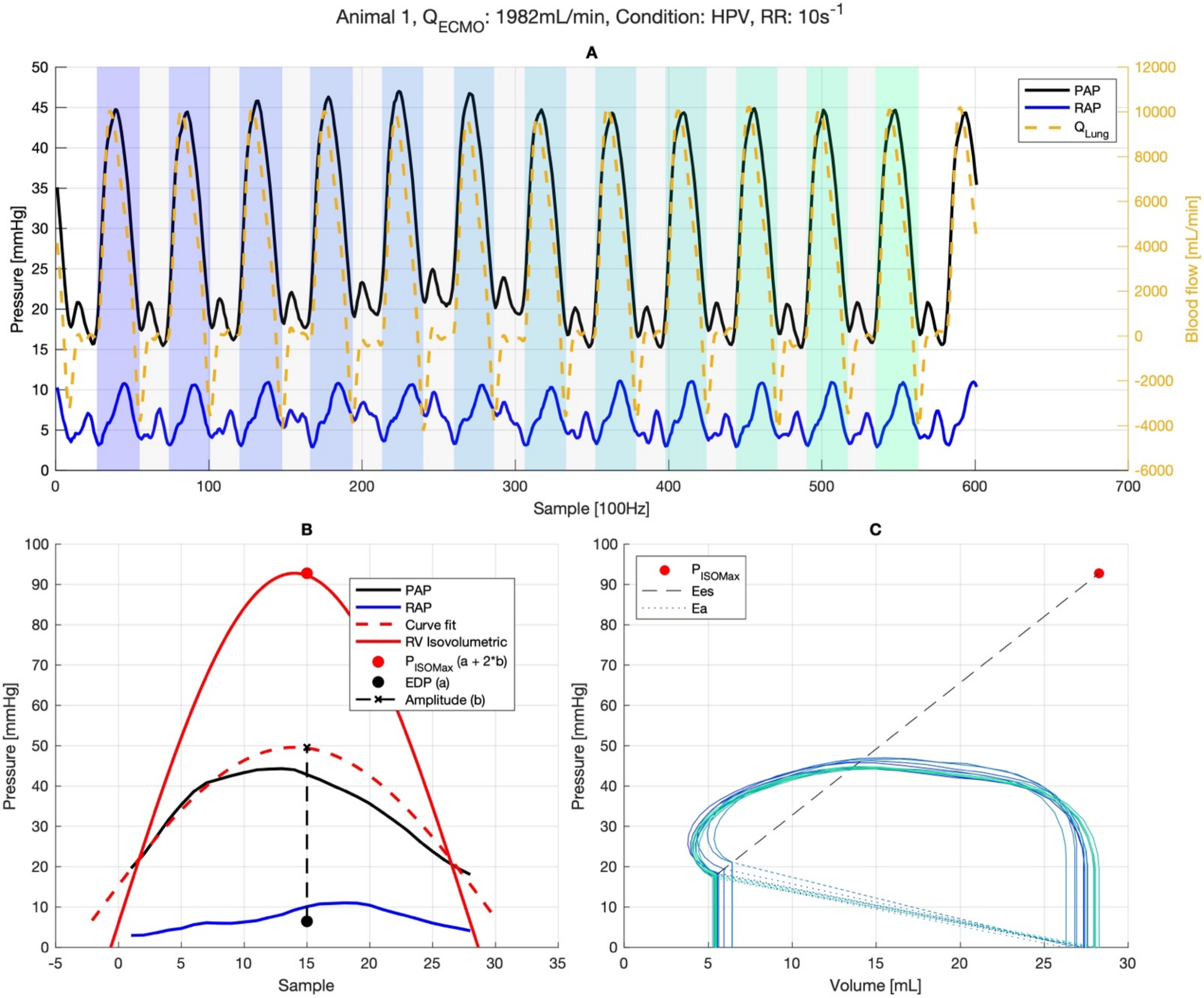
Animal 1, Q_{ECMO}: 1982mL/min, Condition: HPV, RR: $10s^{-1}$
A
PAP
RAP
Q_{Lung}
Pressure [mmHg]
Blood flow [mL/min]
Sample [100Hz]
B
PAP
RAP
Curve fit
RV Isovolumetric
P_{ISOMax} (a + 2*b)
EDP (a)
Amplitude (b)
Pressure [mmHg]
Sample
C
P_{ISOMax}
Ees
Ea
Pressure [mmHg]
Volume [mL]

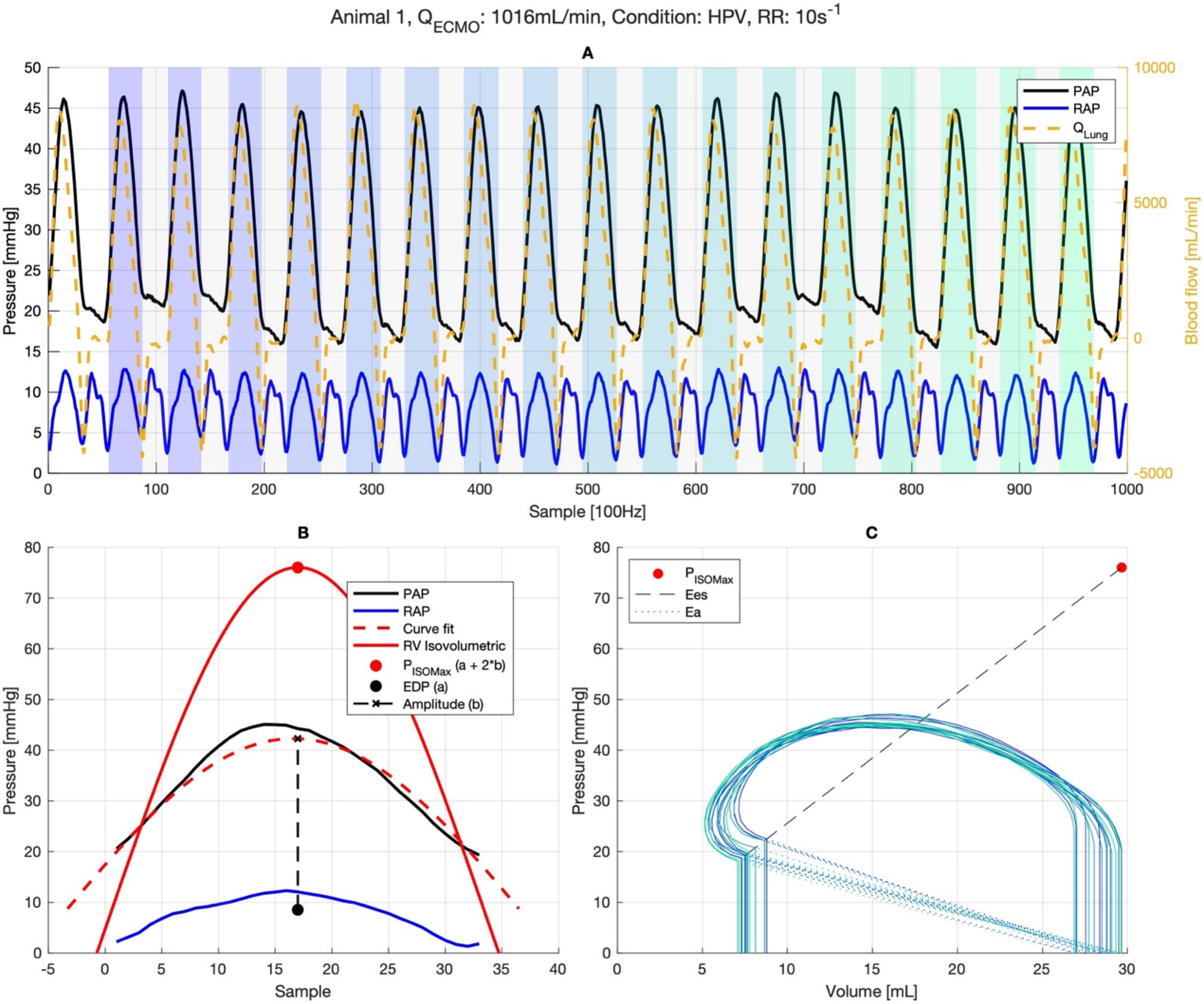

Animal 1, Q_ECMO: 1016mL/min, Condition: HPV, RR: 10s^-1
A
PAP
RAP
Q_Lung
Pressure [mmHg]
Blood flow [mL/min]
Sample [100Hz]
B
PAP
RAP
Curve fit
RV Isovolumetric
P_ISOMax (a + 2*b)
EDP (a)
Amplitude (b)
Pressure [mmHg]
Sample
C
P_ISOMax
Ees
Ea
Pressure [mmHg]
Volume [mL]

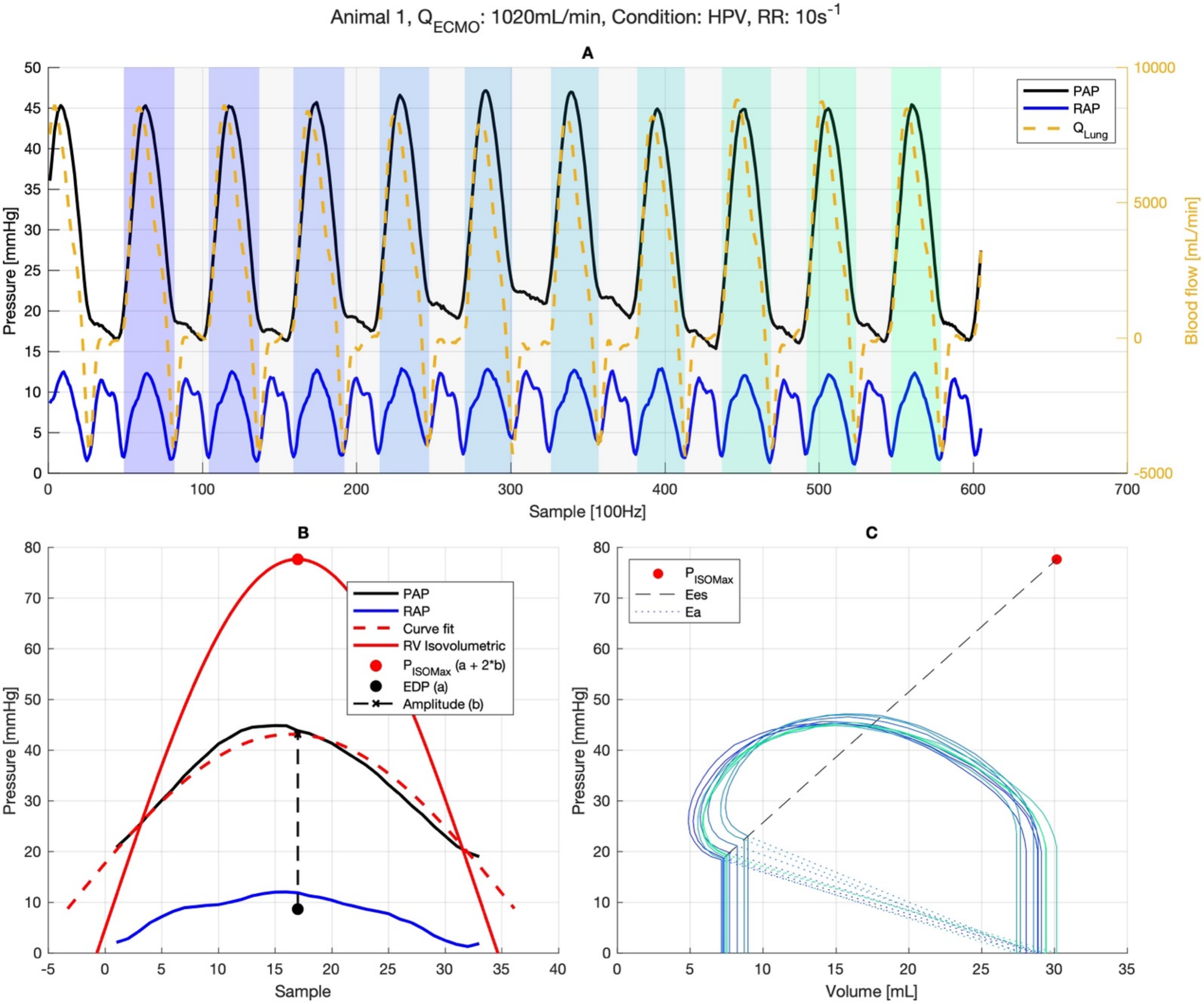

Animal 1, QECMO: 1020mL/min, Condition: HPV, RR: 10s-1
A
PAP
RAP
QLung
Pressure [mmHg]
Blood flow [mL/min]
Sample [100Hz]
B
PAP
RAP
Curve fit
RV Isovolumetric
PISOMax (a + 2*b)
EDP (a)
Amplitude (b)
Pressure [mmHg]
Sample
C
PISOMax
Ees
Ea
Pressure [mmHg]
Volume [mL]

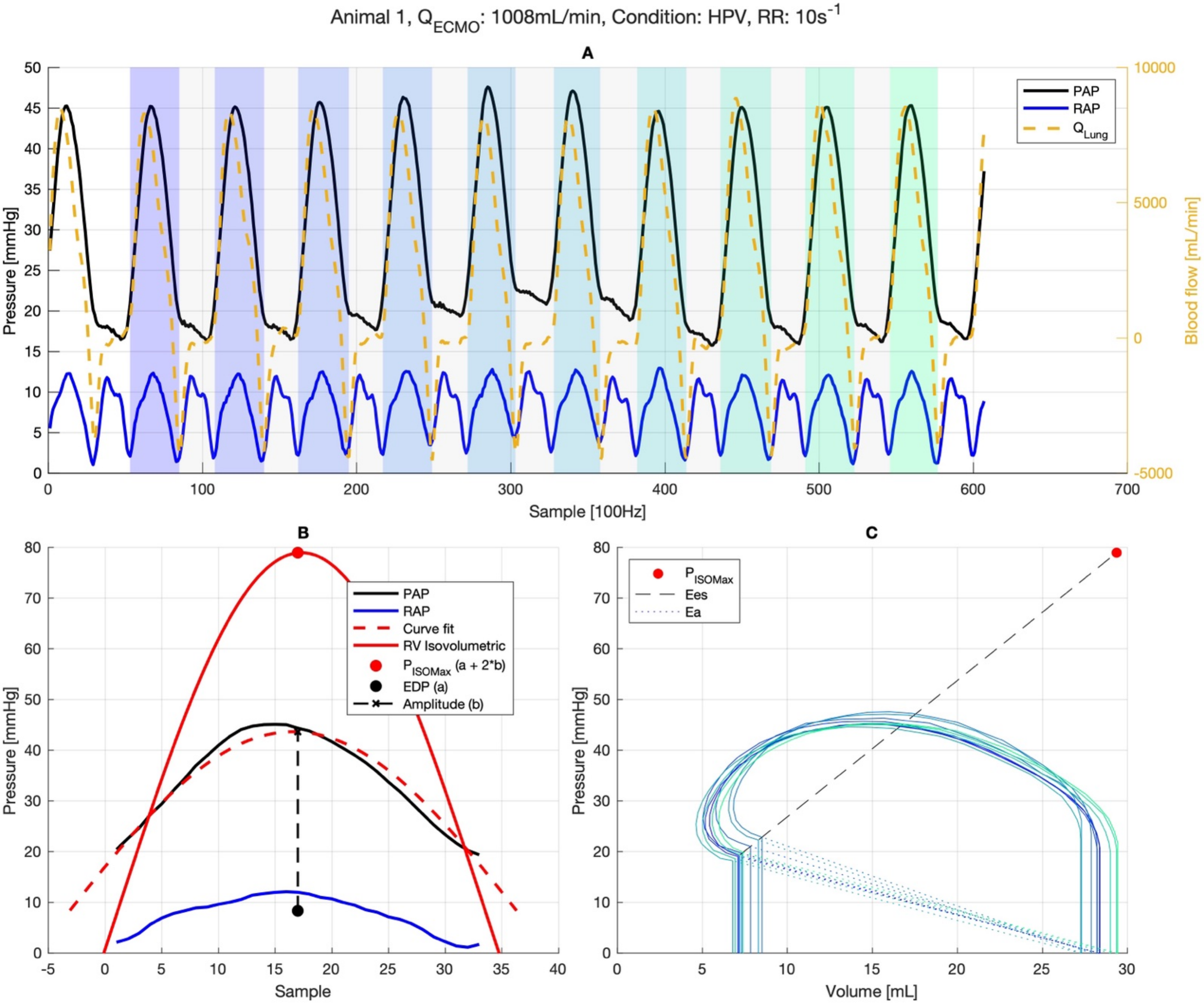

Animal 1, Q_{ECMO}: 1008mL/min, Condition: HPV, RR: $10s^{-1}$
A
Pressure [mmHg]
Blood flow [mL/min]
Sample [100Hz]
PAP
RAP
Q_{Lung}
B
Pressure [mmHg]
Sample
PAP
RAP
Curve fit
RV Isovolumetric
P_{ISOMax} (a + 2*b)
EDP (a)
Amplitude (b)
C
Pressure [mmHg]
Volume [mL]
P_{ISOMax}
Ees
Ea

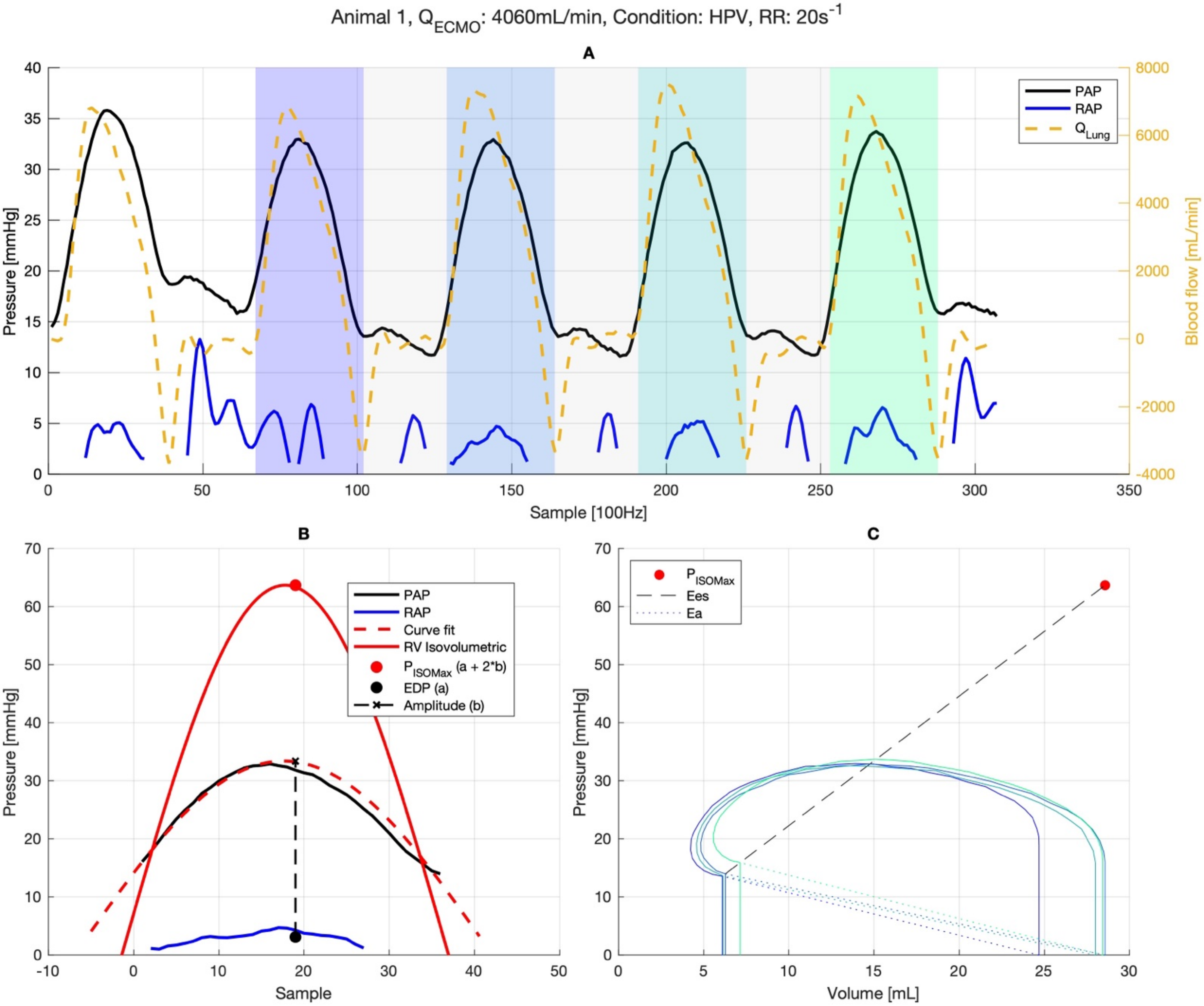

Animal 1, Q_{ECMO}: 4060mL/min, Condition: HPV, RR: 20s^{-1}
A
PAP
RAP
Q_{Lung}
Pressure [mmHg]
Blood flow [mL/min]
Sample [100Hz]
B
PAP
RAP
Curve fit
RV Isovolumetric
P_{ISOMax} (a + 2*b)
EDP (a)
Amplitude (b)
Pressure [mmHg]
Sample
C
P_{ISOMax}
Ees
Ea
Pressure [mmHg]
Volume [mL]

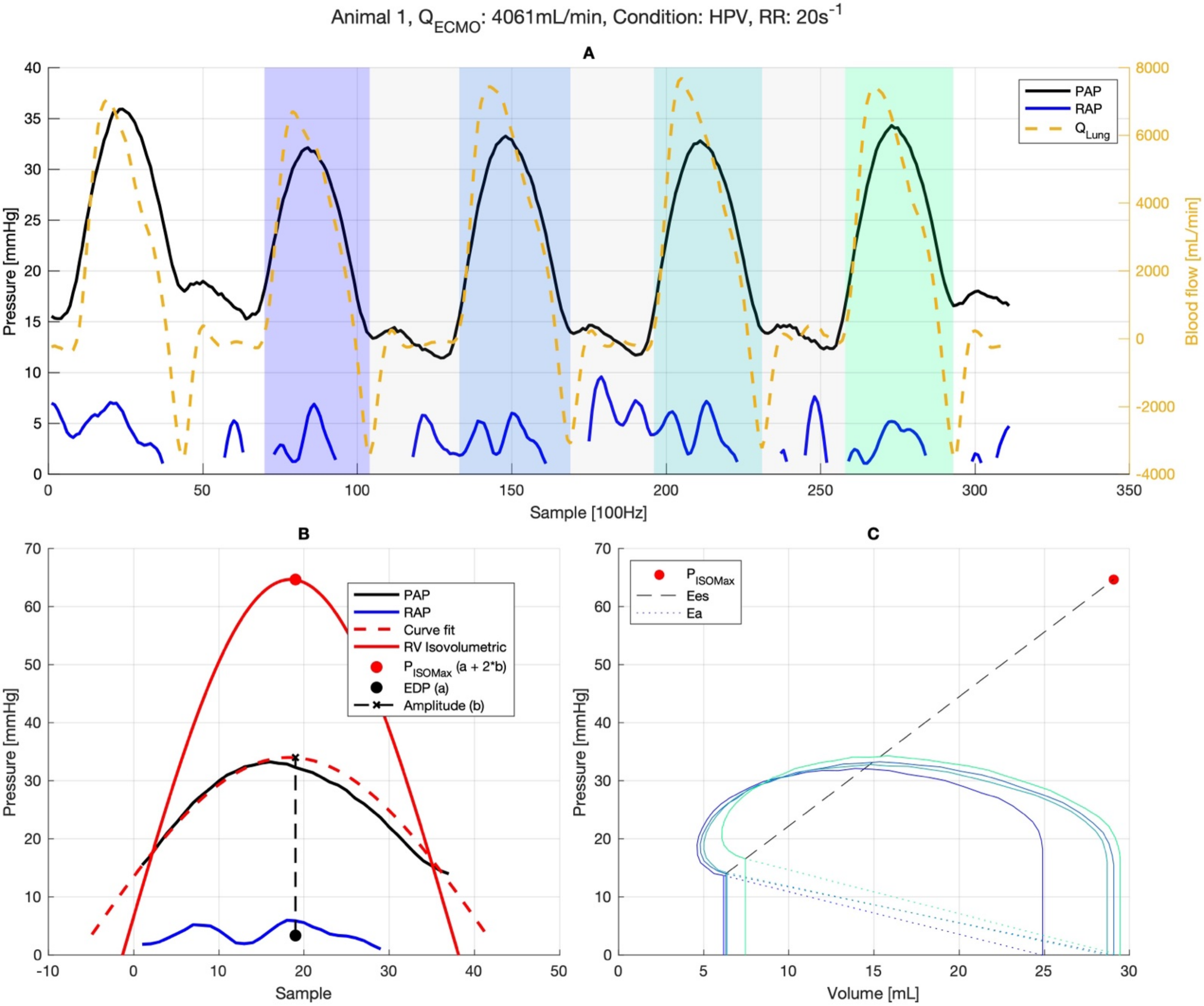

Animal 1, Q_{ECMO}: 4061mL/min, Condition: HPV, RR: $20s^{-1}$
A
PAP
RAP
Q_{Lung}
Pressure [mmHg]
Blood flow [mL/min]
Sample [100Hz]
B
PAP
RAP
Curve fit
RV Isovolumetric
P_{ISOMax} (a + 2*b)
EDP (a)
Amplitude (b)
Pressure [mmHg]
Sample
C
P_{ISOMax}
Ees
Ea
Pressure [mmHg]
Volume [mL]

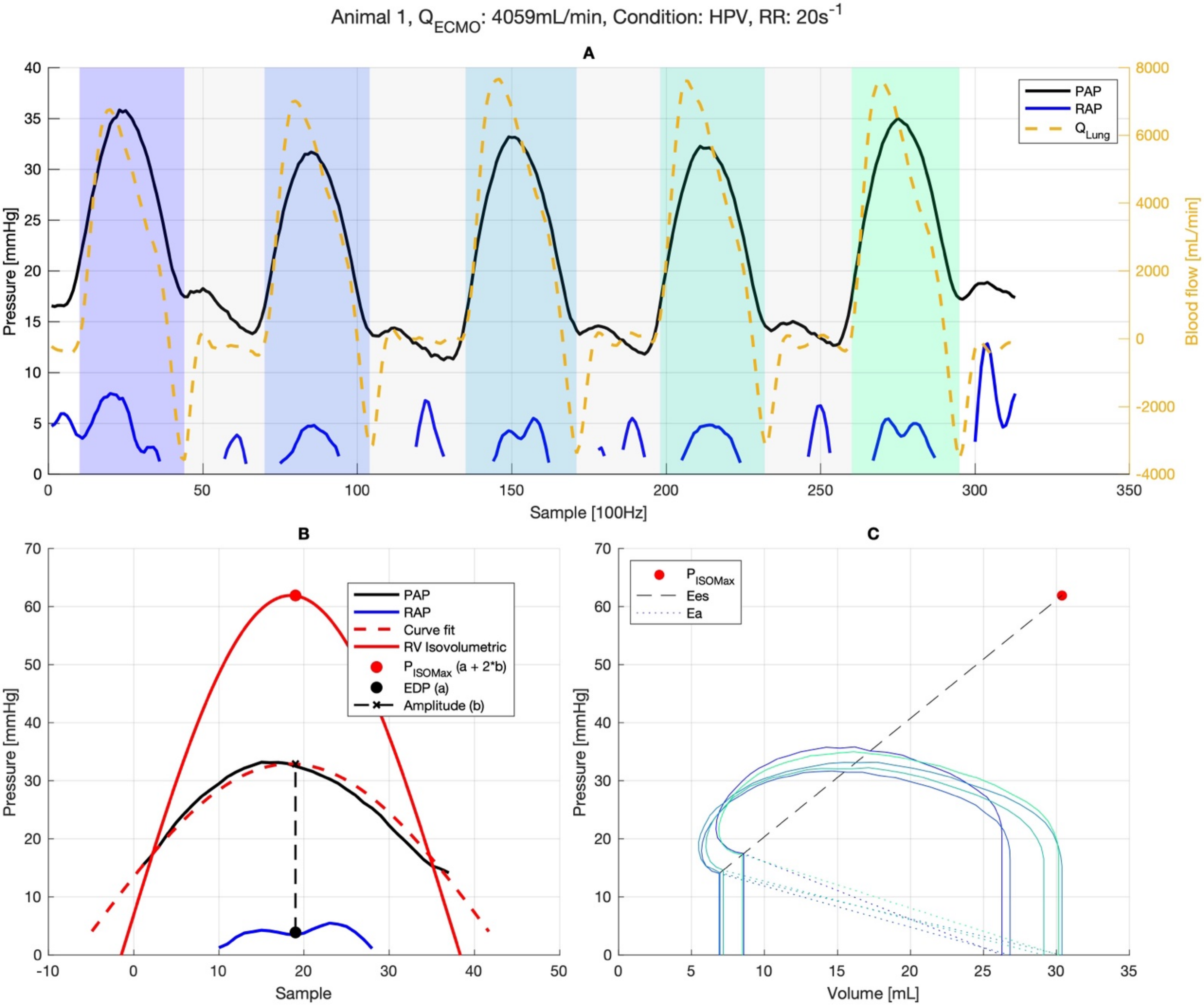
Animal 1, Q_ECMO: 4059mL/min, Condition: HPV, RR: 20s^-1
A
PAP
RAP
Q_Lung
Pressure [mmHg]
Blood flow [mL/min]
Sample [100Hz]
B
Curve fit
RV Isovolumetric
P_ISOMax (a + 2*b)
EDP (a)
Amplitude (b)
Pressure [mmHg]
Sample
C
P_ISOMax
Ees
Ea
Pressure [mmHg]
Volume [mL]

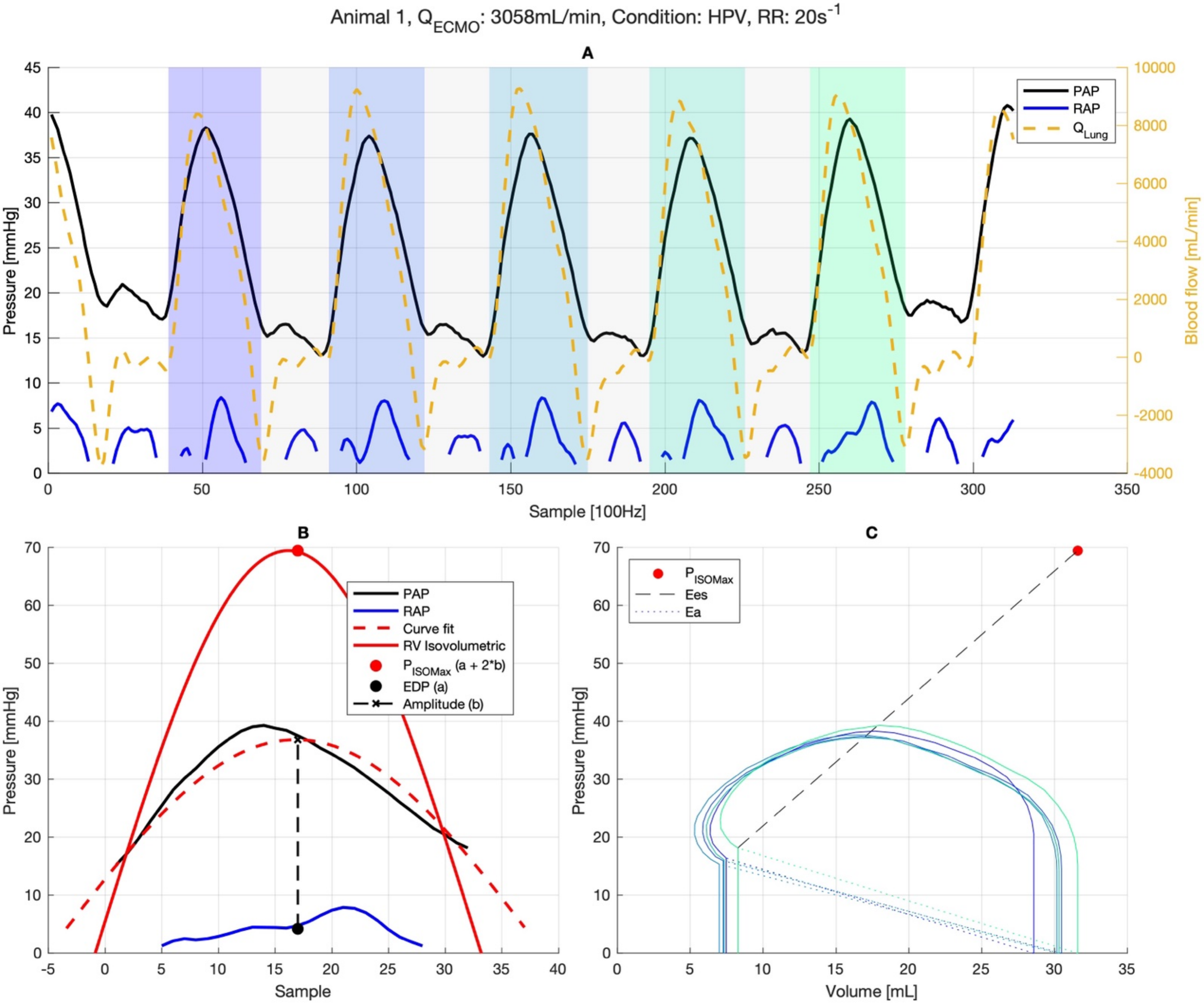

Animal 1, Q_{ECMO}: 3058mL/min, Condition: HPV, RR: $20s^{-1}$
A
PAP
RAP
Q_{Lung}
Pressure [mmHg]
Blood flow [mL/min]
Sample [100Hz]
B
PAP
RAP
Curve fit
RV Isovolumetric
P_{ISOMax} (a + 2*b)
EDP (a)
Amplitude (b)
Pressure [mmHg]
Sample
C
P_{ISOMax}
Ees
Ea
Pressure [mmHg]
Volume [mL]

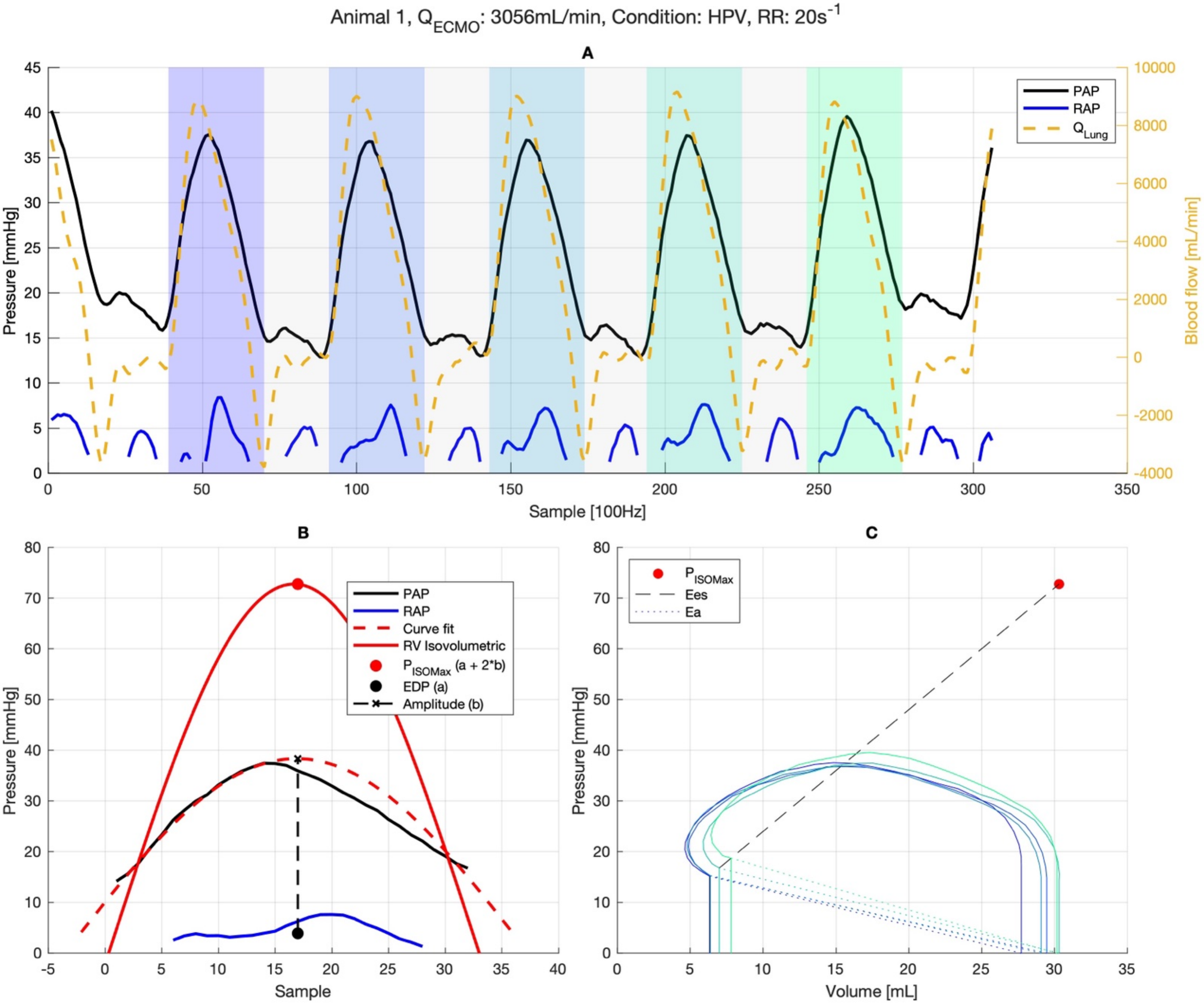

Animal 1, Q_ECMO: 3056mL/min, Condition: HPV, RR: 20s^-1
A
PAP
RAP
Q_Lung
Pressure [mmHg]
Blood flow [mL/min]
Sample [100Hz]
B
PAP
RAP
Curve fit
RV Isovolumetric
P_ISOMax (a + 2*b)
EDP (a)
Amplitude (b)
Pressure [mmHg]
Sample
C
P_ISOMax
Ees
Ea
Pressure [mmHg]
Volume [mL]

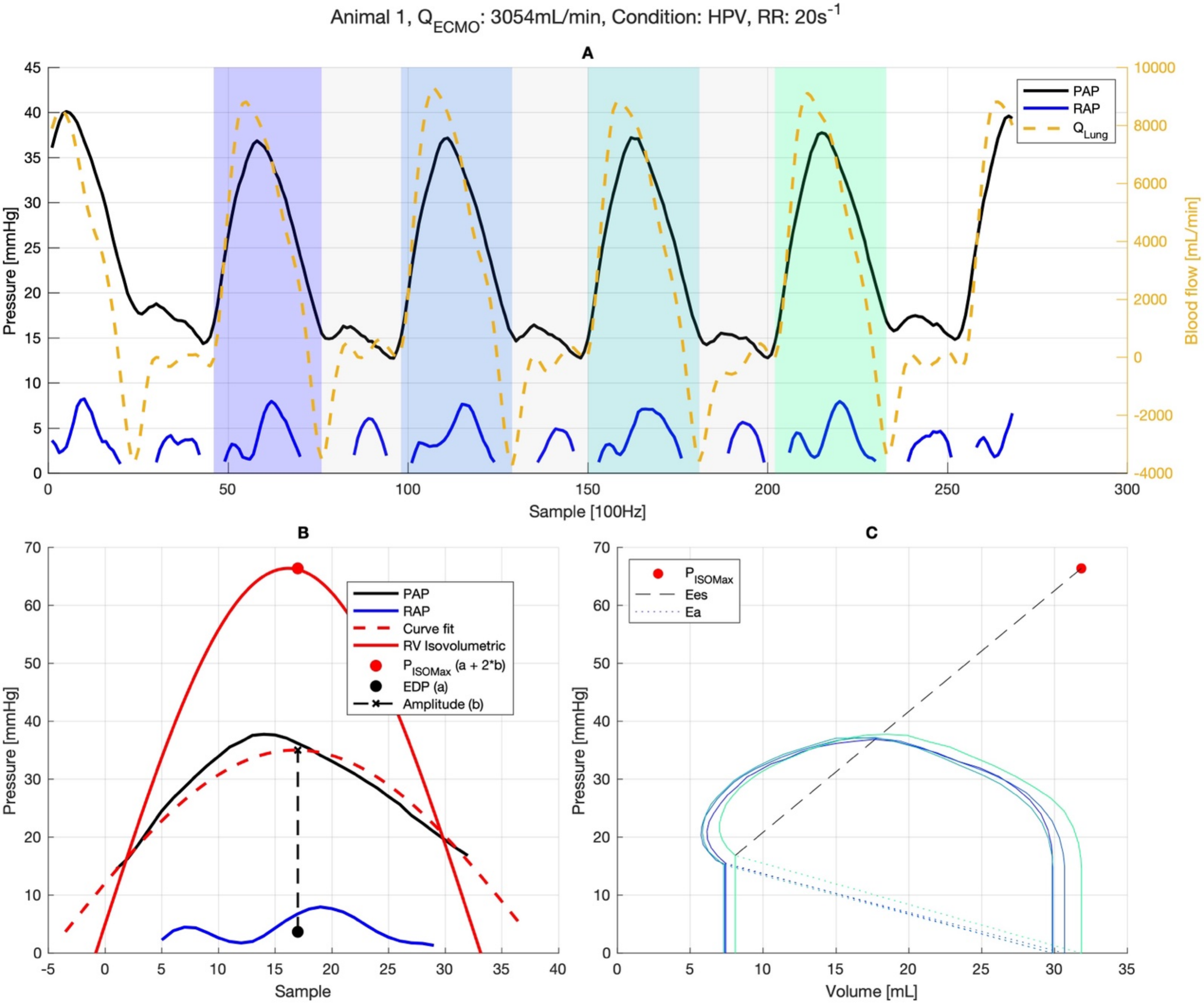

Animal 1, Q_{ECMO}: 3054mL/min, Condition: HPV, RR: $20s^{-1}$
A
PAP
RAP
Q_{Lung}
Pressure [mmHg]
Blood flow [mL/min]
Sample [100Hz]
B
PAP
RAP
Curve fit
RV Isovolumetric
P_{ISOMax} (a + 2*b)
EDP (a)
Amplitude (b)
Pressure [mmHg]
Sample
C
P_{ISOMax}
Ees
Ea
Pressure [mmHg]
Volume [mL]

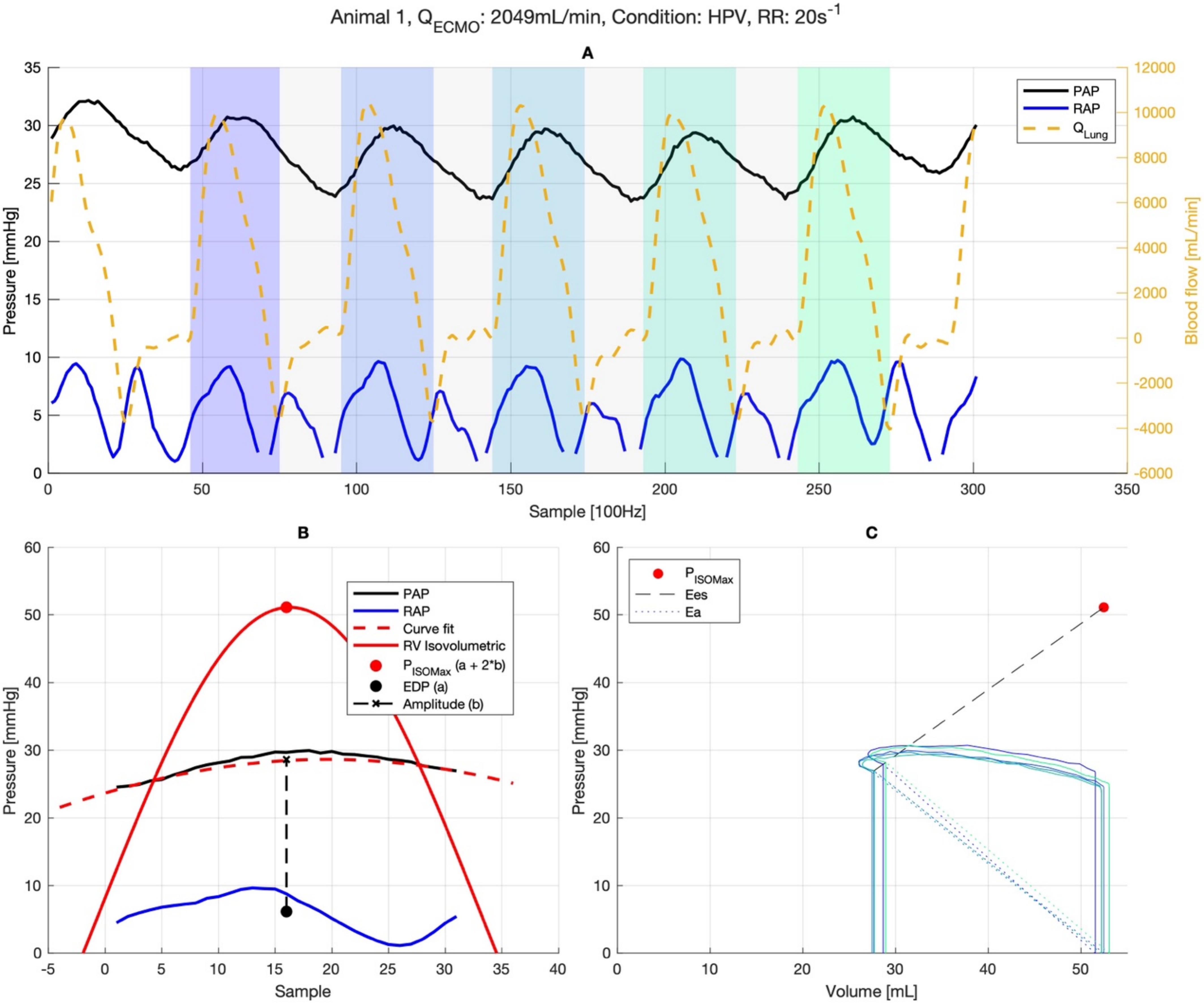

Animal 1, Q_{ECMO}: 2049mL/min, Condition: HPV, RR: $20s^{-1}$
A
PAP
RAP
Q_{Lung}
Pressure [mmHg]
Blood flow [mL/min]
Sample [100Hz]
B
PAP
RAP
Curve fit
RV Isovolumetric
P_{ISOMax} (a + 2*b)
EDP (a)
Amplitude (b)
Pressure [mmHg]
Sample
C
P_{ISOMax}
Ees
Ea
Pressure [mmHg]
Volume [mL]

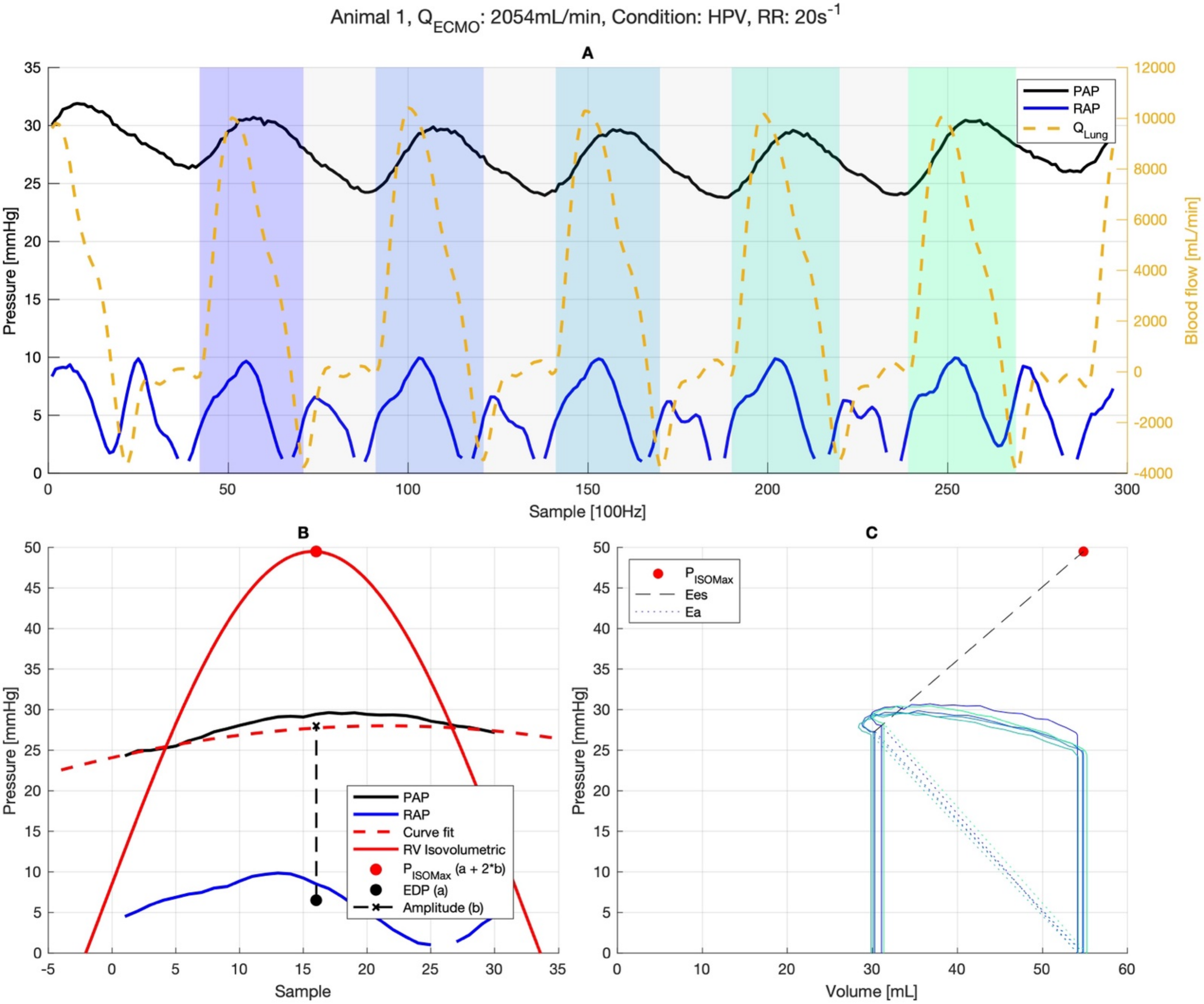

Animal 1, Q_ECMO: 2054mL/min, Condition: HPV, RR: 20s^-1
A
Pressure [mmHg]
Blood flow [mL/min]
Sample [100Hz]
PAP
RAP
Q_Lung
B
Pressure [mmHg]
Sample
PAP
RAP
Curve fit
RV Isovolumetric
P_ISOMax (a + 2*b)
EDP (a)
Amplitude (b)
C
Pressure [mmHg]
Volume [mL]
P_ISOMax
Ees
Ea

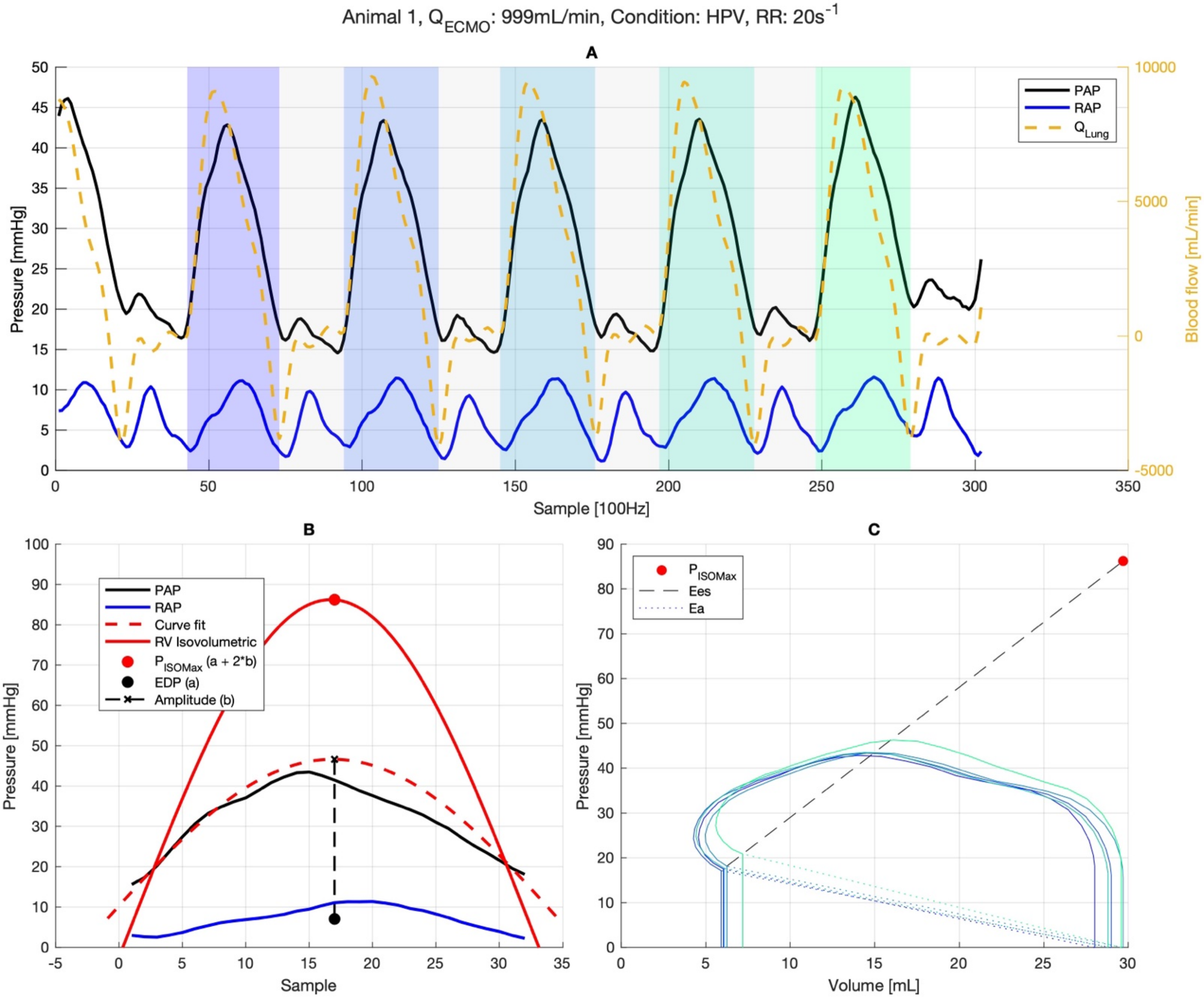

Animal 1, Q_{ECMO}: 999mL/min, Condition: HPV, RR: $20s^{-1}$
A
PAP
RAP
Q_{Lung}
Pressure [mmHg]
Blood flow [mL/min]
Sample [100Hz]
B
PAP
RAP
Curve fit
RV Isovolumetric
P_{ISOMax} (a + 2*b)
EDP (a)
Amplitude (b)
Pressure [mmHg]
Sample
C
P_{ISOMax}
Ees
Ea
Pressure [mmHg]
Volume [mL]

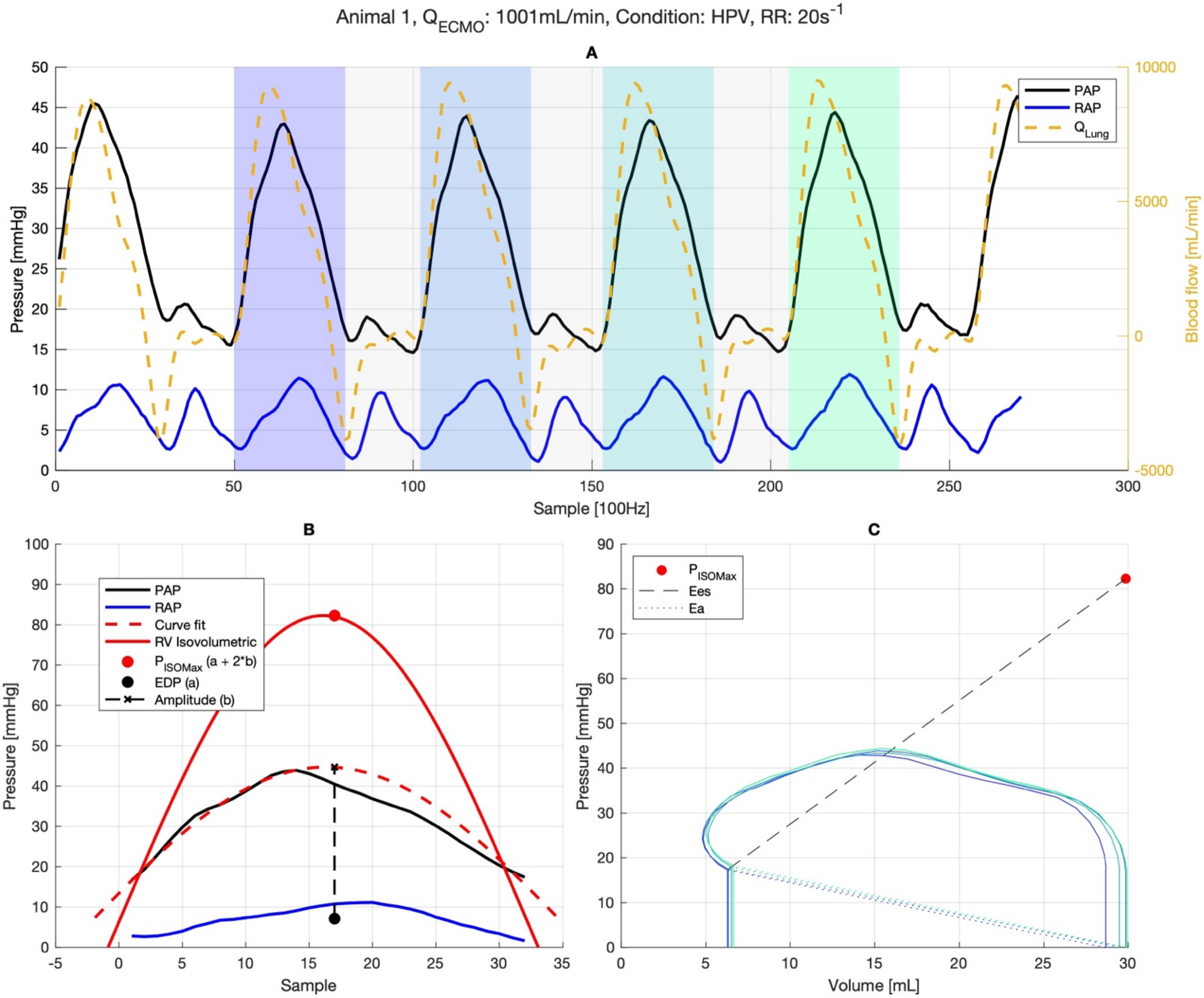

Animal 1, Q_{ECMO}: 1001mL/min, Condition: HPV, RR: $20s^{-1}$
A
PAP
RAP
Q_{Lung}
Pressure [mmHg]
Blood flow [mL/min]
Sample [100Hz]
B
PAP
RAP
Curve fit
RV Isovolumetric
P_{ISOMax} (a + 2*b)
EDP (a)
Amplitude (b)
Pressure [mmHg]
Sample
C
P_{ISOMax}
Ees
Ea
Pressure [mmHg]
Volume [mL]

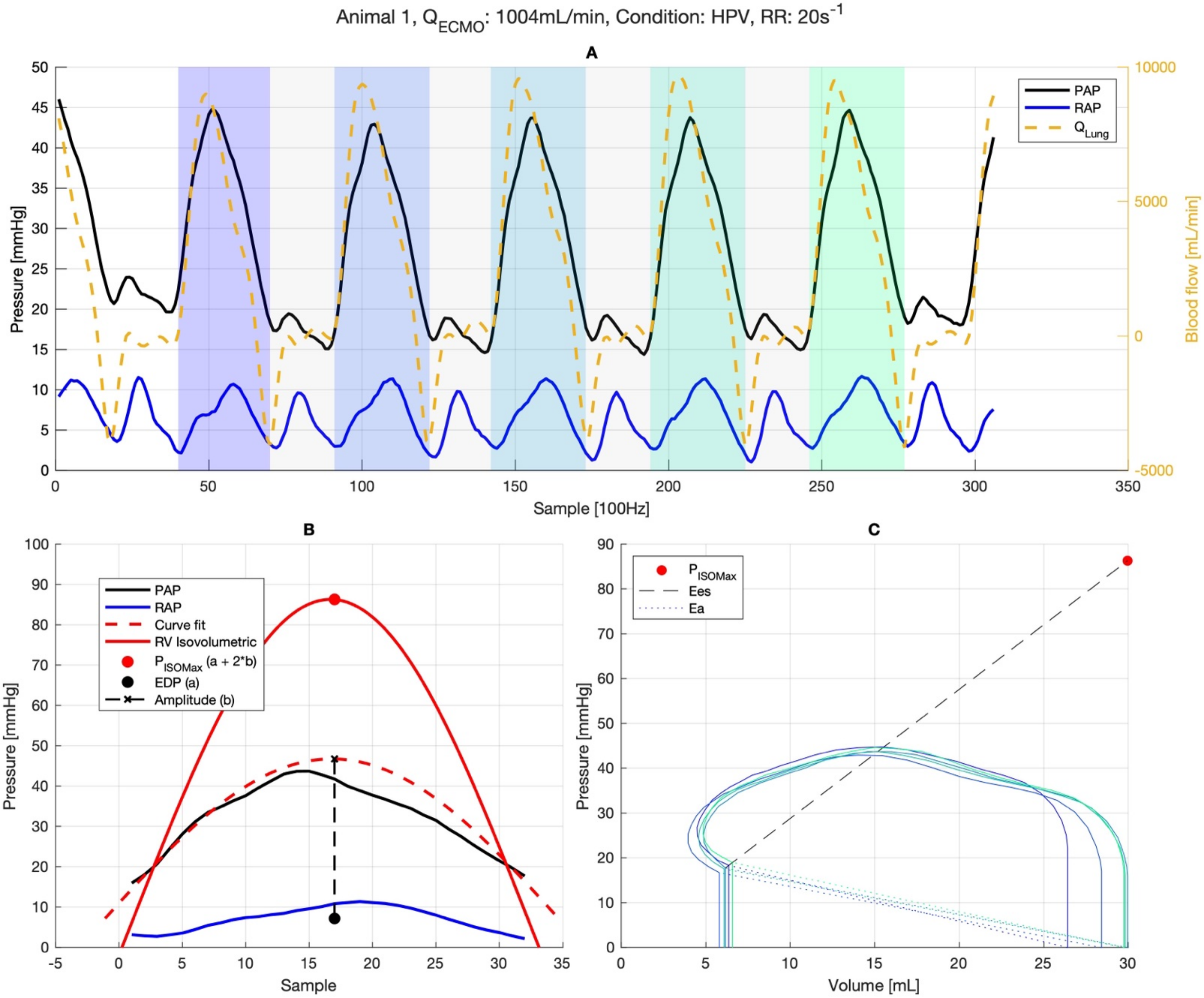

Animal 1, QECMO: 1004mL/min, Condition: HPV, RR: 20s-1
A
PAP
RAP
QLung
Pressure [mmHg]
Blood flow [mL/min]
Sample [100Hz]
B
PAP
RAP
Curve fit
RV Isovolumetric
PISOMax (a + 2*b)
EDP (a)
Amplitude (b)
Pressure [mmHg]
Sample
C
PISOMax
Ees
Ea
Pressure [mmHg]
Volume [mL]

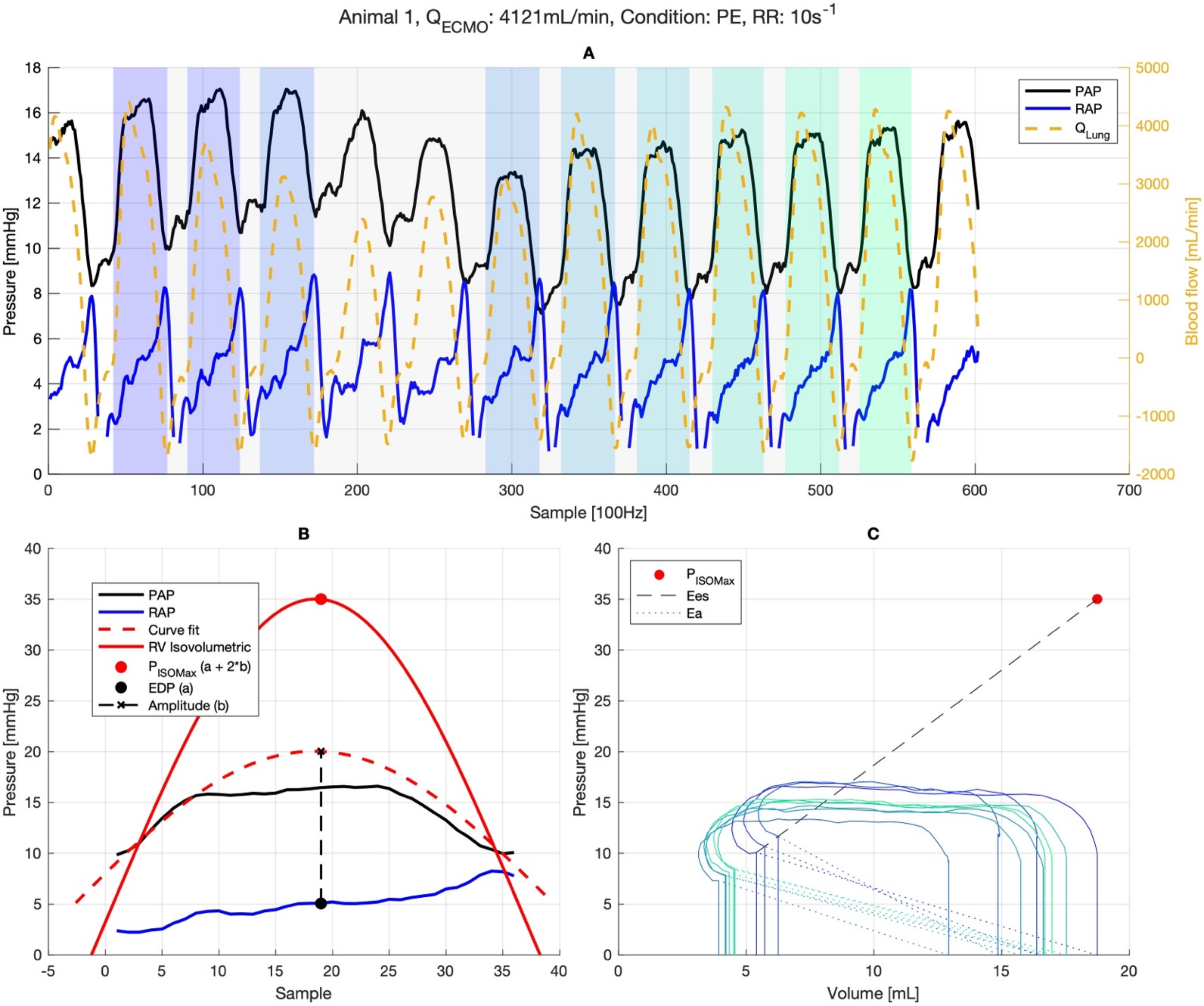

Animal 1, Q_ECMO: 4121mL/min, Condition: PE, RR: 10s^-1
A
PAP
RAP
Q_Lung
Pressure [mmHg]
Blood flow [mL/min]
Sample [100Hz]
B
PAP
RAP
Curve fit
RV Isovolumetric
P_ISOMax (a + 2*b)
EDP (a)
Amplitude (b)
Pressure [mmHg]
Sample
C
P_ISOMax
Ees
Ea
Pressure [mmHg]
Volume [mL]

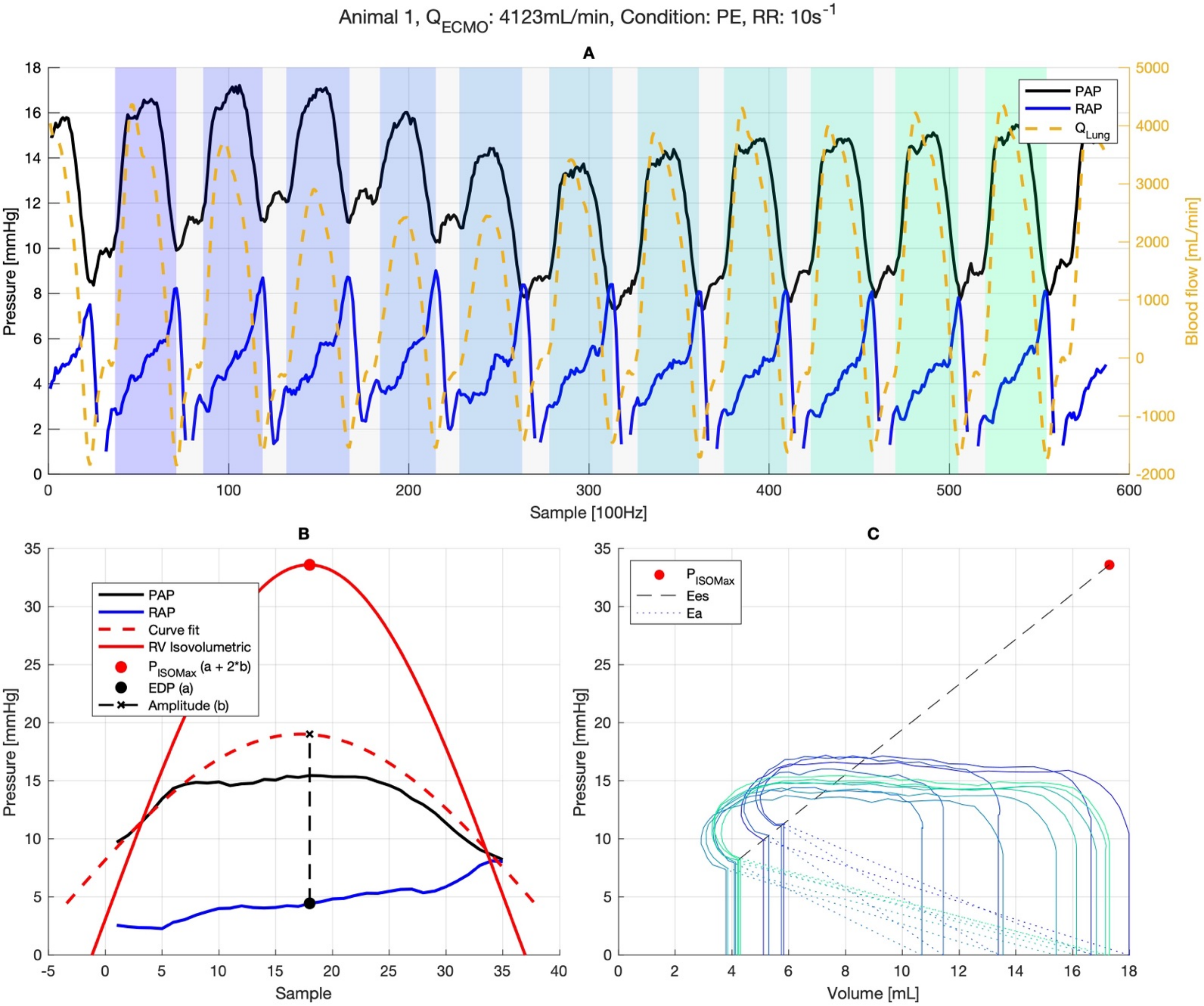

Animal 1, Q_{ECMO}: 4123mL/min, Condition: PE, RR: $10s^{-1}$
A
PAP
RAP
Q_{Lung}
Pressure [mmHg]
Blood flow [mL/min]
Sample [100Hz]
B
PAP
RAP
Curve fit
RV Isovolumetric
P_{ISOMax} (a + 2*b)
EDP (a)
Amplitude (b)
Pressure [mmHg]
Sample
C
P_{ISOMax}
Ees
Ea
Pressure [mmHg]
Volume [mL]

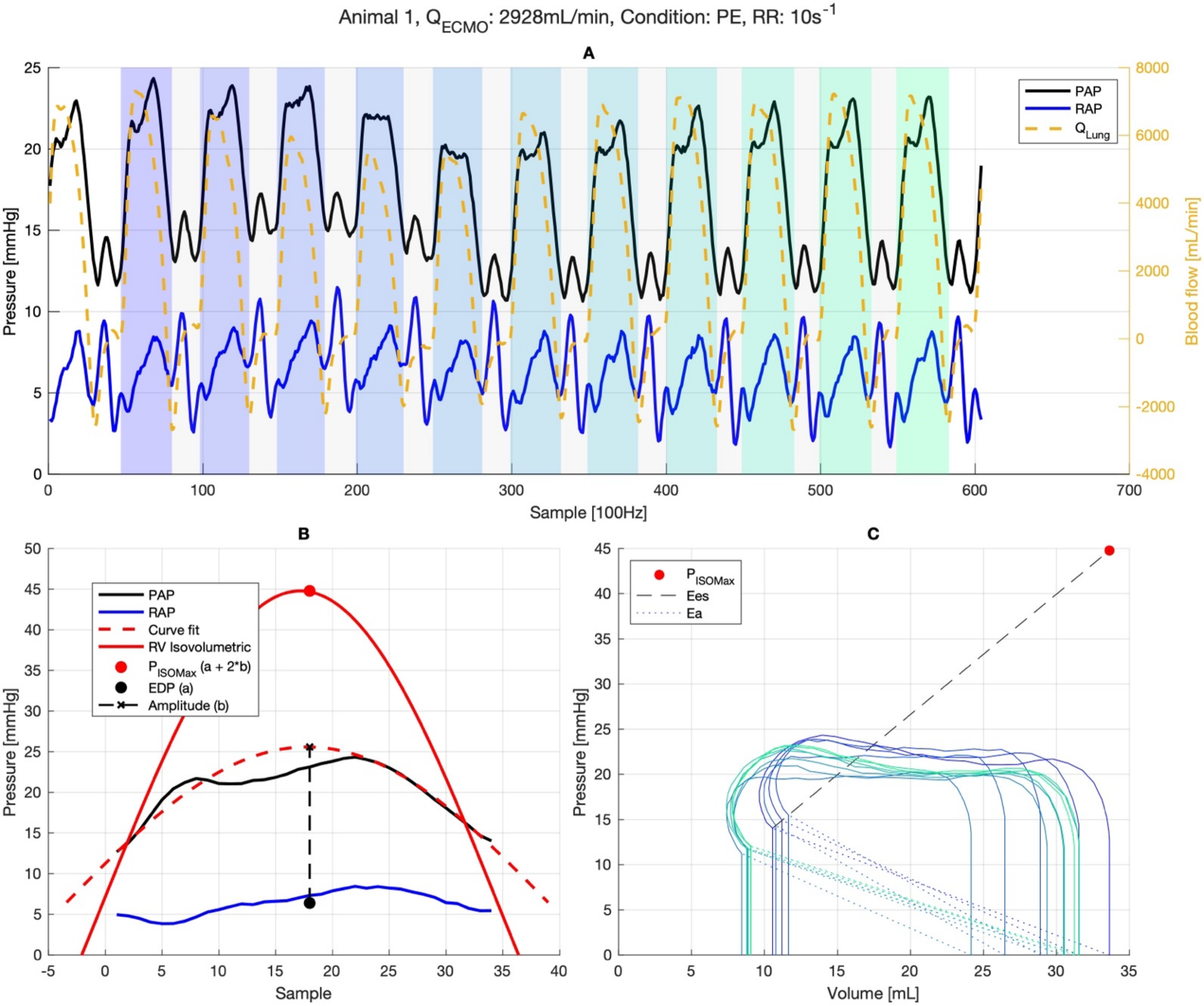

Animal 1, Q_ECMO: 2928mL/min, Condition: PE, RR: 10s^-1
A
Pressure [mmHg]
Blood flow [mL/min]
Sample [100Hz]
PAP
RAP
Q_Lung
B
Pressure [mmHg]
Sample
PAP
RAP
Curve fit
RV Isovolumetric
P_ISOMax (a + 2*b)
EDP (a)
Amplitude (b)
C
Pressure [mmHg]
Volume [mL]
P_ISOMax
Ees
Ea

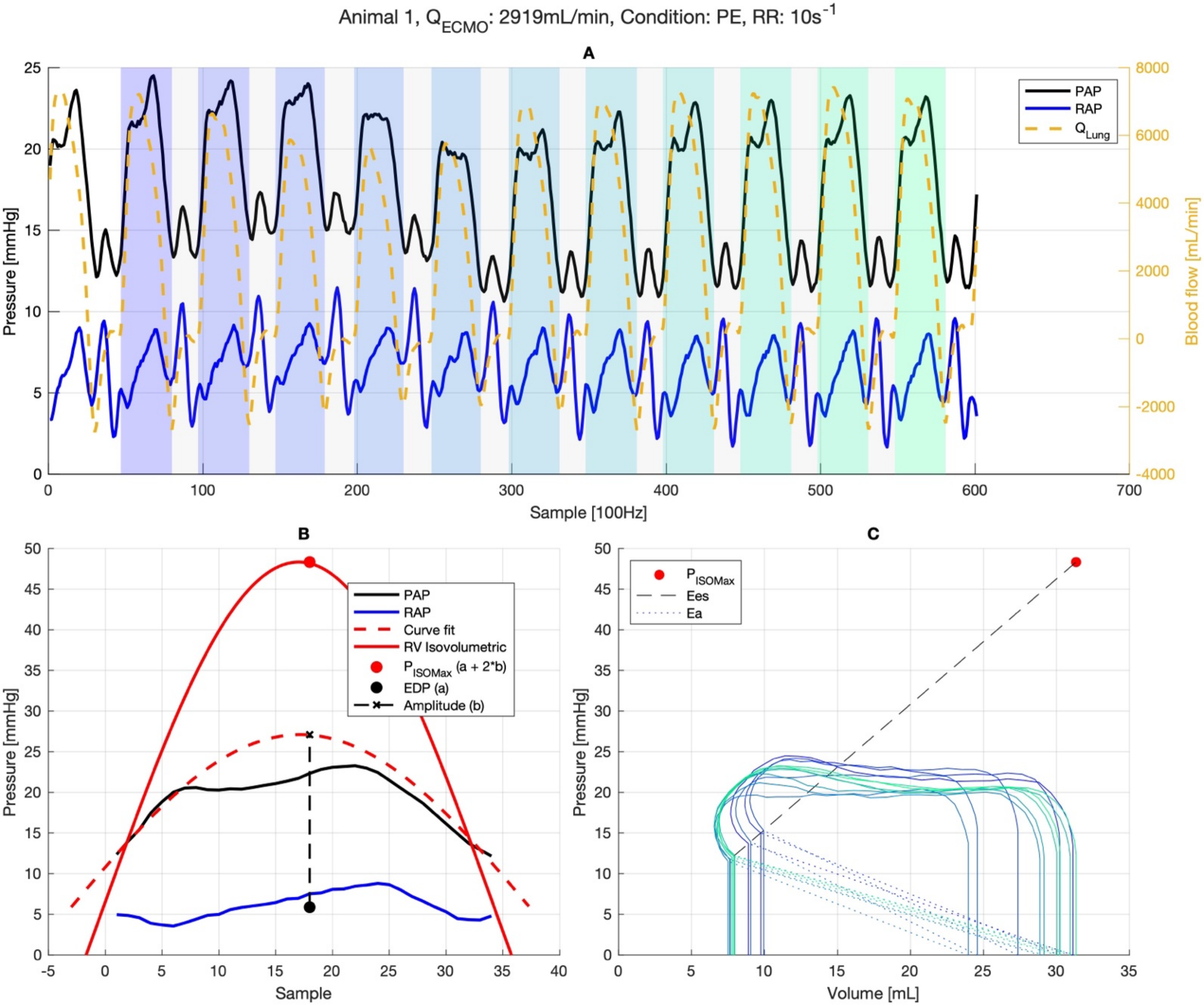
Animal 1, Q_ECMO: 2919mL/min, Condition: PE, RR: 10s^-1
A
PAP
RAP
Q_Lung
Pressure [mmHg]
Blood flow [mL/min]
Sample [100Hz]
B
PAP
RAP
Curve fit
RV Isovolumetric
P_ISOMax (a + 2*b)
EDP (a)
Amplitude (b)
Pressure [mmHg]
Sample
C
P_ISOMax
Ees
Ea
Pressure [mmHg]
Volume [mL]

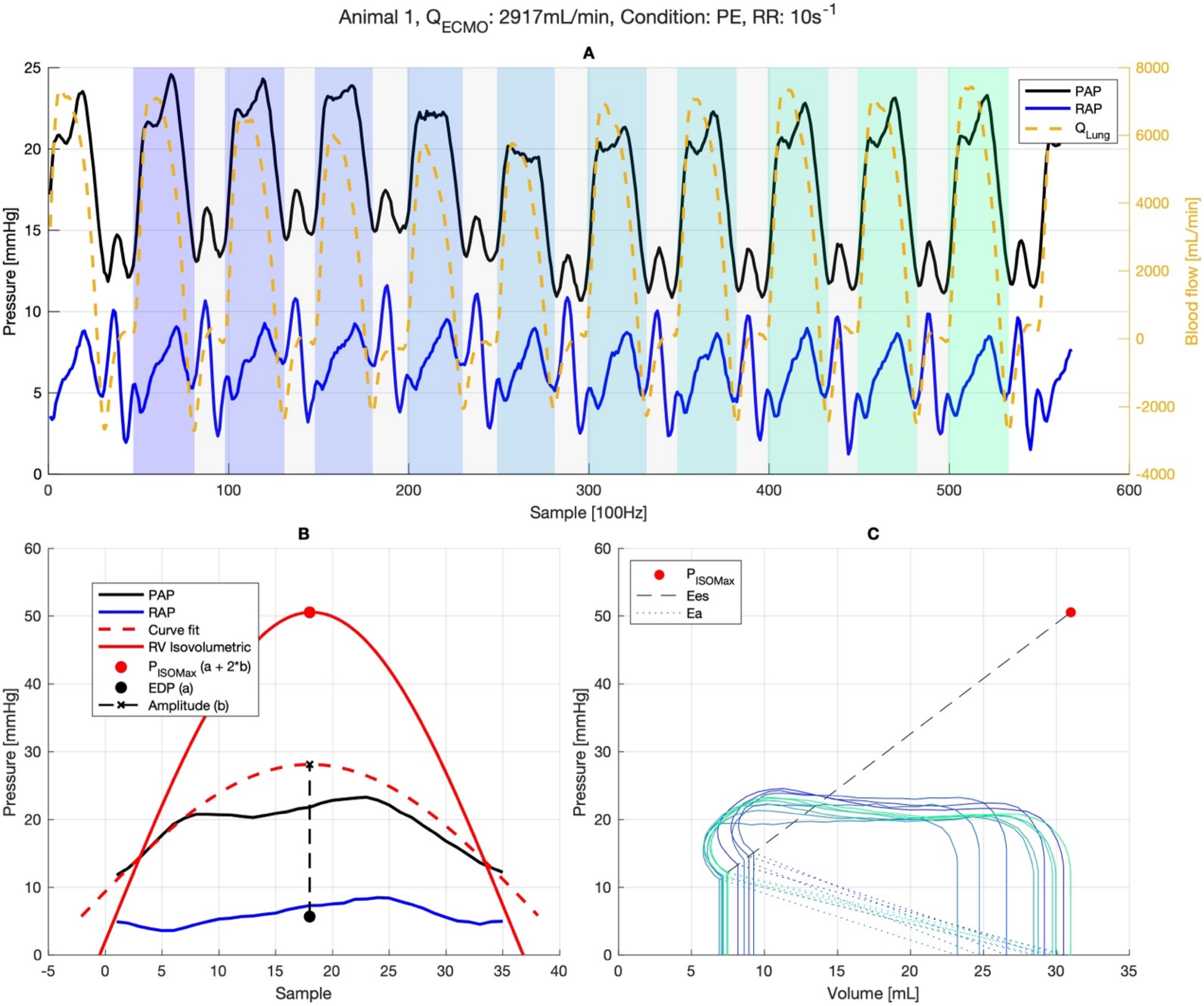

Animal 1, Q_{ECMO}: 2917mL/min, Condition: PE, RR: $10s^{-1}$
A
PAP
RAP
Q_{Lung}
Pressure [mmHg]
Blood flow [mL/min]
Sample [100Hz]
B
PAP
RAP
Curve fit
RV Isovolumetric
P_{ISOMax} (a + 2*b)
EDP (a)
Amplitude (b)
Pressure [mmHg]
Sample
C
P_{ISOMax}
Ees
Ea
Pressure [mmHg]
Volume [mL]

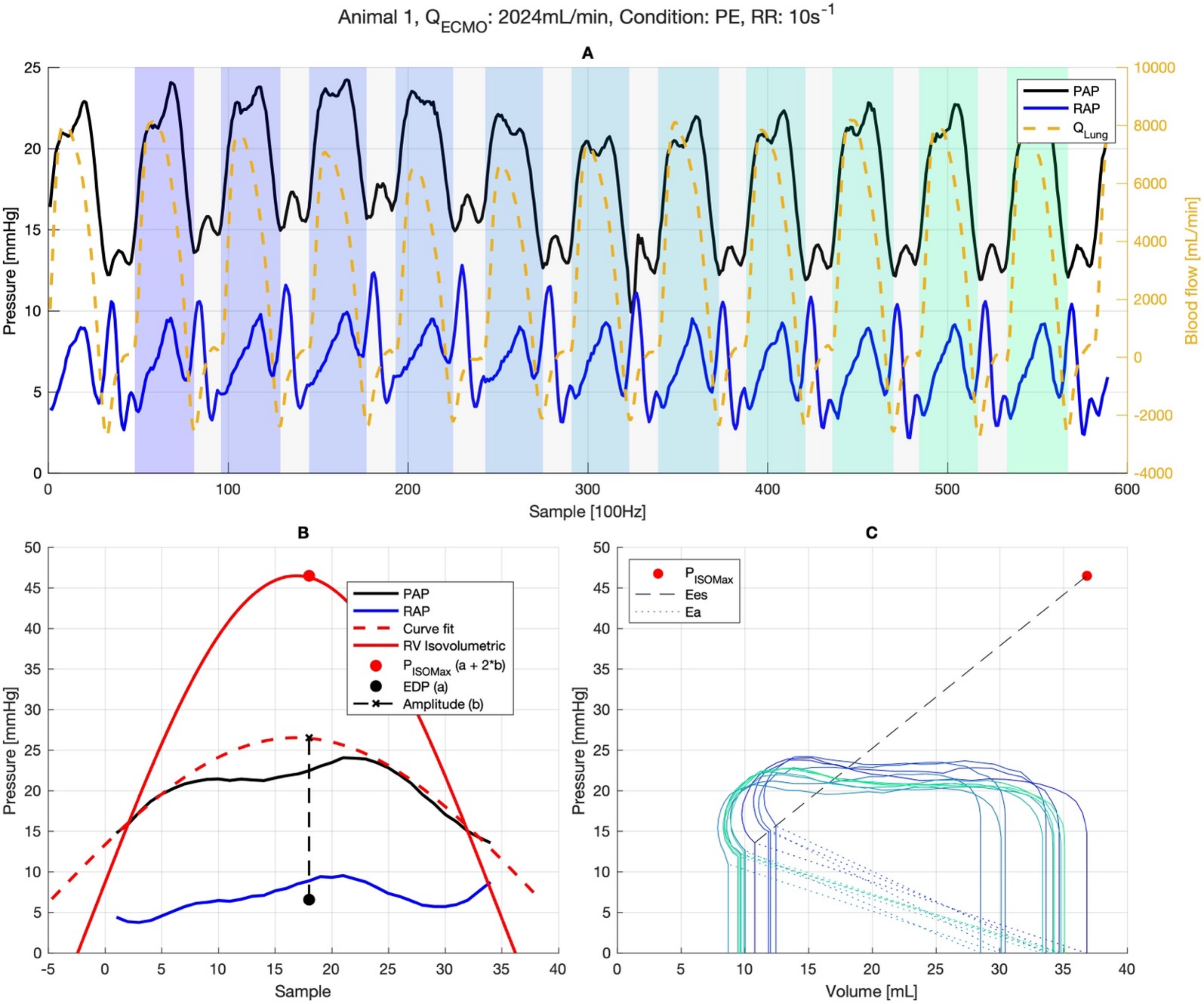

Animal 1, Q_{ECMO}: 2024mL/min, Condition: PE, RR: $10s^{-1}$
A
PAP
RAP
Q_{Lung}
Pressure [mmHg]
Blood flow [mL/min]
Sample [100Hz]
B
PAP
RAP
Curve fit
RV Isovolumetric
P_{ISOMax} (a + 2*b)
EDP (a)
Amplitude (b)
Pressure [mmHg]
Sample
C
P_{ISOMax}
Ees
Ea
Pressure [mmHg]
Volume [mL]

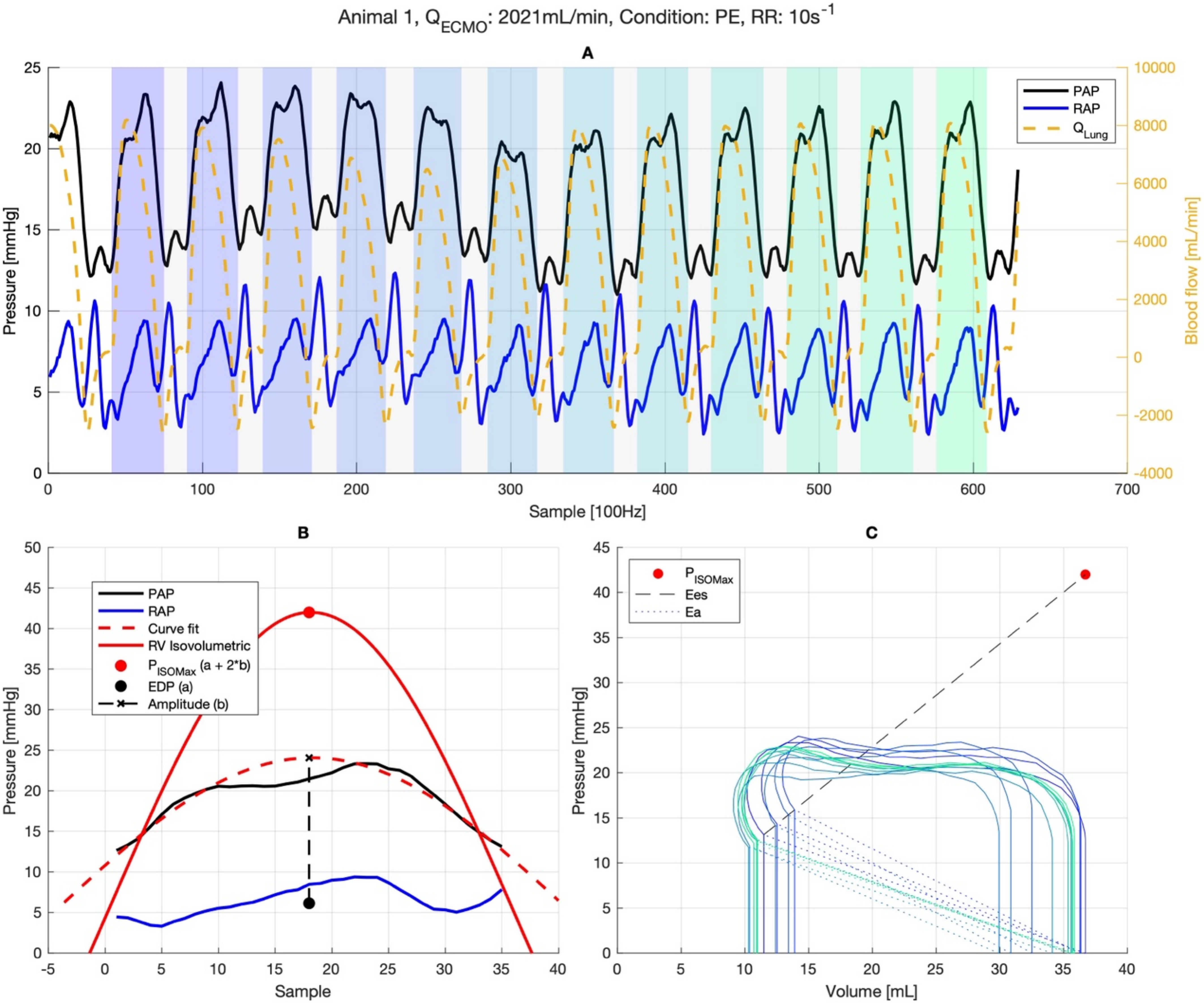

Animal 1, Q_{ECMO}: 2021mL/min, Condition: PE, RR: $10s^{-1}$
A
PAP
RAP
Q_{Lung}
Pressure [mmHg]
Blood flow [mL/min]
Sample [100Hz]
B
PAP
RAP
Curve fit
RV Isovolumetric
P_{ISOMax} (a + 2*b)
EDP (a)
Amplitude (b)
Pressure [mmHg]
Sample
C
P_{ISOMax}
Ees
Ea
Pressure [mmHg]
Volume [mL]

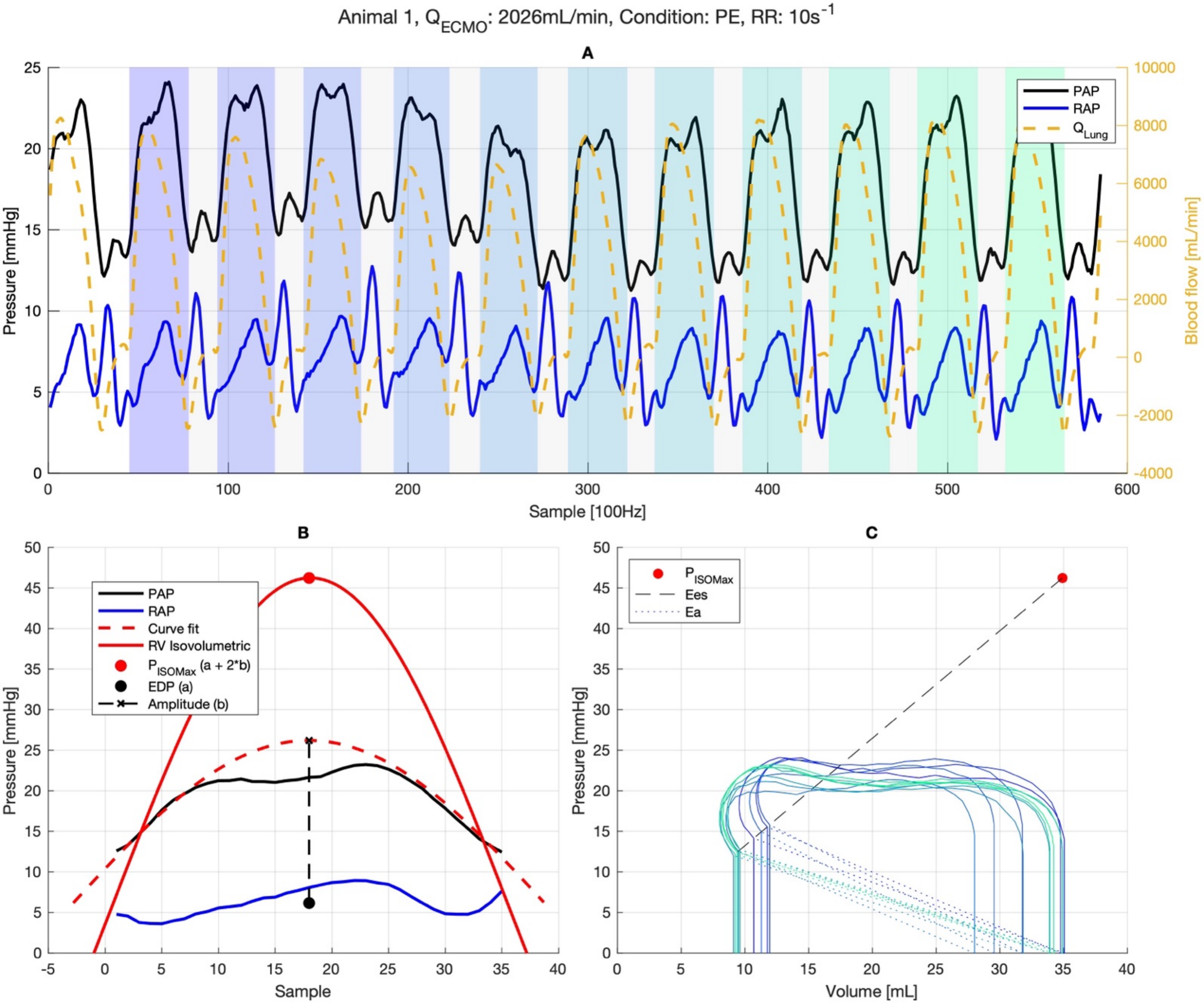
Animal 1, Q_{ECMO}: 2026mL/min, Condition: PE, RR: $10s^{-1}$
A
PAP
RAP
Q_{Lung}
Pressure [mmHg]
Blood flow [mL/min]
Sample [100Hz]
B
PAP
RAP
Curve fit
RV Isovolumetric
P_{ISOMax} (a + 2*b)
EDP (a)
Amplitude (b)
Pressure [mmHg]
Sample
C
P_{ISOMax}
Ees
Ea
Pressure [mmHg]
Volume [mL]

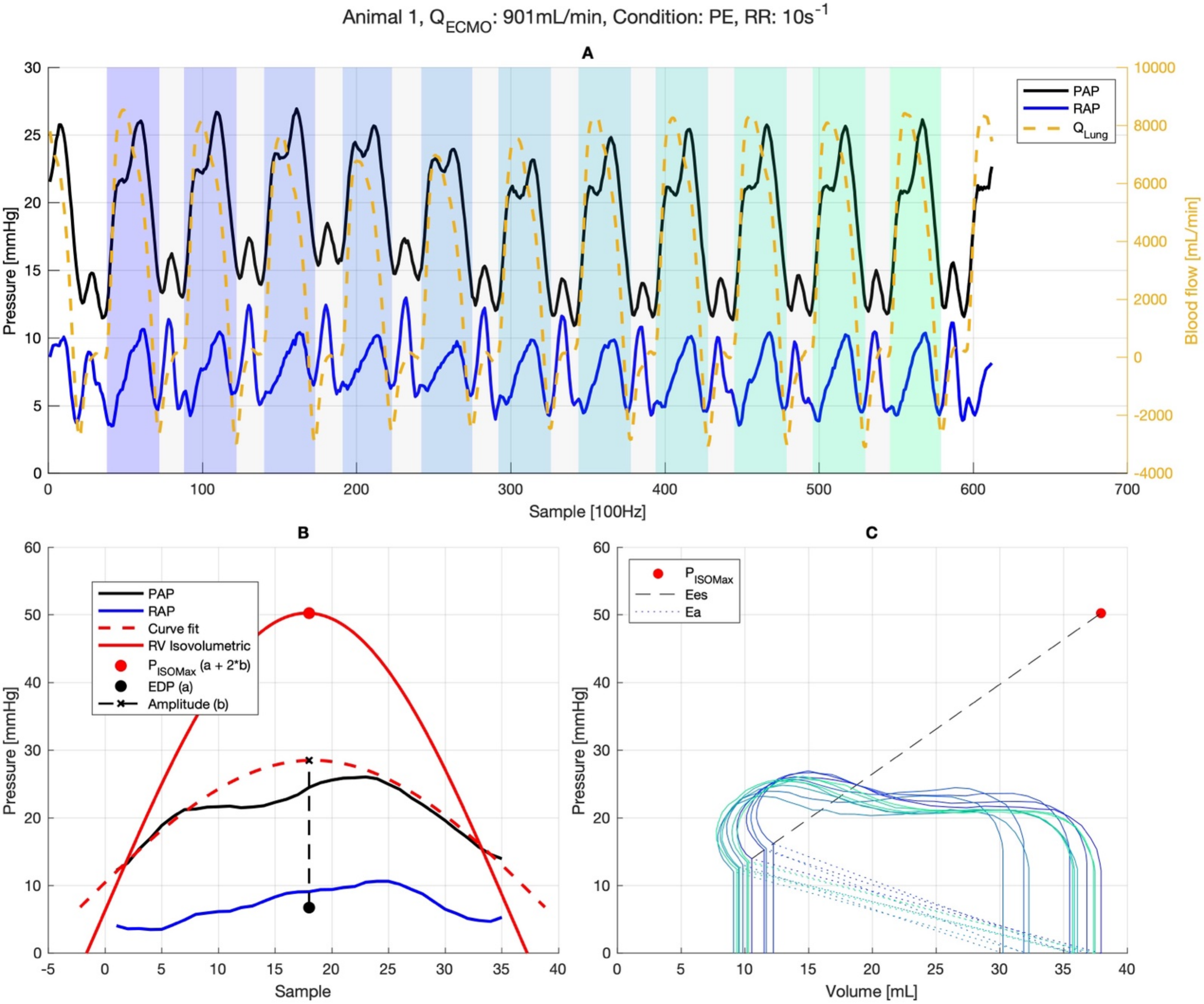

Animal 1, Q_ECMO: 901mL/min, Condition: PE, RR: 10s^-1
A
Pressure [mmHg]
Blood flow [mL/min]
Sample [100Hz]
PAP
RAP
Q_Lung
B
Pressure [mmHg]
Sample
PAP
RAP
Curve fit
RV Isovolumetric
P_ISOMax (a + 2*b)
EDP (a)
Amplitude (b)
C
Pressure [mmHg]
Volume [mL]
P_ISOMax
Ees
Ea

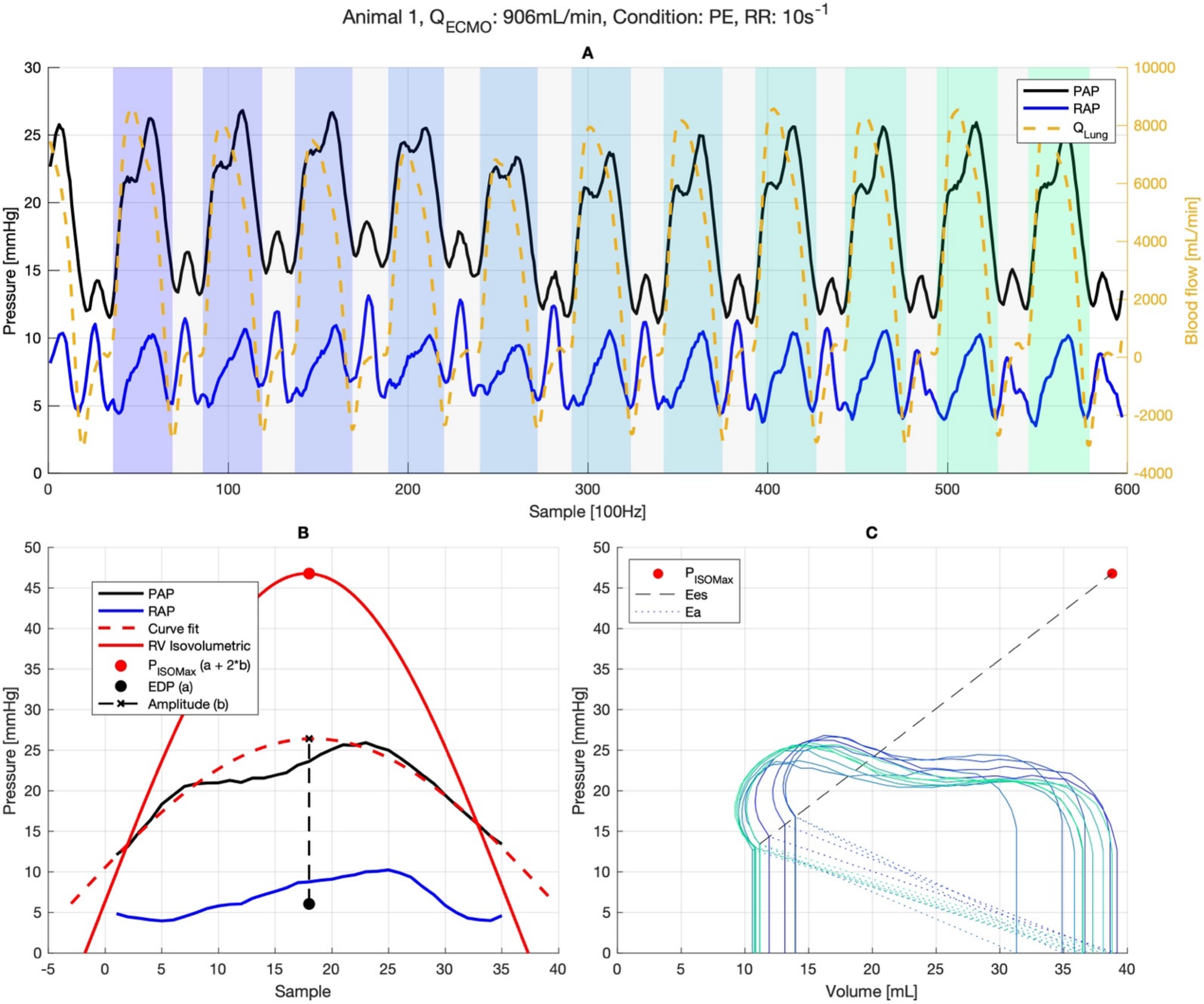

Animal 1, Q_{ECMO}: 906mL/min, Condition: PE, RR: $10s^{-1}$
A
PAP
RAP
Q_{Lung}
Pressure [mmHg]
Blood flow [mL/min]
Sample [100Hz]
B
PAP
RAP
Curve fit
RV Isovolumetric
P_{ISOMax} (a + 2*b)
EDP (a)
Amplitude (b)
Pressure [mmHg]
Sample
C
P_{ISOMax}
Ees
Ea
Pressure [mmHg]
Volume [mL]

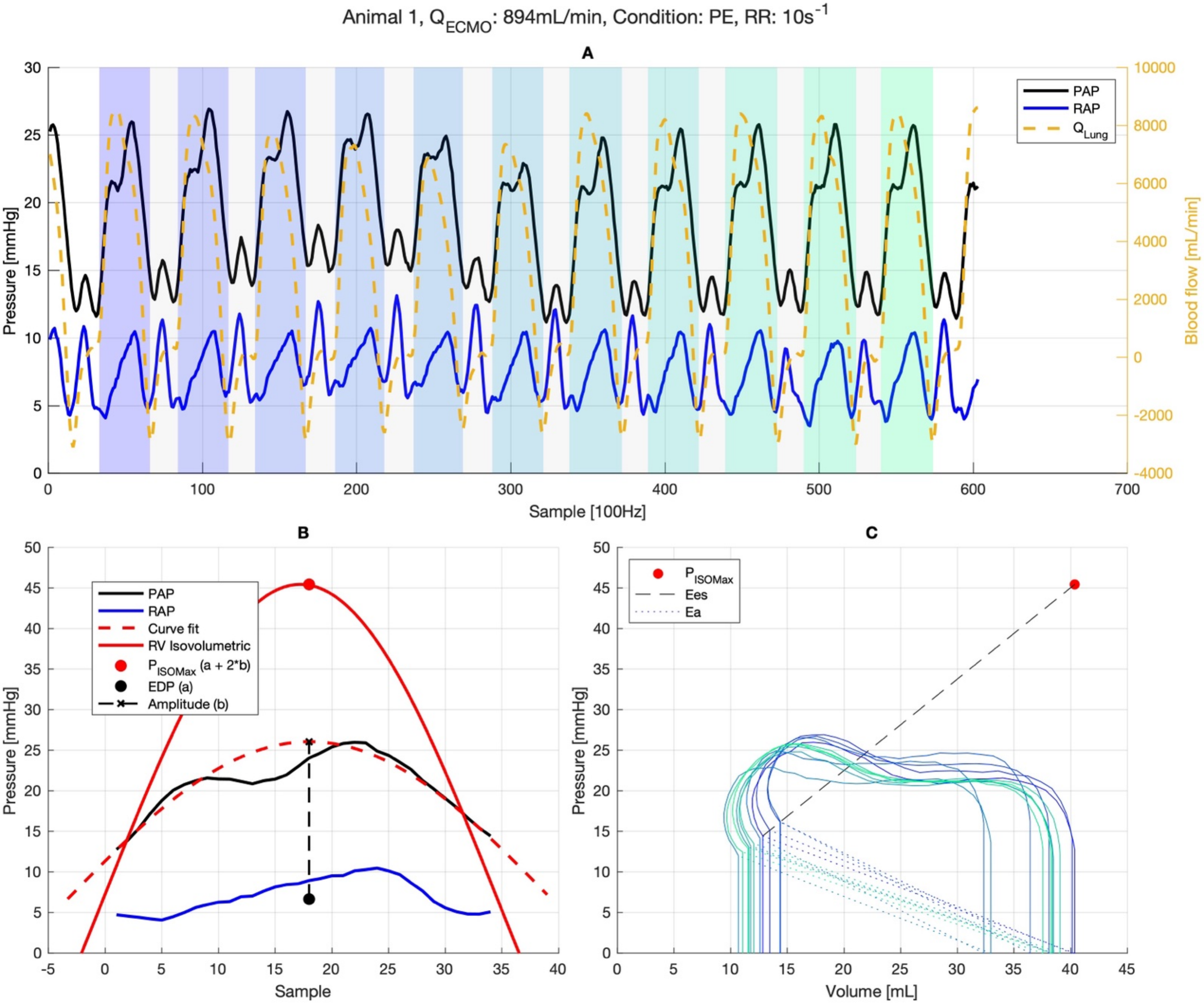

Animal 1, Q_ECMO: 894mL/min, Condition: PE, RR: 10s^-1
A
PAP
RAP
Q_Lung
Pressure [mmHg]
Blood flow [mL/min]
Sample [100Hz]
B
PAP
RAP
Curve fit
RV Isovolumetric
P_ISOMax (a + 2*b)
EDP (a)
Amplitude (b)
Pressure [mmHg]
Sample
C
P_ISOMax
Ees
Ea
Pressure [mmHg]
Volume [mL]

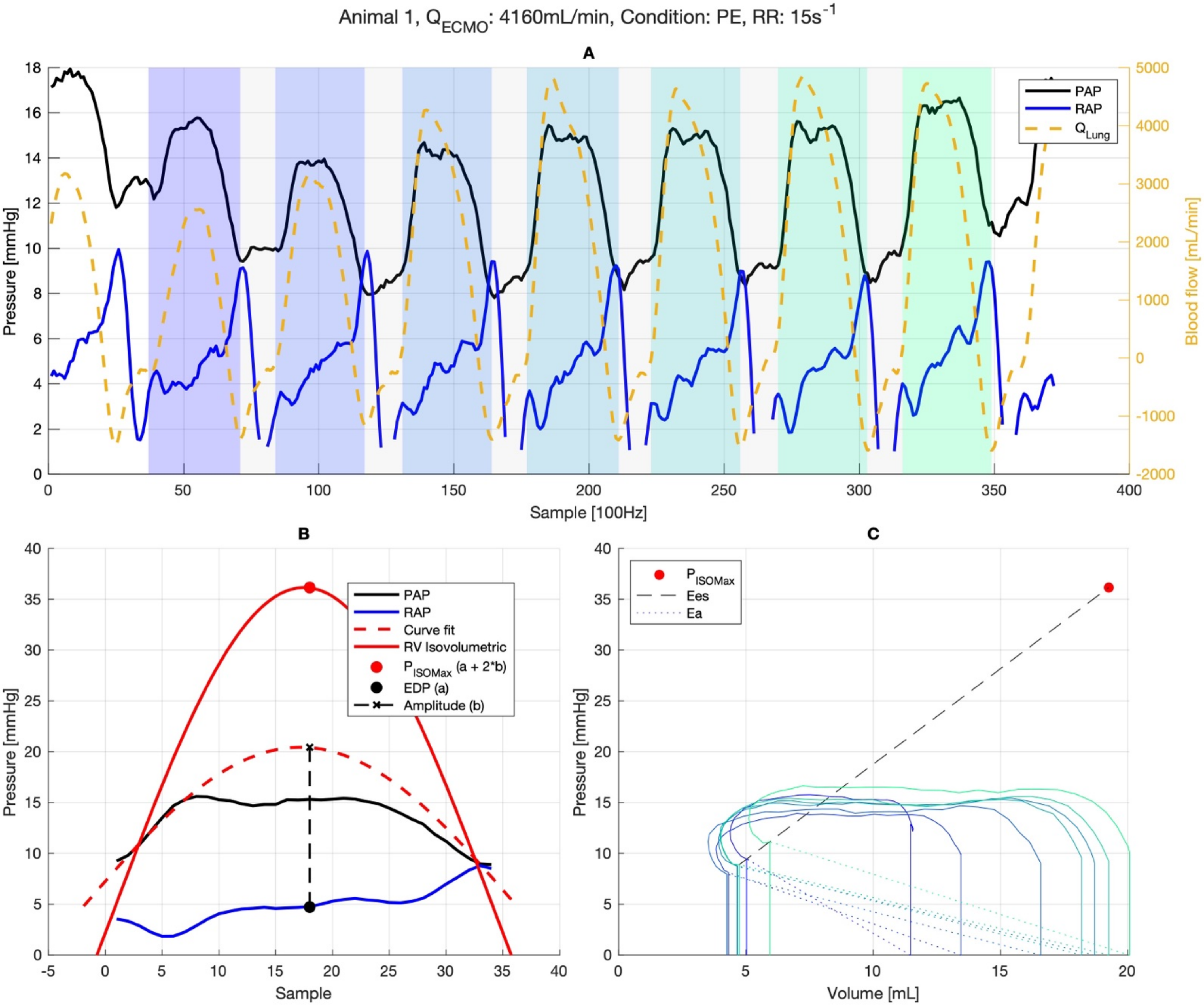

Animal 1, Q_ECMO: 4160mL/min, Condition: PE, RR: 15s^-1
A
PAP
RAP
Q_Lung
Pressure [mmHg]
Blood flow [mL/min]
Sample [100Hz]
B
PAP
RAP
Curve fit
RV Isovolumetric
P_ISOMax (a + 2*b)
EDP (a)
Amplitude (b)
Pressure [mmHg]
Sample
C
P_ISOMax
Ees
Ea
Pressure [mmHg]
Volume [mL]

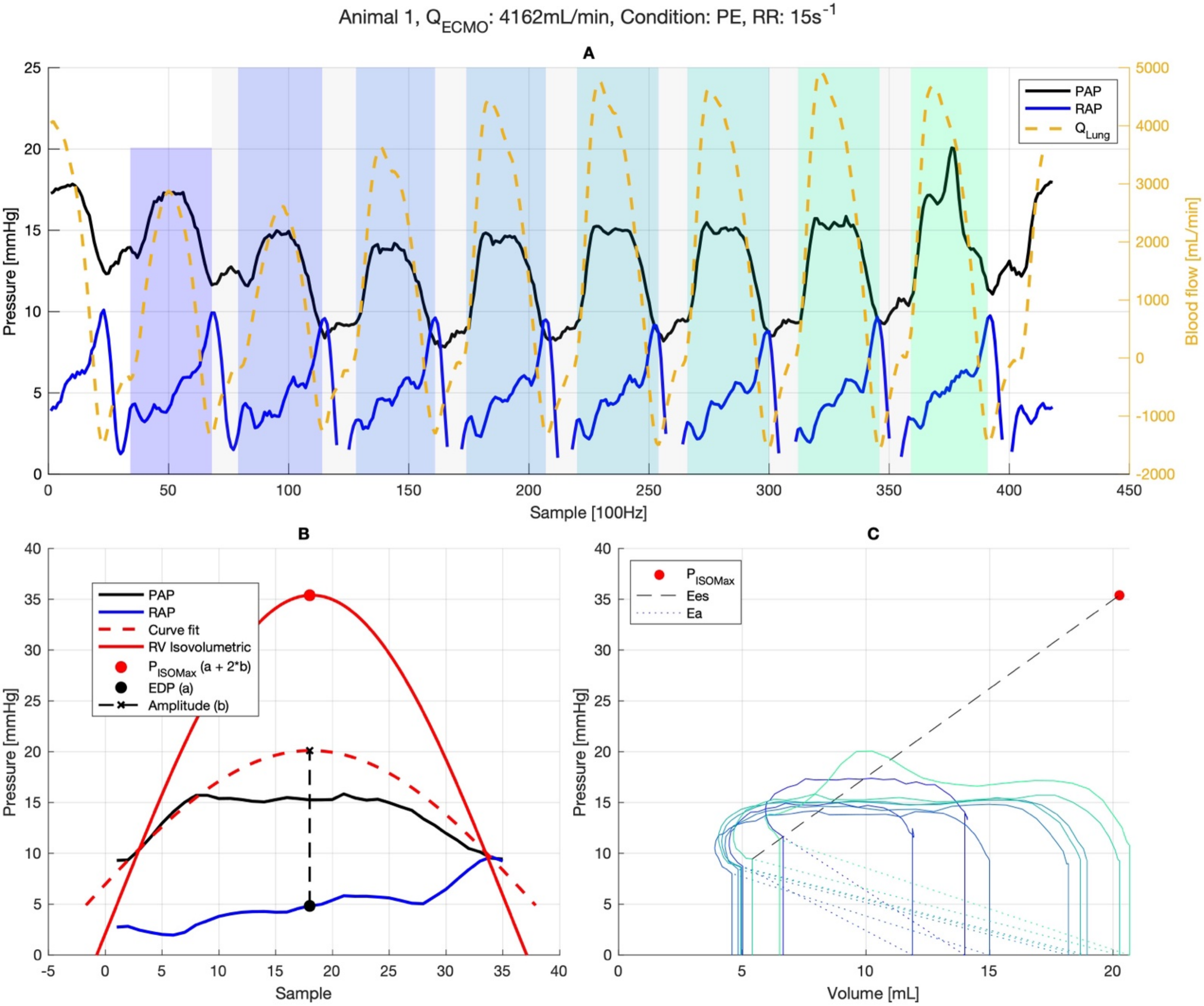

Animal 1, Q_ECMO: 4162mL/min, Condition: PE, RR: 15s^-1
A
PAP
RAP
Q_Lung
Pressure [mmHg]
Blood flow [mL/min]
Sample [100Hz]
B
PAP
RAP
Curve fit
RV Isovolumetric
P_ISOMax (a + 2*b)
EDP (a)
Amplitude (b)
Pressure [mmHg]
Sample
C
P_ISOMax
Ees
Ea
Pressure [mmHg]
Volume [mL]

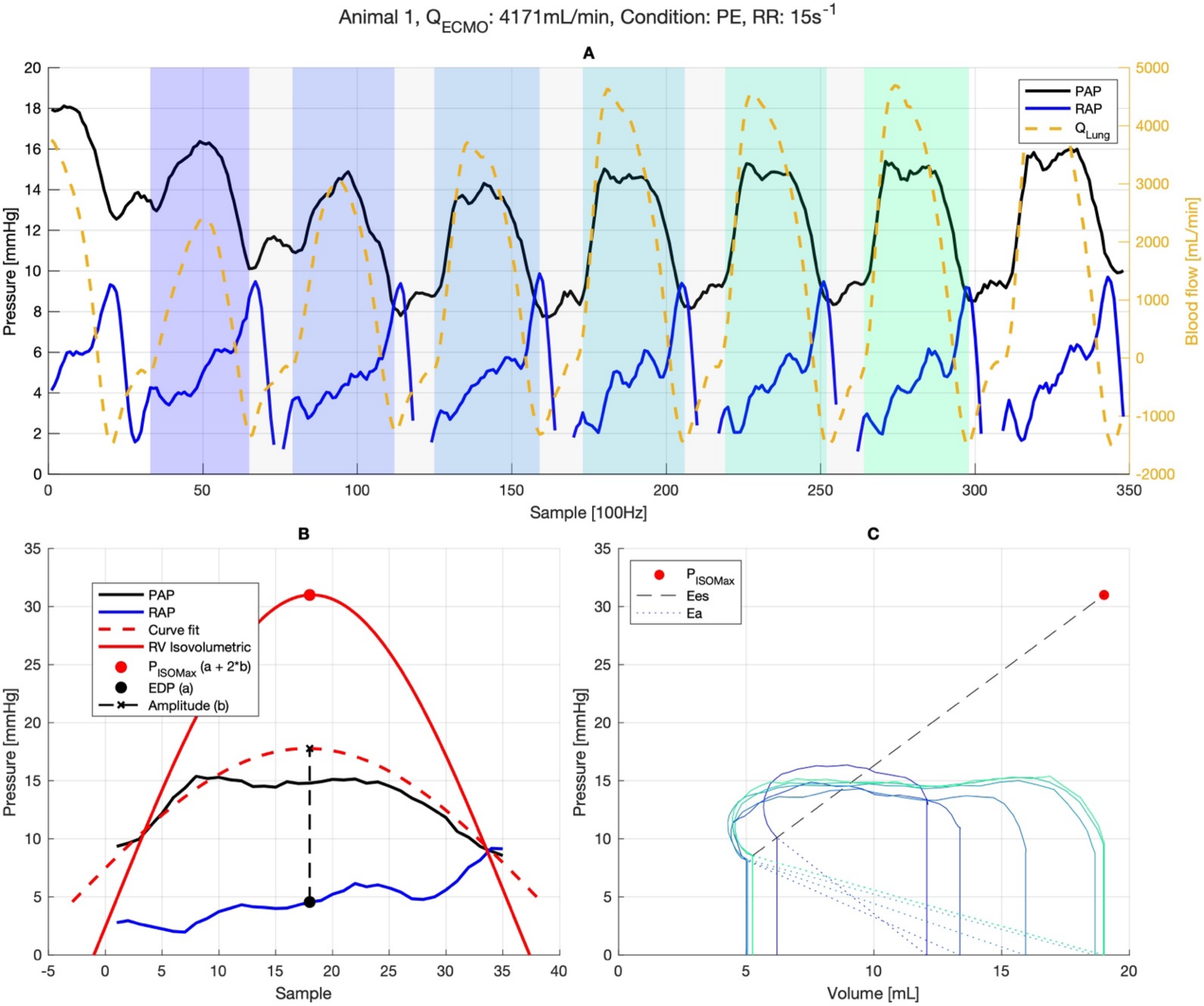

Animal 1, Q_{ECMO}: 4171mL/min, Condition: PE, RR: $15s^{-1}$
A
PAP
RAP
Q_{Lung}
Pressure [mmHg]
Blood flow [mL/min]
Sample [100Hz]
B
PAP
RAP
Curve fit
RV Isovolumetric
P_{ISOMax} (a + 2*b)
EDP (a)
Amplitude (b)
Pressure [mmHg]
Sample
C
P_{ISOMax}
Ees
Ea
Pressure [mmHg]
Volume [mL]

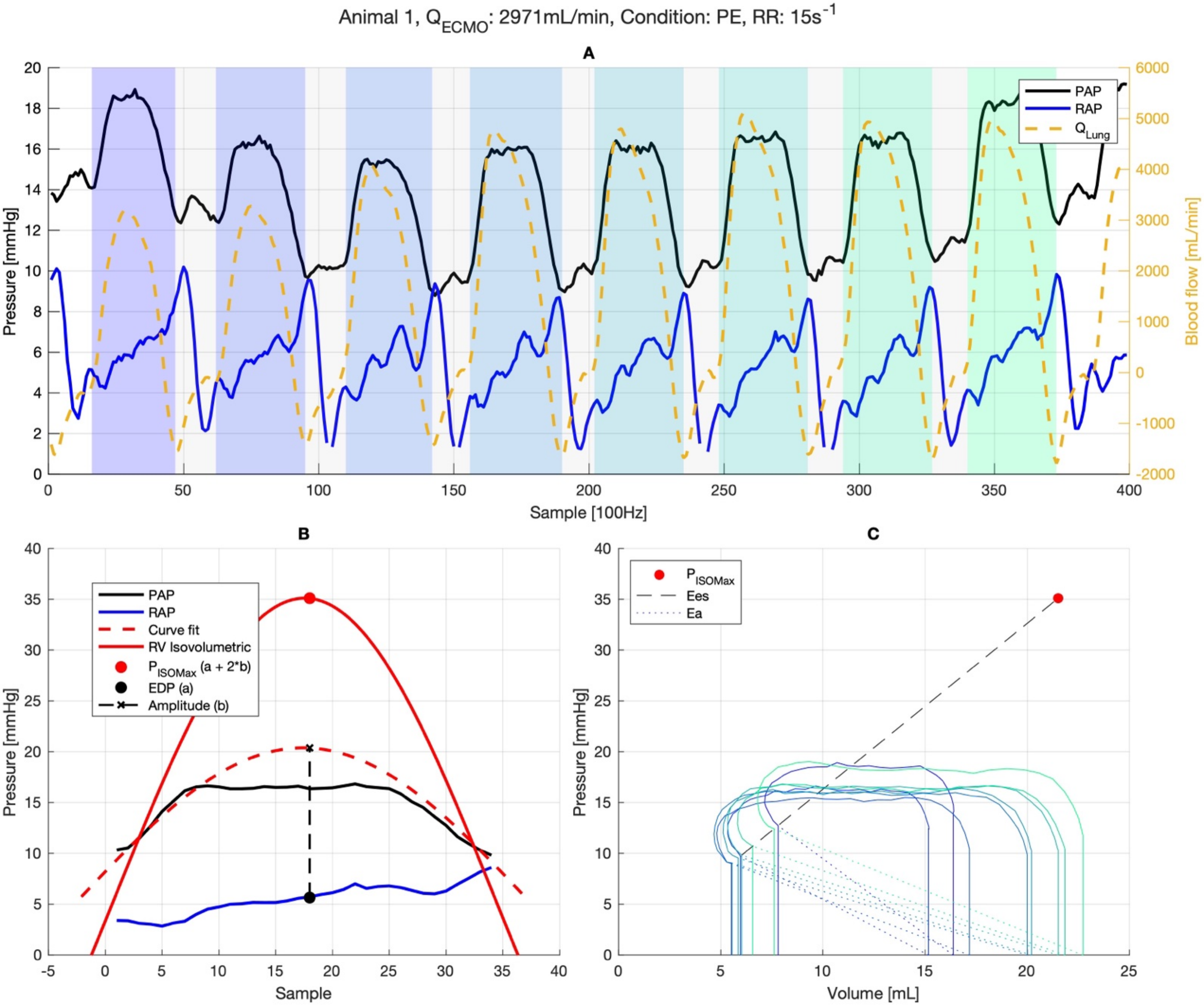

Animal 1, Q_ECMO: 2971mL/min, Condition: PE, RR: 15s^-1
A
PAP
RAP
Q_Lung
Pressure [mmHg]
Blood flow [mL/min]
Sample [100Hz]
B
PAP
RAP
Curve fit
RV Isovolumetric
P_ISOMax (a + 2*b)
EDP (a)
Amplitude (b)
Pressure [mmHg]
Sample
C
P_ISOMax
Ees
Ea
Pressure [mmHg]
Volume [mL]

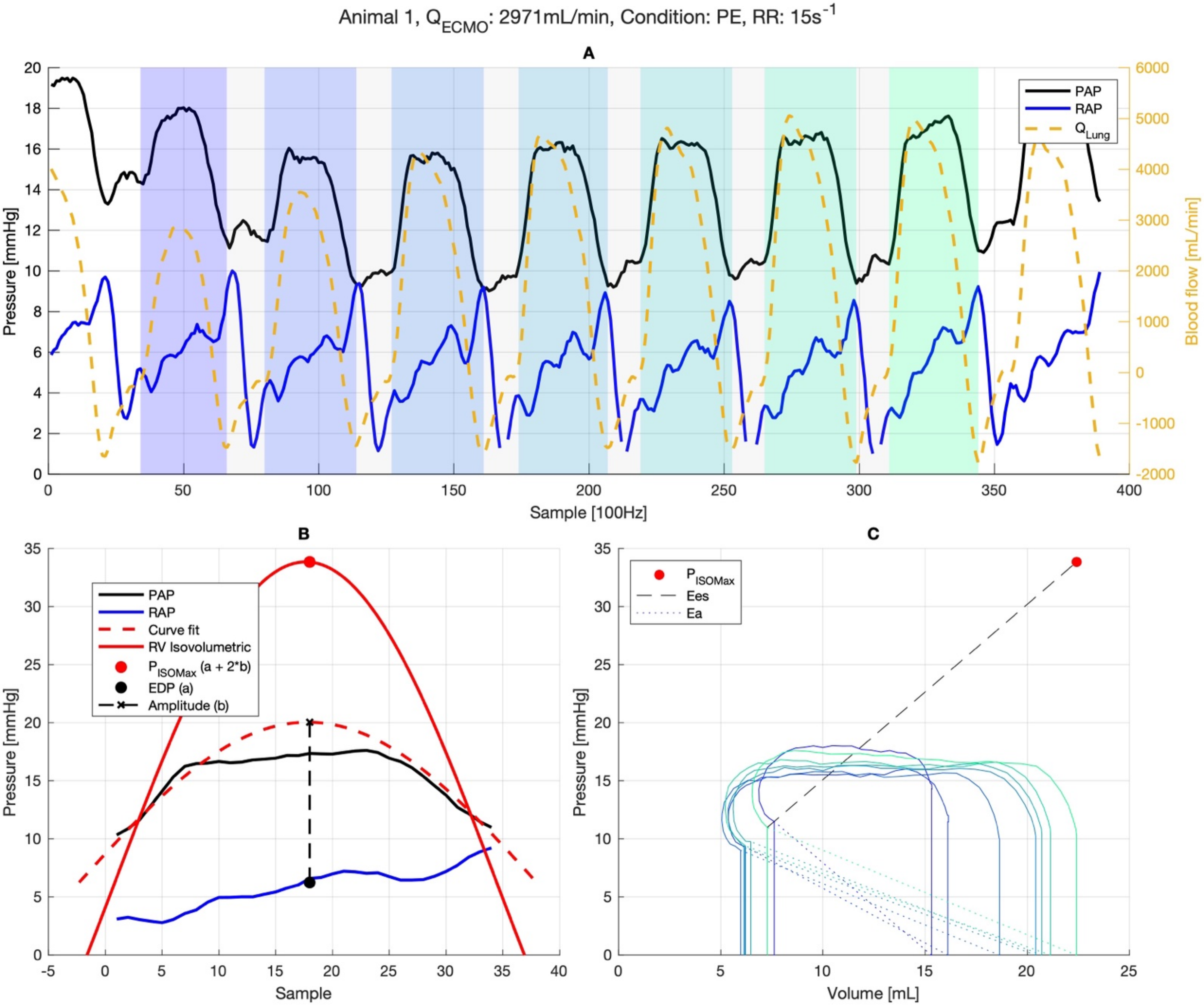
Animal 1, Q_{ECMO}: 2971mL/min, Condition: PE, RR: $15s^{-1}$
A
PAP
RAP
Q_{Lung}
Pressure [mmHg]
Blood flow [mL/min]
Sample [100Hz]
B
PAP
RAP
Curve fit
RV Isovolumetric
P_{ISOMax} (a + 2*b)
EDP (a)
Amplitude (b)
Pressure [mmHg]
Sample
C
P_{ISOMax}
Ees
Ea
Pressure [mmHg]
Volume [mL]

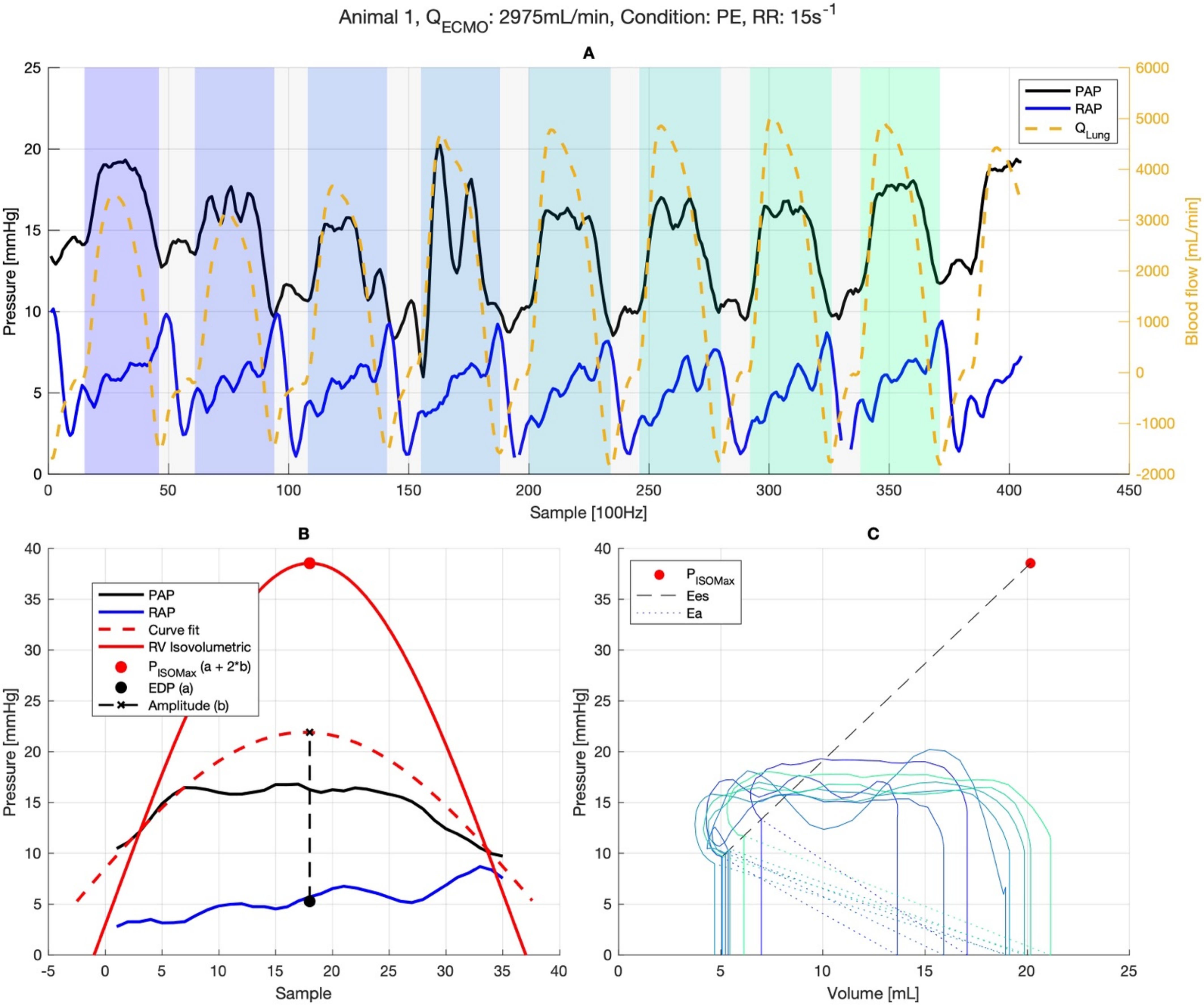

Animal 1, Q_ECMO: 2975mL/min, Condition: PE, RR: 15s^-1
A
PAP
RAP
Q_Lung
Pressure [mmHg]
Blood flow [mL/min]
Sample [100Hz]
B
PAP
RAP
Curve fit
RV Isovolumetric
P_ISOMax (a + 2*b)
EDP (a)
Amplitude (b)
Pressure [mmHg]
Sample
C
P_ISOMax
Ees
Ea
Pressure [mmHg]
Volume [mL]

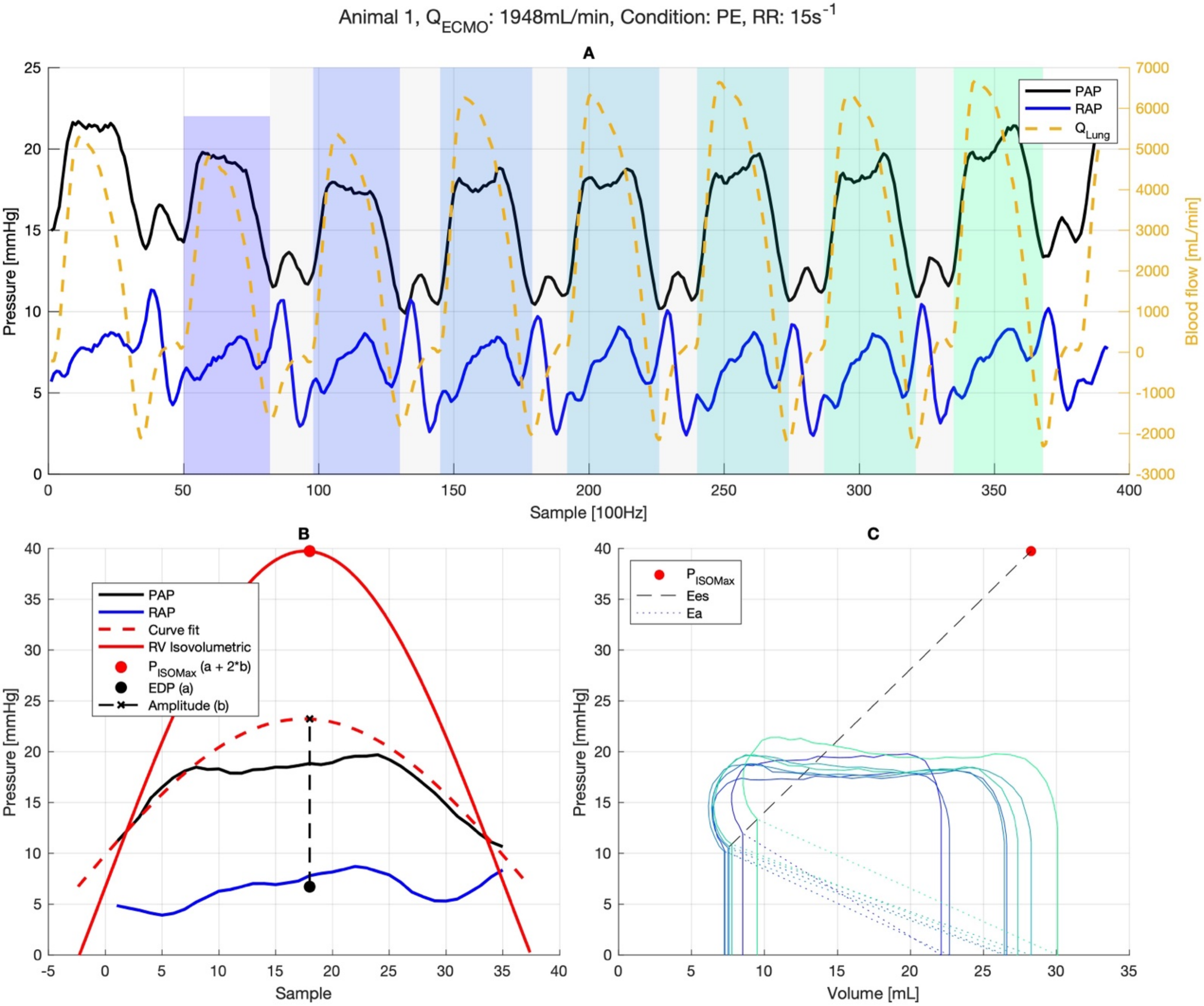

Animal 1, Q_{ECMO}: 1948mL/min, Condition: PE, RR: $15s^{-1}$
A
PAP
RAP
Q_{Lung}
Pressure [mmHg]
Blood flow [mL/min]
Sample [100Hz]
B
PAP
RAP
Curve fit
RV Isovolumetric
P_{ISOMax} (a + 2*b)
EDP (a)
Amplitude (b)
Pressure [mmHg]
Sample
C
P_{ISOMax}
Ees
Ea
Pressure [mmHg]
Volume [mL]

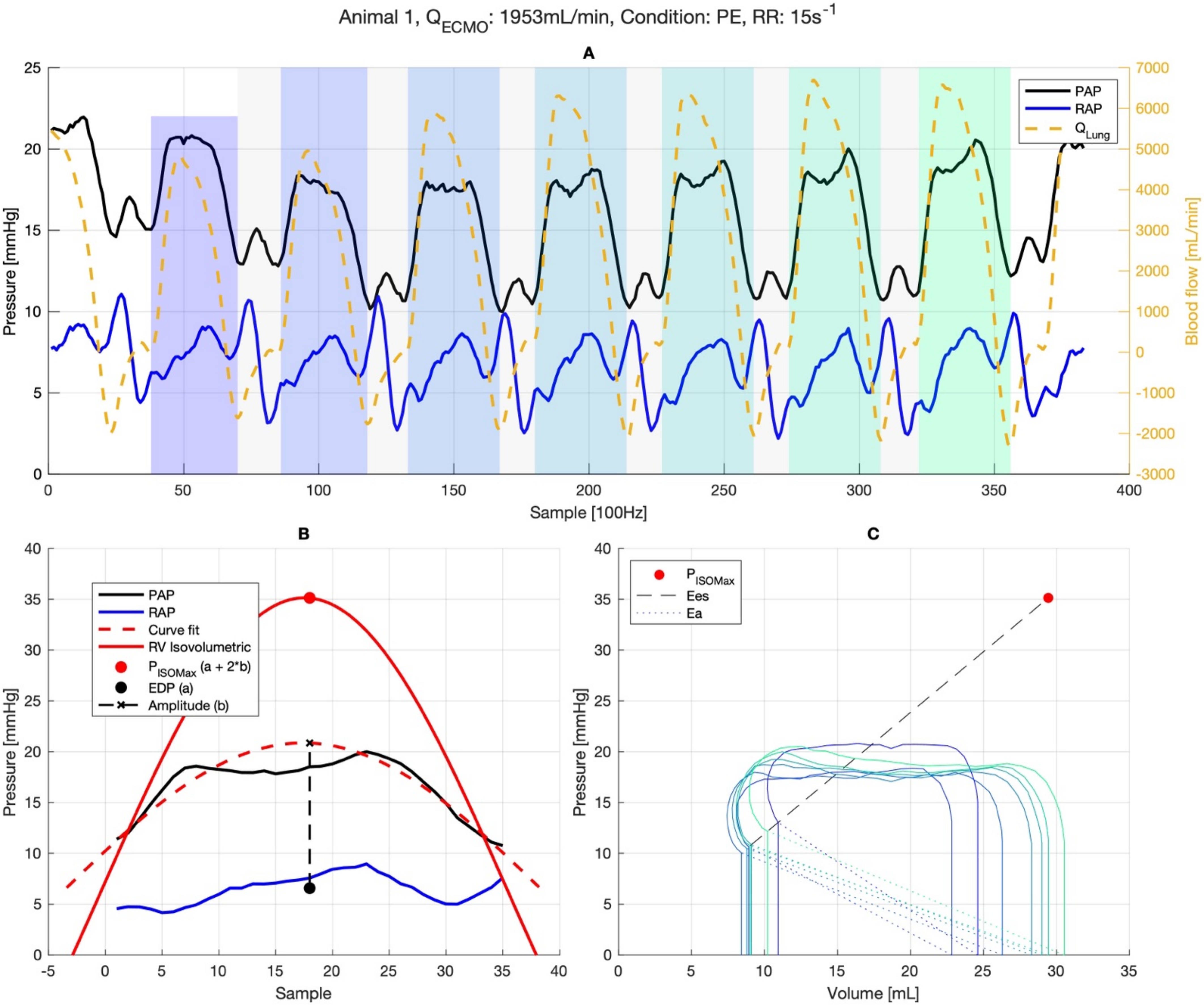

Animal 1, Q_{ECMO}: 1953mL/min, Condition: PE, RR: $15s^{-1}$
A
PAP
RAP
Q_{Lung}
Pressure [mmHg]
Blood flow [mL/min]
Sample [100Hz]
B
PAP
RAP
Curve fit
RV Isovolumetric
P_{ISOMax} (a + 2*b)
EDP (a)
Amplitude (b)
Pressure [mmHg]
Sample
C
P_{ISOMax}
Ees
Ea
Pressure [mmHg]
Volume [mL]

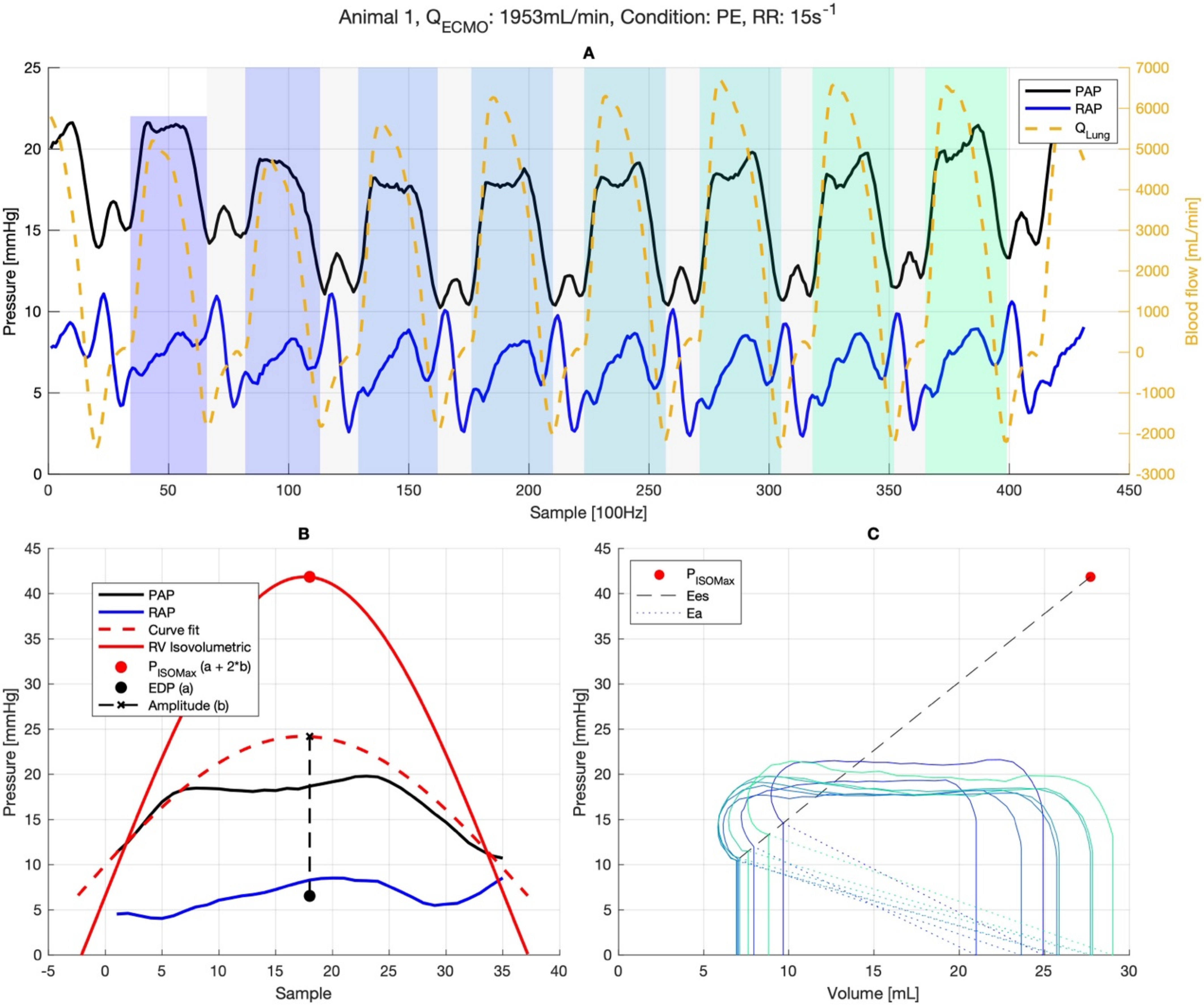

Animal 1, Q_{ECMO}: 1953mL/min, Condition: PE, RR: $15s^{-1}$
A
PAP
RAP
Q_{Lung}
Pressure [mmHg]
Blood flow [mL/min]
Sample [100Hz]
B
PAP
RAP
Curve fit
RV Isovolumetric
P_{ISOMax} (a + 2*b)
EDP (a)
Amplitude (b)
Pressure [mmHg]
Sample
C
P_{ISOMax}
Ees
Ea
Pressure [mmHg]
Volume [mL]

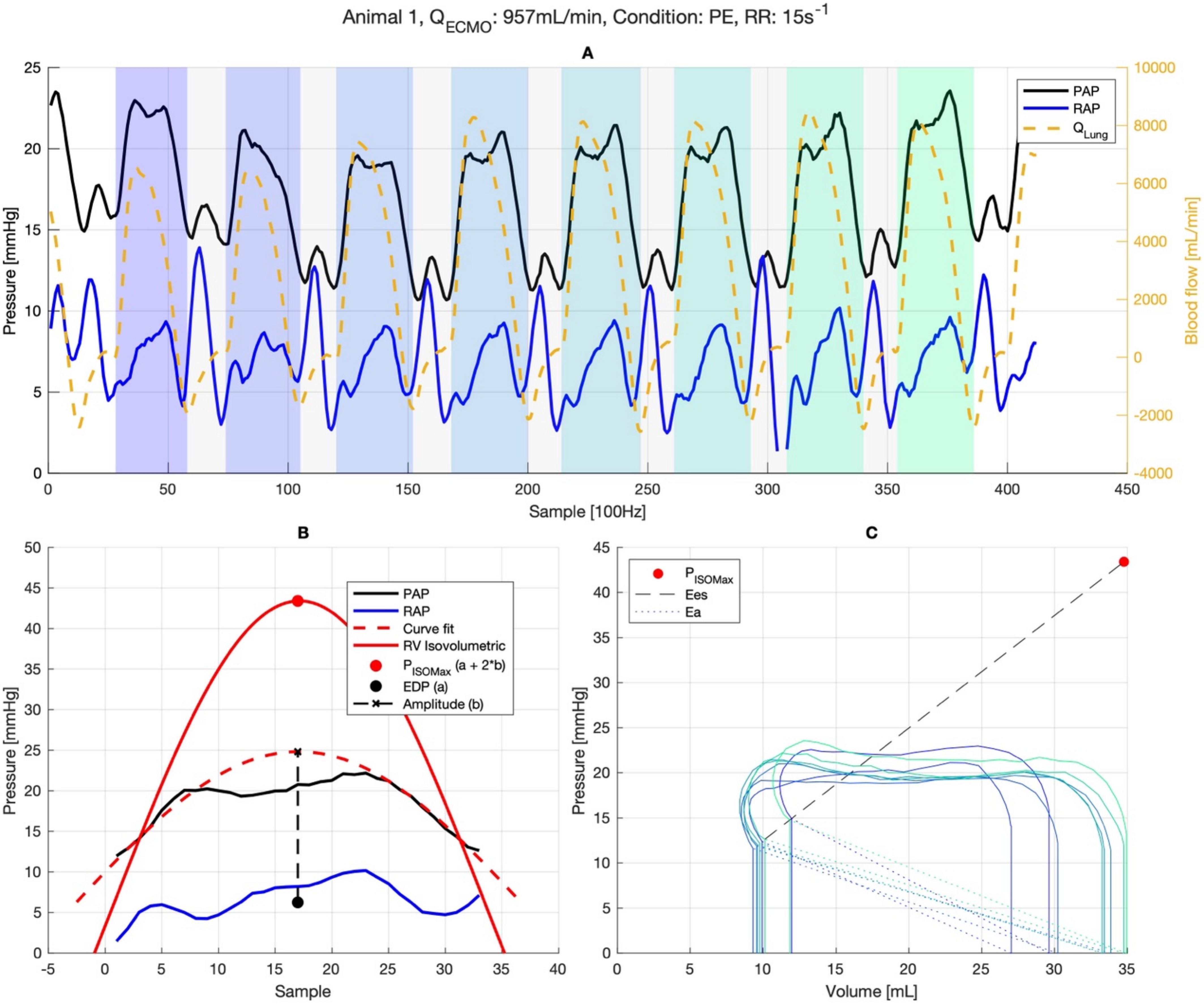

Animal 1, Q_{ECMO}: 957mL/min, Condition: PE, RR: $15s^{-1}$
A
PAP
RAP
Q_{Lung}
Pressure [mmHg]
Blood flow [mL/min]
Sample [100Hz]
B
PAP
RAP
Curve fit
RV Isovolumetric
P_{ISOMax} (a + 2*b)
EDP (a)
Amplitude (b)
Pressure [mmHg]
Sample
C
P_{ISOMax}
Ees
Ea
Pressure [mmHg]
Volume [mL]

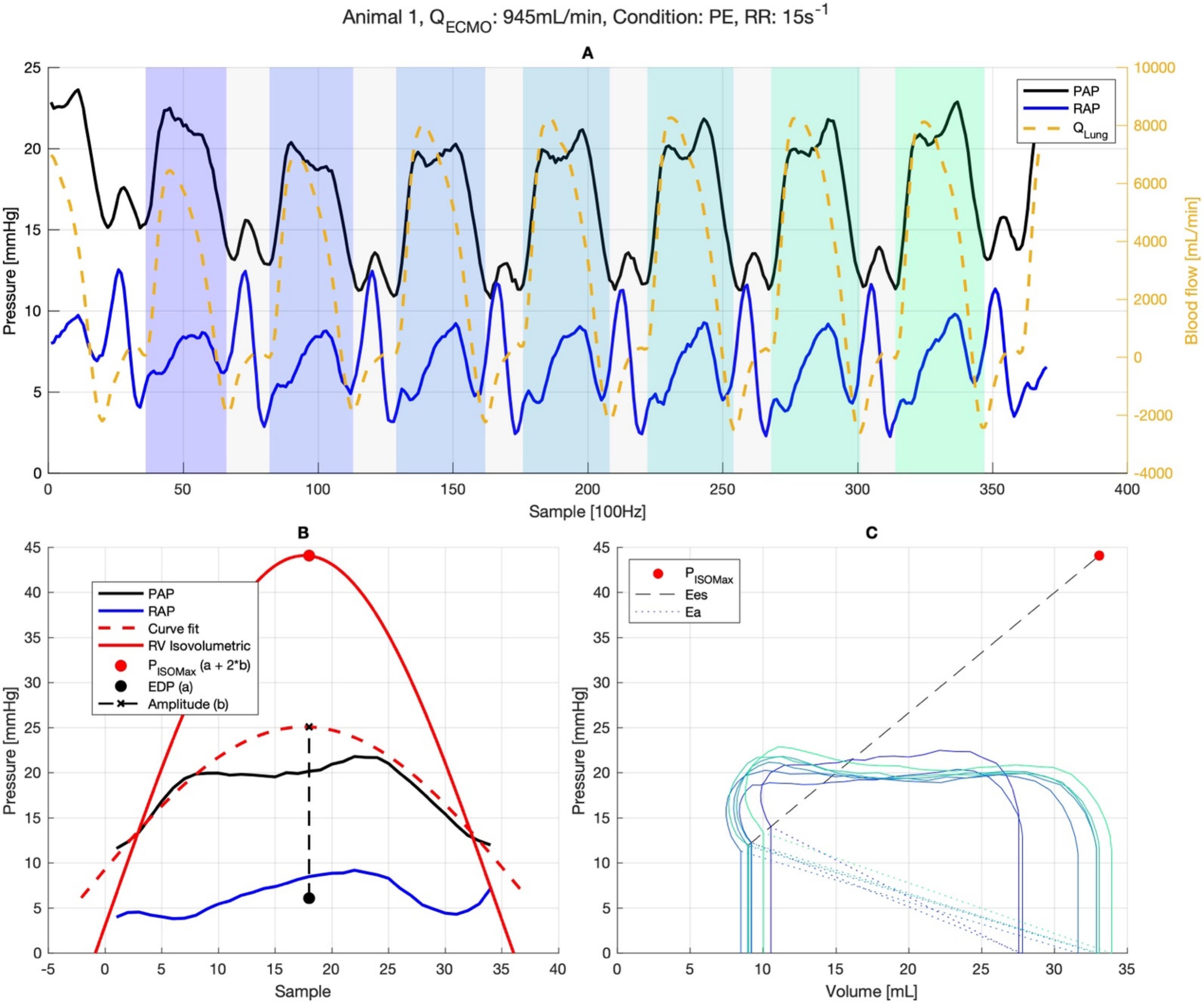
Animal 1, Q_ECMO: 945mL/min, Condition: PE, RR: 15s^-1
A
PAP
RAP
Q_Lung
Pressure [mmHg]
Blood flow [mL/min]
Sample [100Hz]
B
PAP
RAP
Curve fit
RV Isovolumetric
P_ISOMax (a + 2*b)
EDP (a)
Amplitude (b)
Pressure [mmHg]
Sample
C
P_ISOMax
Ees
Ea
Pressure [mmHg]
Volume [mL]

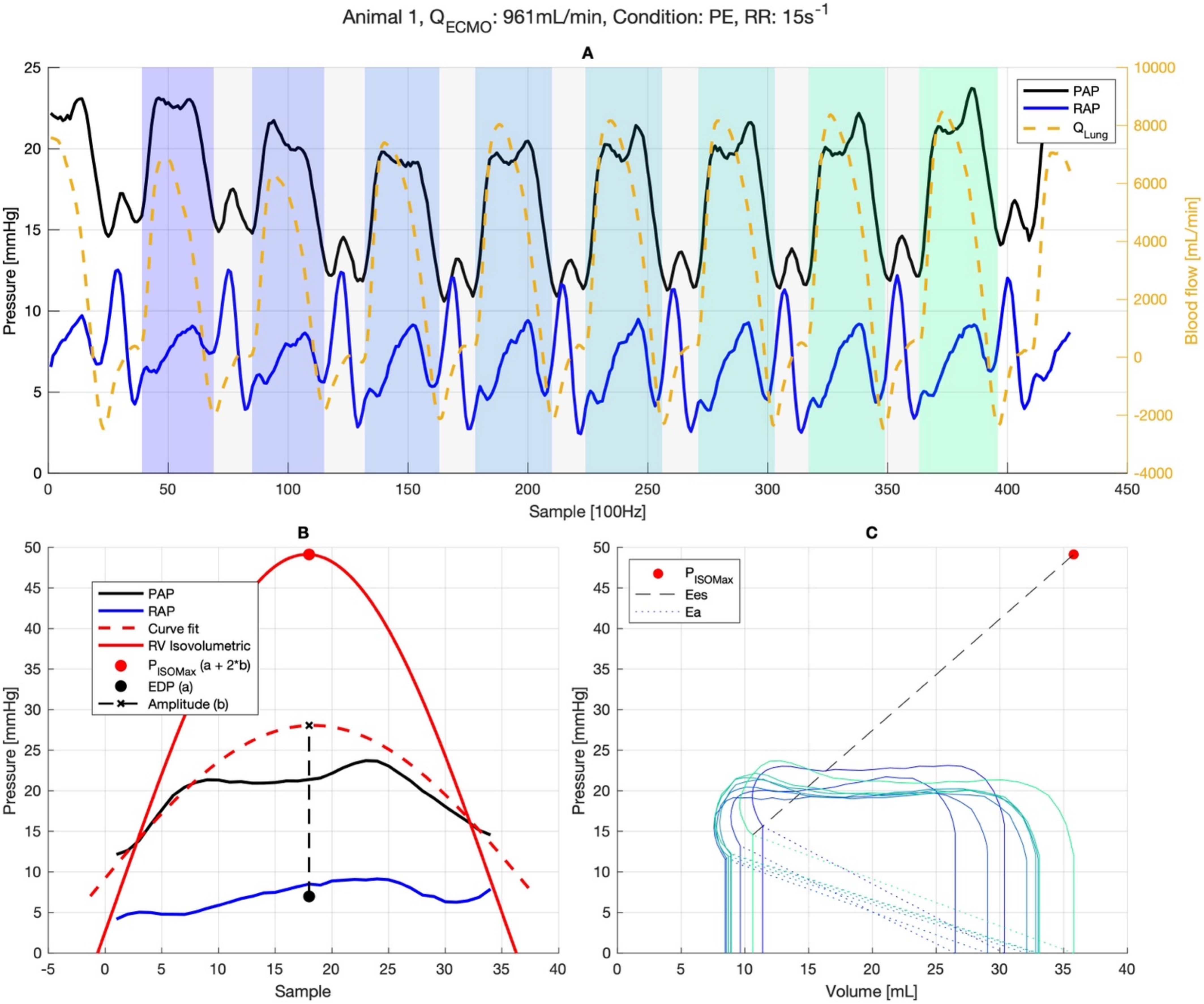

Animal 1, QECMO: 961mL/min, Condition: PE, RR: 15s-1
A
PAP
RAP
QLung
Pressure [mmHg]
Blood flow [mL/min]
Sample [100Hz]
B
PAP
RAP
Curve fit
RV Isovolumetric
PISOMax (a + 2*b)
EDP (a)
Amplitude (b)
Pressure [mmHg]
Sample
C
PISOMax
Ees
Ea
Pressure [mmHg]
Volume [mL]

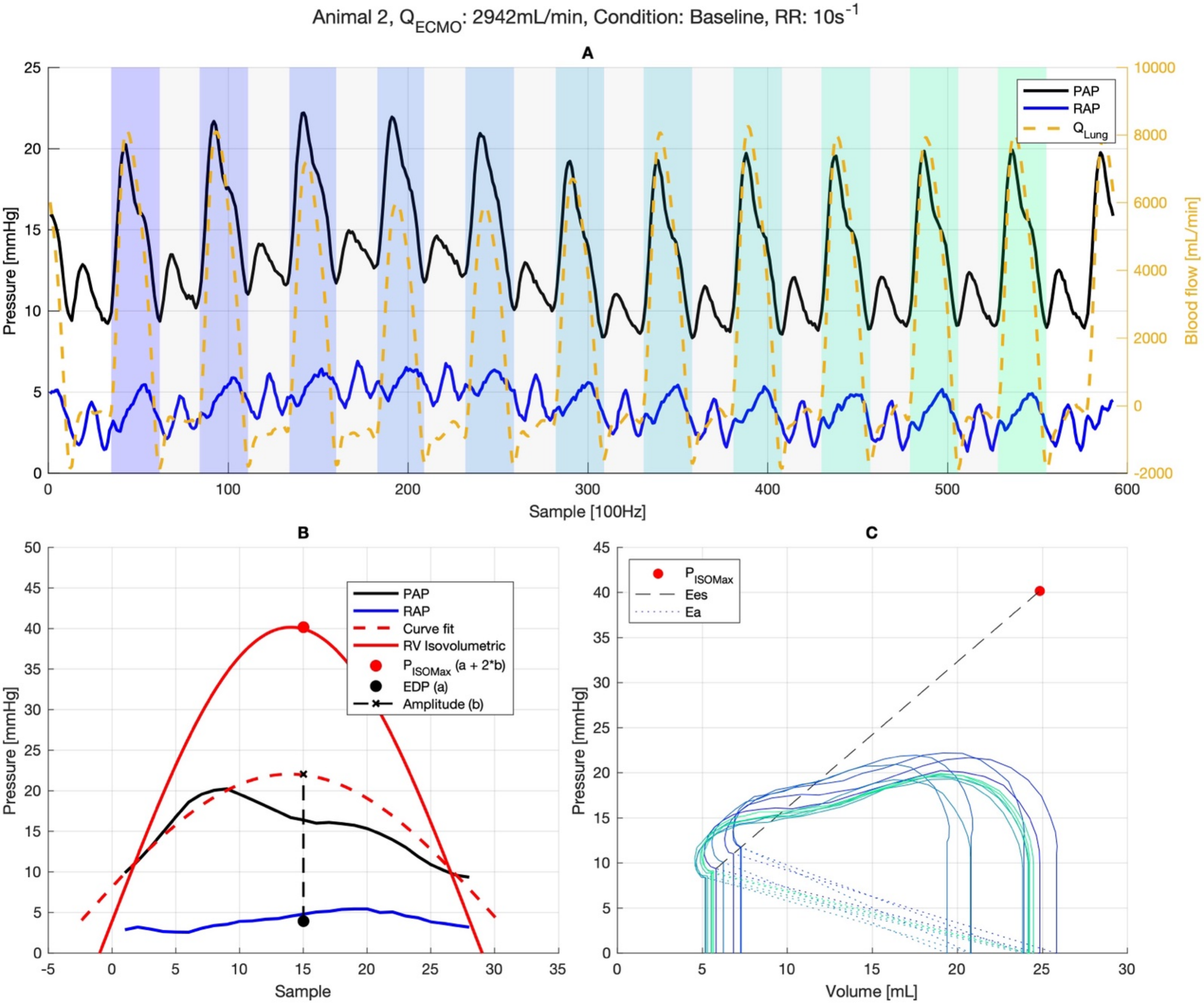

Animal 2, Q_{ECMO}: 2942mL/min, Condition: Baseline, RR: $10s^{-1}$
A
Pressure [mmHg]
Blood flow [mL/min]
Sample [100Hz]
PAP
RAP
Q_{Lung}
B
PAP
RAP
Curve fit
RV Isovolumetric
P_{ISOMax} (a + 2*b)
EDP (a)
Amplitude (b)
Pressure [mmHg]
Sample
C
P_{ISOMax}
Ees
Ea
Pressure [mmHg]
Volume [mL]

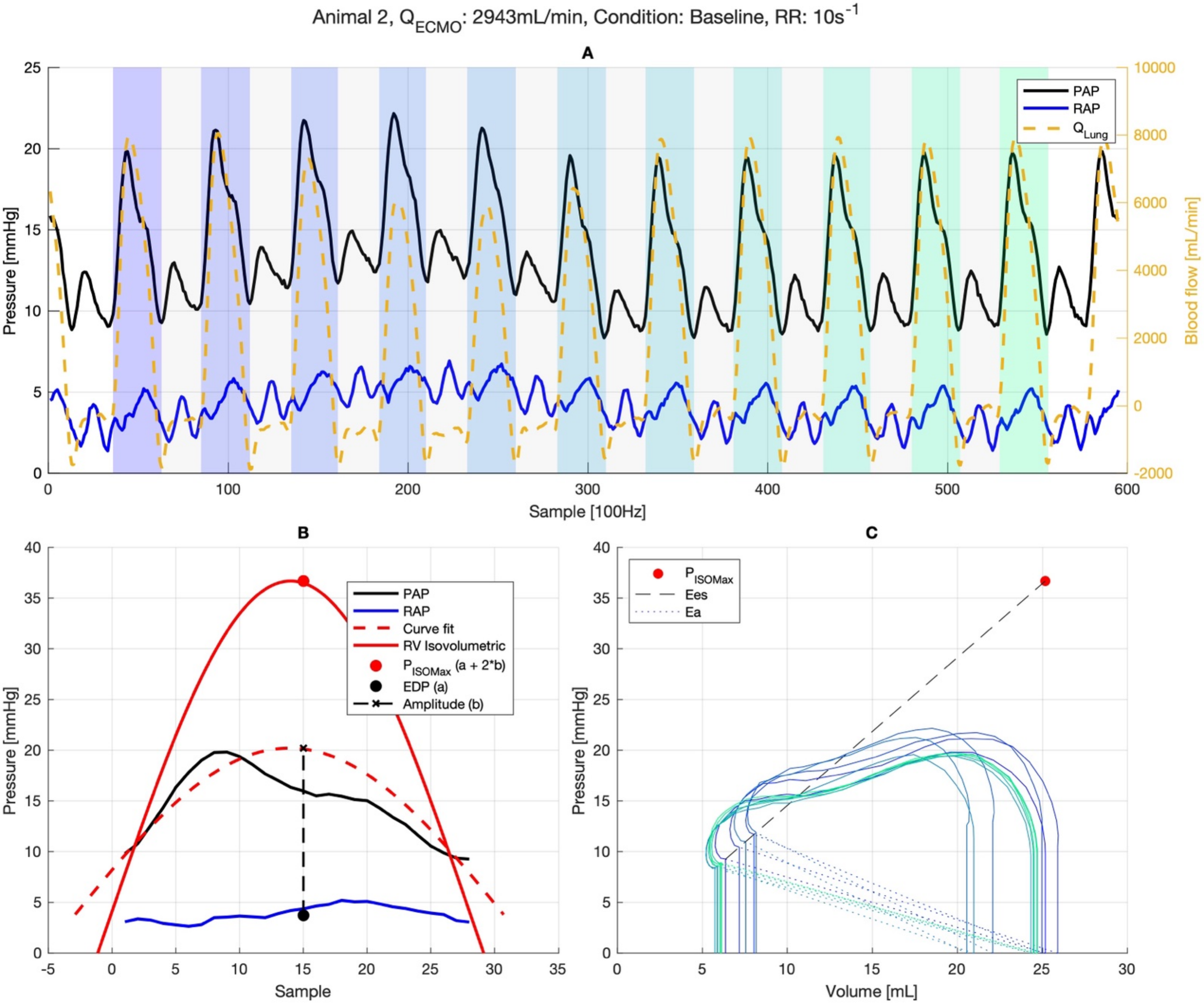

Animal 2, Q_ECMO: 2943mL/min, Condition: Baseline, RR: 10s^-1
A
PAP
RAP
Q_Lung
Pressure [mmHg]
Blood flow [mL/min]
Sample [100Hz]
B
PAP
RAP
Curve fit
RV Isovolumetric
P_ISOMax (a + 2*b)
EDP (a)
Amplitude (b)
Pressure [mmHg]
Sample
C
P_ISOMax
Ees
Ea
Pressure [mmHg]
Volume [mL]

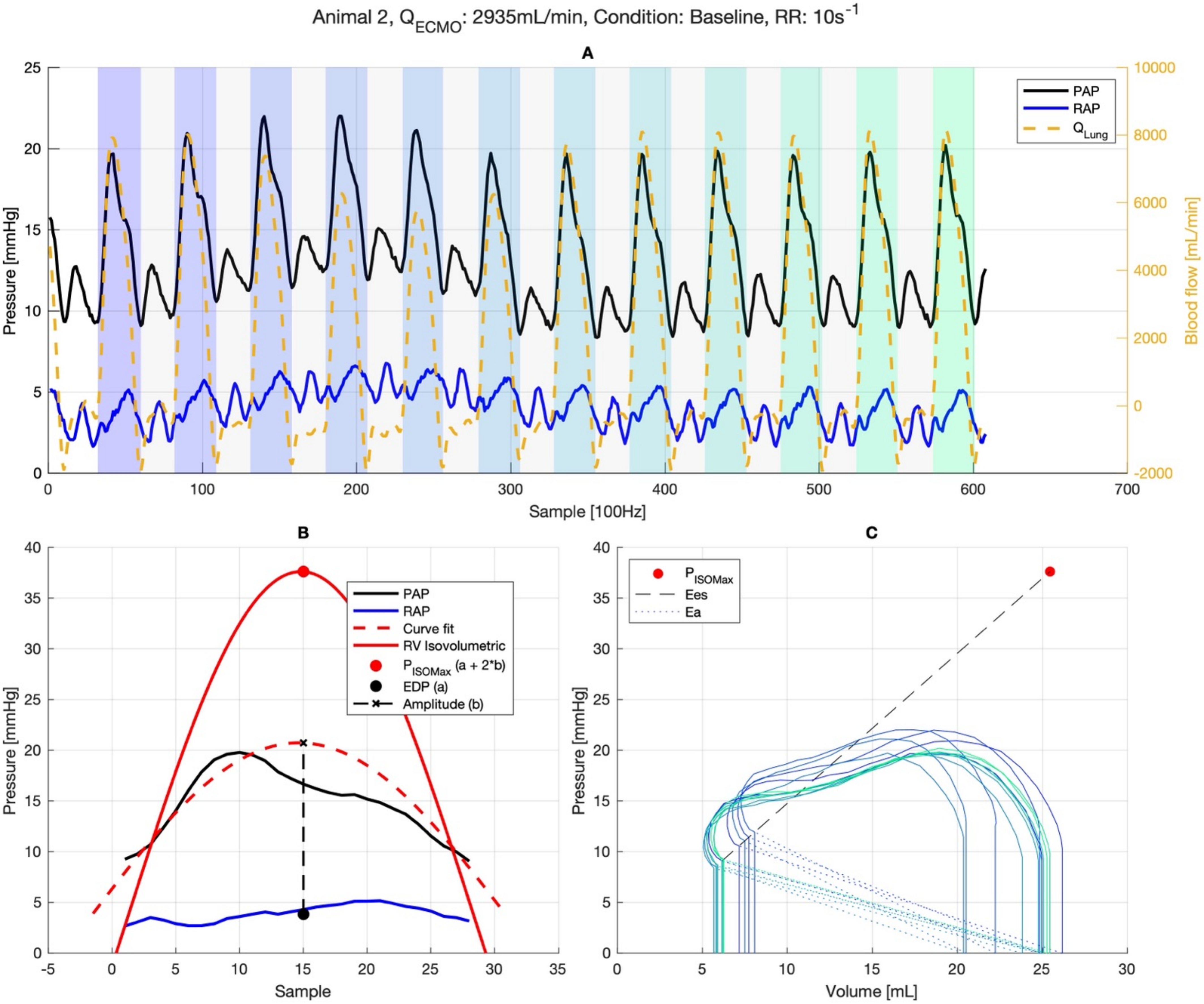

Animal 2, Q_ECMO: 2935mL/min, Condition: Baseline, RR: 10s^-1
A
Pressure [mmHg]
Blood flow [mL/min]
Sample [100Hz]
PAP
RAP
Q_Lung
B
Pressure [mmHg]
Sample
PAP
RAP
Curve fit
RV Isovolumetric
P_ISOMax (a + 2*b)
EDP (a)
Amplitude (b)
C
Pressure [mmHg]
Volume [mL]
P_ISOMax
Ees
Ea

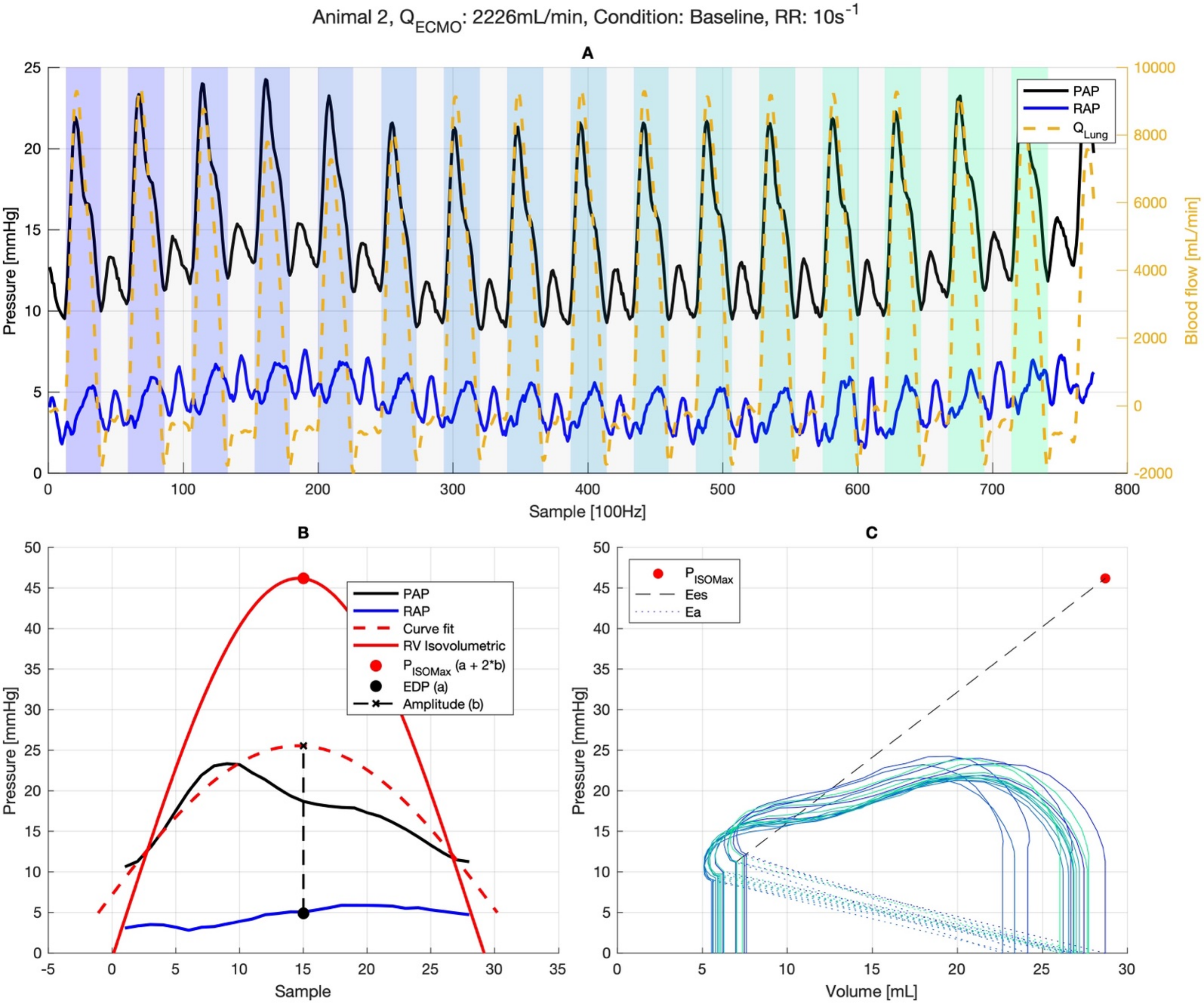

Animal 2, Q_ECMO: 2226mL/min, Condition: Baseline, RR: 10s^-1
A
Pressure [mmHg]
Blood flow [mL/min]
Sample [100Hz]
PAP
RAP
Q_Lung
B
Pressure [mmHg]
Sample
PAP
RAP
Curve fit
RV Isovolumetric
P_ISOMax (a + 2*b)
EDP (a)
Amplitude (b)
C
Pressure [mmHg]
Volume [mL]
P_ISOMax
Ees
Ea

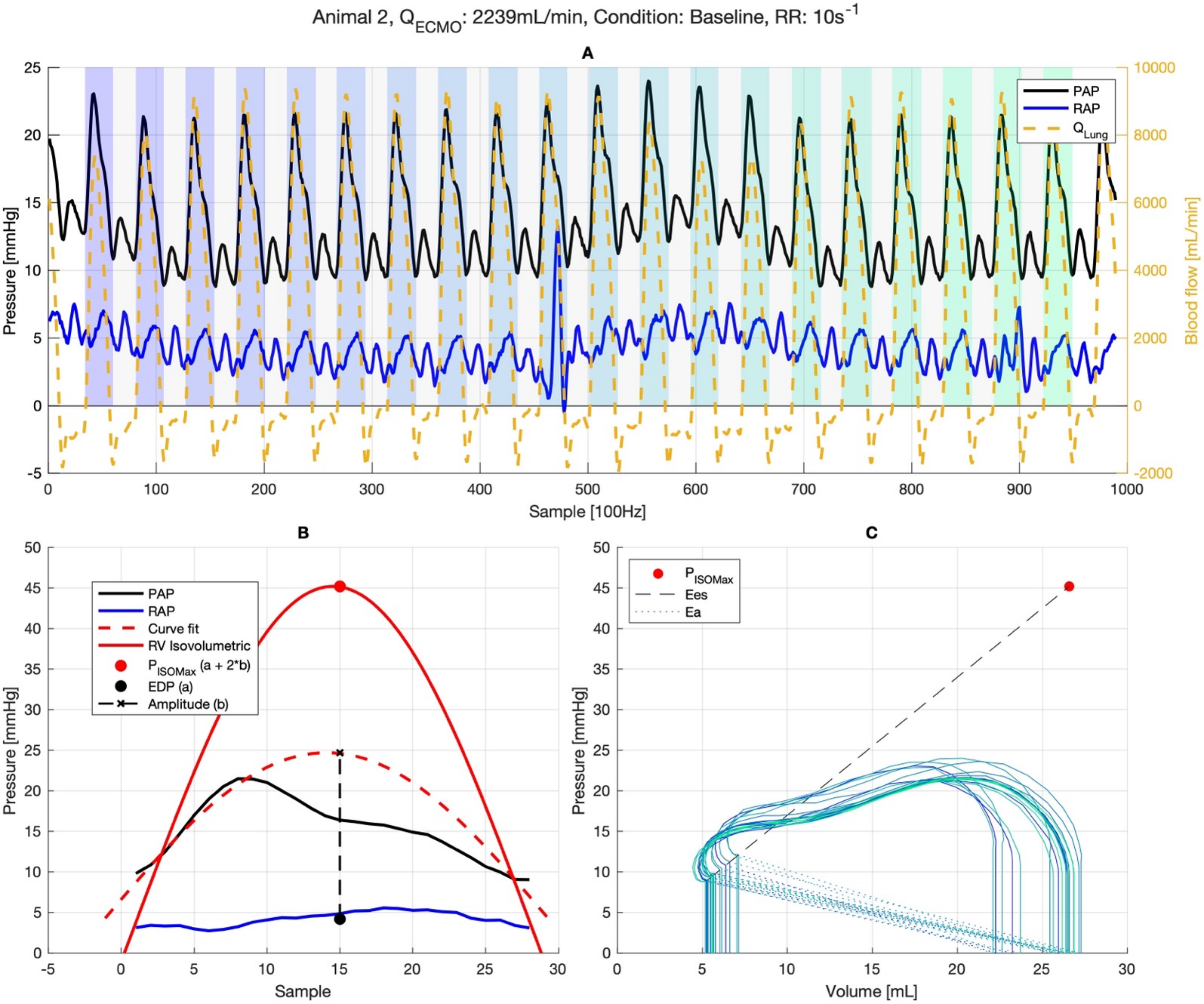

Animal 2, Q_{ECMO}: 2239mL/min, Condition: Baseline, RR: $10s^{-1}$
A
PAP
RAP
Q_{Lung}
Pressure [mmHg]
Blood flow [mL/min]
Sample [100Hz]
B
PAP
RAP
Curve fit
RV Isovolumetric
P_{ISOMax} (a + 2*b)
EDP (a)
Amplitude (b)
Pressure [mmHg]
Sample
C
P_{ISOMax}
Ees
Ea
Pressure [mmHg]
Volume [mL]

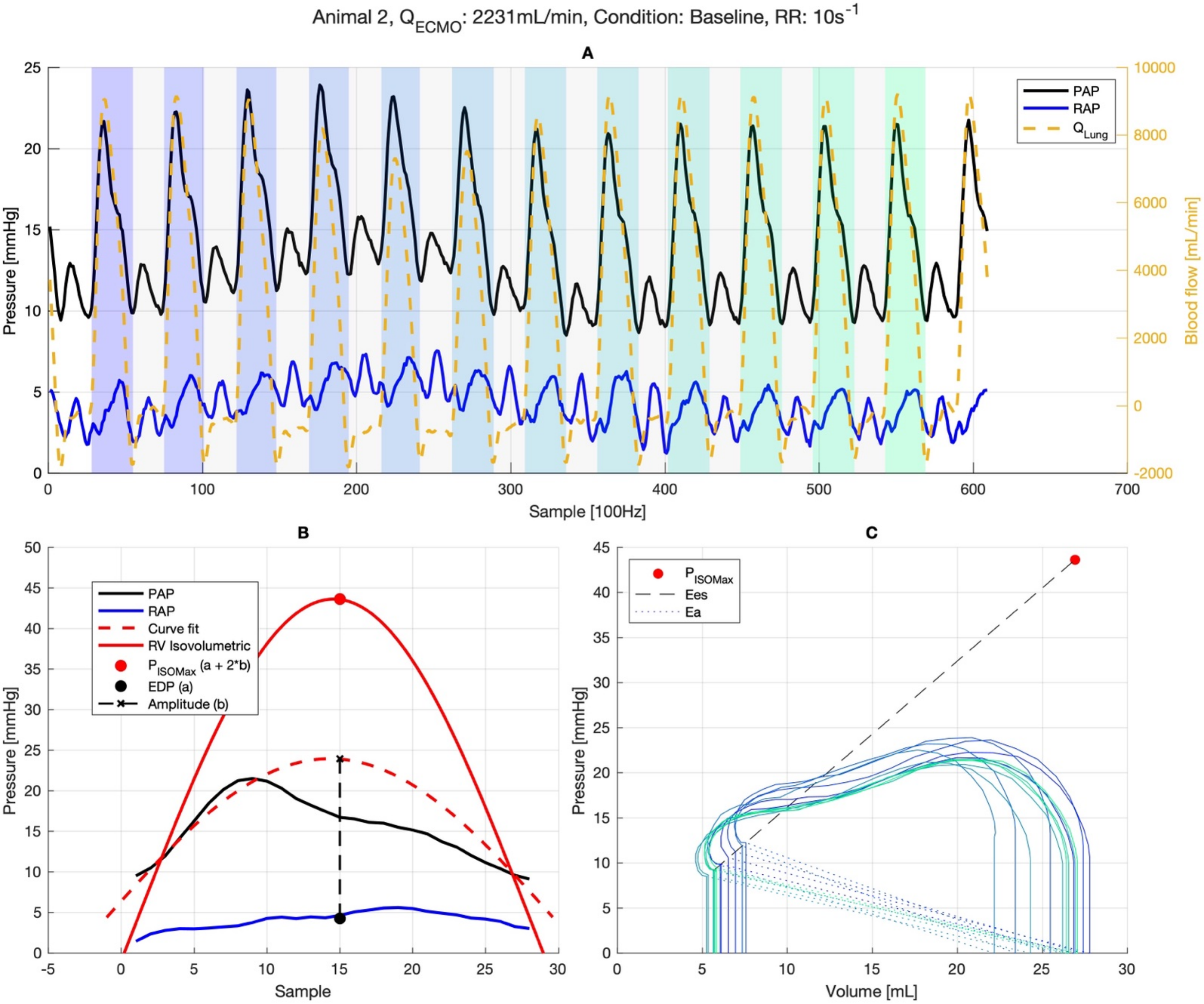

Animal 2, Q_{ECMO}: 2231mL/min, Condition: Baseline, RR: 10s^{-1}
A
PAP
RAP
Q_{Lung}
Pressure [mmHg]
Blood flow [mL/min]
Sample [100Hz]
B
PAP
RAP
Curve fit
RV Isovolumetric
P_{ISOMax} (a + 2*b)
EDP (a)
Amplitude (b)
Pressure [mmHg]
Sample
C
P_{ISOMax}
Ees
Ea
Pressure [mmHg]
Volume [mL]

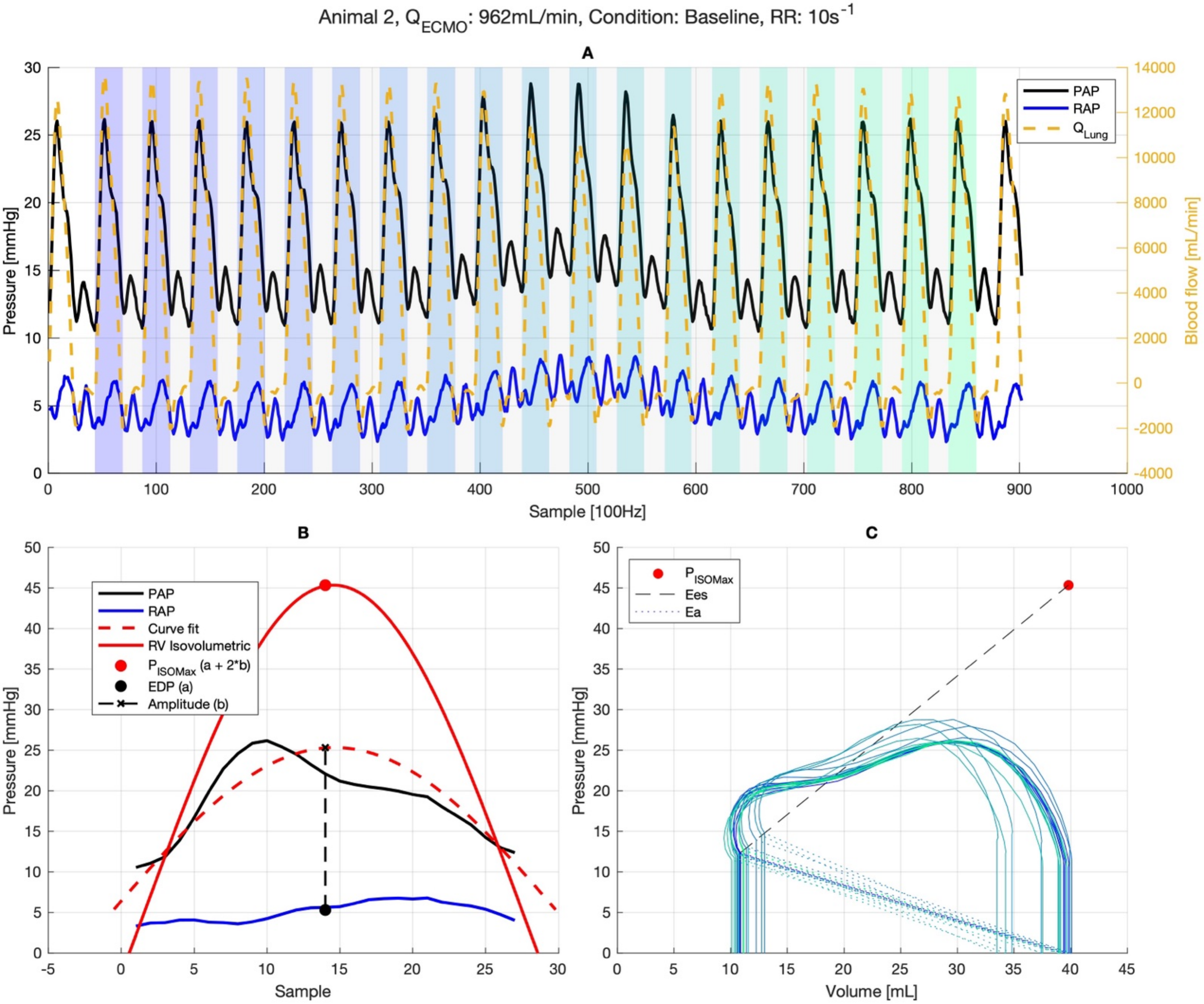
Animal 2, QECMO: 962mL/min, Condition: Baseline, RR: 10s-1
A
PAP
RAP
QLung
Pressure [mmHg]
Blood flow [mL/min]
Sample [100Hz]
B
PAP
RAP
Curve fit
RV Isovolumetric
PISOMax (a + 2*b)
EDP (a)
Amplitude (b)
Pressure [mmHg]
Sample
C
PISOMax
Ees
Ea
Pressure [mmHg]
Volume [mL]

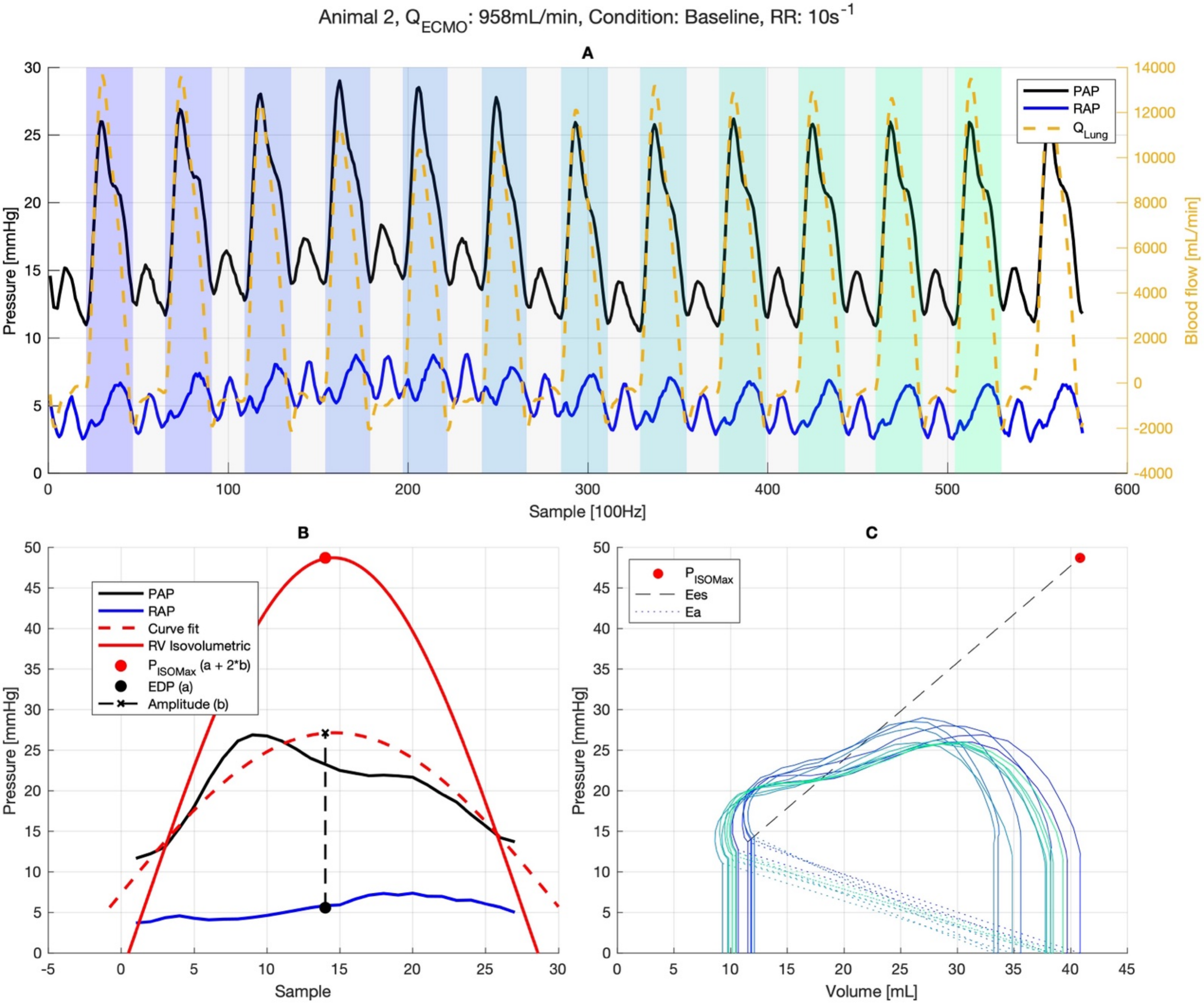

Animal 2, Q_ECMO: 958mL/min, Condition: Baseline, RR: 10s^-1
A
PAP
RAP
Q_Lung
Pressure [mmHg]
Blood flow [mL/min]
Sample [100Hz]
B
PAP
RAP
Curve fit
RV Isovolumetric
P_ISOMax (a + 2*b)
EDP (a)
Amplitude (b)
Pressure [mmHg]
Sample
C
P_ISOMax
Ees
Ea
Pressure [mmHg]
Volume [mL]

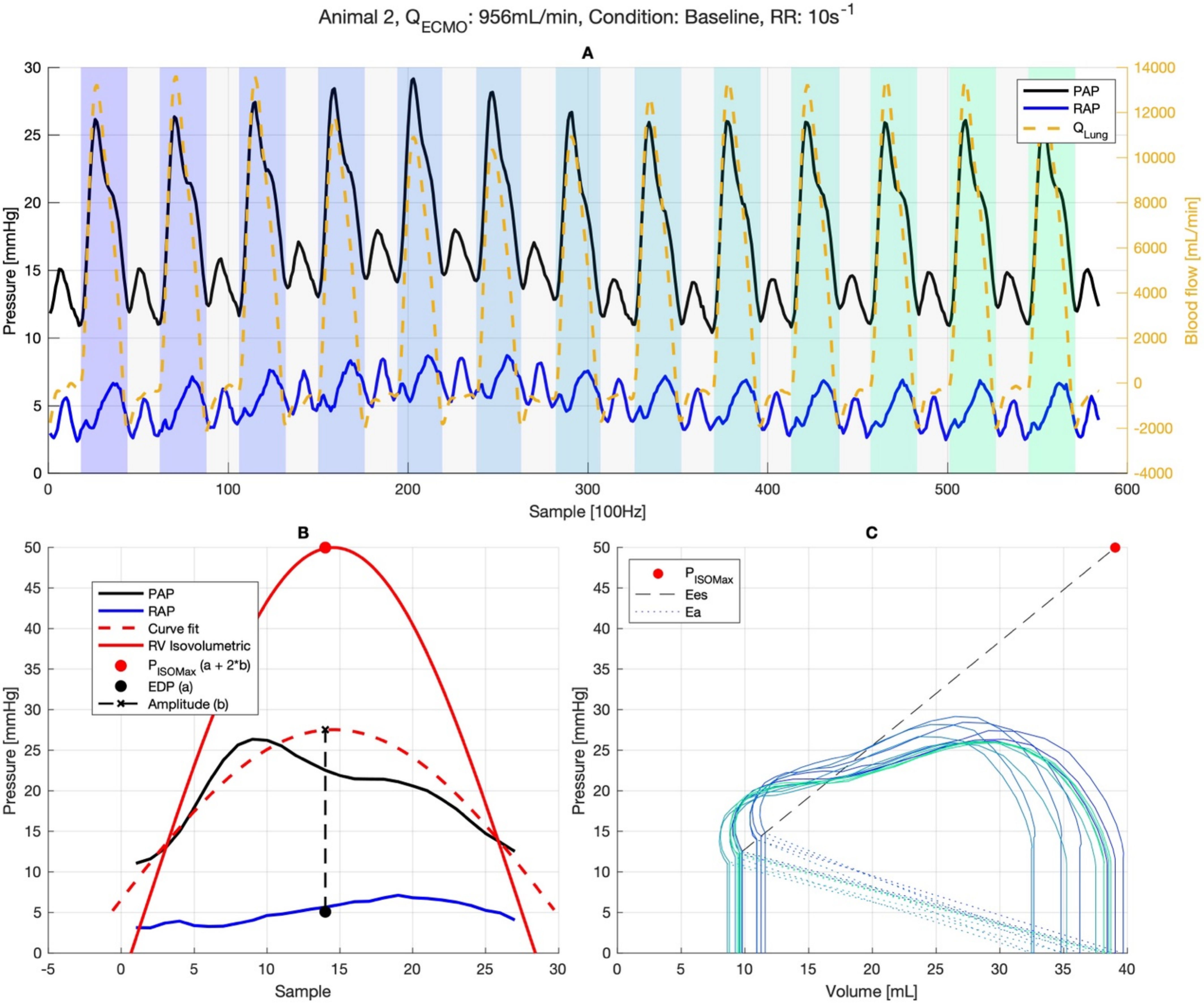
Animal 2, Q_{ECMO}: 956mL/min, Condition: Baseline, RR: $10s^{-1}$
A
PAP
RAP
Q_{Lung}
Pressure [mmHg]
Blood flow [mL/min]
Sample [100Hz]
B
PAP
RAP
Curve fit
RV Isovolumetric
P_{ISOMax} (a + 2*b)
EDP (a)
Amplitude (b)
Pressure [mmHg]
Sample
C
P_{ISOMax}
Ees
Ea
Pressure [mmHg]
Volume [mL]

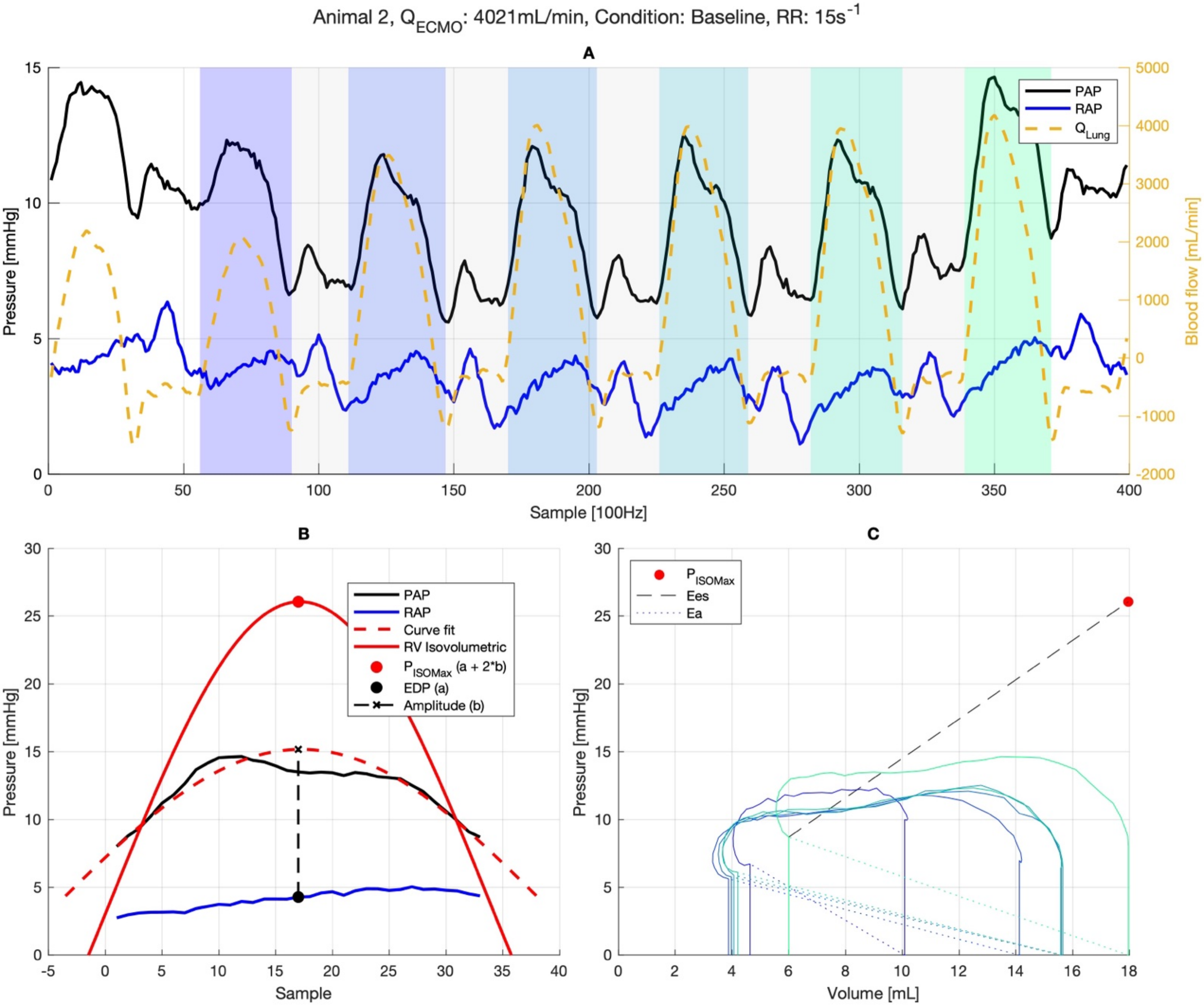

Animal 2, Q_{ECMO}: 4021mL/min, Condition: Baseline, RR: 15s^{-1}
A
PAP
RAP
Q_{Lung}
Pressure [mmHg]
Blood flow [mL/min]
Sample [100Hz]
B
PAP
RAP
Curve fit
RV Isovolumetric
P_{ISOMax} (a + 2*b)
EDP (a)
Amplitude (b)
Pressure [mmHg]
Sample
C
P_{ISOMax}
Ees
Ea
Pressure [mmHg]
Volume [mL]

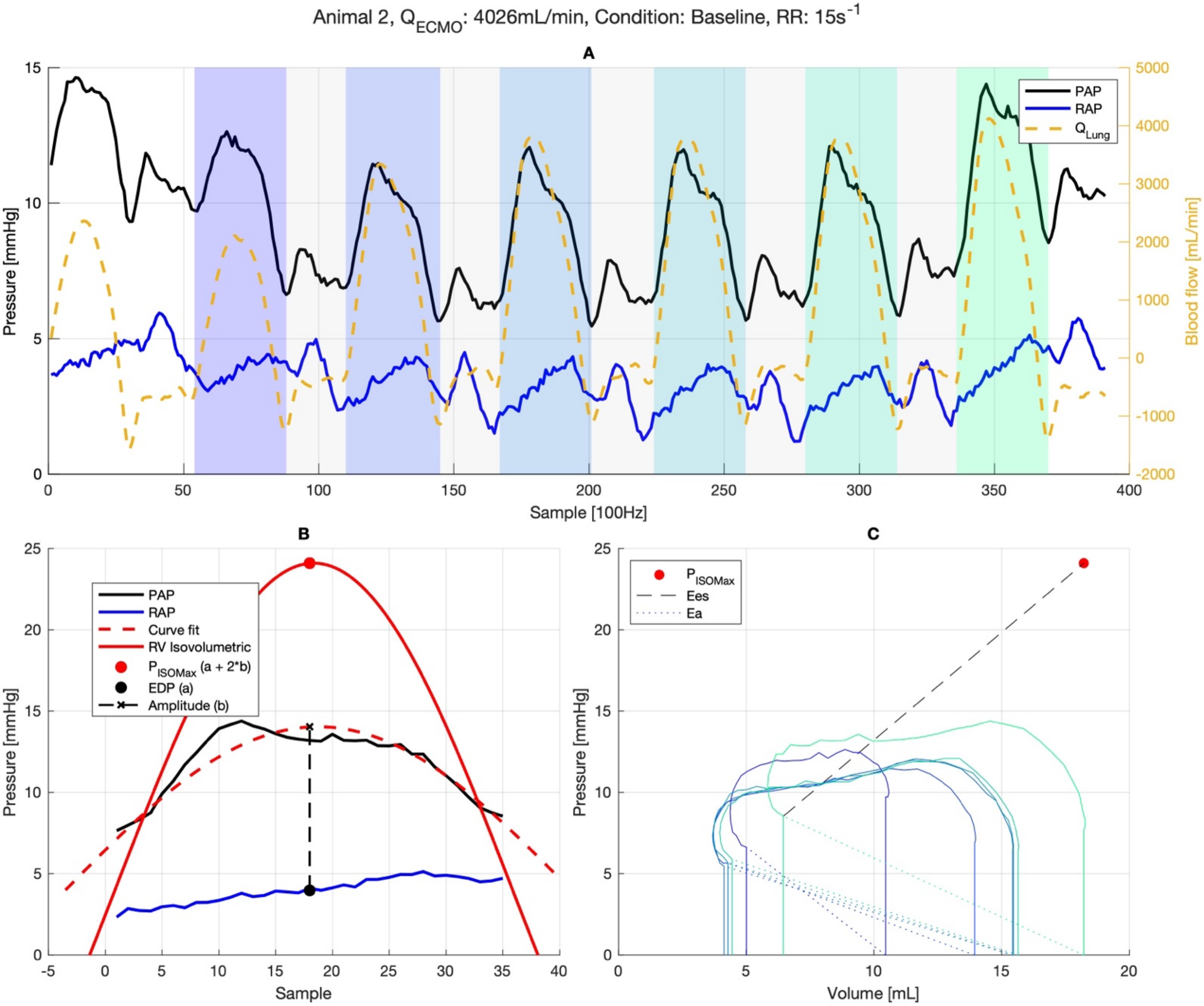

Animal 2, Q_{ECMO}: 4026mL/min, Condition: Baseline, RR: $15s^{-1}$
A
PAP
RAP
Q_{Lung}
Pressure [mmHg]
Blood flow [mL/min]
Sample [100Hz]
B
PAP
RAP
Curve fit
RV Isovolumetric
P_{ISOMax} (a + 2*b)
EDP (a)
Amplitude (b)
Pressure [mmHg]
Sample
C
P_{ISOMax}
Ees
Ea
Pressure [mmHg]
Volume [mL]

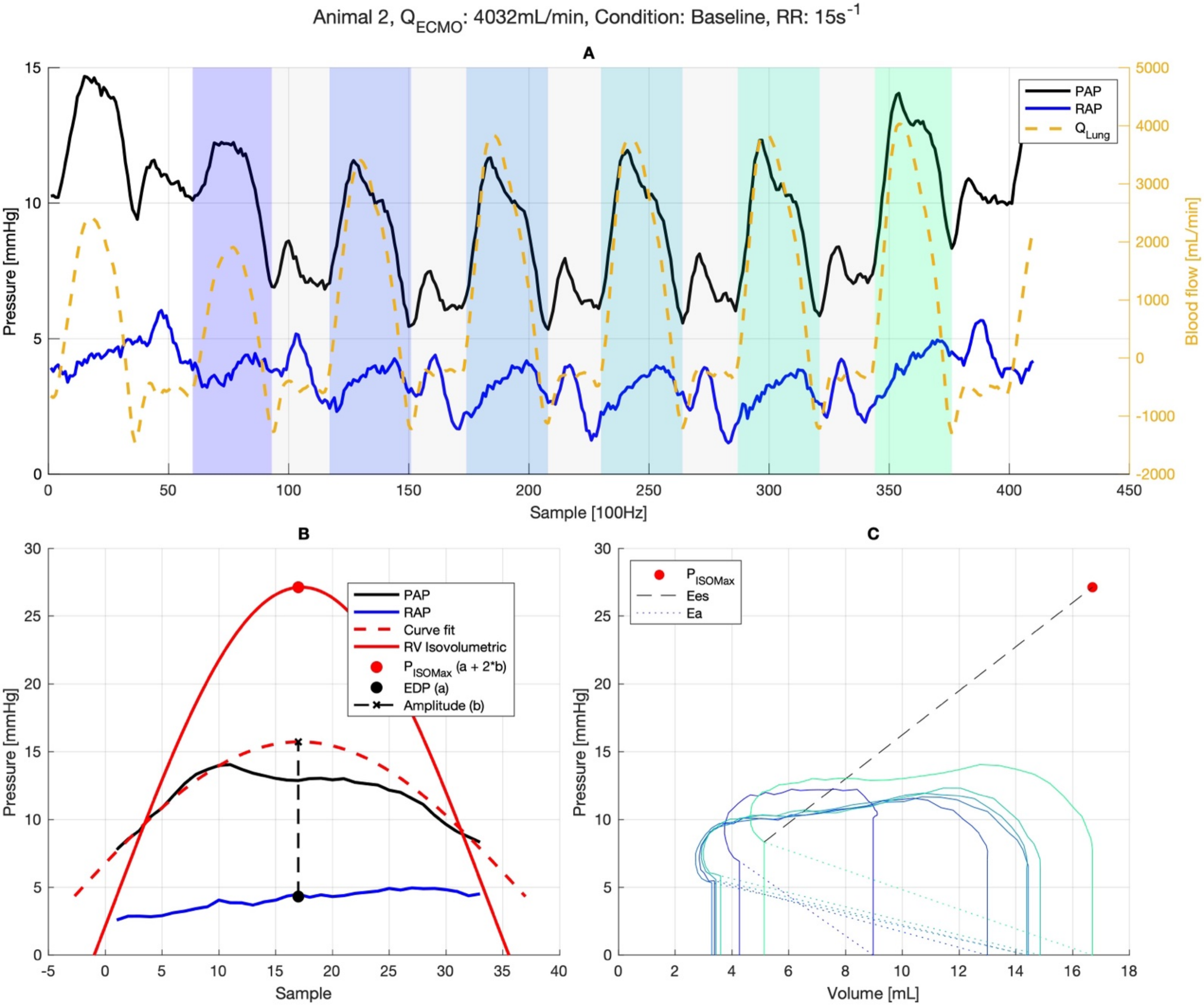

Animal 2, Q_{ECMO}: 4032mL/min, Condition: Baseline, RR: $15s^{-1}$
A
PAP
RAP
Q_{Lung}
Pressure [mmHg]
Blood flow [mL/min]
Sample [100Hz]
B
PAP
RAP
Curve fit
RV Isovolumetric
P_{ISOMax} (a + 2*b)
EDP (a)
Amplitude (b)
Pressure [mmHg]
Sample
C
P_{ISOMax}
Ees
Ea
Pressure [mmHg]
Volume [mL]

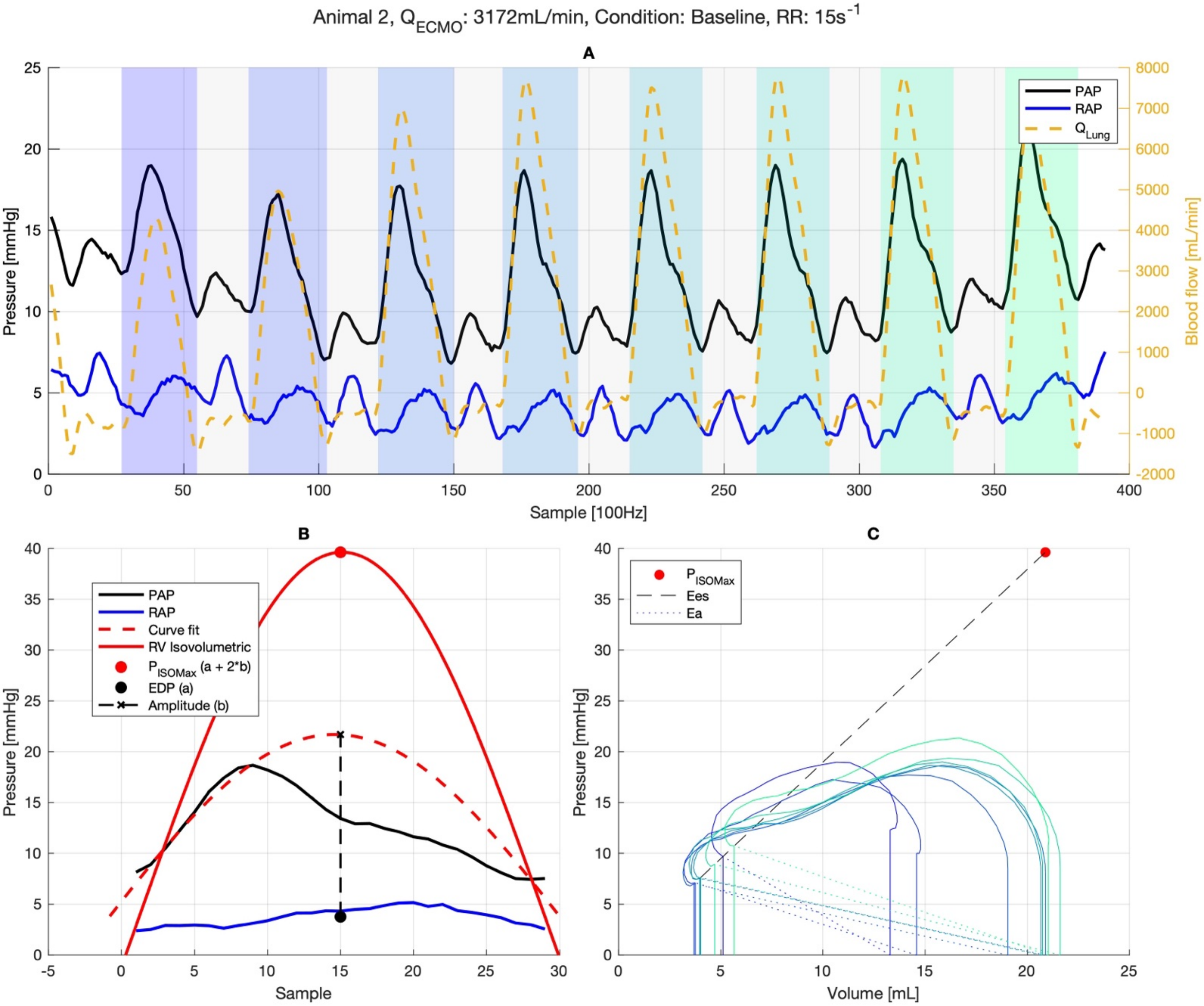
Animal 2, Q_{ECMO}: 3172mL/min, Condition: Baseline, RR: $15s^{-1}$
A
PAP
RAP
Q_{Lung}
Pressure [mmHg]
Blood flow [mL/min]
Sample [100Hz]
B
PAP
RAP
Curve fit
RV Isovolumetric
P_{ISOMax} (a + 2*b)
EDP (a)
Amplitude (b)
Pressure [mmHg]
Sample
C
P_{ISOMax}
Ees
Ea
Pressure [mmHg]
Volume [mL]

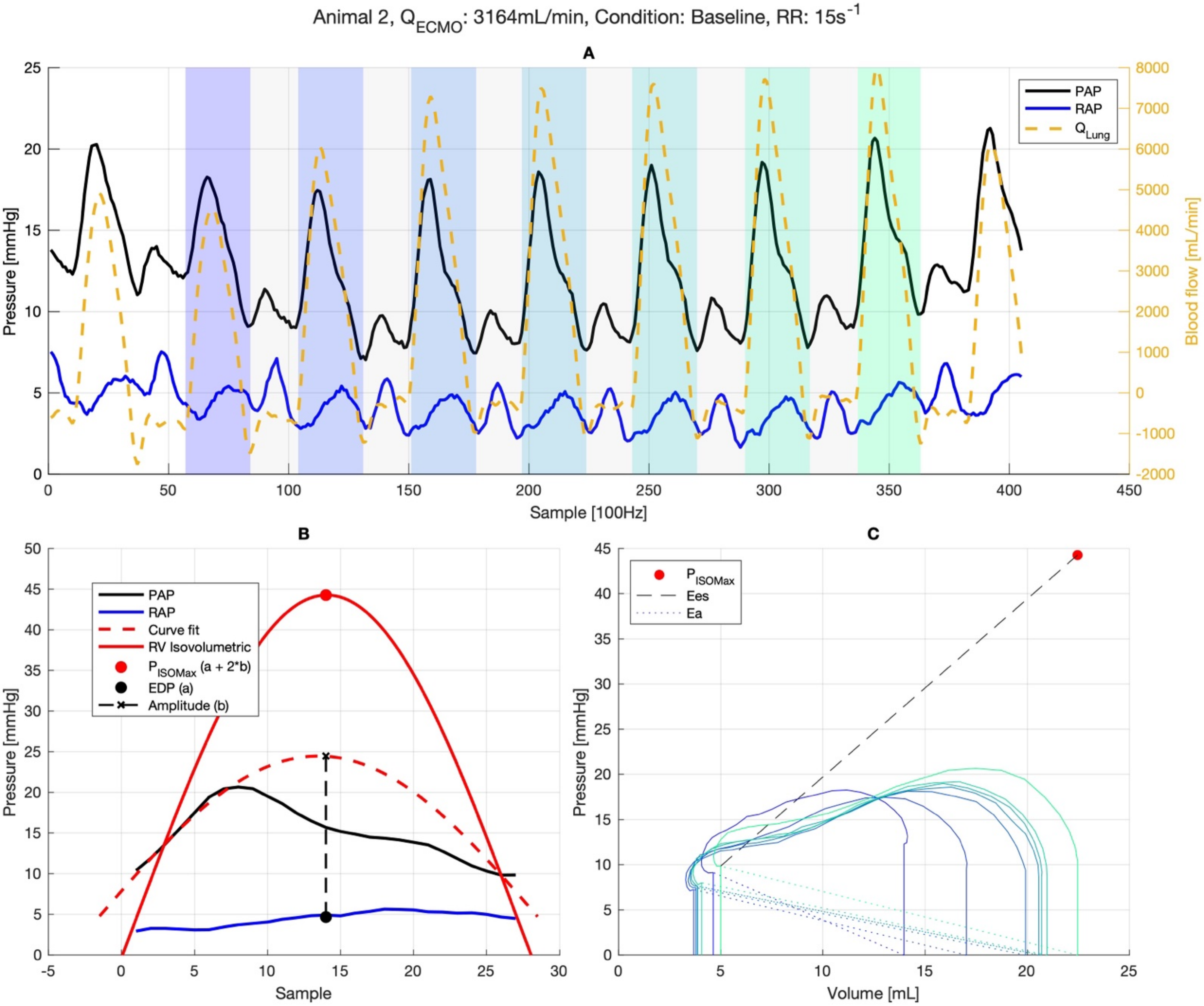

Animal 2, Q_{ECMO}: 3164mL/min, Condition: Baseline, RR: $15s^{-1}$
A
PAP
RAP
Q_{Lung}
Pressure [mmHg]
Blood flow [mL/min]
Sample [100Hz]
B
PAP
RAP
Curve fit
RV Isovolumetric
P_{ISOMax} (a + 2*b)
EDP (a)
Amplitude (b)
Pressure [mmHg]
Sample
C
P_{ISOMax}
Ees
Ea
Pressure [mmHg]
Volume [mL]

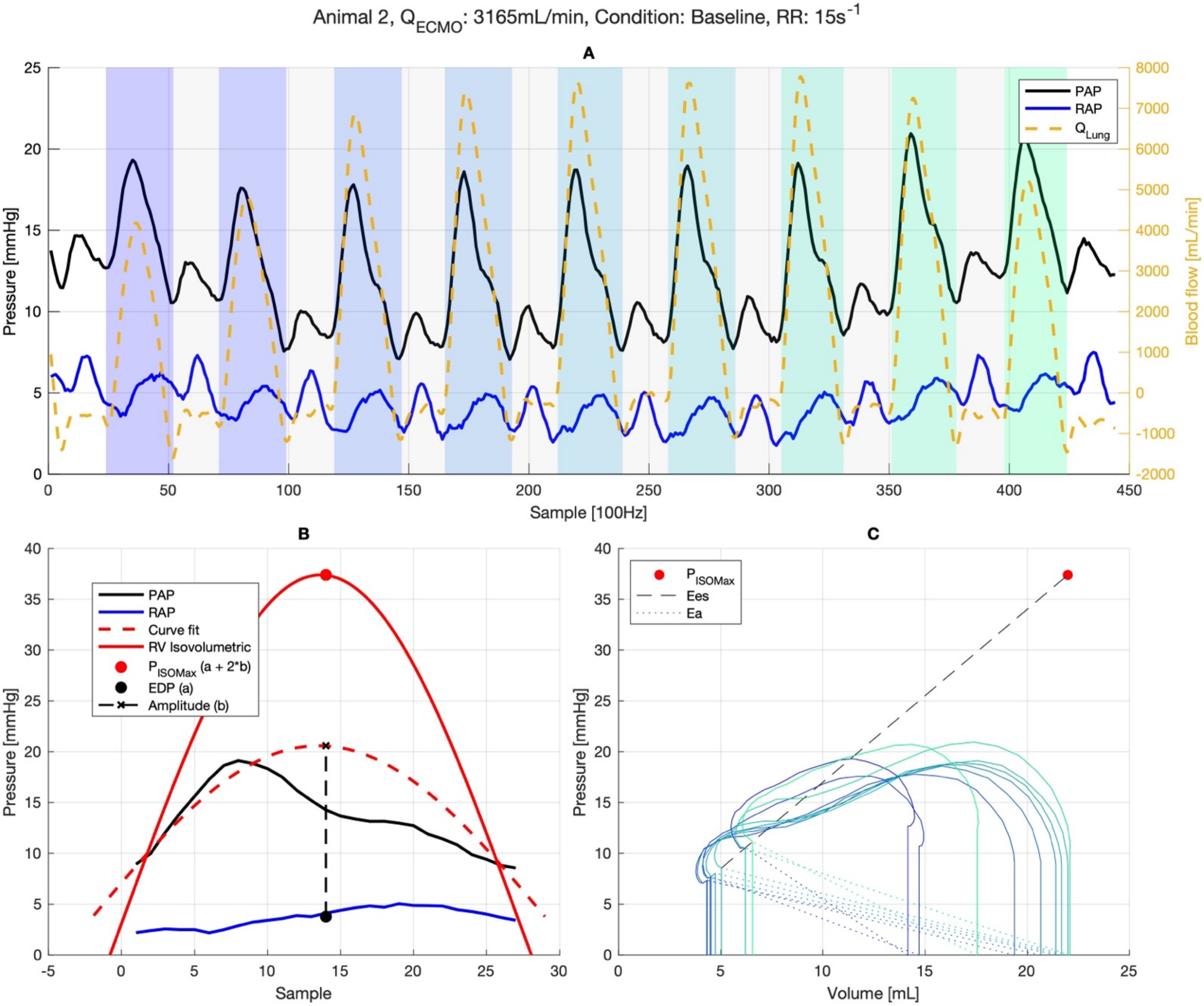

Animal 2, Q_{ECMO}: 3165mL/min, Condition: Baseline, RR: 15s^{-1}
A
PAP
RAP
Q_{Lung}
Pressure [mmHg]
Blood flow [mL/min]
Sample [100Hz]
B
PAP
RAP
Curve fit
RV Isovolumetric
P_{ISOMax} (a + 2*b)
EDP (a)
Amplitude (b)
Pressure [mmHg]
Sample
C
P_{ISOMax}
Ees
Ea
Pressure [mmHg]
Volume [mL]

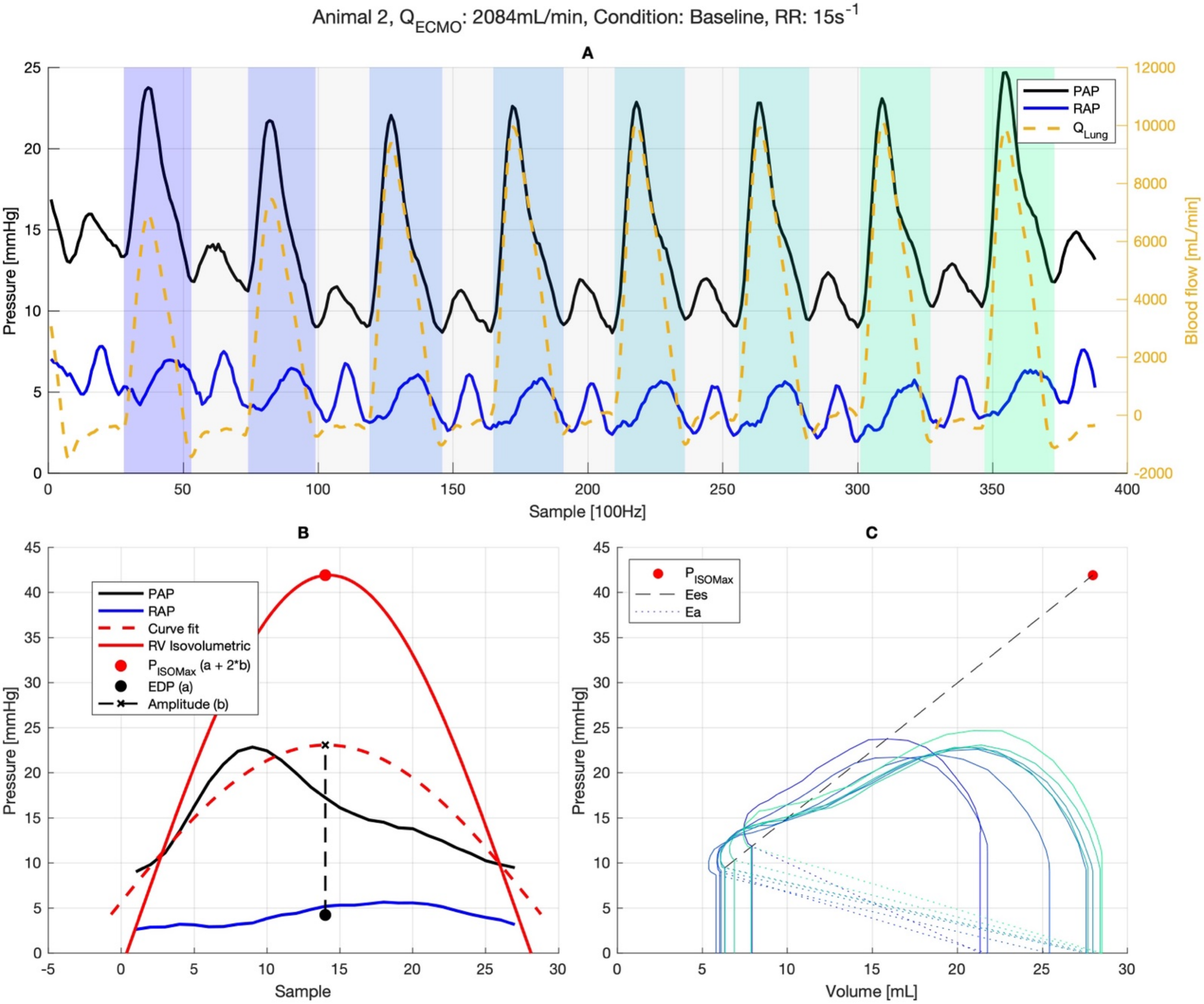

Animal 2, Q_{ECMO}: 2084mL/min, Condition: Baseline, RR: $15s^{-1}$
A
PAP
RAP
Q_{Lung}
Pressure [mmHg]
Blood flow [mL/min]
Sample [100Hz]
B
PAP
RAP
Curve fit
RV Isovolumetric
P_{ISOMax} (a + 2*b)
EDP (a)
Amplitude (b)
Pressure [mmHg]
Sample
C
P_{ISOMax}
Ees
Ea
Pressure [mmHg]
Volume [mL]

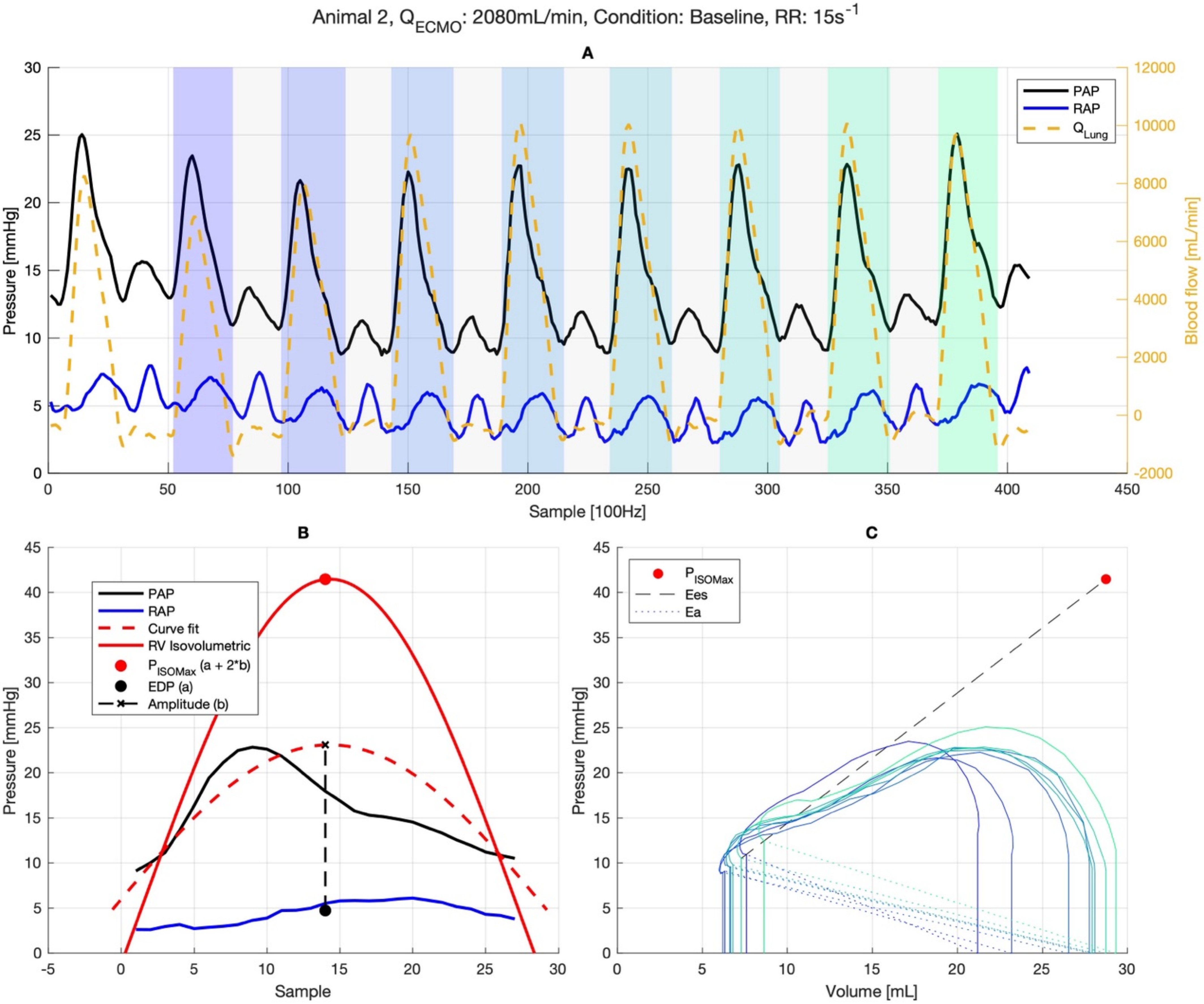

Animal 2, Q_{ECMO}: 2080mL/min, Condition: Baseline, RR: $15s^{-1}$
A
PAP
RAP
Q_{Lung}
Pressure [mmHg]
Blood flow [mL/min]
Sample [100Hz]
B
PAP
RAP
Curve fit
RV Isovolumetric
P_{ISOMax} (a + 2*b)
EDP (a)
Amplitude (b)
Pressure [mmHg]
Sample
C
P_{ISOMax}
Ees
Ea
Pressure [mmHg]
Volume [mL]

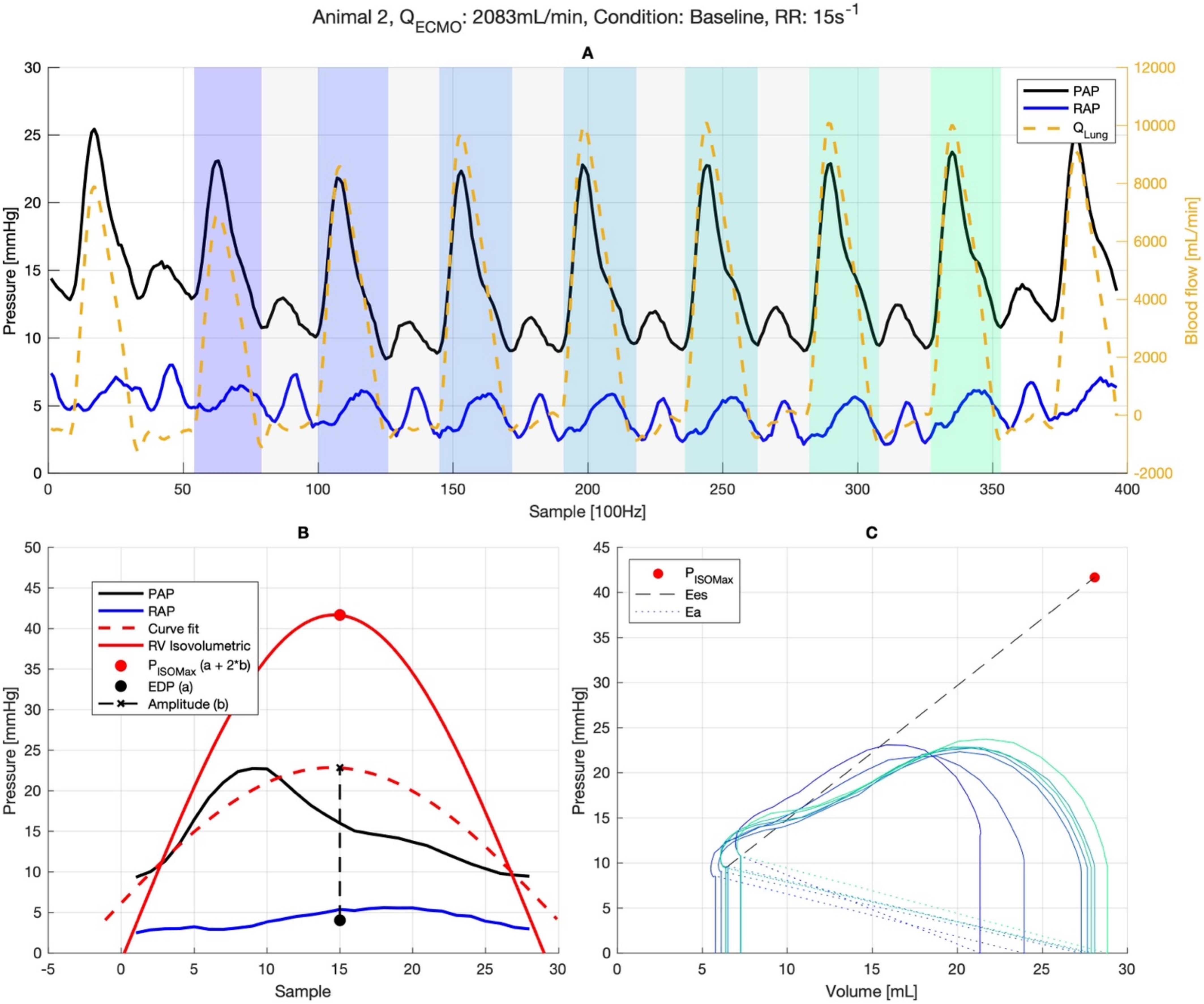

Animal 2, Q_ECMO: 2083mL/min, Condition: Baseline, RR: 15s^-1
A
PAP
RAP
Q_Lung
Pressure [mmHg]
Blood flow [mL/min]
Sample [100Hz]
B
PAP
RAP
Curve fit
RV Isovolumetric
P_ISOMax (a + 2*b)
EDP (a)
Amplitude (b)
Pressure [mmHg]
Sample
C
P_ISOMax
Ees
Ea
Pressure [mmHg]
Volume [mL]

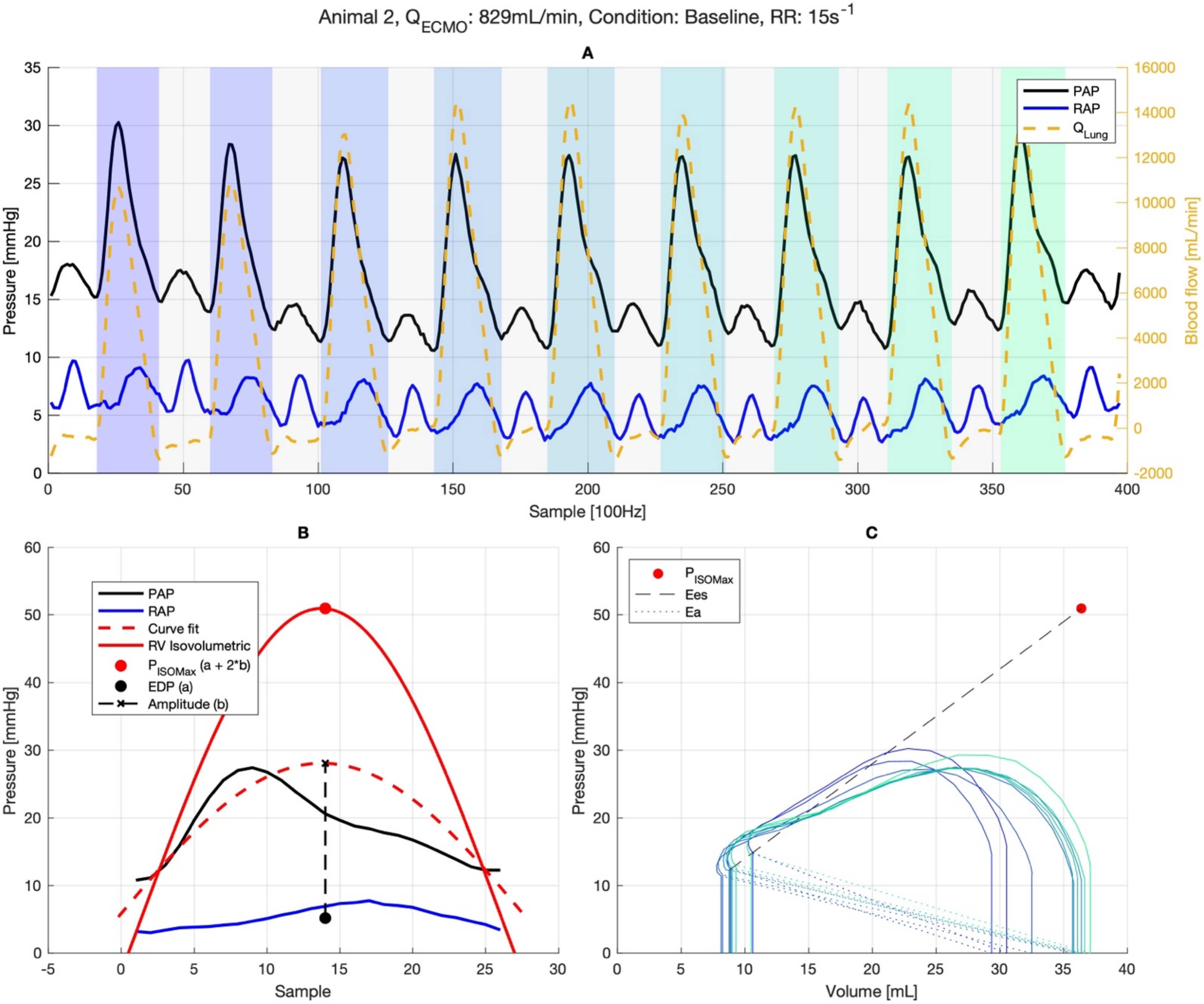
Animal 2, Q_{ECMO}: 829mL/min, Condition: Baseline, RR: $15s^{-1}$
A
Pressure [mmHg]
Blood flow [mL/min]
Sample [100Hz]
PAP
RAP
Q_{Lung}
B
Pressure [mmHg]
Sample
PAP
RAP
Curve fit
RV Isovolumetric
P_{ISOMax} (a + 2*b)
EDP (a)
Amplitude (b)
C
Pressure [mmHg]
Volume [mL]
P_{ISOMax}
Ees
Ea

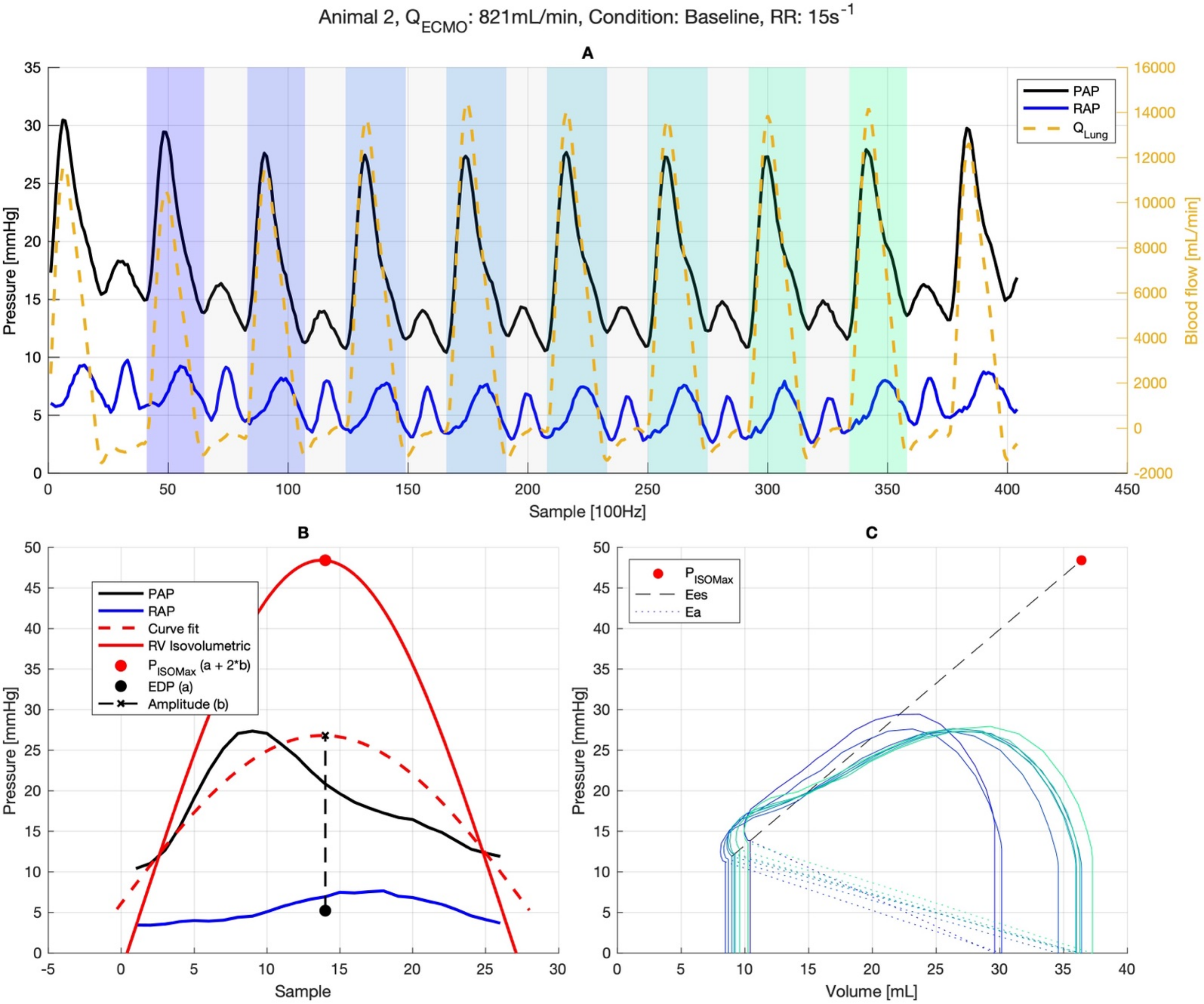

Animal 2, Q_{ECMO}: 821mL/min, Condition: Baseline, RR: $15s^{-1}$
A
Pressure [mmHg]
Blood flow [mL/min]
Sample [100Hz]
PAP
RAP
Q_{Lung}
B
Pressure [mmHg]
Sample
PAP
RAP
Curve fit
RV Isovolumetric
P_{ISOMax} (a + 2*b)
EDP (a)
Amplitude (b)
C
Pressure [mmHg]
Volume [mL]
P_{ISOMax}
Ees
Ea

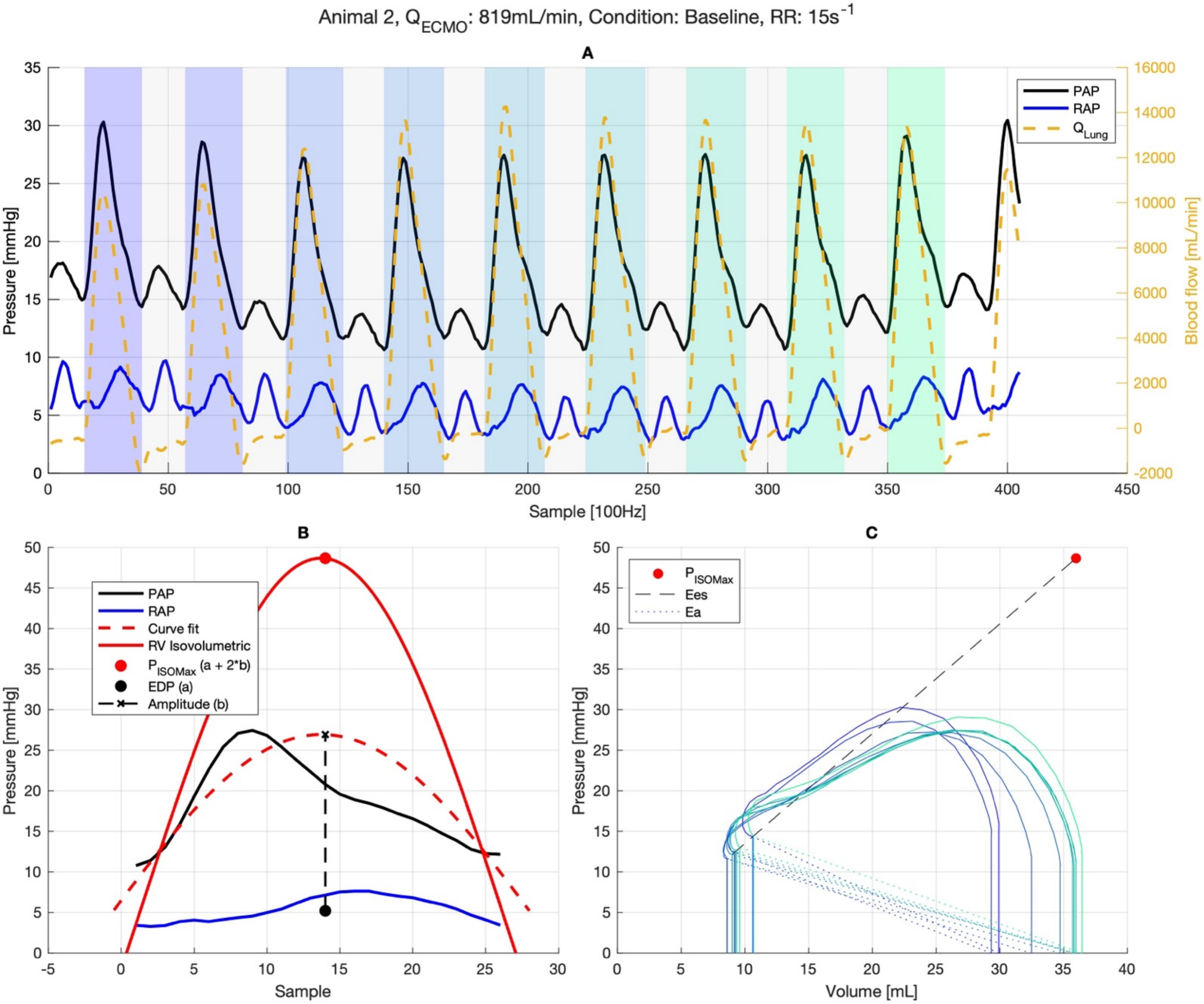
Animal 2, Q_ECMO: 819mL/min, Condition: Baseline, RR: 15s^-1
A
PAP
RAP
Q_Lung
Pressure [mmHg]
Blood flow [mL/min]
Sample [100Hz]
B
PAP
RAP
Curve fit
RV Isovolumetric
P_ISOMax (a + 2*b)
EDP (a)
Amplitude (b)
Pressure [mmHg]
Sample
C
P_ISOMax
Ees
Ea
Pressure [mmHg]
Volume [mL]

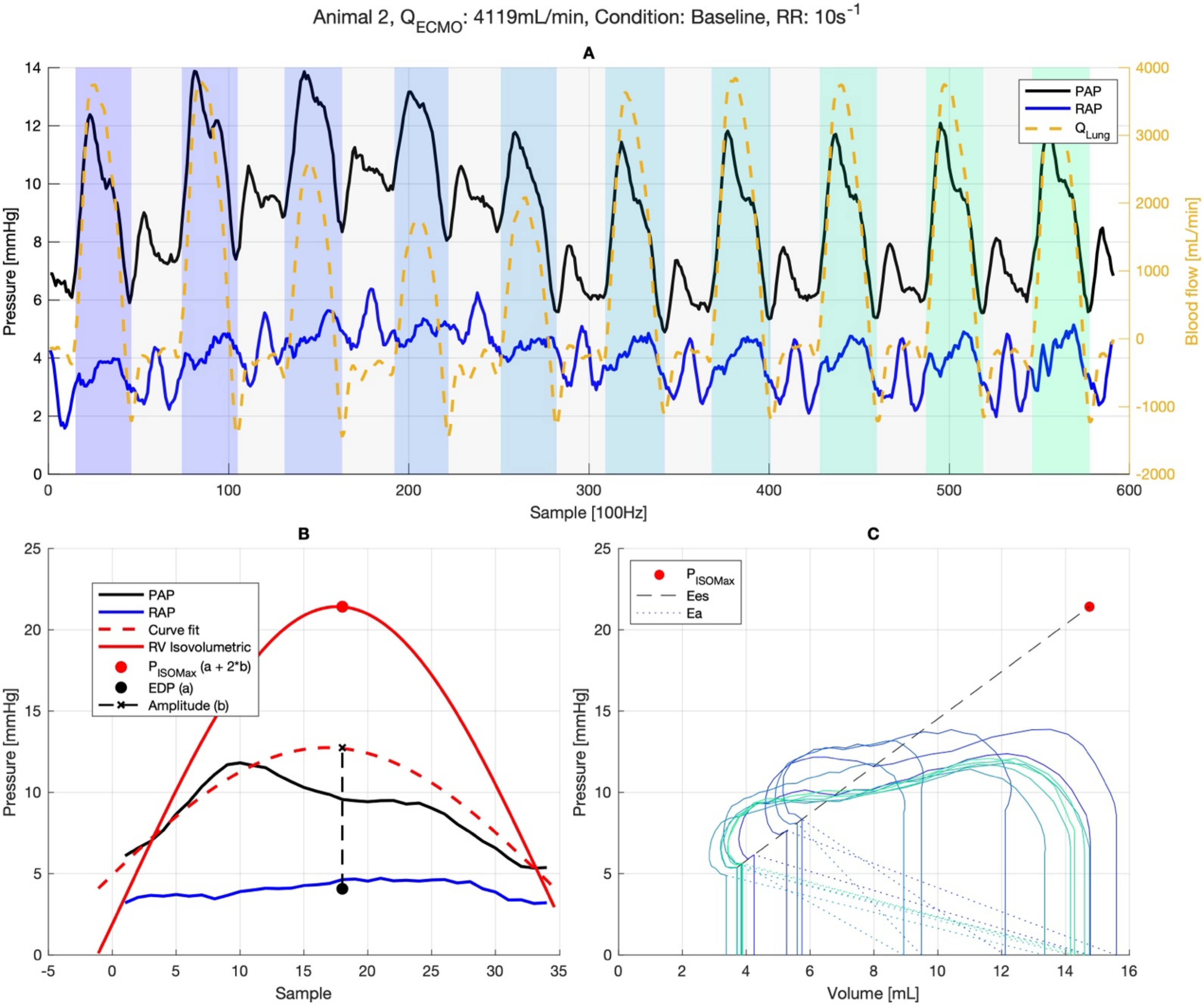

Animal 2, Q_{ECMO}: 4119mL/min, Condition: Baseline, RR: $10s^{-1}$
A
PAP
RAP
Q_{Lung}
Pressure [mmHg]
Blood flow [mL/min]
Sample [100Hz]
B
PAP
RAP
Curve fit
RV Isovolumetric
P_{ISOMax} (a + 2*b)
EDP (a)
Amplitude (b)
Pressure [mmHg]
Sample
C
P_{ISOMax}
Ees
Ea
Pressure [mmHg]
Volume [mL]

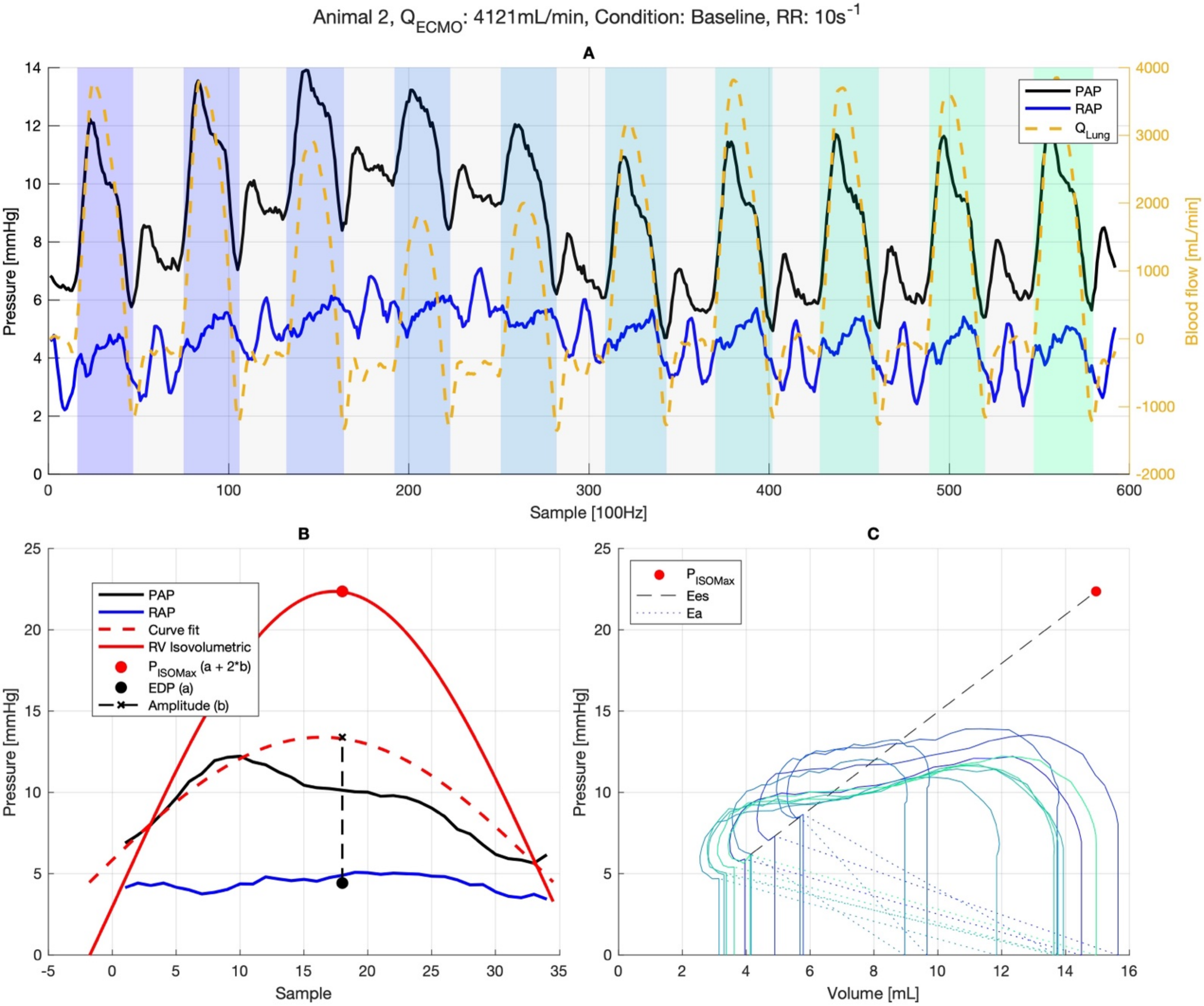

Animal 2, Q_{ECMO}: 4121mL/min, Condition: Baseline, RR: $10s^{-1}$
A
PAP
RAP
Q_{Lung}
Pressure [mmHg]
Blood flow [mL/min]
Sample [100Hz]
B
PAP
RAP
Curve fit
RV Isovolumetric
P_{ISOMax} (a + 2*b)
EDP (a)
Amplitude (b)
Pressure [mmHg]
Sample
C
P_{ISOMax}
Ees
Ea
Pressure [mmHg]
Volume [mL]

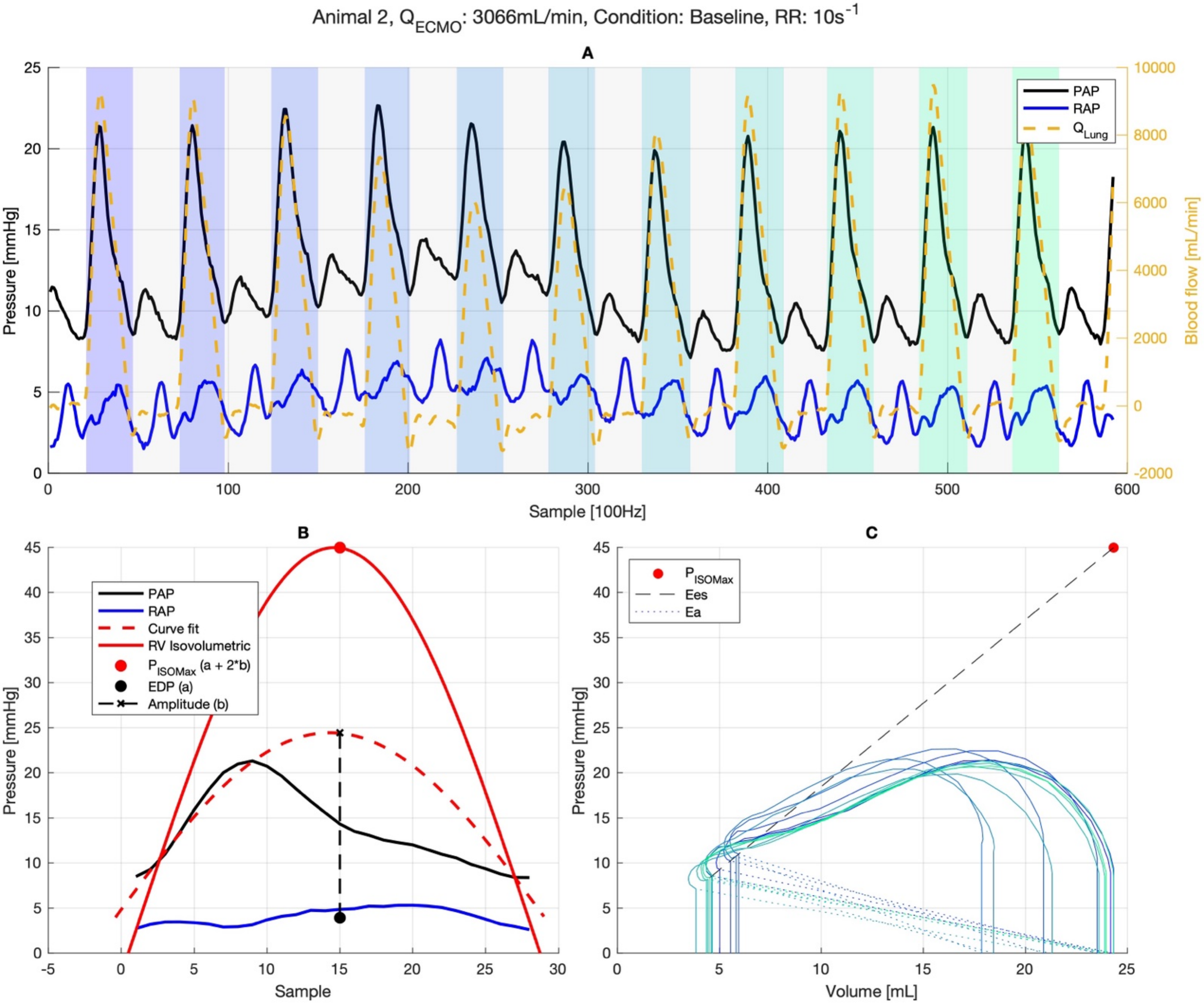

Animal 2, Q_{ECMO}: 3066mL/min, Condition: Baseline, RR: $10s^{-1}$
A
PAP
RAP
Q_{Lung}
Pressure [mmHg]
Blood flow [mL/min]
Sample [100Hz]
B
PAP
RAP
Curve fit
RV Isovolumetric
P_{ISOMax} (a + 2*b)
EDP (a)
Amplitude (b)
Pressure [mmHg]
Sample
C
P_{ISOMax}
Ees
Ea
Pressure [mmHg]
Volume [mL]

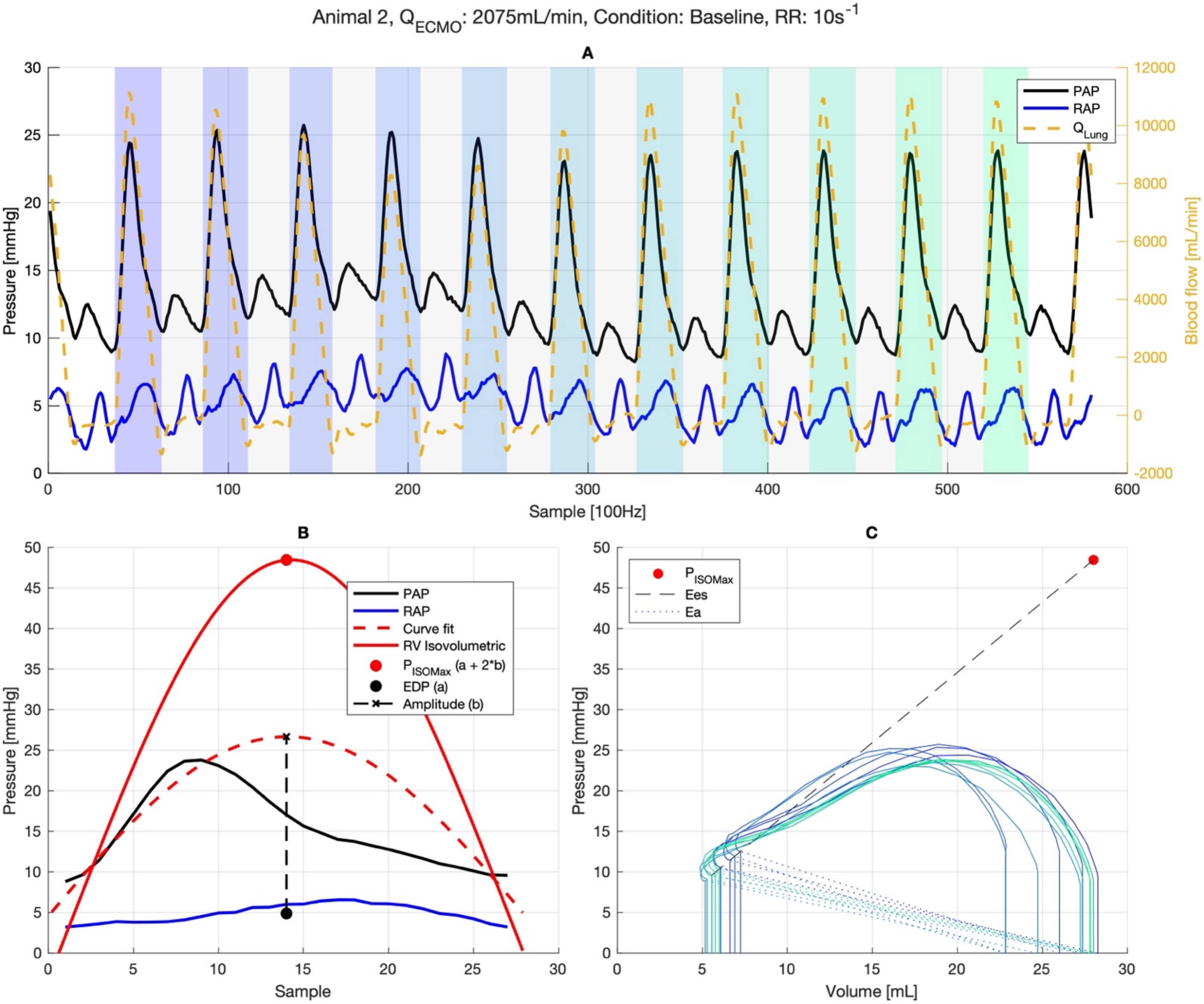

Animal 2, Q_{ECMO}: 2075mL/min, Condition: Baseline, RR: $10s^{-1}$
A
Pressure [mmHg]
Blood flow [mL/min]
Sample [100Hz]
PAP
RAP
Q_{Lung}
B
Pressure [mmHg]
Sample
PAP
RAP
Curve fit
RV Isovolumetric
P_{ISOMax} (a + 2*b)
EDP (a)
Amplitude (b)
C
Pressure [mmHg]
Volume [mL]
P_{ISOMax}
Ees
Ea

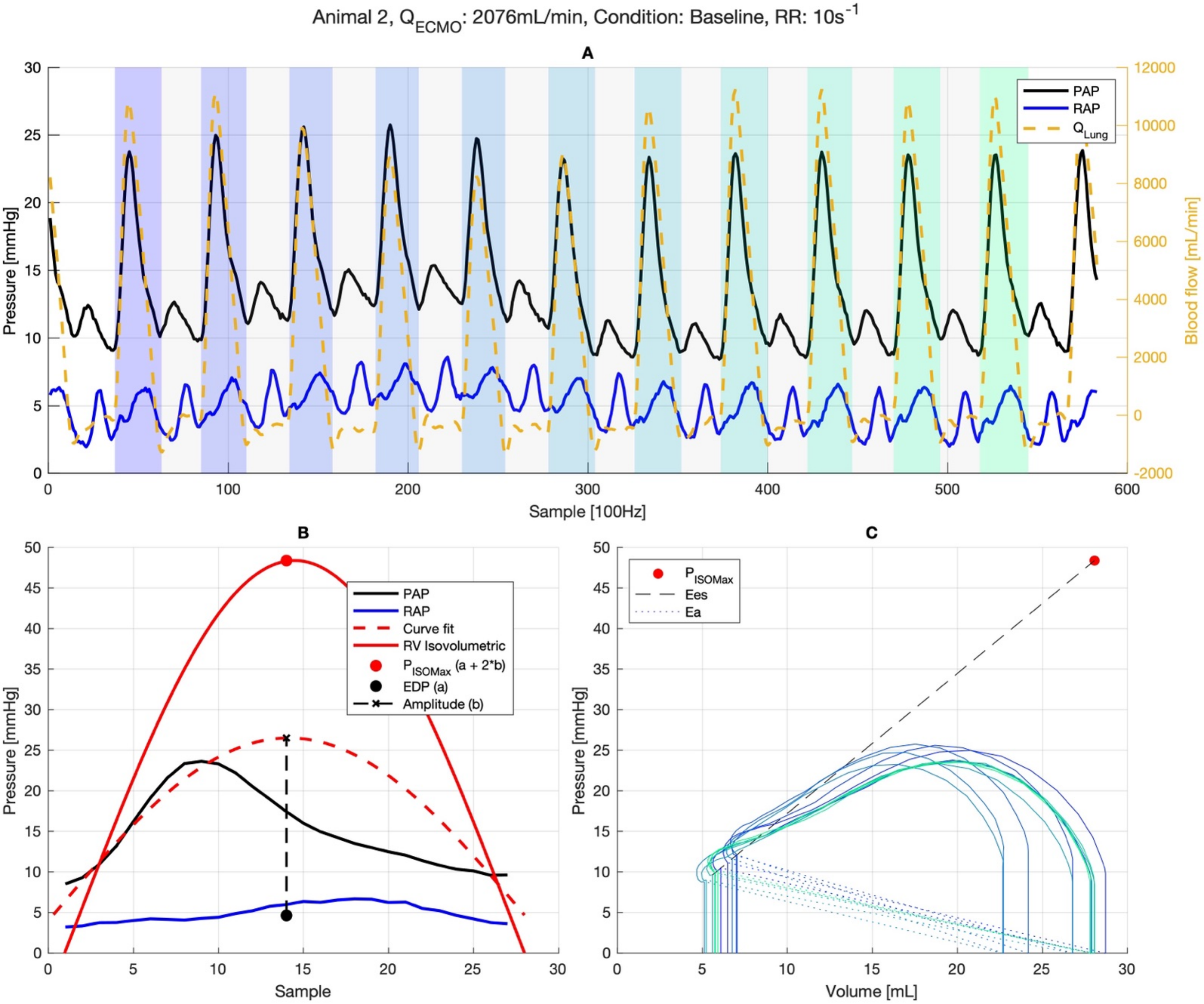

Animal 2, Q_ECMO: 2076mL/min, Condition: Baseline, RR: 10s^-1
A
Pressure [mmHg]
Blood flow [mL/min]
Sample [100Hz]
PAP
RAP
Q_Lung
B
Pressure [mmHg]
Sample
PAP
RAP
Curve fit
RV Isovolumetric
P_ISOMax (a + 2*b)
EDP (a)
Amplitude (b)
C
Pressure [mmHg]
Volume [mL]
P_ISOMax
Ees
Ea

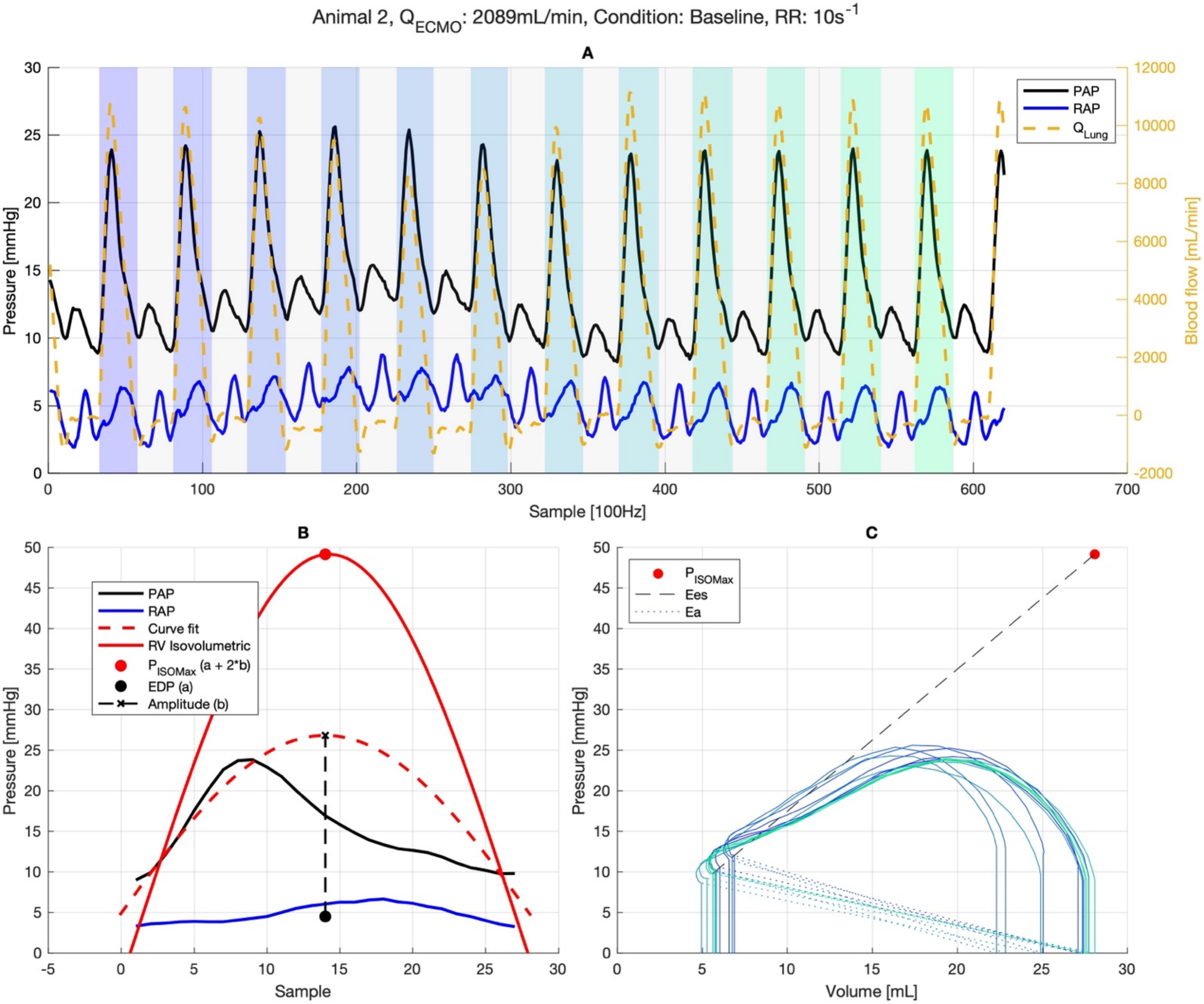

Animal 2, Q_{ECMO}: 2089mL/min, Condition: Baseline, RR: $10s^{-1}$
A
PAP
RAP
Q_{Lung}
Pressure [mmHg]
Blood flow [mL/min]
Sample [100Hz]
B
PAP
RAP
Curve fit
RV Isovolumetric
P_{ISOMax} (a + 2*b)
EDP (a)
Amplitude (b)
Pressure [mmHg]
Sample
C
P_{ISOMax}
Ees
Ea
Pressure [mmHg]
Volume [mL]

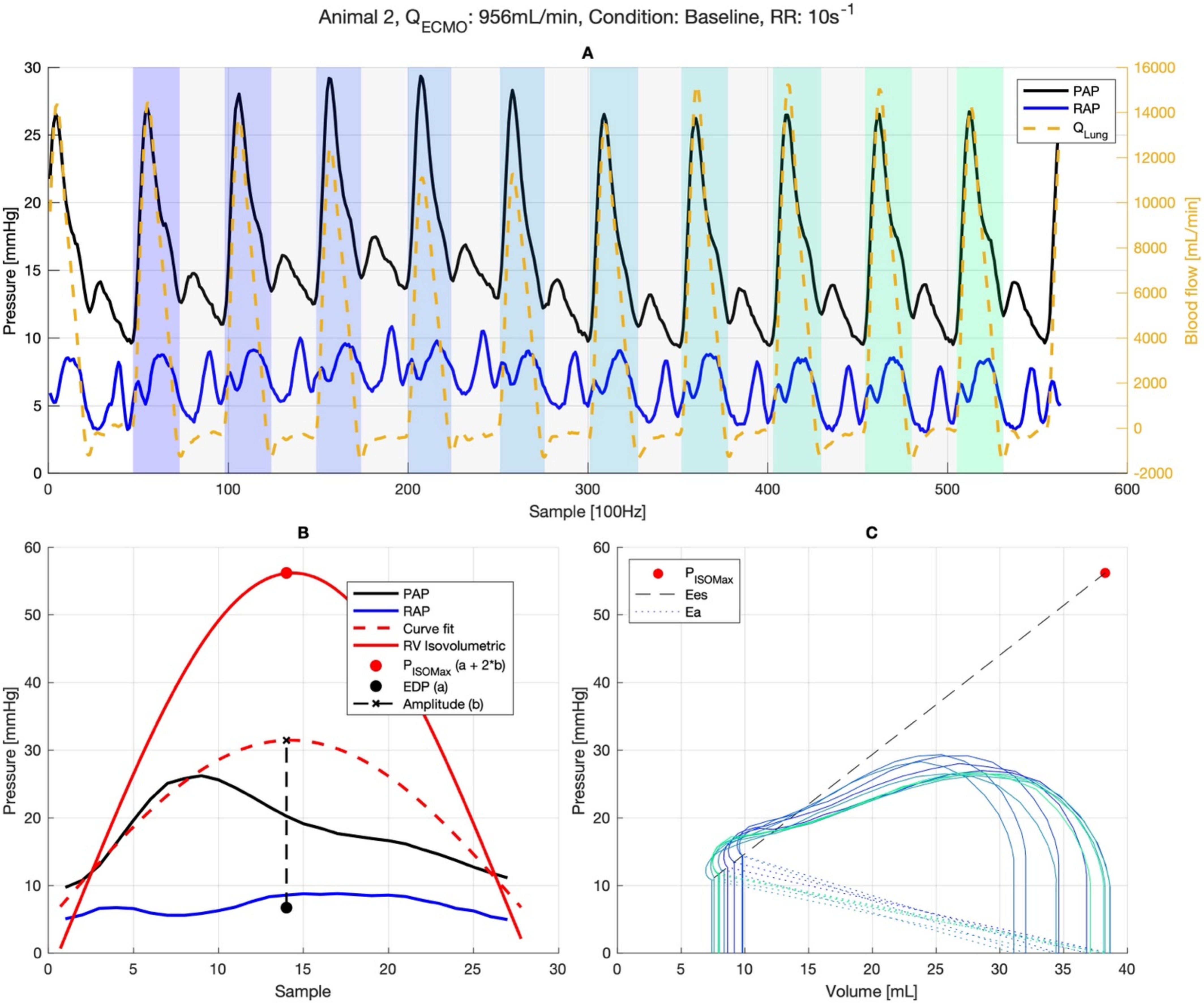

Animal 2, Q_ECMO: 956mL/min, Condition: Baseline, RR: 10s^-1
A
Pressure [mmHg]
Blood flow [mL/min]
Sample [100Hz]
PAP
RAP
Q_Lung
B
Pressure [mmHg]
Sample
PAP
RAP
Curve fit
RV Isovolumetric
P_ISOMax (a + 2*b)
EDP (a)
Amplitude (b)
C
Pressure [mmHg]
Volume [mL]
P_ISOMax
Ees
Ea

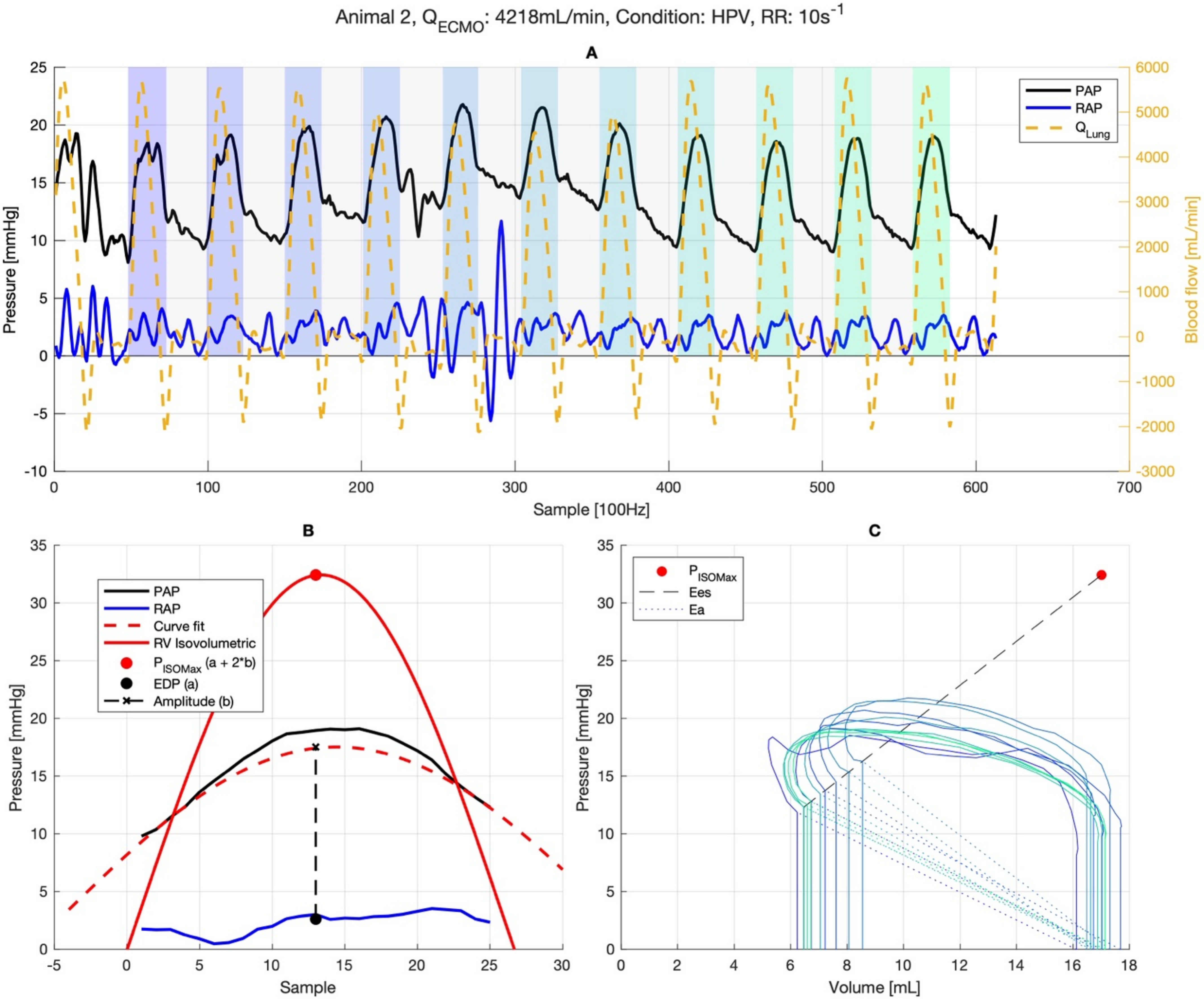

Animal 2, Q_{ECMO}: 4218mL/min, Condition: HPV, RR: $10s^{-1}$
A
Pressure [mmHg]
Blood flow [mL/min]
Sample [100Hz]
PAP
RAP
Q_{Lung}
B
Pressure [mmHg]
Sample
PAP
RAP
Curve fit
RV Isovolumetric
P_{ISOMax} (a + 2*b)
EDP (a)
Amplitude (b)
C
Pressure [mmHg]
Volume [mL]
P_{ISOMax}
Ees
Ea

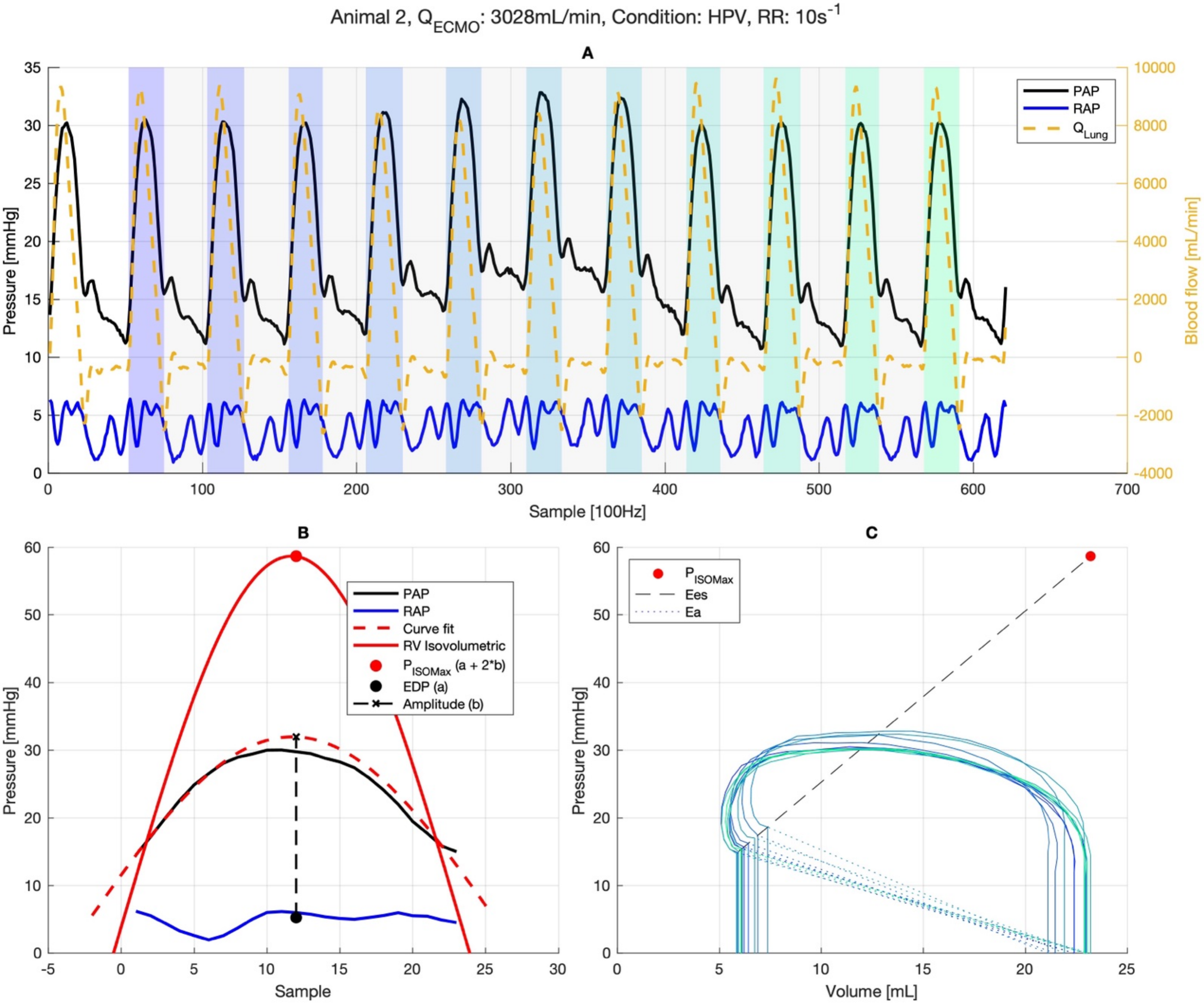

Animal 2, Q_ECMO: 3028mL/min, Condition: HPV, RR: 10s^-1
A
Pressure [mmHg]
Blood flow [mL/min]
Sample [100Hz]
PAP
RAP
Q_Lung
B
Pressure [mmHg]
Sample
PAP
RAP
Curve fit
RV Isovolumetric
P_ISOMax (a + 2*b)
EDP (a)
Amplitude (b)
C
Pressure [mmHg]
Volume [mL]
P_ISOMax
Ees
Ea

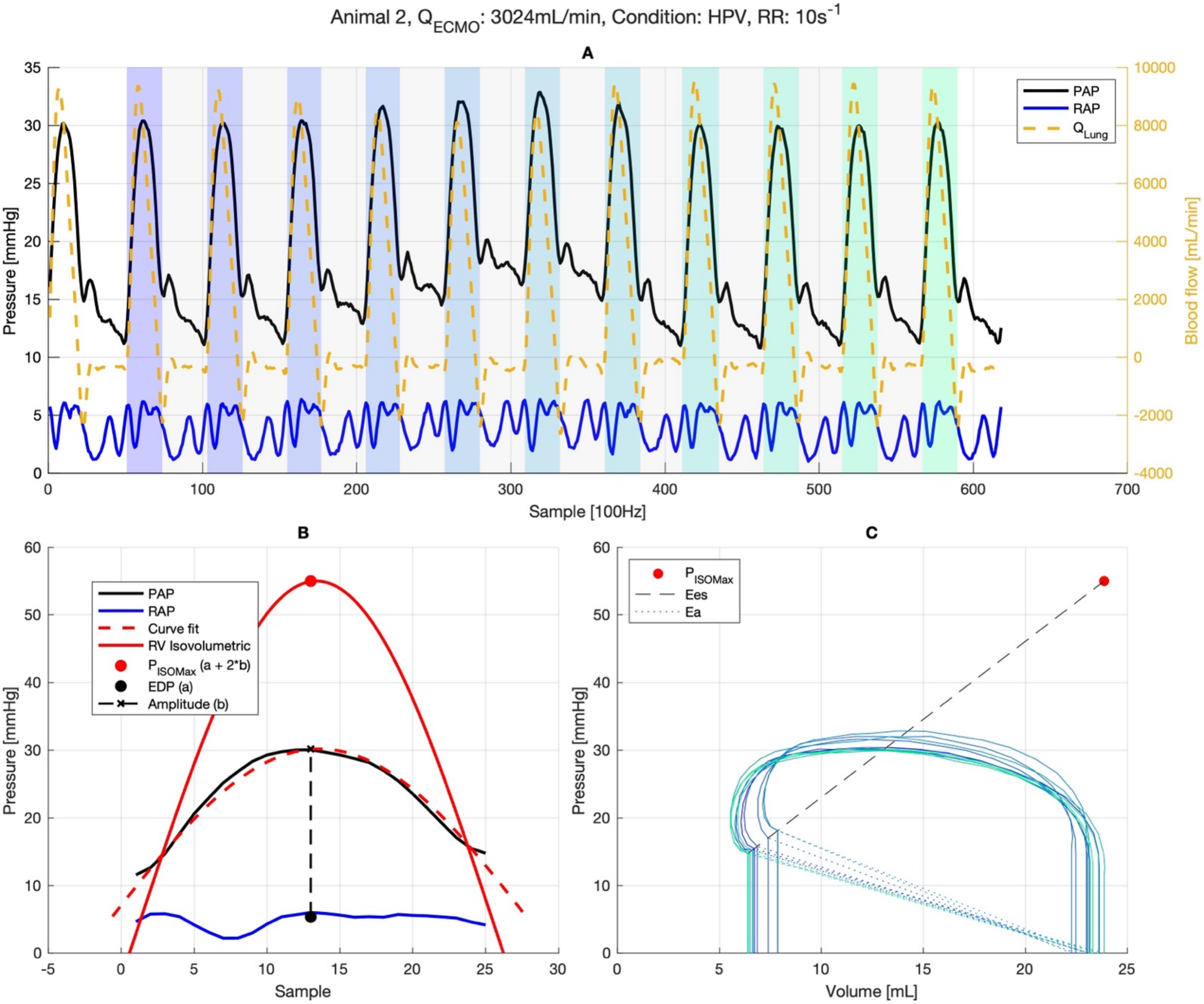

Animal 2, Q_ECMO: 3024mL/min, Condition: HPV, RR: 10s^-1
A
Pressure [mmHg]
Blood flow [mL/min]
Sample [100Hz]
PAP
RAP
Q_Lung
B
PAP
RAP
Curve fit
RV Isovolumetric
P_ISOMax (a + 2*b)
EDP (a)
Amplitude (b)
Pressure [mmHg]
Sample
C
P_ISOMax
Ees
Ea
Pressure [mmHg]
Volume [mL]

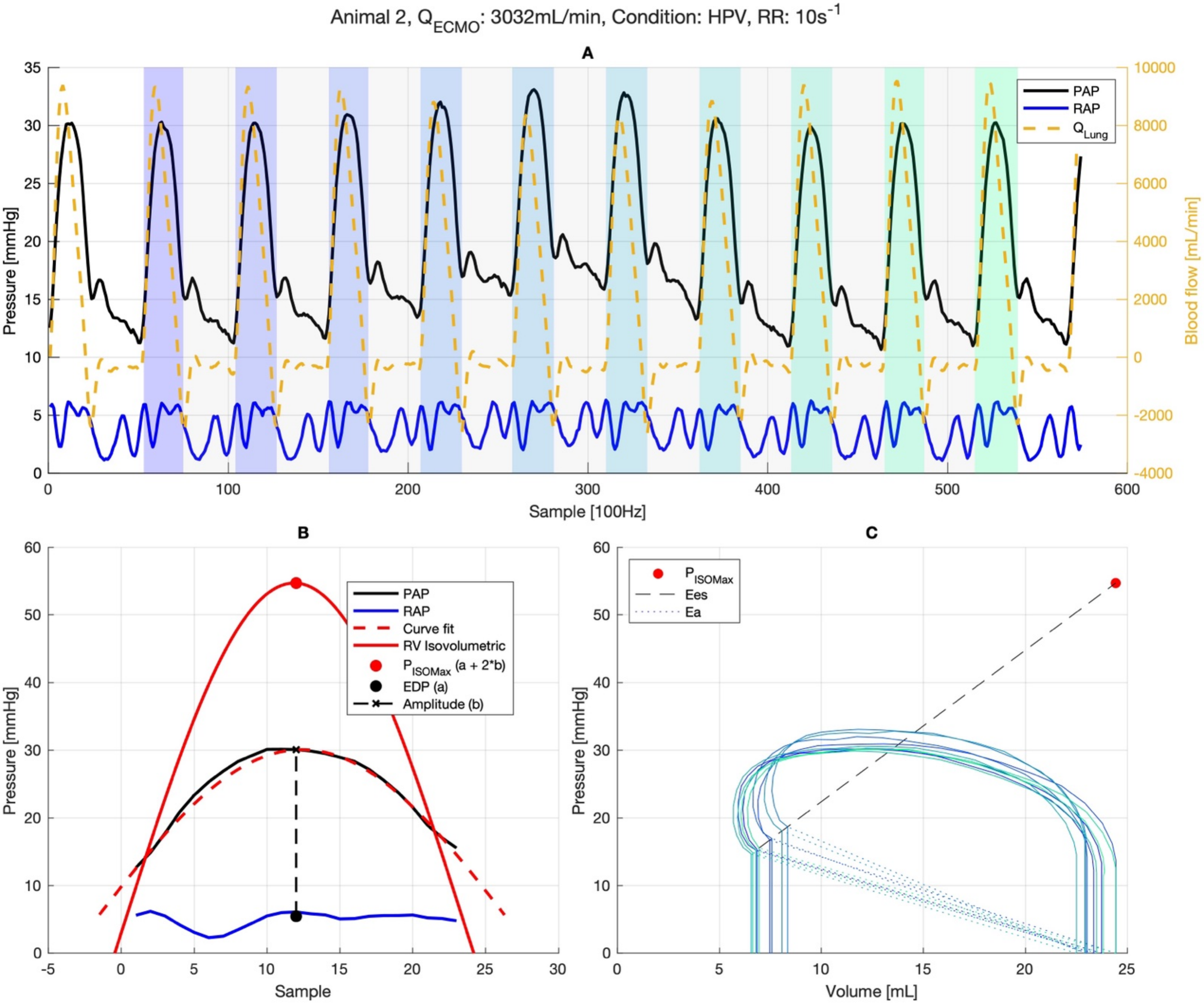

Animal 2, Q_{ECMO}: 3032mL/min, Condition: HPV, RR: $10s^{-1}$
A
PAP
RAP
Q_{Lung}
Pressure [mmHg]
Blood flow [mL/min]
Sample [100Hz]
B
PAP
RAP
Curve fit
RV Isovolumetric
P_{ISOMax} (a + 2*b)
EDP (a)
Amplitude (b)
Pressure [mmHg]
Sample
C
P_{ISOMax}
Ees
Ea
Pressure [mmHg]
Volume [mL]

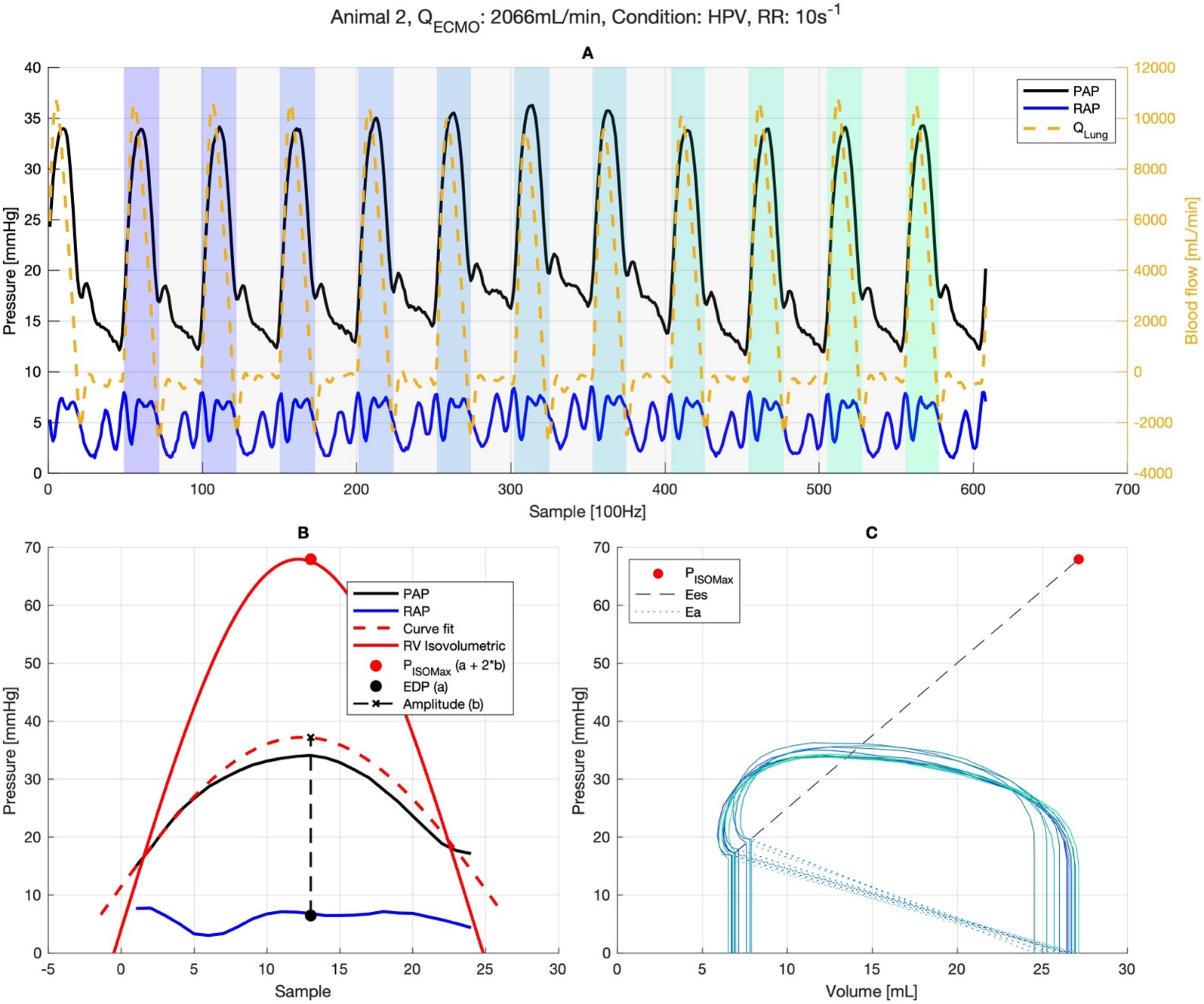

Animal 2, Q_ECMO: 2066mL/min, Condition: HPV, RR: 10s^-1
A
PAP
RAP
Q_Lung
Pressure [mmHg]
Blood flow [mL/min]
Sample [100Hz]
B
PAP
RAP
Curve fit
RV Isovolumetric
P_ISOMax (a + 2*b)
EDP (a)
Amplitude (b)
Pressure [mmHg]
Sample
C
P_ISOMax
Ees
Ea
Pressure [mmHg]
Volume [mL]

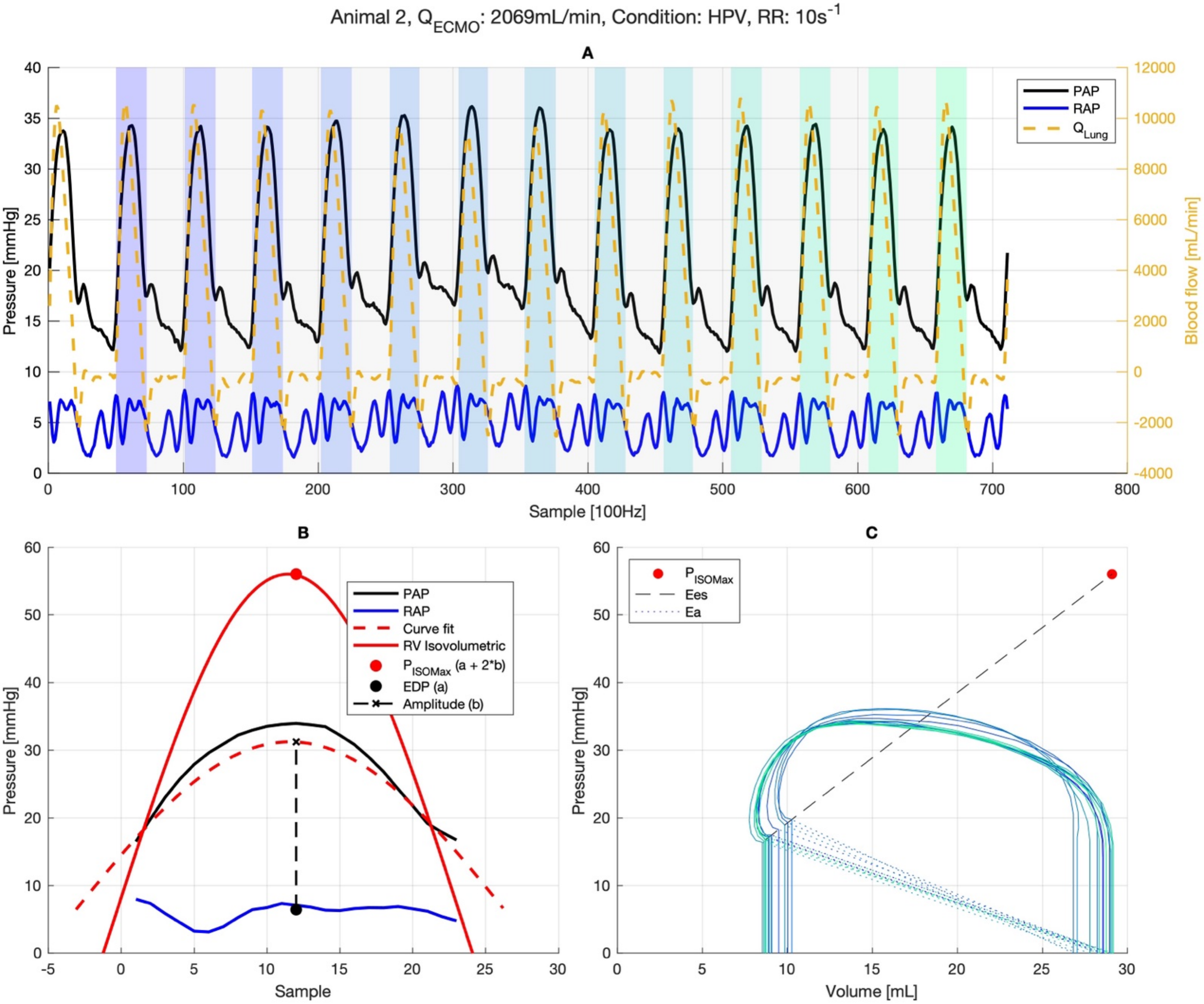

Animal 2, Q_{ECMO}: 2069mL/min, Condition: HPV, RR: 10s^{-1}
A
PAP
RAP
Q_{Lung}
Pressure [mmHg]
Blood flow [mL/min]
Sample [100Hz]
B
PAP
RAP
Curve fit
RV Isovolumetric
P_{ISOMax} (a + 2*b)
EDP (a)
Amplitude (b)
Pressure [mmHg]
Sample
C
P_{ISOMax}
Ees
Ea
Pressure [mmHg]
Volume [mL]

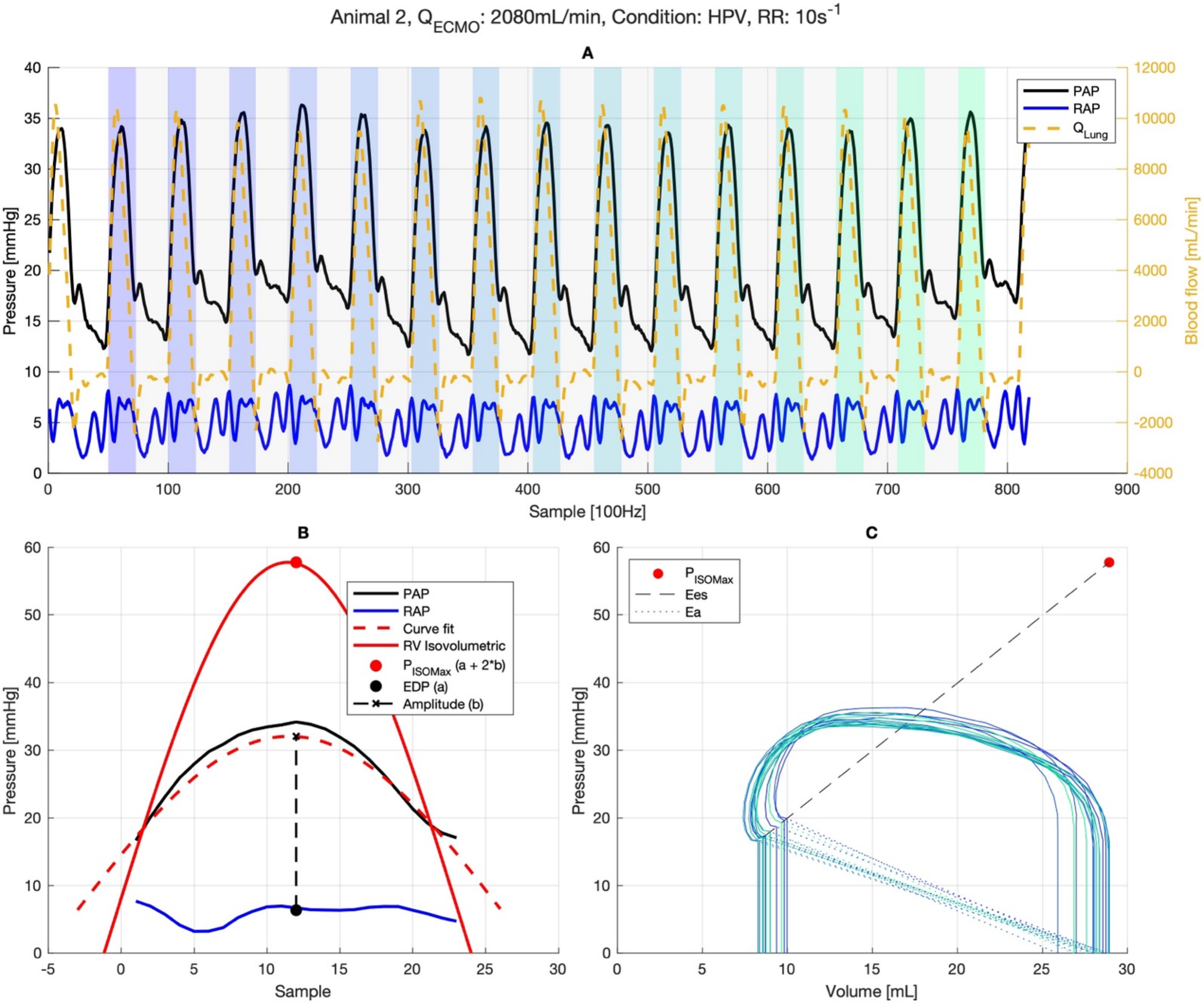

Animal 2, Q_{ECMO}: 2080mL/min, Condition: HPV, RR: $10s^{-1}$
A
PAP
RAP
Q_{Lung}
Pressure [mmHg]
Blood flow [mL/min]
Sample [100Hz]
B
PAP
RAP
Curve fit
RV Isovolumetric
P_{ISOMax} (a + 2*b)
EDP (a)
Amplitude (b)
Pressure [mmHg]
Sample
C
P_{ISOMax}
Ees
Ea
Pressure [mmHg]
Volume [mL]

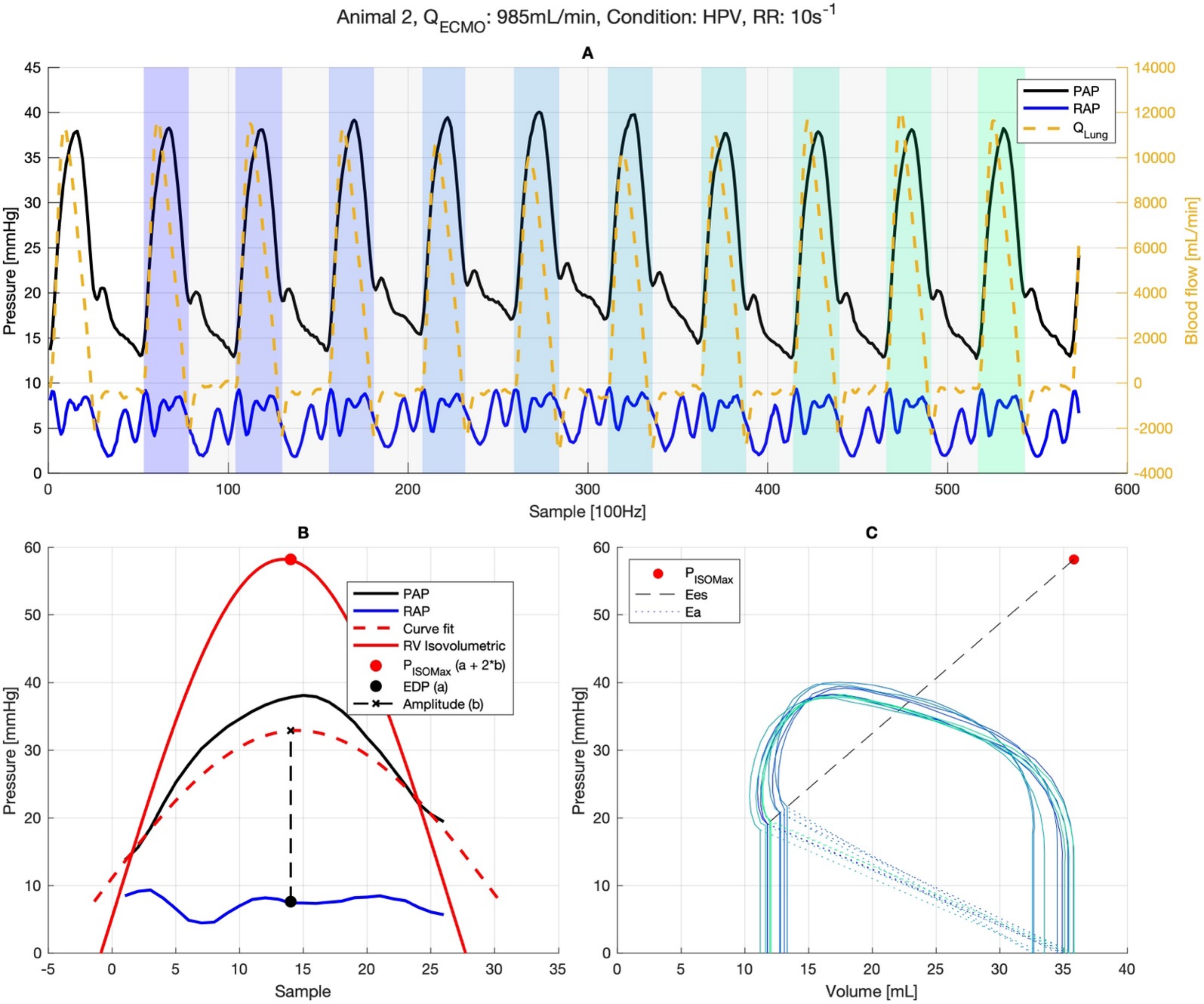

Animal 2, Q_ECMO: 985mL/min, Condition: HPV, RR: 10s^-1
A
PAP
RAP
Q_Lung
Pressure [mmHg]
Blood flow [mL/min]
Sample [100Hz]
B
PAP
RAP
Curve fit
RV Isovolumetric
P_ISOMax (a + 2*b)
EDP (a)
Amplitude (b)
Pressure [mmHg]
Sample
C
P_ISOMax
Ees
Ea
Pressure [mmHg]
Volume [mL]

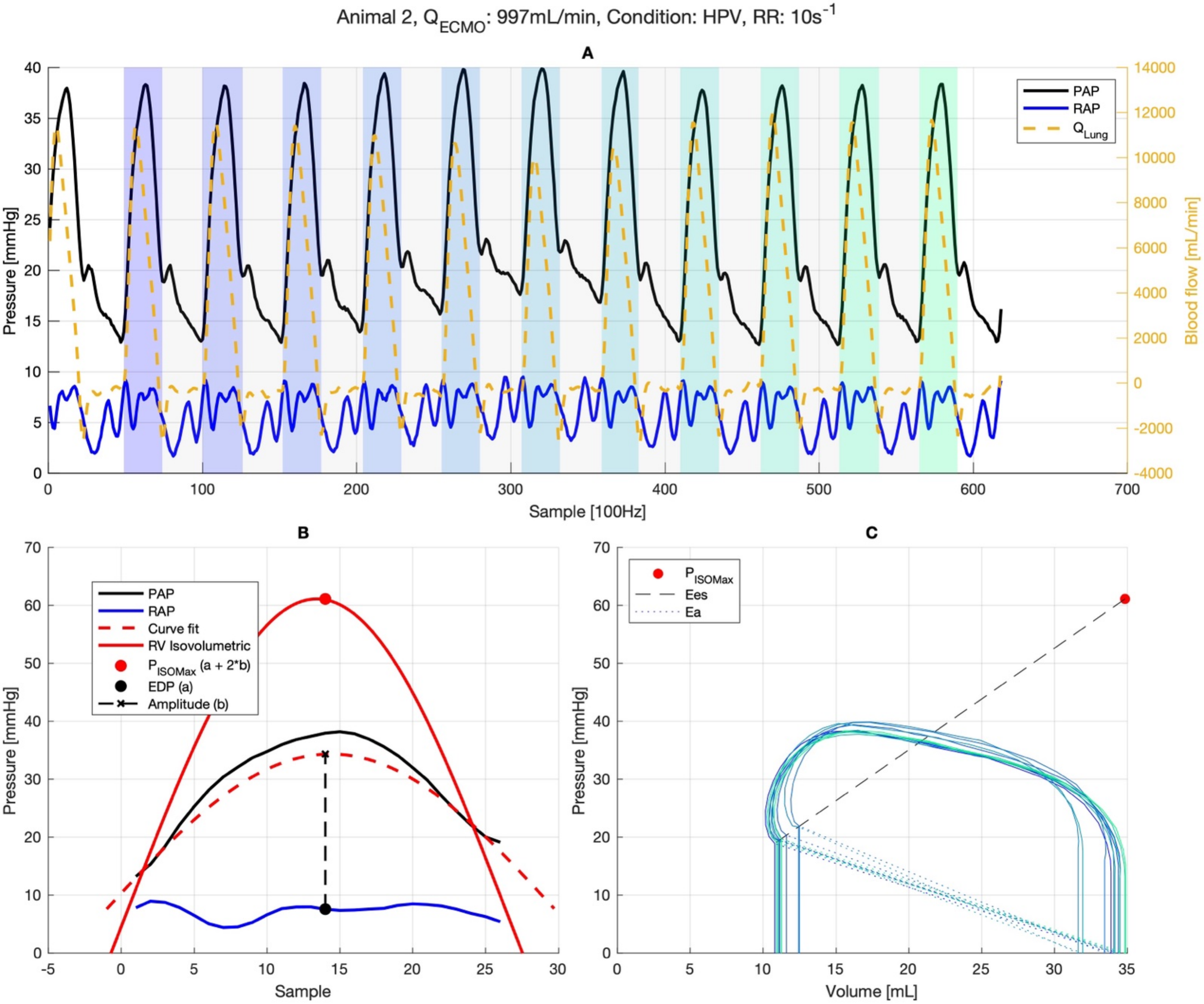
Animal 2, Q_{ECMO}: 997mL/min, Condition: HPV, RR: $10s^{-1}$
A
PAP
RAP
Q_{Lung}
Pressure [mmHg]
Blood flow [mL/min]
Sample [100Hz]
B
PAP
RAP
Curve fit
RV Isovolumetric
P_{ISOMax} (a + 2*b)
EDP (a)
Amplitude (b)
Pressure [mmHg]
Sample
C
P_{ISOMax}
Ees
Ea
Pressure [mmHg]
Volume [mL]

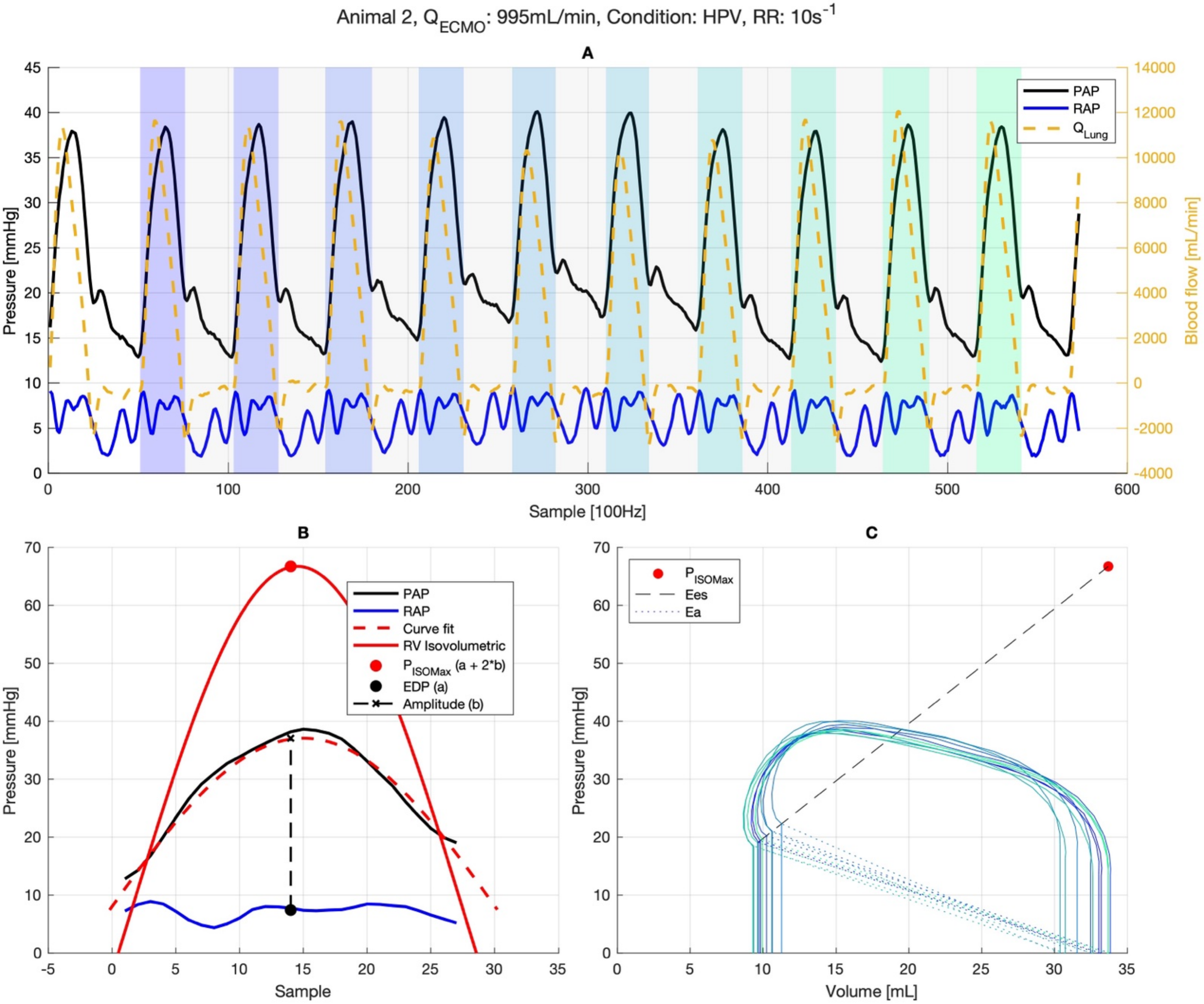

Animal 2, Q_ECMO: 995mL/min, Condition: HPV, RR: 10s^-1
A
Pressure [mmHg]
Blood flow [mL/min]
Sample [100Hz]
PAP
RAP
Q_Lung
B
PAP
RAP
Curve fit
RV Isovolumetric
P_ISOMax (a + 2*b)
EDP (a)
Amplitude (b)
Pressure [mmHg]
Sample
C
P_ISOMax
Ees
Ea
Pressure [mmHg]
Volume [mL]

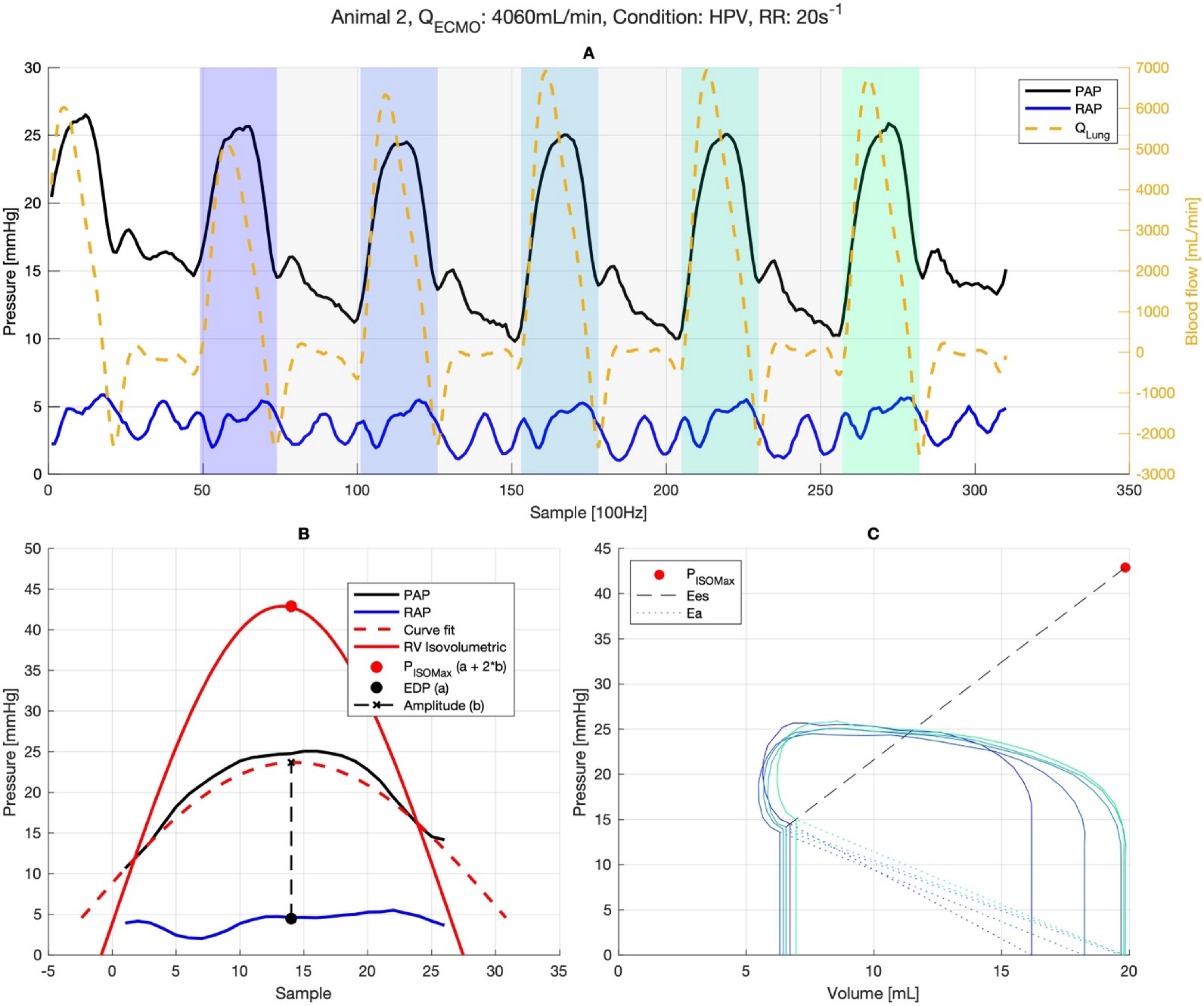

Animal 2, Q_{ECMO}: 4060mL/min, Condition: HPV, RR: 20s^{-1}
A
PAP
RAP
Q_{Lung}
Pressure [mmHg]
Blood flow [mL/min]
Sample [100Hz]
B
PAP
RAP
Curve fit
RV Isovolumetric
P_{ISOMax} (a + 2*b)
EDP (a)
Amplitude (b)
Pressure [mmHg]
Sample
C
P_{ISOMax}
Ees
Ea
Pressure [mmHg]
Volume [mL]

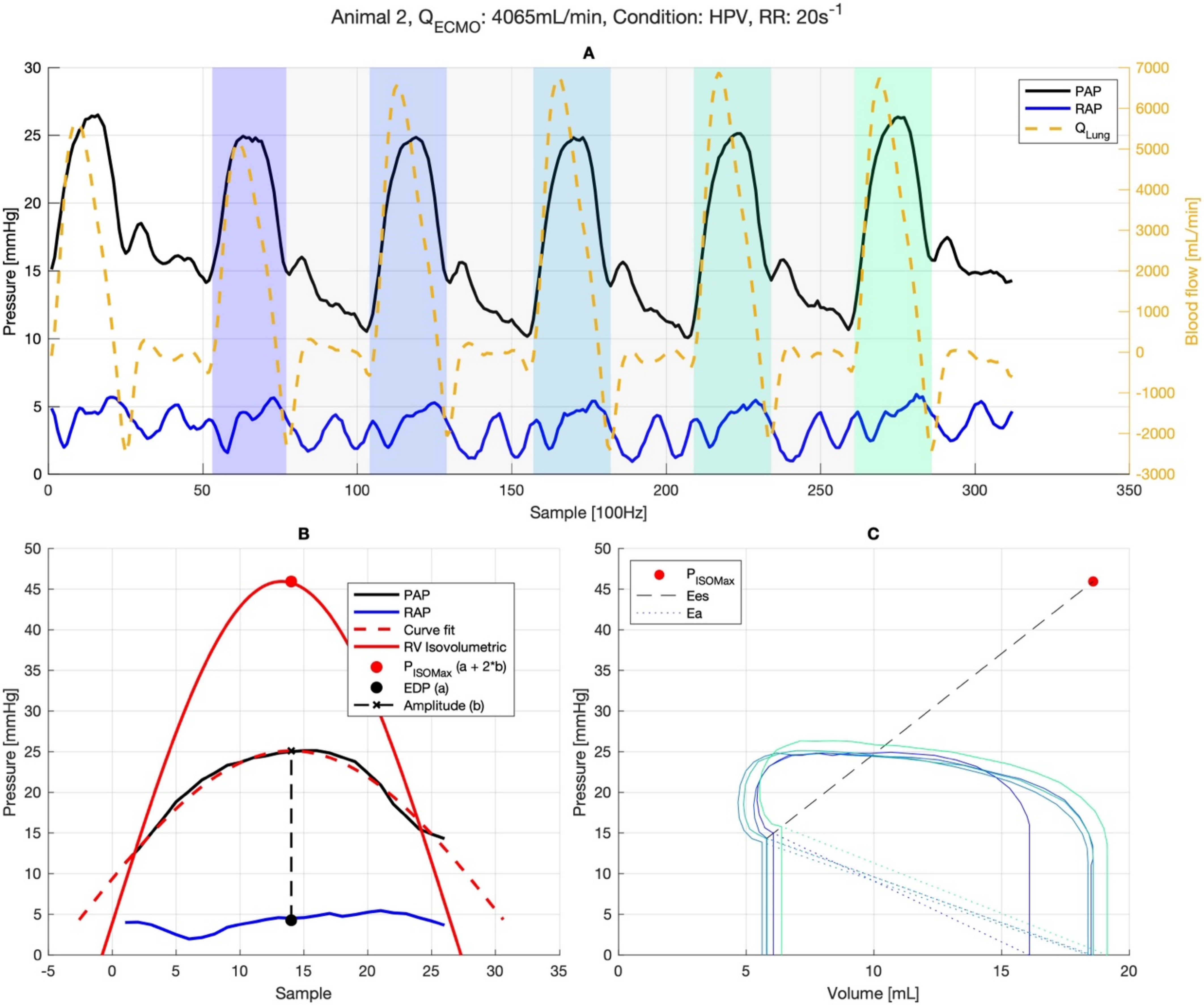

Animal 2, Q_{ECMO}: 4065mL/min, Condition: HPV, RR: 20s^{-1}
A
Pressure [mmHg]
Blood flow [mL/min]
Sample [100Hz]
PAP
RAP
Q_{Lung}
B
Pressure [mmHg]
Sample
PAP
RAP
Curve fit
RV Isovolumetric
P_{ISOMax} (a + 2*b)
EDP (a)
Amplitude (b)
C
Pressure [mmHg]
Volume [mL]
P_{ISOMax}
Ees
Ea

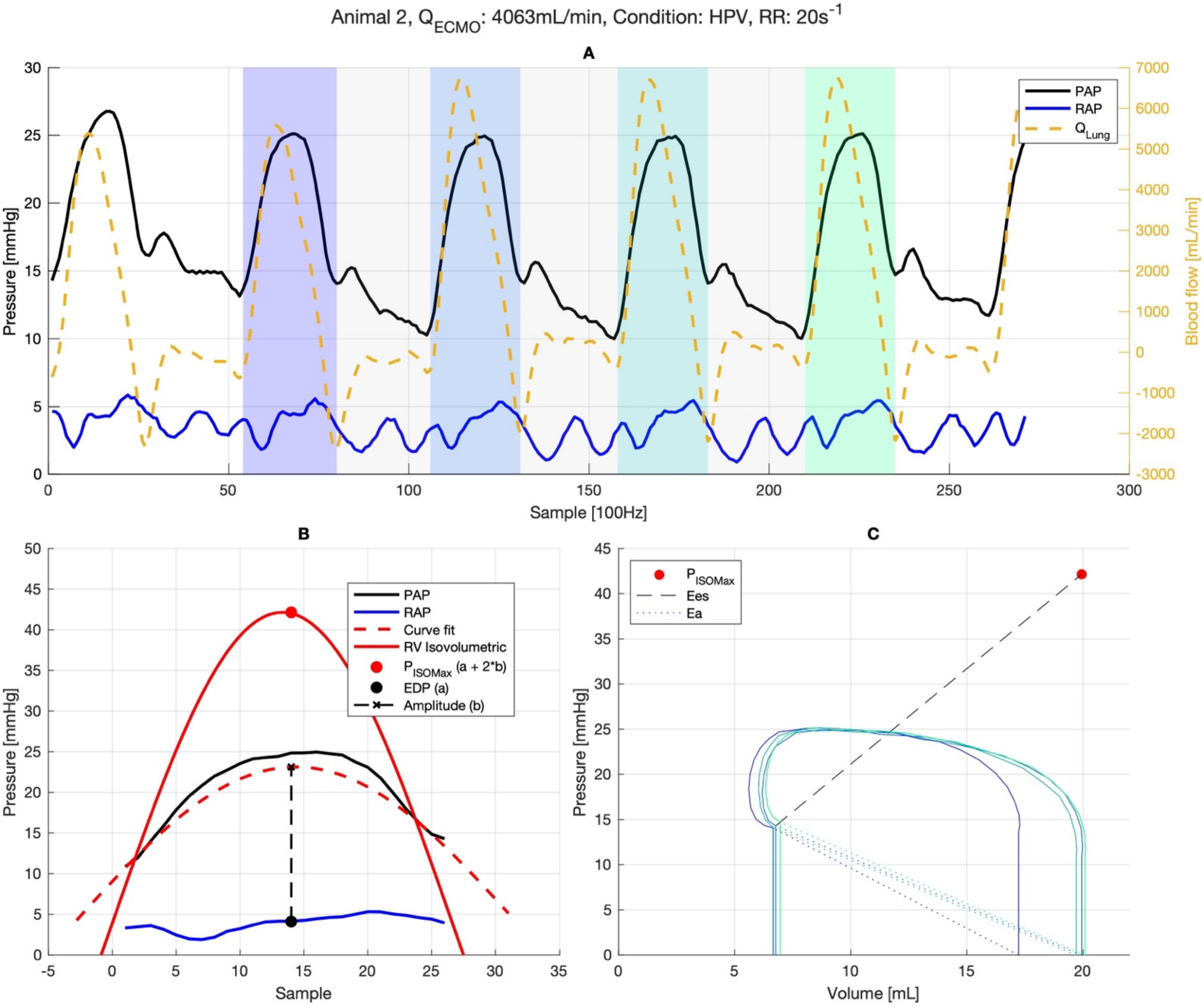

Animal 2, Q_ECMO: 4063mL/min, Condition: HPV, RR: 20s^-1
A
PAP
RAP
Q_Lung
Pressure [mmHg]
Blood flow [mL/min]
Sample [100Hz]
B
PAP
RAP
Curve fit
RV Isovolumetric
P_ISOMax (a + 2*b)
EDP (a)
Amplitude (b)
Pressure [mmHg]
Sample
C
P_ISOMax
Ees
Ea
Pressure [mmHg]
Volume [mL]

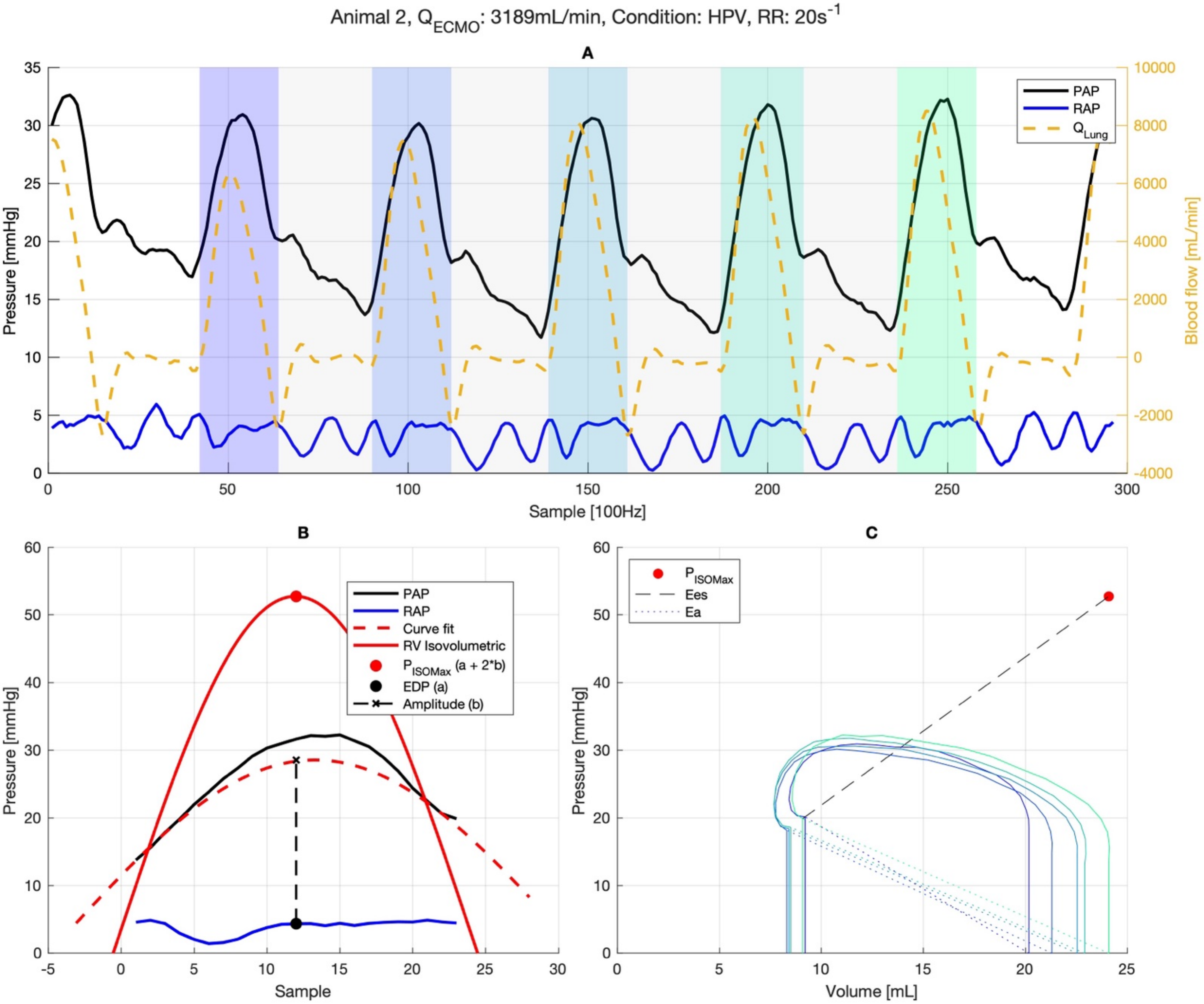

Animal 2, Q ECMO: 3189mL/min, Condition: HPV, RR: 20s-1
A
Pressure [mmHg]
Blood flow [mL/min]
Sample [100Hz]
PAP
RAP
Q Lung
B
Pressure [mmHg]
Sample
PAP
RAP
Curve fit
RV Isovolumetric
P ISOMax (a + 2*b)
EDP (a)
Amplitude (b)
C
Pressure [mmHg]
Volume [mL]
P ISOMax
Ees
Ea

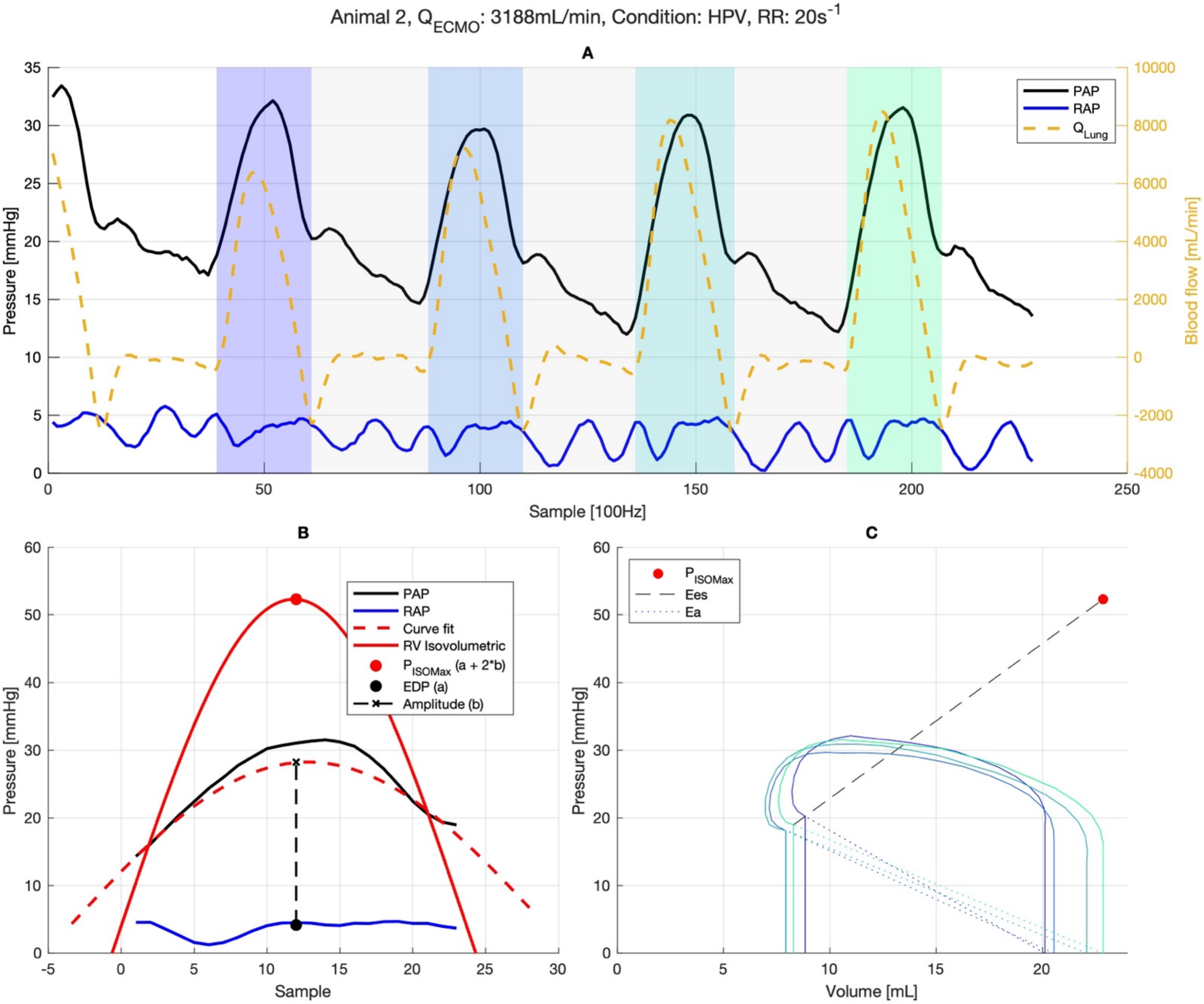

Animal 2, Q_{ECMO}: 3188mL/min, Condition: HPV, RR: $20s^{-1}$
A
PAP
RAP
Q_{Lung}
Pressure [mmHg]
Blood flow [mL/min]
Sample [100Hz]
B
PAP
RAP
Curve fit
RV Isovolumetric
P_{ISOMax} (a + 2*b)
EDP (a)
Amplitude (b)
Pressure [mmHg]
Sample
C
P_{ISOMax}
Ees
Ea
Pressure [mmHg]
Volume [mL]

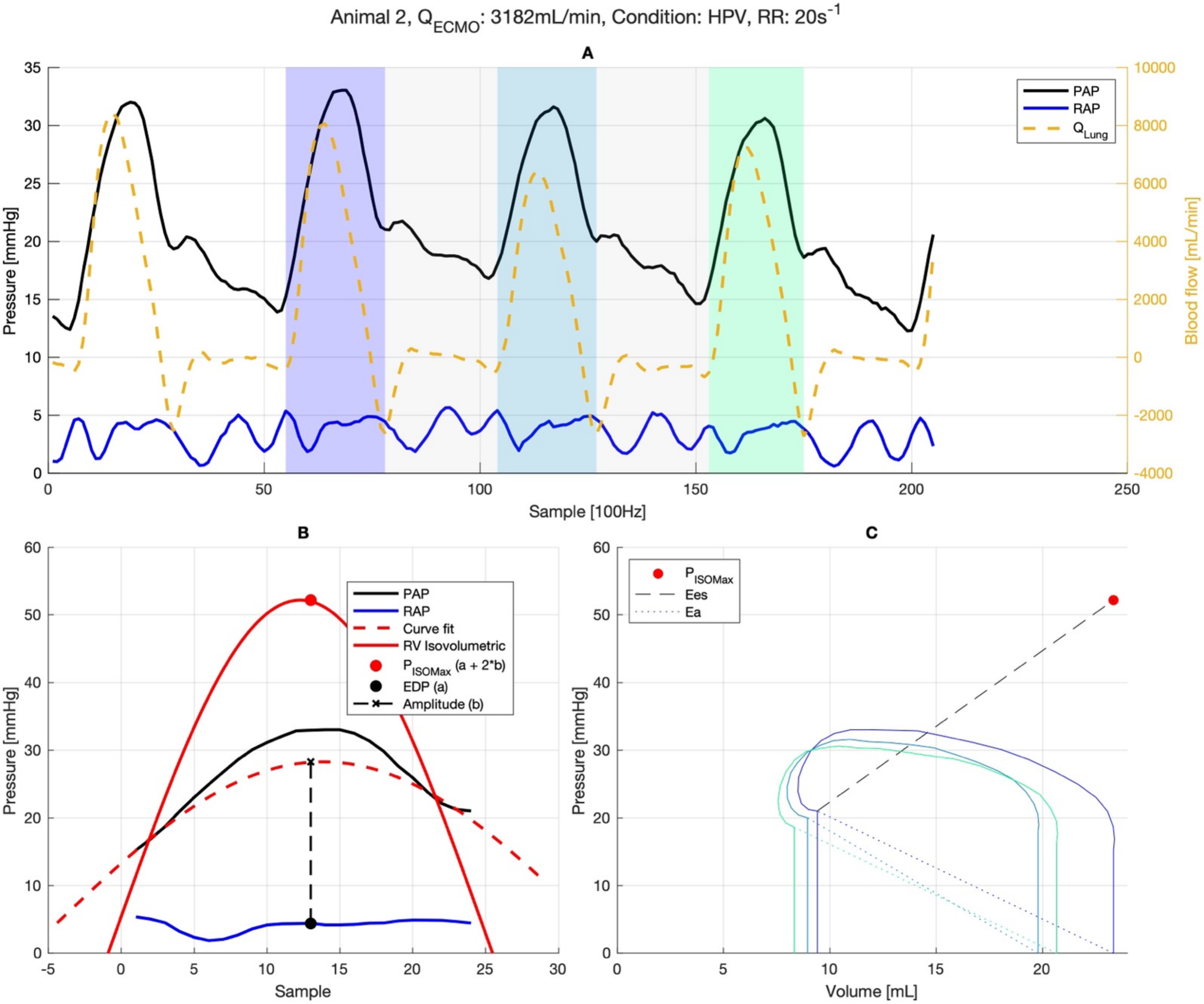

Animal 2, Q_ECMO: 3182mL/min, Condition: HPV, RR: 20s^-1
A
PAP
RAP
Q_Lung
Pressure [mmHg]
Blood flow [mL/min]
Sample [100Hz]
B
PAP
RAP
Curve fit
RV Isovolumetric
P_ISOMax (a + 2*b)
EDP (a)
Amplitude (b)
Pressure [mmHg]
Sample
C
P_ISOMax
Ees
Ea
Pressure [mmHg]
Volume [mL]

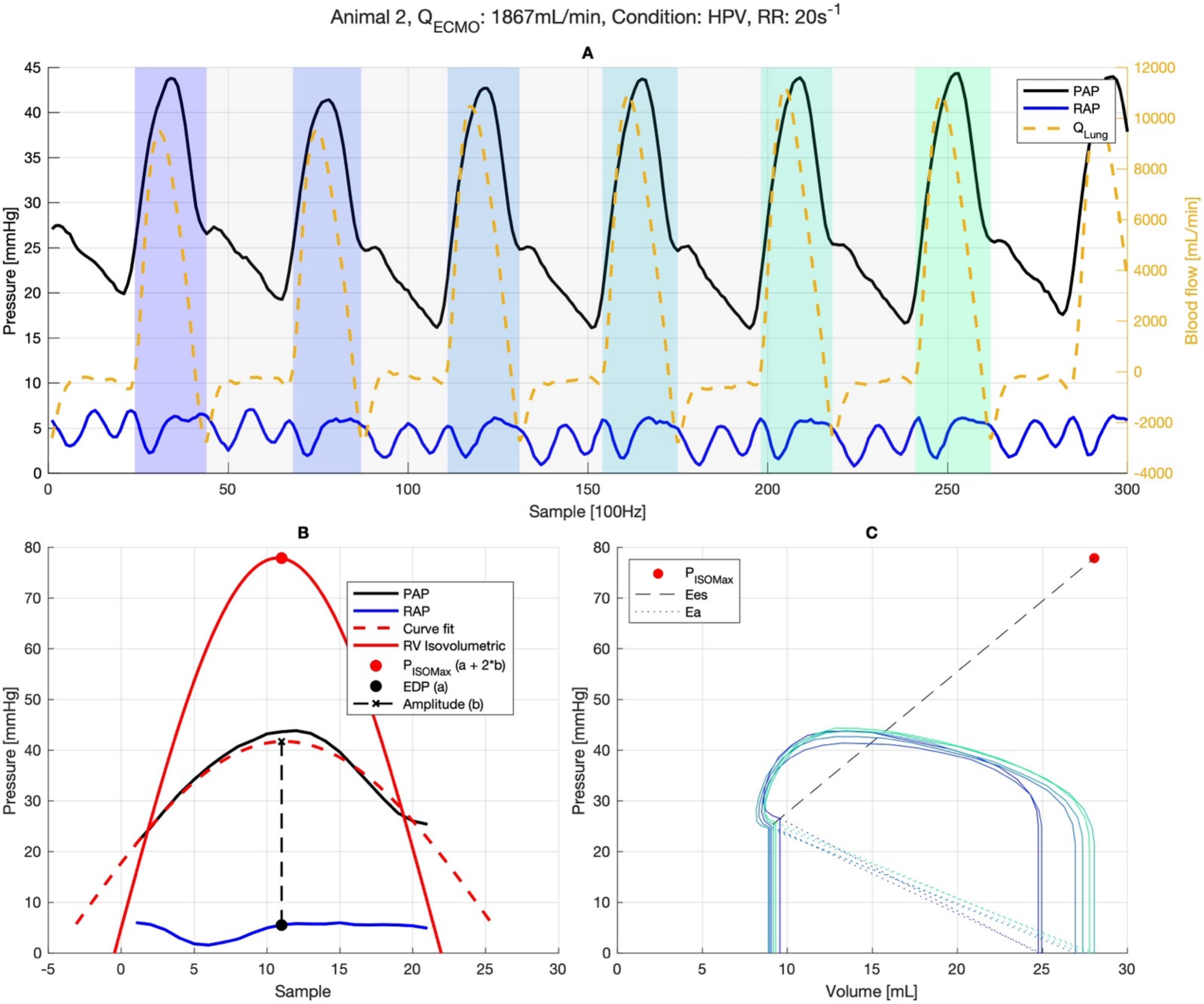

Animal 2, Q_{ECMO}: 1867mL/min, Condition: HPV, RR: 20s^{-1}
A
PAP
RAP
Q_{Lung}
Pressure [mmHg]
Blood flow [mL/min]
Sample [100Hz]
B
PAP
RAP
Curve fit
RV Isovolumetric
P_{ISOMax} (a + 2*b)
EDP (a)
Amplitude (b)
Pressure [mmHg]
Sample
C
P_{ISOMax}
Ees
Ea
Pressure [mmHg]
Volume [mL]

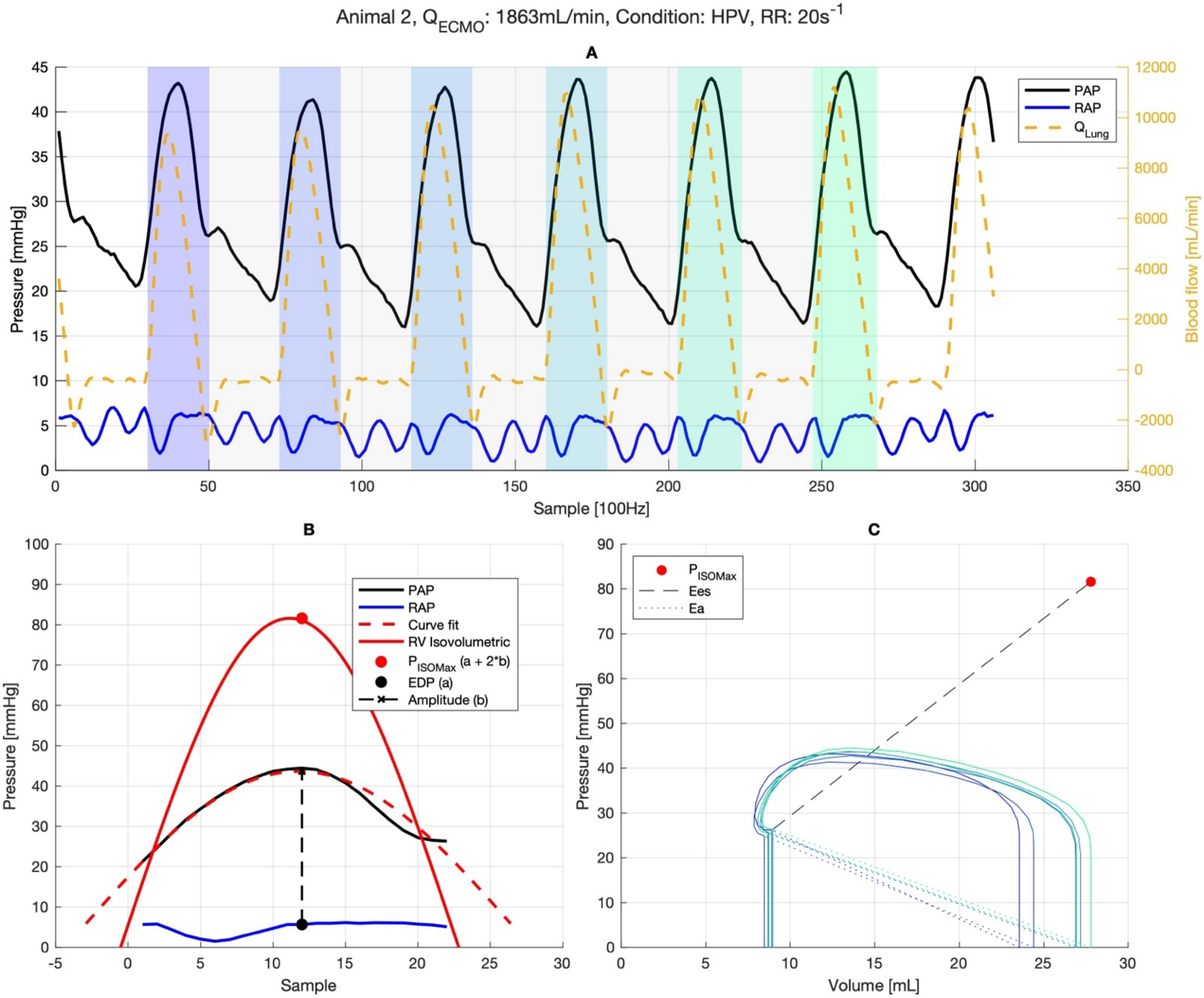

Animal 2, Q_ECMO: 1863mL/min, Condition: HPV, RR: 20s^-1
A
PAP
RAP
Q_Lung
Pressure [mmHg]
Blood flow [mL/min]
Sample [100Hz]
B
PAP
RAP
Curve fit
RV Isovolumetric
P_ISOMax (a + 2*b)
EDP (a)
Amplitude (b)
Pressure [mmHg]
Sample
C
P_ISOMax
Ees
Ea
Pressure [mmHg]
Volume [mL]

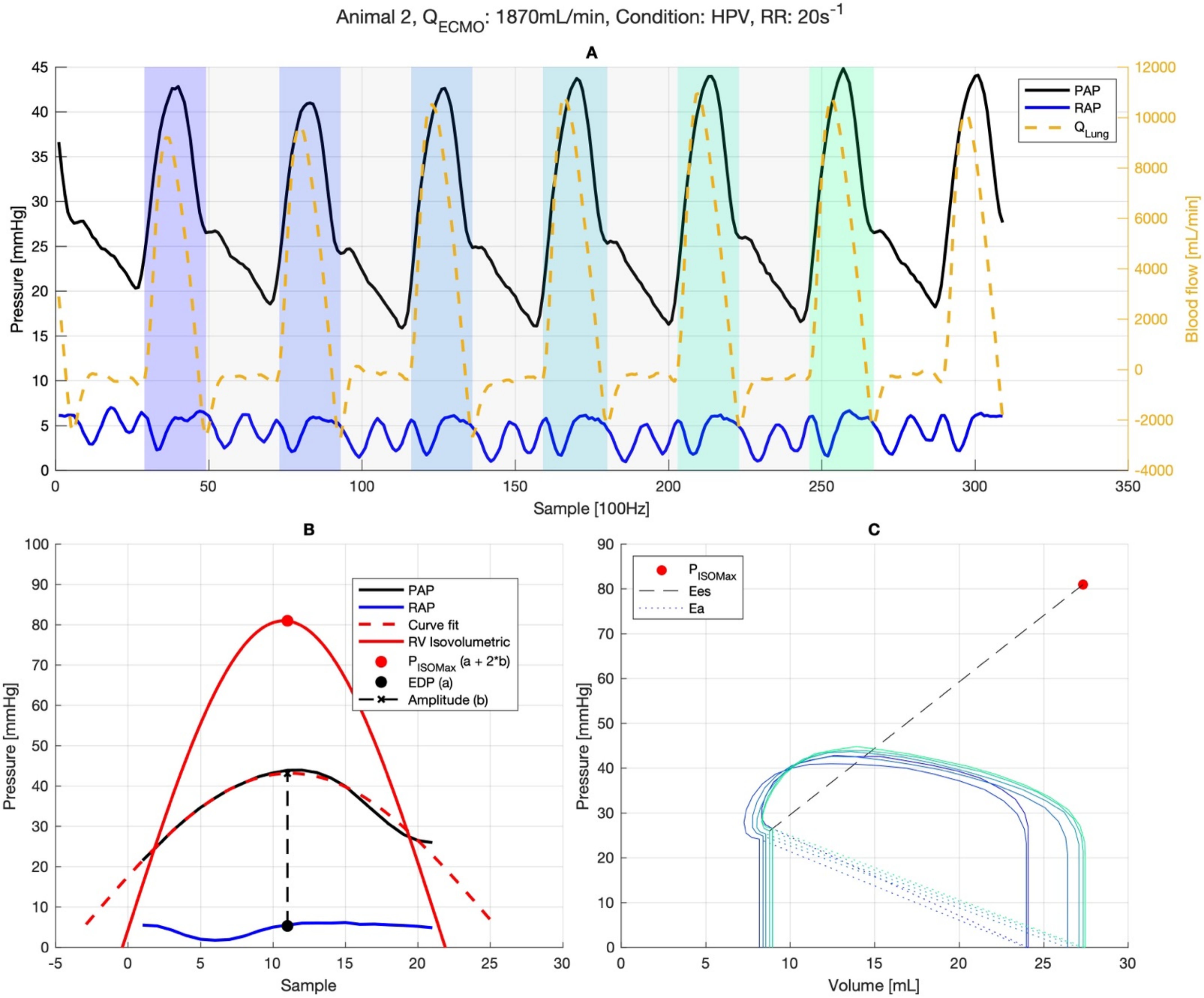

Animal 2, Q_{ECMO}: 1870mL/min, Condition: HPV, RR: $20s^{-1}$
A
PAP
RAP
Q_{Lung}
Pressure [mmHg]
Blood flow [mL/min]
Sample [100Hz]
B
PAP
RAP
Curve fit
RV Isovolumetric
P_{ISOMax} (a + 2*b)
EDP (a)
Amplitude (b)
Pressure [mmHg]
Sample
C
P_{ISOMax}
Ees
Ea
Pressure [mmHg]
Volume [mL]

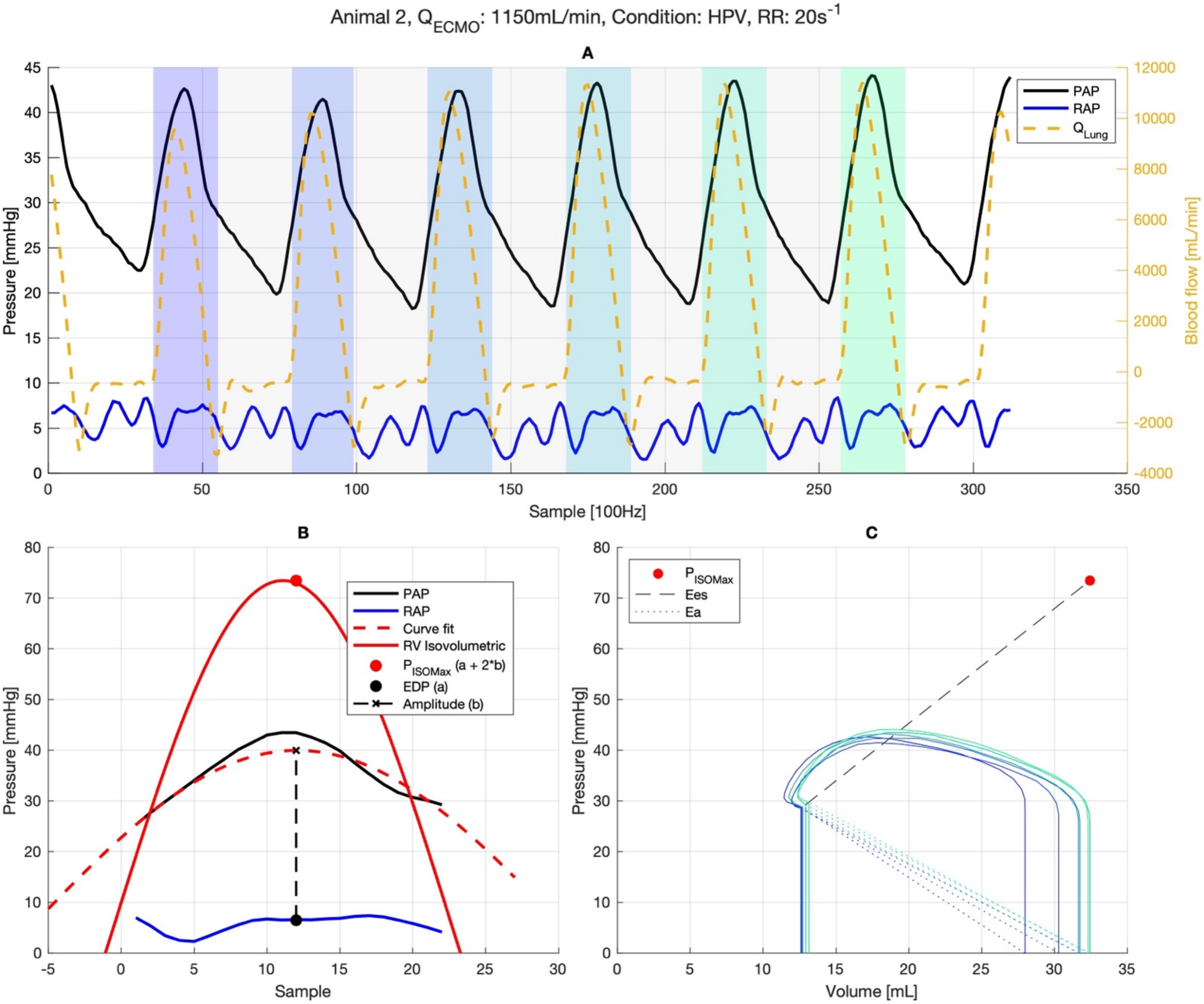

Animal 2, Q ECMO: 1150mL/min, Condition: HPV, RR: 20s-1
A
Pressure [mmHg]
Blood flow [mL/min]
Sample [100Hz]
PAP
RAP
Q Lung
B
PAP
RAP
Curve fit
RV Isovolumetric
P ISOMax (a + 2*b)
EDP (a)
Amplitude (b)
Pressure [mmHg]
Sample
C
P ISOMax
Ees
Ea
Pressure [mmHg]
Volume [mL]

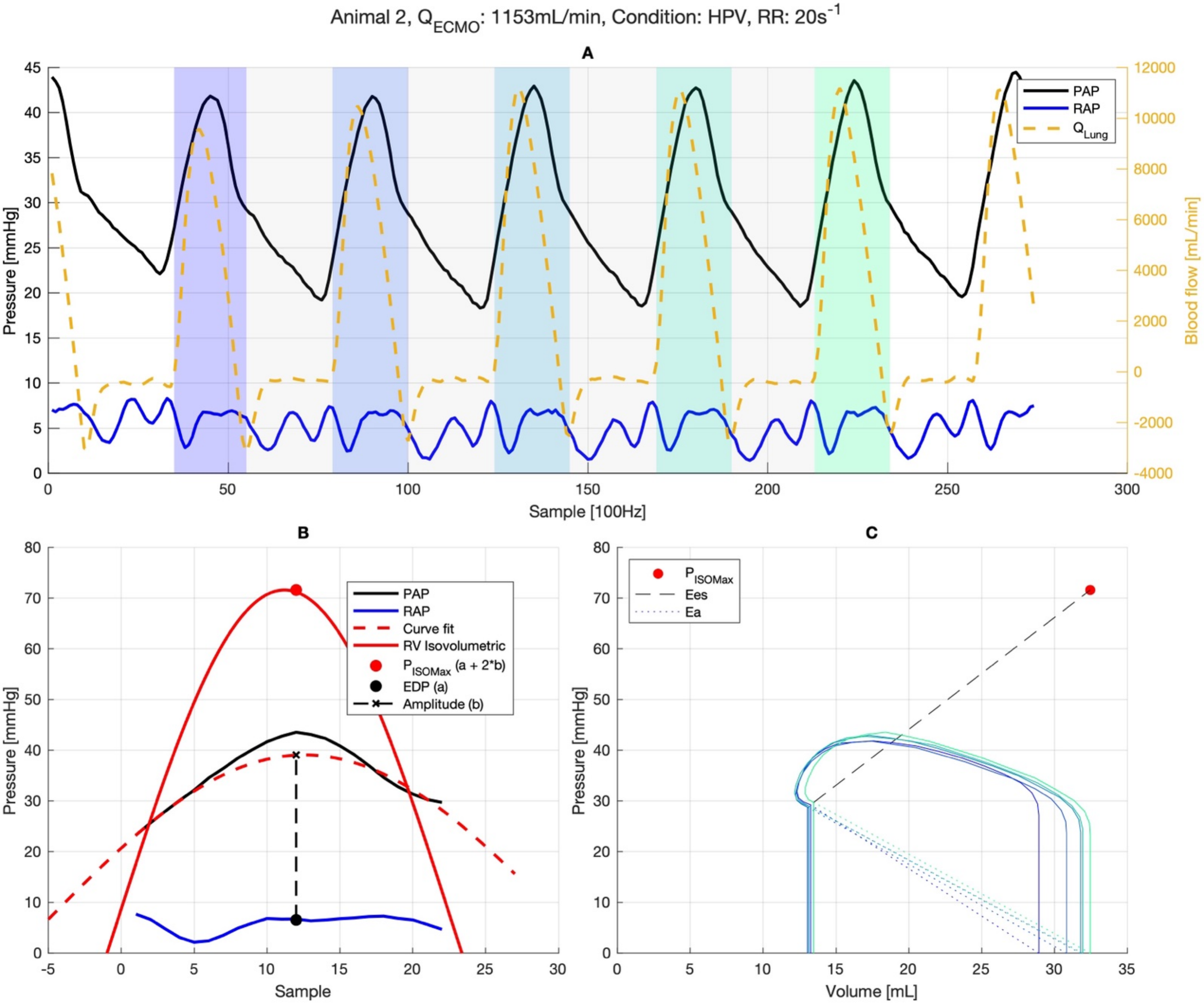

Animal 2, QECMO: 1153mL/min, Condition: HPV, RR: 20s-1
A
PAP
RAP
QLung
Pressure [mmHg]
Blood flow [mL/min]
Sample [100Hz]
B
PAP
RAP
Curve fit
RV Isovolumetric
PISOMax (a + 2*b)
EDP (a)
Amplitude (b)
Pressure [mmHg]
Sample
C
PISOMax
Ees
Ea
Pressure [mmHg]
Volume [mL]

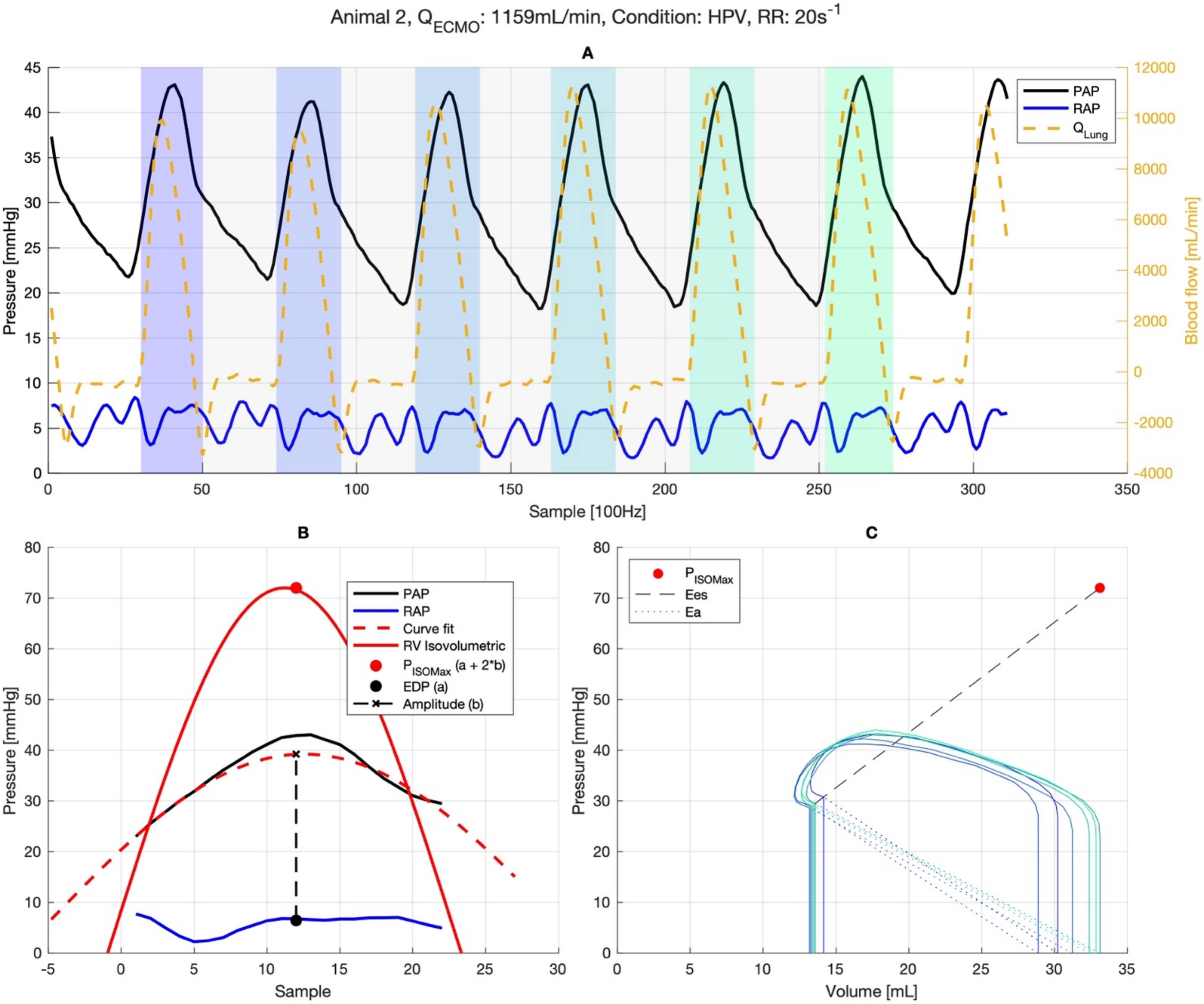

Animal 2, Q_ECMO: 1159mL/min, Condition: HPV, RR: 20s^-1
A
PAP
RAP
Q_Lung
Pressure [mmHg]
Blood flow [mL/min]
Sample [100Hz]
B
PAP
RAP
Curve fit
RV Isovolumetric
P_ISOMax (a + 2*b)
EDP (a)
Amplitude (b)
Pressure [mmHg]
Sample
C
P_ISOMax
Ees
Ea
Pressure [mmHg]
Volume [mL]

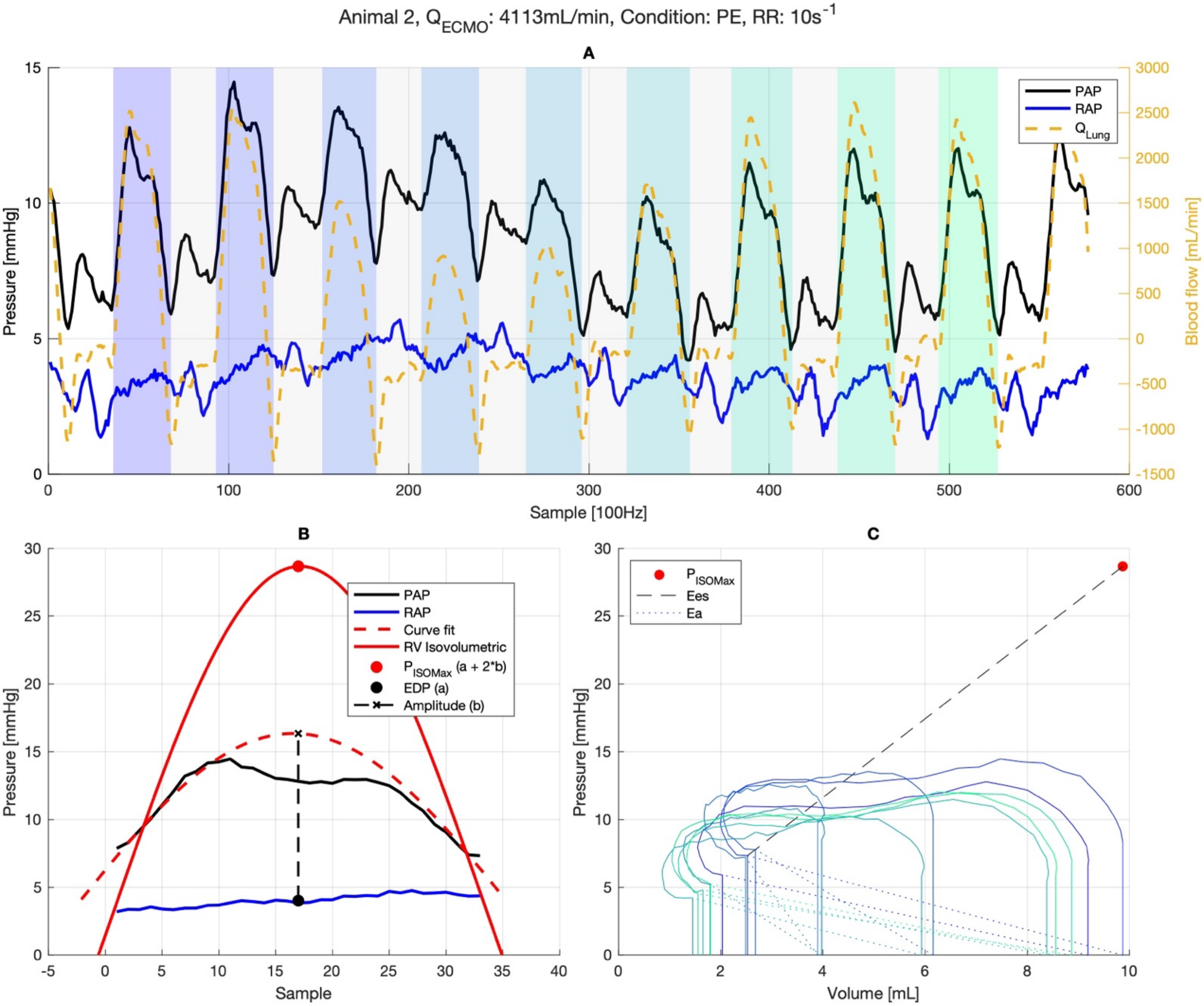

Animal 2, Q_ECMO: 4113mL/min, Condition: PE, RR: 10s^-1
A
PAP
RAP
Q_Lung
Pressure [mmHg]
Blood flow [mL/min]
Sample [100Hz]
B
PAP
RAP
Curve fit
RV Isovolumetric
P_ISOMax (a + 2*b)
EDP (a)
Amplitude (b)
Pressure [mmHg]
Sample
C
P_ISOMax
Ees
Ea
Pressure [mmHg]
Volume [mL]

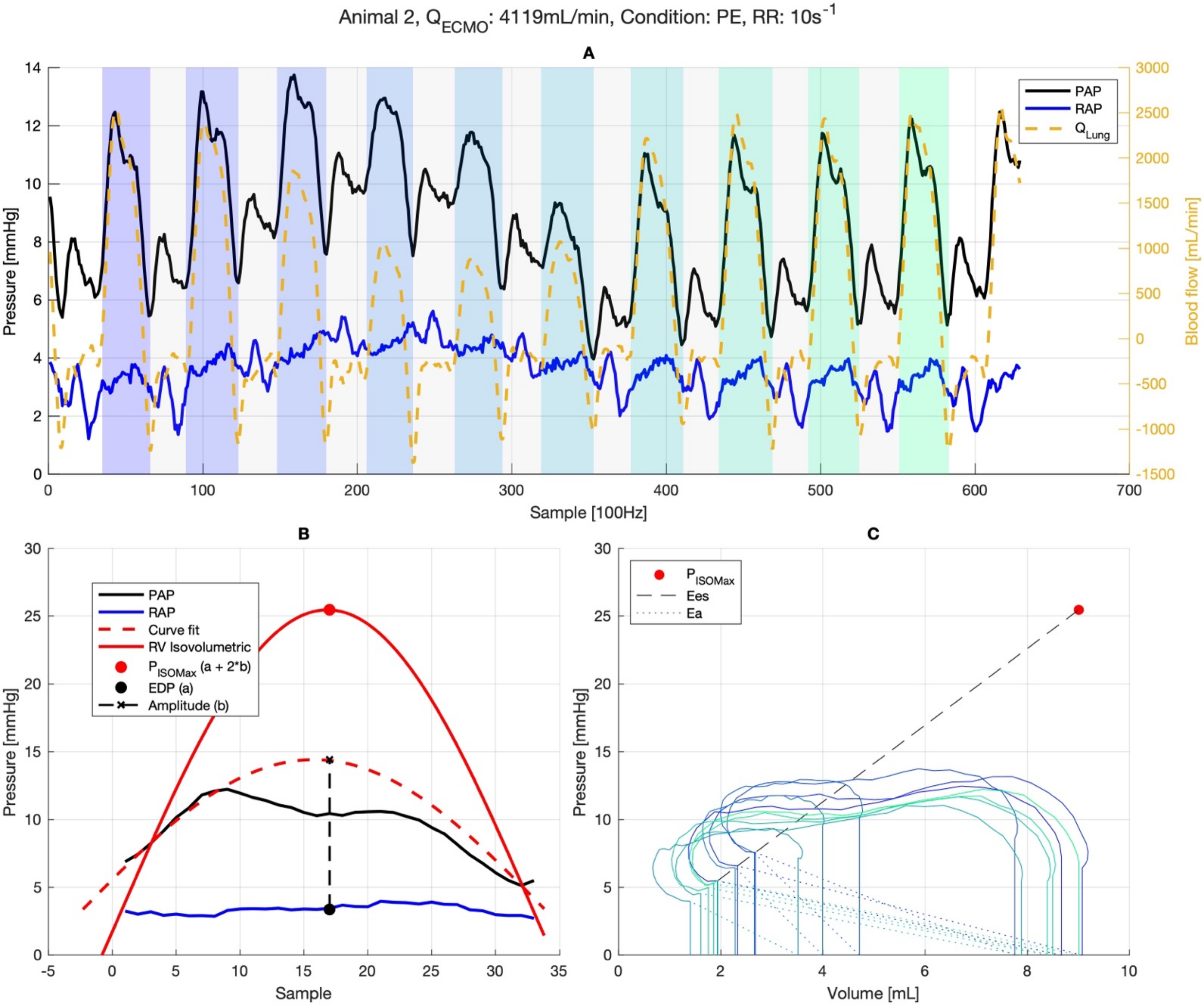

Animal 2, Q_ECMO: 4119mL/min, Condition: PE, RR: 10s^-1
A
Pressure [mmHg]
Blood flow [mL/min]
Sample [100Hz]
PAP
RAP
Q_Lung
B
Pressure [mmHg]
Sample
PAP
RAP
Curve fit
RV Isovolumetric
P_ISOMax (a + 2*b)
EDP (a)
Amplitude (b)
C
Pressure [mmHg]
Volume [mL]
P_ISOMax
Ees
Ea

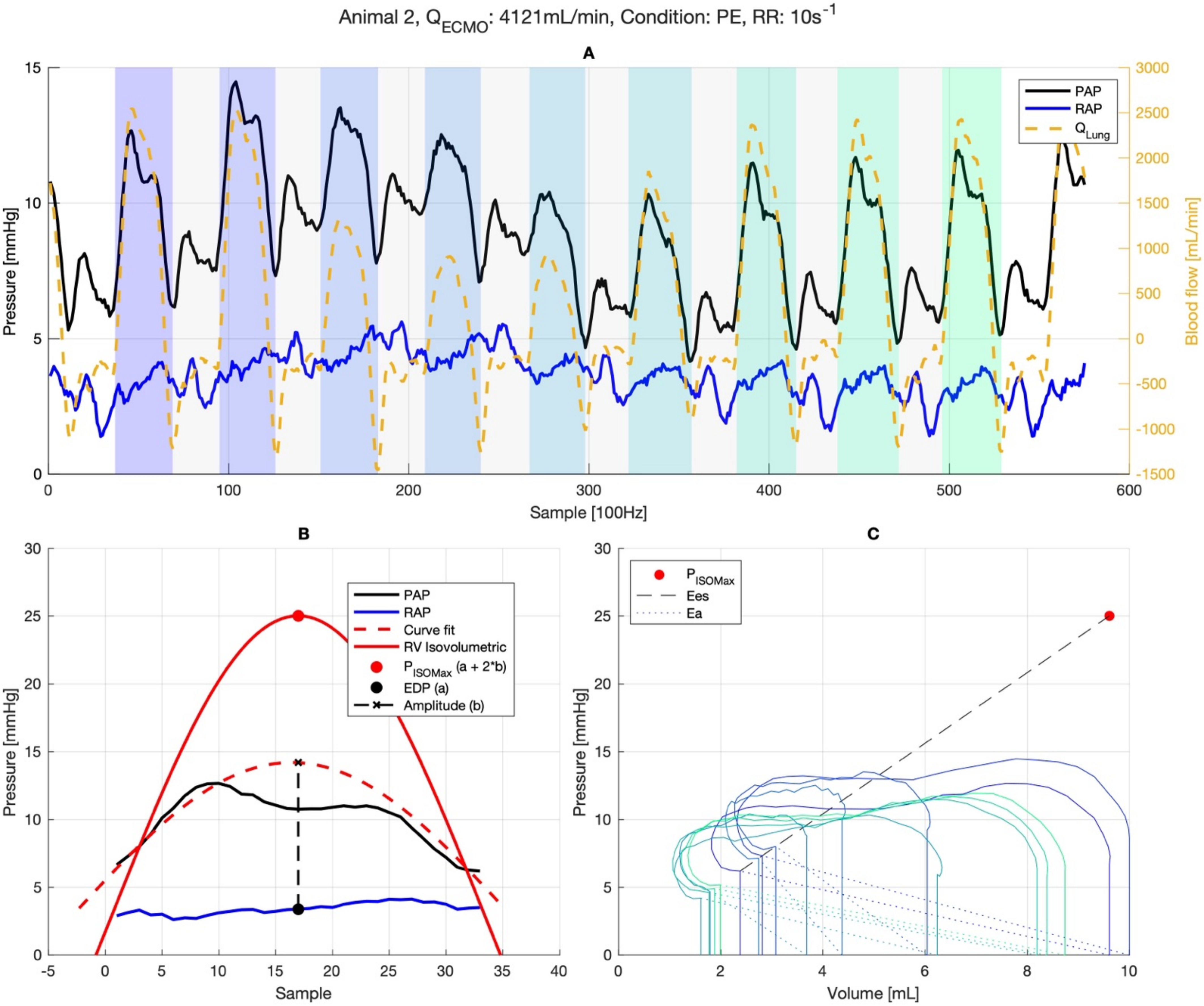

Animal 2, Q_{ECMO}: 4121mL/min, Condition: PE, RR: $10s^{-1}$
A
PAP
RAP
Q_{Lung}
Pressure [mmHg]
Blood flow [mL/min]
Sample [100Hz]
B
PAP
RAP
Curve fit
RV Isovolumetric
P_{ISOMax} (a + 2*b)
EDP (a)
Amplitude (b)
Pressure [mmHg]
Sample
C
P_{ISOMax}
Ees
Ea
Pressure [mmHg]
Volume [mL]

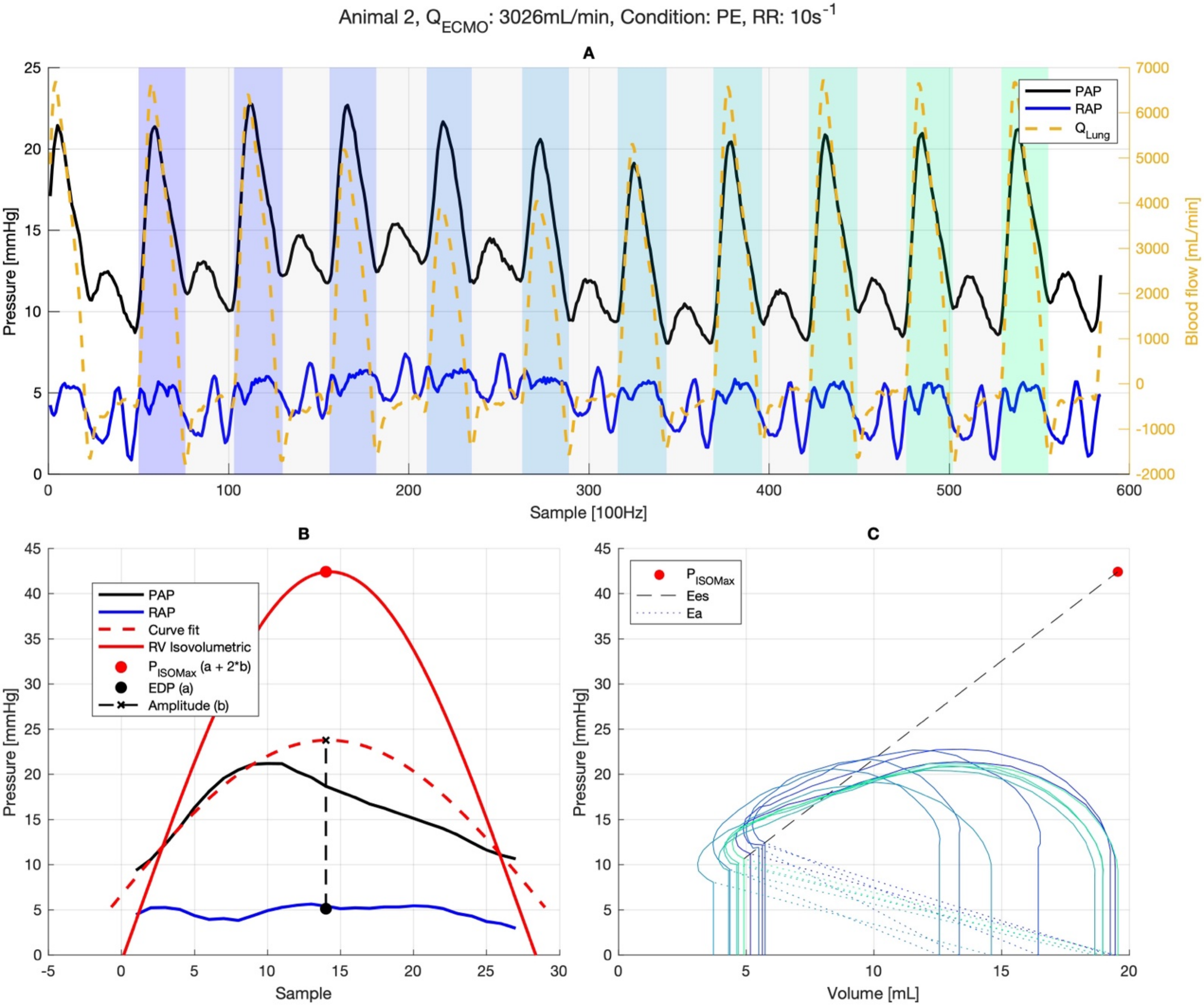

Animal 2, Q_{ECMO}: 3026mL/min, Condition: PE, RR: 10s^{-1}
A
PAP
RAP
Q_{Lung}
Pressure [mmHg]
Blood flow [mL/min]
Sample [100Hz]
B
PAP
RAP
Curve fit
RV Isovolumetric
P_{ISOMax} (a + 2*b)
EDP (a)
Amplitude (b)
Pressure [mmHg]
Sample
C
P_{ISOMax}
Ees
Ea
Pressure [mmHg]
Volume [mL]

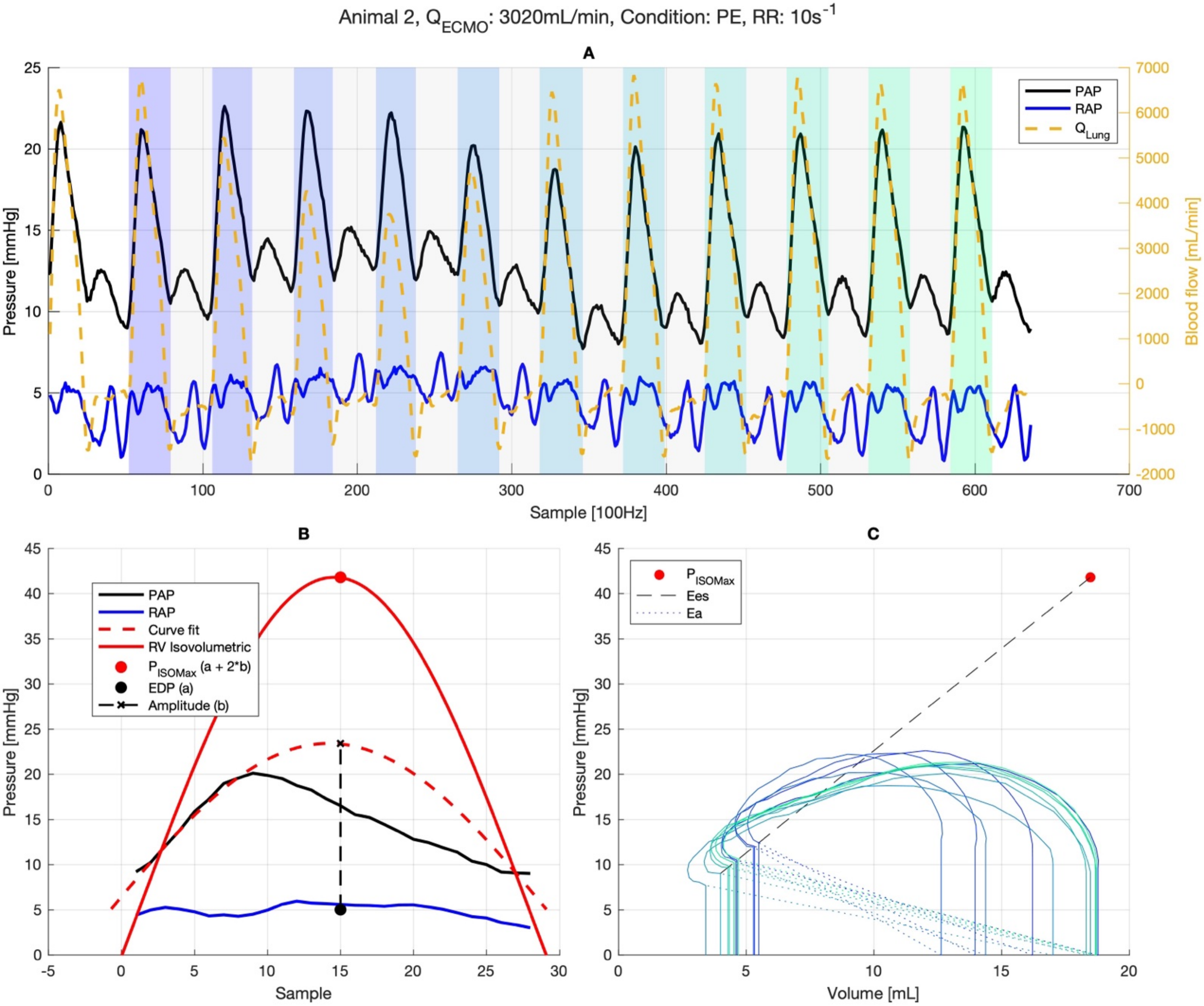

Animal 2, Q_ECMO: 3020mL/min, Condition: PE, RR: 10s^-1
A
PAP
RAP
Q_Lung
Pressure [mmHg]
Blood flow [mL/min]
Sample [100Hz]
B
PAP
RAP
Curve fit
RV Isovolumetric
P_ISOMax (a + 2*b)
EDP (a)
Amplitude (b)
Pressure [mmHg]
Sample
C
P_ISOMax
Ees
Ea
Pressure [mmHg]
Volume [mL]

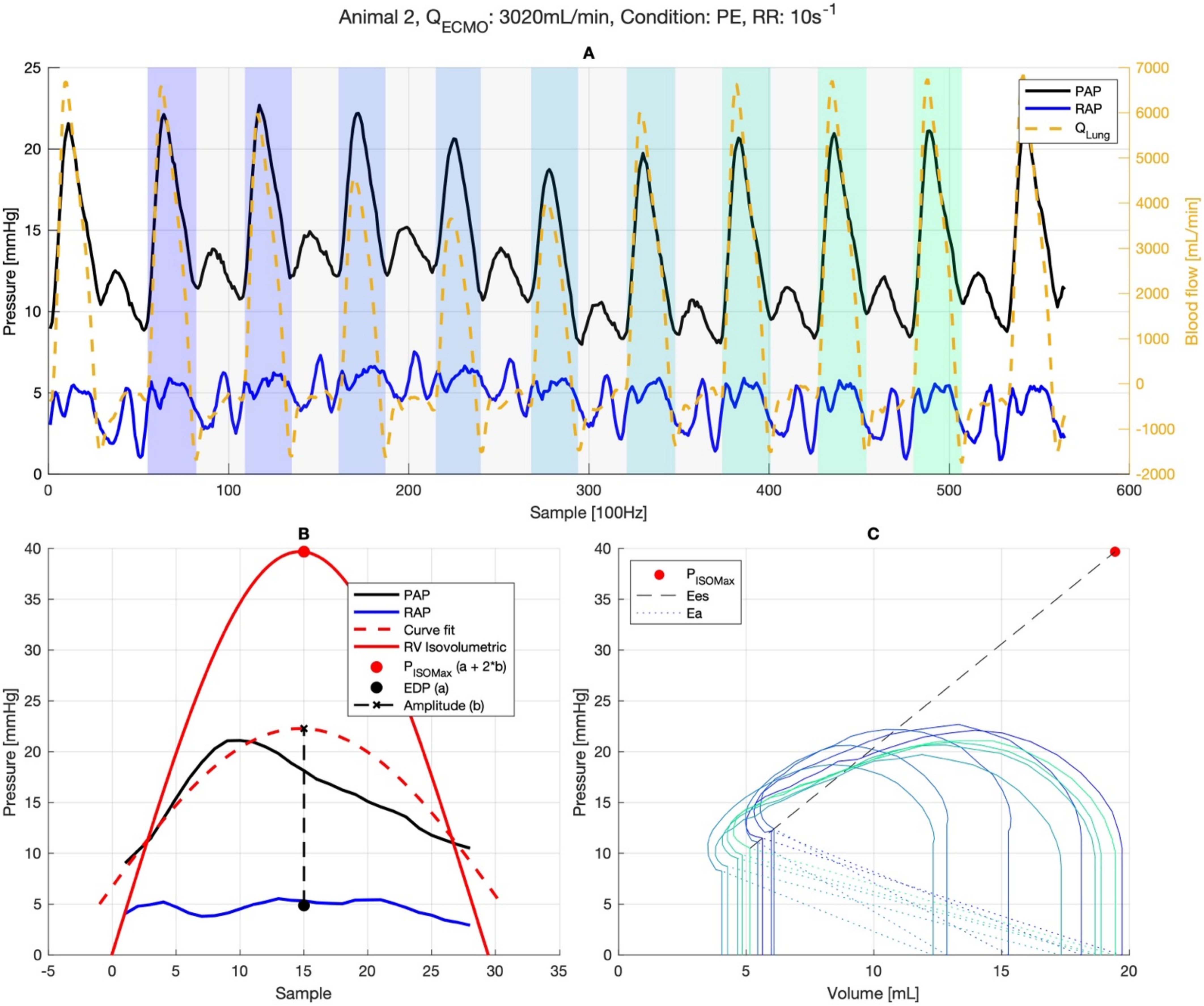

Animal 2, Q_ECMO: 3020mL/min, Condition: PE, RR: 10s^-1
A
Pressure [mmHg]
Blood flow [mL/min]
Sample [100Hz]
PAP
RAP
Q_Lung
B
Pressure [mmHg]
Sample
PAP
RAP
Curve fit
RV Isovolumetric
P_ISOMax (a + 2*b)
EDP (a)
Amplitude (b)
C
Pressure [mmHg]
Volume [mL]
P_ISOMax
Ees
Ea

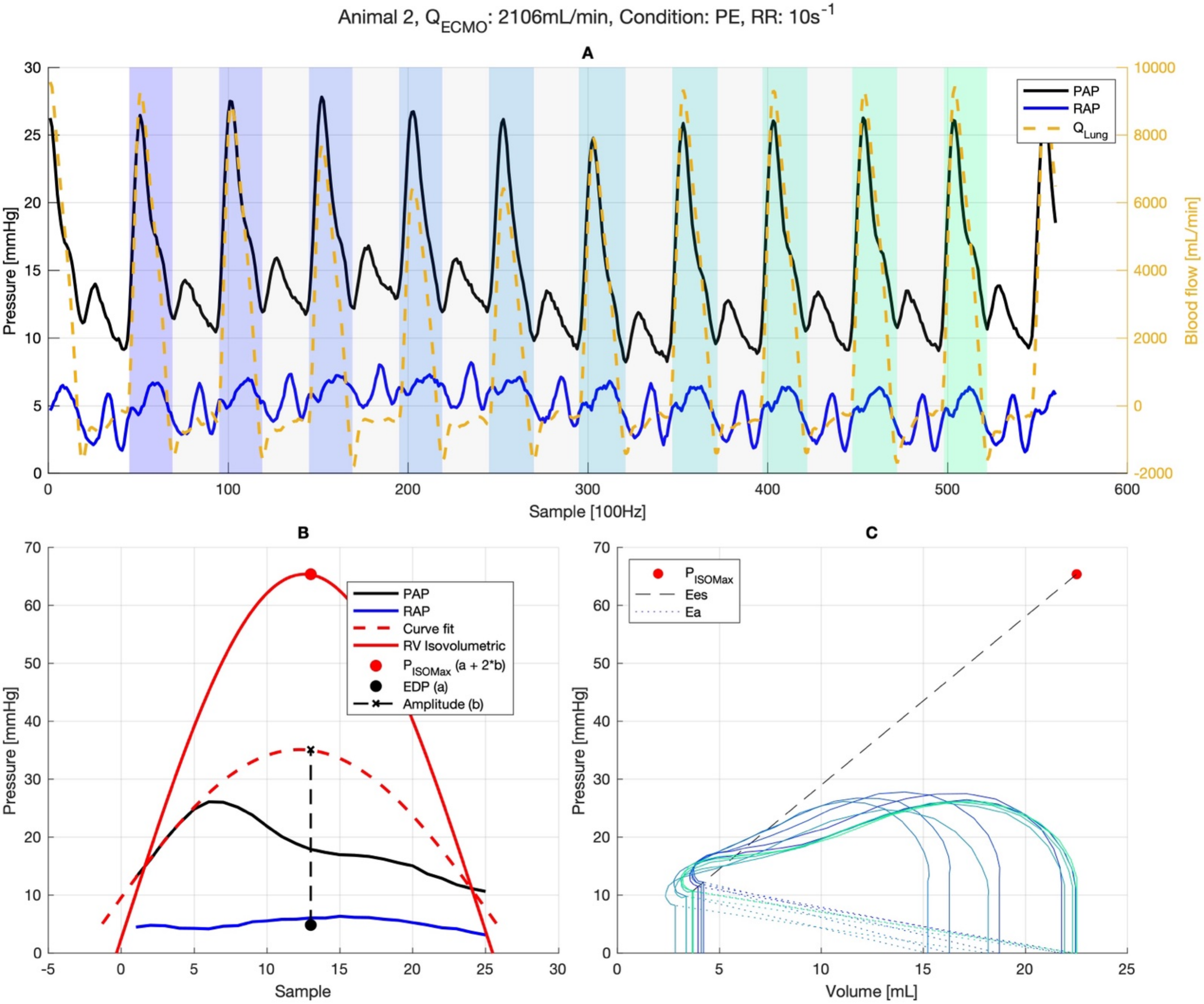

Animal 2, Q_{ECMO}: 2106mL/min, Condition: PE, RR: $10s^{-1}$
A
Pressure [mmHg]
Blood flow [mL/min]
Sample [100Hz]
PAP
RAP
Q_{Lung}
B
Pressure [mmHg]
Sample
PAP
RAP
Curve fit
RV Isovolumetric
P_{ISOMax} (a + 2*b)
EDP (a)
Amplitude (b)
C
Pressure [mmHg]
Volume [mL]
P_{ISOMax}
Ees
Ea

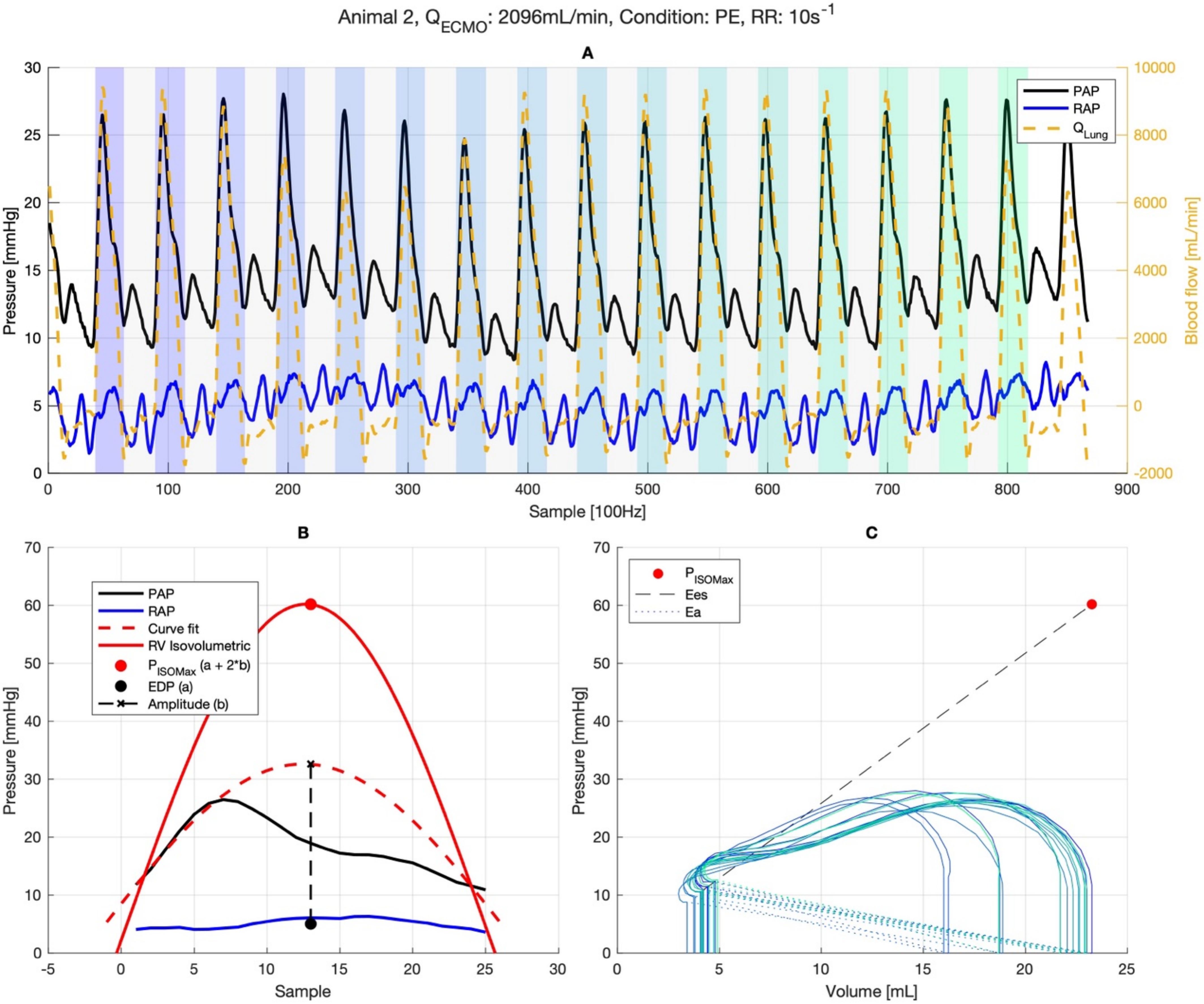

Animal 2, Q_ECMO: 2096mL/min, Condition: PE, RR: 10s^-1
A
Pressure [mmHg]
Blood flow [mL/min]
Sample [100Hz]
PAP
RAP
Q_Lung
B
PAP
RAP
Curve fit
RV Isovolumetric
P_ISOMax (a + 2*b)
EDP (a)
Amplitude (b)
Pressure [mmHg]
Sample
C
P_ISOMax
Ees
Ea
Pressure [mmHg]
Volume [mL]

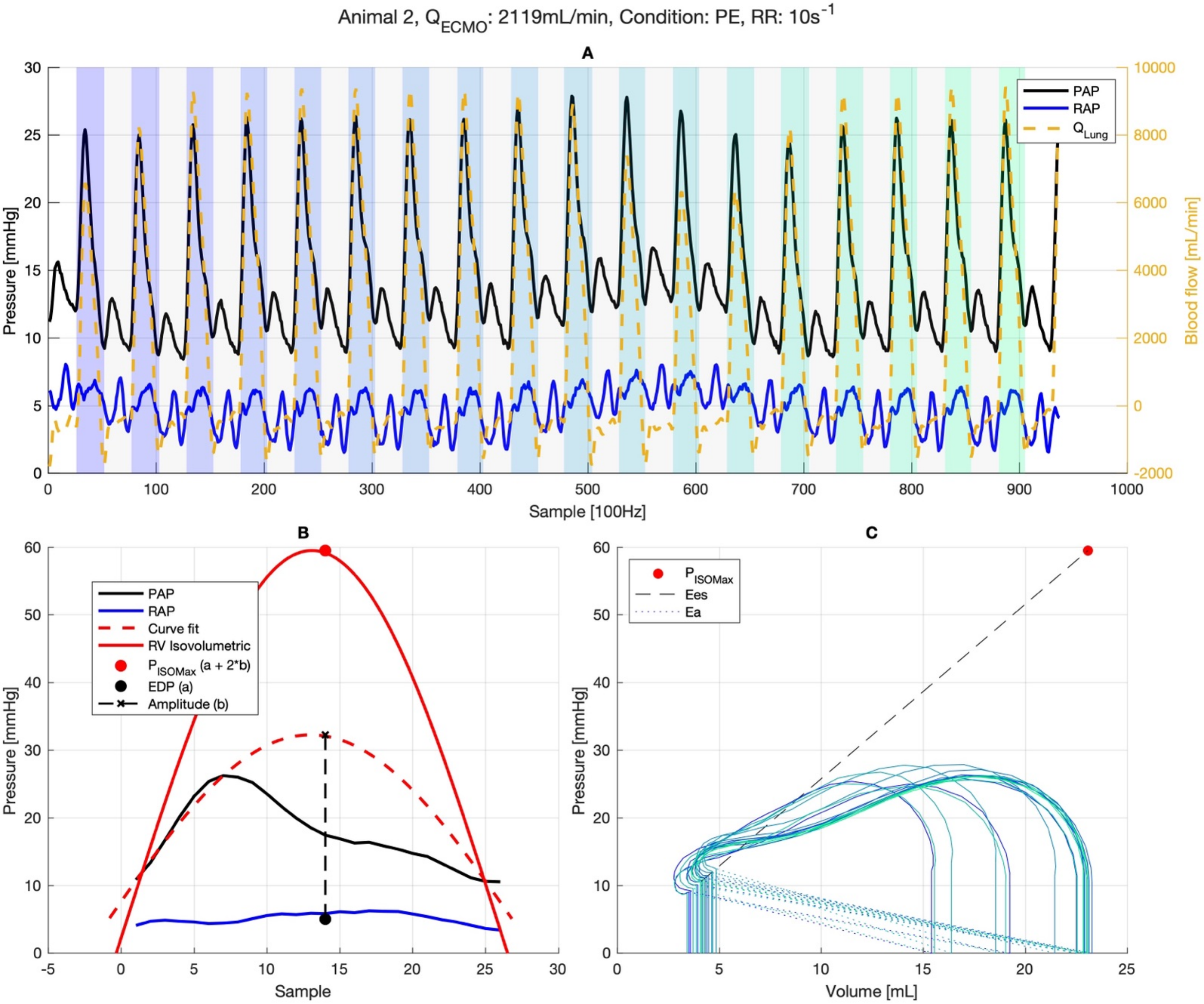

Animal 2, Q_{ECMO}: 2119mL/min, Condition: PE, RR: $10s^{-1}$
A
PAP
RAP
Q_{Lung}
Pressure [mmHg]
Blood flow [mL/min]
Sample [100Hz]
B
PAP
RAP
Curve fit
RV Isovolumetric
P_{ISOMax} (a + 2*b)
EDP (a)
Amplitude (b)
Pressure [mmHg]
Sample
C
P_{ISOMax}
Ees
Ea
Pressure [mmHg]
Volume [mL]

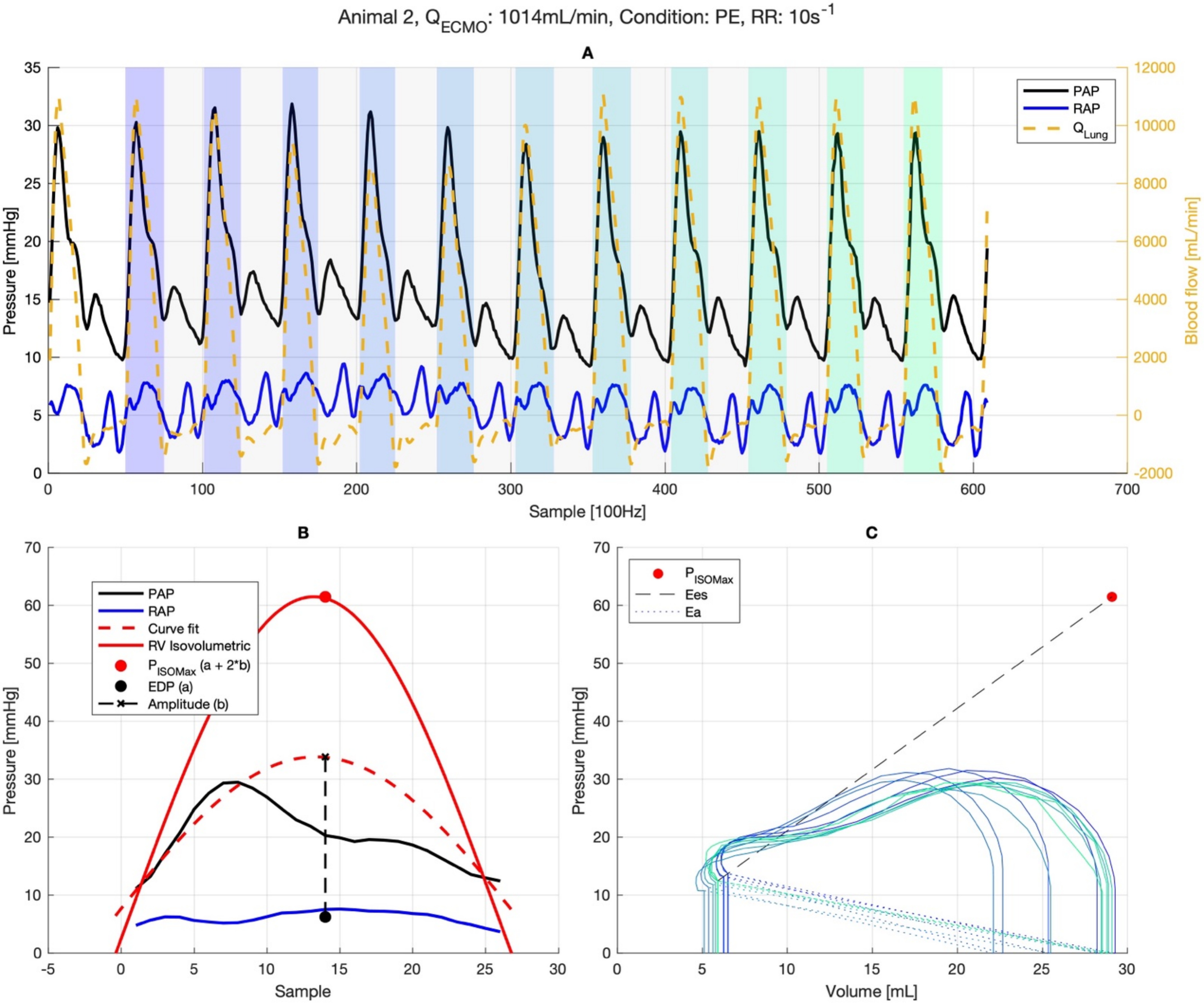

Animal 2, QECMO: 1014mL/min, Condition: PE, RR: 10s-1
A
PAP
RAP
QLung
Pressure [mmHg]
Blood flow [mL/min]
Sample [100Hz]
B
PAP
RAP
Curve fit
RV Isovolumetric
PISOMax (a + 2*b)
EDP (a)
Amplitude (b)
Pressure [mmHg]
Sample
C
PISOMax
Ees
Ea
Pressure [mmHg]
Volume [mL]

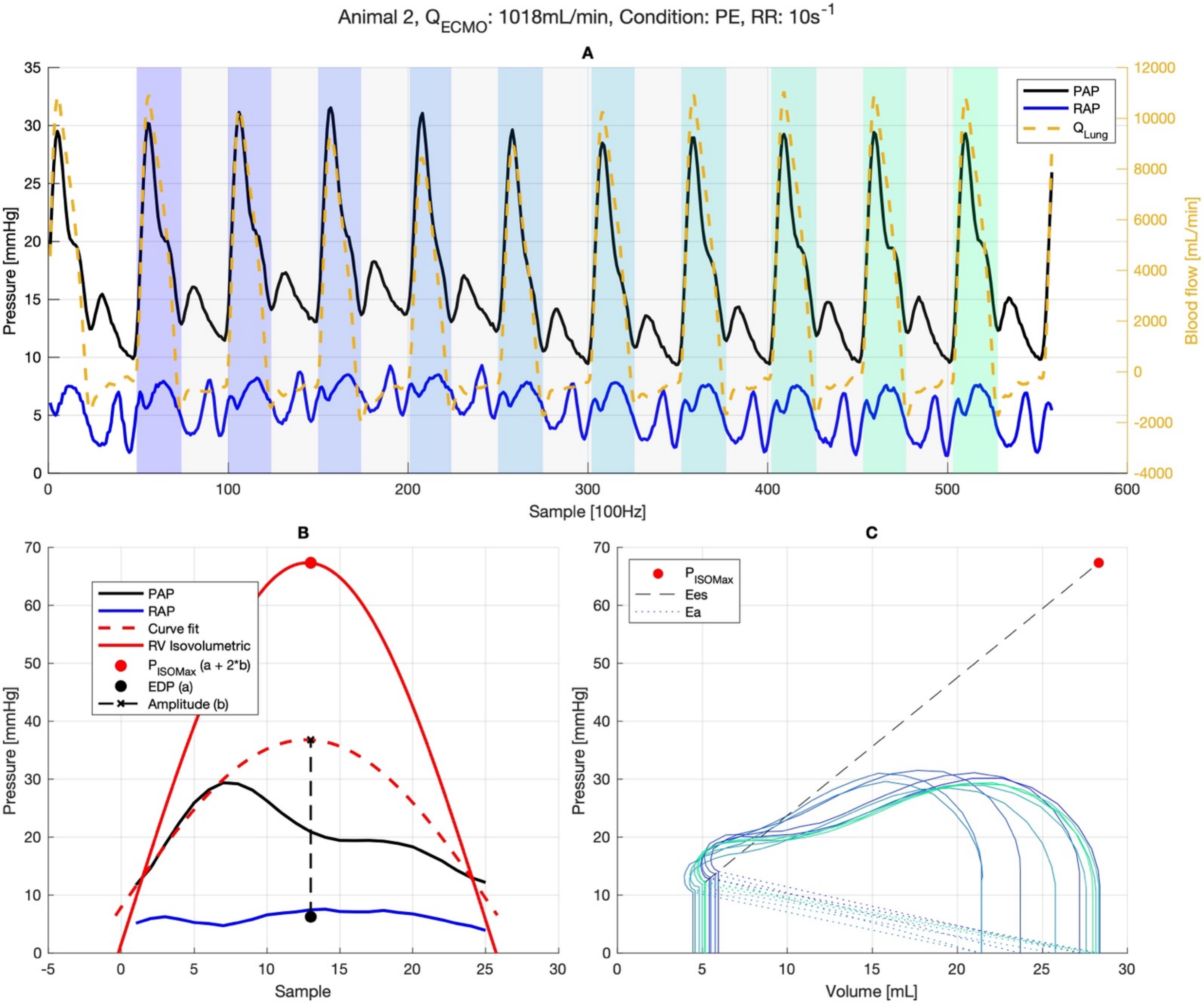

Animal 2, Q_ECMO: 1018mL/min, Condition: PE, RR: 10s^-1
A
Pressure [mmHg]
Blood flow [mL/min]
Sample [100Hz]
PAP
RAP
Q_Lung
B
Pressure [mmHg]
Sample
Curve fit
RV Isovolumetric
P_ISOMax (a + 2*b)
EDP (a)
Amplitude (b)
C
Pressure [mmHg]
Volume [mL]
P_ISOMax
Ees
Ea

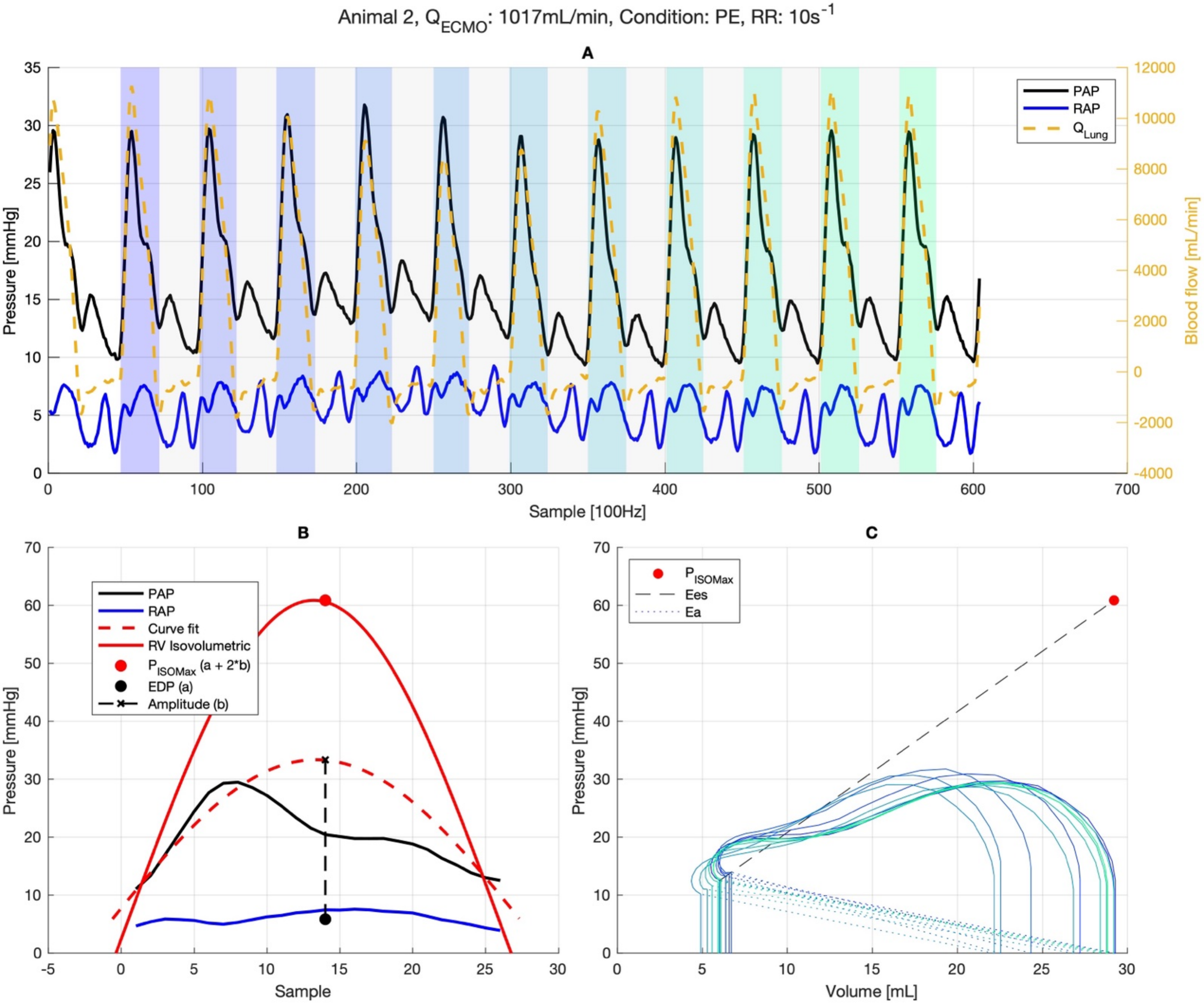

Animal 2, Q_{ECMO}: 1017mL/min, Condition: PE, RR: $10s^{-1}$
A
Pressure [mmHg]
Blood flow [mL/min]
Sample [100Hz]
PAP
RAP
Q_{Lung}
B
PAP
RAP
Curve fit
RV Isovolumetric
P_{ISOMax} (a + 2*b)
EDP (a)
Amplitude (b)
Pressure [mmHg]
Sample
C
P_{ISOMax}
Ees
Ea
Pressure [mmHg]
Volume [mL]

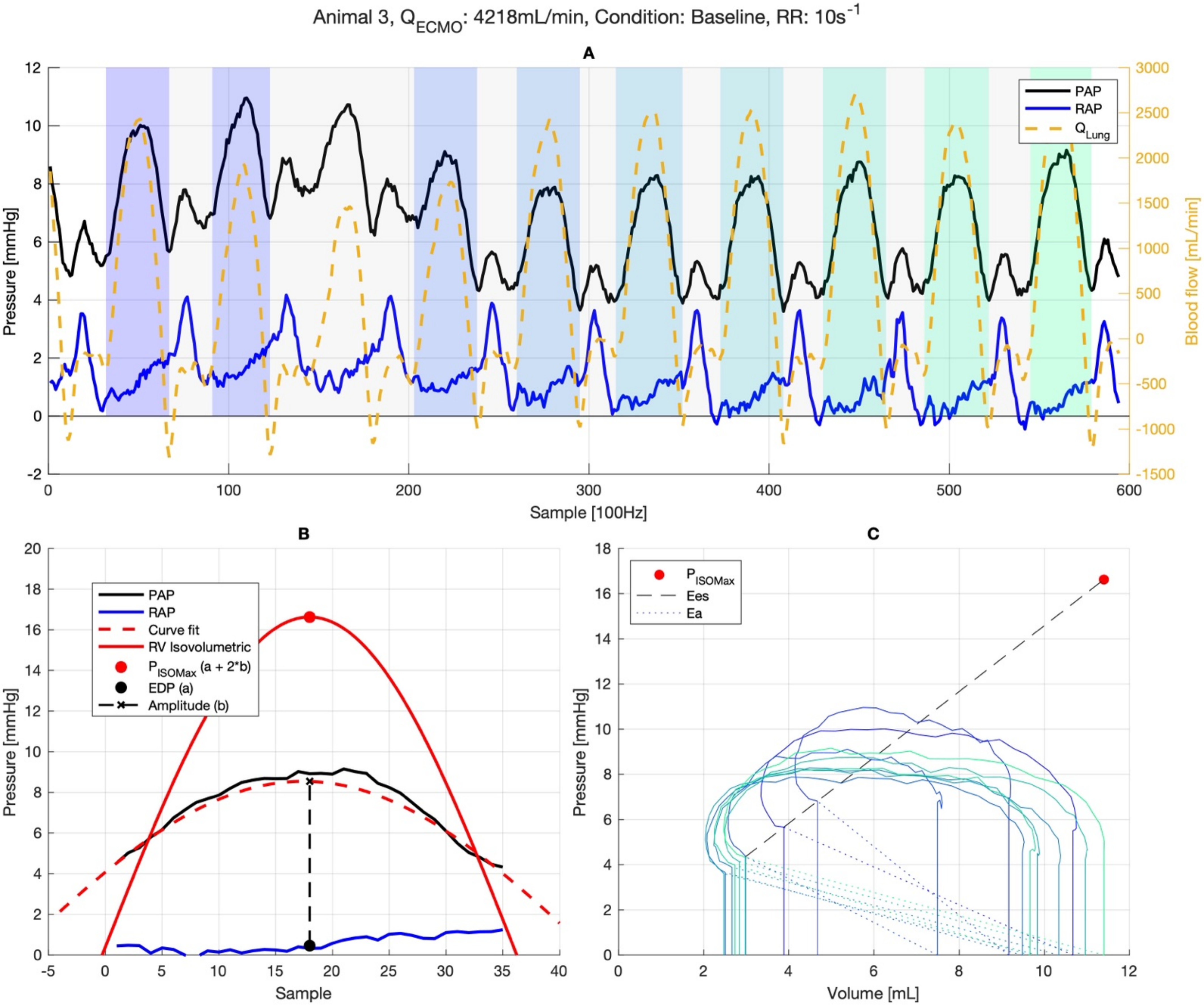

Animal 3, Q_ECMO: 4218mL/min, Condition: Baseline, RR: 10s^-1
A
PAP
RAP
Q_Lung
Pressure [mmHg]
Blood flow [mL/min]
Sample [100Hz]
B
PAP
RAP
Curve fit
RV Isovolumetric
P_ISOMax (a + 2*b)
EDP (a)
Amplitude (b)
Pressure [mmHg]
Sample
C
P_ISOMax
Ees
Ea
Pressure [mmHg]
Volume [mL]

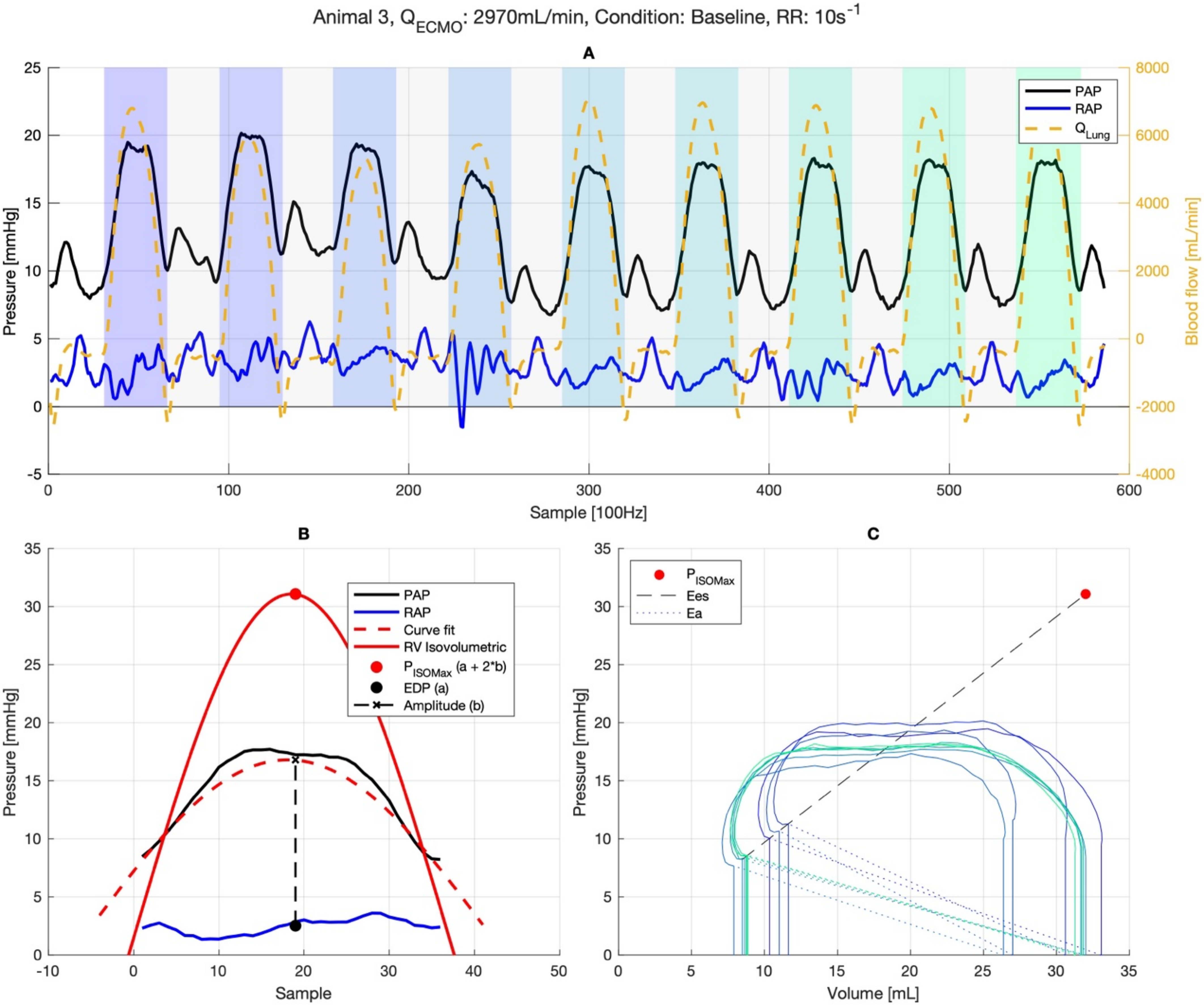

Animal 3, Q_{ECMO}: 2970mL/min, Condition: Baseline, RR: $10s^{-1}$
A
PAP
RAP
Q_{Lung}
Pressure [mmHg]
Blood flow [mL/min]
Sample [100Hz]
B
PAP
RAP
Curve fit
RV Isovolumetric
P_{ISOMax} (a + 2*b)
EDP (a)
Amplitude (b)
Pressure [mmHg]
Sample
C
P_{ISOMax}
Ees
Ea
Pressure [mmHg]
Volume [mL]

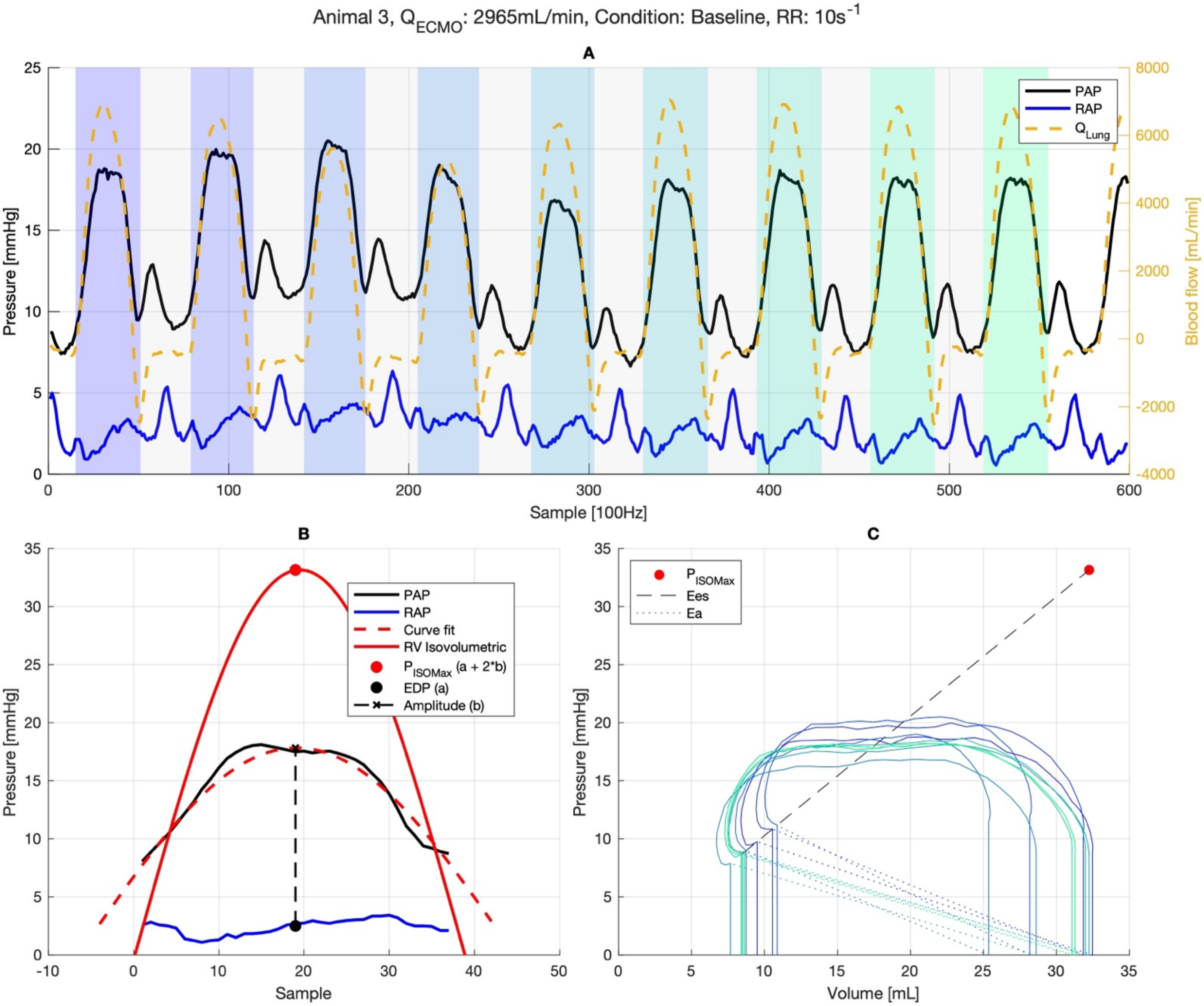

Animal 3, Q_{ECMO}: 2965mL/min, Condition: Baseline, RR: $10s^{-1}$
A
PAP
RAP
Q_{Lung}
Pressure [mmHg]
Blood flow [mL/min]
Sample [100Hz]
B
PAP
RAP
Curve fit
RV Isovolumetric
P_{ISOMax} (a + 2*b)
EDP (a)
Amplitude (b)
Pressure [mmHg]
Sample
C
P_{ISOMax}
Ees
Ea
Pressure [mmHg]
Volume [mL]

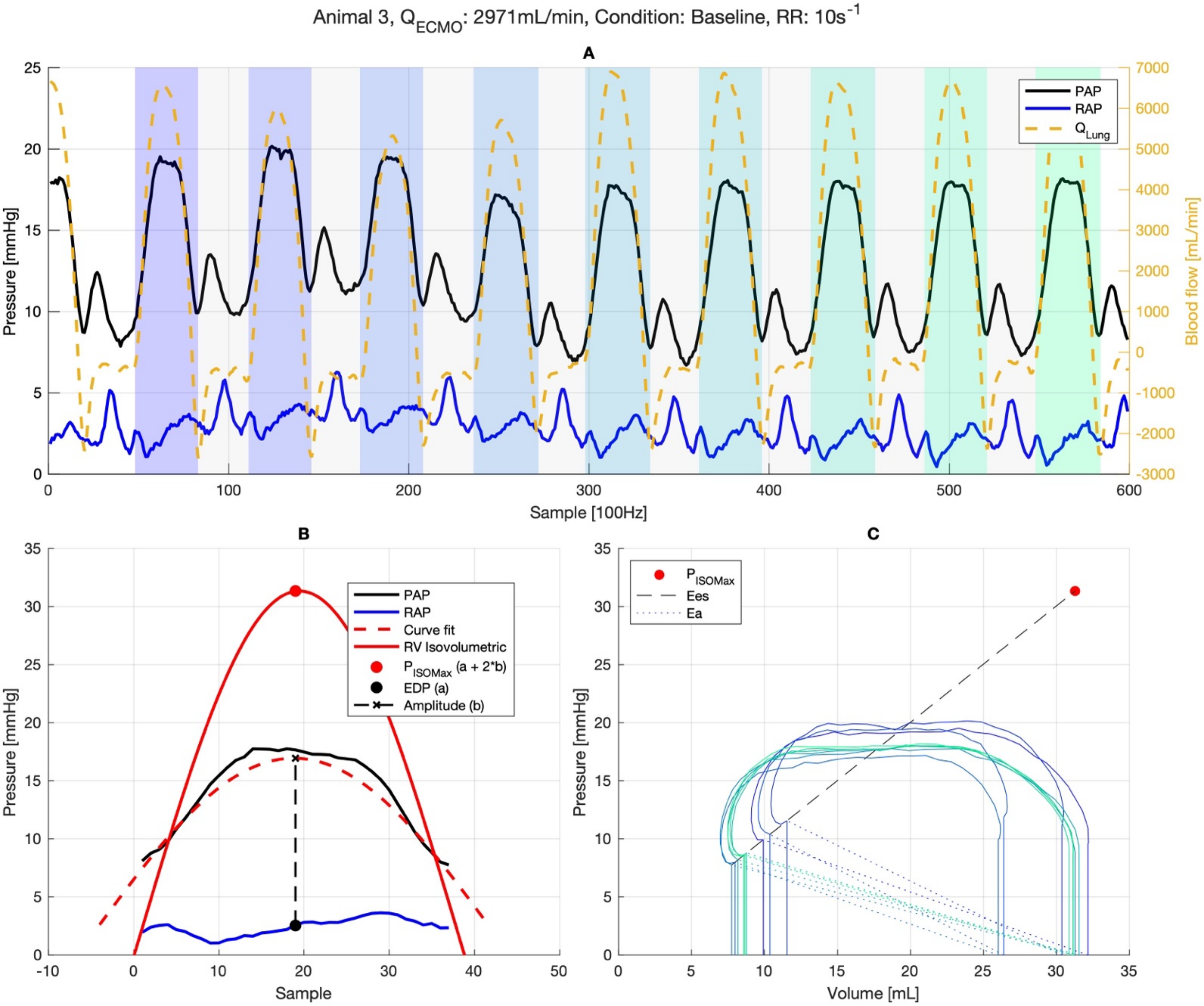
Animal 3, Q_{ECMO}: 2971mL/min, Condition: Baseline, RR: $10s^{-1}$
A
PAP
RAP
Q_{Lung}
Pressure [mmHg]
Blood flow [mL/min]
Sample [100Hz]
B
PAP
RAP
Curve fit
RV Isovolumetric
P_{ISOMax} (a + 2*b)
EDP (a)
Amplitude (b)
Pressure [mmHg]
Sample
C
P_{ISOMax}
Ees
Ea
Pressure [mmHg]
Volume [mL]

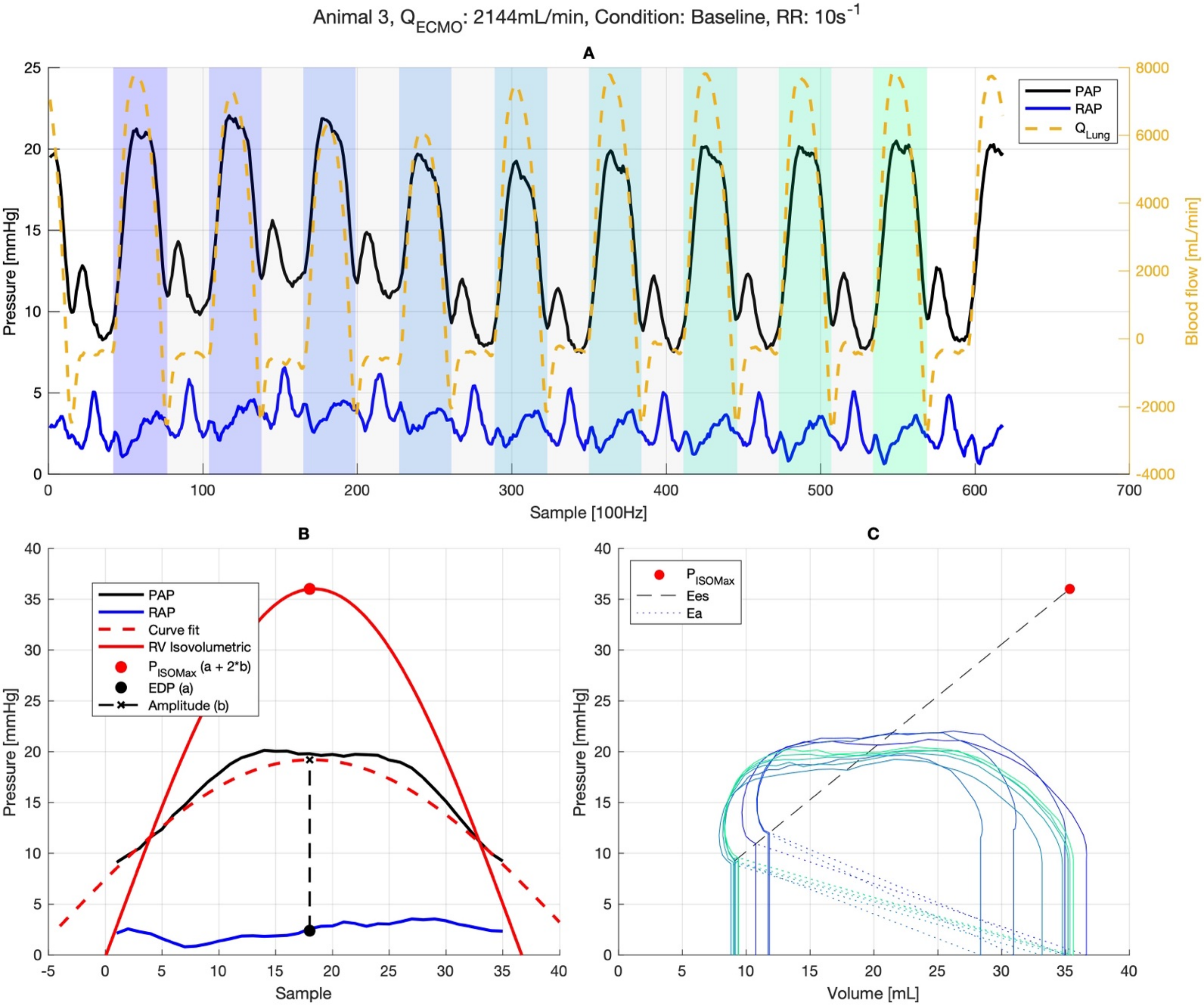

Animal 3, Q_{ECMO}: 2144mL/min, Condition: Baseline, RR: $10s^{-1}$
A
PAP
RAP
Q_{Lung}
Pressure [mmHg]
Blood flow [mL/min]
Sample [100Hz]
B
PAP
RAP
Curve fit
RV Isovolumetric
P_{ISOMax} (a + 2*b)
EDP (a)
Amplitude (b)
Pressure [mmHg]
Sample
C
P_{ISOMax}
Ees
Ea
Pressure [mmHg]
Volume [mL]

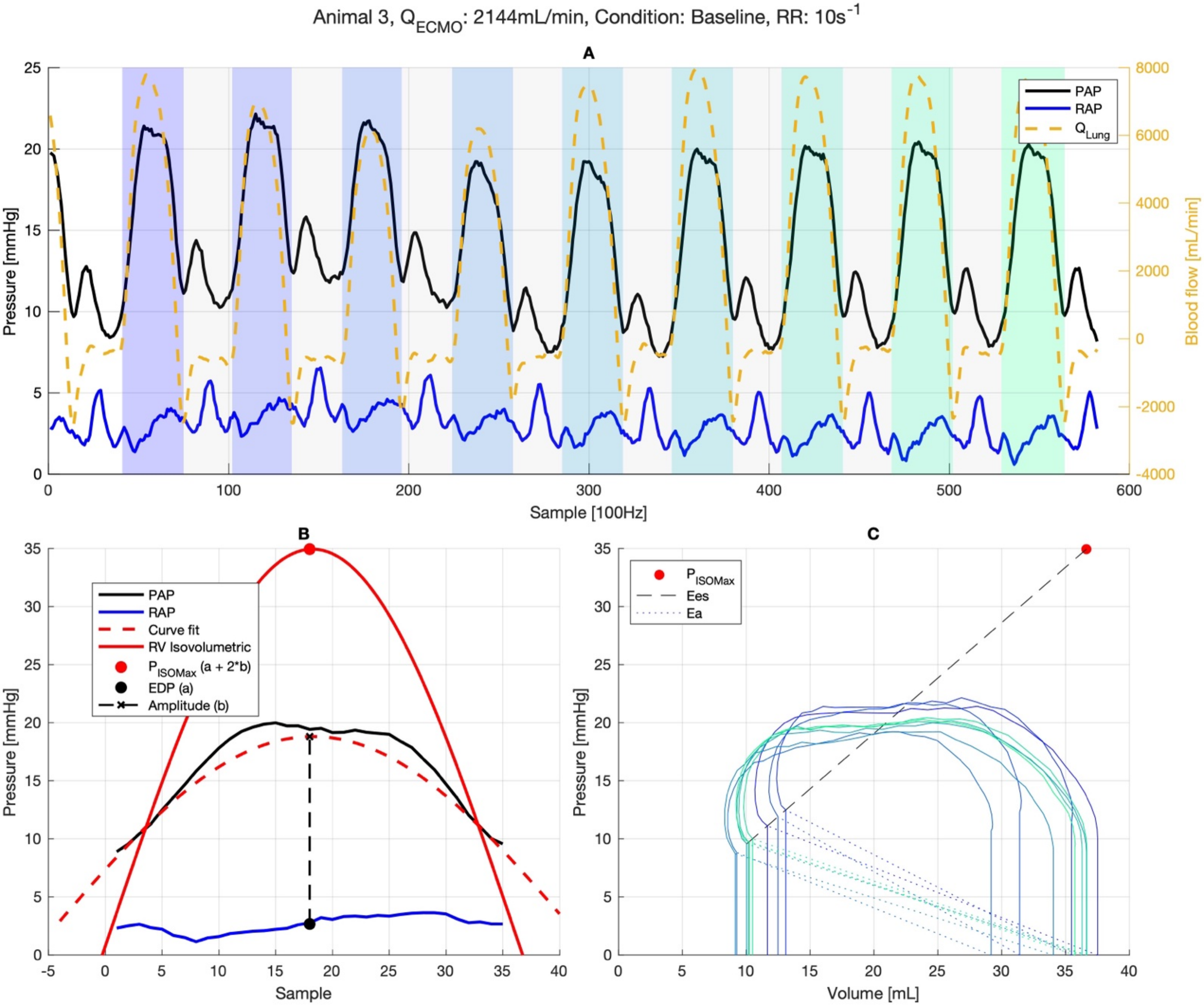

Animal 3, Q_{ECMO}: 2144mL/min, Condition: Baseline, RR: $10s^{-1}$
A
PAP
RAP
Q_{Lung}
Pressure [mmHg]
Blood flow [mL/min]
Sample [100Hz]
B
PAP
RAP
Curve fit
RV Isovolumetric
P_{ISOMax} (a + 2*b)
EDP (a)
Amplitude (b)
Pressure [mmHg]
Sample
C
P_{ISOMax}
Ees
Ea
Pressure [mmHg]
Volume [mL]

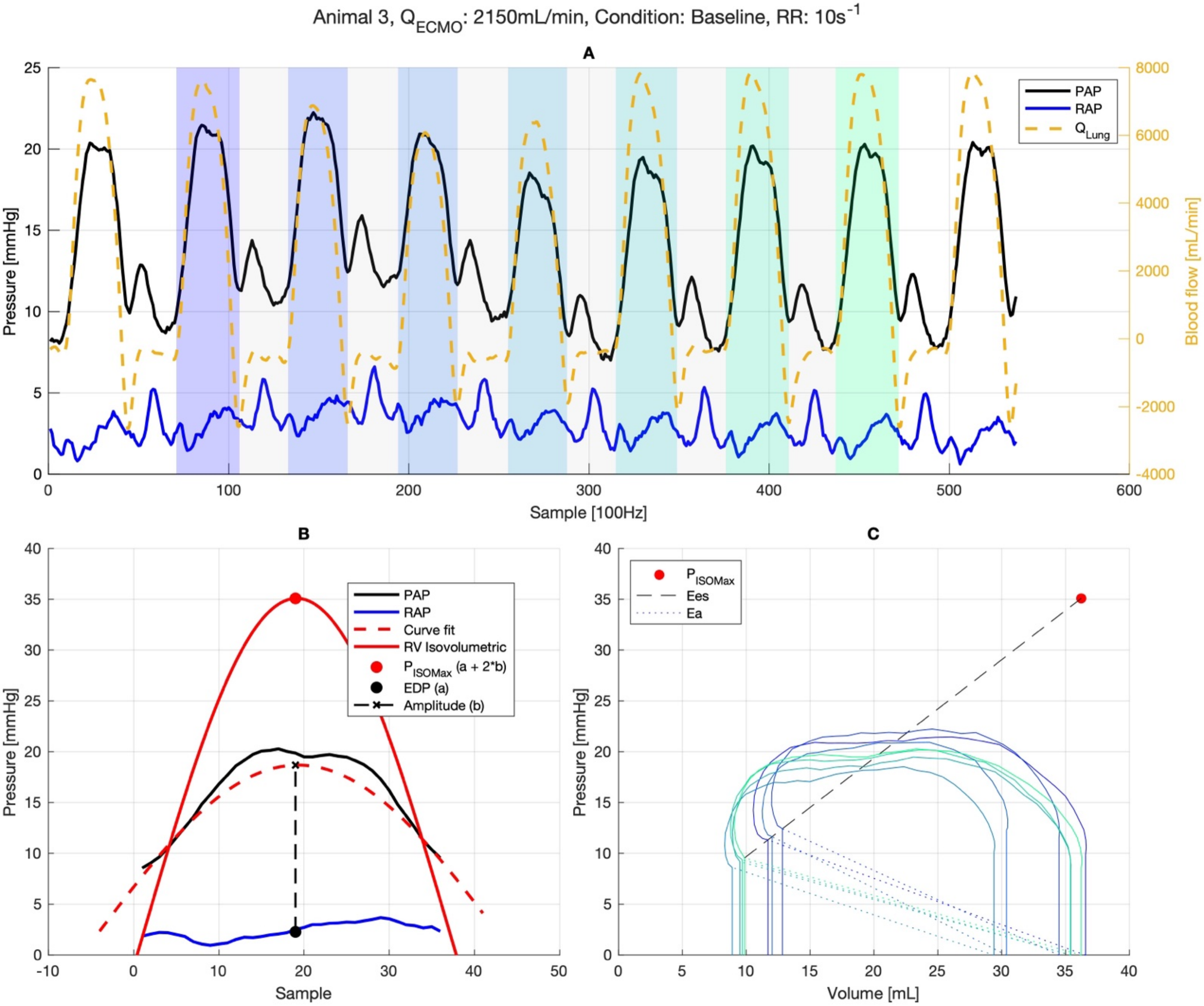
Animal 3, Q_{ECMO}: 2150mL/min, Condition: Baseline, RR: $10s^{-1}$
A
PAP
RAP
Q_{Lung}
Pressure [mmHg]
Blood flow [mL/min]
Sample [100Hz]
B
PAP
RAP
Curve fit
RV Isovolumetric
P_{ISOMax} (a + 2*b)
EDP (a)
Amplitude (b)
Pressure [mmHg]
Sample
C
P_{ISOMax}
Ees
Ea
Pressure [mmHg]
Volume [mL]

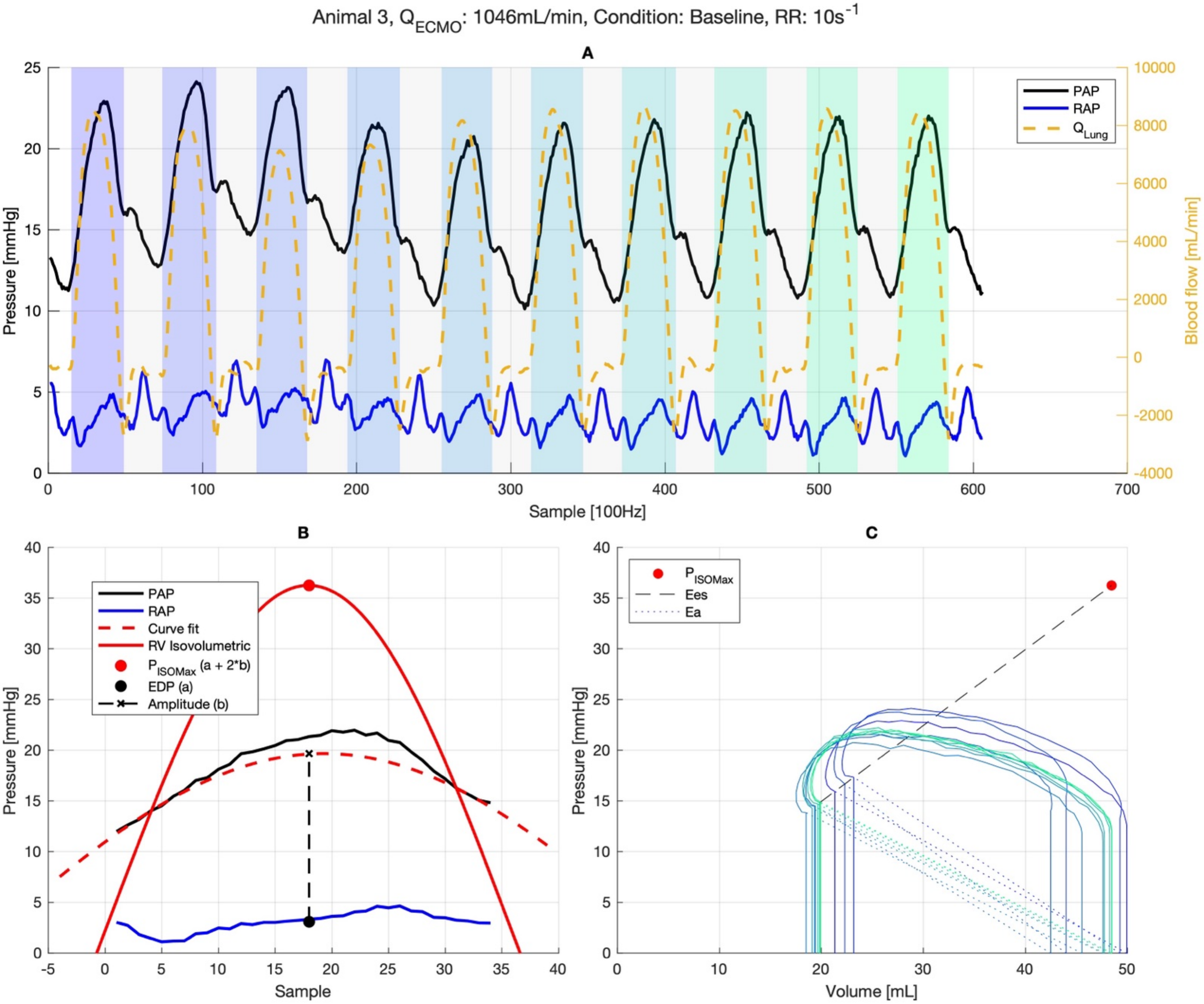

Animal 3, Q_{ECMO}: 1046mL/min, Condition: Baseline, RR: 10s^{-1}
A
Pressure [mmHg]
Blood flow [mL/min]
Sample [100Hz]
PAP
RAP
Q_{Lung}
B
Pressure [mmHg]
Sample
PAP
RAP
Curve fit
RV Isovolumetric
P_{ISOMax} (a + 2*b)
EDP (a)
Amplitude (b)
C
Pressure [mmHg]
Volume [mL]
P_{ISOMax}
Ees
Ea

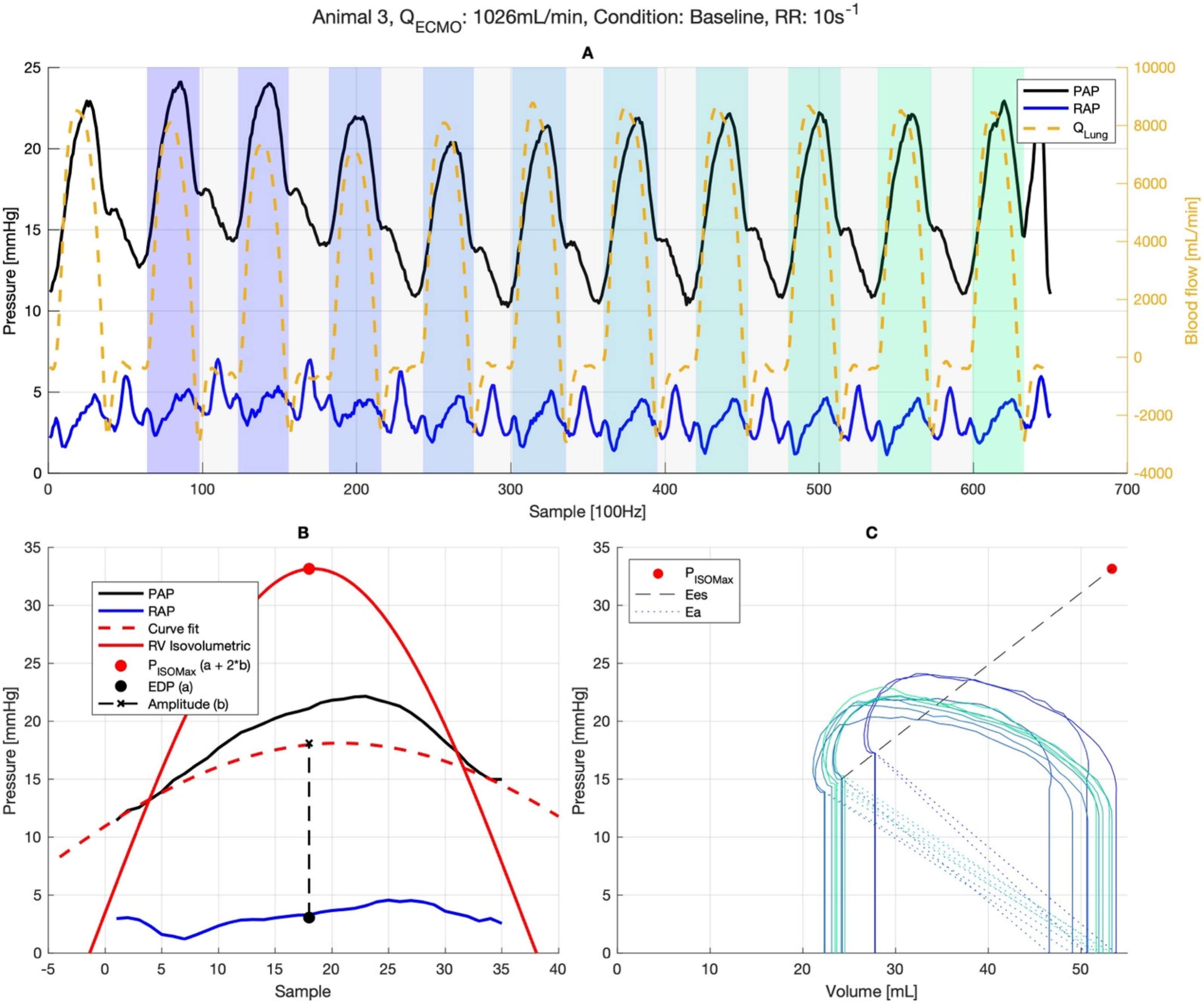

Animal 3, Q_ECMO: 1026mL/min, Condition: Baseline, RR: 10s^-1
A
PAP
RAP
Q_Lung
Pressure [mmHg]
Blood flow [mL/min]
Sample [100Hz]
B
PAP
RAP
Curve fit
RV Isovolumetric
P_ISOMax (a + 2*b)
EDP (a)
Amplitude (b)
Pressure [mmHg]
Sample
C
P_ISOMax
Ees
Ea
Pressure [mmHg]
Volume [mL]

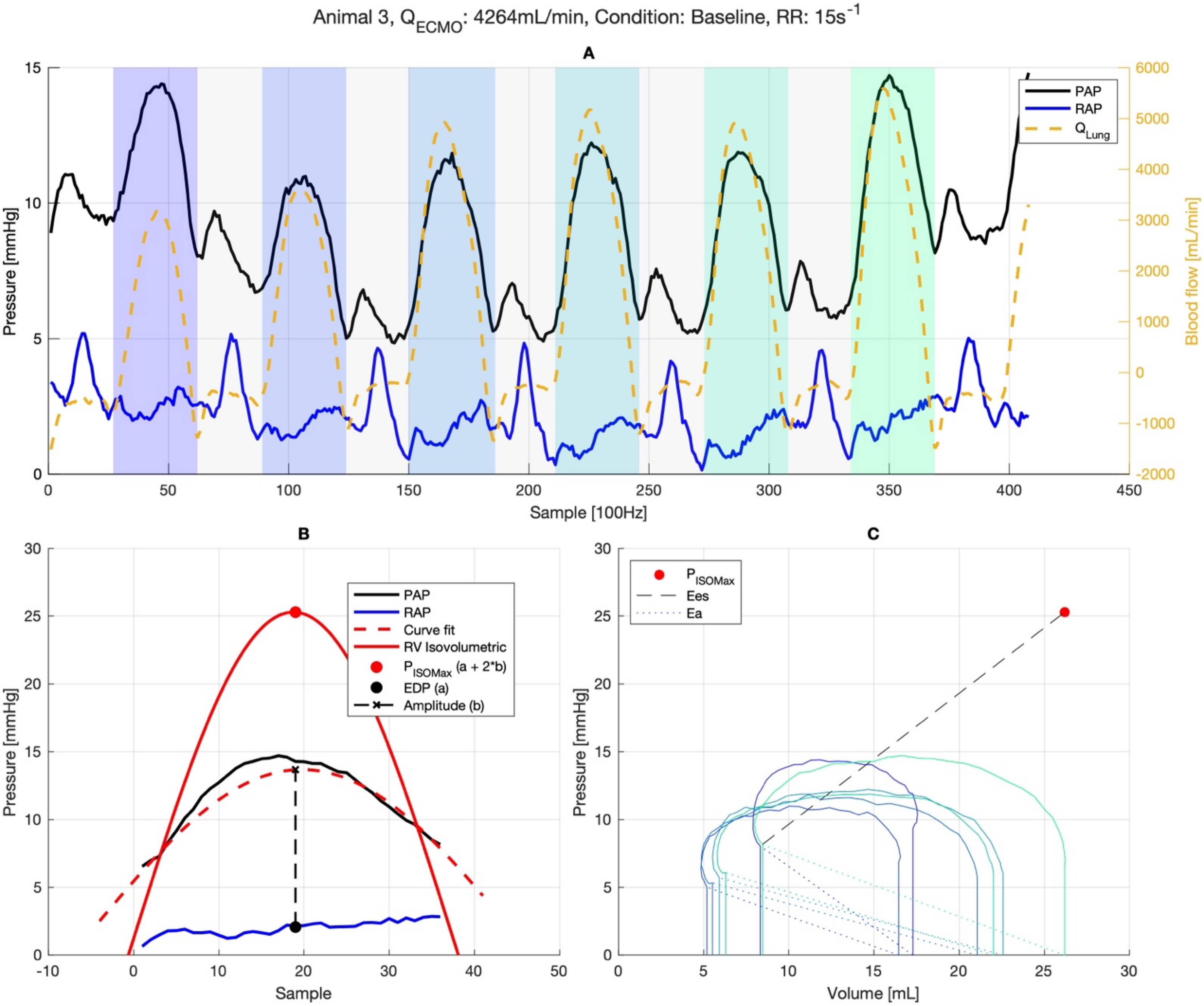

Animal 3, Q_{ECMO}: 4264mL/min, Condition: Baseline, RR: $15s^{-1}$
A
PAP
RAP
Q_{Lung}
Pressure [mmHg]
Blood flow [mL/min]
Sample [100Hz]
B
PAP
RAP
Curve fit
RV Isovolumetric
P_{ISOMax} (a + 2*b)
EDP (a)
Amplitude (b)
Pressure [mmHg]
Sample
C
P_{ISOMax}
Ees
Ea
Pressure [mmHg]
Volume [mL]

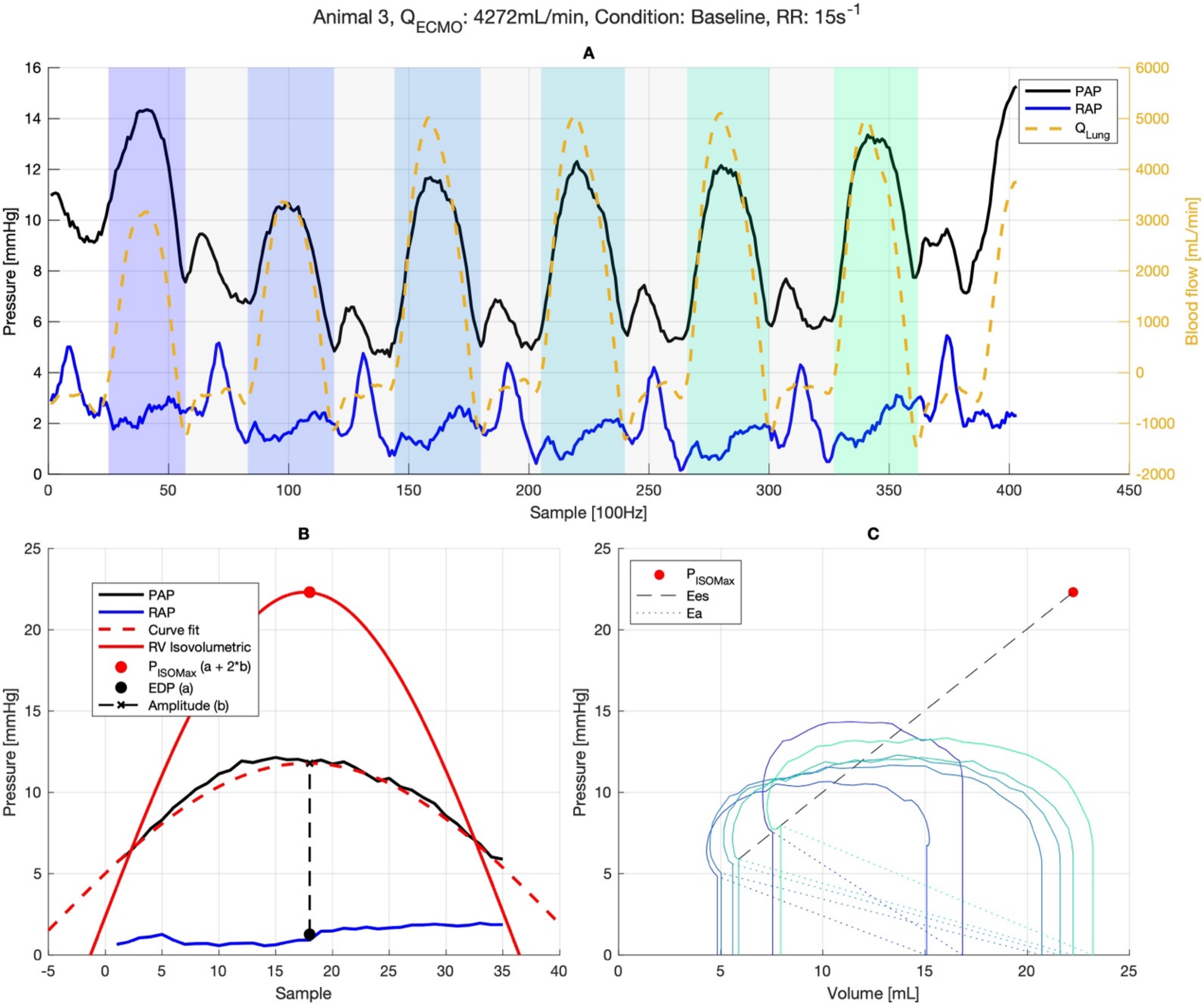

Animal 3, Q_{ECMO}: 4272mL/min, Condition: Baseline, RR: $15s^{-1}$
A
Pressure [mmHg]
Blood flow [mL/min]
Sample [100Hz]
PAP
RAP
Q_{Lung}
B
PAP
RAP
Curve fit
RV Isovolumetric
P_{ISOMax} (a + 2*b)
EDP (a)
Amplitude (b)
Pressure [mmHg]
Sample
C
P_{ISOMax}
Ees
Ea
Pressure [mmHg]
Volume [mL]

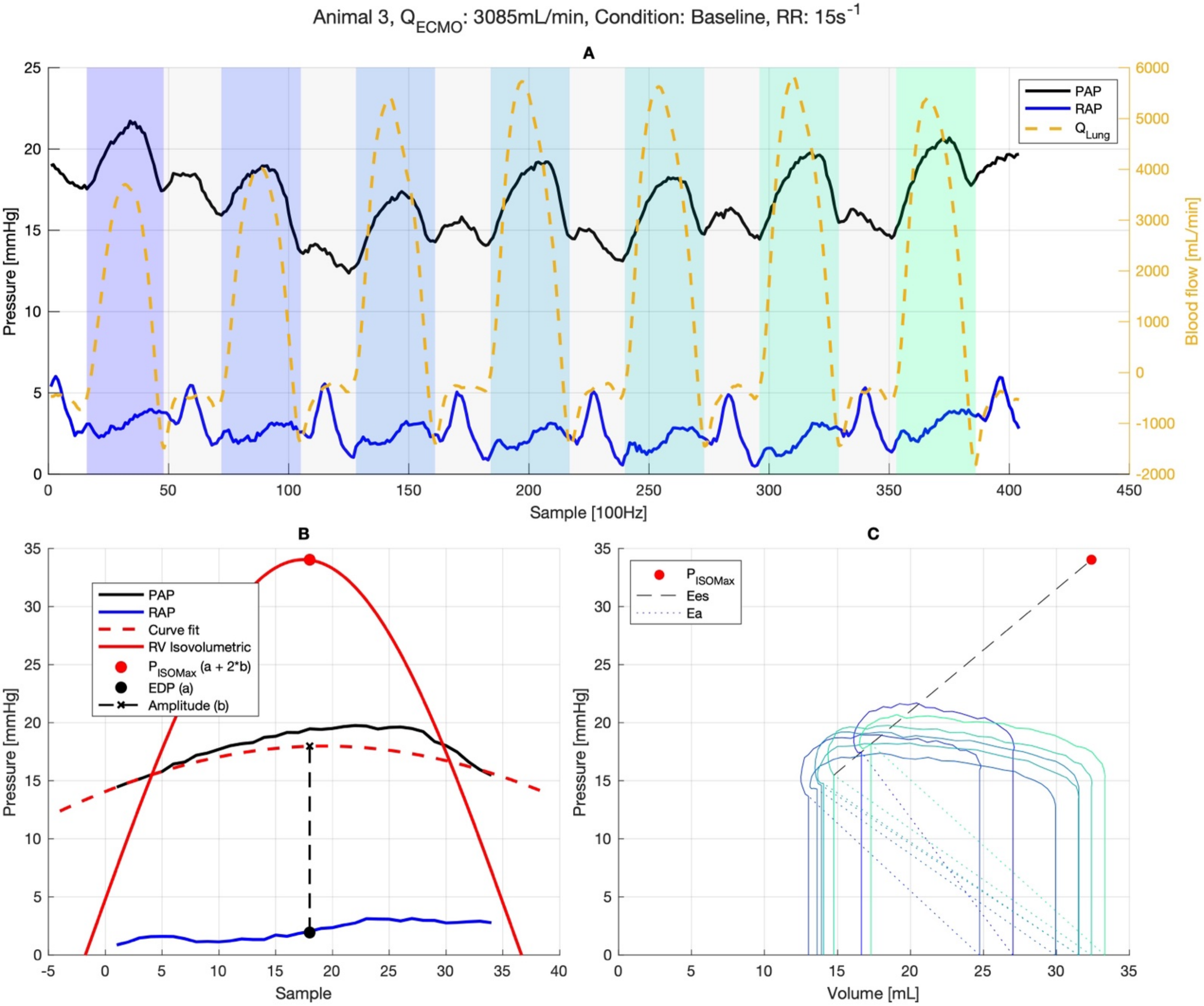

Animal 3, Q_{ECMO}: 3085mL/min, Condition: Baseline, RR: 15s^{-1}
A
PAP
RAP
Q_{Lung}
Pressure [mmHg]
Blood flow [mL/min]
Sample [100Hz]
B
PAP
RAP
Curve fit
RV Isovolumetric
P_{ISOMax} (a + 2*b)
EDP (a)
Amplitude (b)
Pressure [mmHg]
Sample
C
P_{ISOMax}
Ees
Ea
Pressure [mmHg]
Volume [mL]

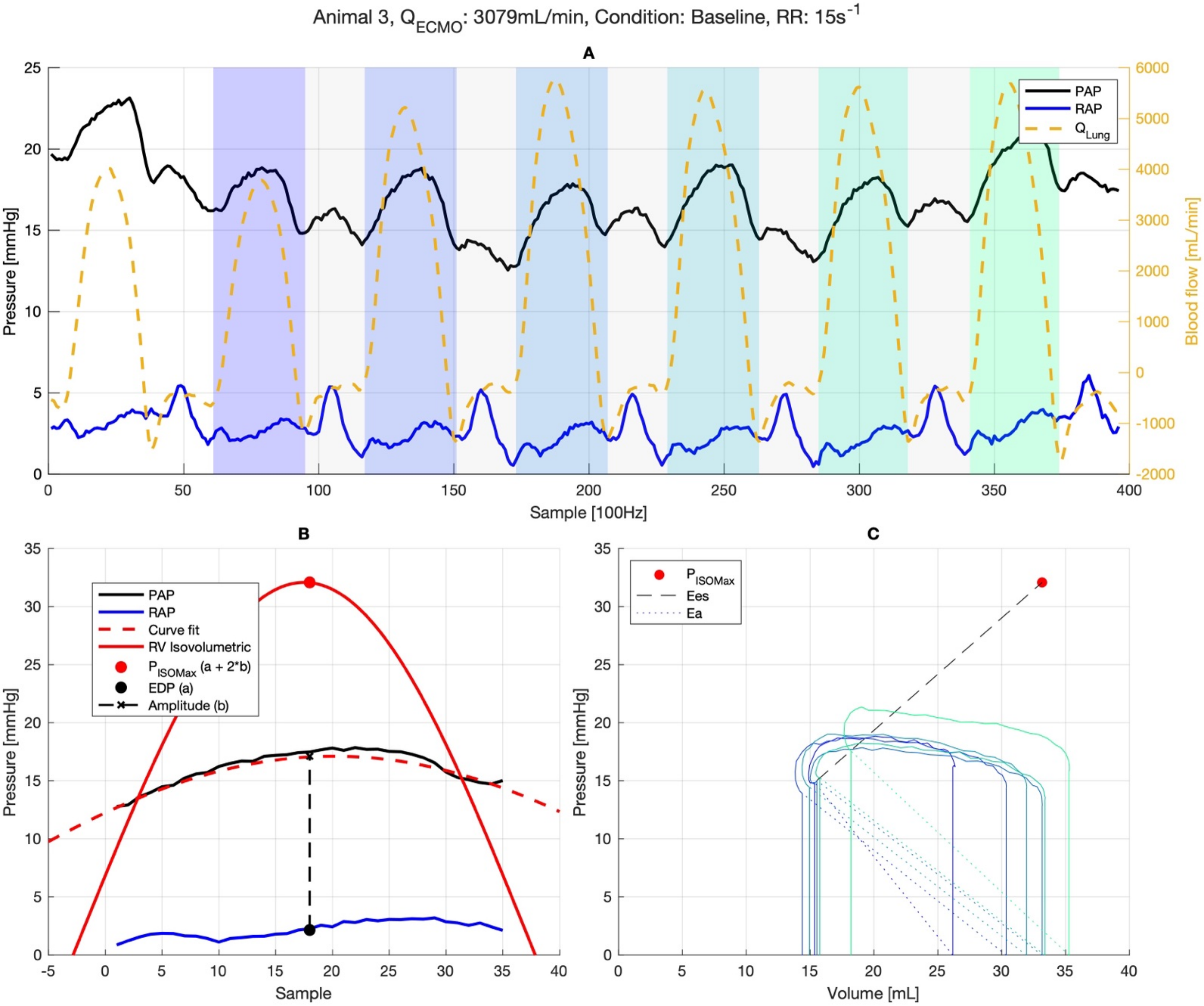
Animal 3, Q_ECMO: 3079mL/min, Condition: Baseline, RR: 15s^-1
A
PAP
RAP
Q_Lung
Pressure [mmHg]
Blood flow [mL/min]
Sample [100Hz]
B
Curve fit
RV Isovolumetric
P_ISOMax (a + 2*b)
EDP (a)
Amplitude (b)
Pressure [mmHg]
Sample
C
P_ISOMax
Ees
Ea
Pressure [mmHg]
Volume [mL]

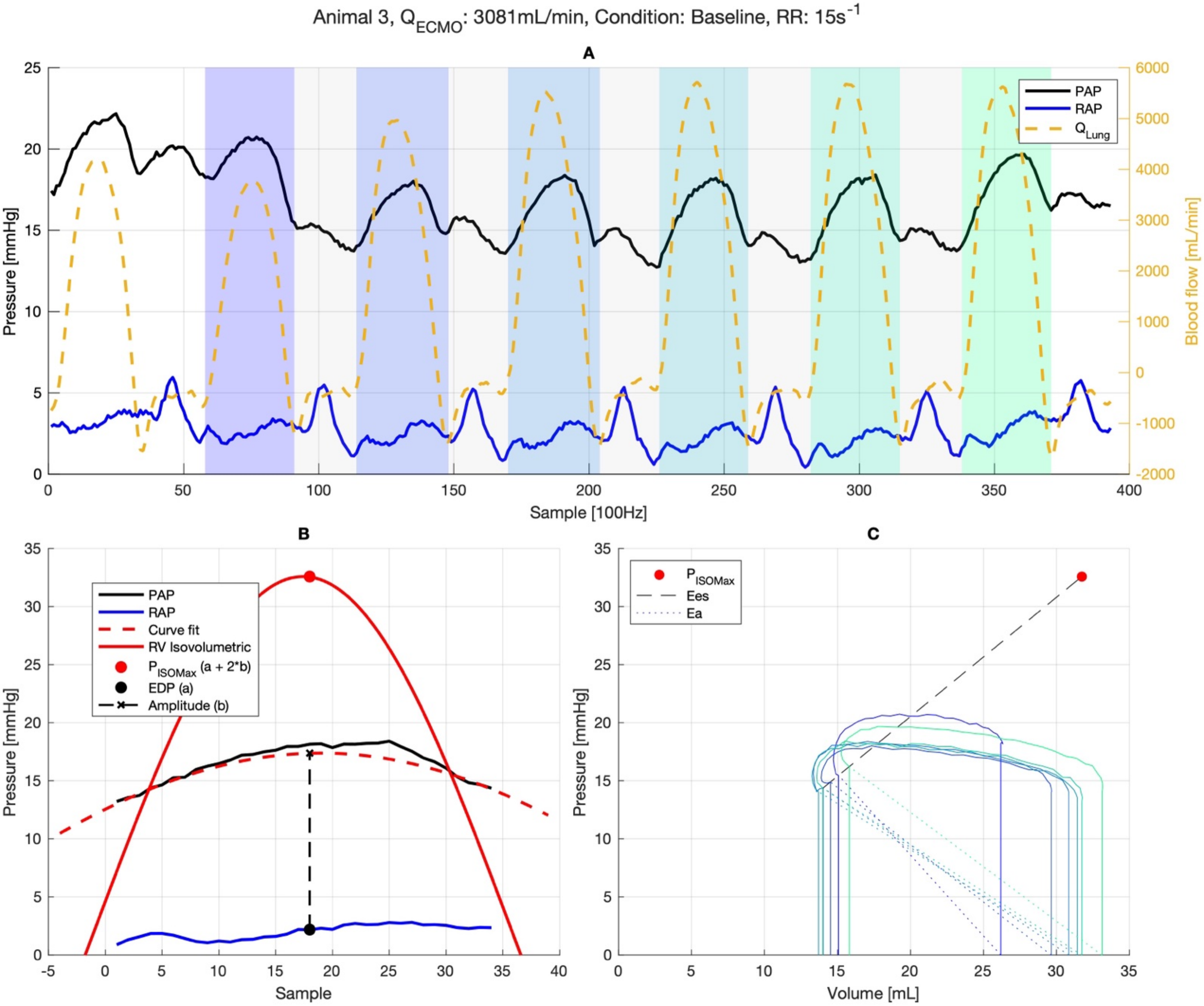

Animal 3, Q_{ECMO}: 3081mL/min, Condition: Baseline, RR: $15s^{-1}$
A
PAP
RAP
Q_{Lung}
Pressure [mmHg]
Blood flow [mL/min]
Sample [100Hz]
B
PAP
RAP
Curve fit
RV Isovolumetric
P_{ISOMax} (a + 2*b)
EDP (a)
Amplitude (b)
Pressure [mmHg]
Sample
C
P_{ISOMax}
Ees
Ea
Pressure [mmHg]
Volume [mL]

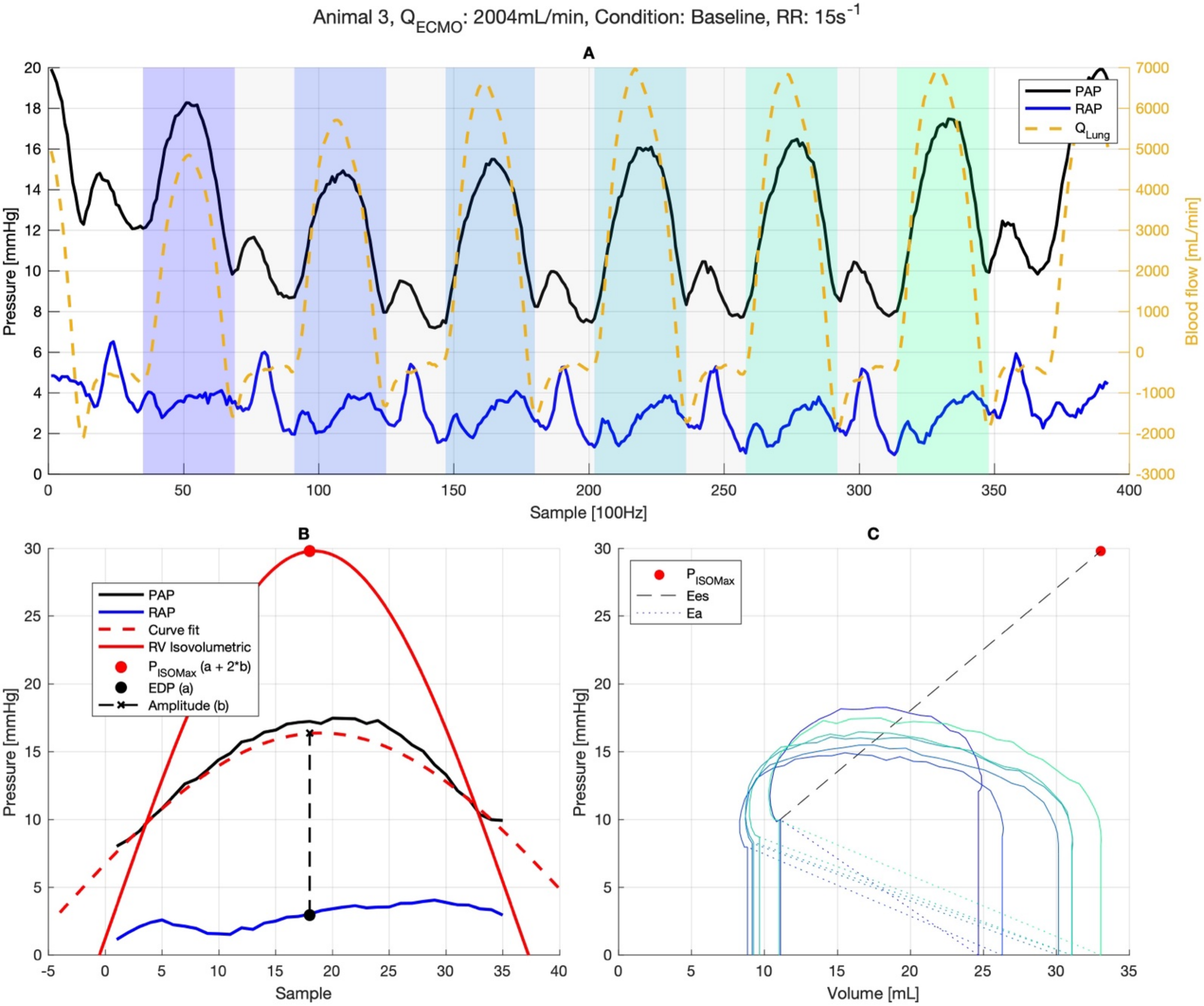

Animal 3, Q_{ECMO}: 2004mL/min, Condition: Baseline, RR: $15s^{-1}$
A
PAP
RAP
Q_{Lung}
Pressure [mmHg]
Blood flow [mL/min]
Sample [100Hz]
B
PAP
RAP
Curve fit
RV Isovolumetric
P_{ISOMax} (a + 2*b)
EDP (a)
Amplitude (b)
Pressure [mmHg]
Sample
C
P_{ISOMax}
Ees
Ea
Pressure [mmHg]
Volume [mL]

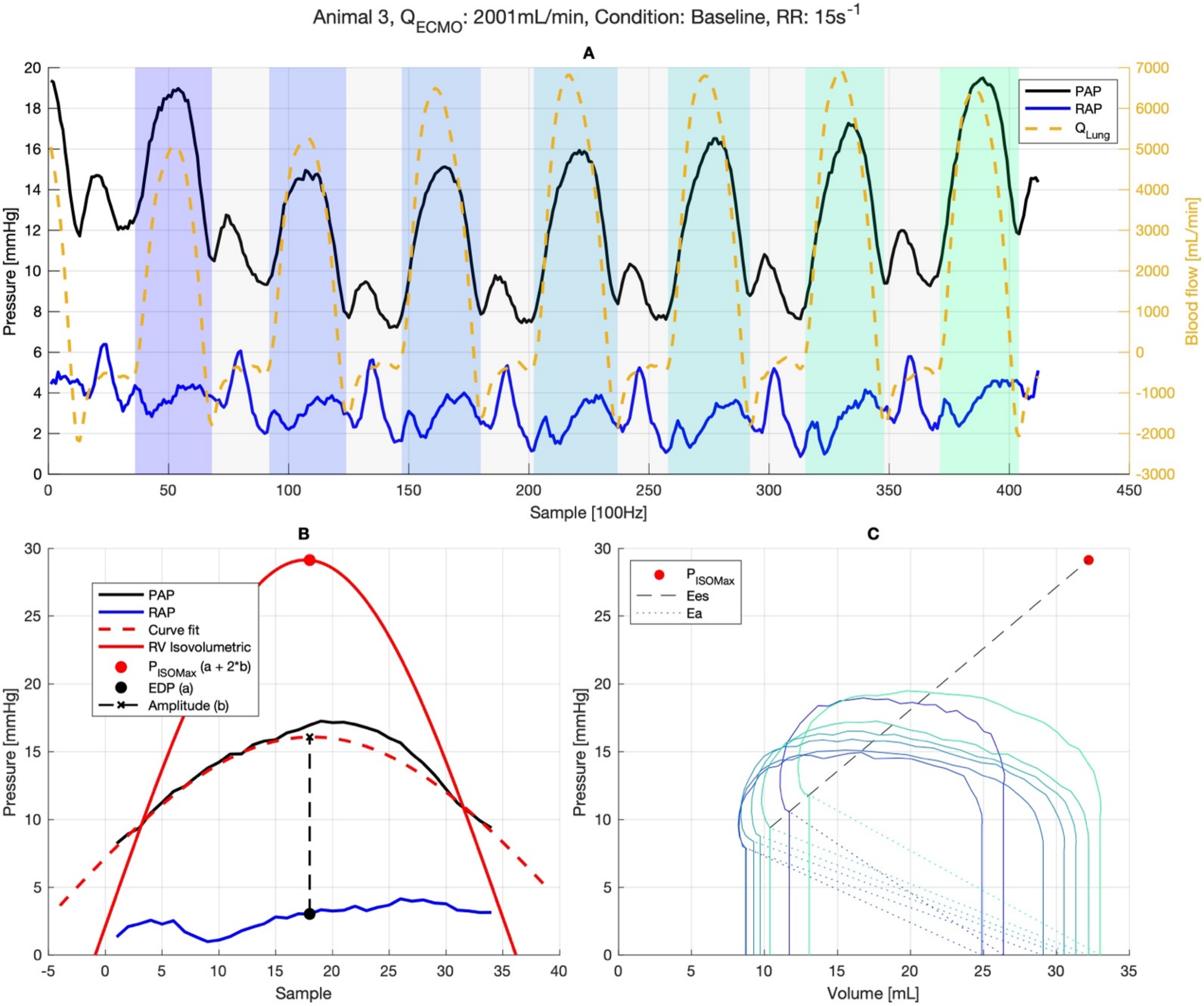

Animal 3, Q_{ECMO}: 2001mL/min, Condition: Baseline, RR: $15s^{-1}$
A
PAP
RAP
Q_{Lung}
Pressure [mmHg]
Blood flow [mL/min]
Sample [100Hz]
B
PAP
RAP
Curve fit
RV Isovolumetric
P_{ISOMax} (a + 2*b)
EDP (a)
Amplitude (b)
Pressure [mmHg]
Sample
C
P_{ISOMax}
Ees
Ea
Pressure [mmHg]
Volume [mL]

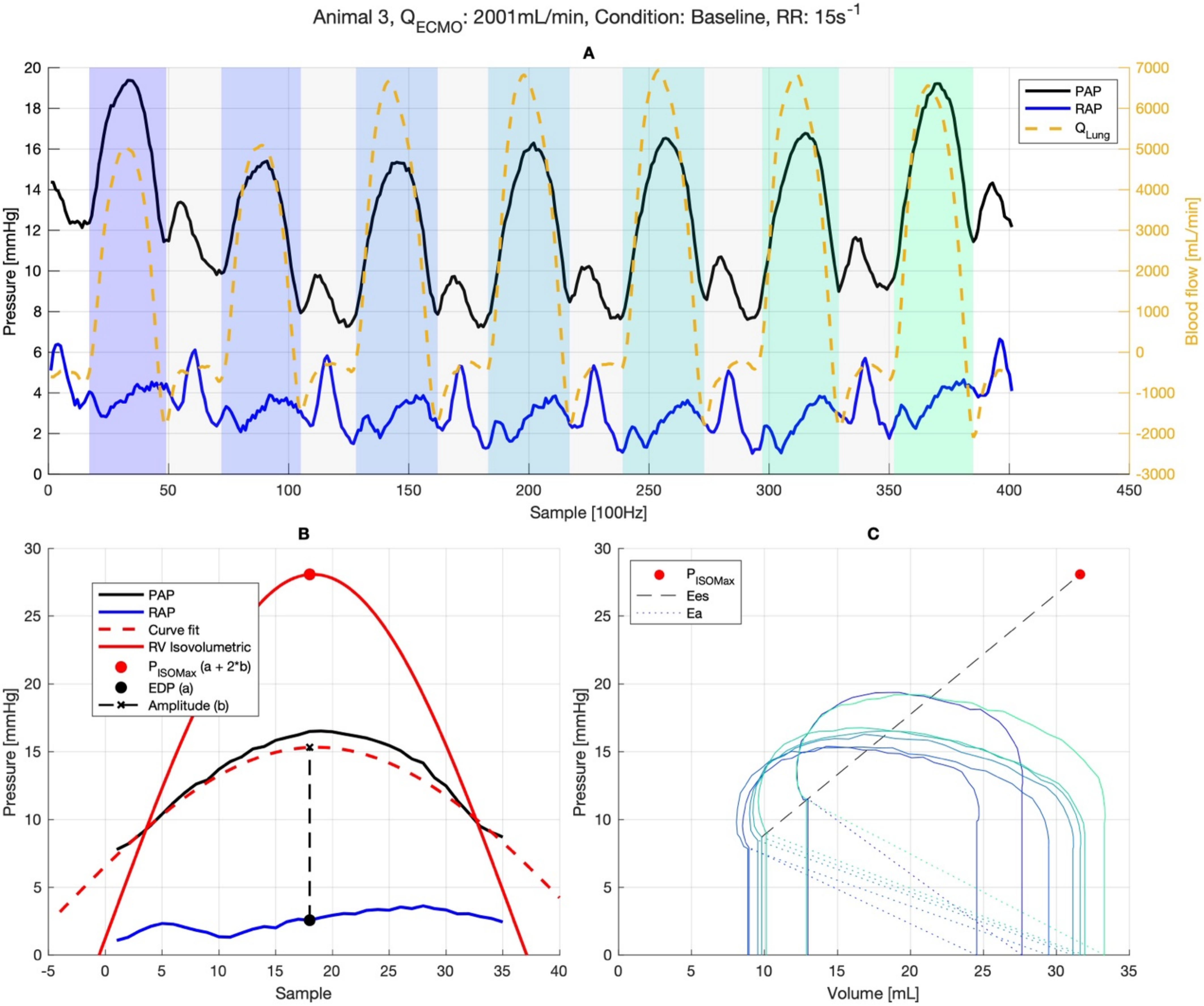

Animal 3, Q ECMO: 2001mL/min, Condition: Baseline, RR: 15s-1
A
PAP
RAP
Q Lung
Pressure [mmHg]
Blood flow [mL/min]
Sample [100Hz]
B
PAP
RAP
Curve fit
RV Isovolumetric
P ISOMax (a + 2*b)
EDP (a)
Amplitude (b)
Pressure [mmHg]
Sample
C
P ISOMax
Ees
Ea
Pressure [mmHg]
Volume [mL]

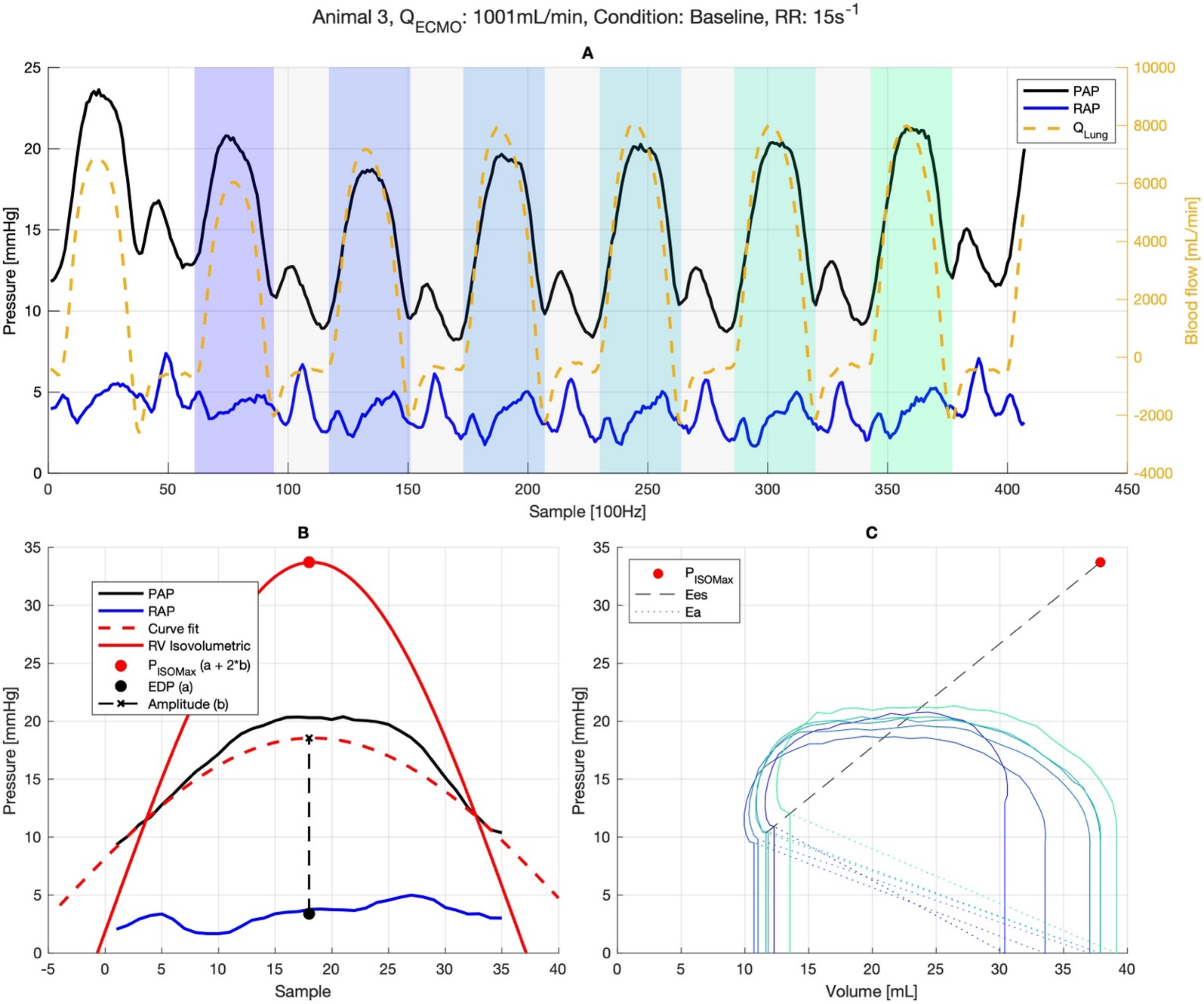

Animal 3, Q_{ECMO}: 1001mL/min, Condition: Baseline, RR: $15s^{-1}$
A
PAP
RAP
Q_{Lung}
Pressure [mmHg]
Blood flow [mL/min]
Sample [100Hz]
B
PAP
RAP
Curve fit
RV Isovolumetric
P_{ISOMax} (a + 2*b)
EDP (a)
Amplitude (b)
Pressure [mmHg]
Sample
C
P_{ISOMax}
Ees
Ea
Pressure [mmHg]
Volume [mL]

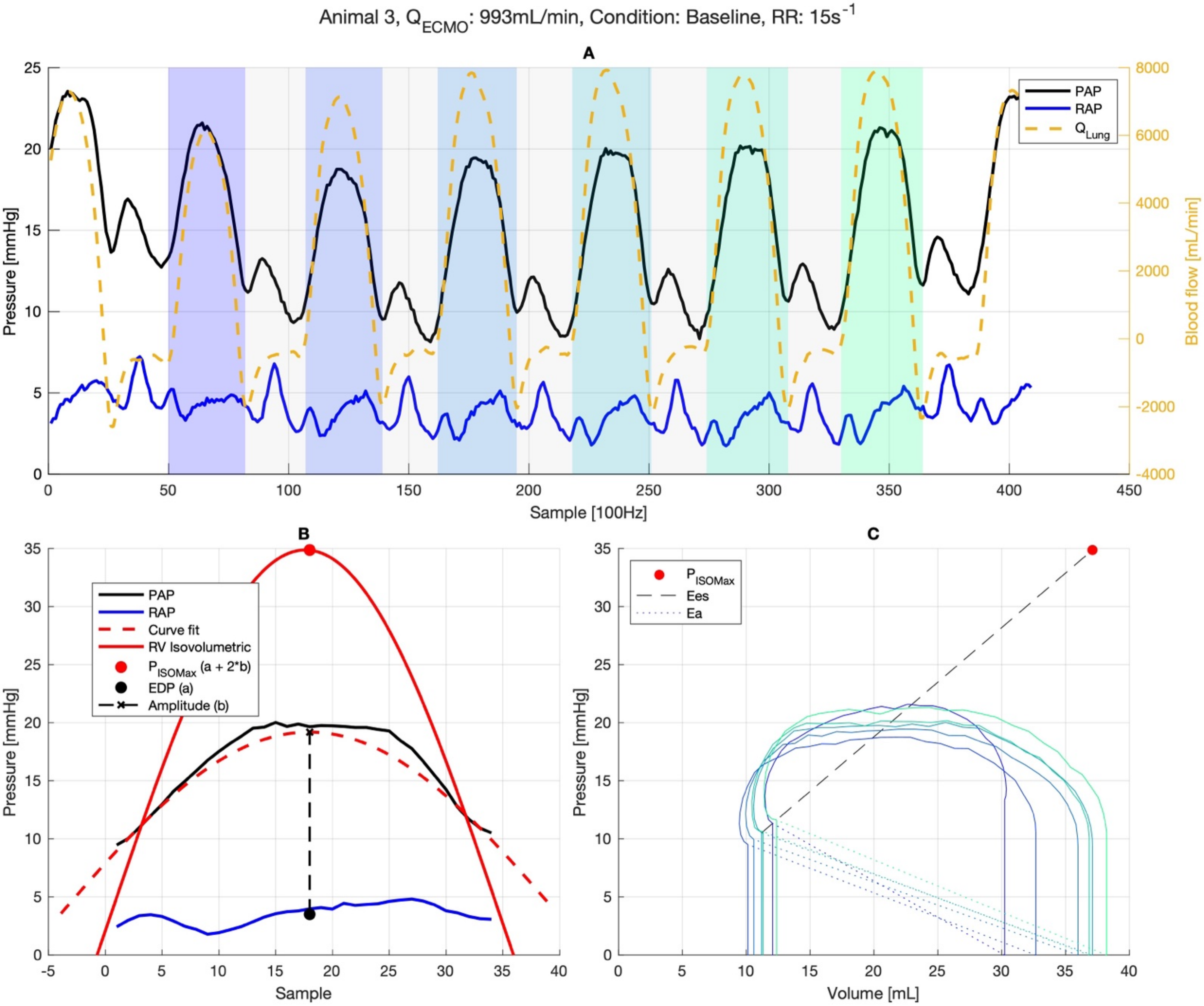

Animal 3, Q_{ECMO}: 993mL/min, Condition: Baseline, RR: $15s^{-1}$
A
Pressure [mmHg]
Blood flow [mL/min]
Sample [100Hz]
PAP
RAP
Q_{Lung}
B
Pressure [mmHg]
Sample
PAP
RAP
Curve fit
RV Isovolumetric
P_{ISOMax} (a + 2*b)
EDP (a)
Amplitude (b)
C
Pressure [mmHg]
Volume [mL]
P_{ISOMax}
Ees
Ea

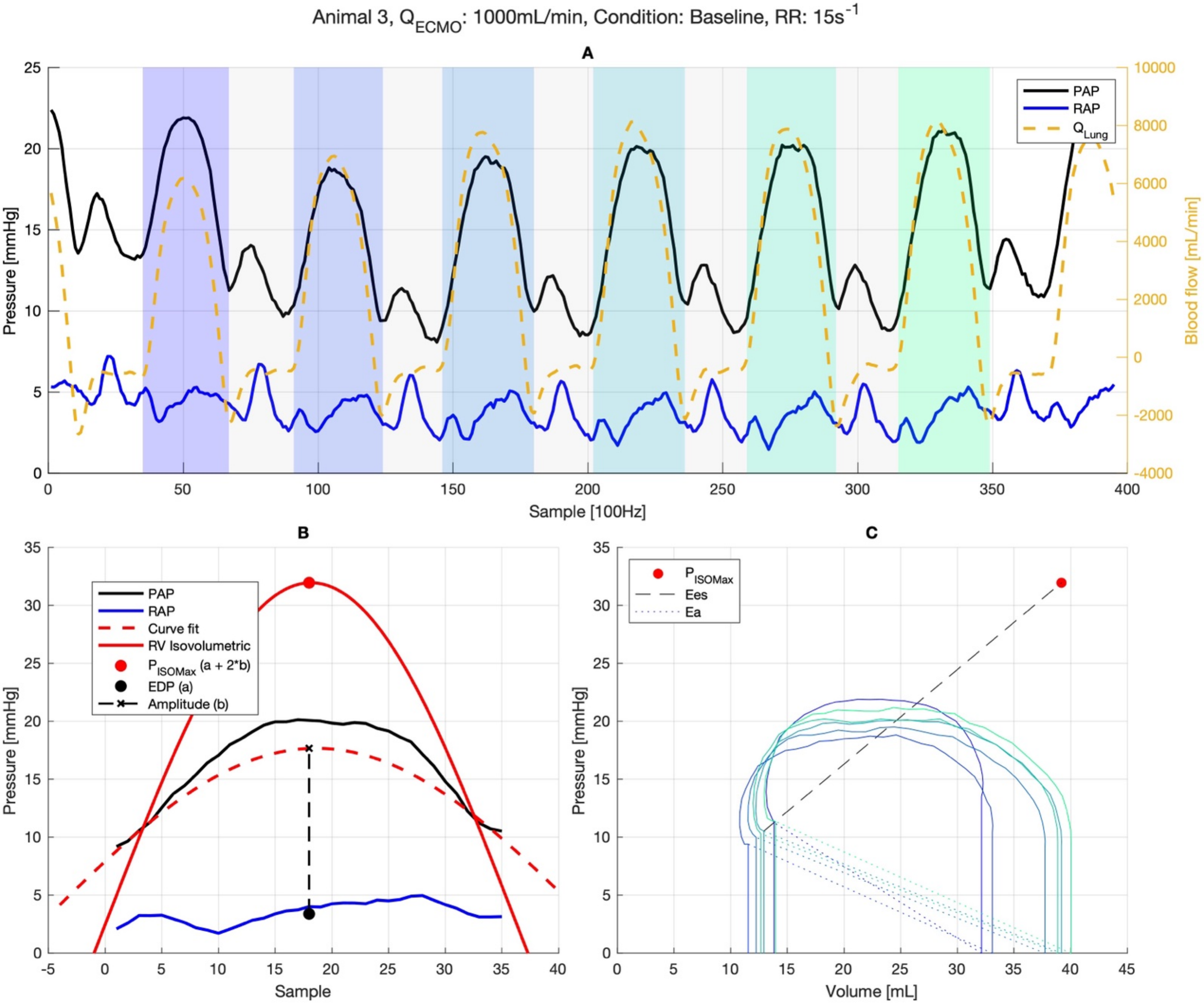

Animal 3, Q_{ECMO}: 1000mL/min, Condition: Baseline, RR: $15s^{-1}$
A
PAP
RAP
Q_{Lung}
Pressure [mmHg]
Blood flow [mL/min]
Sample [100Hz]
B
PAP
RAP
Curve fit
RV Isovolumetric
P_{ISOMax} (a + 2*b)
EDP (a)
Amplitude (b)
Pressure [mmHg]
Sample
C
P_{ISOMax}
Ees
Ea
Pressure [mmHg]
Volume [mL]

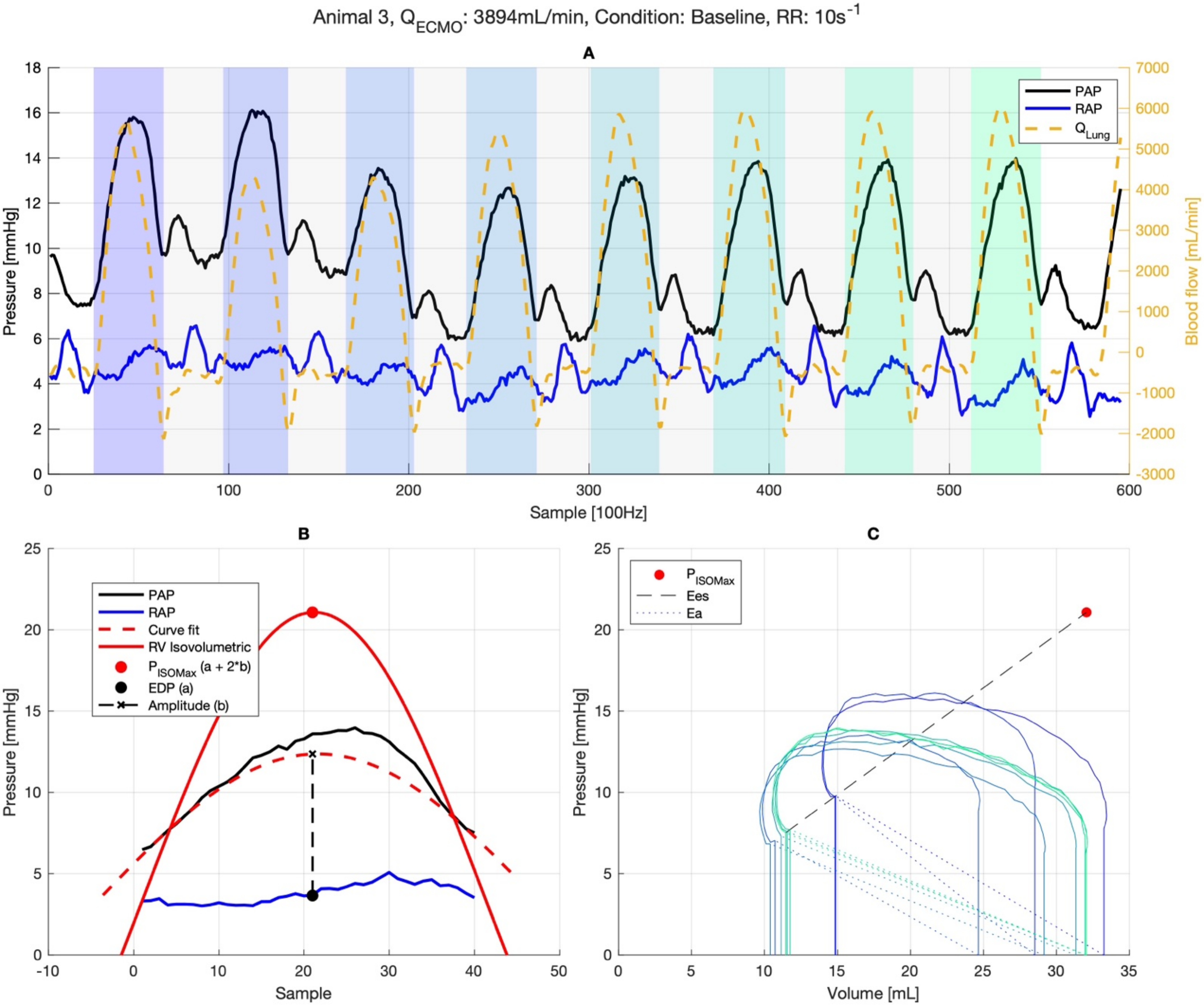

Animal 3, Q_{ECMO}: 3894mL/min, Condition: Baseline, RR: $10s^{-1}$
A
PAP
RAP
Q_{Lung}
Pressure [mmHg]
Blood flow [mL/min]
Sample [100Hz]
B
PAP
RAP
Curve fit
RV Isovolumetric
P_{ISOMax} (a + 2*b)
EDP (a)
Amplitude (b)
Pressure [mmHg]
Sample
C
P_{ISOMax}
Ees
Ea
Pressure [mmHg]
Volume [mL]

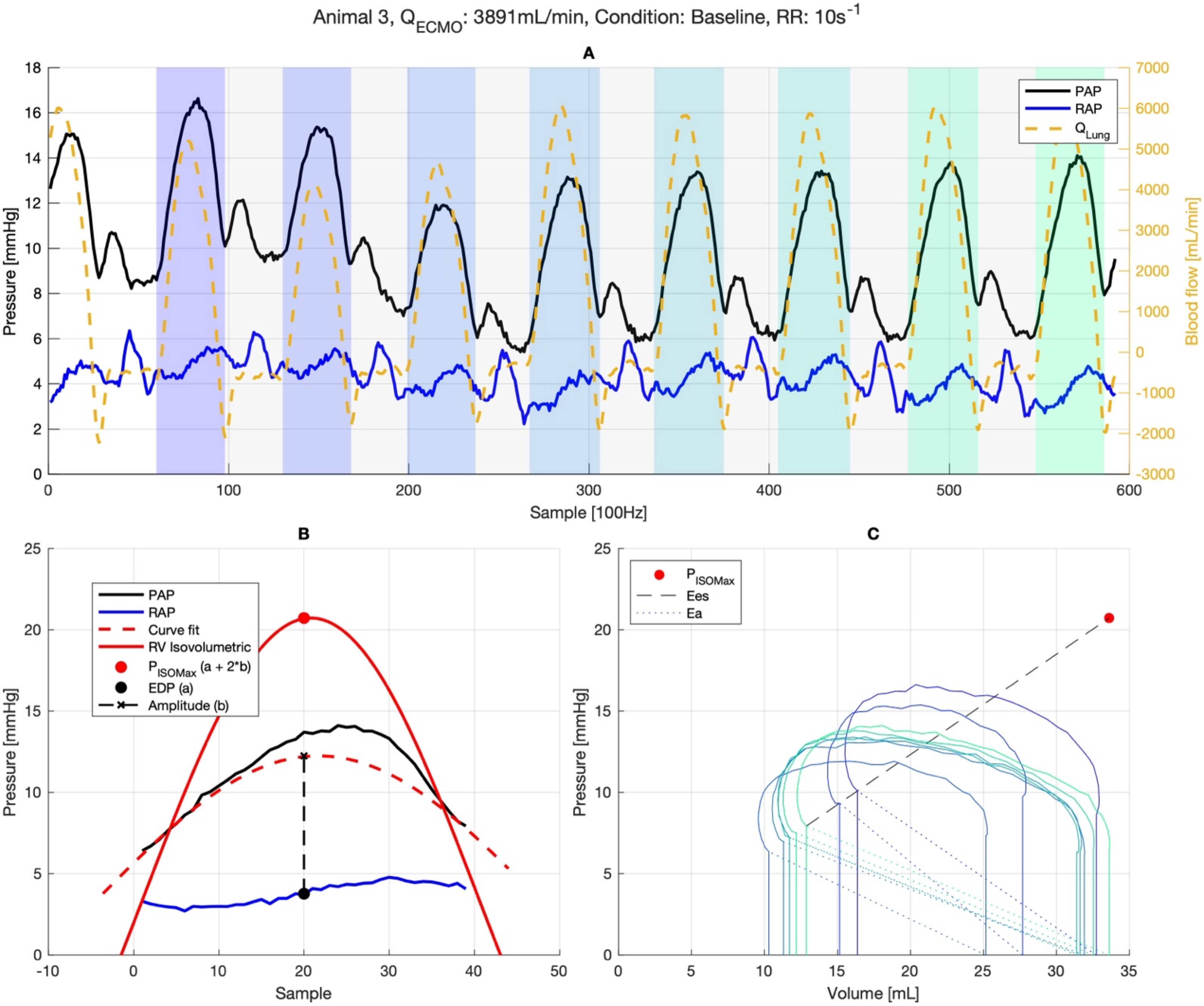
Animal 3, Q_{ECMO}: 3891mL/min, Condition: Baseline, RR: $10s^{-1}$
A
PAP
RAP
Q_{Lung}
Pressure [mmHg]
Blood flow [mL/min]
Sample [100Hz]
B
PAP
RAP
Curve fit
RV Isovolumetric
P_{ISOMax} (a + 2*b)
EDP (a)
Amplitude (b)
Pressure [mmHg]
Sample
C
P_{ISOMax}
Ees
Ea
Pressure [mmHg]
Volume [mL]

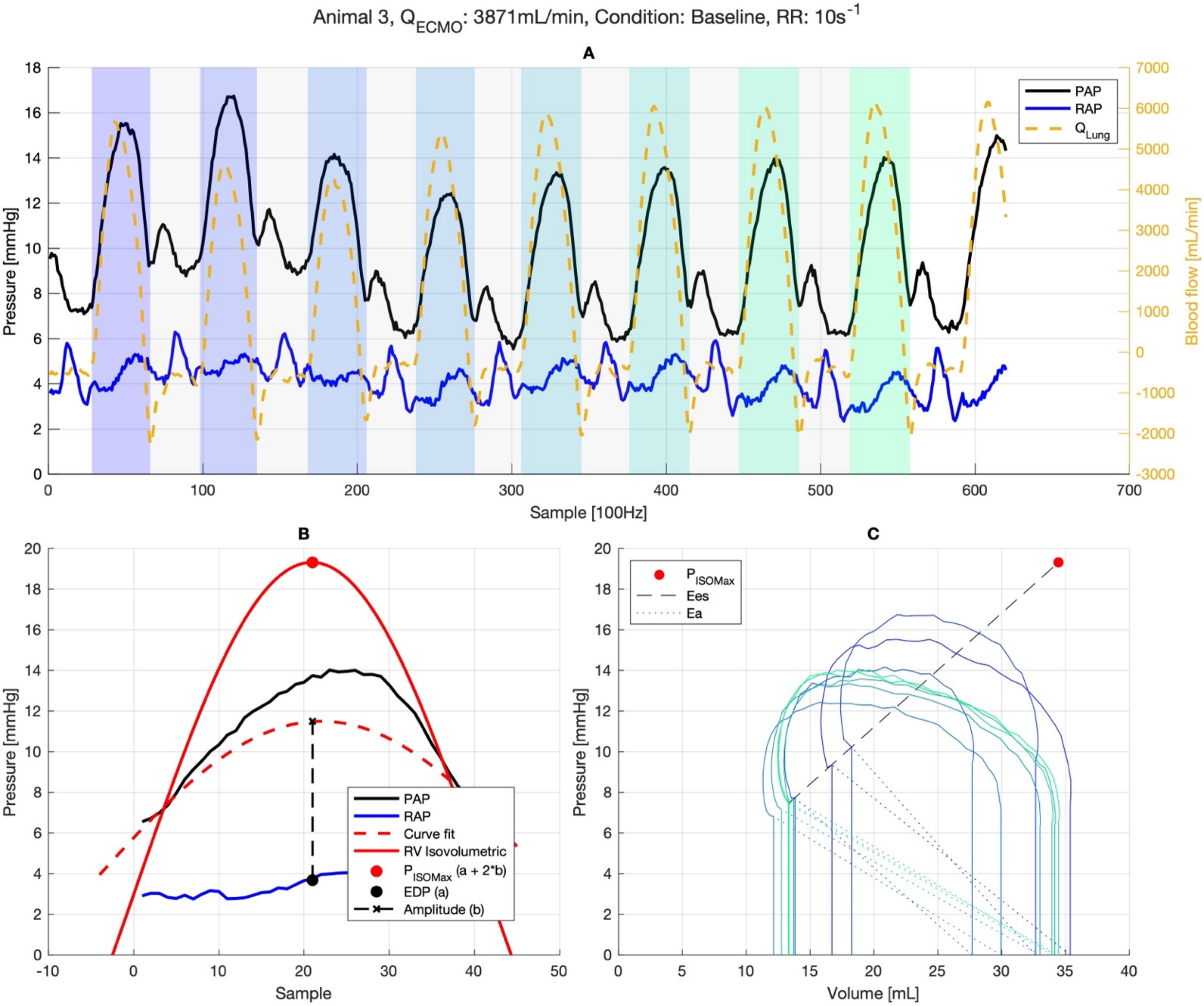

Animal 3, Q_ECMO: 3871mL/min, Condition: Baseline, RR: 10s^-1
A
Pressure [mmHg]
Blood flow [mL/min]
Sample [100Hz]
PAP
RAP
Q_Lung
B
Pressure [mmHg]
Sample
PAP
RAP
Curve fit
RV Isovolumetric
P_ISOMax (a + 2*b)
EDP (a)
Amplitude (b)
C
Pressure [mmHg]
Volume [mL]
P_ISOMax
Ees
Ea

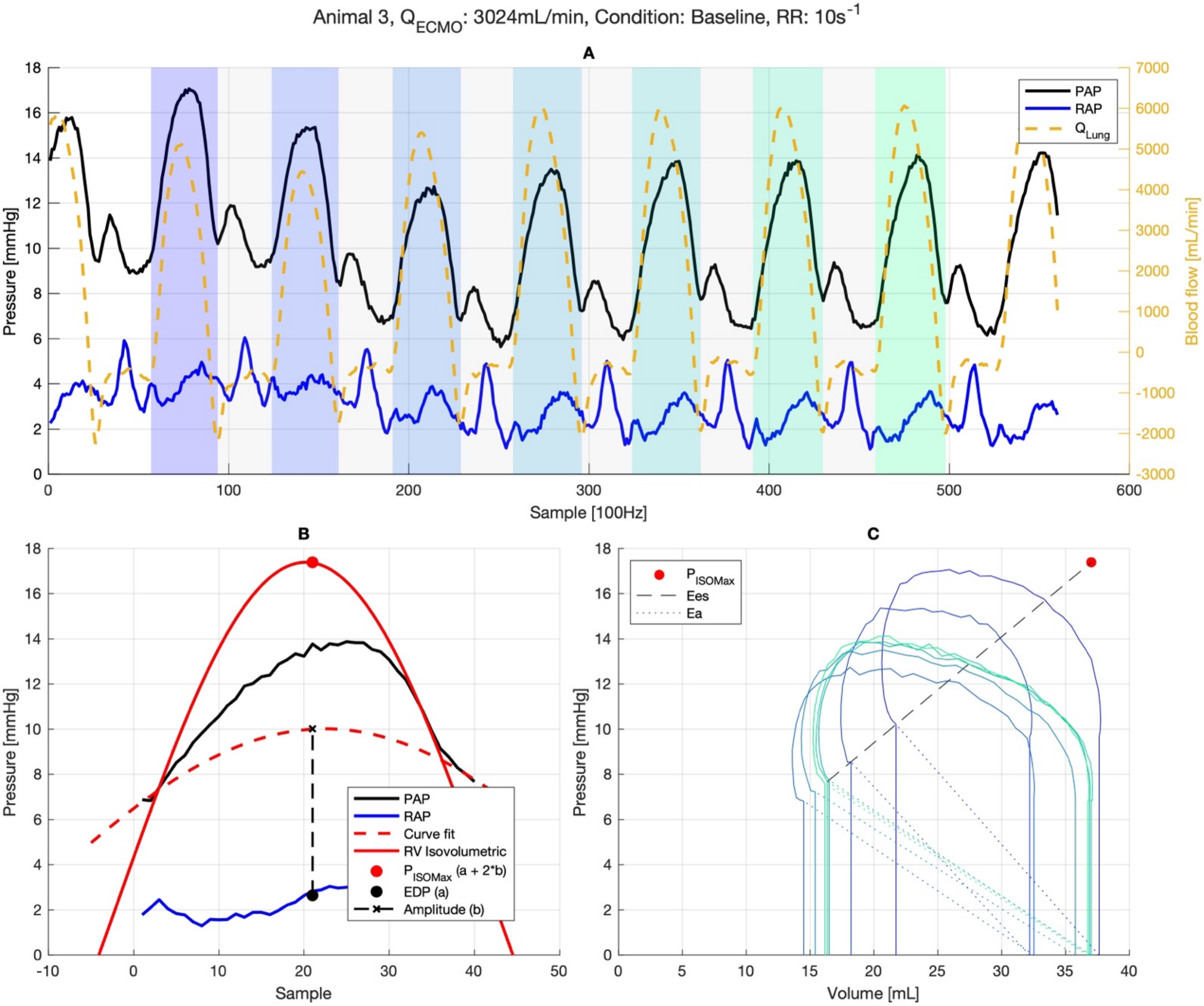

Animal 3, Q_ECMO: 3024mL/min, Condition: Baseline, RR: 10s^-1
A
Pressure [mmHg]
Blood flow [mL/min]
Sample [100Hz]
PAP
RAP
Q_Lung
B
Pressure [mmHg]
Sample
PAP
RAP
Curve fit
RV Isovolumetric
P_ISOMax (a + 2*b)
EDP (a)
Amplitude (b)
C
Pressure [mmHg]
Volume [mL]
P_ISOMax
Ees
Ea

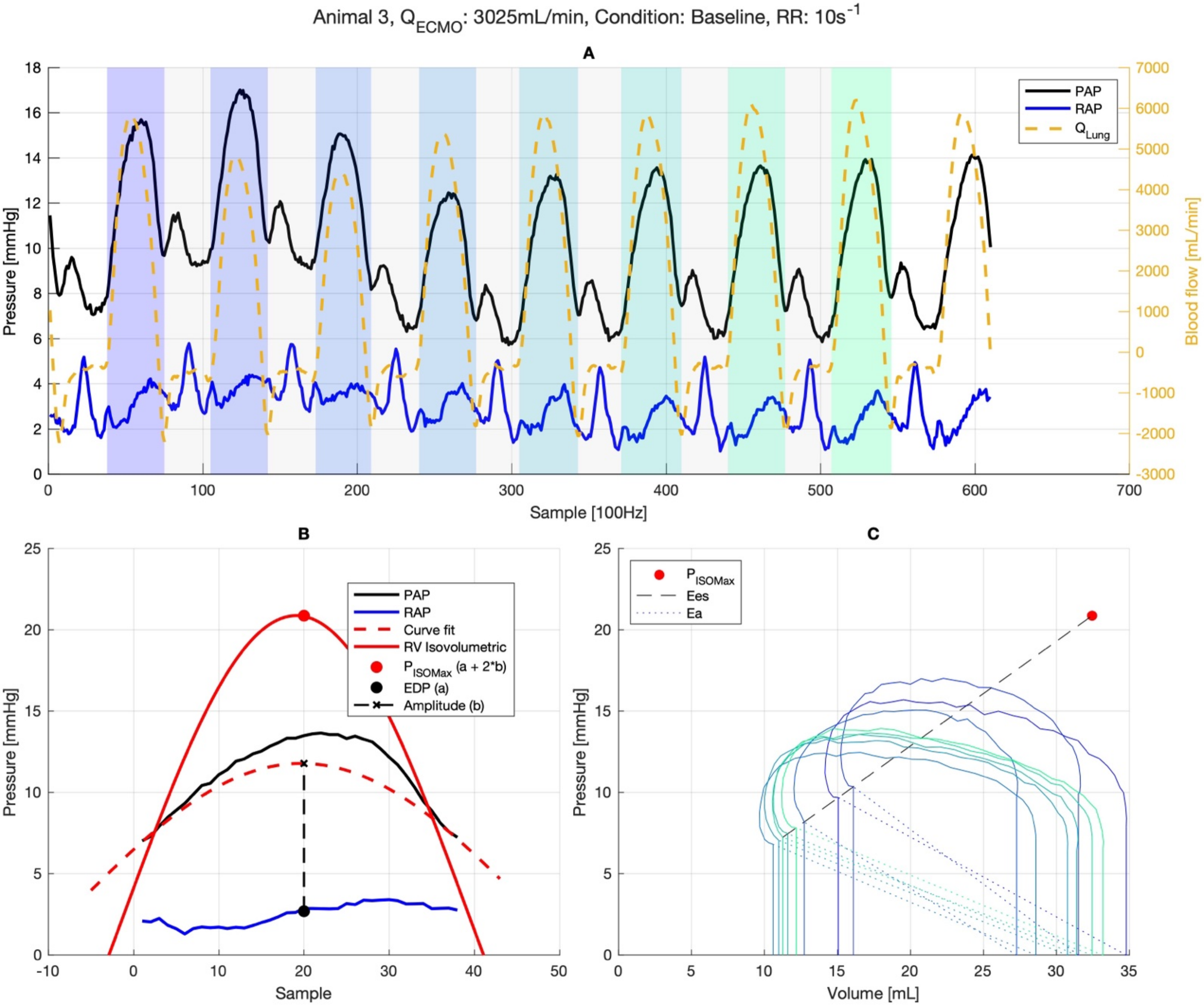

Animal 3, Q_{ECMO}: 3025mL/min, Condition: Baseline, RR: $10s^{-1}$
A
PAP
RAP
Q_{Lung}
Pressure [mmHg]
Blood flow [mL/min]
Sample [100Hz]
B
PAP
RAP
Curve fit
RV Isovolumetric
P_{ISOMax} (a + 2*b)
EDP (a)
Amplitude (b)
Pressure [mmHg]
Sample
C
P_{ISOMax}
Ees
Ea
Pressure [mmHg]
Volume [mL]

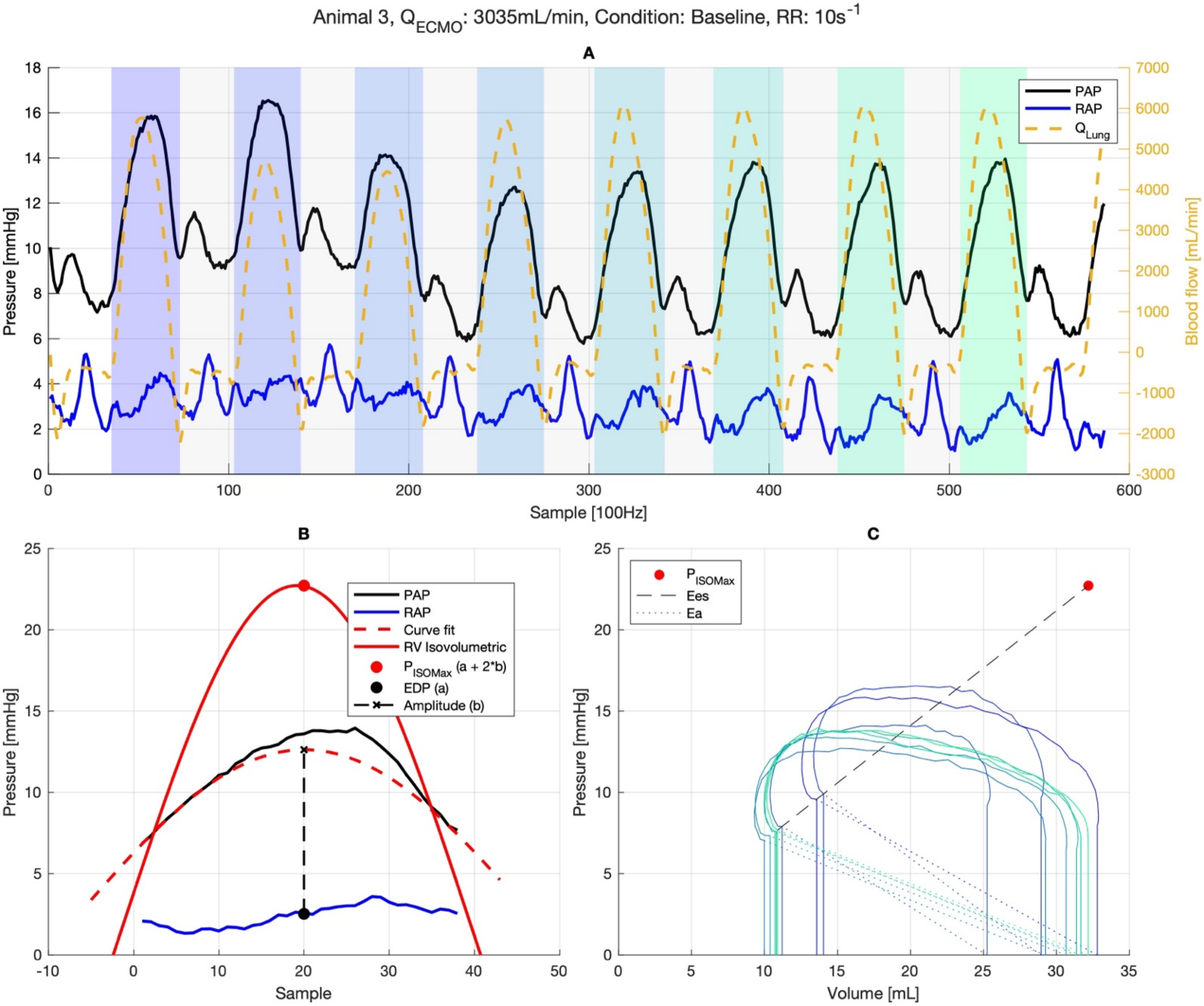

Animal 3, Q_ECMO: 3035mL/min, Condition: Baseline, RR: 10s^-1
A
PAP
RAP
Q_Lung
Pressure [mmHg]
Blood flow [mL/min]
Sample [100Hz]
B
PAP
RAP
Curve fit
RV Isovolumetric
P_ISOMax (a + 2*b)
EDP (a)
Amplitude (b)
Pressure [mmHg]
Sample
C
P_ISOMax
Ees
Ea
Pressure [mmHg]
Volume [mL]

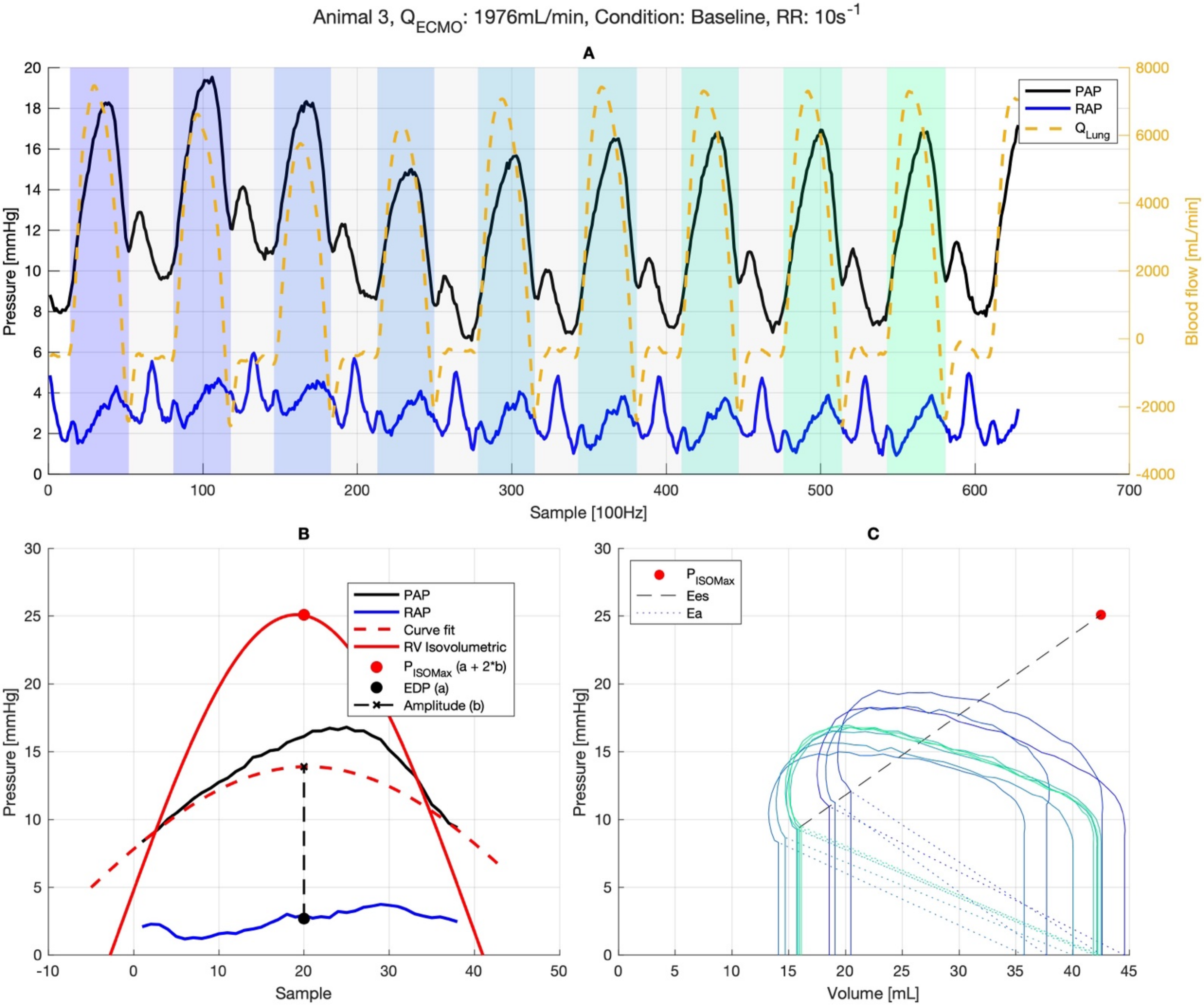

Animal 3, Q_{ECMO}: 1976mL/min, Condition: Baseline, RR: $10s^{-1}$
A
PAP
RAP
Q_{Lung}
Pressure [mmHg]
Blood flow [mL/min]
Sample [100Hz]
B
PAP
RAP
Curve fit
RV Isovolumetric
P_{ISOMax} (a + 2*b)
EDP (a)
Amplitude (b)
Pressure [mmHg]
Sample
C
P_{ISOMax}
Ees
Ea
Pressure [mmHg]
Volume [mL]

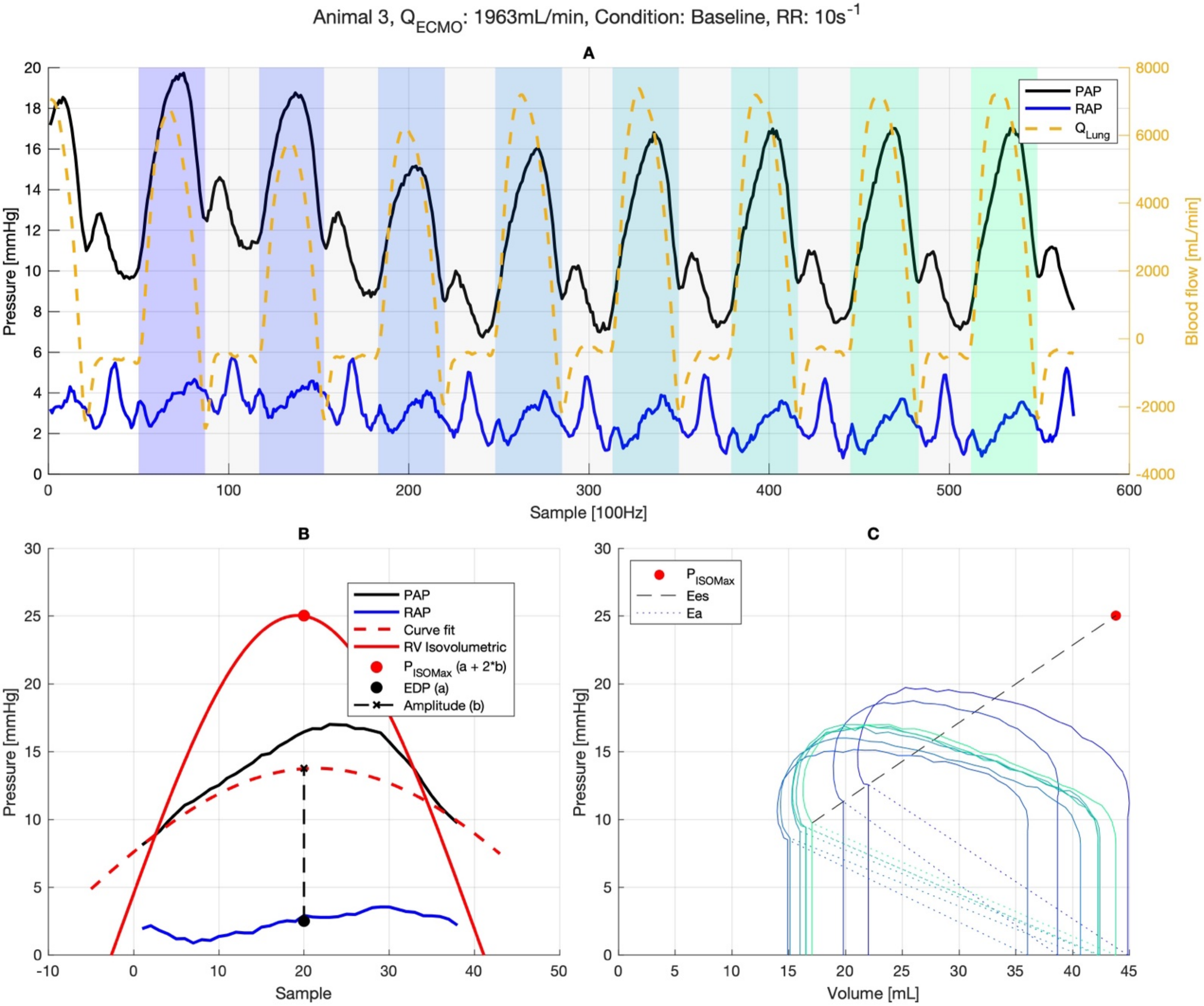

Animal 3, Q_{ECMO}: 1963mL/min, Condition: Baseline, RR: $10s^{-1}$
A
PAP
RAP
Q_{Lung}
Pressure [mmHg]
Blood flow [mL/min]
Sample [100Hz]
B
PAP
RAP
Curve fit
RV Isovolumetric
P_{ISOMax} (a + 2*b)
EDP (a)
Amplitude (b)
Pressure [mmHg]
Sample
C
P_{ISOMax}
Ees
Ea
Pressure [mmHg]
Volume [mL]

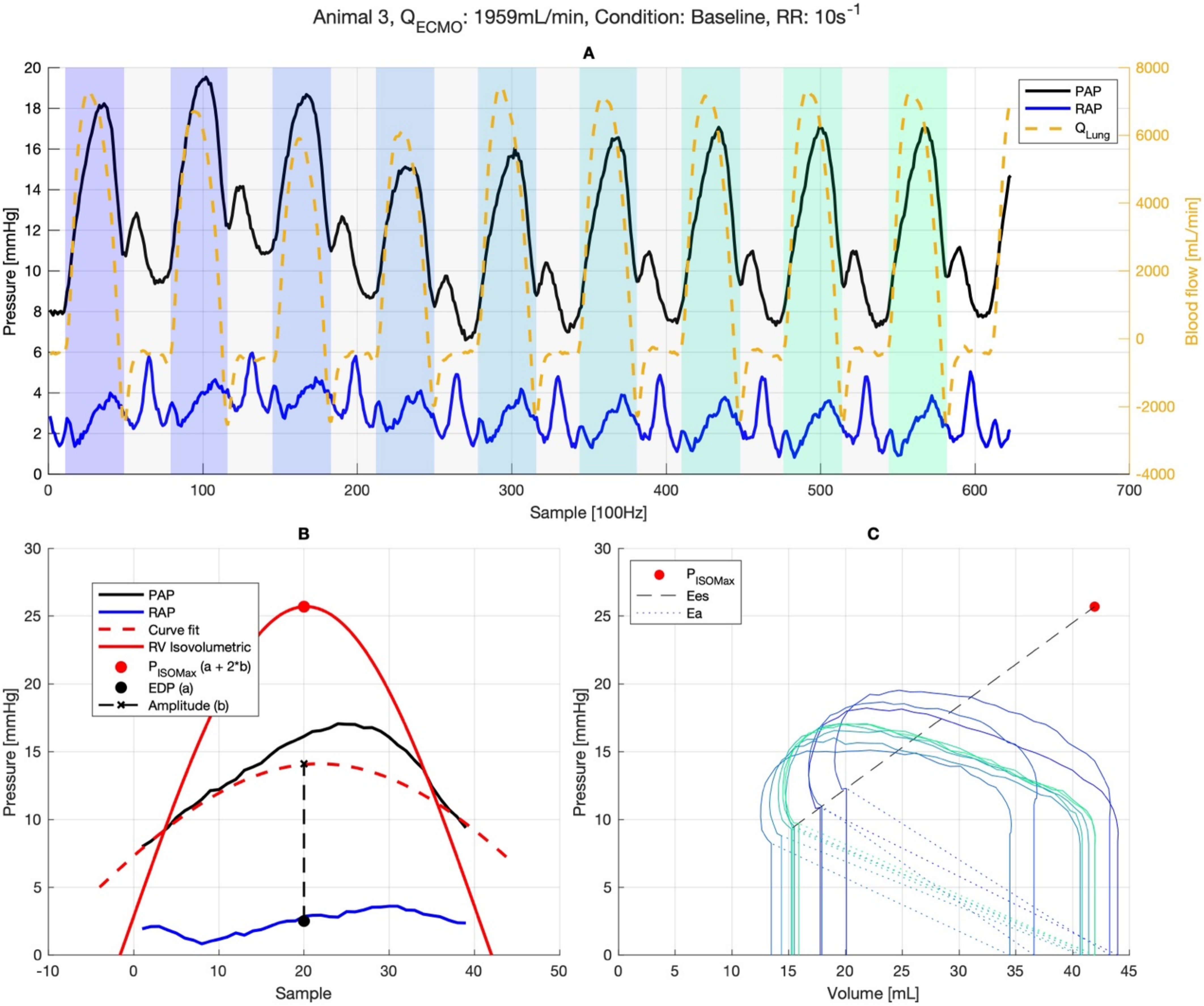

Animal 3, Q_ECMO: 1959mL/min, Condition: Baseline, RR: 10s-1
A
Pressure [mmHg]
Blood flow [mL/min]
Sample [100Hz]
PAP
RAP
Q_Lung
B
Pressure [mmHg]
Sample
PAP
RAP
Curve fit
RV Isovolumetric
P_ISOMax (a + 2*b)
EDP (a)
Amplitude (b)
C
Pressure [mmHg]
Volume [mL]
P_ISOMax
Ees
Ea

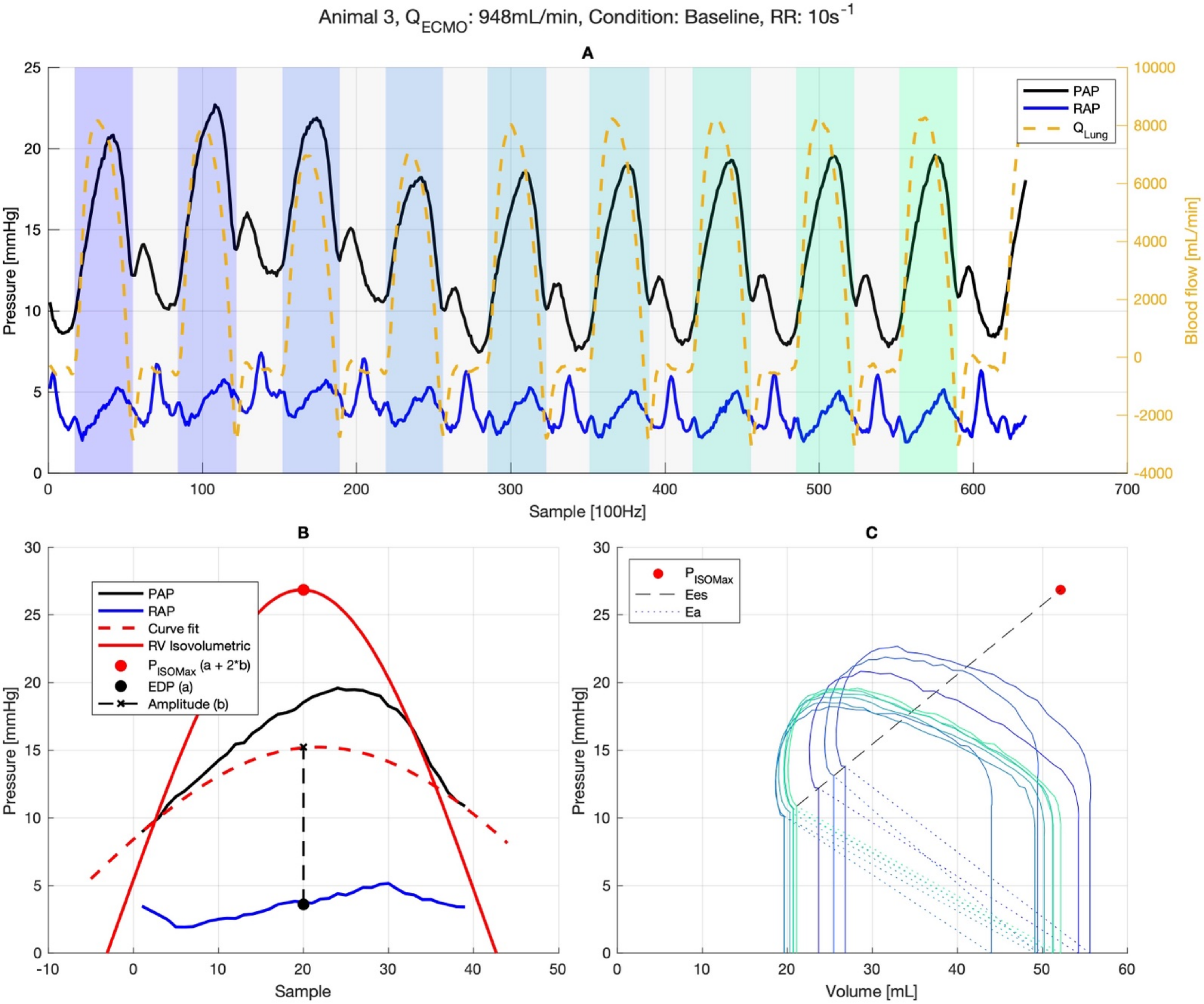

Animal 3, Q_ECMO: 948mL/min, Condition: Baseline, RR: 10s^-1
A
PAP
RAP
Q_Lung
Pressure [mmHg]
Blood flow [mL/min]
Sample [100Hz]
B
PAP
RAP
Curve fit
RV Isovolumetric
P_ISOMax (a + 2*b)
EDP (a)
Amplitude (b)
Pressure [mmHg]
Sample
C
P_ISOMax
Ees
Ea
Pressure [mmHg]
Volume [mL]

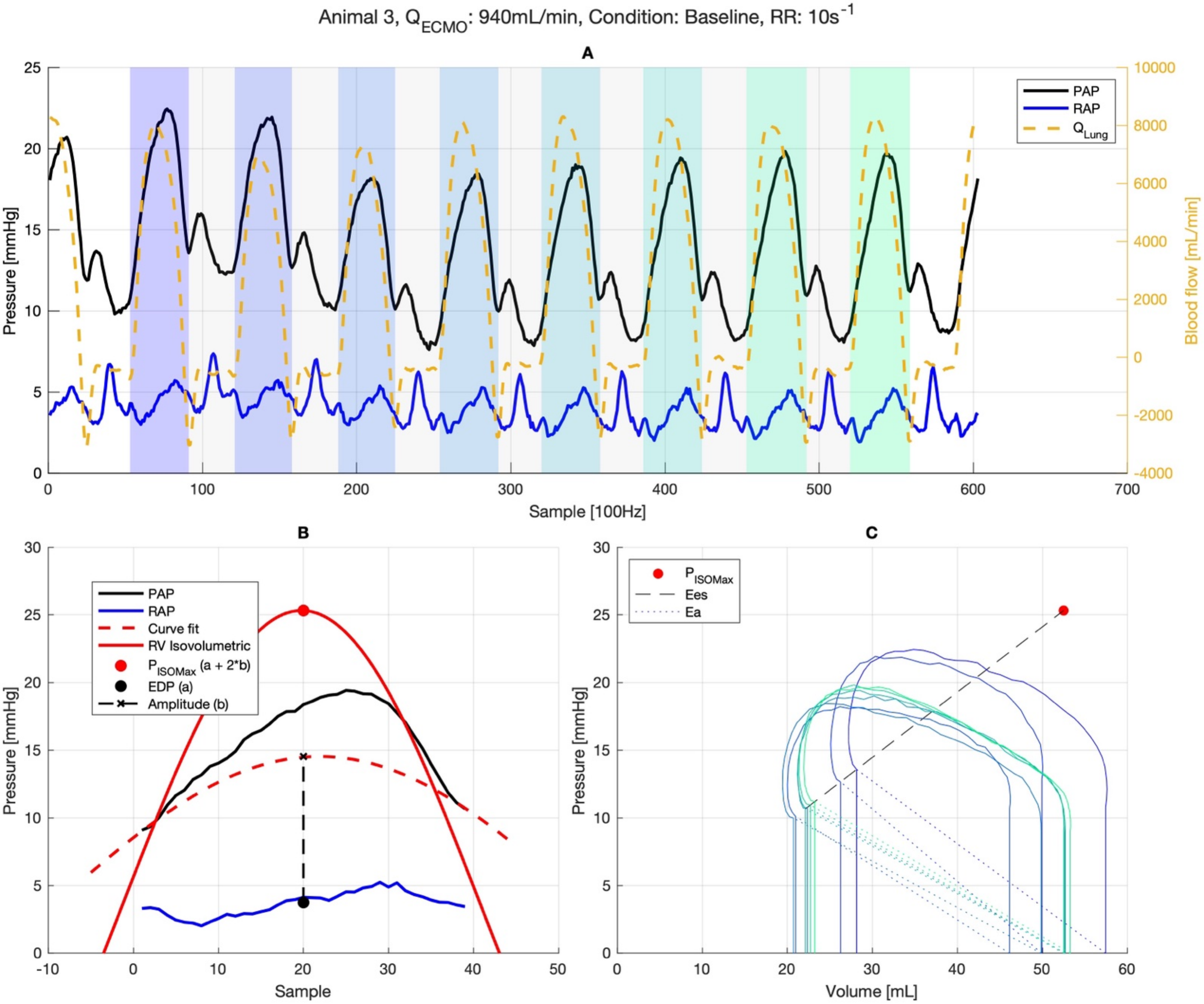
Animal 3, Q_ECMO: 940mL/min, Condition: Baseline, RR: 10s^-1
A
PAP
RAP
Q_Lung
Pressure [mmHg]
Blood flow [mL/min]
Sample [100Hz]
B
PAP
RAP
Curve fit
RV Isovolumetric
P_ISOMax (a + 2*b)
EDP (a)
Amplitude (b)
Pressure [mmHg]
Sample
C
P_ISOMax
Ees
Ea
Pressure [mmHg]
Volume [mL]

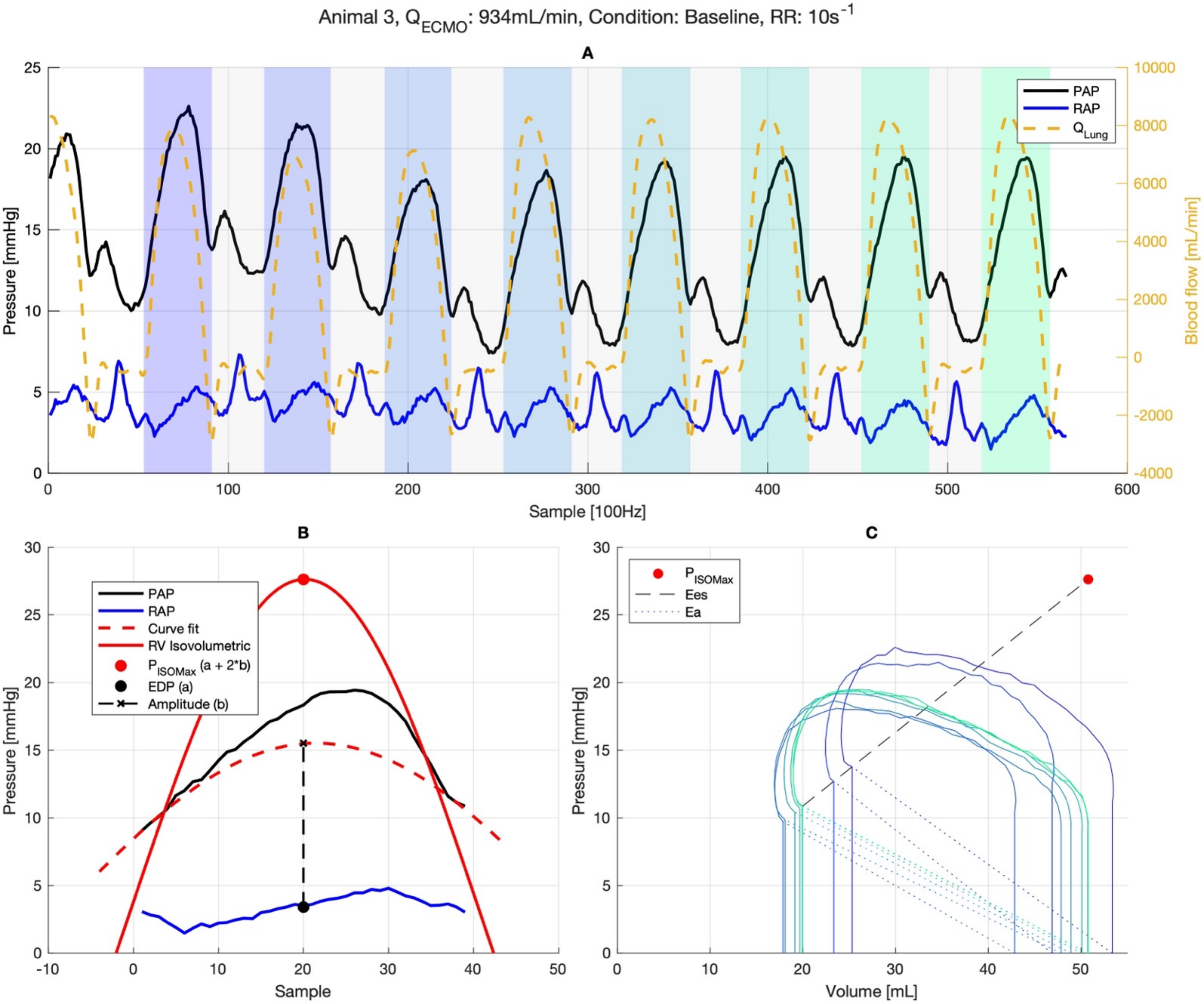

Animal 3, Q_ECMO: 934mL/min, Condition: Baseline, RR: 10s^-1
A
PAP
RAP
Q_Lung
Pressure [mmHg]
Blood flow [mL/min]
Sample [100Hz]
B
PAP
RAP
Curve fit
RV Isovolumetric
P_ISOMax (a + 2*b)
EDP (a)
Amplitude (b)
Pressure [mmHg]
Sample
C
P_ISOMax
Ees
Ea
Pressure [mmHg]
Volume [mL]

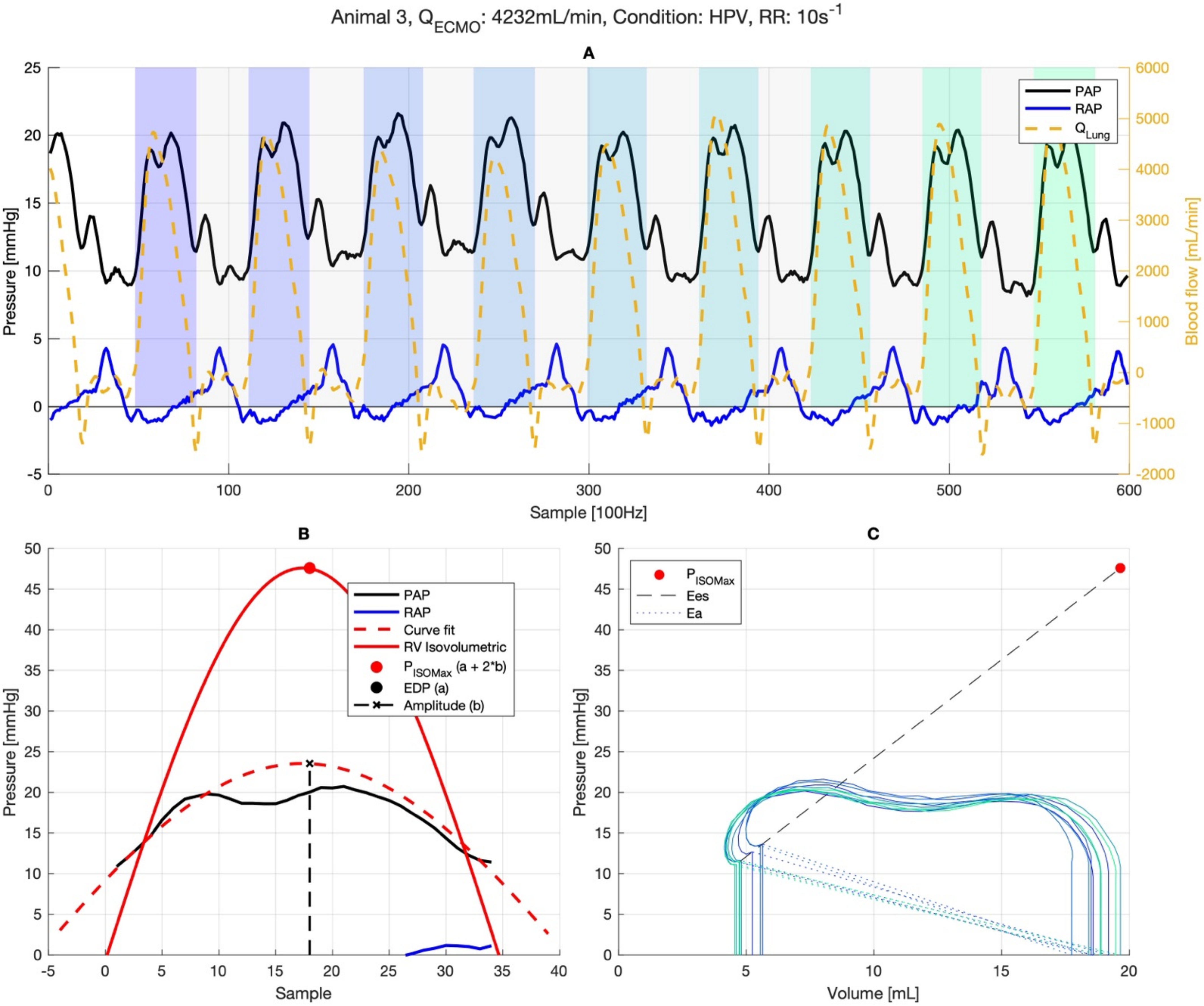

Animal 3, QECMO: 4232mL/min, Condition: HPV, RR: 10s-1
A
PAP
RAP
QLung
Pressure [mmHg]
Blood flow [mL/min]
Sample [100Hz]
B
PAP
RAP
Curve fit
RV Isovolumetric
PISOMax (a + 2*b)
EDP (a)
Amplitude (b)
Pressure [mmHg]
Sample
C
PISOMax
Ees
Ea
Pressure [mmHg]
Volume [mL]

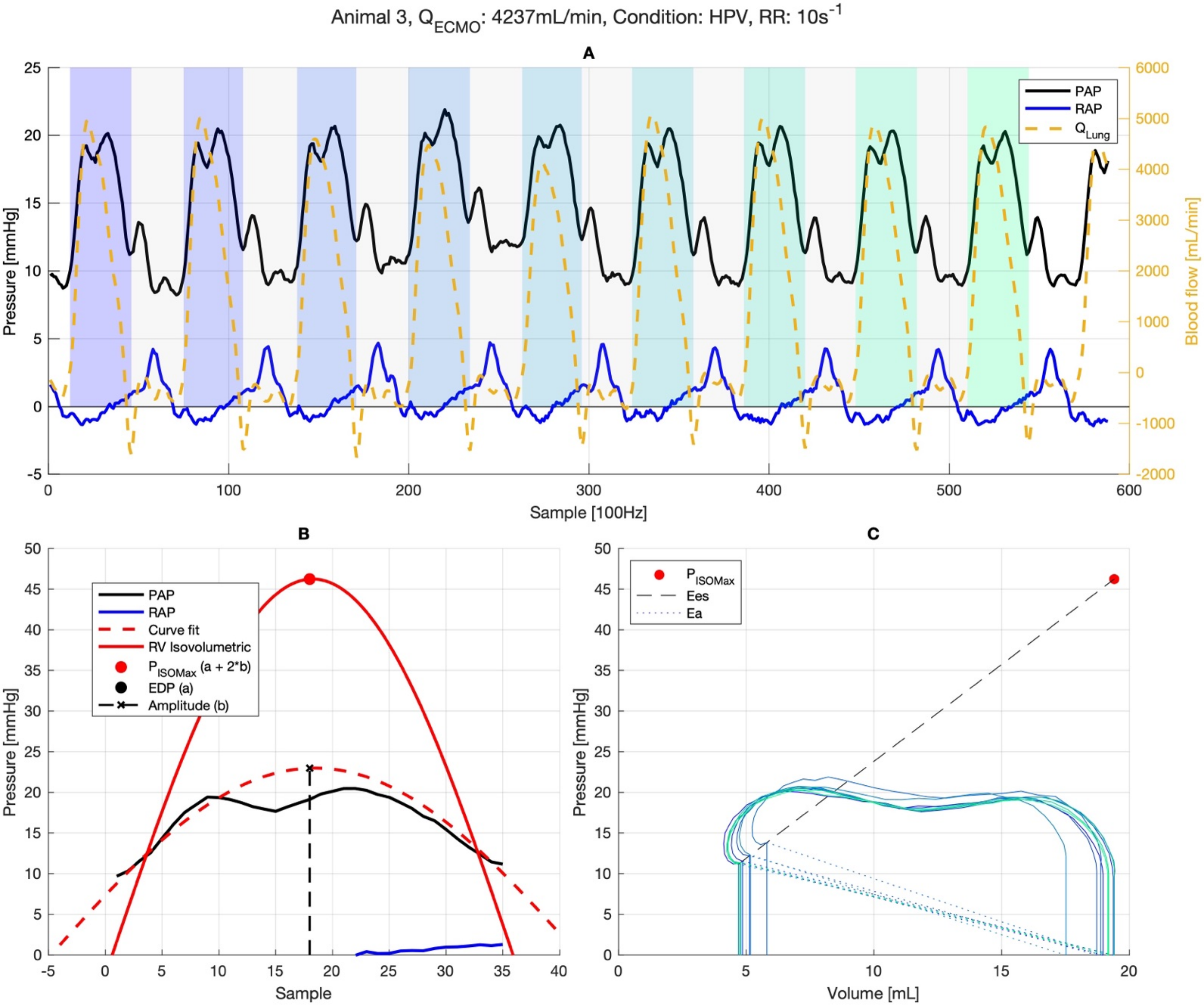

Animal 3, Q_ECMO: 4237mL/min, Condition: HPV, RR: 10s^-1
A
PAP
RAP
Q_Lung
Pressure [mmHg]
Blood flow [mL/min]
Sample [100Hz]
B
PAP
RAP
Curve fit
RV Isovolumetric
P_ISOMax (a + 2*b)
EDP (a)
Amplitude (b)
Pressure [mmHg]
Sample
C
P_ISOMax
Ees
Ea
Pressure [mmHg]
Volume [mL]

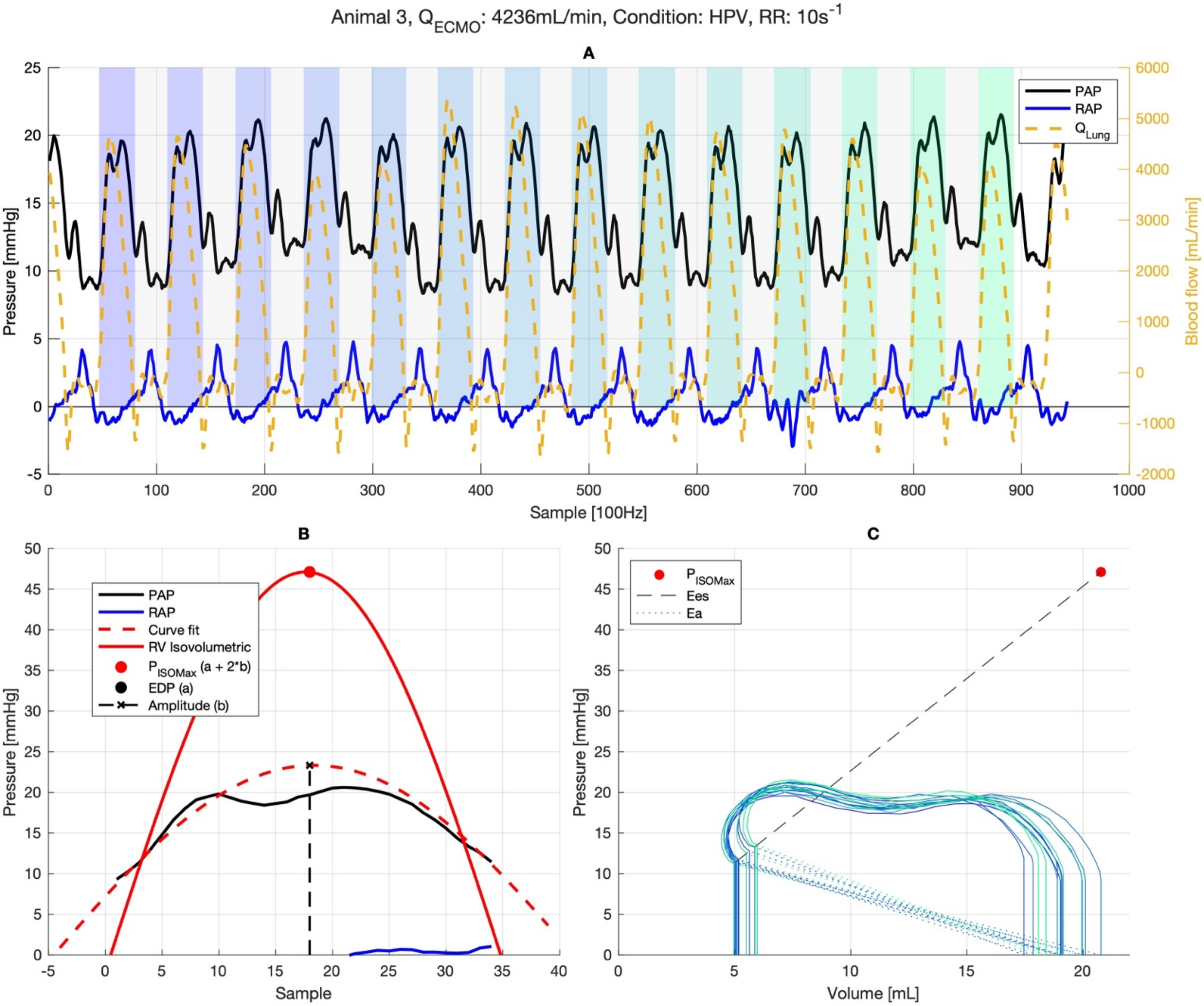

Animal 3, Q_{ECMO}: 4236mL/min, Condition: HPV, RR: $10s^{-1}$
A
PAP
RAP
Q_{Lung}
Pressure [mmHg]
Blood flow [mL/min]
Sample [100Hz]
B
PAP
RAP
Curve fit
RV Isovolumetric
P_{ISOMax} (a + 2*b)
EDP (a)
Amplitude (b)
Pressure [mmHg]
Sample
C
P_{ISOMax}
Ees
Ea
Pressure [mmHg]
Volume [mL]

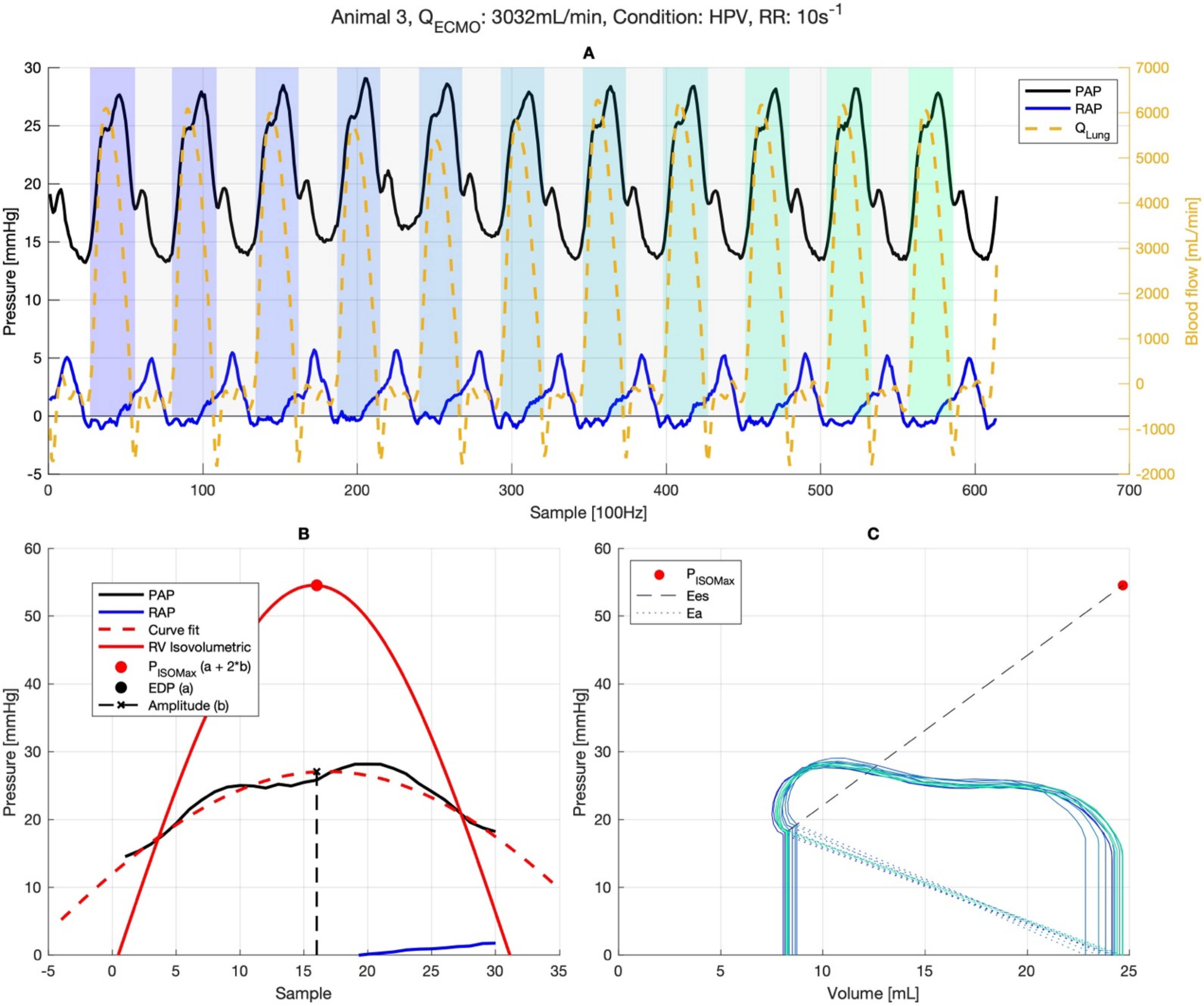

Animal 3, Q_ECMO: 3032mL/min, Condition: HPV, RR: 10s^-1
A
PAP
RAP
Q_Lung
Pressure [mmHg]
Blood flow [mL/min]
Sample [100Hz]
B
PAP
RAP
Curve fit
RV Isovolumetric
P_ISOMax (a + 2*b)
EDP (a)
Amplitude (b)
Pressure [mmHg]
Sample
C
P_ISOMax
Ees
Ea
Pressure [mmHg]
Volume [mL]

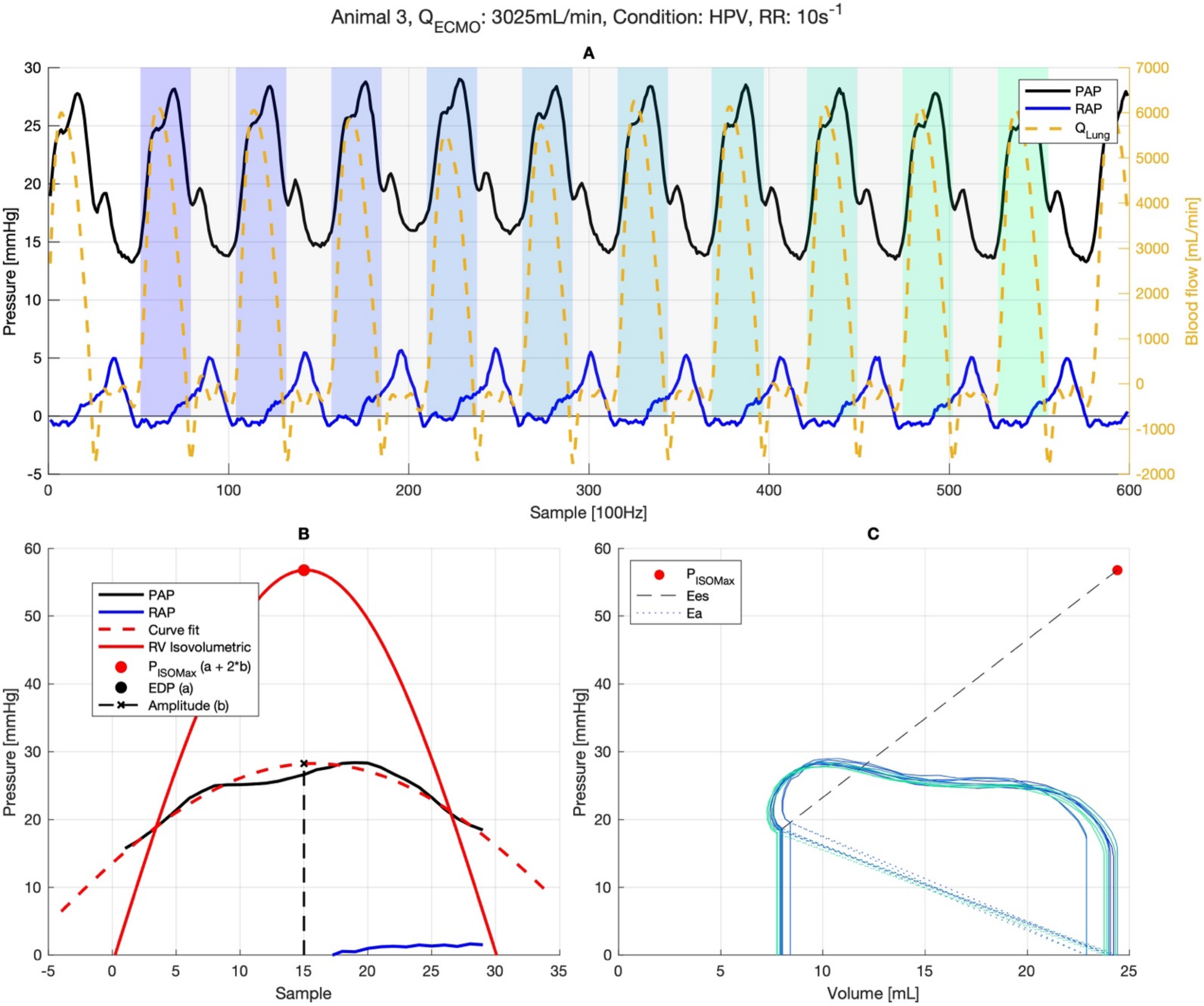

Animal 3, Q_{ECMO}: 3025mL/min, Condition: HPV, RR: $10s^{-1}$
A
PAP
RAP
Q_{Lung}
Pressure [mmHg]
Blood flow [mL/min]
Sample [100Hz]
B
PAP
RAP
Curve fit
RV Isovolumetric
P_{ISOMax} (a + 2*b)
EDP (a)
Amplitude (b)
Pressure [mmHg]
Sample
C
P_{ISOMax}
Ees
Ea
Pressure [mmHg]
Volume [mL]

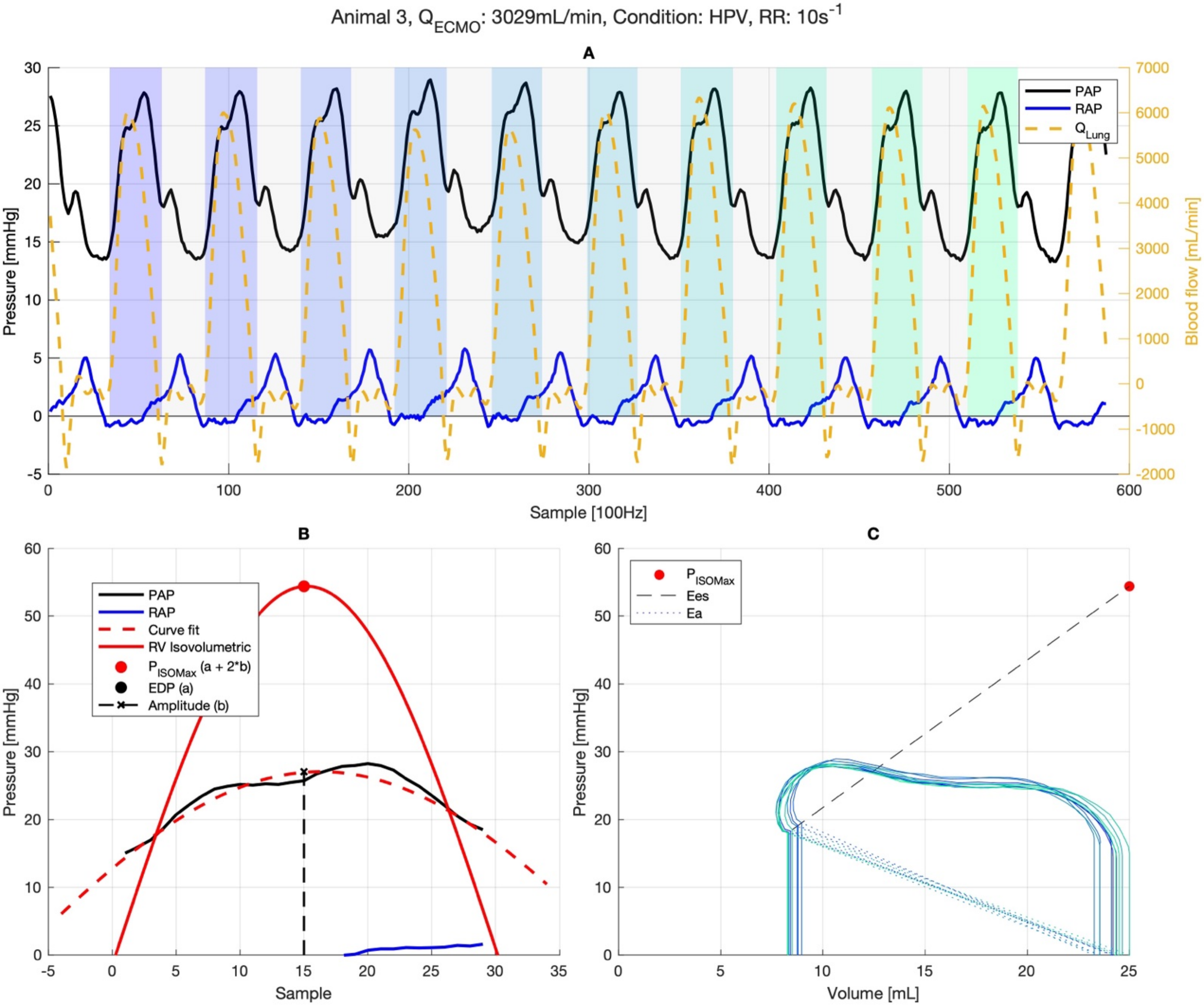

Animal 3, Q_{ECMO}: 3029mL/min, Condition: HPV, RR: $10s^{-1}$
A
Pressure [mmHg]
Blood flow [mL/min]
Sample [100Hz]
PAP
RAP
Q_{Lung}
B
Pressure [mmHg]
Sample
PAP
RAP
Curve fit
RV Isovolumetric
P_{ISOMax} (a + 2*b)
EDP (a)
Amplitude (b)
C
Pressure [mmHg]
Volume [mL]
P_{ISOMax}
Ees
Ea

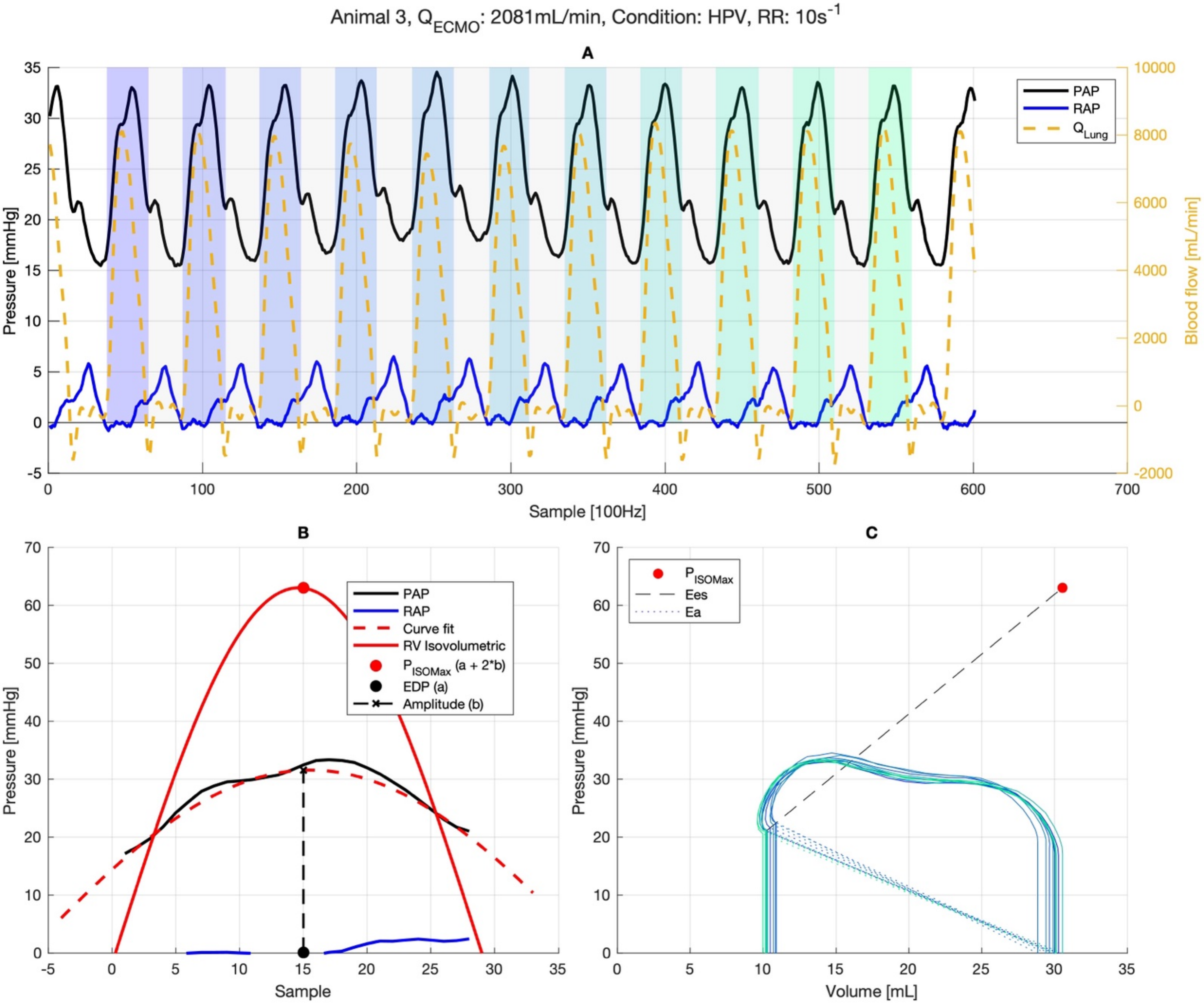

Animal 3, Q_{ECMO}: 2081mL/min, Condition: HPV, RR: $10s^{-1}$
A
PAP
RAP
Q_{Lung}
Pressure [mmHg]
Blood flow [mL/min]
Sample [100Hz]
B
PAP
RAP
Curve fit
RV Isovolumetric
P_{ISOMax} (a + 2*b)
EDP (a)
Amplitude (b)
Pressure [mmHg]
Sample
C
P_{ISOMax}
Ees
Ea
Pressure [mmHg]
Volume [mL]

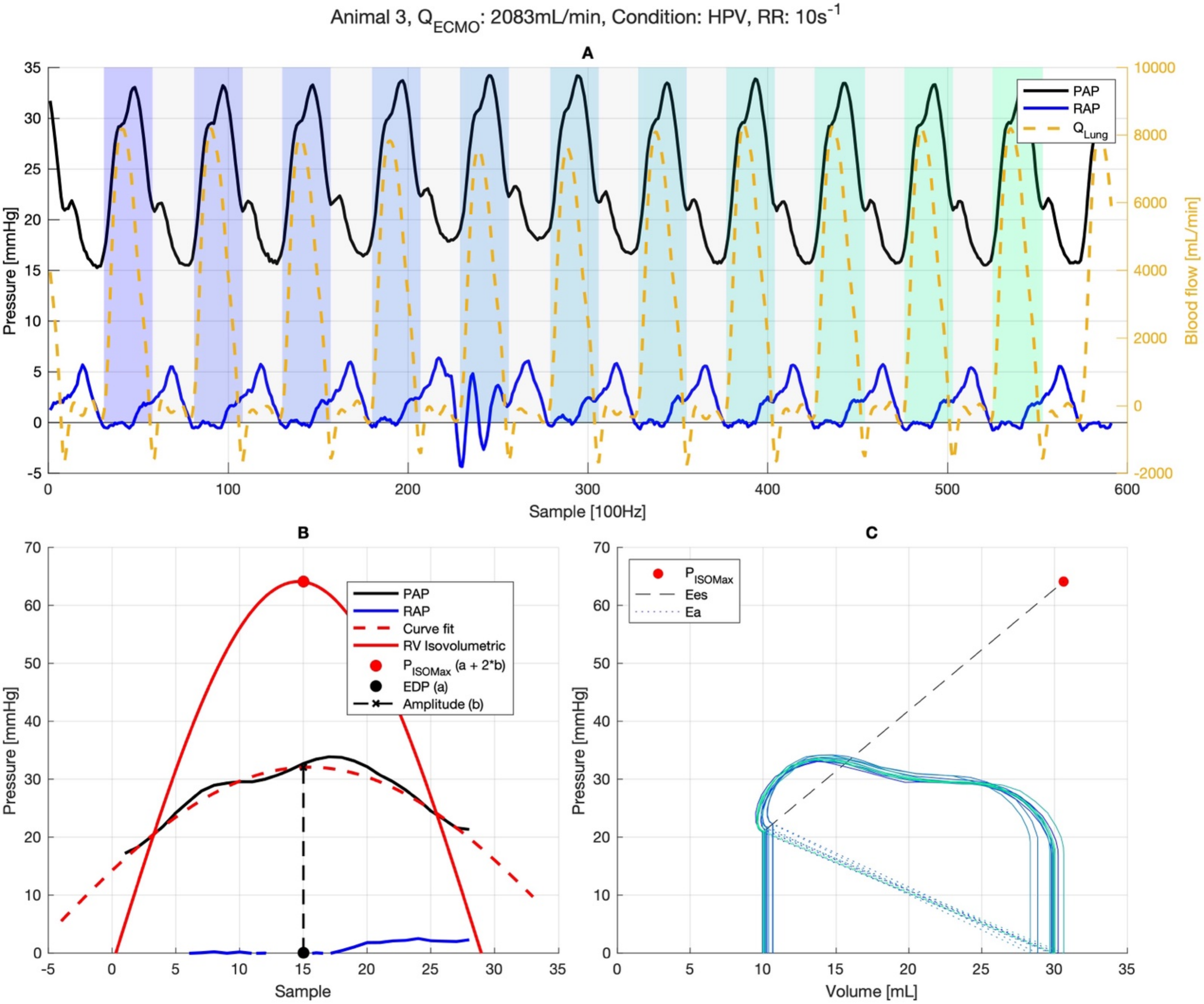

Animal 3, Q_{ECMO}: 2083mL/min, Condition: HPV, RR: 10s^{-1}
A
PAP
RAP
Q_{Lung}
Pressure [mmHg]
Blood flow [mL/min]
Sample [100Hz]
B
PAP
RAP
Curve fit
RV Isovolumetric
P_{ISOMax} (a + 2*b)
EDP (a)
Amplitude (b)
Pressure [mmHg]
Sample
C
P_{ISOMax}
Ees
Ea
Pressure [mmHg]
Volume [mL]

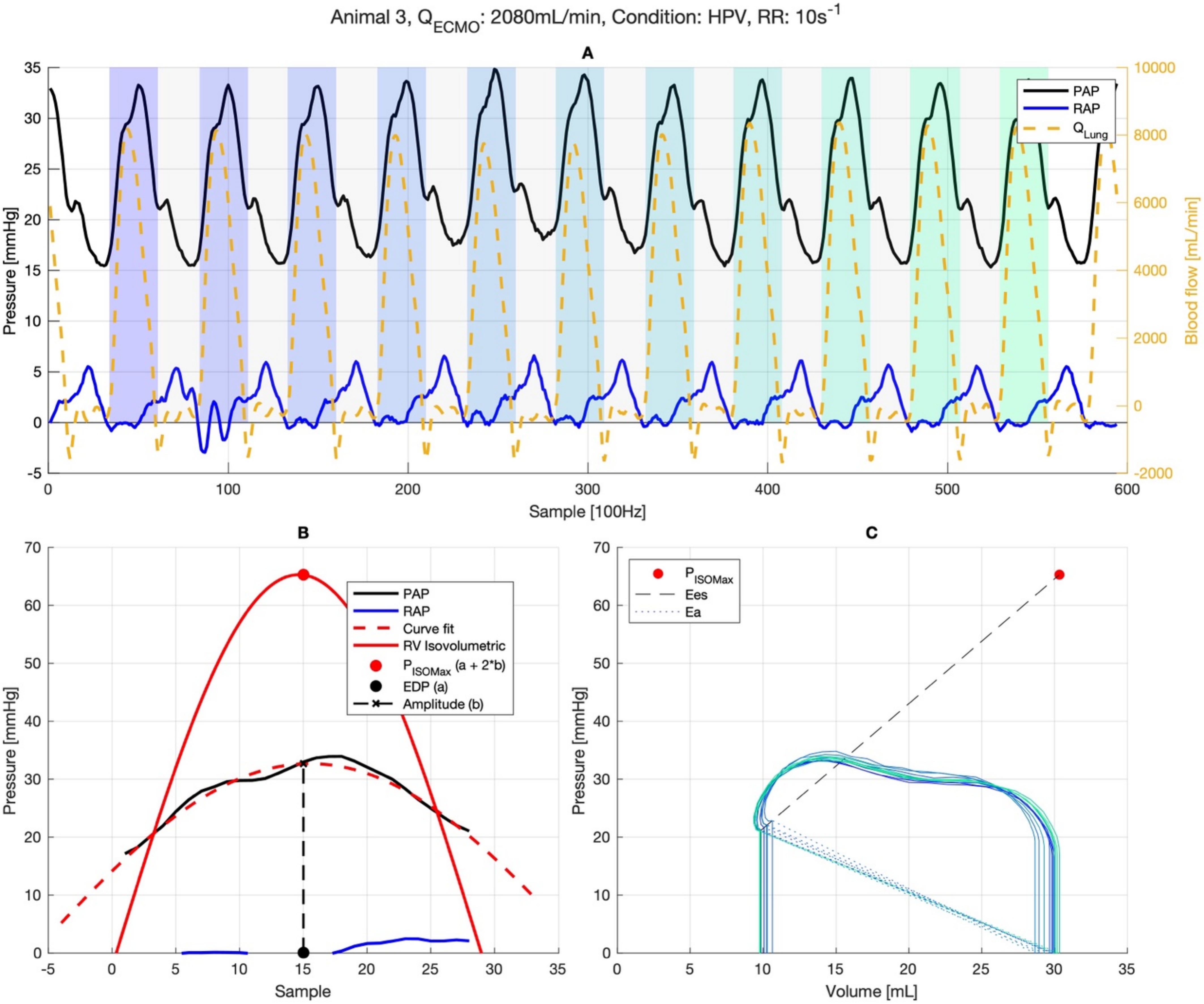

Animal 3, Q_ECMO: 2080mL/min, Condition: HPV, RR: 10s^-1
A
PAP
RAP
Q_Lung
Pressure [mmHg]
Blood flow [mL/min]
Sample [100Hz]
B
PAP
RAP
Curve fit
RV Isovolumetric
P_ISOMax (a + 2*b)
EDP (a)
Amplitude (b)
Pressure [mmHg]
Sample
C
P_ISOMax
Ees
Ea
Pressure [mmHg]
Volume [mL]

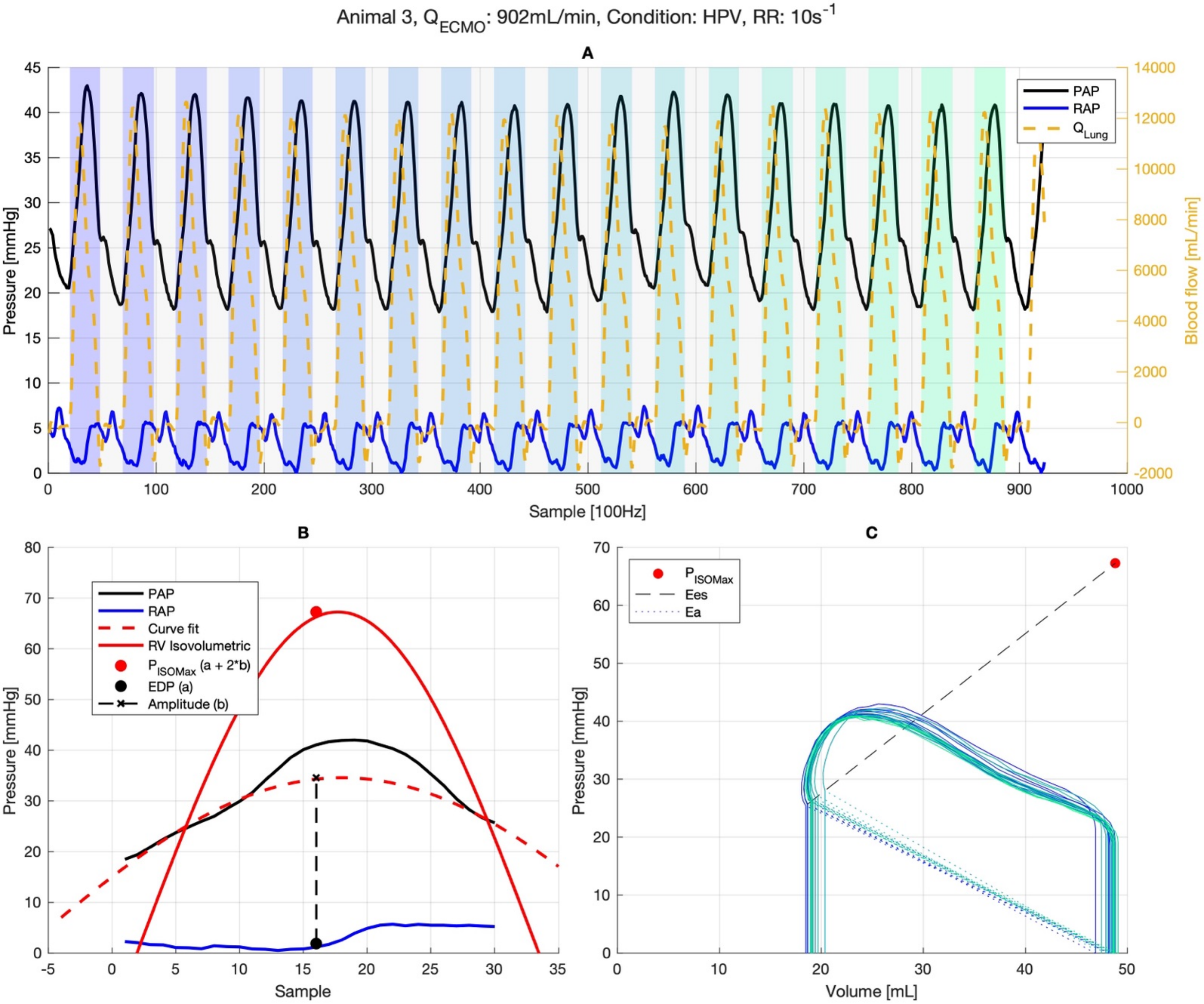

Animal 3, Q_{ECMO}: 902mL/min, Condition: HPV, RR: $10s^{-1}$
A
Pressure [mmHg]
Blood flow [mL/min]
Sample [100Hz]
PAP
RAP
Q_{Lung}
B
Pressure [mmHg]
Sample
PAP
RAP
Curve fit
RV Isovolumetric
P_{ISOMax} (a + 2*b)
EDP (a)
Amplitude (b)
C
Pressure [mmHg]
Volume [mL]
P_{ISOMax}
Ees
Ea

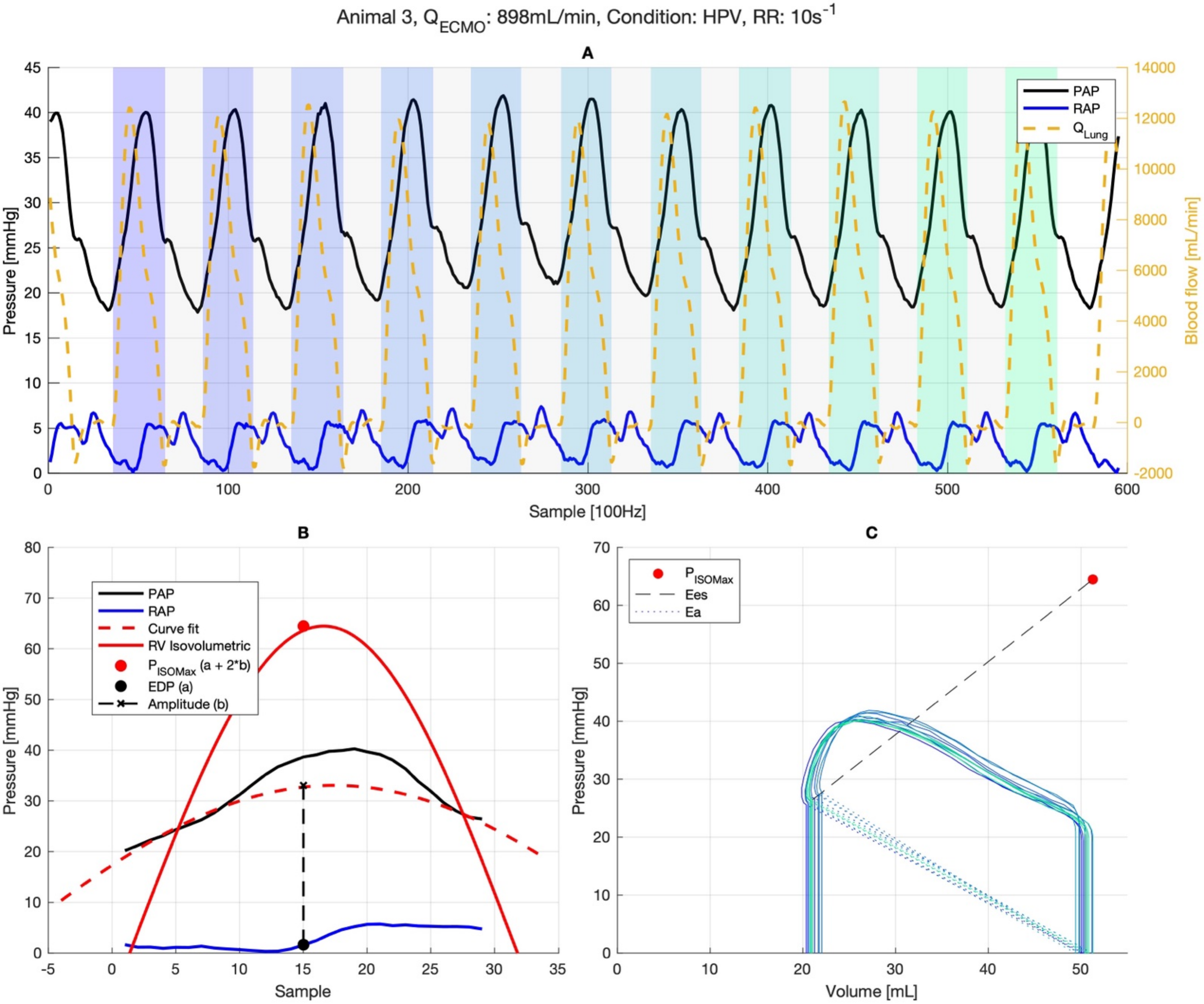
Animal 3, Q_{ECMO}: 898mL/min, Condition: HPV, RR: $10s^{-1}$
A
PAP
RAP
Q_{Lung}
Pressure [mmHg]
Blood flow [mL/min]
Sample [100Hz]
B
PAP
RAP
Curve fit
RV Isovolumetric
P_{ISOMax} (a + 2*b)
EDP (a)
Amplitude (b)
Pressure [mmHg]
Sample
C
P_{ISOMax}
Ees
Ea
Pressure [mmHg]
Volume [mL]

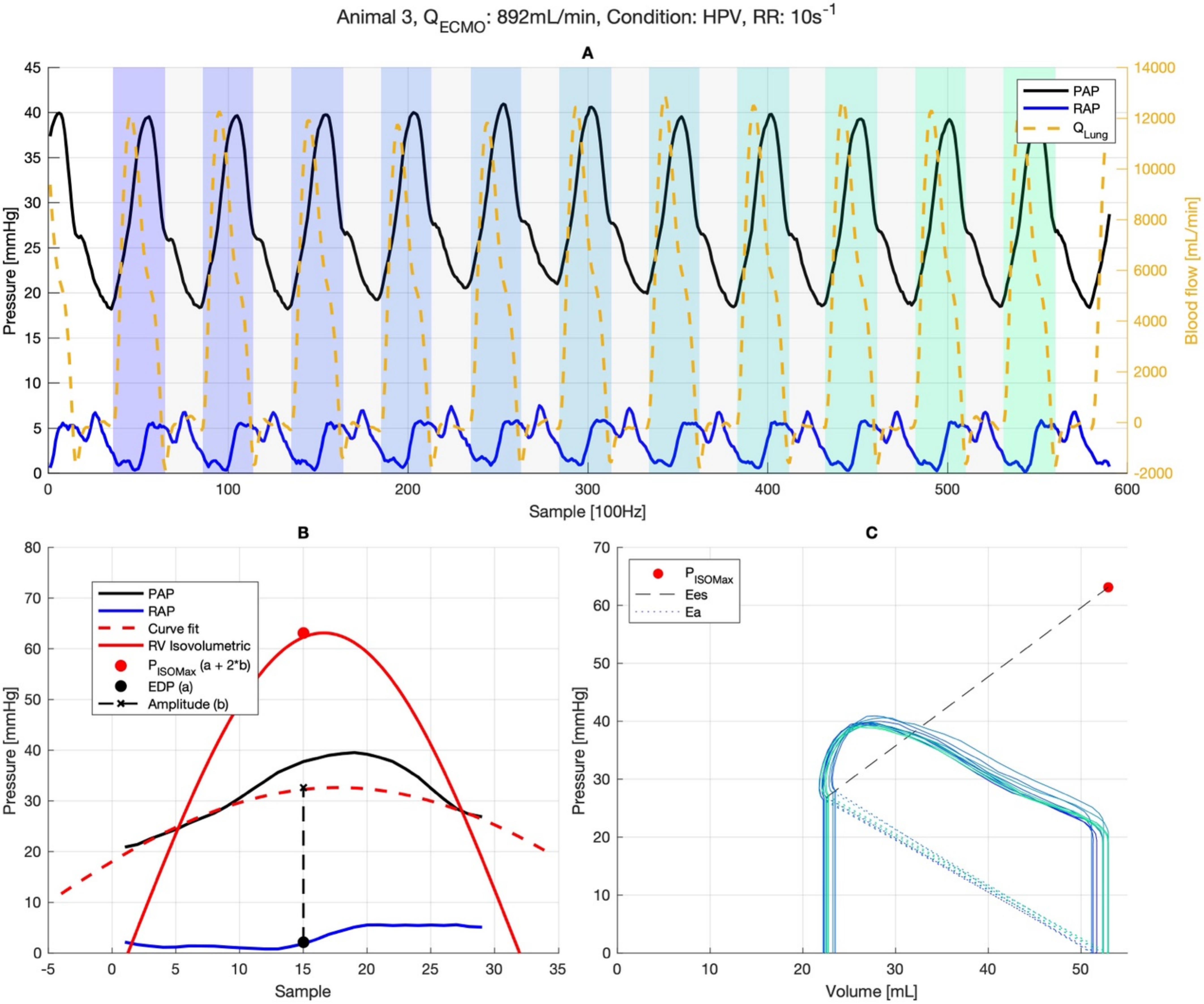

Animal 3, Q_{ECMO}: 892mL/min, Condition: HPV, RR: $10s^{-1}$
A
PAP
RAP
Q_{Lung}
Pressure [mmHg]
Blood flow [mL/min]
Sample [100Hz]
B
PAP
RAP
Curve fit
RV Isovolumetric
P_{ISOMax} (a + 2*b)
EDP (a)
Amplitude (b)
Pressure [mmHg]
Sample
C
P_{ISOMax}
Ees
Ea
Pressure [mmHg]
Volume [mL]

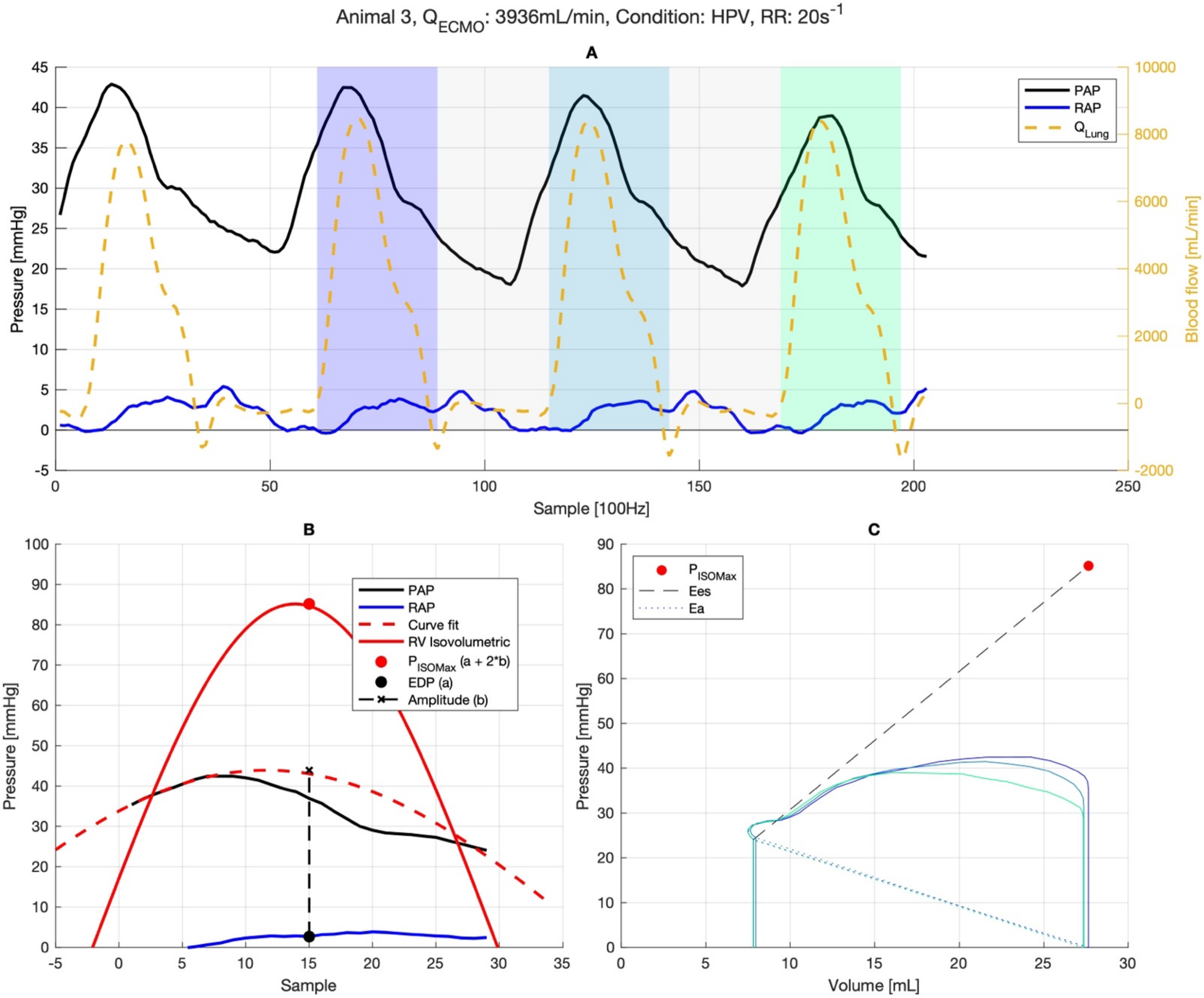
Animal 3, Q_{ECMO}: 3936mL/min, Condition: HPV, RR: $20s^{-1}$
A
Pressure [mmHg]
Blood flow [mL/min]
Sample [100Hz]
PAP
RAP
Q_{Lung}
B
Pressure [mmHg]
Sample
Curve fit
RV Isovolumetric
P_{ISOMax} (a + 2*b)
EDP (a)
Amplitude (b)
C
Pressure [mmHg]
Volume [mL]
P_{ISOMax}
Ees
Ea

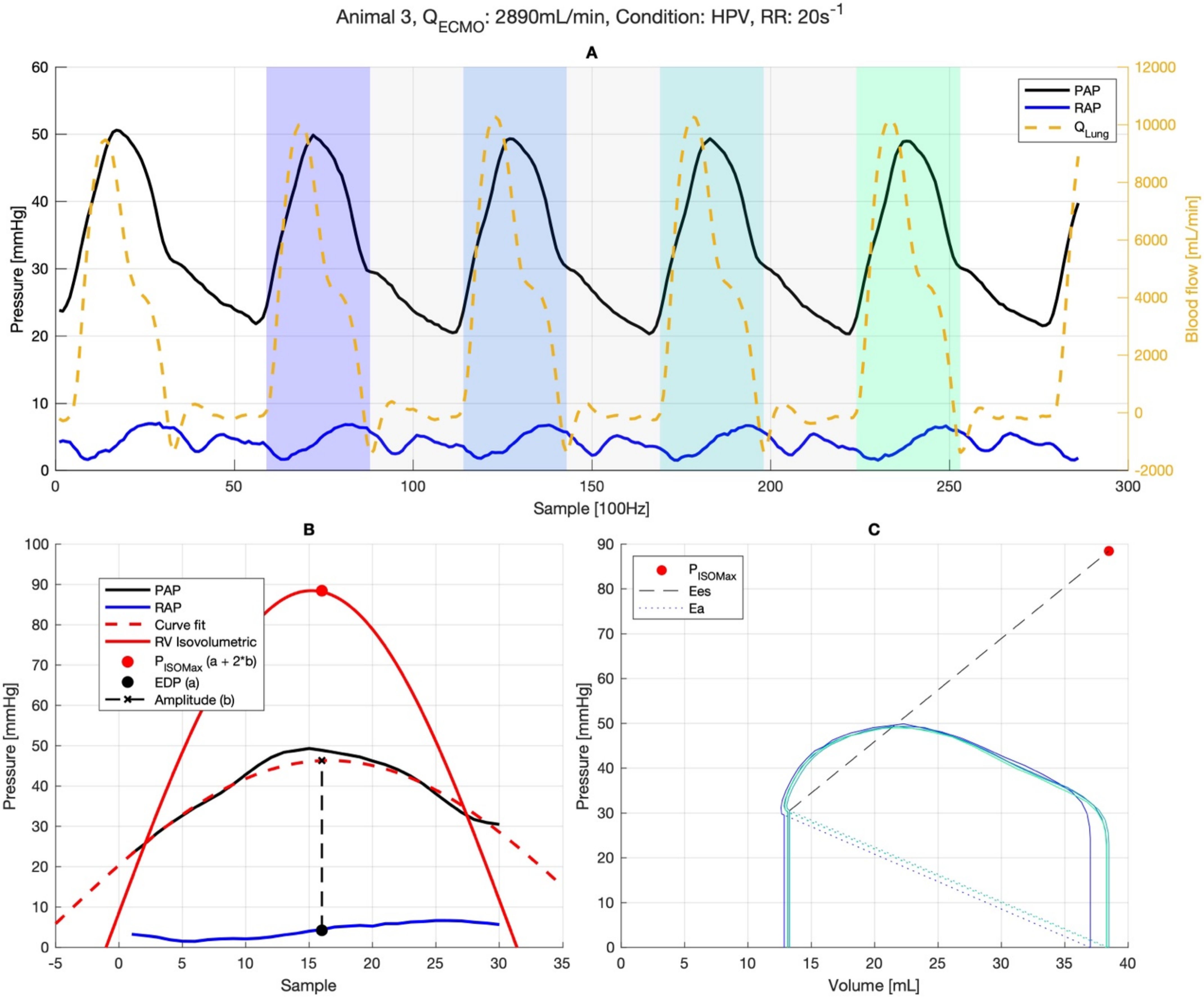

Animal 3, Q_ECMO: 2890mL/min, Condition: HPV, RR: 20s^-1
A
PAP
RAP
Q_Lung
Pressure [mmHg]
Blood flow [mL/min]
Sample [100Hz]
B
PAP
RAP
Curve fit
RV Isovolumetric
P_ISOMax (a + 2*b)
EDP (a)
Amplitude (b)
Pressure [mmHg]
Sample
C
P_ISOMax
Ees
Ea
Pressure [mmHg]
Volume [mL]

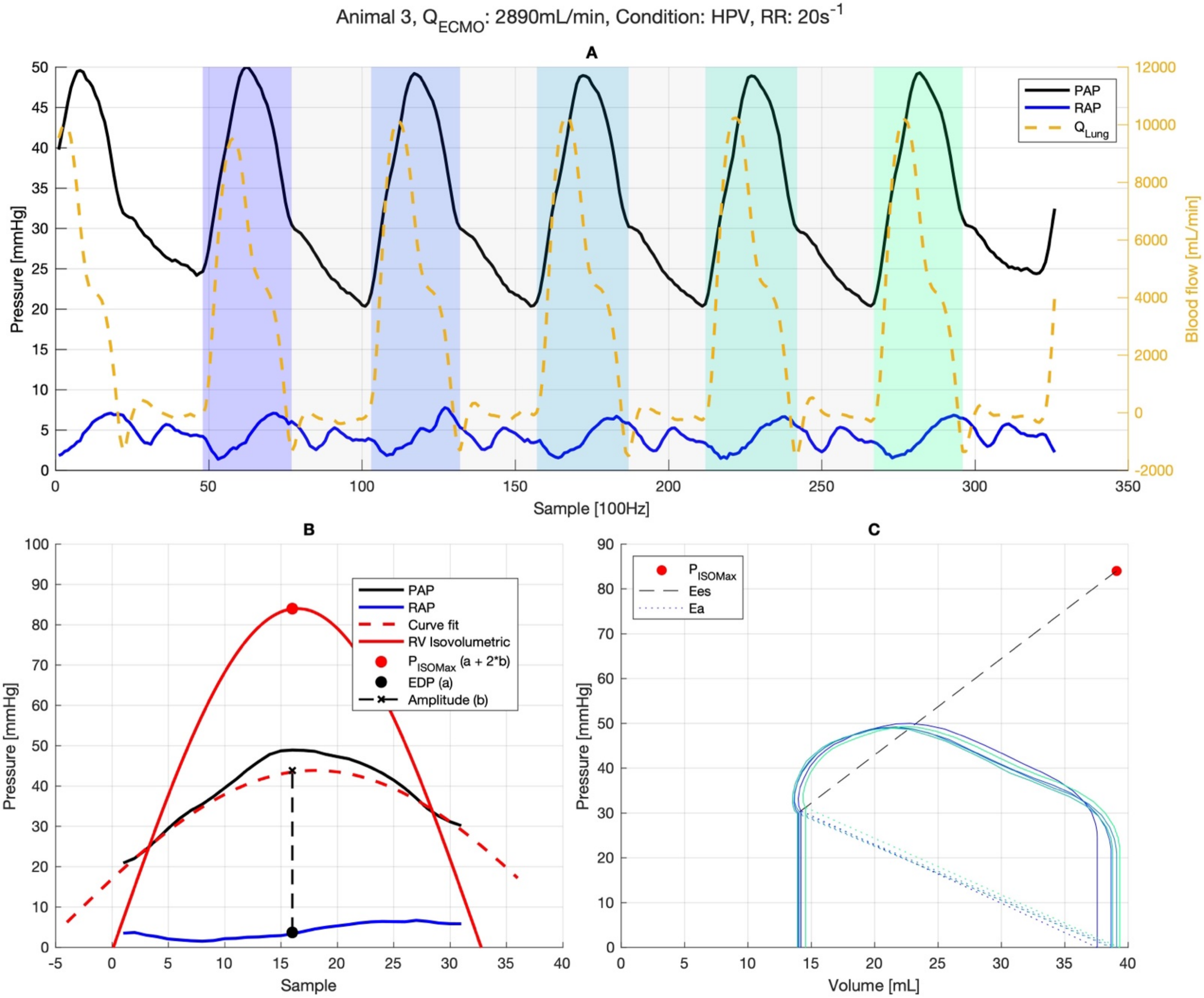

Animal 3, Q_{ECMO}: 2890mL/min, Condition: HPV, RR: $20s^{-1}$
A
PAP
RAP
Q_{Lung}
Pressure [mmHg]
Blood flow [mL/min]
Sample [100Hz]
B
PAP
RAP
Curve fit
RV Isovolumetric
P_{ISOMax} (a + 2*b)
EDP (a)
Amplitude (b)
Pressure [mmHg]
Sample
C
P_{ISOMax}
Ees
Ea
Pressure [mmHg]
Volume [mL]

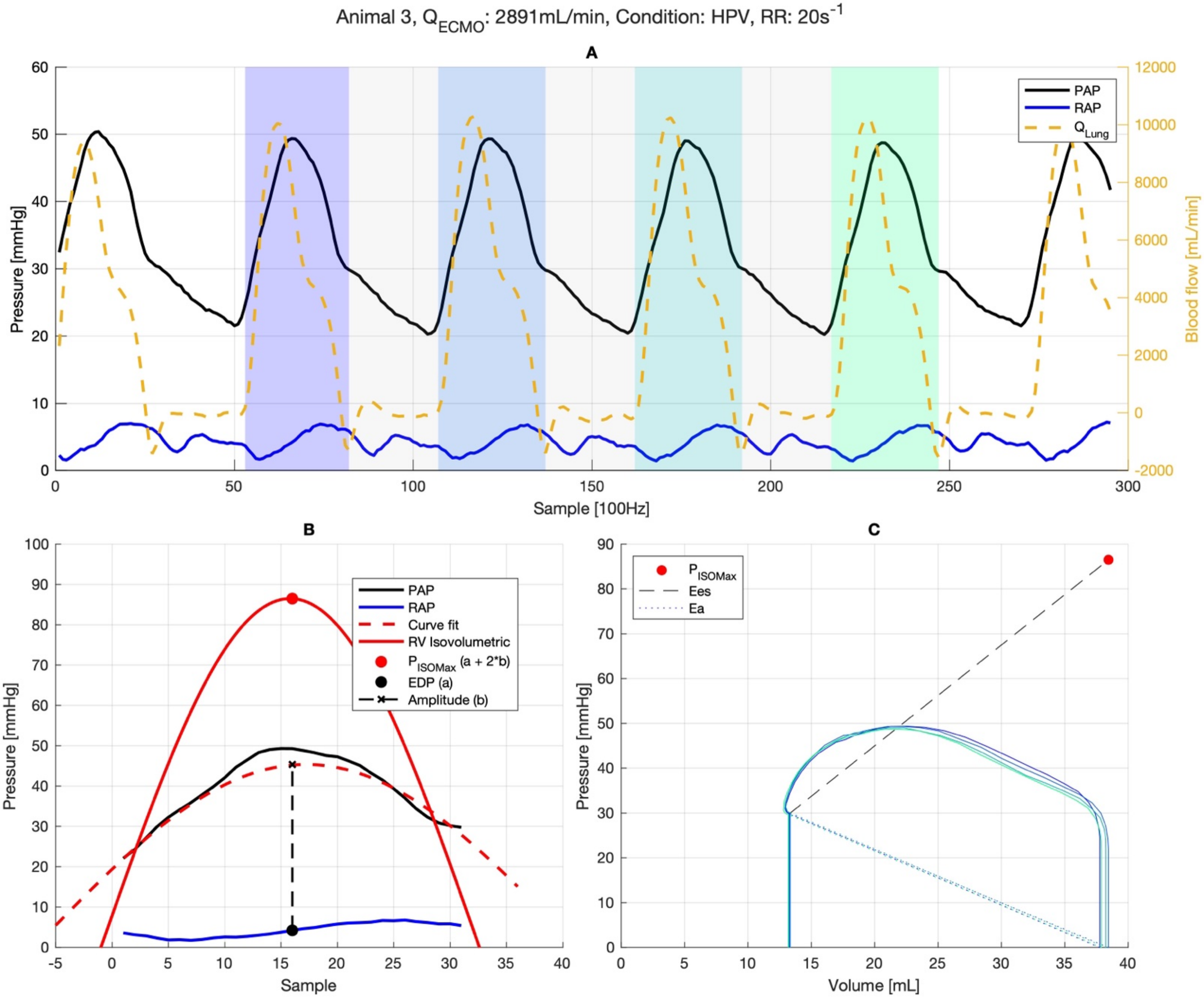

Animal 3, Q_{ECMO}: 2891mL/min, Condition: HPV, RR: 20s^{-1}
A
Pressure [mmHg]
Blood flow [mL/min]
Sample [100Hz]
PAP
RAP
Q_{Lung}
B
Pressure [mmHg]
Sample
PAP
RAP
Curve fit
RV Isovolumetric
P_{ISOMax} (a + 2*b)
EDP (a)
Amplitude (b)
C
Pressure [mmHg]
Volume [mL]
P_{ISOMax}
Ees
Ea

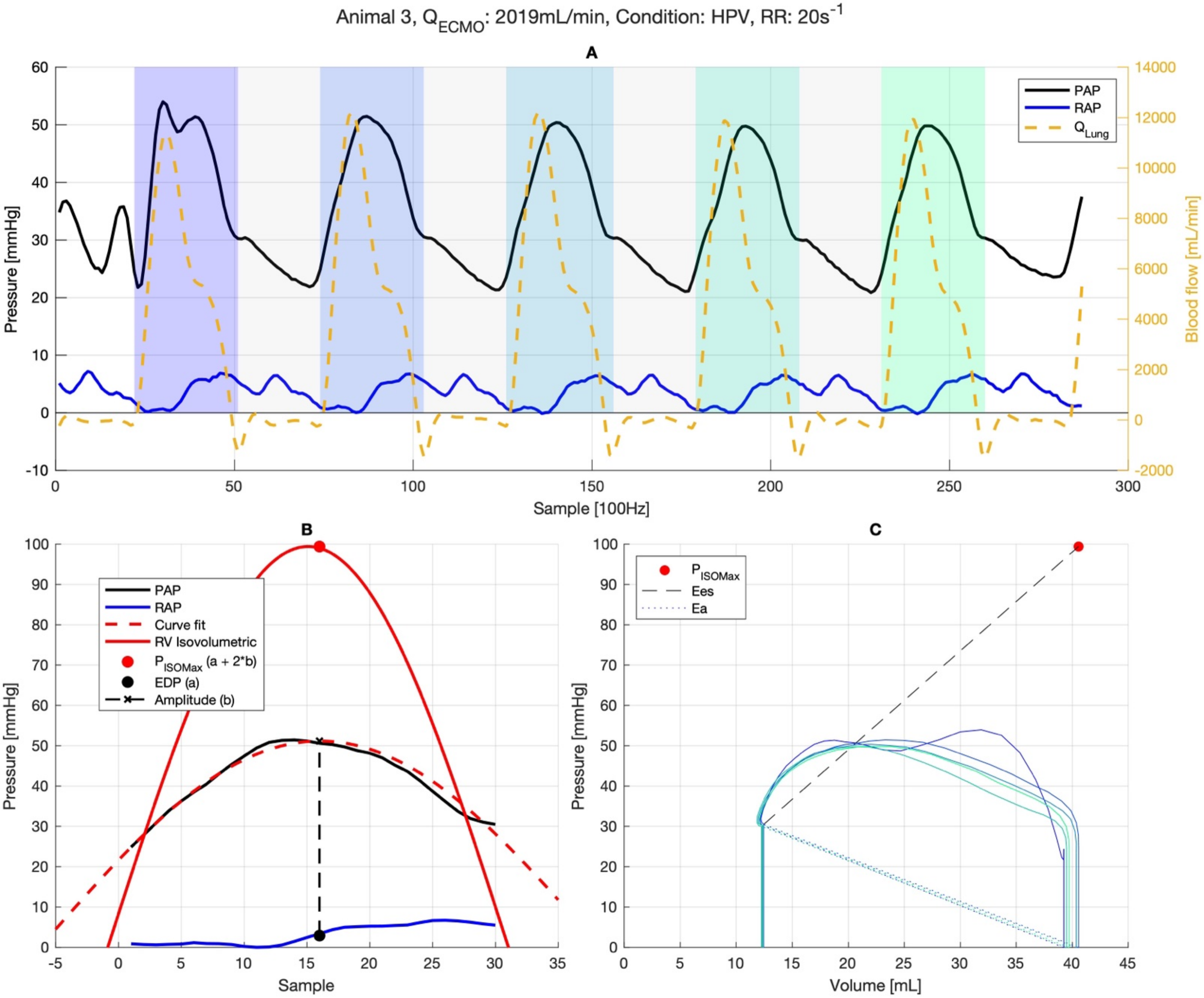

Animal 3, Q_ECMO: 2019mL/min, Condition: HPV, RR: 20s^-1
A
PAP
RAP
Q_Lung
Pressure [mmHg]
Blood flow [mL/min]
Sample [100Hz]
B
PAP
RAP
Curve fit
RV Isovolumetric
P_ISOMax (a + 2*b)
EDP (a)
Amplitude (b)
Pressure [mmHg]
Sample
C
P_ISOMax
Ees
Ea
Pressure [mmHg]
Volume [mL]

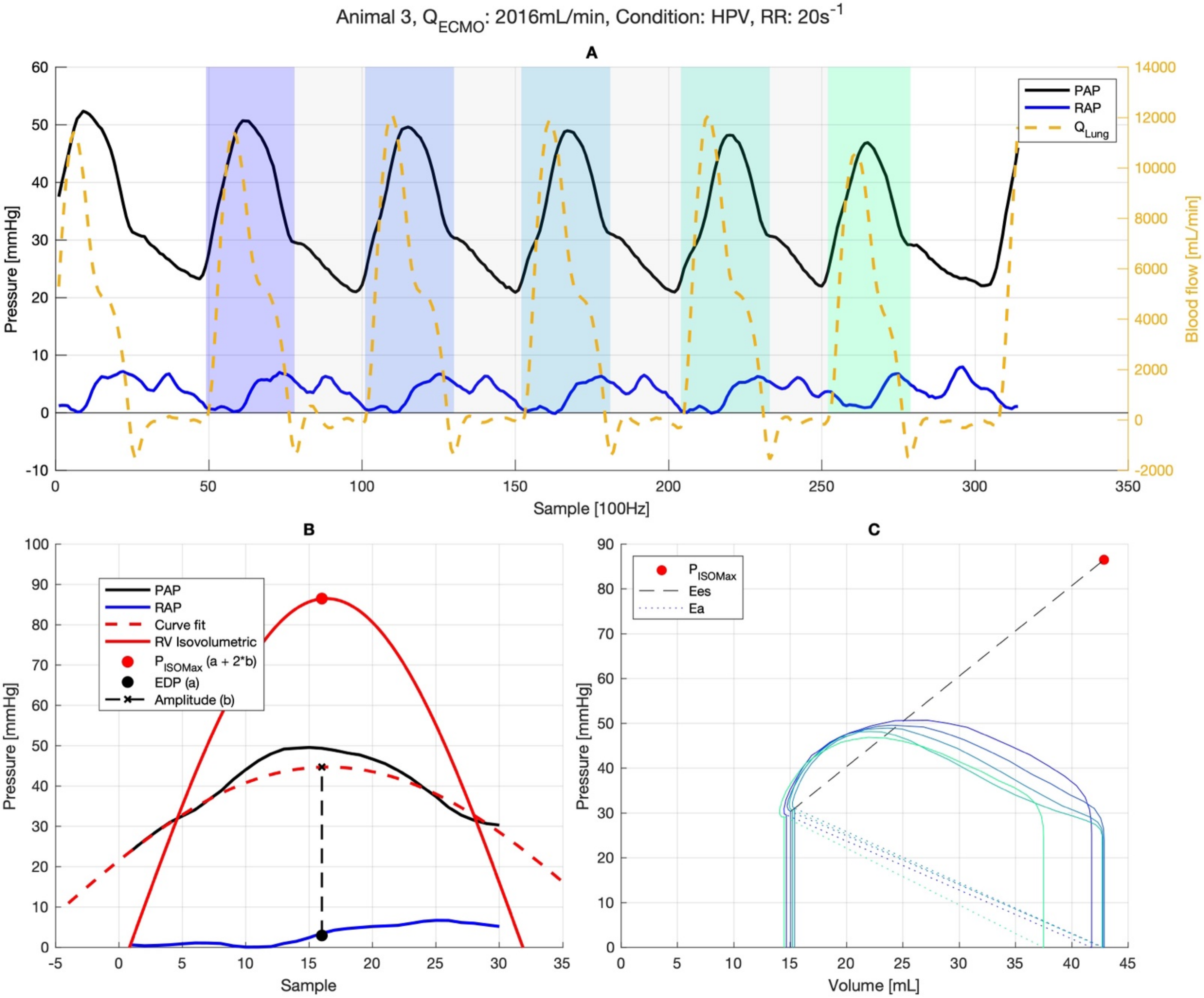

Animal 3, Q_ECMO: 2016mL/min, Condition: HPV, RR: 20s-1
A
Pressure [mmHg]
Blood flow [mL/min]
Sample [100Hz]
PAP
RAP
Q_Lung
B
PAP
RAP
Curve fit
RV Isovolumetric
P_ISOMax (a + 2*b)
EDP (a)
Amplitude (b)
Pressure [mmHg]
Sample
C
P_ISOMax
Ees
Ea
Pressure [mmHg]
Volume [mL]

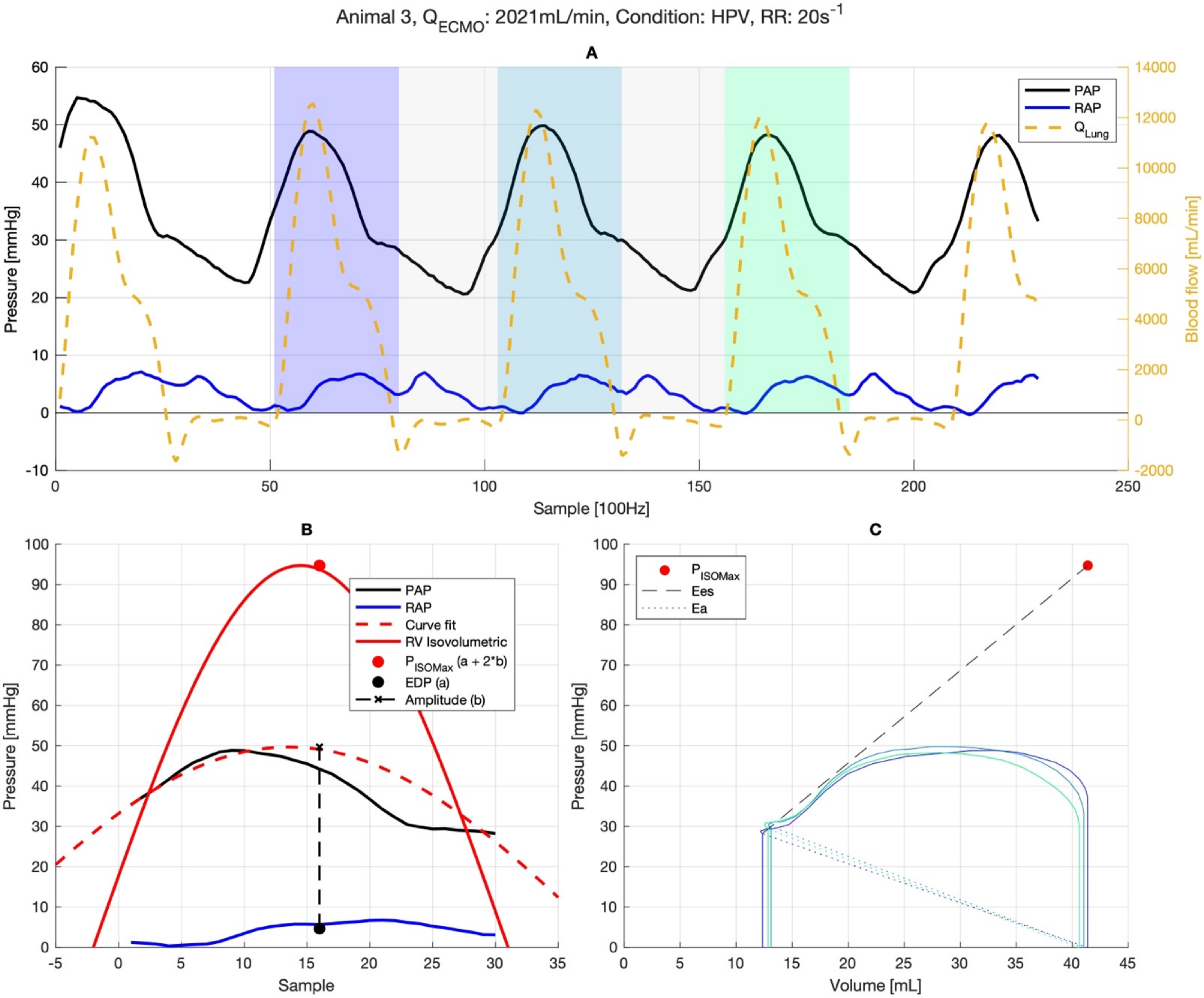

Animal 3, Q_{ECMO}: 2021mL/min, Condition: HPV, RR: 20s^{-1}
A
Pressure [mmHg]
Blood flow [mL/min]
Sample [100Hz]
PAP
RAP
Q_{Lung}
B
Pressure [mmHg]
Sample
PAP
RAP
Curve fit
RV Isovolumetric
P_{ISOMax} (a + 2*b)
EDP (a)
Amplitude (b)
C
Pressure [mmHg]
Volume [mL]
P_{ISOMax}
Ees
Ea

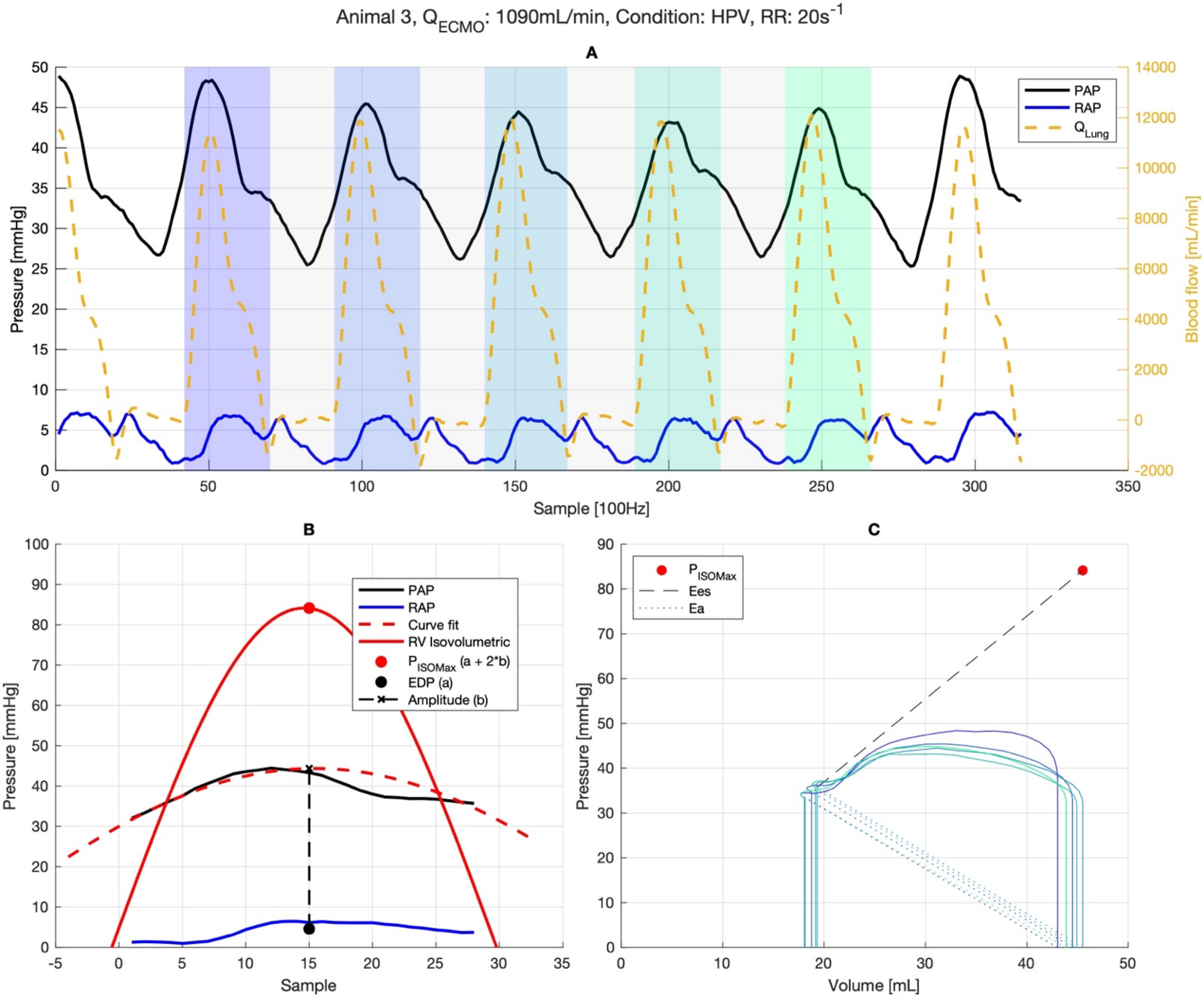

Animal 3, Q_ECMO: 1090mL/min, Condition: HPV, RR: 20s^-1
A
PAP
RAP
Q_Lung
Pressure [mmHg]
Blood flow [mL/min]
Sample [100Hz]
B
PAP
RAP
Curve fit
RV Isovolumetric
P_ISOMax (a + 2*b)
EDP (a)
Amplitude (b)
Pressure [mmHg]
Sample
C
P_ISOMax
Ees
Ea
Pressure [mmHg]
Volume [mL]

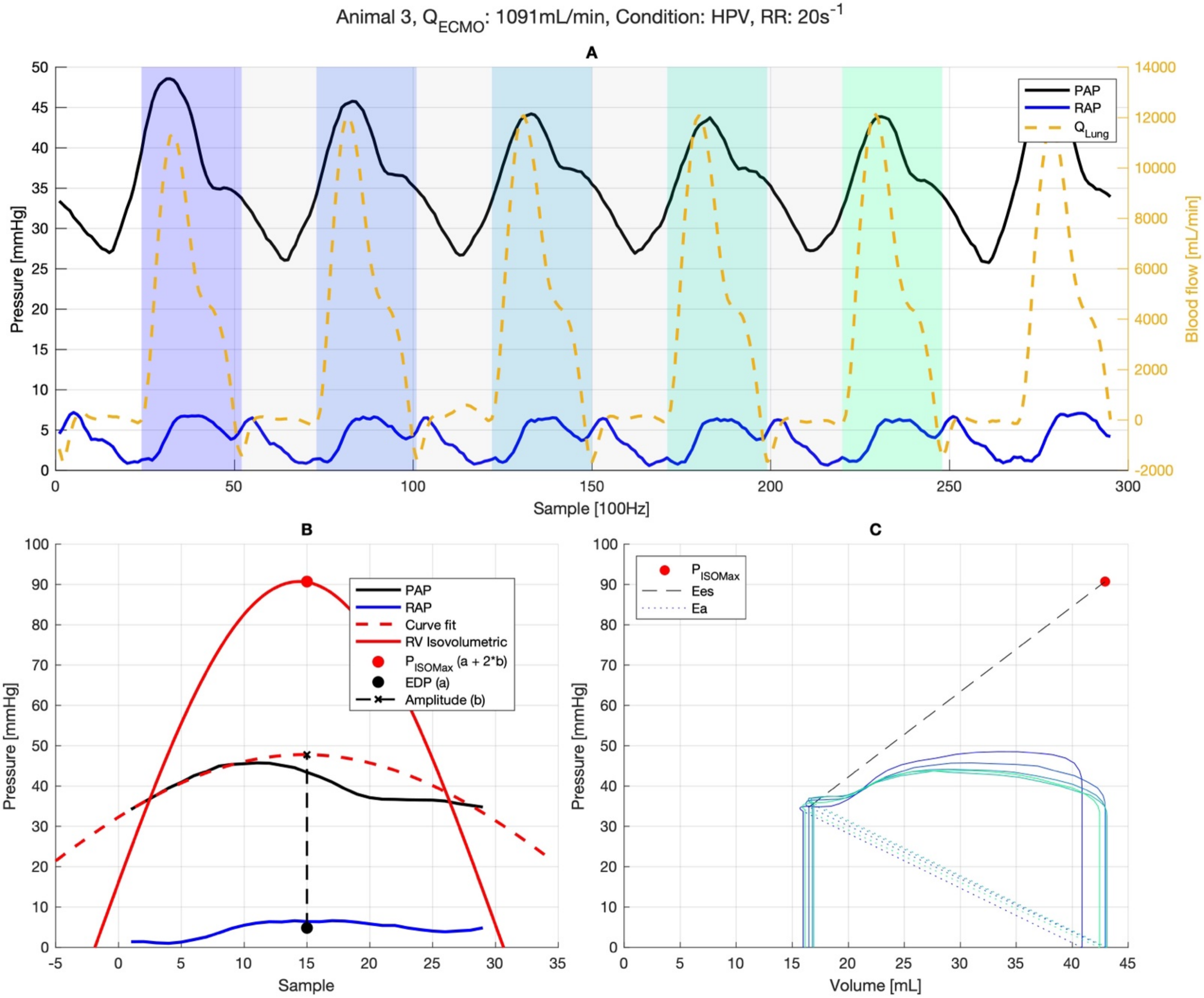

Animal 3, Q_{ECMO}: 1091mL/min, Condition: HPV, RR: $20s^{-1}$
A
PAP
RAP
Q_{Lung}
Pressure [mmHg]
Blood flow [mL/min]
Sample [100Hz]
B
PAP
RAP
Curve fit
RV Isovolumetric
P_{ISOMax} (a + 2*b)
EDP (a)
Amplitude (b)
Pressure [mmHg]
Sample
C
P_{ISOMax}
Ees
Ea
Pressure [mmHg]
Volume [mL]

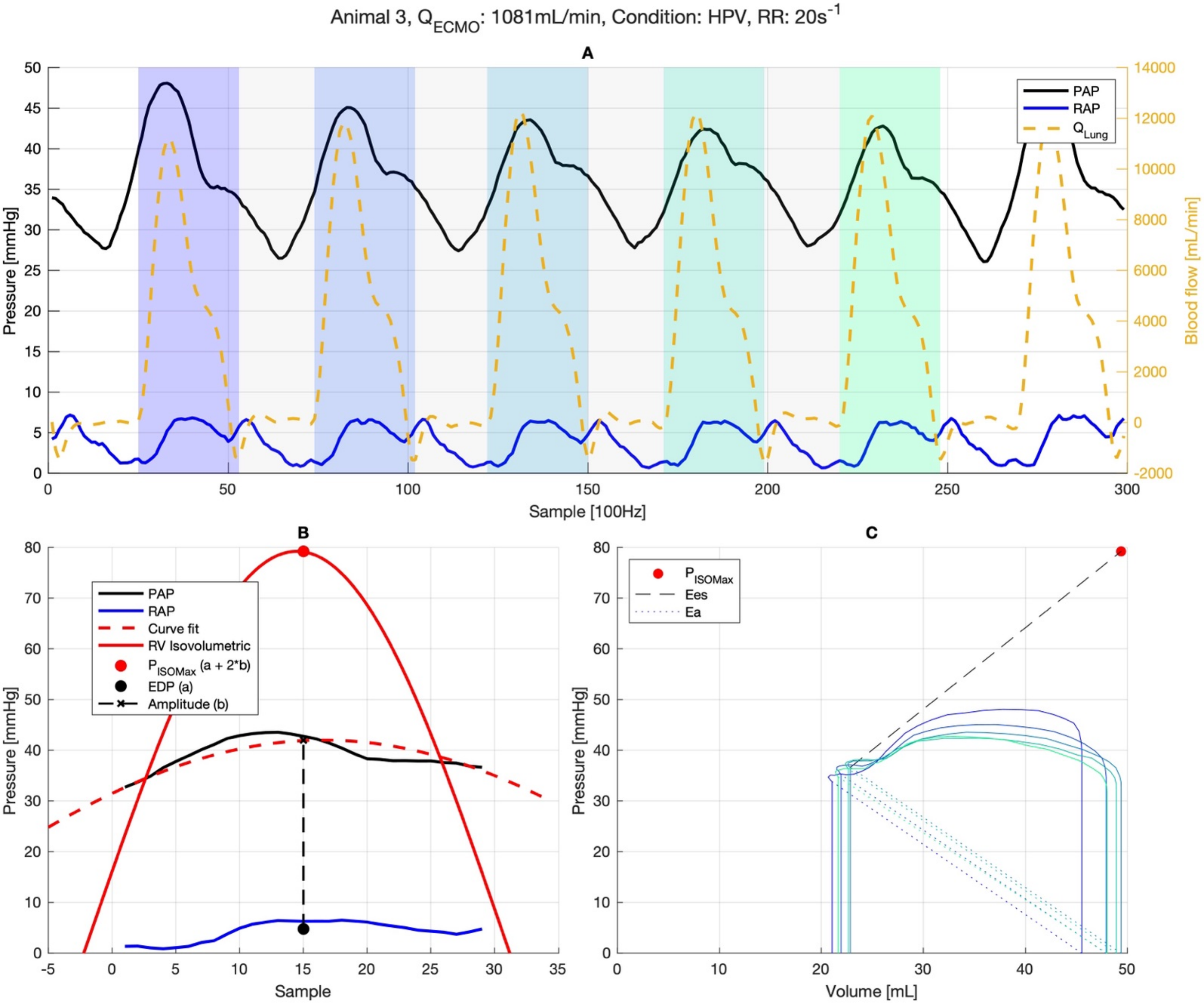

Animal 3, Q_ECMO: 1081mL/min, Condition: HPV, RR: 20s^-1
A
Pressure [mmHg]
Blood flow [mL/min]
Sample [100Hz]
PAP
RAP
Q_Lung
B
Pressure [mmHg]
Sample
Curve fit
RV Isovolumetric
P_ISOMax (a + 2*b)
EDP (a)
Amplitude (b)
C
Pressure [mmHg]
Volume [mL]
P_ISOMax
Ees
Ea

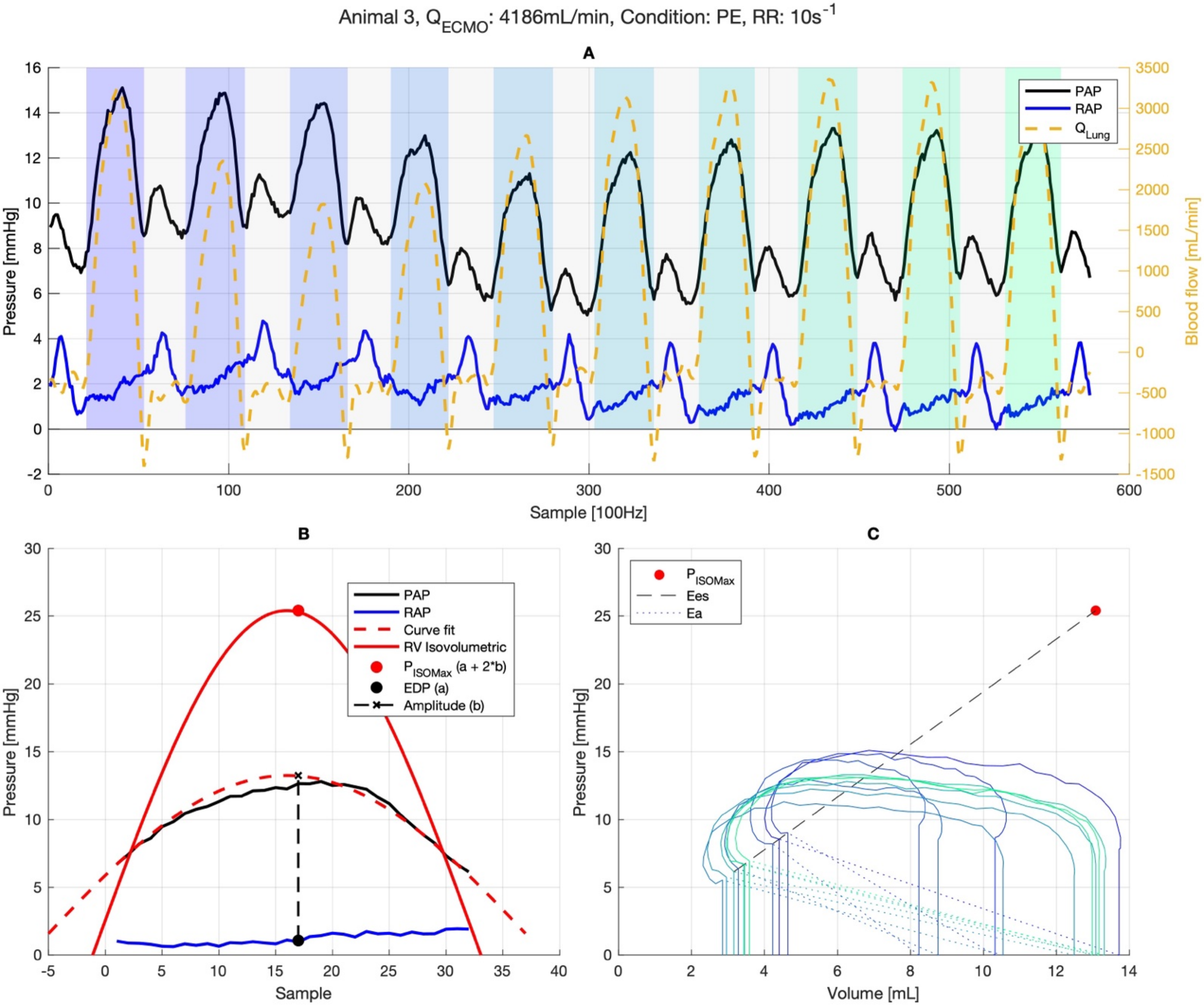

Animal 3, Q_ECMO: 4186mL/min, Condition: PE, RR: 10s^-1
A
PAP
RAP
Q_Lung
Pressure [mmHg]
Blood flow [mL/min]
Sample [100Hz]
B
PAP
RAP
Curve fit
RV Isovolumetric
P_ISOMax (a + 2*b)
EDP (a)
Amplitude (b)
Pressure [mmHg]
Sample
C
P_ISOMax
Ees
Ea
Pressure [mmHg]
Volume [mL]

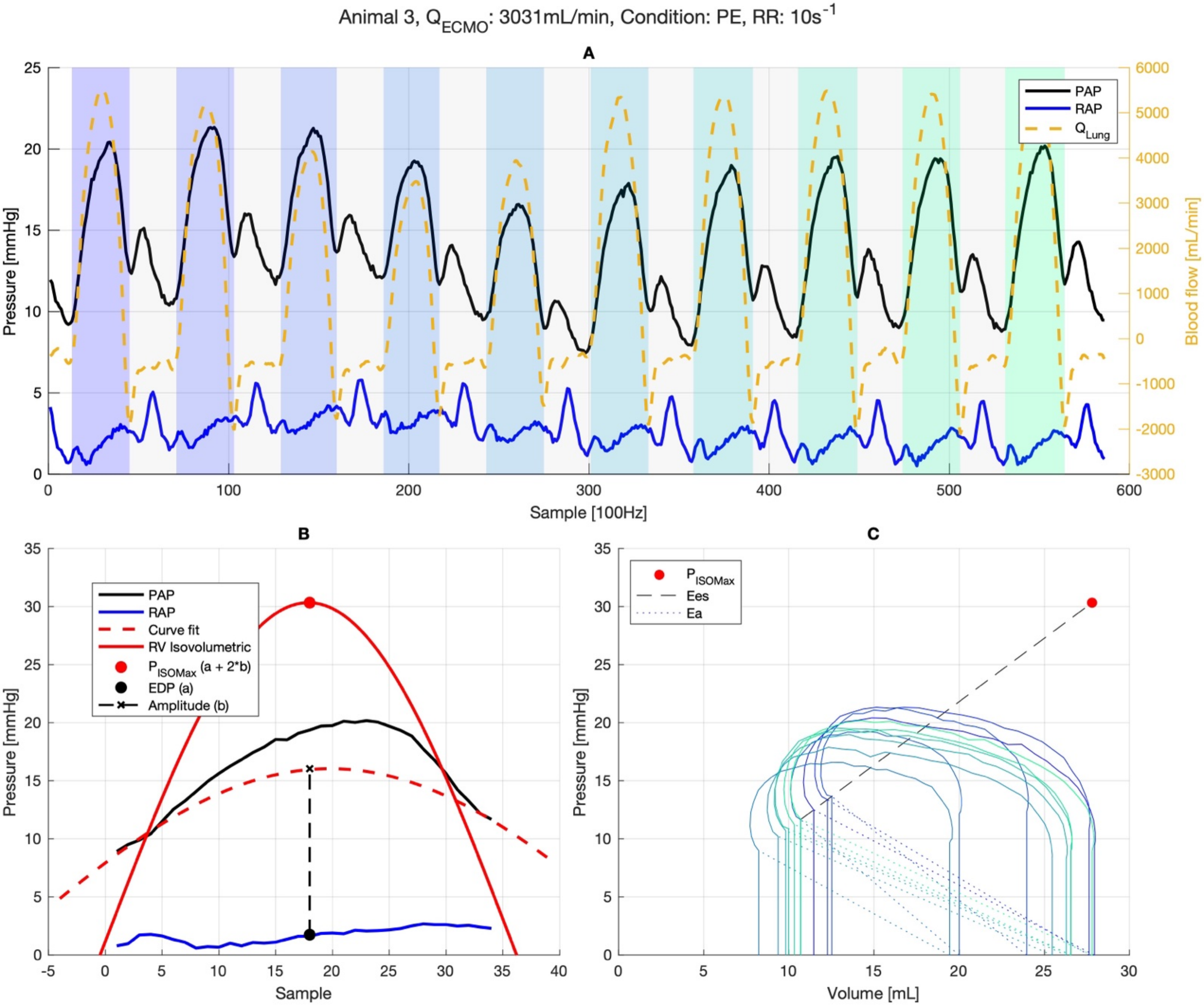

Animal 3, Q_{ECMO}: 3031mL/min, Condition: PE, RR: $10s^{-1}$
A
PAP
RAP
Q_{Lung}
Pressure [mmHg]
Blood flow [mL/min]
Sample [100Hz]
B
PAP
RAP
Curve fit
RV Isovolumetric
P_{ISOMax} (a + 2*b)
EDP (a)
Amplitude (b)
Pressure [mmHg]
Sample
C
P_{ISOMax}
Ees
Ea
Pressure [mmHg]
Volume [mL]

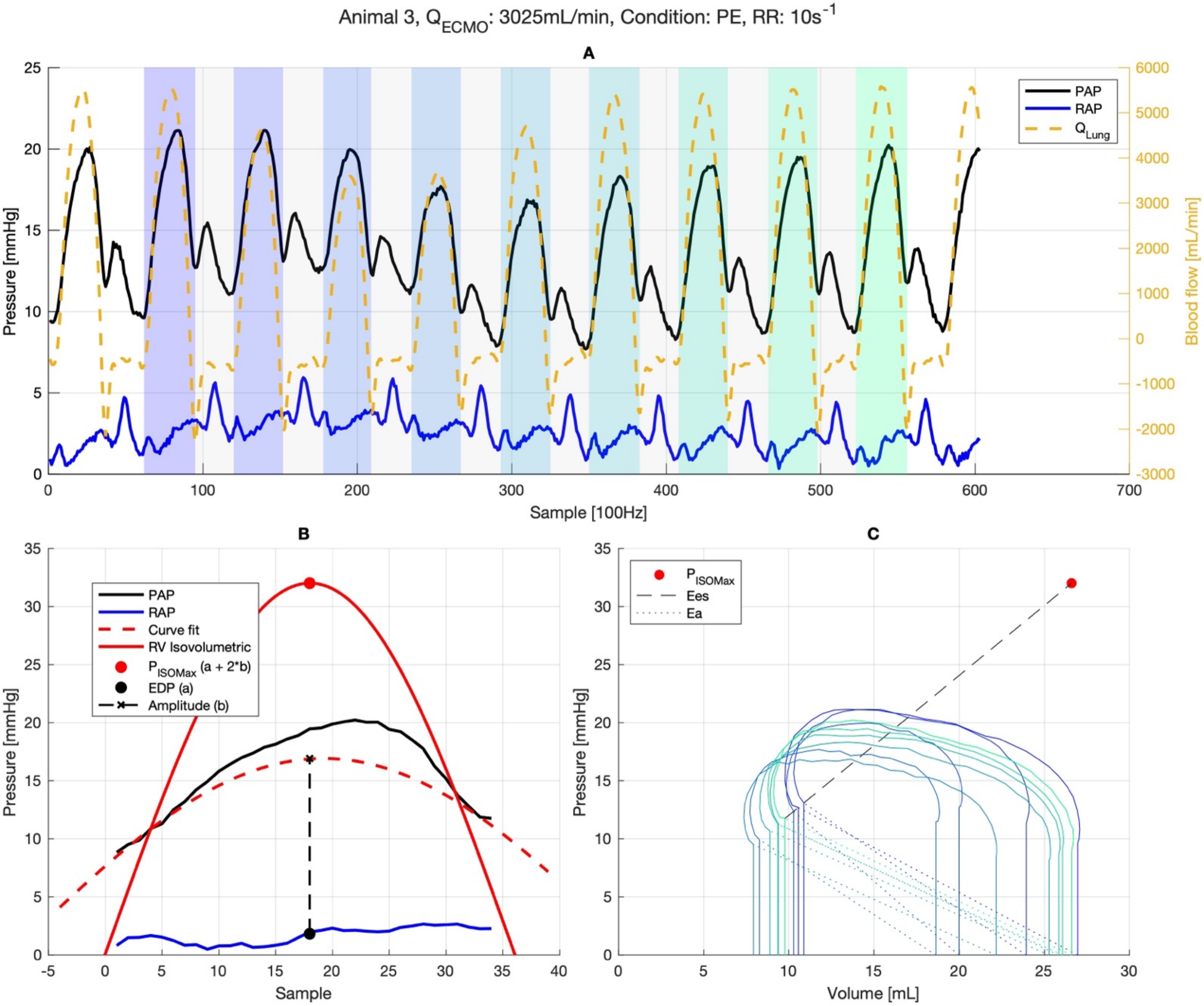

Animal 3, Q_ECMO: 3025mL/min, Condition: PE, RR: 10s^-1
A
PAP
RAP
Q_Lung
Pressure [mmHg]
Blood flow [mL/min]
Sample [100Hz]
B
PAP
RAP
Curve fit
RV Isovolumetric
P_ISOMax (a + 2*b)
EDP (a)
Amplitude (b)
Pressure [mmHg]
Sample
C
P_ISOMax
Ees
Ea
Pressure [mmHg]
Volume [mL]

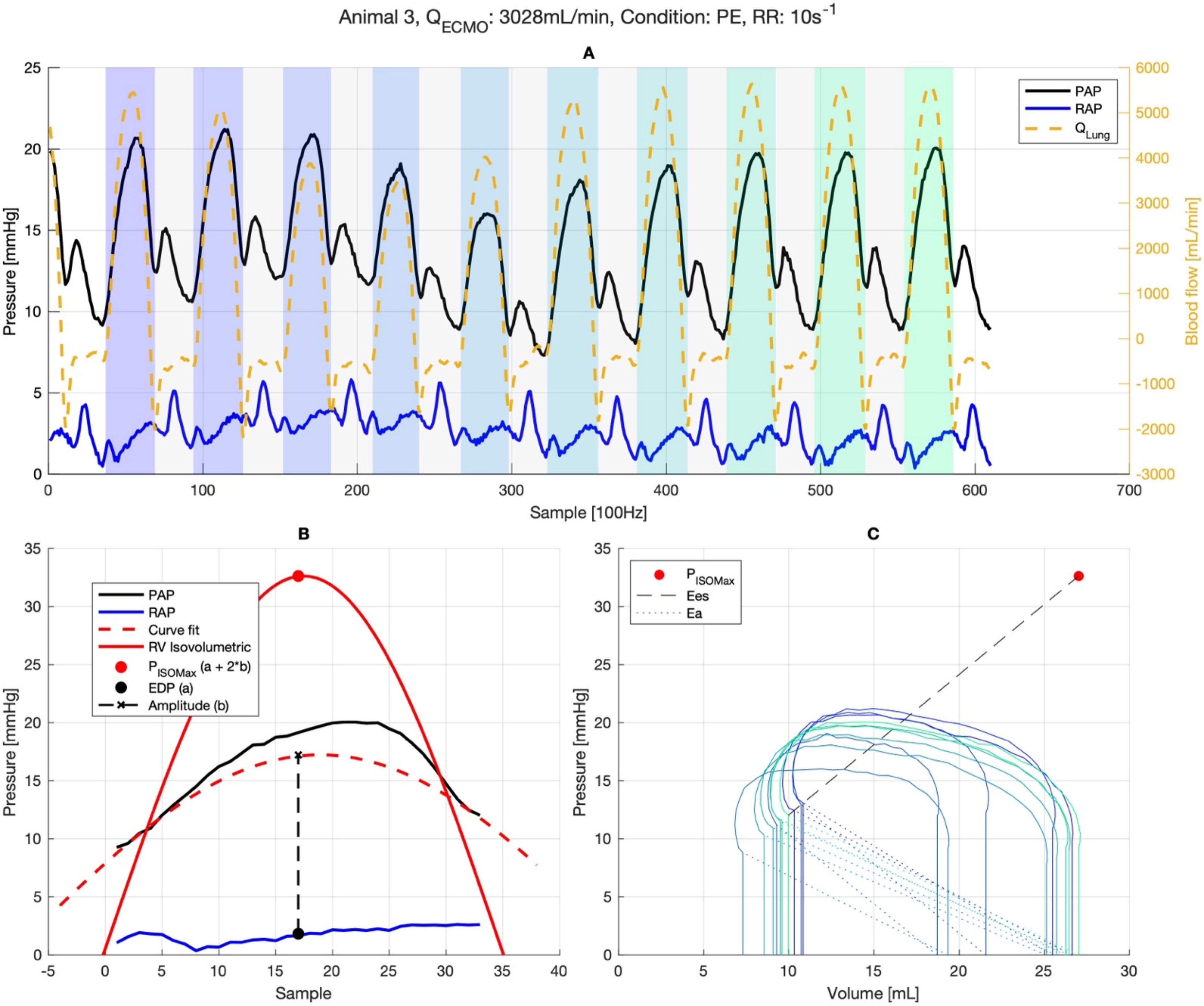

Animal 3, Q_{ECMO}: 3028mL/min, Condition: PE, RR: $10s^{-1}$
A
PAP
RAP
Q_{Lung}
Pressure [mmHg]
Blood flow [mL/min]
Sample [100Hz]
B
PAP
RAP
Curve fit
RV Isovolumetric
P_{ISOMax} (a + 2*b)
EDP (a)
Amplitude (b)
Pressure [mmHg]
Sample
C
P_{ISOMax}
Ees
Ea
Pressure [mmHg]
Volume [mL]

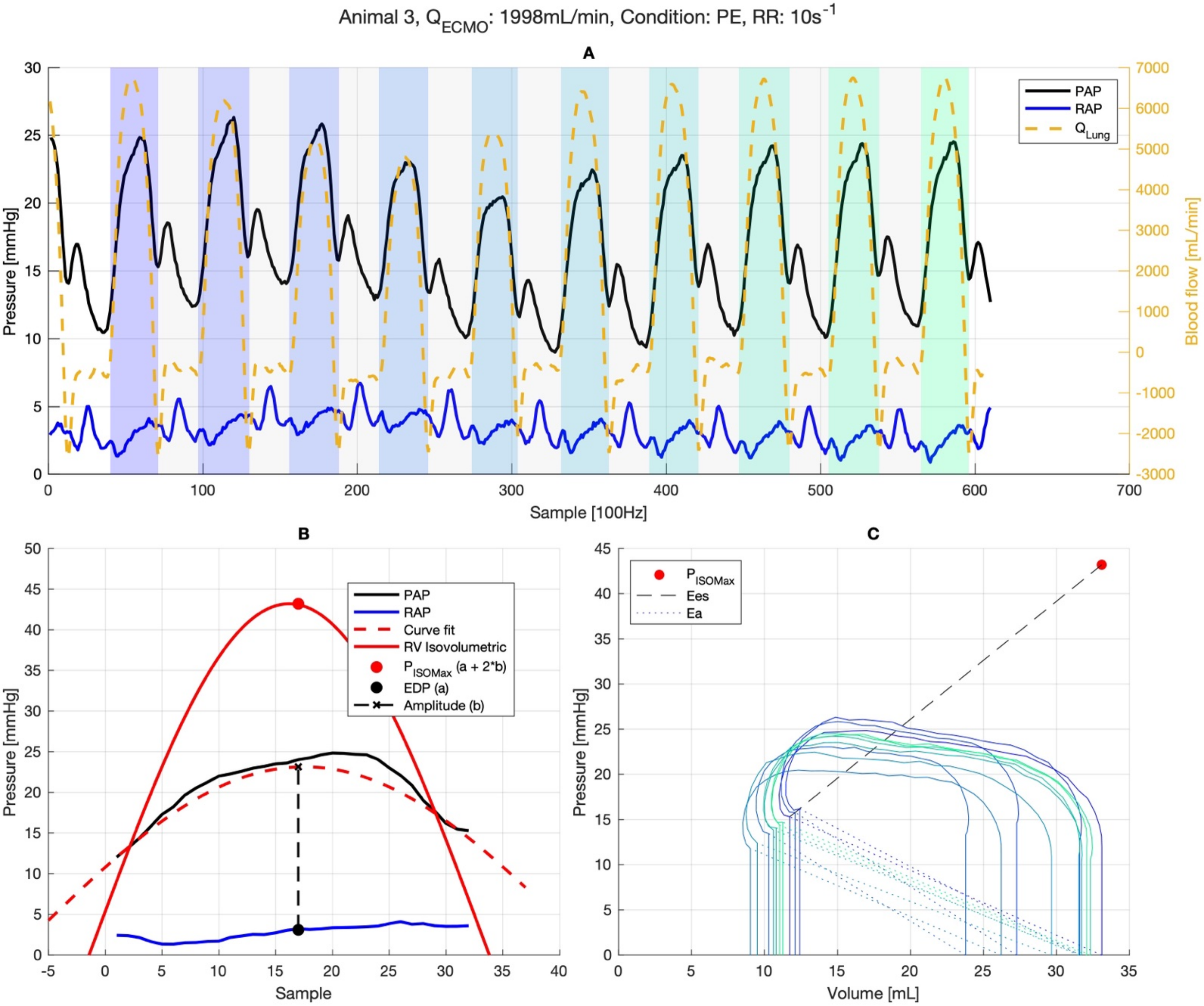

Animal 3, Q_{ECMO}: 1998mL/min, Condition: PE, RR: $10s^{-1}$
A
PAP
RAP
Q_{Lung}
Pressure [mmHg]
Blood flow [mL/min]
Sample [100Hz]
B
PAP
RAP
Curve fit
RV Isovolumetric
P_{ISOMax} (a + 2*b)
EDP (a)
Amplitude (b)
Pressure [mmHg]
Sample
C
P_{ISOMax}
Ees
Ea
Pressure [mmHg]
Volume [mL]

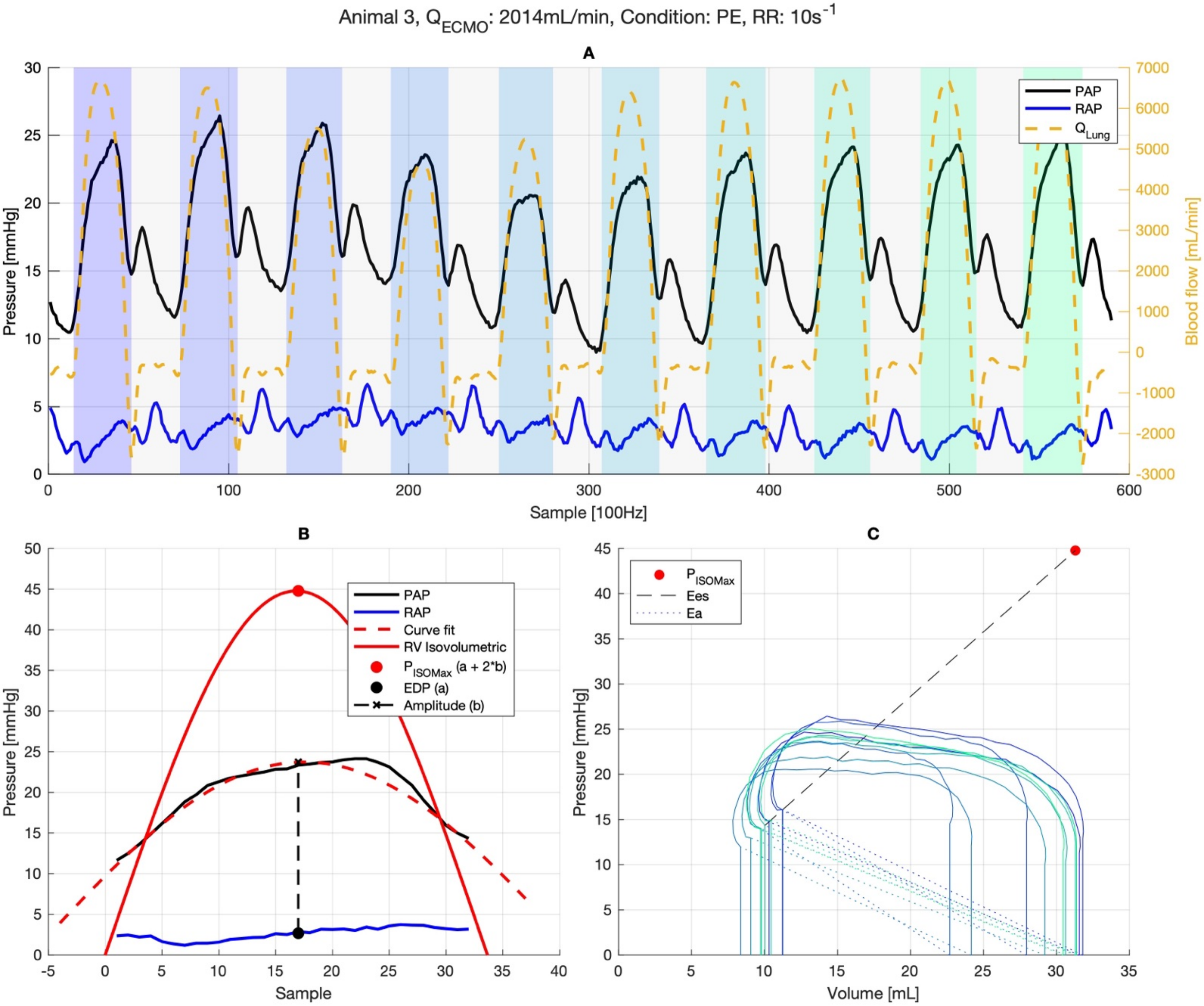

Animal 3, QECMO: 2014mL/min, Condition: PE, RR: 10s-1
A
Pressure [mmHg]
Blood flow [mL/min]
Sample [100Hz]
PAP
RAP
QLung
B
Pressure [mmHg]
Sample
PAP
RAP
Curve fit
RV Isovolumetric
PISOMax (a + 2*b)
EDP (a)
Amplitude (b)
C
Pressure [mmHg]
Volume [mL]
PISOMax
Ees
Ea

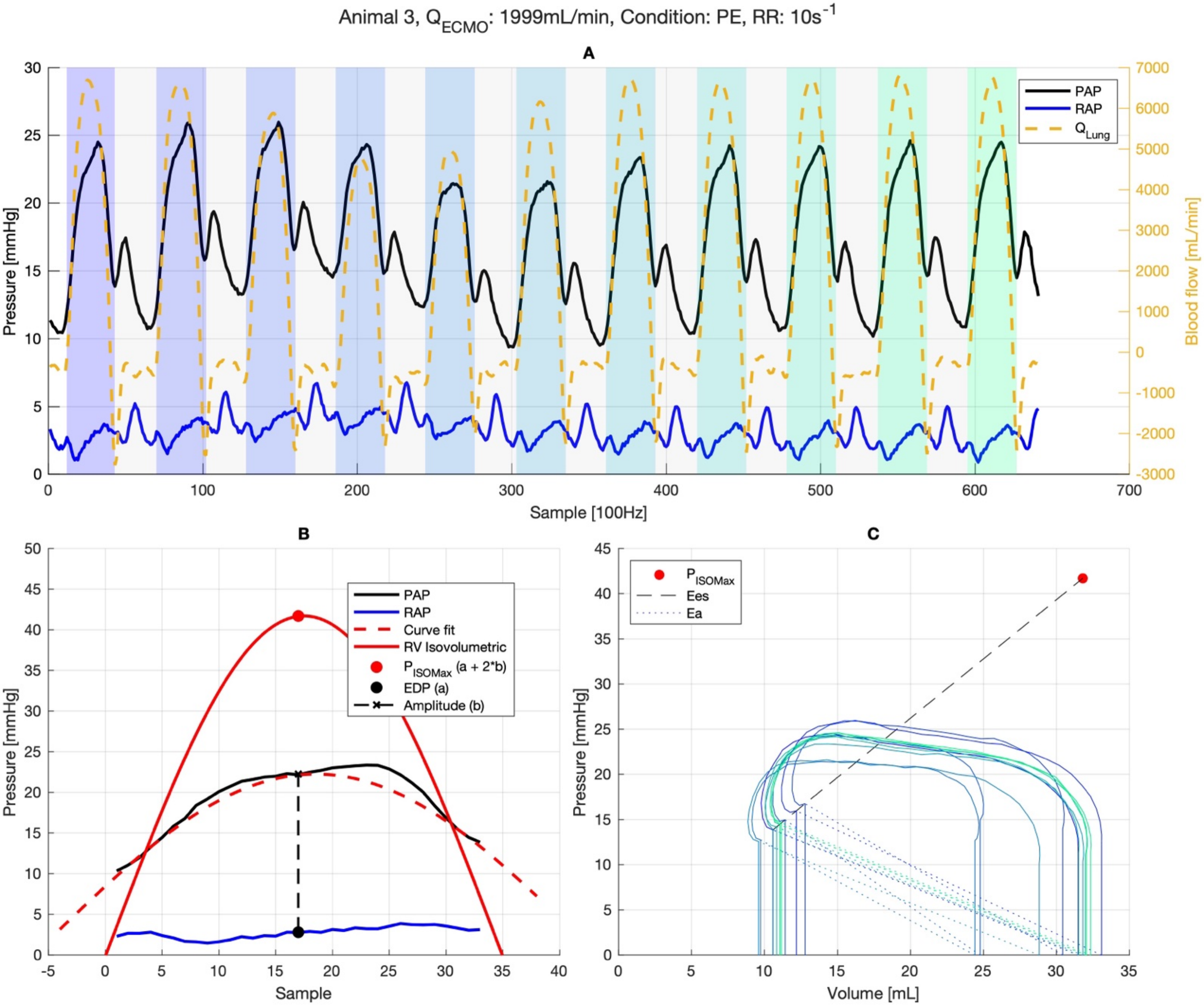

Animal 3, Q_{ECMO}: 1999mL/min, Condition: PE, RR: $10s^{-1}$
A
PAP
RAP
Q_{Lung}
Pressure [mmHg]
Blood flow [mL/min]
Sample [100Hz]
B
PAP
RAP
Curve fit
RV Isovolumetric
P_{ISOMax} (a + 2*b)
EDP (a)
Amplitude (b)
Pressure [mmHg]
Sample
C
P_{ISOMax}
Ees
Ea
Pressure [mmHg]
Volume [mL]

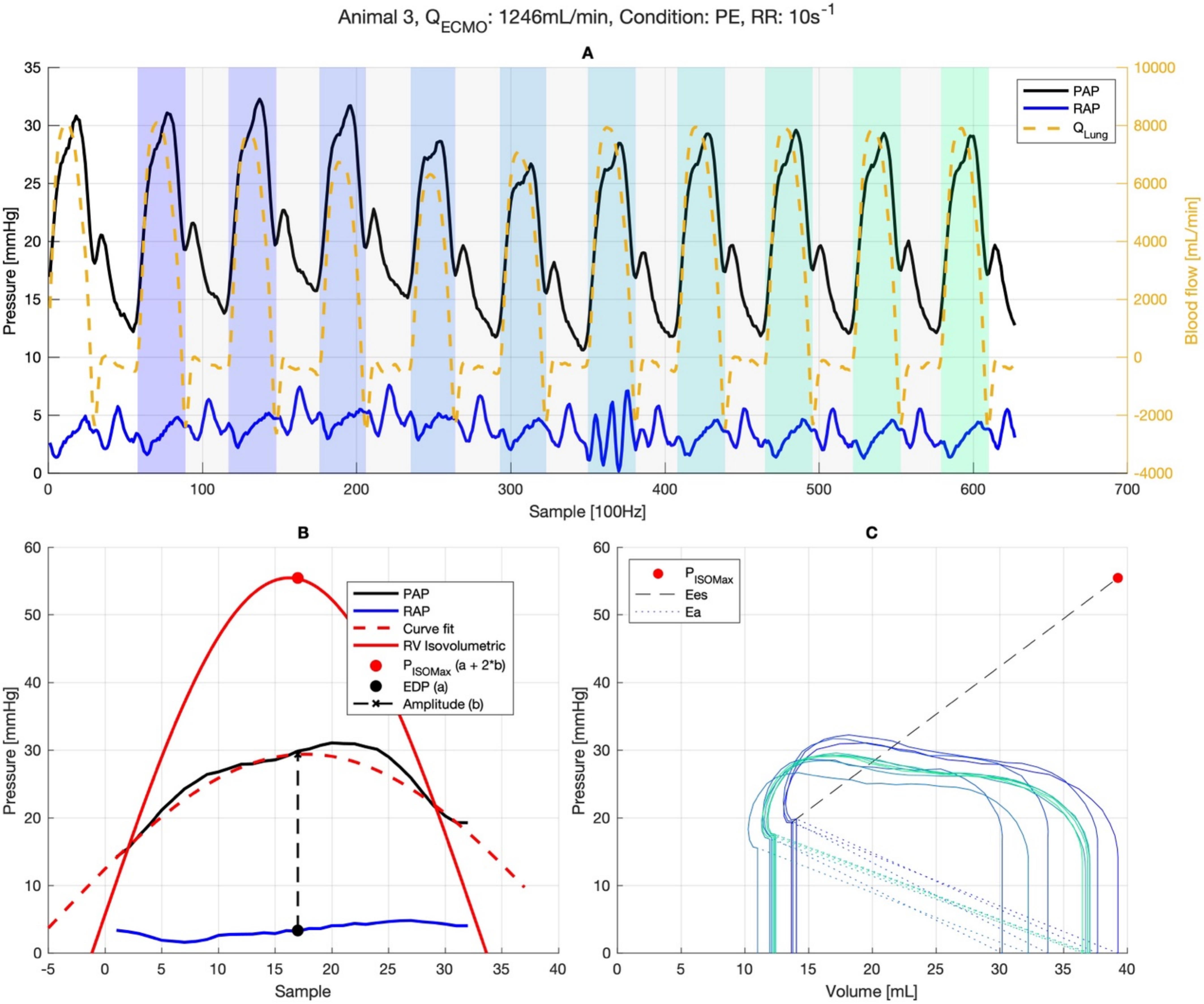

Animal 3, Q_{ECMO}: 1246mL/min, Condition: PE, RR: $10s^{-1}$
A
PAP
RAP
Q_{Lung}
Pressure [mmHg]
Blood flow [mL/min]
Sample [100Hz]
B
PAP
RAP
Curve fit
RV Isovolumetric
P_{ISOMax} (a + 2*b)
EDP (a)
Amplitude (b)
Pressure [mmHg]
Sample
C
P_{ISOMax}
Ees
Ea
Pressure [mmHg]
Volume [mL]

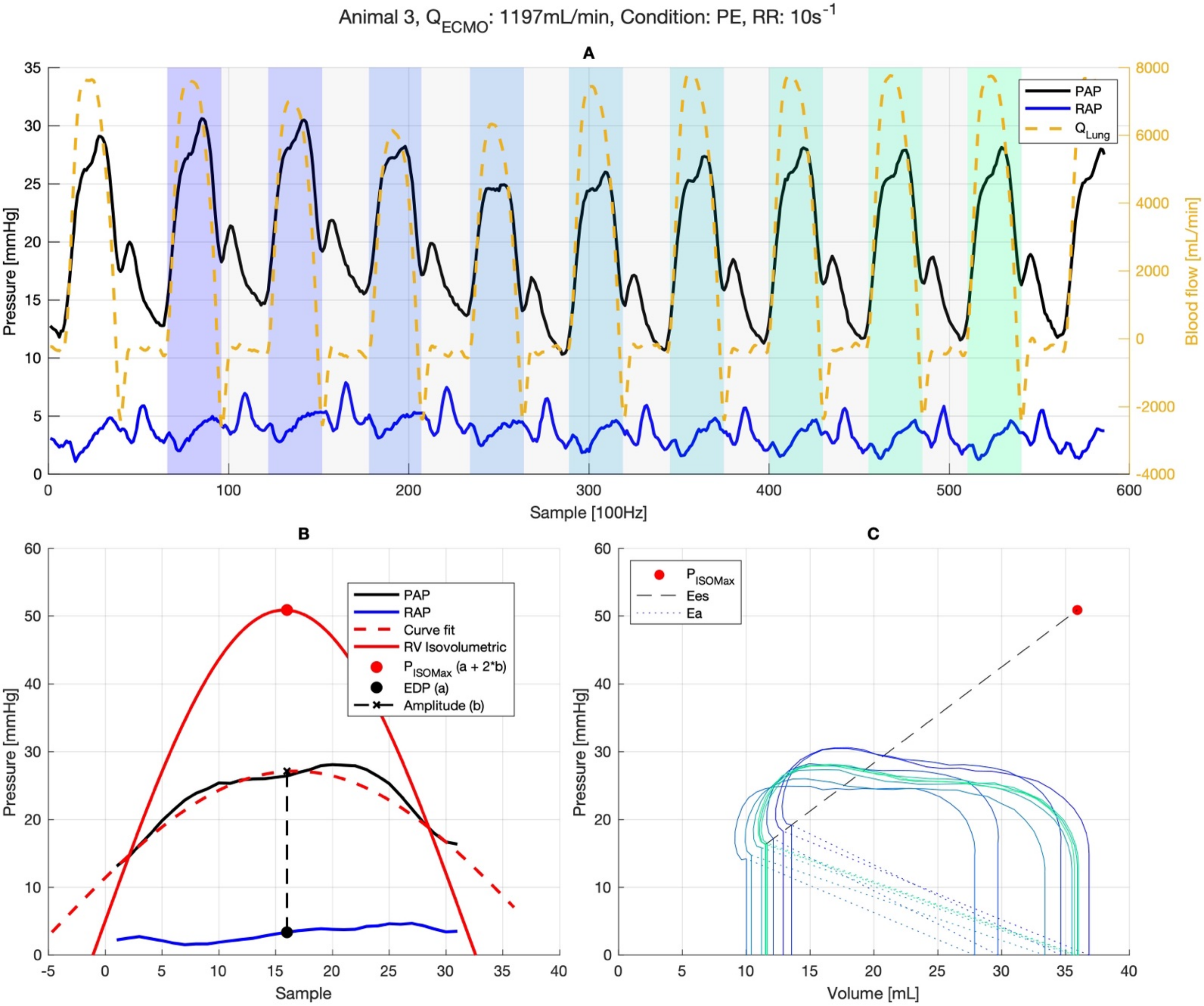

Animal 3, Q_{ECMO}: 1197mL/min, Condition: PE, RR: $10s^{-1}$
A
PAP
RAP
Q_{Lung}
Pressure [mmHg]
Blood flow [mL/min]
Sample [100Hz]
B
PAP
RAP
Curve fit
RV Isovolumetric
P_{ISOMax} (a + 2*b)
EDP (a)
Amplitude (b)
Pressure [mmHg]
Sample
C
P_{ISOMax}
Ees
Ea
Pressure [mmHg]
Volume [mL]

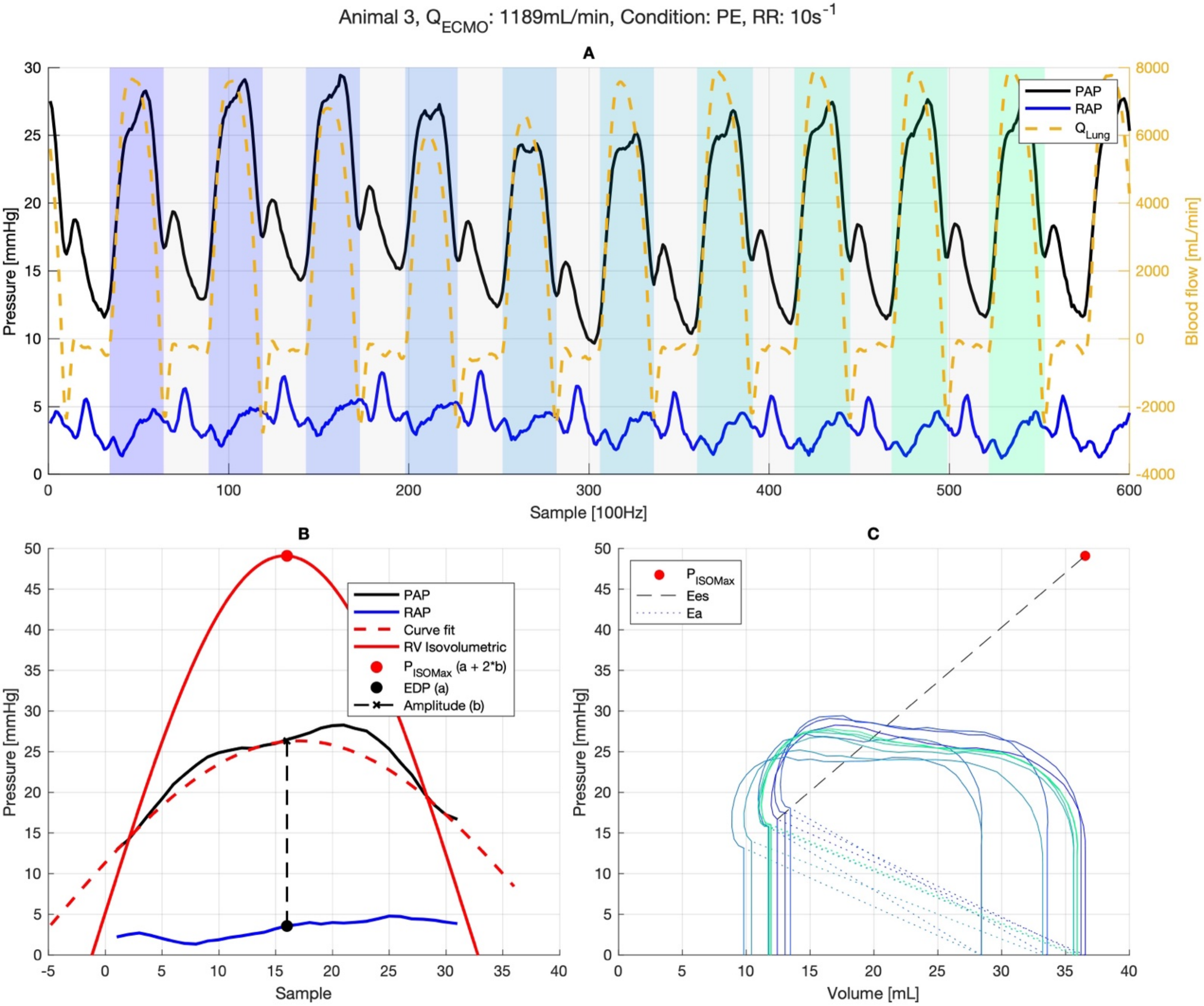

Animal 3, Q_ECMO: 1189mL/min, Condition: PE, RR: 10s^-1
A
PAP
RAP
Q_Lung
Pressure [mmHg]
Blood flow [mL/min]
Sample [100Hz]
B
PAP
RAP
Curve fit
RV Isovolumetric
P_ISOMax (a + 2*b)
EDP (a)
Amplitude (b)
Pressure [mmHg]
Sample
C
P_ISOMax
Ees
Ea
Pressure [mmHg]
Volume [mL]

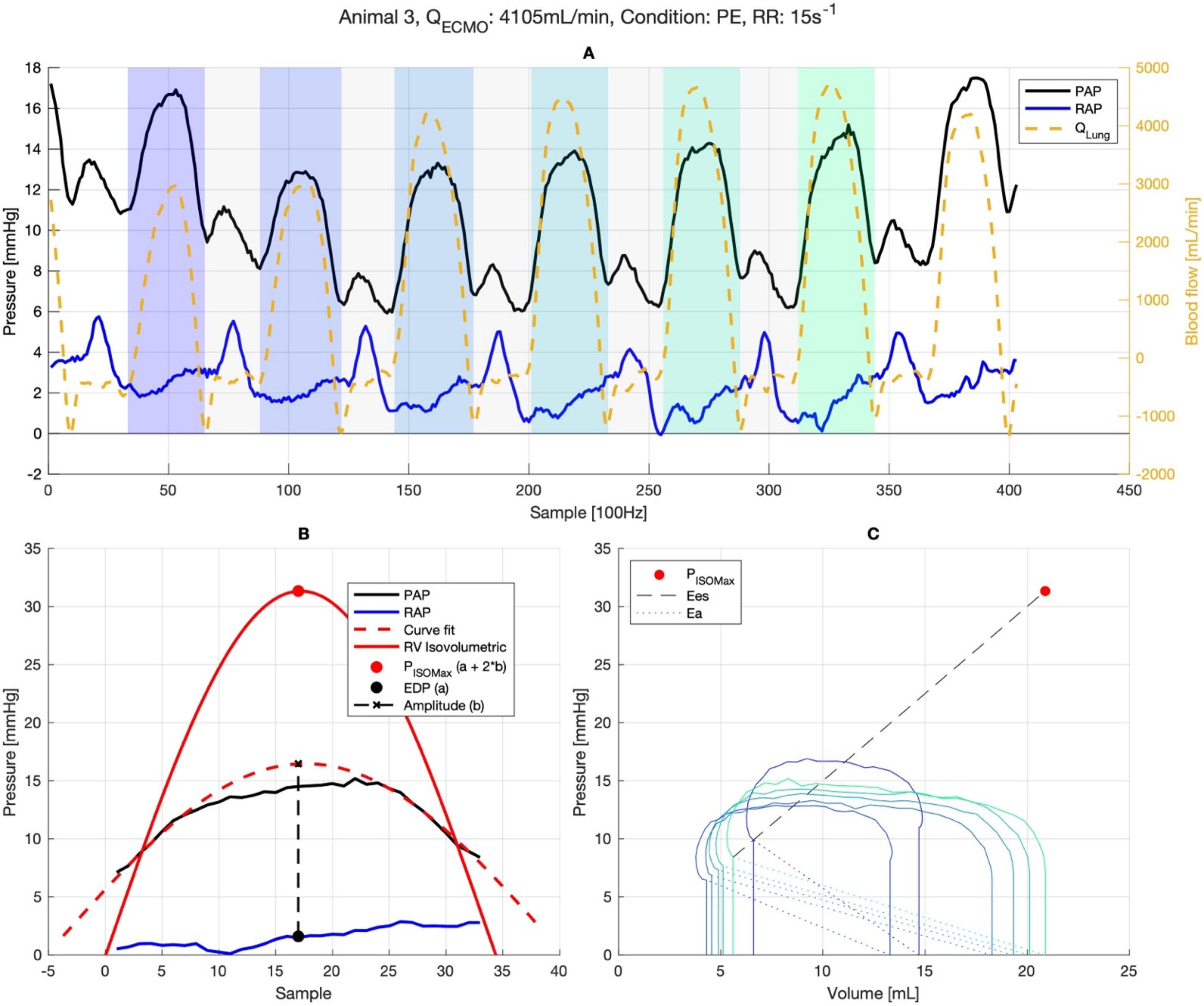

Animal 3, Q_ECMO: 4105mL/min, Condition: PE, RR: 15s^-1
A
PAP
RAP
Q_Lung
Pressure [mmHg]
Blood flow [mL/min]
Sample [100Hz]
B
PAP
RAP
Curve fit
RV Isovolumetric
P_ISOMax (a + 2*b)
EDP (a)
Amplitude (b)
Pressure [mmHg]
Sample
C
P_ISOMax
Ees
Ea
Pressure [mmHg]
Volume [mL]

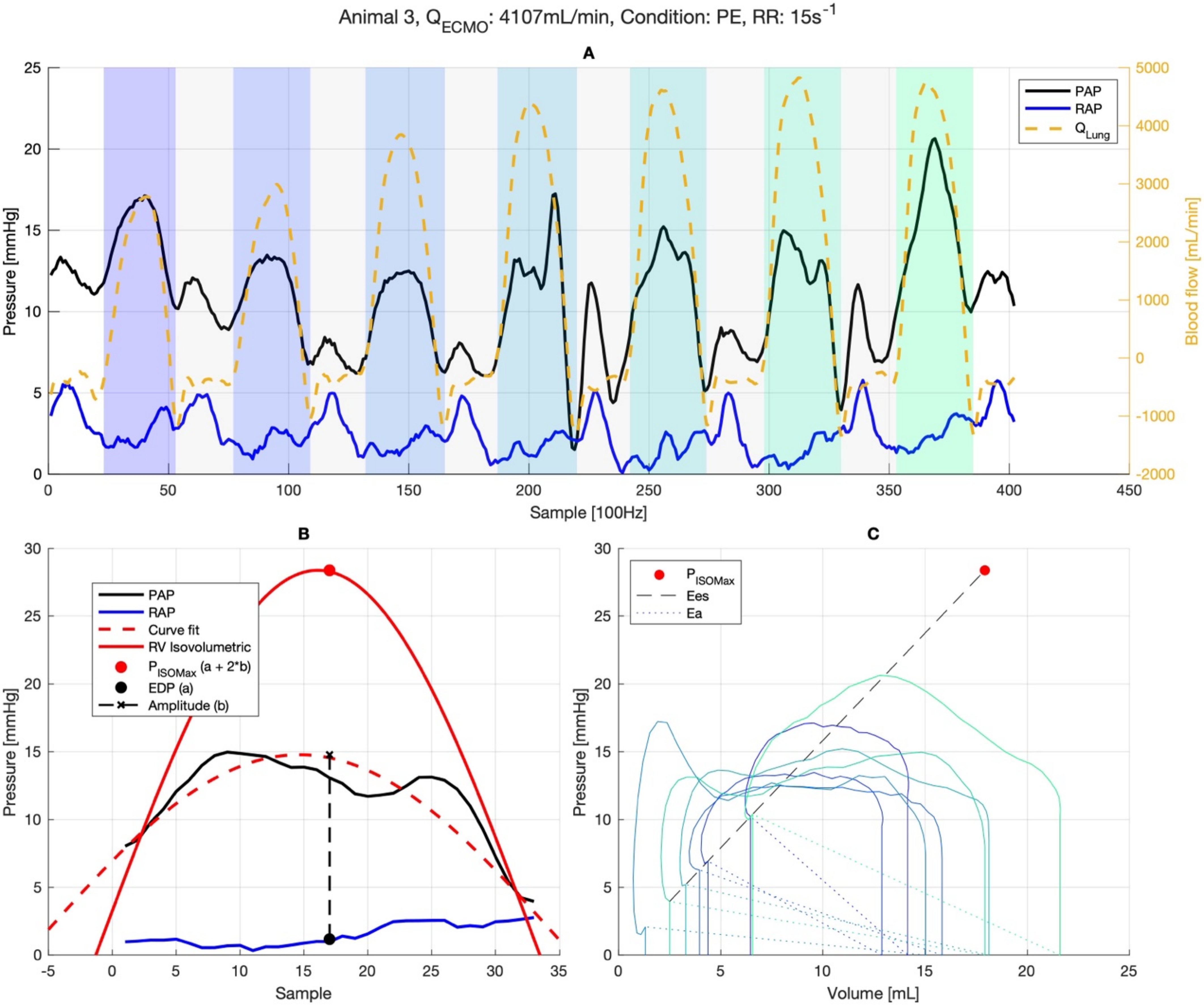

Animal 3, Q_ECMO: 4107mL/min, Condition: PE, RR: 15s^-1
A
PAP
RAP
Q_Lung
Pressure [mmHg]
Blood flow [mL/min]
Sample [100Hz]
B
PAP
RAP
Curve fit
RV Isovolumetric
P_ISOMax (a + 2*b)
EDP (a)
Amplitude (b)
Pressure [mmHg]
Sample
C
P_ISOMax
Ees
Ea
Pressure [mmHg]
Volume [mL]

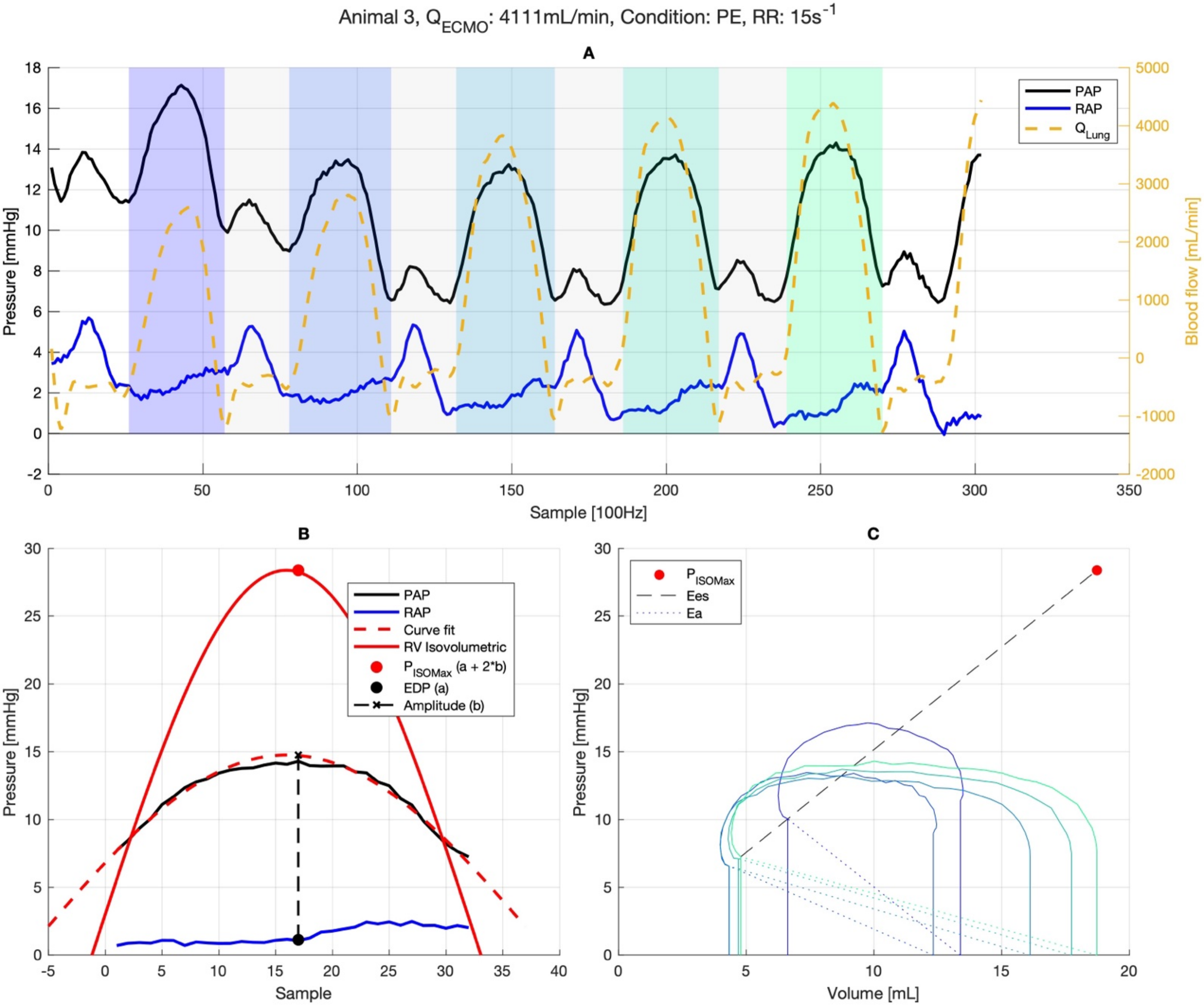

Animal 3, Q_{ECMO}: 4111mL/min, Condition: PE, RR: $15s^{-1}$
A
PAP
RAP
Q_{Lung}
Pressure [mmHg]
Blood flow [mL/min]
Sample [100Hz]
B
PAP
RAP
Curve fit
RV Isovolumetric
P_{ISOMax} (a + 2*b)
EDP (a)
Amplitude (b)
Pressure [mmHg]
Sample
C
P_{ISOMax}
Ees
Ea
Pressure [mmHg]
Volume [mL]

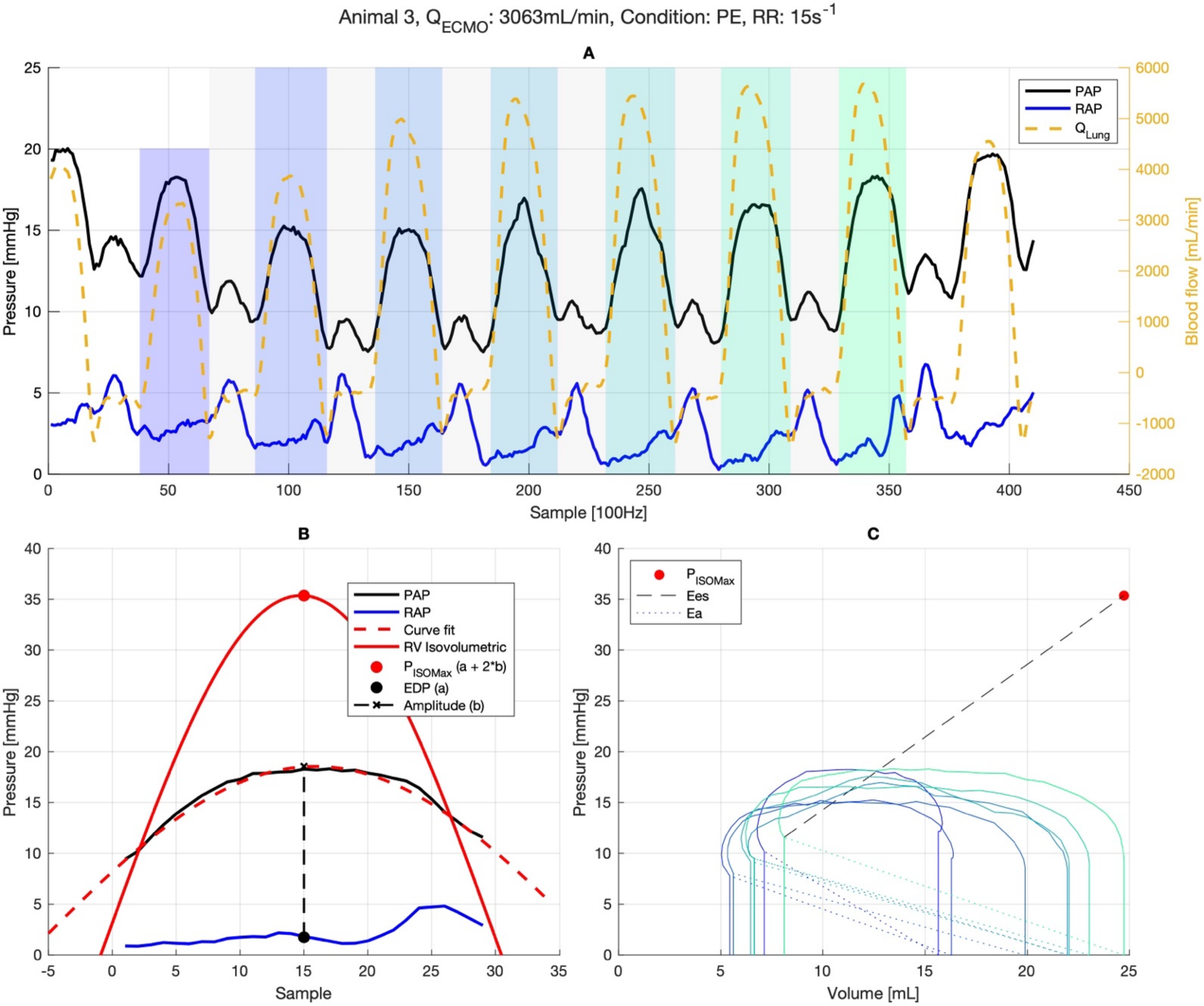

Animal 3, QECMO: 3063mL/min, Condition: PE, RR: 15s-1
A
Pressure [mmHg]
Blood flow [mL/min]
Sample [100Hz]
PAP
RAP
QLung
B
Pressure [mmHg]
Sample
PAP
RAP
Curve fit
RV Isovolumetric
PISOMax (a + 2*b)
EDP (a)
Amplitude (b)
C
Pressure [mmHg]
Volume [mL]
PISOMax
Ees
Ea

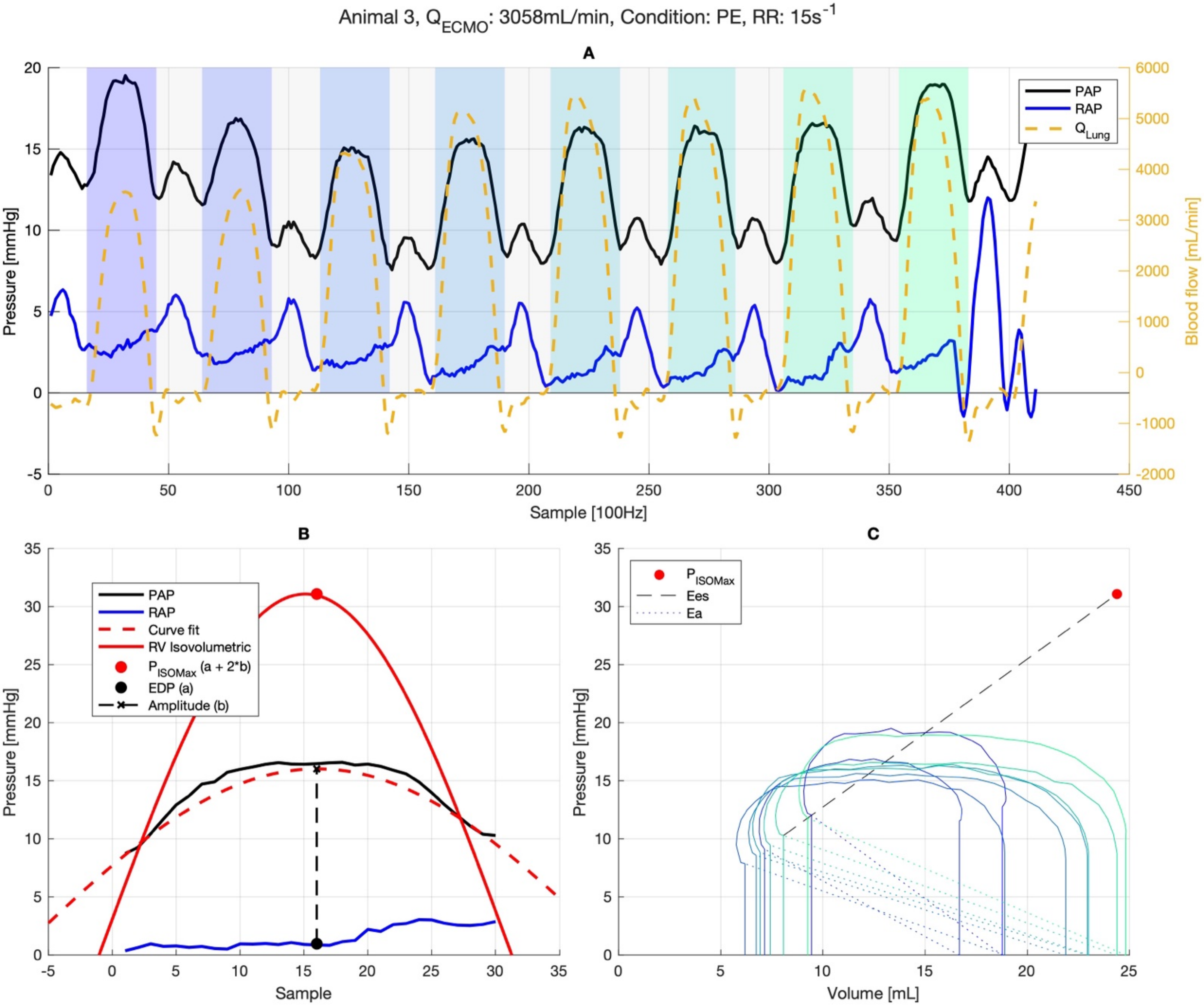

Animal 3, Q_{ECMO}: 3058mL/min, Condition: PE, RR: 15s^{-1}
A
PAP
RAP
Q_{Lung}
Pressure [mmHg]
Blood flow [mL/min]
Sample [100Hz]
B
PAP
RAP
Curve fit
RV Isovolumetric
P_{ISOMax} (a + 2*b)
EDP (a)
Amplitude (b)
Pressure [mmHg]
Sample
C
P_{ISOMax}
Ees
Ea
Pressure [mmHg]
Volume [mL]

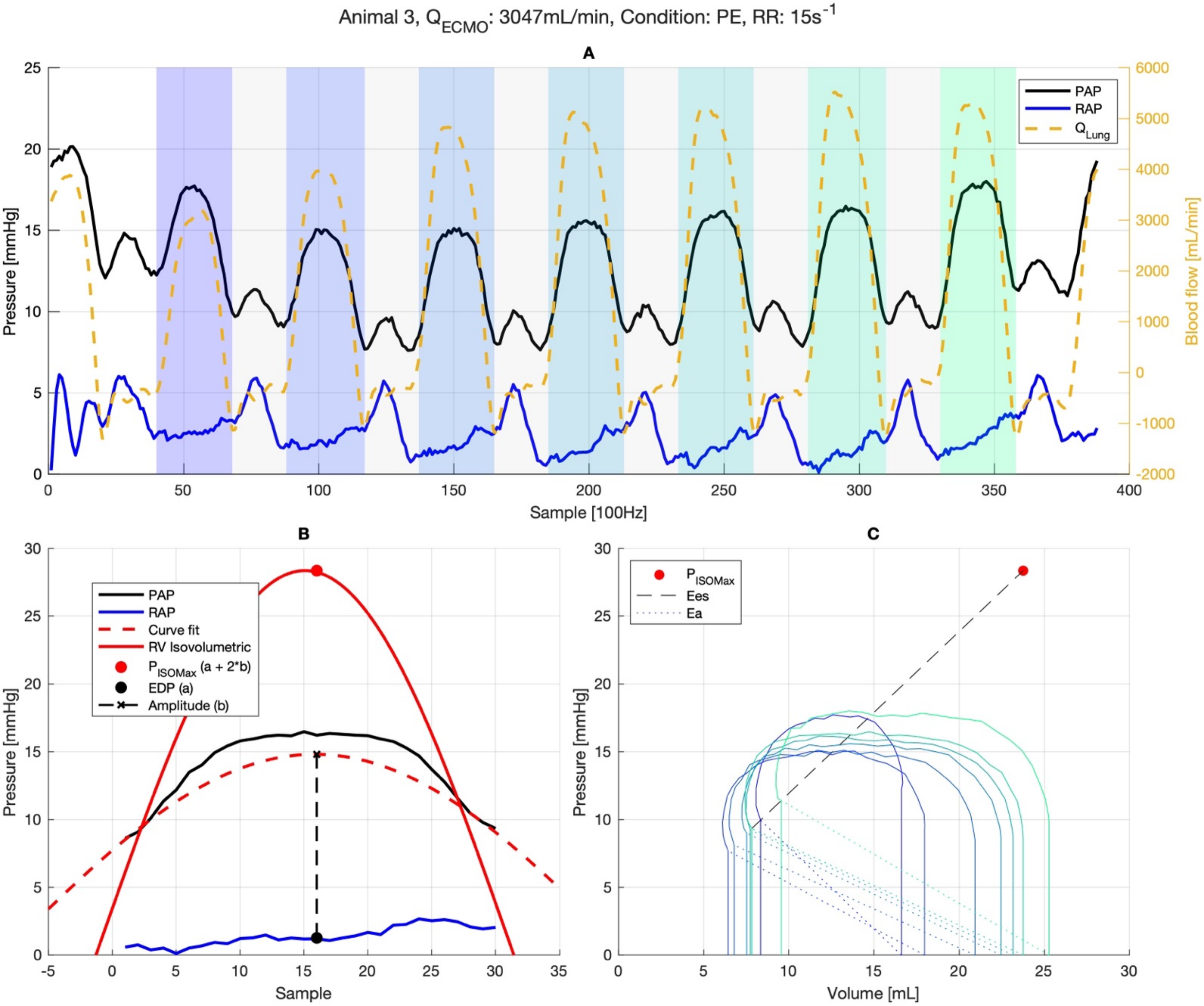

Animal 3, Q_{ECMO}: 3047mL/min, Condition: PE, RR: $15s^{-1}$
A
PAP
RAP
Q_{Lung}
Pressure [mmHg]
Blood flow [mL/min]
Sample [100Hz]
B
PAP
RAP
Curve fit
RV Isovolumetric
P_{ISOMax} (a + 2*b)
EDP (a)
Amplitude (b)
Pressure [mmHg]
Sample
C
P_{ISOMax}
Ees
Ea
Pressure [mmHg]
Volume [mL]

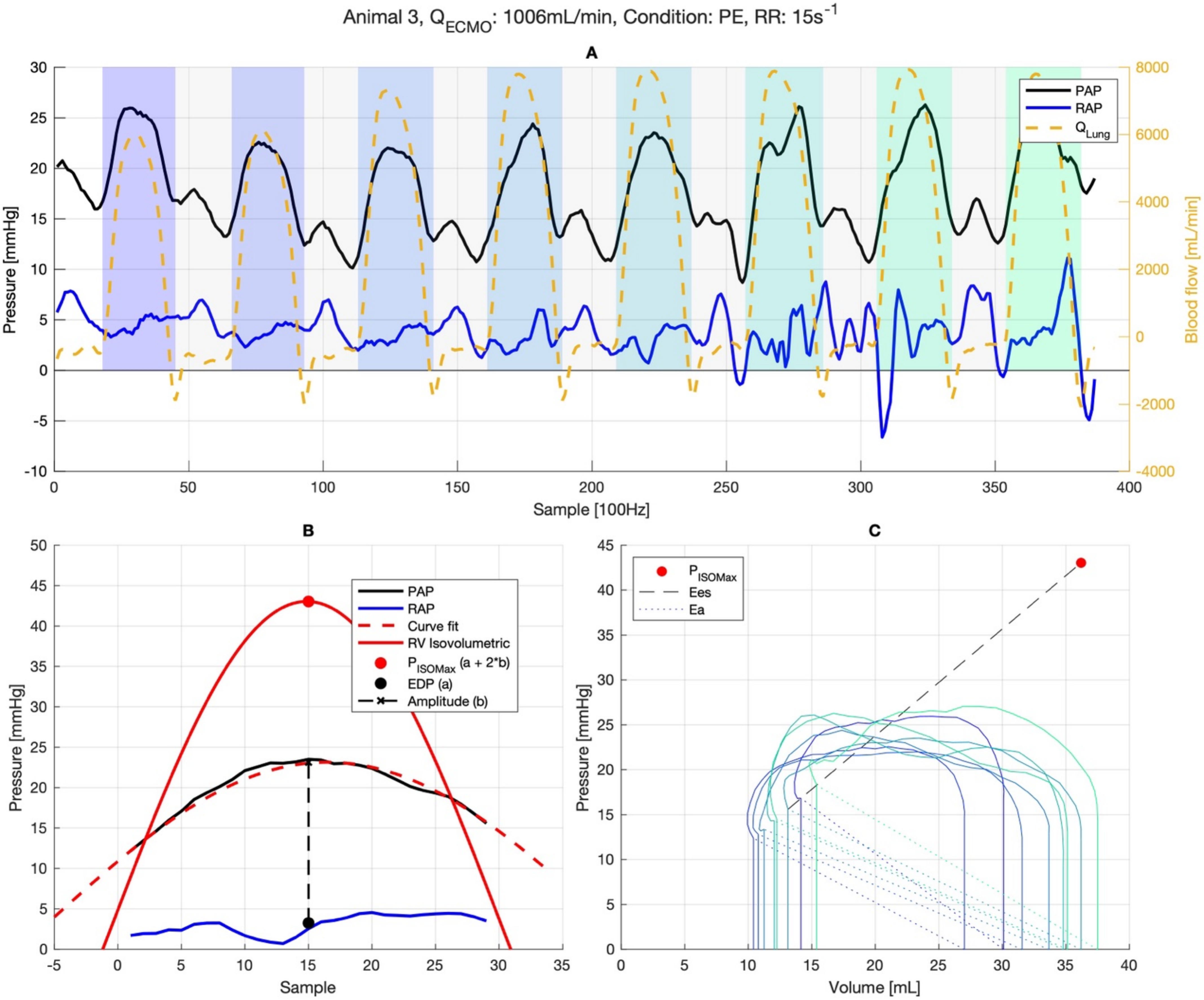

Animal 3, Q_{ECMO}: 1006mL/min, Condition: PE, RR: $15s^{-1}$
A
PAP
RAP
Q_{Lung}
Pressure [mmHg]
Blood flow [mL/min]
Sample [100Hz]
B
PAP
RAP
Curve fit
RV Isovolumetric
P_{ISOMax} (a + 2*b)
EDP (a)
Amplitude (b)
Pressure [mmHg]
Sample
C
P_{ISOMax}
Ees
Ea
Pressure [mmHg]
Volume [mL]

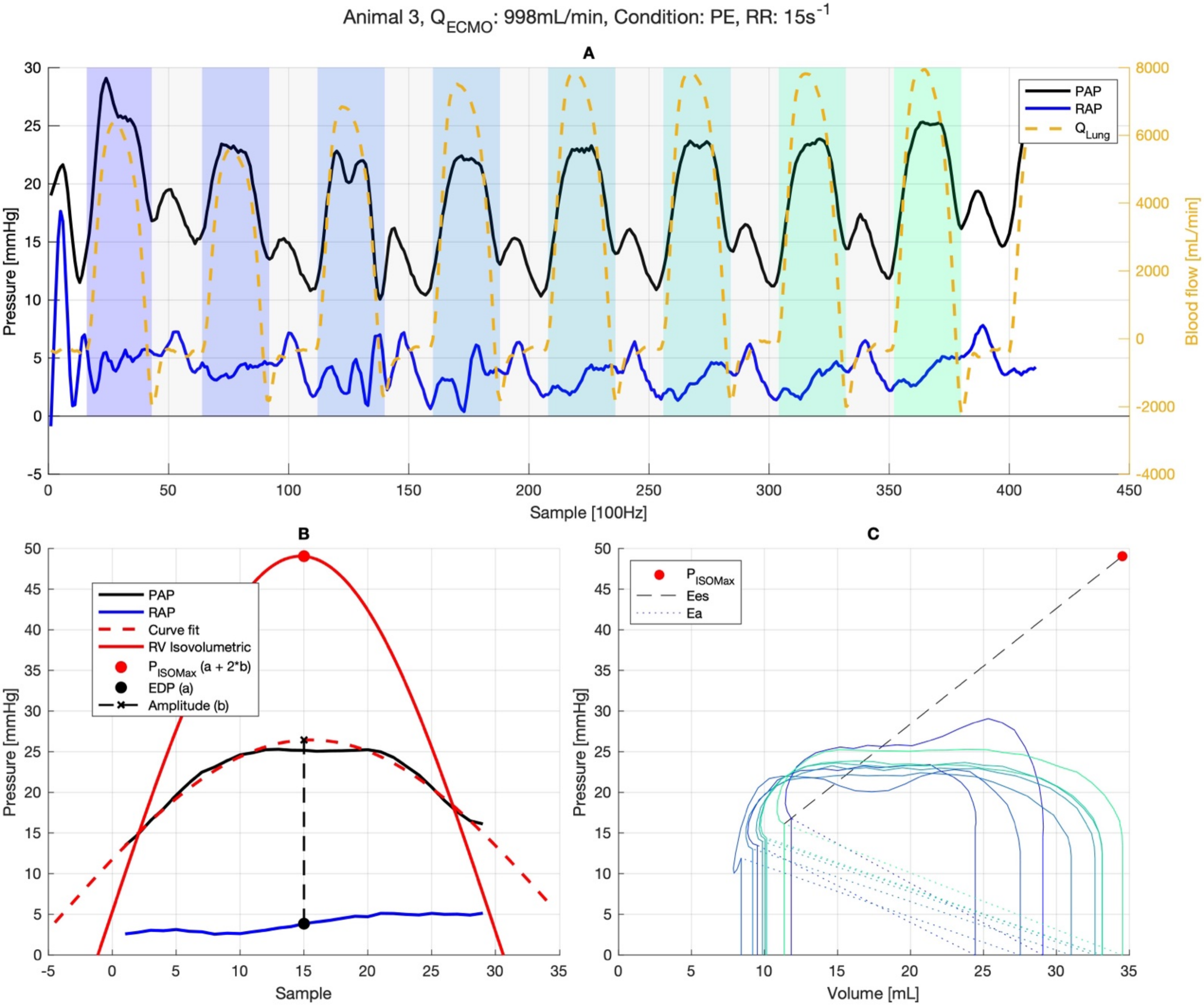

Animal 3, Q_{ECMO}: 998mL/min, Condition: PE, RR: $15s^{-1}$
A
PAP
RAP
Q_{Lung}
Pressure [mmHg]
Blood flow [mL/min]
Sample [100Hz]
B
PAP
RAP
Curve fit
RV Isovolumetric
P_{ISOMax} (a + 2*b)
EDP (a)
Amplitude (b)
Pressure [mmHg]
Sample
C
P_{ISOMax}
Ees
Ea
Pressure [mmHg]
Volume [mL]

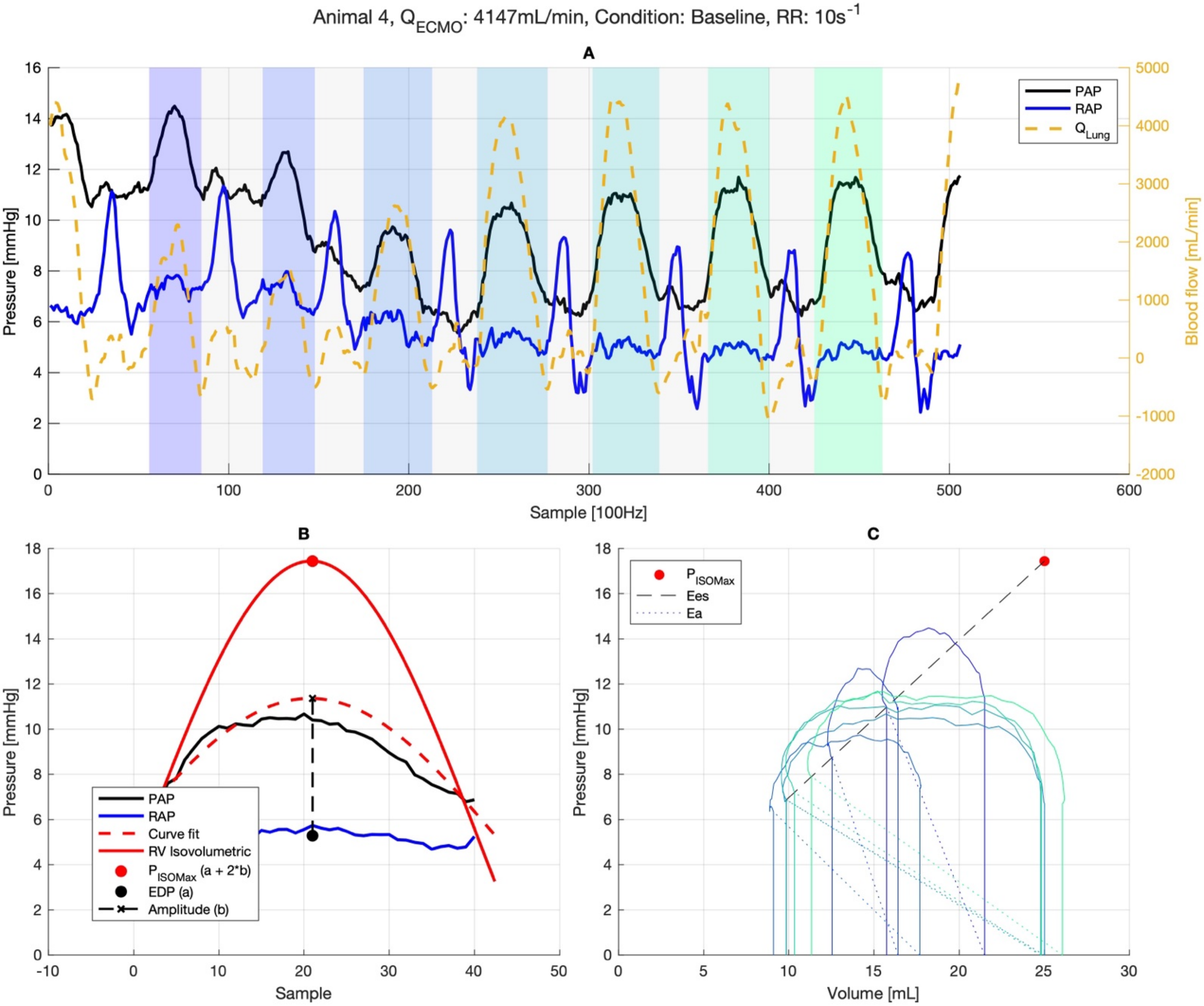

Animal 4, Q_{ECMO}: 4147mL/min, Condition: Baseline, RR: 10s^{-1}
A
Pressure [mmHg]
Blood flow [mL/min]
Sample [100Hz]
PAP
RAP
Q_{Lung}
B
Pressure [mmHg]
Sample
PAP
RAP
Curve fit
RV Isovolumetric
P_{ISOMax} (a + 2*b)
EDP (a)
Amplitude (b)
C
Pressure [mmHg]
Volume [mL]
P_{ISOMax}
Ees
Ea

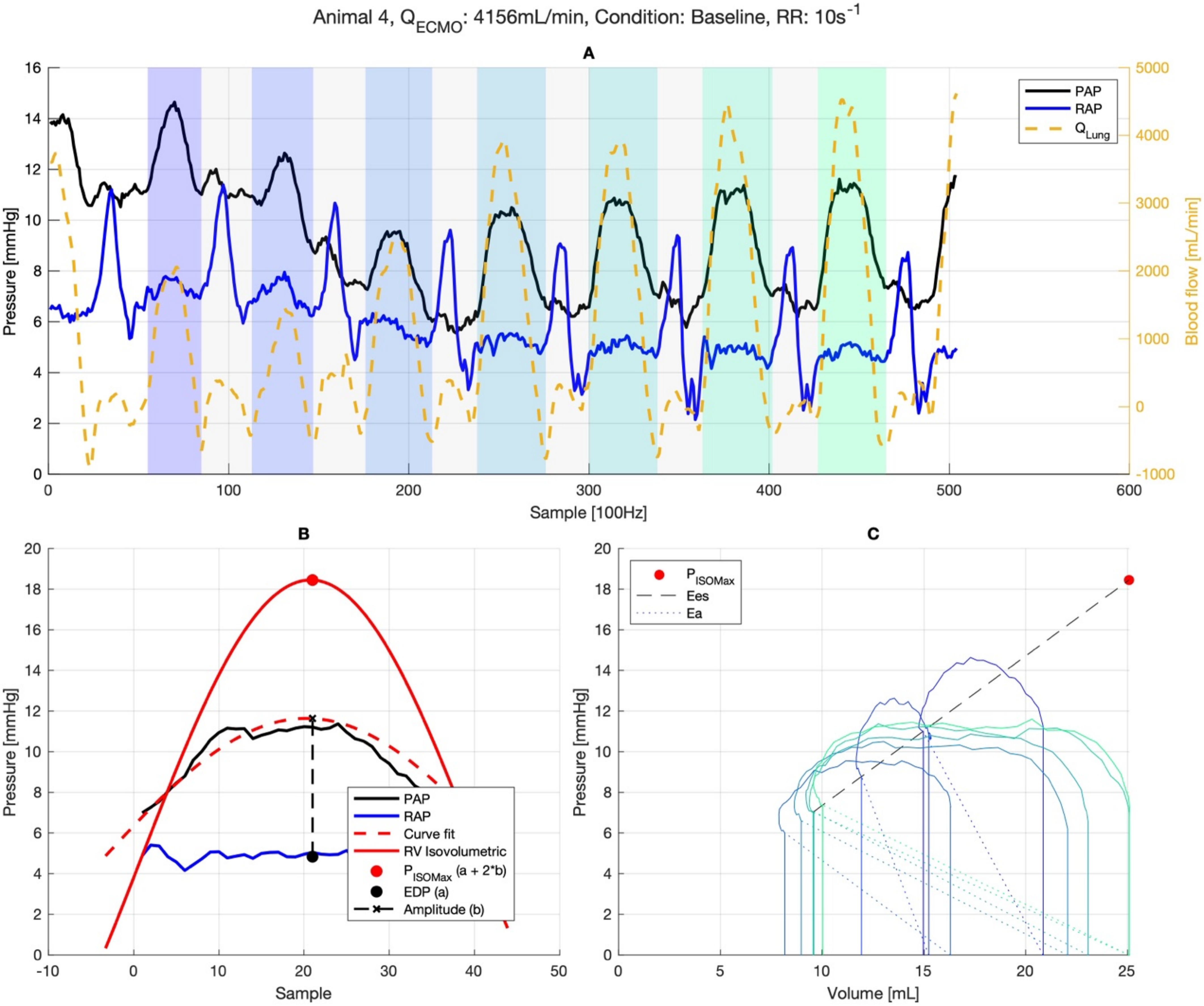

Animal 4, Q_ECMO: 4156mL/min, Condition: Baseline, RR: 10s^-1
A
PAP
RAP
Q_Lung
Pressure [mmHg]
Blood flow [mL/min]
Sample [100Hz]
B
Pressure [mmHg]
Sample
PAP
RAP
Curve fit
RV Isovolumetric
P_ISOMax (a + 2*b)
EDP (a)
Amplitude (b)
C
P_ISOMax
Ees
Ea
Pressure [mmHg]
Volume [mL]

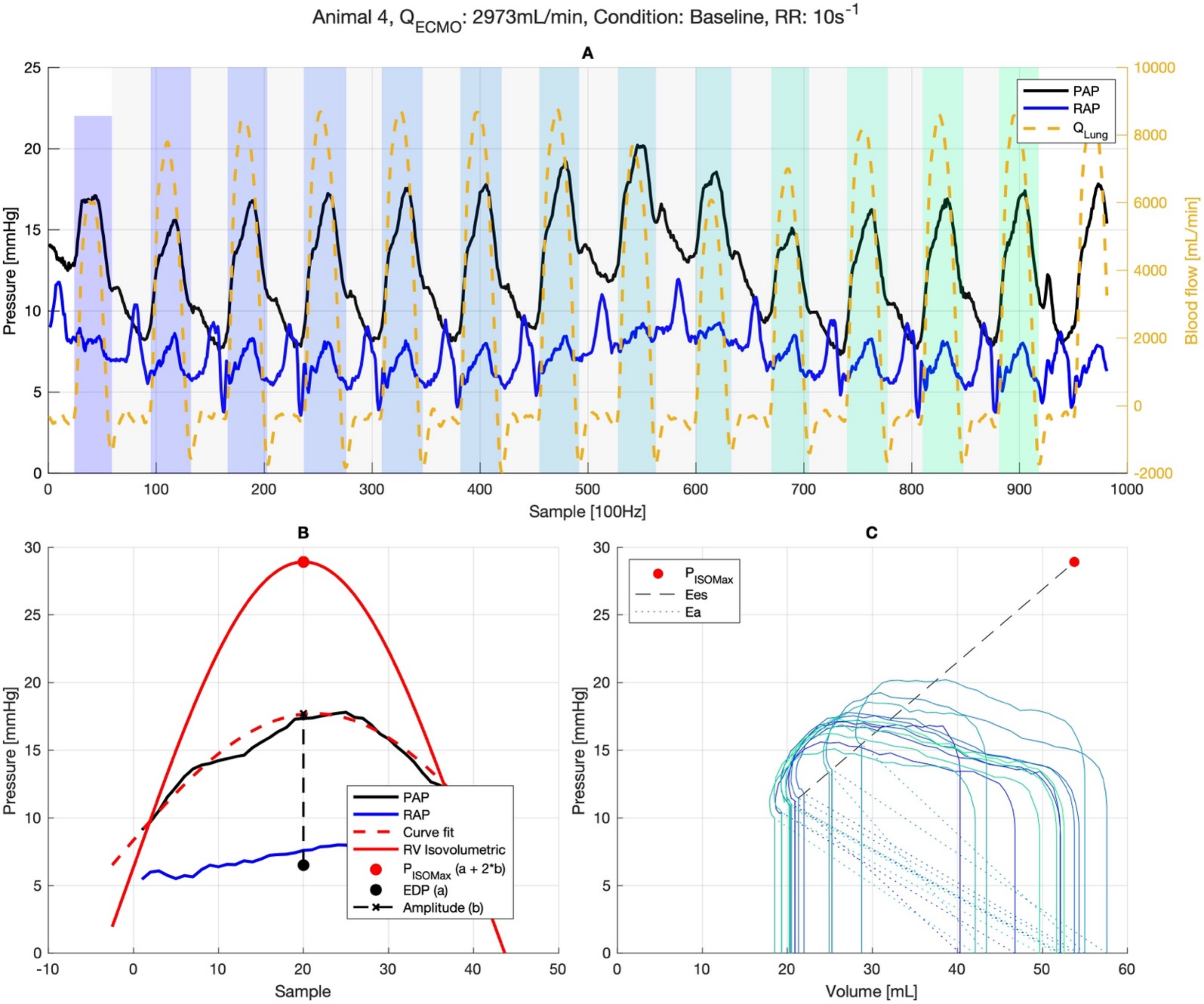

Animal 4, Q_{ECMO}: 2973mL/min, Condition: Baseline, RR: $10s^{-1}$
A
Pressure [mmHg]
Blood flow [mL/min]
Sample [100Hz]
PAP
RAP
Q_{Lung}
B
Pressure [mmHg]
Sample
PAP
RAP
Curve fit
RV Isovolumetric
P_{ISOMax} (a + 2*b)
EDP (a)
Amplitude (b)
C
Pressure [mmHg]
Volume [mL]
P_{ISOMax}
Ees
Ea

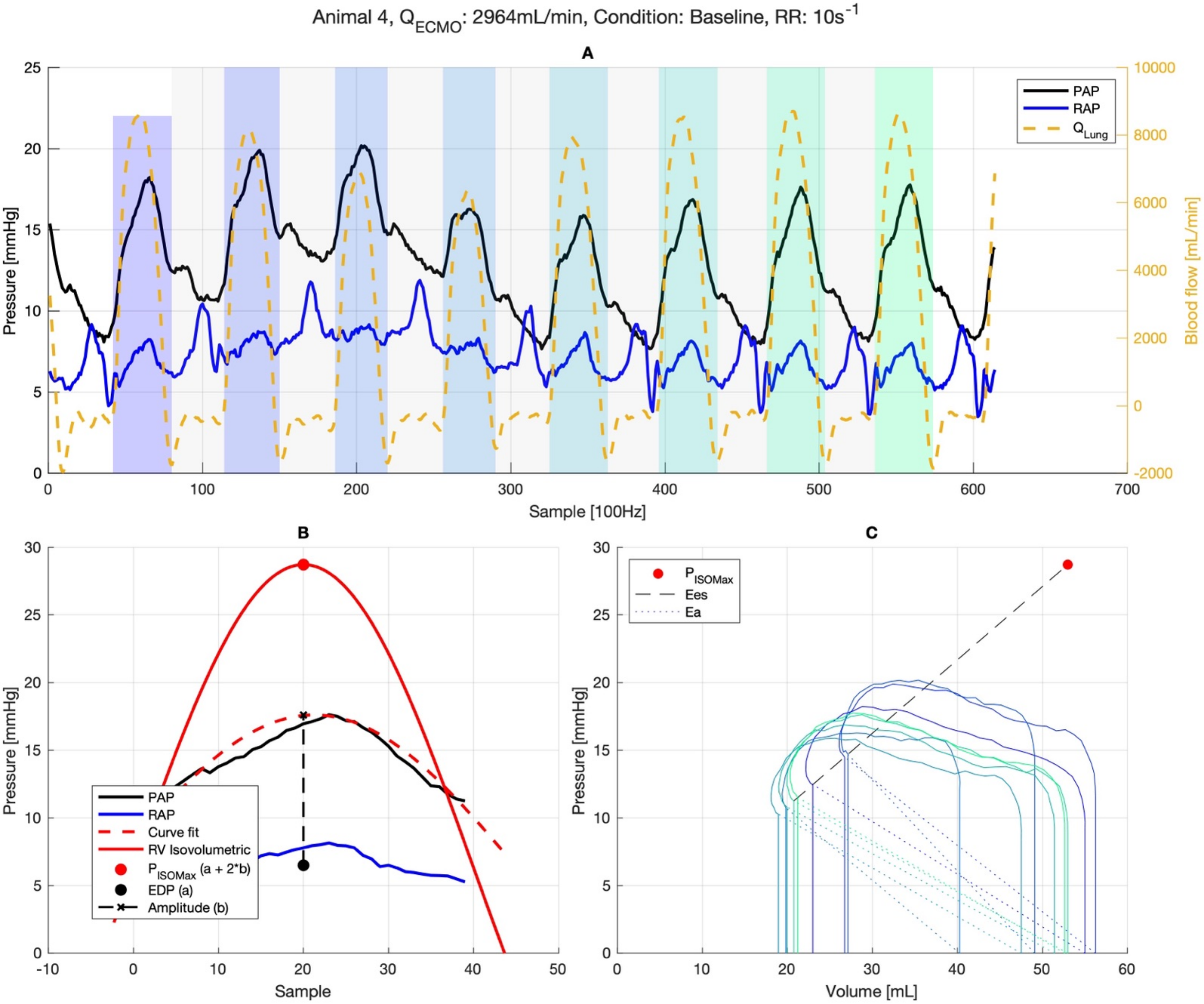

Animal 4, Q_{ECMO}: 2964mL/min, Condition: Baseline, RR: $10s^{-1}$
A
Pressure [mmHg]
Blood flow [mL/min]
Sample [100Hz]
PAP
RAP
Q_{Lung}
B
Pressure [mmHg]
Sample
PAP
RAP
Curve fit
RV Isovolumetric
P_{ISOMax} (a + 2*b)
EDP (a)
Amplitude (b)
C
Pressure [mmHg]
Volume [mL]
P_{ISOMax}
Ees
Ea

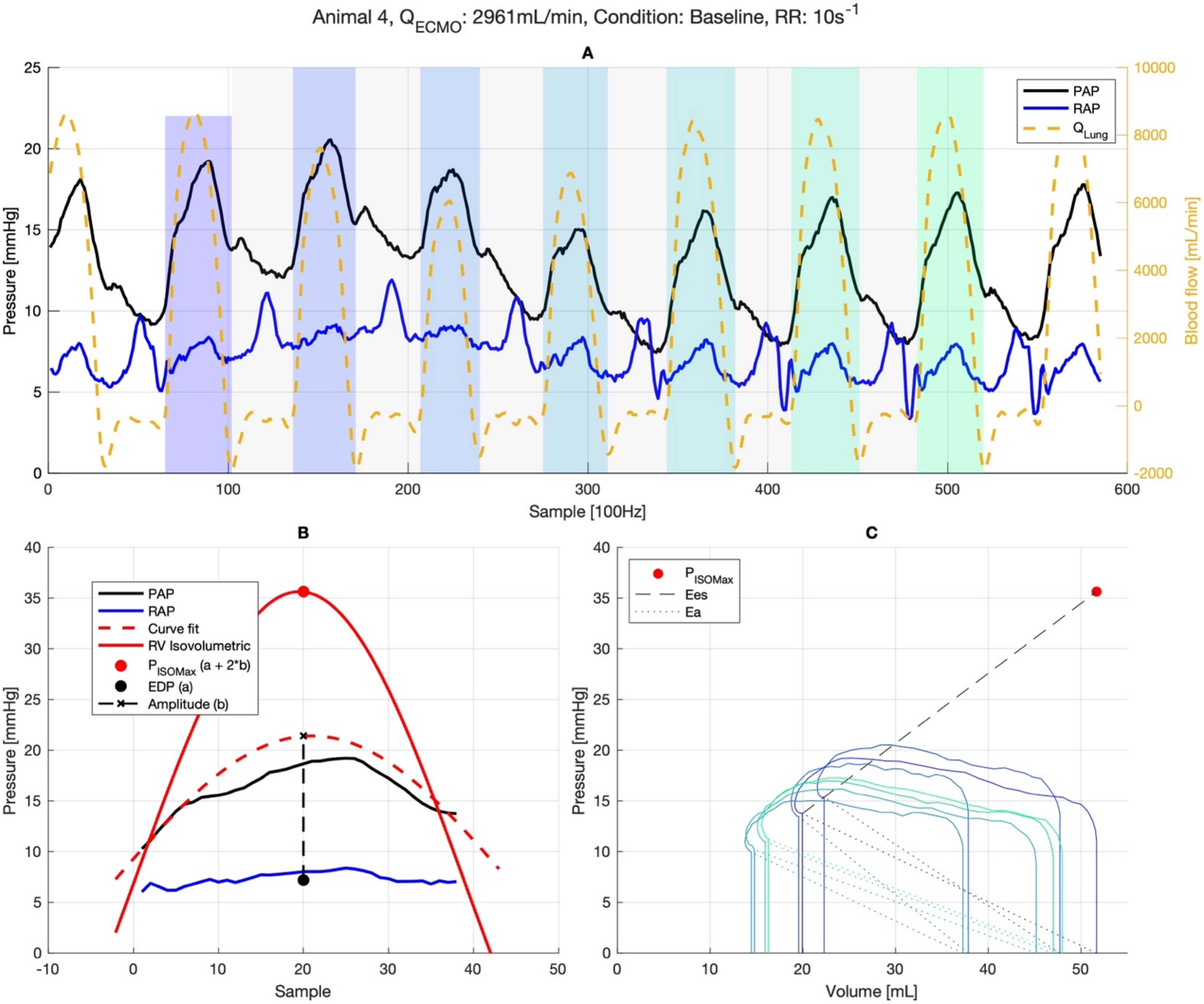

Animal 4, Q_{ECMO}: 2961mL/min, Condition: Baseline, RR: $10s^{-1}$
A
PAP
RAP
Q_{Lung}
Pressure [mmHg]
Blood flow [mL/min]
Sample [100Hz]
B
PAP
RAP
Curve fit
RV Isovolumetric
P_{ISOMax} (a + 2*b)
EDP (a)
Amplitude (b)
Pressure [mmHg]
Sample
C
P_{ISOMax}
Ees
Ea
Pressure [mmHg]
Volume [mL]

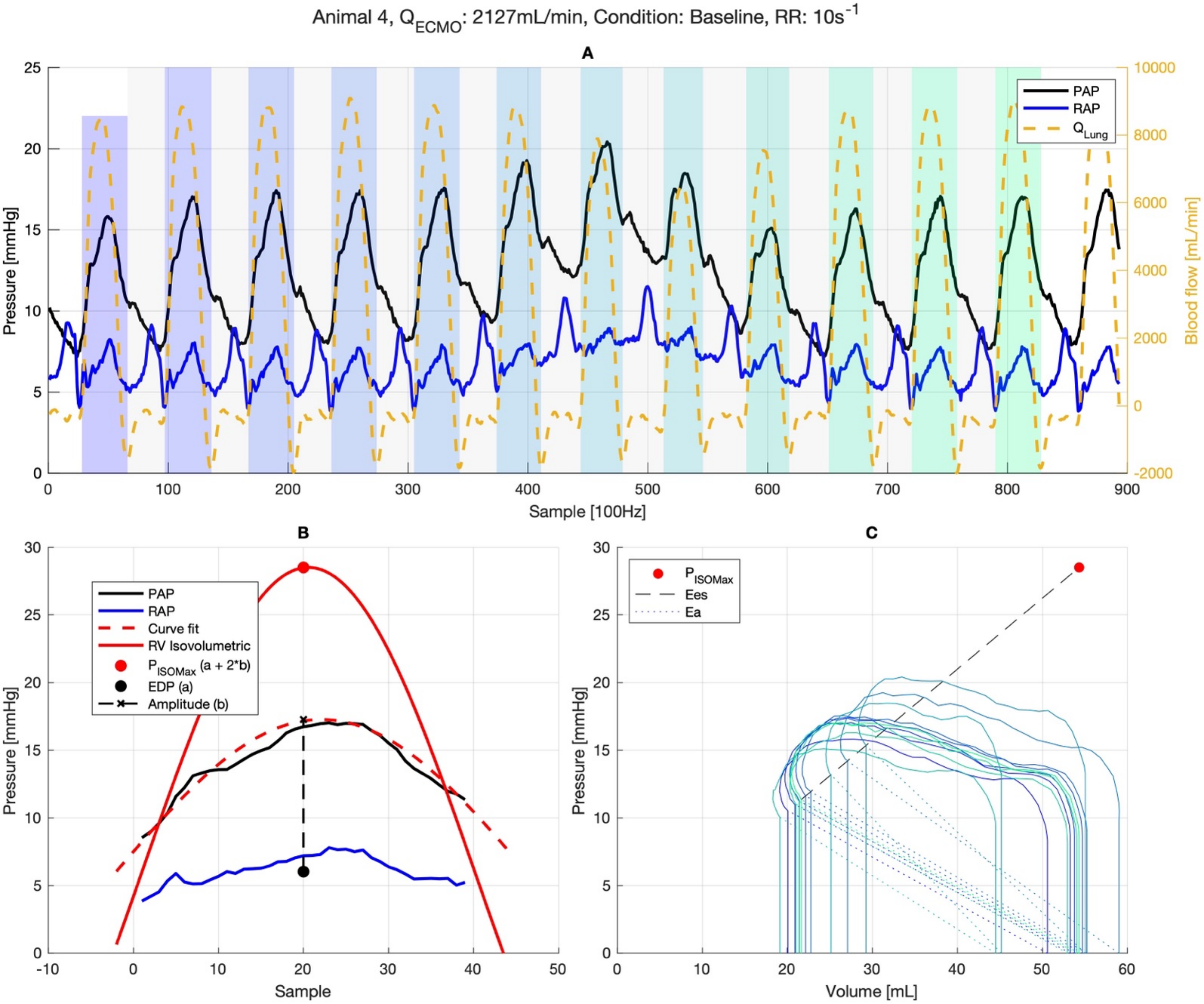

Animal 4, Q ECMO: 2127mL/min, Condition: Baseline, RR: 10s-1
A
PAP
RAP
Q Lung
Pressure [mmHg]
Blood flow [mL/min]
Sample [100Hz]
B
PAP
RAP
Curve fit
RV Isovolumetric
P ISOMax (a + 2*b)
EDP (a)
Amplitude (b)
Pressure [mmHg]
Sample
C
P ISOMax
Ees
Ea
Pressure [mmHg]
Volume [mL]

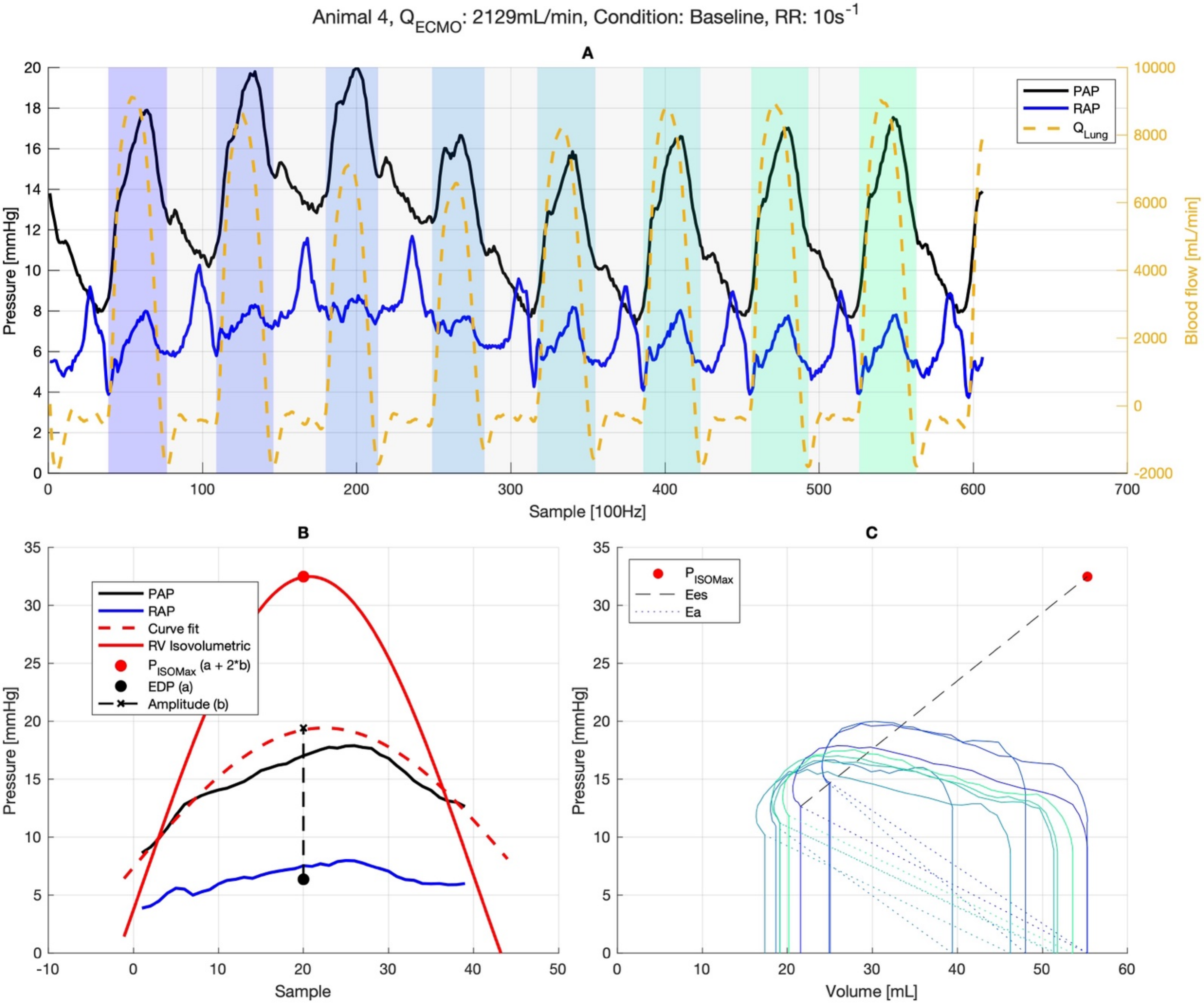

Animal 4, Q_{ECMO}: 2129mL/min, Condition: Baseline, RR: $10s^{-1}$
A
PAP
RAP
Q_{Lung}
Pressure [mmHg]
Blood flow [mL/min]
Sample [100Hz]
B
PAP
RAP
Curve fit
RV Isovolumetric
P_{ISOMax} (a + 2*b)
EDP (a)
Amplitude (b)
Pressure [mmHg]
Sample
C
P_{ISOMax}
Ees
Ea
Pressure [mmHg]
Volume [mL]

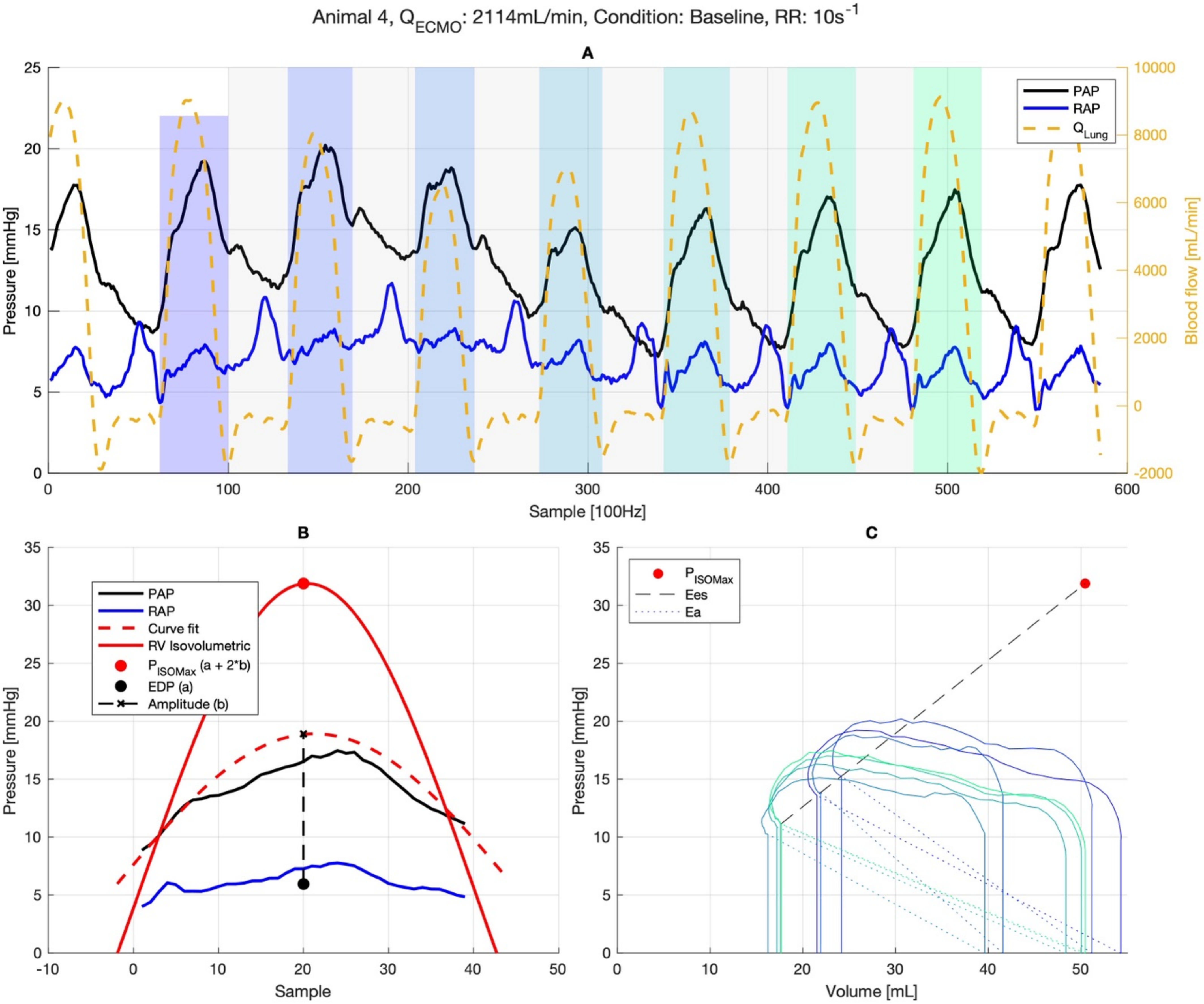

Animal 4, Q_{ECMO}: 2114mL/min, Condition: Baseline, RR: $10s^{-1}$
A
PAP
RAP
Q_{Lung}
Pressure [mmHg]
Blood flow [mL/min]
Sample [100Hz]
B
PAP
RAP
Curve fit
RV Isovolumetric
P_{ISOMax} (a + 2*b)
EDP (a)
Amplitude (b)
Pressure [mmHg]
Sample
C
P_{ISOMax}
Ees
Ea
Pressure [mmHg]
Volume [mL]

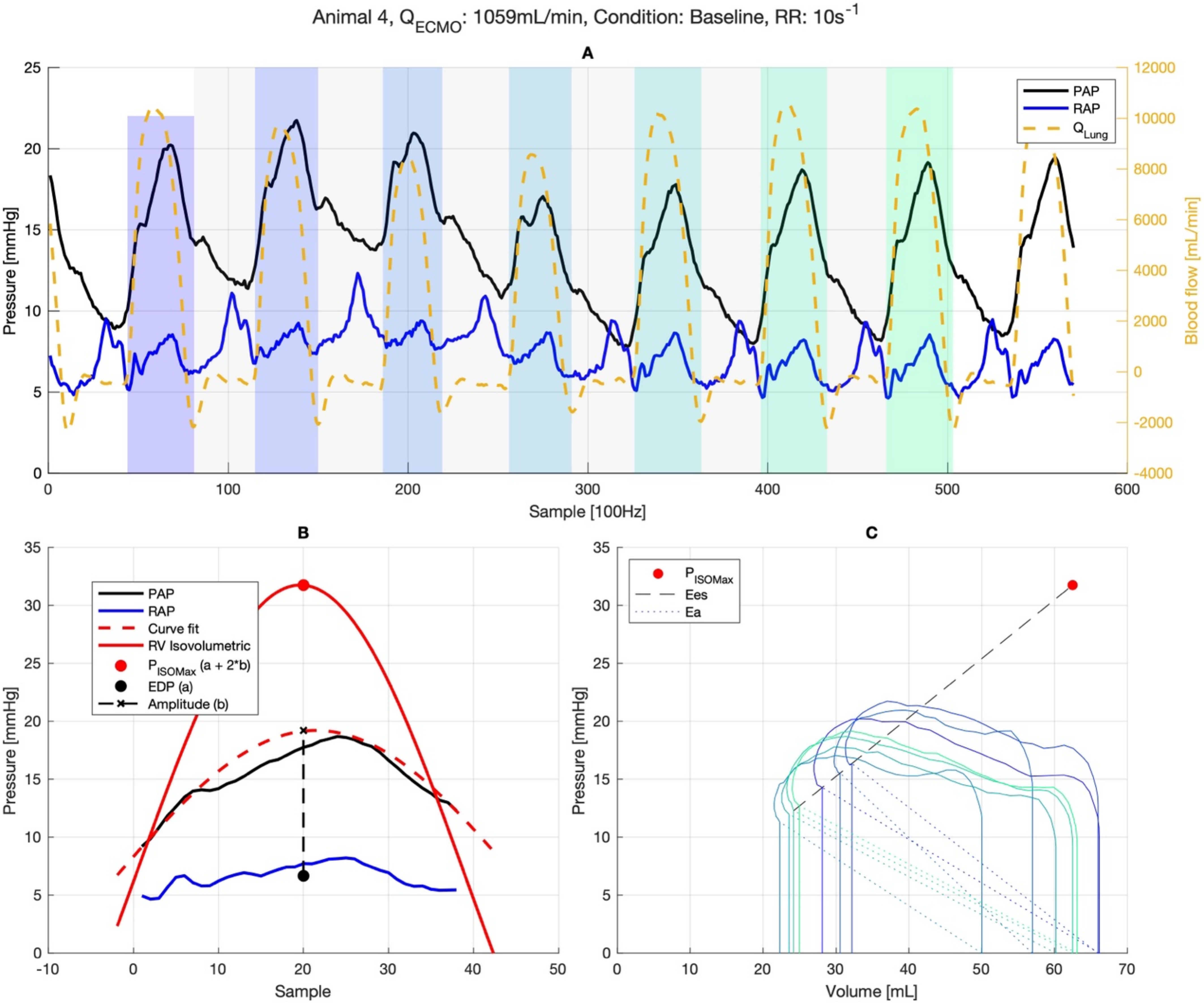

Animal 4, QECMO: 1059mL/min, Condition: Baseline, RR: 10s-1
A
PAP
RAP
QLung
Pressure [mmHg]
Blood flow [mL/min]
Sample [100Hz]
B
PAP
RAP
Curve fit
RV Isovolumetric
PISOMax (a + 2*b)
EDP (a)
Amplitude (b)
Pressure [mmHg]
Sample
C
PISOMax
Ees
Ea
Pressure [mmHg]
Volume [mL]

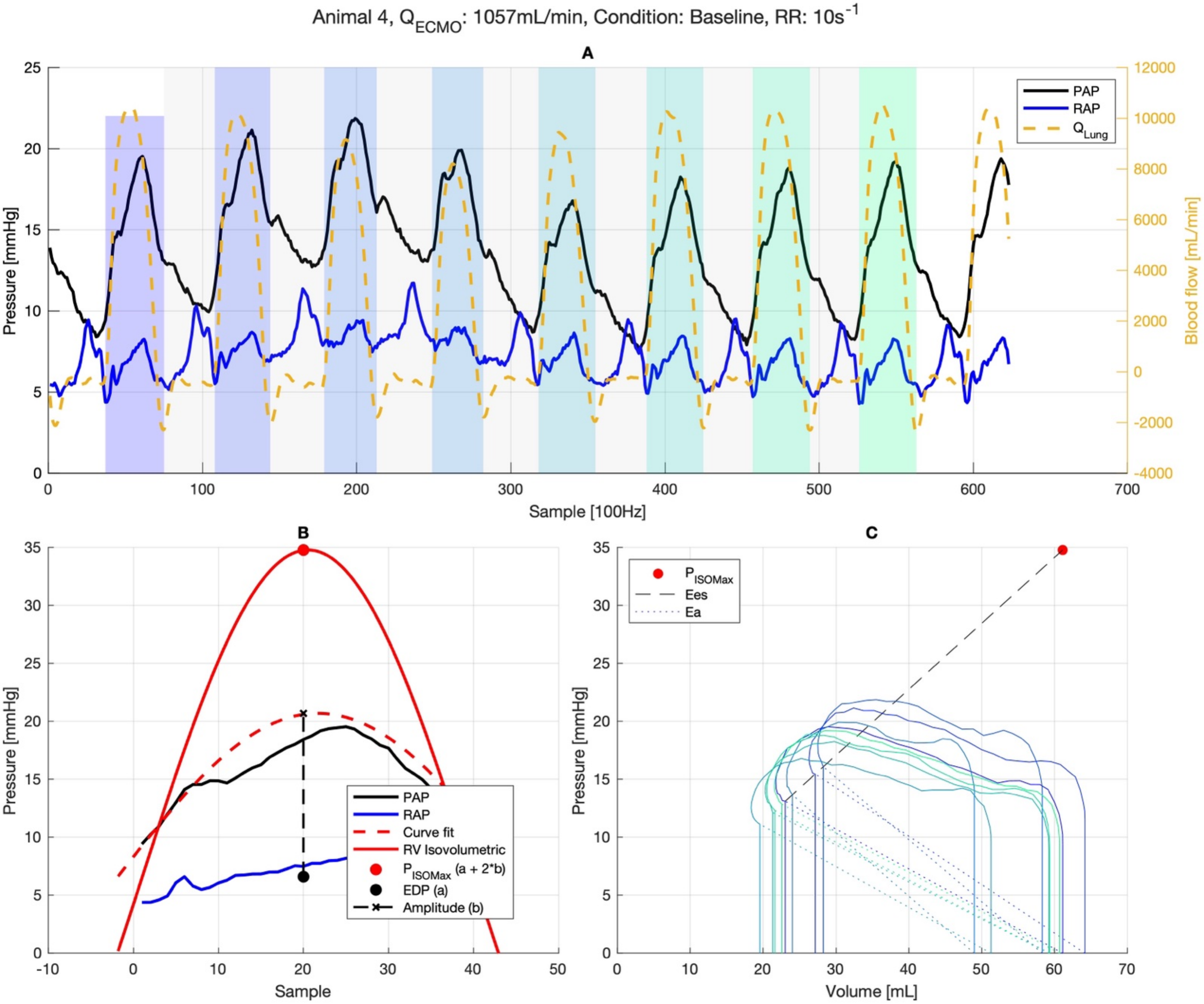
Animal 4, Q_ECMO: 1057mL/min, Condition: Baseline, RR: 10s^-1
A
PAP
RAP
Q_Lung
Pressure [mmHg]
Blood flow [mL/min]
Sample [100Hz]
B
PAP
RAP
Curve fit
RV Isovolumetric
P_ISOMax (a + 2*b)
EDP (a)
Amplitude (b)
Pressure [mmHg]
Sample
C
P_ISOMax
Ees
Ea
Pressure [mmHg]
Volume [mL]

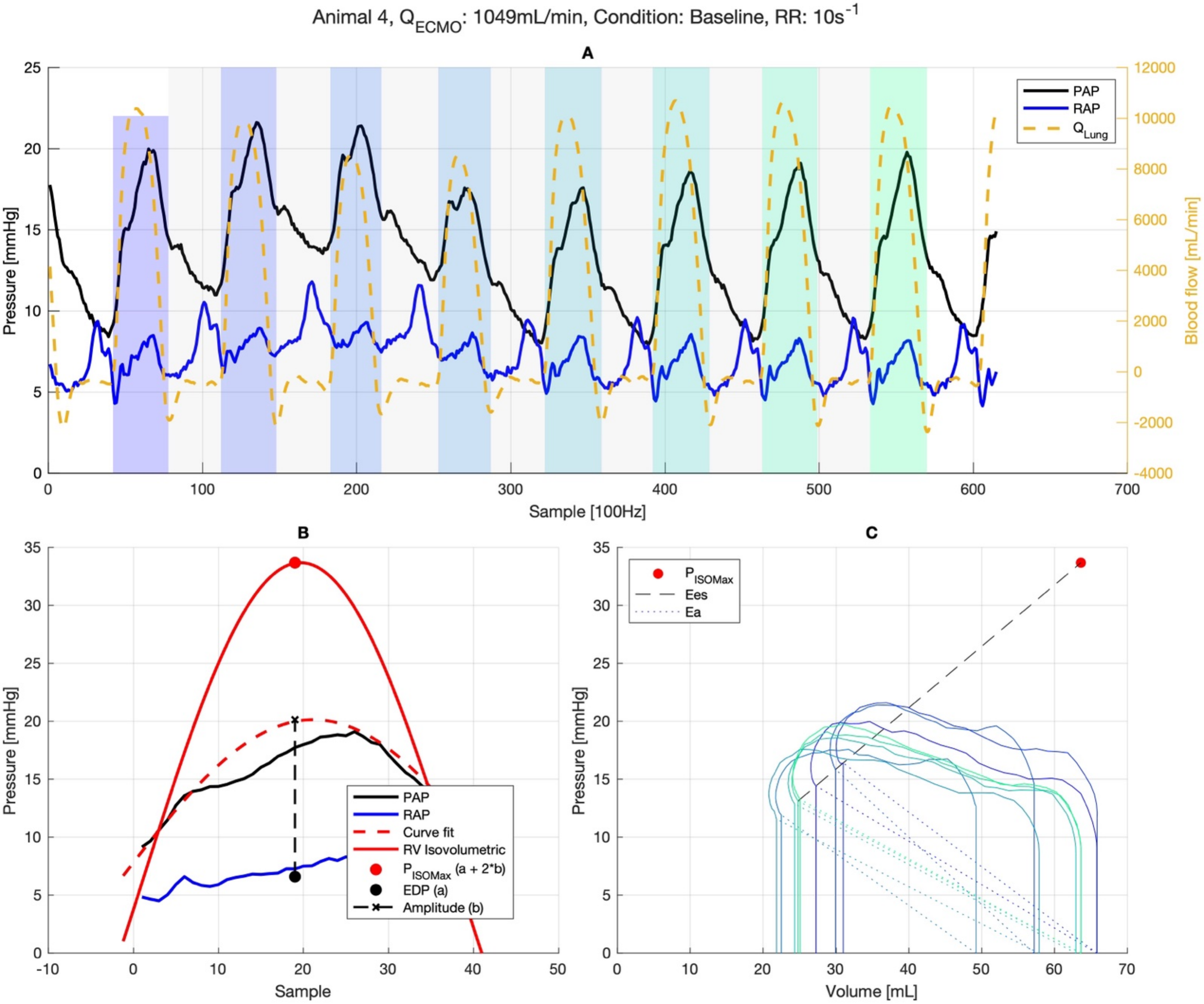

Animal 4, Q_{ECMO}: 1049mL/min, Condition: Baseline, RR: $10s^{-1}$
A
PAP
RAP
Q_{Lung}
Pressure [mmHg]
Blood flow [mL/min]
Sample [100Hz]
B
PAP
RAP
Curve fit
RV Isovolumetric
P_{ISOMax} (a + 2*b)
EDP (a)
Amplitude (b)
Pressure [mmHg]
Sample
C
P_{ISOMax}
Ees
Ea
Pressure [mmHg]
Volume [mL]

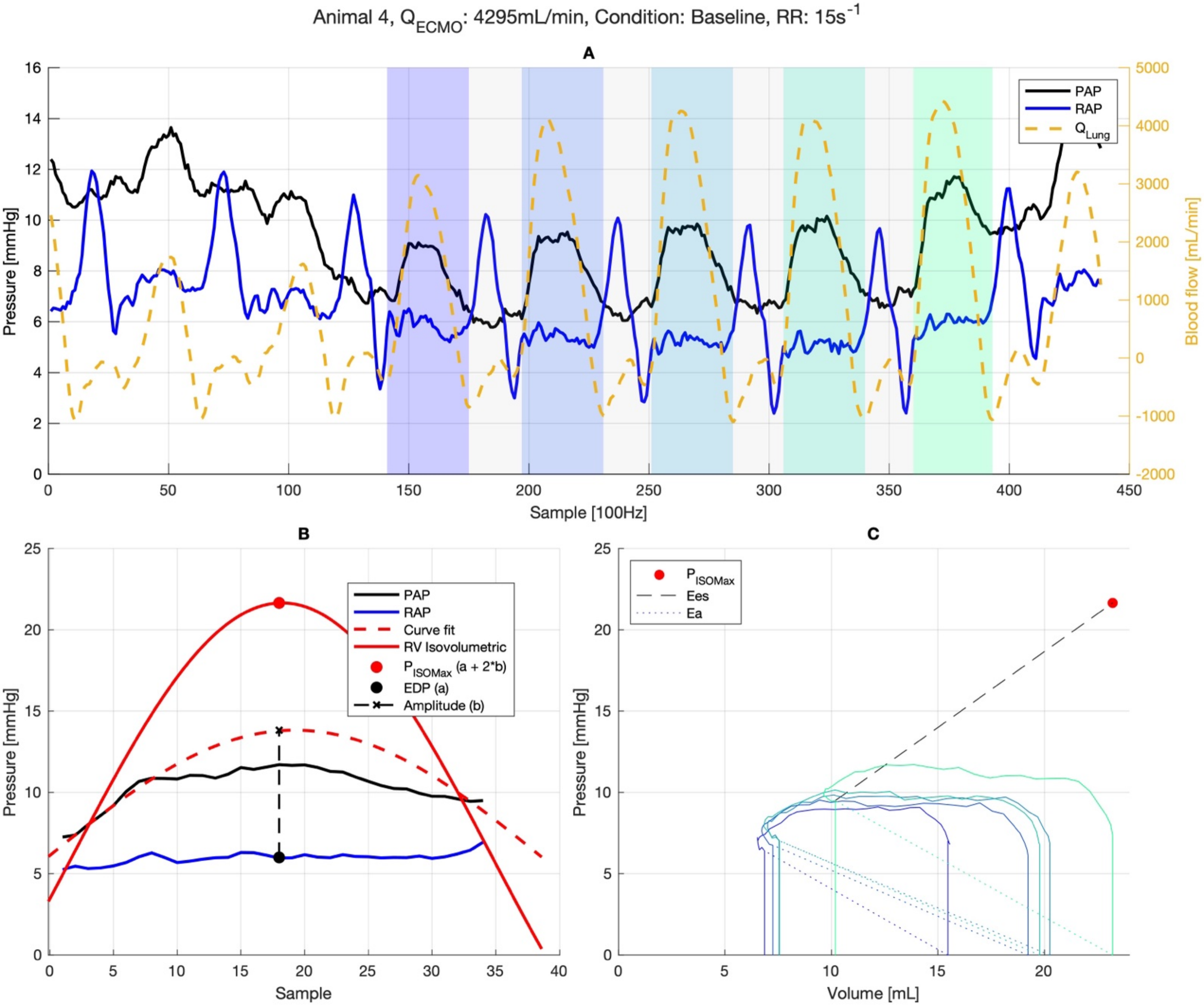

Animal 4, Q_ECMO: 4295mL/min, Condition: Baseline, RR: 15s^-1
A
Pressure [mmHg]
Blood flow [mL/min]
Sample [100Hz]
PAP
RAP
Q_Lung
B
Pressure [mmHg]
Sample
PAP
RAP
Curve fit
RV Isovolumetric
P_ISOMax (a + 2*b)
EDP (a)
Amplitude (b)
C
Pressure [mmHg]
Volume [mL]
P_ISOMax
Ees
Ea

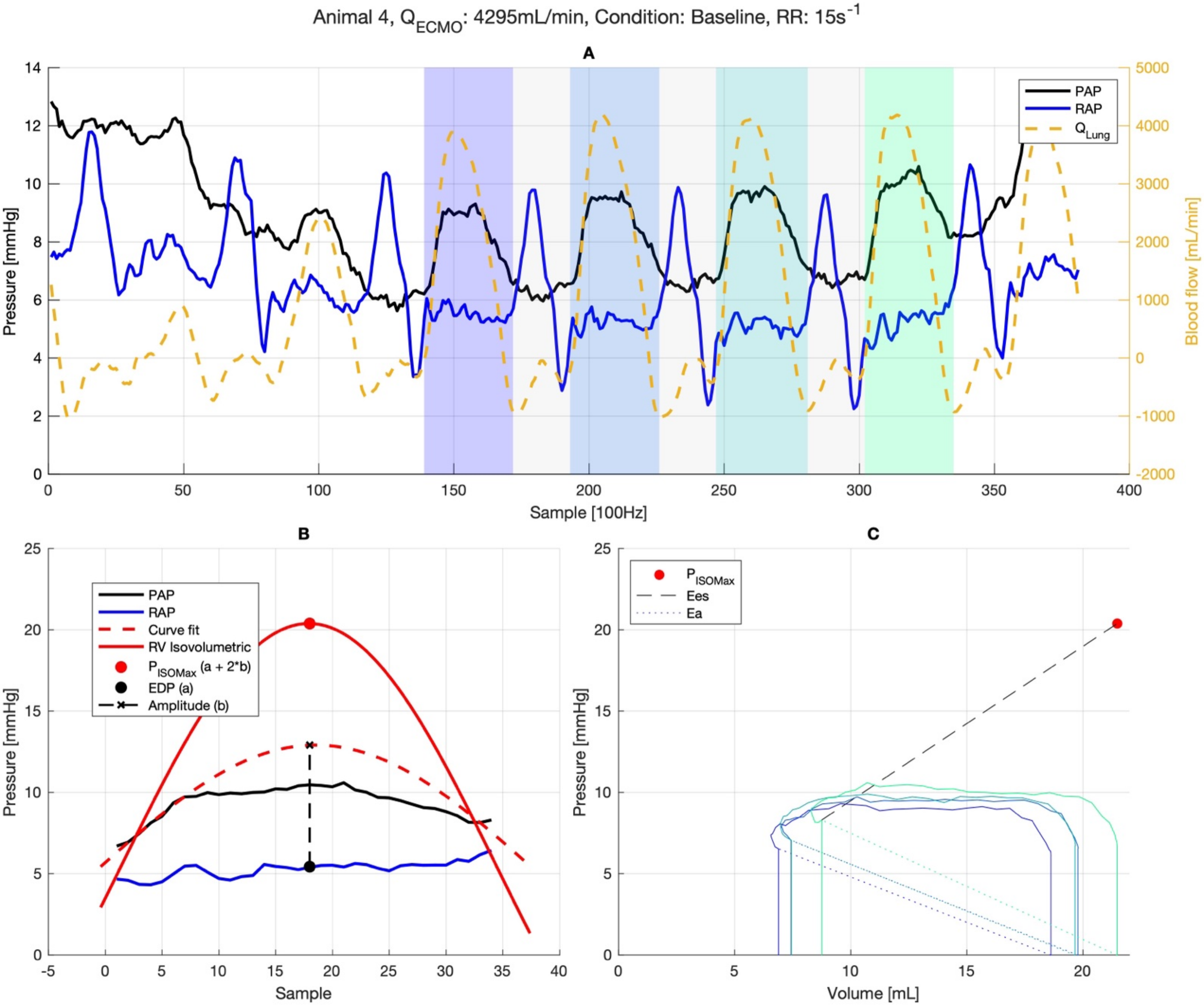

Animal 4, Q_ECMO: 4295mL/min, Condition: Baseline, RR: 15s^-1
A
Pressure [mmHg]
Blood flow [mL/min]
Sample [100Hz]
PAP
RAP
Q_Lung
B
Pressure [mmHg]
Sample
PAP
RAP
Curve fit
RV Isovolumetric
P_ISOMax (a + 2*b)
EDP (a)
Amplitude (b)
C
Pressure [mmHg]
Volume [mL]
P_ISOMax
Ees
Ea

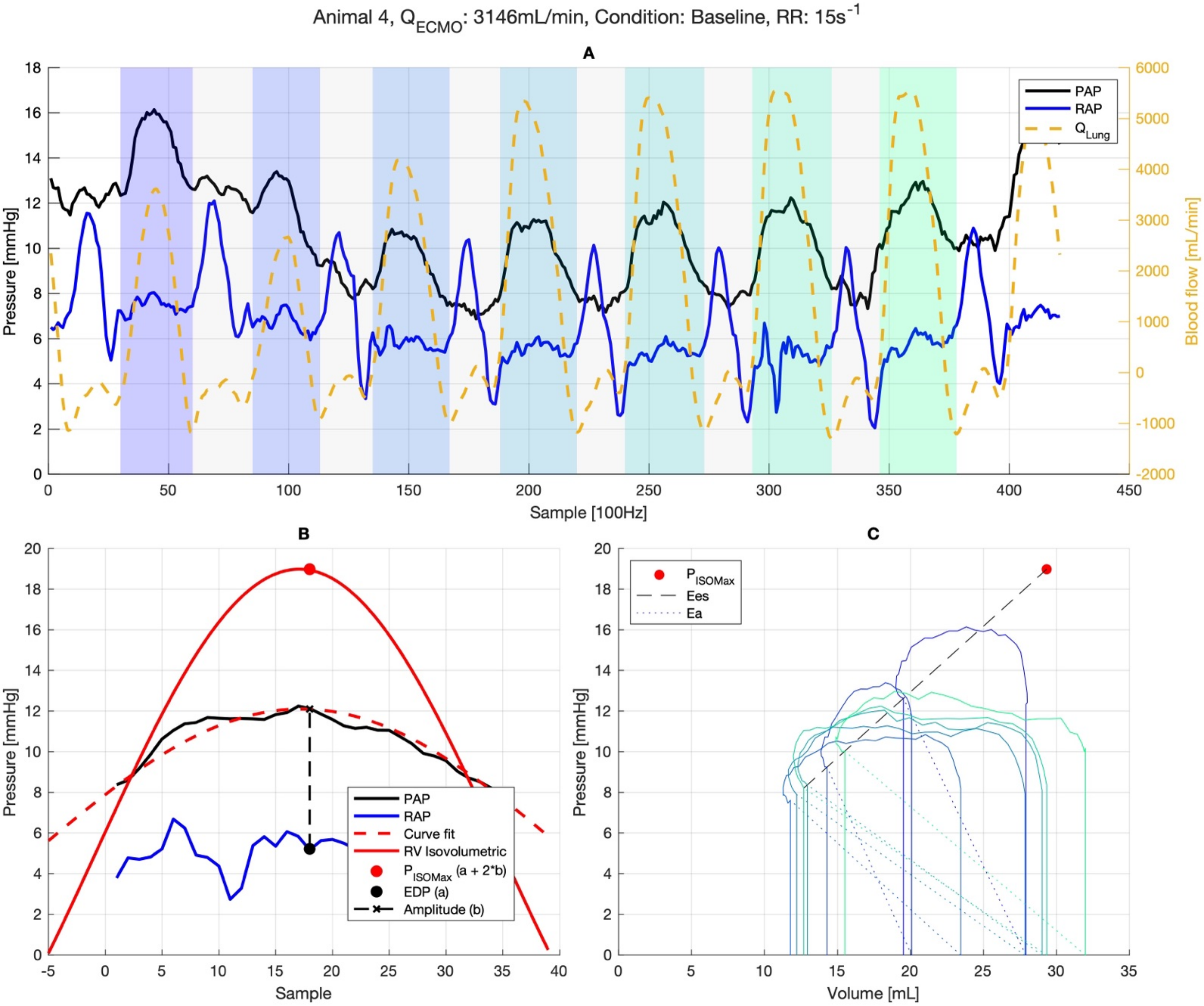

Animal 4, Q_{ECMO}: 3146mL/min, Condition: Baseline, RR: $15s^{-1}$
A
PAP
RAP
Q_{Lung}
Pressure [mmHg]
Blood flow [mL/min]
Sample [100Hz]
B
PAP
RAP
Curve fit
RV Isovolumetric
P_{ISOMax} (a + 2*b)
EDP (a)
Amplitude (b)
Pressure [mmHg]
Sample
C
P_{ISOMax}
Ees
Ea
Pressure [mmHg]
Volume [mL]

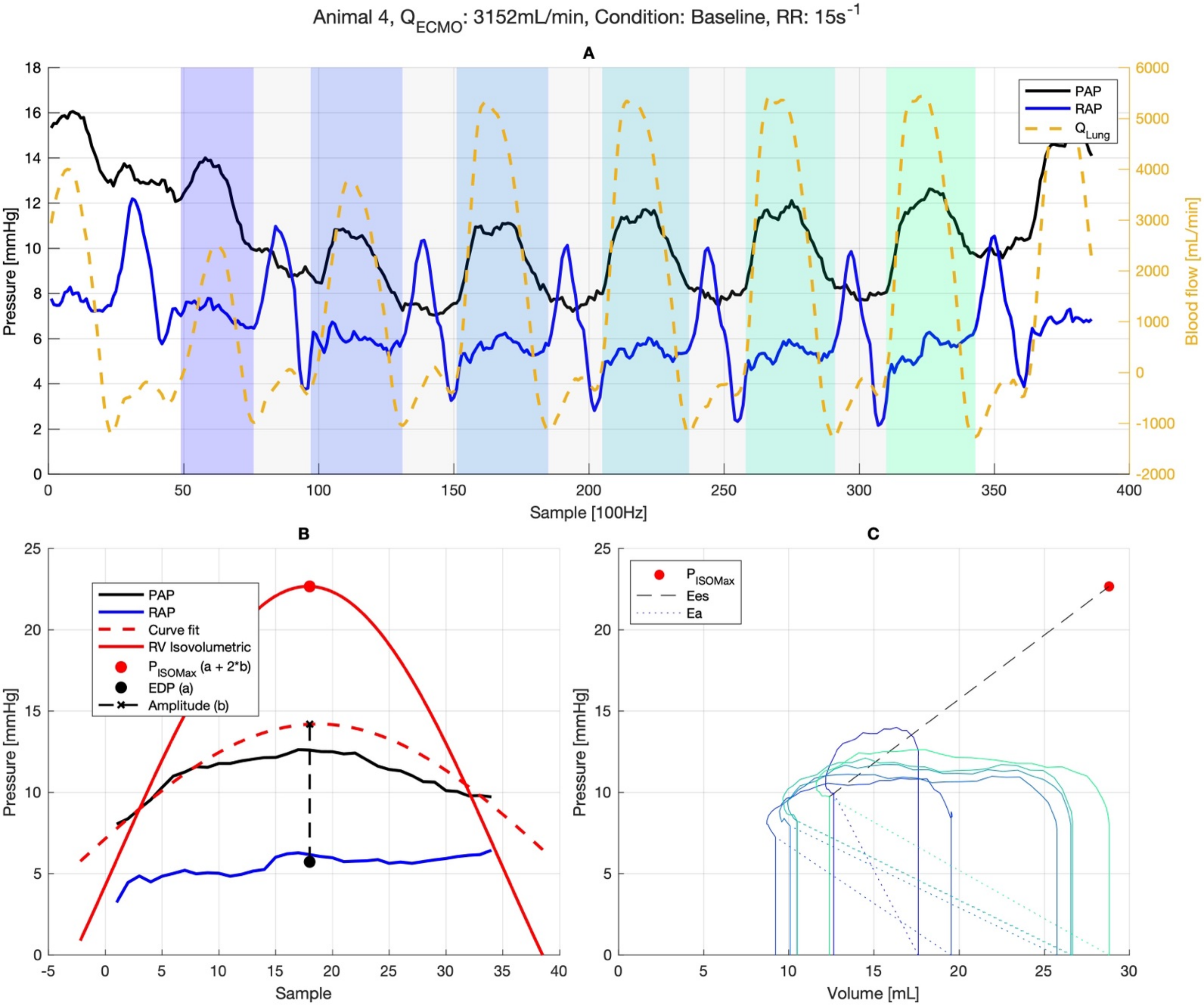

Animal 4, Q_ECMO: 3152mL/min, Condition: Baseline, RR: 15s^-1
A
PAP
RAP
Q_Lung
Pressure [mmHg]
Blood flow [mL/min]
Sample [100Hz]
B
PAP
RAP
Curve fit
RV Isovolumetric
P_ISOMax (a + 2*b)
EDP (a)
Amplitude (b)
Pressure [mmHg]
Sample
C
P_ISOMax
Ees
Ea
Pressure [mmHg]
Volume [mL]

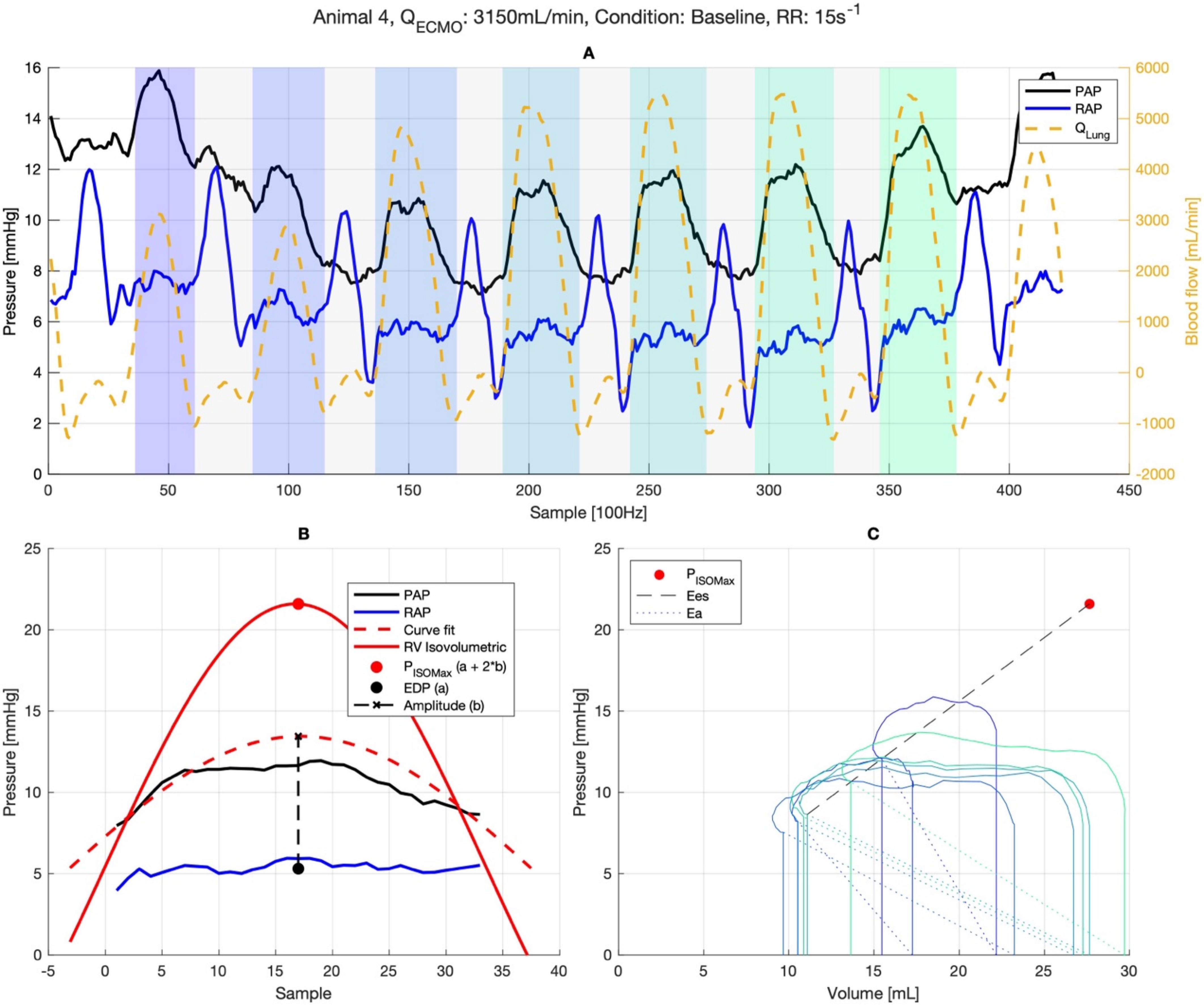

Animal 4, Q_{ECMO}: 3150mL/min, Condition: Baseline, RR: 15s^{-1}
A
PAP
RAP
Q_{Lung}
Pressure [mmHg]
Blood flow [mL/min]
Sample [100Hz]
B
PAP
RAP
Curve fit
RV Isovolumetric
P_{ISOMax} (a + 2*b)
EDP (a)
Amplitude (b)
Pressure [mmHg]
Sample
C
P_{ISOMax}
Ees
Ea
Pressure [mmHg]
Volume [mL]

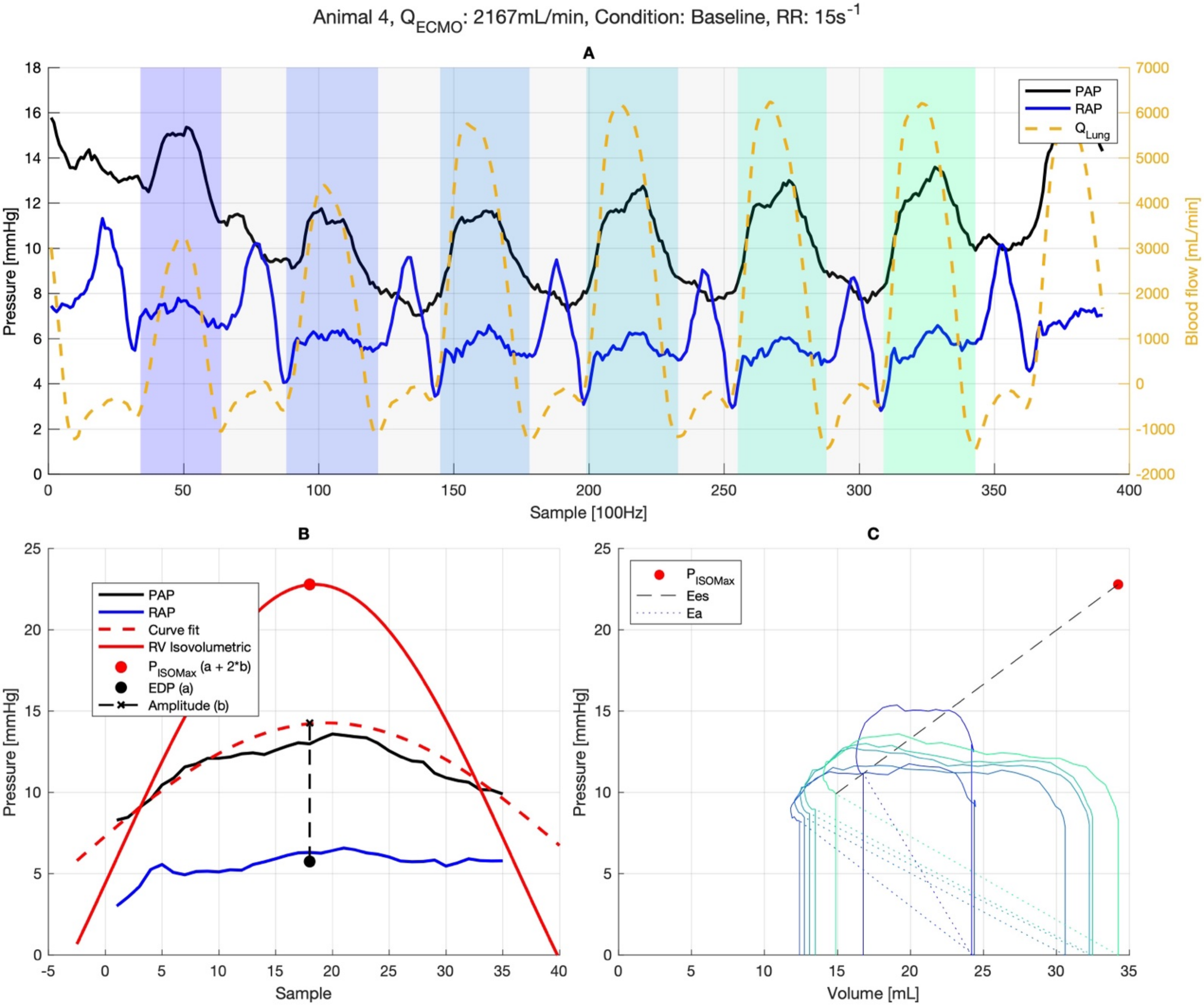

Animal 4, Q_{ECMO}: 2167mL/min, Condition: Baseline, RR: $15s^{-1}$
A
Pressure [mmHg]
Blood flow [mL/min]
Sample [100Hz]
PAP
RAP
Q_{Lung}
B
Pressure [mmHg]
Sample
PAP
RAP
Curve fit
RV Isovolumetric
P_{ISOMax} (a + 2*b)
EDP (a)
Amplitude (b)
C
Pressure [mmHg]
Volume [mL]
P_{ISOMax}
Ees
Ea

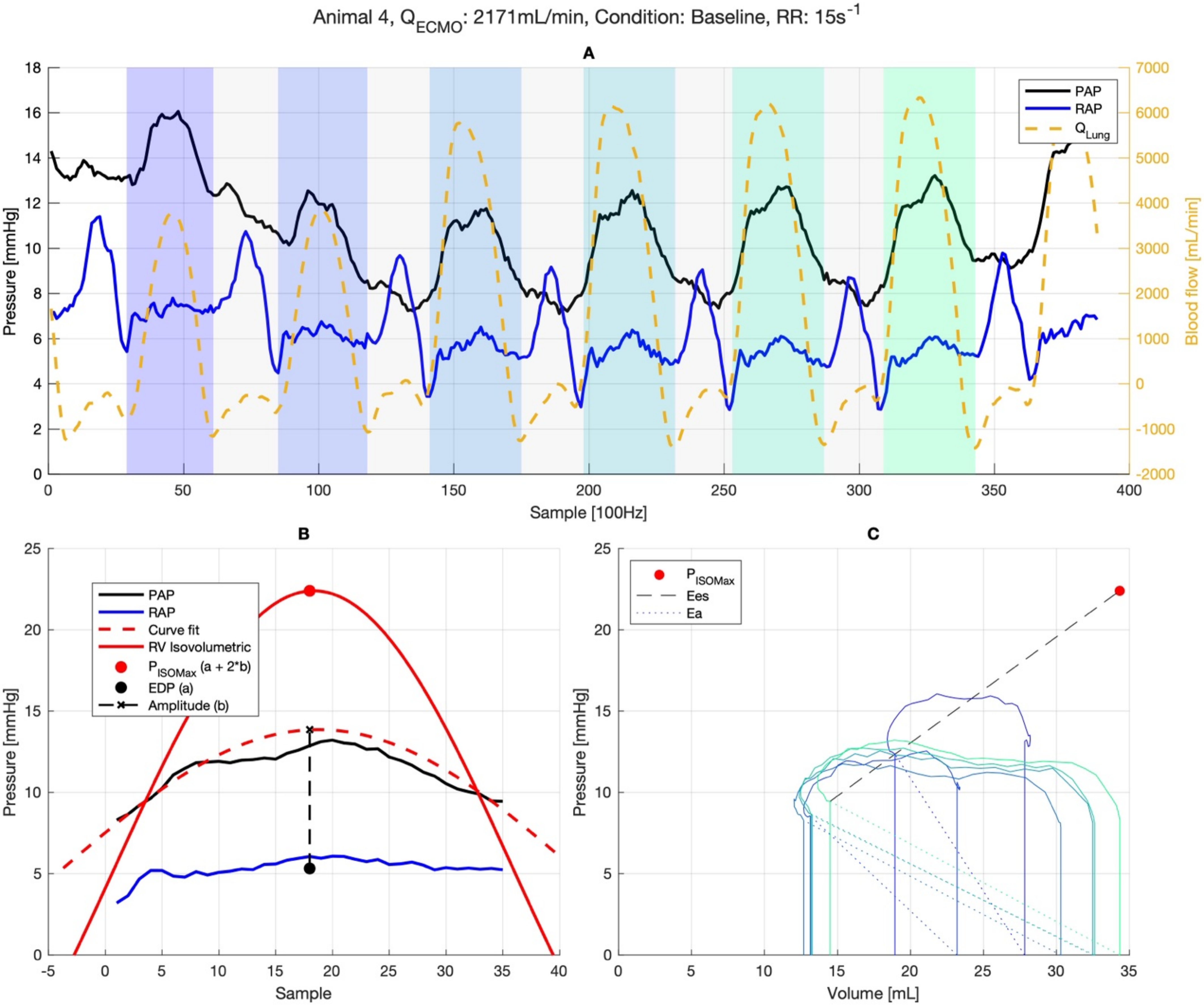

Animal 4, Q_{ECMO}: 2171mL/min, Condition: Baseline, RR: $15s^{-1}$
A
PAP
RAP
Q_{Lung}
Pressure [mmHg]
Blood flow [mL/min]
Sample [100Hz]
B
PAP
RAP
Curve fit
RV Isovolumetric
P_{ISOMax} (a + 2*b)
EDP (a)
Amplitude (b)
Pressure [mmHg]
Sample
C
P_{ISOMax}
Ees
Ea
Pressure [mmHg]
Volume [mL]

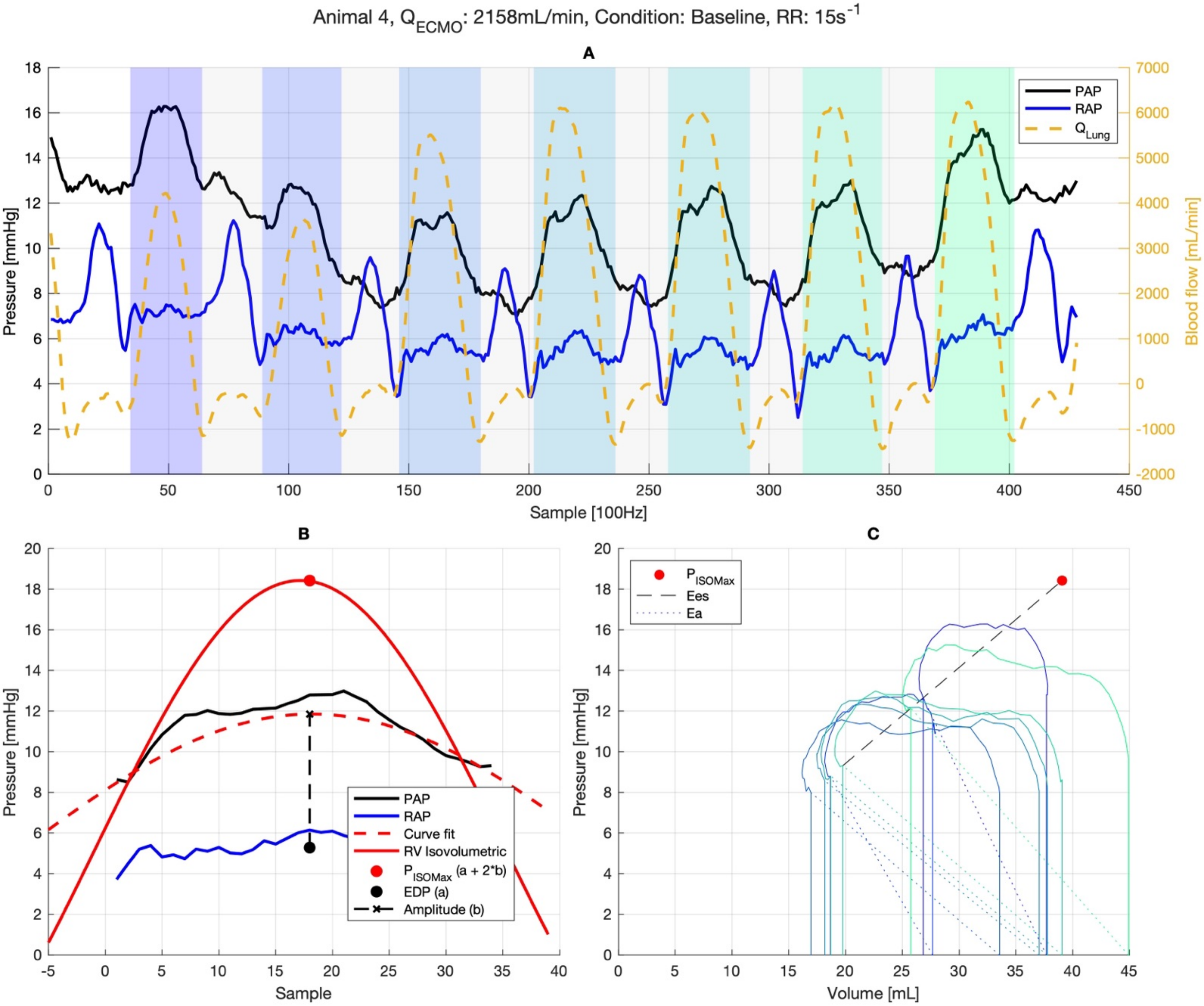

Animal 4, Q_{ECMO}: 2158mL/min, Condition: Baseline, RR: 15s^{-1}
A
PAP
RAP
Q_{Lung}
Pressure [mmHg]
Blood flow [mL/min]
Sample [100Hz]
B
PAP
RAP
Curve fit
RV Isovolumetric
P_{ISOMax} (a + 2*b)
EDP (a)
Amplitude (b)
Pressure [mmHg]
Sample
C
P_{ISOMax}
Ees
Ea
Pressure [mmHg]
Volume [mL]

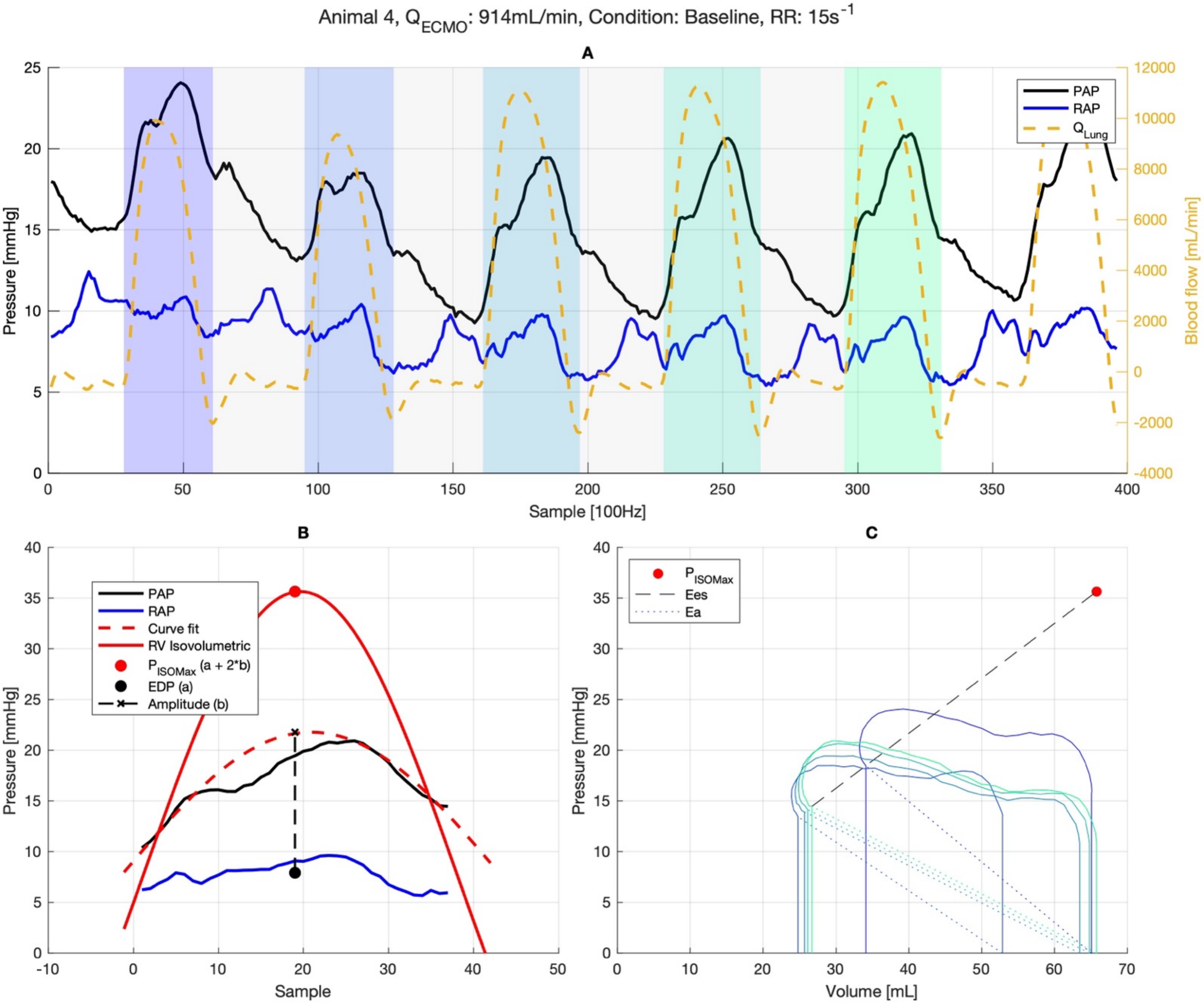

Animal 4, Q_{ECMO}: 914mL/min, Condition: Baseline, RR: $15s^{-1}$
A
PAP
RAP
Q_{Lung}
Pressure [mmHg]
Blood flow [mL/min]
Sample [100Hz]
B
PAP
RAP
Curve fit
RV Isovolumetric
P_{ISOMax} (a + 2*b)
EDP (a)
Amplitude (b)
Pressure [mmHg]
Sample
C
P_{ISOMax}
Ees
Ea
Pressure [mmHg]
Volume [mL]

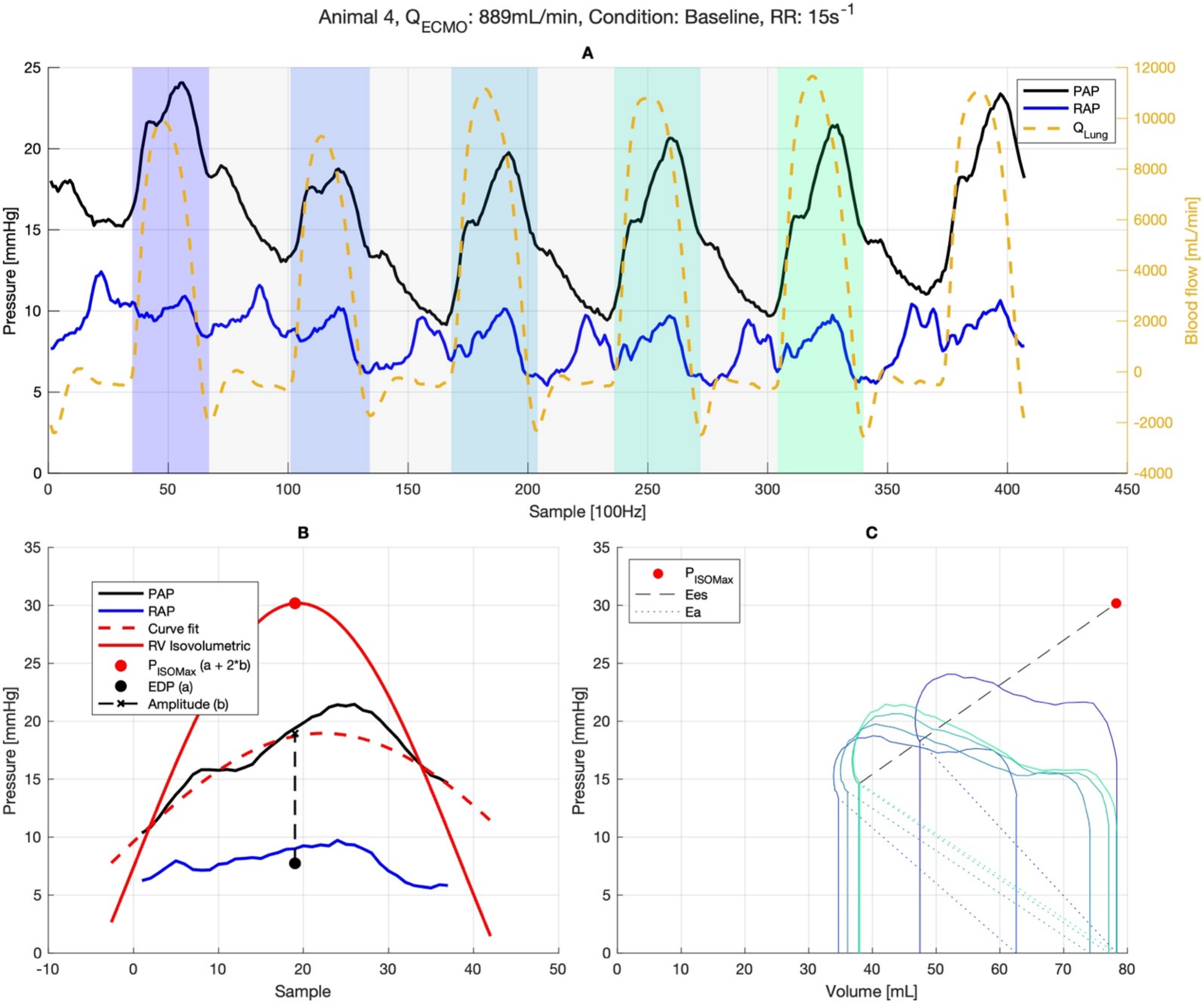

Animal 4, Q_ECMO: 889mL/min, Condition: Baseline, RR: 15s^-1
A
PAP
RAP
Q_Lung
Pressure [mmHg]
Blood flow [mL/min]
Sample [100Hz]
B
PAP
RAP
Curve fit
RV Isovolumetric
P_ISOMax (a + 2*b)
EDP (a)
Amplitude (b)
Pressure [mmHg]
Sample
C
P_ISOMax
Ees
Ea
Pressure [mmHg]
Volume [mL]

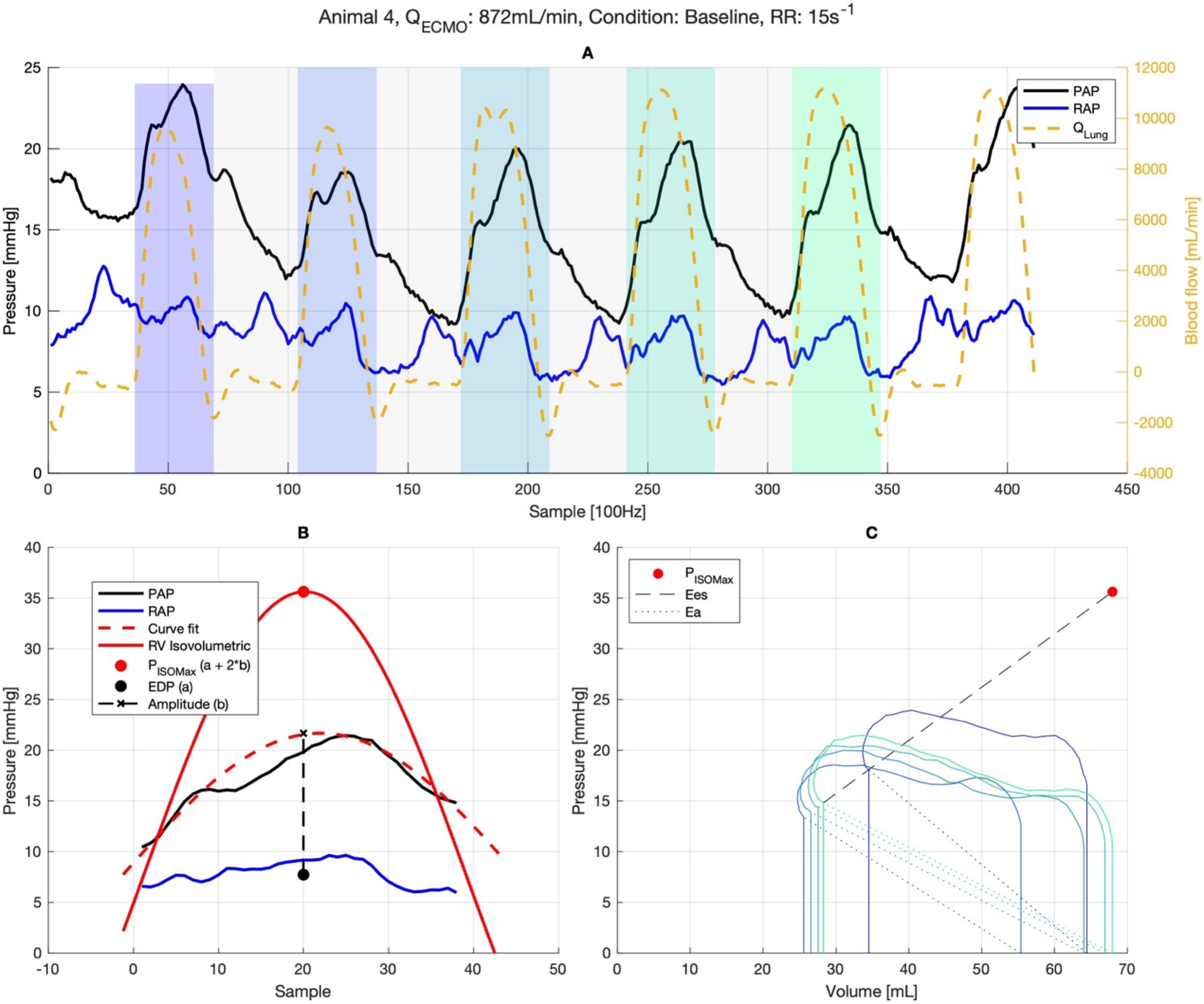
Animal 4, Q_{ECMO}: 872mL/min, Condition: Baseline, RR: 15s^{-1}
A
PAP
RAP
Q_{Lung}
Pressure [mmHg]
Blood flow [mL/min]
Sample [100Hz]
B
PAP
RAP
Curve fit
RV Isovolumetric
P_{ISOMax} (a + 2*b)
EDP (a)
Amplitude (b)
Pressure [mmHg]
Sample
C
P_{ISOMax}
Ees
Ea
Pressure [mmHg]
Volume [mL]

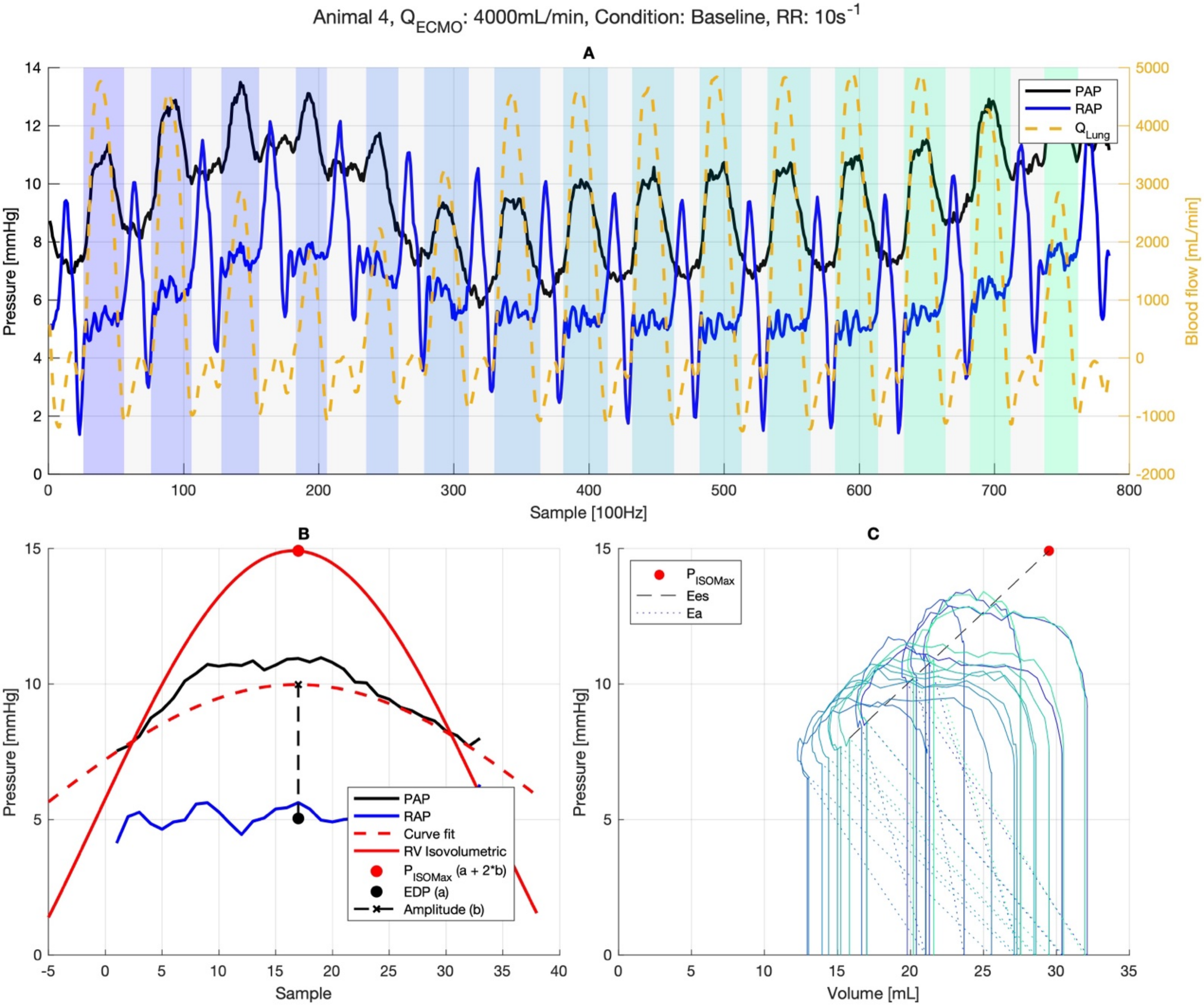

Animal 4, Q_{ECMO}: 4000mL/min, Condition: Baseline, RR: $10s^{-1}$
A
PAP
RAP
Q_{Lung}
Pressure [mmHg]
Blood flow [mL/min]
Sample [100Hz]
B
PAP
RAP
Curve fit
RV Isovolumetric
P_{ISOMax} (a + 2*b)
EDP (a)
Amplitude (b)
Pressure [mmHg]
Sample
C
P_{ISOMax}
Ees
Ea
Pressure [mmHg]
Volume [mL]

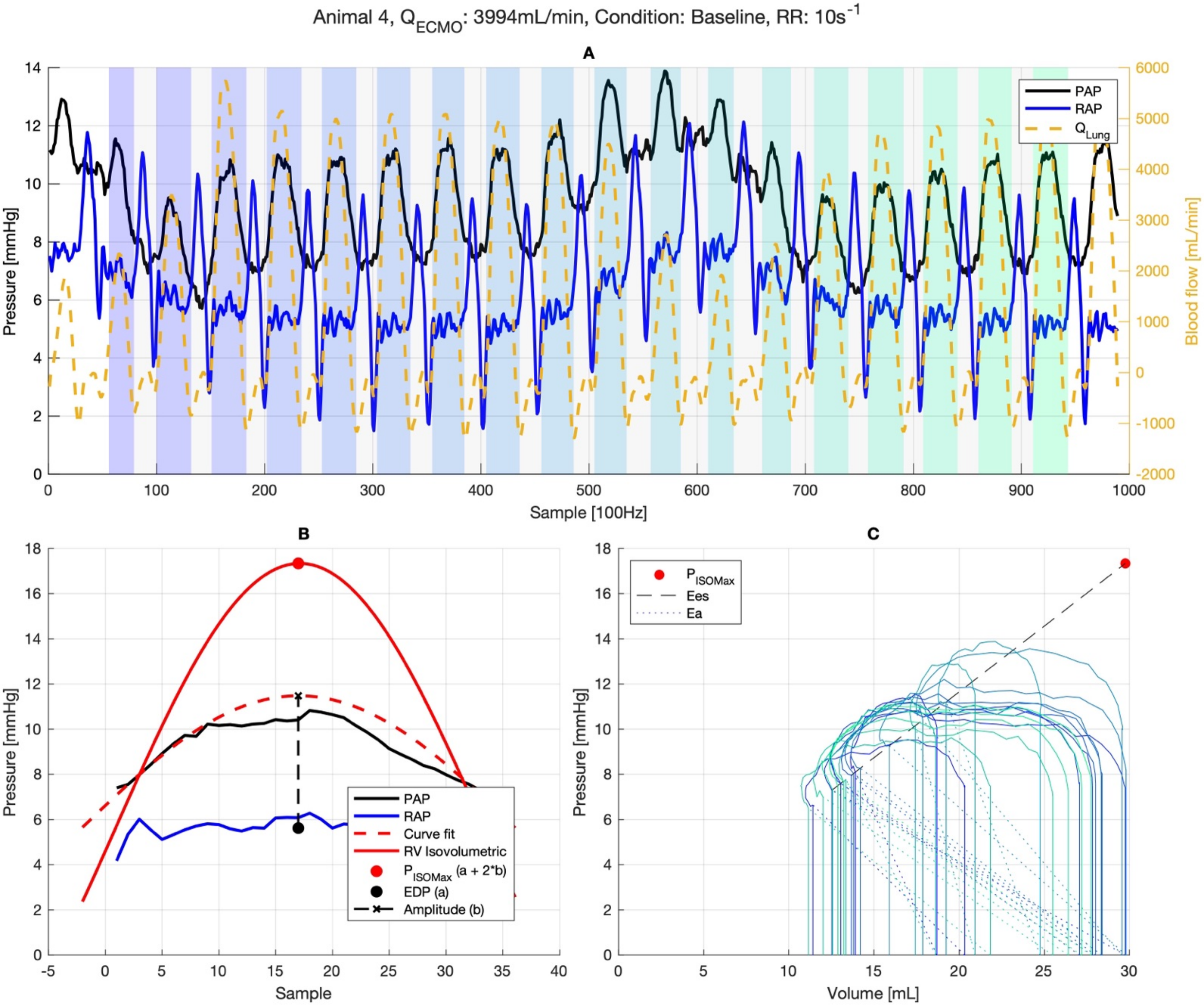

Animal 4, Q_ECMO: 3994mL/min, Condition: Baseline, RR: 10s^-1
A
PAP
RAP
Q_Lung
Pressure [mmHg]
Blood flow [mL/min]
Sample [100Hz]
B
PAP
RAP
Curve fit
RV Isovolumetric
P_ISOMax (a + 2*b)
EDP (a)
Amplitude (b)
Pressure [mmHg]
Sample
C
P_ISOMax
Ees
Ea
Pressure [mmHg]
Volume [mL]

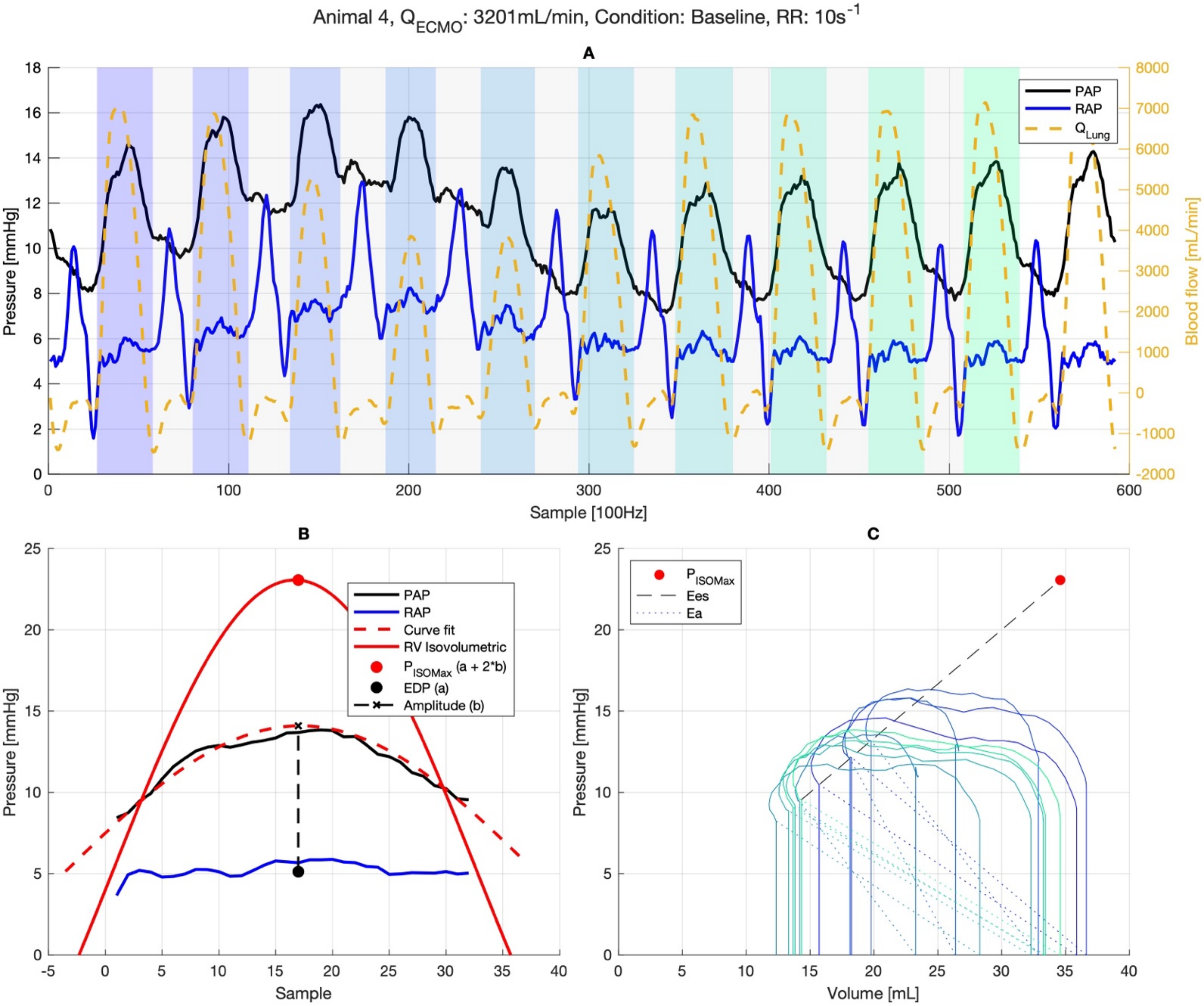

Animal 4, Q_{ECMO}: 3201mL/min, Condition: Baseline, RR: $10s^{-1}$
A
PAP
RAP
Q_{Lung}
Pressure [mmHg]
Blood flow [mL/min]
Sample [100Hz]
B
PAP
RAP
Curve fit
RV Isovolumetric
P_{ISOMax} (a + 2*b)
EDP (a)
Amplitude (b)
Pressure [mmHg]
Sample
C
P_{ISOMax}
Ees
Ea
Pressure [mmHg]
Volume [mL]

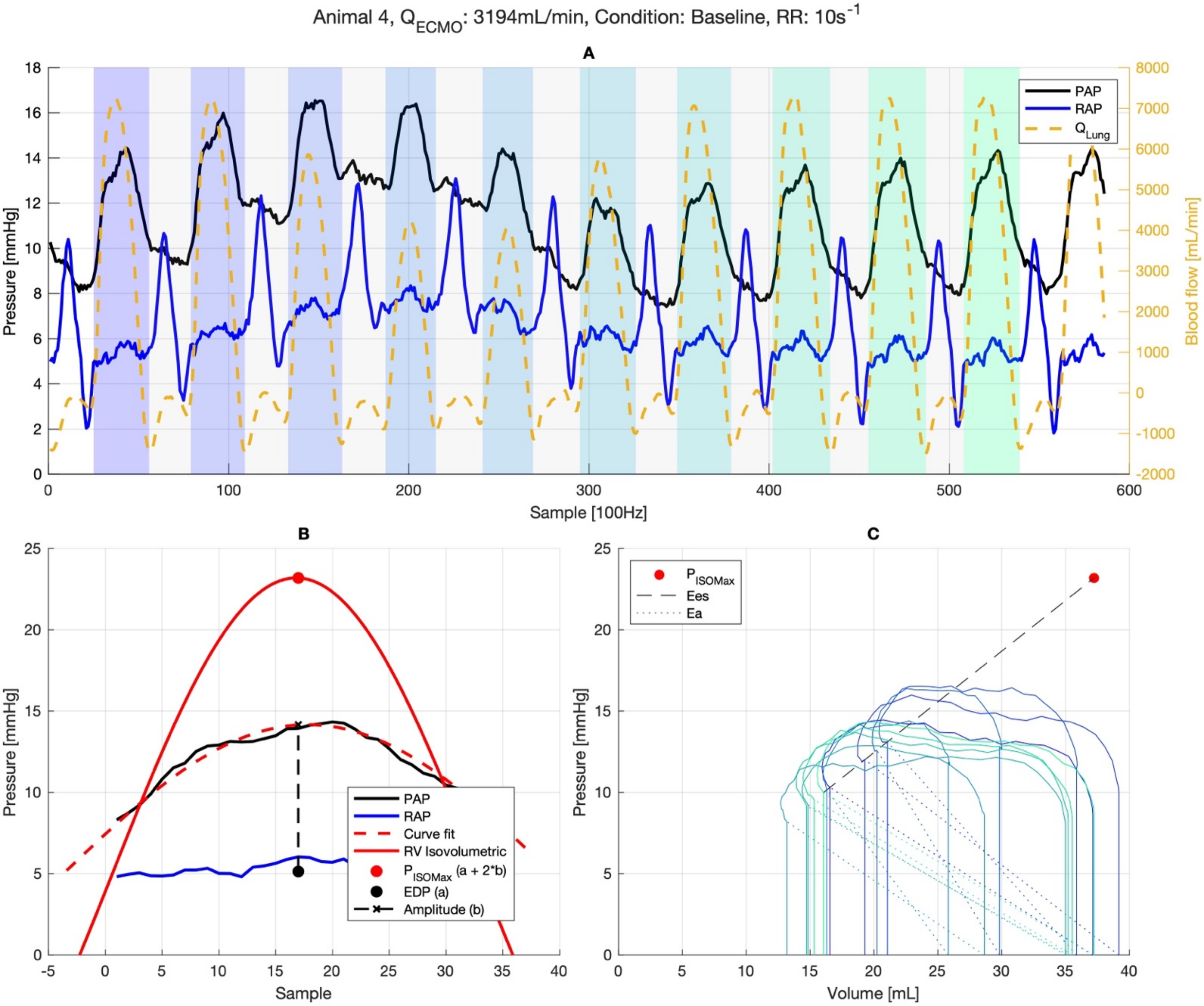

Animal 4, Q_ECMO: 3194mL/min, Condition: Baseline, RR: 10s^-1
A
PAP
RAP
Q_Lung
Pressure [mmHg]
Blood flow [mL/min]
Sample [100Hz]
B
PAP
RAP
Curve fit
RV Isovolumetric
P_ISOMax (a + 2*b)
EDP (a)
Amplitude (b)
Pressure [mmHg]
Sample
C
P_ISOMax
Ees
Ea
Pressure [mmHg]
Volume [mL]

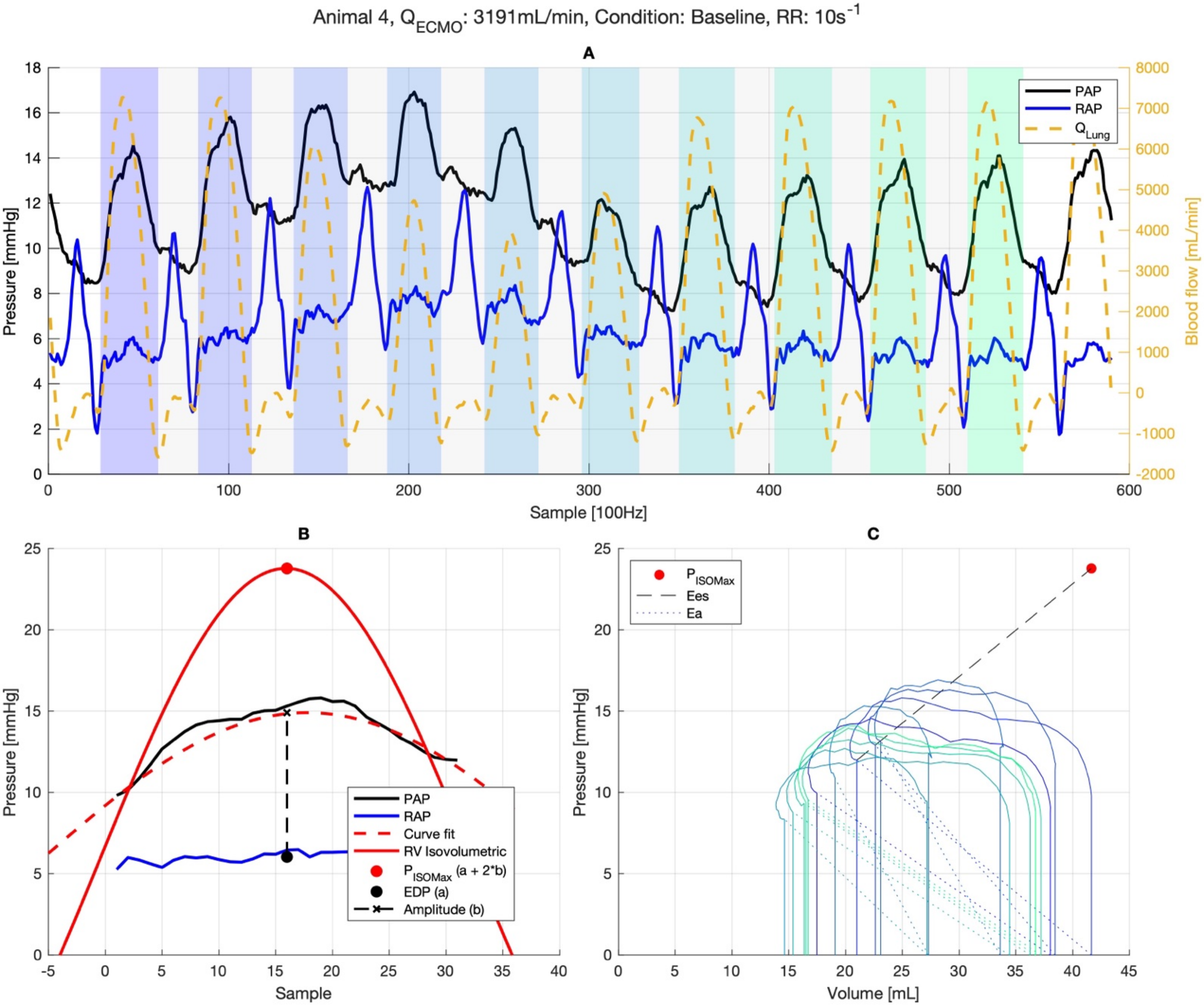

Animal 4, Q_ECMO: 3191mL/min, Condition: Baseline, RR: 10s^-1
A
PAP
RAP
Q_Lung
Pressure [mmHg]
Blood flow [mL/min]
Sample [100Hz]
B
PAP
RAP
Curve fit
RV Isovolumetric
P_ISOMax (a + 2*b)
EDP (a)
Amplitude (b)
Pressure [mmHg]
Sample
C
P_ISOMax
Ees
Ea
Pressure [mmHg]
Volume [mL]

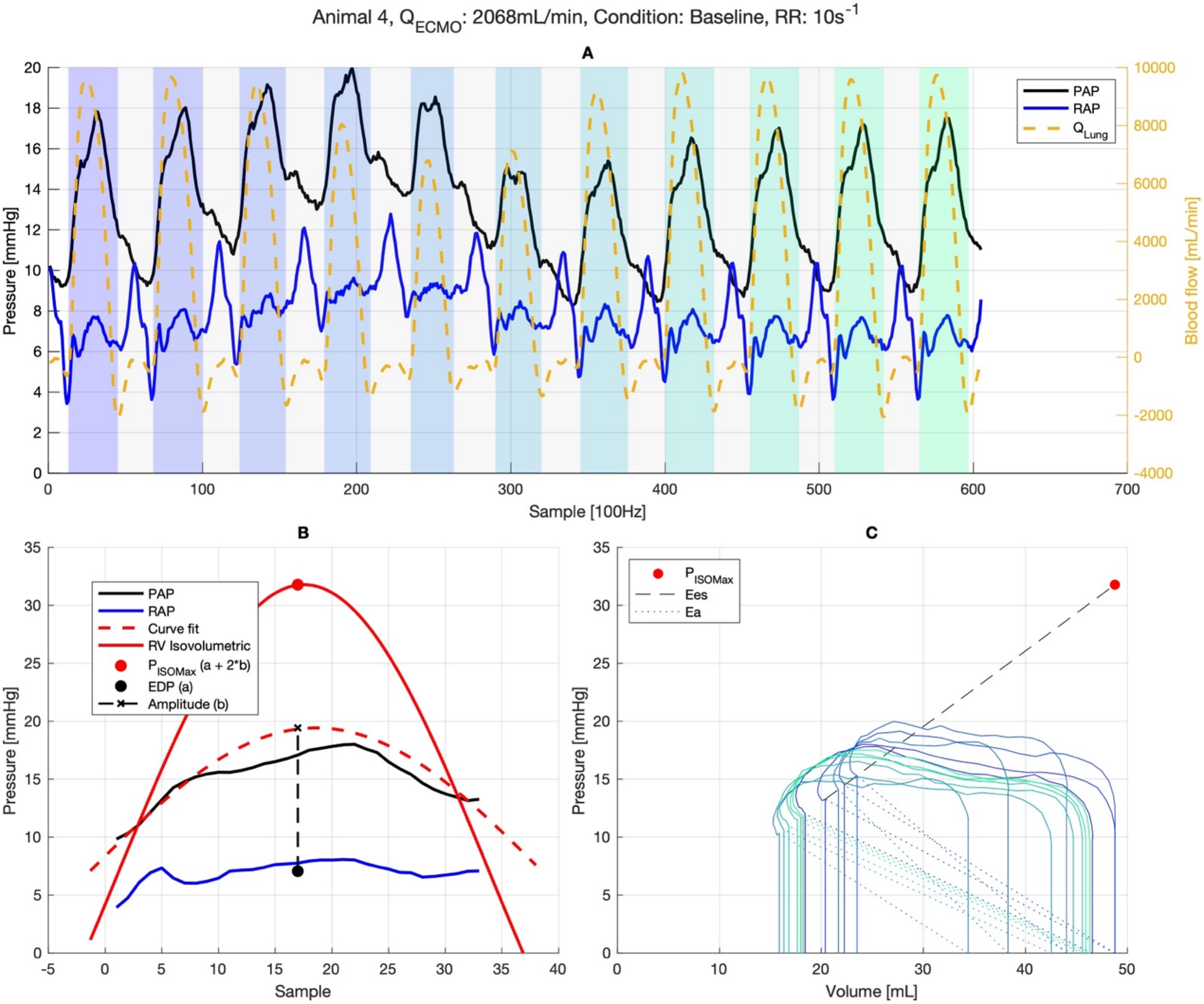

Animal 4, Q_ECMO: 2068mL/min, Condition: Baseline, RR: 10s^-1
A
PAP
RAP
Q_Lung
Pressure [mmHg]
Blood flow [mL/min]
Sample [100Hz]
B
PAP
RAP
Curve fit
RV Isovolumetric
P_ISOMax (a + 2*b)
EDP (a)
Amplitude (b)
Pressure [mmHg]
Sample
C
P_ISOMax
Ees
Ea
Pressure [mmHg]
Volume [mL]

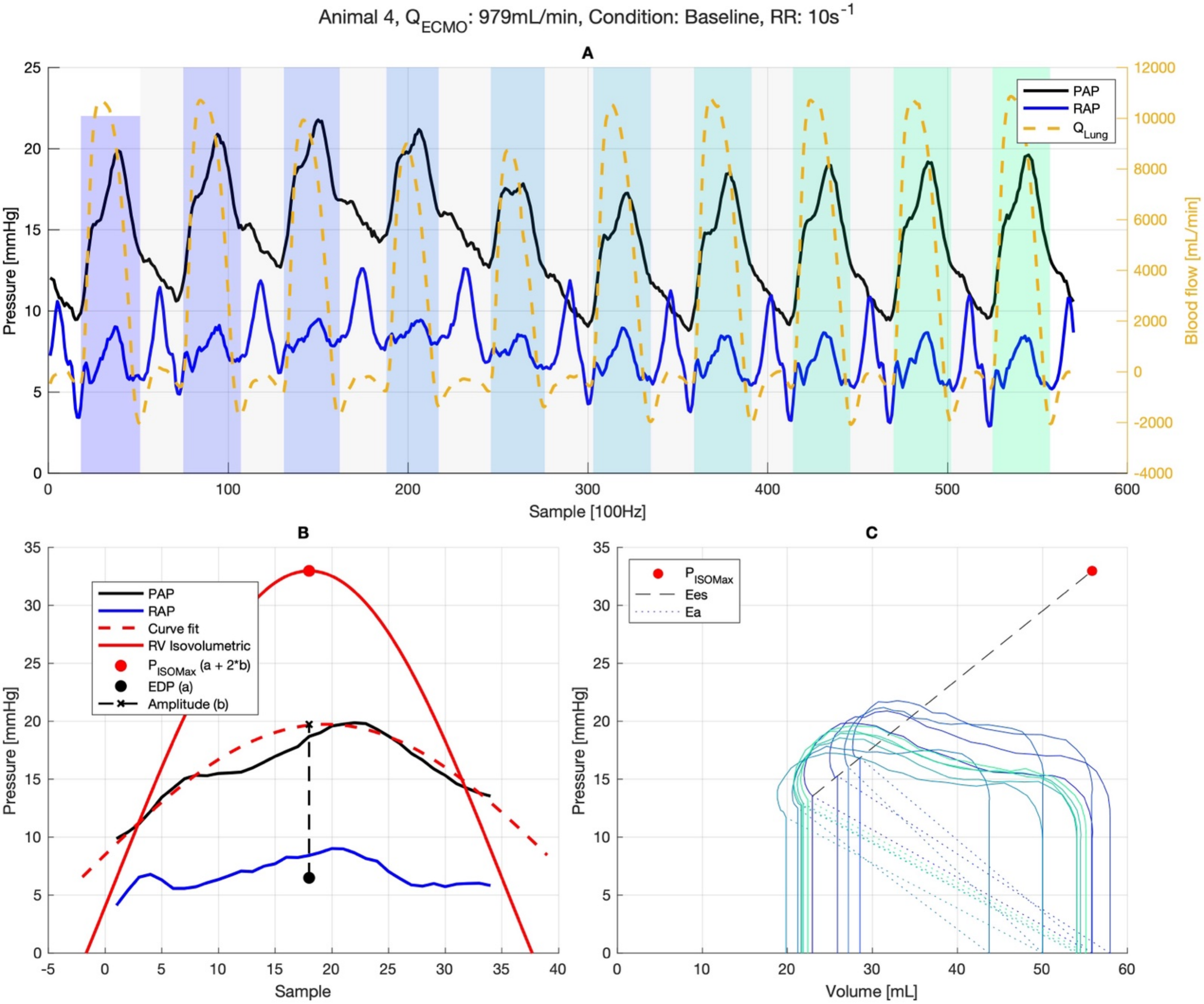
Animal 4, Q_{ECMO}: 979mL/min, Condition: Baseline, RR: $10s^{-1}$
A
PAP
RAP
Q_{Lung}
Pressure [mmHg]
Blood flow [mL/min]
Sample [100Hz]
B
PAP
RAP
Curve fit
RV Isovolumetric
P_{ISOMax} (a + 2*b)
EDP (a)
Amplitude (b)
Pressure [mmHg]
Sample
C
P_{ISOMax}
Ees
Ea
Pressure [mmHg]
Volume [mL]

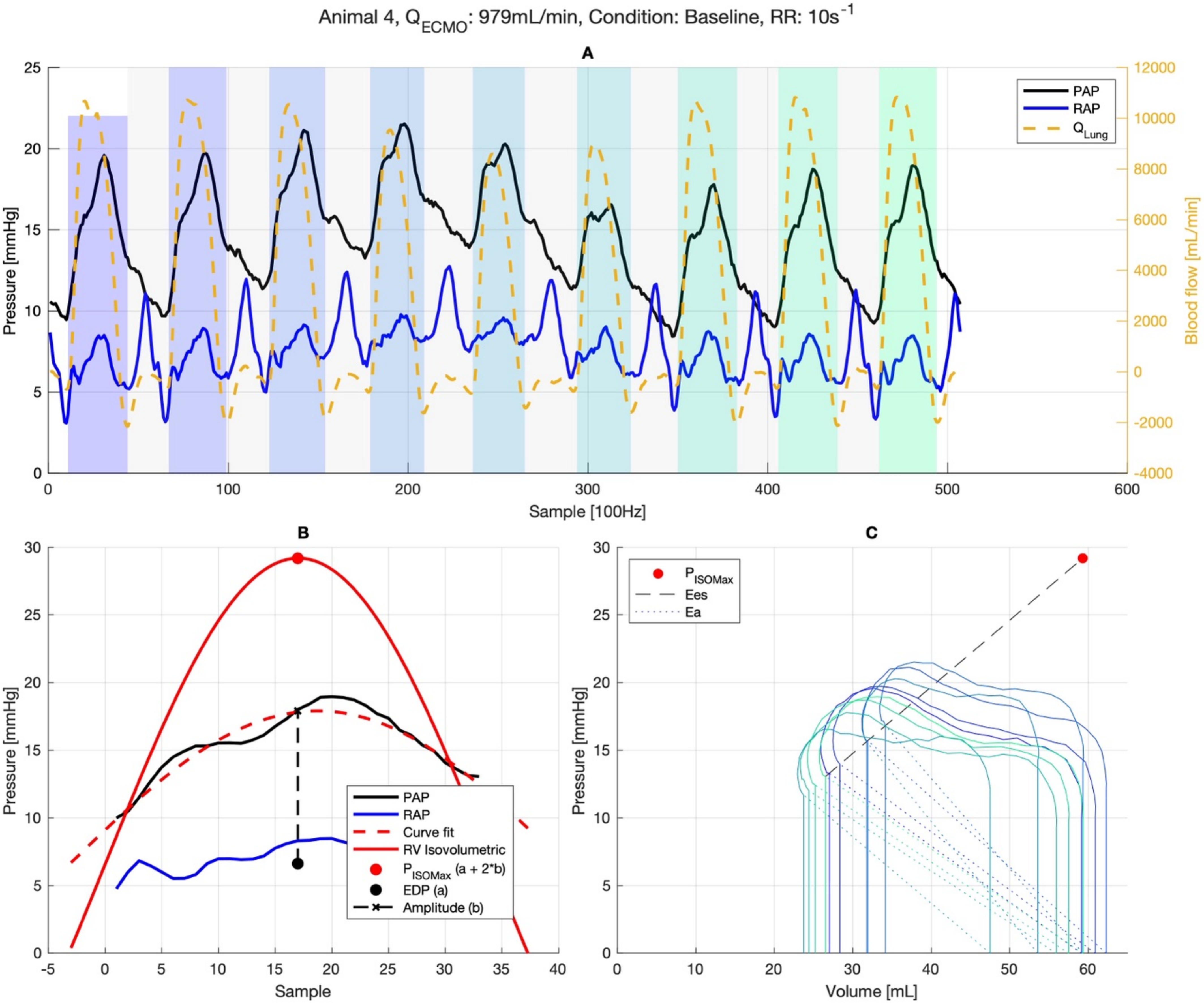

Animal 4, Q_{ECMO}: 979mL/min, Condition: Baseline, RR: $10s^{-1}$
A
Pressure [mmHg]
Blood flow [mL/min]
Sample [100Hz]
PAP
RAP
Q_{Lung}
B
Pressure [mmHg]
Sample
PAP
RAP
Curve fit
RV Isovolumetric
P_{ISOMax} (a + 2*b)
EDP (a)
Amplitude (b)
C
Pressure [mmHg]
Volume [mL]
P_{ISOMax}
Ees
Ea

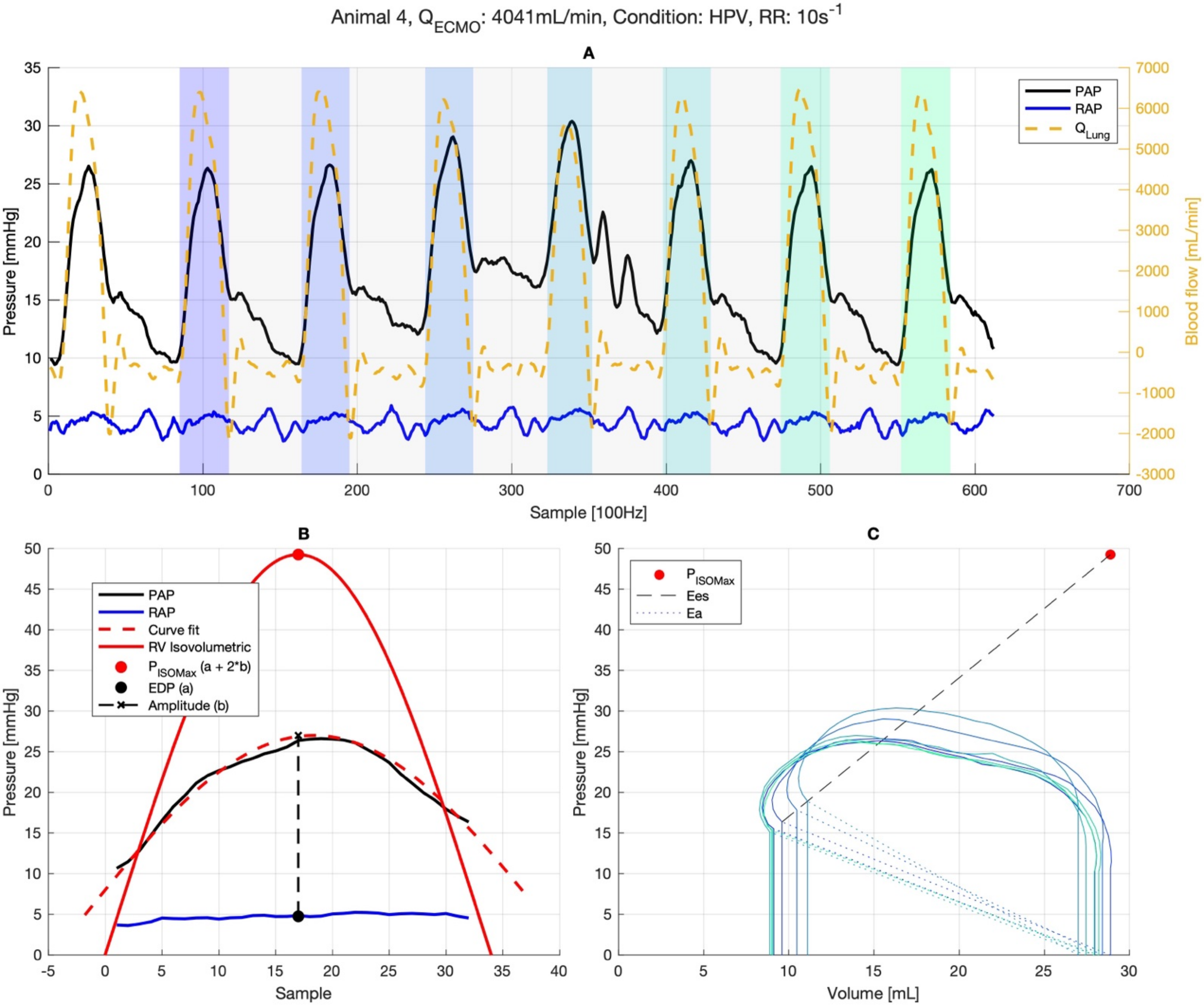

Animal 4, Q_{ECMO}: 4041mL/min, Condition: HPV, RR: $10s^{-1}$
A
PAP
RAP
Q_{Lung}
Pressure [mmHg]
Blood flow [mL/min]
Sample [100Hz]
B
PAP
RAP
Curve fit
RV Isovolumetric
P_{ISOMax} (a + 2*b)
EDP (a)
Amplitude (b)
Pressure [mmHg]
Sample
C
P_{ISOMax}
Ees
Ea
Pressure [mmHg]
Volume [mL]

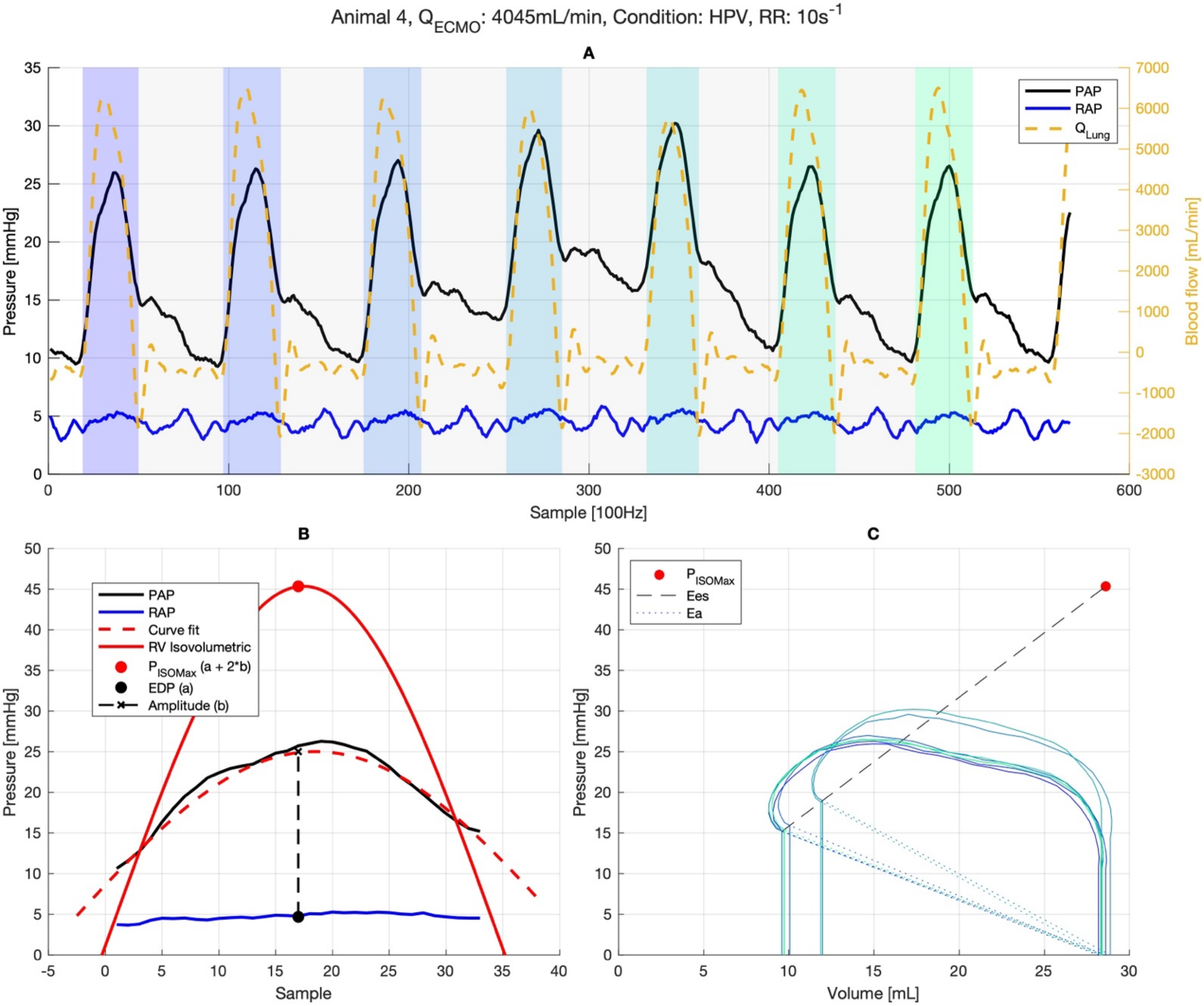

Animal 4, Q_ECMO: 4045mL/min, Condition: HPV, RR: 10s^-1
A
Pressure [mmHg]
Blood flow [mL/min]
Sample [100Hz]
PAP
RAP
Q_Lung
B
Pressure [mmHg]
Sample
PAP
RAP
Curve fit
RV Isovolumetric
P_ISOMax (a + 2*b)
EDP (a)
Amplitude (b)
C
Pressure [mmHg]
Volume [mL]
P_ISOMax
Ees
Ea

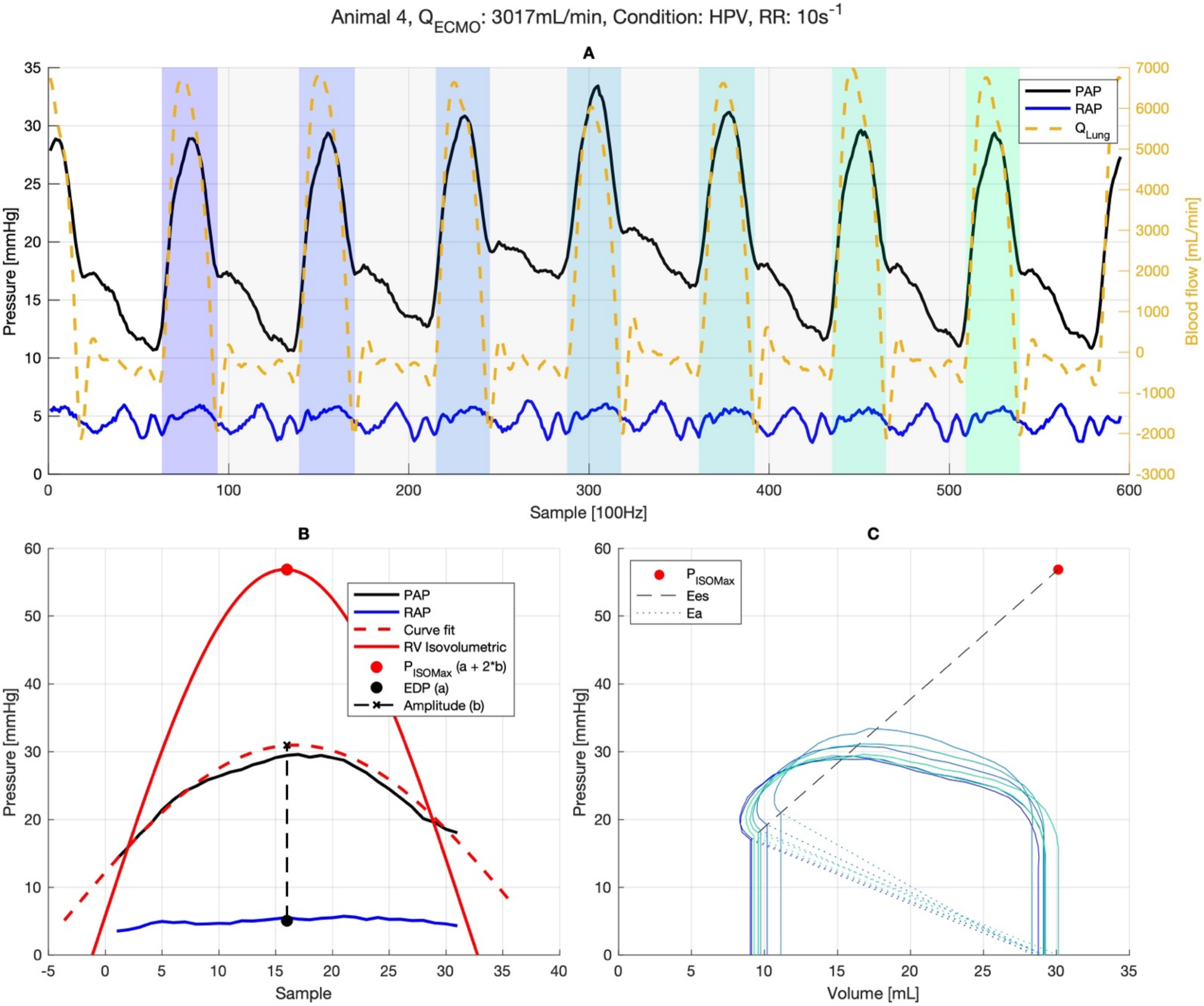

Animal 4, QECMO: 3017mL/min, Condition: HPV, RR: 10s-1
A
Pressure [mmHg]
Blood flow [mL/min]
Sample [100Hz]
PAP
RAP
QLung
B
Pressure [mmHg]
Sample
PAP
RAP
Curve fit
RV Isovolumetric
PISOMax (a + 2*b)
EDP (a)
Amplitude (b)
C
Pressure [mmHg]
Volume [mL]
PISOMax
Ees
Ea

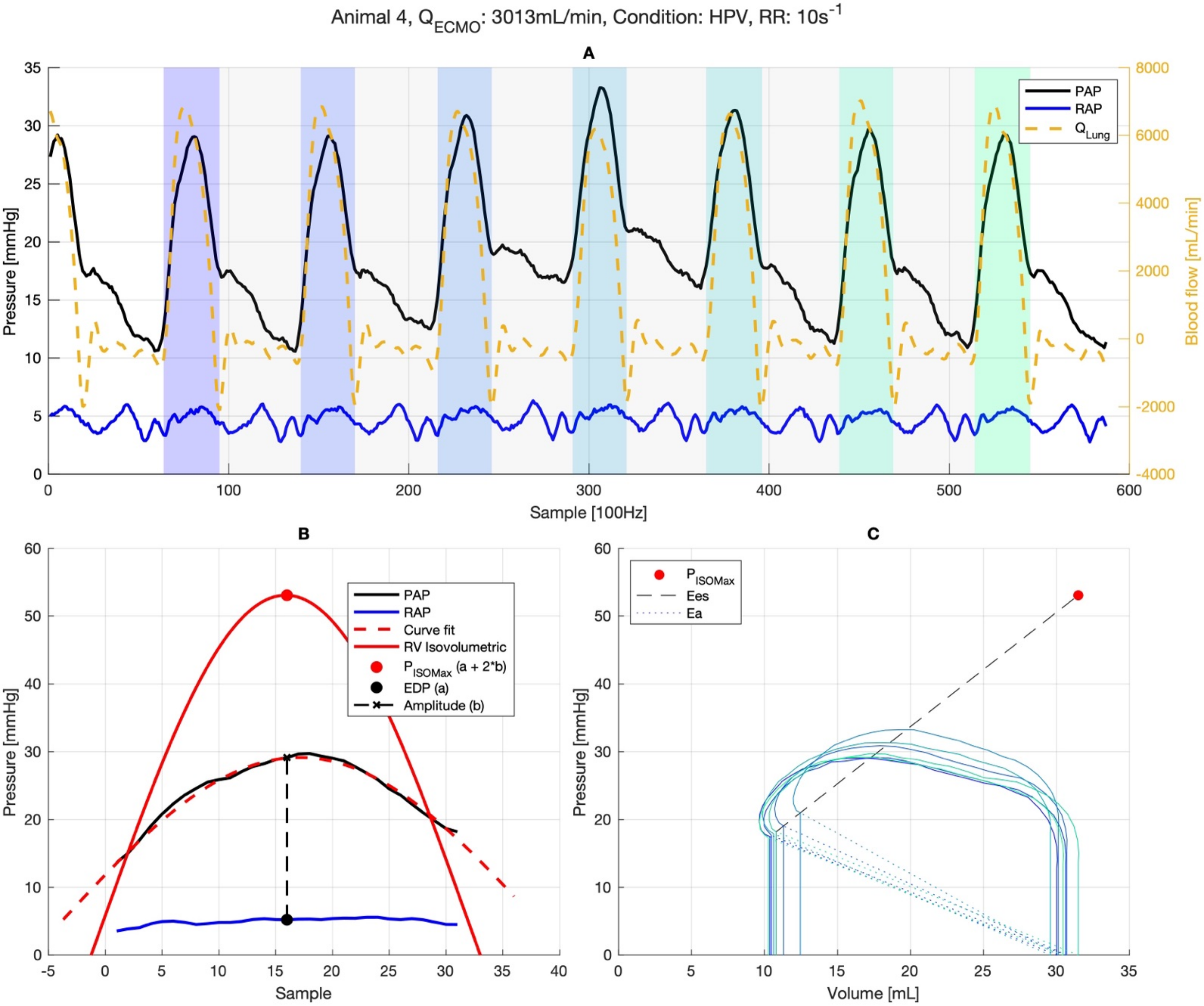

Animal 4, Q_ECMO: 3013mL/min, Condition: HPV, RR: 10s^-1
A
PAP
RAP
Q_Lung
Pressure [mmHg]
Blood flow [mL/min]
Sample [100Hz]
B
PAP
RAP
Curve fit
RV Isovolumetric
P_ISOMax (a + 2*b)
EDP (a)
Amplitude (b)
Pressure [mmHg]
Sample
C
P_ISOMax
Ees
Ea
Pressure [mmHg]
Volume [mL]

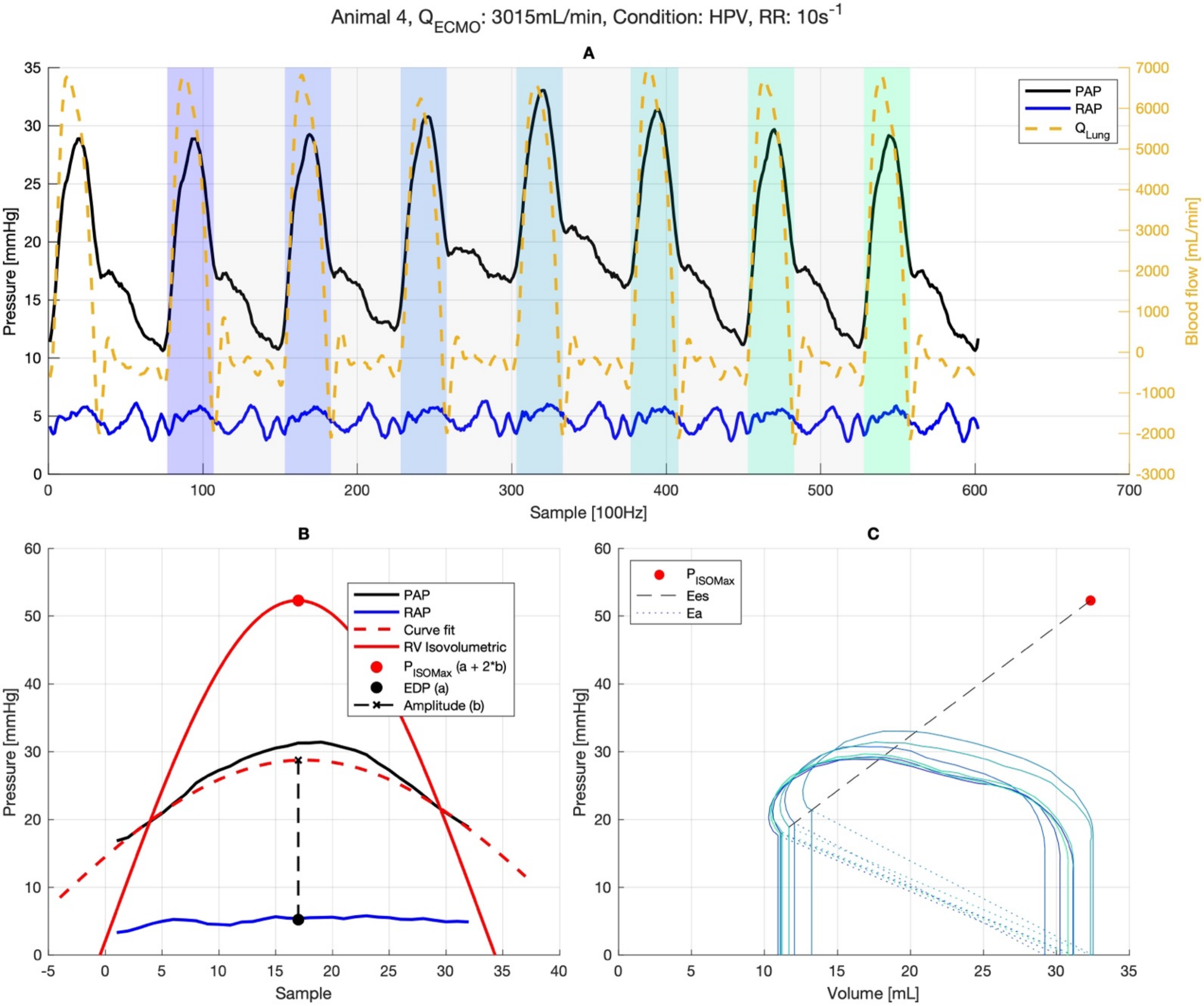

Animal 4, Q_ECMO: 3015mL/min, Condition: HPV, RR: 10s^-1
A
PAP
RAP
Q_Lung
Pressure [mmHg]
Blood flow [mL/min]
Sample [100Hz]
B
PAP
RAP
Curve fit
RV Isovolumetric
P_ISOMax (a + 2*b)
EDP (a)
Amplitude (b)
Pressure [mmHg]
Sample
C
P_ISOMax
Ees
Ea
Pressure [mmHg]
Volume [mL]

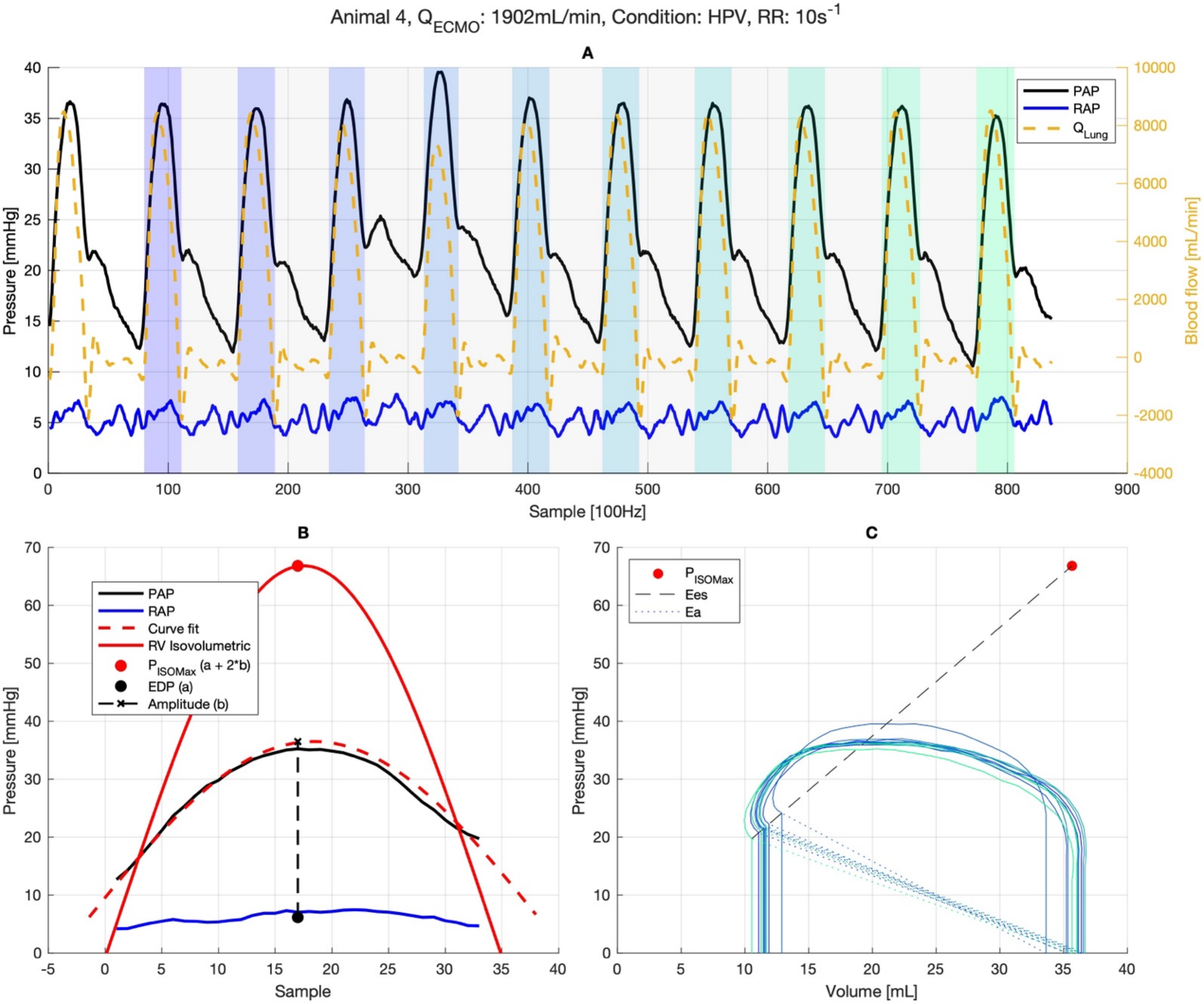

Animal 4, Q_{ECMO}: 1902mL/min, Condition: HPV, RR: 10s^{-1}
A
PAP
RAP
Q_{Lung}
Pressure [mmHg]
Blood flow [mL/min]
Sample [100Hz]
B
PAP
RAP
Curve fit
RV Isovolumetric
P_{ISOMax} (a + 2*b)
EDP (a)
Amplitude (b)
Pressure [mmHg]
Sample
C
P_{ISOMax}
Ees
Ea
Pressure [mmHg]
Volume [mL]

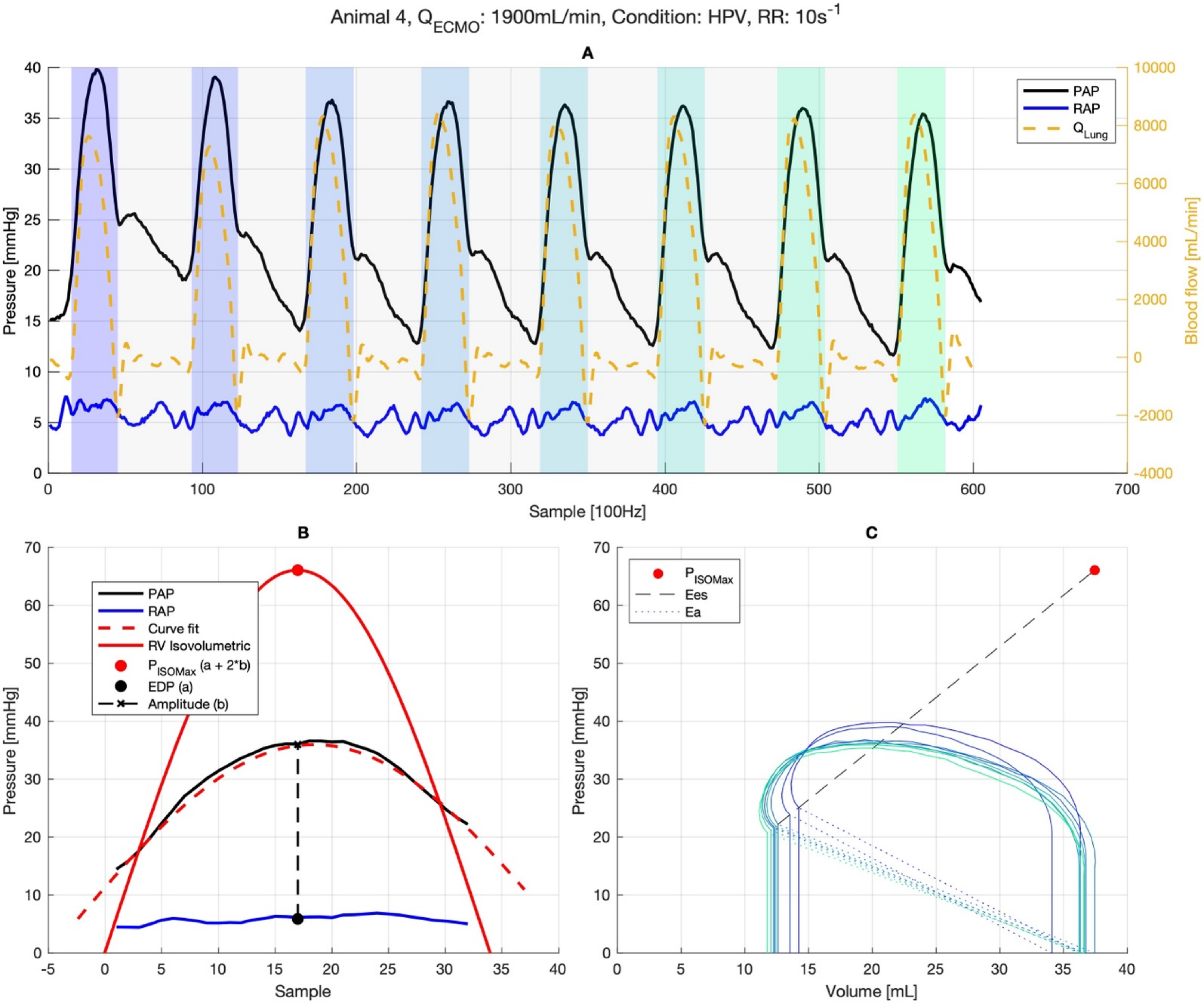

Animal 4, Q_ECMO: 1900mL/min, Condition: HPV, RR: 10s^-1
A
Pressure [mmHg]
Blood flow [mL/min]
Sample [100Hz]
PAP
RAP
Q_Lung
B
PAP
RAP
Curve fit
RV Isovolumetric
P_ISOMax (a + 2*b)
EDP (a)
Amplitude (b)
Pressure [mmHg]
Sample
C
P_ISOMax
Ees
Ea
Pressure [mmHg]
Volume [mL]

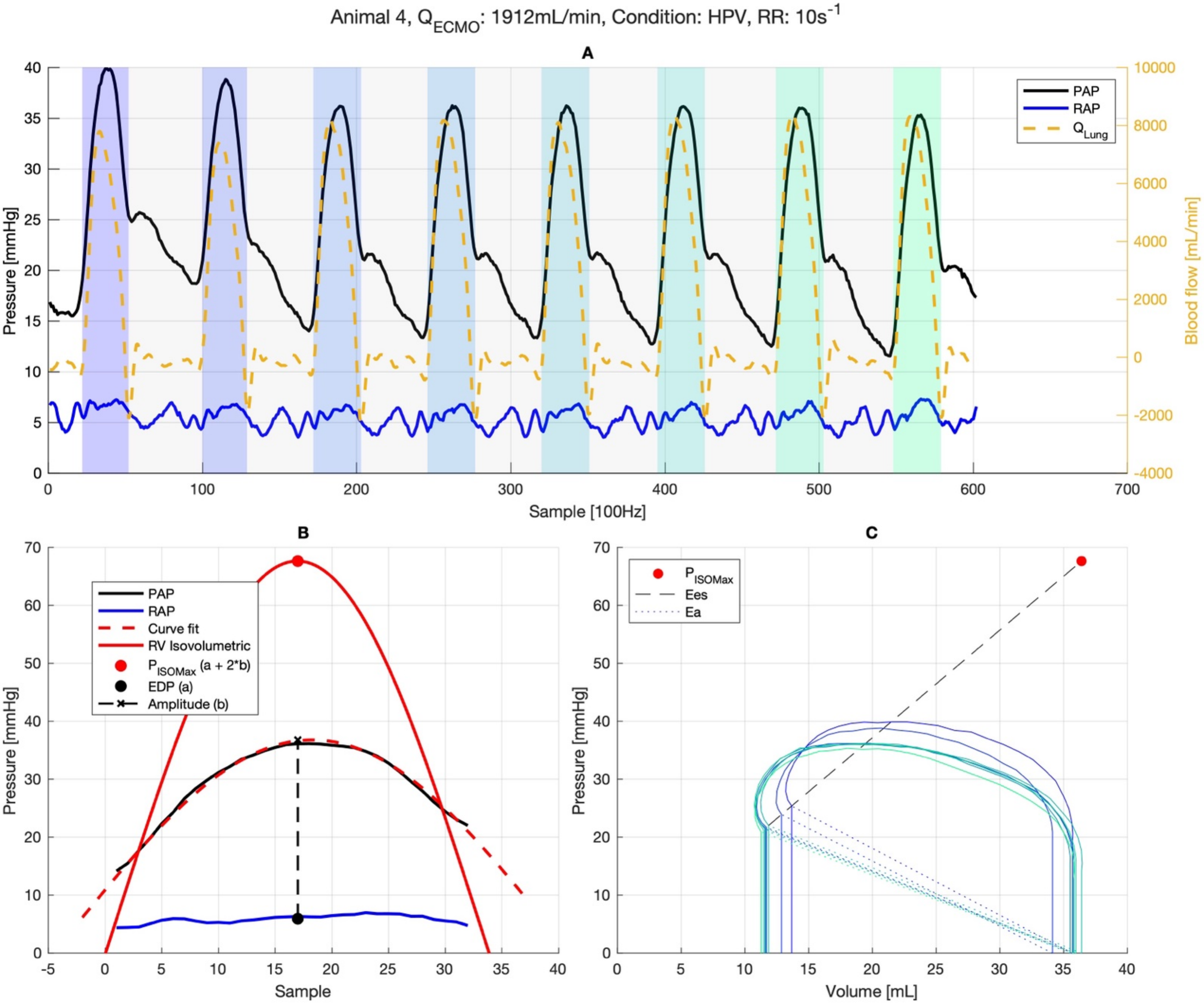

Animal 4, Q_{ECMO}: 1912mL/min, Condition: HPV, RR: $10s^{-1}$
A
Pressure [mmHg]
Blood flow [mL/min]
Sample [100Hz]
PAP
RAP
Q_{Lung}
B
PAP
RAP
Curve fit
RV Isovolumetric
P_{ISOMax} (a + 2*b)
EDP (a)
Amplitude (b)
Pressure [mmHg]
Sample
C
P_{ISOMax}
Ees
Ea
Pressure [mmHg]
Volume [mL]

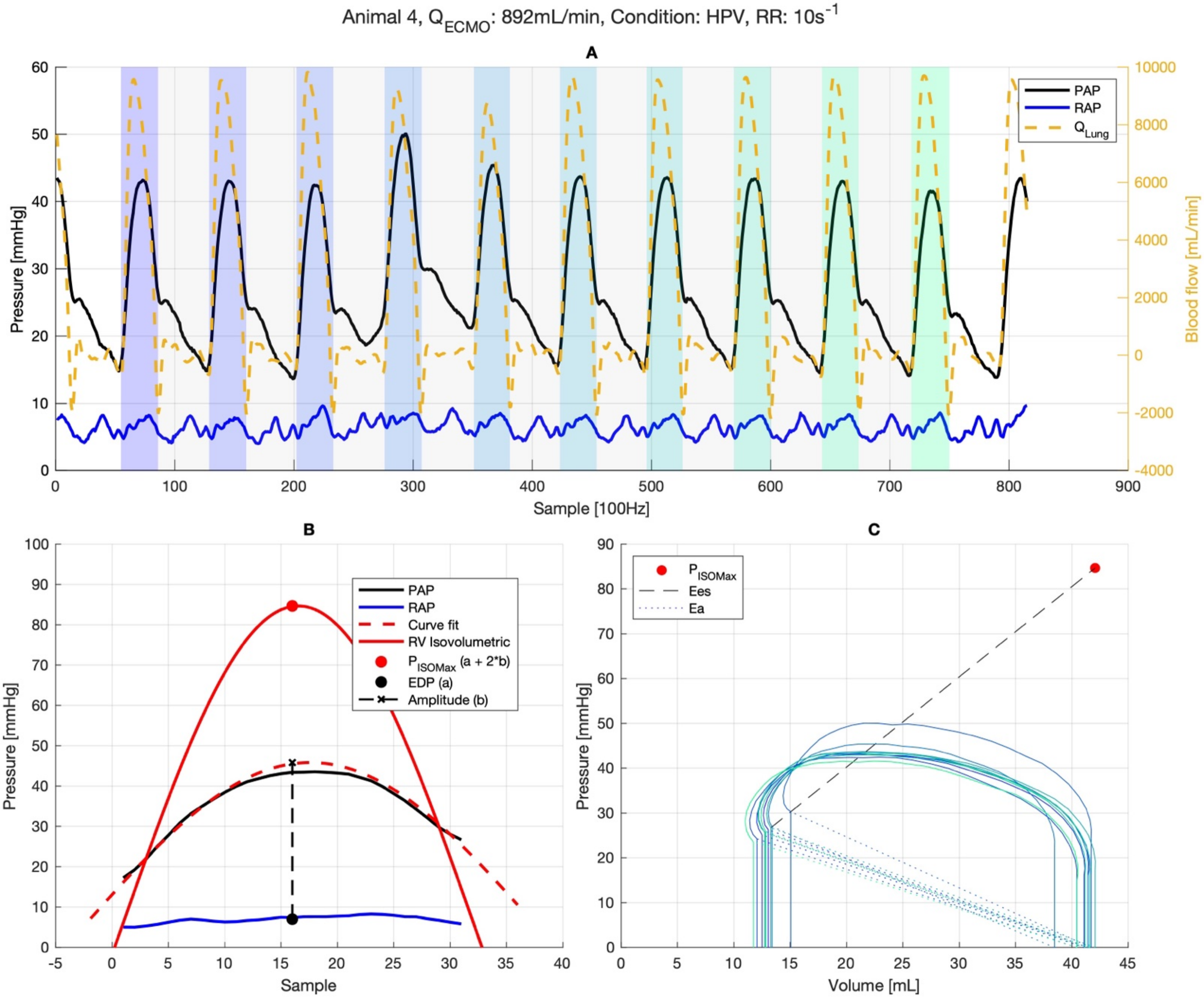

Animal 4, Q_{ECMO}: 892mL/min, Condition: HPV, RR: $10s^{-1}$
A
Pressure [mmHg]
Blood flow [mL/min]
Sample [100Hz]
PAP
RAP
Q_{Lung}
B
Pressure [mmHg]
Sample
PAP
RAP
Curve fit
RV Isovolumetric
P_{ISOMax} (a + 2*b)
EDP (a)
Amplitude (b)
C
Pressure [mmHg]
Volume [mL]
P_{ISOMax}
Ees
Ea

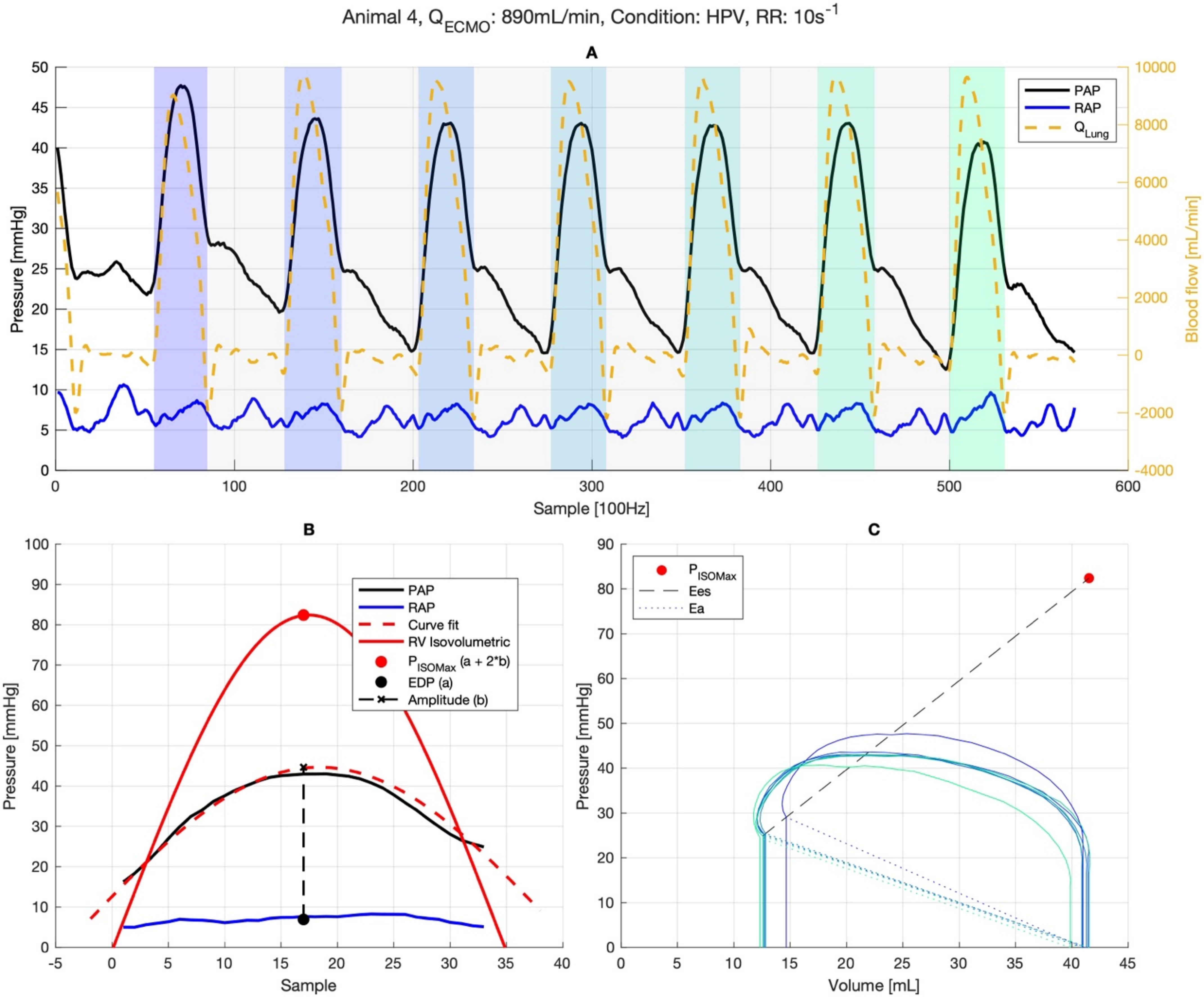

Animal 4, Q_ECMO: 890mL/min, Condition: HPV, RR: 10s^-1
A
PAP
RAP
Q_Lung
Pressure [mmHg]
Blood flow [mL/min]
Sample [100Hz]
B
PAP
RAP
Curve fit
RV Isovolumetric
P_ISOMax (a + 2*b)
EDP (a)
Amplitude (b)
Pressure [mmHg]
Sample
C
P_ISOMax
Ees
Ea
Pressure [mmHg]
Volume [mL]

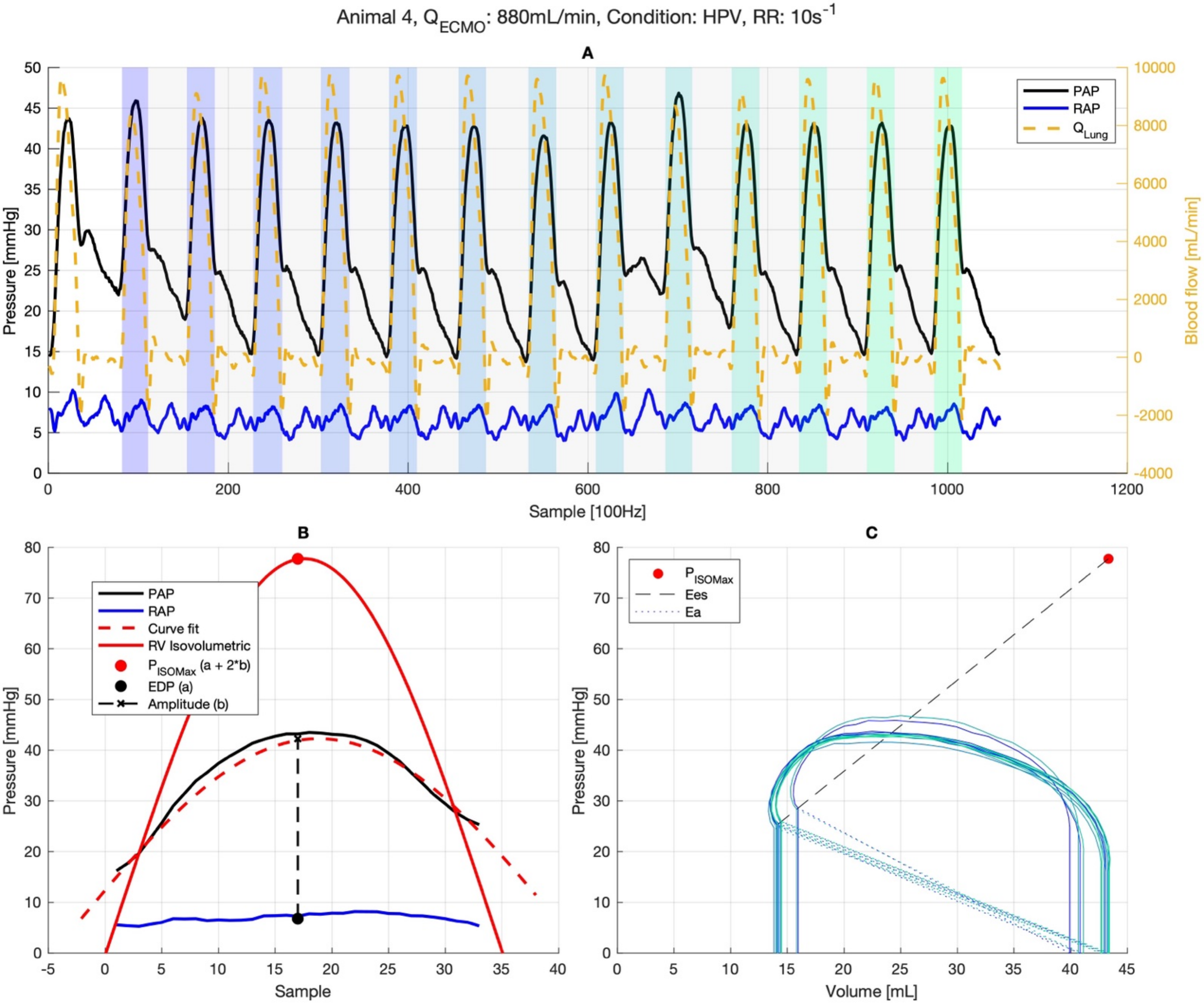
Animal 4, Q_{ECMO}: 880mL/min, Condition: HPV, RR: $10s^{-1}$
A
PAP
RAP
Q_{Lung}
Pressure [mmHg]
Blood flow [mL/min]
Sample [100Hz]
B
PAP
RAP
Curve fit
RV Isovolumetric
P_{ISOMax} (a + 2*b)
EDP (a)
Amplitude (b)
Pressure [mmHg]
Sample
C
P_{ISOMax}
Ees
Ea
Pressure [mmHg]
Volume [mL]

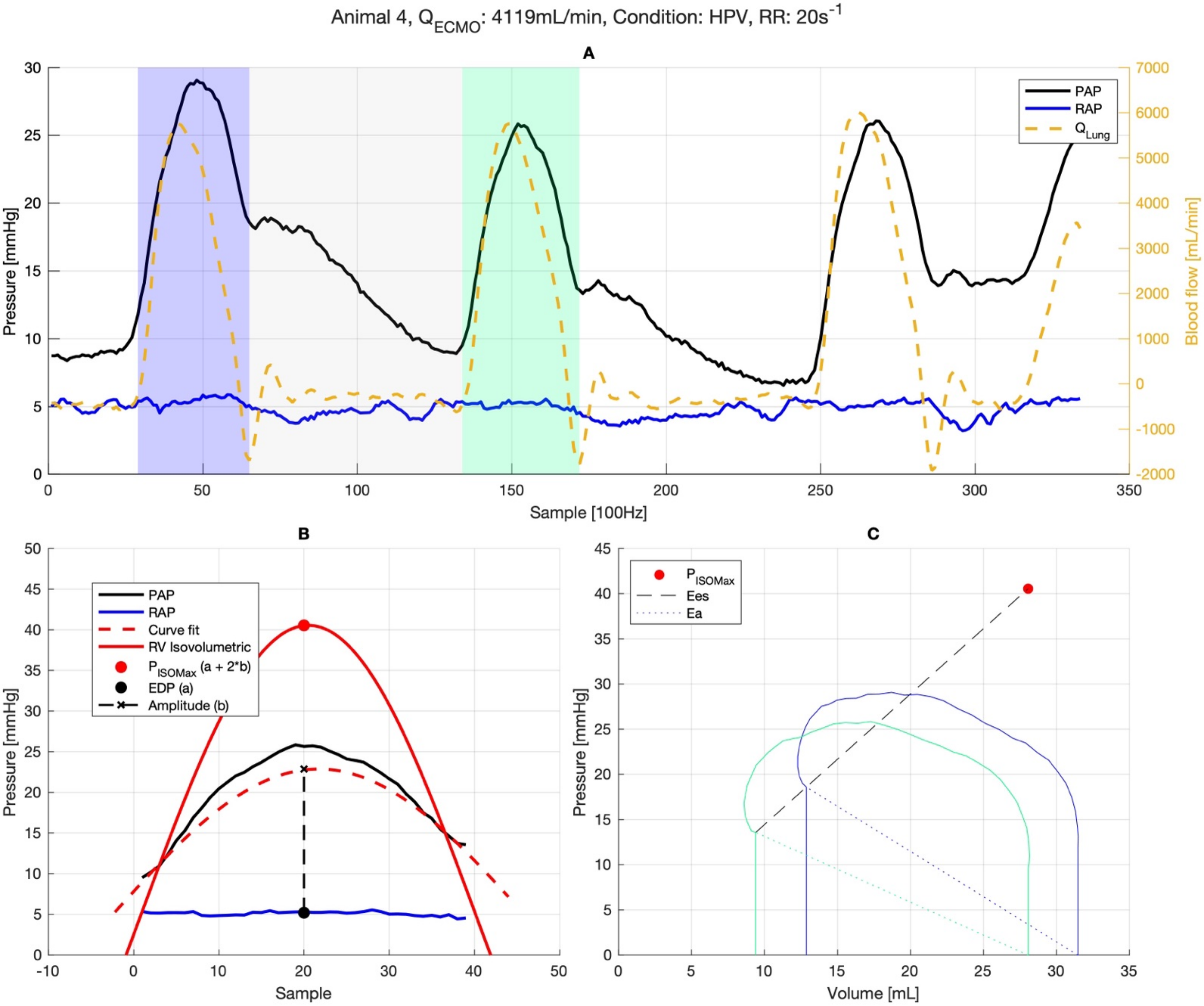

Animal 4, Q_ECMO: 4119mL/min, Condition: HPV, RR: 20s^-1
A
PAP
RAP
Q_Lung
Pressure [mmHg]
Blood flow [mL/min]
Sample [100Hz]
B
PAP
RAP
Curve fit
RV Isovolumetric
P_ISOMax (a + 2*b)
EDP (a)
Amplitude (b)
Pressure [mmHg]
Sample
C
P_ISOMax
Ees
Ea
Pressure [mmHg]
Volume [mL]

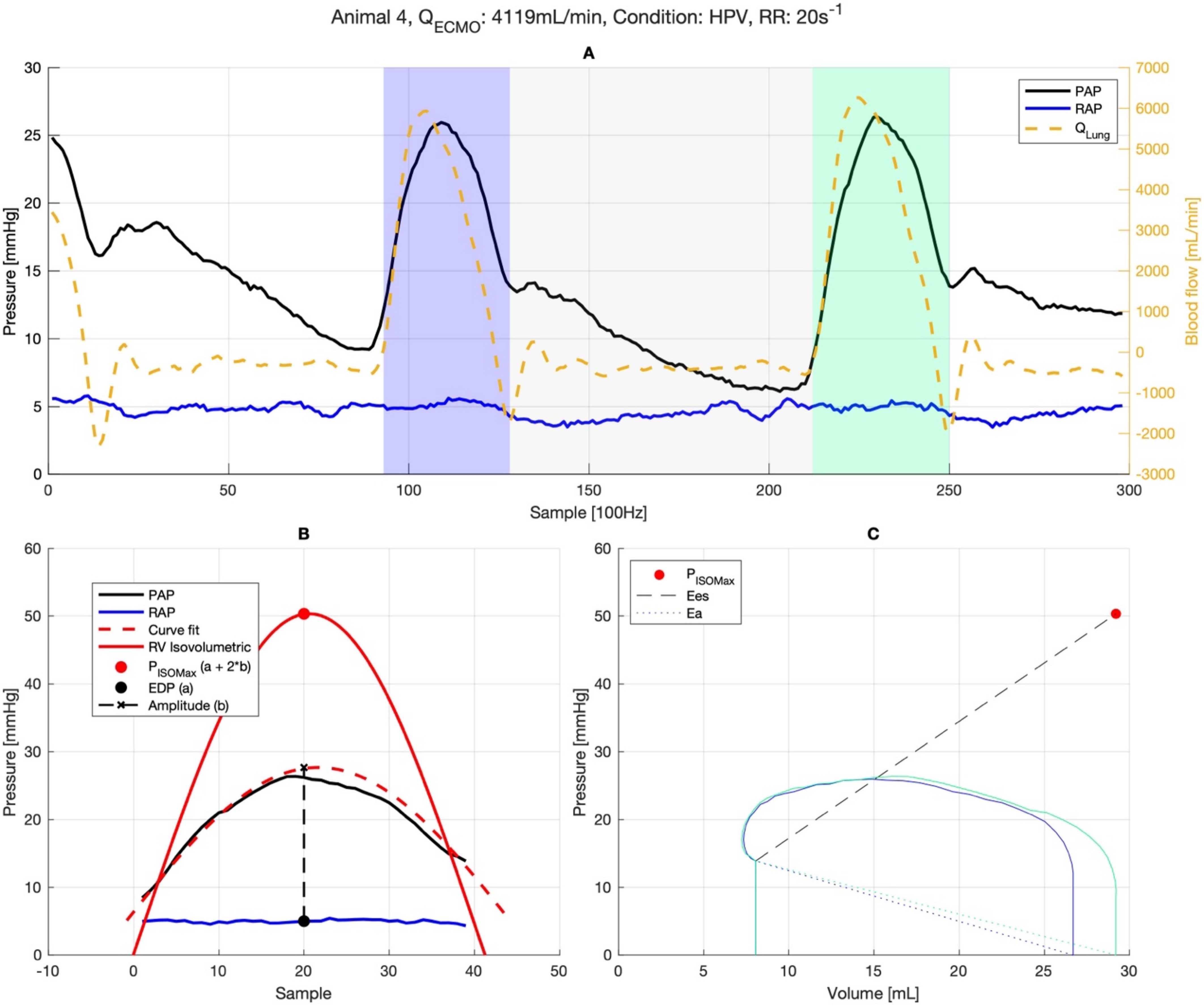

Animal 4, Q_{ECMO}: 4119mL/min, Condition: HPV, RR: $20s^{-1}$
A
PAP
RAP
Q_{Lung}
Pressure [mmHg]
Blood flow [mL/min]
Sample [100Hz]
B
PAP
RAP
Curve fit
RV Isovolumetric
P_{ISOMax} (a + 2*b)
EDP (a)
Amplitude (b)
Pressure [mmHg]
Sample
C
P_{ISOMax}
Ees
Ea
Pressure [mmHg]
Volume [mL]

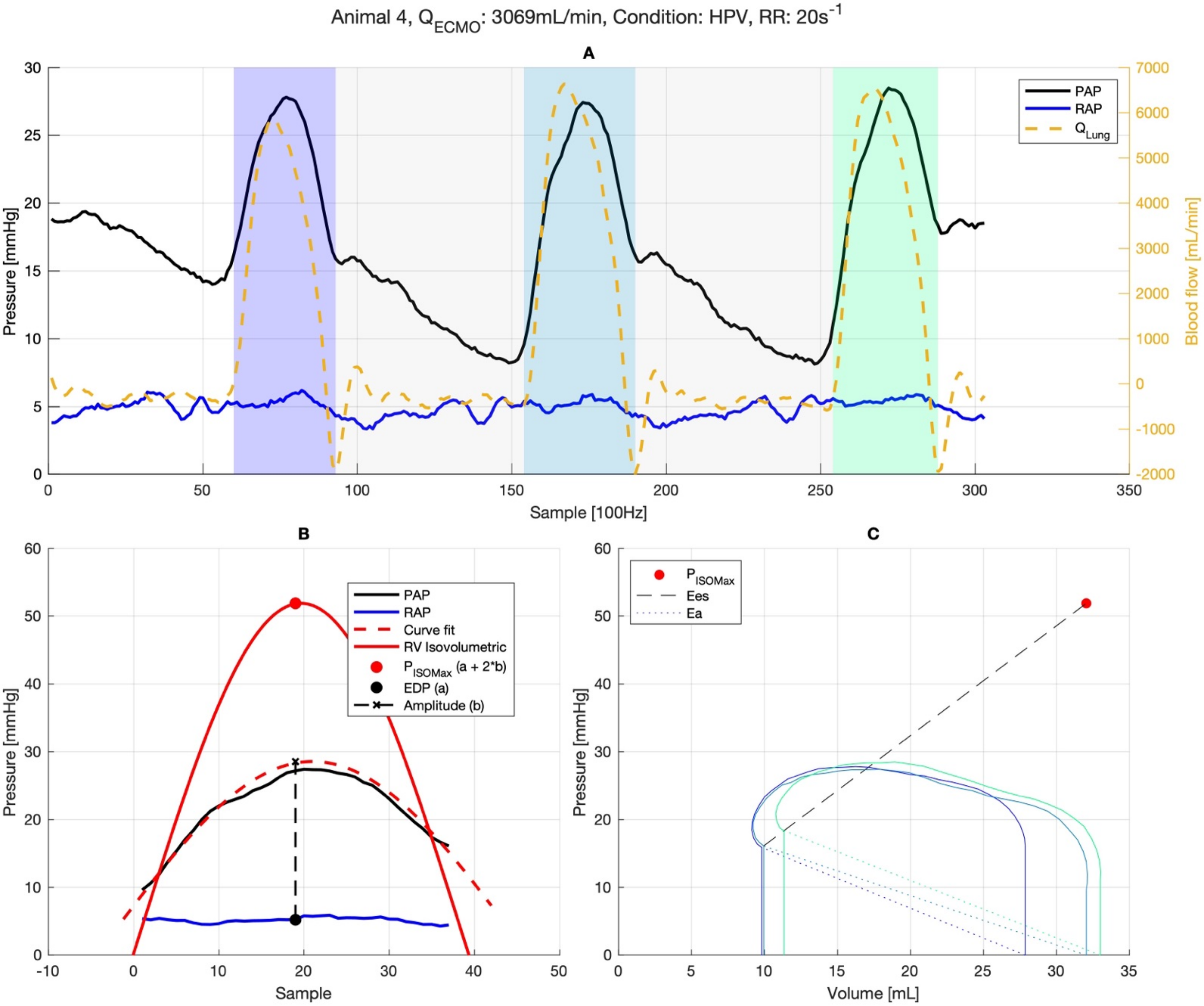

Animal 4, QECMO: 3069mL/min, Condition: HPV, RR: 20s-1
A
PAP
RAP
QLung
Pressure [mmHg]
Blood flow [mL/min]
Sample [100Hz]
B
Curve fit
RV Isovolumetric
PISOMax (a + 2*b)
EDP (a)
Amplitude (b)
Sample
C
PISOMax
Ees
Ea
Volume [mL]

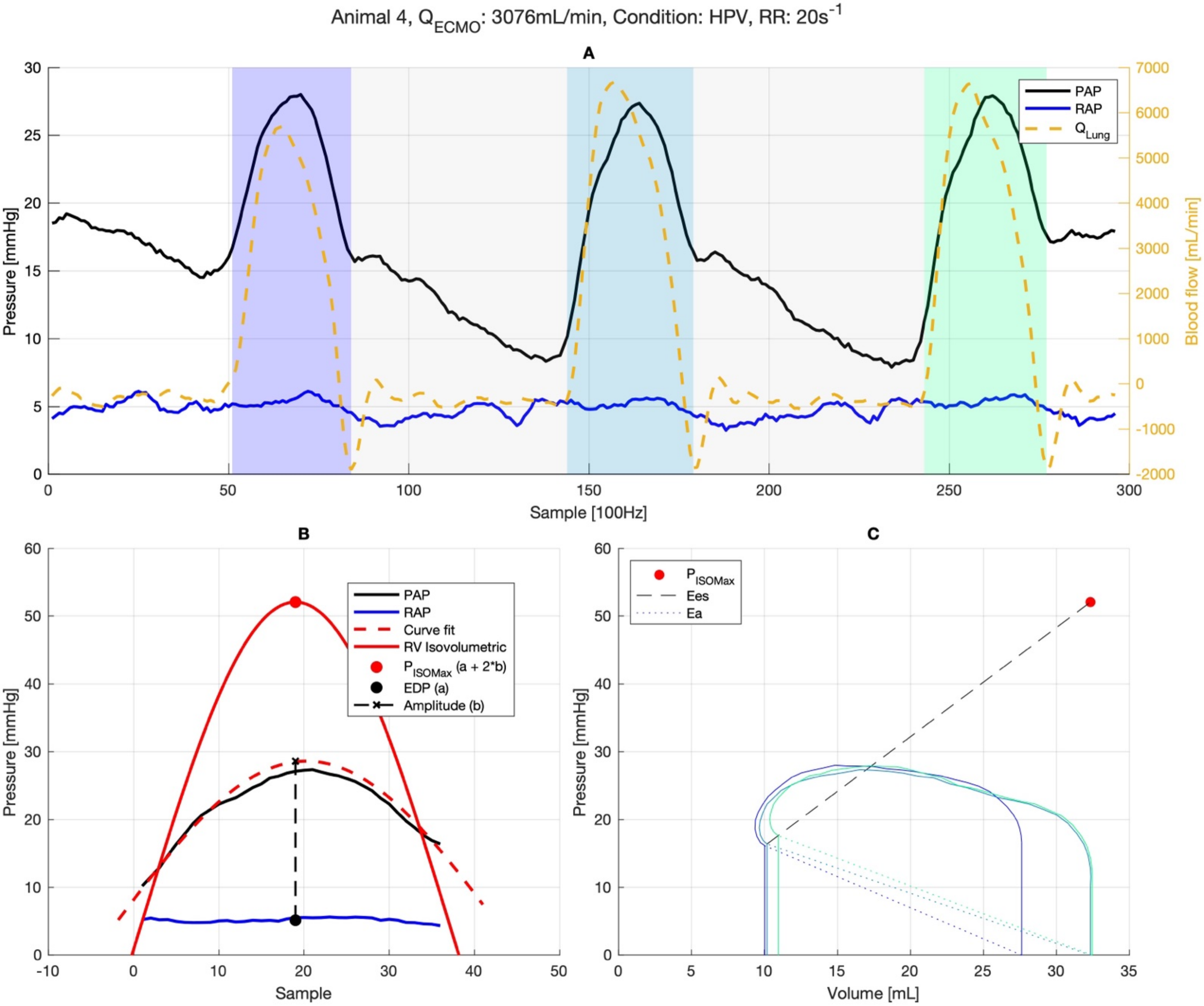

Animal 4, QECMO: 3076mL/min, Condition: HPV, RR: 20s-1
A
PAP
RAP
QLung
Pressure [mmHg]
Blood flow [mL/min]
Sample [100Hz]
B
PAP
RAP
Curve fit
RV Isovolumetric
PISOMax (a + 2*b)
EDP (a)
Amplitude (b)
Pressure [mmHg]
Sample
C
PISOMax
Ees
Ea
Pressure [mmHg]
Volume [mL]

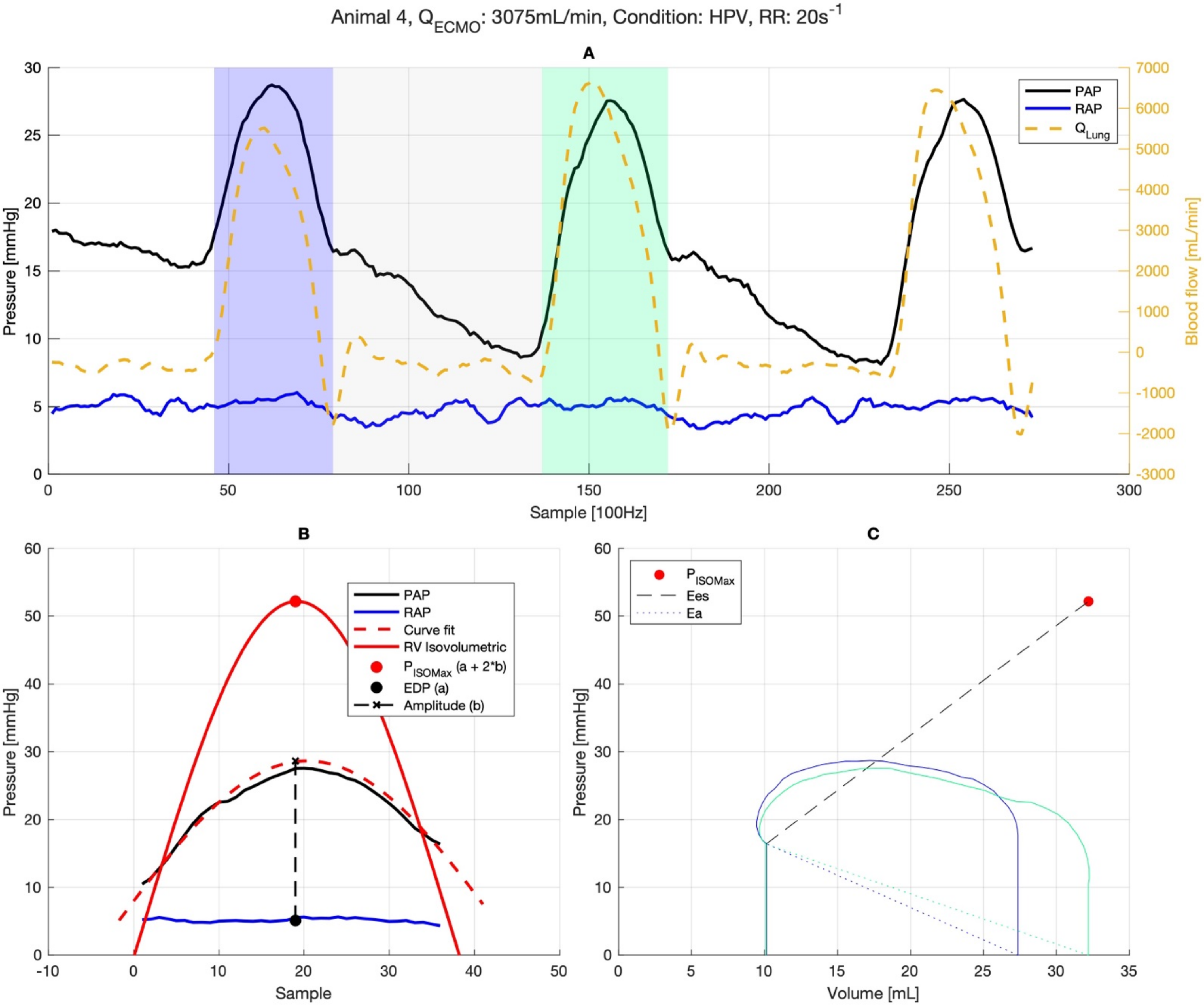

Animal 4, Q_ECMO: 3075mL/min, Condition: HPV, RR: 20s^-1
A
Pressure [mmHg]
Blood flow [mL/min]
Sample [100Hz]
PAP
RAP
Q_Lung
B
Pressure [mmHg]
Sample
PAP
RAP
Curve fit
RV Isovolumetric
P_ISOMax (a + 2*b)
EDP (a)
Amplitude (b)
C
Pressure [mmHg]
Volume [mL]
P_ISOMax
Ees
Ea

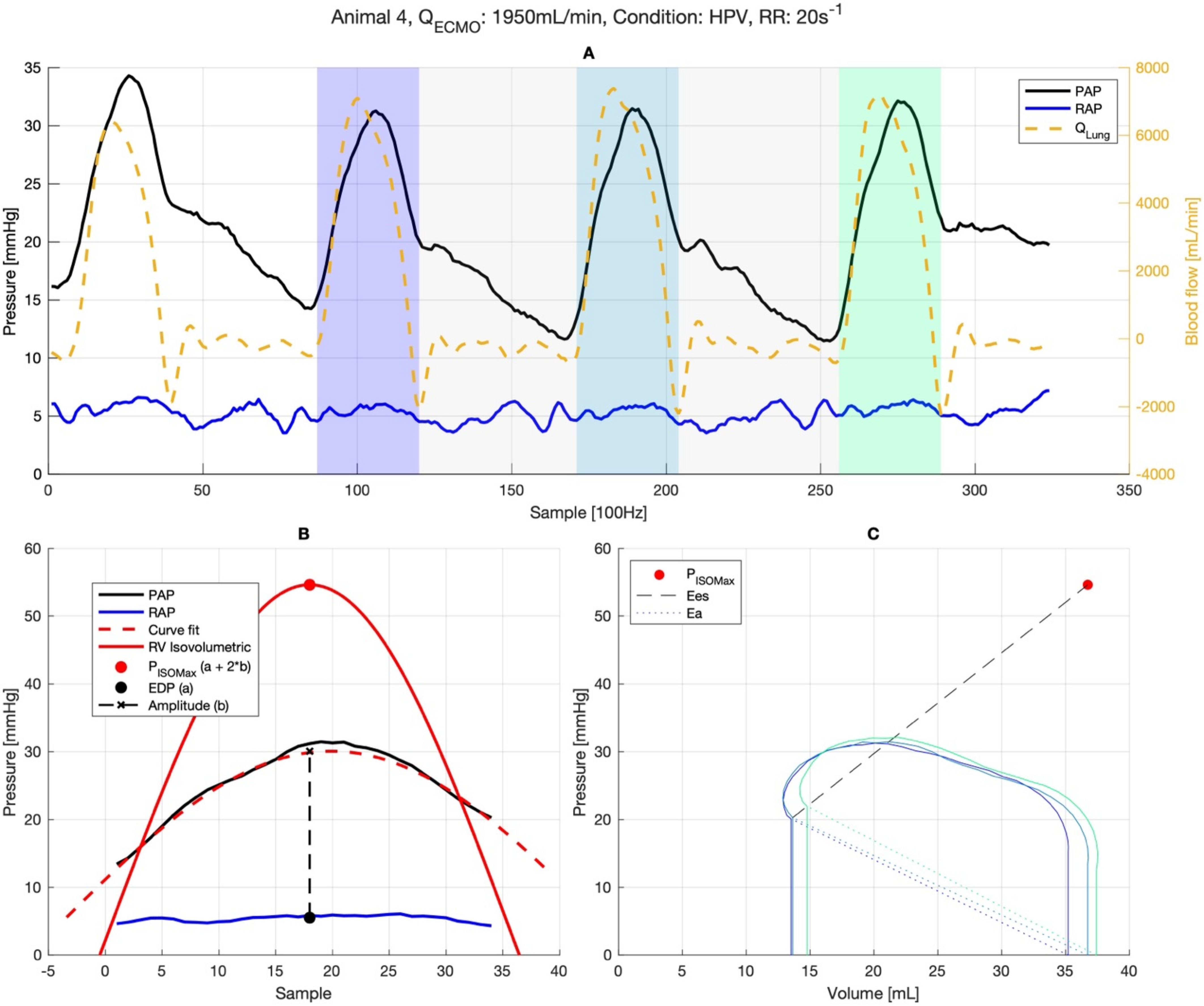

Animal 4, Q_{ECMO}: 1950mL/min, Condition: HPV, RR: $20s^{-1}$
A
PAP
RAP
Q_{Lung}
Pressure [mmHg]
Blood flow [mL/min]
Sample [100Hz]
B
PAP
RAP
Curve fit
RV Isovolumetric
P_{ISOMax} (a + 2*b)
EDP (a)
Amplitude (b)
Pressure [mmHg]
Sample
C
P_{ISOMax}
Ees
Ea
Pressure [mmHg]
Volume [mL]

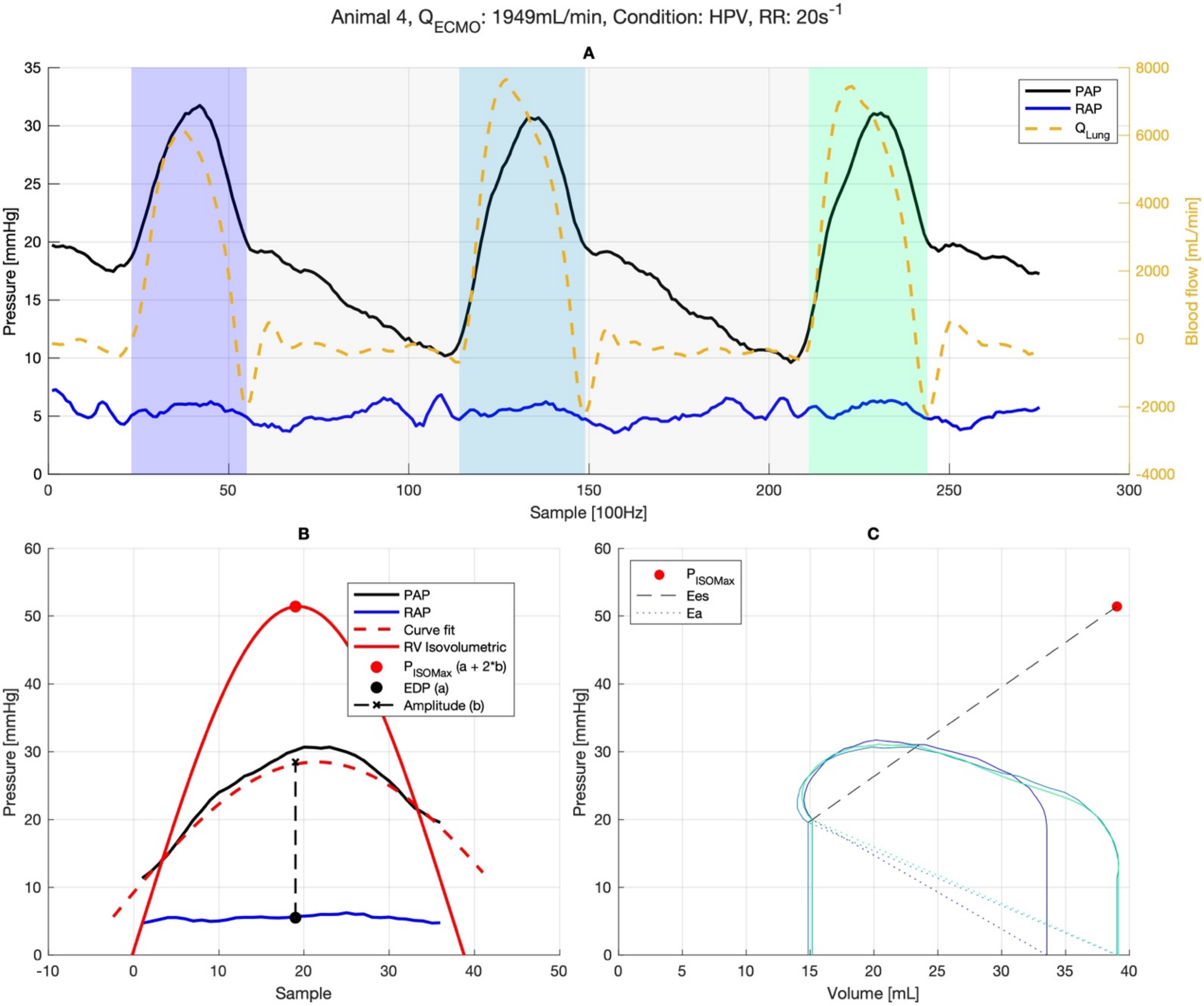

Animal 4, Q_{ECMO}: 1949mL/min, Condition: HPV, RR: $20s^{-1}$
A
PAP
RAP
Q_{Lung}
Pressure [mmHg]
Blood flow [mL/min]
Sample [100Hz]
B
PAP
RAP
Curve fit
RV Isovolumetric
P_{ISOMax} (a + 2*b)
EDP (a)
Amplitude (b)
Pressure [mmHg]
Sample
C
P_{ISOMax}
Ees
Ea
Pressure [mmHg]
Volume [mL]

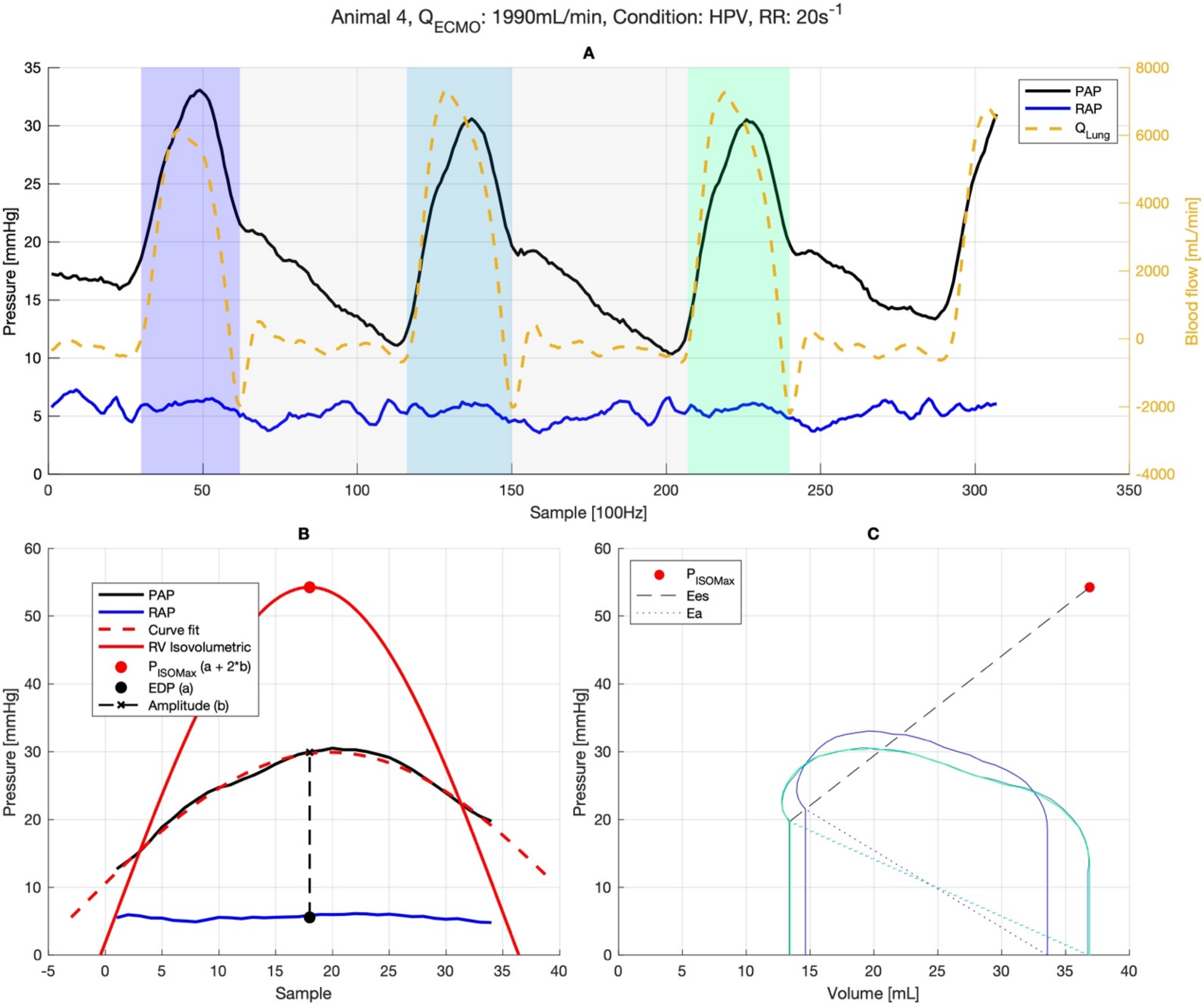

Animal 4, Q_{ECMO}: 1990mL/min, Condition: HPV, RR: $20s^{-1}$
A
PAP
RAP
Q_{Lung}
Pressure [mmHg]
Blood flow [mL/min]
Sample [100Hz]
B
PAP
RAP
Curve fit
RV Isovolumetric
P_{ISOMax} (a + 2*b)
EDP (a)
Amplitude (b)
Pressure [mmHg]
Sample
C
P_{ISOMax}
Ees
Ea
Pressure [mmHg]
Volume [mL]

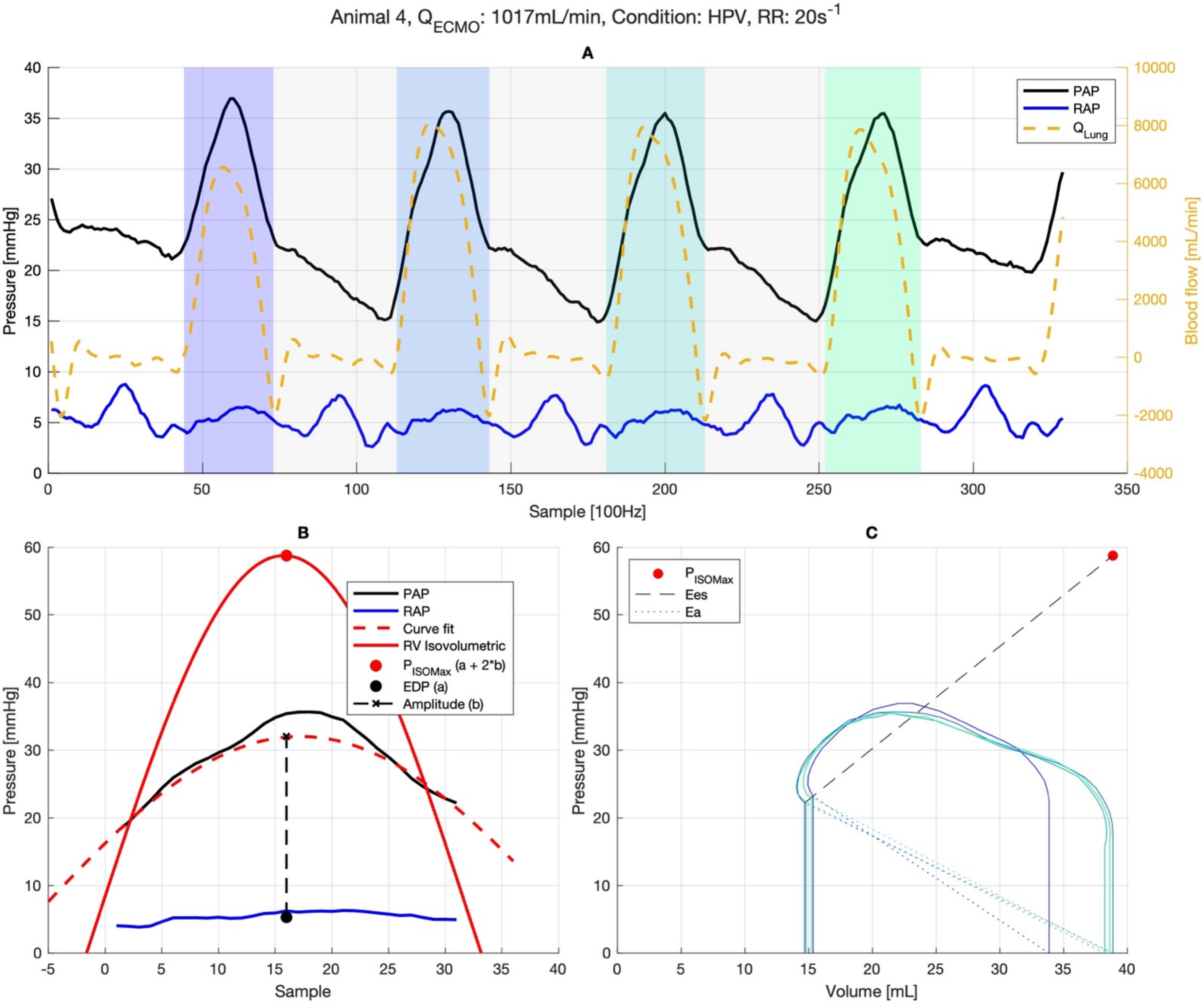

Animal 4, Q_{ECMO}: 1017mL/min, Condition: HPV, RR: $20s^{-1}$
A
PAP
RAP
Q_{Lung}
Pressure [mmHg]
Blood flow [mL/min]
Sample [100Hz]
B
PAP
RAP
Curve fit
RV Isovolumetric
P_{ISOMax} (a + 2*b)
EDP (a)
Amplitude (b)
Pressure [mmHg]
Sample
C
P_{ISOMax}
Ees
Ea
Pressure [mmHg]
Volume [mL]

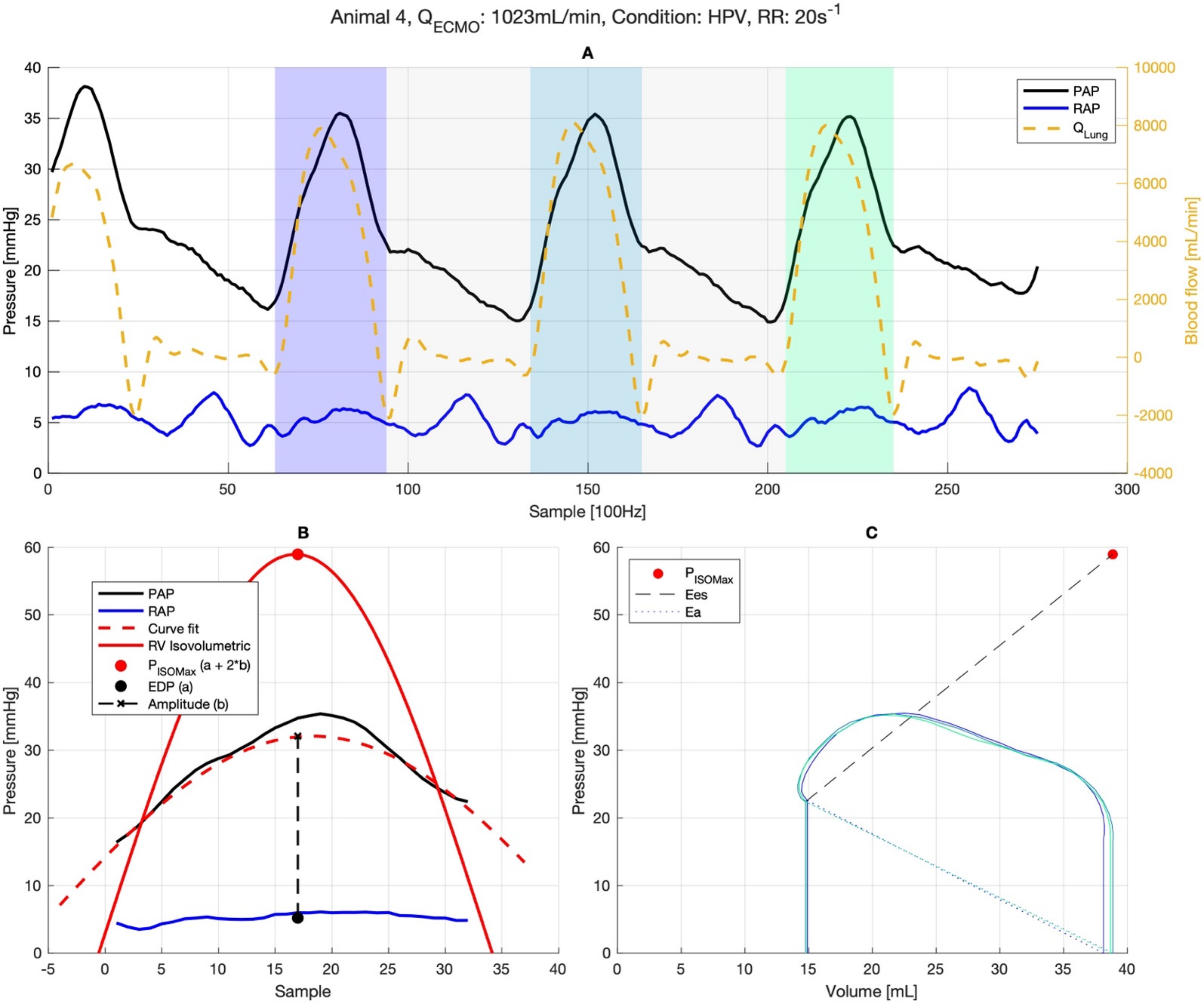

Animal 4, Q_{ECMO}: 1023mL/min, Condition: HPV, RR: 20s^{-1}
A
PAP
RAP
Q_{Lung}
Pressure [mmHg]
Blood flow [mL/min]
Sample [100Hz]
B
PAP
RAP
Curve fit
RV Isovolumetric
P_{ISOMax} (a + 2*b)
EDP (a)
Amplitude (b)
Pressure [mmHg]
Sample
C
P_{ISOMax}
Ees
Ea
Pressure [mmHg]
Volume [mL]

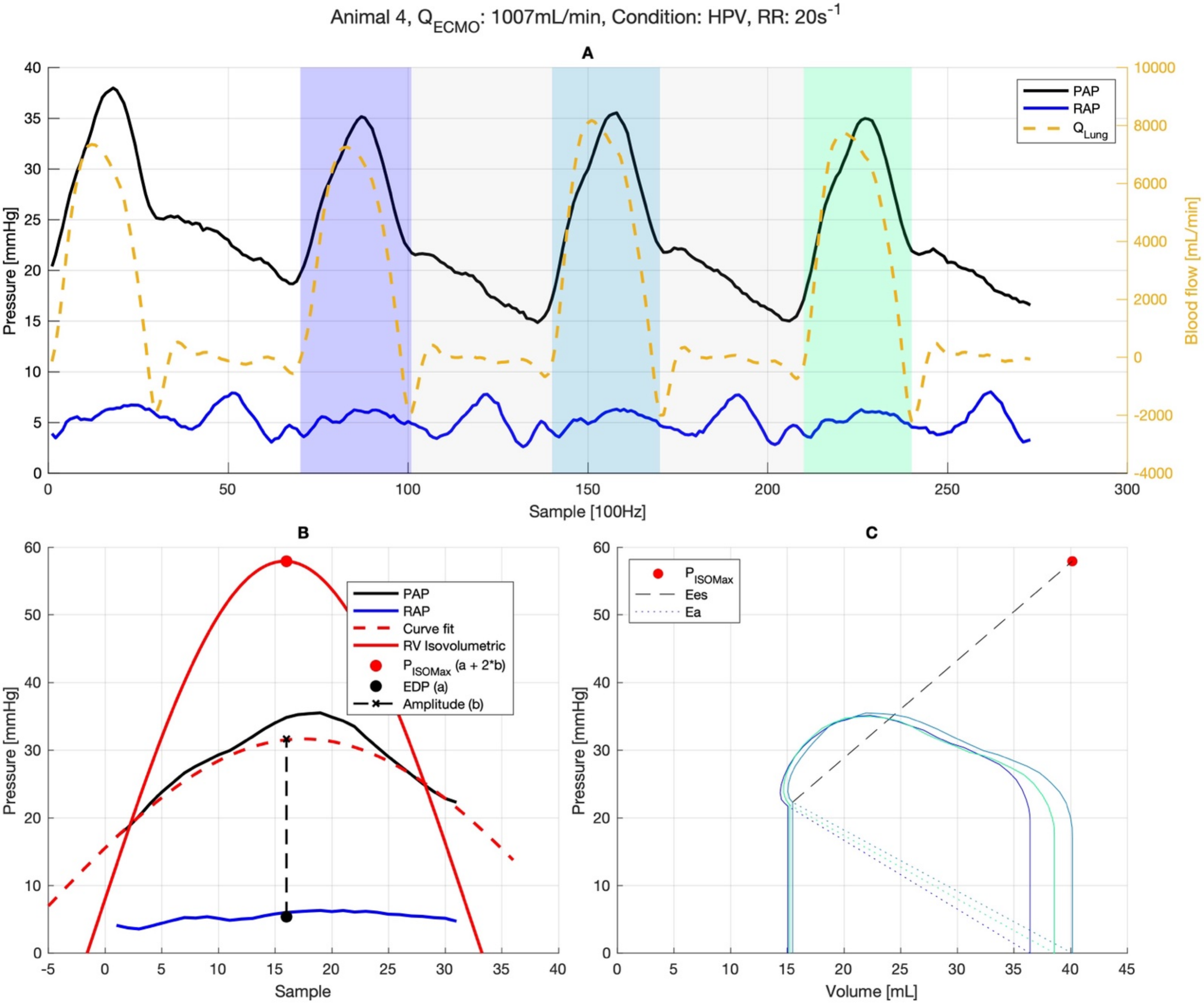

Animal 4, Q_{ECMO}: 1007mL/min, Condition: HPV, RR: $20s^{-1}$
A
PAP
RAP
Q_{Lung}
Pressure [mmHg]
Blood flow [mL/min]
Sample [100Hz]
B
PAP
RAP
Curve fit
RV Isovolumetric
P_{ISOMax} (a + 2*b)
EDP (a)
Amplitude (b)
Pressure [mmHg]
Sample
C
P_{ISOMax}
Ees
Ea
Pressure [mmHg]
Volume [mL]

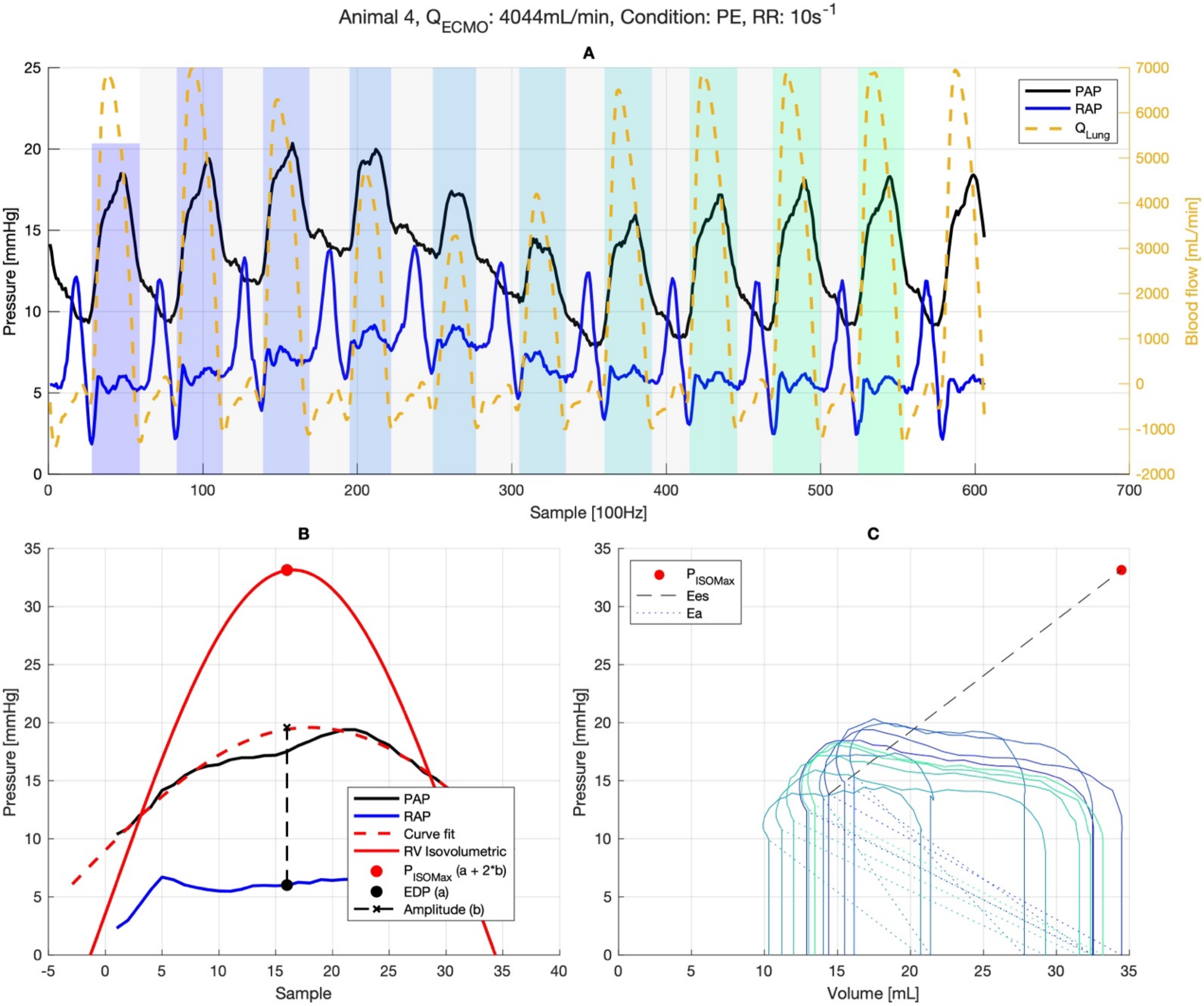
Animal 4, Q_ECMO: 4044mL/min, Condition: PE, RR: 10s^-1
A
PAP
RAP
Q_Lung
Pressure [mmHg]
Blood flow [mL/min]
Sample [100Hz]
B
PAP
RAP
Curve fit
RV Isovolumetric
P_ISOMax (a + 2*b)
EDP (a)
Amplitude (b)
Pressure [mmHg]
Sample
C
P_ISOMax
Ees
Ea
Pressure [mmHg]
Volume [mL]

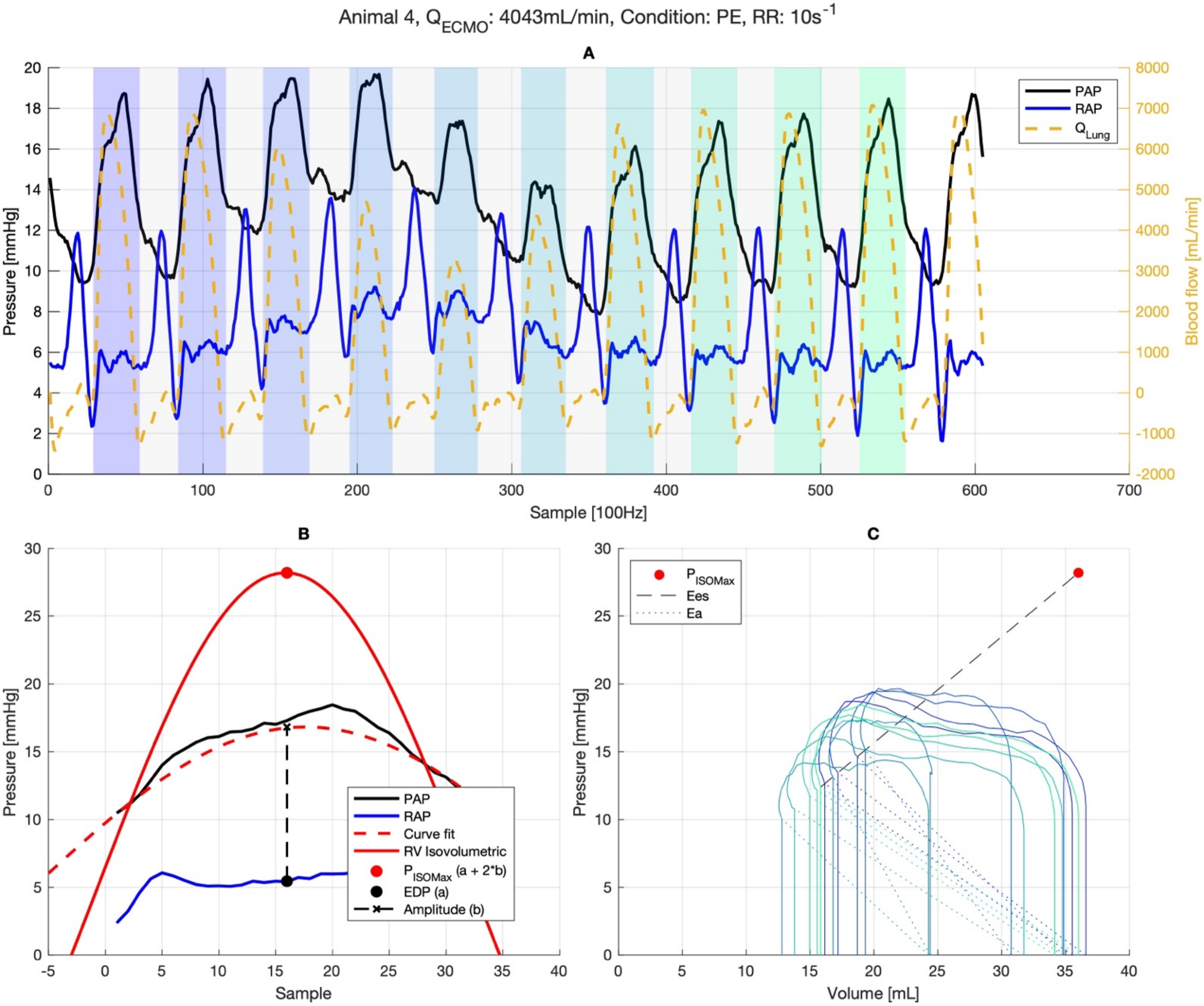

Animal 4, Q_{ECMO}: 4043mL/min, Condition: PE, RR: $10s^{-1}$
A
PAP
RAP
Q_{Lung}
Pressure [mmHg]
Blood flow [mL/min]
Sample [100Hz]
B
PAP
RAP
Curve fit
RV Isovolumetric
P_{ISOMax} (a + 2*b)
EDP (a)
Amplitude (b)
Pressure [mmHg]
Sample
C
P_{ISOMax}
Ees
Ea
Pressure [mmHg]
Volume [mL]

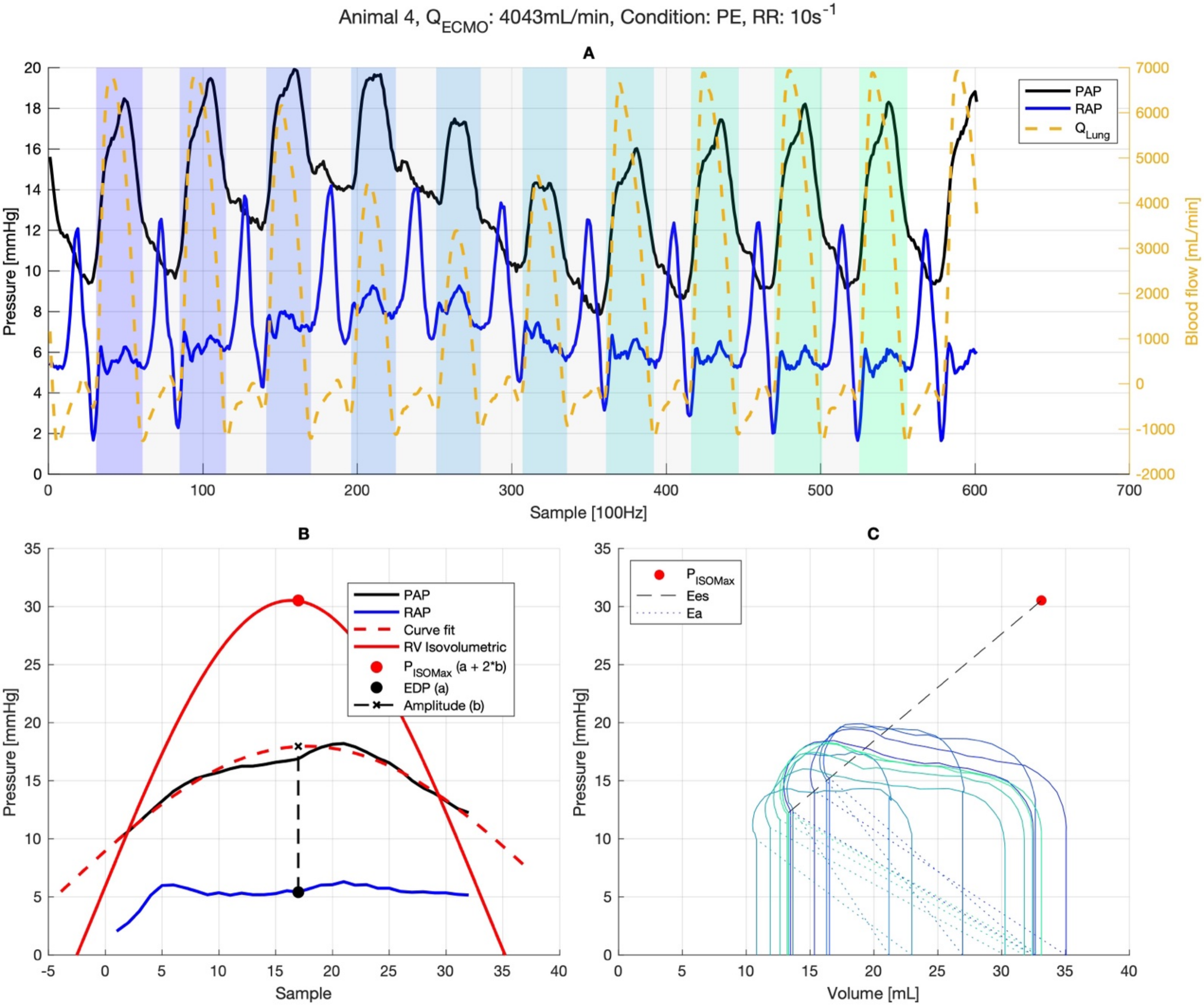

Animal 4, Q_{ECMO}: 4043mL/min, Condition: PE, RR: $10s^{-1}$
A
PAP
RAP
Q_{Lung}
Pressure [mmHg]
Blood flow [mL/min]
Sample [100Hz]
B
PAP
RAP
Curve fit
RV Isovolumetric
P_{ISOMax} (a + 2*b)
EDP (a)
Amplitude (b)
Pressure [mmHg]
Sample
C
P_{ISOMax}
Ees
Ea
Pressure [mmHg]
Volume [mL]

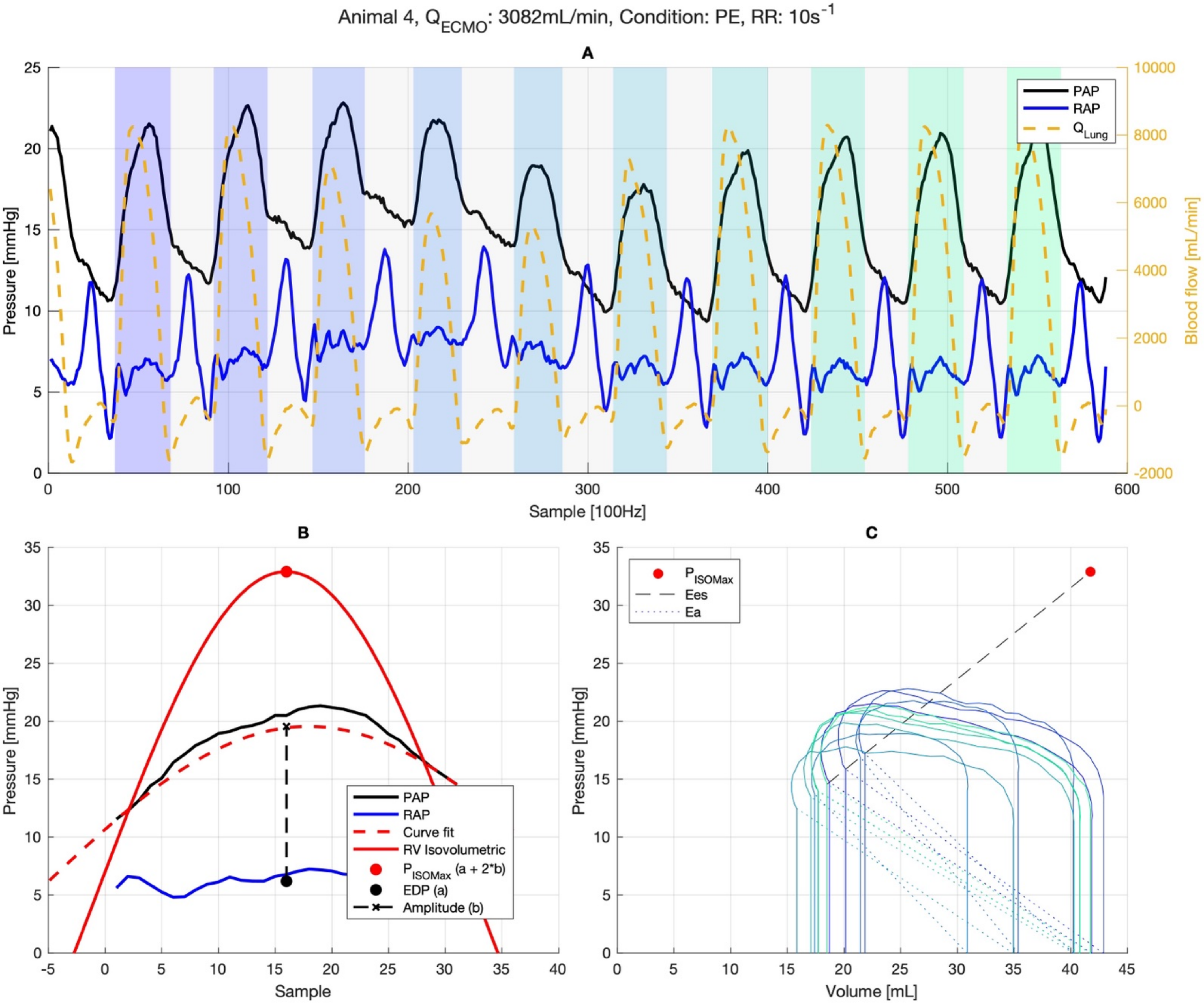

Animal 4, Q_ECMO: 3082mL/min, Condition: PE, RR: 10s^-1
A
PAP
RAP
Q_Lung
Pressure [mmHg]
Blood flow [mL/min]
Sample [100Hz]
B
PAP
RAP
Curve fit
RV Isovolumetric
P_ISOMax (a + 2*b)
EDP (a)
Amplitude (b)
Pressure [mmHg]
Sample
C
P_ISOMax
Ees
Ea
Pressure [mmHg]
Volume [mL]

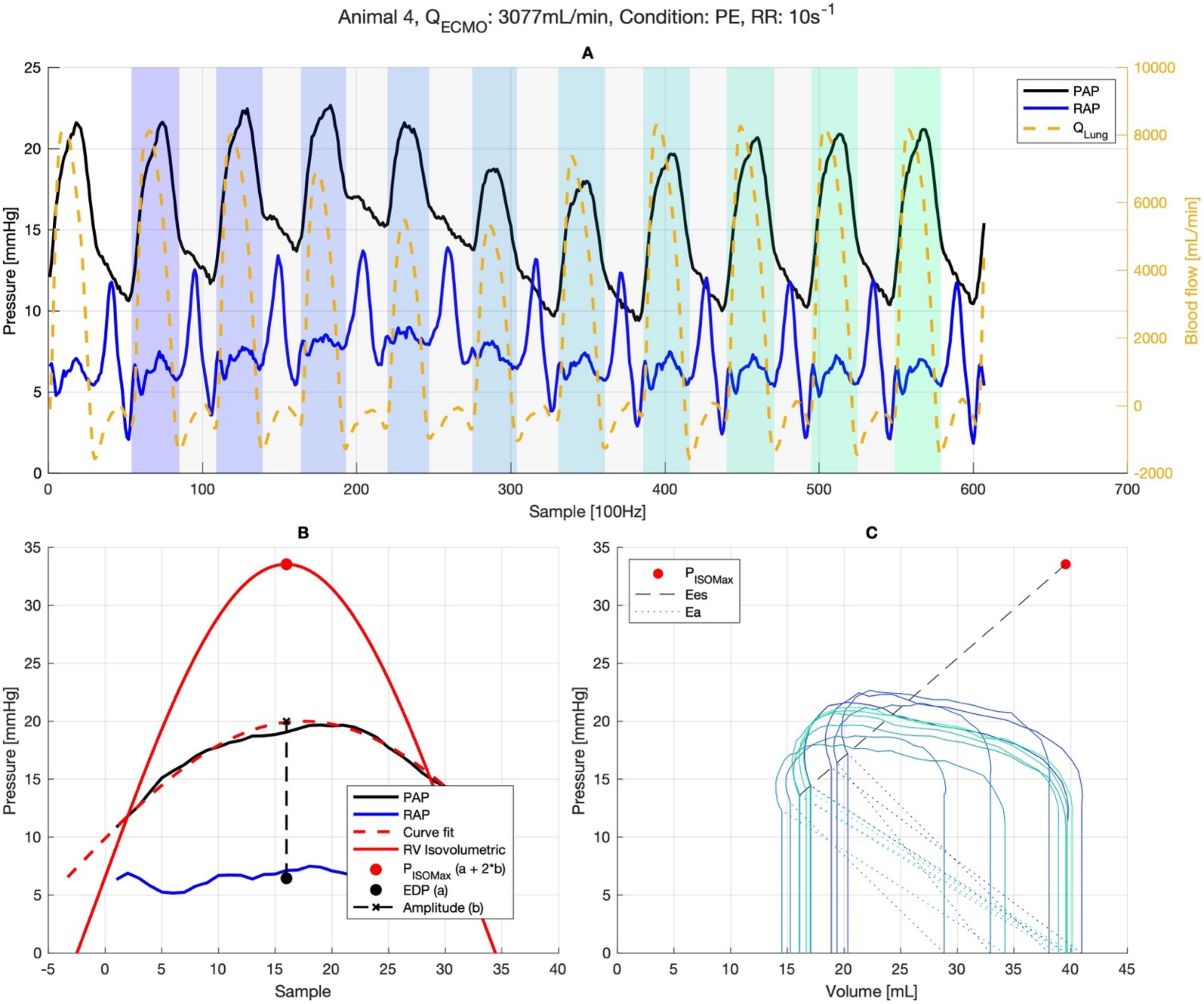

Animal 4, Q_{ECMO}: 3077mL/min, Condition: PE, RR: $10s^{-1}$
A
Pressure [mmHg]
Blood flow [mL/min]
Sample [100Hz]
PAP
RAP
Q_{Lung}
B
Pressure [mmHg]
Sample
PAP
RAP
Curve fit
RV Isovolumetric
P_{ISOMax} (a + 2*b)
EDP (a)
Amplitude (b)
C
Pressure [mmHg]
Volume [mL]
P_{ISOMax}
Ees
Ea

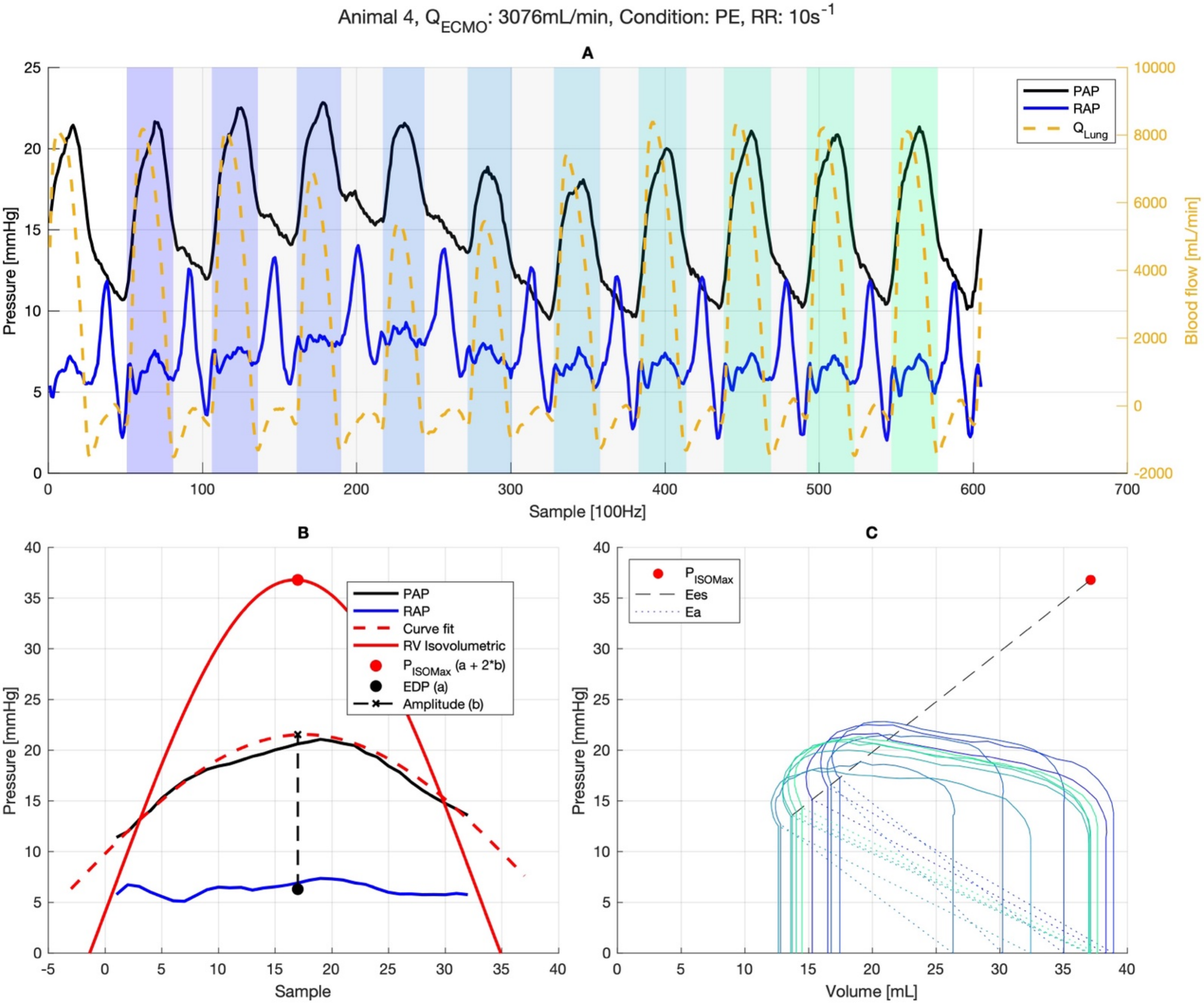

Animal 4, Q_{ECMO}: 3076mL/min, Condition: PE, RR: $10s^{-1}$
A
Pressure [mmHg]
Blood flow [mL/min]
Sample [100Hz]
PAP
RAP
Q_{Lung}
B
Pressure [mmHg]
Sample
PAP
RAP
Curve fit
RV Isovolumetric
P_{ISOMax} (a + 2*b)
EDP (a)
Amplitude (b)
C
Pressure [mmHg]
Volume [mL]
P_{ISOMax}
Ees
Ea

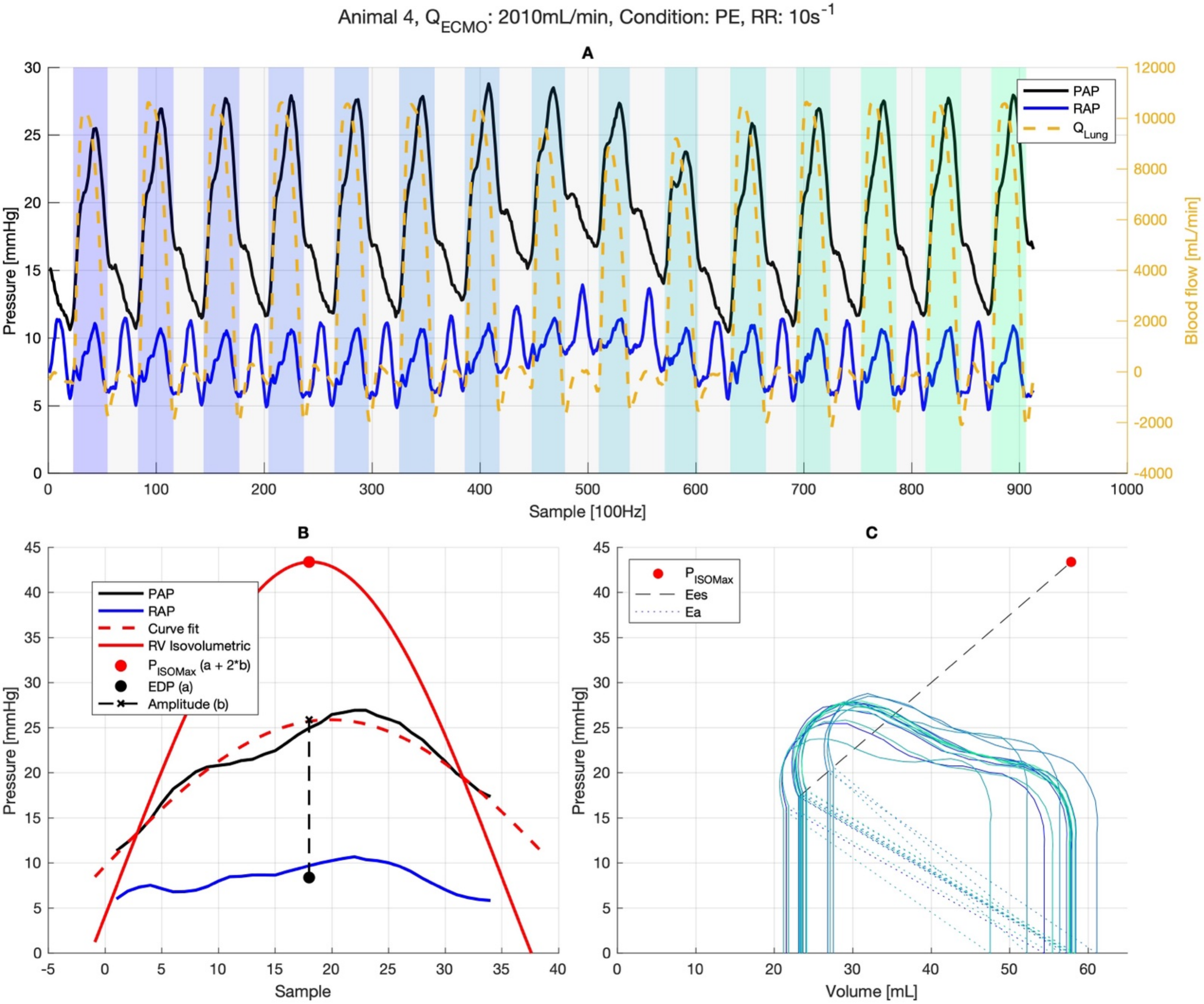

Animal 4, Q_{ECMO}: 2010mL/min, Condition: PE, RR: $10s^{-1}$
A
PAP
RAP
Q_{Lung}
Pressure [mmHg]
Blood flow [mL/min]
Sample [100Hz]
B
PAP
RAP
Curve fit
RV Isovolumetric
P_{ISOMax} (a + 2*b)
EDP (a)
Amplitude (b)
Pressure [mmHg]
Sample
C
P_{ISOMax}
Ees
Ea
Pressure [mmHg]
Volume [mL]

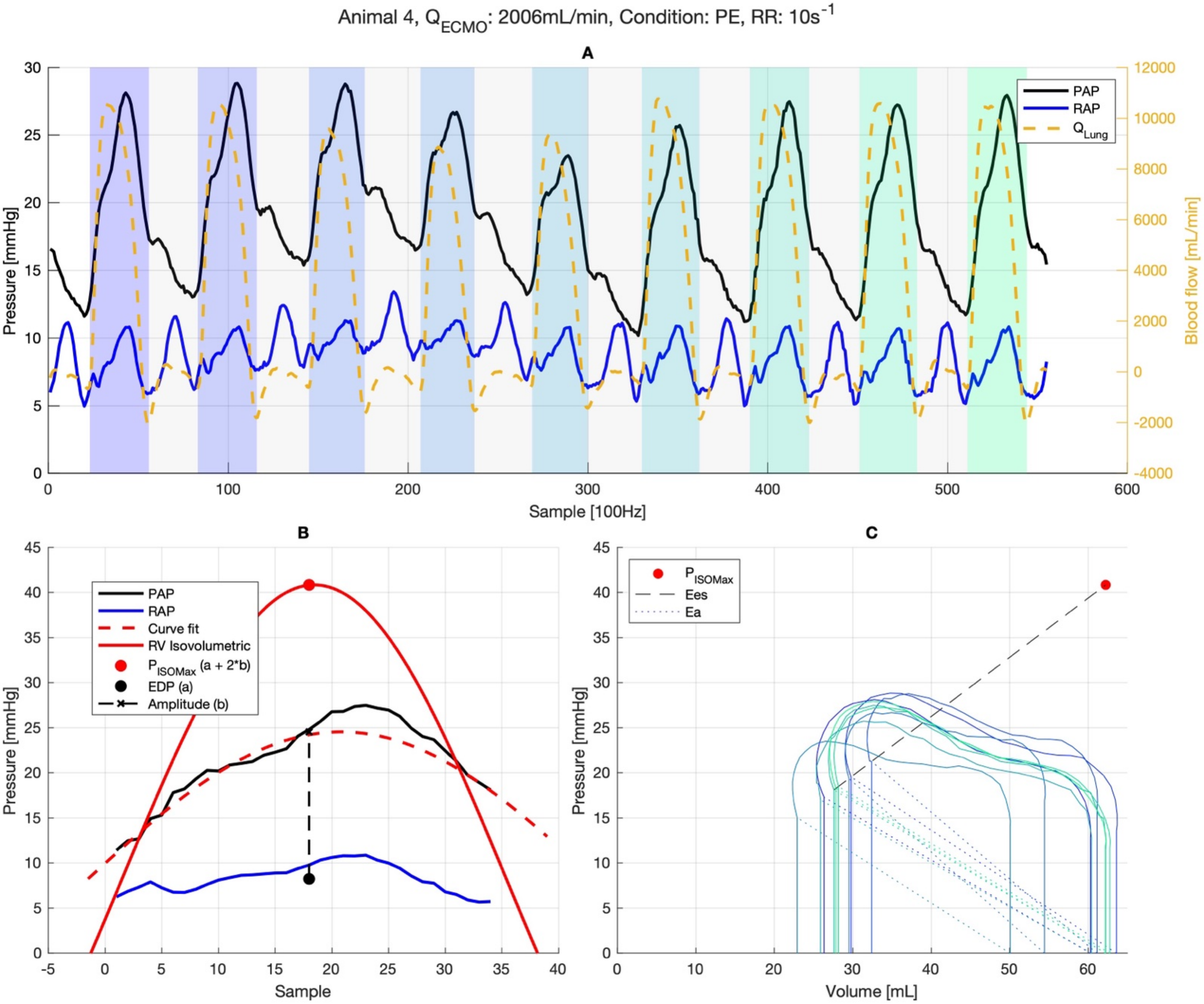

Animal 4, QECMO: 2006mL/min, Condition: PE, RR: 10s-1
A
PAP
RAP
QLung
Pressure [mmHg]
Blood flow [mL/min]
Sample [100Hz]
B
PAP
RAP
Curve fit
RV Isovolumetric
PISOMax (a + 2*b)
EDP (a)
Amplitude (b)
Pressure [mmHg]
Sample
C
PISOMax
Ees
Ea
Pressure [mmHg]
Volume [mL]

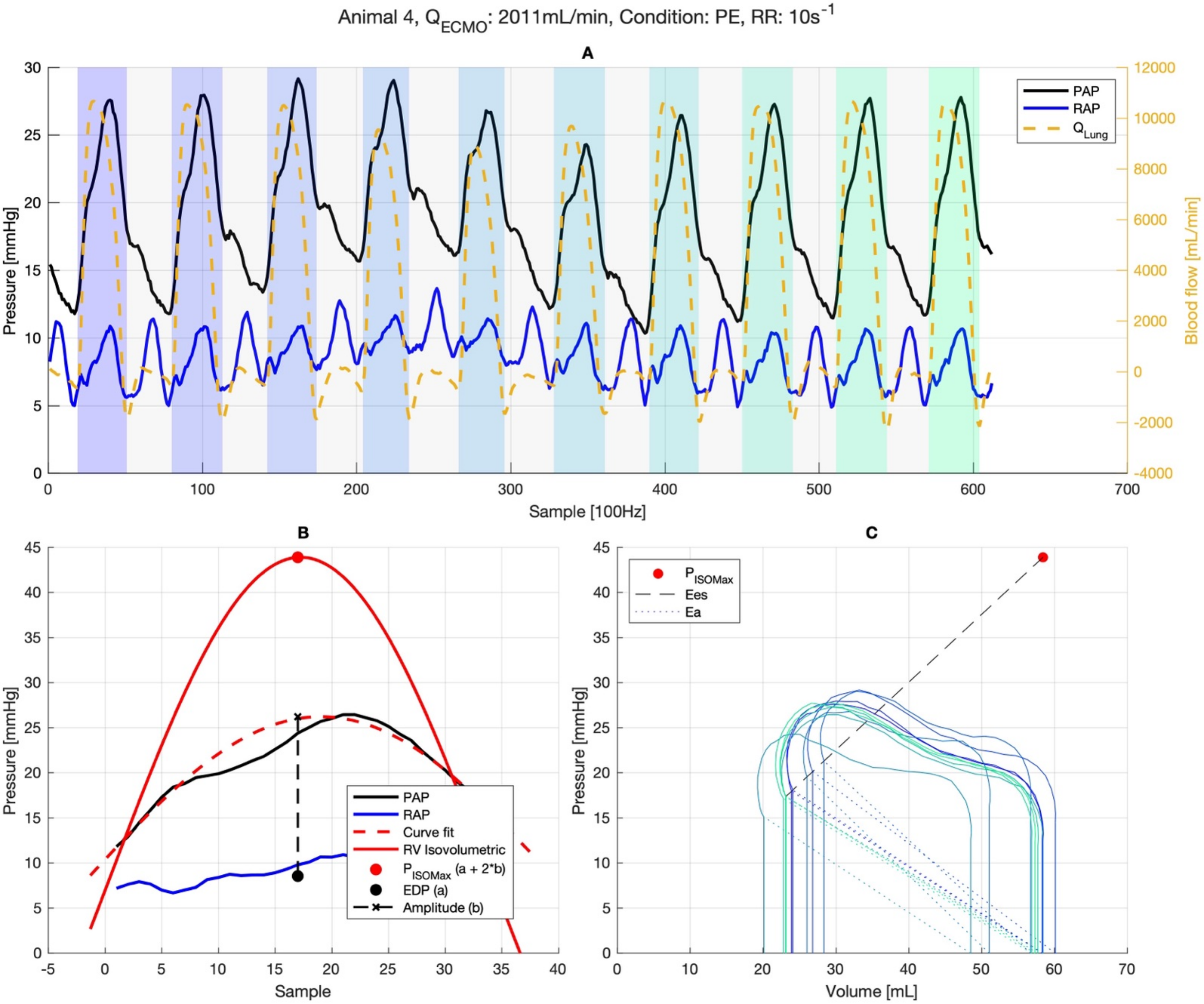

Animal 4, Q_{ECMO}: 2011mL/min, Condition: PE, RR: $10s^{-1}$
A
PAP
RAP
Q_{Lung}
Pressure [mmHg]
Blood flow [mL/min]
Sample [100Hz]
B
PAP
RAP
Curve fit
RV Isovolumetric
P_{ISOMax} (a + 2*b)
EDP (a)
Amplitude (b)
Pressure [mmHg]
Sample
C
P_{ISOMax}
Ees
Ea
Pressure [mmHg]
Volume [mL]

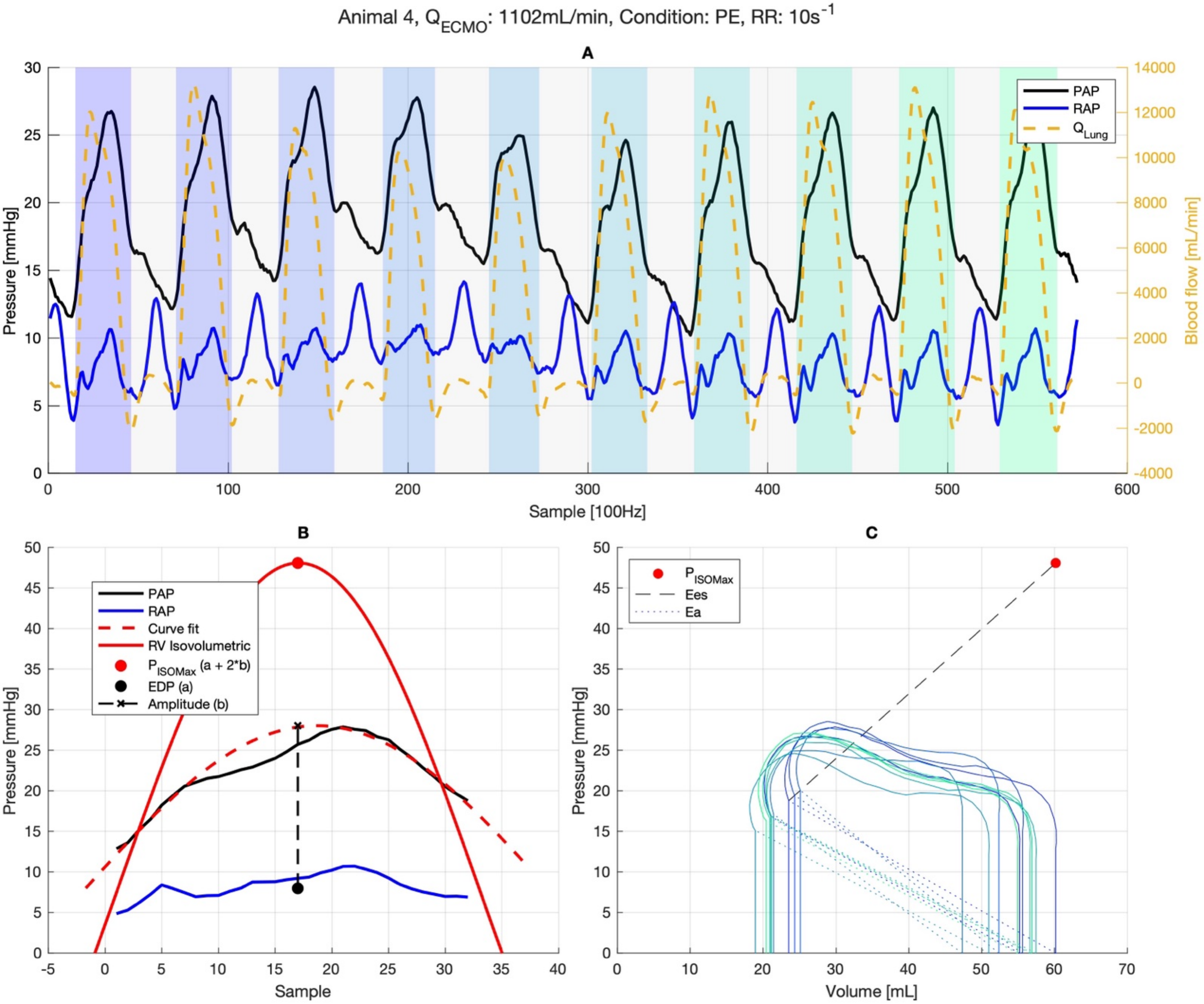

Animal 4, Q_{ECMO}: 1102mL/min, Condition: PE, RR: $10s^{-1}$
A
PAP
RAP
Q_{Lung}
Pressure [mmHg]
Blood flow [mL/min]
Sample [100Hz]
B
PAP
RAP
Curve fit
RV Isovolumetric
P_{ISOMax} (a + 2*b)
EDP (a)
Amplitude (b)
Pressure [mmHg]
Sample
C
P_{ISOMax}
Ees
Ea
Pressure [mmHg]
Volume [mL]

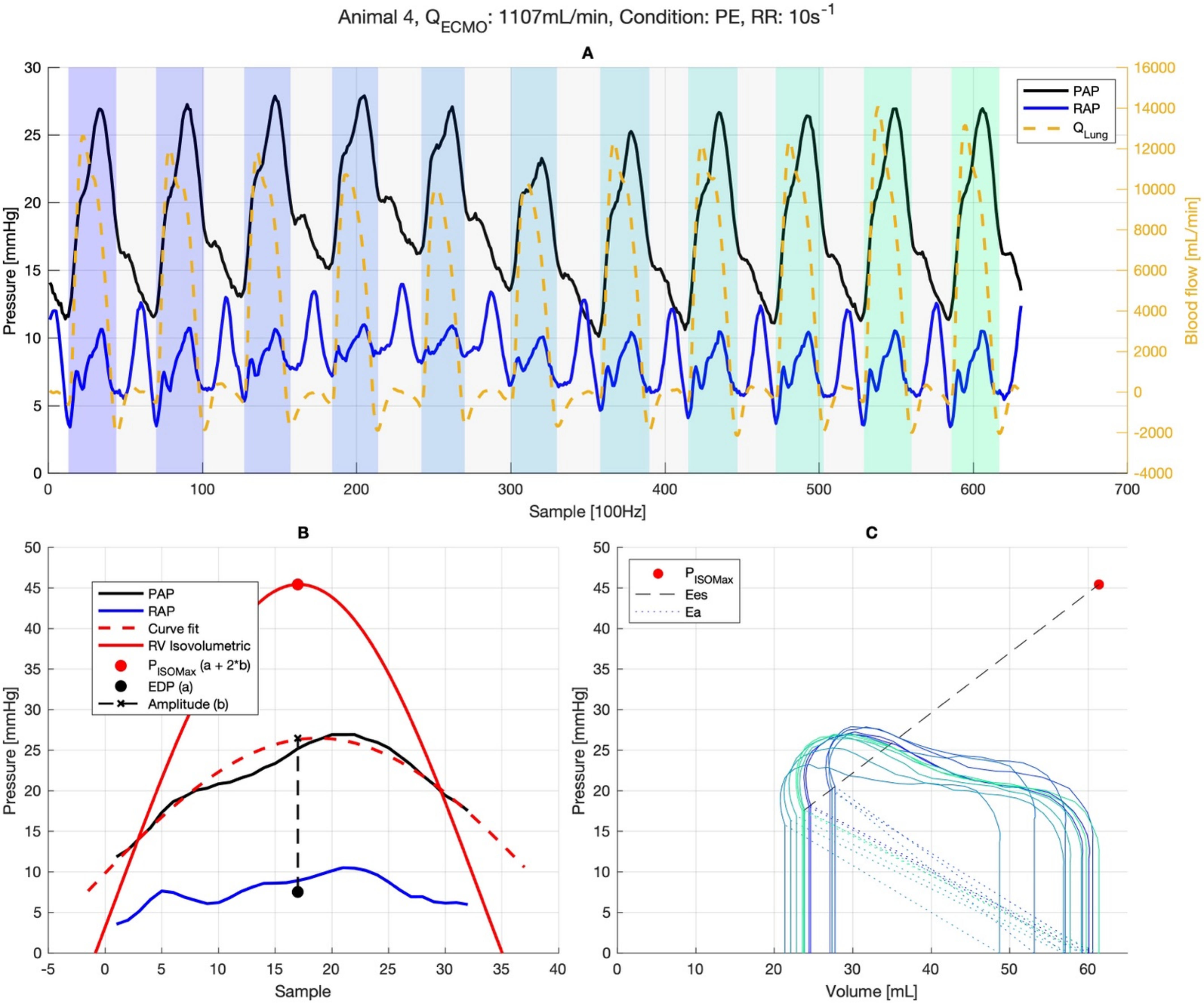
Animal 4, Q_{ECMO}: 1107mL/min, Condition: PE, RR: $10s^{-1}$
A
PAP
RAP
Q_{Lung}
Pressure [mmHg]
Blood flow [mL/min]
Sample [100Hz]
B
PAP
RAP
Curve fit
RV Isovolumetric
P_{ISOMax} (a + 2*b)
EDP (a)
Amplitude (b)
Pressure [mmHg]
Sample
C
P_{ISOMax}
Ees
Ea
Pressure [mmHg]
Volume [mL]

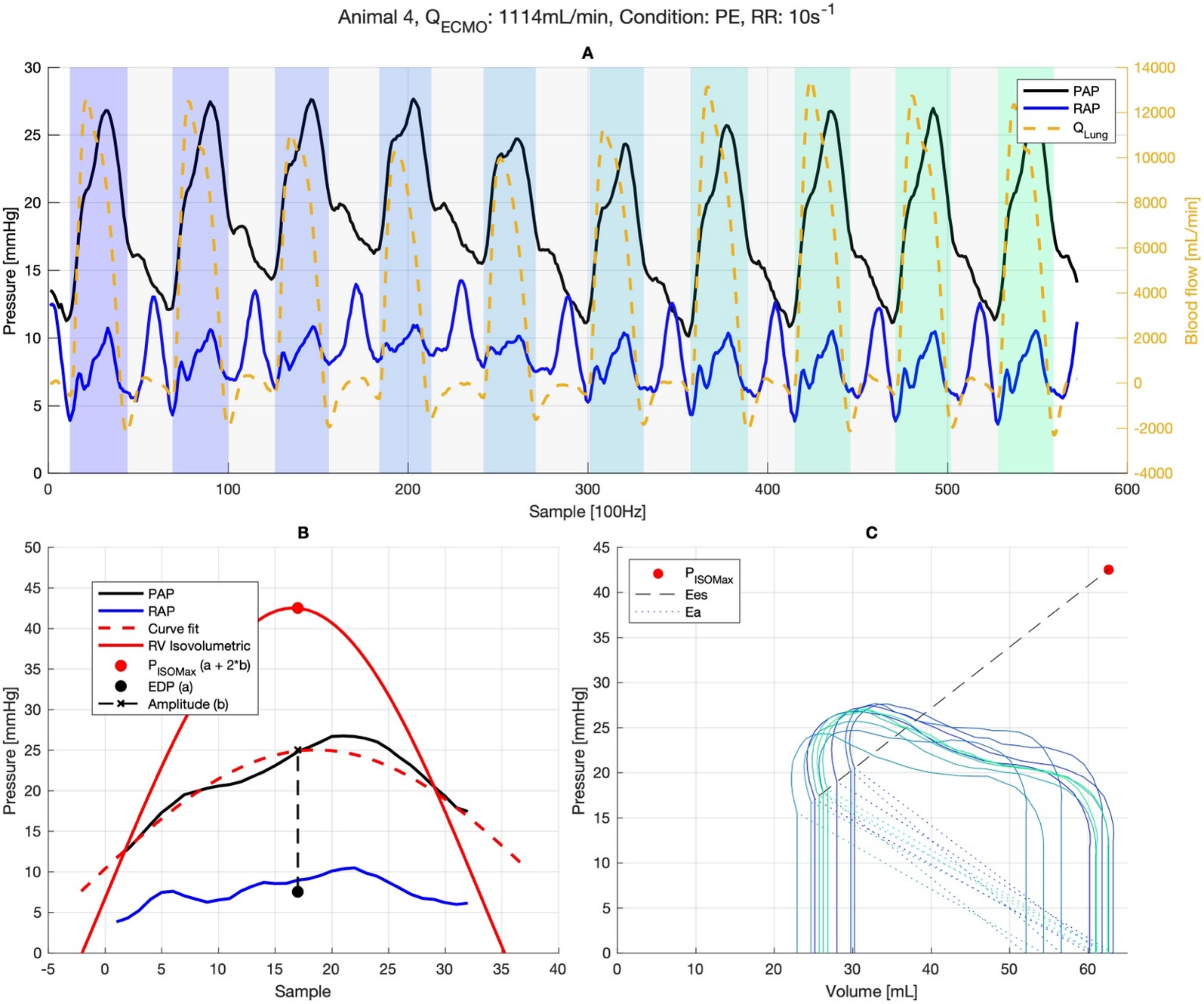
Animal 4, Q_ECMO: 1114mL/min, Condition: PE, RR: 10s^-1
A
PAP
RAP
Q_Lung
Pressure [mmHg]
Blood flow [mL/min]
Sample [100Hz]
B
PAP
RAP
Curve fit
RV Isovolumetric
P_ISOMax (a + 2*b)
EDP (a)
Amplitude (b)
Pressure [mmHg]
Sample
C
P_ISOMax
Ees
Ea
Pressure [mmHg]
Volume [mL]

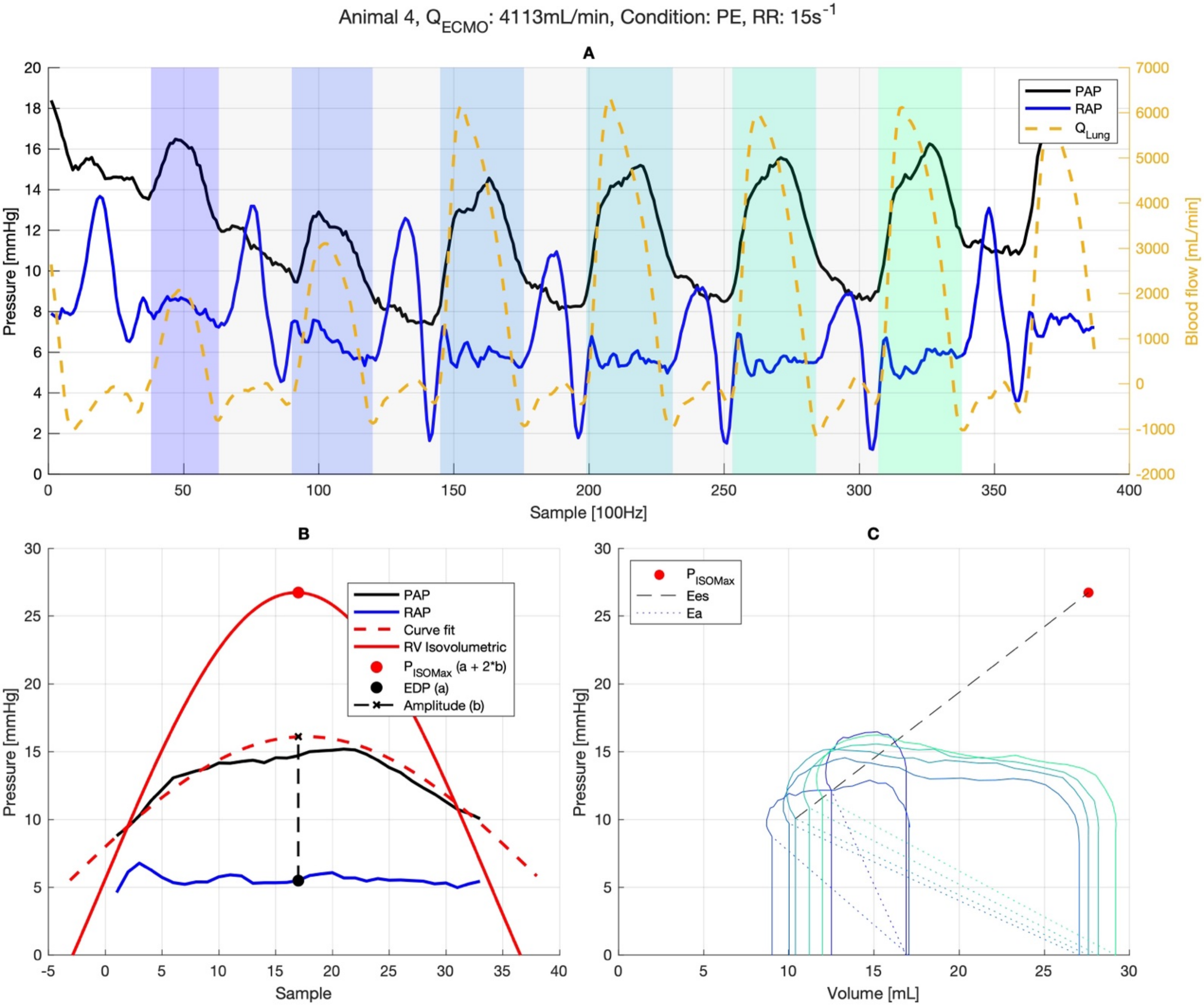

Animal 4, Q_ECMO: 4113mL/min, Condition: PE, RR: 15s^-1
A
PAP
RAP
Q_Lung
Pressure [mmHg]
Blood flow [mL/min]
Sample [100Hz]
B
PAP
RAP
Curve fit
RV Isovolumetric
P_ISOMax (a + 2*b)
EDP (a)
Amplitude (b)
Pressure [mmHg]
Sample
C
P_ISOMax
Ees
Ea
Pressure [mmHg]
Volume [mL]

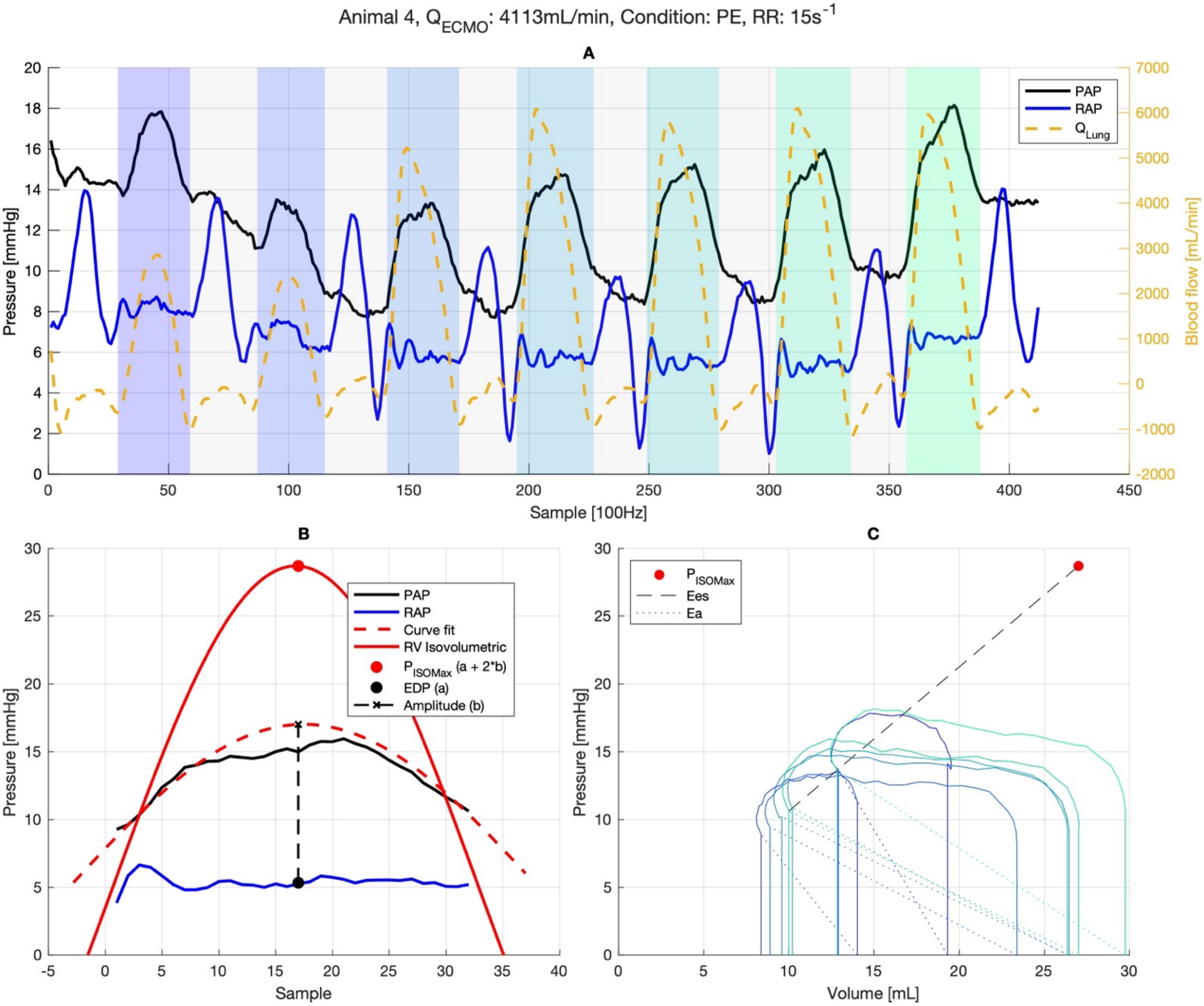

Animal 4, Q_ECMO: 4113mL/min, Condition: PE, RR: 15s^-1
A
PAP
RAP
Q_Lung
Pressure [mmHg]
Blood flow [mL/min]
Sample [100Hz]
B
PAP
RAP
Curve fit
RV Isovolumetric
P_ISOMax (a + 2*b)
EDP (a)
Amplitude (b)
Pressure [mmHg]
Sample
C
P_ISOMax
Ees
Ea
Pressure [mmHg]
Volume [mL]

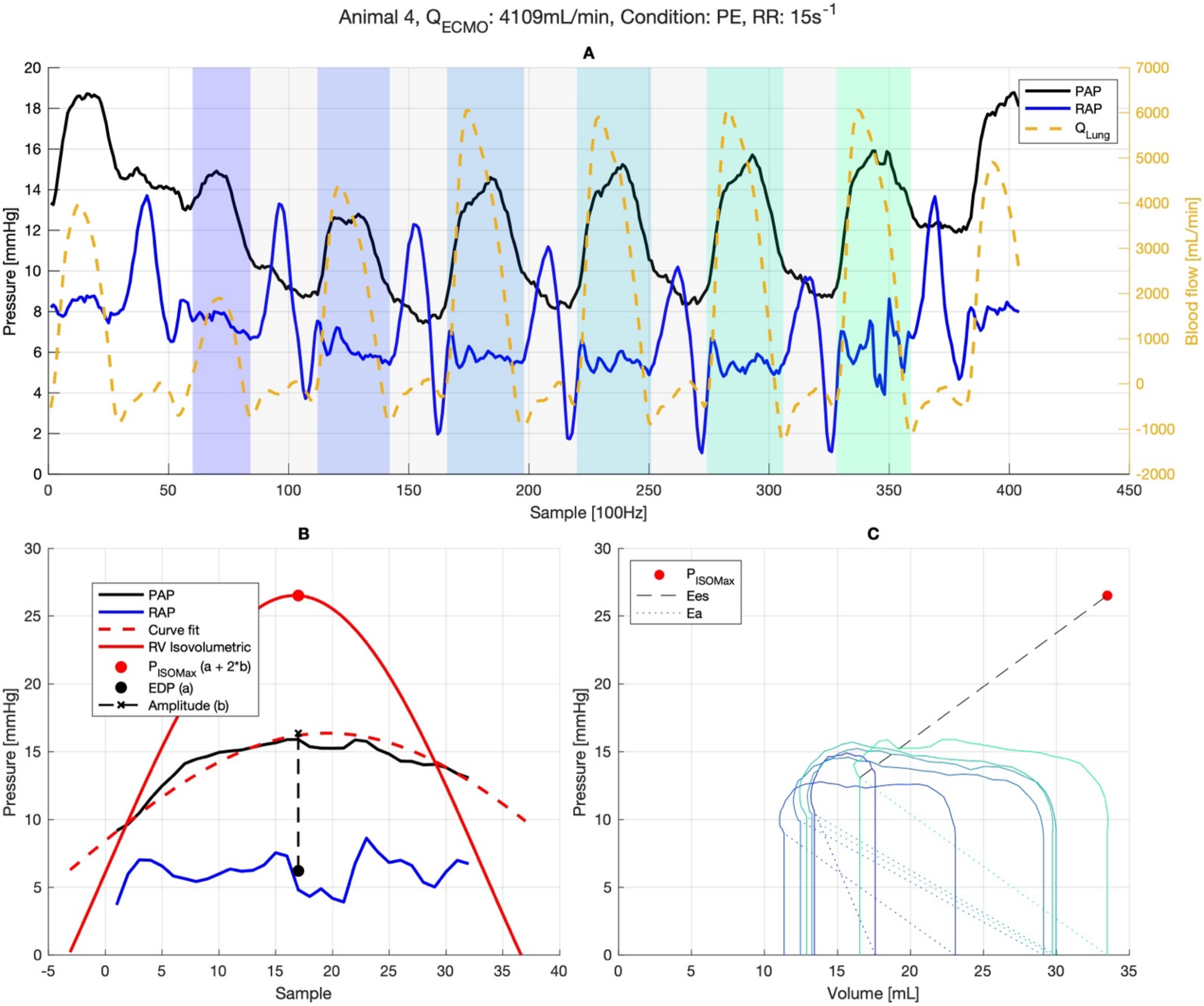

Animal 4, Q_{ECMO}: 4109mL/min, Condition: PE, RR: $15s^{-1}$
A
PAP
RAP
Q_{Lung}
Pressure [mmHg]
Blood flow [mL/min]
Sample [100Hz]
B
PAP
RAP
Curve fit
RV Isovolumetric
P_{ISOMax} (a + 2*b)
EDP (a)
Amplitude (b)
Pressure [mmHg]
Sample
C
P_{ISOMax}
Ees
Ea
Pressure [mmHg]
Volume [mL]

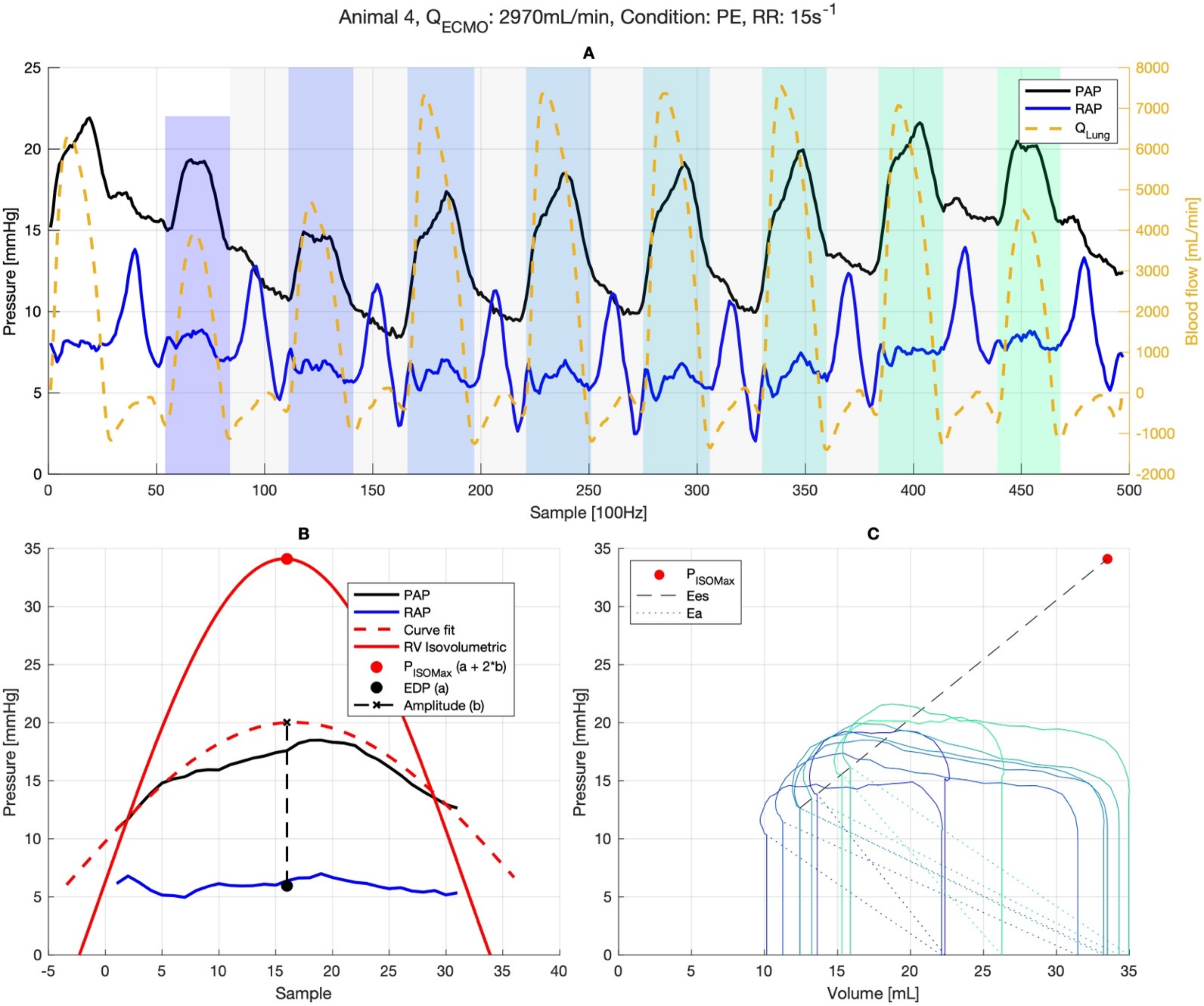

Animal 4, Q_{ECMO}: 2970mL/min, Condition: PE, RR: $15s^{-1}$
A
PAP
RAP
Q_{Lung}
Pressure [mmHg]
Blood flow [mL/min]
Sample [100Hz]
B
PAP
RAP
Curve fit
RV Isovolumetric
P_{ISOMax} (a + 2*b)
EDP (a)
Amplitude (b)
Pressure [mmHg]
Sample
C
P_{ISOMax}
Ees
Ea
Pressure [mmHg]
Volume [mL]

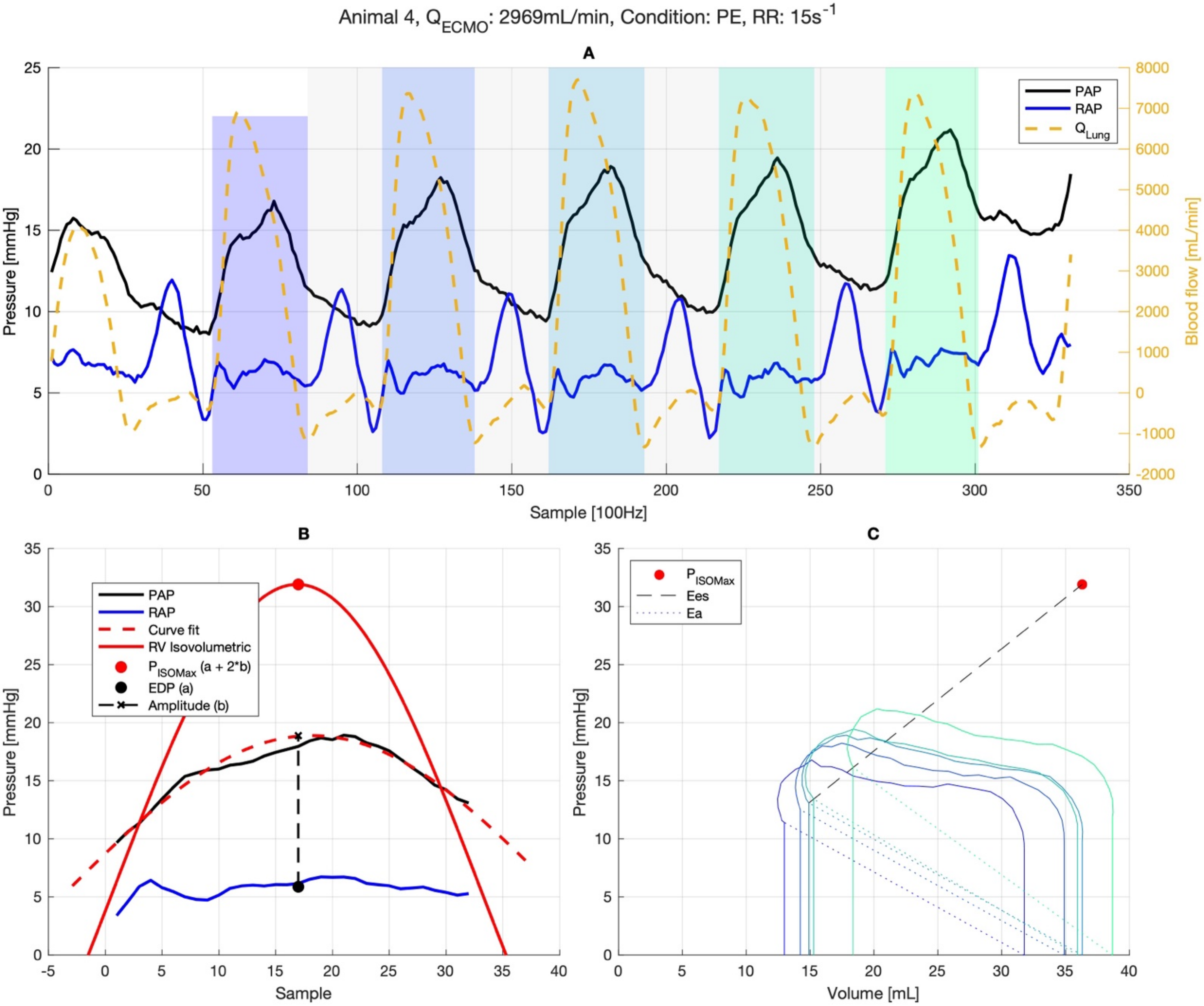

Animal 4, QECMO: 2969mL/min, Condition: PE, RR: 15s-1
A
Pressure [mmHg]
Blood flow [mL/min]
Sample [100Hz]
PAP
RAP
QLung
B
PAP
RAP
Curve fit
RV Isovolumetric
PISOMax (a + 2*b)
EDP (a)
Amplitude (b)
Pressure [mmHg]
Sample
C
PISOMax
Ees
Ea
Pressure [mmHg]
Volume [mL]

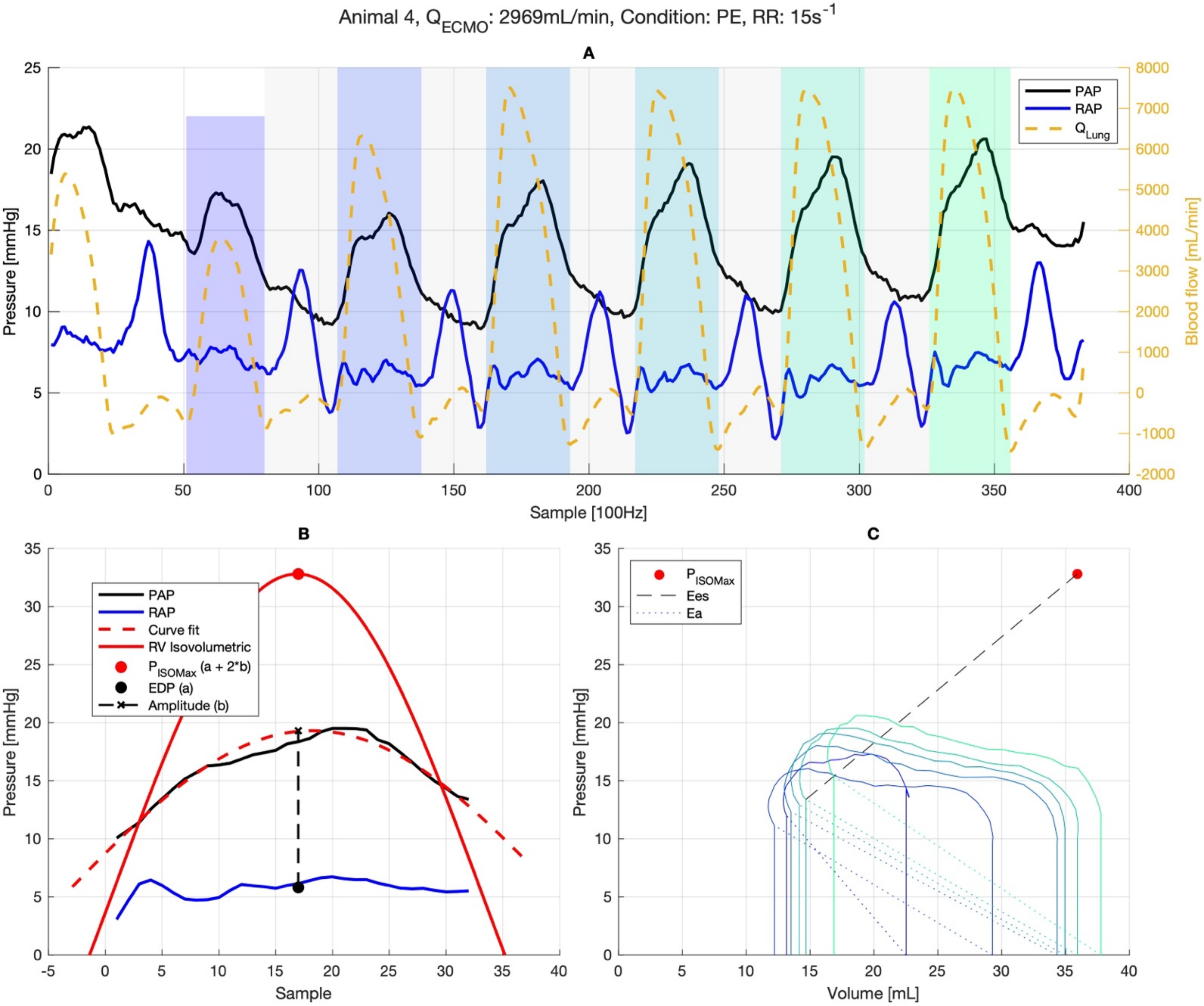

Animal 4, Q_{ECMO}: 2969mL/min, Condition: PE, RR: $15s^{-1}$
A
PAP
RAP
Q_{Lung}
Pressure [mmHg]
Blood flow [mL/min]
Sample [100Hz]
B
PAP
RAP
Curve fit
RV Isovolumetric
P_{ISOMax} (a + 2*b)
EDP (a)
Amplitude (b)
Pressure [mmHg]
Sample
C
P_{ISOMax}
Ees
Ea
Pressure [mmHg]
Volume [mL]

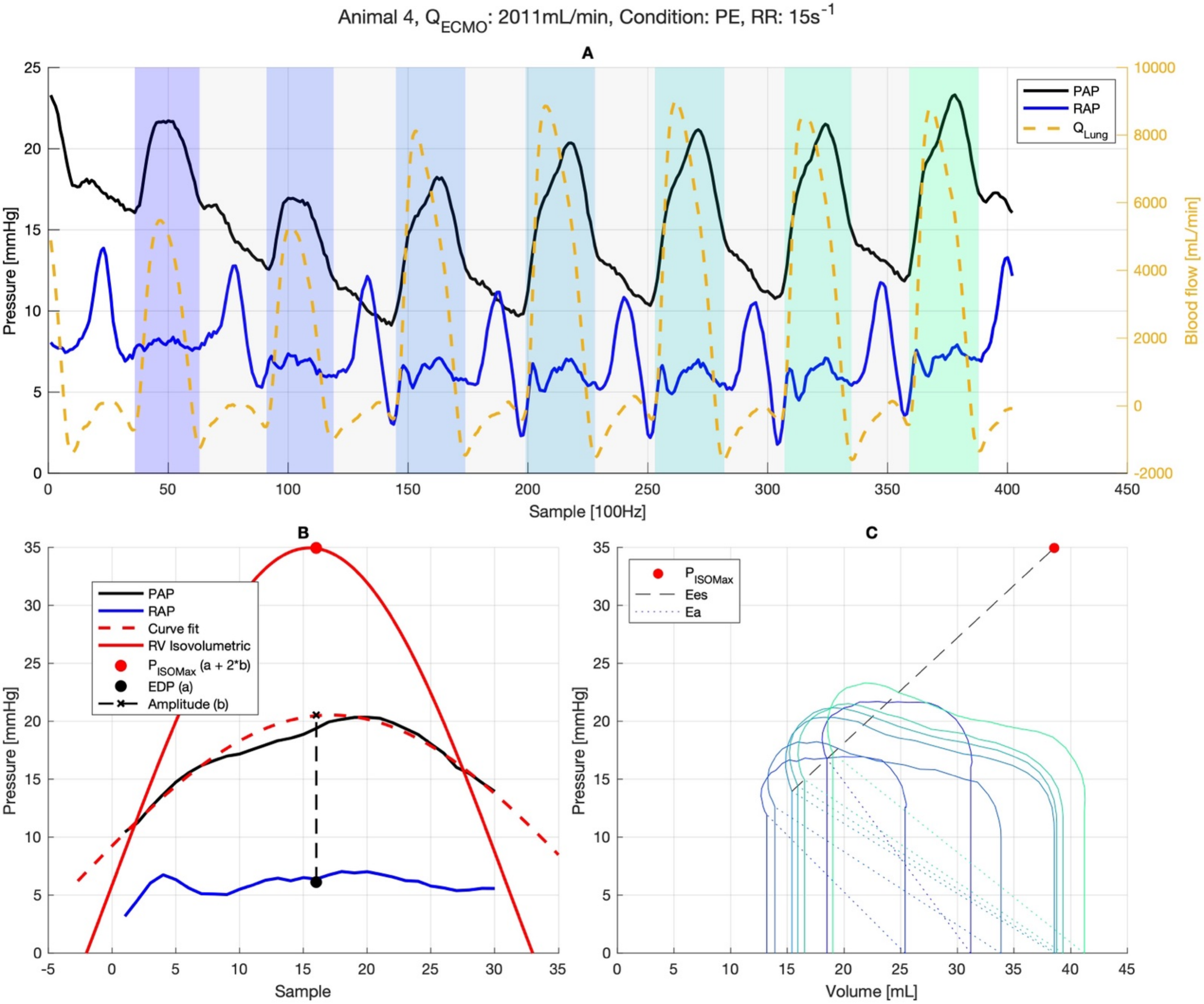

Animal 4, Q_ECMO: 2011mL/min, Condition: PE, RR: 15s^-1
A
Pressure [mmHg]
Blood flow [mL/min]
Sample [100Hz]
PAP
RAP
Q_Lung
B
Pressure [mmHg]
Sample
PAP
RAP
Curve fit
RV Isovolumetric
P_ISOMax (a + 2*b)
EDP (a)
Amplitude (b)
C
Pressure [mmHg]
Volume [mL]
P_ISOMax
Ees
Ea

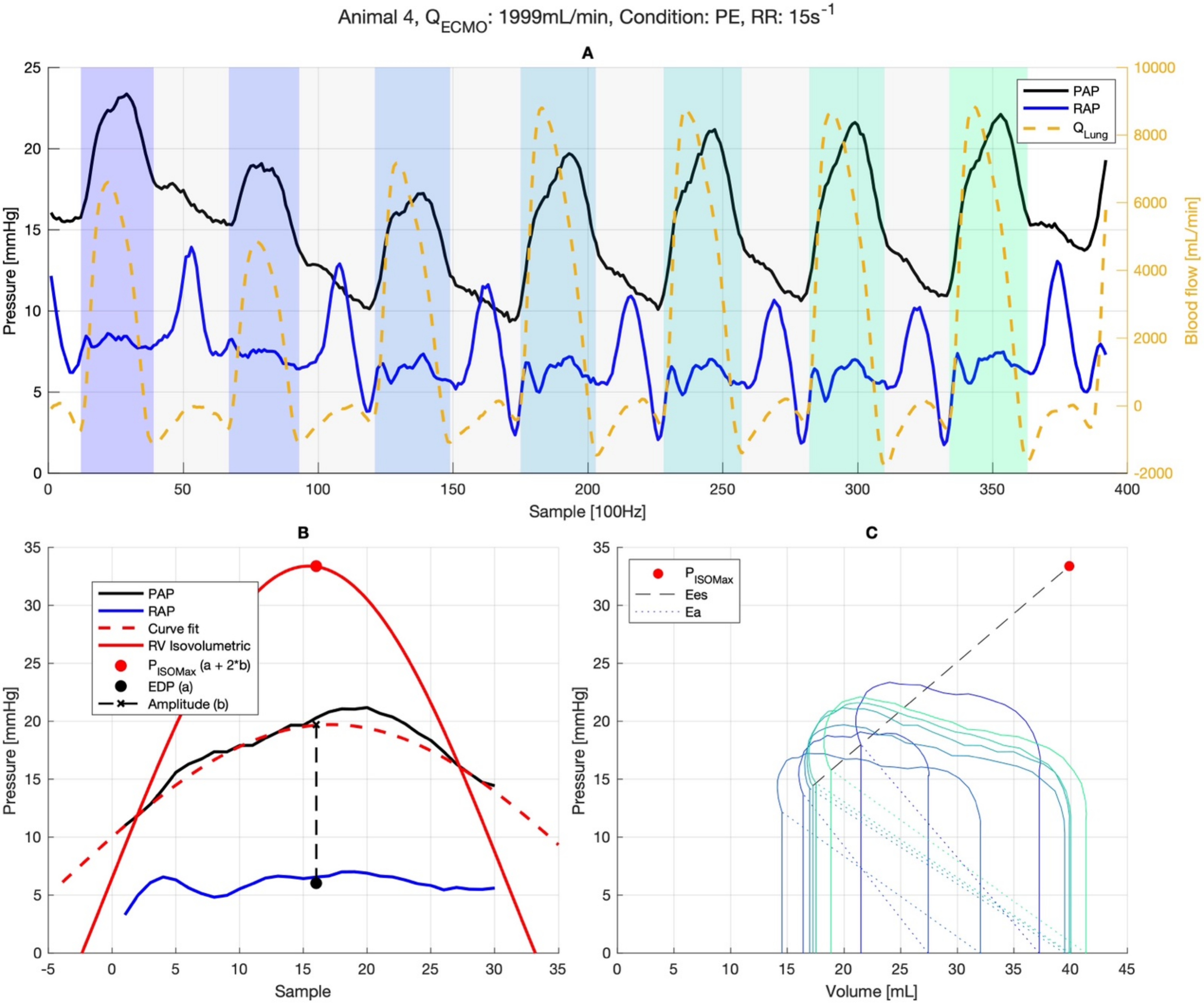

Animal 4, Q_ECMO: 1999mL/min, Condition: PE, RR: 15s^-1
A
PAP
RAP
Q_Lung
Pressure [mmHg]
Blood flow [mL/min]
Sample [100Hz]
B
PAP
RAP
Curve fit
RV Isovolumetric
P_ISOMax (a + 2*b)
EDP (a)
Amplitude (b)
Pressure [mmHg]
Sample
C
P_ISOMax
Ees
Ea
Pressure [mmHg]
Volume [mL]

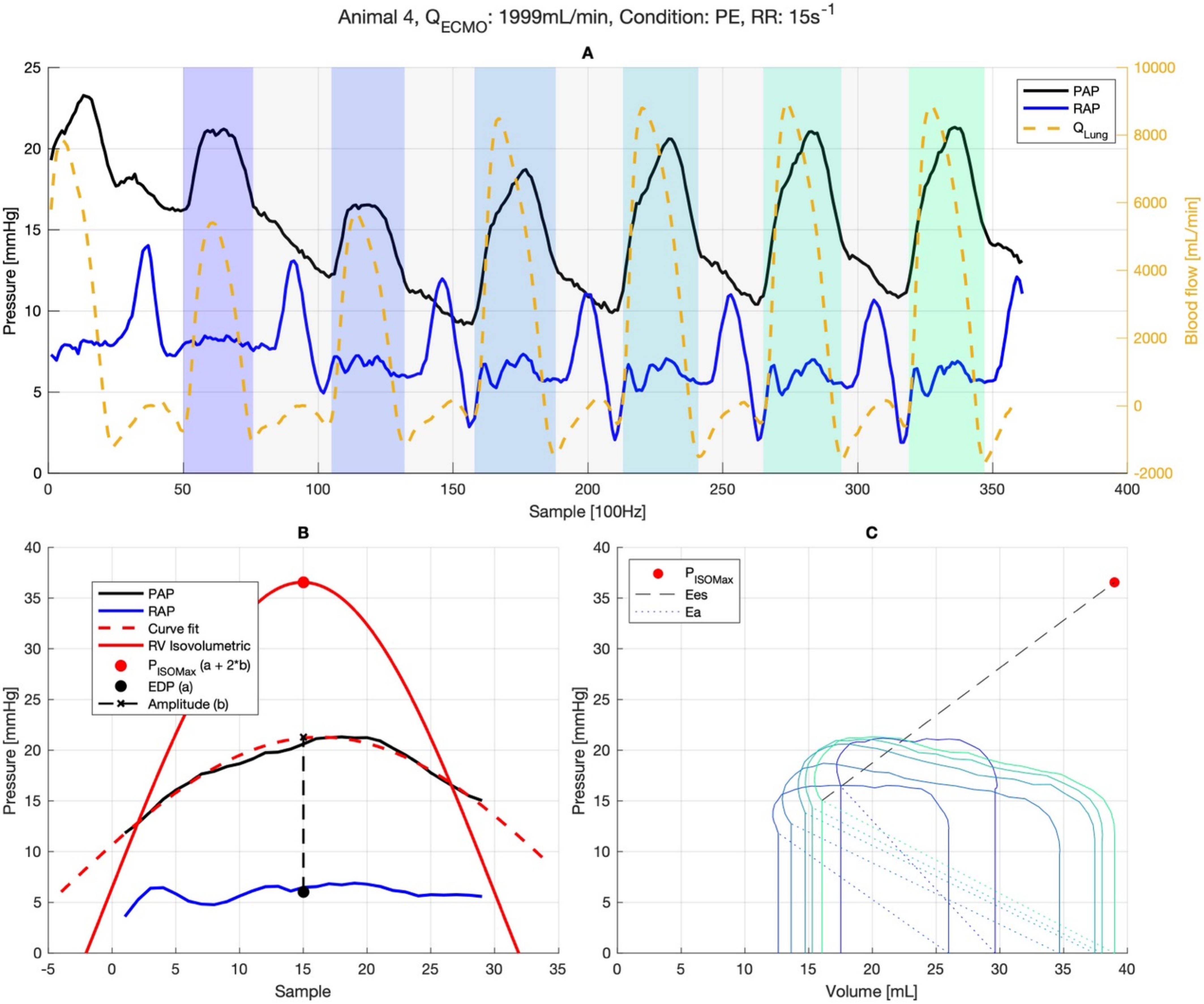

Animal 4, Q_ECMO: 1999mL/min, Condition: PE, RR: 15s^-1
A
PAP
RAP
Q_Lung
Pressure [mmHg]
Blood flow [mL/min]
Sample [100Hz]
B
PAP
RAP
Curve fit
RV Isovolumetric
P_ISOMax (a + 2*b)
EDP (a)
Amplitude (b)
Pressure [mmHg]
Sample
C
P_ISOMax
Ees
Ea
Pressure [mmHg]
Volume [mL]

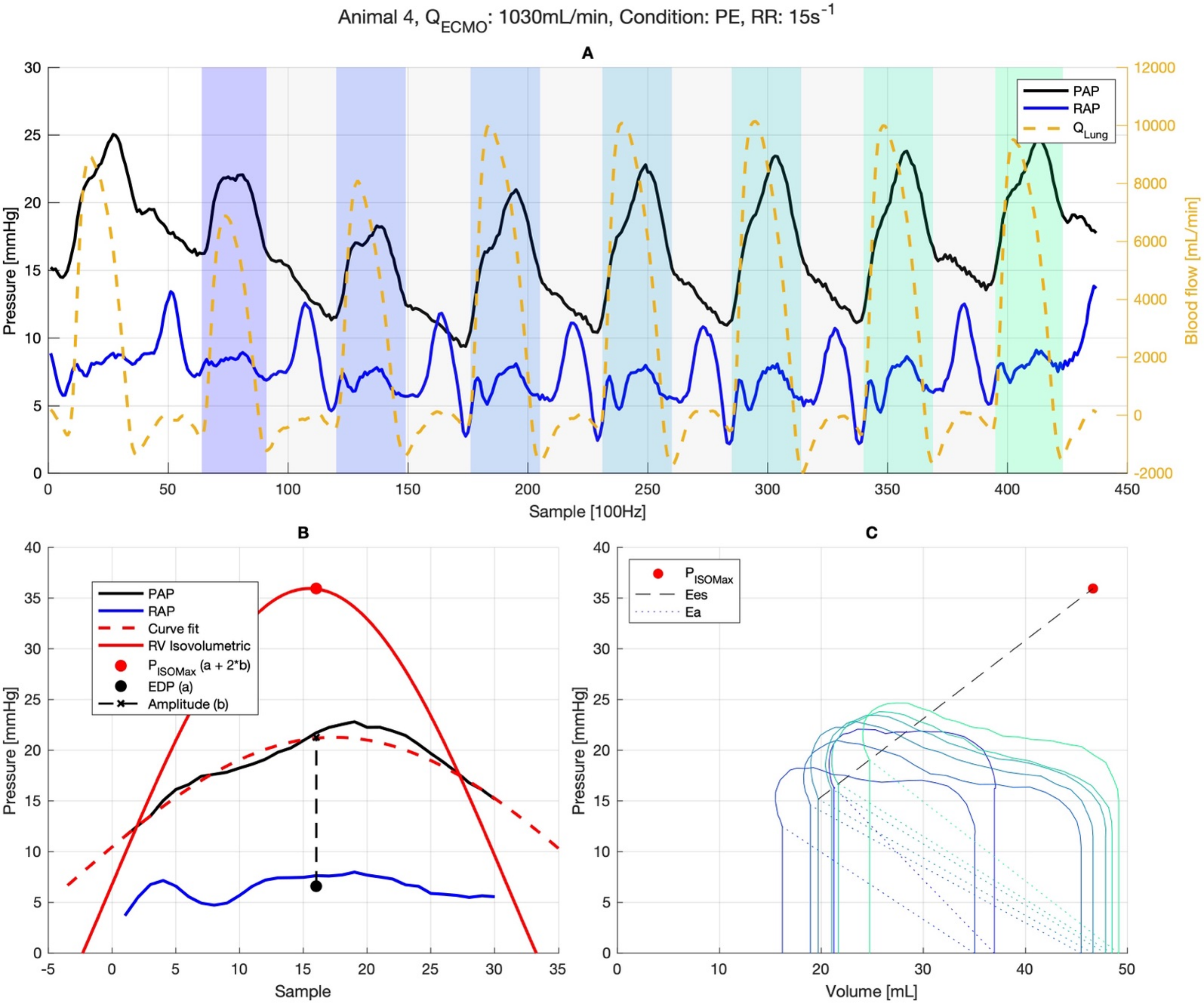

Animal 4, Q_{ECMO}: 1030mL/min, Condition: PE, RR: $15s^{-1}$
A
Pressure [mmHg]
Blood flow [mL/min]
Sample [100Hz]
PAP
RAP
Q_{Lung}
B
PAP
RAP
Curve fit
RV Isovolumetric
P_{ISOMax} (a + 2*b)
EDP (a)
Amplitude (b)
Pressure [mmHg]
Sample
C
P_{ISOMax}
Ees
Ea
Pressure [mmHg]
Volume [mL]

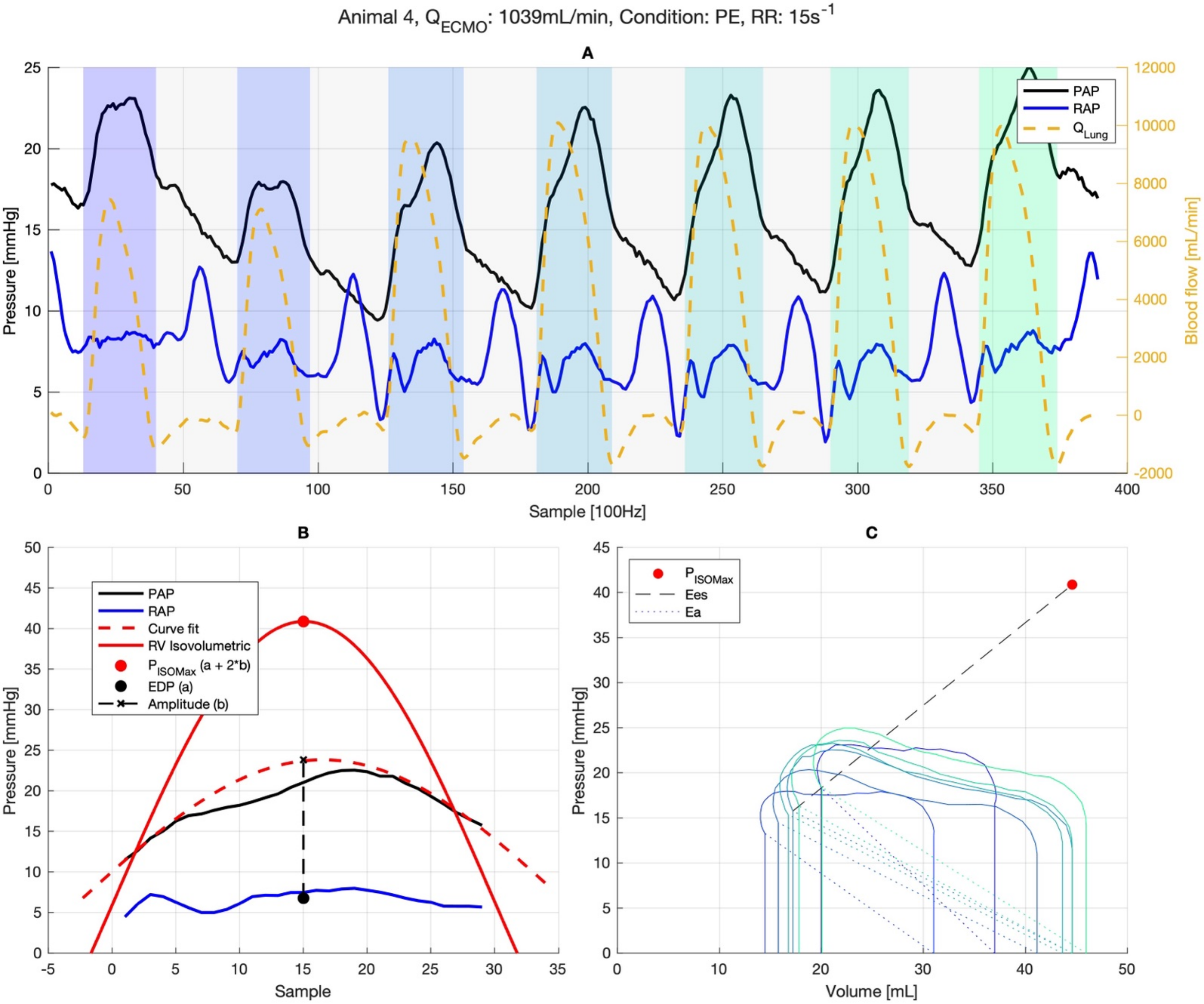
Animal 4, Q_{ECMO}: 1039mL/min, Condition: PE, RR: $15s^{-1}$
A
PAP
RAP
Q_{Lung}
Pressure [mmHg]
Blood flow [mL/min]
Sample [100Hz]
B
PAP
RAP
Curve fit
RV Isovolumetric
P_{ISOMax} (a + 2*b)
EDP (a)
Amplitude (b)
Pressure [mmHg]
Sample
C
P_{ISOMax}
Ees
Ea
Pressure [mmHg]
Volume [mL]

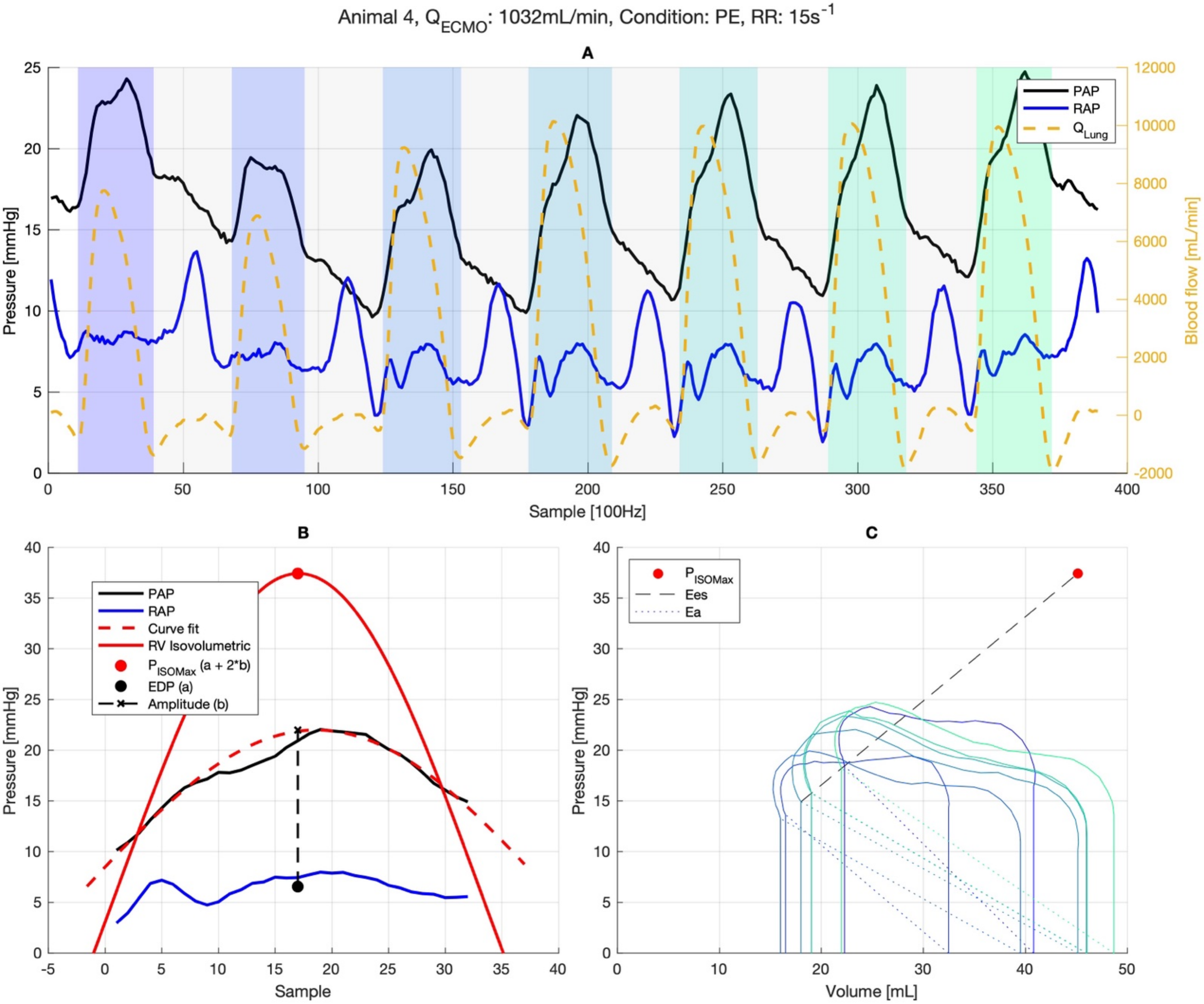
Animal 4, Q_ECMO: 1032mL/min, Condition: PE, RR: 15s-1
A
PAP
RAP
Q_Lung
Pressure [mmHg]
Blood flow [mL/min]
Sample [100Hz]
B
PAP
RAP
Curve fit
RV Isovolumetric
P_ISOMax (a + 2*b)
EDP (a)
Amplitude (b)
Pressure [mmHg]
Sample
C
P_ISOMax
Ees
Ea
Pressure [mmHg]
Volume [mL]

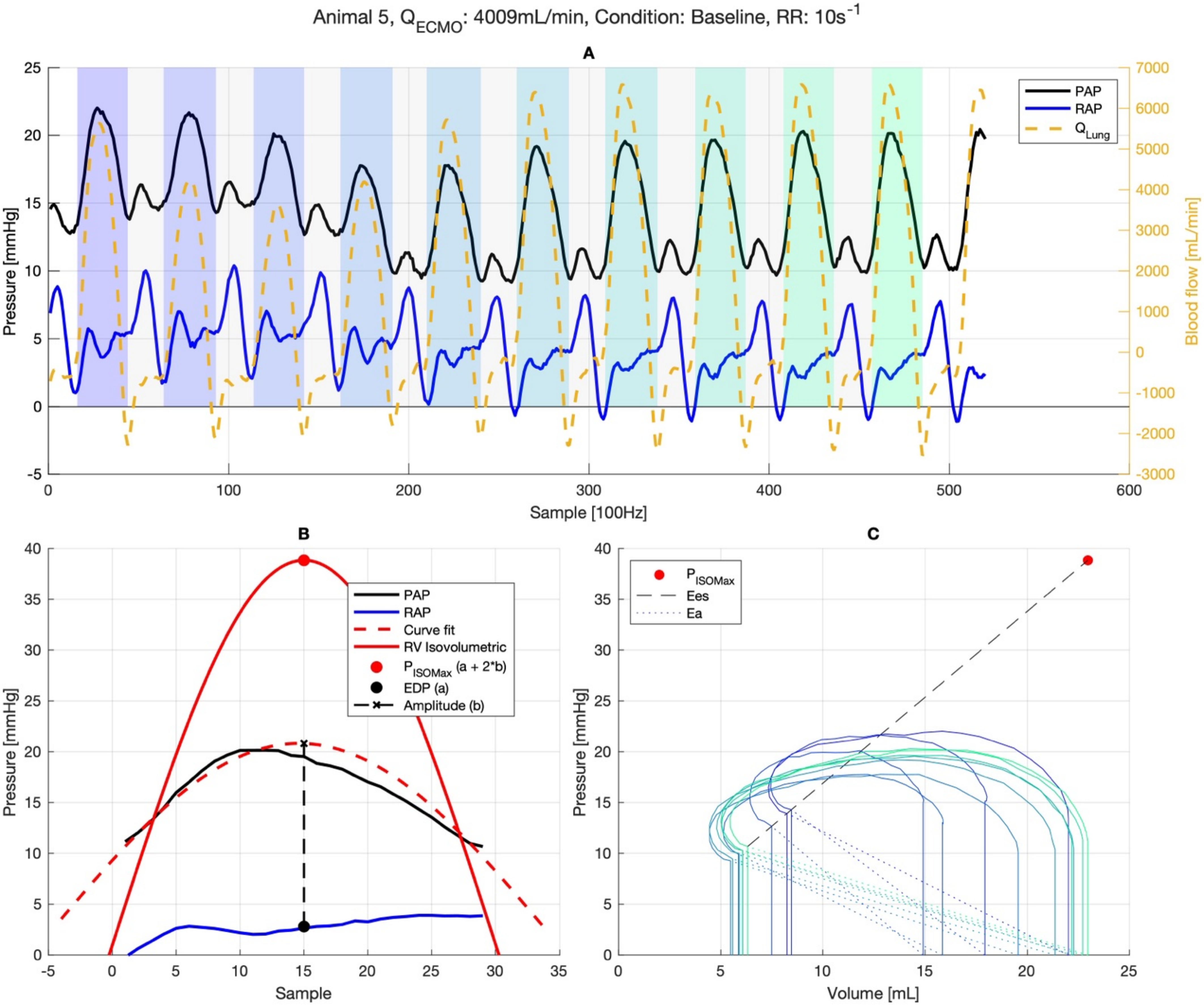

Animal 5, Q_{ECMO}: 4009mL/min, Condition: Baseline, RR: $10s^{-1}$
A
PAP
RAP
Q_{Lung}
Pressure [mmHg]
Blood flow [mL/min]
Sample [100Hz]
B
PAP
RAP
Curve fit
RV Isovolumetric
P_{ISOMax} (a + 2*b)
EDP (a)
Amplitude (b)
Pressure [mmHg]
Sample
C
P_{ISOMax}
Ees
Ea
Pressure [mmHg]
Volume [mL]

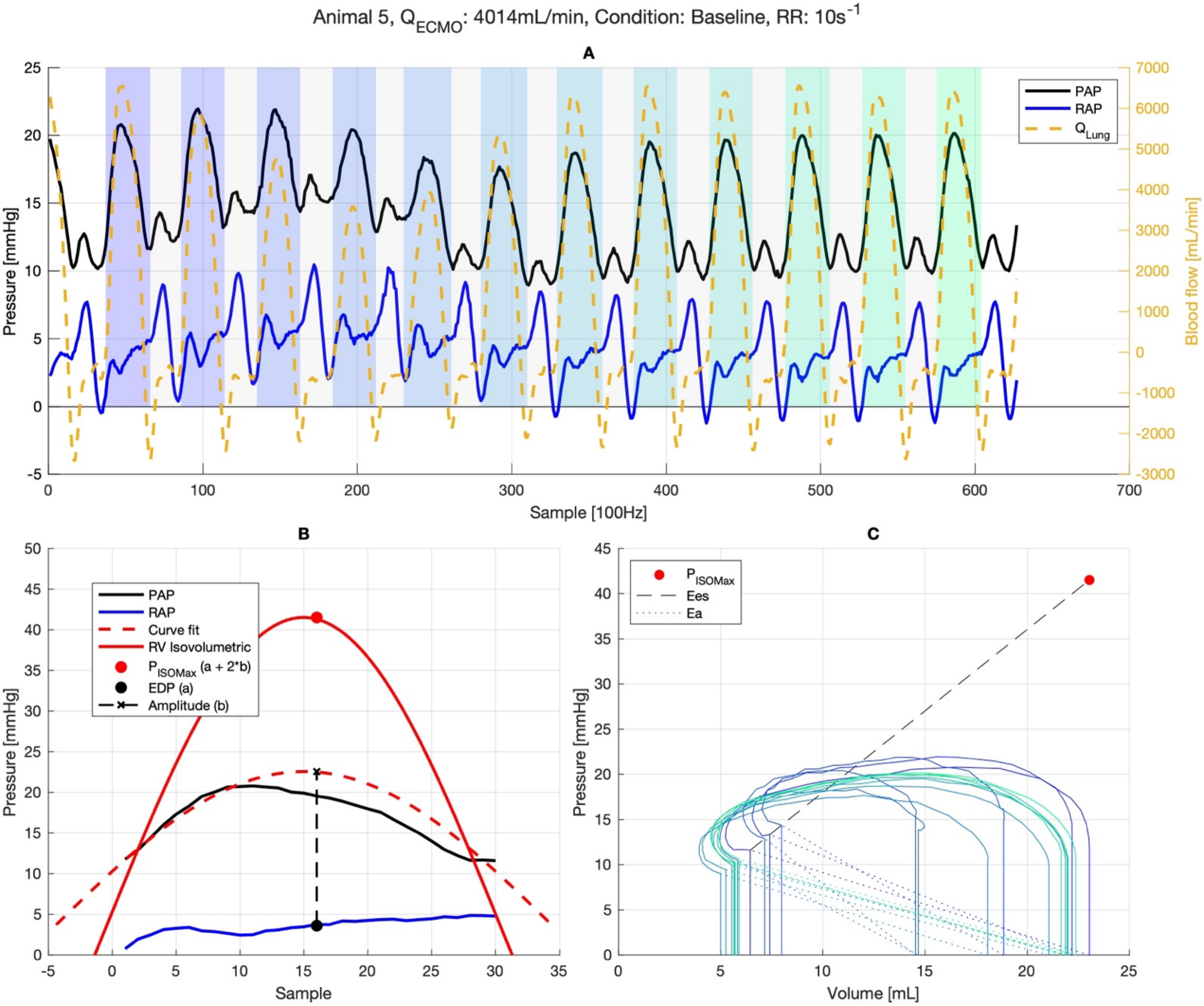

Animal 5, Q_ECMO: 4014mL/min, Condition: Baseline, RR: 10s^-1
A
PAP
RAP
Q_Lung
Pressure [mmHg]
Blood flow [mL/min]
Sample [100Hz]
B
PAP
RAP
Curve fit
RV Isovolumetric
P_ISOMax (a + 2*b)
EDP (a)
Amplitude (b)
Pressure [mmHg]
Sample
C
P_ISOMax
Ees
Ea
Pressure [mmHg]
Volume [mL]

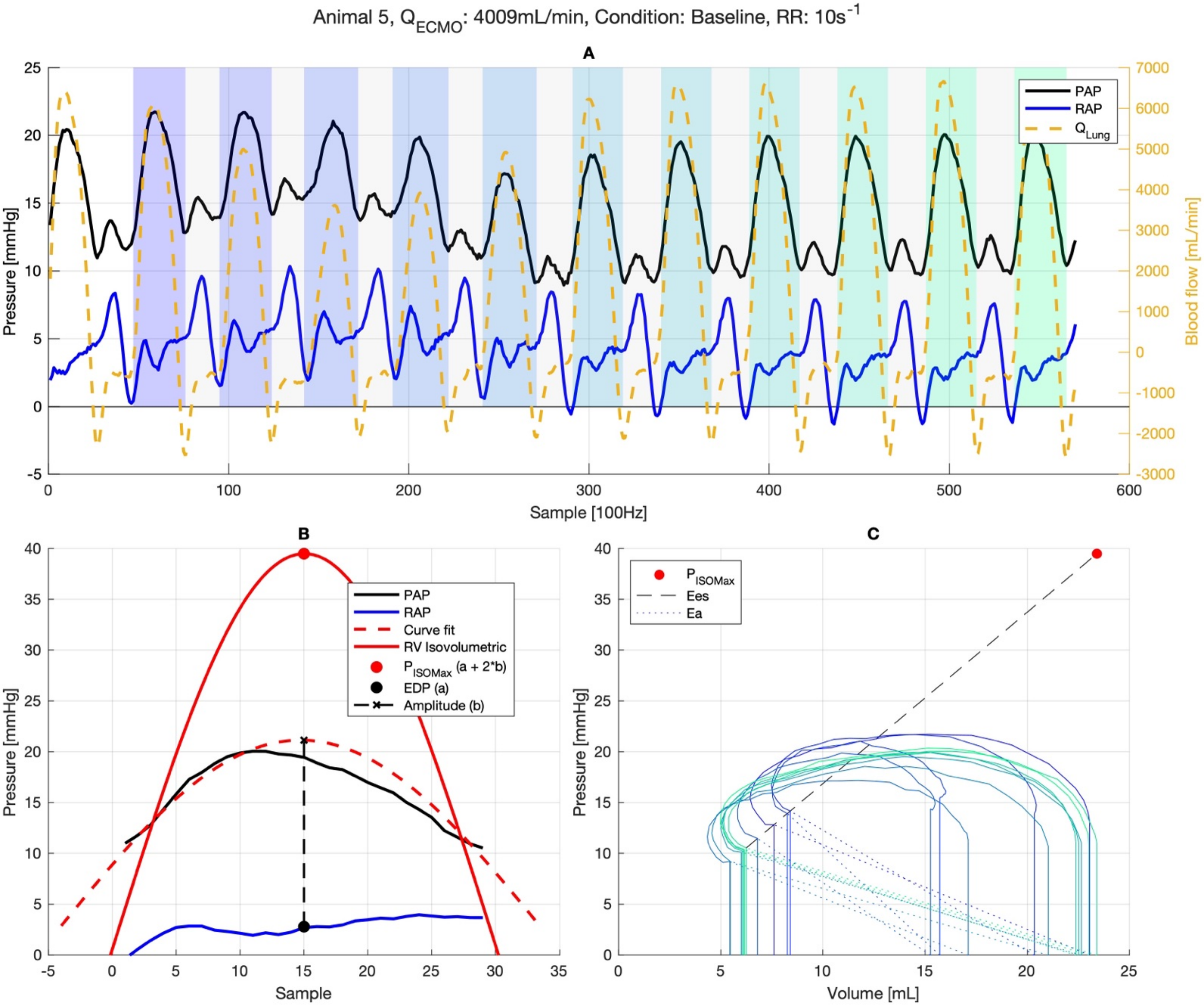

Animal 5, Q_ECMO: 4009mL/min, Condition: Baseline, RR: 10s^-1
A
PAP
RAP
Q_Lung
Pressure [mmHg]
Blood flow [mL/min]
Sample [100Hz]
B
PAP
RAP
Curve fit
RV Isovolumetric
P_ISOMax (a + 2*b)
EDP (a)
Amplitude (b)
Pressure [mmHg]
Sample
C
P_ISOMax
Ees
Ea
Pressure [mmHg]
Volume [mL]

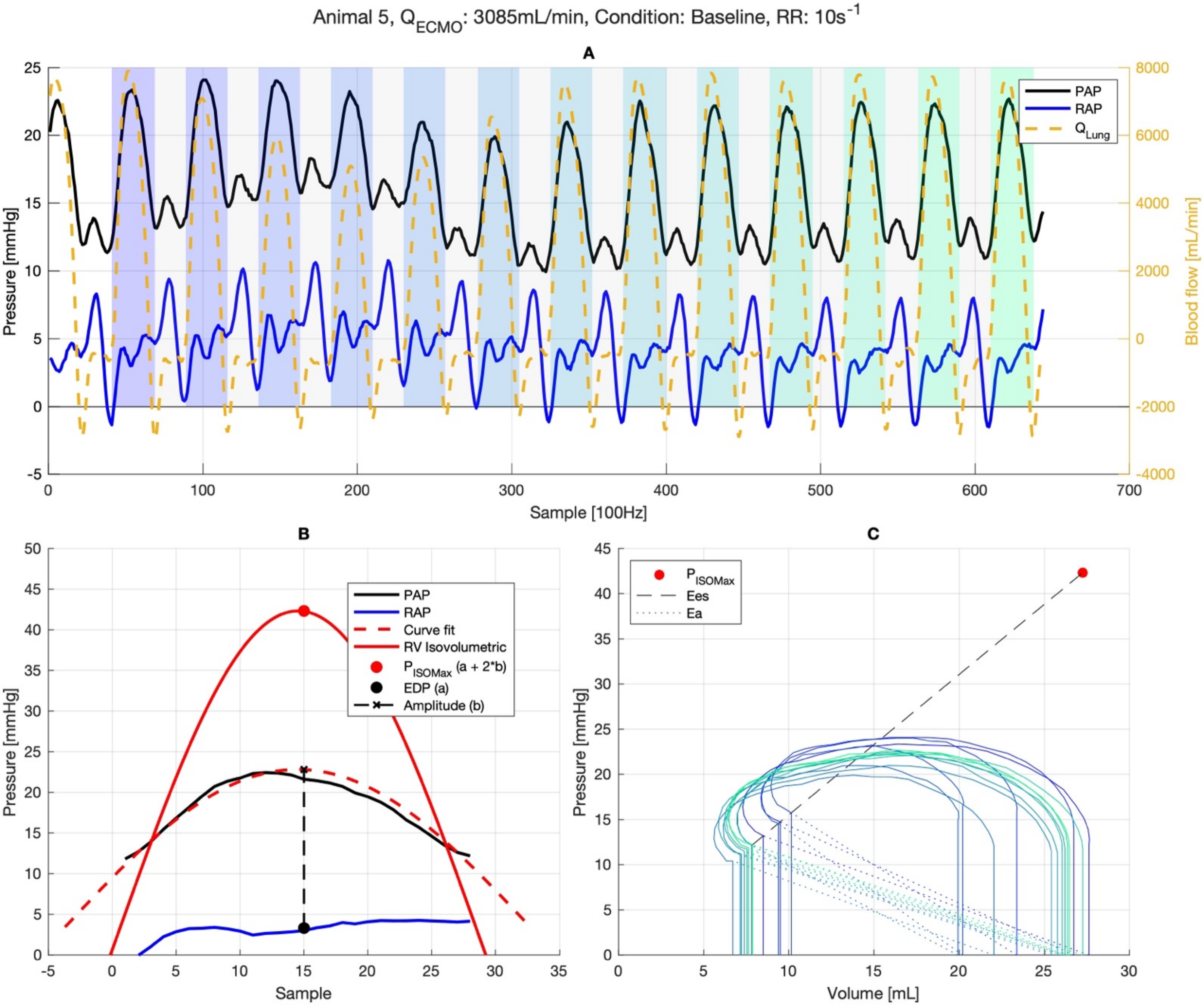

Animal 5, Q_{ECMO}: 3085mL/min, Condition: Baseline, RR: $10s^{-1}$
A
PAP
RAP
Q_{Lung}
Pressure [mmHg]
Blood flow [mL/min]
Sample [100Hz]
B
PAP
RAP
Curve fit
RV Isovolumetric
P_{ISOMax} (a + 2*b)
EDP (a)
Amplitude (b)
Pressure [mmHg]
Sample
C
P_{ISOMax}
Ees
Ea
Pressure [mmHg]
Volume [mL]

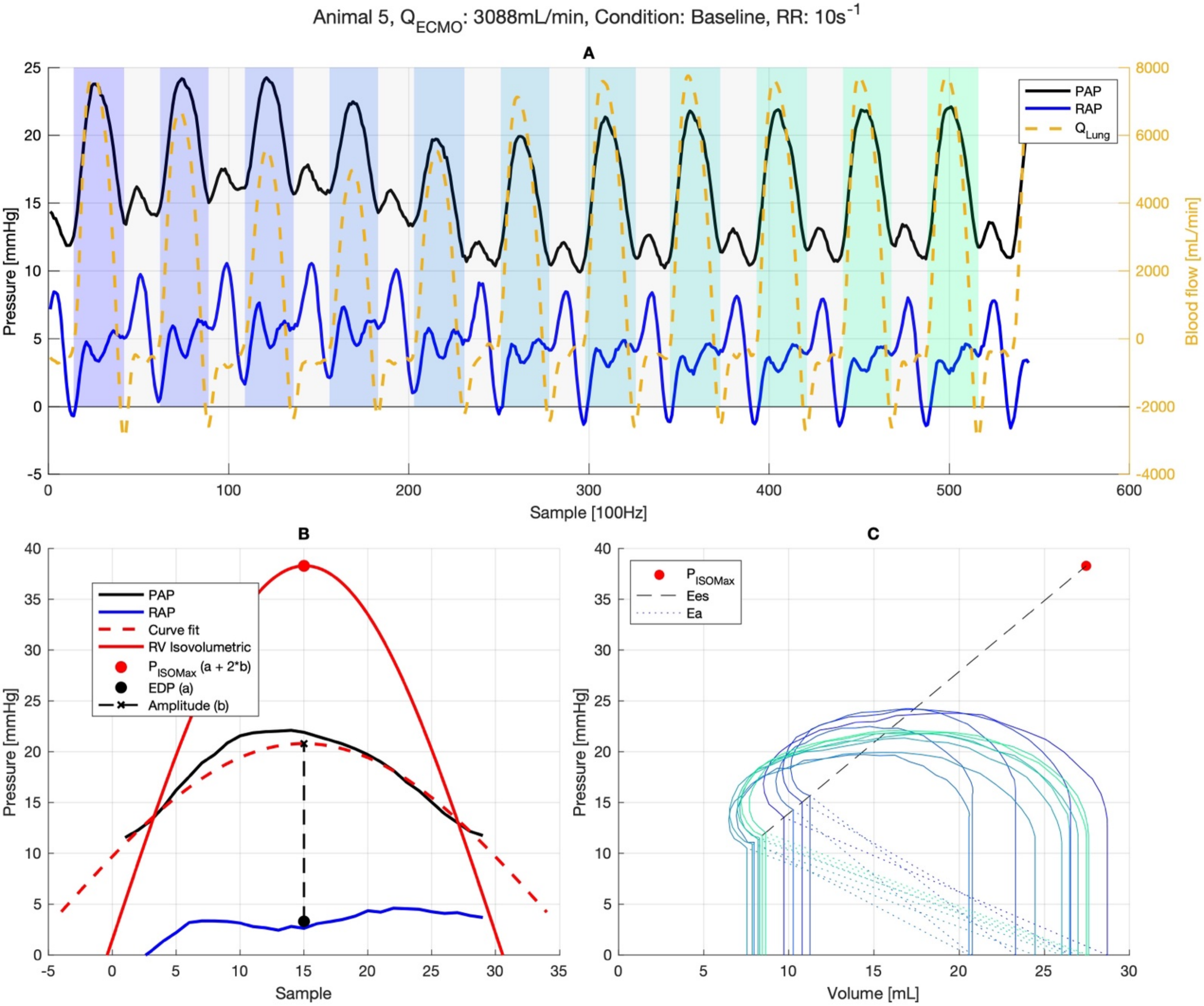

Animal 5, Q_{ECMO}: 3088mL/min, Condition: Baseline, RR: $10s^{-1}$
A
PAP
RAP
Q_{Lung}
Pressure [mmHg]
Blood flow [mL/min]
Sample [100Hz]
B
PAP
RAP
Curve fit
RV Isovolumetric
P_{ISOMax} (a + 2*b)
EDP (a)
Amplitude (b)
Pressure [mmHg]
Sample
C
P_{ISOMax}
Ees
Ea
Pressure [mmHg]
Volume [mL]

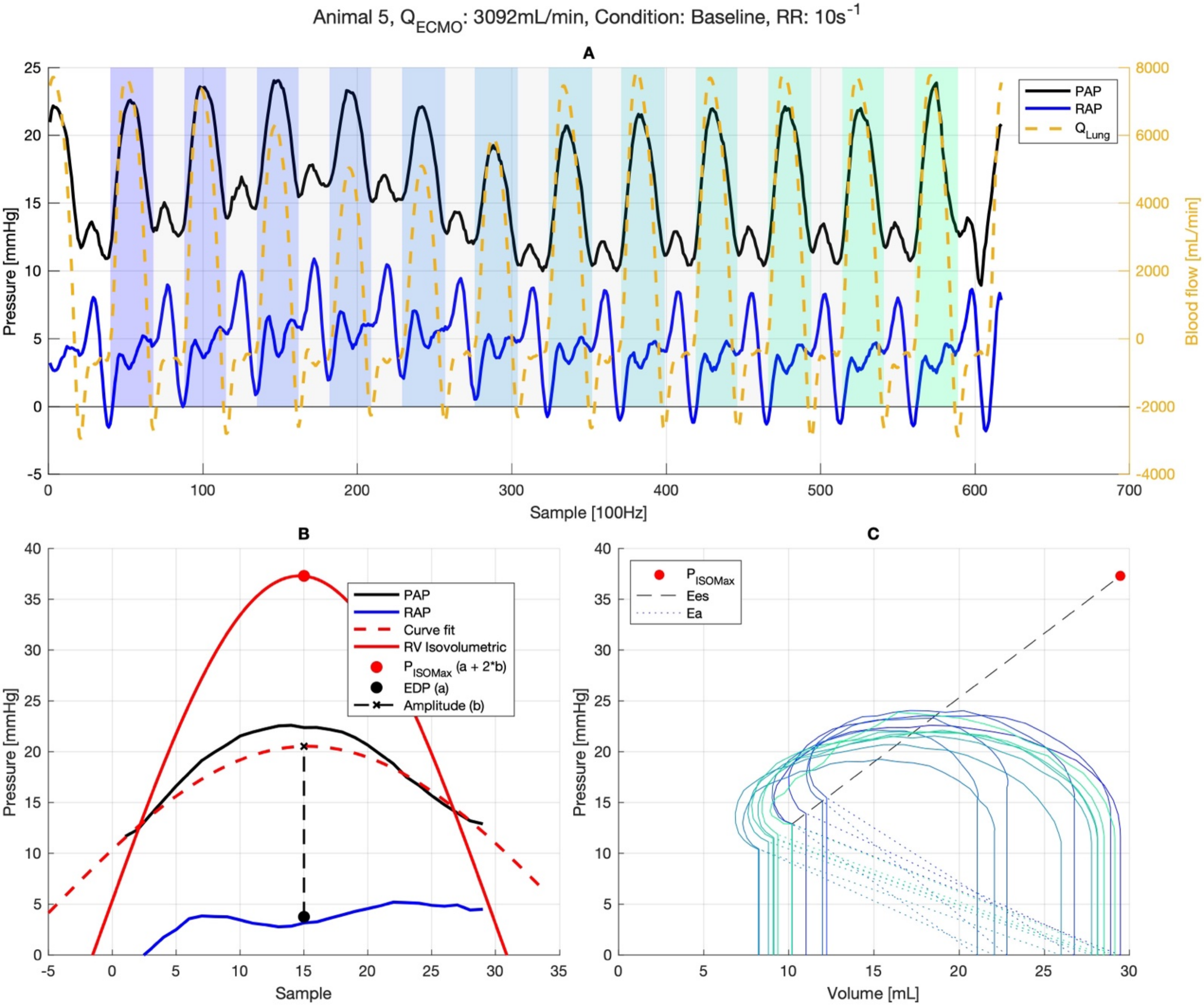

Animal 5, Q_{ECMO}: 3092mL/min, Condition: Baseline, RR: $10s^{-1}$
A
PAP
RAP
Q_{Lung}
Pressure [mmHg]
Blood flow [mL/min]
Sample [100Hz]
B
PAP
RAP
Curve fit
RV Isovolumetric
P_{ISOMax} (a + 2*b)
EDP (a)
Amplitude (b)
Pressure [mmHg]
Sample
C
P_{ISOMax}
Ees
Ea
Pressure [mmHg]
Volume [mL]

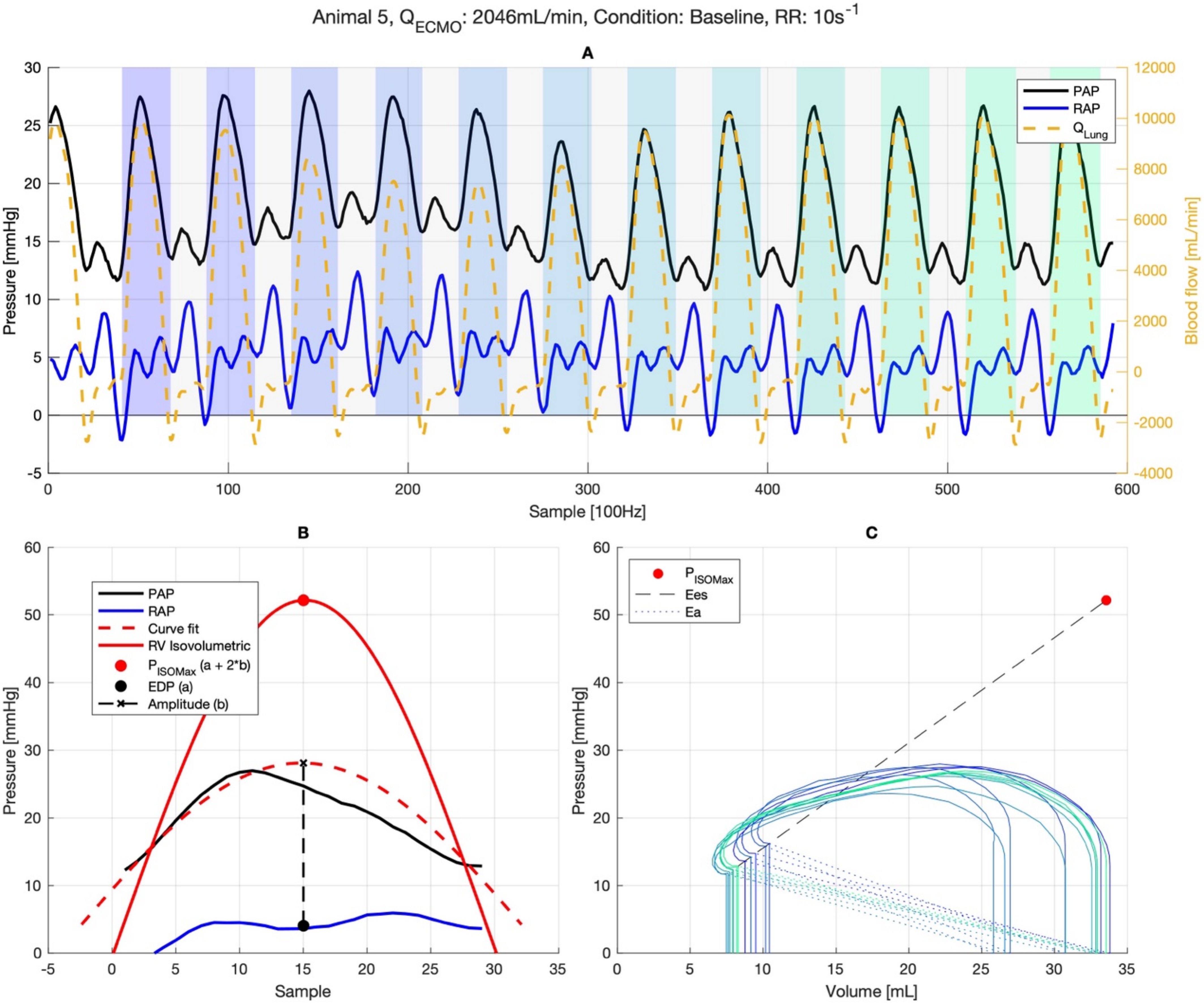
Animal 5, Q_ECMO: 2046mL/min, Condition: Baseline, RR: 10s^-1
A
PAP
RAP
Q_Lung
Pressure [mmHg]
Blood flow [mL/min]
Sample [100Hz]
B
PAP
RAP
Curve fit
RV Isovolumetric
P_ISOMax (a + 2*b)
EDP (a)
Amplitude (b)
Pressure [mmHg]
Sample
C
P_ISOMax
Ees
Ea
Pressure [mmHg]
Volume [mL]

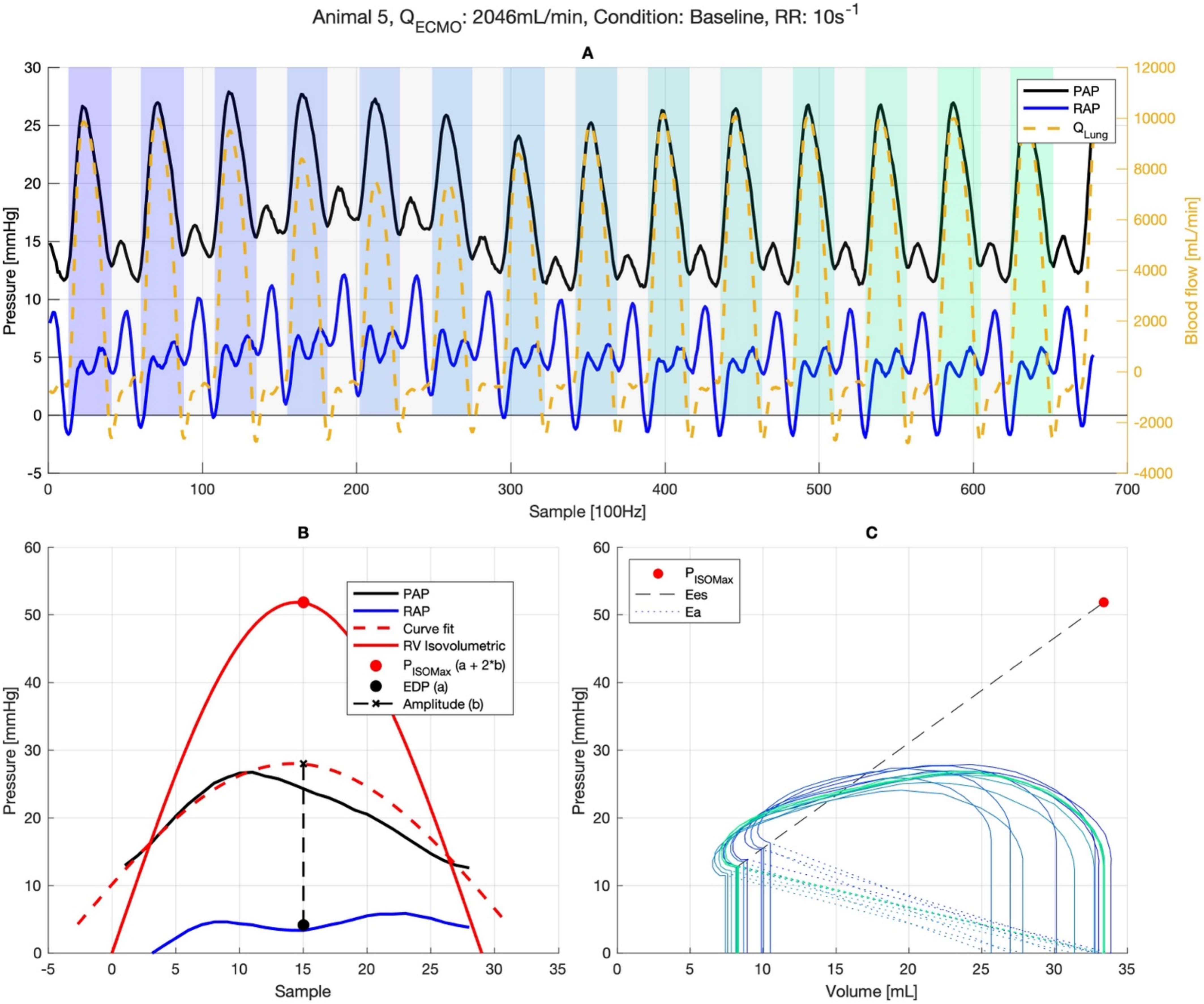

Animal 5, Q_{ECMO}: 2046mL/min, Condition: Baseline, RR: $10s^{-1}$
A
PAP
RAP
Q_{Lung}
Pressure [mmHg]
Blood flow [mL/min]
Sample [100Hz]
B
PAP
RAP
Curve fit
RV Isovolumetric
P_{ISOMax} (a + 2*b)
EDP (a)
Amplitude (b)
Pressure [mmHg]
Sample
C
P_{ISOMax}
Ees
Ea
Pressure [mmHg]
Volume [mL]

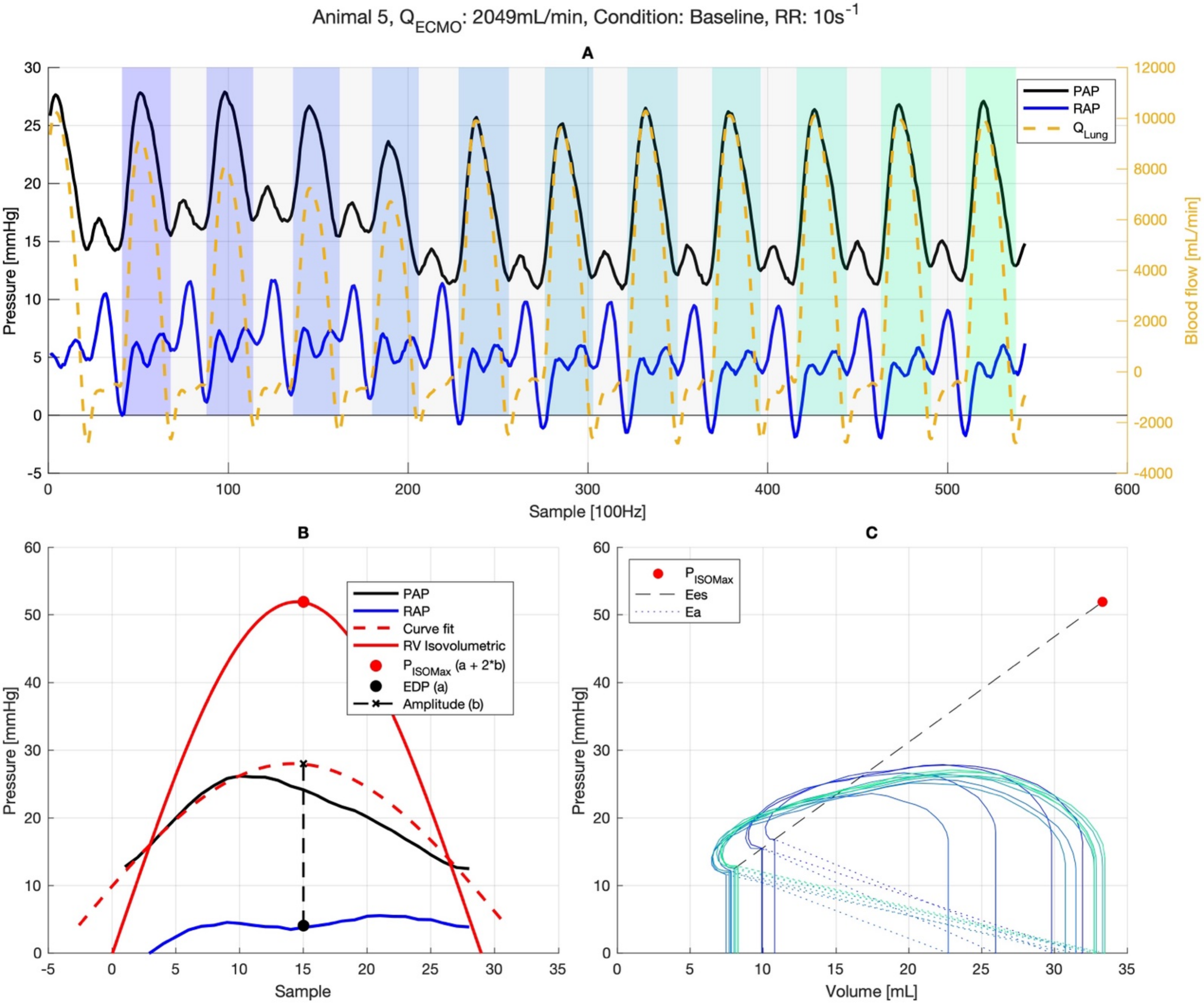
Animal 5, Q_ECMO: 2049mL/min, Condition: Baseline, RR: 10s^-1
A
PAP
RAP
Q_Lung
Pressure [mmHg]
Blood flow [mL/min]
Sample [100Hz]
B
PAP
RAP
Curve fit
RV Isovolumetric
P_ISOMax (a + 2*b)
EDP (a)
Amplitude (b)
Pressure [mmHg]
Sample
C
P_ISOMax
Ees
Ea
Pressure [mmHg]
Volume [mL]

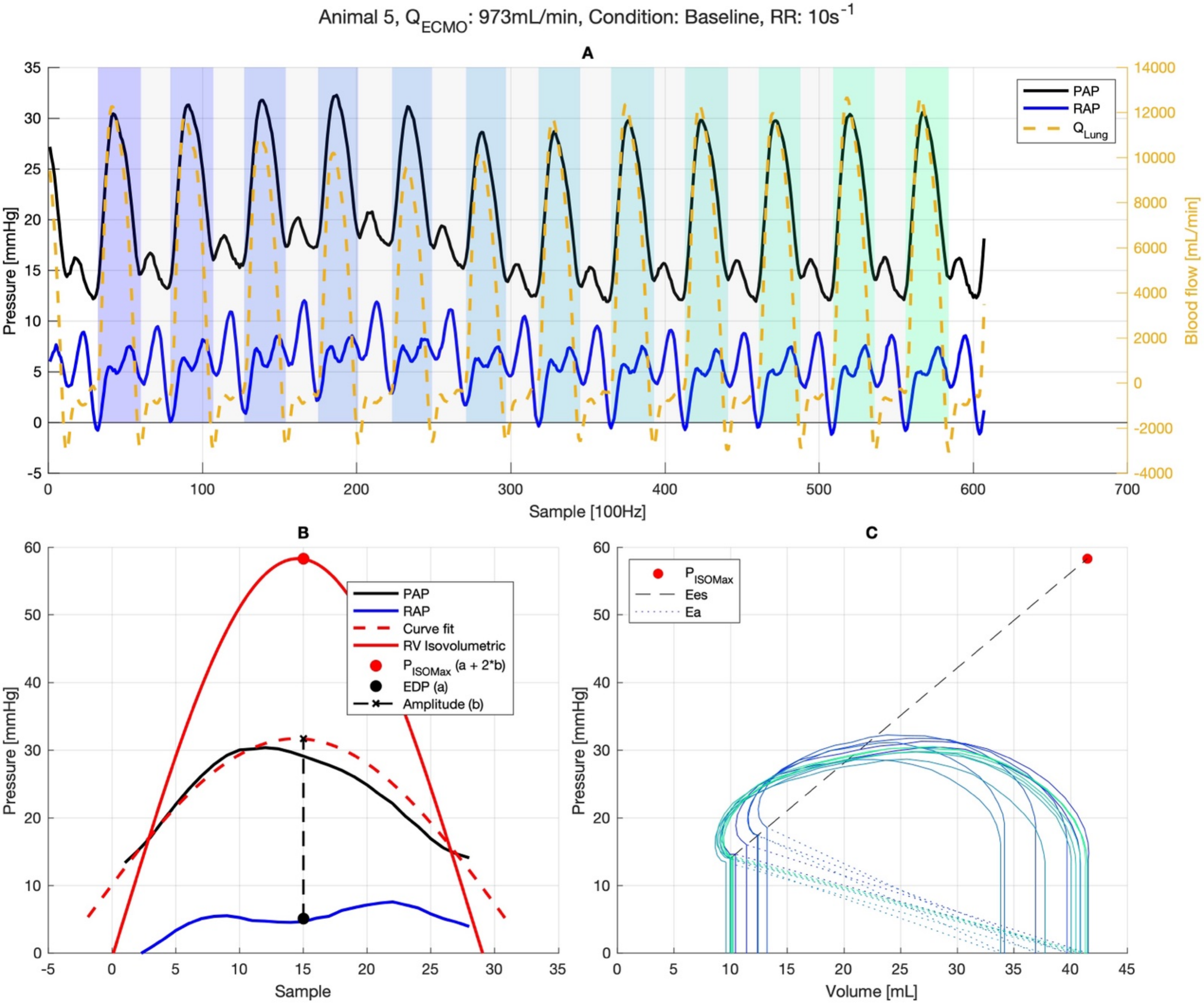

Animal 5, Q_{ECMO}: 973mL/min, Condition: Baseline, RR: $10s^{-1}$
A
PAP
RAP
Q_{Lung}
Pressure [mmHg]
Blood flow [mL/min]
Sample [100Hz]
B
PAP
RAP
Curve fit
RV Isovolumetric
P_{ISOMax} (a + 2*b)
EDP (a)
Amplitude (b)
Pressure [mmHg]
Sample
C
P_{ISOMax}
Ees
Ea
Pressure [mmHg]
Volume [mL]

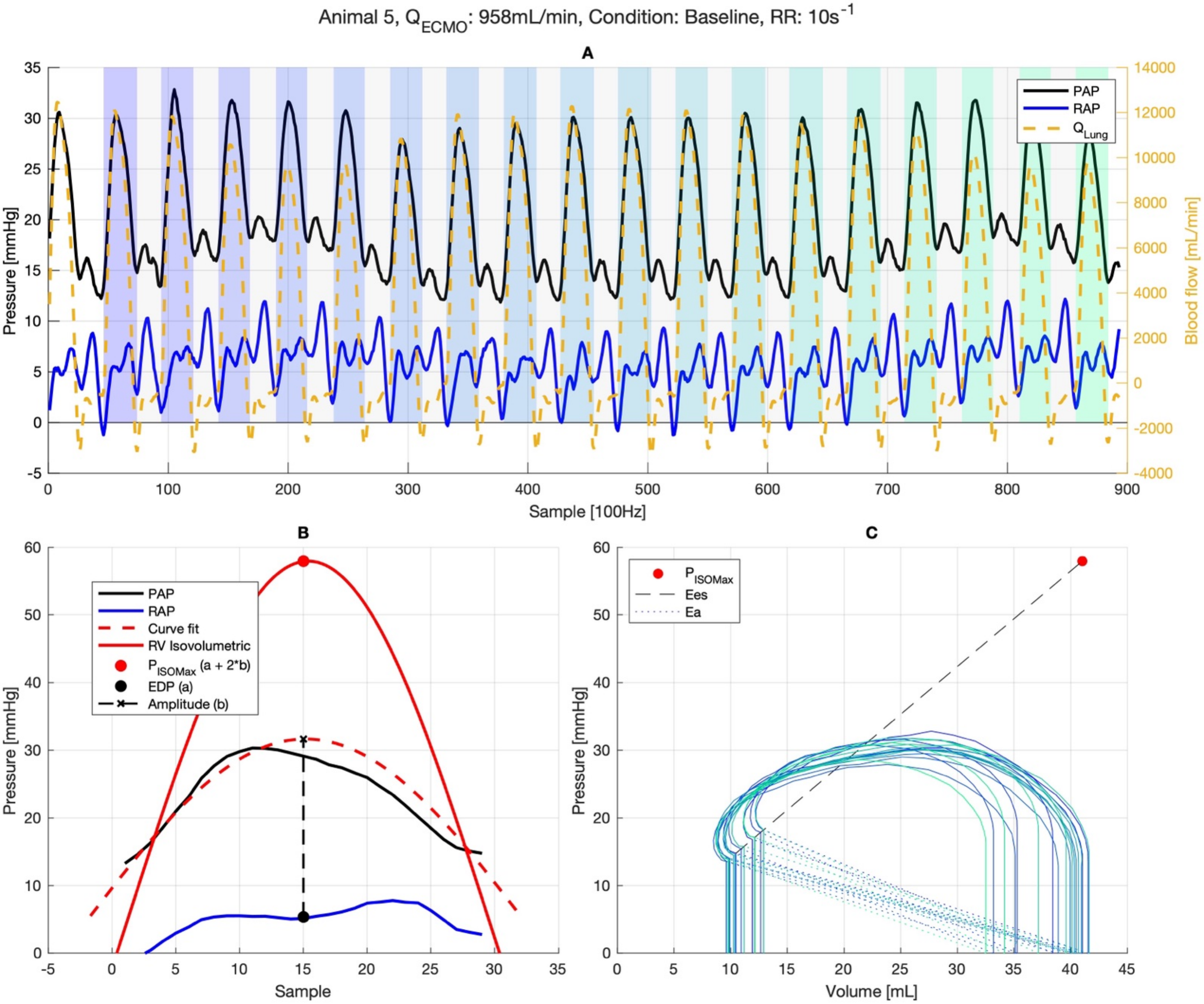
Animal 5, QECMO: 958mL/min, Condition: Baseline, RR: 10s-1
A
PAP
RAP
QLung
Pressure [mmHg]
Blood flow [mL/min]
Sample [100Hz]
B
PAP
RAP
Curve fit
RV Isovolumetric
PISOMax (a + 2*b)
EDP (a)
Amplitude (b)
Pressure [mmHg]
Sample
C
PISOMax
Ees
Ea
Pressure [mmHg]
Volume [mL]

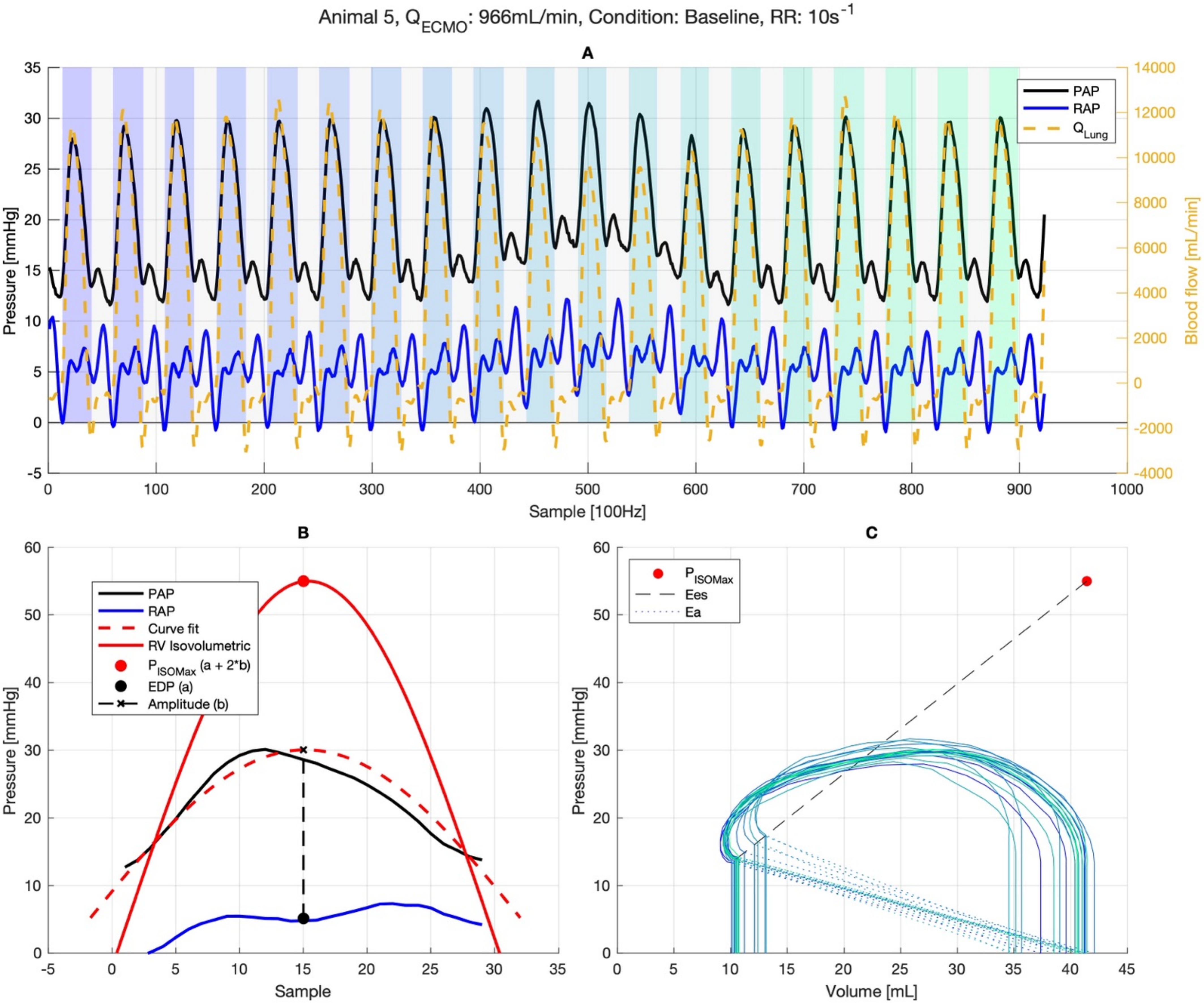

Animal 5, Q_{ECMO}: 966mL/min, Condition: Baseline, RR: $10s^{-1}$
A
PAP
RAP
Q_{Lung}
Pressure [mmHg]
Blood flow [mL/min]
Sample [100Hz]
B
PAP
RAP
Curve fit
RV Isovolumetric
P_{ISOMax} (a + 2*b)
EDP (a)
Amplitude (b)
Pressure [mmHg]
Sample
C
P_{ISOMax}
Ees
Ea
Pressure [mmHg]
Volume [mL]

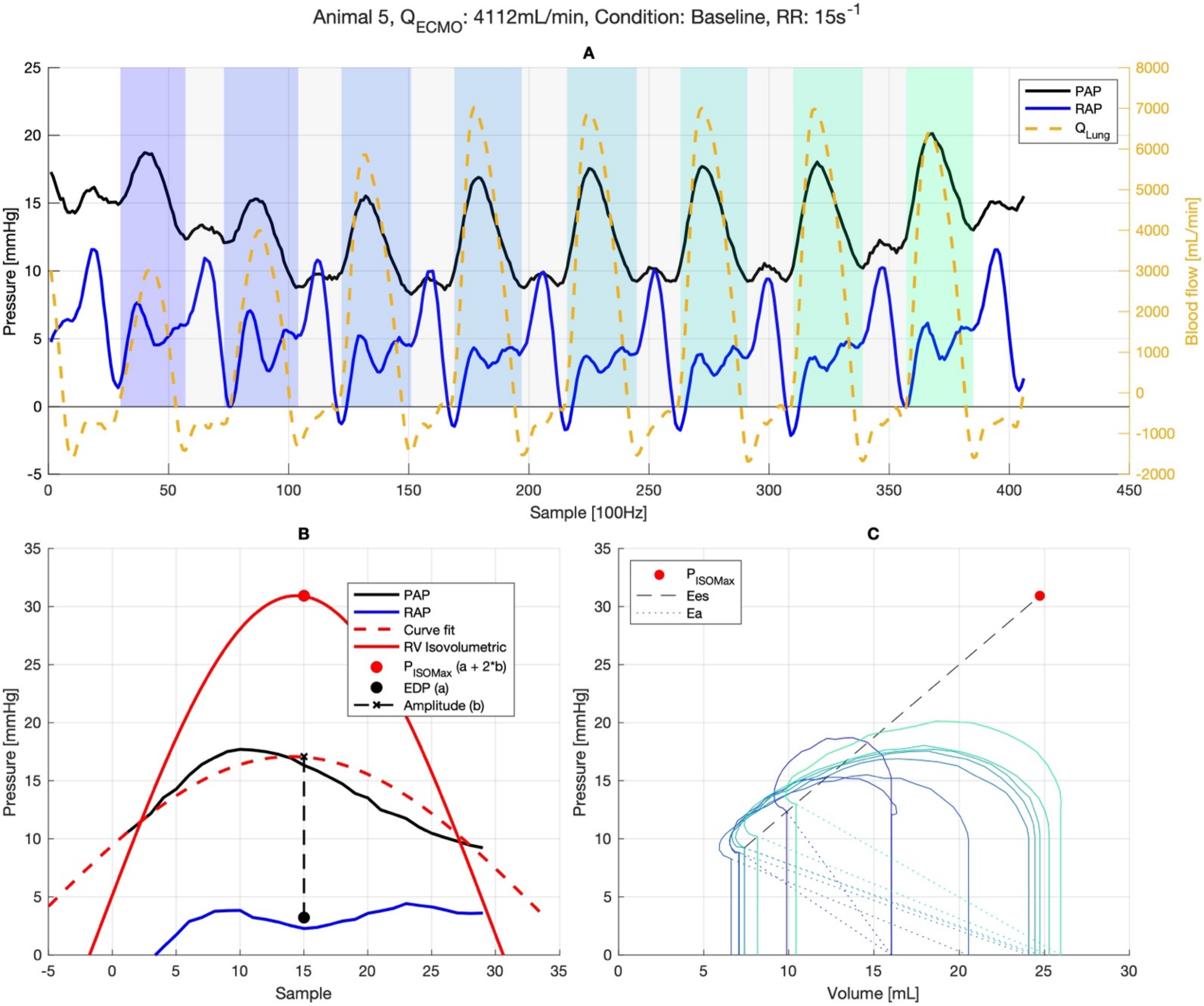

Animal 5, Q_ECMO: 4112mL/min, Condition: Baseline, RR: 15s^-1
A
PAP
RAP
Q_Lung
Pressure [mmHg]
Blood flow [mL/min]
Sample [100Hz]
B
PAP
RAP
Curve fit
RV Isovolumetric
P_ISOMax (a + 2*b)
EDP (a)
Amplitude (b)
Pressure [mmHg]
Sample
C
P_ISOMax
Ees
Ea
Pressure [mmHg]
Volume [mL]

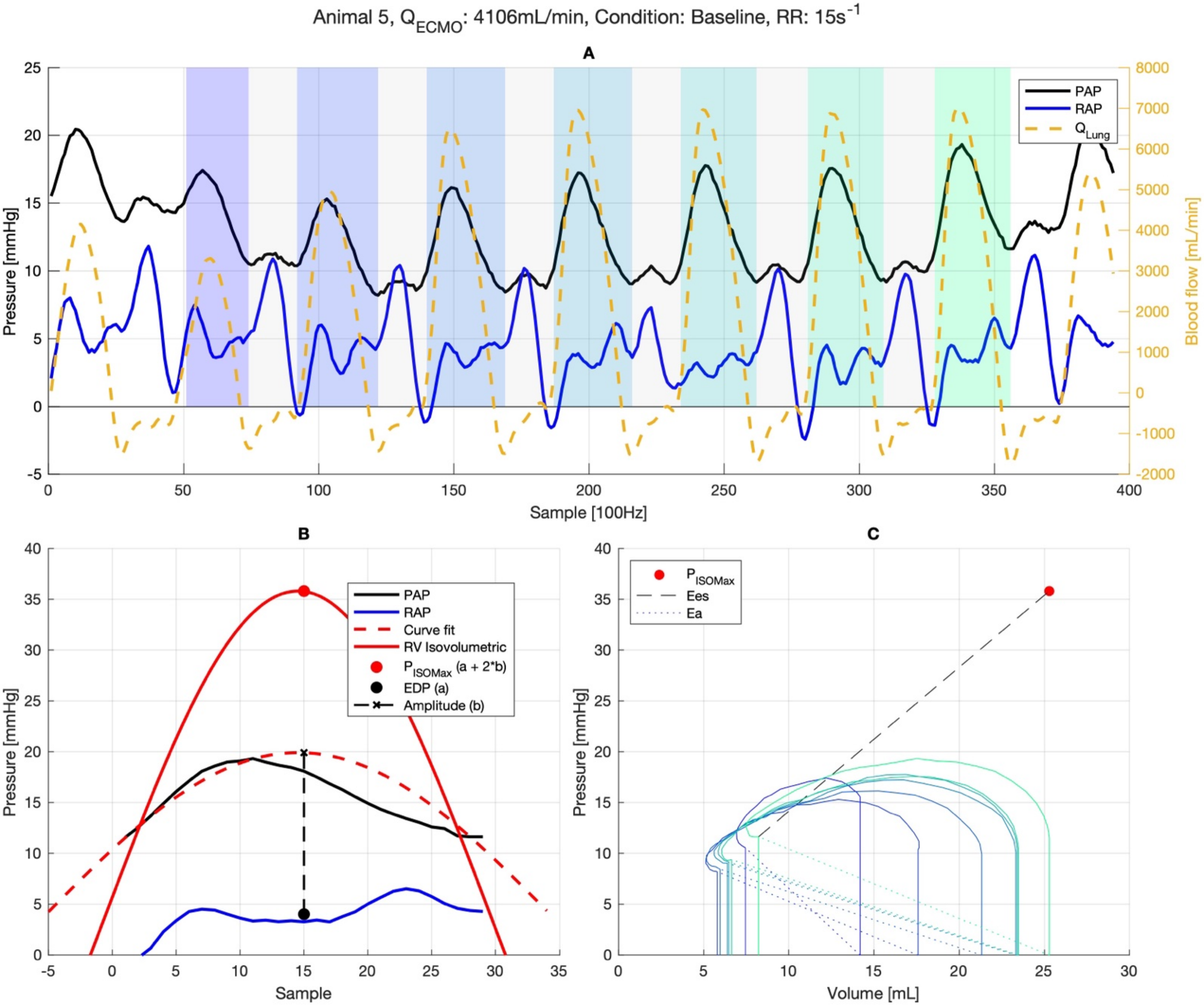

Animal 5, Q_ECMO: 4106mL/min, Condition: Baseline, RR: 15s^-1
A
PAP
RAP
Q_Lung
Pressure [mmHg]
Blood flow [mL/min]
Sample [100Hz]
B
PAP
RAP
Curve fit
RV Isovolumetric
P_ISOMax (a + 2*b)
EDP (a)
Amplitude (b)
Pressure [mmHg]
Sample
C
P_ISOMax
Ees
Ea
Pressure [mmHg]
Volume [mL]

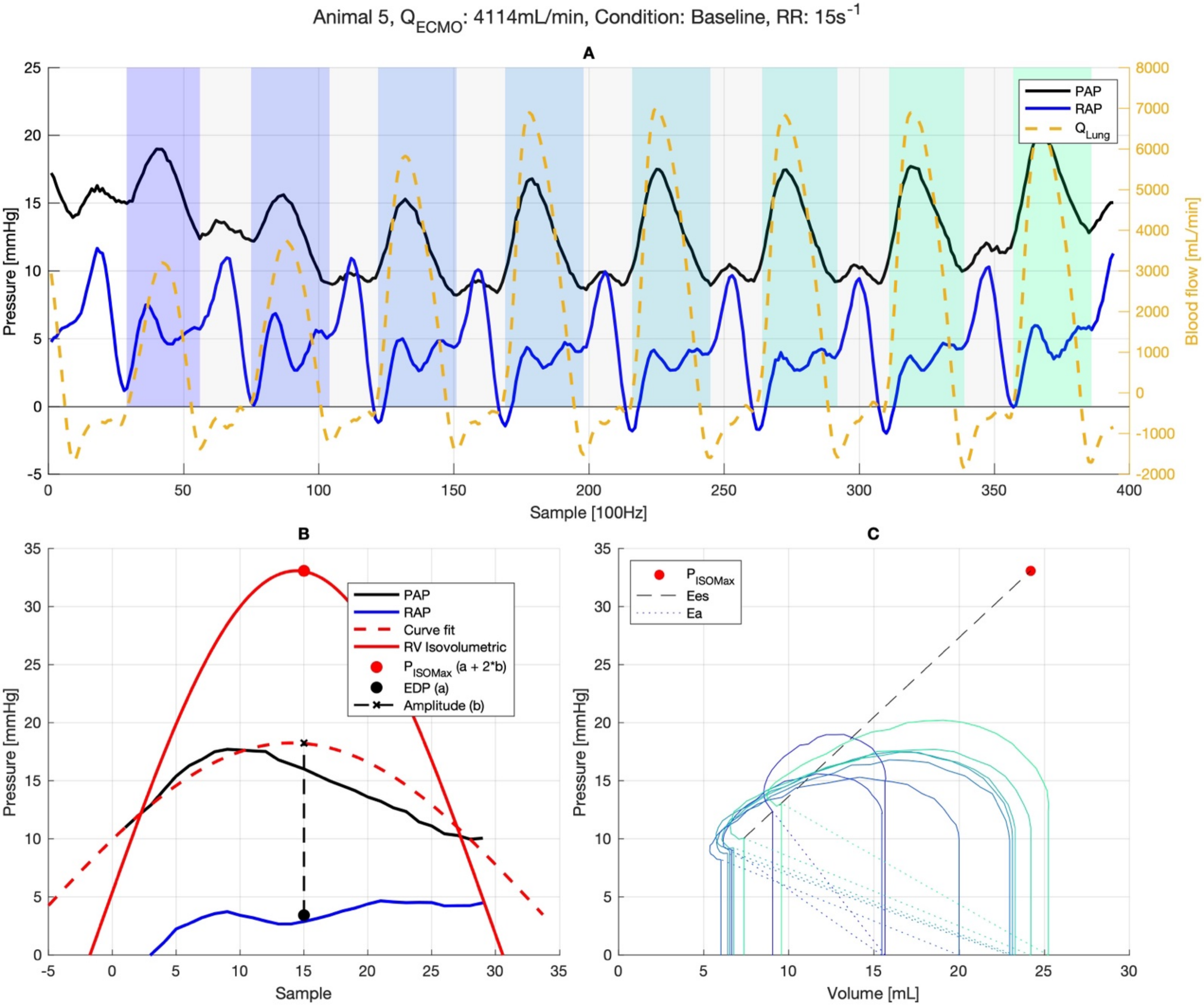

Animal 5, Q_{ECMO}: 4114mL/min, Condition: Baseline, RR: $15s^{-1}$
A
PAP
RAP
Q_{Lung}
Pressure [mmHg]
Blood flow [mL/min]
Sample [100Hz]
B
PAP
RAP
Curve fit
RV Isovolumetric
P_{ISOMax} (a + 2*b)
EDP (a)
Amplitude (b)
Pressure [mmHg]
Sample
C
P_{ISOMax}
Ees
Ea
Pressure [mmHg]
Volume [mL]

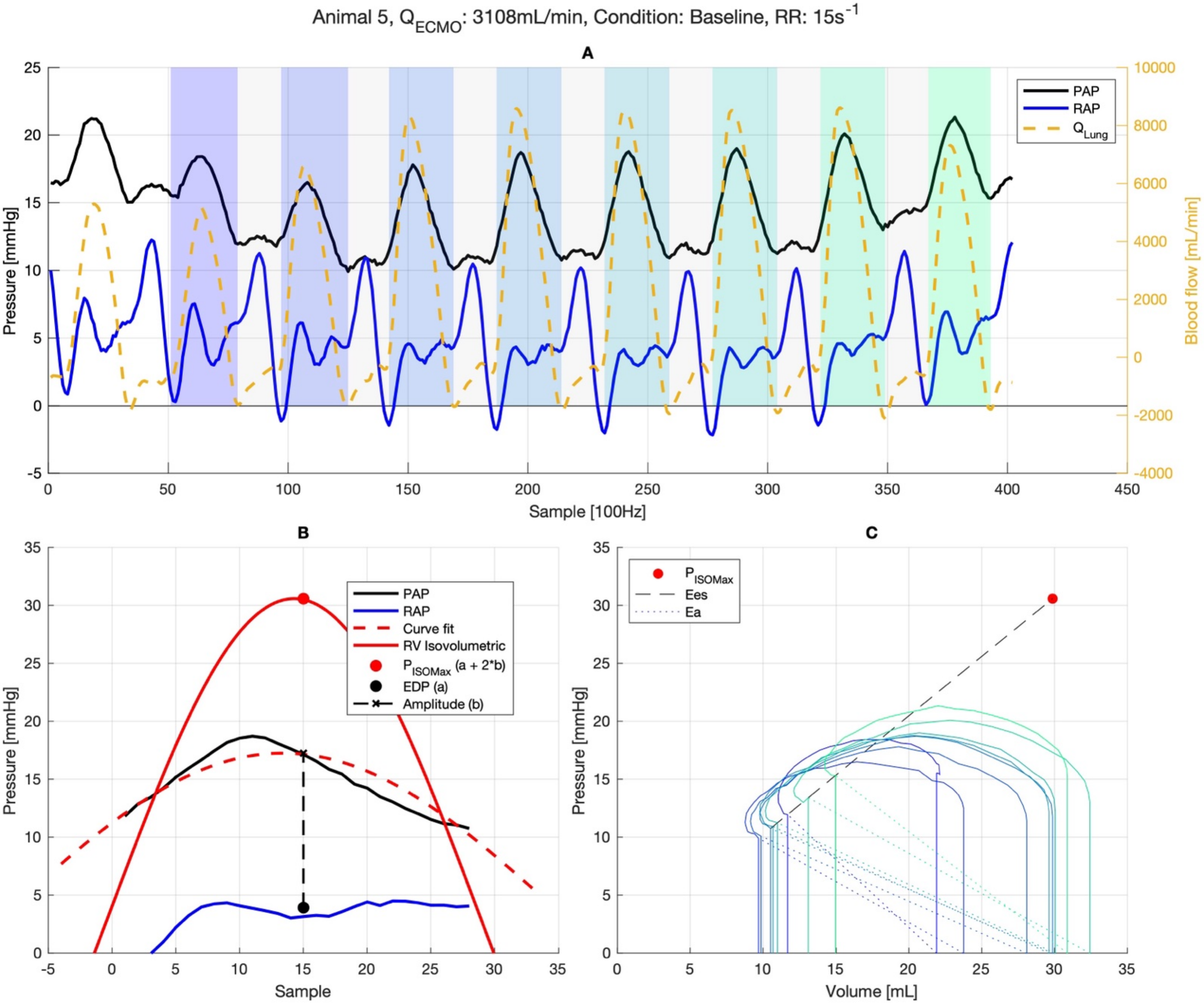

Animal 5, Q_{ECMO}: 3108mL/min, Condition: Baseline, RR: $15s^{-1}$
A
PAP
RAP
Q_{Lung}
Pressure [mmHg]
Blood flow [mL/min]
Sample [100Hz]
B
PAP
RAP
Curve fit
RV Isovolumetric
P_{ISOMax} (a + 2*b)
EDP (a)
Amplitude (b)
Pressure [mmHg]
Sample
C
P_{ISOMax}
Ees
Ea
Pressure [mmHg]
Volume [mL]

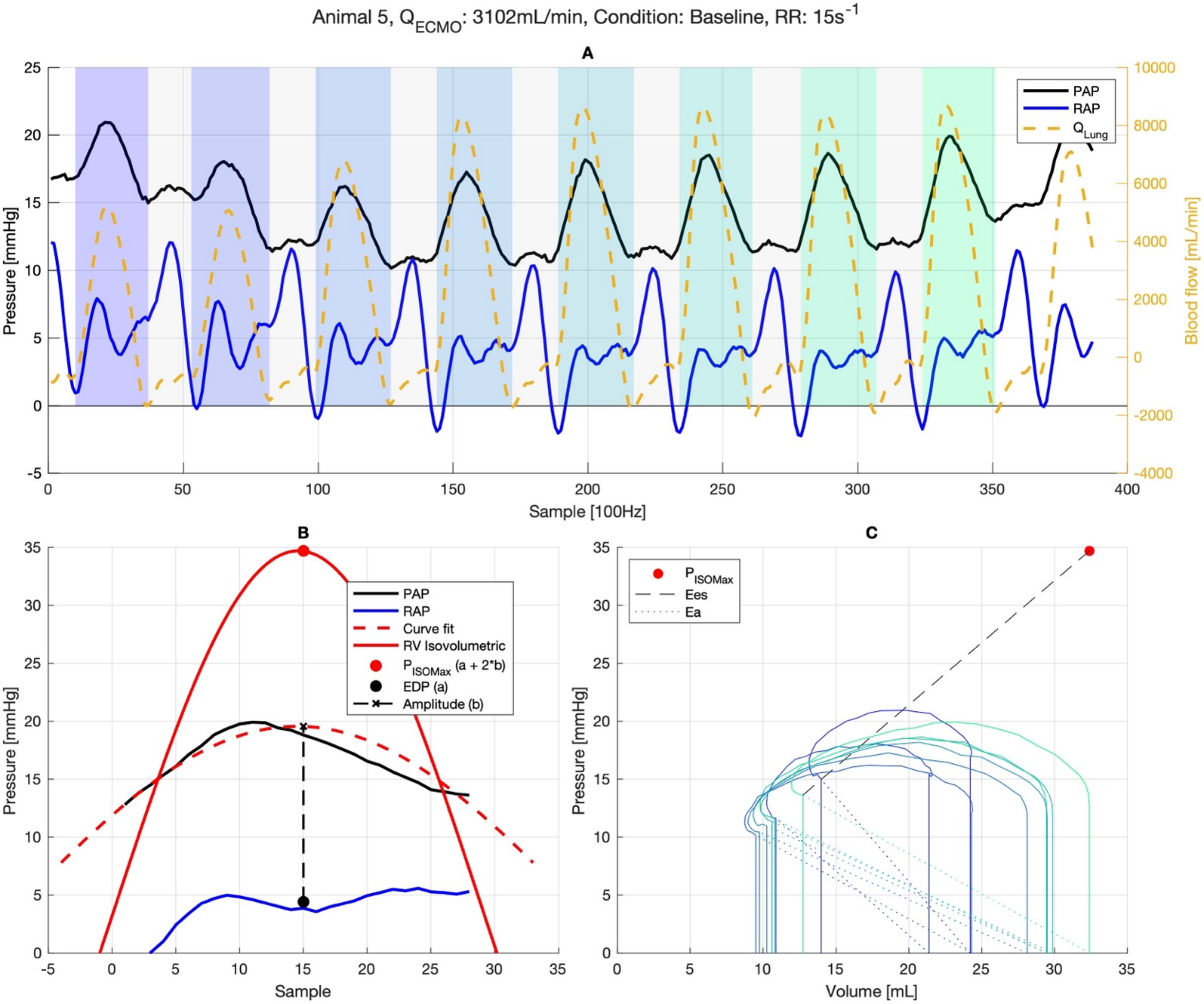

Animal 5, Q_{ECMO}: 3102mL/min, Condition: Baseline, RR: $15s^{-1}$
A
PAP
RAP
Q_{Lung}
Pressure [mmHg]
Blood flow [mL/min]
Sample [100Hz]
B
PAP
RAP
Curve fit
RV Isovolumetric
P_{ISOMax} (a + 2*b)
EDP (a)
Amplitude (b)
Pressure [mmHg]
Sample
C
P_{ISOMax}
Ees
Ea
Pressure [mmHg]
Volume [mL]

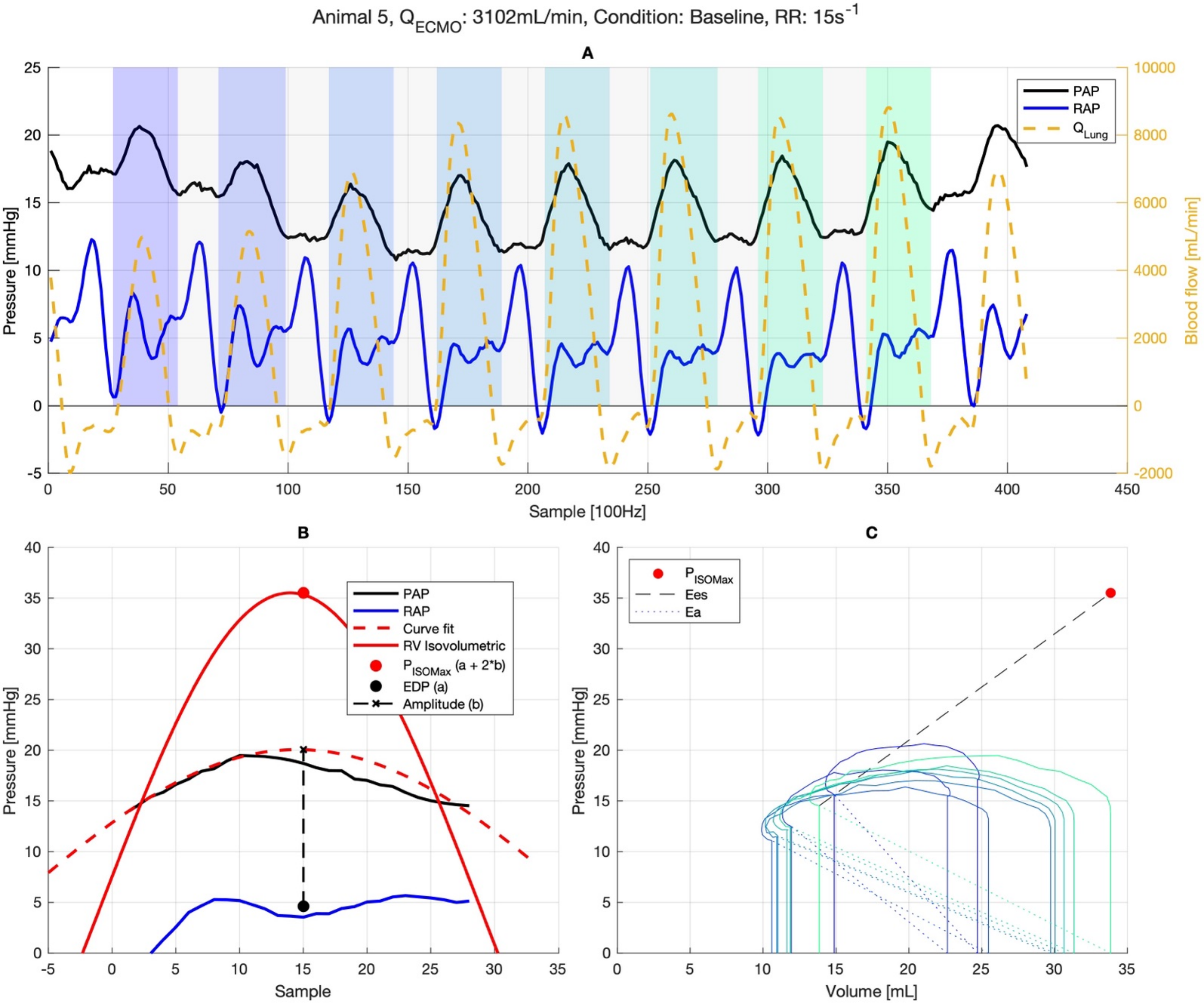
Animal 5, Q_ECMO: 3102mL/min, Condition: Baseline, RR: 15s^-1
A
PAP
RAP
Q_Lung
Pressure [mmHg]
Blood flow [mL/min]
Sample [100Hz]
B
PAP
RAP
Curve fit
RV Isovolumetric
P_ISOMax (a + 2*b)
EDP (a)
Amplitude (b)
Pressure [mmHg]
Sample
C
P_ISOMax
Ees
Ea
Pressure [mmHg]
Volume [mL]

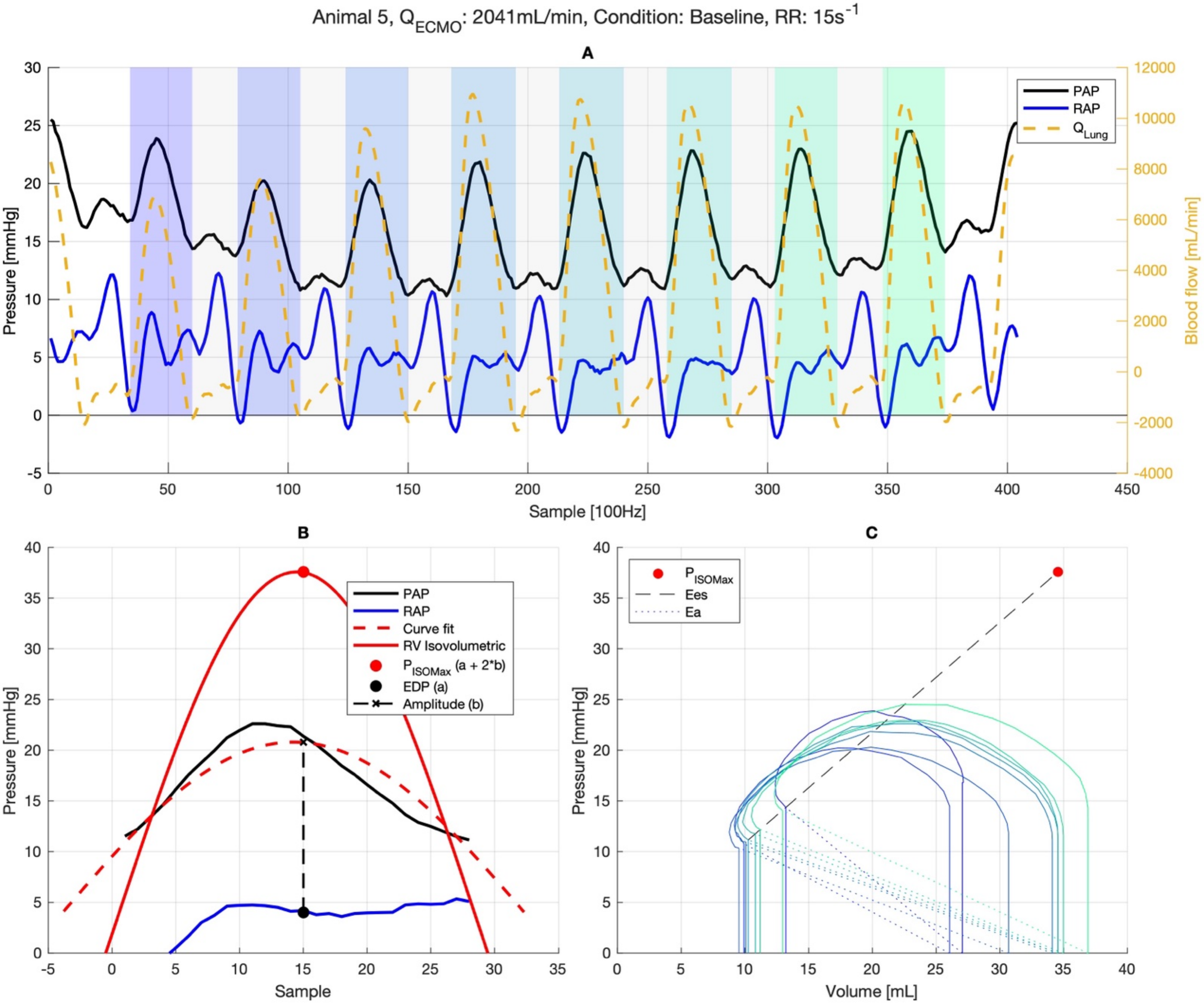

Animal 5, Q_{ECMO}: 2041mL/min, Condition: Baseline, RR: $15s^{-1}$
A
PAP
RAP
Q_{Lung}
Pressure [mmHg]
Blood flow [mL/min]
Sample [100Hz]
B
PAP
RAP
Curve fit
RV Isovolumetric
P_{ISOMax} (a + 2*b)
EDP (a)
Amplitude (b)
Pressure [mmHg]
Sample
C
P_{ISOMax}
Ees
Ea
Pressure [mmHg]
Volume [mL]

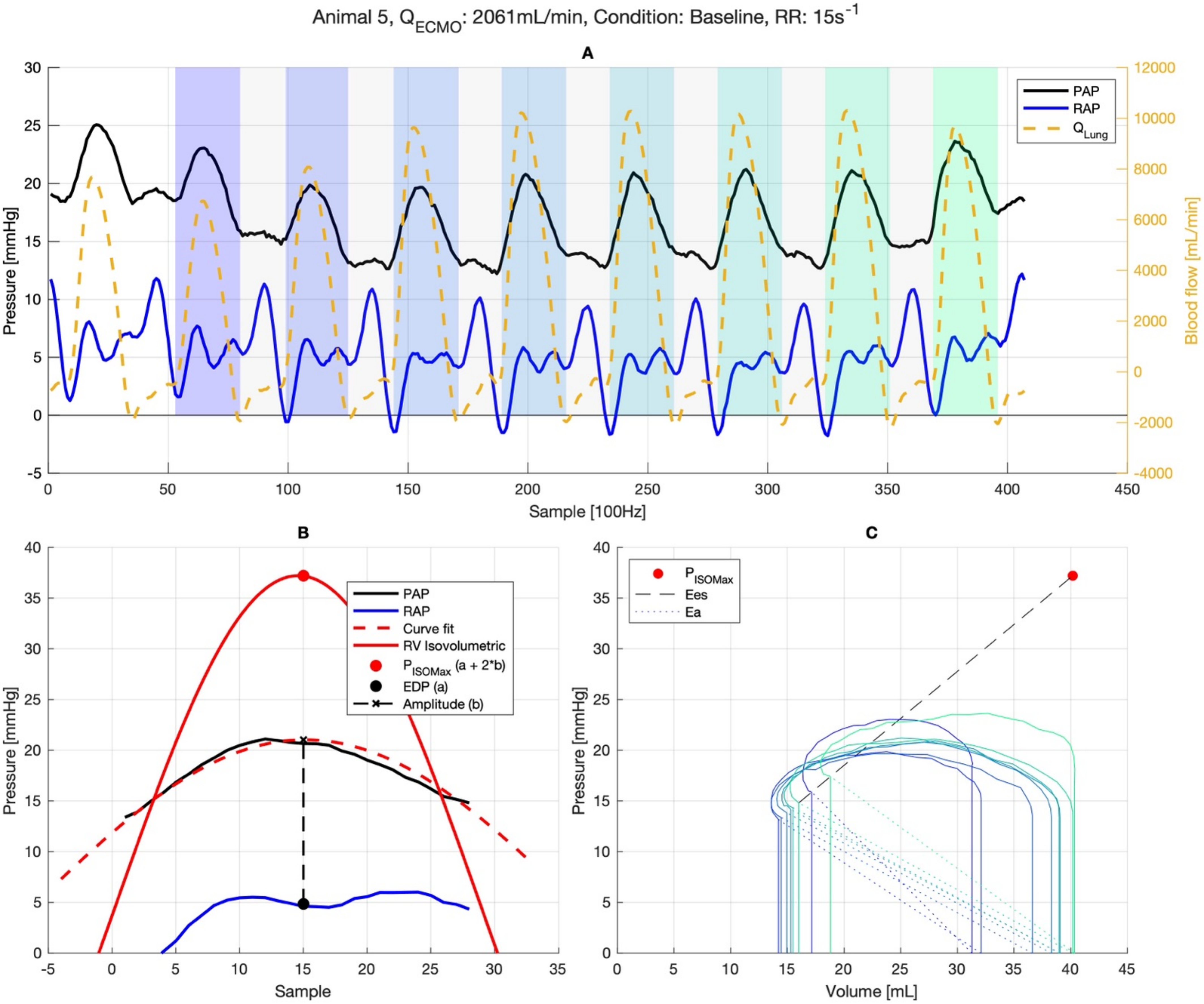

Animal 5, Q_ECMO: 2061mL/min, Condition: Baseline, RR: 15s^-1
A
PAP
RAP
Q_Lung
Pressure [mmHg]
Blood flow [mL/min]
Sample [100Hz]
B
PAP
RAP
Curve fit
RV Isovolumetric
P_ISOMax (a + 2*b)
EDP (a)
Amplitude (b)
Pressure [mmHg]
Sample
C
P_ISOMax
Ees
Ea
Pressure [mmHg]
Volume [mL]

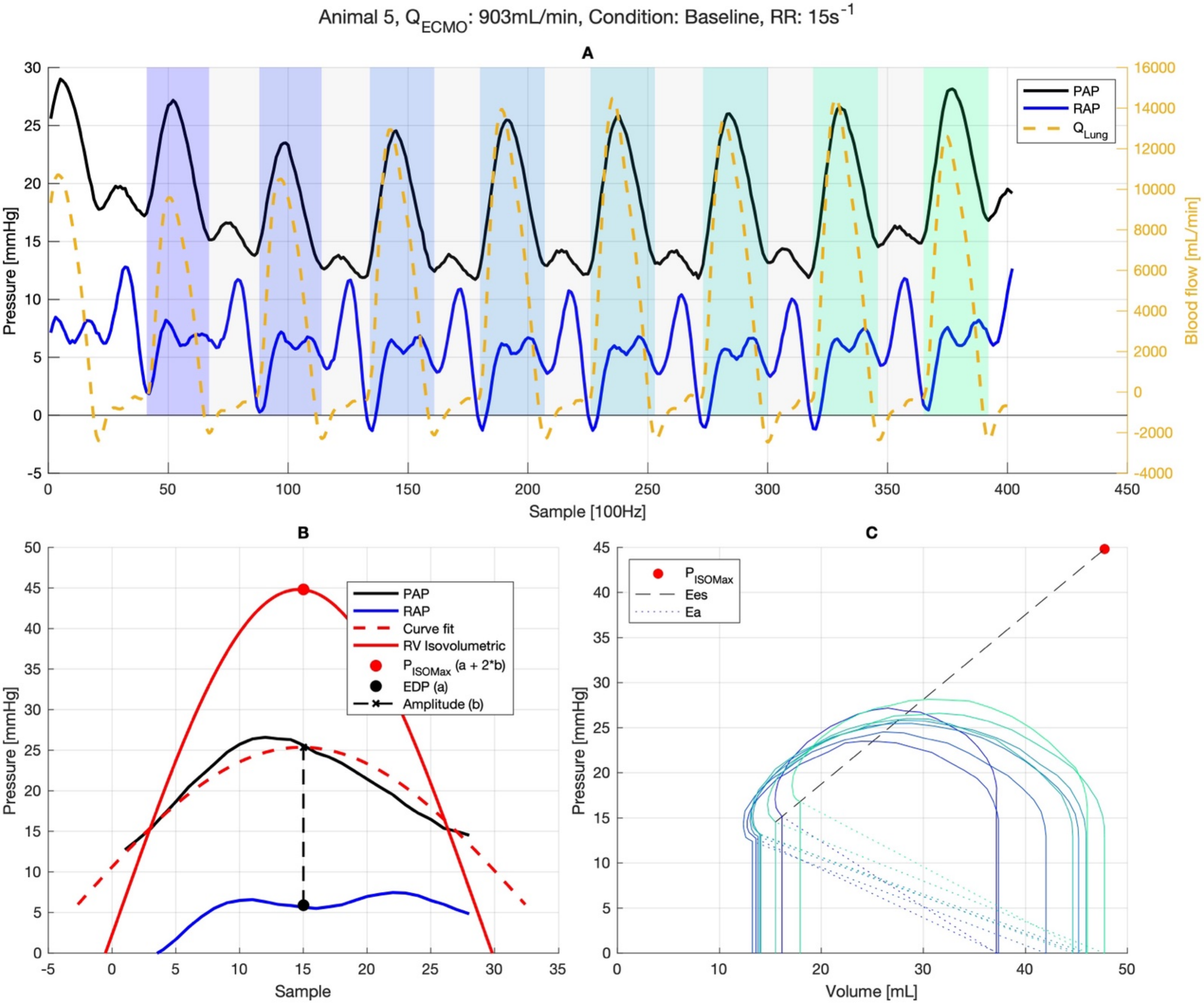

Animal 5, Q_ECMO: 903mL/min, Condition: Baseline, RR: 15s^-1
A
PAP
RAP
Q_Lung
Pressure [mmHg]
Blood flow [mL/min]
Sample [100Hz]
B
PAP
RAP
Curve fit
RV Isovolumetric
P_ISOMax (a + 2*b)
EDP (a)
Amplitude (b)
Pressure [mmHg]
Sample
C
P_ISOMax
Ees
Ea
Pressure [mmHg]
Volume [mL]

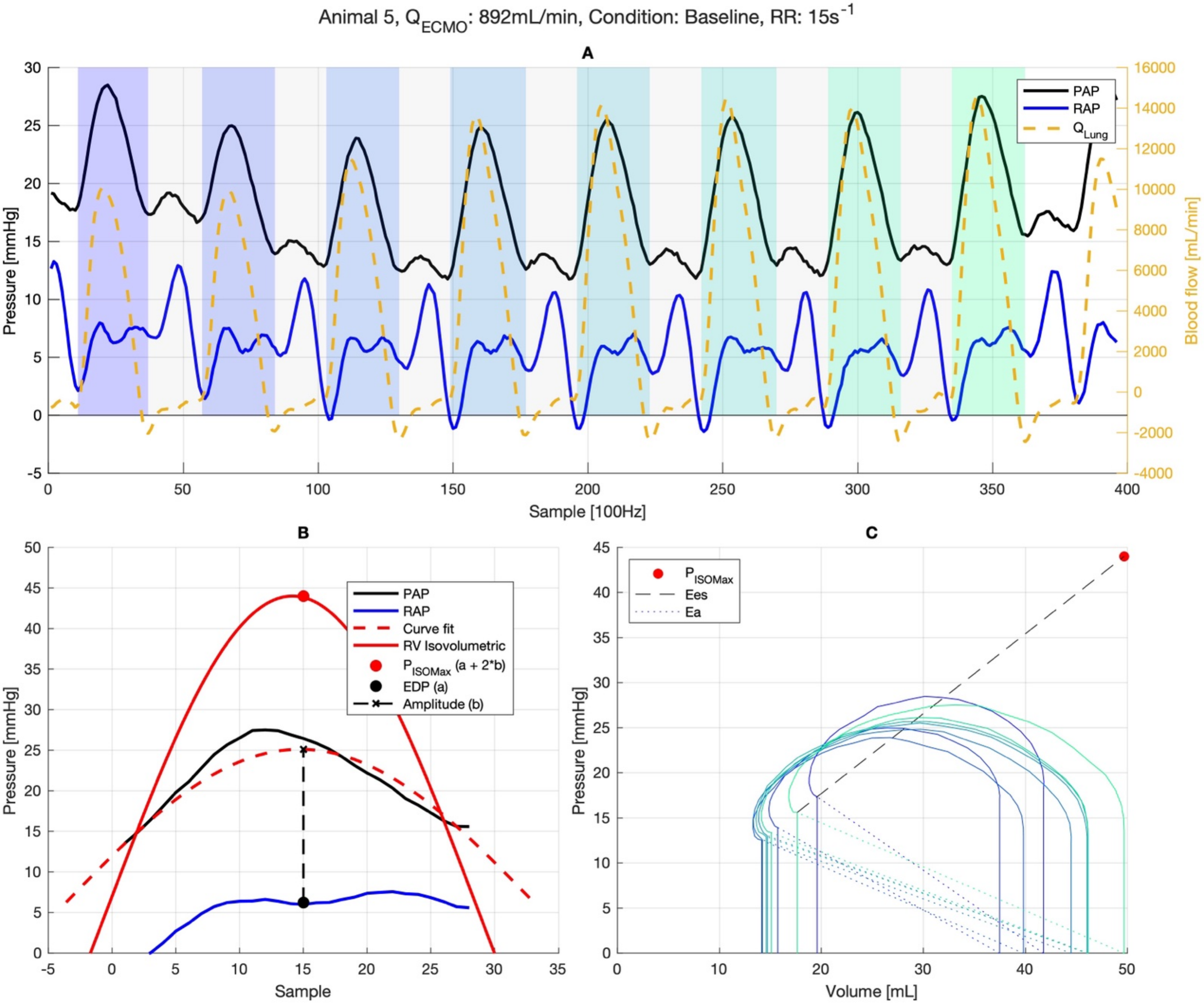

Animal 5, Q_ECMO: 892mL/min, Condition: Baseline, RR: 15s^-1
A
PAP
RAP
Q_Lung
Pressure [mmHg]
Blood flow [mL/min]
Sample [100Hz]
B
PAP
RAP
Curve fit
RV Isovolumetric
P_ISOMax (a + 2*b)
EDP (a)
Amplitude (b)
Pressure [mmHg]
Sample
C
P_ISOMax
Ees
Ea
Pressure [mmHg]
Volume [mL]

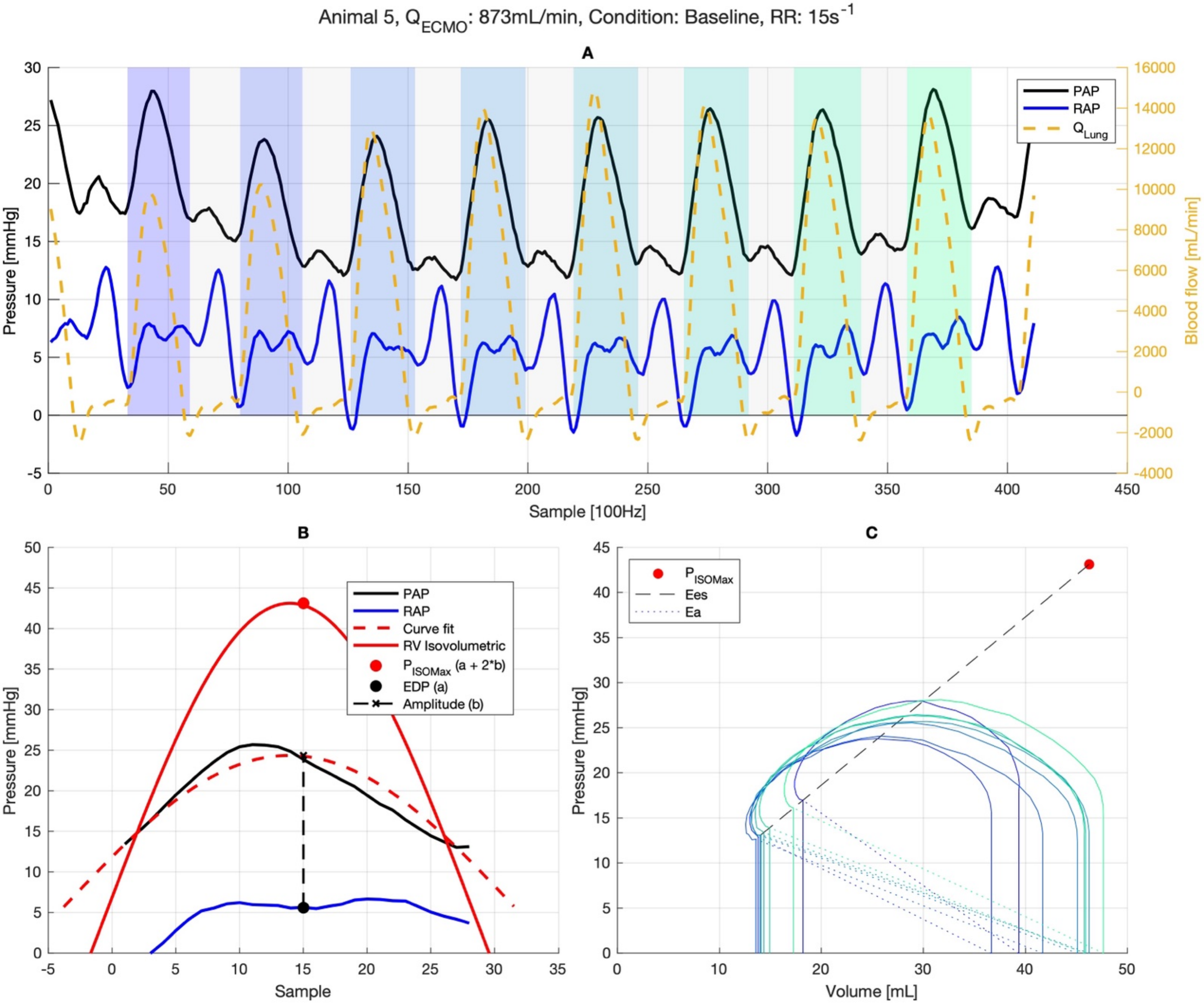

Animal 5, Q_{ECMO}: 873mL/min, Condition: Baseline, RR: $15s^{-1}$
A
PAP
RAP
Q_{Lung}
Pressure [mmHg]
Blood flow [mL/min]
Sample [100Hz]
B
PAP
RAP
Curve fit
RV Isovolumetric
P_{ISOMax} (a + 2*b)
EDP (a)
Amplitude (b)
Pressure [mmHg]
Sample
C
P_{ISOMax}
Ees
Ea
Pressure [mmHg]
Volume [mL]

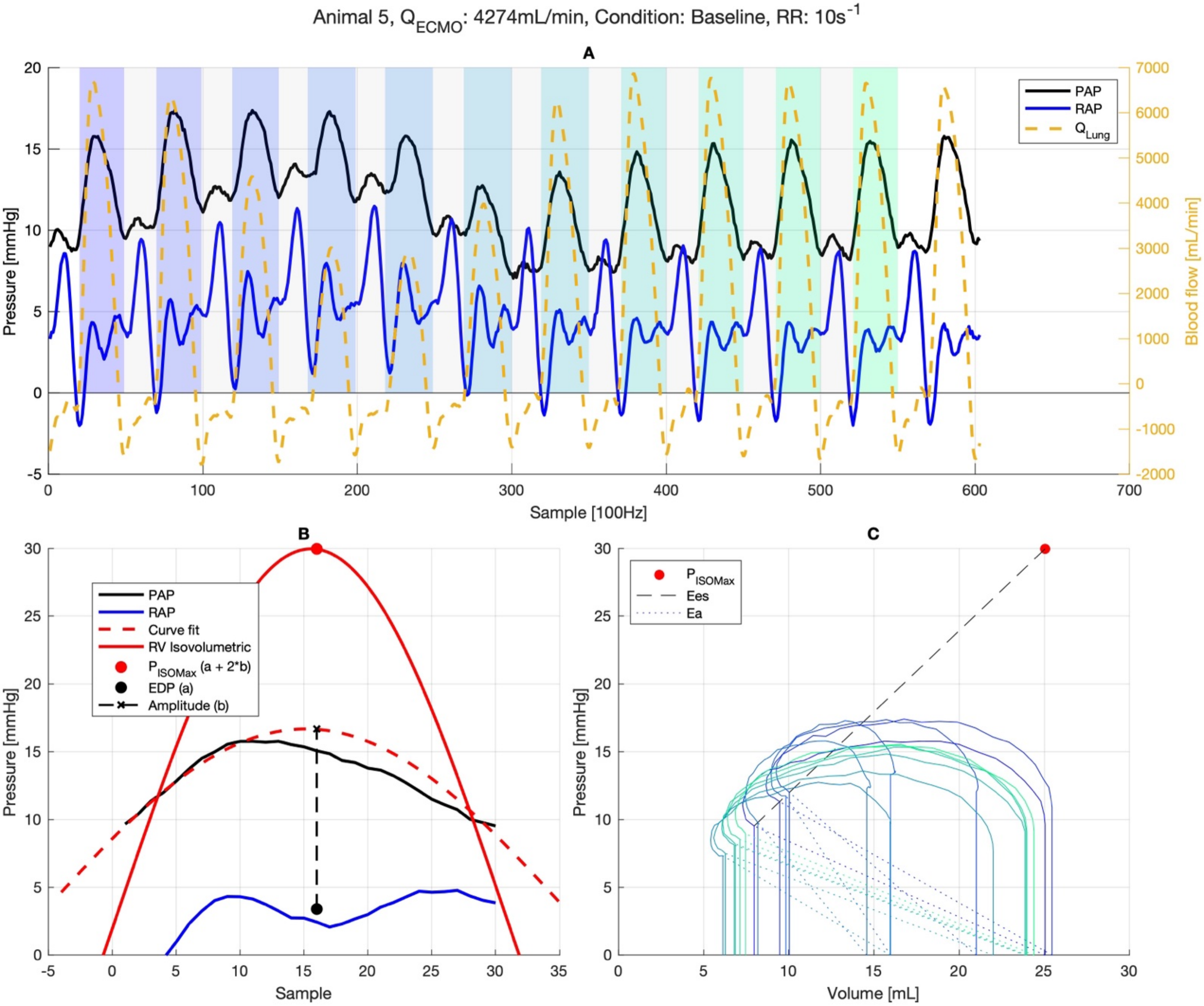

Animal 5, Q_{ECMO}: 4274mL/min, Condition: Baseline, RR: $10s^{-1}$
A
PAP
RAP
Q_{Lung}
Pressure [mmHg]
Blood flow [mL/min]
Sample [100Hz]
B
PAP
RAP
Curve fit
RV Isovolumetric
P_{ISOMax} (a + 2*b)
EDP (a)
Amplitude (b)
Pressure [mmHg]
Sample
C
P_{ISOMax}
Ees
Ea
Pressure [mmHg]
Volume [mL]

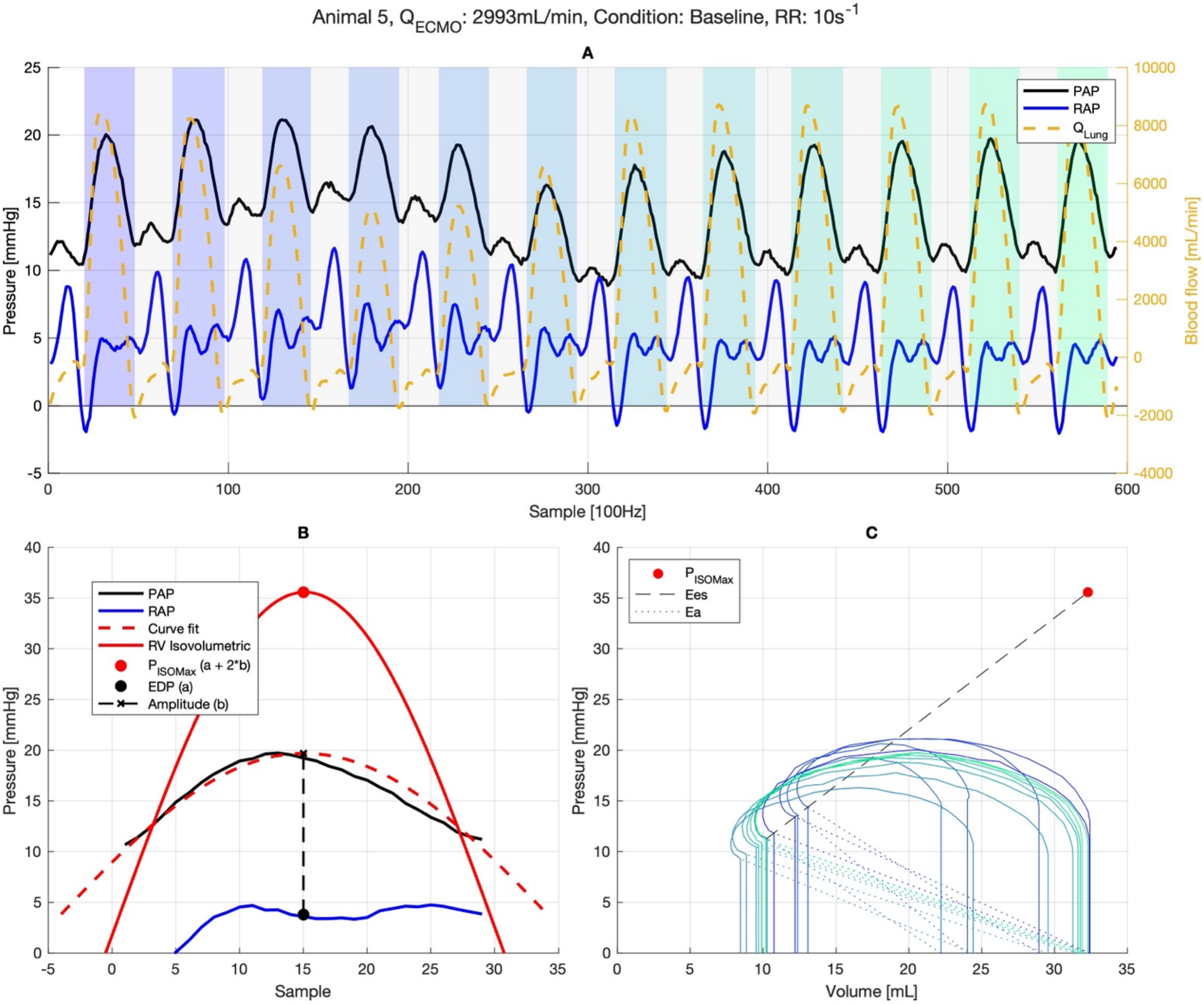

Animal 5, Q_{ECMO}: 2993mL/min, Condition: Baseline, RR: $10s^{-1}$
A
PAP
RAP
Q_{Lung}
Pressure [mmHg]
Blood flow [mL/min]
Sample [100Hz]
B
PAP
RAP
Curve fit
RV Isovolumetric
P_{ISOMax} (a + 2*b)
EDP (a)
Amplitude (b)
Pressure [mmHg]
Sample
C
P_{ISOMax}
Ees
Ea
Pressure [mmHg]
Volume [mL]

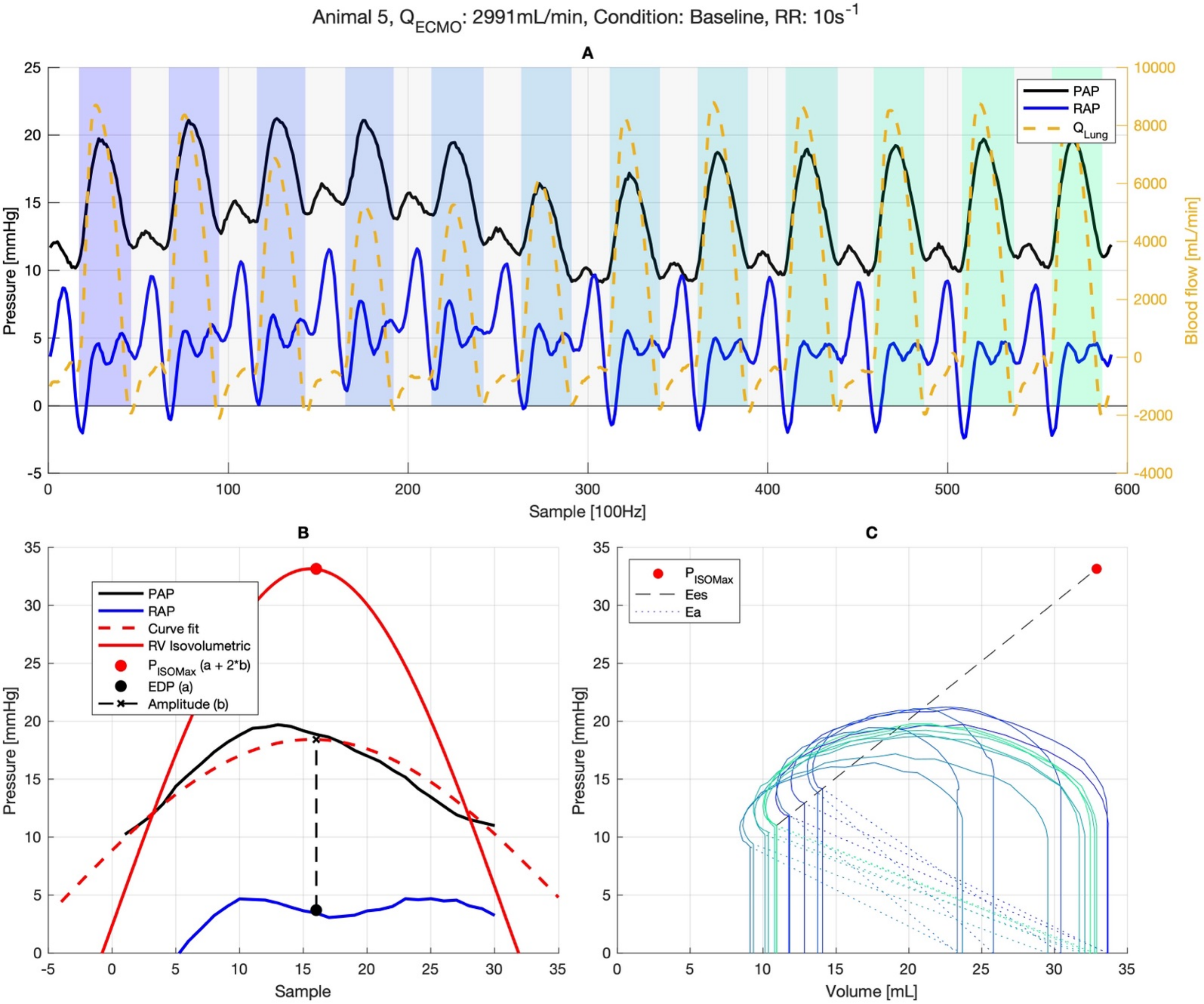

Animal 5, Q_ECMO: 2991mL/min, Condition: Baseline, RR: 10s^-1
A
PAP
RAP
Q_Lung
Pressure [mmHg]
Blood flow [mL/min]
Sample [100Hz]
B
PAP
RAP
Curve fit
RV Isovolumetric
P_ISOMax (a + 2*b)
EDP (a)
Amplitude (b)
Pressure [mmHg]
Sample
C
P_ISOMax
Ees
Ea
Pressure [mmHg]
Volume [mL]

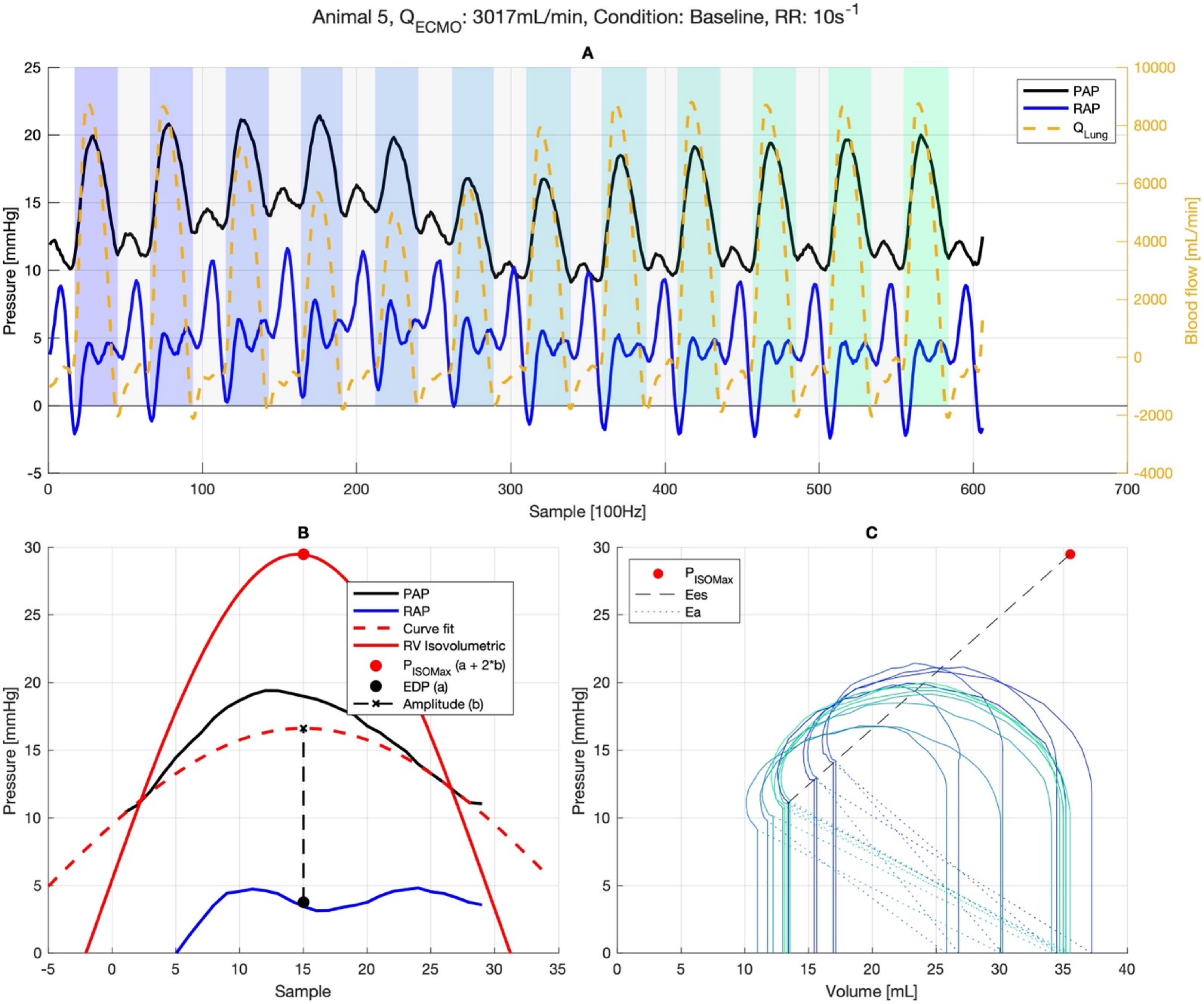

Animal 5, QECMO: 3017mL/min, Condition: Baseline, RR: 10s-1
A
PAP
RAP
QLung
Pressure [mmHg]
Blood flow [mL/min]
Sample [100Hz]
B
PAP
RAP
Curve fit
RV Isovolumetric
PISOMax (a + 2*b)
EDP (a)
Amplitude (b)
Pressure [mmHg]
Sample
C
PISOMax
Ees
Ea
Pressure [mmHg]
Volume [mL]

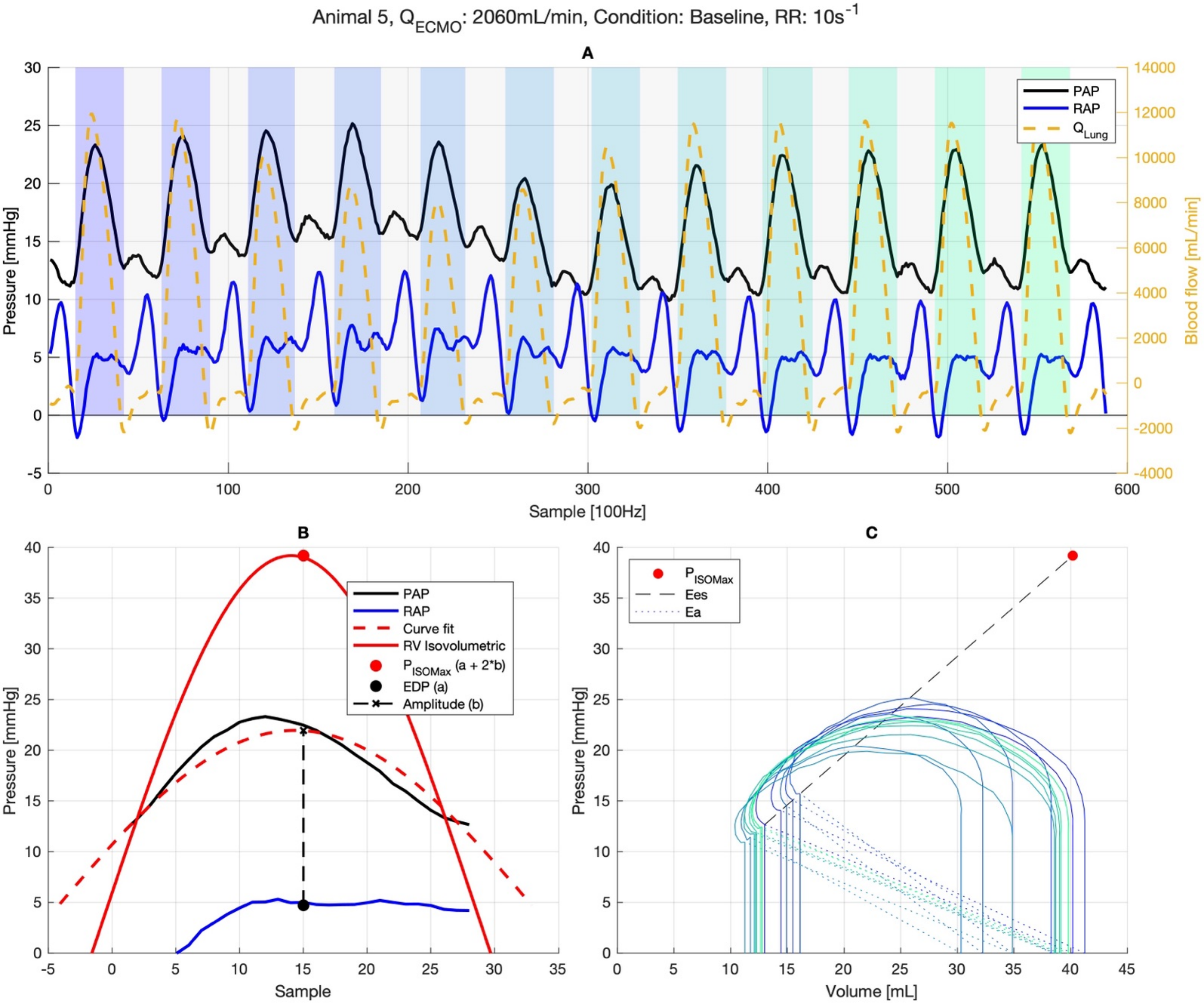

Animal 5, Q_ECMO: 2060mL/min, Condition: Baseline, RR: 10s^-1
A
PAP
RAP
Q_Lung
Pressure [mmHg]
Blood flow [mL/min]
Sample [100Hz]
B
PAP
RAP
Curve fit
RV Isovolumetric
P_ISOMax (a + 2*b)
EDP (a)
Amplitude (b)
Pressure [mmHg]
Sample
C
P_ISOMax
Ees
Ea
Pressure [mmHg]
Volume [mL]

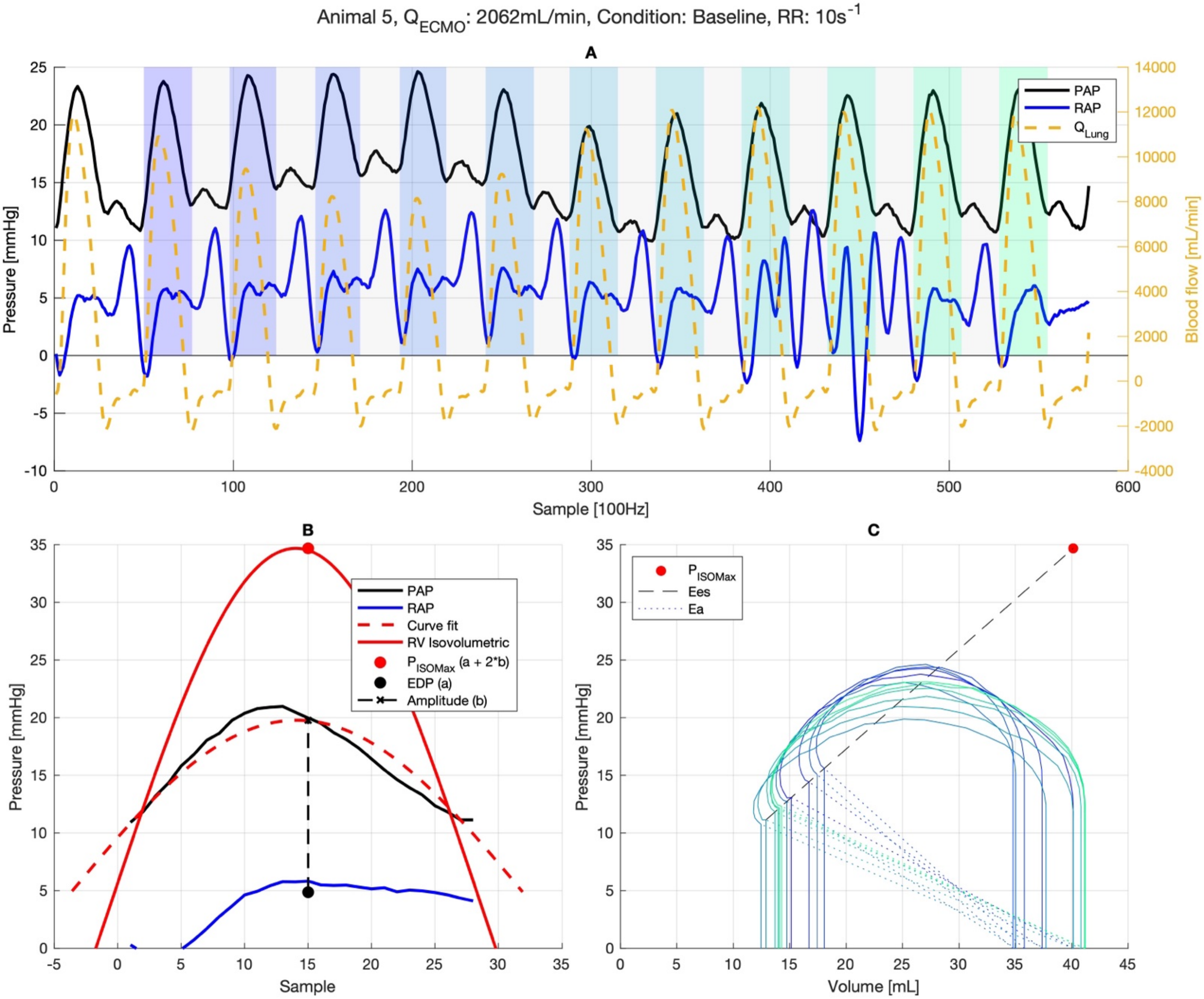

Animal 5, Q_{ECMO}: 2062mL/min, Condition: Baseline, RR: $10s^{-1}$
A
PAP
RAP
Q_{Lung}
Pressure [mmHg]
Blood flow [mL/min]
Sample [100Hz]
B
PAP
RAP
Curve fit
RV Isovolumetric
P_{ISOMax} (a + 2*b)
EDP (a)
Amplitude (b)
Pressure [mmHg]
Sample
C
P_{ISOMax}
Ees
Ea
Pressure [mmHg]
Volume [mL]

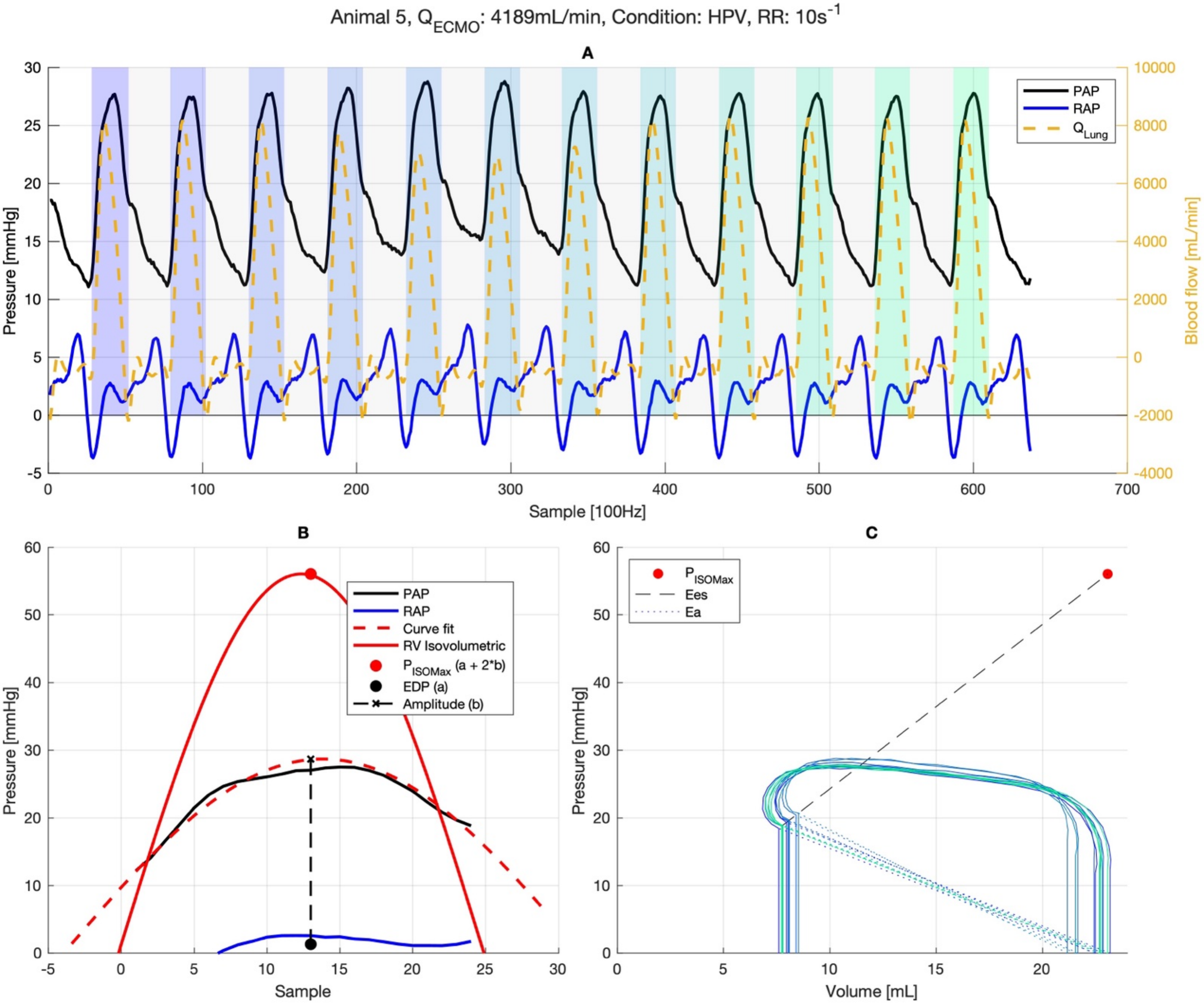

Animal 5, Q_{ECMO}: 4189mL/min, Condition: HPV, RR: $10s^{-1}$
A
PAP
RAP
Q_{Lung}
Pressure [mmHg]
Blood flow [mL/min]
Sample [100Hz]
B
PAP
RAP
Curve fit
RV Isovolumetric
P_{ISOMax} (a + 2*b)
EDP (a)
Amplitude (b)
Pressure [mmHg]
Sample
C
P_{ISOMax}
Ees
Ea
Pressure [mmHg]
Volume [mL]

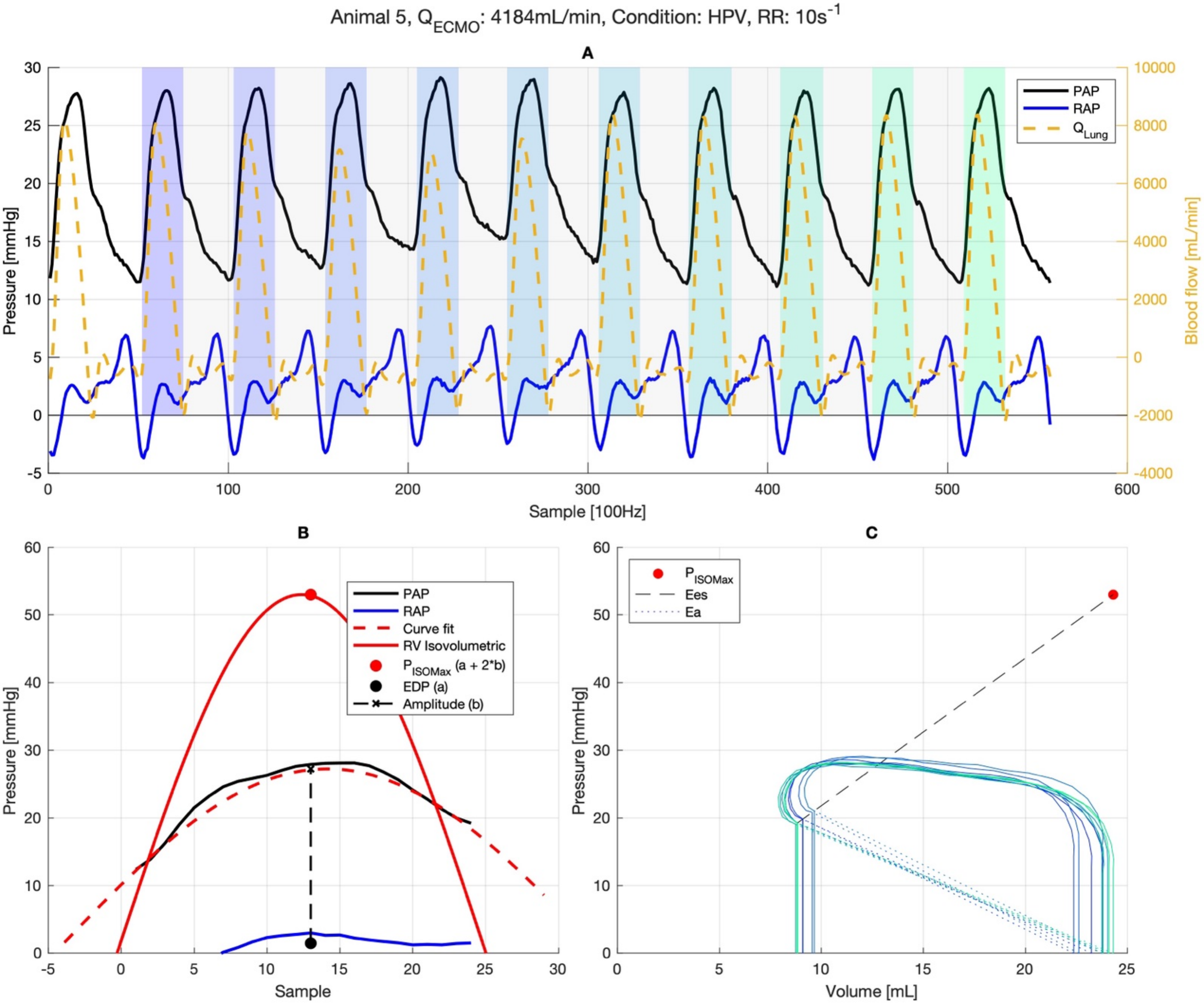
Animal 5, Q_{ECMO}: 4184mL/min, Condition: HPV, RR: $10s^{-1}$
A
PAP
RAP
Q_{Lung}
Pressure [mmHg]
Blood flow [mL/min]
Sample [100Hz]
B
PAP
RAP
Curve fit
RV Isovolumetric
P_{ISOMax} (a + 2*b)
EDP (a)
Amplitude (b)
Pressure [mmHg]
Sample
C
P_{ISOMax}
Ees
Ea
Pressure [mmHg]
Volume [mL]

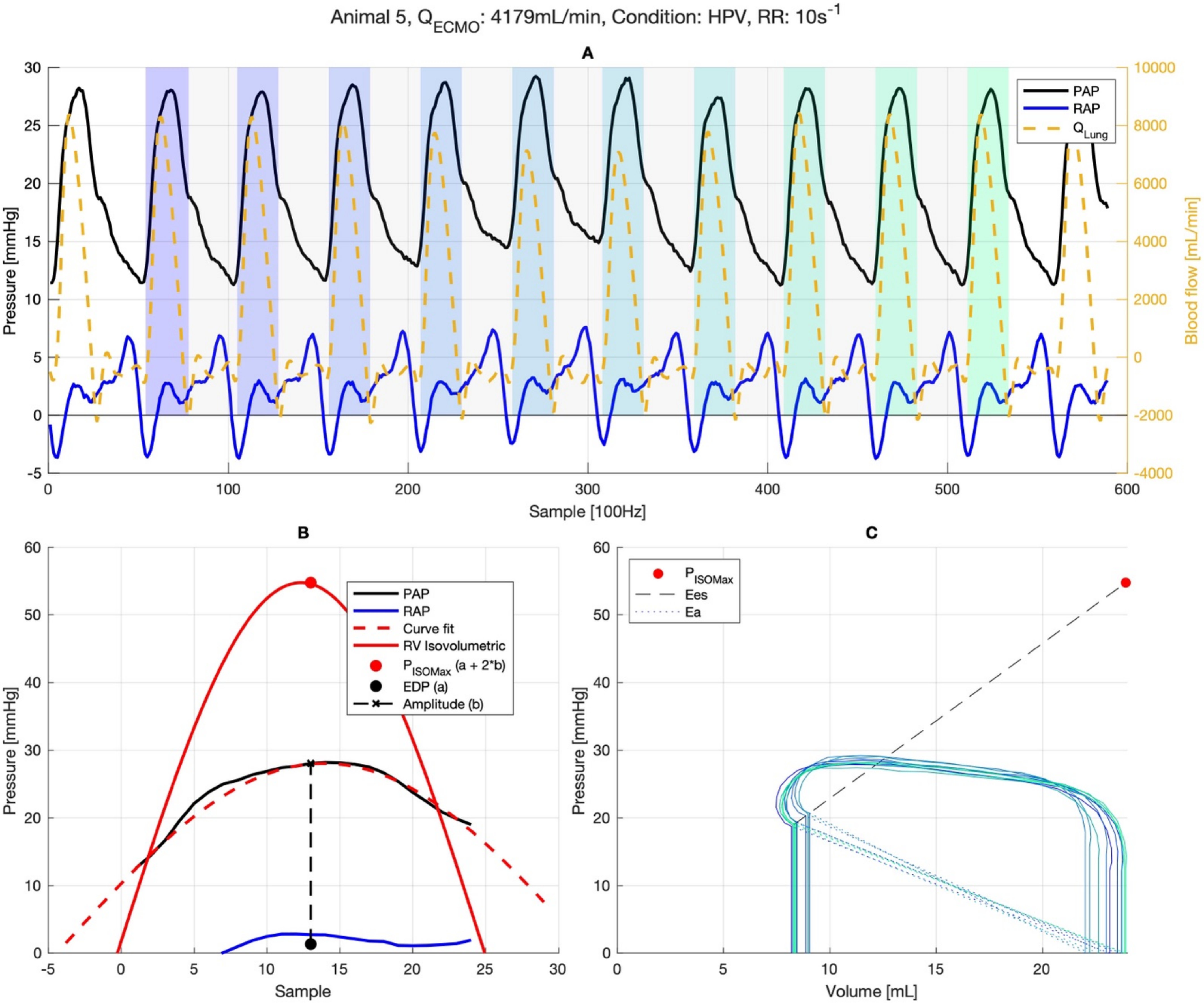

Animal 5, Q_ECMO: 4179mL/min, Condition: HPV, RR: 10s^-1
A
PAP
RAP
Q_Lung
Pressure [mmHg]
Blood flow [mL/min]
Sample [100Hz]
B
PAP
RAP
Curve fit
RV Isovolumetric
P_ISOMax (a + 2*b)
EDP (a)
Amplitude (b)
Pressure [mmHg]
Sample
C
P_ISOMax
Ees
Ea
Pressure [mmHg]
Volume [mL]

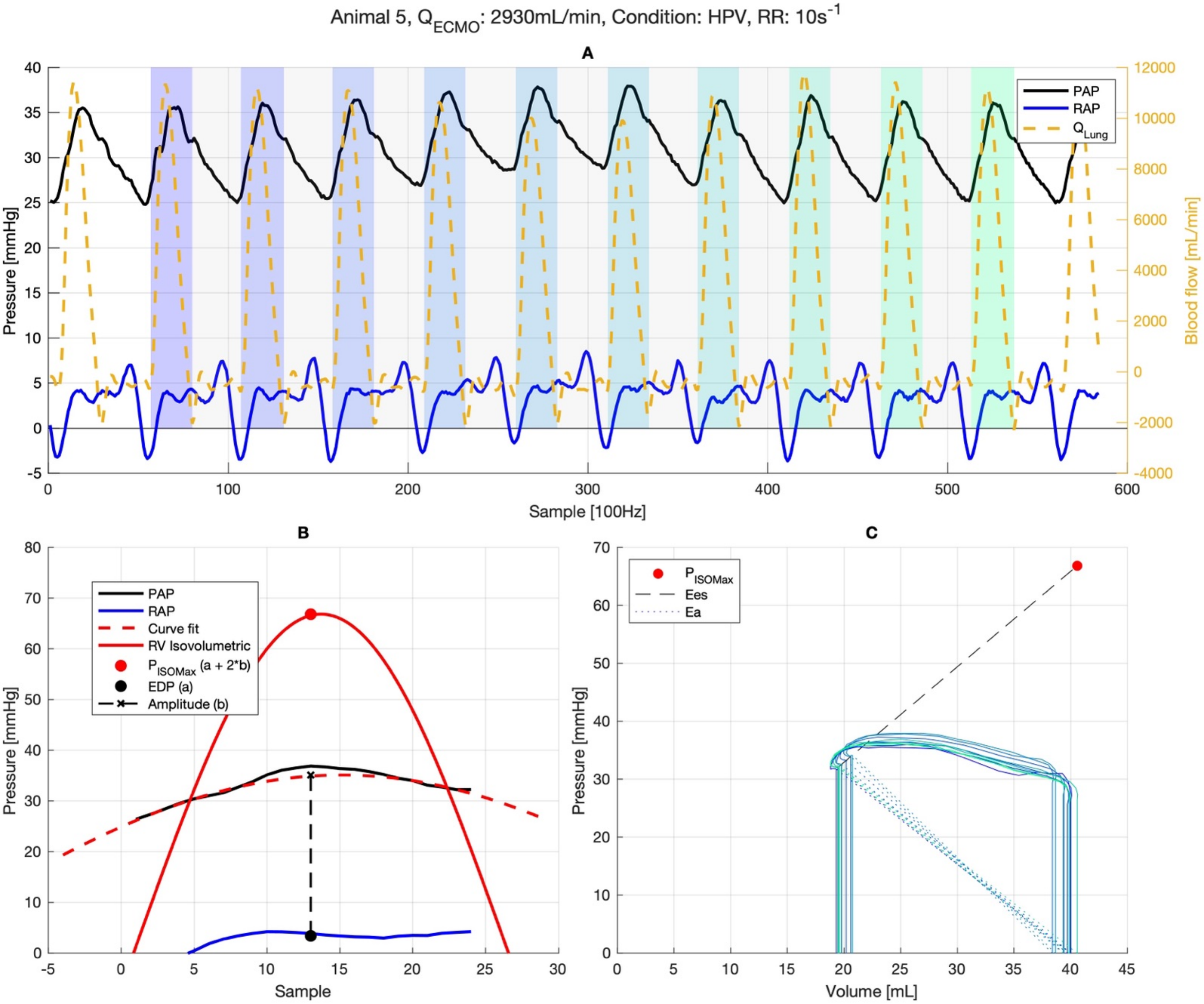

Animal 5, Q_ECMO: 2930mL/min, Condition: HPV, RR: 10s^-1
A
Pressure [mmHg]
Blood flow [mL/min]
Sample [100Hz]
PAP
RAP
Q_Lung
B
PAP
RAP
Curve fit
RV Isovolumetric
P_ISOMax (a + 2*b)
EDP (a)
Amplitude (b)
Pressure [mmHg]
Sample
C
P_ISOMax
Ees
Ea
Pressure [mmHg]
Volume [mL]

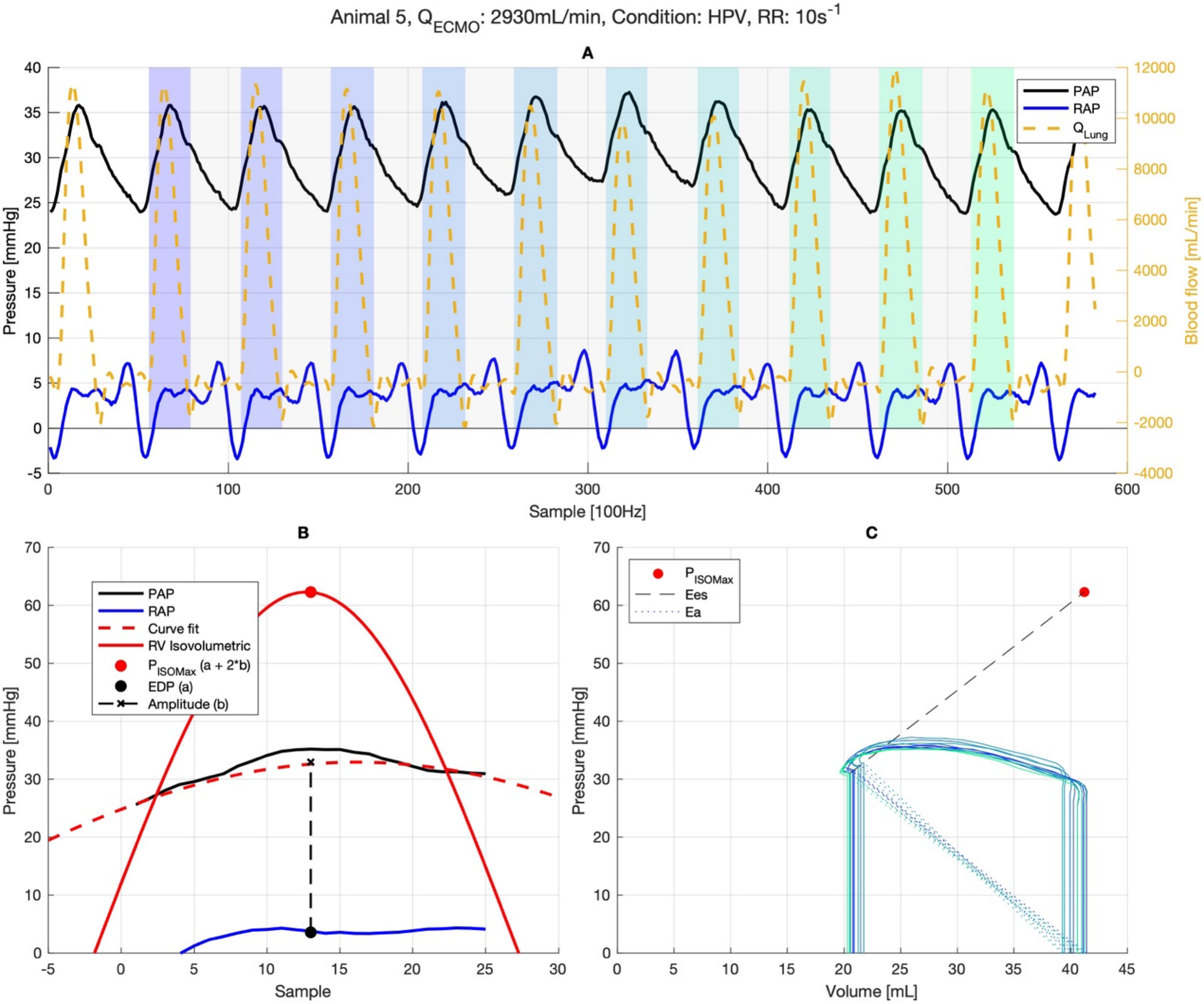

Animal 5, Q_ECMO: 2930mL/min, Condition: HPV, RR: 10s^-1
A
PAP
RAP
Q_Lung
Pressure [mmHg]
Blood flow [mL/min]
Sample [100Hz]
B
PAP
RAP
Curve fit
RV Isovolumetric
P_ISOMax (a + 2*b)
EDP (a)
Amplitude (b)
Pressure [mmHg]
Sample
C
P_ISOMax
Ees
Ea
Pressure [mmHg]
Volume [mL]

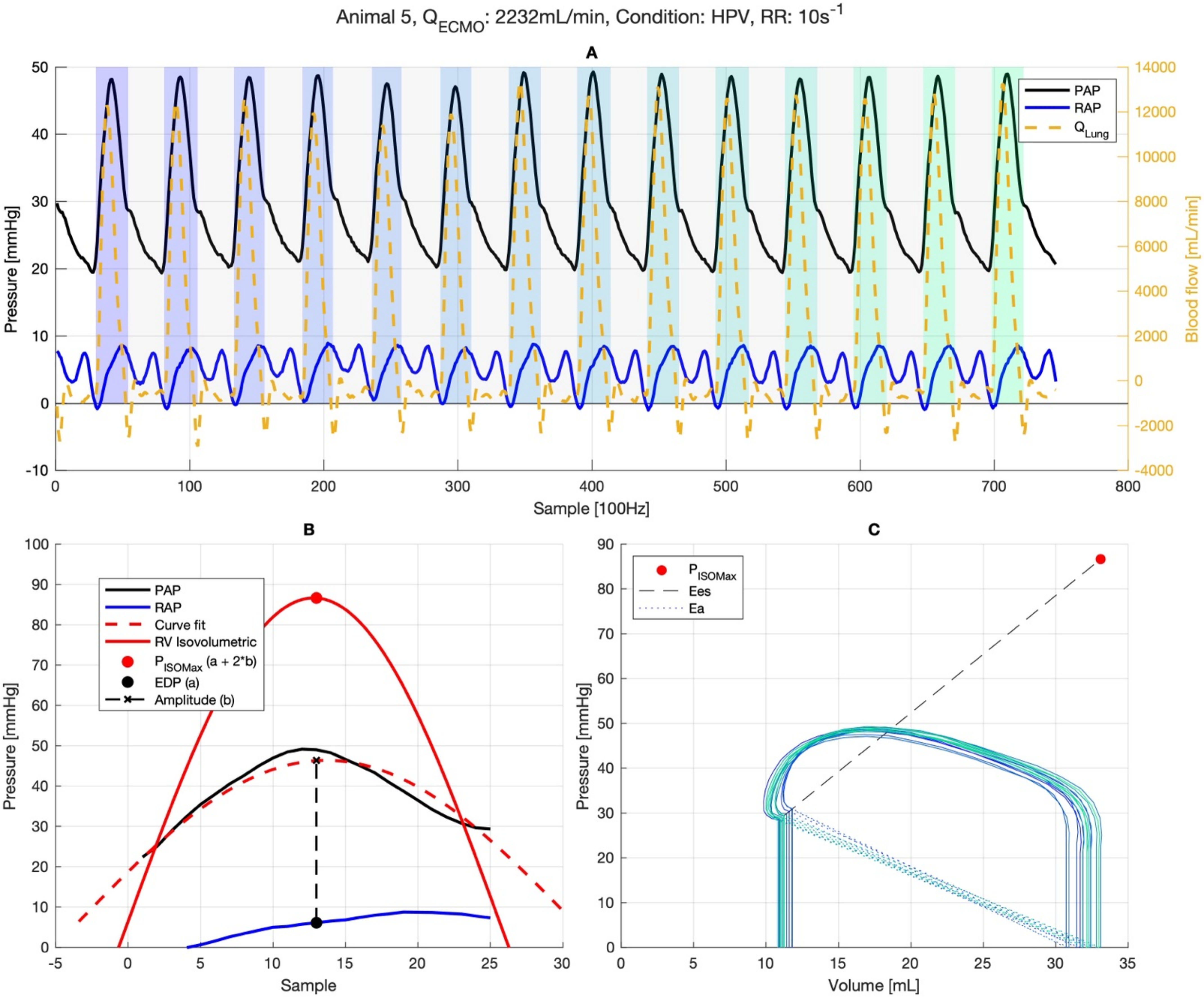

Animal 5, Q_ECMO: 2232mL/min, Condition: HPV, RR: 10s^-1
A
Pressure [mmHg]
Blood flow [mL/min]
Sample [100Hz]
PAP
RAP
Q_Lung
B
Pressure [mmHg]
Sample
PAP
RAP
Curve fit
RV Isovolumetric
P_ISOMax (a + 2*b)
EDP (a)
Amplitude (b)
C
Pressure [mmHg]
Volume [mL]
P_ISOMax
Ees
Ea

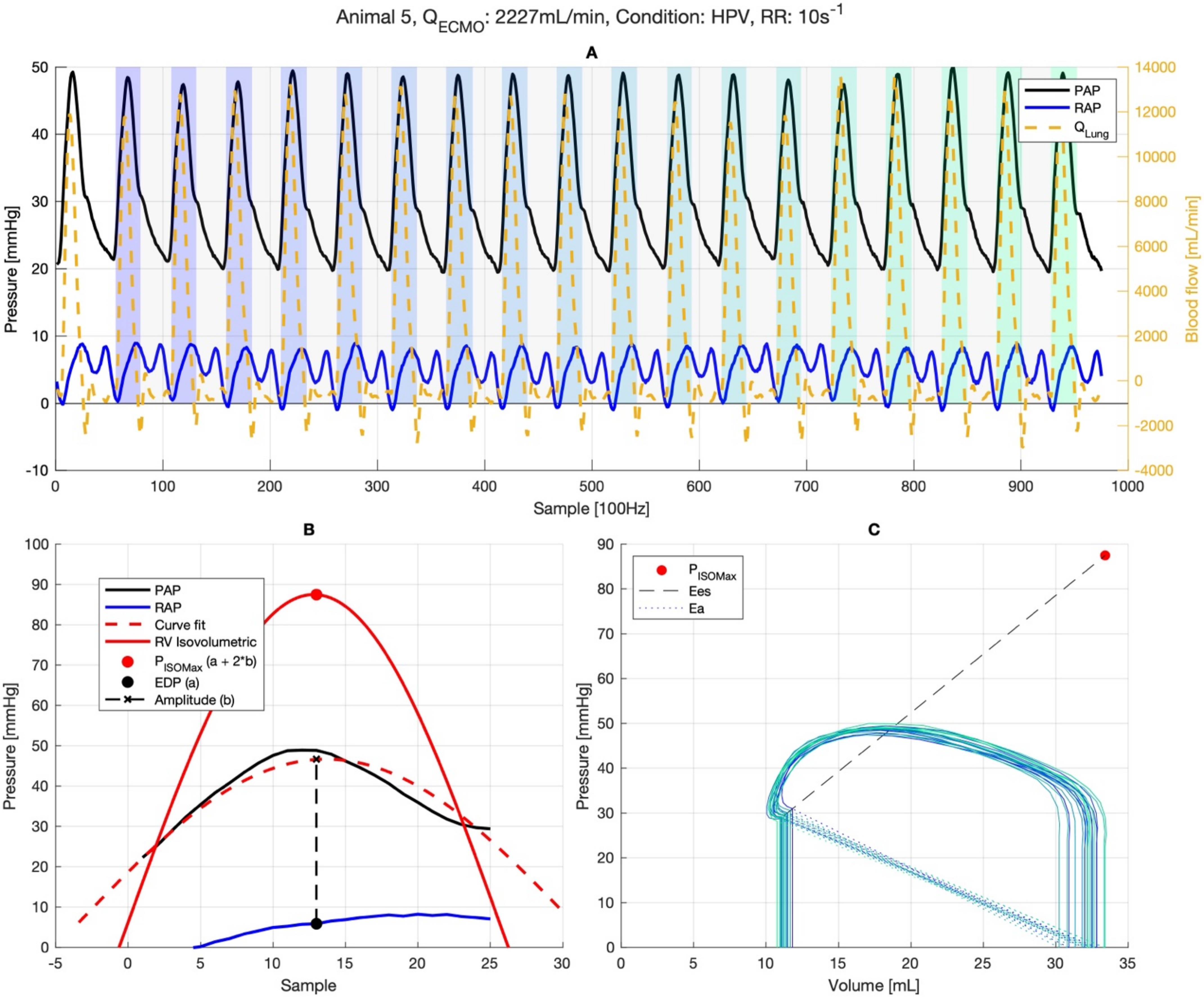

Animal 5, Q_ECMO: 2227mL/min, Condition: HPV, RR: 10s-1
A
PAP
RAP
Q_Lung
Pressure [mmHg]
Blood flow [mL/min]
Sample [100Hz]
B
PAP
RAP
Curve fit
RV Isovolumetric
P_ISOMax (a + 2*b)
EDP (a)
Amplitude (b)
Pressure [mmHg]
Sample
C
P_ISOMax
Ees
Ea
Pressure [mmHg]
Volume [mL]

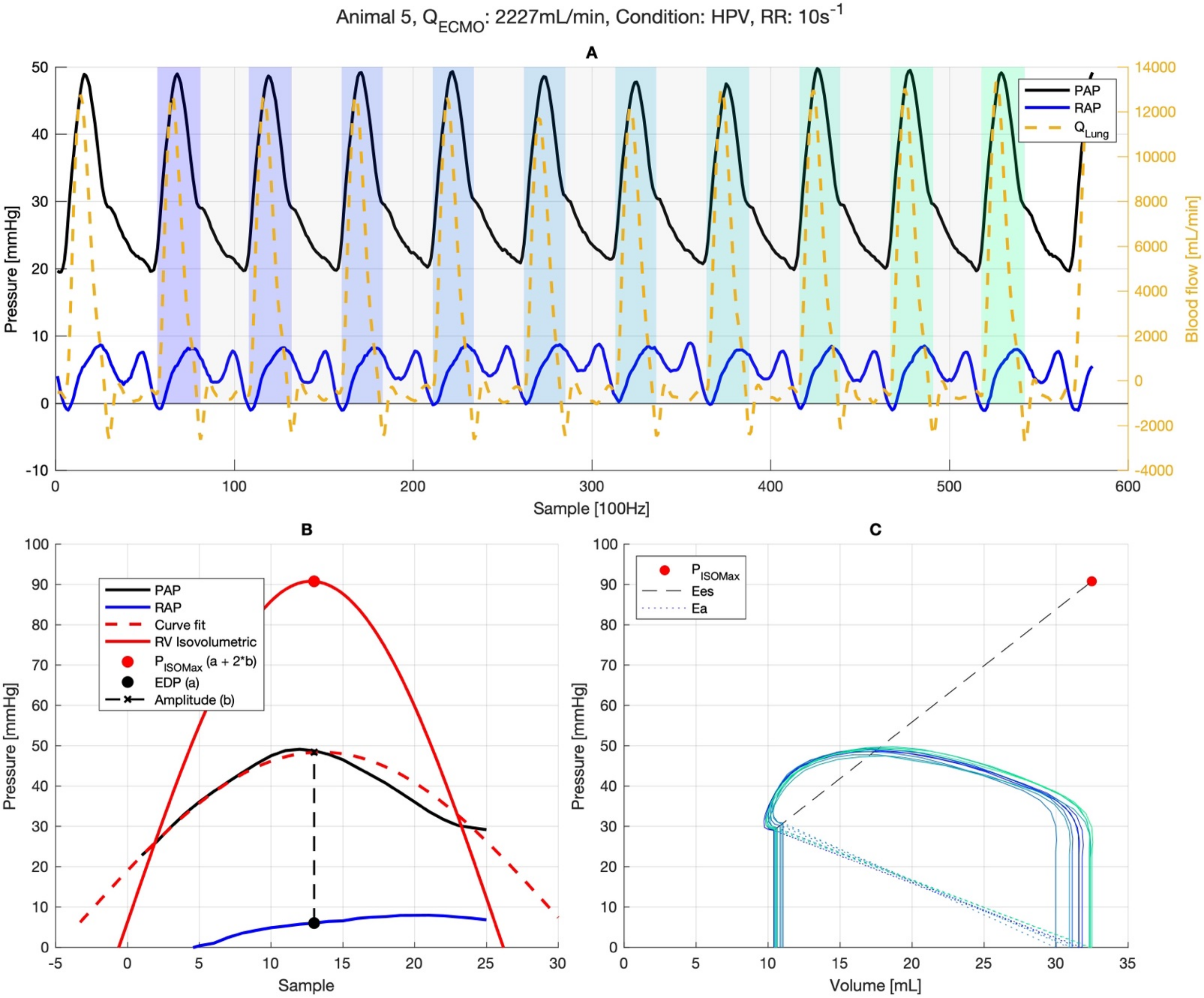

Animal 5, Q_ECMO: 2227mL/min, Condition: HPV, RR: 10s^-1
A
Pressure [mmHg]
Blood flow [mL/min]
Sample [100Hz]
PAP
RAP
Q_Lung
B
Curve fit
RV Isovolumetric
P_ISOMax (a + 2*b)
EDP (a)
Amplitude (b)
Sample
C
P_ISOMax
Ees
Ea
Volume [mL]

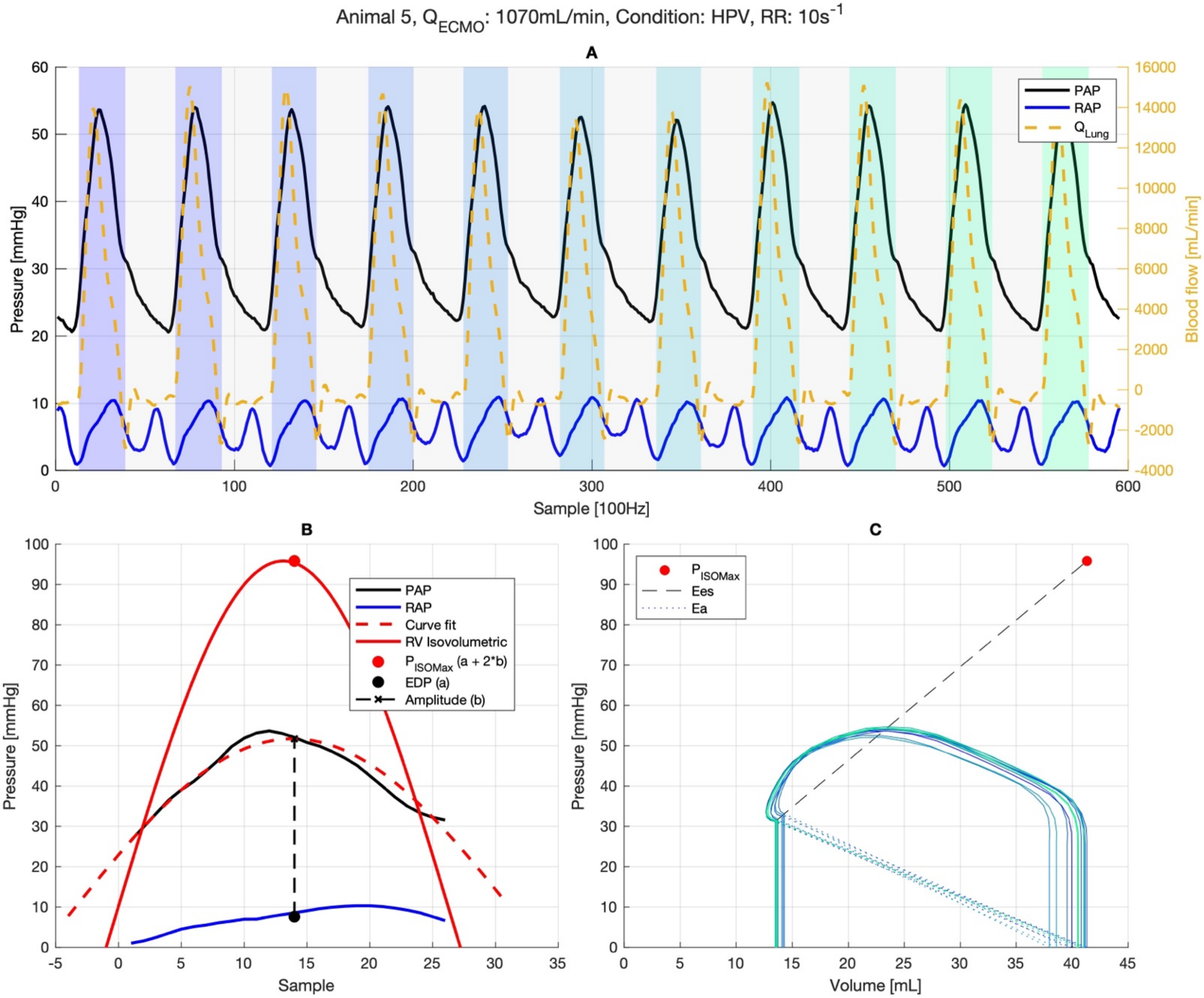

Animal 5, Q_{ECMO}: 1070mL/min, Condition: HPV, RR: $10s^{-1}$
A
Pressure [mmHg]
Blood flow [mL/min]
Sample [100Hz]
PAP
RAP
Q_{Lung}
B
Pressure [mmHg]
Sample
PAP
RAP
Curve fit
RV Isovolumetric
P_{ISOMax} (a + 2*b)
EDP (a)
Amplitude (b)
C
Pressure [mmHg]
Volume [mL]
P_{ISOMax}
Ees
Ea

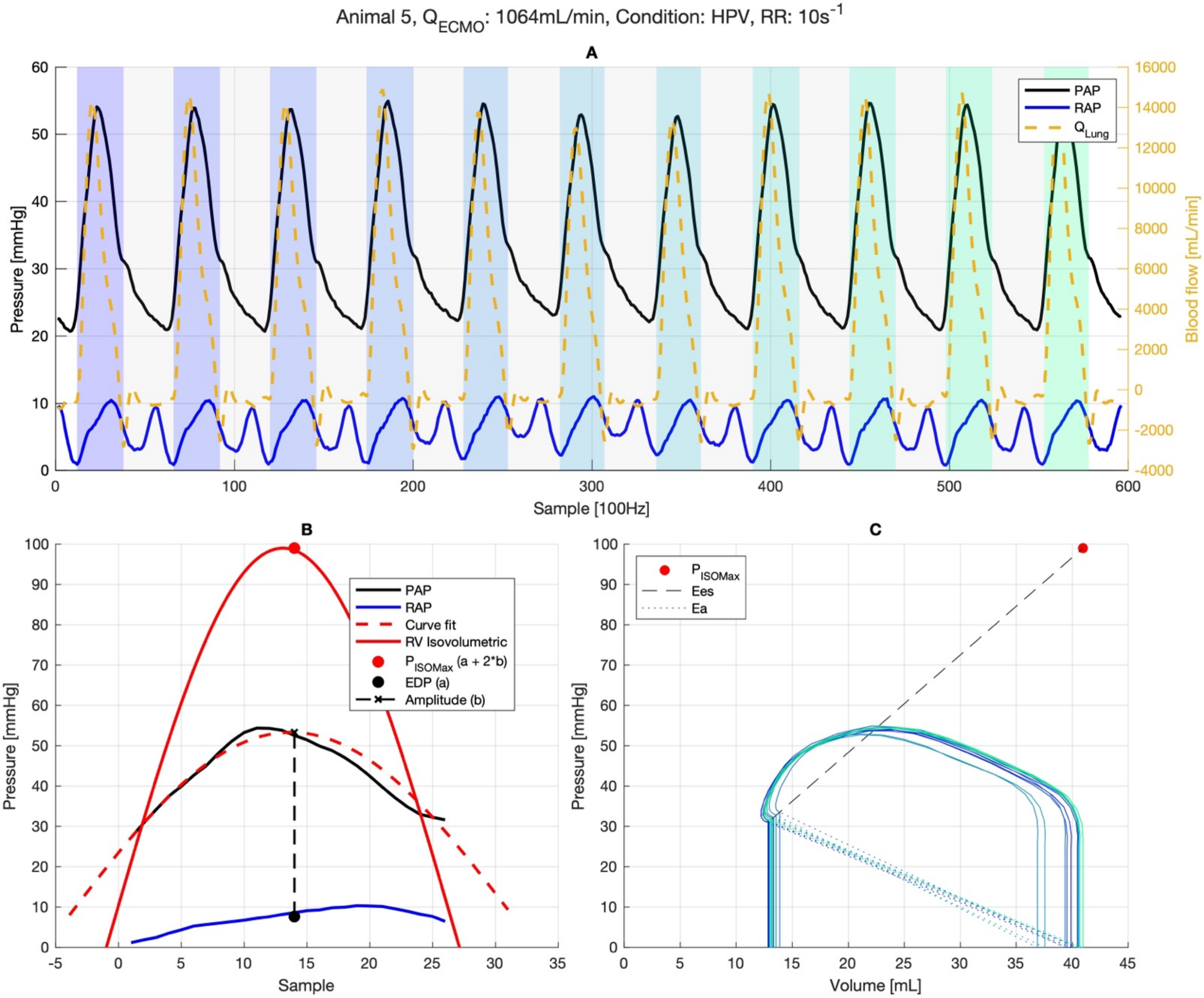

Animal 5, Q_{ECMO}: 1064mL/min, Condition: HPV, RR: $10s^{-1}$
A
Pressure [mmHg]
Blood flow [mL/min]
Sample [100Hz]
PAP
RAP
Q_{Lung}
B
PAP
RAP
Curve fit
RV Isovolumetric
P_{ISOMax} (a + 2*b)
EDP (a)
Amplitude (b)
Pressure [mmHg]
Sample
C
P_{ISOMax}
Ees
Ea
Pressure [mmHg]
Volume [mL]

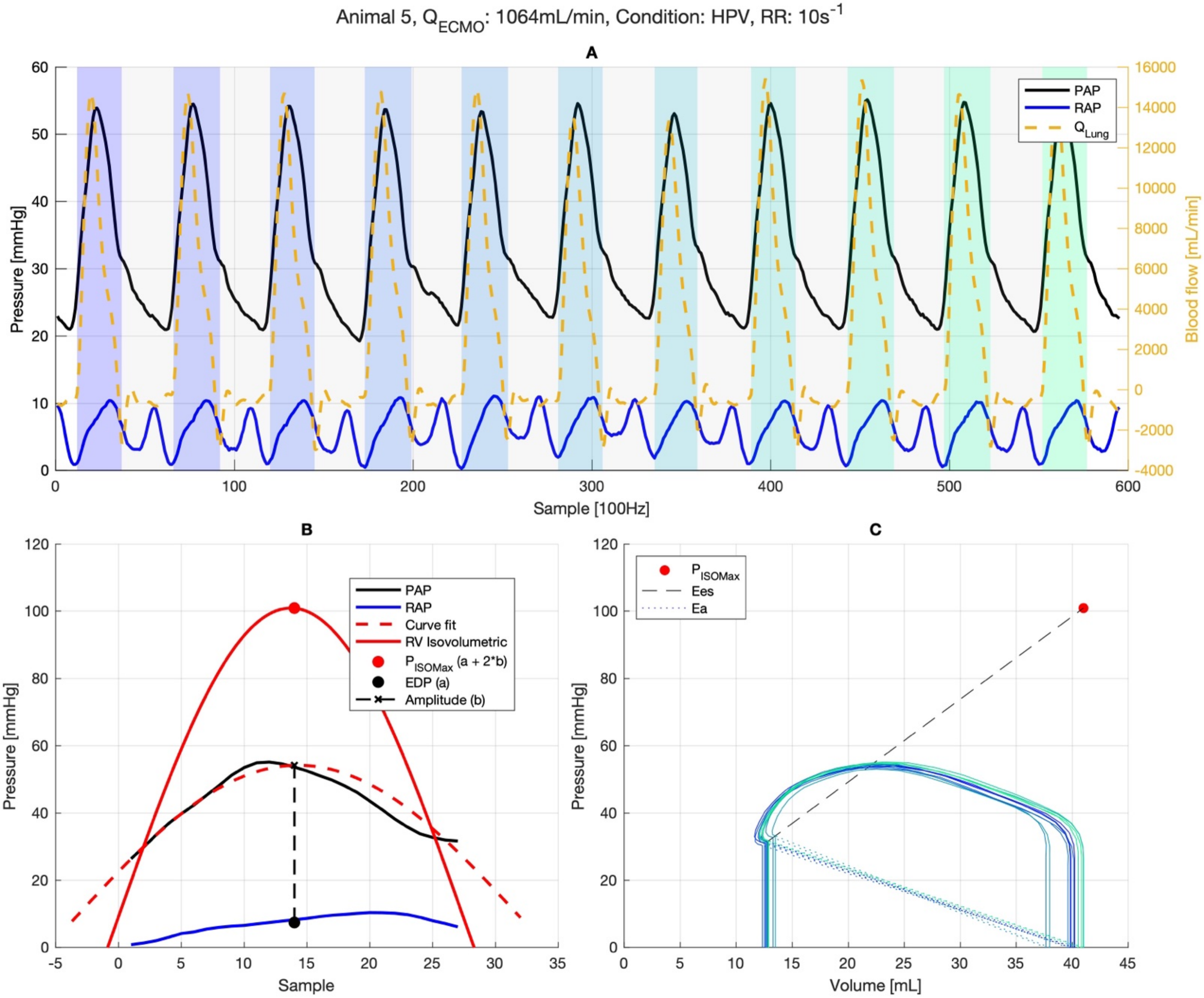
Animal 5, Q_ECMO: 1064mL/min, Condition: HPV, RR: 10s^-1
A
Pressure [mmHg]
Blood flow [mL/min]
Sample [100Hz]
PAP
RAP
Q_Lung
B
PAP
RAP
Curve fit
RV Isovolumetric
P_ISOMax (a + 2*b)
EDP (a)
Amplitude (b)
Pressure [mmHg]
Sample
C
P_ISOMax
Ees
Ea
Pressure [mmHg]
Volume [mL]

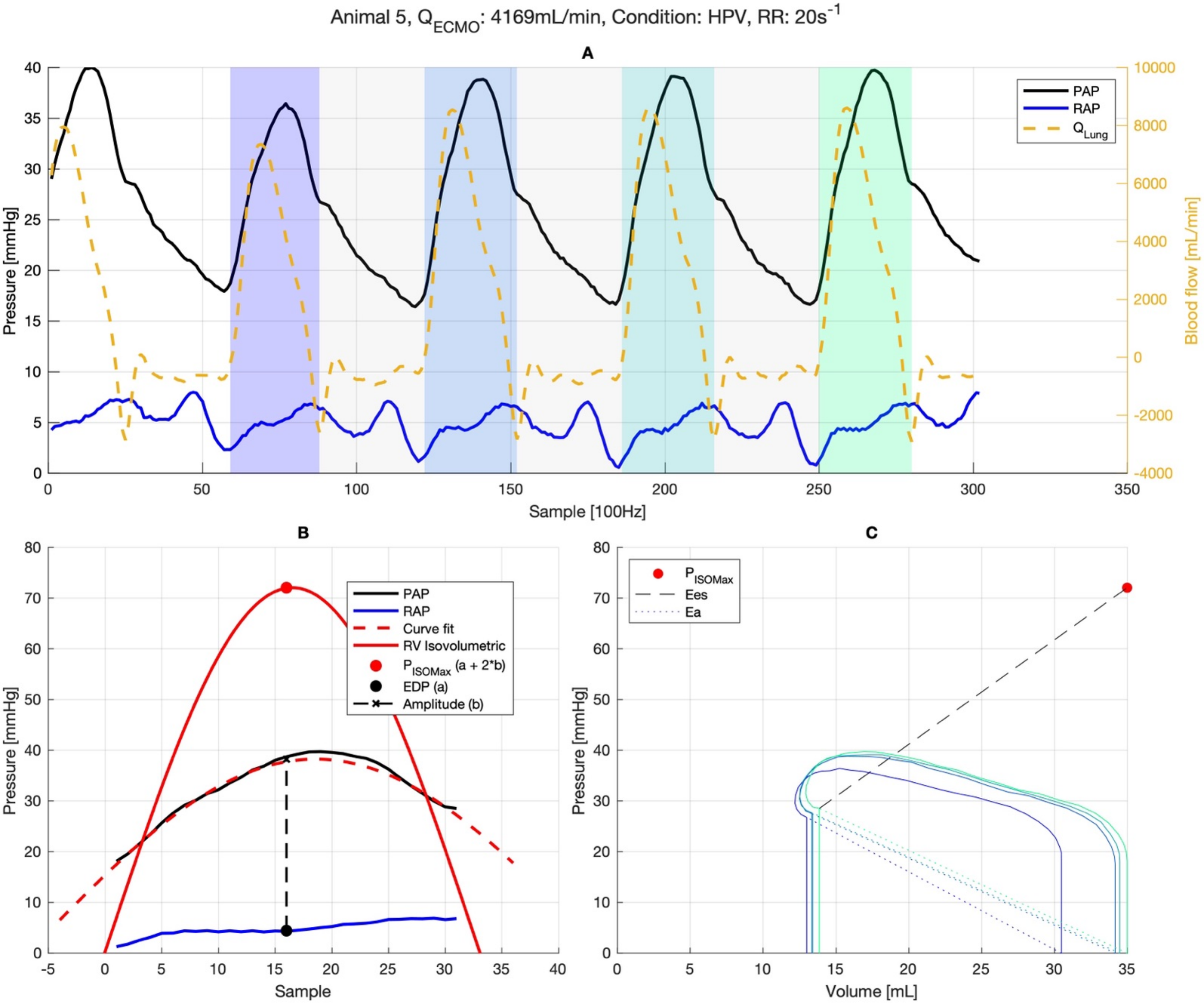

Animal 5, Q_{ECMO}: 4169mL/min, Condition: HPV, RR: $20s^{-1}$
A
PAP
RAP
Q_{Lung}
Pressure [mmHg]
Blood flow [mL/min]
Sample [100Hz]
B
PAP
RAP
Curve fit
RV Isovolumetric
P_{ISOMax} (a + 2*b)
EDP (a)
Amplitude (b)
Pressure [mmHg]
Sample
C
P_{ISOMax}
Ees
Ea
Pressure [mmHg]
Volume [mL]

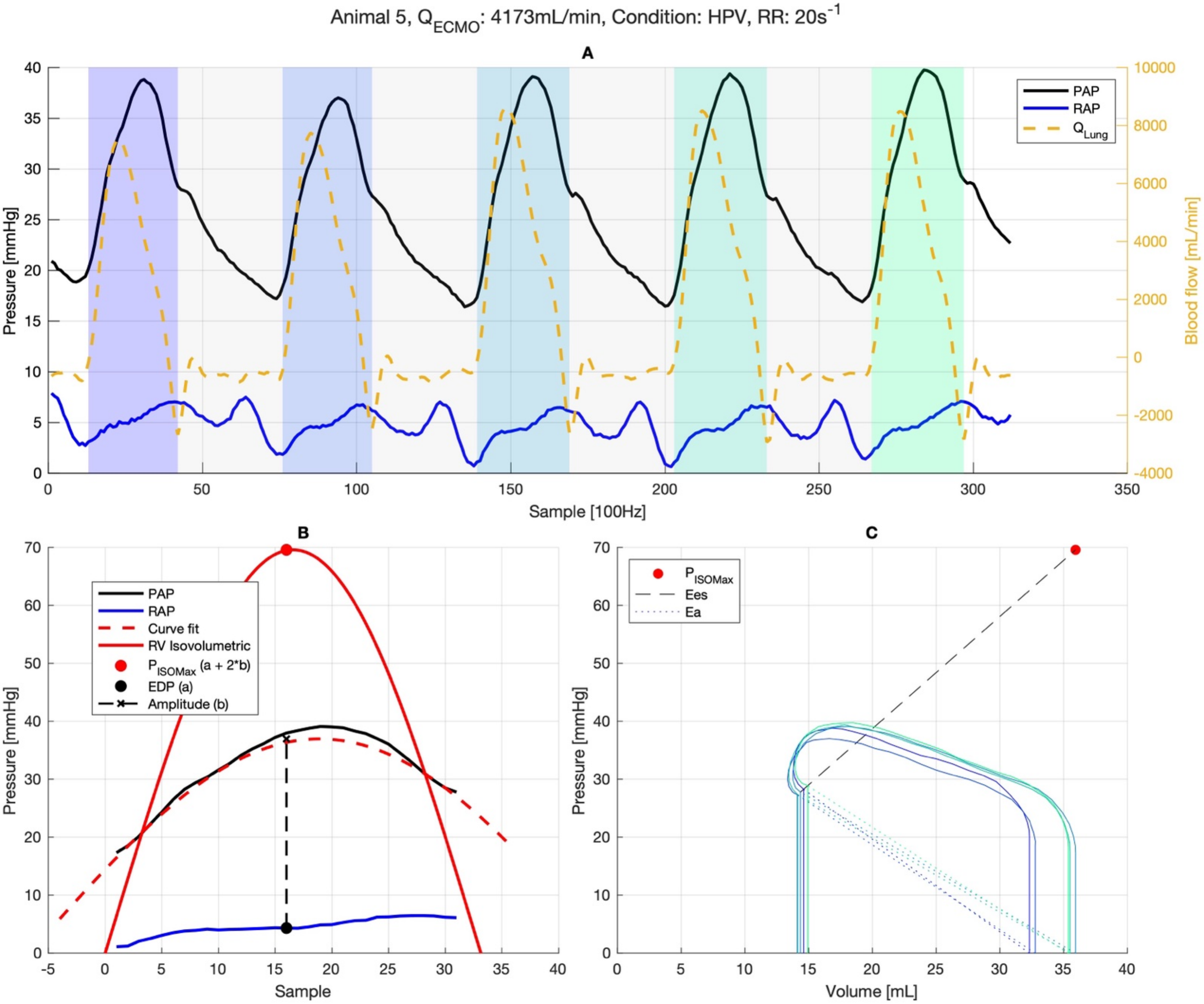

Animal 5, Q_{ECMO}: 4173mL/min, Condition: HPV, RR: $20s^{-1}$
A
PAP
RAP
Q_{Lung}
Pressure [mmHg]
Blood flow [mL/min]
Sample [100Hz]
B
PAP
RAP
Curve fit
RV Isovolumetric
P_{ISOMax} (a + 2*b)
EDP (a)
Amplitude (b)
Pressure [mmHg]
Sample
C
P_{ISOMax}
Ees
Ea
Pressure [mmHg]
Volume [mL]

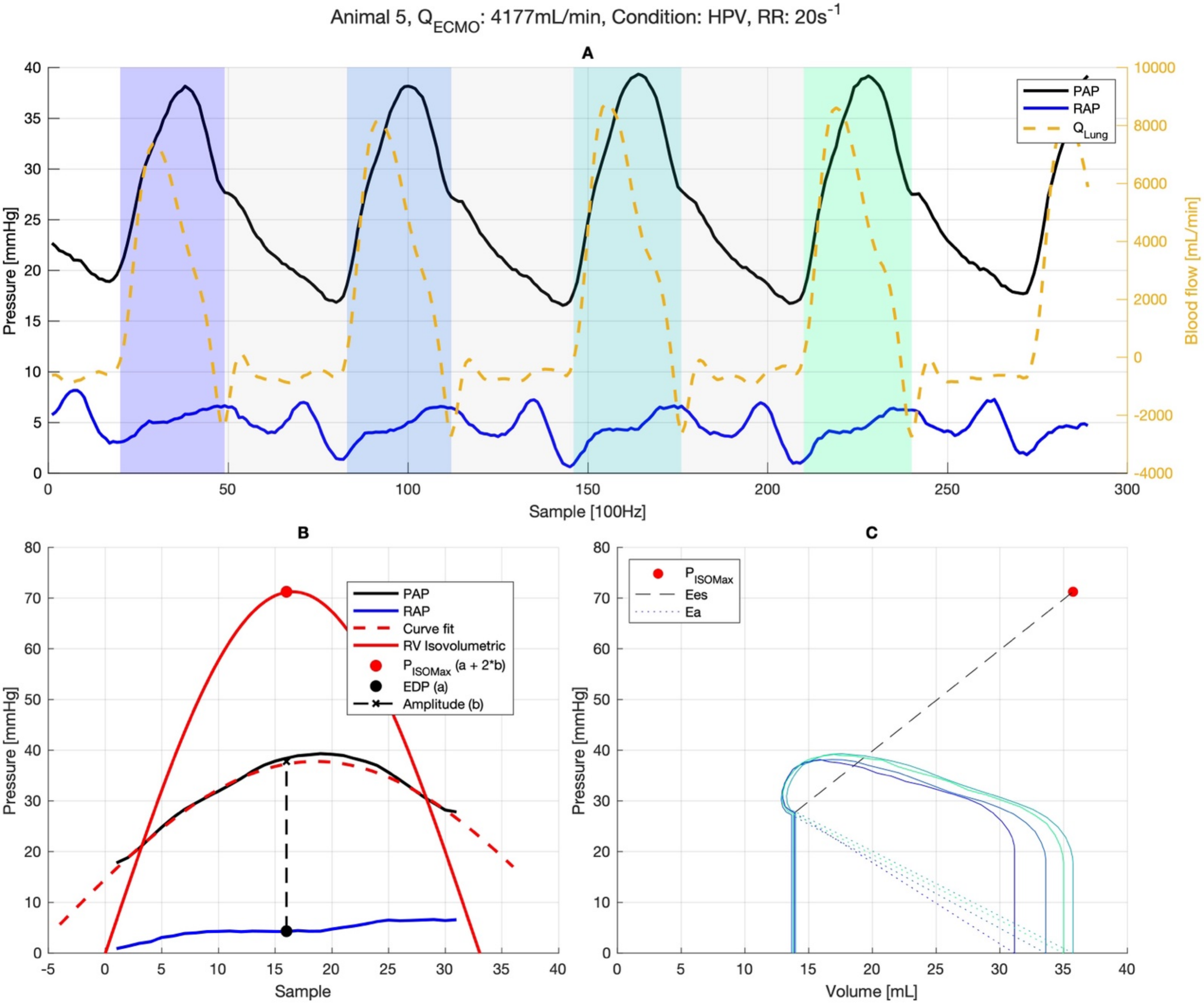

Animal 5, Q_{ECMO}: 4177mL/min, Condition: HPV, RR: $20s^{-1}$
A
Pressure [mmHg]
Blood flow [mL/min]
Sample [100Hz]
PAP
RAP
Q_{Lung}
B
Pressure [mmHg]
Sample
PAP
RAP
Curve fit
RV Isovolumetric
P_{ISOMax} (a + 2*b)
EDP (a)
Amplitude (b)
C
Pressure [mmHg]
Volume [mL]
P_{ISOMax}
Ees
Ea

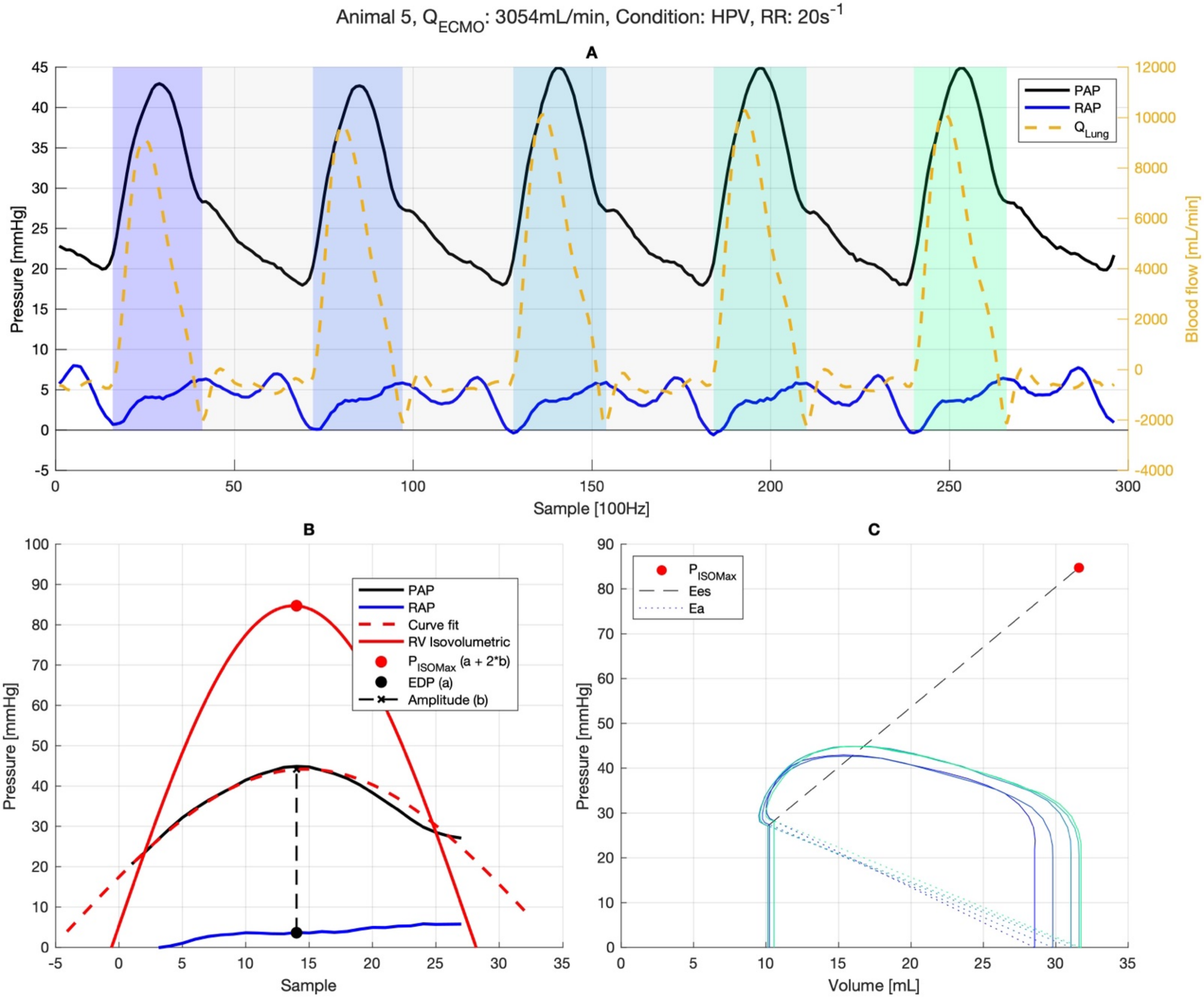

Animal 5, Q_{ECMO}: 3054mL/min, Condition: HPV, RR: $20s^{-1}$
A
PAP
RAP
Q_{Lung}
Pressure [mmHg]
Blood flow [mL/min]
Sample [100Hz]
B
PAP
RAP
Curve fit
RV Isovolumetric
P_{ISOMax} (a + 2*b)
EDP (a)
Amplitude (b)
Pressure [mmHg]
Sample
C
P_{ISOMax}
Ees
Ea
Pressure [mmHg]
Volume [mL]

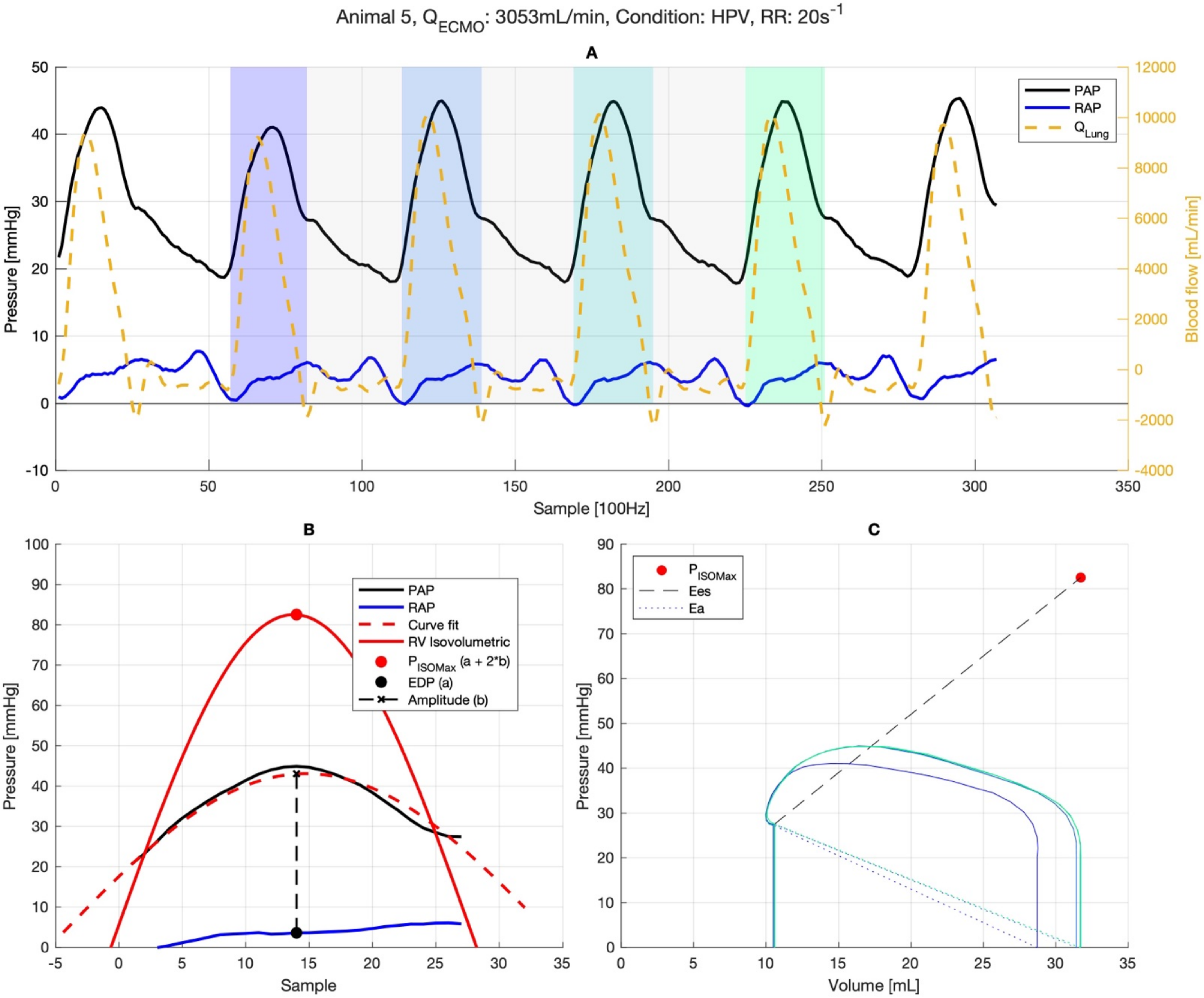

Animal 5, Q_{ECMO}: 3053mL/min, Condition: HPV, RR: $20s^{-1}$
A
Pressure [mmHg]
Blood flow [mL/min]
Sample [100Hz]
PAP
RAP
Q_{Lung}
B
Pressure [mmHg]
Sample
PAP
RAP
Curve fit
RV Isovolumetric
P_{ISOMax} (a + 2*b)
EDP (a)
Amplitude (b)
C
Pressure [mmHg]
Volume [mL]
P_{ISOMax}
Ees
Ea

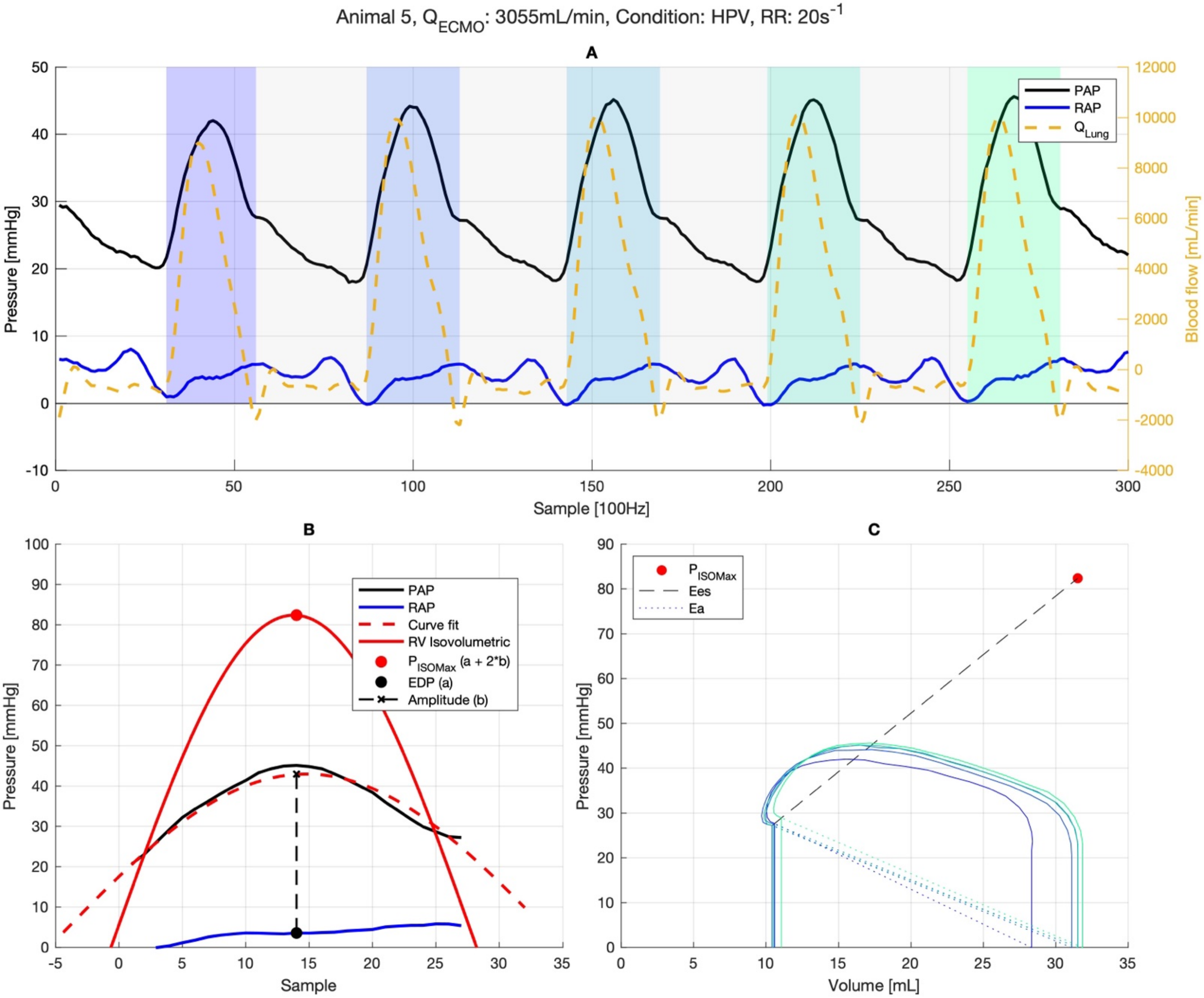

Animal 5, Q_{ECMO}: 3055mL/min, Condition: HPV, RR: 20s^{-1}
A
PAP
RAP
Q_{Lung}
Pressure [mmHg]
Blood flow [mL/min]
Sample [100Hz]
B
PAP
RAP
Curve fit
RV Isovolumetric
P_{ISOMax} (a + 2*b)
EDP (a)
Amplitude (b)
Pressure [mmHg]
Sample
C
P_{ISOMax}
Ees
Ea
Pressure [mmHg]
Volume [mL]

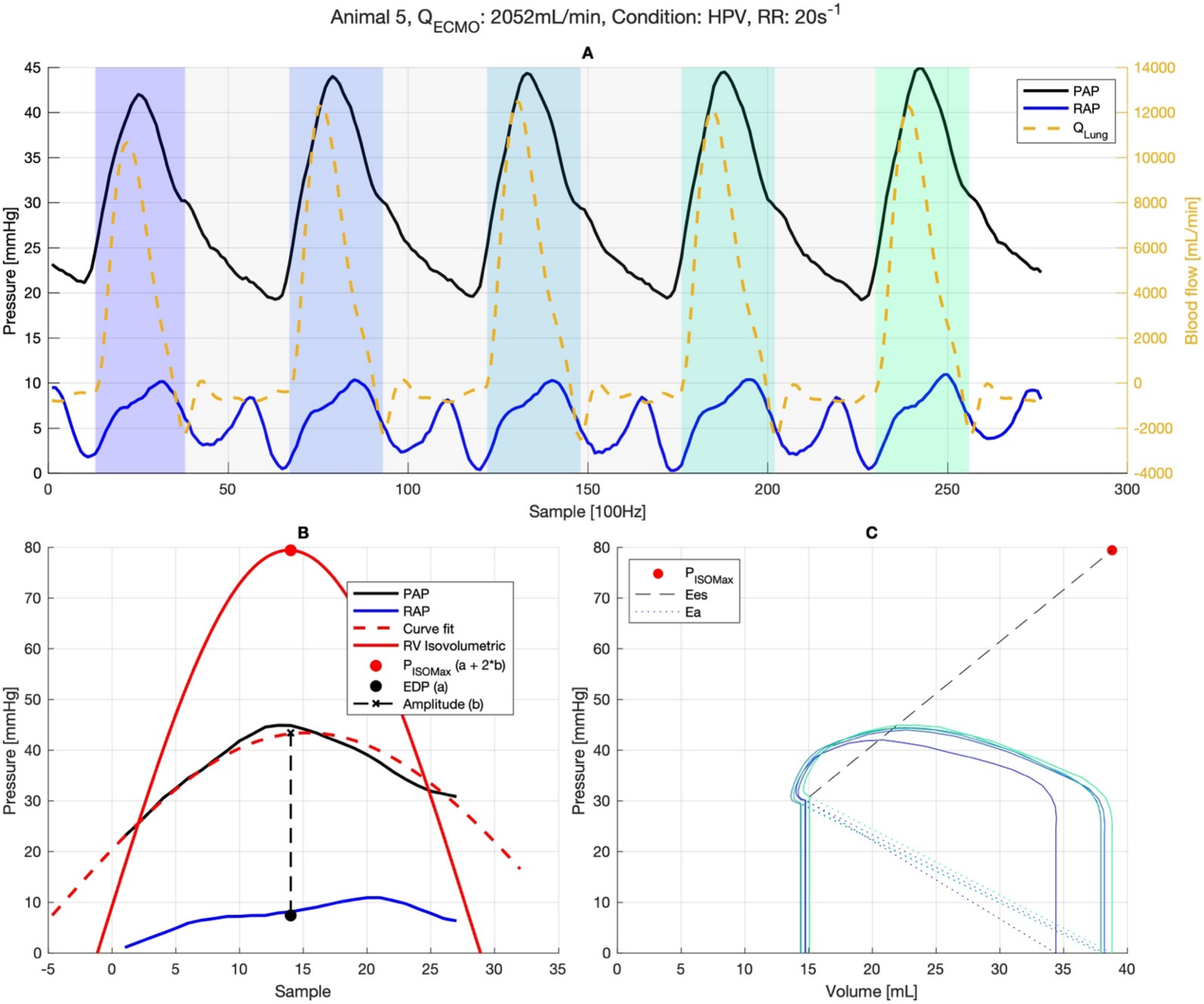
Animal 5, Q_ECMO: 2052mL/min, Condition: HPV, RR: 20s-1
A
PAP
RAP
Q_Lung
Pressure [mmHg]
Blood flow [mL/min]
Sample [100Hz]
B
PAP
RAP
Curve fit
RV Isovolumetric
P_ISOMax (a + 2*b)
EDP (a)
Amplitude (b)
Pressure [mmHg]
Sample
C
P_ISOMax
Ees
Ea
Pressure [mmHg]
Volume [mL]

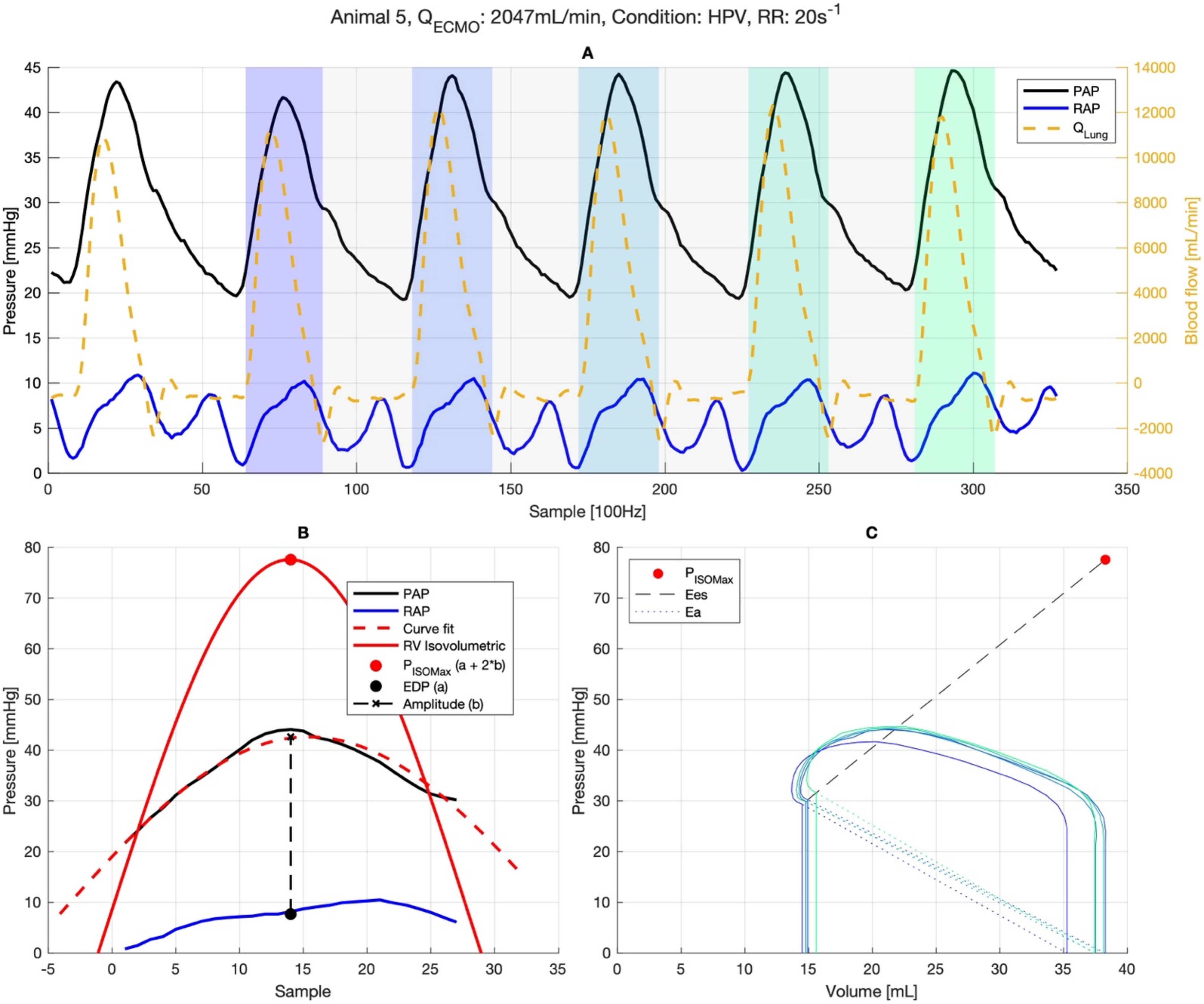

Animal 5, Q_{ECMO}: 2047mL/min, Condition: HPV, RR: $20s^{-1}$
A
PAP
RAP
Q_{Lung}
Pressure [mmHg]
Blood flow [mL/min]
Sample [100Hz]
B
PAP
RAP
Curve fit
RV Isovolumetric
P_{ISOMax} (a + 2*b)
EDP (a)
Amplitude (b)
Pressure [mmHg]
Sample
C
P_{ISOMax}
Ees
Ea
Pressure [mmHg]
Volume [mL]

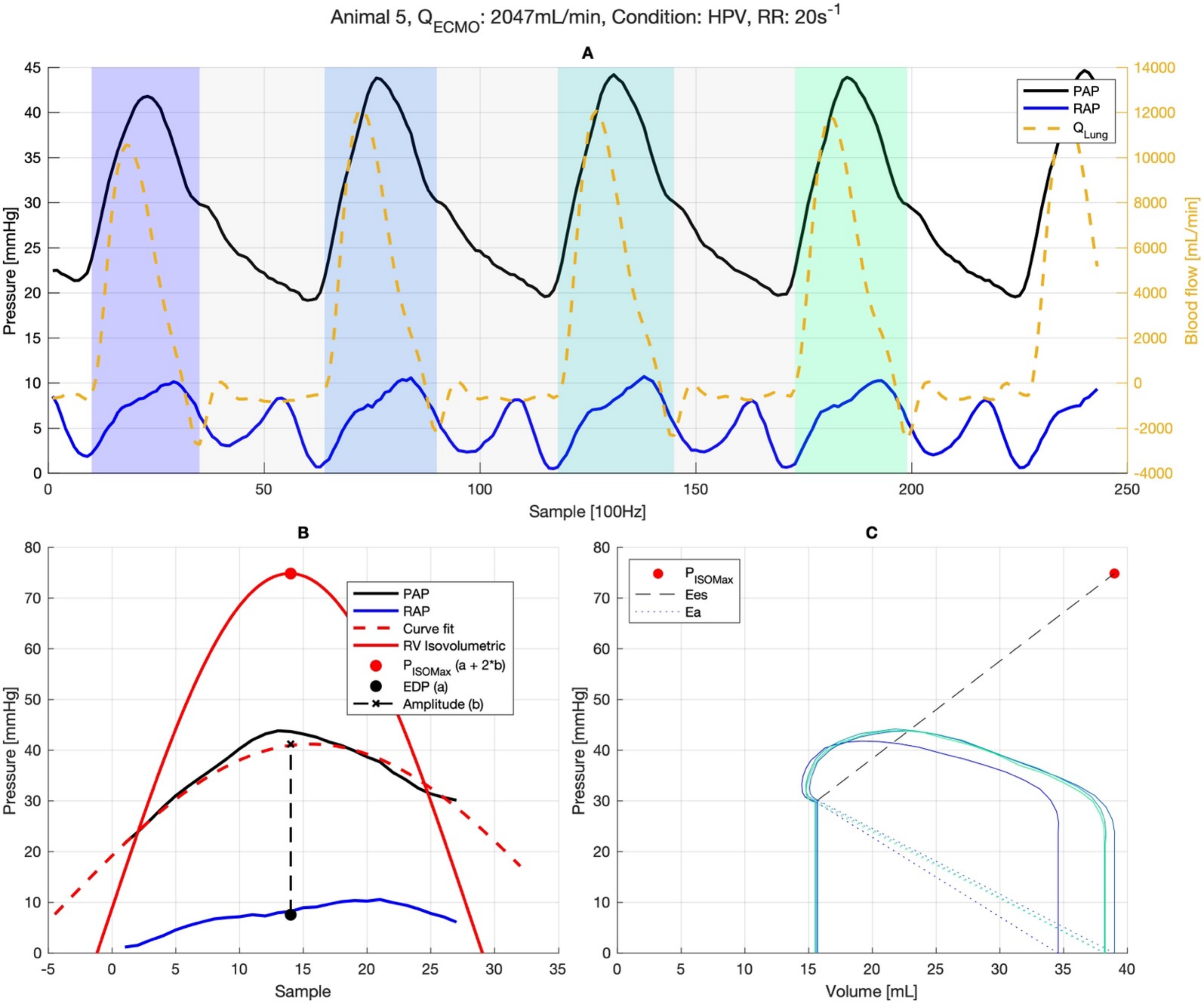

Animal 5, Q_ECMO: 2047mL/min, Condition: HPV, RR: 20s^-1
A
PAP
RAP
Q_Lung
Pressure [mmHg]
Blood flow [mL/min]
Sample [100Hz]
B
PAP
RAP
Curve fit
RV Isovolumetric
P_ISOMax (a + 2*b)
EDP (a)
Amplitude (b)
Pressure [mmHg]
Sample
C
P_ISOMax
Ees
Ea
Pressure [mmHg]
Volume [mL]

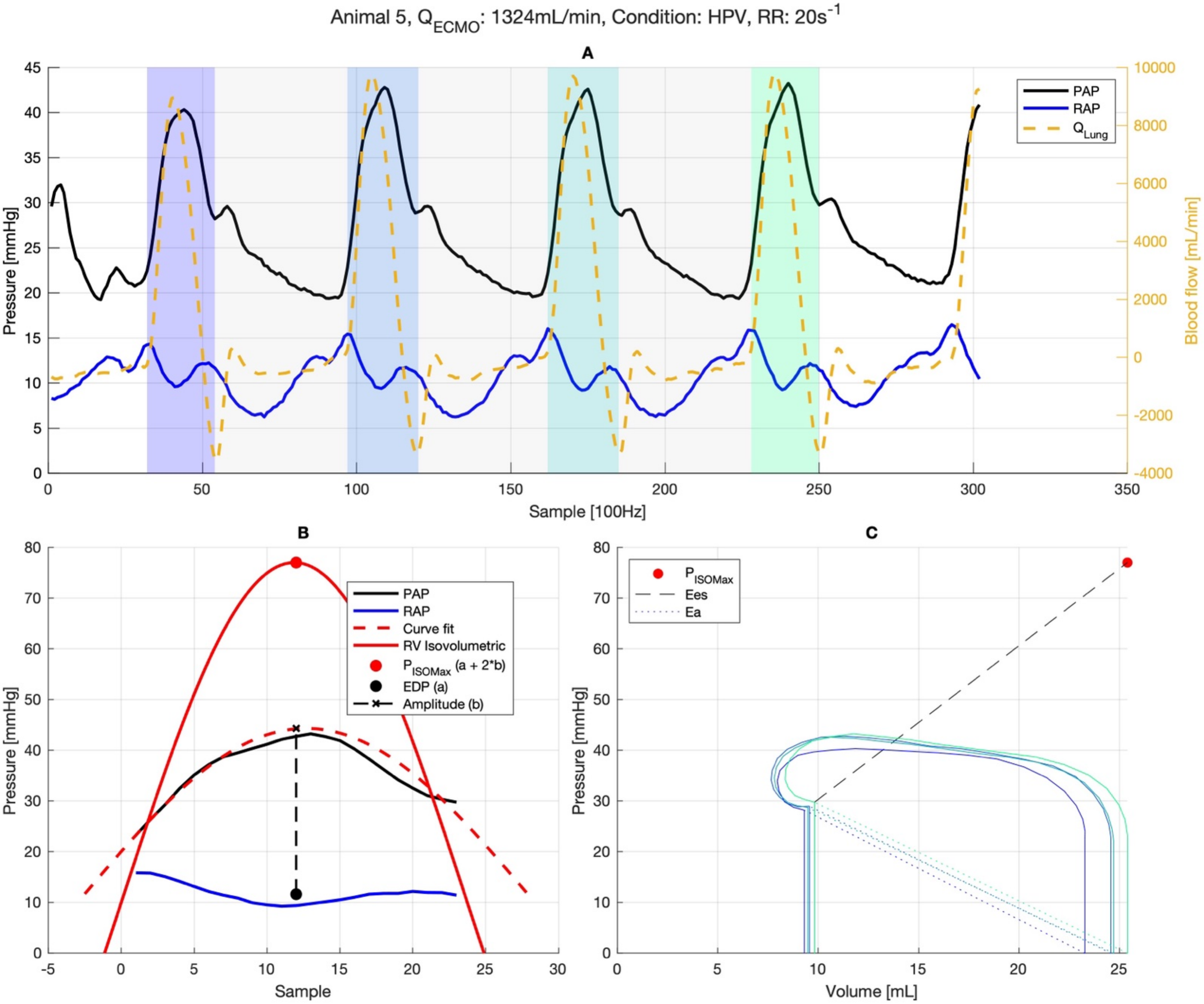

Animal 5, Q_{ECMO}: 1324mL/min, Condition: HPV, RR: $20s^{-1}$
A
PAP
RAP
Q_{Lung}
Pressure [mmHg]
Blood flow [mL/min]
Sample [100Hz]
B
PAP
RAP
Curve fit
RV Isovolumetric
P_{ISOMax} (a + 2*b)
EDP (a)
Amplitude (b)
Pressure [mmHg]
Sample
C
P_{ISOMax}
Ees
Ea
Pressure [mmHg]
Volume [mL]

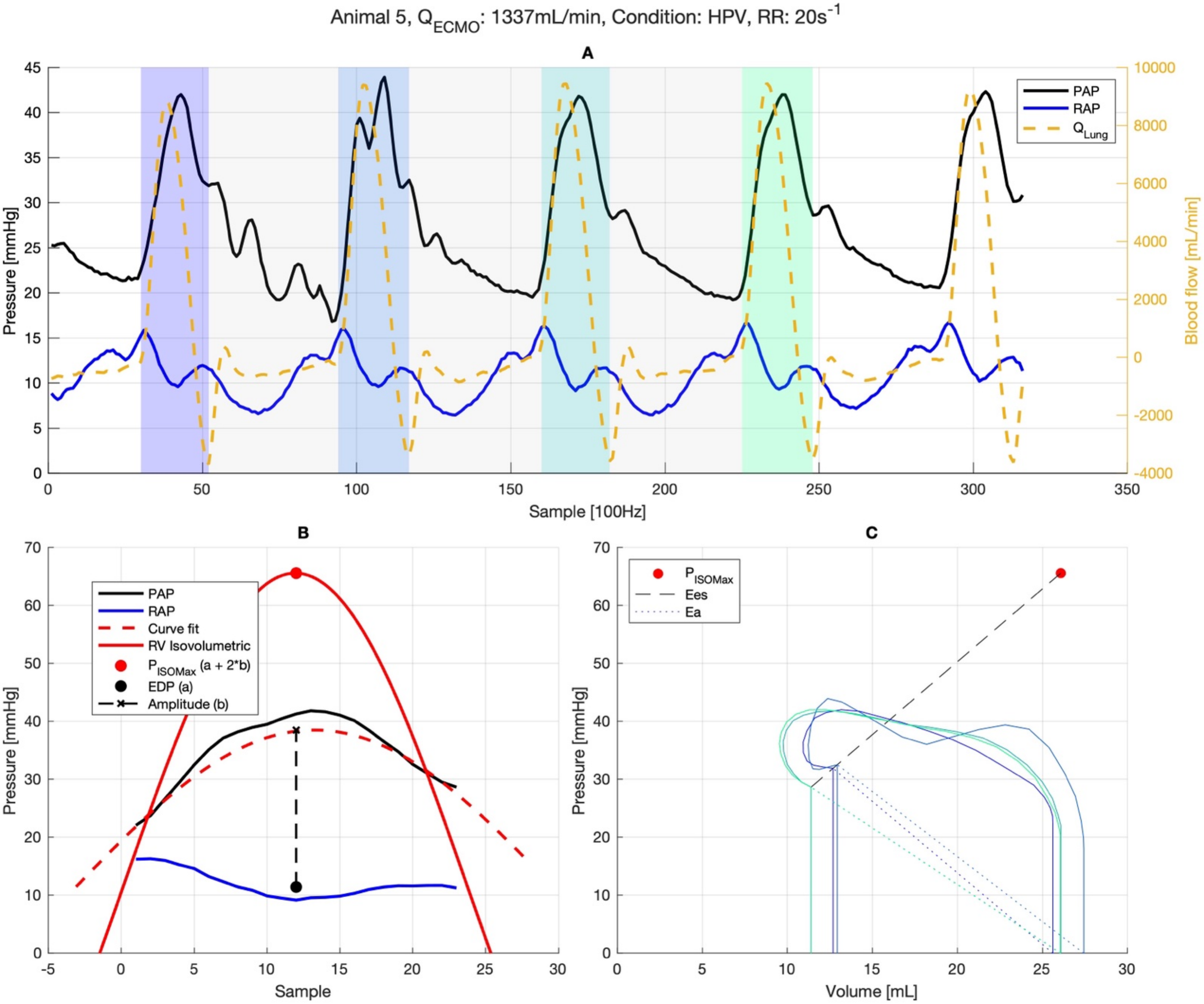

Animal 5, Q_{ECMO}: 1337mL/min, Condition: HPV, RR: $20s^{-1}$
A
PAP
RAP
Q_{Lung}
Pressure [mmHg]
Blood flow [mL/min]
Sample [100Hz]
B
PAP
RAP
Curve fit
RV Isovolumetric
P_{ISOMax} (a + 2*b)
EDP (a)
Amplitude (b)
Pressure [mmHg]
Sample
C
P_{ISOMax}
Ees
Ea
Pressure [mmHg]
Volume [mL]

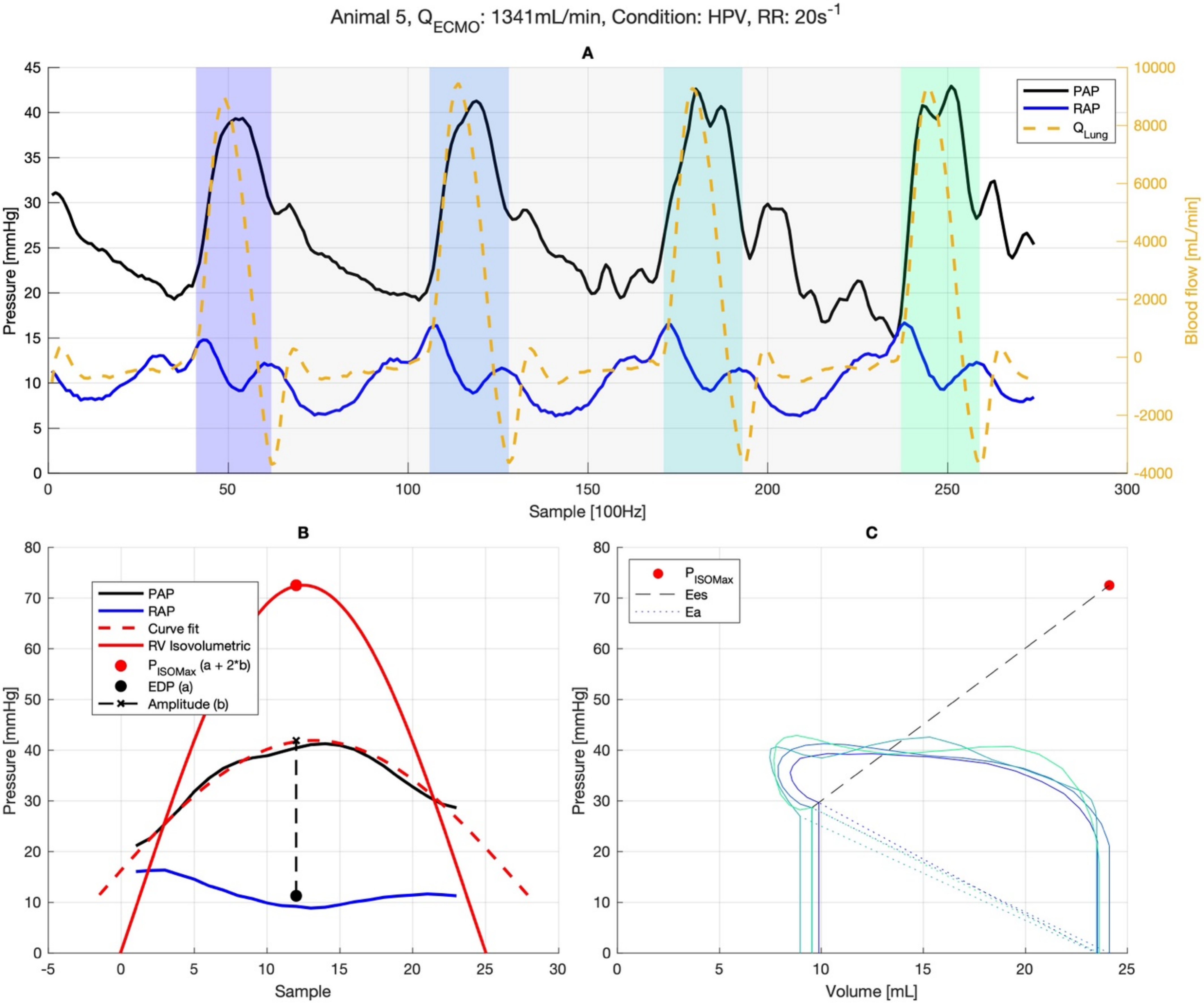
Animal 5, Q_{ECMO}: 1341mL/min, Condition: HPV, RR: 20s^{-1}
A
PAP
RAP
Q_{Lung}
Pressure [mmHg]
Blood flow [mL/min]
Sample [100Hz]
B
PAP
RAP
Curve fit
RV Isovolumetric
P_{ISOMax} (a + 2*b)
EDP (a)
Amplitude (b)
Pressure [mmHg]
Sample
C
P_{ISOMax}
Ees
Ea
Pressure [mmHg]
Volume [mL]

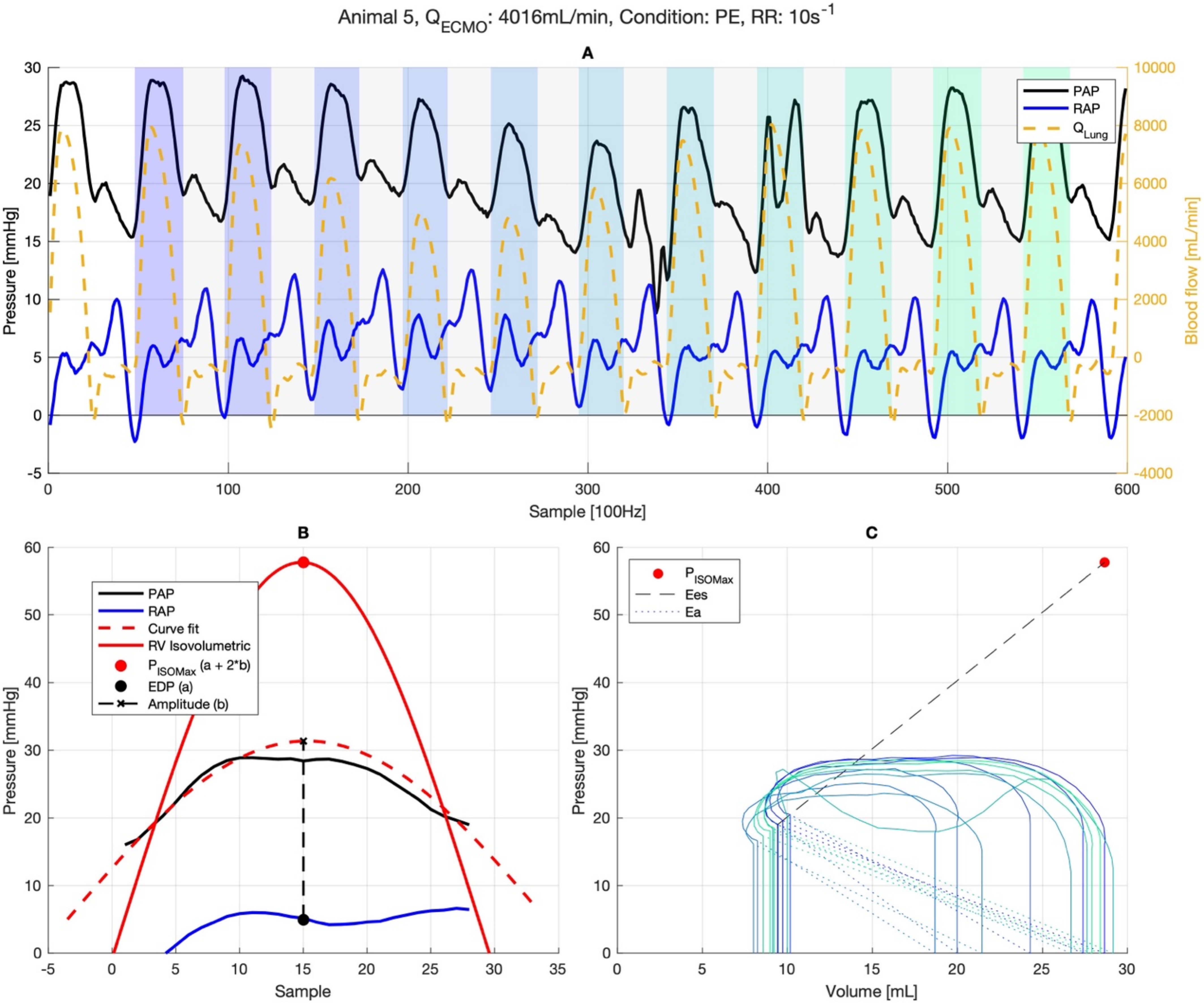

Animal 5, Q_{ECMO}: 4016mL/min, Condition: PE, RR: $10s^{-1}$
A
PAP
RAP
Q_{Lung}
Pressure [mmHg]
Blood flow [mL/min]
Sample [100Hz]
B
PAP
RAP
Curve fit
RV Isovolumetric
P_{ISOMax} (a + 2*b)
EDP (a)
Amplitude (b)
Pressure [mmHg]
Sample
C
P_{ISOMax}
Ees
Ea
Pressure [mmHg]
Volume [mL]

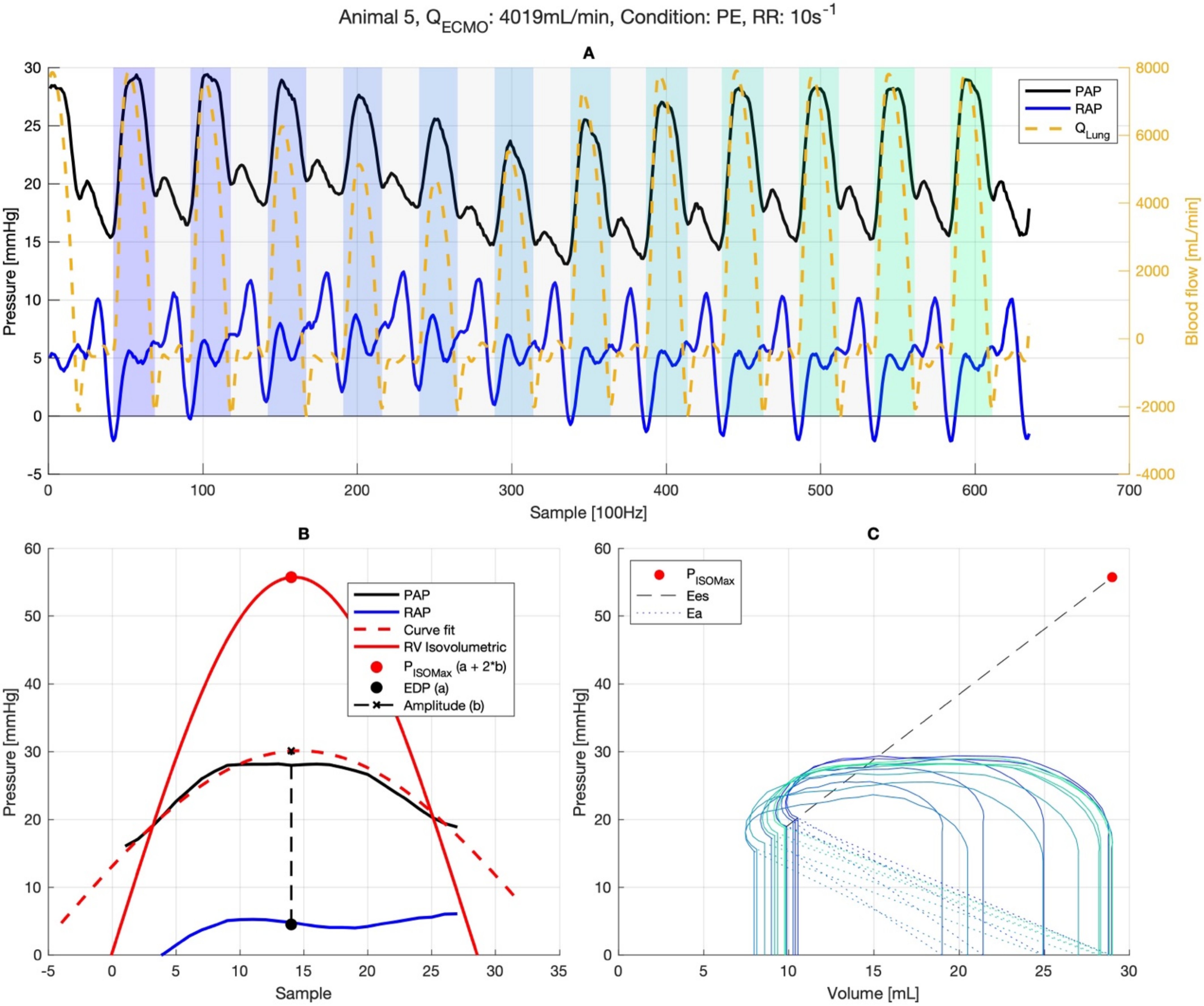

Animal 5, Q_{ECMO}: 4019mL/min, Condition: PE, RR: 10s^{-1}
A
PAP
RAP
Q_{Lung}
Pressure [mmHg]
Blood flow [mL/min]
Sample [100Hz]
B
PAP
RAP
Curve fit
RV Isovolumetric
P_{ISOMax} (a + 2*b)
EDP (a)
Amplitude (b)
Pressure [mmHg]
Sample
C
P_{ISOMax}
Ees
Ea
Pressure [mmHg]
Volume [mL]

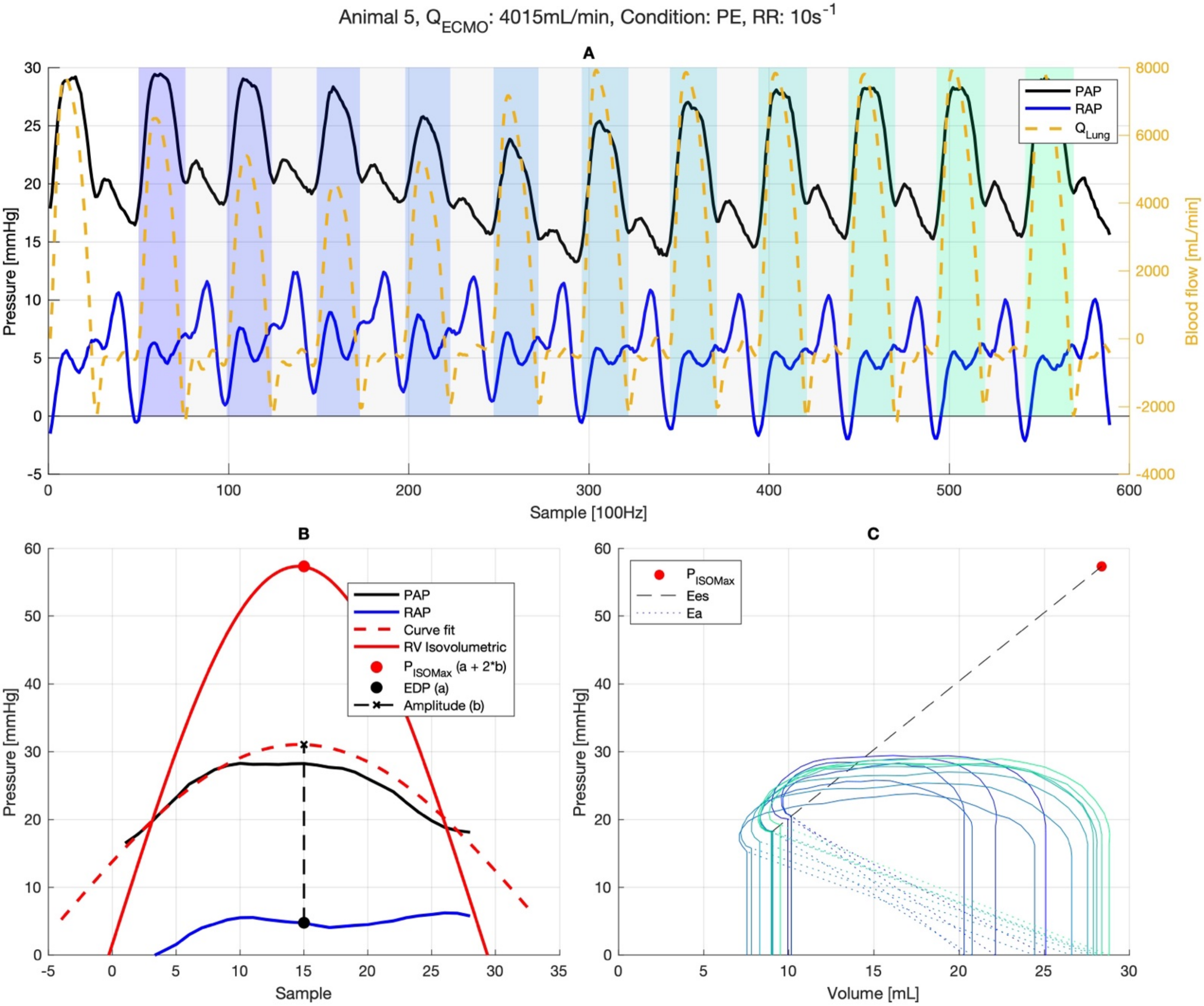

Animal 5, Q_ECMO: 4015mL/min, Condition: PE, RR: 10s^-1
A
PAP
RAP
Q_Lung
Pressure [mmHg]
Blood flow [mL/min]
Sample [100Hz]
B
PAP
RAP
Curve fit
RV Isovolumetric
P_ISOMax (a + 2*b)
EDP (a)
Amplitude (b)
Pressure [mmHg]
Sample
C
P_ISOMax
Ees
Ea
Pressure [mmHg]
Volume [mL]

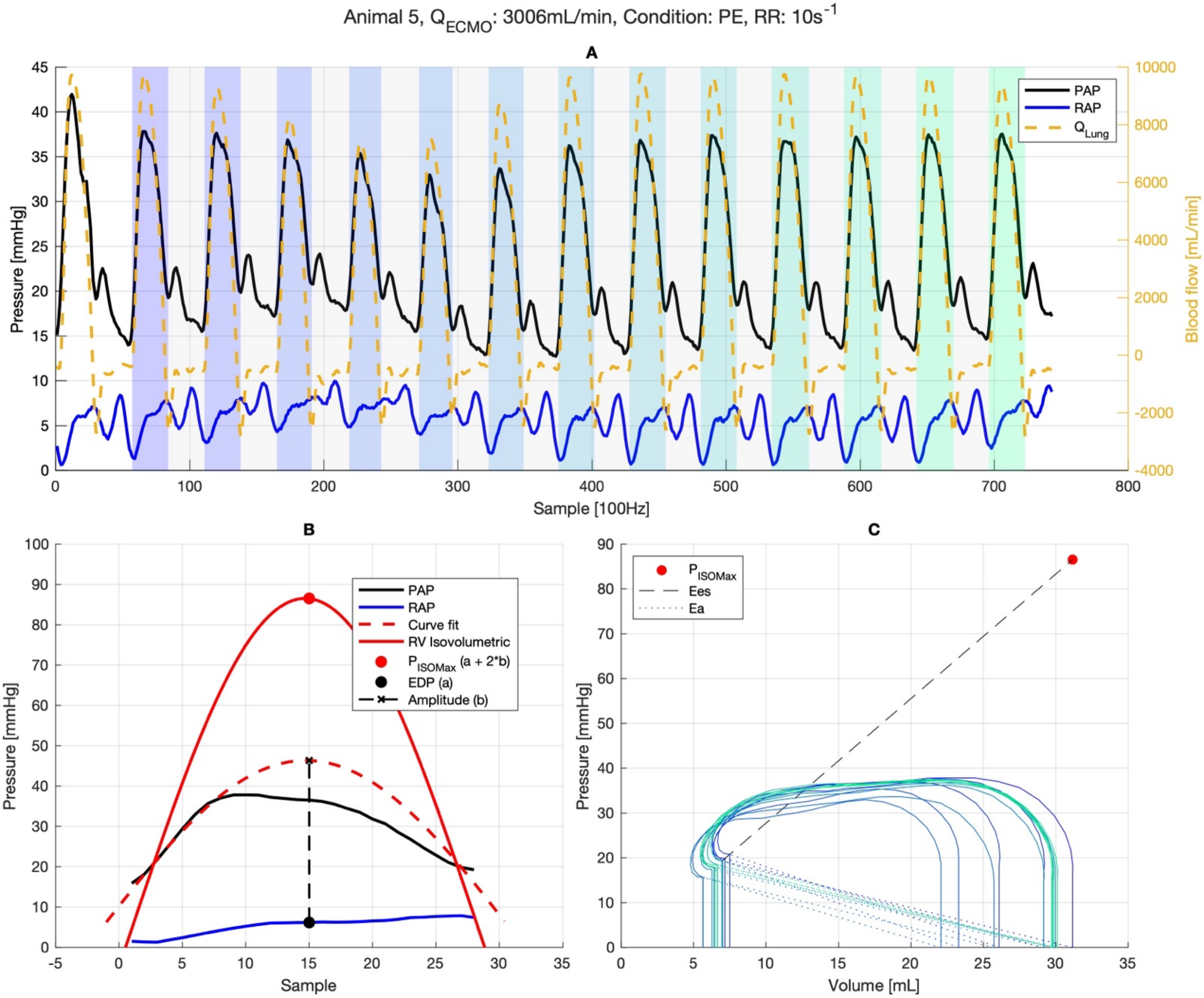

Animal 5, Q_ECMO: 3006mL/min, Condition: PE, RR: 10s^-1
A
PAP
RAP
Q_Lung
Pressure [mmHg]
Blood flow [mL/min]
Sample [100Hz]
B
PAP
RAP
Curve fit
RV Isovolumetric
P_ISOMax (a + 2*b)
EDP (a)
Amplitude (b)
Pressure [mmHg]
Sample
C
P_ISOMax
Ees
Ea
Pressure [mmHg]
Volume [mL]

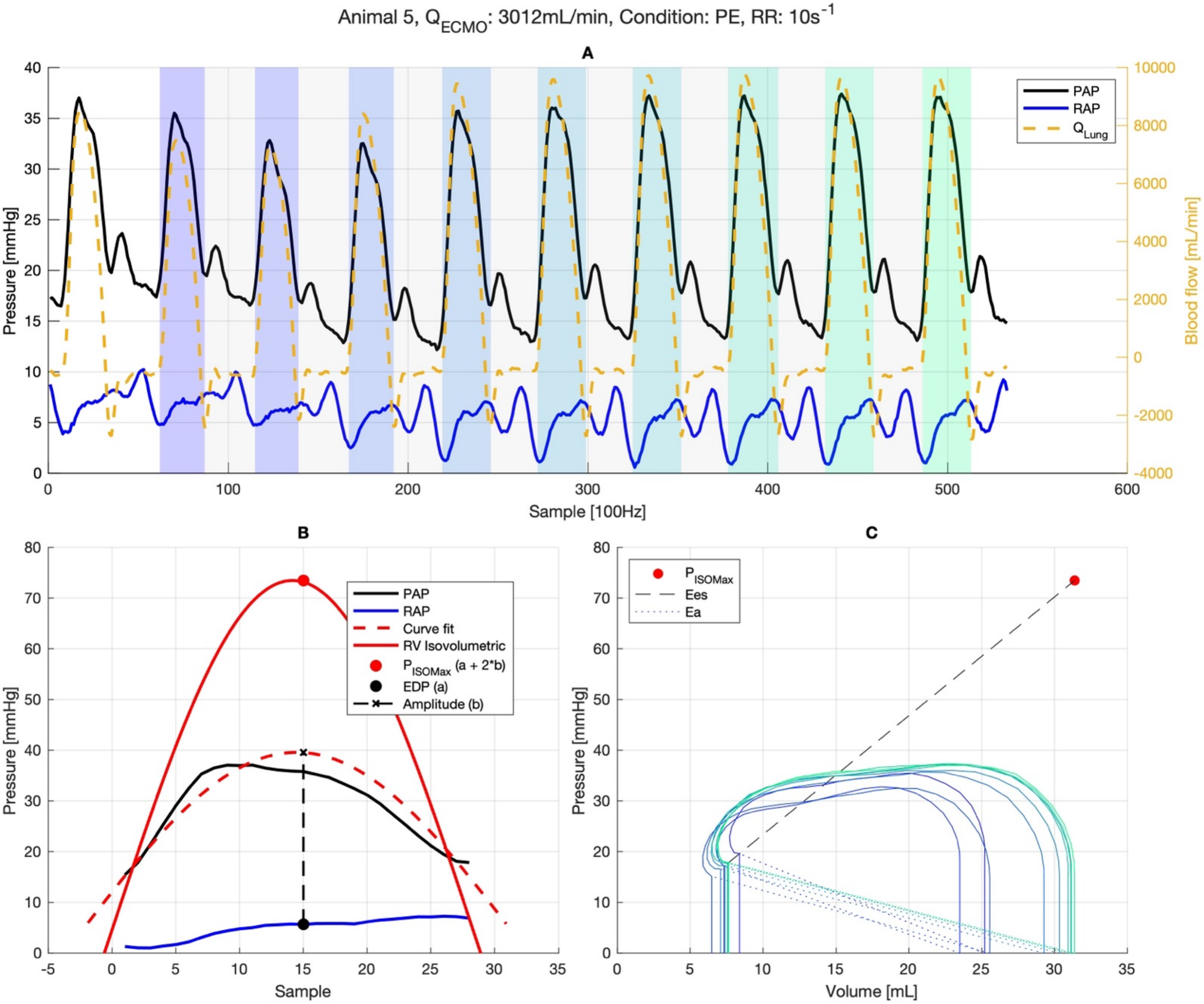

Animal 5, Q_ECMO: 3012mL/min, Condition: PE, RR: 10s^-1
A
PAP
RAP
Q_Lung
Pressure [mmHg]
Blood flow [mL/min]
Sample [100Hz]
B
PAP
RAP
Curve fit
RV Isovolumetric
P_ISOMax (a + 2*b)
EDP (a)
Amplitude (b)
Pressure [mmHg]
Sample
C
P_ISOMax
Ees
Ea
Pressure [mmHg]
Volume [mL]

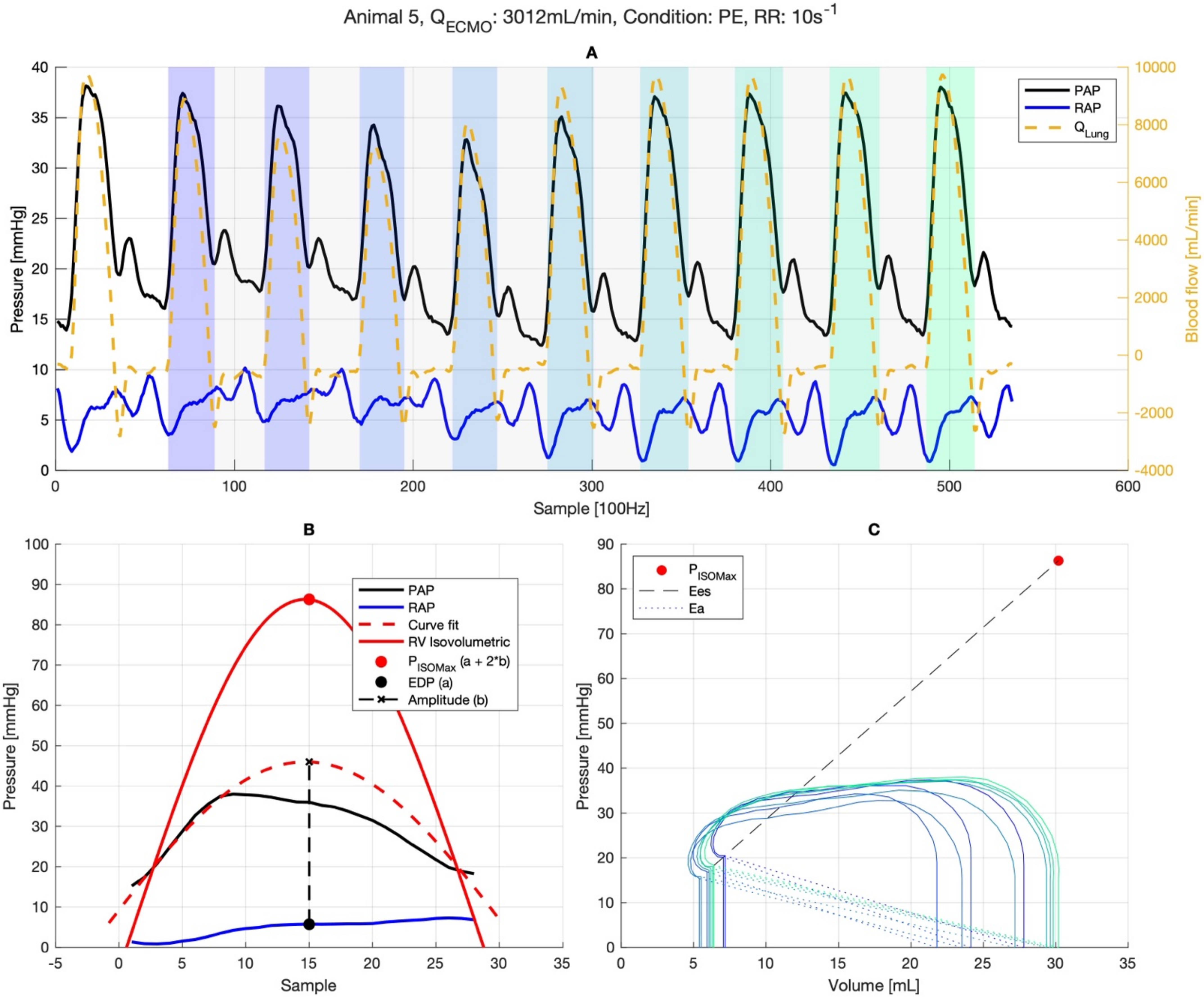

Animal 5, Q_{ECMO}: 3012mL/min, Condition: PE, RR: $10s^{-1}$
A
Pressure [mmHg]
Blood flow [mL/min]
Sample [100Hz]
PAP
RAP
Q_{Lung}
B
Pressure [mmHg]
Sample
PAP
RAP
Curve fit
RV Isovolumetric
P_{ISOMax} (a + 2*b)
EDP (a)
Amplitude (b)
C
Pressure [mmHg]
Volume [mL]
P_{ISOMax}
Ees
Ea

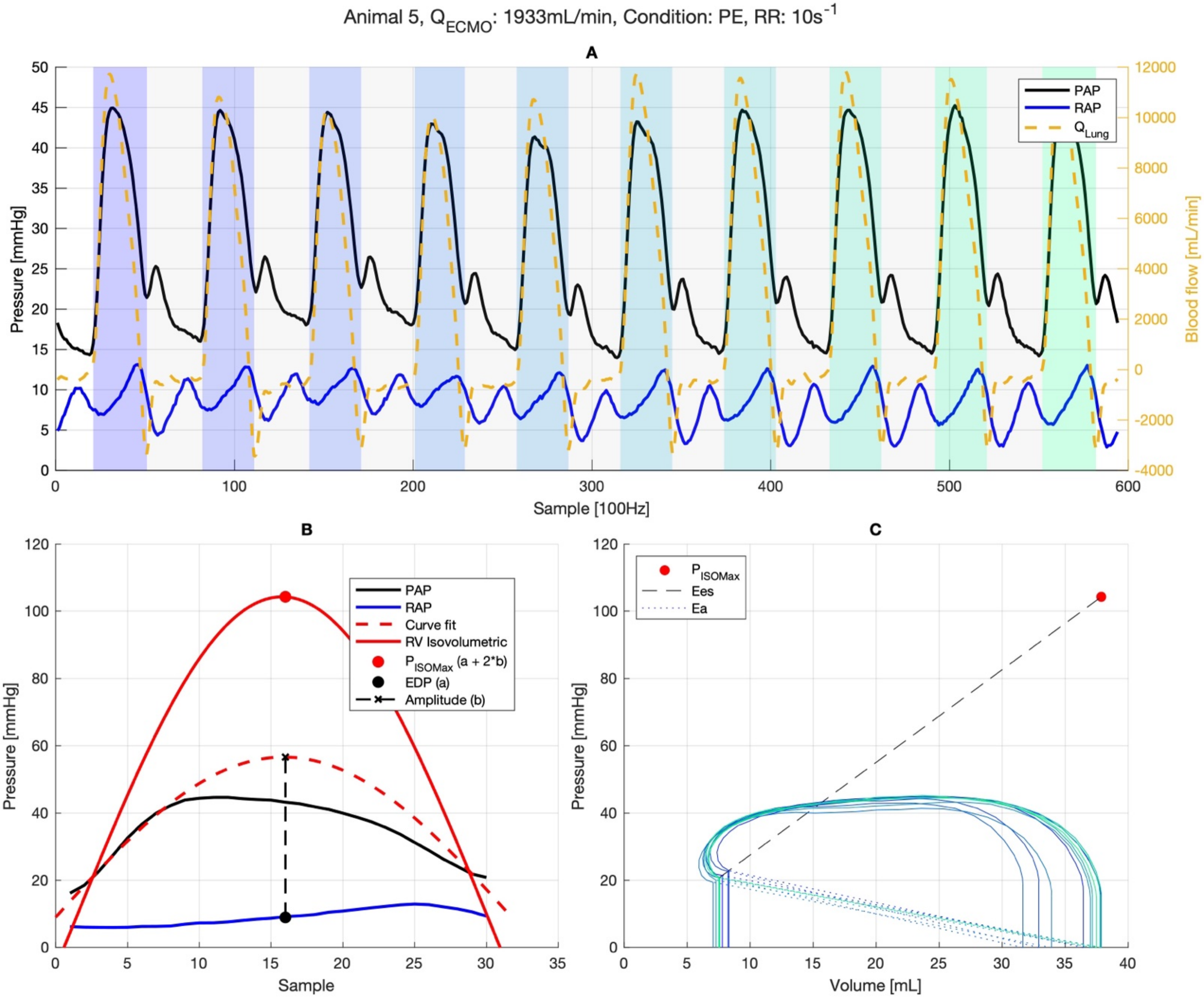

Animal 5, Q_{ECMO}: 1933mL/min, Condition: PE, RR: $10s^{-1}$
A
PAP
RAP
Q_{Lung}
Pressure [mmHg]
Blood flow [mL/min]
Sample [100Hz]
B
PAP
RAP
Curve fit
RV Isovolumetric
P_{ISOMax} (a + 2*b)
EDP (a)
Amplitude (b)
Pressure [mmHg]
Sample
C
P_{ISOMax}
Ees
Ea
Pressure [mmHg]
Volume [mL]

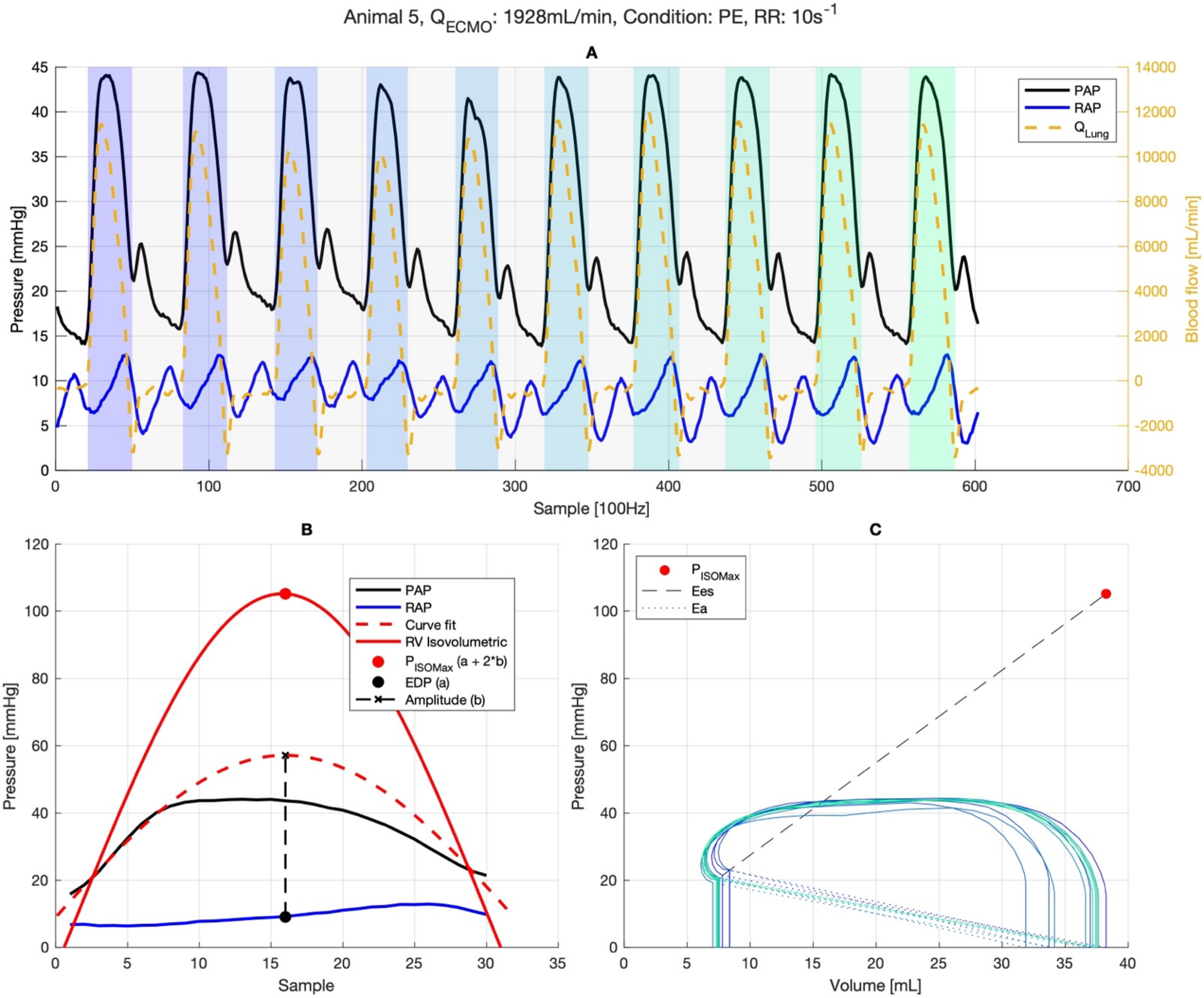

Animal 5, Q_{ECMO}: 1928mL/min, Condition: PE, RR: $10s^{-1}$
A
Pressure [mmHg]
Blood flow [mL/min]
Sample [100Hz]
PAP
RAP
Q_{Lung}
B
Pressure [mmHg]
Sample
PAP
RAP
Curve fit
RV Isovolumetric
P_{ISOMax} (a + 2*b)
EDP (a)
Amplitude (b)
C
Pressure [mmHg]
Volume [mL]
P_{ISOMax}
Ees
Ea

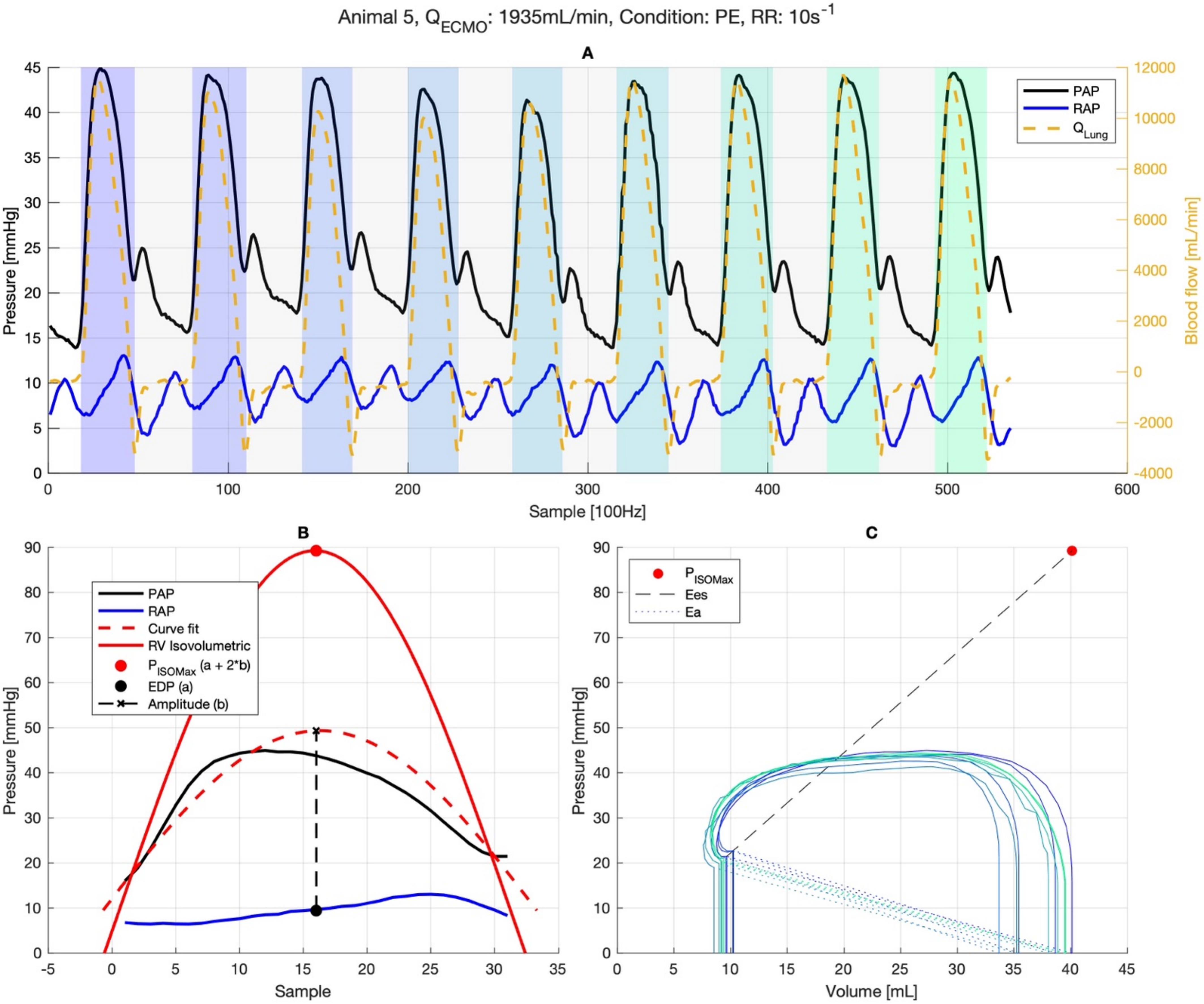

Animal 5, Q_{ECMO}: 1935mL/min, Condition: PE, RR: $10s^{-1}$
A
PAP
RAP
Q_{Lung}
Pressure [mmHg]
Blood flow [mL/min]
Sample [100Hz]
B
PAP
RAP
Curve fit
RV Isovolumetric
P_{ISOMax} (a + 2*b)
EDP (a)
Amplitude (b)
Pressure [mmHg]
Sample
C
P_{ISOMax}
Ees
Ea
Pressure [mmHg]
Volume [mL]

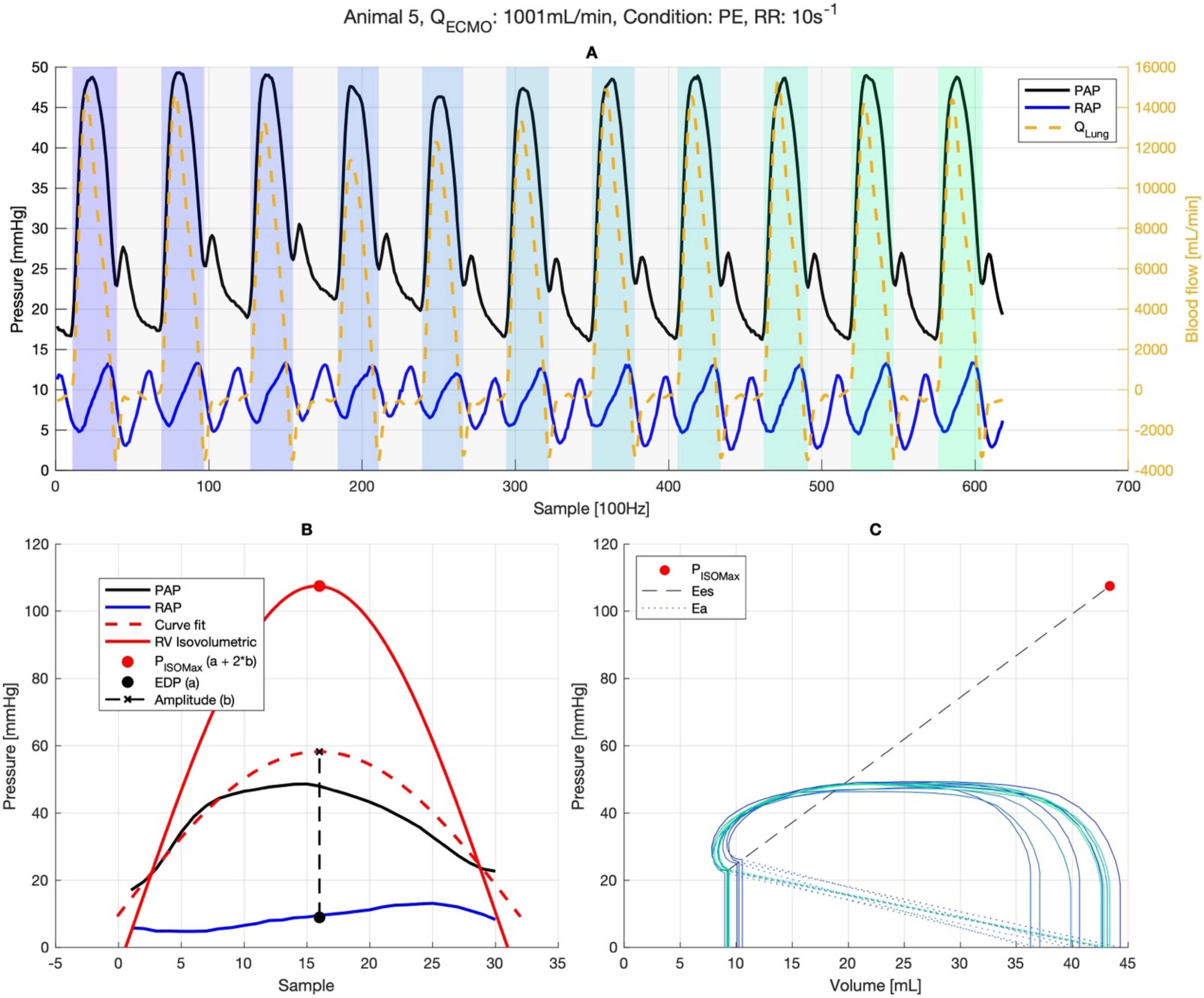

Animal 5, Q_{ECMO}: 1001mL/min, Condition: PE, RR: $10s^{-1}$
A
PAP
RAP
Q_{Lung}
Pressure [mmHg]
Blood flow [mL/min]
Sample [100Hz]
B
PAP
RAP
Curve fit
RV Isovolumetric
P_{ISOMax} (a + 2*b)
EDP (a)
Amplitude (b)
Pressure [mmHg]
Sample
C
P_{ISOMax}
Ees
Ea
Pressure [mmHg]
Volume [mL]

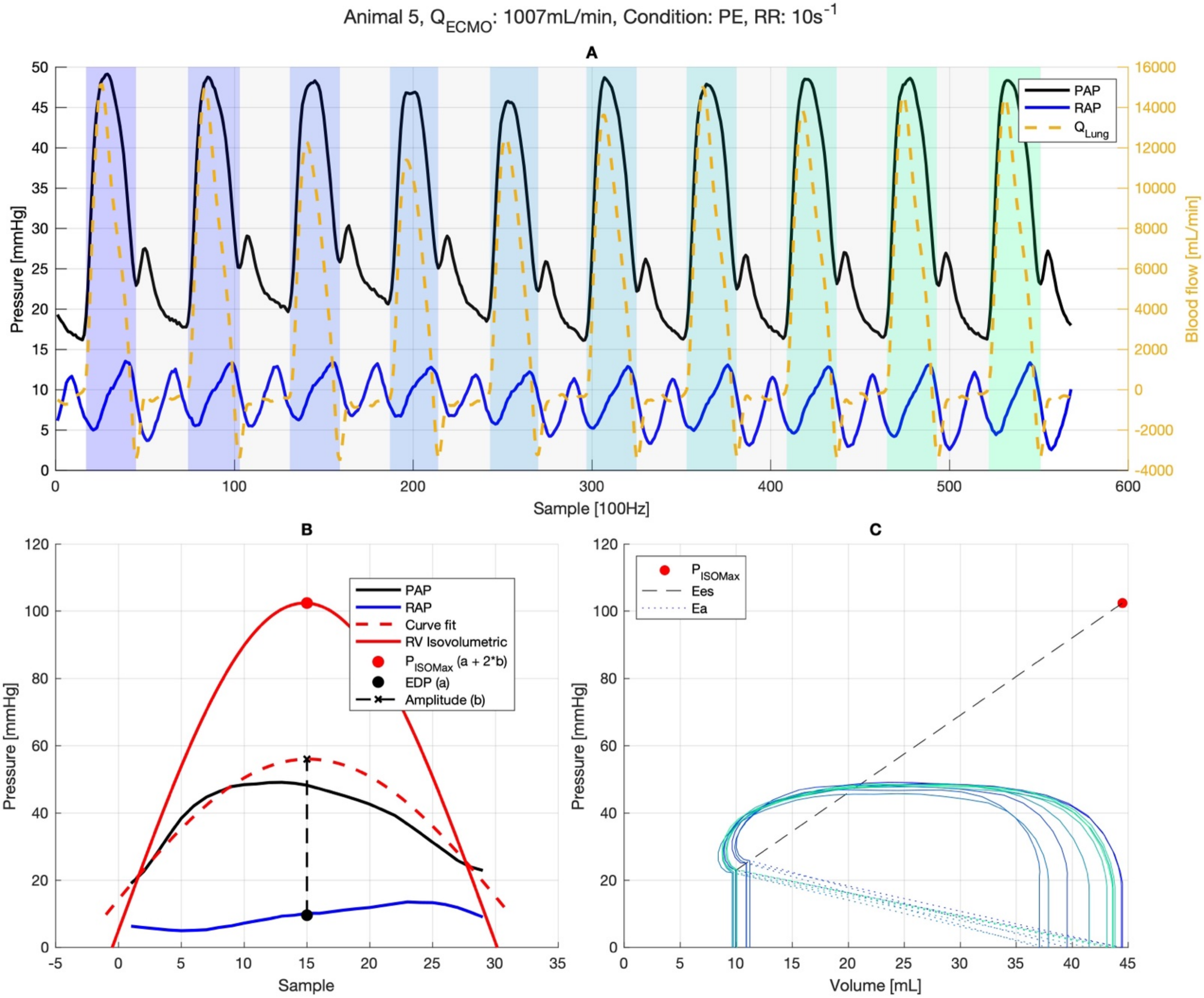

Animal 5, Q_{ECMO}: 1007mL/min, Condition: PE, RR: $10s^{-1}$
A
PAP
RAP
Q_{Lung}
Pressure [mmHg]
Blood flow [mL/min]
Sample [100Hz]
B
PAP
RAP
Curve fit
RV Isovolumetric
P_{ISOMax} (a + 2*b)
EDP (a)
Amplitude (b)
Pressure [mmHg]
Sample
C
P_{ISOMax}
Ees
Ea
Pressure [mmHg]
Volume [mL]

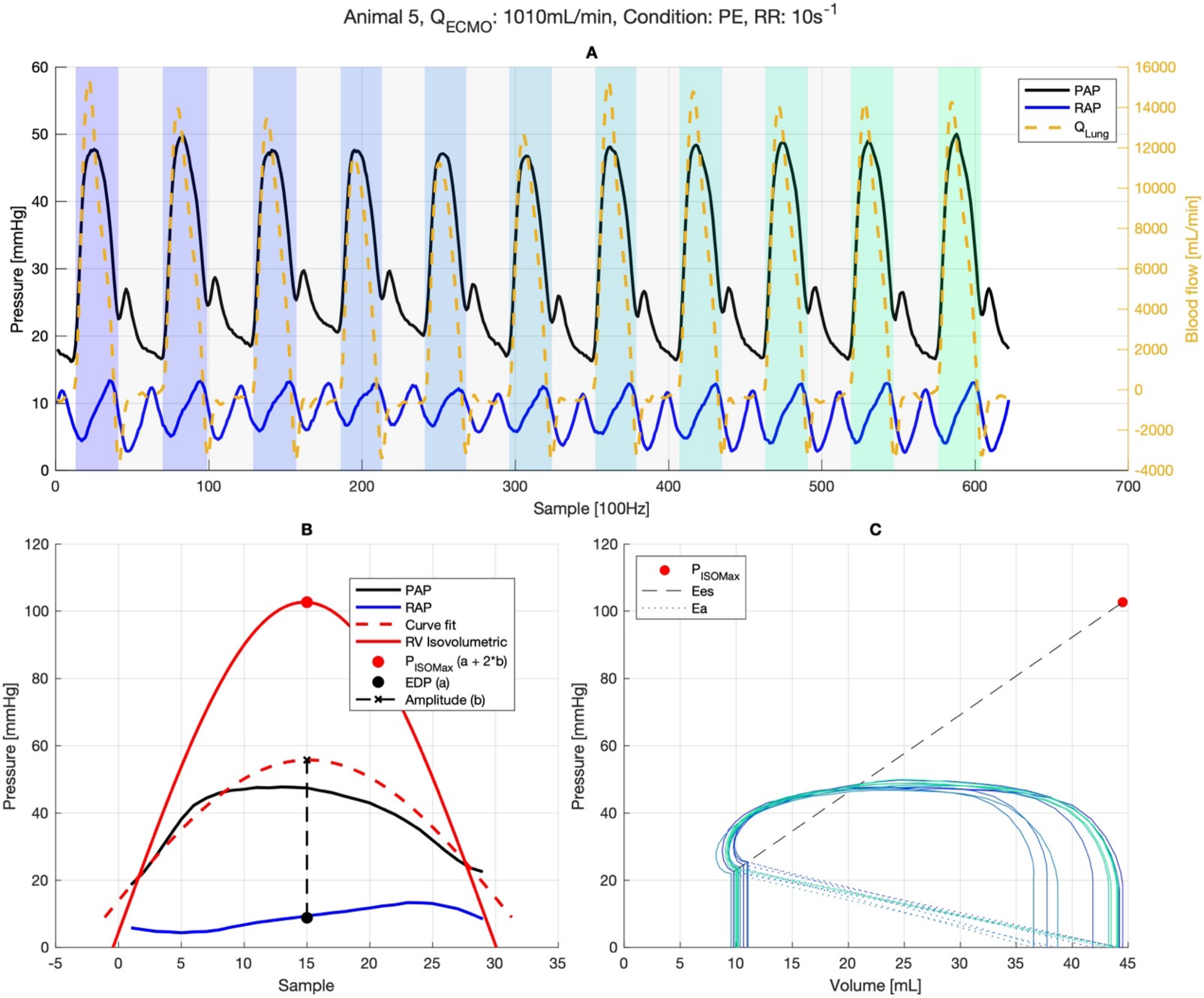

Animal 5, Q_ECMO: 1010mL/min, Condition: PE, RR: 10s^-1
A
Pressure [mmHg]
Blood flow [mL/min]
Sample [100Hz]
PAP
RAP
Q_Lung
B
PAP
RAP
Curve fit
RV Isovolumetric
P_ISOMax (a + 2*b)
EDP (a)
Amplitude (b)
Pressure [mmHg]
Sample
C
P_ISOMax
Ees
Ea
Pressure [mmHg]
Volume [mL]

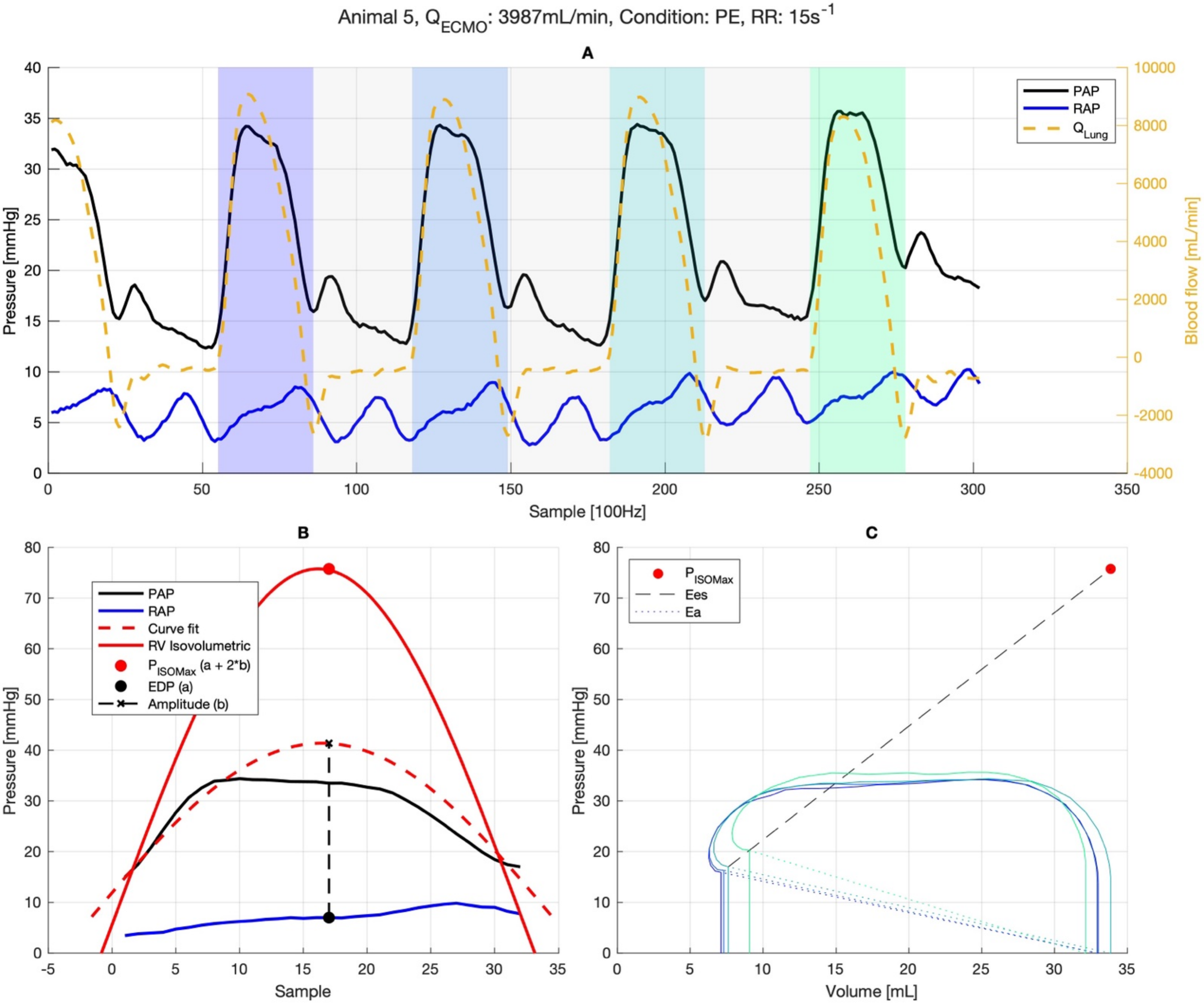

Animal 5, Q_{ECMO}: 3987mL/min, Condition: PE, RR: 15s^{-1}
A
PAP
RAP
Q_{Lung}
Pressure [mmHg]
Blood flow [mL/min]
Sample [100Hz]
B
PAP
RAP
Curve fit
RV Isovolumetric
P_{ISOMax} (a + 2*b)
EDP (a)
Amplitude (b)
Pressure [mmHg]
Sample
C
P_{ISOMax}
Ees
Ea
Pressure [mmHg]
Volume [mL]

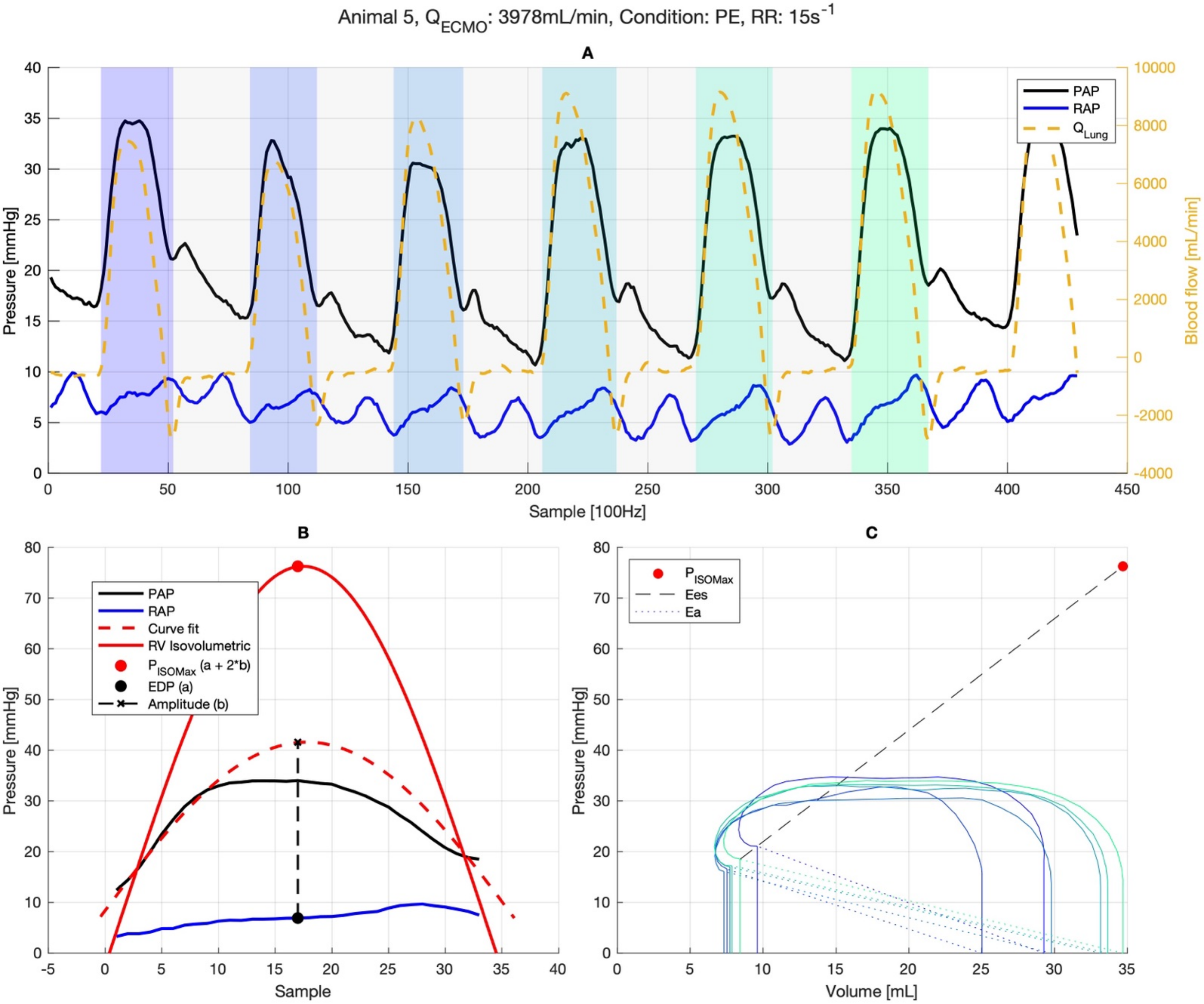

Animal 5, Q_{ECMO}: 3978mL/min, Condition: PE, RR: $15s^{-1}$
A
PAP
RAP
Q_{Lung}
Pressure [mmHg]
Blood flow [mL/min]
Sample [100Hz]
B
PAP
RAP
Curve fit
RV Isovolumetric
P_{ISOMax} (a + 2*b)
EDP (a)
Amplitude (b)
Pressure [mmHg]
Sample
C
P_{ISOMax}
Ees
Ea
Pressure [mmHg]
Volume [mL]

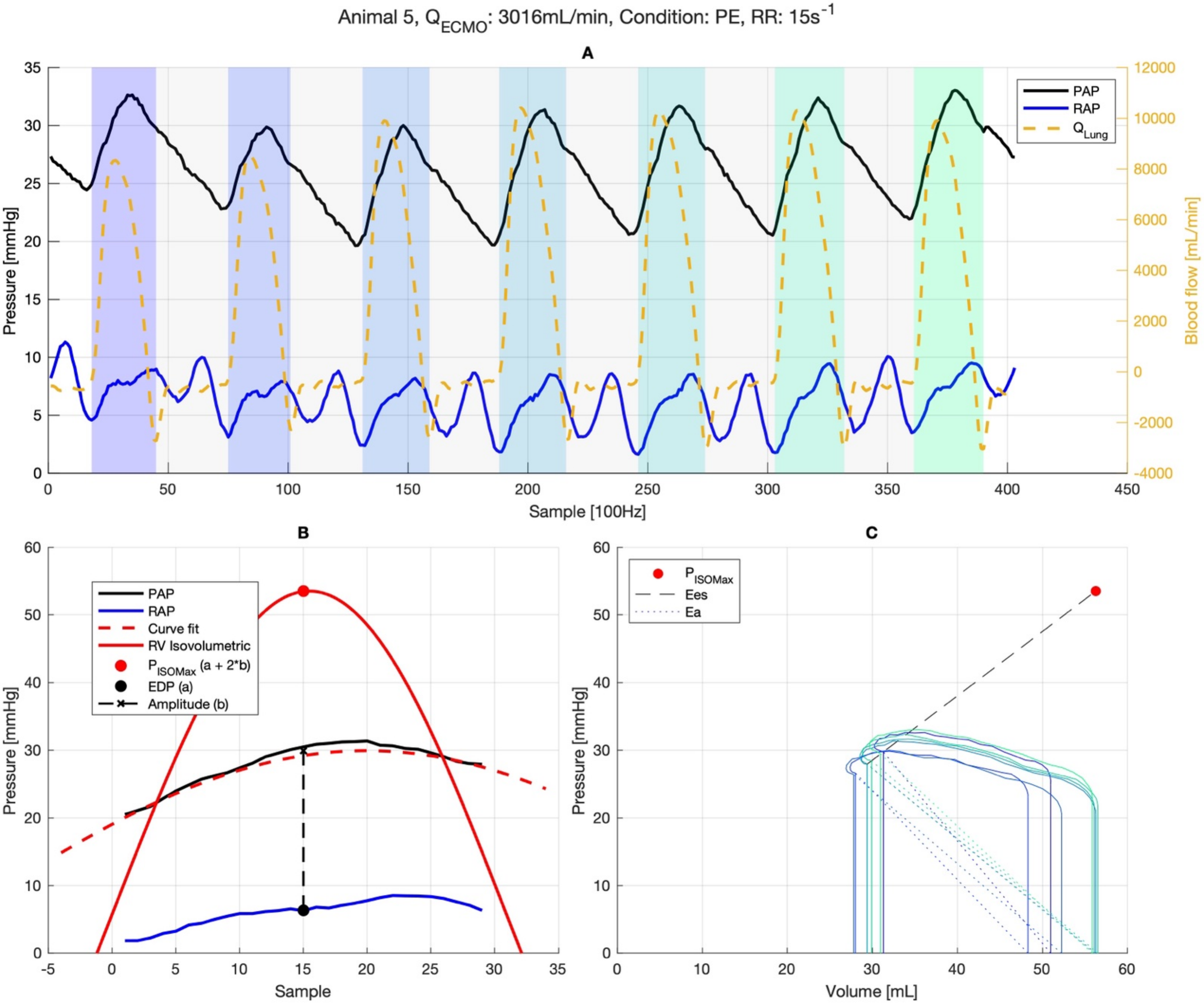

Animal 5, Q_ECMO: 3016mL/min, Condition: PE, RR: 15s^-1
A
PAP
RAP
Q_Lung
Pressure [mmHg]
Blood flow [mL/min]
Sample [100Hz]
B
PAP
RAP
Curve fit
RV Isovolumetric
P_ISOMax (a + 2*b)
EDP (a)
Amplitude (b)
Pressure [mmHg]
Sample
C
P_ISOMax
Ees
Ea
Pressure [mmHg]
Volume [mL]

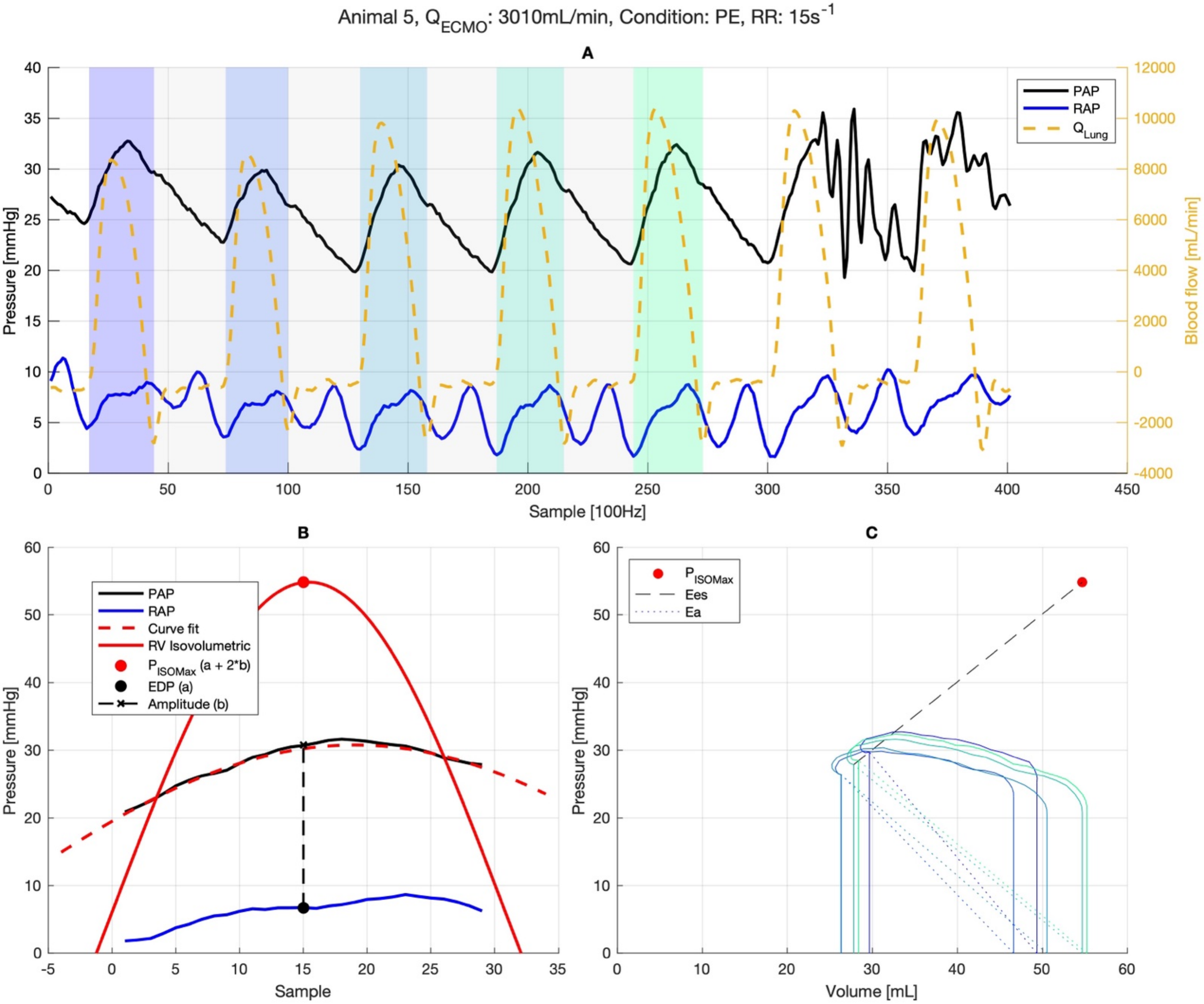

Animal 5, Q_ECMO: 3010mL/min, Condition: PE, RR: 15s^-1
A
Pressure [mmHg]
Blood flow [mL/min]
Sample [100Hz]
PAP
RAP
Q_Lung
B
PAP
RAP
Curve fit
RV Isovolumetric
P_ISOMax (a + 2*b)
EDP (a)
Amplitude (b)
Pressure [mmHg]
Sample
C
P_ISOMax
Ees
Ea
Pressure [mmHg]
Volume [mL]

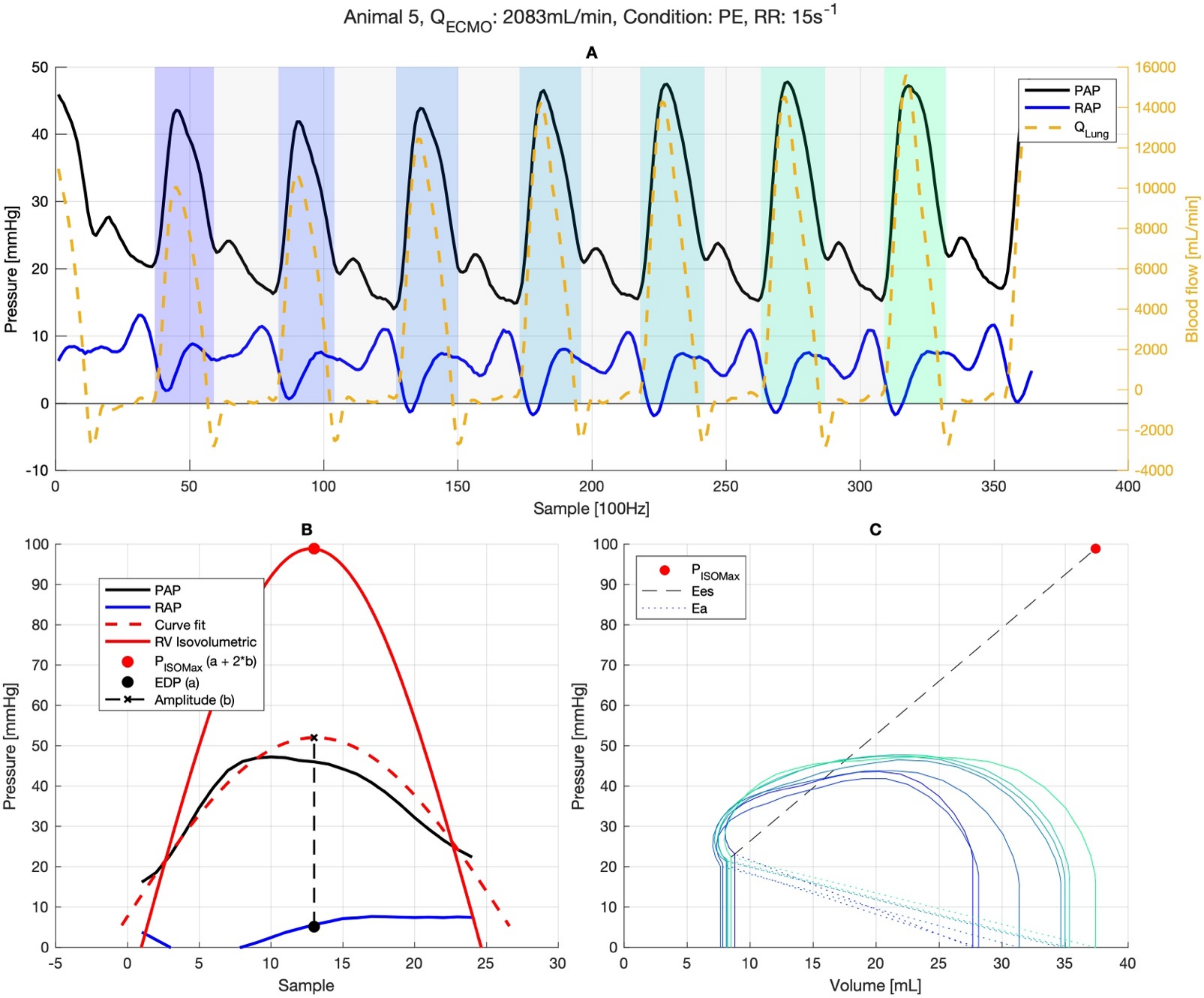
Animal 5, Q_{ECMO}: 2083mL/min, Condition: PE, RR: $15s^{-1}$
A
PAP
RAP
Q_{Lung}
Pressure [mmHg]
Blood flow [mL/min]
Sample [100Hz]
B
PAP
RAP
Curve fit
RV Isovolumetric
P_{ISOMax} (a + 2*b)
EDP (a)
Amplitude (b)
Pressure [mmHg]
Sample
C
P_{ISOMax}
Ees
Ea
Pressure [mmHg]
Volume [mL]

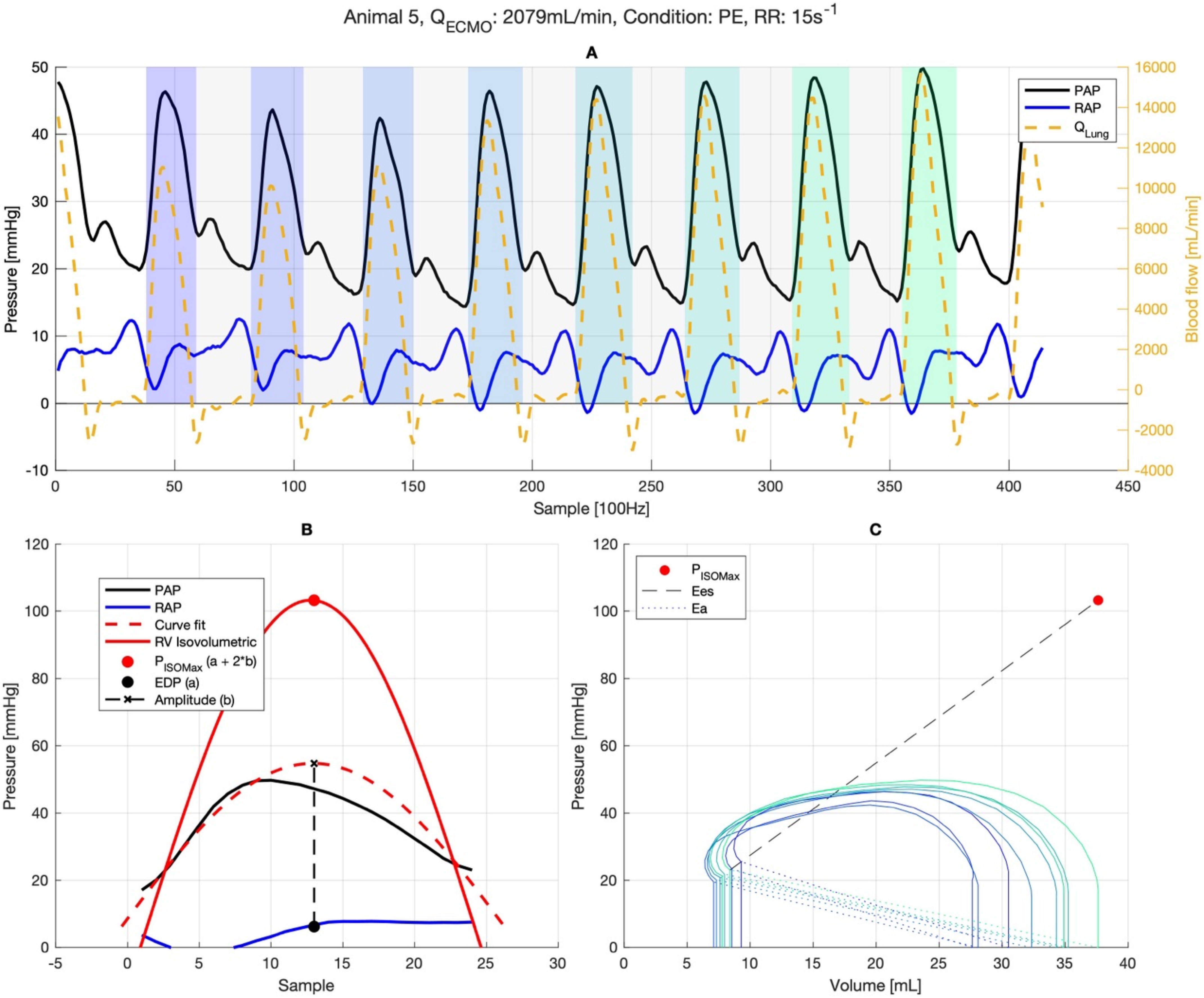

Animal 5, Q_ECMO: 2079mL/min, Condition: PE, RR: 15s^-1
A
PAP
RAP
Q_Lung
Pressure [mmHg]
Blood flow [mL/min]
Sample [100Hz]
B
PAP
RAP
Curve fit
RV Isovolumetric
P_ISOMax (a + 2*b)
EDP (a)
Amplitude (b)
Pressure [mmHg]
Sample
C
P_ISOMax
Ees
Ea
Pressure [mmHg]
Volume [mL]

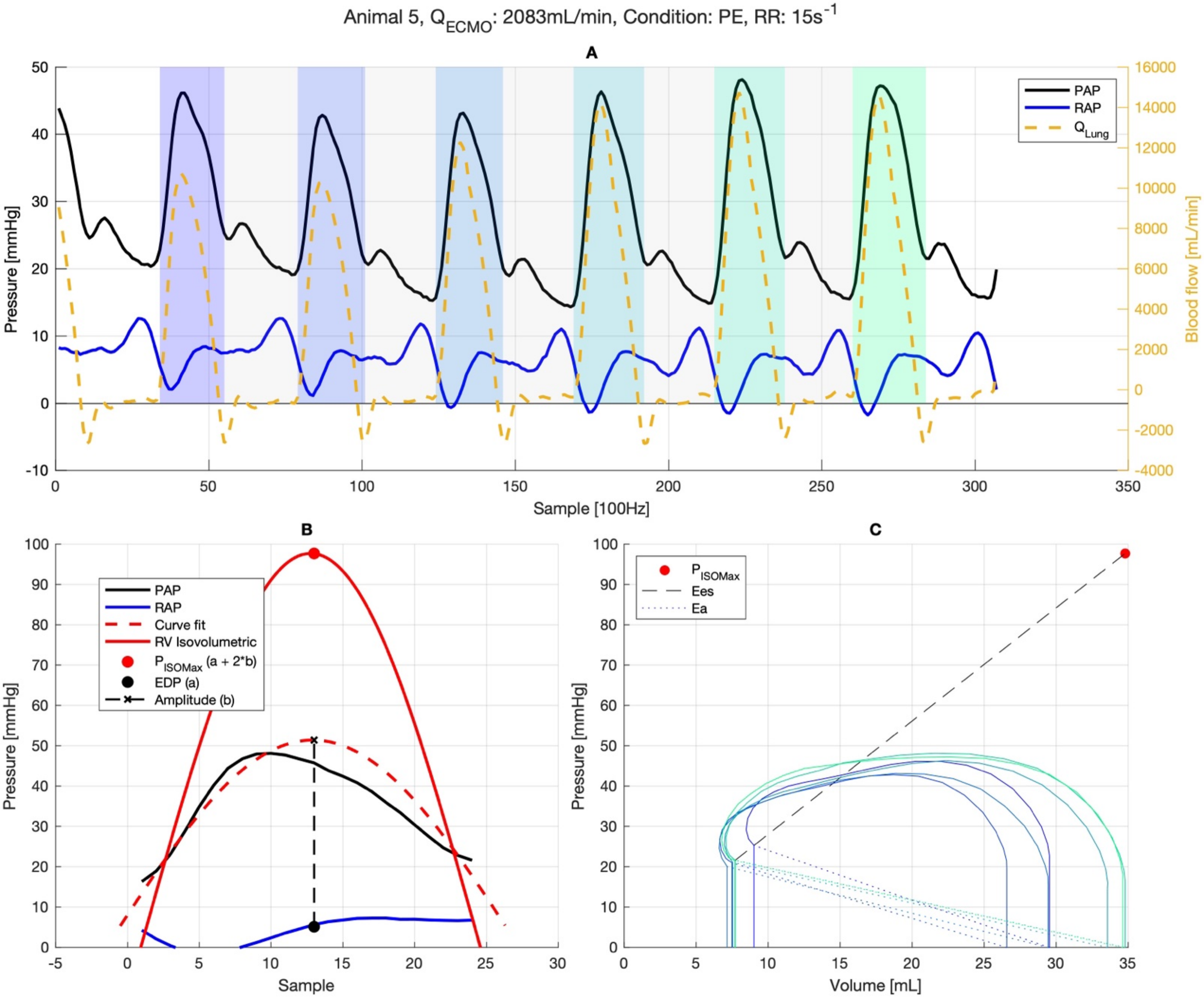

Animal 5, Q_ECMO: 2083mL/min, Condition: PE, RR: 15s^-1
A
Pressure [mmHg]
Blood flow [mL/min]
Sample [100Hz]
PAP
RAP
Q_Lung
B
Pressure [mmHg]
Sample
PAP
RAP
Curve fit
RV Isovolumetric
P_ISOMax (a + 2*b)
EDP (a)
Amplitude (b)
C
Pressure [mmHg]
Volume [mL]
P_ISOMax
Ees
Ea

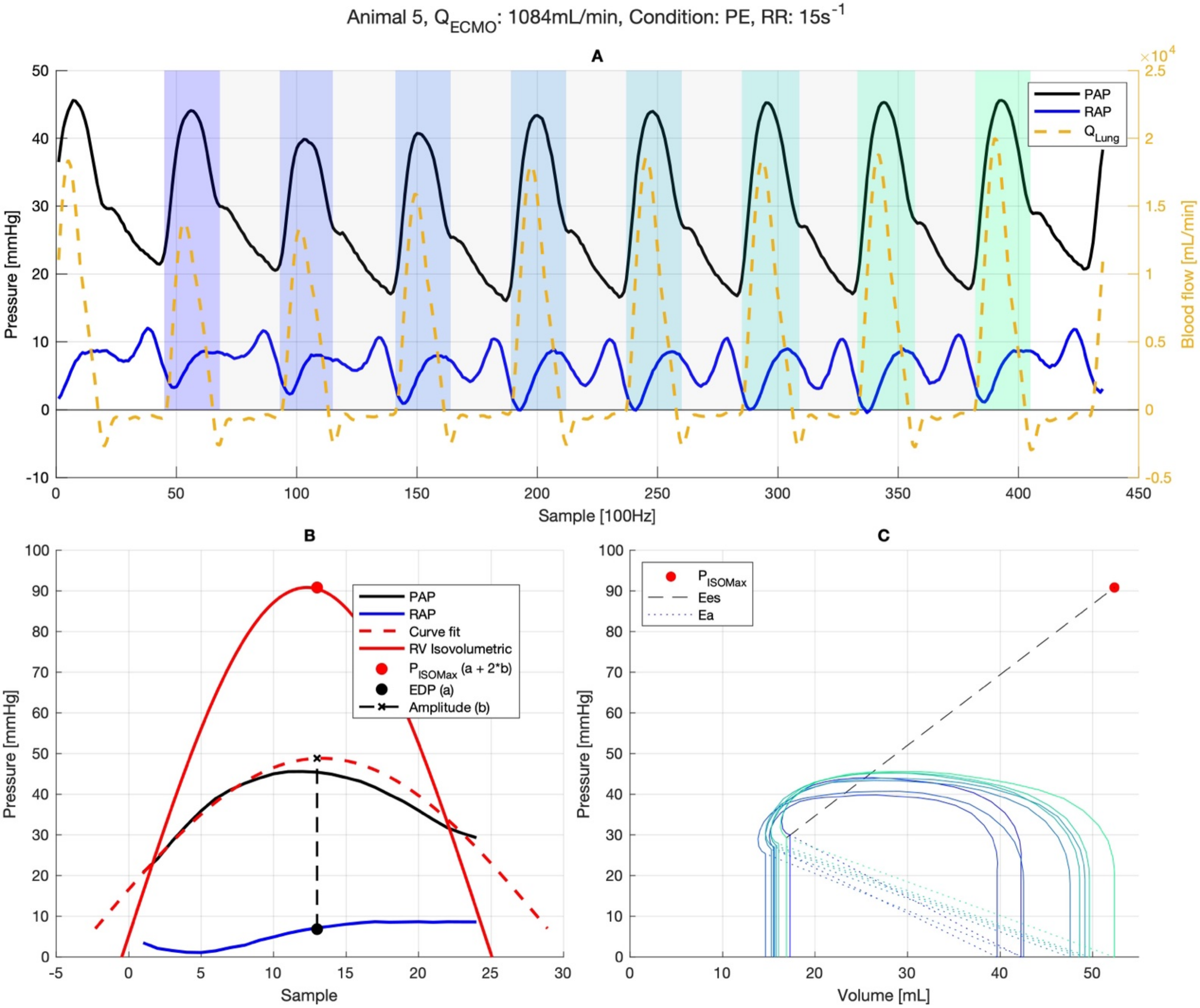

Animal 5, Q_{ECMO}: 1084mL/min, Condition: PE, RR: $15s^{-1}$
A
PAP
RAP
Q_{Lung}
Pressure [mmHg]
Blood flow [mL/min]
$\times 10^4$
Sample [100Hz]
B
PAP
RAP
Curve fit
RV Isovolumetric
P_{ISOMax} (a + 2*b)
EDP (a)
Amplitude (b)
Pressure [mmHg]
Sample
C
P_{ISOMax}
Ees
Ea
Pressure [mmHg]
Volume [mL]

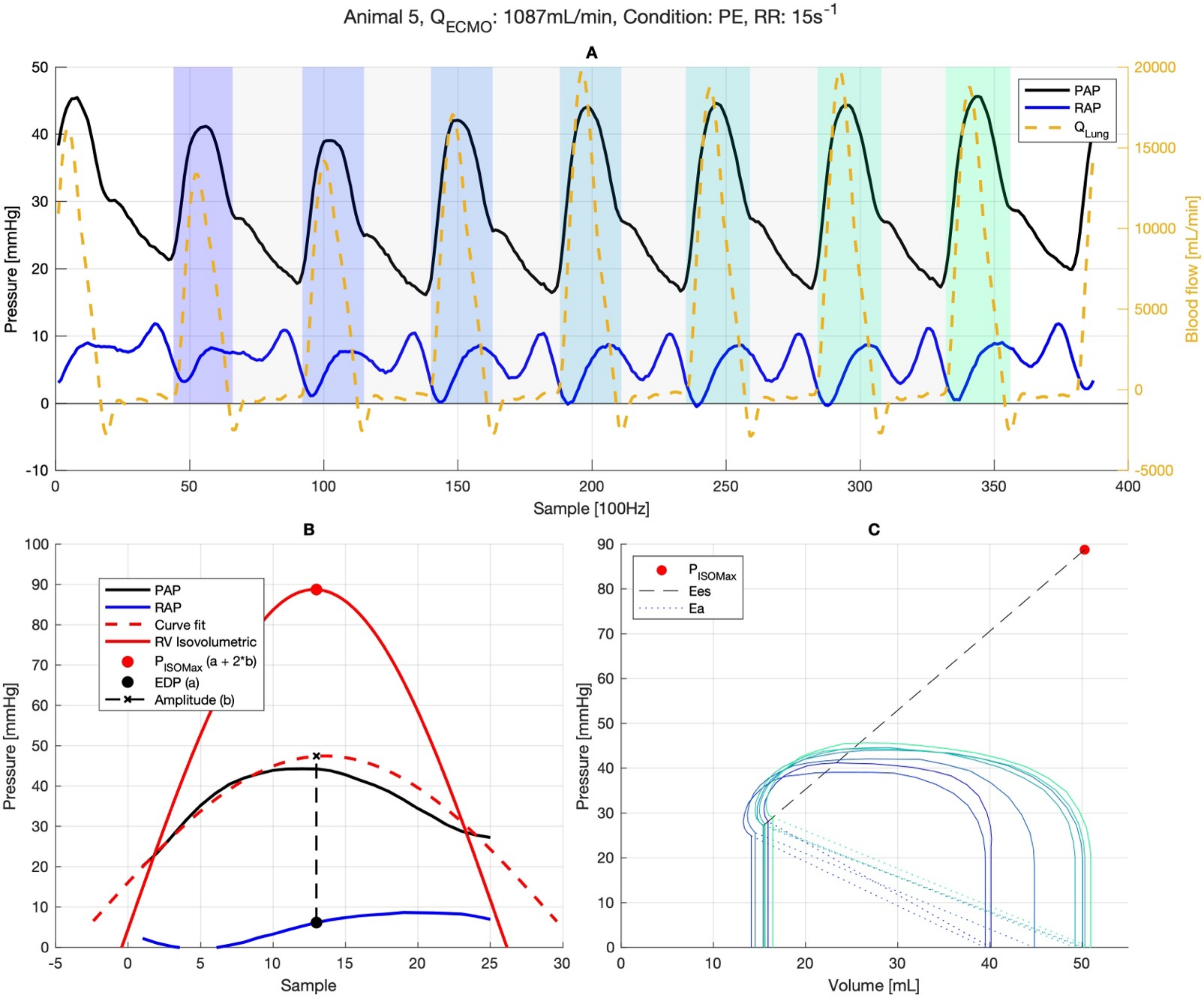

Animal 5, Q_ECMO: 1087mL/min, Condition: PE, RR: 15s^-1
A
Pressure [mmHg]
Blood flow [mL/min]
Sample [100Hz]
PAP
RAP
Q_Lung
B
PAP
RAP
Curve fit
RV Isovolumetric
P_ISOMax (a + 2*b)
EDP (a)
Amplitude (b)
Pressure [mmHg]
Sample
C
P_ISOMax
Ees
Ea
Pressure [mmHg]
Volume [mL]

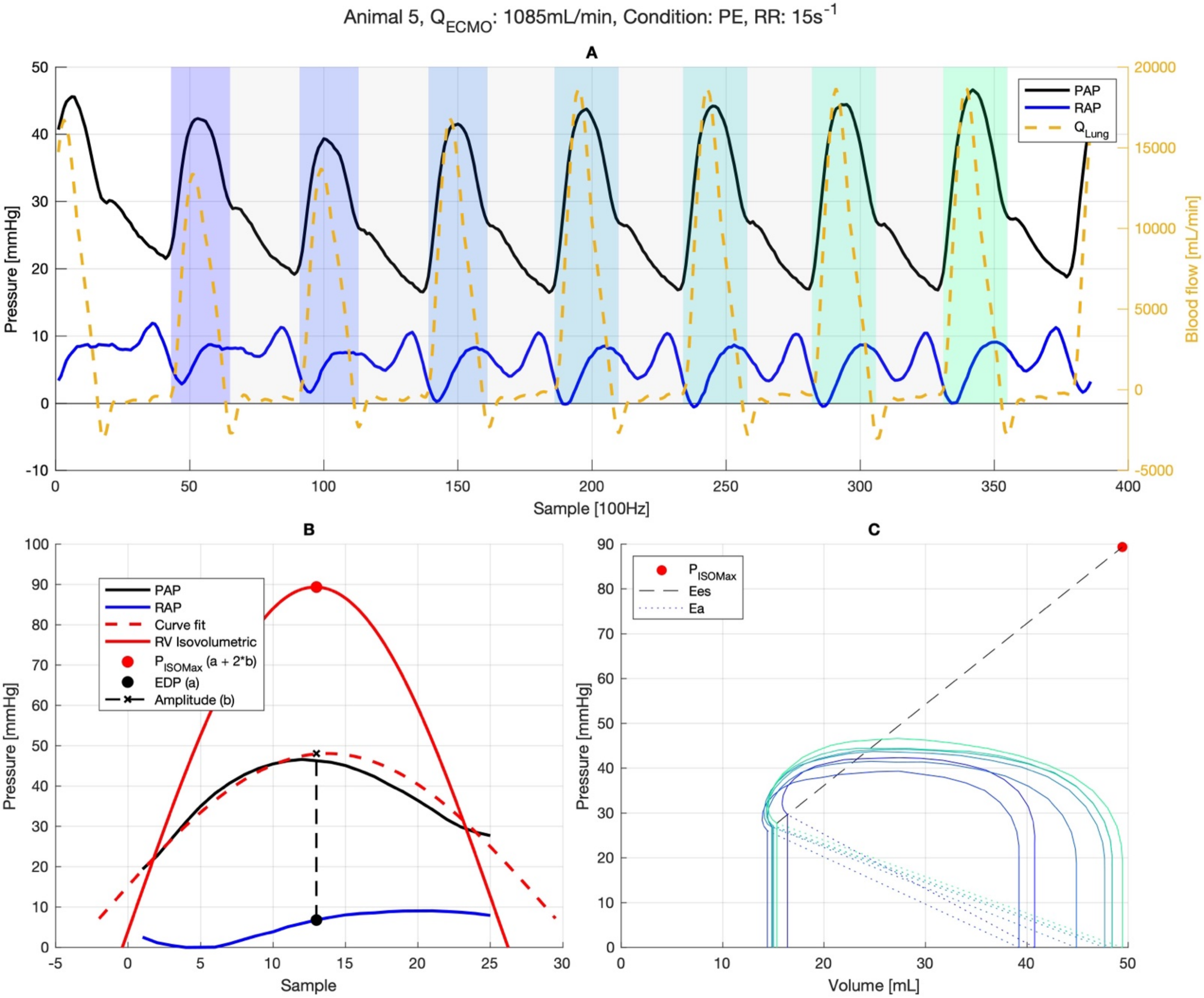

Animal 5, Q_ECMO: 1085mL/min, Condition: PE, RR: 15s-1
A
PAP
RAP
Q_Lung
Pressure [mmHg]
Blood flow [mL/min]
Sample [100Hz]
B
PAP
RAP
Curve fit
RV Isovolumetric
P_ISOMax (a + 2*b)
EDP (a)
Amplitude (b)
Pressure [mmHg]
Sample
C
P_ISOMax
Ees
Ea
Pressure [mmHg]
Volume [mL]

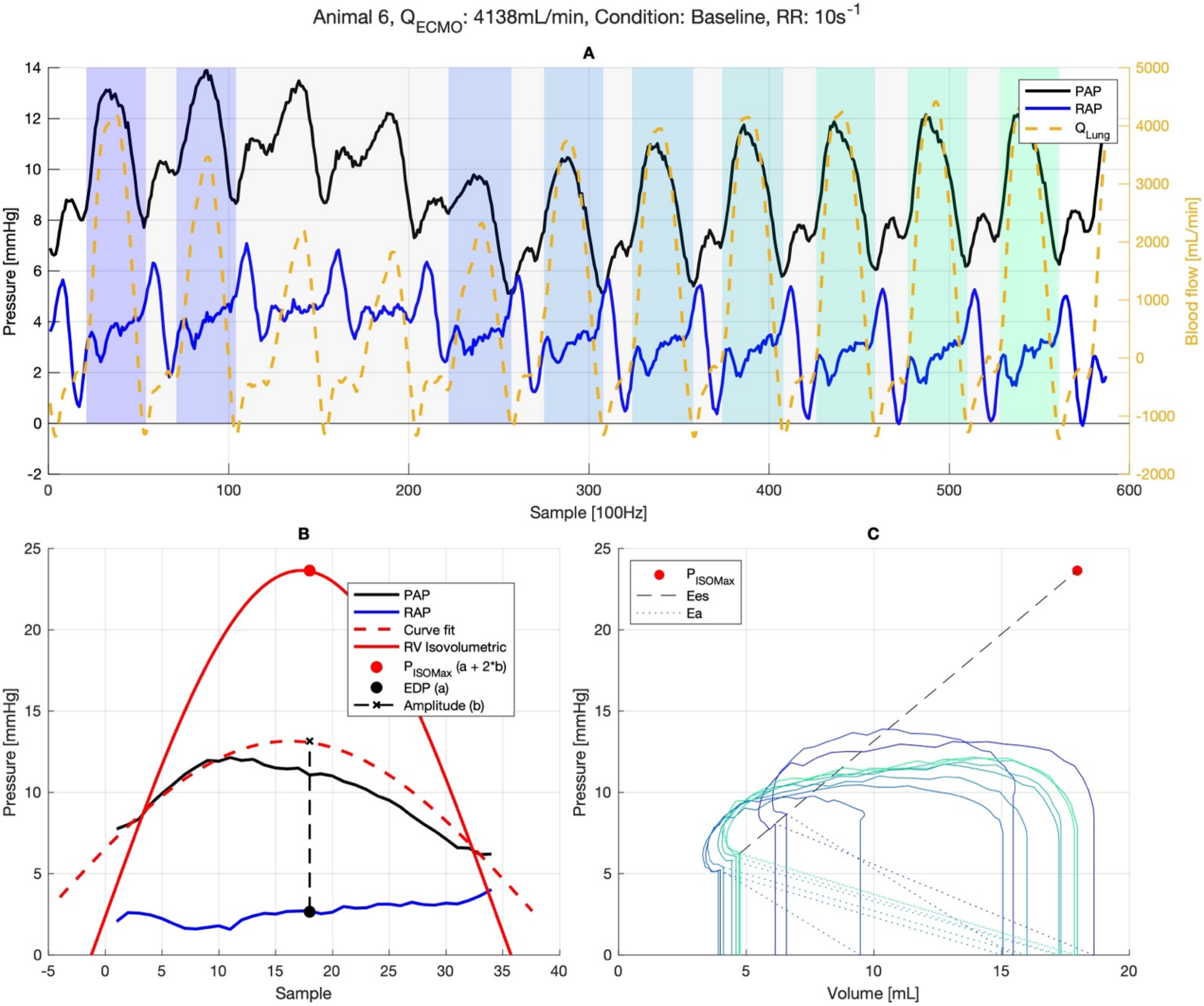

Animal 6, Q_{ECMO}: 4138mL/min, Condition: Baseline, RR: $10s^{-1}$
A
PAP
RAP
Q_{Lung}
Pressure [mmHg]
Blood flow [mL/min]
Sample [100Hz]
B
PAP
RAP
Curve fit
RV Isovolumetric
P_{ISOMax} (a + 2*b)
EDP (a)
Amplitude (b)
Pressure [mmHg]
Sample
C
P_{ISOMax}
Ees
Ea
Pressure [mmHg]
Volume [mL]

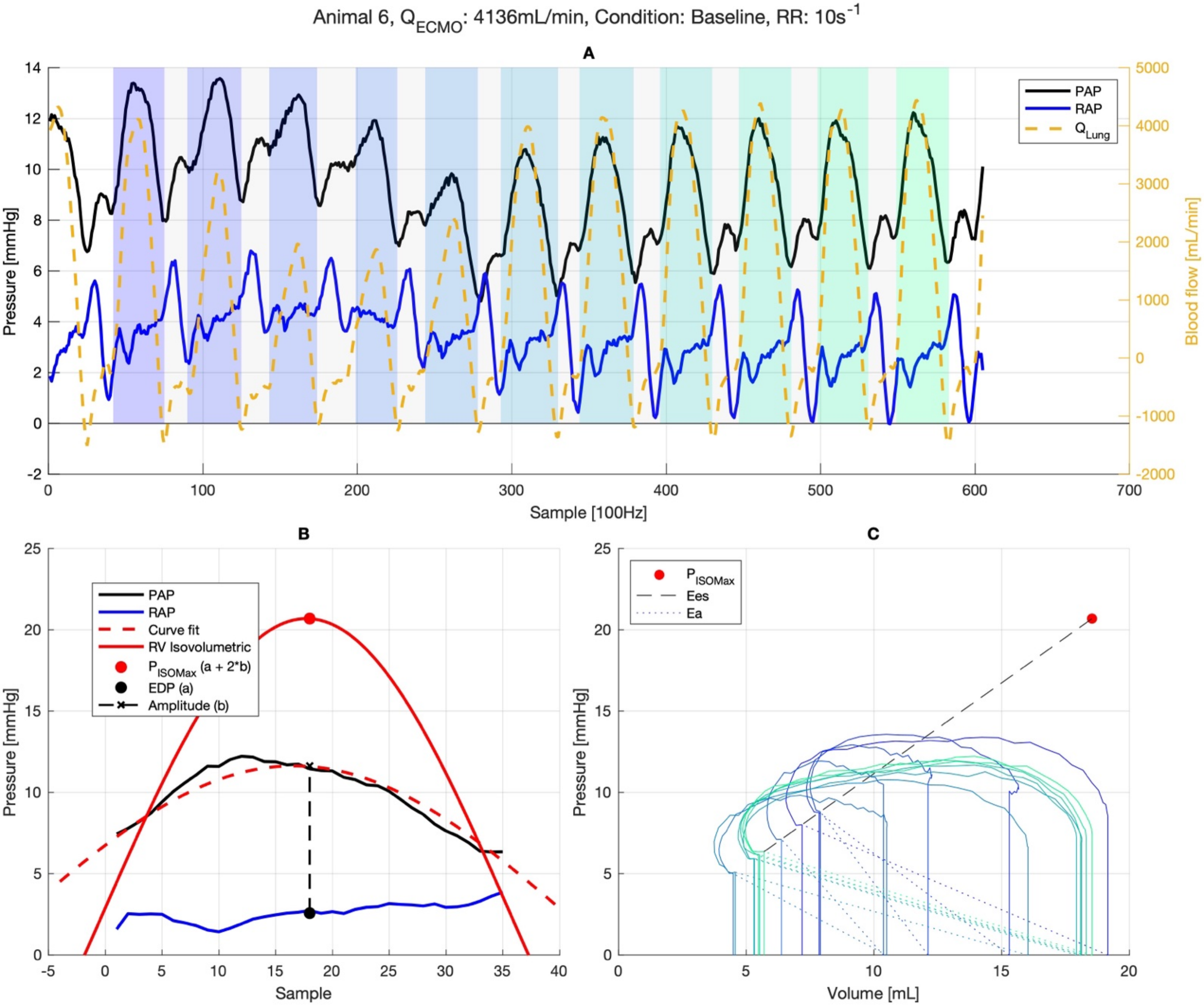

Animal 6, Q_ECMO: 4136mL/min, Condition: Baseline, RR: 10s^-1
A
PAP
RAP
Q_Lung
Pressure [mmHg]
Blood flow [mL/min]
Sample [100Hz]
B
PAP
RAP
Curve fit
RV Isovolumetric
P_ISOMax (a + 2*b)
EDP (a)
Amplitude (b)
Pressure [mmHg]
Sample
C
P_ISOMax
Ees
Ea
Pressure [mmHg]
Volume [mL]

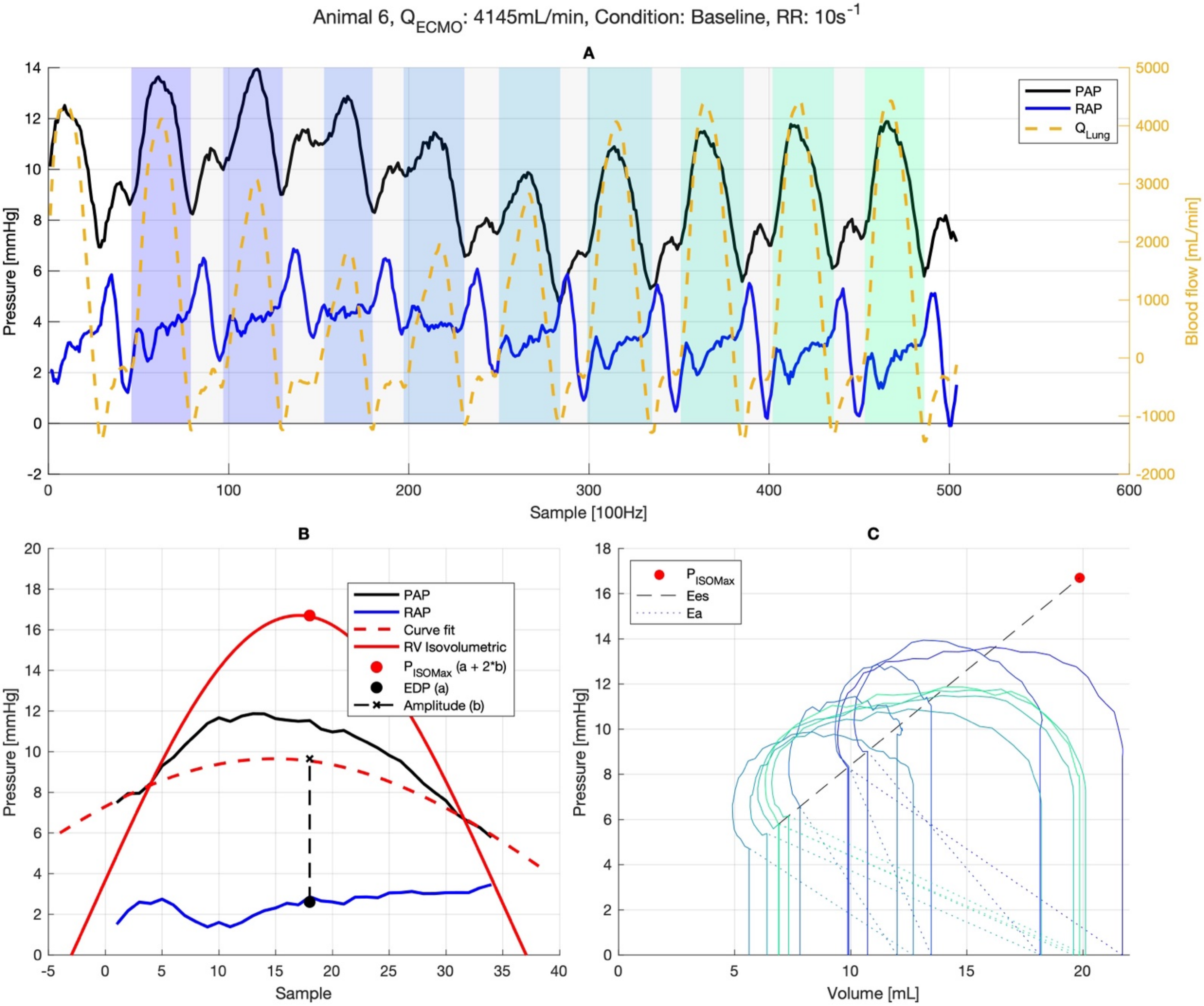

Animal 6, Q ECMO: 4145mL/min, Condition: Baseline, RR: 10s-1
A
PAP
RAP
Q Lung
Pressure [mmHg]
Blood flow [mL/min]
Sample [100Hz]
B
PAP
RAP
Curve fit
RV Isovolumetric
P ISOMax (a + 2*b)
EDP (a)
Amplitude (b)
Pressure [mmHg]
Sample
C
P ISOMax
Ees
Ea
Pressure [mmHg]
Volume [mL]

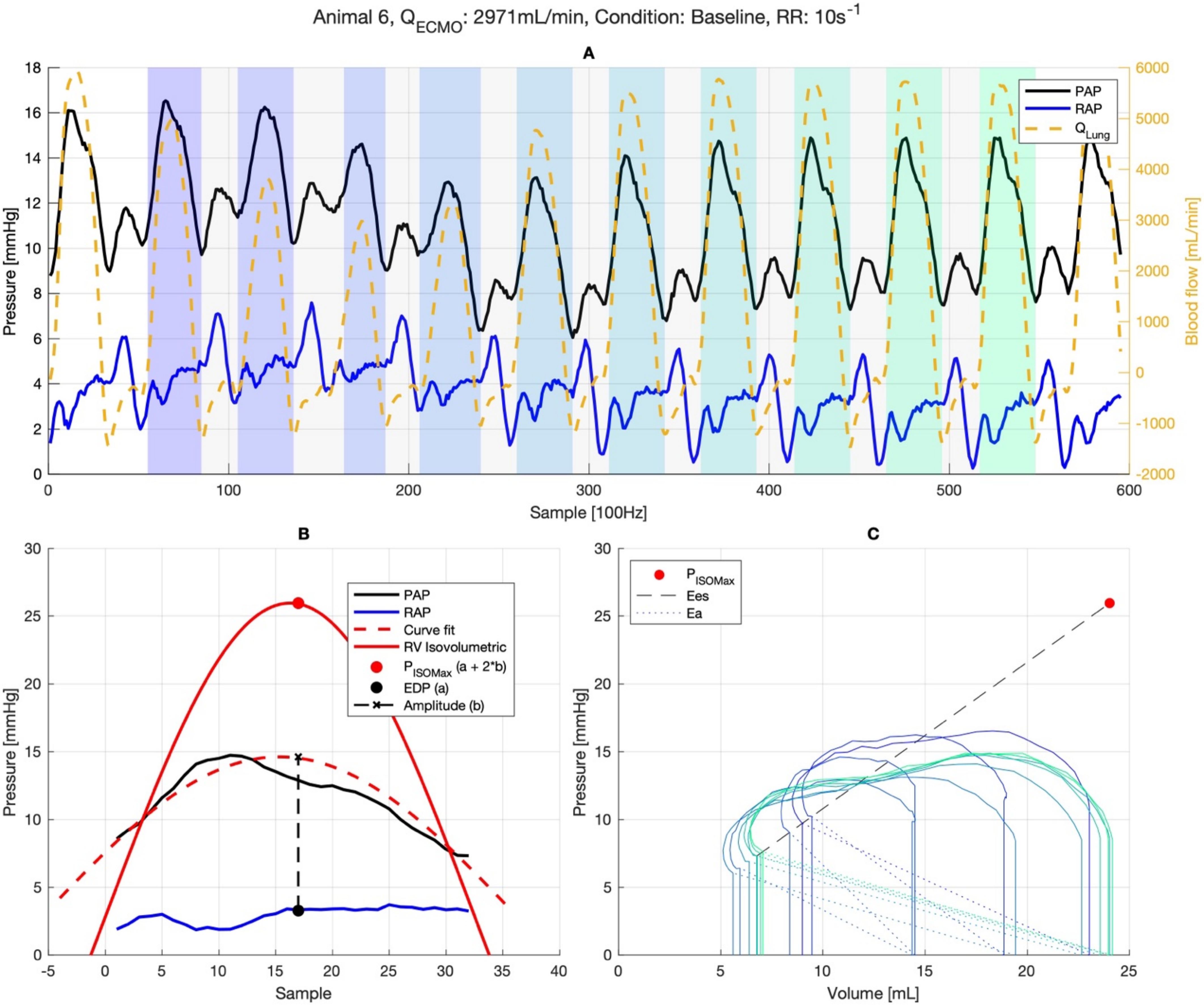

Animal 6, Q_{ECMO}: 2971mL/min, Condition: Baseline, RR: $10s^{-1}$
A
PAP
RAP
Q_{Lung}
Pressure [mmHg]
Blood flow [mL/min]
Sample [100Hz]
B
PAP
RAP
Curve fit
RV Isovolumetric
P_{ISOMax} (a + 2*b)
EDP (a)
Amplitude (b)
Pressure [mmHg]
Sample
C
P_{ISOMax}
Ees
Ea
Pressure [mmHg]
Volume [mL]

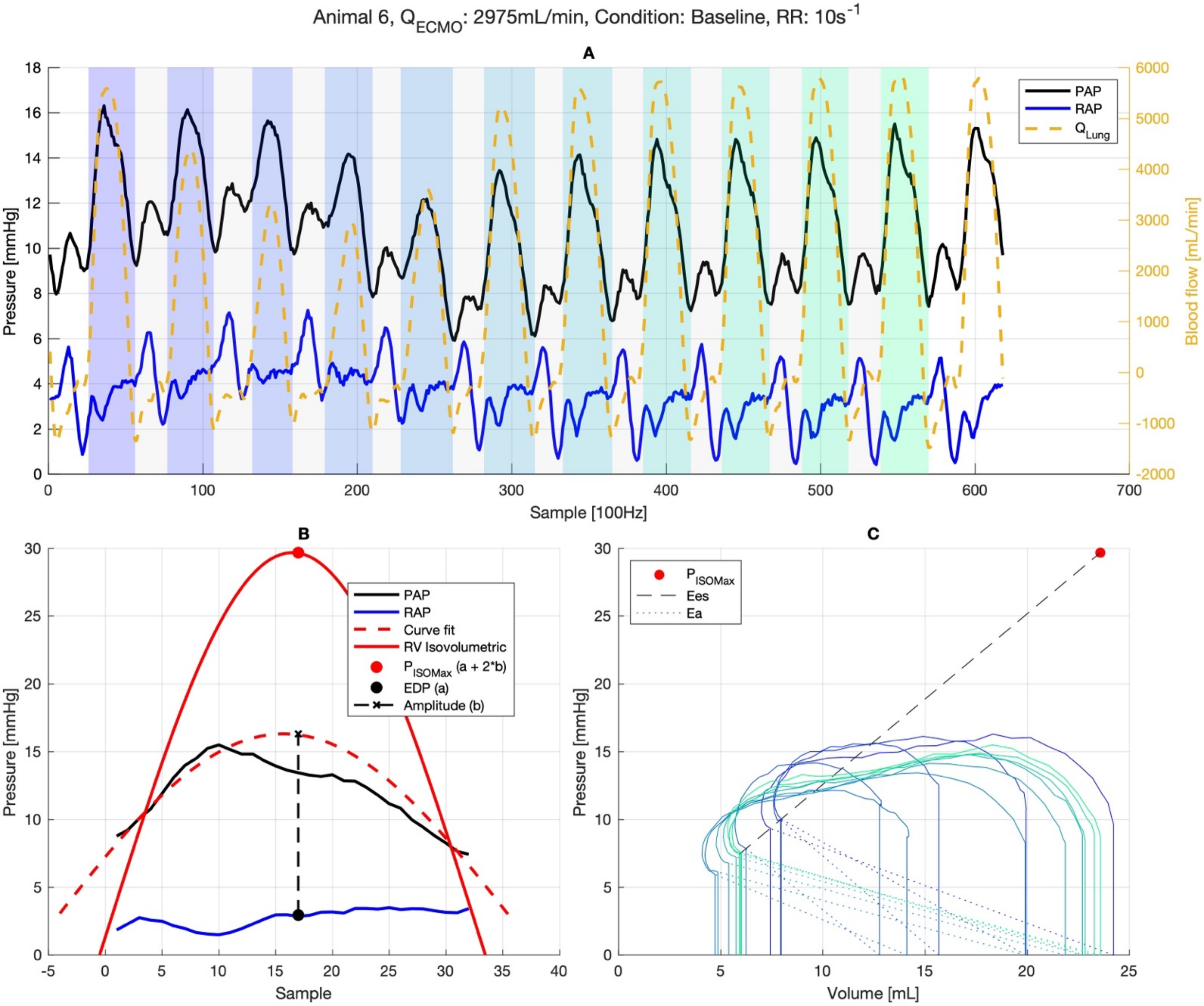

Animal 6, Q_ECMO: 2975mL/min, Condition: Baseline, RR: 10s^-1
A
Pressure [mmHg]
Blood flow [mL/min]
Sample [100Hz]
PAP
RAP
Q_Lung
B
Pressure [mmHg]
Sample
PAP
RAP
Curve fit
RV Isovolumetric
P_ISOMax (a + 2*b)
EDP (a)
Amplitude (b)
C
Pressure [mmHg]
Volume [mL]
P_ISOMax
Ees
Ea

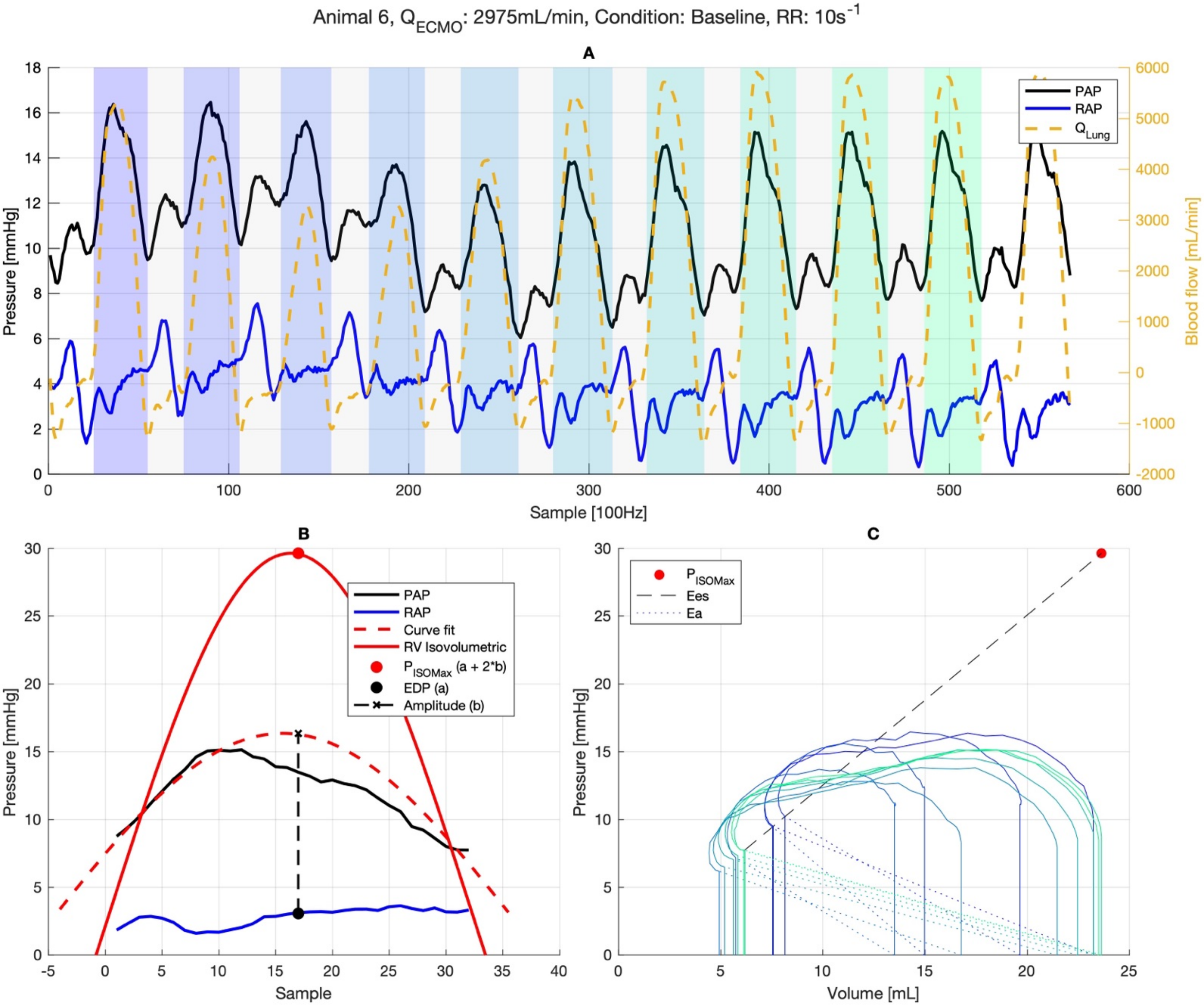

Animal 6, Q_{ECMO}: 2975mL/min, Condition: Baseline, RR: $10s^{-1}$
A
Pressure [mmHg]
Blood flow [mL/min]
Sample [100Hz]
PAP
RAP
Q_{Lung}
B
Pressure [mmHg]
Sample
PAP
RAP
Curve fit
RV Isovolumetric
P_{ISOMax} (a + 2*b)
EDP (a)
Amplitude (b)
C
Pressure [mmHg]
Volume [mL]
P_{ISOMax}
Ees
Ea

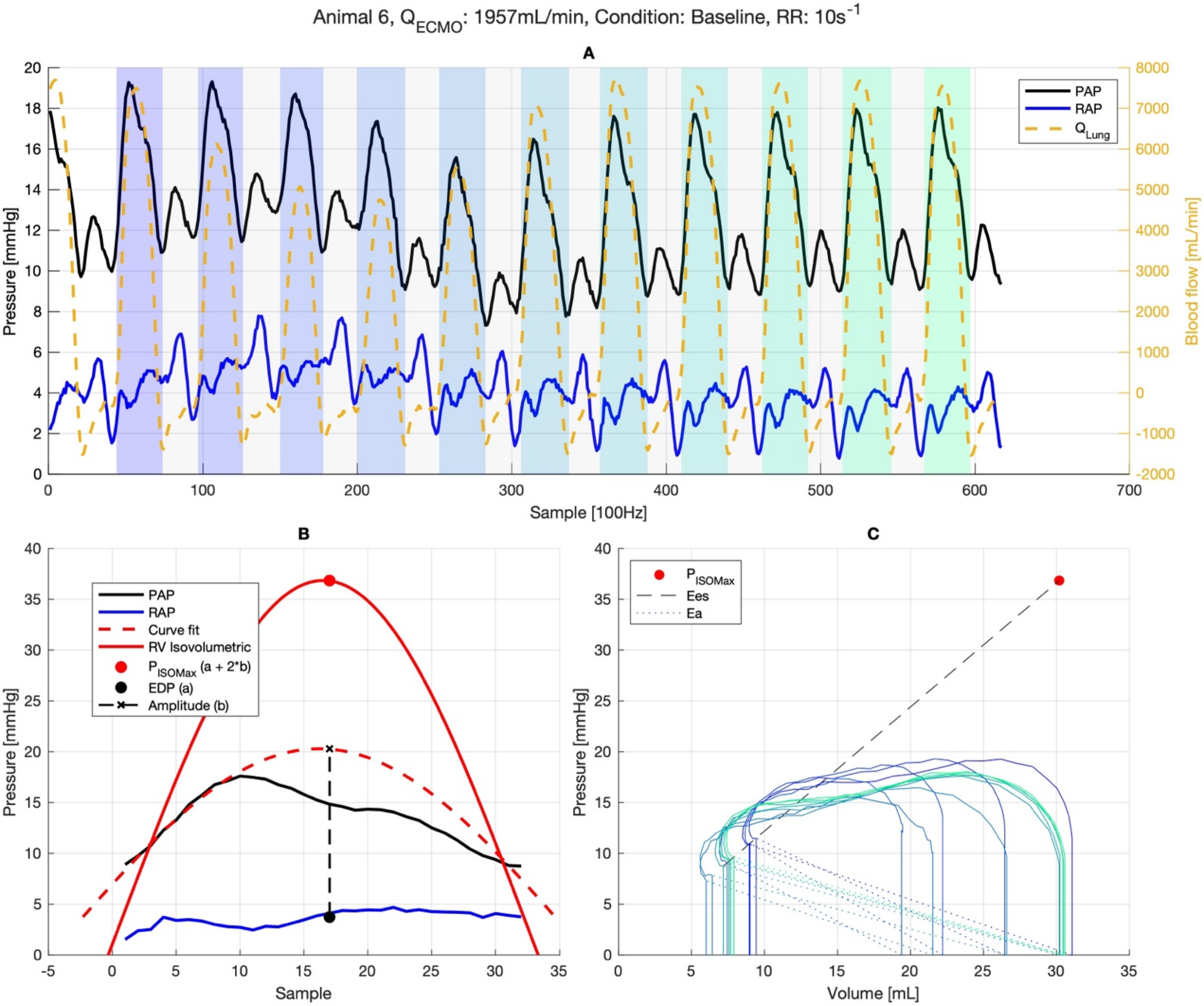

Animal 6, Q_ECMO: 1957mL/min, Condition: Baseline, RR: 10s^-1
A
Pressure [mmHg]
Blood flow [mL/min]
Sample [100Hz]
PAP
RAP
Q_Lung
B
PAP
RAP
Curve fit
RV Isovolumetric
P_ISOMax (a + 2*b)
EDP (a)
Amplitude (b)
Pressure [mmHg]
Sample
C
P_ISOMax
Ees
Ea
Pressure [mmHg]
Volume [mL]

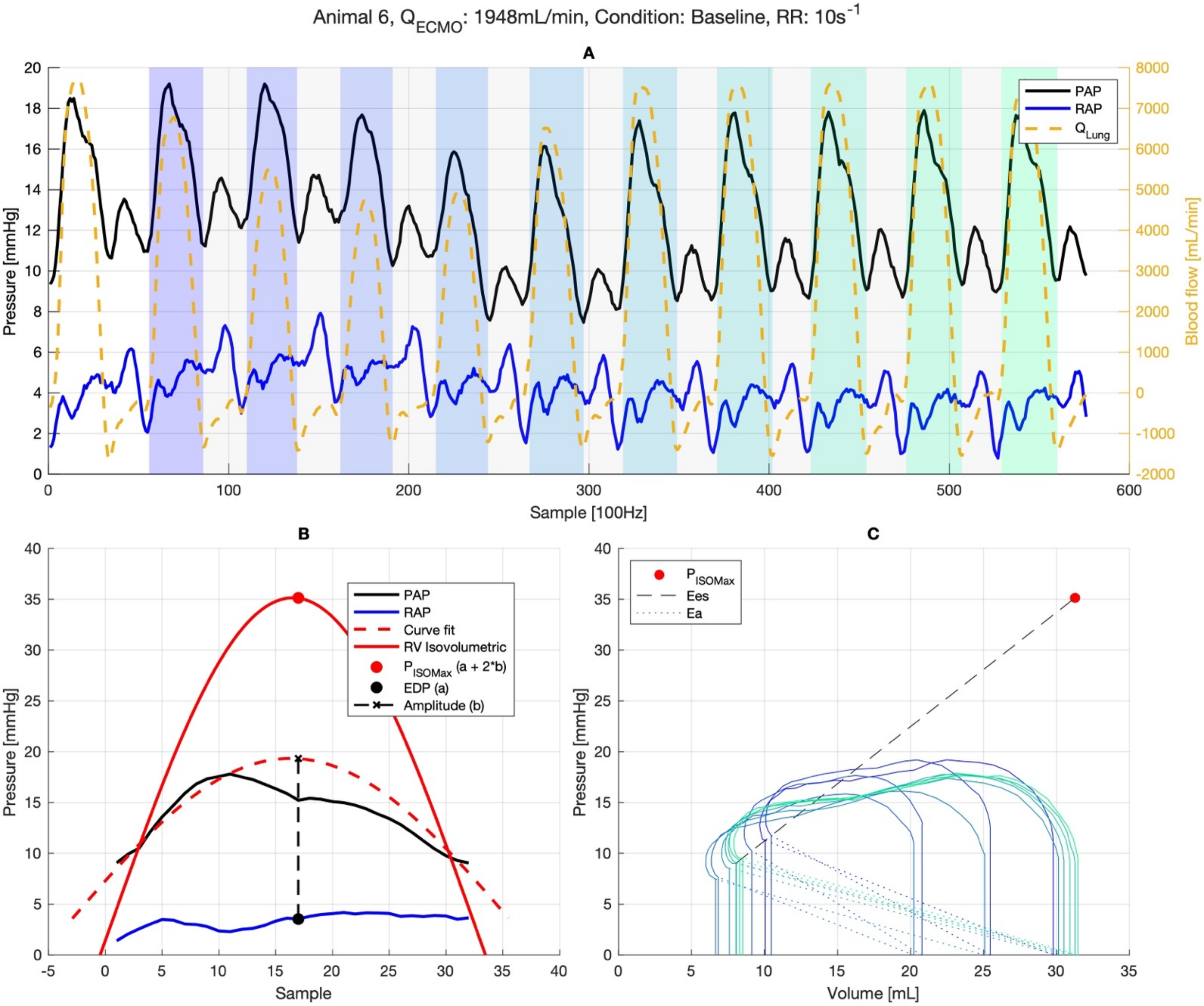

Animal 6, Q_ECMO: 1948mL/min, Condition: Baseline, RR: 10s^-1
A
Pressure [mmHg]
Blood flow [mL/min]
Sample [100Hz]
PAP
RAP
Q_Lung
B
Pressure [mmHg]
Sample
PAP
RAP
Curve fit
RV Isovolumetric
P_ISOMax (a + 2*b)
EDP (a)
Amplitude (b)
C
Pressure [mmHg]
Volume [mL]
P_ISOMax
Ees
Ea

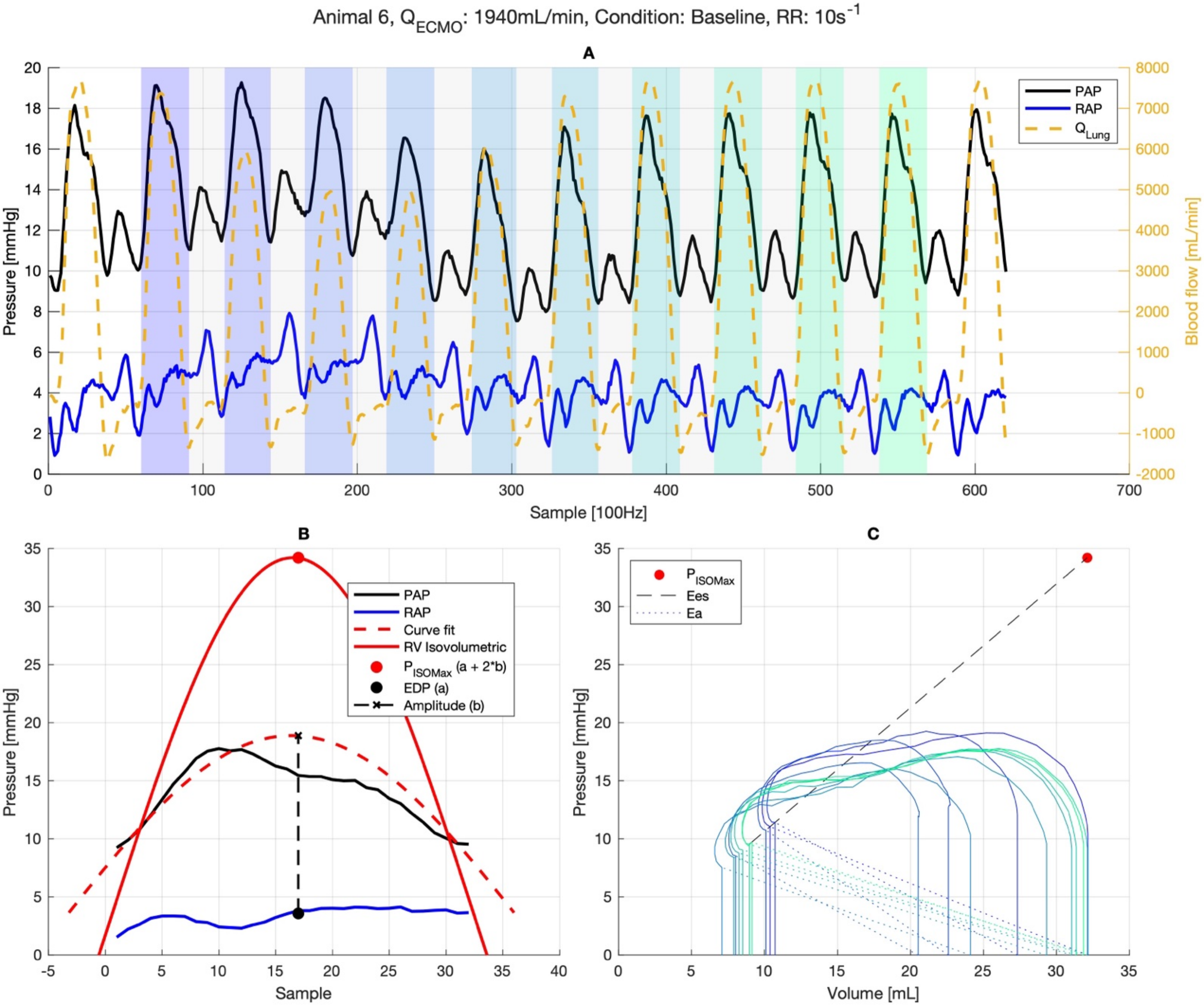

Animal 6, Q_{ECMO}: 1940mL/min, Condition: Baseline, RR: $10s^{-1}$
A
Pressure [mmHg]
Blood flow [mL/min]
Sample [100Hz]
PAP
RAP
Q_{Lung}
B
Pressure [mmHg]
Sample
PAP
RAP
Curve fit
RV Isovolumetric
P_{ISOMax} (a + 2*b)
EDP (a)
Amplitude (b)
C
Pressure [mmHg]
Volume [mL]
P_{ISOMax}
Ees
Ea

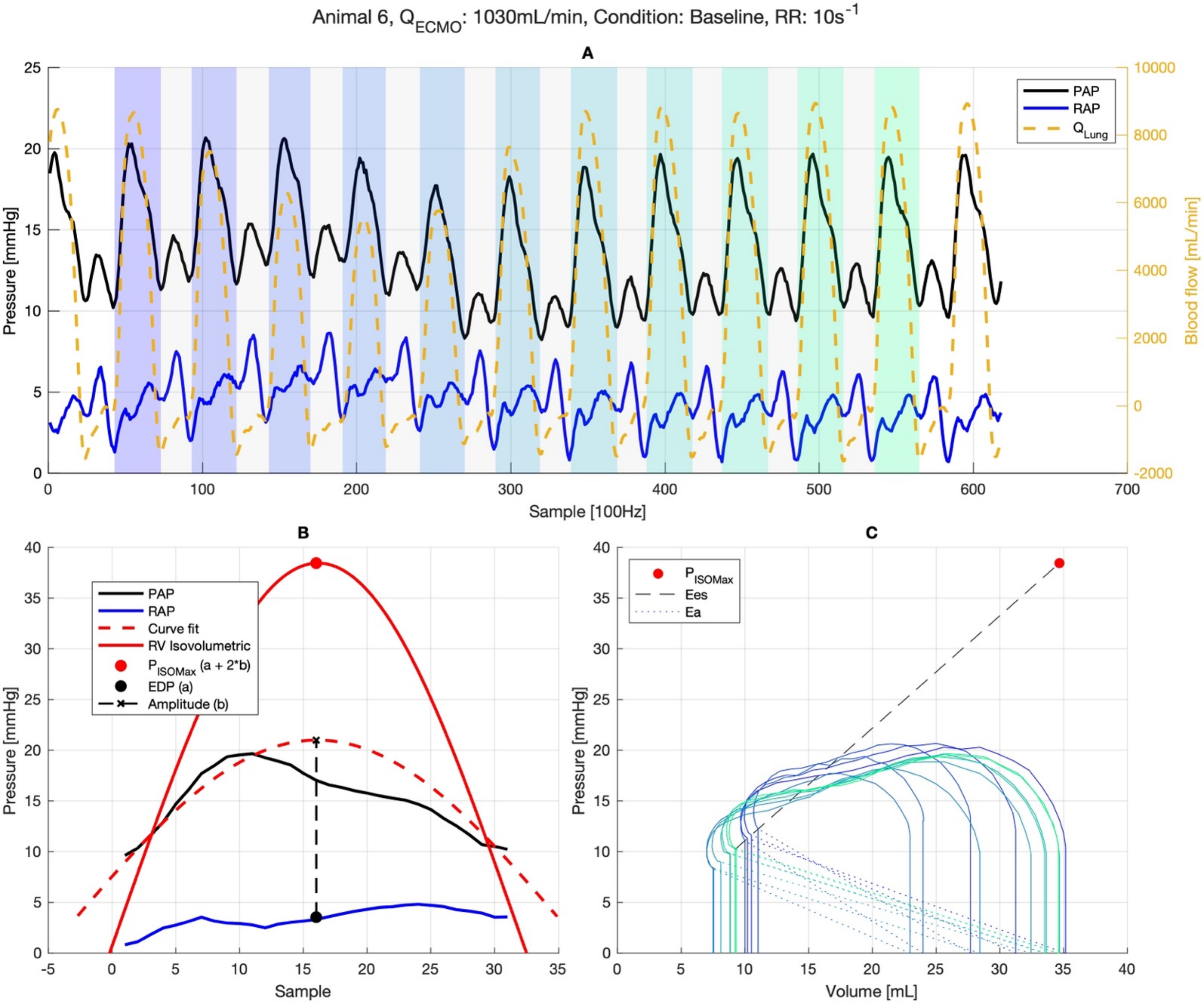

Animal 6, Q_{ECMO}: 1030mL/min, Condition: Baseline, RR: $10s^{-1}$
A
PAP
RAP
Q_{Lung}
Pressure [mmHg]
Blood flow [mL/min]
Sample [100Hz]
B
PAP
RAP
Curve fit
RV Isovolumetric
P_{ISOMax} (a + 2*b)
EDP (a)
Amplitude (b)
Pressure [mmHg]
Sample
C
P_{ISOMax}
Ees
Ea
Pressure [mmHg]
Volume [mL]

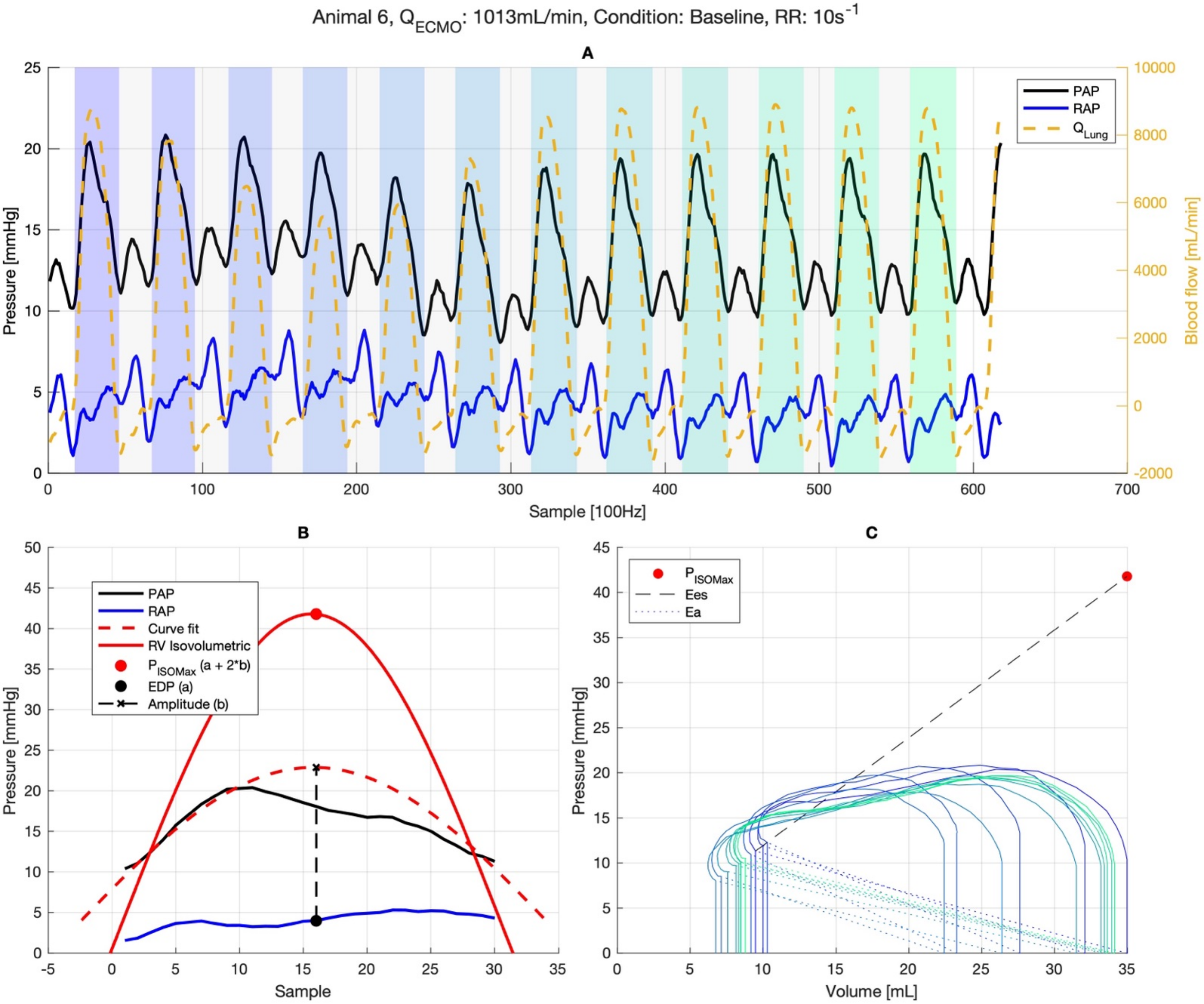

Animal 6, Q_{ECMO}: 1013mL/min, Condition: Baseline, RR: $10s^{-1}$
A
PAP
RAP
Q_{Lung}
Pressure [mmHg]
Blood flow [mL/min]
Sample [100Hz]
B
PAP
RAP
Curve fit
RV Isovolumetric
P_{ISOMax} (a + 2*b)
EDP (a)
Amplitude (b)
Pressure [mmHg]
Sample
C
P_{ISOMax}
Ees
Ea
Pressure [mmHg]
Volume [mL]

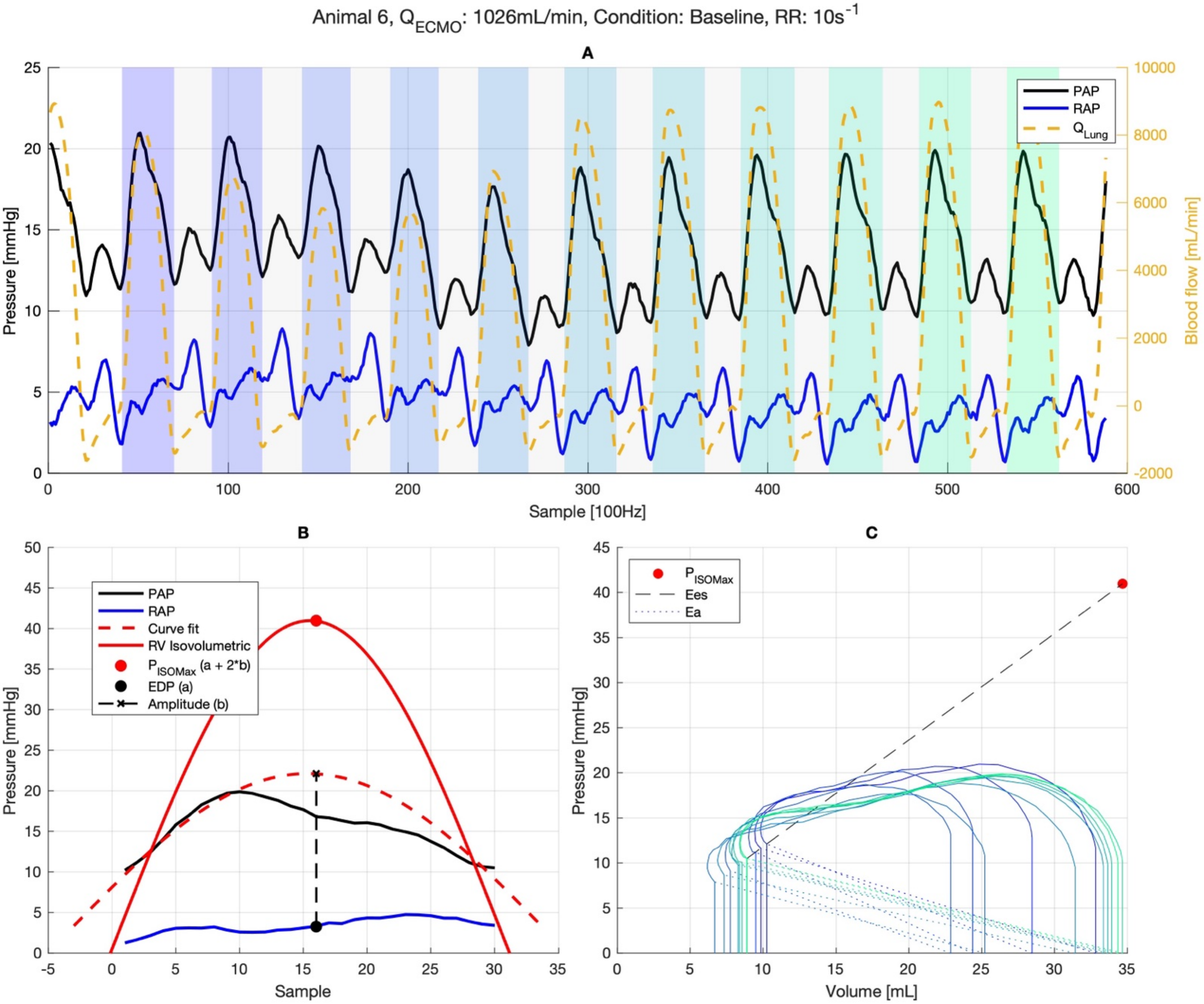

Animal 6, QECMO: 1026mL/min, Condition: Baseline, RR: 10s-1
A
PAP
RAP
QLung
Pressure [mmHg]
Blood flow [mL/min]
Sample [100Hz]
B
PAP
RAP
Curve fit
RV Isovolumetric
PISOMax (a + 2*b)
EDP (a)
Amplitude (b)
Pressure [mmHg]
Sample
C
PISOMax
Ees
Ea
Pressure [mmHg]
Volume [mL]

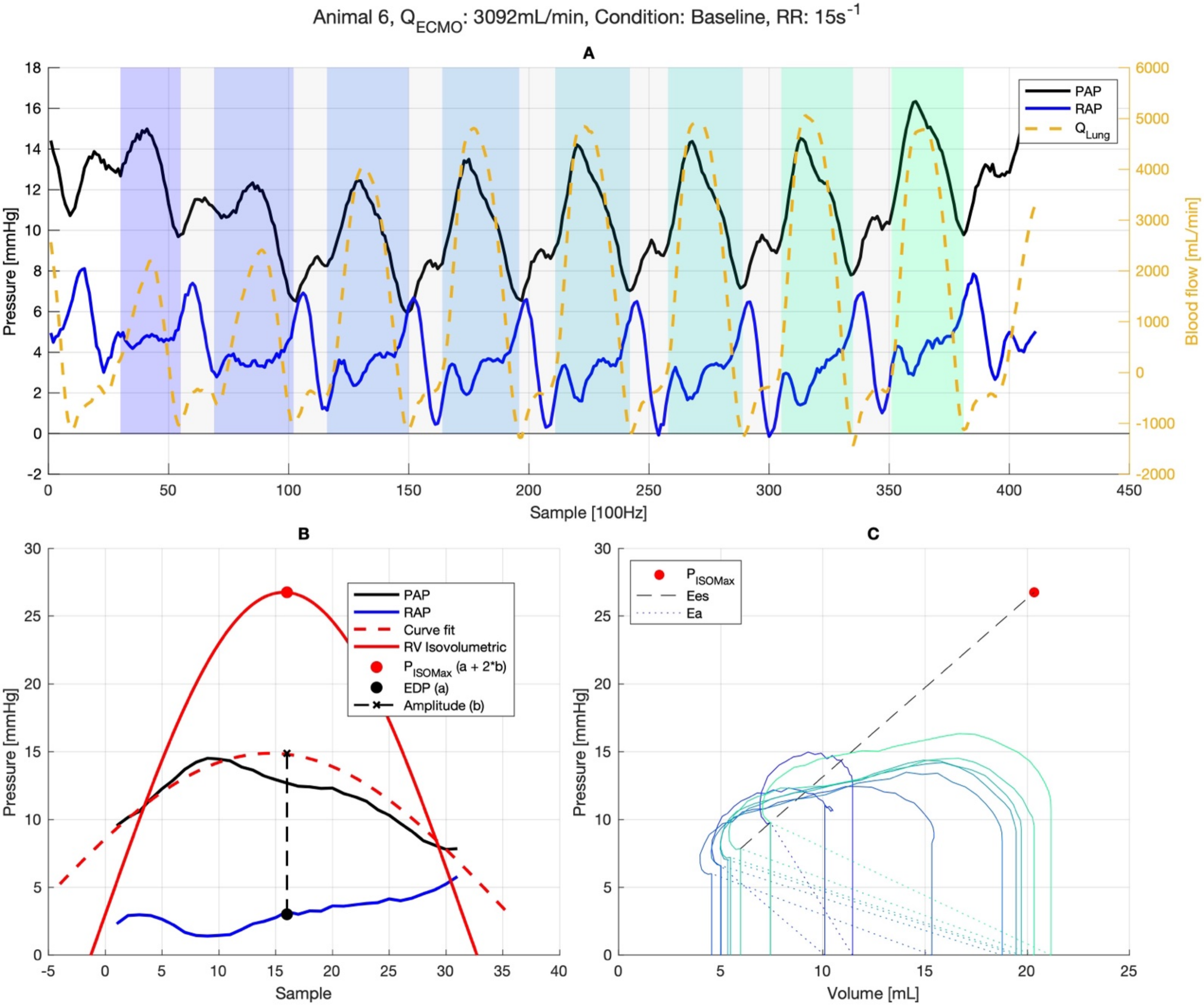

Animal 6, QECMO: 3092mL/min, Condition: Baseline, RR: 15s-1
A
Pressure [mmHg]
Blood flow [mL/min]
Sample [100Hz]
PAP
RAP
QLung
B
Pressure [mmHg]
Sample
PAP
RAP
Curve fit
RV Isovolumetric
PISOMax (a + 2*b)
EDP (a)
Amplitude (b)
C
Pressure [mmHg]
Volume [mL]
PISOMax
Ees
Ea

Animal 6, Q_{ECMO}: 3077mL/min, Condition: Baseline, RR: $15s^{-1}$

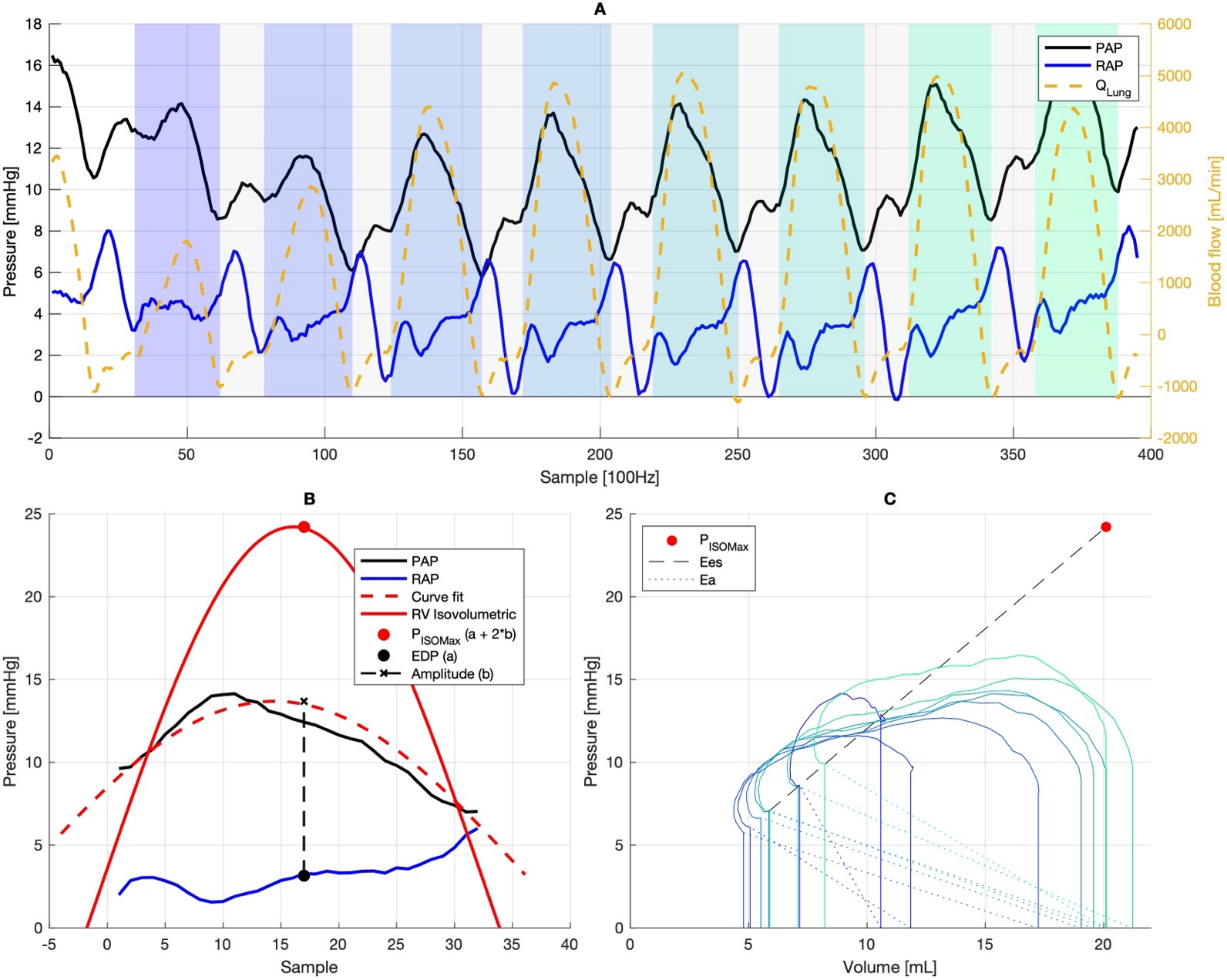

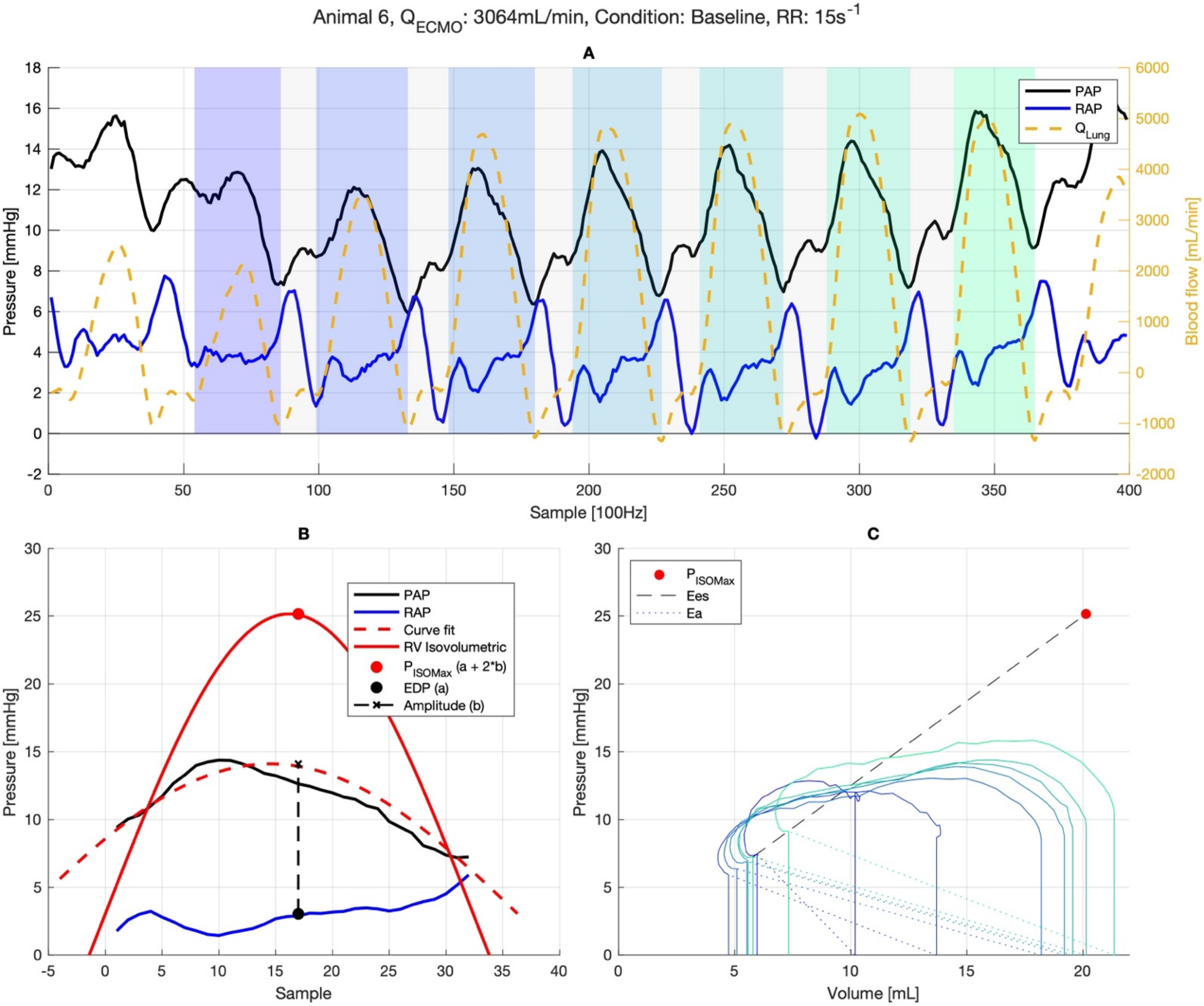

Animal 6, Q_{ECMO}: 3064mL/min, Condition: Baseline, RR: $15s^{-1}$
A
PAP
RAP
Q_{Lung}
Pressure [mmHg]
Blood flow [mL/min]
Sample [100Hz]
B
PAP
RAP
Curve fit
RV Isovolumetric
P_{ISOMax} (a + 2*b)
EDP (a)
Amplitude (b)
Pressure [mmHg]
Sample
C
P_{ISOMax}
Ees
Ea
Pressure [mmHg]
Volume [mL]

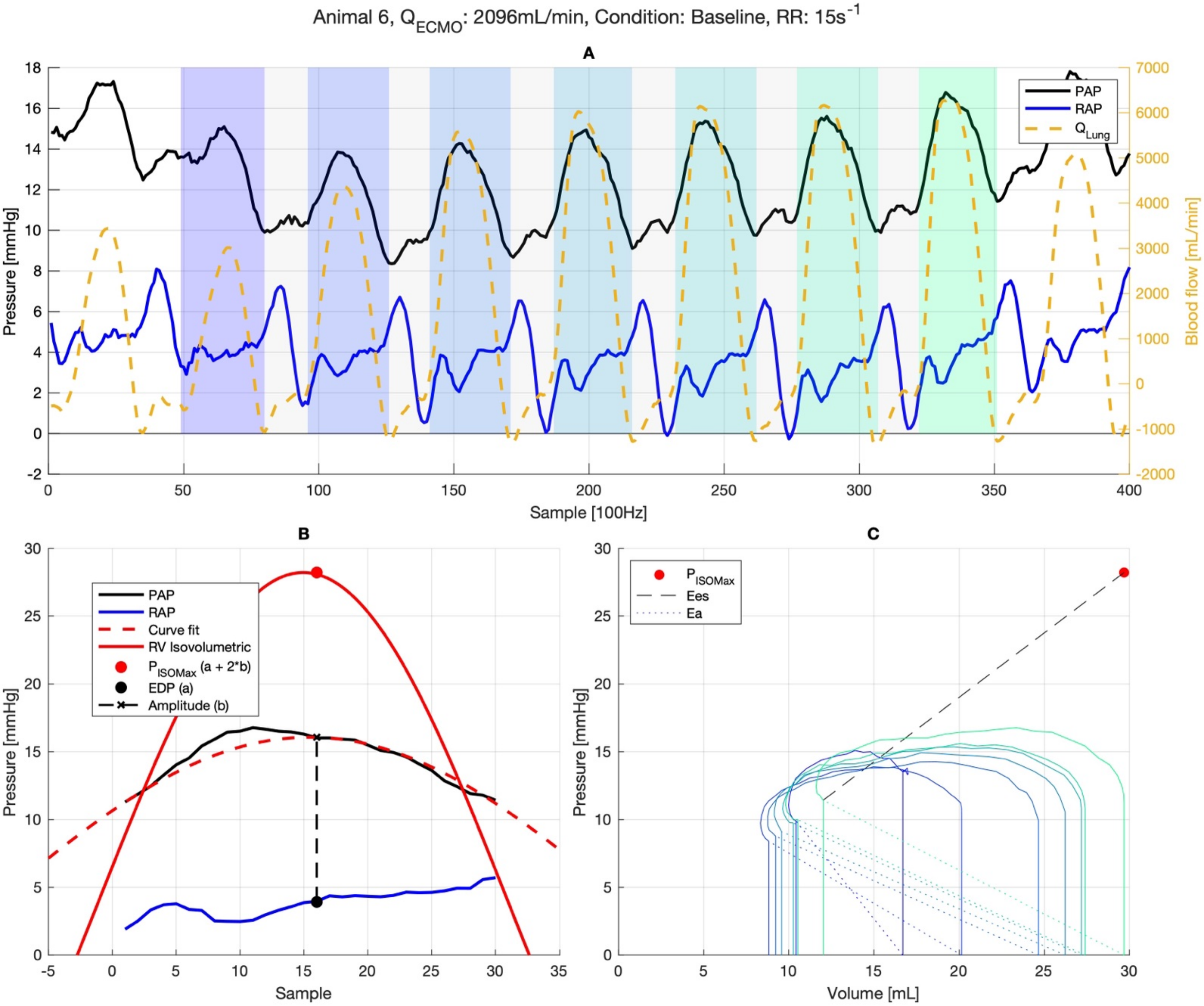

Animal 6, Q_{ECMO}: 2096mL/min, Condition: Baseline, RR: $15s^{-1}$
A
PAP
RAP
Q_{Lung}
Pressure [mmHg]
Blood flow [mL/min]
Sample [100Hz]
B
PAP
RAP
Curve fit
RV Isovolumetric
P_{ISOMax} (a + 2*b)
EDP (a)
Amplitude (b)
Pressure [mmHg]
Sample
C
P_{ISOMax}
Ees
Ea
Pressure [mmHg]
Volume [mL]

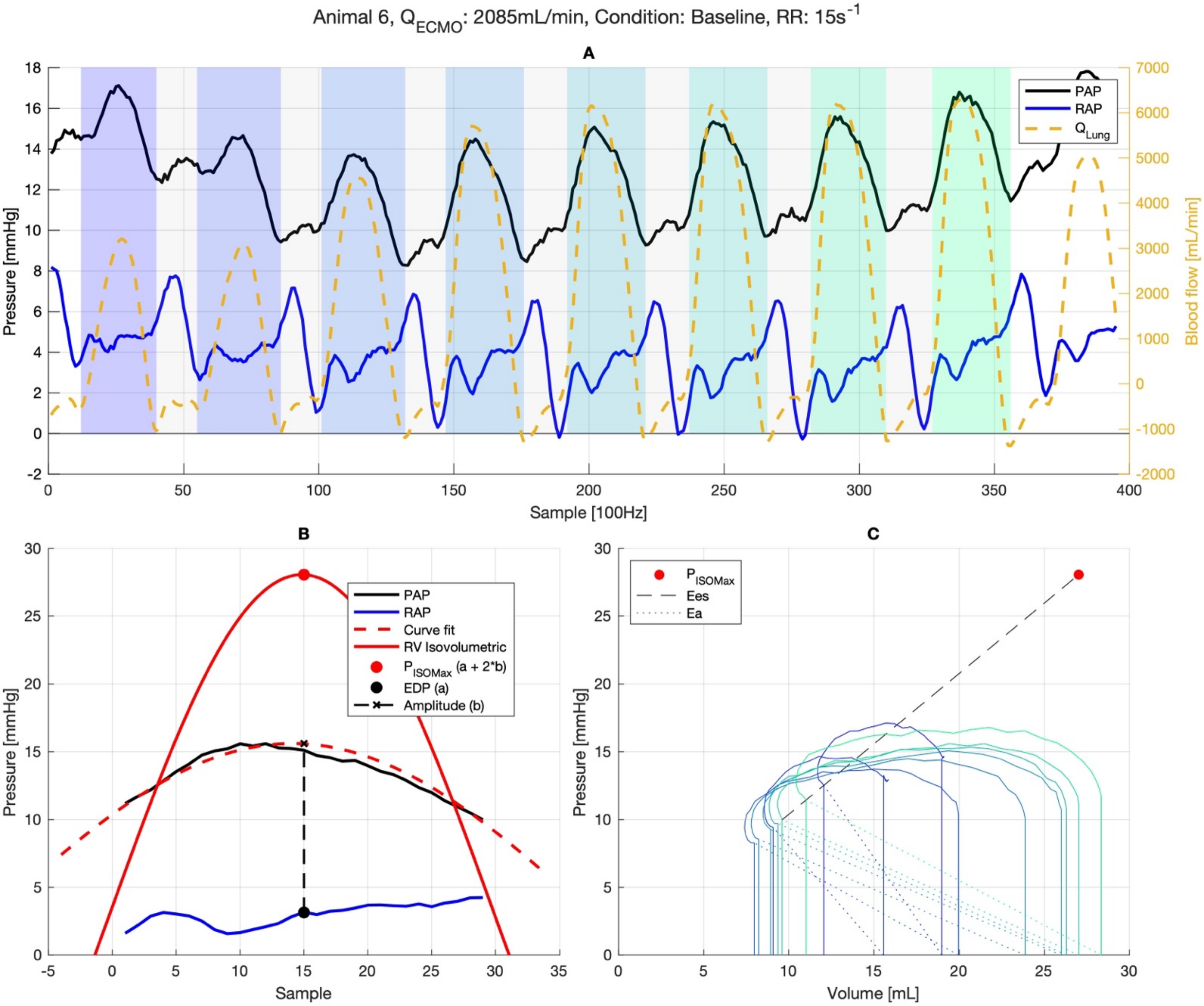

Animal 6, Q_{ECMO}: 2085mL/min, Condition: Baseline, RR: $15s^{-1}$
A
PAP
RAP
Q_{Lung}
Pressure [mmHg]
Blood flow [mL/min]
Sample [100Hz]
B
PAP
RAP
Curve fit
RV Isovolumetric
P_{ISOMax} (a + 2*b)
EDP (a)
Amplitude (b)
Pressure [mmHg]
Sample
C
P_{ISOMax}
Ees
Ea
Pressure [mmHg]
Volume [mL]

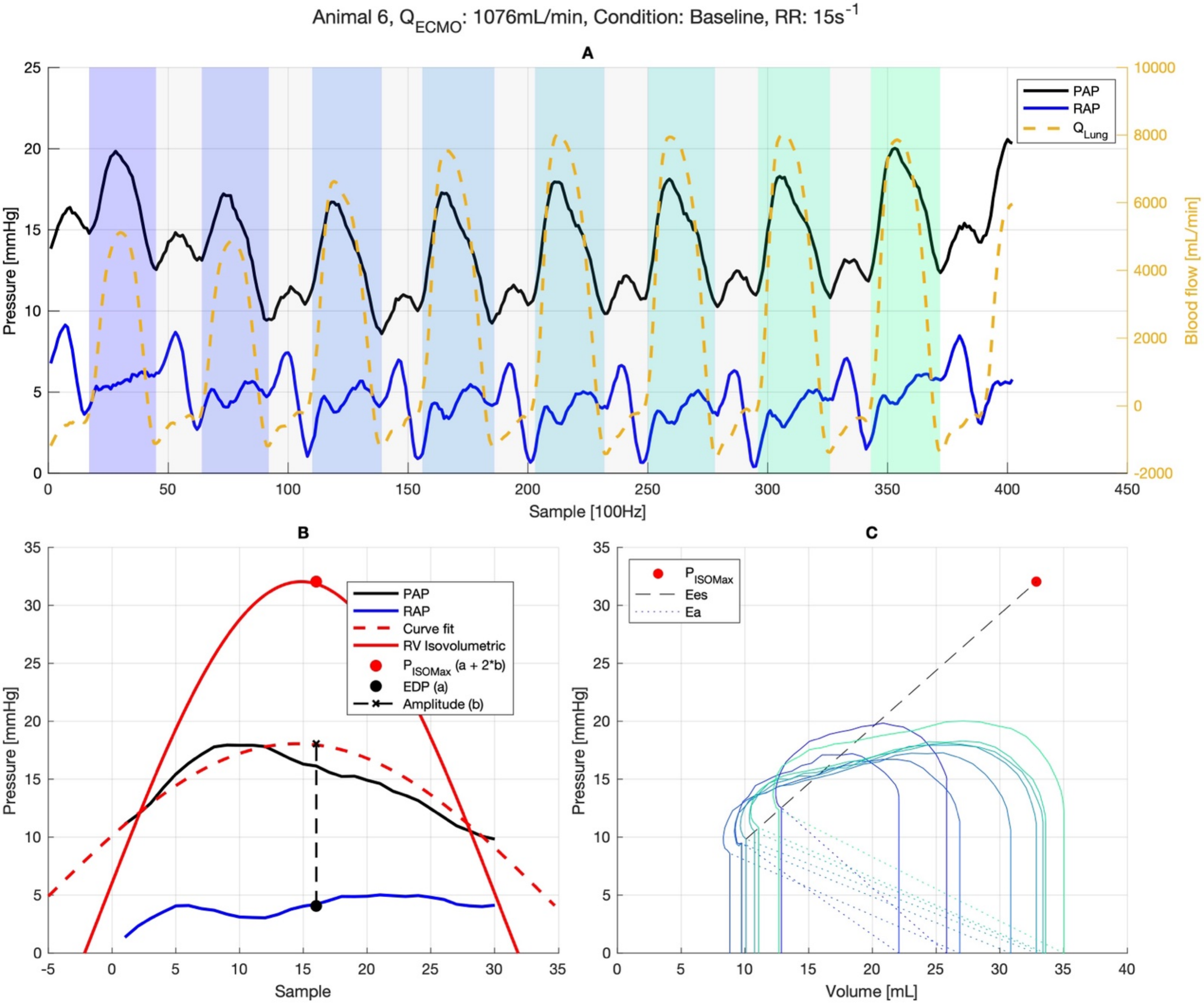
Animal 6, Q_ECMO: 1076mL/min, Condition: Baseline, RR: 15s^-1
A
PAP
RAP
Q_Lung
Pressure [mmHg]
Blood flow [mL/min]
Sample [100Hz]
B
PAP
RAP
Curve fit
RV Isovolumetric
P_ISOMax (a + 2*b)
EDP (a)
Amplitude (b)
Pressure [mmHg]
Sample
C
P_ISOMax
Ees
Ea
Pressure [mmHg]
Volume [mL]

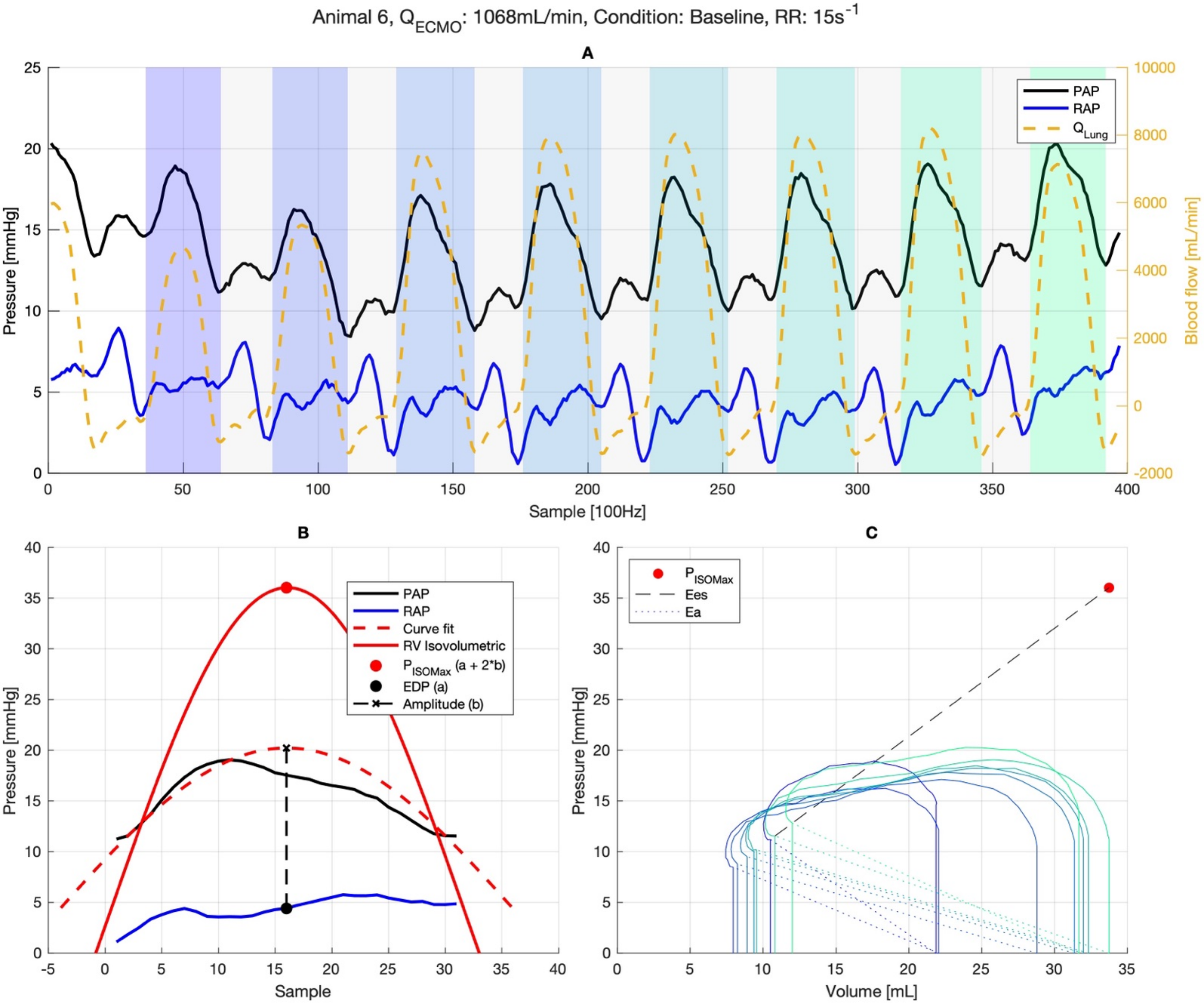

Animal 6, Q_{ECMO}: 1068mL/min, Condition: Baseline, RR: $15s^{-1}$
A
PAP
RAP
Q_{Lung}
Pressure [mmHg]
Blood flow [mL/min]
Sample [100Hz]
B
PAP
RAP
Curve fit
RV Isovolumetric
P_{ISOMax} (a + 2*b)
EDP (a)
Amplitude (b)
Pressure [mmHg]
Sample
C
P_{ISOMax}
Ees
Ea
Pressure [mmHg]
Volume [mL]

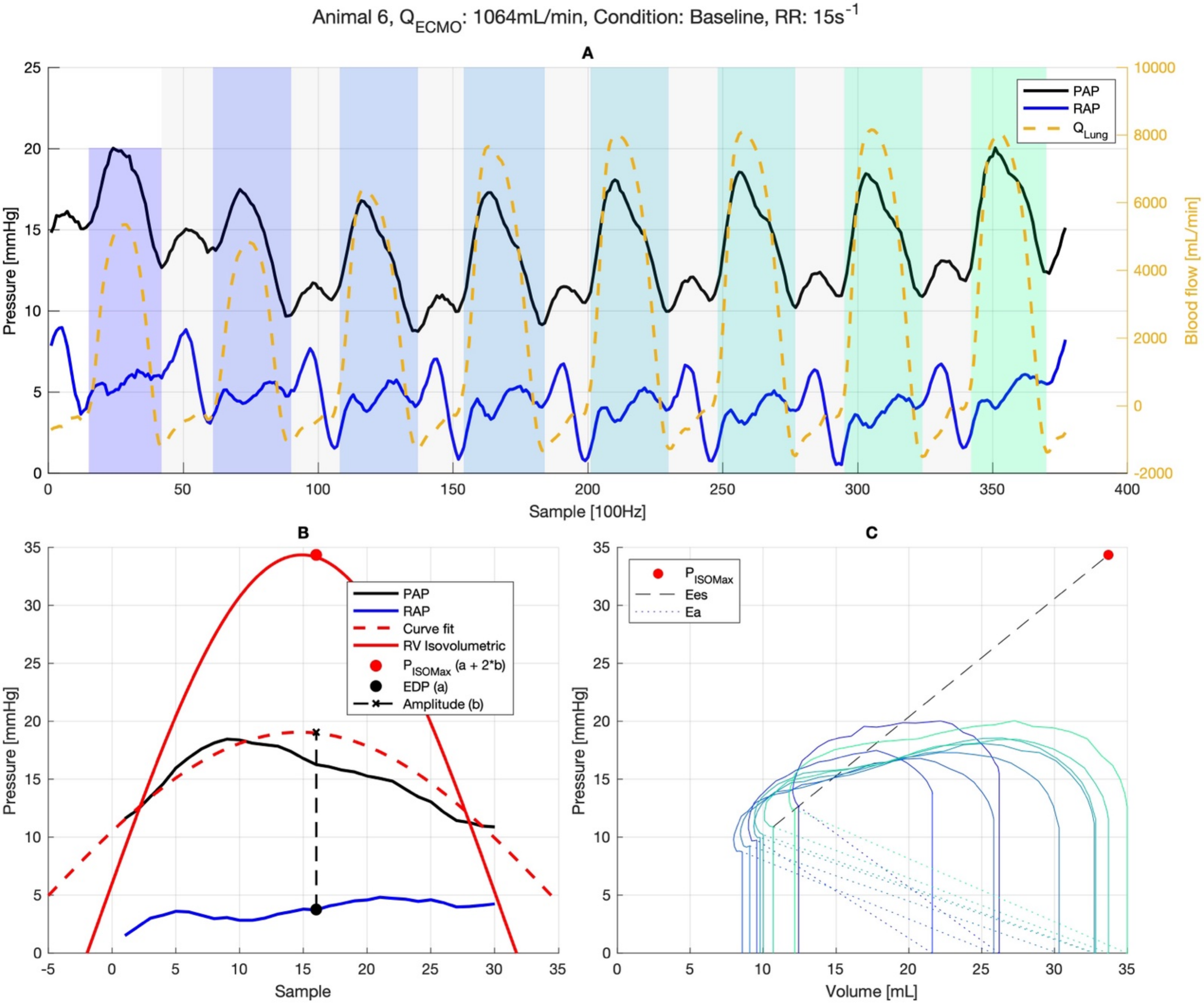

Animal 6, Q_ECMO: 1064mL/min, Condition: Baseline, RR: 15s^-1
A
Pressure [mmHg]
Blood flow [mL/min]
Sample [100Hz]
PAP
RAP
Q_Lung
B
PAP
RAP
Curve fit
RV Isovolumetric
P_ISOMax (a + 2*b)
EDP (a)
Amplitude (b)
Pressure [mmHg]
Sample
C
P_ISOMax
Ees
Ea
Pressure [mmHg]
Volume [mL]

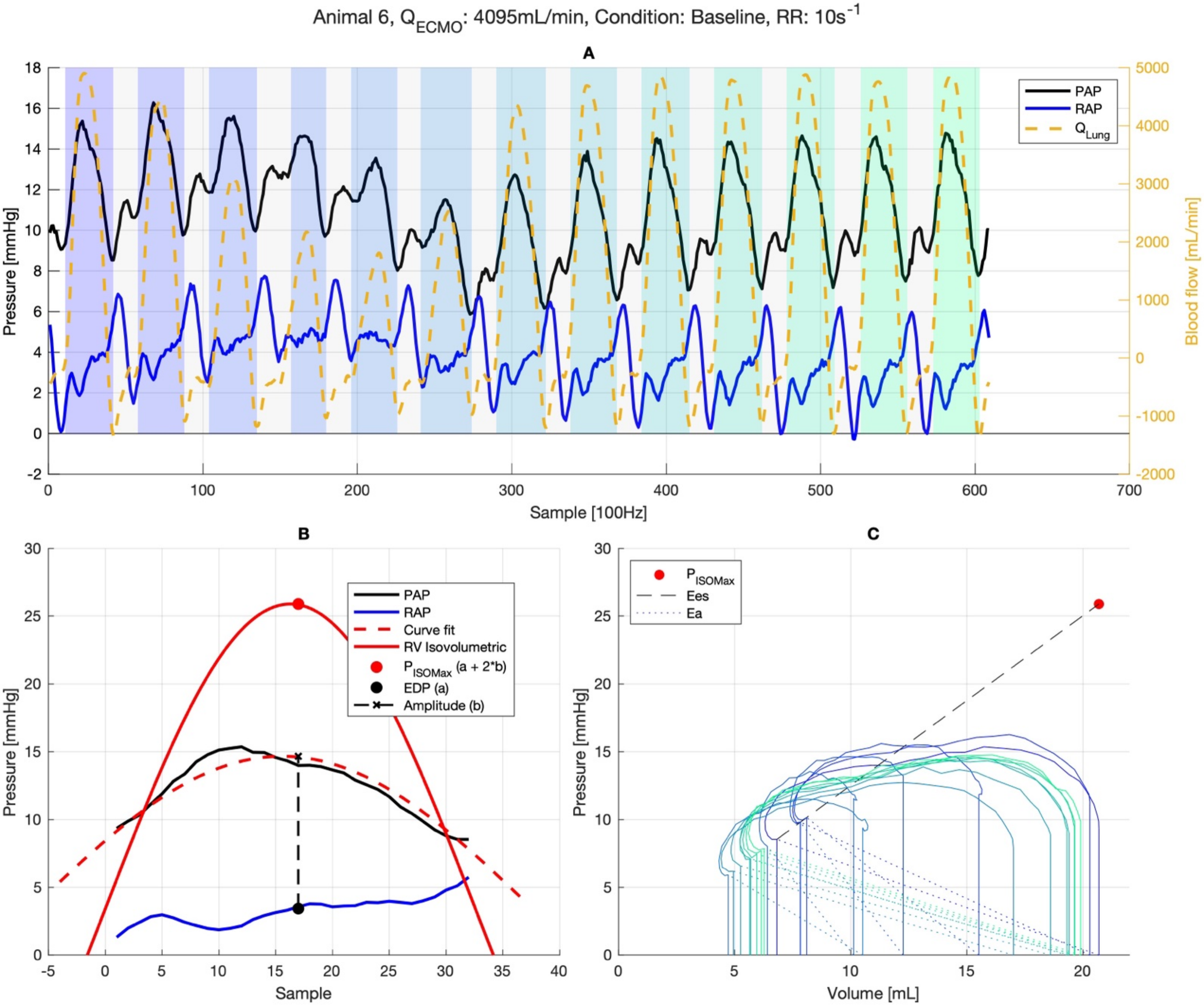
Animal 6, Q_ECMO: 4095mL/min, Condition: Baseline, RR: 10s^-1
A
Pressure [mmHg]
Blood flow [mL/min]
Sample [100Hz]
PAP
RAP
Q_Lung
B
Pressure [mmHg]
Sample
PAP
RAP
Curve fit
RV Isovolumetric
P_ISOMax (a + 2*b)
EDP (a)
Amplitude (b)
C
Pressure [mmHg]
Volume [mL]
P_ISOMax
Ees
Ea

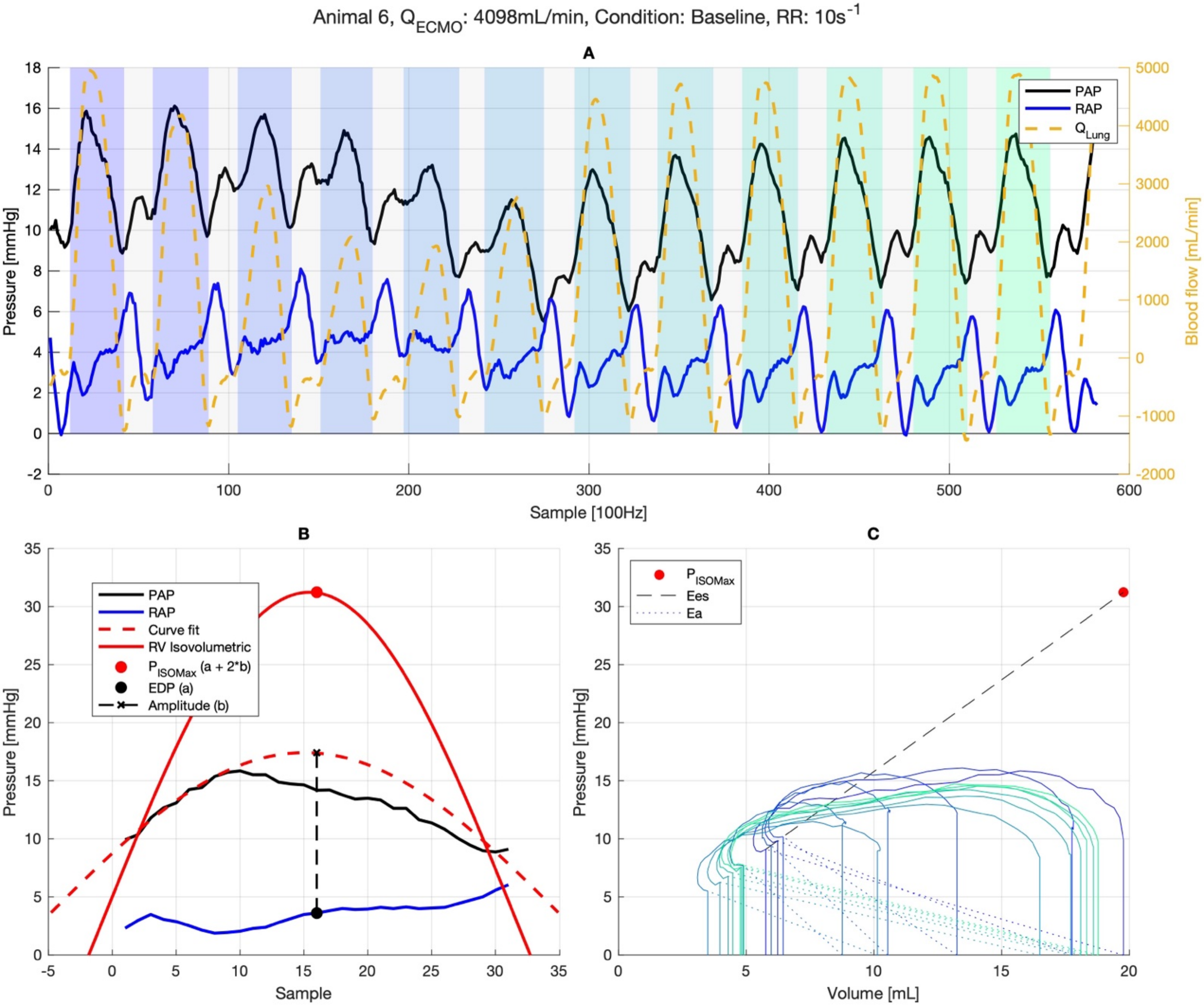

Animal 6, Q_ECMO: 4098mL/min, Condition: Baseline, RR: 10s^-1
A
PAP
RAP
Q_Lung
Pressure [mmHg]
Blood flow [mL/min]
Sample [100Hz]
B
PAP
RAP
Curve fit
RV Isovolumetric
P_ISOMax (a + 2*b)
EDP (a)
Amplitude (b)
Pressure [mmHg]
Sample
C
P_ISOMax
Ees
Ea
Pressure [mmHg]
Volume [mL]

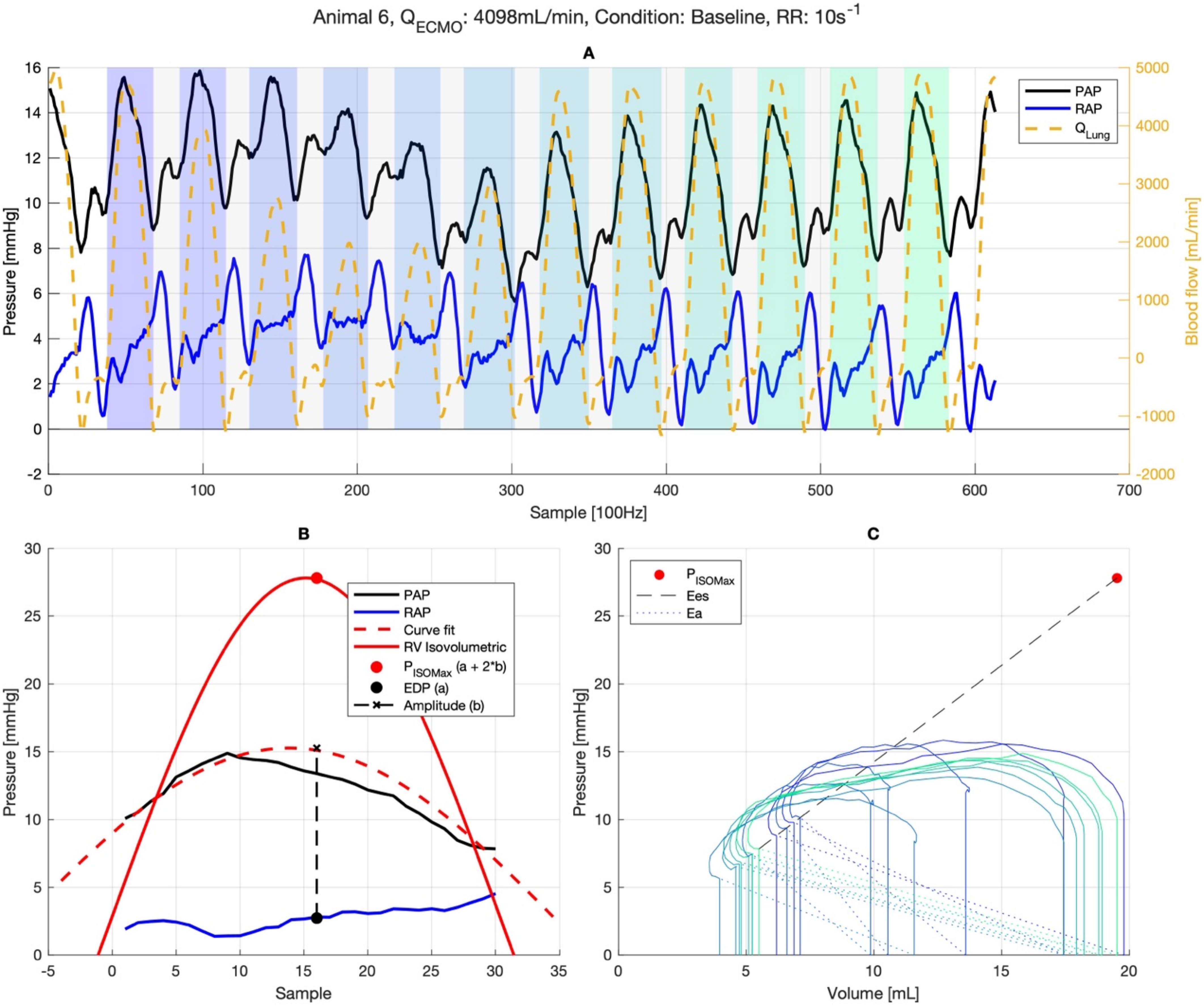

Animal 6, Q_ECMO: 4098mL/min, Condition: Baseline, RR: 10s^-1
A
PAP
RAP
Q_Lung
Pressure [mmHg]
Blood flow [mL/min]
Sample [100Hz]
B
PAP
RAP
Curve fit
RV Isovolumetric
P_ISOMax (a + 2*b)
EDP (a)
Amplitude (b)
Pressure [mmHg]
Sample
C
P_ISOMax
Ees
Ea
Pressure [mmHg]
Volume [mL]

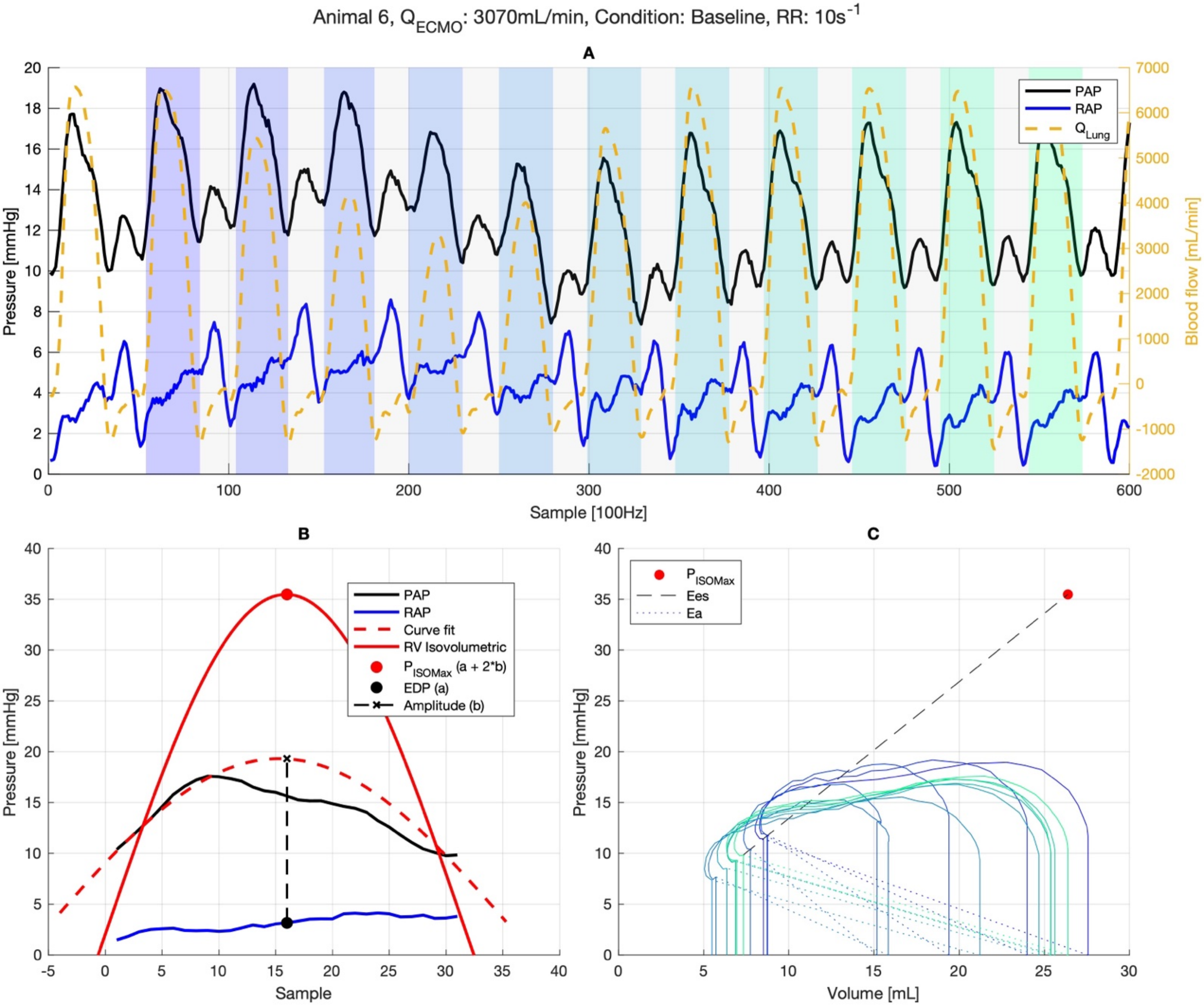

Animal 6, Q_ECMO: 3070mL/min, Condition: Baseline, RR: 10s^-1
A
PAP
RAP
Q_Lung
Pressure [mmHg]
Blood flow [mL/min]
Sample [100Hz]
B
PAP
RAP
Curve fit
RV Isovolumetric
P_ISOMax (a + 2*b)
EDP (a)
Amplitude (b)
Pressure [mmHg]
Sample
C
P_ISOMax
Ees
Ea
Pressure [mmHg]
Volume [mL]

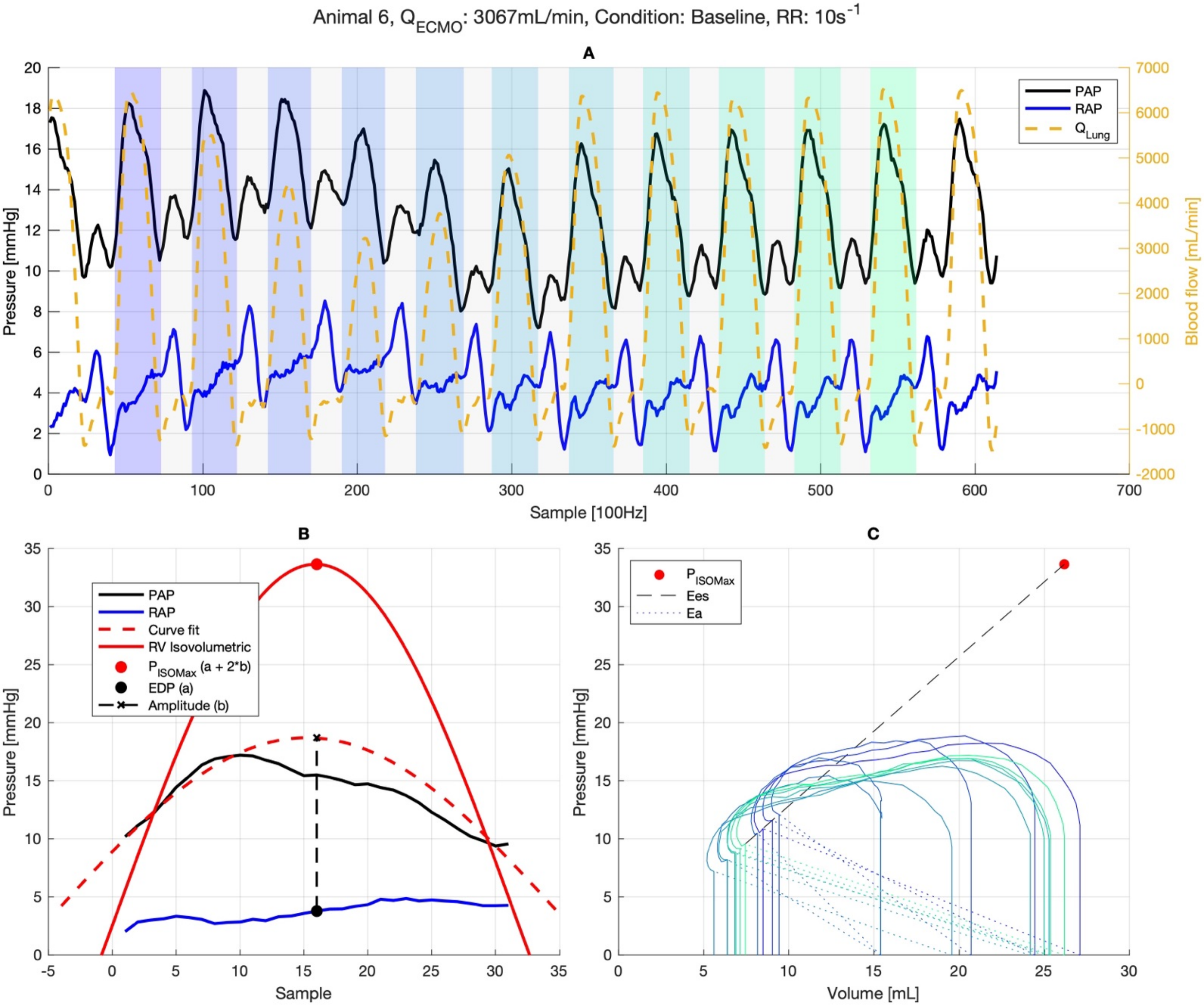

Animal 6, QECMO: 3067mL/min, Condition: Baseline, RR: 10s-1
A
PAP
RAP
QLung
Pressure [mmHg]
Blood flow [mL/min]
Sample [100Hz]
B
PAP
RAP
Curve fit
RV Isovolumetric
PISOMax (a + 2*b)
EDP (a)
Amplitude (b)
Pressure [mmHg]
Sample
C
PISOMax
Ees
Ea
Pressure [mmHg]
Volume [mL]

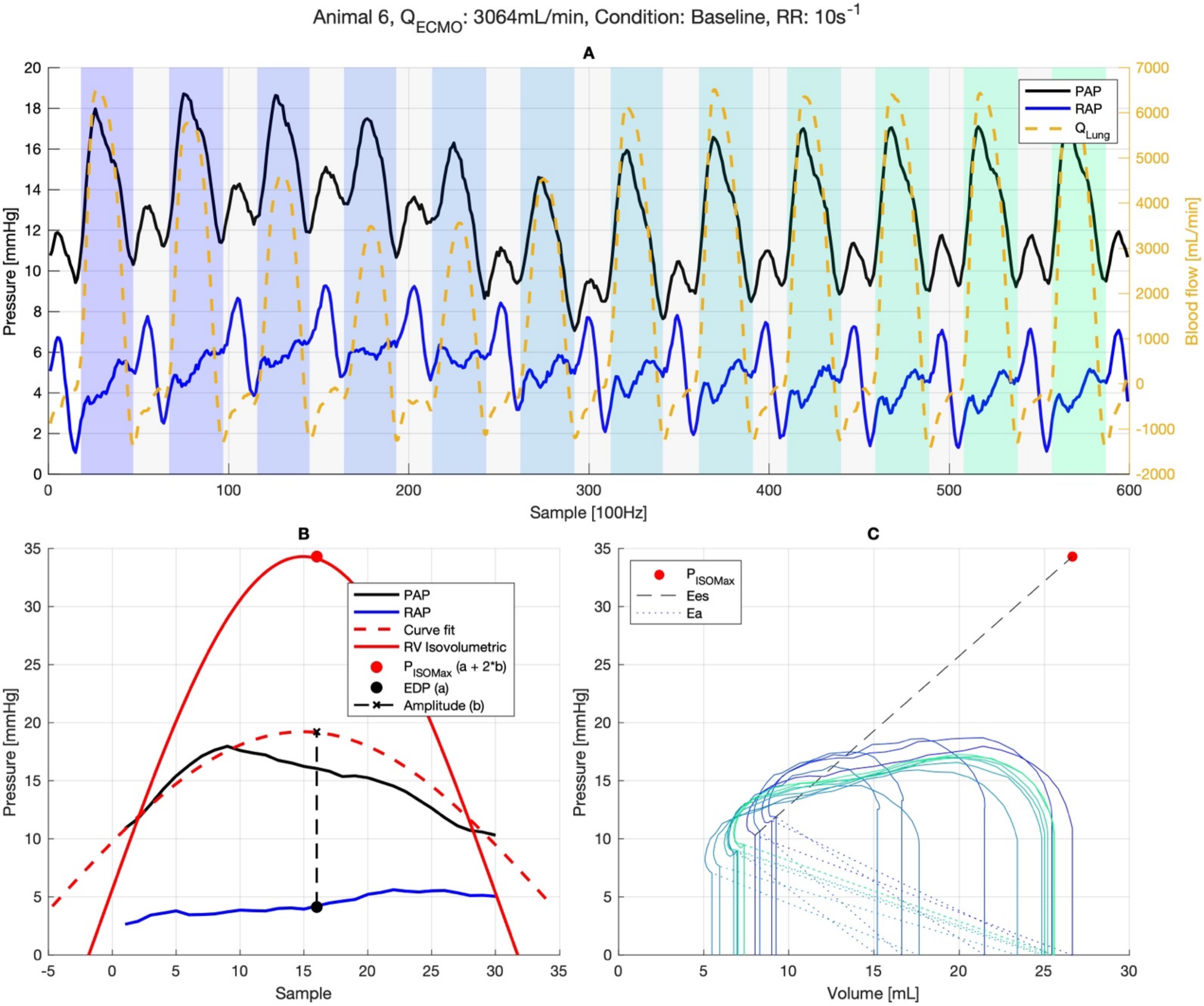

Animal 6, Q_ECMO: 3064mL/min, Condition: Baseline, RR: 10s^-1
A
PAP
RAP
Q_Lung
Pressure [mmHg]
Blood flow [mL/min]
Sample [100Hz]
B
PAP
RAP
Curve fit
RV Isovolumetric
P_ISOMax (a + 2*b)
EDP (a)
Amplitude (b)
Pressure [mmHg]
Sample
C
P_ISOMax
Ees
Ea
Pressure [mmHg]
Volume [mL]

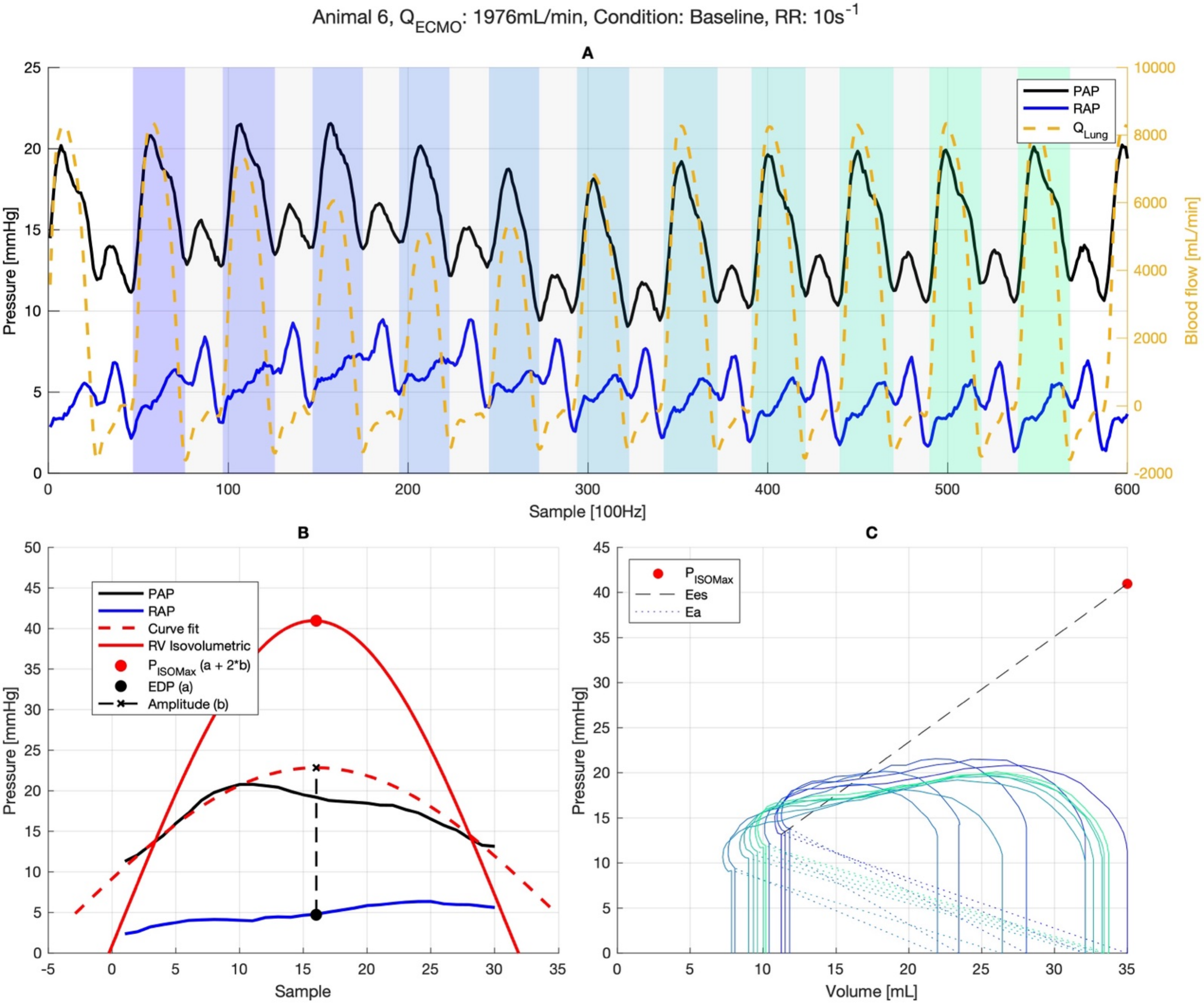

Animal 6, Q_{ECMO}: 1976mL/min, Condition: Baseline, RR: $10s^{-1}$
A
PAP
RAP
Q_{Lung}
Pressure [mmHg]
Blood flow [mL/min]
Sample [100Hz]
B
PAP
RAP
Curve fit
RV Isovolumetric
P_{ISOMax} (a + 2*b)
EDP (a)
Amplitude (b)
Pressure [mmHg]
Sample
C
P_{ISOMax}
Ees
Ea
Pressure [mmHg]
Volume [mL]

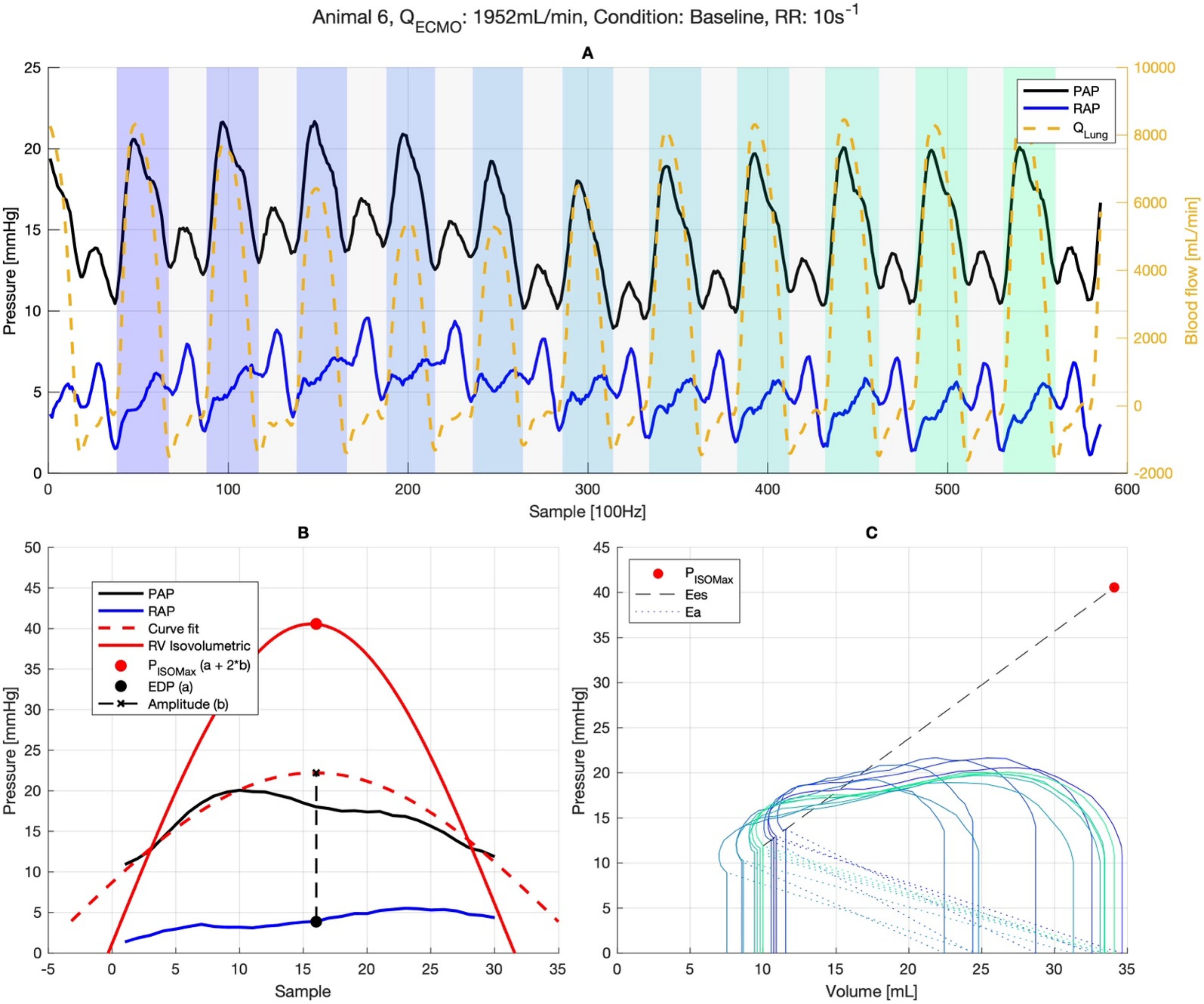

Animal 6, Q_{ECMO}: 1952mL/min, Condition: Baseline, RR: $10s^{-1}$
A
PAP
RAP
Q_{Lung}
Pressure [mmHg]
Blood flow [mL/min]
Sample [100Hz]
B
PAP
RAP
Curve fit
RV Isovolumetric
P_{ISOMax} (a + 2*b)
EDP (a)
Amplitude (b)
Pressure [mmHg]
Sample
C
P_{ISOMax}
Ees
Ea
Pressure [mmHg]
Volume [mL]

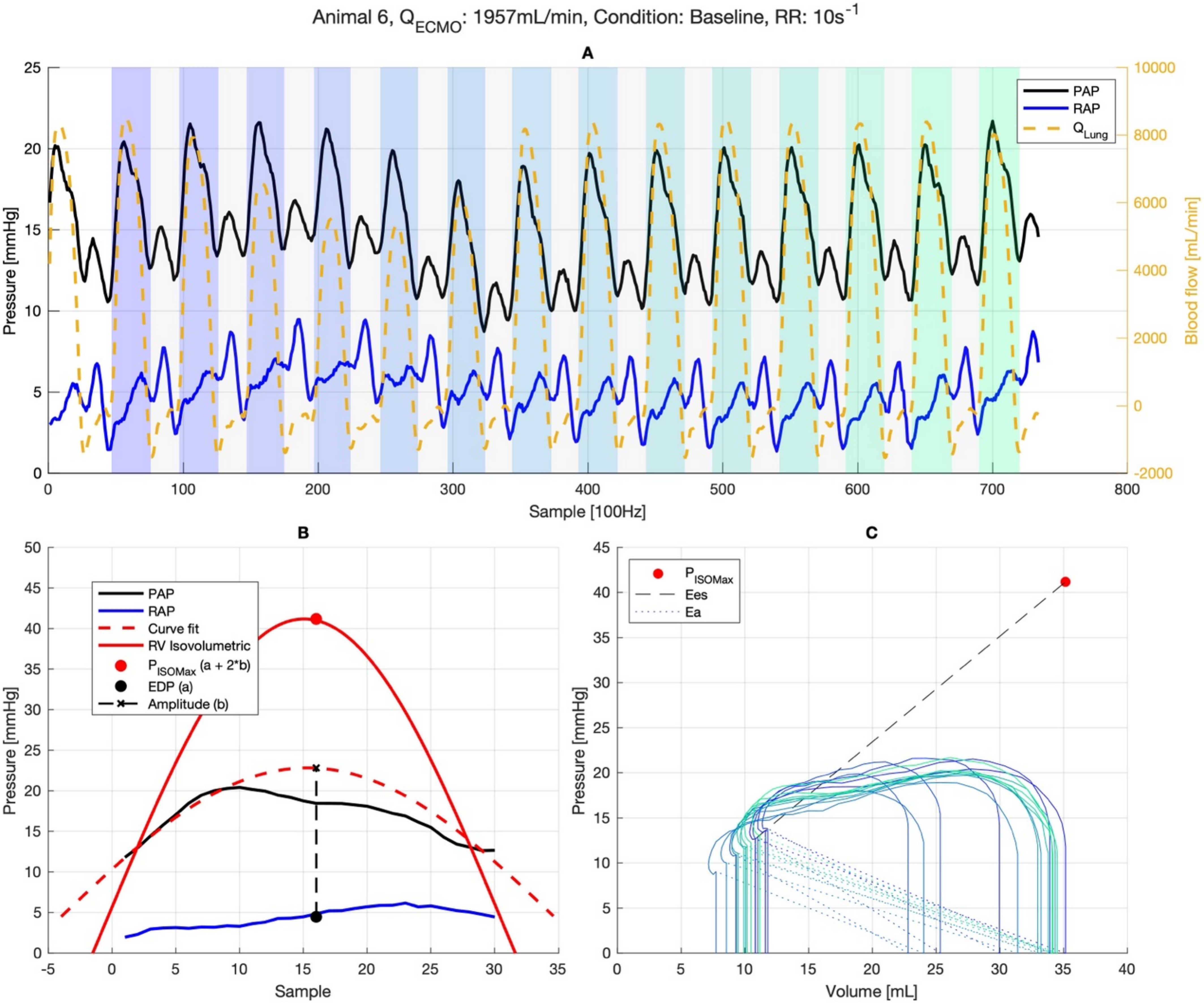
Animal 6, Q_{ECMO}: 1957mL/min, Condition: Baseline, RR: $10s^{-1}$
A
PAP
RAP
Q_{Lung}
Pressure [mmHg]
Blood flow [mL/min]
Sample [100Hz]
B
PAP
RAP
Curve fit
RV Isovolumetric
P_{ISOMax} (a + 2*b)
EDP (a)
Amplitude (b)
Pressure [mmHg]
Sample
C
P_{ISOMax}
Ees
Ea
Pressure [mmHg]
Volume [mL]

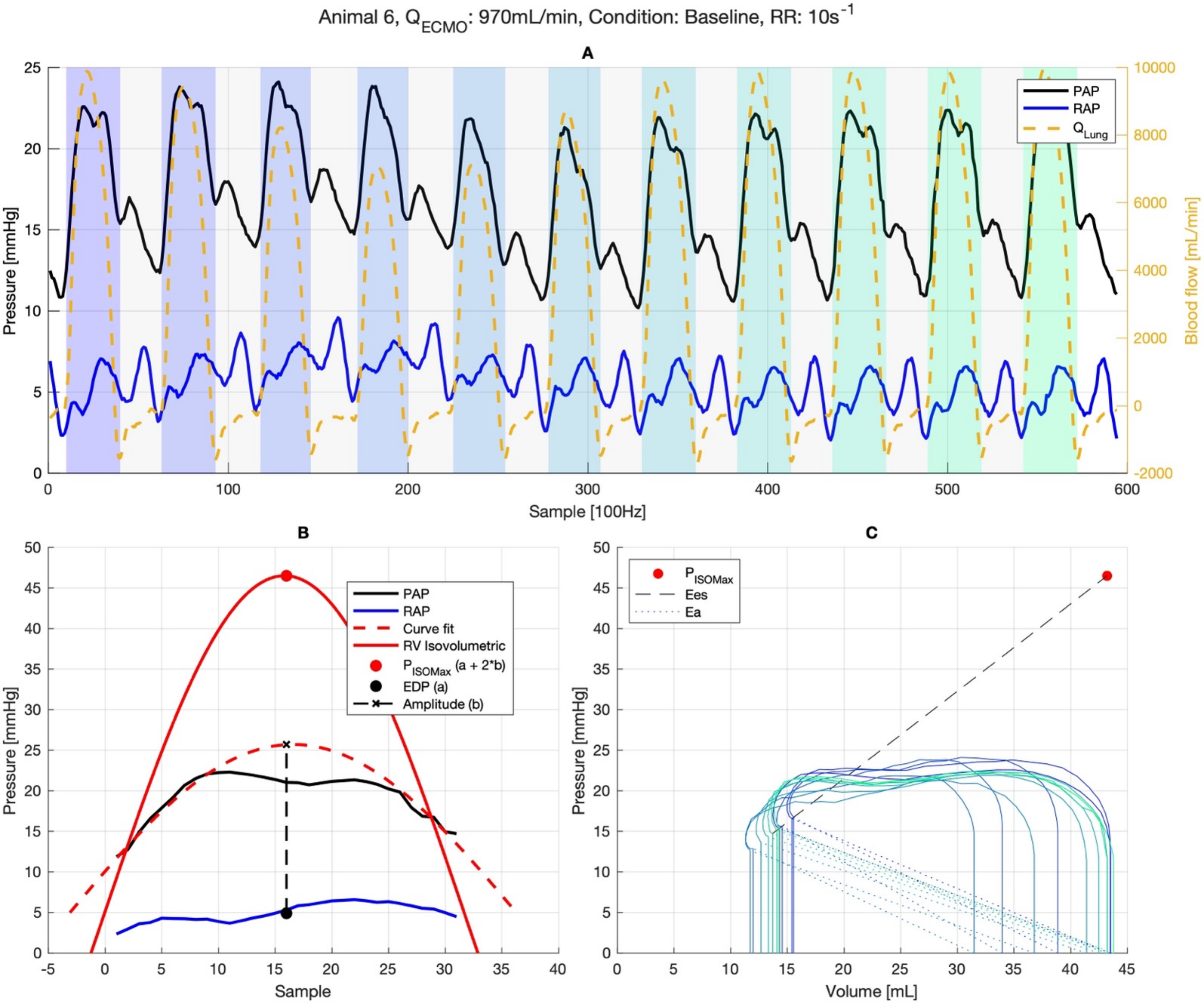
Animal 6, Q_ECMO: 970mL/min, Condition: Baseline, RR: 10s^-1
A
Pressure [mmHg]
Blood flow [mL/min]
Sample [100Hz]
PAP
RAP
Q_Lung
B
Pressure [mmHg]
Sample
PAP
RAP
Curve fit
RV Isovolumetric
P_ISOMax (a + 2*b)
EDP (a)
Amplitude (b)
C
Pressure [mmHg]
Volume [mL]
P_ISOMax
Ees
Ea

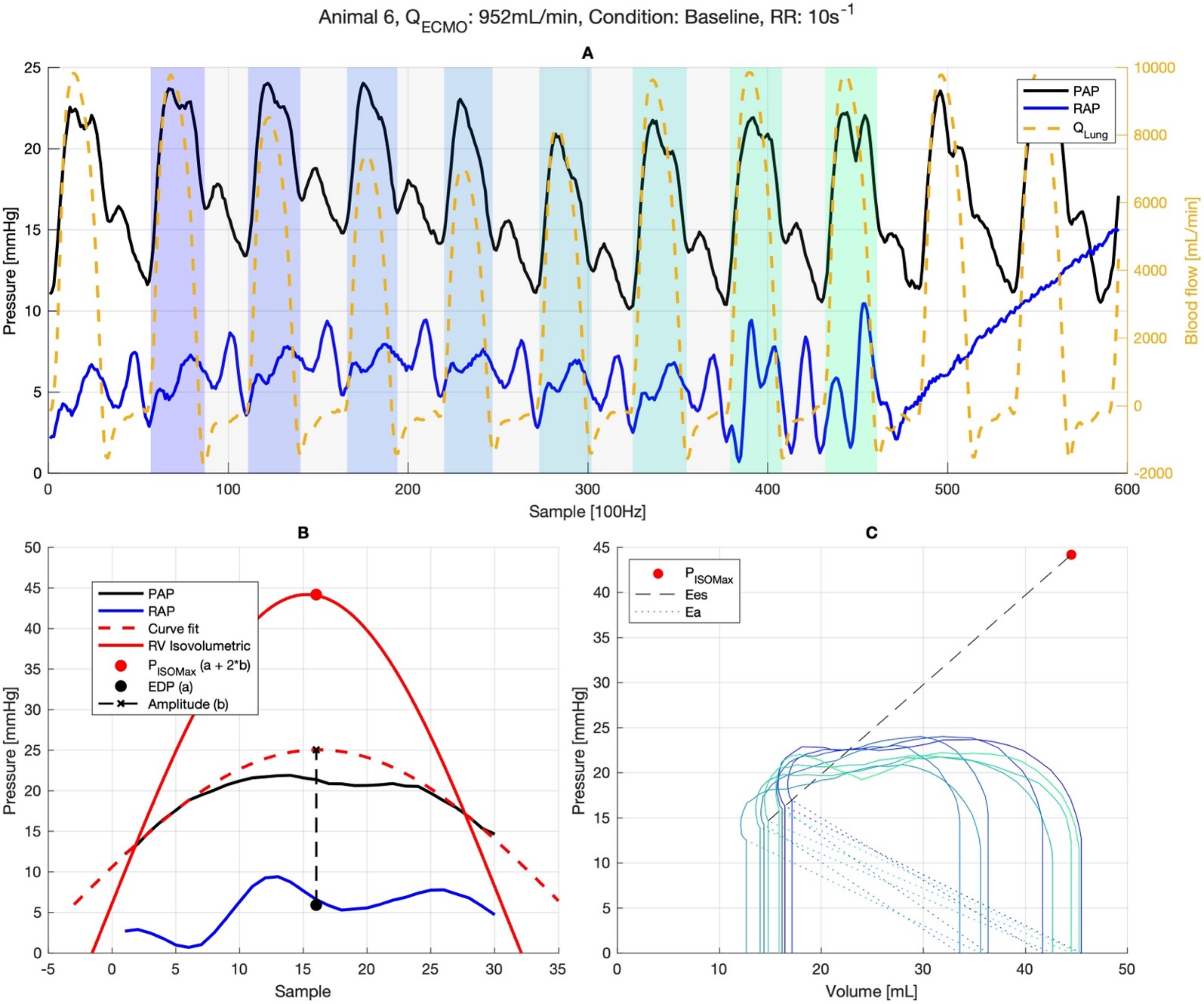

Animal 6, Q_ECMO: 952mL/min, Condition: Baseline, RR: 10s^-1
A
PAP
RAP
Q_Lung
Pressure [mmHg]
Blood flow [mL/min]
Sample [100Hz]
B
PAP
RAP
Curve fit
RV Isovolumetric
P_ISOMax (a + 2*b)
EDP (a)
Amplitude (b)
Pressure [mmHg]
Sample
C
P_ISOMax
Ees
Ea
Pressure [mmHg]
Volume [mL]

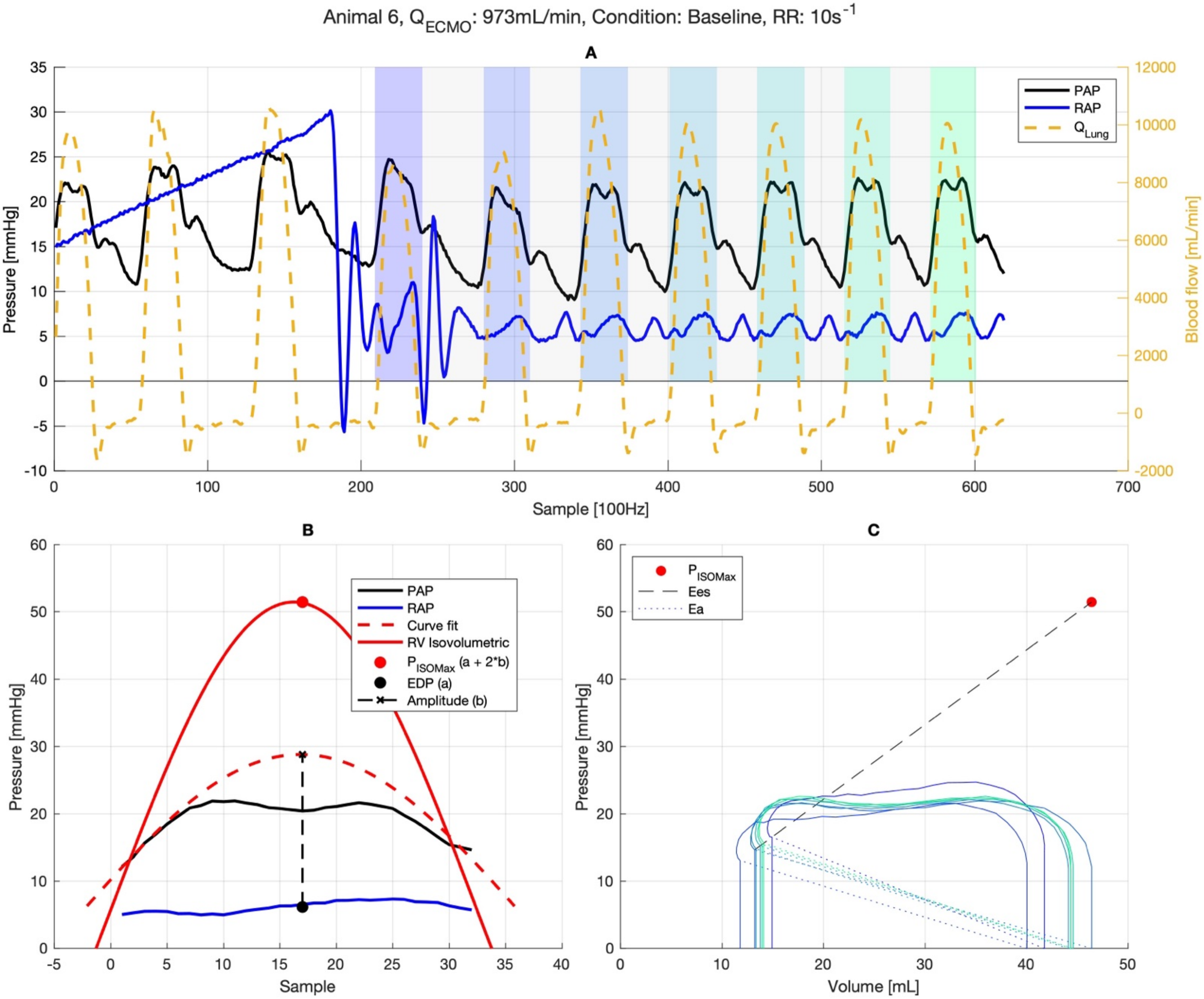

Animal 6, Q_{ECMO}: 973mL/min, Condition: Baseline, RR: 10s^{-1}
A
PAP
RAP
Q_{Lung}
Pressure [mmHg]
Blood flow [mL/min]
Sample [100Hz]
B
PAP
RAP
Curve fit
RV Isovolumetric
P_{ISOMax} (a + 2*b)
EDP (a)
Amplitude (b)
Pressure [mmHg]
Sample
C
P_{ISOMax}
Ees
Ea
Pressure [mmHg]
Volume [mL]

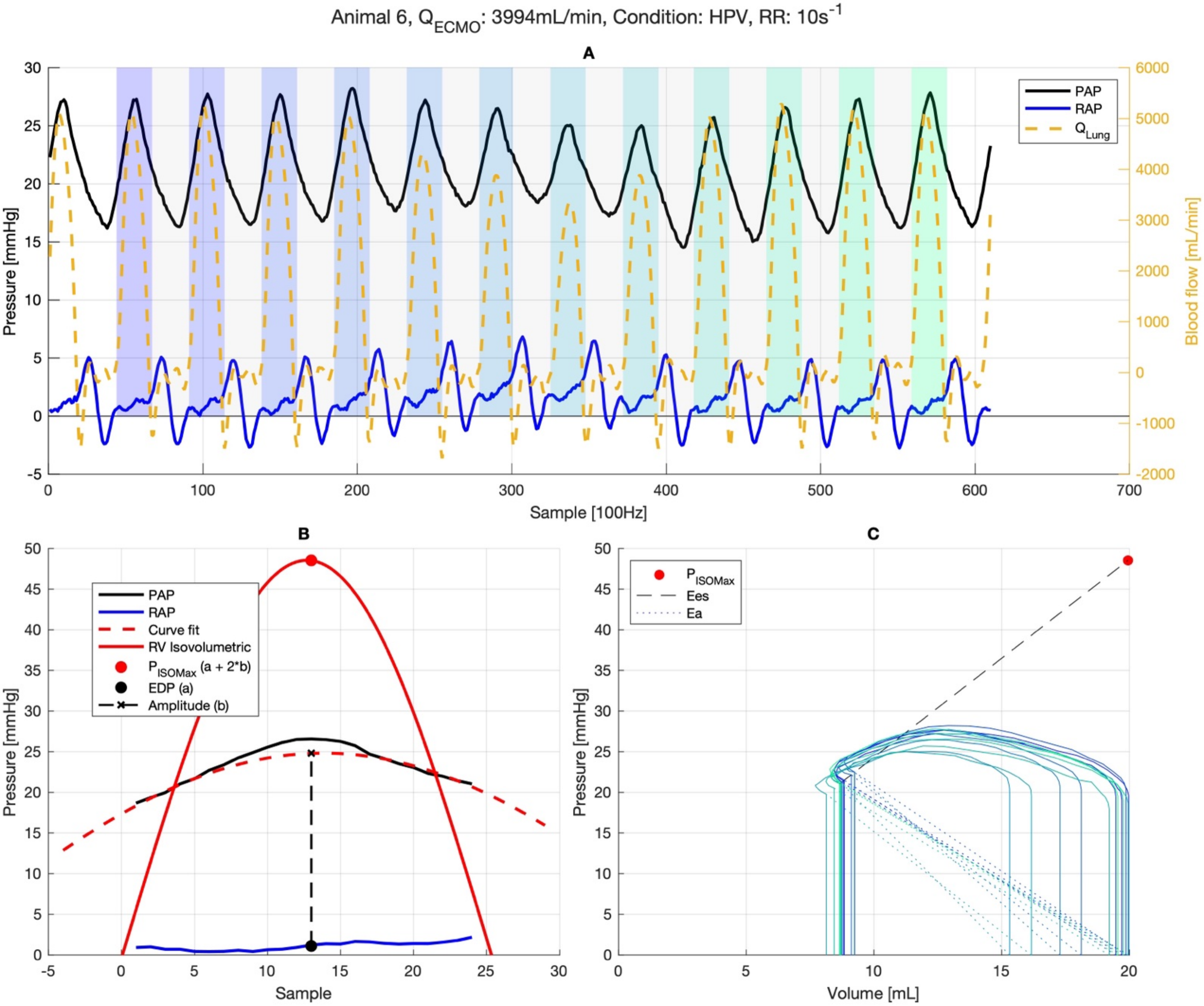

Animal 6, Q_ECMO: 3994mL/min, Condition: HPV, RR: 10s^-1
A
Pressure [mmHg]
Blood flow [mL/min]
Sample [100Hz]
PAP
RAP
Q_Lung
30
25
20
15
10
5
0
-5
6000
5000
4000
3000
2000
1000
0
-1000
-2000
0
100
200
300
400
500
600
700
B
Pressure [mmHg]
Sample
PAP
RAP
Curve fit
RV Isovolumetric
P_ISOMax (a + 2*b)
EDP (a)
Amplitude (b)
50
45
40
35
30
25
20
15
10
5
0
-5
0
5
10
15
20
25
30
C
Pressure [mmHg]
Volume [mL]
P_ISOMax
Ees
Ea
50
45
40
35
30
25
20
15
10
5
0
0
5
10
15
20

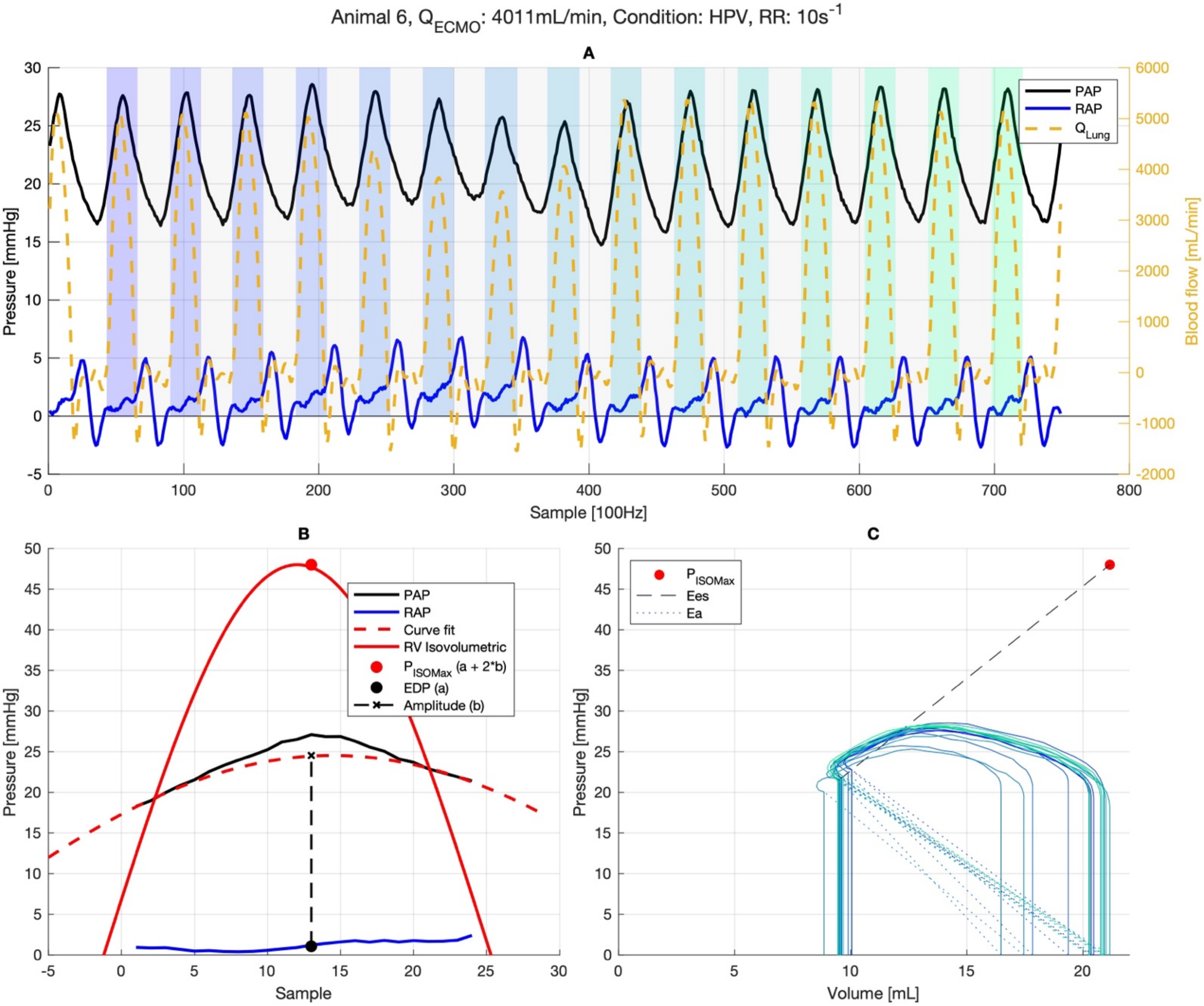

Animal 6, Q_{ECMO}: 4011mL/min, Condition: HPV, RR: 10s^{-1}
A
PAP
RAP
Q_{Lung}
Pressure [mmHg]
Blood flow [mL/min]
Sample [100Hz]
B
PAP
RAP
Curve fit
RV Isovolumetric
P_{ISOMax} (a + 2*b)
EDP (a)
Amplitude (b)
Pressure [mmHg]
Sample
C
P_{ISOMax}
Ees
Ea
Pressure [mmHg]
Volume [mL]

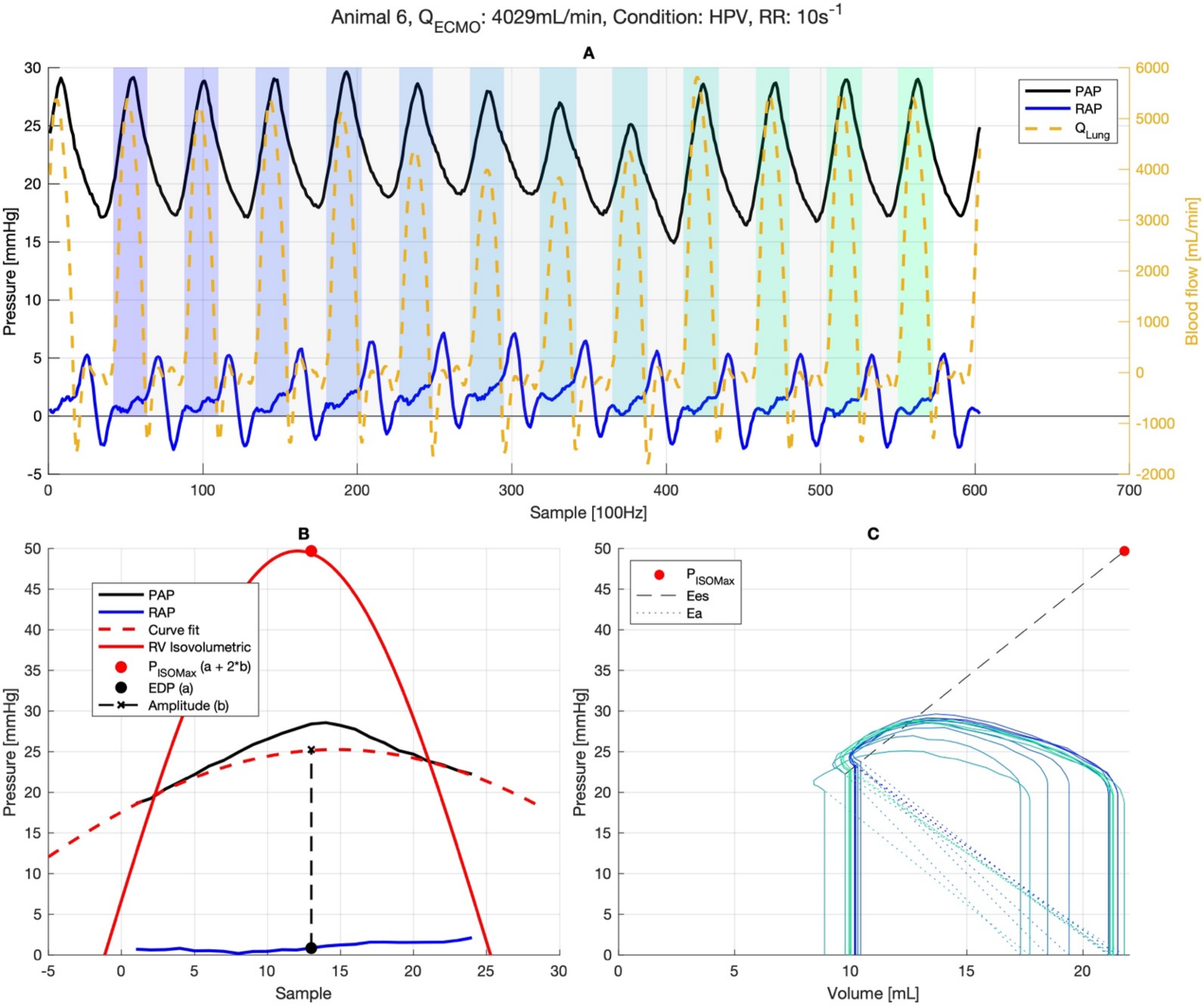

Animal 6, Q$_{ECMO}$: 4029mL/min, Condition: HPV, RR: 10s^{-1}
A
PAP
RAP
Q$_{Lung}$
Pressure [mmHg]
Blood flow [mL/min]
Sample [100Hz]
B
PAP
RAP
Curve fit
RV Isovolumetric
P$_{ISOMax}$ (a + 2*b)
EDP (a)
Amplitude (b)
Pressure [mmHg]
Sample
C
P$_{ISOMax}$
Ees
Ea
Pressure [mmHg]
Volume [mL]

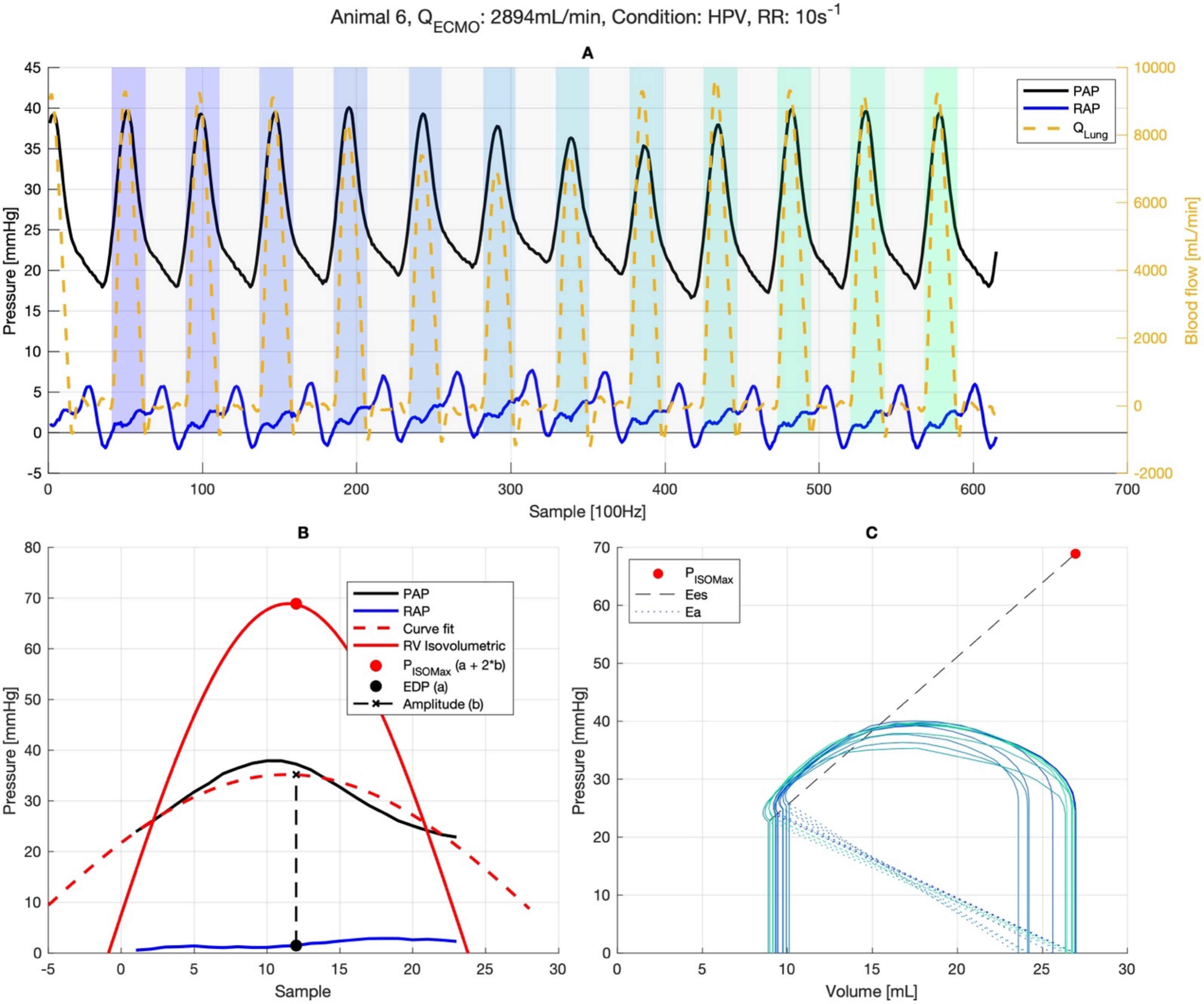

Animal 6, Q_{ECMO}: 2894mL/min, Condition: HPV, RR: $10s^{-1}$
A
PAP
RAP
Q_{Lung}
Pressure [mmHg]
Blood flow [mL/min]
Sample [100Hz]
B
PAP
RAP
Curve fit
RV Isovolumetric
P_{ISOMax} (a + 2*b)
EDP (a)
Amplitude (b)
Pressure [mmHg]
Sample
C
P_{ISOMax}
Ees
Ea
Pressure [mmHg]
Volume [mL]

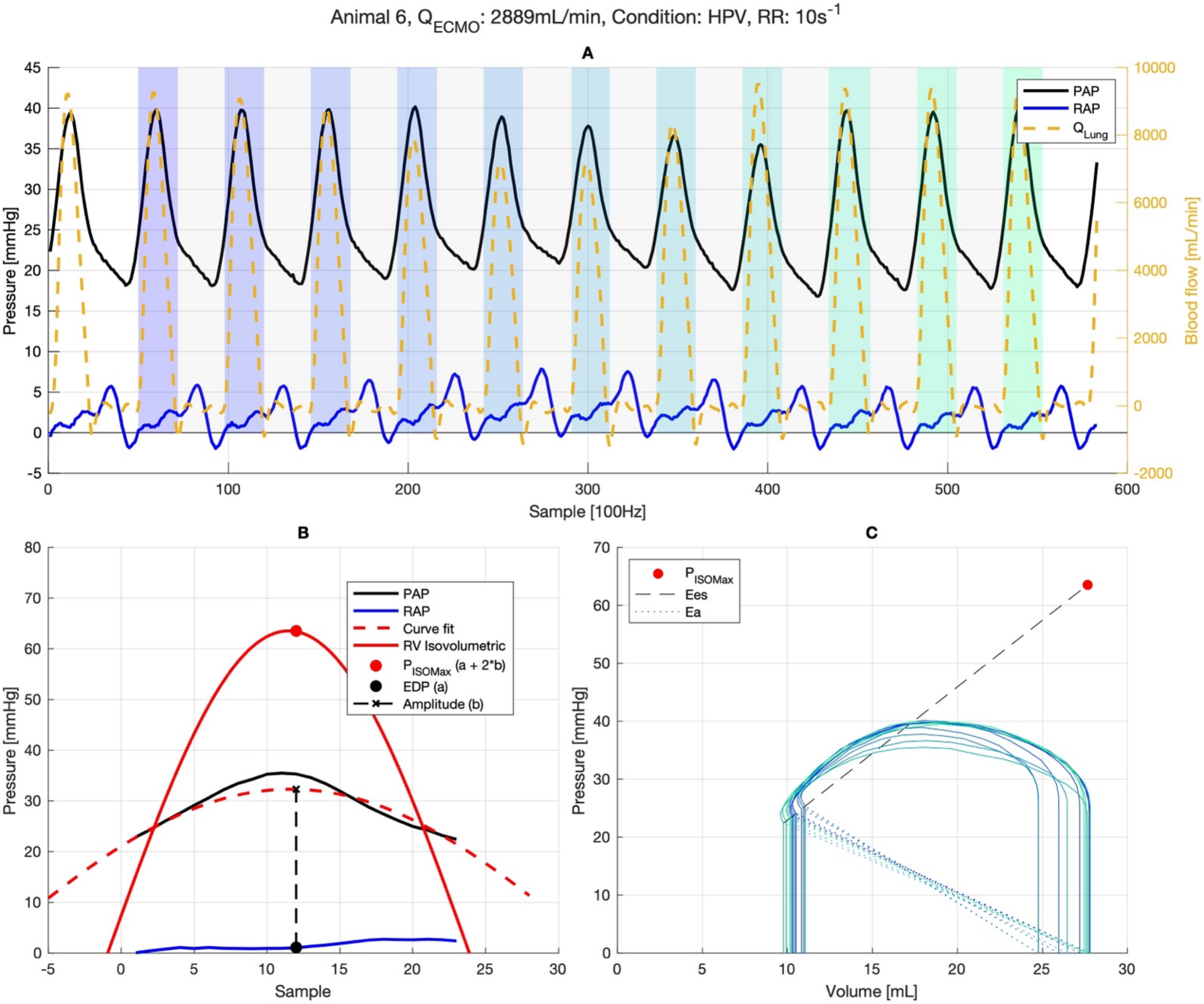

Animal 6, Q_{ECMO}: 2889mL/min, Condition: HPV, RR: $10s^{-1}$
A
PAP
RAP
Q_{Lung}
Pressure [mmHg]
Blood flow [mL/min]
Sample [100Hz]
B
PAP
RAP
Curve fit
RV Isovolumetric
P_{ISOMax} (a + 2*b)
EDP (a)
Amplitude (b)
Pressure [mmHg]
Sample
C
P_{ISOMax}
Ees
Ea
Pressure [mmHg]
Volume [mL]

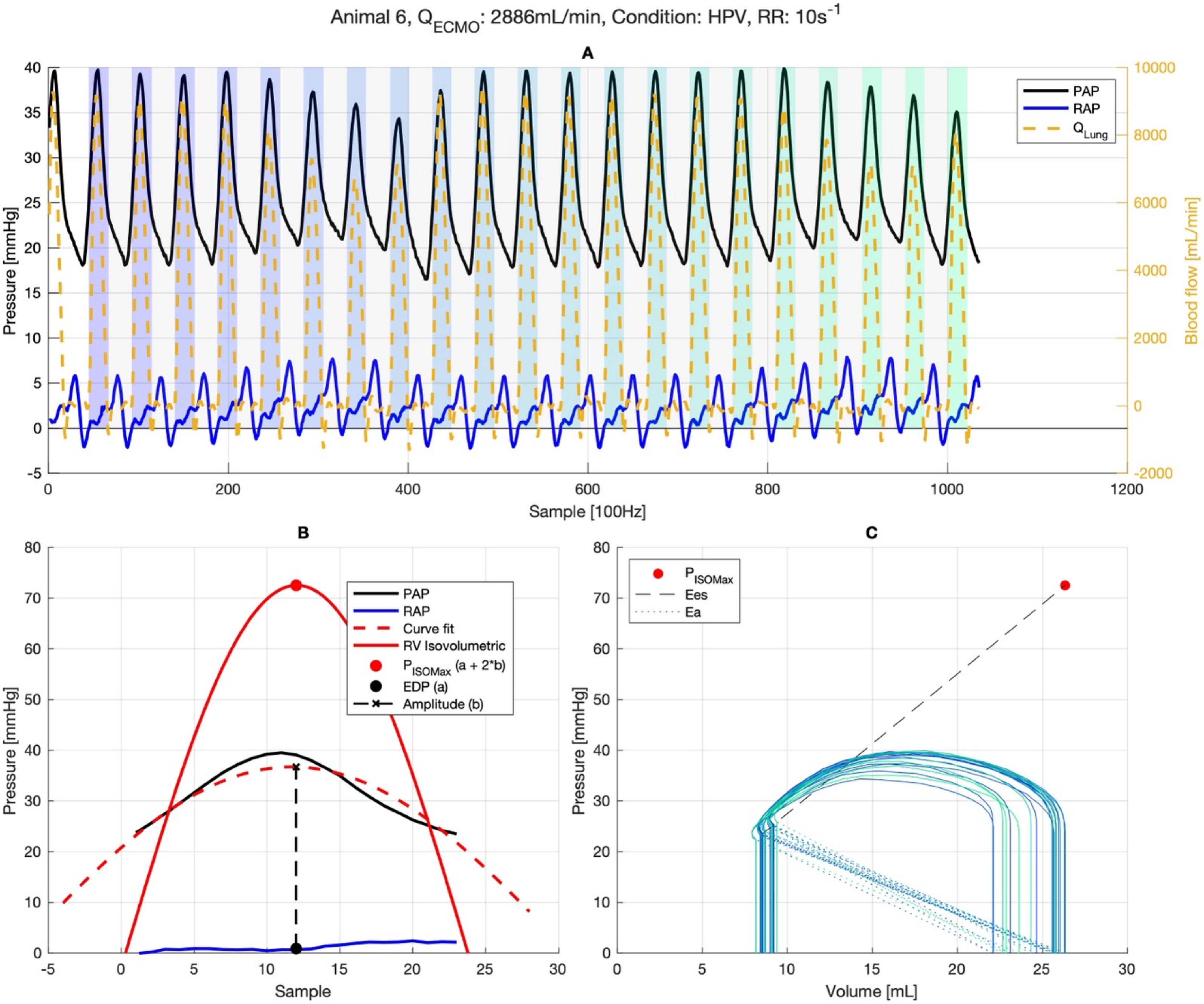

Animal 6, Q_{ECMO}: 2886mL/min, Condition: HPV, RR: $10s^{-1}$
A
Pressure [mmHg]
Blood flow [mL/min]
Sample [100Hz]
PAP
RAP
Q_{Lung}
B
PAP
RAP
Curve fit
RV Isovolumetric
P_{ISOMax} (a + 2*b)
EDP (a)
Amplitude (b)
Pressure [mmHg]
Sample
C
P_{ISOMax}
Ees
Ea
Pressure [mmHg]
Volume [mL]

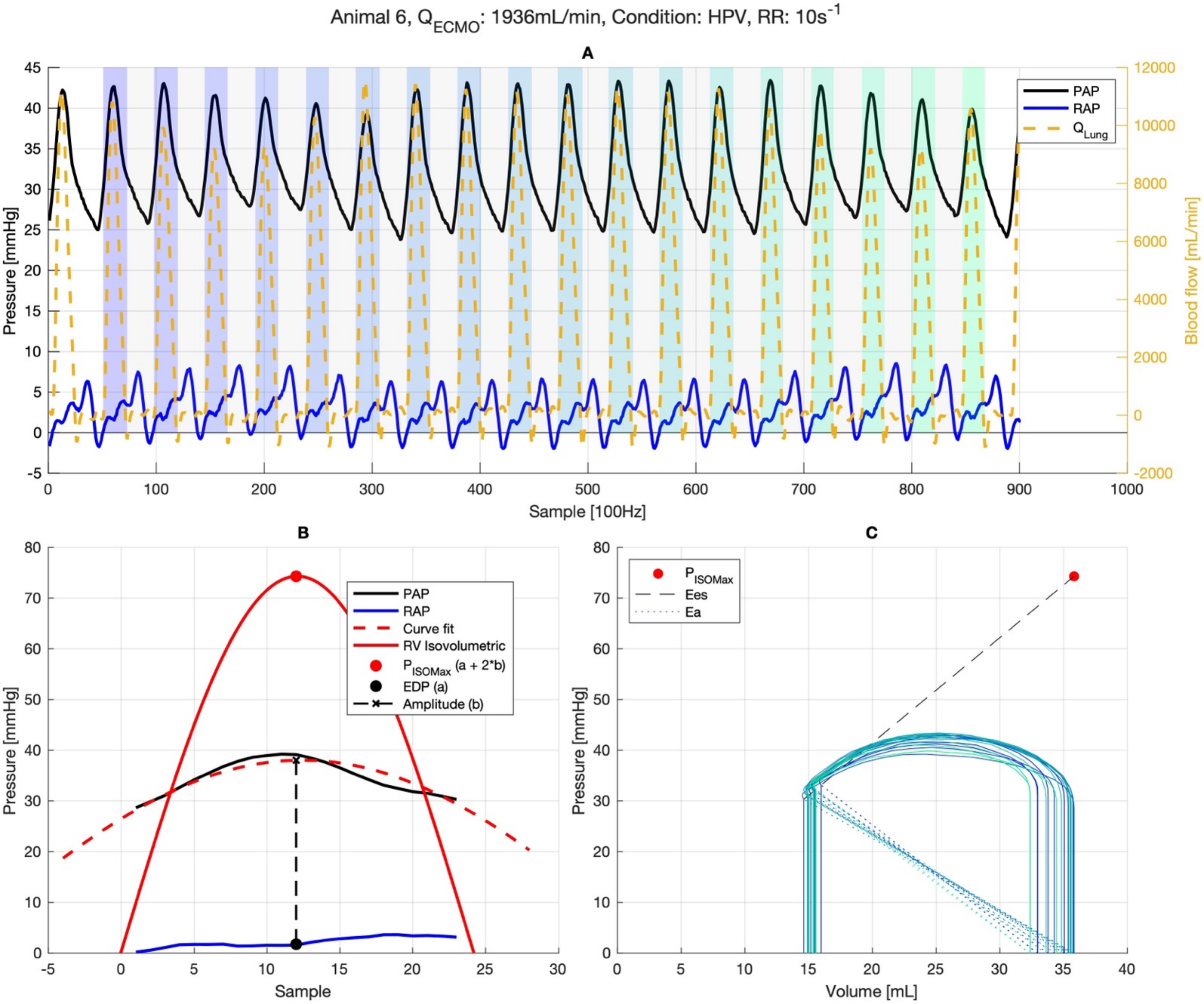

Animal 6, Q_ECMO: 1936mL/min, Condition: HPV, RR: 10s^-1
A
Pressure [mmHg]
Blood flow [mL/min]
Sample [100Hz]
PAP
RAP
Q_Lung
B
Pressure [mmHg]
Sample
PAP
RAP
Curve fit
RV Isovolumetric
P_ISOMax (a + 2*b)
EDP (a)
Amplitude (b)
C
Pressure [mmHg]
Volume [mL]
P_ISOMax
Ees
Ea

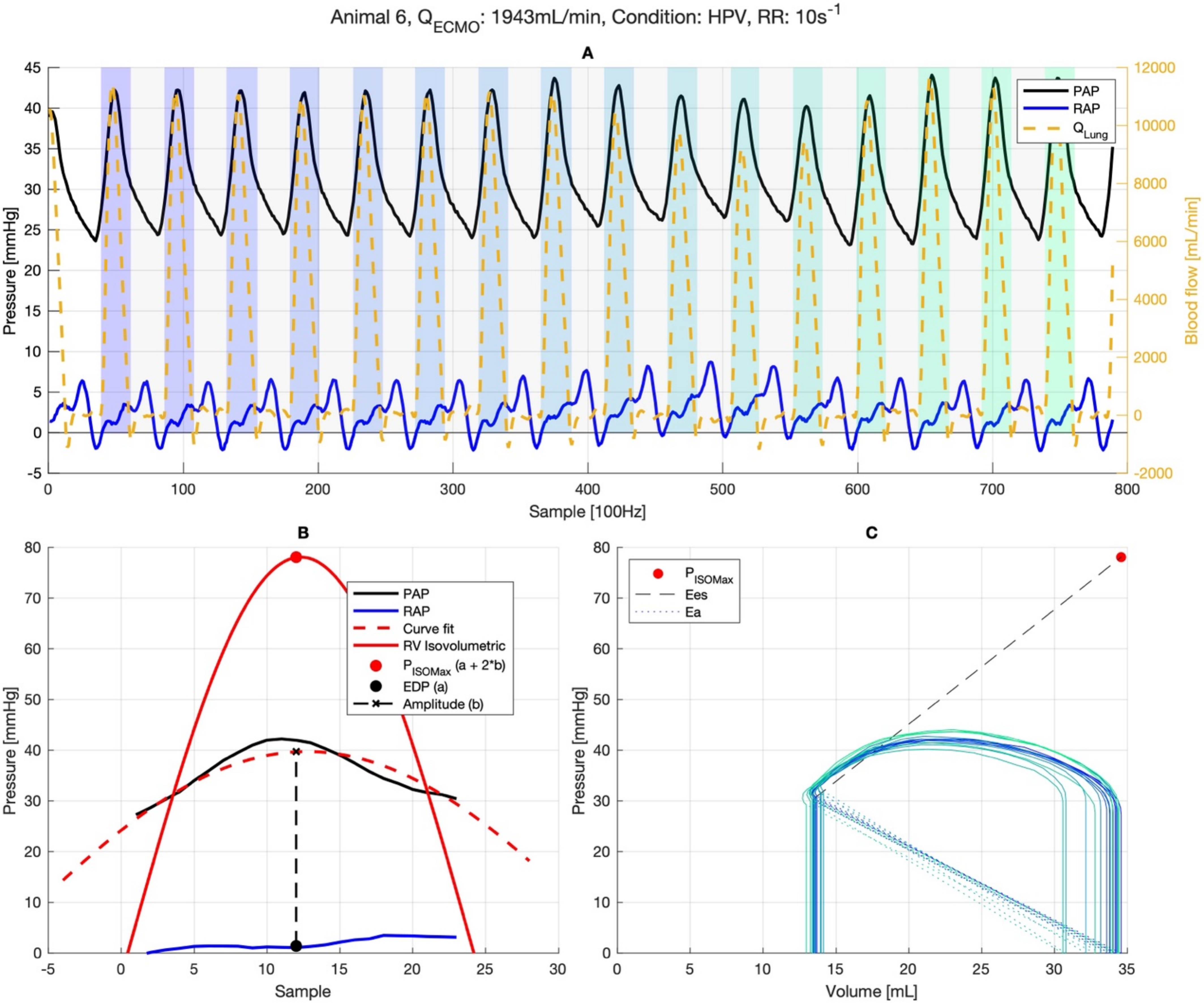

Animal 6, Q_{ECMO}: 1943mL/min, Condition: HPV, RR: $10s^{-1}$
A
PAP
RAP
Q_{Lung}
Pressure [mmHg]
Blood flow [mL/min]
Sample [100Hz]
B
PAP
RAP
Curve fit
RV Isovolumetric
P_{ISOMax} (a + 2*b)
EDP (a)
Amplitude (b)
Pressure [mmHg]
Sample
C
P_{ISOMax}
Ees
Ea
Pressure [mmHg]
Volume [mL]

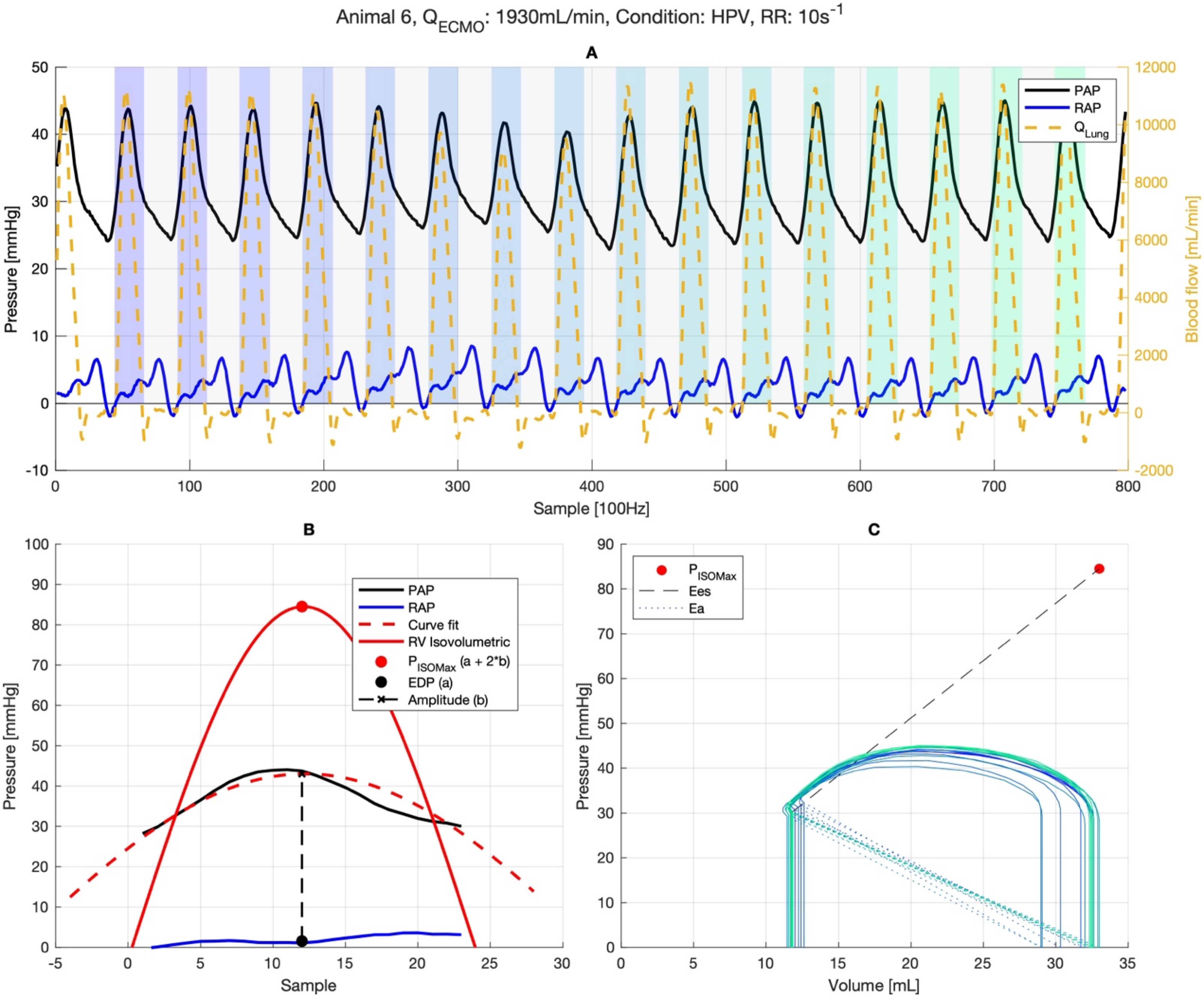

Animal 6, QECMO: 1930mL/min, Condition: HPV, RR: 10s-1
A
PAP
RAP
QLung
Pressure [mmHg]
Blood flow [mL/min]
Sample [100Hz]
B
PAP
RAP
Curve fit
RV Isovolumetric
PISOMax (a + 2*b)
EDP (a)
Amplitude (b)
Pressure [mmHg]
Sample
C
PISOMax
Ees
Ea
Pressure [mmHg]
Volume [mL]

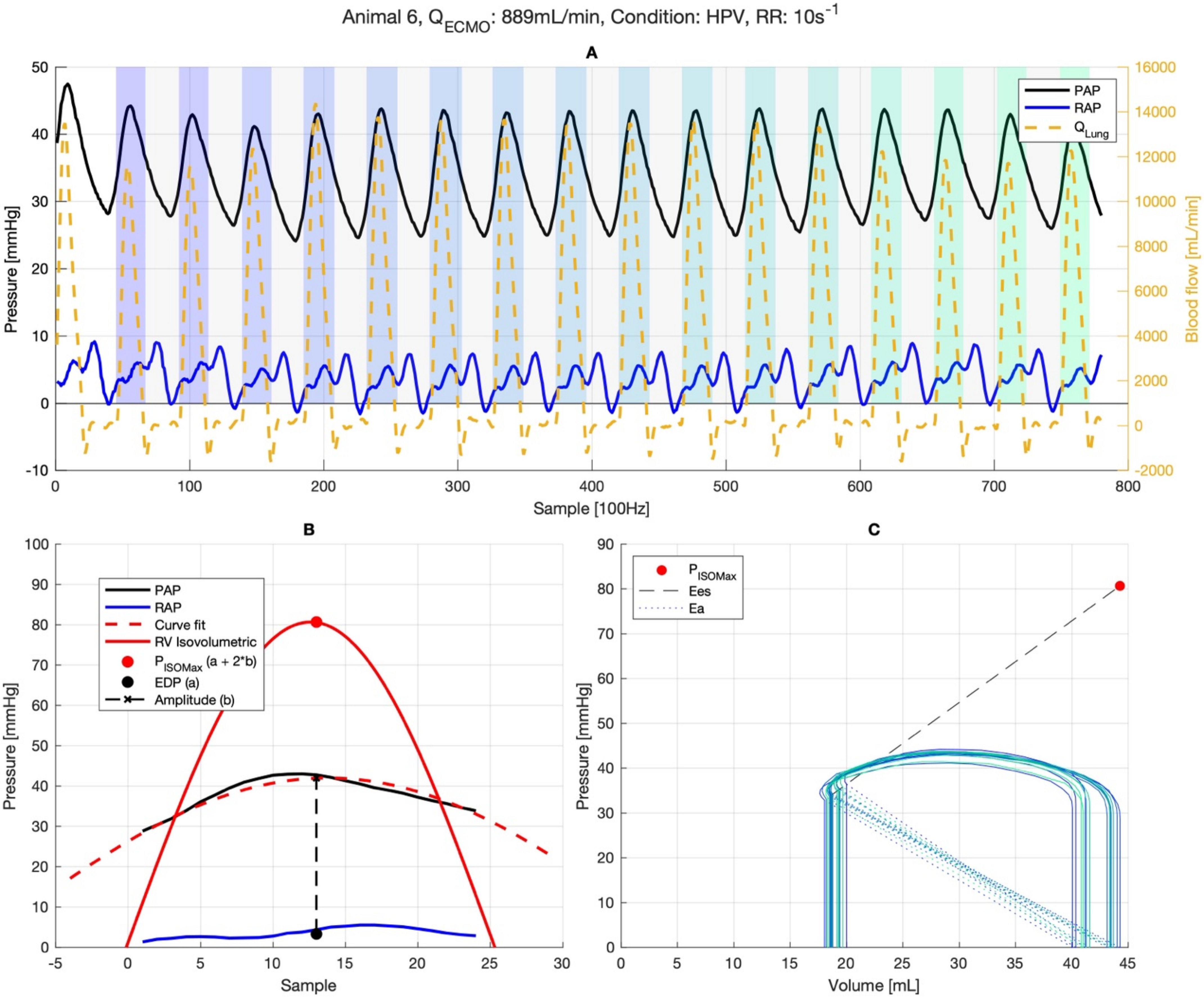

Animal 6, Q_{ECMO}: 889mL/min, Condition: HPV, RR: $10s^{-1}$
A
PAP
RAP
Q_{Lung}
Pressure [mmHg]
Blood flow [mL/min]
Sample [100Hz]
B
PAP
RAP
Curve fit
RV Isovolumetric
P_{ISOMax} (a + 2*b)
EDP (a)
Amplitude (b)
Pressure [mmHg]
Sample
C
P_{ISOMax}
Ees
Ea
Pressure [mmHg]
Volume [mL]

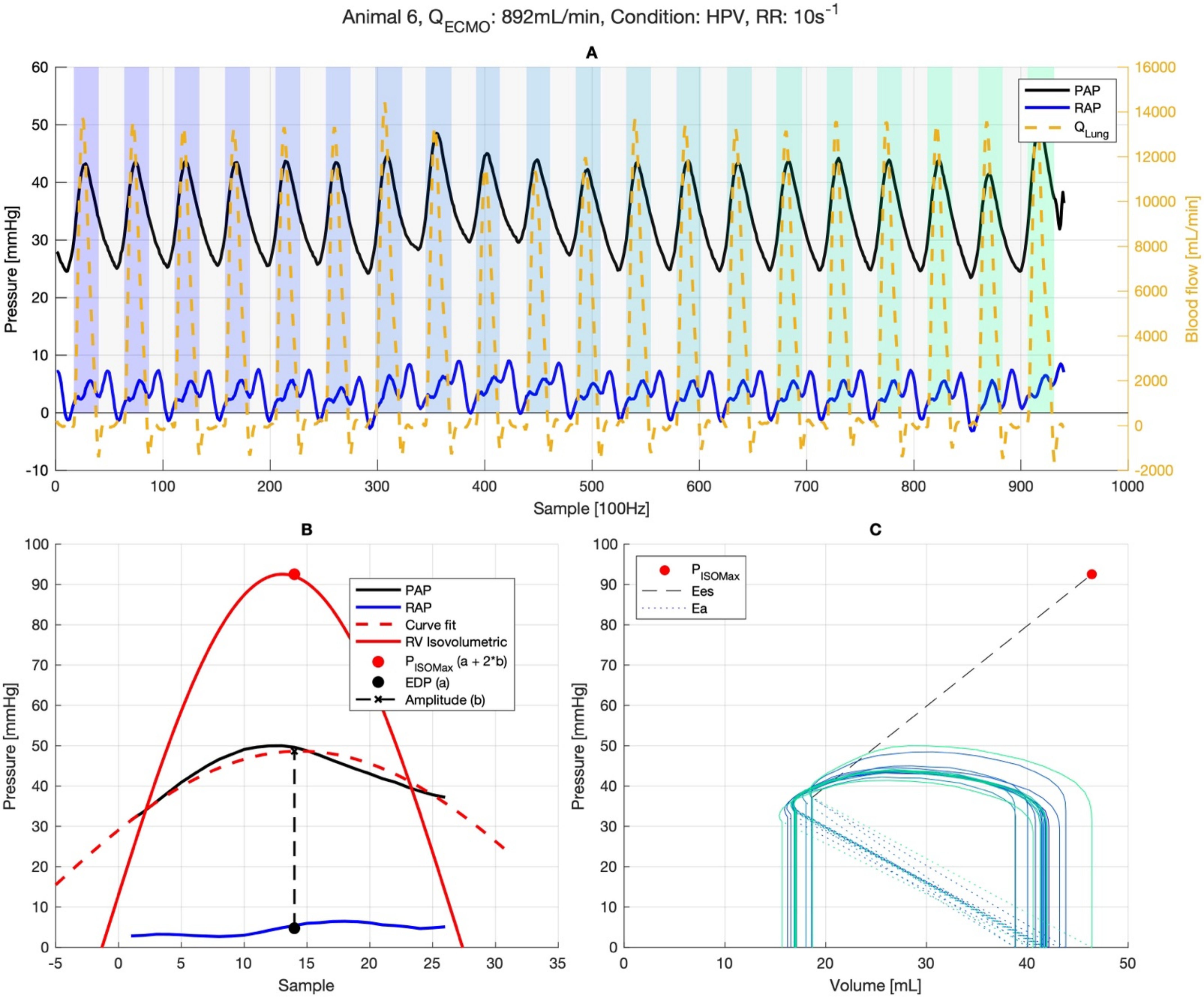

Animal 6, Q_ECMO: 892mL/min, Condition: HPV, RR: 10s^-1
A
PAP
RAP
Q_Lung
Pressure [mmHg]
Blood flow [mL/min]
Sample [100Hz]
B
PAP
RAP
Curve fit
RV Isovolumetric
P_ISOMax (a + 2*b)
EDP (a)
Amplitude (b)
Pressure [mmHg]
Sample
C
P_ISOMax
Ees
Ea
Pressure [mmHg]
Volume [mL]

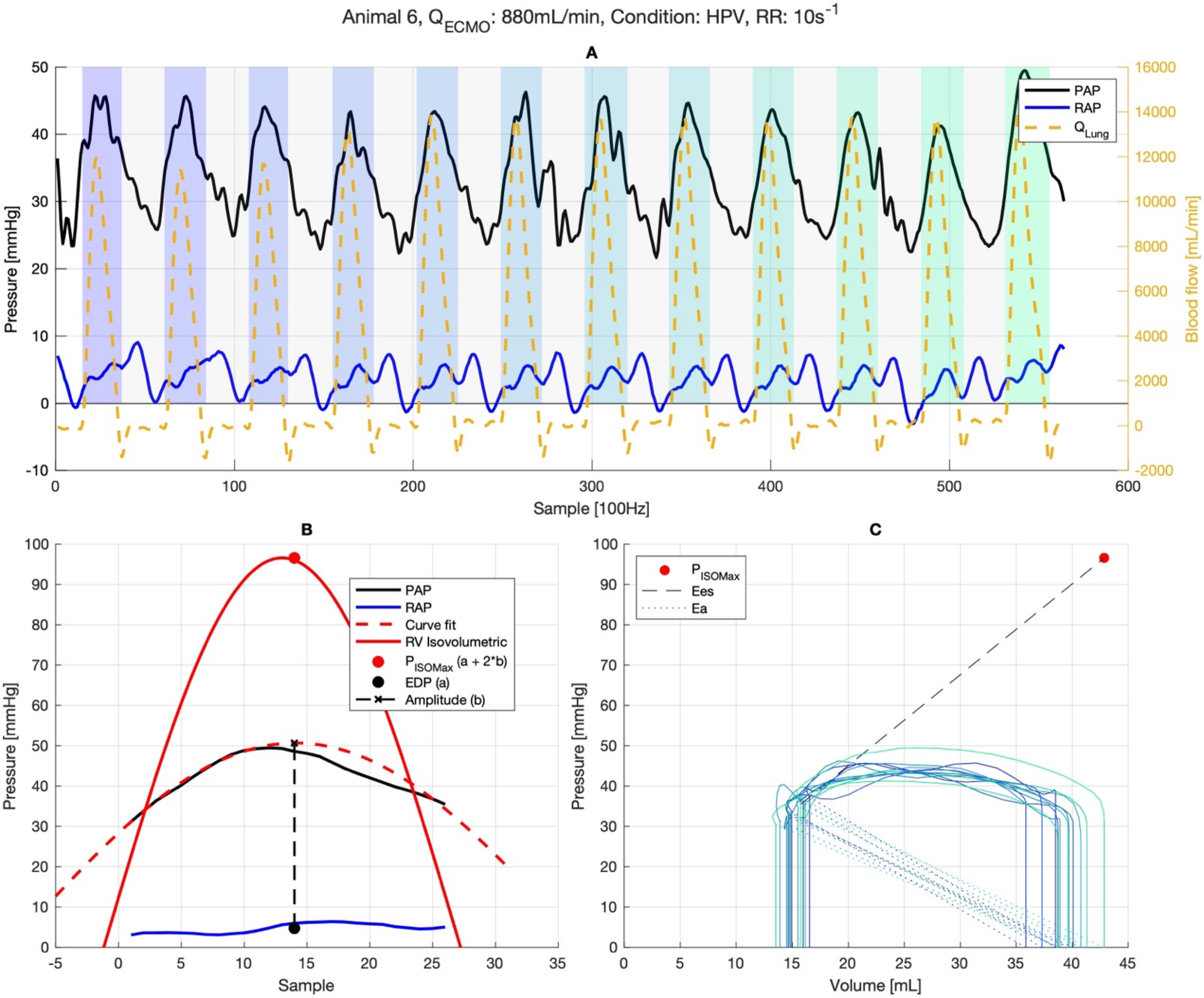

Animal 6, Q_{ECMO}: 880mL/min, Condition: HPV, RR: $10s^{-1}$
A
Pressure [mmHg]
Blood flow [mL/min]
Sample [100Hz]
PAP
RAP
Q_{Lung}
B
Pressure [mmHg]
Sample
PAP
RAP
Curve fit
RV Isovolumetric
P_{ISOMax} (a + 2*b)
EDP (a)
Amplitude (b)
C
Pressure [mmHg]
Volume [mL]
P_{ISOMax}
Ees
Ea

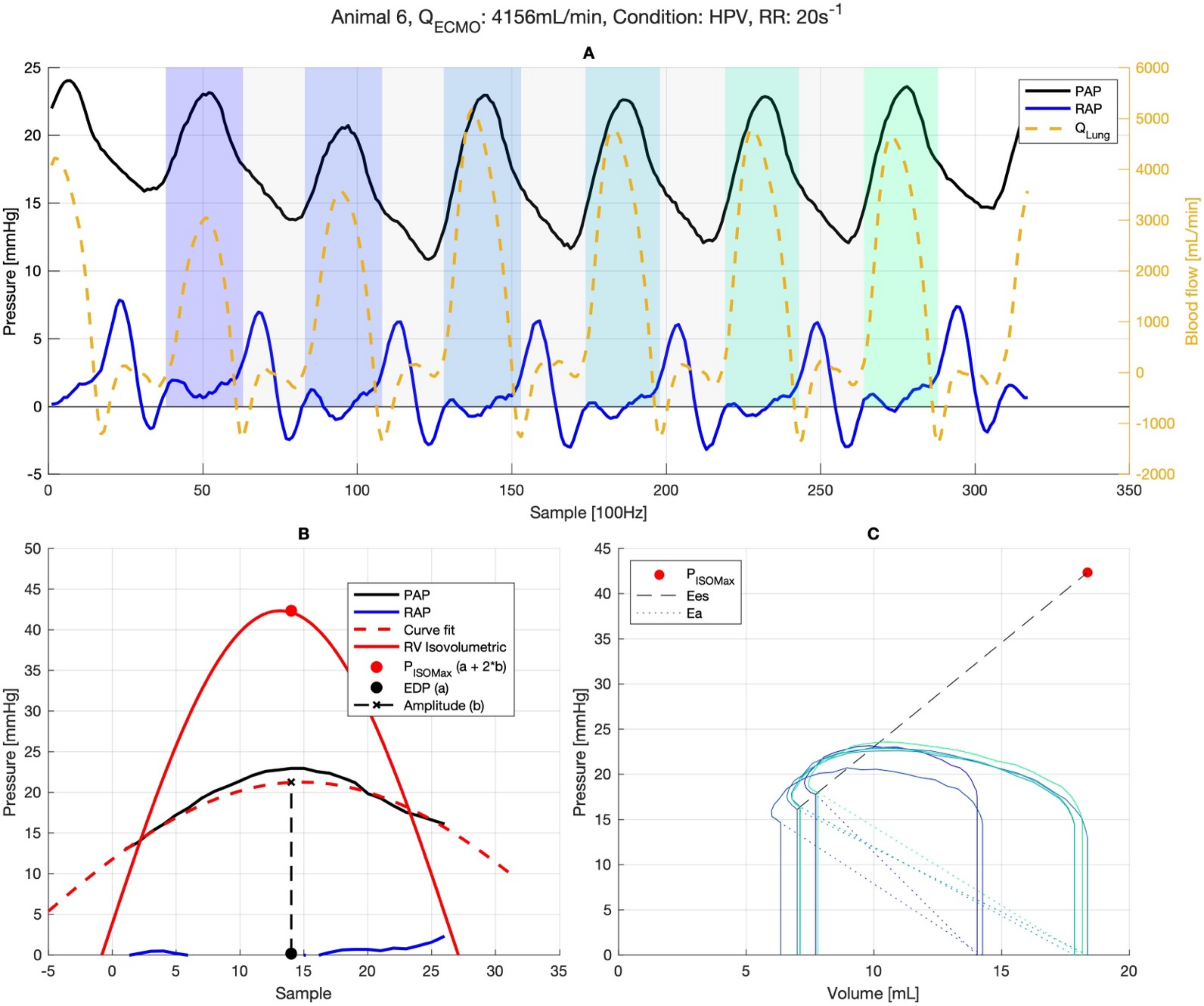

Animal 6, Q_ECMO: 4156mL/min, Condition: HPV, RR: 20s^-1
A
PAP
RAP
Q_Lung
Pressure [mmHg]
Blood flow [mL/min]
Sample [100Hz]
B
PAP
RAP
Curve fit
RV Isovolumetric
P_ISOMax (a + 2*b)
EDP (a)
Amplitude (b)
Pressure [mmHg]
Sample
C
P_ISOMax
Ees
Ea
Pressure [mmHg]
Volume [mL]

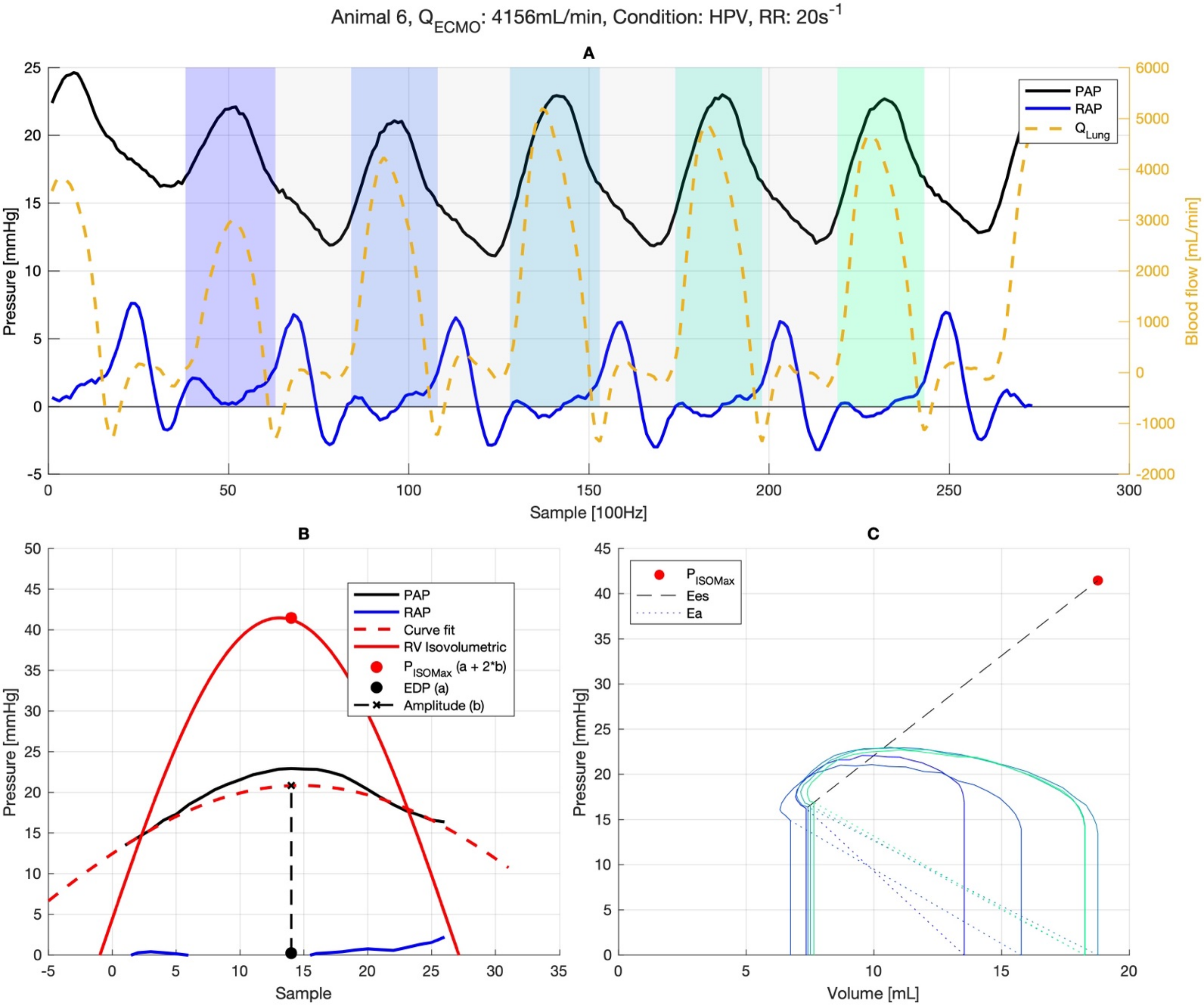
Animal 6, QECMO: 4156mL/min, Condition: HPV, RR: 20s-1
A
PAP
RAP
QLung
Pressure [mmHg]
Blood flow [mL/min]
Sample [100Hz]
B
PAP
RAP
Curve fit
RV Isovolumetric
PISOMax (a + 2*b)
EDP (a)
Amplitude (b)
Pressure [mmHg]
Sample
C
PISOMax
Ees
Ea
Pressure [mmHg]
Volume [mL]

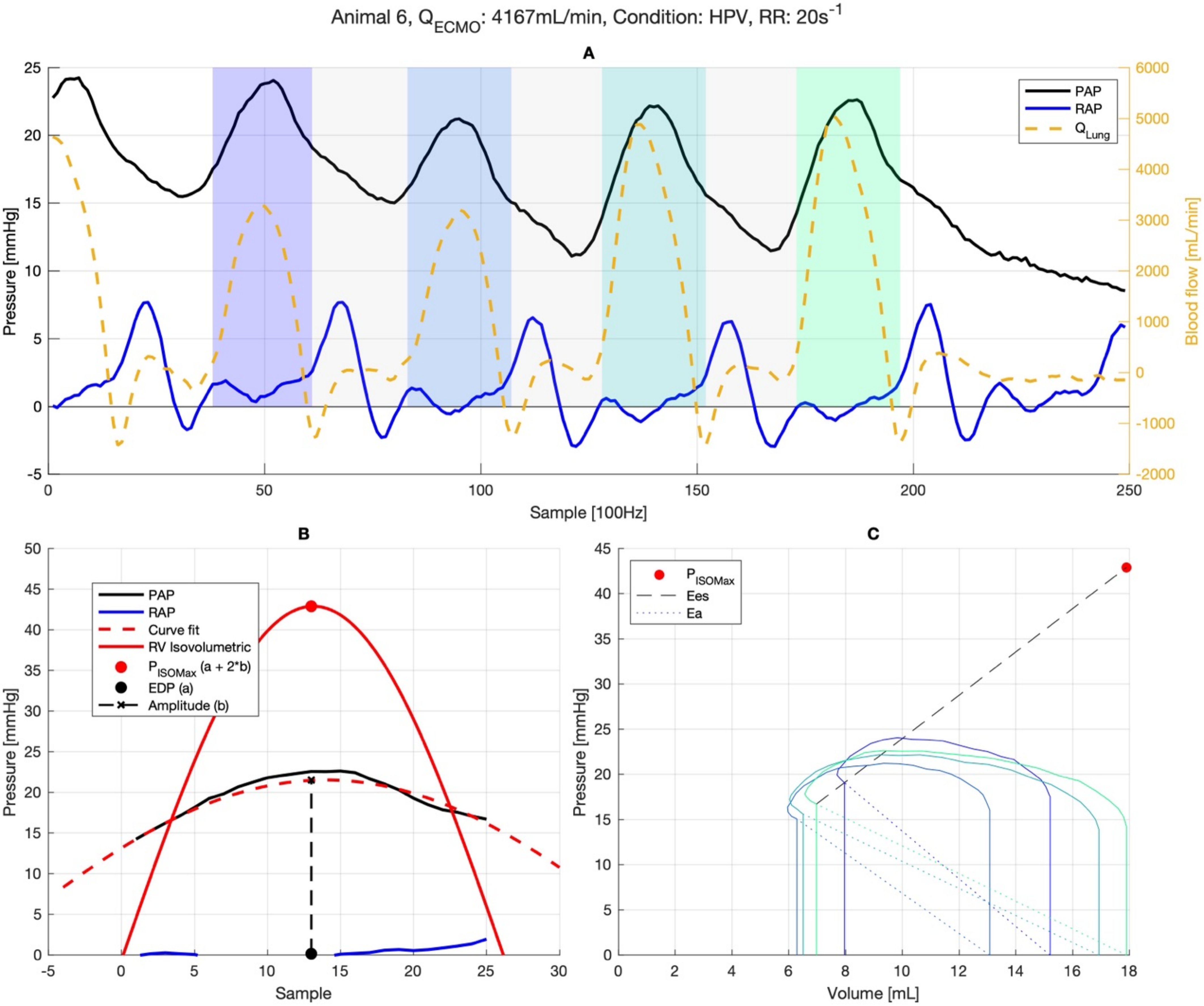

Animal 6, QECMO: 4167mL/min, Condition: HPV, RR: 20s-1
A
PAP
RAP
QLung
Pressure [mmHg]
Blood flow [mL/min]
Sample [100Hz]
B
PAP
RAP
Curve fit
RV Isovolumetric
PISOMax (a + 2*b)
EDP (a)
Amplitude (b)
Pressure [mmHg]
Sample
C
PISOMax
Ees
Ea
Pressure [mmHg]
Volume [mL]

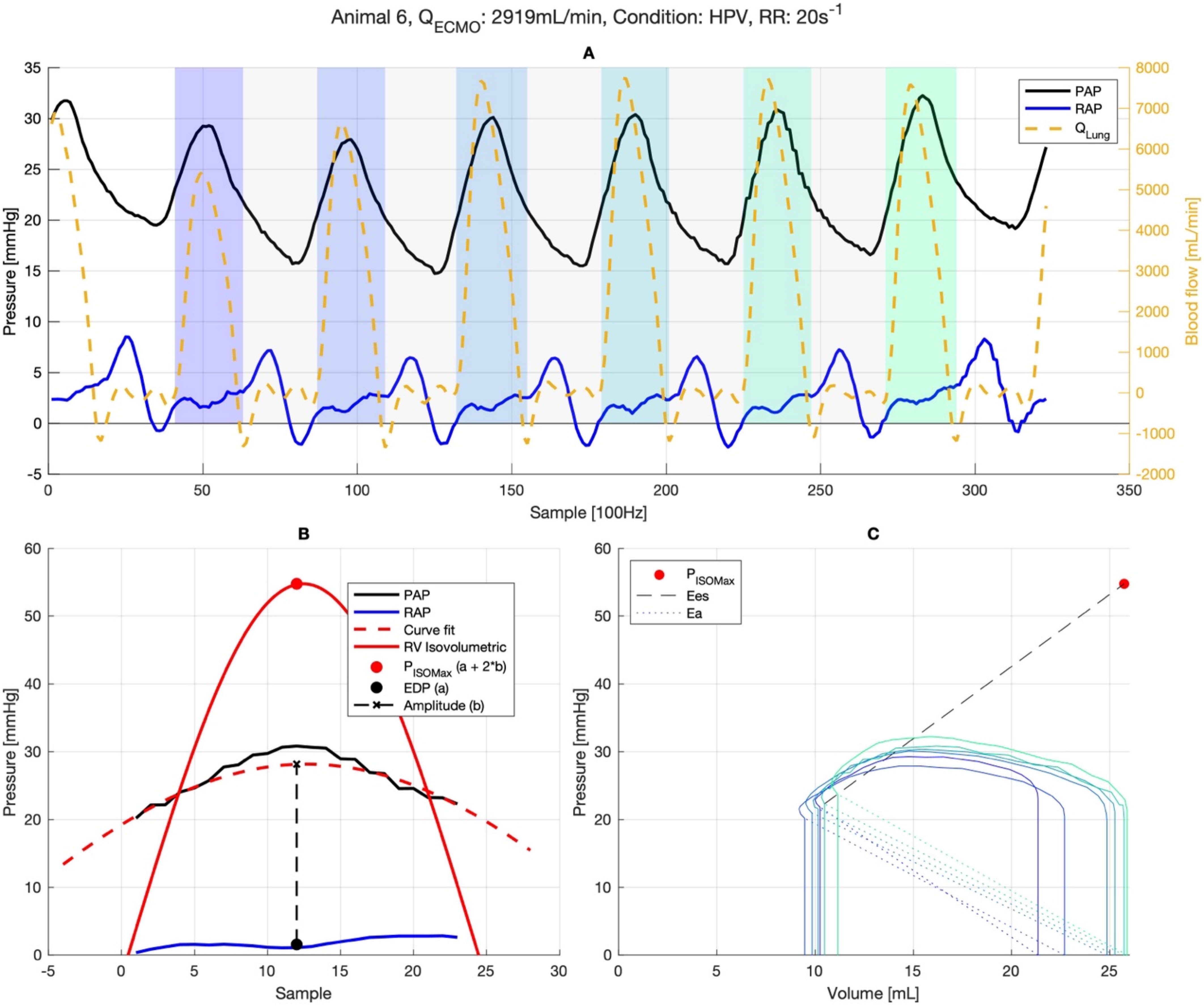

Animal 6, Q_{ECMO}: 2919mL/min, Condition: HPV, RR: $20s^{-1}$
A
PAP
RAP
Q_{Lung}
Pressure [mmHg]
Blood flow [mL/min]
Sample [100Hz]
B
PAP
RAP
Curve fit
RV Isovolumetric
P_{ISOMax} (a + 2*b)
EDP (a)
Amplitude (b)
Pressure [mmHg]
Sample
C
P_{ISOMax}
Ees
Ea
Pressure [mmHg]
Volume [mL]

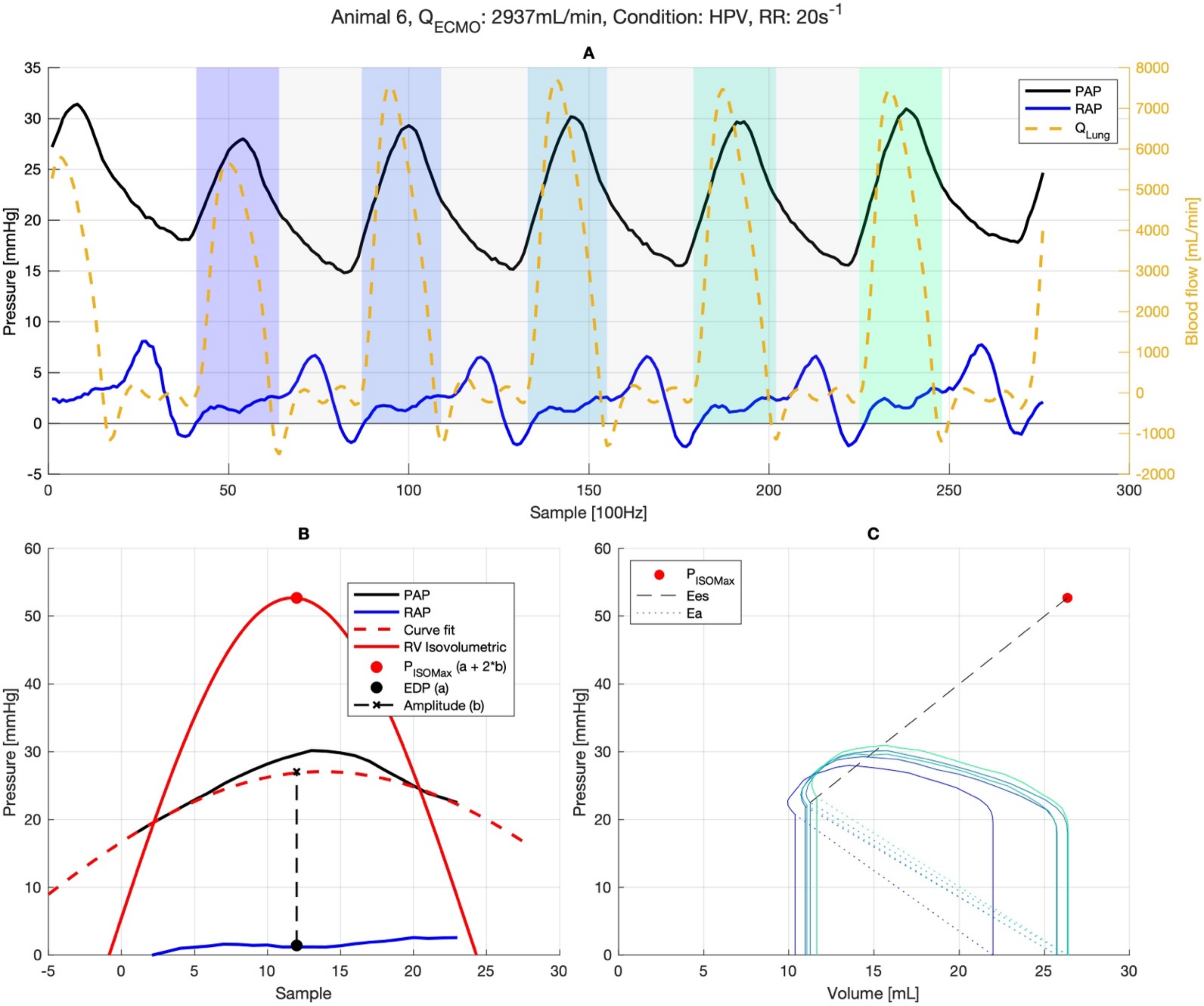

Animal 6, Q_ECMO: 2937mL/min, Condition: HPV, RR: 20s^-1
A
Pressure [mmHg]
Blood flow [mL/min]
Sample [100Hz]
PAP
RAP
Q_Lung
B
PAP
RAP
Curve fit
RV Isovolumetric
P_ISOMax (a + 2*b)
EDP (a)
Amplitude (b)
Pressure [mmHg]
Sample
C
P_ISOMax
Ees
Ea
Pressure [mmHg]
Volume [mL]

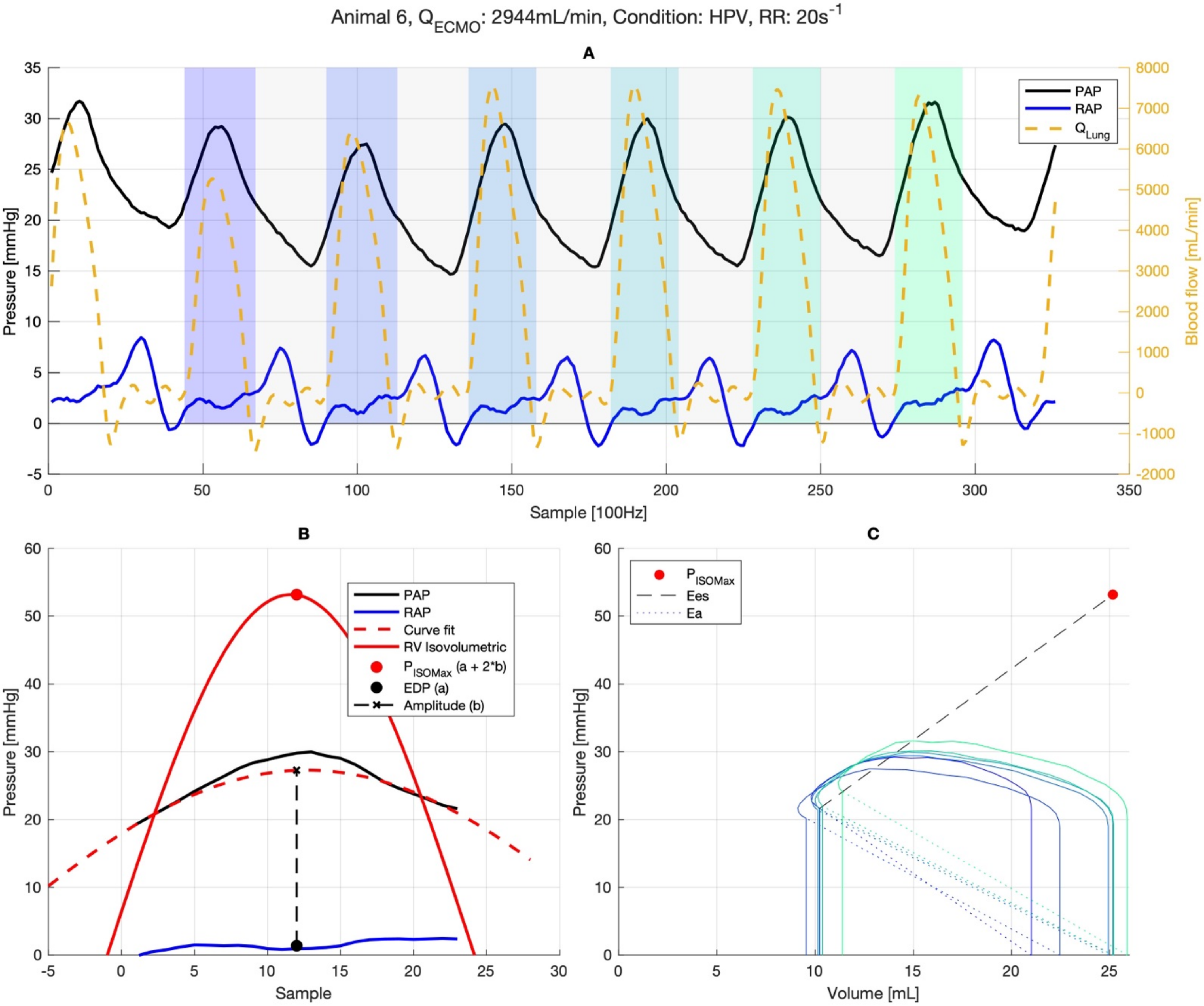

Animal 6, Q_{ECMO}: 2944mL/min, Condition: HPV, RR: $20s^{-1}$
A
Pressure [mmHg]
Blood flow [mL/min]
Sample [100Hz]
PAP
RAP
Q_{Lung}
B
Pressure [mmHg]
Sample
PAP
RAP
Curve fit
RV Isovolumetric
P_{ISOMax} (a + 2*b)
EDP (a)
Amplitude (b)
C
Pressure [mmHg]
Volume [mL]
P_{ISOMax}
Ees
Ea

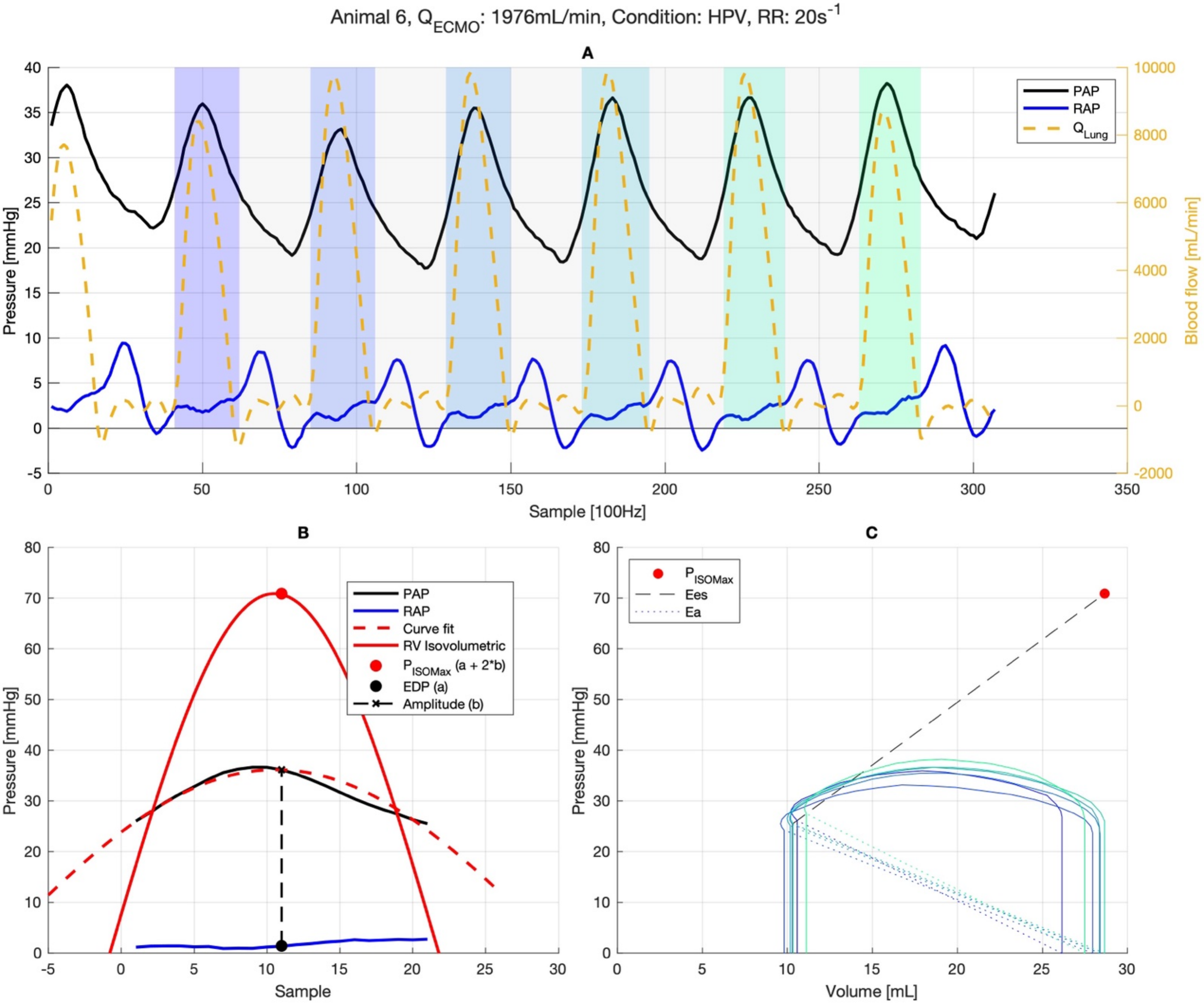

Animal 6, QECMO: 1976mL/min, Condition: HPV, RR: 20s-1
A
Pressure [mmHg]
Blood flow [mL/min]
Sample [100Hz]
PAP
RAP
QLung
B
PAP
RAP
Curve fit
RV Isovolumetric
PISOMax (a + 2*b)
EDP (a)
Amplitude (b)
Pressure [mmHg]
Sample
C
PISOMax
Ees
Ea
Pressure [mmHg]
Volume [mL]

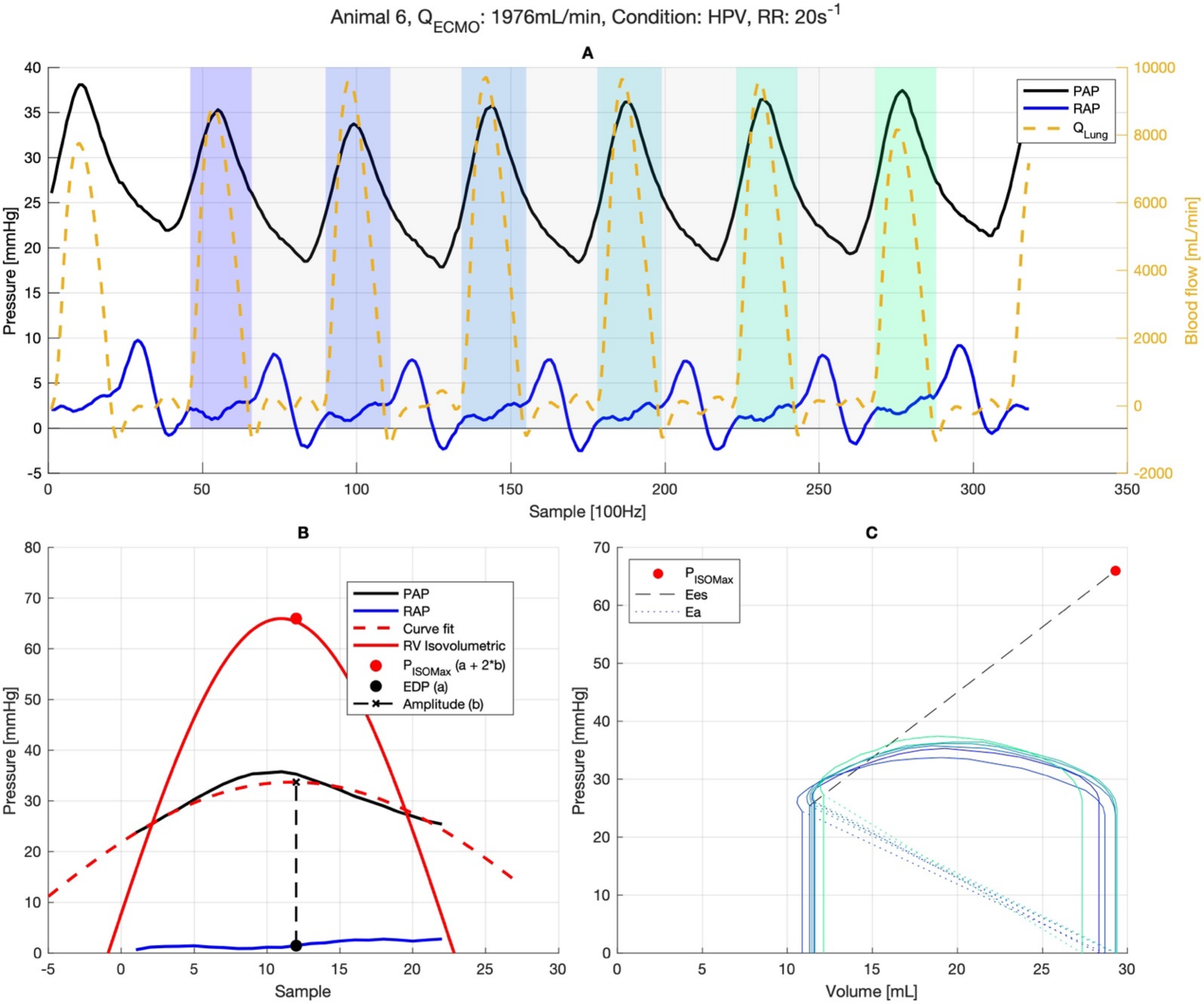

Animal 6, Q_ECMO: 1976mL/min, Condition: HPV, RR: 20s^-1
A
PAP
RAP
Q_Lung
Pressure [mmHg]
Blood flow [mL/min]
Sample [100Hz]
B
PAP
RAP
Curve fit
RV Isovolumetric
P_ISOMax (a + 2*b)
EDP (a)
Amplitude (b)
Pressure [mmHg]
Sample
C
P_ISOMax
Ees
Ea
Pressure [mmHg]
Volume [mL]

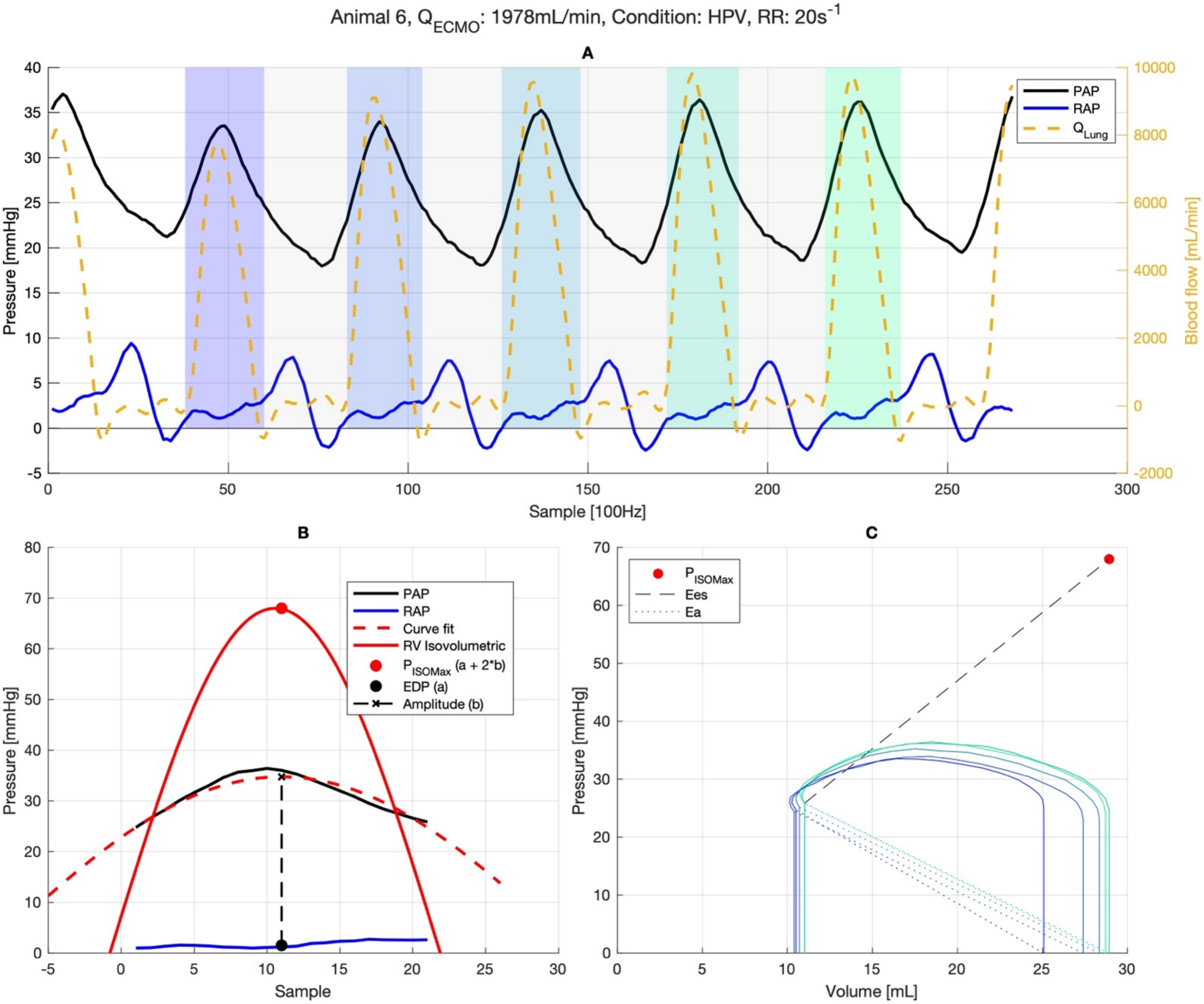

Animal 6, Q_ECMO: 1978mL/min, Condition: HPV, RR: 20s^-1
A
PAP
RAP
Q_Lung
Pressure [mmHg]
Blood flow [mL/min]
Sample [100Hz]
B
PAP
RAP
Curve fit
RV Isovolumetric
P_ISOMax (a + 2*b)
EDP (a)
Amplitude (b)
Pressure [mmHg]
Sample
C
P_ISOMax
Ees
Ea
Pressure [mmHg]
Volume [mL]

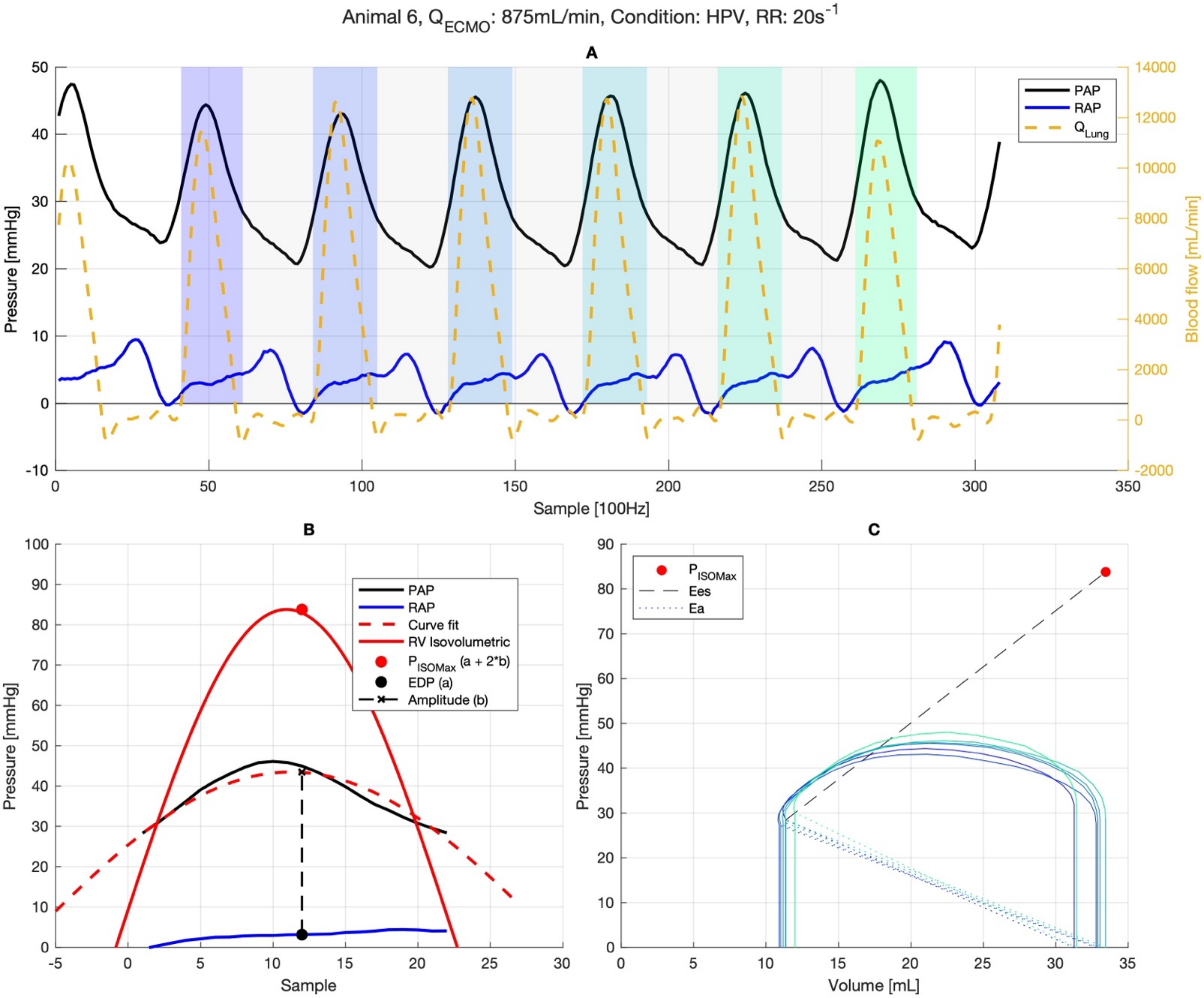

Animal 6, Q_{ECMO}: 875mL/min, Condition: HPV, RR: $20s^{-1}$
A
Pressure [mmHg]
Blood flow [mL/min]
Sample [100Hz]
PAP
RAP
Q_{Lung}
B
Pressure [mmHg]
Sample
PAP
RAP
Curve fit
RV Isovolumetric
P_{ISOMax} (a + 2*b)
EDP (a)
Amplitude (b)
C
Pressure [mmHg]
Volume [mL]
P_{ISOMax}
Ees
Ea

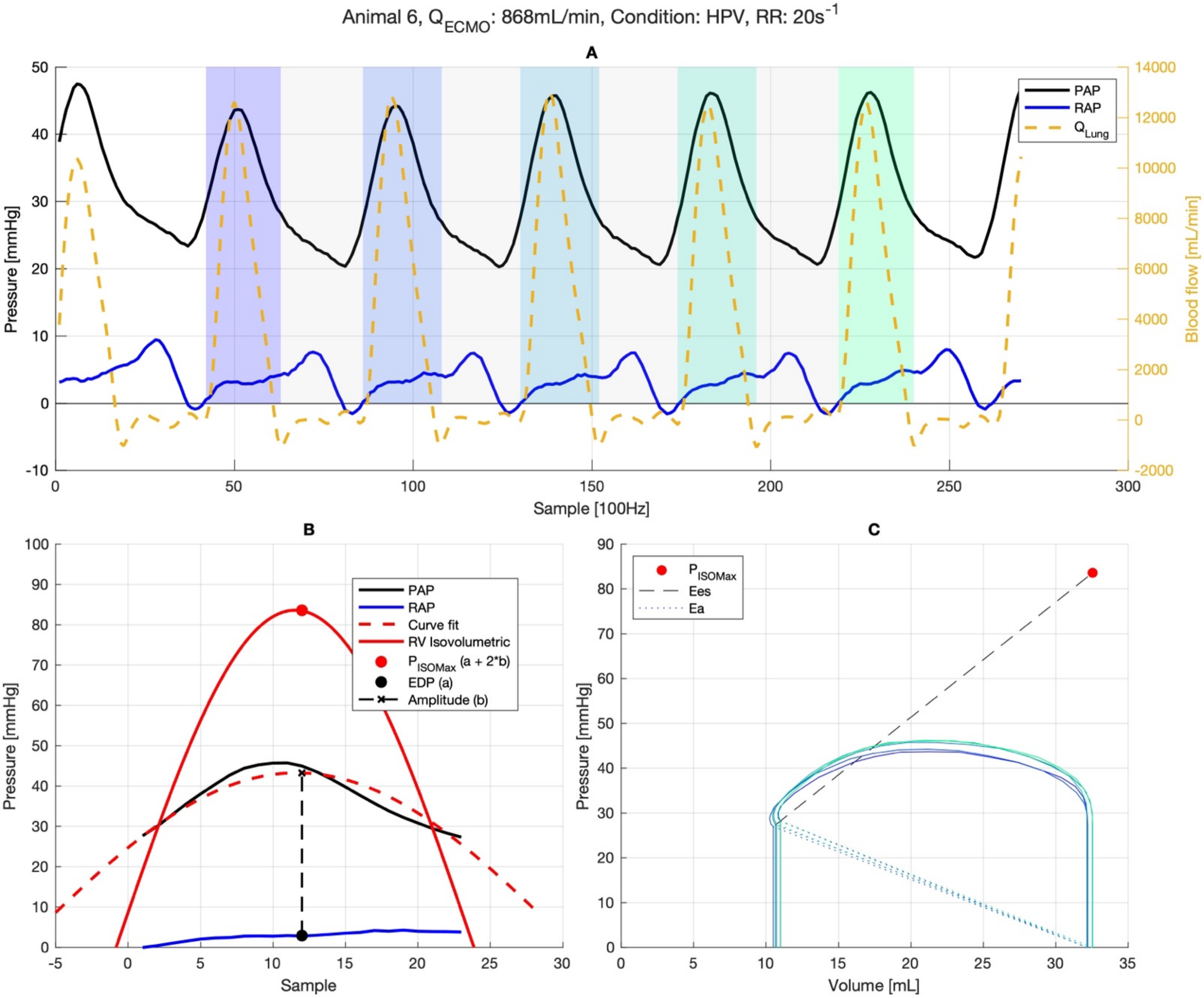

Animal 6, Q_{ECMO}: 868mL/min, Condition: HPV, RR: $20s^{-1}$
A
PAP
RAP
Q_{Lung}
Pressure [mmHg]
Blood flow [mL/min]
Sample [100Hz]
B
PAP
RAP
Curve fit
RV Isovolumetric
P_{ISOMax} (a + 2*b)
EDP (a)
Amplitude (b)
Pressure [mmHg]
Sample
C
P_{ISOMax}
Ees
Ea
Pressure [mmHg]
Volume [mL]

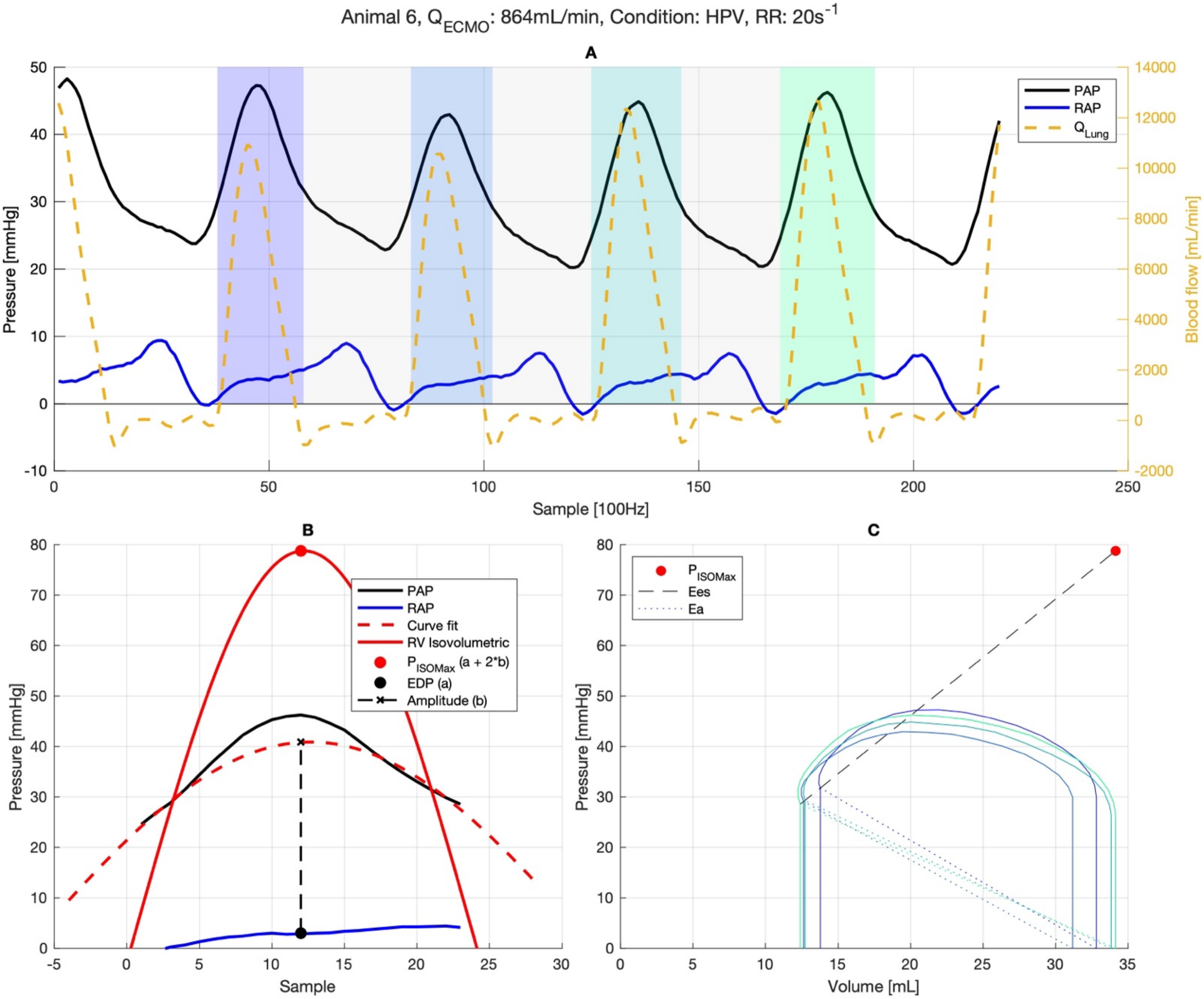
Animal 6, Q_{ECMO}: 864mL/min, Condition: HPV, RR: $20s^{-1}$
A
PAP
RAP
Q_{Lung}
Pressure [mmHg]
Blood flow [mL/min]
Sample [100Hz]
B
PAP
RAP
Curve fit
RV Isovolumetric
P_{ISOMax} (a + 2*b)
EDP (a)
Amplitude (b)
Pressure [mmHg]
Sample
C
P_{ISOMax}
Ees
Ea
Pressure [mmHg]
Volume [mL]

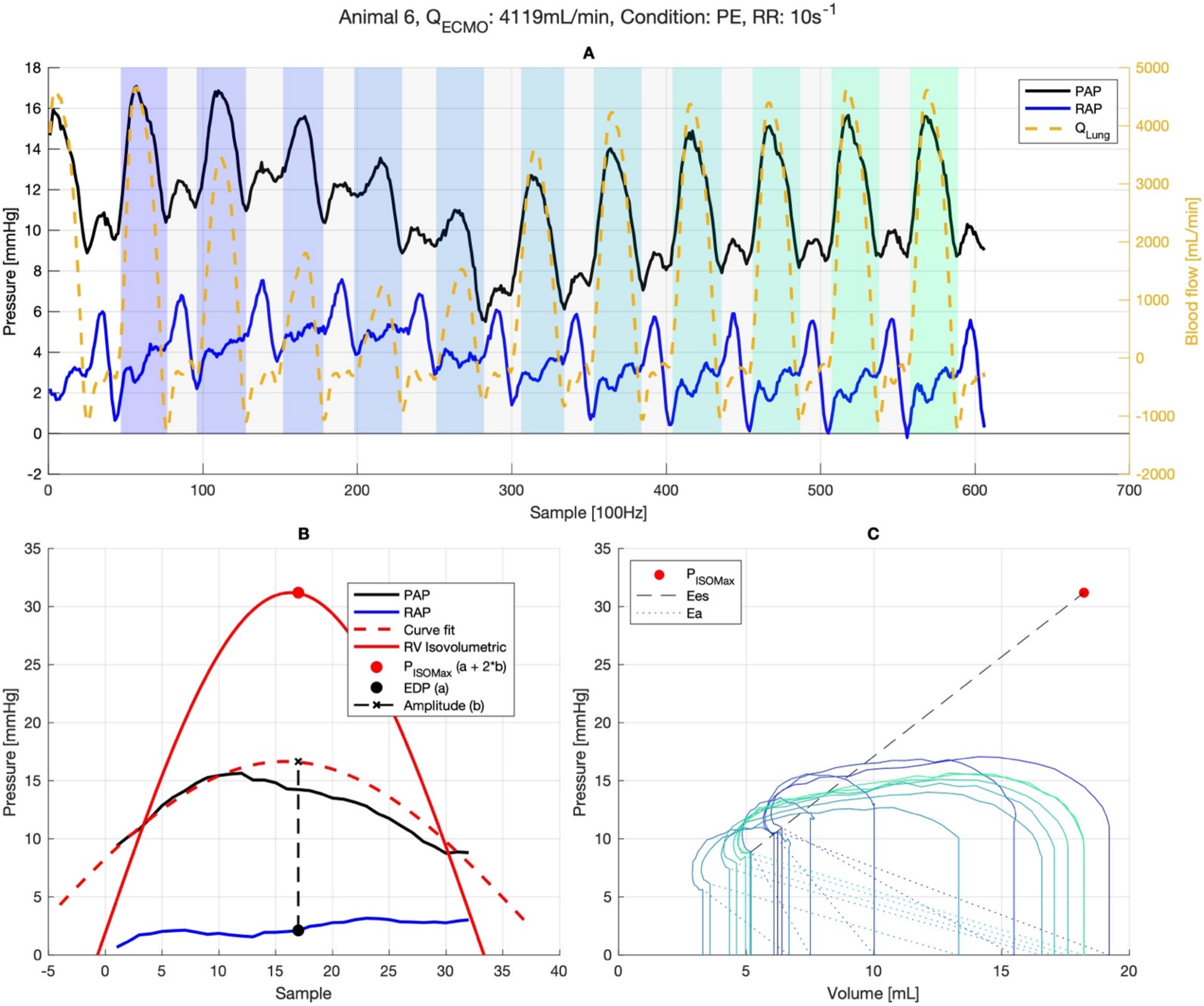

Animal 6, Q_{ECMO}: 4119mL/min, Condition: PE, RR: $10s^{-1}$
A
PAP
RAP
Q_{Lung}
Pressure [mmHg]
Blood flow [mL/min]
Sample [100Hz]
B
PAP
RAP
Curve fit
RV Isovolumetric
P_{ISOMax} (a + 2*b)
EDP (a)
Amplitude (b)
Pressure [mmHg]
Sample
C
P_{ISOMax}
Ees
Ea
Pressure [mmHg]
Volume [mL]

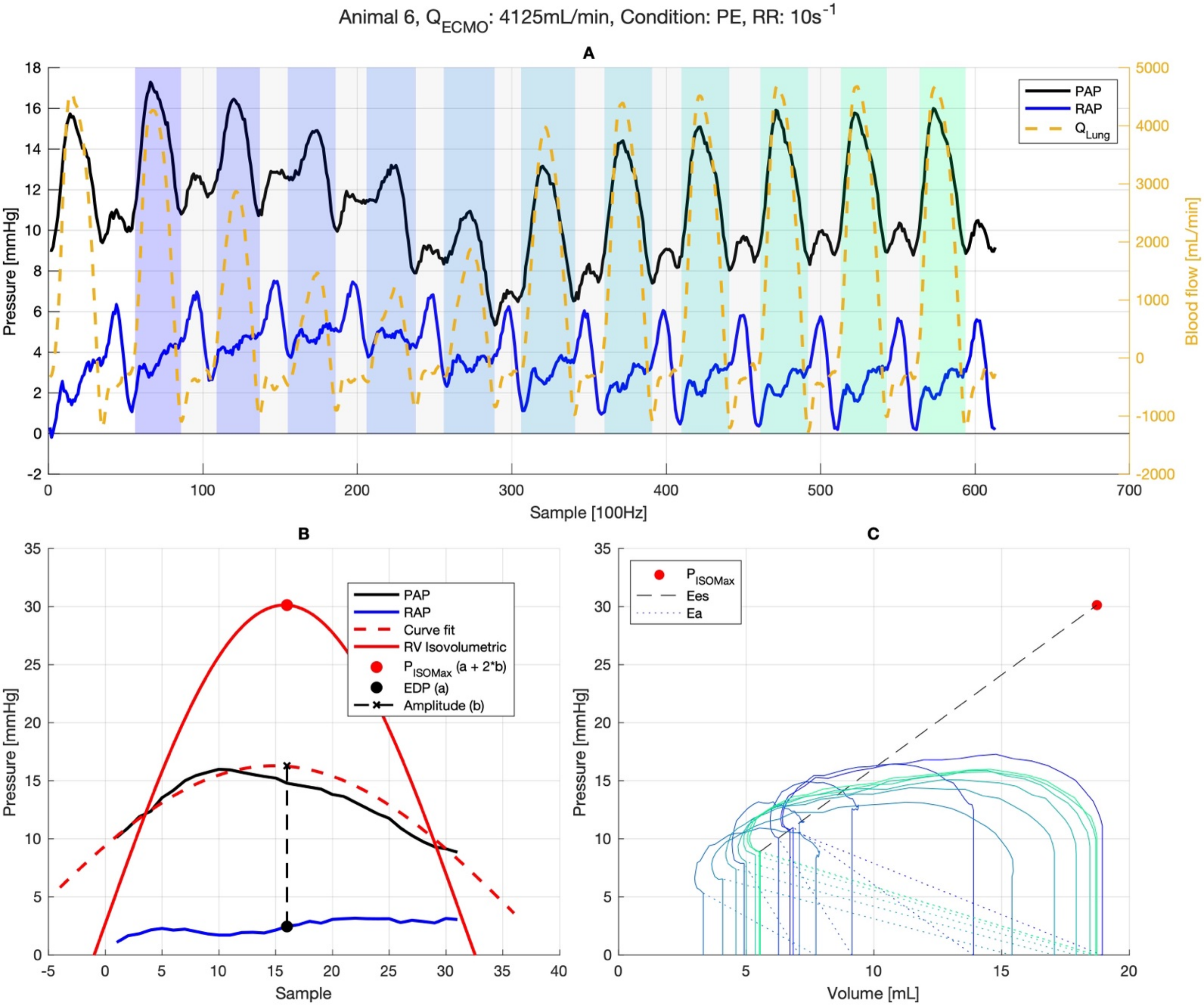

Animal 6, QECMO: 4125mL/min, Condition: PE, RR: 10s-1
A
Pressure [mmHg]
Blood flow [mL/min]
Sample [100Hz]
PAP
RAP
QLung
B
Pressure [mmHg]
Sample
PAP
RAP
Curve fit
RV Isovolumetric
PISOMax (a + 2*b)
EDP (a)
Amplitude (b)
C
Pressure [mmHg]
Volume [mL]
PISOMax
Ees
Ea

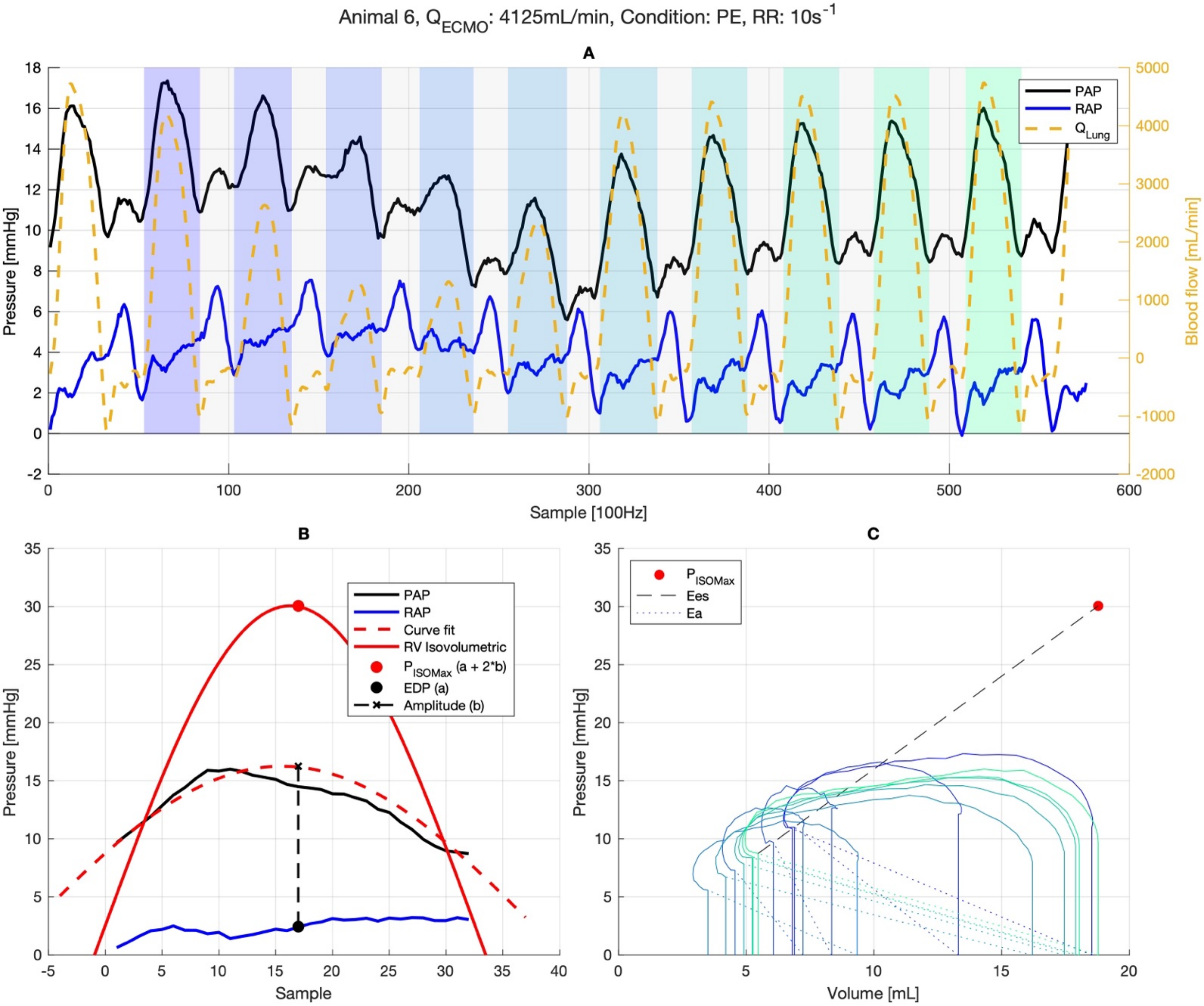

Animal 6, Q_{ECMO}: 4125mL/min, Condition: PE, RR: $10s^{-1}$
A
PAP
RAP
Q_{Lung}
Pressure [mmHg]
Blood flow [mL/min]
Sample [100Hz]
B
PAP
RAP
Curve fit
RV Isovolumetric
P_{ISOMax} (a + 2*b)
EDP (a)
Amplitude (b)
Pressure [mmHg]
Sample
C
P_{ISOMax}
Ees
Ea
Pressure [mmHg]
Volume [mL]

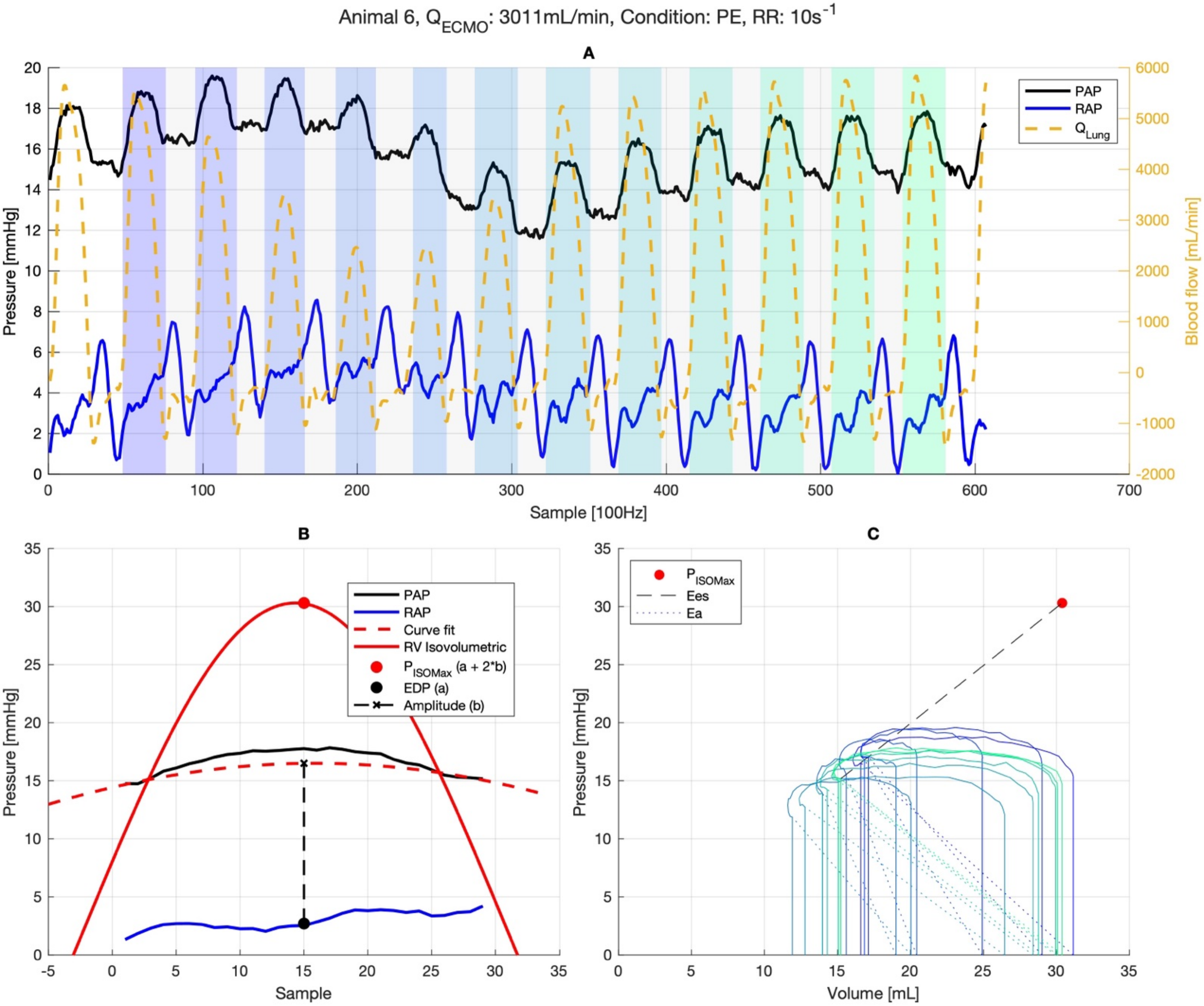

Animal 6, Q_{ECMO}: 3011mL/min, Condition: PE, RR: $10s^{-1}$
A
PAP
RAP
Q_{Lung}
Pressure [mmHg]
Blood flow [mL/min]
Sample [100Hz]
B
PAP
RAP
Curve fit
RV Isovolumetric
P_{ISOMax} (a + 2*b)
EDP (a)
Amplitude (b)
Pressure [mmHg]
Sample
C
P_{ISOMax}
Ees
Ea
Pressure [mmHg]
Volume [mL]

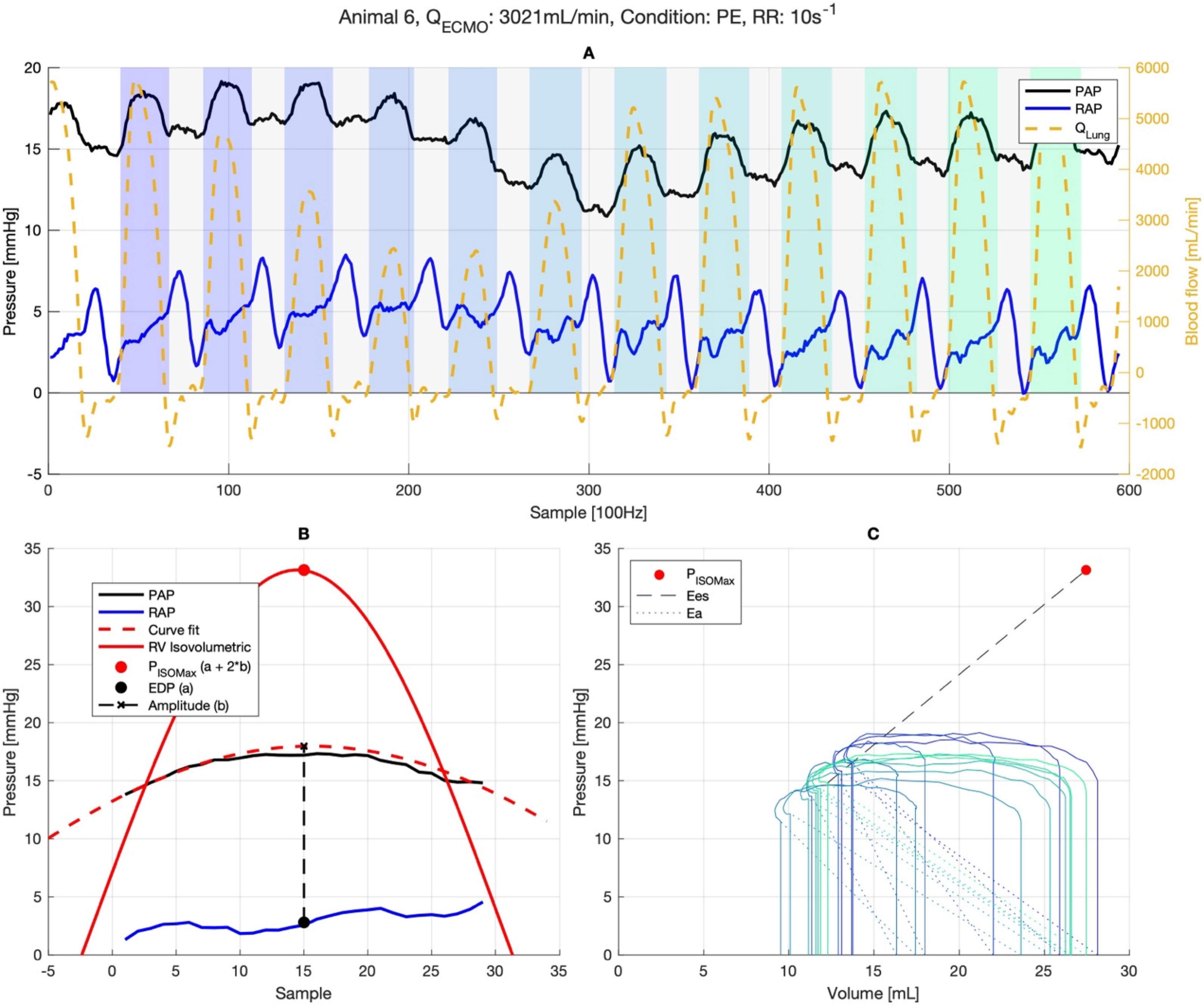

Animal 6, Q_ECMO: 3021mL/min, Condition: PE, RR: 10s^-1
A
PAP
RAP
Q_Lung
Pressure [mmHg]
Blood flow [mL/min]
Sample [100Hz]
B
PAP
RAP
Curve fit
RV Isovolumetric
P_ISOMax (a + 2*b)
EDP (a)
Amplitude (b)
Pressure [mmHg]
Sample
C
P_ISOMax
Ees
Ea
Pressure [mmHg]
Volume [mL]

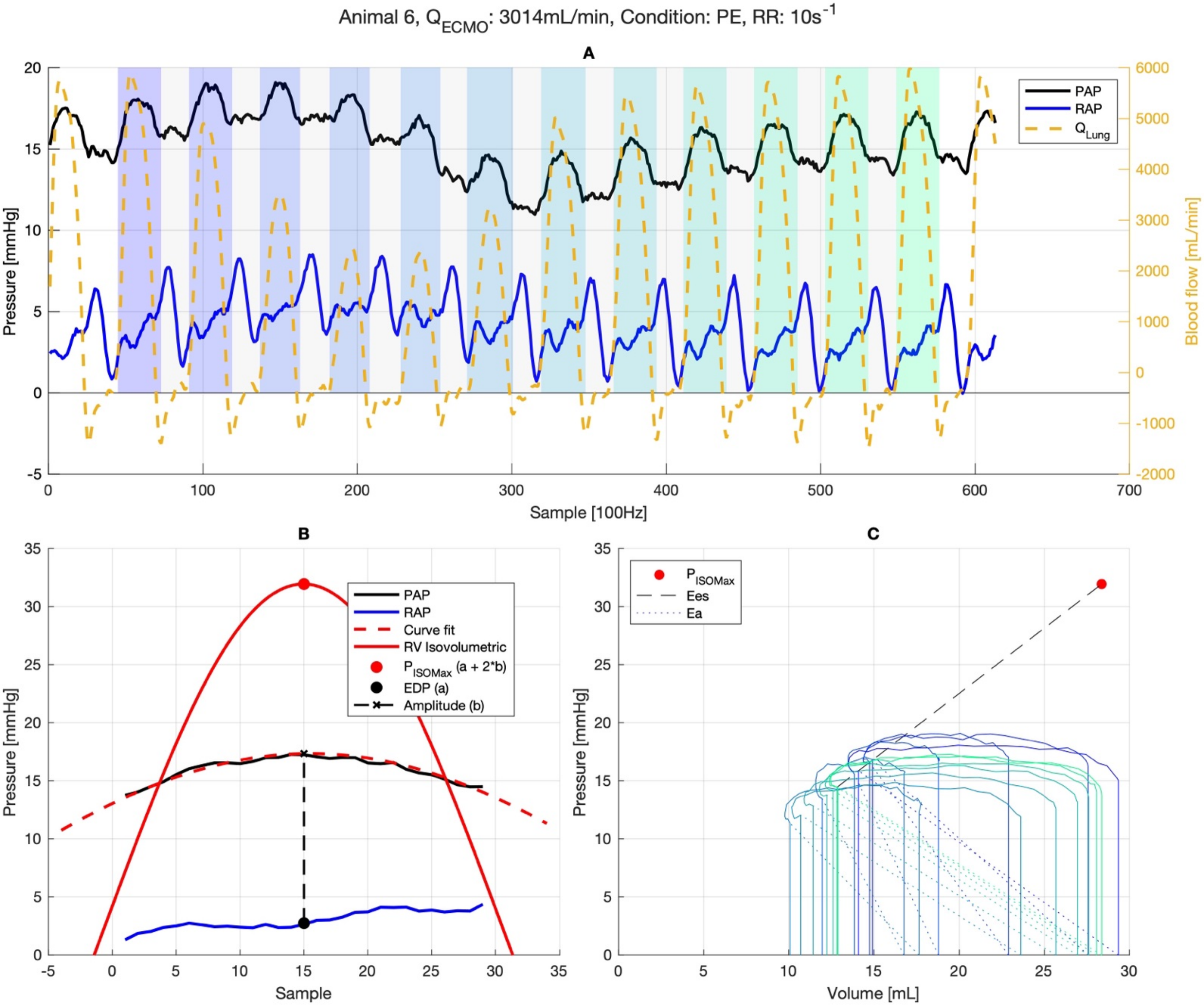

Animal 6, Q_ECMO: 3014mL/min, Condition: PE, RR: 10s^-1
A
PAP
RAP
Q_Lung
Pressure [mmHg]
Blood flow [mL/min]
Sample [100Hz]
B
PAP
RAP
Curve fit
RV Isovolumetric
P_ISOMax (a + 2*b)
EDP (a)
Amplitude (b)
Pressure [mmHg]
Sample
C
P_ISOMax
Ees
Ea
Pressure [mmHg]
Volume [mL]

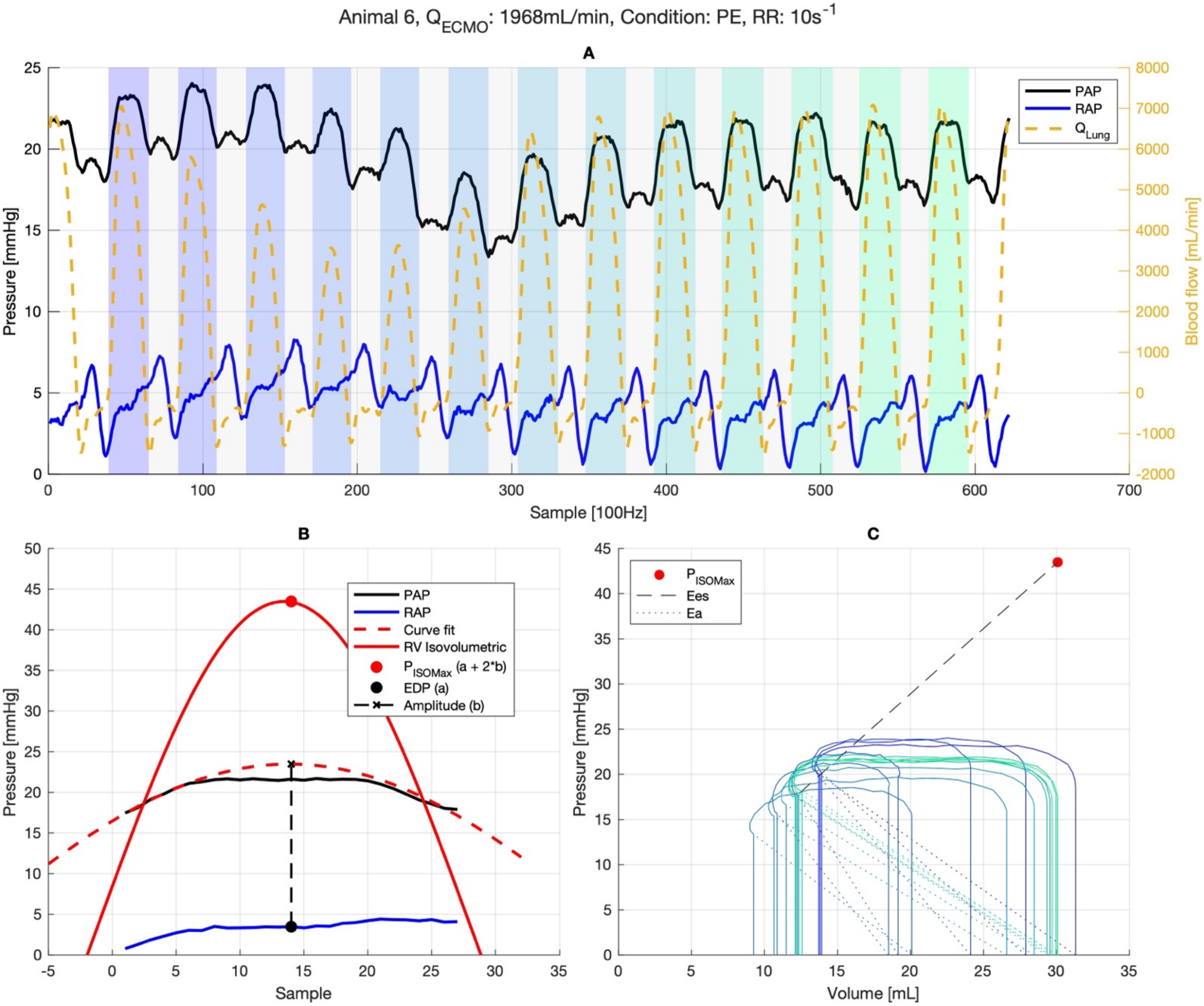

Animal 6, Q ECMO: 1968mL/min, Condition: PE, RR: 10s-1
A
Pressure [mmHg]
Blood flow [mL/min]
Sample [100Hz]
PAP
RAP
Q Lung
B
Pressure [mmHg]
Sample
PAP
RAP
Curve fit
RV Isovolumetric
P ISOMax (a + 2*b)
EDP (a)
Amplitude (b)
C
Pressure [mmHg]
Volume [mL]
P ISOMax
Ees
Ea

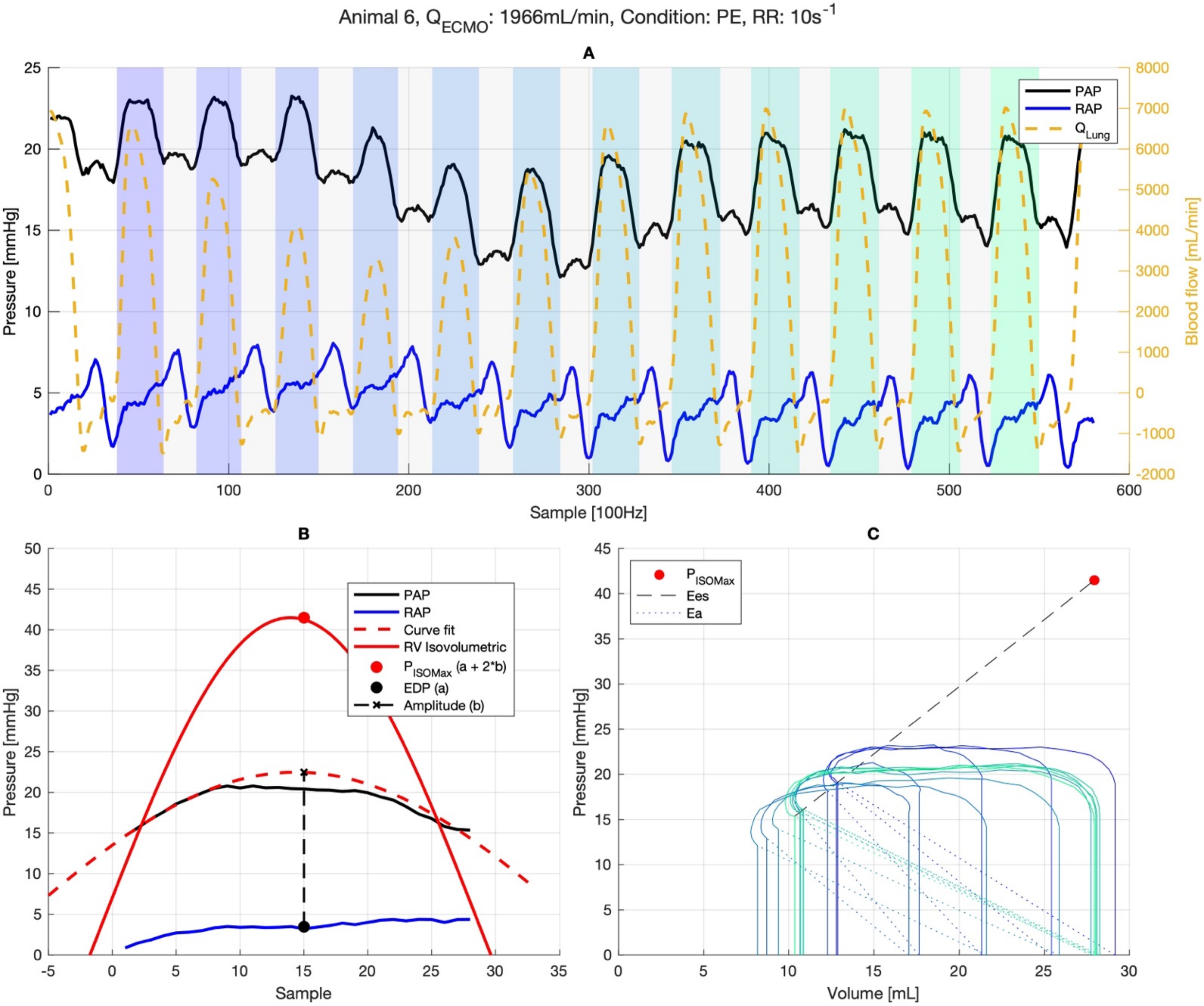
Animal 6, Q_{ECMO}: 1966mL/min, Condition: PE, RR: $10s^{-1}$
A
PAP
RAP
Q_{Lung}
Pressure [mmHg]
Blood flow [mL/min]
Sample [100Hz]
B
PAP
RAP
Curve fit
RV Isovolumetric
P_{ISOMax} (a + 2*b)
EDP (a)
Amplitude (b)
Pressure [mmHg]
Sample
C
P_{ISOMax}
Ees
Ea
Pressure [mmHg]
Volume [mL]

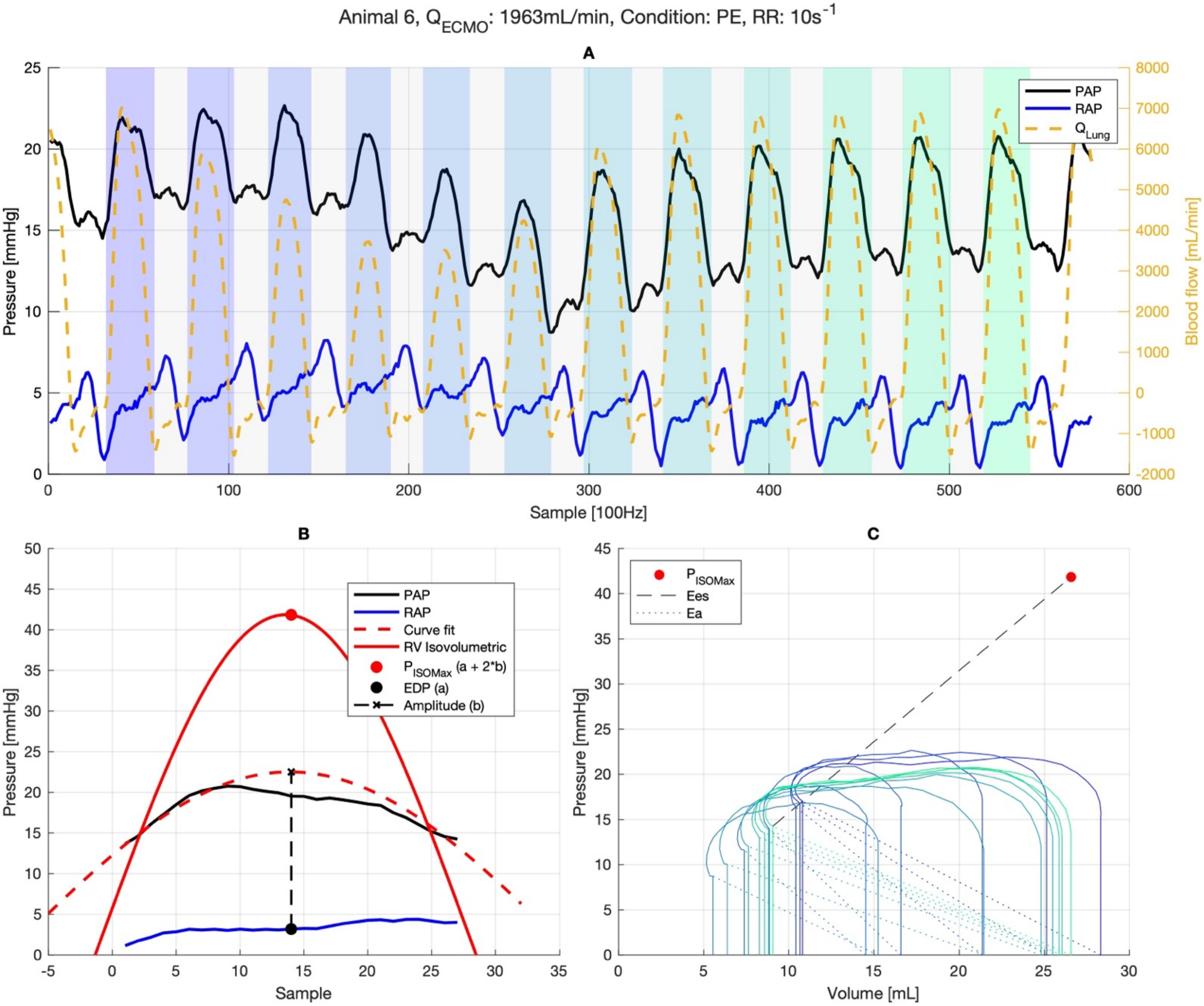
Animal 6, Q_{ECMO}: 1963mL/min, Condition: PE, RR: $10s^{-1}$
A
Pressure [mmHg]
Blood flow [mL/min]
Sample [100Hz]
PAP
RAP
Q_{Lung}
B
Pressure [mmHg]
Sample
PAP
RAP
Curve fit
RV Isovolumetric
P_{ISOMax} (a + 2*b)
EDP (a)
Amplitude (b)
C
Pressure [mmHg]
Volume [mL]
P_{ISOMax}
Ees
Ea

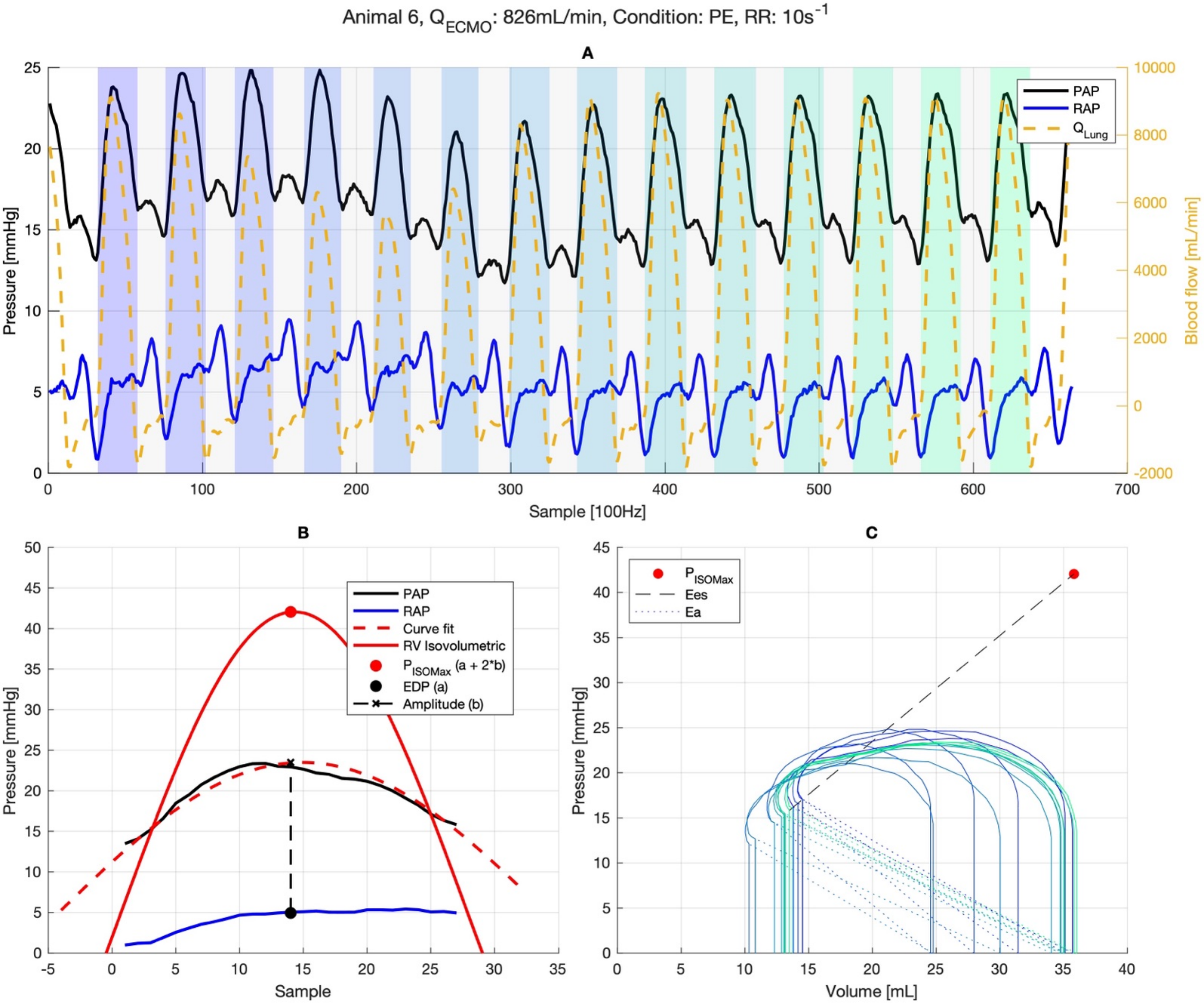

Animal 6, Q_ECMO: 826mL/min, Condition: PE, RR: 10s^-1
A
Pressure [mmHg]
Blood flow [mL/min]
Sample [100Hz]
PAP
RAP
Q_Lung
B
Pressure [mmHg]
Sample
PAP
RAP
Curve fit
RV Isovolumetric
P_ISOMax (a + 2*b)
EDP (a)
Amplitude (b)
C
Pressure [mmHg]
Volume [mL]
P_ISOMax
Ees
Ea

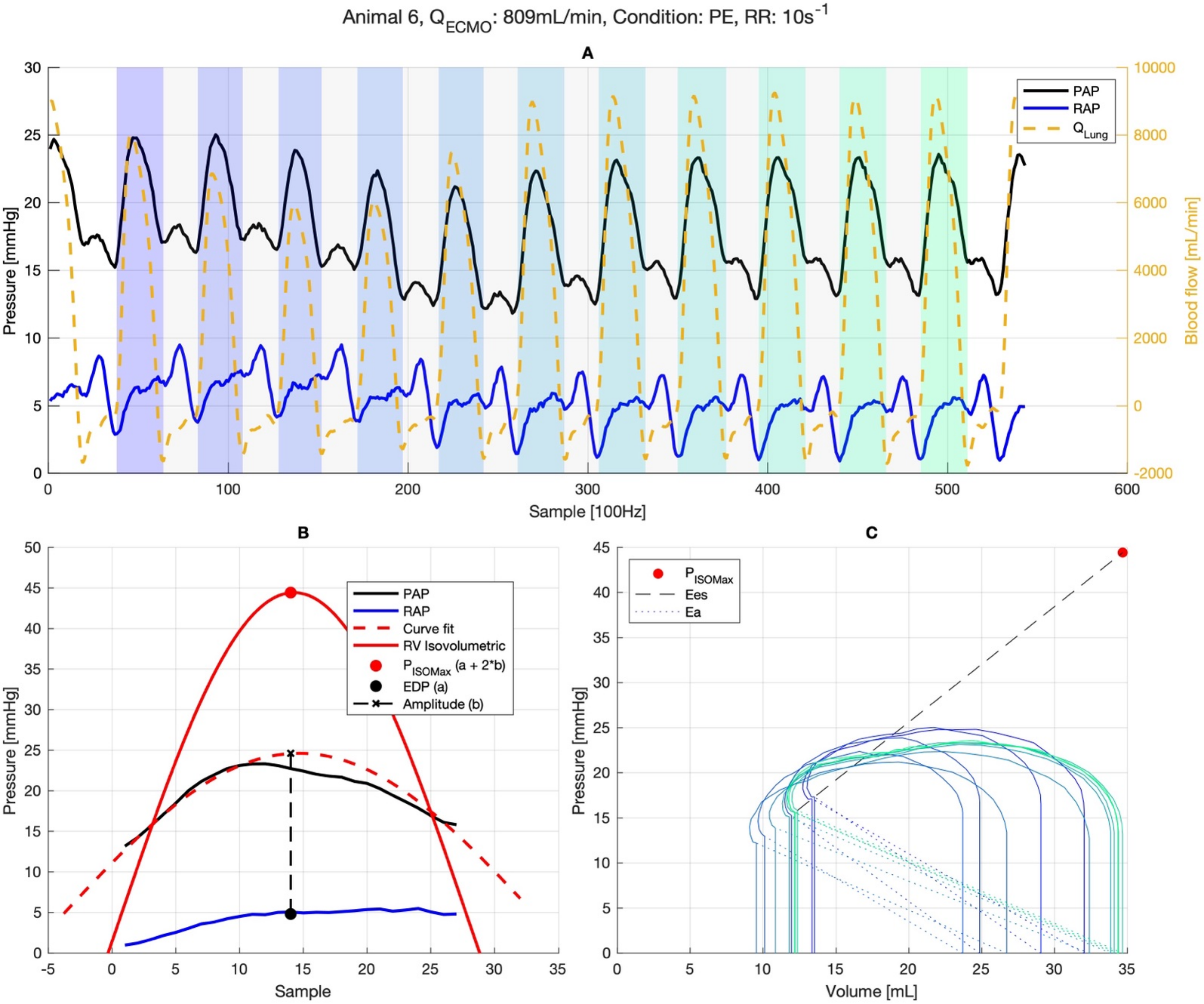

Animal 6, Q_{ECMO}: 809mL/min, Condition: PE, RR: 10s^{-1}
A
PAP
RAP
Q_{Lung}
Pressure [mmHg]
Blood flow [mL/min]
Sample [100Hz]
B
PAP
RAP
Curve fit
RV Isovolumetric
P_{ISOMax} (a + 2*b)
EDP (a)
Amplitude (b)
Pressure [mmHg]
Sample
C
P_{ISOMax}
Ees
Ea
Pressure [mmHg]
Volume [mL]

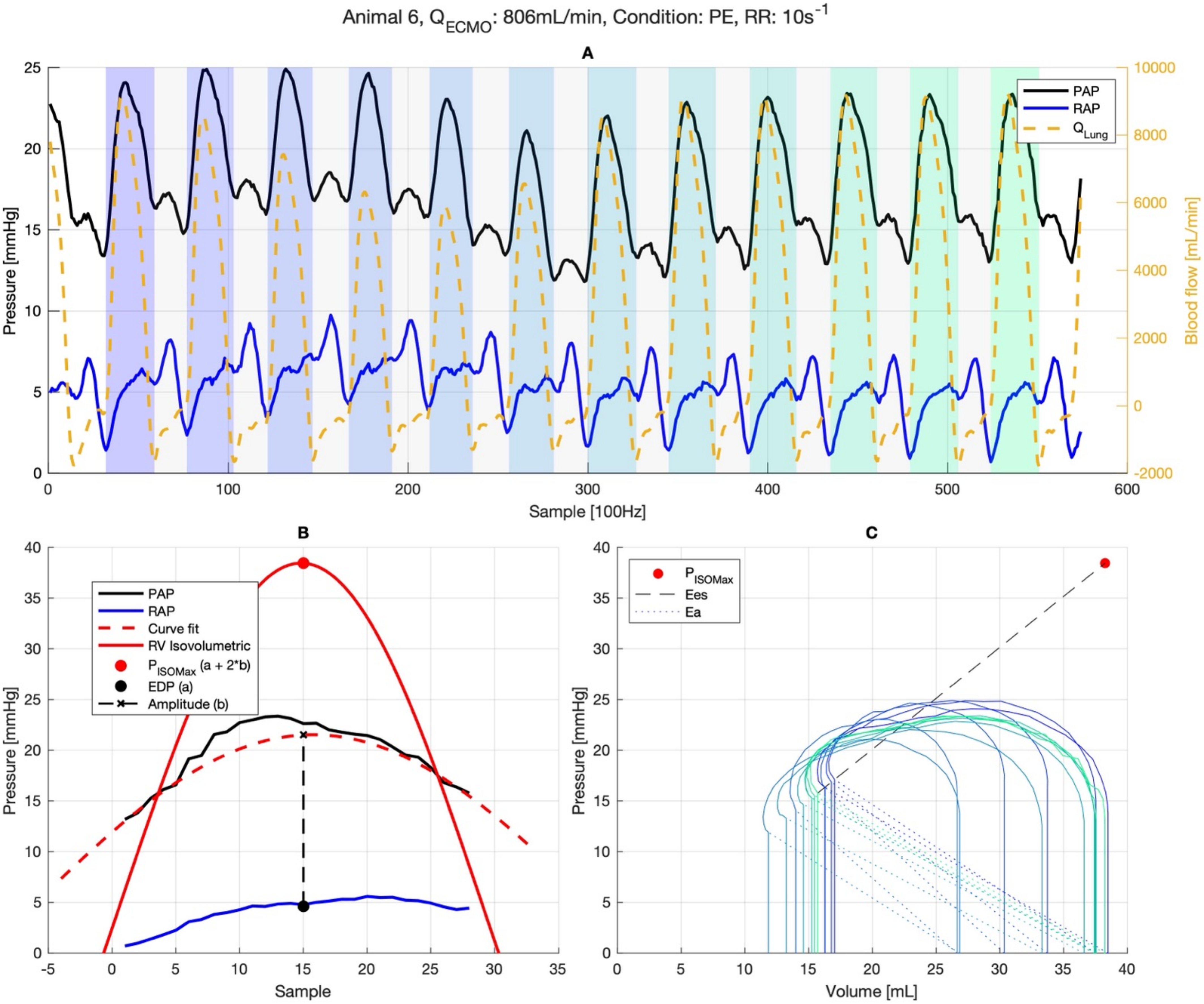
Animal 6, Q_{ECMO}: 806mL/min, Condition: PE, RR: $10s^{-1}$
A
PAP
RAP
Q_{Lung}
Pressure [mmHg]
Blood flow [mL/min]
Sample [100Hz]
B
PAP
RAP
Curve fit
RV Isovolumetric
P_{ISOMax} (a + 2*b)
EDP (a)
Amplitude (b)
Pressure [mmHg]
Sample
C
P_{ISOMax}
Ees
Ea
Pressure [mmHg]
Volume [mL]

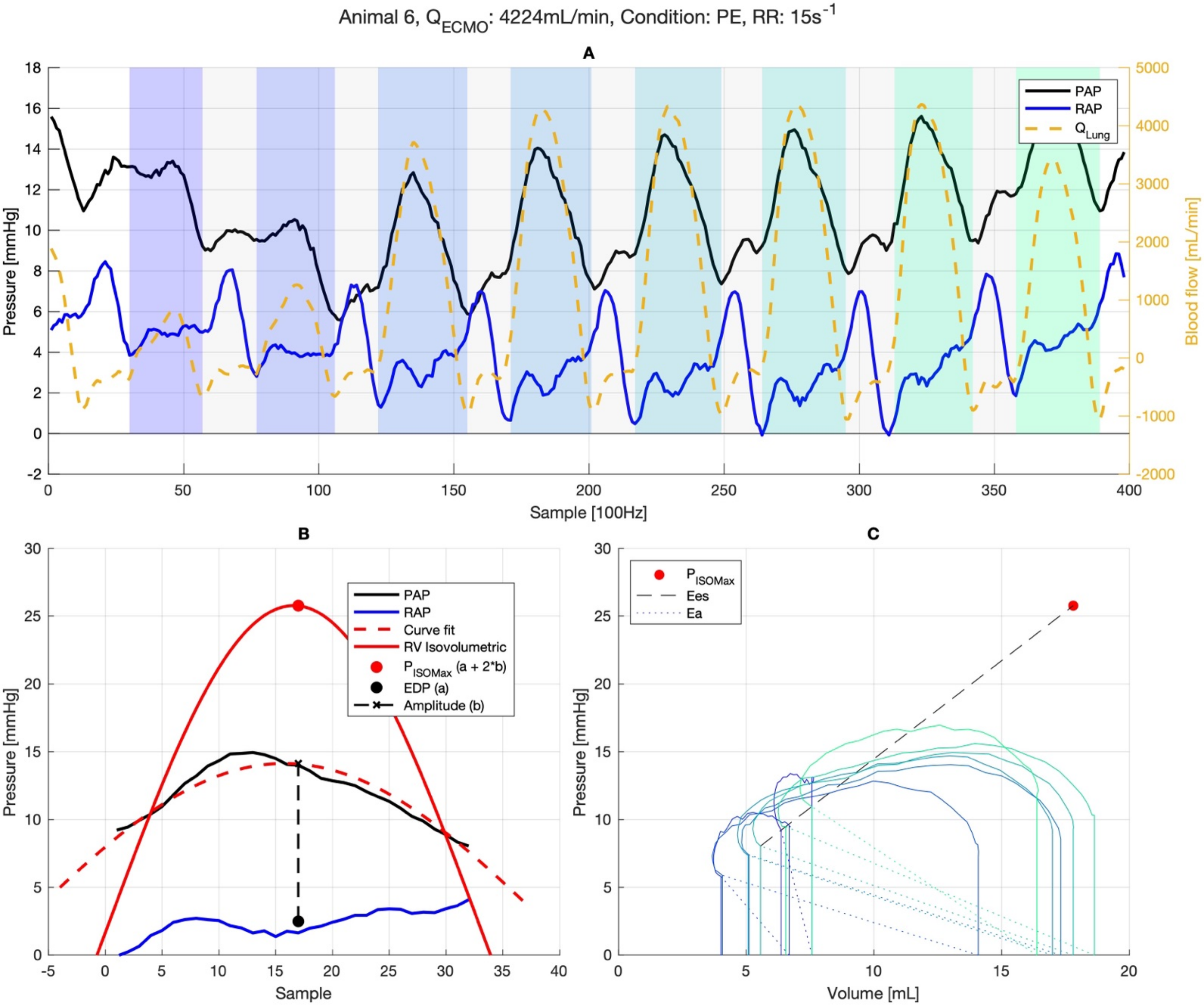

Animal 6, Q_ECMO: 4224mL/min, Condition: PE, RR: 15s^-1
A
PAP
RAP
Q_Lung
Pressure [mmHg]
Blood flow [mL/min]
Sample [100Hz]
B
PAP
RAP
Curve fit
RV Isovolumetric
P_ISOMax (a + 2*b)
EDP (a)
Amplitude (b)
Pressure [mmHg]
Sample
C
P_ISOMax
Ees
Ea
Pressure [mmHg]
Volume [mL]

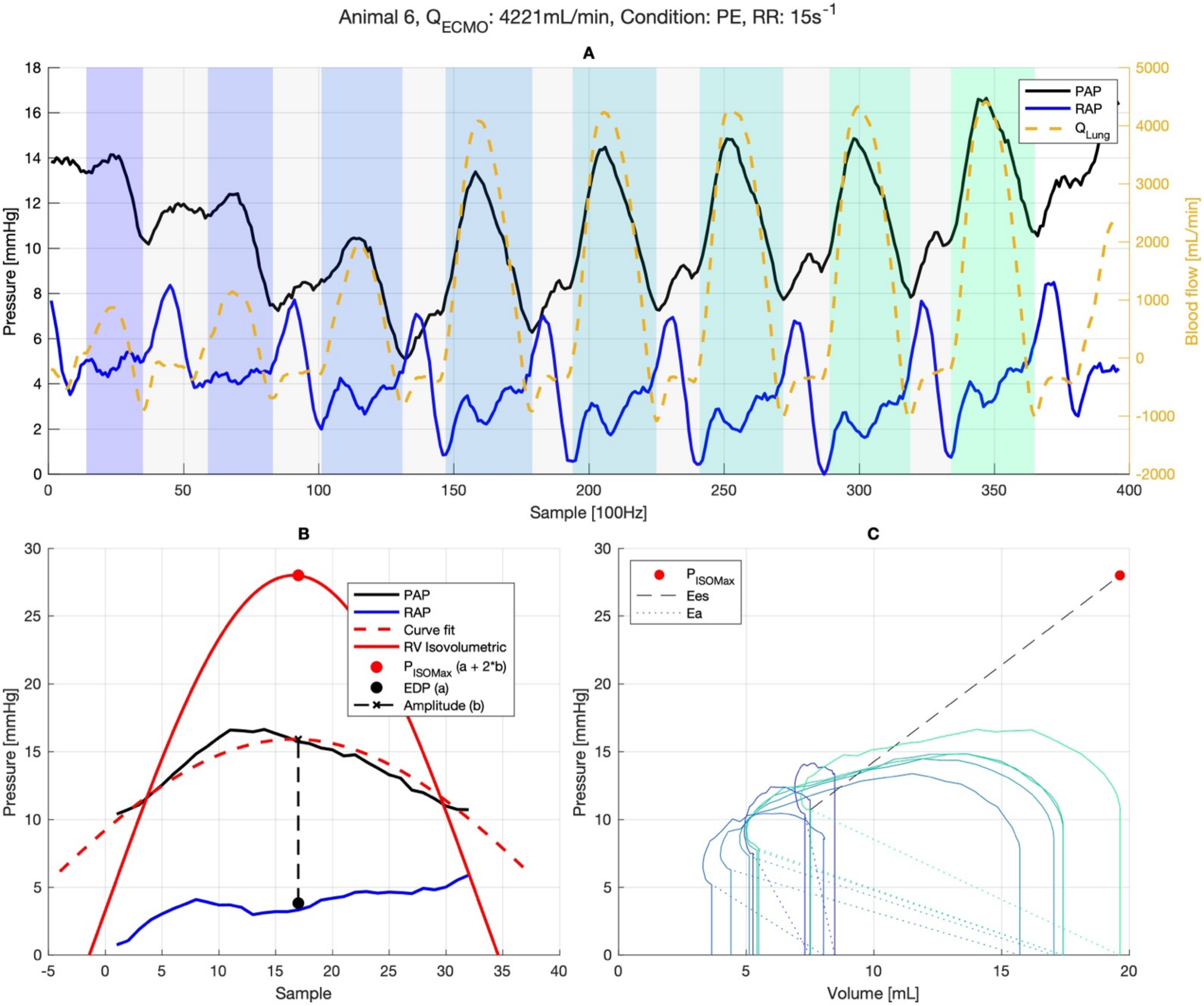
Animal 6, Q_ECMO: 4221mL/min, Condition: PE, RR: 15s^-1
A
PAP
RAP
Q_Lung
Pressure [mmHg]
Blood flow [mL/min]
Sample [100Hz]
B
PAP
RAP
Curve fit
RV Isovolumetric
P_ISOMax (a + 2*b)
EDP (a)
Amplitude (b)
Pressure [mmHg]
Sample
C
P_ISOMax
Ees
Ea
Pressure [mmHg]
Volume [mL]

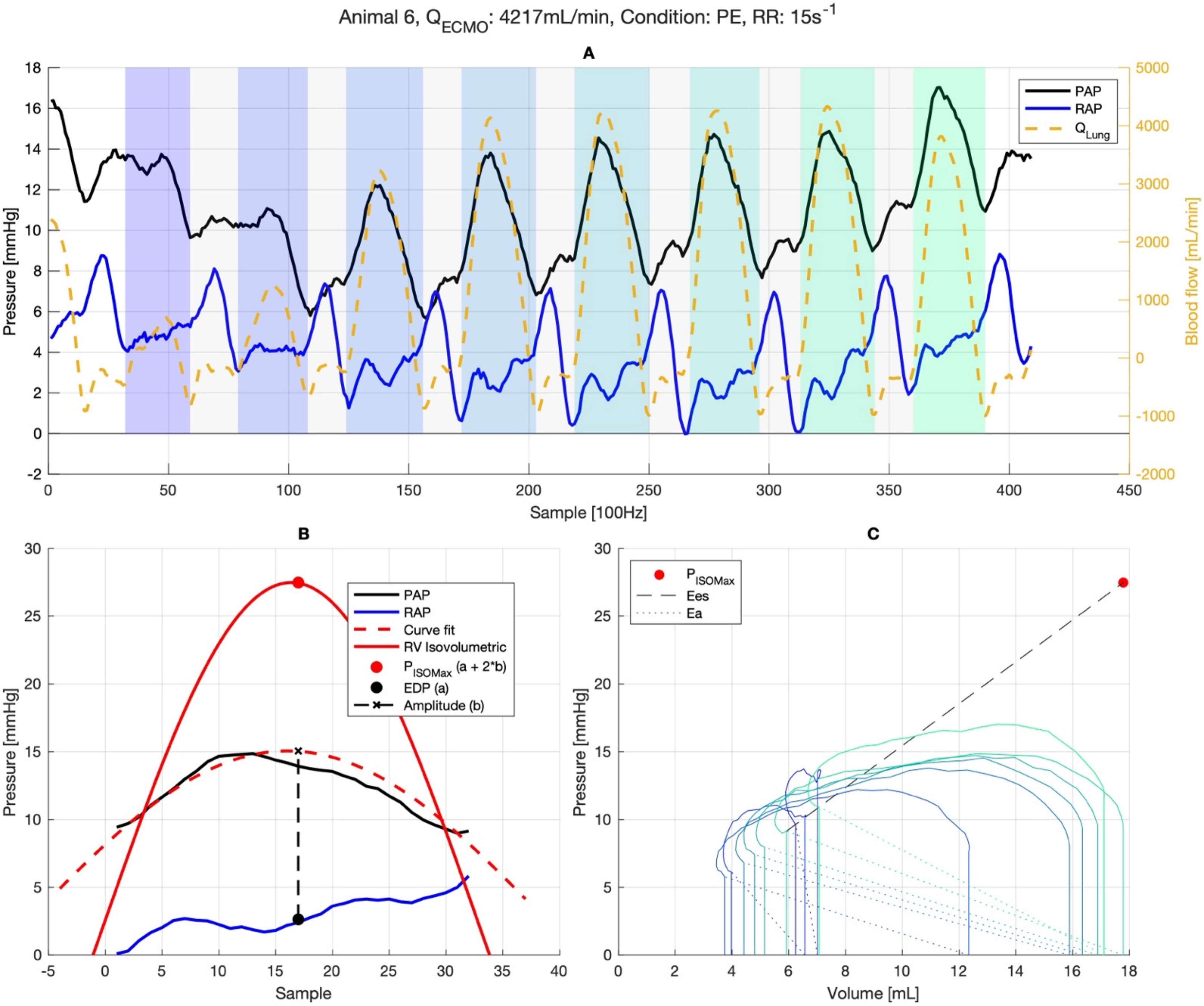

Animal 6, Q_ECMO: 4217mL/min, Condition: PE, RR: 15s^-1
A
PAP
RAP
Q_Lung
Pressure [mmHg]
Blood flow [mL/min]
Sample [100Hz]
B
PAP
RAP
Curve fit
RV Isovolumetric
P_ISOMax (a + 2*b)
EDP (a)
Amplitude (b)
Pressure [mmHg]
Sample
C
P_ISOMax
Ees
Ea
Pressure [mmHg]
Volume [mL]

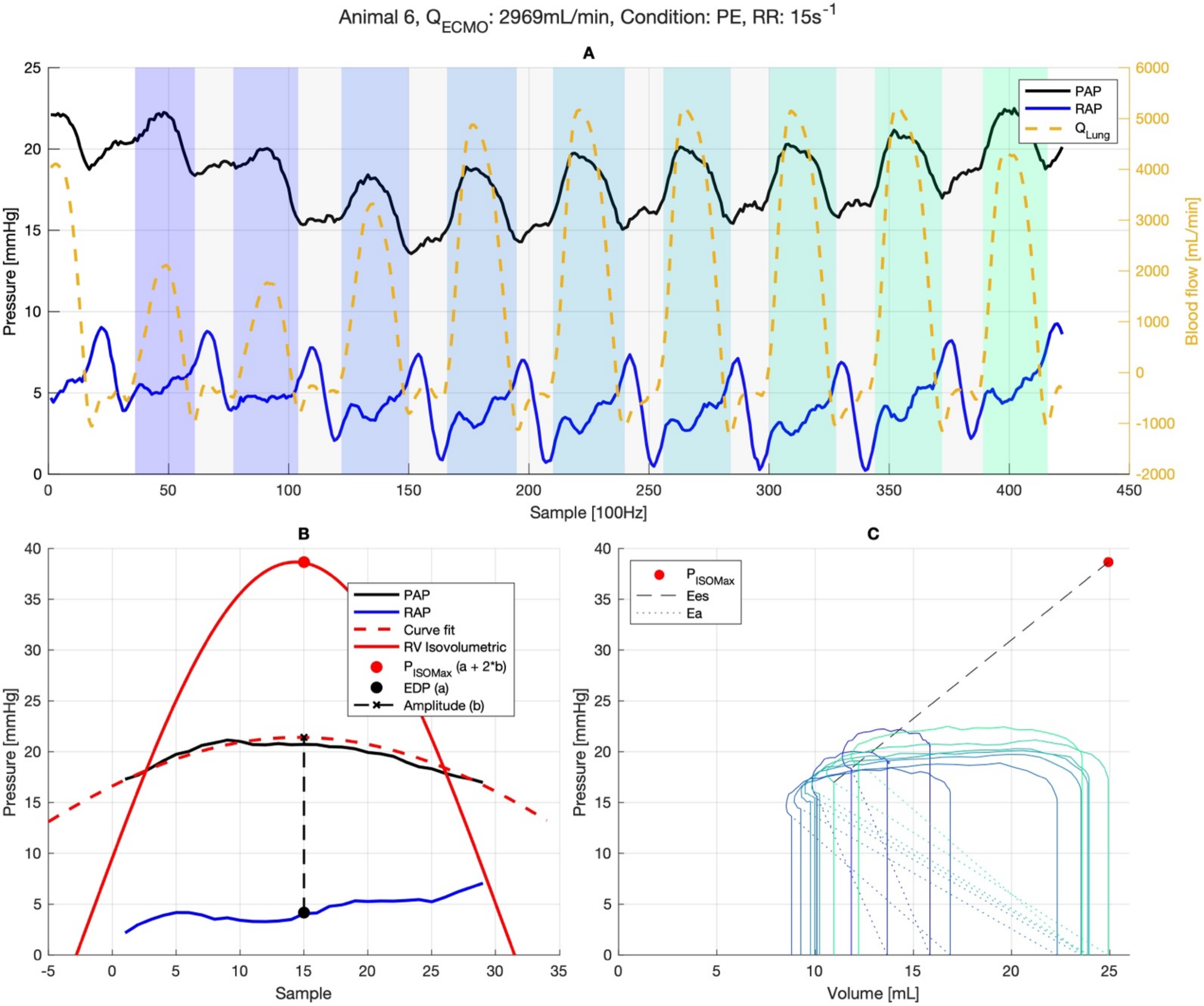

Animal 6, Q_{ECMO}: 2969mL/min, Condition: PE, RR: $15s^{-1}$
A
PAP
RAP
Q_{Lung}
Pressure [mmHg]
Blood flow [mL/min]
Sample [100Hz]
B
PAP
RAP
Curve fit
RV Isovolumetric
P_{ISOMax} (a + 2*b)
EDP (a)
Amplitude (b)
Pressure [mmHg]
Sample
C
P_{ISOMax}
Ees
Ea
Pressure [mmHg]
Volume [mL]

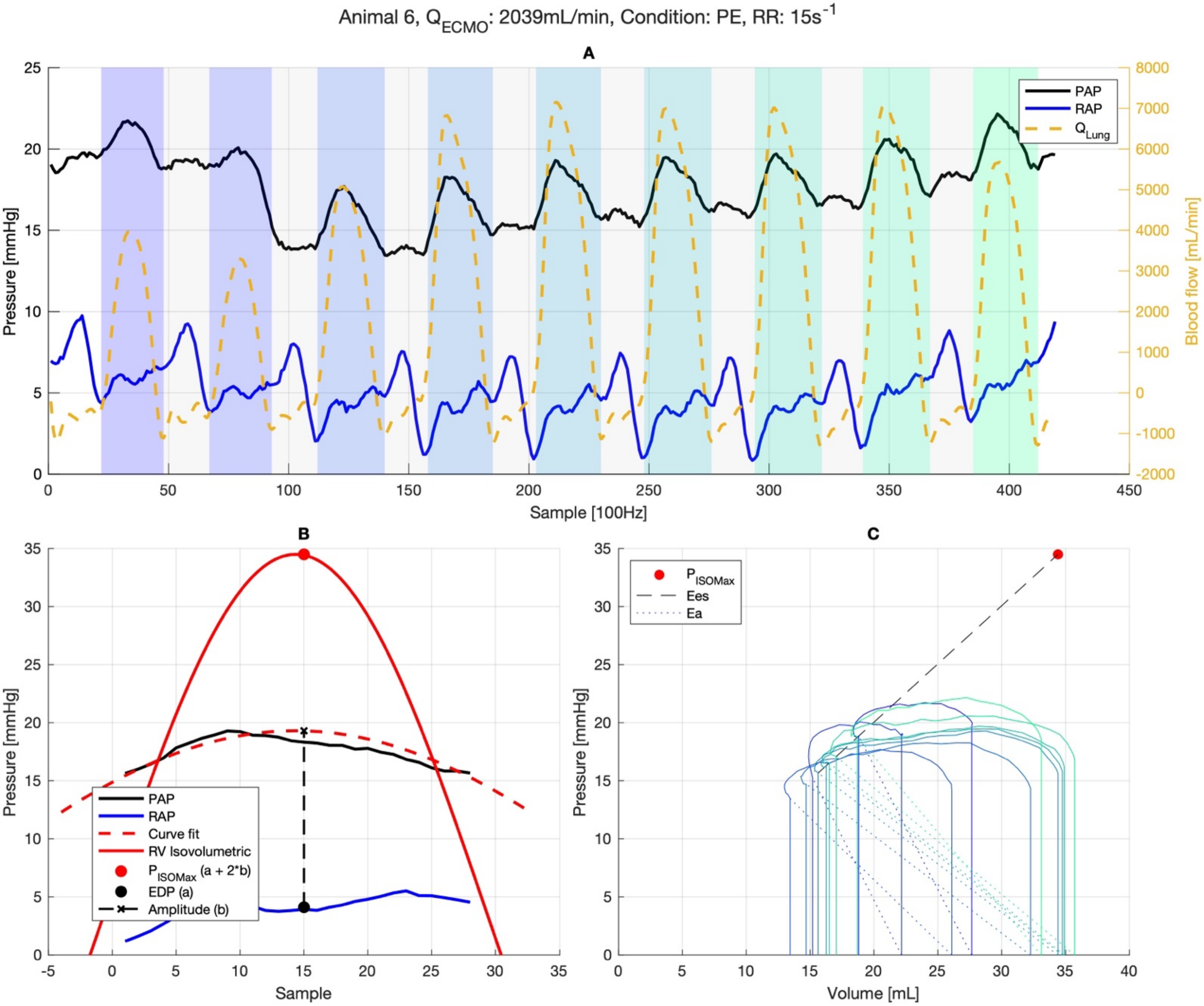

Animal 6, Q_{ECMO}: 2039mL/min, Condition: PE, RR: $15s^{-1}$
A
PAP
RAP
Q_{Lung}
Pressure [mmHg]
Blood flow [mL/min]
Sample [100Hz]
B
PAP
RAP
Curve fit
RV Isovolumetric
P_{ISOMax} (a + 2*b)
EDP (a)
Amplitude (b)
Pressure [mmHg]
Sample
C
P_{ISOMax}
Ees
Ea
Pressure [mmHg]
Volume [mL]

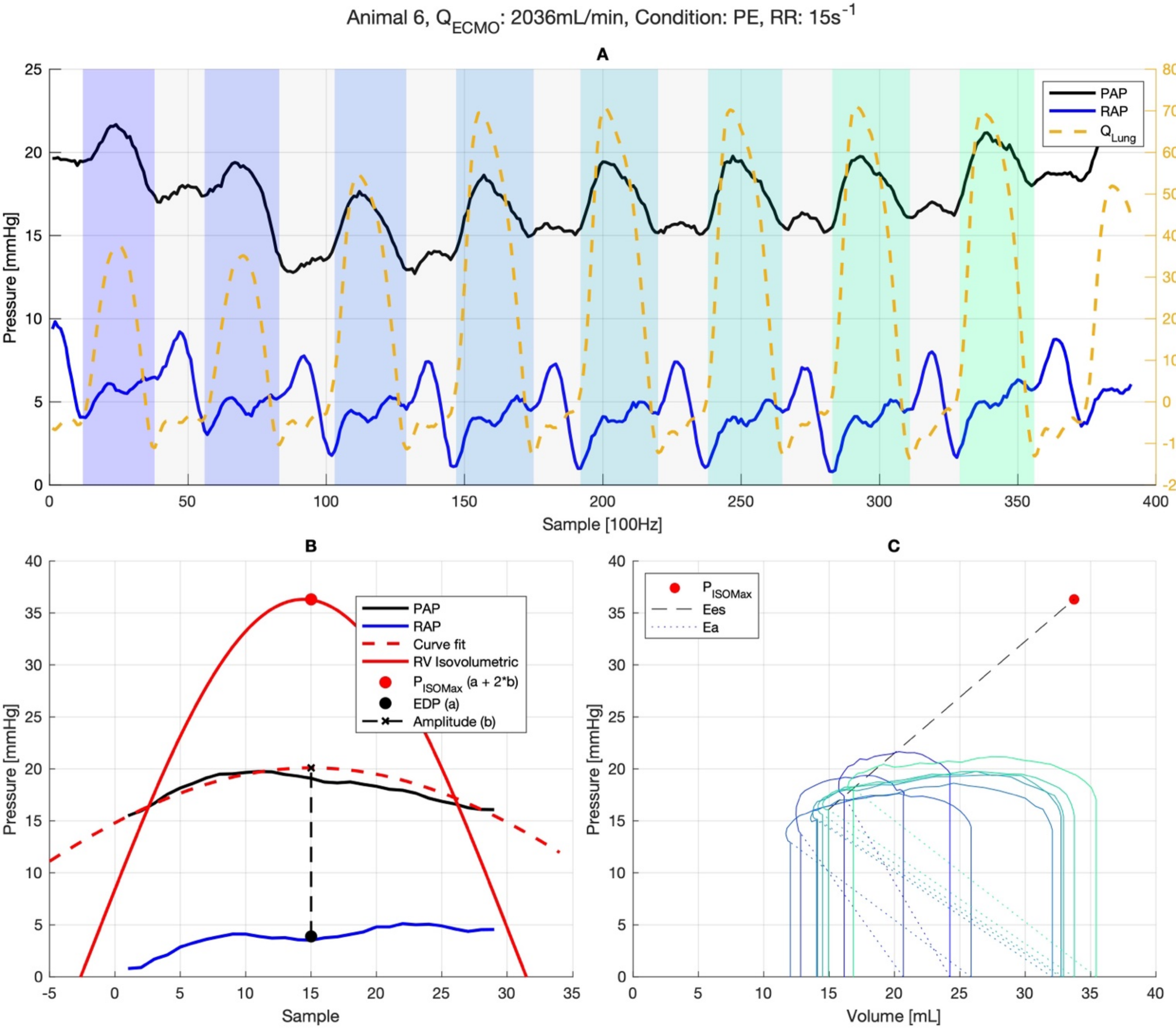

Animal 6, Q_ECMO: 2036mL/min, Condition: PE, RR: 15s^-1
A
PAP
RAP
Q_Lung
Pressure [mmHg]
Blood flow [mL/min]
Sample [100Hz]
B
PAP
RAP
Curve fit
RV Isovolumetric
P_ISOMax (a + 2*b)
EDP (a)
Amplitude (b)
Pressure [mmHg]
Sample
C
P_ISOMax
Ees
Ea
Pressure [mmHg]
Volume [mL]

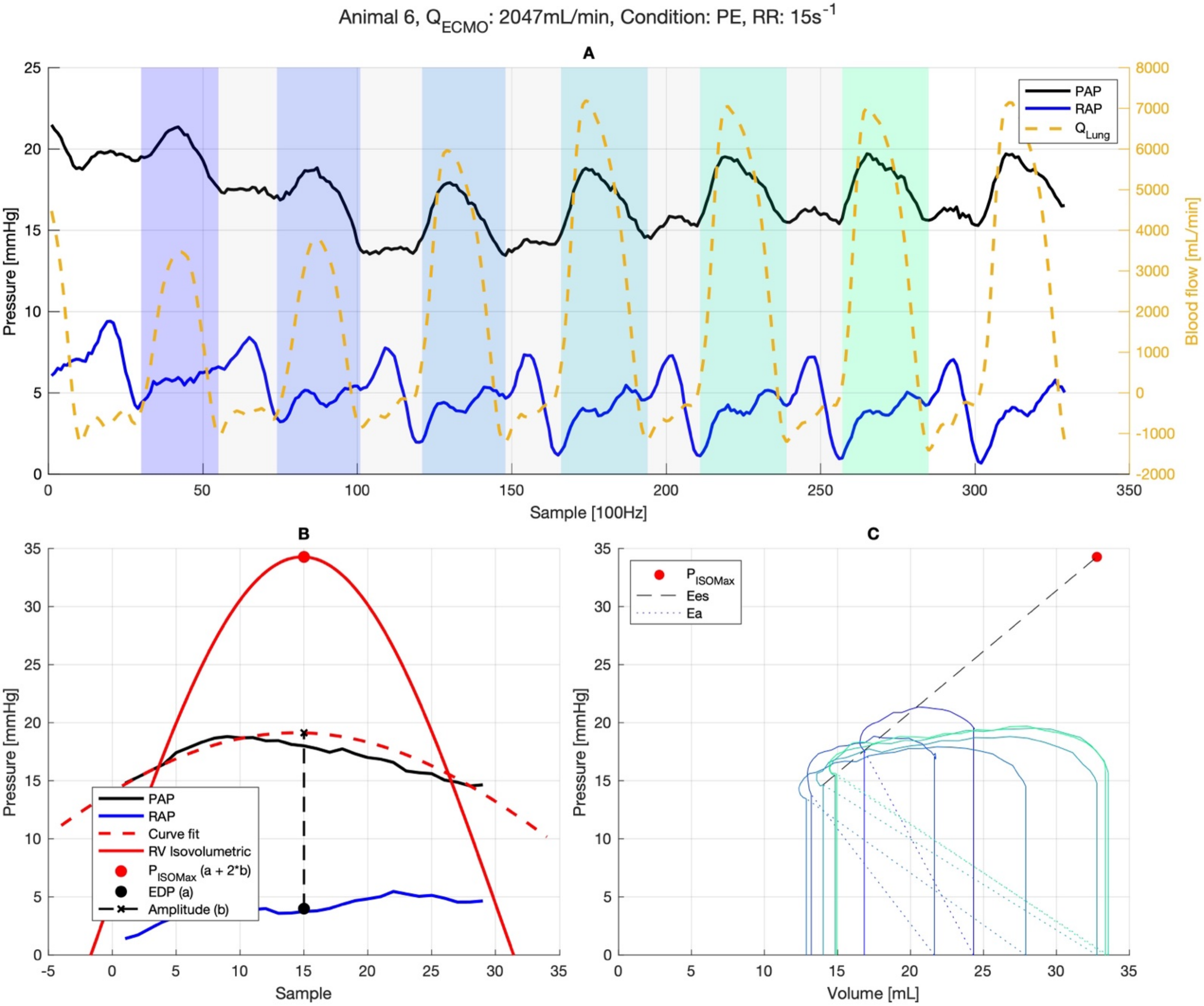

Animal 6, Q_{ECMO}: 2047mL/min, Condition: PE, RR: $15s^{-1}$
A
PAP
RAP
Q_{Lung}
Pressure [mmHg]
Blood flow [mL/min]
Sample [100Hz]
B
PAP
RAP
Curve fit
RV Isovolumetric
P_{ISOMax} (a + 2*b)
EDP (a)
Amplitude (b)
Pressure [mmHg]
Sample
C
P_{ISOMax}
Ees
Ea
Pressure [mmHg]
Volume [mL]

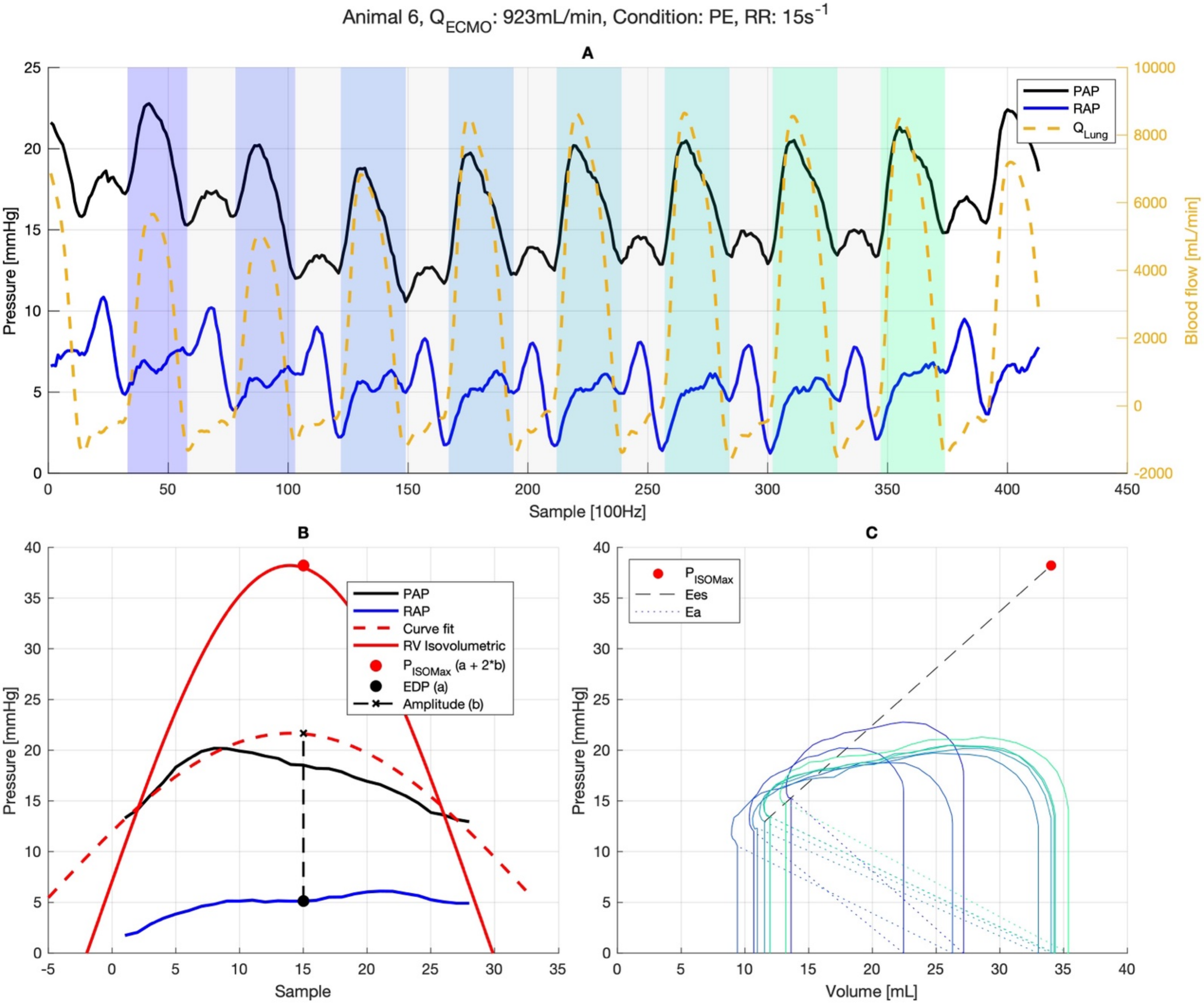

Animal 6, Q_{ECMO}: 923mL/min, Condition: PE, RR: $15s^{-1}$
A
PAP
RAP
Q_{Lung}
Pressure [mmHg]
Blood flow [mL/min]
Sample [100Hz]
B
PAP
RAP
Curve fit
RV Isovolumetric
P_{ISOMax} (a + 2*b)
EDP (a)
Amplitude (b)
Pressure [mmHg]
Sample
C
P_{ISOMax}
Ees
Ea
Pressure [mmHg]
Volume [mL]

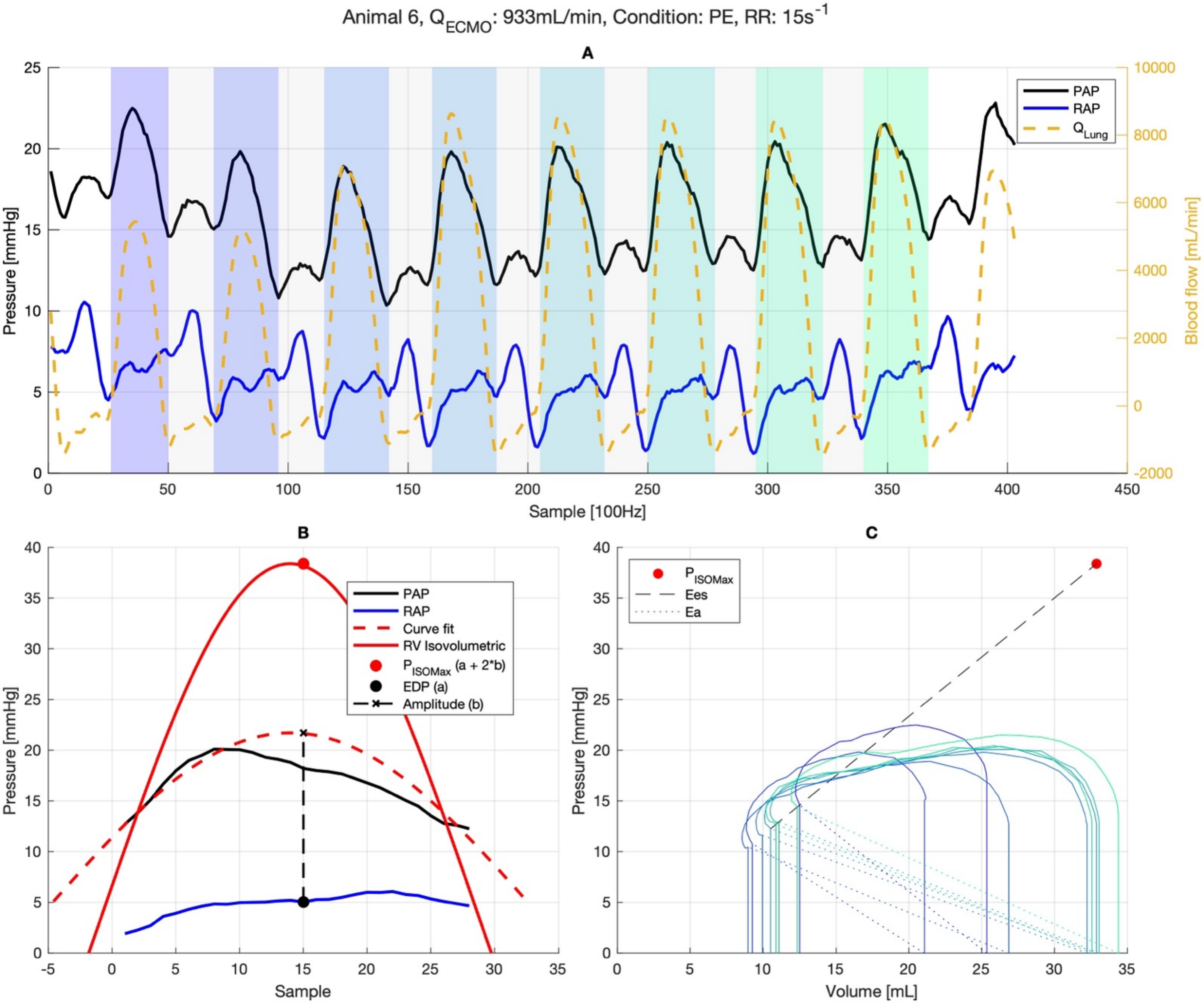

Animal 6, Q_{ECMO}: 933mL/min, Condition: PE, RR: $15s^{-1}$
A
PAP
RAP
Q_{Lung}
Pressure [mmHg]
Blood flow [mL/min]
Sample [100Hz]
B
PAP
RAP
Curve fit
RV Isovolumetric
P_{ISOMax} (a + 2*b)
EDP (a)
Amplitude (b)
Pressure [mmHg]
Sample
C
P_{ISOMax}
Ees
Ea
Pressure [mmHg]
Volume [mL]

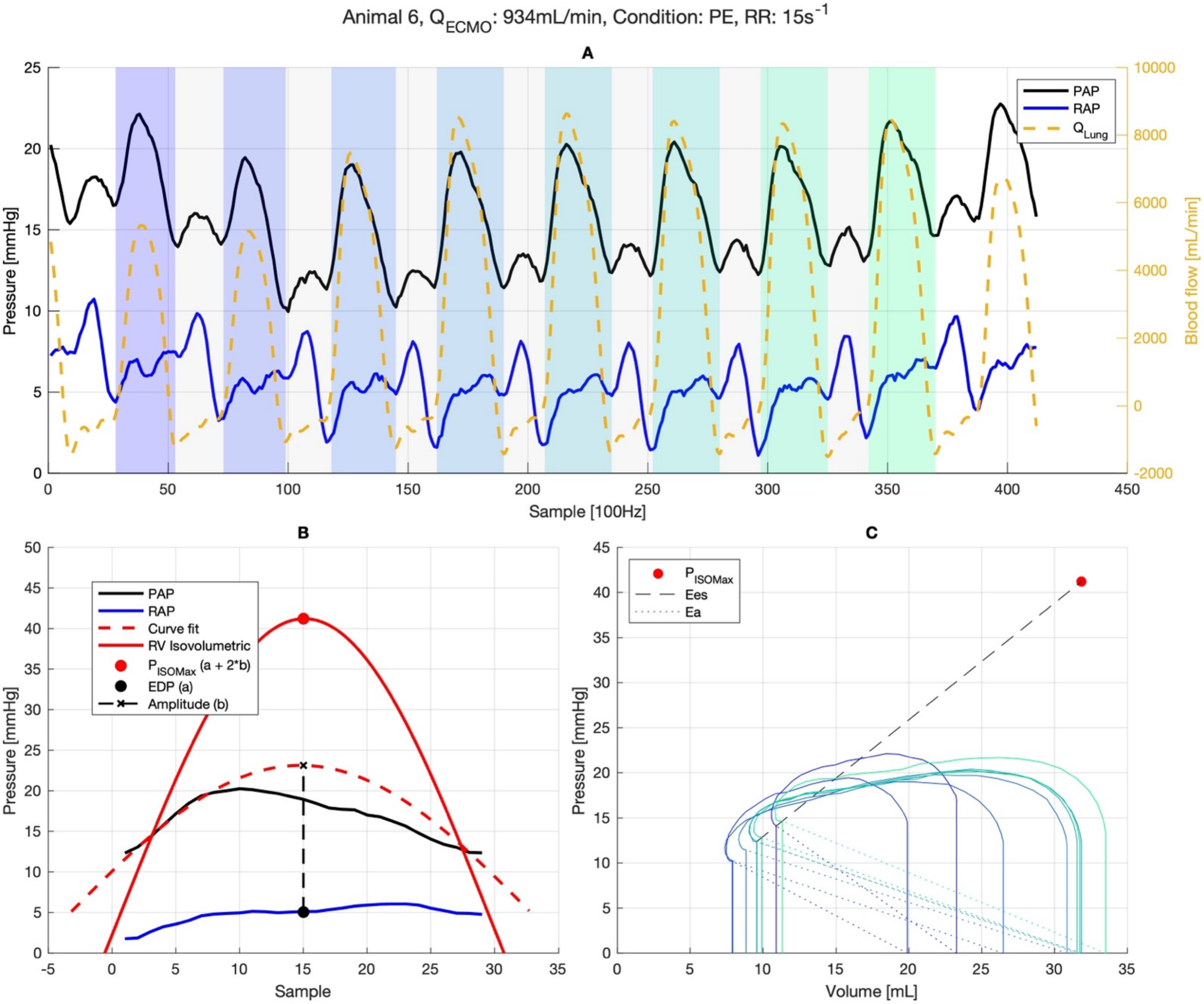

Animal 6, Q_{ECMO}: 934mL/min, Condition: PE, RR: $15s^{-1}$
A
PAP
RAP
Q_{Lung}
Pressure [mmHg]
Blood flow [mL/min]
Sample [100Hz]
B
PAP
RAP
Curve fit
RV Isovolumetric
P_{ISOMax} (a + 2*b)
EDP (a)
Amplitude (b)
Pressure [mmHg]
Sample
C
P_{ISOMax}
Ees
Ea
Pressure [mmHg]
Volume [mL]

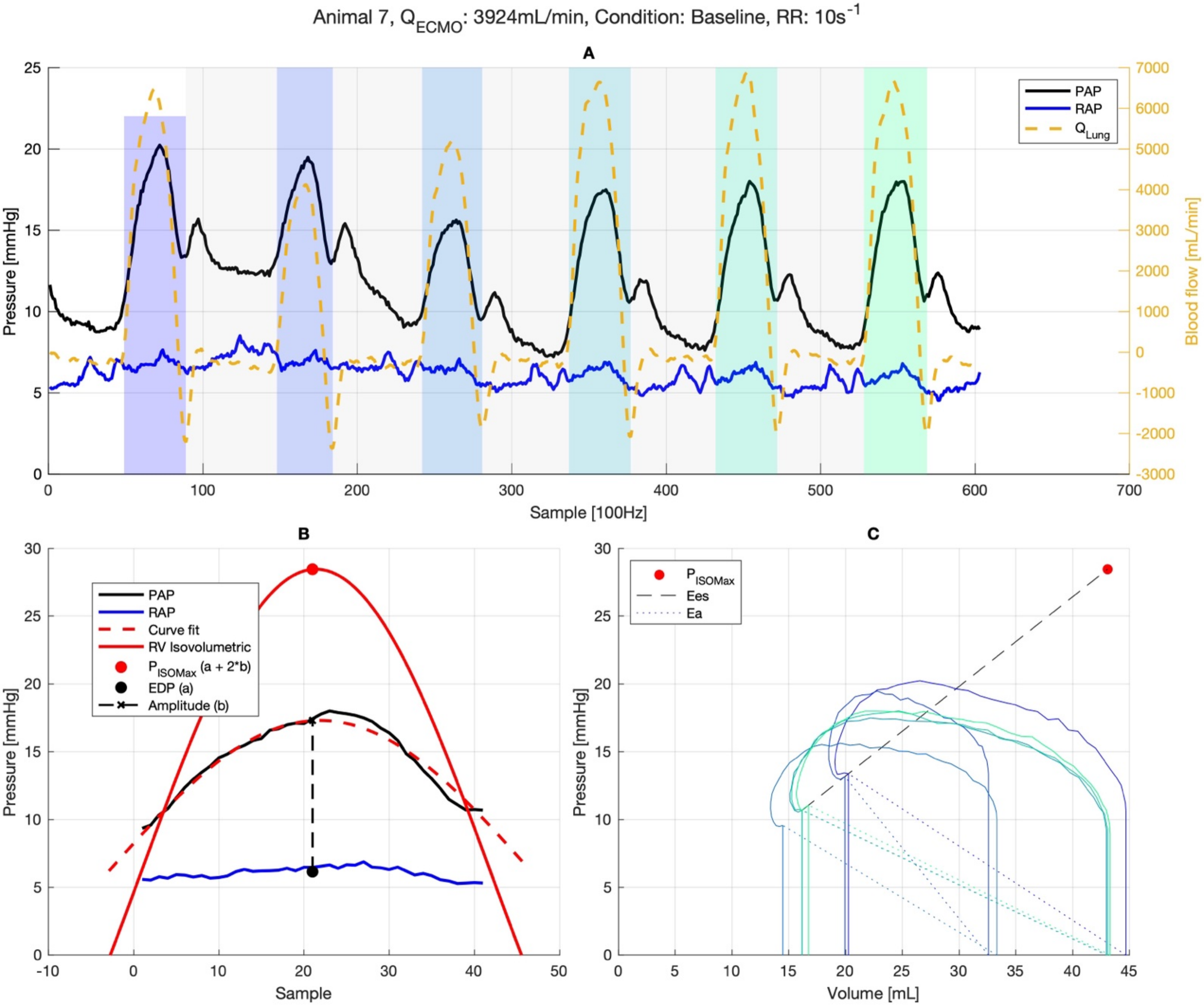

Animal 7, Q_{ECMO}: 3924mL/min, Condition: Baseline, RR: $10s^{-1}$
A
PAP
RAP
Q_{Lung}
Pressure [mmHg]
Blood flow [mL/min]
Sample [100Hz]
B
PAP
RAP
Curve fit
RV Isovolumetric
P_{ISOMax} (a + 2*b)
EDP (a)
Amplitude (b)
Pressure [mmHg]
Sample
C
P_{ISOMax}
Ees
Ea
Pressure [mmHg]
Volume [mL]

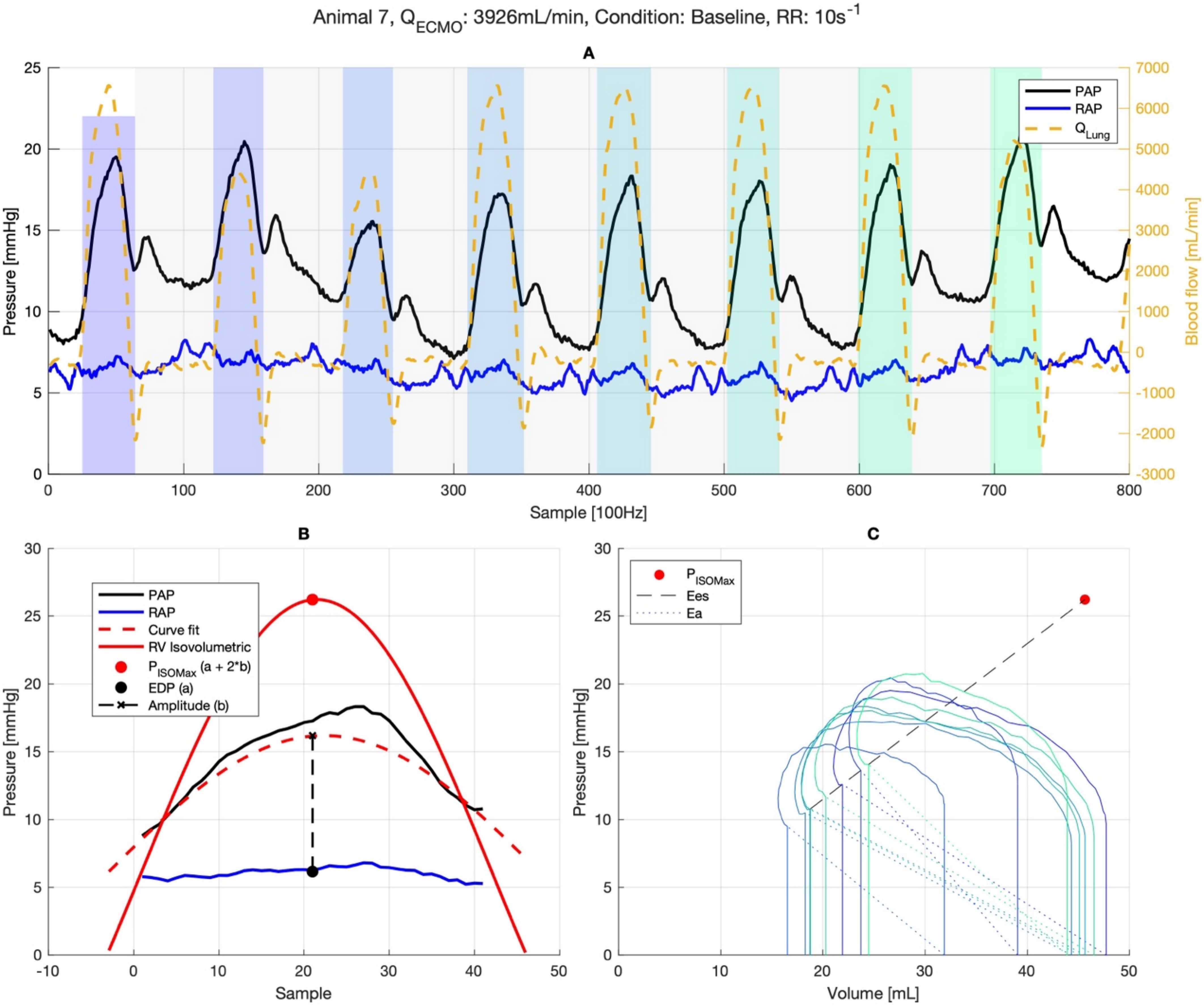

Animal 7, Q_{ECMO}: 3926mL/min, Condition: Baseline, RR: $10s^{-1}$
A
PAP
RAP
Q_{Lung}
Pressure [mmHg]
Blood flow [mL/min]
Sample [100Hz]
B
PAP
RAP
Curve fit
RV Isovolumetric
P_{ISOMax} (a + 2*b)
EDP (a)
Amplitude (b)
Pressure [mmHg]
Sample
C
P_{ISOMax}
Ees
Ea
Pressure [mmHg]
Volume [mL]

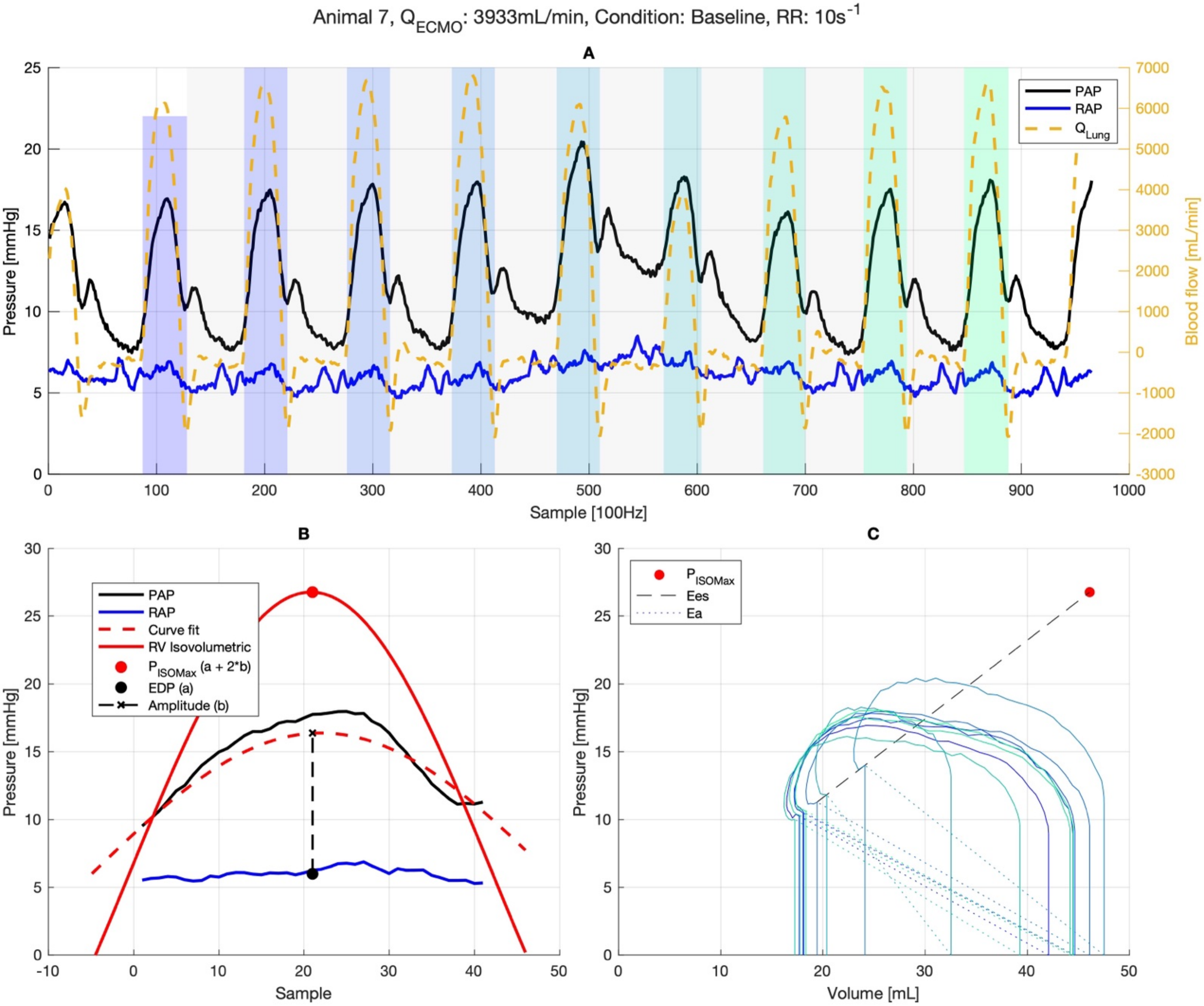

Animal 7, Q_{ECMO}: 3933mL/min, Condition: Baseline, RR: $10s^{-1}$
A
PAP
RAP
Q_{Lung}
Pressure [mmHg]
Blood flow [mL/min]
Sample [100Hz]
B
PAP
RAP
Curve fit
RV Isovolumetric
P_{ISOMax} (a + 2*b)
EDP (a)
Amplitude (b)
Pressure [mmHg]
Sample
C
P_{ISOMax}
Ees
Ea
Pressure [mmHg]
Volume [mL]

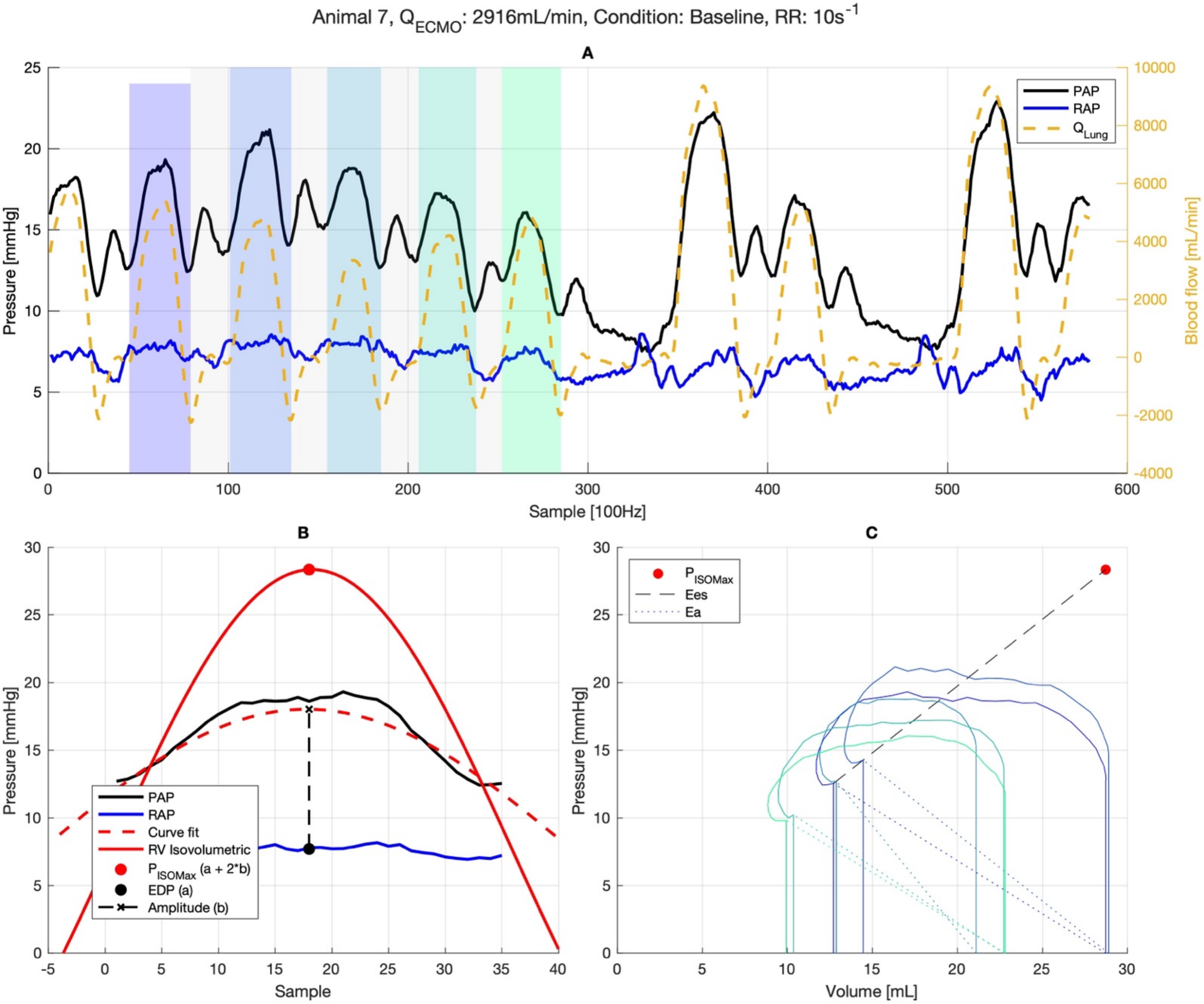

Animal 7, QECMO: 2916mL/min, Condition: Baseline, RR: 10s-1
A
PAP
RAP
QLung
Pressure [mmHg]
Blood flow [mL/min]
Sample [100Hz]
B
PAP
RAP
Curve fit
RV Isovolumetric
PISOMax (a + 2*b)
EDP (a)
Amplitude (b)
Pressure [mmHg]
Sample
C
PISOMax
Ees
Ea
Pressure [mmHg]
Volume [mL]

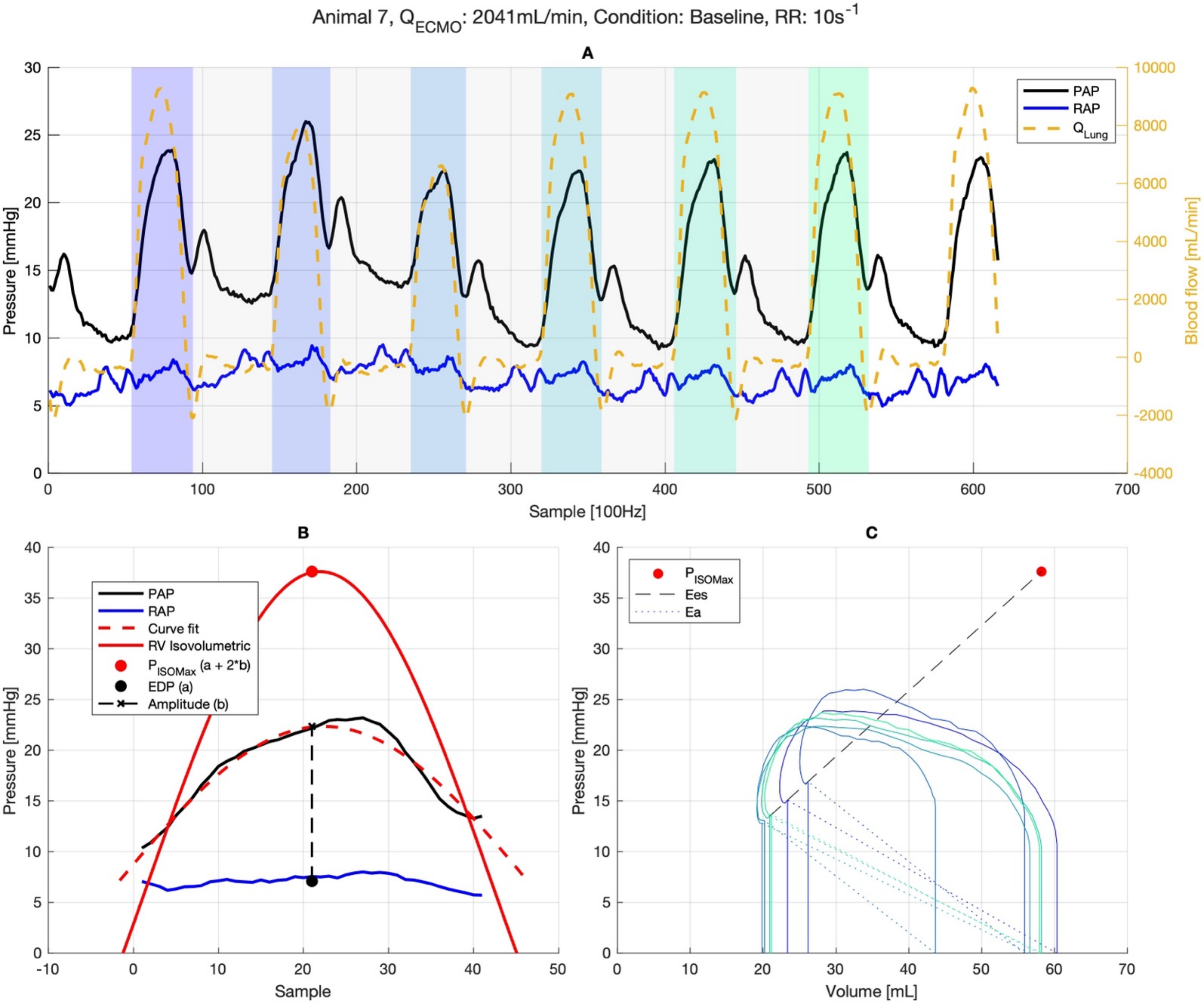

Animal 7, Q_ECMO: 2041mL/min, Condition: Baseline, RR: 10s^-1
A
Pressure [mmHg]
Blood flow [mL/min]
Sample [100Hz]
PAP
RAP
Q_Lung
B
PAP
RAP
Curve fit
RV Isovolumetric
P_ISOMax (a + 2*b)
EDP (a)
Amplitude (b)
Pressure [mmHg]
Sample
C
P_ISOMax
Ees
Ea
Pressure [mmHg]
Volume [mL]

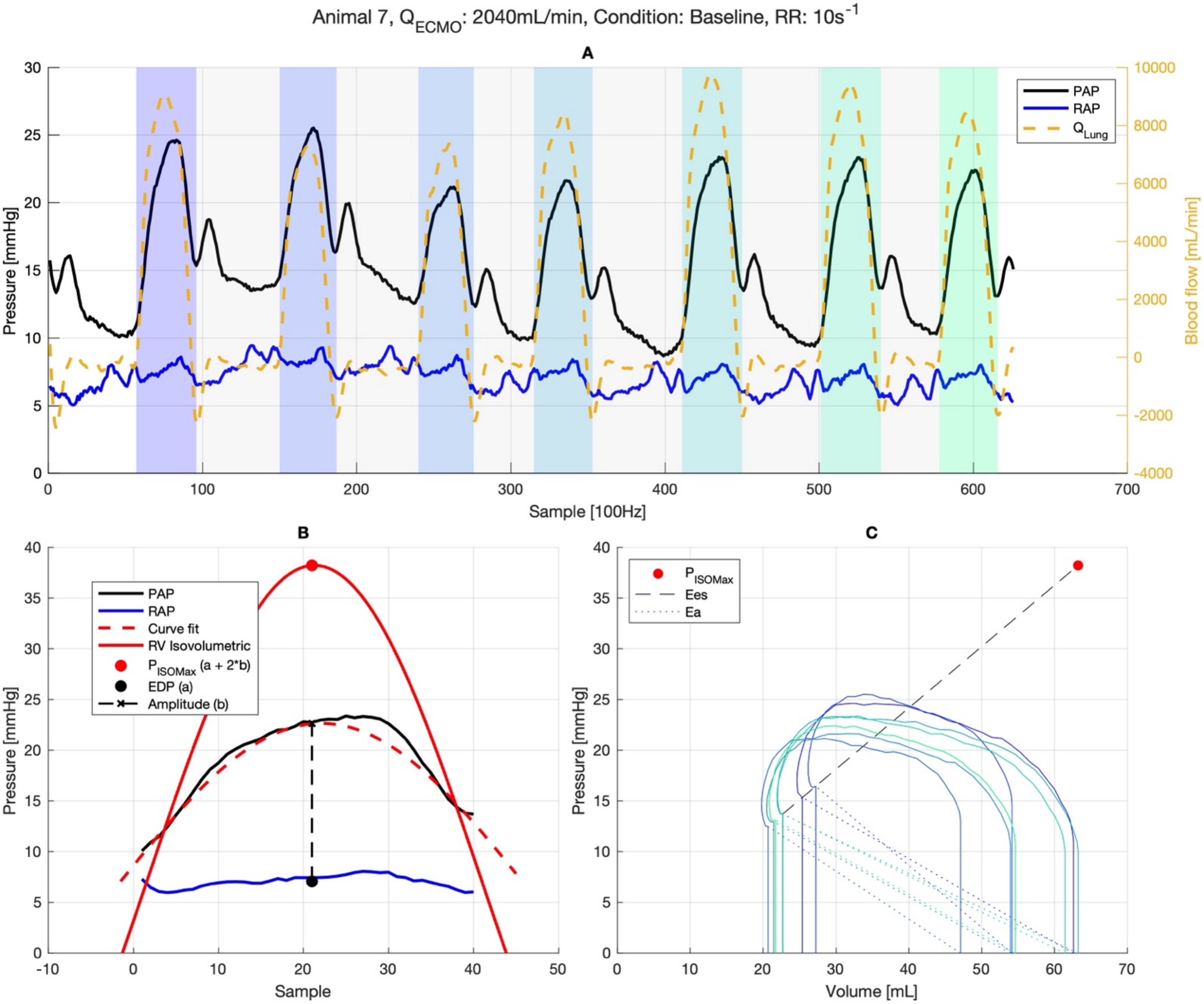
Animal 7, Q_{ECMO}: 2040mL/min, Condition: Baseline, RR: $10s^{-1}$
A
Pressure [mmHg]
Blood flow [mL/min]
Sample [100Hz]
PAP
RAP
Q_{Lung}
B
Pressure [mmHg]
Sample
PAP
RAP
Curve fit
RV Isovolumetric
P_{ISOMax} (a + 2*b)
EDP (a)
Amplitude (b)
C
Pressure [mmHg]
Volume [mL]
P_{ISOMax}
Ees
Ea

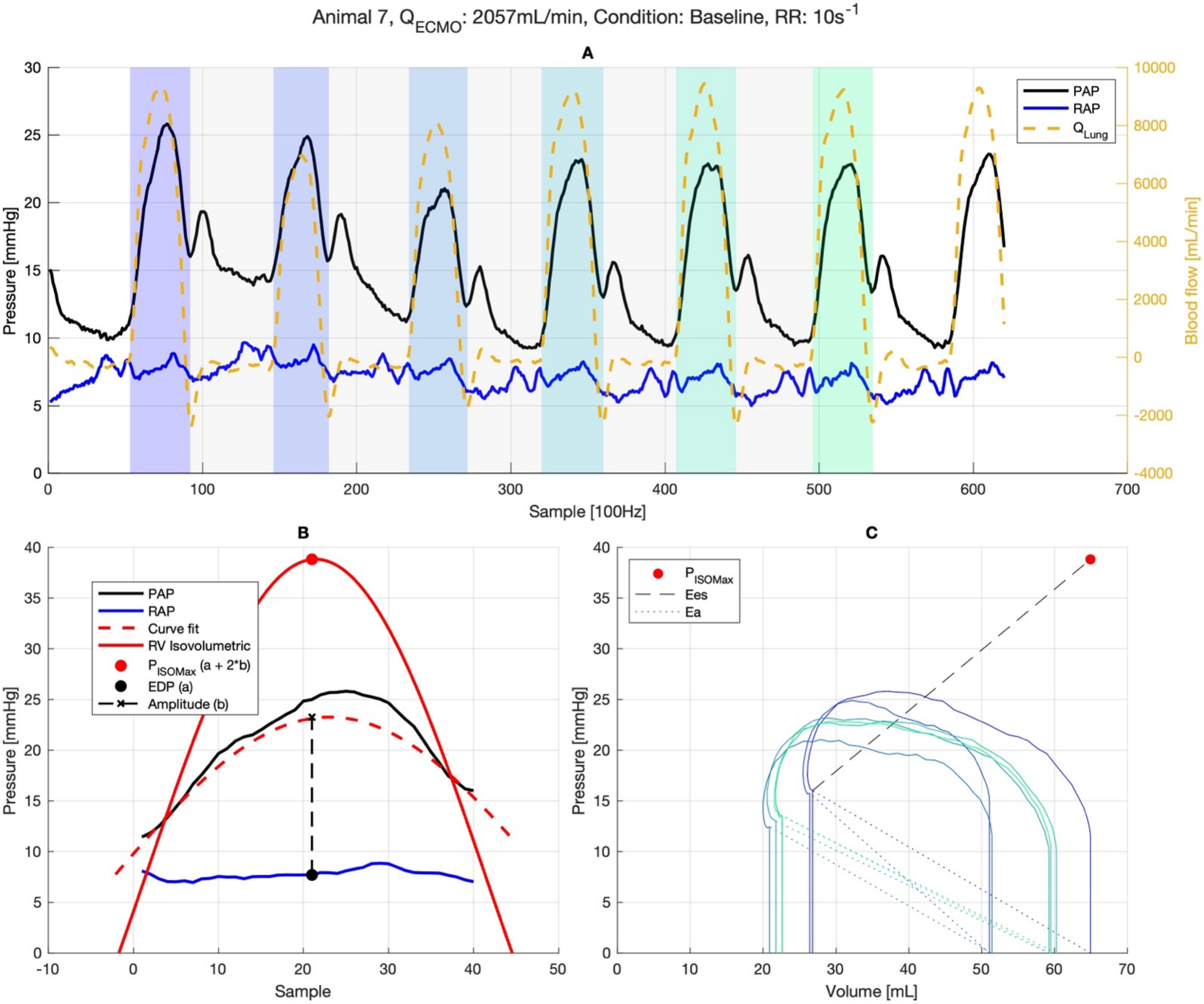

Animal 7, Q_{ECMO}: 2057mL/min, Condition: Baseline, RR: $10s^{-1}$
A
PAP
RAP
Q_{Lung}
Pressure [mmHg]
Blood flow [mL/min]
Sample [100Hz]
B
PAP
RAP
Curve fit
RV Isovolumetric
P_{ISOMax} (a + 2*b)
EDP (a)
Amplitude (b)
Pressure [mmHg]
Sample
C
P_{ISOMax}
Ees
Ea
Pressure [mmHg]
Volume [mL]

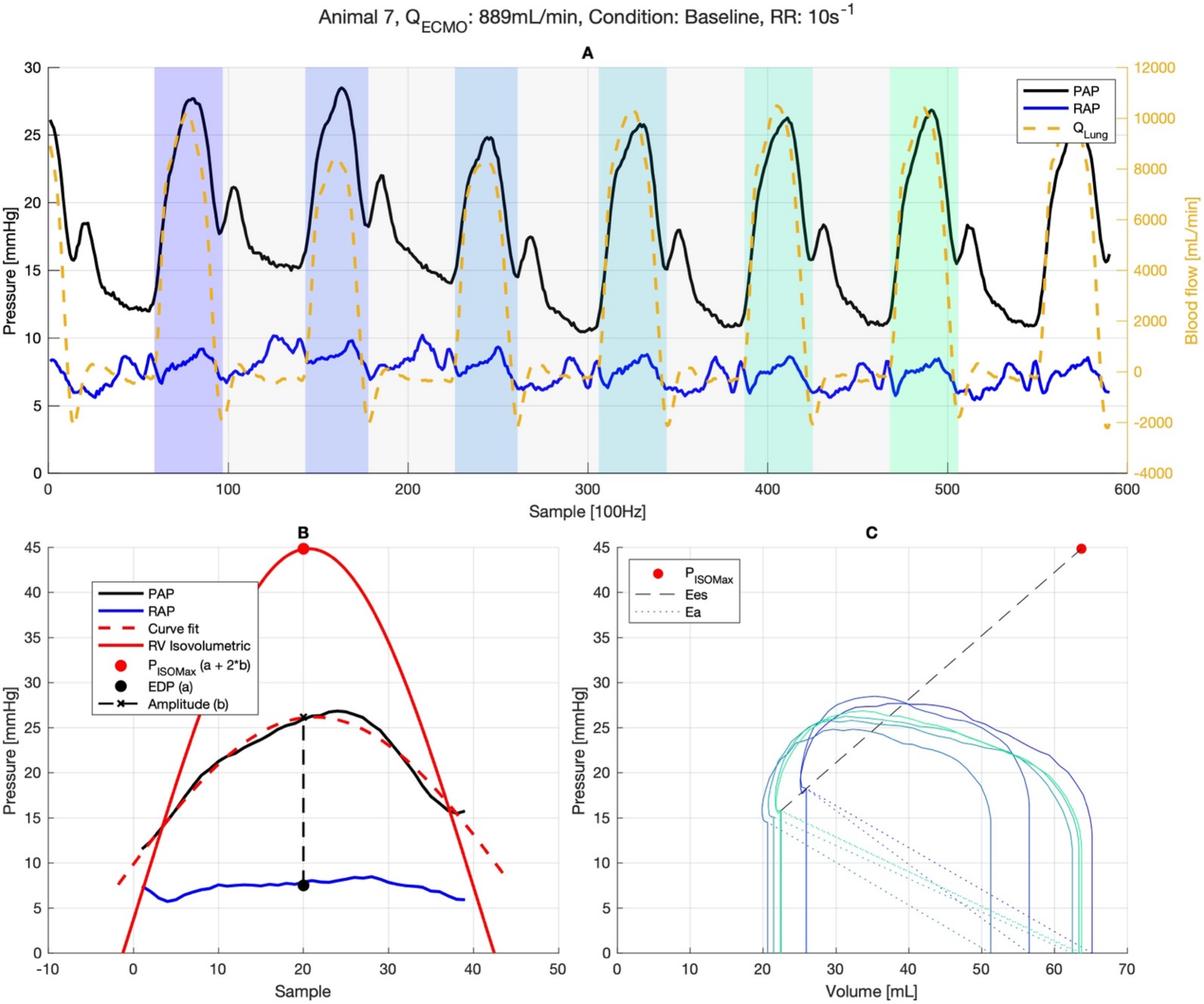

Animal 7, Q_{ECMO}: 889mL/min, Condition: Baseline, RR: $10s^{-1}$
A
PAP
RAP
Q_{Lung}
Pressure [mmHg]
Blood flow [mL/min]
Sample [100Hz]
B
PAP
RAP
Curve fit
RV Isovolumetric
P_{ISOMax} (a + 2*b)
EDP (a)
Amplitude (b)
Pressure [mmHg]
Sample
C
P_{ISOMax}
Ees
Ea
Pressure [mmHg]
Volume [mL]

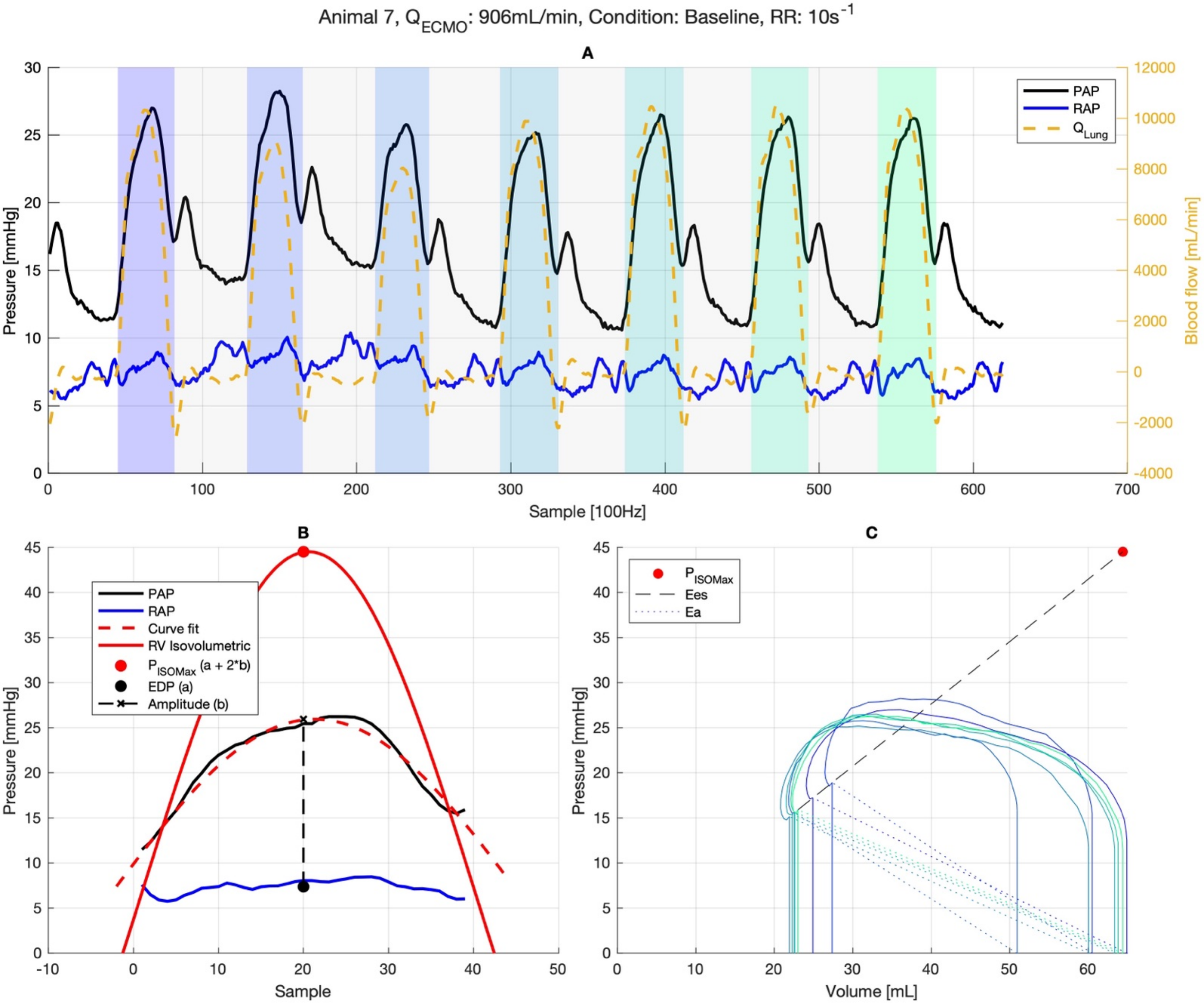

Animal 7, Q_ECMO: 906mL/min, Condition: Baseline, RR: 10s^-1
A
PAP
RAP
Q_Lung
Pressure [mmHg]
Blood flow [mL/min]
Sample [100Hz]
B
PAP
RAP
Curve fit
RV Isovolumetric
P_ISOMax (a + 2*b)
EDP (a)
Amplitude (b)
Pressure [mmHg]
Sample
C
P_ISOMax
Ees
Ea
Pressure [mmHg]
Volume [mL]

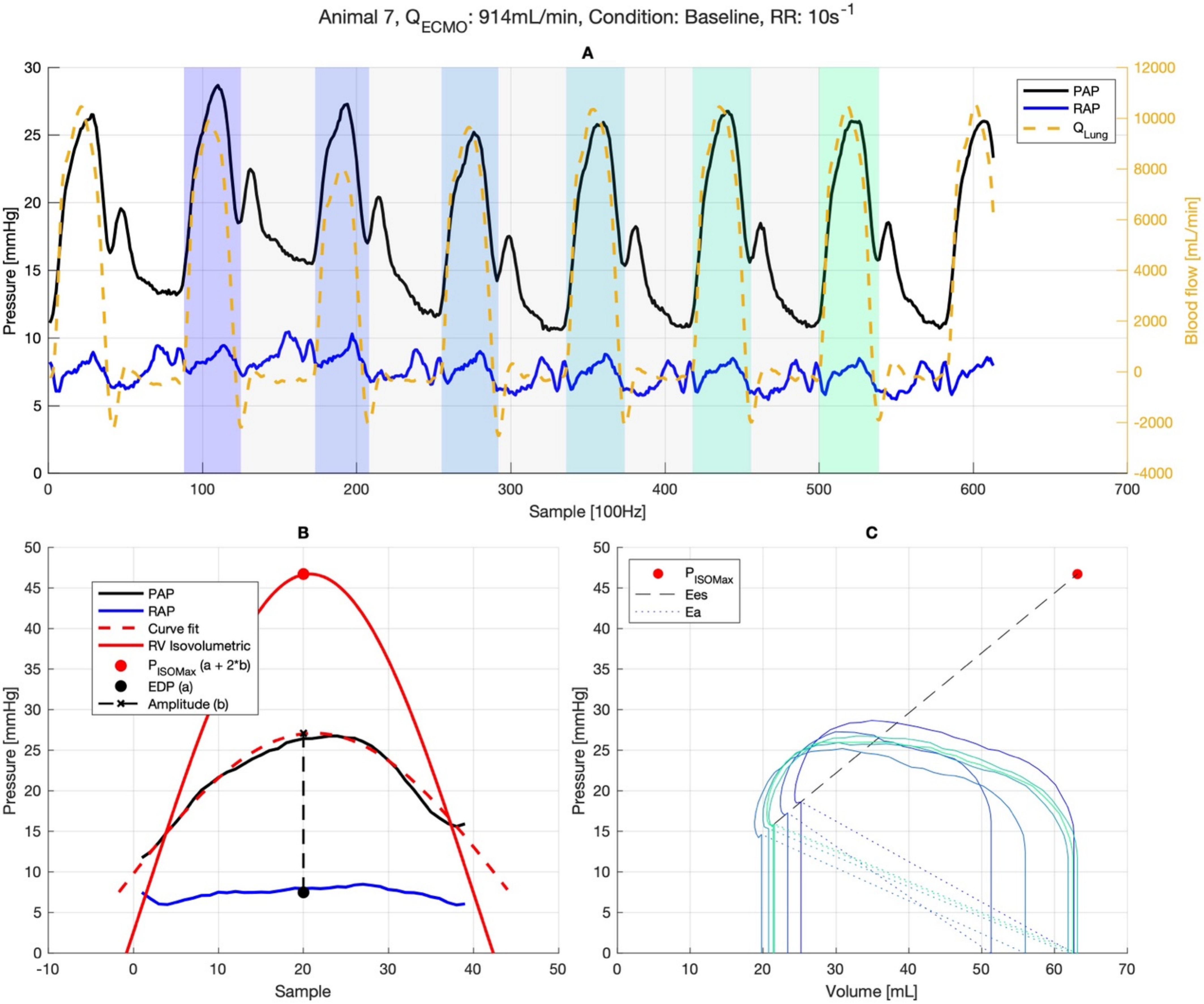
Animal 7, Q_{ECMO}: 914mL/min, Condition: Baseline, RR: $10s^{-1}$
A
Pressure [mmHg]
Blood flow [mL/min]
Sample [100Hz]
PAP
RAP
Q_{Lung}
B
PAP
RAP
Curve fit
RV Isovolumetric
P_{ISOMax} (a + 2*b)
EDP (a)
Amplitude (b)
Pressure [mmHg]
Sample
C
P_{ISOMax}
Ees
Ea
Pressure [mmHg]
Volume [mL]

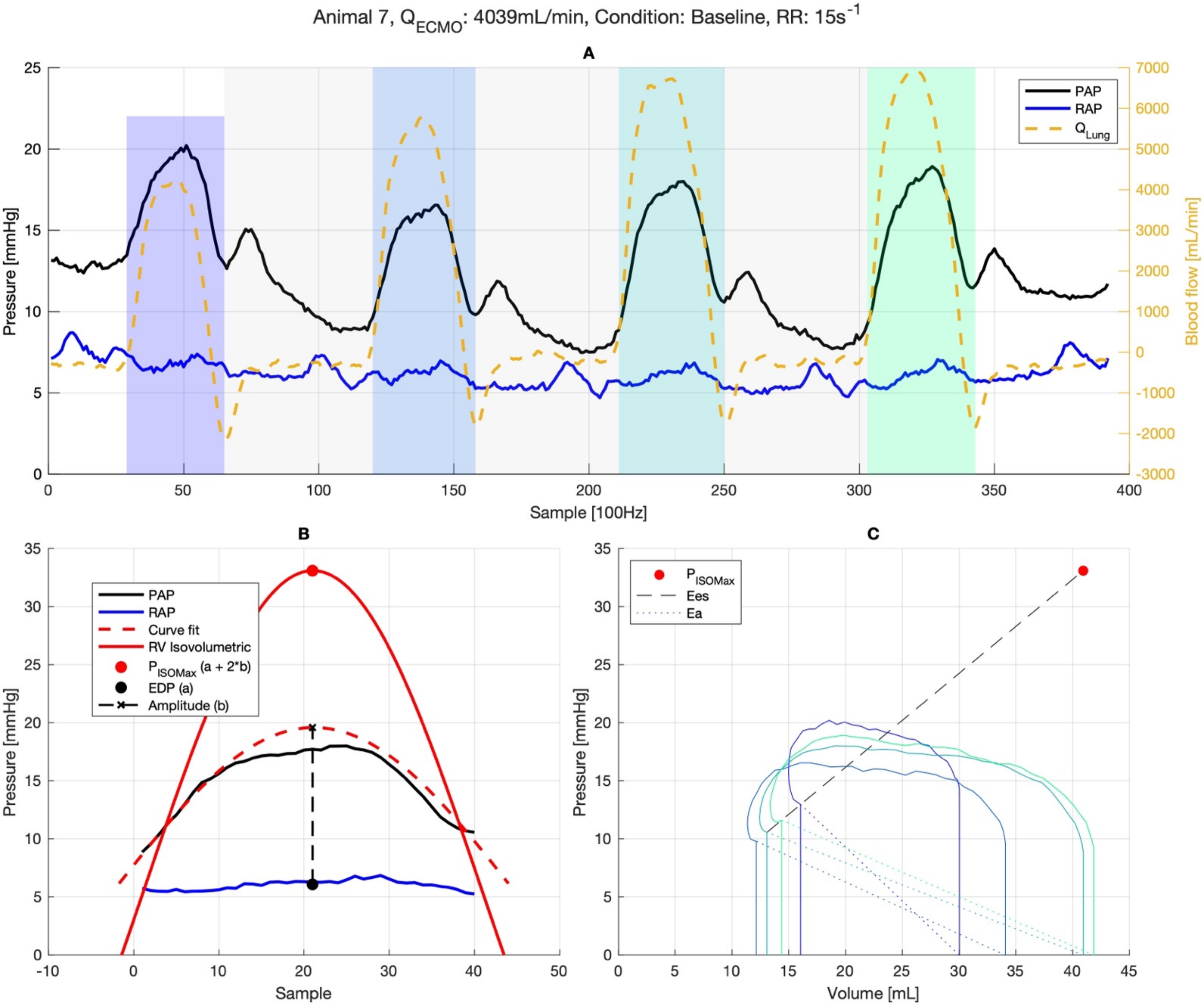

Animal 7, Q_ECMO: 4039mL/min, Condition: Baseline, RR: 15s^-1
A
PAP
RAP
Q_Lung
Pressure [mmHg]
Blood flow [mL/min]
Sample [100Hz]
B
PAP
RAP
Curve fit
RV Isovolumetric
P_ISOMax (a + 2*b)
EDP (a)
Amplitude (b)
Pressure [mmHg]
Sample
C
P_ISOMax
Ees
Ea
Pressure [mmHg]
Volume [mL]

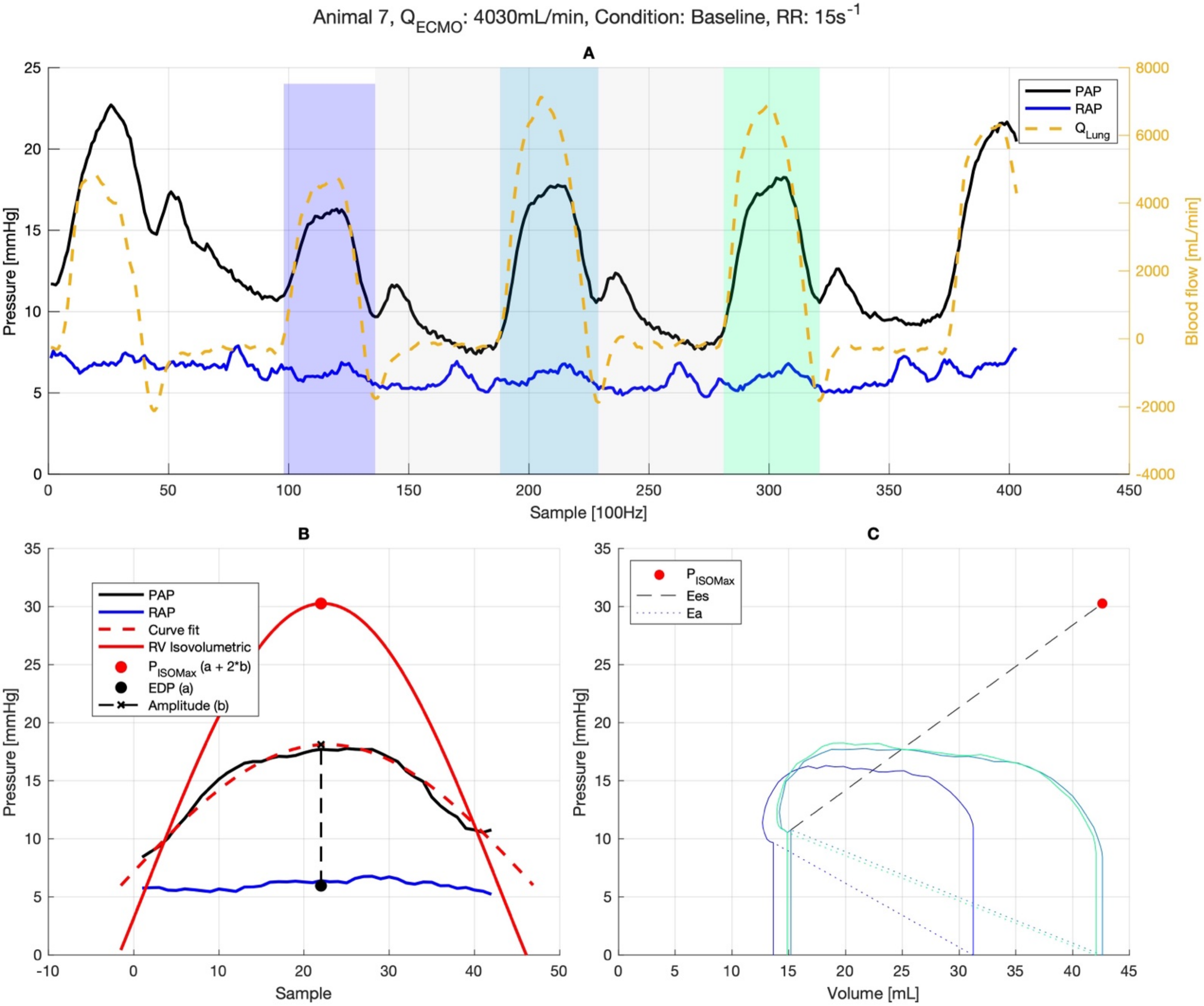

Animal 7, Q_{ECMO}: 4030mL/min, Condition: Baseline, RR: $15s^{-1}$
A
PAP
RAP
Q_{Lung}
Pressure [mmHg]
Blood flow [mL/min]
Sample [100Hz]
B
PAP
RAP
Curve fit
RV Isovolumetric
P_{ISOMax} (a + 2*b)
EDP (a)
Amplitude (b)
Pressure [mmHg]
Sample
C
P_{ISOMax}
Ees
Ea
Pressure [mmHg]
Volume [mL]

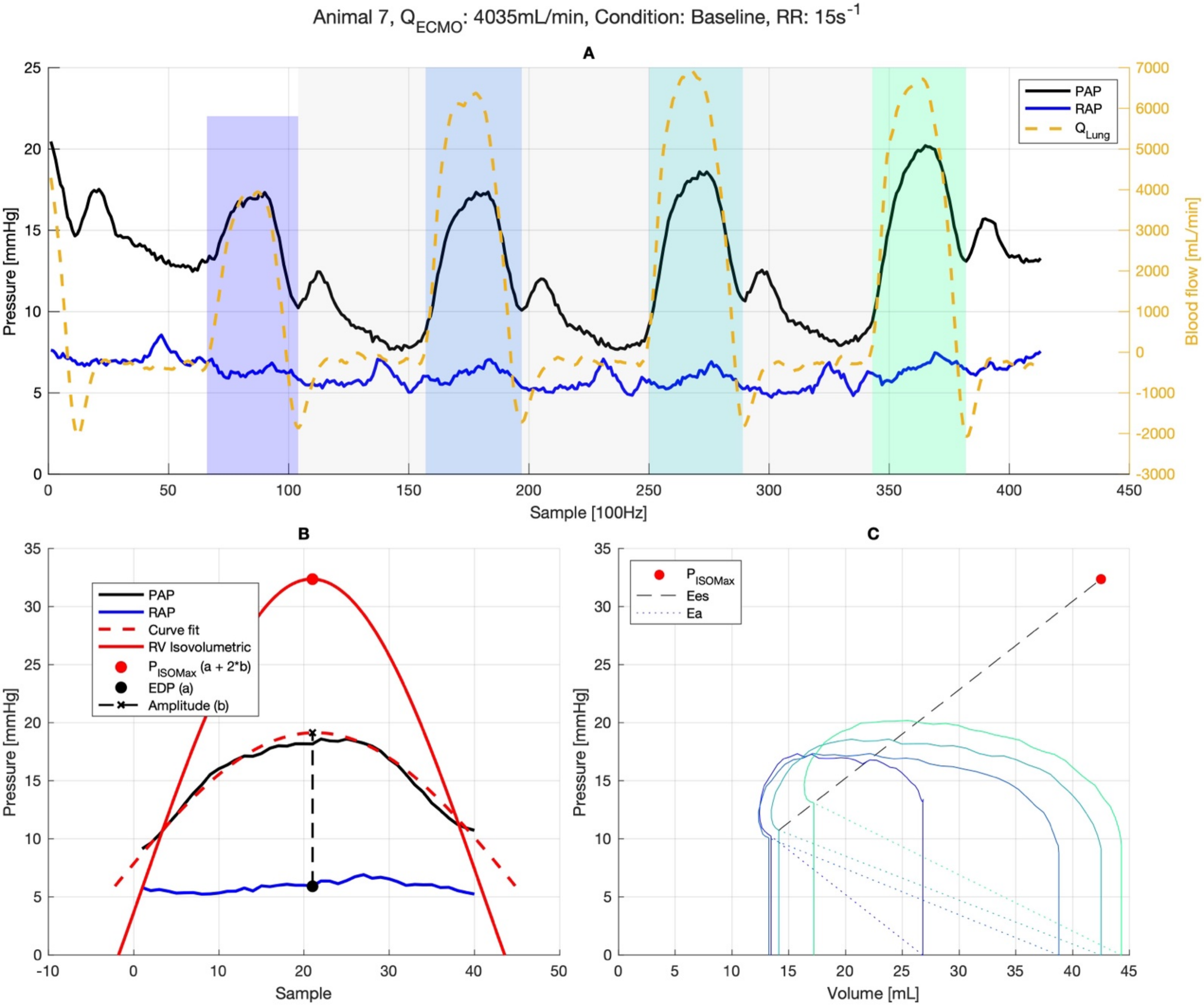

Animal 7, Q_ECMO: 4035mL/min, Condition: Baseline, RR: 15s^-1
A
Pressure [mmHg]
Blood flow [mL/min]
Sample [100Hz]
PAP
RAP
Q_Lung
B
Pressure [mmHg]
Sample
PAP
RAP
Curve fit
RV Isovolumetric
P_ISOMax (a + 2*b)
EDP (a)
Amplitude (b)
C
Pressure [mmHg]
Volume [mL]
P_ISOMax
Ees
Ea

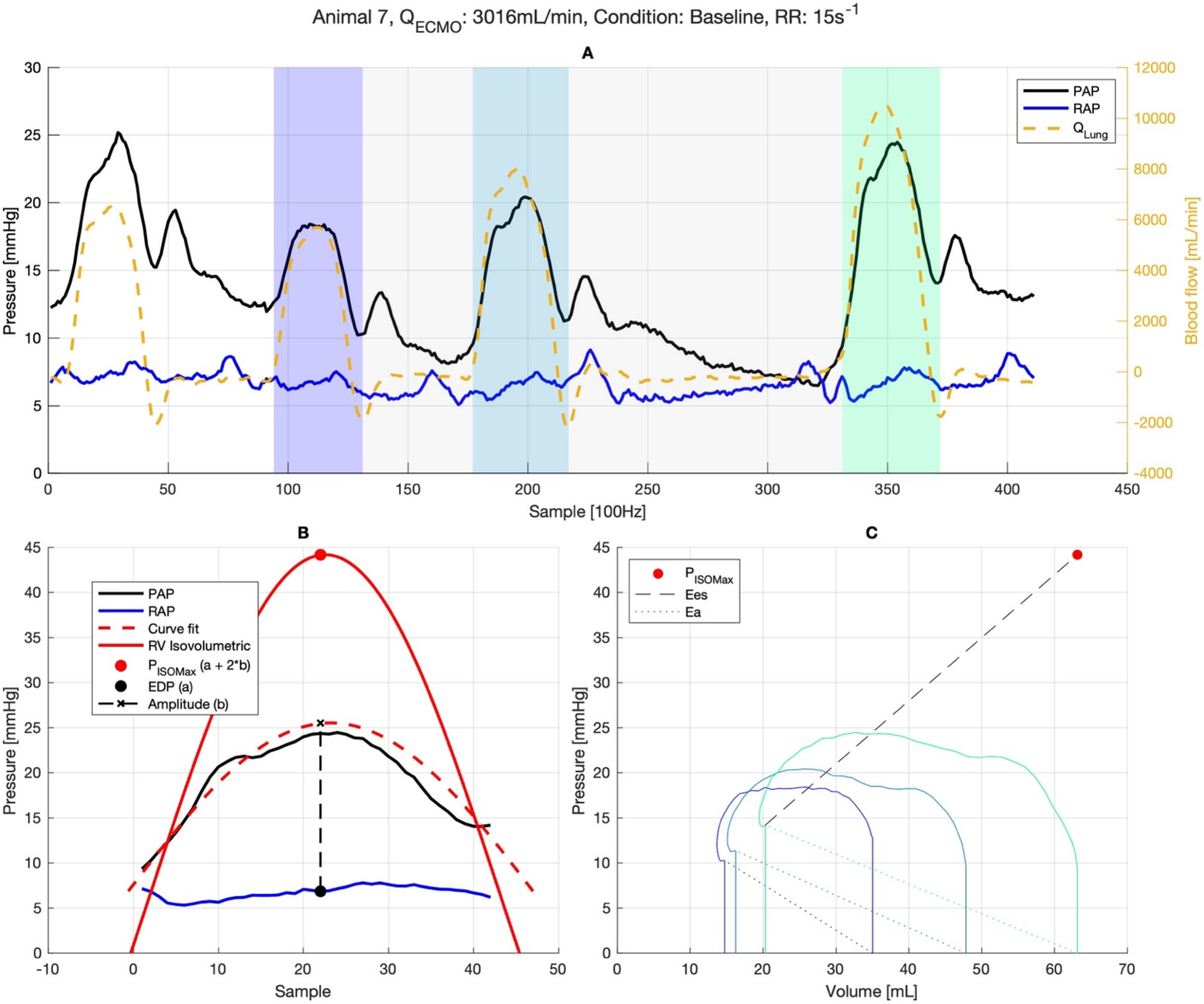

Animal 7, Q_{ECMO}: 3016mL/min, Condition: Baseline, RR: $15s^{-1}$
A
Pressure [mmHg]
Blood flow [mL/min]
Sample [100Hz]
PAP
RAP
Q_{Lung}
B
Pressure [mmHg]
Sample
PAP
RAP
Curve fit
RV Isovolumetric
P_{ISOMax} (a + 2*b)
EDP (a)
Amplitude (b)
C
Pressure [mmHg]
Volume [mL]
P_{ISOMax}
Ees
Ea

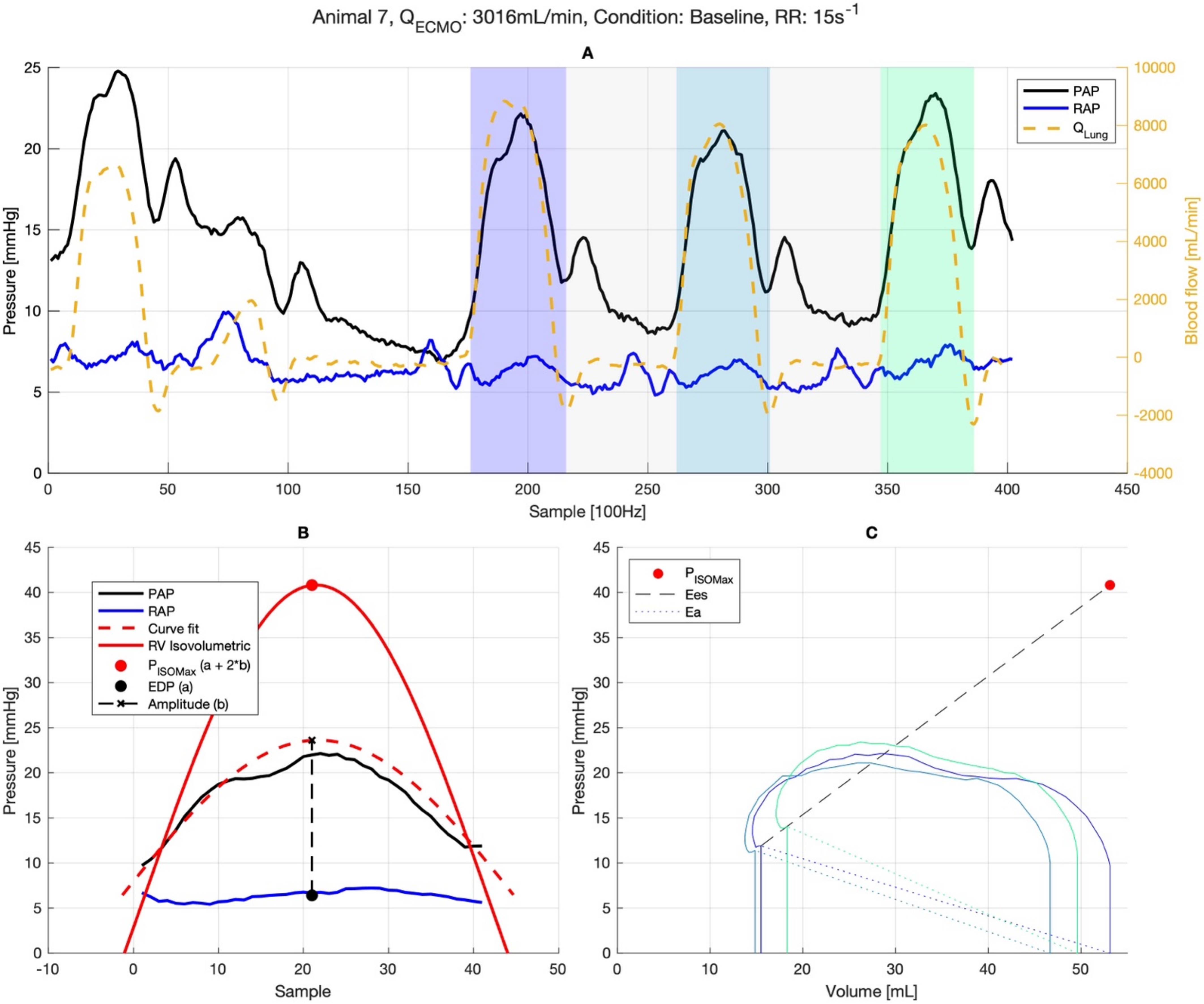
Animal 7, Q_ECMO: 3016mL/min, Condition: Baseline, RR: 15s^-1
A
PAP
RAP
Q_Lung
Pressure [mmHg]
Blood flow [mL/min]
Sample [100Hz]
B
PAP
RAP
Curve fit
RV Isovolumetric
P_ISOMax (a + 2*b)
EDP (a)
Amplitude (b)
Pressure [mmHg]
Sample
C
P_ISOMax
Ees
Ea
Pressure [mmHg]
Volume [mL]

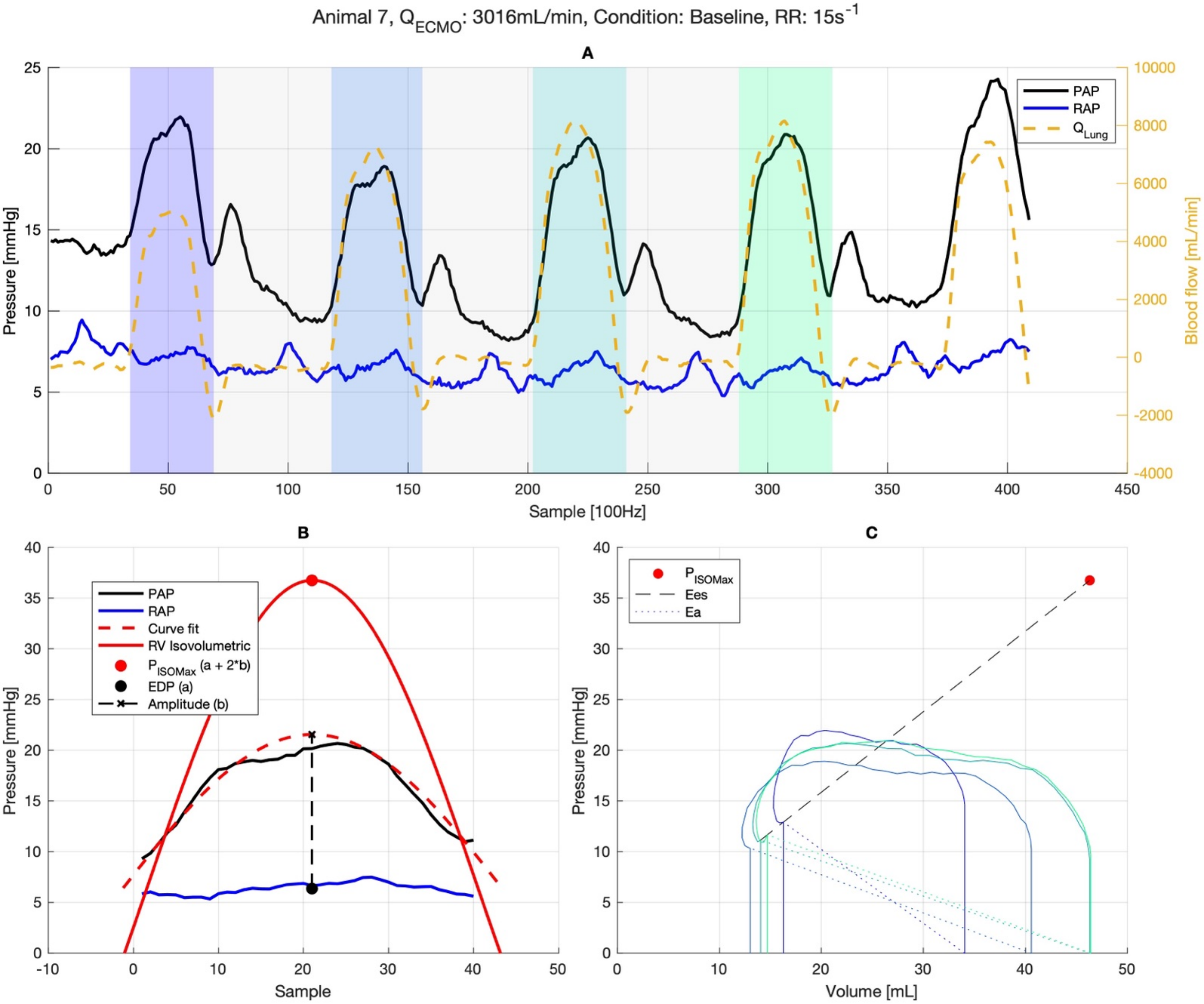

Animal 7, Q_ECMO: 3016mL/min, Condition: Baseline, RR: 15s^-1
A
PAP
RAP
Q_Lung
Pressure [mmHg]
Blood flow [mL/min]
Sample [100Hz]
B
PAP
RAP
Curve fit
RV Isovolumetric
P_ISOMax (a + 2*b)
EDP (a)
Amplitude (b)
Pressure [mmHg]
Sample
C
P_ISOMax
Ees
Ea
Pressure [mmHg]
Volume [mL]

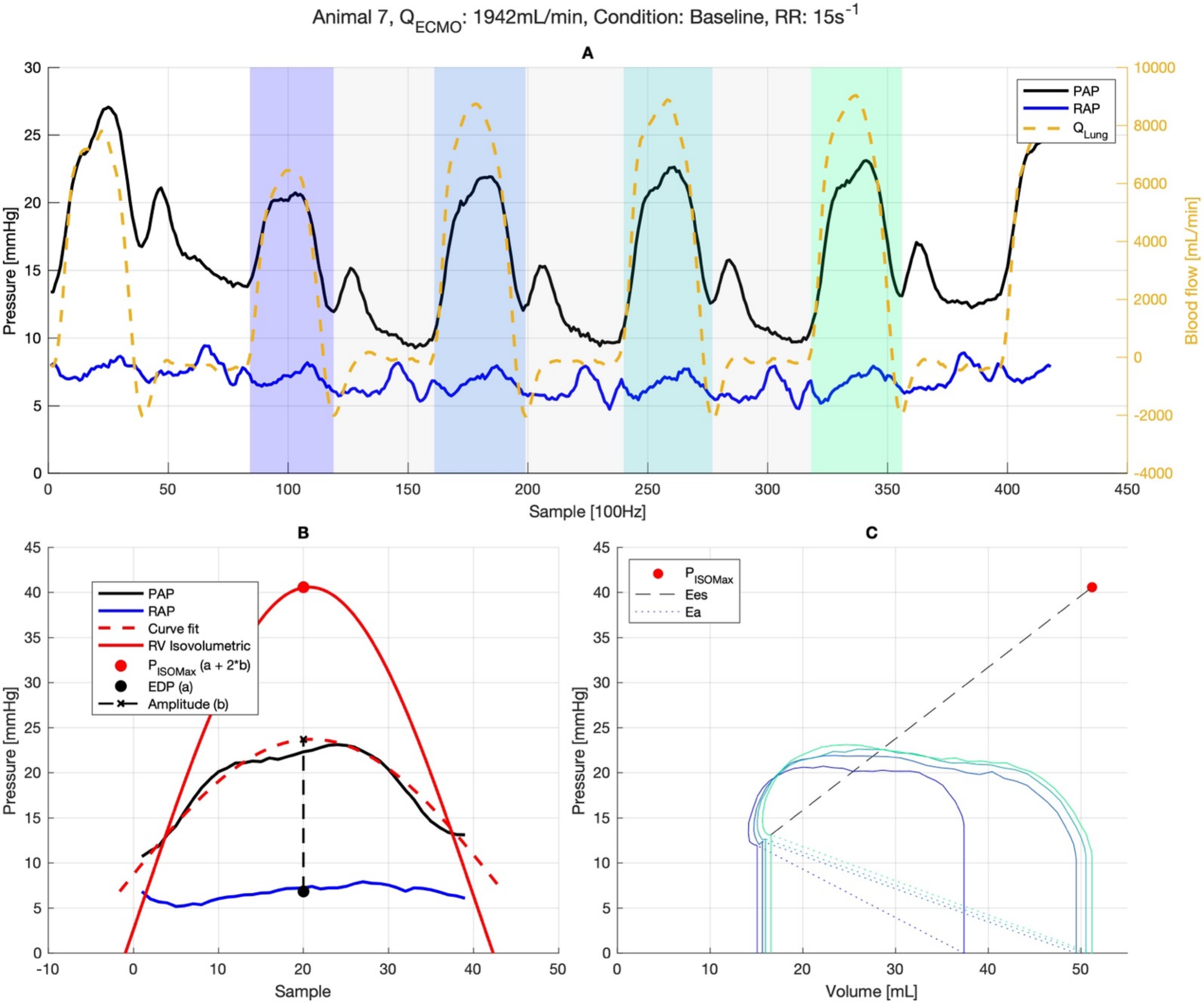
Animal 7, Q_ECMO: 1942mL/min, Condition: Baseline, RR: 15s^-1
A
PAP
RAP
Q_Lung
Pressure [mmHg]
Blood flow [mL/min]
Sample [100Hz]
B
PAP
RAP
Curve fit
RV Isovolumetric
P_ISOMax (a + 2*b)
EDP (a)
Amplitude (b)
Pressure [mmHg]
Sample
C
P_ISOMax
Ees
Ea
Pressure [mmHg]
Volume [mL]

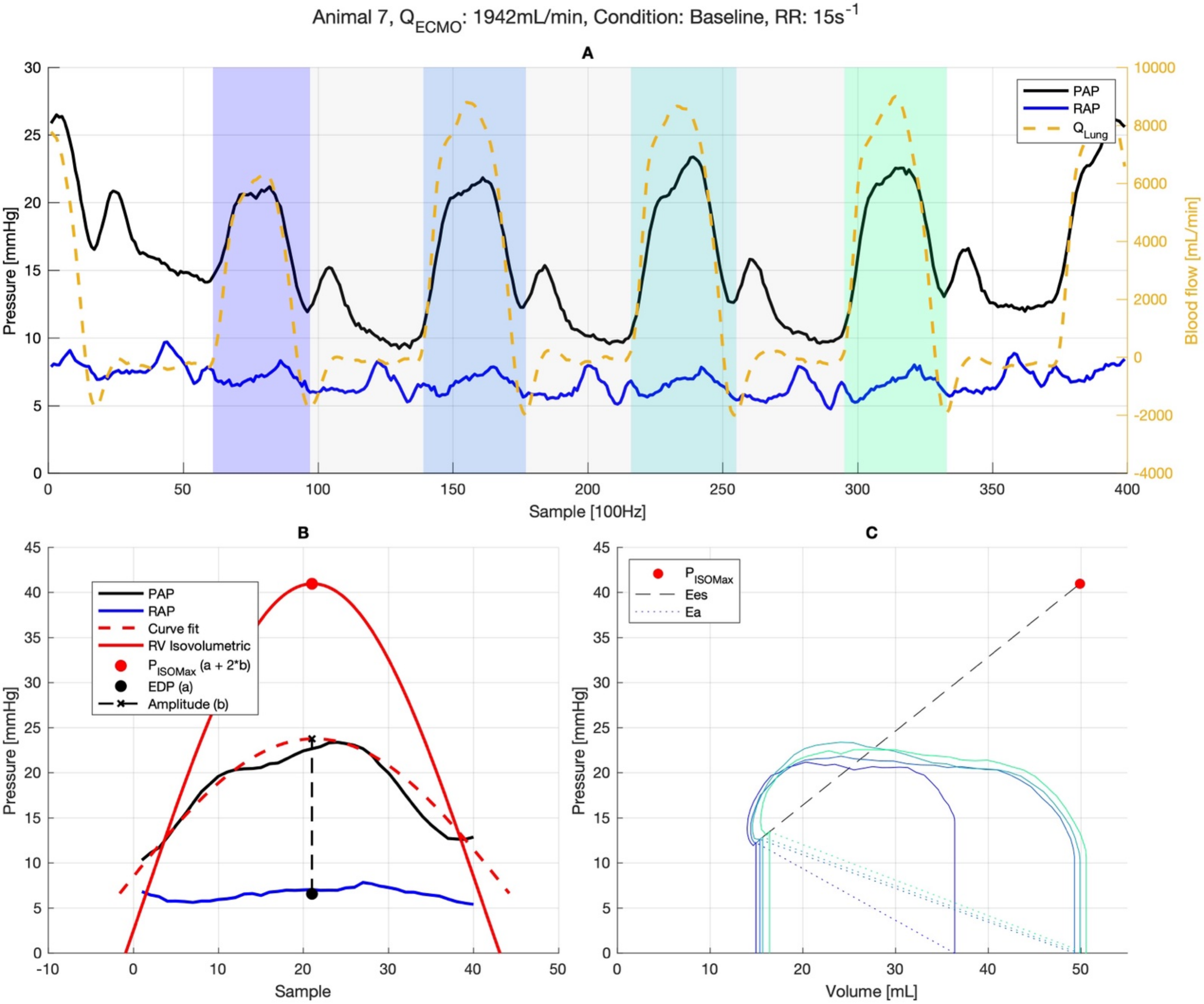

Animal 7, Q_ECMO: 1942mL/min, Condition: Baseline, RR: 15s^-1
A
PAP
RAP
Q_Lung
Pressure [mmHg]
Blood flow [mL/min]
Sample [100Hz]
B
PAP
RAP
Curve fit
RV Isovolumetric
P_ISOMax (a + 2*b)
EDP (a)
Amplitude (b)
Pressure [mmHg]
Sample
C
P_ISOMax
Ees
Ea
Pressure [mmHg]
Volume [mL]

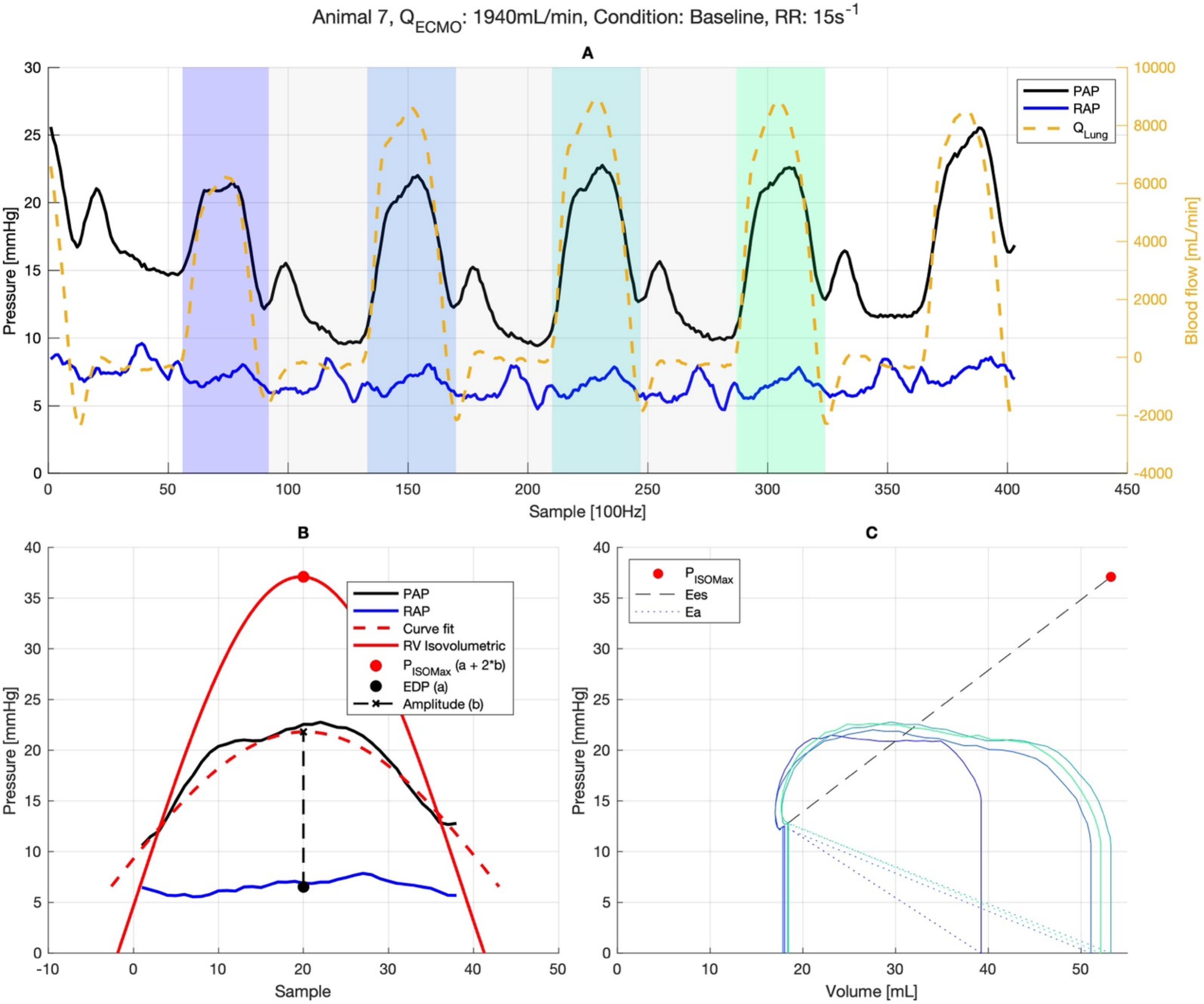
Animal 7, Q_{ECMO}: 1940mL/min, Condition: Baseline, RR: $15s^{-1}$
A
PAP
RAP
Q_{Lung}
Pressure [mmHg]
Blood flow [mL/min]
Sample [100Hz]
B
PAP
RAP
Curve fit
RV Isovolumetric
P_{ISOMax} (a + 2*b)
EDP (a)
Amplitude (b)
Pressure [mmHg]
Sample
C
P_{ISOMax}
Ees
Ea
Pressure [mmHg]
Volume [mL]

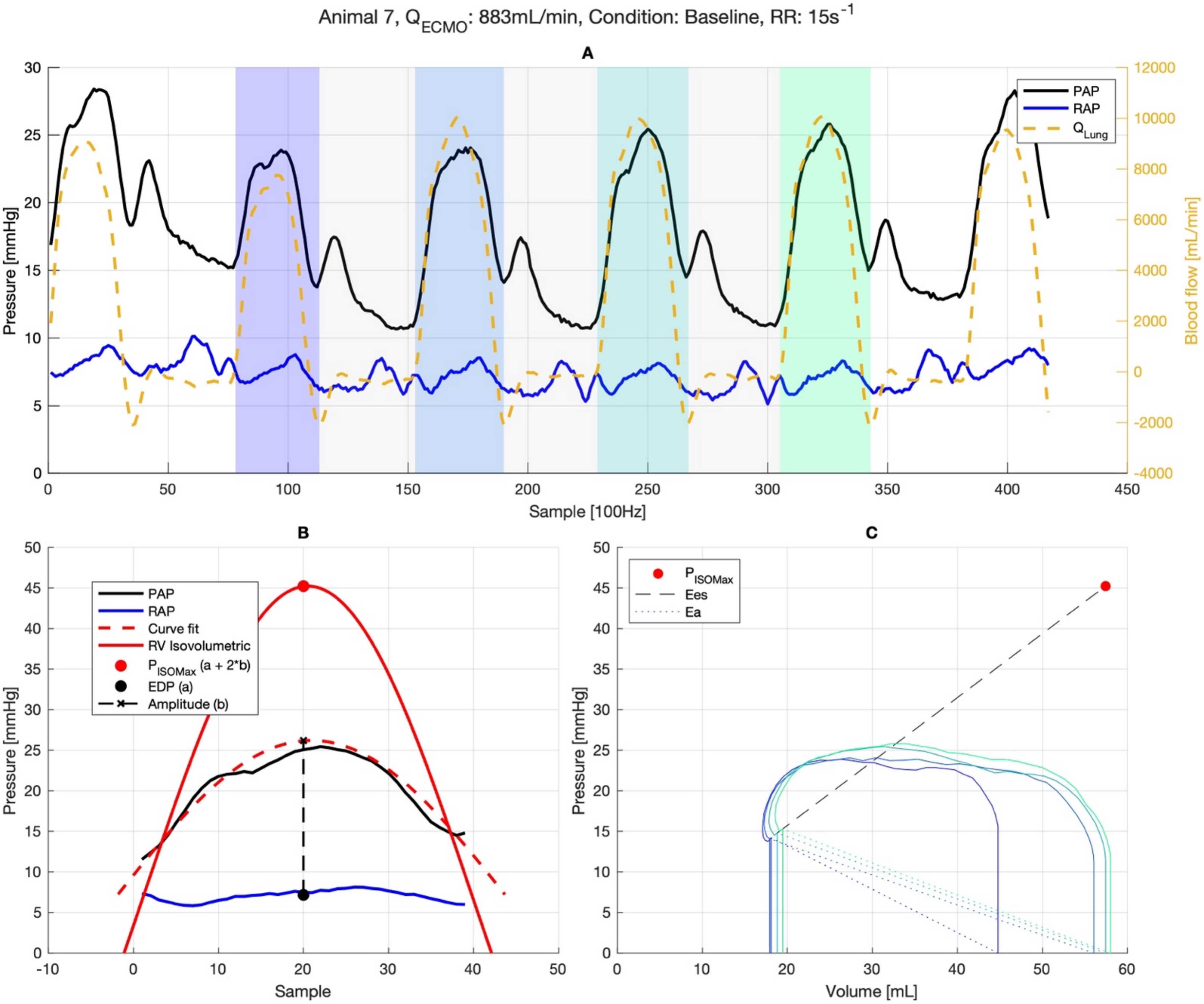

Animal 7, Q_{ECMO}: 883mL/min, Condition: Baseline, RR: $15s^{-1}$
A
Pressure [mmHg]
Blood flow [mL/min]
Sample [100Hz]
PAP
RAP
Q_{Lung}
B
Pressure [mmHg]
Sample
PAP
RAP
Curve fit
RV Isovolumetric
P_{ISOMax} (a + 2*b)
EDP (a)
Amplitude (b)
C
Pressure [mmHg]
Volume [mL]
P_{ISOMax}
Ees
Ea

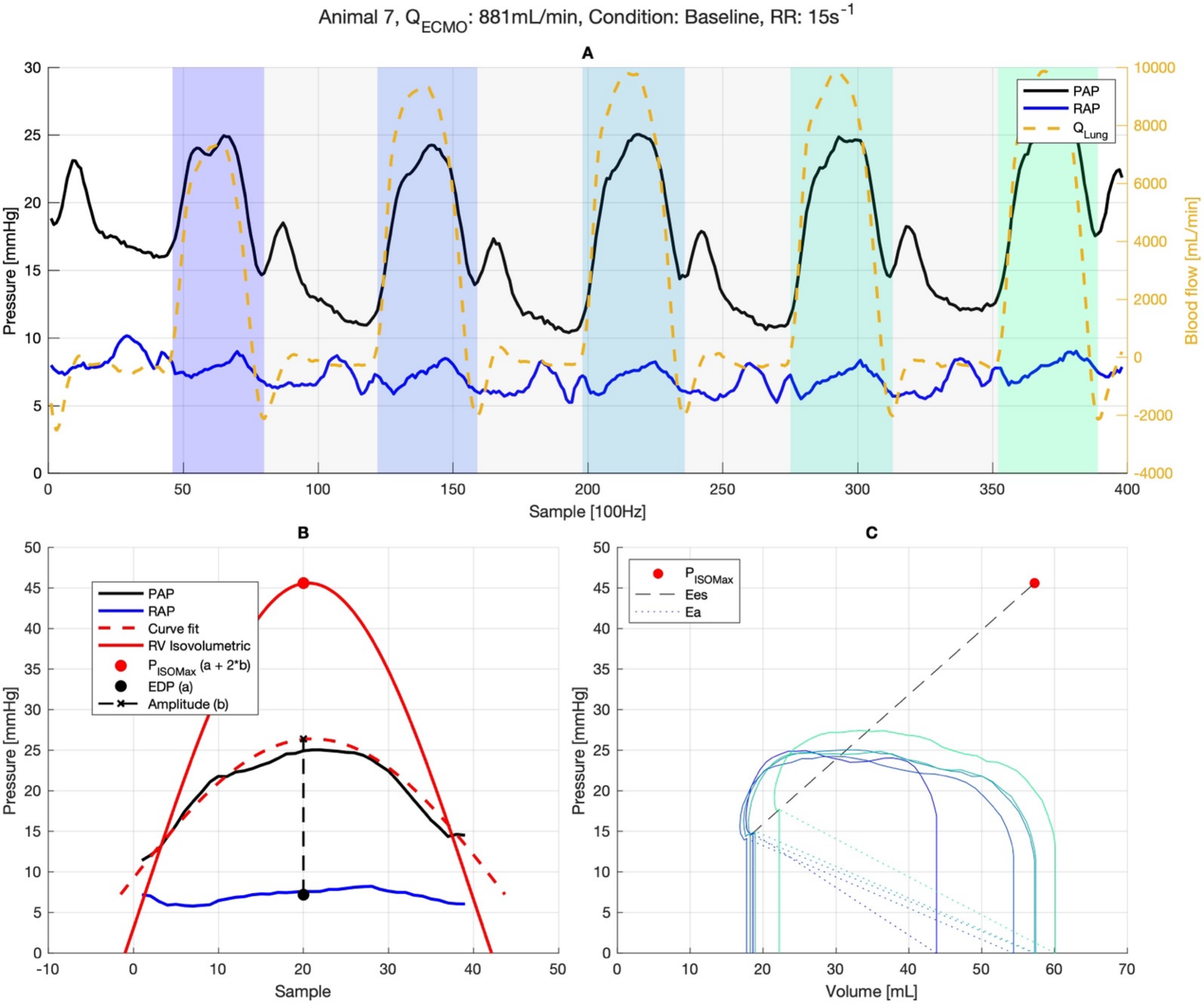
Animal 7, Q_{ECMO}: 881mL/min, Condition: Baseline, RR: $15s^{-1}$
A
PAP
RAP
Q_{Lung}
Pressure [mmHg]
Blood flow [mL/min]
Sample [100Hz]
B
PAP
RAP
Curve fit
RV Isovolumetric
P_{ISOMax} (a + 2*b)
EDP (a)
Amplitude (b)
Pressure [mmHg]
Sample
C
P_{ISOMax}
Ees
Ea
Pressure [mmHg]
Volume [mL]

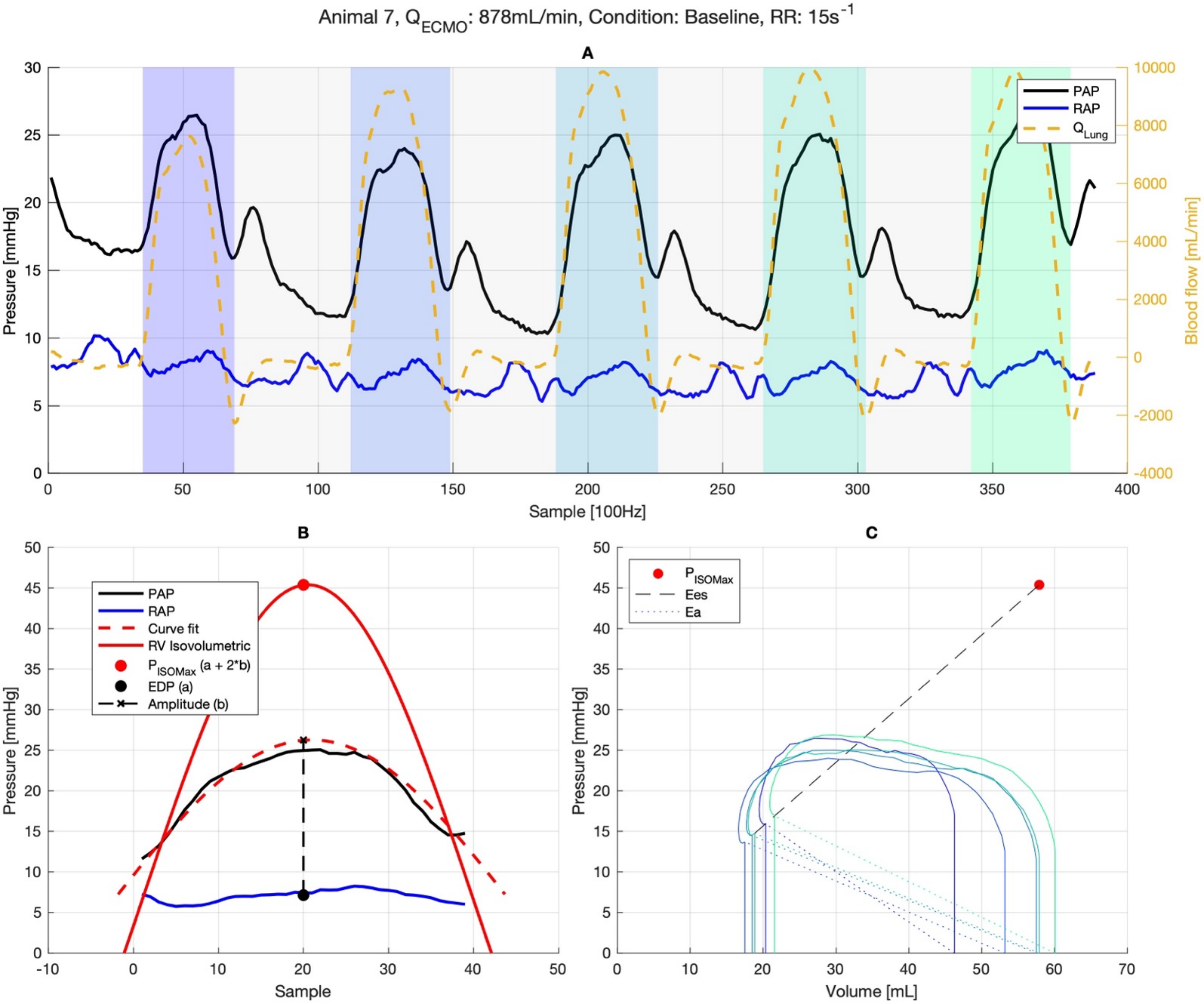

Animal 7, QECMO: 878mL/min, Condition: Baseline, RR: 15s-1
A
Pressure [mmHg]
Blood flow [mL/min]
Sample [100Hz]
PAP
RAP
QLung
B
Pressure [mmHg]
Sample
PAP
RAP
Curve fit
RV Isovolumetric
PISOMax (a + 2*b)
EDP (a)
Amplitude (b)
C
Pressure [mmHg]
Volume [mL]
PISOMax
Ees
Ea

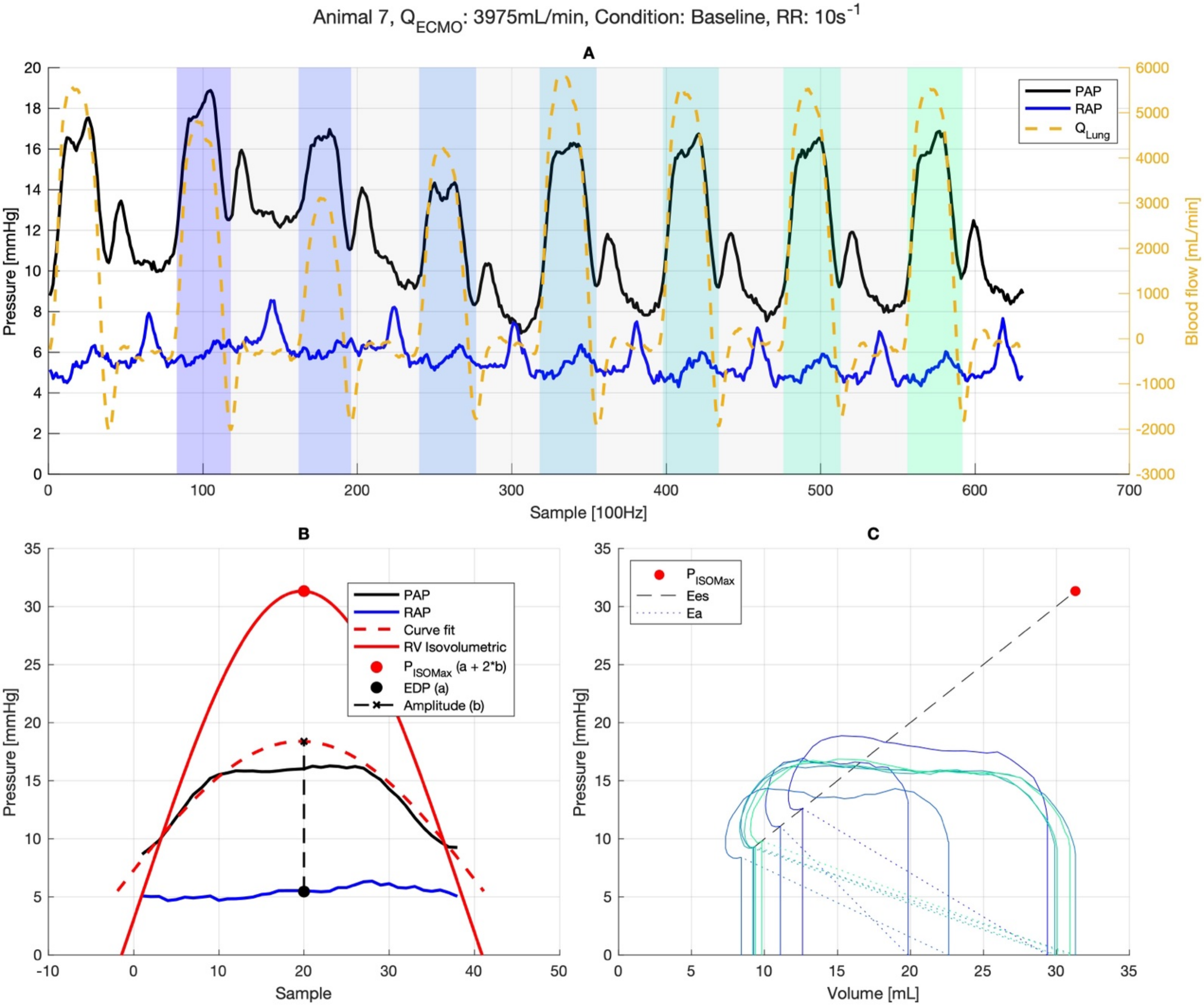

Animal 7, Q_{ECMO}: 3975mL/min, Condition: Baseline, RR: $10s^{-1}$
A
PAP
RAP
Q_{Lung}
Pressure [mmHg]
Blood flow [mL/min]
Sample [100Hz]
B
PAP
RAP
Curve fit
RV Isovolumetric
P_{ISOMax} (a + 2*b)
EDP (a)
Amplitude (b)
Pressure [mmHg]
Sample
C
P_{ISOMax}
Ees
Ea
Pressure [mmHg]
Volume [mL]

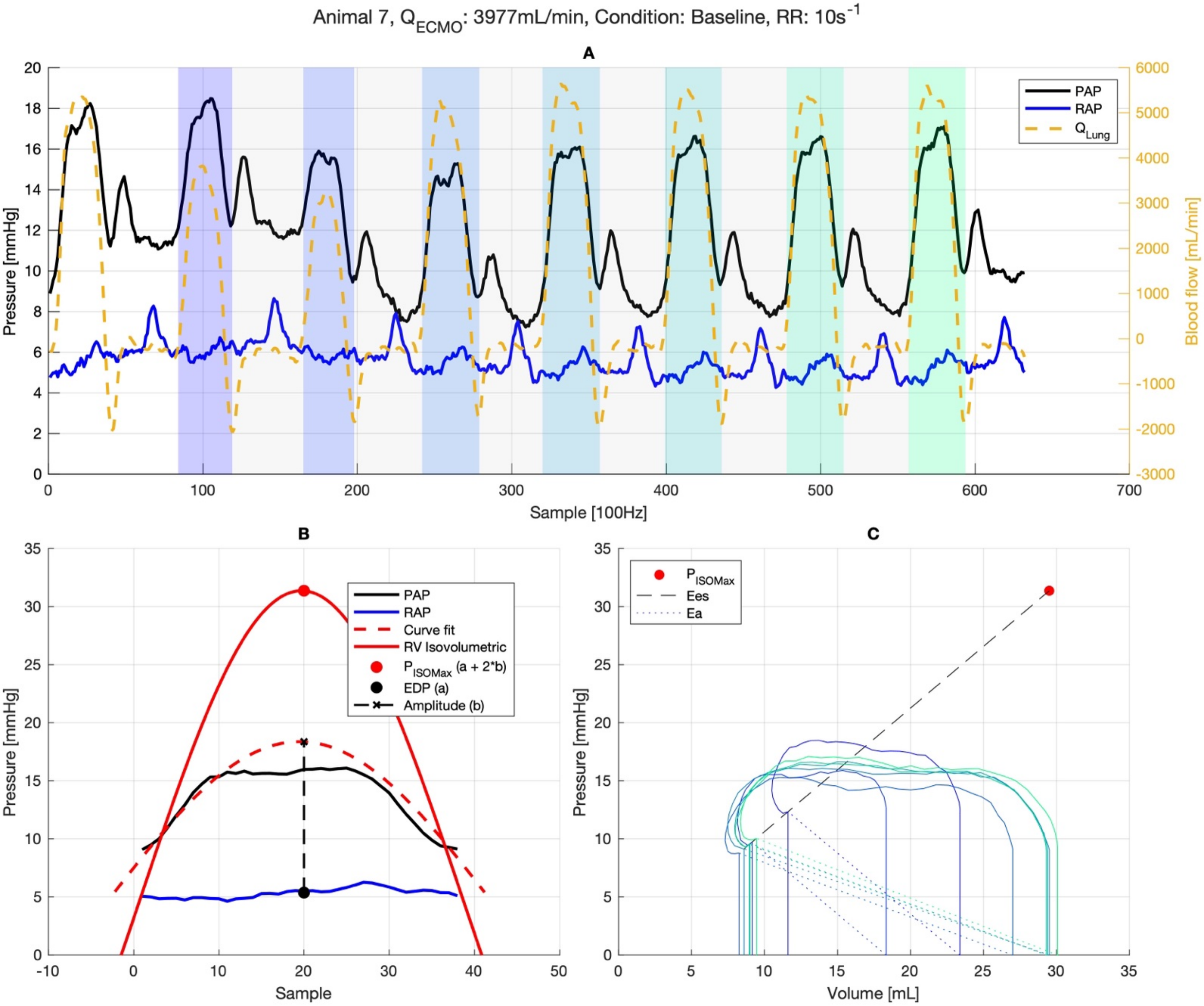

Animal 7, Q_{ECMO}: 3977mL/min, Condition: Baseline, RR: $10s^{-1}$
A
PAP
RAP
Q_{Lung}
Pressure [mmHg]
Blood flow [mL/min]
Sample [100Hz]
B
PAP
RAP
Curve fit
RV Isovolumetric
P_{ISOMax} (a + 2*b)
EDP (a)
Amplitude (b)
Pressure [mmHg]
Sample
C
P_{ISOMax}
Ees
Ea
Pressure [mmHg]
Volume [mL]

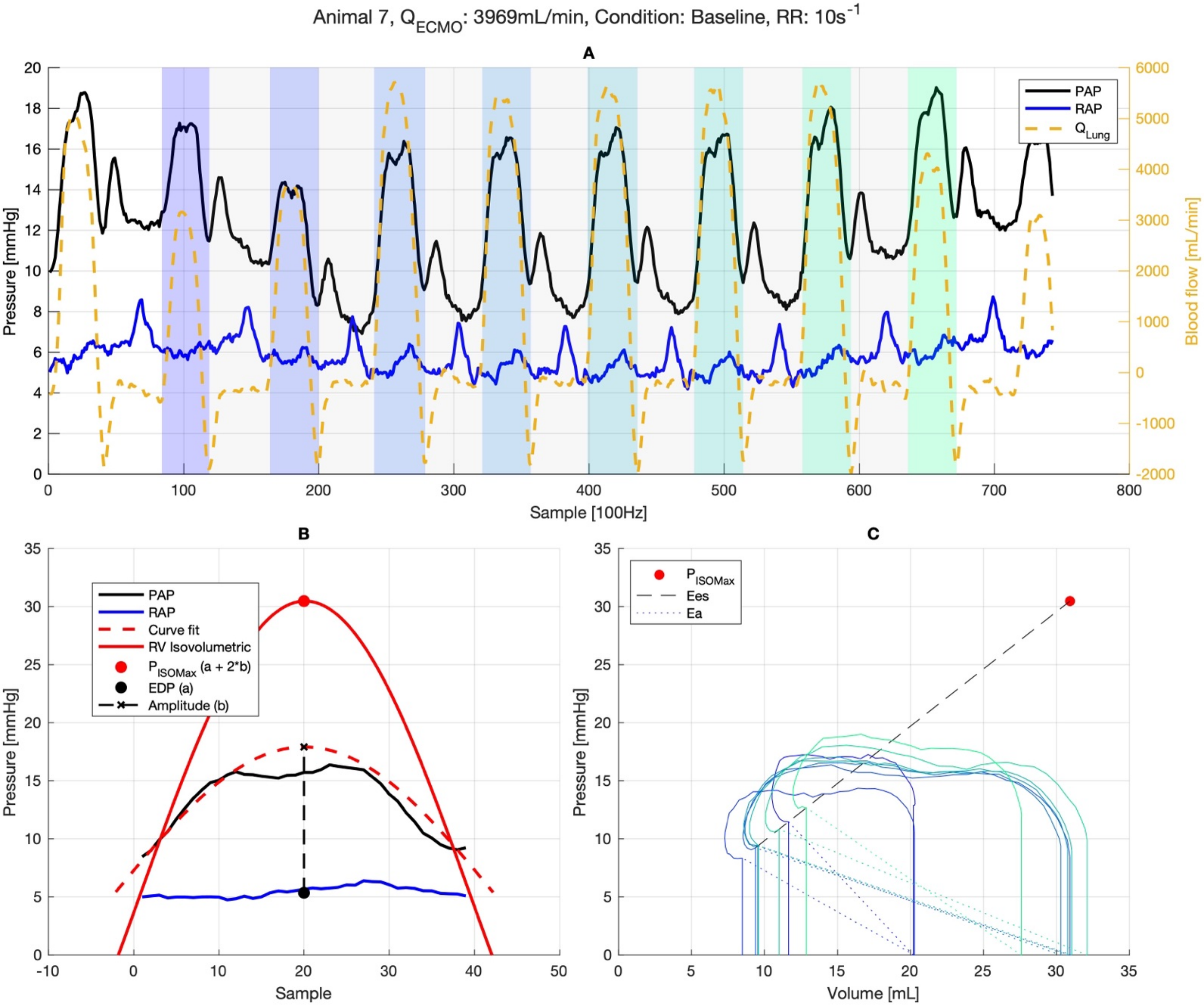
Animal 7, Q_{ECMO}: 3969mL/min, Condition: Baseline, RR: $10s^{-1}$
A
PAP
RAP
Q_{Lung}
Pressure [mmHg]
Blood flow [mL/min]
Sample [100Hz]
B
PAP
RAP
Curve fit
RV Isovolumetric
P_{ISOMax} (a + 2*b)
EDP (a)
Amplitude (b)
Pressure [mmHg]
Sample
C
P_{ISOMax}
Ees
Ea
Pressure [mmHg]
Volume [mL]

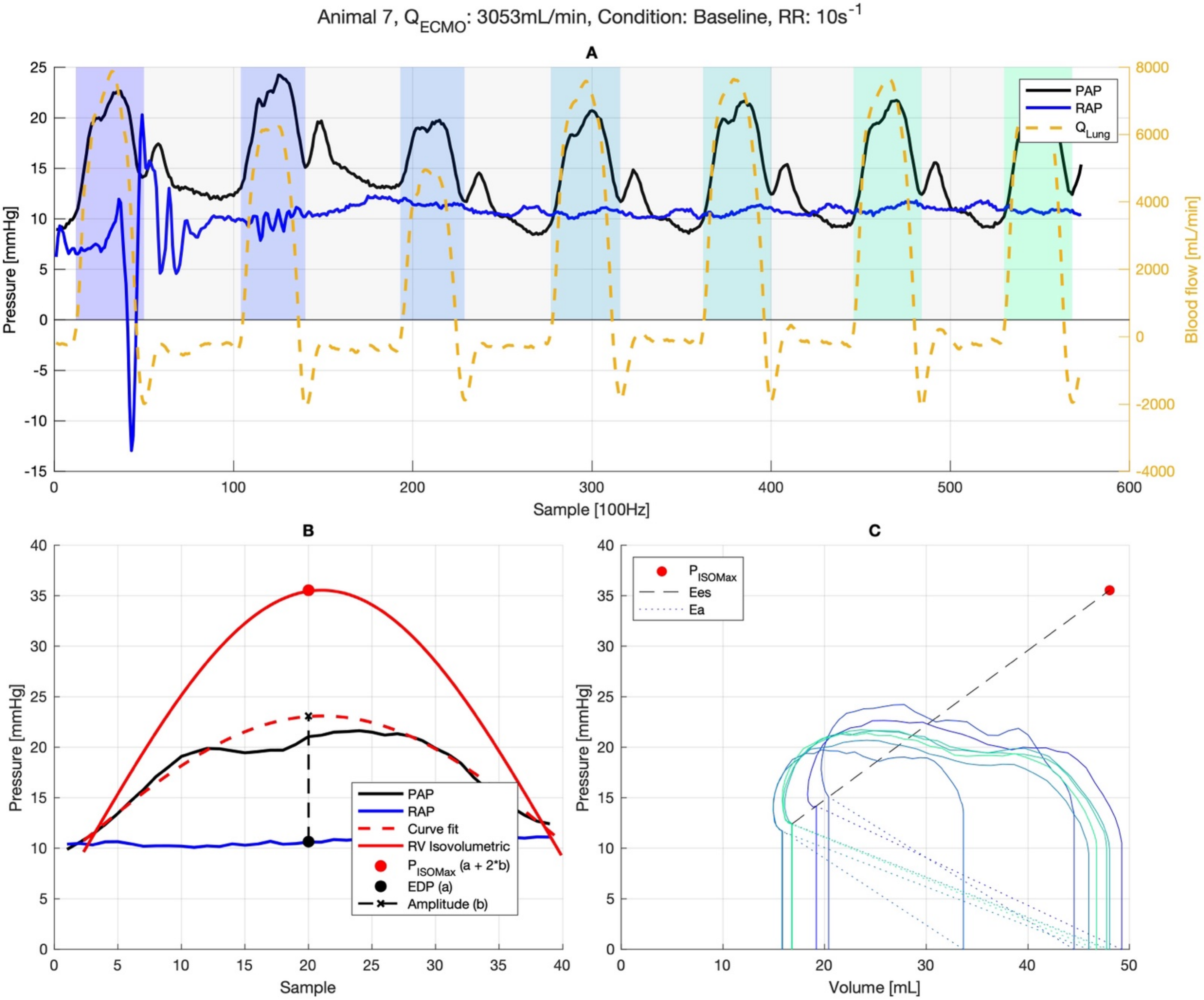

Animal 7, Q_{ECMO}: 3053mL/min, Condition: Baseline, RR: $10s^{-1}$
A
PAP
RAP
Q_{Lung}
Pressure [mmHg]
Blood flow [mL/min]
Sample [100Hz]
B
Pressure [mmHg]
Sample
PAP
RAP
Curve fit
RV Isovolumetric
P_{ISOMax} (a + 2*b)
EDP (a)
Amplitude (b)
C
P_{ISOMax}
Ees
Ea
Pressure [mmHg]
Volume [mL]

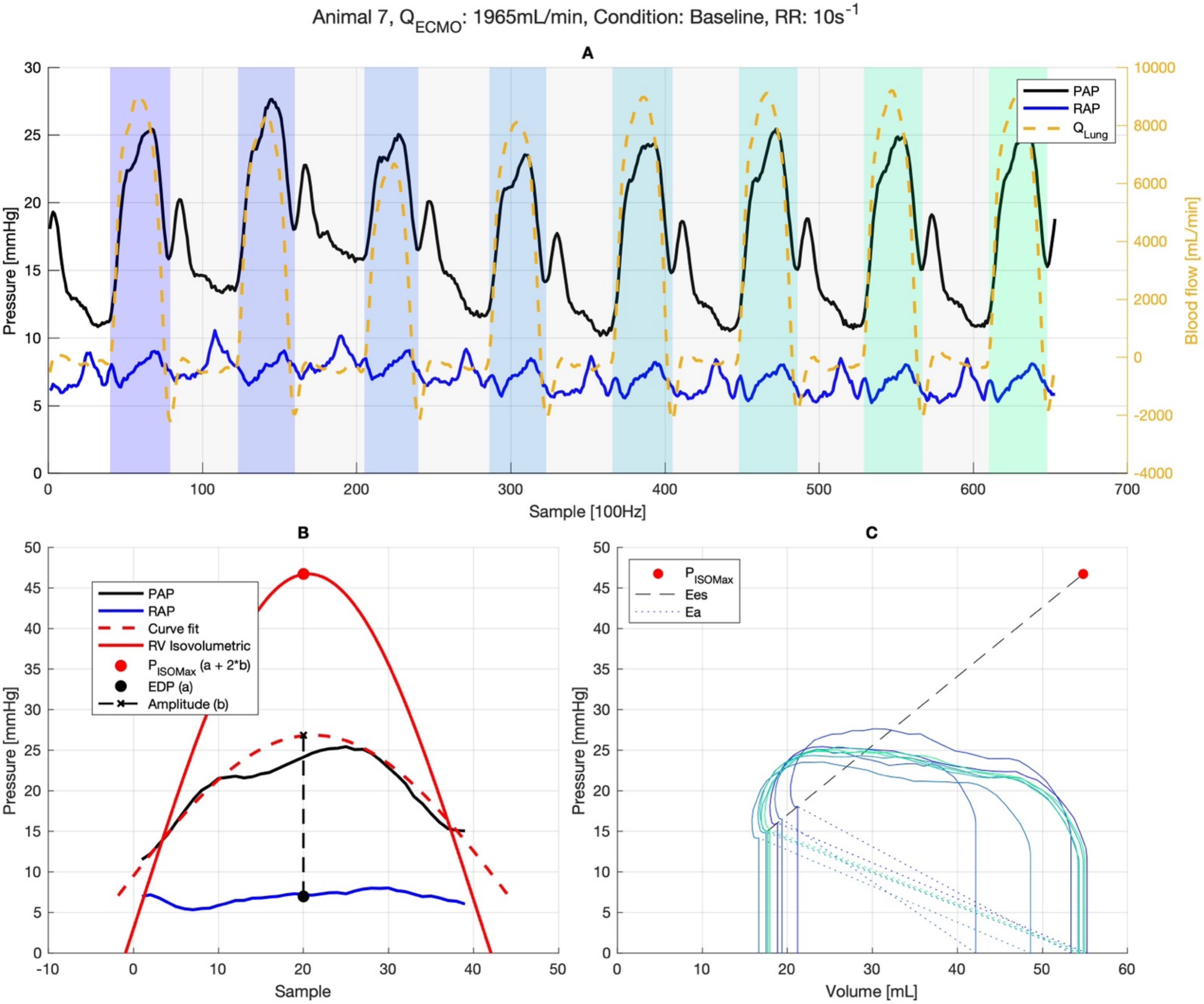

Animal 7, Q_{ECMO}: 1965mL/min, Condition: Baseline, RR: 10s^{-1}
A
PAP
RAP
Q_{Lung}
Pressure [mmHg]
Blood flow [mL/min]
Sample [100Hz]
B
PAP
RAP
Curve fit
RV Isovolumetric
P_{ISOMax} (a + 2*b)
EDP (a)
Amplitude (b)
Pressure [mmHg]
Sample
C
P_{ISOMax}
Ees
Ea
Pressure [mmHg]
Volume [mL]

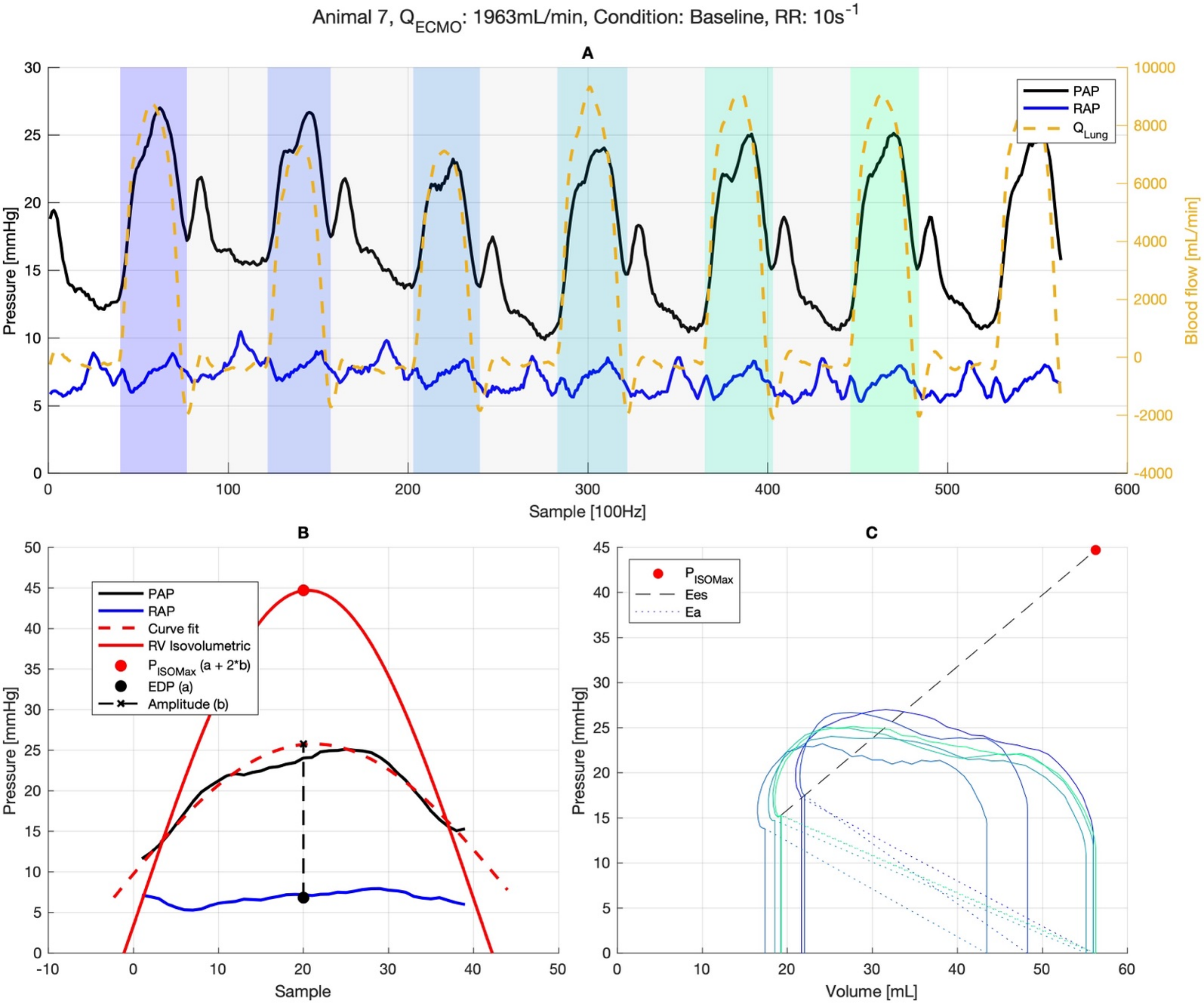
Animal 7, Q_ECMO: 1963mL/min, Condition: Baseline, RR: 10s^-1
A
PAP
RAP
Q_Lung
Pressure [mmHg]
Blood flow [mL/min]
Sample [100Hz]
B
PAP
RAP
Curve fit
RV Isovolumetric
P_ISOMax (a + 2*b)
EDP (a)
Amplitude (b)
Pressure [mmHg]
Sample
C
P_ISOMax
Ees
Ea
Pressure [mmHg]
Volume [mL]

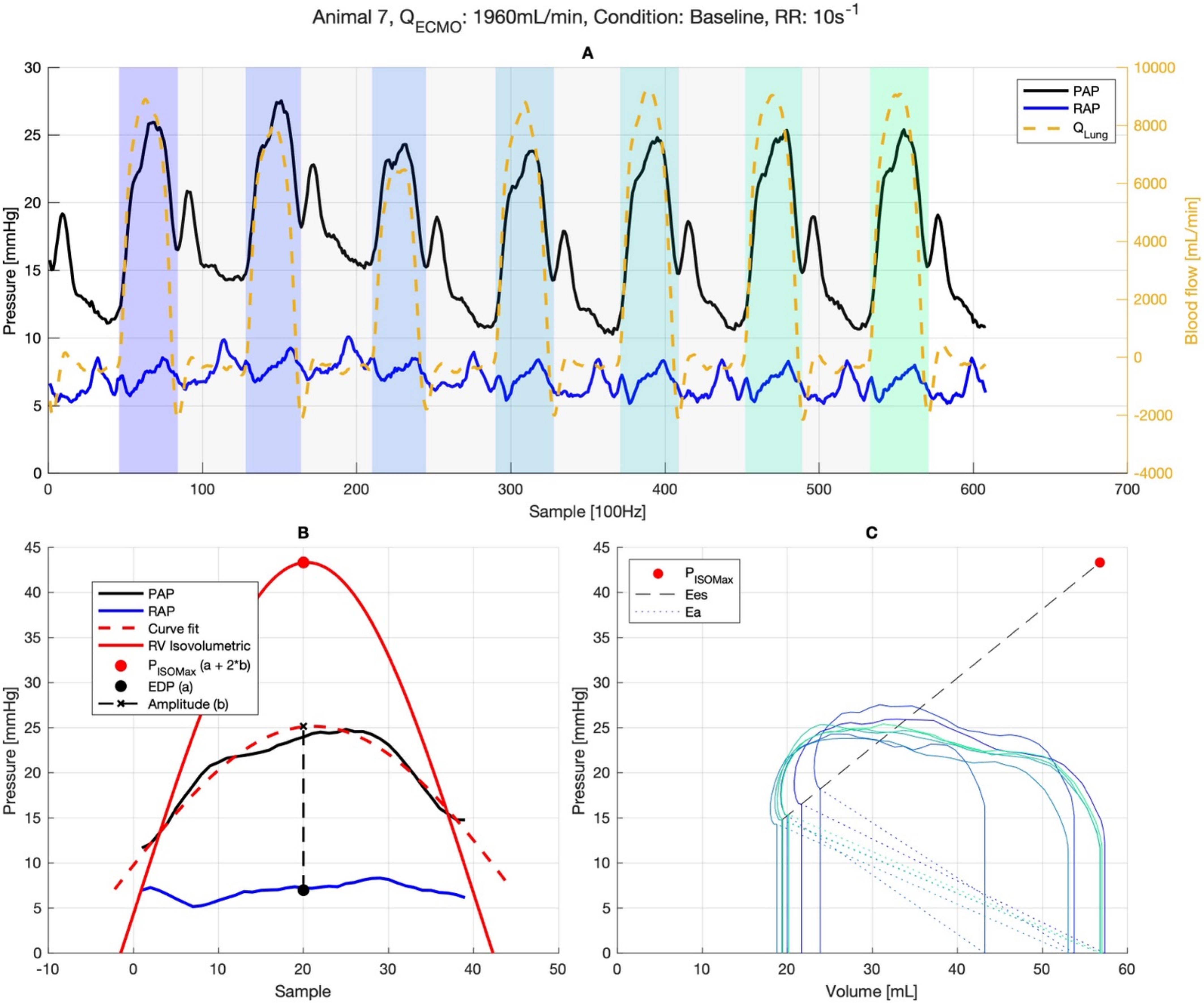

Animal 7, Q_{ECMO}: 1960mL/min, Condition: Baseline, RR: $10s^{-1}$
A
PAP
RAP
Q_{Lung}
Pressure [mmHg]
Blood flow [mL/min]
Sample [100Hz]
B
PAP
RAP
Curve fit
RV Isovolumetric
P_{ISOMax} (a + 2*b)
EDP (a)
Amplitude (b)
Pressure [mmHg]
Sample
C
P_{ISOMax}
Ees
Ea
Pressure [mmHg]
Volume [mL]

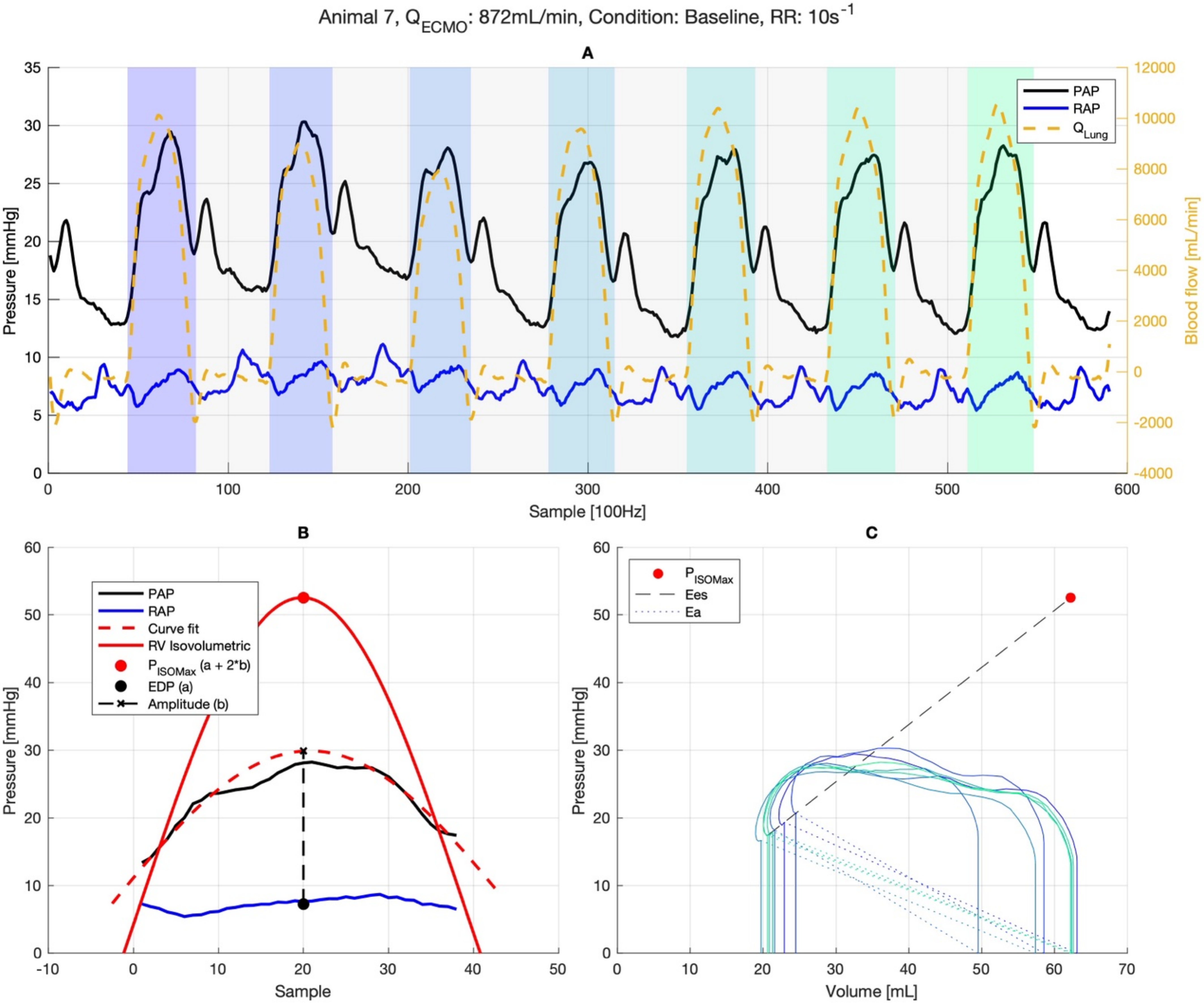

Animal 7, Q_{ECMO}: 872mL/min, Condition: Baseline, RR: $10s^{-1}$
A
PAP
RAP
Q_{Lung}
Pressure [mmHg]
Blood flow [mL/min]
Sample [100Hz]
B
PAP
RAP
Curve fit
RV Isovolumetric
P_{ISOMax} (a + 2*b)
EDP (a)
Amplitude (b)
Pressure [mmHg]
Sample
C
P_{ISOMax}
Ees
Ea
Pressure [mmHg]
Volume [mL]

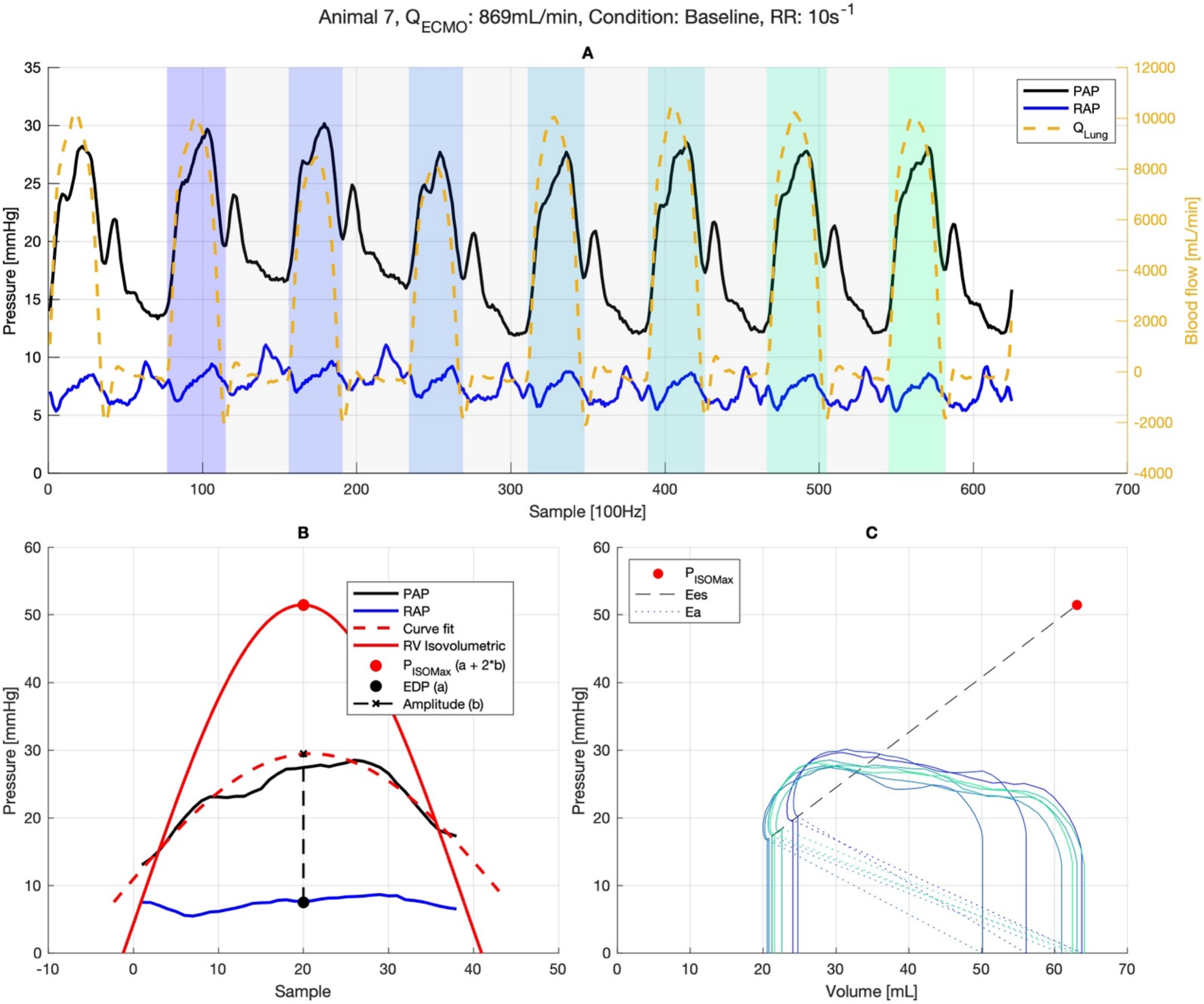
Animal 7, QECMO: 869mL/min, Condition: Baseline, RR: 10s-1
A
PAP
RAP
QLung
Pressure [mmHg]
Blood flow [mL/min]
Sample [100Hz]
B
PAP
RAP
Curve fit
RV Isovolumetric
PISOMax (a + 2*b)
EDP (a)
Amplitude (b)
Pressure [mmHg]
Sample
C
PISOMax
Ees
Ea
Pressure [mmHg]
Volume [mL]

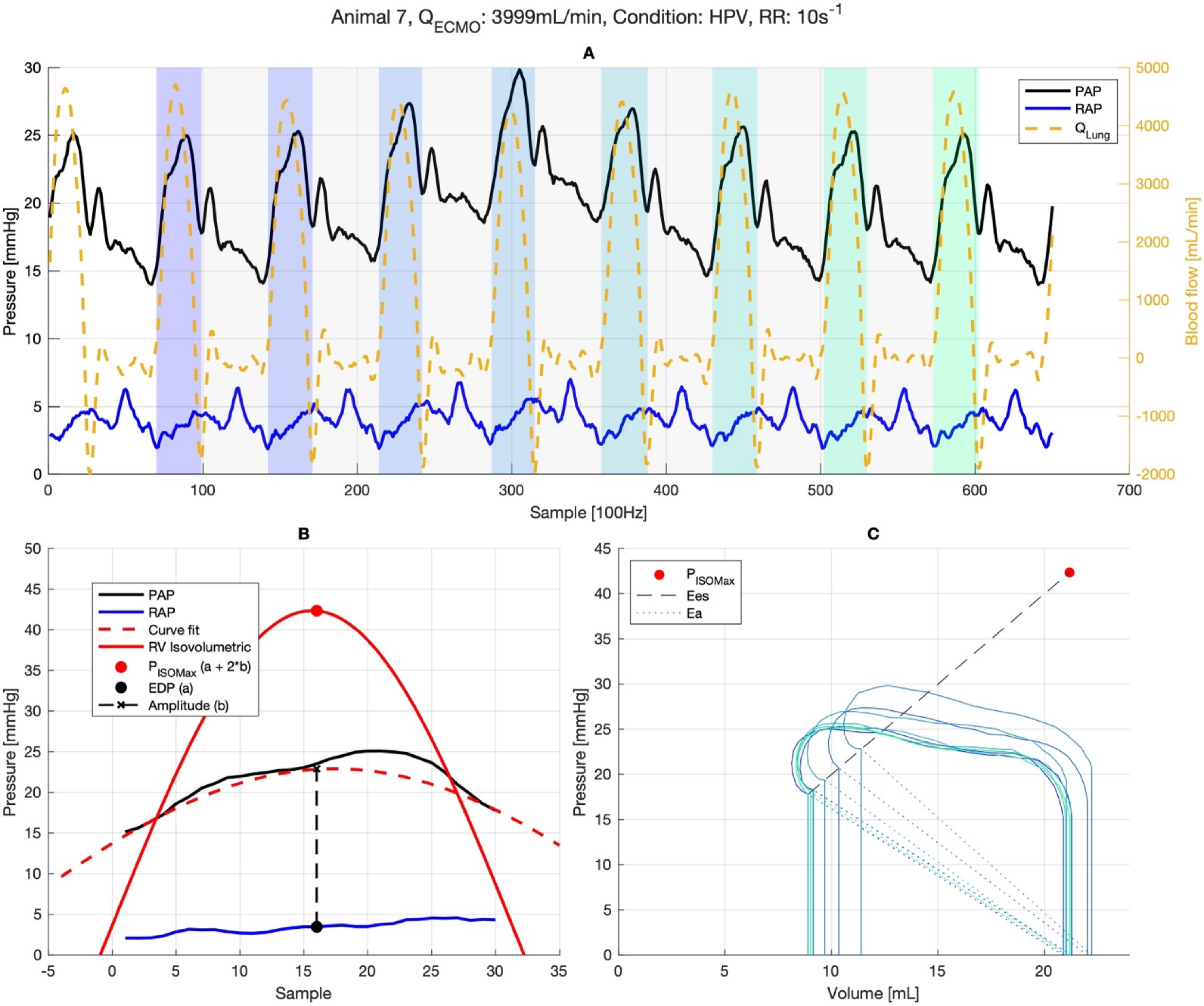

Animal 7, Q_{ECMO}: 3999mL/min, Condition: HPV, RR: $10s^{-1}$
A
PAP
RAP
Q_{Lung}
Pressure [mmHg]
Blood flow [mL/min]
Sample [100Hz]
B
PAP
RAP
Curve fit
RV Isovolumetric
P_{ISOMax} (a + 2*b)
EDP (a)
Amplitude (b)
Pressure [mmHg]
Sample
C
P_{ISOMax}
Ees
Ea
Pressure [mmHg]
Volume [mL]

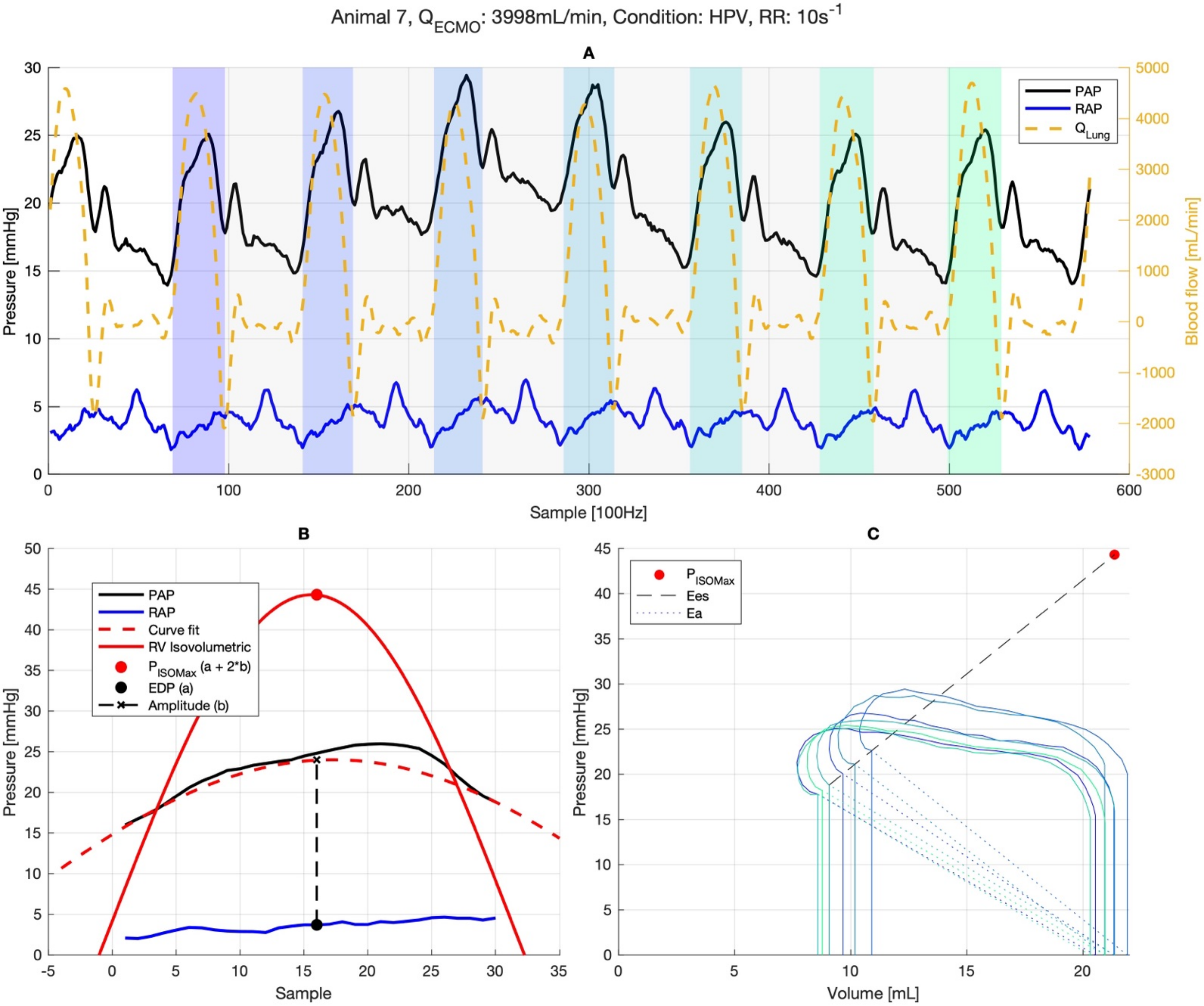

Animal 7, Q_{ECMO}: 3998mL/min, Condition: HPV, RR: 10s^{-1}
A
PAP
RAP
Q_{Lung}
Pressure [mmHg]
Blood flow [mL/min]
Sample [100Hz]
B
PAP
RAP
Curve fit
RV Isovolumetric
P_{ISOMax} (a + 2*b)
EDP (a)
Amplitude (b)
Pressure [mmHg]
Sample
C
P_{ISOMax}
Ees
Ea
Pressure [mmHg]
Volume [mL]

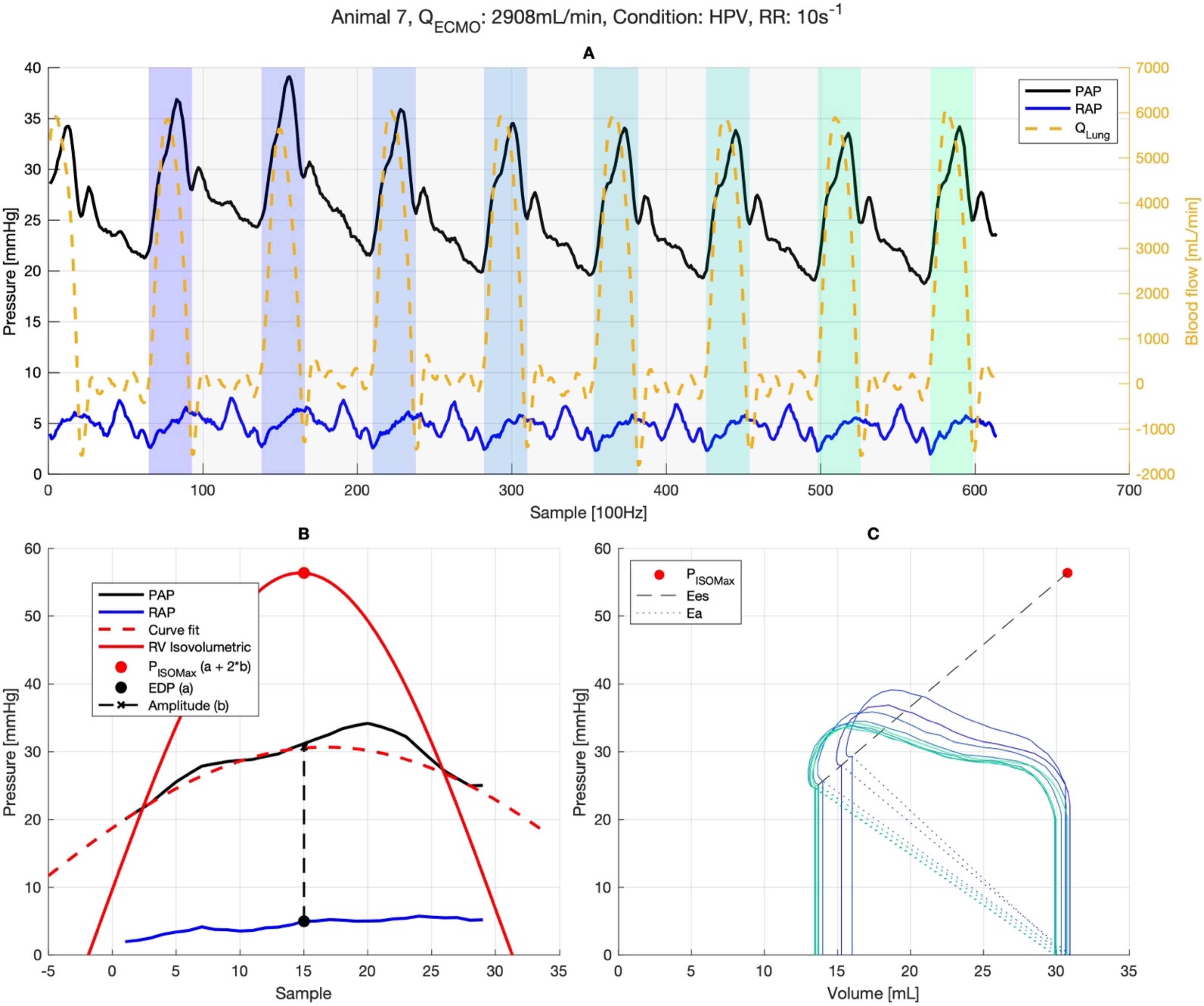

Animal 7, Q_{ECMO}: 2908mL/min, Condition: HPV, RR: $10s^{-1}$
A
PAP
RAP
Q_{Lung}
Pressure [mmHg]
Blood flow [mL/min]
Sample [100Hz]
B
PAP
RAP
Curve fit
RV Isovolumetric
P_{ISOMax} (a + 2*b)
EDP (a)
Amplitude (b)
Pressure [mmHg]
Sample
C
P_{ISOMax}
Ees
Ea
Pressure [mmHg]
Volume [mL]

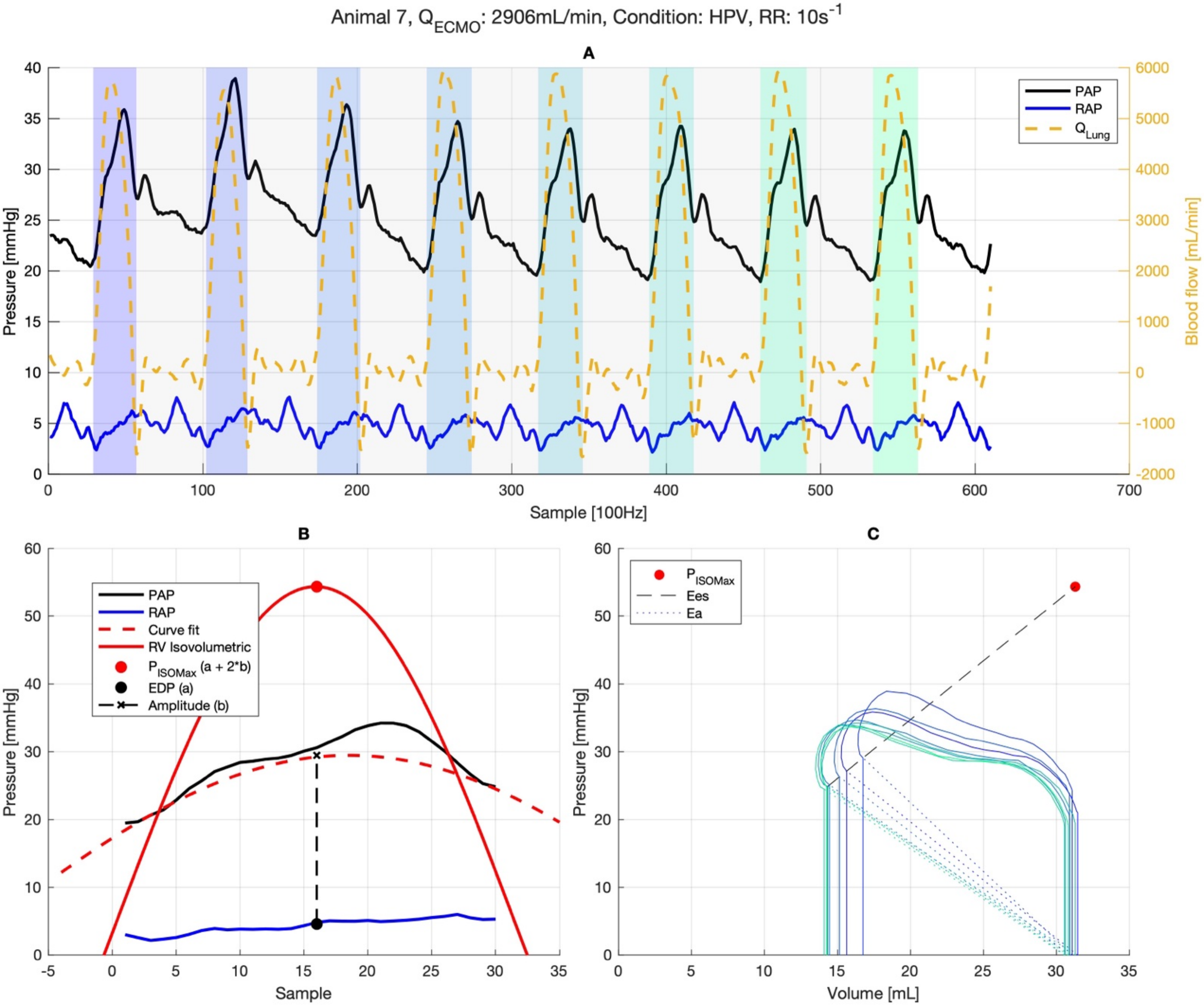

Animal 7, Q_ECMO: 2906mL/min, Condition: HPV, RR: 10s^-1
A
PAP
RAP
Q_Lung
Pressure [mmHg]
Blood flow [mL/min]
Sample [100Hz]
B
PAP
RAP
Curve fit
RV Isovolumetric
P_ISOMax (a + 2*b)
EDP (a)
Amplitude (b)
Pressure [mmHg]
Sample
C
P_ISOMax
Ees
Ea
Pressure [mmHg]
Volume [mL]

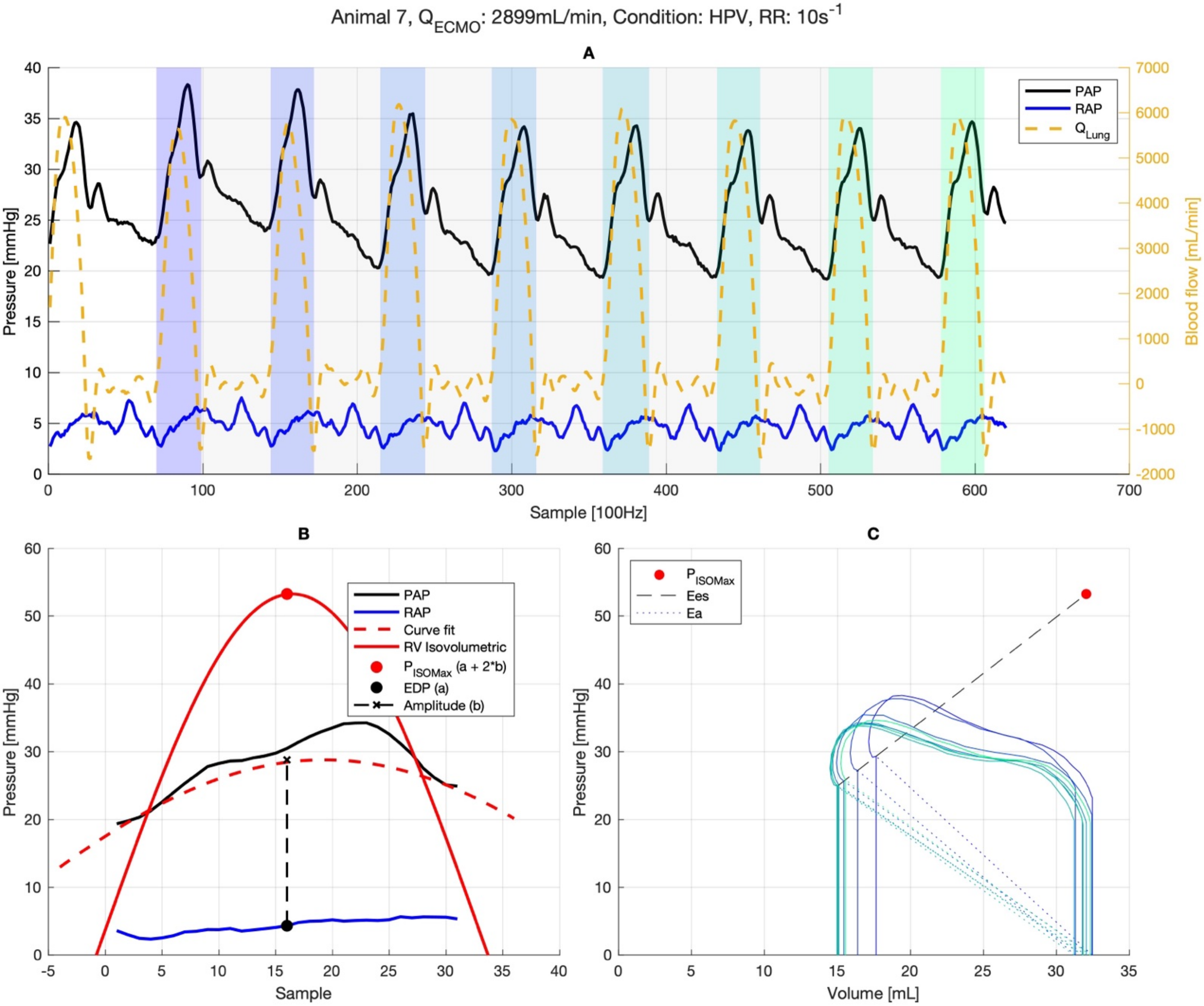

Animal 7, Q_{ECMO}: 2899mL/min, Condition: HPV, RR: 10s^{-1}
A
PAP
RAP
Q_{Lung}
Pressure [mmHg]
Blood flow [mL/min]
Sample [100Hz]
B
PAP
RAP
Curve fit
RV Isovolumetric
P_{ISOMax} (a + 2*b)
EDP (a)
Amplitude (b)
Pressure [mmHg]
Sample
C
P_{ISOMax}
Ees
Ea
Pressure [mmHg]
Volume [mL]

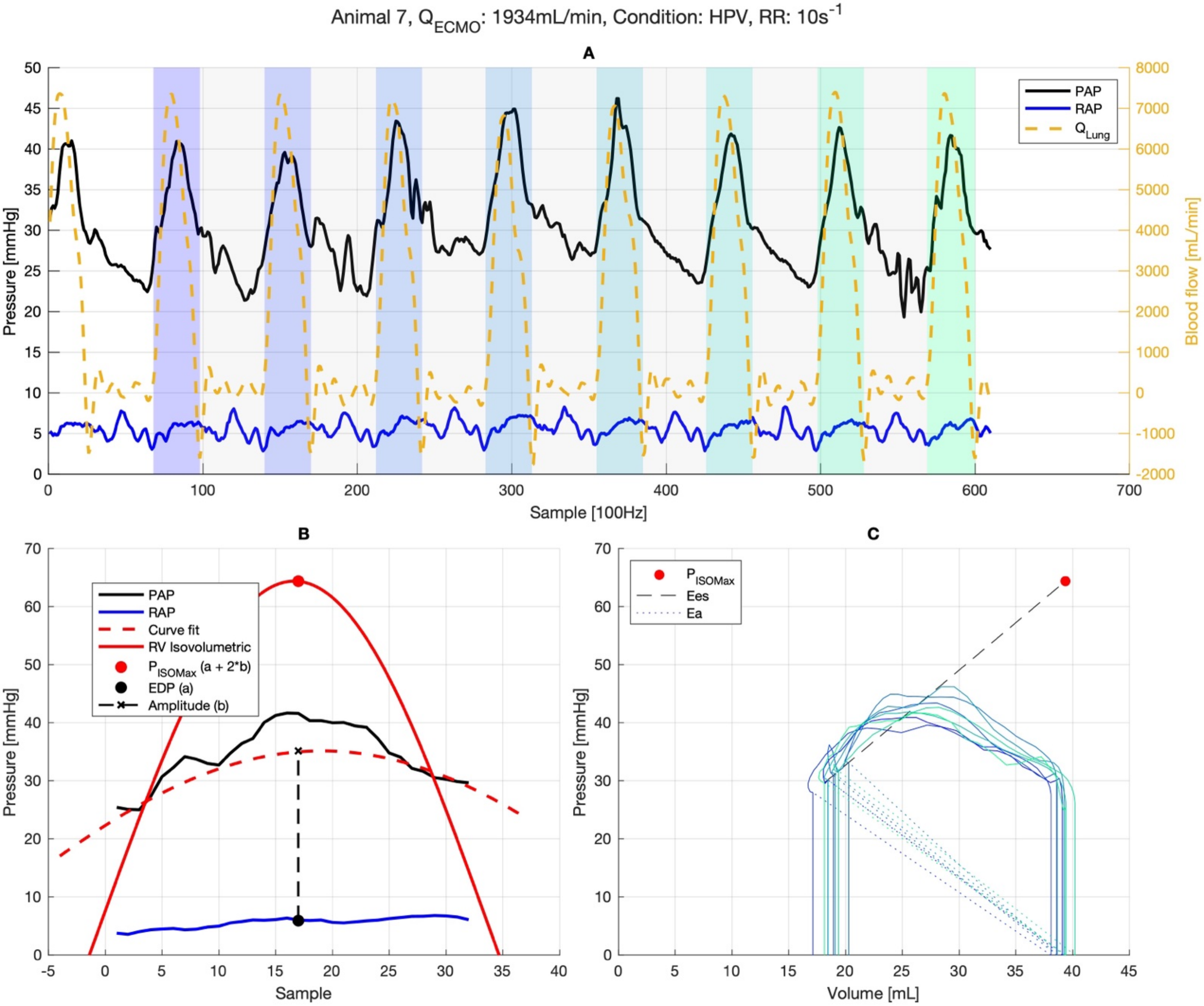

Animal 7, QECMO: 1934mL/min, Condition: HPV, RR: 10s-1
A
PAP
RAP
QLung
Pressure [mmHg]
Blood flow [mL/min]
Sample [100Hz]
B
PAP
RAP
Curve fit
RV Isovolumetric
PISOMax (a + 2*b)
EDP (a)
Amplitude (b)
Pressure [mmHg]
Sample
C
PISOMax
Ees
Ea
Pressure [mmHg]
Volume [mL]

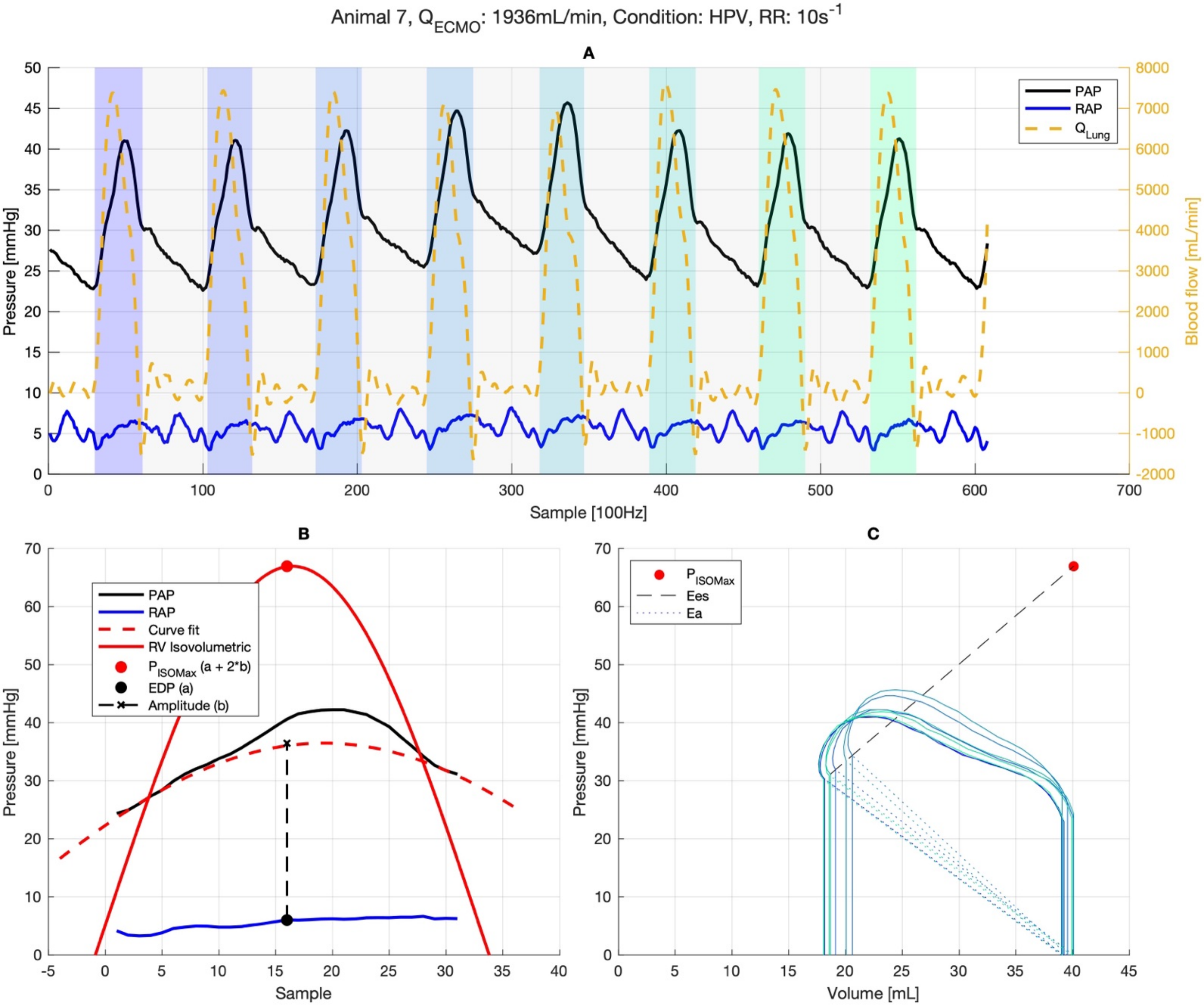

Animal 7, Q_ECMO: 1936mL/min, Condition: HPV, RR: 10s^-1
A
PAP
RAP
Q_Lung
Pressure [mmHg]
Blood flow [mL/min]
Sample [100Hz]
B
PAP
RAP
Curve fit
RV Isovolumetric
P_ISOMax (a + 2*b)
EDP (a)
Amplitude (b)
Pressure [mmHg]
Sample
C
P_ISOMax
Ees
Ea
Pressure [mmHg]
Volume [mL]

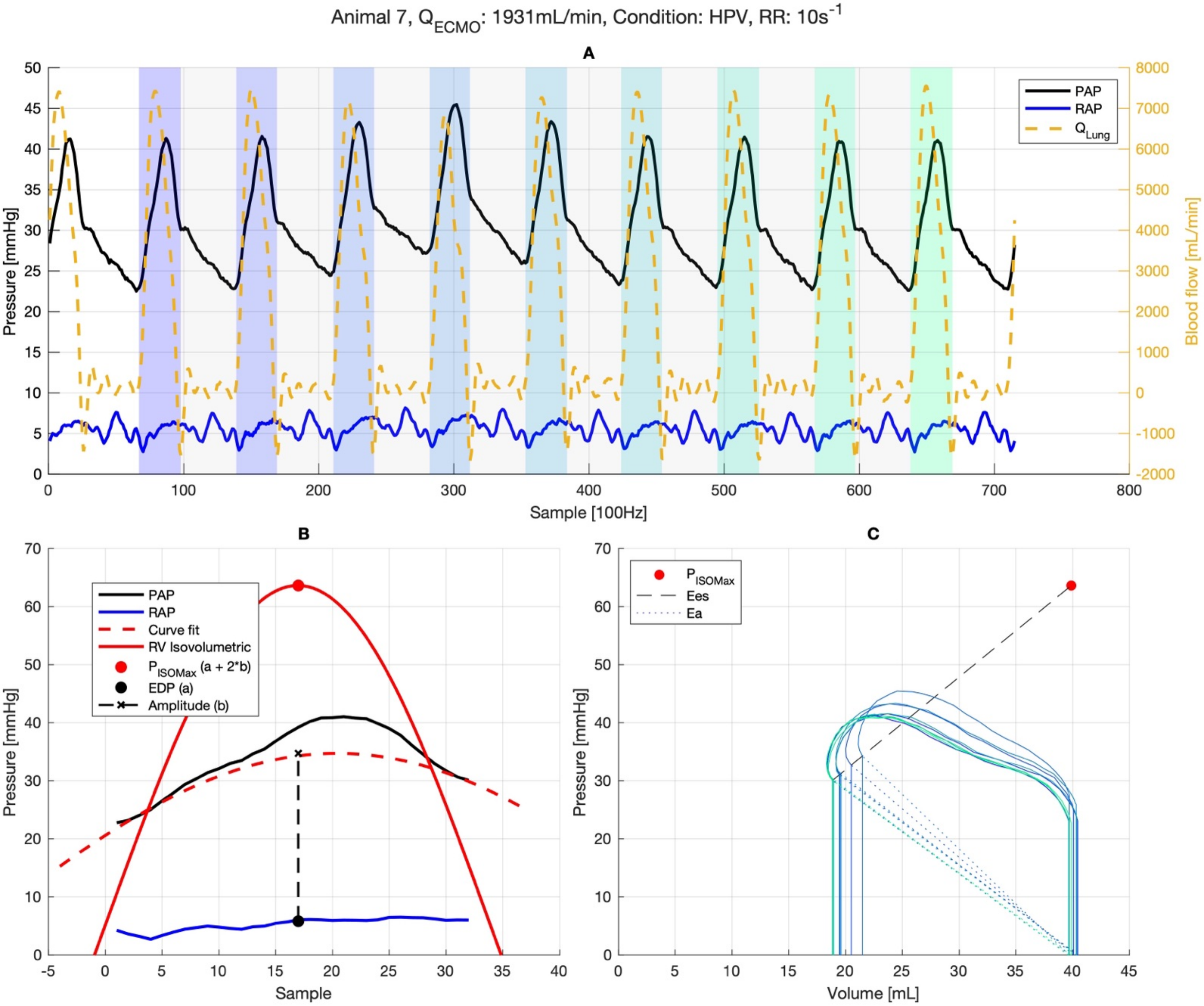

Animal 7, Q_{ECMO}: 1931mL/min, Condition: HPV, RR: $10s^{-1}$
A
PAP
RAP
Q_{Lung}
Pressure [mmHg]
Blood flow [mL/min]
Sample [100Hz]
B
PAP
RAP
Curve fit
RV Isovolumetric
P_{ISOMax} (a + 2*b)
EDP (a)
Amplitude (b)
Pressure [mmHg]
Sample
C
P_{ISOMax}
Ees
Ea
Pressure [mmHg]
Volume [mL]

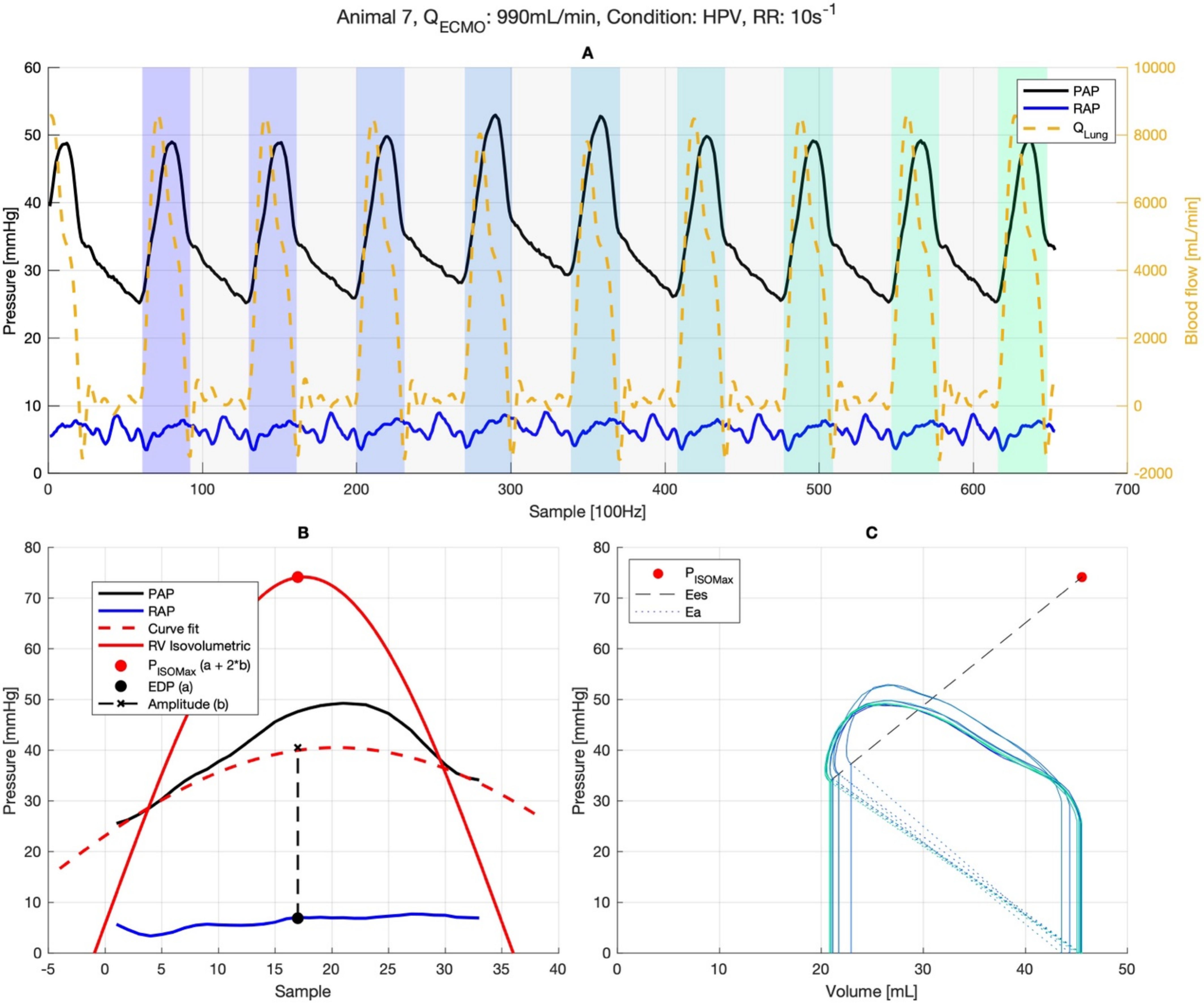

Animal 7, Q_{ECMO}: 990mL/min, Condition: HPV, RR: $10s^{-1}$
A
PAP
RAP
Q_{Lung}
Pressure [mmHg]
Blood flow [mL/min]
Sample [100Hz]
B
PAP
RAP
Curve fit
RV Isovolumetric
P_{ISOMax} (a + 2*b)
EDP (a)
Amplitude (b)
Pressure [mmHg]
Sample
C
P_{ISOMax}
Ees
Ea
Pressure [mmHg]
Volume [mL]

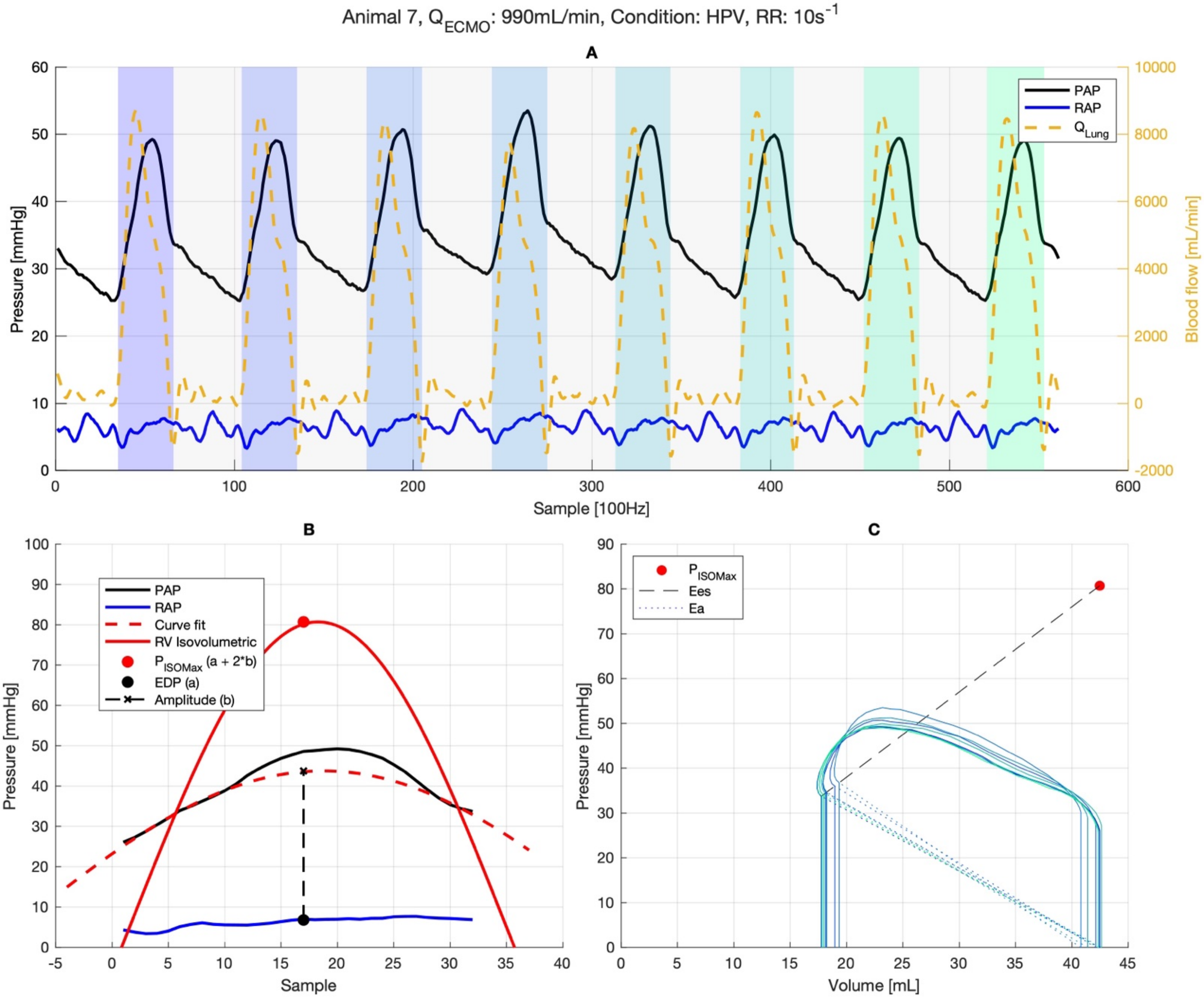

Animal 7, Q_{ECMO}: 990mL/min, Condition: HPV, RR: $10s^{-1}$
A
PAP
RAP
Q_{Lung}
Pressure [mmHg]
Blood flow [mL/min]
Sample [100Hz]
B
PAP
RAP
Curve fit
RV Isovolumetric
P_{ISOMax} (a + 2*b)
EDP (a)
Amplitude (b)
Pressure [mmHg]
Sample
C
P_{ISOMax}
Ees
Ea
Pressure [mmHg]
Volume [mL]

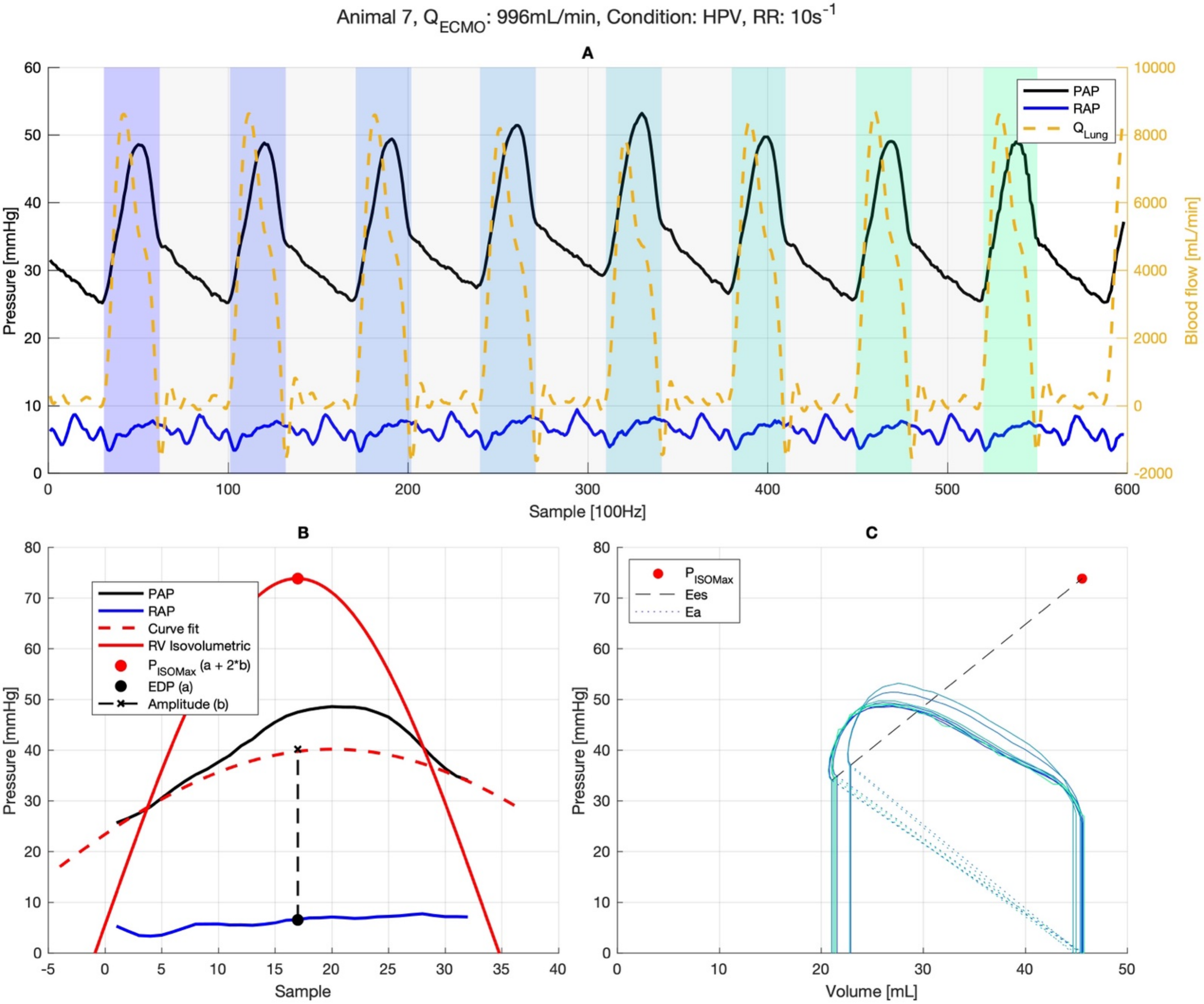

Animal 7, Q_ECMO: 996mL/min, Condition: HPV, RR: 10s^-1
A
Pressure [mmHg]
Blood flow [mL/min]
Sample [100Hz]
PAP
RAP
Q_Lung
B
PAP
RAP
Curve fit
RV Isovolumetric
P_ISOMax (a + 2*b)
EDP (a)
Amplitude (b)
Pressure [mmHg]
Sample
C
P_ISOMax
Ees
Ea
Pressure [mmHg]
Volume [mL]

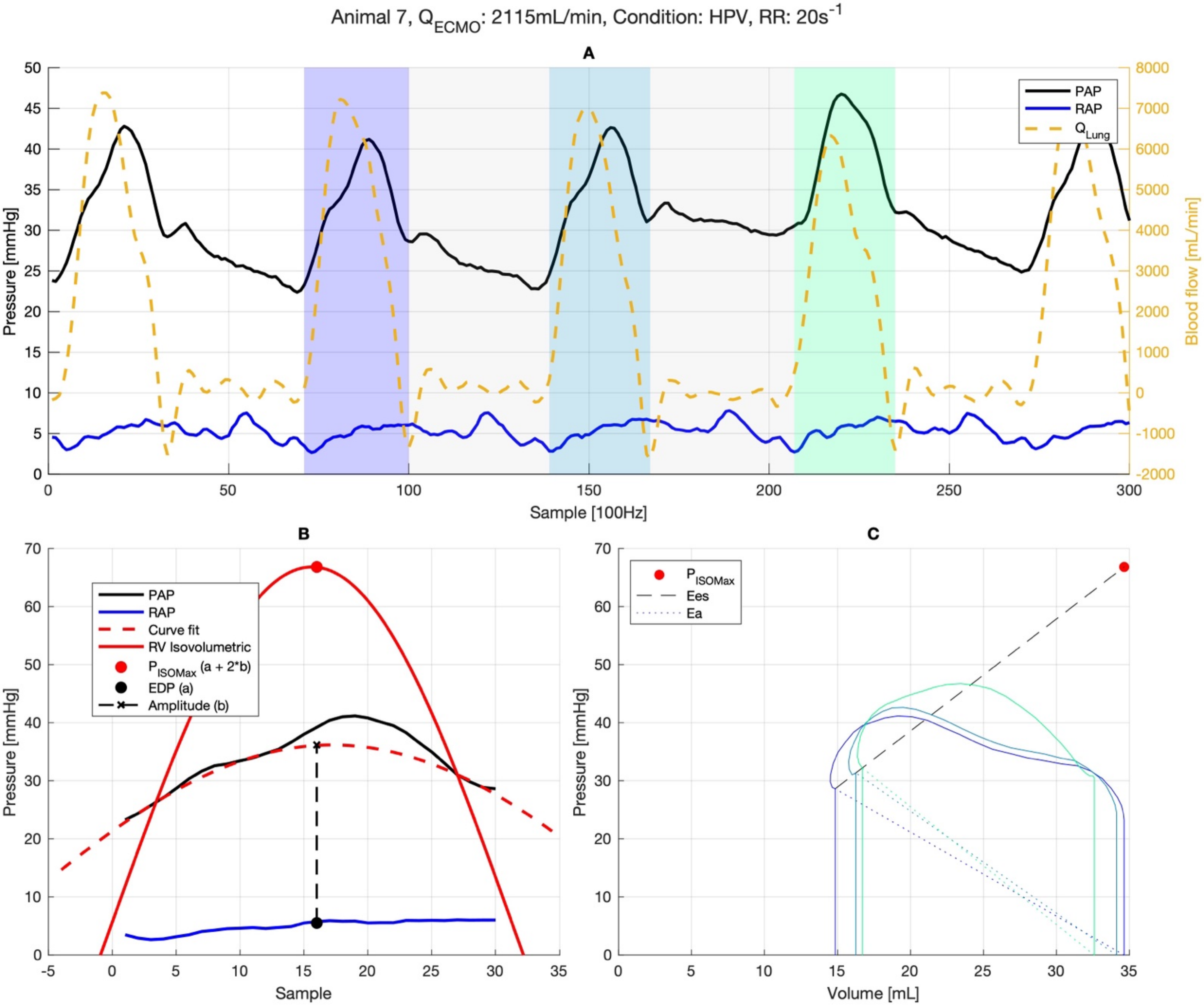

Animal 7, Q_ECMO: 2115mL/min, Condition: HPV, RR: 20s^-1
A
PAP
RAP
Q_Lung
Pressure [mmHg]
Blood flow [mL/min]
Sample [100Hz]
B
PAP
RAP
Curve fit
RV Isovolumetric
P_ISOMax (a + 2*b)
EDP (a)
Amplitude (b)
Pressure [mmHg]
Sample
C
P_ISOMax
Ees
Ea
Pressure [mmHg]
Volume [mL]

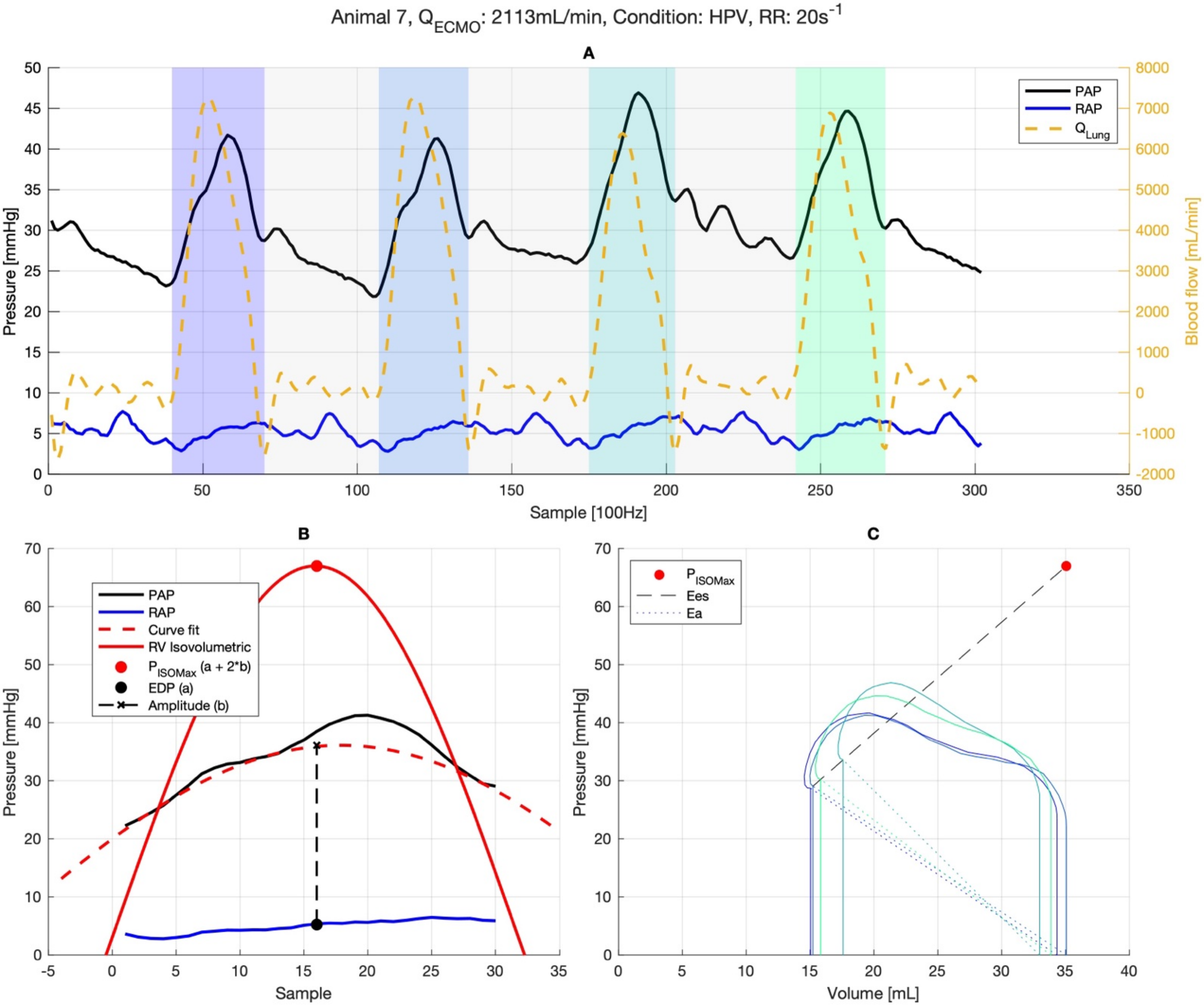
Animal 7, Q_{ECMO}: 2113mL/min, Condition: HPV, RR: $20s^{-1}$
A
PAP
RAP
Q_{Lung}
Pressure [mmHg]
Blood flow [mL/min]
Sample [100Hz]
B
PAP
RAP
Curve fit
RV Isovolumetric
P_{ISOMax} (a + 2*b)
EDP (a)
Amplitude (b)
Pressure [mmHg]
Sample
C
P_{ISOMax}
Ees
Ea
Pressure [mmHg]
Volume [mL]

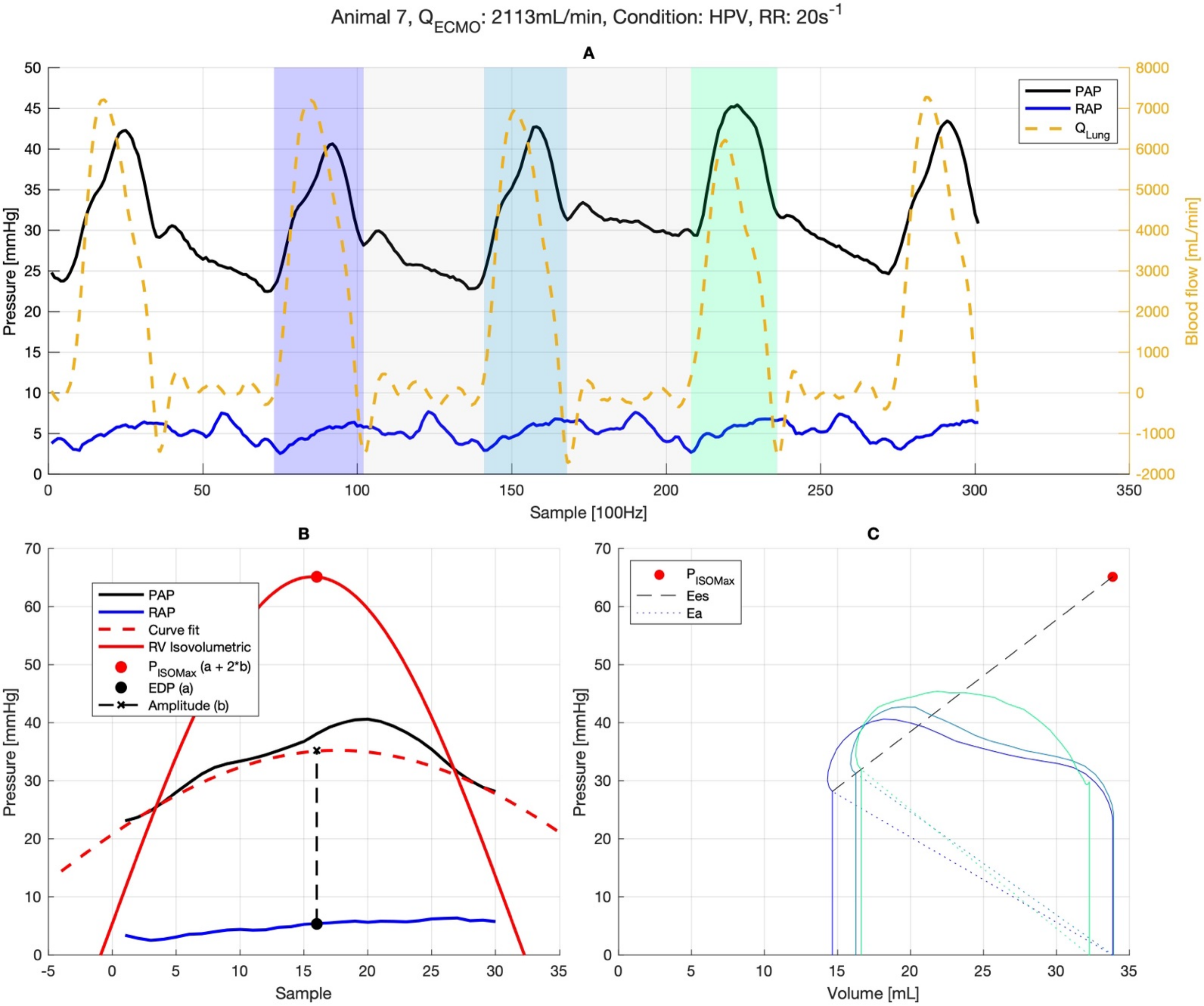

Animal 7, QECMO: 2113mL/min, Condition: HPV, RR: 20s-1
A
Pressure [mmHg]
Blood flow [mL/min]
Sample [100Hz]
PAP
RAP
QLung
B
PAP
RAP
Curve fit
RV Isovolumetric
PISOMax (a + 2*b)
EDP (a)
Amplitude (b)
Pressure [mmHg]
Sample
C
PISOMax
Ees
Ea
Pressure [mmHg]
Volume [mL]

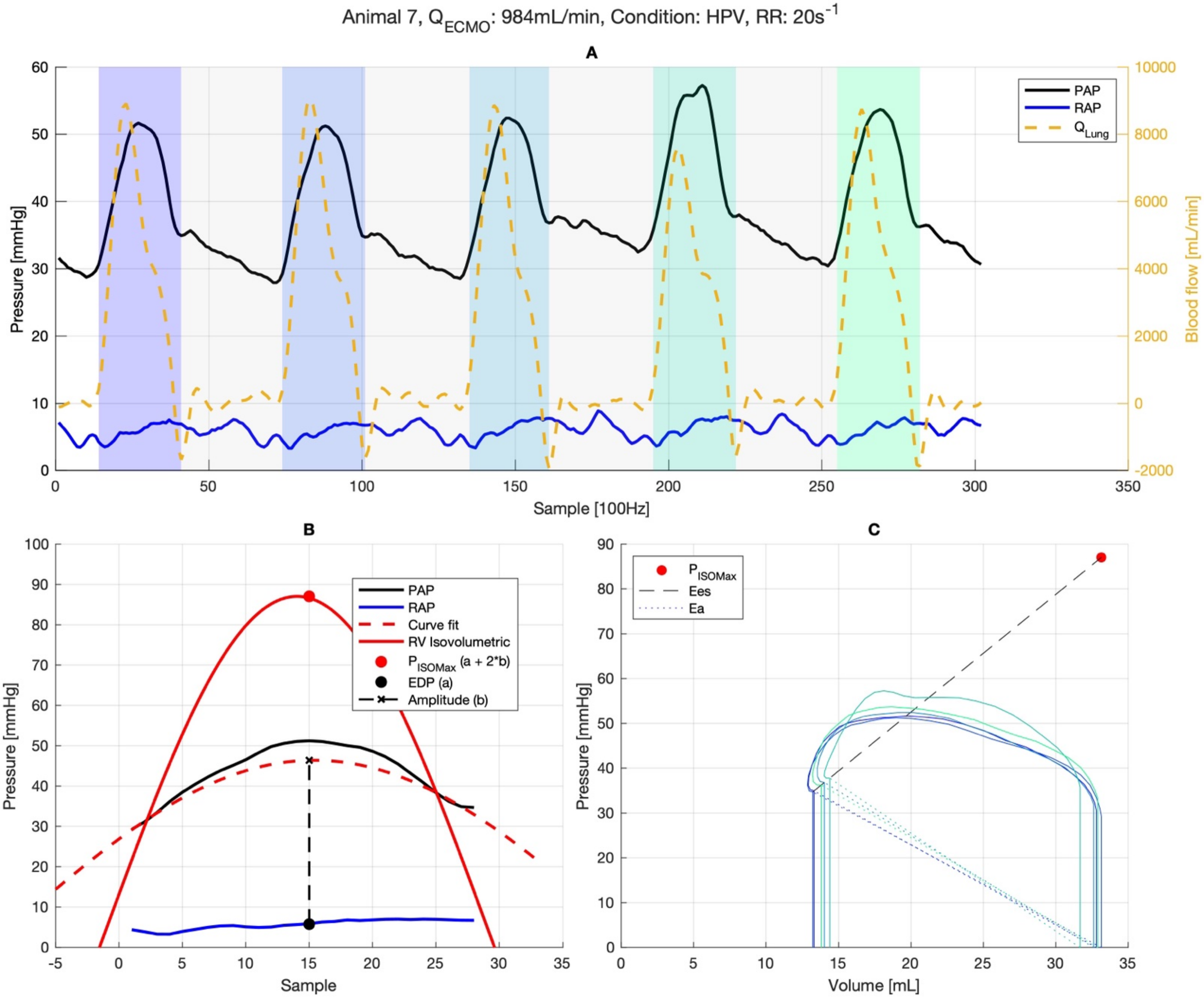

Animal 7, Q_{ECMO}: 984mL/min, Condition: HPV, RR: $20s^{-1}$
A
PAP
RAP
Q_{Lung}
Pressure [mmHg]
Blood flow [mL/min]
Sample [100Hz]
B
PAP
RAP
Curve fit
RV Isovolumetric
P_{ISOMax} (a + 2*b)
EDP (a)
Amplitude (b)
Pressure [mmHg]
Sample
C
P_{ISOMax}
Ees
Ea
Pressure [mmHg]
Volume [mL]

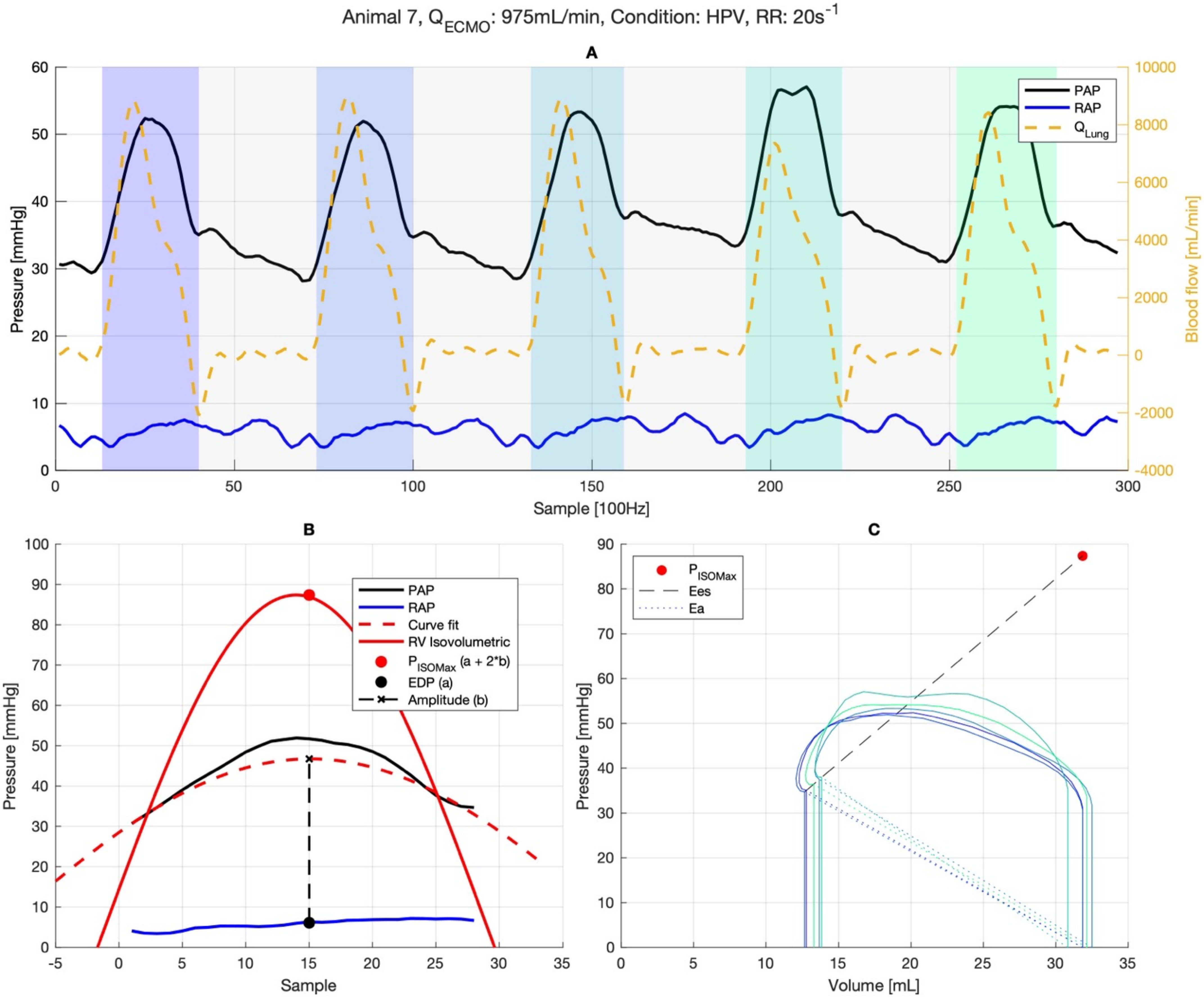
Animal 7, Q_{ECMO}: 975mL/min, Condition: HPV, RR: $20s^{-1}$
A
PAP
RAP
Q_{Lung}
Pressure [mmHg]
Blood flow [mL/min]
Sample [100Hz]
B
PAP
RAP
Curve fit
RV Isovolumetric
P_{ISOMax} (a + 2*b)
EDP (a)
Amplitude (b)
Pressure [mmHg]
Sample
C
P_{ISOMax}
Ees
Ea
Pressure [mmHg]
Volume [mL]

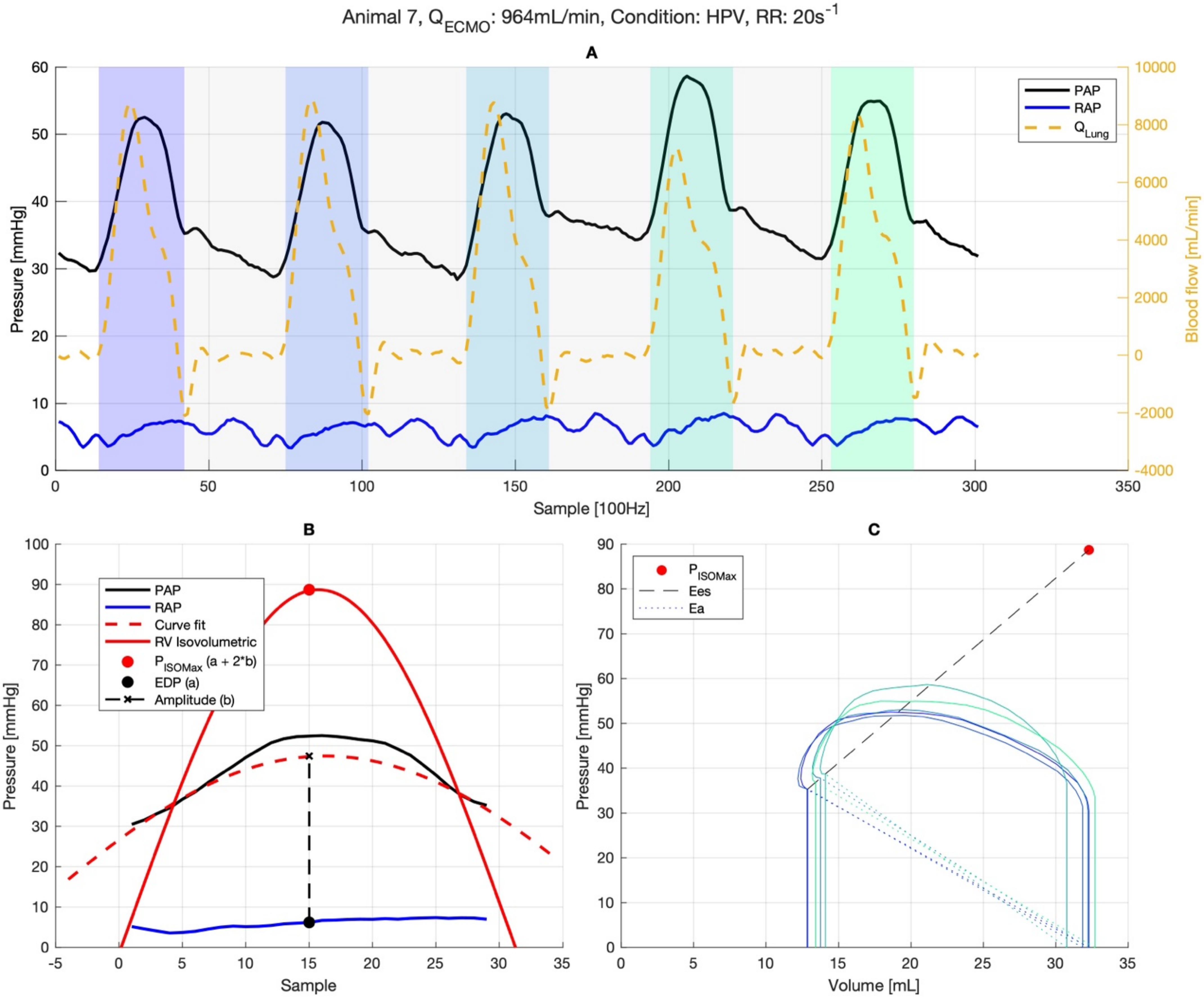

Animal 7, QECMO: 964mL/min, Condition: HPV, RR: 20s-1
A
Pressure [mmHg]
Blood flow [mL/min]
Sample [100Hz]
PAP
RAP
QLung
B
PAP
RAP
Curve fit
RV Isovolumetric
PISOMax (a + 2*b)
EDP (a)
Amplitude (b)
Pressure [mmHg]
Sample
C
PISOMax
Ees
Ea
Pressure [mmHg]
Volume [mL]

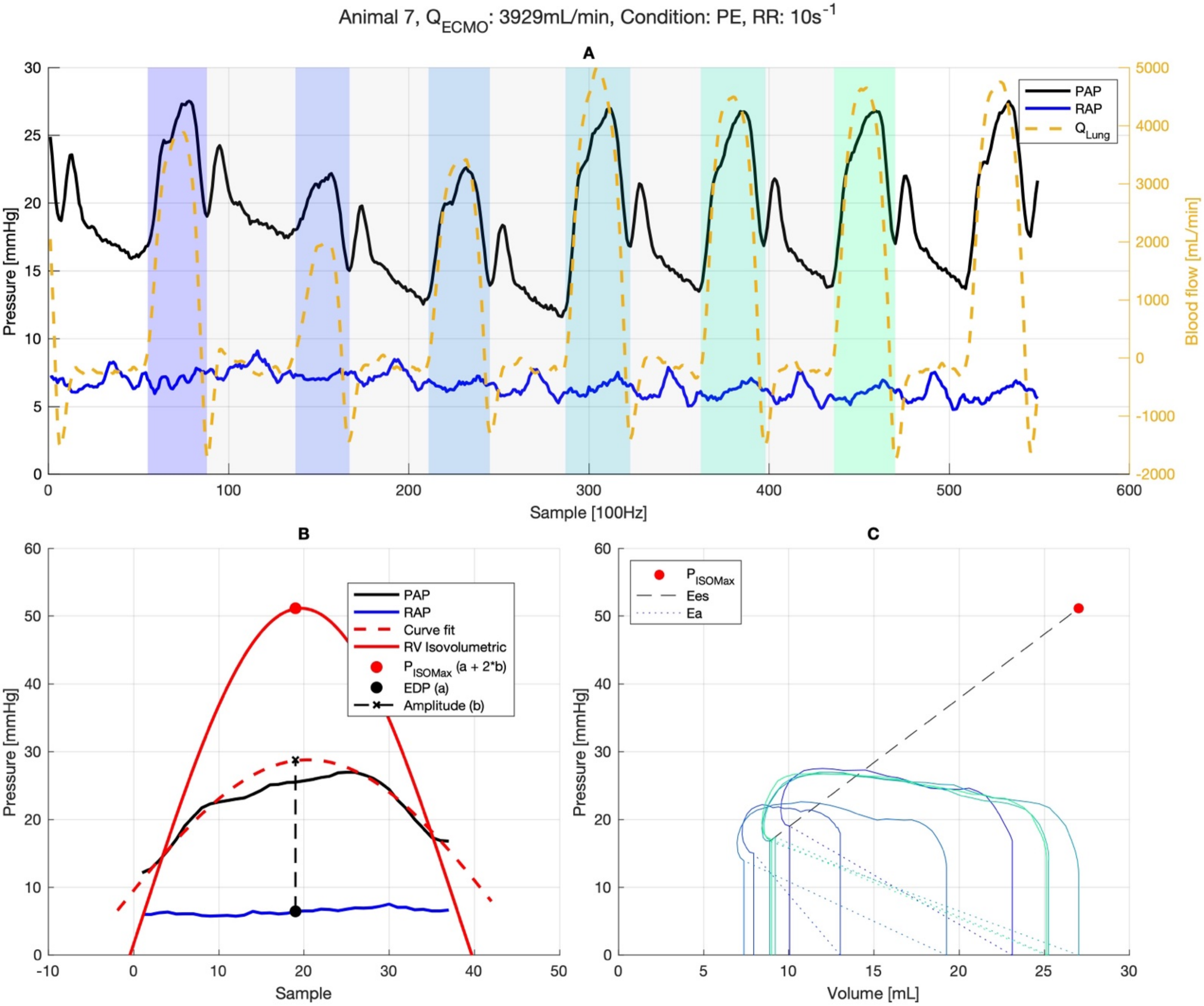

Animal 7, Q_{ECMO}: 3929mL/min, Condition: PE, RR: $10s^{-1}$
A
PAP
RAP
Q_{Lung}
Pressure [mmHg]
Blood flow [mL/min]
Sample [100Hz]
B
PAP
RAP
Curve fit
RV Isovolumetric
P_{ISOMax} (a + 2*b)
EDP (a)
Amplitude (b)
Pressure [mmHg]
Sample
C
P_{ISOMax}
Ees
Ea
Pressure [mmHg]
Volume [mL]

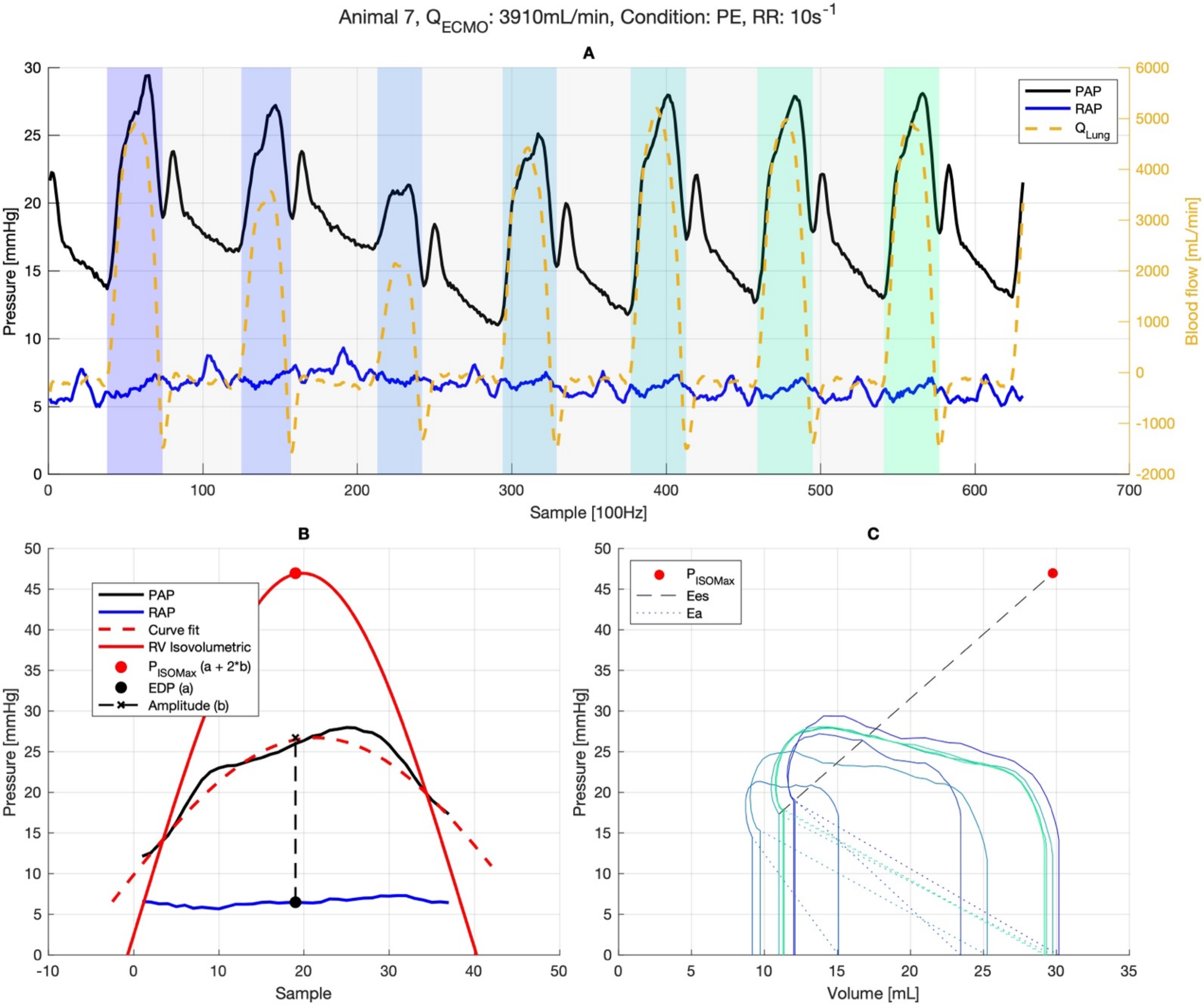

Animal 7, Q_{ECMO}: 3910mL/min, Condition: PE, RR: $10s^{-1}$
A
PAP
RAP
Q_{Lung}
Pressure [mmHg]
Blood flow [mL/min]
Sample [100Hz]
B
PAP
RAP
Curve fit
RV Isovolumetric
P_{ISOMax} (a + 2*b)
EDP (a)
Amplitude (b)
Pressure [mmHg]
Sample
C
P_{ISOMax}
Ees
Ea
Pressure [mmHg]
Volume [mL]

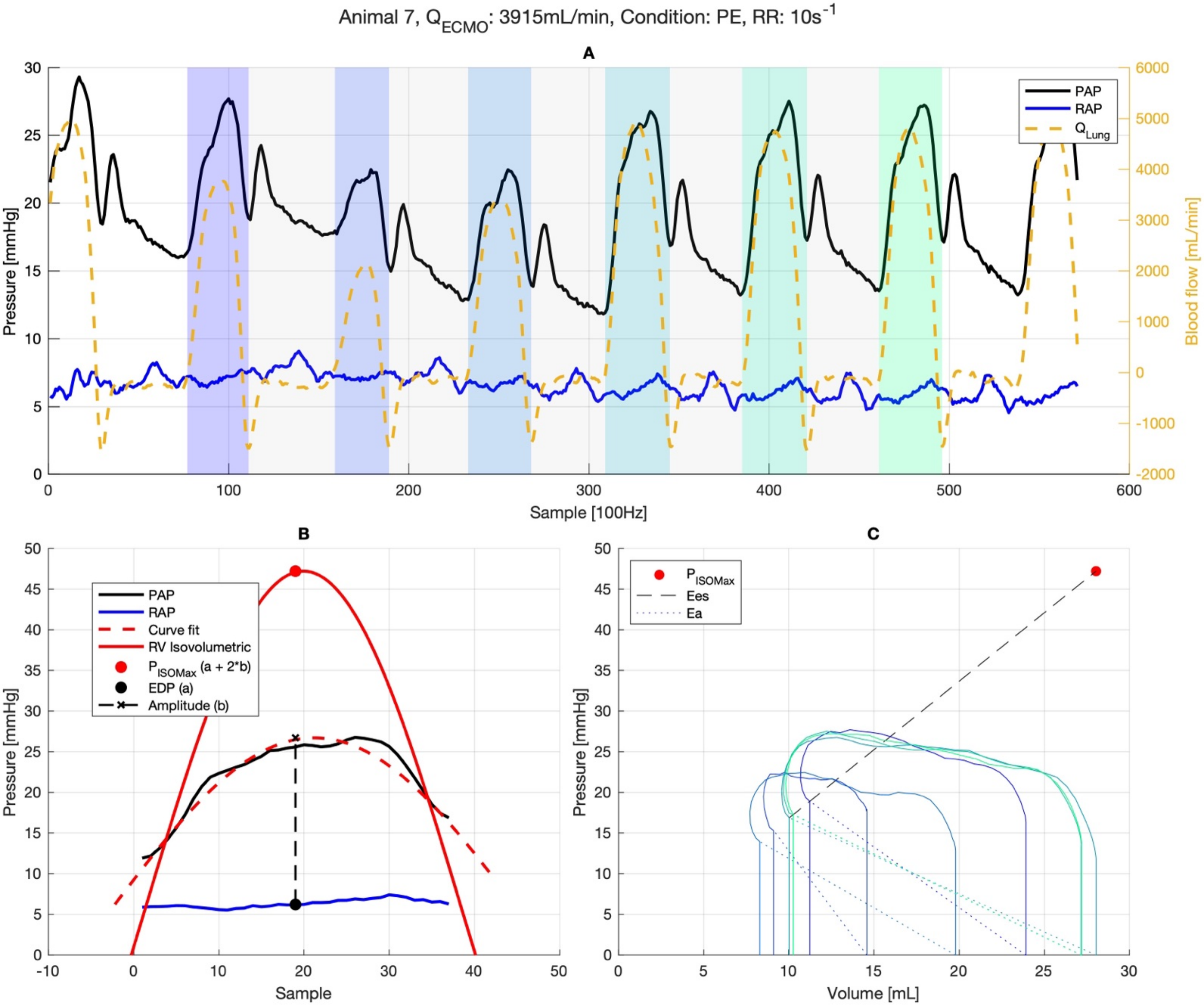

Animal 7, Q_{ECMO}: 3915mL/min, Condition: PE, RR: $10s^{-1}$
A
PAP
RAP
Q_{Lung}
Pressure [mmHg]
Blood flow [mL/min]
Sample [100Hz]
B
PAP
RAP
Curve fit
RV Isovolumetric
P_{ISOMax} (a + 2*b)
EDP (a)
Amplitude (b)
Pressure [mmHg]
Sample
C
P_{ISOMax}
Ees
Ea
Pressure [mmHg]
Volume [mL]

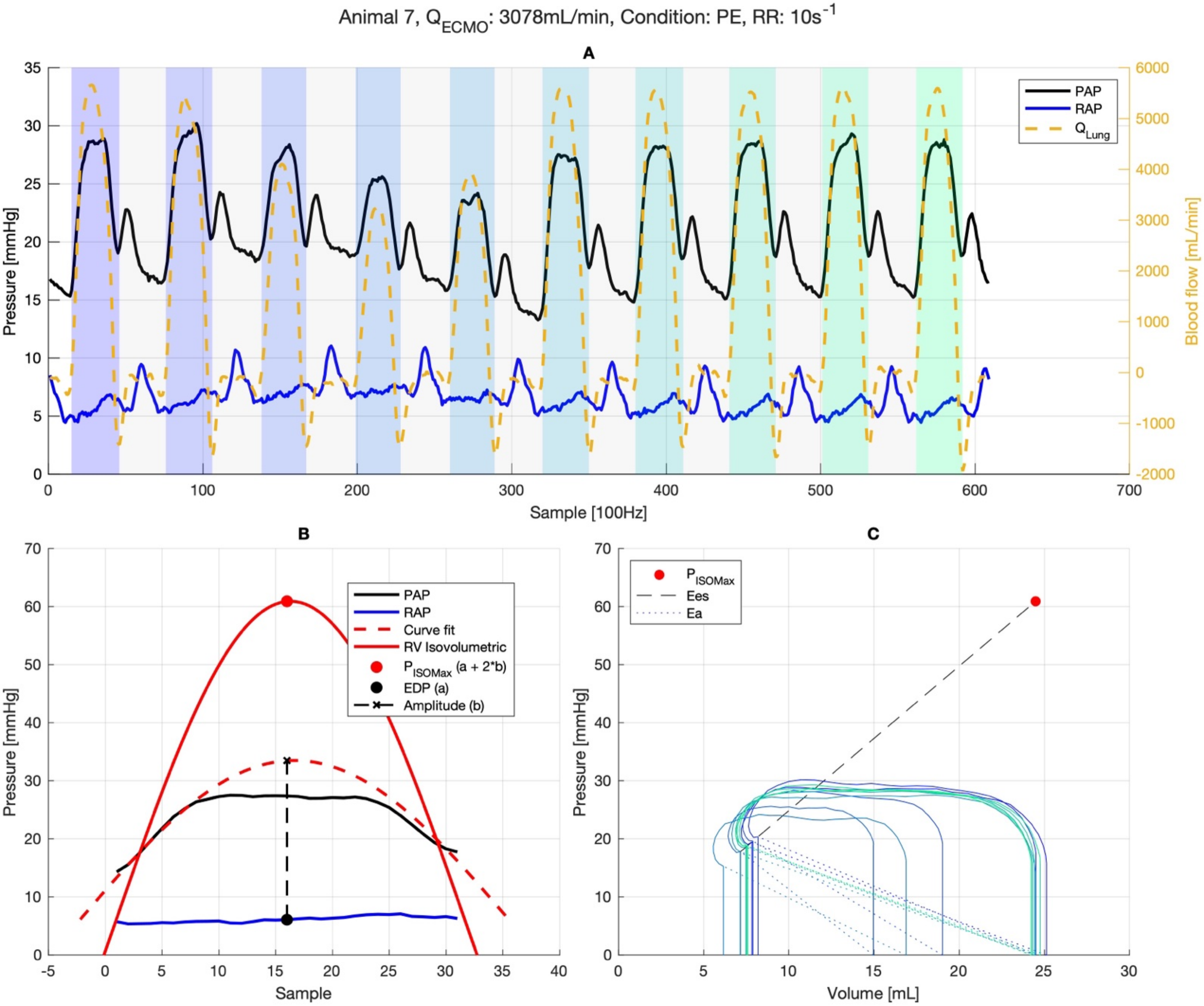

Animal 7, Q_ECMO: 3078mL/min, Condition: PE, RR: 10s^-1
A
PAP
RAP
Q_Lung
Pressure [mmHg]
Blood flow [mL/min]
Sample [100Hz]
B
PAP
RAP
Curve fit
RV Isovolumetric
P_ISOMax (a + 2*b)
EDP (a)
Amplitude (b)
Pressure [mmHg]
Sample
C
P_ISOMax
Ees
Ea
Pressure [mmHg]
Volume [mL]

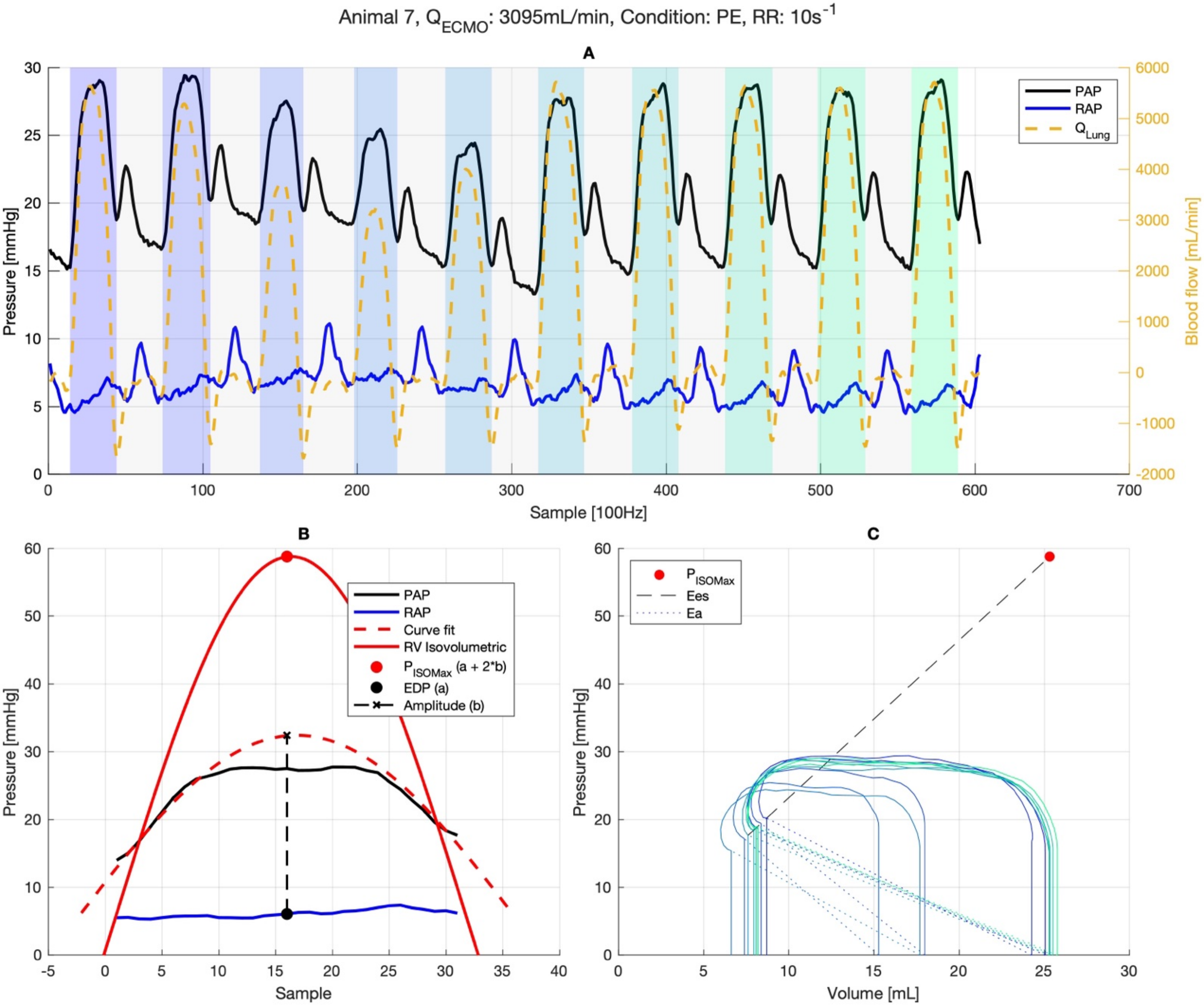

Animal 7, Q_{ECMO}: 3095mL/min, Condition: PE, RR: $10s^{-1}$
A
Pressure [mmHg]
Blood flow [mL/min]
Sample [100Hz]
PAP
RAP
Q_{Lung}
B
Pressure [mmHg]
Sample
PAP
RAP
Curve fit
RV Isovolumetric
P_{ISOMax} (a + 2*b)
EDP (a)
Amplitude (b)
C
Pressure [mmHg]
Volume [mL]
P_{ISOMax}
Ees
Ea

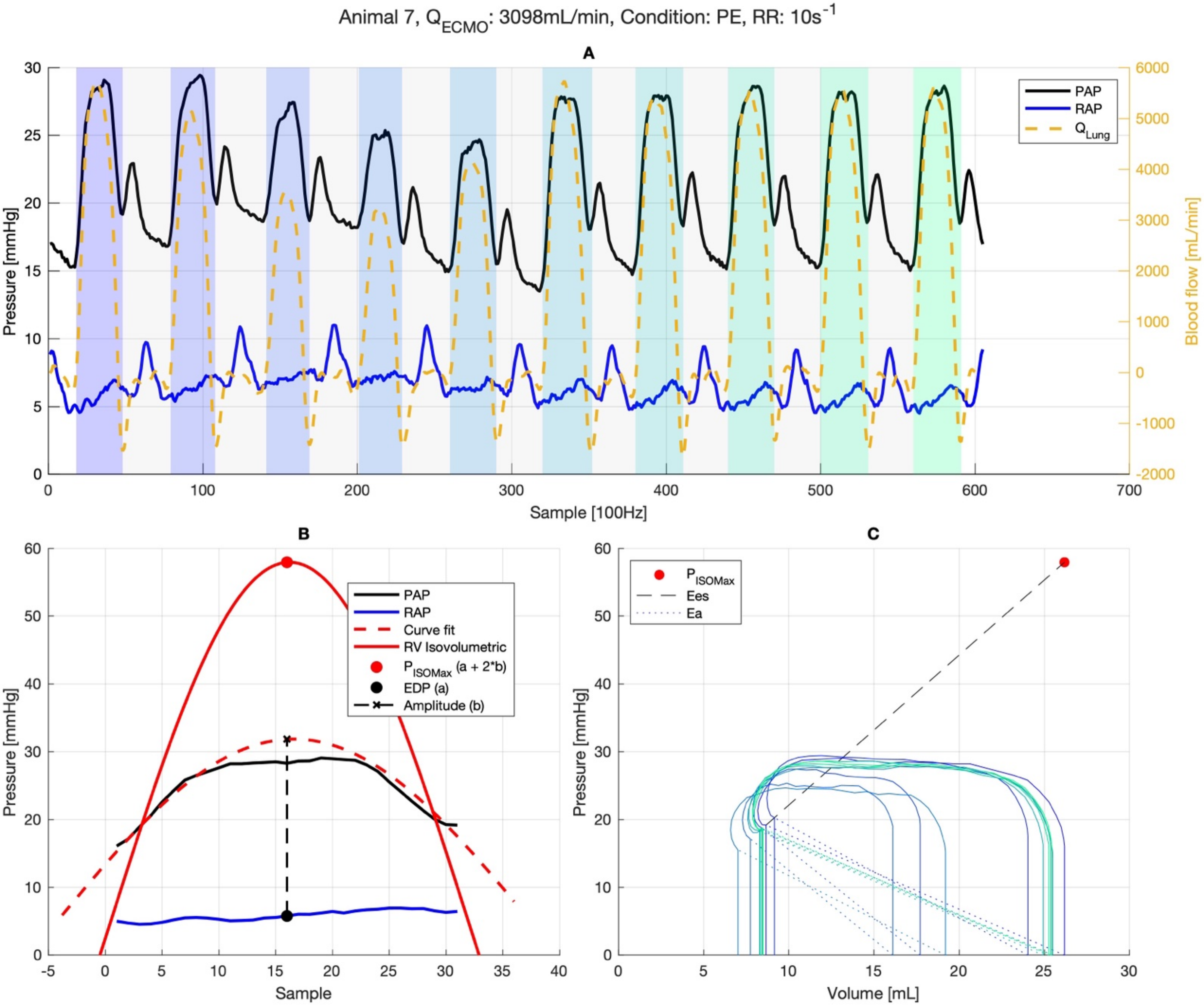

Animal 7, Q_{ECMO}: 3098mL/min, Condition: PE, RR: 10s^{-1}
A
Pressure [mmHg]
Blood flow [mL/min]
Sample [100Hz]
PAP
RAP
Q_{Lung}
B
Pressure [mmHg]
Sample
PAP
RAP
Curve fit
RV Isovolumetric
P_{ISOMax} (a + 2*b)
EDP (a)
Amplitude (b)
C
Pressure [mmHg]
Volume [mL]
P_{ISOMax}
Ees
Ea

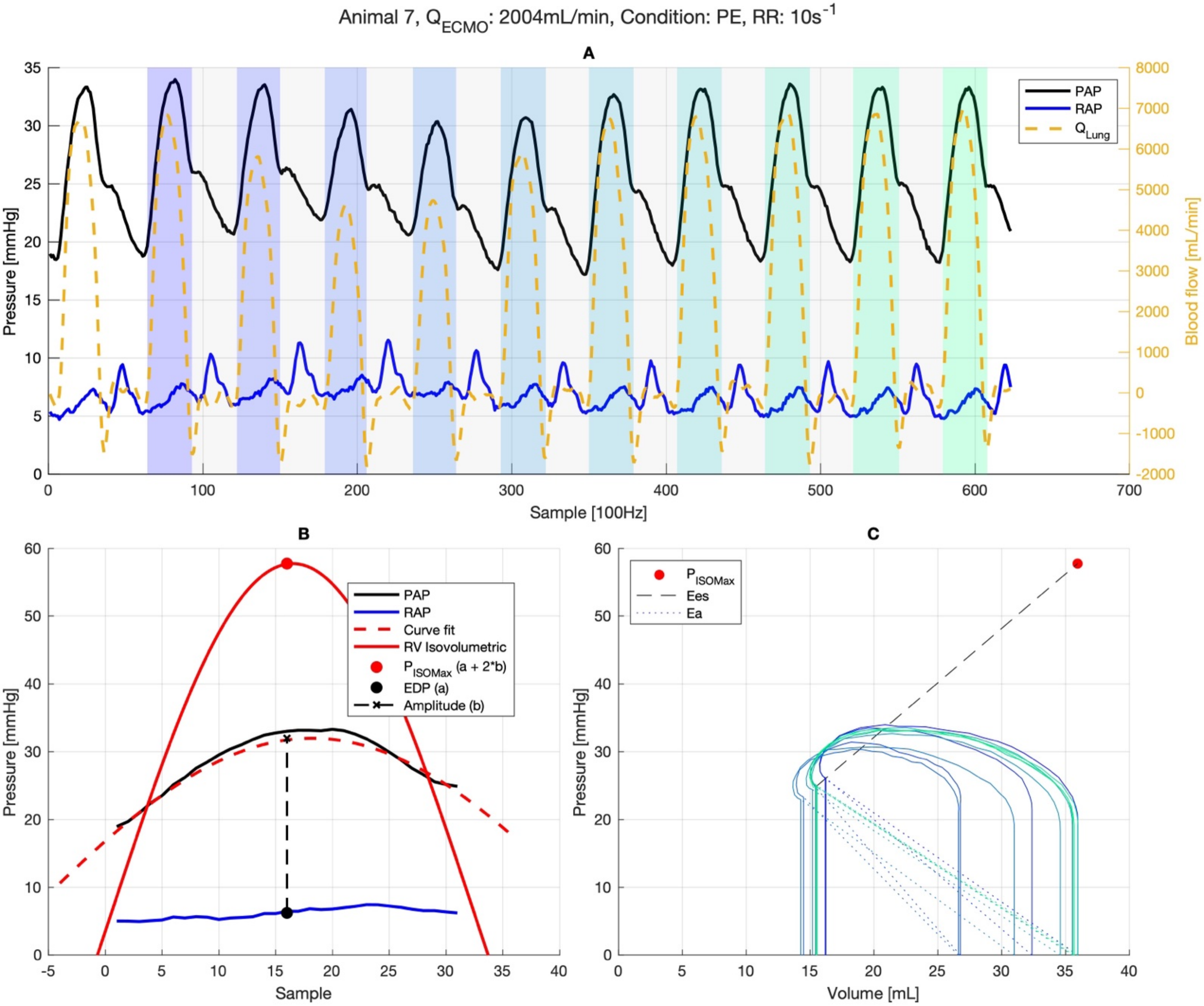
Animal 7, Q_ECMO: 2004mL/min, Condition: PE, RR: 10s^-1
A
PAP
RAP
Q_Lung
Pressure [mmHg]
Blood flow [mL/min]
Sample [100Hz]
B
PAP
RAP
Curve fit
RV Isovolumetric
P_ISOMax (a + 2*b)
EDP (a)
Amplitude (b)
Pressure [mmHg]
Sample
C
P_ISOMax
Ees
Ea
Pressure [mmHg]
Volume [mL]

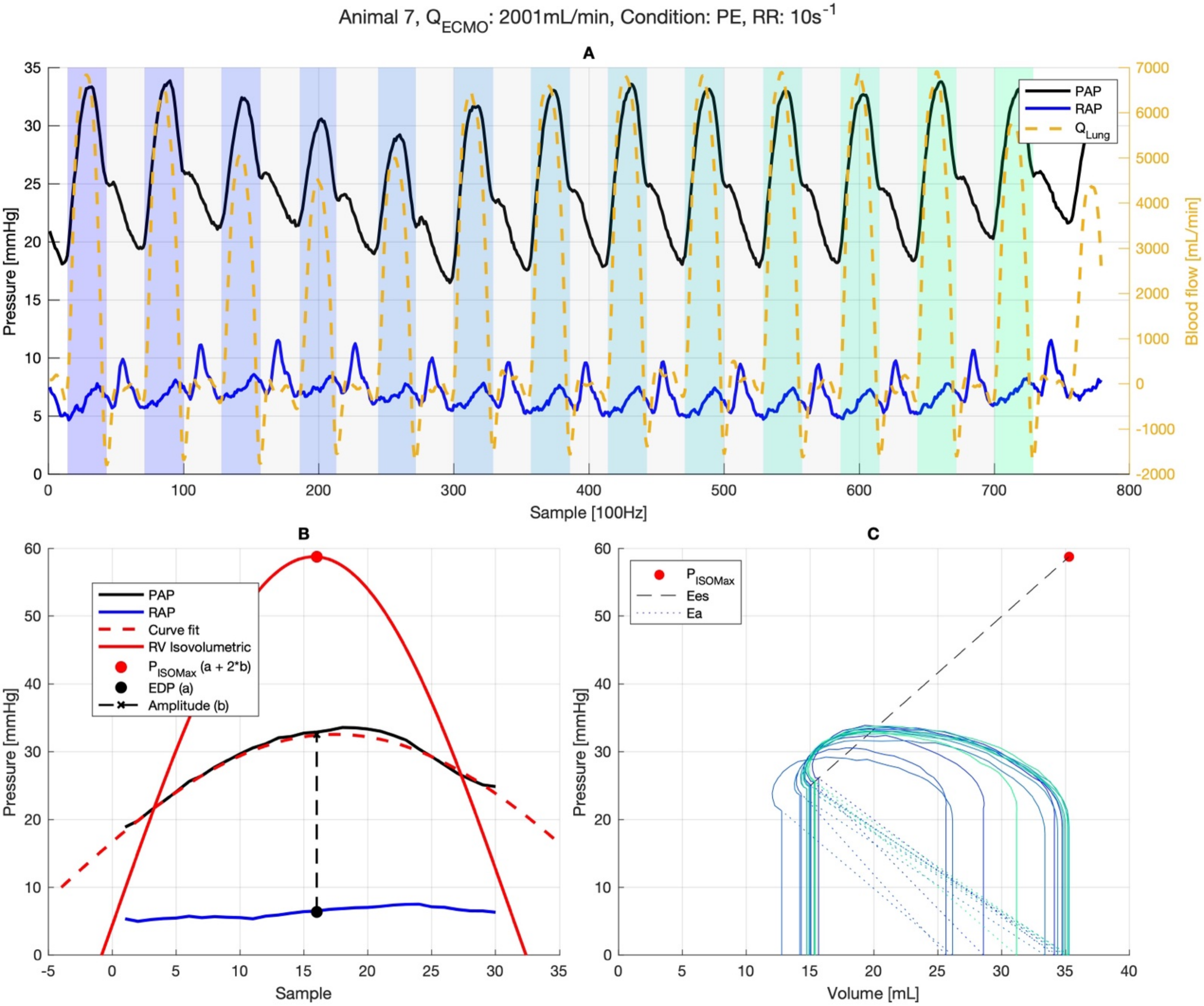

Animal 7, Q_{ECMO}: 2001mL/min, Condition: PE, RR: $10s^{-1}$
A
PAP
RAP
Q_{Lung}
Pressure [mmHg]
Blood flow [mL/min]
Sample [100Hz]
B
PAP
RAP
Curve fit
RV Isovolumetric
P_{ISOMax} (a + 2*b)
EDP (a)
Amplitude (b)
Pressure [mmHg]
Sample
C
P_{ISOMax}
Ees
Ea
Pressure [mmHg]
Volume [mL]

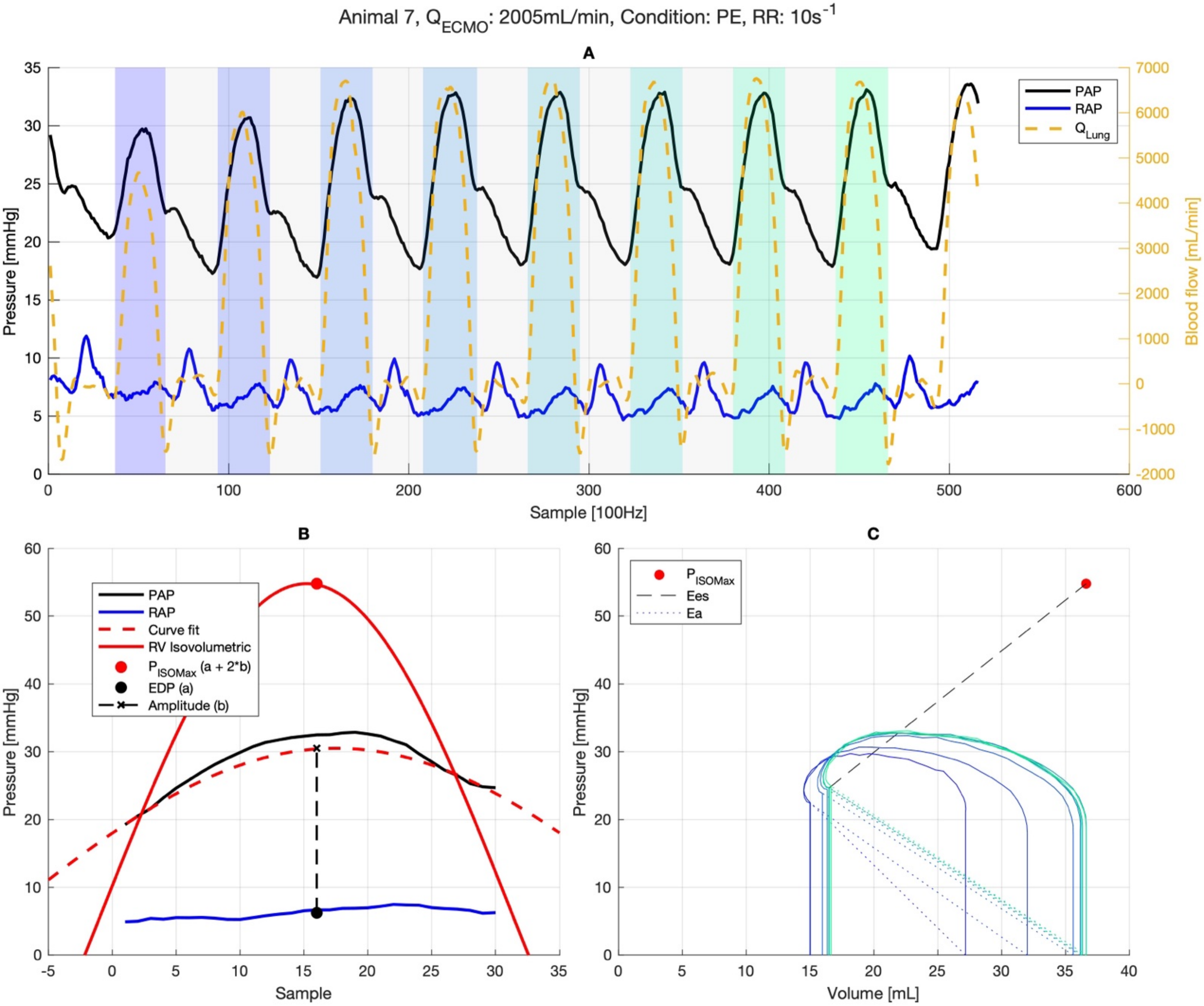

Animal 7, Q_{ECMO}: 2005mL/min, Condition: PE, RR: 10s^{-1}
A
PAP
RAP
Q_{Lung}
Pressure [mmHg]
Blood flow [mL/min]
Sample [100Hz]
B
PAP
RAP
Curve fit
RV Isovolumetric
P_{ISOMax} (a + 2*b)
EDP (a)
Amplitude (b)
Pressure [mmHg]
Sample
C
P_{ISOMax}
Ees
Ea
Pressure [mmHg]
Volume [mL]

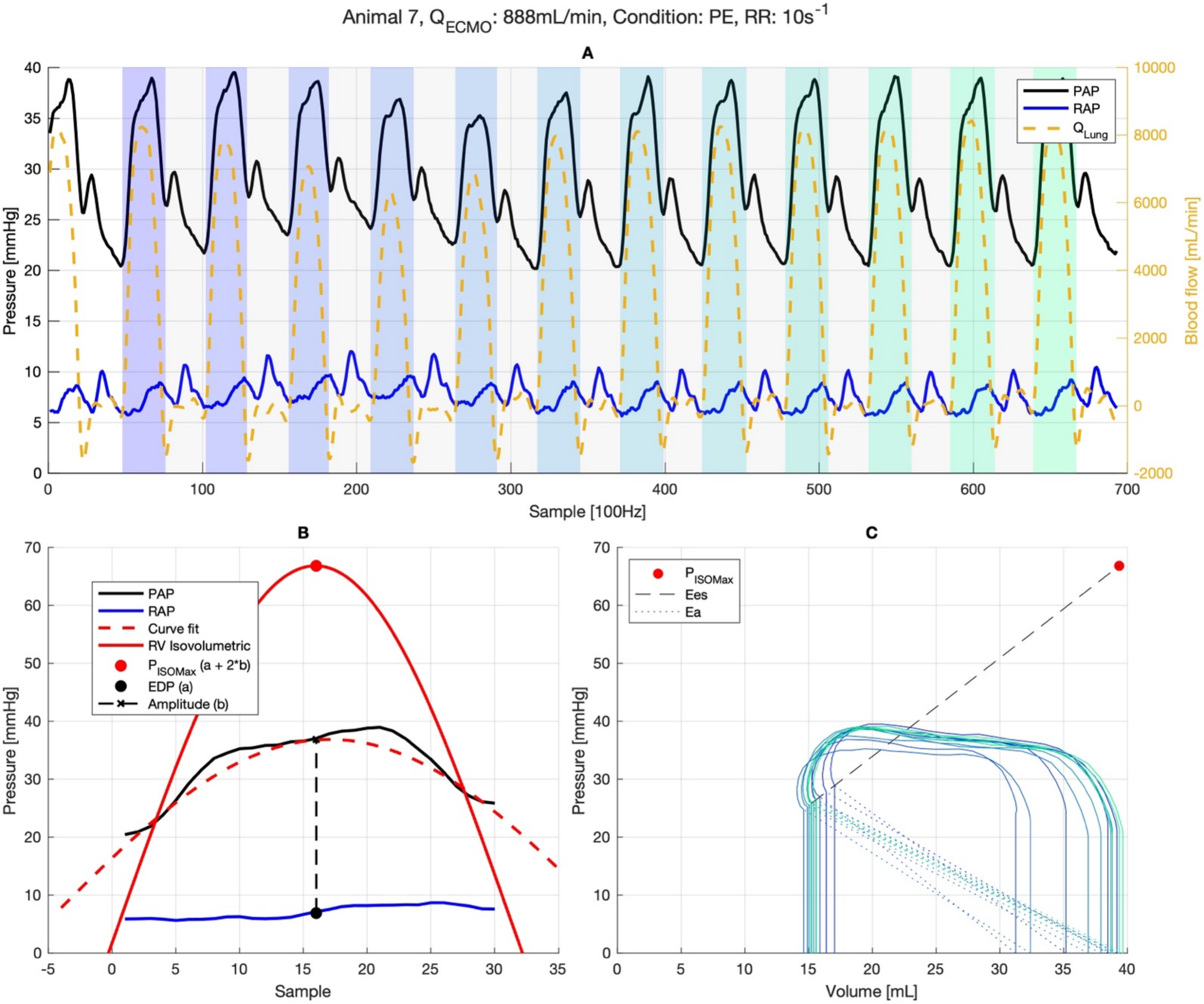

Animal 7, Q_{ECMO}: 888mL/min, Condition: PE, RR: $10s^{-1}$
A
PAP
RAP
Q_{Lung}
Pressure [mmHg]
Blood flow [mL/min]
Sample [100Hz]
B
Curve fit
RV Isovolumetric
P_{ISOMax} (a + 2*b)
EDP (a)
Amplitude (b)
Pressure [mmHg]
Sample
C
P_{ISOMax}
Ees
Ea
Pressure [mmHg]
Volume [mL]

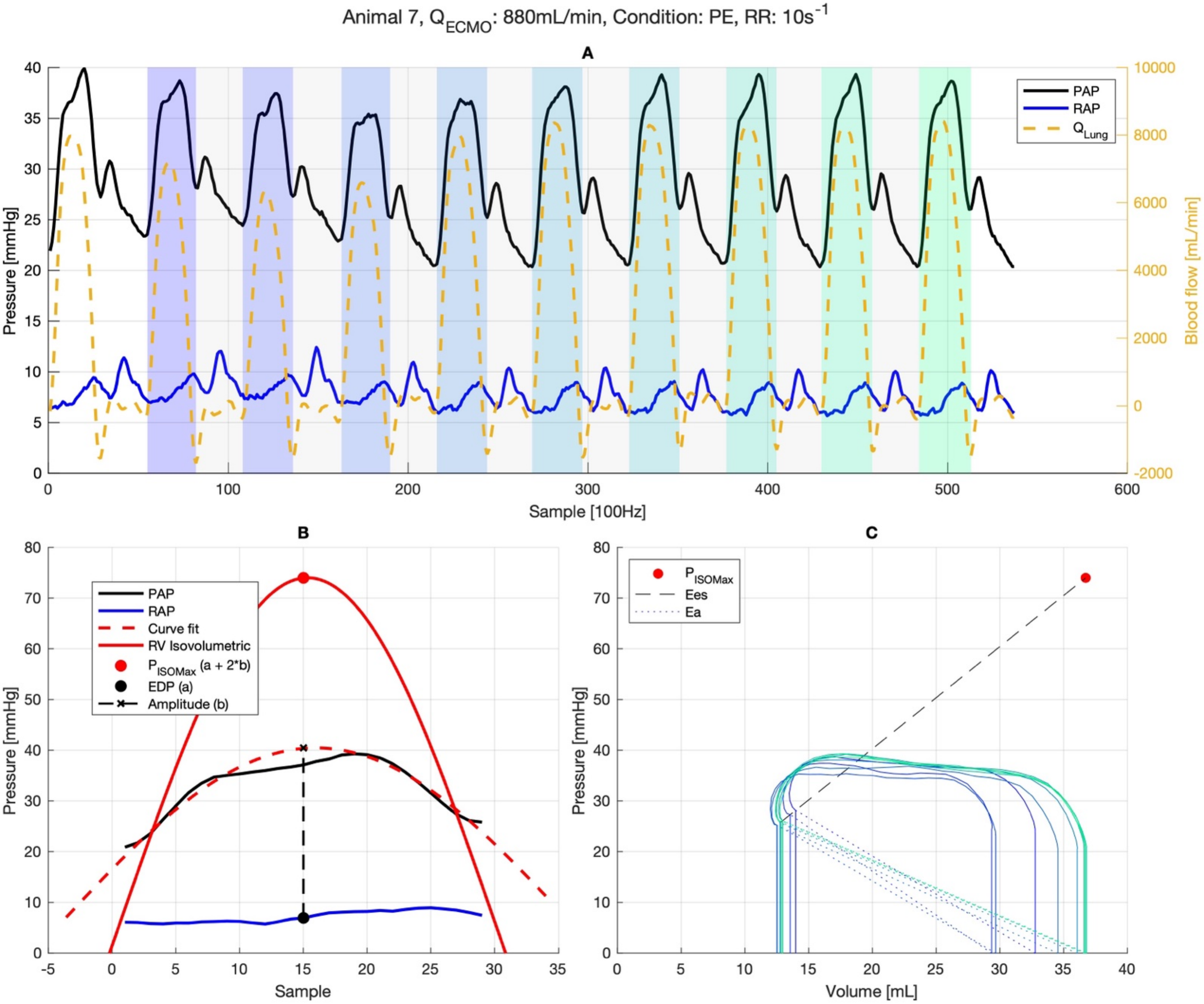

Animal 7, Q_{ECMO}: 880mL/min, Condition: PE, RR: $10s^{-1}$
A
PAP
RAP
Q_{Lung}
Pressure [mmHg]
Blood flow [mL/min]
Sample [100Hz]
B
PAP
RAP
Curve fit
RV Isovolumetric
P_{ISOMax} (a + 2*b)
EDP (a)
Amplitude (b)
Pressure [mmHg]
Sample
C
P_{ISOMax}
Ees
Ea
Pressure [mmHg]
Volume [mL]

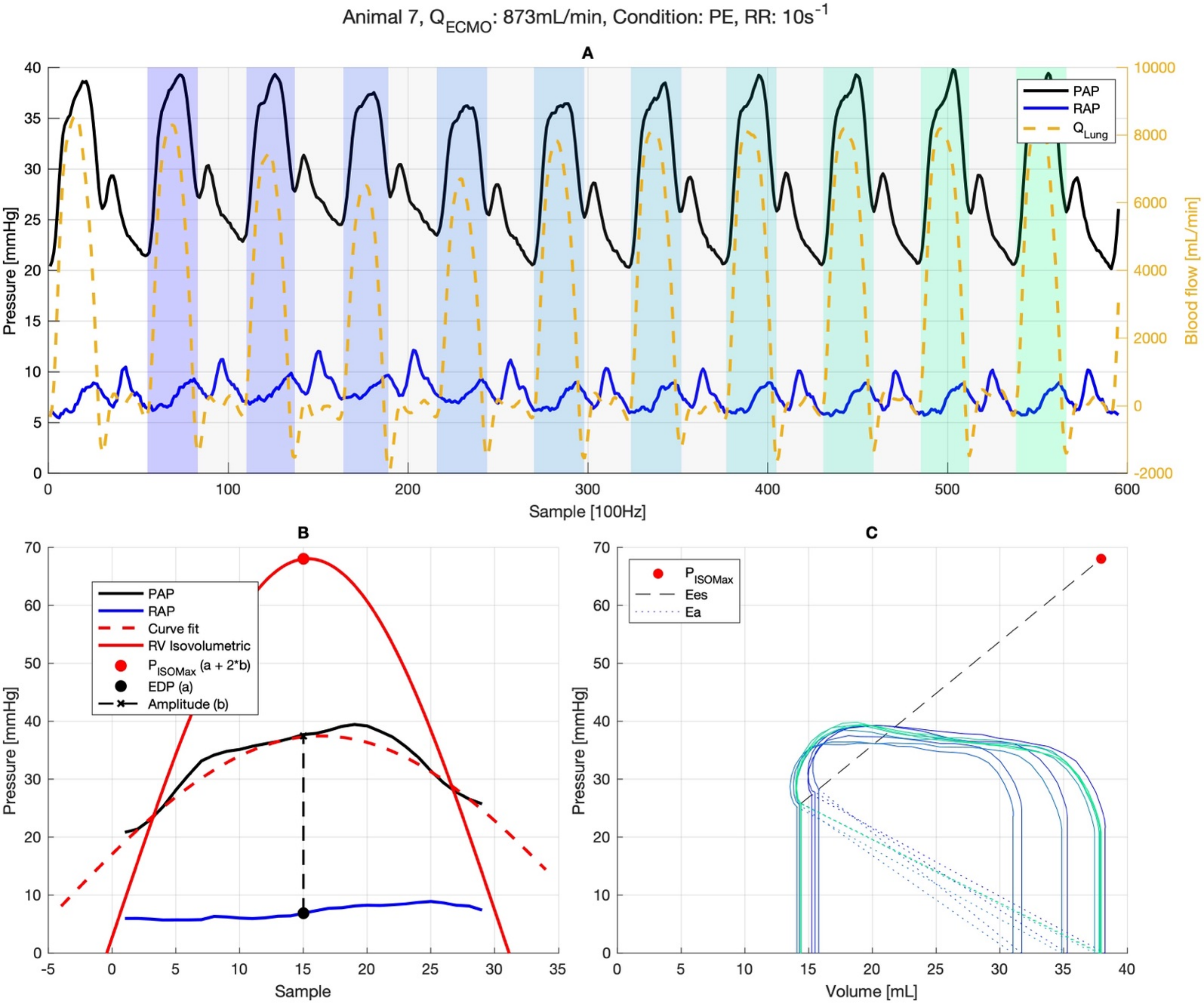

Animal 7, Q_{ECMO}: 873mL/min, Condition: PE, RR: $10s^{-1}$
A
PAP
RAP
Q_{Lung}
Pressure [mmHg]
Blood flow [mL/min]
Sample [100Hz]
B
PAP
RAP
Curve fit
RV Isovolumetric
P_{ISOMax} (a + 2*b)
EDP (a)
Amplitude (b)
Pressure [mmHg]
Sample
C
P_{ISOMax}
Ees
Ea
Pressure [mmHg]
Volume [mL]

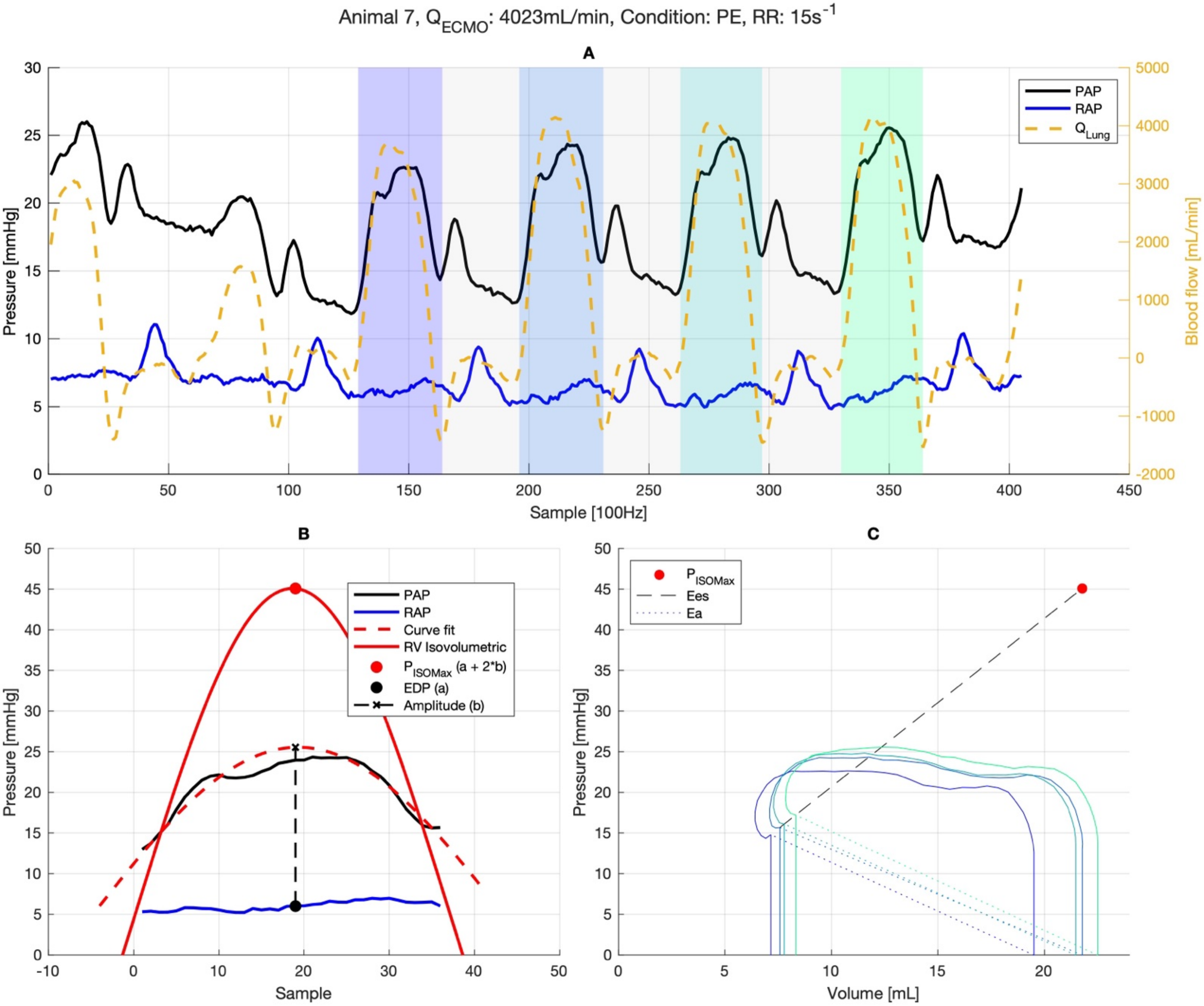

Animal 7, Q_ECMO: 4023mL/min, Condition: PE, RR: 15s^-1
A
PAP
RAP
Q_Lung
Pressure [mmHg]
Blood flow [mL/min]
Sample [100Hz]
B
PAP
RAP
Curve fit
RV Isovolumetric
P_ISOMax (a + 2*b)
EDP (a)
Amplitude (b)
Pressure [mmHg]
Sample
C
P_ISOMax
Ees
Ea
Pressure [mmHg]
Volume [mL]

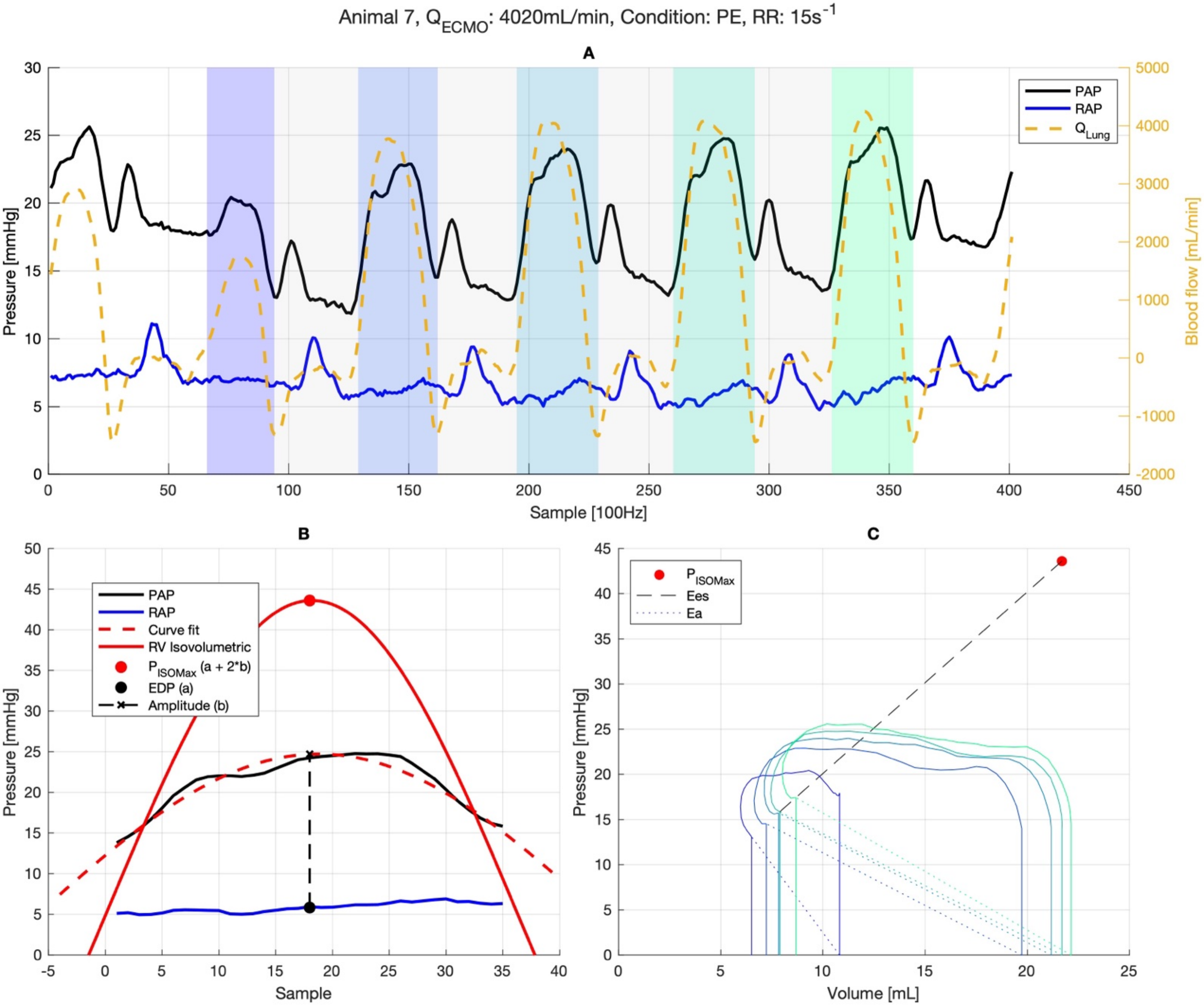

Animal 7, Q_{ECMO}: 4020mL/min, Condition: PE, RR: $15s^{-1}$
A
PAP
RAP
Q_{Lung}
Pressure [mmHg]
Blood flow [mL/min]
Sample [100Hz]
B
PAP
RAP
Curve fit
RV Isovolumetric
P_{ISOMax} (a + 2*b)
EDP (a)
Amplitude (b)
Pressure [mmHg]
Sample
C
P_{ISOMax}
Ees
Ea
Pressure [mmHg]
Volume [mL]

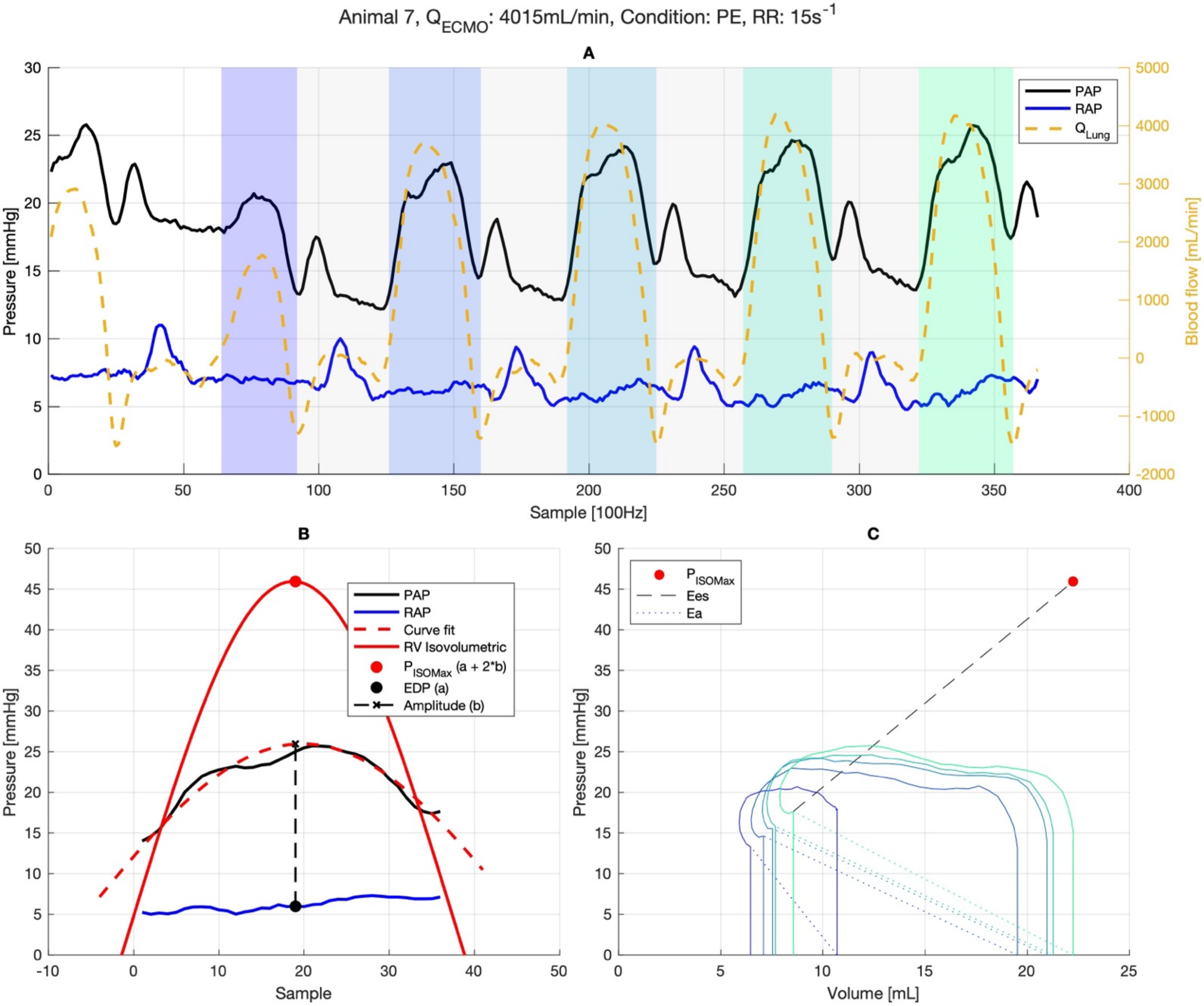

Animal 7, Q_ECMO: 4015mL/min, Condition: PE, RR: 15s^-1
A
Pressure [mmHg]
Blood flow [mL/min]
Sample [100Hz]
PAP
RAP
Q_Lung
B
Pressure [mmHg]
Sample
PAP
RAP
Curve fit
RV Isovolumetric
P_ISOMax (a + 2*b)
EDP (a)
Amplitude (b)
C
Pressure [mmHg]
Volume [mL]
P_ISOMax
Ees
Ea

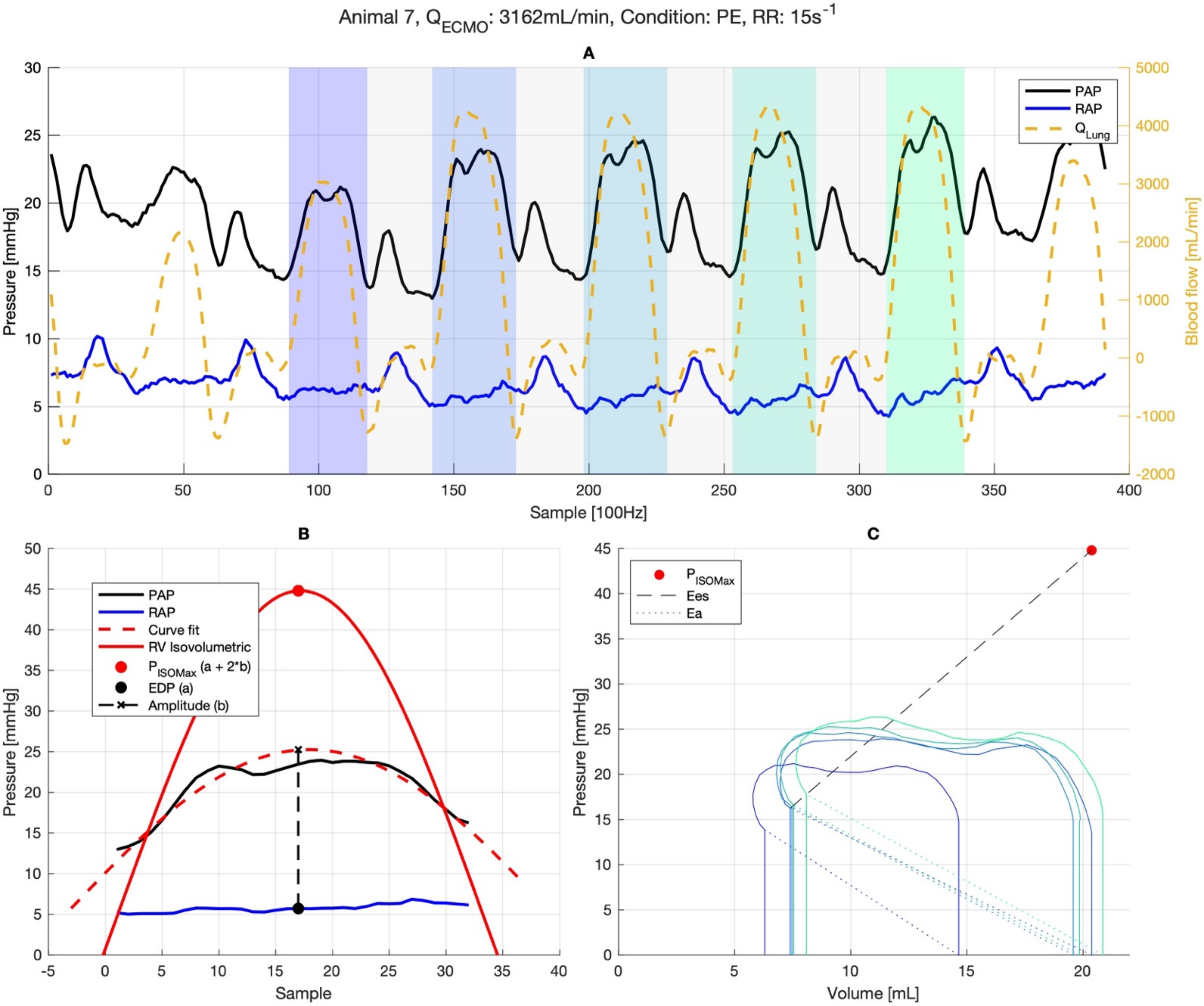

Animal 7, Q_{ECMO}: 3162mL/min, Condition: PE, RR: $15s^{-1}$
A
PAP
RAP
Q_{Lung}
Pressure [mmHg]
Blood flow [mL/min]
Sample [100Hz]
B
PAP
RAP
Curve fit
RV Isovolumetric
P_{ISOMax} (a + 2*b)
EDP (a)
Amplitude (b)
Pressure [mmHg]
Sample
C
P_{ISOMax}
Ees
Ea
Pressure [mmHg]
Volume [mL]

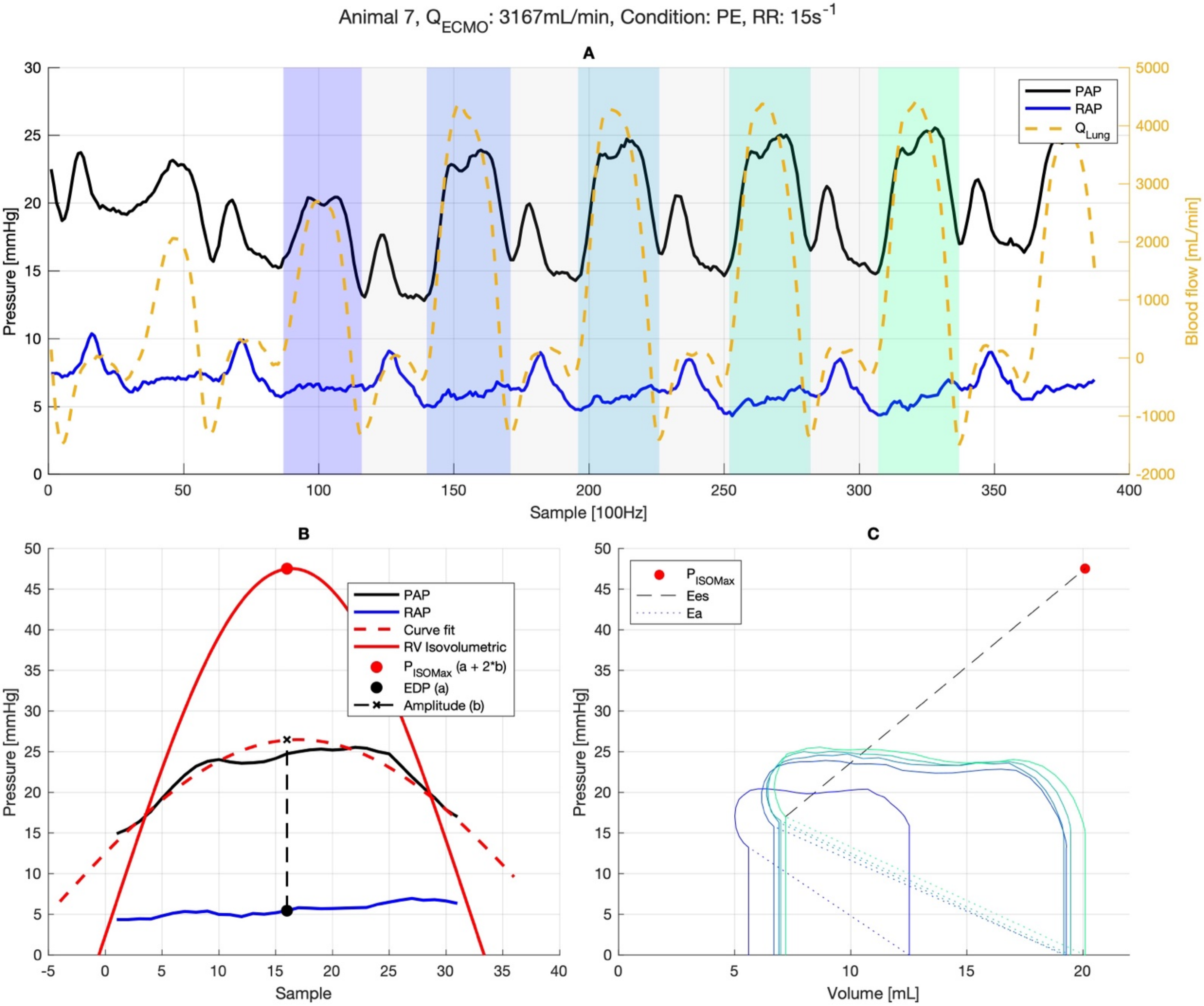

Animal 7, Q_{ECMO}: 3167mL/min, Condition: PE, RR: $15s^{-1}$
A
PAP
RAP
Q_{Lung}
Pressure [mmHg]
Blood flow [mL/min]
Sample [100Hz]
B
PAP
RAP
Curve fit
RV Isovolumetric
P_{ISOMax} (a + 2*b)
EDP (a)
Amplitude (b)
Pressure [mmHg]
Sample
C
P_{ISOMax}
Ees
Ea
Pressure [mmHg]
Volume [mL]

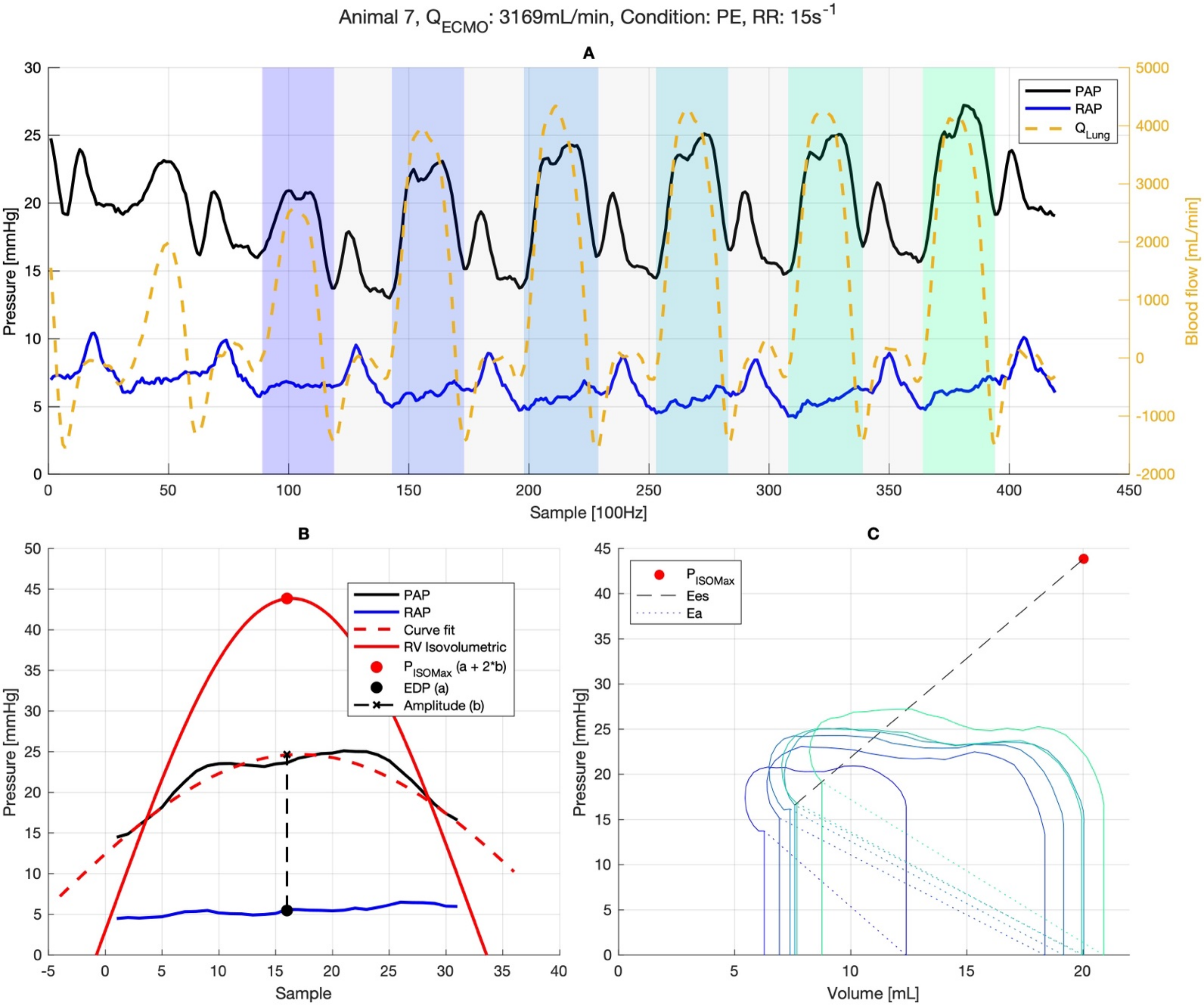
Animal 7, QECMO: 3169mL/min, Condition: PE, RR: 15s-1
A
Pressure [mmHg]
Blood flow [mL/min]
Sample [100Hz]
PAP
RAP
QLung
B
Pressure [mmHg]
Sample
PAP
RAP
Curve fit
RV Isovolumetric
PISOMax (a + 2*b)
EDP (a)
Amplitude (b)
C
Pressure [mmHg]
Volume [mL]
PISOMax
Ees
Ea

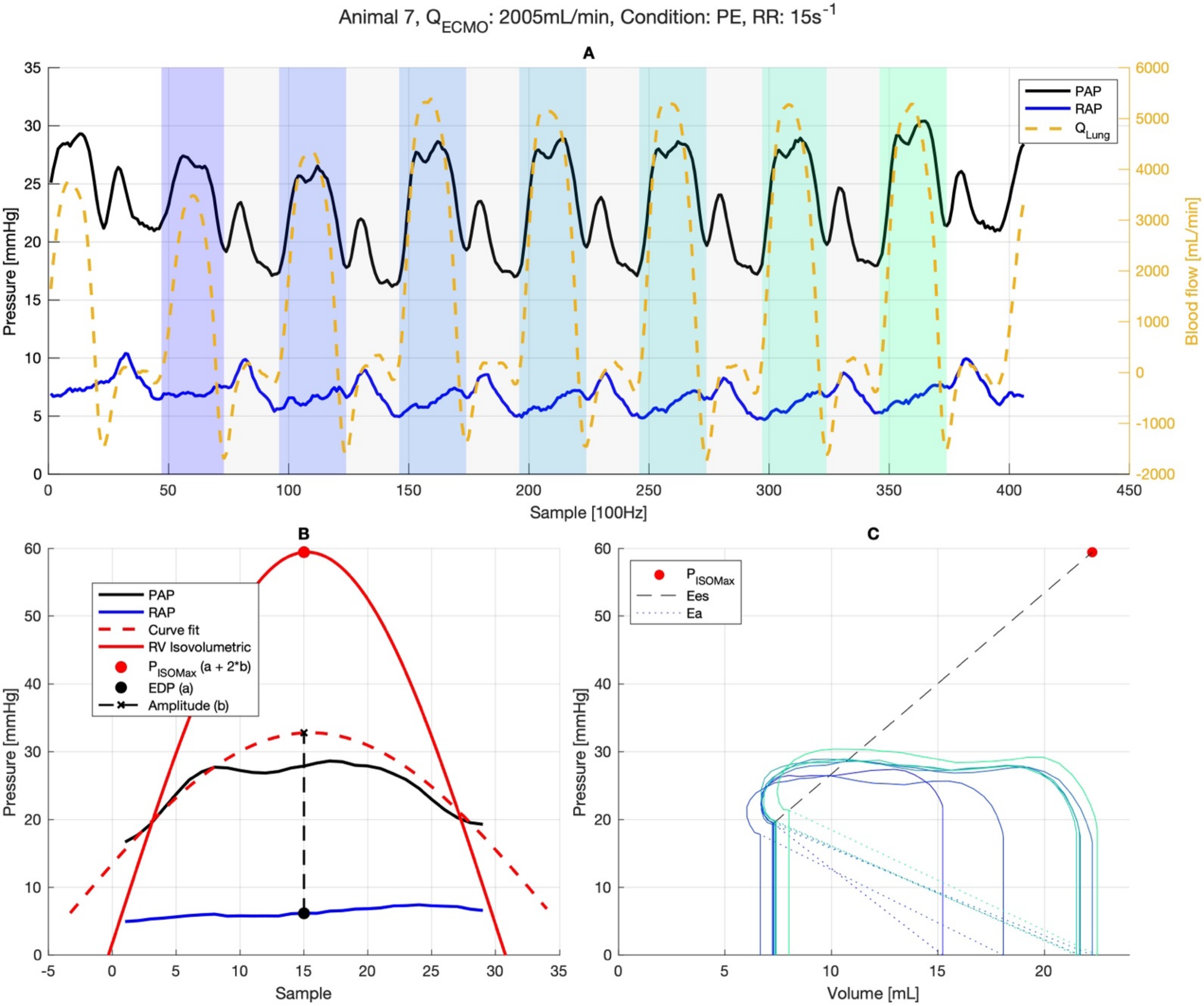

Animal 7, Q_{ECMO}: 2005mL/min, Condition: PE, RR: $15s^{-1}$
A
Pressure [mmHg]
Blood flow [mL/min]
Sample [100Hz]
PAP
RAP
Q_{Lung}
B
PAP
RAP
Curve fit
RV Isovolumetric
P_{ISOMax} (a + 2*b)
EDP (a)
Amplitude (b)
Pressure [mmHg]
Sample
C
P_{ISOMax}
Ees
Ea
Pressure [mmHg]
Volume [mL]

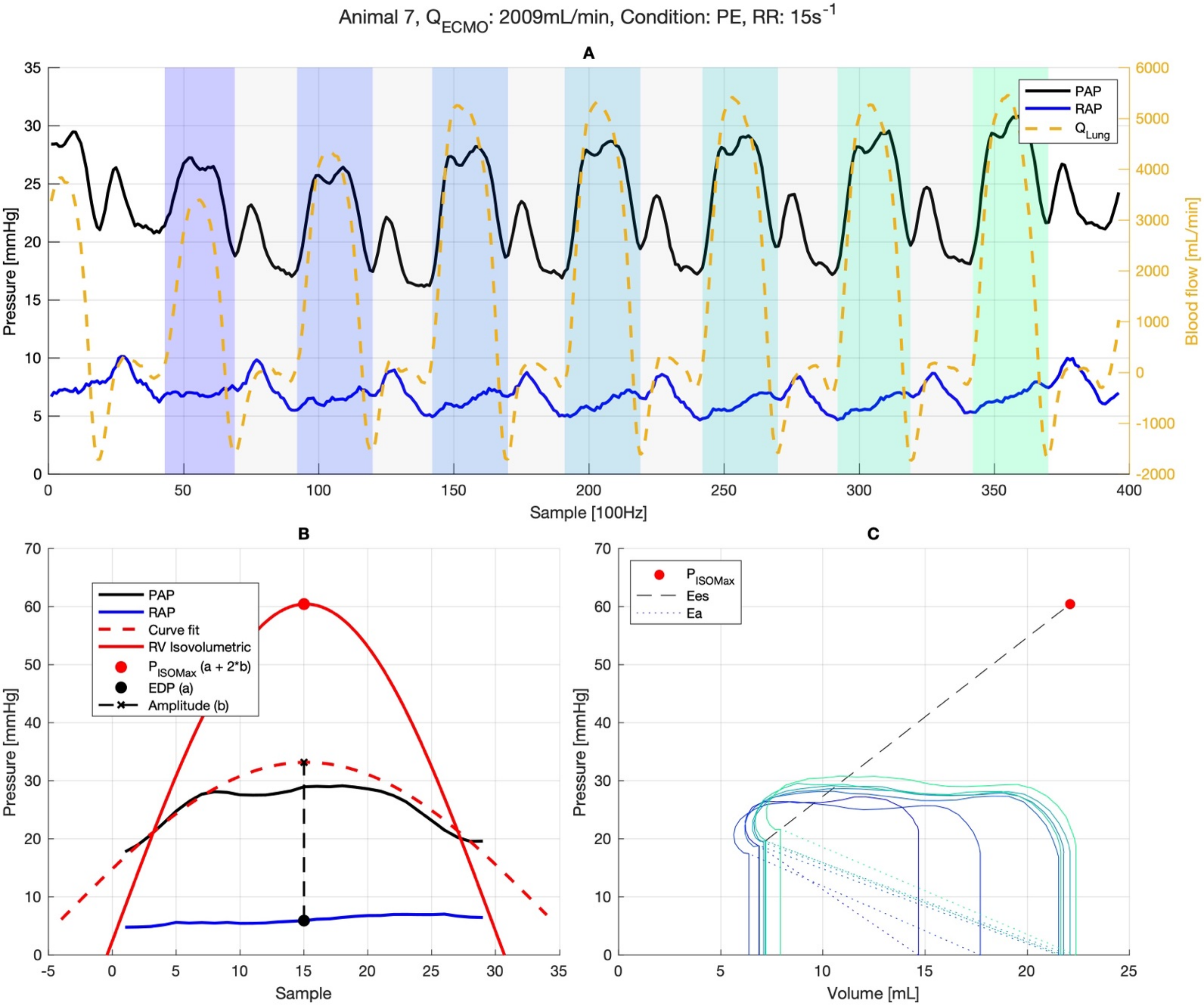

Animal 7, Q_{ECMO}: 2009mL/min, Condition: PE, RR: $15s^{-1}$
A
PAP
RAP
Q_{Lung}
Pressure [mmHg]
Blood flow [mL/min]
Sample [100Hz]
B
PAP
RAP
Curve fit
RV Isovolumetric
P_{ISOMax} (a + 2*b)
EDP (a)
Amplitude (b)
Pressure [mmHg]
Sample
C
P_{ISOMax}
Ees
Ea
Pressure [mmHg]
Volume [mL]

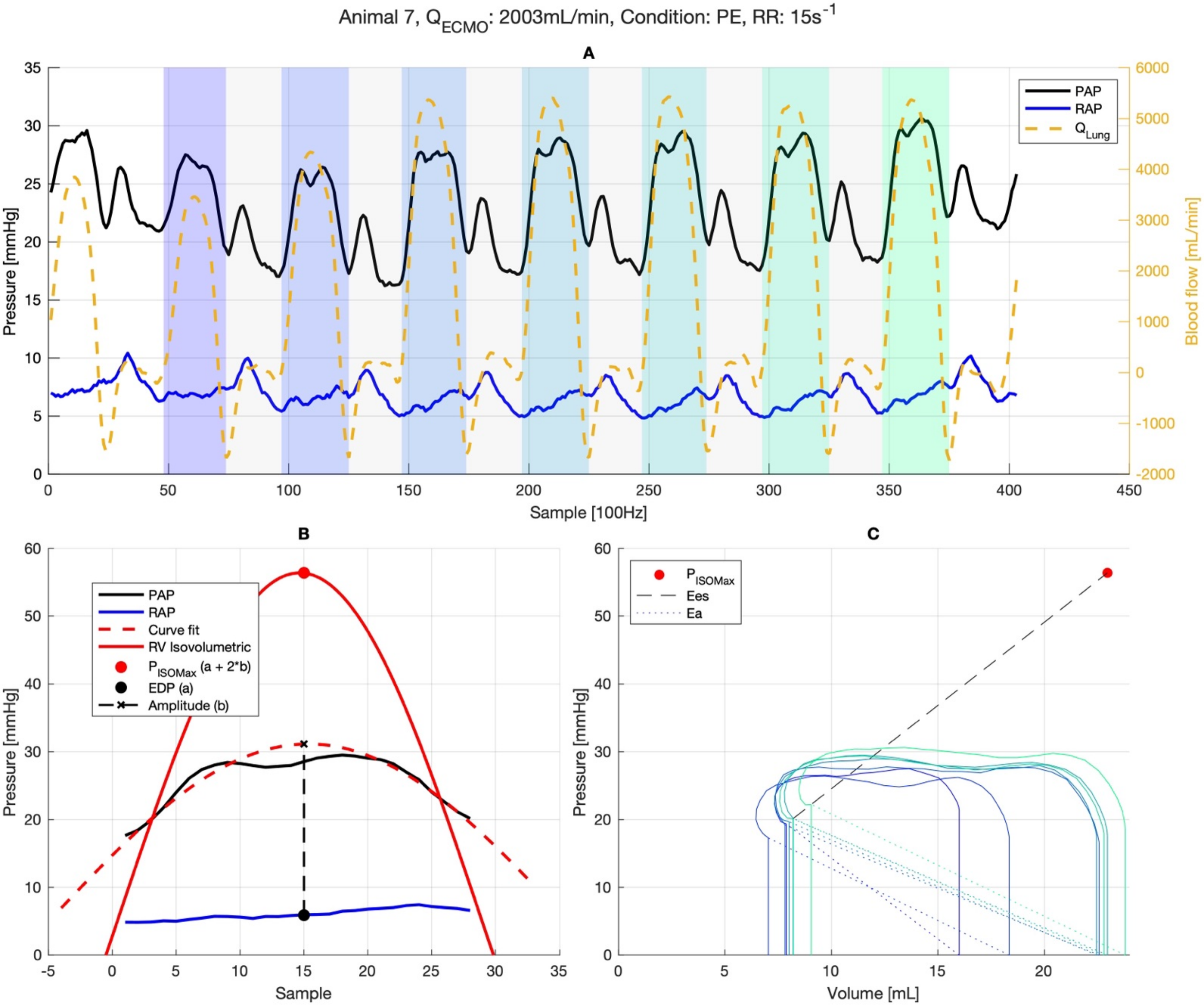

Animal 7, Q_{ECMO}: 2003mL/min, Condition: PE, RR: $15s^{-1}$
A
PAP
RAP
Q_{Lung}
Pressure [mmHg]
Blood flow [mL/min]
Sample [100Hz]
B
PAP
RAP
Curve fit
RV Isovolumetric
P_{ISOMax} (a + 2*b)
EDP (a)
Amplitude (b)
Pressure [mmHg]
Sample
C
P_{ISOMax}
Ees
Ea
Pressure [mmHg]
Volume [mL]

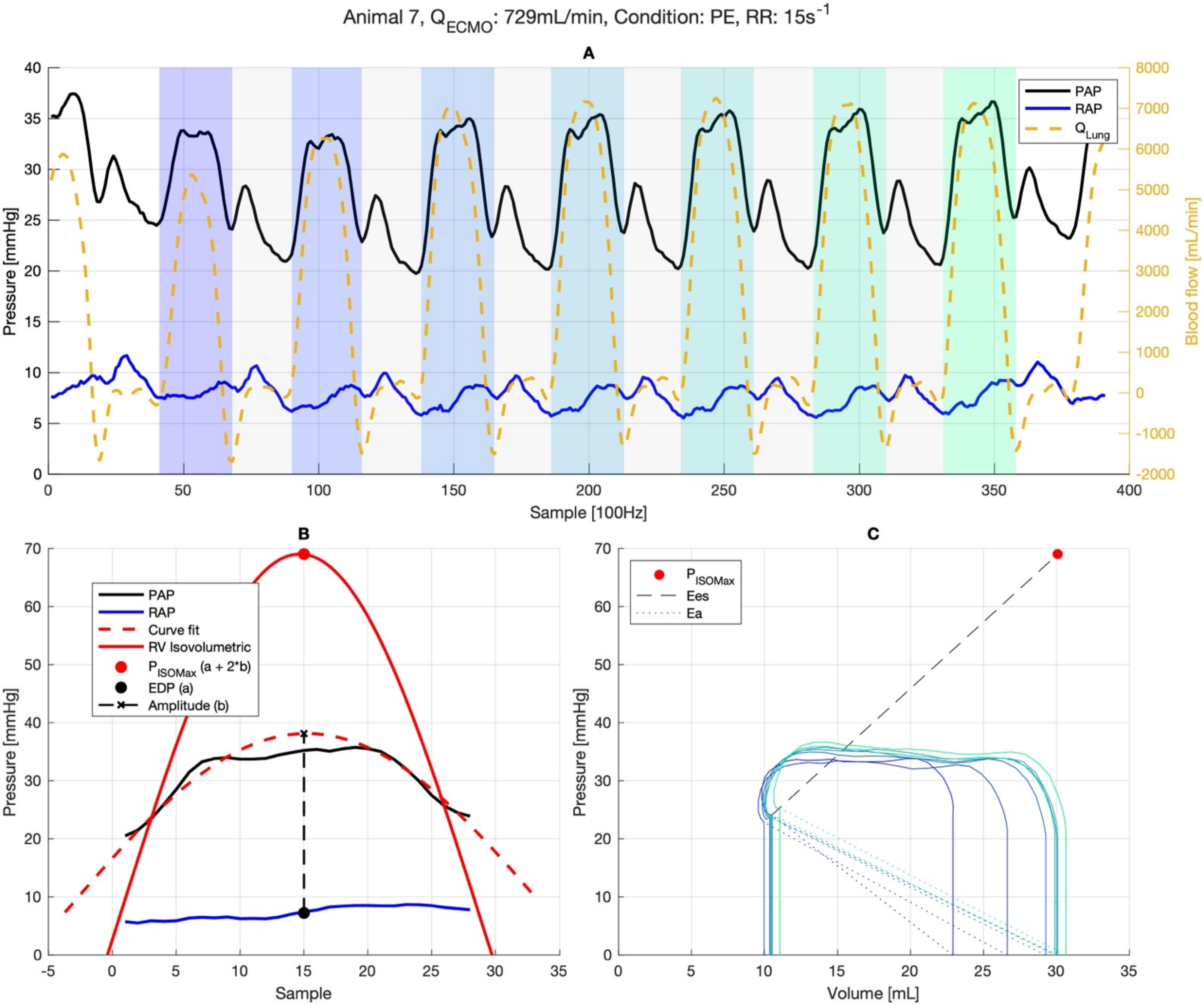

Animal 7, Q_{ECMO}: 729mL/min, Condition: PE, RR: $15s^{-1}$
A
PAP
RAP
Q_{Lung}
Pressure [mmHg]
Blood flow [mL/min]
Sample [100Hz]
B
PAP
RAP
Curve fit
RV Isovolumetric
P_{ISOMax} (a + 2*b)
EDP (a)
Amplitude (b)
Pressure [mmHg]
Sample
C
P_{ISOMax}
Ees
Ea
Pressure [mmHg]
Volume [mL]

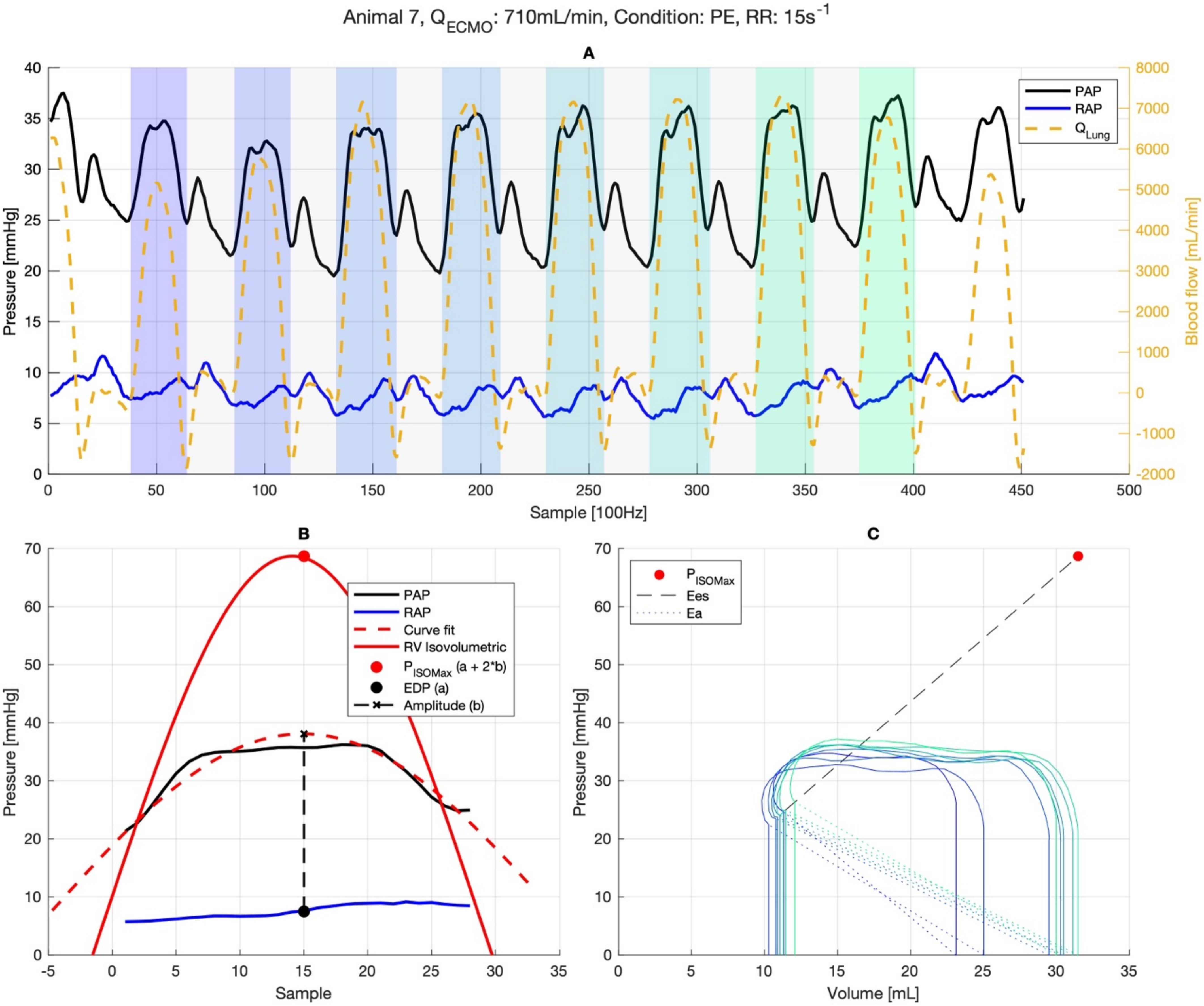

Animal 7, Q_ECMO: 710mL/min, Condition: PE, RR: 15s^-1
A
PAP
RAP
Q_Lung
Pressure [mmHg]
Blood flow [mL/min]
Sample [100Hz]
B
PAP
RAP
Curve fit
RV Isovolumetric
P_ISOMax (a + 2*b)
EDP (a)
Amplitude (b)
Pressure [mmHg]
Sample
C
P_ISOMax
Ees
Ea
Pressure [mmHg]
Volume [mL]

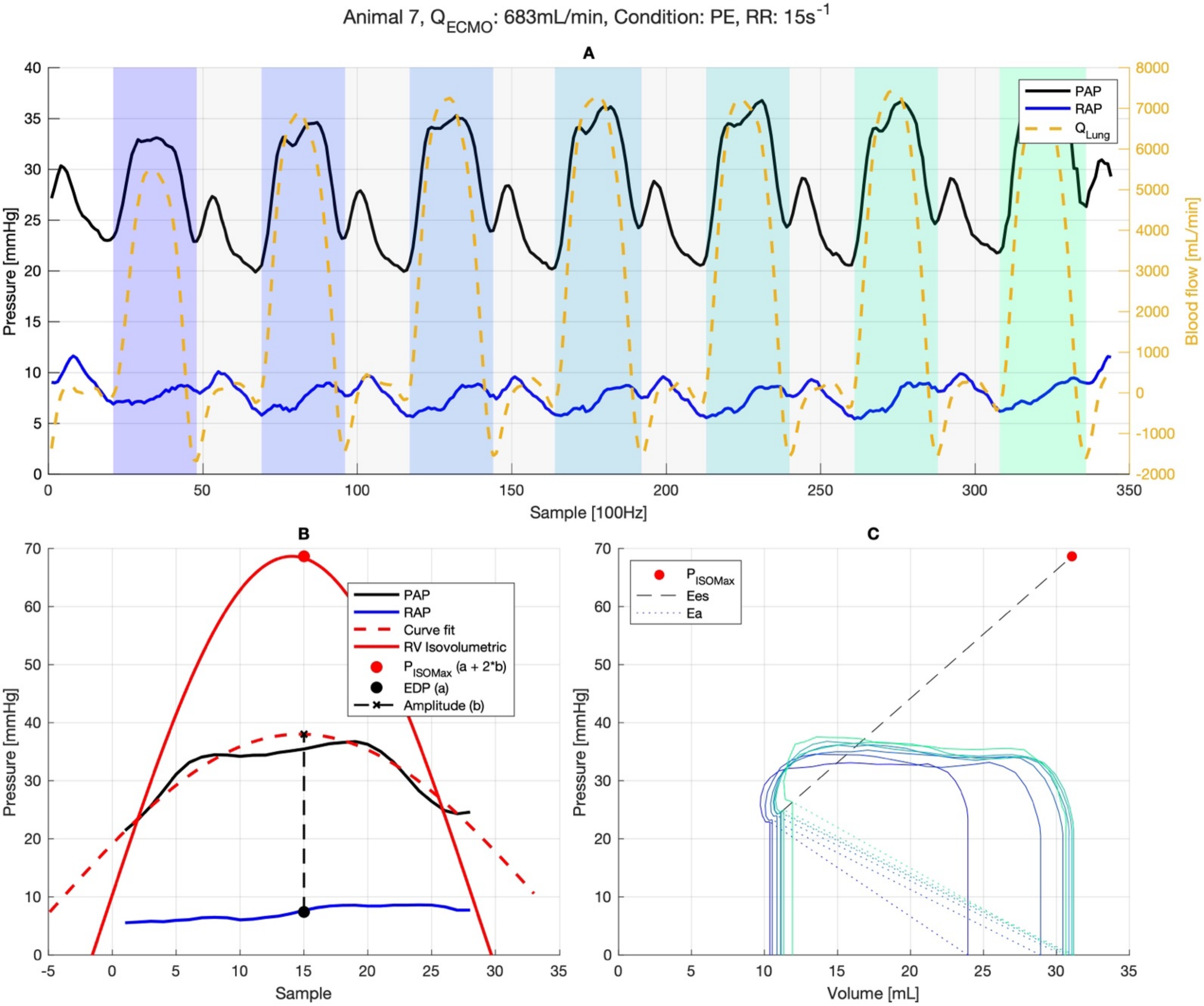

Animal 7, Q_{ECMO}: 683mL/min, Condition: PE, RR: 15s^{-1}
A
Pressure [mmHg]
Blood flow [mL/min]
Sample [100Hz]
PAP
RAP
Q_{Lung}
B
Pressure [mmHg]
Sample
PAP
RAP
Curve fit
RV Isovolumetric
P_{ISOMax} (a + 2*b)
EDP (a)
Amplitude (b)
C
Pressure [mmHg]
Volume [mL]
P_{ISOMax}
Ees
Ea

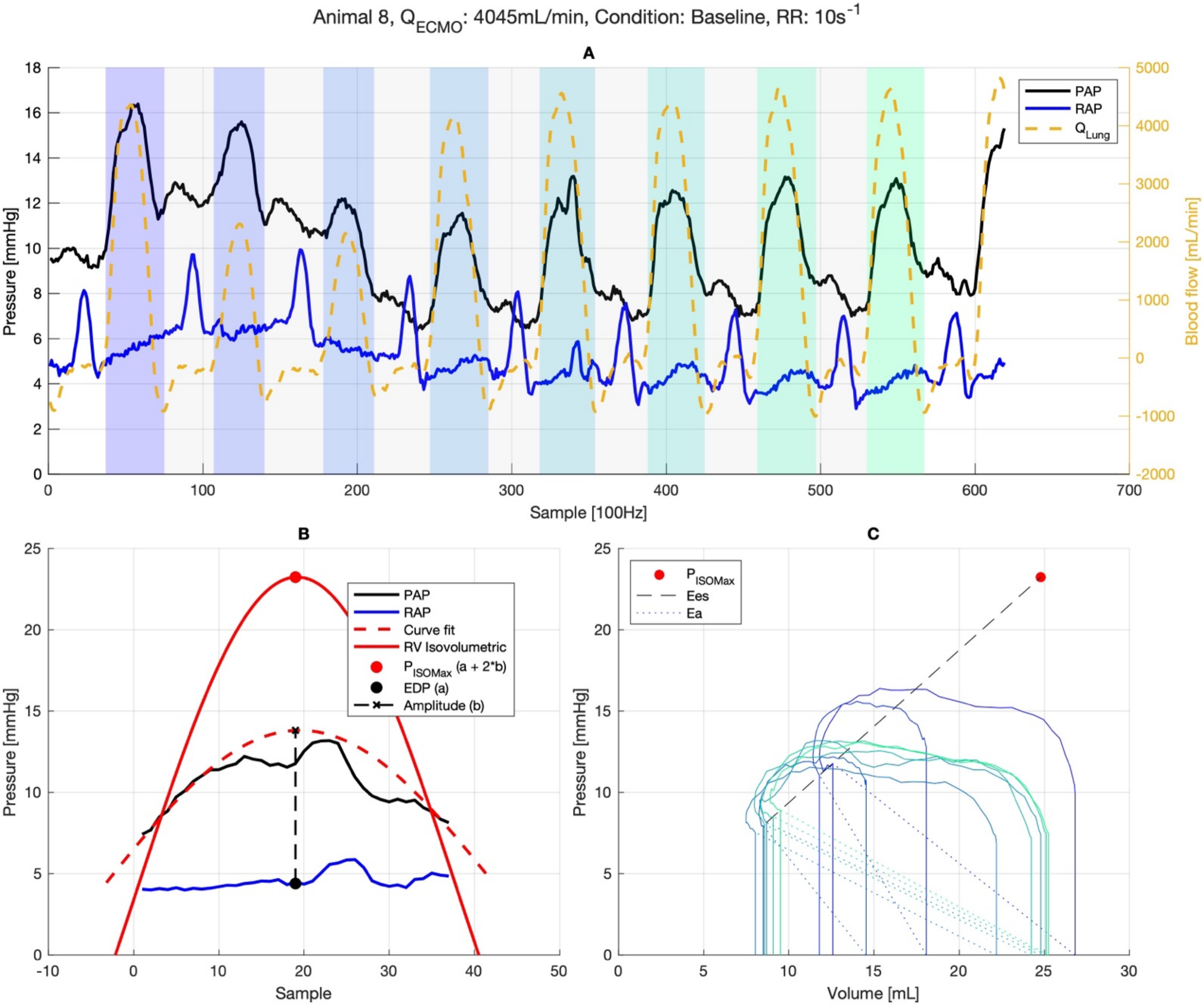

Animal 8, Q_{ECMO}: 4045mL/min, Condition: Baseline, RR: $10s^{-1}$
A
PAP
RAP
Q_{Lung}
Pressure [mmHg]
Blood flow [mL/min]
Sample [100Hz]
B
PAP
RAP
Curve fit
RV Isovolumetric
P_{ISOMax} (a + 2*b)
EDP (a)
Amplitude (b)
Pressure [mmHg]
Sample
C
P_{ISOMax}
Ees
Ea
Pressure [mmHg]
Volume [mL]

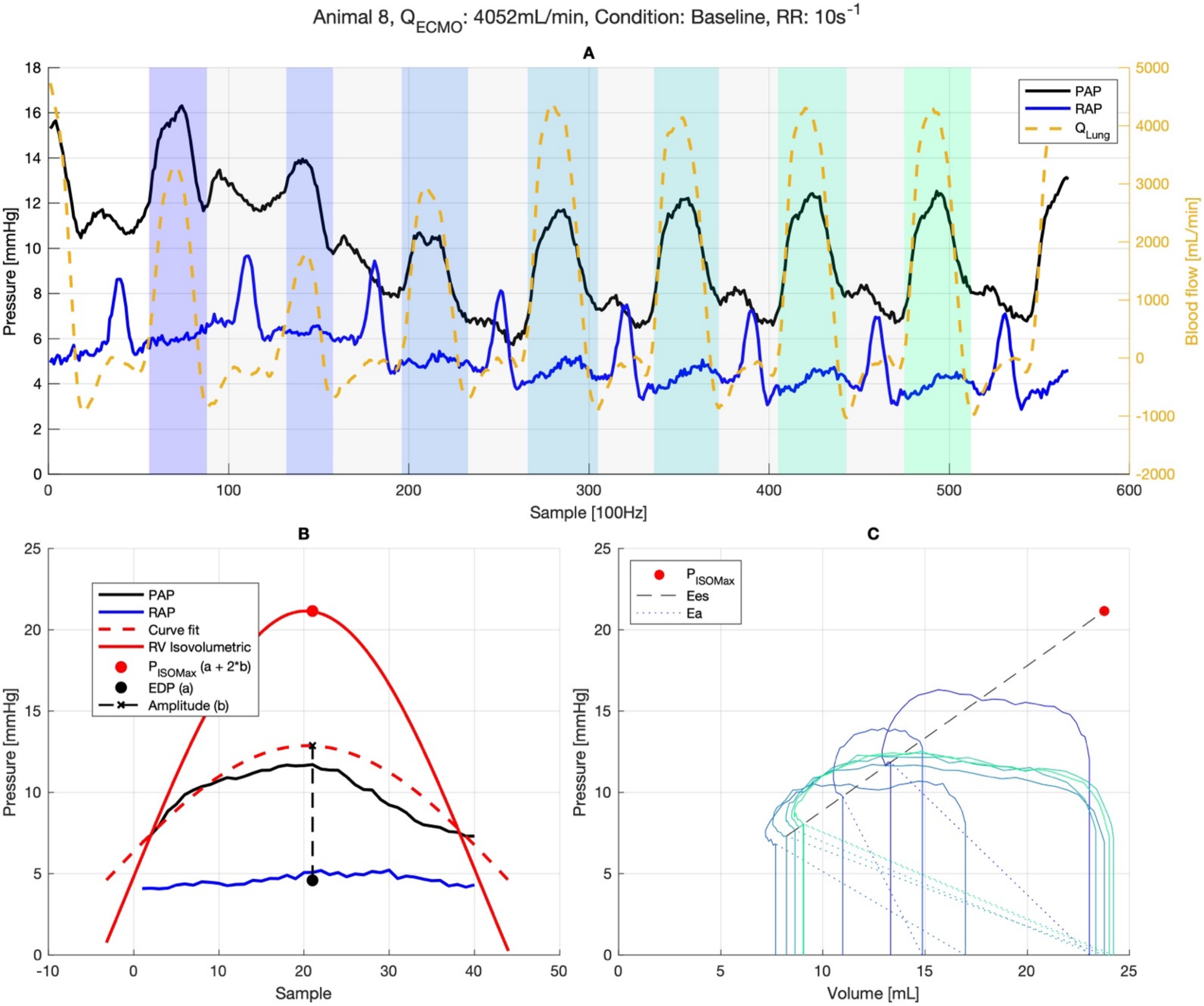

Animal 8, Q_{ECMO}: 4052mL/min, Condition: Baseline, RR: $10s^{-1}$
A
PAP
RAP
Q_{Lung}
Pressure [mmHg]
Blood flow [mL/min]
Sample [100Hz]
B
PAP
RAP
Curve fit
RV Isovolumetric
P_{ISOMax} (a + 2*b)
EDP (a)
Amplitude (b)
Pressure [mmHg]
Sample
C
P_{ISOMax}
Ees
Ea
Pressure [mmHg]
Volume [mL]

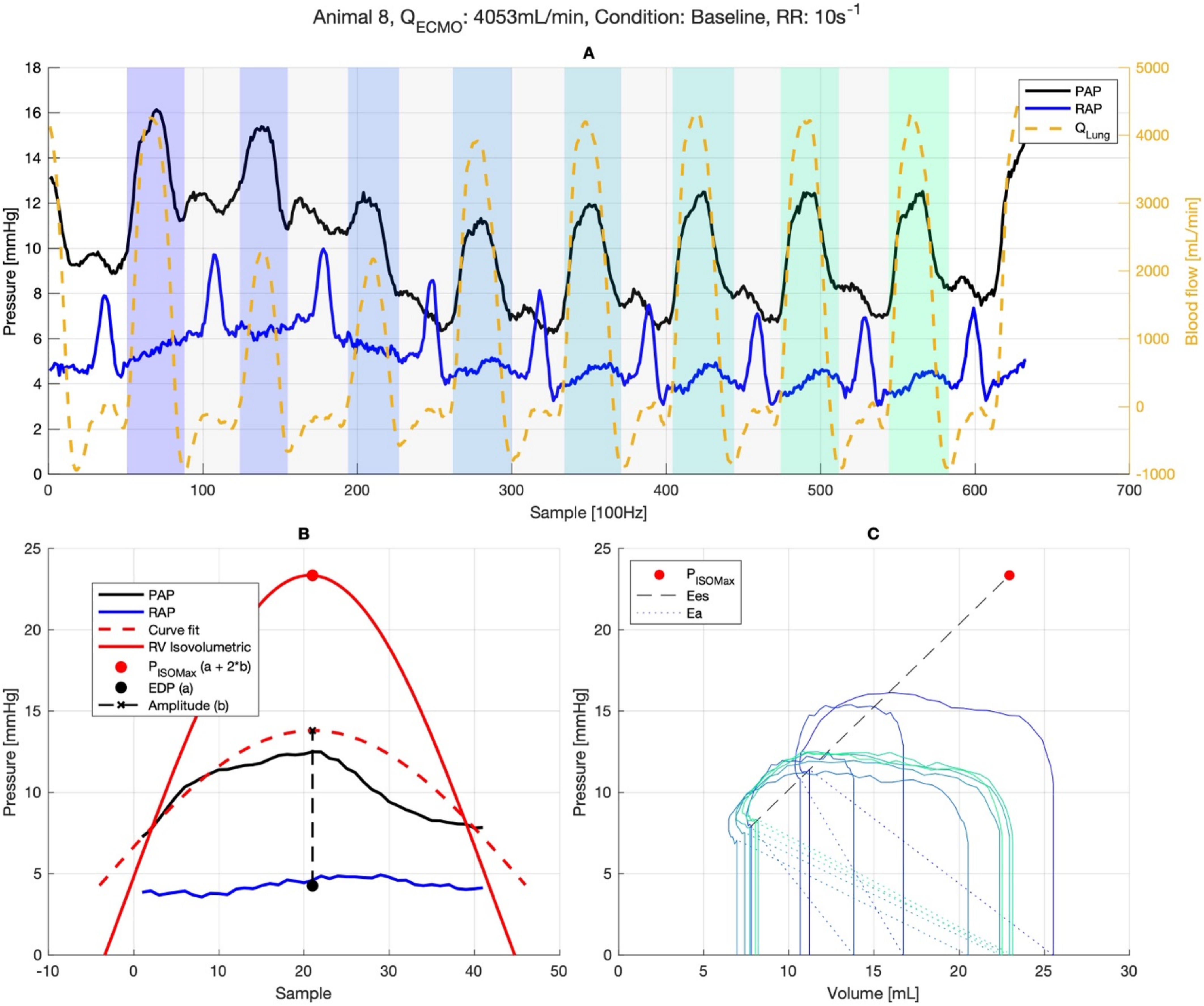

Animal 8, Q_{ECMO}: 4053mL/min, Condition: Baseline, RR: $10s^{-1}$
A
PAP
RAP
Q_{Lung}
Pressure [mmHg]
Blood flow [mL/min]
Sample [100Hz]
B
PAP
RAP
Curve fit
RV Isovolumetric
P_{ISOMax} (a + 2*b)
EDP (a)
Amplitude (b)
Pressure [mmHg]
Sample
C
P_{ISOMax}
Ees
Ea
Pressure [mmHg]
Volume [mL]

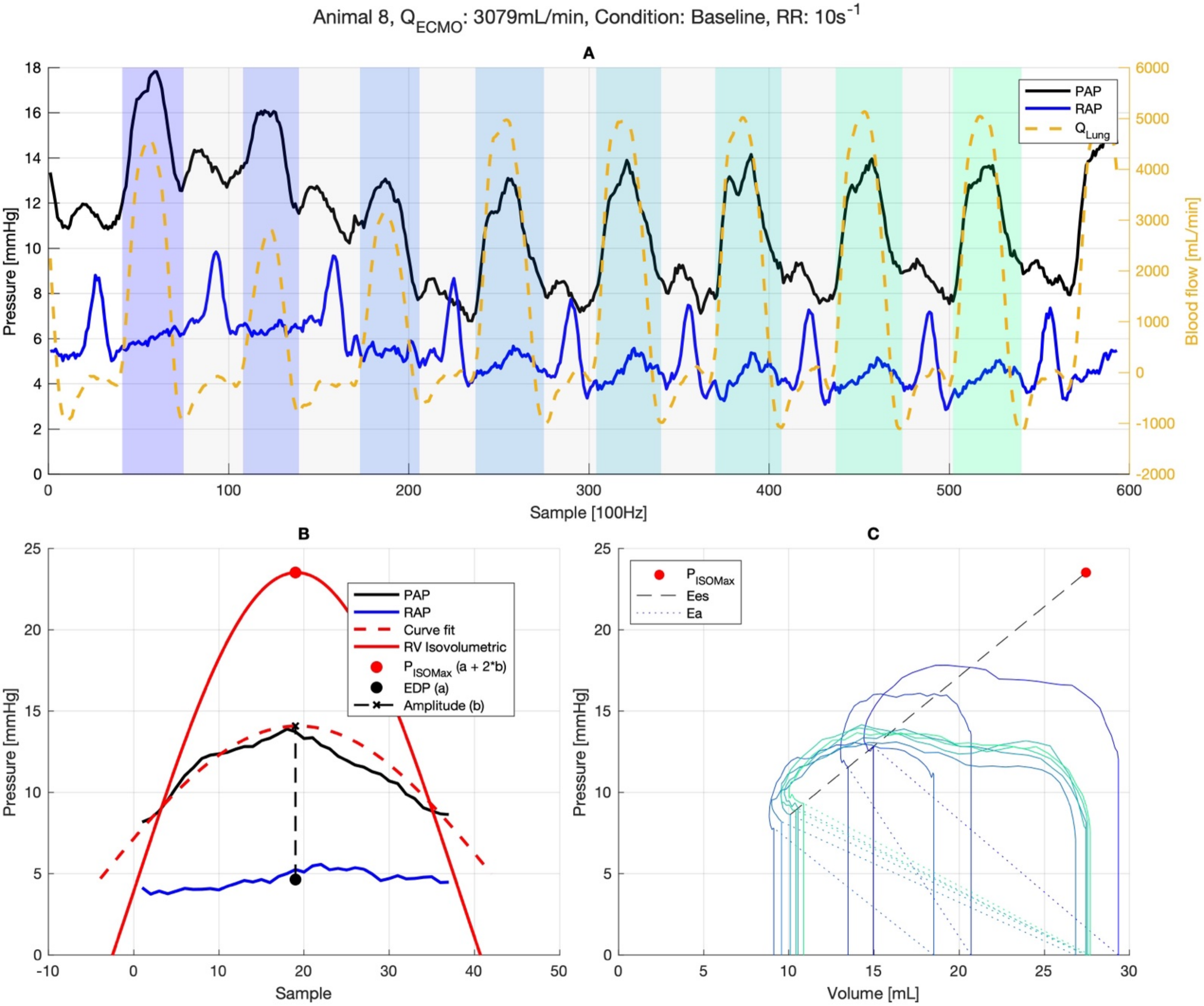

Animal 8, Q_{ECMO}: 3079mL/min, Condition: Baseline, RR: $10s^{-1}$
A
PAP
RAP
Q_{Lung}
Pressure [mmHg]
Blood flow [mL/min]
Sample [100Hz]
B
PAP
RAP
Curve fit
RV Isovolumetric
P_{ISOMax} (a + 2*b)
EDP (a)
Amplitude (b)
Pressure [mmHg]
Sample
C
P_{ISOMax}
Ees
Ea
Pressure [mmHg]
Volume [mL]

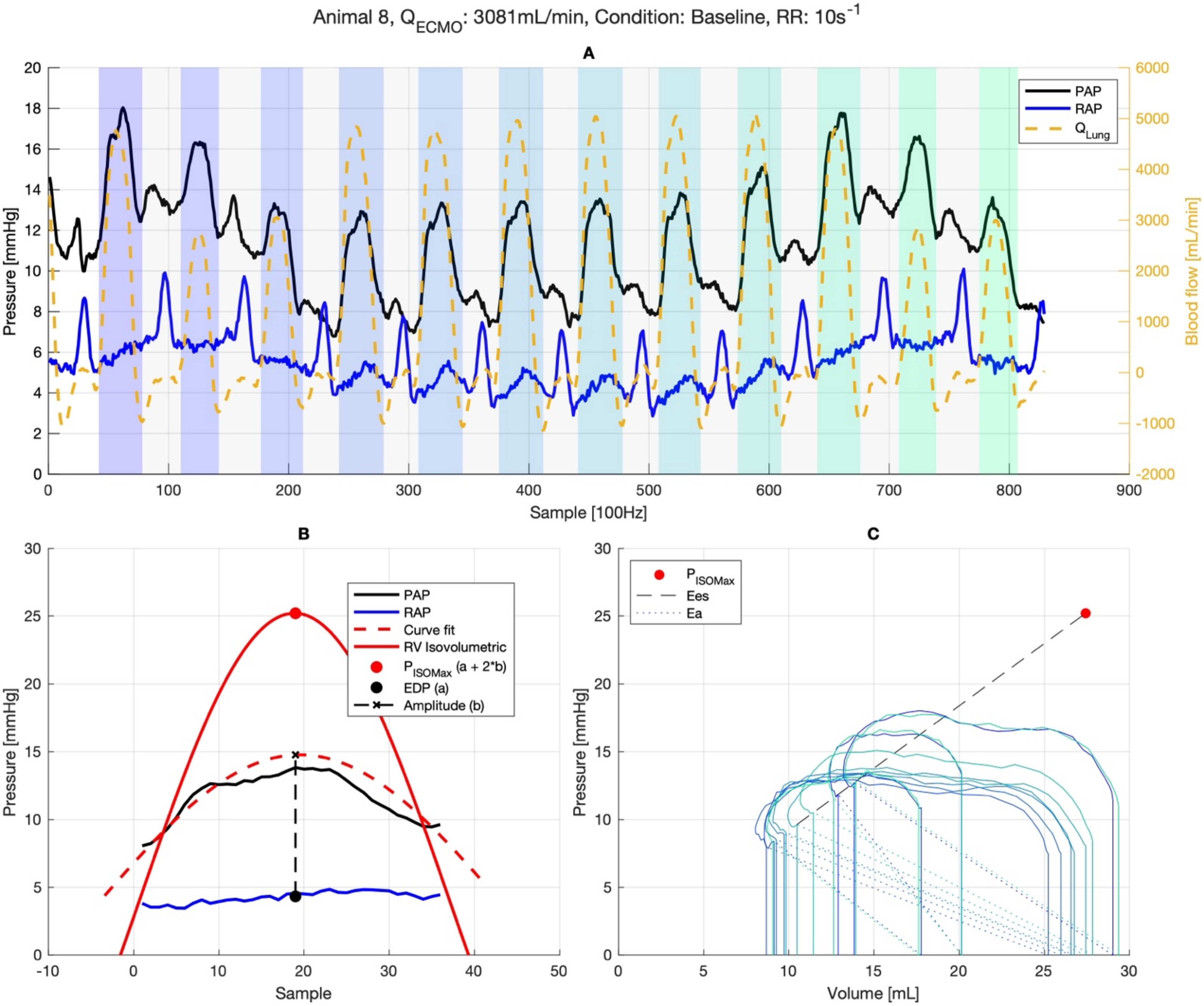
Animal 8, Q_{ECMO}: 3081mL/min, Condition: Baseline, RR: $10s^{-1}$
A
PAP
RAP
Q_{Lung}
Pressure [mmHg]
Blood flow [mL/min]
Sample [100Hz]
B
PAP
RAP
Curve fit
RV Isovolumetric
P_{ISOMax} (a + 2*b)
EDP (a)
Amplitude (b)
Pressure [mmHg]
Sample
C
P_{ISOMax}
Ees
Ea
Pressure [mmHg]
Volume [mL]

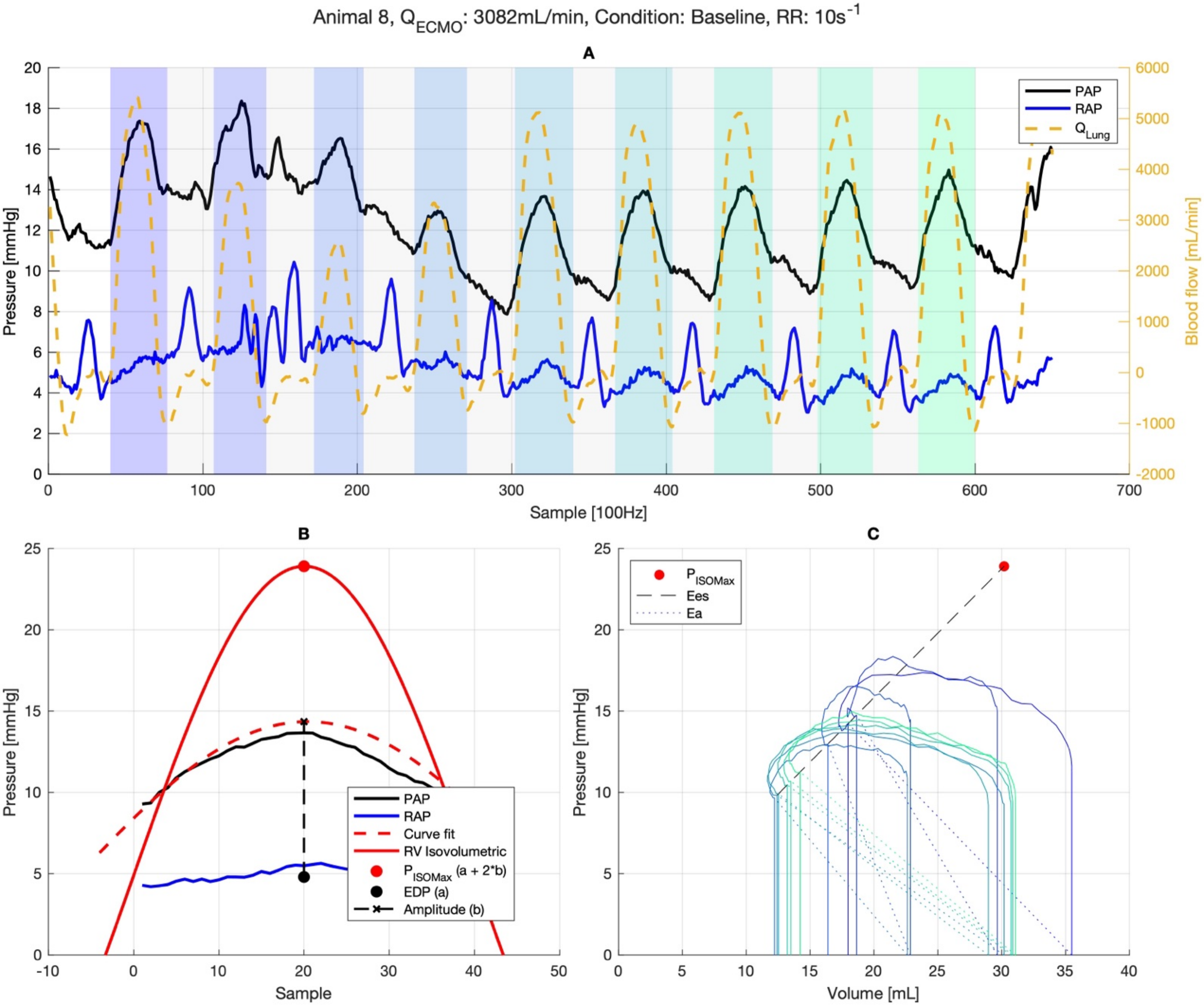

Animal 8, QECMO: 3082mL/min, Condition: Baseline, RR: 10s-1
A
PAP
RAP
QLung
Pressure [mmHg]
Blood flow [mL/min]
Sample [100Hz]
B
PAP
RAP
Curve fit
RV Isovolumetric
PISOMax (a + 2*b)
EDP (a)
Amplitude (b)
Pressure [mmHg]
Sample
C
PISOMax
Ees
Ea
Pressure [mmHg]
Volume [mL]

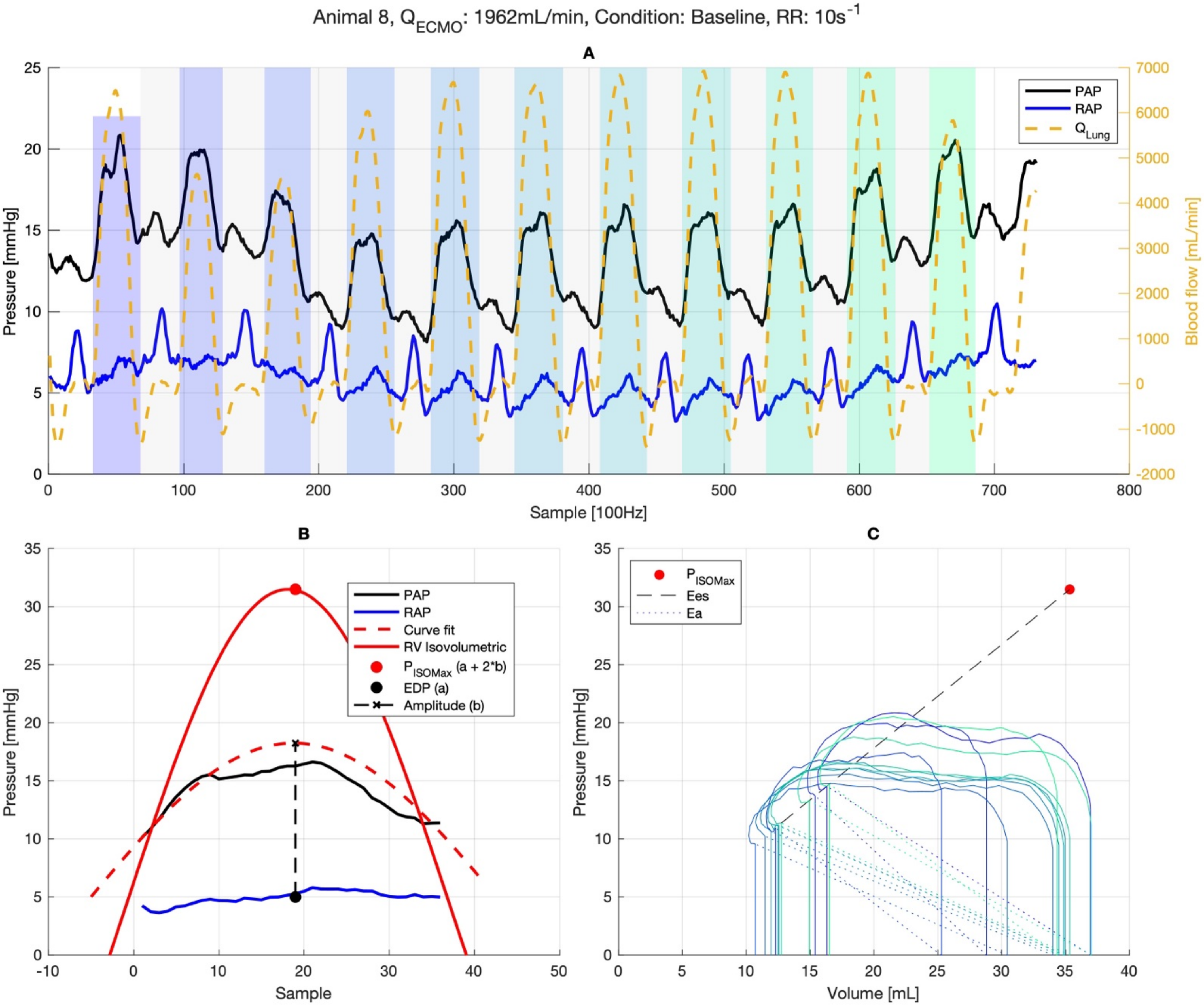

Animal 8, Q_ECMO: 1962mL/min, Condition: Baseline, RR: 10s^-1
A
PAP
RAP
Q_Lung
Pressure [mmHg]
Blood flow [mL/min]
Sample [100Hz]
B
PAP
RAP
Curve fit
RV Isovolumetric
P_ISOMax (a + 2*b)
EDP (a)
Amplitude (b)
Pressure [mmHg]
Sample
C
P_ISOMax
Ees
Ea
Pressure [mmHg]
Volume [mL]

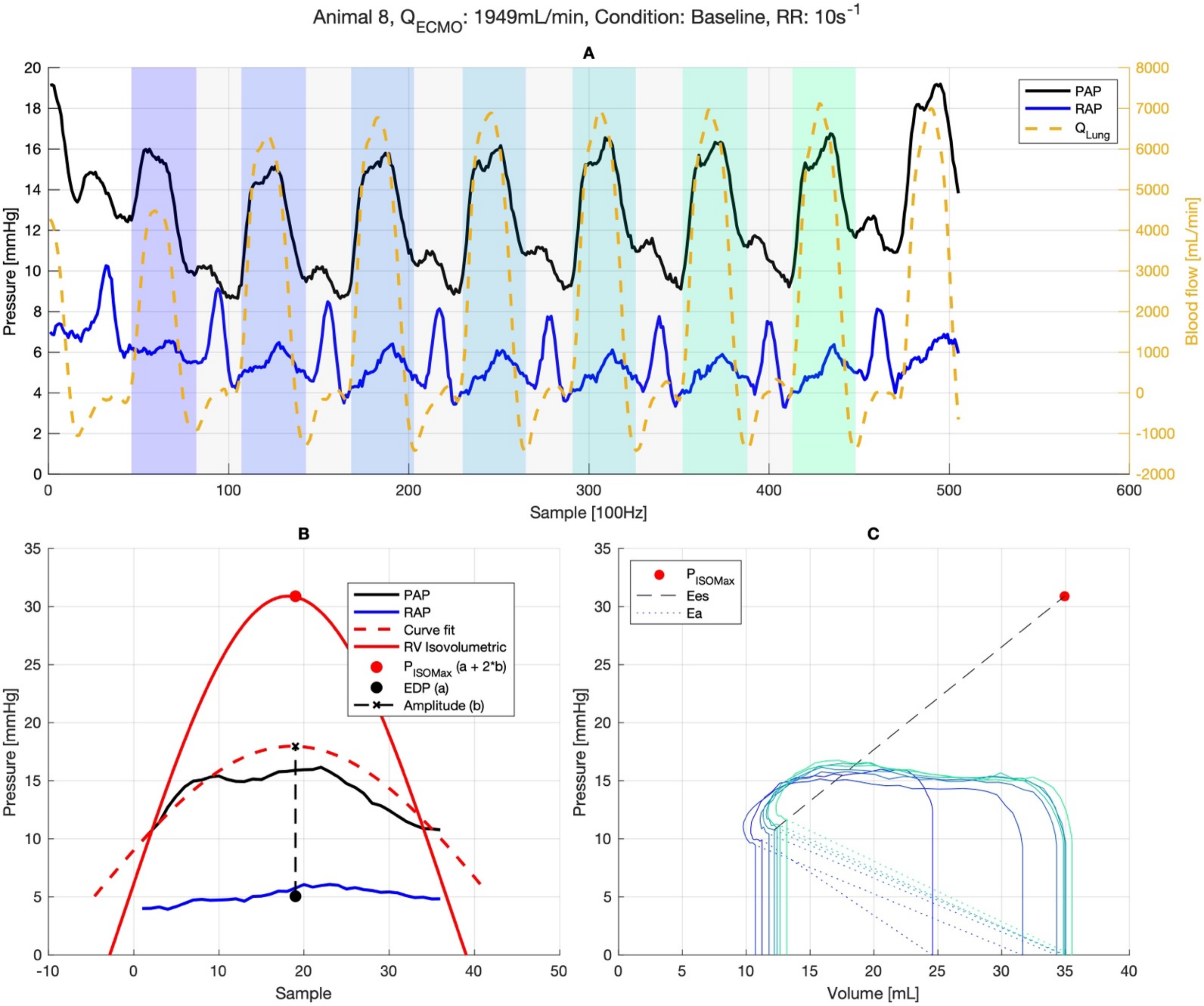

Animal 8, Q_{ECMO}: 1949mL/min, Condition: Baseline, RR: $10s^{-1}$
A
PAP
RAP
Q_{Lung}
Pressure [mmHg]
Blood flow [mL/min]
Sample [100Hz]
B
PAP
RAP
Curve fit
RV Isovolumetric
P_{ISOMax} (a + 2*b)
EDP (a)
Amplitude (b)
Pressure [mmHg]
Sample
C
P_{ISOMax}
Ees
Ea
Pressure [mmHg]
Volume [mL]

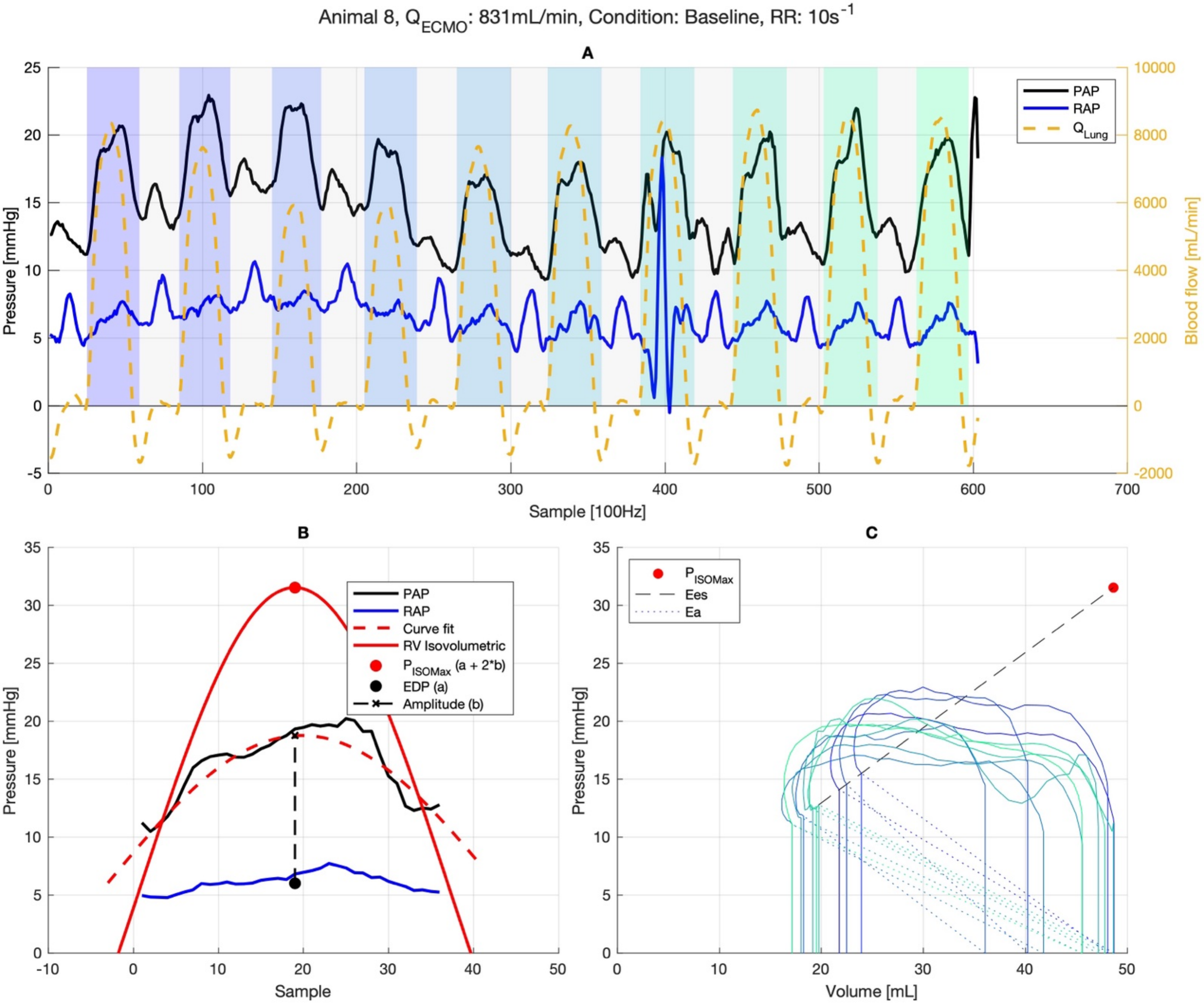

Animal 8, Q_{ECMO}: 831mL/min, Condition: Baseline, RR: $10s^{-1}$
A
PAP
RAP
Q_{Lung}
Pressure [mmHg]
Blood flow [mL/min]
Sample [100Hz]
B
PAP
RAP
Curve fit
RV Isovolumetric
P_{ISOMax} (a + 2*b)
EDP (a)
Amplitude (b)
Pressure [mmHg]
Sample
C
P_{ISOMax}
Ees
Ea
Pressure [mmHg]
Volume [mL]

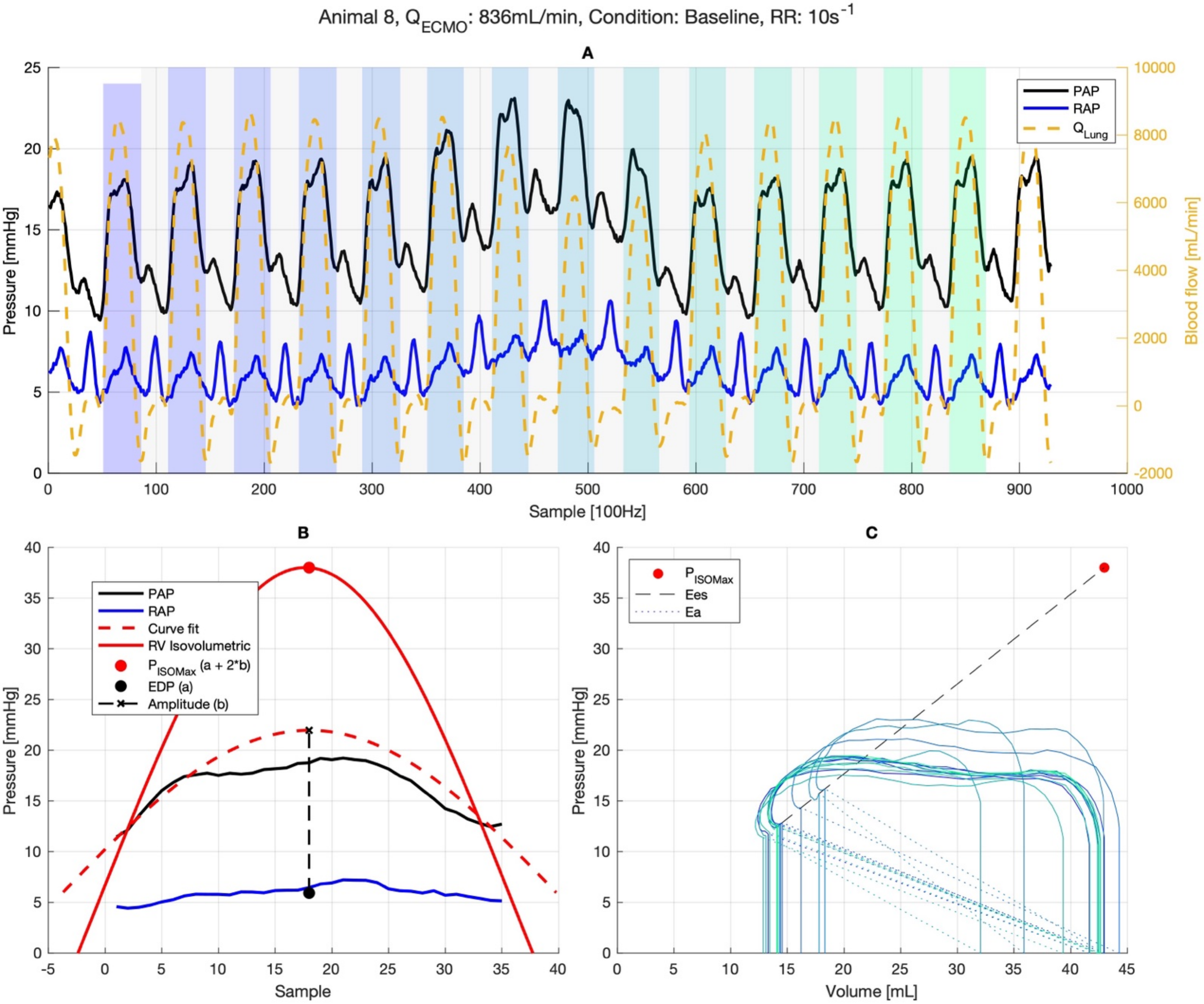

Animal 8, Q_{ECMO}: 836mL/min, Condition: Baseline, RR: $10s^{-1}$
A
PAP
RAP
Q_{Lung}
Pressure [mmHg]
Blood flow [mL/min]
Sample [100Hz]
B
PAP
RAP
Curve fit
RV Isovolumetric
P_{ISOMax} (a + 2*b)
EDP (a)
Amplitude (b)
Pressure [mmHg]
Sample
C
P_{ISOMax}
Ees
Ea
Pressure [mmHg]
Volume [mL]

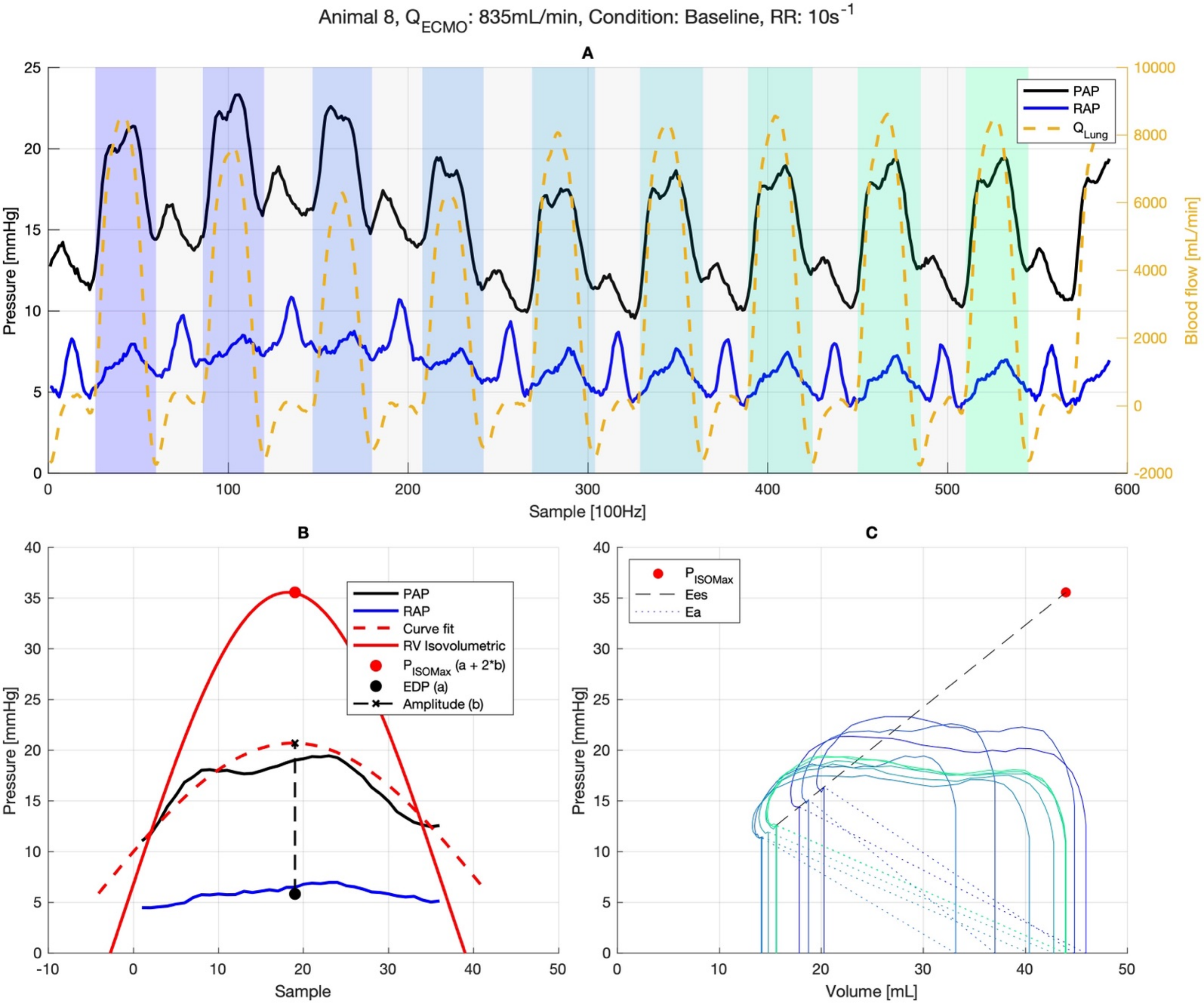

Animal 8, Q_ECMO: 835mL/min, Condition: Baseline, RR: 10s^-1
A
PAP
RAP
Q_Lung
Pressure [mmHg]
Blood flow [mL/min]
Sample [100Hz]
B
PAP
RAP
Curve fit
RV Isovolumetric
P_ISOMax (a + 2*b)
EDP (a)
Amplitude (b)
Pressure [mmHg]
Sample
C
P_ISOMax
Ees
Ea
Pressure [mmHg]
Volume [mL]

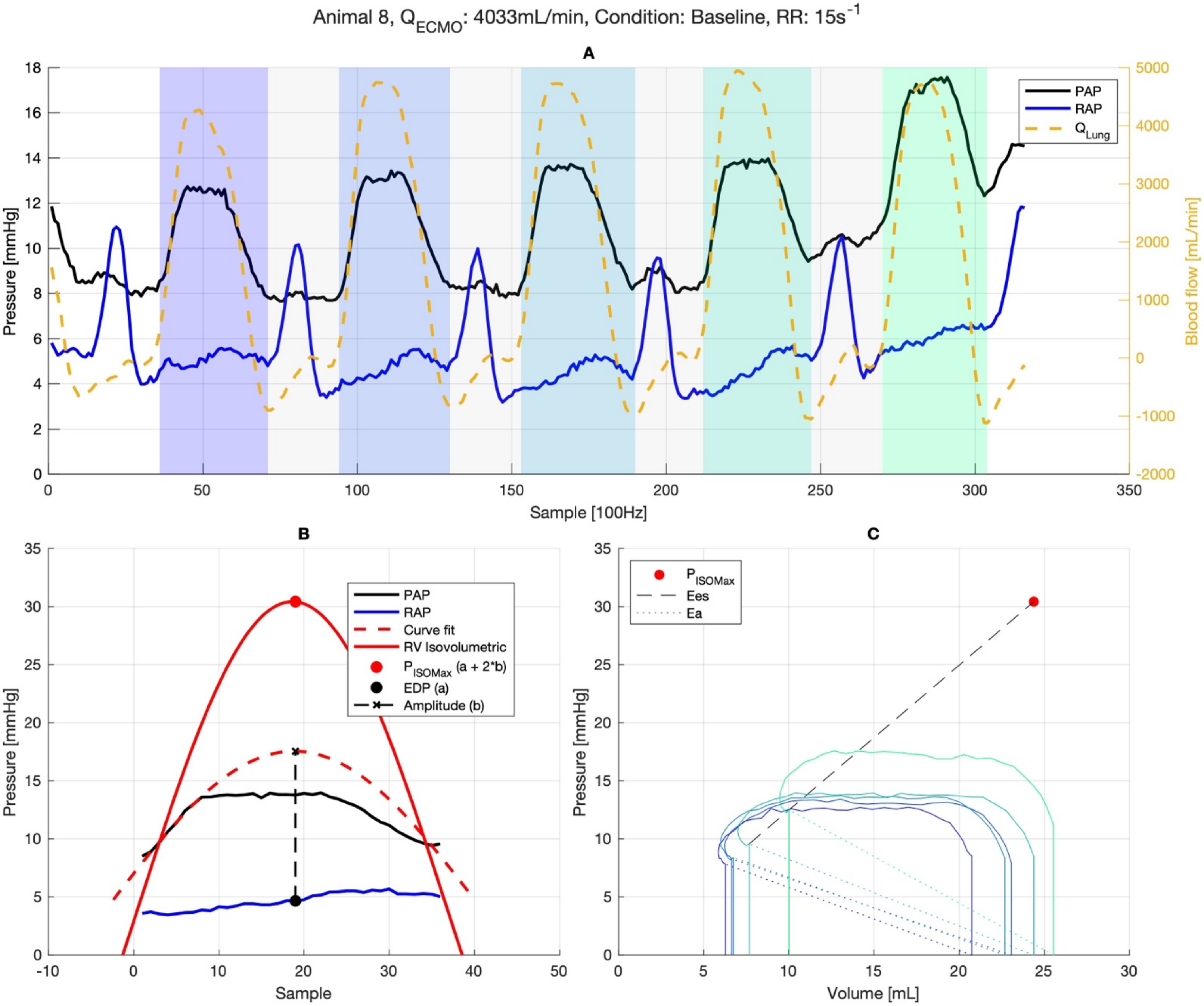
Animal 8, Q_ECMO: 4033mL/min, Condition: Baseline, RR: 15s^-1
A
PAP
RAP
Q_Lung
Pressure [mmHg]
Blood flow [mL/min]
Sample [100Hz]
B
PAP
RAP
Curve fit
RV Isovolumetric
P_ISOMax (a + 2*b)
EDP (a)
Amplitude (b)
Pressure [mmHg]
Sample
C
P_ISOMax
Ees
Ea
Pressure [mmHg]
Volume [mL]

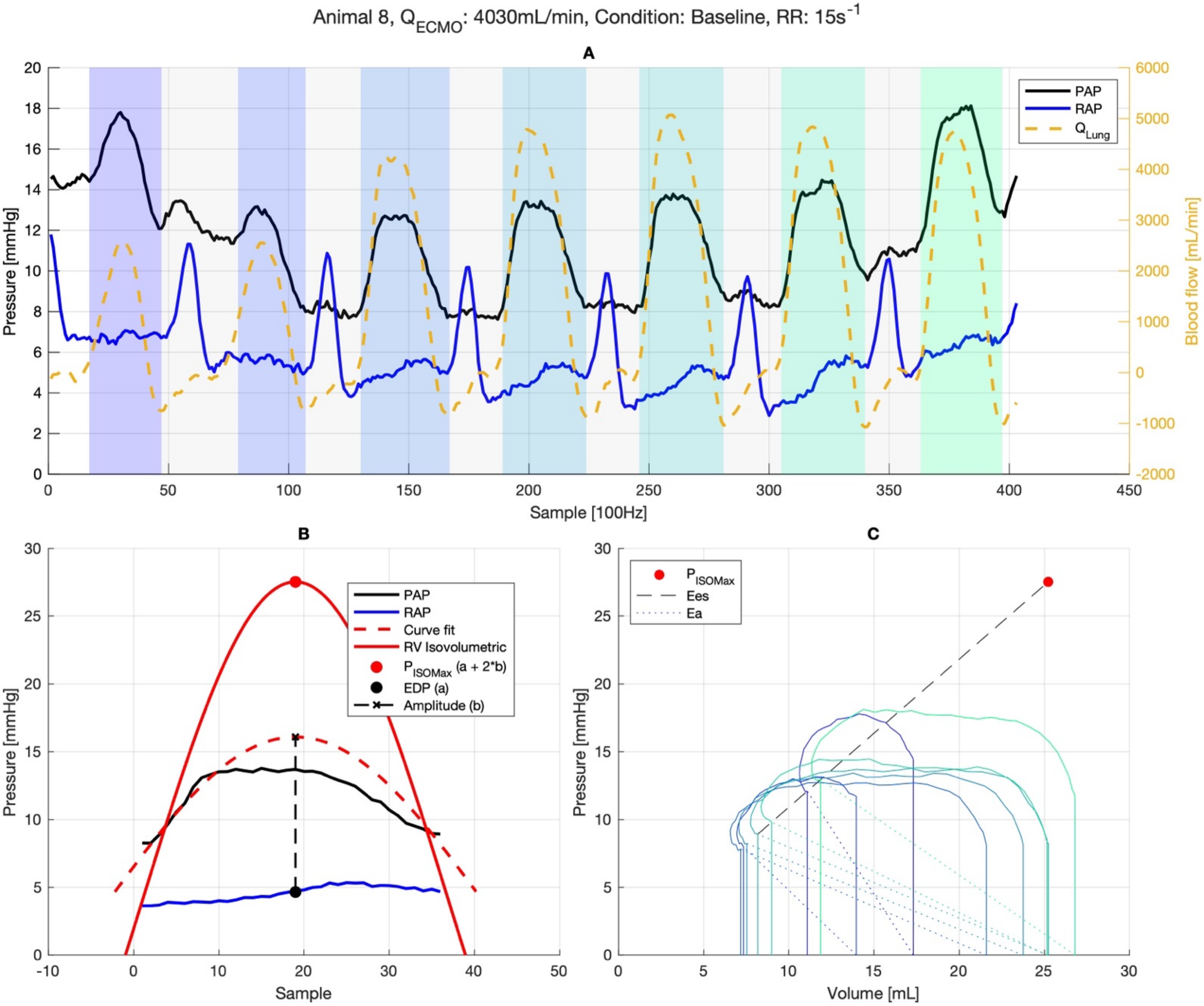

Animal 8, Q_{ECMO}: 4030mL/min, Condition: Baseline, RR: $15s^{-1}$
A
PAP
RAP
Q_{Lung}
Pressure [mmHg]
Blood flow [mL/min]
Sample [100Hz]
B
PAP
RAP
Curve fit
RV Isovolumetric
P_{ISOMax} (a + 2*b)
EDP (a)
Amplitude (b)
Pressure [mmHg]
Sample
C
P_{ISOMax}
Ees
Ea
Pressure [mmHg]
Volume [mL]

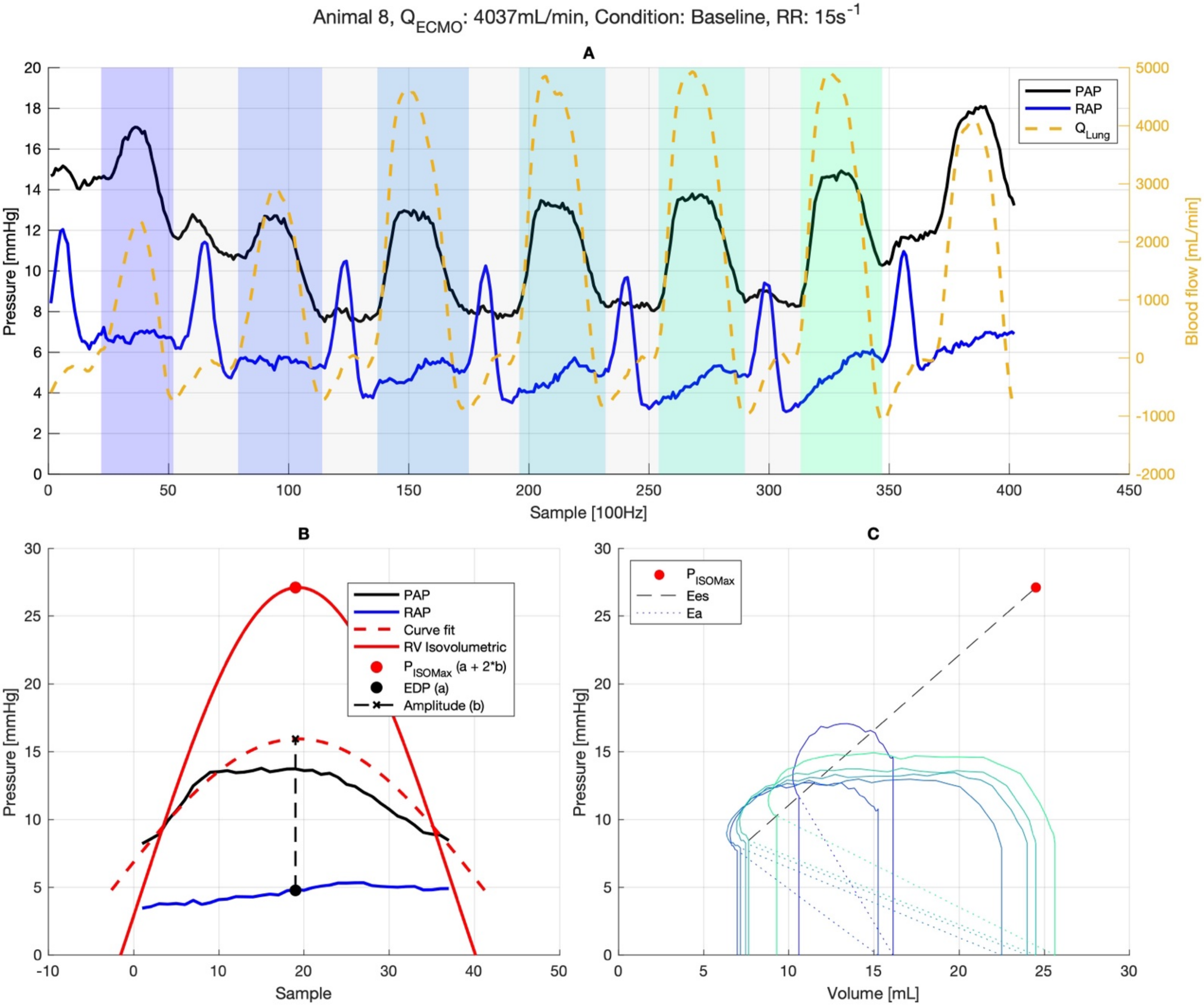

Animal 8, Q_ECMO: 4037mL/min, Condition: Baseline, RR: 15s^-1
A
PAP
RAP
Q_Lung
Pressure [mmHg]
Blood flow [mL/min]
Sample [100Hz]
B
PAP
RAP
Curve fit
RV Isovolumetric
P_ISOMax (a + 2*b)
EDP (a)
Amplitude (b)
Pressure [mmHg]
Sample
C
P_ISOMax
Ees
Ea
Pressure [mmHg]
Volume [mL]

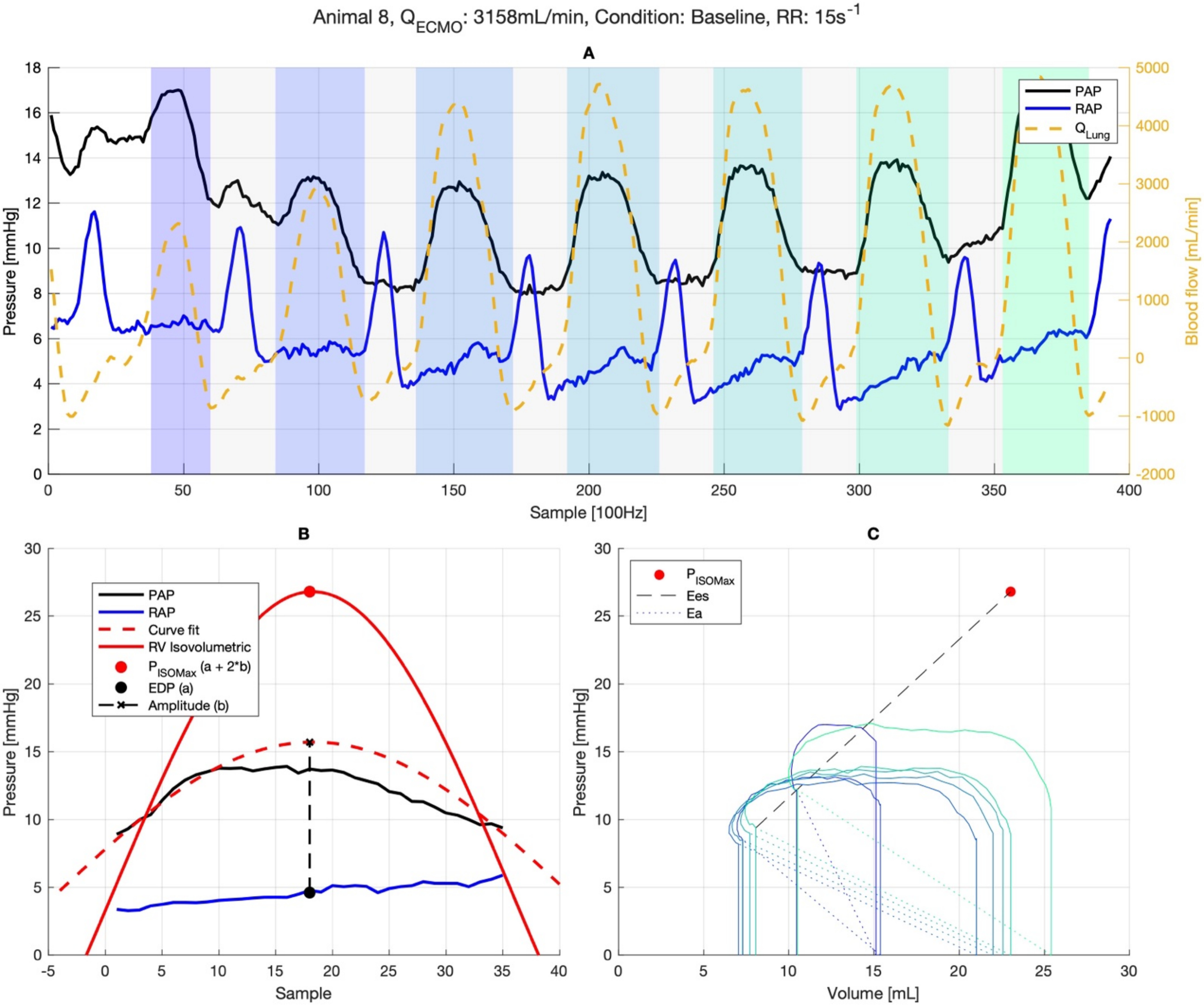

Animal 8, Q_ECMO: 3158mL/min, Condition: Baseline, RR: 15s^-1
A
PAP
RAP
Q_Lung
Pressure [mmHg]
Blood flow [mL/min]
Sample [100Hz]
B
PAP
RAP
Curve fit
RV Isovolumetric
P_ISOMax (a + 2*b)
EDP (a)
Amplitude (b)
Pressure [mmHg]
Sample
C
P_ISOMax
Ees
Ea
Pressure [mmHg]
Volume [mL]

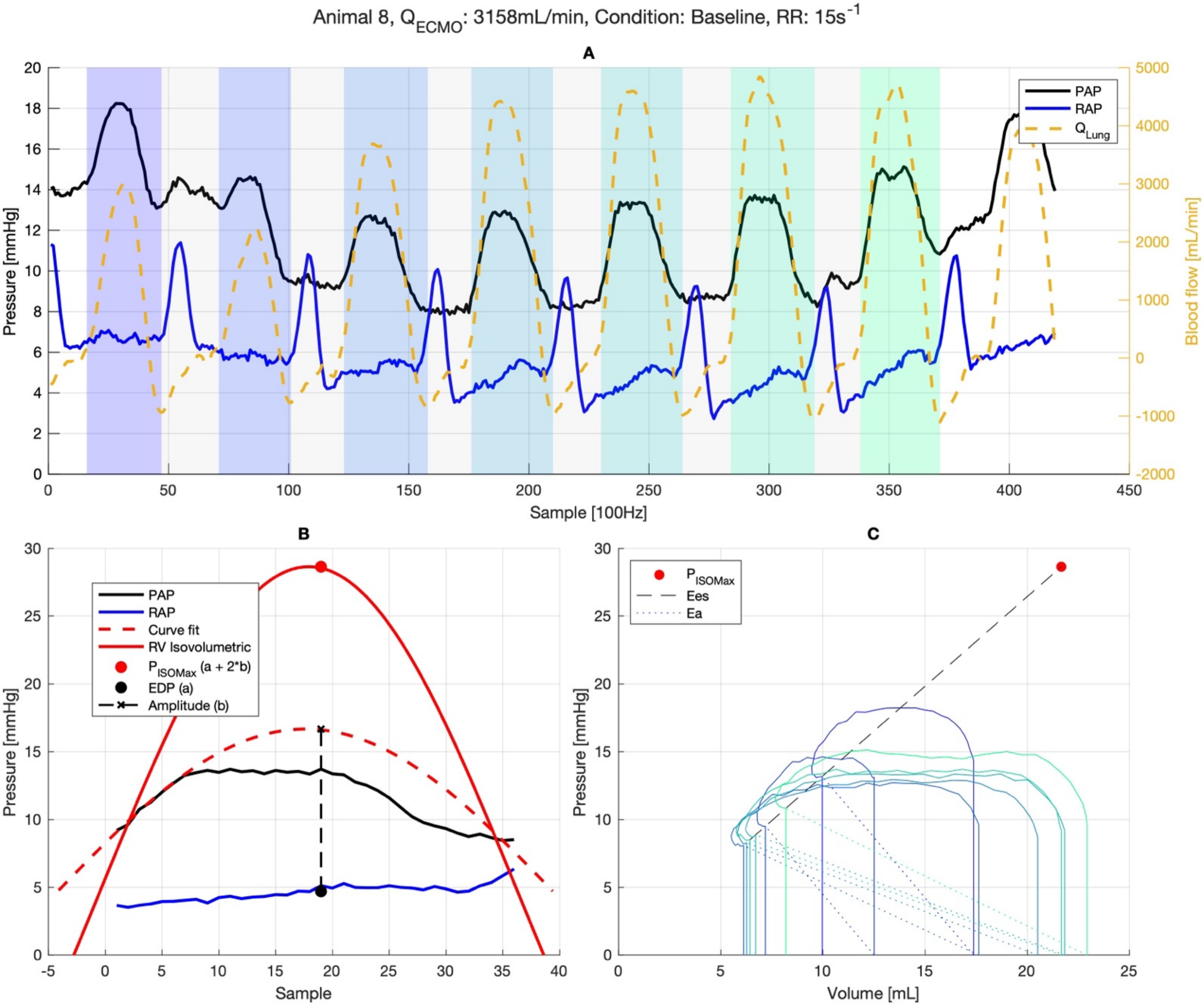

Animal 8, Q_ECMO: 3158mL/min, Condition: Baseline, RR: 15s^-1
A
PAP
RAP
Q_Lung
Pressure [mmHg]
Blood flow [mL/min]
Sample [100Hz]
B
PAP
RAP
Curve fit
RV Isovolumetric
P_ISOMax (a + 2*b)
EDP (a)
Amplitude (b)
Pressure [mmHg]
Sample
C
P_ISOMax
Ees
Ea
Pressure [mmHg]
Volume [mL]

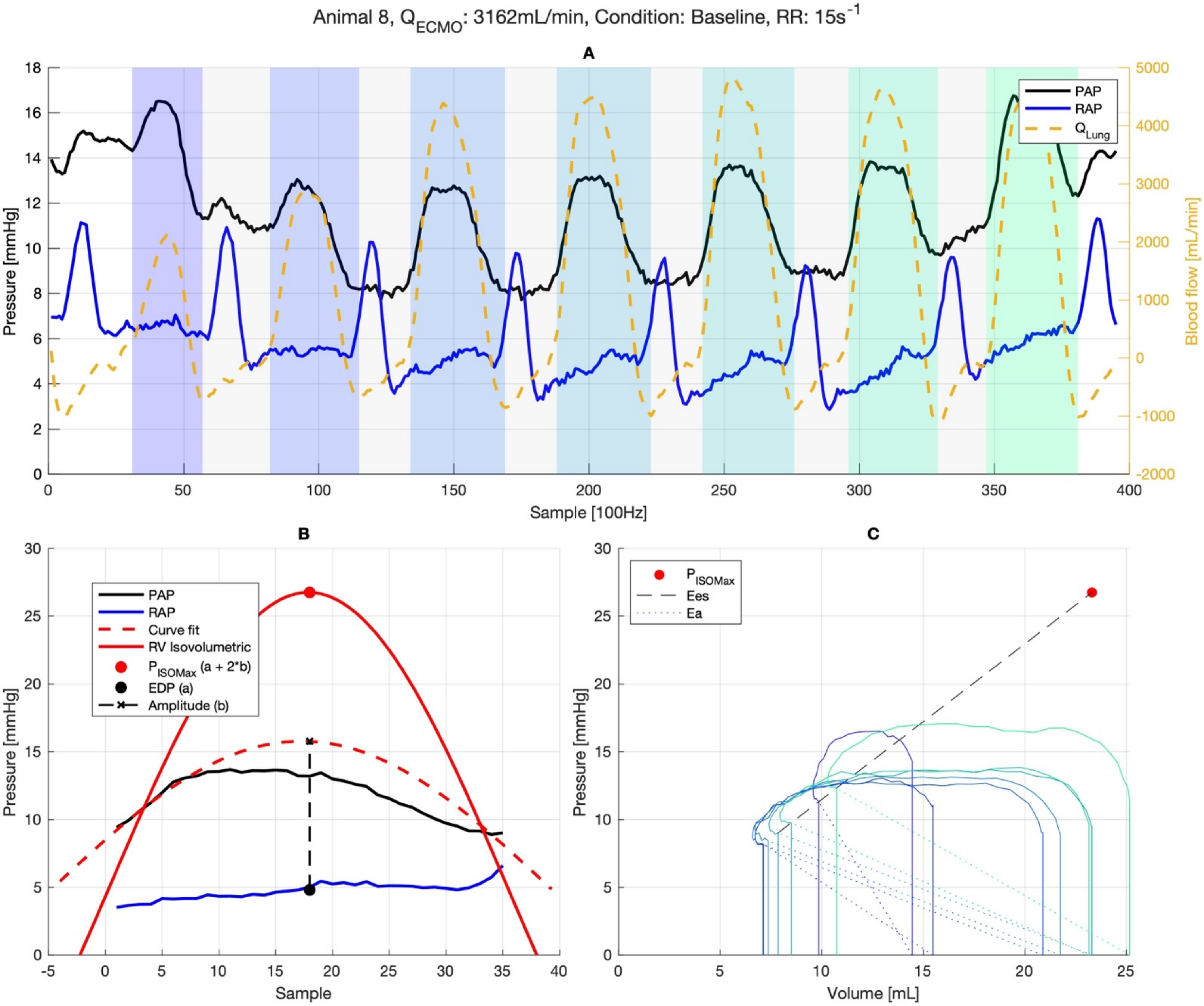

Animal 8, Q_ECMO: 3162mL/min, Condition: Baseline, RR: 15s^-1
A
PAP
RAP
Q_Lung
Pressure [mmHg]
Blood flow [mL/min]
Sample [100Hz]
B
PAP
RAP
Curve fit
RV Isovolumetric
P_ISOMax (a + 2*b)
EDP (a)
Amplitude (b)
Pressure [mmHg]
Sample
C
P_ISOMax
Ees
Ea
Pressure [mmHg]
Volume [mL]

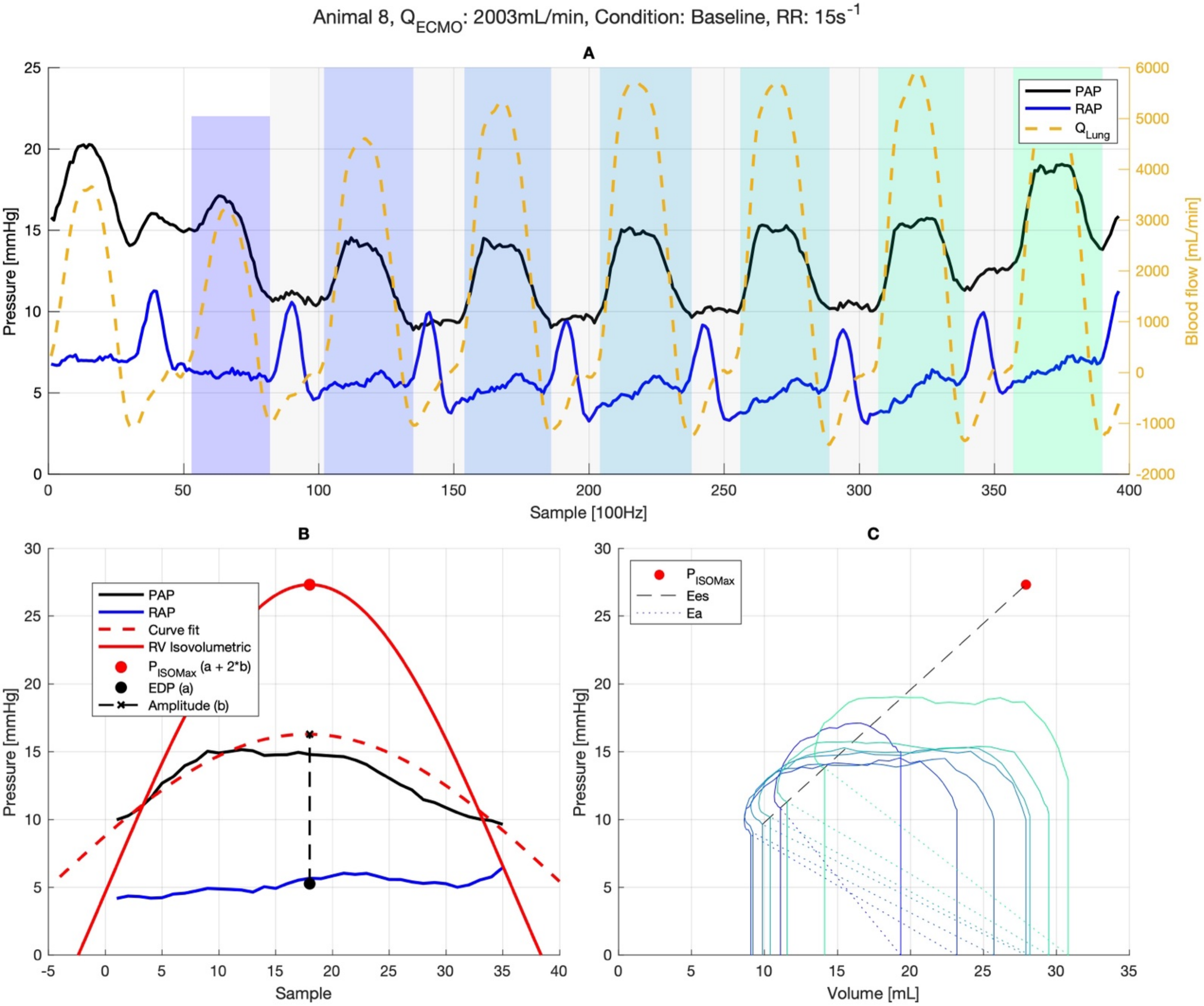
Animal 8, Q_{ECMO}: 2003mL/min, Condition: Baseline, RR: $15s^{-1}$
A
PAP
RAP
Q_{Lung}
Pressure [mmHg]
Blood flow [mL/min]
Sample [100Hz]
B
PAP
RAP
Curve fit
RV Isovolumetric
P_{ISOMax} (a + 2*b)
EDP (a)
Amplitude (b)
Pressure [mmHg]
Sample
C
P_{ISOMax}
Ees
Ea
Pressure [mmHg]
Volume [mL]

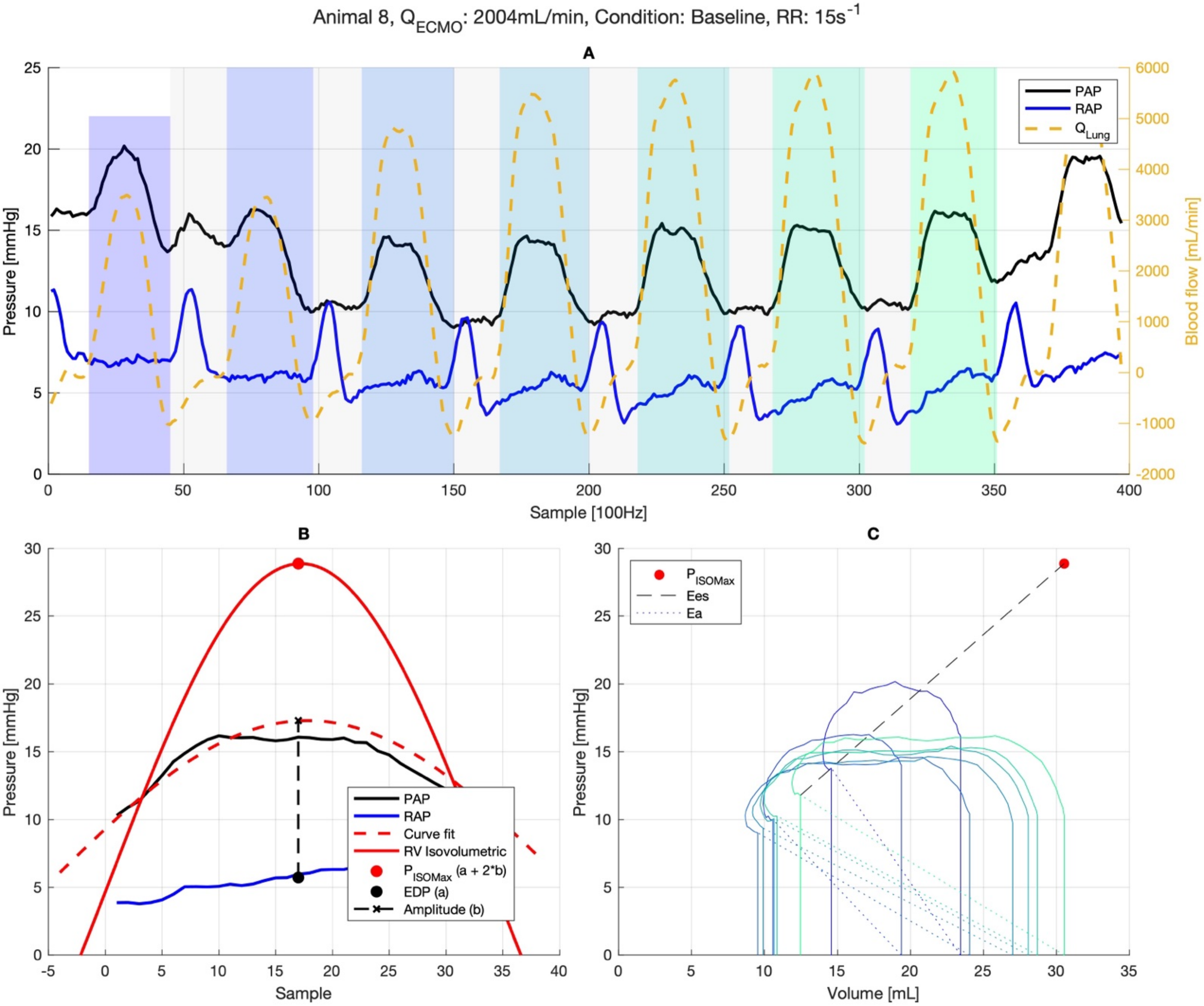

Animal 8, Q_{ECMO}: 2004mL/min, Condition: Baseline, RR: $15s^{-1}$
A
PAP
RAP
Q_{Lung}
Pressure [mmHg]
Blood flow [mL/min]
Sample [100Hz]
B
PAP
RAP
Curve fit
RV Isovolumetric
P_{ISOMax} (a + 2*b)
EDP (a)
Amplitude (b)
Pressure [mmHg]
Sample
C
P_{ISOMax}
Ees
Ea
Pressure [mmHg]
Volume [mL]

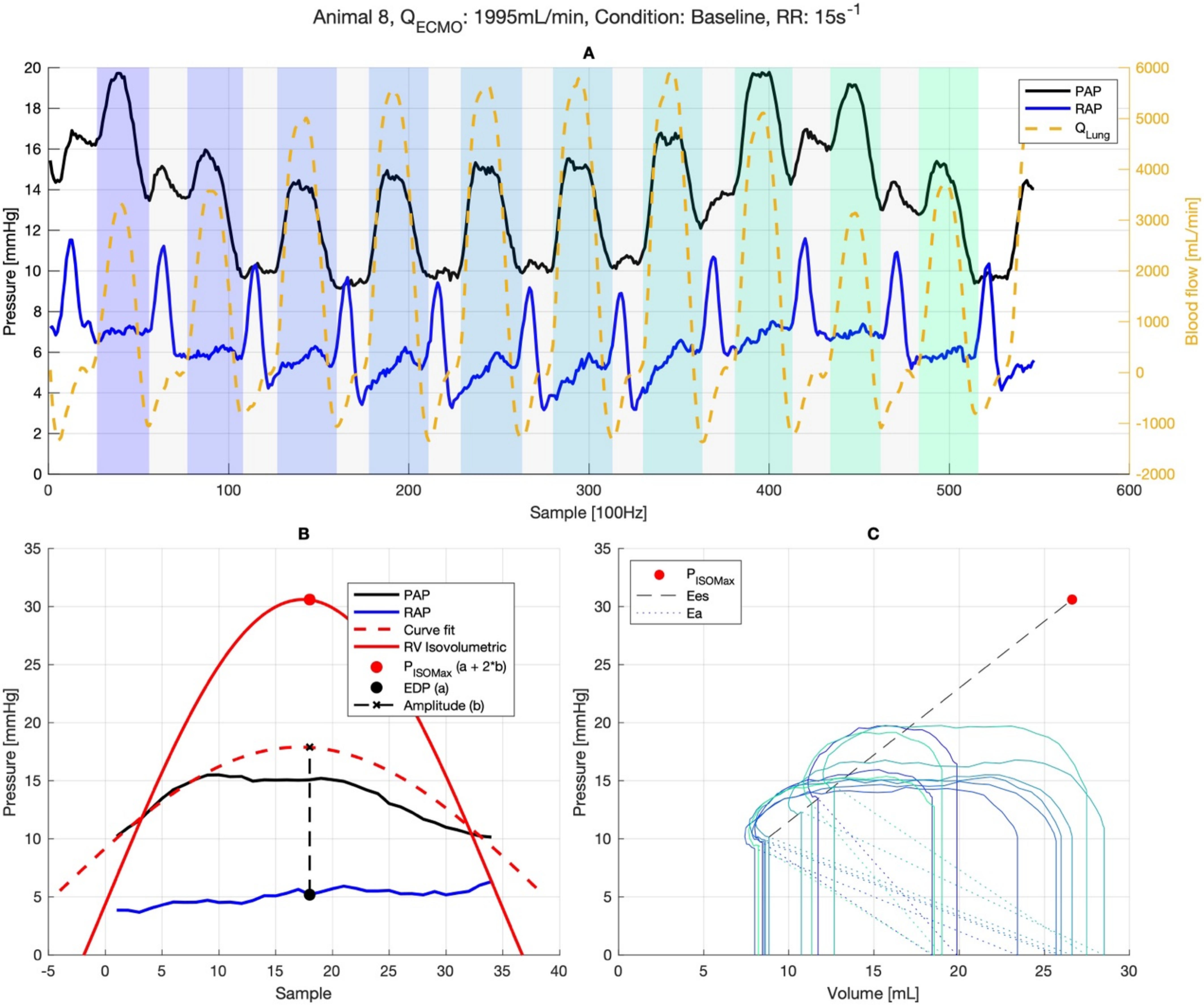
Animal 8, Q_{ECMO}: 1995mL/min, Condition: Baseline, RR: $15s^{-1}$
A
PAP
RAP
Q_{Lung}
Pressure [mmHg]
Blood flow [mL/min]
Sample [100Hz]
B
PAP
RAP
Curve fit
RV Isovolumetric
P_{ISOMax} (a + 2*b)
EDP (a)
Amplitude (b)
Pressure [mmHg]
Sample
C
P_{ISOMax}
Ees
Ea
Pressure [mmHg]
Volume [mL]

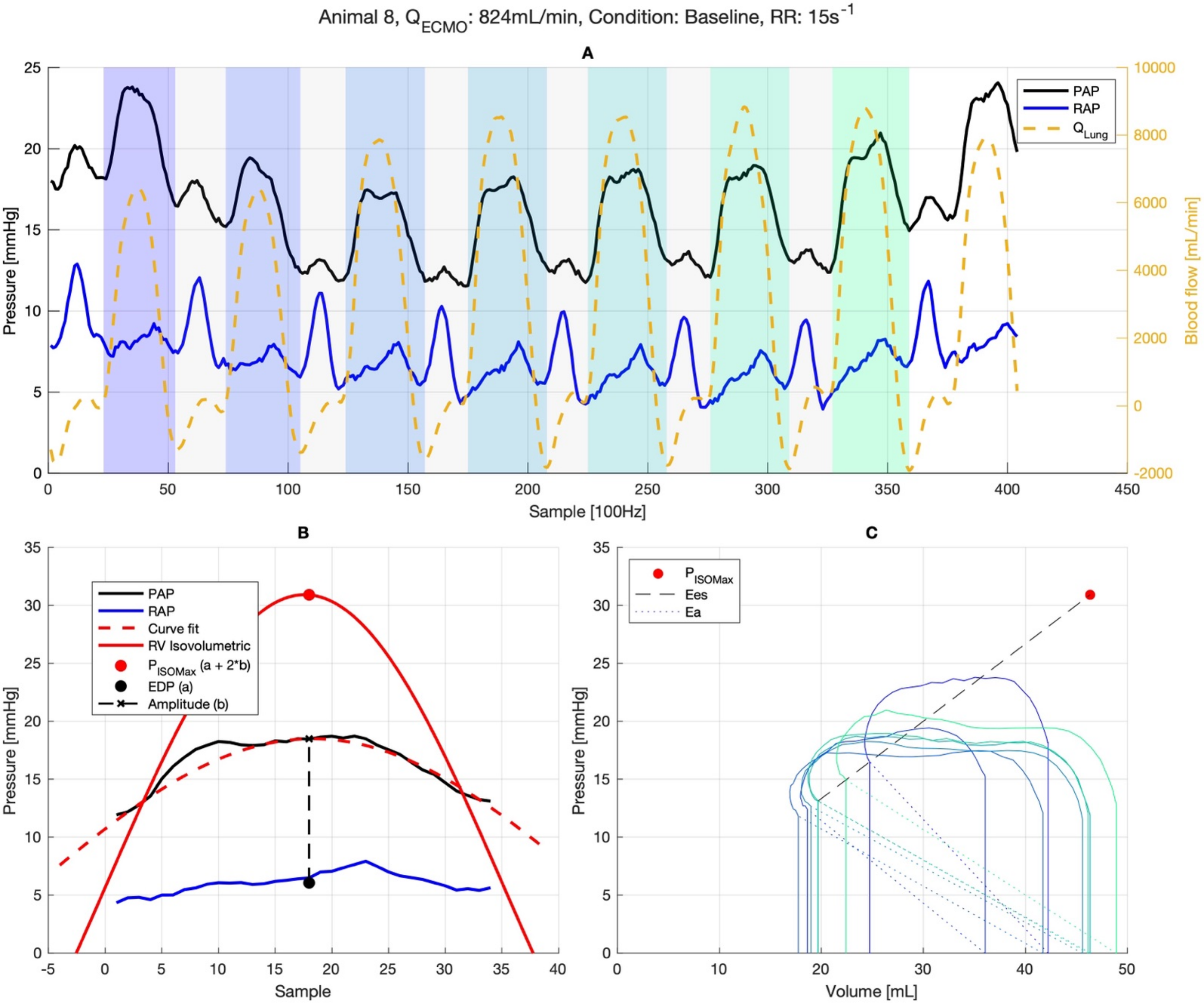

Animal 8, Q_ECMO: 824mL/min, Condition: Baseline, RR: 15s^-1
A
PAP
RAP
Q_Lung
Pressure [mmHg]
Blood flow [mL/min]
Sample [100Hz]
B
PAP
RAP
Curve fit
RV Isovolumetric
P_ISOMax (a + 2*b)
EDP (a)
Amplitude (b)
Pressure [mmHg]
Sample
C
P_ISOMax
Ees
Ea
Pressure [mmHg]
Volume [mL]

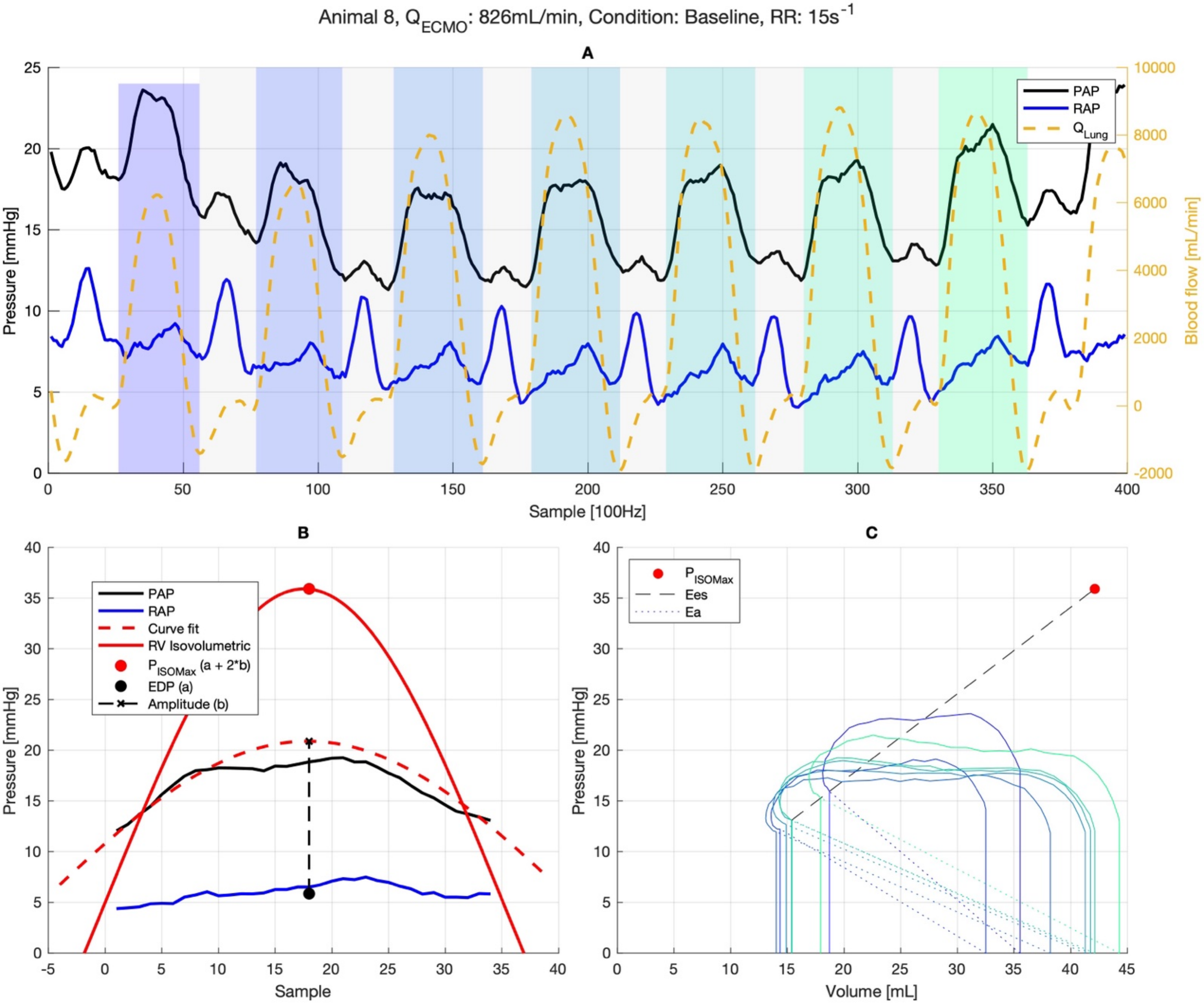
Animal 8, Q_{ECMO}: 826mL/min, Condition: Baseline, RR: $15s^{-1}$
A
PAP
RAP
Q_{Lung}
Pressure [mmHg]
Blood flow [mL/min]
Sample [100Hz]
B
PAP
RAP
Curve fit
RV Isovolumetric
P_{ISOMax} (a + 2*b)
EDP (a)
Amplitude (b)
Pressure [mmHg]
Sample
C
P_{ISOMax}
Ees
Ea
Pressure [mmHg]
Volume [mL]

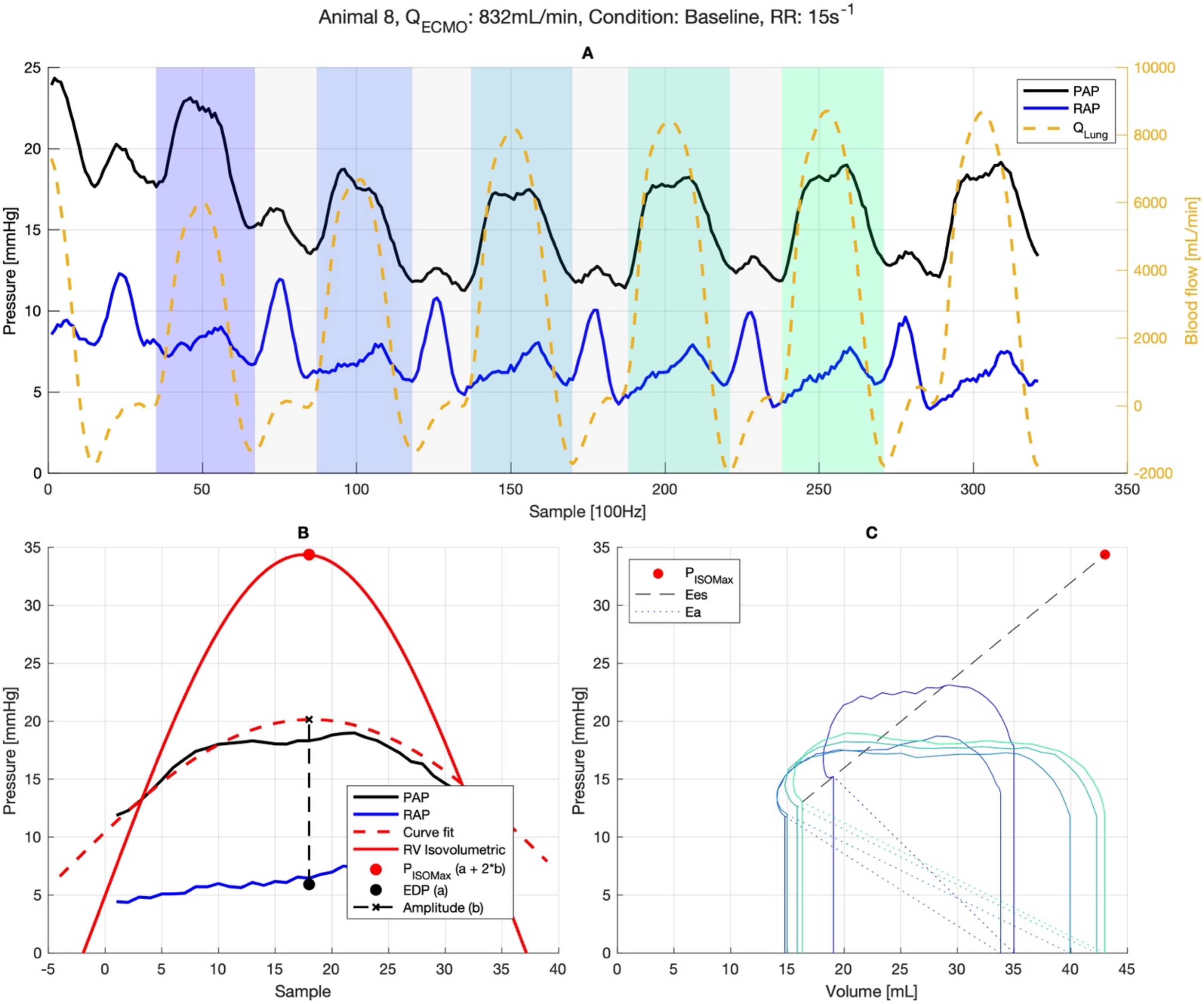

Animal 8, Q_{ECMO}: 832mL/min, Condition: Baseline, RR: $15s^{-1}$
A
Pressure [mmHg]
Blood flow [mL/min]
Sample [100Hz]
PAP
RAP
Q_{Lung}
B
Pressure [mmHg]
Sample
PAP
RAP
Curve fit
RV Isovolumetric
P_{ISOMax} (a + 2*b)
EDP (a)
Amplitude (b)
C
Pressure [mmHg]
Volume [mL]
P_{ISOMax}
Ees
Ea

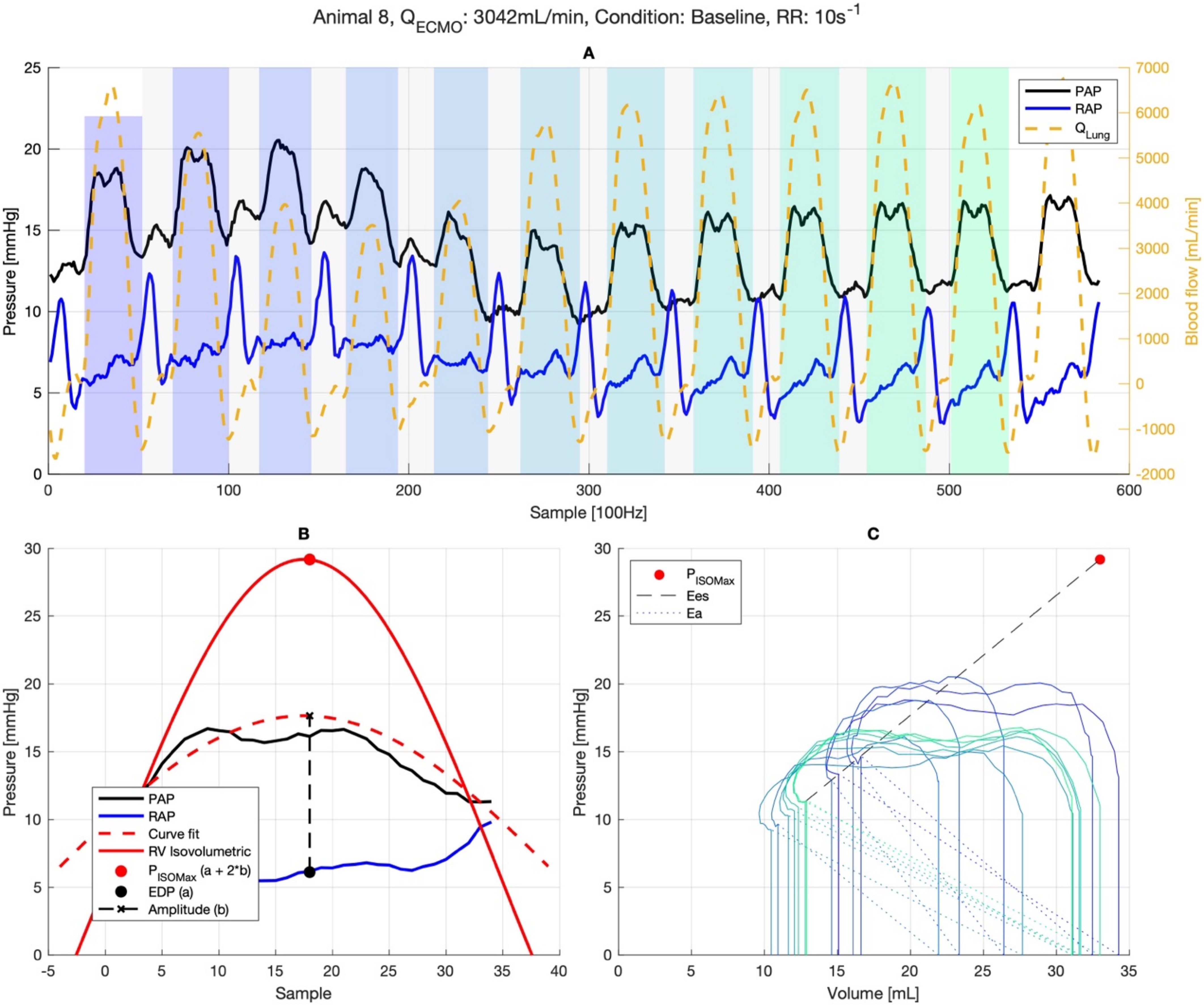

Animal 8, Q_ECMO: 3042mL/min, Condition: Baseline, RR: 10s^-1
A
PAP
RAP
Q_Lung
Pressure [mmHg]
Blood flow [mL/min]
Sample [100Hz]
B
PAP
RAP
Curve fit
RV Isovolumetric
P_ISOMax (a + 2*b)
EDP (a)
Amplitude (b)
Pressure [mmHg]
Sample
C
P_ISOMax
Ees
Ea
Pressure [mmHg]
Volume [mL]

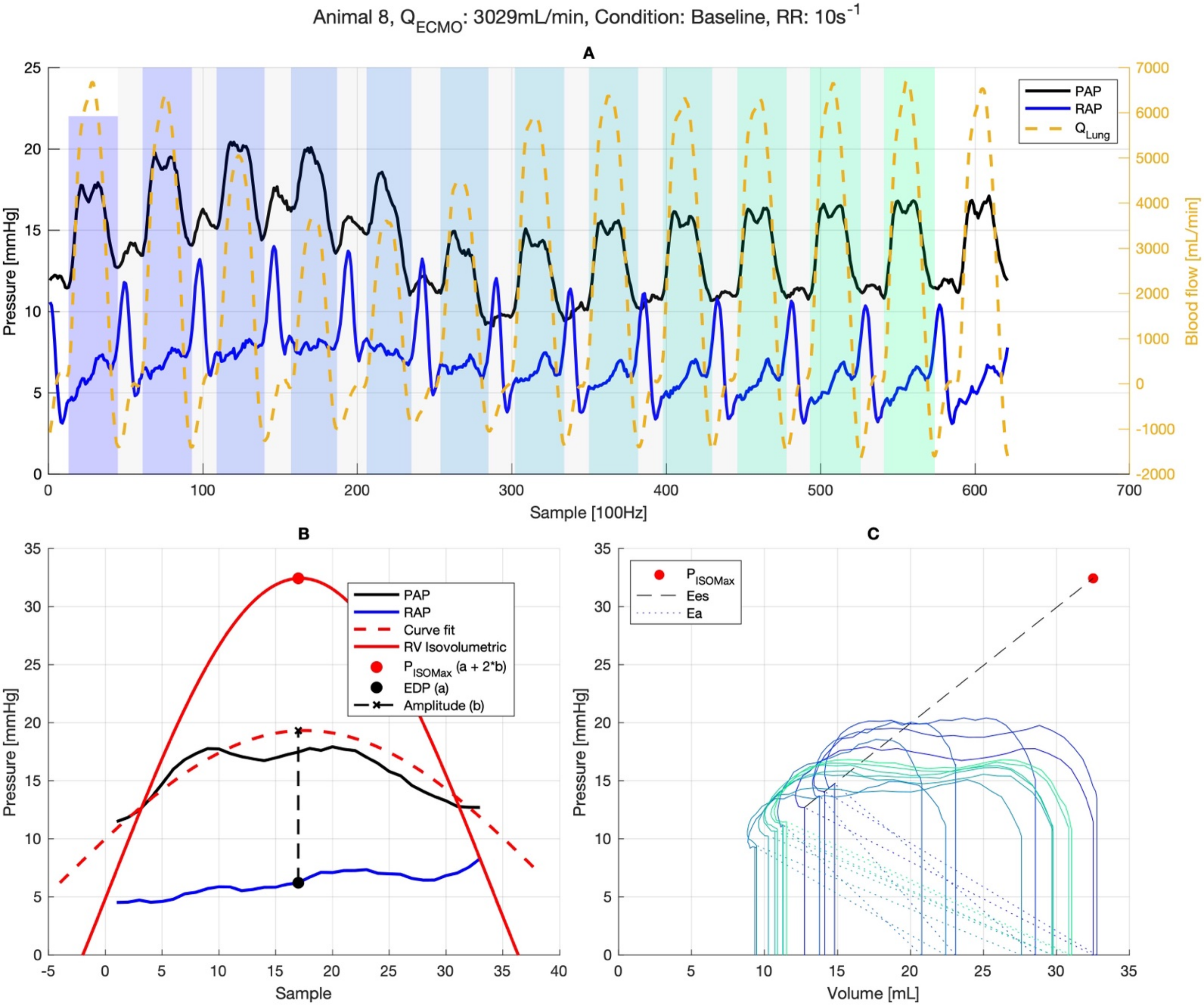

Animal 8, Q_ECMO: 3029mL/min, Condition: Baseline, RR: 10s^-1
A
PAP
RAP
Q_Lung
Pressure [mmHg]
Blood flow [mL/min]
Sample [100Hz]
B
PAP
RAP
Curve fit
RV Isovolumetric
P_ISOMax (a + 2*b)
EDP (a)
Amplitude (b)
Pressure [mmHg]
Sample
C
P_ISOMax
Ees
Ea
Pressure [mmHg]
Volume [mL]

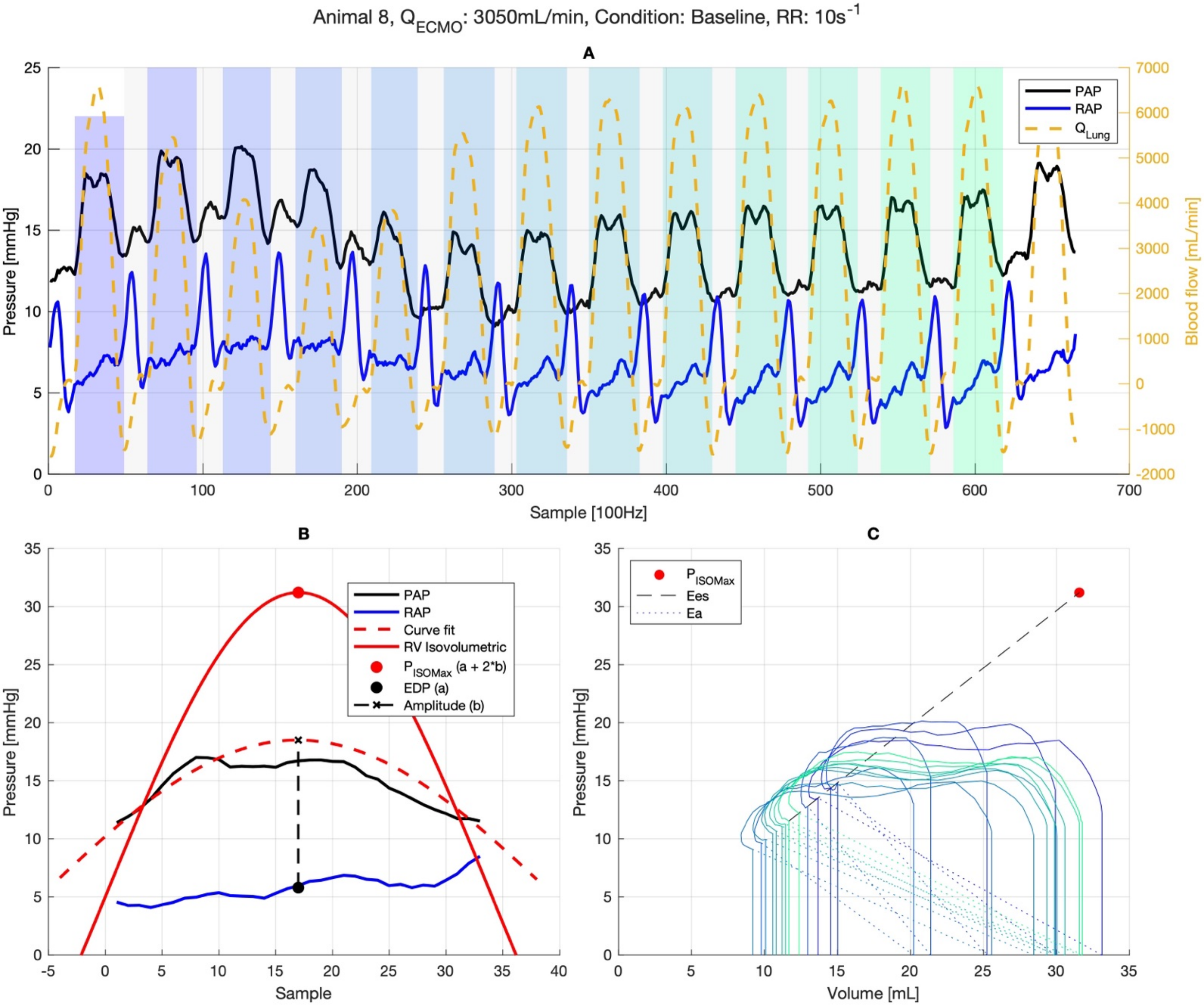
Animal 8, QECMO: 3050mL/min, Condition: Baseline, RR: 10s-1
A
PAP
RAP
QLung
Pressure [mmHg]
Blood flow [mL/min]
Sample [100Hz]
B
PAP
RAP
Curve fit
RV Isovolumetric
PISOMax (a + 2*b)
EDP (a)
Amplitude (b)
Pressure [mmHg]
Sample
C
PISOMax
Ees
Ea
Pressure [mmHg]
Volume [mL]

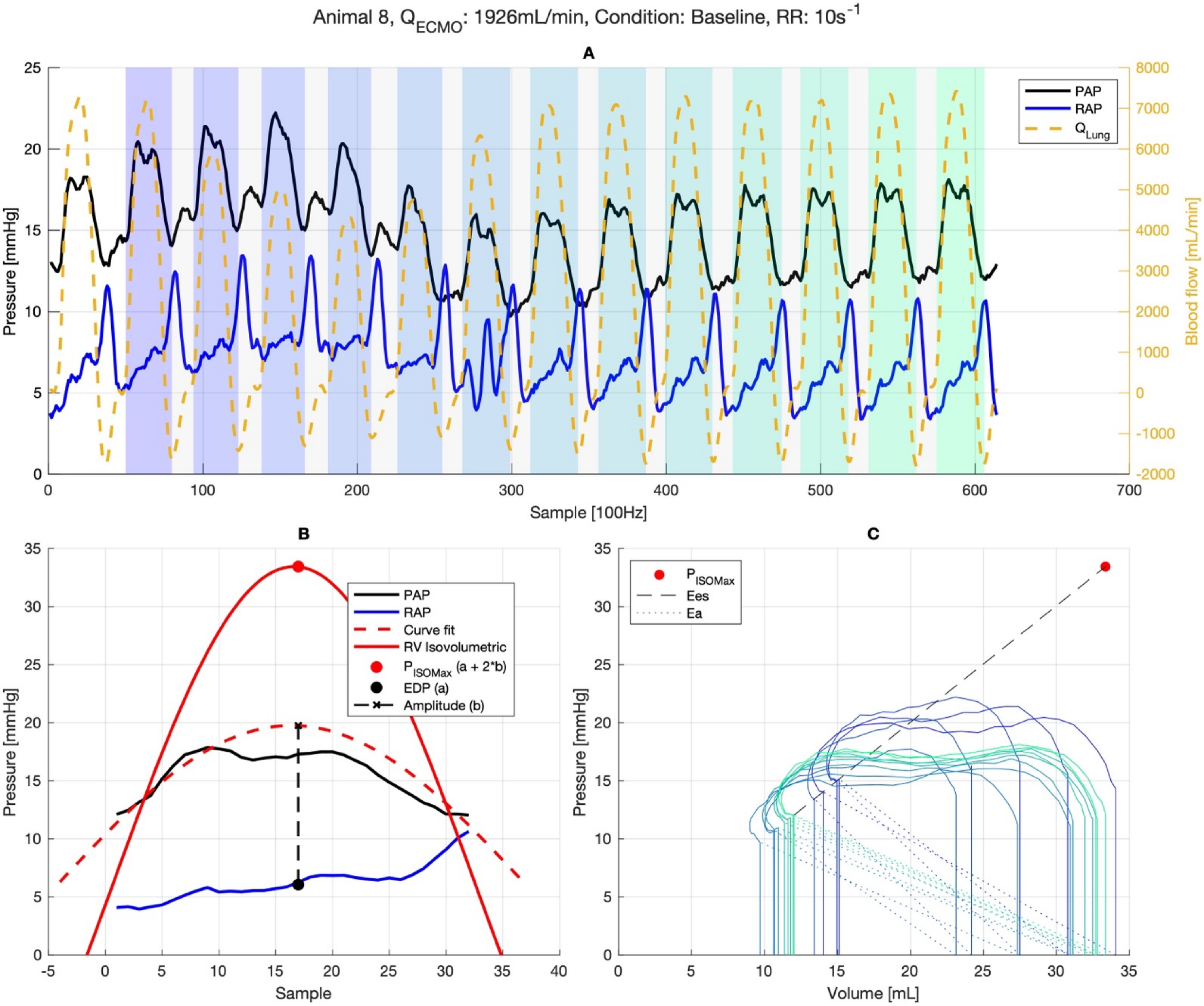

Animal 8, Q_ECMO: 1926mL/min, Condition: Baseline, RR: 10s^-1
A
PAP
RAP
Q_Lung
Pressure [mmHg]
Blood flow [mL/min]
Sample [100Hz]
B
PAP
RAP
Curve fit
RV Isovolumetric
P_ISOMax (a + 2*b)
EDP (a)
Amplitude (b)
Pressure [mmHg]
Sample
C
P_ISOMax
Ees
Ea
Pressure [mmHg]
Volume [mL]

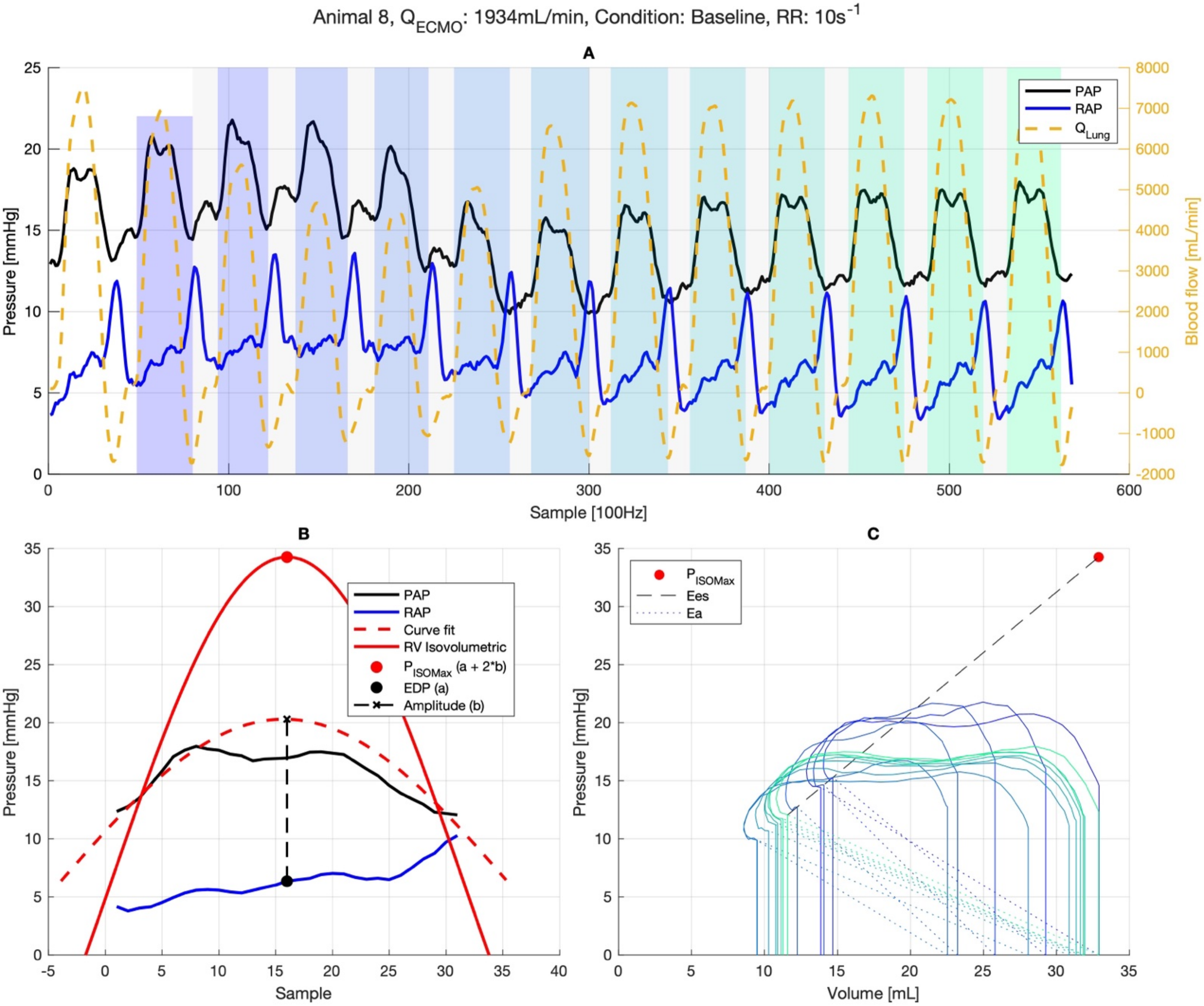

Animal 8, Q_ECMO: 1934mL/min, Condition: Baseline, RR: 10s^-1
A
Pressure [mmHg]
Blood flow [mL/min]
Sample [100Hz]
PAP
RAP
Q_Lung
B
PAP
RAP
Curve fit
RV Isovolumetric
P_ISOMax (a + 2*b)
EDP (a)
Amplitude (b)
Pressure [mmHg]
Sample
C
P_ISOMax
Ees
Ea
Pressure [mmHg]
Volume [mL]

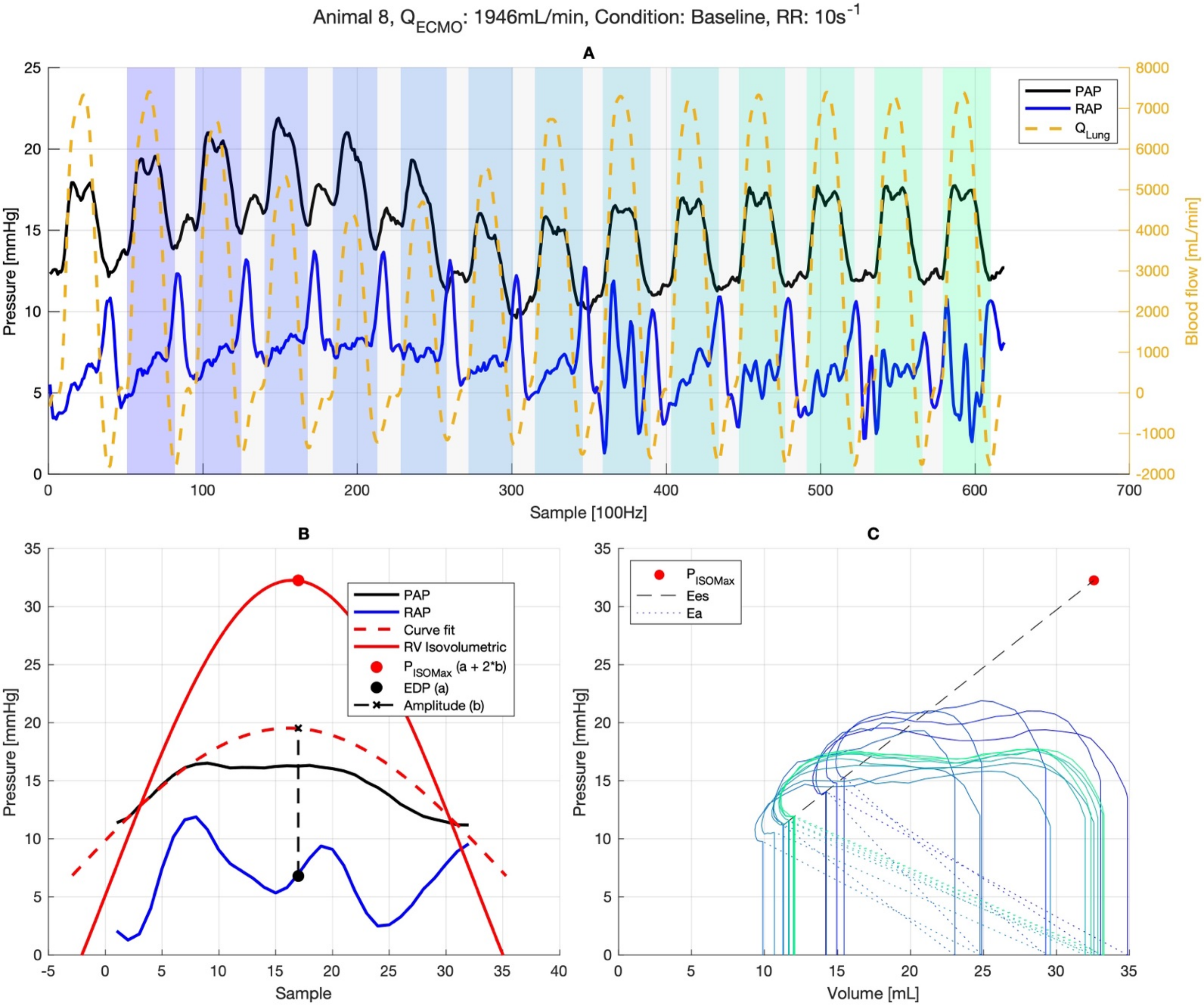

Animal 8, Q_{ECMO}: 1946mL/min, Condition: Baseline, RR: $10s^{-1}$
A
PAP
RAP
Q_{Lung}
Pressure [mmHg]
Blood flow [mL/min]
Sample [100Hz]
B
PAP
RAP
Curve fit
RV Isovolumetric
P_{ISOMax} (a + 2*b)
EDP (a)
Amplitude (b)
Pressure [mmHg]
Sample
C
P_{ISOMax}
Ees
Ea
Pressure [mmHg]
Volume [mL]

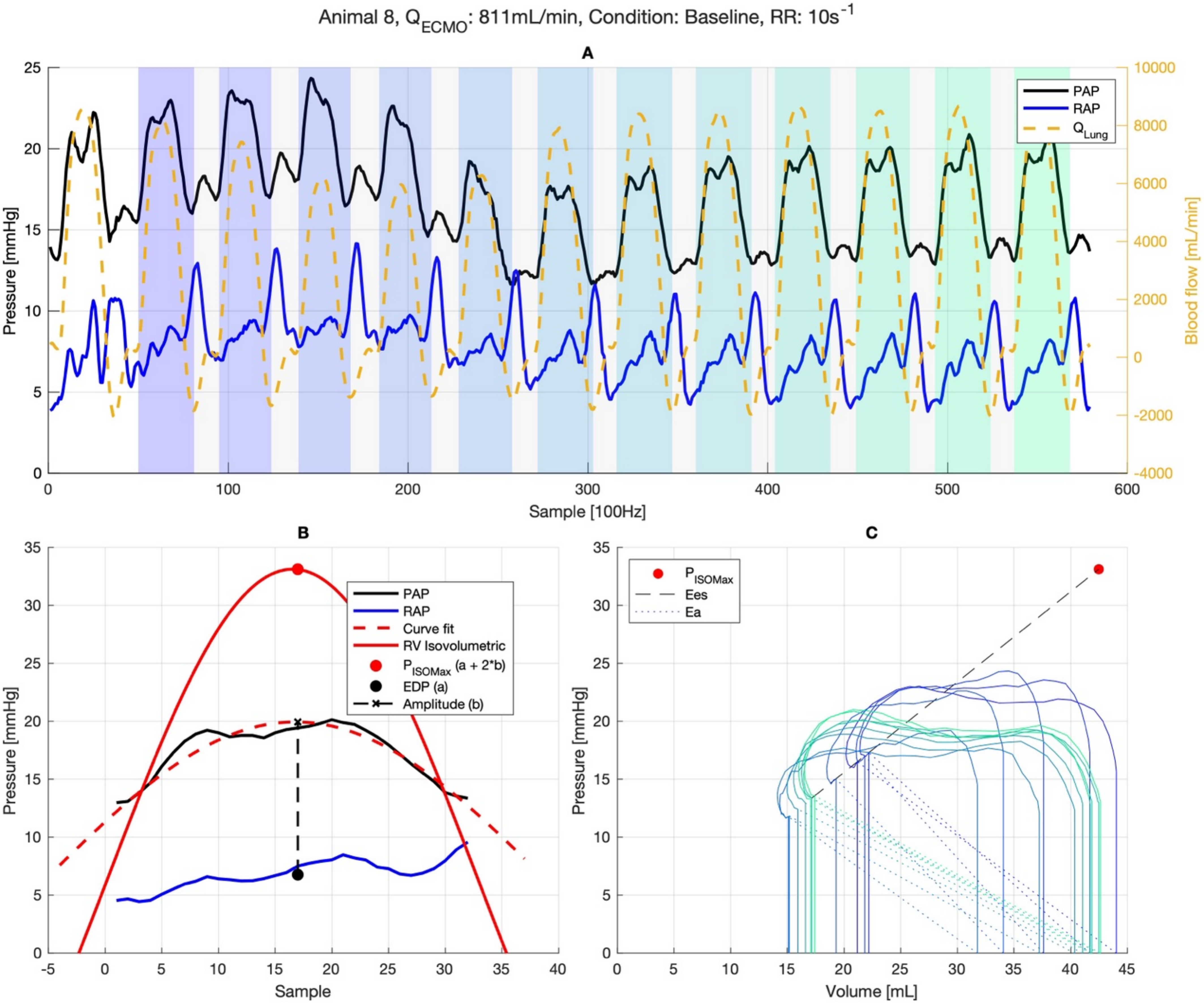

Animal 8, Q_{ECMO}: 811mL/min, Condition: Baseline, RR: $10s^{-1}$
A
PAP
RAP
Q_{Lung}
Pressure [mmHg]
Blood flow [mL/min]
Sample [100Hz]
B
PAP
RAP
Curve fit
RV Isovolumetric
P_{ISOMax} (a + 2*b)
EDP (a)
Amplitude (b)
Pressure [mmHg]
Sample
C
P_{ISOMax}
Ees
Ea
Pressure [mmHg]
Volume [mL]

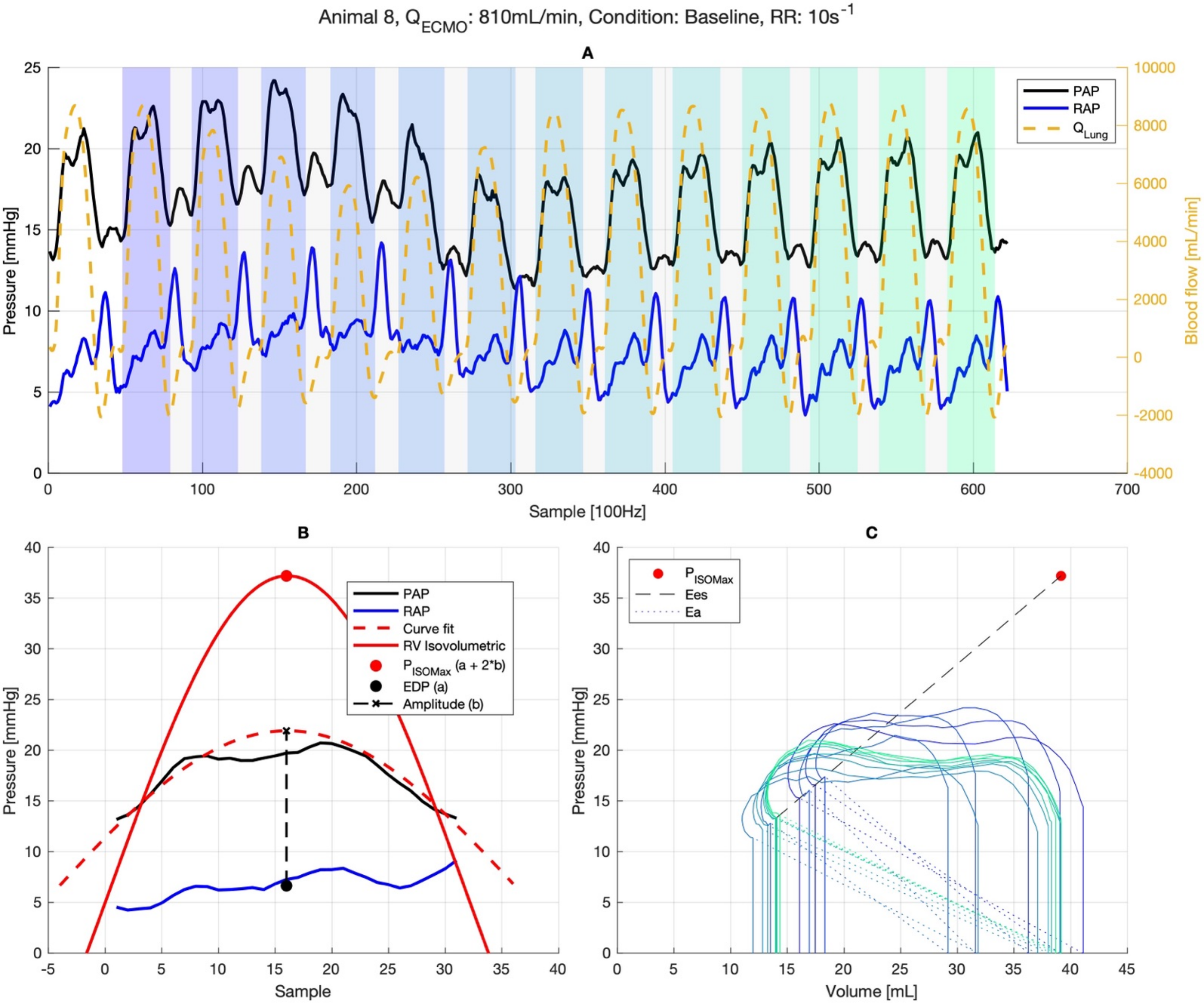

Animal 8, Q_{ECMO}: 810mL/min, Condition: Baseline, RR: $10s^{-1}$
A
PAP
RAP
Q_{Lung}
Pressure [mmHg]
Blood flow [mL/min]
Sample [100Hz]
B
PAP
RAP
Curve fit
RV Isovolumetric
P_{ISOMax} (a + 2*b)
EDP (a)
Amplitude (b)
Pressure [mmHg]
Sample
C
P_{ISOMax}
Ees
Ea
Pressure [mmHg]
Volume [mL]

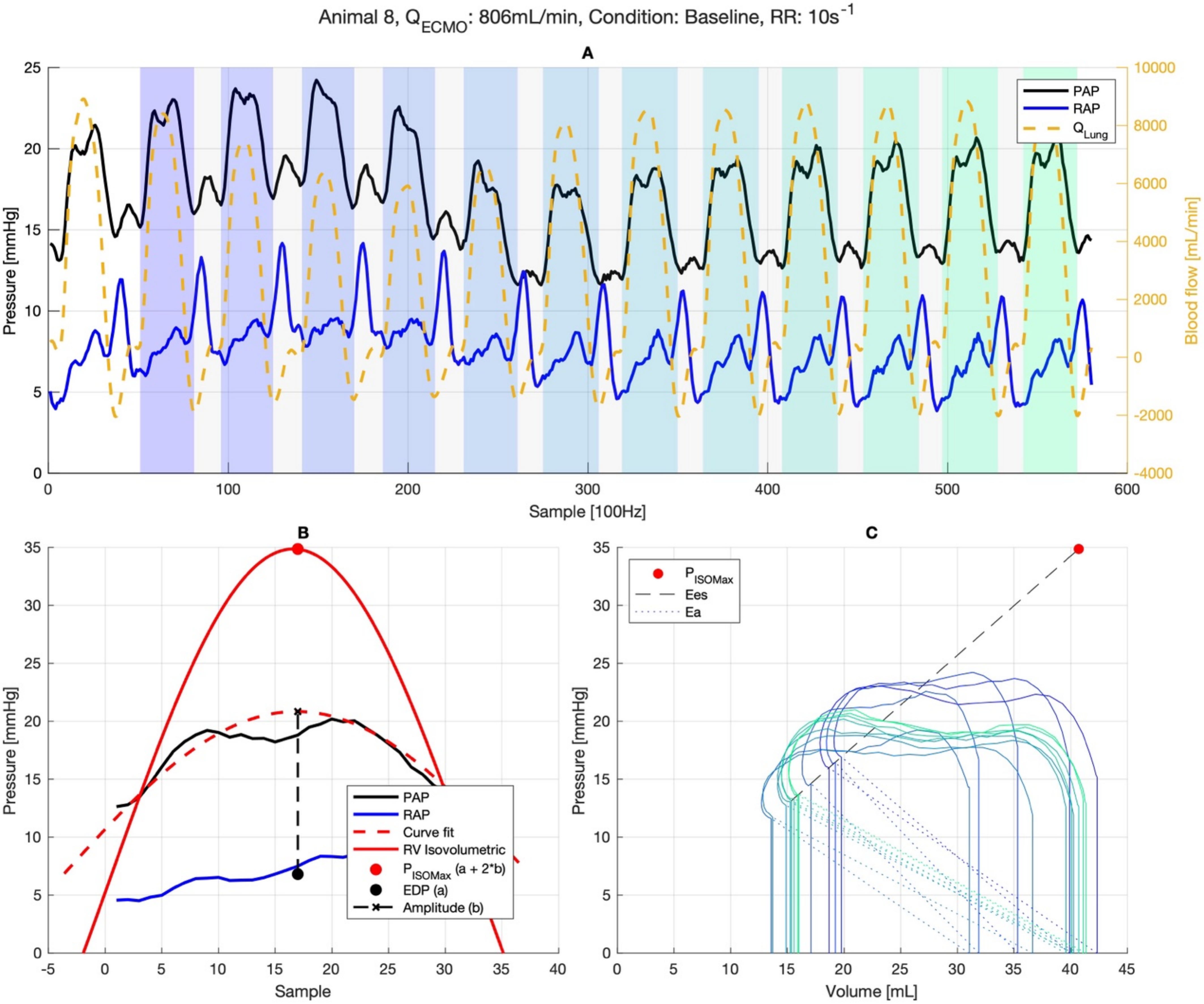

Animal 8, Q_{ECMO}: 806mL/min, Condition: Baseline, RR: $10s^{-1}$
A
PAP
RAP
Q_{Lung}
Pressure [mmHg]
Blood flow [mL/min]
Sample [100Hz]
B
PAP
RAP
Curve fit
RV Isovolumetric
P_{ISOMax} (a + 2*b)
EDP (a)
Amplitude (b)
Pressure [mmHg]
Sample
C
P_{ISOMax}
Ees
Ea
Pressure [mmHg]
Volume [mL]

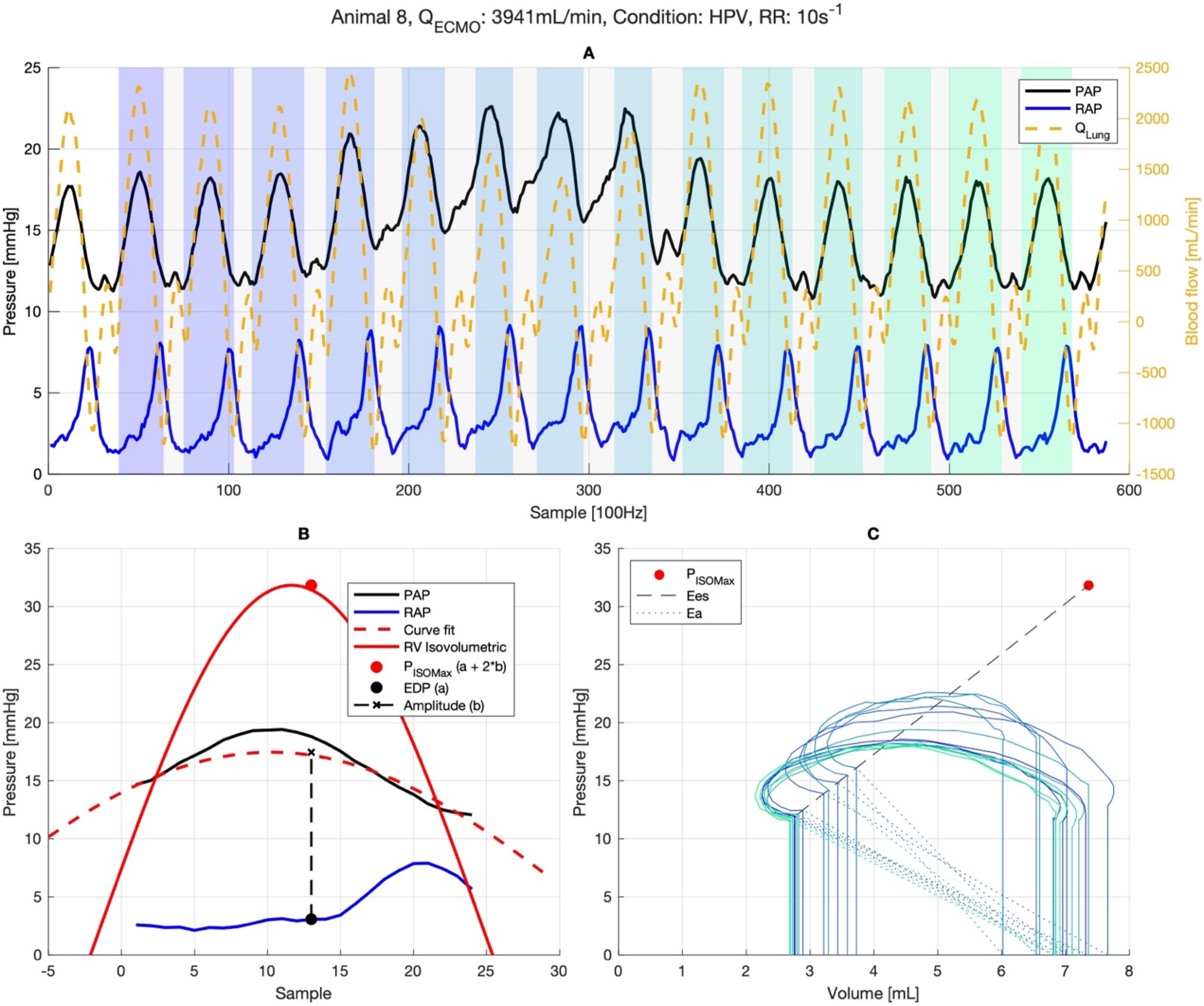
Animal 8, Q_{ECMO}: 3941mL/min, Condition: HPV, RR: $10s^{-1}$
A
PAP
RAP
Q_{Lung}
Pressure [mmHg]
Blood flow [mL/min]
Sample [100Hz]
B
PAP
RAP
Curve fit
RV Isovolumetric
P_{ISOMax} (a + 2*b)
EDP (a)
Amplitude (b)
Pressure [mmHg]
Sample
C
P_{ISOMax}
Ees
Ea
Pressure [mmHg]
Volume [mL]

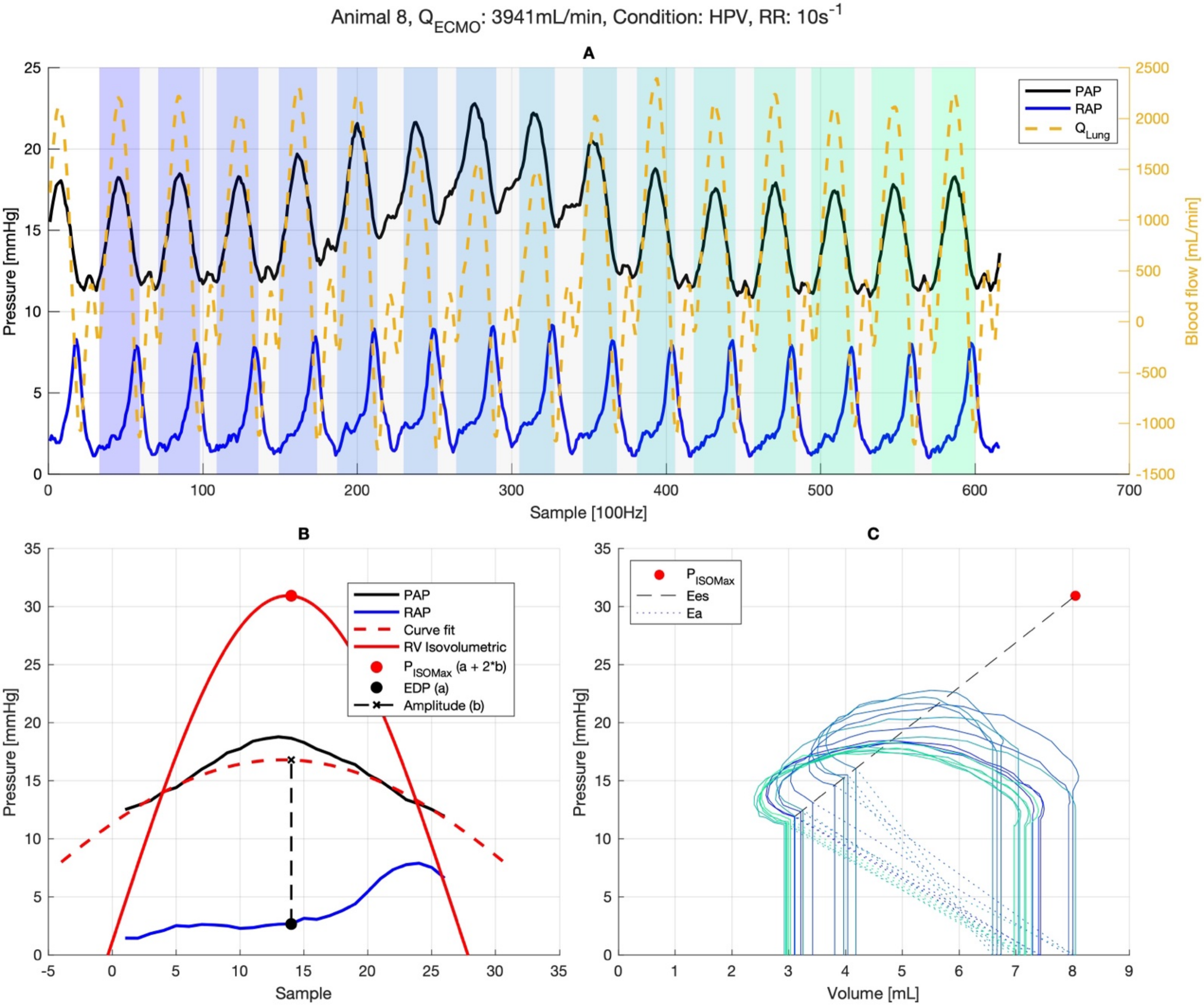

Animal 8, Q_{ECMO}: 3941mL/min, Condition: HPV, RR: $10s^{-1}$
A
PAP
RAP
Q_{Lung}
Pressure [mmHg]
Blood flow [mL/min]
Sample [100Hz]
B
PAP
RAP
Curve fit
RV Isovolumetric
P_{ISOMax} (a + 2*b)
EDP (a)
Amplitude (b)
Pressure [mmHg]
Sample
C
P_{ISOMax}
Ees
Ea
Pressure [mmHg]
Volume [mL]

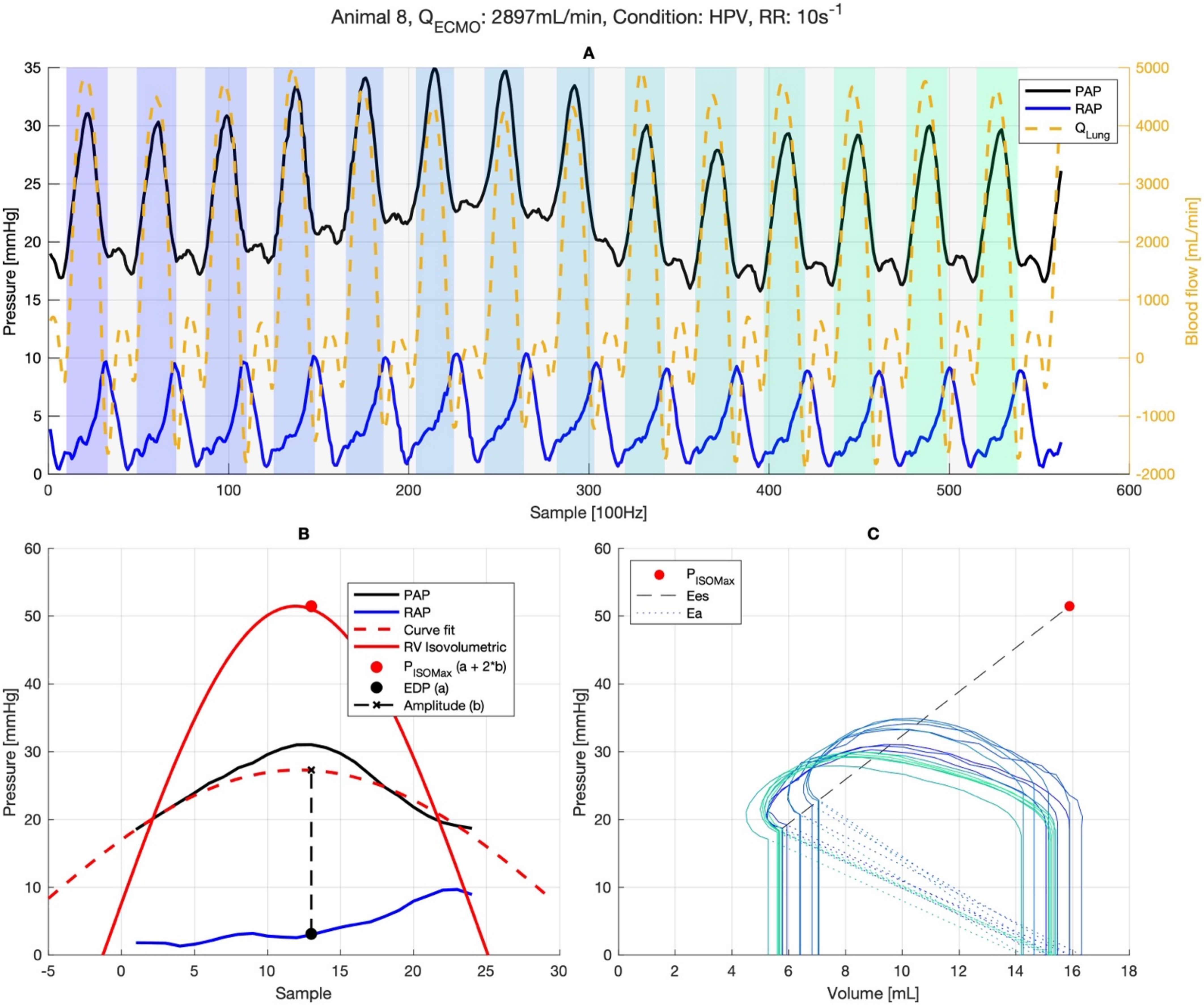

Animal 8, Q_ECMO: 2897mL/min, Condition: HPV, RR: 10s^-1
A
Pressure [mmHg]
Blood flow [mL/min]
Sample [100Hz]
PAP
RAP
Q_Lung
B
Pressure [mmHg]
Sample
PAP
RAP
Curve fit
RV Isovolumetric
P_ISOMax (a + 2*b)
EDP (a)
Amplitude (b)
C
Pressure [mmHg]
Volume [mL]
P_ISOMax
Ees
Ea

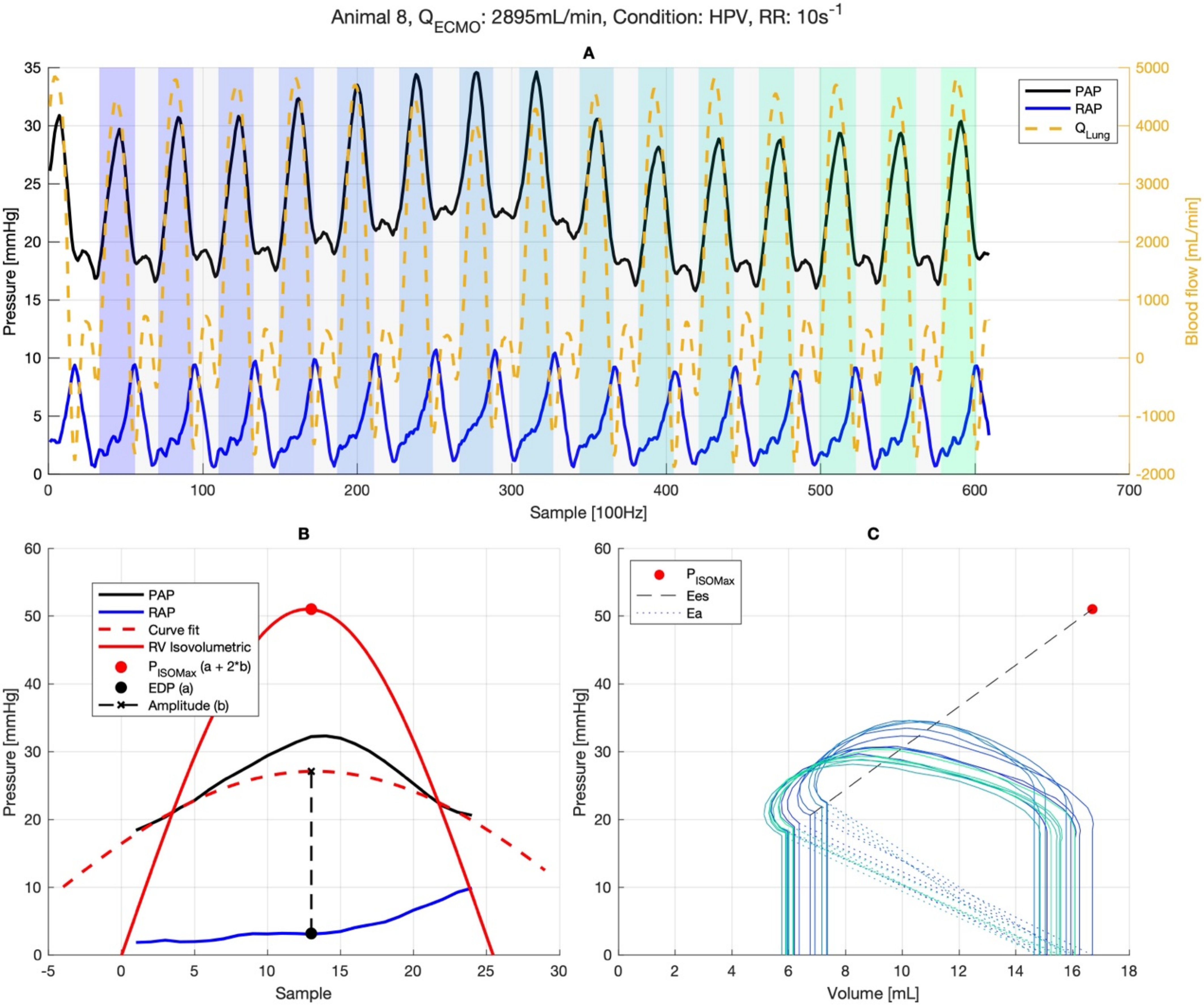

Animal 8, Q_{ECMO}: 2895mL/min, Condition: HPV, RR: $10s^{-1}$
A
PAP
RAP
Q_{Lung}
Pressure [mmHg]
Blood flow [mL/min]
Sample [100Hz]
B
PAP
RAP
Curve fit
RV Isovolumetric
P_{ISOMax} (a + 2*b)
EDP (a)
Amplitude (b)
Pressure [mmHg]
Sample
C
P_{ISOMax}
Ees
Ea
Pressure [mmHg]
Volume [mL]

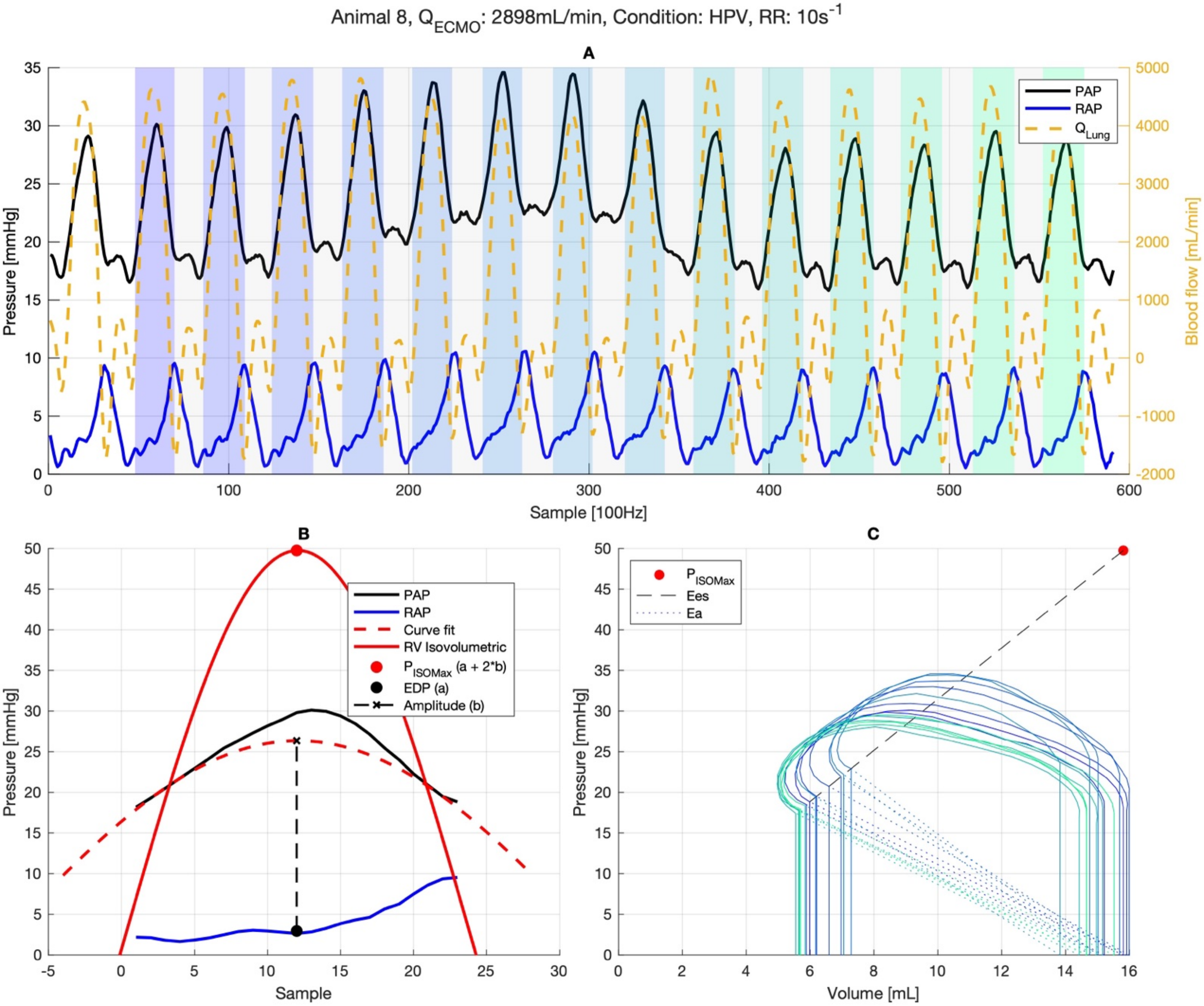

Animal 8, Q_{ECMO}: 2898mL/min, Condition: HPV, RR: $10s^{-1}$
A
PAP
RAP
Q_{Lung}
Pressure [mmHg]
Blood flow [mL/min]
Sample [100Hz]
B
PAP
RAP
Curve fit
RV Isovolumetric
P_{ISOMax} (a + 2*b)
EDP (a)
Amplitude (b)
Pressure [mmHg]
Sample
C
P_{ISOMax}
Ees
Ea
Pressure [mmHg]
Volume [mL]

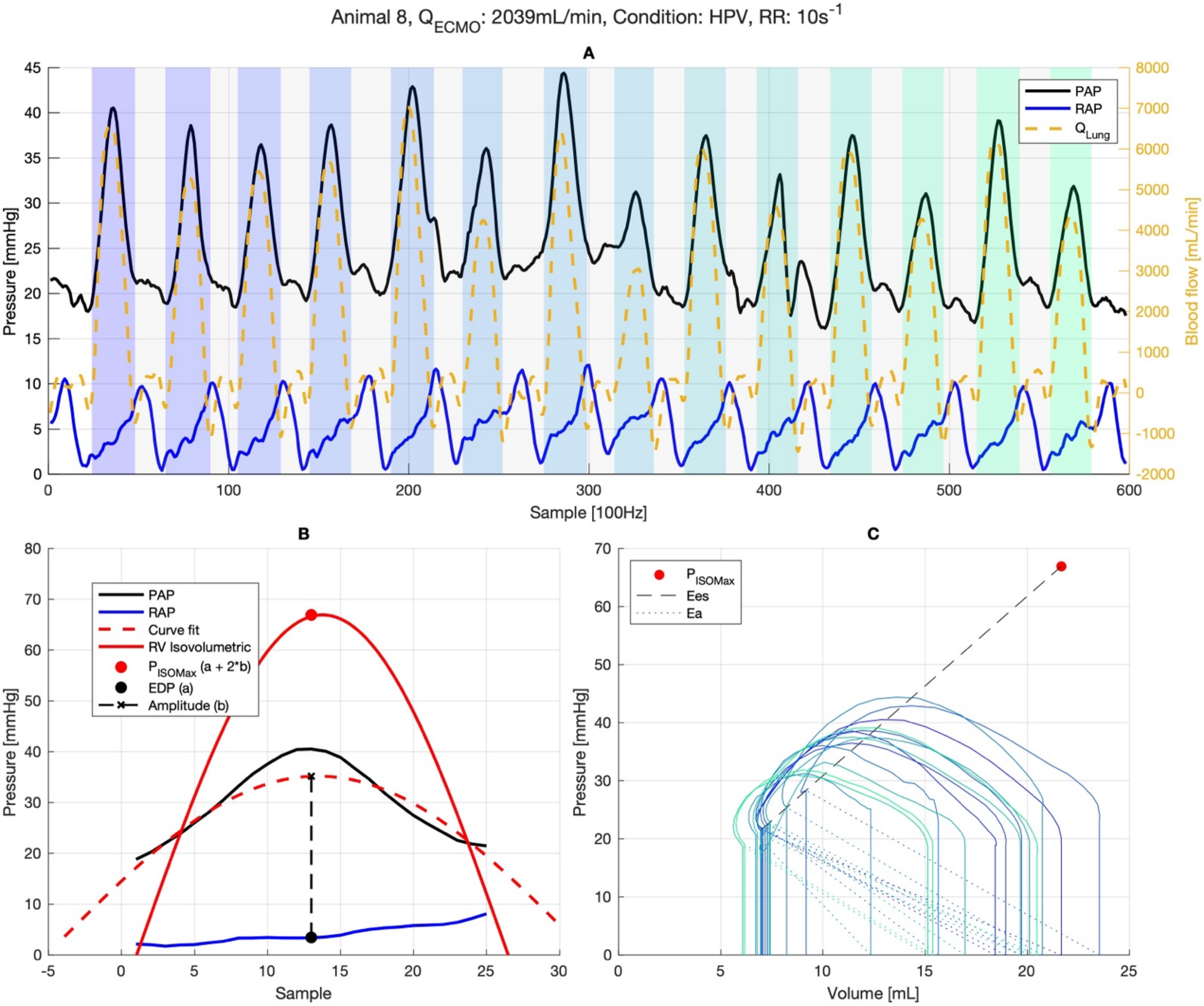

Animal 8, Q_{ECMO}: 2039mL/min, Condition: HPV, RR: $10s^{-1}$
A
Pressure [mmHg]
Blood flow [mL/min]
Sample [100Hz]
PAP
RAP
Q_{Lung}
B
Pressure [mmHg]
Sample
PAP
RAP
Curve fit
RV Isovolumetric
P_{ISOMax} (a + 2*b)
EDP (a)
Amplitude (b)
C
Pressure [mmHg]
Volume [mL]
P_{ISOMax}
Ees
Ea

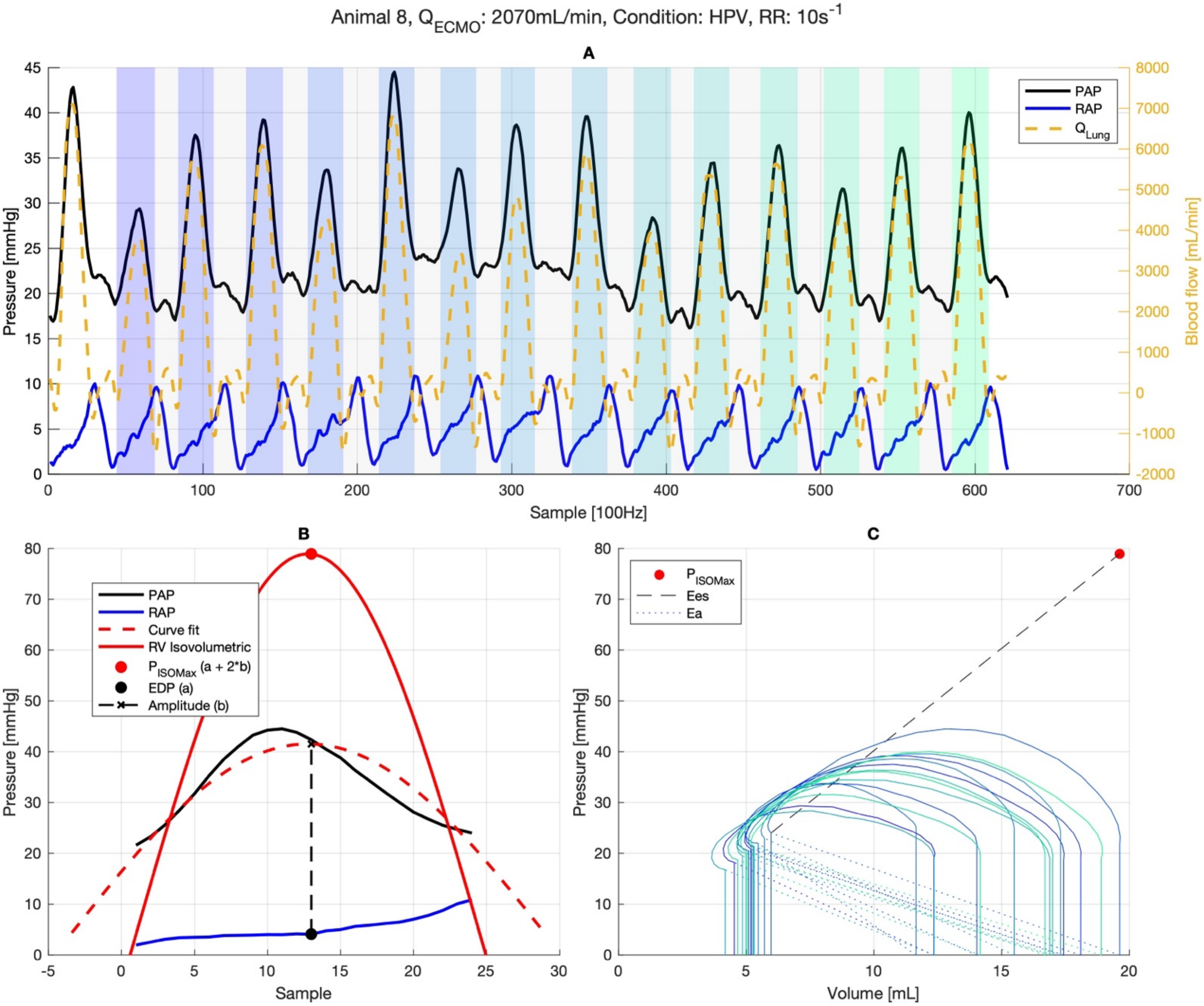

Animal 8, Q_ECMO: 2070mL/min, Condition: HPV, RR: 10s^-1
A
PAP
RAP
Q_Lung
Pressure [mmHg]
Blood flow [mL/min]
Sample [100Hz]
B
PAP
RAP
Curve fit
RV Isovolumetric
P_ISOMax (a + 2*b)
EDP (a)
Amplitude (b)
Pressure [mmHg]
Sample
C
P_ISOMax
Ees
Ea
Pressure [mmHg]
Volume [mL]

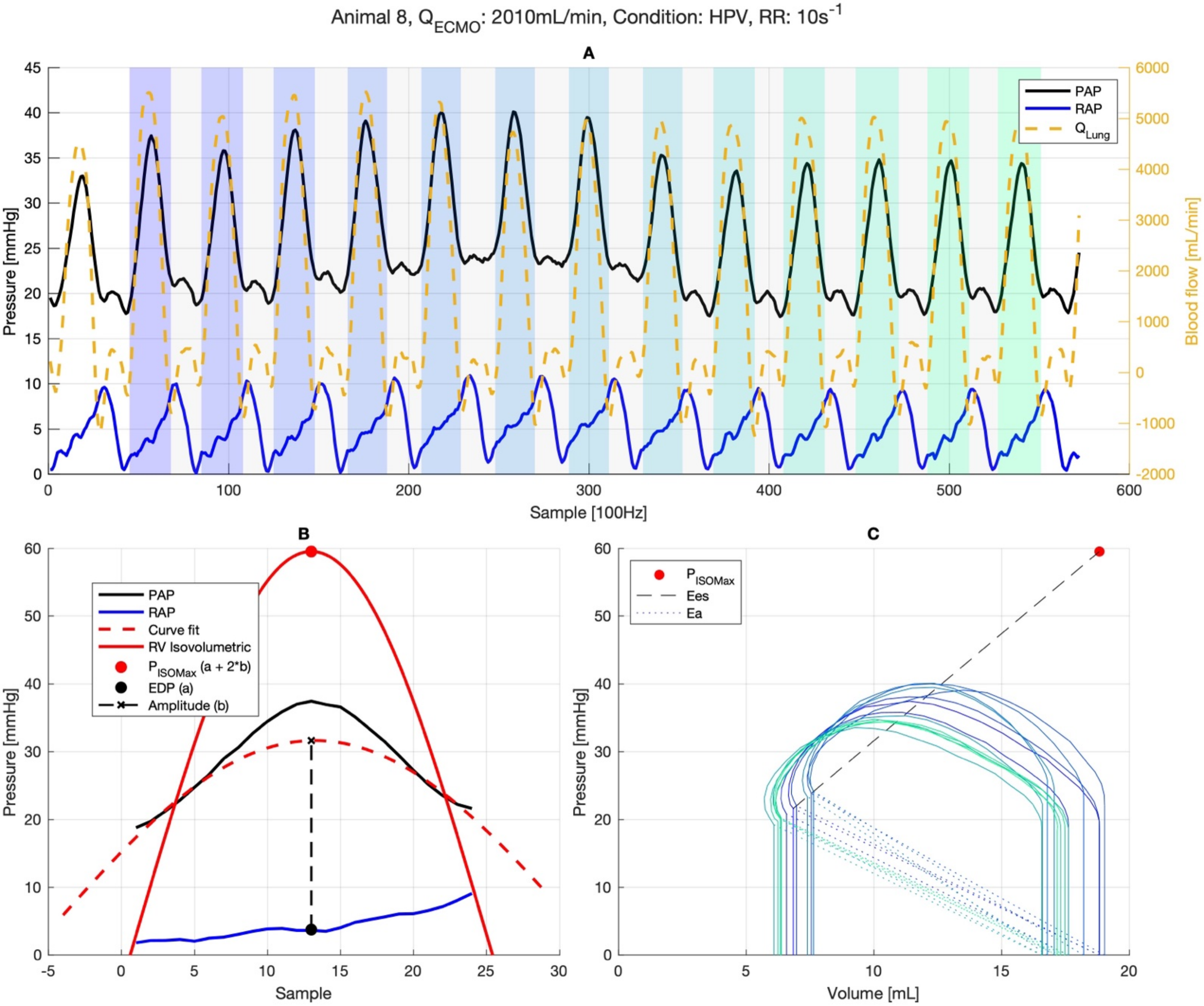

Animal 8, Q_{ECMO}: 2010mL/min, Condition: HPV, RR: 10s^{-1}
A
PAP
RAP
Q_{Lung}
Pressure [mmHg]
Blood flow [mL/min]
Sample [100Hz]
B
PAP
RAP
Curve fit
RV Isovolumetric
P_{ISOMax} (a + 2*b)
EDP (a)
Amplitude (b)
Pressure [mmHg]
Sample
C
P_{ISOMax}
Ees
Ea
Pressure [mmHg]
Volume [mL]

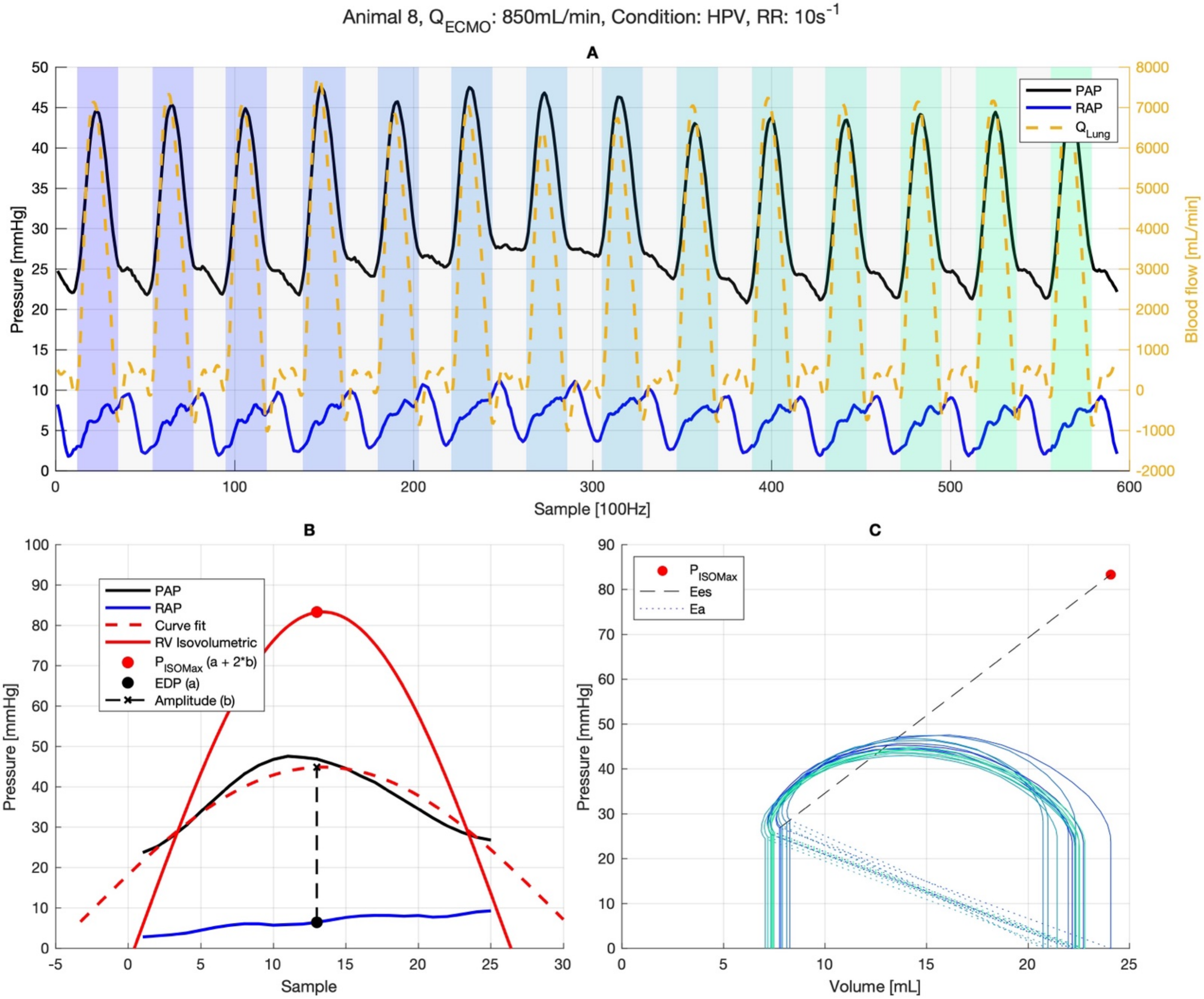

Animal 8, Q_{ECMO}: 850mL/min, Condition: HPV, RR: $10s^{-1}$
A
PAP
RAP
Q_{Lung}
Pressure [mmHg]
Blood flow [mL/min]
Sample [100Hz]
B
PAP
RAP
Curve fit
RV Isovolumetric
P_{ISOMax} (a + 2*b)
EDP (a)
Amplitude (b)
Pressure [mmHg]
Sample
C
P_{ISOMax}
Ees
Ea
Pressure [mmHg]
Volume [mL]

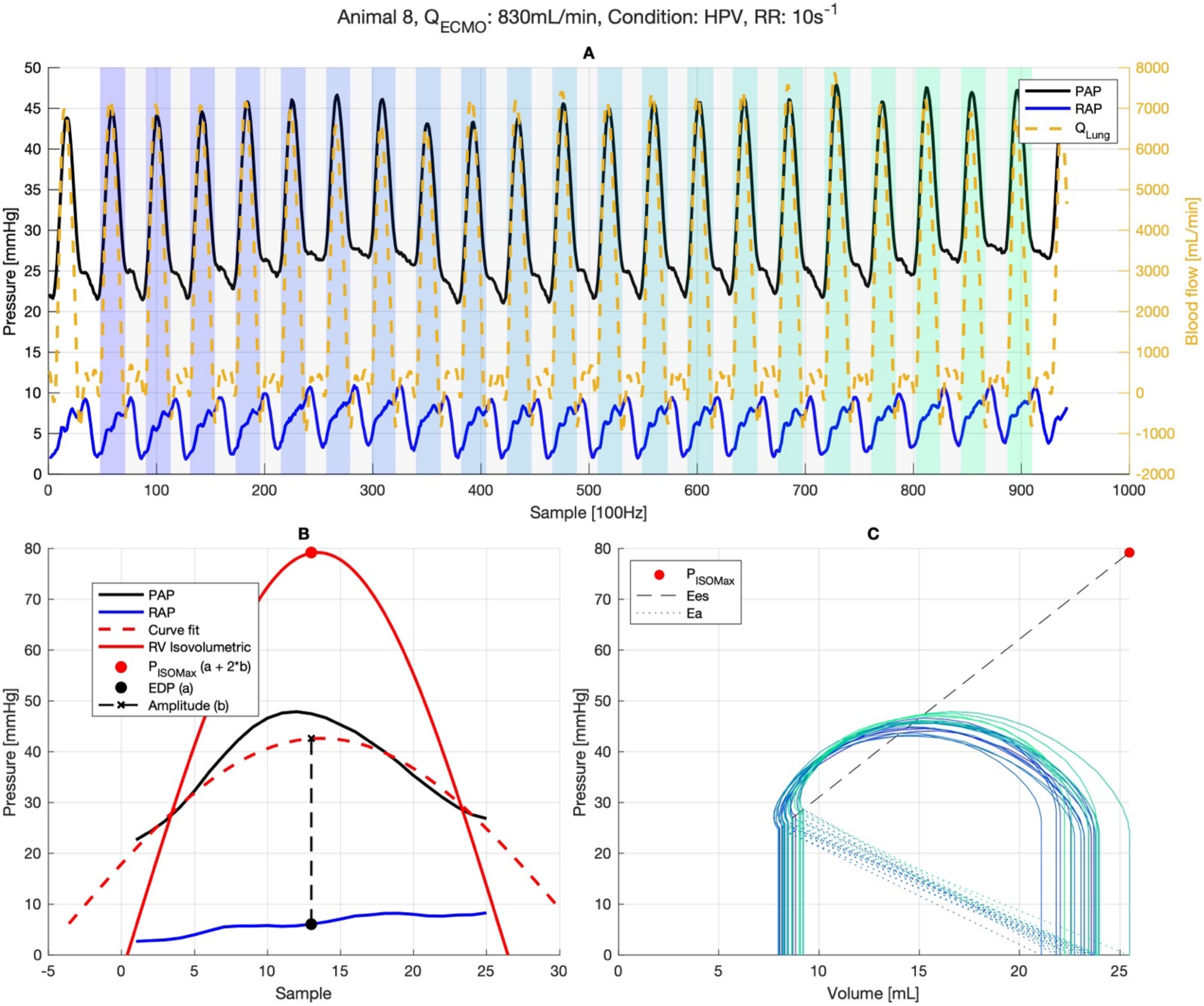
Animal 8, Q_{ECMO}: 830mL/min, Condition: HPV, RR: $10s^{-1}$
A
PAP
RAP
Q_{Lung}
Pressure [mmHg]
Blood flow [mL/min]
Sample [100Hz]
B
PAP
RAP
Curve fit
RV Isovolumetric
P_{ISOMax} (a + 2*b)
EDP (a)
Amplitude (b)
Pressure [mmHg]
Sample
C
P_{ISOMax}
Ees
Ea
Pressure [mmHg]
Volume [mL]

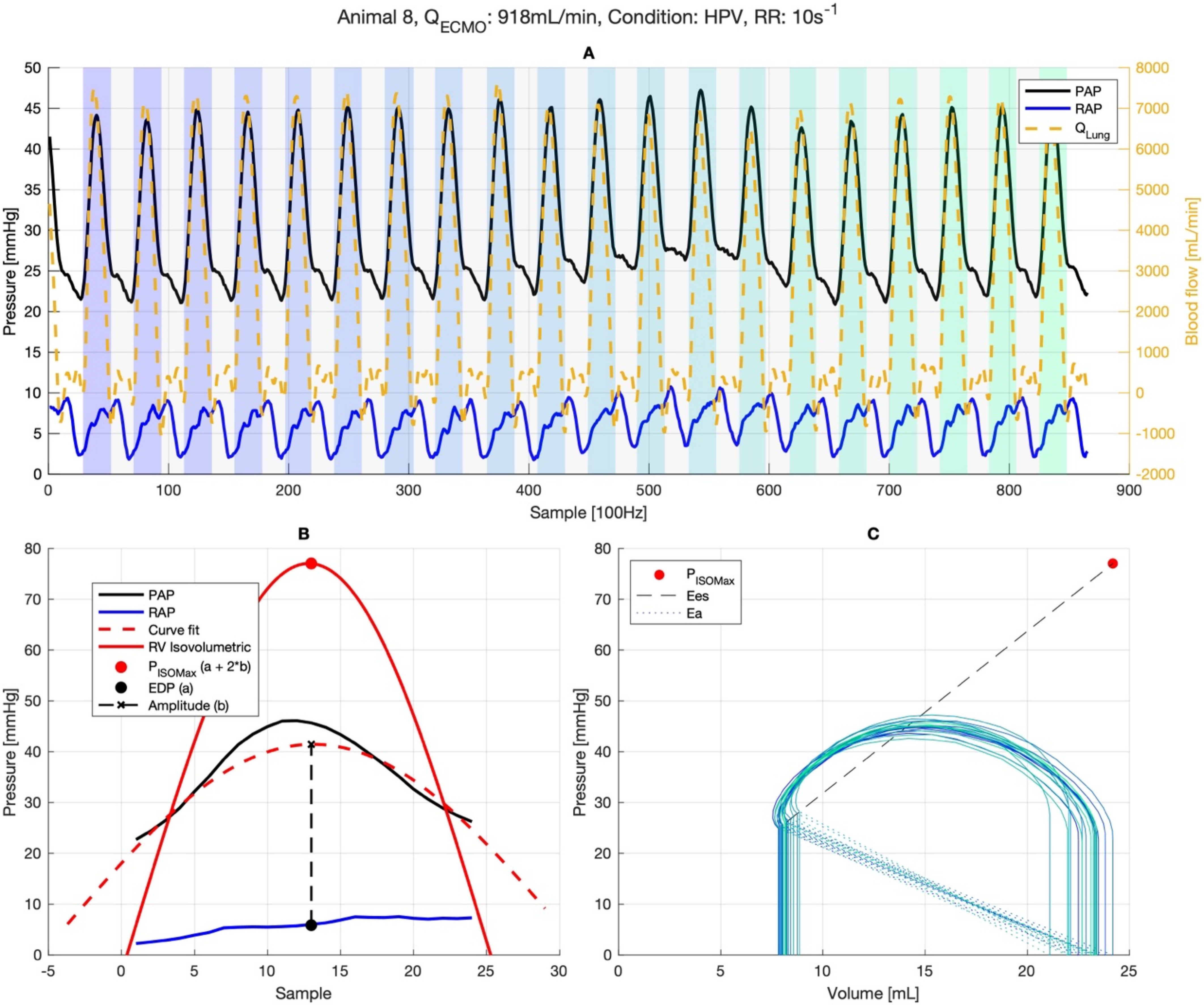

Animal 8, Q_{ECMO}: 918mL/min, Condition: HPV, RR: $10s^{-1}$
A
PAP
RAP
Q_{Lung}
Pressure [mmHg]
Blood flow [mL/min]
Sample [100Hz]
B
PAP
RAP
Curve fit
RV Isovolumetric
P_{ISOMax} (a + 2*b)
EDP (a)
Amplitude (b)
Pressure [mmHg]
Sample
C
P_{ISOMax}
Ees
Ea
Pressure [mmHg]
Volume [mL]

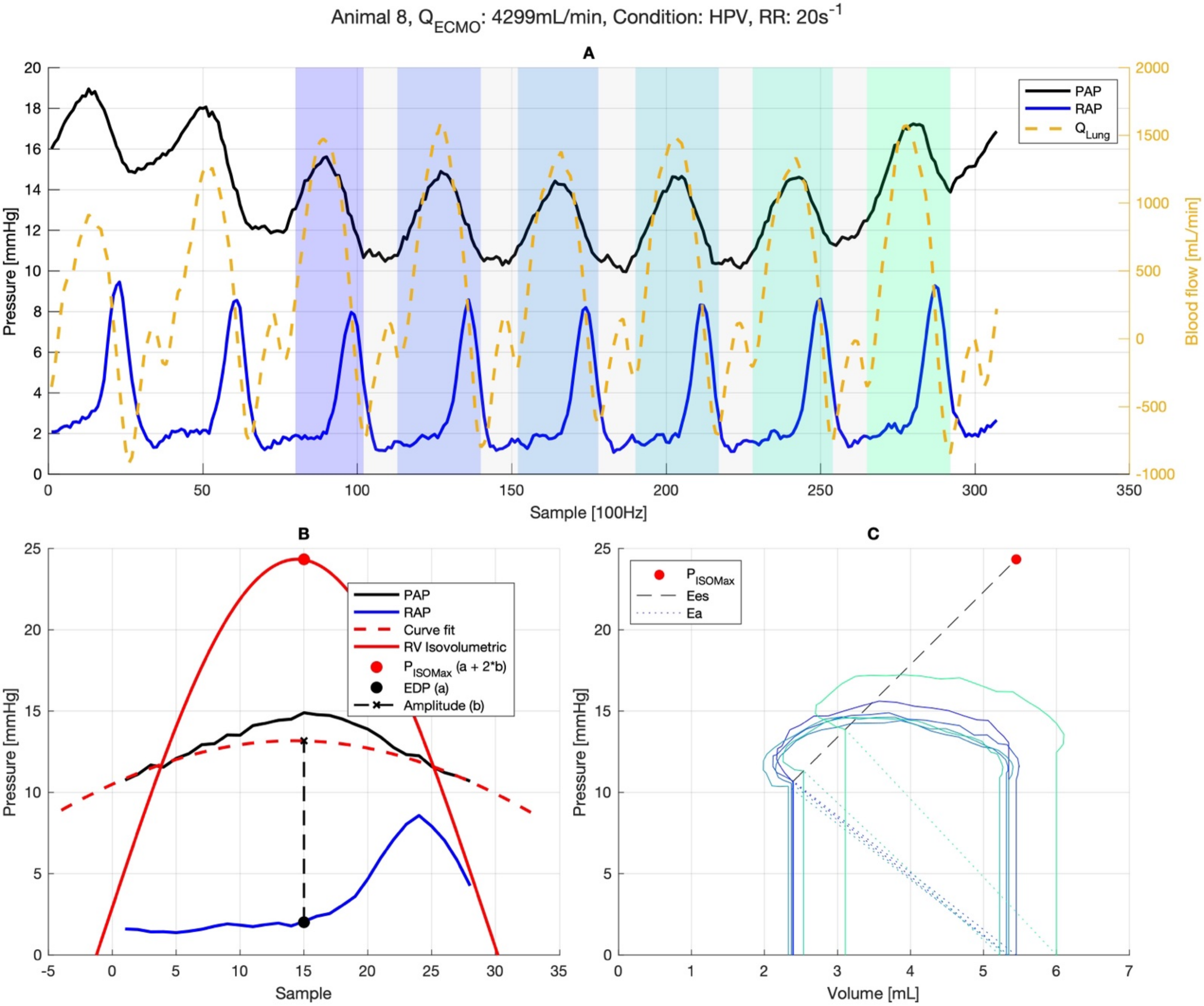

Animal 8, QECMO: 4299mL/min, Condition: HPV, RR: 20s-1
A
PAP
RAP
QLung
Pressure [mmHg]
Blood flow [mL/min]
Sample [100Hz]
B
PAP
RAP
Curve fit
RV Isovolumetric
PISOMax (a + 2*b)
EDP (a)
Amplitude (b)
Pressure [mmHg]
Sample
C
PISOMax
Ees
Ea
Pressure [mmHg]
Volume [mL]

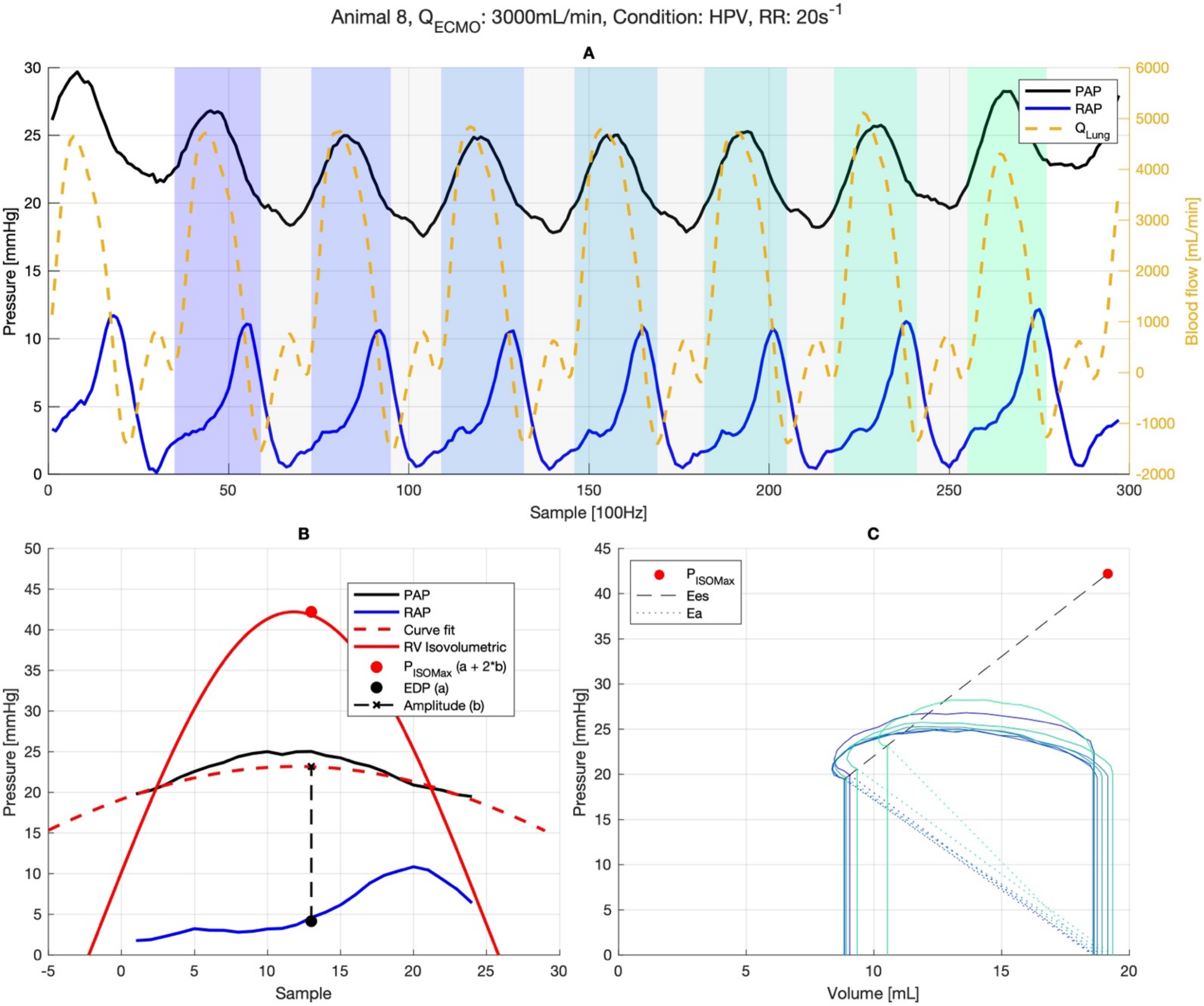

Animal 8, Q_{ECMO}: 3000mL/min, Condition: HPV, RR: 20s^{-1}
A
PAP
RAP
Q_{Lung}
Pressure [mmHg]
Blood flow [mL/min]
Sample [100Hz]
B
PAP
RAP
Curve fit
RV Isovolumetric
P_{ISOMax} (a + 2*b)
EDP (a)
Amplitude (b)
Pressure [mmHg]
Sample
C
P_{ISOMax}
Ees
Ea
Pressure [mmHg]
Volume [mL]

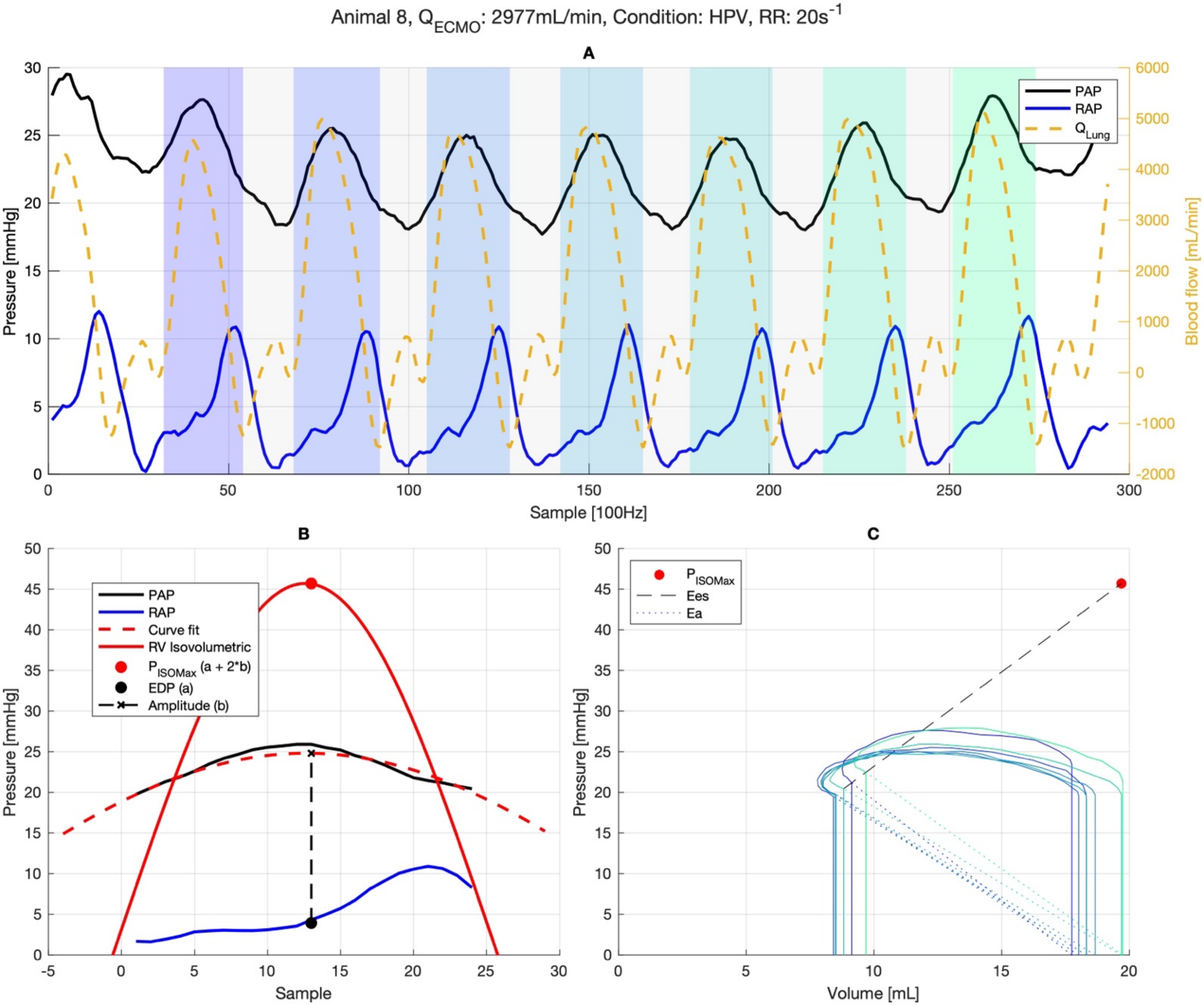

Animal 8, QECMO: 2977mL/min, Condition: HPV, RR: 20s-1
A
PAP
RAP
QLung
Pressure [mmHg]
Blood flow [mL/min]
Sample [100Hz]
B
PAP
RAP
Curve fit
RV Isovolumetric
PISOMax (a + 2*b)
EDP (a)
Amplitude (b)
Pressure [mmHg]
Sample
C
PISOMax
Ees
Ea
Pressure [mmHg]
Volume [mL]

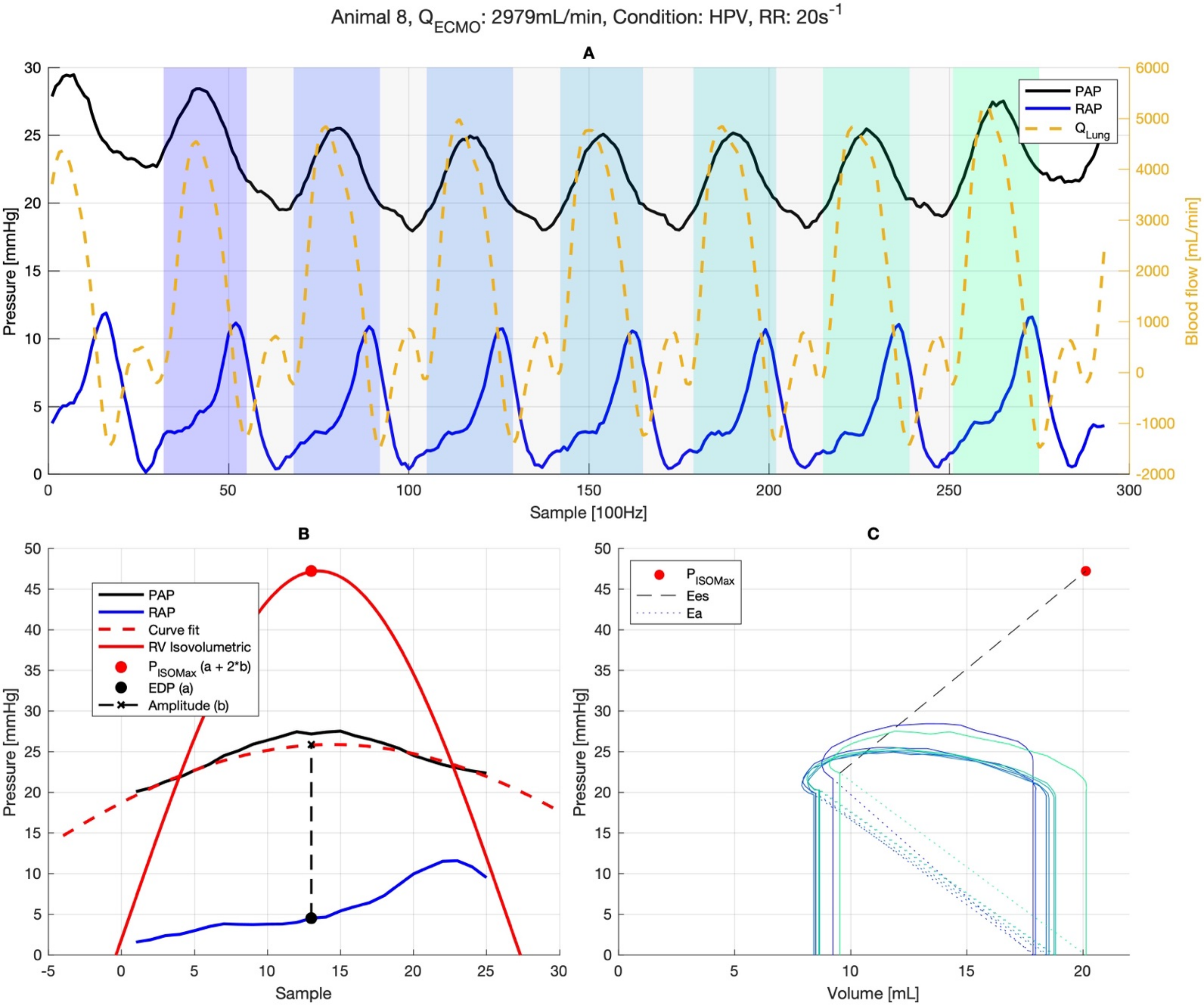

Animal 8, Q_{ECMO}: 2979mL/min, Condition: HPV, RR: $20s^{-1}$
A
PAP
RAP
Q_{Lung}
Pressure [mmHg]
Blood flow [mL/min]
Sample [100Hz]
B
PAP
RAP
Curve fit
RV Isovolumetric
P_{ISOMax} (a + 2*b)
EDP (a)
Amplitude (b)
Pressure [mmHg]
Sample
C
P_{ISOMax}
Ees
Ea
Pressure [mmHg]
Volume [mL]

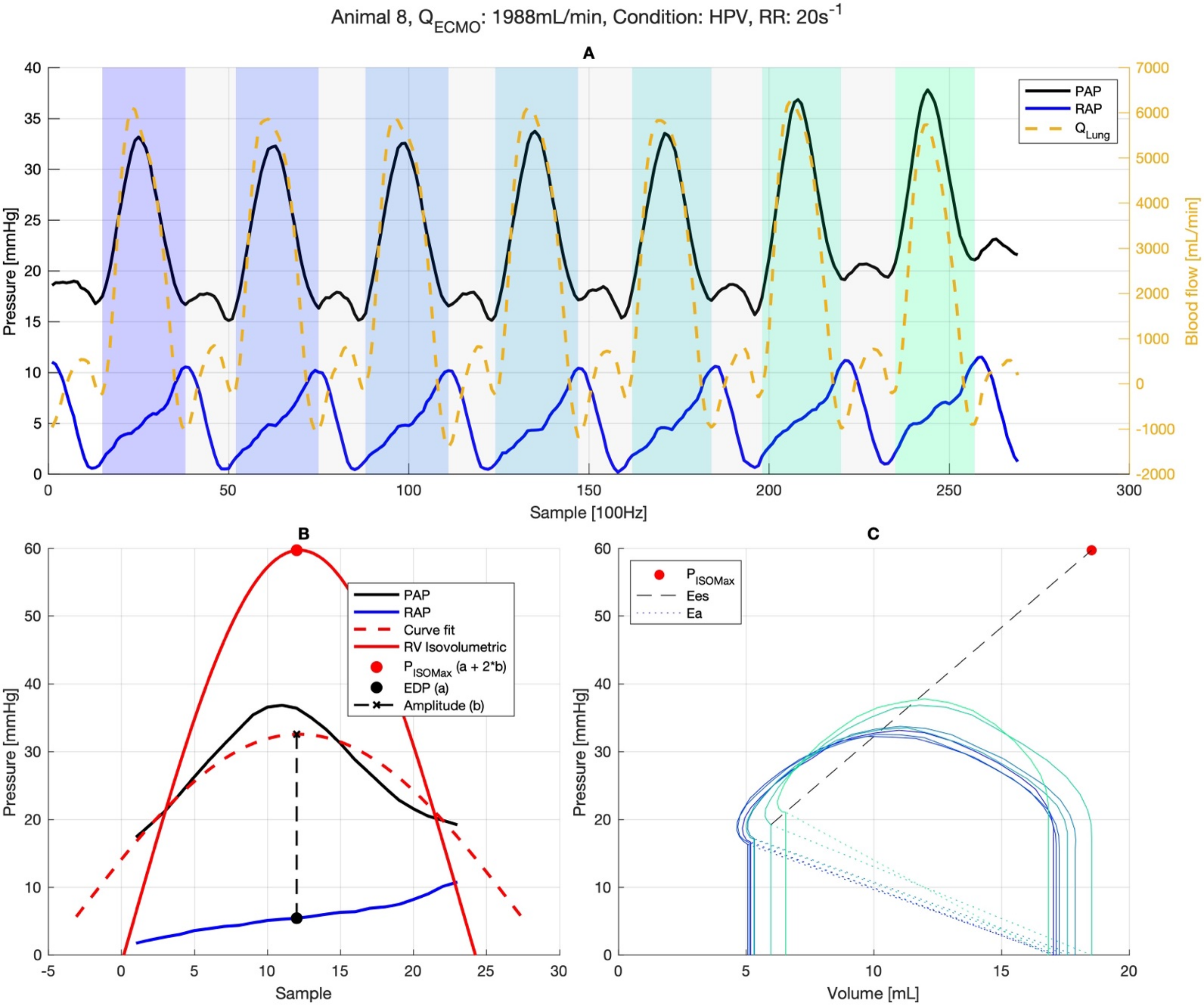

Animal 8, Q_{ECMO}: 1988mL/min, Condition: HPV, RR: 20s^{-1}
A
Pressure [mmHg]
Blood flow [mL/min]
Sample [100Hz]
PAP
RAP
Q_{Lung}
B
Pressure [mmHg]
Sample
PAP
RAP
Curve fit
RV Isovolumetric
P_{ISOMax} (a + 2*b)
EDP (a)
Amplitude (b)
C
Pressure [mmHg]
Volume [mL]
P_{ISOMax}
Ees
Ea

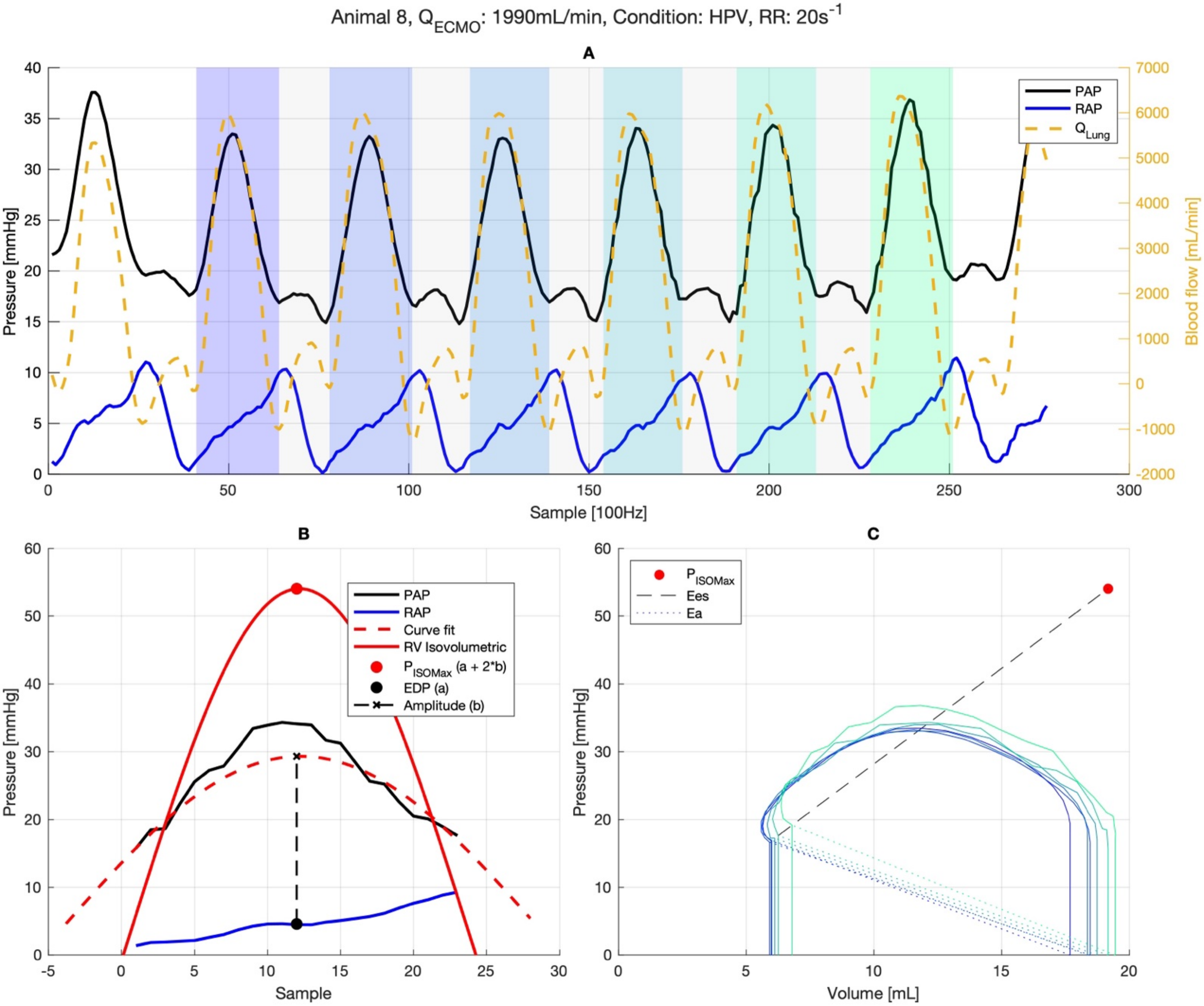
Animal 8, Q_ECMO: 1990mL/min, Condition: HPV, RR: 20s^-1
A
Pressure [mmHg]
Blood flow [mL/min]
Sample [100Hz]
PAP
RAP
Q_Lung
B
Pressure [mmHg]
Sample
PAP
RAP
Curve fit
RV Isovolumetric
P_ISOMax (a + 2*b)
EDP (a)
Amplitude (b)
C
Pressure [mmHg]
Volume [mL]
P_ISOMax
Ees
Ea

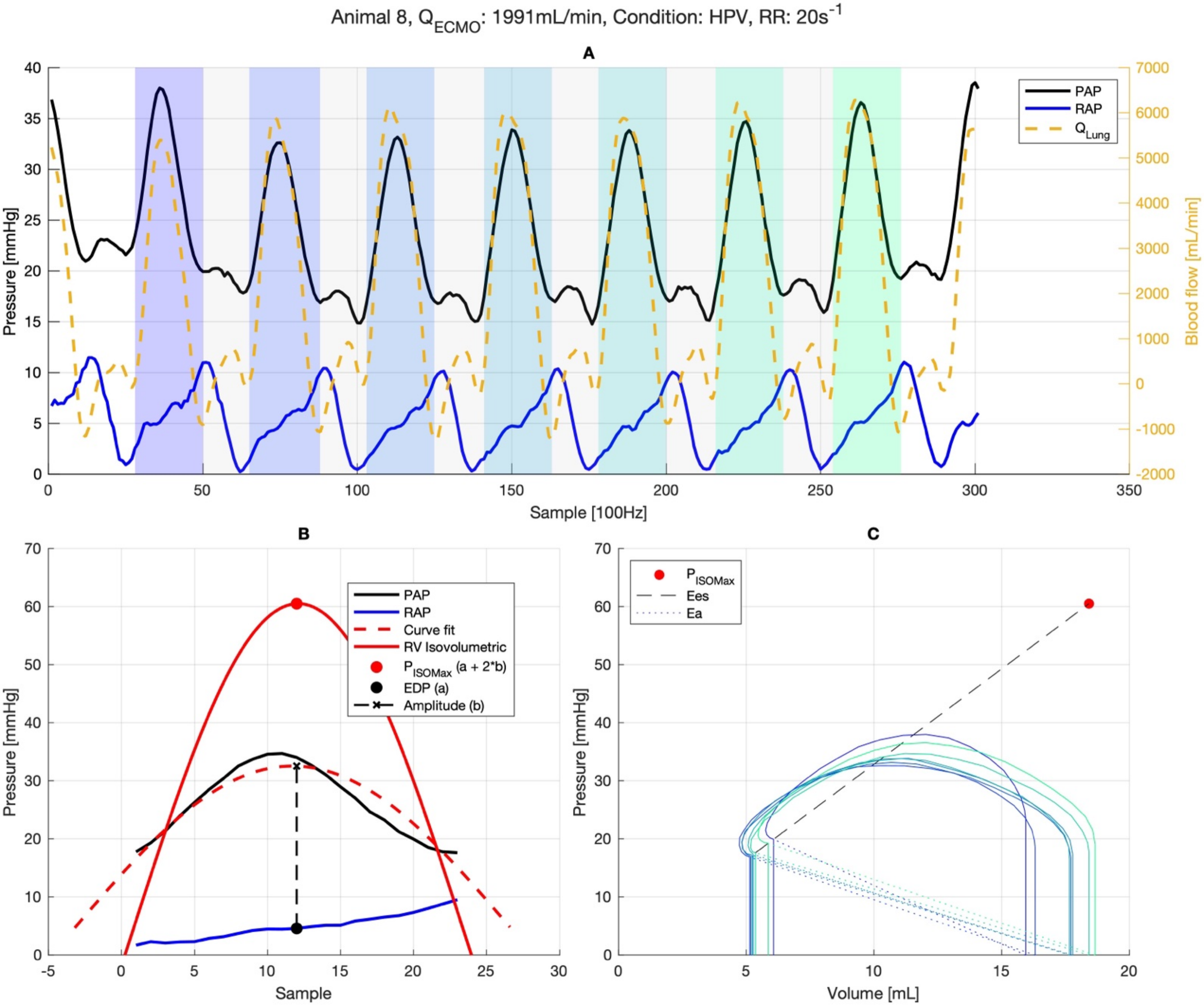

Animal 8, Q_{ECMO}: 1991mL/min, Condition: HPV, RR: $20s^{-1}$
A
PAP
RAP
Q_{Lung}
Pressure [mmHg]
Blood flow [mL/min]
Sample [100Hz]
B
PAP
RAP
Curve fit
RV Isovolumetric
P_{ISOMax} (a + 2*b)
EDP (a)
Amplitude (b)
Pressure [mmHg]
Sample
C
P_{ISOMax}
Ees
Ea
Pressure [mmHg]
Volume [mL]

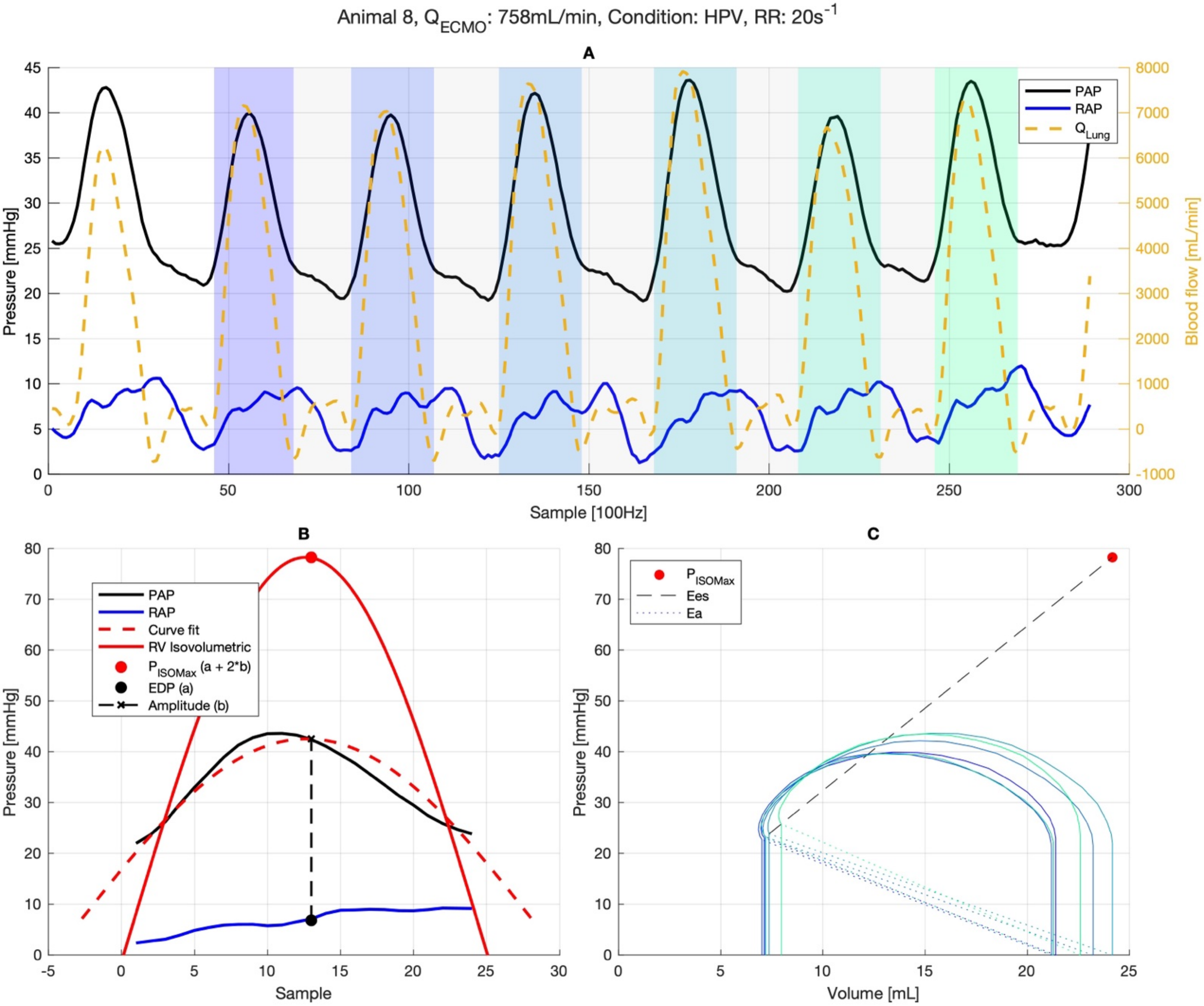

Animal 8, Q_ECMO: 758mL/min, Condition: HPV, RR: 20s^-1
A
PAP
RAP
Q_Lung
Pressure [mmHg]
Blood flow [mL/min]
Sample [100Hz]
B
PAP
RAP
Curve fit
RV Isovolumetric
P_ISOMax (a + 2*b)
EDP (a)
Amplitude (b)
Pressure [mmHg]
Sample
C
P_ISOMax
Ees
Ea
Pressure [mmHg]
Volume [mL]

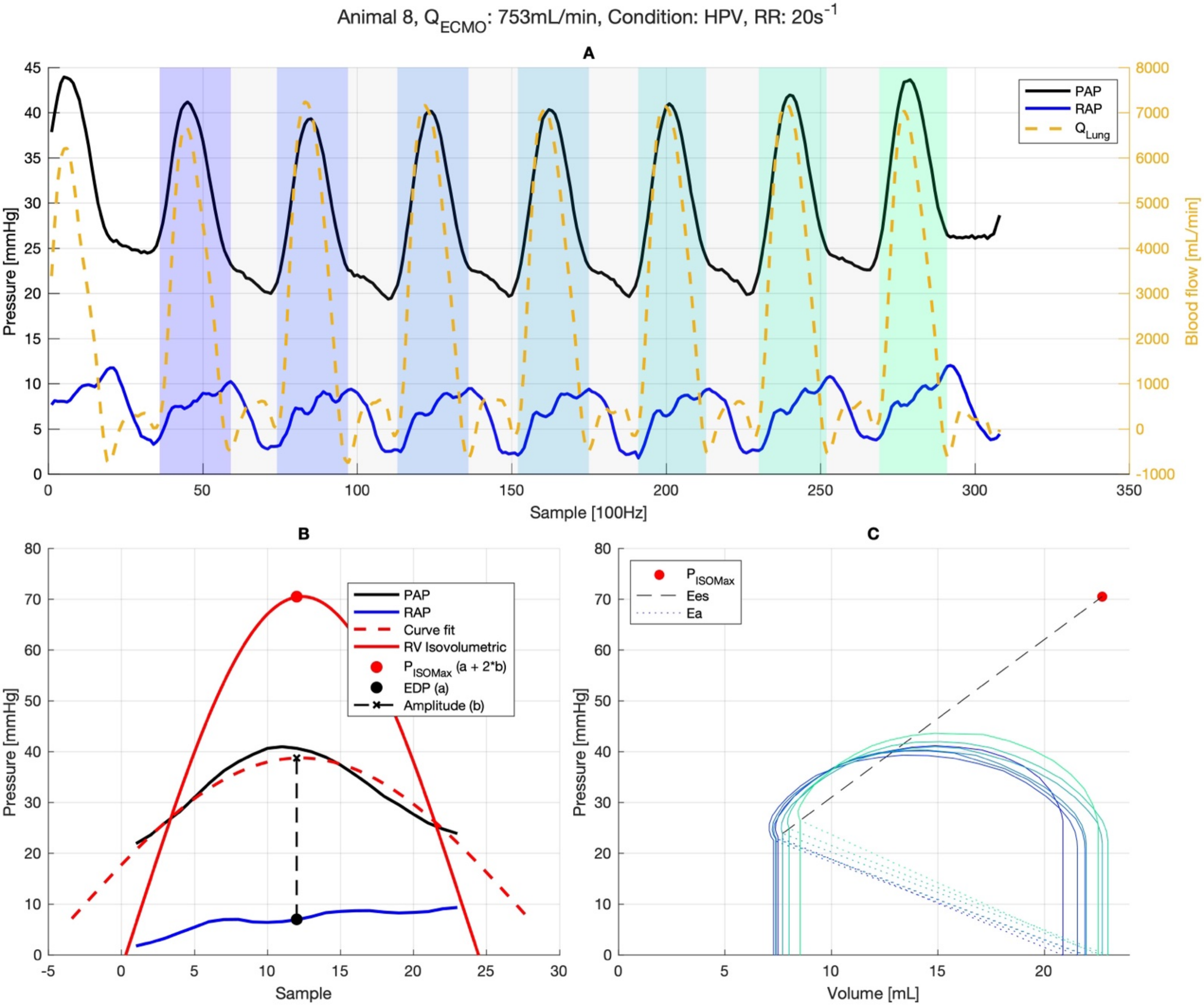

Animal 8, Q_{ECMO}: 753mL/min, Condition: HPV, RR: 20s^{-1}
A
PAP
RAP
Q_{Lung}
Pressure [mmHg]
Blood flow [mL/min]
Sample [100Hz]
B
PAP
RAP
Curve fit
RV Isovolumetric
P_{ISOMax} (a + 2*b)
EDP (a)
Amplitude (b)
Pressure [mmHg]
Sample
C
P_{ISOMax}
Ees
Ea
Pressure [mmHg]
Volume [mL]

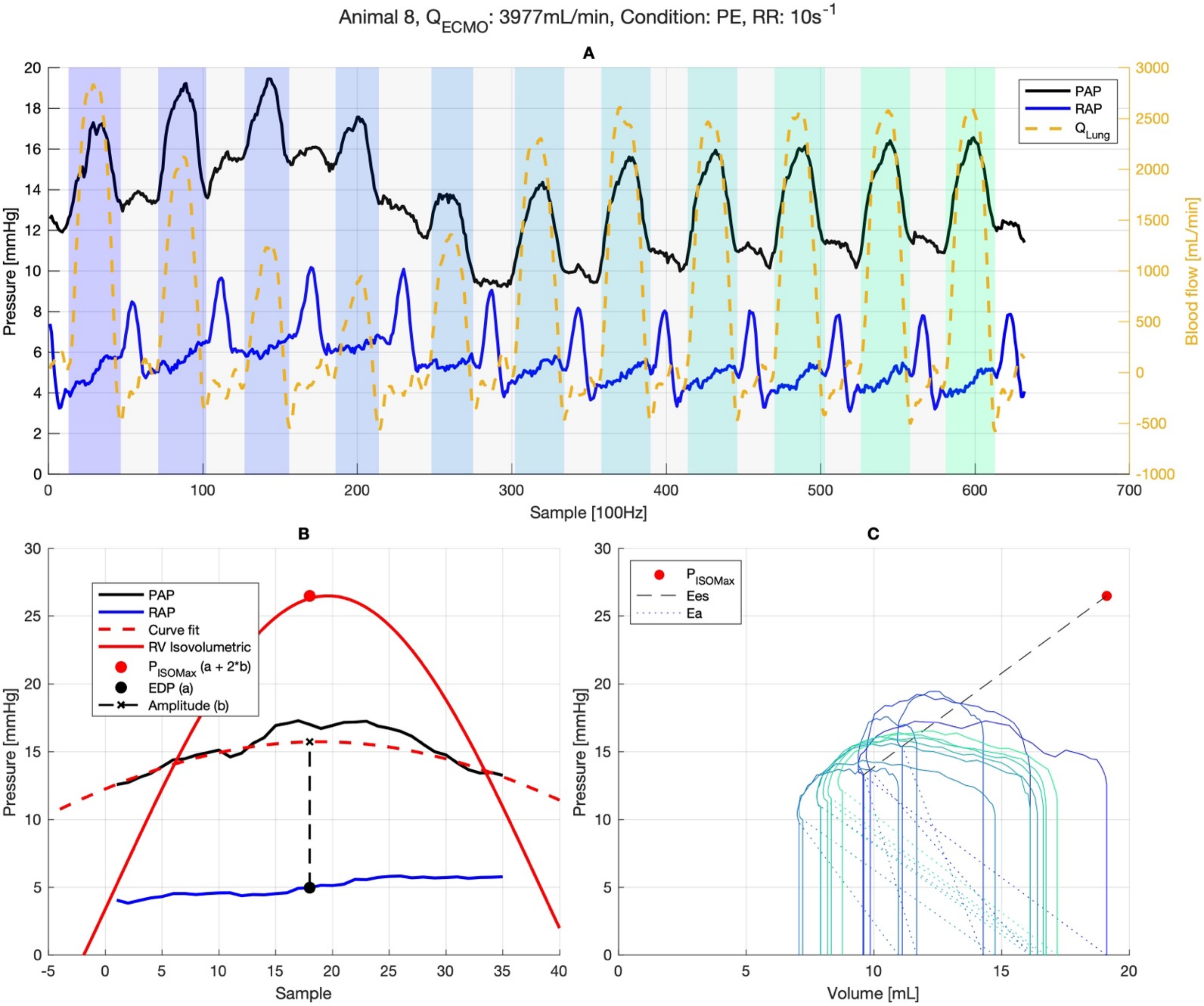

Animal 8, Q_{ECMO}: 3977mL/min, Condition: PE, RR: $10s^{-1}$
A
PAP
RAP
Q_{Lung}
Pressure [mmHg]
Blood flow [mL/min]
Sample [100Hz]
B
PAP
RAP
Curve fit
RV Isovolumetric
P_{ISOMax} (a + 2*b)
EDP (a)
Amplitude (b)
Pressure [mmHg]
Sample
C
P_{ISOMax}
Ees
Ea
Pressure [mmHg]
Volume [mL]

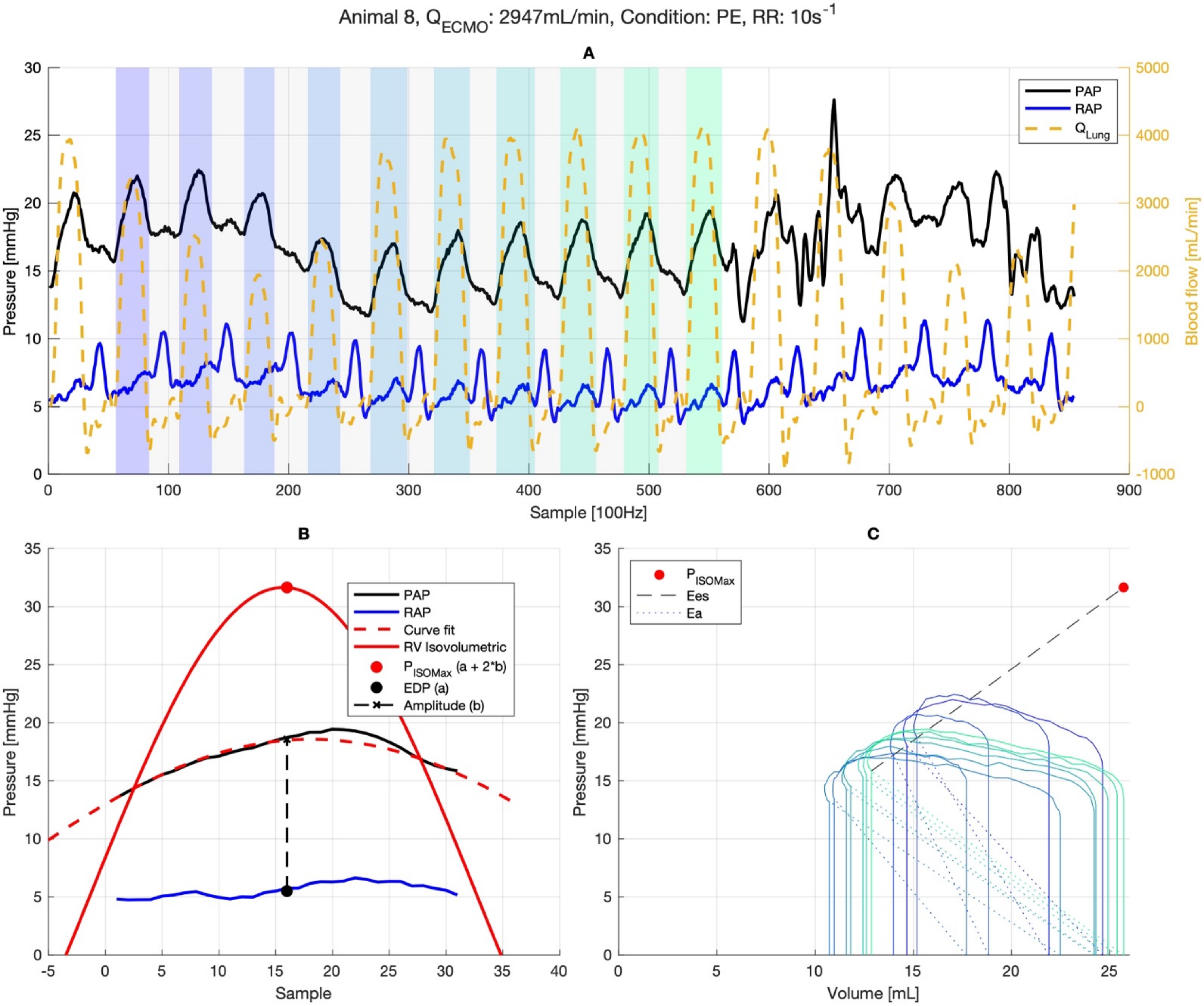

Animal 8, Q_ECMO: 2947mL/min, Condition: PE, RR: 10s^-1
A
PAP
RAP
Q_Lung
Pressure [mmHg]
Blood flow [mL/min]
Sample [100Hz]
B
PAP
RAP
Curve fit
RV Isovolumetric
P_ISOMax (a + 2*b)
EDP (a)
Amplitude (b)
Pressure [mmHg]
Sample
C
P_ISOMax
Ees
Ea
Pressure [mmHg]
Volume [mL]

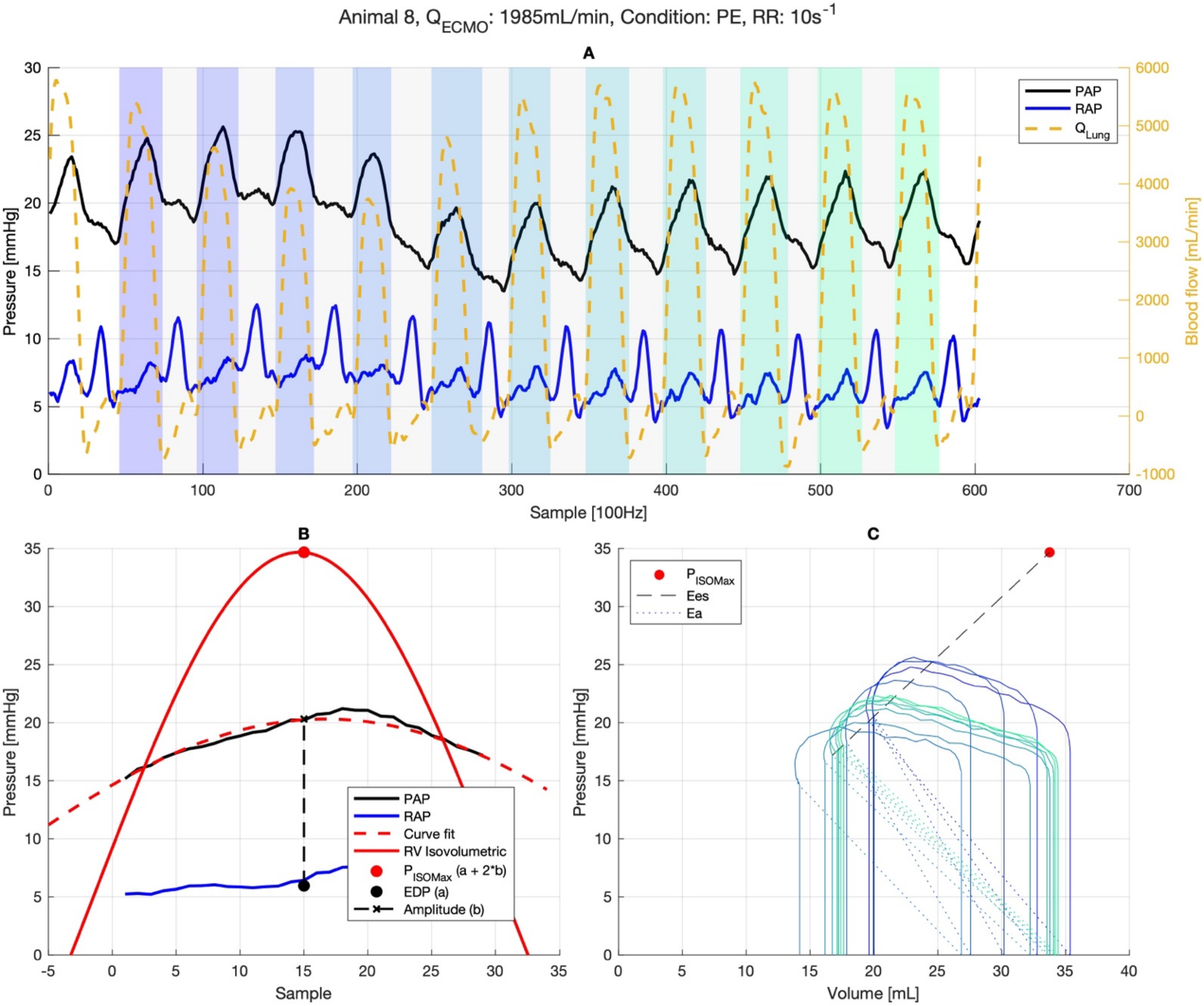

Animal 8, Q_{ECMO}: 1985mL/min, Condition: PE, RR: $10s^{-1}$
A
PAP
RAP
Q_{Lung}
Pressure [mmHg]
Blood flow [mL/min]
Sample [100Hz]
B
PAP
RAP
Curve fit
RV Isovolumetric
P_{ISOMax} (a + 2*b)
EDP (a)
Amplitude (b)
Pressure [mmHg]
Sample
C
P_{ISOMax}
Ees
Ea
Pressure [mmHg]
Volume [mL]

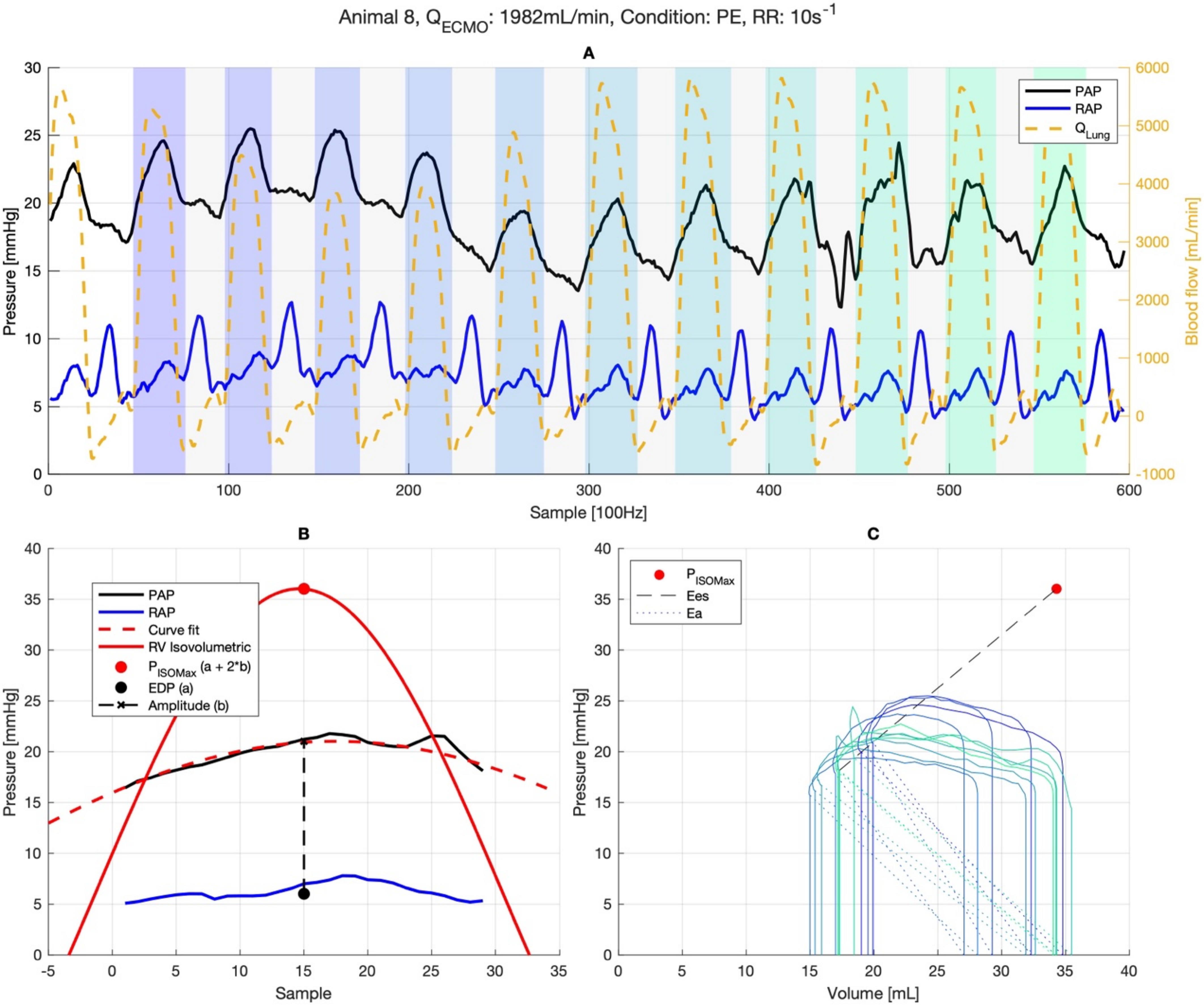

Animal 8, Q_{ECMO}: 1982mL/min, Condition: PE, RR: $10s^{-1}$
A
PAP
RAP
Q_{Lung}
Pressure [mmHg]
Blood flow [mL/min]
Sample [100Hz]
B
PAP
RAP
Curve fit
RV Isovolumetric
P_{ISOMax} (a + 2*b)
EDP (a)
Amplitude (b)
Pressure [mmHg]
Sample
C
P_{ISOMax}
Ees
Ea
Pressure [mmHg]
Volume [mL]

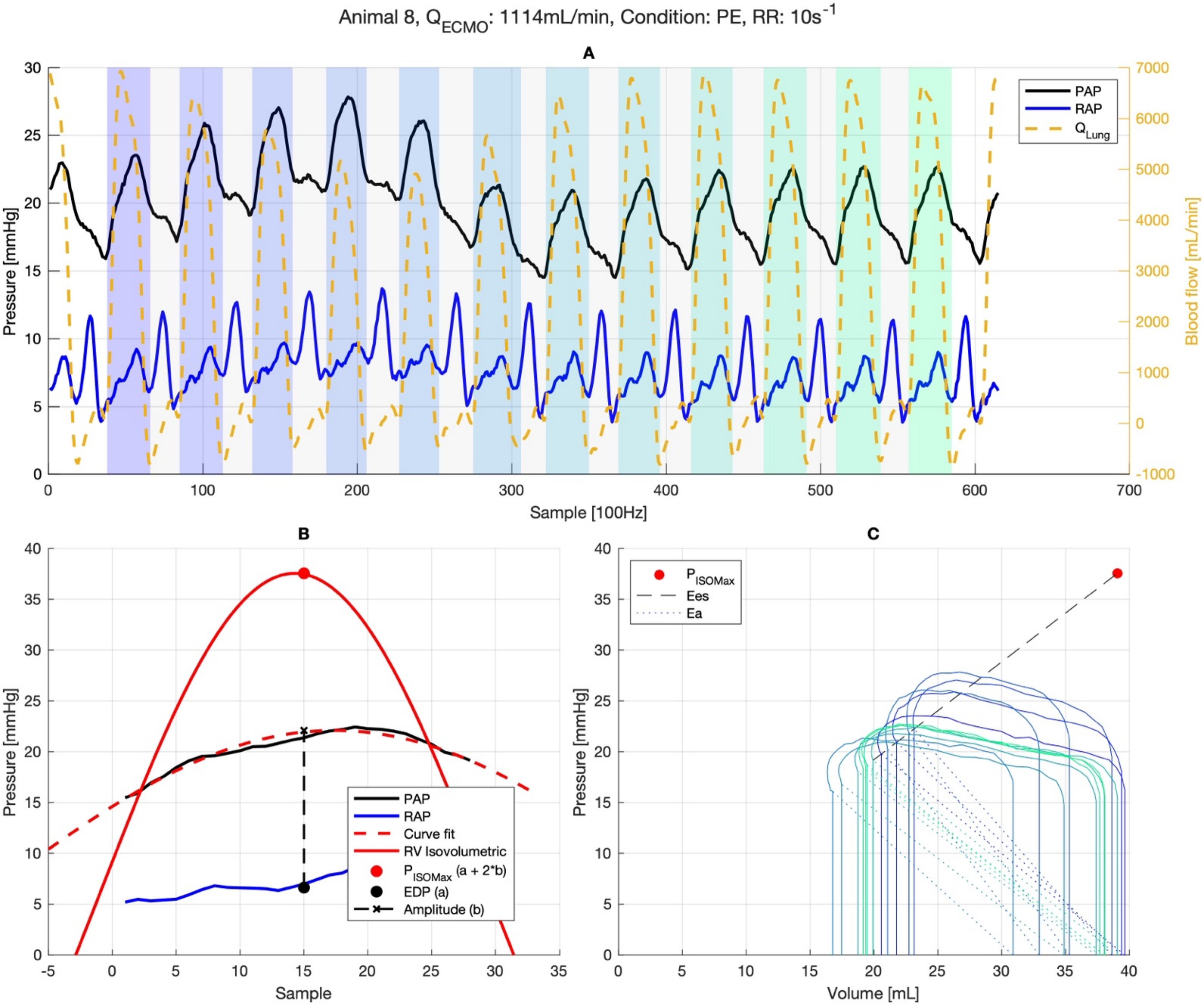

Animal 8, QECMO: 1114mL/min, Condition: PE, RR: 10s-1
A
Pressure [mmHg]
Blood flow [mL/min]
Sample [100Hz]
PAP
RAP
QLung
B
Pressure [mmHg]
Sample
PAP
RAP
Curve fit
RV Isovolumetric
PISOMax (a + 2*b)
EDP (a)
Amplitude (b)
C
Pressure [mmHg]
Volume [mL]
PISOMax
Ees
Ea

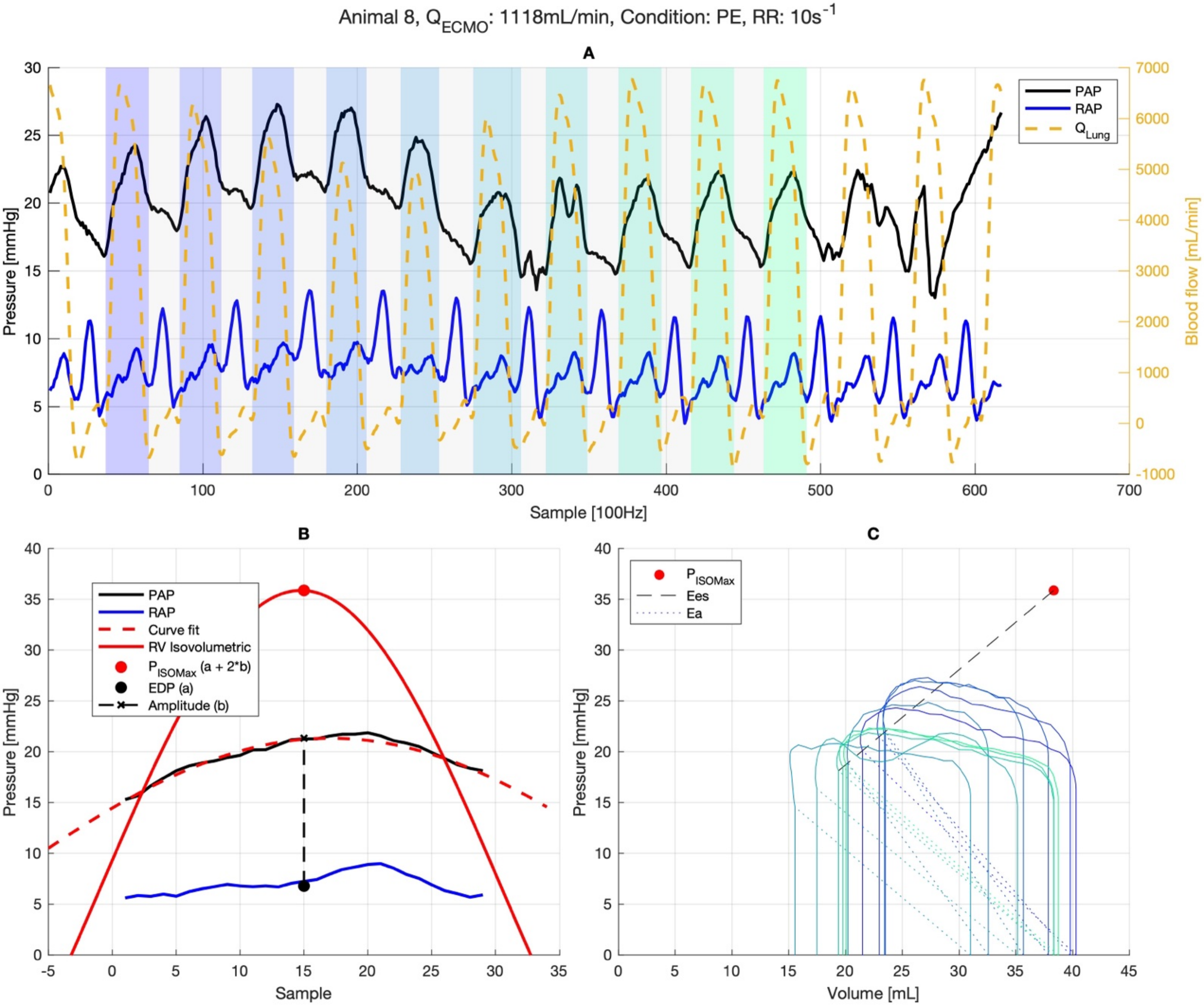

Animal 8, Q_ECMO: 1118mL/min, Condition: PE, RR: 10s^-1
A
PAP
RAP
Q_Lung
Pressure [mmHg]
Blood flow [mL/min]
Sample [100Hz]
B
PAP
RAP
Curve fit
RV Isovolumetric
P_ISOMax (a + 2*b)
EDP (a)
Amplitude (b)
Pressure [mmHg]
Sample
C
P_ISOMax
Ees
Ea
Pressure [mmHg]
Volume [mL]

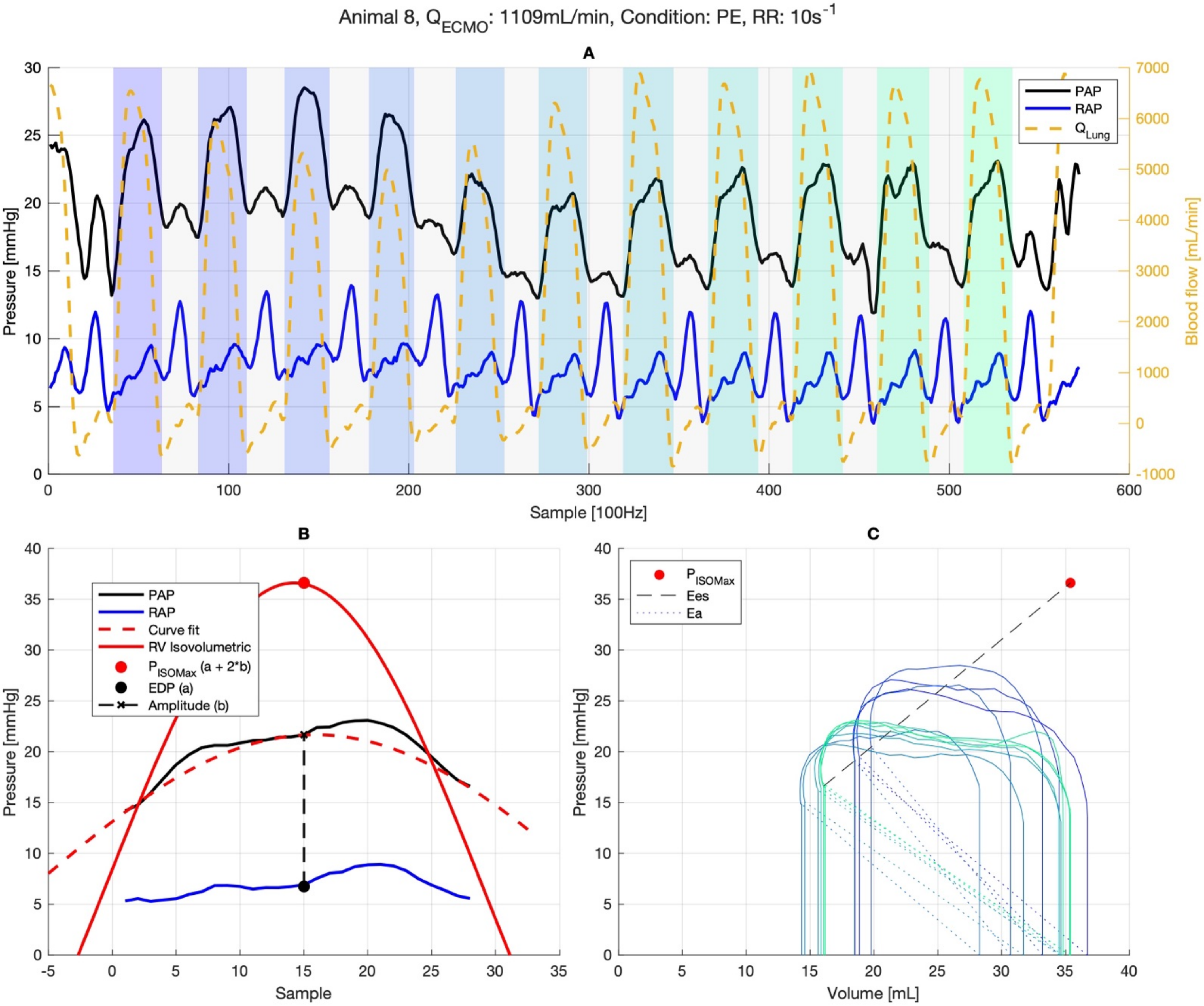

Animal 8, Q_{ECMO}: 1109mL/min, Condition: PE, RR: $10s^{-1}$
A
PAP
RAP
Q_{Lung}
Pressure [mmHg]
Blood flow [mL/min]
Sample [100Hz]
B
PAP
RAP
Curve fit
RV Isovolumetric
P_{ISOMax} (a + 2*b)
EDP (a)
Amplitude (b)
Pressure [mmHg]
Sample
C
P_{ISOMax}
Ees
Ea
Pressure [mmHg]
Volume [mL]

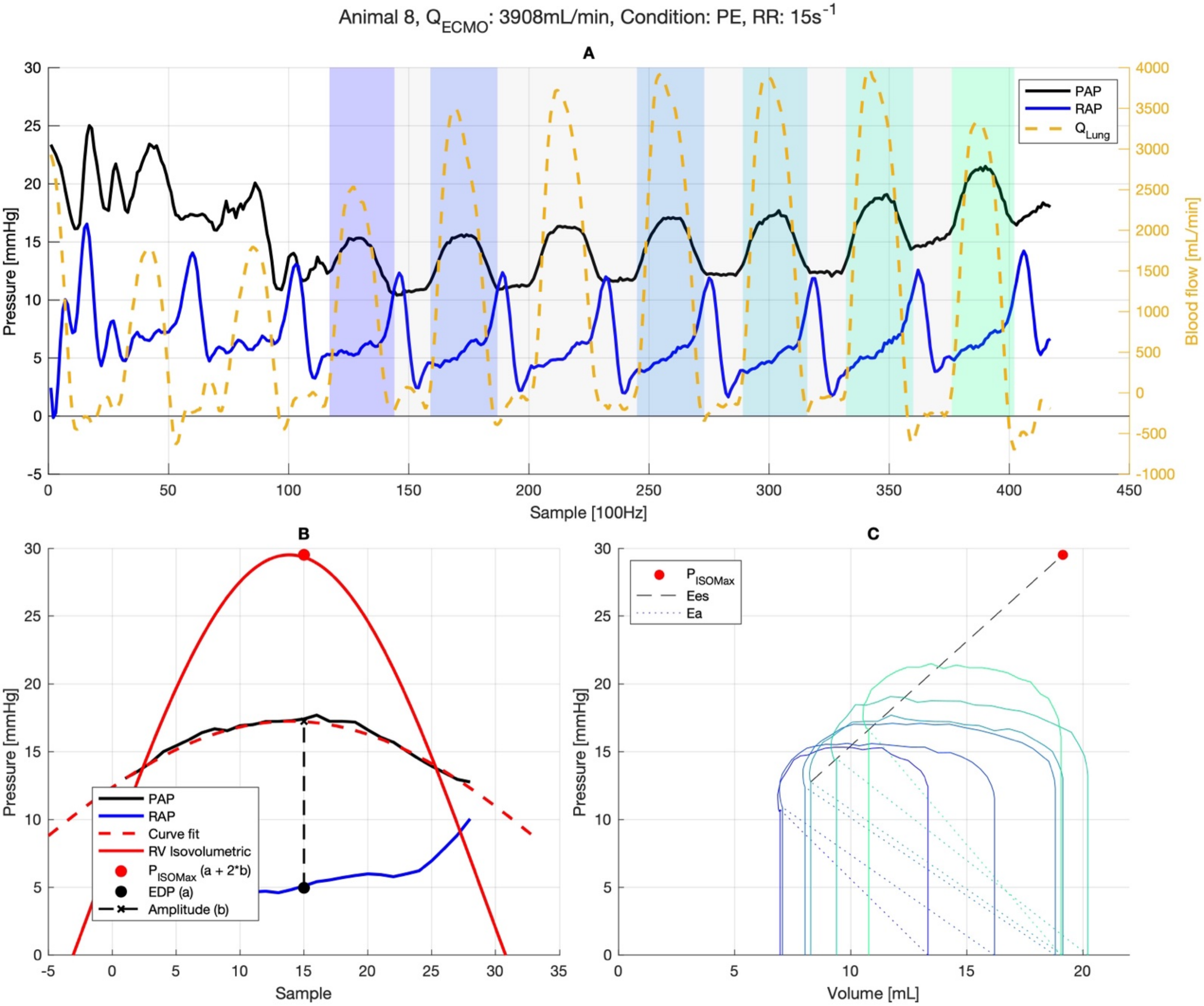
Animal 8, Q_ECMO: 3908mL/min, Condition: PE, RR: 15s^-1
A
PAP
RAP
Q_Lung
Pressure [mmHg]
Blood flow [mL/min]
Sample [100Hz]
B
PAP
RAP
Curve fit
RV Isovolumetric
P_ISOMax (a + 2*b)
EDP (a)
Amplitude (b)
Pressure [mmHg]
Sample
C
P_ISOMax
Ees
Ea
Pressure [mmHg]
Volume [mL]

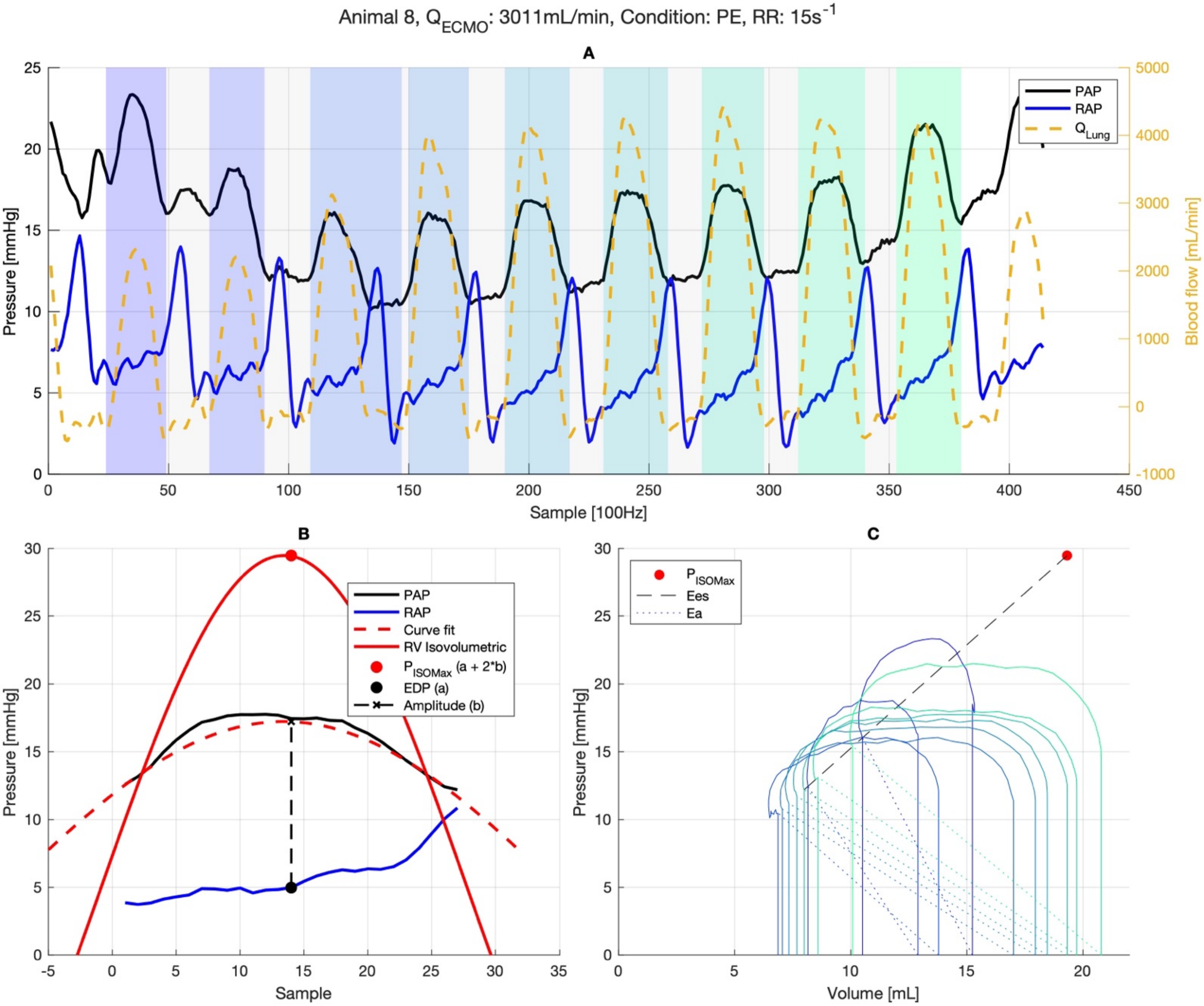

Animal 8, QECMO: 3011mL/min, Condition: PE, RR: 15s-1
A
PAP
RAP
QLung
Pressure [mmHg]
Blood flow [mL/min]
Sample [100Hz]
B
PAP
RAP
Curve fit
RV Isovolumetric
PISOMax (a + 2*b)
EDP (a)
Amplitude (b)
Pressure [mmHg]
Sample
C
PISOMax
Ees
Ea
Pressure [mmHg]
Volume [mL]

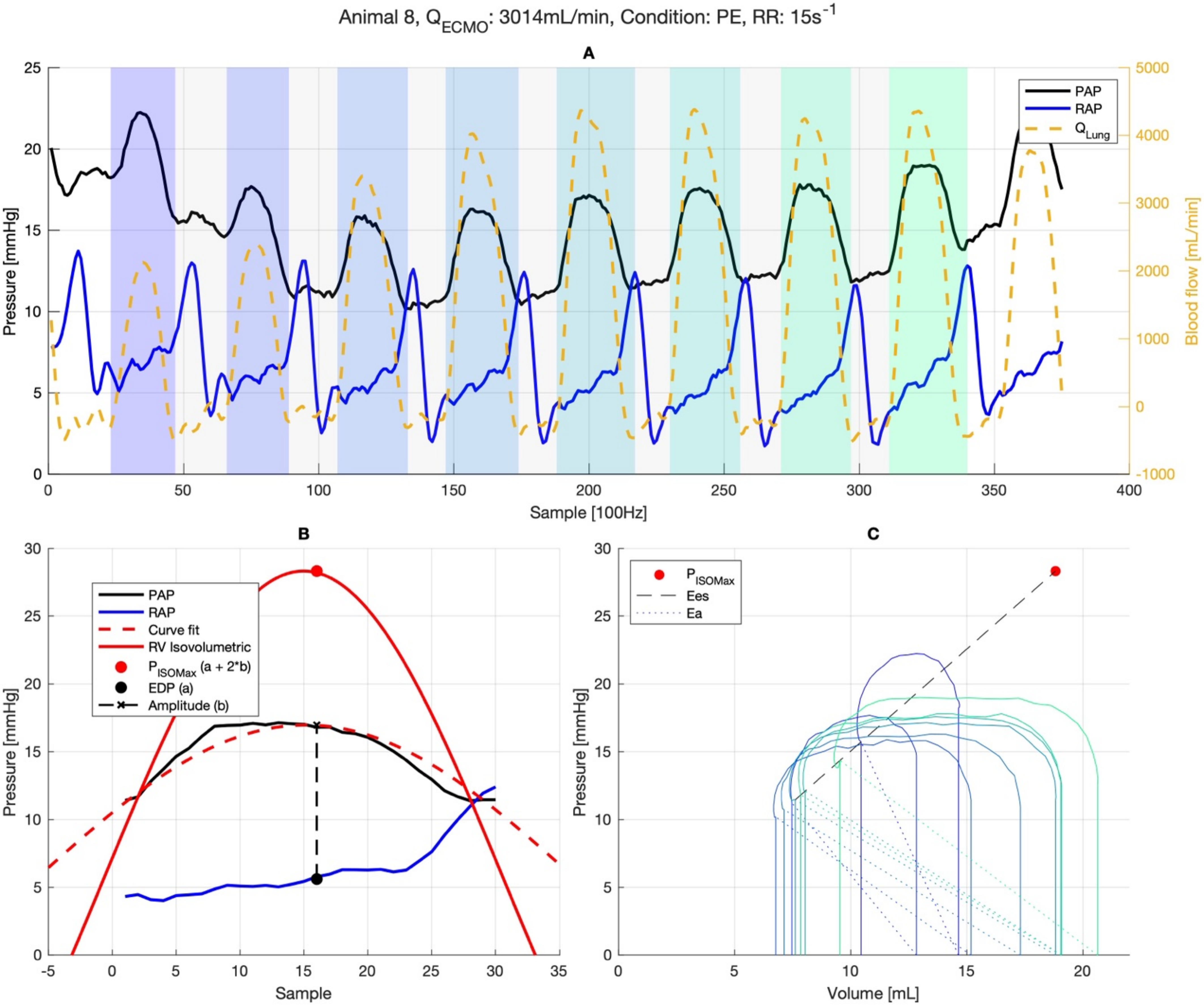

Animal 8, Q_ECMO: 3014mL/min, Condition: PE, RR: 15s^-1
A
PAP
RAP
Q_Lung
Pressure [mmHg]
Blood flow [mL/min]
Sample [100Hz]
B
PAP
RAP
Curve fit
RV Isovolumetric
P_ISOMax (a + 2*b)
EDP (a)
Amplitude (b)
Pressure [mmHg]
Sample
C
P_ISOMax
Ees
Ea
Pressure [mmHg]
Volume [mL]

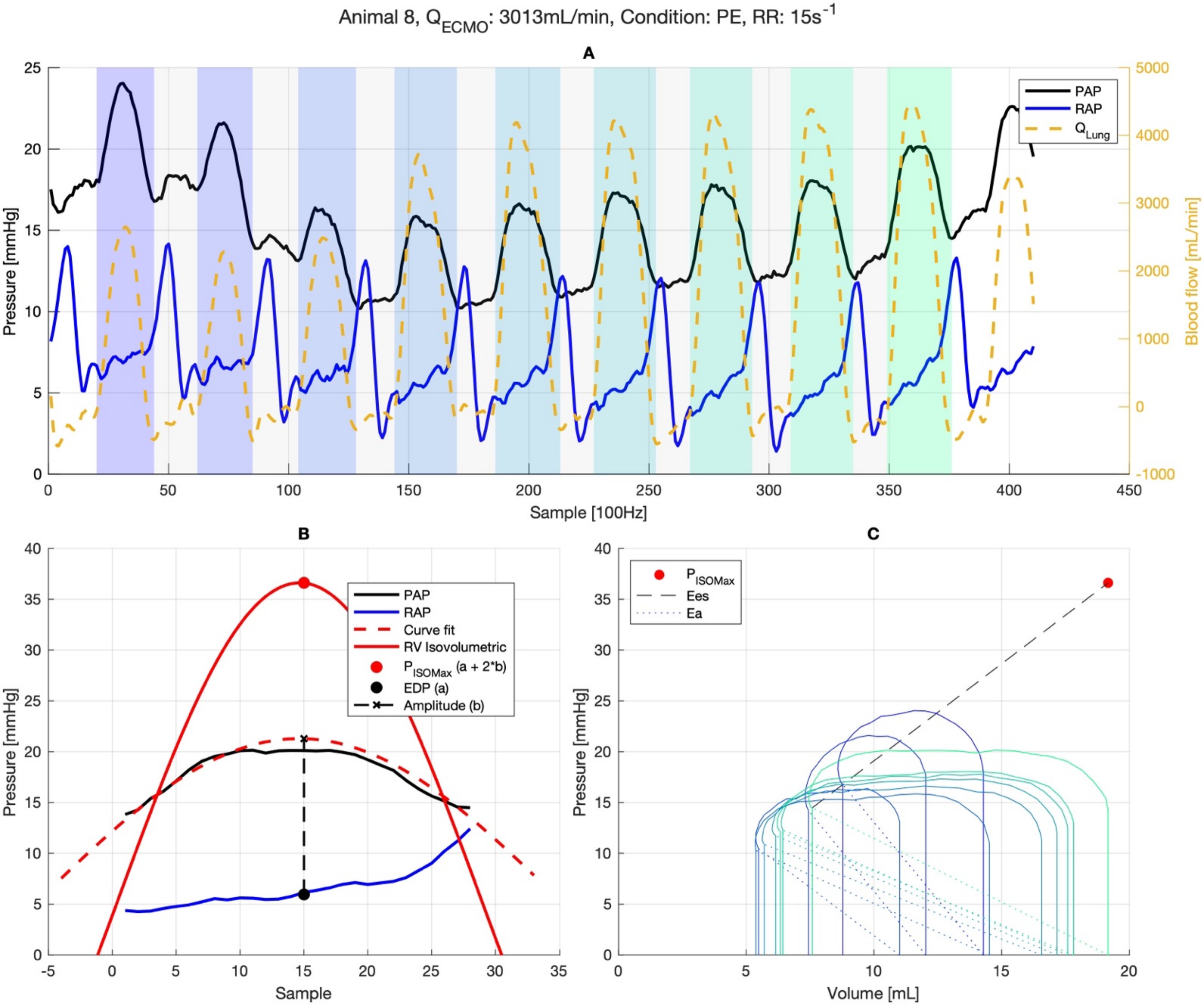

Animal 8, Q_ECMO: 3013mL/min, Condition: PE, RR: 15s^-1
A
PAP
RAP
Q_Lung
Pressure [mmHg]
Blood flow [mL/min]
Sample [100Hz]
B
PAP
RAP
Curve fit
RV Isovolumetric
P_ISOMax (a + 2*b)
EDP (a)
Amplitude (b)
Pressure [mmHg]
Sample
C
P_ISOMax
Ees
Ea
Pressure [mmHg]
Volume [mL]

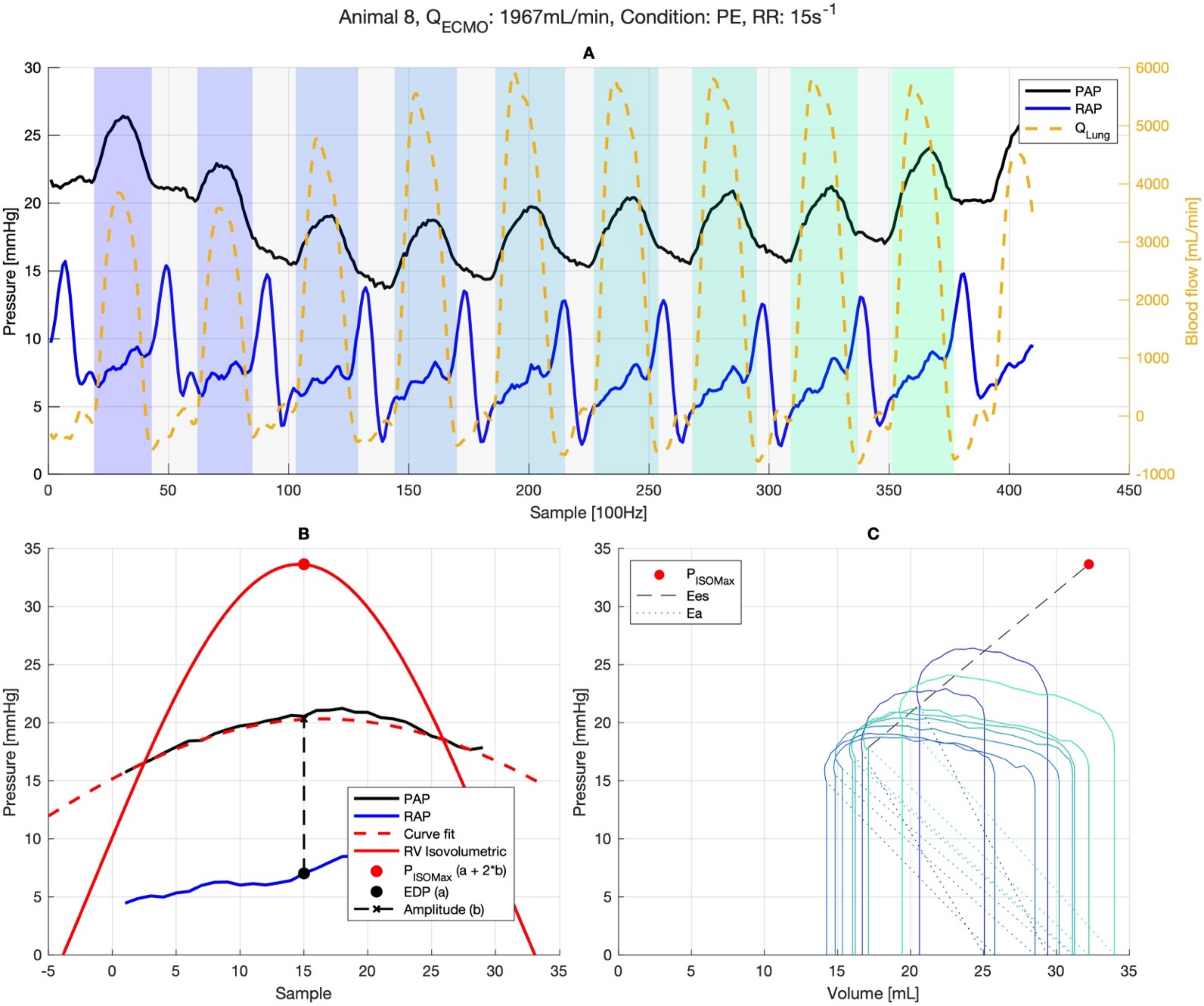
Animal 8, Q_ECMO: 1967mL/min, Condition: PE, RR: 15s^-1
A
PAP
RAP
Q_Lung
Pressure [mmHg]
Blood flow [mL/min]
Sample [100Hz]
B
PAP
RAP
Curve fit
RV Isovolumetric
P_ISOMax (a + 2*b)
EDP (a)
Amplitude (b)
Pressure [mmHg]
Sample
C
P_ISOMax
Ees
Ea
Pressure [mmHg]
Volume [mL]

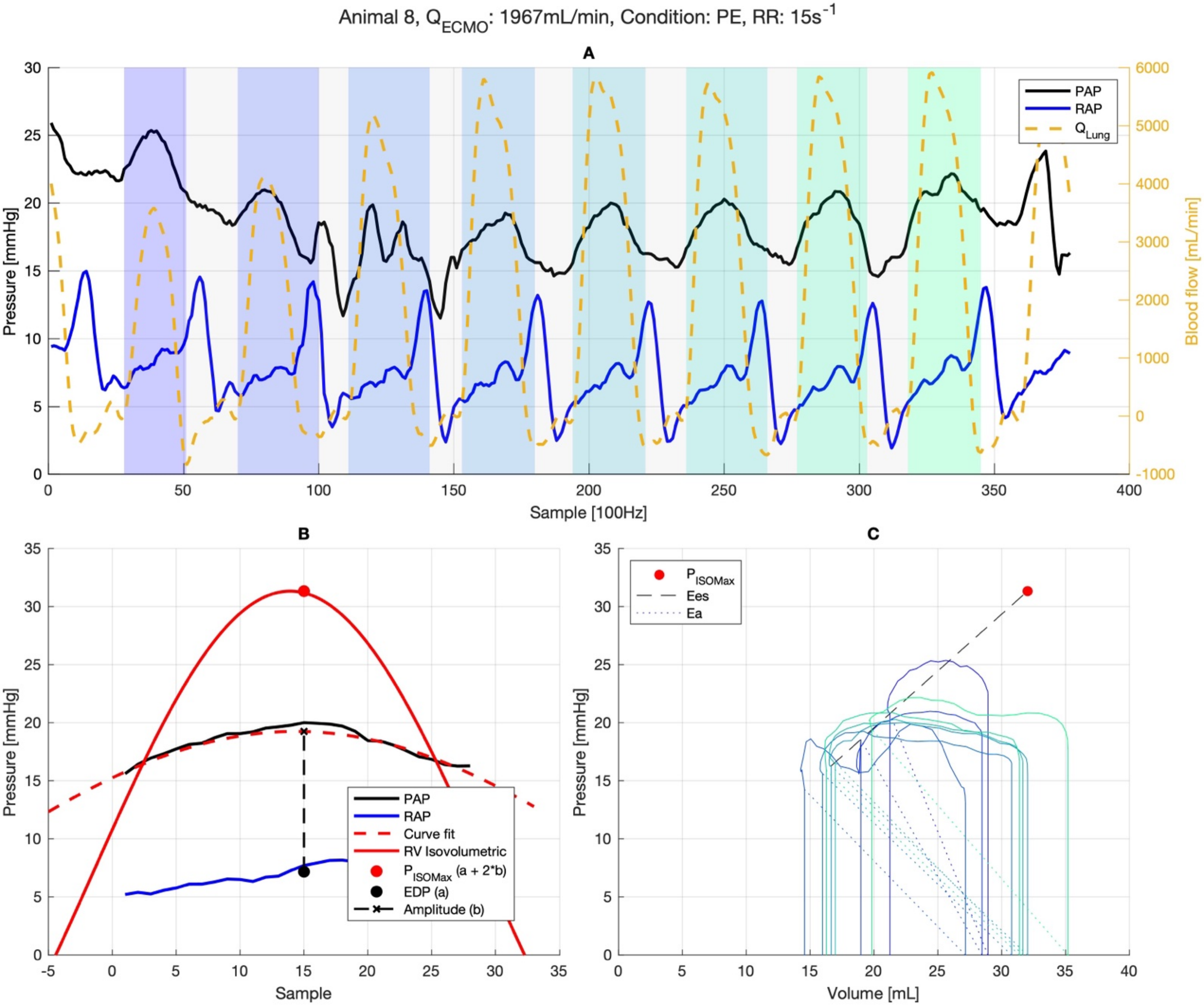
Animal 8, Q_ECMO: 1967mL/min, Condition: PE, RR: 15s^-1
A
PAP
RAP
Q_Lung
Pressure [mmHg]
Blood flow [mL/min]
Sample [100Hz]
B
PAP
RAP
Curve fit
RV Isovolumetric
P_ISOMax (a + 2*b)
EDP (a)
Amplitude (b)
Pressure [mmHg]
Sample
C
P_ISOMax
Ees
Ea
Pressure [mmHg]
Volume [mL]

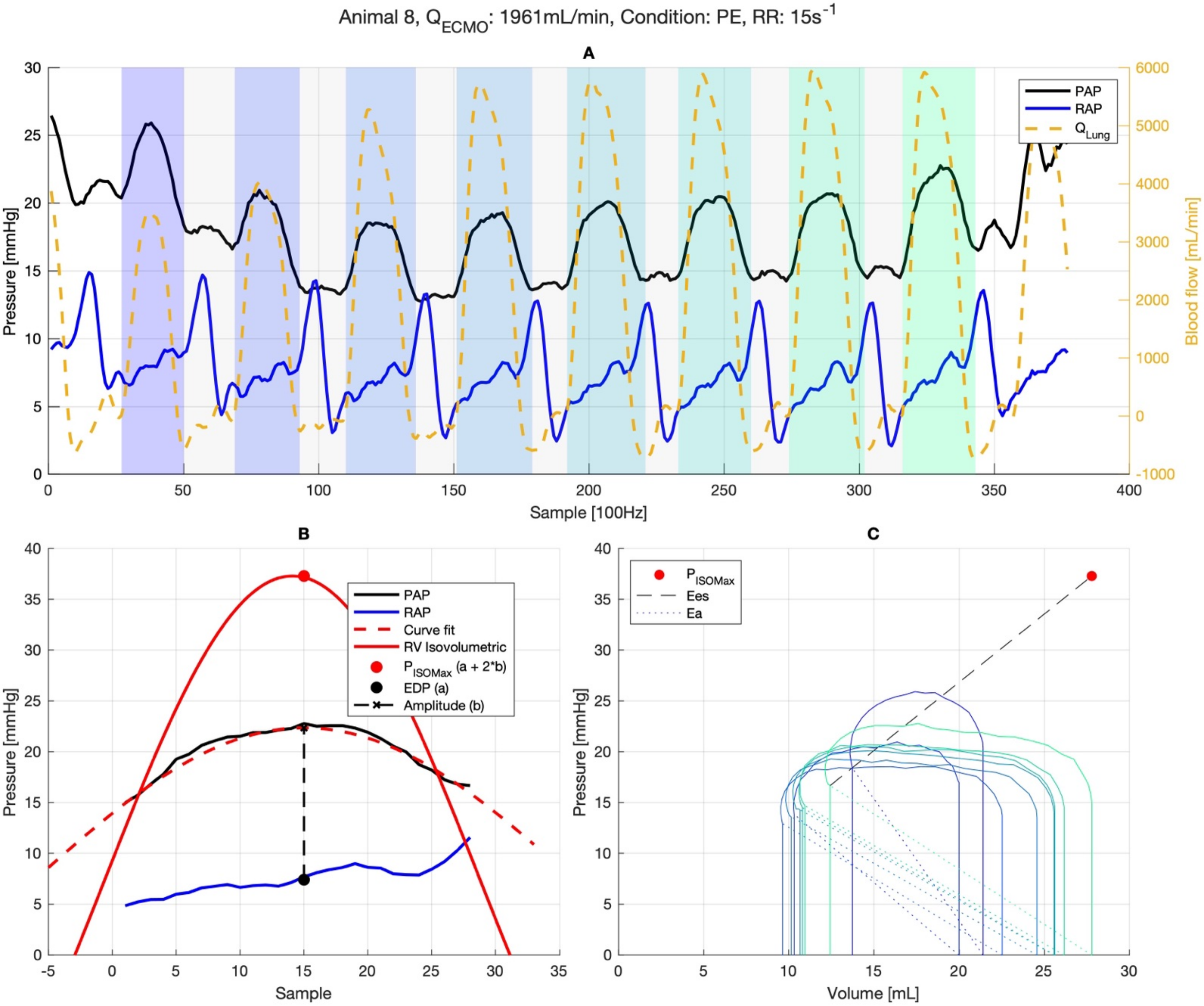

Animal 8, QECMO: 1961mL/min, Condition: PE, RR: 15s-1
A
PAP
RAP
QLung
Pressure [mmHg]
Blood flow [mL/min]
Sample [100Hz]
B
PAP
RAP
Curve fit
RV Isovolumetric
PISOMax (a + 2*b)
EDP (a)
Amplitude (b)
Pressure [mmHg]
Sample
C
PISOMax
Ees
Ea
Pressure [mmHg]
Volume [mL]

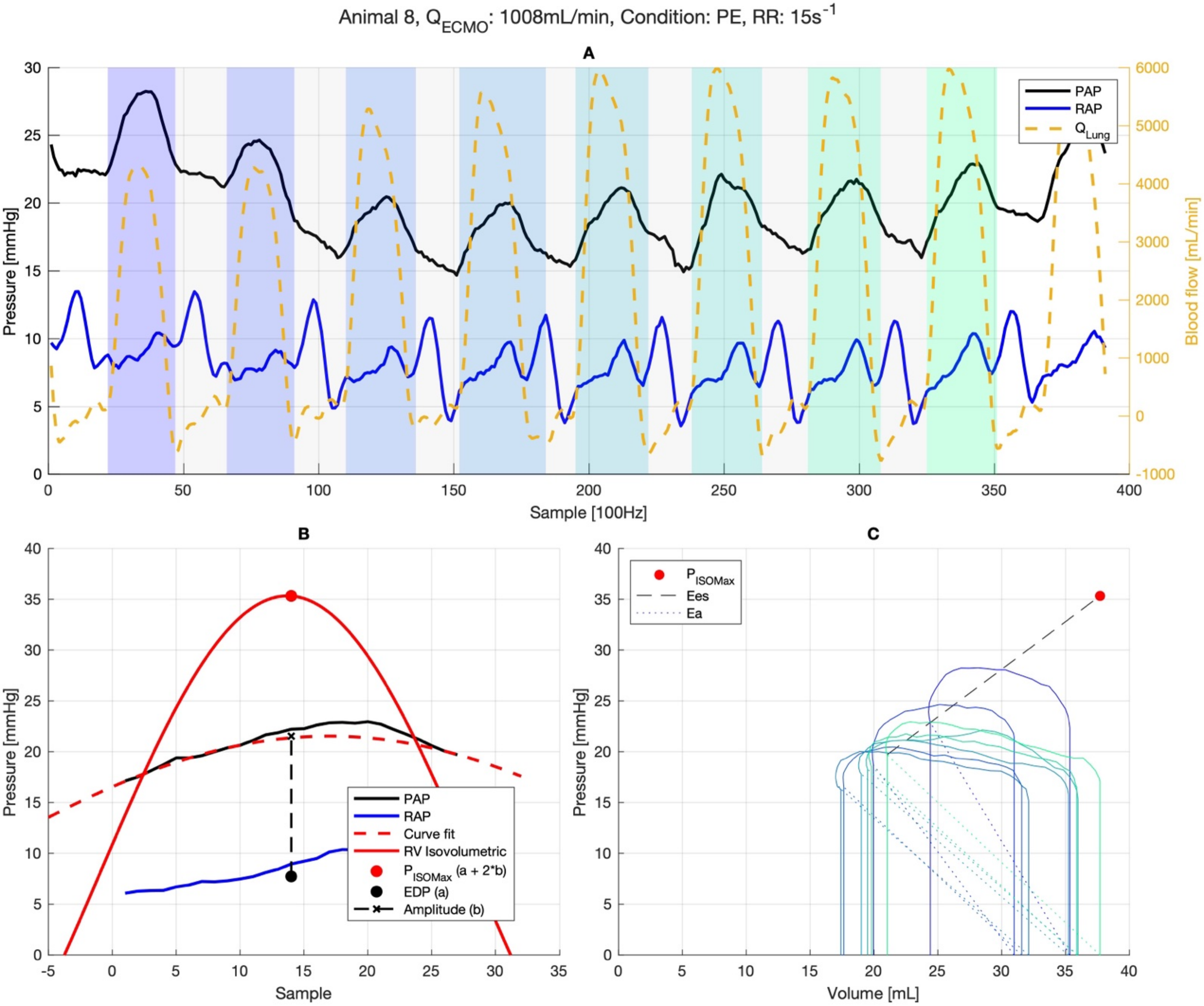

Animal 8, Q_ECMO: 1008mL/min, Condition: PE, RR: 15s^-1
A
PAP
RAP
Q_Lung
Pressure [mmHg]
Blood flow [mL/min]
Sample [100Hz]
B
PAP
RAP
Curve fit
RV Isovolumetric
P_ISOMax (a + 2*b)
EDP (a)
Amplitude (b)
Pressure [mmHg]
Sample
C
P_ISOMax
Ees
Ea
Pressure [mmHg]
Volume [mL]

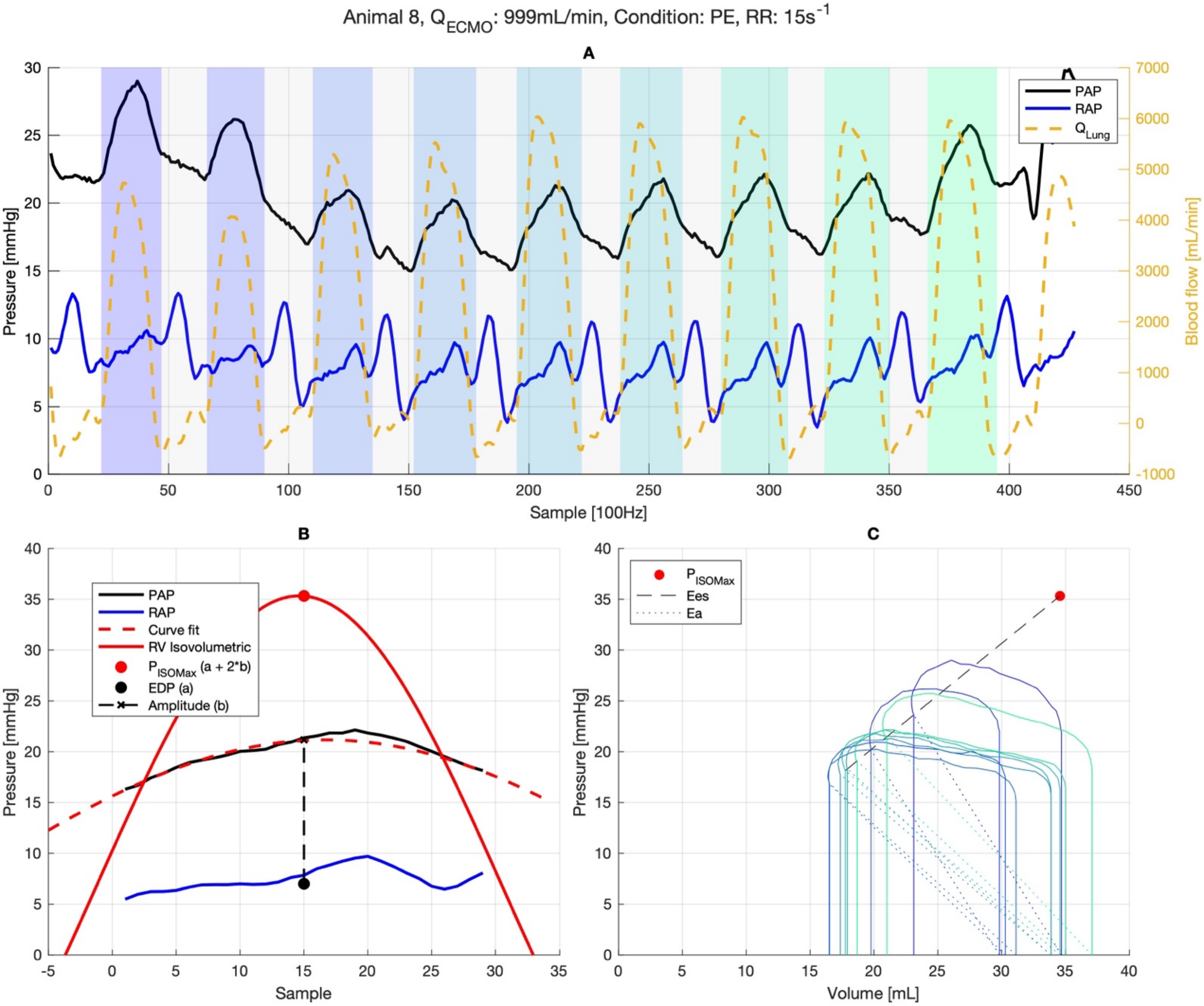

Animal 8, Q_ECMO: 999mL/min, Condition: PE, RR: 15s^-1
A
Pressure [mmHg]
Blood flow [mL/min]
Sample [100Hz]
PAP
RAP
Q_Lung
B
Pressure [mmHg]
Sample
PAP
RAP
Curve fit
RV Isovolumetric
P_ISOMax (a + 2*b)
EDP (a)
Amplitude (b)
C
Pressure [mmHg]
Volume [mL]
P_ISOMax
Ees
Ea

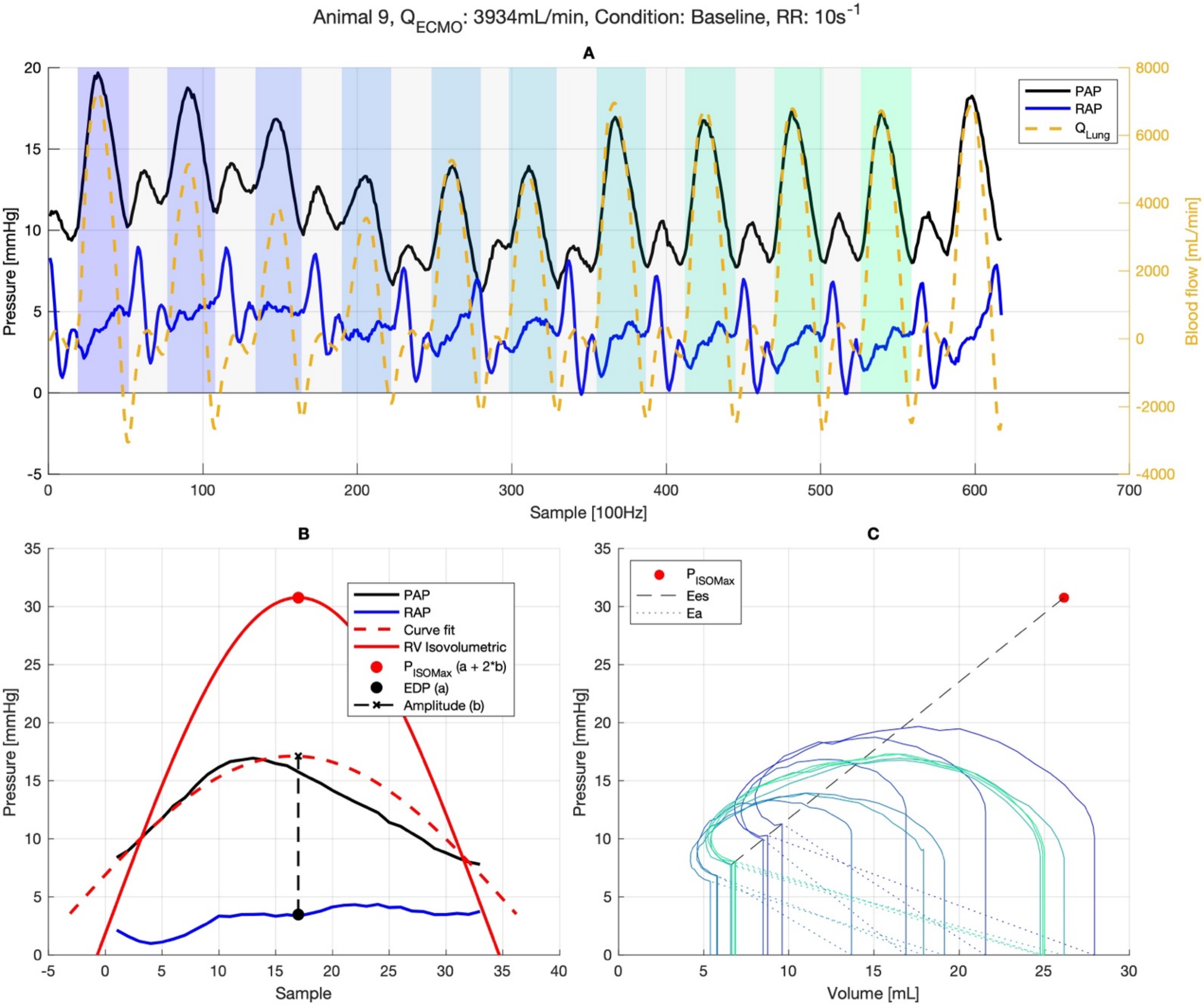

Animal 9, Q_{ECMO}: 3934mL/min, Condition: Baseline, RR: $10s^{-1}$
A
PAP
RAP
Q_{Lung}
Pressure [mmHg]
Blood flow [mL/min]
Sample [100Hz]
B
PAP
RAP
Curve fit
RV Isovolumetric
P_{ISOMax} (a + 2*b)
EDP (a)
Amplitude (b)
Pressure [mmHg]
Sample
C
P_{ISOMax}
Ees
Ea
Pressure [mmHg]
Volume [mL]

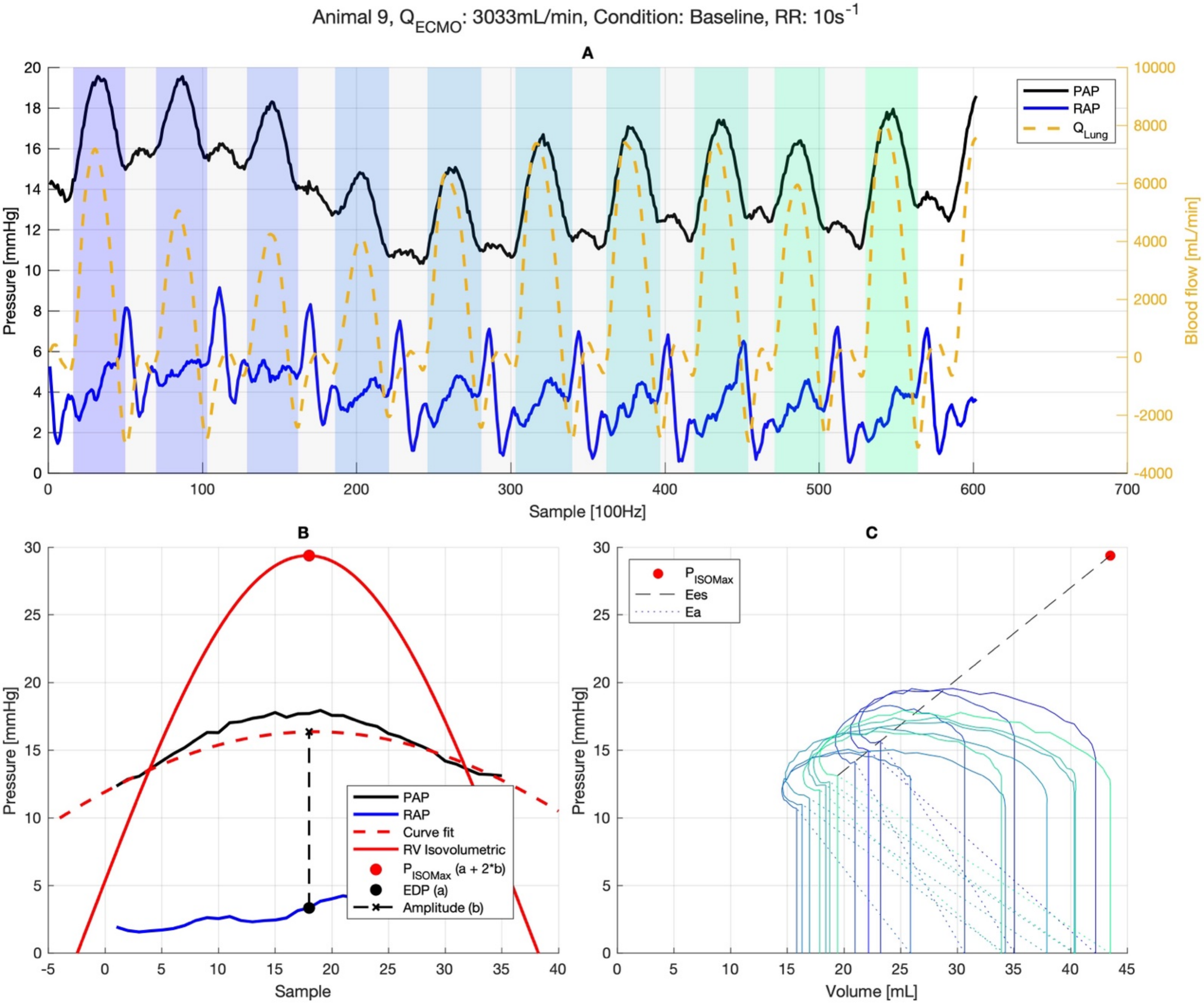

Animal 9, Q_ECMO: 3033mL/min, Condition: Baseline, RR: 10s^-1
A
PAP
RAP
Q_Lung
Pressure [mmHg]
Blood flow [mL/min]
Sample [100Hz]
B
PAP
RAP
Curve fit
RV Isovolumetric
P_ISOMax (a + 2*b)
EDP (a)
Amplitude (b)
Pressure [mmHg]
Sample
C
P_ISOMax
Ees
Ea
Pressure [mmHg]
Volume [mL]

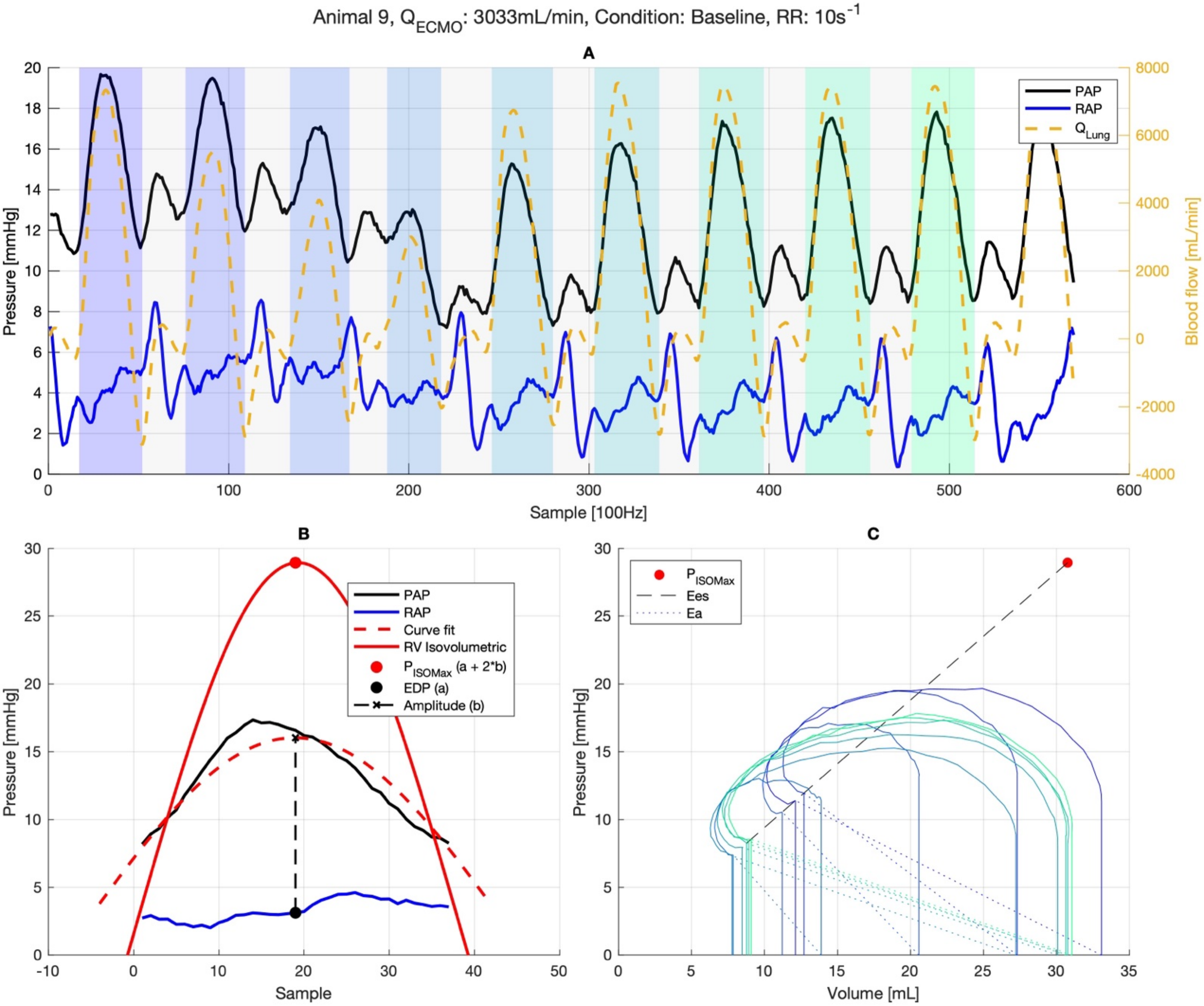

Animal 9, Q_ECMO: 3033mL/min, Condition: Baseline, RR: 10s^-1
A
PAP
RAP
Q_Lung
Pressure [mmHg]
Blood flow [mL/min]
Sample [100Hz]
B
PAP
RAP
Curve fit
RV Isovolumetric
P_ISOMax (a + 2*b)
EDP (a)
Amplitude (b)
Pressure [mmHg]
Sample
C
P_ISOMax
Ees
Ea
Pressure [mmHg]
Volume [mL]

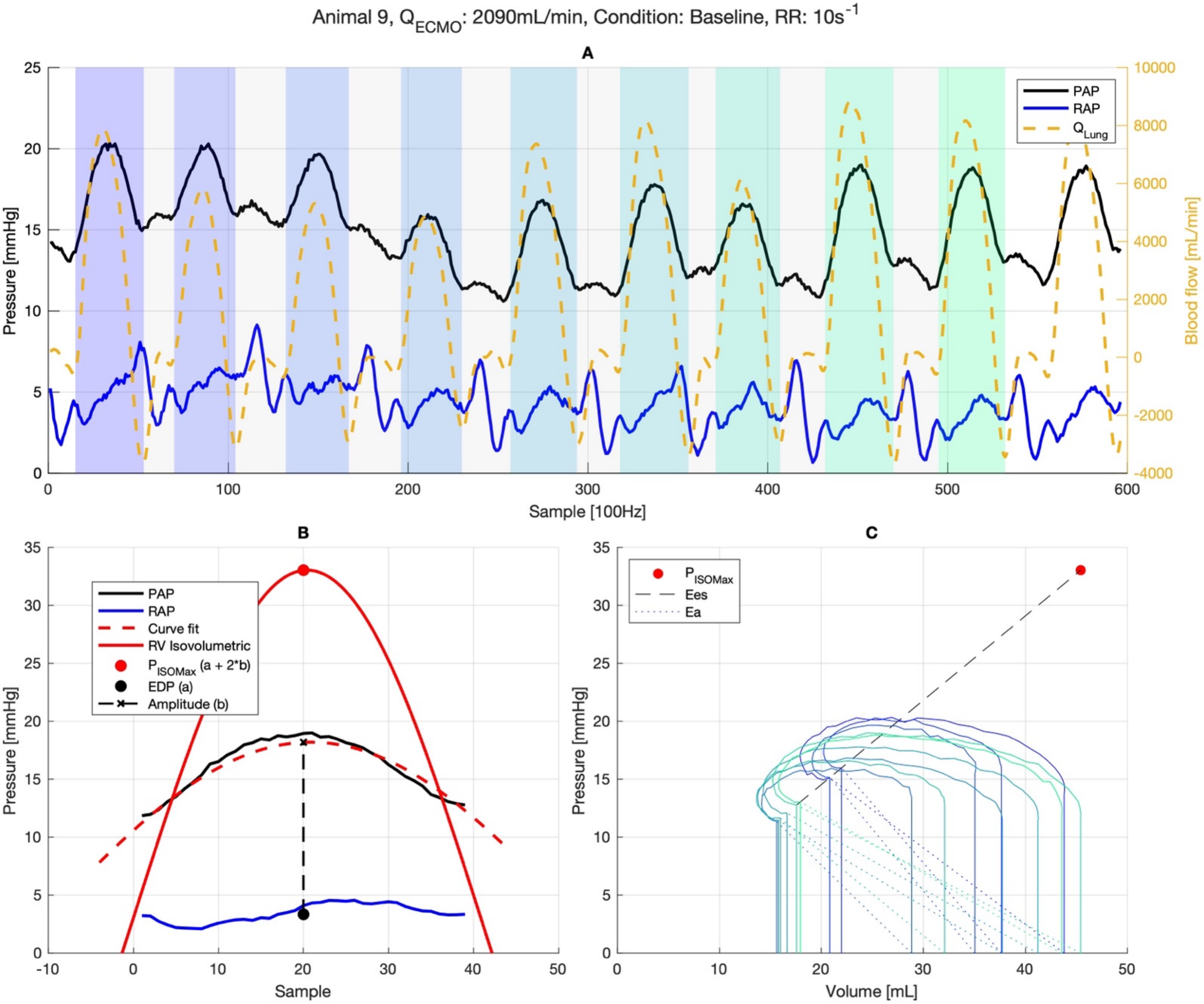
Animal 9, QECMO: 2090mL/min, Condition: Baseline, RR: 10s-1
A
PAP
RAP
QLung
Pressure [mmHg]
Blood flow [mL/min]
Sample [100Hz]
B
PAP
RAP
Curve fit
RV Isovolumetric
PISOMax (a + 2*b)
EDP (a)
Amplitude (b)
Pressure [mmHg]
Sample
C
PISOMax
Ees
Ea
Pressure [mmHg]
Volume [mL]

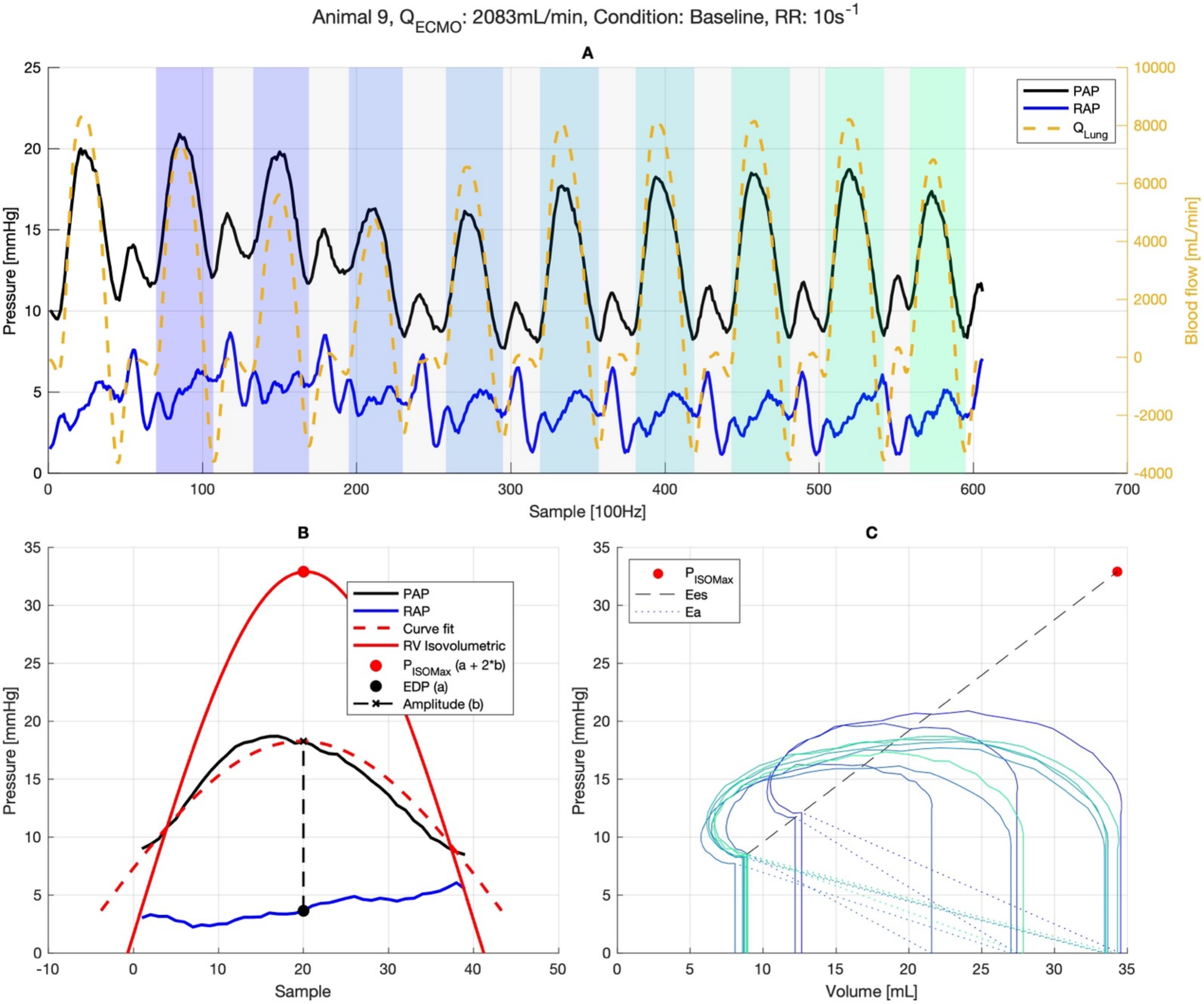

Animal 9, Q_{ECMO}: 2083mL/min, Condition: Baseline, RR: $10s^{-1}$
A
PAP
RAP
Q_{Lung}
Pressure [mmHg]
Blood flow [mL/min]
Sample [100Hz]
B
PAP
RAP
Curve fit
RV Isovolumetric
P_{ISOMax} (a + 2*b)
EDP (a)
Amplitude (b)
Pressure [mmHg]
Sample
C
P_{ISOMax}
Ees
Ea
Pressure [mmHg]
Volume [mL]

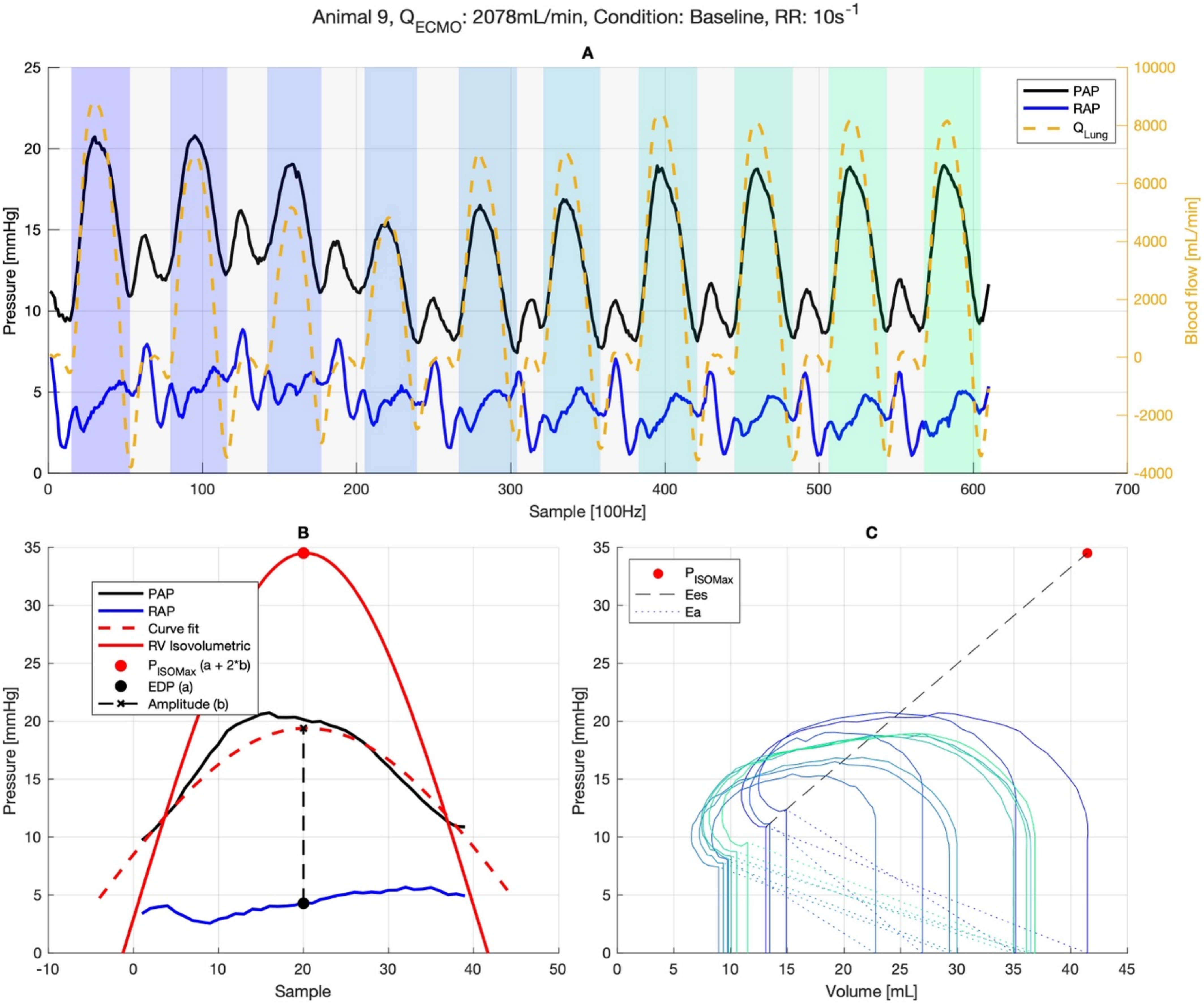
Animal 9, Q_{ECMO}: 2078mL/min, Condition: Baseline, RR: $10s^{-1}$
A
PAP
RAP
Q_{Lung}
Pressure [mmHg]
Blood flow [mL/min]
Sample [100Hz]
B
PAP
RAP
Curve fit
RV Isovolumetric
P_{ISOMax} (a + 2*b)
EDP (a)
Amplitude (b)
Pressure [mmHg]
Sample
C
P_{ISOMax}
Ees
Ea
Pressure [mmHg]
Volume [mL]

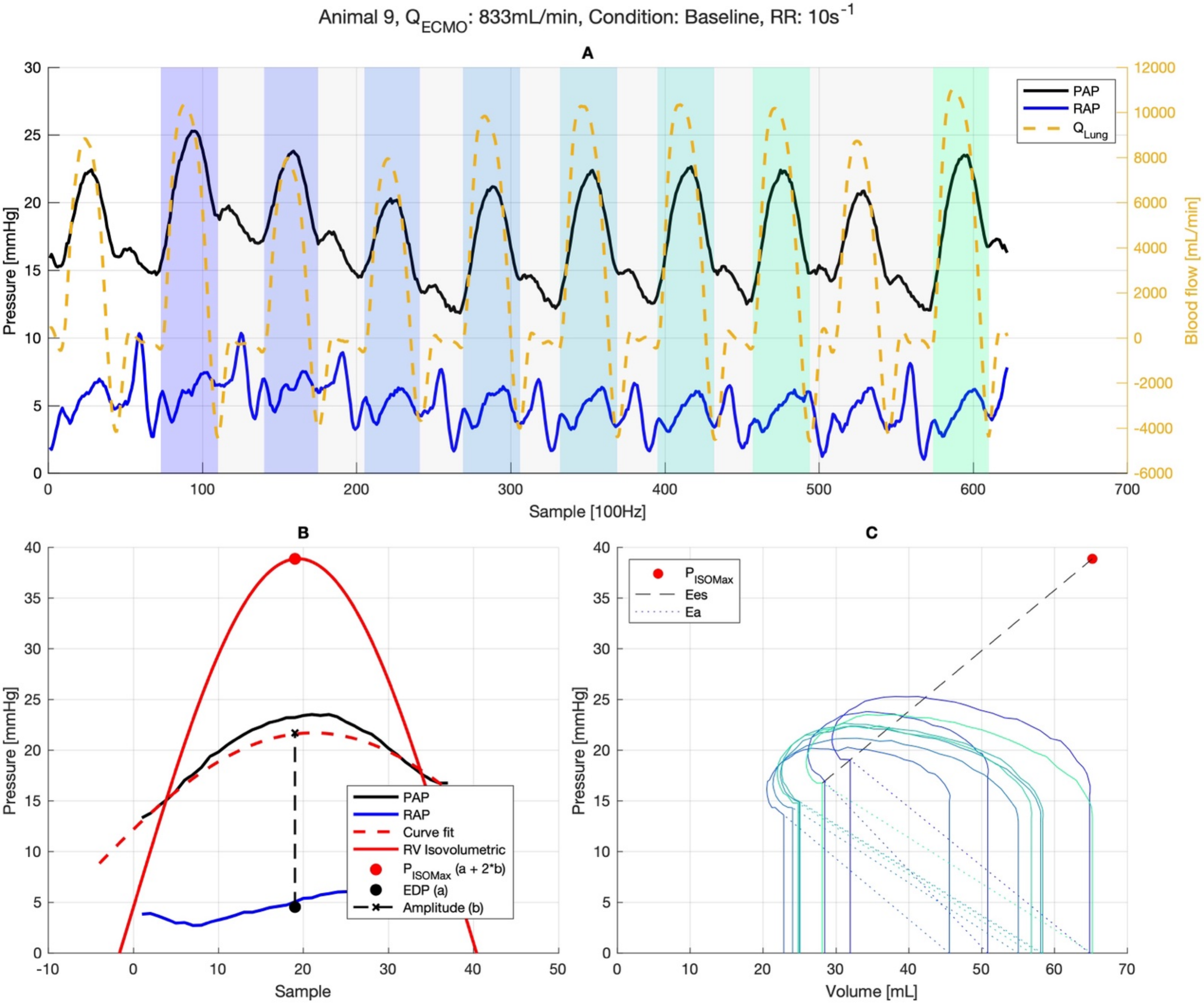

Animal 9, Q_{ECMO}: 833mL/min, Condition: Baseline, RR: $10s^{-1}$
A
Pressure [mmHg]
Blood flow [mL/min]
Sample [100Hz]
PAP
RAP
Q_{Lung}
B
Pressure [mmHg]
Sample
PAP
RAP
Curve fit
RV Isovolumetric
P_{ISOMax} (a + 2*b)
EDP (a)
Amplitude (b)
C
Pressure [mmHg]
Volume [mL]
P_{ISOMax}
Ees
Ea

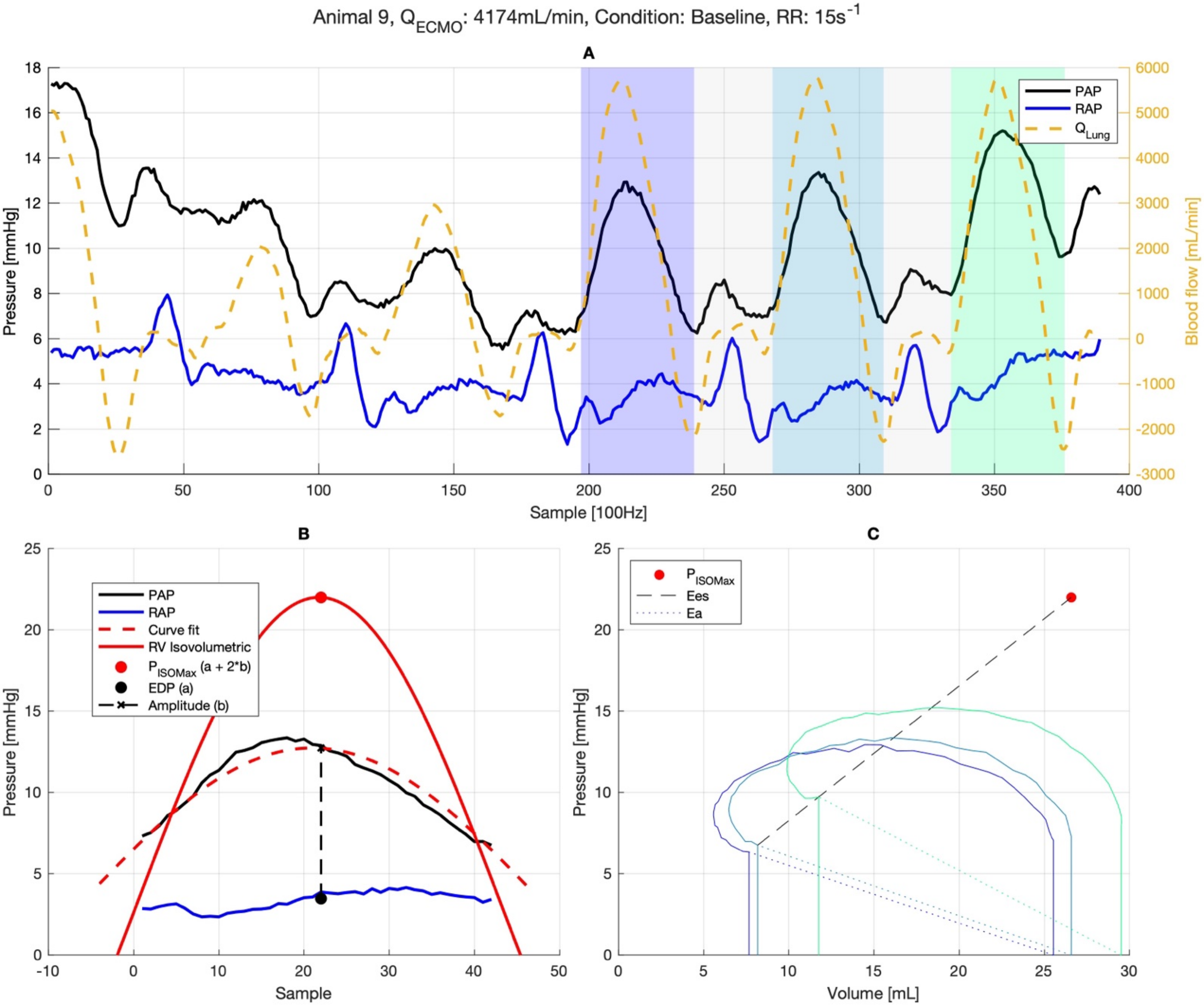

Animal 9, Q_{ECMO}: 4174mL/min, Condition: Baseline, RR: $15s^{-1}$
A
PAP
RAP
Q_{Lung}
Pressure [mmHg]
Blood flow [mL/min]
Sample [100Hz]
B
PAP
RAP
Curve fit
RV Isovolumetric
P_{ISOMax} (a + 2*b)
EDP (a)
Amplitude (b)
Pressure [mmHg]
Sample
C
P_{ISOMax}
Ees
Ea
Pressure [mmHg]
Volume [mL]

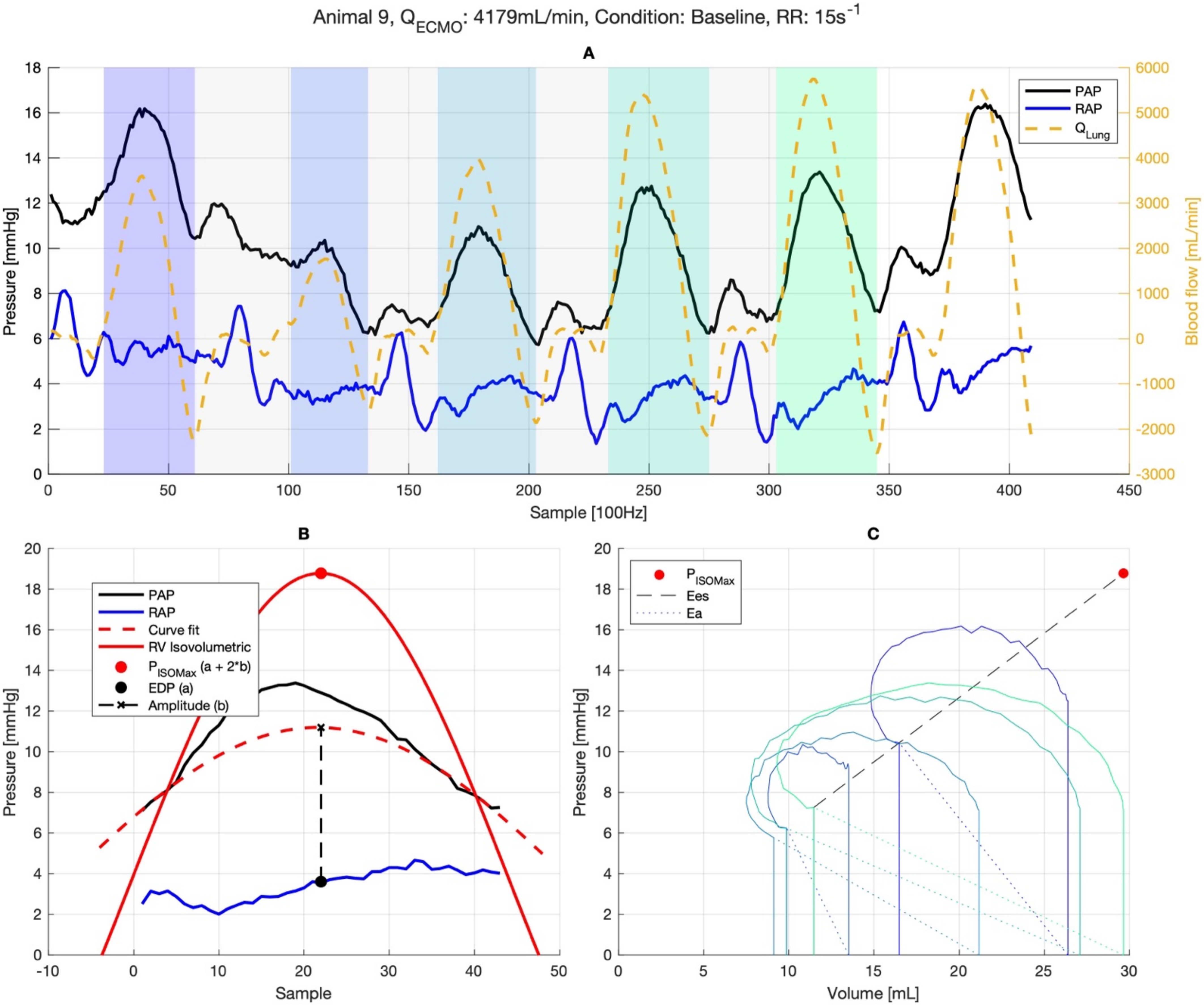

Animal 9, Q_ECMO: 4179mL/min, Condition: Baseline, RR: 15s^-1
A
PAP
RAP
Q_Lung
Pressure [mmHg]
Blood flow [mL/min]
Sample [100Hz]
B
PAP
RAP
Curve fit
RV Isovolumetric
P_ISOMax (a + 2*b)
EDP (a)
Amplitude (b)
Pressure [mmHg]
Sample
C
P_ISOMax
Ees
Ea
Pressure [mmHg]
Volume [mL]

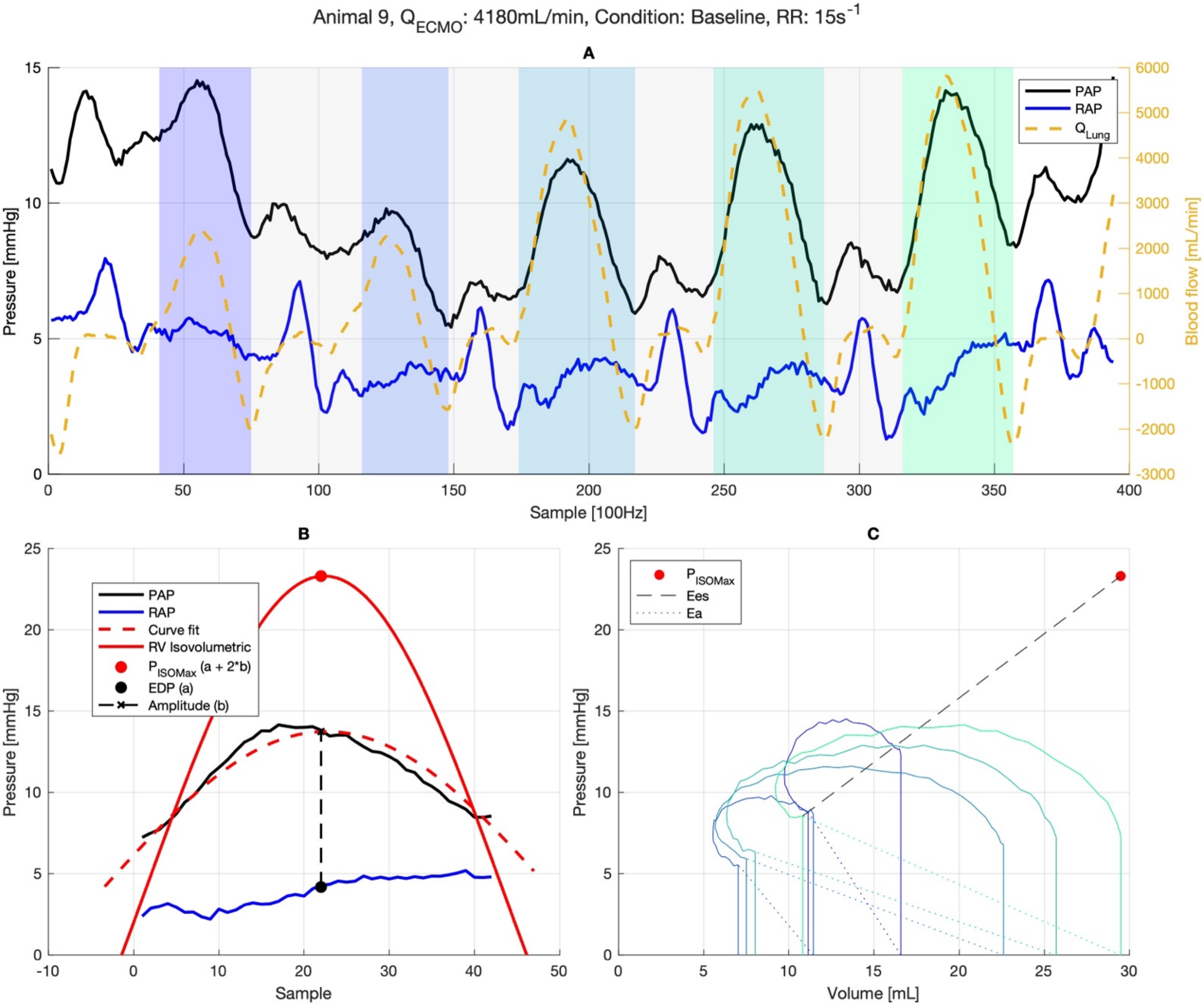

Animal 9, Q_{ECMO}: 4180mL/min, Condition: Baseline, RR: $15s^{-1}$
A
Pressure [mmHg]
Blood flow [mL/min]
Sample [100Hz]
PAP
RAP
Q_{Lung}
B
Pressure [mmHg]
Sample
PAP
RAP
Curve fit
RV Isovolumetric
P_{ISOMax} (a + 2*b)
EDP (a)
Amplitude (b)
C
Pressure [mmHg]
Volume [mL]
P_{ISOMax}
Ees
Ea

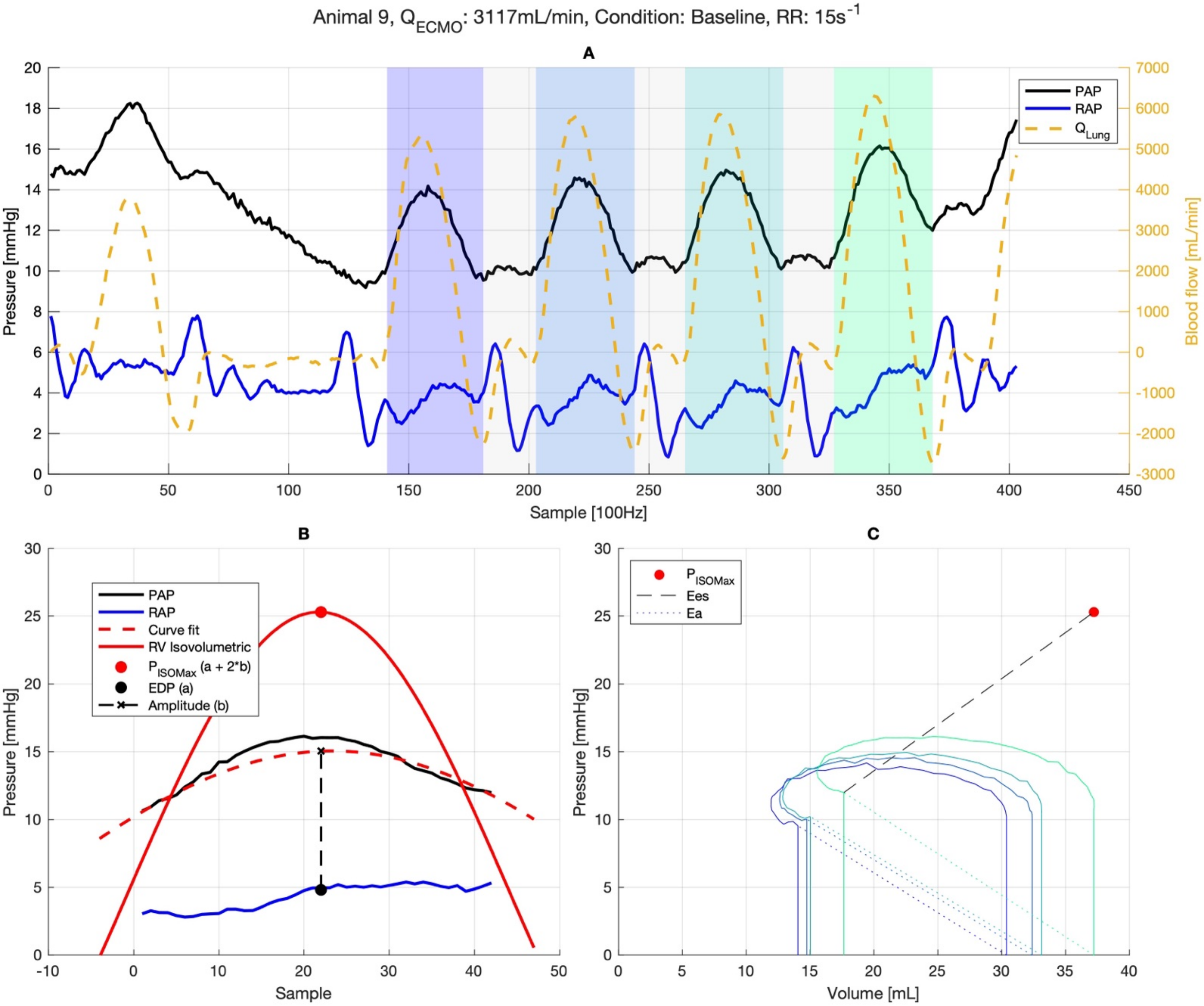

Animal 9, Q_ECMO: 3117mL/min, Condition: Baseline, RR: 15s^-1
A
PAP
RAP
Q_Lung
Pressure [mmHg]
Blood flow [mL/min]
Sample [100Hz]
B
PAP
RAP
Curve fit
RV Isovolumetric
P_ISOMax (a + 2*b)
EDP (a)
Amplitude (b)
Pressure [mmHg]
Sample
C
P_ISOMax
Ees
Ea
Pressure [mmHg]
Volume [mL]

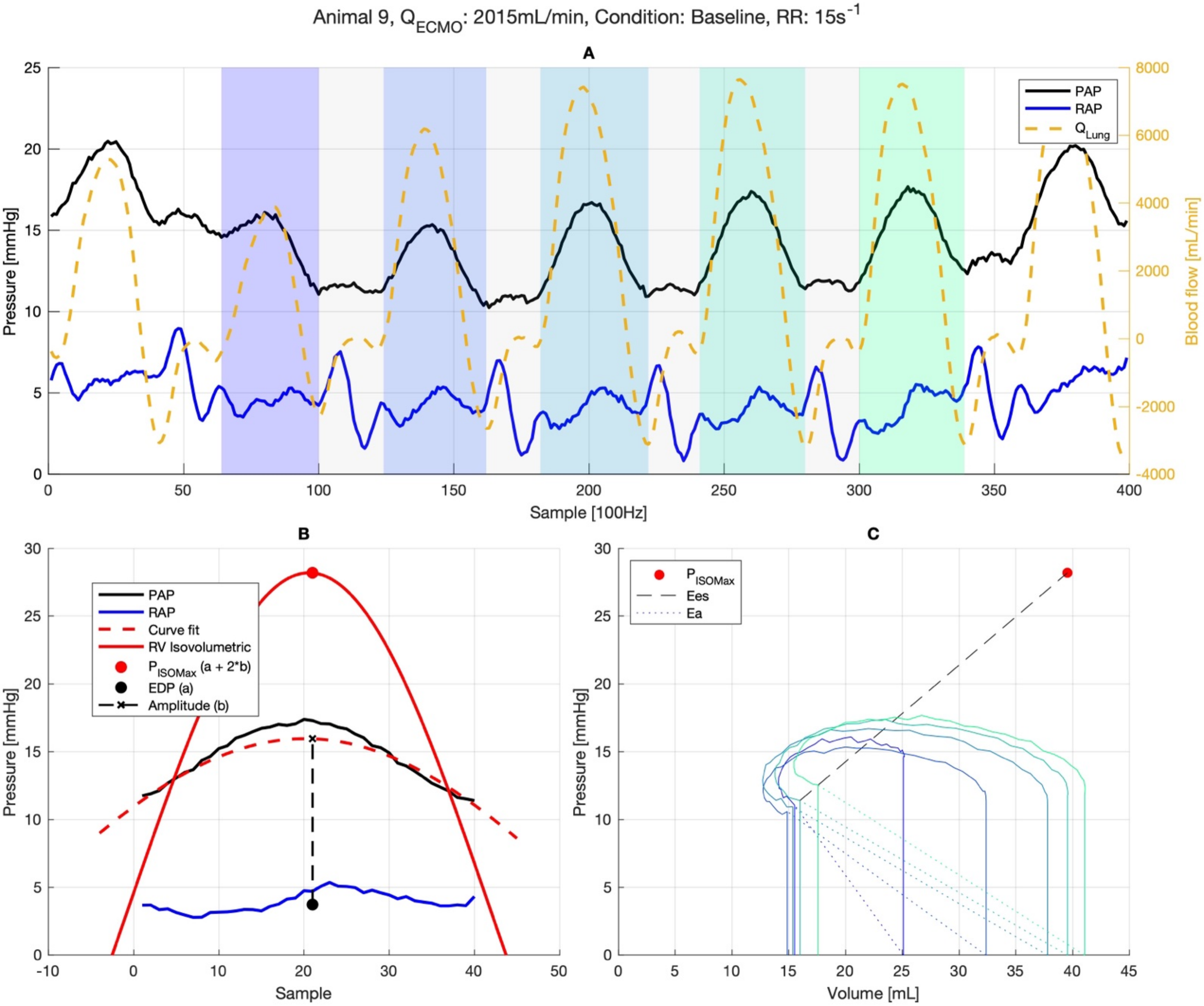

Animal 9, Q_{ECMO}: 2015mL/min, Condition: Baseline, RR: $15s^{-1}$
A
Pressure [mmHg]
Blood flow [mL/min]
Sample [100Hz]
PAP
RAP
Q_{Lung}
B
Pressure [mmHg]
Sample
PAP
RAP
Curve fit
RV Isovolumetric
P_{ISOMax} (a + 2*b)
EDP (a)
Amplitude (b)
C
Pressure [mmHg]
Volume [mL]
P_{ISOMax}
Ees
Ea

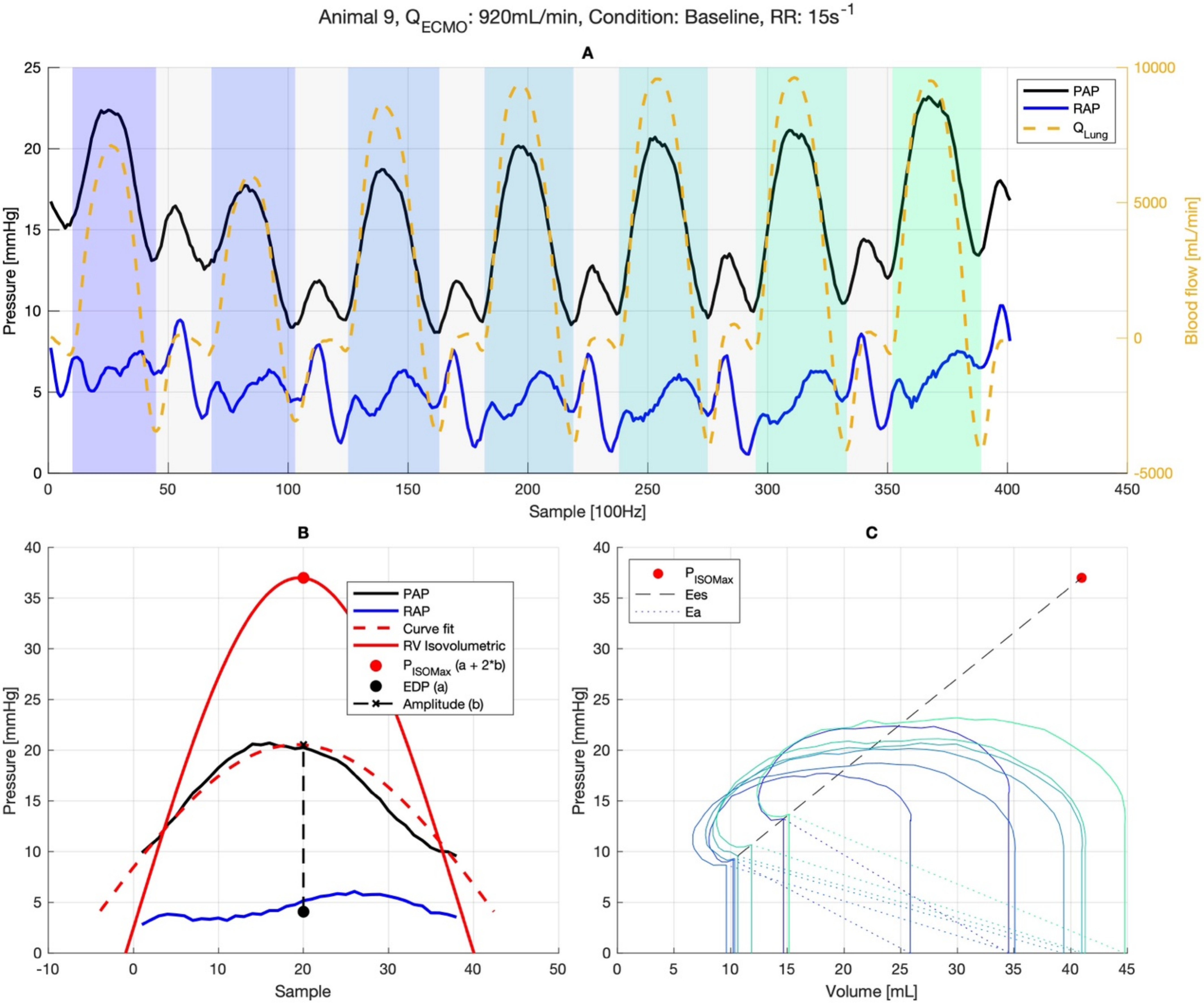

Animal 9, Q_ECMO: 920mL/min, Condition: Baseline, RR: 15s^-1
A
PAP
RAP
Q_Lung
Pressure [mmHg]
Blood flow [mL/min]
Sample [100Hz]
B
PAP
RAP
Curve fit
RV Isovolumetric
P_ISOMax (a + 2*b)
EDP (a)
Amplitude (b)
Pressure [mmHg]
Sample
C
P_ISOMax
Ees
Ea
Pressure [mmHg]
Volume [mL]

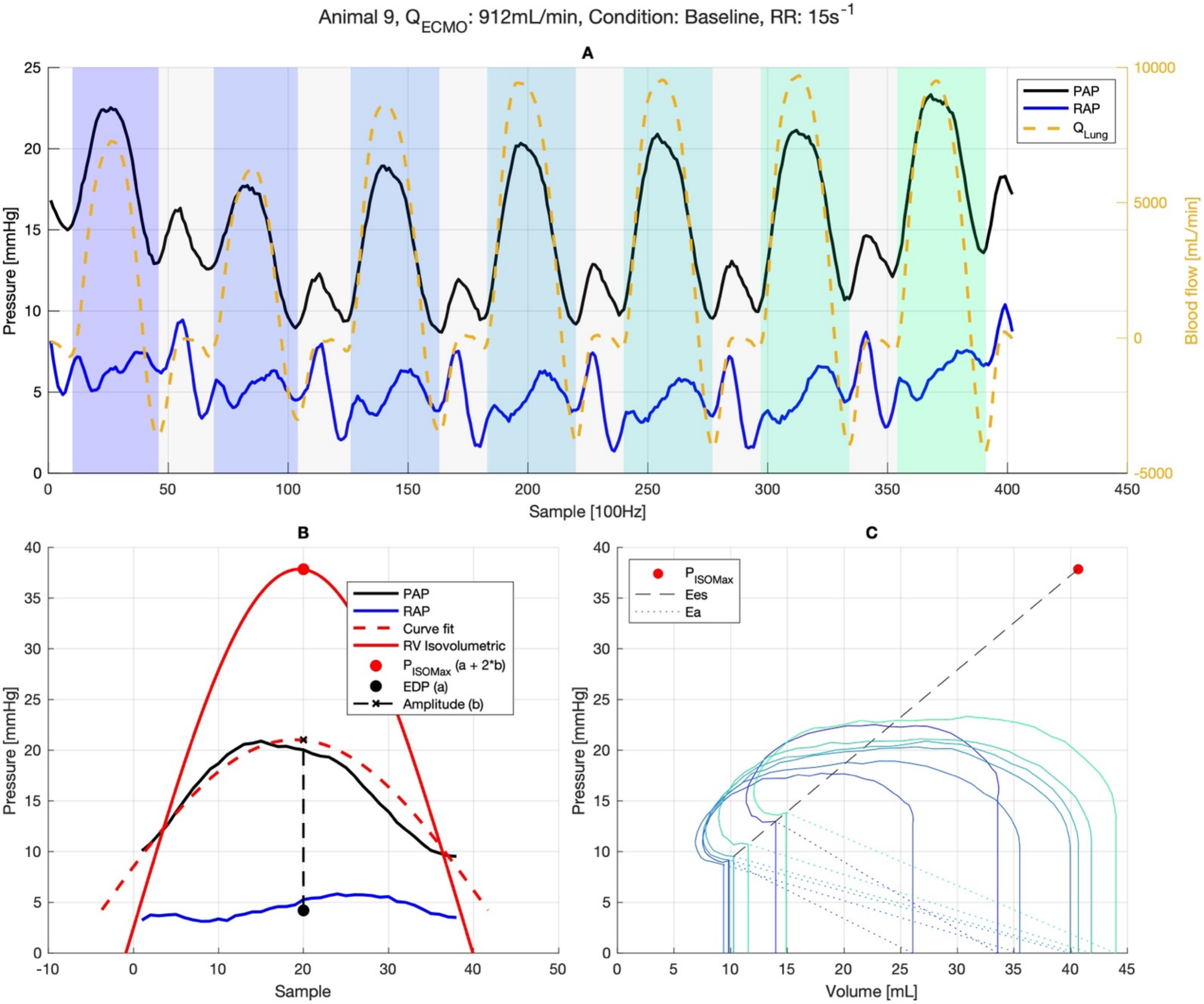

Animal 9, Q_ECMO: 912mL/min, Condition: Baseline, RR: 15s^-1
A
PAP
RAP
Q_Lung
Pressure [mmHg]
Blood flow [mL/min]
Sample [100Hz]
B
PAP
RAP
Curve fit
RV Isovolumetric
P_ISOMax (a + 2*b)
EDP (a)
Amplitude (b)
Pressure [mmHg]
Sample
C
P_ISOMax
Ees
Ea
Pressure [mmHg]
Volume [mL]

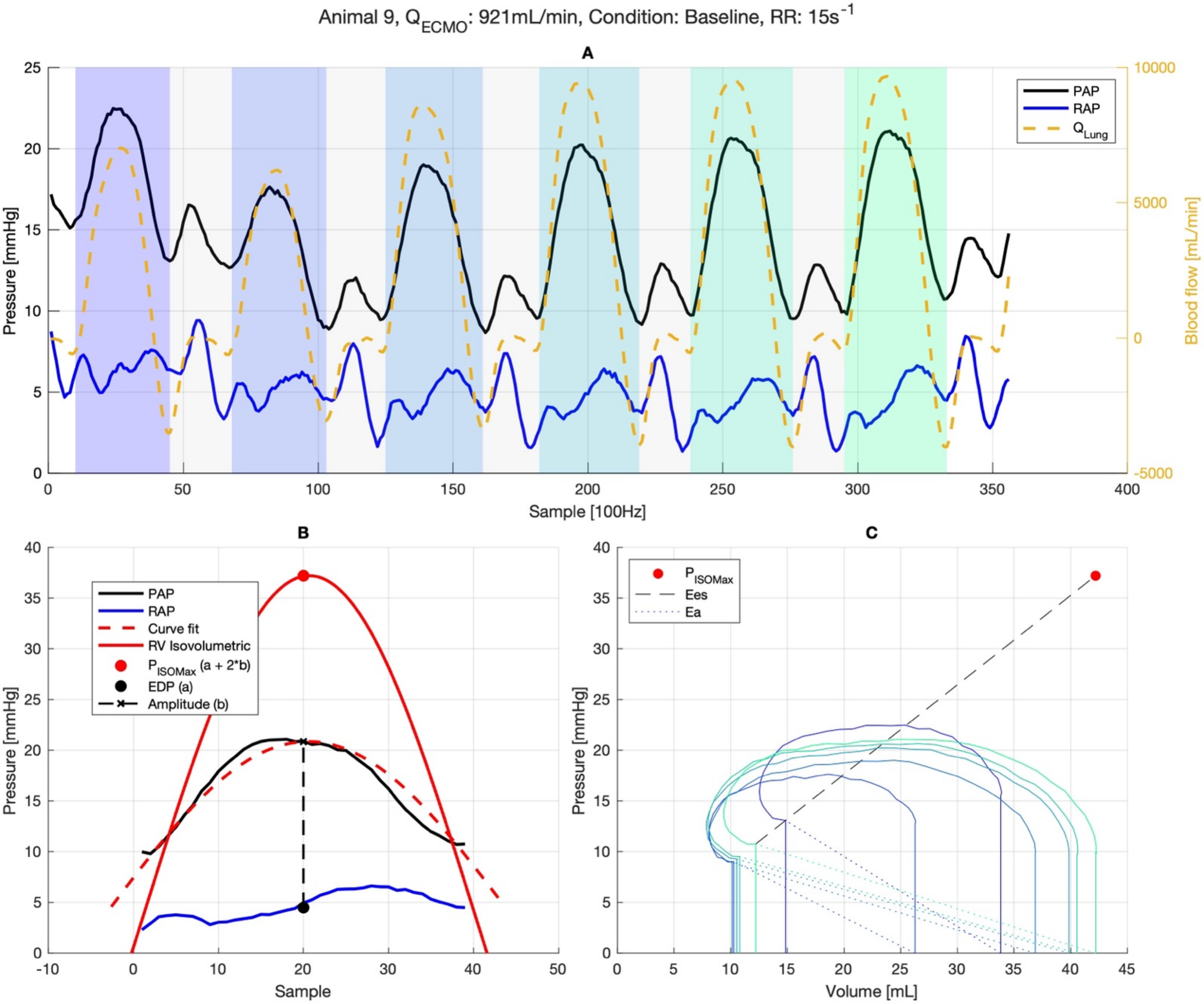
Animal 9, Q_{ECMO}: 921mL/min, Condition: Baseline, RR: $15s^{-1}$
A
Pressure [mmHg]
Blood flow [mL/min]
Sample [100Hz]
PAP
RAP
Q_{Lung}
B
Pressure [mmHg]
Sample
PAP
RAP
Curve fit
RV Isovolumetric
P_{ISOMax} (a + 2*b)
EDP (a)
Amplitude (b)
C
Pressure [mmHg]
Volume [mL]
P_{ISOMax}
Ees
Ea

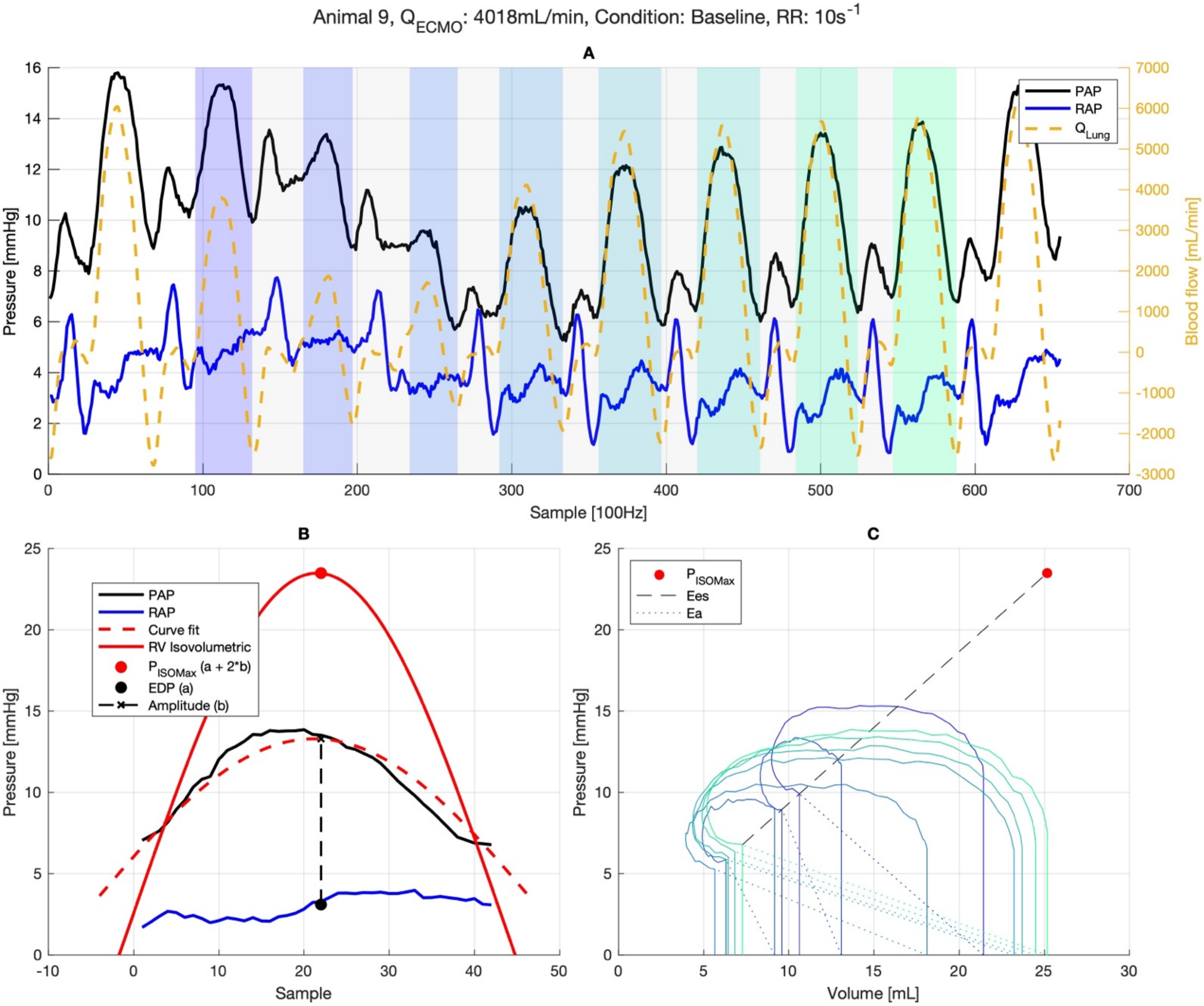

Animal 9, Q_{ECMO}: 4018mL/min, Condition: Baseline, RR: $10s^{-1}$
A
PAP
RAP
Q_{Lung}
Pressure [mmHg]
Blood flow [mL/min]
Sample [100Hz]
B
PAP
RAP
Curve fit
RV Isovolumetric
P_{ISOMax} (a + 2*b)
EDP (a)
Amplitude (b)
Pressure [mmHg]
Sample
C
P_{ISOMax}
Ees
Ea
Pressure [mmHg]
Volume [mL]

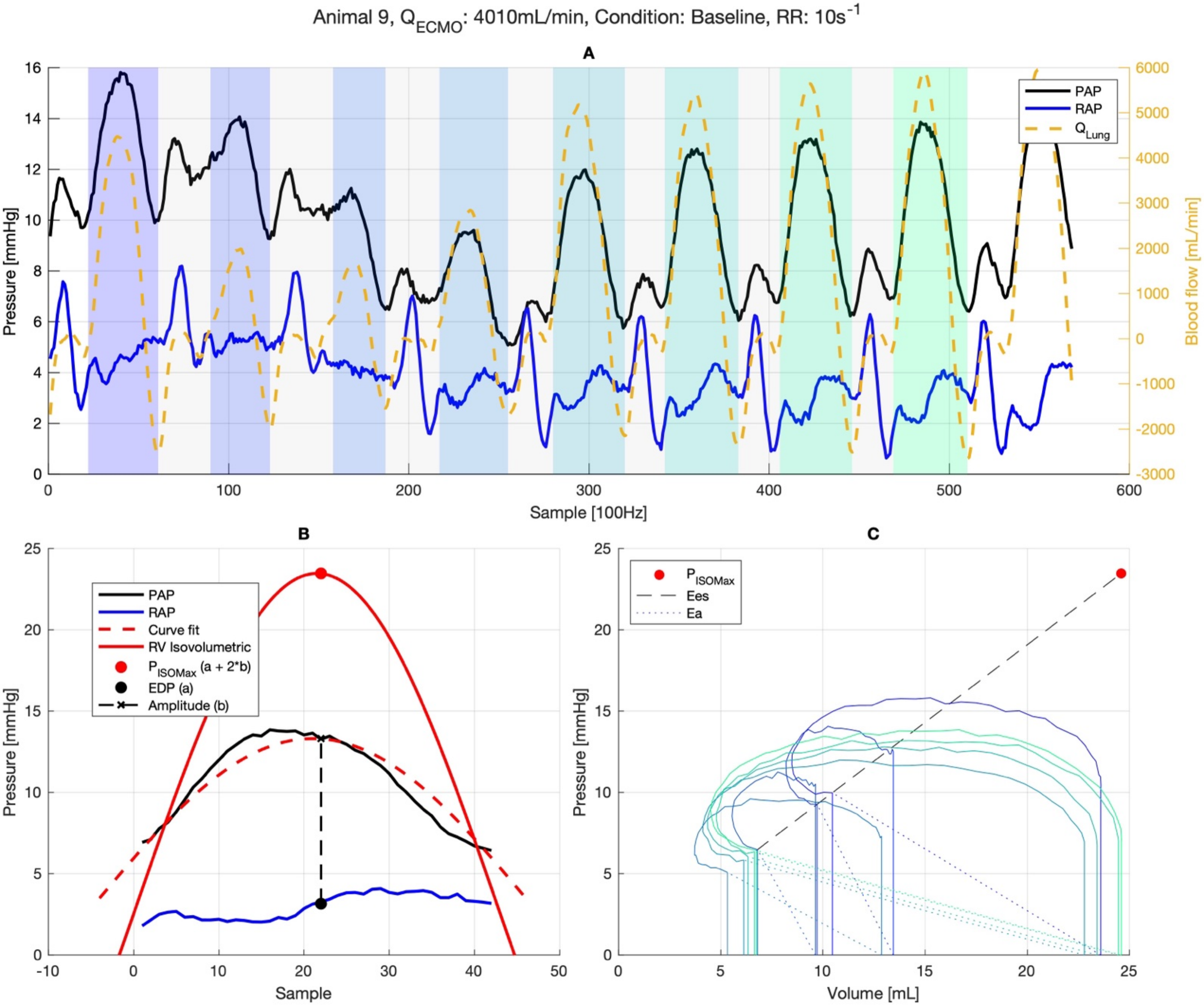

Animal 9, Q_{ECMO}: 4010mL/min, Condition: Baseline, RR: $10s^{-1}$
A
PAP
RAP
Q_{Lung}
Pressure [mmHg]
Blood flow [mL/min]
Sample [100Hz]
B
PAP
RAP
Curve fit
RV Isovolumetric
P_{ISOMax} (a + 2*b)
EDP (a)
Amplitude (b)
Pressure [mmHg]
Sample
C
P_{ISOMax}
Ees
Ea
Pressure [mmHg]
Volume [mL]

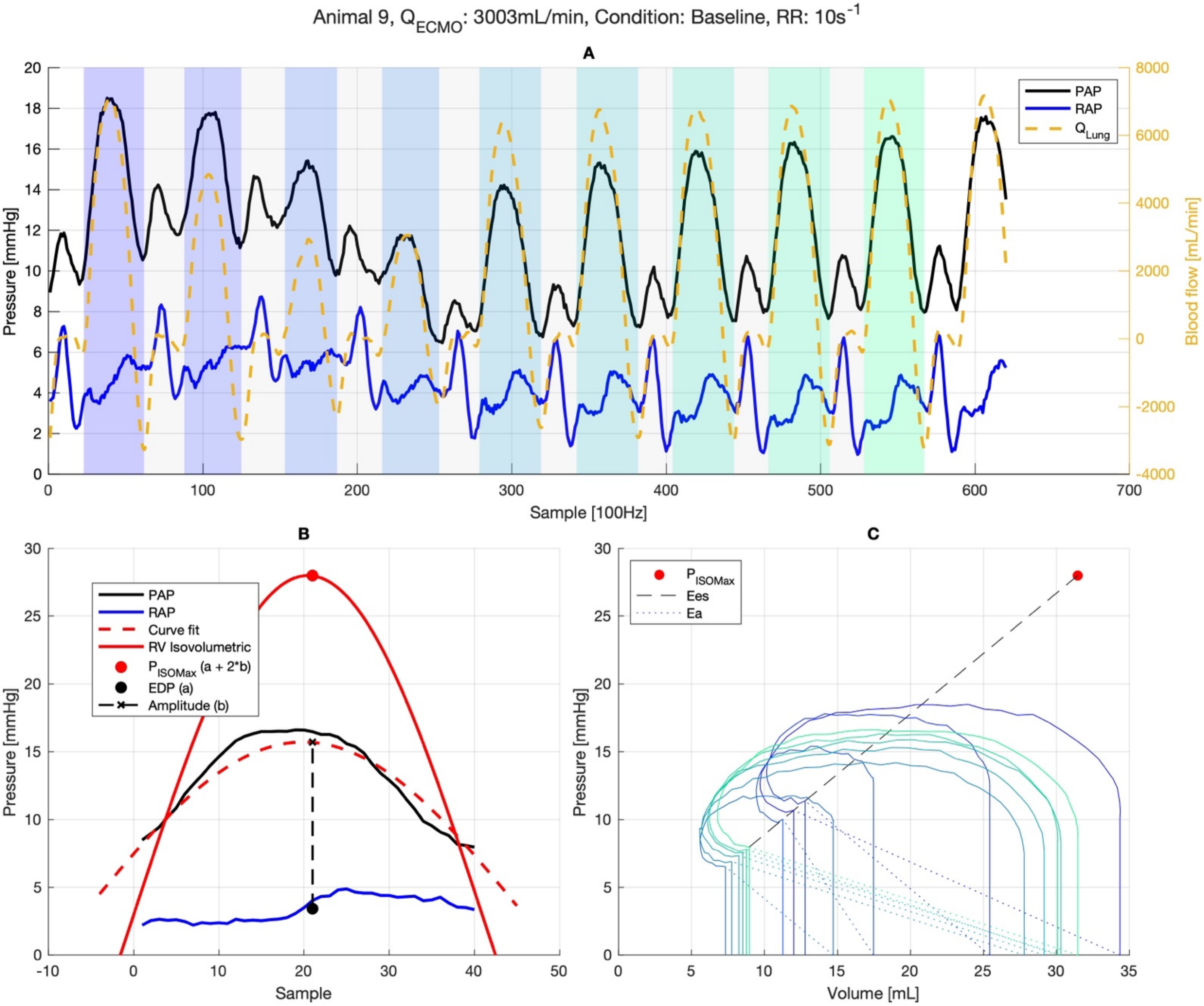

Animal 9, QECMO: 3003mL/min, Condition: Baseline, RR: 10s-1
A
Pressure [mmHg]
Blood flow [mL/min]
Sample [100Hz]
PAP
RAP
QLung
B
PAP
RAP
Curve fit
RV Isovolumetric
PISOMax (a + 2*b)
EDP (a)
Amplitude (b)
Pressure [mmHg]
Sample
C
PISOMax
Ees
Ea
Pressure [mmHg]
Volume [mL]

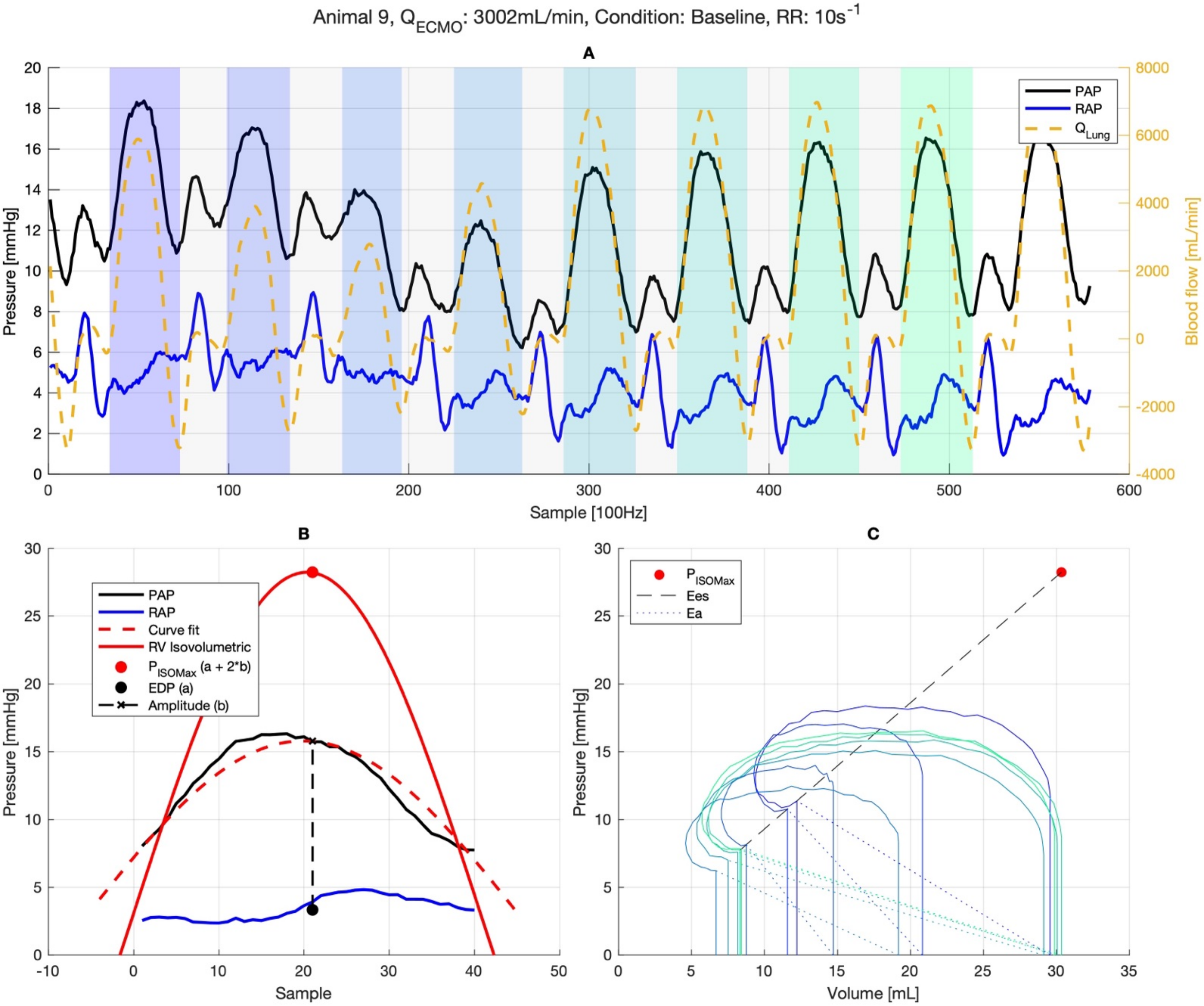

Animal 9, Q_{ECMO}: 3002mL/min, Condition: Baseline, RR: $10s^{-1}$
A
PAP
RAP
Q_{Lung}
Pressure [mmHg]
Blood flow [mL/min]
Sample [100Hz]
B
PAP
RAP
Curve fit
RV Isovolumetric
P_{ISOMax} (a + 2*b)
EDP (a)
Amplitude (b)
Pressure [mmHg]
Sample
C
P_{ISOMax}
Ees
Ea
Pressure [mmHg]
Volume [mL]

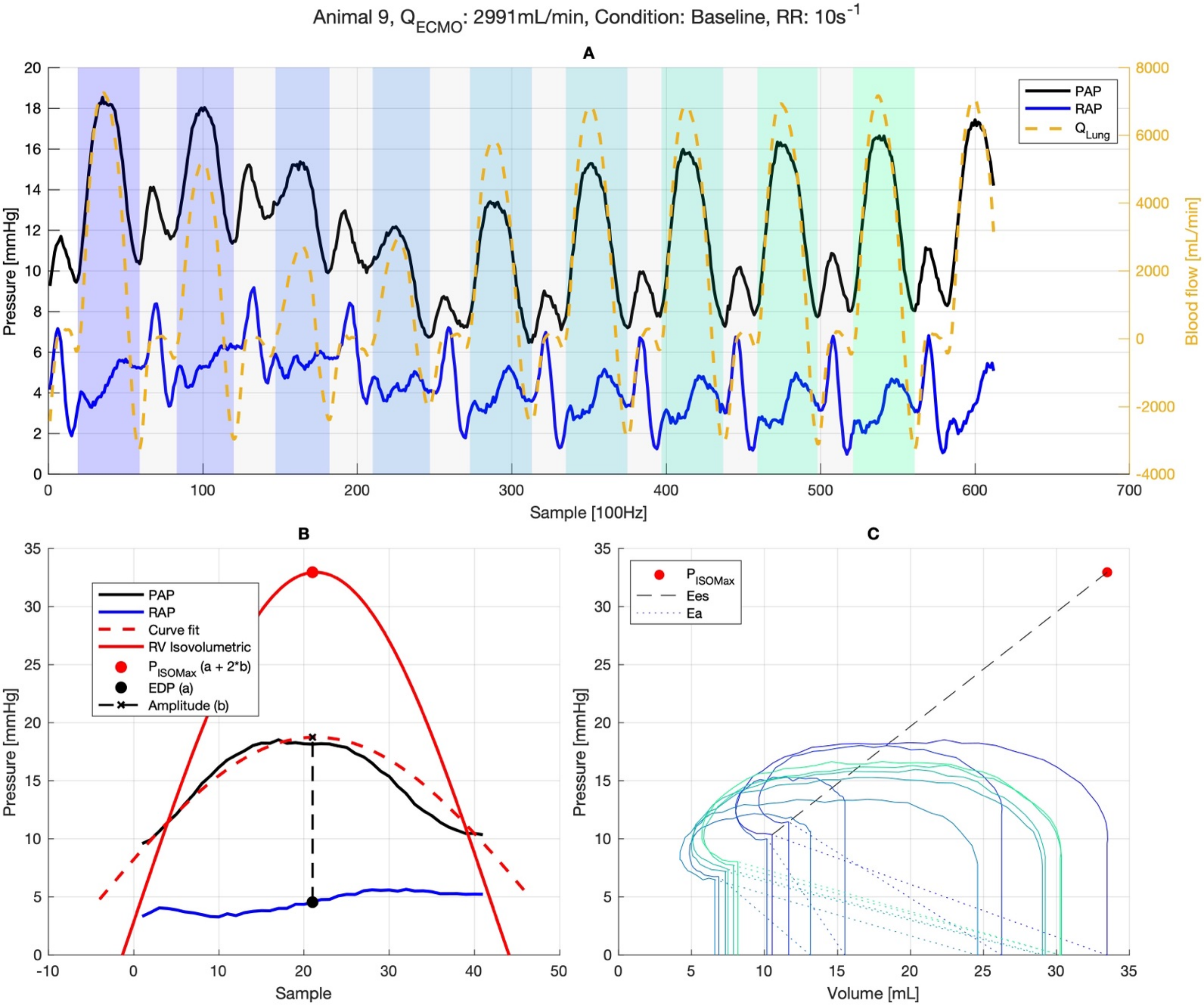

Animal 9, QECMO: 2991mL/min, Condition: Baseline, RR: 10s-1
A
PAP
RAP
QLung
Pressure [mmHg]
Blood flow [mL/min]
Sample [100Hz]
B
PAP
RAP
Curve fit
RV Isovolumetric
PISOMax (a + 2*b)
EDP (a)
Amplitude (b)
Pressure [mmHg]
Sample
C
PISOMax
Ees
Ea
Pressure [mmHg]
Volume [mL]

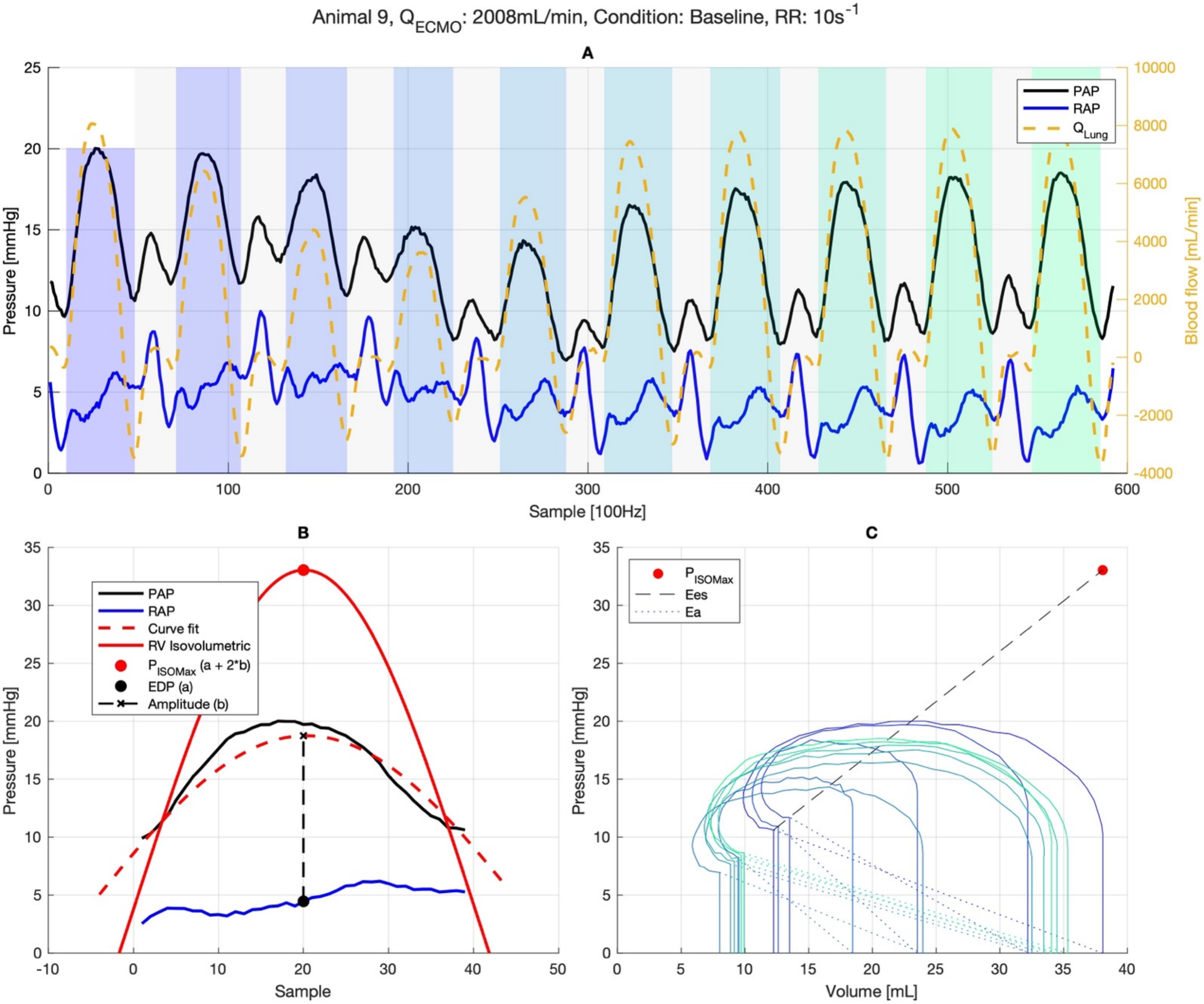

Animal 9, Q_ECMO: 2008mL/min, Condition: Baseline, RR: 10s^-1
A
PAP
RAP
Q_Lung
Pressure [mmHg]
Blood flow [mL/min]
Sample [100Hz]
B
PAP
RAP
Curve fit
RV Isovolumetric
P_ISOMax (a + 2*b)
EDP (a)
Amplitude (b)
Pressure [mmHg]
Sample
C
P_ISOMax
Ees
Ea
Pressure [mmHg]
Volume [mL]

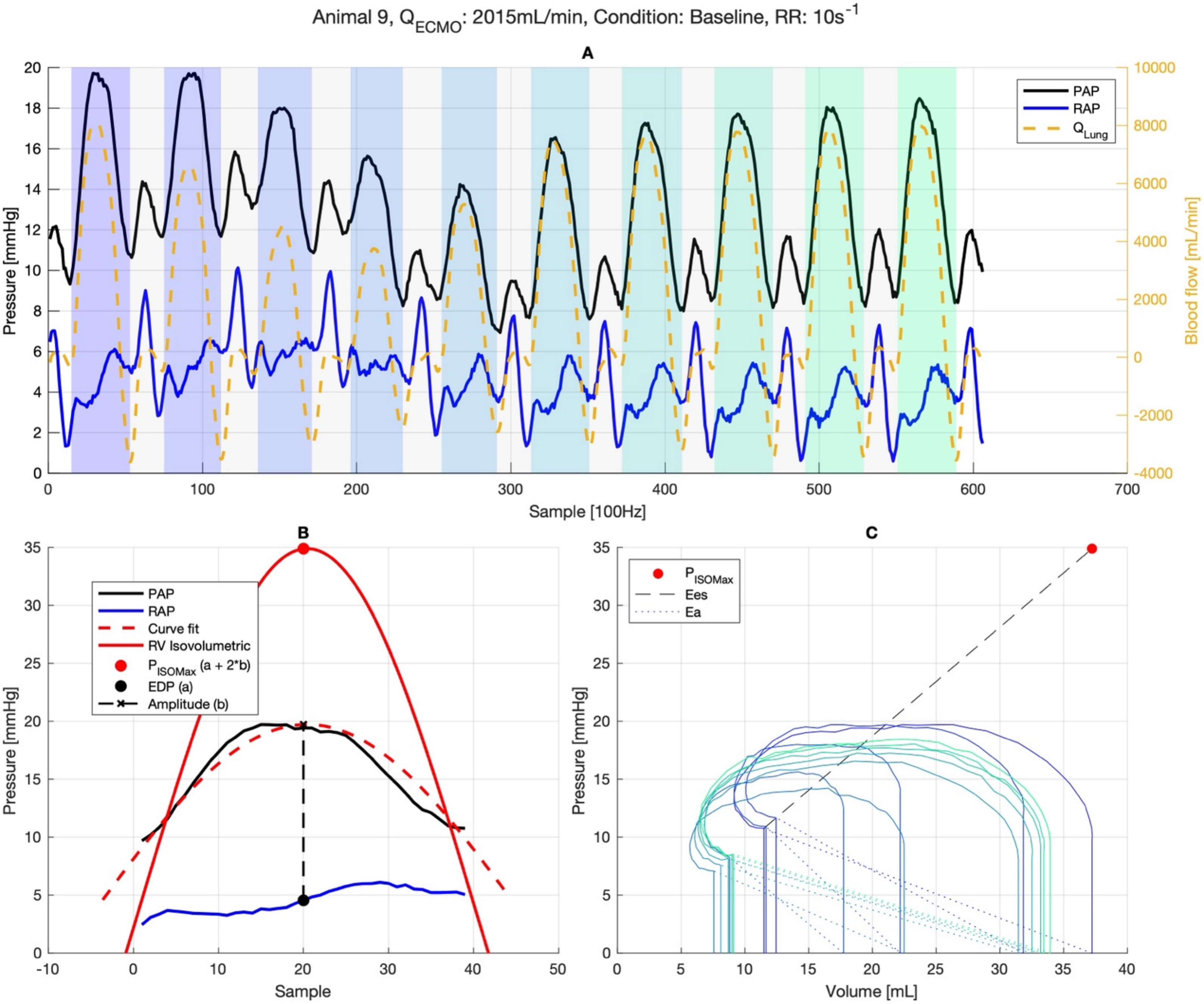

Animal 9, Q_{ECMO}: 2015mL/min, Condition: Baseline, RR: $10s^{-1}$
A
PAP
RAP
Q_{Lung}
Pressure [mmHg]
Blood flow [mL/min]
Sample [100Hz]
B
PAP
RAP
Curve fit
RV Isovolumetric
P_{ISOMax} (a + 2*b)
EDP (a)
Amplitude (b)
Pressure [mmHg]
Sample
C
P_{ISOMax}
Ees
Ea
Pressure [mmHg]
Volume [mL]

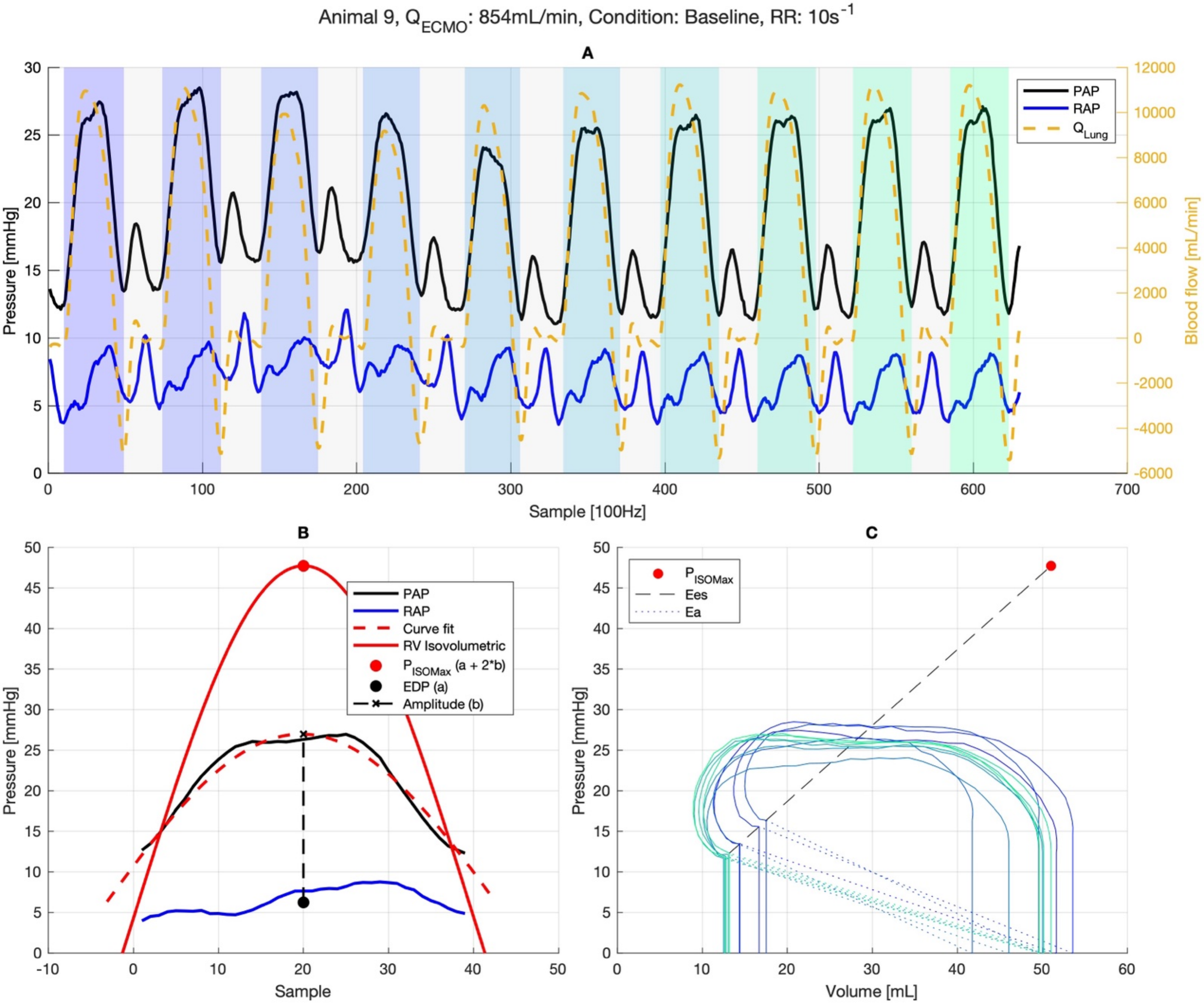

Animal 9, Q_{ECMO}: 854mL/min, Condition: Baseline, RR: $10s^{-1}$
A
Pressure [mmHg]
Blood flow [mL/min]
Sample [100Hz]
PAP
RAP
Q_{Lung}
B
Pressure [mmHg]
Sample
PAP
RAP
Curve fit
RV Isovolumetric
P_{ISOMax} (a + 2*b)
EDP (a)
Amplitude (b)
C
Pressure [mmHg]
Volume [mL]
P_{ISOMax}
Ees
Ea

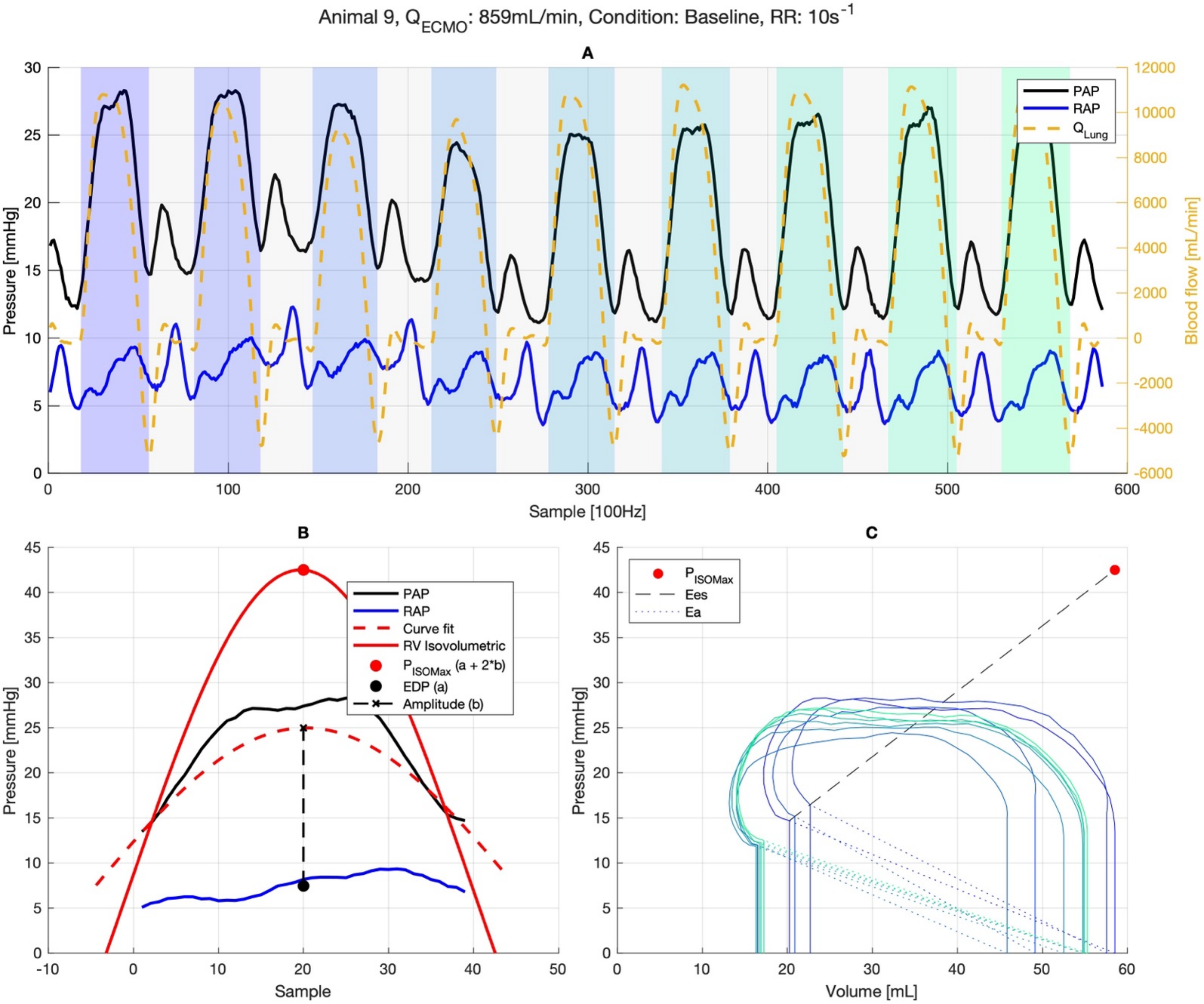

Animal 9, QECMO: 859mL/min, Condition: Baseline, RR: 10s-1
A
PAP
RAP
QLung
Pressure [mmHg]
Blood flow [mL/min]
Sample [100Hz]
B
PAP
RAP
Curve fit
RV Isovolumetric
PISOMax (a + 2*b)
EDP (a)
Amplitude (b)
Pressure [mmHg]
Sample
C
PISOMax
Ees
Ea
Pressure [mmHg]
Volume [mL]

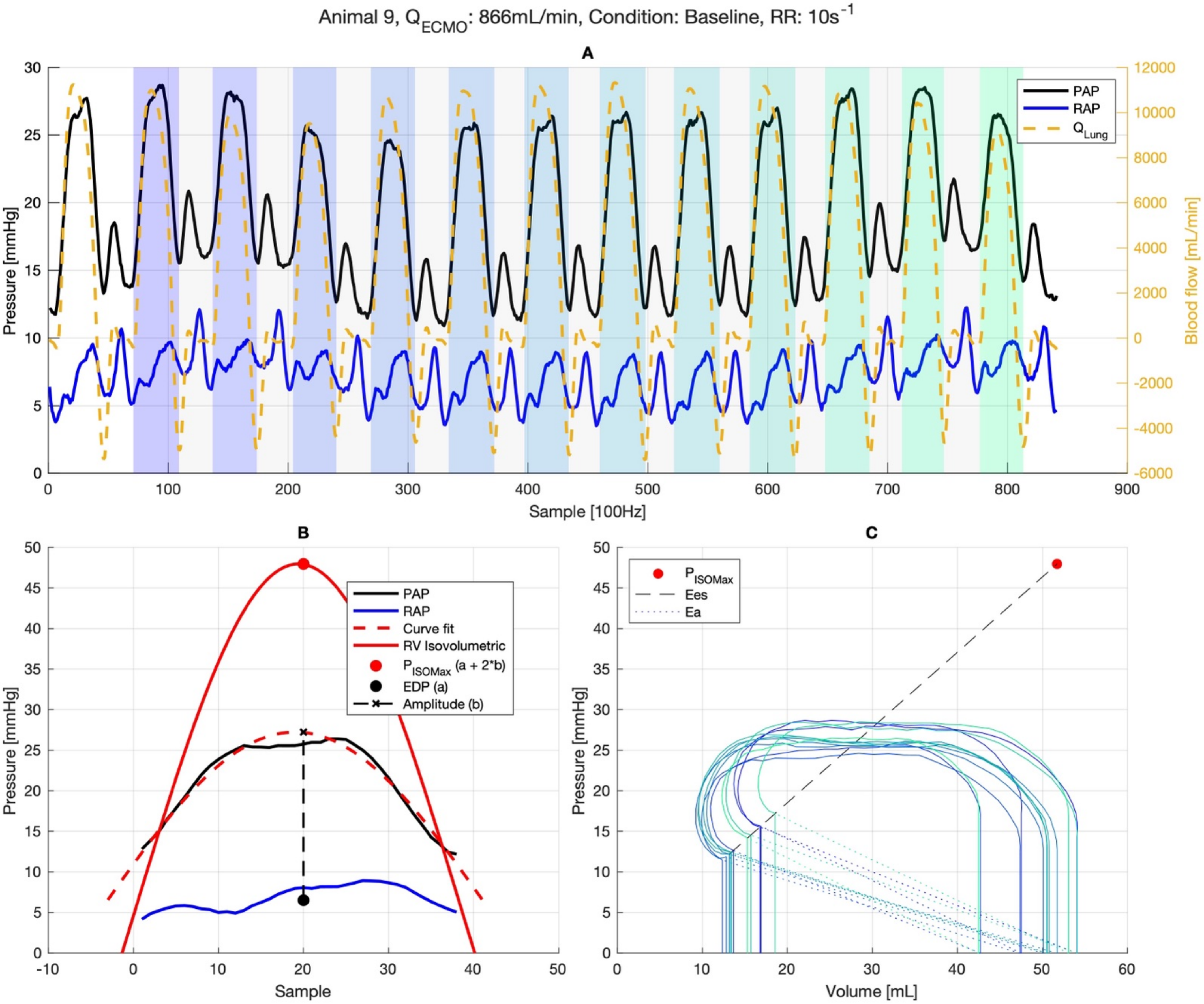

Animal 9, Q_ECMO: 866mL/min, Condition: Baseline, RR: 10s^-1
A
PAP
RAP
Q_Lung
Pressure [mmHg]
Blood flow [mL/min]
Sample [100Hz]
B
PAP
RAP
Curve fit
RV Isovolumetric
P_ISOMax (a + 2*b)
EDP (a)
Amplitude (b)
Pressure [mmHg]
Sample
C
P_ISOMax
Ees
Ea
Pressure [mmHg]
Volume [mL]

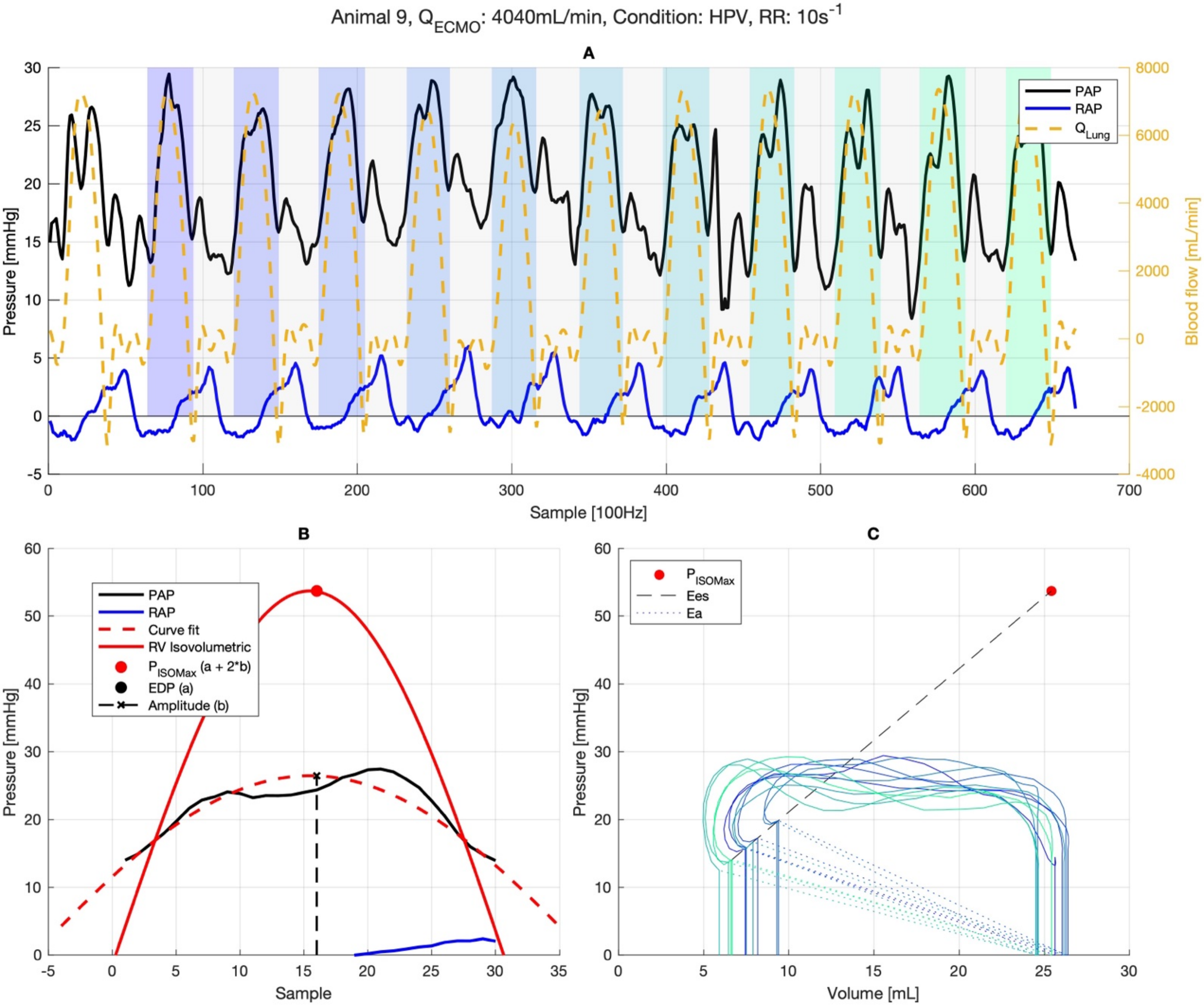

Animal 9, Q_{ECMO}: 4040mL/min, Condition: HPV, RR: $10s^{-1}$
A
PAP
RAP
Q_{Lung}
Pressure [mmHg]
Blood flow [mL/min]
Sample [100Hz]
B
PAP
RAP
Curve fit
RV Isovolumetric
P_{ISOMax} (a + 2*b)
EDP (a)
Amplitude (b)
Pressure [mmHg]
Sample
C
P_{ISOMax}
Ees
Ea
Pressure [mmHg]
Volume [mL]

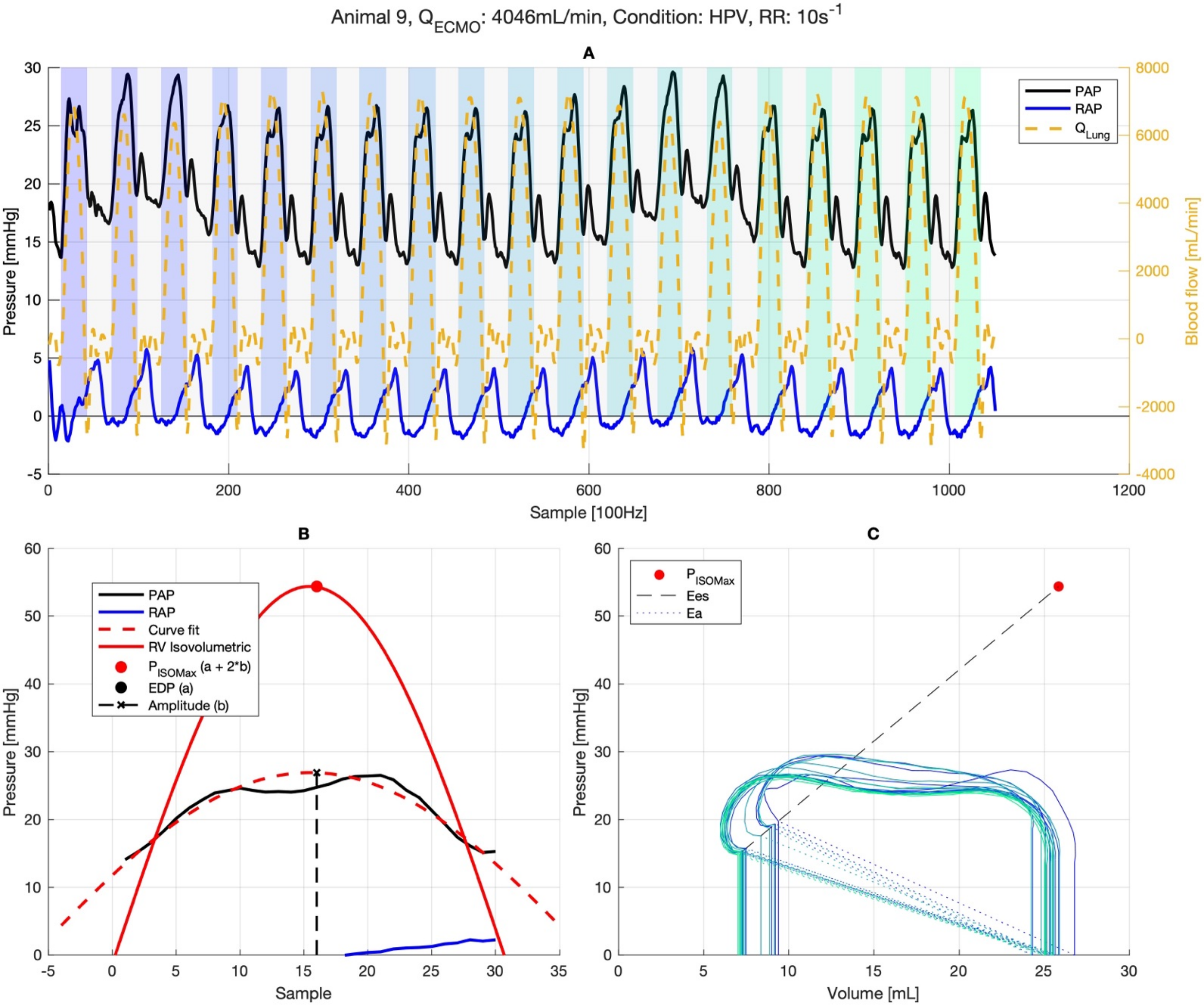
Animal 9, Q_ECMO: 4046mL/min, Condition: HPV, RR: 10s^-1
A
PAP
RAP
Q_Lung
Pressure [mmHg]
Blood flow [mL/min]
Sample [100Hz]
B
PAP
RAP
Curve fit
RV Isovolumetric
P_ISOMax (a + 2*b)
EDP (a)
Amplitude (b)
Pressure [mmHg]
Sample
C
P_ISOMax
Ees
Ea
Pressure [mmHg]
Volume [mL]

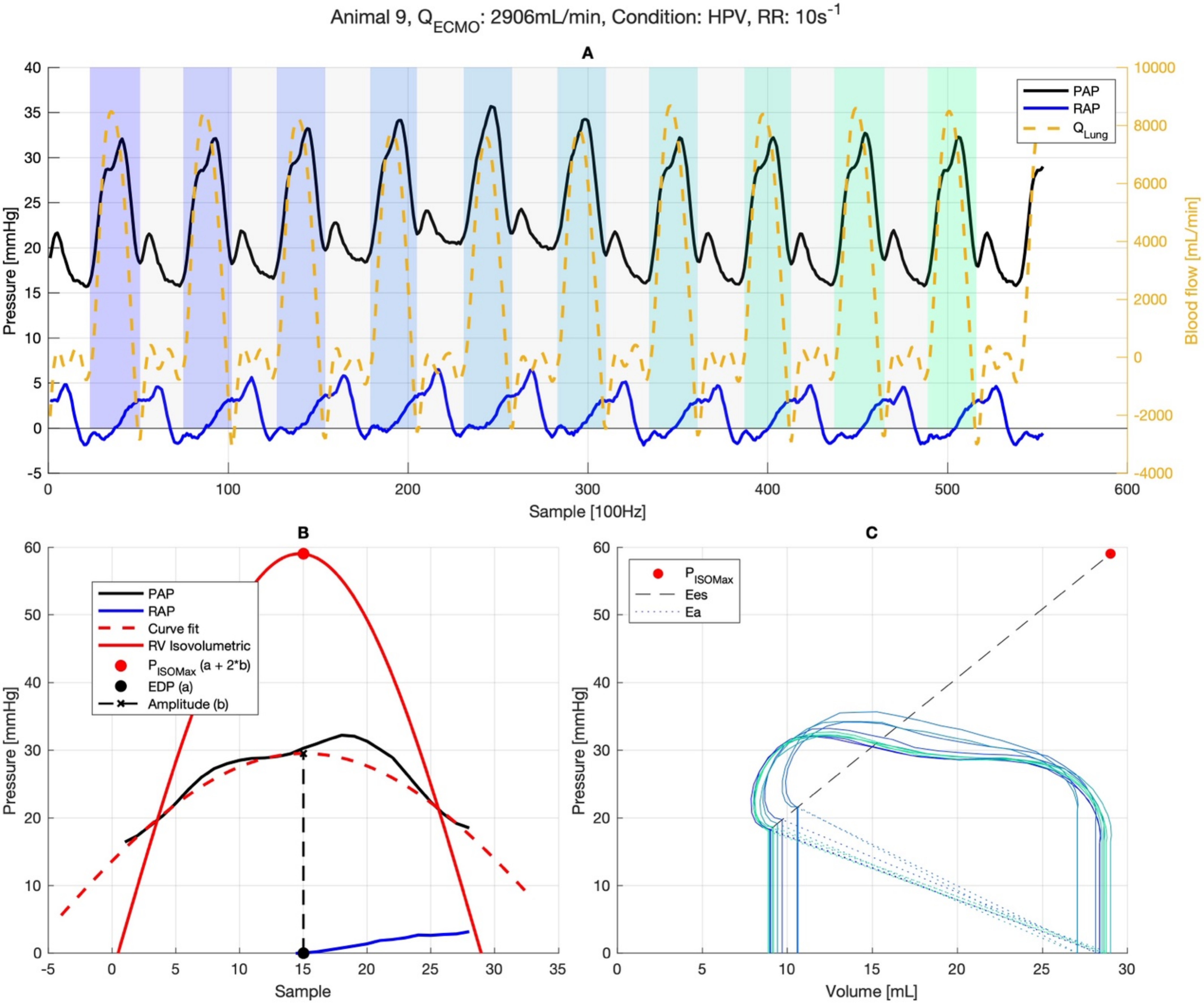

Animal 9, Q_{ECMO}: 2906mL/min, Condition: HPV, RR: $10s^{-1}$
A
PAP
RAP
Q_{Lung}
Pressure [mmHg]
Blood flow [mL/min]
Sample [100Hz]
B
PAP
RAP
Curve fit
RV Isovolumetric
P_{ISOMax} (a + 2*b)
EDP (a)
Amplitude (b)
Pressure [mmHg]
Sample
C
P_{ISOMax}
Ees
Ea
Pressure [mmHg]
Volume [mL]

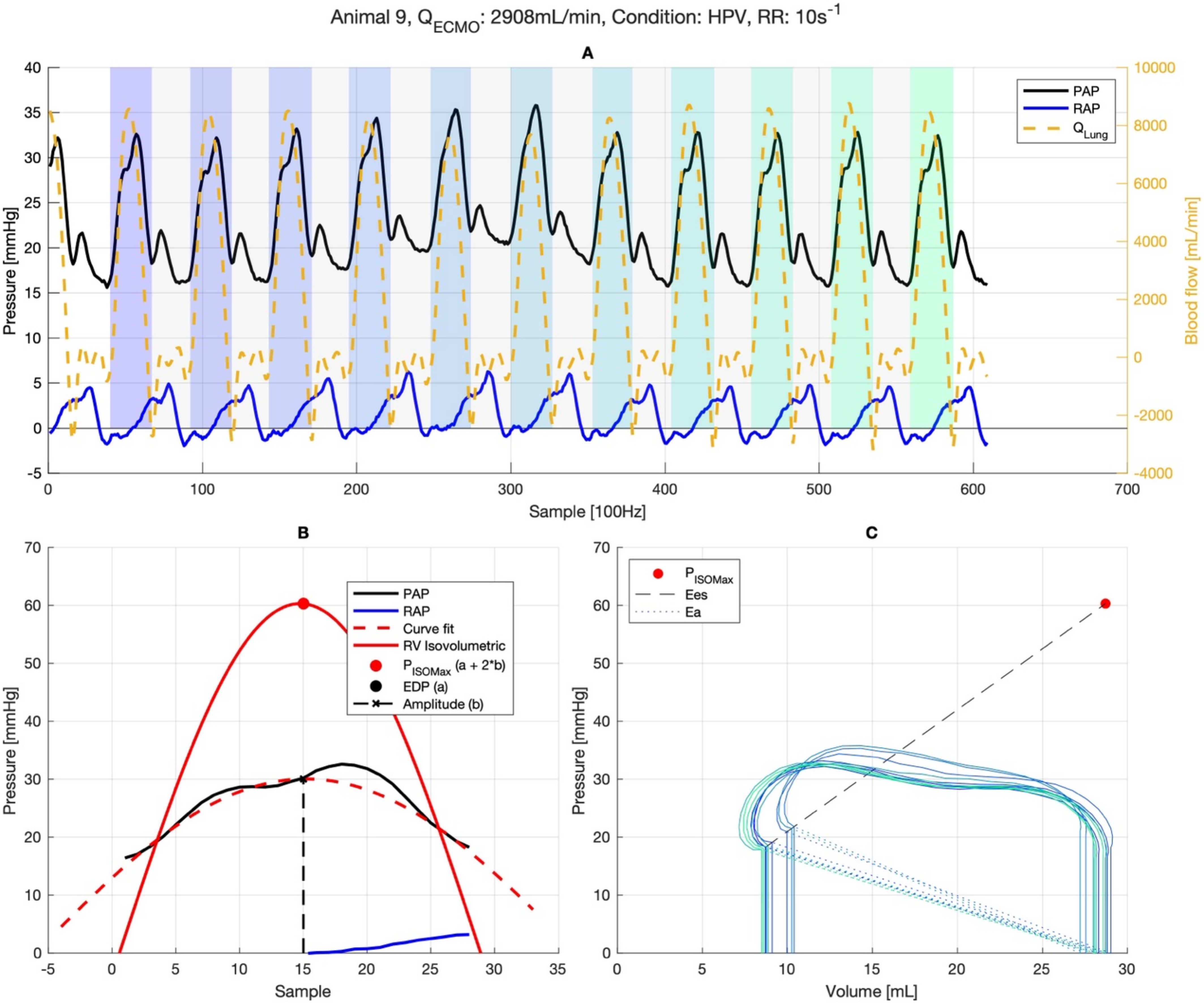

Animal 9, Q_ECMO: 2908mL/min, Condition: HPV, RR: 10s^-1
A
Pressure [mmHg]
Blood flow [mL/min]
Sample [100Hz]
PAP
RAP
Q_Lung
B
Pressure [mmHg]
Sample
PAP
RAP
Curve fit
RV Isovolumetric
P_ISOMax (a + 2*b)
EDP (a)
Amplitude (b)
C
Pressure [mmHg]
Volume [mL]
P_ISOMax
Ees
Ea

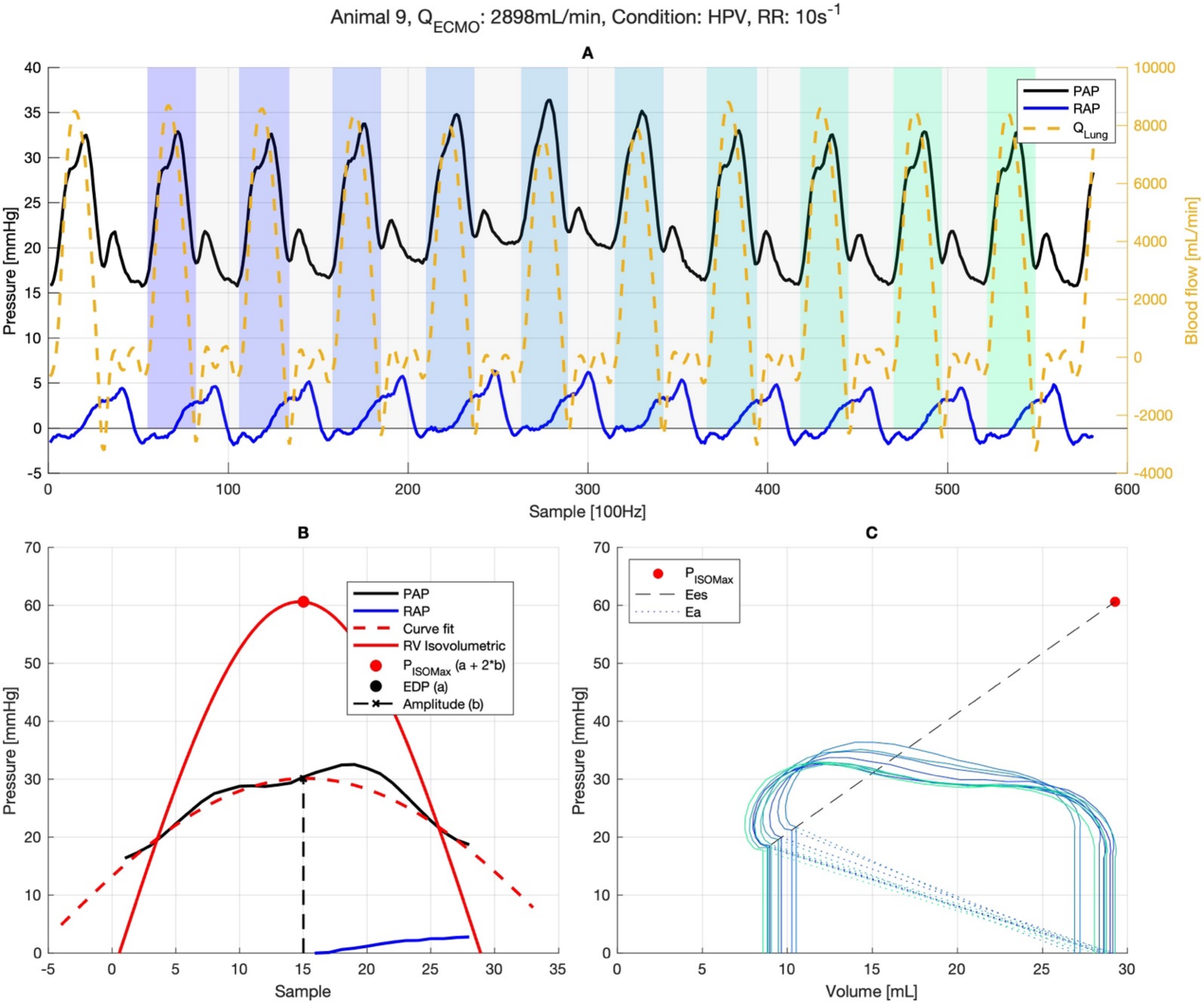

Animal 9, Q_{ECMO}: 2898mL/min, Condition: HPV, RR: $10s^{-1}$
A
PAP
RAP
Q_{Lung}
Pressure [mmHg]
Blood flow [mL/min]
Sample [100Hz]
B
PAP
RAP
Curve fit
RV Isovolumetric
P_{ISOMax} (a + 2*b)
EDP (a)
Amplitude (b)
Pressure [mmHg]
Sample
C
P_{ISOMax}
Ees
Ea
Pressure [mmHg]
Volume [mL]

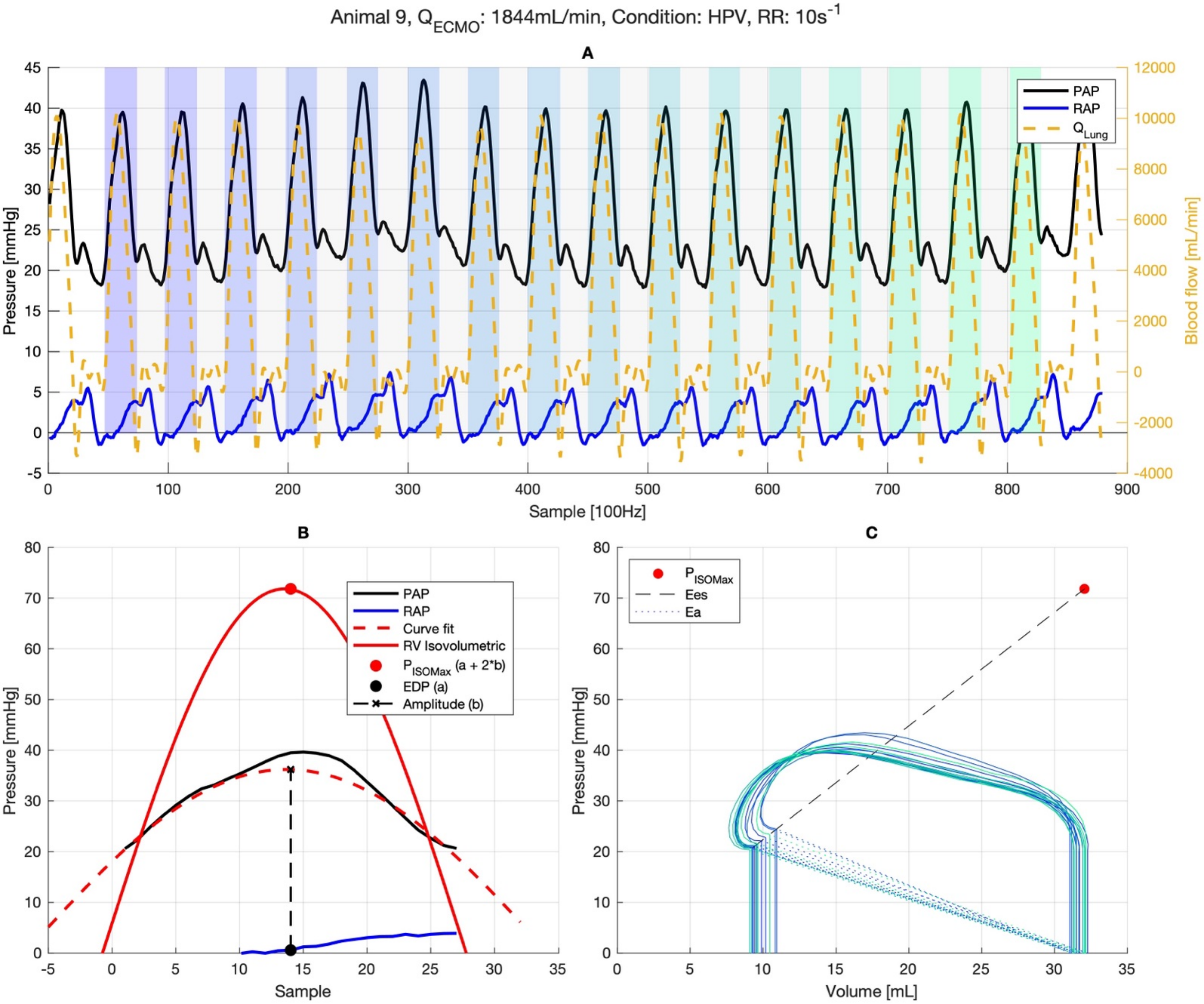

Animal 9, Q_{ECMO}: 1844mL/min, Condition: HPV, RR: $10s^{-1}$
A
Pressure [mmHg]
Blood flow [mL/min]
Sample [100Hz]
PAP
RAP
Q_{Lung}
B
Pressure [mmHg]
Sample
PAP
RAP
Curve fit
RV Isovolumetric
P_{ISOMax} (a + 2*b)
EDP (a)
Amplitude (b)
C
Pressure [mmHg]
Volume [mL]
P_{ISOMax}
Ees
Ea

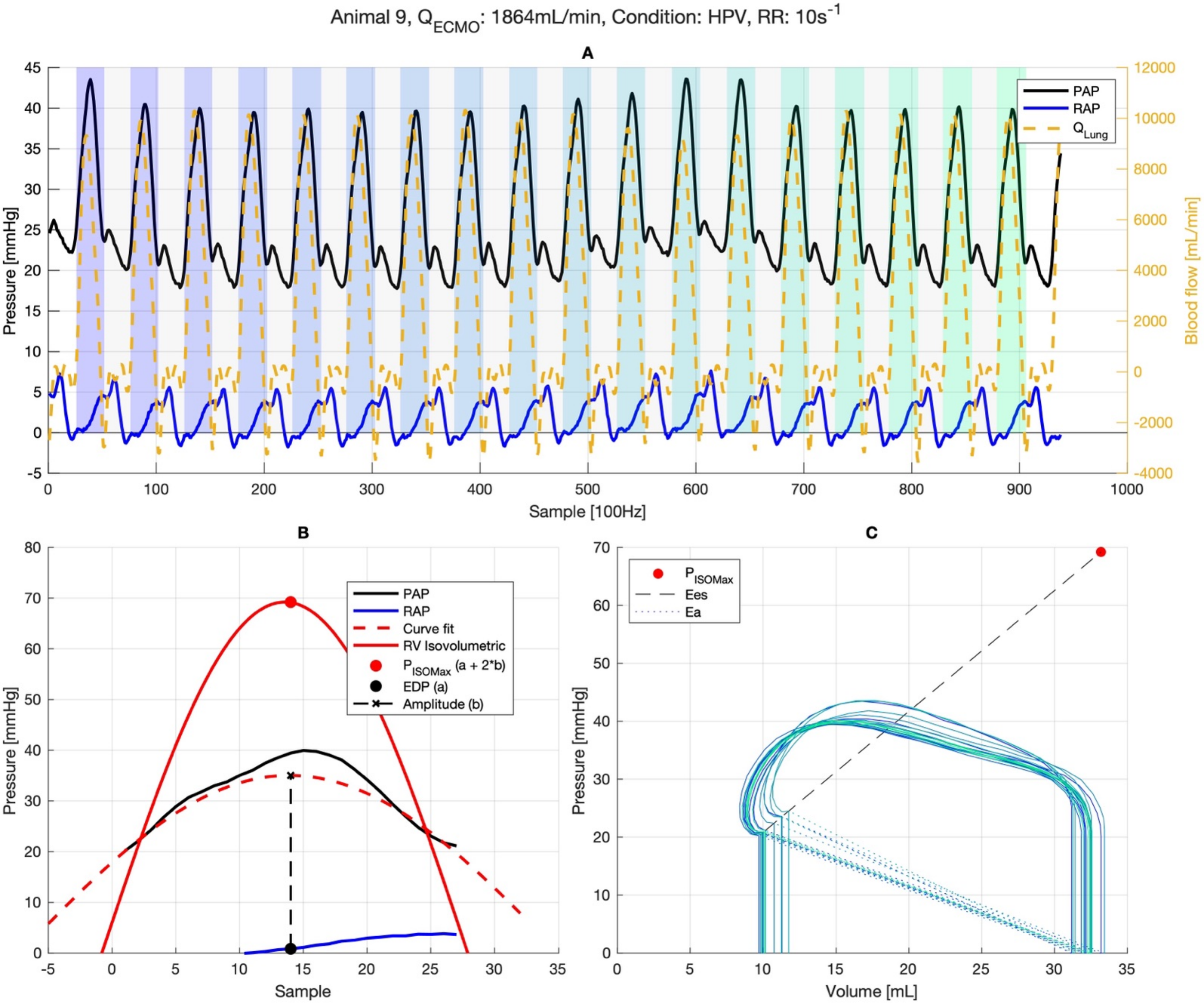

Animal 9, Q_ECMO: 1864mL/min, Condition: HPV, RR: 10s^-1
A
Pressure [mmHg]
Blood flow [mL/min]
Sample [100Hz]
PAP
RAP
Q_Lung
B
Pressure [mmHg]
Sample
PAP
RAP
Curve fit
RV Isovolumetric
P_ISOMax (a + 2*b)
EDP (a)
Amplitude (b)
C
Pressure [mmHg]
Volume [mL]
P_ISOMax
Ees
Ea

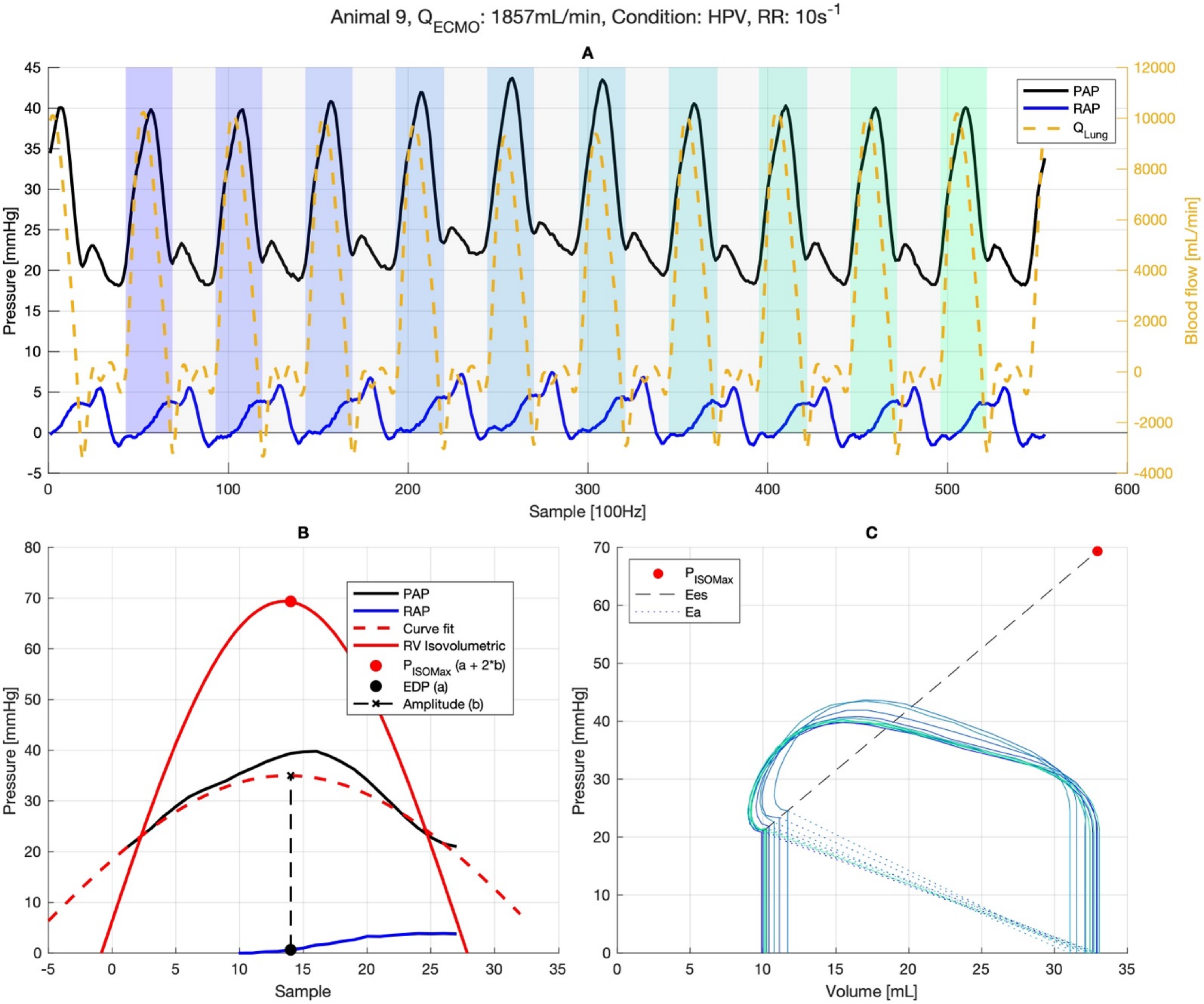

Animal 9, Q_{ECMO}: 1857mL/min, Condition: HPV, RR: $10s^{-1}$
A
PAP
RAP
Q_{Lung}
Pressure [mmHg]
Blood flow [mL/min]
Sample [100Hz]
B
PAP
RAP
Curve fit
RV Isovolumetric
P_{ISOMax} (a + 2*b)
EDP (a)
Amplitude (b)
Pressure [mmHg]
Sample
C
P_{ISOMax}
Ees
Ea
Pressure [mmHg]
Volume [mL]

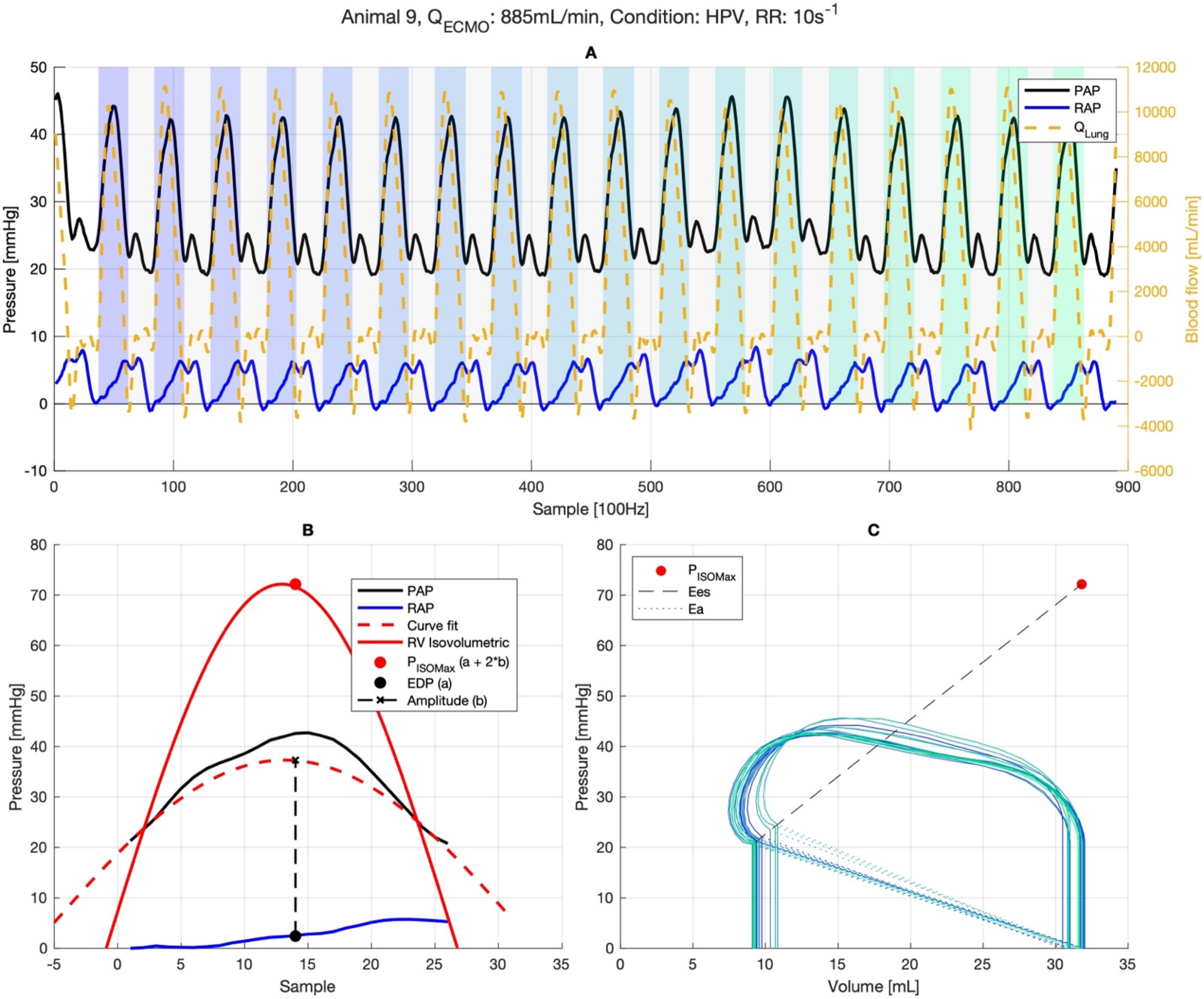

Animal 9, Q_{ECMO}: 885mL/min, Condition: HPV, RR: $10s^{-1}$
A
PAP
RAP
Q_{Lung}
Pressure [mmHg]
Blood flow [mL/min]
Sample [100Hz]
B
PAP
RAP
Curve fit
RV Isovolumetric
P_{ISOMax} (a + 2*b)
EDP (a)
Amplitude (b)
Pressure [mmHg]
Sample
C
P_{ISOMax}
Ees
Ea
Pressure [mmHg]
Volume [mL]

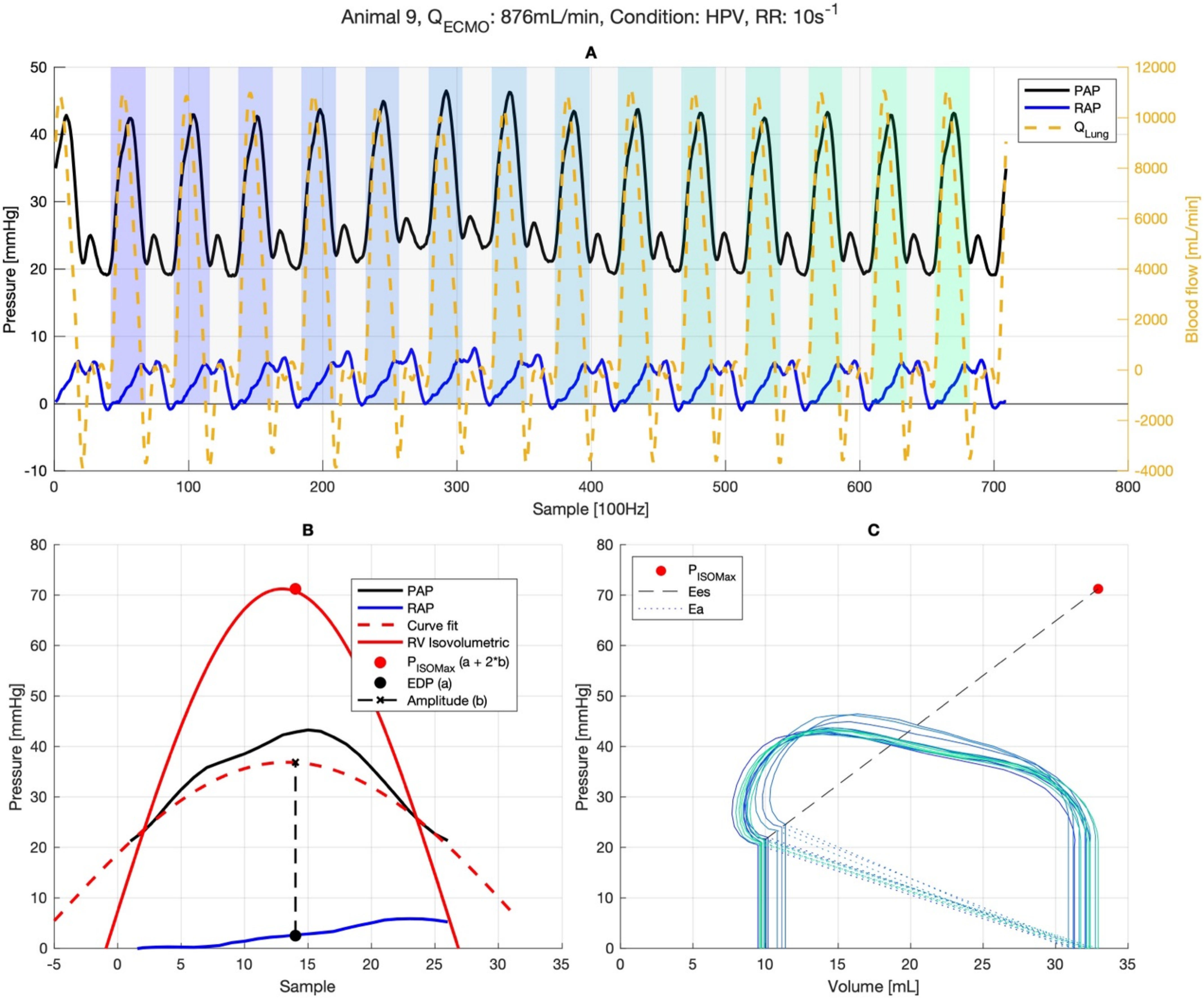

Animal 9, Q_{ECMO}: 876mL/min, Condition: HPV, RR: $10s^{-1}$
A
PAP
RAP
Q_{Lung}
Pressure [mmHg]
Blood flow [mL/min]
Sample [100Hz]
B
PAP
RAP
Curve fit
RV Isovolumetric
P_{ISOMax} (a + 2*b)
EDP (a)
Amplitude (b)
Pressure [mmHg]
Sample
C
P_{ISOMax}
Ees
Ea
Pressure [mmHg]
Volume [mL]

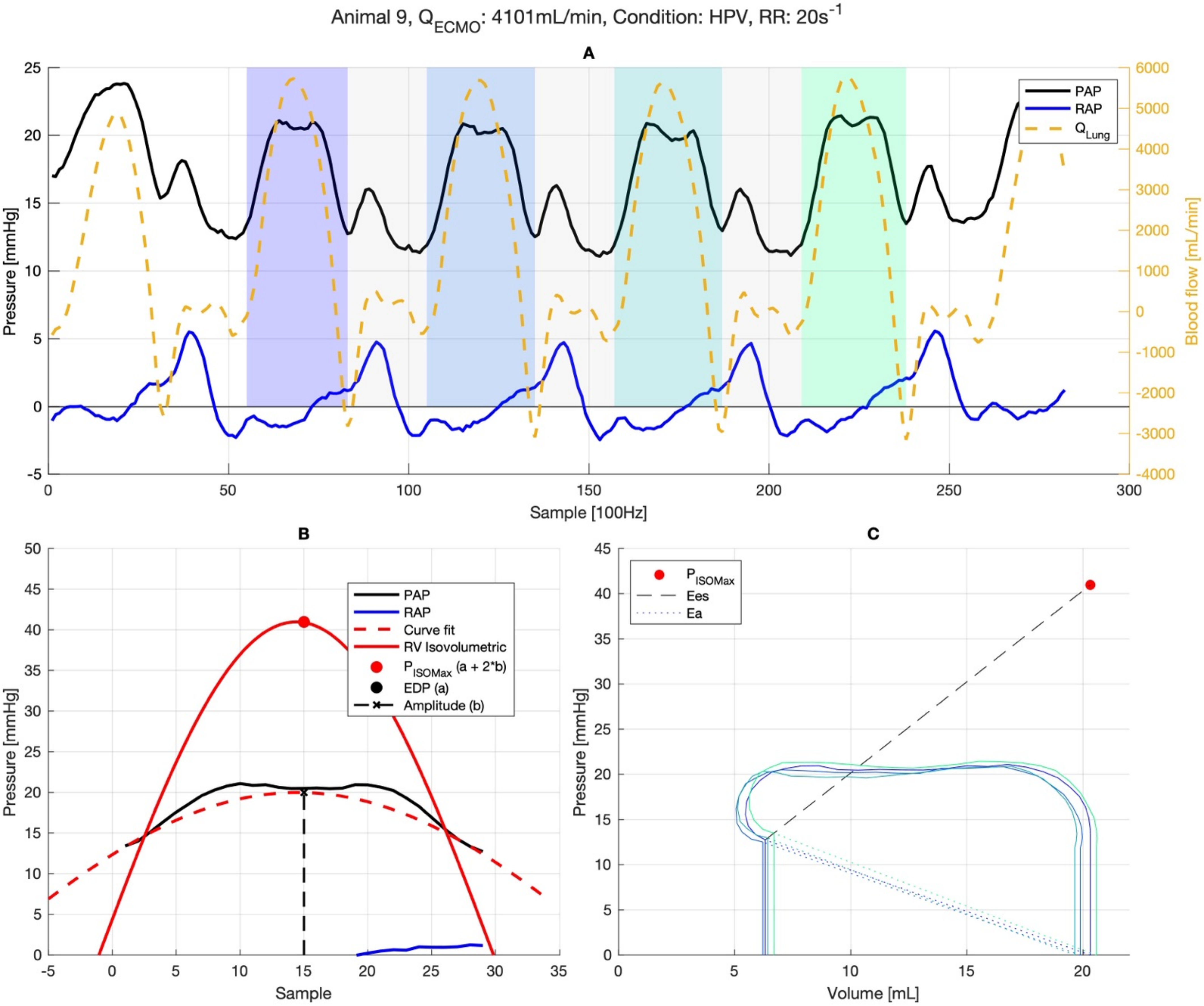

Animal 9, Q_{ECMO}: 4101mL/min, Condition: HPV, RR: $20s^{-1}$
A
Pressure [mmHg]
Blood flow [mL/min]
Sample [100Hz]
PAP
RAP
Q_{Lung}
B
Pressure [mmHg]
Sample
PAP
RAP
Curve fit
RV Isovolumetric
P_{ISOMax} (a + 2*b)
EDP (a)
Amplitude (b)
C
Pressure [mmHg]
Volume [mL]
P_{ISOMax}
Ees
Ea

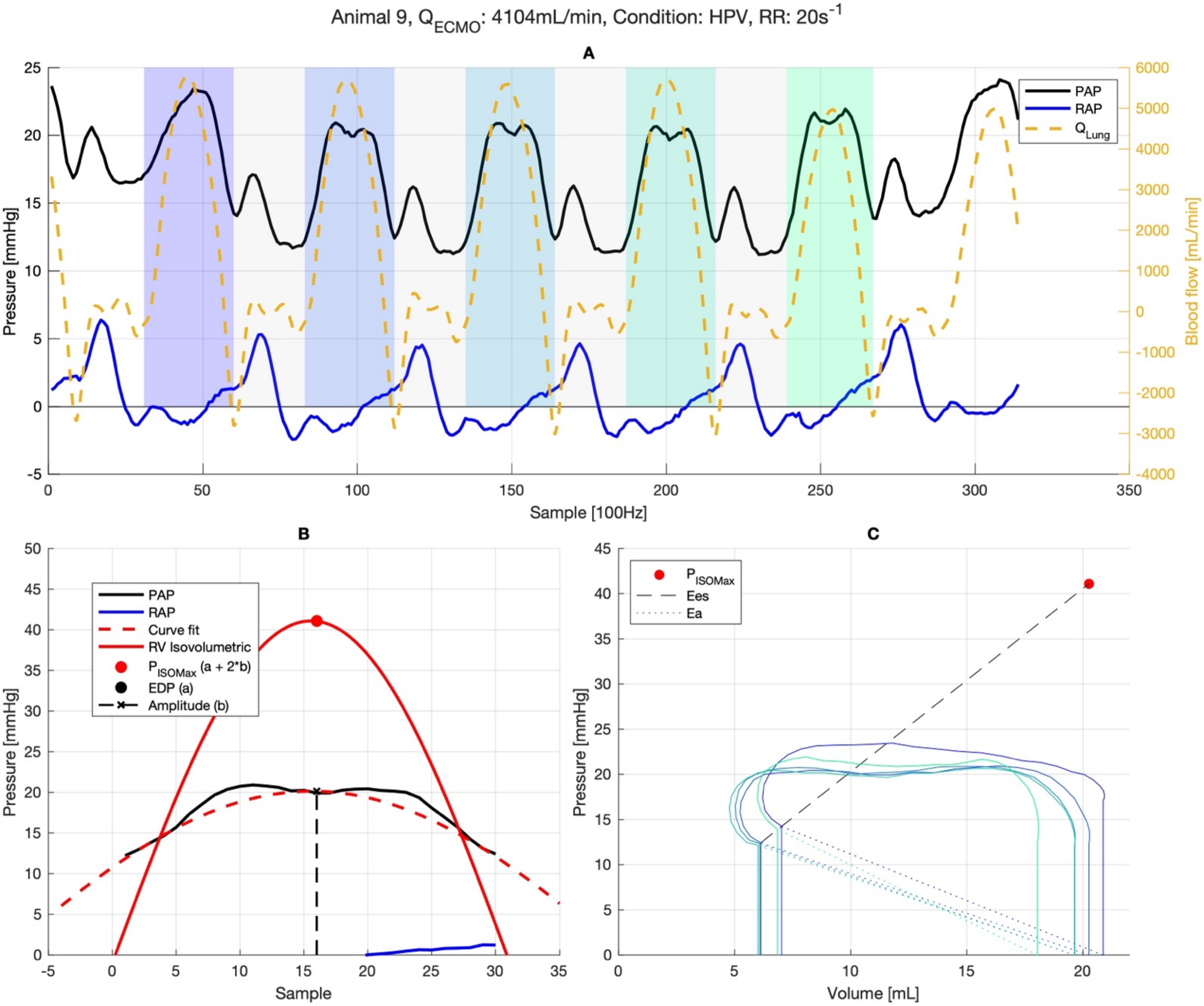

Animal 9, Q_ECMO: 4104mL/min, Condition: HPV, RR: 20s^-1
A
PAP
RAP
Q_Lung
Pressure [mmHg]
Blood flow [mL/min]
Sample [100Hz]
B
PAP
RAP
Curve fit
RV Isovolumetric
P_ISOMax (a + 2*b)
EDP (a)
Amplitude (b)
Pressure [mmHg]
Sample
C
P_ISOMax
Ees
Ea
Pressure [mmHg]
Volume [mL]

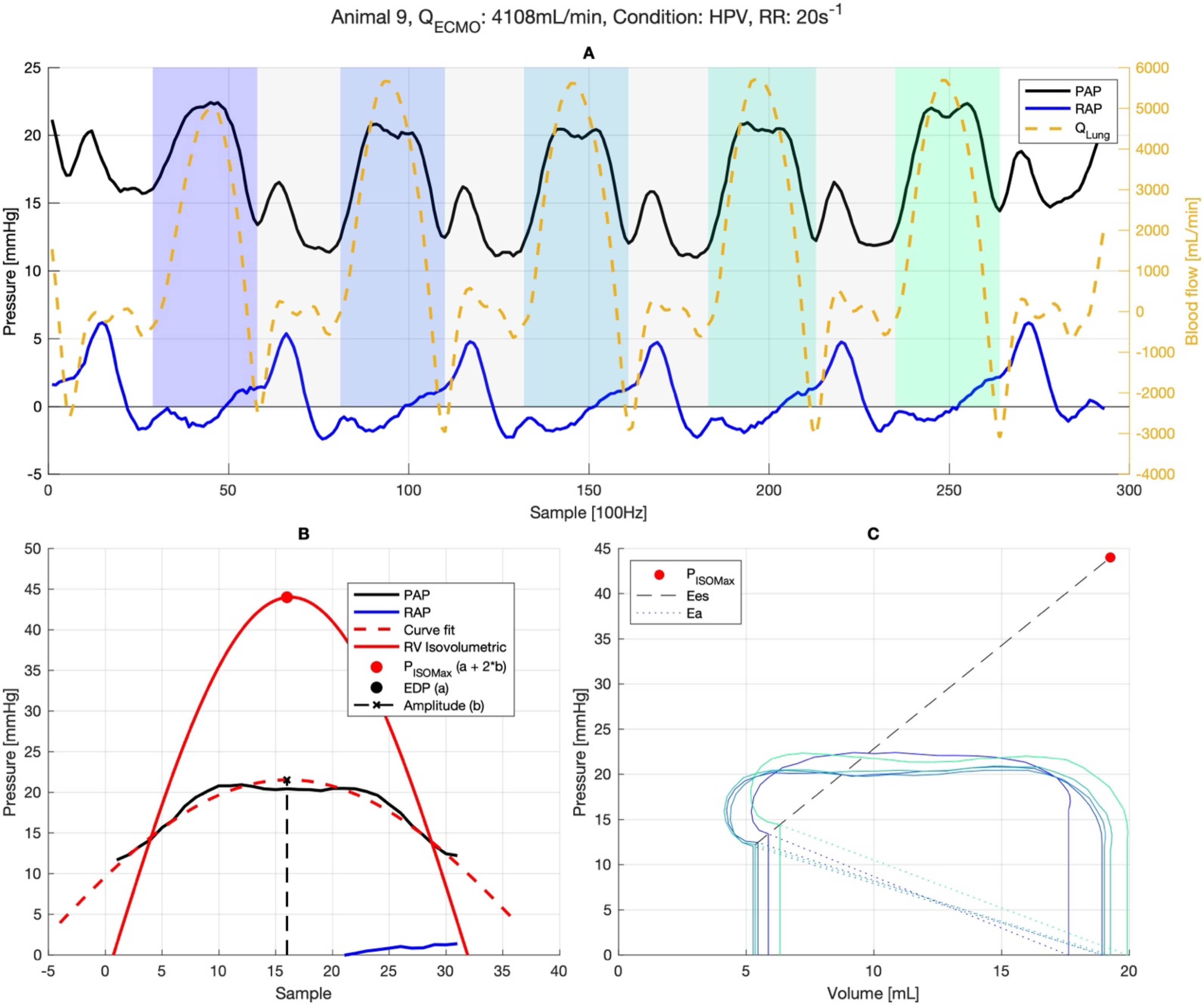

Animal 9, Q_{ECMO}: 4108mL/min, Condition: HPV, RR: $20s^{-1}$
A
PAP
RAP
Q_{Lung}
Pressure [mmHg]
Blood flow [mL/min]
Sample [100Hz]
B
PAP
RAP
Curve fit
RV Isovolumetric
P_{ISOMax} (a + 2*b)
EDP (a)
Amplitude (b)
Pressure [mmHg]
Sample
C
P_{ISOMax}
Ees
Ea
Pressure [mmHg]
Volume [mL]

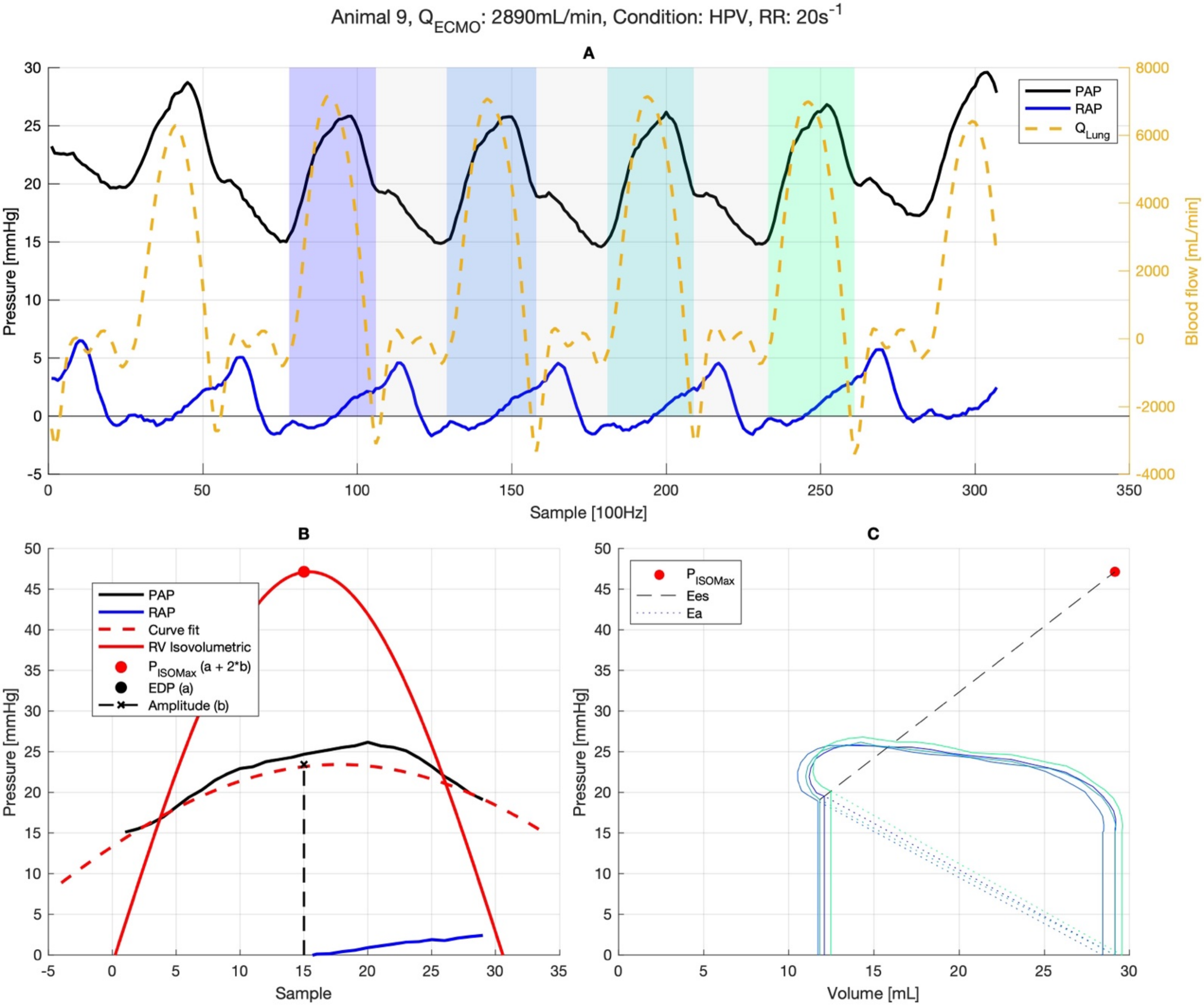

Animal 9, Q_ECMO: 2890mL/min, Condition: HPV, RR: 20s^-1
A
PAP
RAP
Q_Lung
Pressure [mmHg]
Blood flow [mL/min]
Sample [100Hz]
B
PAP
RAP
Curve fit
RV Isovolumetric
P_ISOMax (a + 2*b)
EDP (a)
Amplitude (b)
Pressure [mmHg]
Sample
C
P_ISOMax
Ees
Ea
Pressure [mmHg]
Volume [mL]

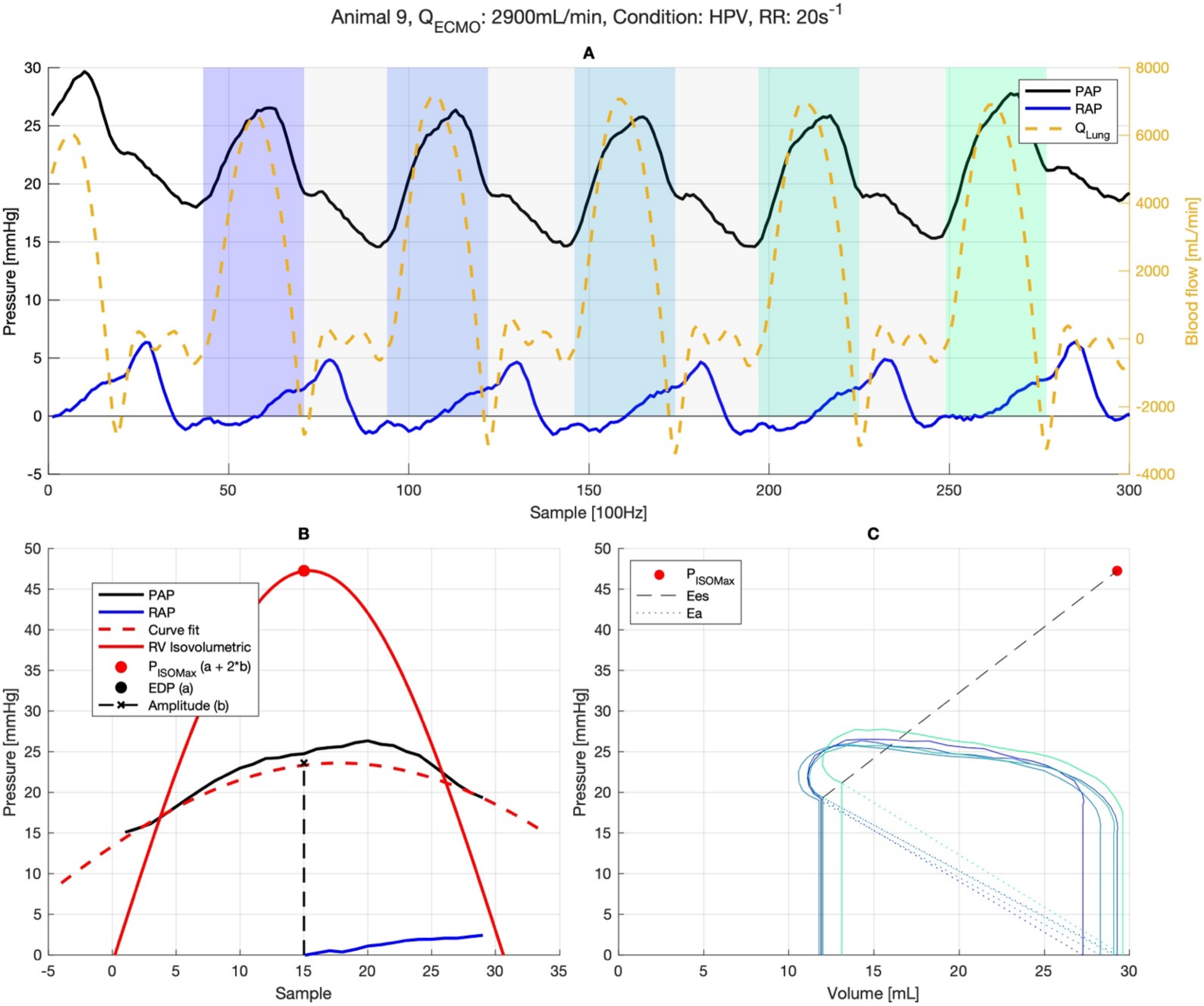

Animal 9, Q_{ECMO}: 2900mL/min, Condition: HPV, RR: $20s^{-1}$
A
PAP
RAP
Q_{Lung}
Pressure [mmHg]
Blood flow [mL/min]
Sample [100Hz]
B
PAP
RAP
Curve fit
RV Isovolumetric
P_{ISOMax} (a + 2*b)
EDP (a)
Amplitude (b)
Pressure [mmHg]
Sample
C
P_{ISOMax}
Ees
Ea
Pressure [mmHg]
Volume [mL]

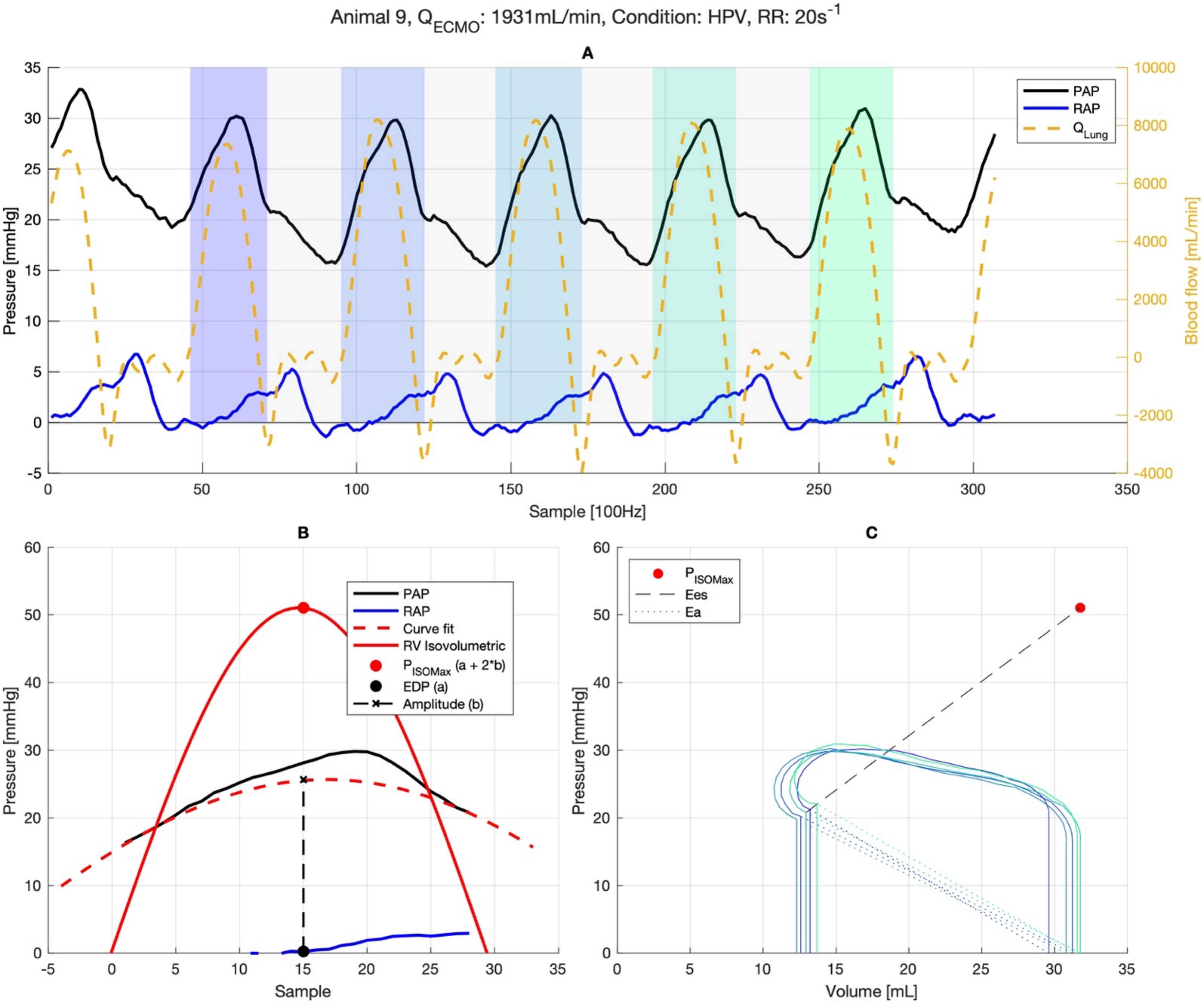

Animal 9, Q_{ECMO}: 1931mL/min, Condition: HPV, RR: $20s^{-1}$
A
PAP
RAP
Q_{Lung}
Pressure [mmHg]
Blood flow [mL/min]
Sample [100Hz]
B
PAP
RAP
Curve fit
RV Isovolumetric
P_{ISOMax} (a + 2*b)
EDP (a)
Amplitude (b)
Pressure [mmHg]
Sample
C
P_{ISOMax}
Ees
Ea
Pressure [mmHg]
Volume [mL]

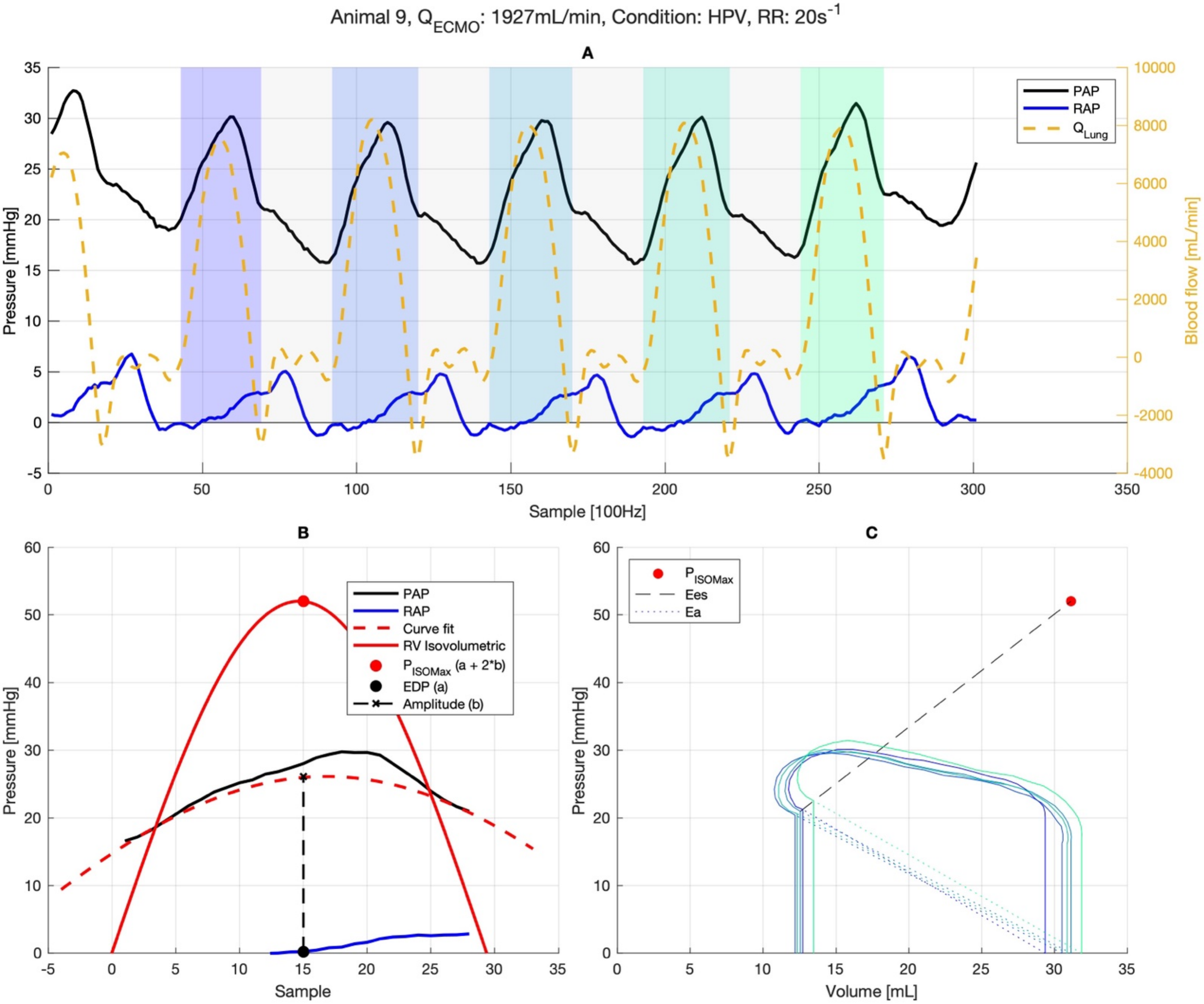

Animal 9, Q_ECMO: 1927mL/min, Condition: HPV, RR: 20s^-1
A
Pressure [mmHg]
Blood flow [mL/min]
Sample [100Hz]
PAP
RAP
Q_Lung
B
Pressure [mmHg]
Sample
PAP
RAP
Curve fit
RV Isovolumetric
P_ISOMax (a + 2*b)
EDP (a)
Amplitude (b)
C
Pressure [mmHg]
Volume [mL]
P_ISOMax
Ees
Ea

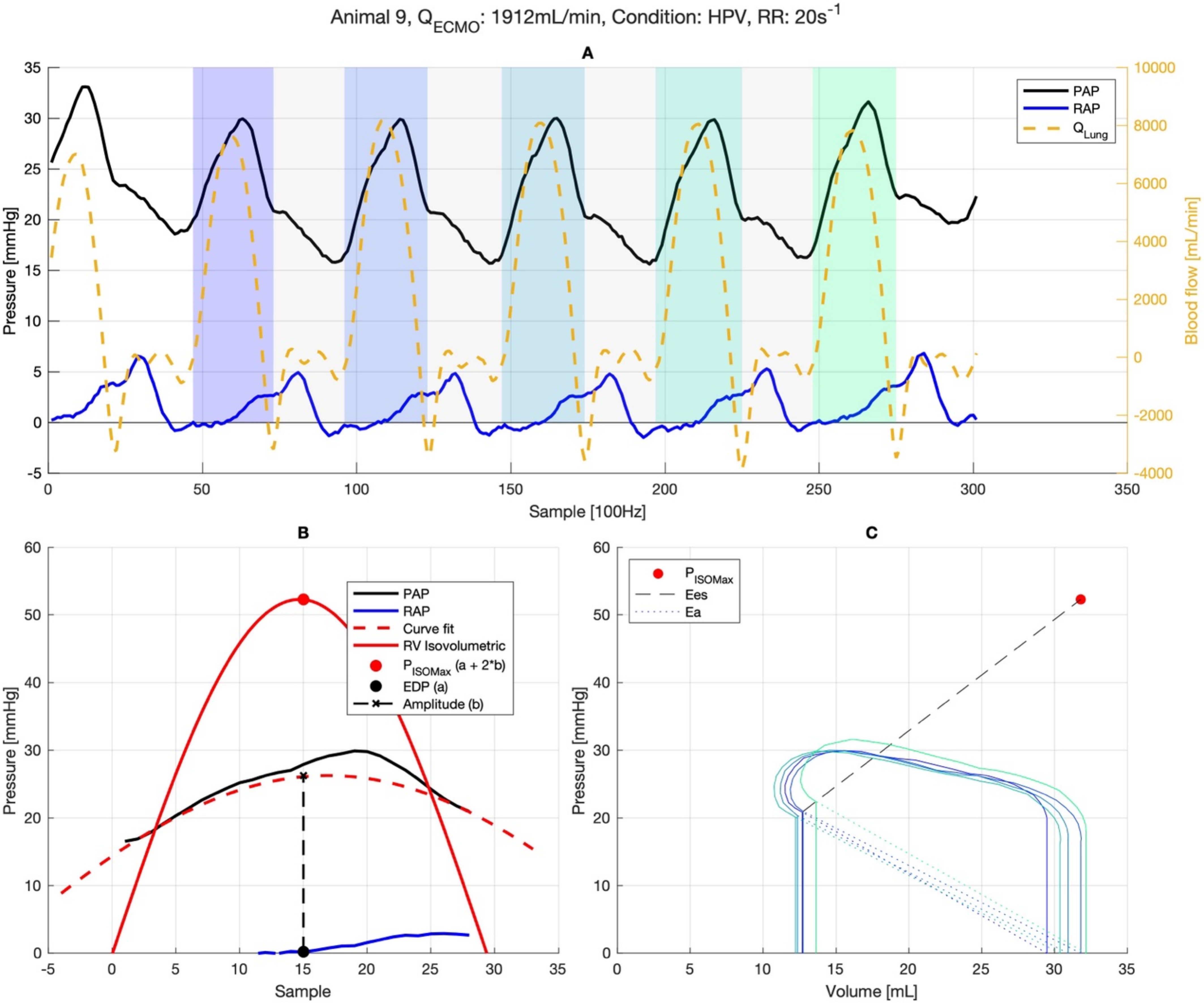

Animal 9, Q_{ECMO}: 1912mL/min, Condition: HPV, RR: $20s^{-1}$
A
Pressure [mmHg]
Blood flow [mL/min]
Sample [100Hz]
PAP
RAP
Q_{Lung}
B
Pressure [mmHg]
Sample
PAP
RAP
Curve fit
RV Isovolumetric
P_{ISOMax} (a + 2*b)
EDP (a)
Amplitude (b)
C
Pressure [mmHg]
Volume [mL]
P_{ISOMax}
Ees
Ea

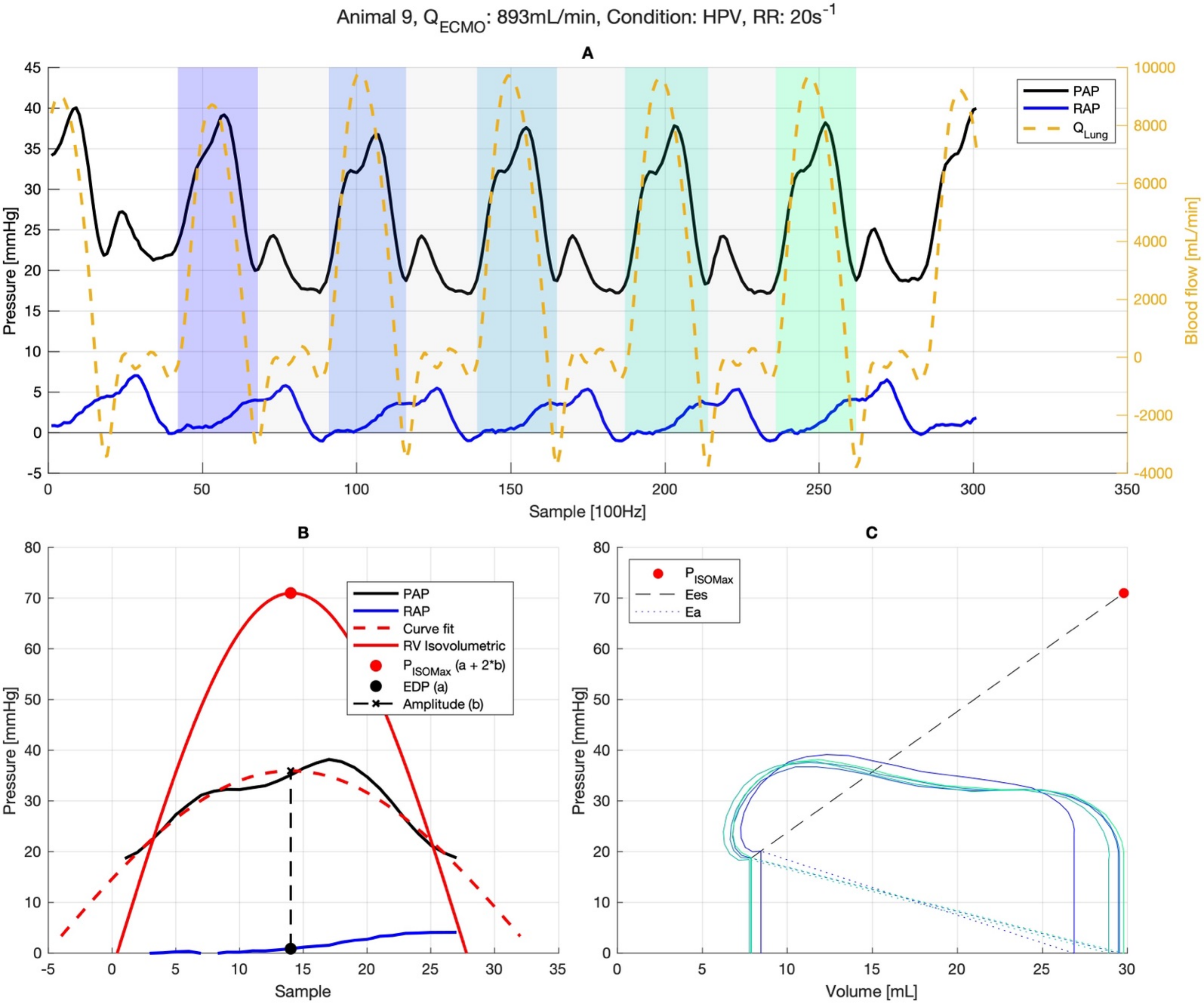

Animal 9, Q_ECMO: 893mL/min, Condition: HPV, RR: 20s^-1
A
PAP
RAP
Q_Lung
Pressure [mmHg]
Blood flow [mL/min]
Sample [100Hz]
B
PAP
RAP
Curve fit
RV Isovolumetric
P_ISOMax (a + 2*b)
EDP (a)
Amplitude (b)
Pressure [mmHg]
Sample
C
P_ISOMax
Ees
Ea
Pressure [mmHg]
Volume [mL]

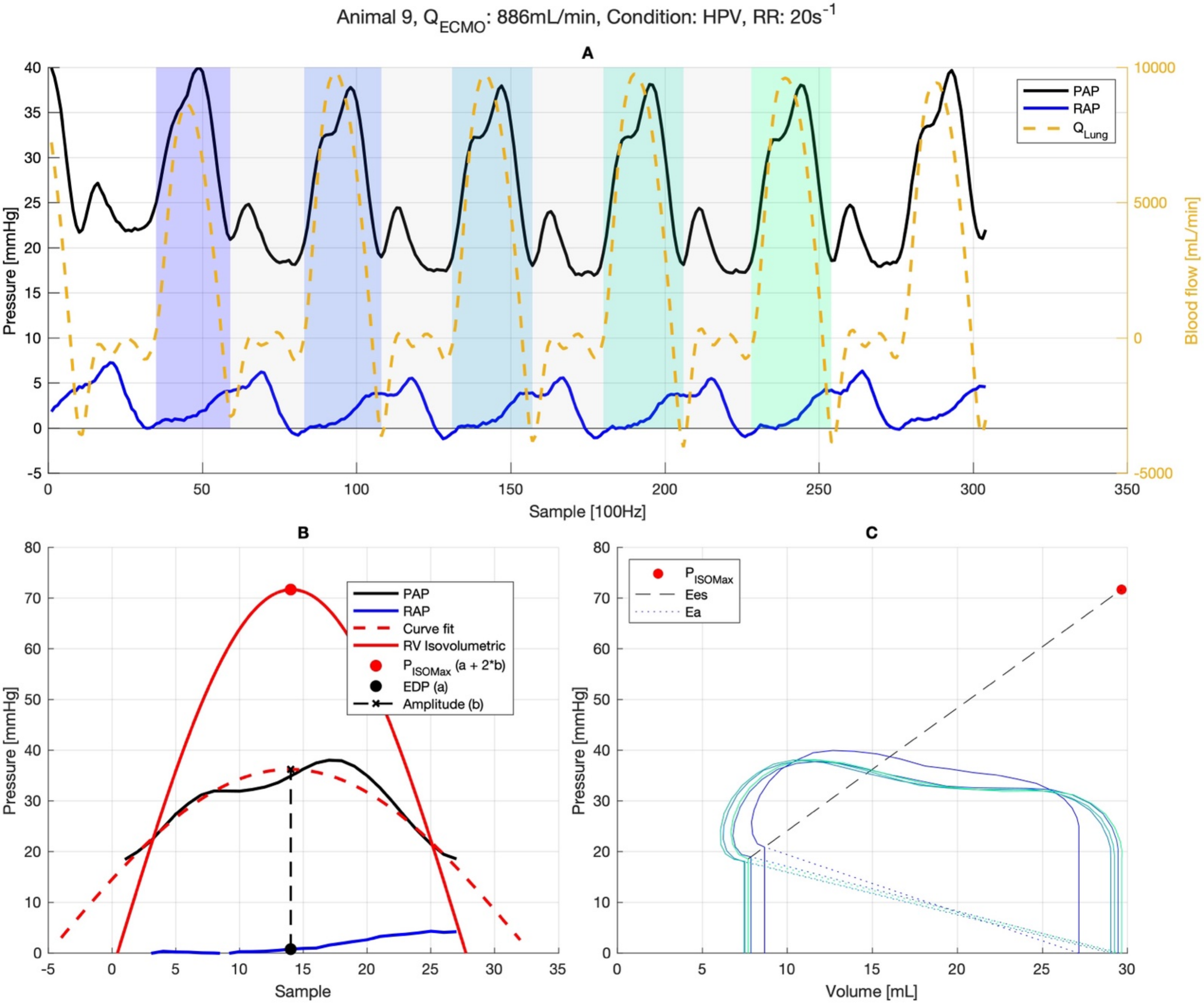

Animal 9, Q_ECMO: 886mL/min, Condition: HPV, RR: 20s^-1
A
PAP
RAP
Q_Lung
Pressure [mmHg]
Blood flow [mL/min]
Sample [100Hz]
B
PAP
RAP
Curve fit
RV Isovolumetric
P_ISOMax (a + 2*b)
EDP (a)
Amplitude (b)
Pressure [mmHg]
Sample
C
P_ISOMax
Ees
Ea
Pressure [mmHg]
Volume [mL]

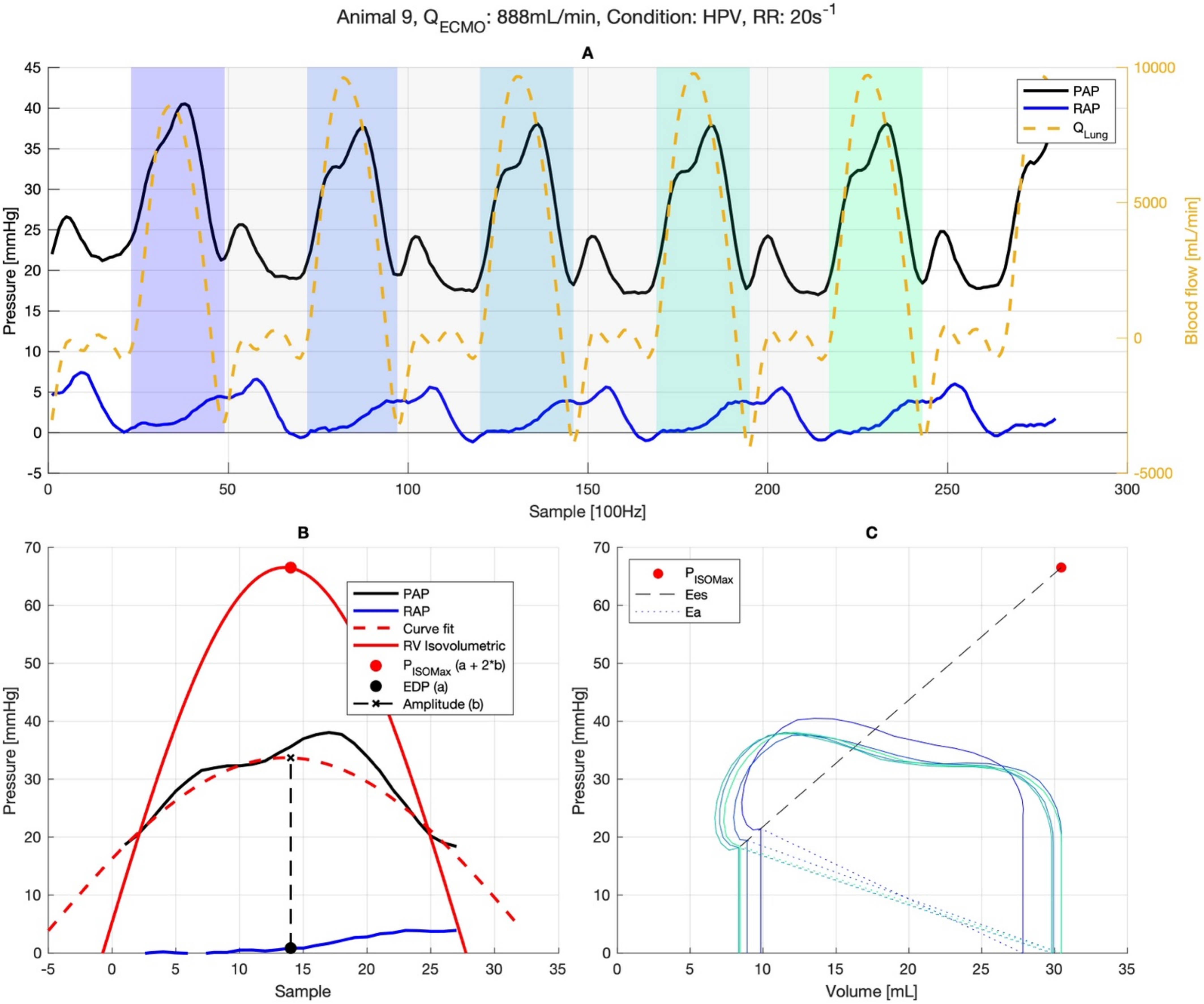
Animal 9, Q_{ECMO}: 888mL/min, Condition: HPV, RR: $20s^{-1}$
A
PAP
RAP
Q_{Lung}
Pressure [mmHg]
Blood flow [mL/min]
Sample [100Hz]
B
PAP
RAP
Curve fit
RV Isovolumetric
P_{ISOMax} (a + 2*b)
EDP (a)
Amplitude (b)
Pressure [mmHg]
Sample
C
P_{ISOMax}
Ees
Ea
Pressure [mmHg]
Volume [mL]

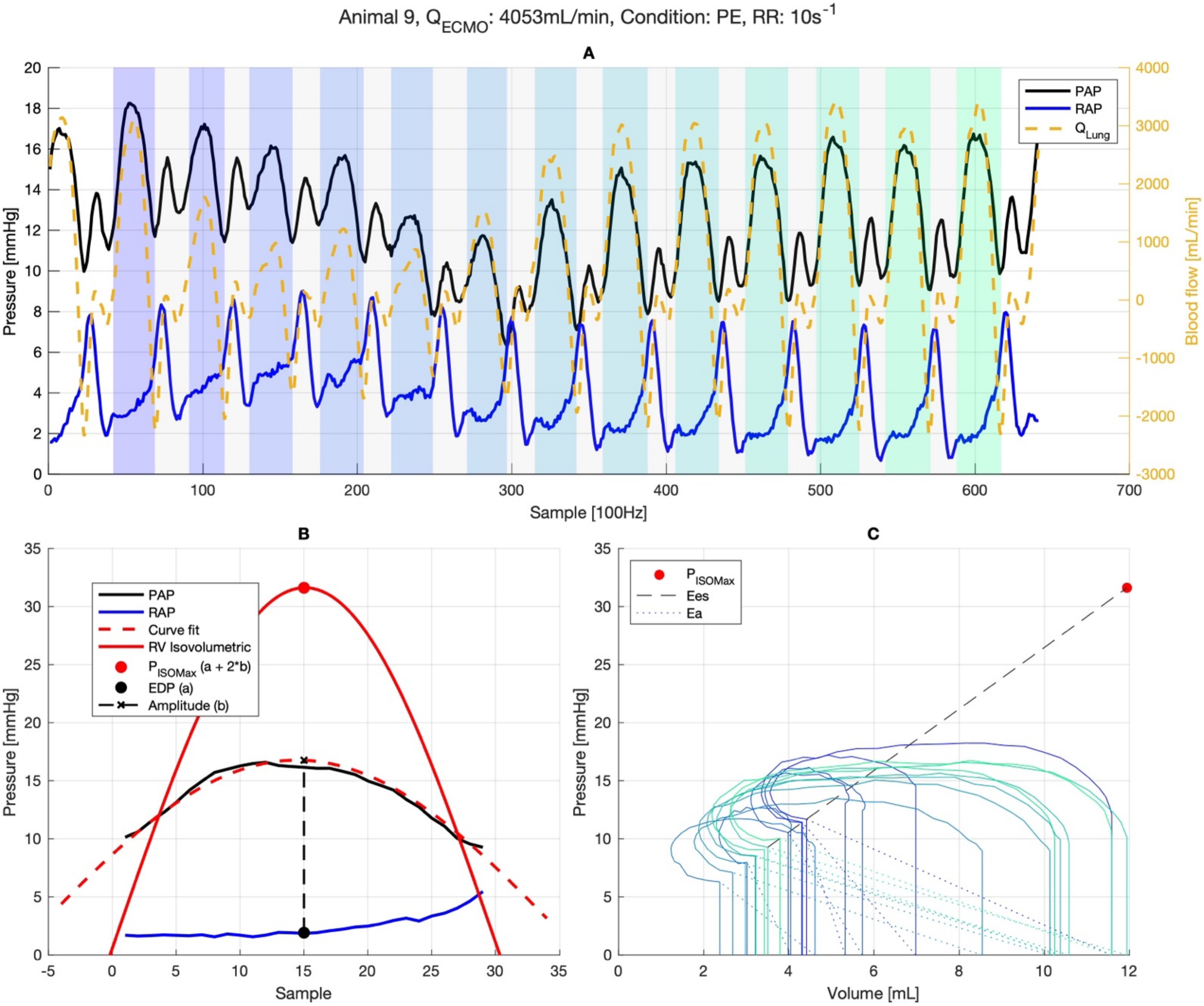

Animal 9, Q_ECMO: 4053mL/min, Condition: PE, RR: 10s^-1
A
PAP
RAP
Q_Lung
Pressure [mmHg]
Blood flow [mL/min]
Sample [100Hz]
B
PAP
RAP
Curve fit
RV Isovolumetric
P_ISOMax (a + 2*b)
EDP (a)
Amplitude (b)
Pressure [mmHg]
Sample
C
P_ISOMax
Ees
Ea
Pressure [mmHg]
Volume [mL]

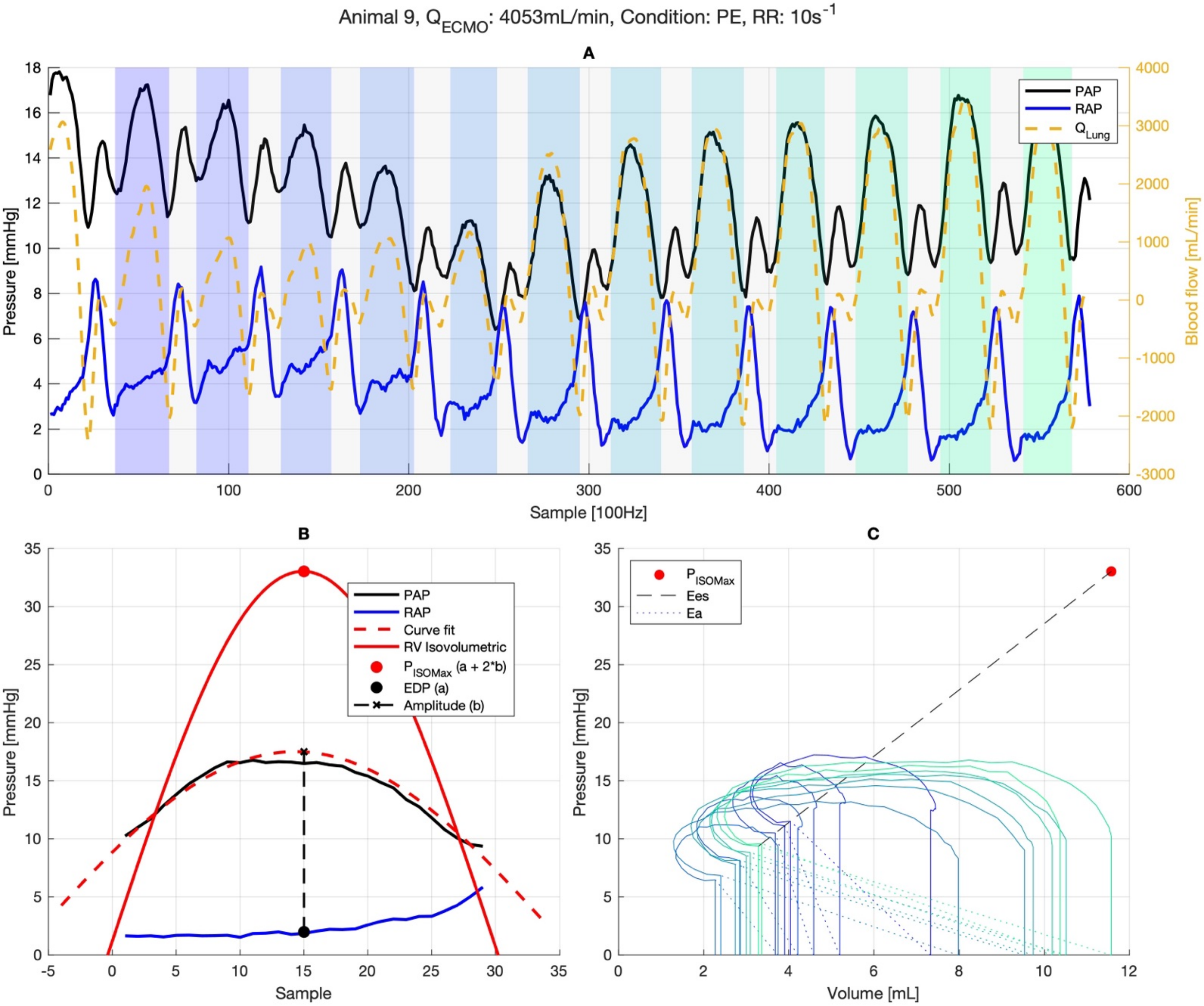

Animal 9, Q_ECMO: 4053mL/min, Condition: PE, RR: 10s^-1
A
PAP
RAP
Q_Lung
Pressure [mmHg]
Blood flow [mL/min]
Sample [100Hz]
B
PAP
RAP
Curve fit
RV Isovolumetric
P_ISOMax (a + 2*b)
EDP (a)
Amplitude (b)
Pressure [mmHg]
Sample
C
P_ISOMax
Ees
Ea
Pressure [mmHg]
Volume [mL]

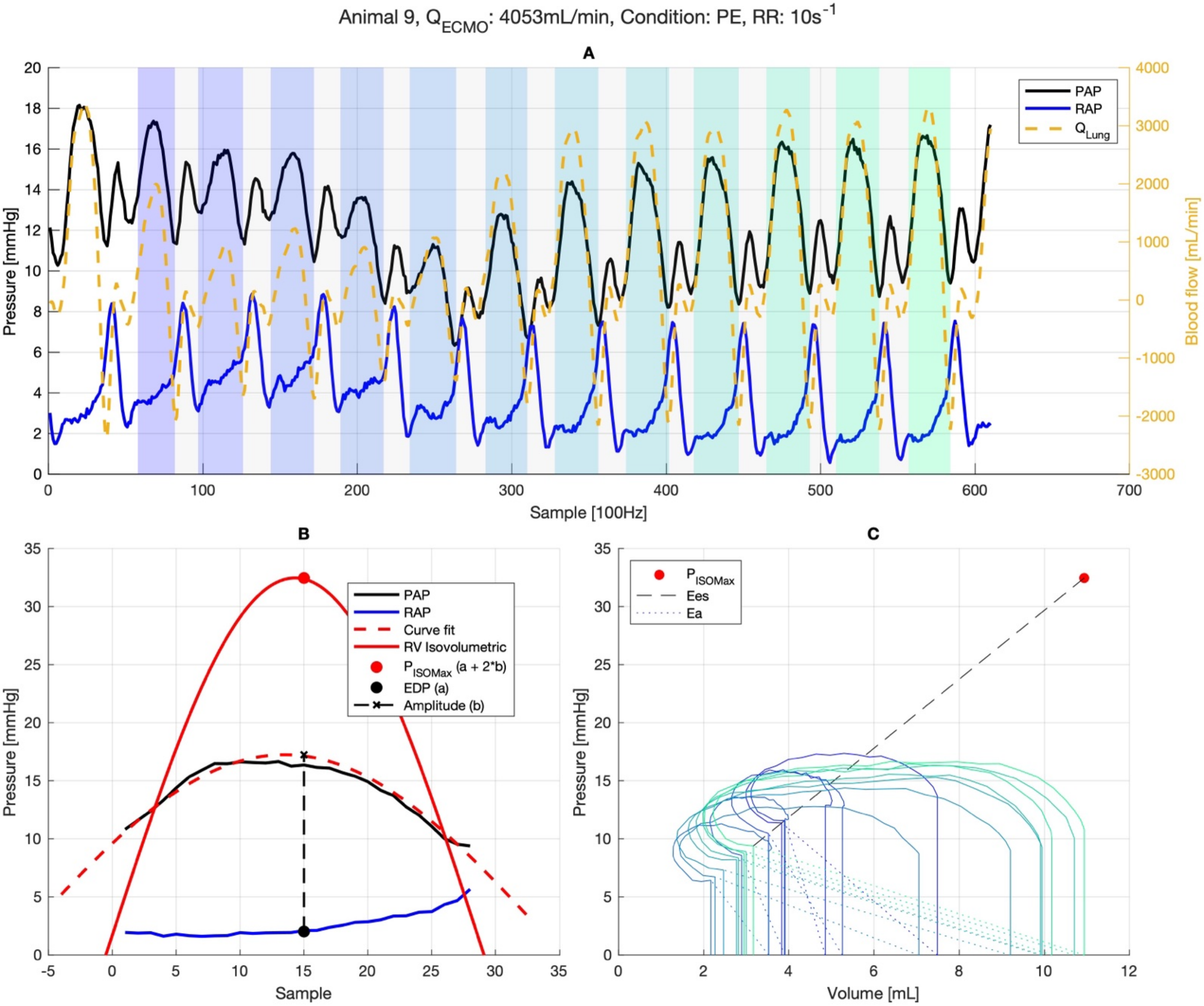
Animal 9, Q_{ECMO}: 4053mL/min, Condition: PE, RR: $10s^{-1}$
A
PAP
RAP
Q_{Lung}
Pressure [mmHg]
Blood flow [mL/min]
Sample [100Hz]
B
PAP
RAP
Curve fit
RV Isovolumetric
P_{ISOMax} (a + 2*b)
EDP (a)
Amplitude (b)
Pressure [mmHg]
Sample
C
P_{ISOMax}
Ees
Ea
Pressure [mmHg]
Volume [mL]

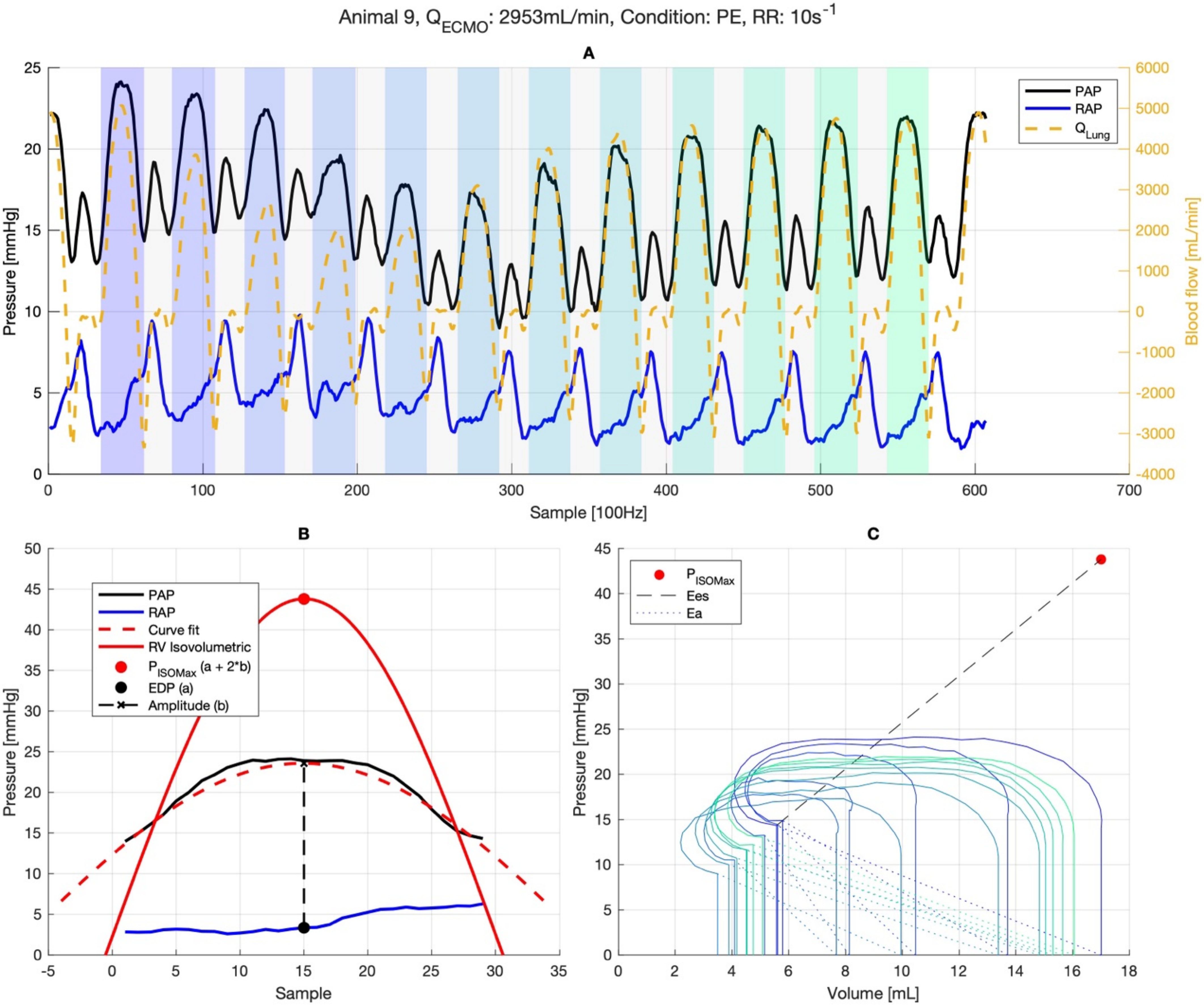

Animal 9, Q_{ECMO}: 2953mL/min, Condition: PE, RR: $10s^{-1}$
A
PAP
RAP
Q_{Lung}
Pressure [mmHg]
Blood flow [mL/min]
Sample [100Hz]
B
PAP
RAP
Curve fit
RV Isovolumetric
P_{ISOMax} (a + 2*b)
EDP (a)
Amplitude (b)
Pressure [mmHg]
Sample
C
P_{ISOMax}
Ees
Ea
Pressure [mmHg]
Volume [mL]

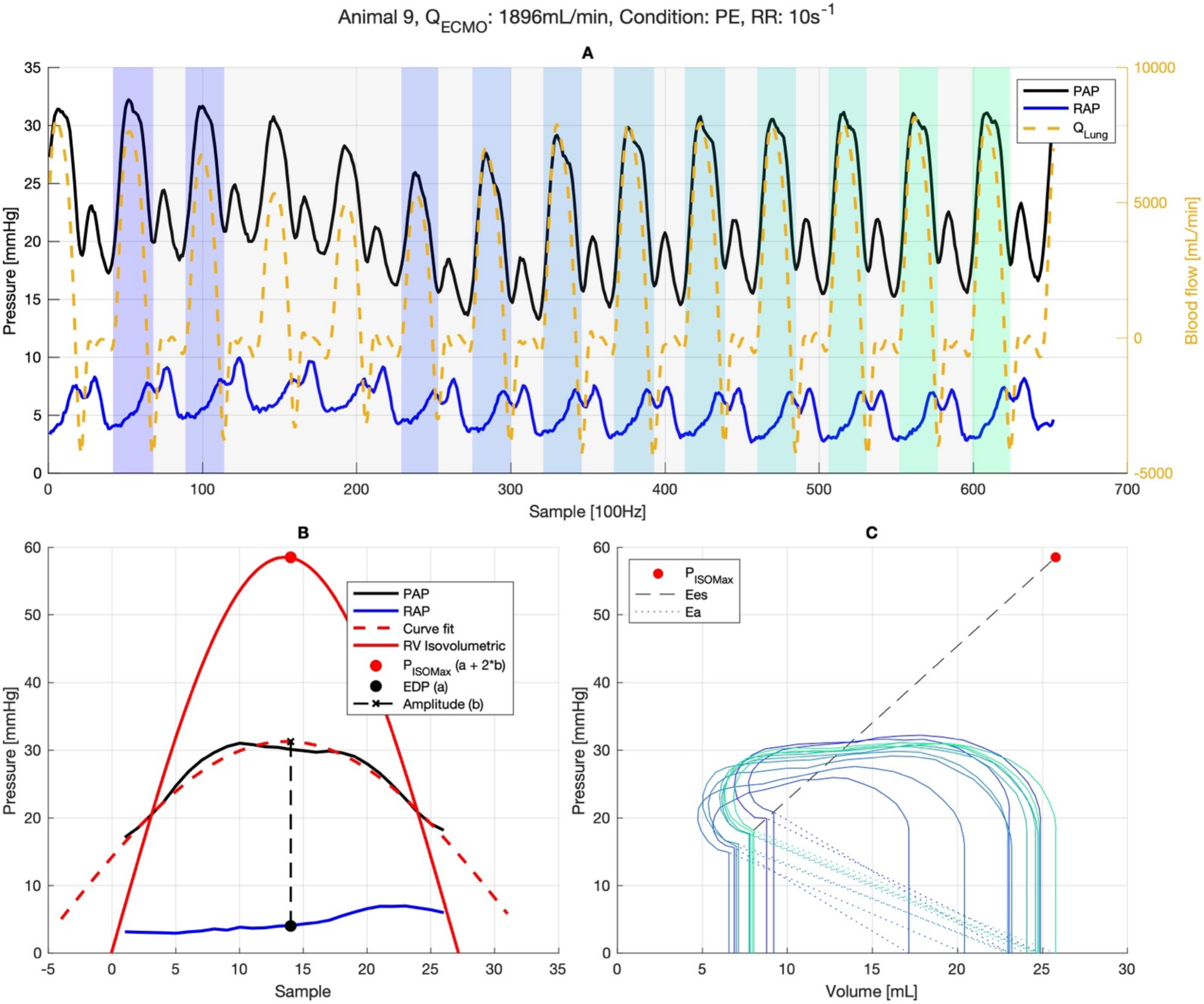

Animal 9, Q_{ECMO}: 1896mL/min, Condition: PE, RR: $10s^{-1}$
A
PAP
RAP
Q_{Lung}
Pressure [mmHg]
Blood flow [mL/min]
Sample [100Hz]
B
PAP
RAP
Curve fit
RV Isovolumetric
P_{ISOMax} (a + 2*b)
EDP (a)
Amplitude (b)
Pressure [mmHg]
Sample
C
P_{ISOMax}
Ees
Ea
Pressure [mmHg]
Volume [mL]

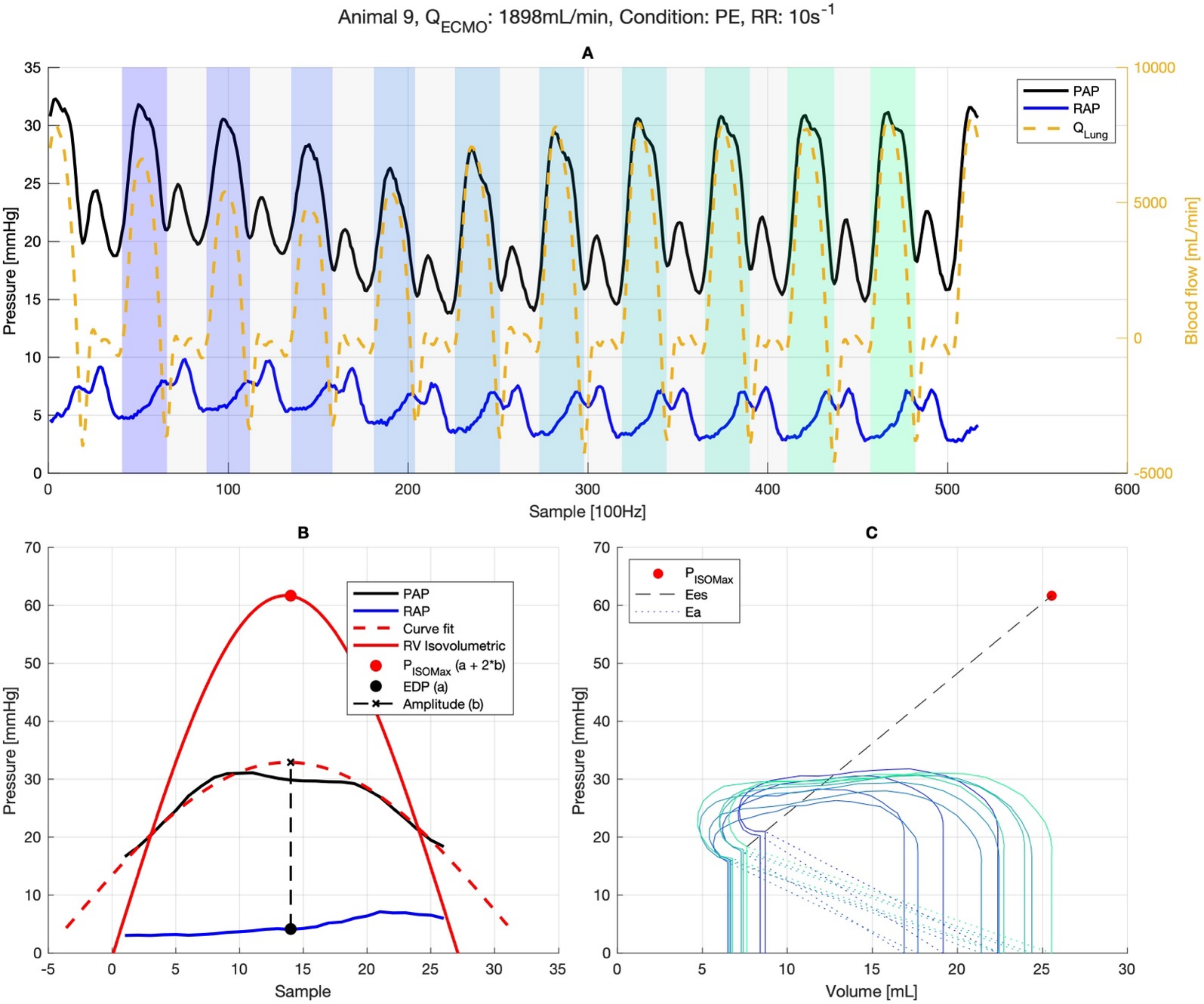

Animal 9, Q_{ECMO}: 1898mL/min, Condition: PE, RR: 10s^{-1}
A
PAP
RAP
Q_{Lung}
Pressure [mmHg]
Blood flow [mL/min]
Sample [100Hz]
B
PAP
RAP
Curve fit
RV Isovolumetric
P_{ISOMax} (a + 2*b)
EDP (a)
Amplitude (b)
Pressure [mmHg]
Sample
C
P_{ISOMax}
Ees
Ea
Pressure [mmHg]
Volume [mL]

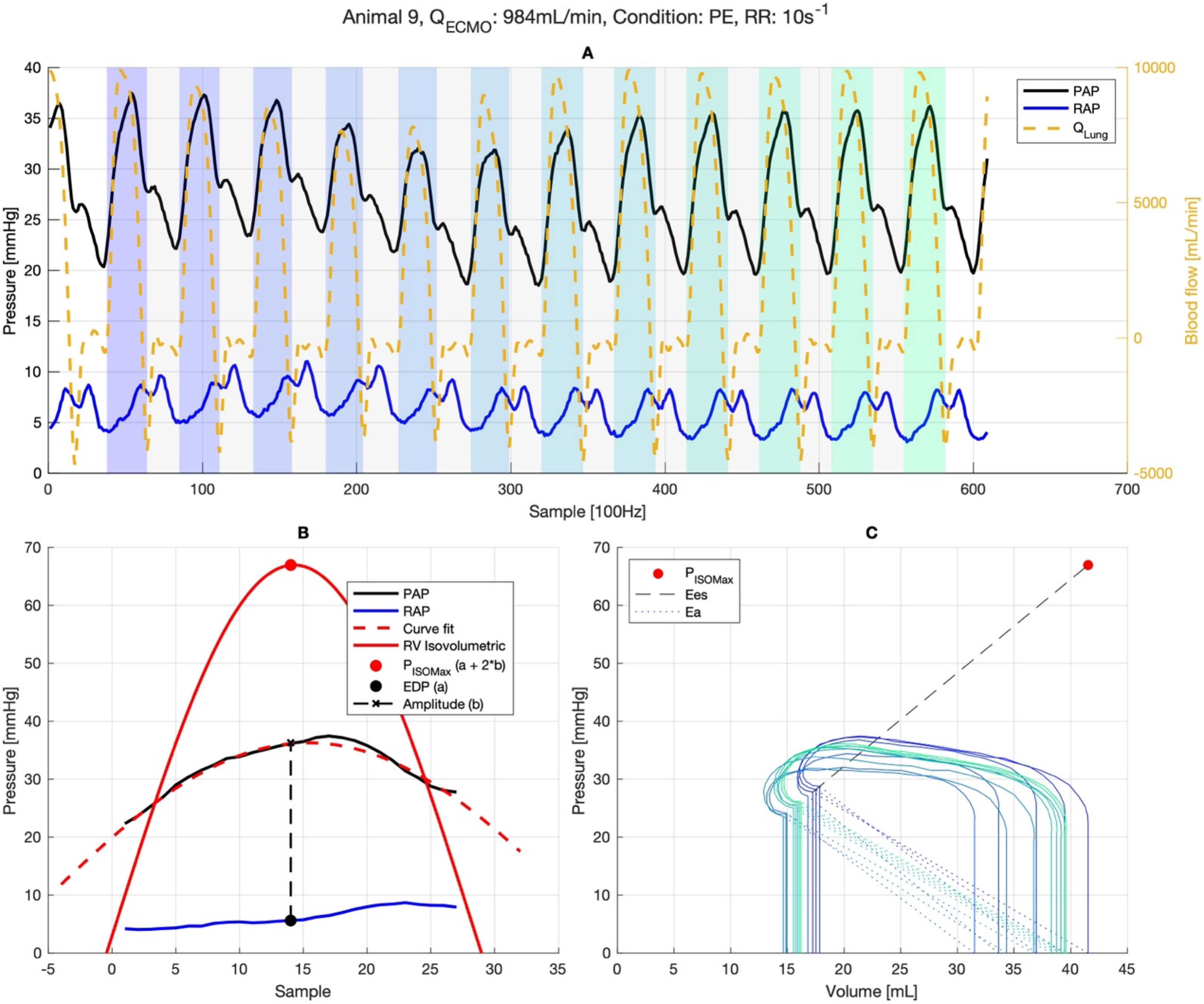

Animal 9, Q_ECMO: 984mL/min, Condition: PE, RR: 10s^-1
A
PAP
RAP
Q_Lung
Pressure [mmHg]
Blood flow [mL/min]
Sample [100Hz]
B
PAP
RAP
Curve fit
RV Isovolumetric
P_ISOMax (a + 2*b)
EDP (a)
Amplitude (b)
Pressure [mmHg]
Sample
C
P_ISOMax
Ees
Ea
Pressure [mmHg]
Volume [mL]

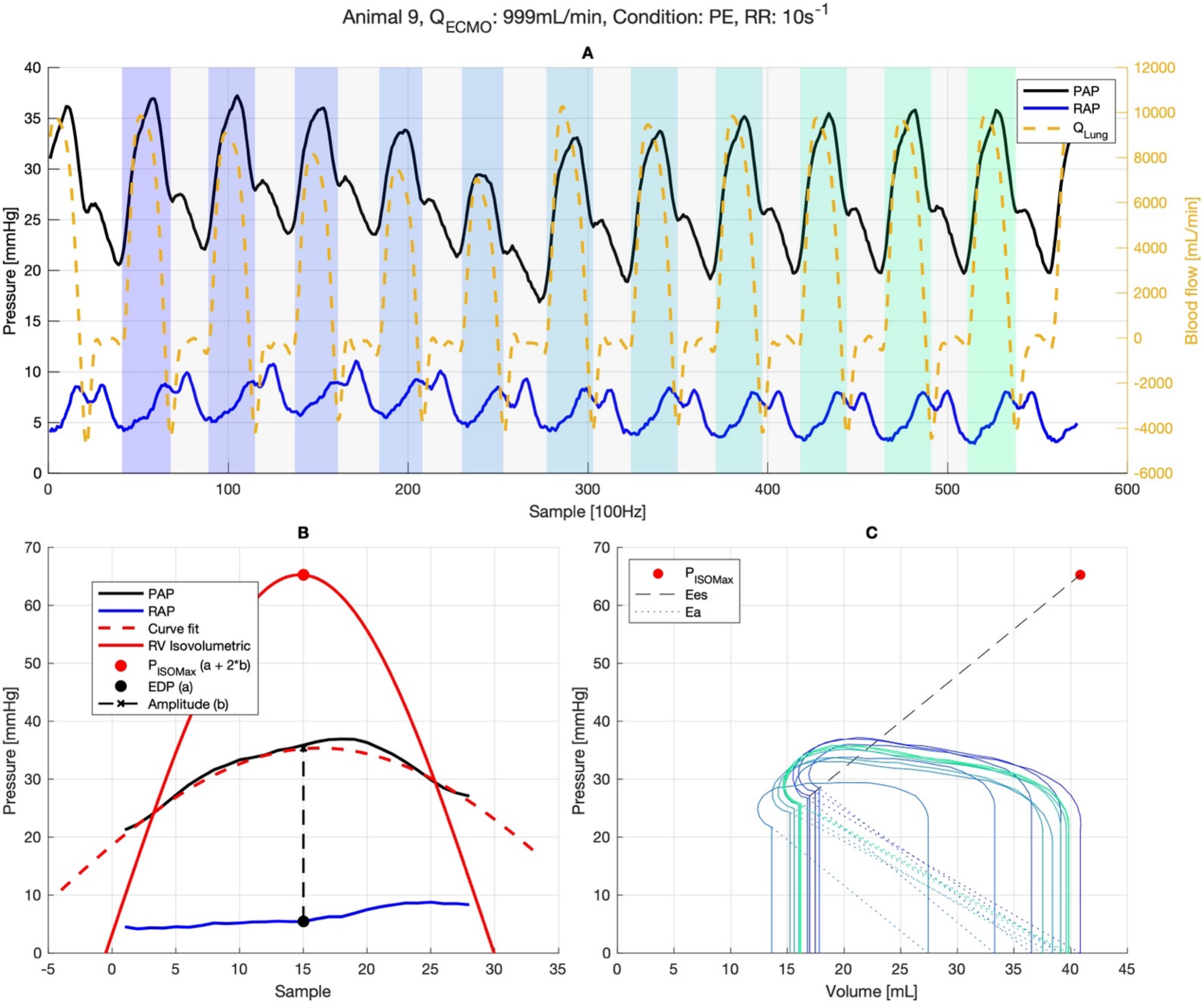

Animal 9, Q_{ECMO}: 999mL/min, Condition: PE, RR: $10s^{-1}$
A
PAP
RAP
Q_{Lung}
Pressure [mmHg]
Blood flow [mL/min]
Sample [100Hz]
B
PAP
RAP
Curve fit
RV Isovolumetric
P_{ISOMax} (a + 2*b)
EDP (a)
Amplitude (b)
Pressure [mmHg]
Sample
C
P_{ISOMax}
Ees
Ea
Pressure [mmHg]
Volume [mL]

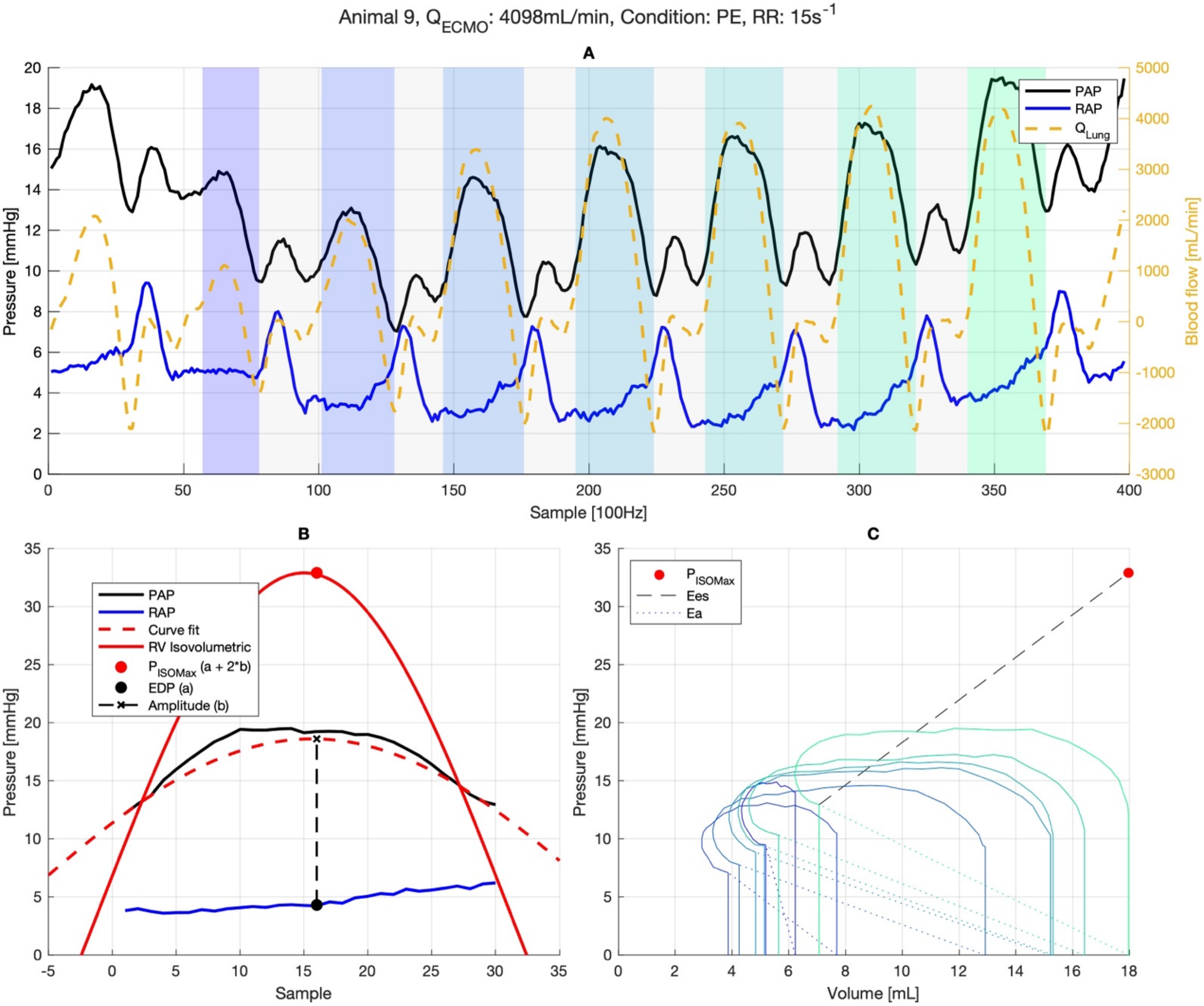

Animal 9, Q_{ECMO}: 4098mL/min, Condition: PE, RR: $15s^{-1}$
A
PAP
RAP
Q_{Lung}
Pressure [mmHg]
Blood flow [mL/min]
Sample [100Hz]
B
PAP
RAP
Curve fit
RV Isovolumetric
P_{ISOMax} (a + 2*b)
EDP (a)
Amplitude (b)
Pressure [mmHg]
Sample
C
P_{ISOMax}
Ees
Ea
Pressure [mmHg]
Volume [mL]

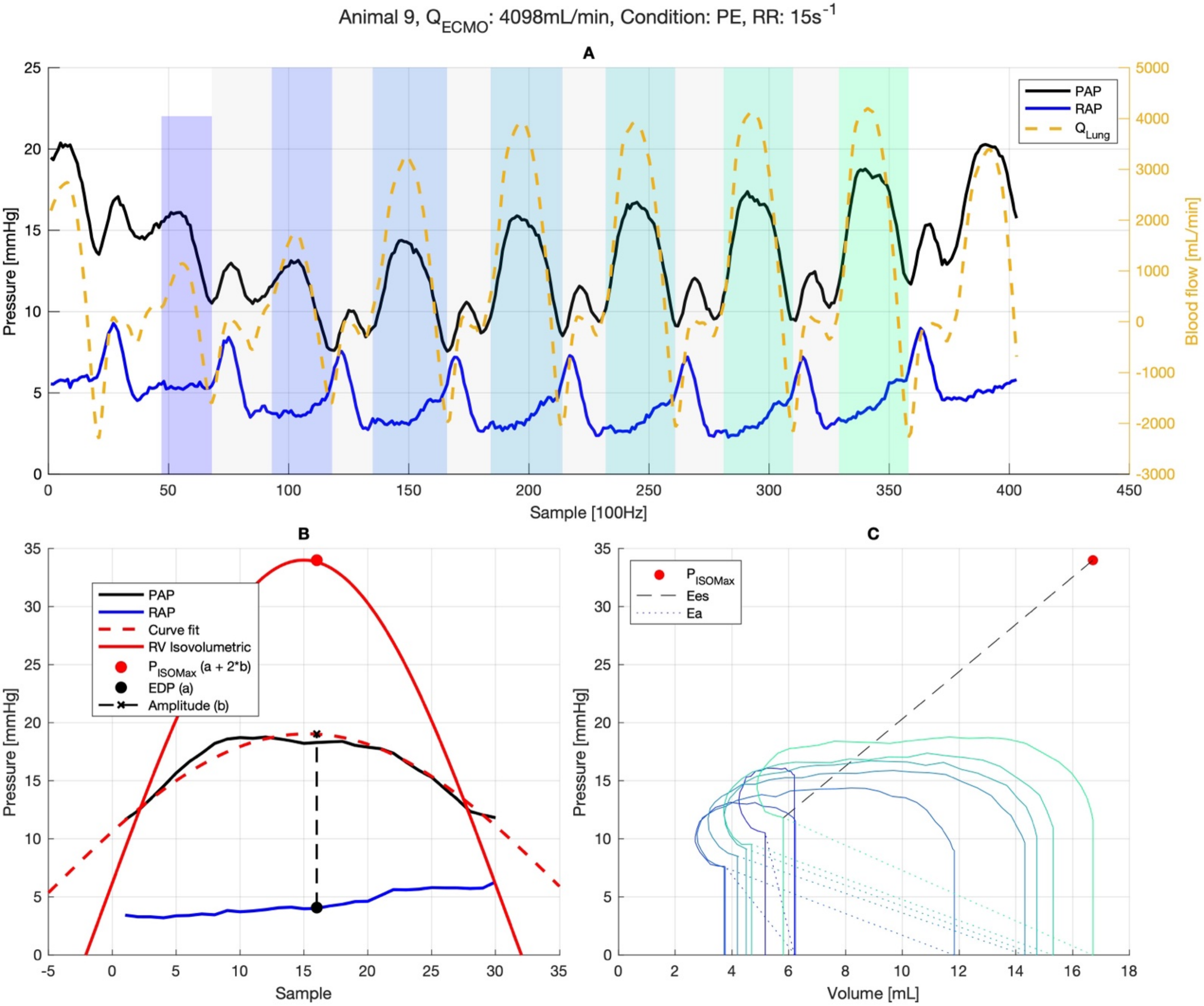

Animal 9, Q_{ECMO}: 4098mL/min, Condition: PE, RR: 15s^{-1}
A
Pressure [mmHg]
Blood flow [mL/min]
Sample [100Hz]
PAP
RAP
Q_{Lung}
B
Pressure [mmHg]
Sample
Curve fit
RV Isovolumetric
P_{ISOMax} (a + 2*b)
EDP (a)
Amplitude (b)
C
Pressure [mmHg]
Volume [mL]
Ees
Ea

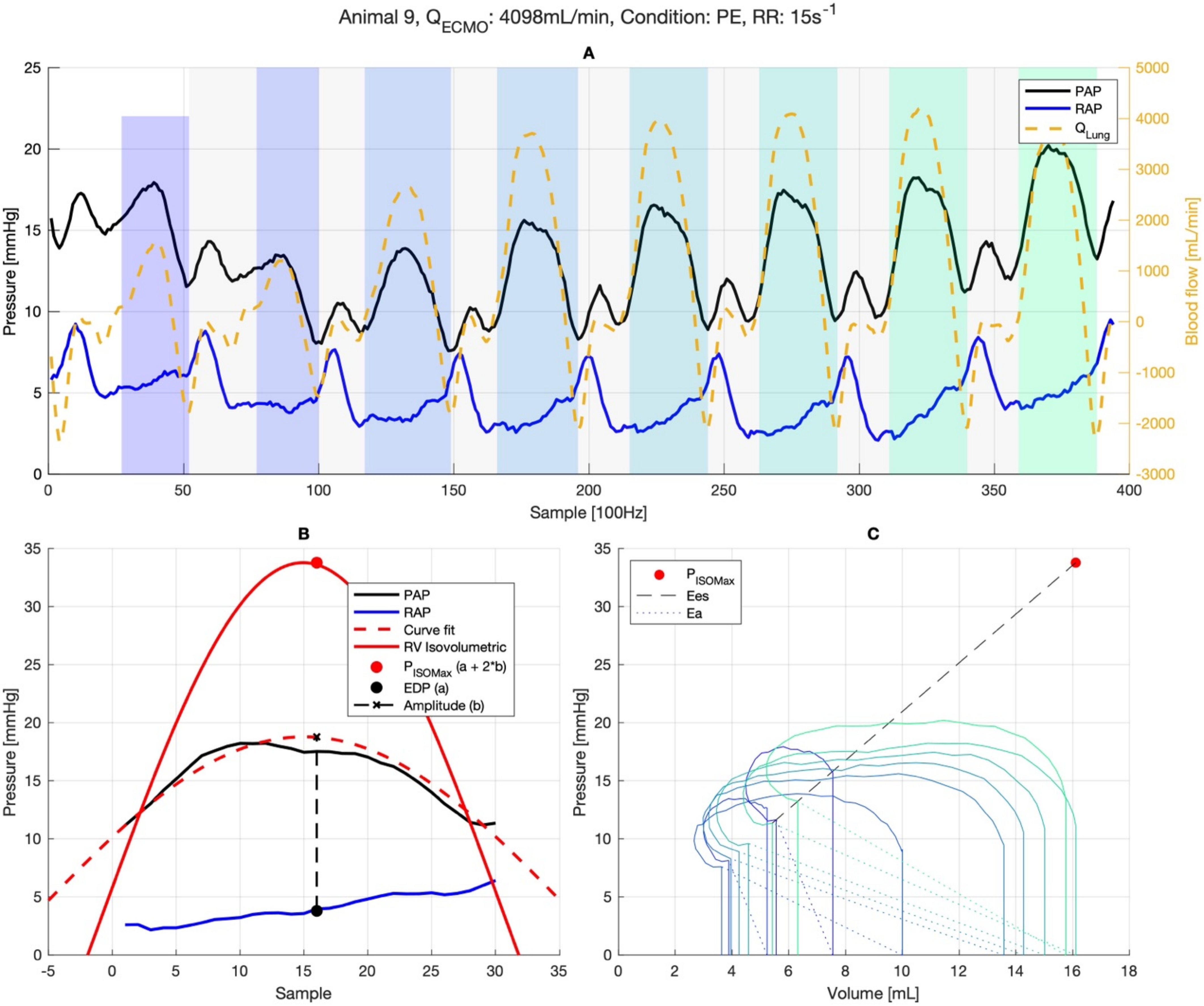

Animal 9, Q_{ECMO}: 4098mL/min, Condition: PE, RR: $15s^{-1}$
A
PAP
RAP
Q_{Lung}
Pressure [mmHg]
Blood flow [mL/min]
Sample [100Hz]
B
PAP
RAP
Curve fit
RV Isovolumetric
P_{ISOMax} (a + 2*b)
EDP (a)
Amplitude (b)
Pressure [mmHg]
Sample
C
P_{ISOMax}
Ees
Ea
Pressure [mmHg]
Volume [mL]

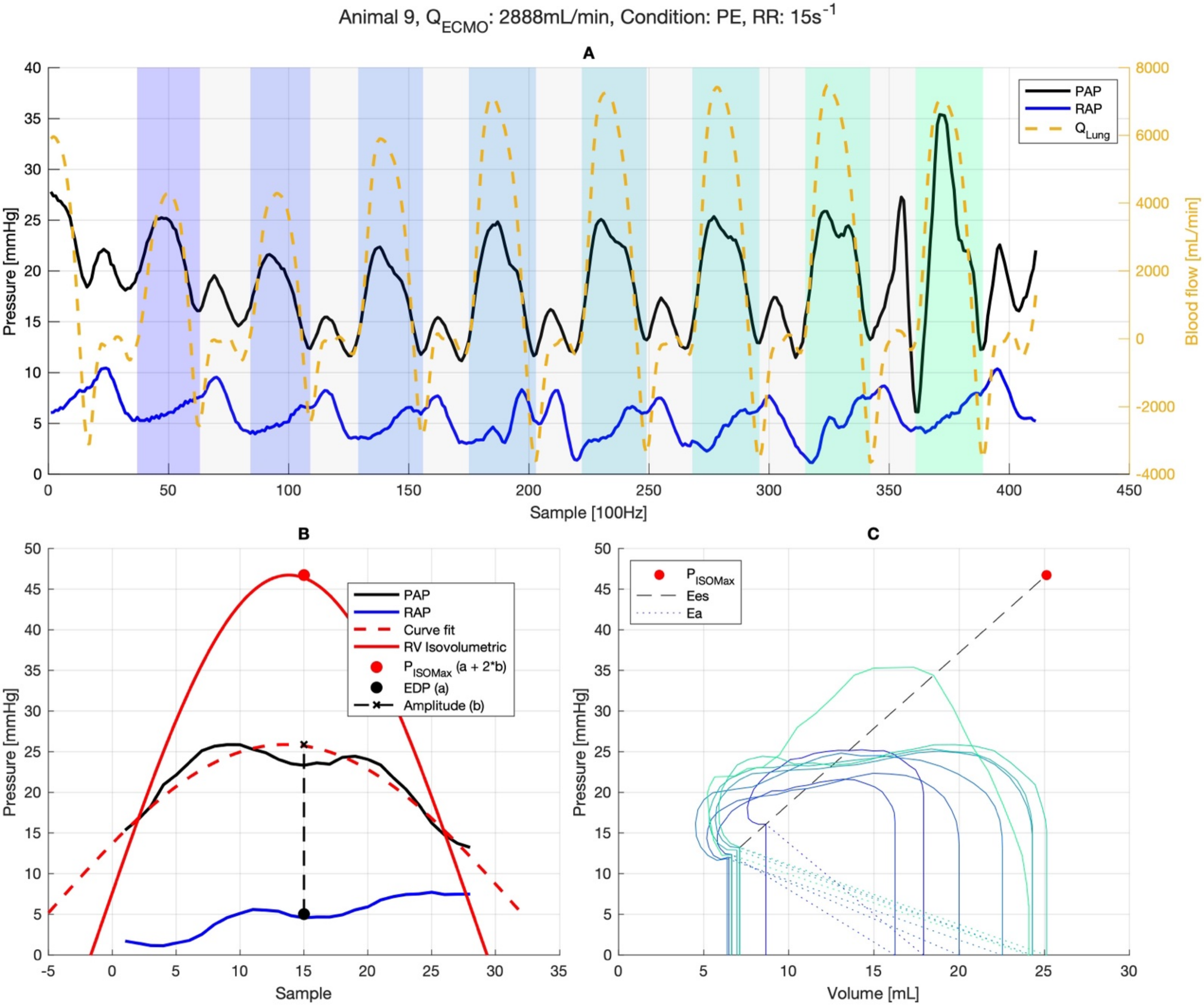

Animal 9, Q_{ECMO}: 2888mL/min, Condition: PE, RR: $15s^{-1}$
A
PAP
RAP
Q_{Lung}
Pressure [mmHg]
Blood flow [mL/min]
Sample [100Hz]
B
PAP
RAP
Curve fit
RV Isovolumetric
P_{ISOMax} (a + 2*b)
EDP (a)
Amplitude (b)
Pressure [mmHg]
Sample
C
P_{ISOMax}
Ees
Ea
Pressure [mmHg]
Volume [mL]

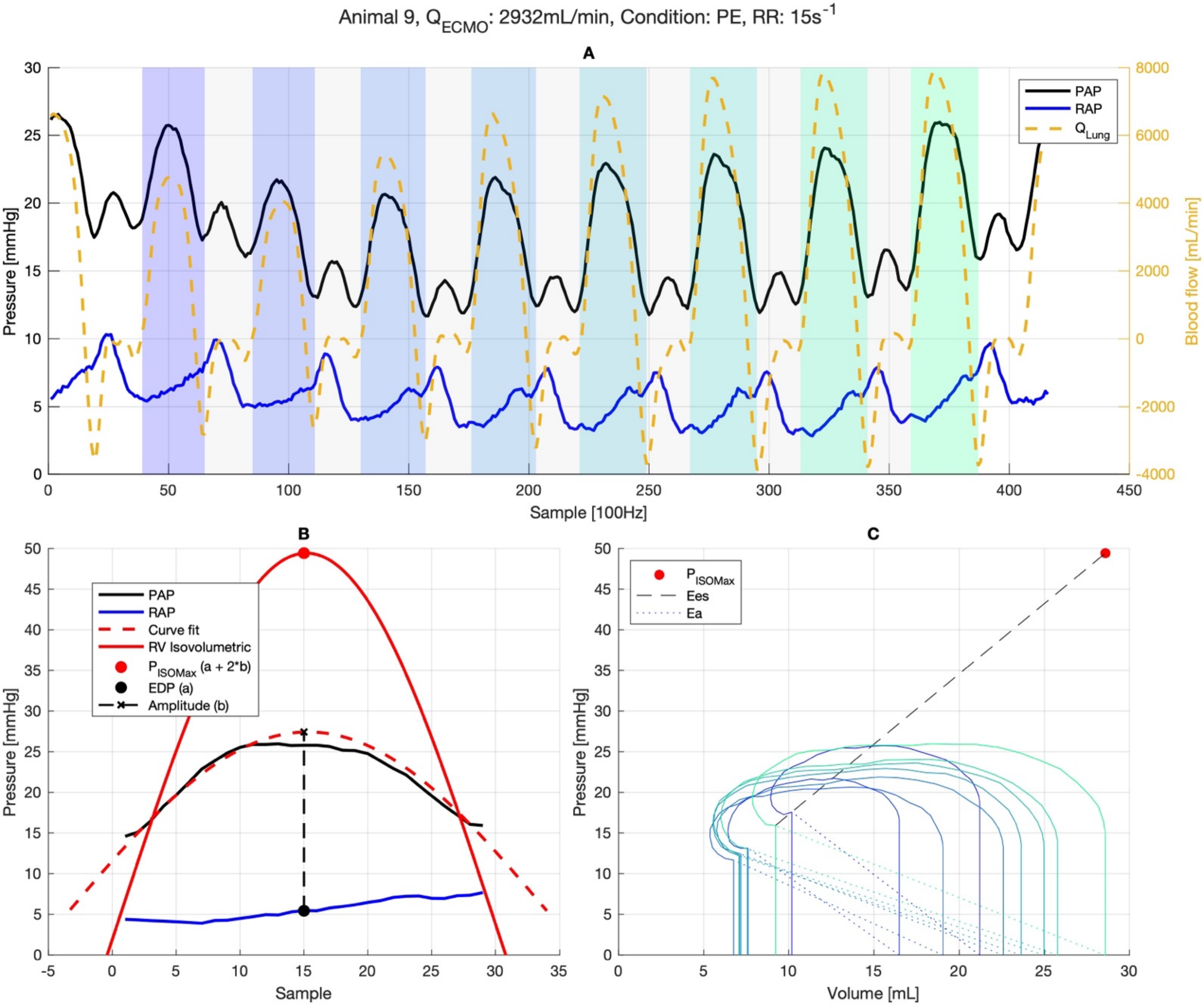

Animal 9, Q_{ECMO}: 2932mL/min, Condition: PE, RR: $15s^{-1}$
A
PAP
RAP
Q_{Lung}
Pressure [mmHg]
Blood flow [mL/min]
Sample [100Hz]
B
PAP
RAP
Curve fit
RV Isovolumetric
P_{ISOMax} (a + 2*b)
EDP (a)
Amplitude (b)
Pressure [mmHg]
Sample
C
P_{ISOMax}
Ees
Ea
Pressure [mmHg]
Volume [mL]

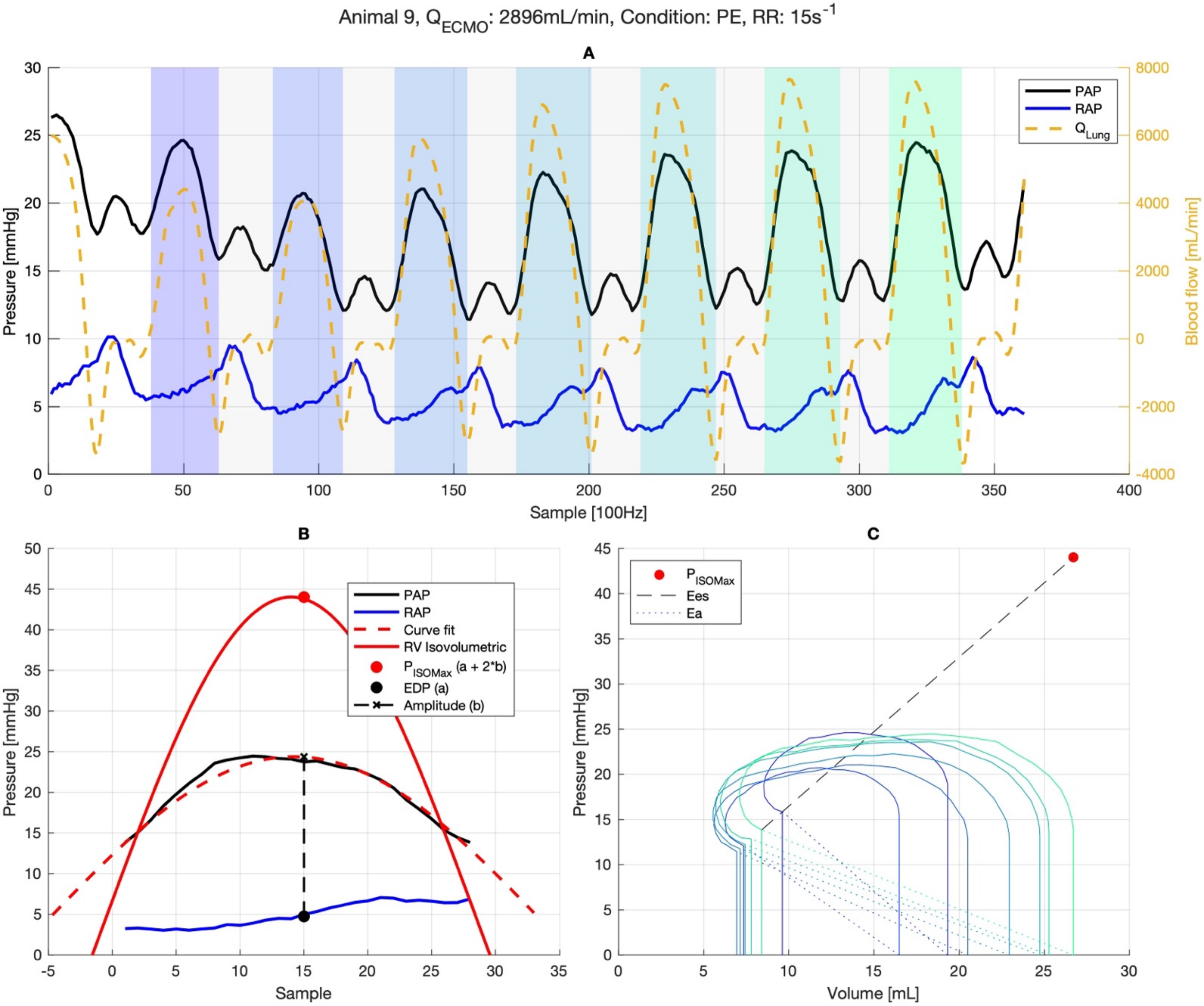
Animal 9, Q_{ECMO}: 2896mL/min, Condition: PE, RR: $15s^{-1}$
A
Pressure [mmHg]
Blood flow [mL/min]
Sample [100Hz]
PAP
RAP
Q_{Lung}
B
Pressure [mmHg]
Sample
PAP
RAP
Curve fit
RV Isovolumetric
P_{ISOMax} (a + 2*b)
EDP (a)
Amplitude (b)
C
Pressure [mmHg]
Volume [mL]
P_{ISOMax}
Ees
Ea

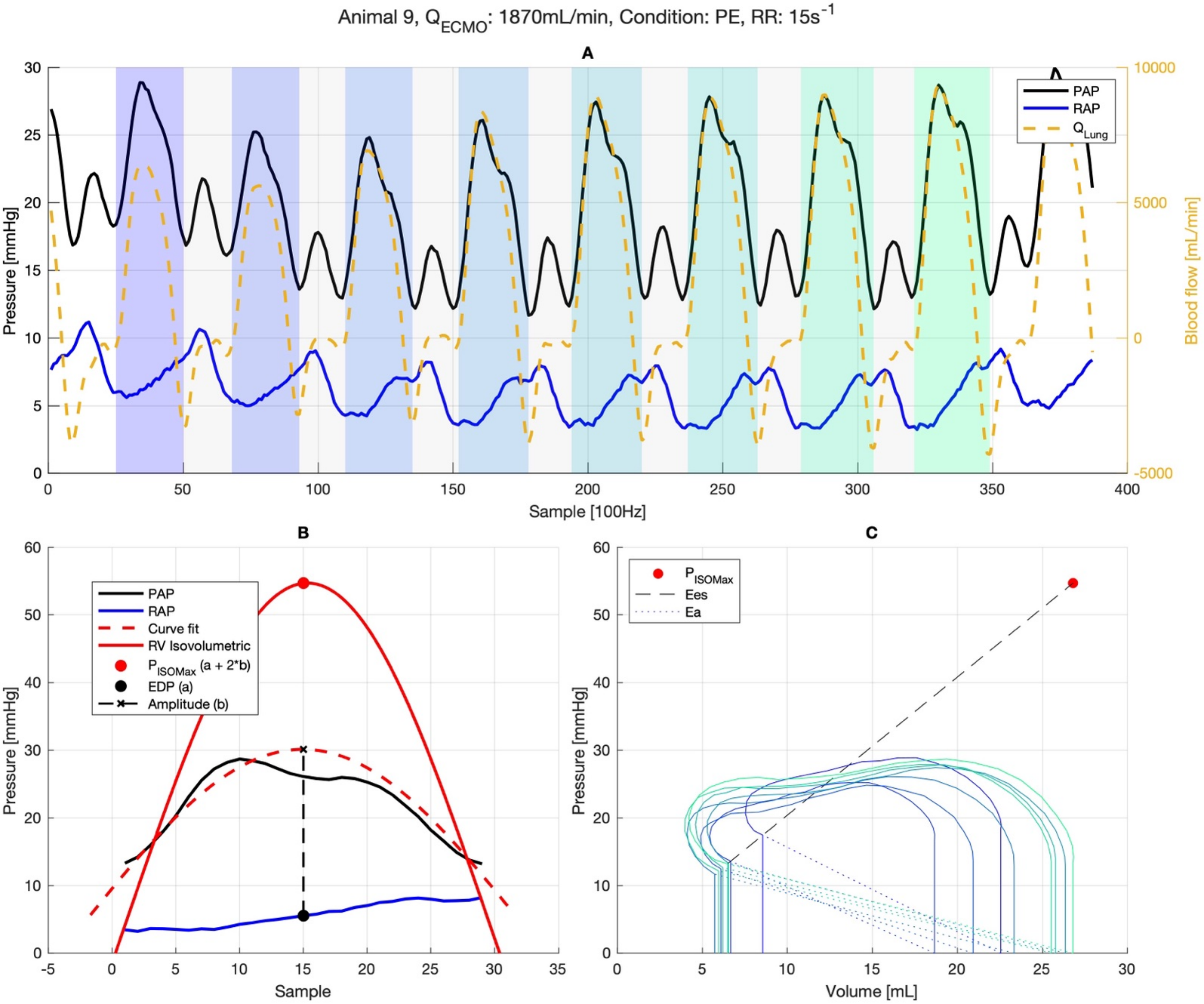
Animal 9, Q_ECMO: 1870mL/min, Condition: PE, RR: 15s^-1
A
Pressure [mmHg]
Blood flow [mL/min]
Sample [100Hz]
PAP
RAP
Q_Lung
B
PAP
RAP
Curve fit
RV Isovolumetric
P_ISOMax (a + 2*b)
EDP (a)
Amplitude (b)
Pressure [mmHg]
Sample
C
P_ISOMax
Ees
Ea
Pressure [mmHg]
Volume [mL]

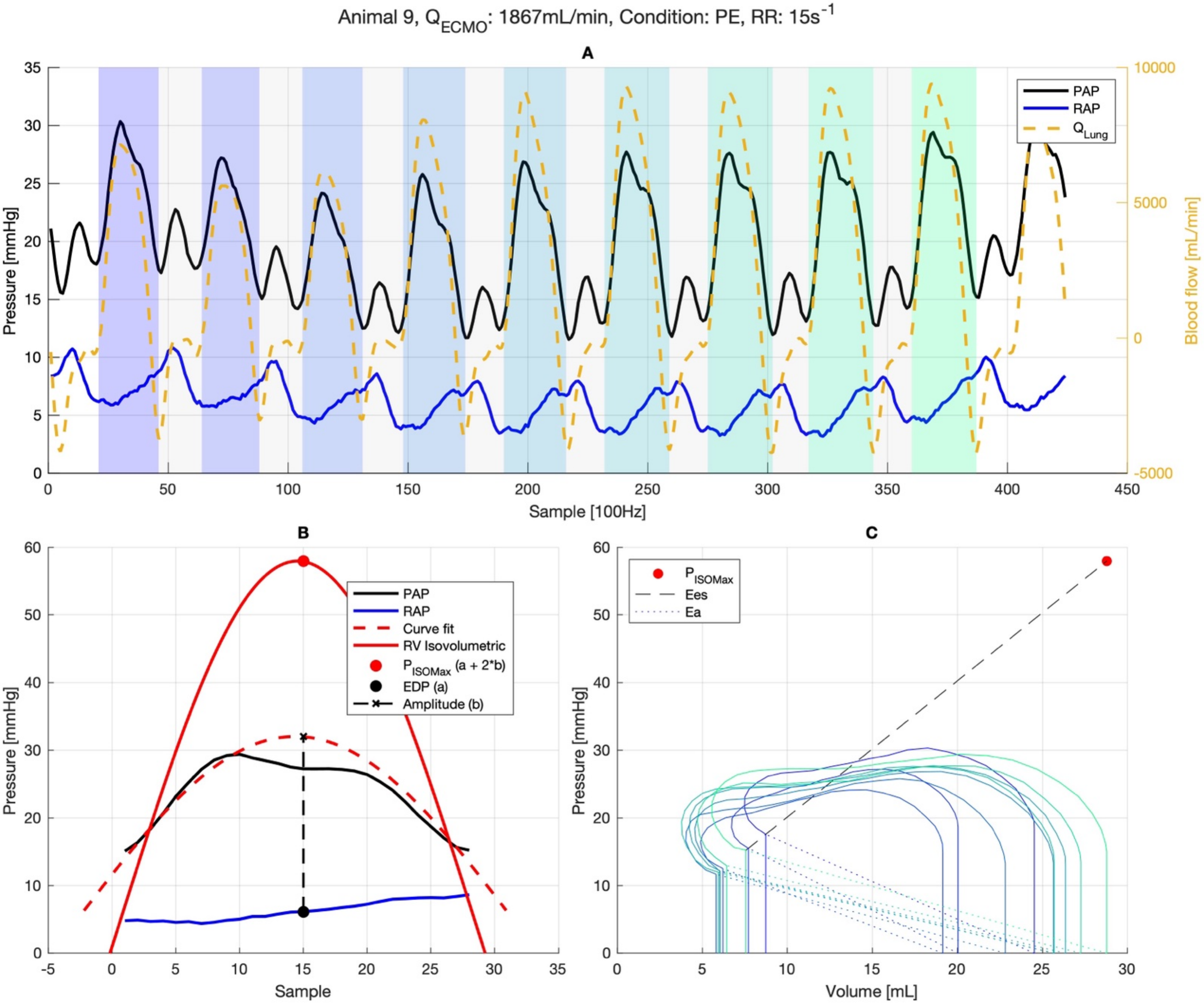

Animal 9, Q_{ECMO}: 1867mL/min, Condition: PE, RR: $15s^{-1}$
A
Pressure [mmHg]
Blood flow [mL/min]
Sample [100Hz]
PAP
RAP
Q_{Lung}
B
Pressure [mmHg]
Sample
PAP
RAP
Curve fit
RV Isovolumetric
P_{ISOMax} (a + 2*b)
EDP (a)
Amplitude (b)
C
Pressure [mmHg]
Volume [mL]
P_{ISOMax}
Ees
Ea

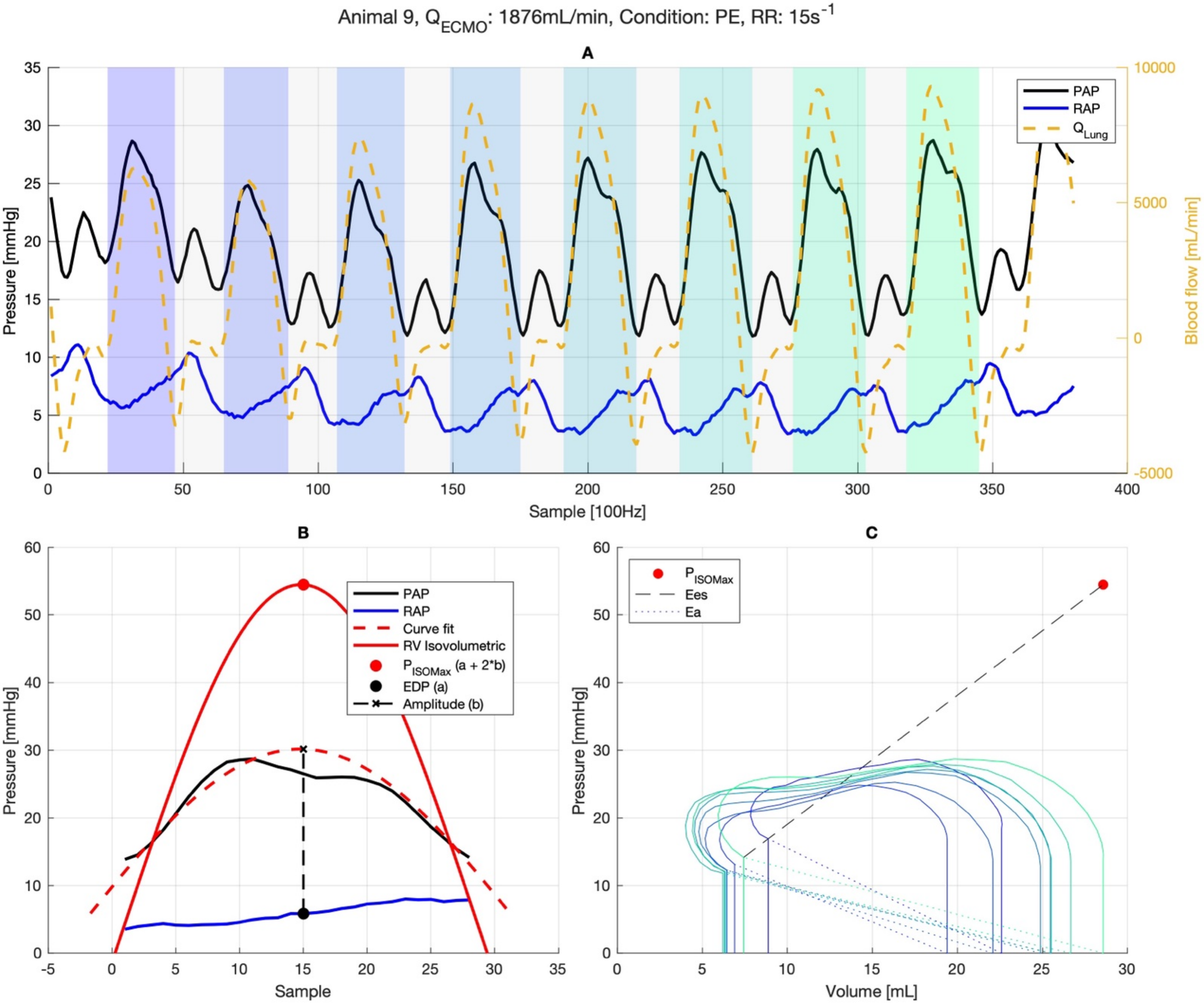

Animal 9, QECMO: 1876mL/min, Condition: PE, RR: 15s-1
A
Pressure [mmHg]
Blood flow [mL/min]
Sample [100Hz]
PAP
RAP
QLung
B
PAP
RAP
Curve fit
RV Isovolumetric
PISOMax (a + 2*b)
EDP (a)
Amplitude (b)
Pressure [mmHg]
Sample
C
PISOMax
Ees
Ea
Pressure [mmHg]
Volume [mL]

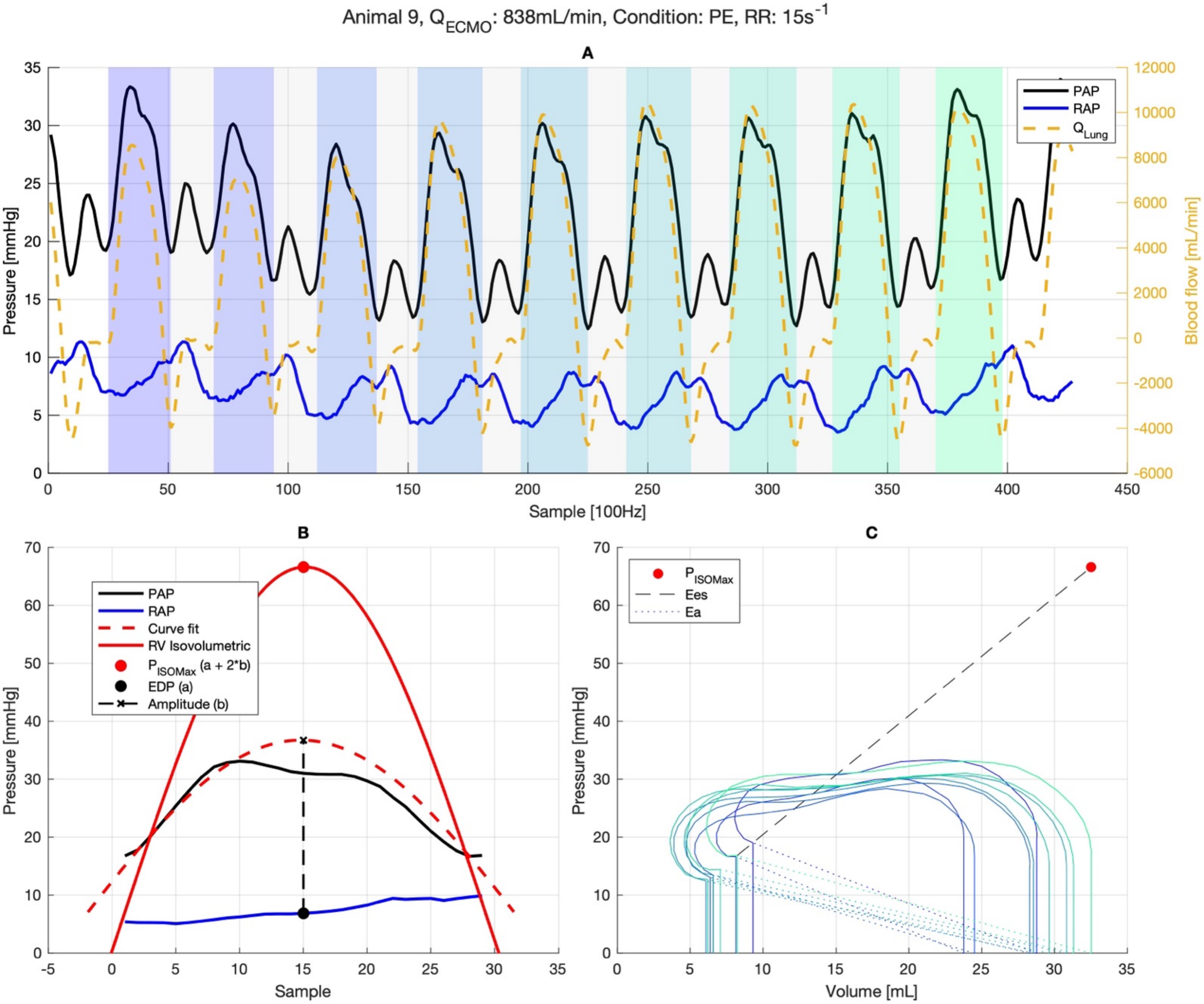

Animal 9, Q_ECMO: 838mL/min, Condition: PE, RR: 15s^-1
A
Pressure [mmHg]
Blood flow [mL/min]
Sample [100Hz]
PAP
RAP
Q_Lung
B
PAP
RAP
Curve fit
RV Isovolumetric
P_ISOMax (a + 2*b)
EDP (a)
Amplitude (b)
Pressure [mmHg]
Sample
C
P_ISOMax
Ees
Ea
Pressure [mmHg]
Volume [mL]

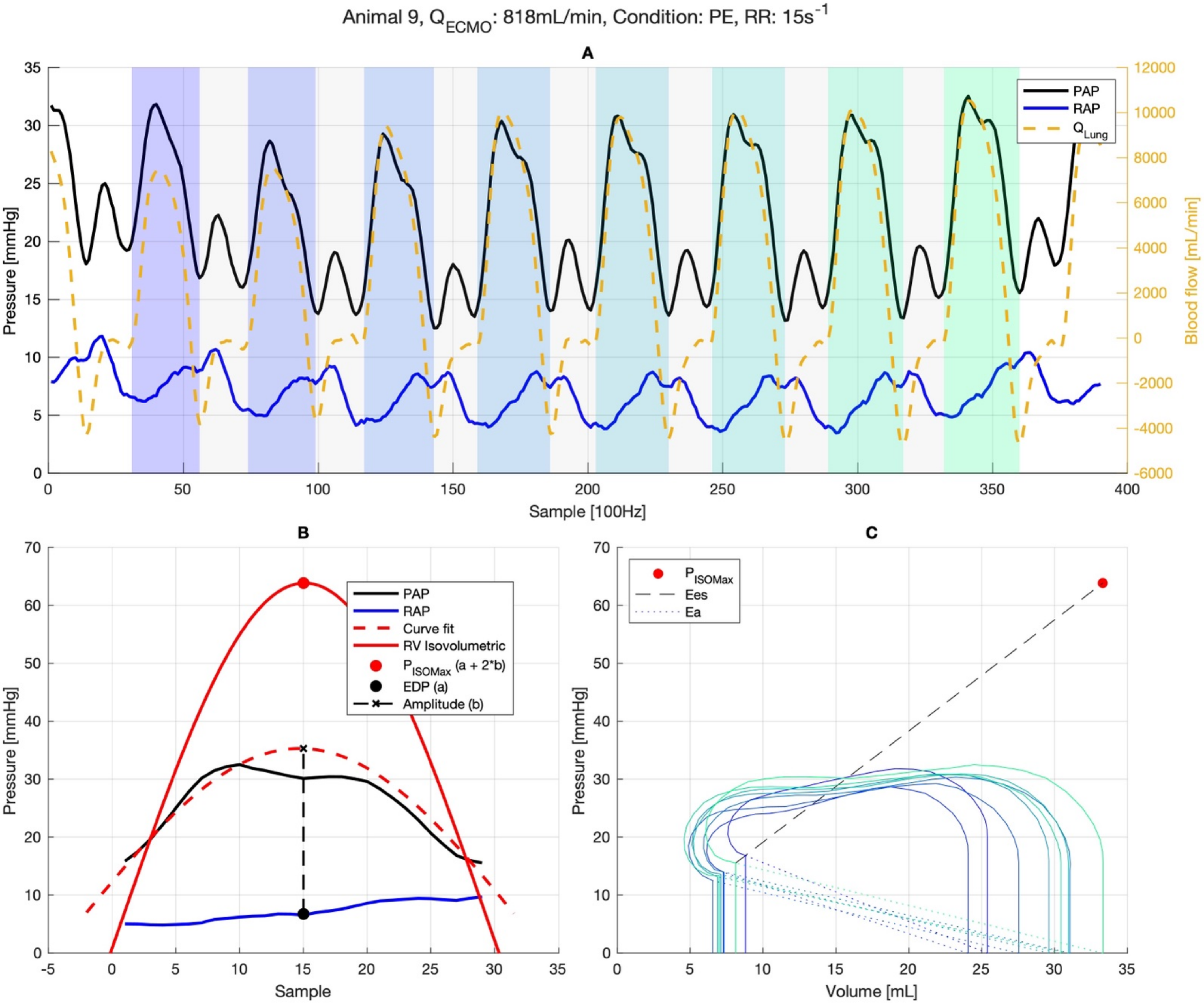

Animal 9, Q_{ECMO}: 818mL/min, Condition: PE, RR: 15s^{-1}
A
Pressure [mmHg]
Blood flow [mL/min]
Sample [100Hz]
PAP
RAP
Q_{Lung}
B
Pressure [mmHg]
Sample
PAP
RAP
Curve fit
RV Isovolumetric
P_{ISOMax} (a + 2*b)
EDP (a)
Amplitude (b)
C
Pressure [mmHg]
Volume [mL]
P_{ISOMax}
Ees
Ea

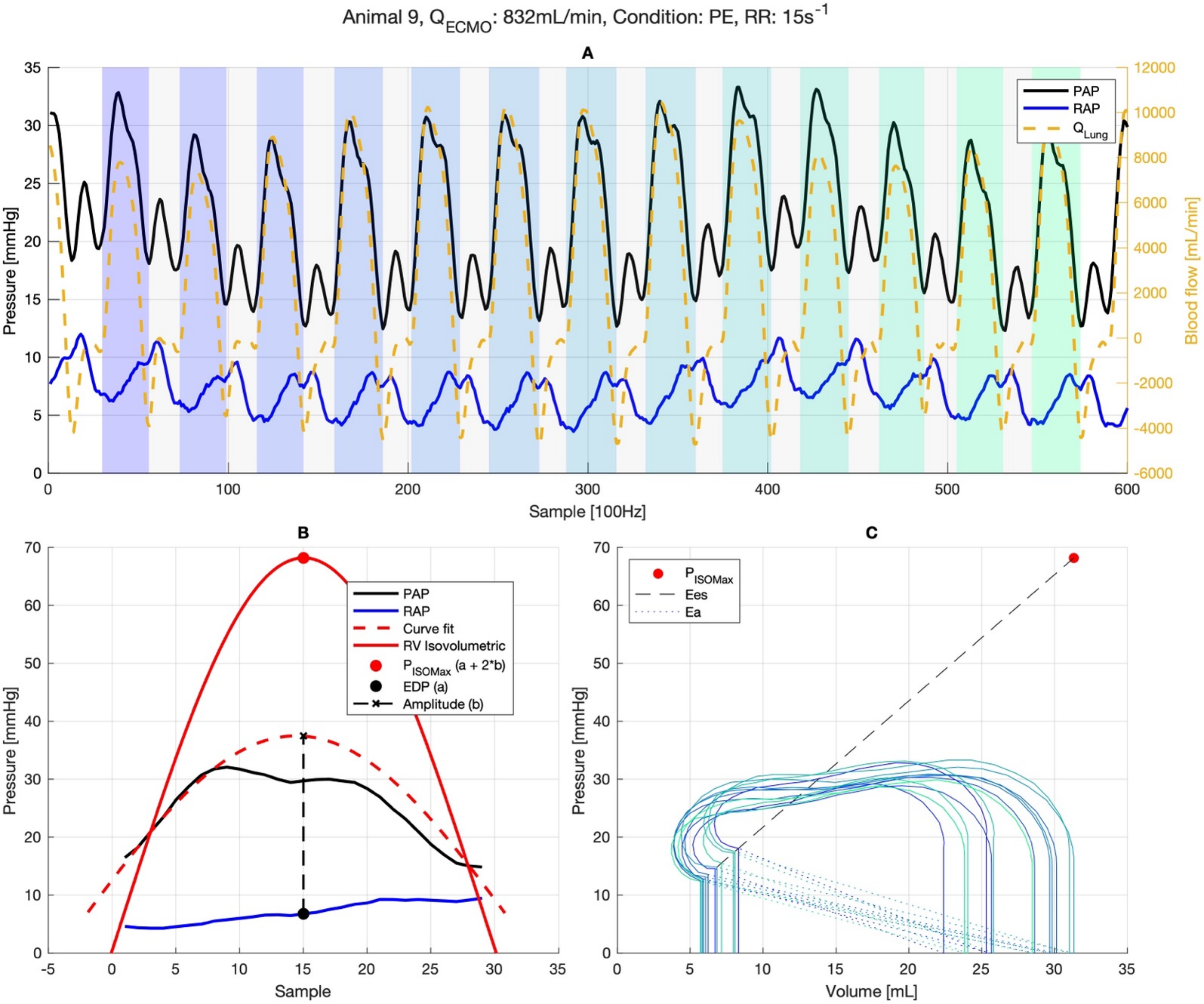

Animal 9, Q_{ECMO}: 832mL/min, Condition: PE, RR: $15s^{-1}$
A
PAP
RAP
Q_{Lung}
Pressure [mmHg]
Blood flow [mL/min]
Sample [100Hz]
B
PAP
RAP
Curve fit
RV Isovolumetric
P_{ISOMax} (a + 2*b)
EDP (a)
Amplitude (b)
Pressure [mmHg]
Sample
C
P_{ISOMax}
Ees
Ea
Pressure [mmHg]
Volume [mL]

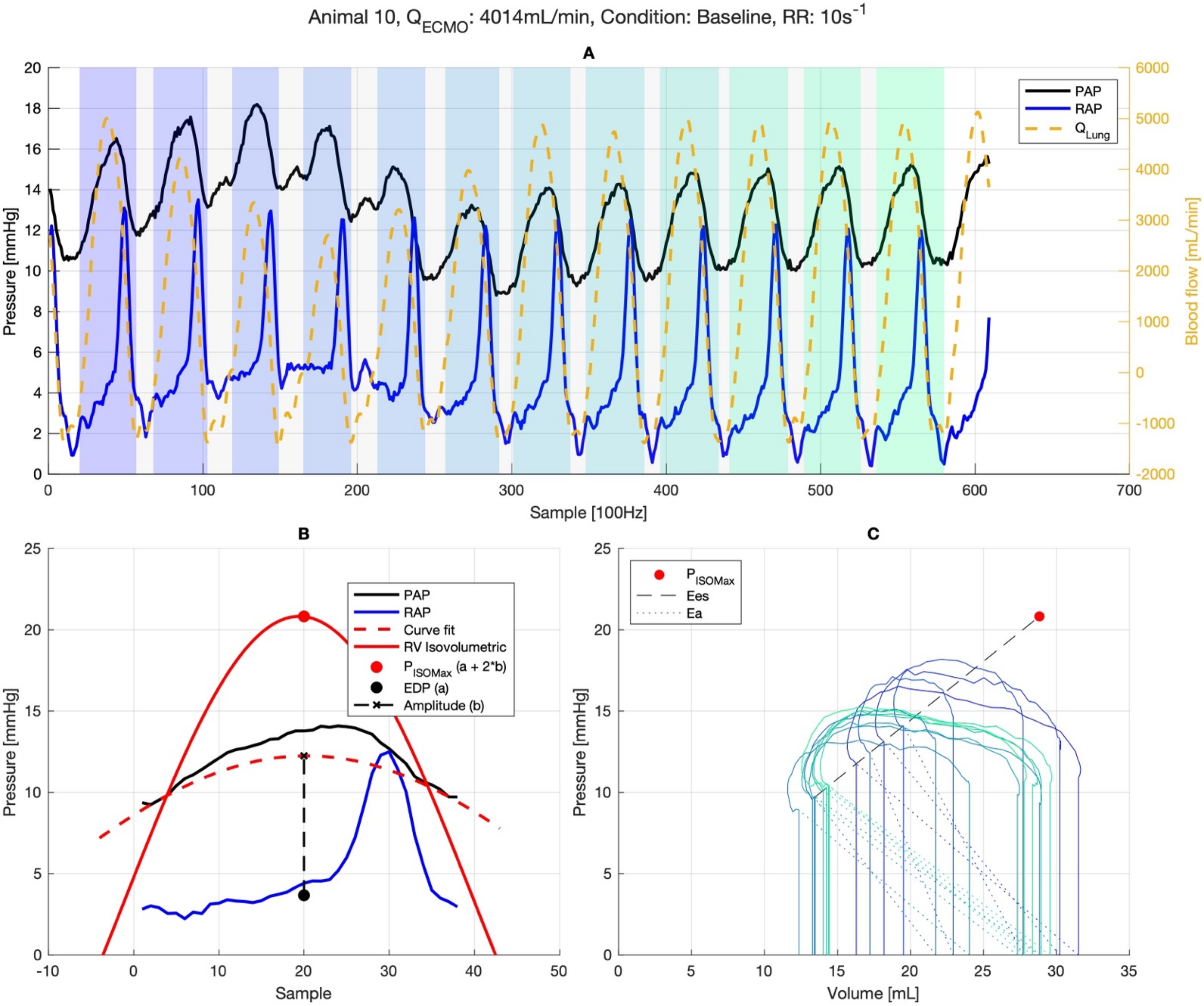

Animal 10, Q_ECMO: 4014mL/min, Condition: Baseline, RR: 10s-1
A
Pressure [mmHg]
Blood flow [mL/min]
Sample [100Hz]
PAP
RAP
Q_Lung
B
PAP
RAP
Curve fit
RV Isovolumetric
P_ISOMax (a + 2*b)
EDP (a)
Amplitude (b)
Pressure [mmHg]
Sample
C
P_ISOMax
Ees
Ea
Pressure [mmHg]
Volume [mL]

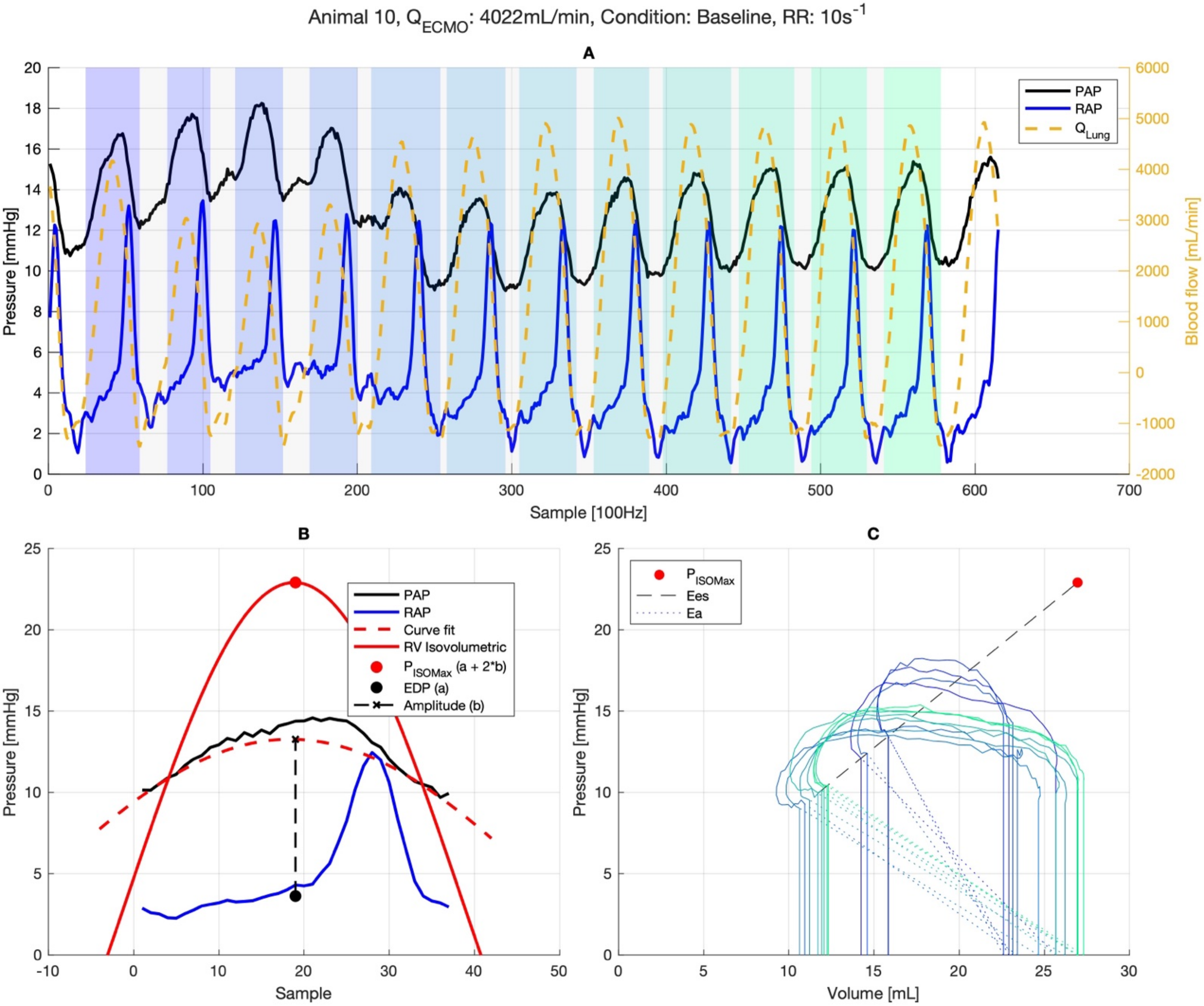

Animal 10, Q_ECMO: 4022mL/min, Condition: Baseline, RR: 10s^-1
A
PAP
RAP
Q_Lung
Pressure [mmHg]
Blood flow [mL/min]
Sample [100Hz]
B
Curve fit
RV Isovolumetric
P_ISOMax (a + 2*b)
EDP (a)
Amplitude (b)
Sample
C
P_ISOMax
Ees
Ea
Volume [mL]

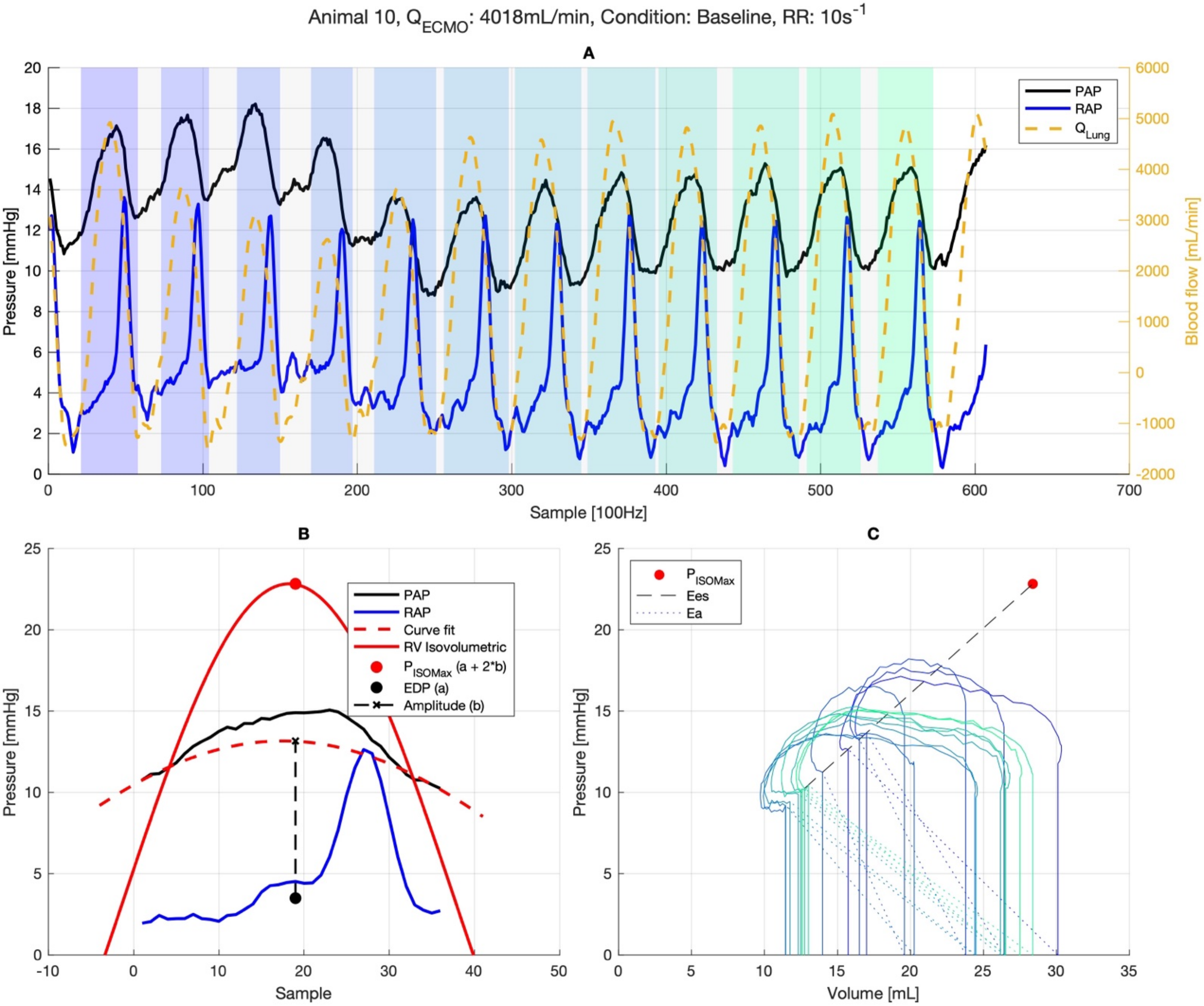

Animal 10, Q_{ECMO}: 4018mL/min, Condition: Baseline, RR: $10s^{-1}$
A
PAP
RAP
Q_{Lung}
Pressure [mmHg]
Blood flow [mL/min]
Sample [100Hz]
B
PAP
RAP
Curve fit
RV Isovolumetric
P_{ISOMax} (a + 2*b)
EDP (a)
Amplitude (b)
Pressure [mmHg]
Sample
C
P_{ISOMax}
Ees
Ea
Pressure [mmHg]
Volume [mL]

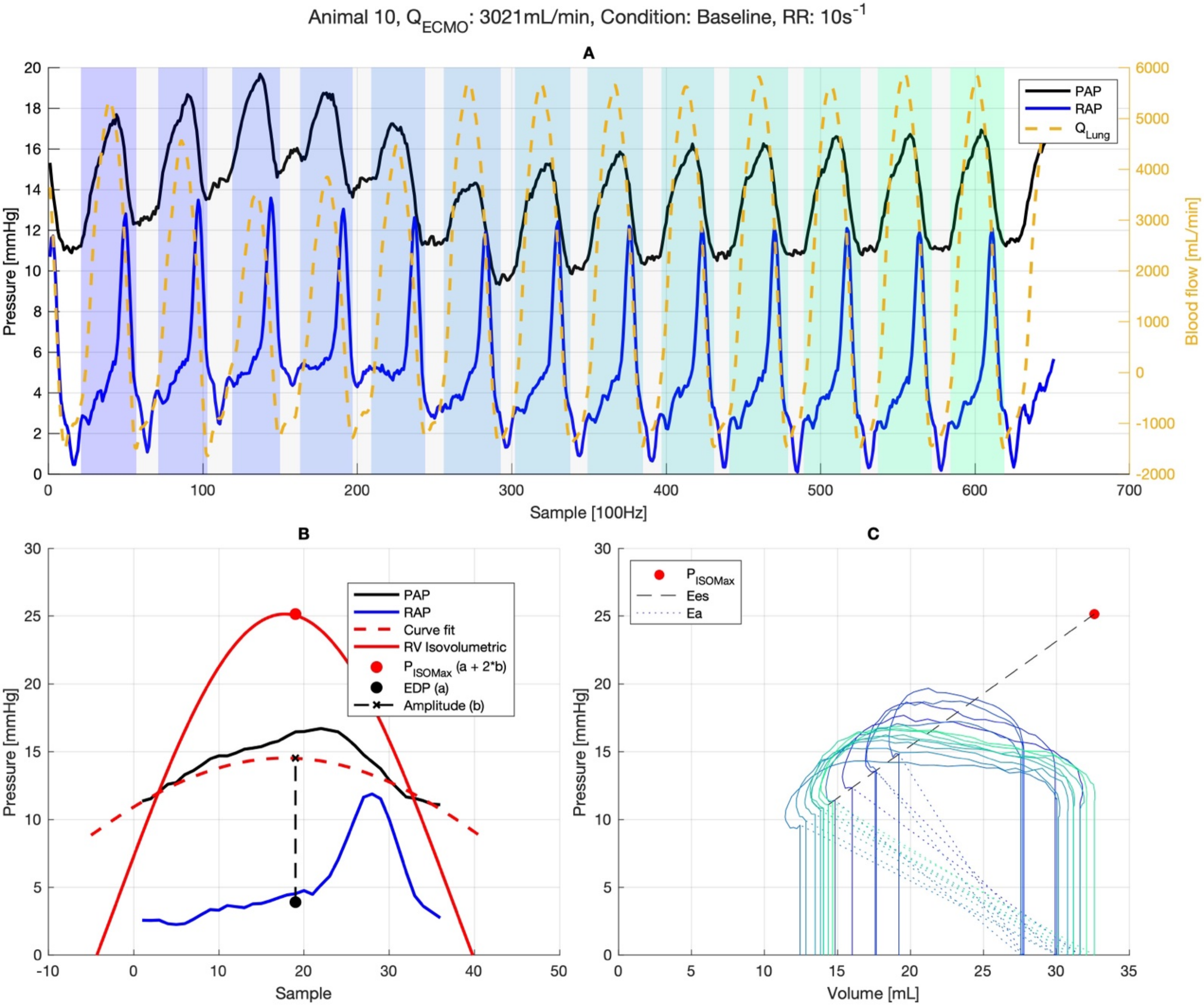

Animal 10, Q_{ECMO}: 3021mL/min, Condition: Baseline, RR: $10s^{-1}$
A
PAP
RAP
Q_{Lung}
Pressure [mmHg]
Blood flow [mL/min]
Sample [100Hz]
B
PAP
RAP
Curve fit
RV Isovolumetric
P_{ISOMax} (a + 2*b)
EDP (a)
Amplitude (b)
Pressure [mmHg]
Sample
C
P_{ISOMax}
Ees
Ea
Pressure [mmHg]
Volume [mL]

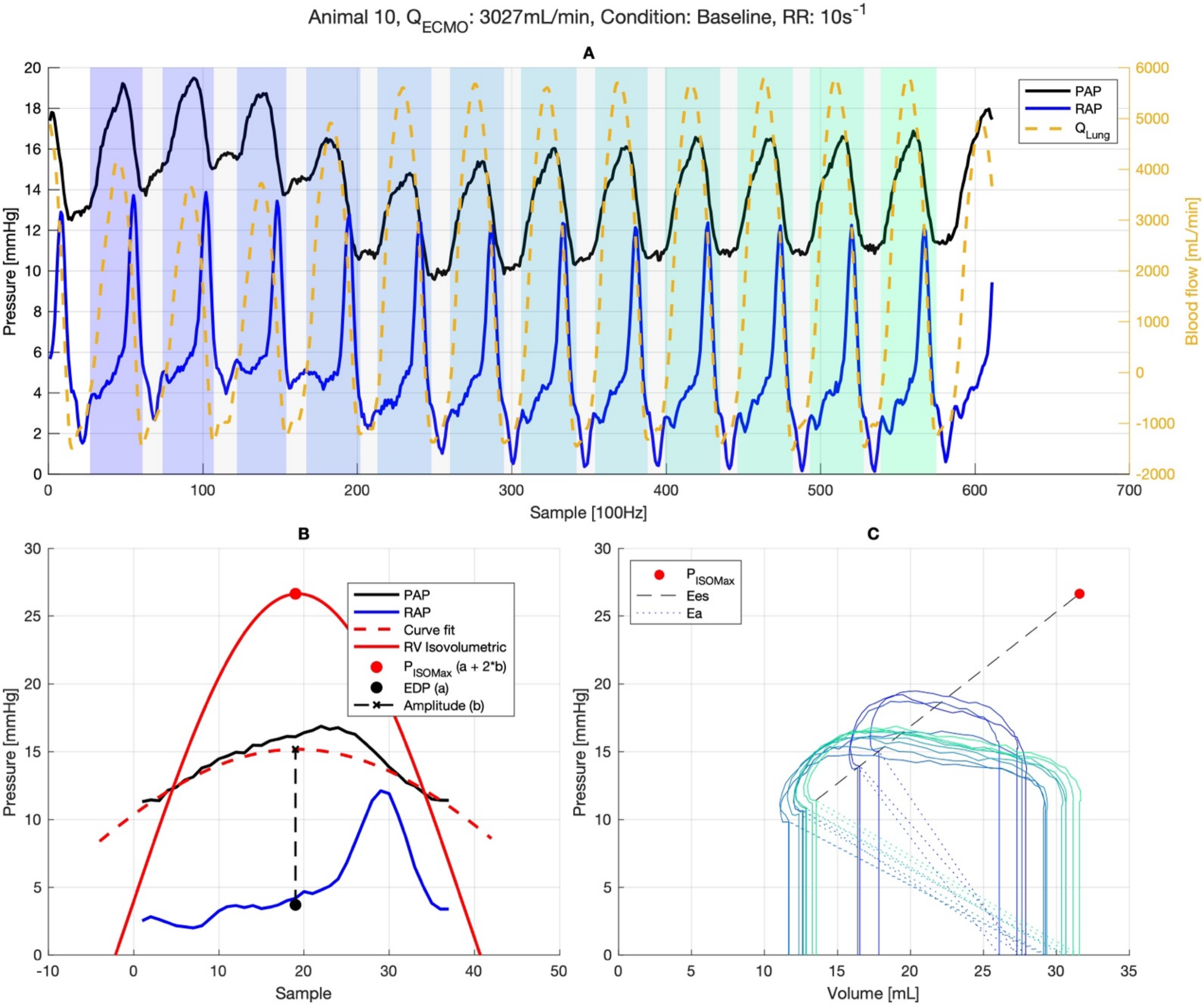
Animal 10, Q_{ECMO}: 3027mL/min, Condition: Baseline, RR: $10s^{-1}$
A
PAP
RAP
Q_{Lung}
Pressure [mmHg]
Blood flow [mL/min]
Sample [100Hz]
B
PAP
RAP
Curve fit
RV Isovolumetric
P_{ISOMax} (a + 2*b)
EDP (a)
Amplitude (b)
Pressure [mmHg]
Sample
C
P_{ISOMax}
Ees
Ea
Pressure [mmHg]
Volume [mL]

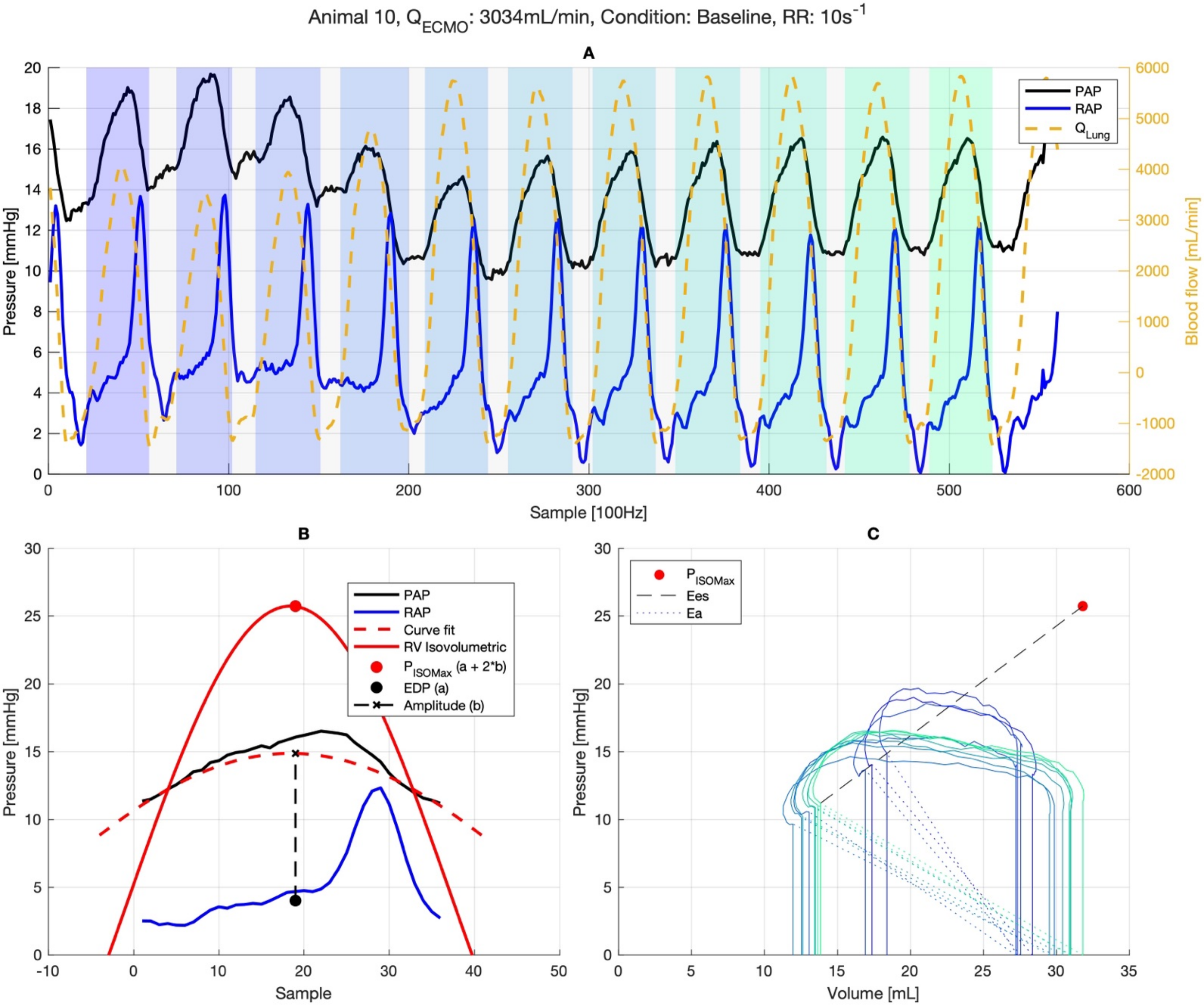

Animal 10, Q_{ECMO}: 3034mL/min, Condition: Baseline, RR: $10s^{-1}$
A
PAP
RAP
Q_{Lung}
Pressure [mmHg]
Blood flow [mL/min]
Sample [100Hz]
B
PAP
RAP
Curve fit
RV Isovolumetric
P_{ISOMax} (a + 2*b)
EDP (a)
Amplitude (b)
Pressure [mmHg]
Sample
C
P_{ISOMax}
Ees
Ea
Pressure [mmHg]
Volume [mL]

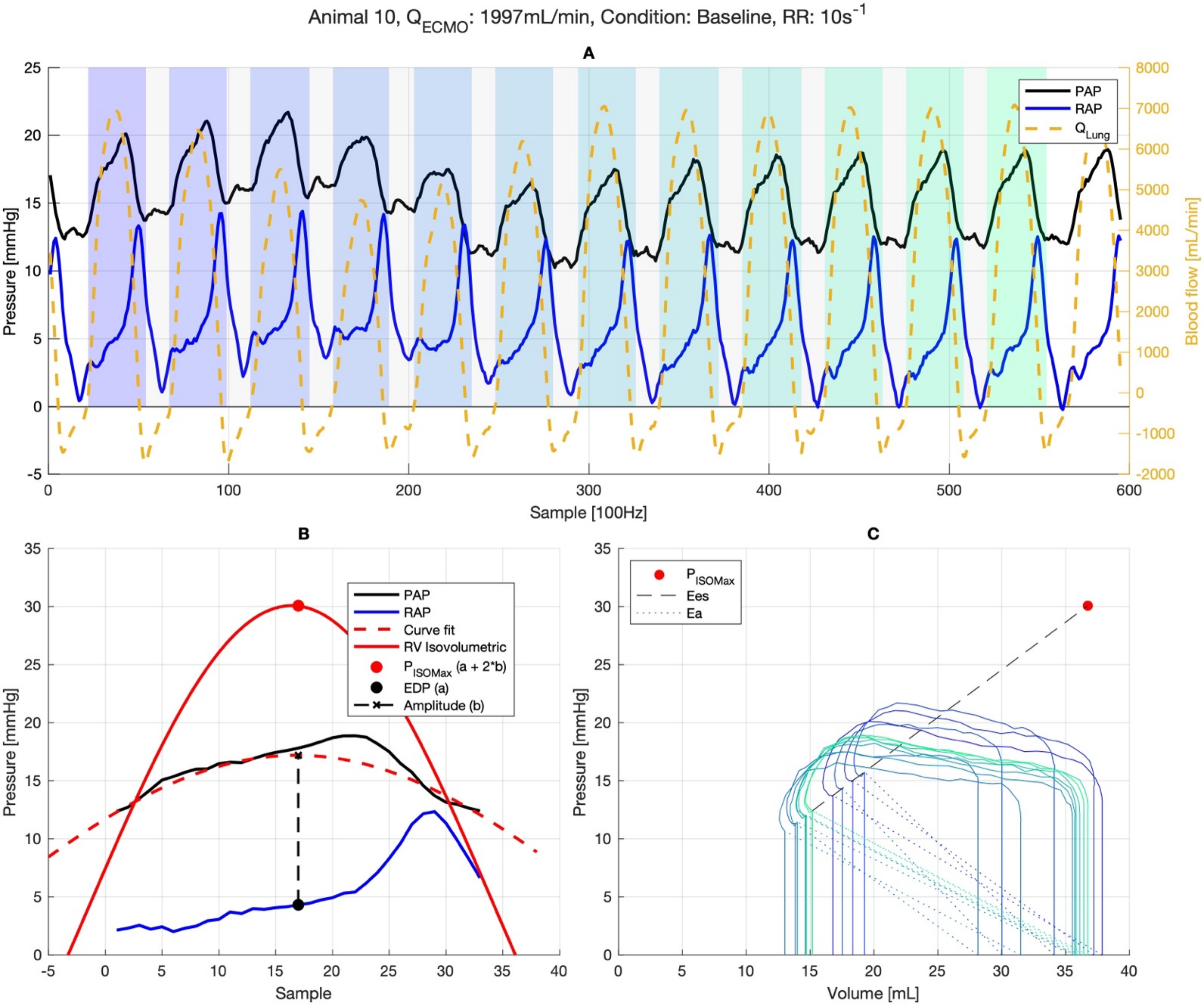

Animal 10, Q_ECMO: 1997mL/min, Condition: Baseline, RR: 10s^-1
A
Pressure [mmHg]
Blood flow [mL/min]
Sample [100Hz]
PAP
RAP
Q_Lung
B
Pressure [mmHg]
Sample
PAP
RAP
Curve fit
RV Isovolumetric
P_ISOMax (a + 2*b)
EDP (a)
Amplitude (b)
C
Pressure [mmHg]
Volume [mL]
P_ISOMax
Ees
Ea

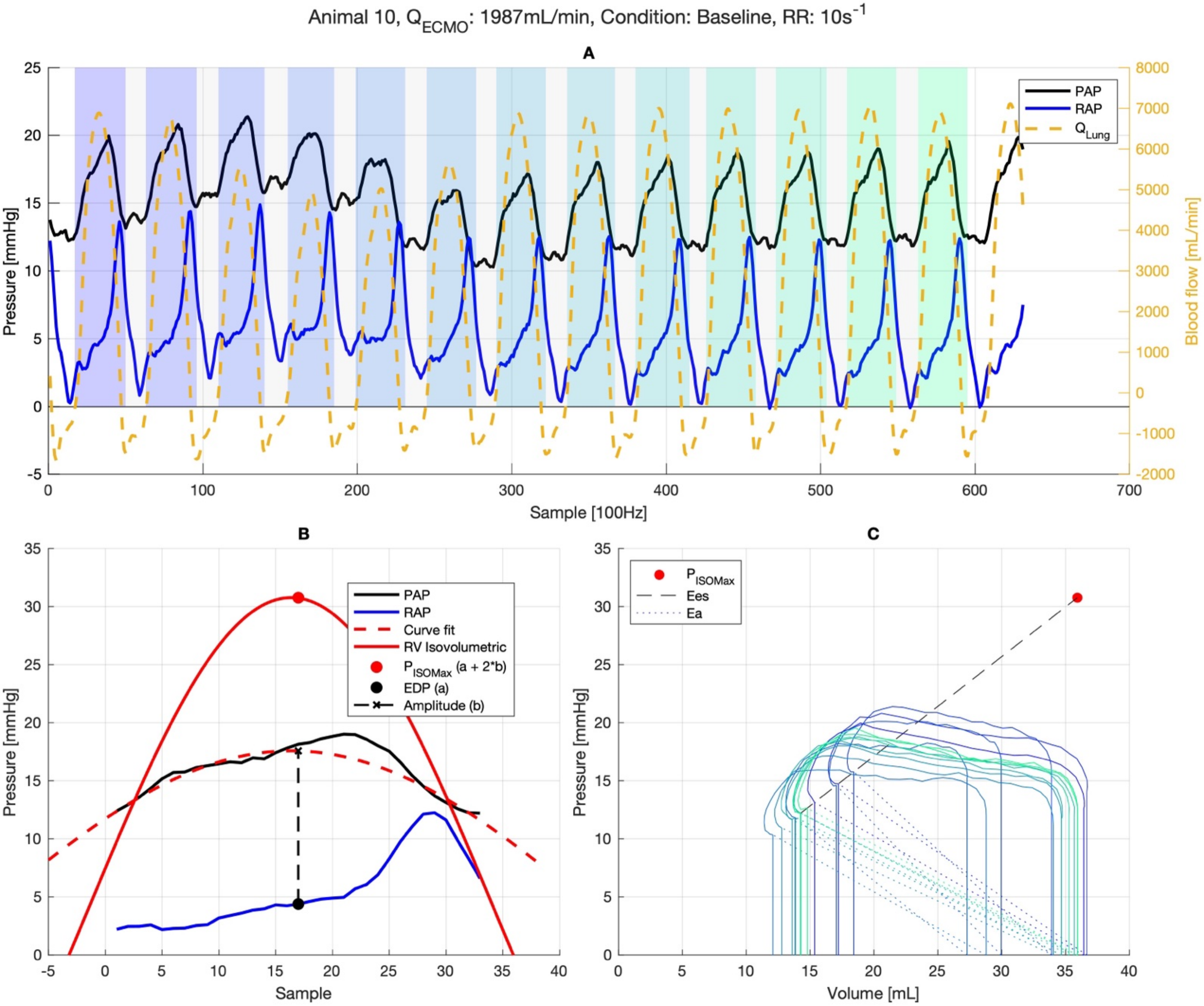

Animal 10, Q_{ECMO}: 1987mL/min, Condition: Baseline, RR: 10s^{-1}
A
PAP
RAP
Q_{Lung}
Pressure [mmHg]
Blood flow [mL/min]
Sample [100Hz]
B
PAP
RAP
Curve fit
RV Isovolumetric
P_{ISOMax} (a + 2*b)
EDP (a)
Amplitude (b)
Pressure [mmHg]
Sample
C
P_{ISOMax}
Ees
Ea
Pressure [mmHg]
Volume [mL]

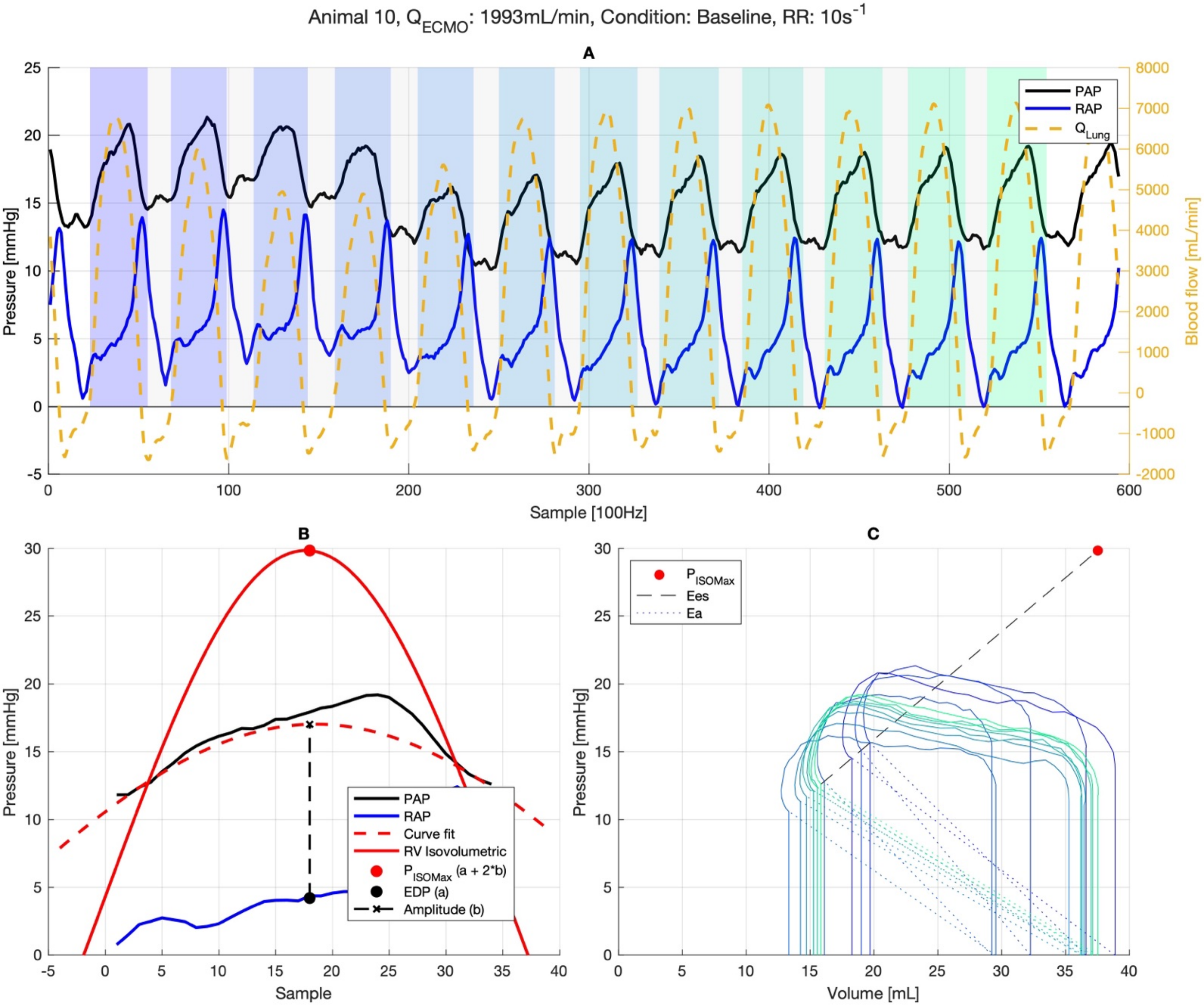

Animal 10, Q_{ECMO}: 1993mL/min, Condition: Baseline, RR: $10s^{-1}$
A
PAP
RAP
Q_{Lung}
Pressure [mmHg]
Blood flow [mL/min]
Sample [100Hz]
B
PAP
RAP
Curve fit
RV Isovolumetric
P_{ISOMax} (a + 2*b)
EDP (a)
Amplitude (b)
Pressure [mmHg]
Sample
C
P_{ISOMax}
Ees
Ea
Pressure [mmHg]
Volume [mL]

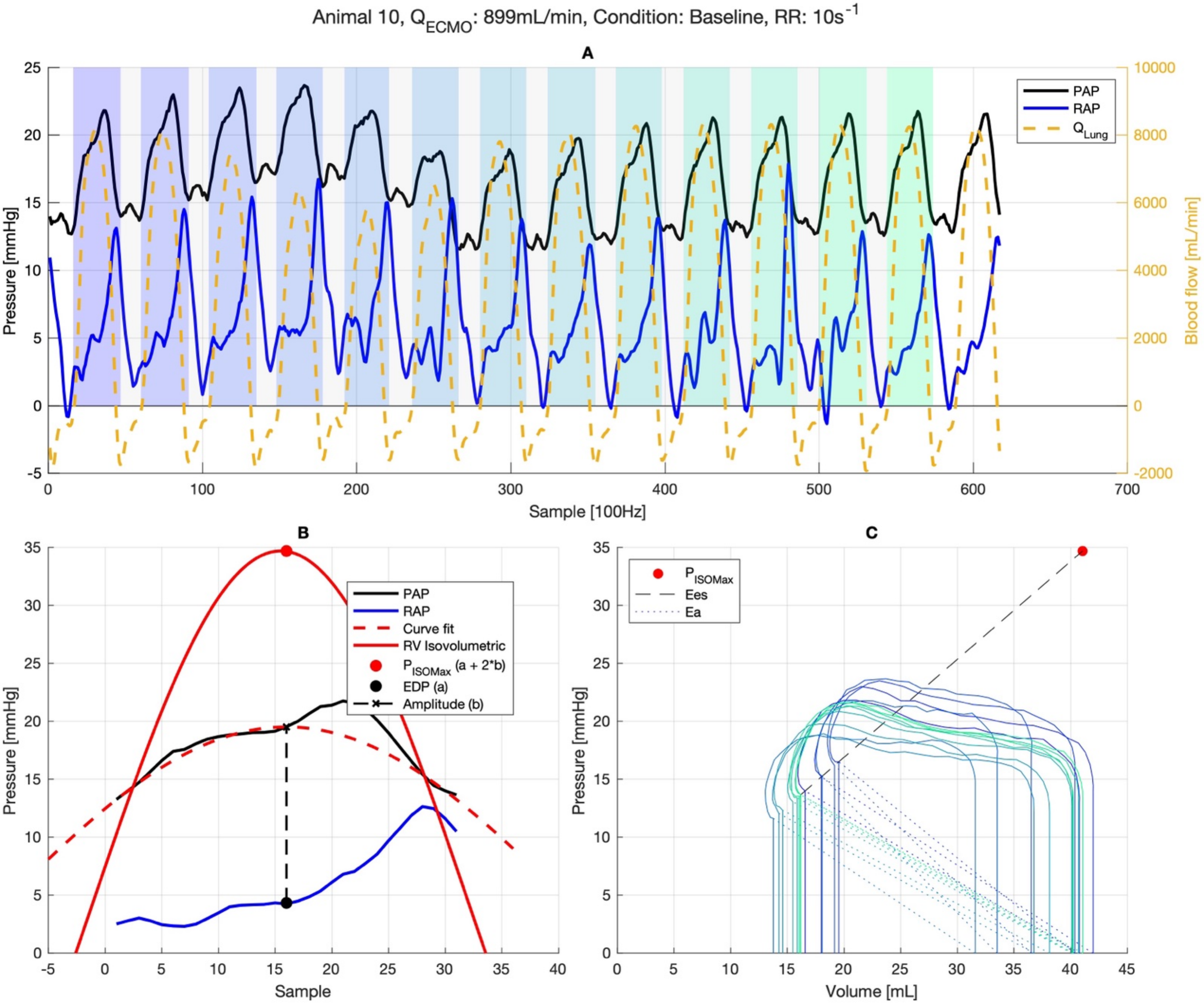

Animal 10, Q_{ECMO}: 899mL/min, Condition: Baseline, RR: $10s^{-1}$
A
PAP
RAP
Q_{Lung}
Pressure [mmHg]
Blood flow [mL/min]
Sample [100Hz]
B
PAP
RAP
Curve fit
RV Isovolumetric
P_{ISOMax} (a + 2*b)
EDP (a)
Amplitude (b)
Pressure [mmHg]
Sample
C
P_{ISOMax}
Ees
Ea
Pressure [mmHg]
Volume [mL]

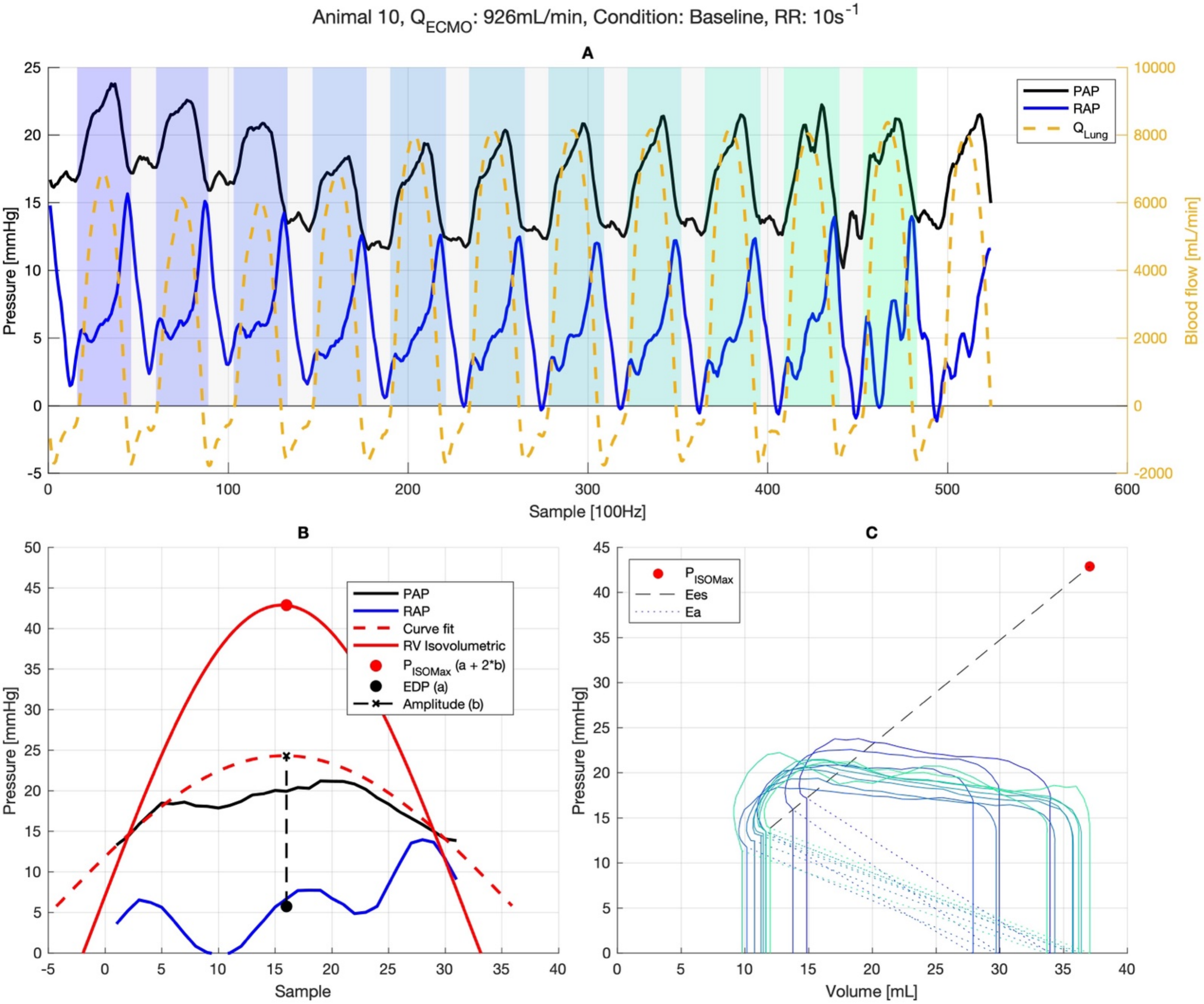

Animal 10, Q_{ECMO}: 926mL/min, Condition: Baseline, RR: $10s^{-1}$
A
PAP
RAP
Q_{Lung}
Pressure [mmHg]
Blood flow [mL/min]
Sample [100Hz]
B
PAP
RAP
Curve fit
RV Isovolumetric
P_{ISOMax} (a + 2*b)
EDP (a)
Amplitude (b)
Pressure [mmHg]
Sample
C
P_{ISOMax}
Ees
Ea
Pressure [mmHg]
Volume [mL]

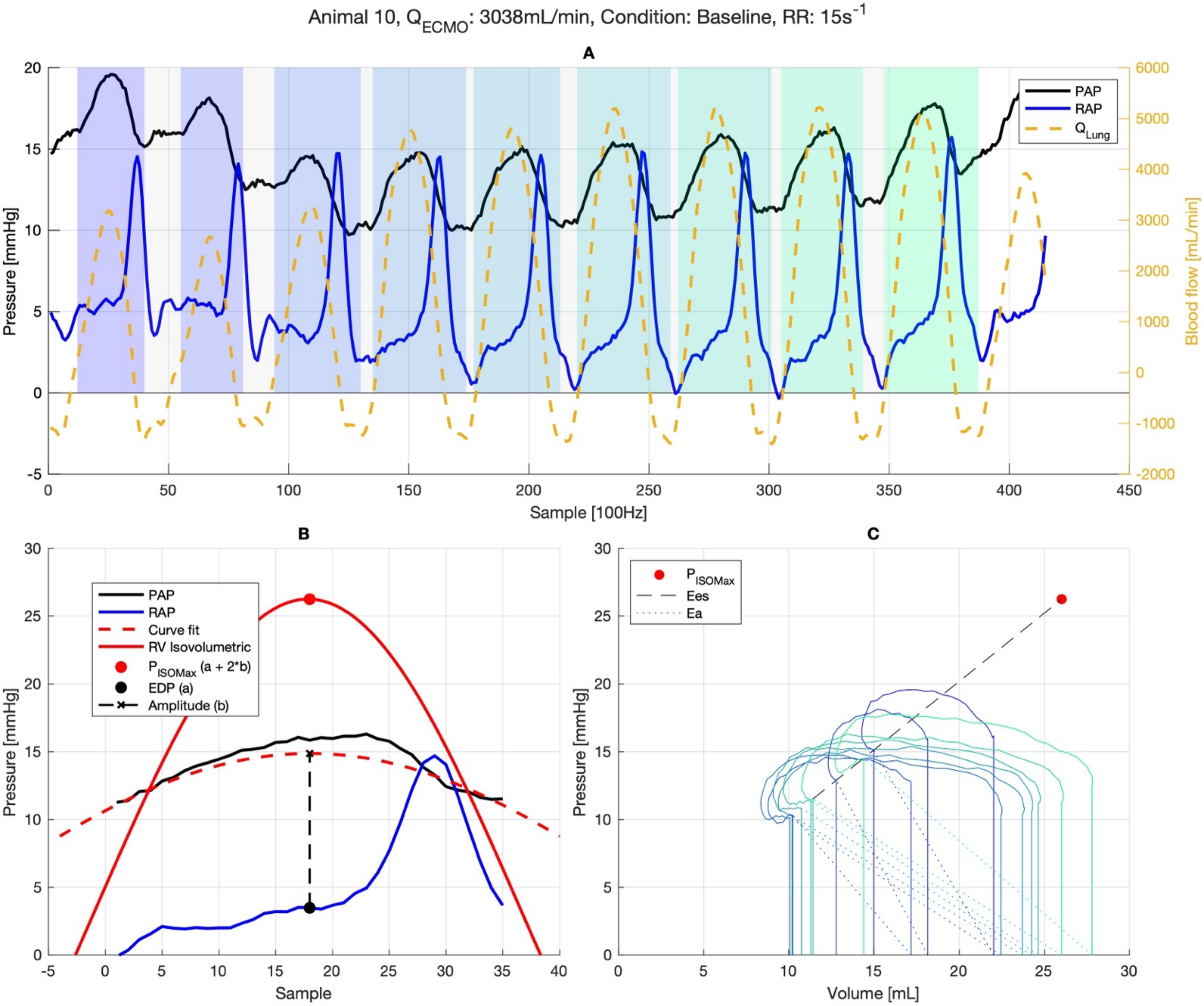

Animal 10, Q_ECMO: 3038mL/min, Condition: Baseline, RR: 15s^-1
A
PAP
RAP
Q_Lung
Pressure [mmHg]
Blood flow [mL/min]
Sample [100Hz]
B
PAP
RAP
Curve fit
RV Isovolumetric
P_ISOMax (a + 2*b)
EDP (a)
Amplitude (b)
Pressure [mmHg]
Sample
C
P_ISOMax
Ees
Ea
Pressure [mmHg]
Volume [mL]

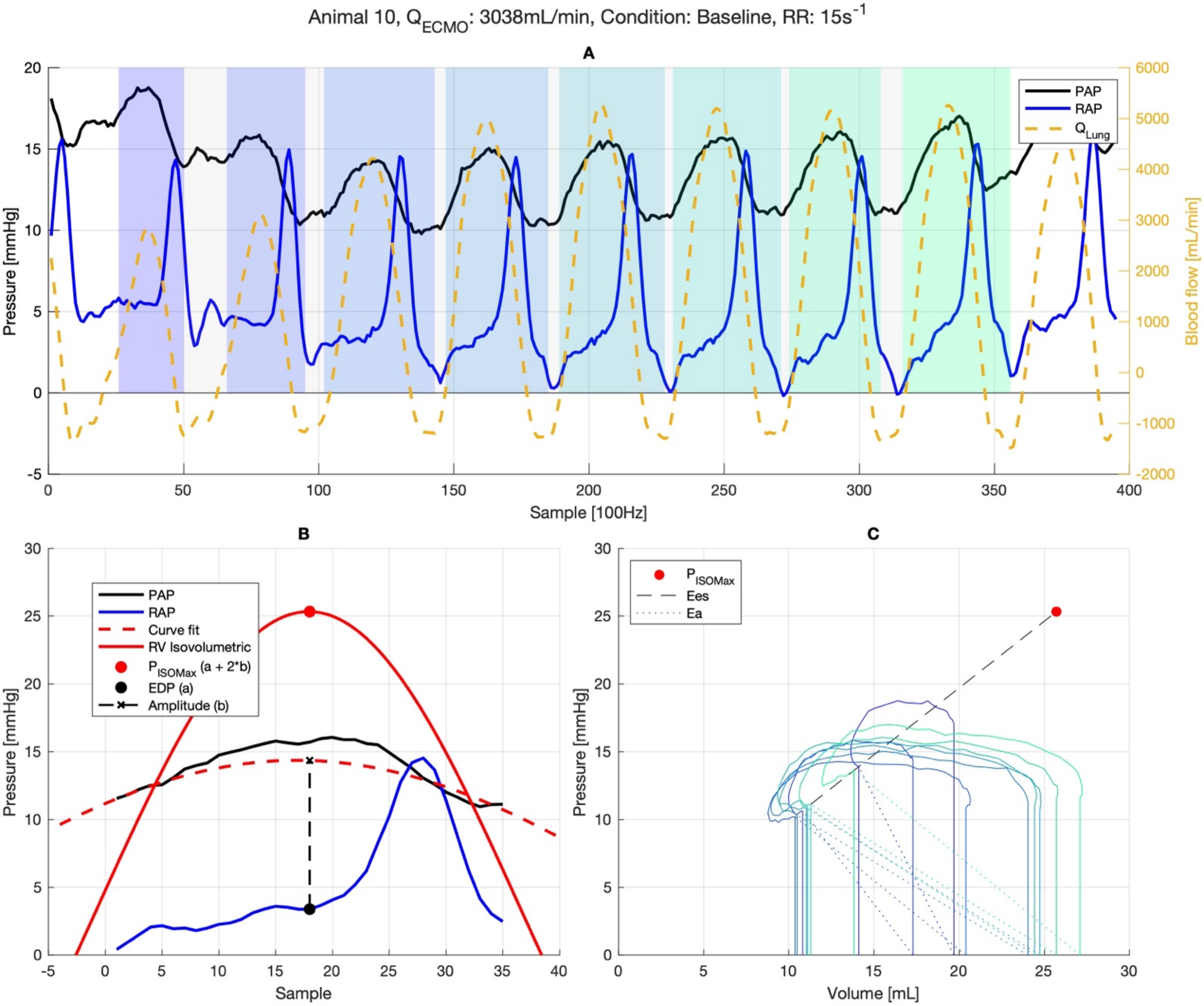

Animal 10, Q_{ECMO}: 3038mL/min, Condition: Baseline, RR: $15s^{-1}$
A
PAP
RAP
Q_{Lung}
Pressure [mmHg]
Blood flow [mL/min]
Sample [100Hz]
B
PAP
RAP
Curve fit
RV Isovolumetric
P_{ISOMax} (a + 2*b)
EDP (a)
Amplitude (b)
Pressure [mmHg]
Sample
C
P_{ISOMax}
Ees
Ea
Pressure [mmHg]
Volume [mL]

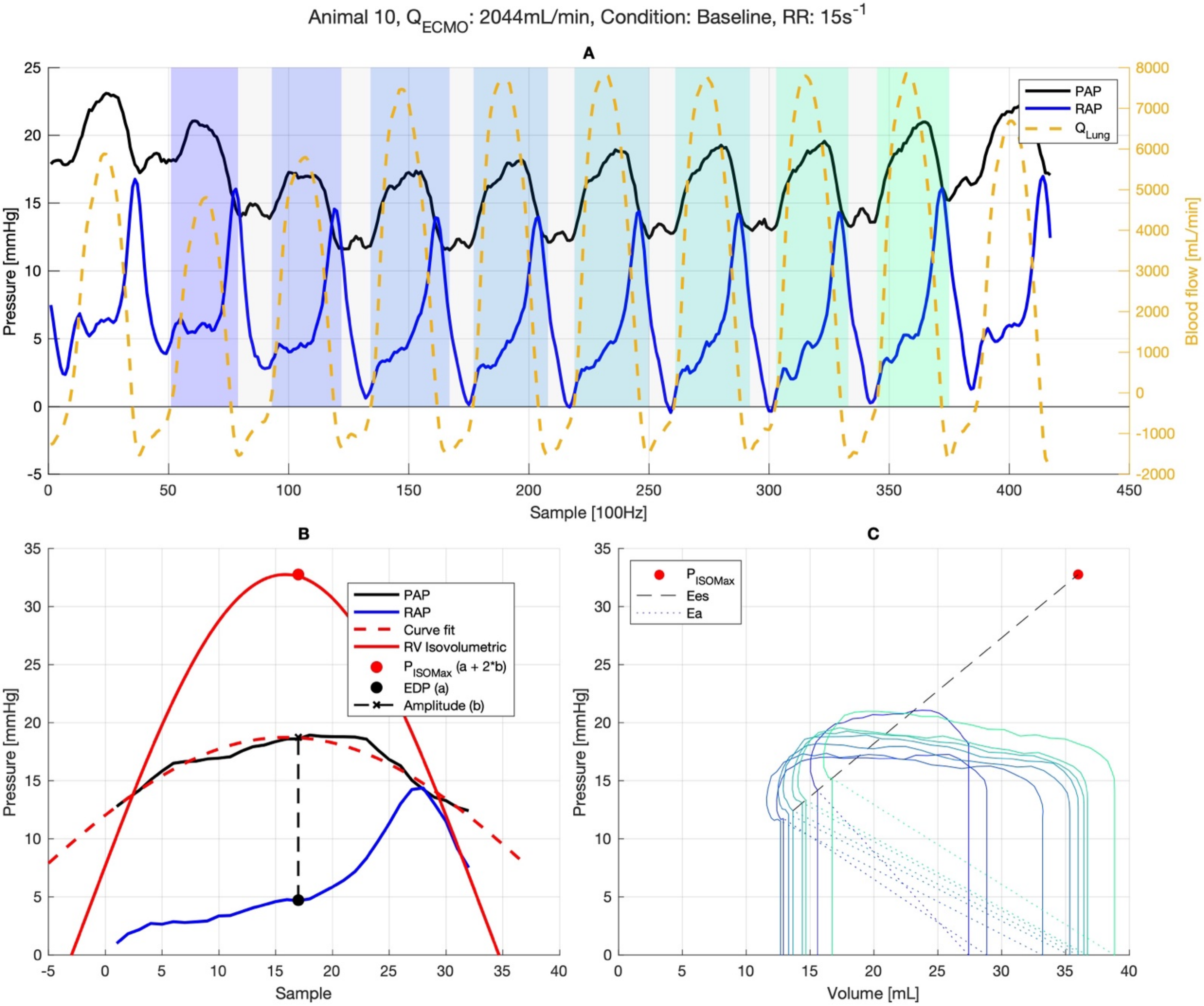

Animal 10, QECMO: 2044mL/min, Condition: Baseline, RR: 15s-1
A
PAP
RAP
QLung
Pressure [mmHg]
Blood flow [mL/min]
Sample [100Hz]
B
PAP
RAP
Curve fit
RV Isovolumetric
PISOMax (a + 2*b)
EDP (a)
Amplitude (b)
Pressure [mmHg]
Sample
C
PISOMax
Ees
Ea
Pressure [mmHg]
Volume [mL]

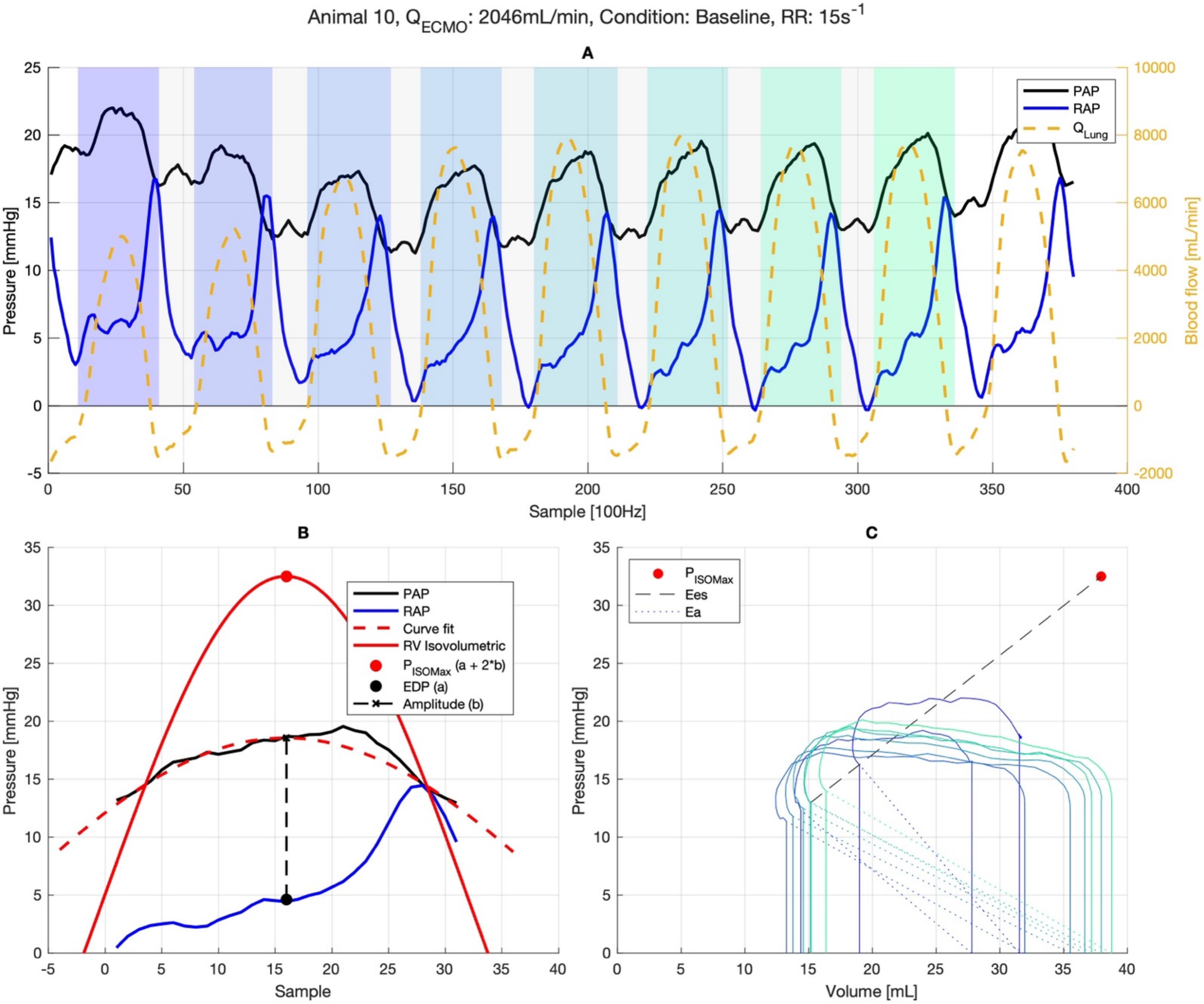

Animal 10, Q_{ECMO}: 2046mL/min, Condition: Baseline, RR: $15s^{-1}$
A
PAP
RAP
Q_{Lung}
Pressure [mmHg]
Blood flow [mL/min]
Sample [100Hz]
B
PAP
RAP
Curve fit
RV Isovolumetric
P_{ISOMax} (a + 2*b)
EDP (a)
Amplitude (b)
Pressure [mmHg]
Sample
C
P_{ISOMax}
Ees
Ea
Pressure [mmHg]
Volume [mL]

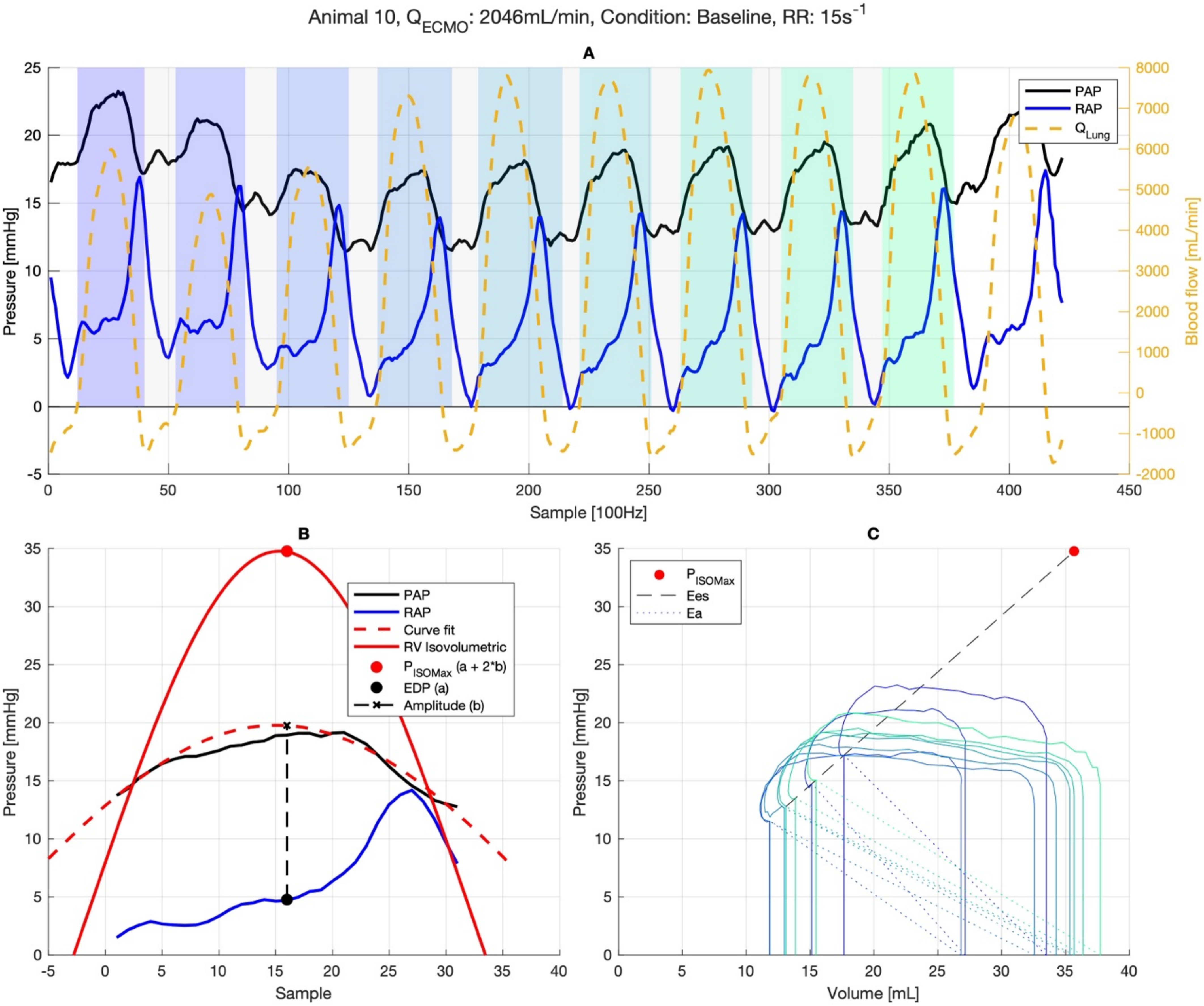

Animal 10, Q_ECMO: 2046mL/min, Condition: Baseline, RR: 15s^-1
A
Pressure [mmHg]
Blood flow [mL/min]
Sample [100Hz]
PAP
RAP
Q_Lung
B
Pressure [mmHg]
Sample
PAP
RAP
Curve fit
RV Isovolumetric
P_ISOMax (a + 2*b)
EDP (a)
Amplitude (b)
C
Pressure [mmHg]
Volume [mL]
P_ISOMax
Ees
Ea

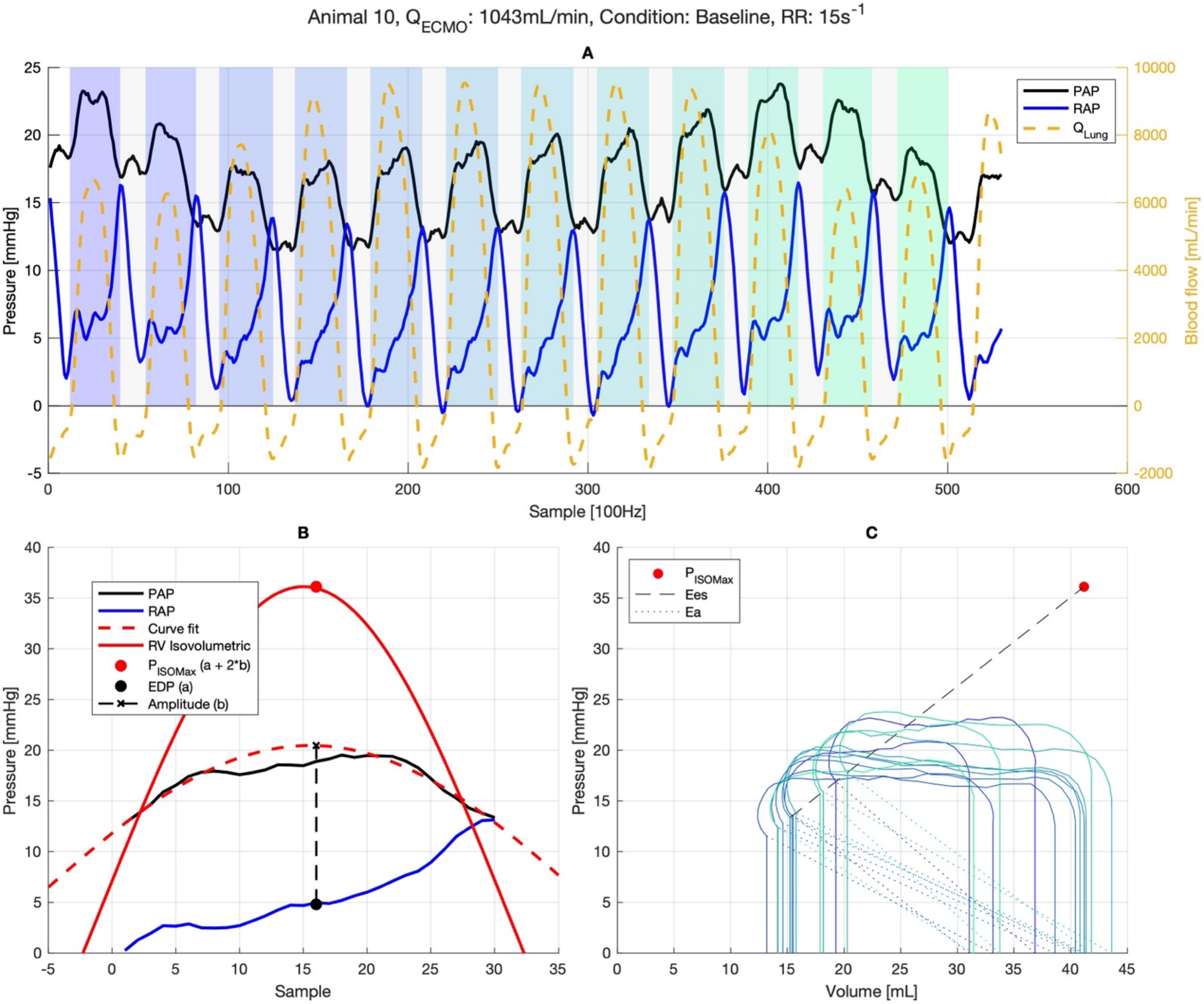

Animal 10, Q_{ECMO}: 1043mL/min, Condition: Baseline, RR: $15s^{-1}$
A
PAP
RAP
Q_{Lung}
Pressure [mmHg]
Blood flow [mL/min]
Sample [100Hz]
B
PAP
RAP
Curve fit
RV Isovolumetric
P_{ISOMax} (a + 2*b)
EDP (a)
Amplitude (b)
Pressure [mmHg]
Sample
C
P_{ISOMax}
Ees
Ea
Pressure [mmHg]
Volume [mL]

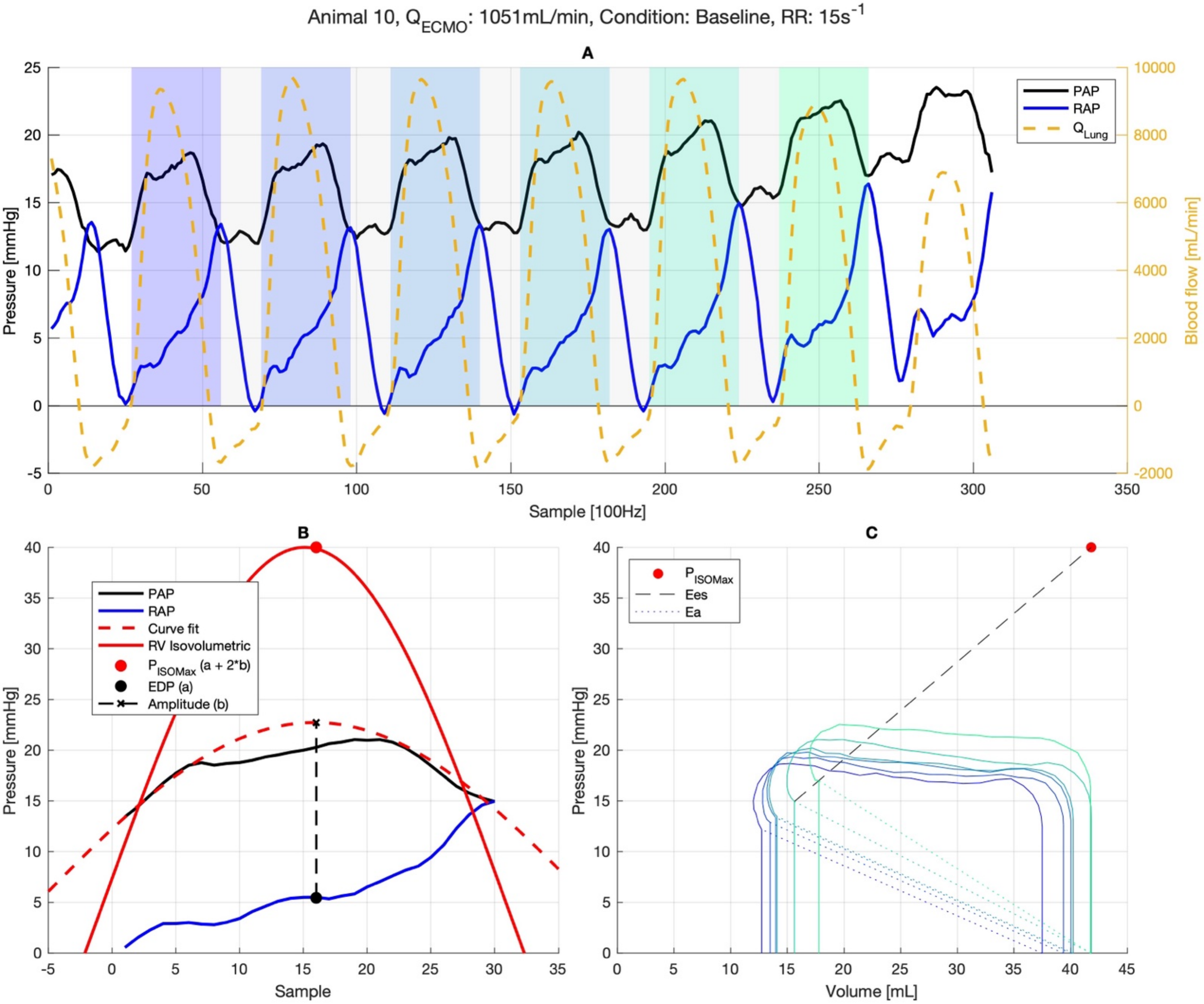

Animal 10, Q_{ECMO}: 1051mL/min, Condition: Baseline, RR: $15s^{-1}$
A
PAP
RAP
Q_{Lung}
Pressure [mmHg]
Blood flow [mL/min]
Sample [100Hz]
B
PAP
RAP
Curve fit
RV Isovolumetric
P_{ISOMax} (a + 2*b)
EDP (a)
Amplitude (b)
Pressure [mmHg]
Sample
C
P_{ISOMax}
Ees
Ea
Pressure [mmHg]
Volume [mL]

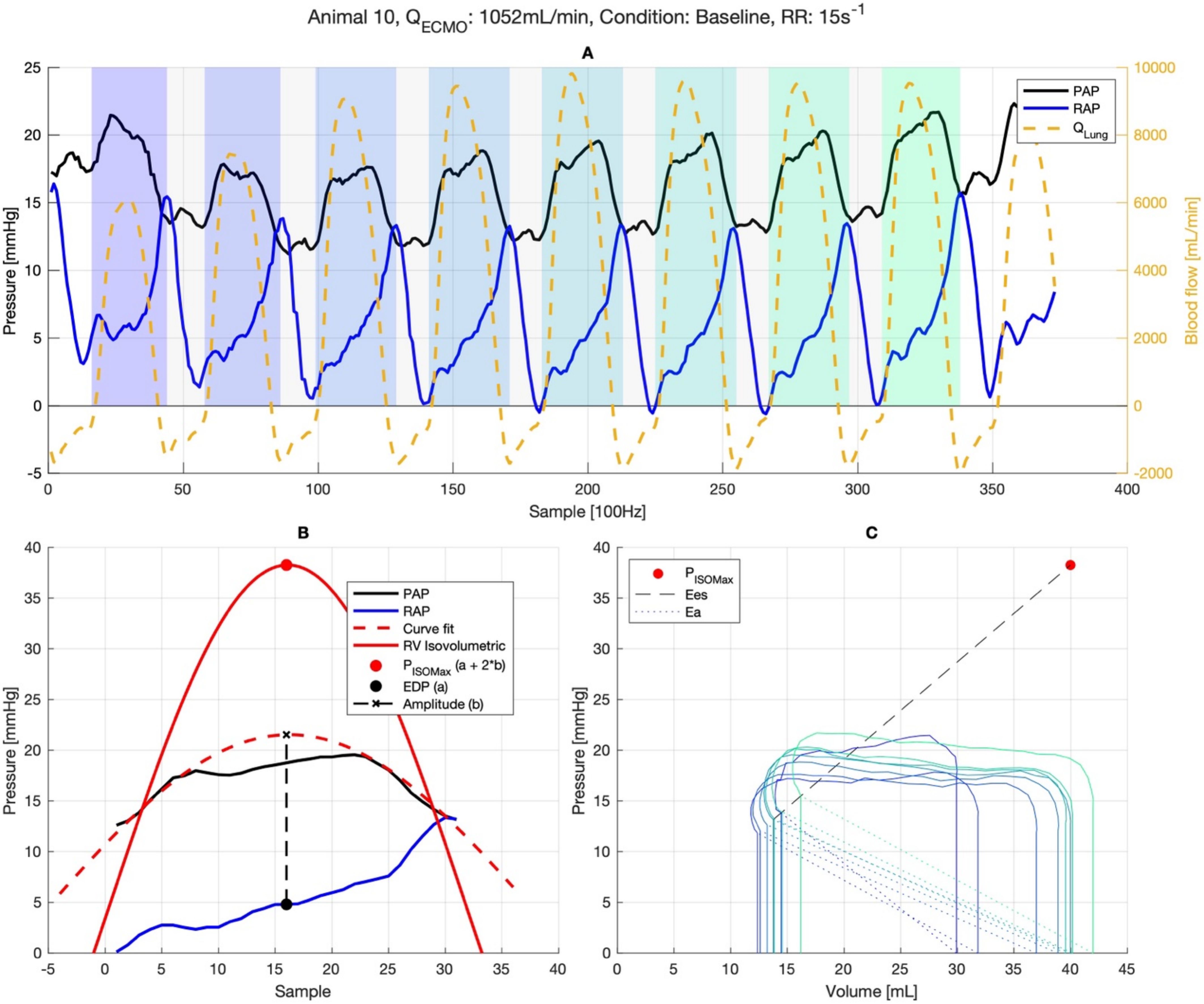

Animal 10, Q_{ECMO}: 1052mL/min, Condition: Baseline, RR: $15s^{-1}$
A
PAP
RAP
Q_{Lung}
Pressure [mmHg]
Blood flow [mL/min]
Sample [100Hz]
B
PAP
RAP
Curve fit
RV Isovolumetric
P_{ISOMax} (a + 2*b)
EDP (a)
Amplitude (b)
Pressure [mmHg]
Sample
C
P_{ISOMax}
Ees
Ea
Pressure [mmHg]
Volume [mL]

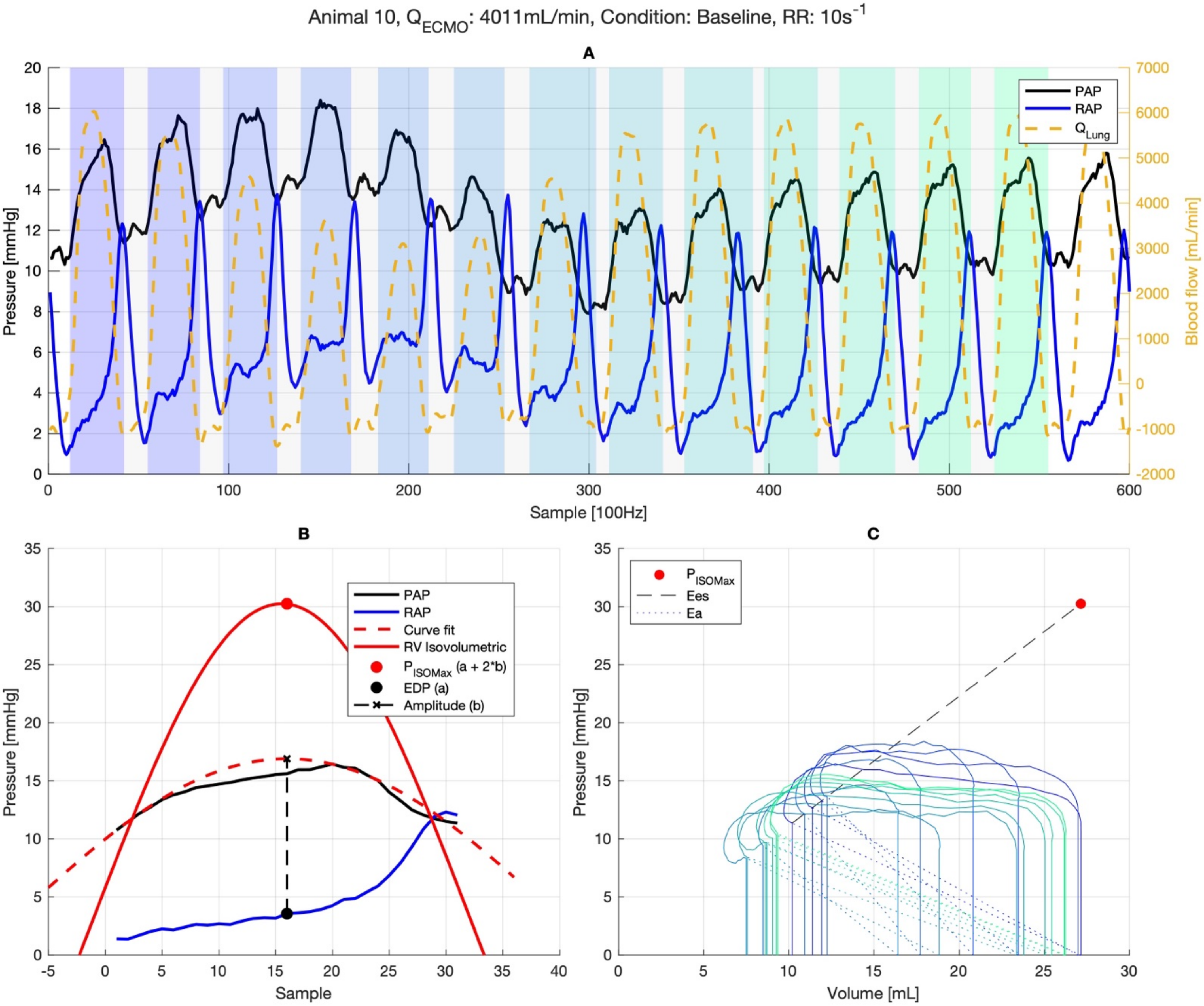

Animal 10, Q_ECMO: 4011mL/min, Condition: Baseline, RR: 10s^-1
A
PAP
RAP
Q_Lung
Pressure [mmHg]
Blood flow [mL/min]
Sample [100Hz]
B
PAP
RAP
Curve fit
RV Isovolumetric
P_ISOMax (a + 2*b)
EDP (a)
Amplitude (b)
Pressure [mmHg]
Sample
C
P_ISOMax
Ees
Ea
Pressure [mmHg]
Volume [mL]

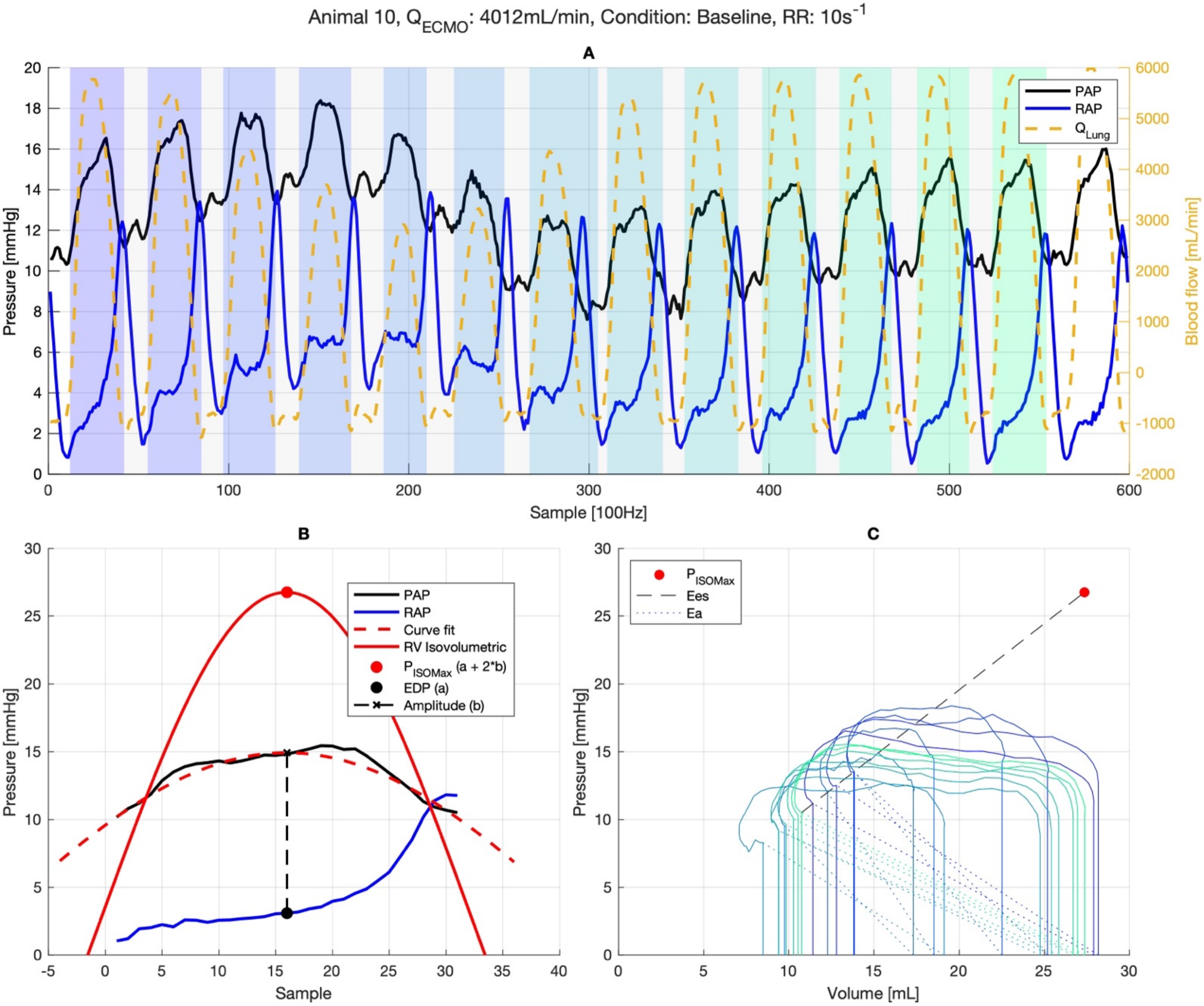

Animal 10, Q_{ECMO}: 4012mL/min, Condition: Baseline, RR: $10s^{-1}$
A
PAP
RAP
Q_{Lung}
Pressure [mmHg]
Blood flow [mL/min]
Sample [100Hz]
B
PAP
RAP
Curve fit
RV Isovolumetric
P_{ISOMax} (a + 2*b)
EDP (a)
Amplitude (b)
Pressure [mmHg]
Sample
C
P_{ISOMax}
Ees
Ea
Pressure [mmHg]
Volume [mL]

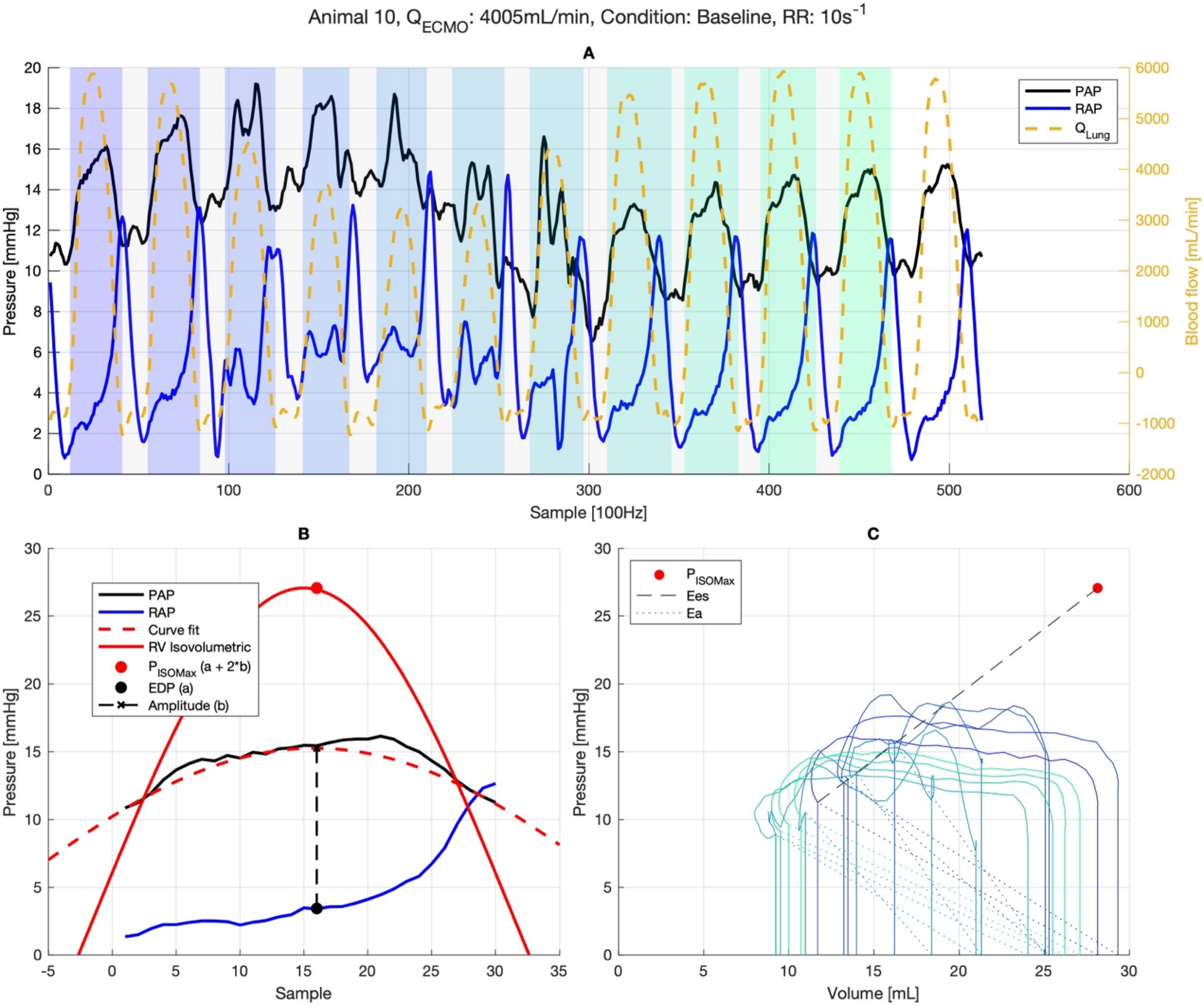
Animal 10, Q_{ECMO}: 4005mL/min, Condition: Baseline, RR: $10s^{-1}$
A
PAP
RAP
Q_{Lung}
Pressure [mmHg]
Blood flow [mL/min]
Sample [100Hz]
B
PAP
RAP
Curve fit
RV Isovolumetric
P_{ISOMax} (a + 2*b)
EDP (a)
Amplitude (b)
Pressure [mmHg]
Sample
C
P_{ISOMax}
Ees
Ea
Pressure [mmHg]
Volume [mL]

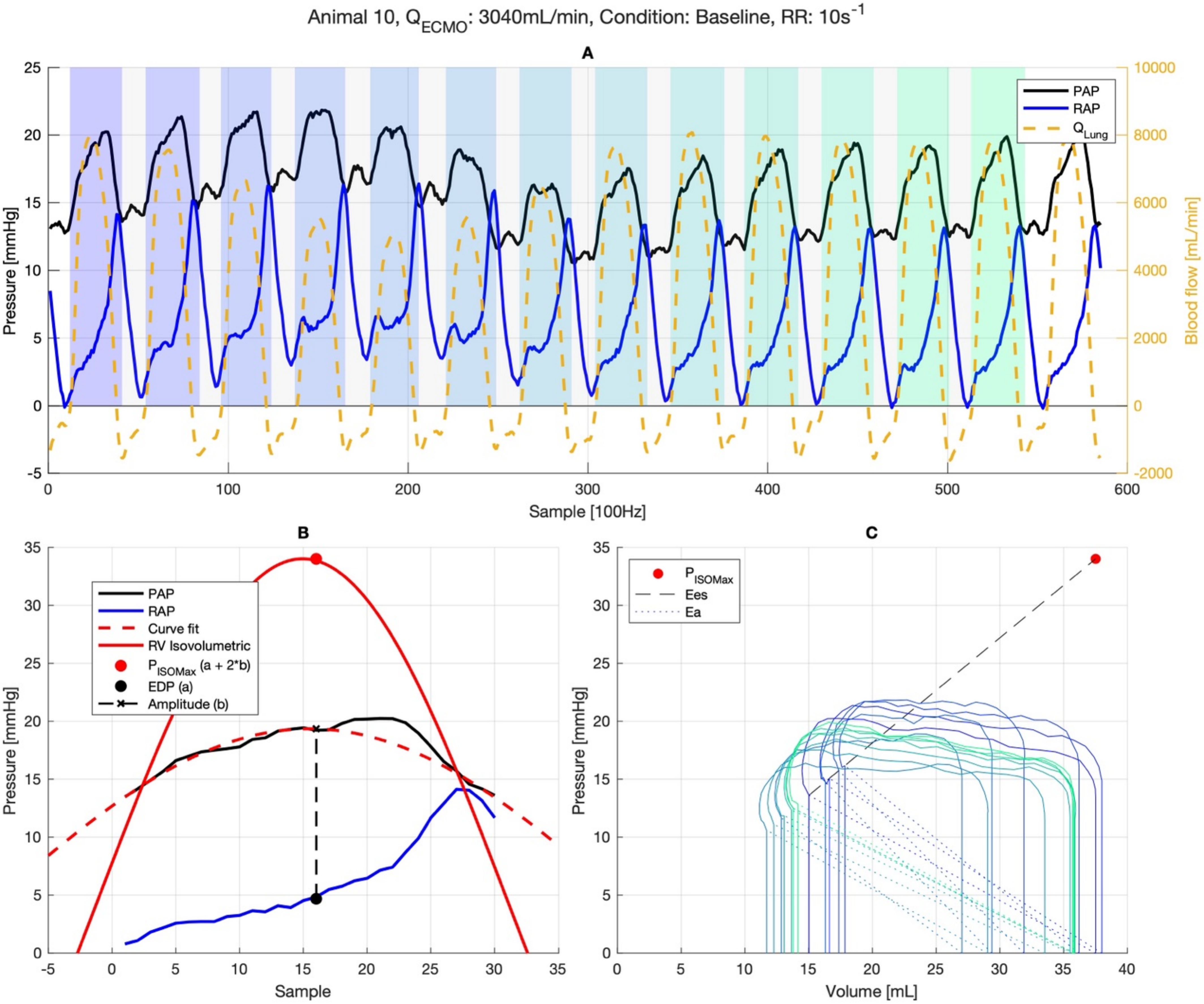

Animal 10, Q_{ECMO}: 3040mL/min, Condition: Baseline, RR: $10s^{-1}$
A
PAP
RAP
Q_{Lung}
Pressure [mmHg]
Blood flow [mL/min]
Sample [100Hz]
B
PAP
RAP
Curve fit
RV Isovolumetric
P_{ISOMax} (a + 2*b)
EDP (a)
Amplitude (b)
Pressure [mmHg]
Sample
C
P_{ISOMax}
Ees
Ea
Pressure [mmHg]
Volume [mL]

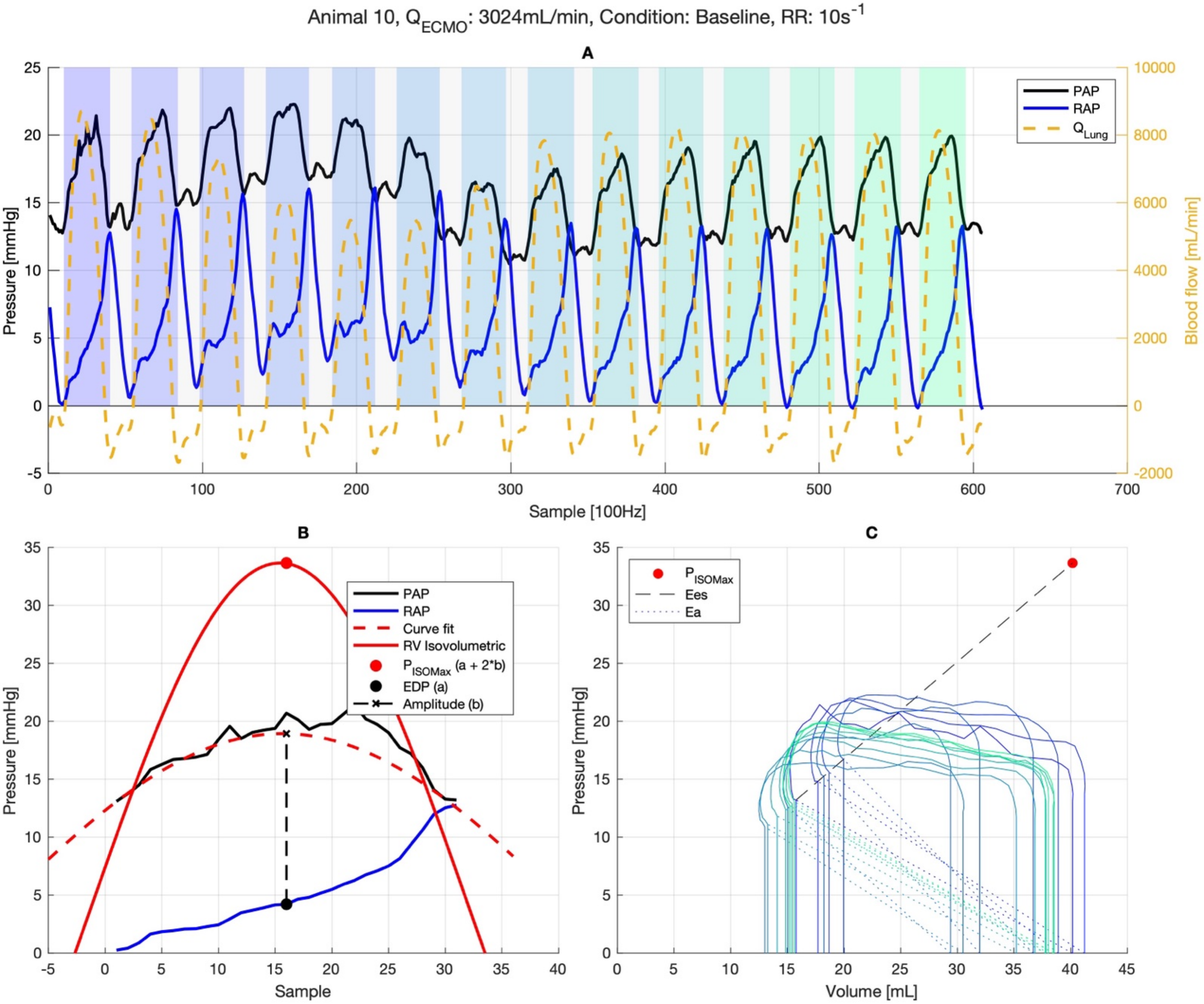

Animal 10, Q_{ECMO}: 3024mL/min, Condition: Baseline, RR: $10s^{-1}$
A
PAP
RAP
Q_{Lung}
Pressure [mmHg]
Blood flow [mL/min]
Sample [100Hz]
B
PAP
RAP
Curve fit
RV Isovolumetric
P_{ISOMax} (a + 2*b)
EDP (a)
Amplitude (b)
Pressure [mmHg]
Sample
C
P_{ISOMax}
Ees
Ea
Pressure [mmHg]
Volume [mL]

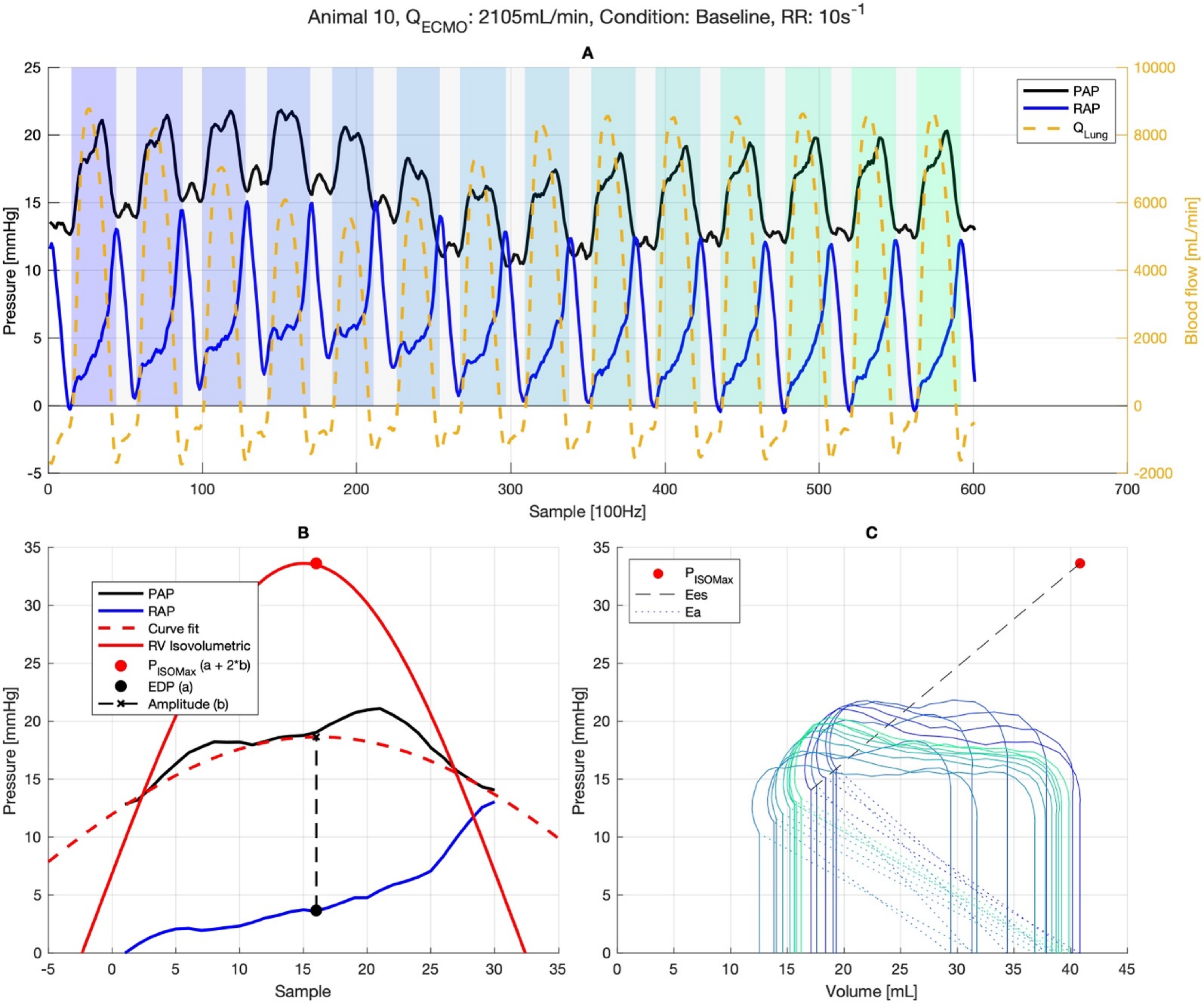

Animal 10, Q_{ECMO}: 2105mL/min, Condition: Baseline, RR: $10s^{-1}$
A
PAP
RAP
Q_{Lung}
Pressure [mmHg]
Blood flow [mL/min]
Sample [100Hz]
B
PAP
RAP
Curve fit
RV Isovolumetric
P_{ISOMax} (a + 2*b)
EDP (a)
Amplitude (b)
Pressure [mmHg]
Sample
C
P_{ISOMax}
Ees
Ea
Pressure [mmHg]
Volume [mL]

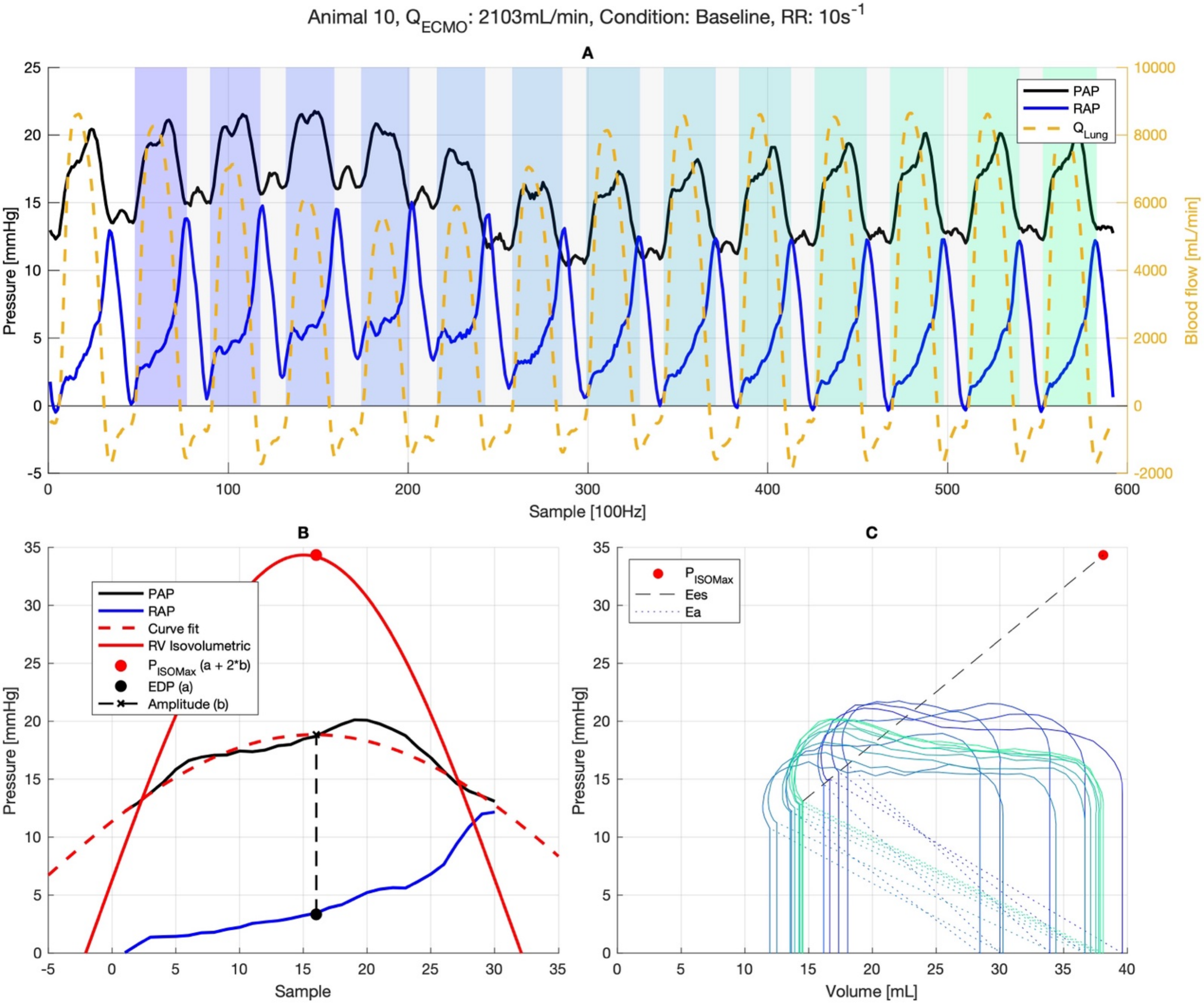

Animal 10, Q_ECMO: 2103mL/min, Condition: Baseline, RR: 10s^-1
A
PAP
RAP
Q_Lung
Pressure [mmHg]
Blood flow [mL/min]
Sample [100Hz]
B
PAP
RAP
Curve fit
RV Isovolumetric
P_ISOMax (a + 2*b)
EDP (a)
Amplitude (b)
Pressure [mmHg]
Sample
C
P_ISOMax
Ees
Ea
Pressure [mmHg]
Volume [mL]

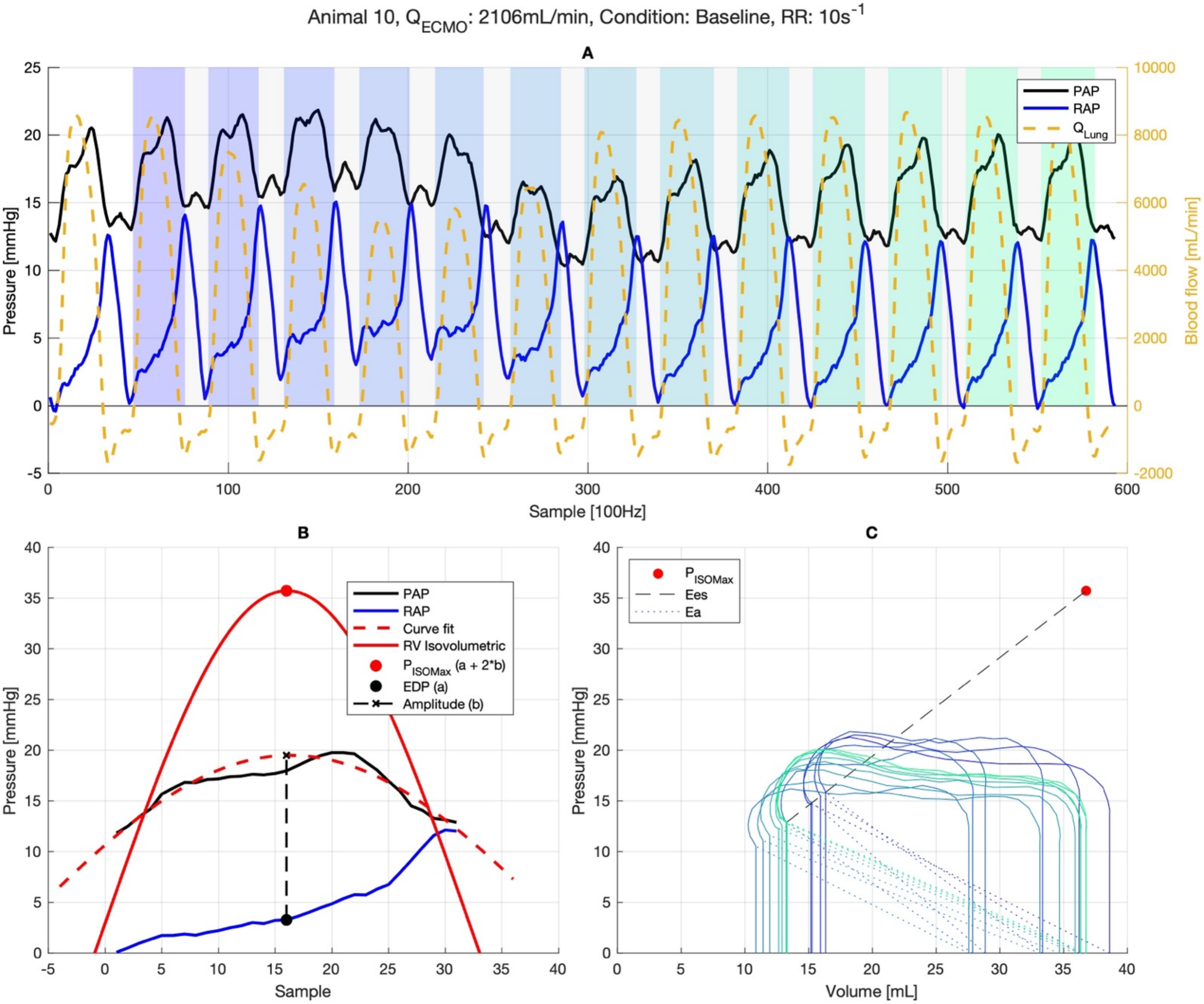

Animal 10, Q_{ECMO}: 2106mL/min, Condition: Baseline, RR: $10s^{-1}$
A
PAP
RAP
Q_{Lung}
Pressure [mmHg]
Blood flow [mL/min]
Sample [100Hz]
B
PAP
RAP
Curve fit
RV Isovolumetric
P_{ISOMax} (a + 2*b)
EDP (a)
Amplitude (b)
Pressure [mmHg]
Sample
C
P_{ISOMax}
Ees
Ea
Pressure [mmHg]
Volume [mL]

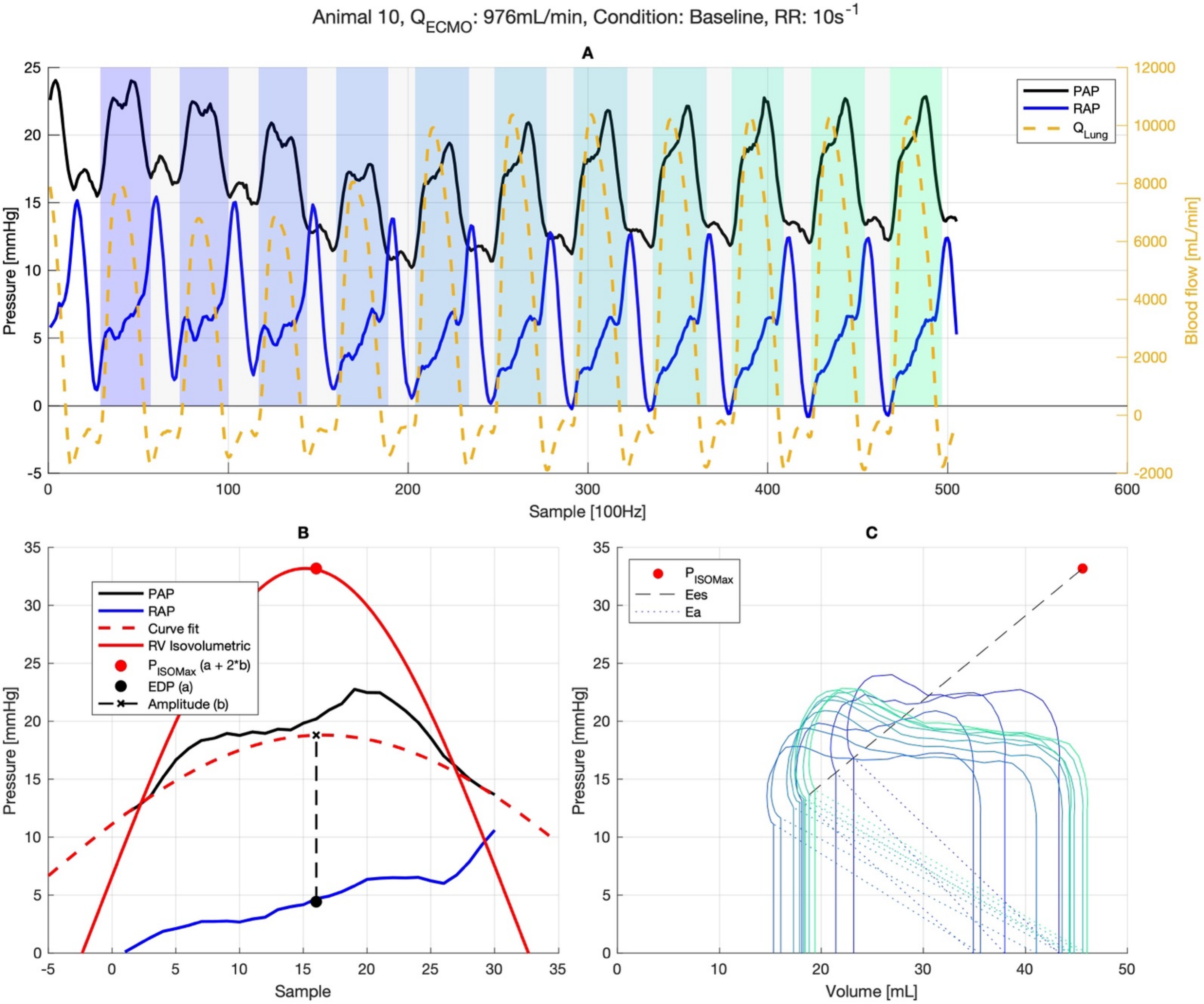

Animal 10, Q_{ECMO}: 976mL/min, Condition: Baseline, RR: $10s^{-1}$
A
PAP
RAP
Q_{Lung}
Pressure [mmHg]
Blood flow [mL/min]
Sample [100Hz]
B
PAP
RAP
Curve fit
RV Isovolumetric
P_{ISOMax} (a + 2*b)
EDP (a)
Amplitude (b)
Pressure [mmHg]
Sample
C
P_{ISOMax}
Ees
Ea
Pressure [mmHg]
Volume [mL]

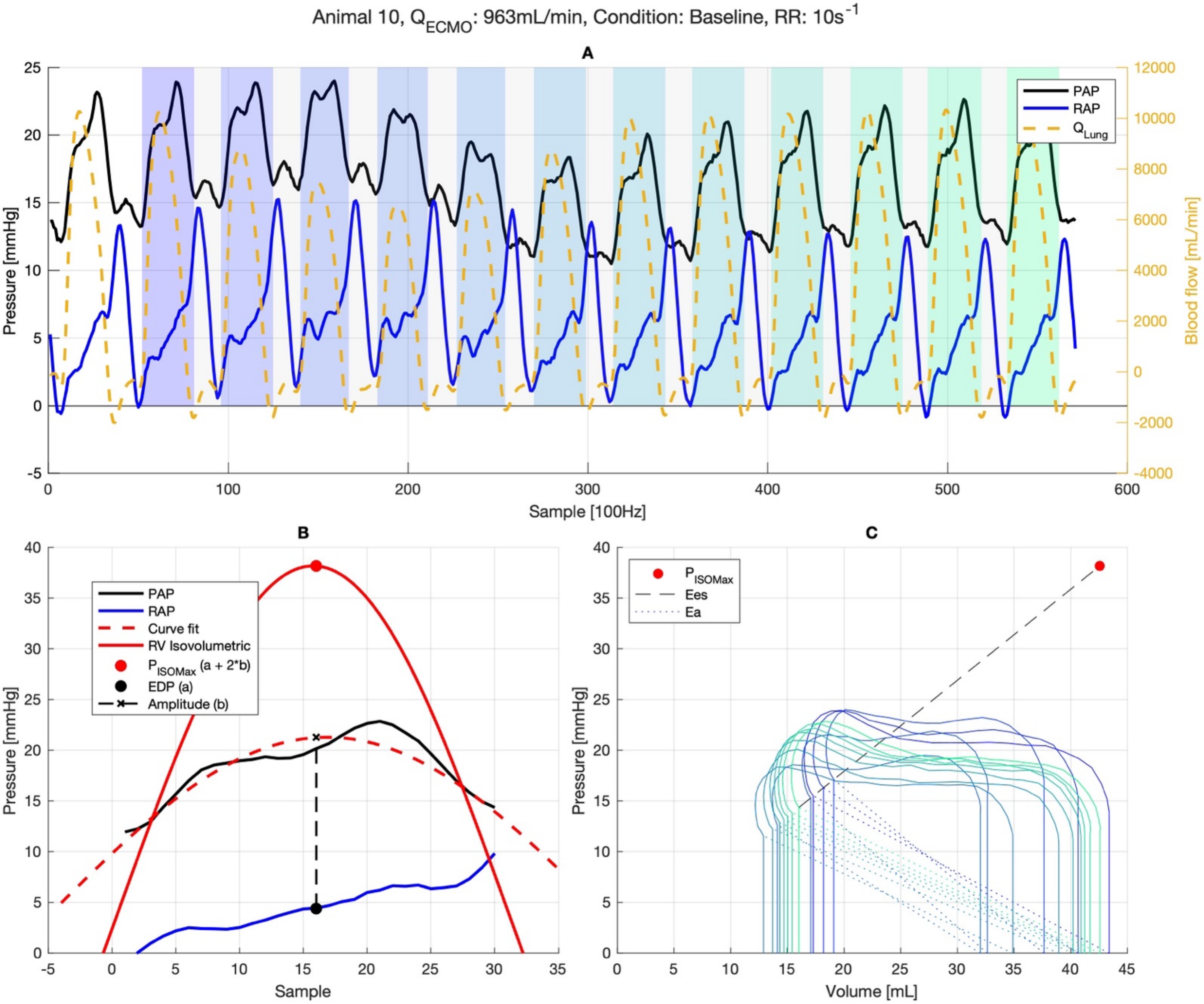

Animal 10, Q_{ECMO}: 963mL/min, Condition: Baseline, RR: $10s^{-1}$
A
Pressure [mmHg]
Blood flow [mL/min]
Sample [100Hz]
PAP
RAP
Q_{Lung}
B
Pressure [mmHg]
Sample
PAP
RAP
Curve fit
RV Isovolumetric
P_{ISOMax} (a + 2*b)
EDP (a)
Amplitude (b)
C
Pressure [mmHg]
Volume [mL]
P_{ISOMax}
Ees
Ea

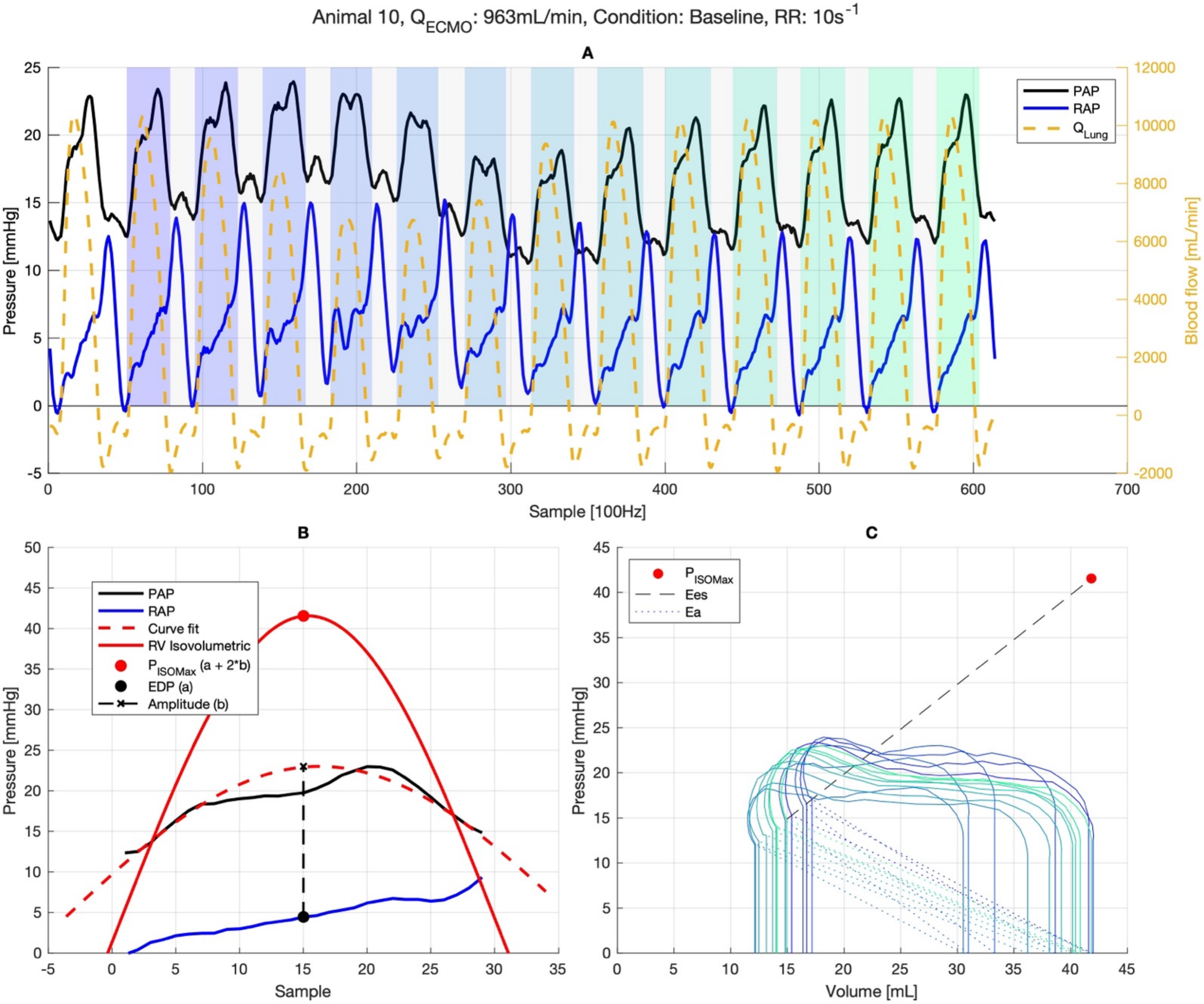

Animal 10, Q_{ECMO}: 963mL/min, Condition: Baseline, RR: $10s^{-1}$
A
PAP
RAP
Q_{Lung}
Pressure [mmHg]
Blood flow [mL/min]
Sample [100Hz]
B
PAP
RAP
Curve fit
RV Isovolumetric
P_{ISOMax} (a + 2*b)
EDP (a)
Amplitude (b)
Pressure [mmHg]
Sample
C
P_{ISOMax}
Ees
Ea
Pressure [mmHg]
Volume [mL]

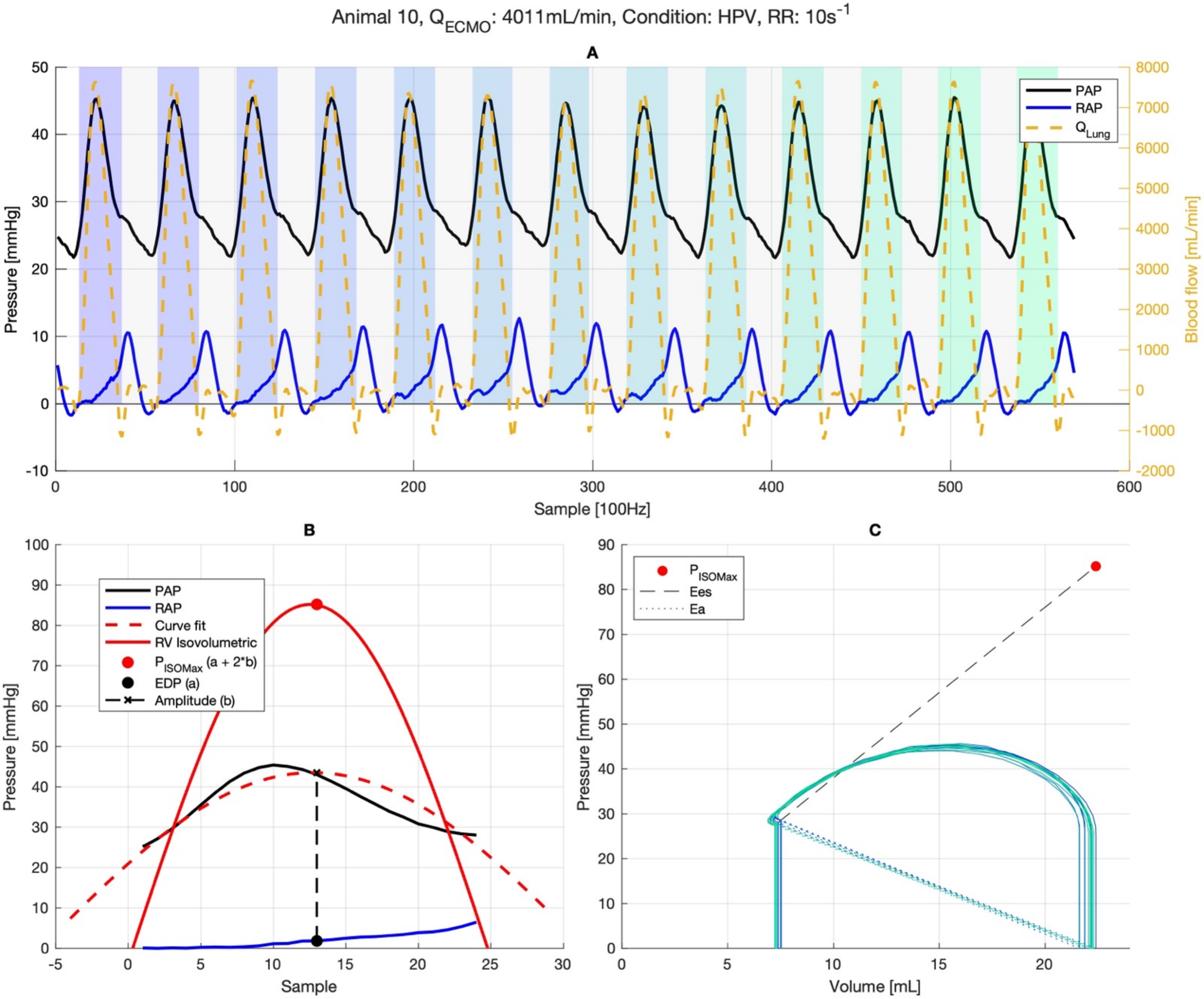

Animal 10, Q_ECMO: 4011mL/min, Condition: HPV, RR: 10s^-1
A
Pressure [mmHg]
Blood flow [mL/min]
Sample [100Hz]
PAP
RAP
Q_Lung
B
PAP
RAP
Curve fit
RV Isovolumetric
P_ISOMax (a + 2*b)
EDP (a)
Amplitude (b)
Pressure [mmHg]
Sample
C
P_ISOMax
Ees
Ea
Pressure [mmHg]
Volume [mL]

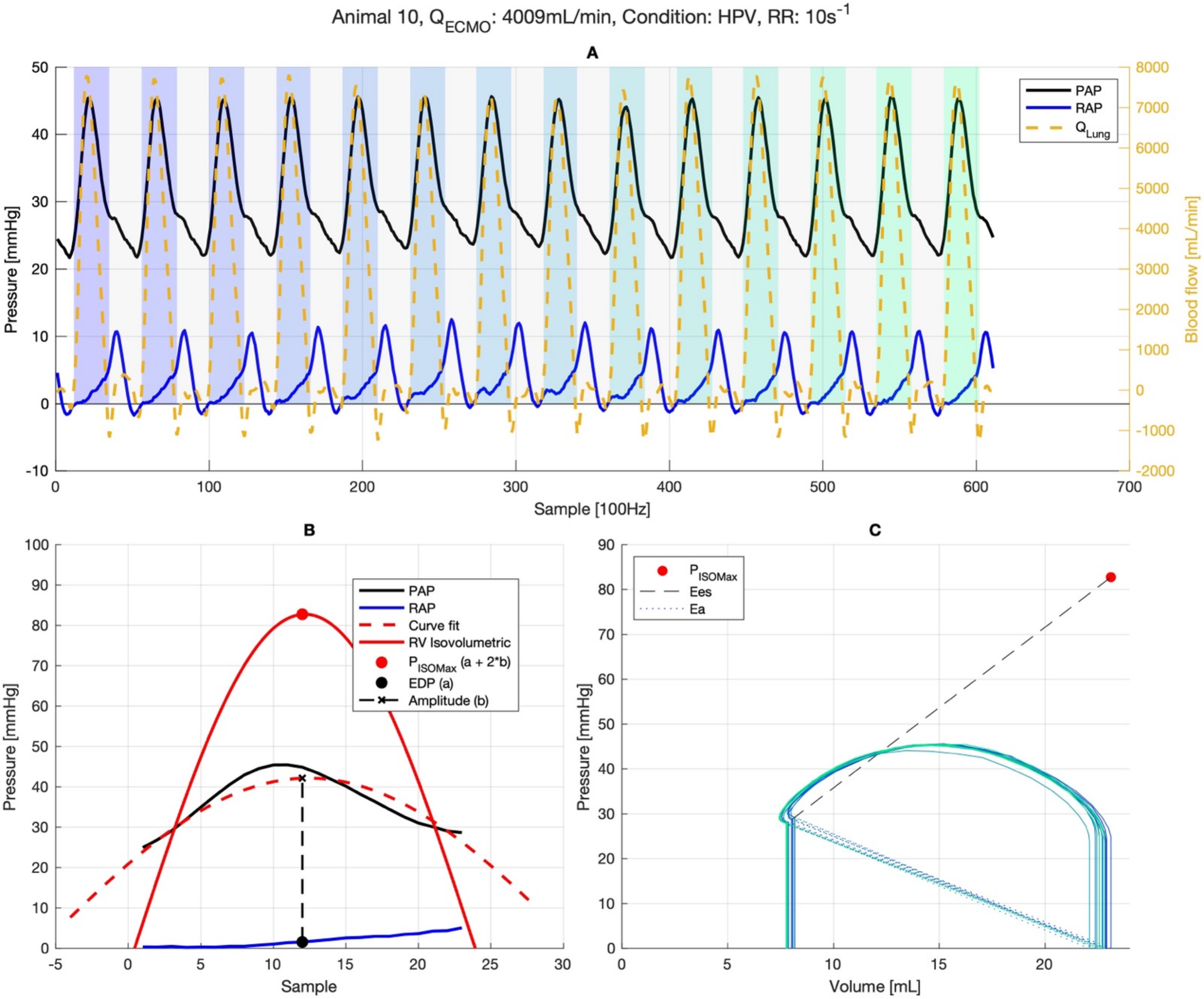

Animal 10, QECMO: 4009mL/min, Condition: HPV, RR: 10s-1
A
Pressure [mmHg]
Blood flow [mL/min]
Sample [100Hz]
PAP
RAP
QLung
B
Pressure [mmHg]
Sample
PAP
RAP
Curve fit
RV Isovolumetric
PISOMax (a + 2*b)
EDP (a)
Amplitude (b)
C
Pressure [mmHg]
Volume [mL]
PISOMax
Ees
Ea

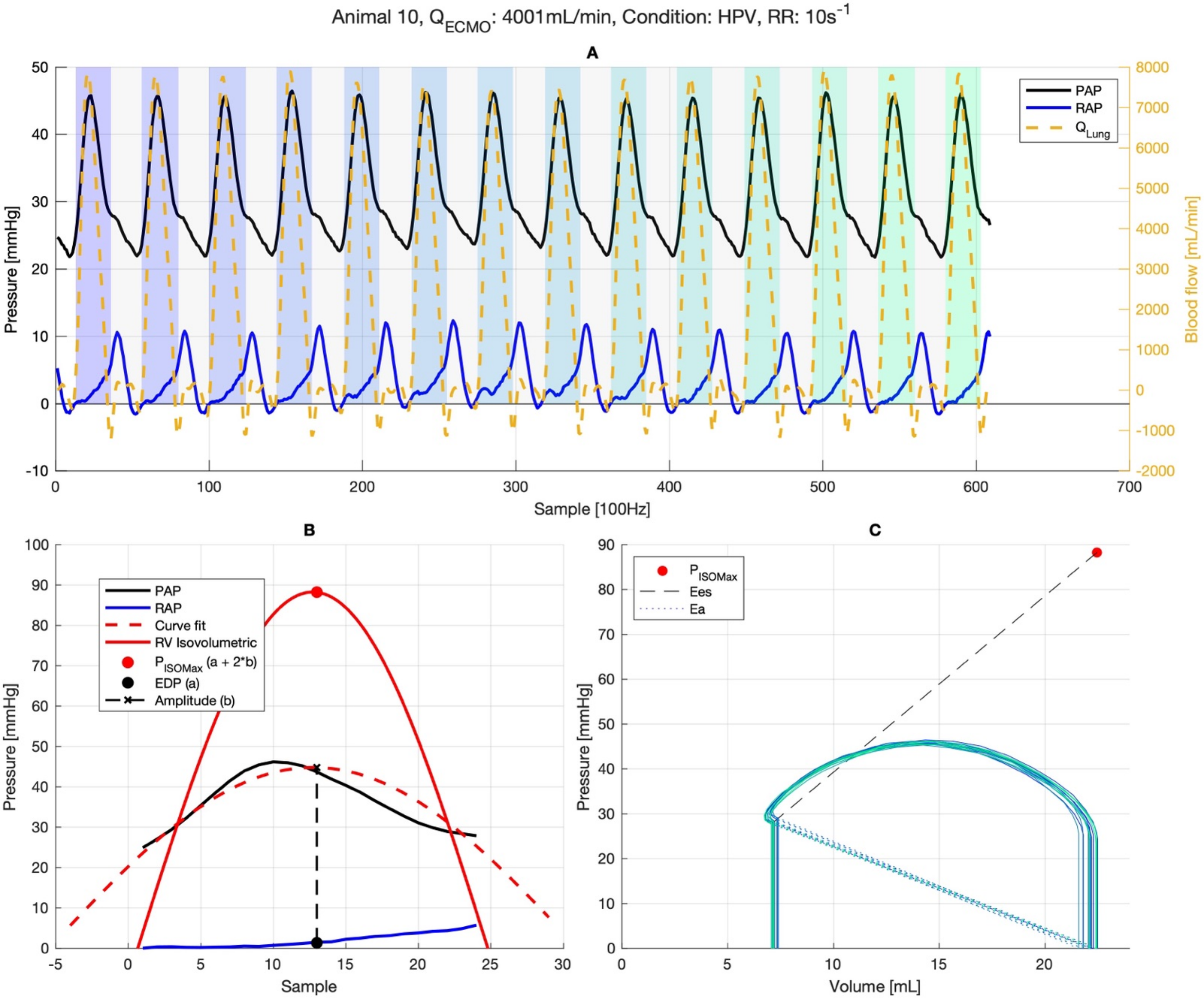

Animal 10, Q_{ECMO}: 4001mL/min, Condition: HPV, RR: $10s^{-1}$
A
PAP
RAP
Q_{Lung}
Pressure [mmHg]
Blood flow [mL/min]
Sample [100Hz]
B
PAP
RAP
Curve fit
RV Isovolumetric
P_{ISOMax} (a + 2*b)
EDP (a)
Amplitude (b)
Pressure [mmHg]
Sample
C
P_{ISOMax}
Ees
Ea
Pressure [mmHg]
Volume [mL]

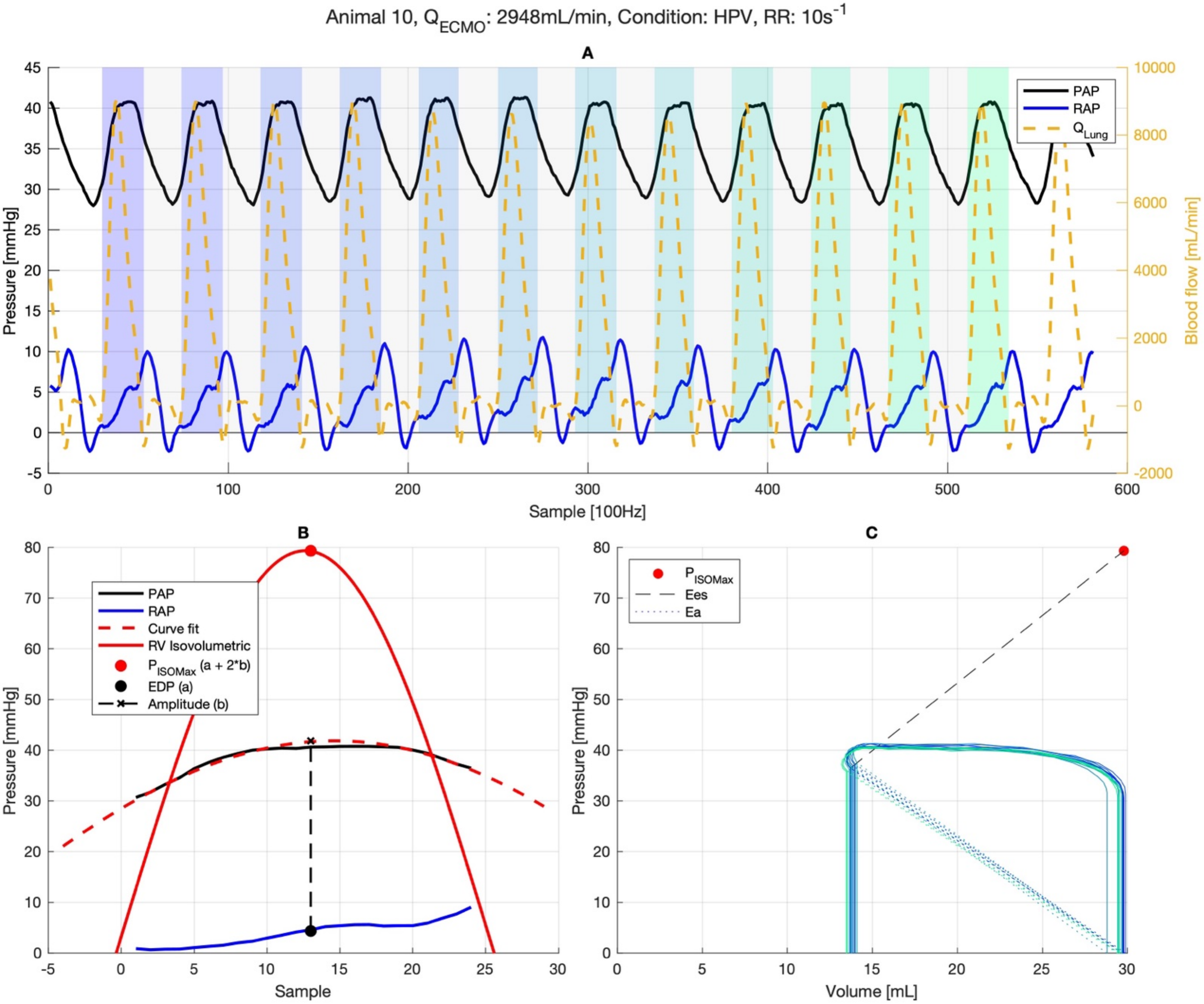

Animal 10, Q_{ECMO}: 2948mL/min, Condition: HPV, RR: 10s^{-1}
A
PAP
RAP
Q_{Lung}
Pressure [mmHg]
Blood flow [mL/min]
Sample [100Hz]
B
PAP
RAP
Curve fit
RV Isovolumetric
P_{ISOMax} (a + 2*b)
EDP (a)
Amplitude (b)
Pressure [mmHg]
Sample
C
P_{ISOMax}
Ees
Ea
Pressure [mmHg]
Volume [mL]

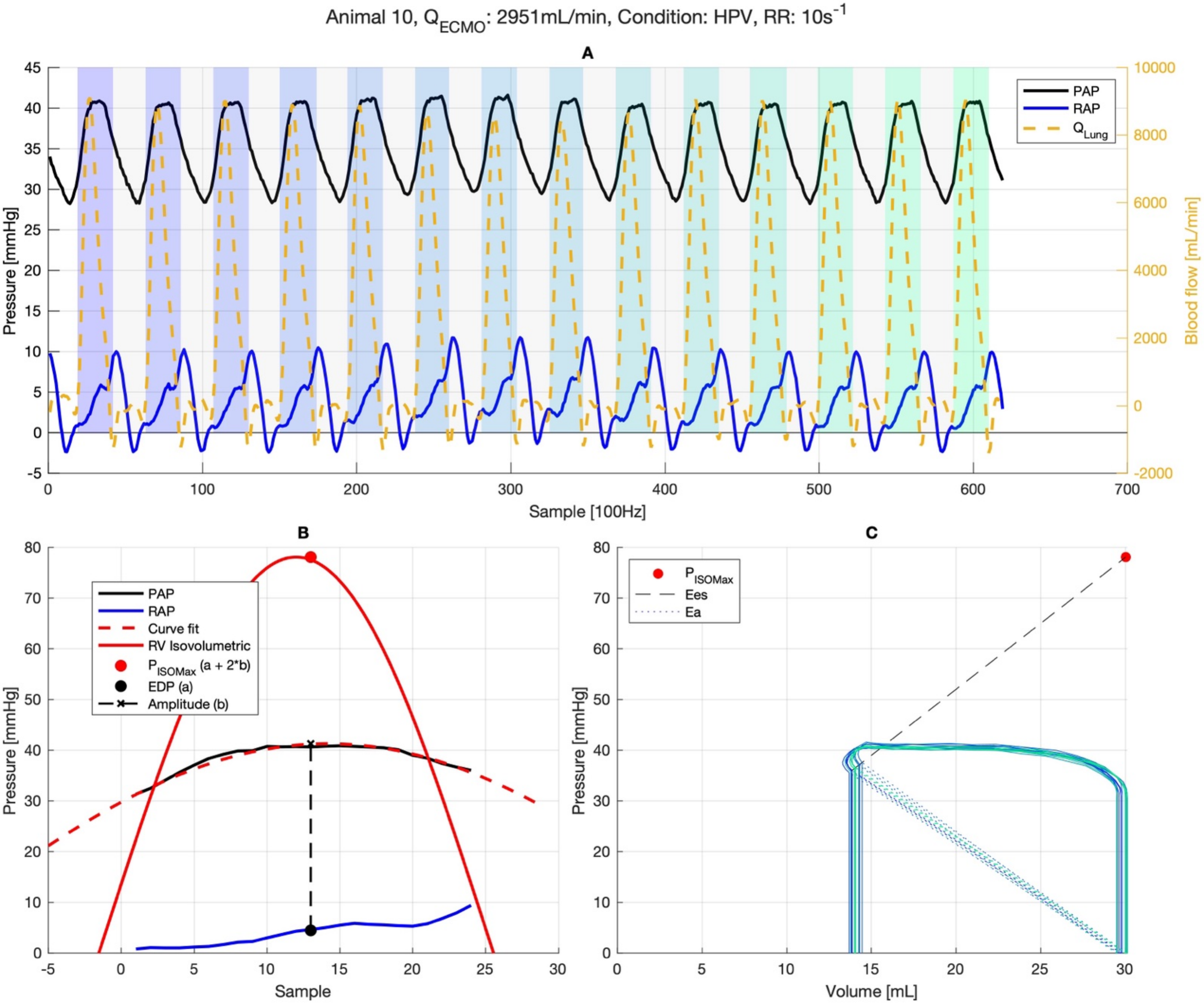

Animal 10, Q_ECMO: 2951mL/min, Condition: HPV, RR: 10s^-1
A
Pressure [mmHg]
Blood flow [mL/min]
Sample [100Hz]
PAP
RAP
Q_Lung
B
PAP
RAP
Curve fit
RV Isovolumetric
P_ISOMax (a + 2*b)
EDP (a)
Amplitude (b)
Pressure [mmHg]
Sample
C
P_ISOMax
Ees
Ea
Pressure [mmHg]
Volume [mL]

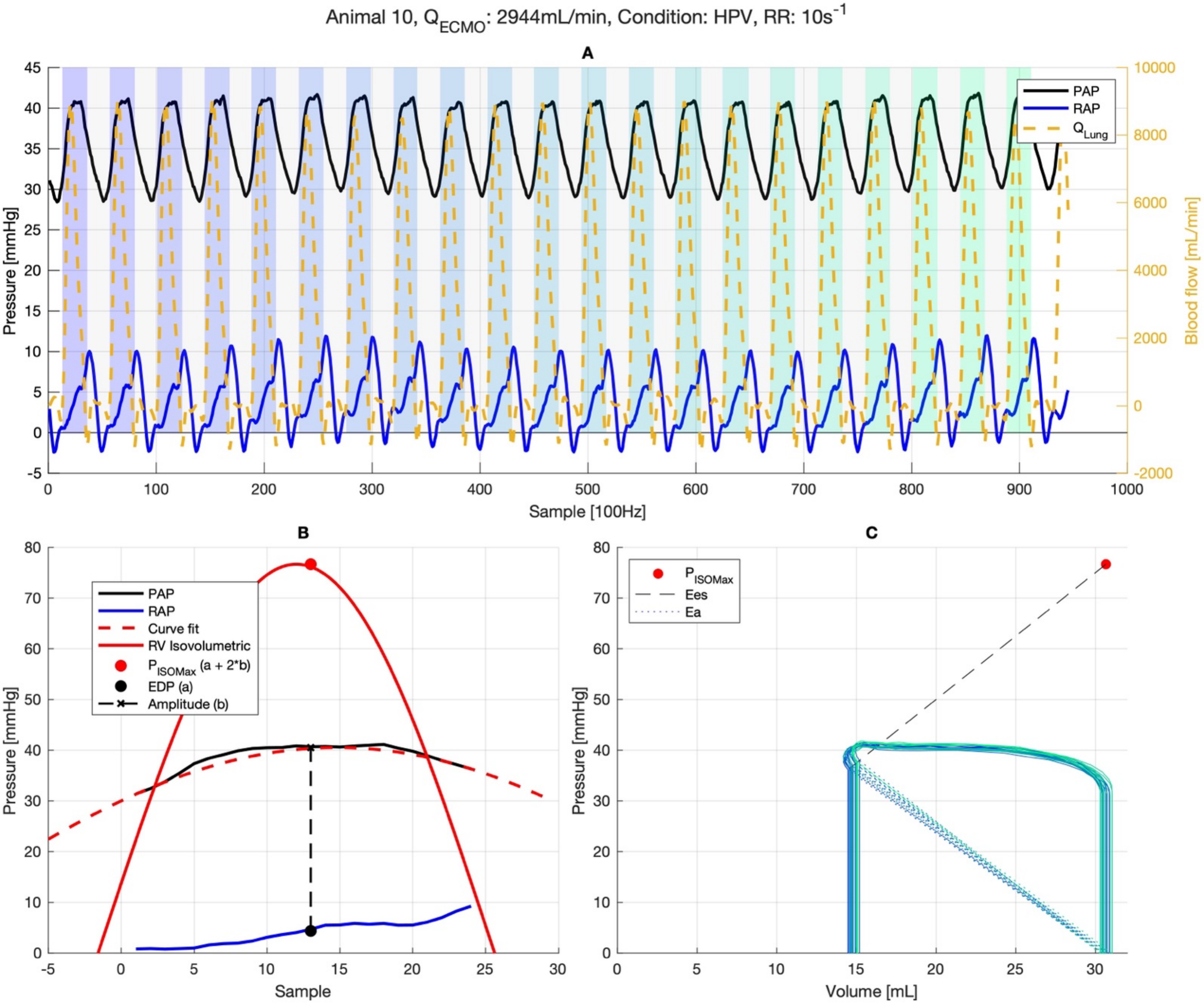
Animal 10, Q_{ECMO}: 2944mL/min, Condition: HPV, RR: $10s^{-1}$
A
PAP
RAP
Q_{Lung}
Pressure [mmHg]
Blood flow [mL/min]
Sample [100Hz]
B
PAP
RAP
Curve fit
RV Isovolumetric
P_{ISOMax} (a + 2*b)
EDP (a)
Amplitude (b)
Pressure [mmHg]
Sample
C
P_{ISOMax}
Ees
Ea
Pressure [mmHg]
Volume [mL]

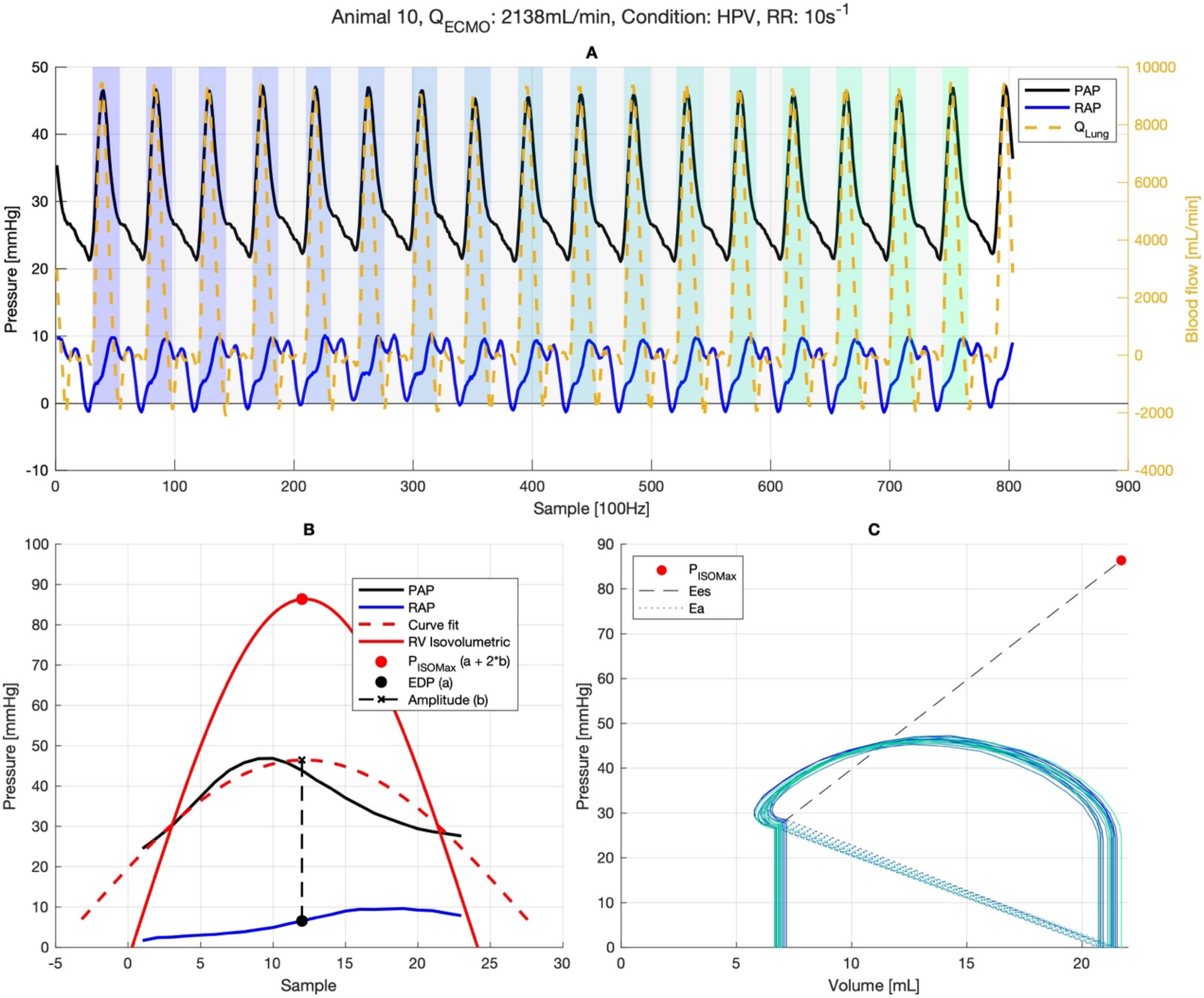

Animal 10, Q_ECMO: 2138mL/min, Condition: HPV, RR: 10s^-1
A
PAP
RAP
Q_Lung
Pressure [mmHg]
Blood flow [mL/min]
Sample [100Hz]
B
PAP
RAP
Curve fit
RV Isovolumetric
P_ISOMax (a + 2*b)
EDP (a)
Amplitude (b)
Pressure [mmHg]
Sample
C
P_ISOMax
Ees
Ea
Pressure [mmHg]
Volume [mL]

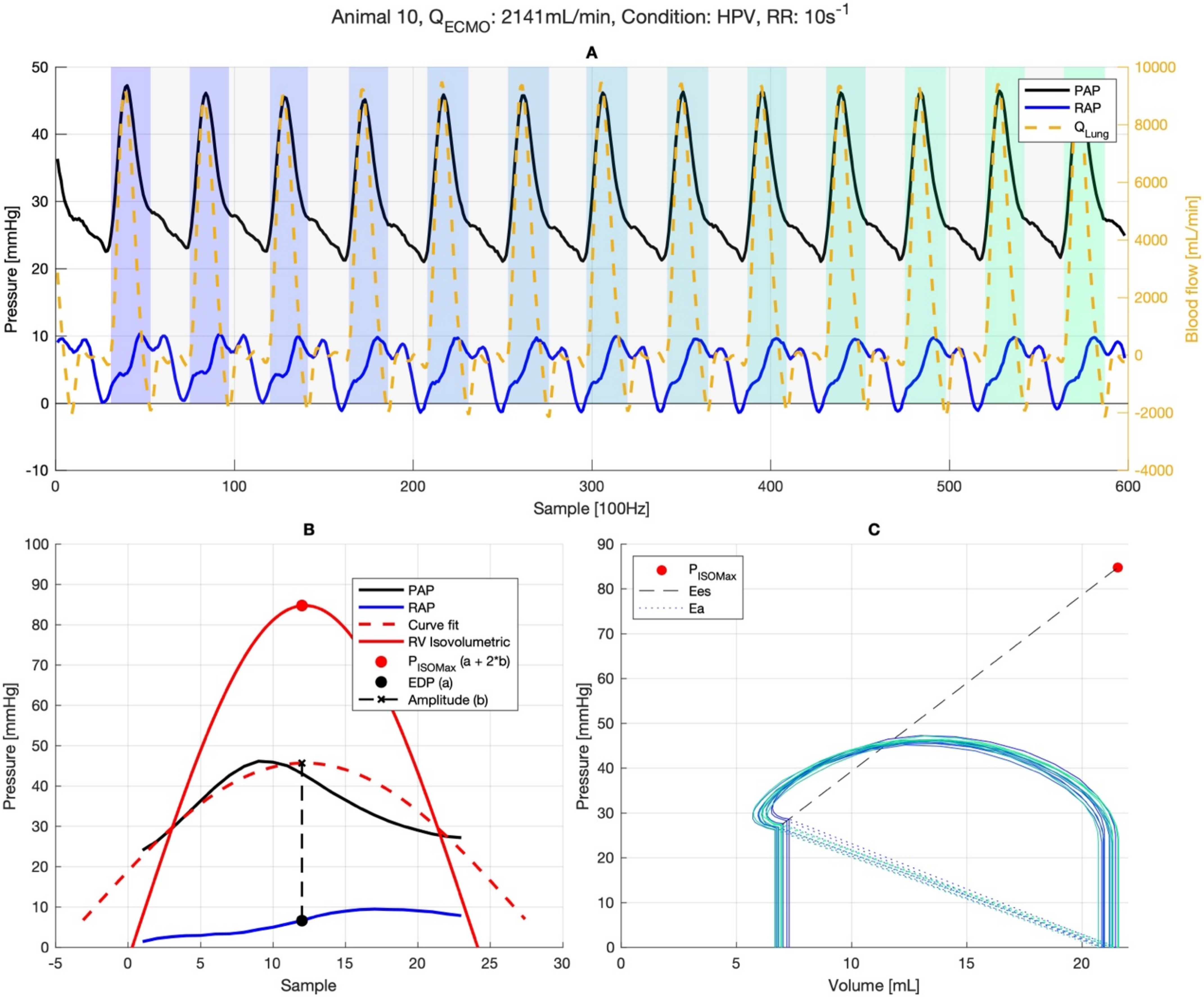

Animal 10, Q_{ECMO}: 2141mL/min, Condition: HPV, RR: $10s^{-1}$
A
Pressure [mmHg]
Blood flow [mL/min]
Sample [100Hz]
PAP
RAP
Q_{Lung}
B
Pressure [mmHg]
Sample
PAP
RAP
Curve fit
RV Isovolumetric
P_{ISOMax} (a + 2*b)
EDP (a)
Amplitude (b)
C
Pressure [mmHg]
Volume [mL]
P_{ISOMax}
Ees
Ea

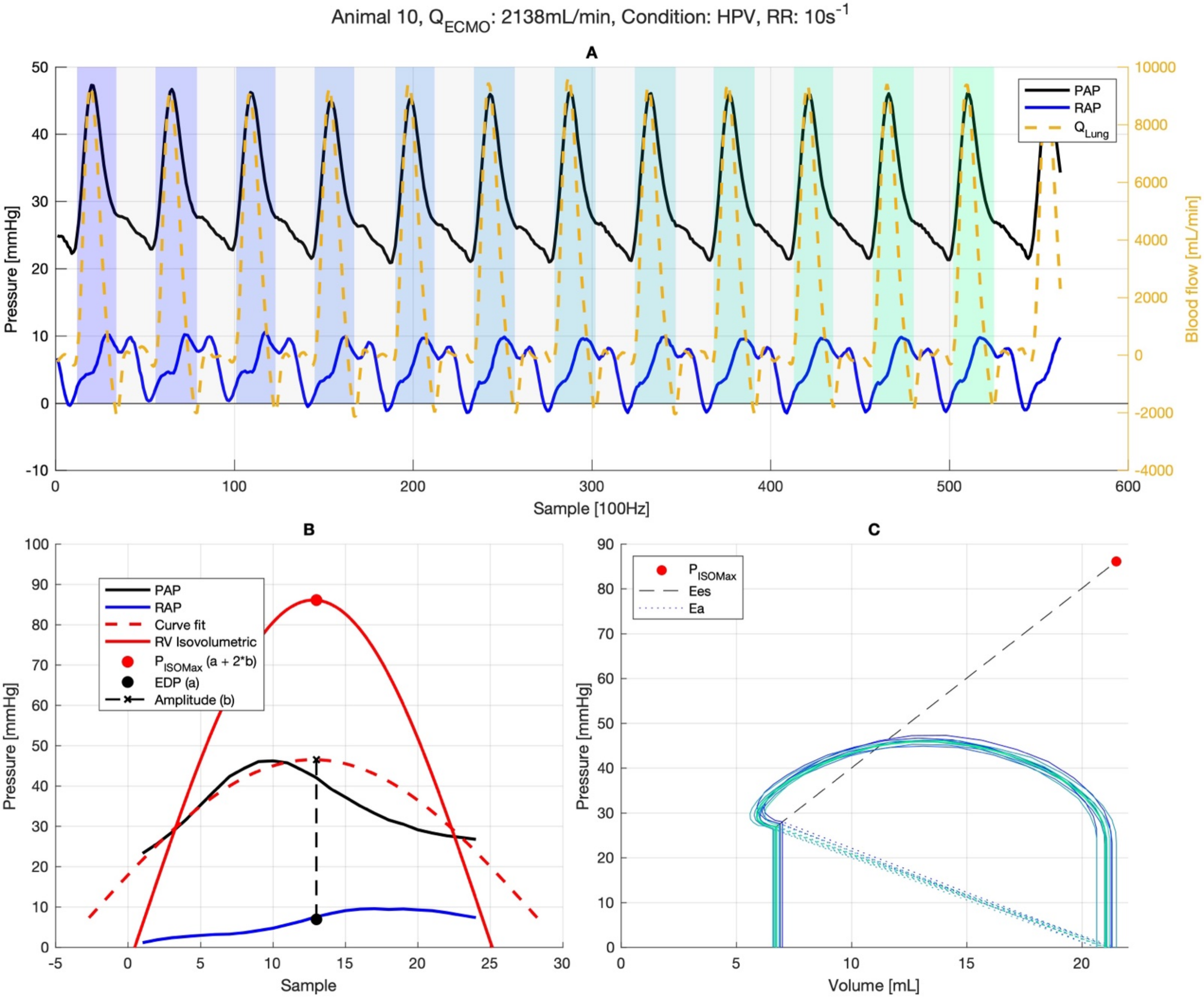

Animal 10, Q_ECMO: 2138mL/min, Condition: HPV, RR: 10s^-1
A
PAP
RAP
Q_Lung
Pressure [mmHg]
Blood flow [mL/min]
Sample [100Hz]
B
PAP
RAP
Curve fit
RV Isovolumetric
P_ISOMax (a + 2*b)
EDP (a)
Amplitude (b)
Pressure [mmHg]
Sample
C
P_ISOMax
Ees
Ea
Pressure [mmHg]
Volume [mL]

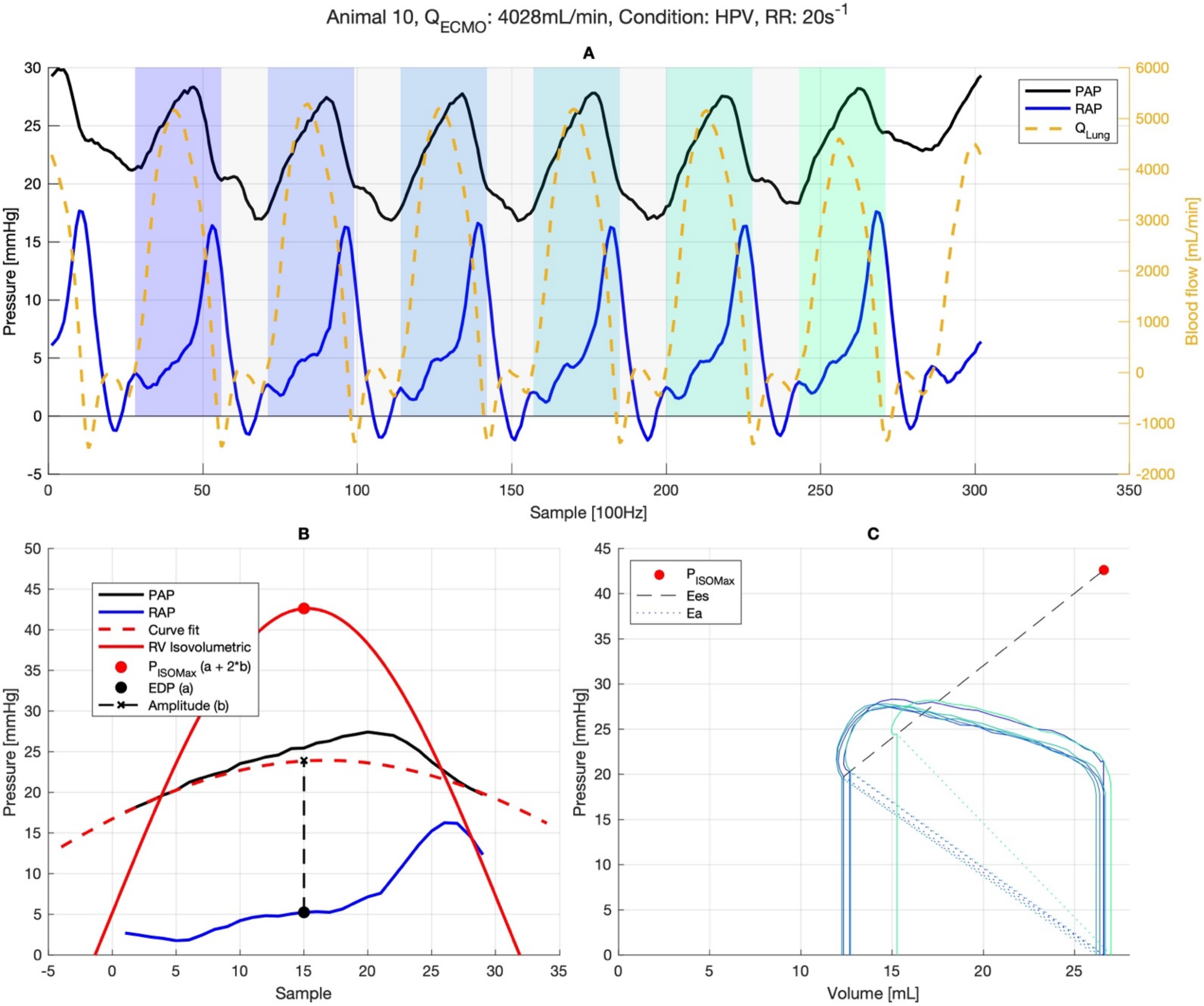

Animal 10, Q_{ECMO}: 4028mL/min, Condition: HPV, RR: 20s^{-1}
A
PAP
RAP
Q_{Lung}
Pressure [mmHg]
Blood flow [mL/min]
Sample [100Hz]
B
PAP
RAP
Curve fit
RV Isovolumetric
P_{ISOMax} (a + 2*b)
EDP (a)
Amplitude (b)
Pressure [mmHg]
Sample
C
P_{ISOMax}
Ees
Ea
Pressure [mmHg]
Volume [mL]

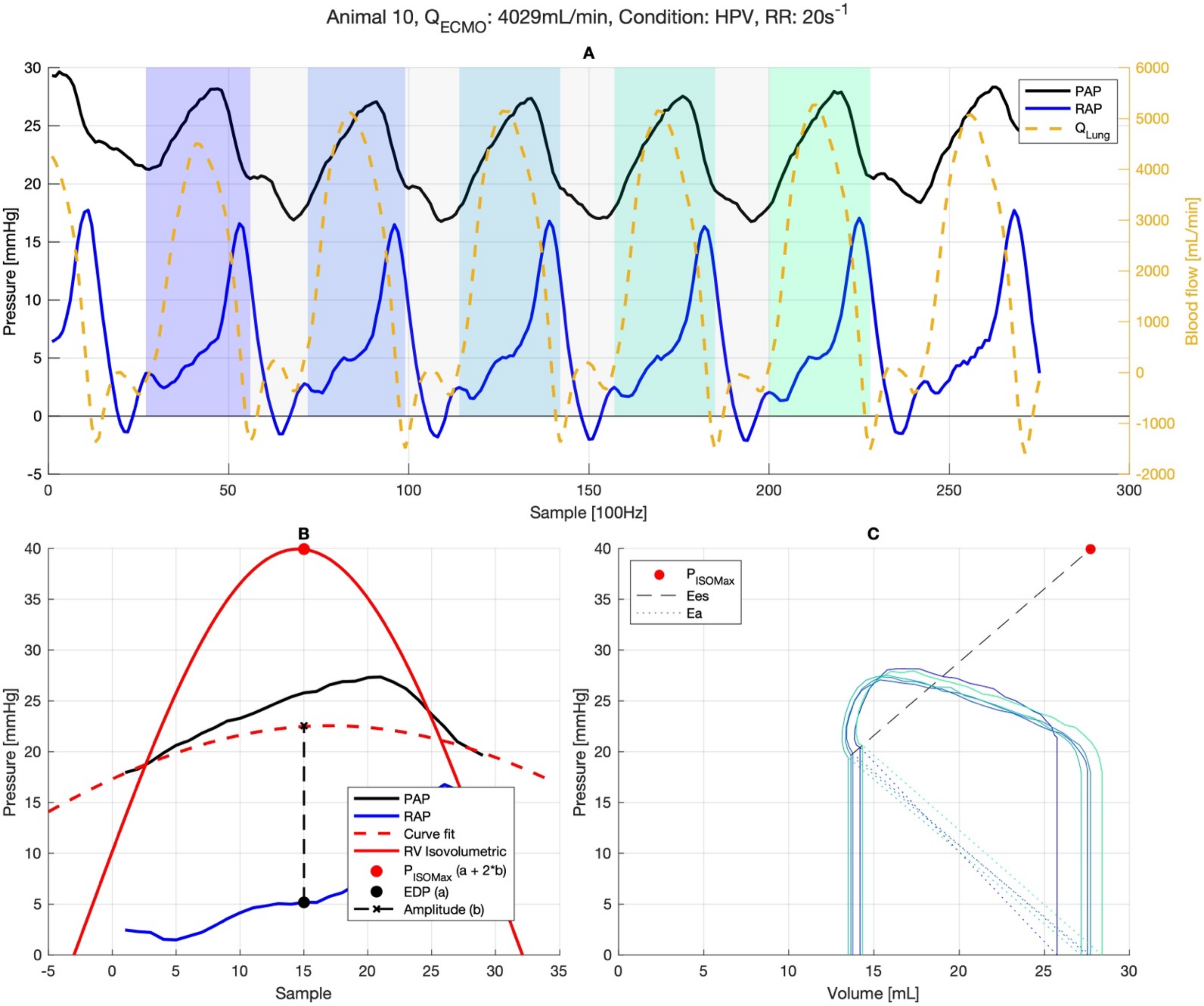

Animal 10, Q_{ECMO}: 4029mL/min, Condition: HPV, RR: $20s^{-1}$
A
PAP
RAP
Q_{Lung}
Pressure [mmHg]
Blood flow [mL/min]
Sample [100Hz]
B
PAP
RAP
Curve fit
RV Isovolumetric
P_{ISOMax} (a + 2*b)
EDP (a)
Amplitude (b)
Pressure [mmHg]
Sample
C
P_{ISOMax}
Ees
Ea
Pressure [mmHg]
Volume [mL]

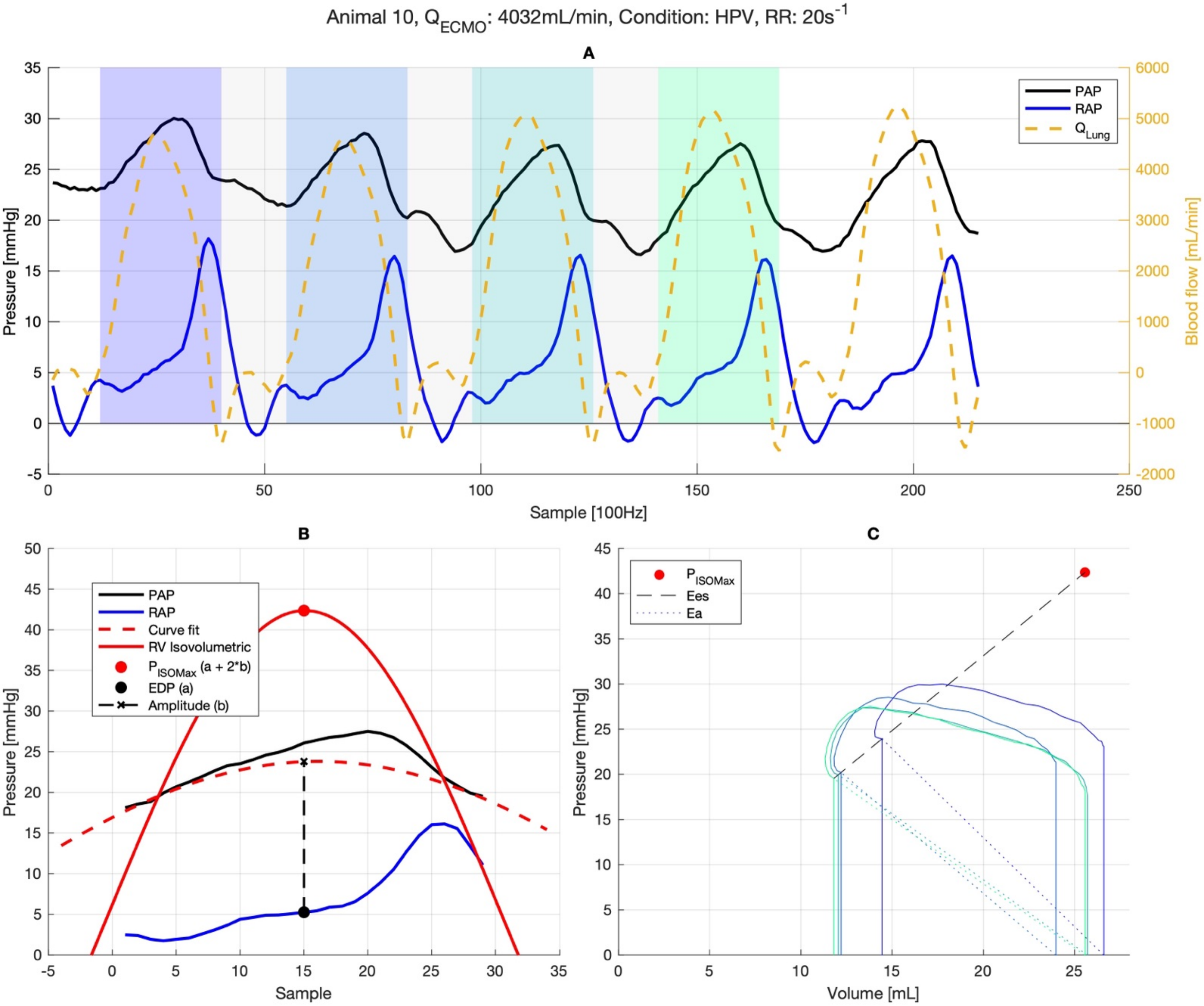
Animal 10, Q_ECMO: 4032mL/min, Condition: HPV, RR: 20s^-1
A
PAP
RAP
Q_Lung
Pressure [mmHg]
Blood flow [mL/min]
Sample [100Hz]
B
PAP
RAP
Curve fit
RV Isovolumetric
P_ISOMax (a + 2*b)
EDP (a)
Amplitude (b)
Pressure [mmHg]
Sample
C
P_ISOMax
Ees
Ea
Pressure [mmHg]
Volume [mL]

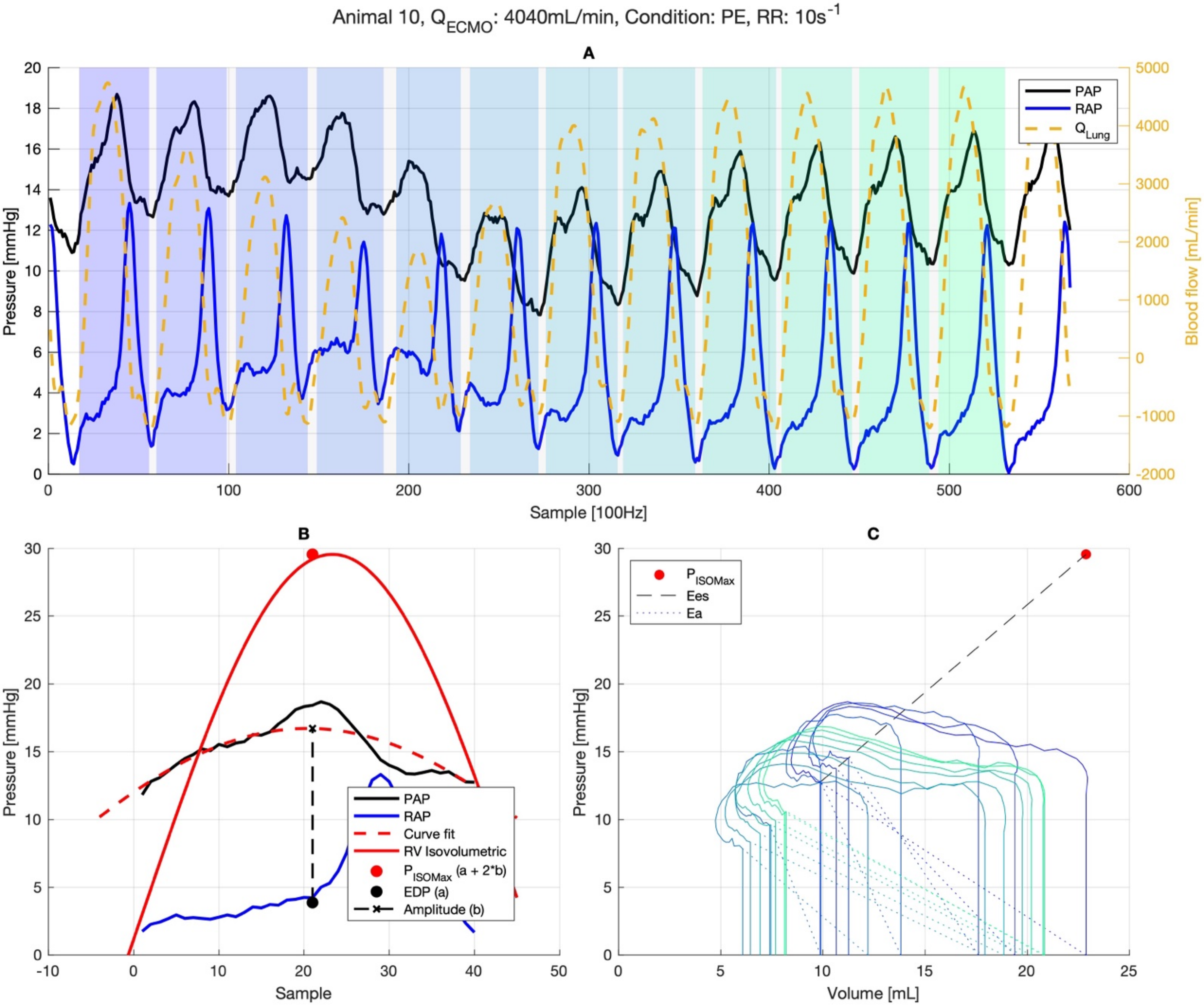
Animal 10, Q_ECMO: 4040mL/min, Condition: PE, RR: 10s^-1
A
PAP
RAP
Q_Lung
Pressure [mmHg]
Blood flow [mL/min]
Sample [100Hz]
B
PAP
RAP
Curve fit
RV Isovolumetric
P_ISOMax (a + 2*b)
EDP (a)
Amplitude (b)
Pressure [mmHg]
Sample
C
P_ISOMax
Ees
Ea
Pressure [mmHg]
Volume [mL]

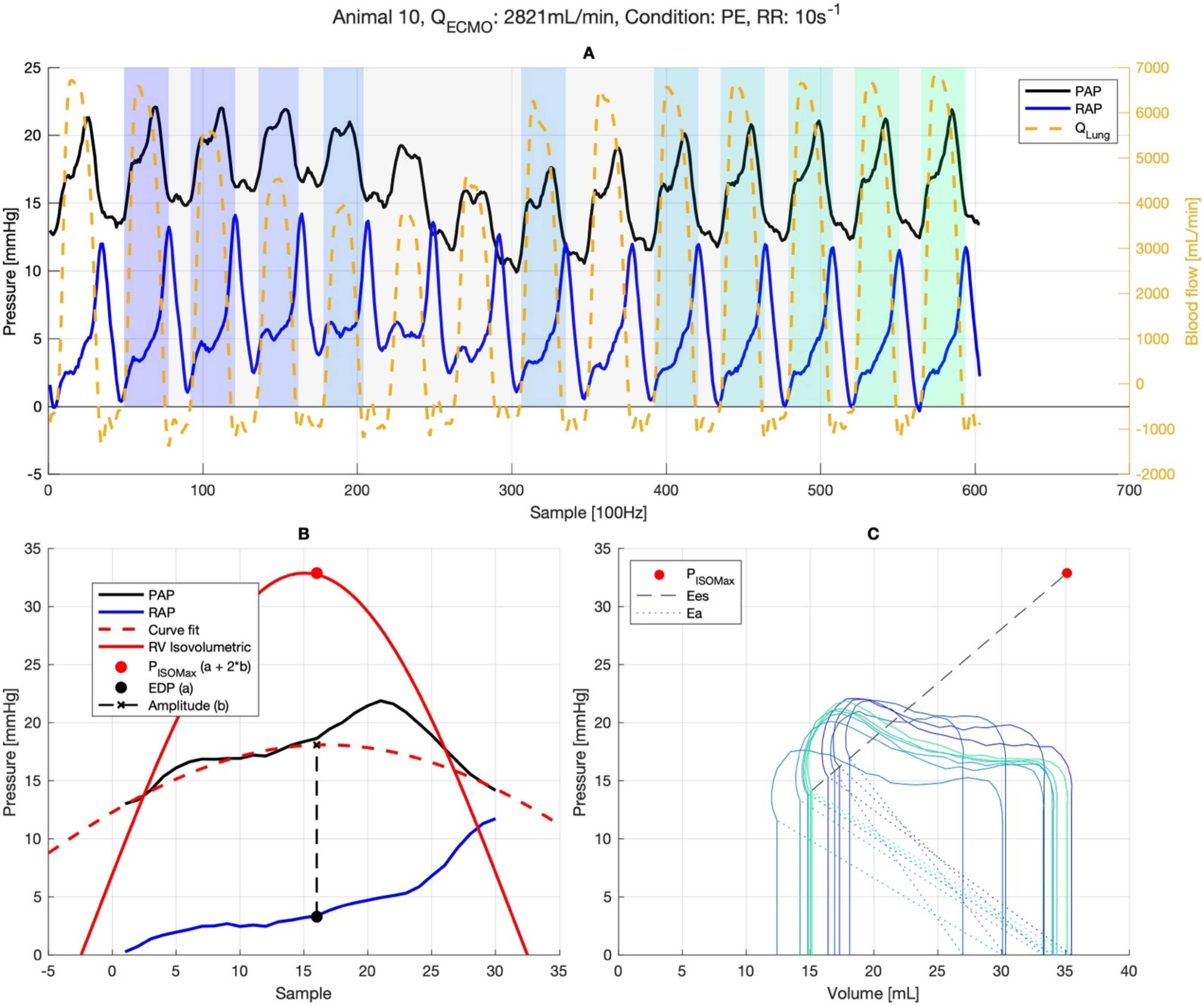
Animal 10, Q_{ECMO}: 2821mL/min, Condition: PE, RR: $10s^{-1}$
A
PAP
RAP
Q_{Lung}
Pressure [mmHg]
Blood flow [mL/min]
Sample [100Hz]
B
PAP
RAP
Curve fit
RV Isovolumetric
P_{ISOMax} (a + 2*b)
EDP (a)
Amplitude (b)
Pressure [mmHg]
Sample
C
P_{ISOMax}
Ees
Ea
Pressure [mmHg]
Volume [mL]

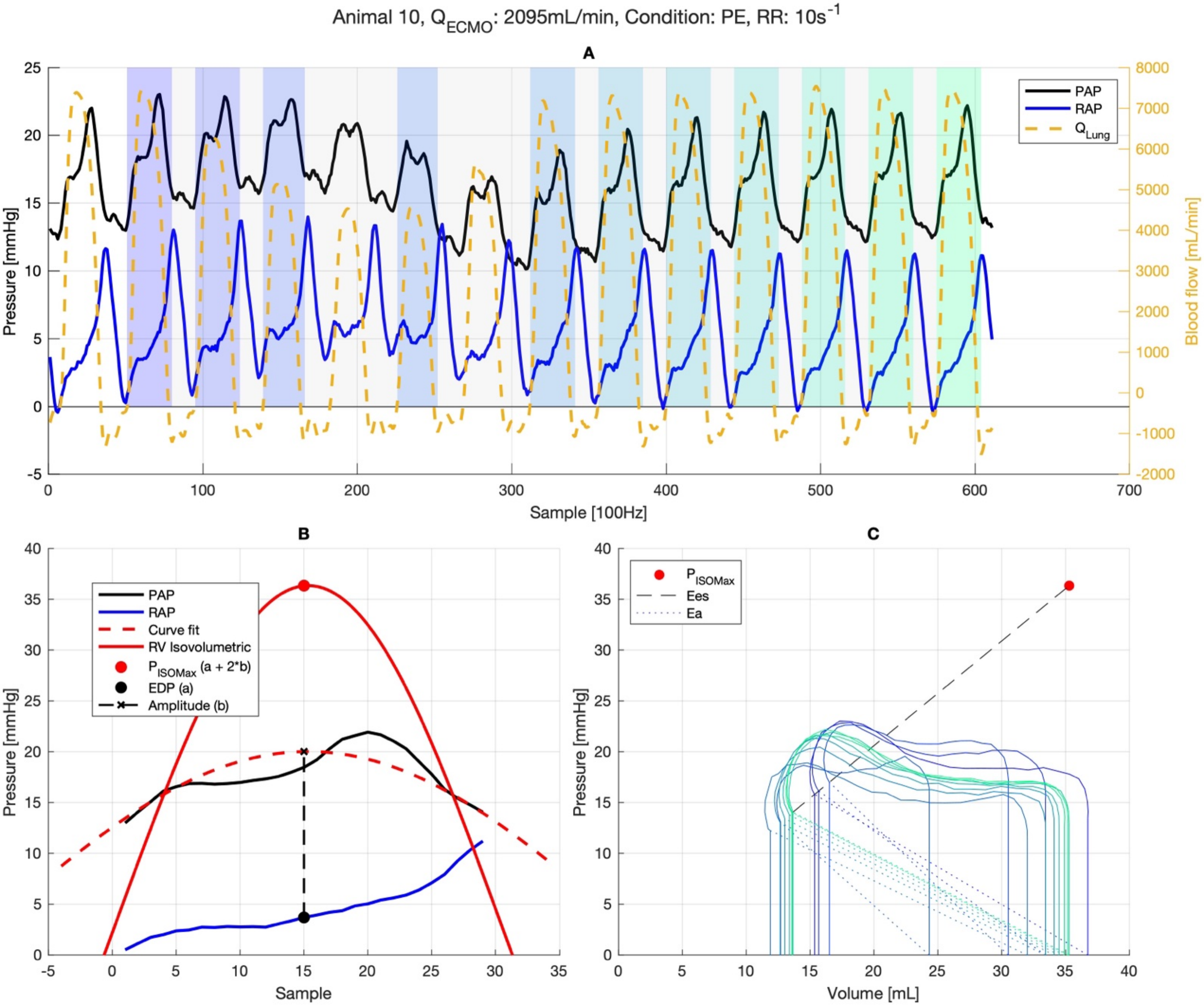
Animal 10, Q_ECMO: 2095mL/min, Condition: PE, RR: 10s^-1
A
PAP
RAP
Q_Lung
Pressure [mmHg]
Blood flow [mL/min]
Sample [100Hz]
B
PAP
RAP
Curve fit
RV Isovolumetric
P_ISOMax (a + 2*b)
EDP (a)
Amplitude (b)
Pressure [mmHg]
Sample
C
P_ISOMax
Ees
Ea
Pressure [mmHg]
Volume [mL]

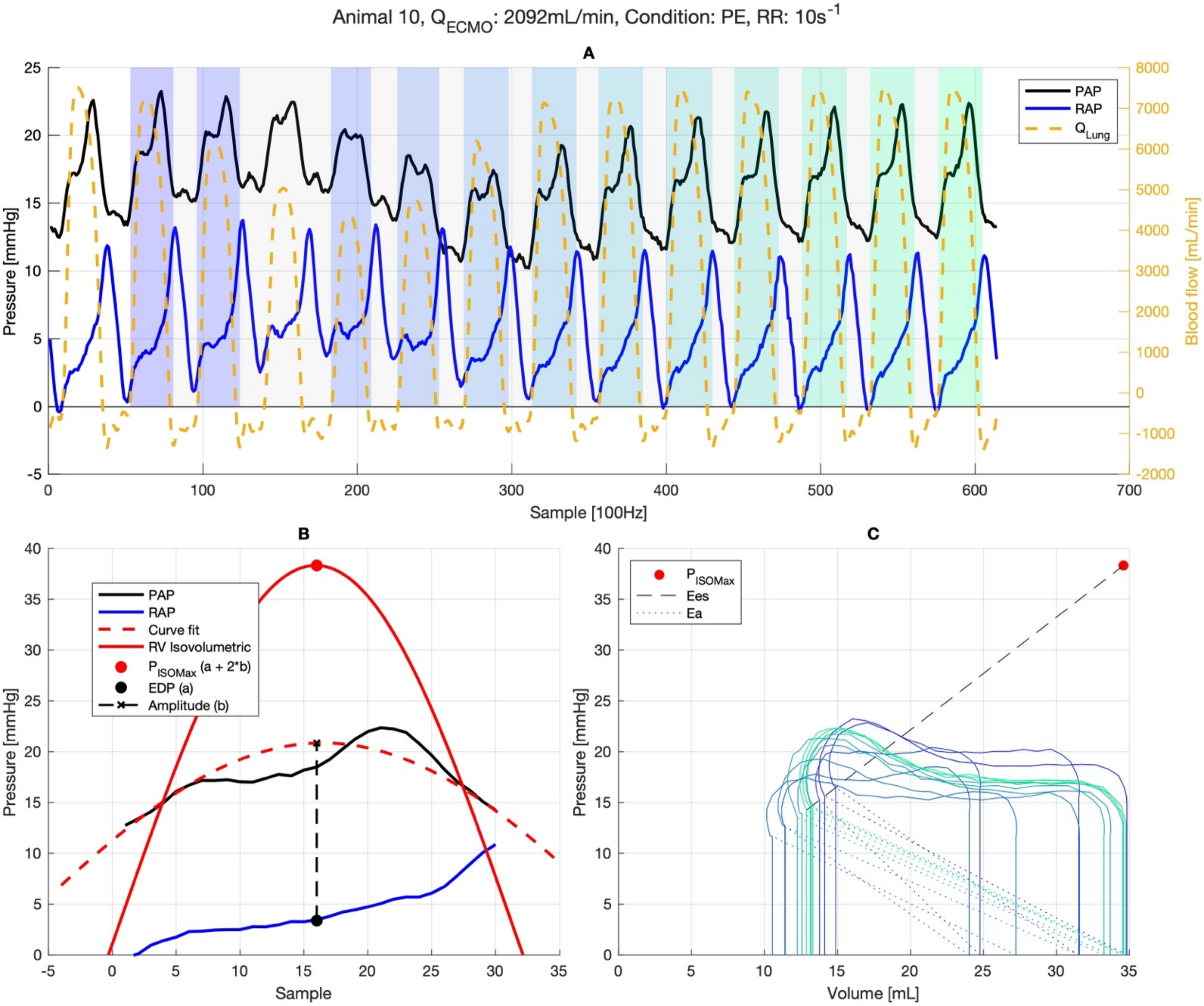

Animal 10, Q_{ECMO}: 2092mL/min, Condition: PE, RR: $10s^{-1}$
A
PAP
RAP
Q_{Lung}
Pressure [mmHg]
Blood flow [mL/min]
Sample [100Hz]
B
PAP
RAP
Curve fit
RV Isovolumetric
P_{ISOMax} (a + 2*b)
EDP (a)
Amplitude (b)
Pressure [mmHg]
Sample
C
P_{ISOMax}
Ees
Ea
Pressure [mmHg]
Volume [mL]

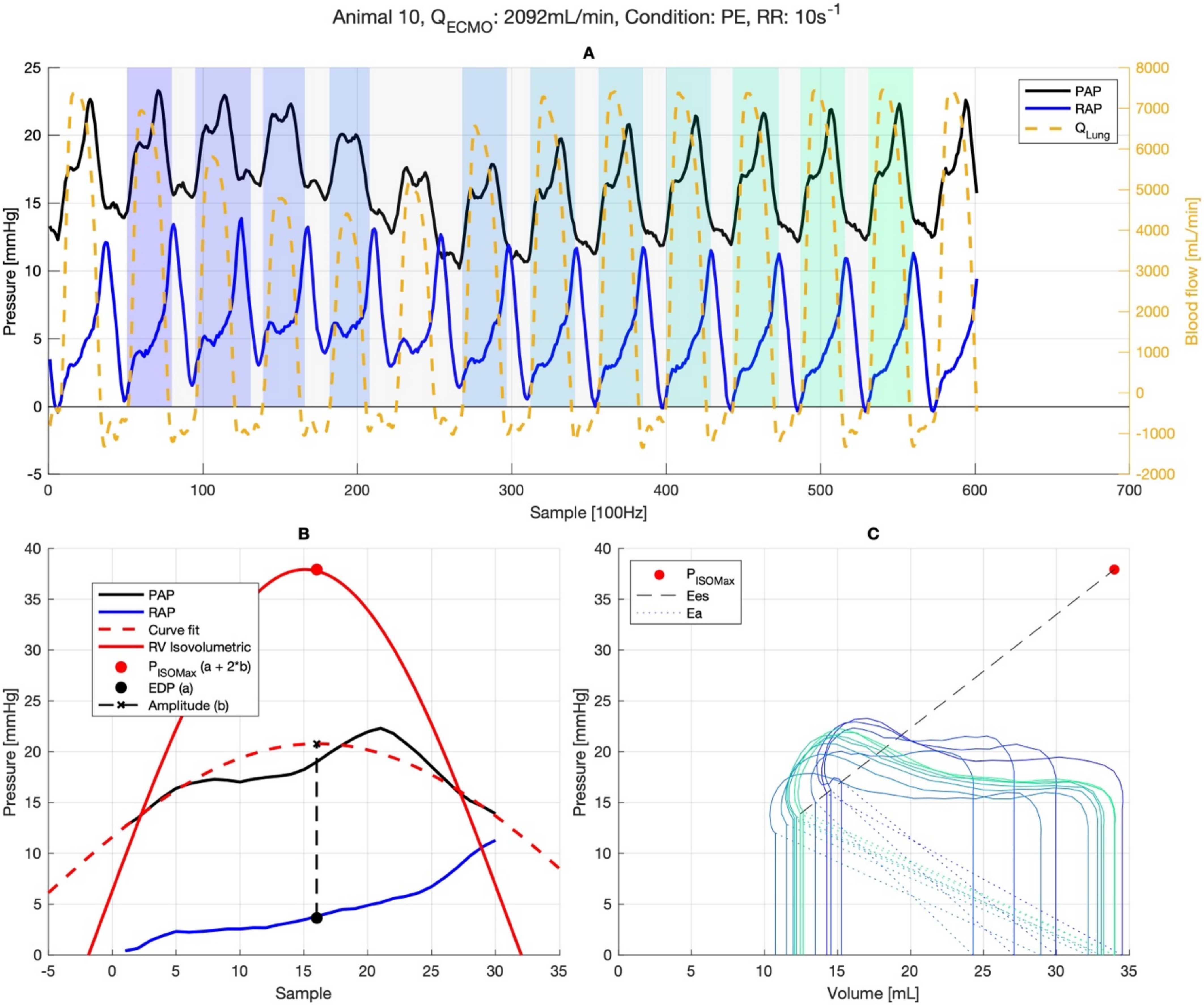

Animal 10, Q_{ECMO}: 2092mL/min, Condition: PE, RR: $10s^{-1}$
A
PAP
RAP
Q_{Lung}
Pressure [mmHg]
Blood flow [mL/min]
Sample [100Hz]
B
PAP
RAP
Curve fit
RV Isovolumetric
P_{ISOMax} (a + 2*b)
EDP (a)
Amplitude (b)
Pressure [mmHg]
Sample
C
P_{ISOMax}
Ees
Ea
Pressure [mmHg]
Volume [mL]

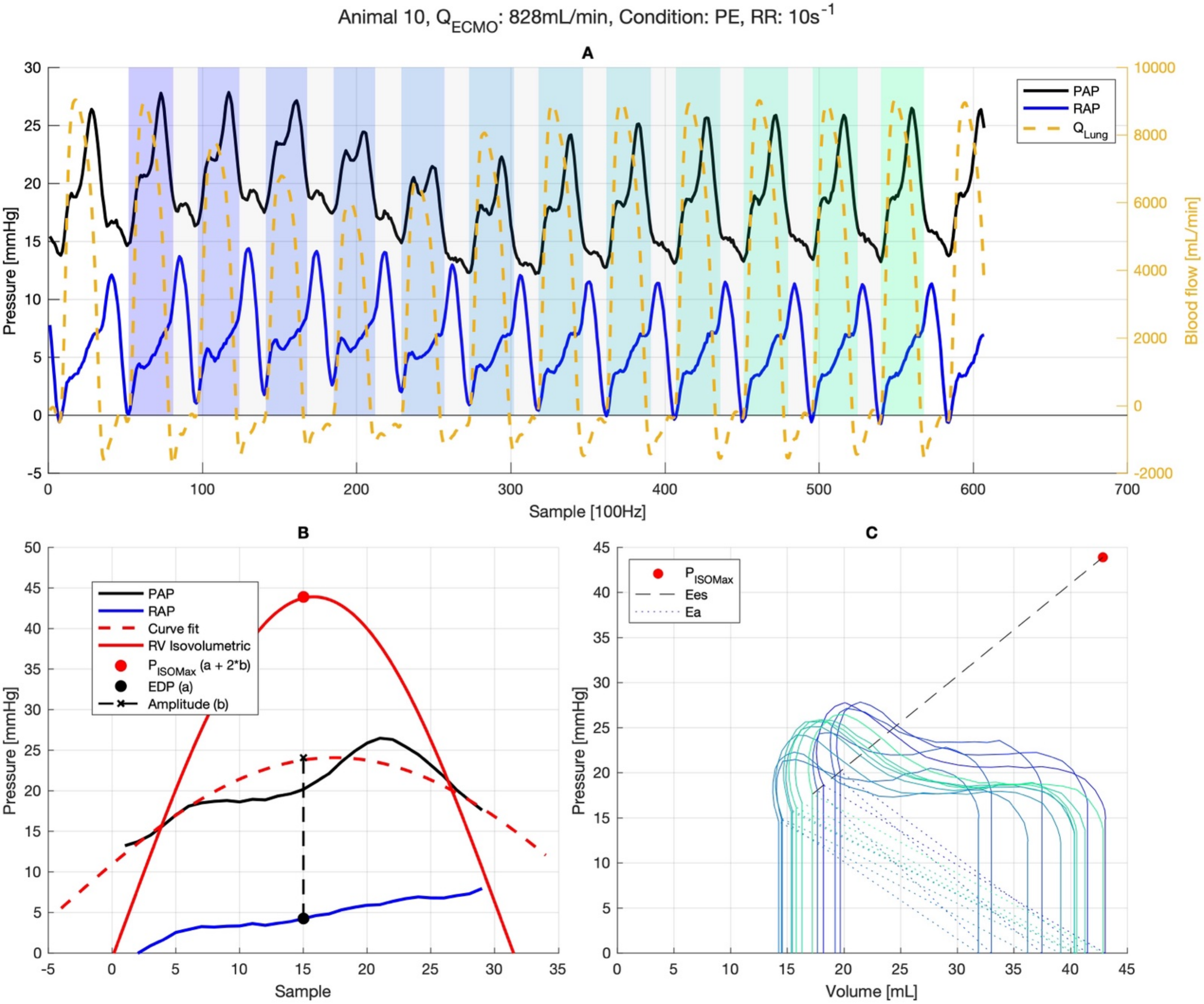

Animal 10, Q_ECMO: 828mL/min, Condition: PE, RR: 10s^-1
A
PAP
RAP
Q_Lung
Pressure [mmHg]
Blood flow [mL/min]
Sample [100Hz]
B
PAP
RAP
Curve fit
RV Isovolumetric
P_ISOMax (a + 2*b)
EDP (a)
Amplitude (b)
Pressure [mmHg]
Sample
C
P_ISOMax
Ees
Ea
Pressure [mmHg]
Volume [mL]

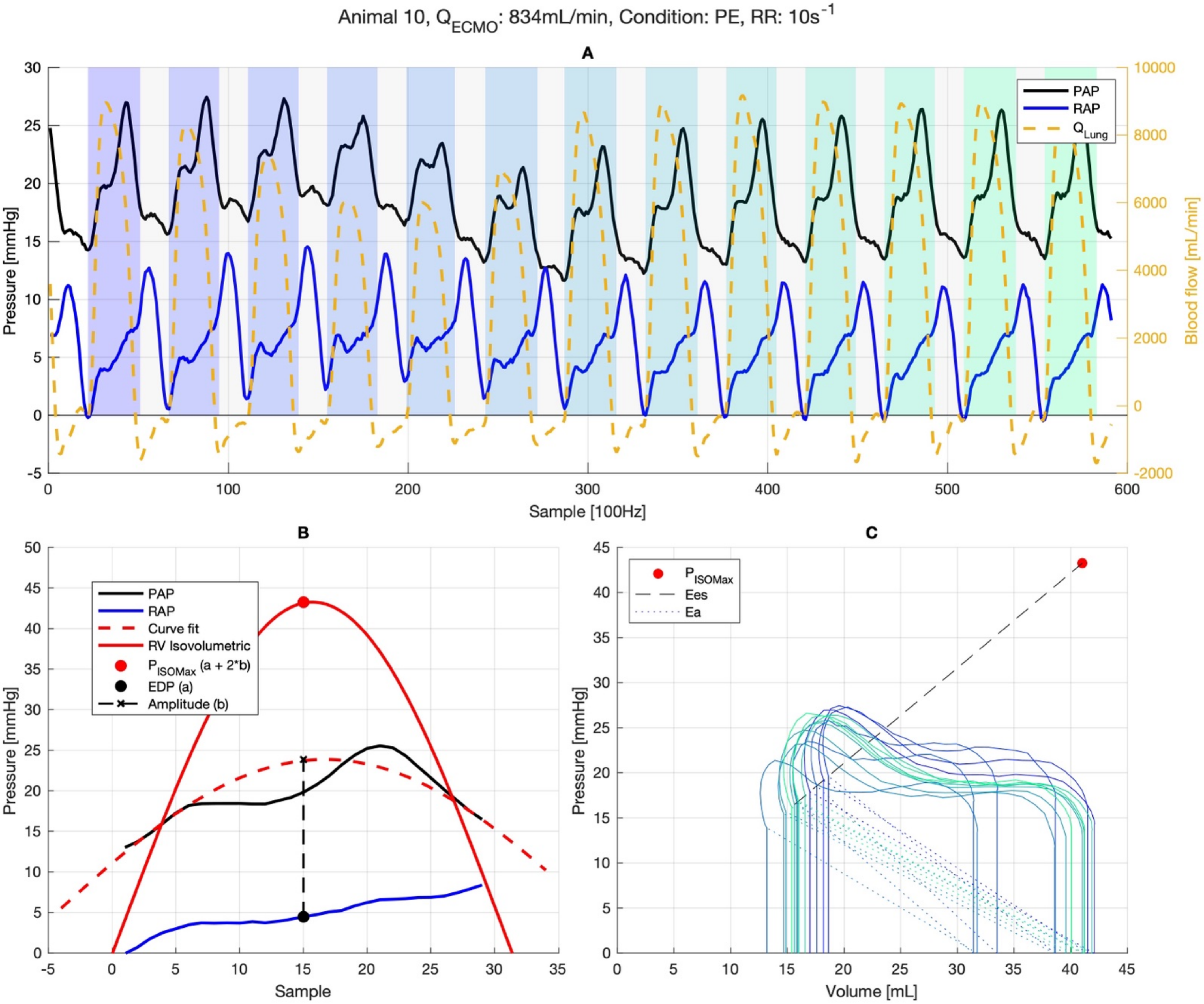

Animal 10, Q_ECMO: 834mL/min, Condition: PE, RR: 10s^-1
A
PAP
RAP
Q_Lung
Pressure [mmHg]
Blood flow [mL/min]
Sample [100Hz]
B
PAP
RAP
Curve fit
RV Isovolumetric
P_ISOMax (a + 2*b)
EDP (a)
Amplitude (b)
Pressure [mmHg]
Sample
C
P_ISOMax
Ees
Ea
Pressure [mmHg]
Volume [mL]

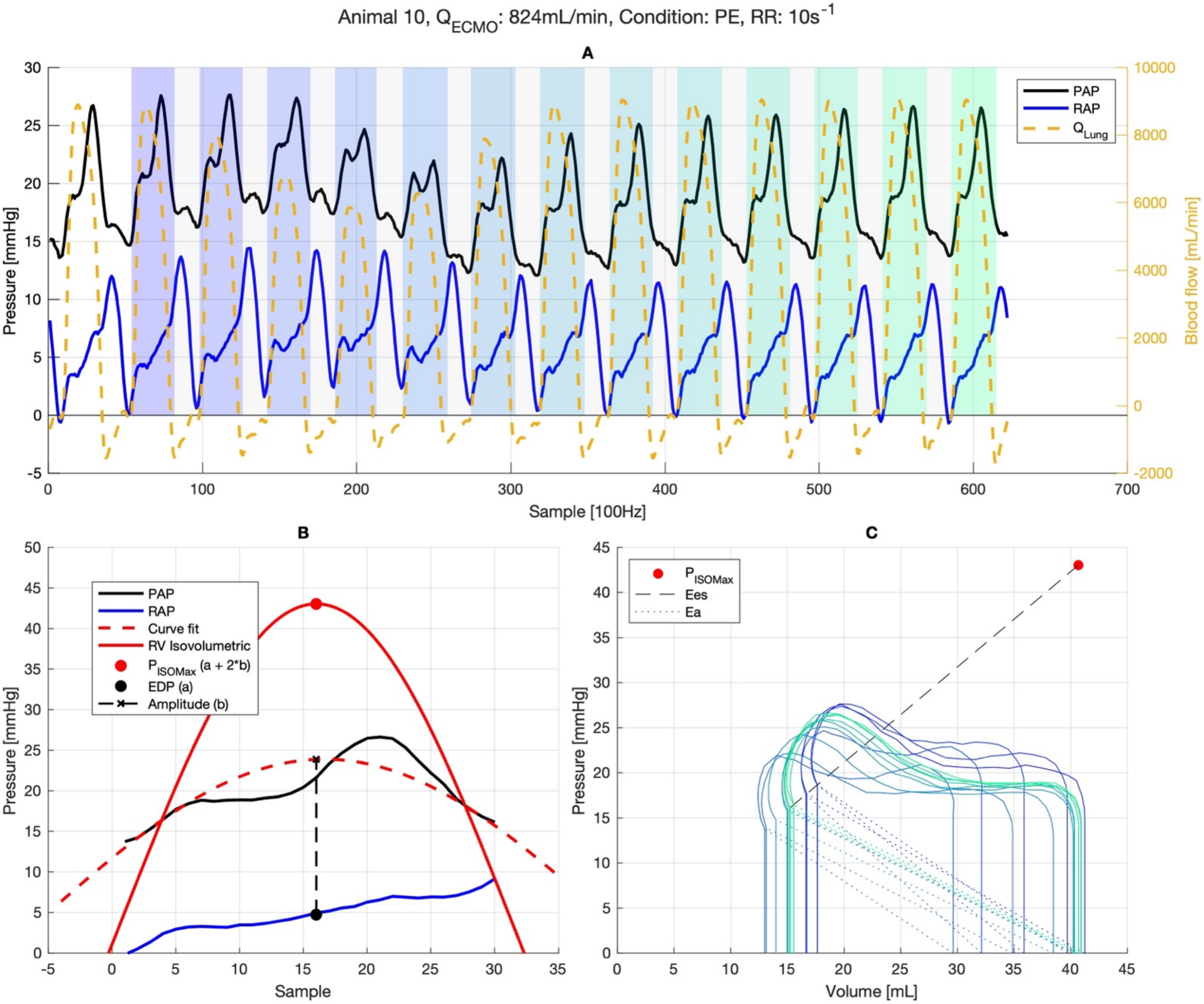

Animal 10, Q_ECMO: 824mL/min, Condition: PE, RR: 10s^-1
A
PAP
RAP
Q_Lung
Pressure [mmHg]
Blood flow [mL/min]
Sample [100Hz]
B
PAP
RAP
Curve fit
RV Isovolumetric
P_ISOMax (a + 2*b)
EDP (a)
Amplitude (b)
Pressure [mmHg]
Sample
C
P_ISOMax
Ees
Ea
Pressure [mmHg]
Volume [mL]

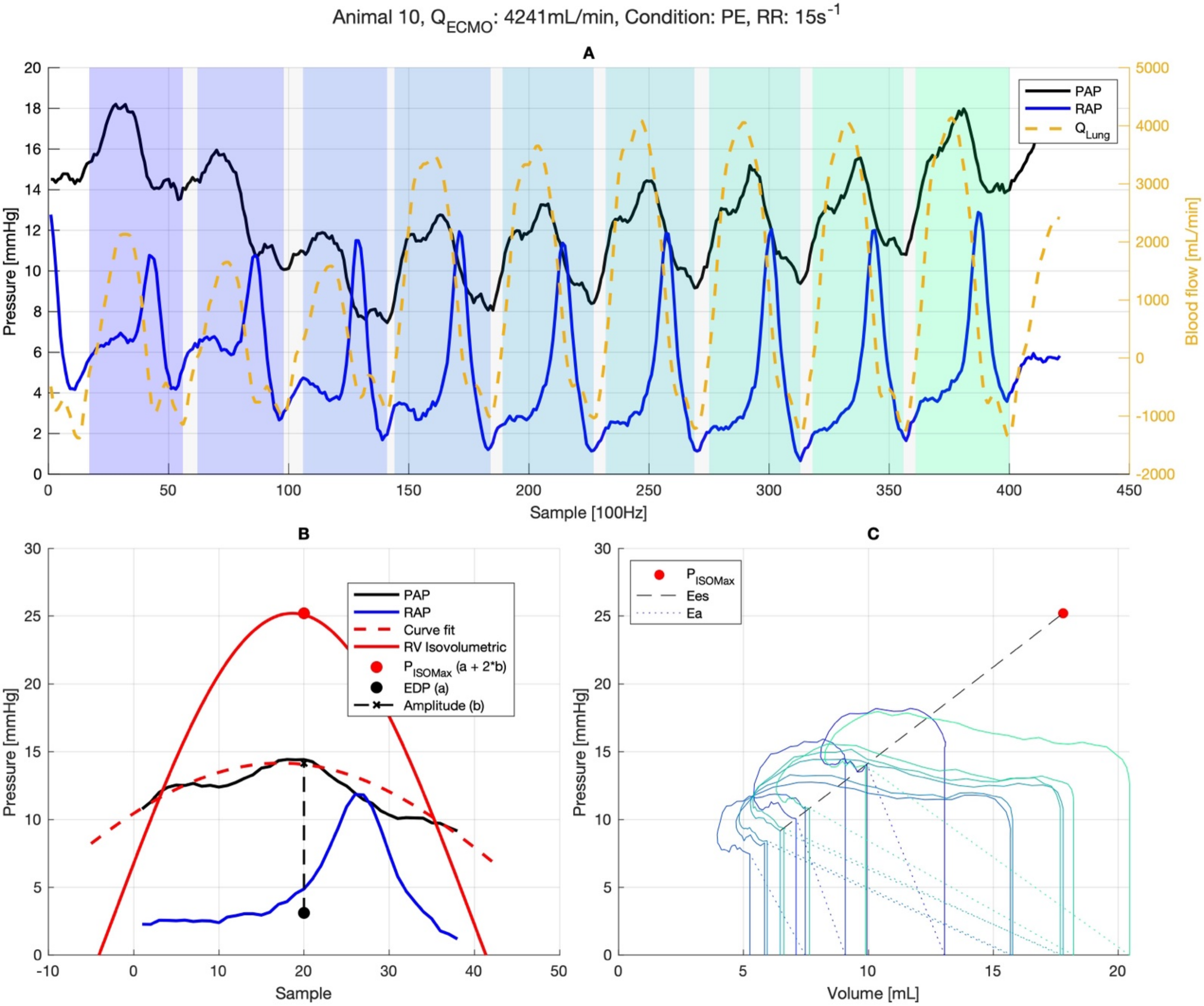

Animal 10, Q_{ECMO}: 4241mL/min, Condition: PE, RR: $15s^{-1}$
A
PAP
RAP
Q_{Lung}
Pressure [mmHg]
Blood flow [mL/min]
Sample [100Hz]
B
PAP
RAP
Curve fit
RV Isovolumetric
P_{ISOMax} (a + 2*b)
EDP (a)
Amplitude (b)
Pressure [mmHg]
Sample
C
P_{ISOMax}
Ees
Ea
Pressure [mmHg]
Volume [mL]

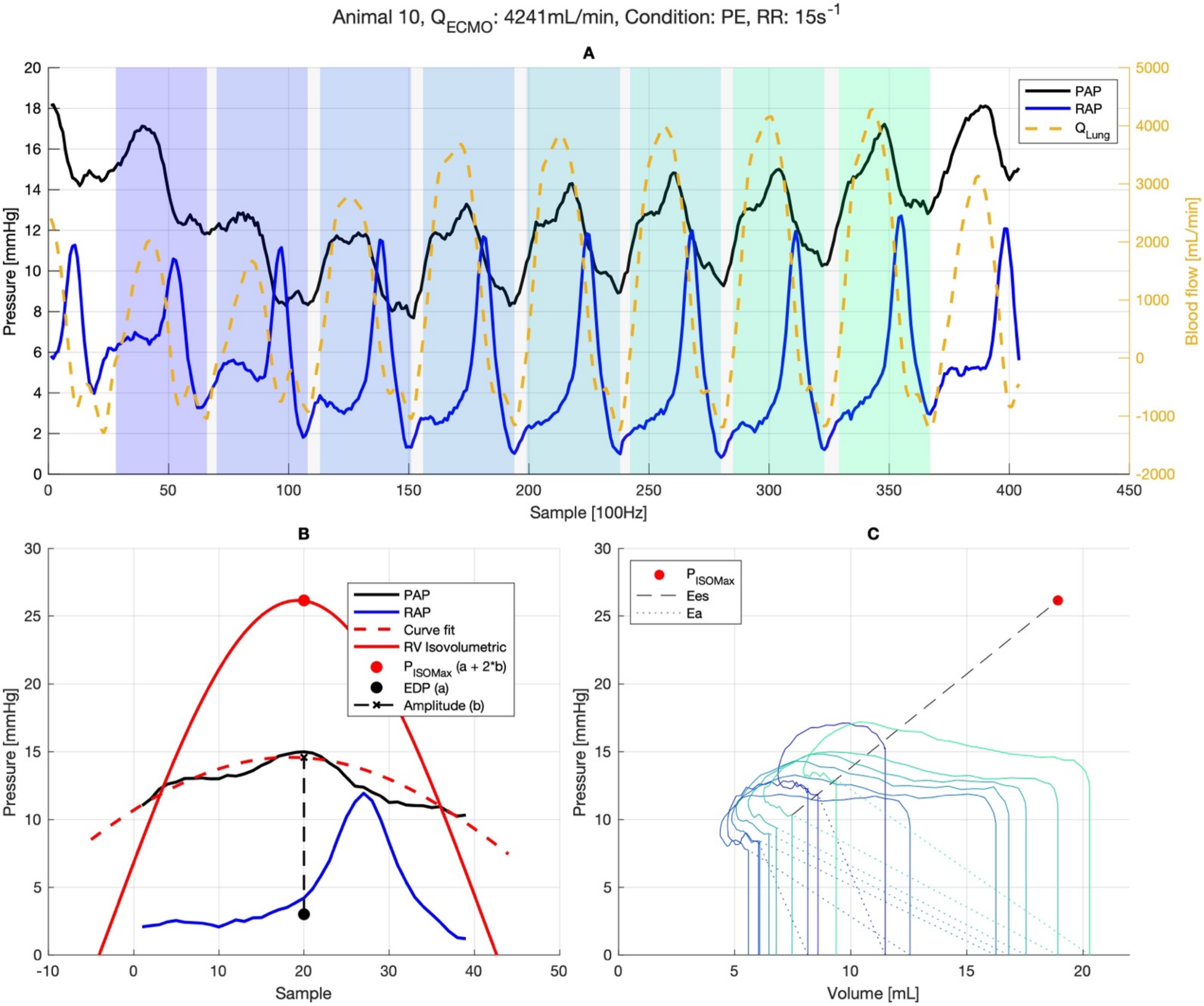

Animal 10, Q_{ECMO}: 4241mL/min, Condition: PE, RR: $15s^{-1}$
A
Pressure [mmHg]
Blood flow [mL/min]
Sample [100Hz]
PAP
RAP
Q_{Lung}
B
Pressure [mmHg]
Sample
PAP
RAP
Curve fit
RV Isovolumetric
P_{ISOMax} (a + 2*b)
EDP (a)
Amplitude (b)
C
Pressure [mmHg]
Volume [mL]
P_{ISOMax}
Ees
Ea

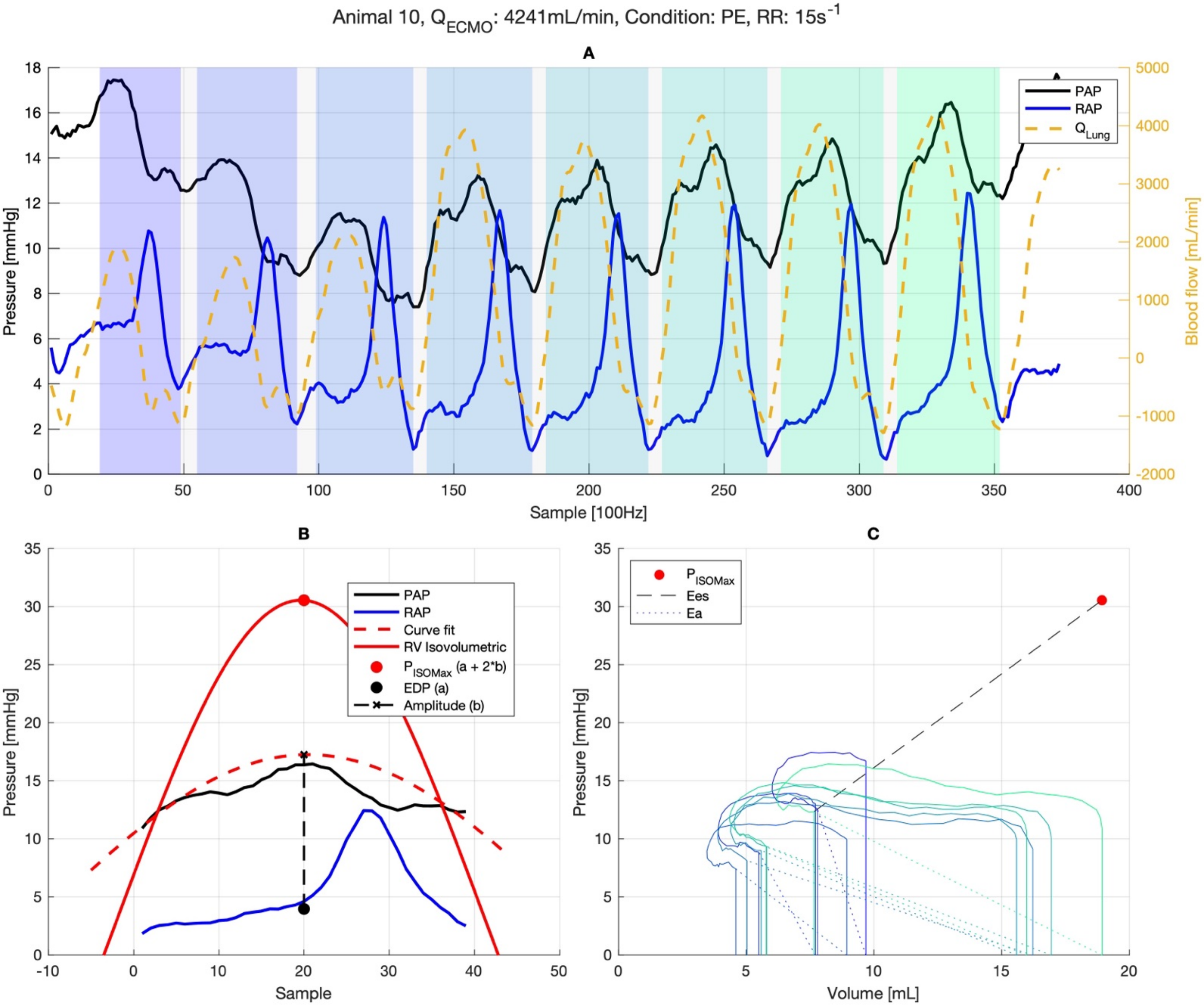
Animal 10, Q_ECMO: 4241mL/min, Condition: PE, RR: 15s^-1
A
Pressure [mmHg]
Blood flow [mL/min]
Sample [100Hz]
PAP
RAP
Q_Lung
B
PAP
RAP
Curve fit
RV Isovolumetric
P_ISOMax (a + 2*b)
EDP (a)
Amplitude (b)
Pressure [mmHg]
Sample
C
P_ISOMax
Ees
Ea
Pressure [mmHg]
Volume [mL]

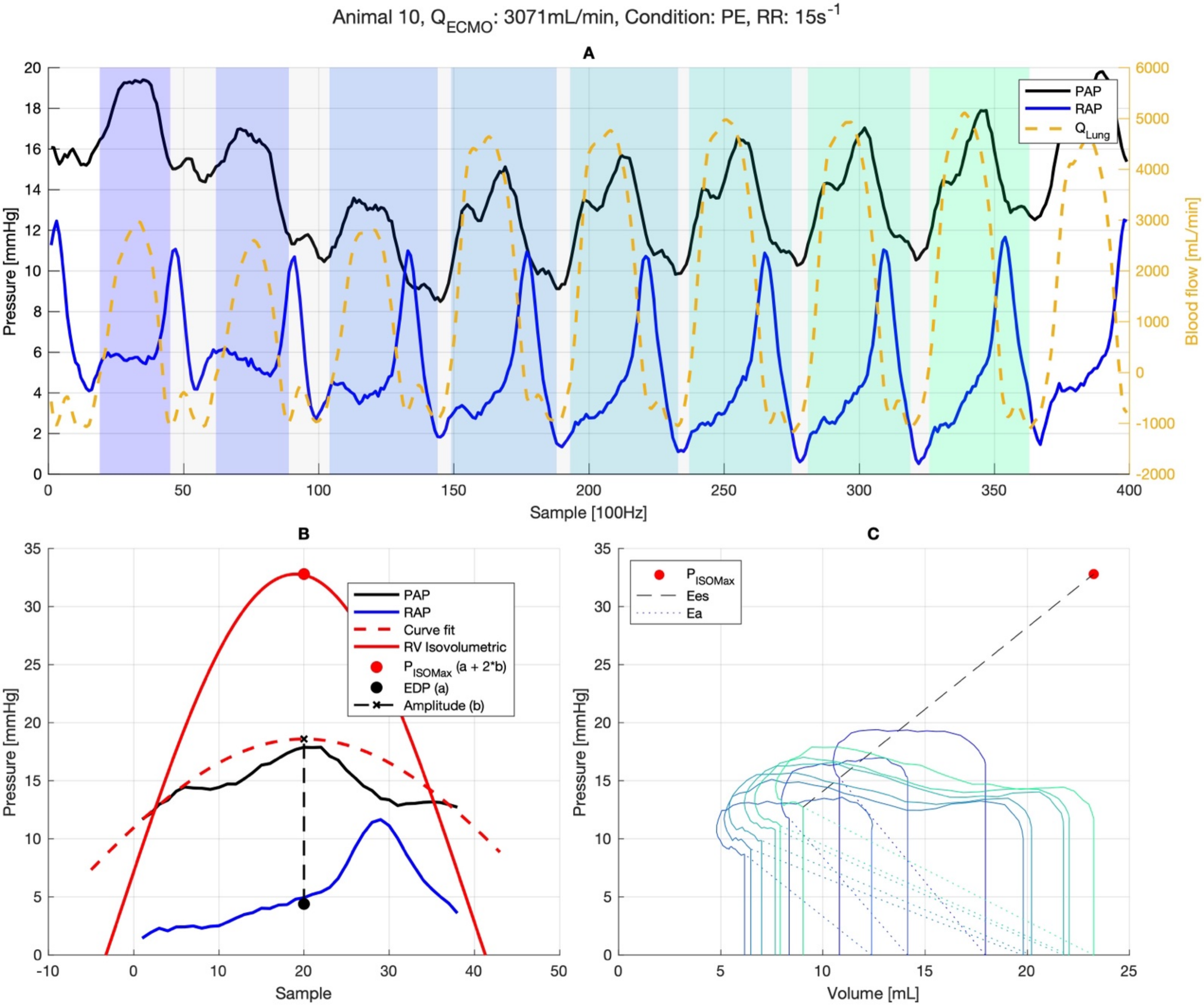

Animal 10, Q_{ECMO}: 3071mL/min, Condition: PE, RR: $15s^{-1}$
A
PAP
RAP
Q_{Lung}
Pressure [mmHg]
Blood flow [mL/min]
Sample [100Hz]
B
PAP
RAP
Curve fit
RV Isovolumetric
P_{ISOMax} (a + 2*b)
EDP (a)
Amplitude (b)
Pressure [mmHg]
Sample
C
P_{ISOMax}
Ees
Ea
Pressure [mmHg]
Volume [mL]

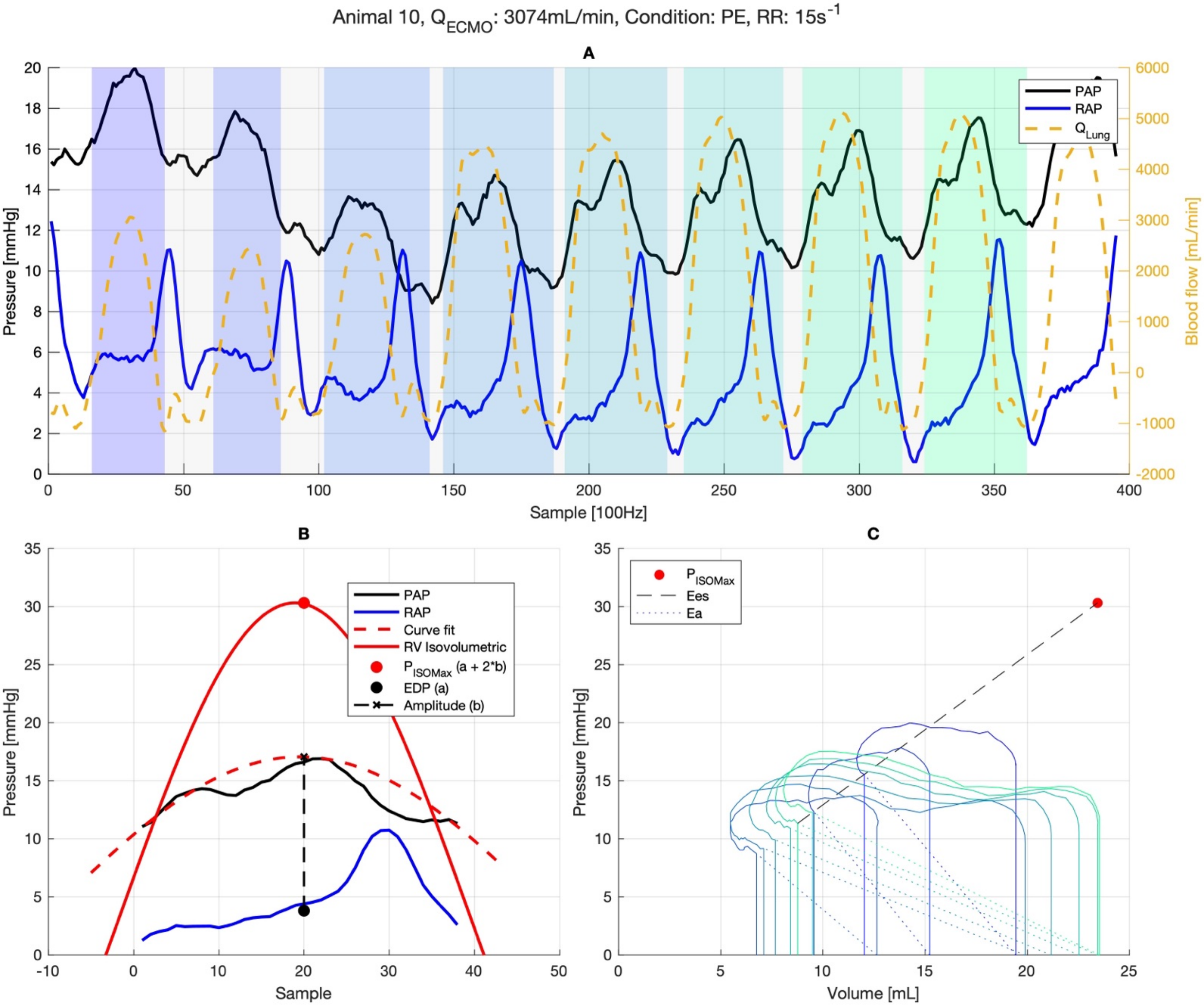

Animal 10, Q_ECMO: 3074mL/min, Condition: PE, RR: 15s^-1
A
PAP
RAP
Q_Lung
Pressure [mmHg]
Blood flow [mL/min]
Sample [100Hz]
B
PAP
RAP
Curve fit
RV Isovolumetric
P_ISOMax (a + 2*b)
EDP (a)
Amplitude (b)
Pressure [mmHg]
Sample
C
P_ISOMax
Ees
Ea
Pressure [mmHg]
Volume [mL]

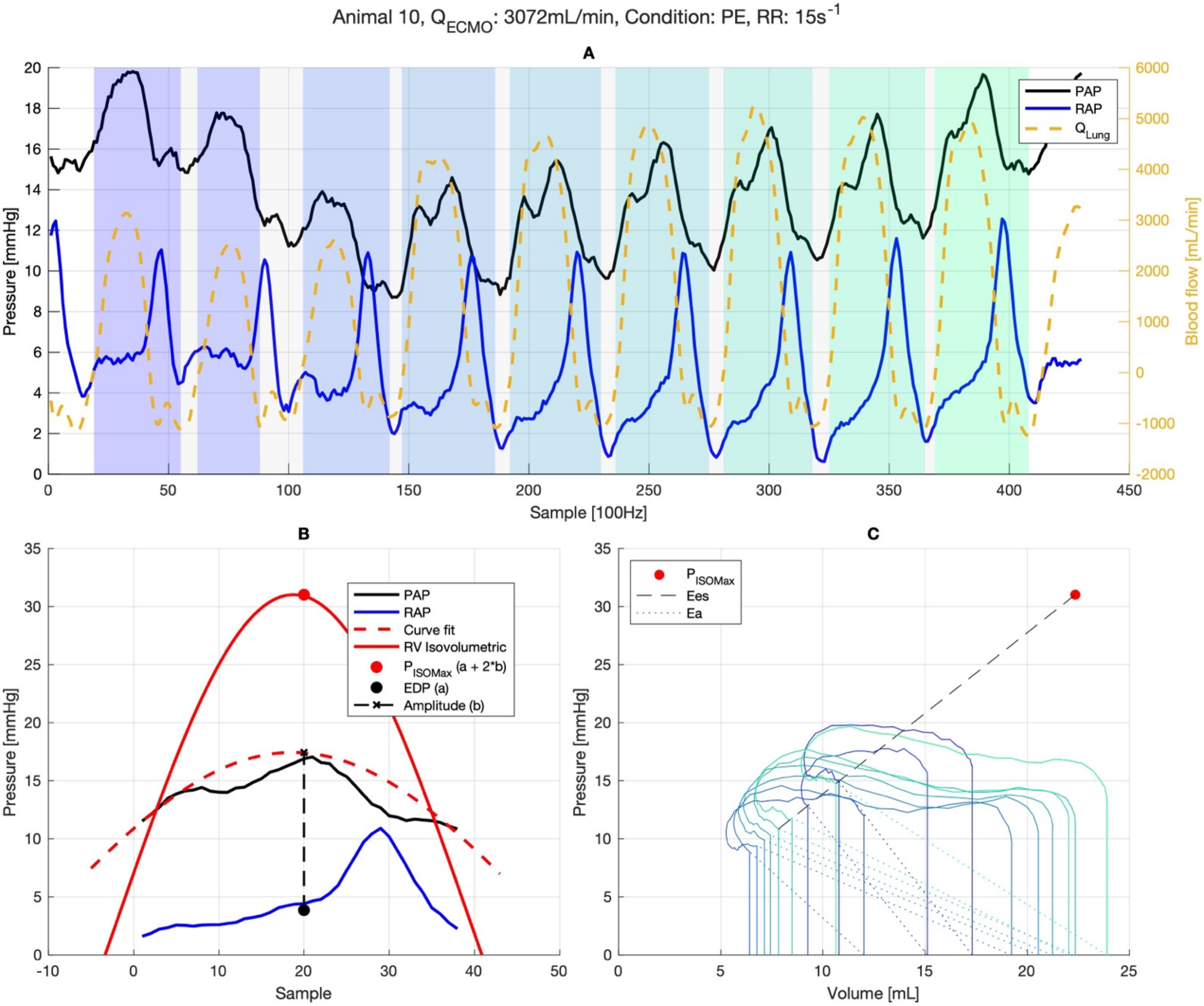

Animal 10, Q_{ECMO}: 3072mL/min, Condition: PE, RR: 15s^{-1}
A
PAP
RAP
Q_{Lung}
Pressure [mmHg]
Blood flow [mL/min]
Sample [100Hz]
B
PAP
RAP
Curve fit
RV Isovolumetric
P_{ISOMax} (a + 2*b)
EDP (a)
Amplitude (b)
Pressure [mmHg]
Sample
C
P_{ISOMax}
Ees
Ea
Pressure [mmHg]
Volume [mL]

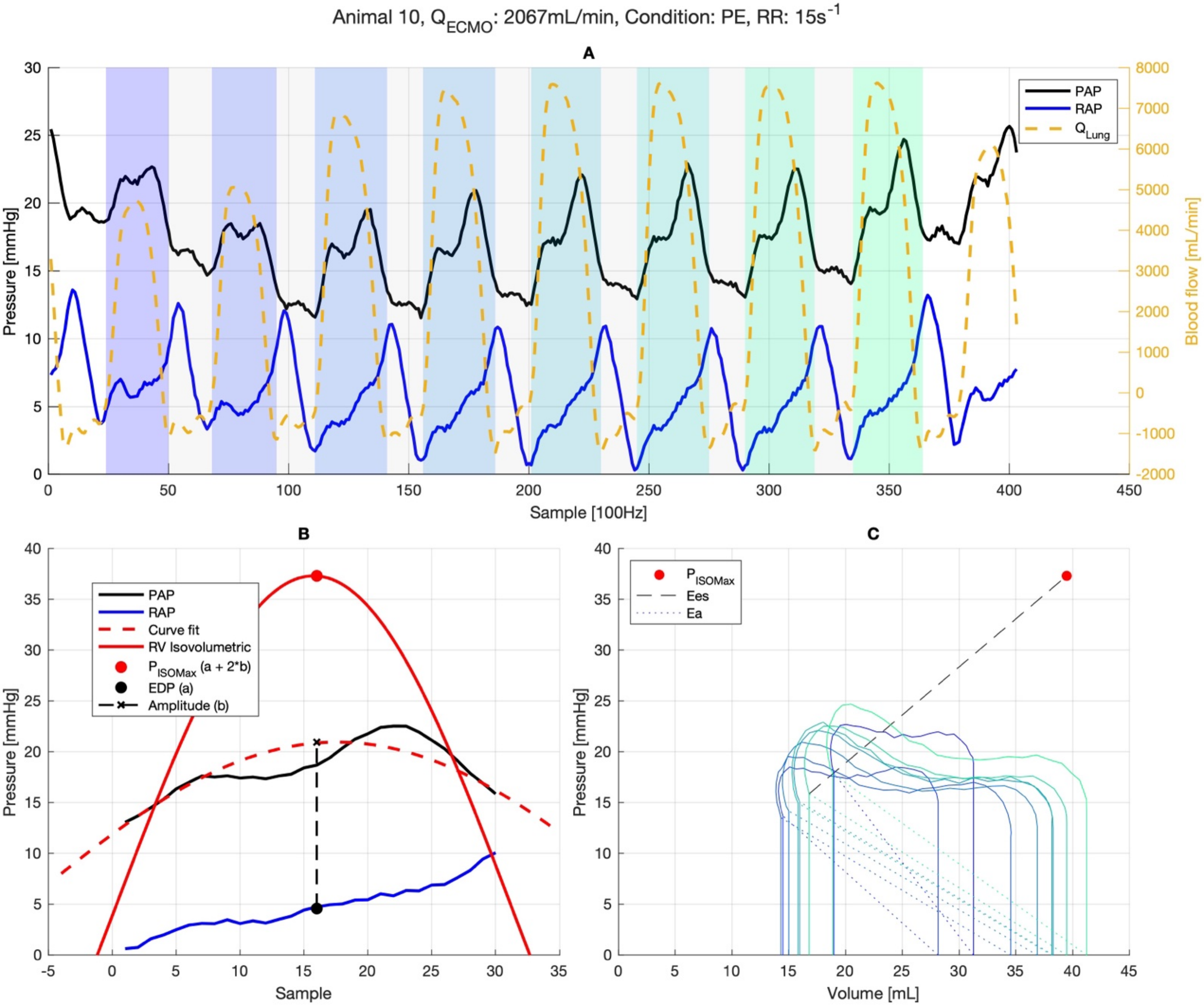

Animal 10, Q_{ECMO}: 2067mL/min, Condition: PE, RR: $15s^{-1}$
A
Pressure [mmHg]
Blood flow [mL/min]
Sample [100Hz]
PAP
RAP
Q_{Lung}
B
PAP
RAP
Curve fit
RV Isovolumetric
P_{ISOMax} (a + 2*b)
EDP (a)
Amplitude (b)
Pressure [mmHg]
Sample
C
P_{ISOMax}
Ees
Ea
Pressure [mmHg]
Volume [mL]

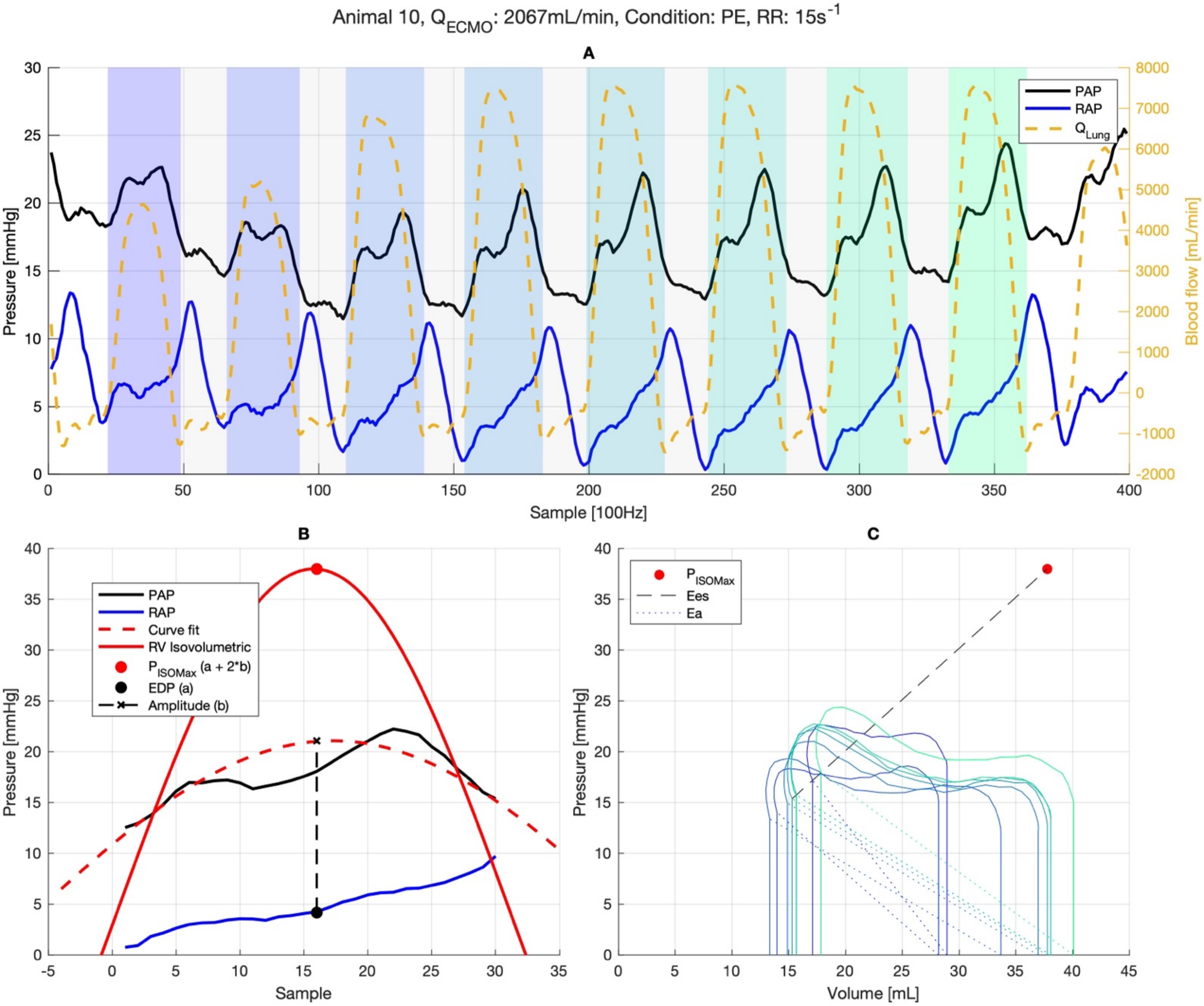

Animal 10, Q_ECMO: 2067mL/min, Condition: PE, RR: 15s^-1
A
PAP
RAP
Q_Lung
Pressure [mmHg]
Blood flow [mL/min]
Sample [100Hz]
B
PAP
RAP
Curve fit
RV Isovolumetric
P_ISOMax (a + 2*b)
EDP (a)
Amplitude (b)
Pressure [mmHg]
Sample
C
P_ISOMax
Ees
Ea
Pressure [mmHg]
Volume [mL]

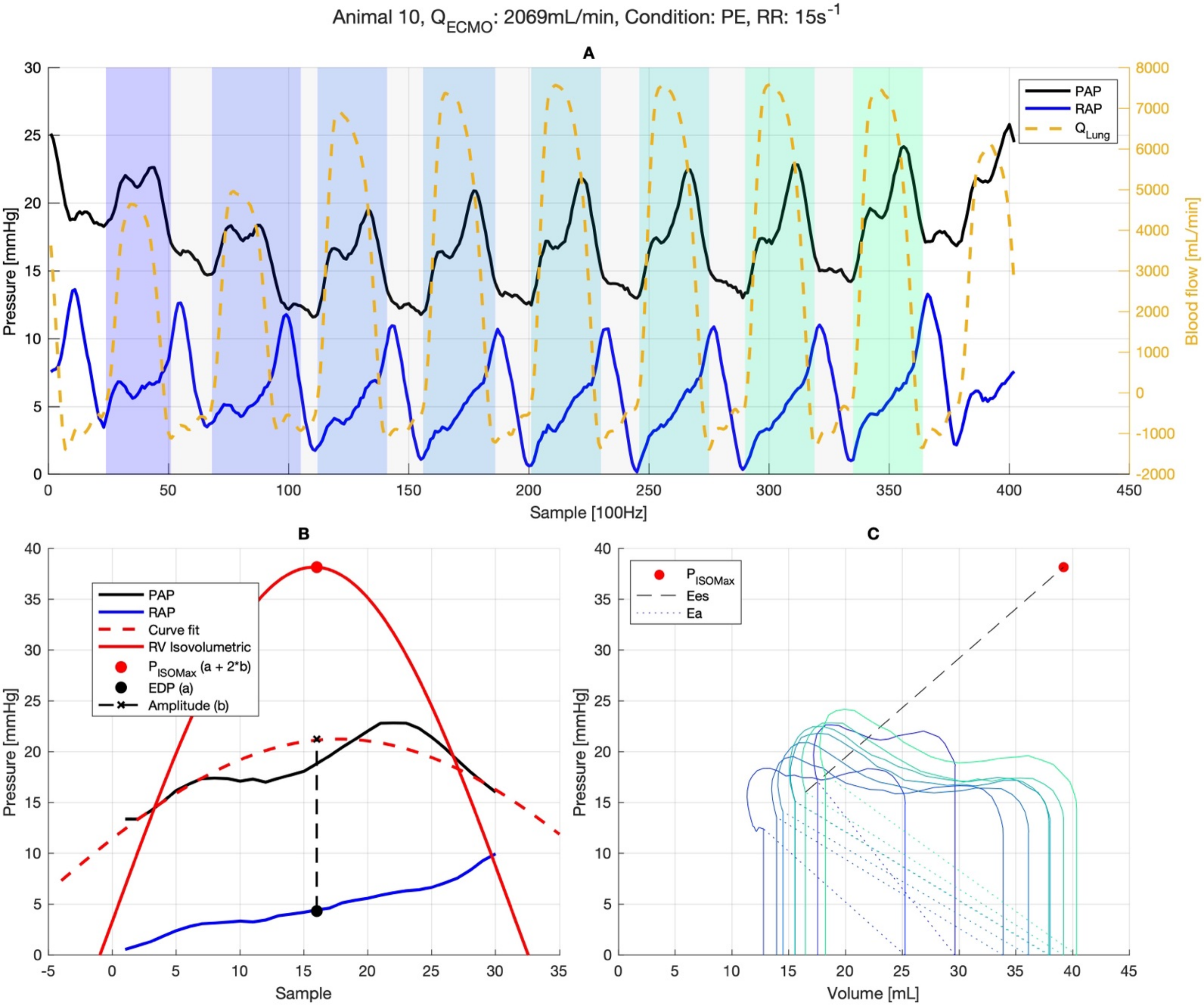
Animal 10, Q_{ECMO}: 2069mL/min, Condition: PE, RR: $15s^{-1}$
A
PAP
RAP
Q_{Lung}
Pressure [mmHg]
Blood flow [mL/min]
Sample [100Hz]
B
PAP
RAP
Curve fit
RV Isovolumetric
P_{ISOMax} (a + 2*b)
EDP (a)
Amplitude (b)
Pressure [mmHg]
Sample
C
P_{ISOMax}
Ees
Ea
Pressure [mmHg]
Volume [mL]

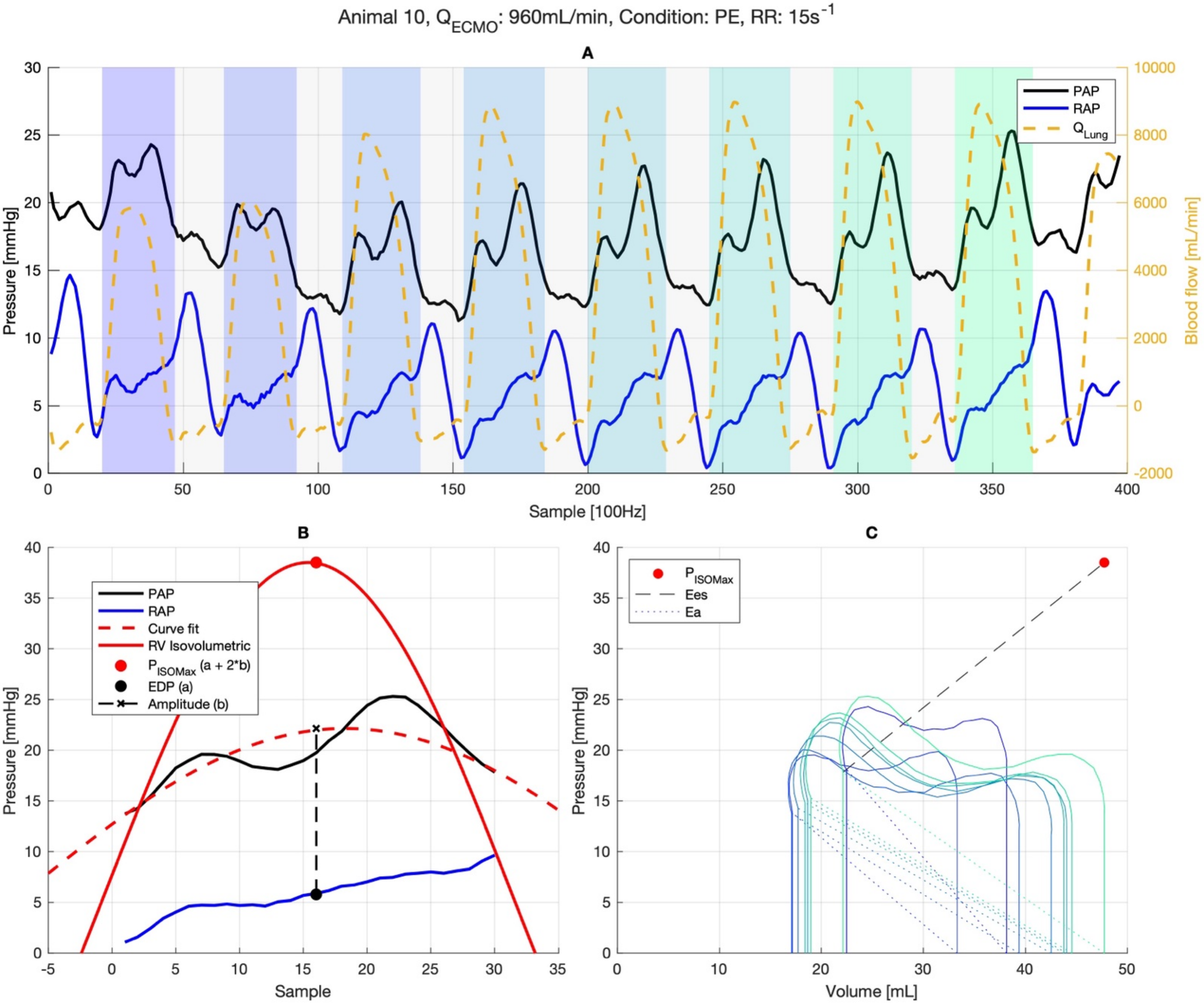

Animal 10, Q_{ECMO}: 960mL/min, Condition: PE, RR: $15s^{-1}$
A
Pressure [mmHg]
Blood flow [mL/min]
Sample [100Hz]
PAP
RAP
Q_{Lung}
B
Pressure [mmHg]
Sample
PAP
RAP
Curve fit
RV Isovolumetric
P_{ISOMax} (a + 2*b)
EDP (a)
Amplitude (b)
C
Pressure [mmHg]
Volume [mL]
P_{ISOMax}
Ees
Ea

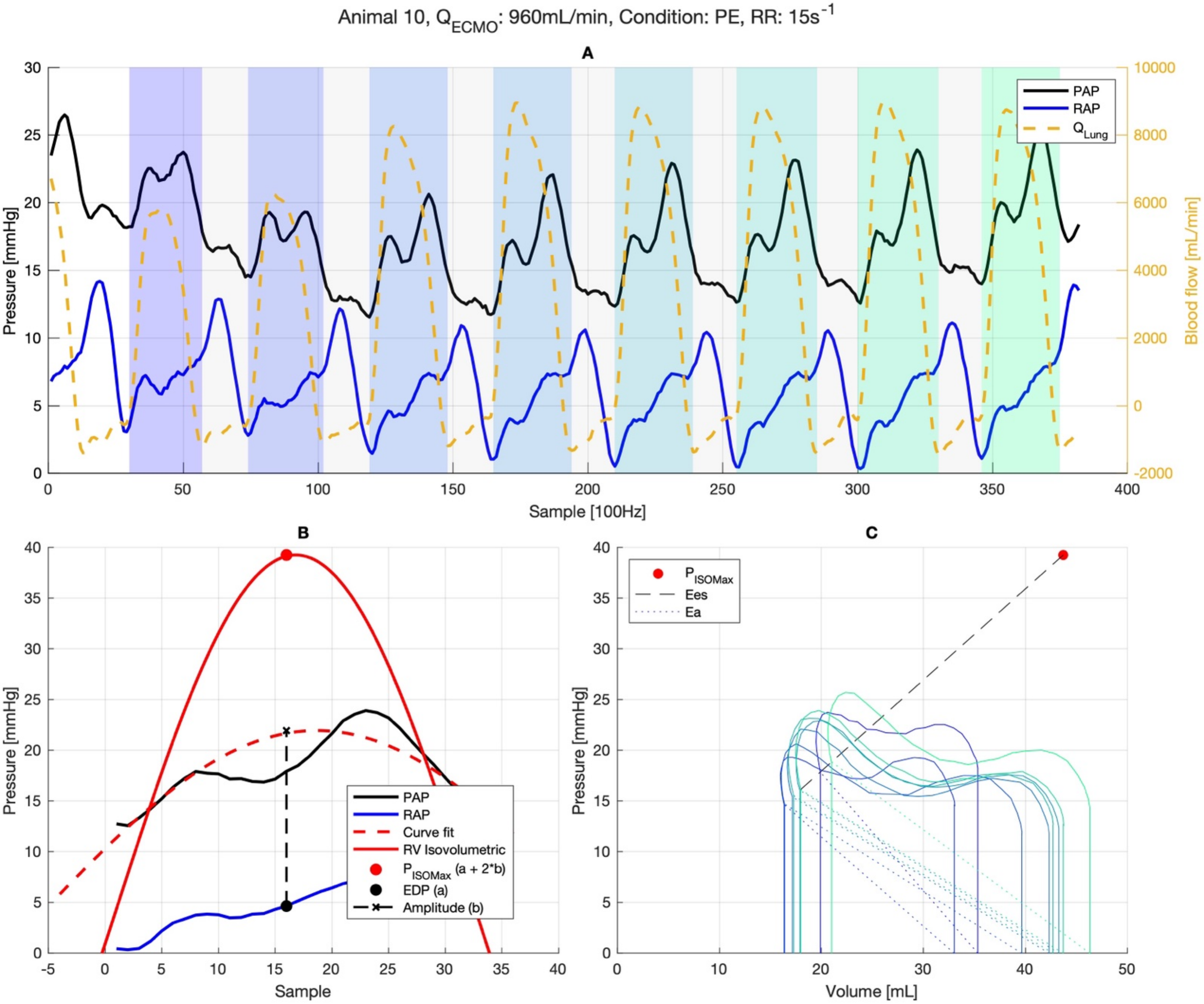

Animal 10, QECMO: 960mL/min, Condition: PE, RR: 15s-1
A
PAP
RAP
QLung
Pressure [mmHg]
Blood flow [mL/min]
Sample [100Hz]
B
PAP
RAP
Curve fit
RV Isovolumetric
PISOMax (a + 2*b)
EDP (a)
Amplitude (b)
Pressure [mmHg]
Sample
C
PISOMax
Ees
Ea
Pressure [mmHg]
Volume [mL]

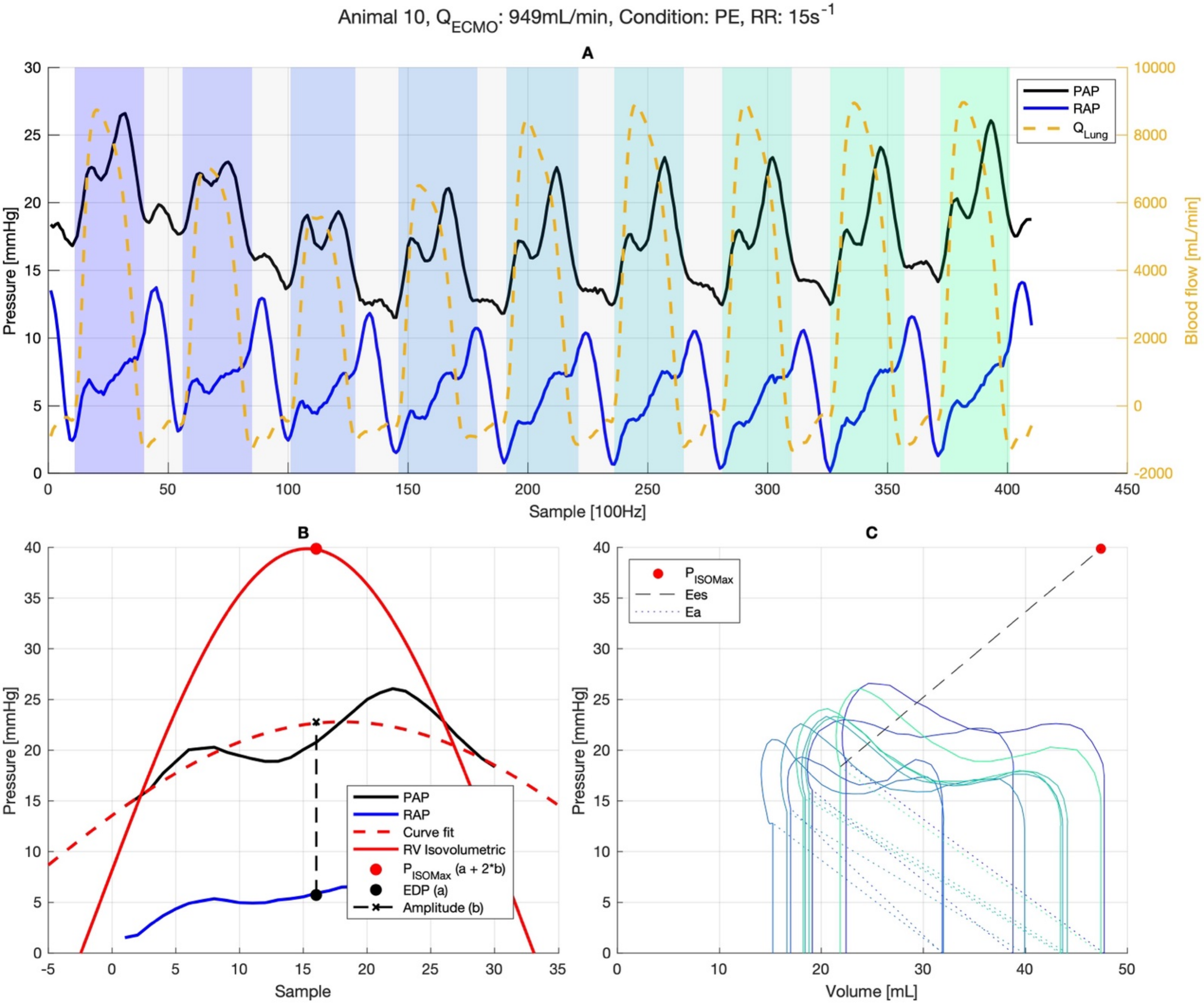

Animal 10, Q_{ECMO}: 949mL/min, Condition: PE, RR: $15s^{-1}$
A
Pressure [mmHg]
Blood flow [mL/min]
Sample [100Hz]
PAP
RAP
Q_{Lung}
B
Pressure [mmHg]
Sample
PAP
RAP
Curve fit
RV Isovolumetric
P_{ISOMax} (a + 2*b)
EDP (a)
Amplitude (b)
C
Pressure [mmHg]
Volume [mL]
P_{ISOMax}
Ees
Ea

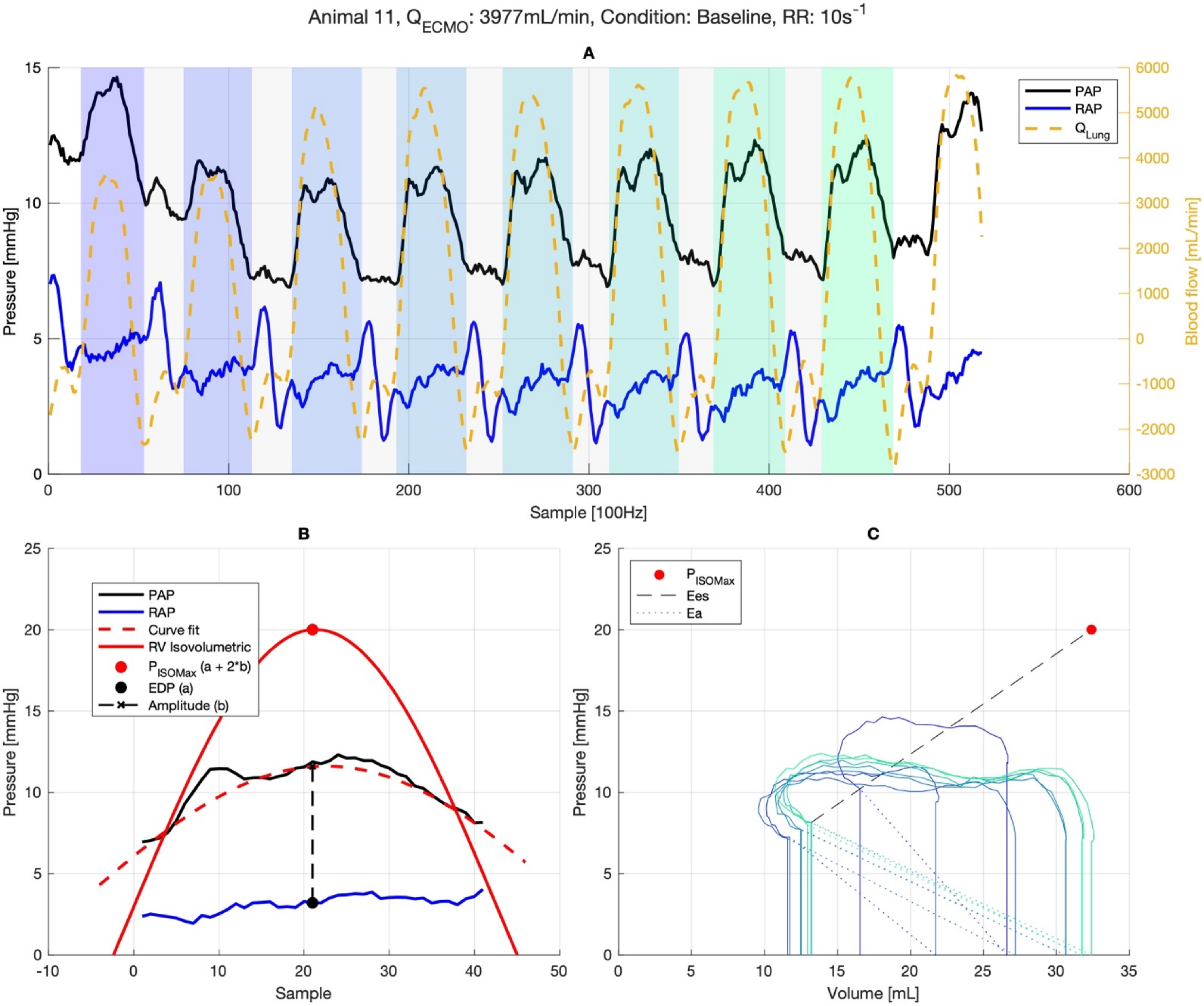

Animal 11, Q_{ECMO}: 3977mL/min, Condition: Baseline, RR: $10s^{-1}$
A
PAP
RAP
Q_{Lung}
Pressure [mmHg]
Blood flow [mL/min]
Sample [100Hz]
B
PAP
RAP
Curve fit
RV Isovolumetric
P_{ISOMax} (a + 2*b)
EDP (a)
Amplitude (b)
Pressure [mmHg]
Sample
C
P_{ISOMax}
Ees
Ea
Pressure [mmHg]
Volume [mL]

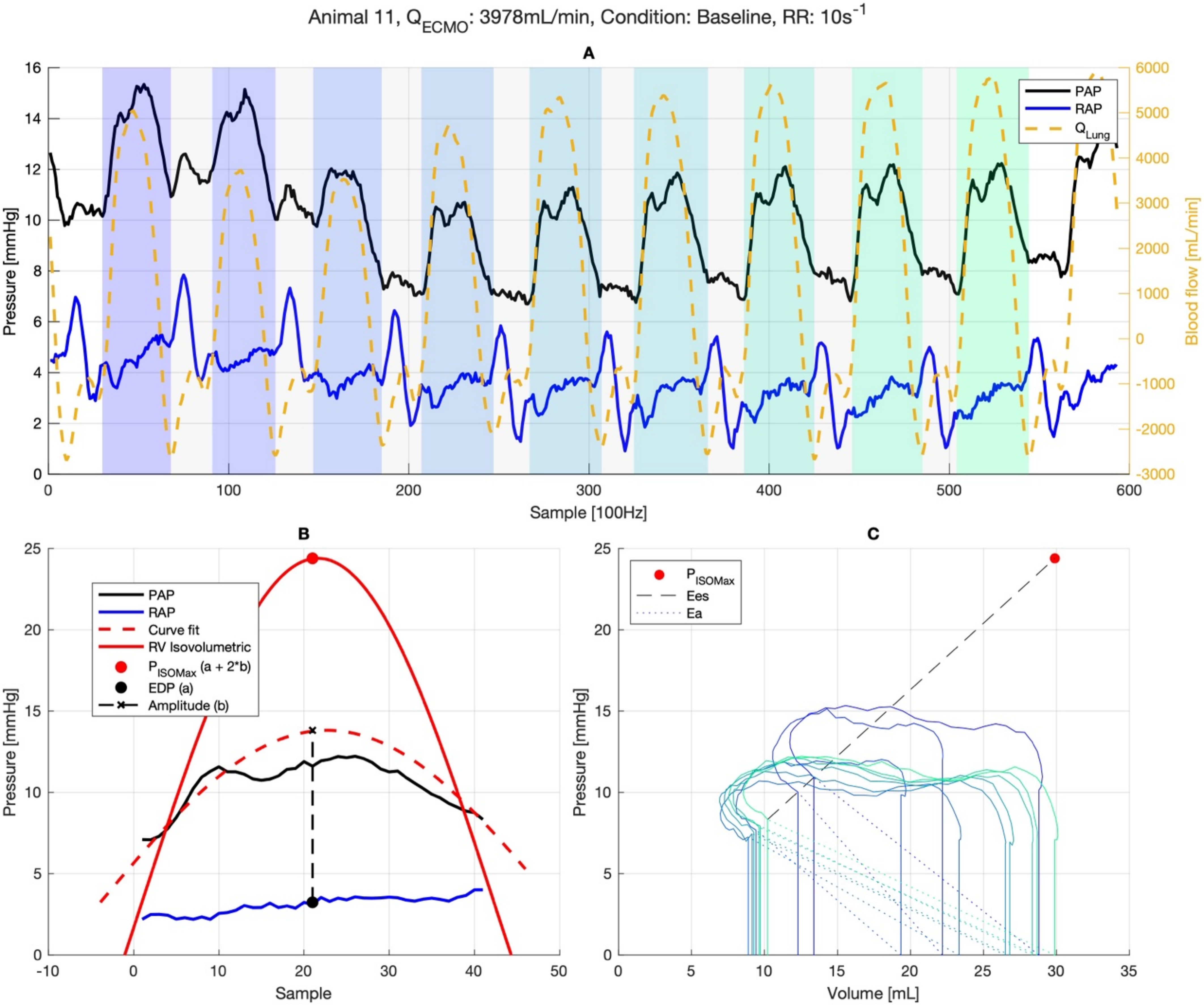

Animal 11, Q_{ECMO}: 3978mL/min, Condition: Baseline, RR: $10s^{-1}$
A
Pressure [mmHg]
Blood flow [mL/min]
Sample [100Hz]
PAP
RAP
Q_{Lung}
B
Pressure [mmHg]
Sample
PAP
RAP
Curve fit
RV Isovolumetric
P_{ISOMax} (a + 2*b)
EDP (a)
Amplitude (b)
C
Pressure [mmHg]
Volume [mL]
P_{ISOMax}
Ees
Ea

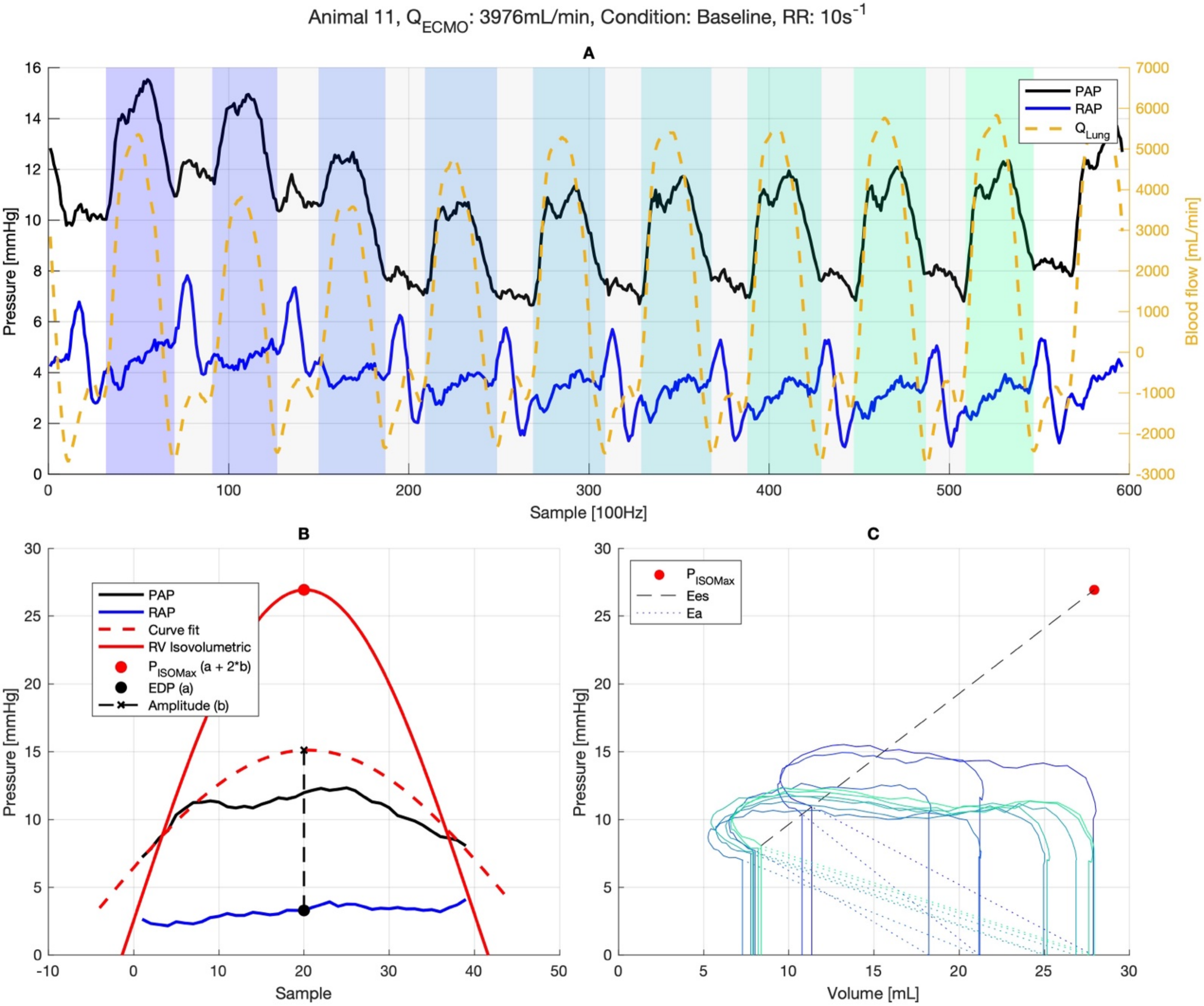

Animal 11, Q_{ECMO}: 3976mL/min, Condition: Baseline, RR: $10s^{-1}$
A
PAP
RAP
Q_{Lung}
Pressure [mmHg]
Blood flow [mL/min]
Sample [100Hz]
B
PAP
RAP
Curve fit
RV Isovolumetric
P_{ISOMax} (a + 2*b)
EDP (a)
Amplitude (b)
Pressure [mmHg]
Sample
C
P_{ISOMax}
Ees
Ea
Pressure [mmHg]
Volume [mL]

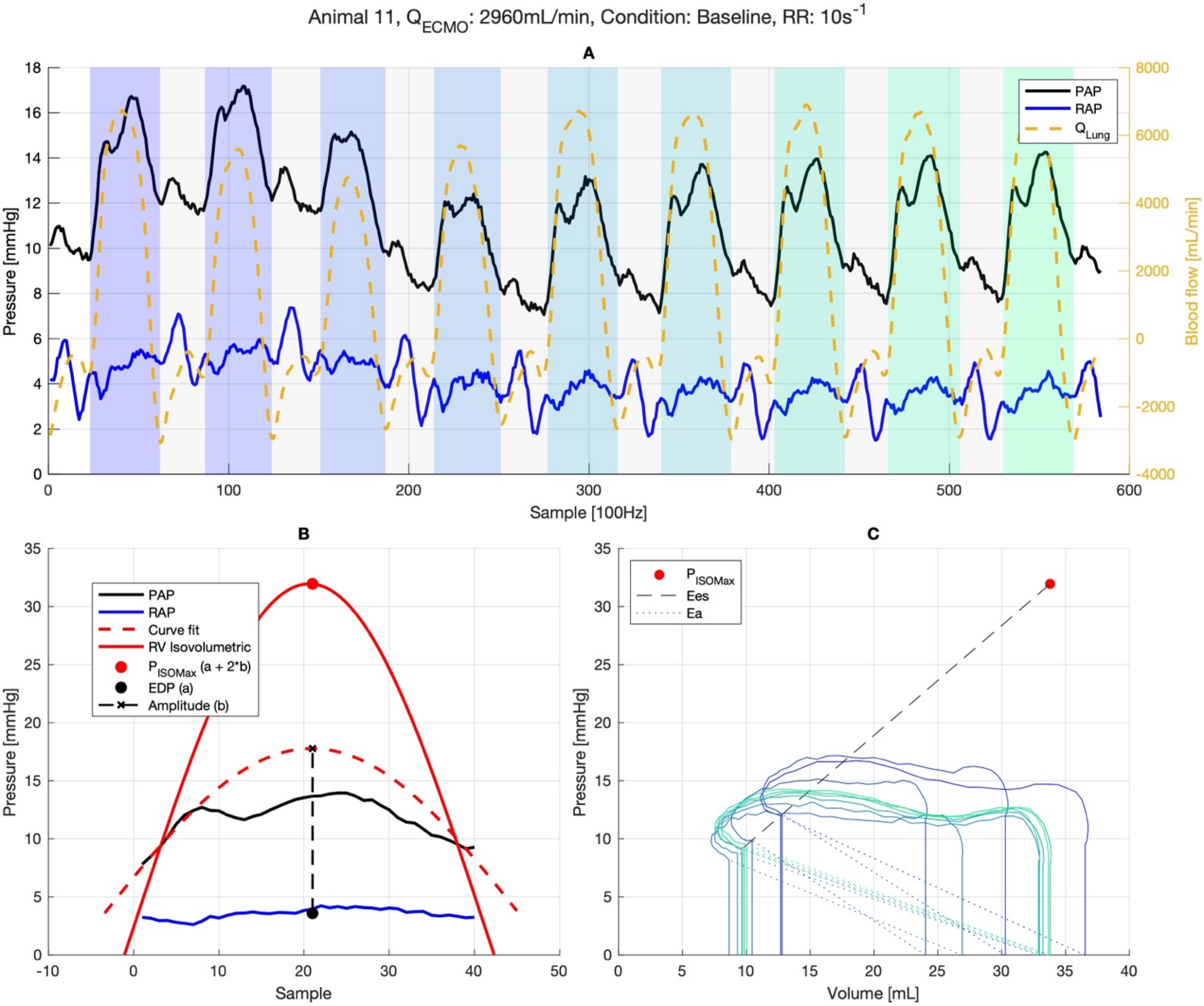

Animal 11, Q_{ECMO}: 2960mL/min, Condition: Baseline, RR: 10s^{-1}
A
PAP
RAP
Q_{Lung}
Pressure [mmHg]
Blood flow [mL/min]
Sample [100Hz]
B
PAP
RAP
Curve fit
RV Isovolumetric
P_{ISOMax} (a + 2*b)
EDP (a)
Amplitude (b)
Pressure [mmHg]
Sample
C
P_{ISOMax}
Ees
Ea
Pressure [mmHg]
Volume [mL]

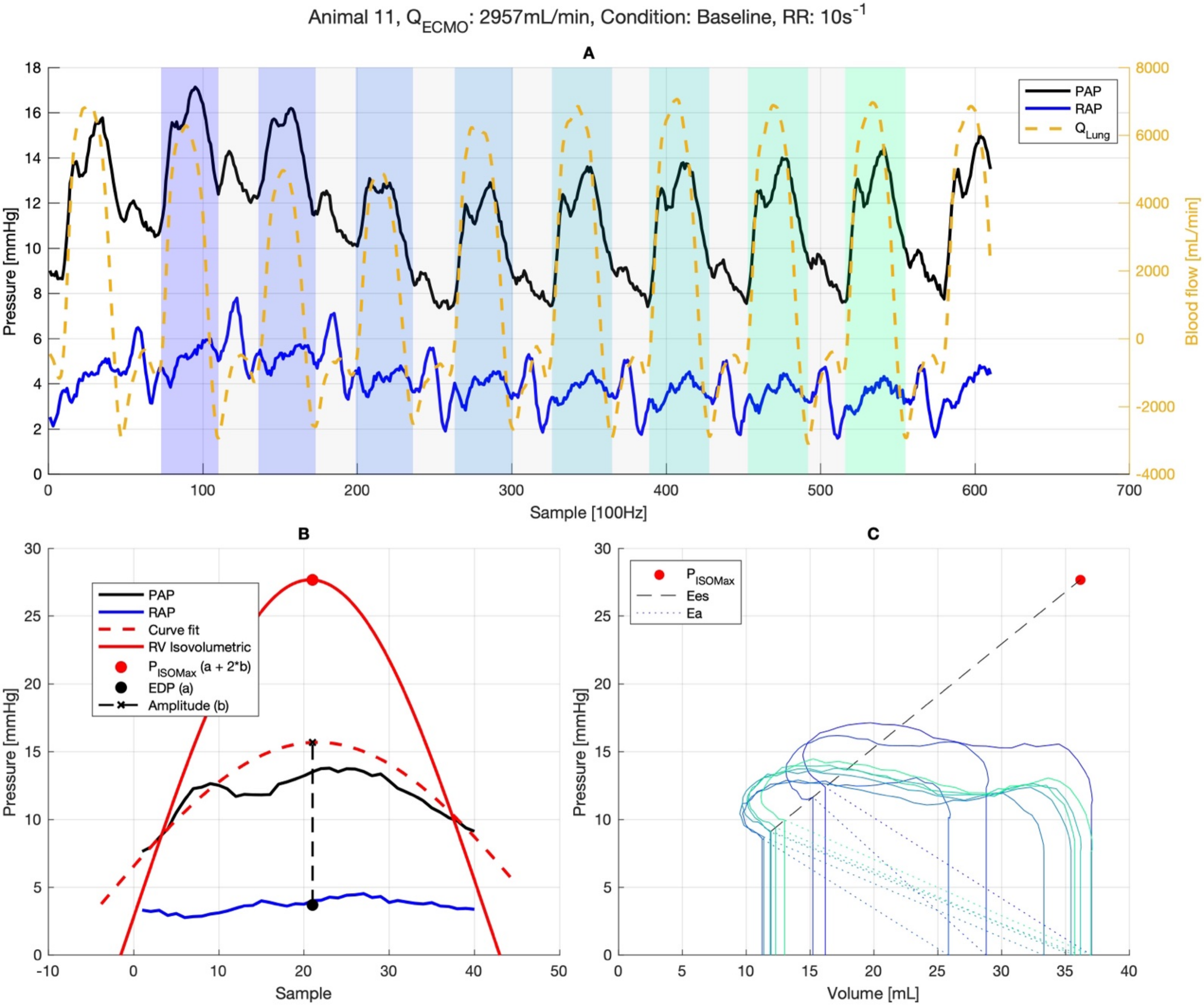

Animal 11, Q_{ECMO}: 2957mL/min, Condition: Baseline, RR: $10s^{-1}$
A
PAP
RAP
Q_{Lung}
Pressure [mmHg]
Blood flow [mL/min]
Sample [100Hz]
B
PAP
RAP
Curve fit
RV Isovolumetric
P_{ISOMax} (a + 2*b)
EDP (a)
Amplitude (b)
Pressure [mmHg]
Sample
C
P_{ISOMax}
Ees
Ea
Pressure [mmHg]
Volume [mL]

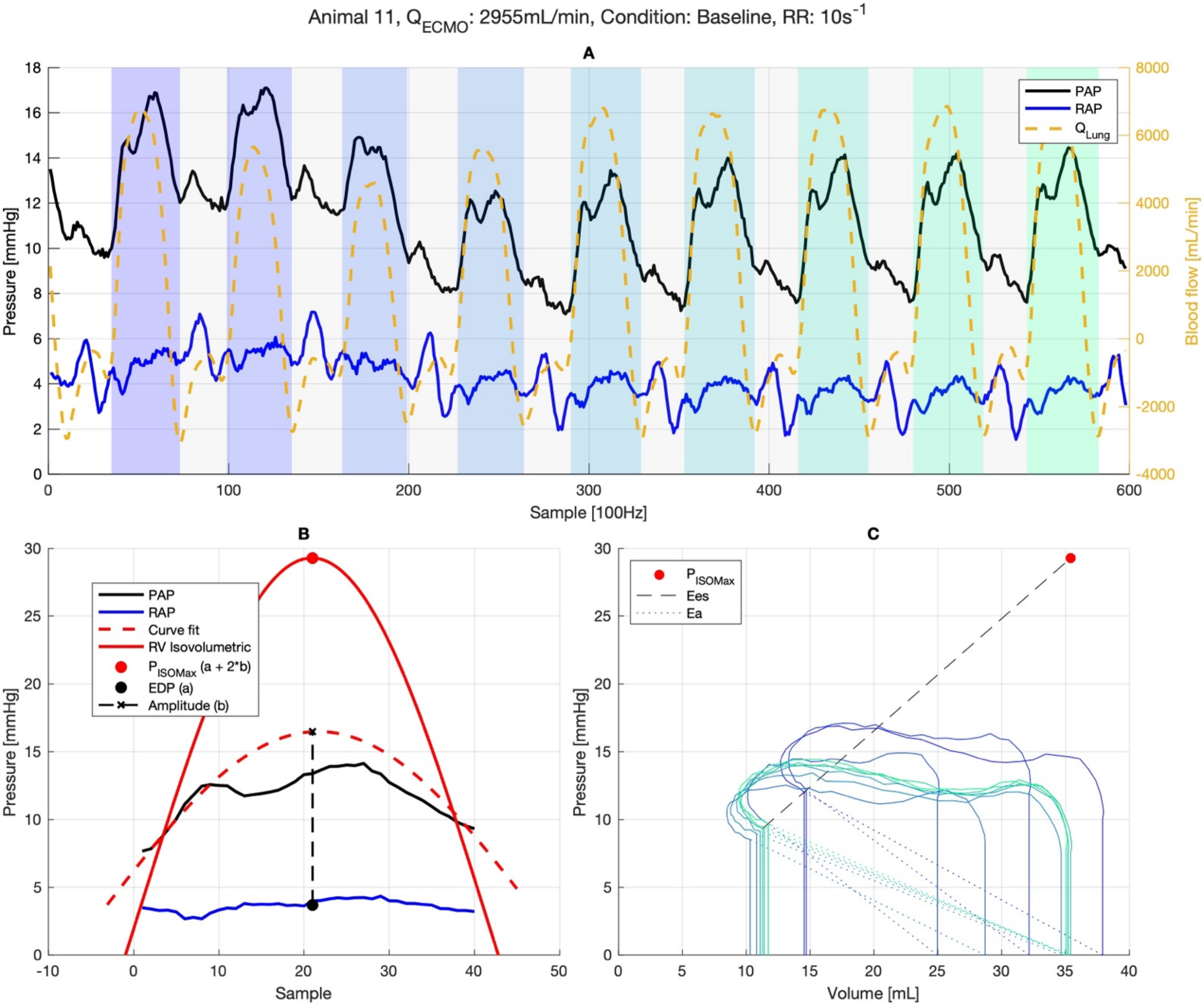

Animal 11, Q_{ECMO}: 2955mL/min, Condition: Baseline, RR: 10s-1
A
PAP
RAP
QLung
Pressure [mmHg]
Blood flow [mL/min]
Sample [100Hz]
B
PAP
RAP
Curve fit
RV Isovolumetric
PISOMax (a + 2*b)
EDP (a)
Amplitude (b)
Pressure [mmHg]
Sample
C
PISOMax
Ees
Ea
Pressure [mmHg]
Volume [mL]

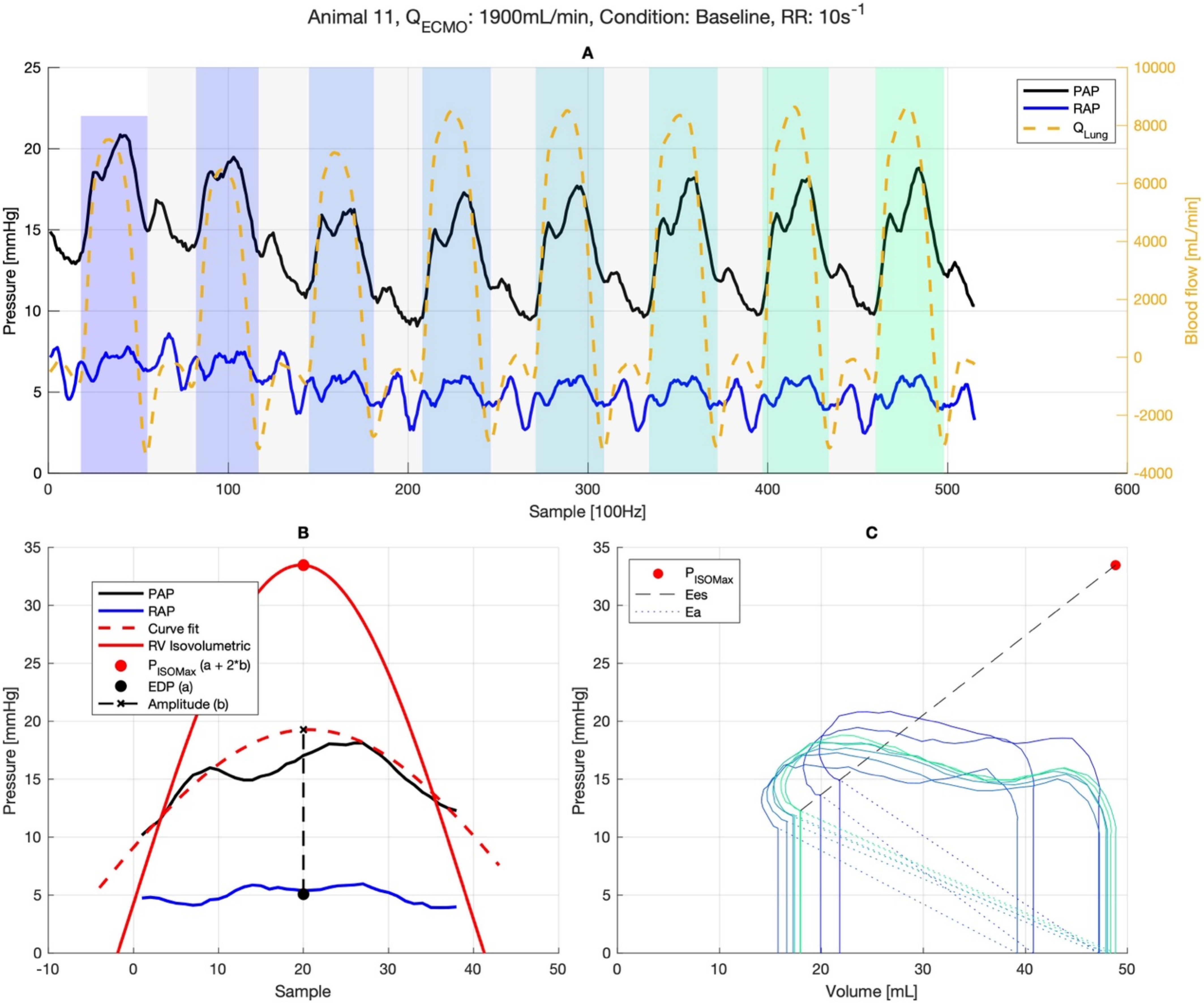

Animal 11, Q_{ECMO}: 1900mL/min, Condition: Baseline, RR: $10s^{-1}$
A
PAP
RAP
Q_{Lung}
Pressure [mmHg]
Blood flow [mL/min]
Sample [100Hz]
B
PAP
RAP
Curve fit
RV Isovolumetric
P_{ISOMax} (a + 2*b)
EDP (a)
Amplitude (b)
Pressure [mmHg]
Sample
C
P_{ISOMax}
Ees
Ea
Pressure [mmHg]
Volume [mL]

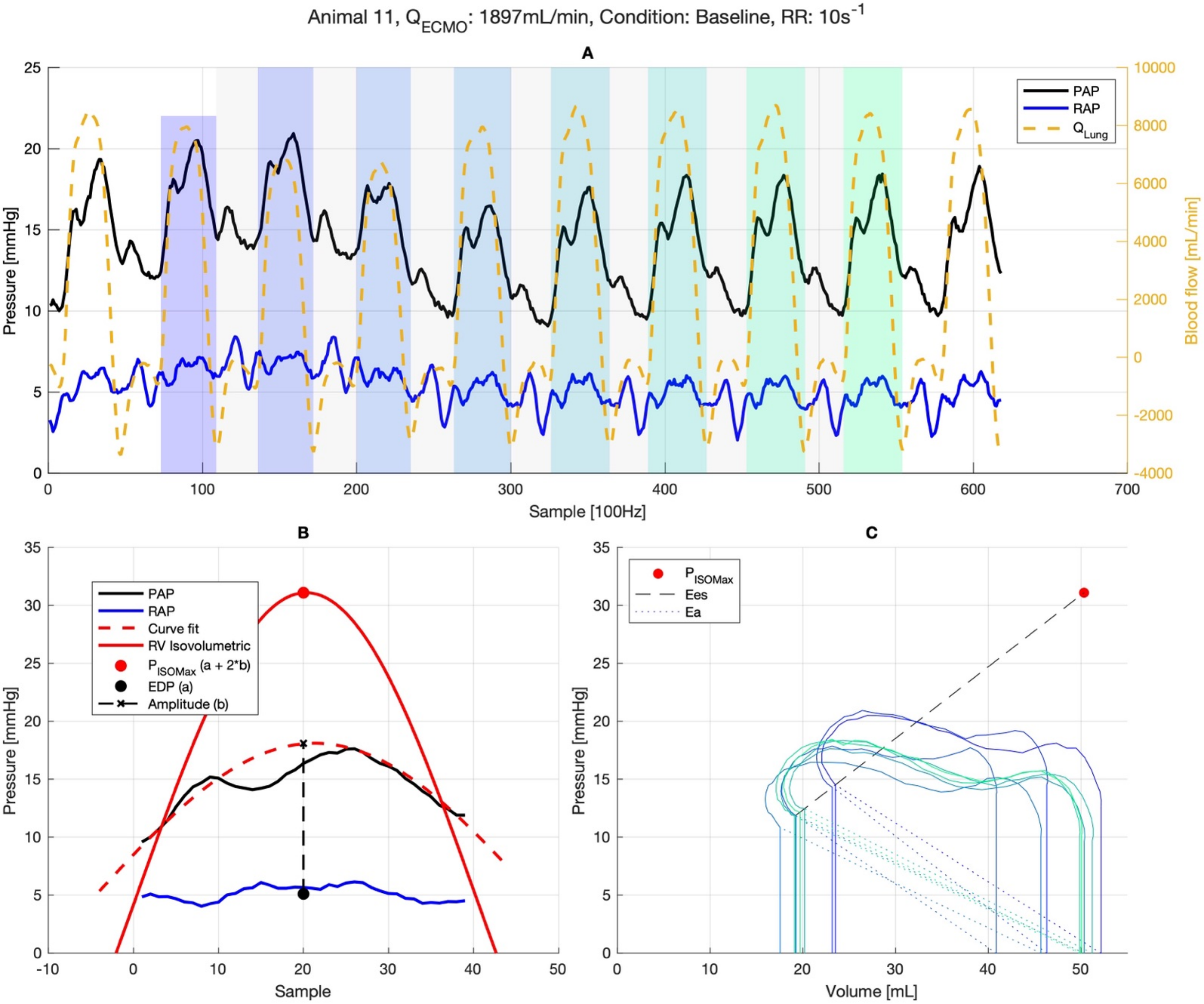

Animal 11, Q_ECMO: 1897mL/min, Condition: Baseline, RR: 10s^-1
A
PAP
RAP
Q_Lung
Pressure [mmHg]
Blood flow [mL/min]
Sample [100Hz]
B
PAP
RAP
Curve fit
RV Isovolumetric
P_ISOMax (a + 2*b)
EDP (a)
Amplitude (b)
Pressure [mmHg]
Sample
C
P_ISOMax
Ees
Ea
Pressure [mmHg]
Volume [mL]

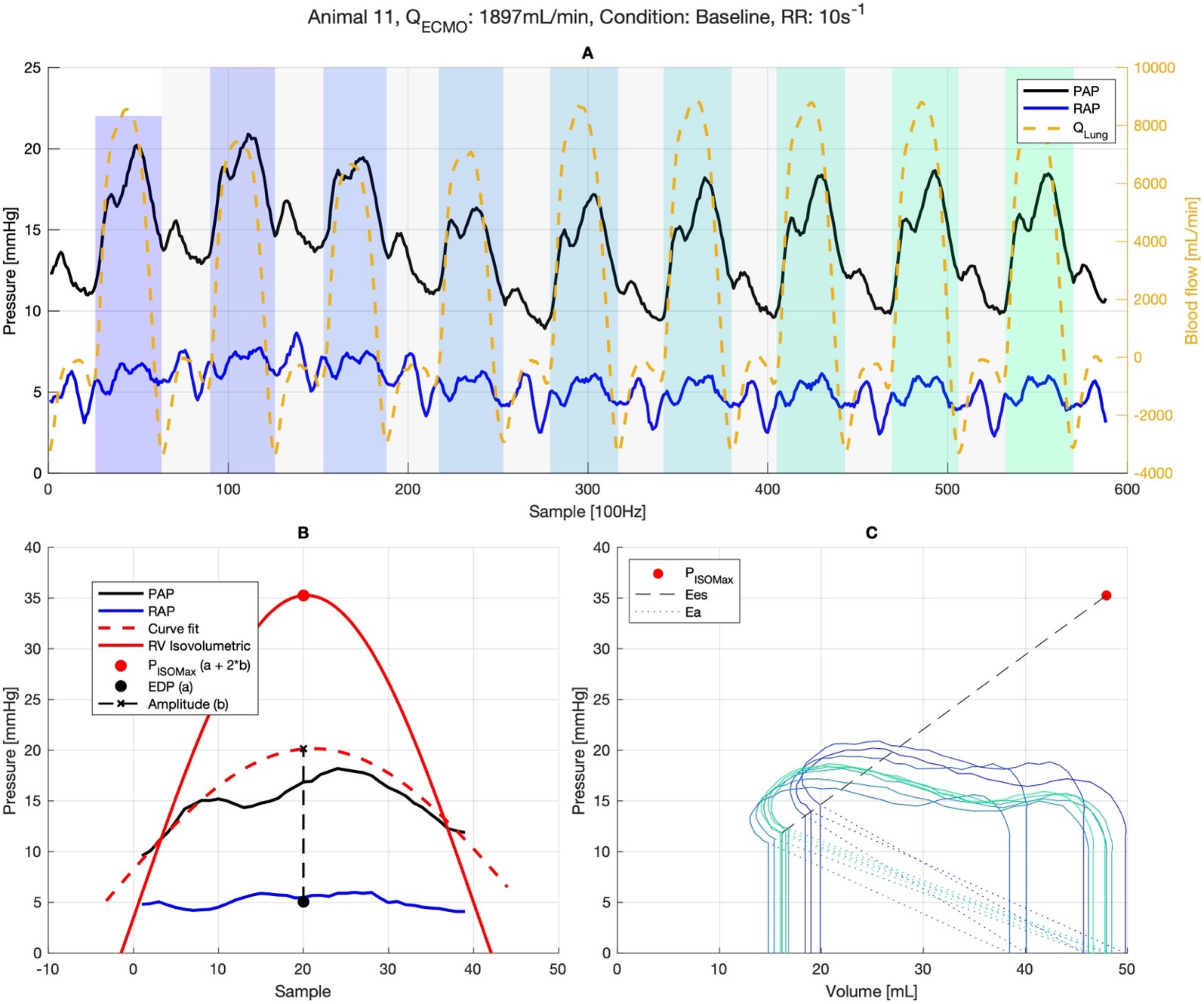

Animal 11, Q_{ECMO}: 1897mL/min, Condition: Baseline, RR: $10s^{-1}$
A
PAP
RAP
Q_{Lung}
Pressure [mmHg]
Blood flow [mL/min]
Sample [100Hz]
B
PAP
RAP
Curve fit
RV Isovolumetric
P_{ISOMax} (a + 2*b)
EDP (a)
Amplitude (b)
Pressure [mmHg]
Sample
C
P_{ISOMax}
Ees
Ea
Pressure [mmHg]
Volume [mL]

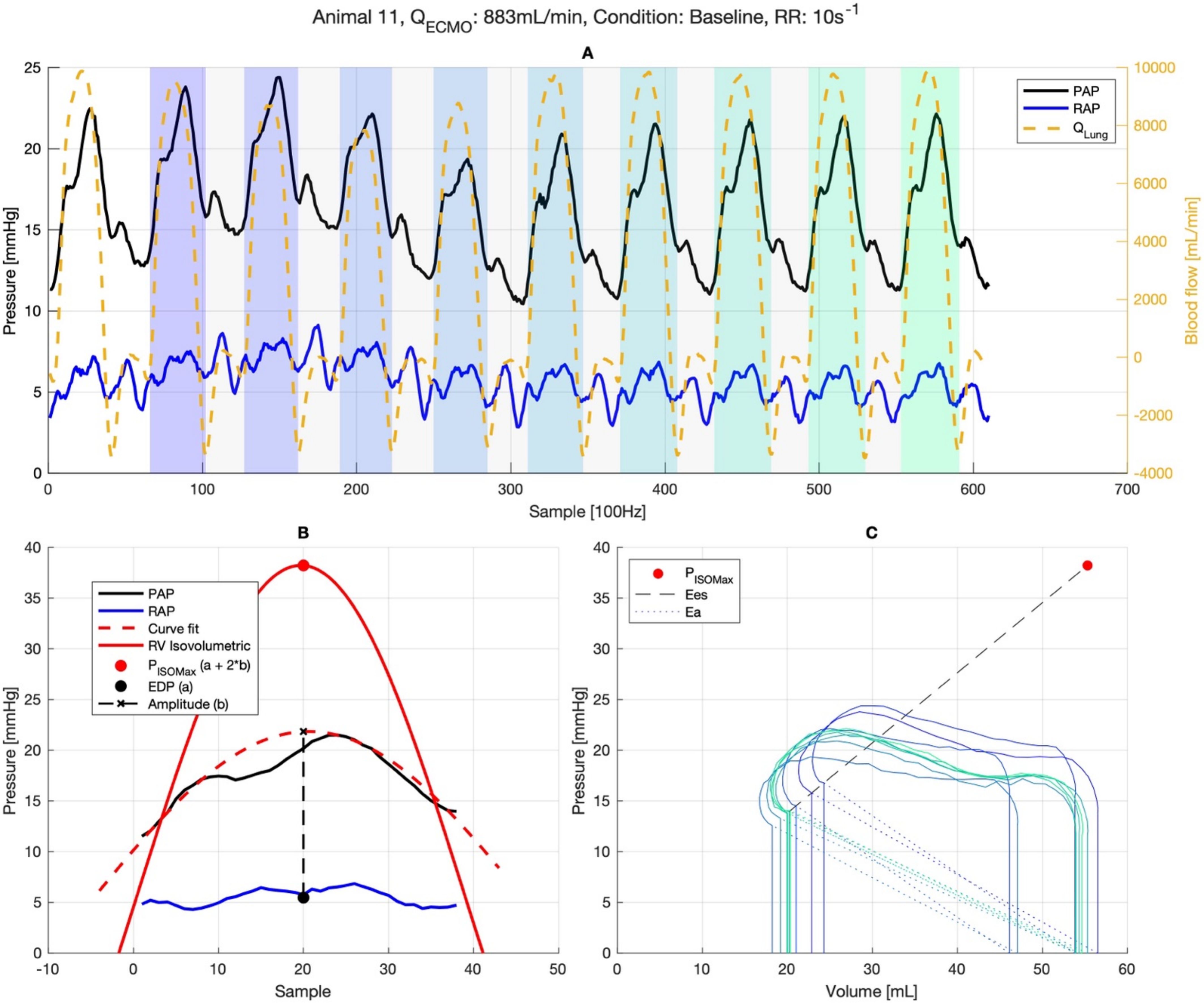

Animal 11, Q_{ECMO}: 883mL/min, Condition: Baseline, RR: $10s^{-1}$
A
PAP
RAP
Q_{Lung}
Pressure [mmHg]
Blood flow [mL/min]
Sample [100Hz]
B
PAP
RAP
Curve fit
RV Isovolumetric
P_{ISOMax} (a + 2*b)
EDP (a)
Amplitude (b)
Pressure [mmHg]
Sample
C
P_{ISOMax}
Ees
Ea
Pressure [mmHg]
Volume [mL]

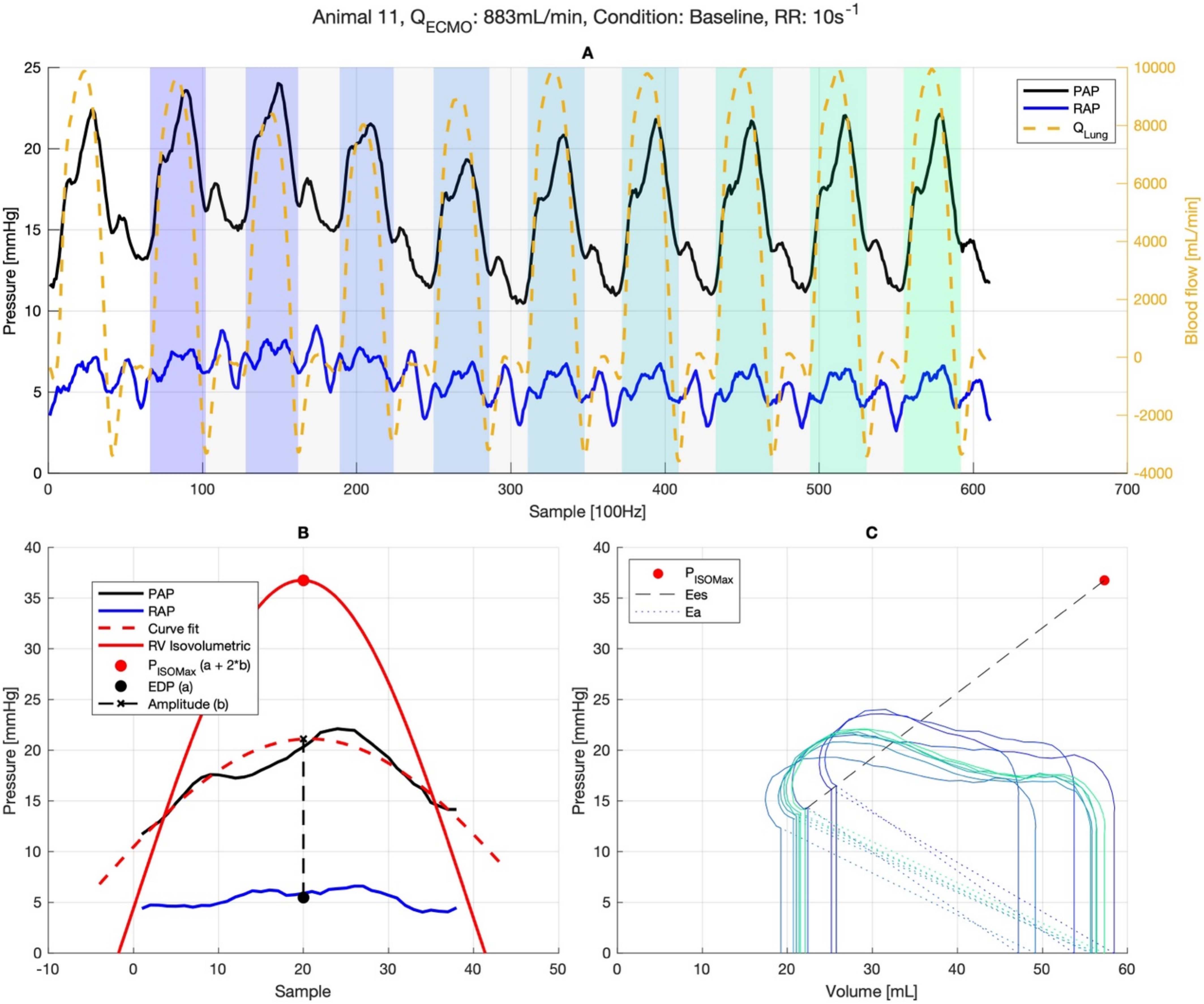

Animal 11, Q_{ECMO}: 883mL/min, Condition: Baseline, RR: $10s^{-1}$
A
Pressure [mmHg]
Blood flow [mL/min]
Sample [100Hz]
PAP
RAP
Q_{Lung}
B
Pressure [mmHg]
Sample
PAP
RAP
Curve fit
RV Isovolumetric
P_{ISOMax} (a + 2*b)
EDP (a)
Amplitude (b)
C
Pressure [mmHg]
Volume [mL]
P_{ISOMax}
Ees
Ea

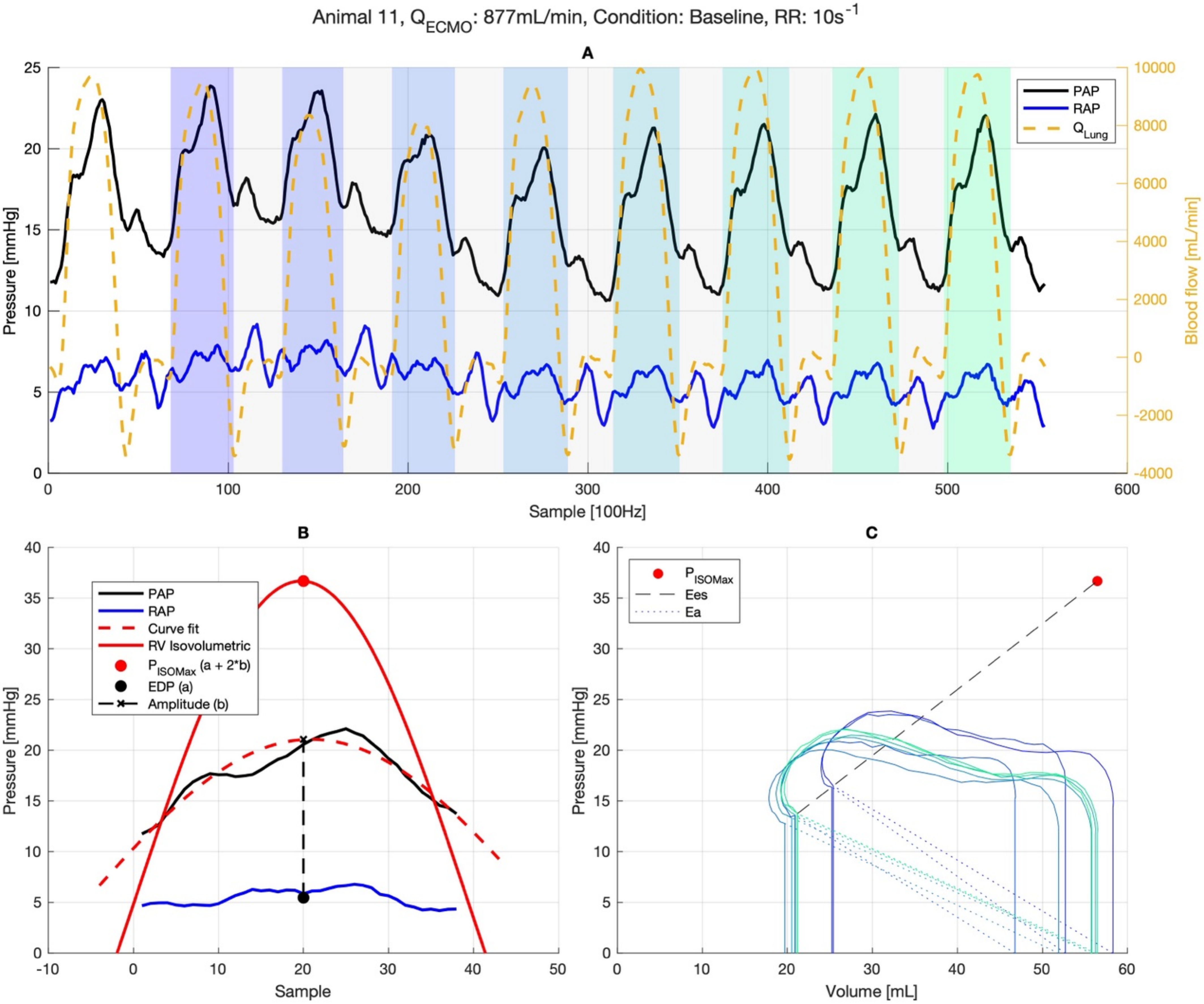

Animal 11, Q_{ECMO}: 877mL/min, Condition: Baseline, RR: $10s^{-1}$
A
PAP
RAP
Q_{Lung}
Pressure [mmHg]
Blood flow [mL/min]
Sample [100Hz]
B
PAP
RAP
Curve fit
RV Isovolumetric
P_{ISOMax} (a + 2*b)
EDP (a)
Amplitude (b)
Pressure [mmHg]
Sample
C
P_{ISOMax}
Ees
Ea
Pressure [mmHg]
Volume [mL]

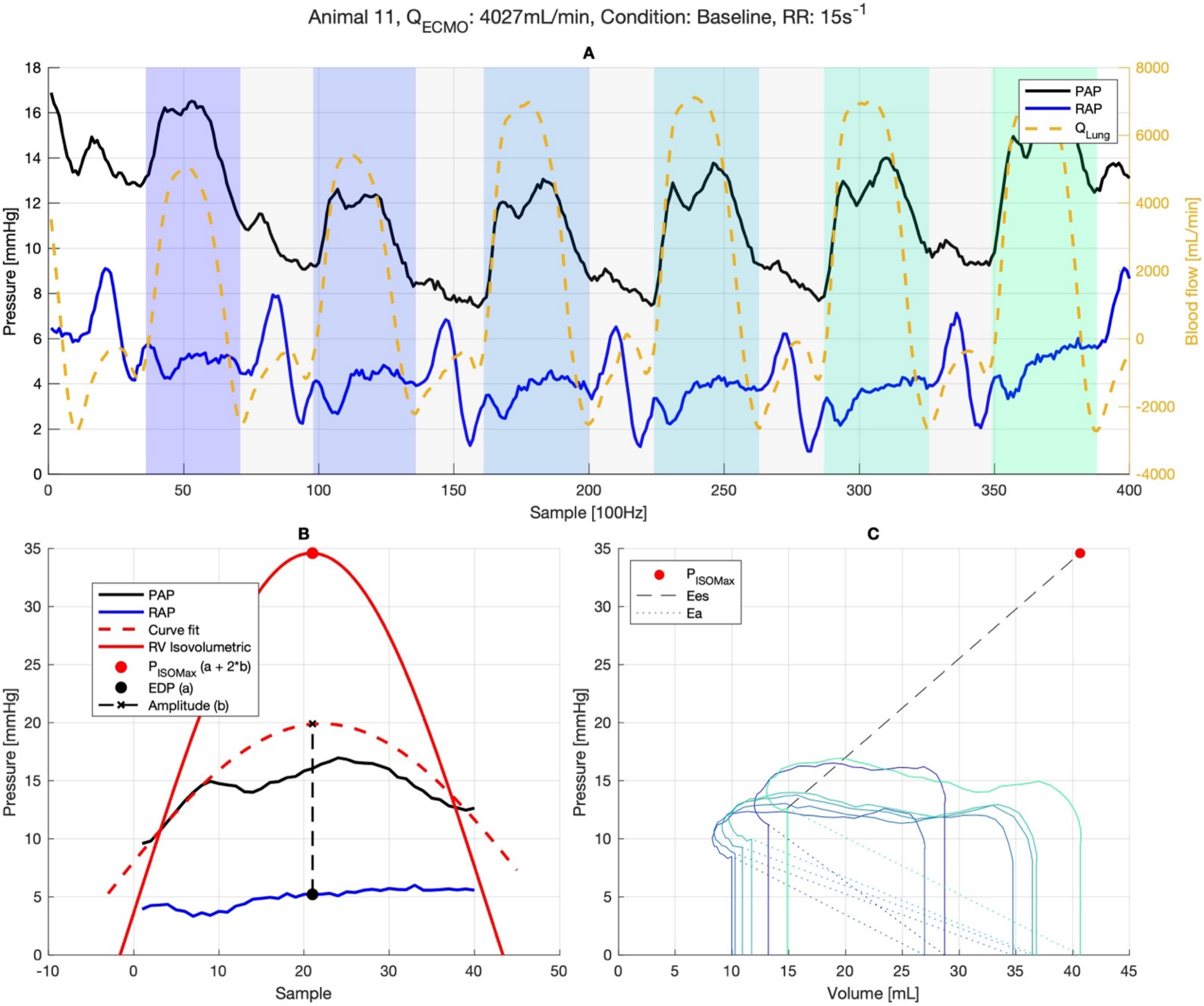

Animal 11, Q_{ECMO}: 4027mL/min, Condition: Baseline, RR: $15s^{-1}$
A
PAP
RAP
Q_{Lung}
Pressure [mmHg]
Blood flow [mL/min]
Sample [100Hz]
B
PAP
RAP
Curve fit
RV Isovolumetric
P_{ISOMax} (a + 2*b)
EDP (a)
Amplitude (b)
Pressure [mmHg]
Sample
C
P_{ISOMax}
Ees
Ea
Pressure [mmHg]
Volume [mL]

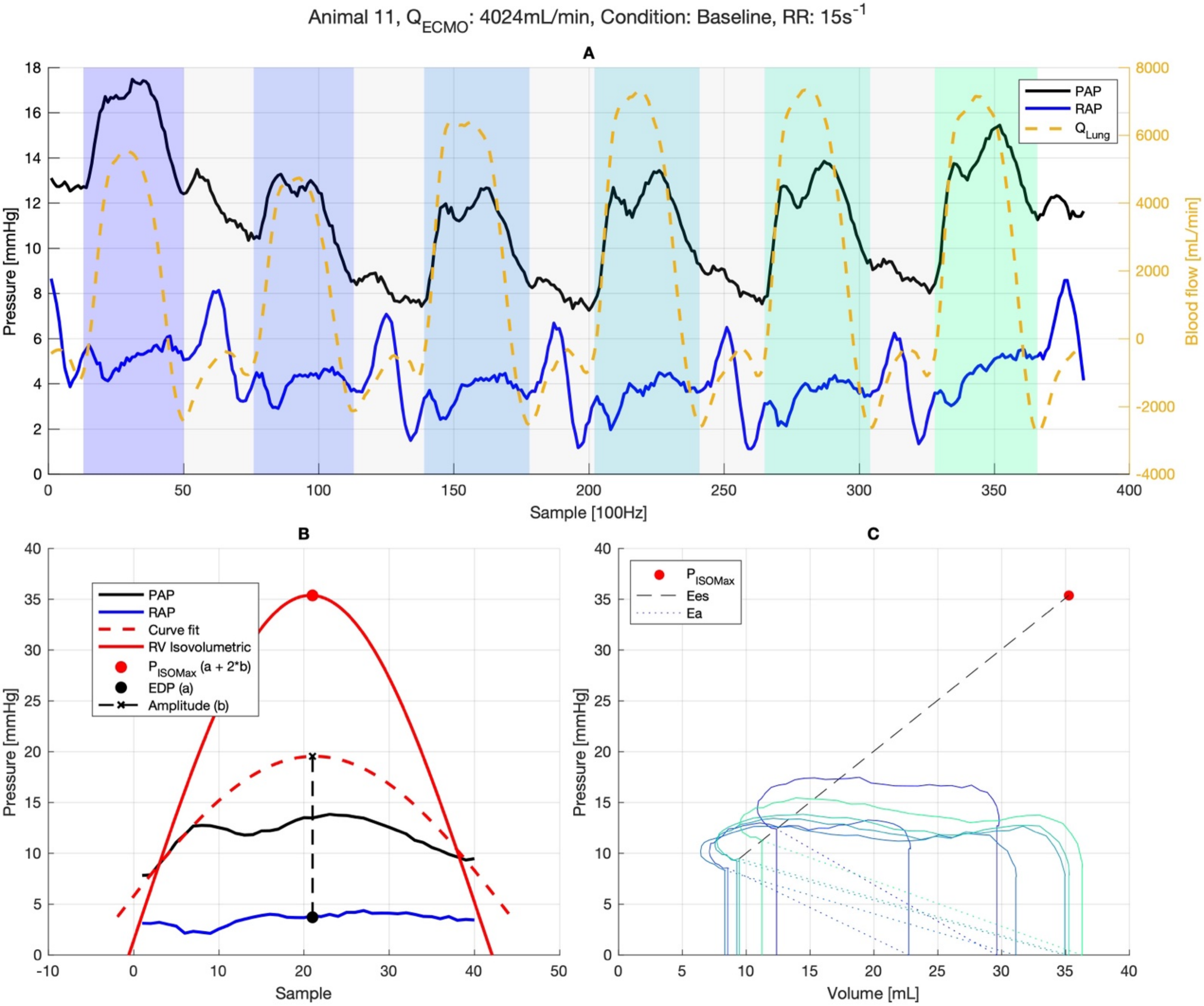

Animal 11, Q_{ECMO}: 4024mL/min, Condition: Baseline, RR: $15s^{-1}$
A
PAP
RAP
Q_{Lung}
Pressure [mmHg]
Blood flow [mL/min]
Sample [100Hz]
B
PAP
RAP
Curve fit
RV Isovolumetric
P_{ISOMax} (a + 2*b)
EDP (a)
Amplitude (b)
Pressure [mmHg]
Sample
C
P_{ISOMax}
Ees
Ea
Pressure [mmHg]
Volume [mL]

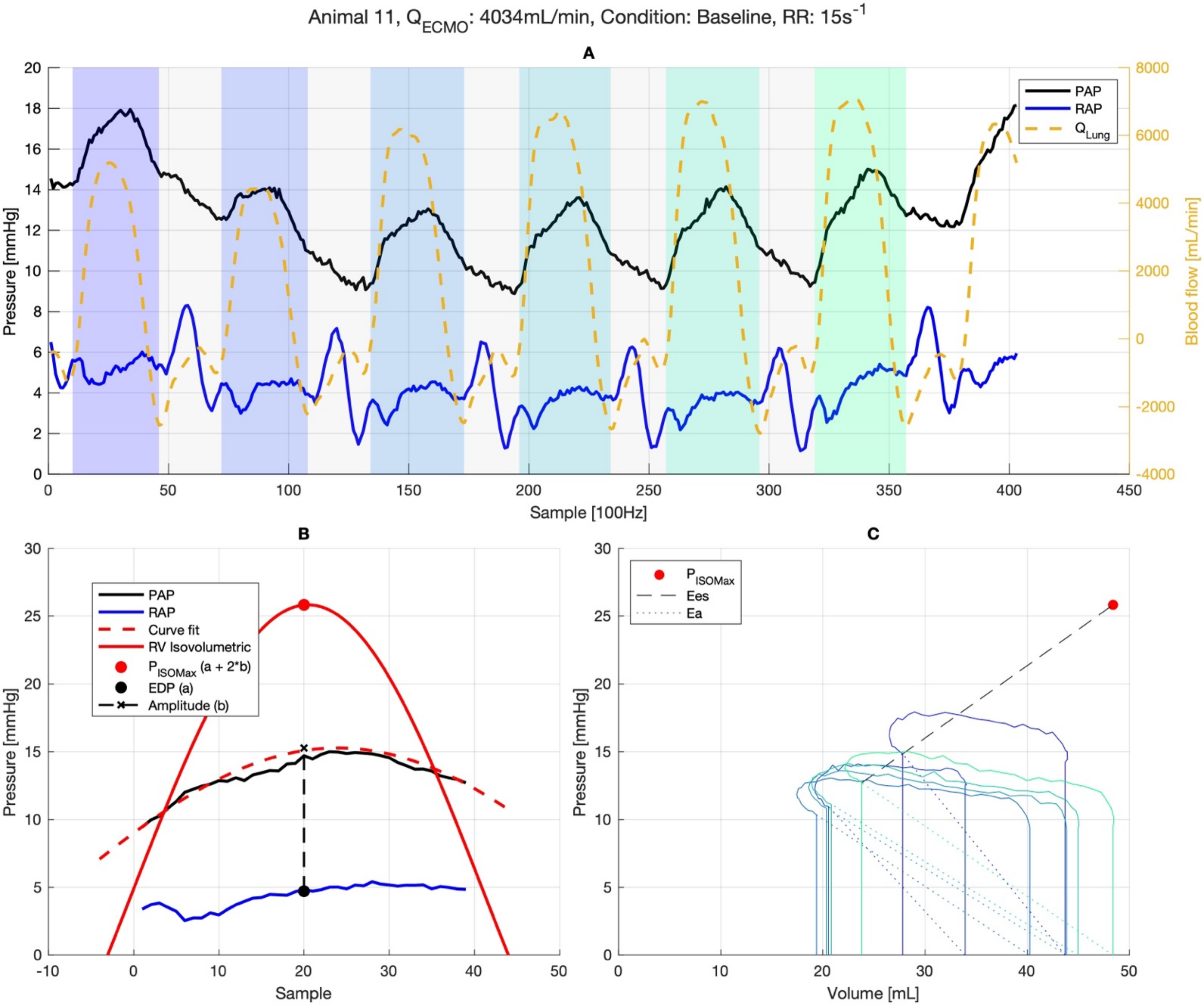
Animal 11, Q_{ECMO}: 4034mL/min, Condition: Baseline, RR: 15s^{-1}
A
PAP
RAP
Q_{Lung}
Pressure [mmHg]
Blood flow [mL/min]
Sample [100Hz]
B
PAP
RAP
Curve fit
RV Isovolumetric
P_{ISOMax} (a + 2*b)
EDP (a)
Amplitude (b)
Pressure [mmHg]
Sample
C
P_{ISOMax}
Ees
Ea
Pressure [mmHg]
Volume [mL]

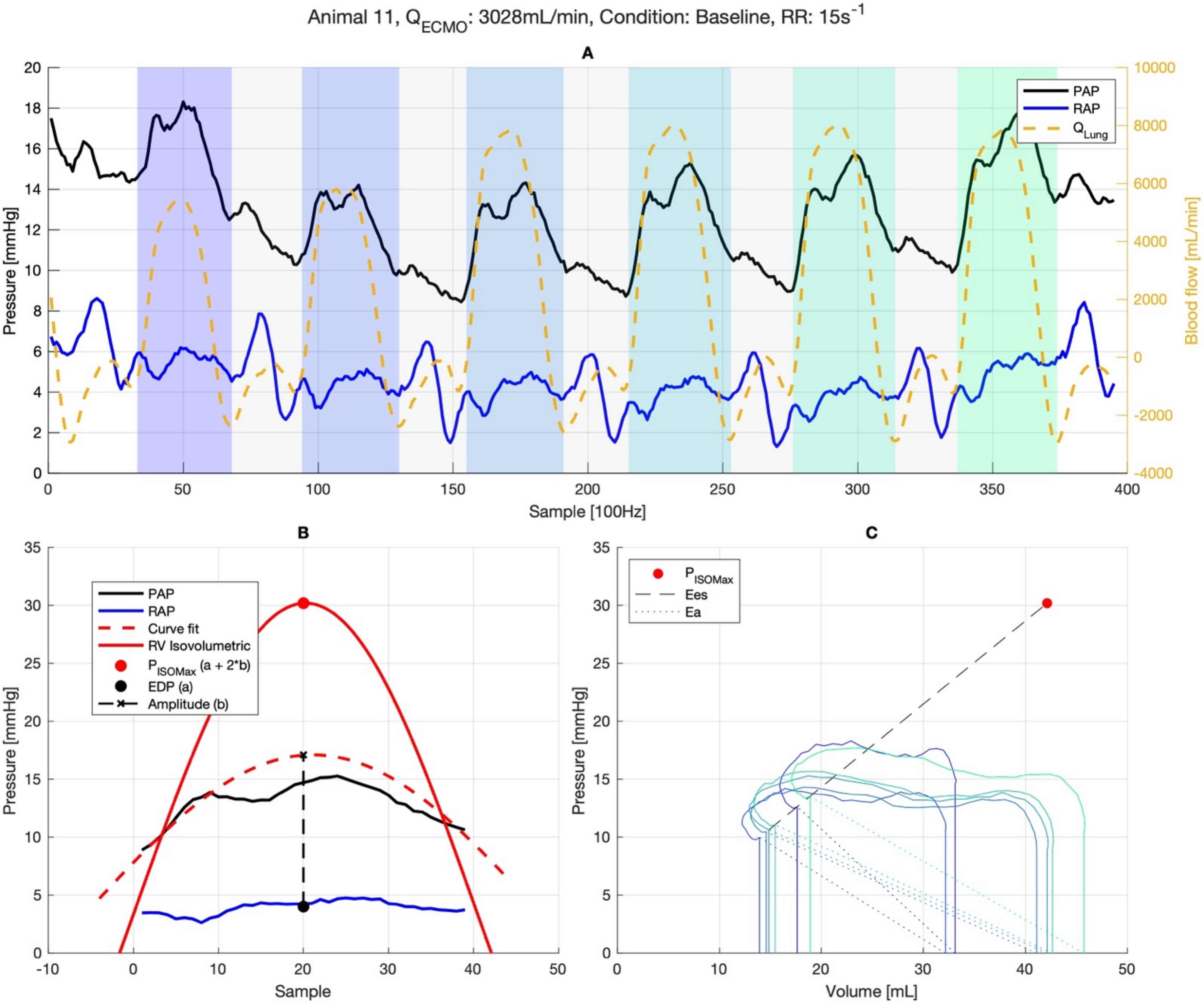

Animal 11, Q_{ECMO}: 3028mL/min, Condition: Baseline, RR: $15s^{-1}$
A
PAP
RAP
Q_{Lung}
Pressure [mmHg]
Blood flow [mL/min]
Sample [100Hz]
B
PAP
RAP
Curve fit
RV Isovolumetric
P_{ISOMax} (a + 2*b)
EDP (a)
Amplitude (b)
Pressure [mmHg]
Sample
C
P_{ISOMax}
Ees
Ea
Pressure [mmHg]
Volume [mL]

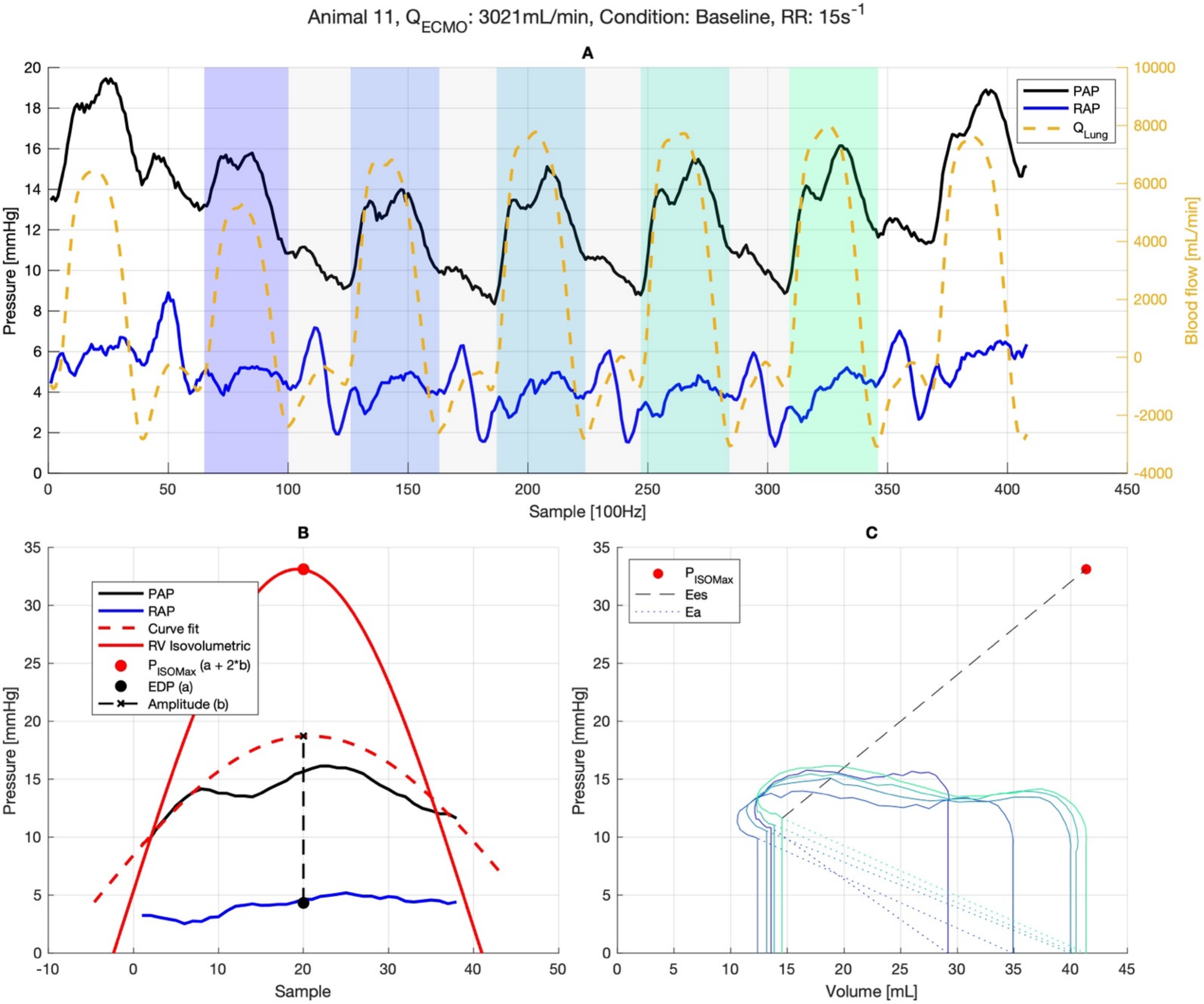

Animal 11, Q_ECMO: 3021mL/min, Condition: Baseline, RR: 15s^-1
A
Pressure [mmHg]
Blood flow [mL/min]
Sample [100Hz]
PAP
RAP
Q_Lung
B
Pressure [mmHg]
Sample
PAP
RAP
Curve fit
RV Isovolumetric
P_ISOMax (a + 2*b)
EDP (a)
Amplitude (b)
C
Pressure [mmHg]
Volume [mL]
P_ISOMax
Ees
Ea

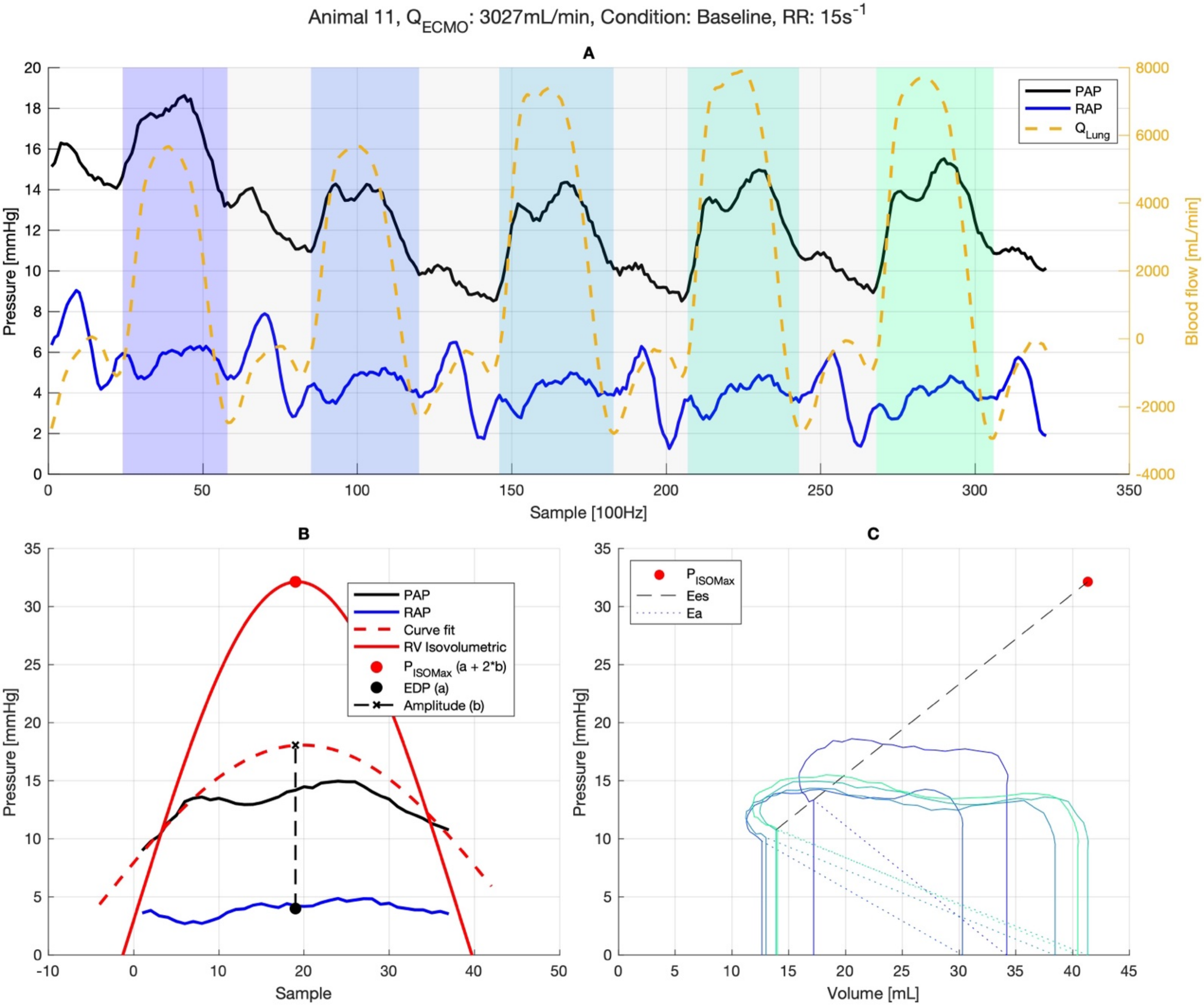

Animal 11, Q_ECMO: 3027mL/min, Condition: Baseline, RR: 15s^-1
A
PAP
RAP
Q_Lung
Pressure [mmHg]
Blood flow [mL/min]
Sample [100Hz]
B
PAP
RAP
Curve fit
RV Isovolumetric
P_ISOMax (a + 2*b)
EDP (a)
Amplitude (b)
Pressure [mmHg]
Sample
C
P_ISOMax
Ees
Ea
Pressure [mmHg]
Volume [mL]

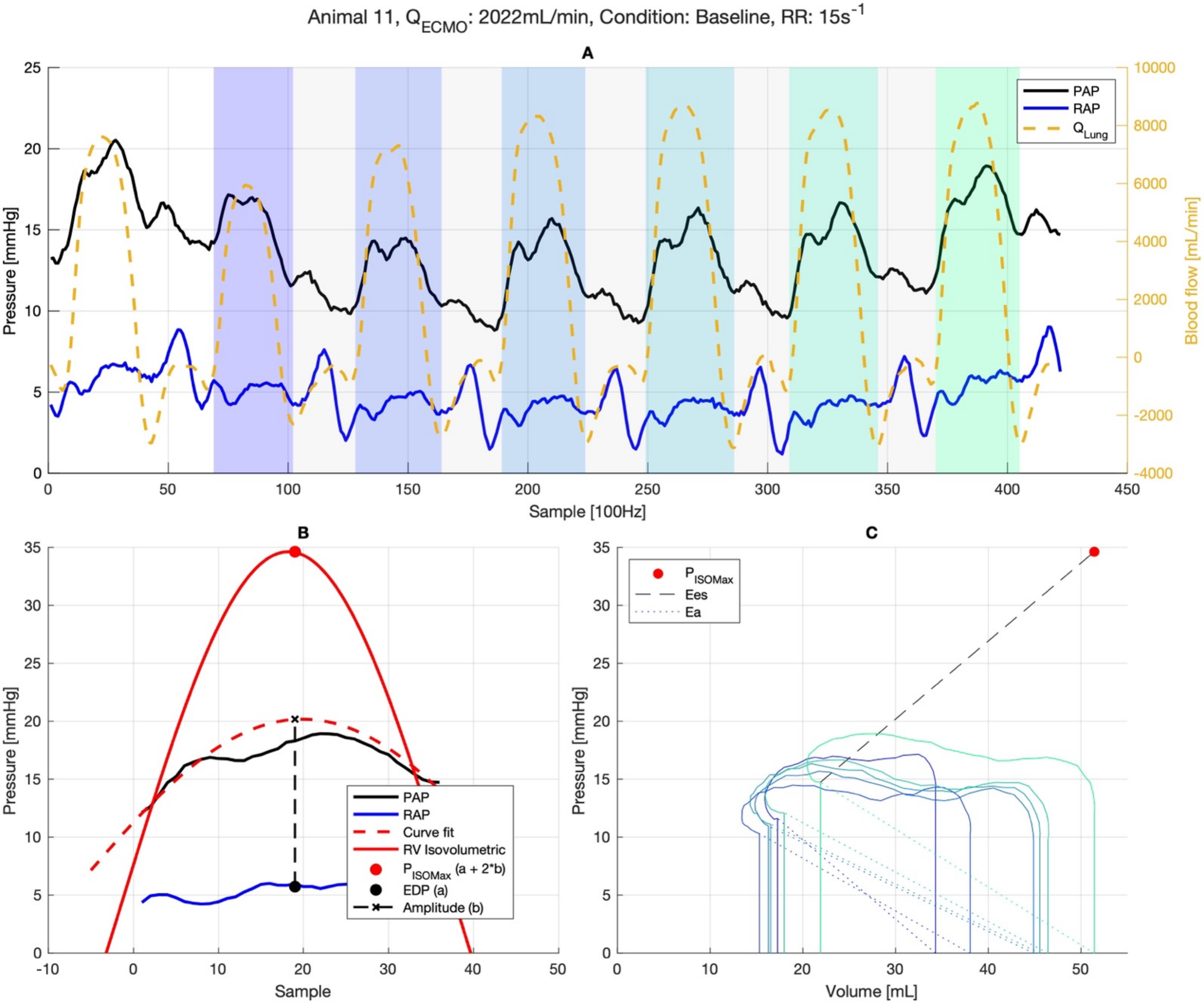
Animal 11, QECMO: 2022mL/min, Condition: Baseline, RR: 15s-1
A
PAP
RAP
QLung
Pressure [mmHg]
Blood flow [mL/min]
Sample [100Hz]
B
PAP
RAP
Curve fit
RV Isovolumetric
PISOMax (a + 2*b)
EDP (a)
Amplitude (b)
Pressure [mmHg]
Sample
C
PISOMax
Ees
Ea
Pressure [mmHg]
Volume [mL]

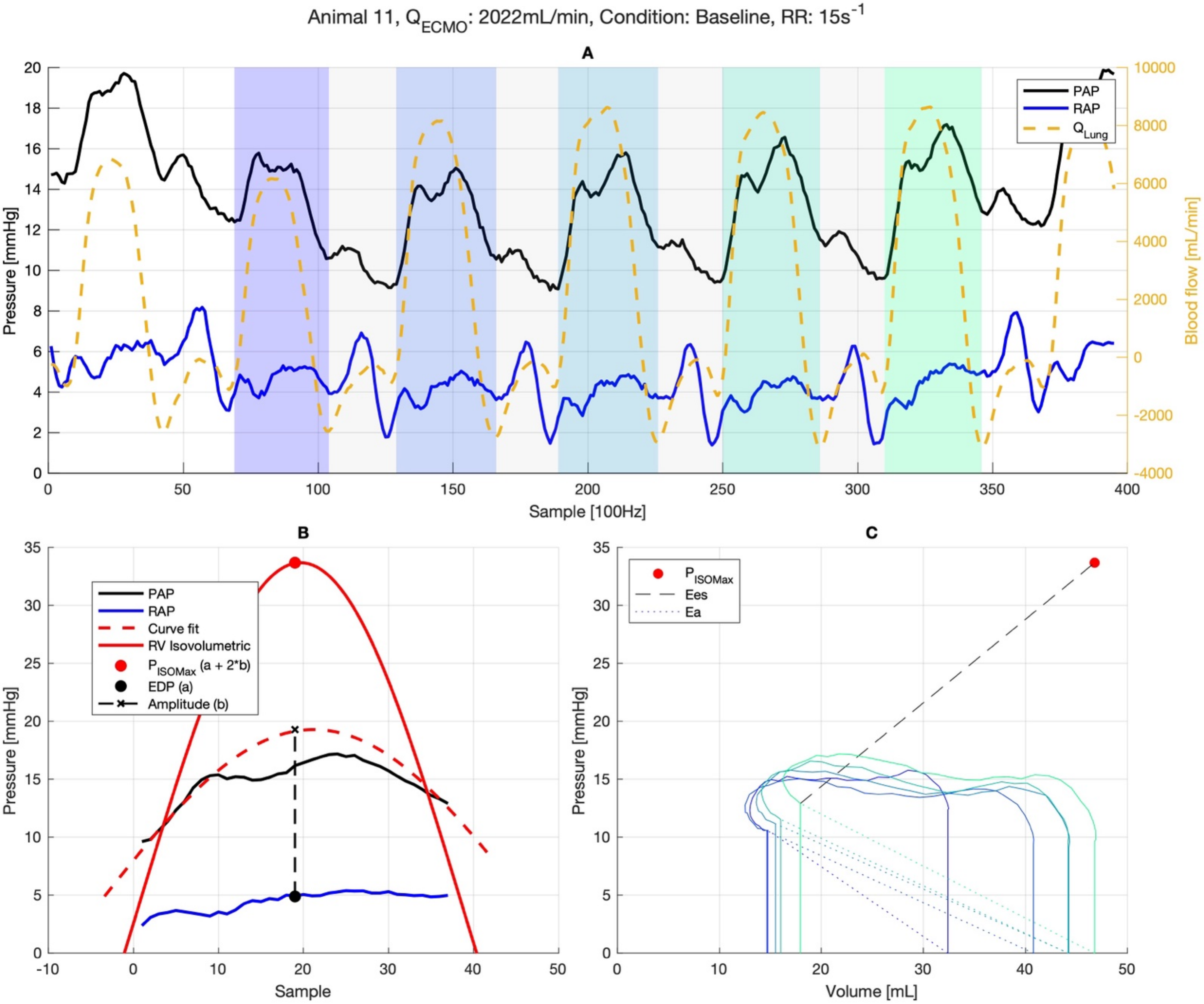

Animal 11, Q_{ECMO}: 2022mL/min, Condition: Baseline, RR: $15s^{-1}$
A
PAP
RAP
Q_{Lung}
Pressure [mmHg]
Blood flow [mL/min]
Sample [100Hz]
B
PAP
RAP
Curve fit
RV Isovolumetric
P_{ISOMax} (a + 2*b)
EDP (a)
Amplitude (b)
Pressure [mmHg]
Sample
C
P_{ISOMax}
Ees
Ea
Pressure [mmHg]
Volume [mL]

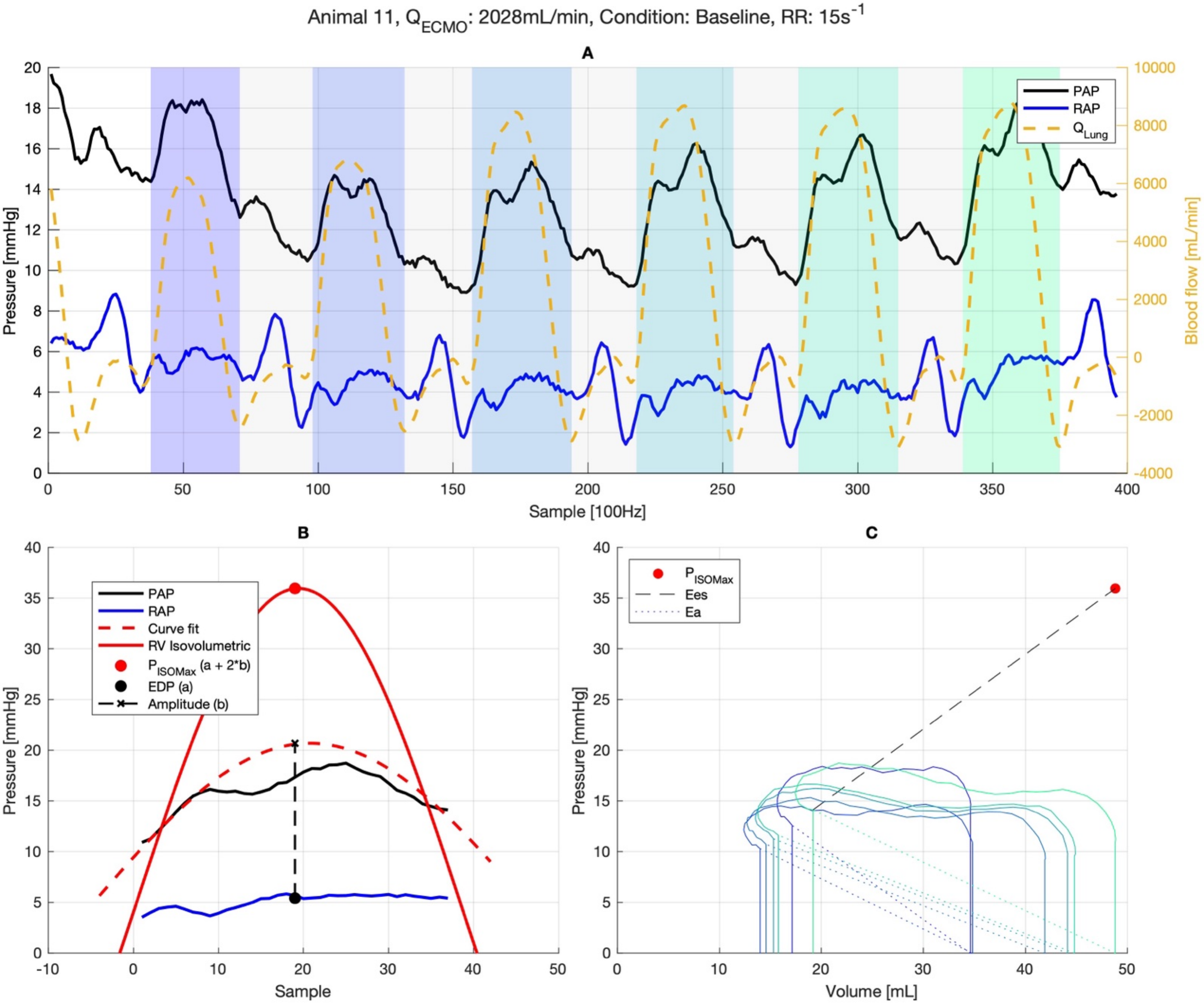
Animal 11, Q_{ECMO}: 2028mL/min, Condition: Baseline, RR: 15s^{-1}
A
PAP
RAP
Q_{Lung}
Pressure [mmHg]
Blood flow [mL/min]
Sample [100Hz]
B
PAP
RAP
Curve fit
RV Isovolumetric
P_{ISOMax} (a + 2*b)
EDP (a)
Amplitude (b)
Pressure [mmHg]
Sample
C
P_{ISOMax}
Ees
Ea
Pressure [mmHg]
Volume [mL]

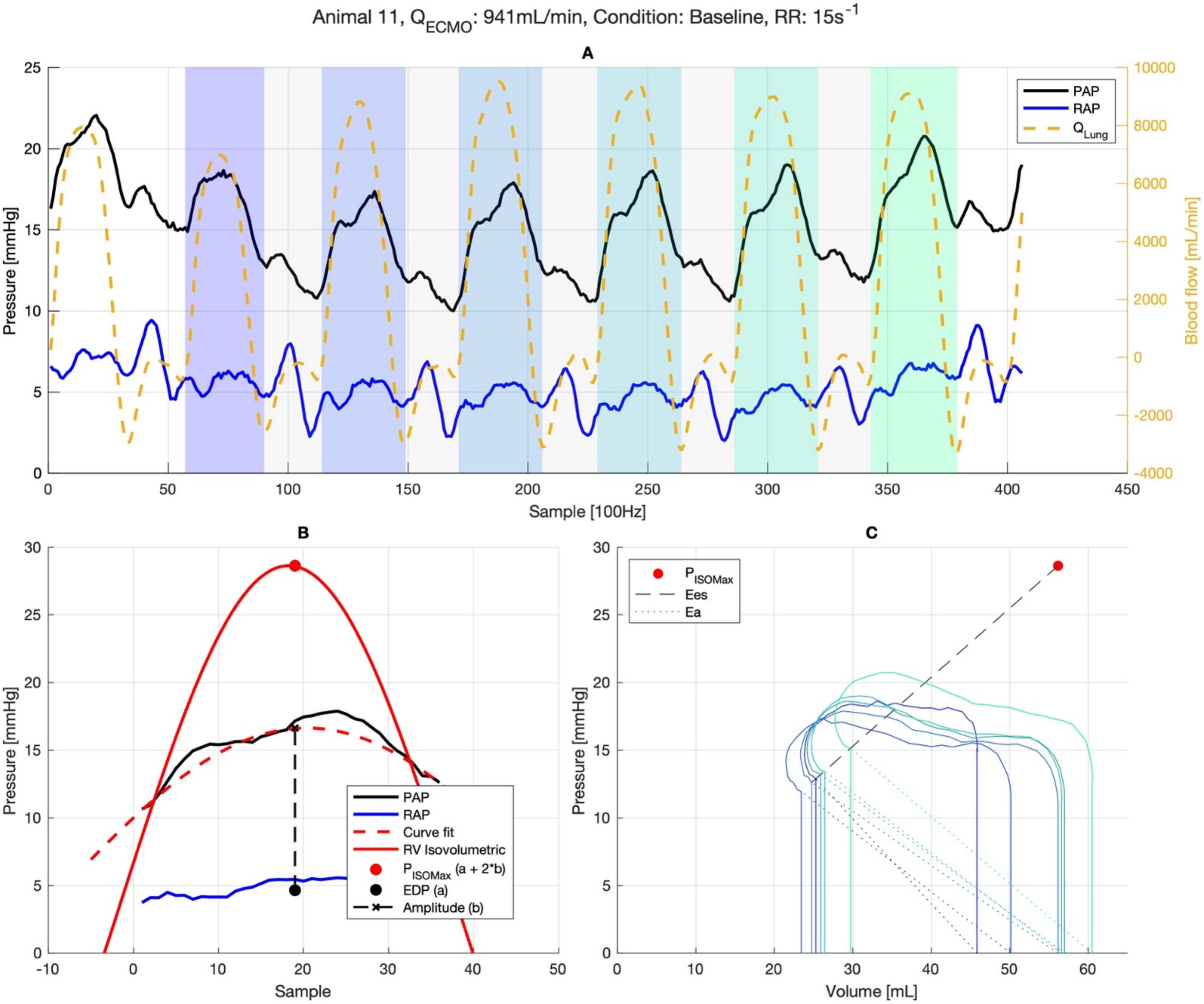

Animal 11, Q_{ECMO}: 941mL/min, Condition: Baseline, RR: 15s^{-1}
A
PAP
RAP
Q_{Lung}
Pressure [mmHg]
Blood flow [mL/min]
Sample [100Hz]
B
PAP
RAP
Curve fit
RV Isovolumetric
P_{ISOMax} (a + 2*b)
EDP (a)
Amplitude (b)
Pressure [mmHg]
Sample
C
P_{ISOMax}
Ees
Ea
Pressure [mmHg]
Volume [mL]

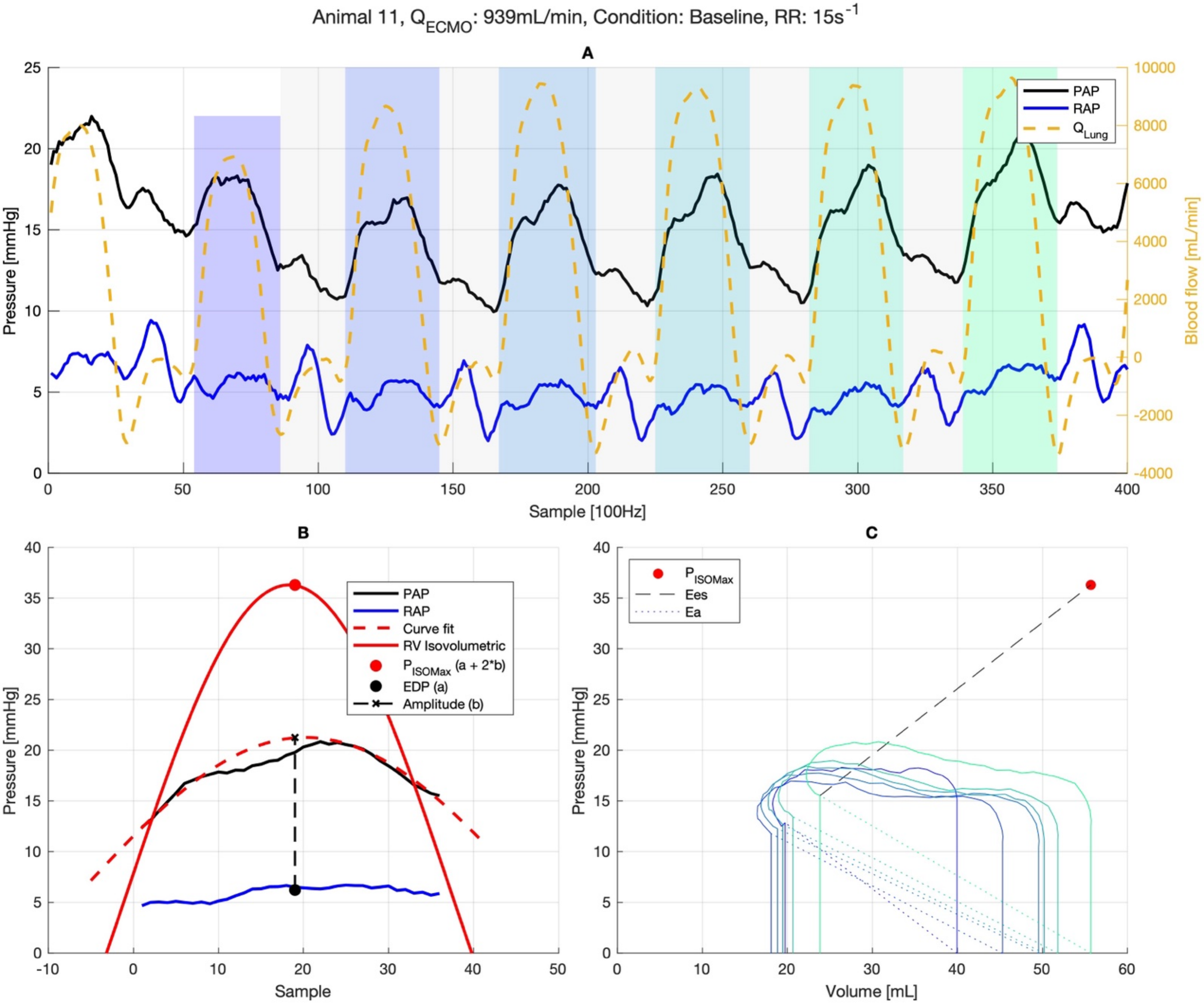

Animal 11, Q ECMO: 939mL/min, Condition: Baseline, RR: 15s-1
A
PAP
RAP
Q Lung
Pressure [mmHg]
Blood flow [mL/min]
Sample [100Hz]
B
PAP
RAP
Curve fit
RV Isovolumetric
P ISOMax (a + 2*b)
EDP (a)
Amplitude (b)
Pressure [mmHg]
Sample
C
P ISOMax
Ees
Ea
Pressure [mmHg]
Volume [mL]

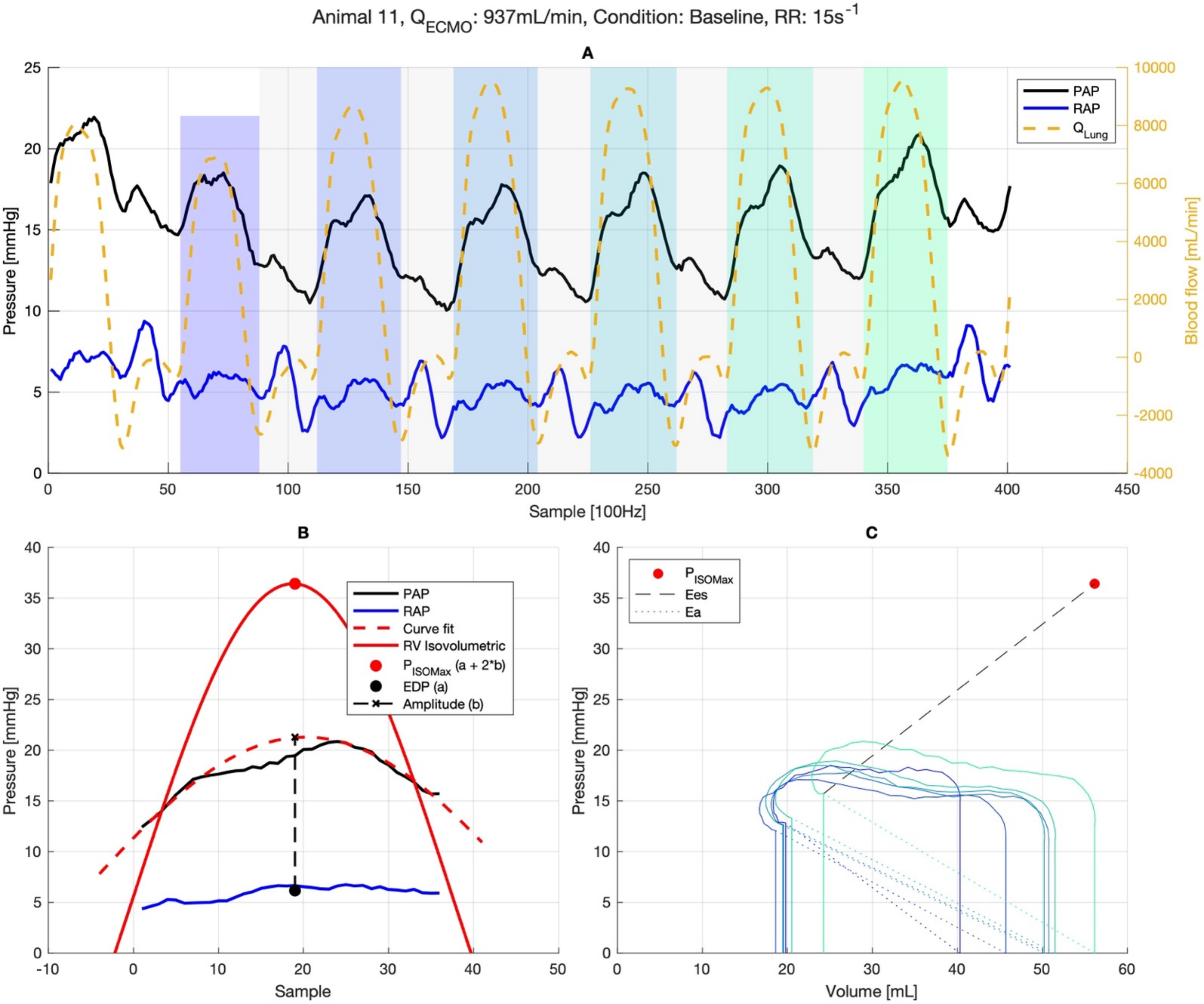

Animal 11, Q_{ECMO}: 937mL/min, Condition: Baseline, RR: 15s^{-1}
A
PAP
RAP
Q_{Lung}
Pressure [mmHg]
Blood flow [mL/min]
Sample [100Hz]
B
PAP
RAP
Curve fit
RV Isovolumetric
P_{ISOMax} (a + 2*b)
EDP (a)
Amplitude (b)
Pressure [mmHg]
Sample
C
P_{ISOMax}
Ees
Ea
Pressure [mmHg]
Volume [mL]

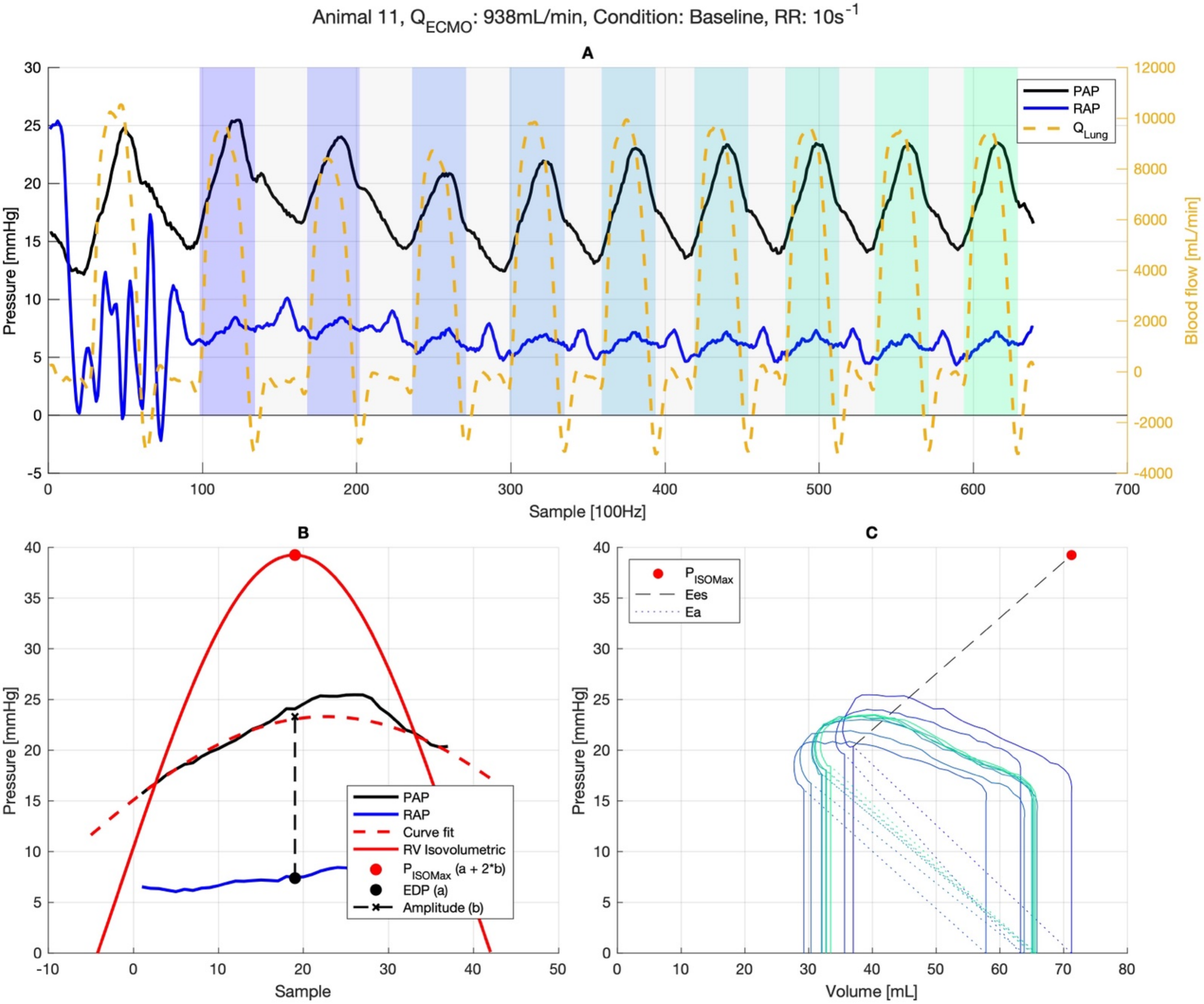

Animal 11, Q_{ECMO}: 938mL/min, Condition: Baseline, RR: $10s^{-1}$
A
PAP
RAP
Q_{Lung}
Pressure [mmHg]
Blood flow [mL/min]
Sample [100Hz]
B
PAP
RAP
Curve fit
RV Isovolumetric
P_{ISOMax} (a + 2*b)
EDP (a)
Amplitude (b)
Pressure [mmHg]
Sample
C
P_{ISOMax}
Ees
Ea
Pressure [mmHg]
Volume [mL]

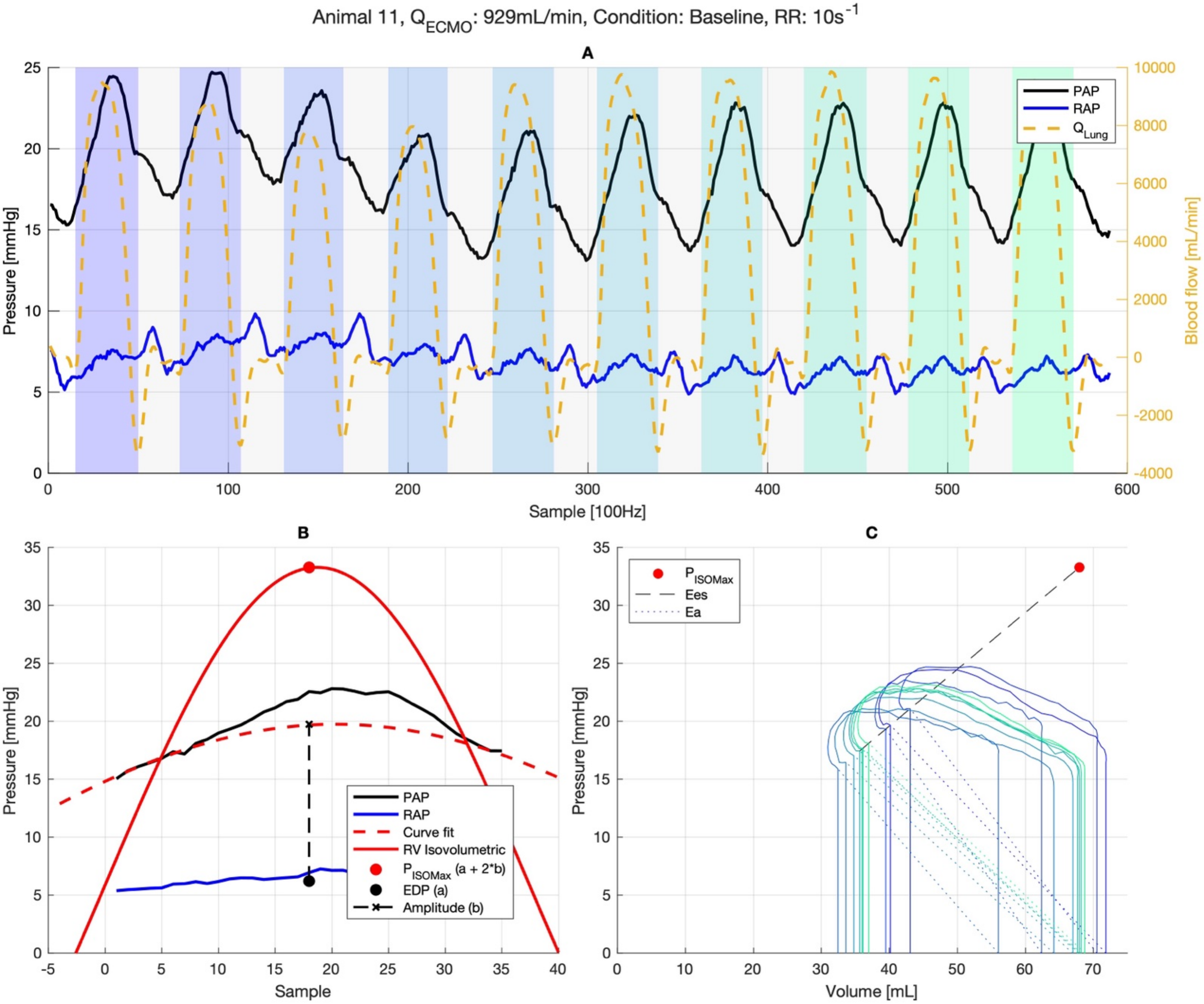

Animal 11, Q_{ECMO}: 929mL/min, Condition: Baseline, RR: $10s^{-1}$
A
PAP
RAP
Q_{Lung}
Pressure [mmHg]
Blood flow [mL/min]
Sample [100Hz]
B
PAP
RAP
Curve fit
RV Isovolumetric
P_{ISOMax} (a + 2*b)
EDP (a)
Amplitude (b)
Pressure [mmHg]
Sample
C
P_{ISOMax}
Ees
Ea
Pressure [mmHg]
Volume [mL]

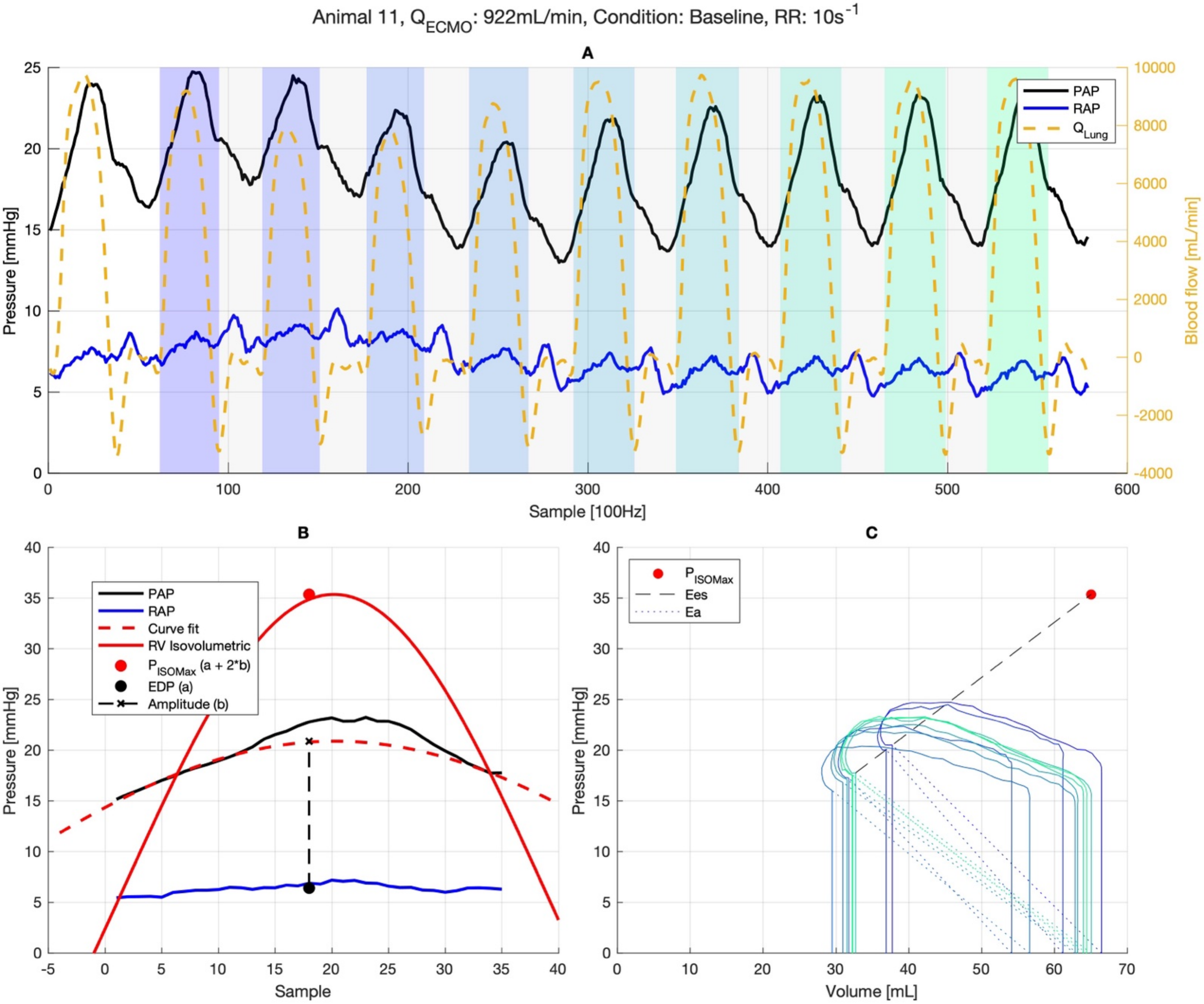

Animal 11, Q_{ECMO}: 922mL/min, Condition: Baseline, RR: $10s^{-1}$
A
PAP
RAP
Q_{Lung}
Pressure [mmHg]
Blood flow [mL/min]
Sample [100Hz]
B
PAP
RAP
Curve fit
RV Isovolumetric
P_{ISOMax} (a + 2*b)
EDP (a)
Amplitude (b)
Pressure [mmHg]
Sample
C
P_{ISOMax}
Ees
Ea
Pressure [mmHg]
Volume [mL]

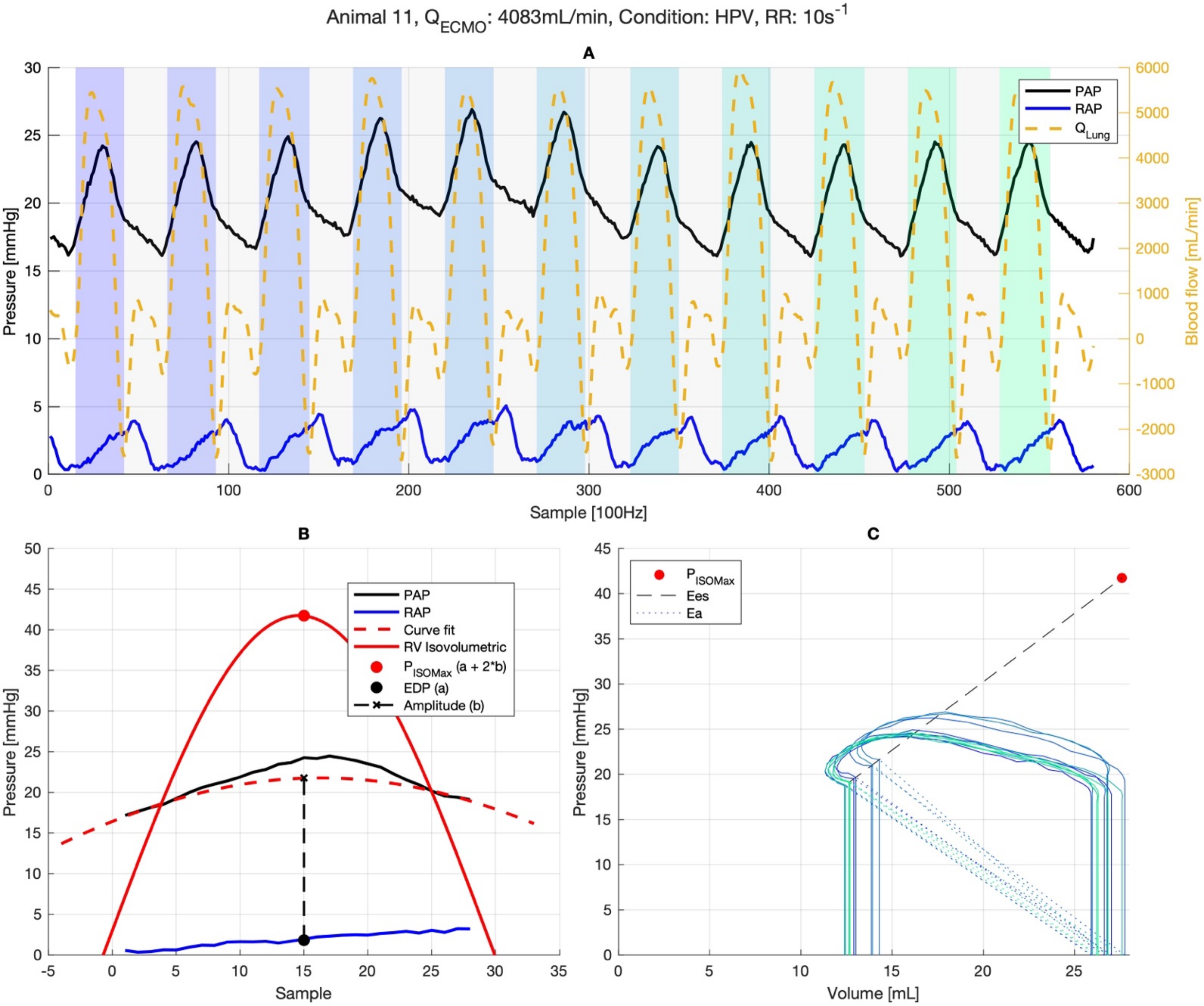

Animal 11, Q_{ECMO}: 4083mL/min, Condition: HPV, RR: 10s^{-1}
A
PAP
RAP
Q_{Lung}
Pressure [mmHg]
Blood flow [mL/min]
Sample [100Hz]
B
PAP
RAP
Curve fit
RV Isovolumetric
P_{ISOMax} (a + 2*b)
EDP (a)
Amplitude (b)
Pressure [mmHg]
Sample
C
P_{ISOMax}
Ees
Ea
Pressure [mmHg]
Volume [mL]

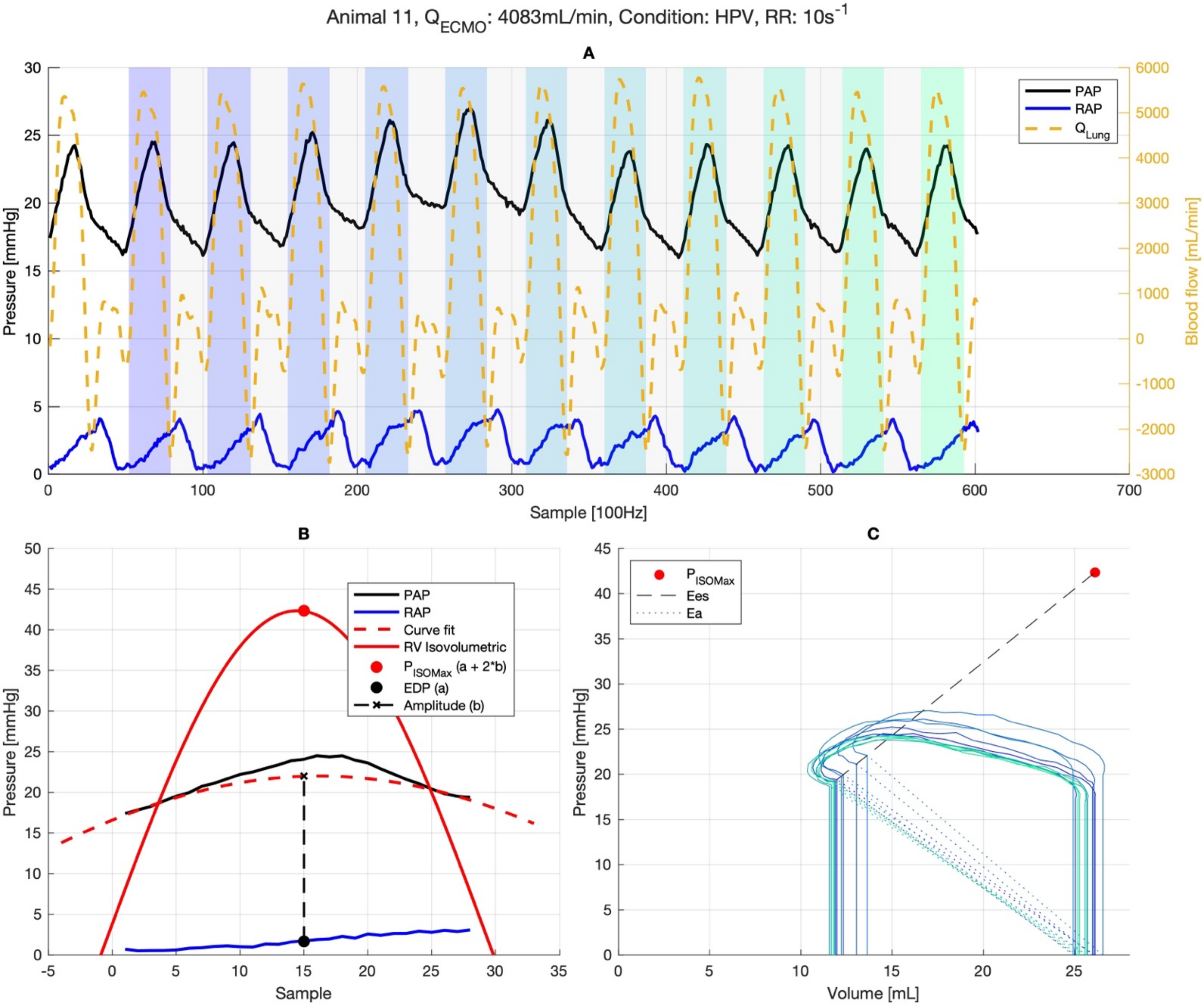

Animal 11, Q_{ECMO}: 4083mL/min, Condition: HPV, RR: $10s^{-1}$
A
PAP
RAP
Q_{Lung}
Pressure [mmHg]
Blood flow [mL/min]
Sample [100Hz]
B
PAP
RAP
Curve fit
RV Isovolumetric
P_{ISOMax} (a + 2*b)
EDP (a)
Amplitude (b)
Pressure [mmHg]
Sample
C
P_{ISOMax}
Ees
Ea
Pressure [mmHg]
Volume [mL]

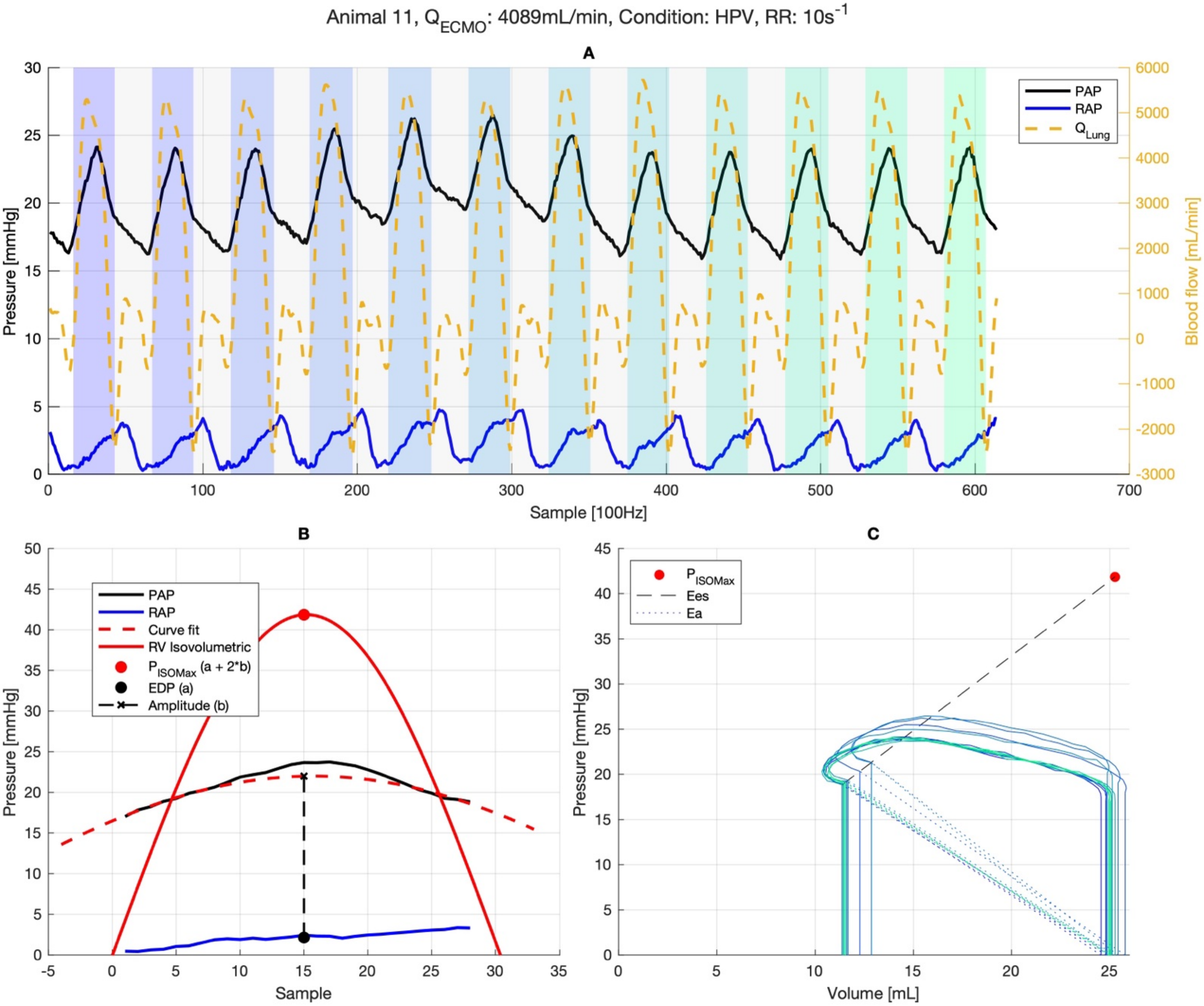

Animal 11, Q_ECMO: 4089mL/min, Condition: HPV, RR: 10s^-1
A
Pressure [mmHg]
Blood flow [mL/min]
Sample [100Hz]
PAP
RAP
Q_Lung
B
PAP
RAP
Curve fit
RV Isovolumetric
P_ISOMax (a + 2*b)
EDP (a)
Amplitude (b)
Pressure [mmHg]
Sample
C
P_ISOMax
Ees
Ea
Pressure [mmHg]
Volume [mL]

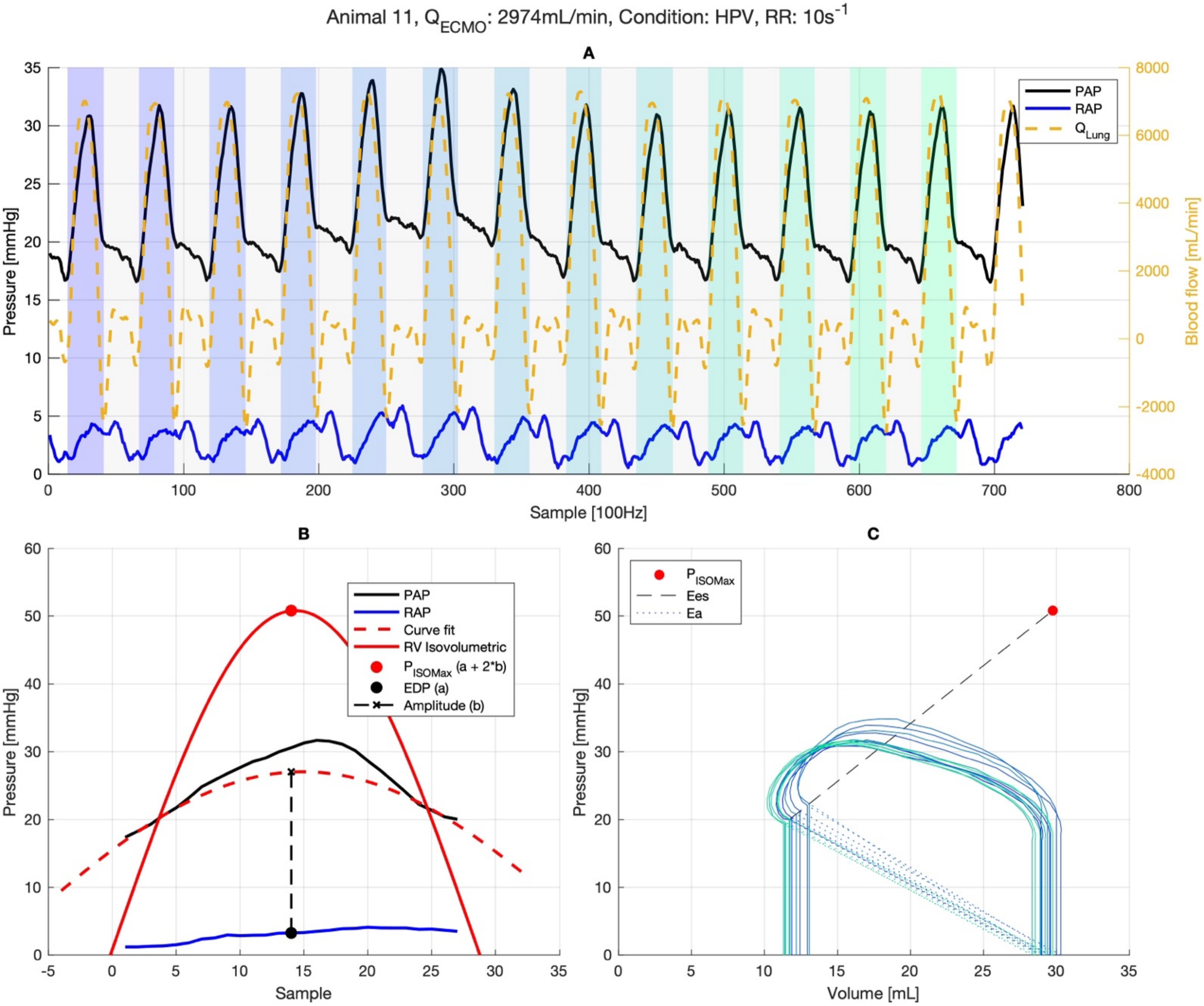

Animal 11, Q_{ECMO}: 2974mL/min, Condition: HPV, RR: 10s^{-1}
A
PAP
RAP
Q_{Lung}
Pressure [mmHg]
Blood flow [mL/min]
Sample [100Hz]
B
PAP
RAP
Curve fit
RV Isovolumetric
P_{ISOMax} (a + 2*b)
EDP (a)
Amplitude (b)
Pressure [mmHg]
Sample
C
P_{ISOMax}
Ees
Ea
Pressure [mmHg]
Volume [mL]

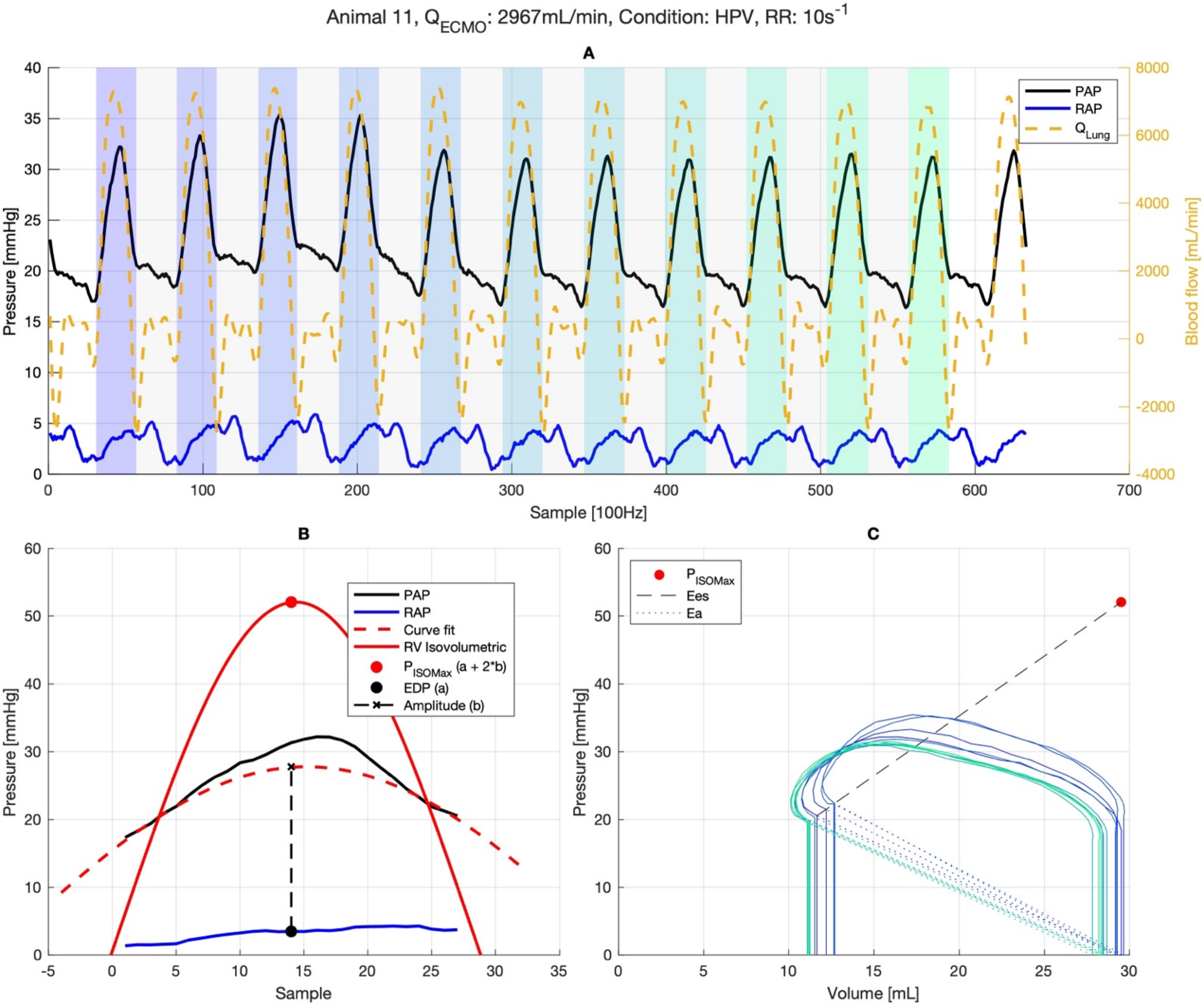

Animal 11, Q_ECMO: 2967mL/min, Condition: HPV, RR: 10s^-1
A
Pressure [mmHg]
Blood flow [mL/min]
Sample [100Hz]
PAP
RAP
Q_Lung
B
Pressure [mmHg]
Sample
PAP
RAP
Curve fit
RV Isovolumetric
P_ISOMax (a + 2*b)
EDP (a)
Amplitude (b)
C
Pressure [mmHg]
Volume [mL]
P_ISOMax
Ees
Ea

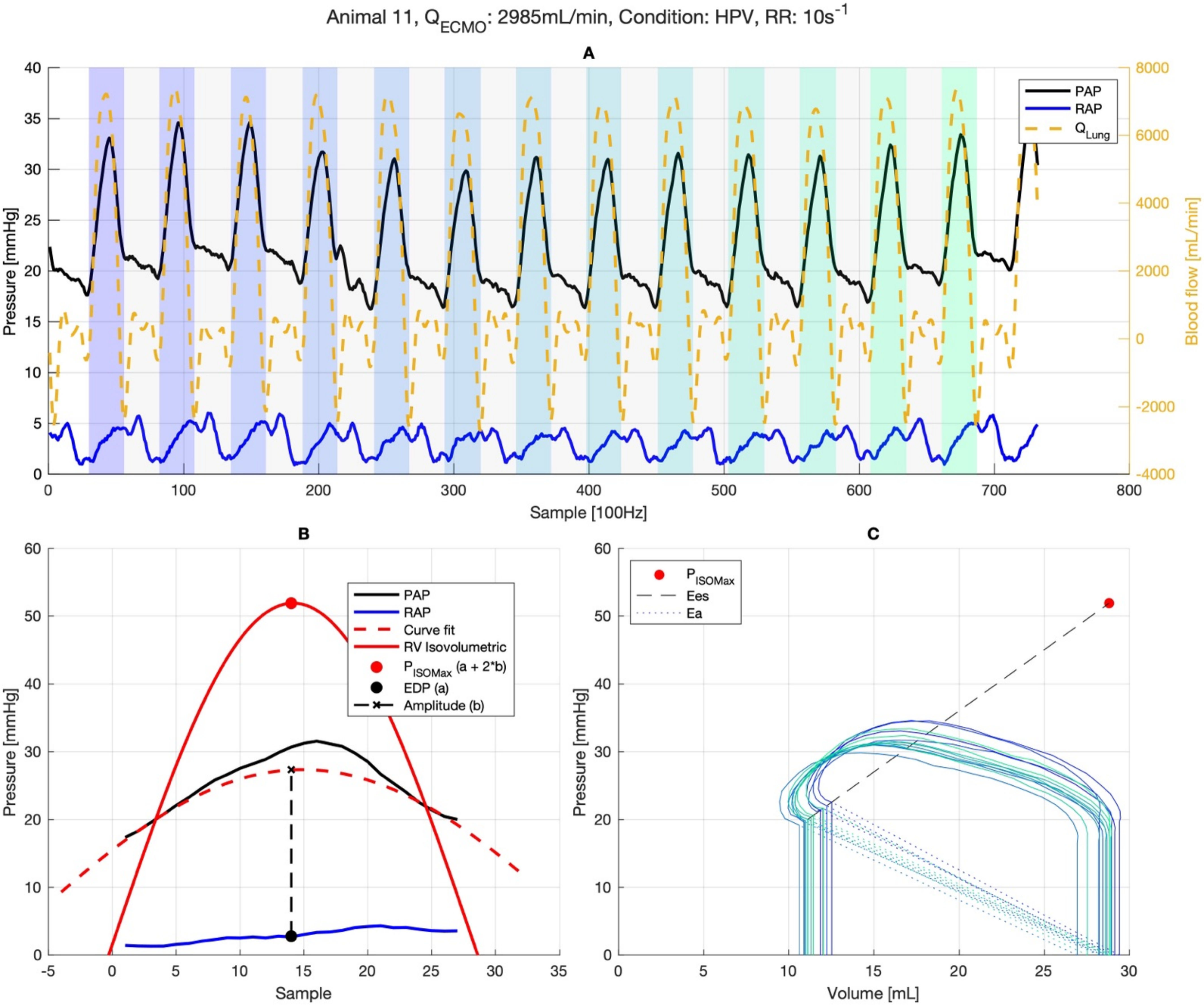
Animal 11, Q_{ECMO}: 2985mL/min, Condition: HPV, RR: $10s^{-1}$
A
PAP
RAP
Q_{Lung}
Pressure [mmHg]
Blood flow [mL/min]
Sample [100Hz]
B
PAP
RAP
Curve fit
RV Isovolumetric
P_{ISOMax} (a + 2*b)
EDP (a)
Amplitude (b)
Pressure [mmHg]
Sample
C
P_{ISOMax}
Ees
Ea
Pressure [mmHg]
Volume [mL]

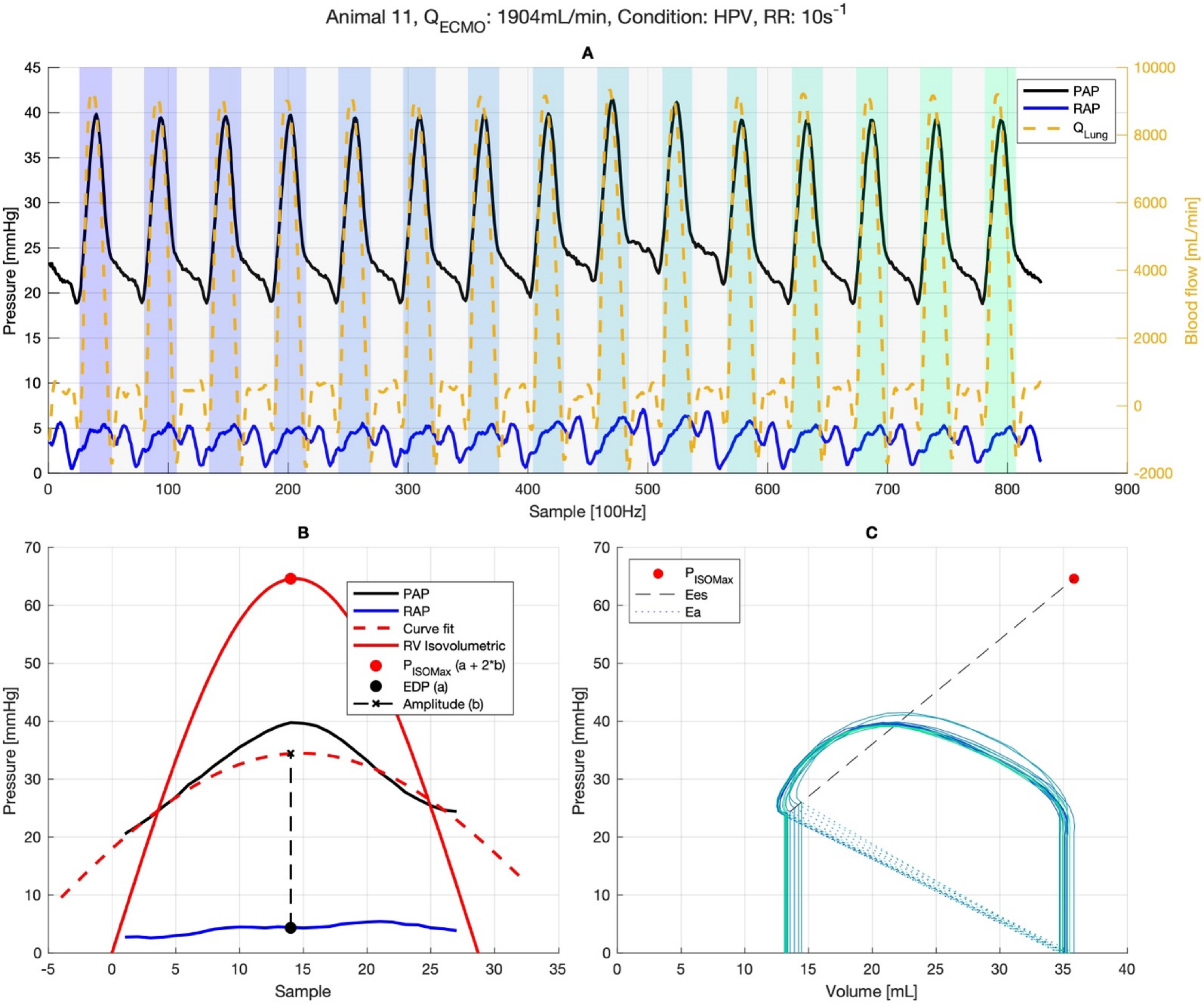

Animal 11, Q_{ECMO}: 1904mL/min, Condition: HPV, RR: $10s^{-1}$
A
Pressure [mmHg]
Blood flow [mL/min]
Sample [100Hz]
PAP
RAP
Q_{Lung}
B
Pressure [mmHg]
Sample
PAP
RAP
Curve fit
RV Isovolumetric
P_{ISOMax} (a + 2*b)
EDP (a)
Amplitude (b)
C
Pressure [mmHg]
Volume [mL]
P_{ISOMax}
Ees
Ea

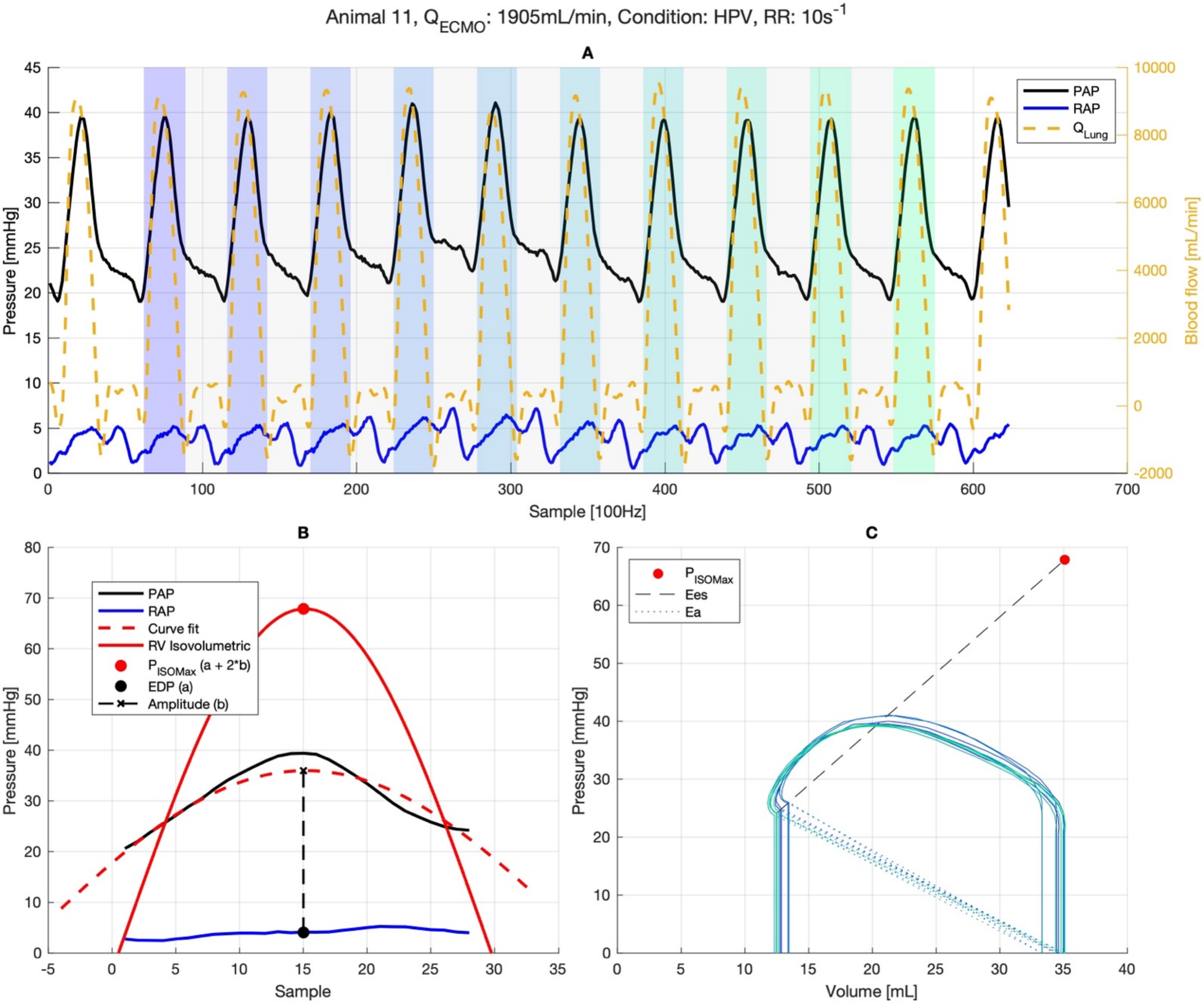

Animal 11, Q_{ECMO}: 1905mL/min, Condition: HPV, RR: $10s^{-1}$
A
Pressure [mmHg]
Blood flow [mL/min]
Sample [100Hz]
PAP
RAP
Q_{Lung}
B
PAP
RAP
Curve fit
RV Isovolumetric
P_{ISOMax} (a + 2*b)
EDP (a)
Amplitude (b)
Pressure [mmHg]
Sample
C
P_{ISOMax}
Ees
Ea
Pressure [mmHg]
Volume [mL]

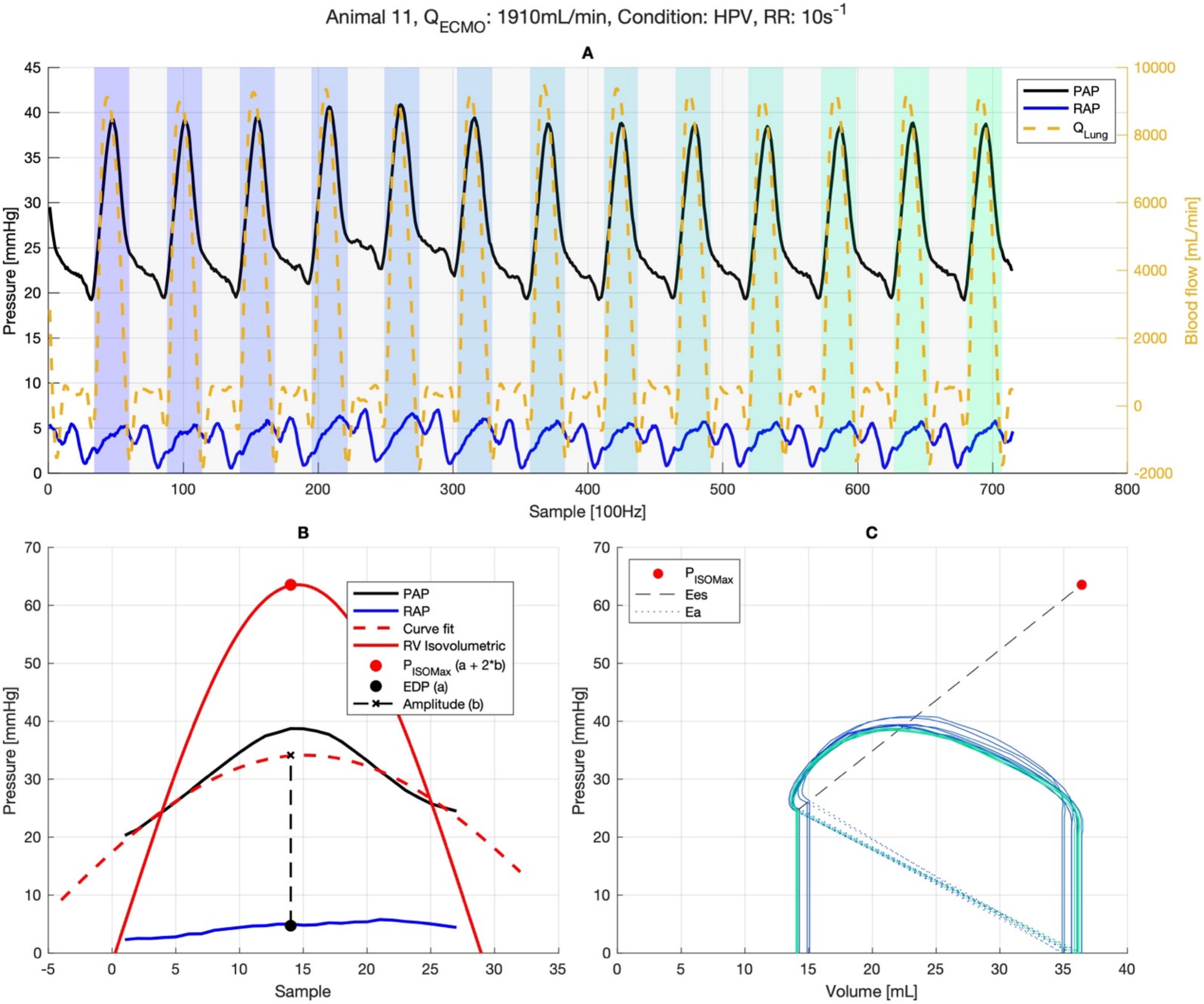

Animal 11, Q_{ECMO}: 1910mL/min, Condition: HPV, RR: $10s^{-1}$
A
PAP
RAP
Q_{Lung}
Pressure [mmHg]
Blood flow [mL/min]
Sample [100Hz]
B
PAP
RAP
Curve fit
RV Isovolumetric
P_{ISOMax} (a + 2*b)
EDP (a)
Amplitude (b)
Pressure [mmHg]
Sample
C
P_{ISOMax}
Ees
Ea
Pressure [mmHg]
Volume [mL]

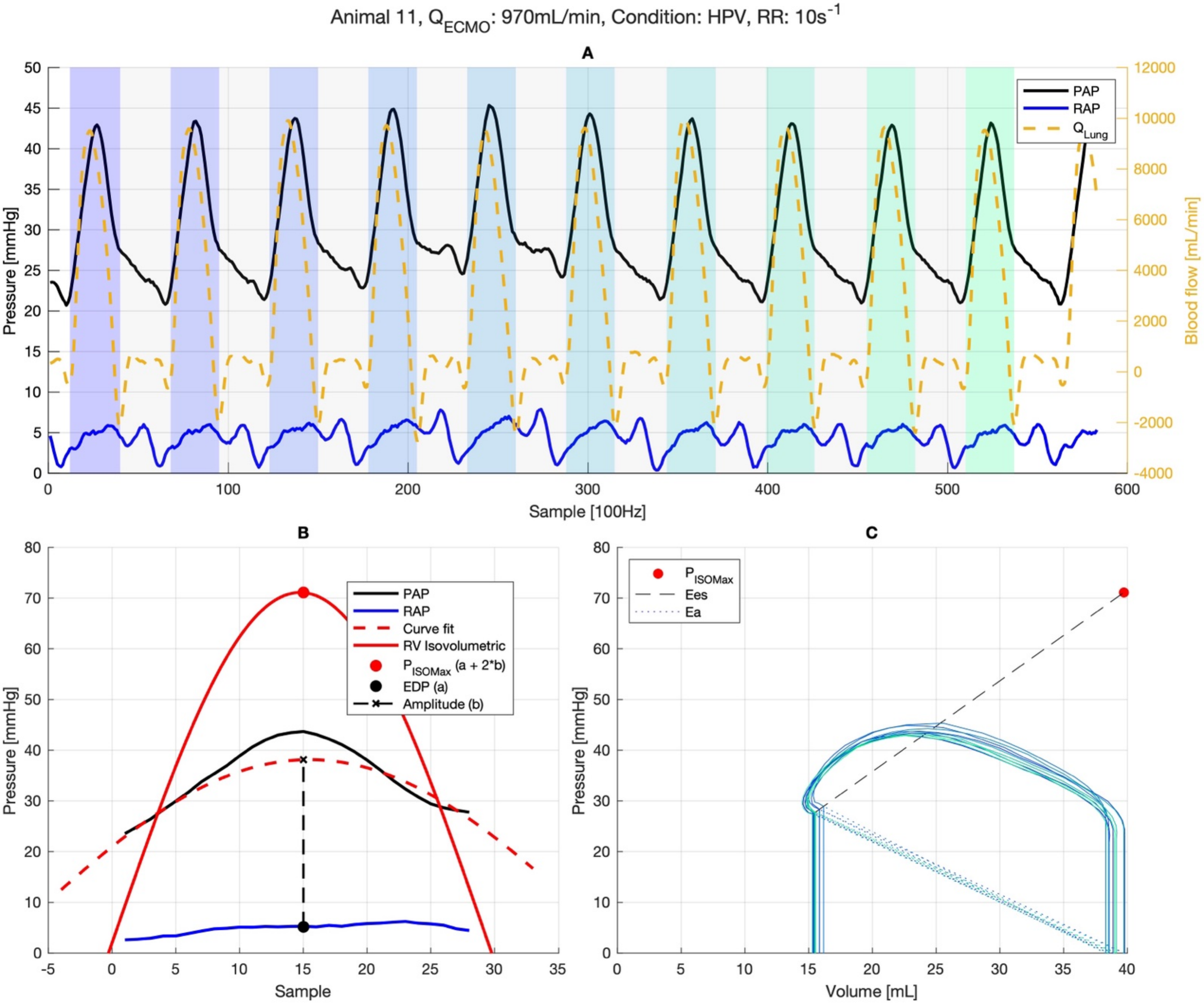

Animal 11, Q_{ECMO}: 970mL/min, Condition: HPV, RR: $10s^{-1}$
A
PAP
RAP
Q_{Lung}
Pressure [mmHg]
Blood flow [mL/min]
Sample [100Hz]
B
PAP
RAP
Curve fit
RV Isovolumetric
P_{ISOMax} (a + 2*b)
EDP (a)
Amplitude (b)
Pressure [mmHg]
Sample
C
P_{ISOMax}
Ees
Ea
Pressure [mmHg]
Volume [mL]

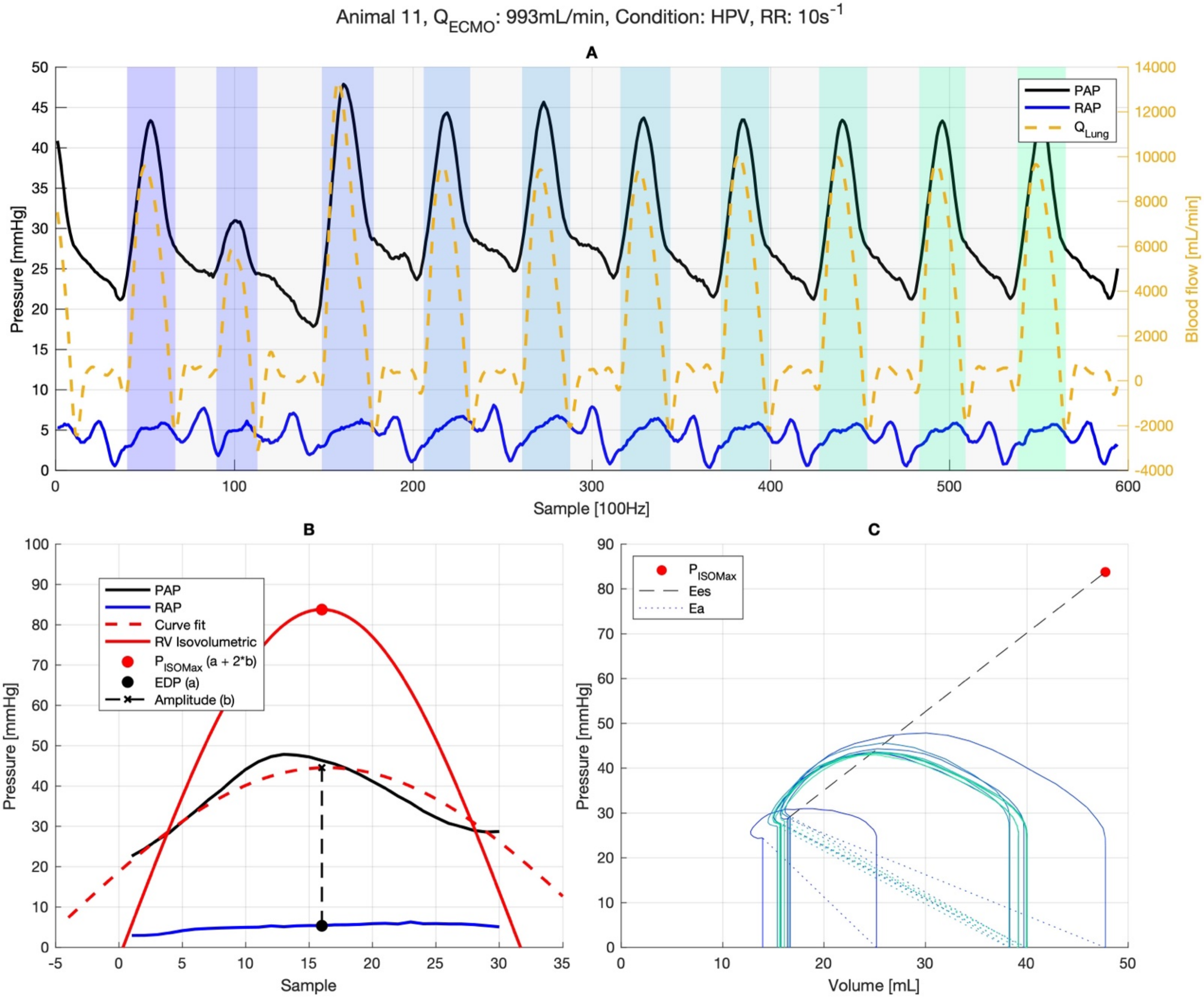

Animal 11, QECMO: 993mL/min, Condition: HPV, RR: 10s-1
A
Pressure [mmHg]
Blood flow [mL/min]
Sample [100Hz]
PAP
RAP
QLung
B
PAP
RAP
Curve fit
RV Isovolumetric
PISOMax (a + 2*b)
EDP (a)
Amplitude (b)
Pressure [mmHg]
Sample
C
PISOMax
Ees
Ea
Pressure [mmHg]
Volume [mL]

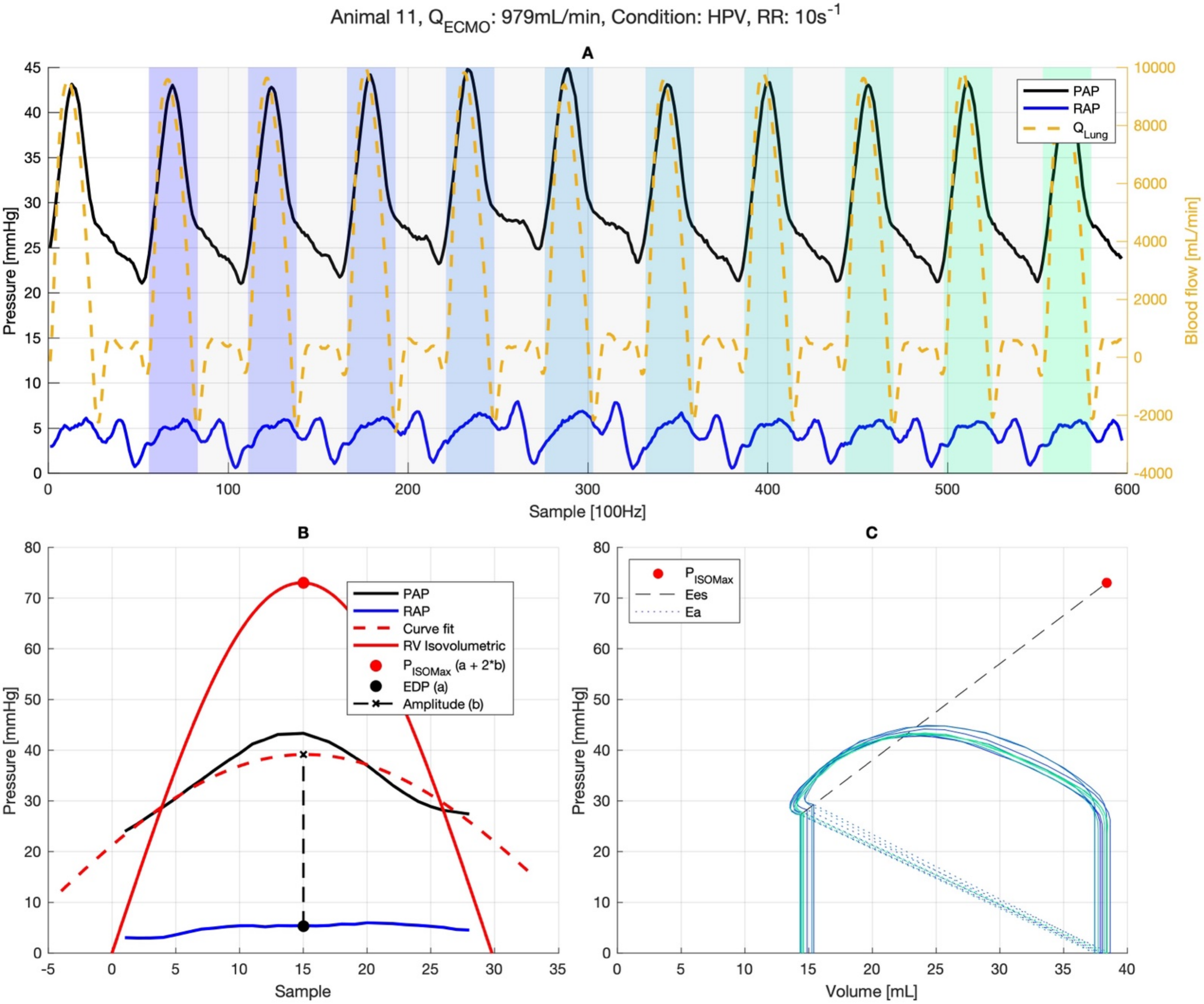

Animal 11, Q_ECMO: 979mL/min, Condition: HPV, RR: 10s^-1
A
PAP
RAP
Q_Lung
Pressure [mmHg]
Blood flow [mL/min]
Sample [100Hz]
B
PAP
RAP
Curve fit
RV Isovolumetric
P_ISOMax (a + 2*b)
EDP (a)
Amplitude (b)
Pressure [mmHg]
Sample
C
P_ISOMax
Ees
Ea
Pressure [mmHg]
Volume [mL]

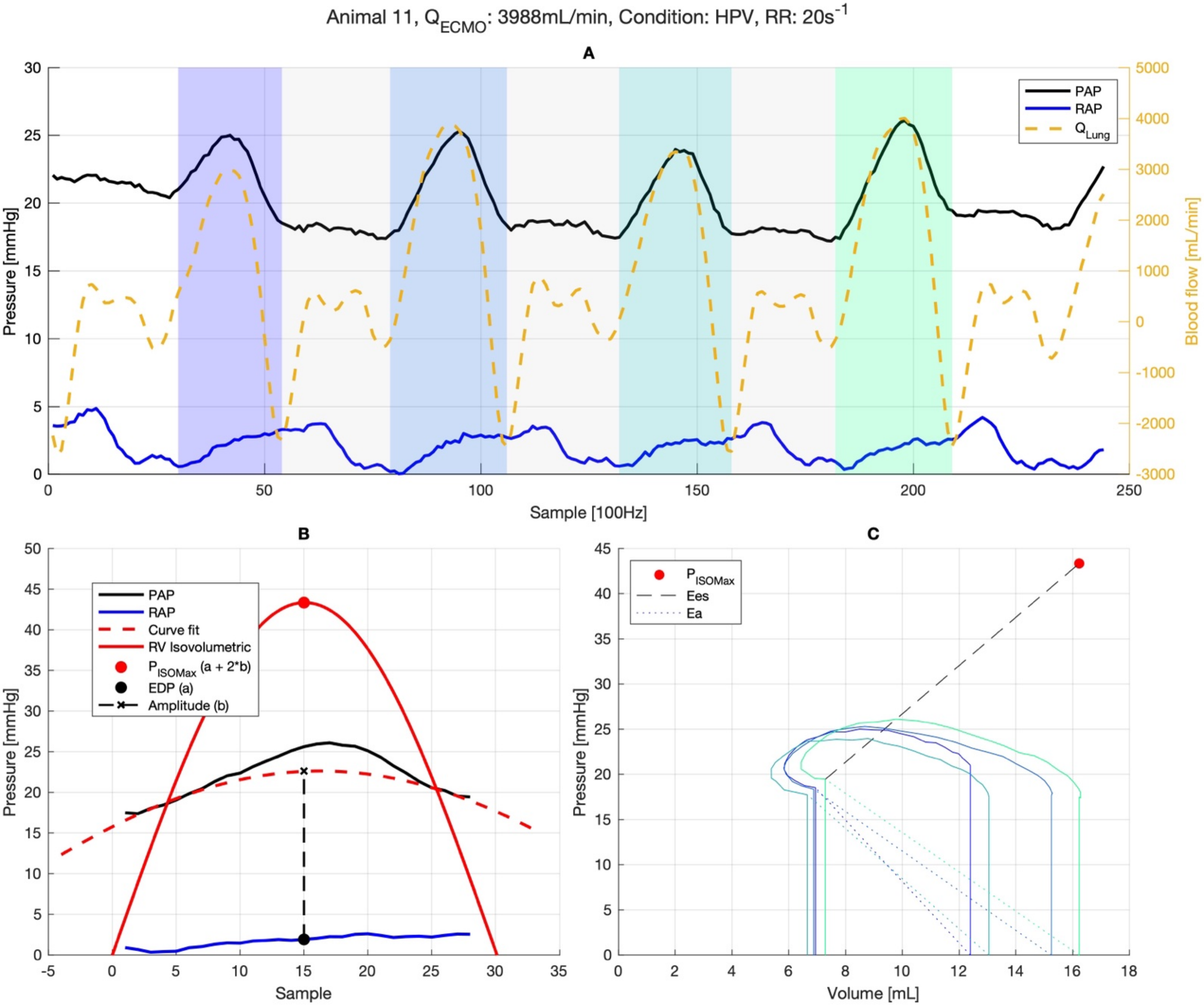

Animal 11, Q_{ECMO}: 3988mL/min, Condition: HPV, RR: $20s^{-1}$
A
PAP
RAP
Q_{Lung}
Pressure [mmHg]
Blood flow [mL/min]
Sample [100Hz]
B
PAP
RAP
Curve fit
RV Isovolumetric
P_{ISOMax} (a + 2*b)
EDP (a)
Amplitude (b)
Pressure [mmHg]
Sample
C
P_{ISOMax}
Ees
Ea
Pressure [mmHg]
Volume [mL]

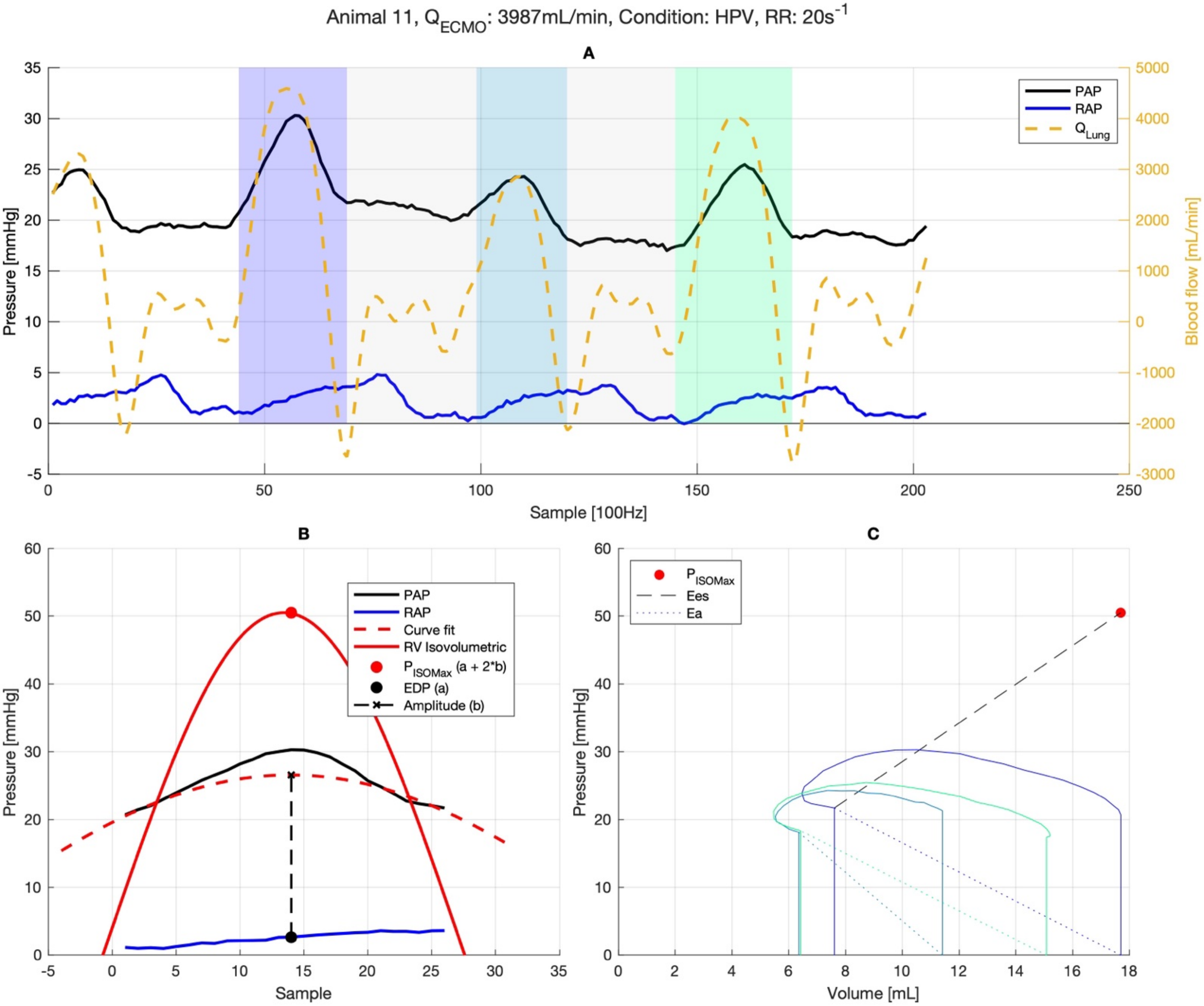

Animal 11, Q_{ECMO}: 3987mL/min, Condition: HPV, RR: 20s^{-1}
A
PAP
RAP
Q_{Lung}
Pressure [mmHg]
Blood flow [mL/min]
Sample [100Hz]
B
PAP
RAP
Curve fit
RV Isovolumetric
P_{ISOMax} (a + 2*b)
EDP (a)
Amplitude (b)
Pressure [mmHg]
Sample
C
P_{ISOMax}
Ees
Ea
Pressure [mmHg]
Volume [mL]

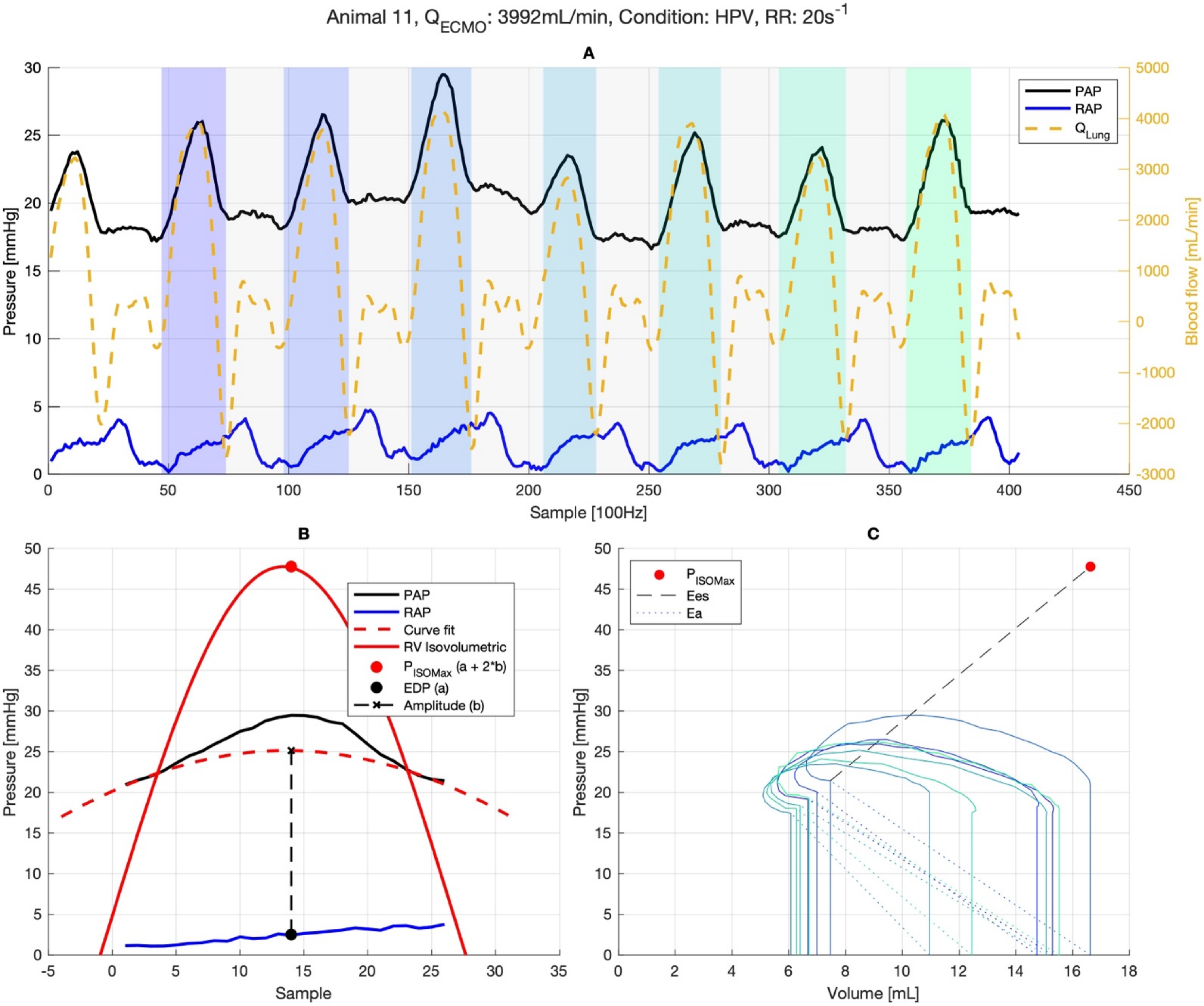

Animal 11, Q_{ECMO}: 3992mL/min, Condition: HPV, RR: $20s^{-1}$
A
PAP
RAP
Q_{Lung}
Pressure [mmHg]
Blood flow [mL/min]
Sample [100Hz]
B
PAP
RAP
Curve fit
RV Isovolumetric
P_{ISOMax} (a + 2*b)
EDP (a)
Amplitude (b)
Pressure [mmHg]
Sample
C
P_{ISOMax}
Ees
Ea
Pressure [mmHg]
Volume [mL]

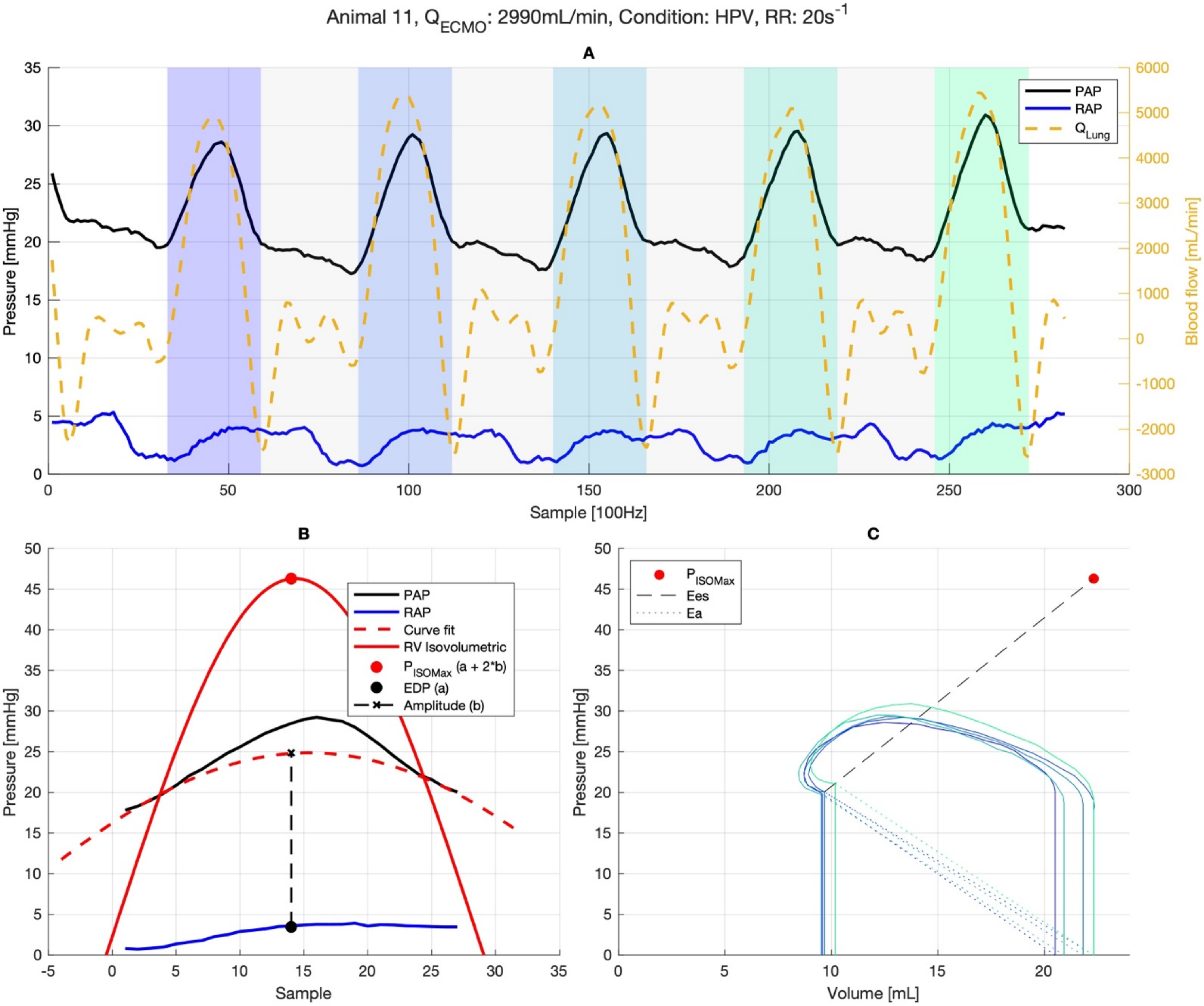

Animal 11, Q_ECMO: 2990mL/min, Condition: HPV, RR: 20s^-1
A
Pressure [mmHg]
Blood flow [mL/min]
Sample [100Hz]
PAP
RAP
Q_Lung
B
Pressure [mmHg]
Sample
PAP
RAP
Curve fit
RV Isovolumetric
P_ISOMax (a + 2*b)
EDP (a)
Amplitude (b)
C
Pressure [mmHg]
Volume [mL]
P_ISOMax
Ees
Ea

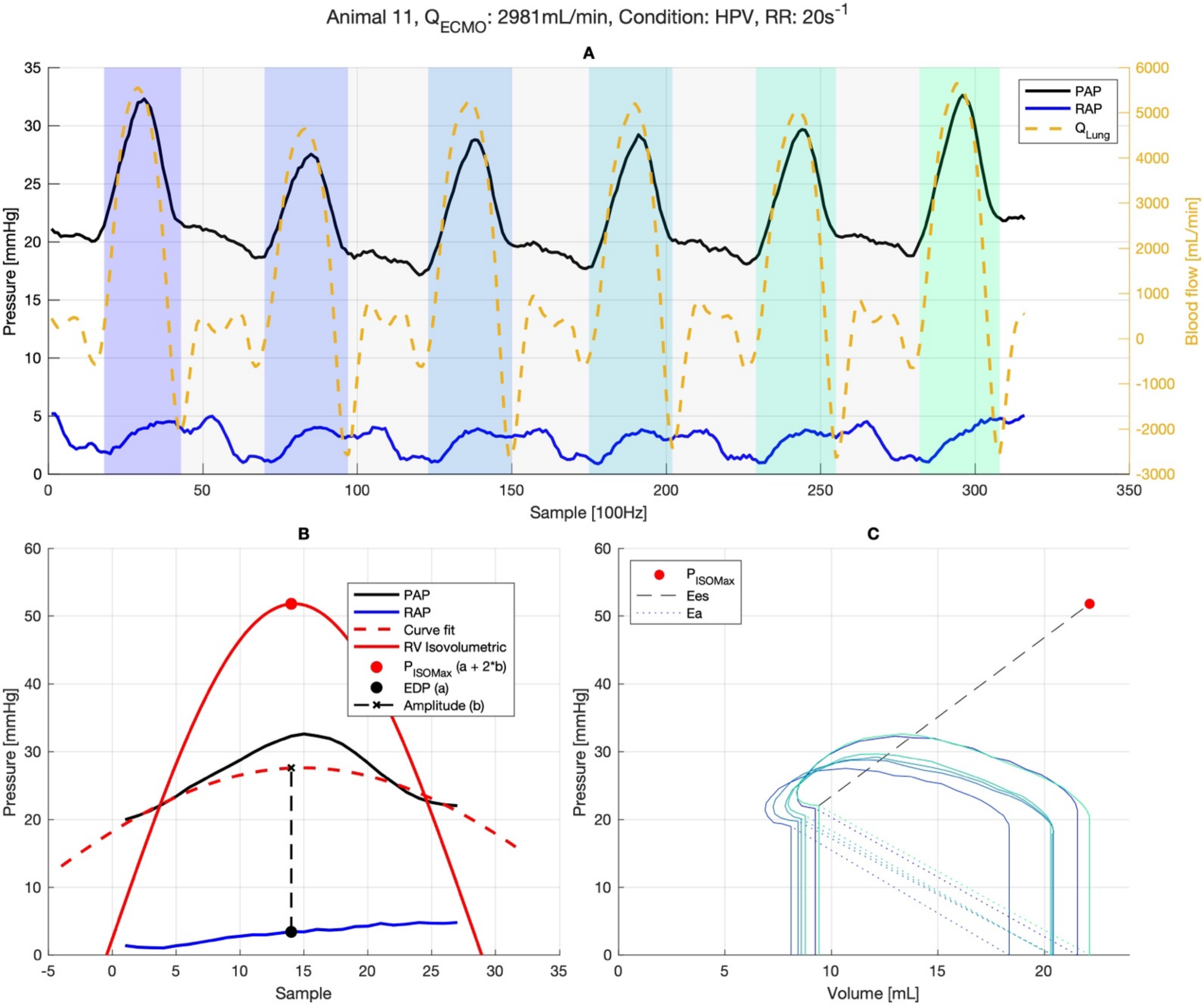

Animal 11, Q_ECMO: 2981mL/min, Condition: HPV, RR: 20s^-1
A
PAP
RAP
Q_Lung
Pressure [mmHg]
Blood flow [mL/min]
Sample [100Hz]
B
PAP
RAP
Curve fit
RV Isovolumetric
P_ISOMax (a + 2*b)
EDP (a)
Amplitude (b)
Pressure [mmHg]
Sample
C
P_ISOMax
Ees
Ea
Pressure [mmHg]
Volume [mL]

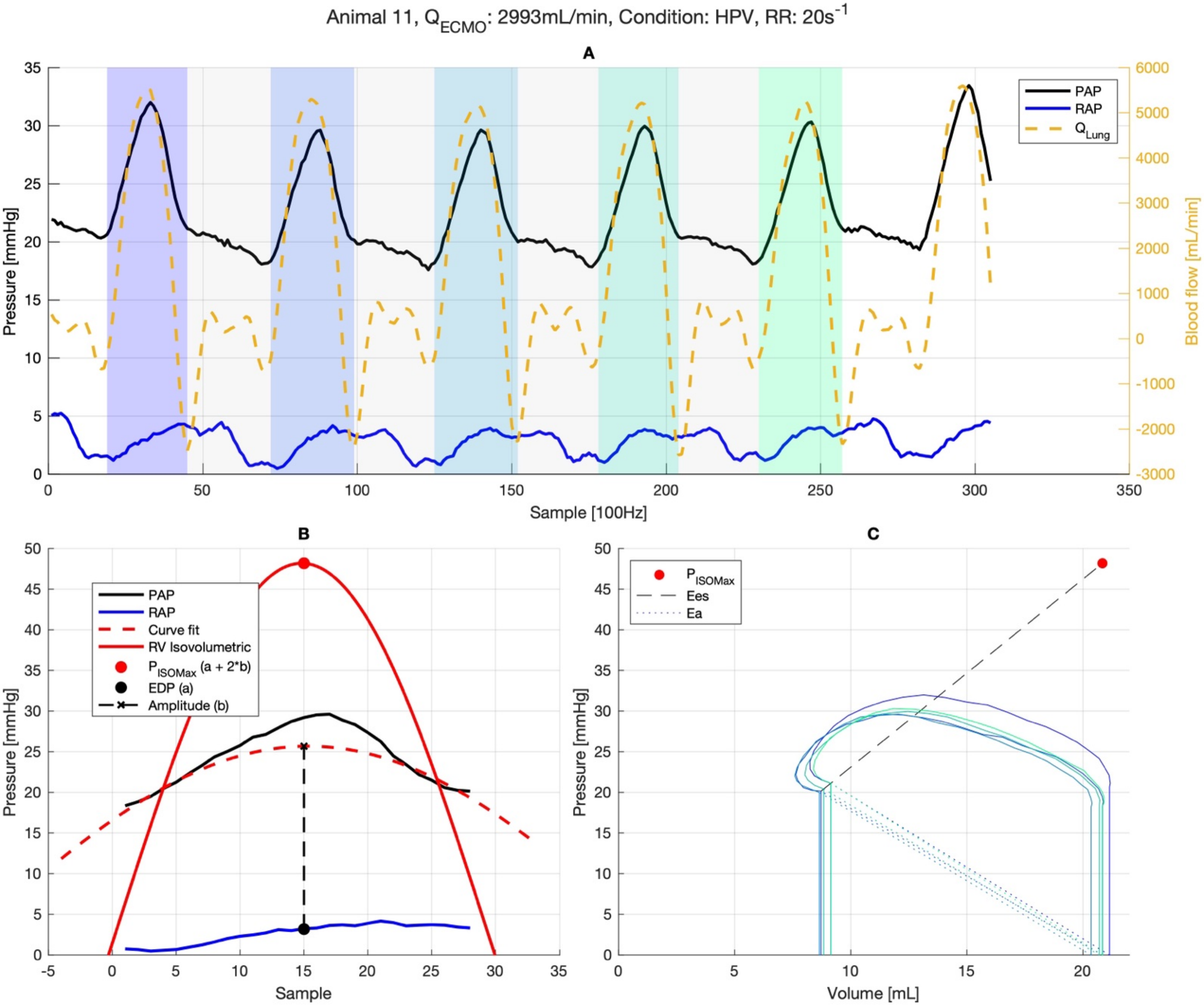

Animal 11, Q_ECMO: 2993mL/min, Condition: HPV, RR: 20s^-1
A
Pressure [mmHg]
Blood flow [mL/min]
Sample [100Hz]
PAP
RAP
Q_Lung
B
PAP
RAP
Curve fit
RV Isovolumetric
P_ISOMax (a + 2*b)
EDP (a)
Amplitude (b)
Pressure [mmHg]
Sample
C
P_ISOMax
Ees
Ea
Pressure [mmHg]
Volume [mL]

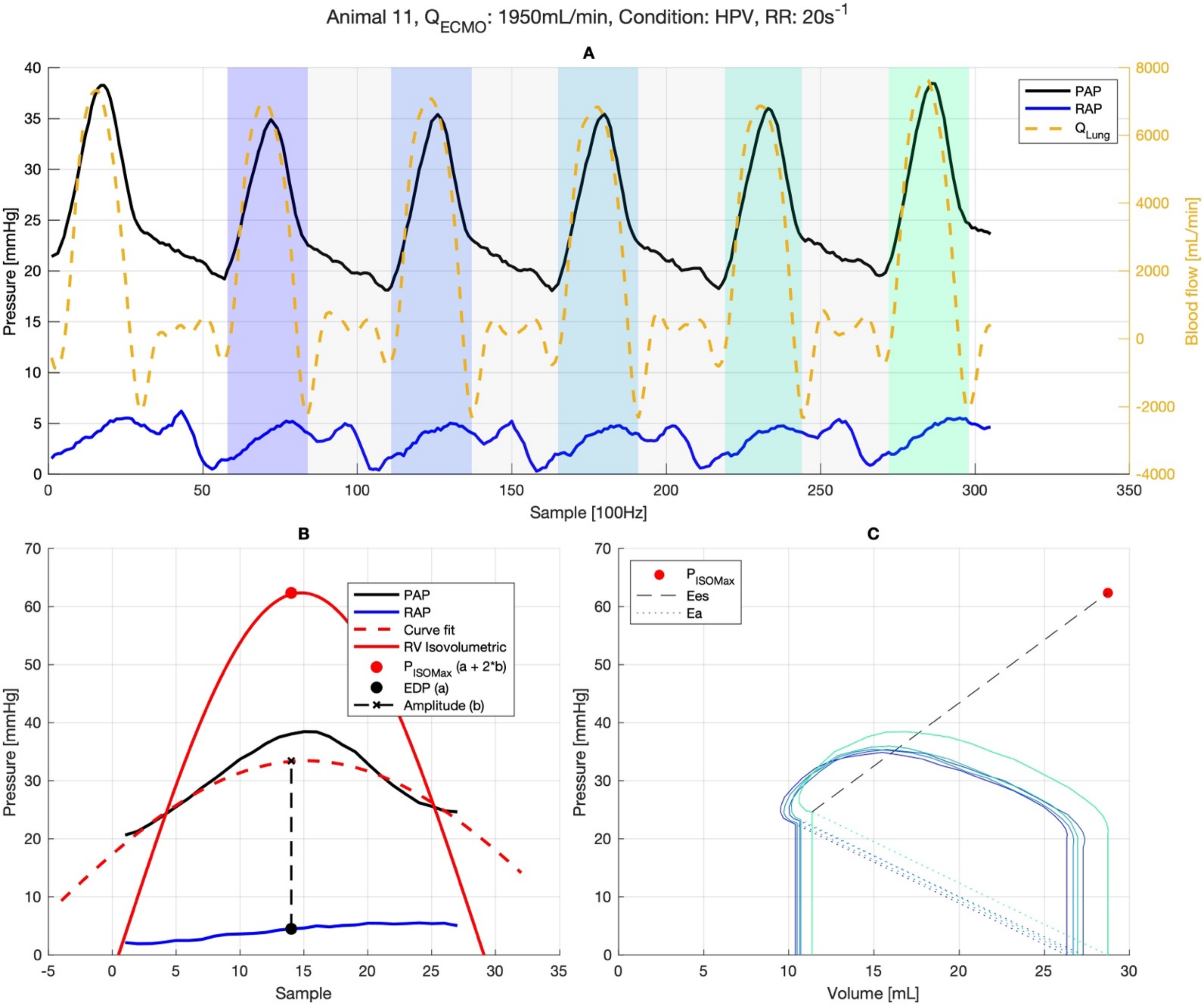

Animal 11, Q_ECMO: 1950mL/min, Condition: HPV, RR: 20s^-1
A
Pressure [mmHg]
Blood flow [mL/min]
Sample [100Hz]
PAP
RAP
Q_Lung
B
Pressure [mmHg]
Sample
Curve fit
RV Isovolumetric
P_ISOMax (a + 2*b)
EDP (a)
Amplitude (b)
C
Pressure [mmHg]
Volume [mL]
P_ISOMax
Ees
Ea

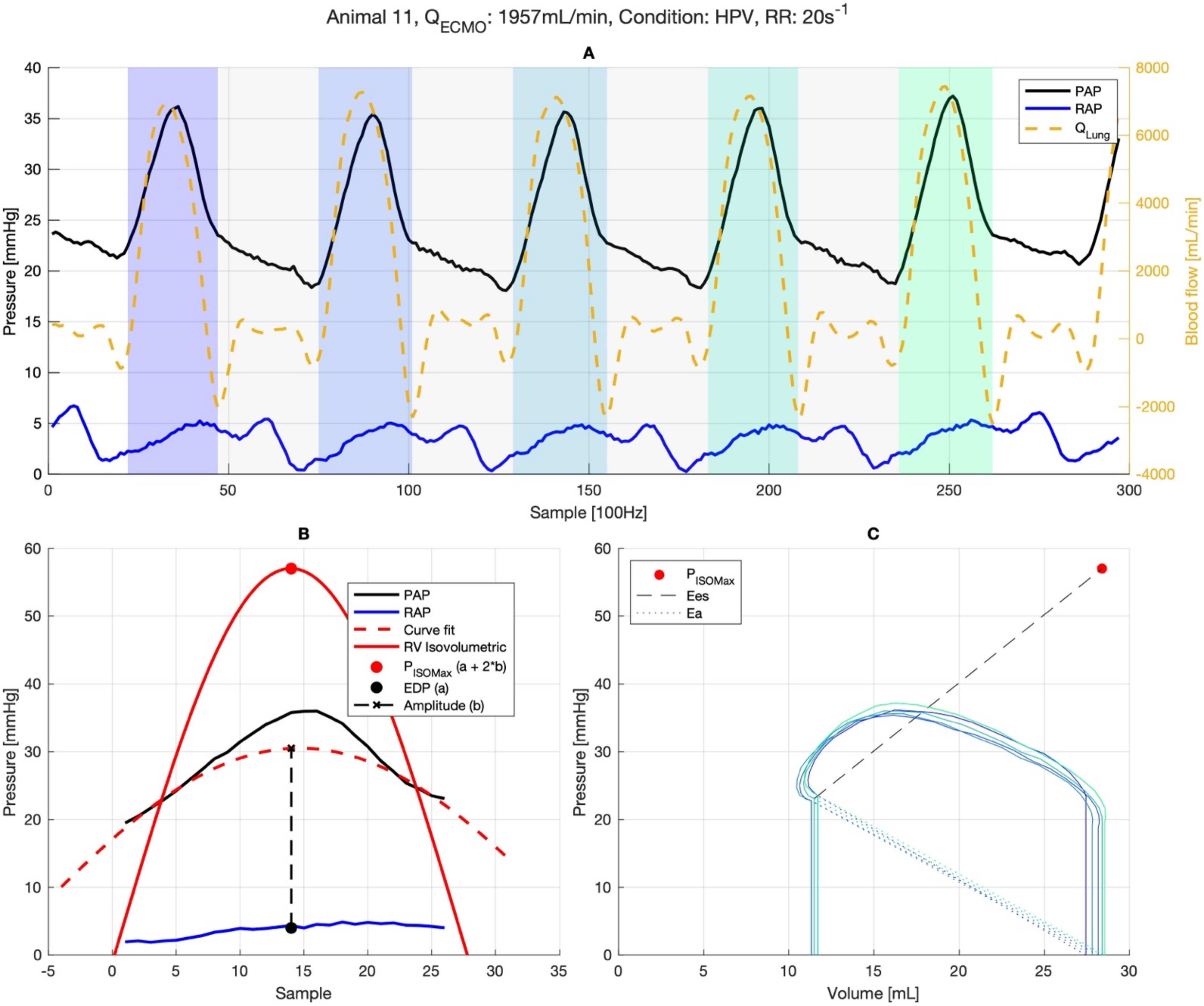
Animal 11, Q_ECMO: 1957mL/min, Condition: HPV, RR: 20s^-1
A
PAP
RAP
Q_Lung
Pressure [mmHg]
Blood flow [mL/min]
Sample [100Hz]
B
PAP
RAP
Curve fit
RV Isovolumetric
P_ISOMax (a + 2*b)
EDP (a)
Amplitude (b)
Pressure [mmHg]
Sample
C
P_ISOMax
Ees
Ea
Pressure [mmHg]
Volume [mL]

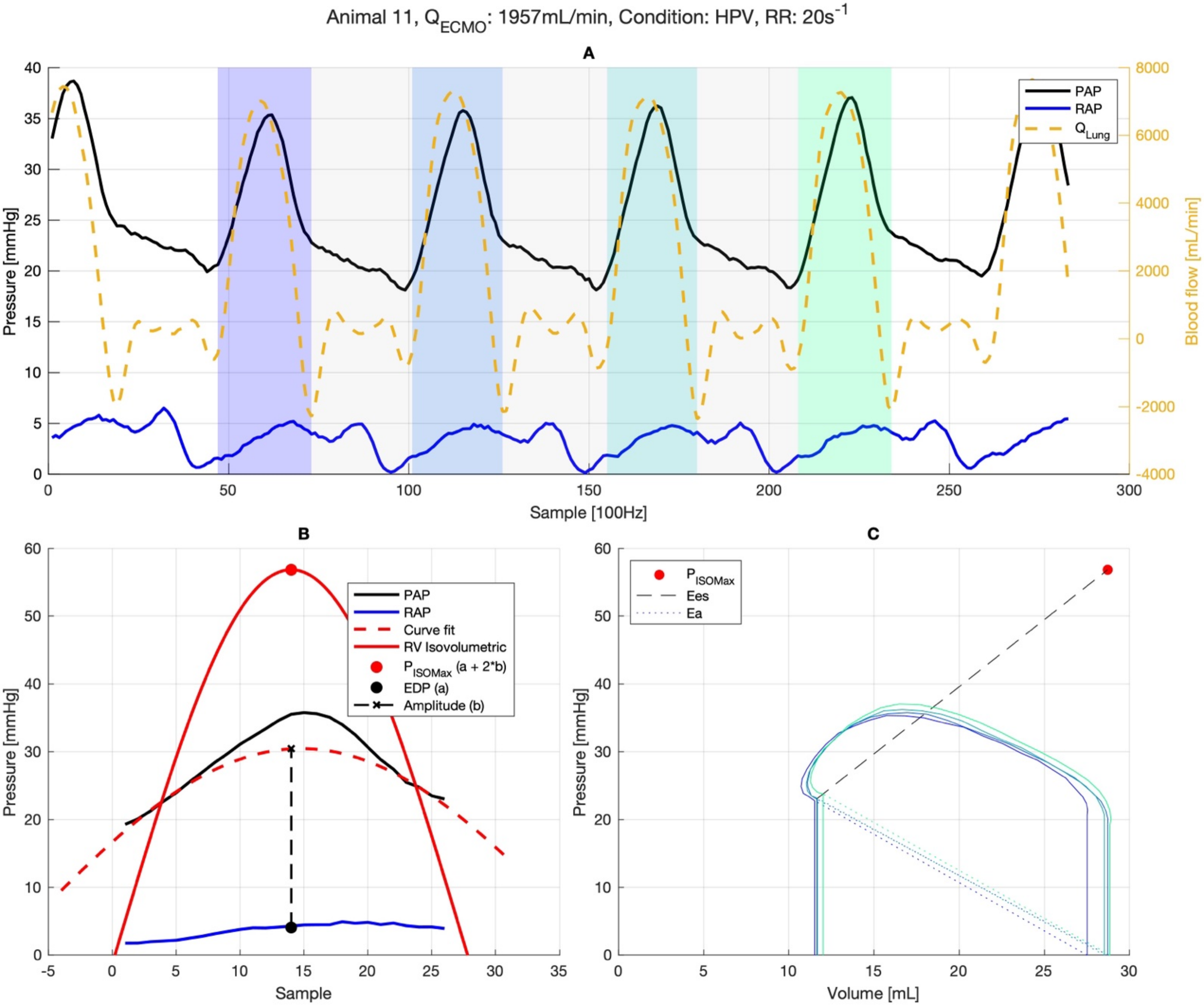

Animal 11, Q_{ECMO}: 1957mL/min, Condition: HPV, RR: 20s^{-1}
A
PAP
RAP
Q_{Lung}
Pressure [mmHg]
Blood flow [mL/min]
Sample [100Hz]
B
PAP
RAP
Curve fit
RV Isovolumetric
P_{ISOMax} (a + 2*b)
EDP (a)
Amplitude (b)
Pressure [mmHg]
Sample
C
P_{ISOMax}
Ees
Ea
Pressure [mmHg]
Volume [mL]

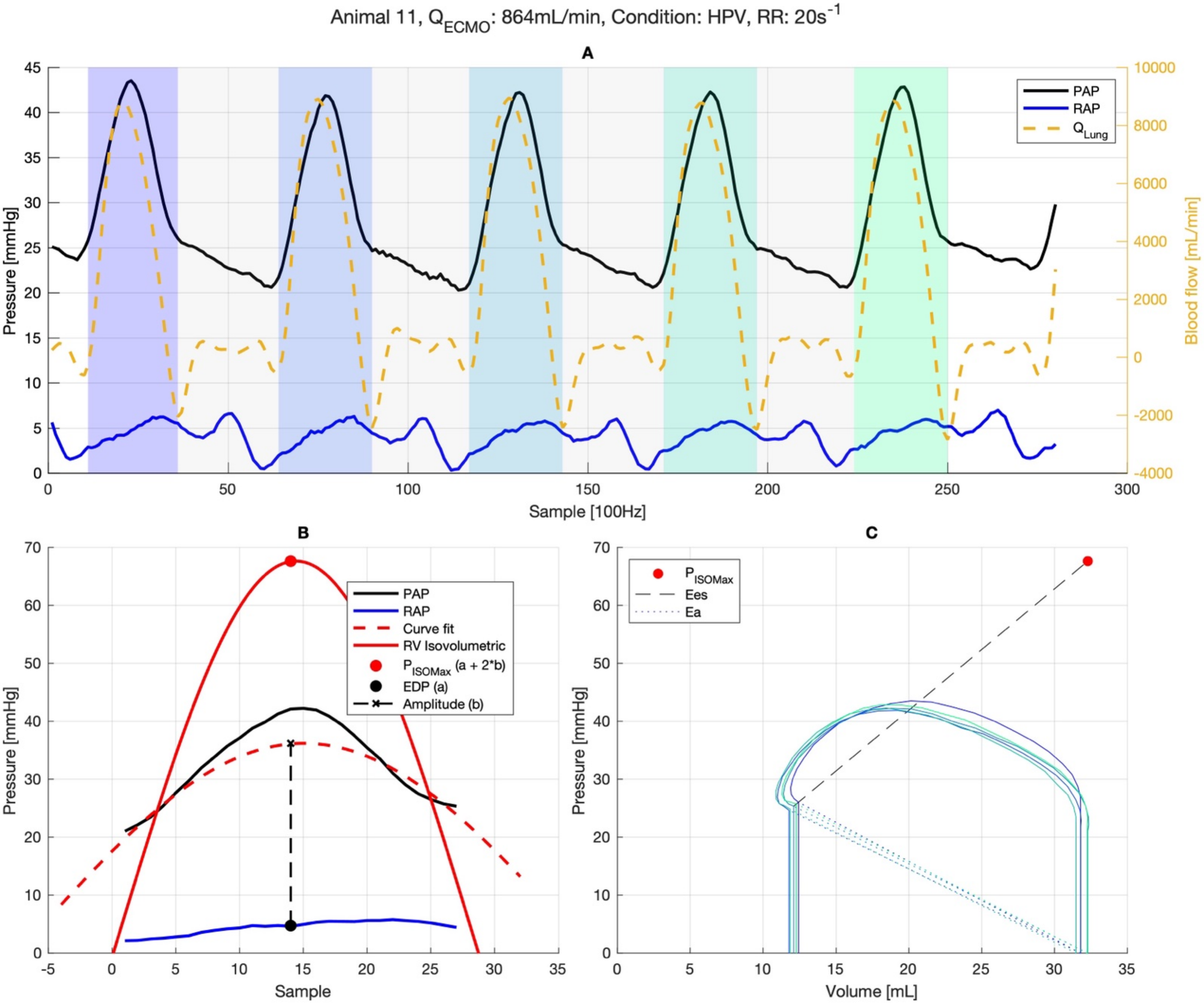

Animal 11, Q_{ECMO}: 864mL/min, Condition: HPV, RR: 20s^{-1}
A
PAP
RAP
Q_{Lung}
Pressure [mmHg]
Blood flow [mL/min]
Sample [100Hz]
B
PAP
RAP
Curve fit
RV Isovolumetric
P_{ISOMax} (a + 2*b)
EDP (a)
Amplitude (b)
Pressure [mmHg]
Sample
C
P_{ISOMax}
Ees
Ea
Pressure [mmHg]
Volume [mL]

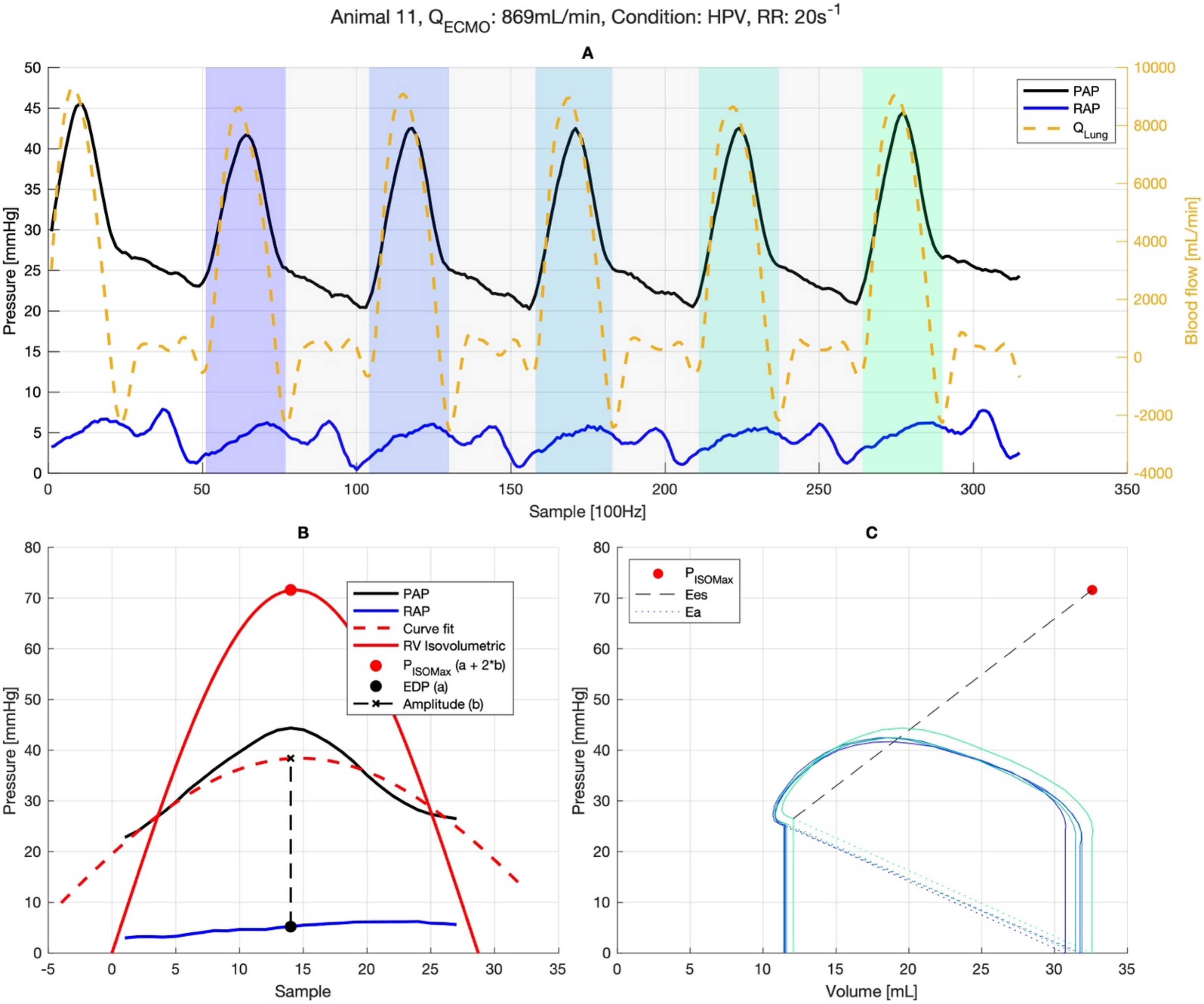

Animal 11, Q_ECMO: 869mL/min, Condition: HPV, RR: 20s^-1
A
PAP
RAP
Q_Lung
Pressure [mmHg]
Blood flow [mL/min]
Sample [100Hz]
B
PAP
RAP
Curve fit
RV Isovolumetric
P_ISOMax (a + 2*b)
EDP (a)
Amplitude (b)
Pressure [mmHg]
Sample
C
P_ISOMax
Ees
Ea
Pressure [mmHg]
Volume [mL]

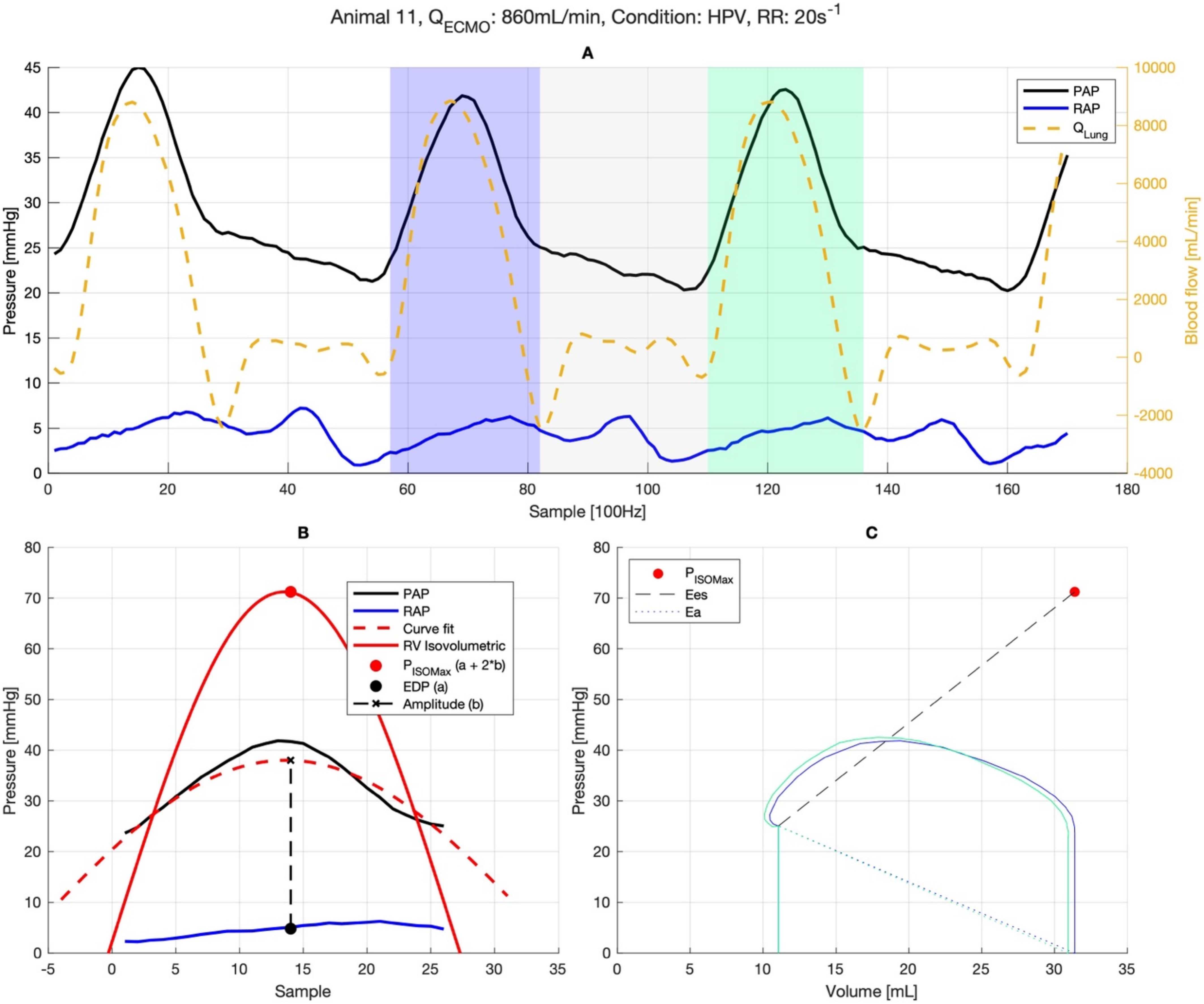

Animal 11, Q_{ECMO}: 860mL/min, Condition: HPV, RR: 20s^{-1}
A
PAP
RAP
Q_{Lung}
Pressure [mmHg]
Blood flow [mL/min]
Sample [100Hz]
B
PAP
RAP
Curve fit
RV Isovolumetric
P_{ISOMax} (a + 2*b)
EDP (a)
Amplitude (b)
Pressure [mmHg]
Sample
C
P_{ISOMax}
Ees
Ea
Pressure [mmHg]
Volume [mL]

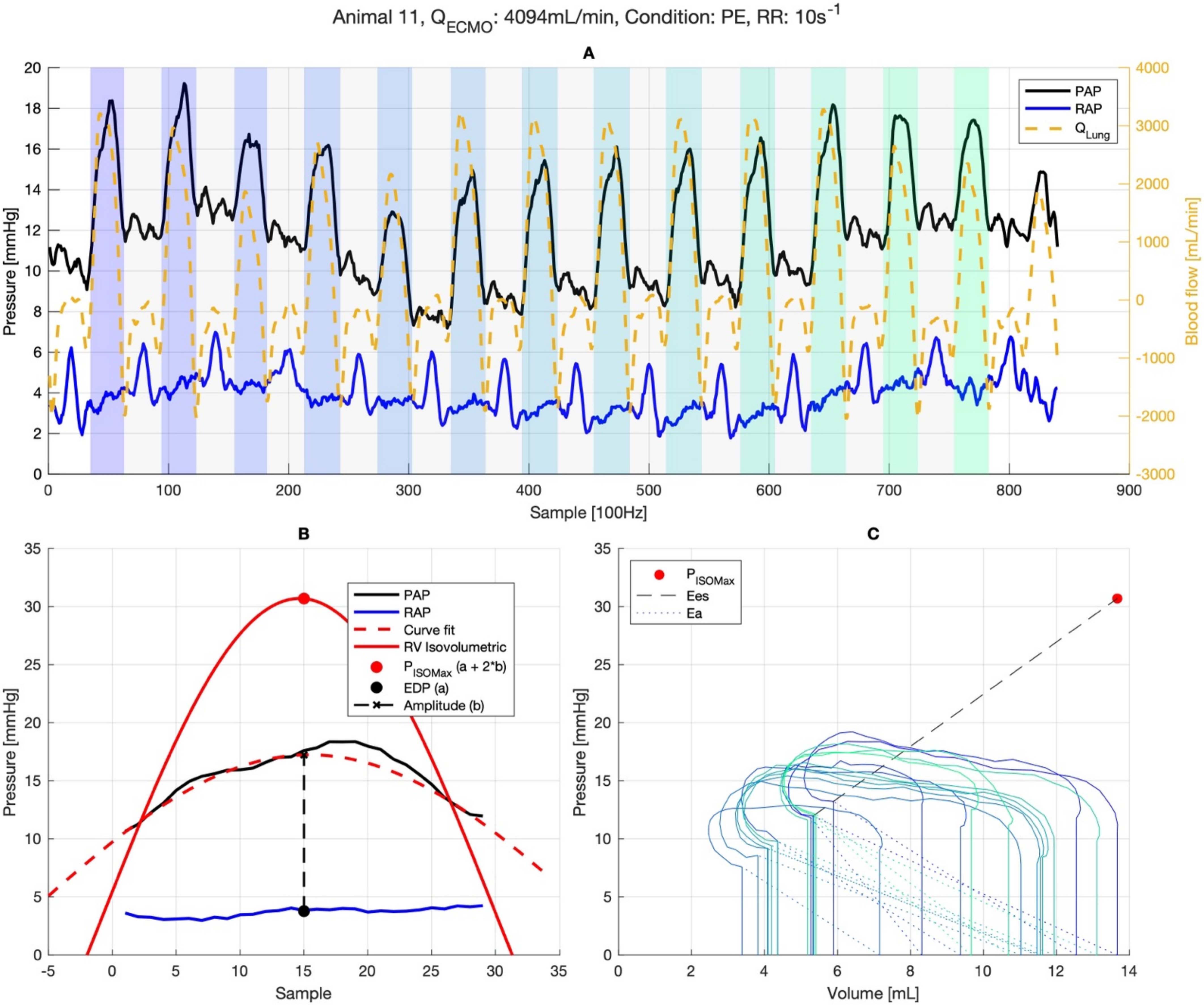

Animal 11, QECMO: 4094mL/min, Condition: PE, RR: 10s-1
A
PAP
RAP
QLung
Pressure [mmHg]
Blood flow [mL/min]
Sample [100Hz]
B
PAP
RAP
Curve fit
RV Isovolumetric
PISOMax (a + 2*b)
EDP (a)
Amplitude (b)
Pressure [mmHg]
Sample
C
PISOMax
Ees
Ea
Pressure [mmHg]
Volume [mL]

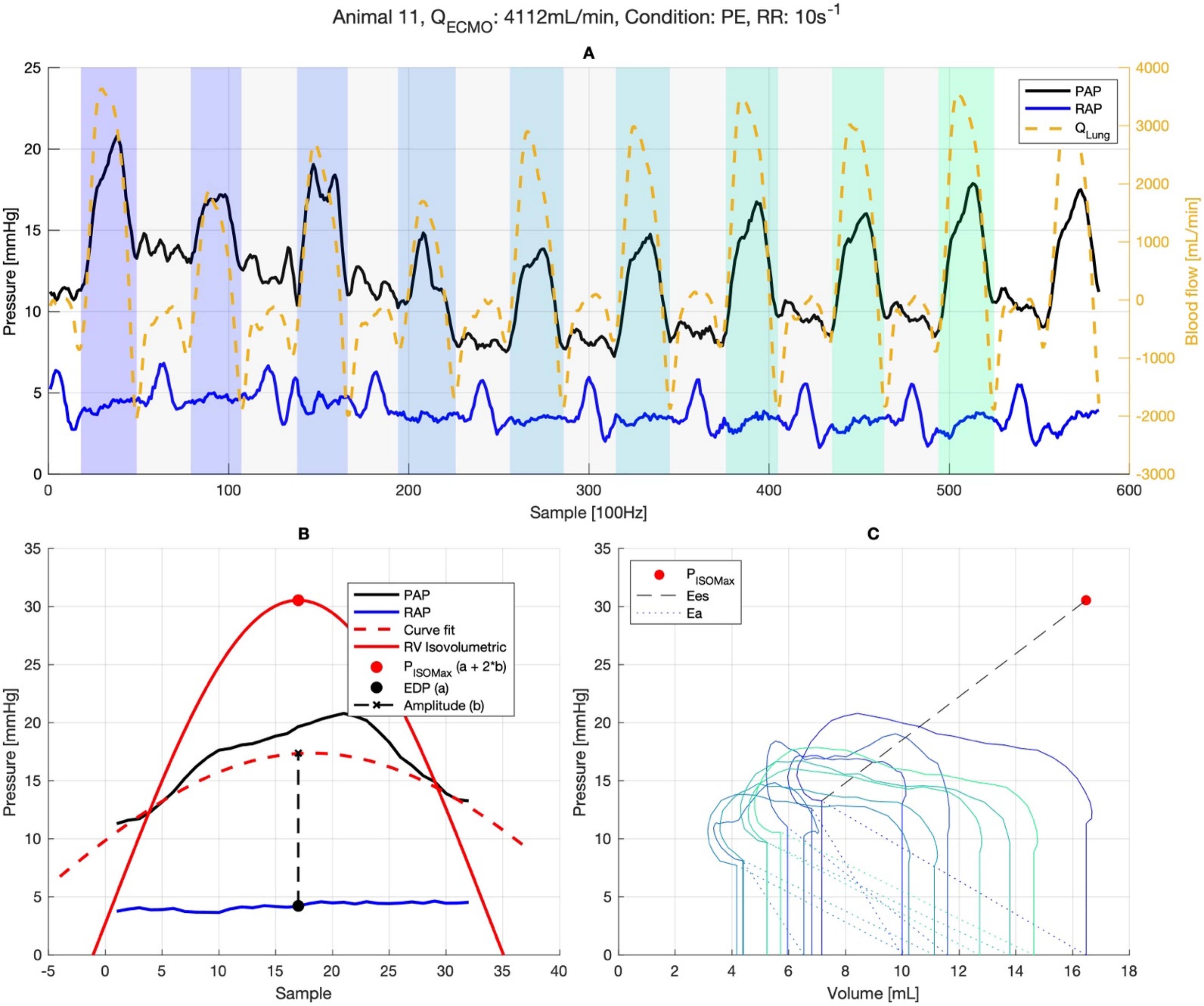

Animal 11, Q_{ECMO}: 4112mL/min, Condition: PE, RR: 10s^{-1}
A
PAP
RAP
Q_{Lung}
Pressure [mmHg]
Blood flow [mL/min]
Sample [100Hz]
B
PAP
RAP
Curve fit
RV Isovolumetric
P_{ISOMax} (a + 2*b)
EDP (a)
Amplitude (b)
Pressure [mmHg]
Sample
C
P_{ISOMax}
Ees
Ea
Pressure [mmHg]
Volume [mL]

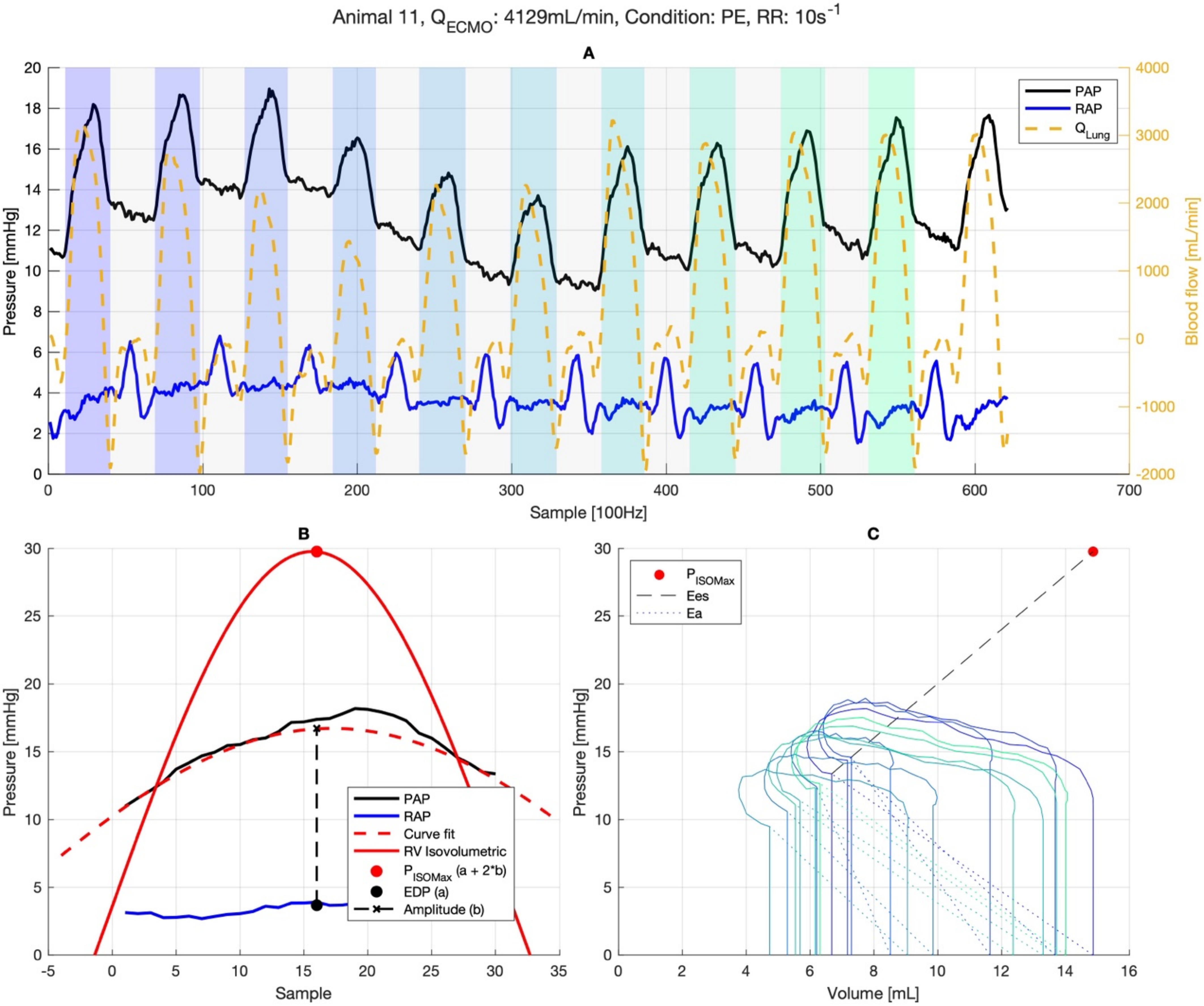

Animal 11, Q_{ECMO}: 4129mL/min, Condition: PE, RR: $10s^{-1}$
A
PAP
RAP
Q_{Lung}
Pressure [mmHg]
Blood flow [mL/min]
Sample [100Hz]
B
PAP
RAP
Curve fit
RV Isovolumetric
P_{ISOMax} (a + 2*b)
EDP (a)
Amplitude (b)
Pressure [mmHg]
Sample
C
P_{ISOMax}
Ees
Ea
Pressure [mmHg]
Volume [mL]

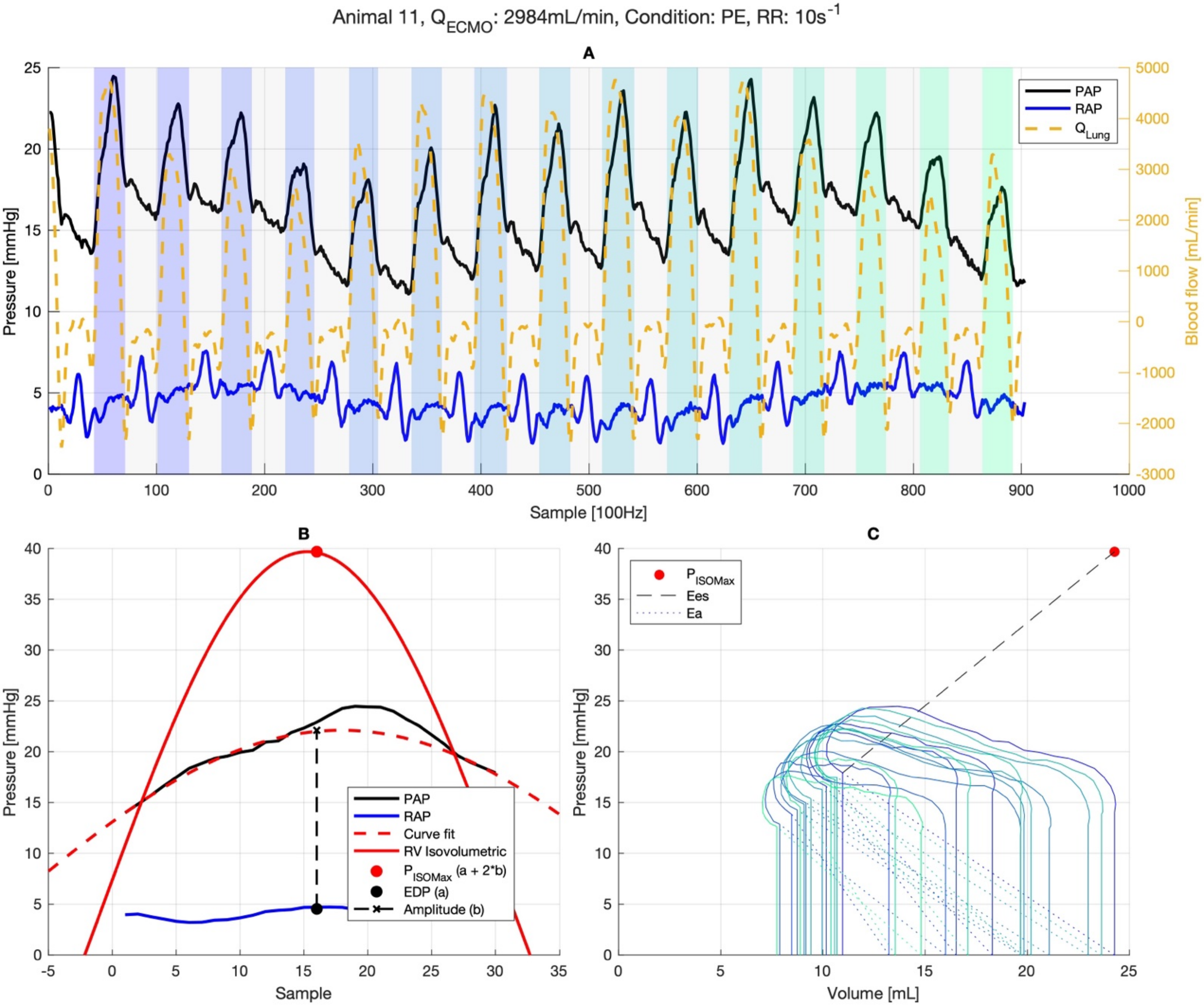

Animal 11, Q_{ECMO}: 2984mL/min, Condition: PE, RR: $10s^{-1}$
A
Pressure [mmHg]
Blood flow [mL/min]
Sample [100Hz]
PAP
RAP
Q_{Lung}
B
Pressure [mmHg]
Sample
PAP
RAP
Curve fit
RV Isovolumetric
P_{ISOMax} (a + 2*b)
EDP (a)
Amplitude (b)
C
Pressure [mmHg]
Volume [mL]
P_{ISOMax}
Ees
Ea

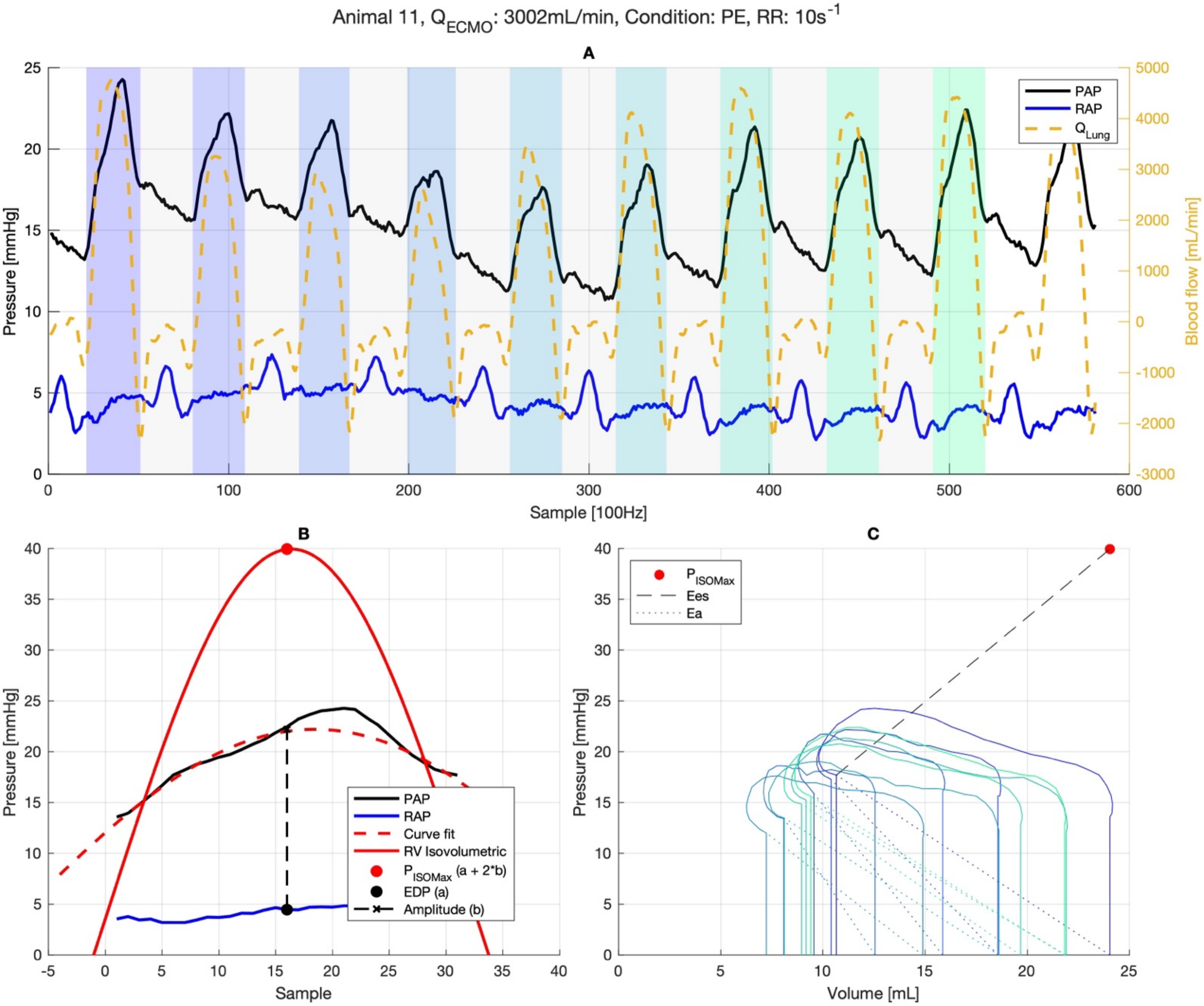

Animal 11, Q_{ECMO}: 3002mL/min, Condition: PE, RR: $10s^{-1}$
A
Pressure [mmHg]
Blood flow [mL/min]
Sample [100Hz]
PAP
RAP
Q_{Lung}
B
Sample
Pressure [mmHg]
PAP
RAP
Curve fit
RV Isovolumetric
P_{ISOMax} (a + 2*b)
EDP (a)
Amplitude (b)
C
Volume [mL]
Pressure [mmHg]
P_{ISOMax}
Ees
Ea

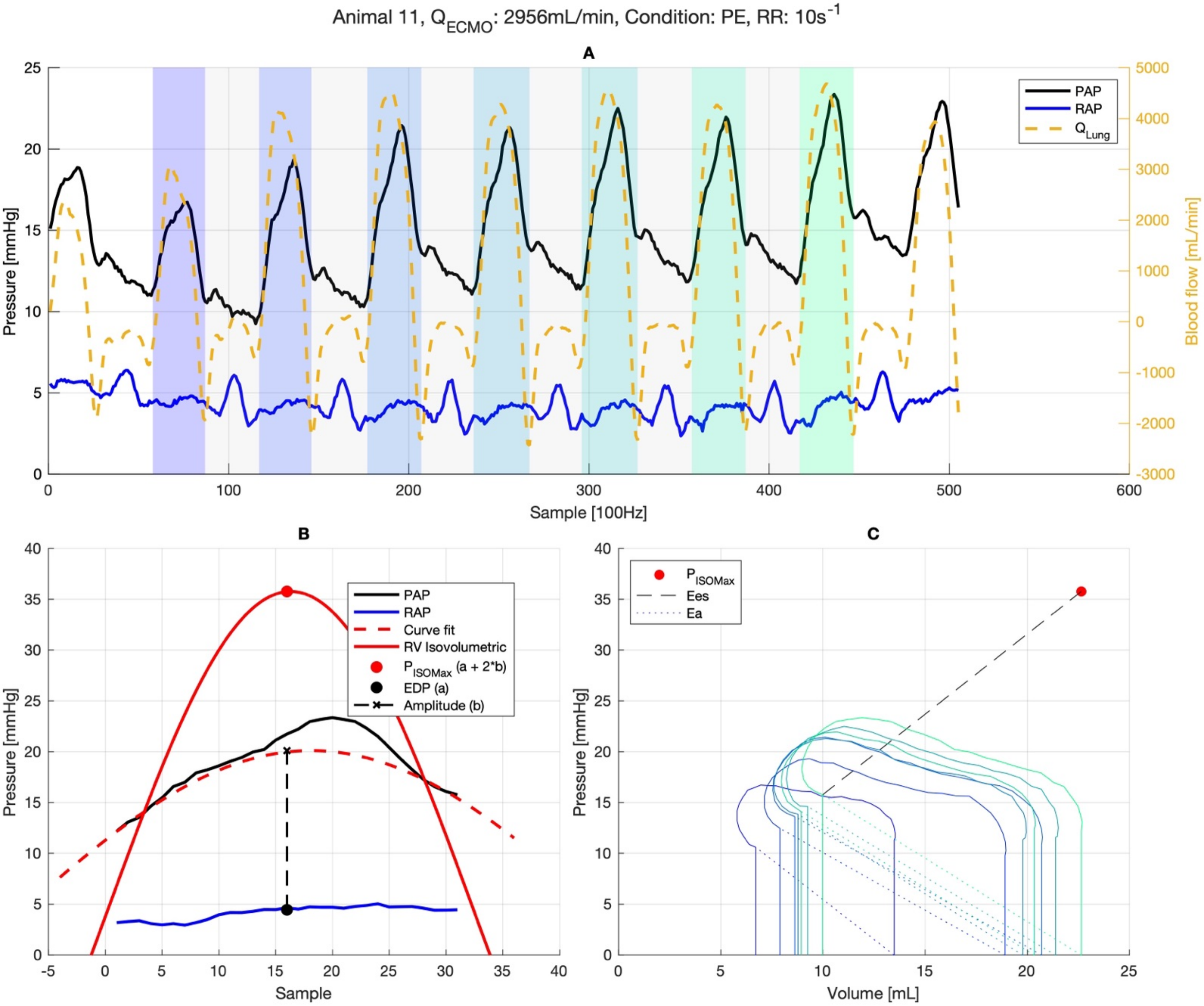

Animal 11, Q_{ECMO}: 2956mL/min, Condition: PE, RR: $10s^{-1}$
A
PAP
RAP
Q_{Lung}
Pressure [mmHg]
Blood flow [mL/min]
Sample [100Hz]
B
PAP
RAP
Curve fit
RV Isovolumetric
P_{ISOMax} (a + 2*b)
EDP (a)
Amplitude (b)
Pressure [mmHg]
Sample
C
P_{ISOMax}
Ees
Ea
Pressure [mmHg]
Volume [mL]

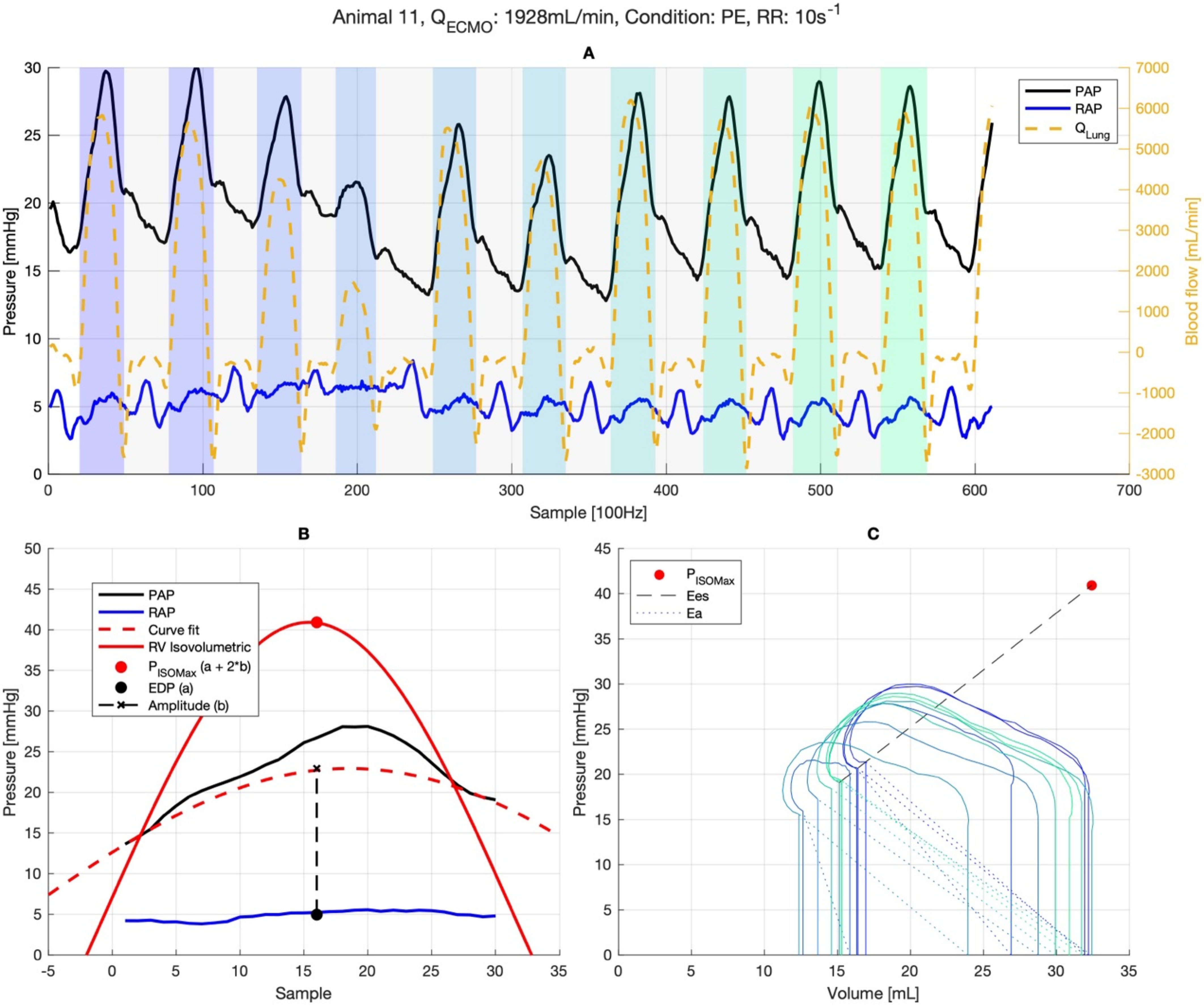

Animal 11, Q_{ECMO}: 1928mL/min, Condition: PE, RR: $10s^{-1}$
A
Pressure [mmHg]
Blood flow [mL/min]
Sample [100Hz]
PAP
RAP
Q_{Lung}
B
PAP
RAP
Curve fit
RV Isovolumetric
P_{ISOMax} (a + 2*b)
EDP (a)
Amplitude (b)
Pressure [mmHg]
Sample
C
P_{ISOMax}
Ees
Ea
Pressure [mmHg]
Volume [mL]

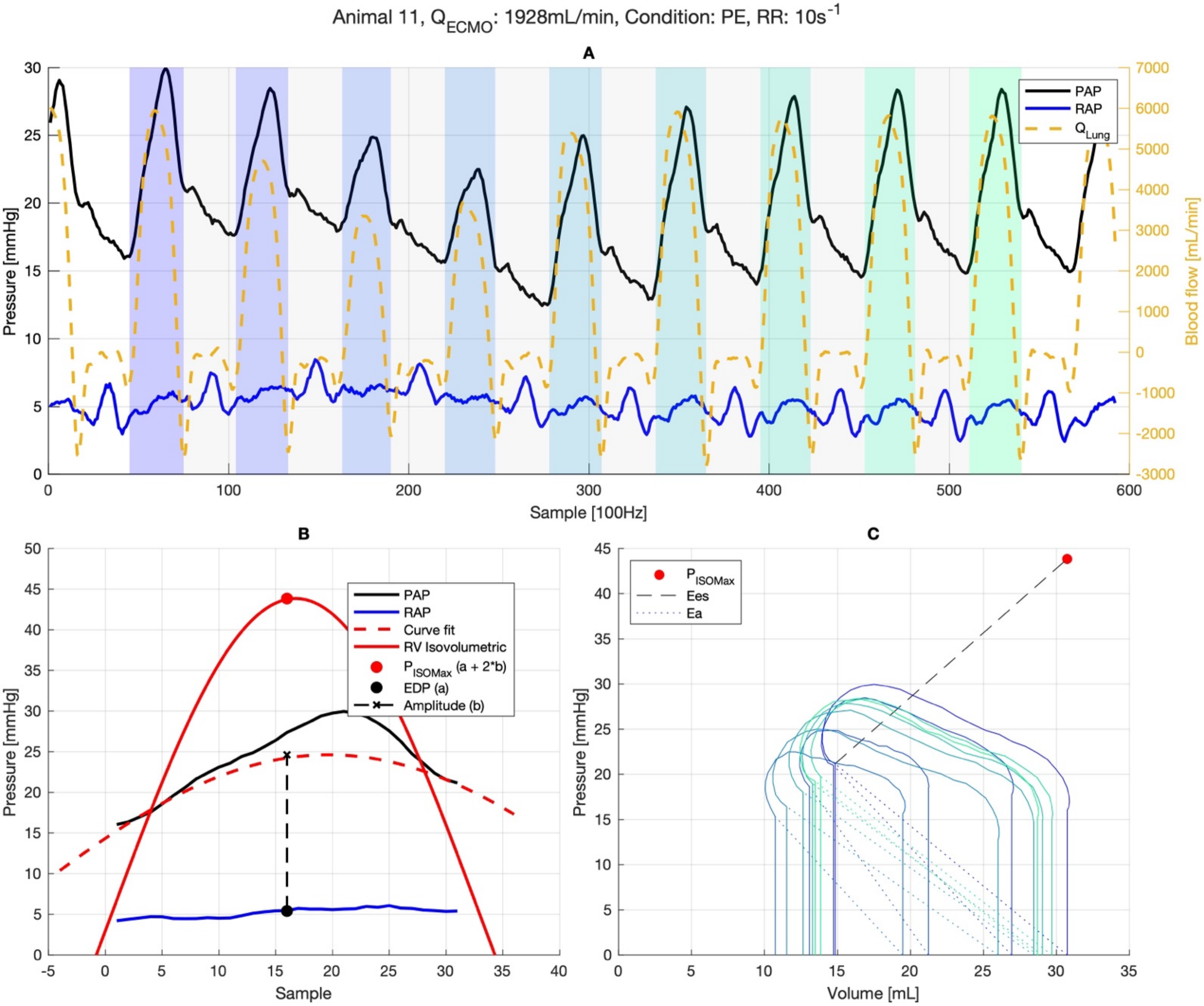

Animal 11, Q_ECMO: 1928mL/min, Condition: PE, RR: 10s^-1
A
PAP
RAP
Q_Lung
Pressure [mmHg]
Blood flow [mL/min]
Sample [100Hz]
B
PAP
RAP
Curve fit
RV Isovolumetric
P_ISOMax (a + 2*b)
EDP (a)
Amplitude (b)
Pressure [mmHg]
Sample
C
P_ISOMax
Ees
Ea
Pressure [mmHg]
Volume [mL]

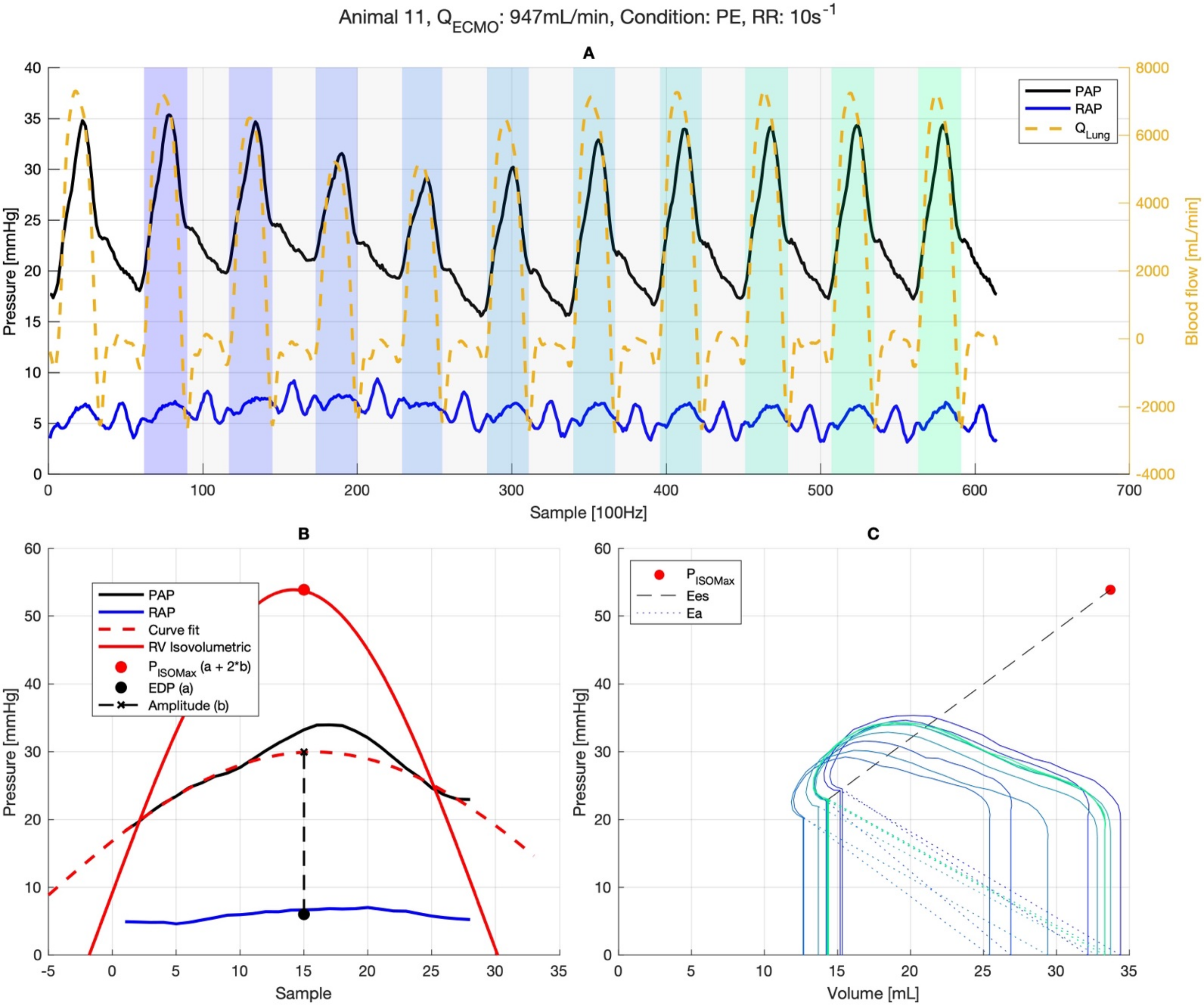
Animal 11, Q ECMO: 947mL/min, Condition: PE, RR: 10s-1
A
Pressure [mmHg]
Blood flow [mL/min]
Sample [100Hz]
PAP
RAP
Q Lung
B
Pressure [mmHg]
Sample
PAP
RAP
Curve fit
RV Isovolumetric
P ISOMax (a + 2*b)
EDP (a)
Amplitude (b)
C
Pressure [mmHg]
Volume [mL]
P ISOMax
Ees
Ea

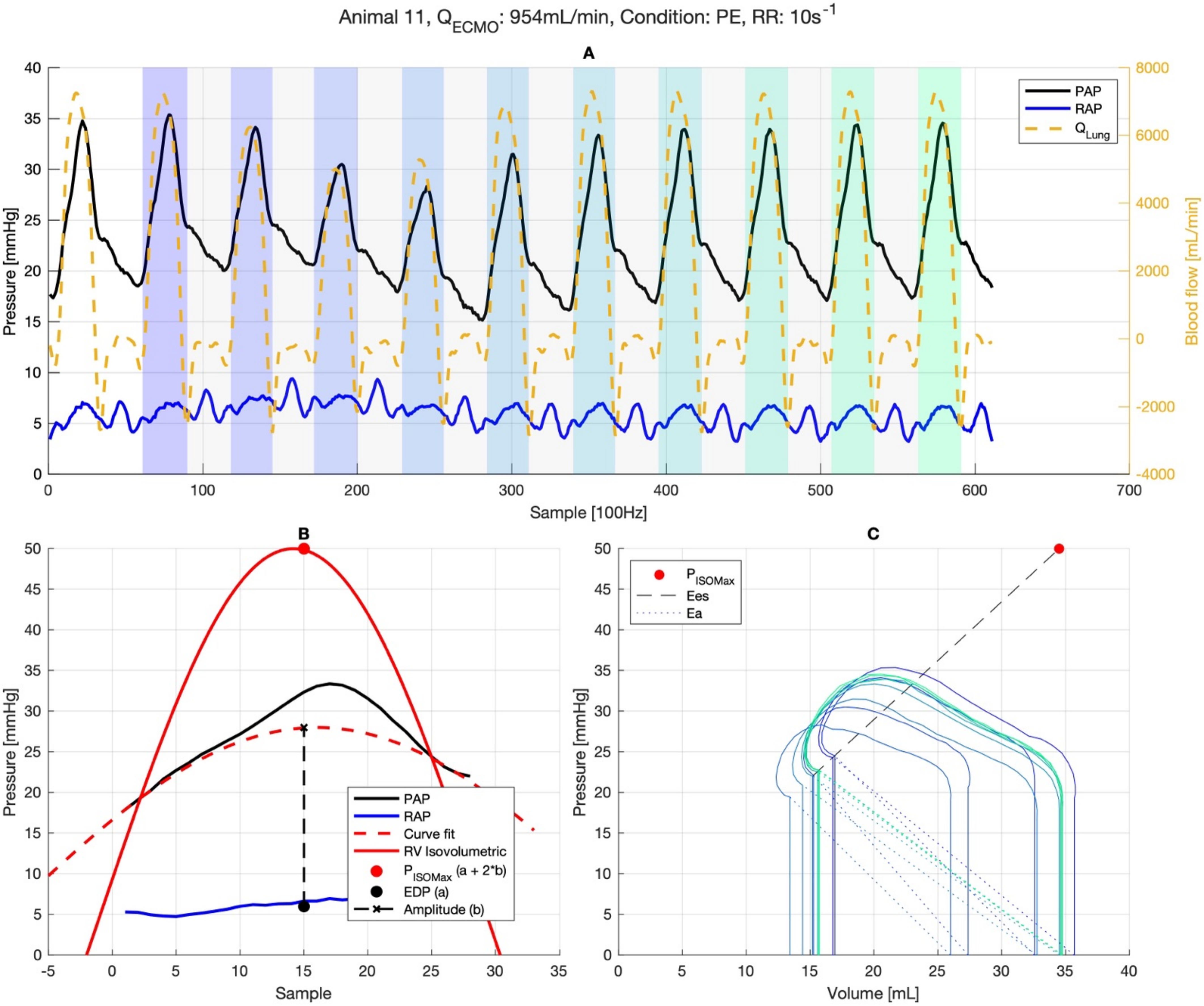

Animal 11, Q_{ECMO}: 954mL/min, Condition: PE, RR: $10s^{-1}$
A
PAP
RAP
Q_{Lung}
Pressure [mmHg]
Blood flow [mL/min]
Sample [100Hz]
B
PAP
RAP
Curve fit
RV Isovolumetric
P_{ISOMax} (a + 2*b)
EDP (a)
Amplitude (b)
Pressure [mmHg]
Sample
C
P_{ISOMax}
Ees
Ea
Pressure [mmHg]
Volume [mL]

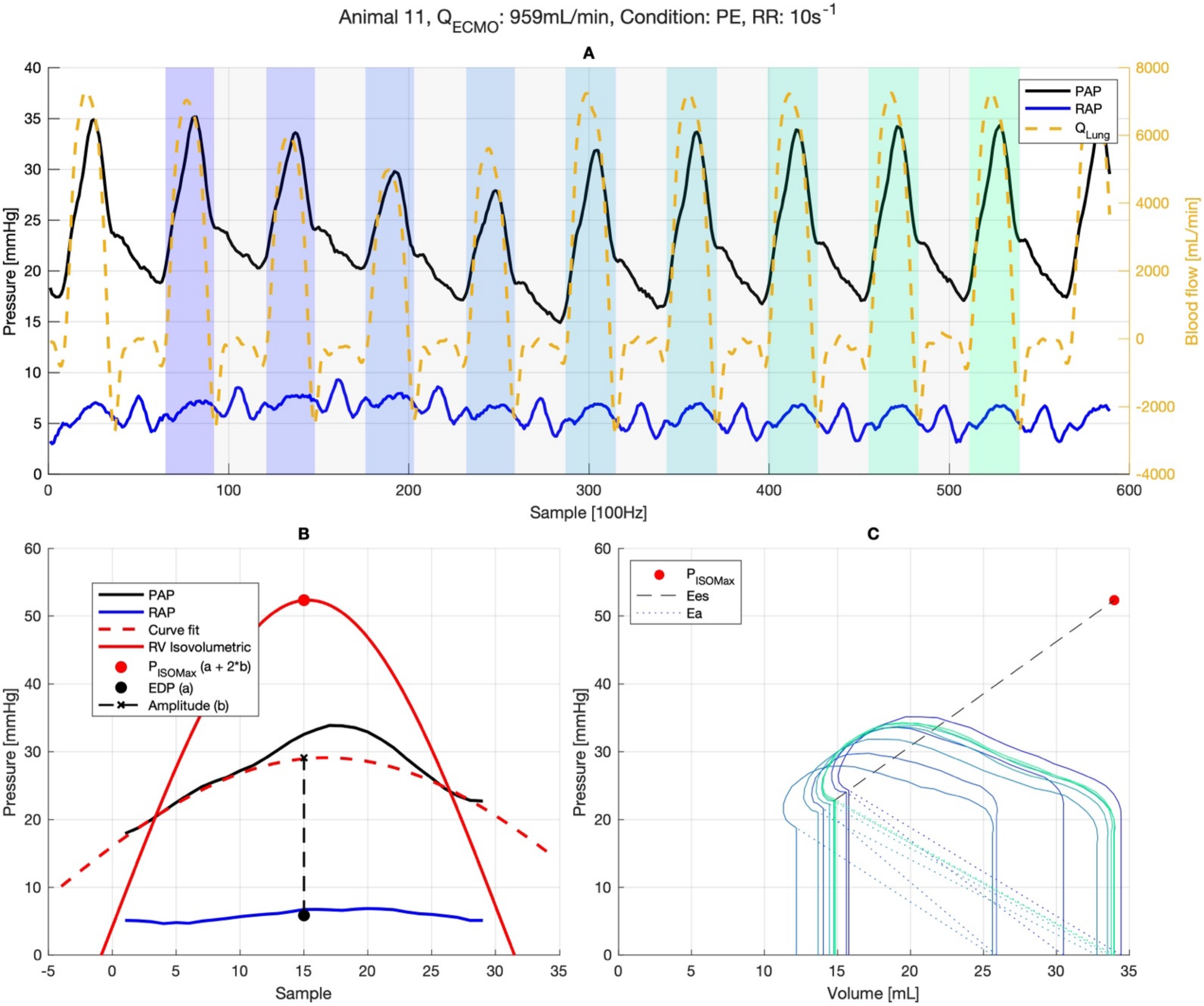

Animal 11, QECMO: 959mL/min, Condition: PE, RR: 10s-1
A
Pressure [mmHg]
Blood flow [mL/min]
Sample [100Hz]
PAP
RAP
QLung
B
PAP
RAP
Curve fit
RV Isovolumetric
PISOMax (a + 2*b)
EDP (a)
Amplitude (b)
Pressure [mmHg]
Sample
C
PISOMax
Ees
Ea
Pressure [mmHg]
Volume [mL]

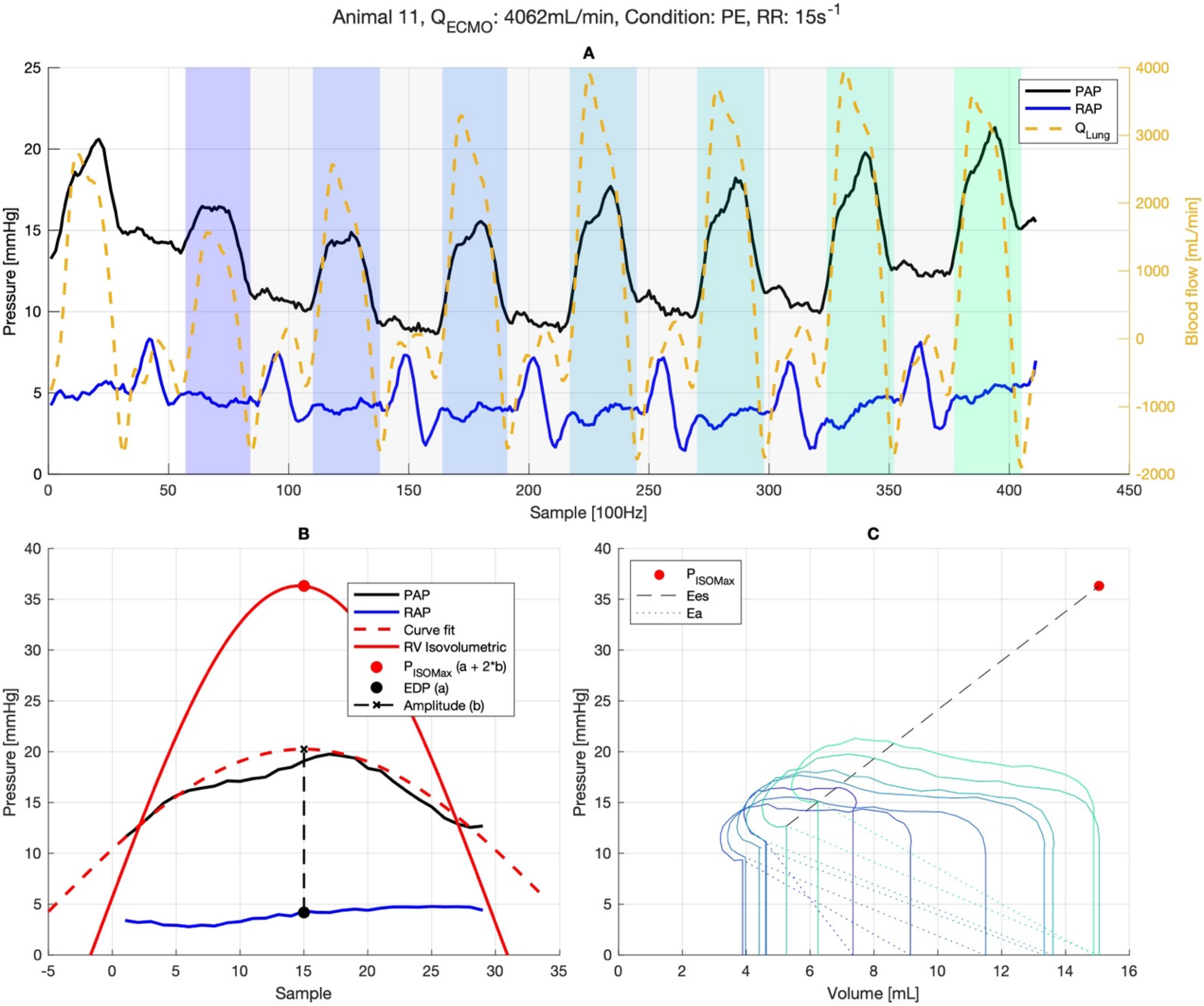

Animal 11, Q_ECMO: 4062mL/min, Condition: PE, RR: 15s^-1
A
Pressure [mmHg]
Blood flow [mL/min]
Sample [100Hz]
PAP
RAP
Q_Lung
B
Pressure [mmHg]
Sample
PAP
RAP
Curve fit
RV Isovolumetric
P_ISOMax (a + 2*b)
EDP (a)
Amplitude (b)
C
Pressure [mmHg]
Volume [mL]
P_ISOMax
Ees
Ea

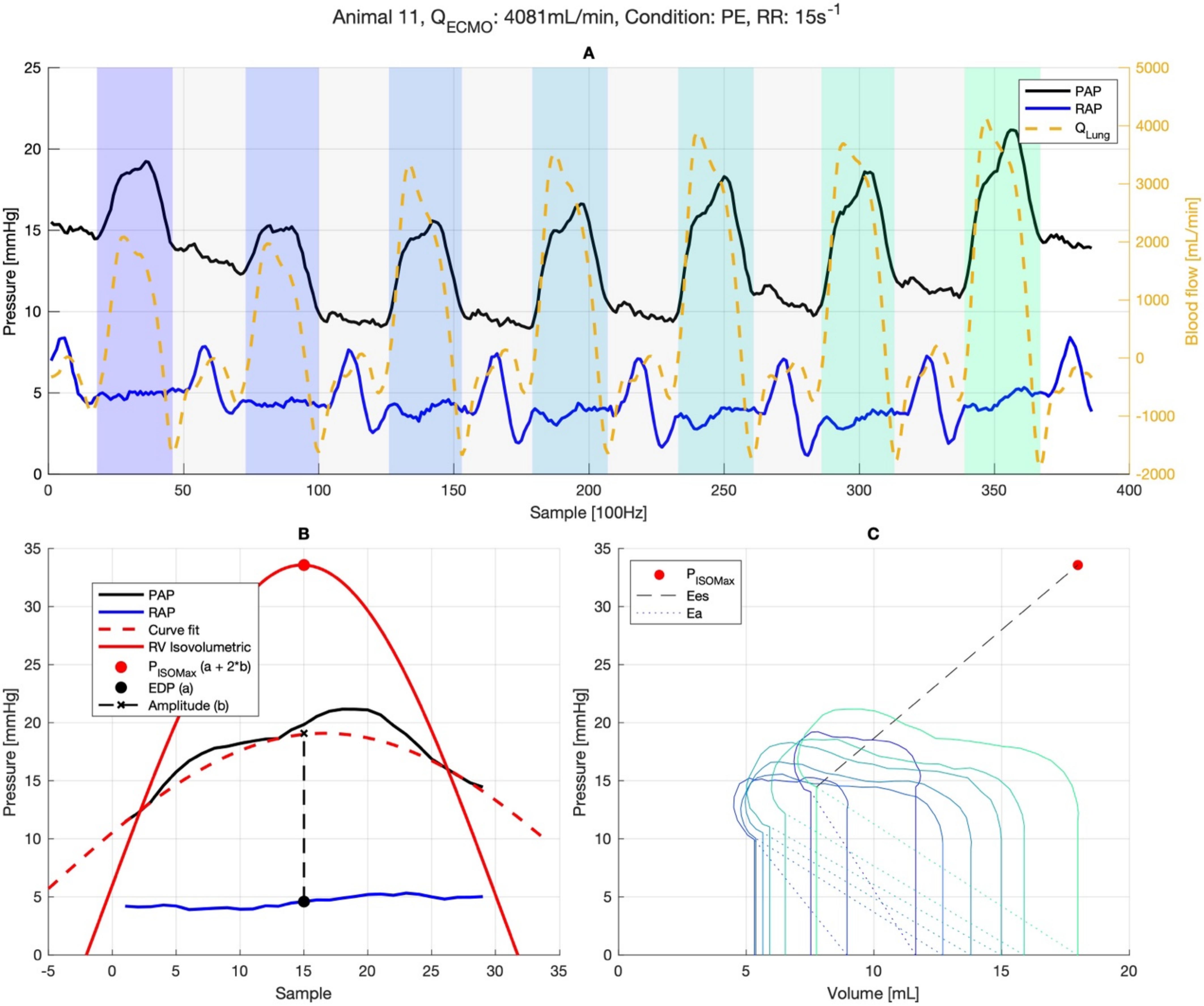

Animal 11, Q_{ECMO}: 4081mL/min, Condition: PE, RR: $15s^{-1}$
A
Pressure [mmHg]
Blood flow [mL/min]
Sample [100Hz]
PAP
RAP
Q_{Lung}
B
Pressure [mmHg]
Sample
PAP
RAP
Curve fit
RV Isovolumetric
P_{ISOMax} (a + 2*b)
EDP (a)
Amplitude (b)
C
Pressure [mmHg]
Volume [mL]
P_{ISOMax}
Ees
Ea

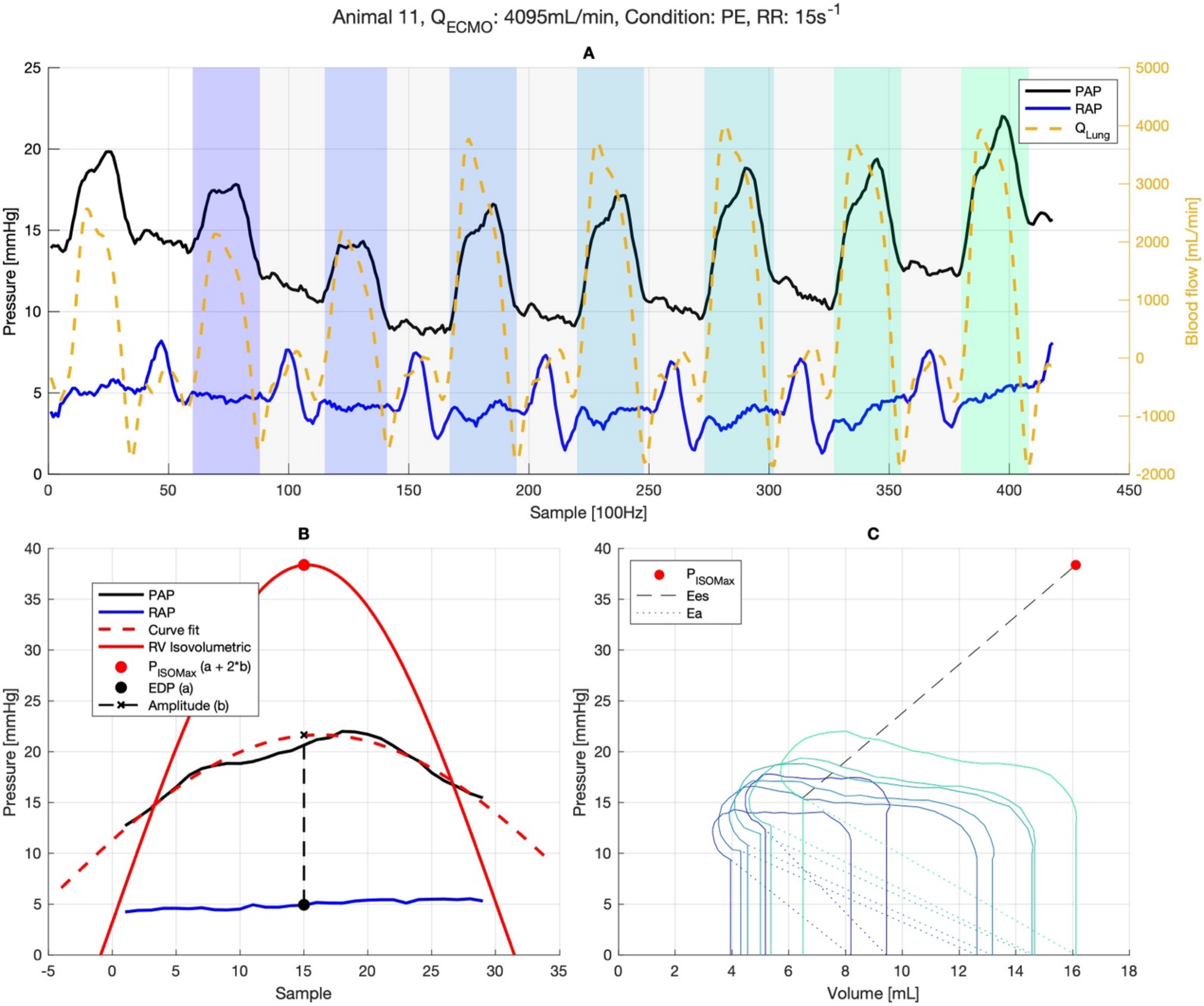

Animal 11, Q_{ECMO}: 4095mL/min, Condition: PE, RR: $15s^{-1}$
A
PAP
RAP
Q_{Lung}
Pressure [mmHg]
Blood flow [mL/min]
Sample [100Hz]
B
PAP
RAP
Curve fit
RV Isovolumetric
P_{ISOMax} (a + 2*b)
EDP (a)
Amplitude (b)
Pressure [mmHg]
Sample
C
P_{ISOMax}
Ees
Ea
Pressure [mmHg]
Volume [mL]

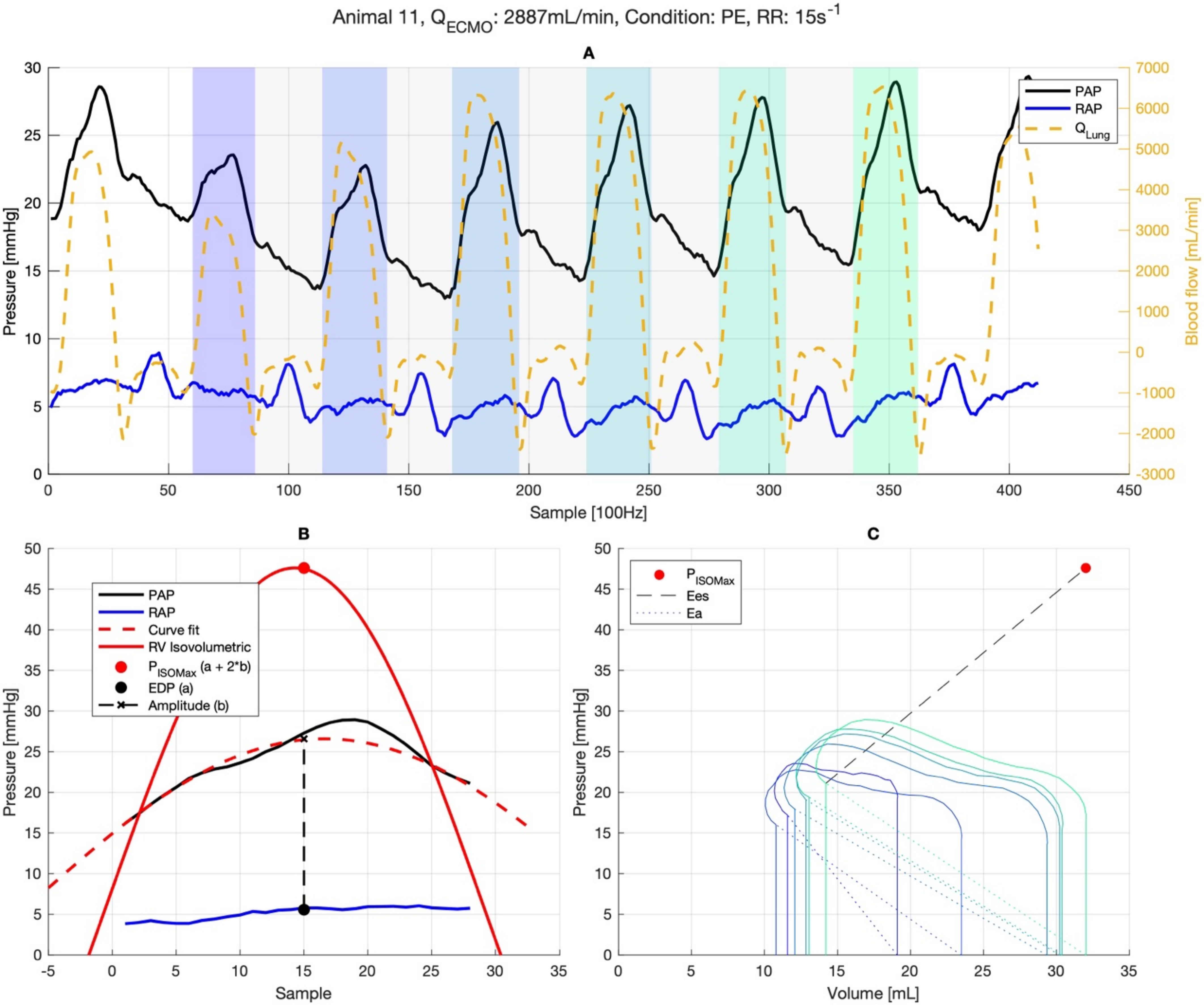

Animal 11, Q_{ECMO}: 2887mL/min, Condition: PE, RR: 15s^{-1}
A
PAP
RAP
Q_{Lung}
Pressure [mmHg]
Blood flow [mL/min]
Sample [100Hz]
B
PAP
RAP
Curve fit
RV Isovolumetric
P_{ISOMax} (a + 2*b)
EDP (a)
Amplitude (b)
Pressure [mmHg]
Sample
C
P_{ISOMax}
Ees
Ea
Pressure [mmHg]
Volume [mL]

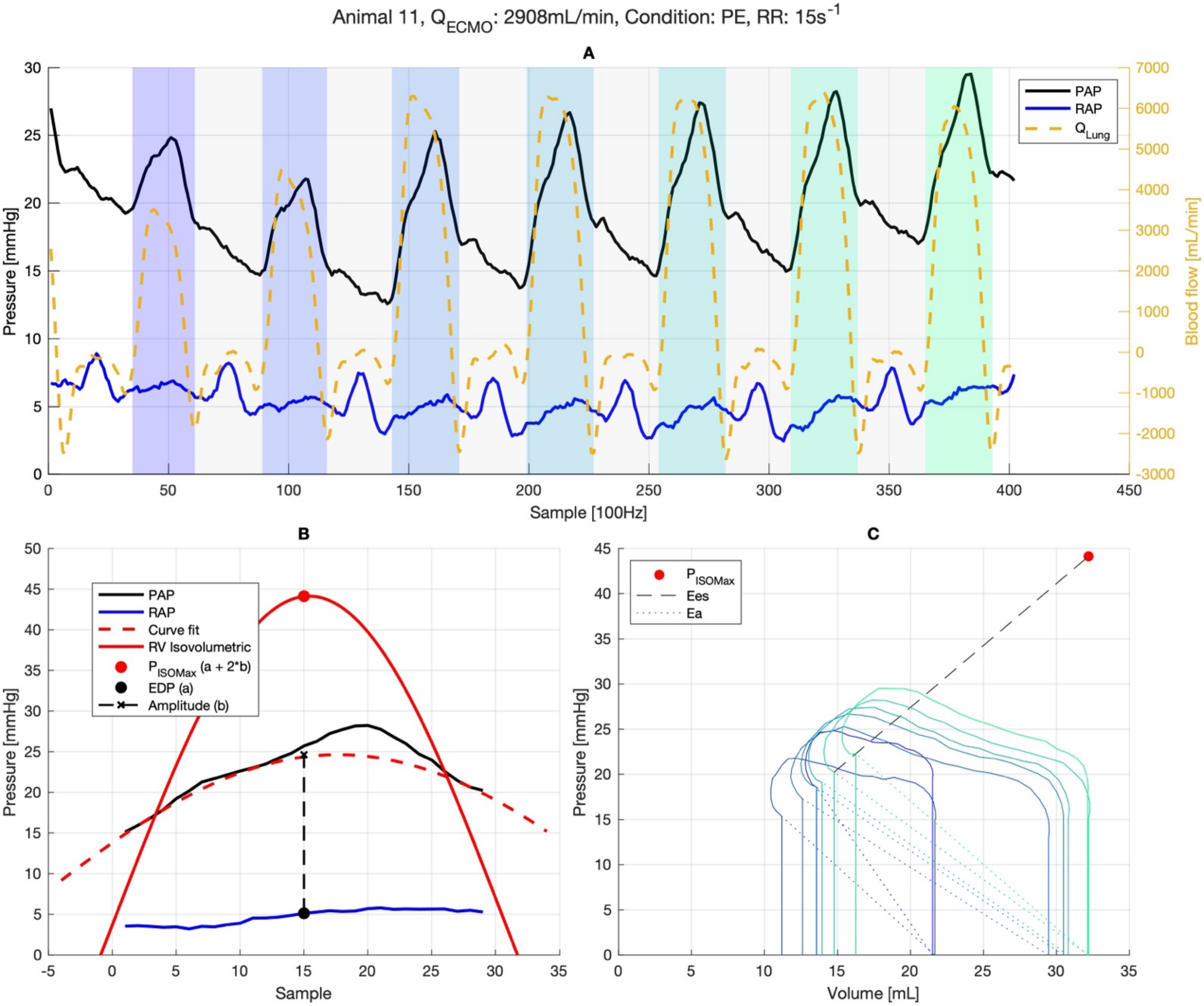

Animal 11, Q_ECMO: 2908mL/min, Condition: PE, RR: 15s^-1
A
PAP
RAP
Q_Lung
Pressure [mmHg]
Blood flow [mL/min]
Sample [100Hz]
B
PAP
RAP
Curve fit
RV Isovolumetric
P_ISOMax (a + 2*b)
EDP (a)
Amplitude (b)
Pressure [mmHg]
Sample
C
P_ISOMax
Ees
Ea
Pressure [mmHg]
Volume [mL]

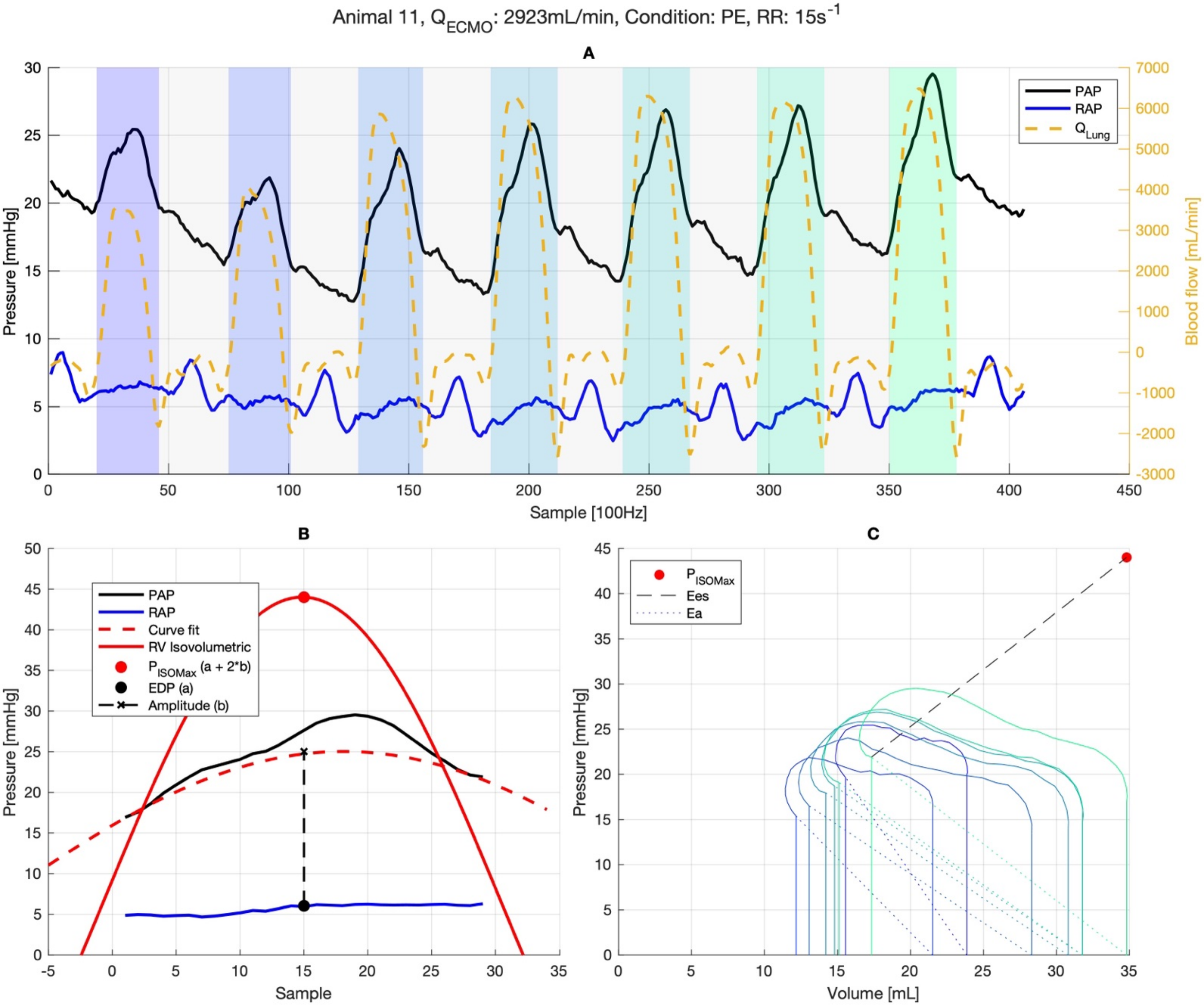

Animal 11, Q_{ECMO}: 2923mL/min, Condition: PE, RR: $15s^{-1}$
A
PAP
RAP
Q_{Lung}
Pressure [mmHg]
Blood flow [mL/min]
Sample [100Hz]
B
PAP
RAP
Curve fit
RV Isovolumetric
P_{ISOMax} (a + 2*b)
EDP (a)
Amplitude (b)
Pressure [mmHg]
Sample
C
P_{ISOMax}
Ees
Ea
Pressure [mmHg]
Volume [mL]

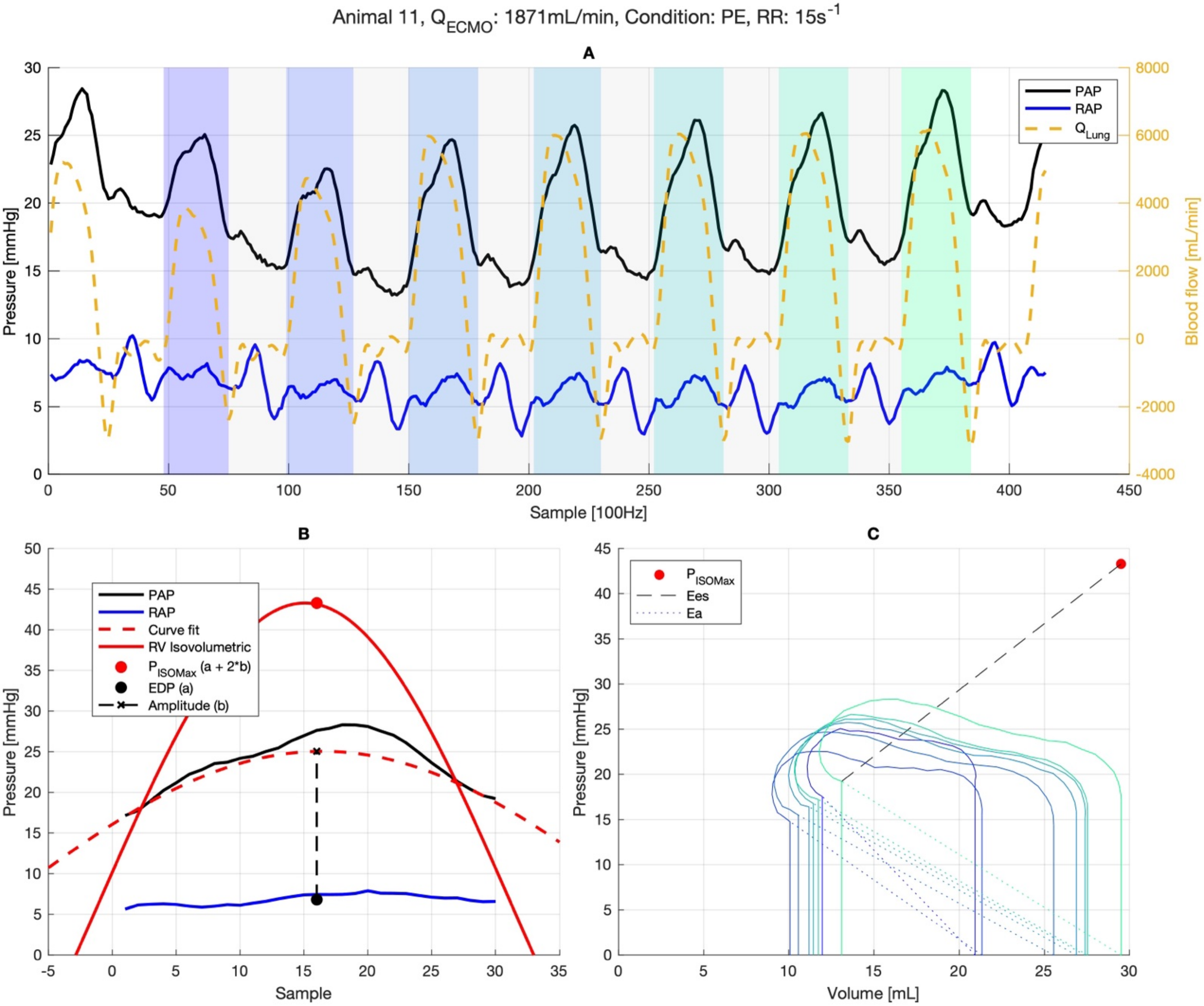

Animal 11, Q_ECMO: 1871mL/min, Condition: PE, RR: 15s^-1
A
Pressure [mmHg]
Blood flow [mL/min]
Sample [100Hz]
PAP
RAP
Q_Lung
B
Pressure [mmHg]
Sample
PAP
RAP
Curve fit
RV Isovolumetric
P_ISOMax (a + 2*b)
EDP (a)
Amplitude (b)
C
Pressure [mmHg]
Volume [mL]
P_ISOMax
Ees
Ea

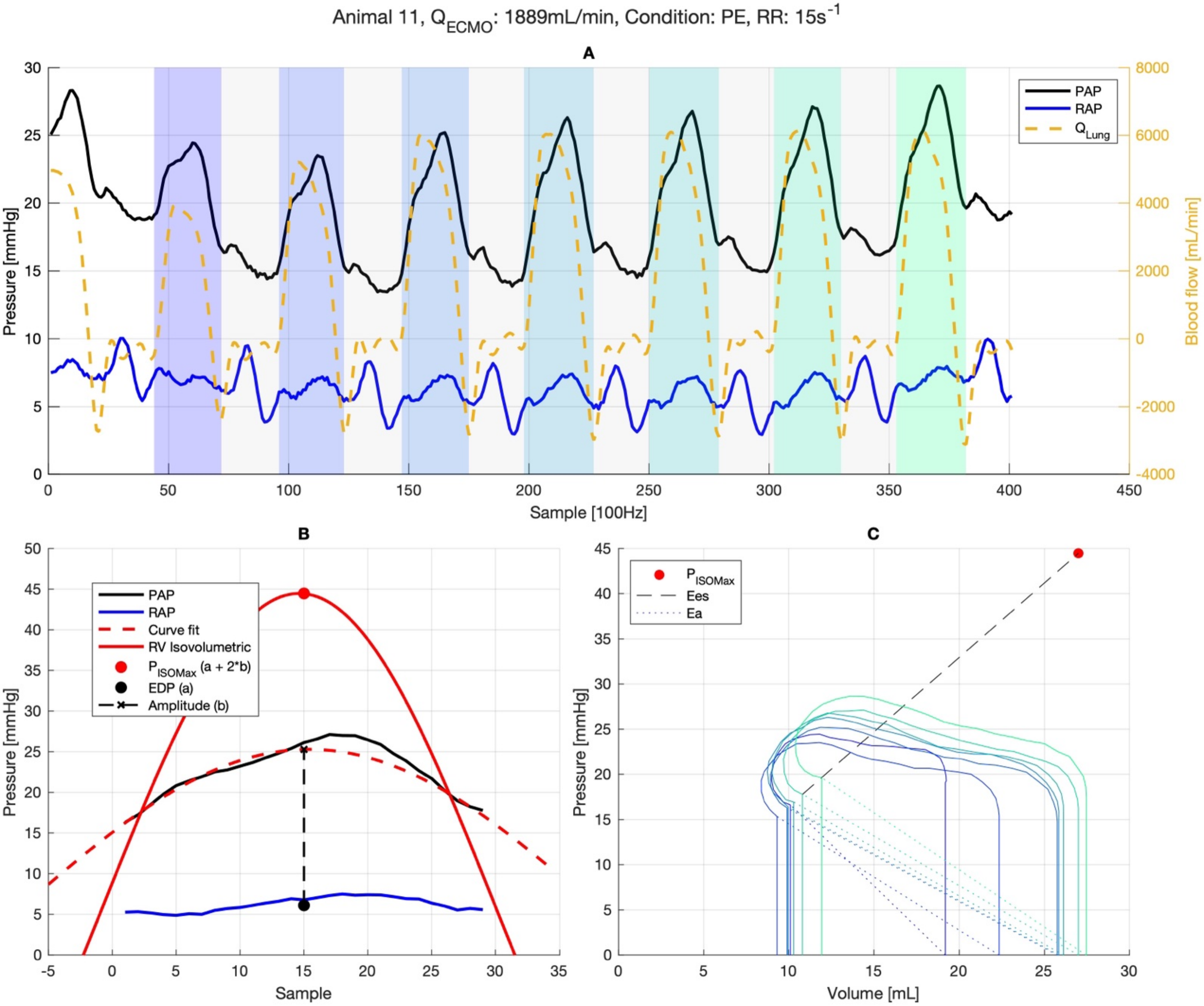

Animal 11, Q_{ECMO}: 1889mL/min, Condition: PE, RR: $15s^{-1}$
A
PAP
RAP
Q_{Lung}
Pressure [mmHg]
Blood flow [mL/min]
Sample [100Hz]
B
PAP
RAP
Curve fit
RV Isovolumetric
P_{ISOMax} (a + 2*b)
EDP (a)
Amplitude (b)
Pressure [mmHg]
Sample
C
P_{ISOMax}
Ees
Ea
Pressure [mmHg]
Volume [mL]

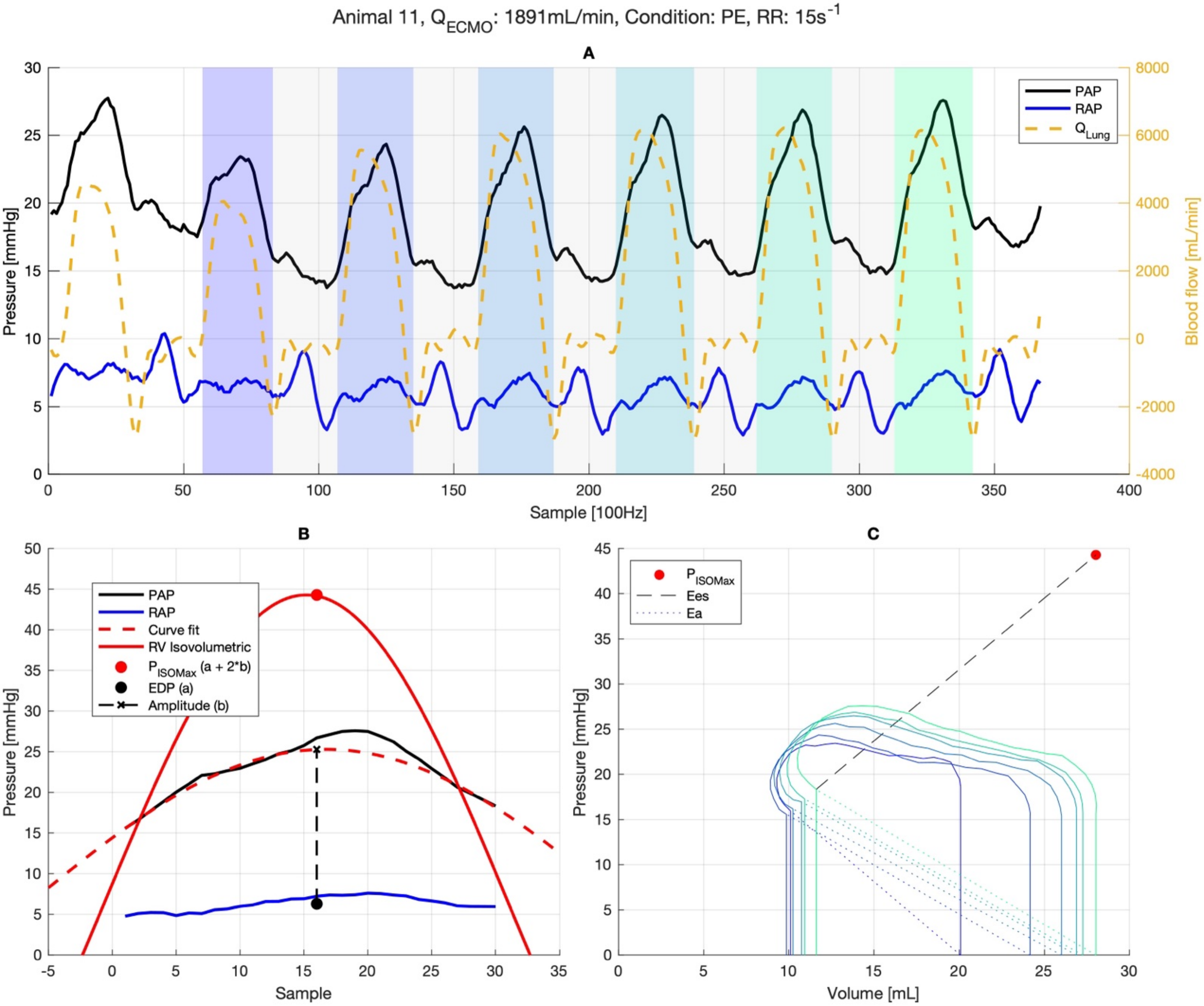

Animal 11, Q_ECMO: 1891mL/min, Condition: PE, RR: 15s^-1
A
PAP
RAP
Q_Lung
Pressure [mmHg]
Blood flow [mL/min]
Sample [100Hz]
B
PAP
RAP
Curve fit
RV Isovolumetric
P_ISOMax (a + 2*b)
EDP (a)
Amplitude (b)
Pressure [mmHg]
Sample
C
P_ISOMax
Ees
Ea
Pressure [mmHg]
Volume [mL]

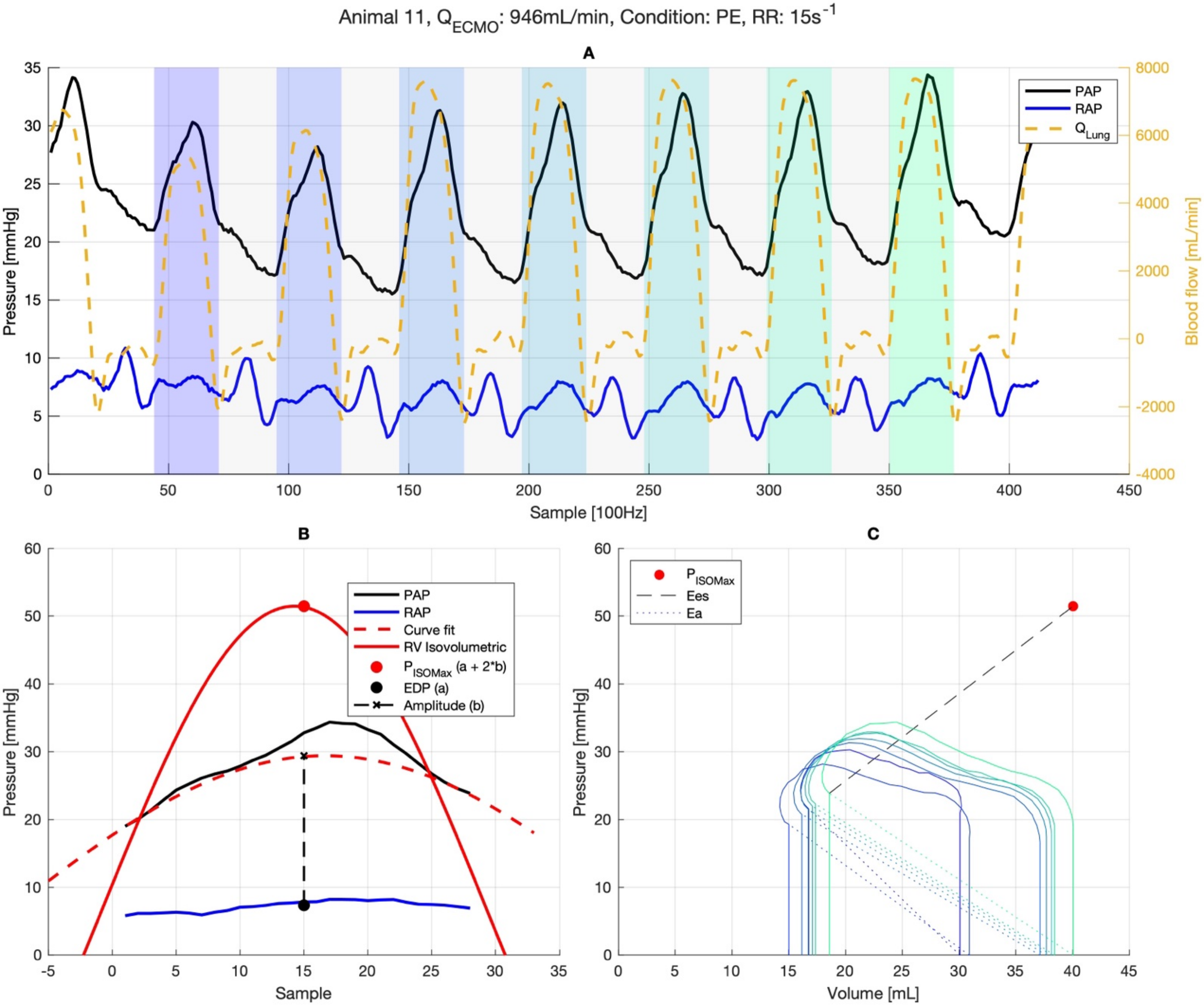

Animal 11, QECMO: 946mL/min, Condition: PE, RR: 15s-1
A
PAP
RAP
QLung
Pressure [mmHg]
Blood flow [mL/min]
Sample [100Hz]
B
PAP
RAP
Curve fit
RV Isovolumetric
PISOMax (a + 2*b)
EDP (a)
Amplitude (b)
Pressure [mmHg]
Sample
C
PISOMax
Ees
Ea
Pressure [mmHg]
Volume [mL]

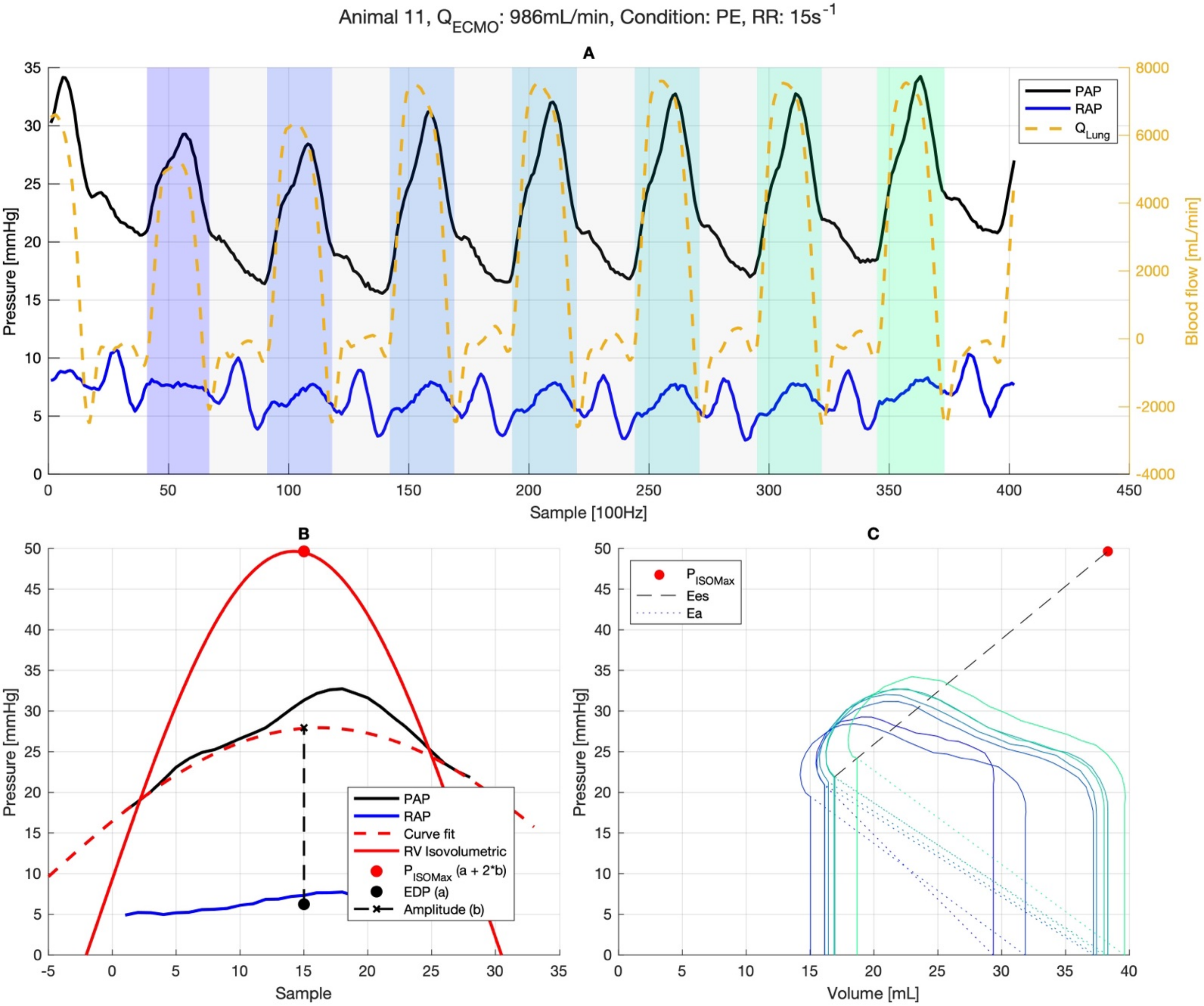

Animal 11, Q_{ECMO}: 986mL/min, Condition: PE, RR: $15s^{-1}$
A
Pressure [mmHg]
Sample [100Hz]
Blood flow [mL/min]
PAP
RAP
Q_{Lung}
B
Pressure [mmHg]
Sample
PAP
RAP
Curve fit
RV Isovolumetric
P_{ISOMax} (a + 2*b)
EDP (a)
Amplitude (b)
C
Pressure [mmHg]
Volume [mL]
P_{ISOMax}
Ees
Ea

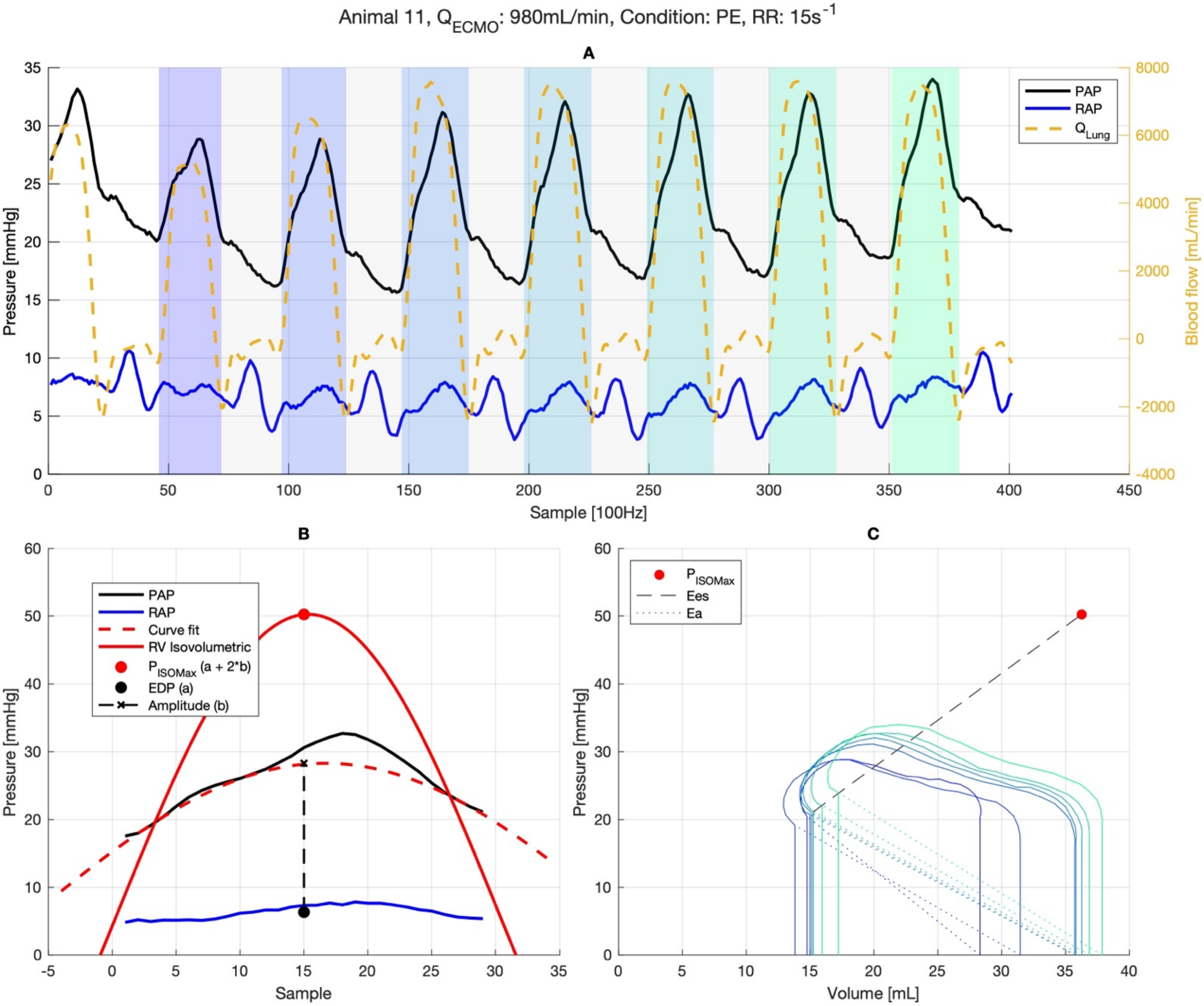

Animal 11, Q_{ECMO}: 980mL/min, Condition: PE, RR: $15s^{-1}$
A
Pressure [mmHg]
Blood flow [mL/min]
Sample [100Hz]
PAP
RAP
Q_{Lung}
B
PAP
RAP
Curve fit
RV Isovolumetric
P_{ISOMax} (a + 2*b)
EDP (a)
Amplitude (b)
Pressure [mmHg]
Sample
C
P_{ISOMax}
Ees
Ea
Pressure [mmHg]
Volume [mL]

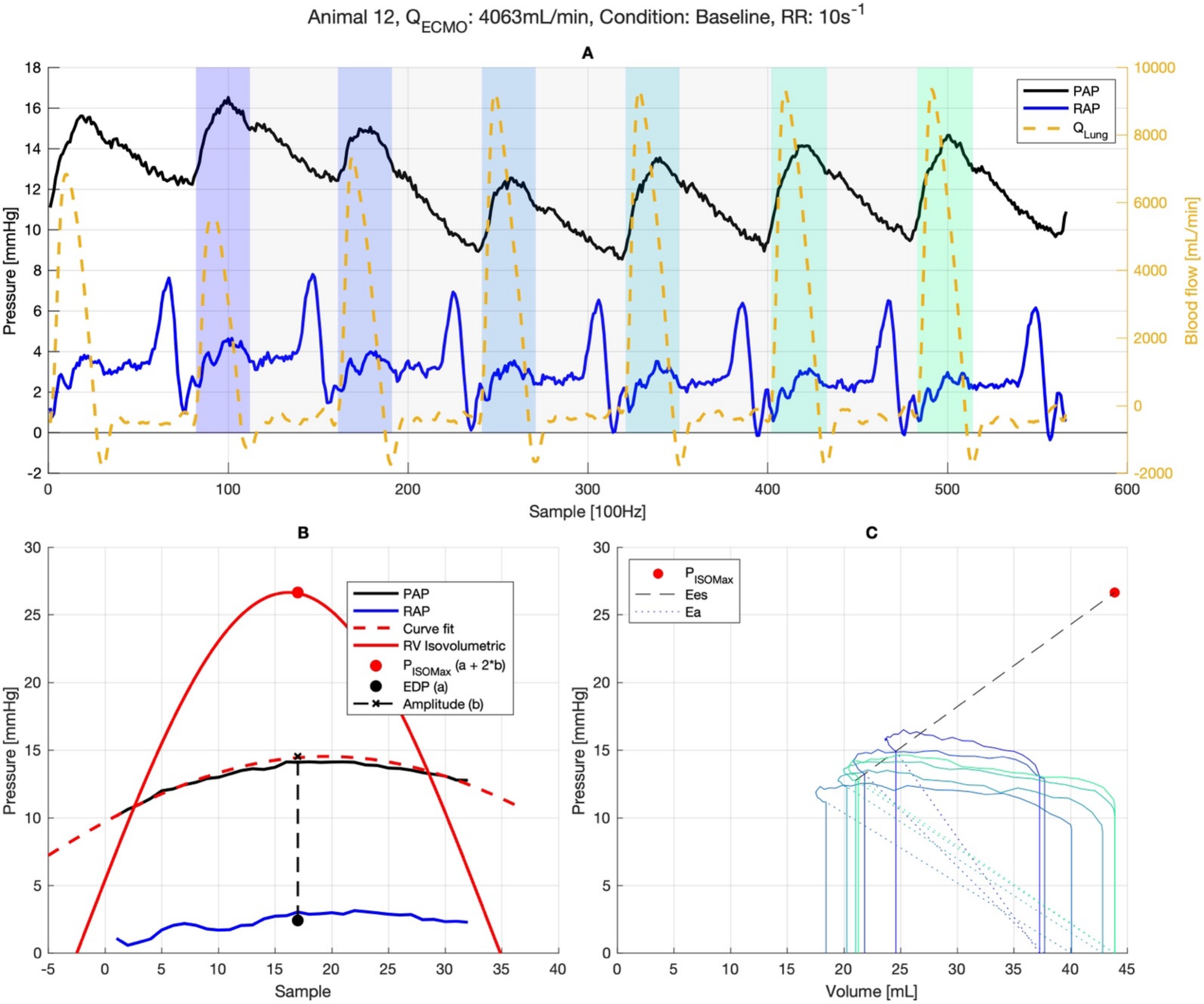
Animal 12, Q_{ECMO}: 4063mL/min, Condition: Baseline, RR: $10s^{-1}$
A
PAP
RAP
Q_{Lung}
Pressure [mmHg]
Blood flow [mL/min]
Sample [100Hz]
B
PAP
RAP
Curve fit
RV Isovolumetric
P_{ISOMax} (a + 2*b)
EDP (a)
Amplitude (b)
Pressure [mmHg]
Sample
C
P_{ISOMax}
Ees
Ea
Pressure [mmHg]
Volume [mL]

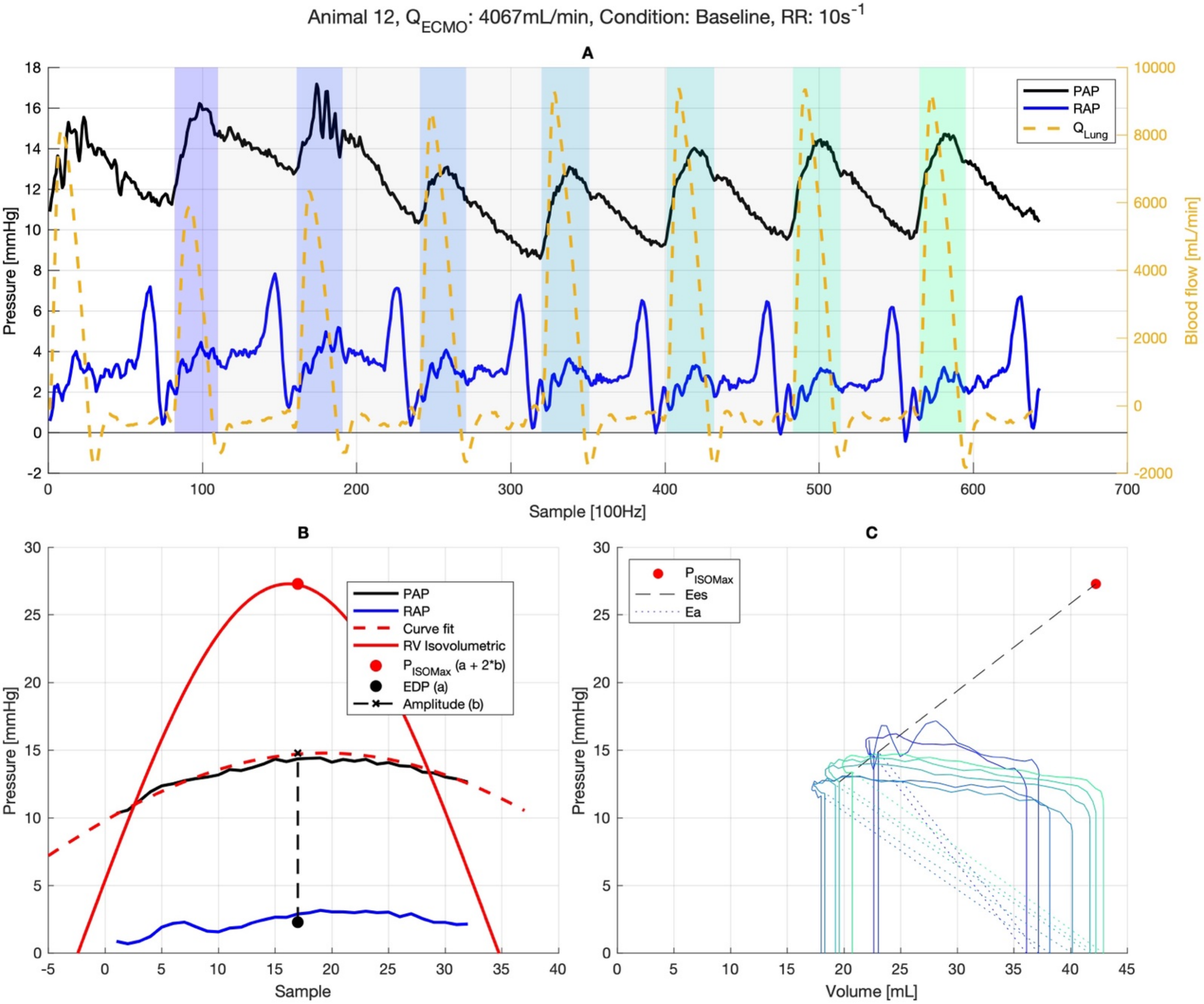

Animal 12, Q_{ECMO}: 4067mL/min, Condition: Baseline, RR: $10s^{-1}$
A
PAP
RAP
Q_{Lung}
Pressure [mmHg]
Blood flow [mL/min]
Sample [100Hz]
B
PAP
RAP
Curve fit
RV Isovolumetric
P_{ISOMax} (a + 2*b)
EDP (a)
Amplitude (b)
Pressure [mmHg]
Sample
C
P_{ISOMax}
Ees
Ea
Pressure [mmHg]
Volume [mL]

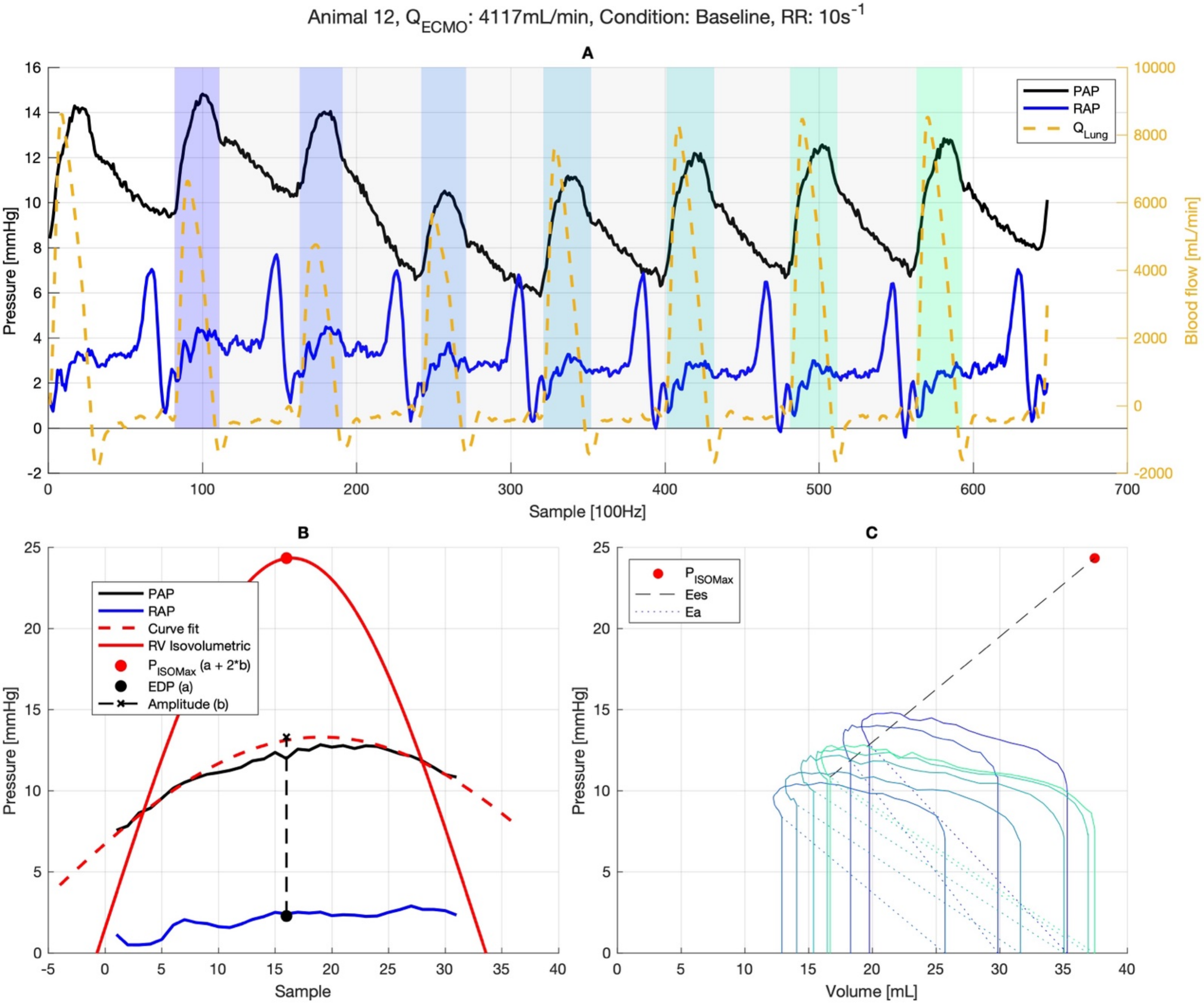
Animal 12, Q_ECMO: 4117mL/min, Condition: Baseline, RR: 10s^-1
A
PAP
RAP
Q_Lung
Pressure [mmHg]
Blood flow [mL/min]
Sample [100Hz]
B
PAP
RAP
Curve fit
RV Isovolumetric
P_ISOMax (a + 2*b)
EDP (a)
Amplitude (b)
Pressure [mmHg]
Sample
C
P_ISOMax
Ees
Ea
Pressure [mmHg]
Volume [mL]

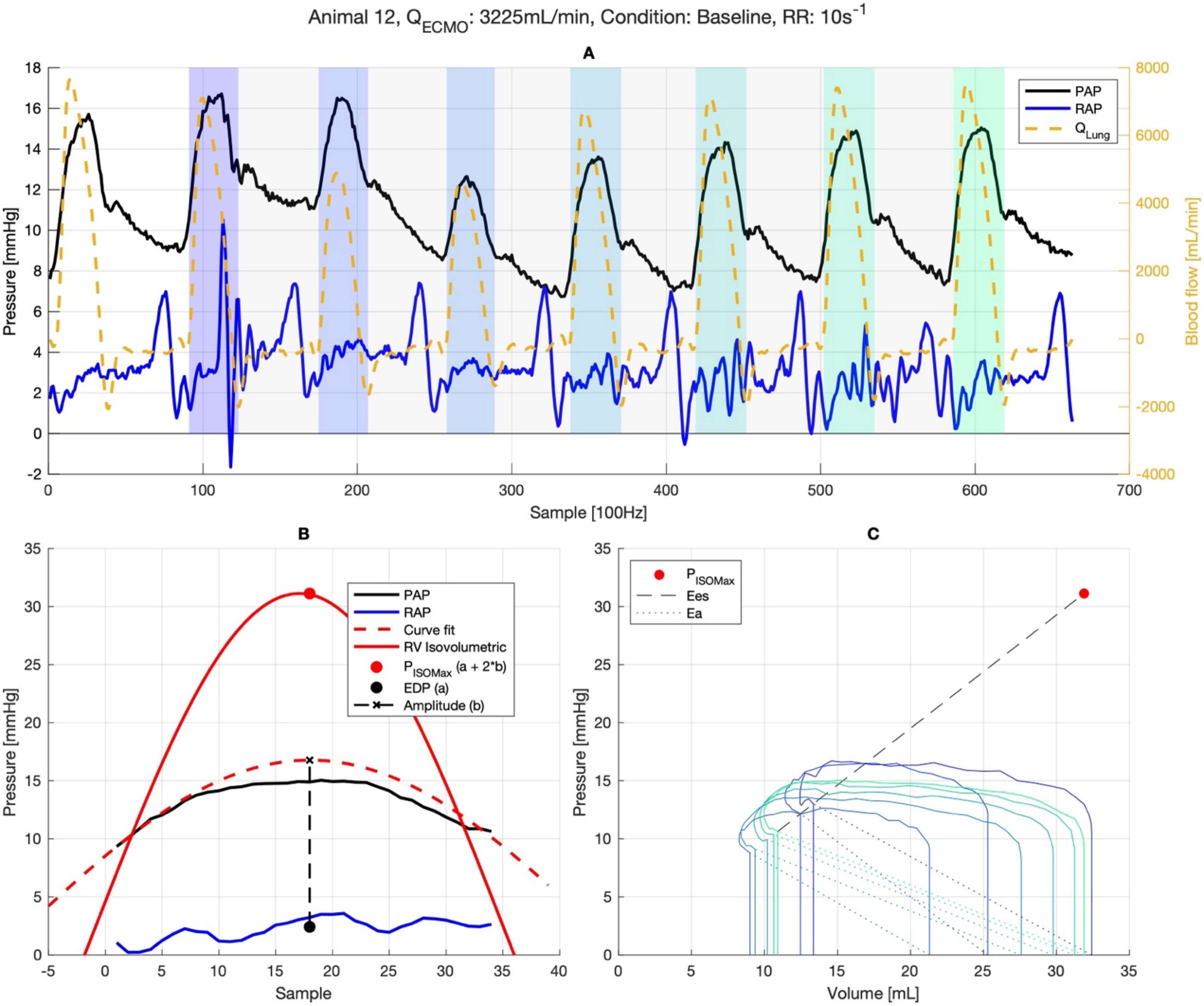

Animal 12, Q_{ECMO}: 3225mL/min, Condition: Baseline, RR: $10s^{-1}$
A
PAP
RAP
Q_{Lung}
Pressure [mmHg]
Blood flow [mL/min]
Sample [100Hz]
B
PAP
RAP
Curve fit
RV Isovolumetric
P_{ISOMax} (a + 2*b)
EDP (a)
Amplitude (b)
Pressure [mmHg]
Sample
C
P_{ISOMax}
Ees
Ea
Pressure [mmHg]
Volume [mL]

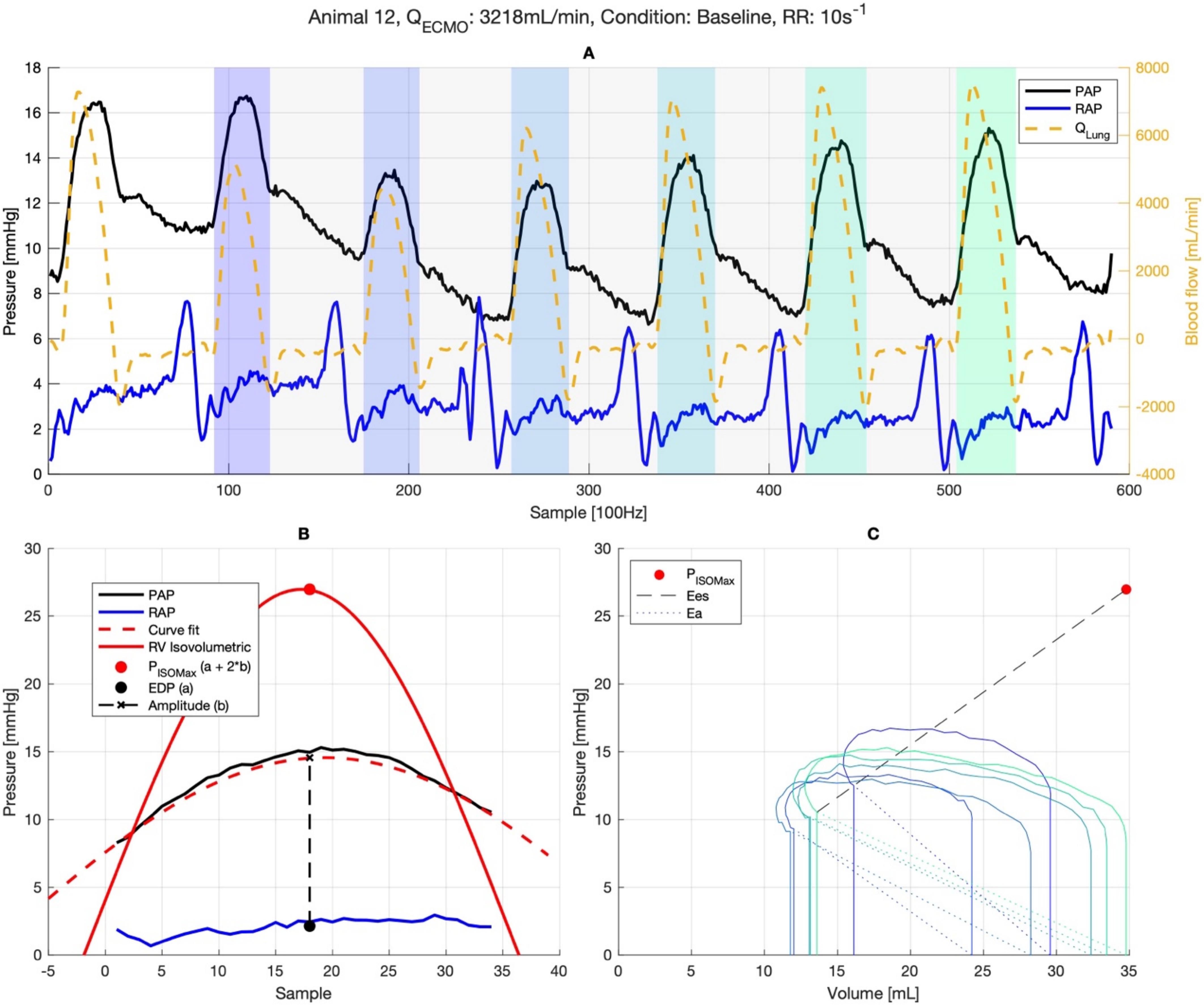

Animal 12, Q_{ECMO}: 3218mL/min, Condition: Baseline, RR: $10s^{-1}$
A
PAP
RAP
Q_{Lung}
Pressure [mmHg]
Blood flow [mL/min]
Sample [100Hz]
B
PAP
RAP
Curve fit
RV Isovolumetric
P_{ISOMax} (a + 2*b)
EDP (a)
Amplitude (b)
Pressure [mmHg]
Sample
C
P_{ISOMax}
Ees
Ea
Pressure [mmHg]
Volume [mL]

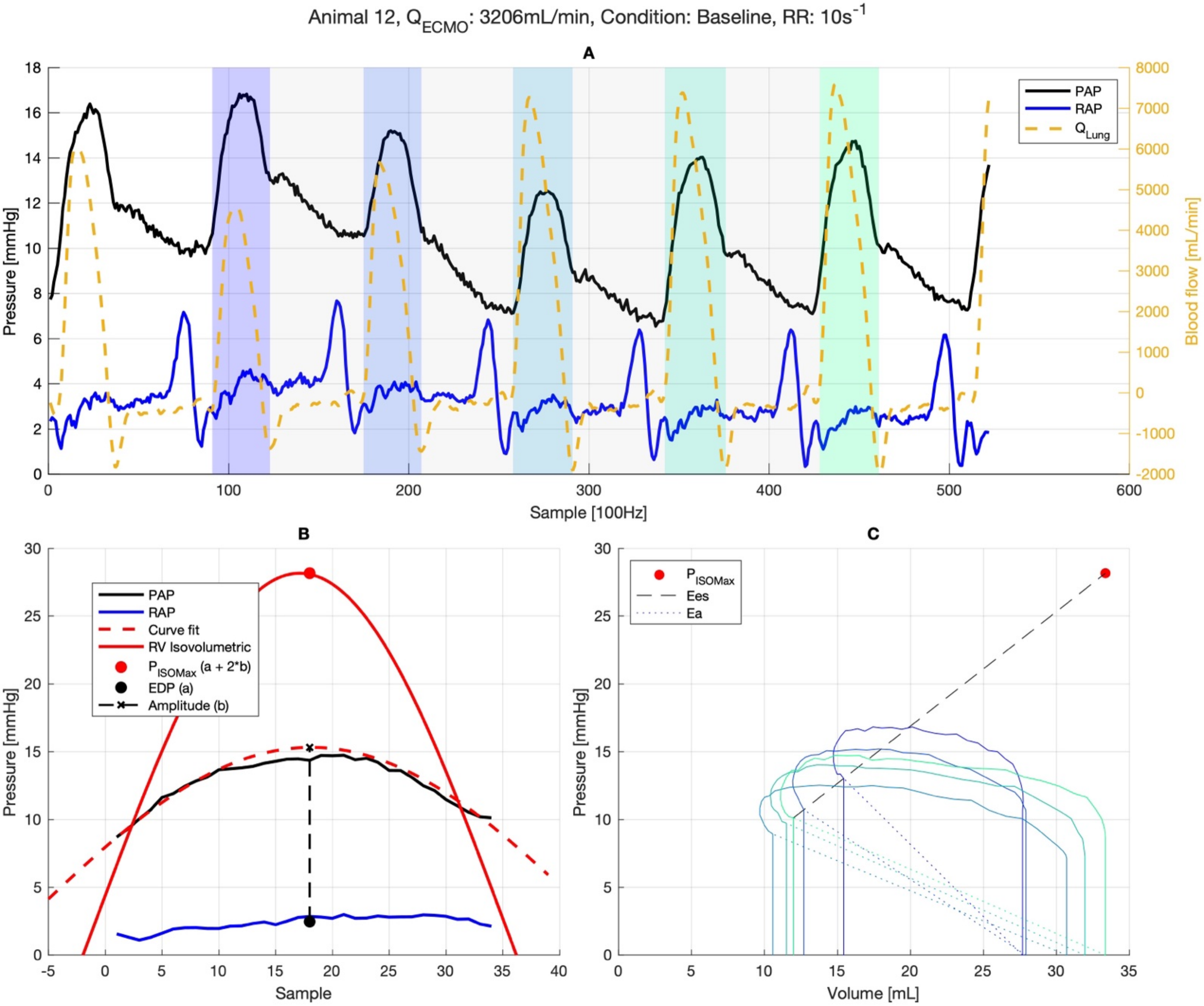
Animal 12, QECMO: 3206mL/min, Condition: Baseline, RR: 10s-1
A
PAP
RAP
QLung
Pressure [mmHg]
Blood flow [mL/min]
Sample [100Hz]
B
PAP
RAP
Curve fit
RV Isovolumetric
PISOMax (a + 2*b)
EDP (a)
Amplitude (b)
Pressure [mmHg]
Sample
C
PISOMax
Ees
Ea
Pressure [mmHg]
Volume [mL]

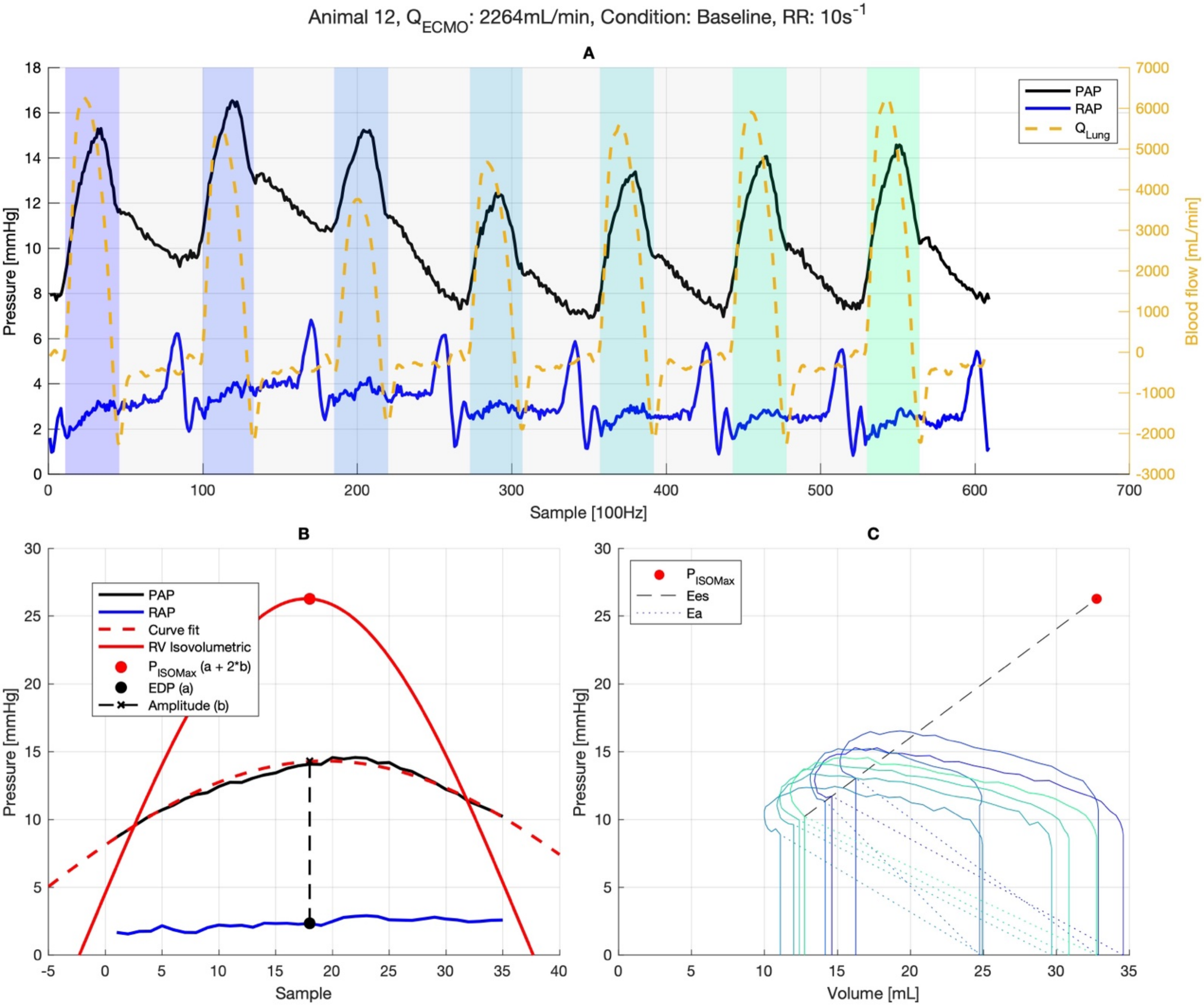

Animal 12, Q_{ECMO}: 2264mL/min, Condition: Baseline, RR: $10s^{-1}$
A
PAP
RAP
Q_{Lung}
Pressure [mmHg]
Blood flow [mL/min]
Sample [100Hz]
B
PAP
RAP
Curve fit
RV Isovolumetric
P_{ISOMax} (a + 2*b)
EDP (a)
Amplitude (b)
Pressure [mmHg]
Sample
C
P_{ISOMax}
Ees
Ea
Pressure [mmHg]
Volume [mL]

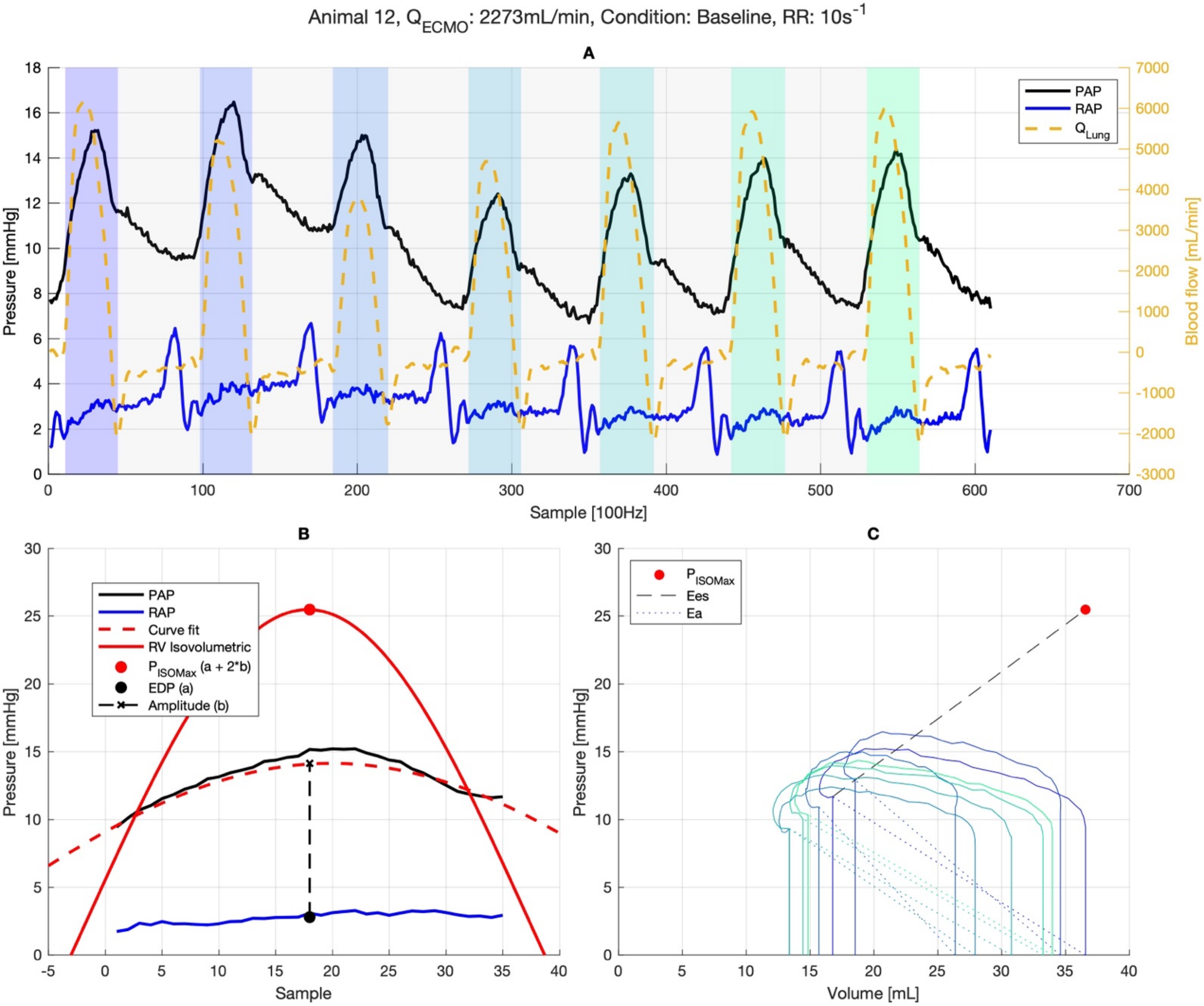

Animal 12, Q_{ECMO}: 2273mL/min, Condition: Baseline, RR: $10s^{-1}$
A
PAP
RAP
Q_{Lung}
Pressure [mmHg]
Blood flow [mL/min]
Sample [100Hz]
B
PAP
RAP
Curve fit
RV Isovolumetric
P_{ISOMax} (a + 2*b)
EDP (a)
Amplitude (b)
Pressure [mmHg]
Sample
C
P_{ISOMax}
Ees
Ea
Pressure [mmHg]
Volume [mL]

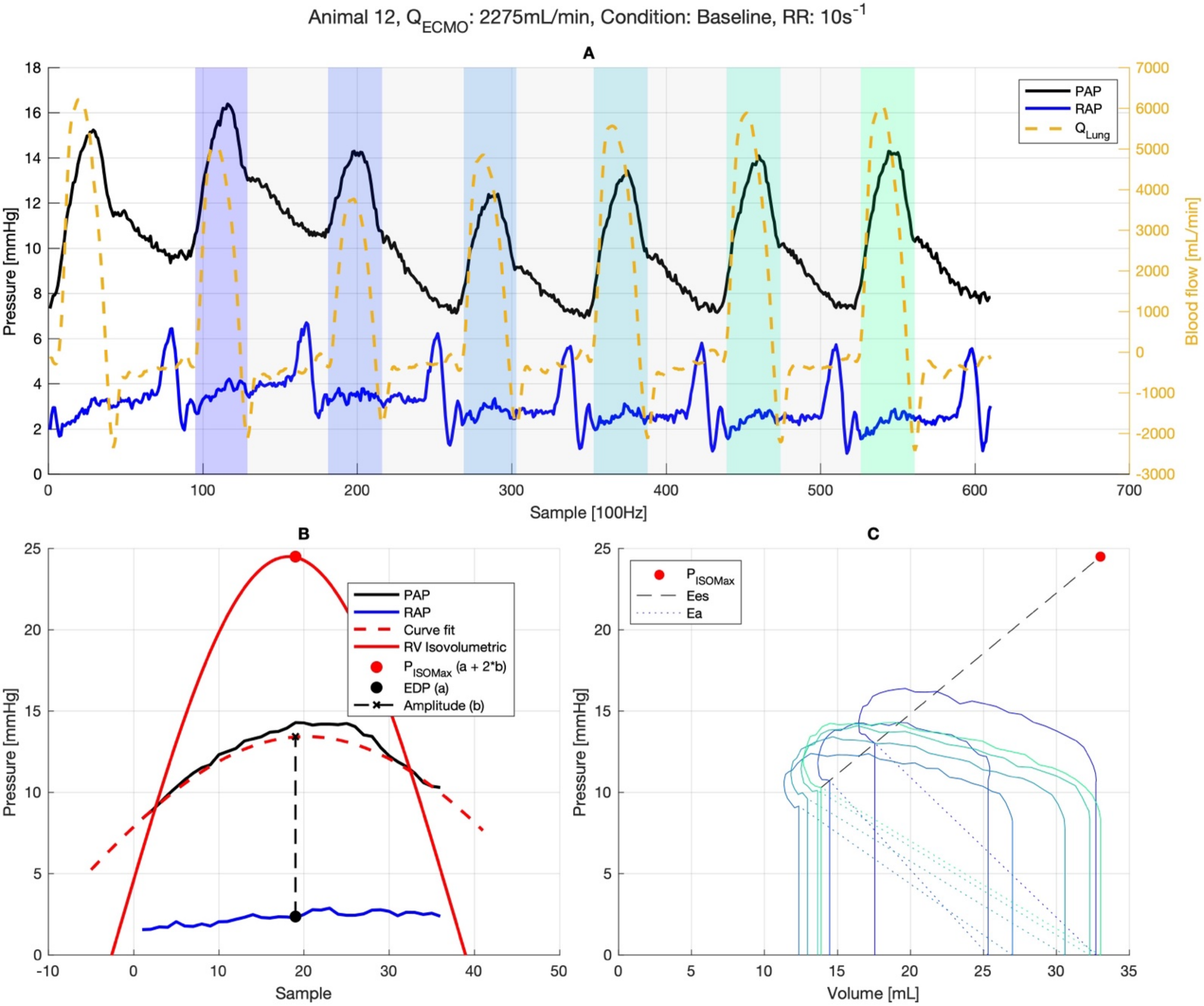

Animal 12, Q_{ECMO}: 2275mL/min, Condition: Baseline, RR: $10s^{-1}$
A
PAP
RAP
Q_{Lung}
Pressure [mmHg]
Blood flow [mL/min]
Sample [100Hz]
B
PAP
RAP
Curve fit
RV Isovolumetric
P_{ISOMax} (a + 2*b)
EDP (a)
Amplitude (b)
Pressure [mmHg]
Sample
C
P_{ISOMax}
Ees
Ea
Pressure [mmHg]
Volume [mL]

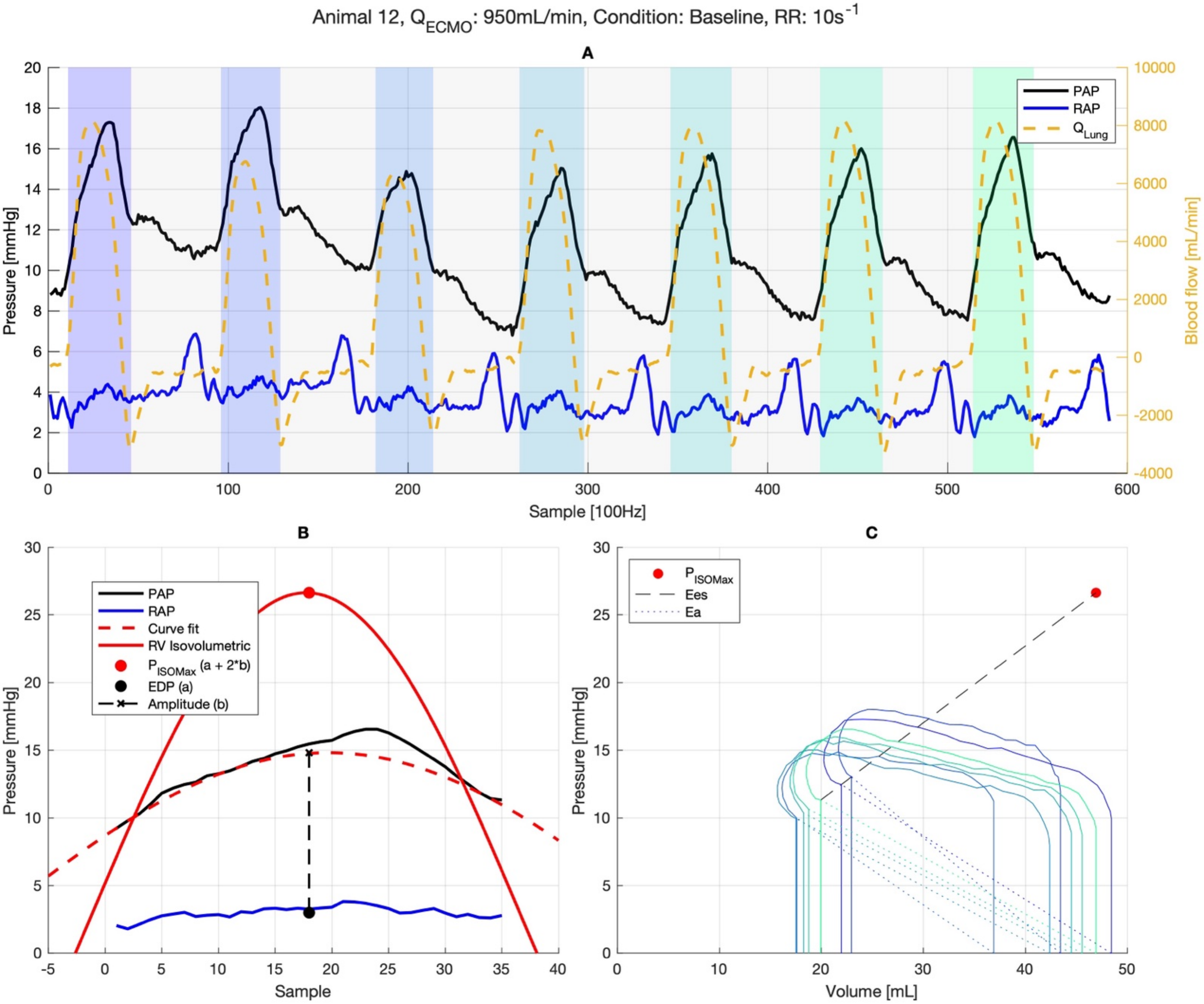
Animal 12, Q_{ECMO}: 950mL/min, Condition: Baseline, RR: $10s^{-1}$
A
PAP
RAP
Q_{Lung}
Pressure [mmHg]
Blood flow [mL/min]
Sample [100Hz]
B
PAP
RAP
Curve fit
RV Isovolumetric
P_{ISOMax} (a + 2*b)
EDP (a)
Amplitude (b)
Pressure [mmHg]
Sample
C
P_{ISOMax}
Ees
Ea
Pressure [mmHg]
Volume [mL]

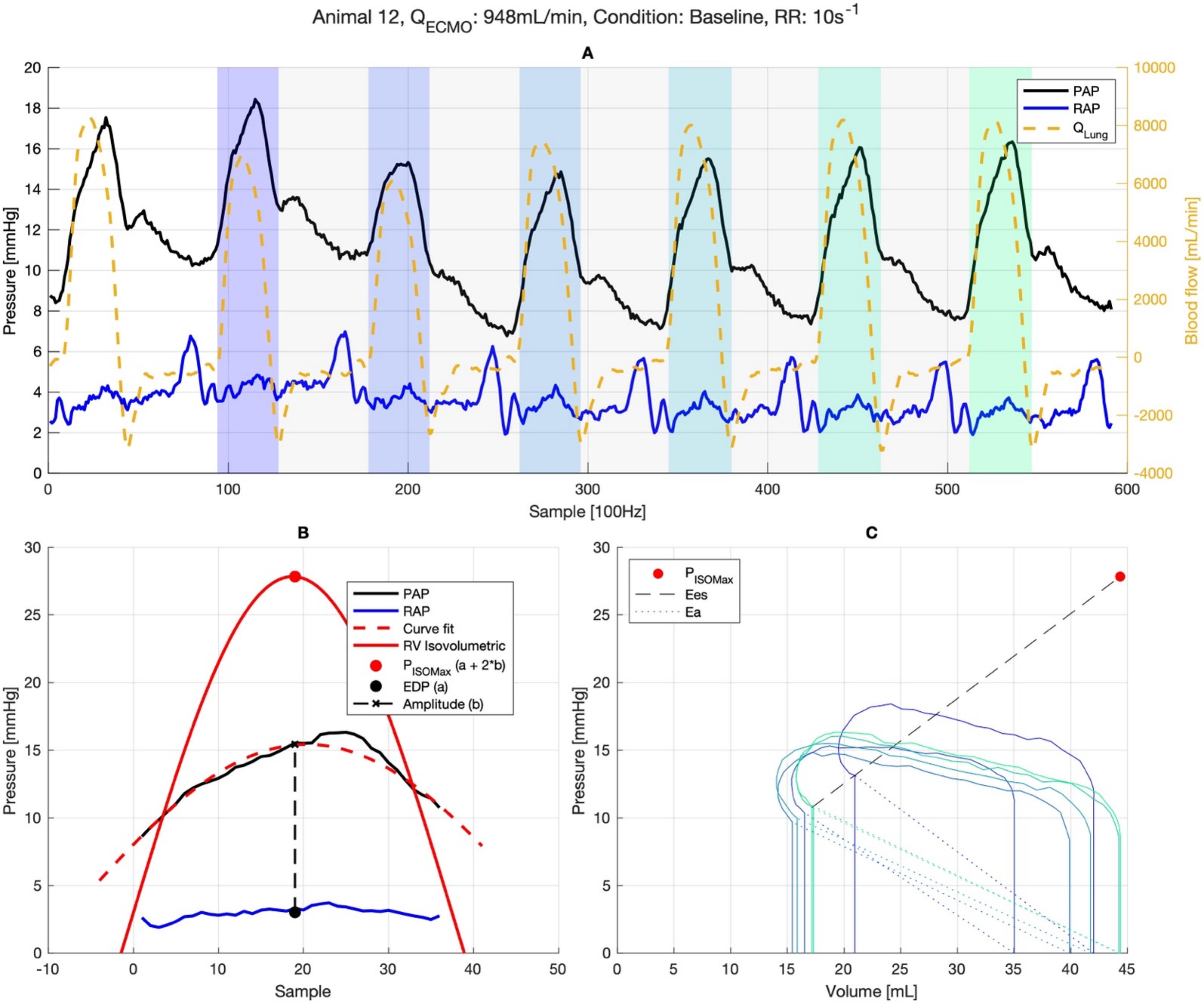

Animal 12, Q_{ECMO}: 948mL/min, Condition: Baseline, RR: $10s^{-1}$
A
Pressure [mmHg]
Blood flow [mL/min]
Sample [100Hz]
PAP
RAP
Q_{Lung}
B
Pressure [mmHg]
Sample
PAP
RAP
Curve fit
RV Isovolumetric
P_{ISOMax} (a + 2*b)
EDP (a)
Amplitude (b)
C
Pressure [mmHg]
Volume [mL]
P_{ISOMax}
Ees
Ea

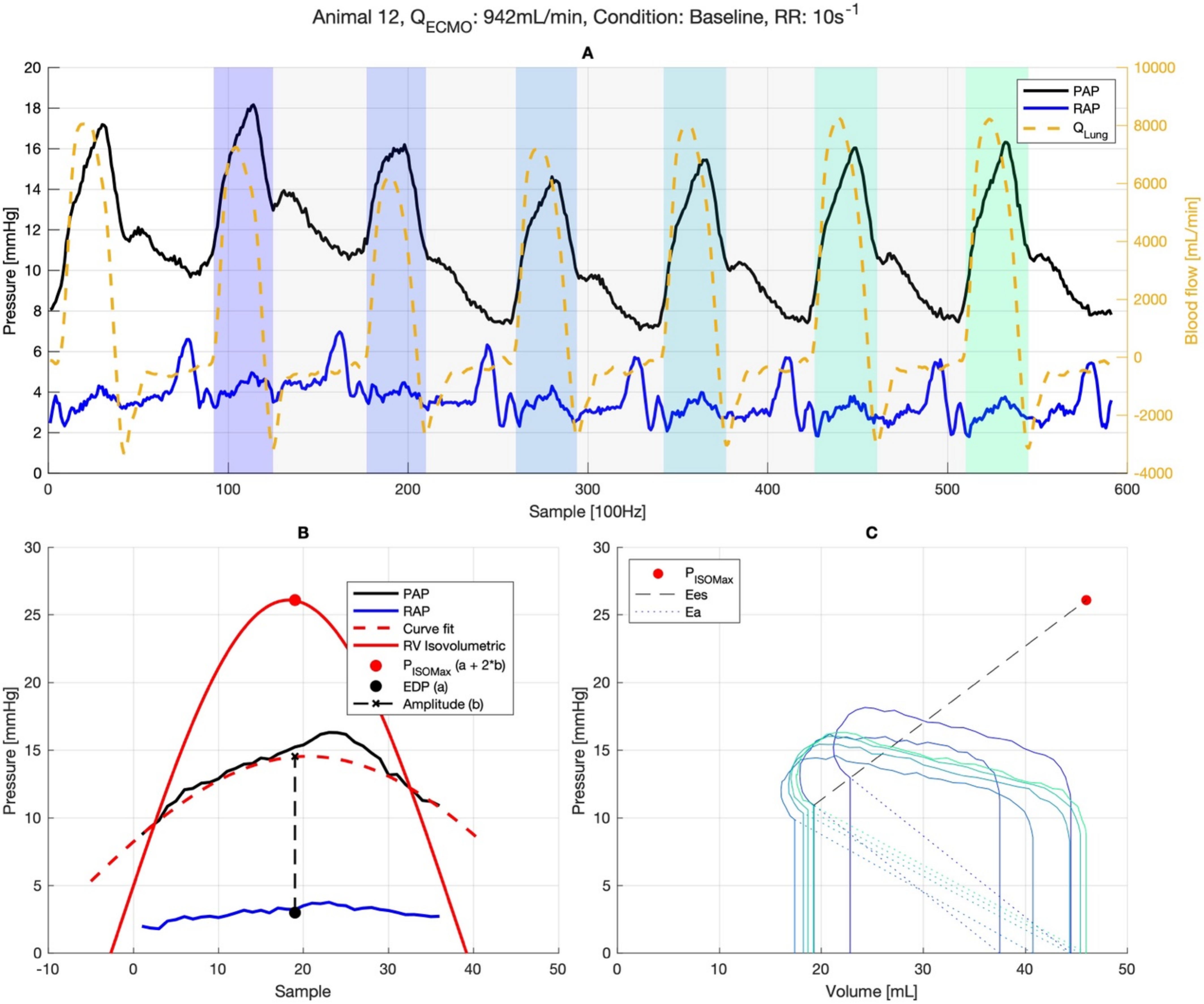

Animal 12, Q_ECMO: 942mL/min, Condition: Baseline, RR: 10s^-1
A
Pressure [mmHg]
Blood flow [mL/min]
Sample [100Hz]
PAP
RAP
Q_Lung
B
Pressure [mmHg]
Sample
PAP
RAP
Curve fit
RV Isovolumetric
P_ISOMax (a + 2*b)
EDP (a)
Amplitude (b)
C
Pressure [mmHg]
Volume [mL]
P_ISOMax
Ees
Ea

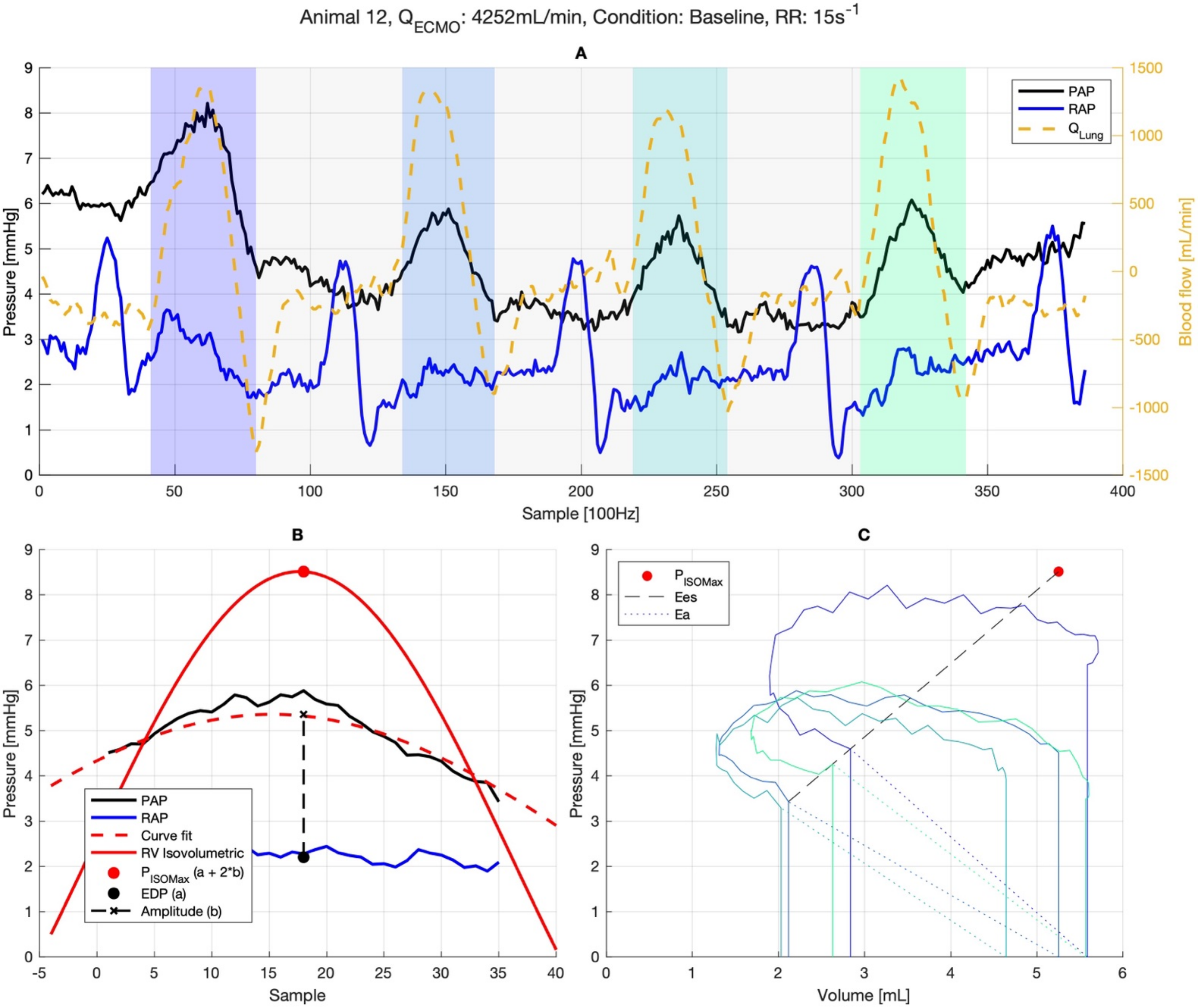

Animal 12, QECMO: 4252mL/min, Condition: Baseline, RR: 15s-1
A
PAP
RAP
QLung
Pressure [mmHg]
Blood flow [mL/min]
Sample [100Hz]
B
PAP
RAP
Curve fit
RV Isovolumetric
PISOMax (a + 2*b)
EDP (a)
Amplitude (b)
Pressure [mmHg]
Sample
C
PISOMax
Ees
Ea
Pressure [mmHg]
Volume [mL]

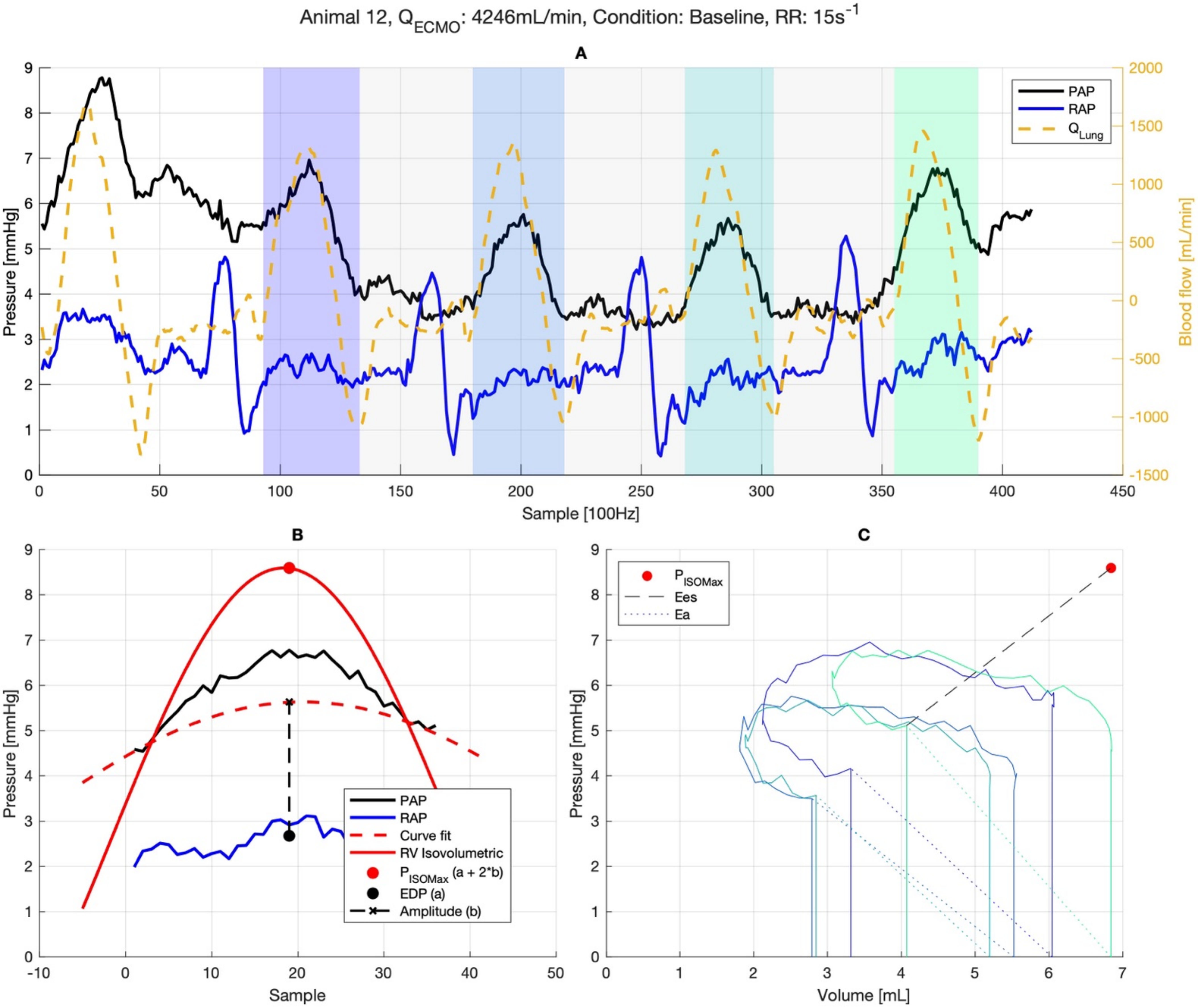
Animal 12, Q_{ECMO}: 4246mL/min, Condition: Baseline, RR: $15s^{-1}$
A
PAP
RAP
Q_{Lung}
Pressure [mmHg]
Blood flow [mL/min]
Sample [100Hz]
B
PAP
RAP
Curve fit
RV Isovolumetric
P_{ISOMax} (a + 2*b)
EDP (a)
Amplitude (b)
Pressure [mmHg]
Sample
C
P_{ISOMax}
Ees
Ea
Pressure [mmHg]
Volume [mL]

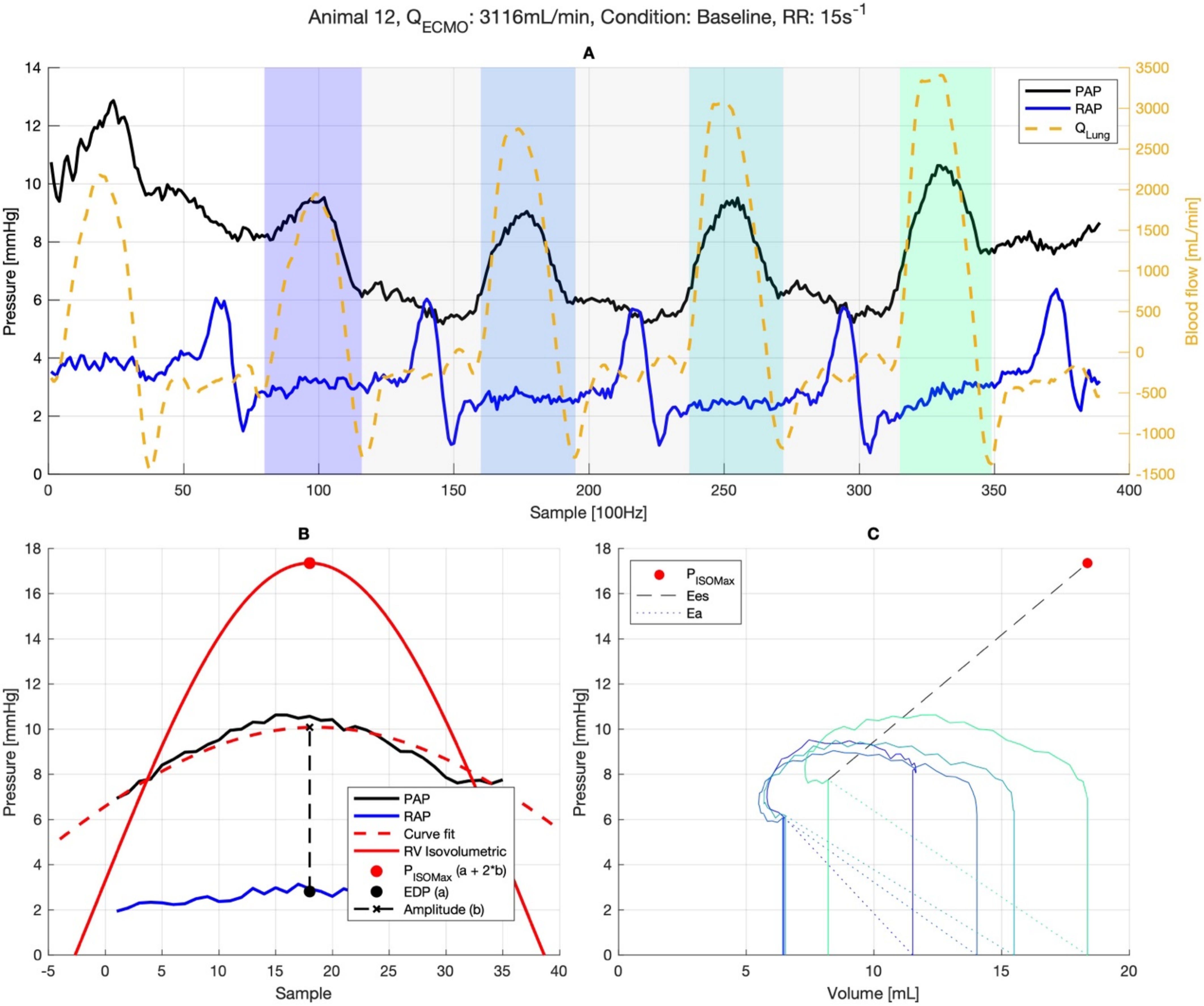

Animal 12, Q_{ECMO}: 3116mL/min, Condition: Baseline, RR: $15s^{-1}$
A
PAP
RAP
Q_{Lung}
Pressure [mmHg]
Blood flow [mL/min]
Sample [100Hz]
B
PAP
RAP
Curve fit
RV Isovolumetric
P_{ISOMax} (a + 2*b)
EDP (a)
Amplitude (b)
Pressure [mmHg]
Sample
C
P_{ISOMax}
Ees
Ea
Pressure [mmHg]
Volume [mL]

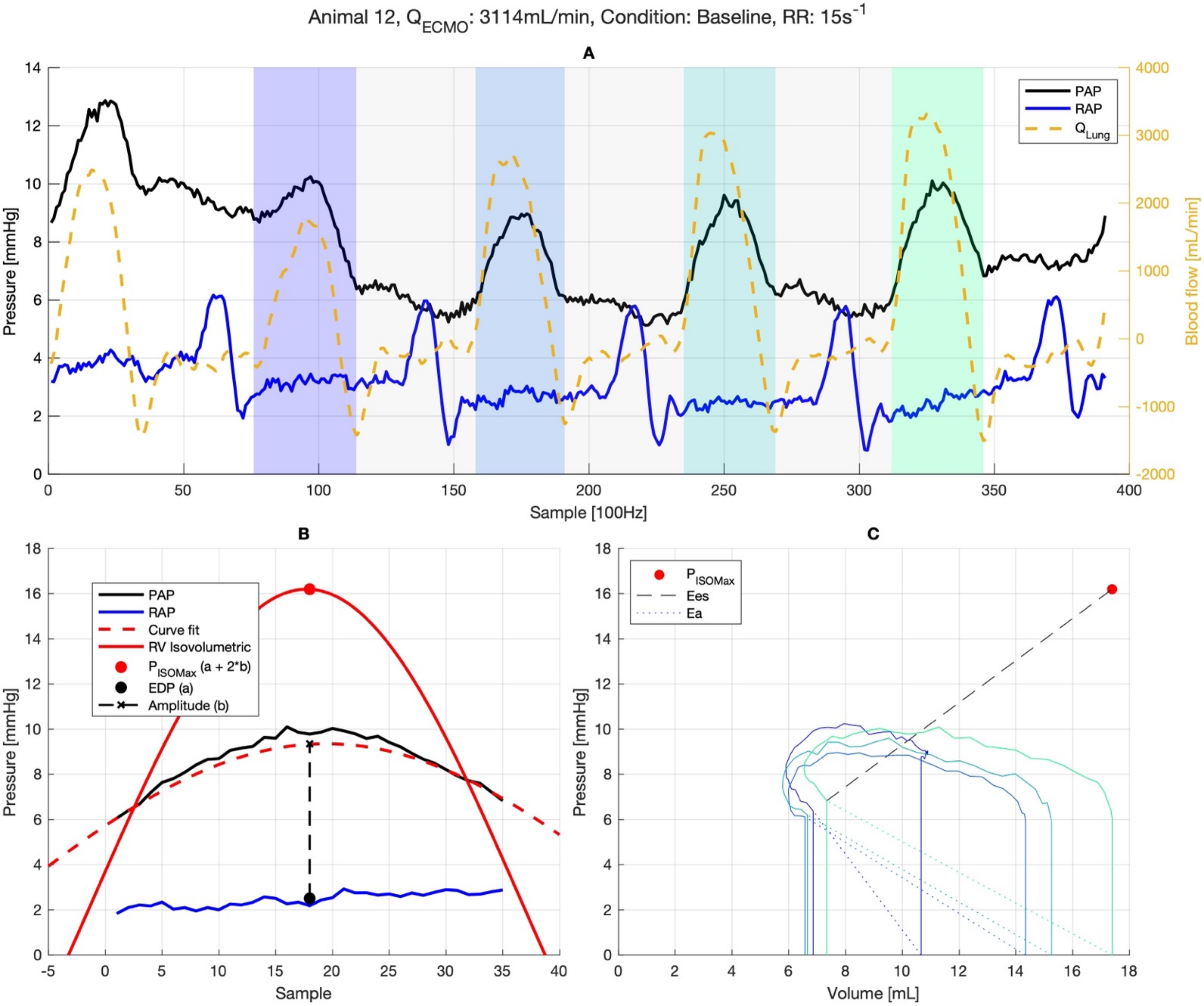

Animal 12, QECMO: 3114mL/min, Condition: Baseline, RR: 15s-1
A
PAP
RAP
QLung
Pressure [mmHg]
Blood flow [mL/min]
Sample [100Hz]
B
PAP
RAP
Curve fit
RV Isovolumetric
PISOMax (a + 2*b)
EDP (a)
Amplitude (b)
Pressure [mmHg]
Sample
C
PISOMax
Ees
Ea
Pressure [mmHg]
Volume [mL]

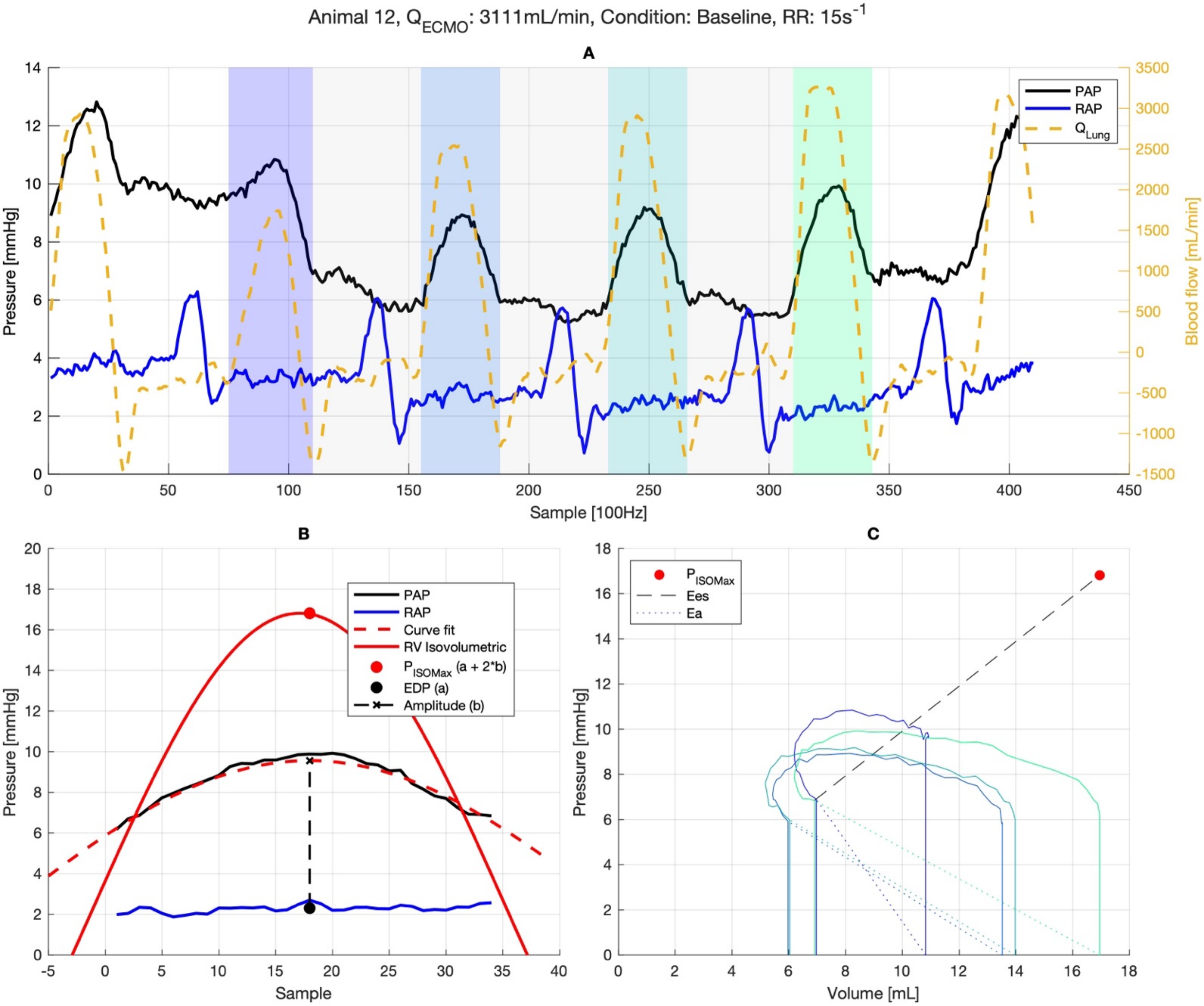

Animal 12, Q_{ECMO}: 3111mL/min, Condition: Baseline, RR: 15s^{-1}
A
PAP
RAP
Q_{Lung}
Pressure [mmHg]
Blood flow [mL/min]
Sample [100Hz]
B
PAP
RAP
Curve fit
RV Isovolumetric
P_{ISOMax} (a + 2*b)
EDP (a)
Amplitude (b)
Pressure [mmHg]
Sample
C
P_{ISOMax}
Ees
Ea
Pressure [mmHg]
Volume [mL]

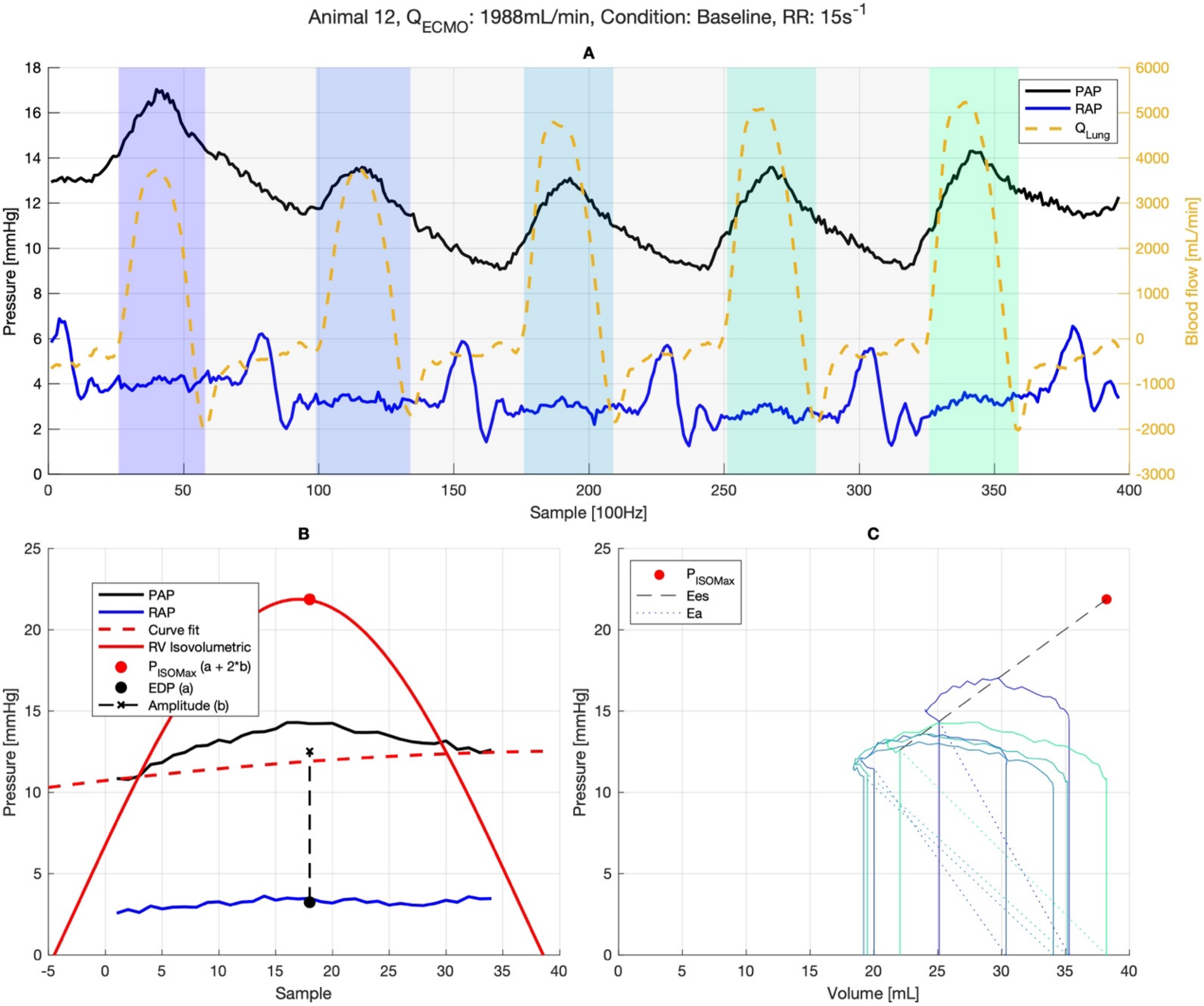

Animal 12, Q_{ECMO}: 1988mL/min, Condition: Baseline, RR: 15s^{-1}
A
Pressure [mmHg]
Blood flow [mL/min]
Sample [100Hz]
PAP
RAP
Q_{Lung}
B
PAP
RAP
Curve fit
RV Isovolumetric
P_{ISOMax} (a + 2*b)
EDP (a)
Amplitude (b)
Pressure [mmHg]
Sample
C
P_{ISOMax}
Ees
Ea
Pressure [mmHg]
Volume [mL]

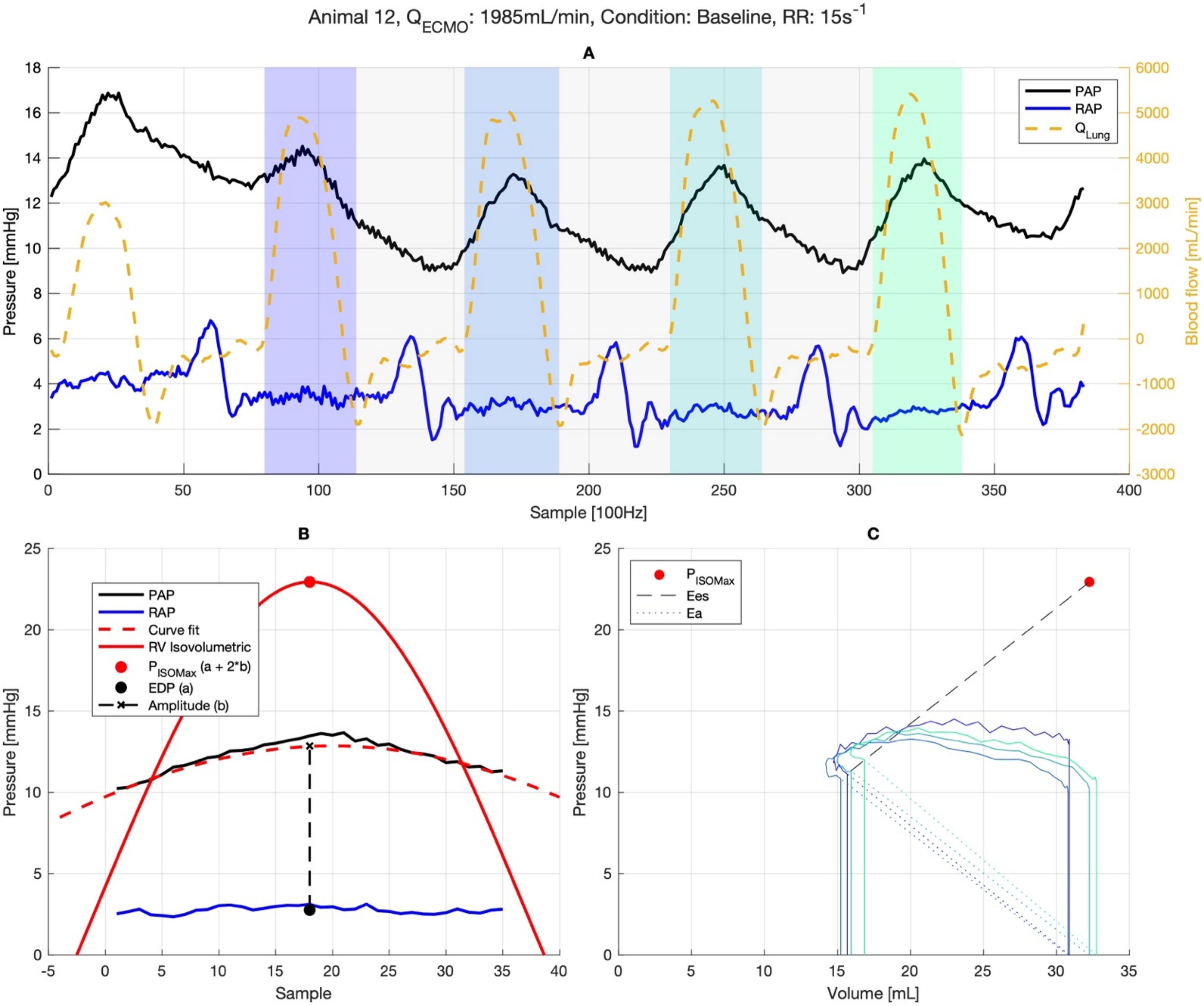

Animal 12, Q_ECMO: 1985mL/min, Condition: Baseline, RR: 15s^-1
A
PAP
RAP
Q_Lung
Pressure [mmHg]
Blood flow [mL/min]
Sample [100Hz]
B
PAP
RAP
Curve fit
RV Isovolumetric
P_ISOMax (a + 2*b)
EDP (a)
Amplitude (b)
Pressure [mmHg]
Sample
C
P_ISOMax
Ees
Ea
Pressure [mmHg]
Volume [mL]

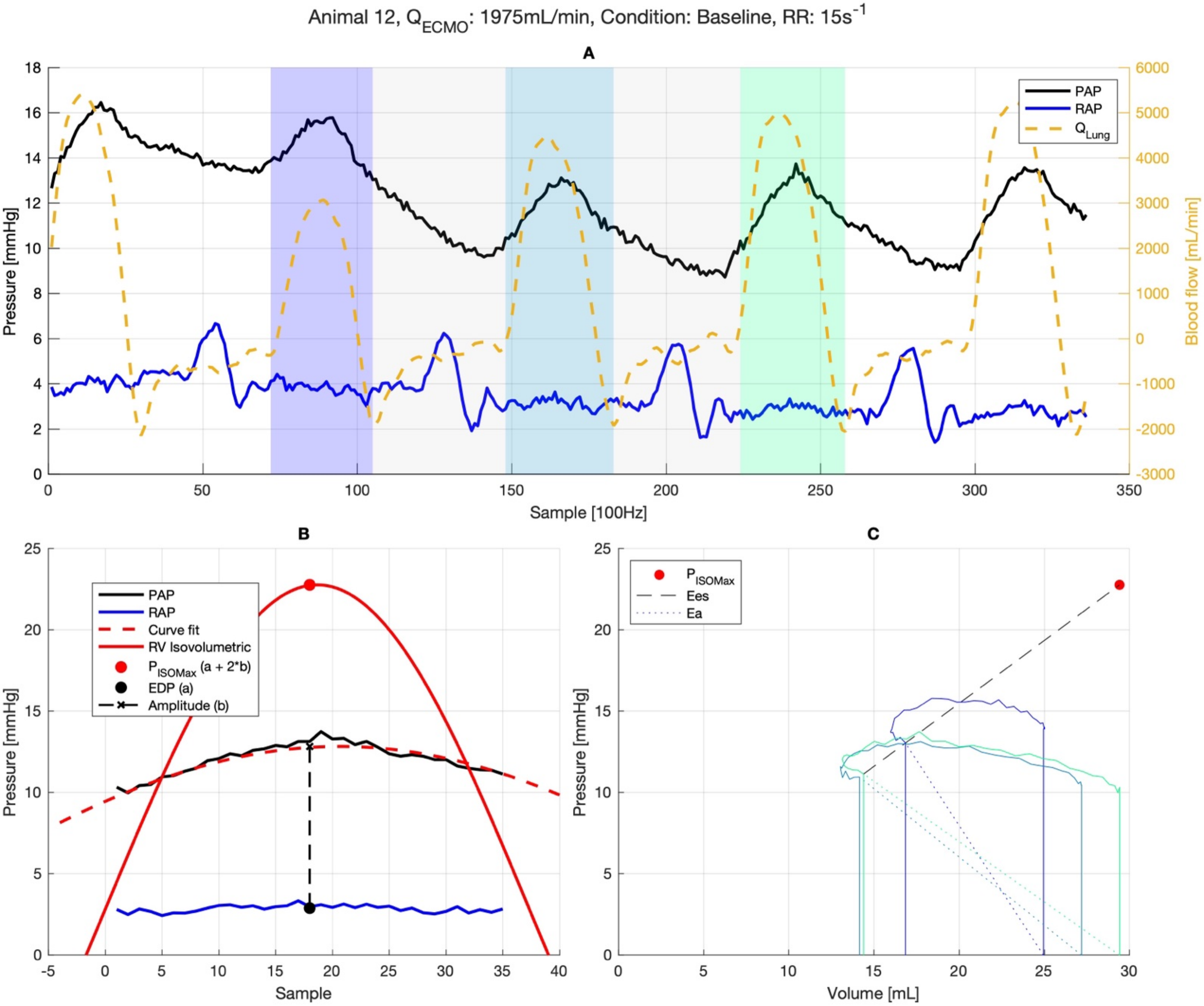

Animal 12, Q_{ECMO}: 1975mL/min, Condition: Baseline, RR: $15s^{-1}$
A
PAP
RAP
Q_{Lung}
Pressure [mmHg]
Blood flow [mL/min]
Sample [100Hz]
B
PAP
RAP
Curve fit
RV Isovolumetric
P_{ISOMax} (a + 2*b)
EDP (a)
Amplitude (b)
Pressure [mmHg]
Sample
C
P_{ISOMax}
Ees
Ea
Pressure [mmHg]
Volume [mL]

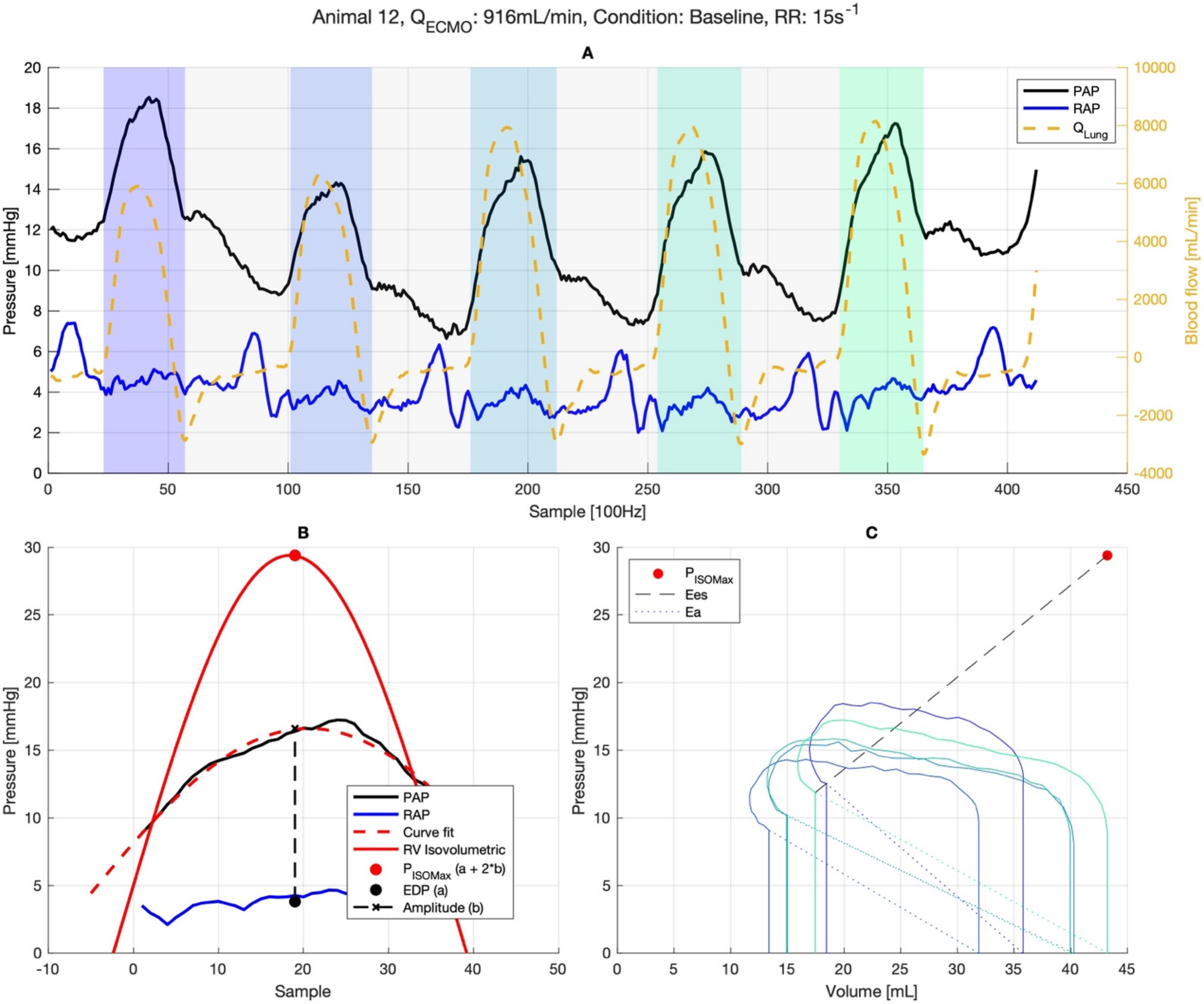

Animal 12, Q_{ECMO}: 916mL/min, Condition: Baseline, RR: $15s^{-1}$
A
PAP
RAP
Q_{Lung}
Pressure [mmHg]
Blood flow [mL/min]
Sample [100Hz]
B
PAP
RAP
Curve fit
RV Isovolumetric
P_{ISOMax} (a + 2*b)
EDP (a)
Amplitude (b)
Pressure [mmHg]
Sample
C
P_{ISOMax}
Ees
Ea
Pressure [mmHg]
Volume [mL]

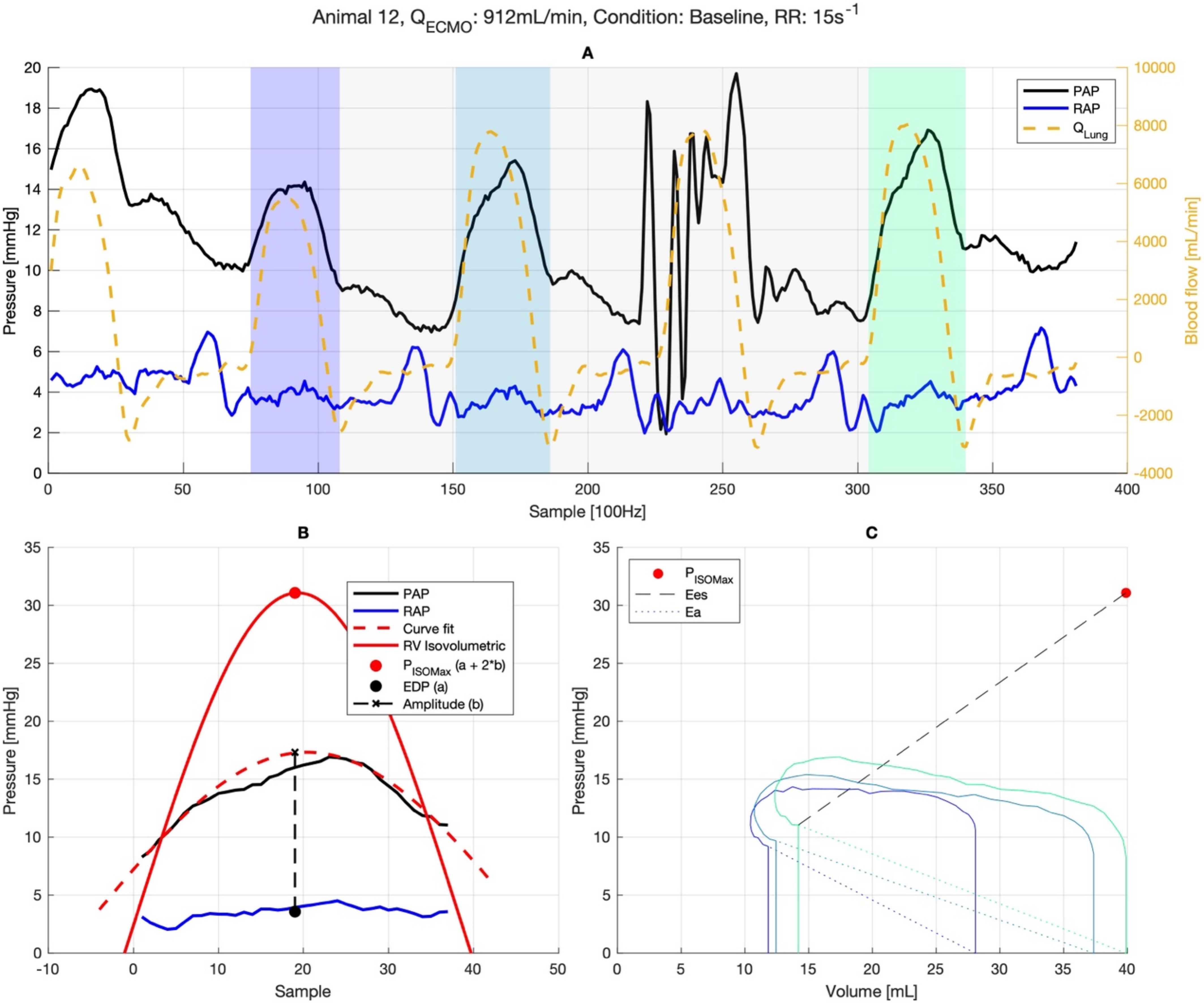

Animal 12, Q_{ECMO}: 912mL/min, Condition: Baseline, RR: $15s^{-1}$
A
PAP
RAP
Q_{Lung}
Pressure [mmHg]
Blood flow [mL/min]
Sample [100Hz]
B
PAP
RAP
Curve fit
RV Isovolumetric
P_{ISOMax} (a + 2*b)
EDP (a)
Amplitude (b)
Pressure [mmHg]
Sample
C
P_{ISOMax}
Ees
Ea
Pressure [mmHg]
Volume [mL]

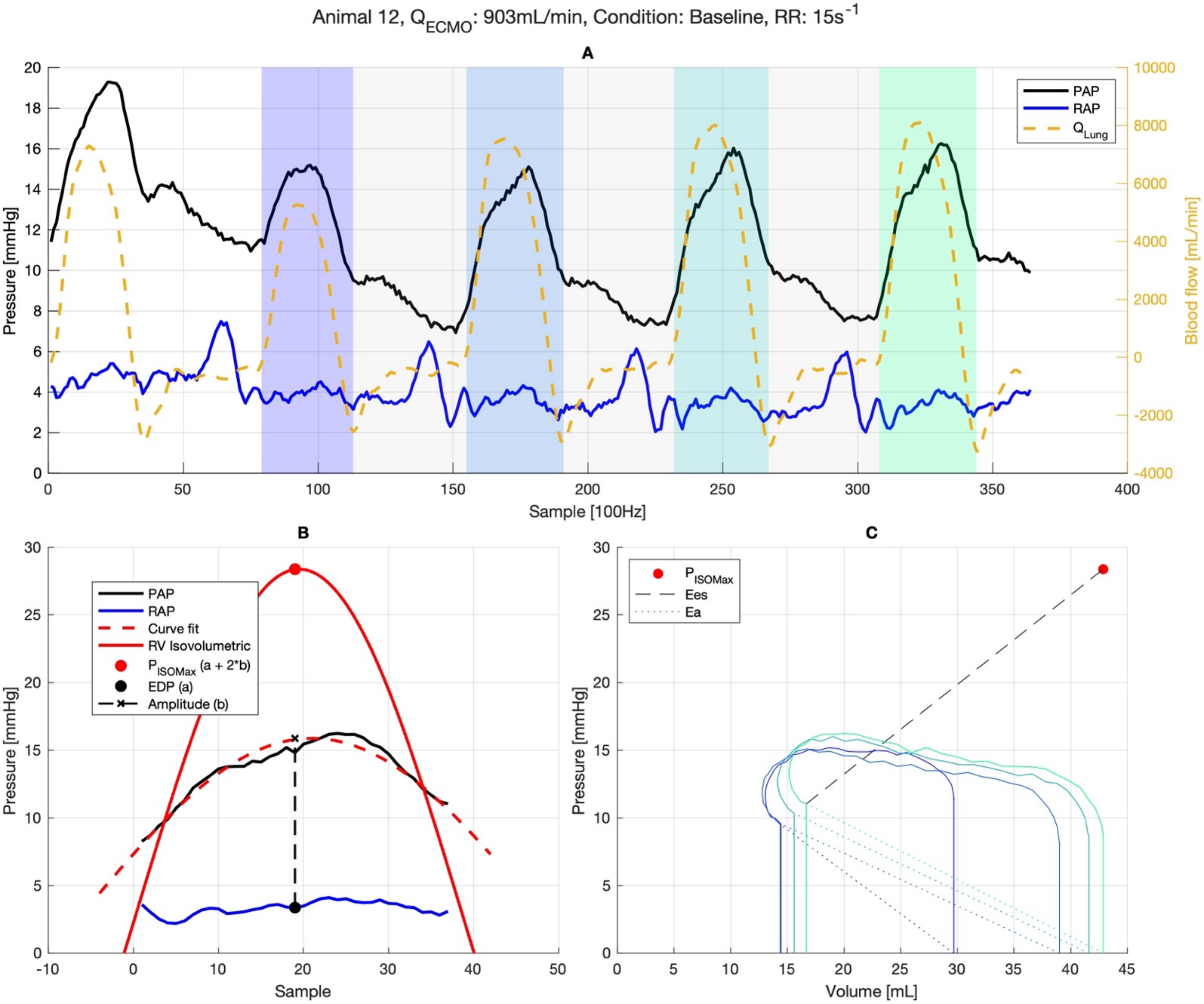

Animal 12, Q_{ECMO}: 903mL/min, Condition: Baseline, RR: $15s^{-1}$
A
PAP
RAP
Q_{Lung}
Pressure [mmHg]
Blood flow [mL/min]
Sample [100Hz]
B
PAP
RAP
Curve fit
RV Isovolumetric
P_{ISOMax} (a + 2*b)
EDP (a)
Amplitude (b)
Pressure [mmHg]
Sample
C
P_{ISOMax}
Ees
Ea
Pressure [mmHg]
Volume [mL]

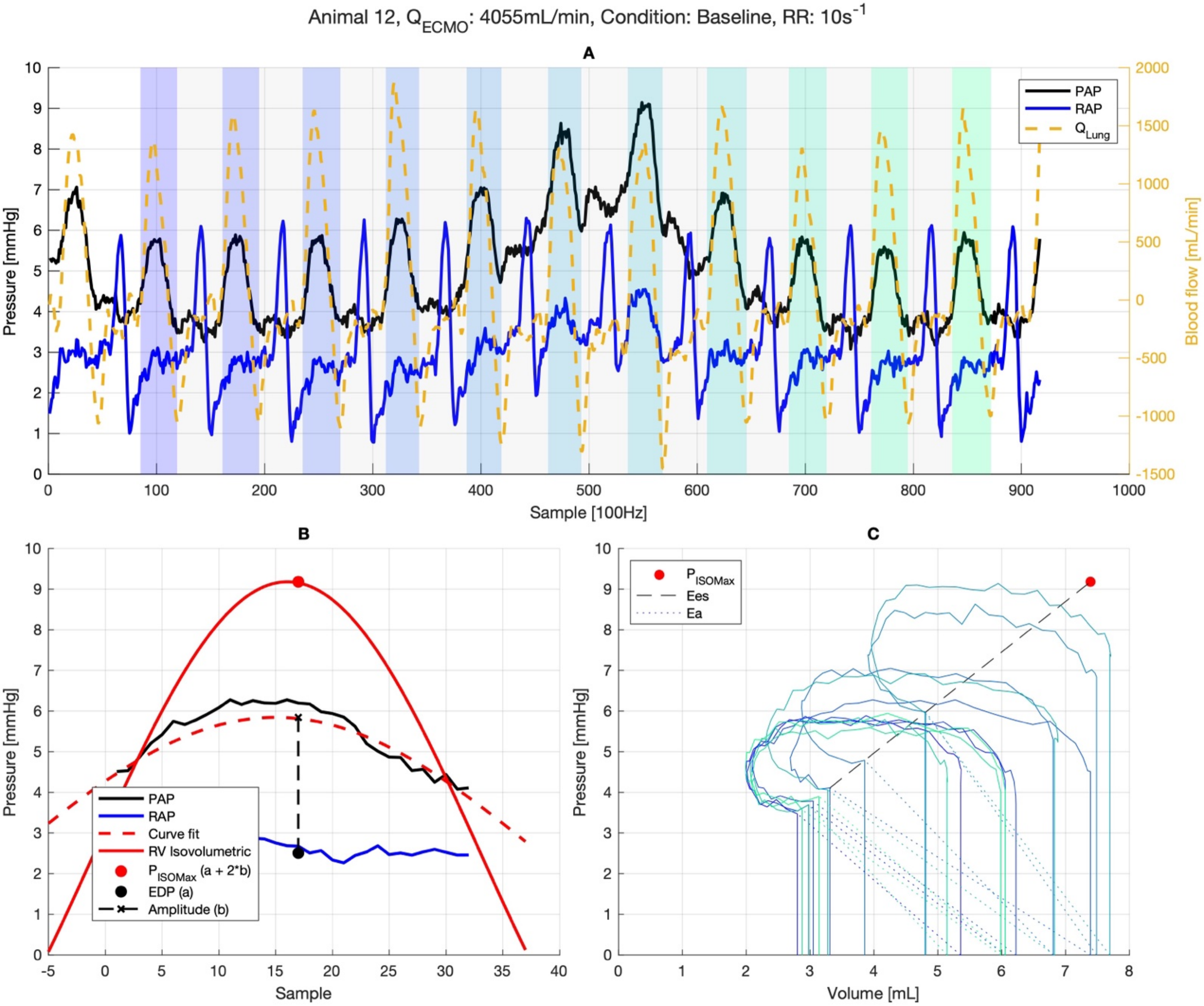
Animal 12, Q_{ECMO}: 4055mL/min, Condition: Baseline, RR: $10s^{-1}$
A
PAP
RAP
Q_{Lung}
Pressure [mmHg]
Blood flow [mL/min]
Sample [100Hz]
B
PAP
RAP
Curve fit
RV Isovolumetric
P_{ISOMax} (a + 2*b)
EDP (a)
Amplitude (b)
Pressure [mmHg]
Sample
C
P_{ISOMax}
Ees
Ea
Pressure [mmHg]
Volume [mL]

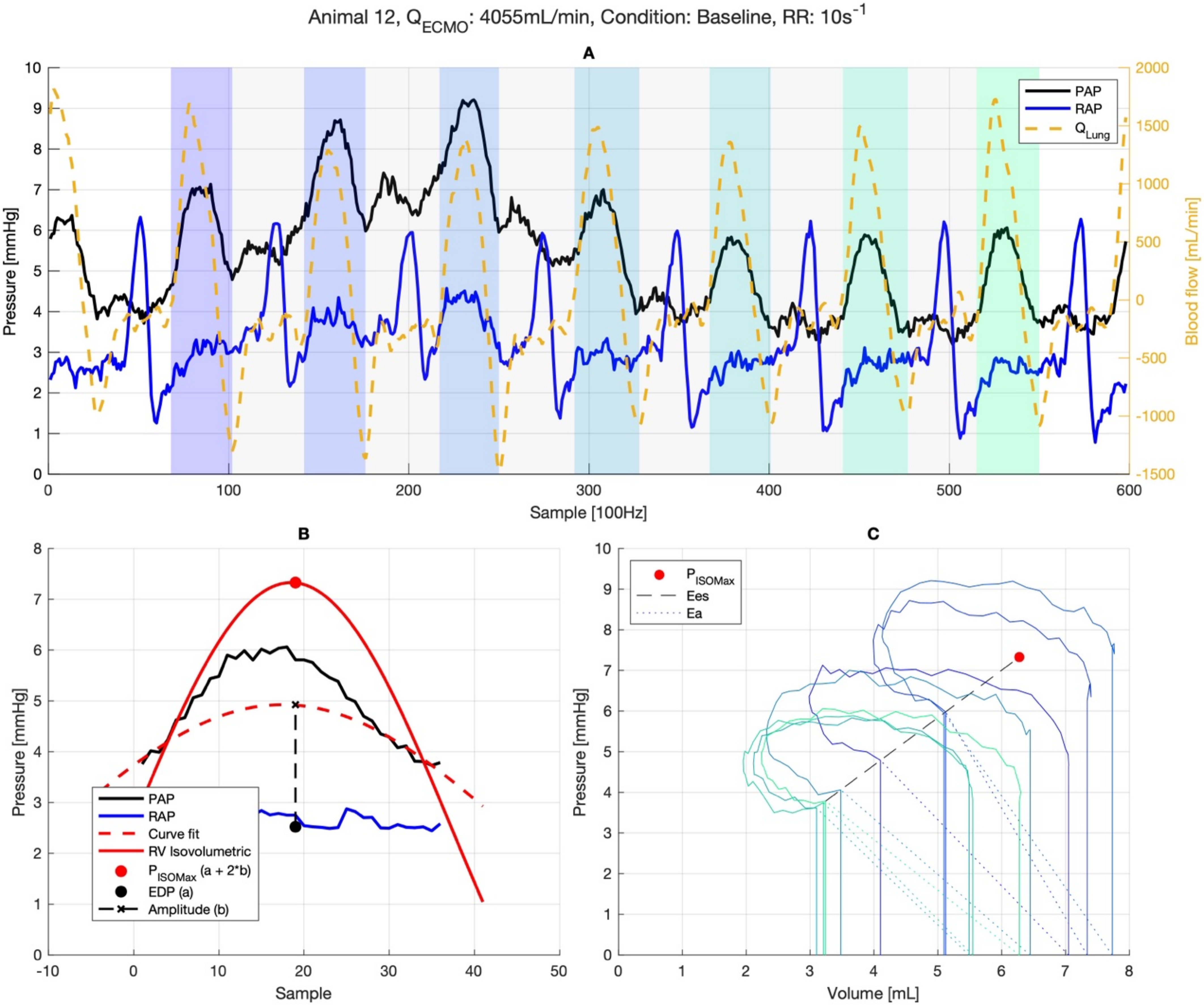

Animal 12, Q_{ECMO}: 4055mL/min, Condition: Baseline, RR: $10s^{-1}$
A
Pressure [mmHg]
Blood flow [mL/min]
Sample [100Hz]
PAP
RAP
Q_{Lung}
B
Pressure [mmHg]
Sample
PAP
RAP
Curve fit
RV Isovolumetric
P_{ISOMax} (a + 2*b)
EDP (a)
Amplitude (b)
C
Pressure [mmHg]
Volume [mL]
P_{ISOMax}
Ees
Ea

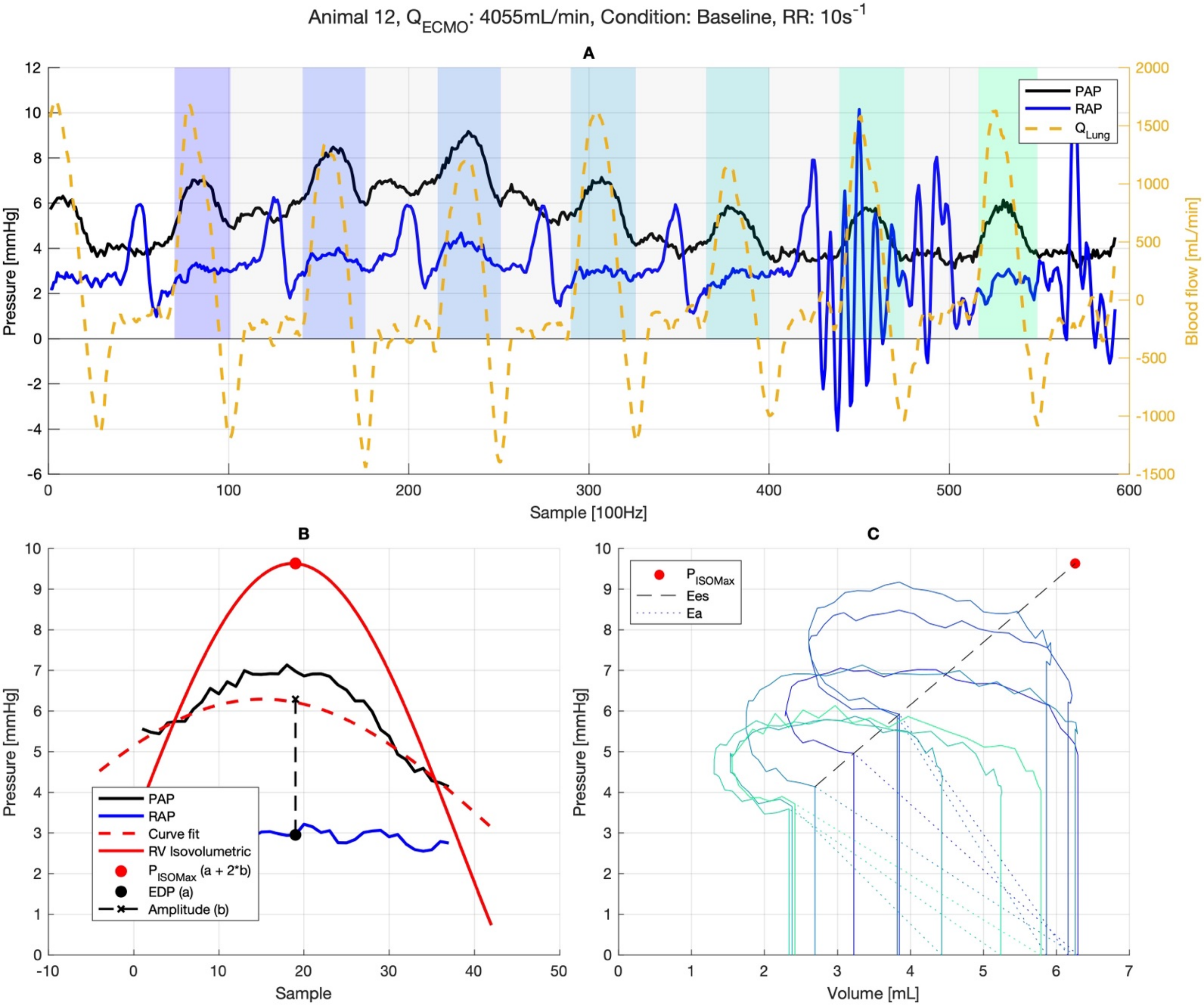

Animal 12, QECMO: 4055mL/min, Condition: Baseline, RR: 10s-1
A
PAP
RAP
QLung
Pressure [mmHg]
Blood flow [mL/min]
Sample [100Hz]
B
PAP
RAP
Curve fit
RV Isovolumetric
PISOMax (a + 2*b)
EDP (a)
Amplitude (b)
Pressure [mmHg]
Sample
C
PISOMax
Ees
Ea
Pressure [mmHg]
Volume [mL]

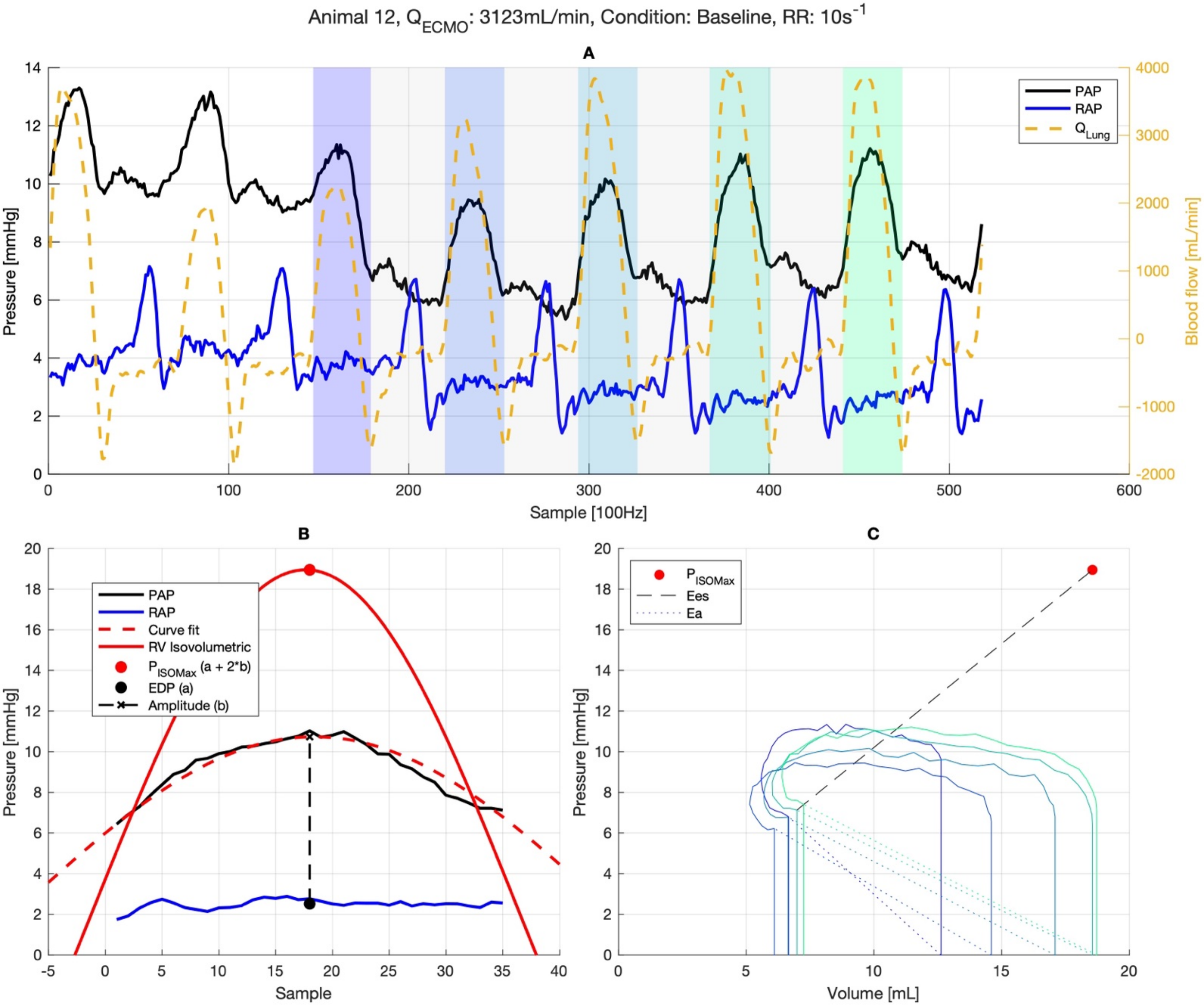

Animal 12, Q_ECMO: 3123mL/min, Condition: Baseline, RR: 10s^-1
A
PAP
RAP
Q_Lung
Pressure [mmHg]
Blood flow [mL/min]
Sample [100Hz]
B
PAP
RAP
Curve fit
RV Isovolumetric
P_ISOMax (a + 2*b)
EDP (a)
Amplitude (b)
Pressure [mmHg]
Sample
C
P_ISOMax
Ees
Ea
Pressure [mmHg]
Volume [mL]

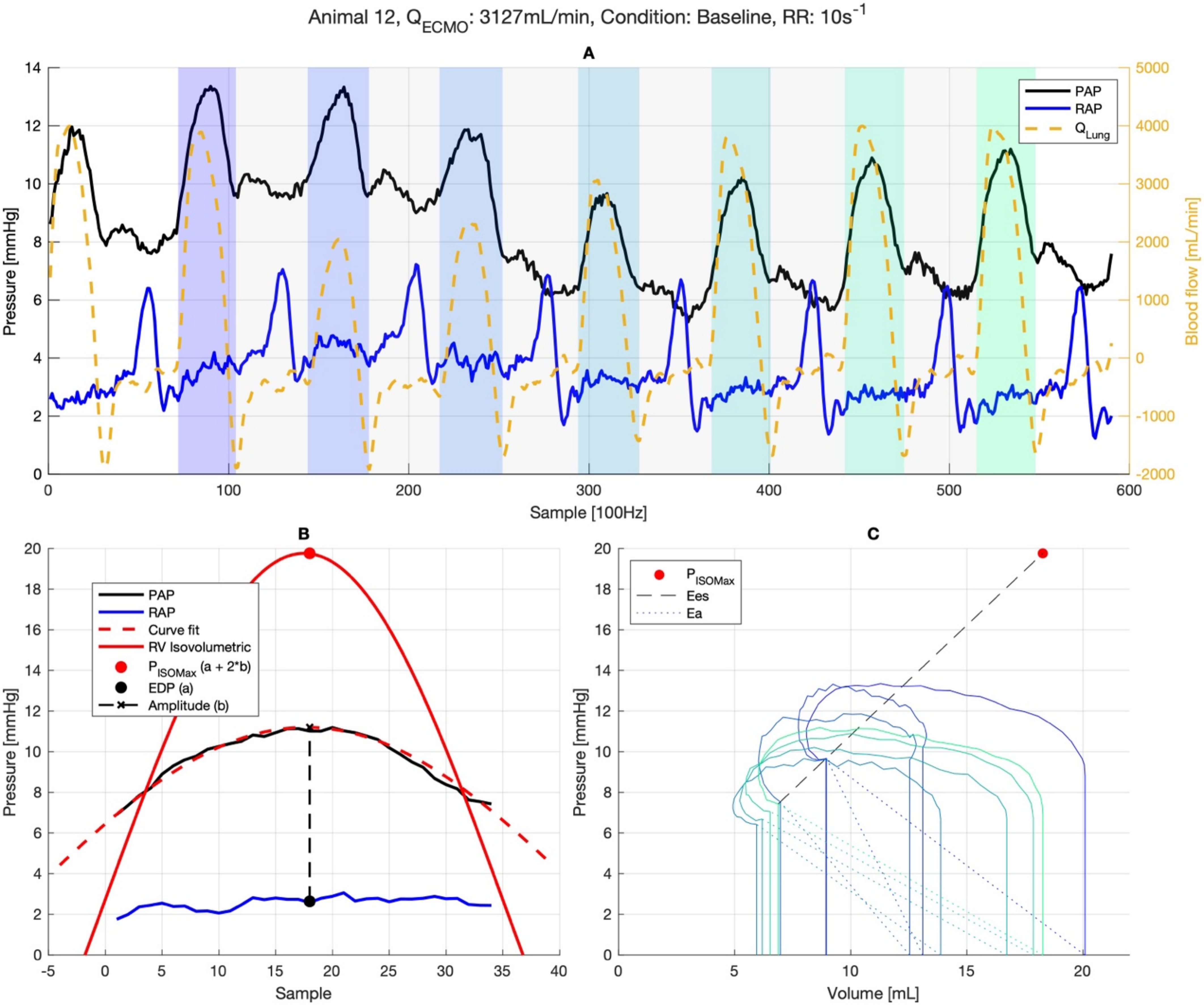
Animal 12, Q_ECMO: 3127mL/min, Condition: Baseline, RR: 10s^-1
A
PAP
RAP
Q_Lung
Pressure [mmHg]
Blood flow [mL/min]
Sample [100Hz]
B
PAP
RAP
Curve fit
RV Isovolumetric
P_ISOMax (a + 2*b)
EDP (a)
Amplitude (b)
Pressure [mmHg]
Sample
C
P_ISOMax
Ees
Ea
Pressure [mmHg]
Volume [mL]

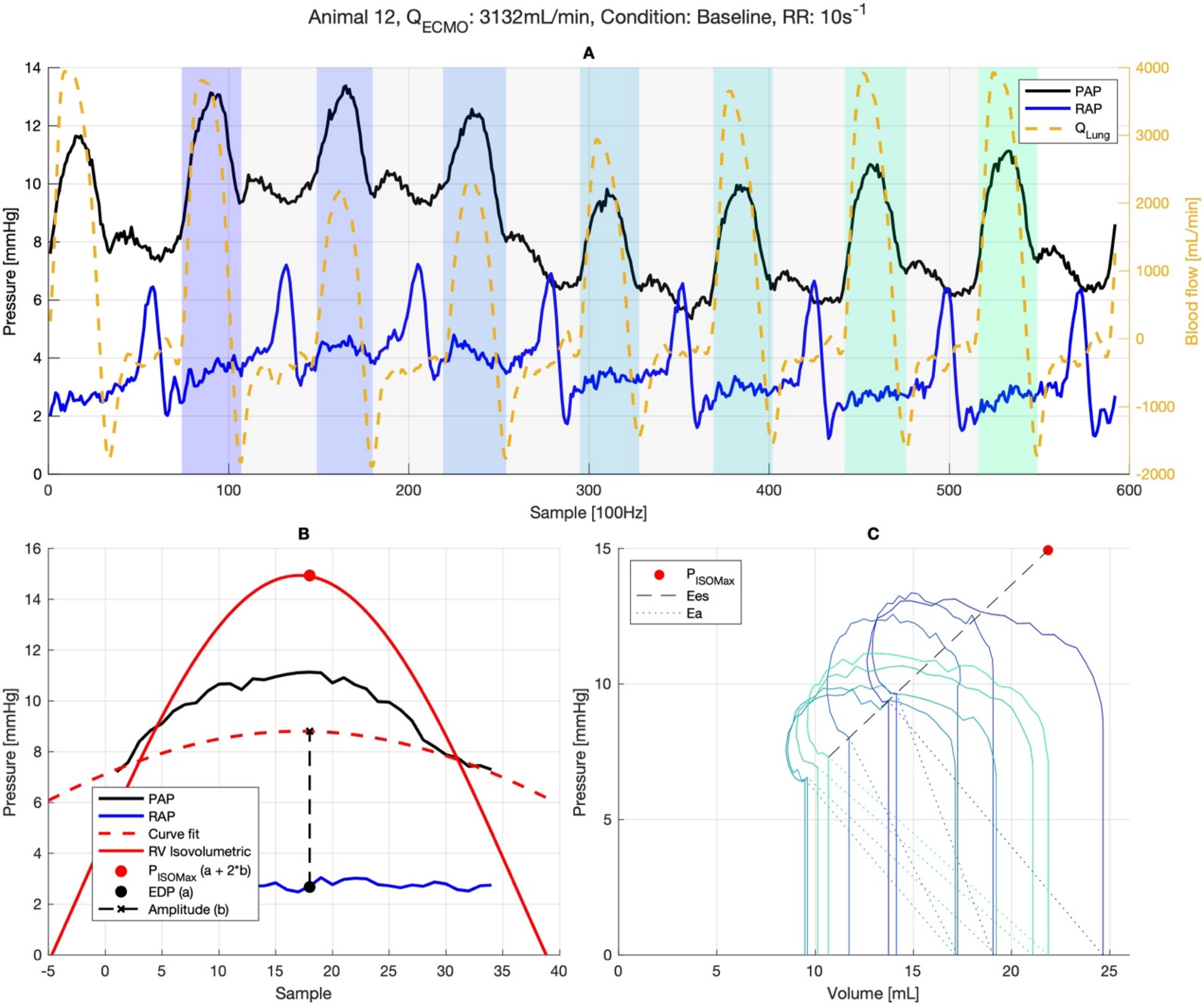

Animal 12, Q_{ECMO}: 3132mL/min, Condition: Baseline, RR: $10s^{-1}$
A
Pressure [mmHg]
Blood flow [mL/min]
Sample [100Hz]
PAP
RAP
Q_{Lung}
B
Pressure [mmHg]
Sample
PAP
RAP
Curve fit
RV Isovolumetric
P_{ISOMax} (a + 2*b)
EDP (a)
Amplitude (b)
C
Pressure [mmHg]
Volume [mL]
P_{ISOMax}
Ees
Ea

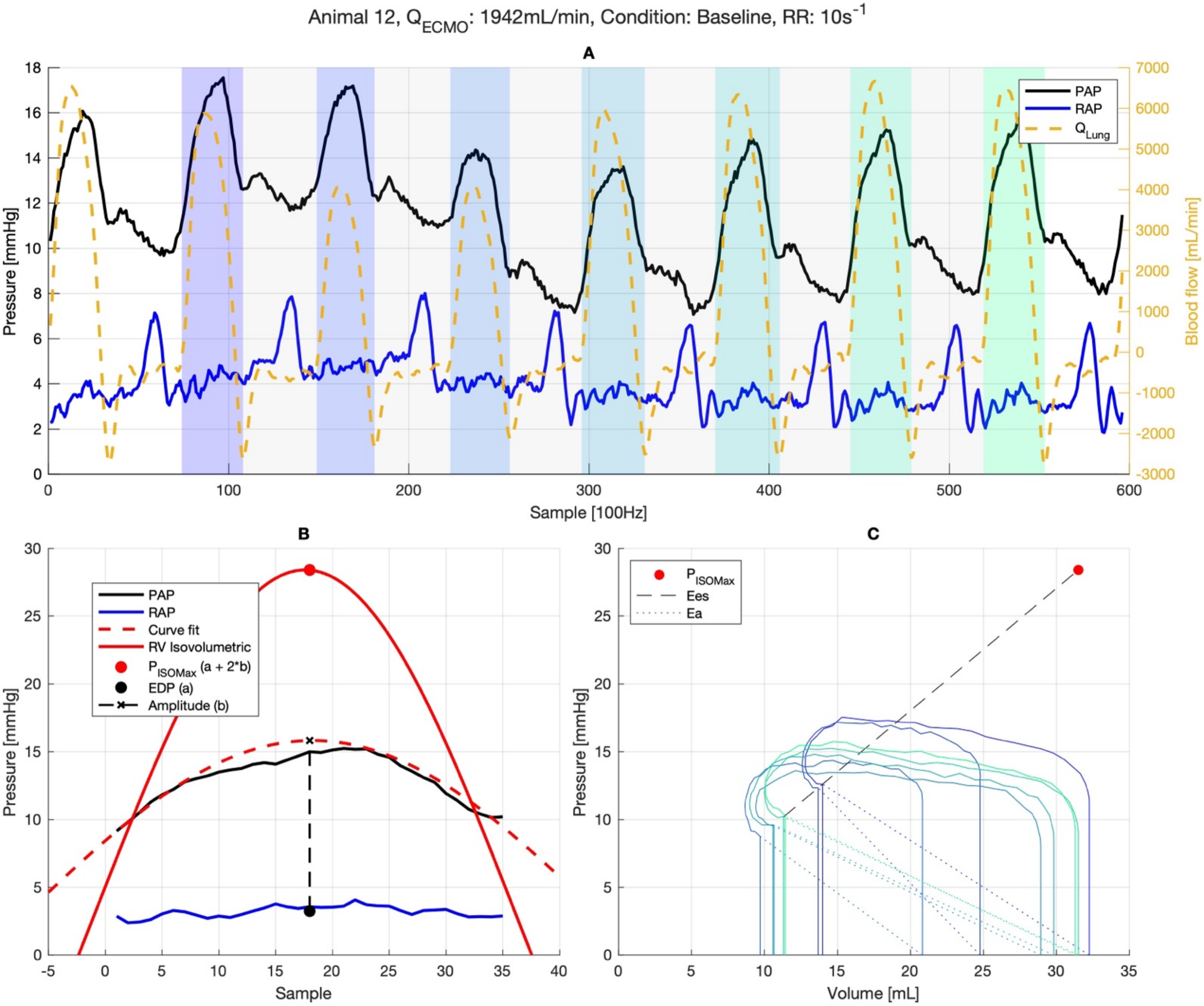

Animal 12, Q_{ECMO}: 1942mL/min, Condition: Baseline, RR: $10s^{-1}$
A
PAP
RAP
Q_{Lung}
Pressure [mmHg]
Blood flow [mL/min]
Sample [100Hz]
B
PAP
RAP
Curve fit
RV Isovolumetric
P_{ISOMax} (a + 2*b)
EDP (a)
Amplitude (b)
Pressure [mmHg]
Sample
C
P_{ISOMax}
Ees
Ea
Pressure [mmHg]
Volume [mL]

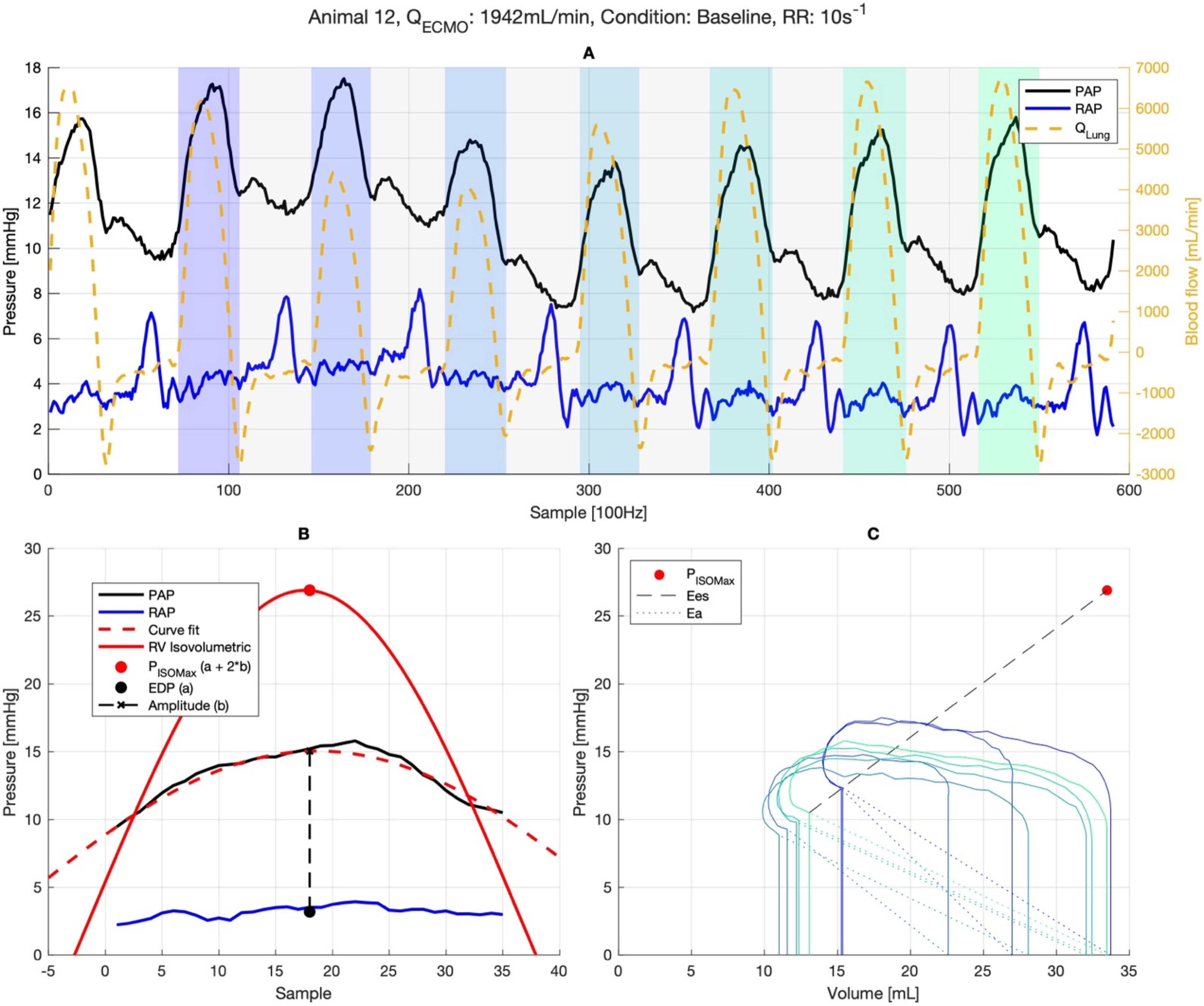

Animal 12, Q_{ECMO}: 1942mL/min, Condition: Baseline, RR: $10s^{-1}$
A
Pressure [mmHg]
Blood flow [mL/min]
Sample [100Hz]
PAP
RAP
Q_{Lung}
B
Pressure [mmHg]
Sample
PAP
RAP
Curve fit
RV Isovolumetric
P_{ISOMax} (a + 2*b)
EDP (a)
Amplitude (b)
C
Pressure [mmHg]
Volume [mL]
P_{ISOMax}
Ees
Ea

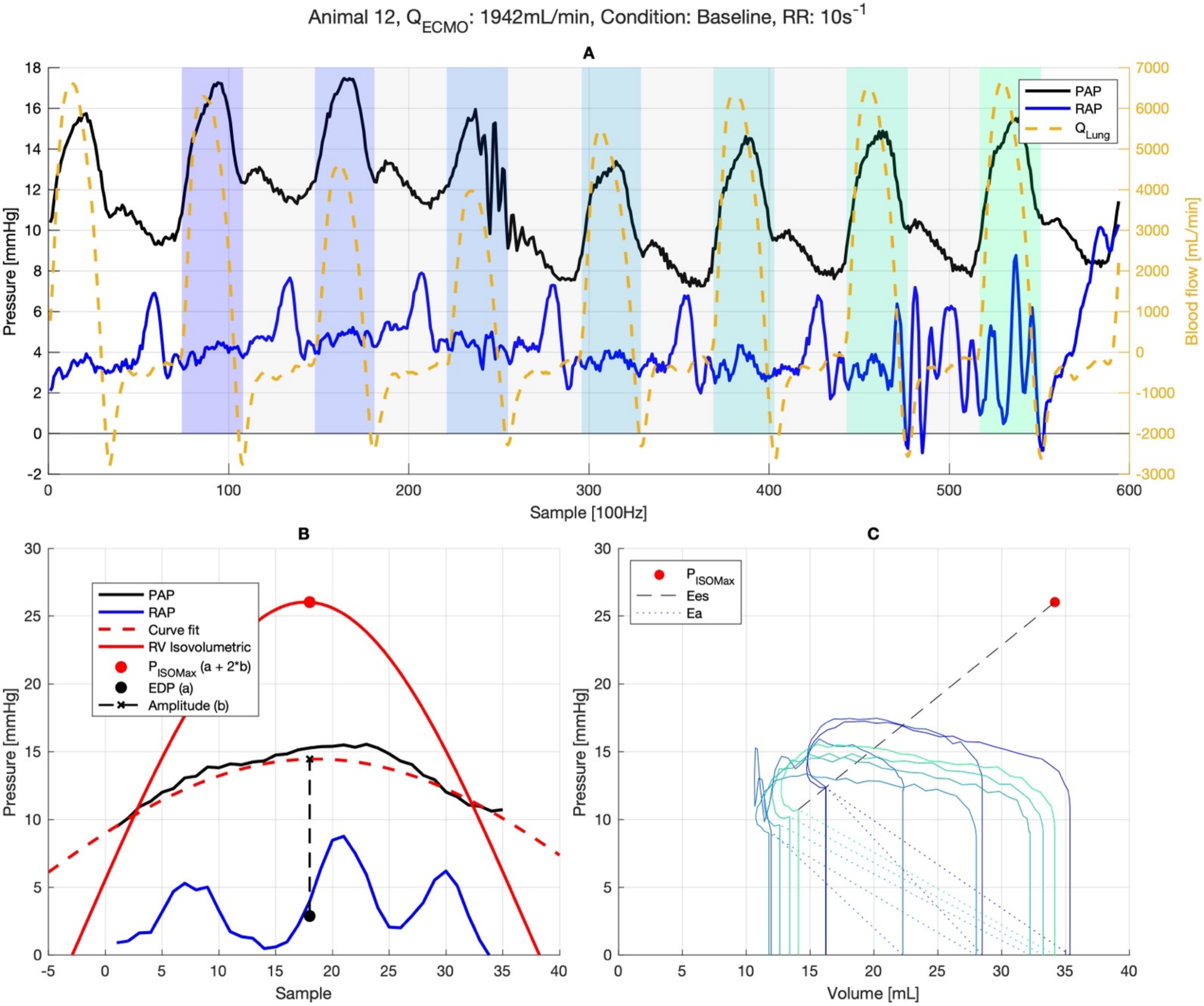
Animal 12, Q_{ECMO}: 1942mL/min, Condition: Baseline, RR: $10s^{-1}$
A
PAP
RAP
Q_{Lung}
Pressure [mmHg]
Blood flow [mL/min]
Sample [100Hz]
B
PAP
RAP
Curve fit
RV Isovolumetric
P_{ISOMax} (a + 2*b)
EDP (a)
Amplitude (b)
Pressure [mmHg]
Sample
C
P_{ISOMax}
Ees
Ea
Pressure [mmHg]
Volume [mL]

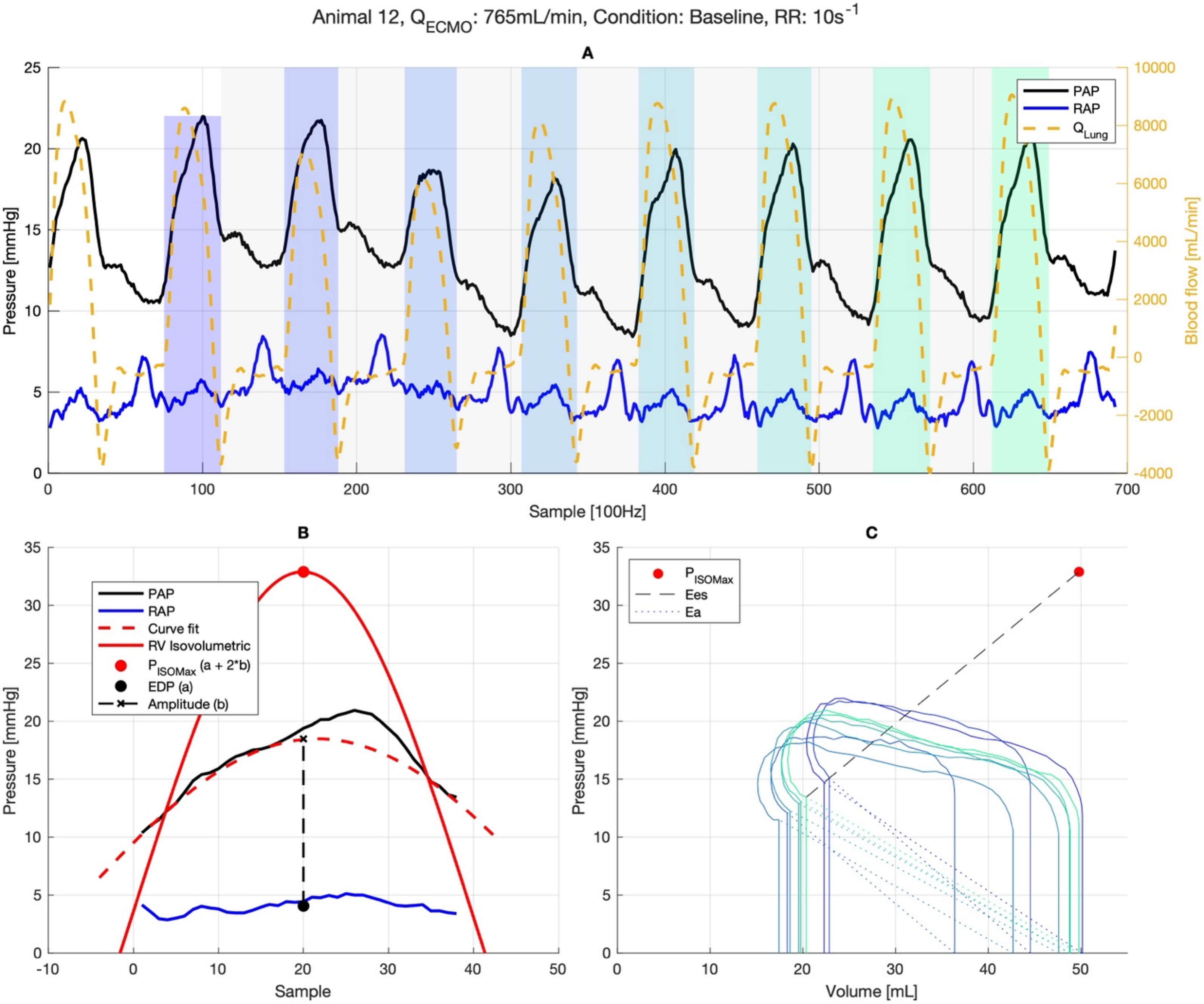

Animal 12, Q_{ECMO}: 765mL/min, Condition: Baseline, RR: $10s^{-1}$
A
PAP
RAP
Q_{Lung}
Pressure [mmHg]
Blood flow [mL/min]
Sample [100Hz]
B
PAP
RAP
Curve fit
RV Isovolumetric
P_{ISOMax} (a + 2*b)
EDP (a)
Amplitude (b)
Pressure [mmHg]
Sample
C
P_{ISOMax}
Ees
Ea
Pressure [mmHg]
Volume [mL]

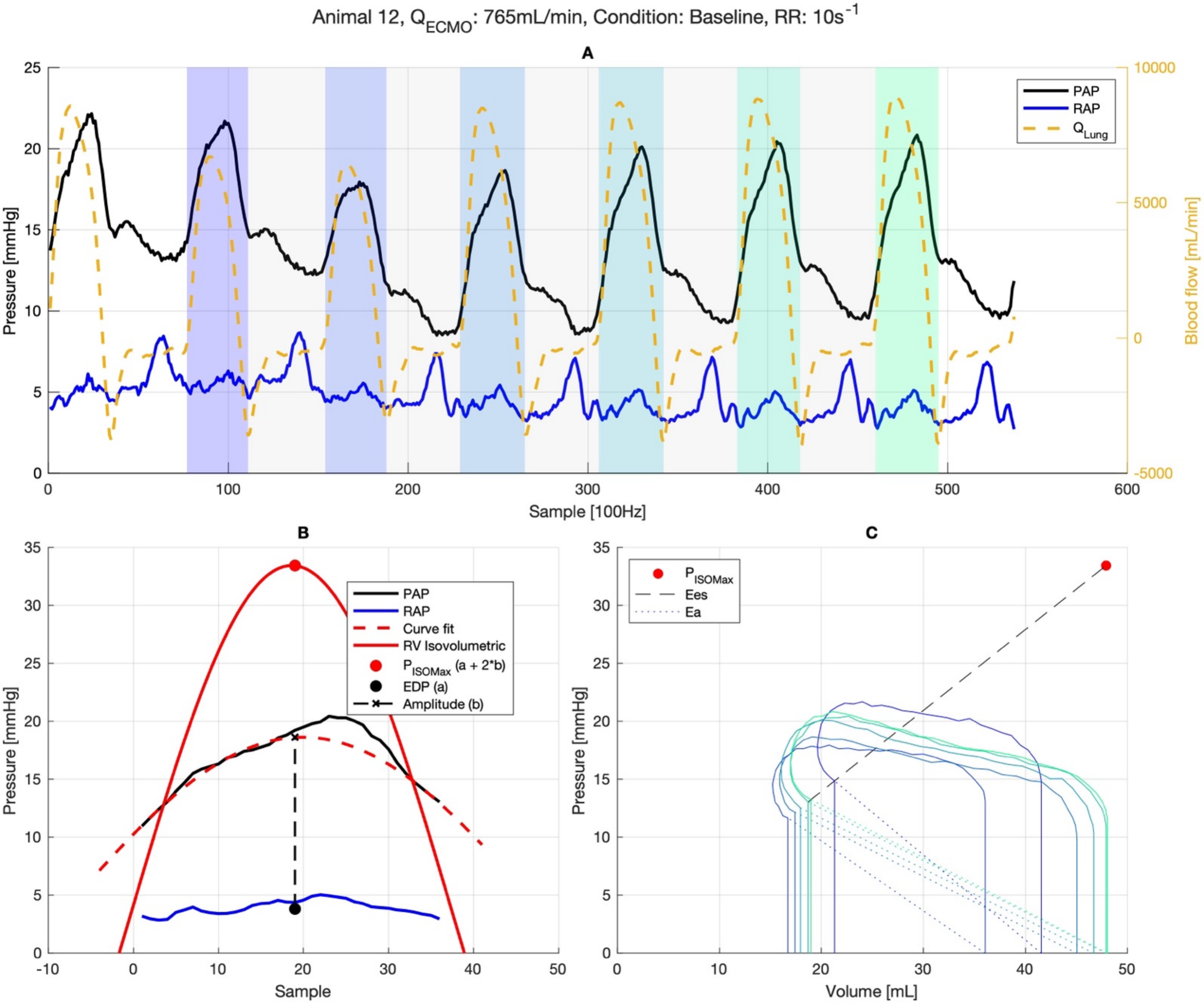

Animal 12, QECMO: 765mL/min, Condition: Baseline, RR: 10s-1
A
PAP
RAP
QLung
Pressure [mmHg]
Blood flow [mL/min]
Sample [100Hz]
B
PAP
RAP
Curve fit
RV Isovolumetric
PISOMax (a + 2*b)
EDP (a)
Amplitude (b)
Pressure [mmHg]
Sample
C
PISOMax
Ees
Ea
Pressure [mmHg]
Volume [mL]

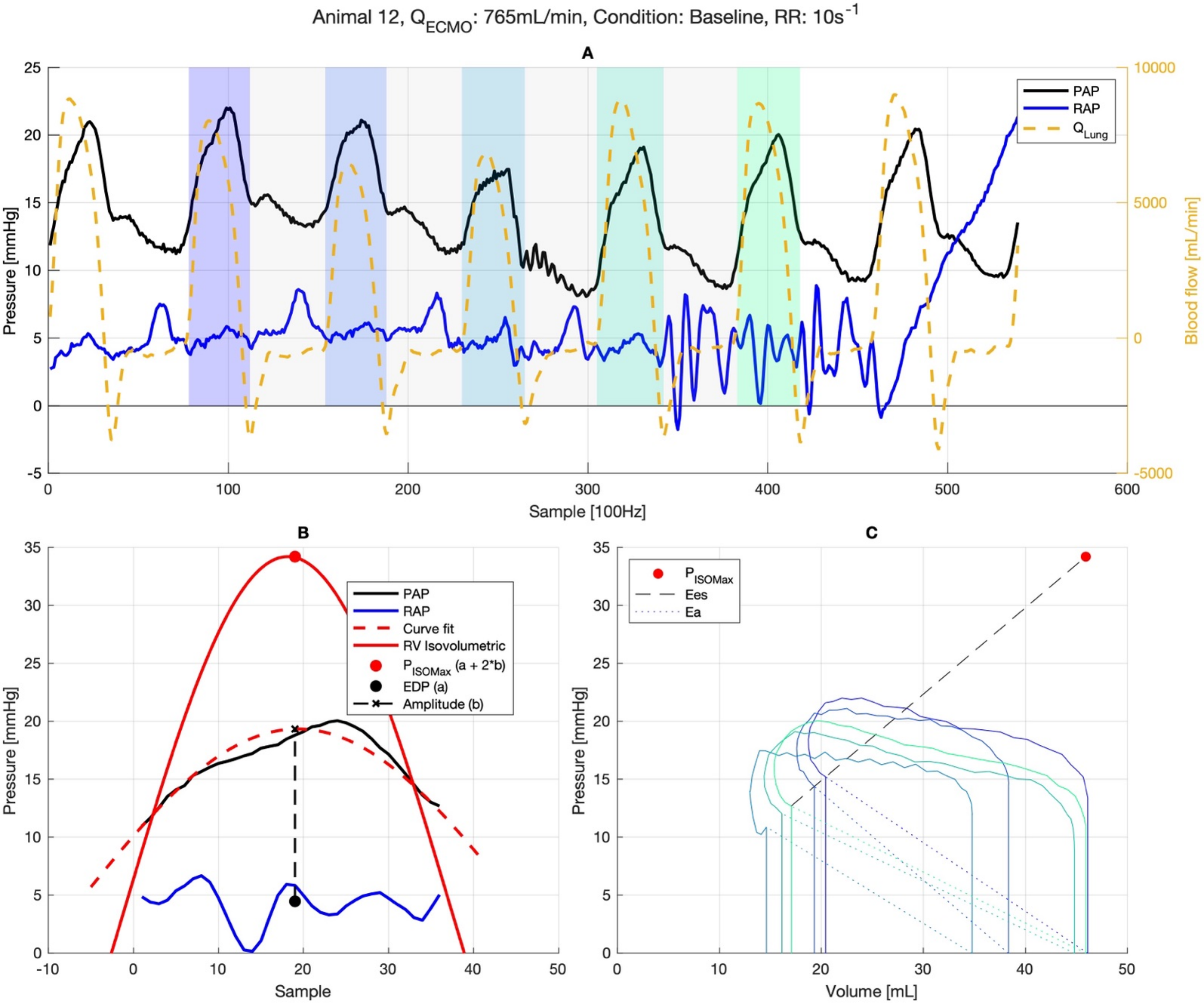

Animal 12, Q_ECMO: 765mL/min, Condition: Baseline, RR: 10s^-1
A
PAP
RAP
Q_Lung
Pressure [mmHg]
Blood flow [mL/min]
Sample [100Hz]
B
PAP
RAP
Curve fit
RV Isovolumetric
P_ISOMax (a + 2*b)
EDP (a)
Amplitude (b)
Pressure [mmHg]
Sample
C
P_ISOMax
Ees
Ea
Pressure [mmHg]
Volume [mL]

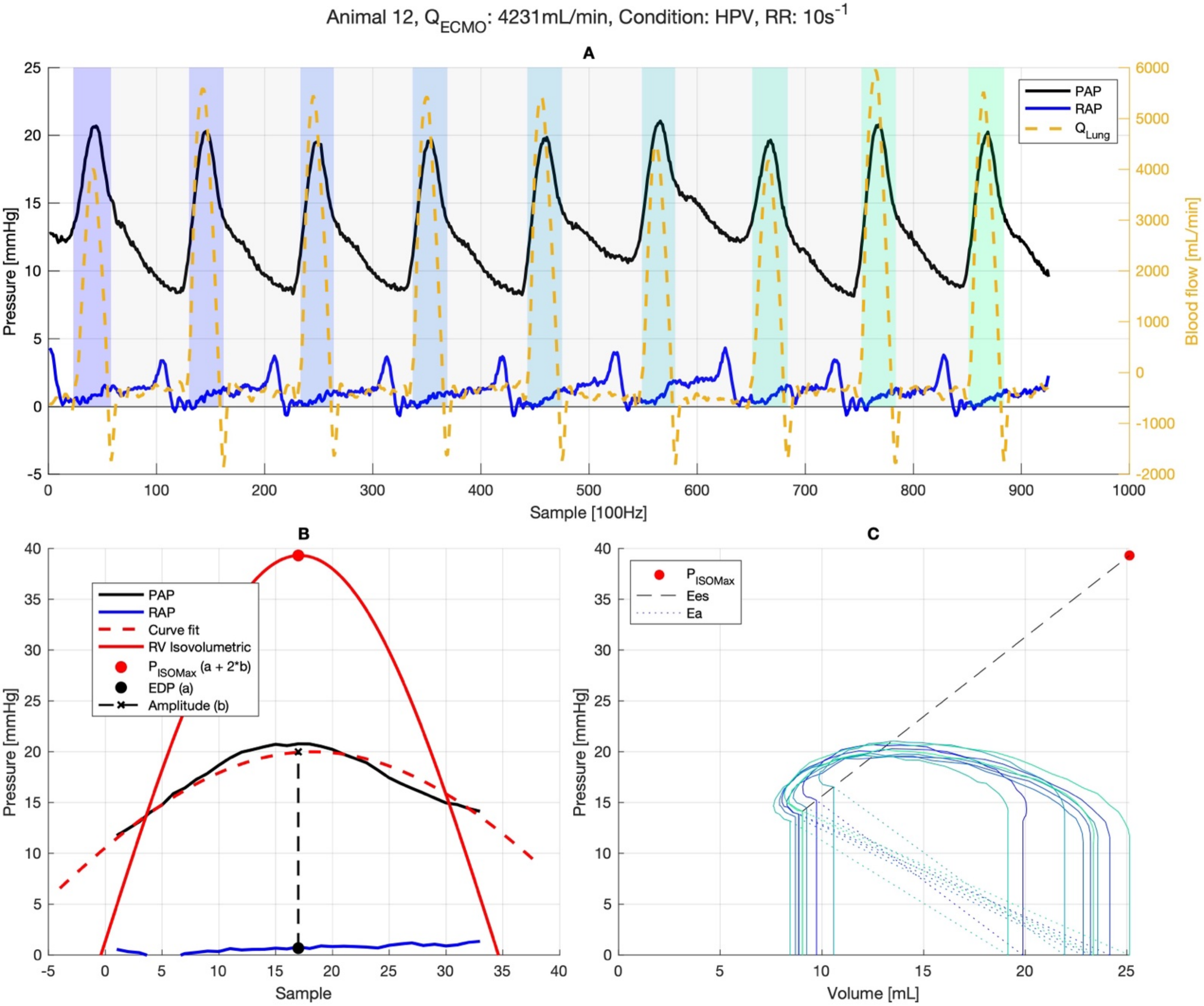

Animal 12, Q_{ECMO}: 4231mL/min, Condition: HPV, RR: $10s^{-1}$
A
PAP
RAP
Q_{Lung}
Pressure [mmHg]
Blood flow [mL/min]
Sample [100Hz]
B
PAP
RAP
Curve fit
RV Isovolumetric
P_{ISOMax} (a + 2*b)
EDP (a)
Amplitude (b)
Pressure [mmHg]
Sample
C
P_{ISOMax}
Ees
Ea
Pressure [mmHg]
Volume [mL]

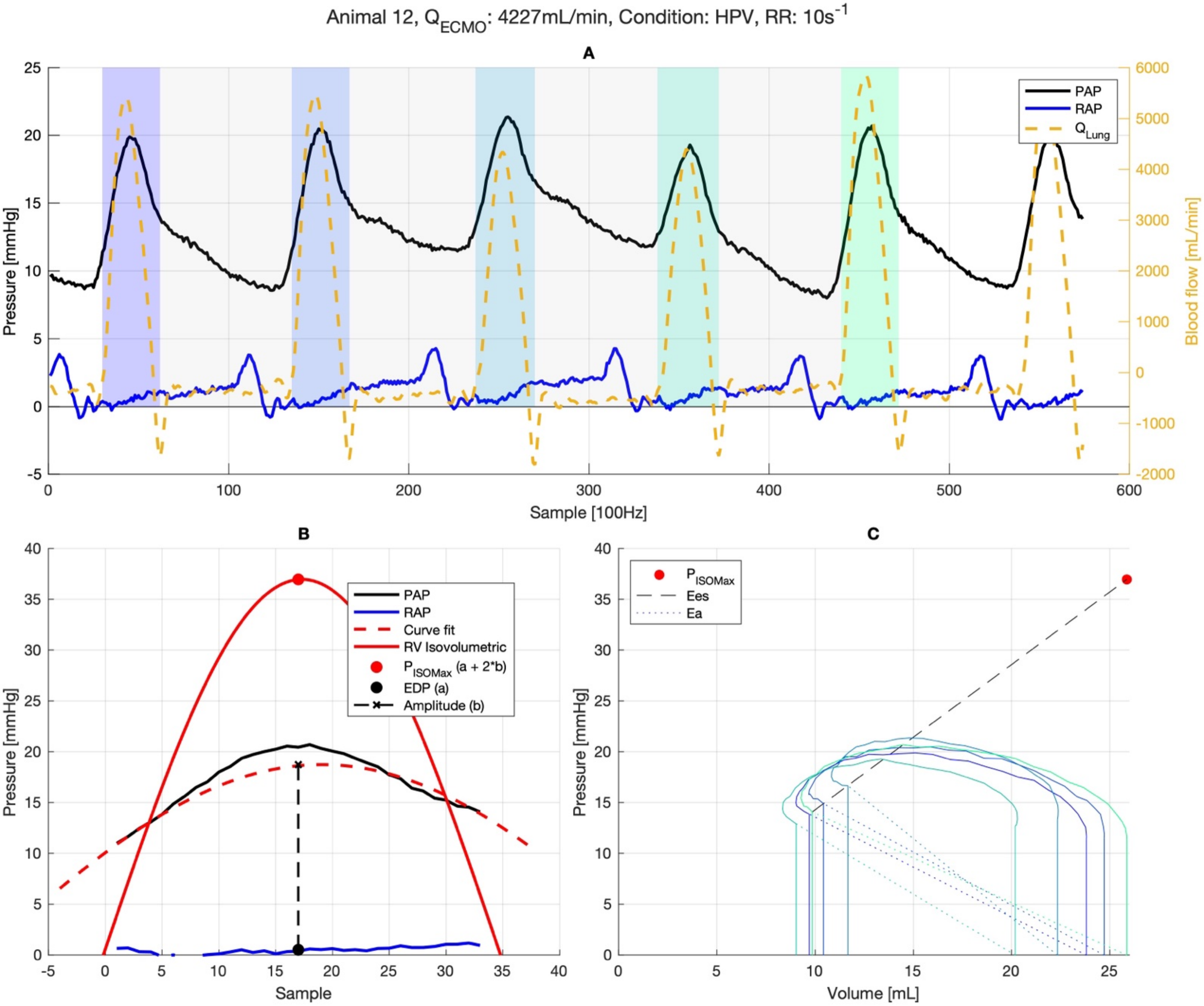
Animal 12, Q_ECMO: 4227mL/min, Condition: HPV, RR: 10s^-1
A
Pressure [mmHg]
Blood flow [mL/min]
Sample [100Hz]
PAP
RAP
Q_Lung
B
Pressure [mmHg]
Sample
PAP
RAP
Curve fit
RV Isovolumetric
P_ISOMax (a + 2*b)
EDP (a)
Amplitude (b)
C
Pressure [mmHg]
Volume [mL]
P_ISOMax
Ees
Ea

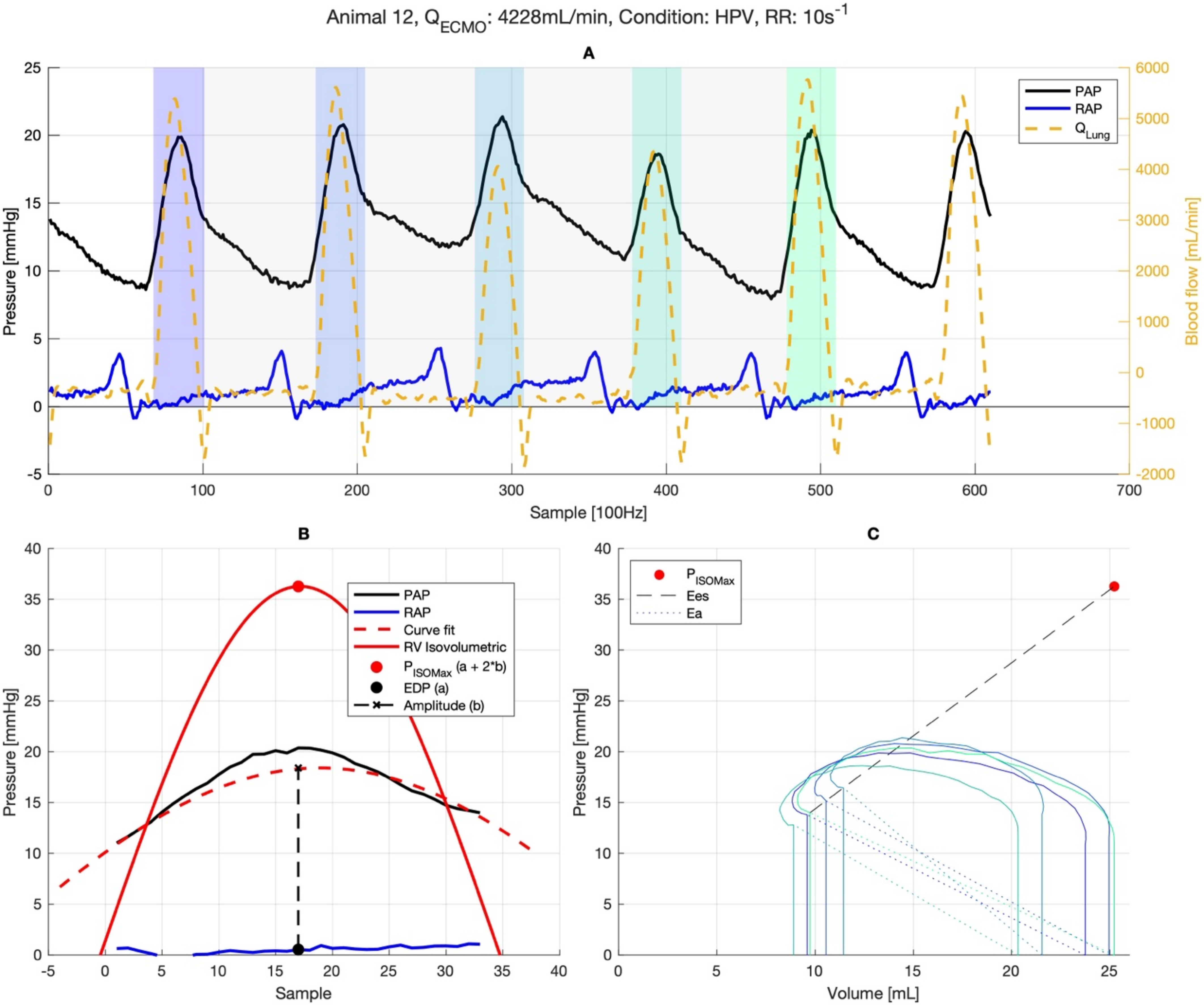

Animal 12, Q_{ECMO}: 4228mL/min, Condition: HPV, RR: $10s^{-1}$
A
PAP
RAP
Q_{Lung}
Pressure [mmHg]
Blood flow [mL/min]
Sample [100Hz]
B
PAP
RAP
Curve fit
RV Isovolumetric
P_{ISOMax} (a + 2*b)
EDP (a)
Amplitude (b)
Pressure [mmHg]
Sample
C
P_{ISOMax}
Ees
Ea
Pressure [mmHg]
Volume [mL]

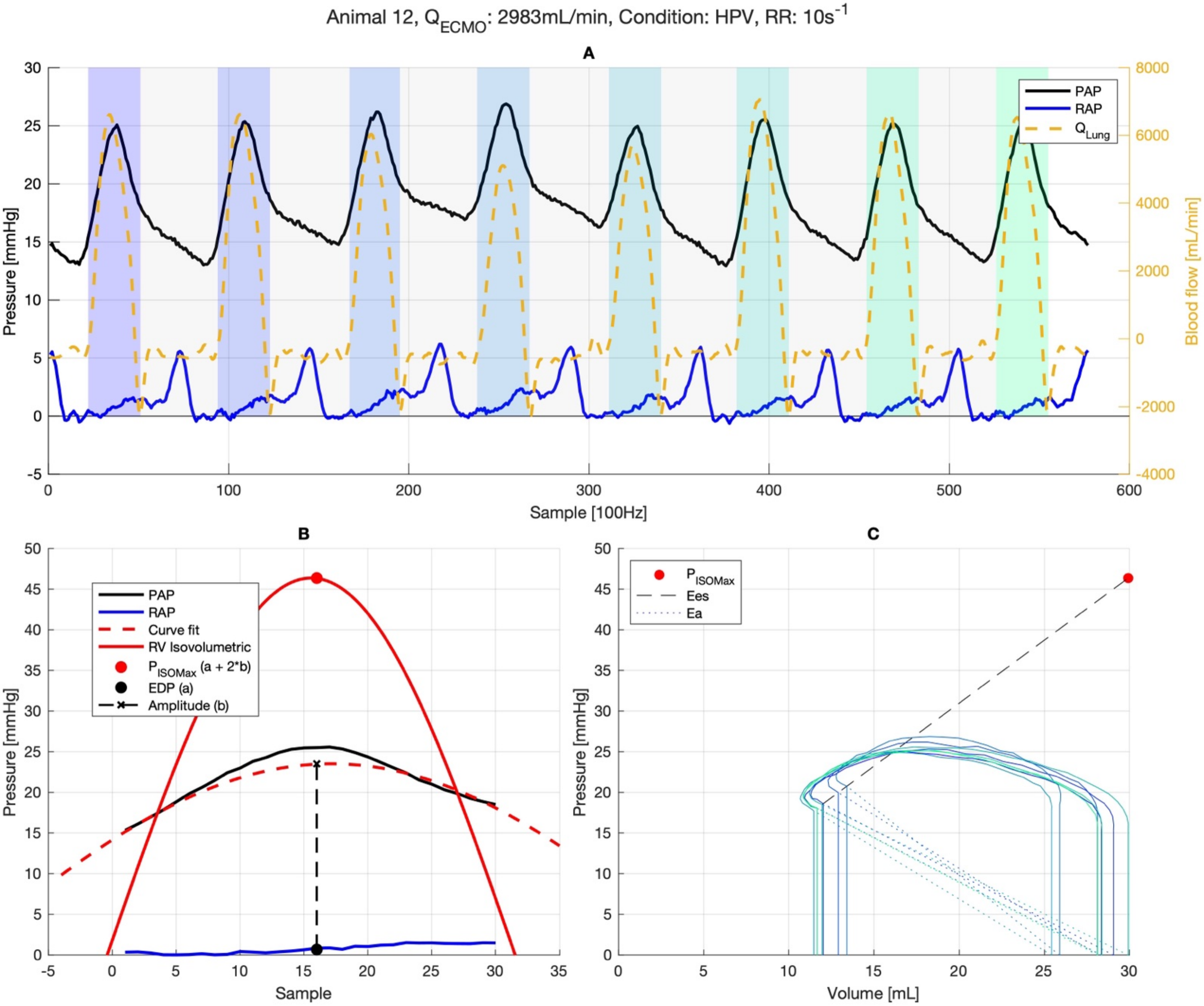
Animal 12, QECMO: 2983mL/min, Condition: HPV, RR: 10s-1
A
PAP
RAP
QLung
Pressure [mmHg]
Blood flow [mL/min]
Sample [100Hz]
B
PAP
RAP
Curve fit
RV Isovolumetric
PISOMax (a + 2*b)
EDP (a)
Amplitude (b)
Pressure [mmHg]
Sample
C
PISOMax
Ees
Ea
Pressure [mmHg]
Volume [mL]

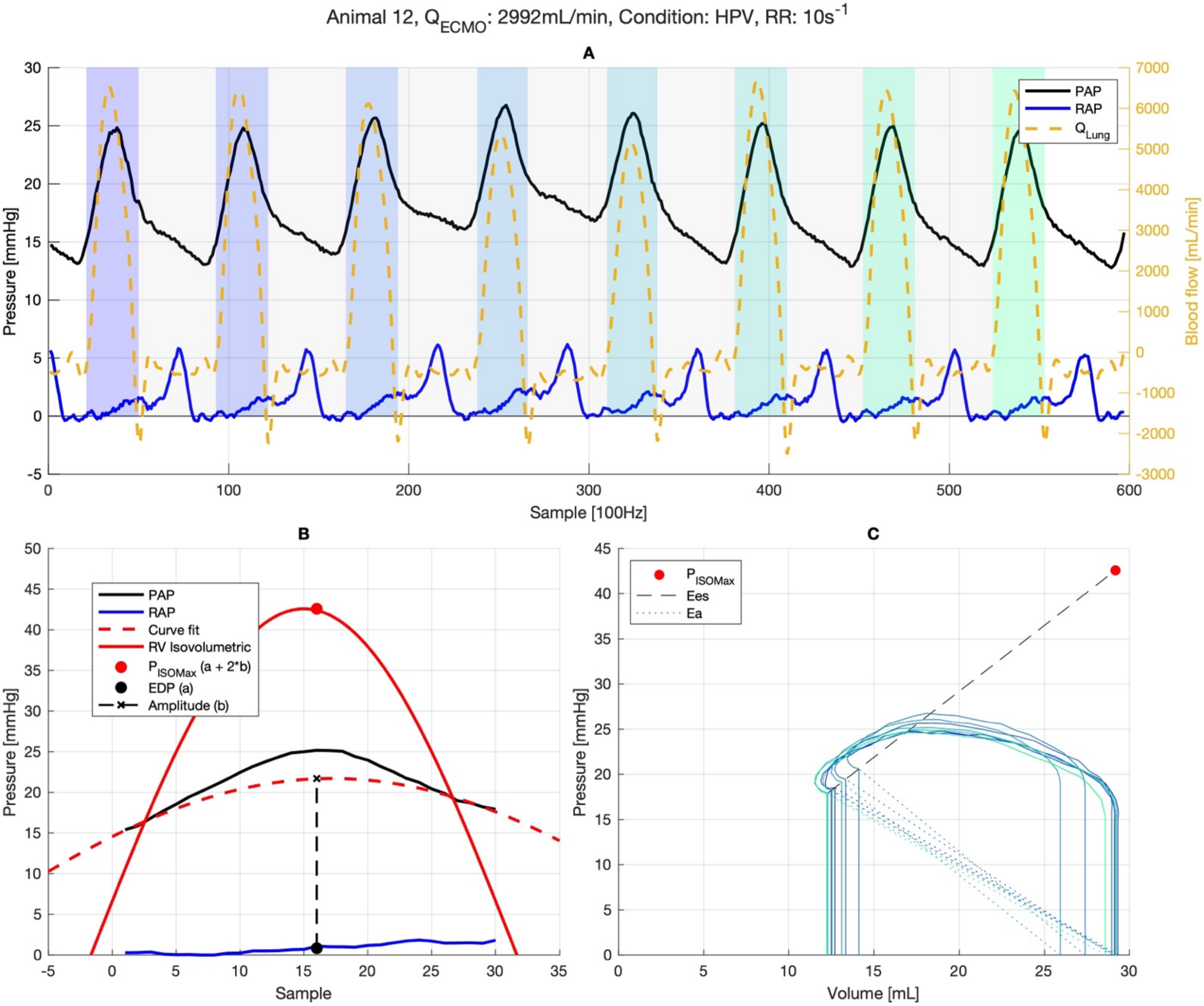

Animal 12, Q_{ECMO}: 2992mL/min, Condition: HPV, RR: $10s^{-1}$
A
PAP
RAP
Q_{Lung}
Pressure [mmHg]
Blood flow [mL/min]
Sample [100Hz]
B
PAP
RAP
Curve fit
RV Isovolumetric
P_{ISOMax} (a + 2*b)
EDP (a)
Amplitude (b)
Pressure [mmHg]
Sample
C
P_{ISOMax}
Ees
Ea
Pressure [mmHg]
Volume [mL]

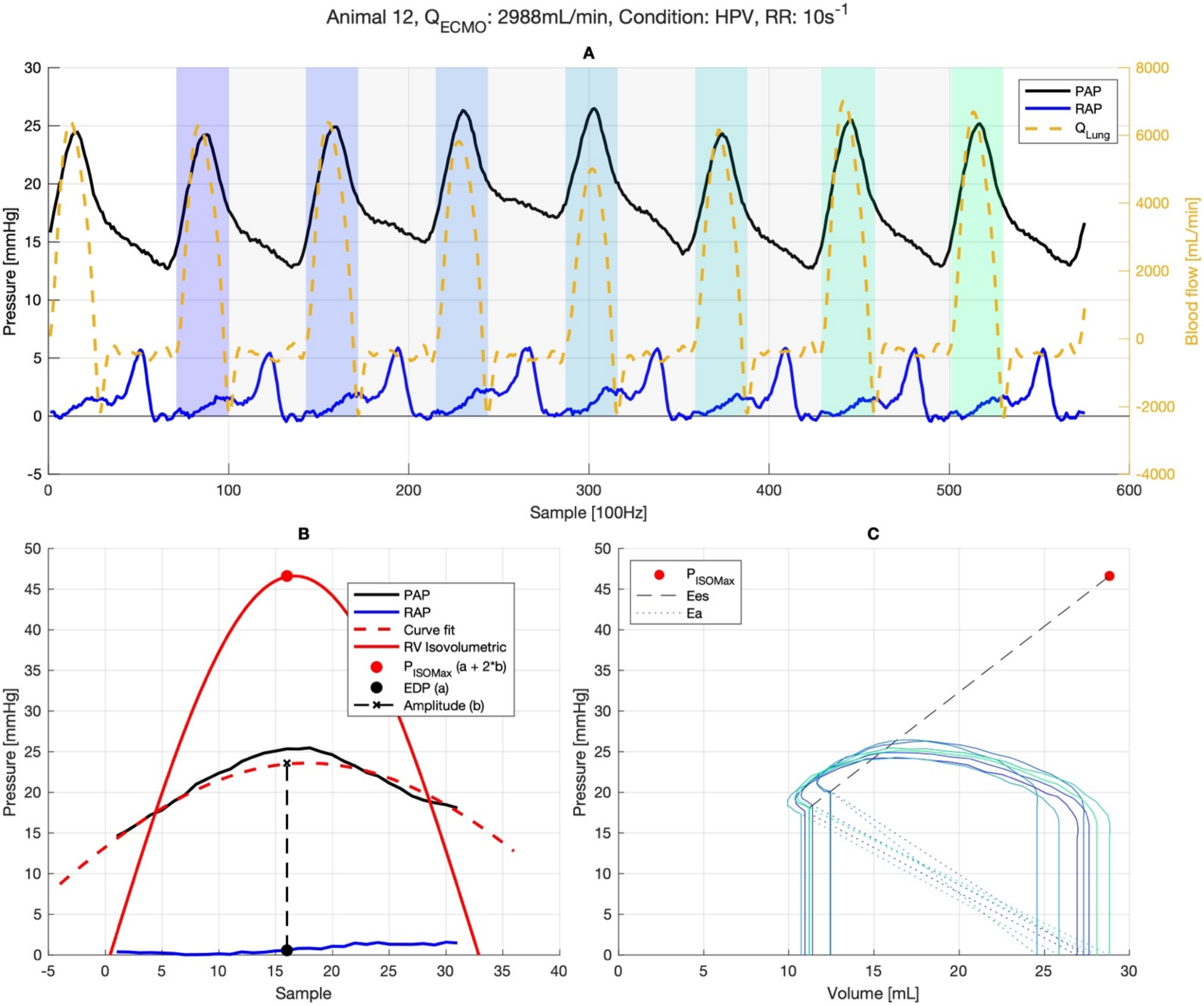
Animal 12, QECMO: 2988mL/min, Condition: HPV, RR: 10s-1
A
PAP
RAP
QLung
Pressure [mmHg]
Blood flow [mL/min]
Sample [100Hz]
B
PAP
RAP
Curve fit
RV Isovolumetric
PISOMax (a + 2*b)
EDP (a)
Amplitude (b)
Pressure [mmHg]
Sample
C
PISOMax
Ees
Ea
Pressure [mmHg]
Volume [mL]

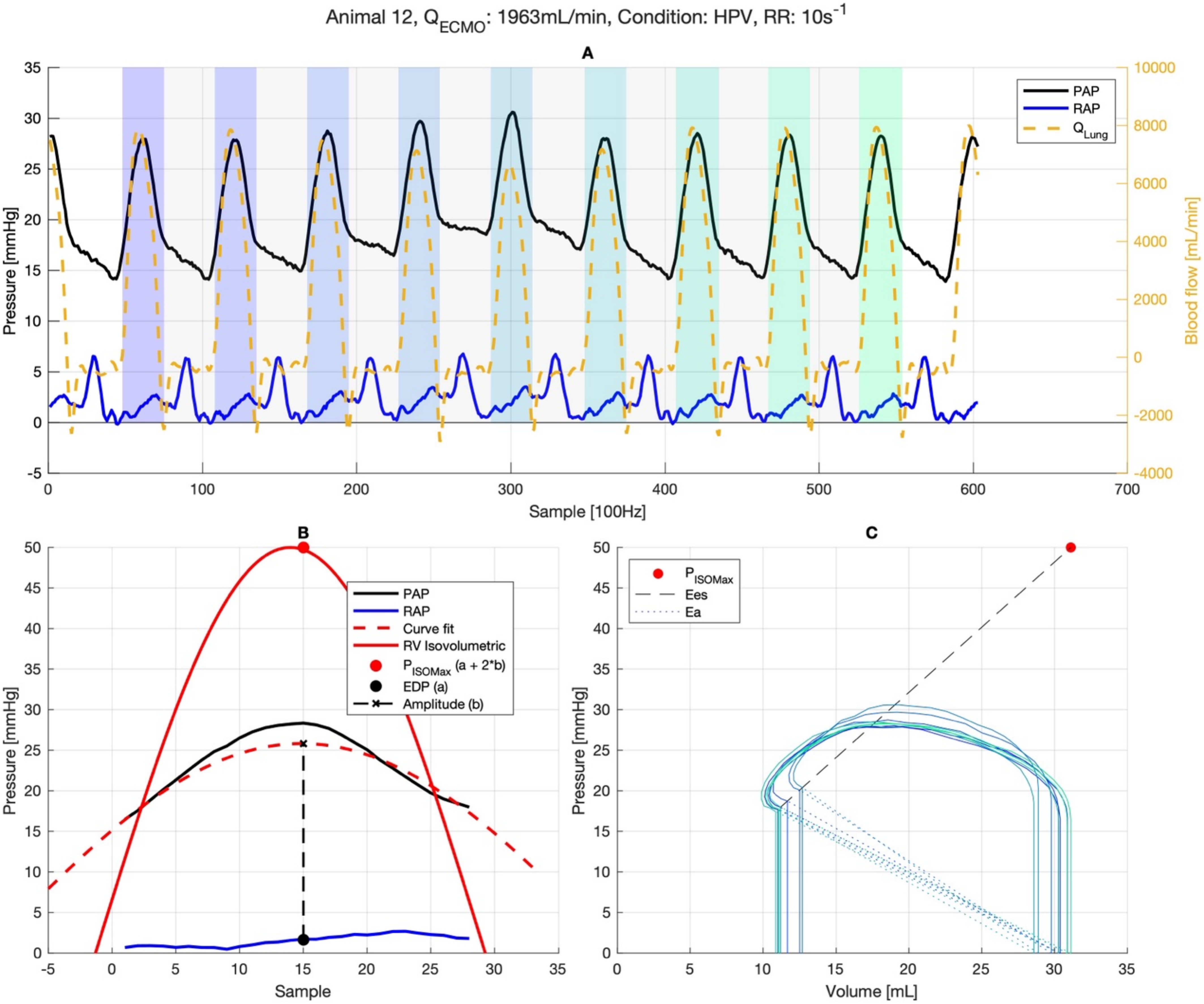

Animal 12, Q_ECMO: 1963mL/min, Condition: HPV, RR: 10s^-1
A
Pressure [mmHg]
Blood flow [mL/min]
Sample [100Hz]
PAP
RAP
Q_Lung
B
Pressure [mmHg]
Sample
PAP
RAP
Curve fit
RV Isovolumetric
P_ISOMax (a + 2*b)
EDP (a)
Amplitude (b)
C
Pressure [mmHg]
Volume [mL]
P_ISOMax
Ees
Ea

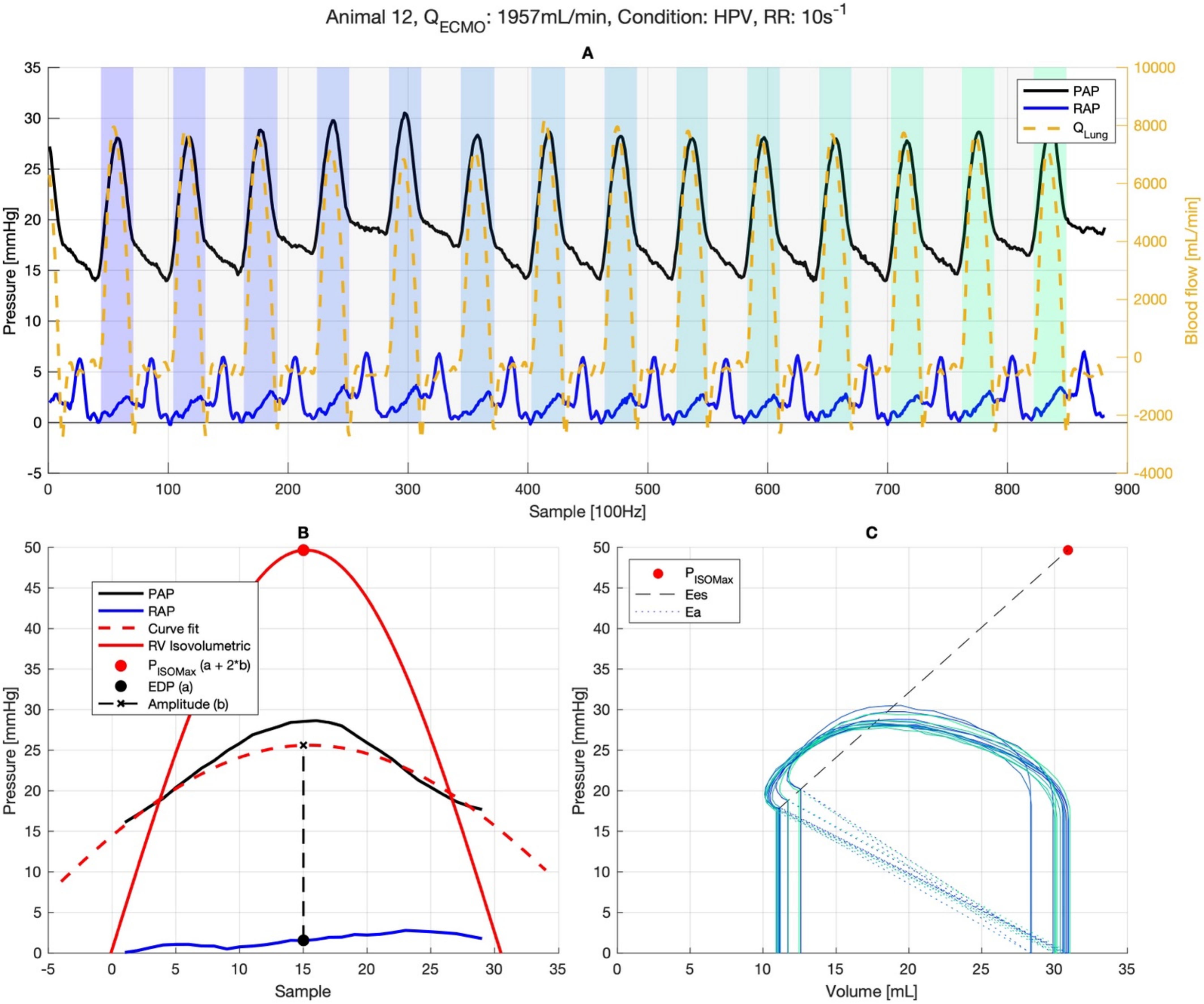

Animal 12, Q_ECMO: 1957mL/min, Condition: HPV, RR: 10s^-1
A
PAP
RAP
Q_Lung
Pressure [mmHg]
Blood flow [mL/min]
Sample [100Hz]
B
PAP
RAP
Curve fit
RV Isovolumetric
P_ISOMax (a + 2*b)
EDP (a)
Amplitude (b)
Pressure [mmHg]
Sample
C
P_ISOMax
Ees
Ea
Pressure [mmHg]
Volume [mL]

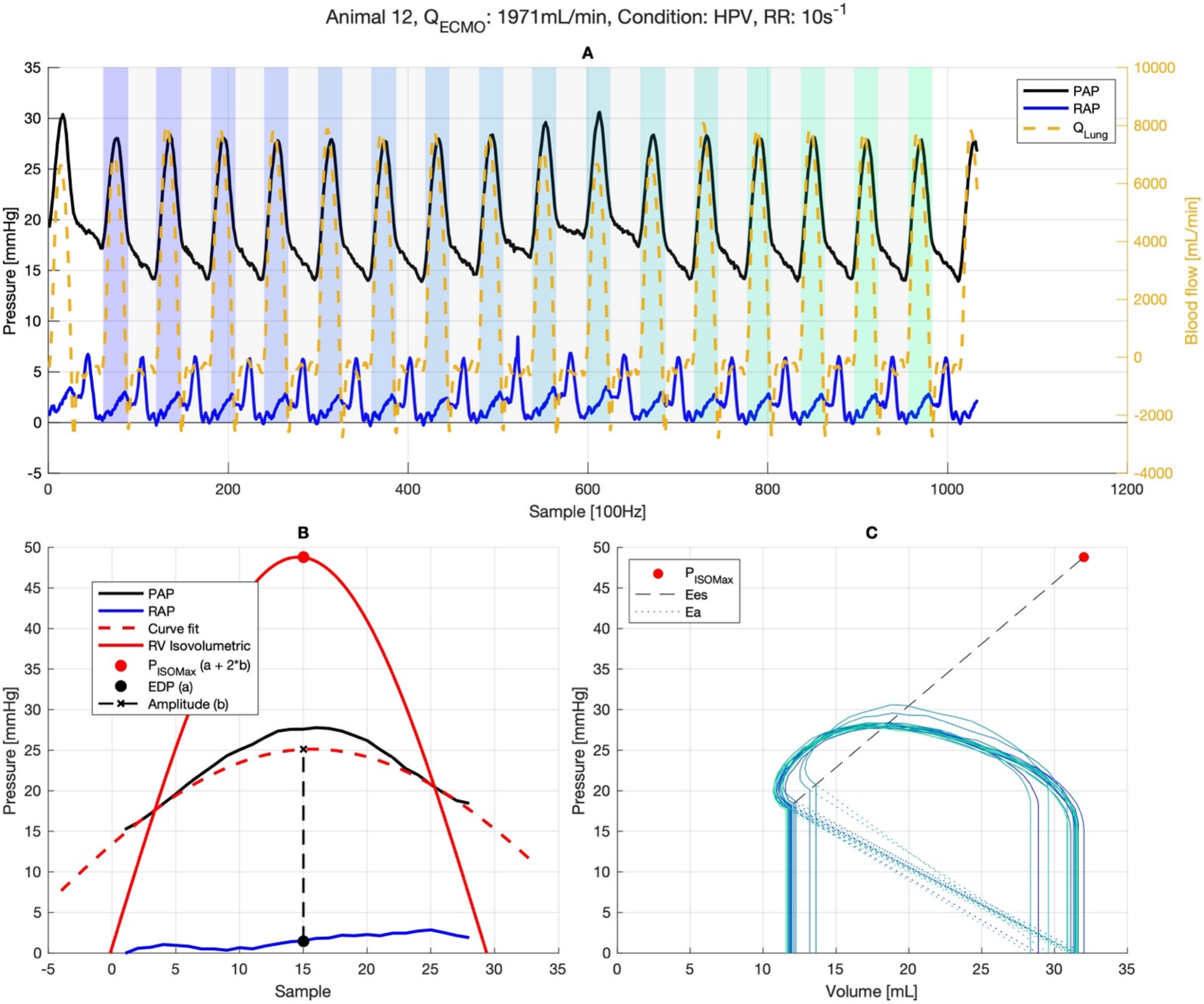

Animal 12, Q_{ECMO}: 1971mL/min, Condition: HPV, RR: 10s^{-1}
A
PAP
RAP
Q_{Lung}
Pressure [mmHg]
Blood flow [mL/min]
Sample [100Hz]
B
PAP
RAP
Curve fit
RV Isovolumetric
P_{ISOMax} (a + 2*b)
EDP (a)
Amplitude (b)
Pressure [mmHg]
Sample
C
P_{ISOMax}
Ees
Ea
Pressure [mmHg]
Volume [mL]

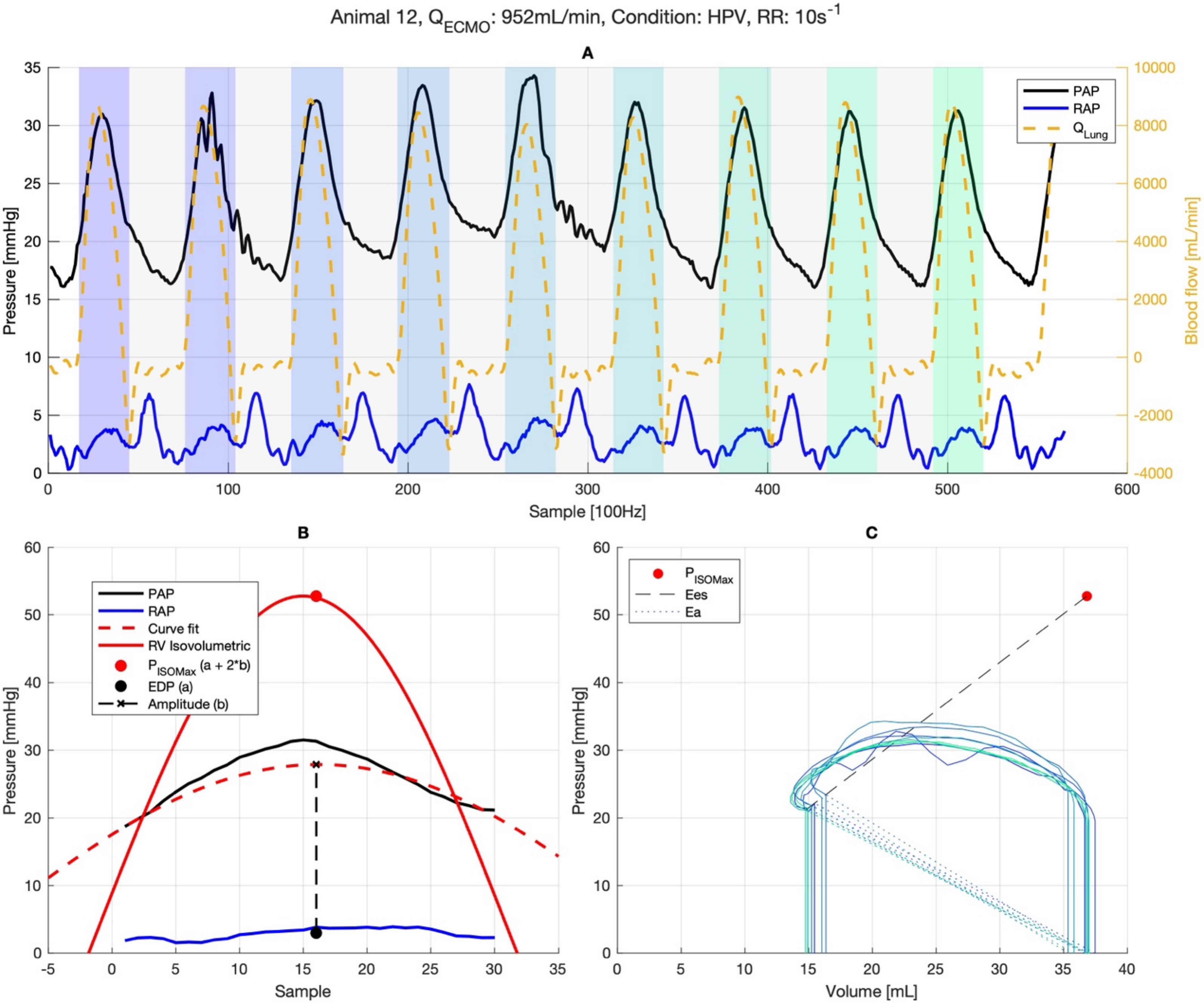

Animal 12, Q_{ECMO}: 952mL/min, Condition: HPV, RR: 10s^{-1}
A
PAP
RAP
Q_{Lung}
Pressure [mmHg]
Blood flow [mL/min]
Sample [100Hz]
B
PAP
RAP
Curve fit
RV Isovolumetric
P_{ISOMax} (a + 2*b)
EDP (a)
Amplitude (b)
Pressure [mmHg]
Sample
C
P_{ISOMax}
Ees
Ea
Pressure [mmHg]
Volume [mL]

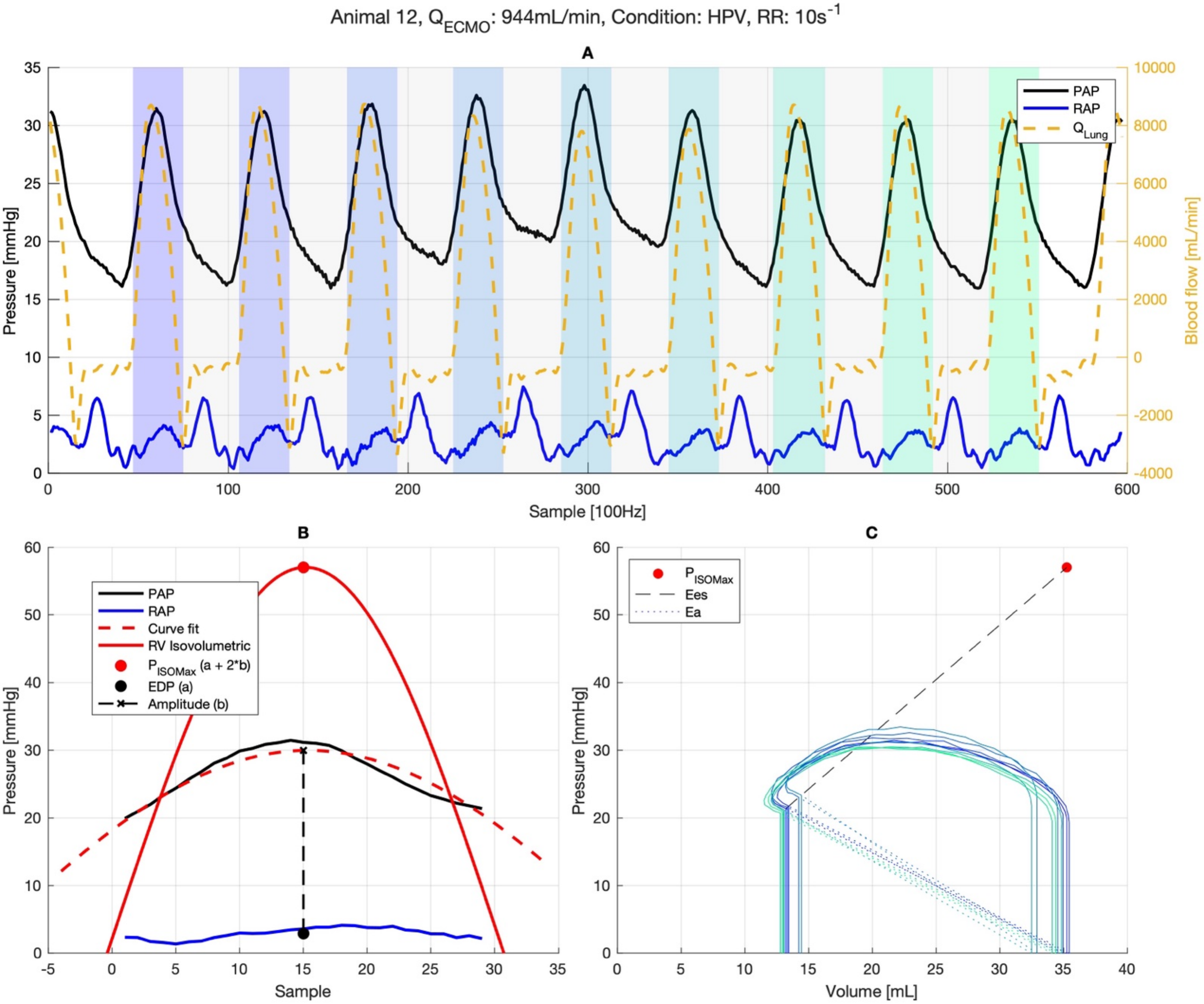

Animal 12, Q_{ECMO}: 944mL/min, Condition: HPV, RR: $10s^{-1}$
A
PAP
RAP
Q_{Lung}
Pressure [mmHg]
Blood flow [mL/min]
Sample [100Hz]
B
PAP
RAP
Curve fit
RV Isovolumetric
P_{ISOMax} (a + 2*b)
EDP (a)
Amplitude (b)
Pressure [mmHg]
Sample
C
P_{ISOMax}
Ees
Ea
Pressure [mmHg]
Volume [mL]

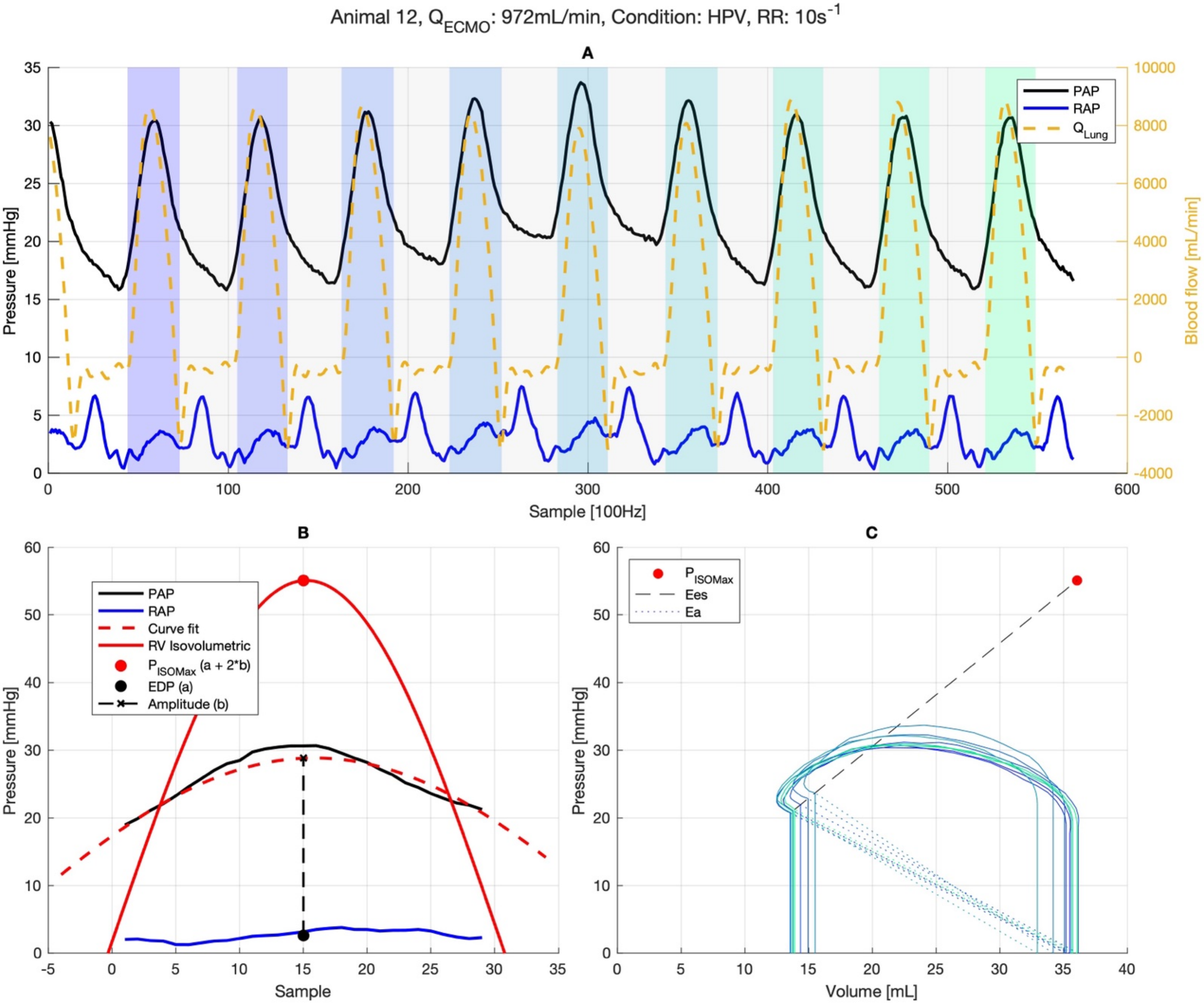
Animal 12, Q_{ECMO}: 972mL/min, Condition: HPV, RR: 10s^{-1}
A
PAP
RAP
Q_{Lung}
Pressure [mmHg]
Blood flow [mL/min]
Sample [100Hz]
B
PAP
RAP
Curve fit
RV Isovolumetric
P_{ISOMax} (a + 2*b)
EDP (a)
Amplitude (b)
Pressure [mmHg]
Sample
C
P_{ISOMax}
Ees
Ea
Pressure [mmHg]
Volume [mL]

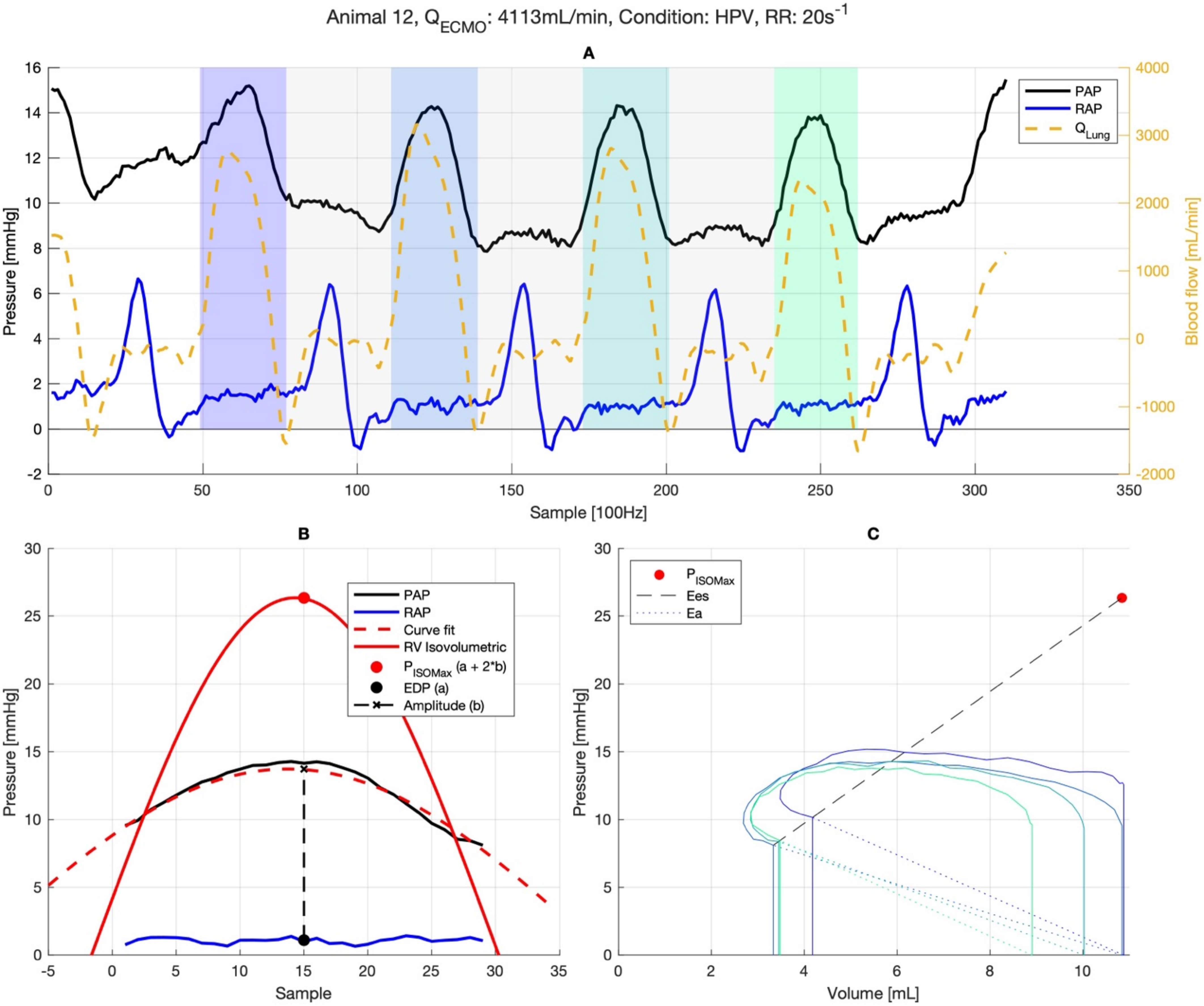

Animal 12, Q_{ECMO}: 4113mL/min, Condition: HPV, RR: 20s^{-1}
A
PAP
RAP
Q_{Lung}
Pressure [mmHg]
Blood flow [mL/min]
Sample [100Hz]
B
PAP
RAP
Curve fit
RV Isovolumetric
P_{ISOMax} (a + 2*b)
EDP (a)
Amplitude (b)
Pressure [mmHg]
Sample
C
P_{ISOMax}
Ees
Ea
Pressure [mmHg]
Volume [mL]

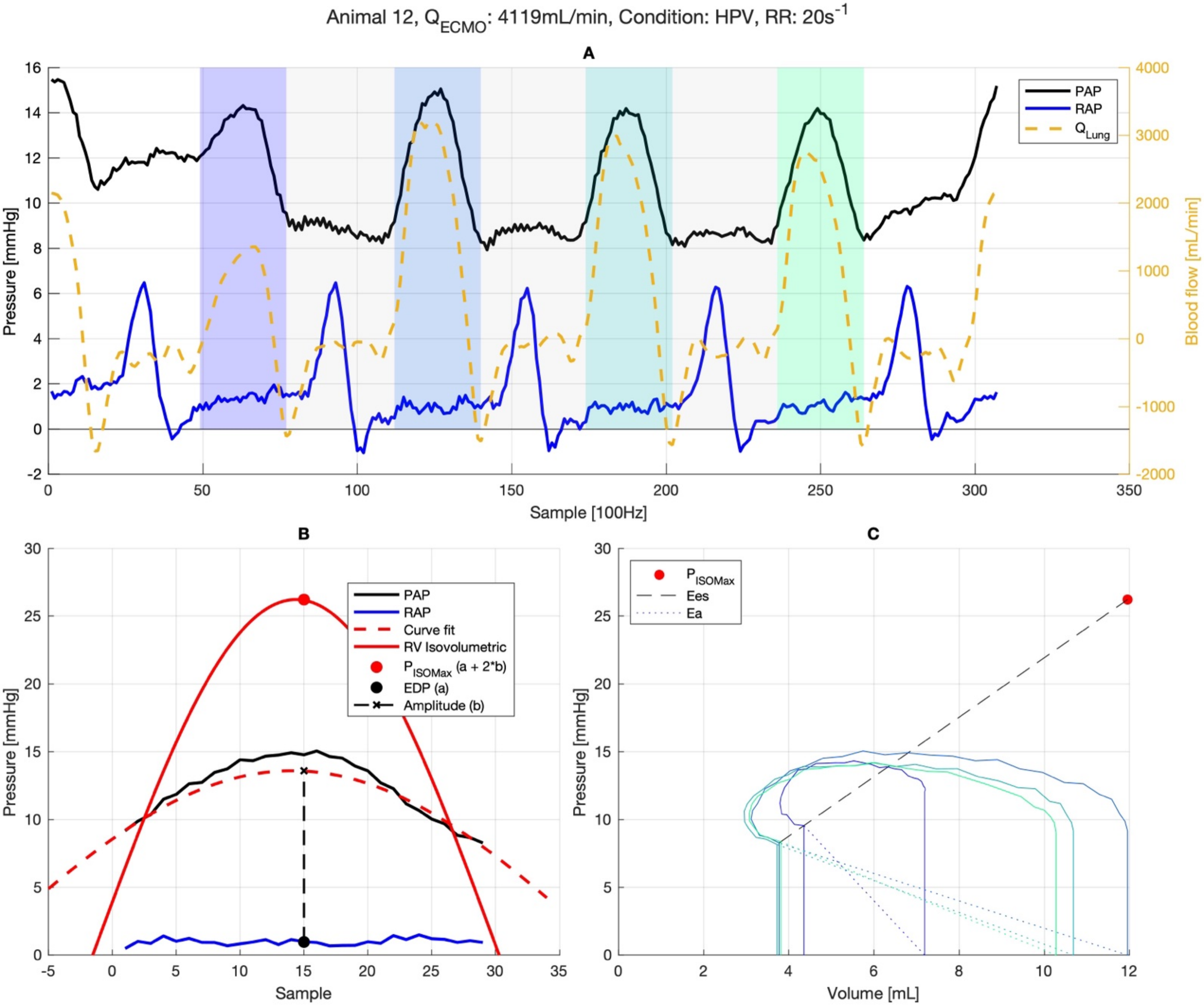

Animal 12, Q_{ECMO}: 4119mL/min, Condition: HPV, RR: $20s^{-1}$
A
Pressure [mmHg]
Blood flow [mL/min]
Sample [100Hz]
PAP
RAP
Q_{Lung}
B
Pressure [mmHg]
Sample
PAP
RAP
Curve fit
RV Isovolumetric
P_{ISOMax} (a + 2*b)
EDP (a)
Amplitude (b)
C
Pressure [mmHg]
Volume [mL]
P_{ISOMax}
Ees
Ea

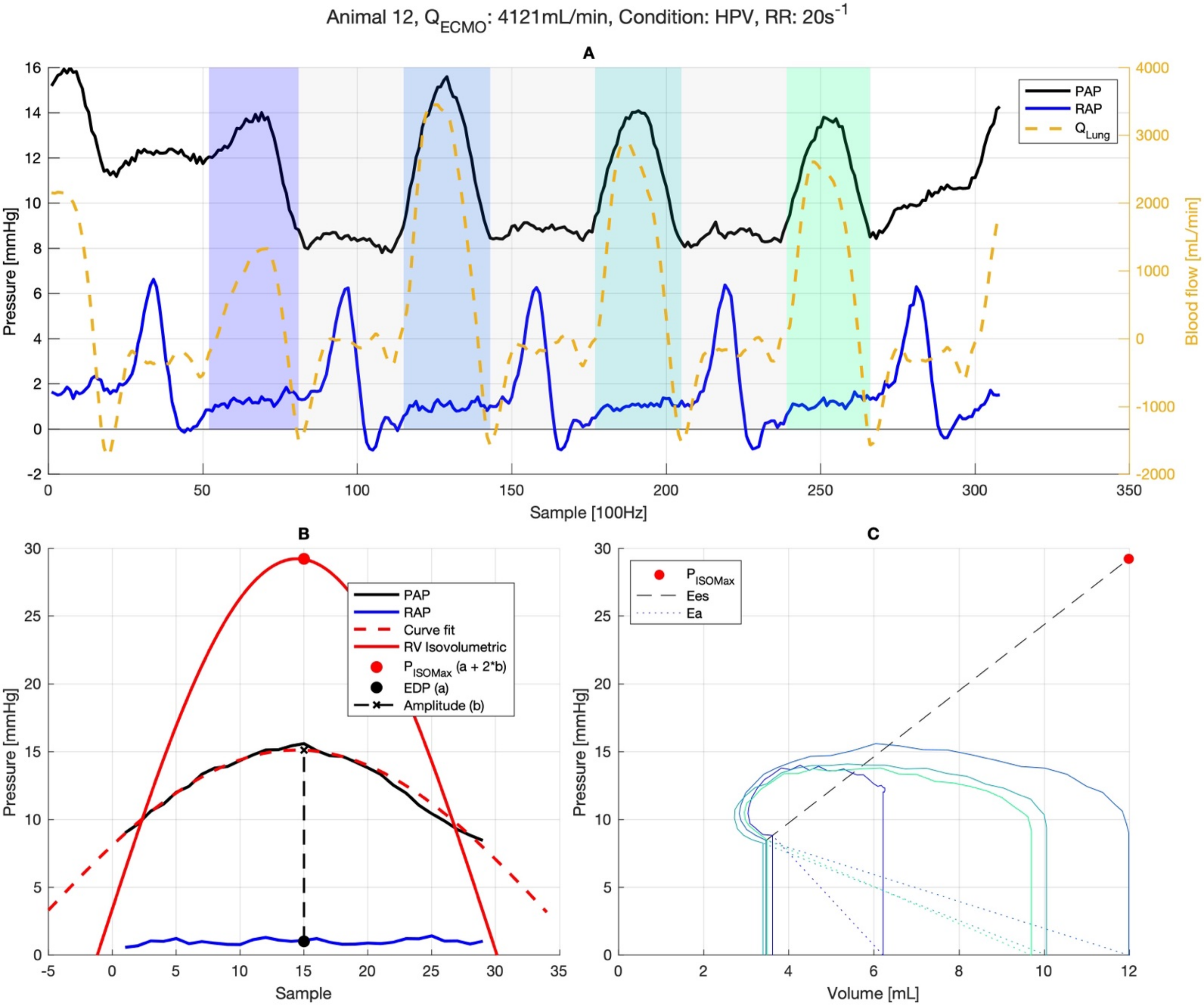

Animal 12, Q_ECMO: 4121mL/min, Condition: HPV, RR: 20s^-1
A
PAP
RAP
Q_Lung
Pressure [mmHg]
Blood flow [mL/min]
Sample [100Hz]
B
PAP
RAP
Curve fit
RV Isovolumetric
P_ISOMax (a + 2*b)
EDP (a)
Amplitude (b)
Pressure [mmHg]
Sample
C
P_ISOMax
Ees
Ea
Pressure [mmHg]
Volume [mL]

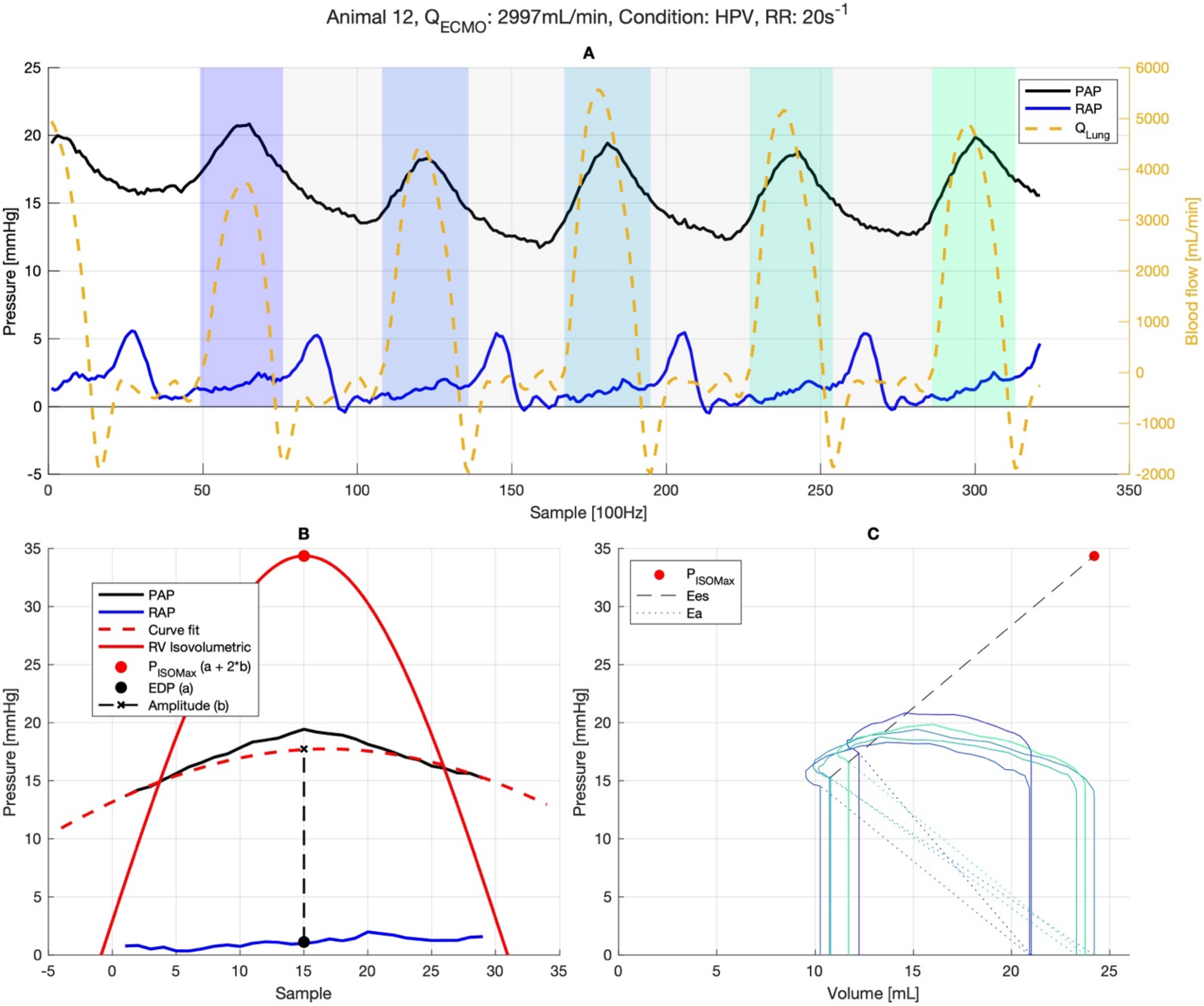

Animal 12, Q_{ECMO}: 2997mL/min, Condition: HPV, RR: 20s^{-1}
A
PAP
RAP
Q_{Lung}
Pressure [mmHg]
Blood flow [mL/min]
Sample [100Hz]
B
PAP
RAP
Curve fit
RV Isovolumetric
P_{ISOMax} (a + 2*b)
EDP (a)
Amplitude (b)
Pressure [mmHg]
Sample
C
P_{ISOMax}
Ees
Ea
Pressure [mmHg]
Volume [mL]

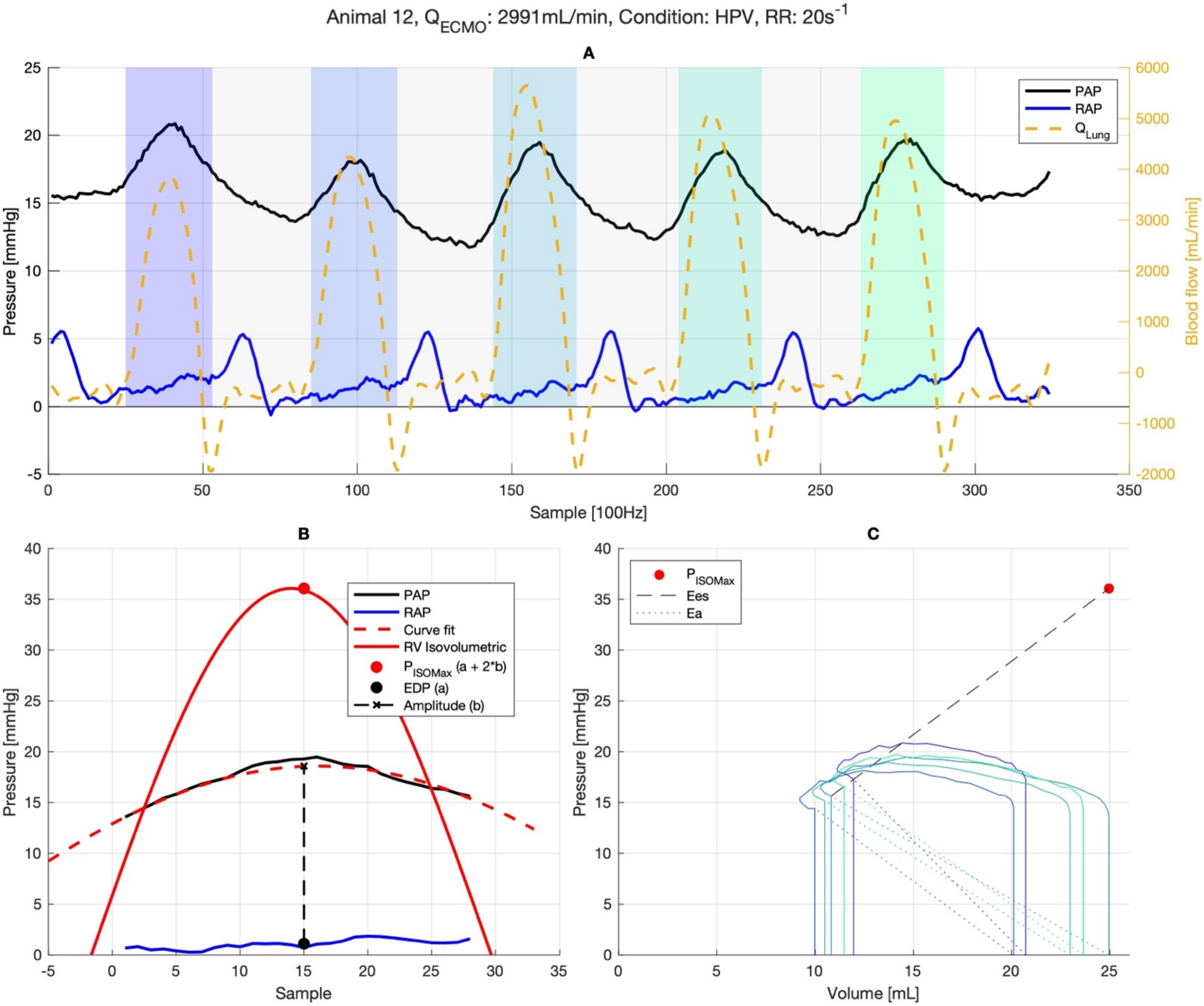

Animal 12, Q_{ECMO}: 2991mL/min, Condition: HPV, RR: $20s^{-1}$
A
PAP
RAP
Q_{Lung}
Pressure [mmHg]
Blood flow [mL/min]
Sample [100Hz]
B
PAP
RAP
Curve fit
RV Isovolumetric
P_{ISOMax} (a + 2*b)
EDP (a)
Amplitude (b)
Pressure [mmHg]
Sample
C
P_{ISOMax}
Ees
Ea
Pressure [mmHg]
Volume [mL]

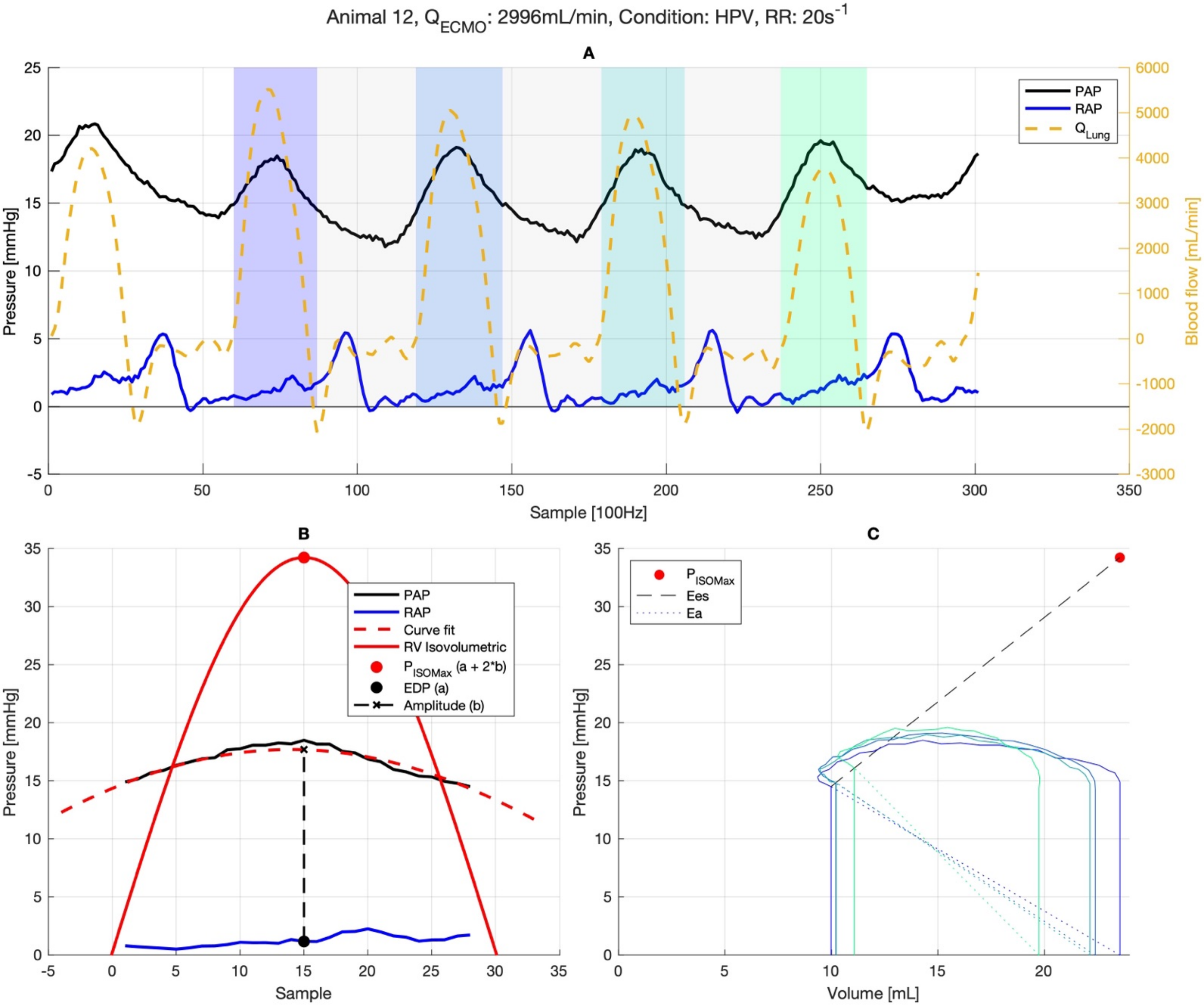

Animal 12, Q_ECMO: 2996mL/min, Condition: HPV, RR: 20s^-1
A
Pressure [mmHg]
Blood flow [mL/min]
Sample [100Hz]
PAP
RAP
Q_Lung
B
Pressure [mmHg]
Sample
PAP
RAP
Curve fit
RV Isovolumetric
P_ISOMax (a + 2*b)
EDP (a)
Amplitude (b)
C
Pressure [mmHg]
Volume [mL]
P_ISOMax
Ees
Ea

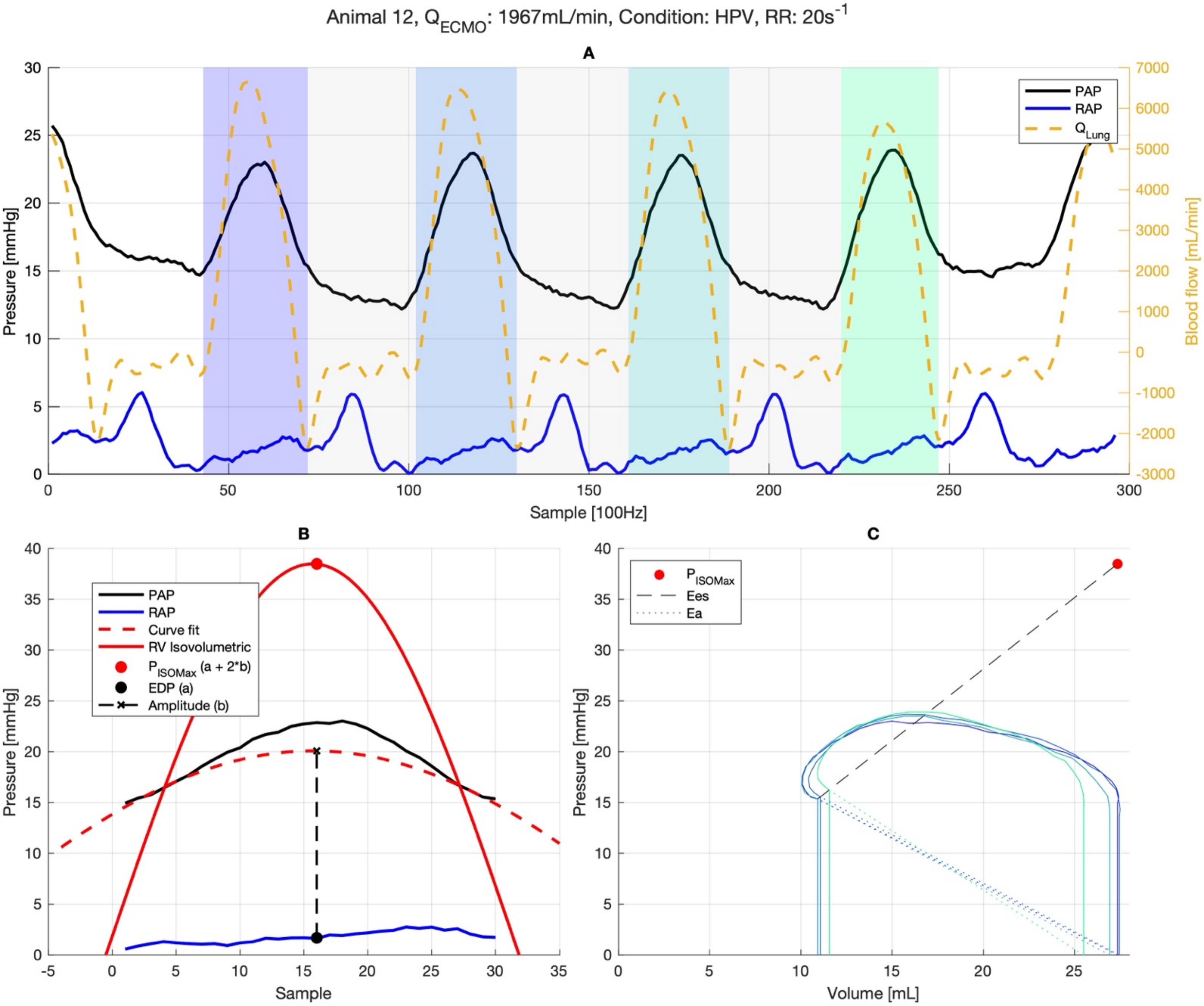
Animal 12, Q_{ECMO}: 1967mL/min, Condition: HPV, RR: $20s^{-1}$
A
PAP
RAP
Q_{Lung}
Pressure [mmHg]
Blood flow [mL/min]
Sample [100Hz]
B
PAP
RAP
Curve fit
RV Isovolumetric
P_{ISOMax} (a + 2*b)
EDP (a)
Amplitude (b)
Pressure [mmHg]
Sample
C
P_{ISOMax}
Ees
Ea
Pressure [mmHg]
Volume [mL]

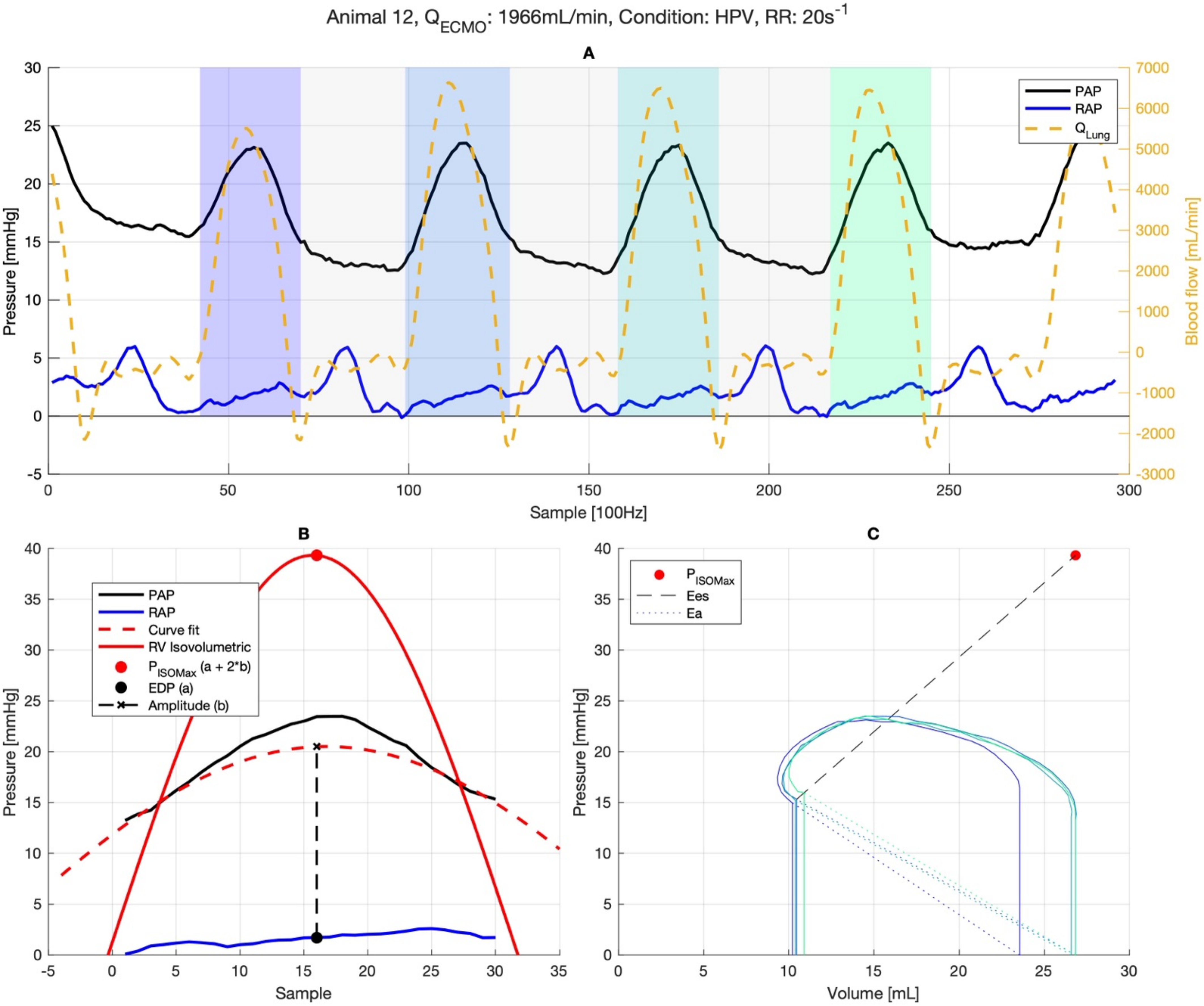

Animal 12, Q_ECMO: 1966mL/min, Condition: HPV, RR: 20s^-1
A
PAP
RAP
Q_Lung
Pressure [mmHg]
Blood flow [mL/min]
Sample [100Hz]
B
PAP
RAP
Curve fit
RV Isovolumetric
P_ISOMax (a + 2*b)
EDP (a)
Amplitude (b)
Pressure [mmHg]
Sample
C
P_ISOMax
Ees
Ea
Pressure [mmHg]
Volume [mL]

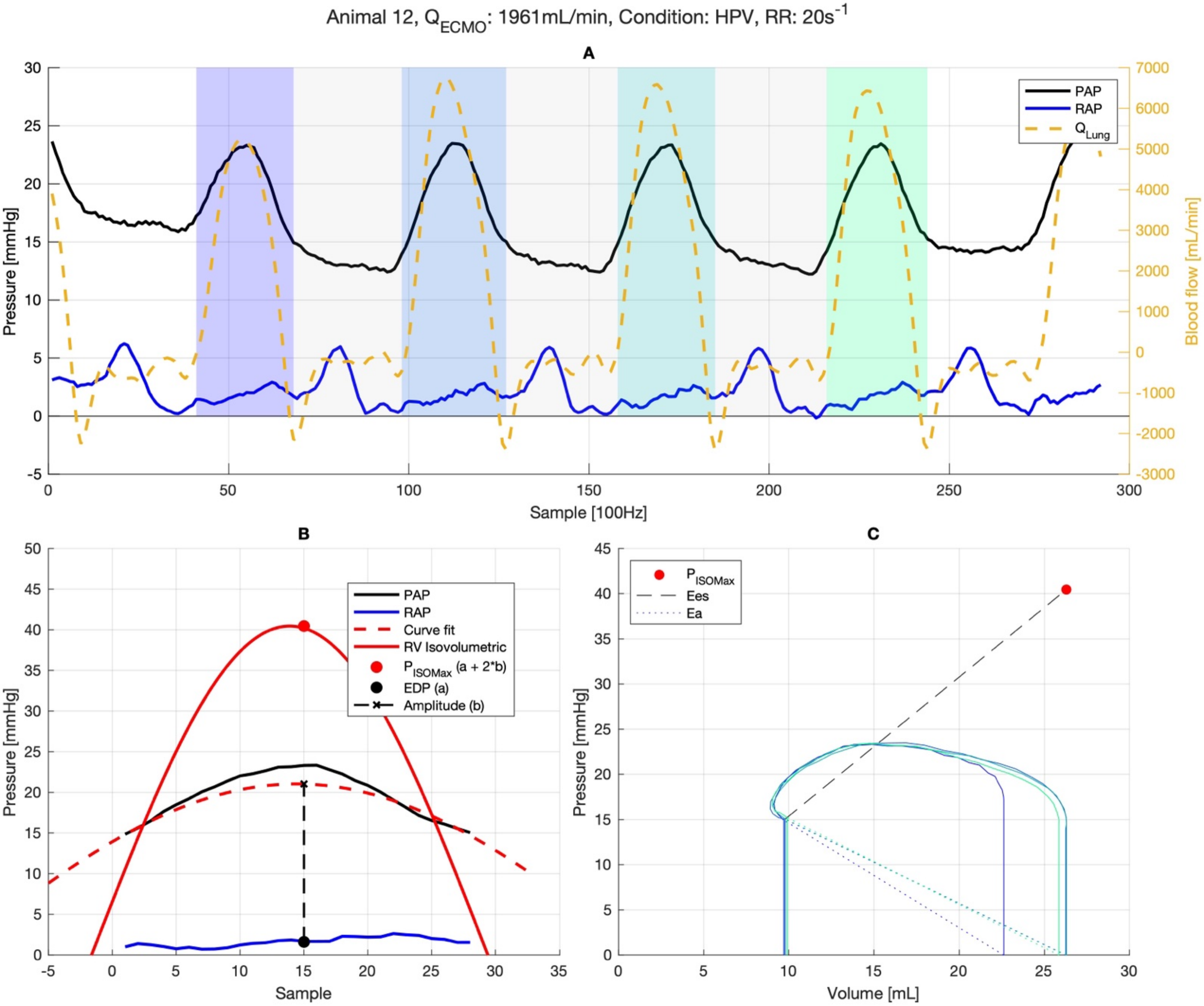

Animal 12, Q_{ECMO}: 1961mL/min, Condition: HPV, RR: $20s^{-1}$
A
PAP
RAP
Q_{Lung}
Pressure [mmHg]
Blood flow [mL/min]
Sample [100Hz]
B
PAP
RAP
Curve fit
RV Isovolumetric
P_{ISOMax} (a + 2*b)
EDP (a)
Amplitude (b)
Pressure [mmHg]
Sample
C
P_{ISOMax}
Ees
Ea
Pressure [mmHg]
Volume [mL]

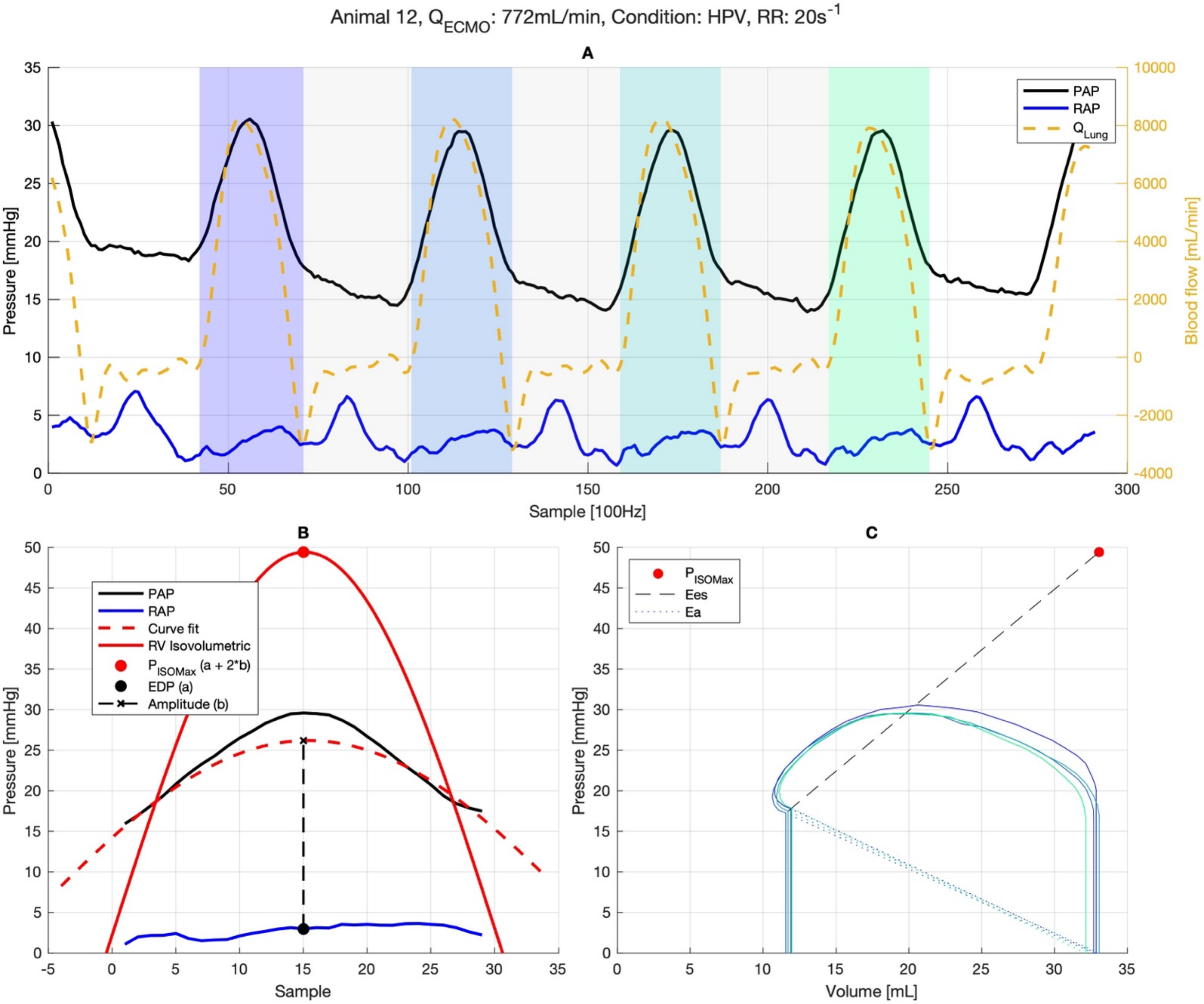

Animal 12, Q_{ECMO}: 772mL/min, Condition: HPV, RR: $20s^{-1}$
A
PAP
RAP
Q_{Lung}
Pressure [mmHg]
Blood flow [mL/min]
Sample [100Hz]
B
PAP
RAP
Curve fit
RV Isovolumetric
P_{ISOMax} (a + 2*b)
EDP (a)
Amplitude (b)
Pressure [mmHg]
Sample
C
P_{ISOMax}
Ees
Ea
Pressure [mmHg]
Volume [mL]

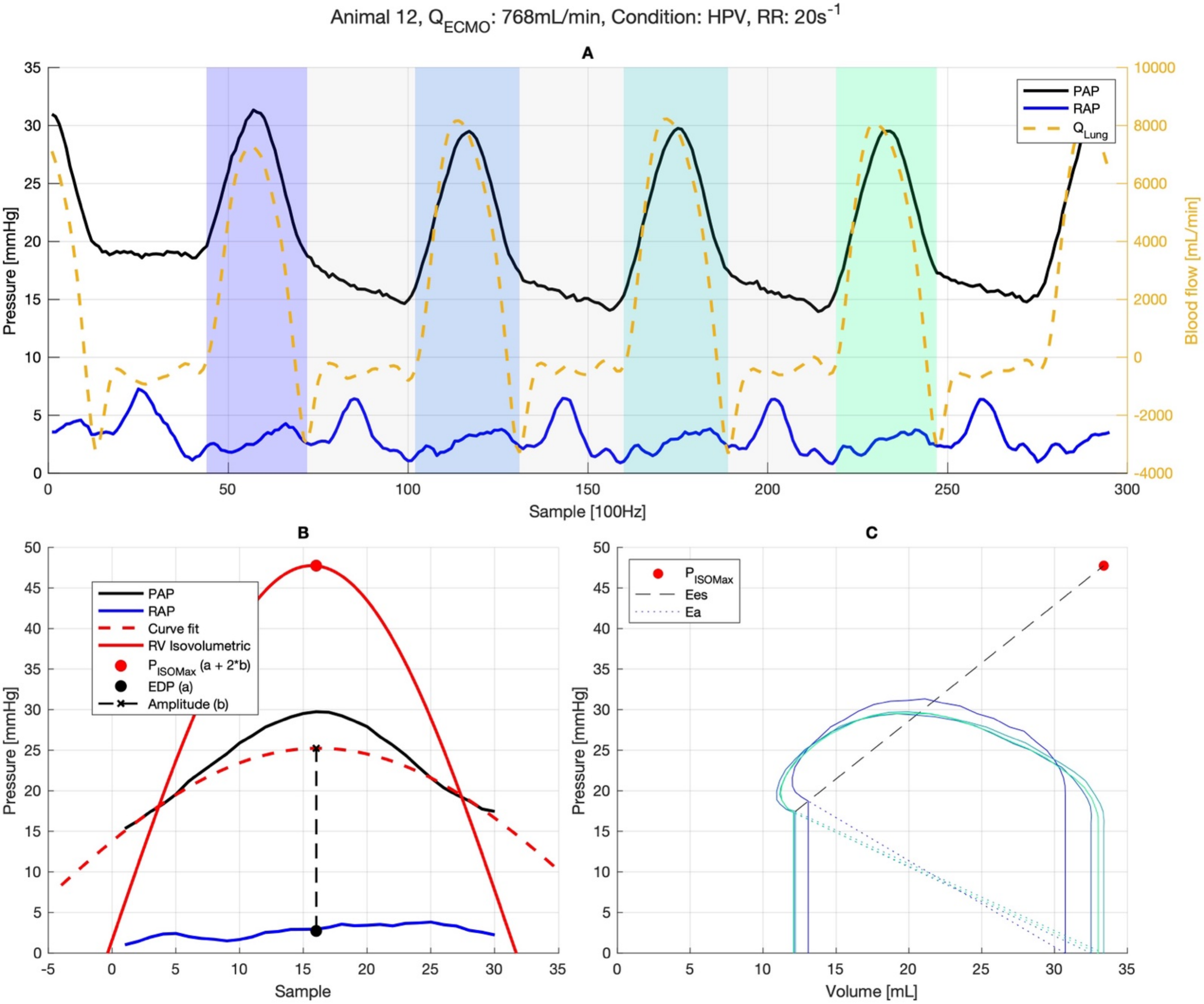

Animal 12, Q_ECMO: 768mL/min, Condition: HPV, RR: 20s^-1
A
PAP
RAP
Q_Lung
Pressure [mmHg]
Blood flow [mL/min]
Sample [100Hz]
B
PAP
RAP
Curve fit
RV Isovolumetric
P_ISOMax (a + 2*b)
EDP (a)
Amplitude (b)
Pressure [mmHg]
Sample
C
P_ISOMax
Ees
Ea
Pressure [mmHg]
Volume [mL]

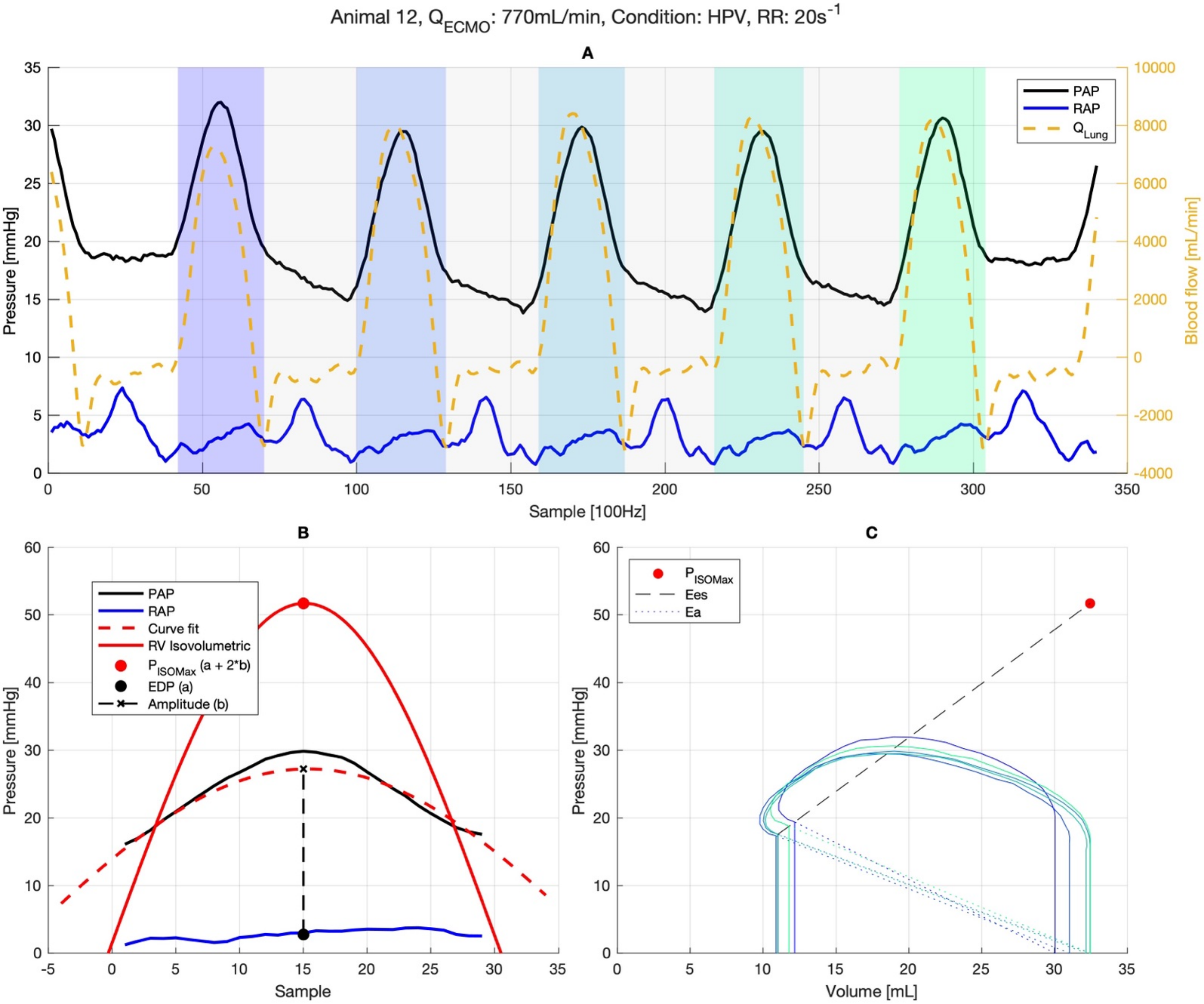

Animal 12, Q_{ECMO}: 770mL/min, Condition: HPV, RR: $20s^{-1}$
A
PAP
RAP
Q_{Lung}
Pressure [mmHg]
Blood flow [mL/min]
Sample [100Hz]
B
PAP
RAP
Curve fit
RV Isovolumetric
P_{ISOMax} (a + 2*b)
EDP (a)
Amplitude (b)
Pressure [mmHg]
Sample
C
P_{ISOMax}
Ees
Ea
Pressure [mmHg]
Volume [mL]

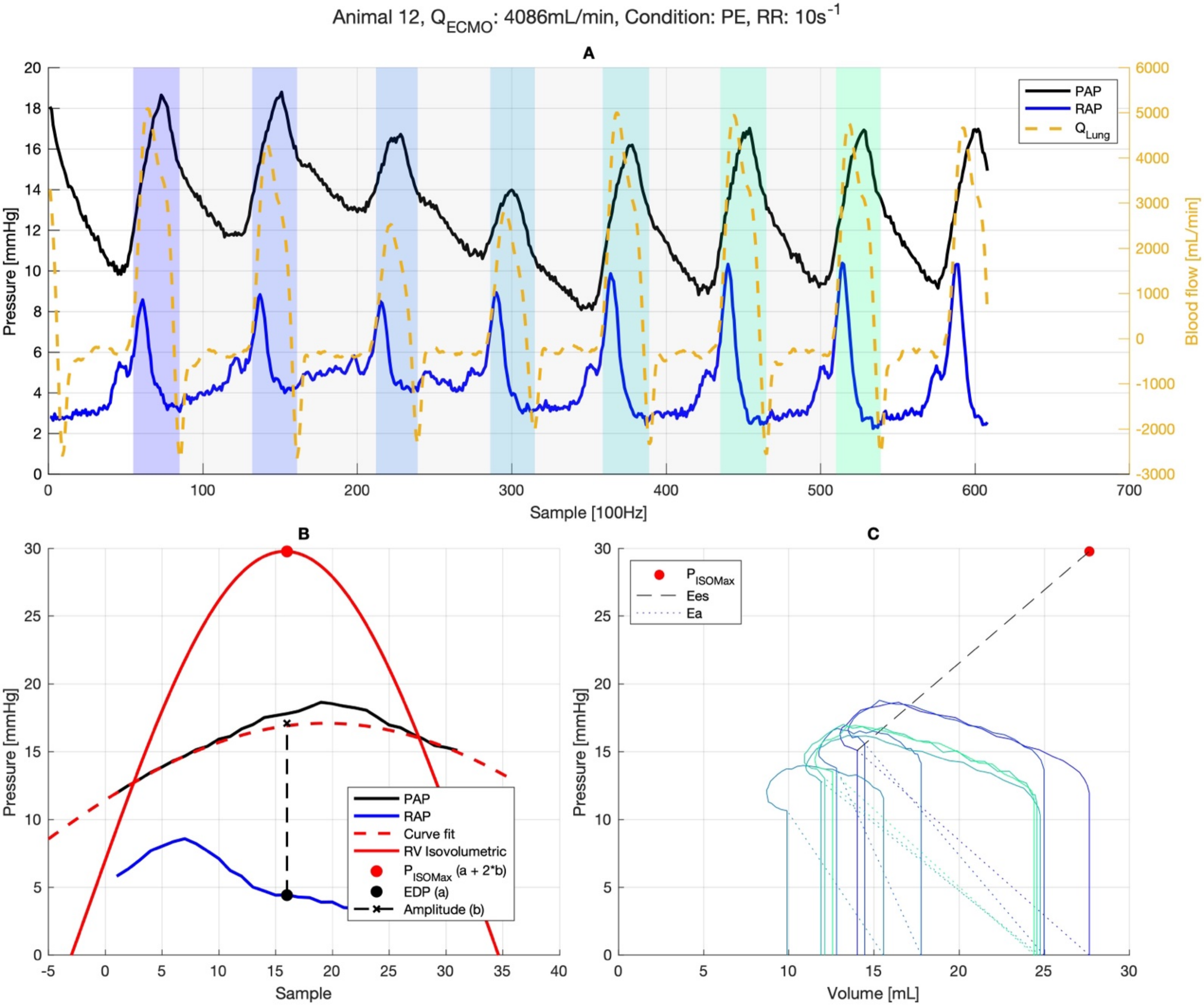

Animal 12, Q_{ECMO}: 4086mL/min, Condition: PE, RR: $10s^{-1}$
A
Pressure [mmHg]
Blood flow [mL/min]
Sample [100Hz]
PAP
RAP
Q_{Lung}
B
Pressure [mmHg]
Sample
PAP
RAP
Curve fit
RV Isovolumetric
P_{ISOMax} (a + 2*b)
EDP (a)
Amplitude (b)
C
Pressure [mmHg]
Volume [mL]
P_{ISOMax}
Ees
Ea

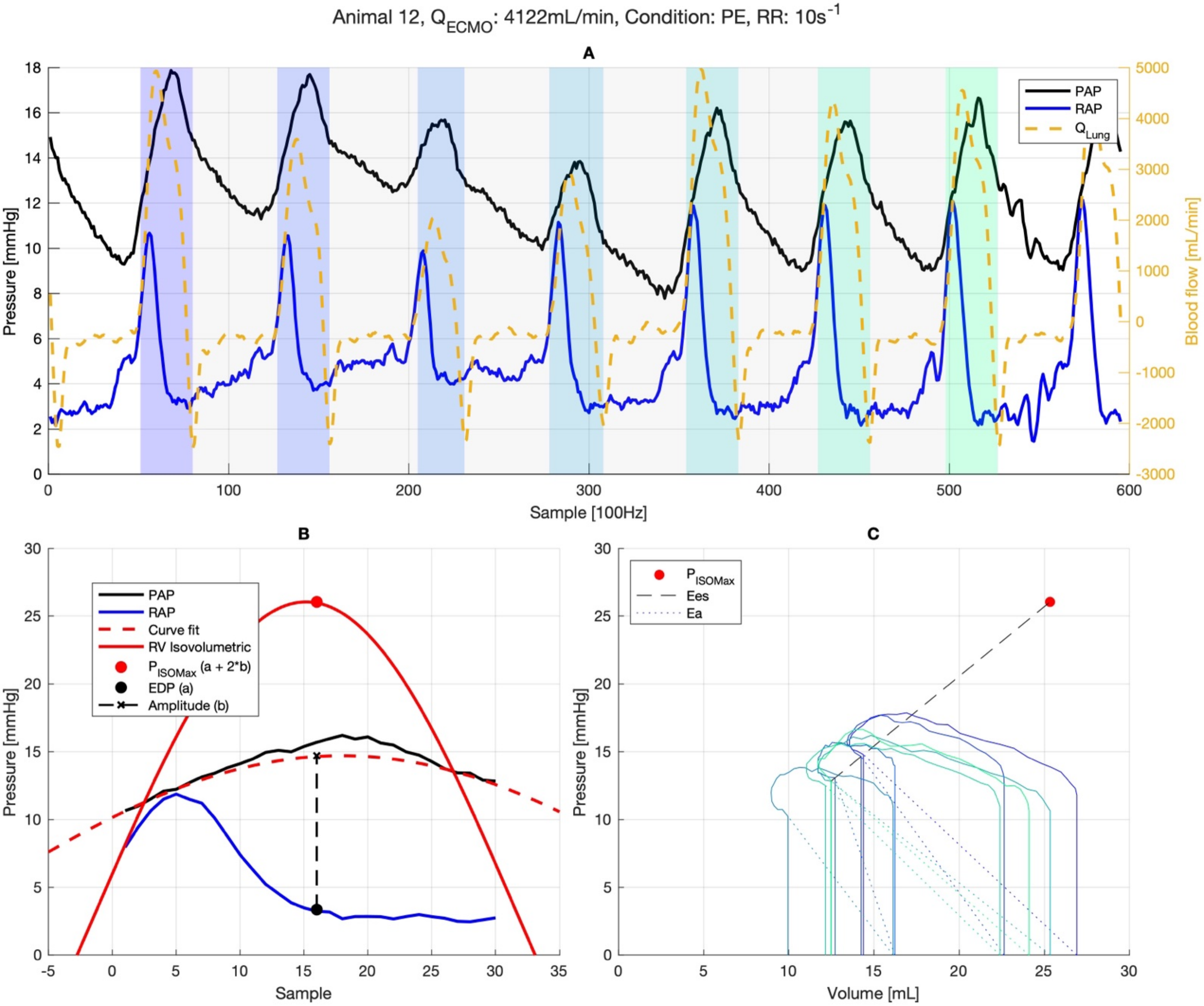

Animal 12, Q_{ECMO}: 4122mL/min, Condition: PE, RR: $10s^{-1}$
A
PAP
RAP
Q_{Lung}
Pressure [mmHg]
Blood flow [mL/min]
Sample [100Hz]
B
PAP
RAP
Curve fit
RV Isovolumetric
P_{ISOMax} (a + 2*b)
EDP (a)
Amplitude (b)
Pressure [mmHg]
Sample
C
P_{ISOMax}
Ees
Ea
Pressure [mmHg]
Volume [mL]

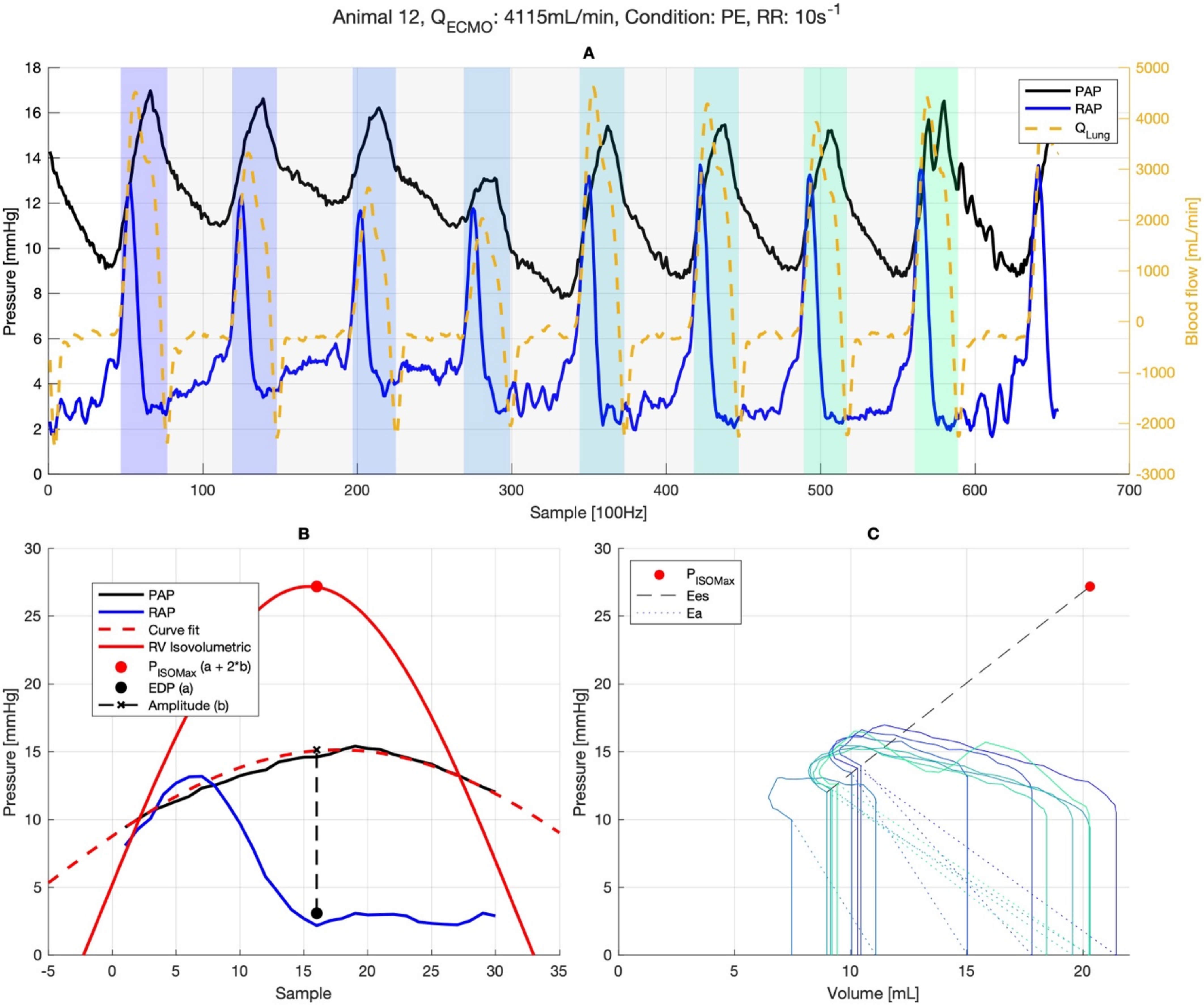

Animal 12, Q_{ECMO}: 4115mL/min, Condition: PE, RR: $10s^{-1}$
A
PAP
RAP
Q_{Lung}
Pressure [mmHg]
Blood flow [mL/min]
Sample [100Hz]
B
PAP
RAP
Curve fit
RV Isovolumetric
P_{ISOMax} (a + 2*b)
EDP (a)
Amplitude (b)
Pressure [mmHg]
Sample
C
P_{ISOMax}
Ees
Ea
Pressure [mmHg]
Volume [mL]

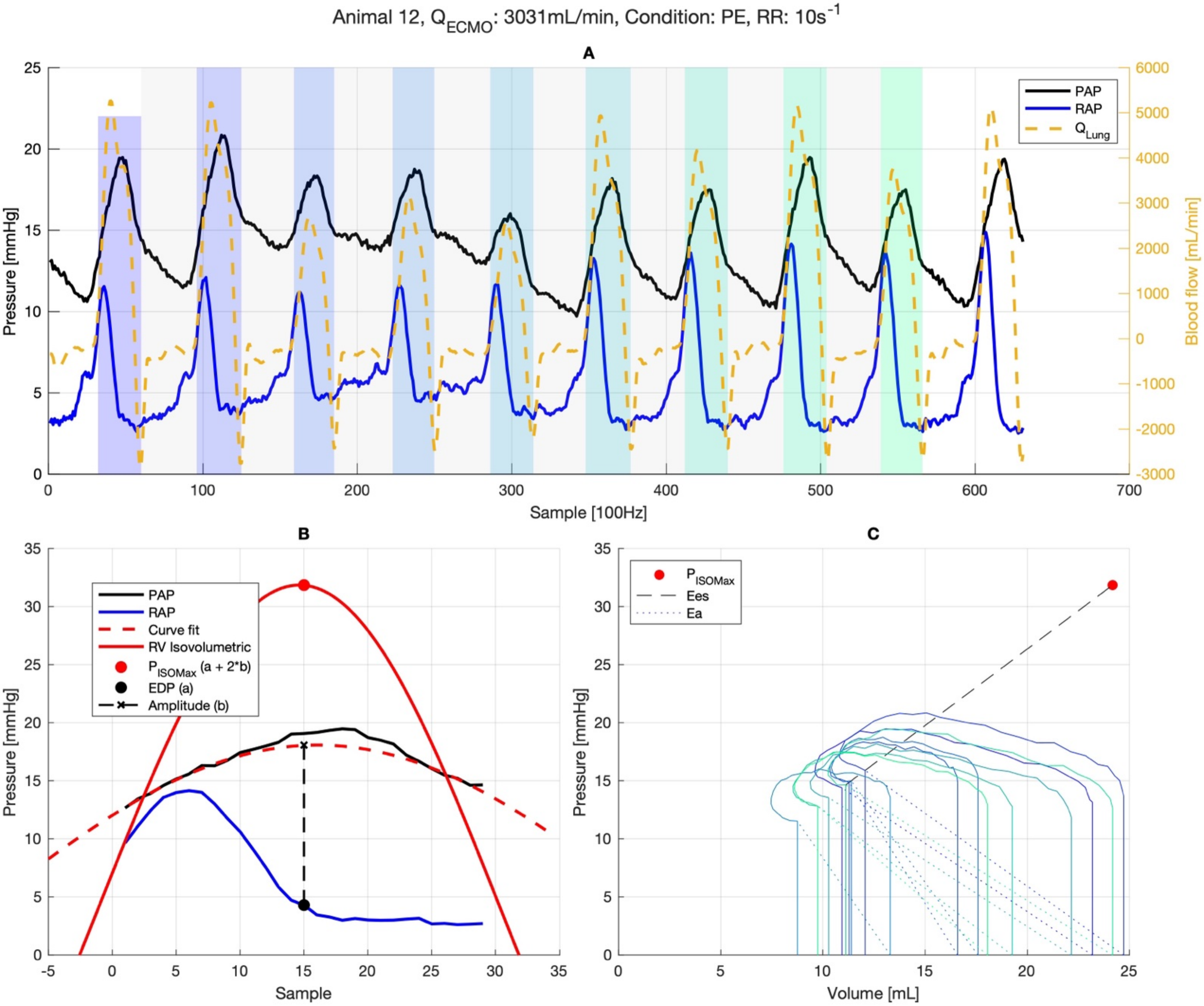

Animal 12, Q_{ECMO}: 3031mL/min, Condition: PE, RR: $10s^{-1}$
A
PAP
RAP
Q_{Lung}
Pressure [mmHg]
Blood flow [mL/min]
Sample [100Hz]
B
PAP
RAP
Curve fit
RV Isovolumetric
P_{ISOMax} (a + 2*b)
EDP (a)
Amplitude (b)
Pressure [mmHg]
Sample
C
P_{ISOMax}
Ees
Ea
Pressure [mmHg]
Volume [mL]

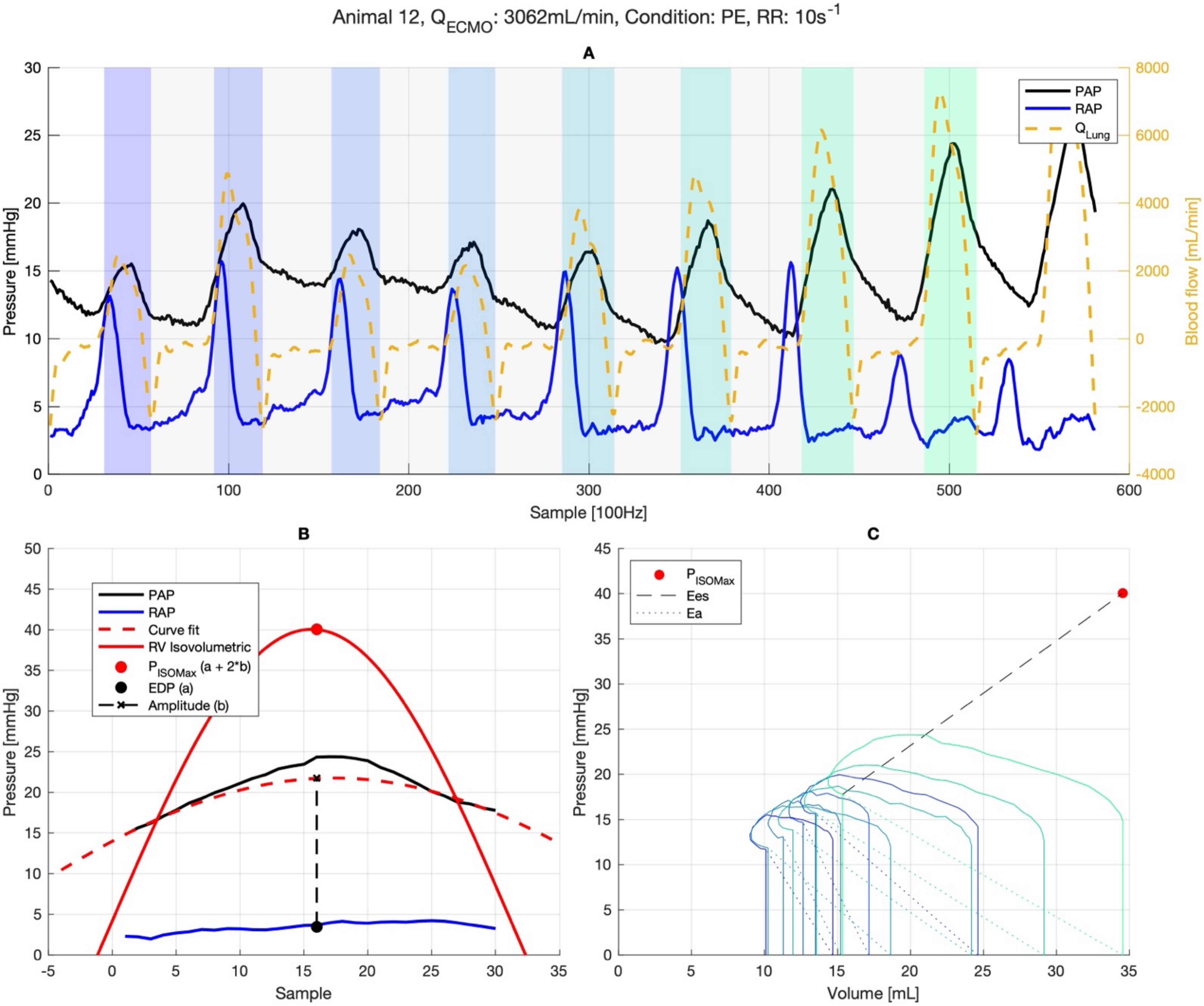

Animal 12, Q_{ECMO}: 3062mL/min, Condition: PE, RR: $10s^{-1}$
A
PAP
RAP
Q_{Lung}
Pressure [mmHg]
Blood flow [mL/min]
Sample [100Hz]
B
PAP
RAP
Curve fit
RV Isovolumetric
P_{ISOMax} (a + 2*b)
EDP (a)
Amplitude (b)
Pressure [mmHg]
Sample
C
P_{ISOMax}
Ees
Ea
Pressure [mmHg]
Volume [mL]

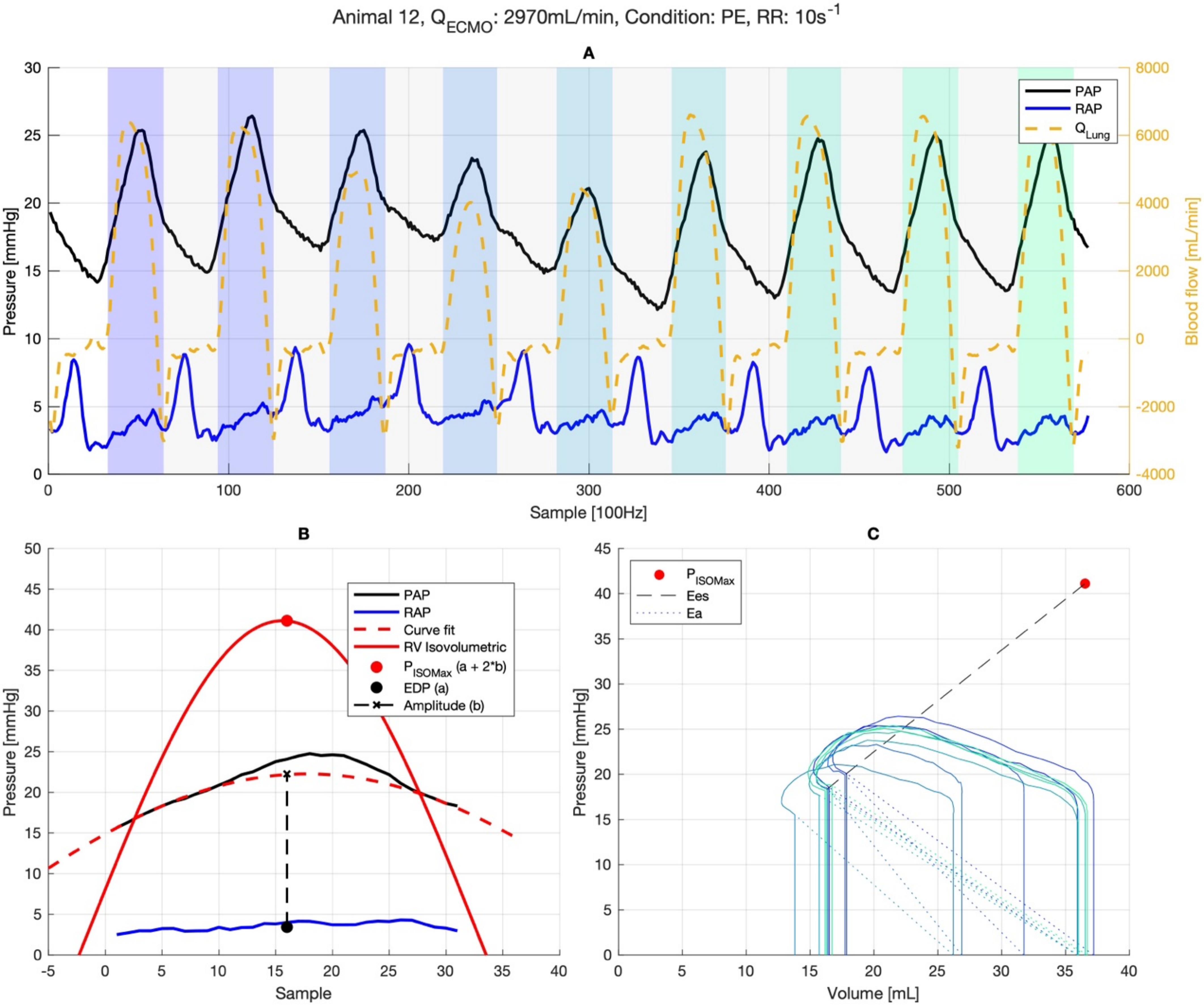

Animal 12, Q_{ECMO}: 2970mL/min, Condition: PE, RR: $10s^{-1}$
A
PAP
RAP
Q_{Lung}
Pressure [mmHg]
Blood flow [mL/min]
Sample [100Hz]
B
PAP
RAP
Curve fit
RV Isovolumetric
P_{ISOMax} (a + 2*b)
EDP (a)
Amplitude (b)
Pressure [mmHg]
Sample
C
P_{ISOMax}
Ees
Ea
Pressure [mmHg]
Volume [mL]

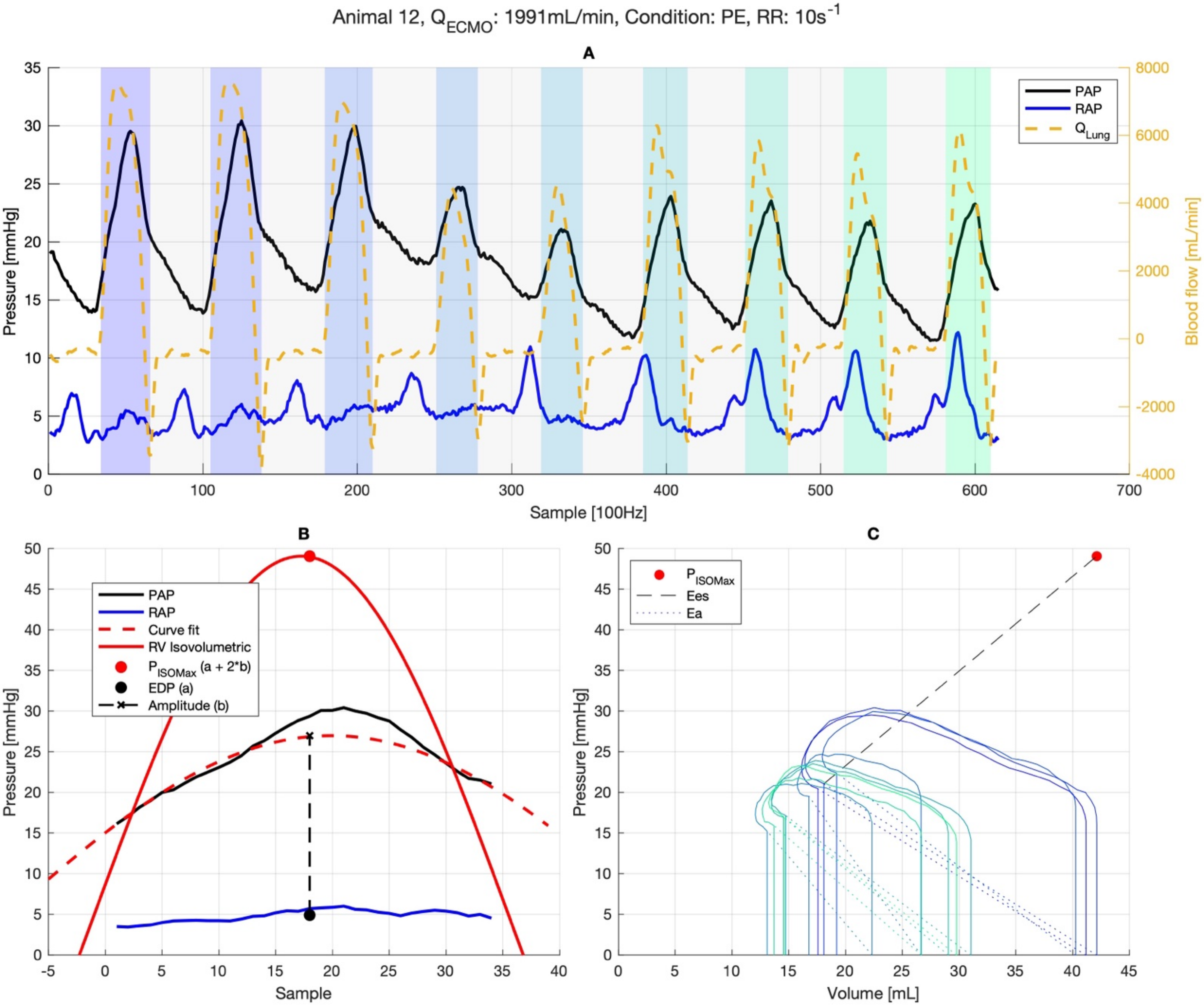

Animal 12, Q_{ECMO}: 1991mL/min, Condition: PE, RR: $10s^{-1}$
A
Pressure [mmHg]
Blood flow [mL/min]
Sample [100Hz]
PAP
RAP
Q_{Lung}
B
Pressure [mmHg]
Sample
PAP
RAP
Curve fit
RV Isovolumetric
P_{ISOMax} (a + 2*b)
EDP (a)
Amplitude (b)
C
Pressure [mmHg]
Volume [mL]
P_{ISOMax}
Ees
Ea

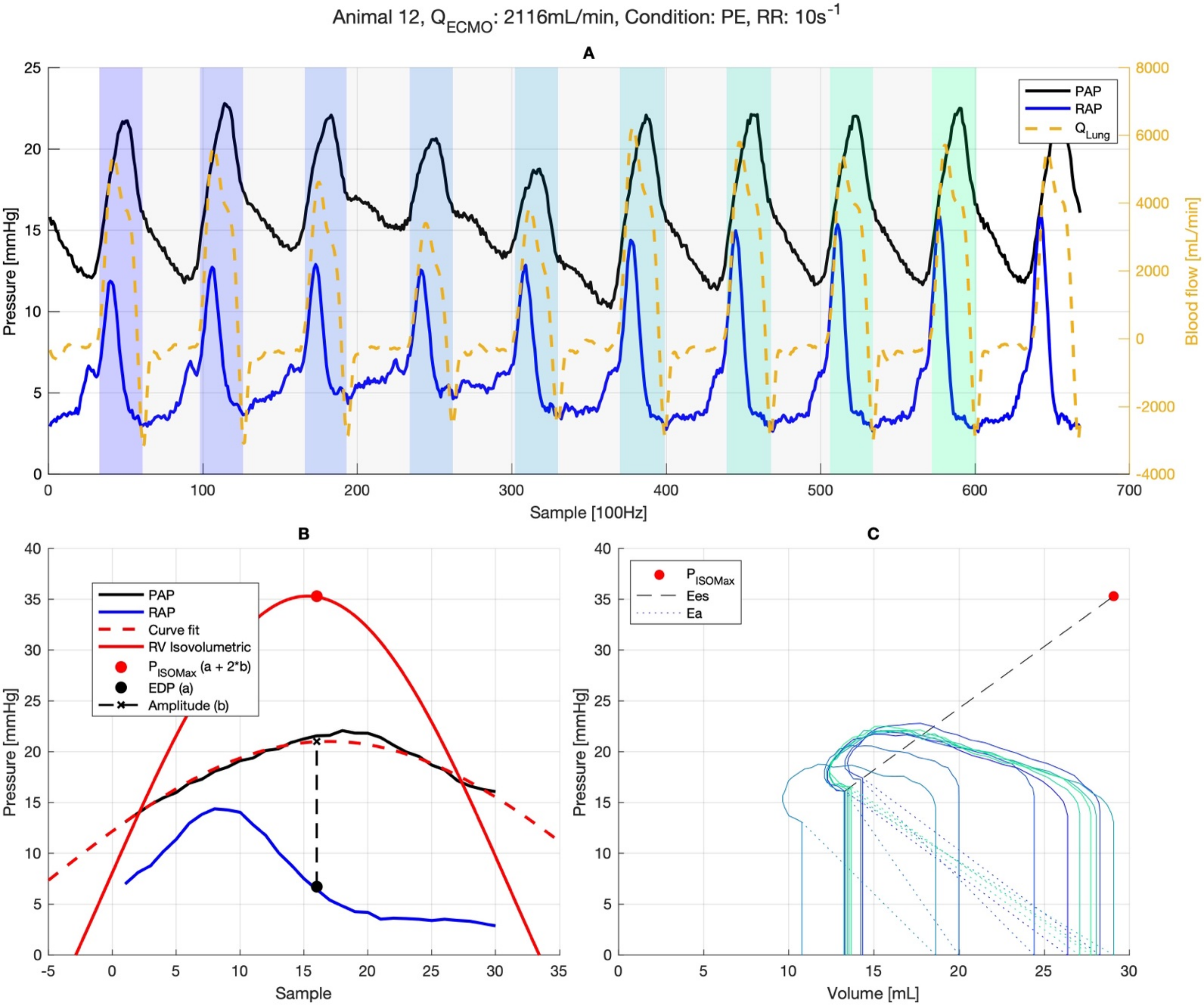

Animal 12, Q_{ECMO}: 2116mL/min, Condition: PE, RR: $10s^{-1}$
A
PAP
RAP
Q_{Lung}
Pressure [mmHg]
Blood flow [mL/min]
Sample [100Hz]
B
PAP
RAP
Curve fit
RV Isovolumetric
P_{ISOMax} (a + 2*b)
EDP (a)
Amplitude (b)
Pressure [mmHg]
Sample
C
P_{ISOMax}
Ees
Ea
Pressure [mmHg]
Volume [mL]

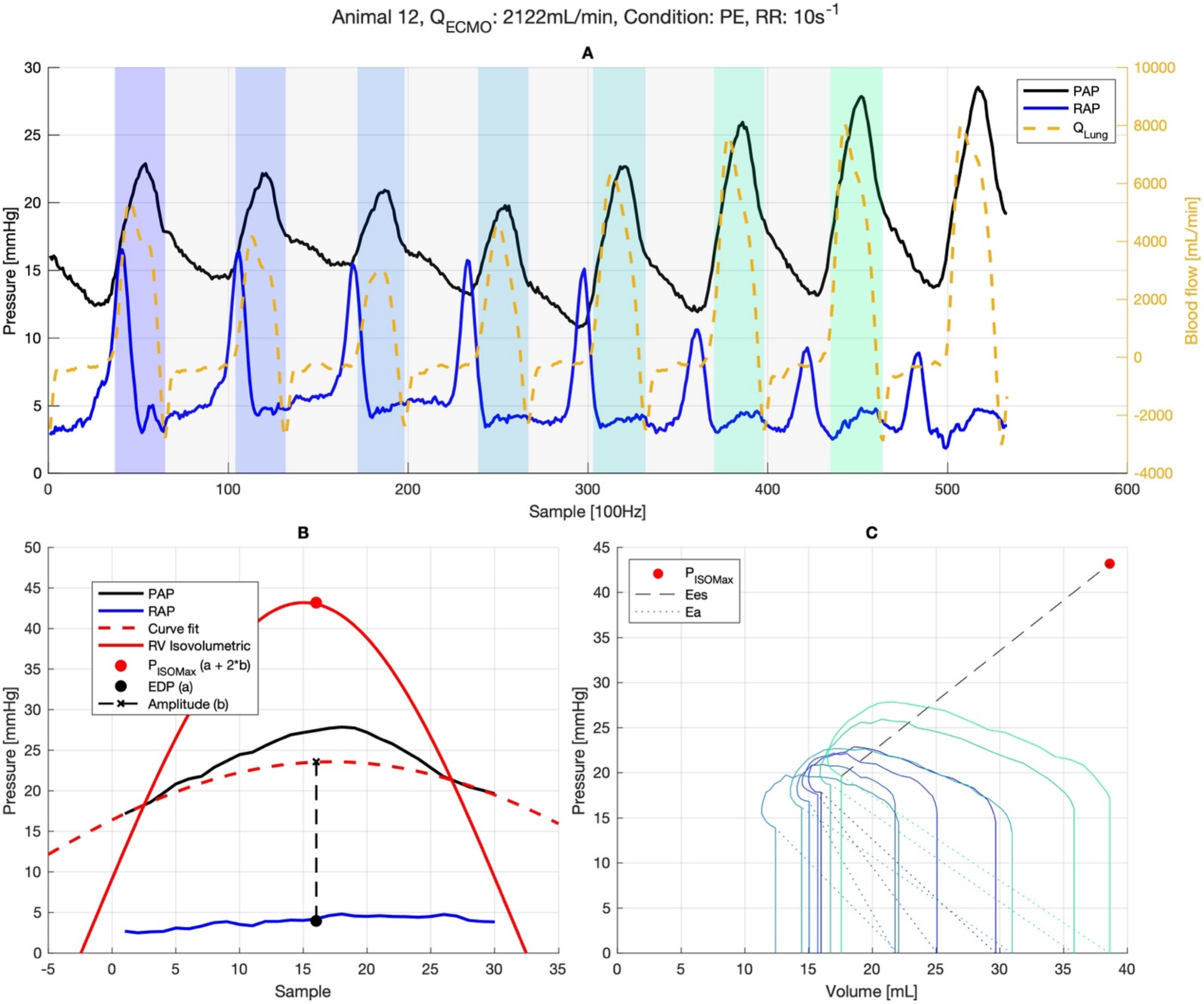

Animal 12, Q_{ECMO}: 2122mL/min, Condition: PE, RR: $10s^{-1}$
A
PAP
RAP
Q_{Lung}
Pressure [mmHg]
Blood flow [mL/min]
Sample [100Hz]
B
PAP
RAP
Curve fit
RV Isovolumetric
P_{ISOMax} (a + 2*b)
EDP (a)
Amplitude (b)
Pressure [mmHg]
Sample
C
P_{ISOMax}
Ees
Ea
Pressure [mmHg]
Volume [mL]

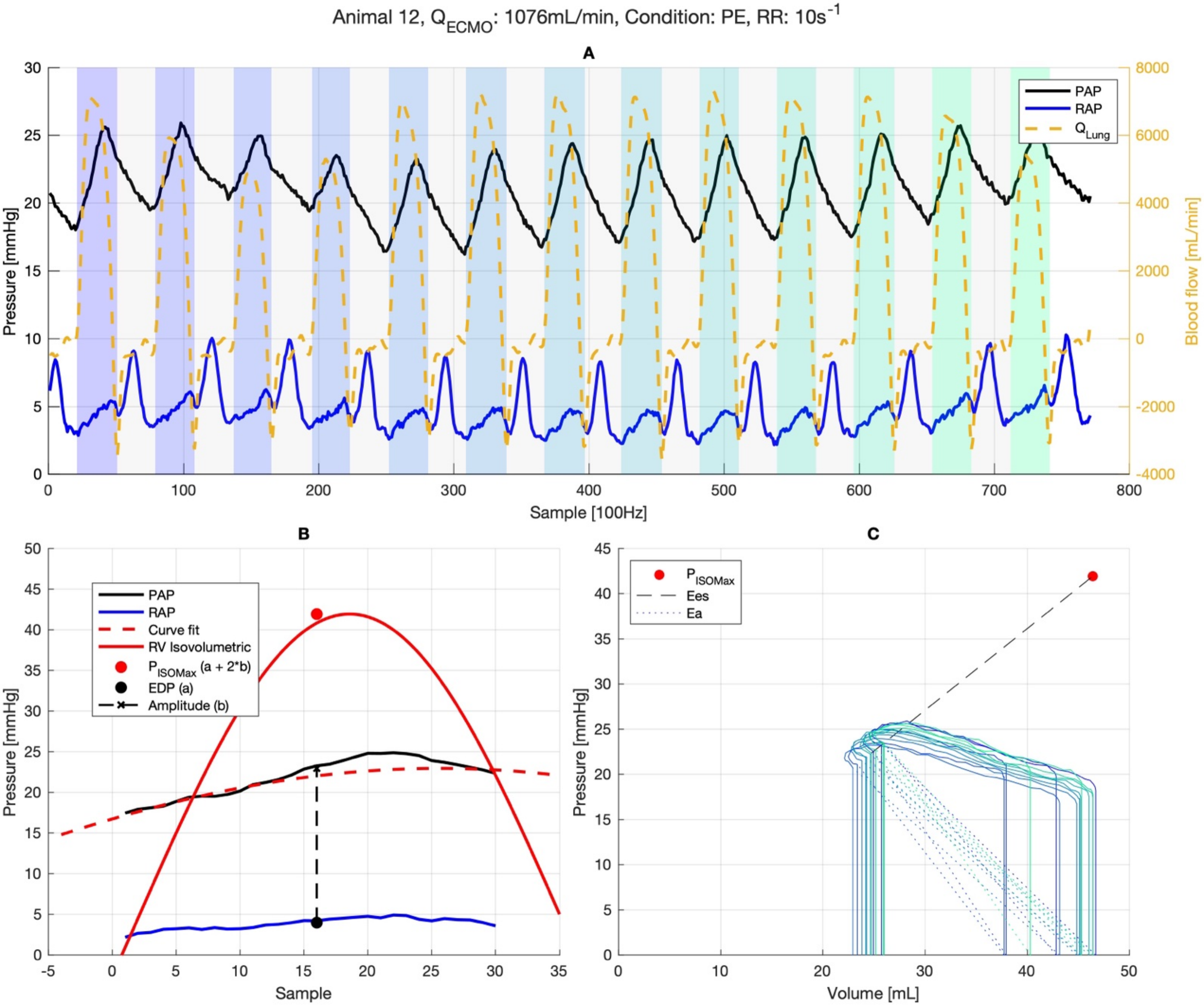

Animal 12, Q_ECMO: 1076mL/min, Condition: PE, RR: 10s^-1
A
PAP
RAP
Q_Lung
Pressure [mmHg]
Blood flow [mL/min]
Sample [100Hz]
B
PAP
RAP
Curve fit
RV Isovolumetric
P_ISOMax (a + 2*b)
EDP (a)
Amplitude (b)
Pressure [mmHg]
Sample
C
P_ISOMax
Ees
Ea
Pressure [mmHg]
Volume [mL]

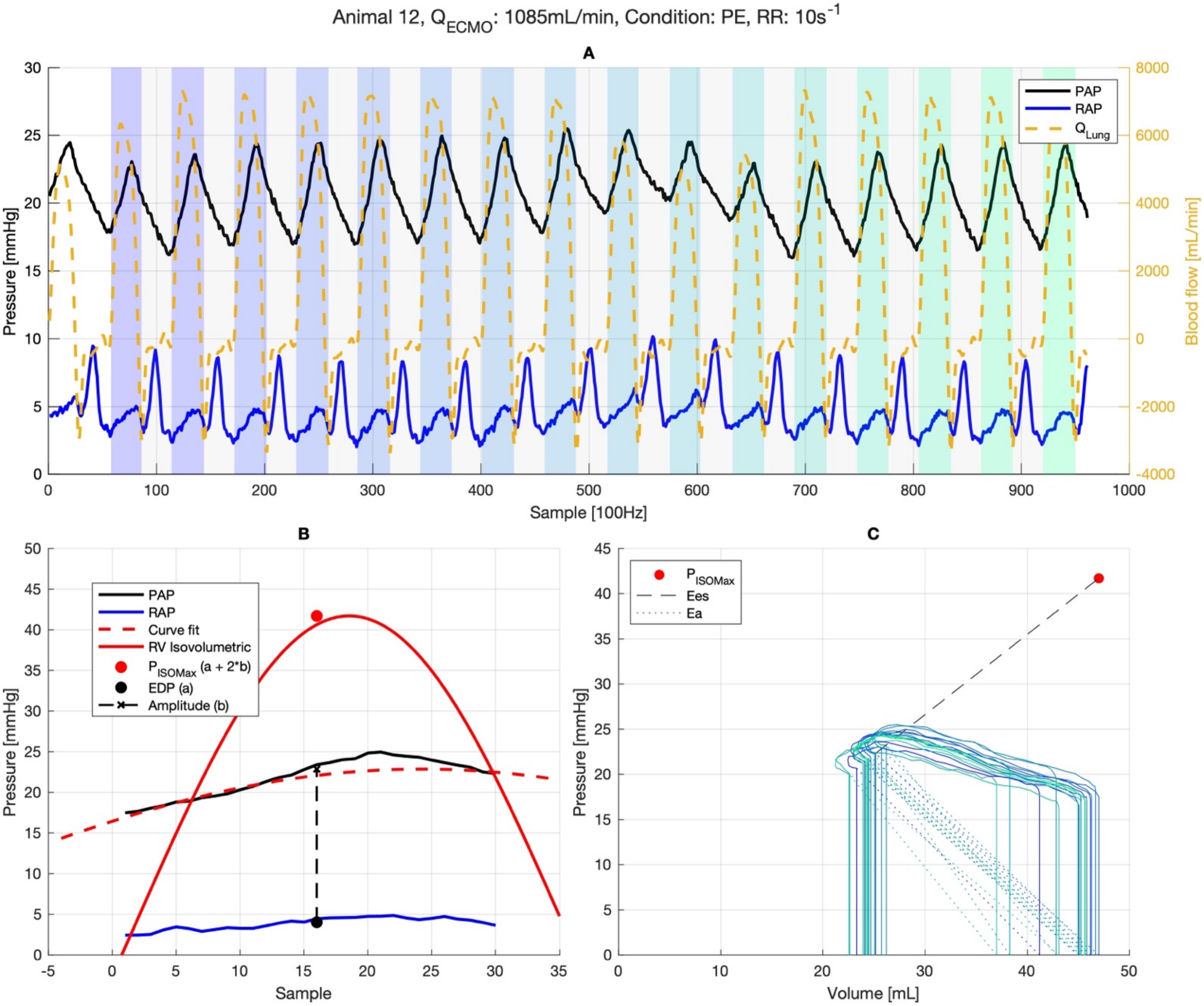

Animal 12, Q_ECMO: 1085mL/min, Condition: PE, RR: 10s^-1
A
Pressure [mmHg]
Blood flow [mL/min]
Sample [100Hz]
PAP
RAP
Q_Lung
B
Pressure [mmHg]
Sample
PAP
RAP
Curve fit
RV Isovolumetric
P_ISOMax (a + 2*b)
EDP (a)
Amplitude (b)
C
Pressure [mmHg]
Volume [mL]
P_ISOMax
Ees
Ea

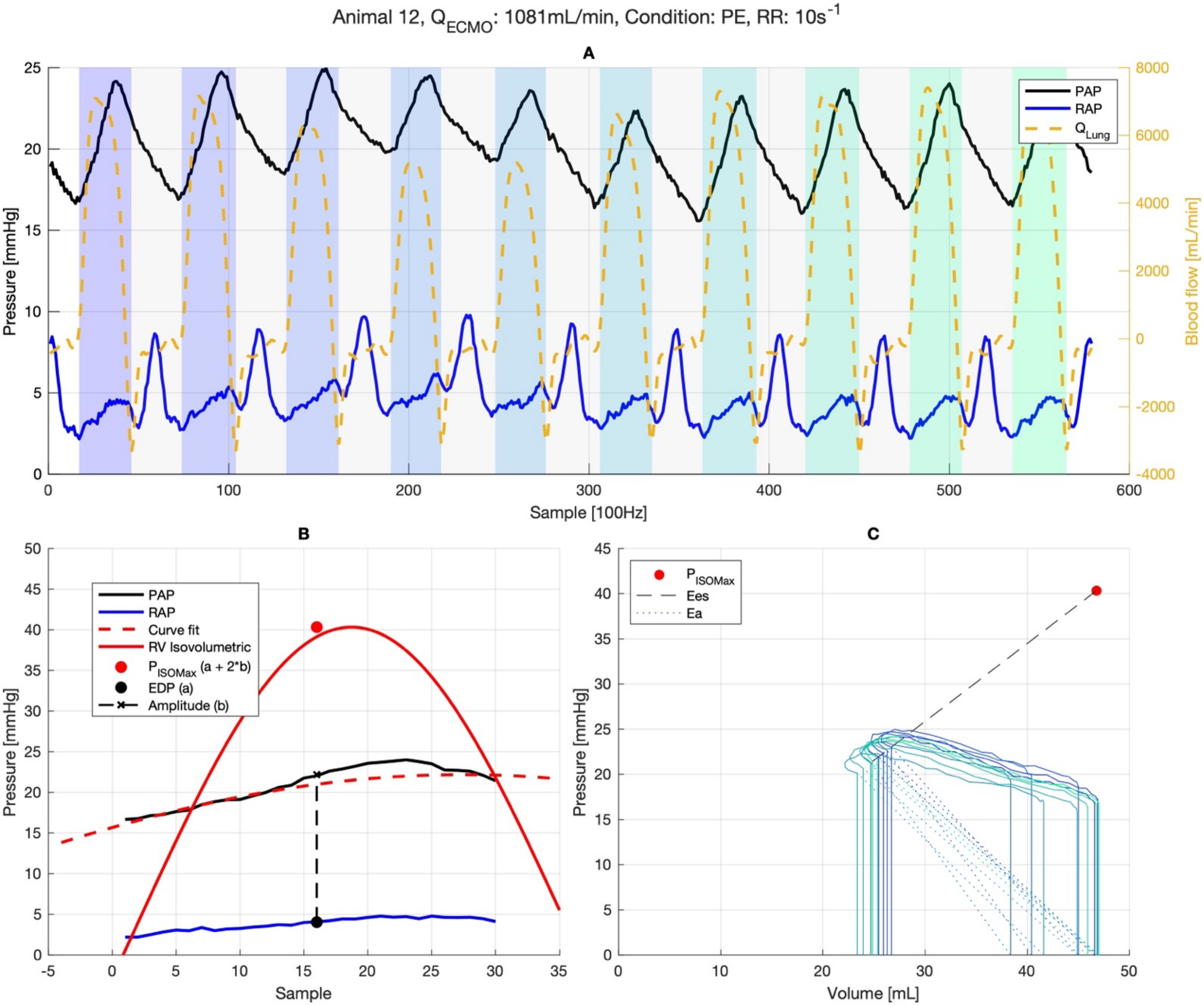

Animal 12, Q_{ECMO}: 1081mL/min, Condition: PE, RR: $10s^{-1}$
A
PAP
RAP
Q_{Lung}
Pressure [mmHg]
Blood flow [mL/min]
Sample [100Hz]
B
PAP
RAP
Curve fit
RV Isovolumetric
P_{ISOMax} (a + 2*b)
EDP (a)
Amplitude (b)
Pressure [mmHg]
Sample
C
P_{ISOMax}
Ees
Ea
Pressure [mmHg]
Volume [mL]

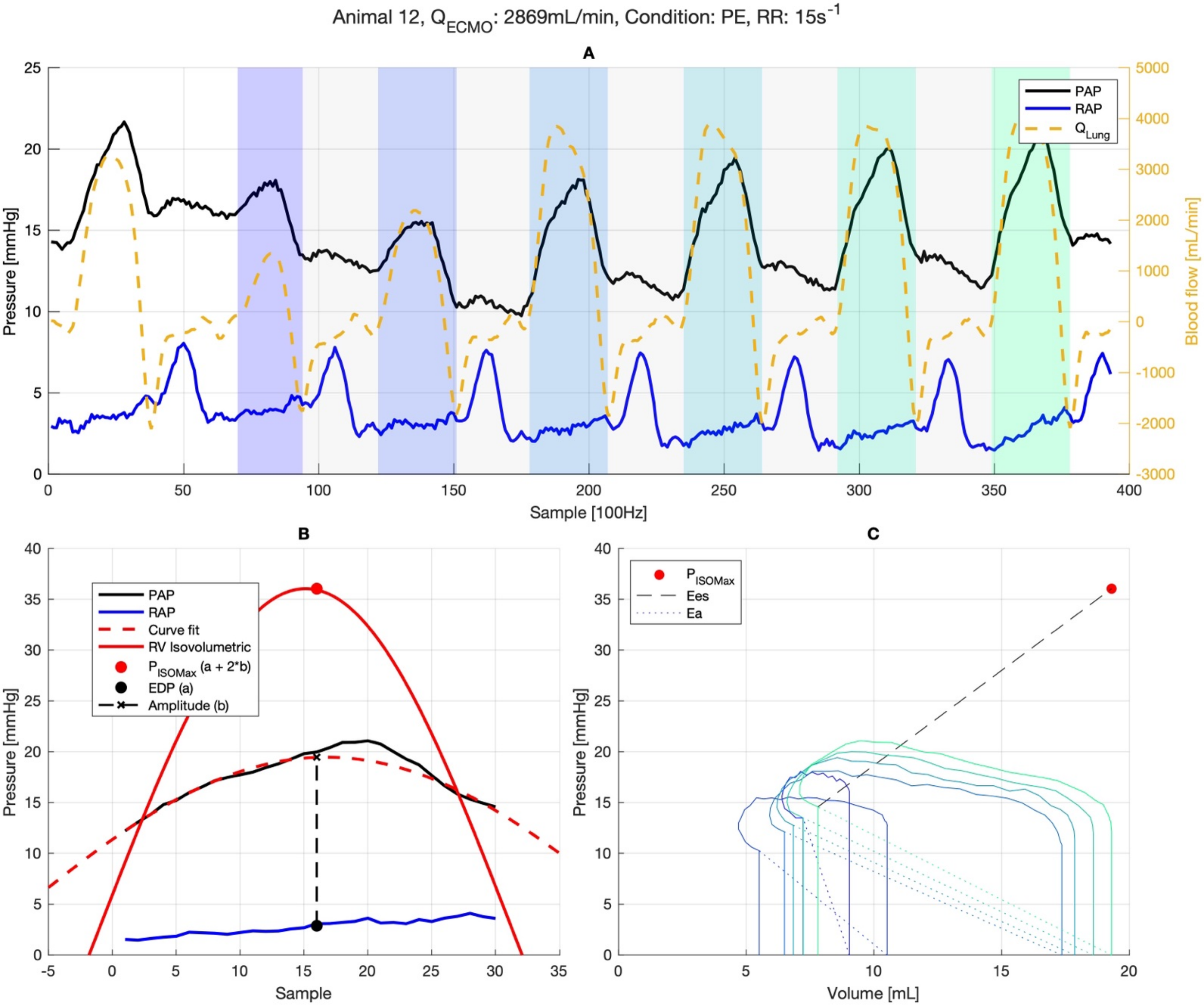

Animal 12, Q_{ECMO}: 2869mL/min, Condition: PE, RR: $15s^{-1}$
A
PAP
RAP
Q_{Lung}
Pressure [mmHg]
Blood flow [mL/min]
Sample [100Hz]
B
PAP
RAP
Curve fit
RV Isovolumetric
P_{ISOMax} (a + 2*b)
EDP (a)
Amplitude (b)
Pressure [mmHg]
Sample
C
P_{ISOMax}
Ees
Ea
Pressure [mmHg]
Volume [mL]

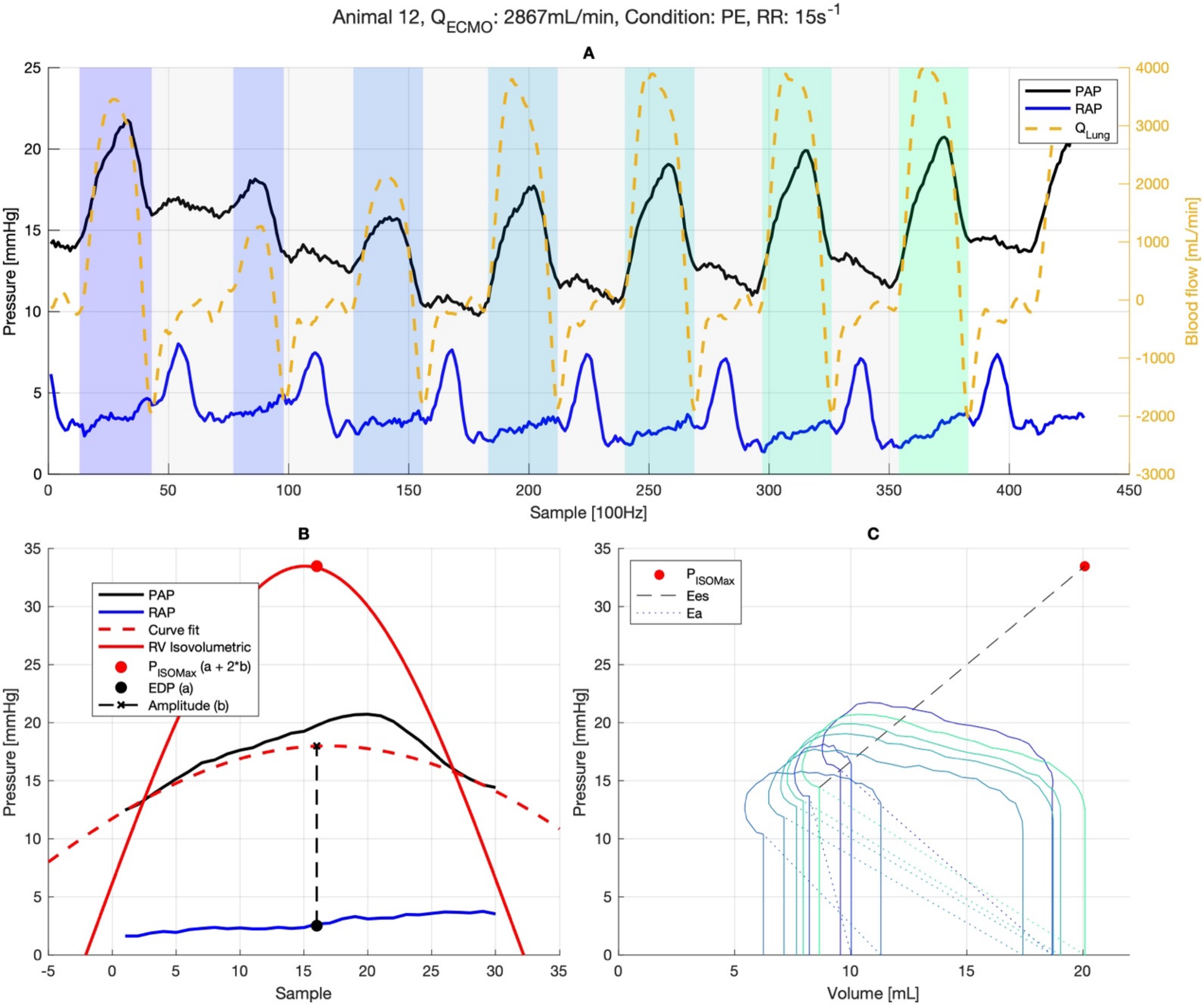

Animal 12, Q_{ECMO}: 2867mL/min, Condition: PE, RR: 15s^{-1}
A
Pressure [mmHg]
Blood flow [mL/min]
Sample [100Hz]
PAP
RAP
Q_{Lung}
B
Pressure [mmHg]
Sample
PAP
RAP
Curve fit
RV Isovolumetric
P_{ISOMax} (a + 2*b)
EDP (a)
Amplitude (b)
C
Pressure [mmHg]
Volume [mL]
P_{ISOMax}
Ees
Ea

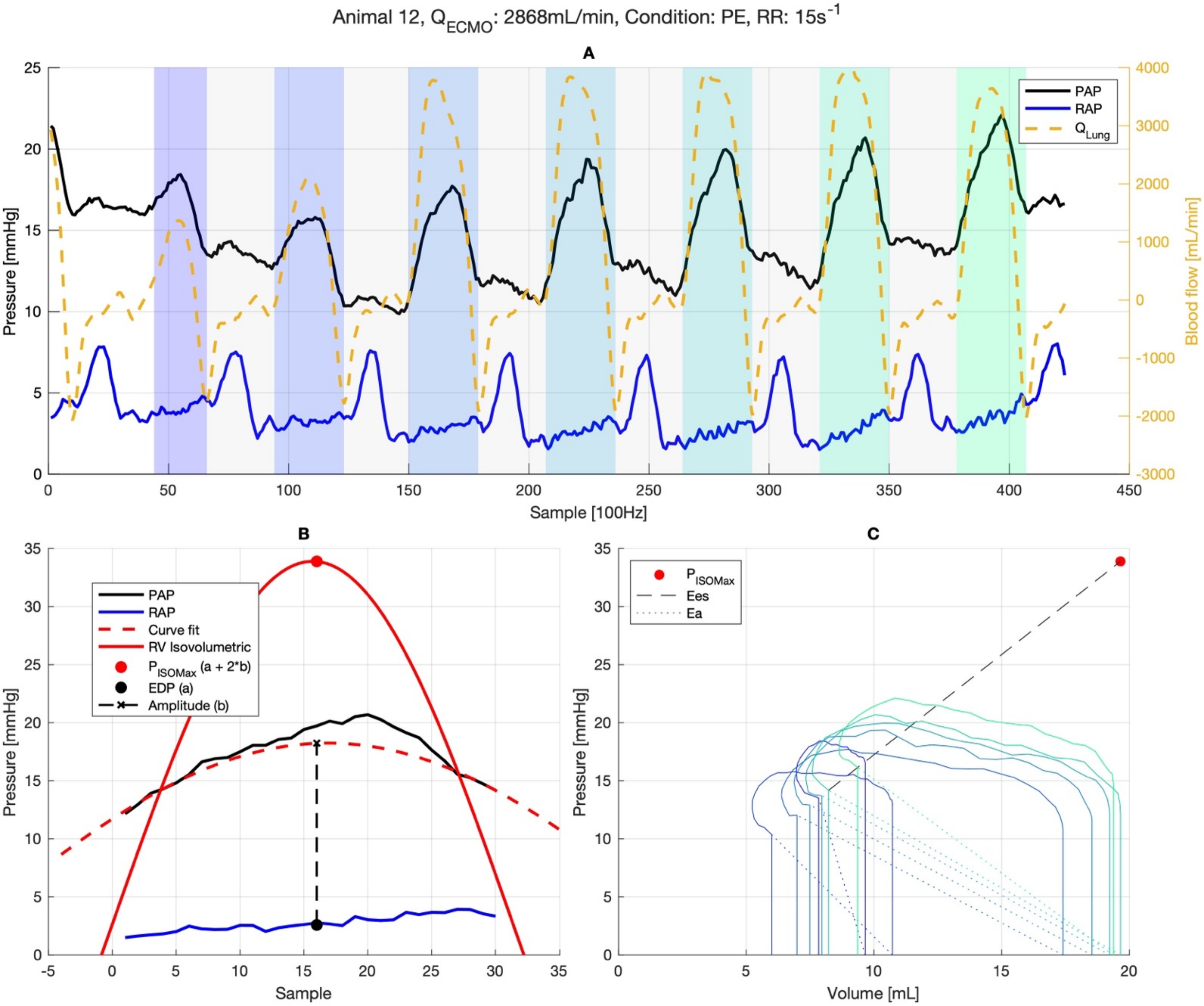

Animal 12, Q_{ECMO}: 2868mL/min, Condition: PE, RR: 15s^{-1}
A
PAP
RAP
Q_{Lung}
Pressure [mmHg]
Blood flow [mL/min]
Sample [100Hz]
B
PAP
RAP
Curve fit
RV Isovolumetric
P_{ISOMax} (a + 2*b)
EDP (a)
Amplitude (b)
Pressure [mmHg]
Sample
C
P_{ISOMax}
Ees
Ea
Pressure [mmHg]
Volume [mL]

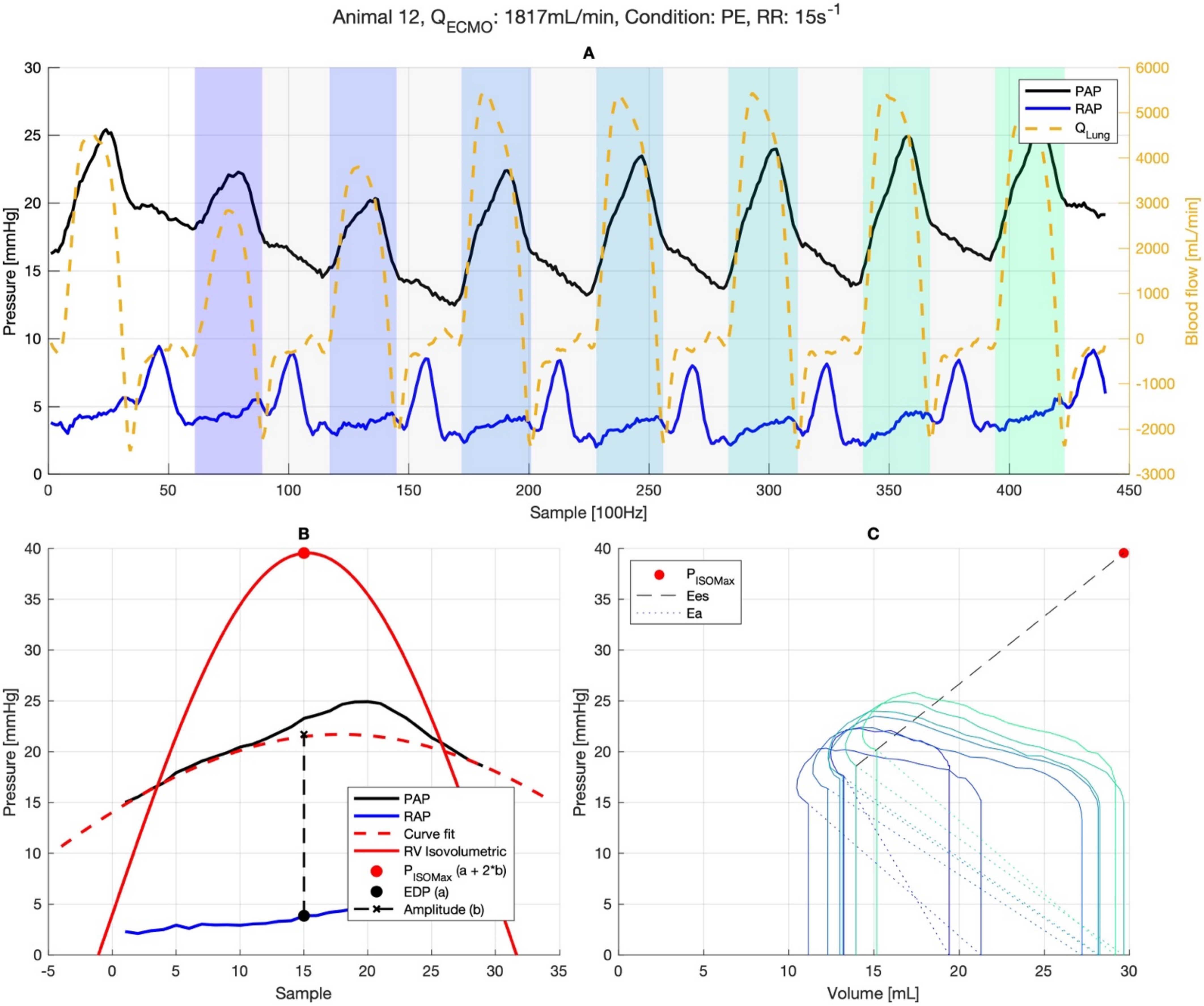

Animal 12, Q_{ECMO}: 1817mL/min, Condition: PE, RR: $15s^{-1}$
A
PAP
RAP
Q_{Lung}
Pressure [mmHg]
Blood flow [mL/min]
Sample [100Hz]
B
Pressure [mmHg]
Sample
PAP
RAP
Curve fit
RV Isovolumetric
P_{ISOMax} (a + 2*b)
EDP (a)
Amplitude (b)
C
P_{ISOMax}
Ees
Ea
Pressure [mmHg]
Volume [mL]

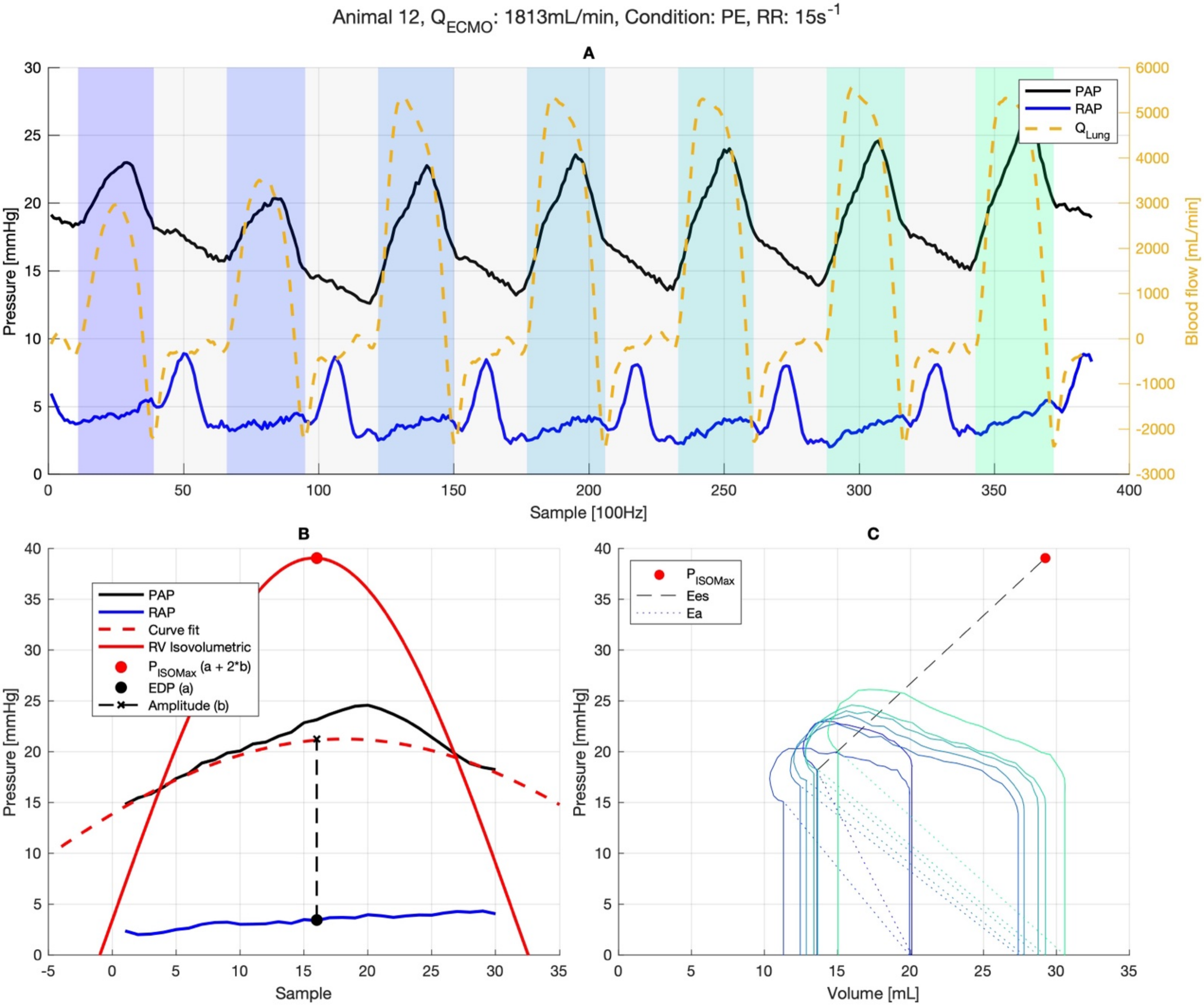

Animal 12, Q_{ECMO}: 1813mL/min, Condition: PE, RR: $15s^{-1}$
A
PAP
RAP
Q_{Lung}
Pressure [mmHg]
Blood flow [mL/min]
Sample [100Hz]
B
PAP
RAP
Curve fit
RV Isovolumetric
P_{ISOMax} (a + 2*b)
EDP (a)
Amplitude (b)
Pressure [mmHg]
Sample
C
P_{ISOMax}
Ees
Ea
Pressure [mmHg]
Volume [mL]

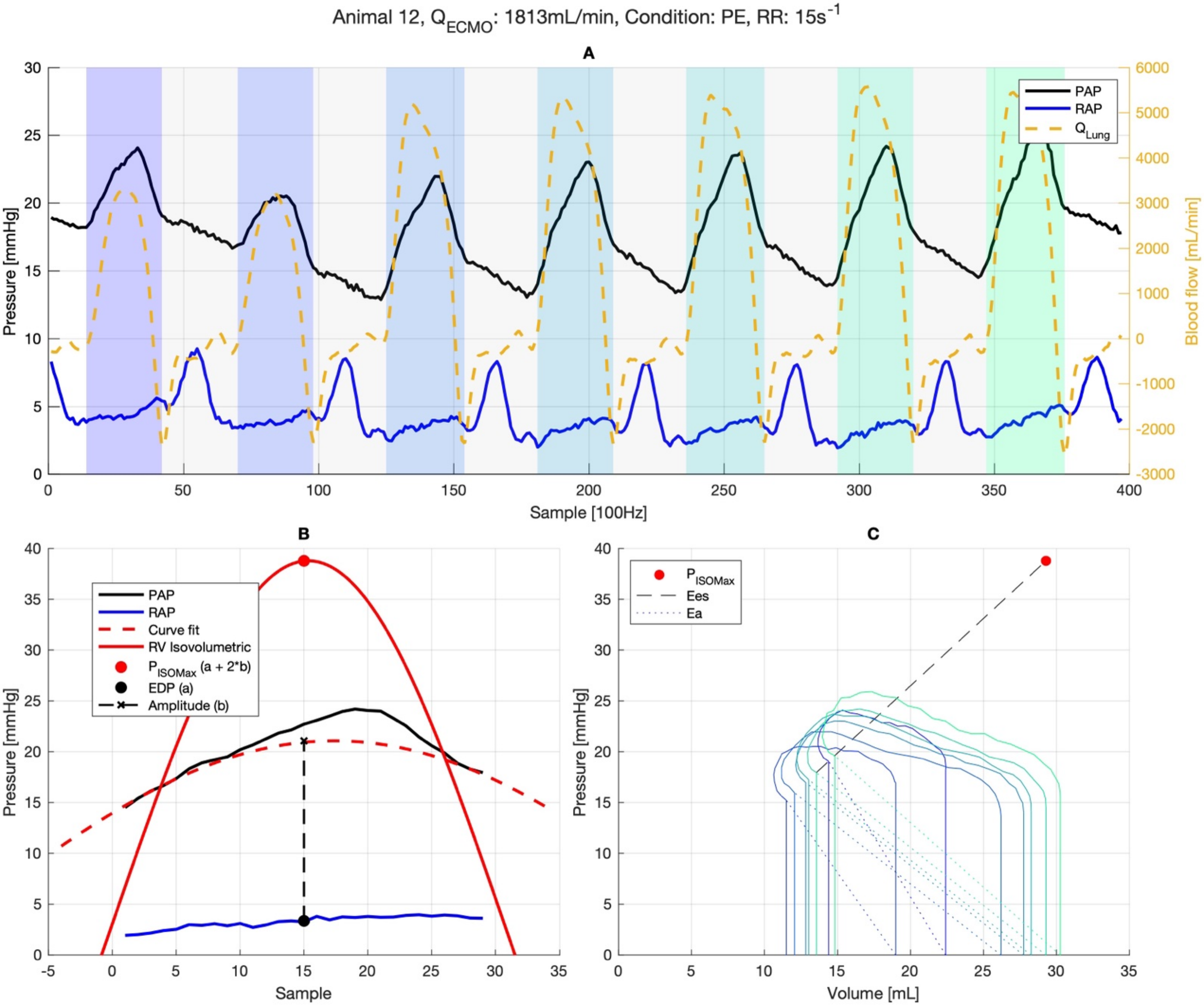

Animal 12, Q_{ECMO}: 1813mL/min, Condition: PE, RR: $15s^{-1}$
A
PAP
RAP
Q_{Lung}
Pressure [mmHg]
Blood flow [mL/min]
Sample [100Hz]
B
PAP
RAP
Curve fit
RV Isovolumetric
P_{ISOMax} (a + 2*b)
EDP (a)
Amplitude (b)
Pressure [mmHg]
Sample
C
P_{ISOMax}
Ees
Ea
Pressure [mmHg]
Volume [mL]

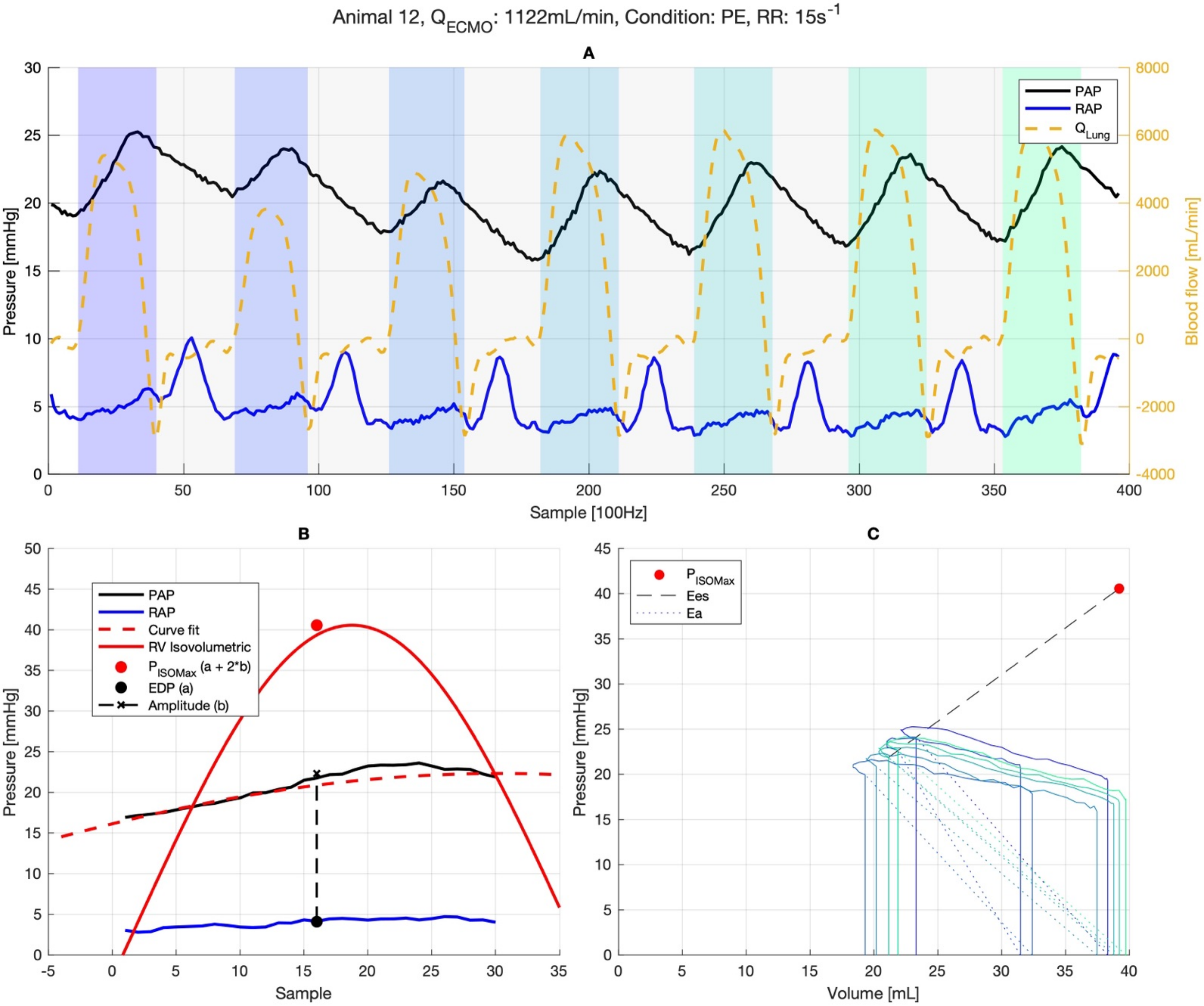

Animal 12, Q_{ECMO}: 1122mL/min, Condition: PE, RR: $15s^{-1}$
A
PAP
RAP
Q_{Lung}
Pressure [mmHg]
Blood flow [mL/min]
Sample [100Hz]
B
PAP
RAP
Curve fit
RV Isovolumetric
P_{ISOMax} (a + 2*b)
EDP (a)
Amplitude (b)
Pressure [mmHg]
Sample
C
P_{ISOMax}
Ees
Ea
Pressure [mmHg]
Volume [mL]

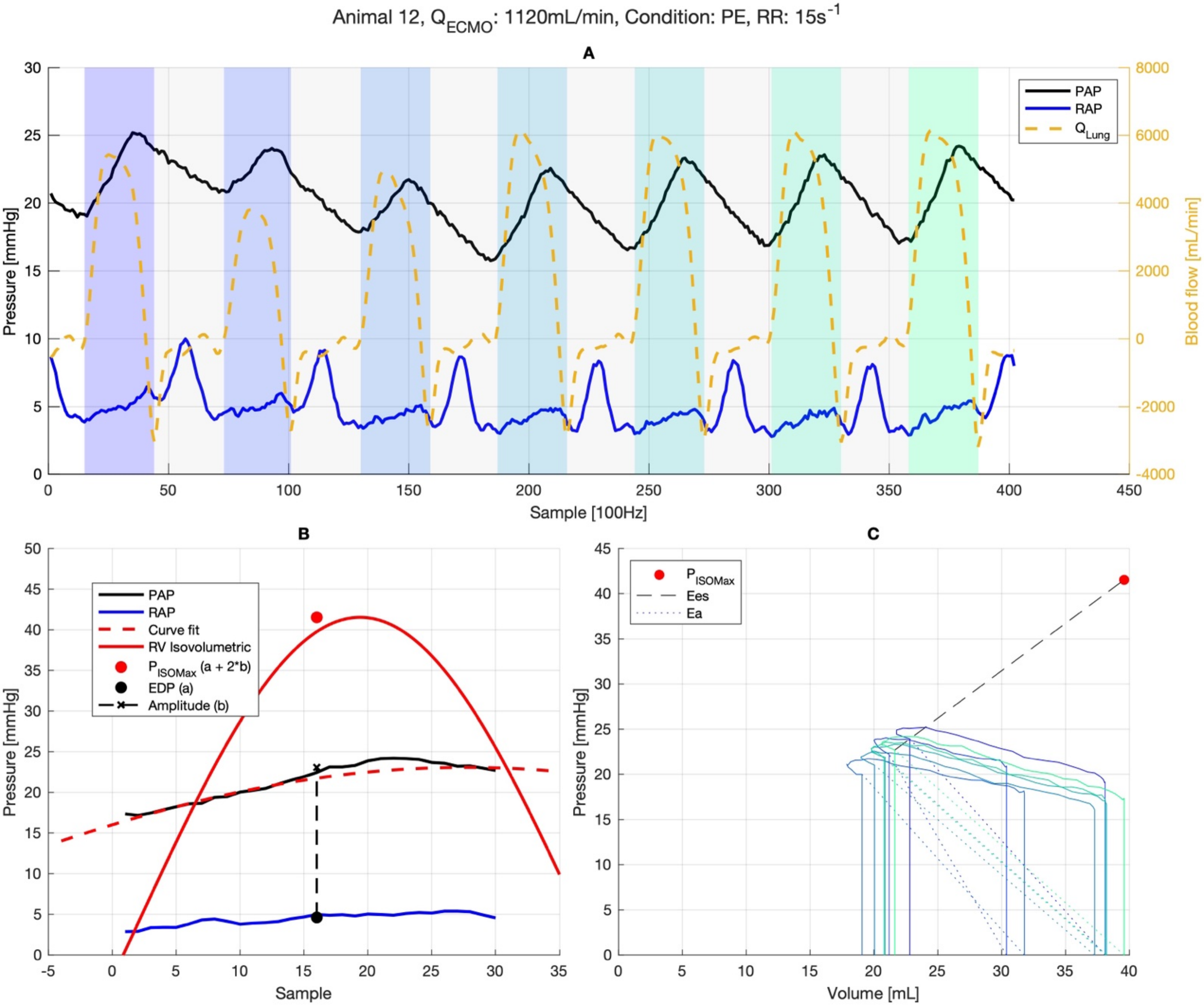
Animal 12, Q_ECMO: 1120mL/min, Condition: PE, RR: 15s^-1
A
PAP
RAP
Q_Lung
Pressure [mmHg]
Blood flow [mL/min]
Sample [100Hz]
B
PAP
RAP
Curve fit
RV Isovolumetric
P_ISOMax (a + 2*b)
EDP (a)
Amplitude (b)
Pressure [mmHg]
Sample
C
P_ISOMax
Ees
Ea
Pressure [mmHg]
Volume [mL]

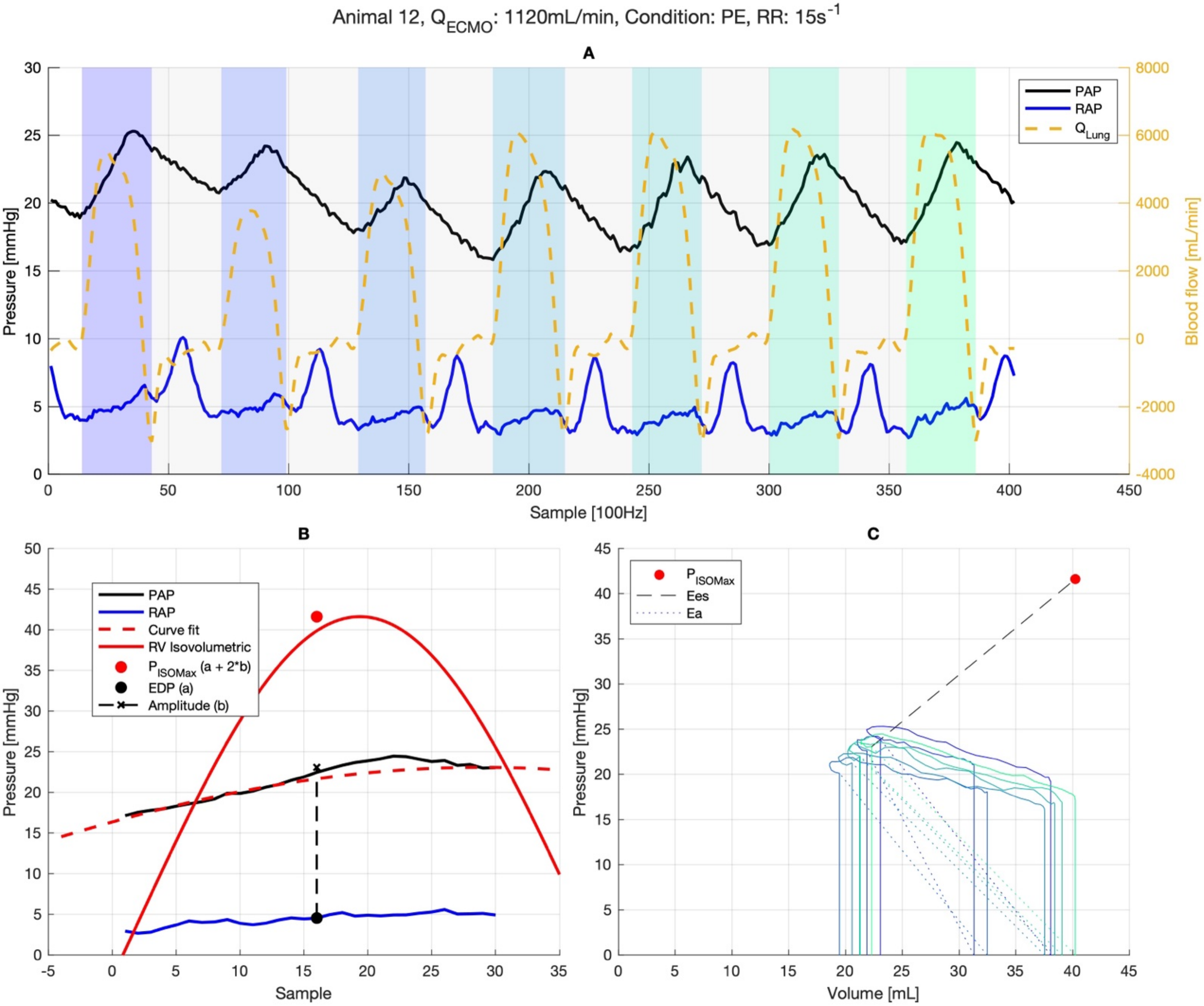

Animal 12, Q_ECMO: 1120mL/min, Condition: PE, RR: 15s^-1
A
Pressure [mmHg]
Blood flow [mL/min]
Sample [100Hz]
PAP
RAP
Q_Lung
B
Pressure [mmHg]
Sample
Curve fit
RV Isovolumetric
P_ISOMax (a + 2*b)
EDP (a)
Amplitude (b)
C
P_ISOMax
Ees
Ea
Pressure [mmHg]
Volume [mL]

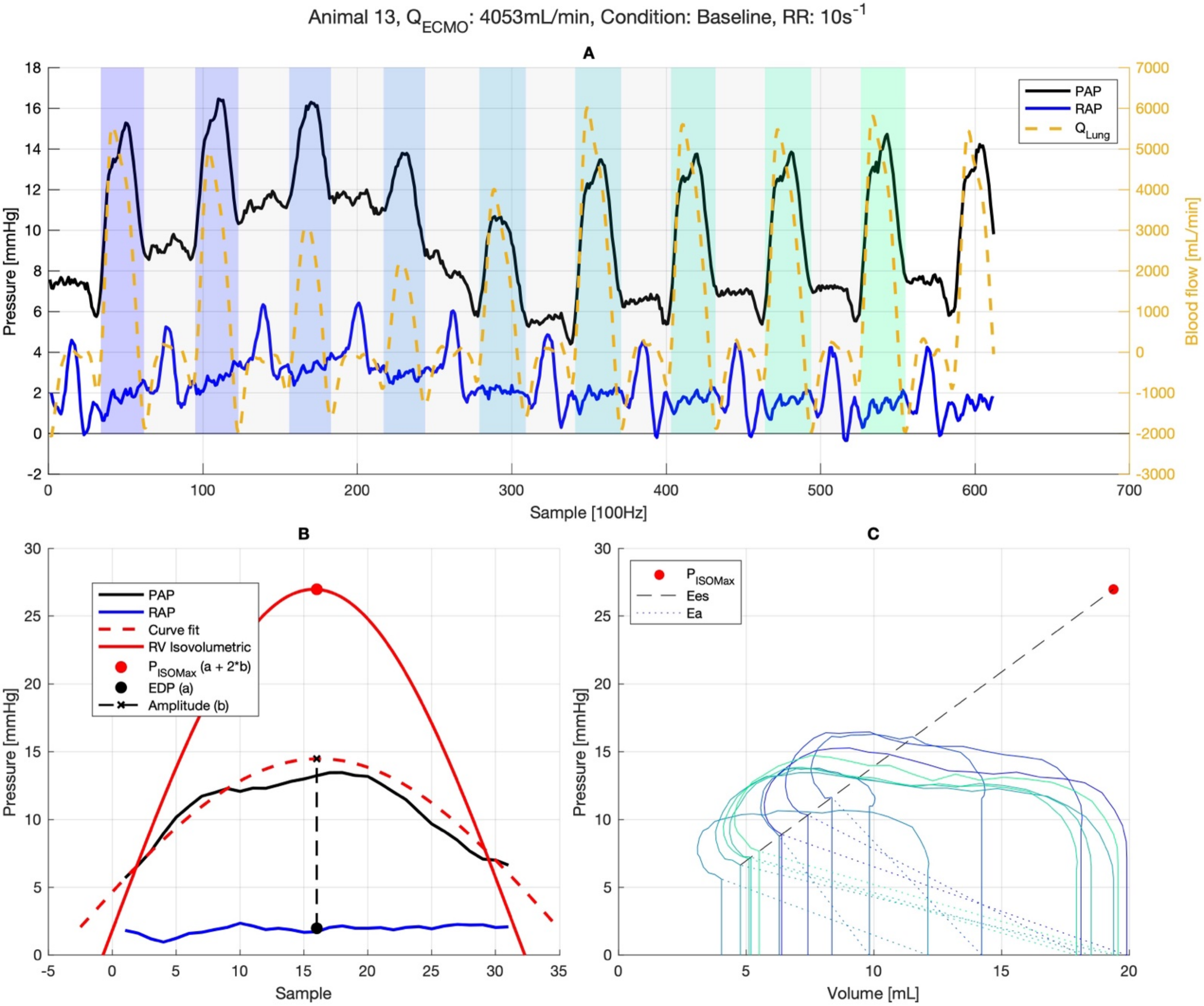

Animal 13, Q_ECMO: 4053mL/min, Condition: Baseline, RR: 10s^-1
A
Pressure [mmHg]
Blood flow [mL/min]
Sample [100Hz]
PAP
RAP
Q_Lung
B
Pressure [mmHg]
Sample
Curve fit
RV Isovolumetric
P_ISOMax (a + 2*b)
EDP (a)
Amplitude (b)
C
Pressure [mmHg]
Volume [mL]
P_ISOMax
Ees
Ea

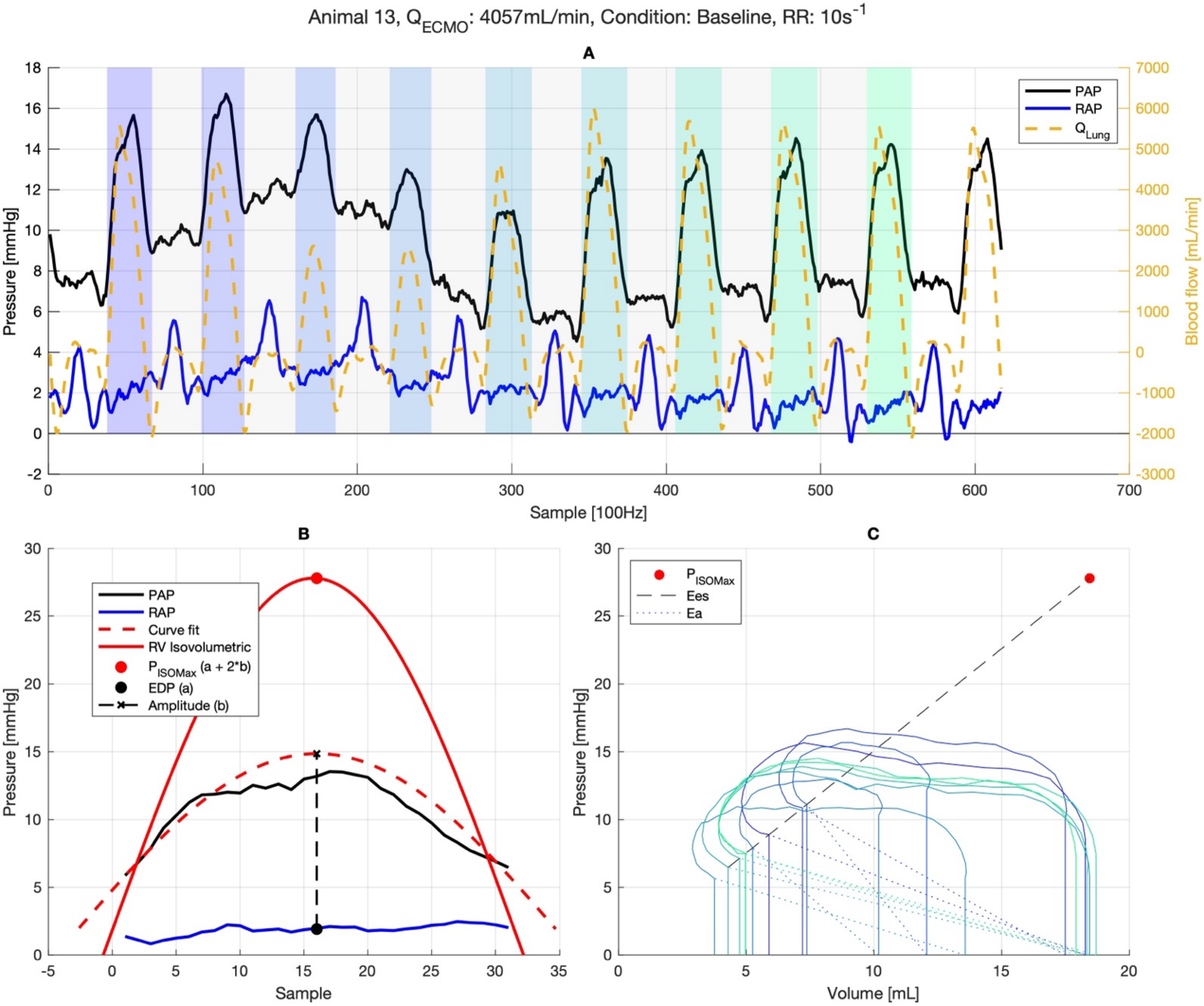

Animal 13, Q_{ECMO}: 4057mL/min, Condition: Baseline, RR: $10s^{-1}$
A
PAP
RAP
Q_{Lung}
Pressure [mmHg]
Blood flow [mL/min]
Sample [100Hz]
B
PAP
RAP
Curve fit
RV Isovolumetric
P_{ISOMax} (a + 2*b)
EDP (a)
Amplitude (b)
Pressure [mmHg]
Sample
C
P_{ISOMax}
Ees
Ea
Pressure [mmHg]
Volume [mL]

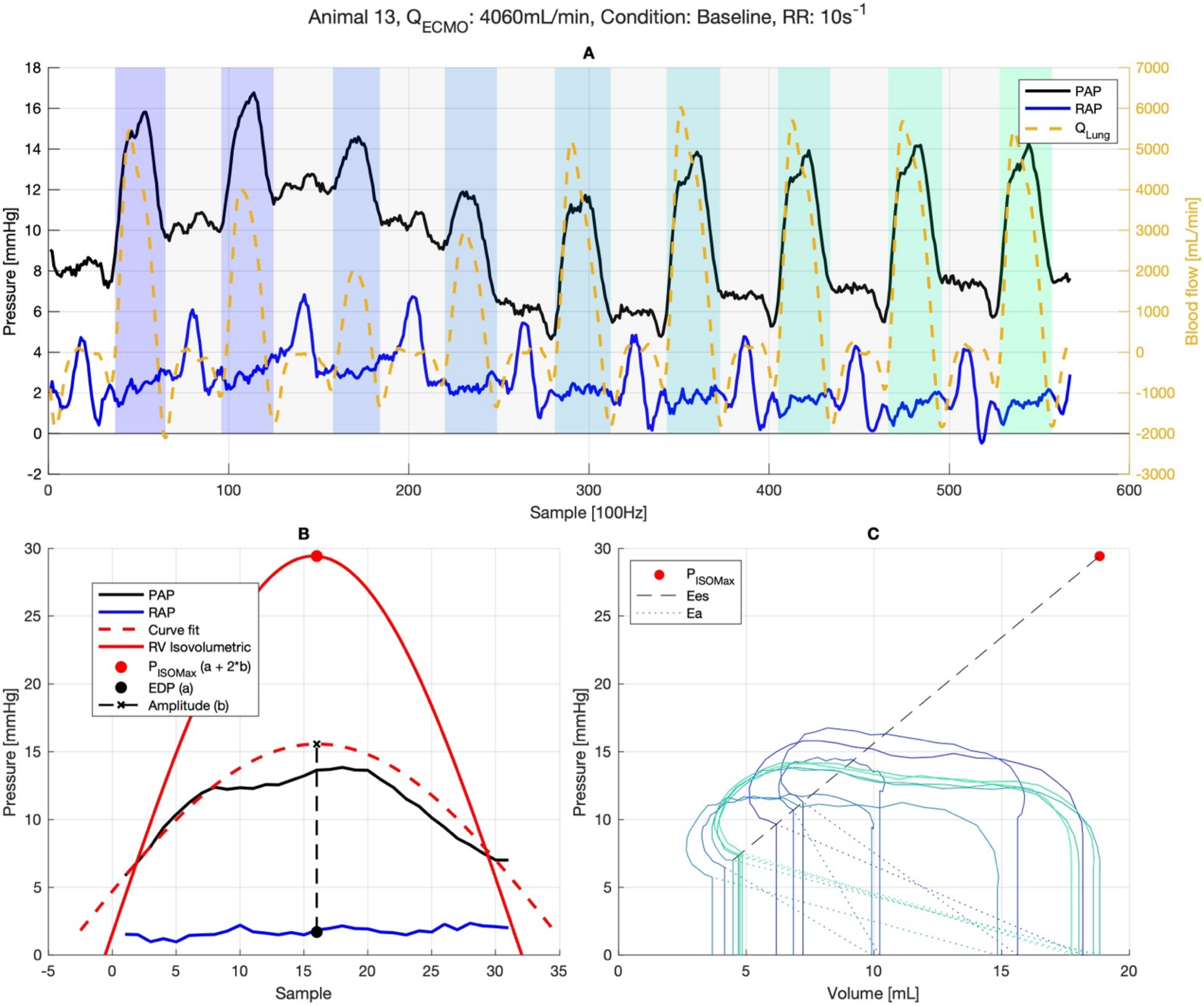

Animal 13, Q_{ECMO}: 4060mL/min, Condition: Baseline, RR: $10s^{-1}$
A
PAP
RAP
Q_{Lung}
Pressure [mmHg]
Blood flow [mL/min]
Sample [100Hz]
B
PAP
RAP
Curve fit
RV Isovolumetric
P_{ISOMax} (a + 2*b)
EDP (a)
Amplitude (b)
Pressure [mmHg]
Sample
C
P_{ISOMax}
Ees
Ea
Pressure [mmHg]
Volume [mL]

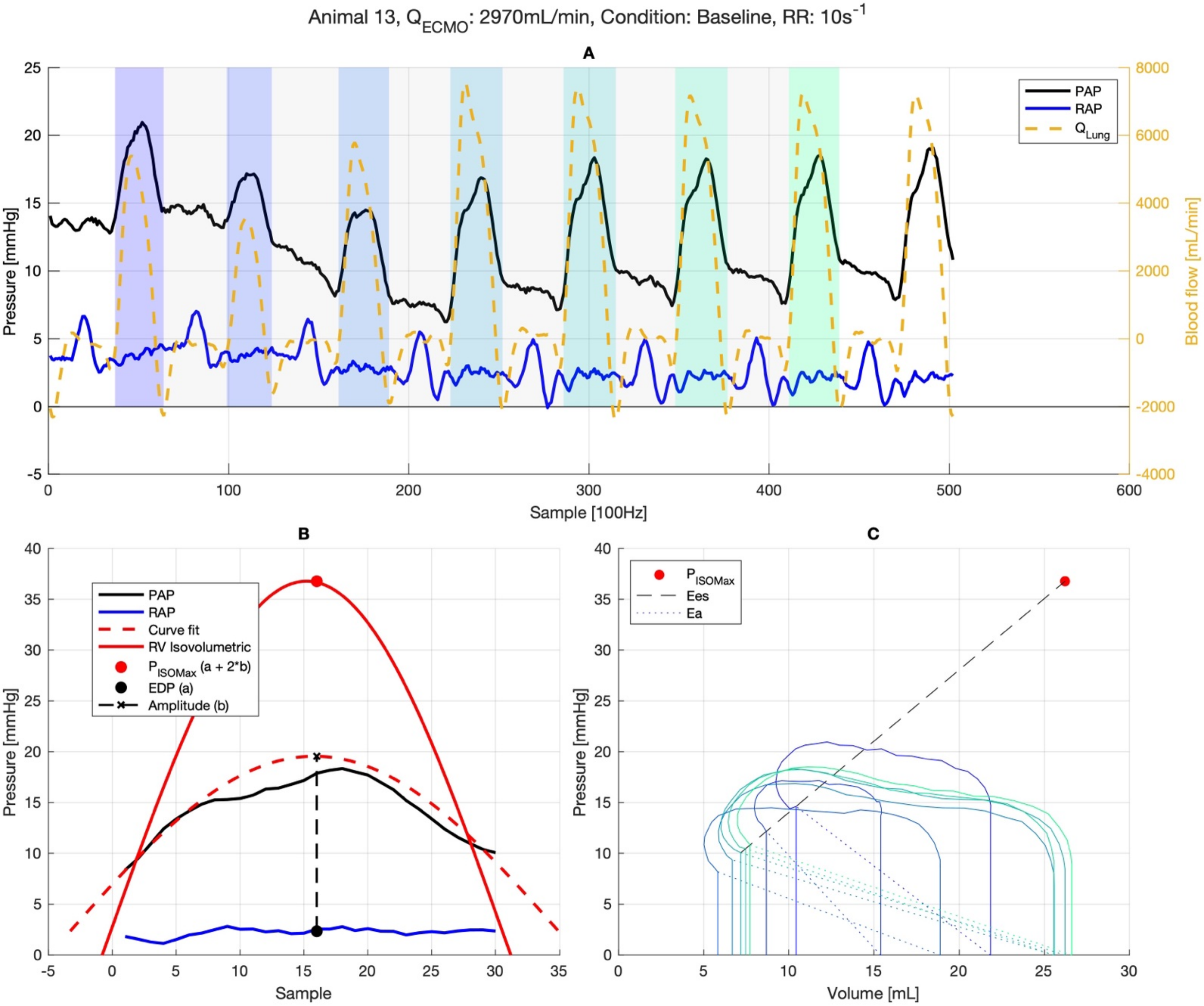

Animal 13, Q_{ECMO}: 2970mL/min, Condition: Baseline, RR: $10s^{-1}$
A
Pressure [mmHg]
Blood flow [mL/min]
Sample [100Hz]
PAP
RAP
Q_{Lung}
B
Pressure [mmHg]
Sample
PAP
RAP
Curve fit
RV Isovolumetric
P_{ISOMax} (a + 2*b)
EDP (a)
Amplitude (b)
C
Pressure [mmHg]
Volume [mL]
P_{ISOMax}
Ees
Ea

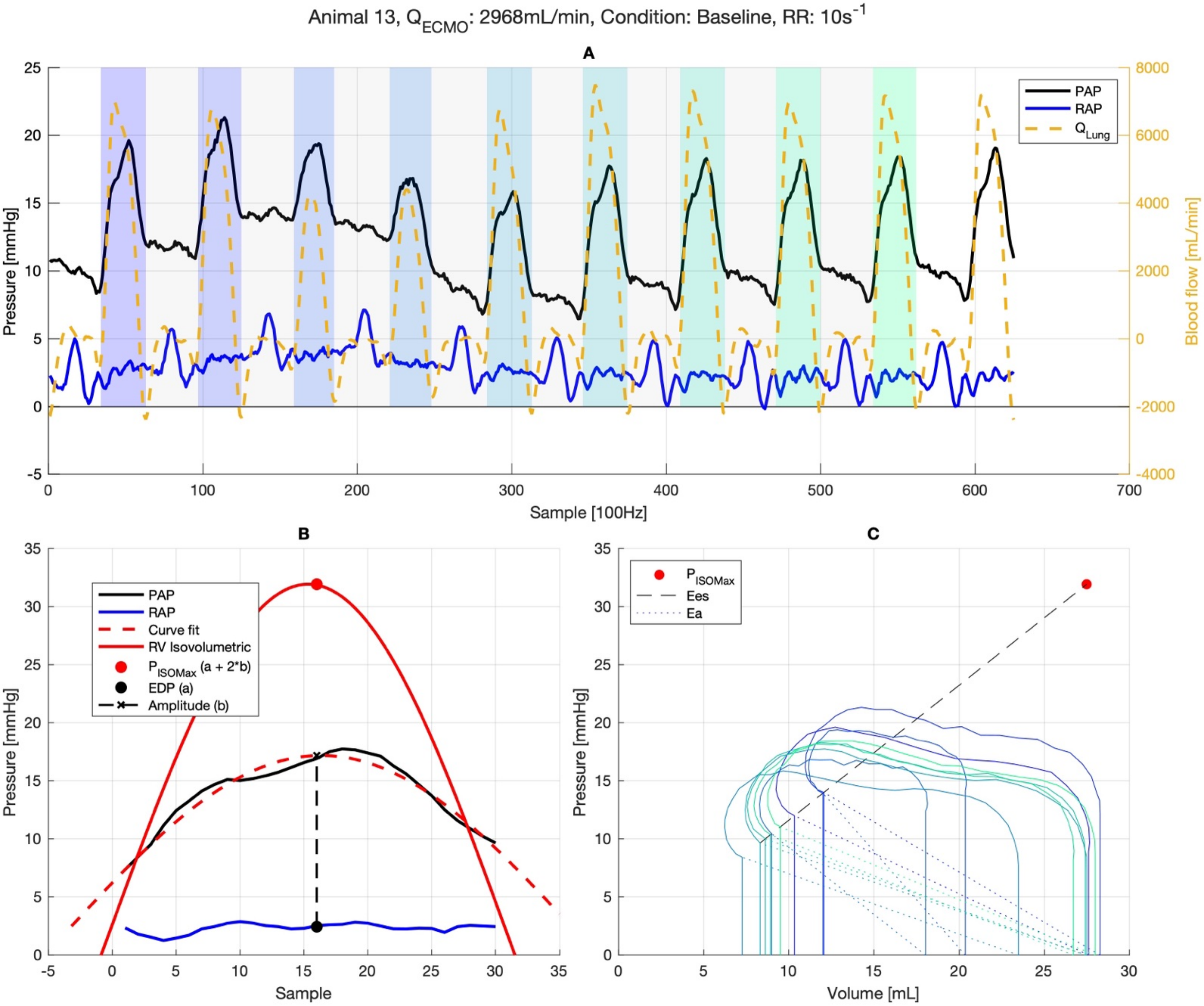
Animal 13, Q_ECMO: 2968mL/min, Condition: Baseline, RR: 10s^-1
A
PAP
RAP
Q_Lung
Pressure [mmHg]
Blood flow [mL/min]
Sample [100Hz]
B
PAP
RAP
Curve fit
RV Isovolumetric
P_ISOMax (a + 2*b)
EDP (a)
Amplitude (b)
Pressure [mmHg]
Sample
C
P_ISOMax
Ees
Ea
Pressure [mmHg]
Volume [mL]

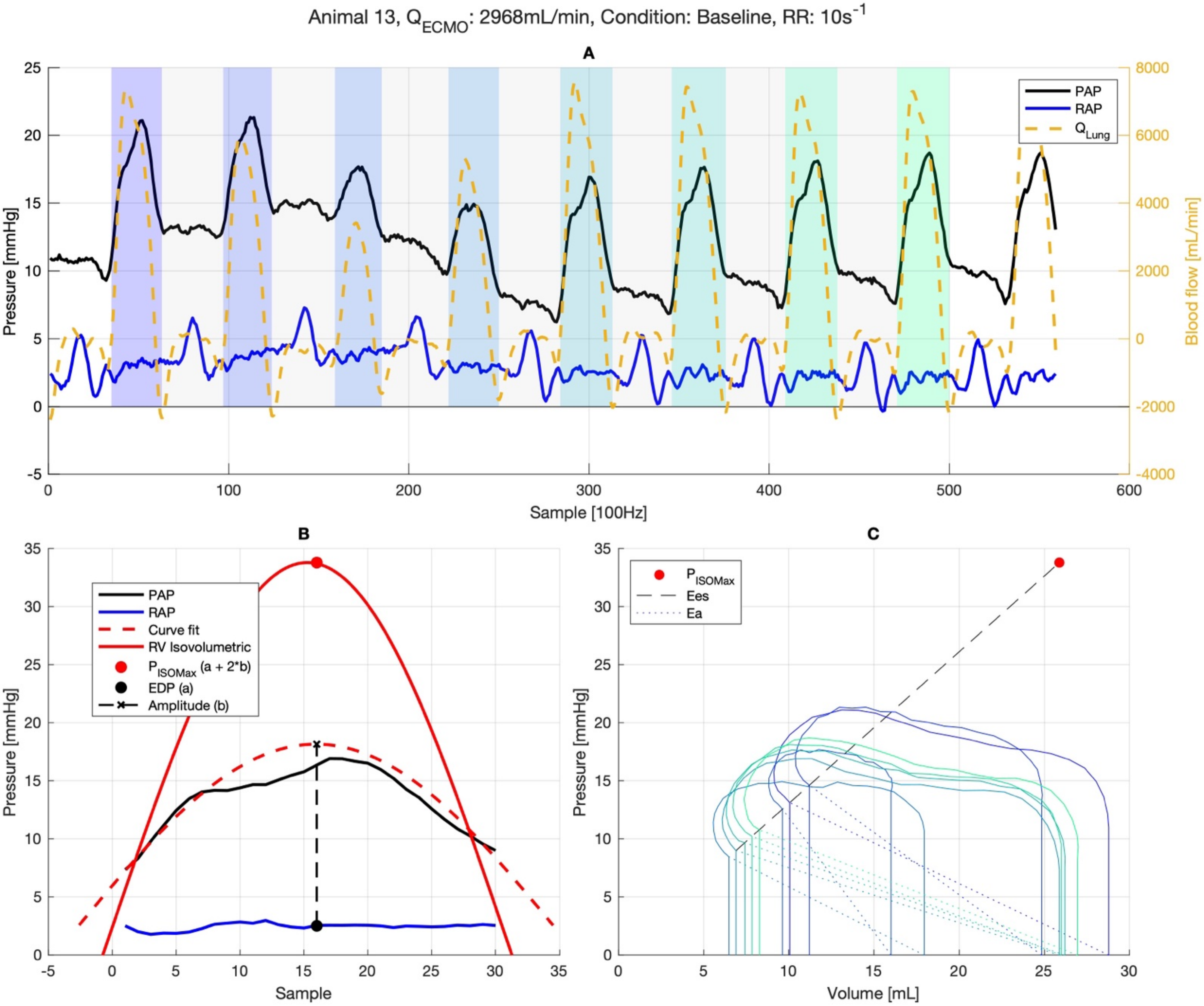

Animal 13, Q_{ECMO}: 2968mL/min, Condition: Baseline, RR: 10s^{-1}
A
PAP
RAP
Q_{Lung}
Pressure [mmHg]
Blood flow [mL/min]
Sample [100Hz]
B
PAP
RAP
Curve fit
RV Isovolumetric
P_{ISOMax} (a + 2*b)
EDP (a)
Amplitude (b)
Pressure [mmHg]
Sample
C
P_{ISOMax}
Ees
Ea
Pressure [mmHg]
Volume [mL]

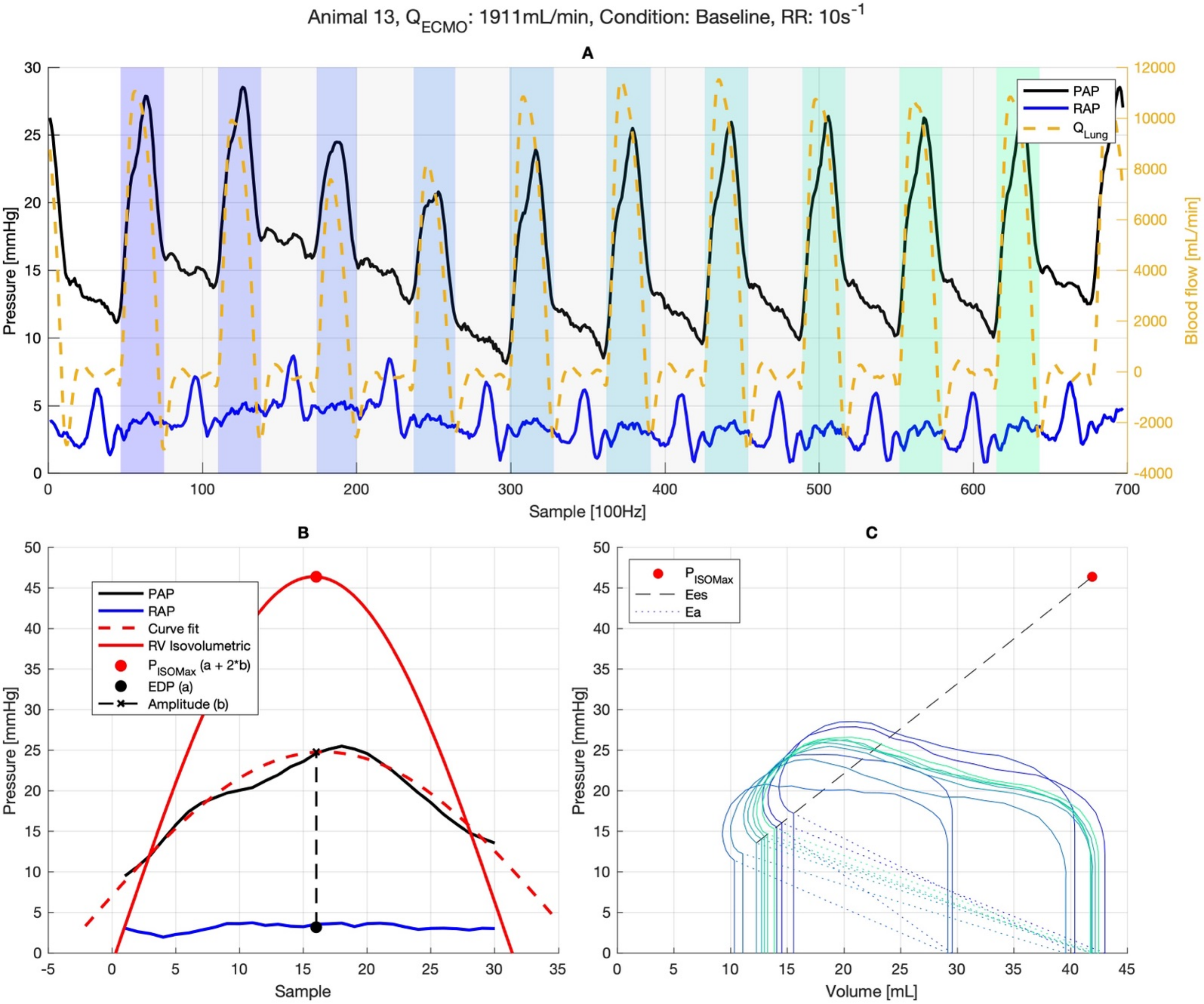

Animal 13, Q_ECMO: 1911mL/min, Condition: Baseline, RR: 10s^-1
A
PAP
RAP
Q_Lung
Pressure [mmHg]
Blood flow [mL/min]
Sample [100Hz]
B
PAP
RAP
Curve fit
RV Isovolumetric
P_ISOMax (a + 2*b)
EDP (a)
Amplitude (b)
Pressure [mmHg]
Sample
C
P_ISOMax
Ees
Ea
Pressure [mmHg]
Volume [mL]

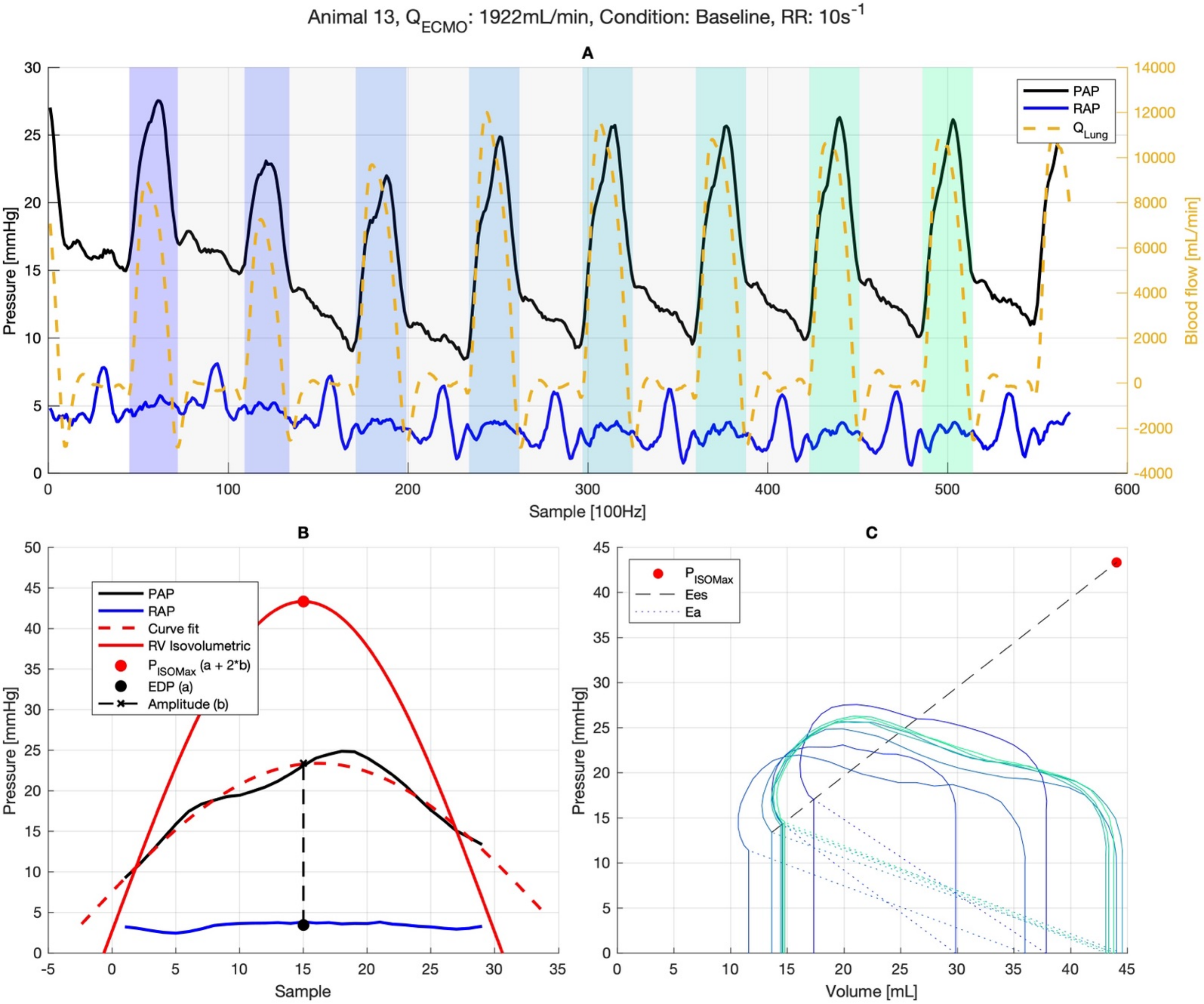
Animal 13, Q_ECMO: 1922mL/min, Condition: Baseline, RR: 10s^-1
A
PAP
RAP
Q_Lung
Pressure [mmHg]
Blood flow [mL/min]
Sample [100Hz]
B
PAP
RAP
Curve fit
RV Isovolumetric
P_ISOMax (a + 2*b)
EDP (a)
Amplitude (b)
Pressure [mmHg]
Sample
C
P_ISOMax
Ees
Ea
Pressure [mmHg]
Volume [mL]

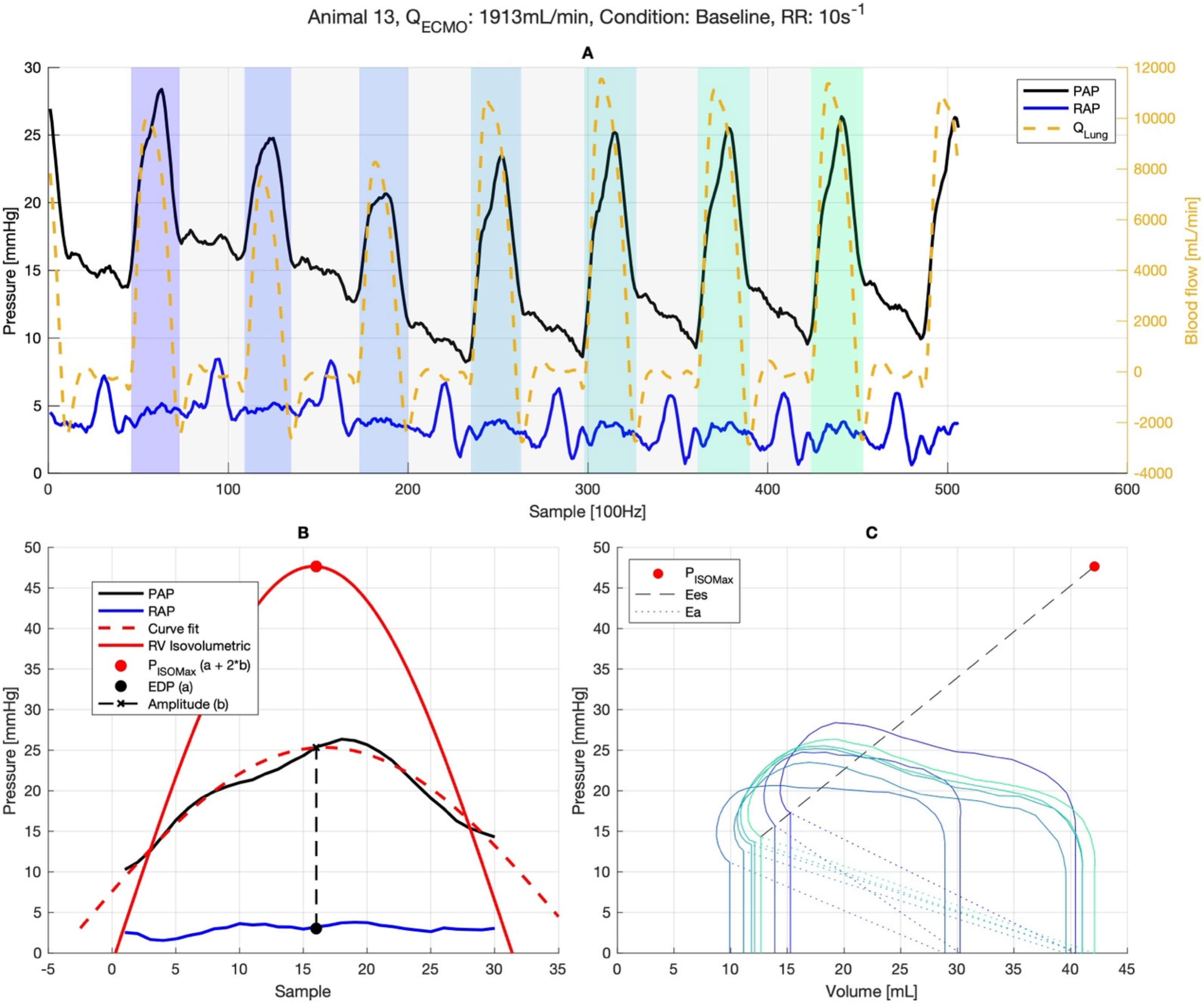
Animal 13, Q_ECMO: 1913mL/min, Condition: Baseline, RR: 10s^-1
A
PAP
RAP
Q_Lung
Pressure [mmHg]
Blood flow [mL/min]
Sample [100Hz]
B
PAP
RAP
Curve fit
RV Isovolumetric
P_ISOMax (a + 2*b)
EDP (a)
Amplitude (b)
Pressure [mmHg]
Sample
C
P_ISOMax
Ees
Ea
Pressure [mmHg]
Volume [mL]

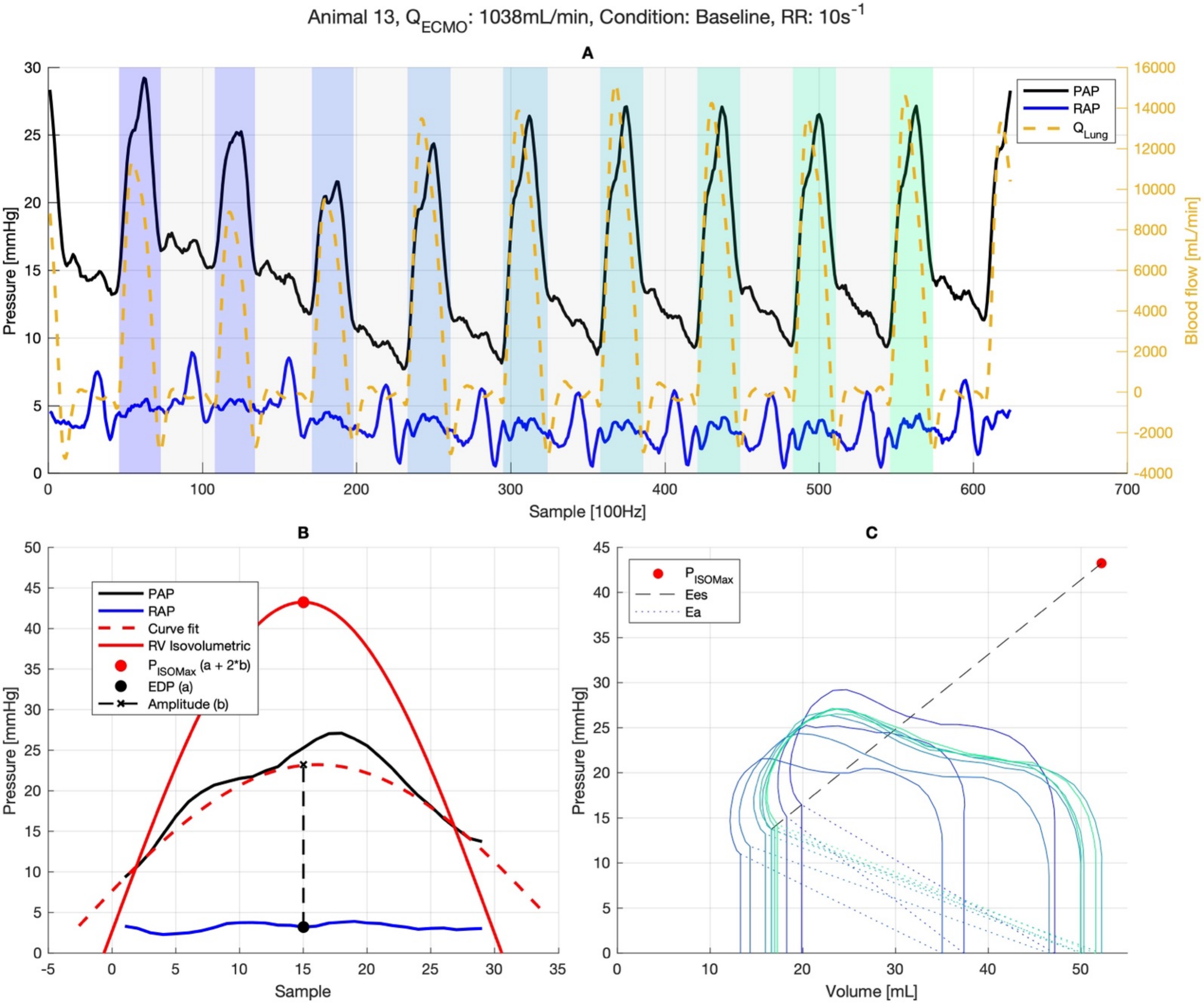

Animal 13, Q_ECMO: 1038mL/min, Condition: Baseline, RR: 10s^-1
A
PAP
RAP
Q_Lung
Pressure [mmHg]
Blood flow [mL/min]
Sample [100Hz]
B
PAP
RAP
Curve fit
RV Isovolumetric
P_ISOMax (a + 2*b)
EDP (a)
Amplitude (b)
Pressure [mmHg]
Sample
C
P_ISOMax
Ees
Ea
Pressure [mmHg]
Volume [mL]

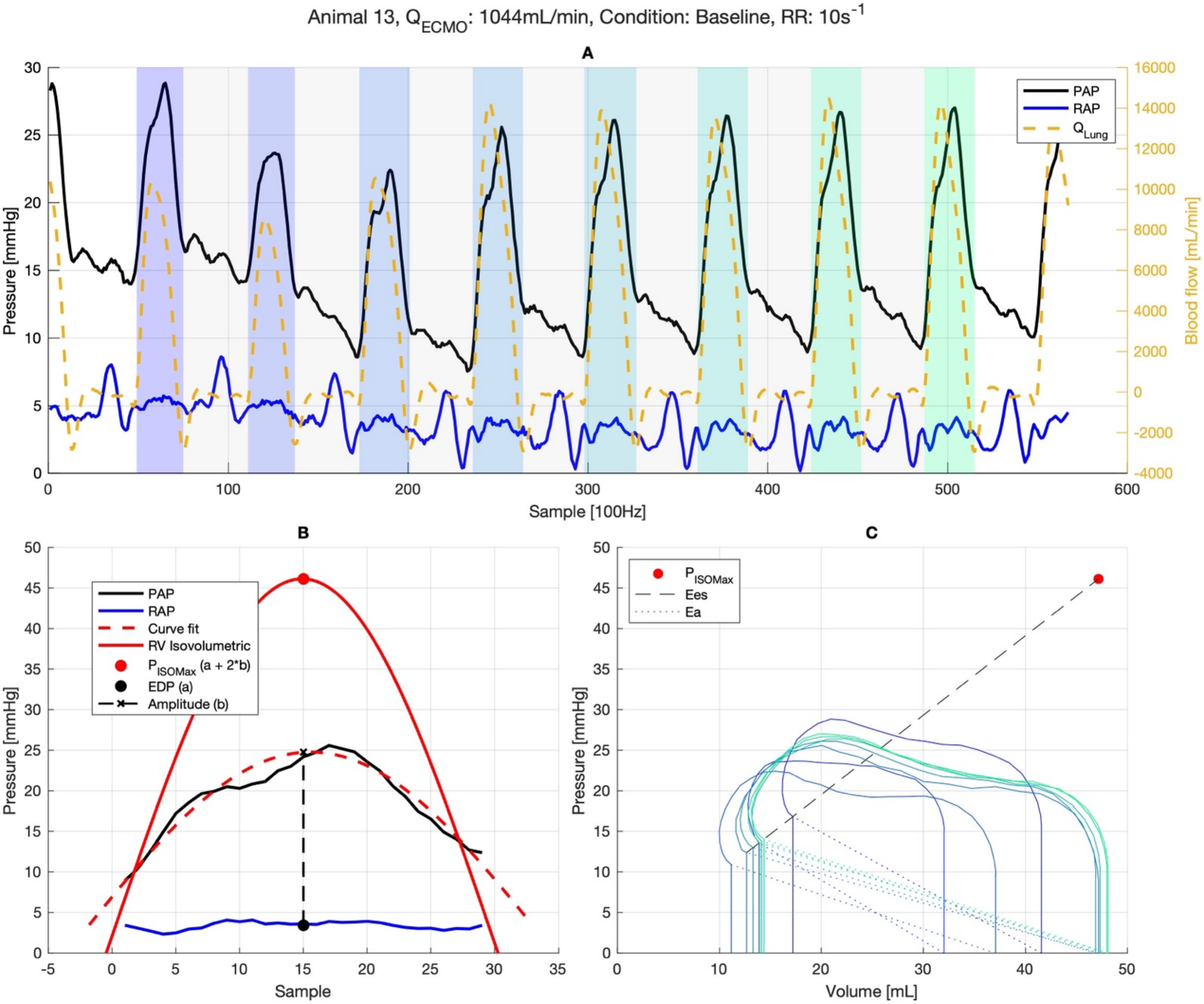

Animal 13, QECMO: 1044mL/min, Condition: Baseline, RR: 10s-1
A
PAP
RAP
QLung
Pressure [mmHg]
Blood flow [mL/min]
Sample [100Hz]
B
PAP
RAP
Curve fit
RV Isovolumetric
PISOMax (a + 2*b)
EDP (a)
Amplitude (b)
Pressure [mmHg]
Sample
C
PISOMax
Ees
Ea
Pressure [mmHg]
Volume [mL]

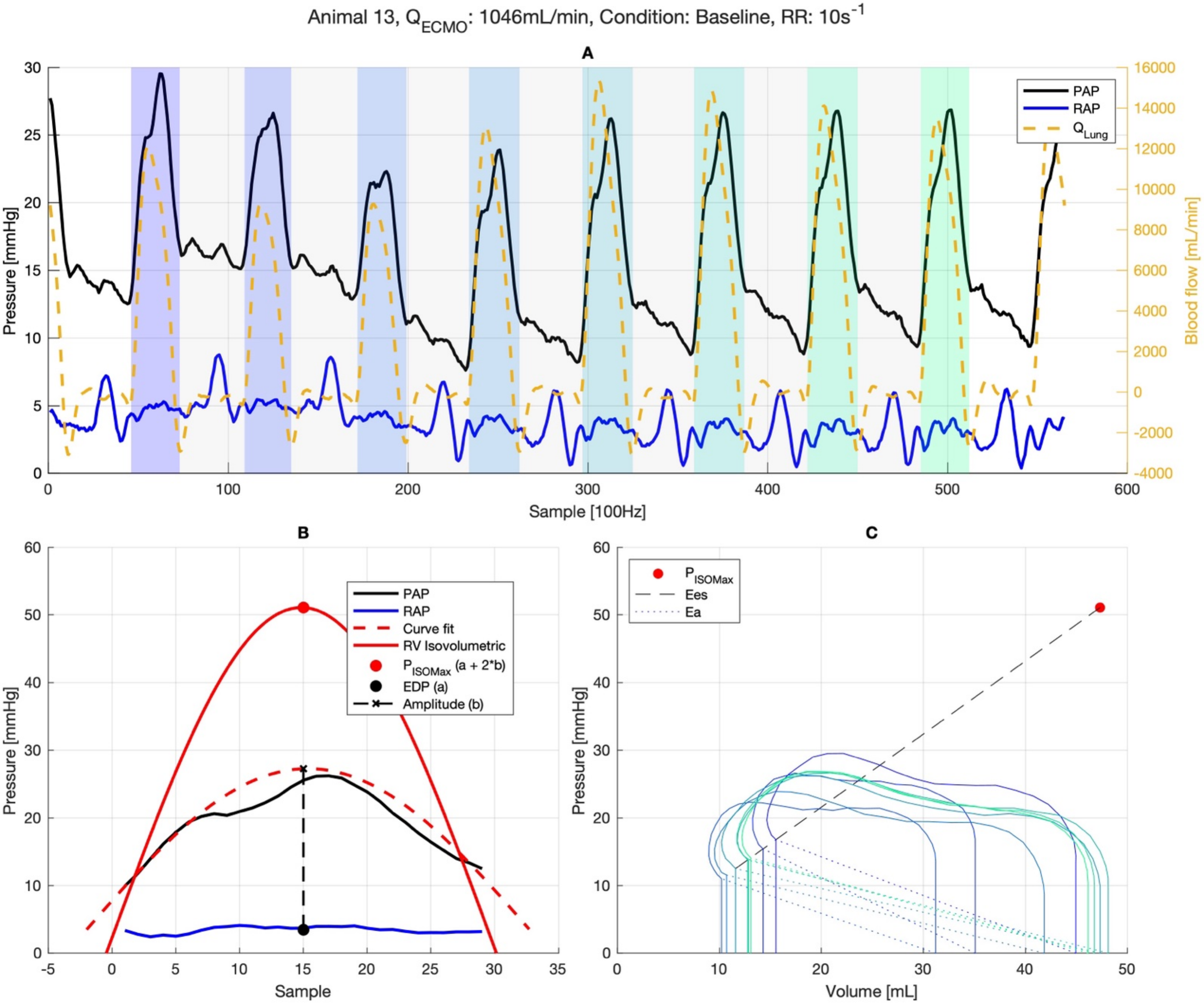

Animal 13, Q_ECMO: 1046mL/min, Condition: Baseline, RR: 10s^-1
A
PAP
RAP
Q_Lung
Pressure [mmHg]
Blood flow [mL/min]
Sample [100Hz]
B
PAP
RAP
Curve fit
RV Isovolumetric
P_ISOMax (a + 2*b)
EDP (a)
Amplitude (b)
Pressure [mmHg]
Sample
C
P_ISOMax
Ees
Ea
Pressure [mmHg]
Volume [mL]

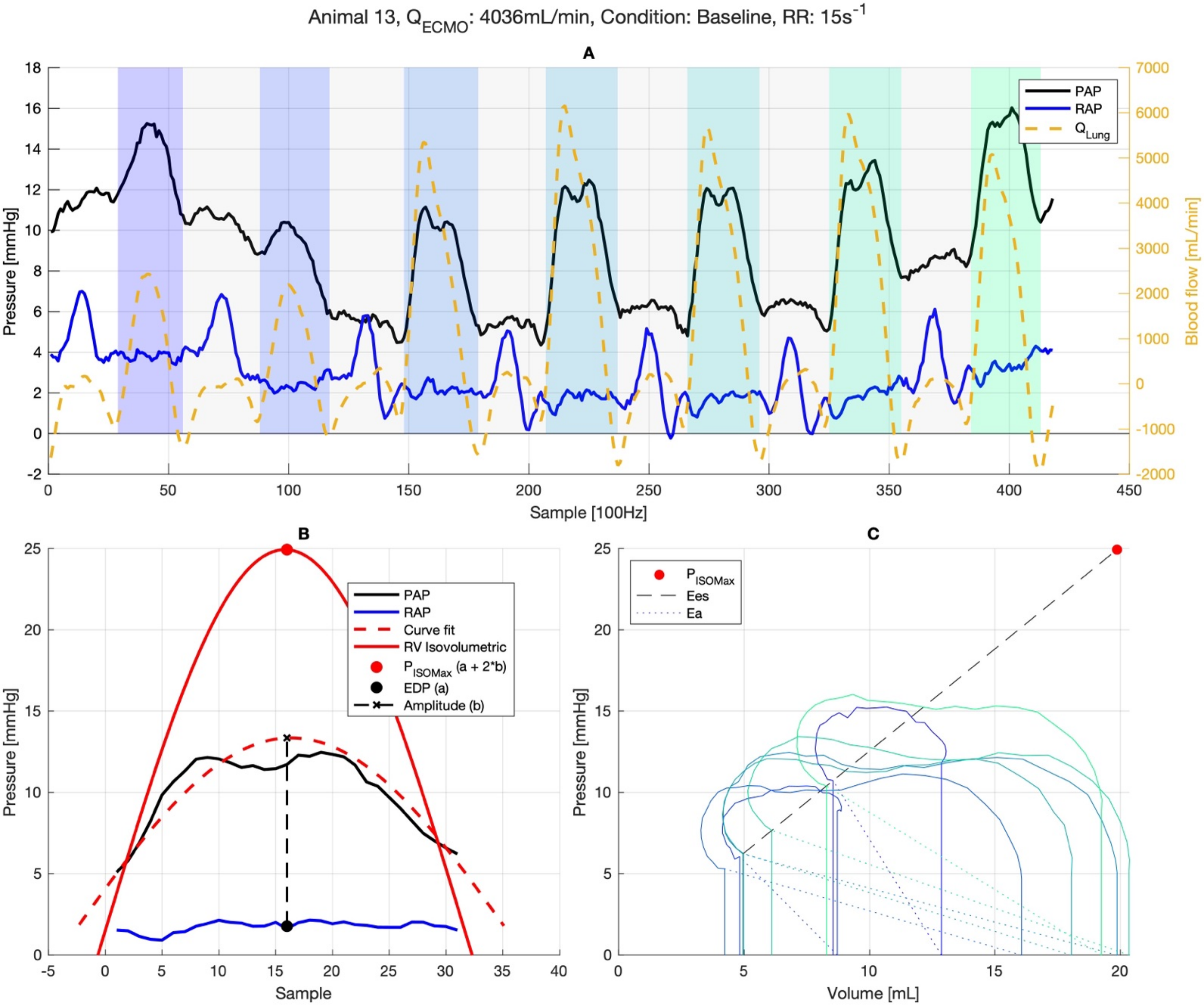

Animal 13, Q_{ECMO}: 4036mL/min, Condition: Baseline, RR: $15s^{-1}$
A
PAP
RAP
Q_{Lung}
Pressure [mmHg]
Blood flow [mL/min]
Sample [100Hz]
B
PAP
RAP
Curve fit
RV Isovolumetric
P_{ISOMax} (a + 2*b)
EDP (a)
Amplitude (b)
Pressure [mmHg]
Sample
C
P_{ISOMax}
Ees
Ea
Pressure [mmHg]
Volume [mL]

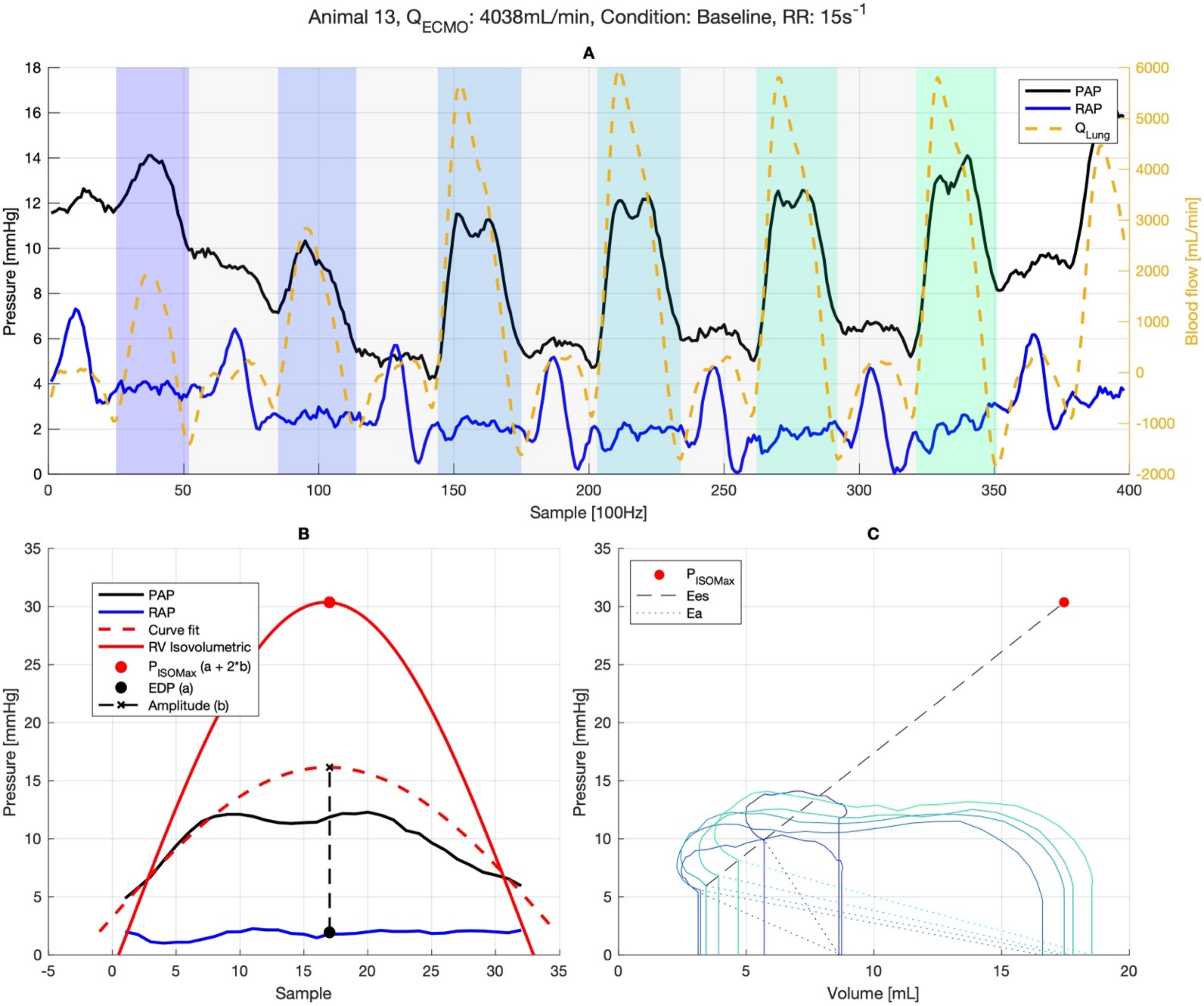

Animal 13, Q_ECMO: 4038mL/min, Condition: Baseline, RR: 15s^-1
A
PAP
RAP
Q_Lung
Pressure [mmHg]
Blood flow [mL/min]
Sample [100Hz]
B
PAP
RAP
Curve fit
RV Isovolumetric
P_ISOMax (a + 2*b)
EDP (a)
Amplitude (b)
Pressure [mmHg]
Sample
C
P_ISOMax
Ees
Ea
Pressure [mmHg]
Volume [mL]

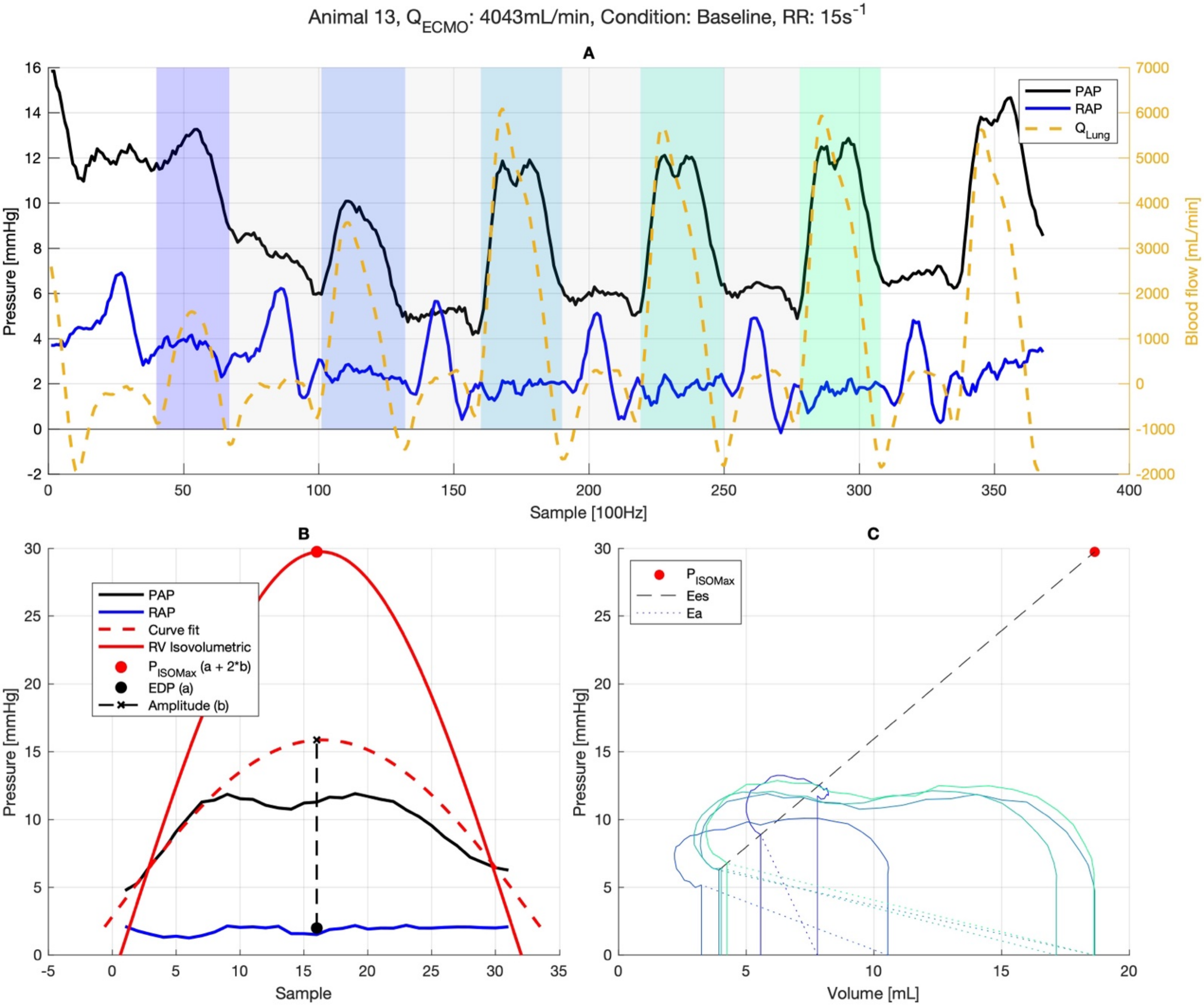

Animal 13, Q_ECMO: 4043mL/min, Condition: Baseline, RR: 15s^-1
A
PAP
RAP
Q_Lung
Pressure [mmHg]
Blood flow [mL/min]
Sample [100Hz]
B
PAP
RAP
Curve fit
RV Isovolumetric
P_ISOMax (a + 2*b)
EDP (a)
Amplitude (b)
Pressure [mmHg]
Sample
C
P_ISOMax
Ees
Ea
Pressure [mmHg]
Volume [mL]

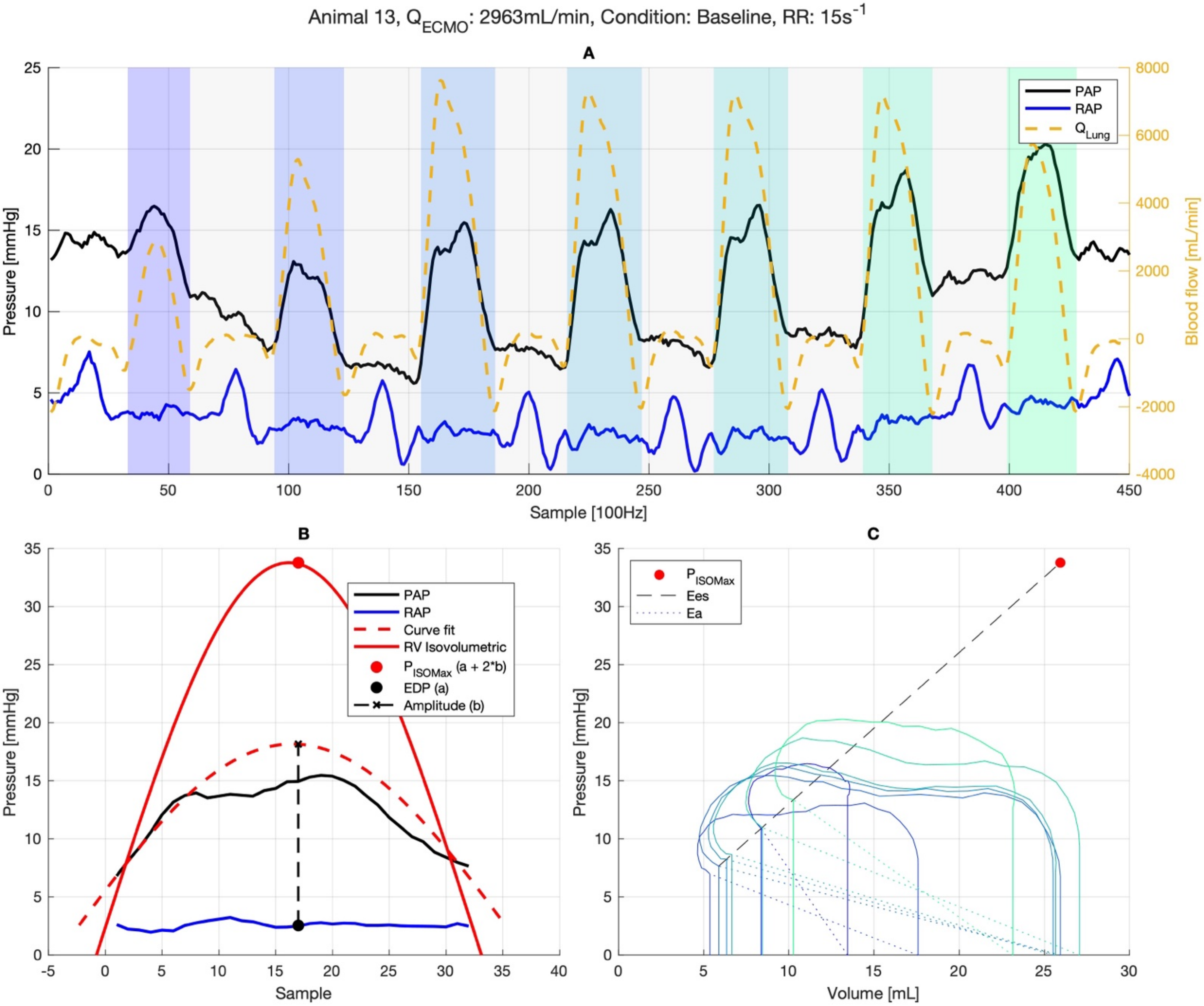

Animal 13, Q_{ECMO}: 2963mL/min, Condition: Baseline, RR: $15s^{-1}$
A
PAP
RAP
Q_{Lung}
Pressure [mmHg]
Blood flow [mL/min]
Sample [100Hz]
B
PAP
RAP
Curve fit
RV Isovolumetric
P_{ISOMax} (a + 2*b)
EDP (a)
Amplitude (b)
Pressure [mmHg]
Sample
C
P_{ISOMax}
Ees
Ea
Pressure [mmHg]
Volume [mL]

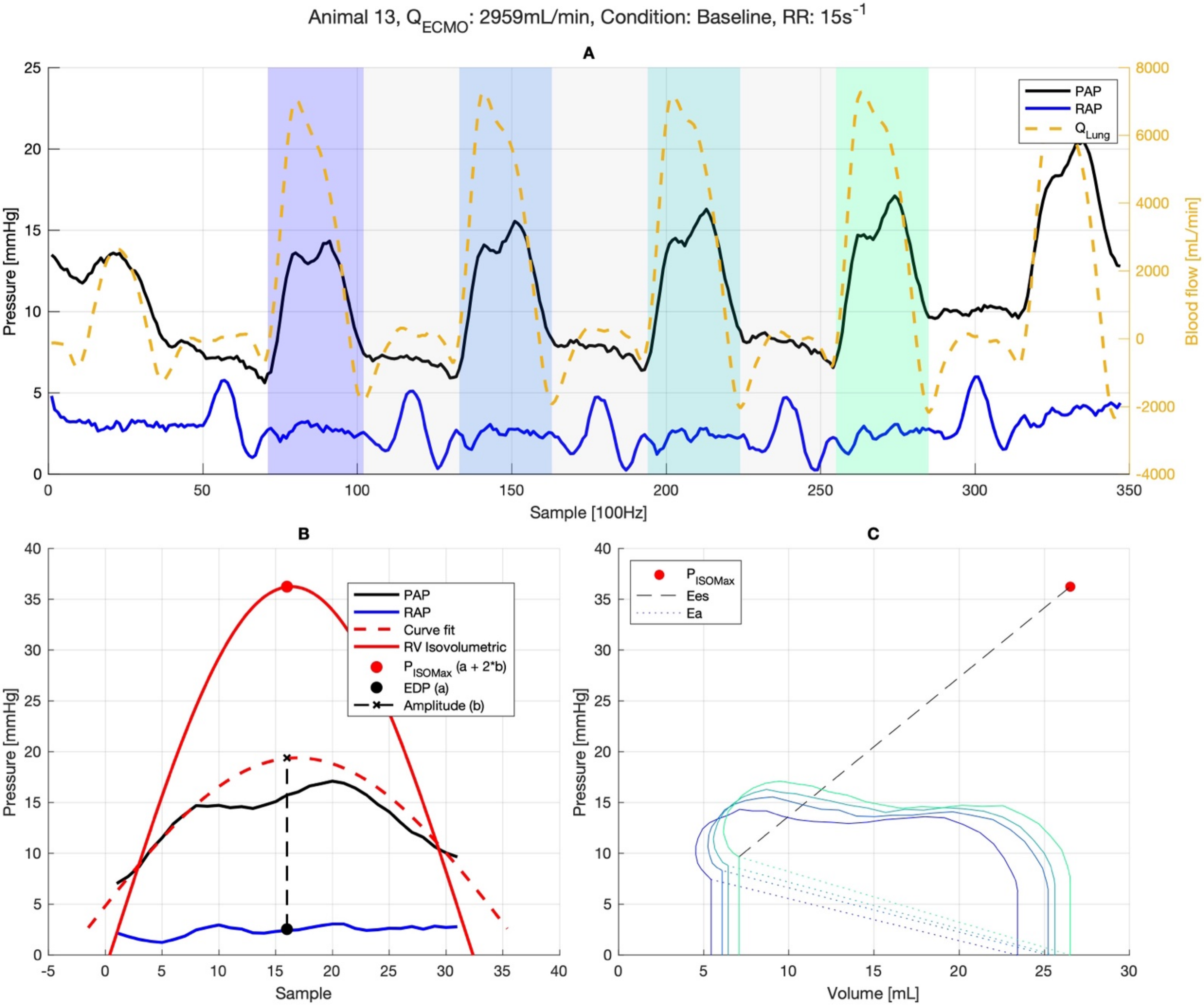

Animal 13, Q_{ECMO}: 2959mL/min, Condition: Baseline, RR: $15s^{-1}$
A
PAP
RAP
Q_{Lung}
Pressure [mmHg]
Blood flow [mL/min]
Sample [100Hz]
B
PAP
RAP
Curve fit
RV Isovolumetric
P_{ISOMax} (a + 2*b)
EDP (a)
Amplitude (b)
Pressure [mmHg]
Sample
C
P_{ISOMax}
Ees
Ea
Pressure [mmHg]
Volume [mL]

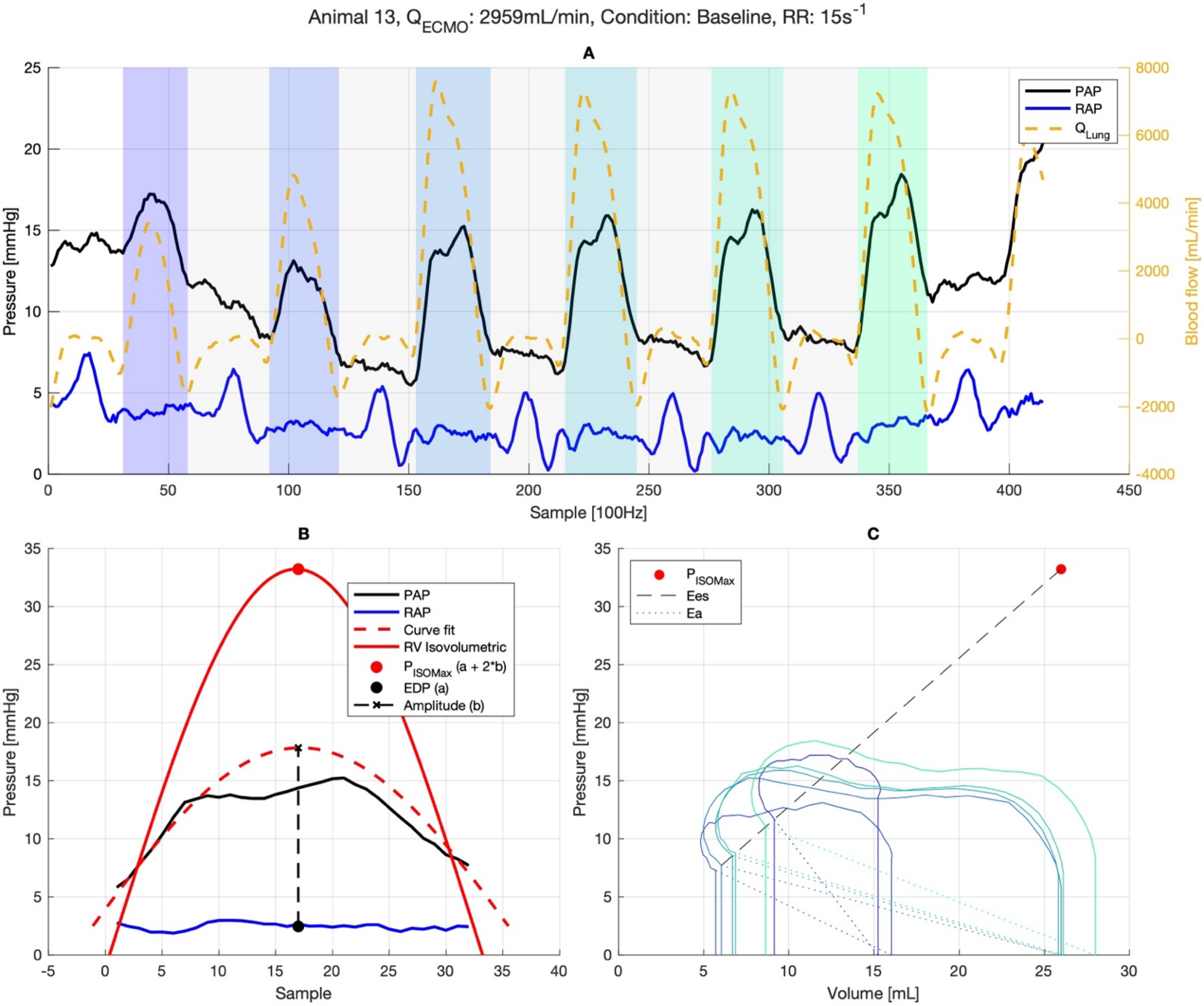

Animal 13, Q_ECMO: 2959mL/min, Condition: Baseline, RR: 15s^-1
A
PAP
RAP
Q_Lung
Pressure [mmHg]
Blood flow [mL/min]
Sample [100Hz]
B
PAP
RAP
Curve fit
RV Isovolumetric
P_ISOMax (a + 2*b)
EDP (a)
Amplitude (b)
Pressure [mmHg]
Sample
C
P_ISOMax
Ees
Ea
Pressure [mmHg]
Volume [mL]

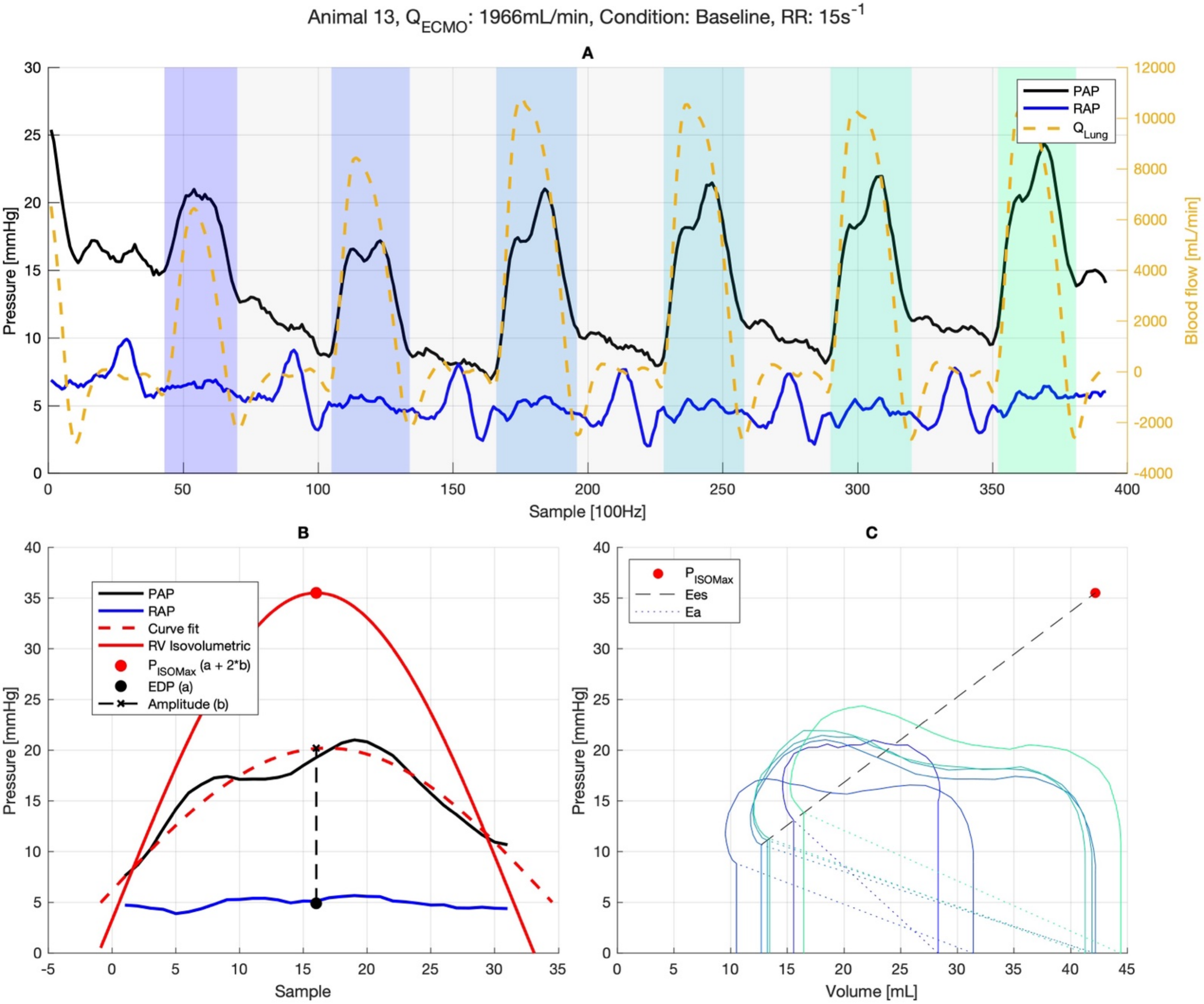

Animal 13, Q_ECMO: 1966mL/min, Condition: Baseline, RR: 15s^-1
A
PAP
RAP
Q_Lung
Pressure [mmHg]
Blood flow [mL/min]
Sample [100Hz]
B
PAP
RAP
Curve fit
RV Isovolumetric
P_ISOMax (a + 2*b)
EDP (a)
Amplitude (b)
Pressure [mmHg]
Sample
C
P_ISOMax
Ees
Ea
Pressure [mmHg]
Volume [mL]

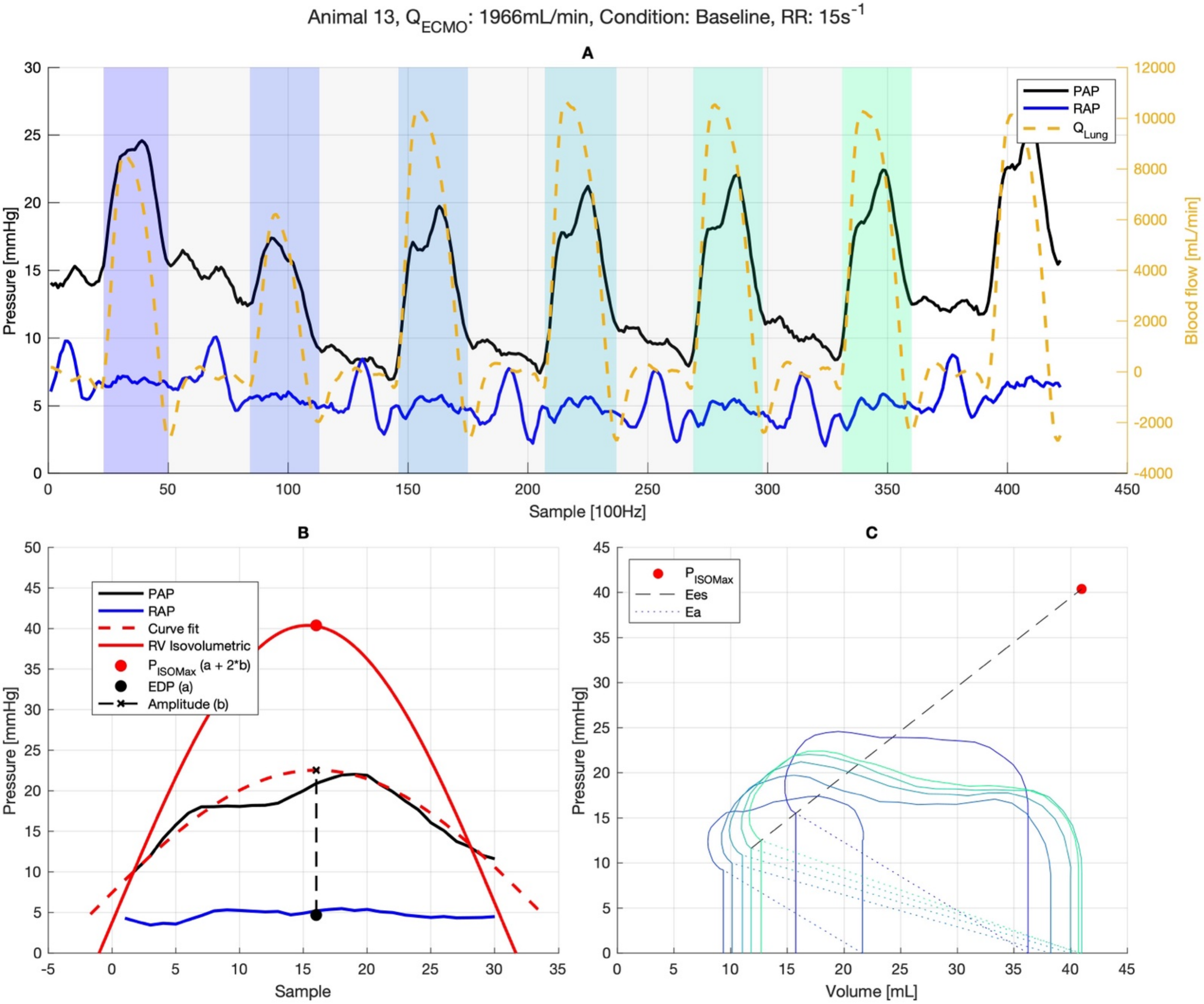

Animal 13, Q_ECMO: 1966mL/min, Condition: Baseline, RR: 15s^-1
A
PAP
RAP
Q_Lung
Pressure [mmHg]
Blood flow [mL/min]
Sample [100Hz]
B
PAP
RAP
Curve fit
RV Isovolumetric
P_ISOMax (a + 2*b)
EDP (a)
Amplitude (b)
Pressure [mmHg]
Sample
C
P_ISOMax
Ees
Ea
Pressure [mmHg]
Volume [mL]

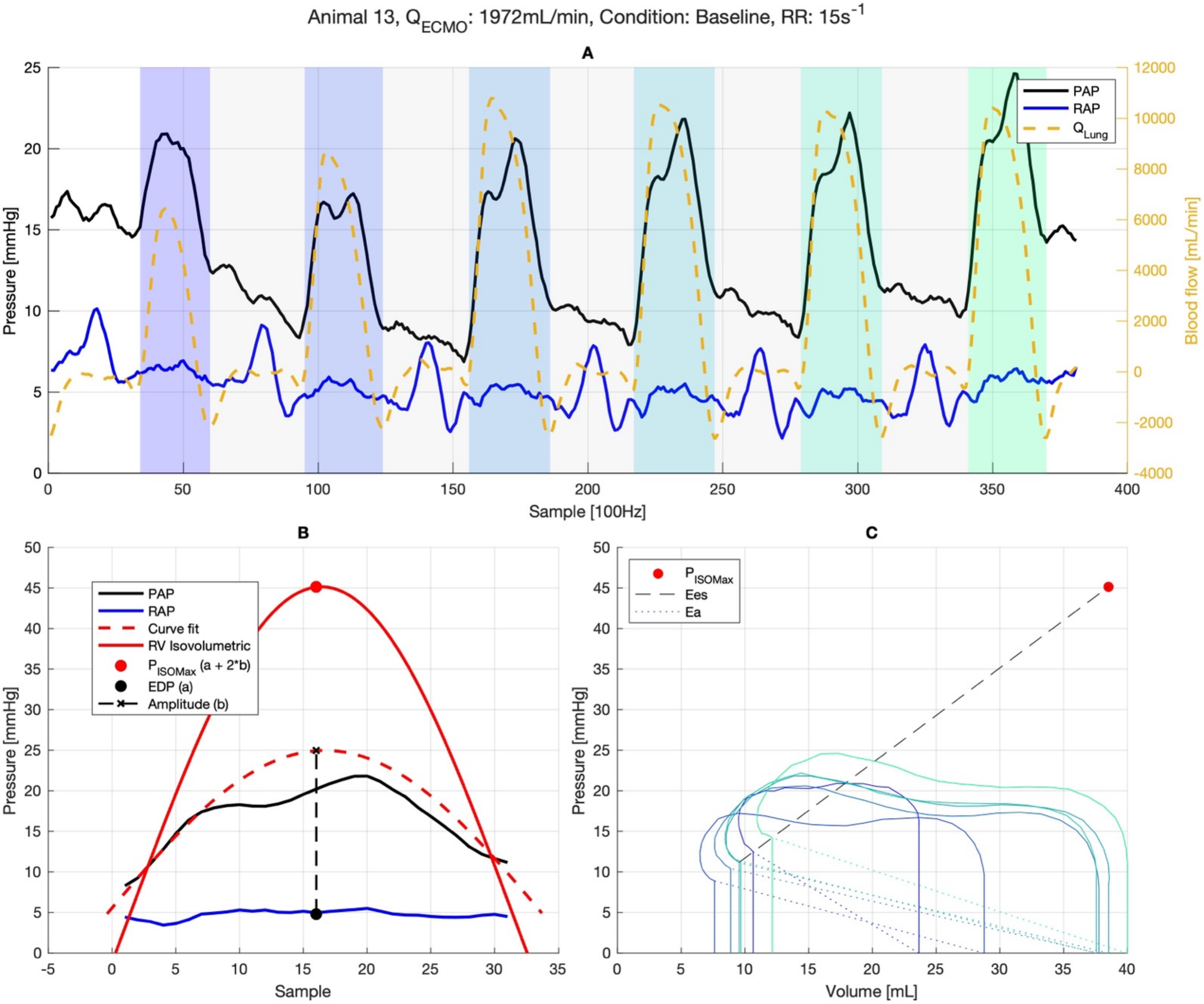

Animal 13, Q_ECMO: 1972mL/min, Condition: Baseline, RR: 15s^-1
A
PAP
RAP
Q_Lung
Pressure [mmHg]
Blood flow [mL/min]
Sample [100Hz]
B
PAP
RAP
Curve fit
RV Isovolumetric
P_ISOMax (a + 2*b)
EDP (a)
Amplitude (b)
Pressure [mmHg]
Sample
C
P_ISOMax
Ees
Ea
Pressure [mmHg]
Volume [mL]

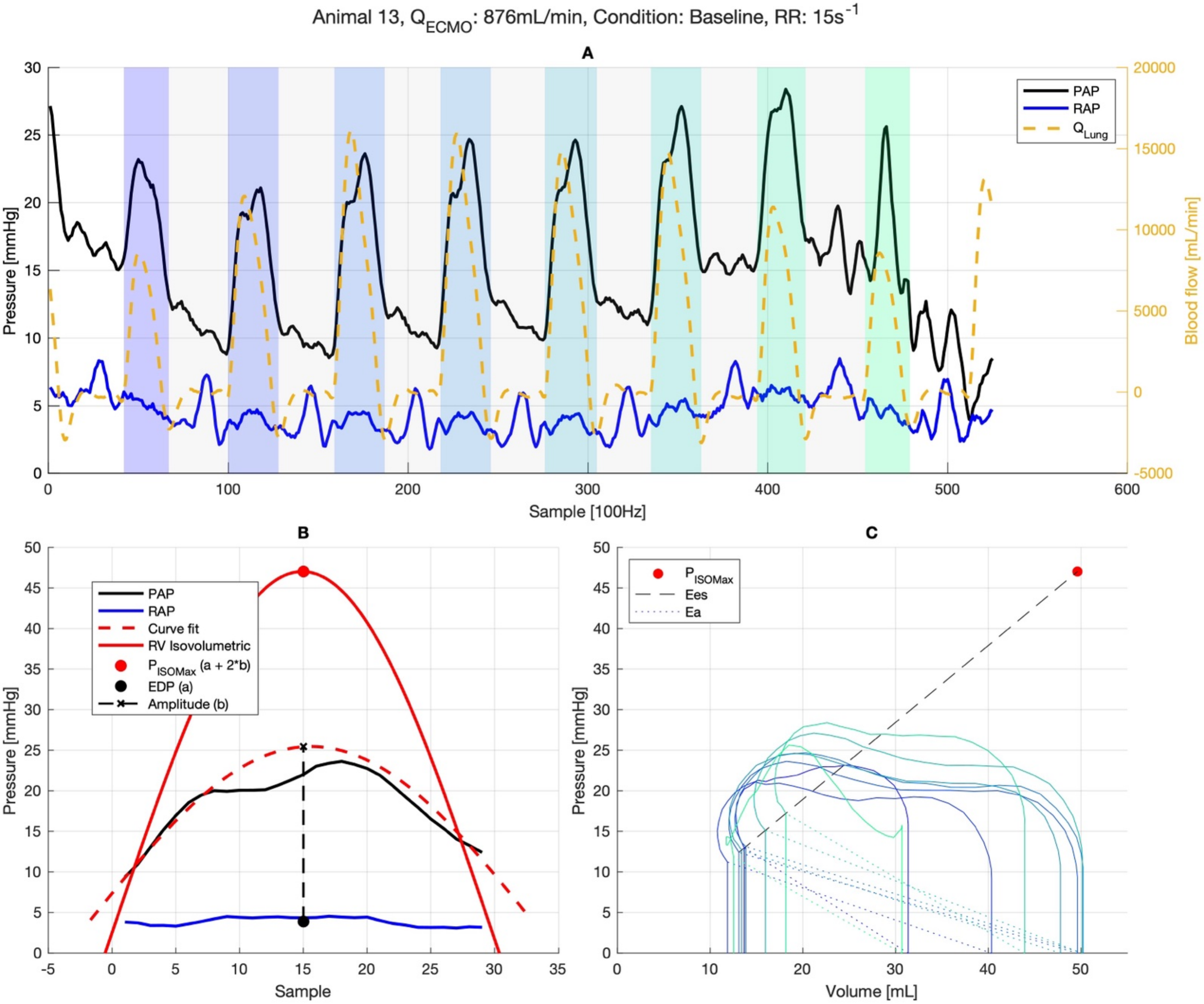

Animal 13, Q_{ECMO}: 876mL/min, Condition: Baseline, RR: 15s^{-1}
A
PAP
RAP
Q_{Lung}
Pressure [mmHg]
Blood flow [mL/min]
Sample [100Hz]
B
PAP
RAP
Curve fit
RV Isovolumetric
P_{ISOMax} (a + 2*b)
EDP (a)
Amplitude (b)
Pressure [mmHg]
Sample
C
P_{ISOMax}
Ees
Ea
Pressure [mmHg]
Volume [mL]

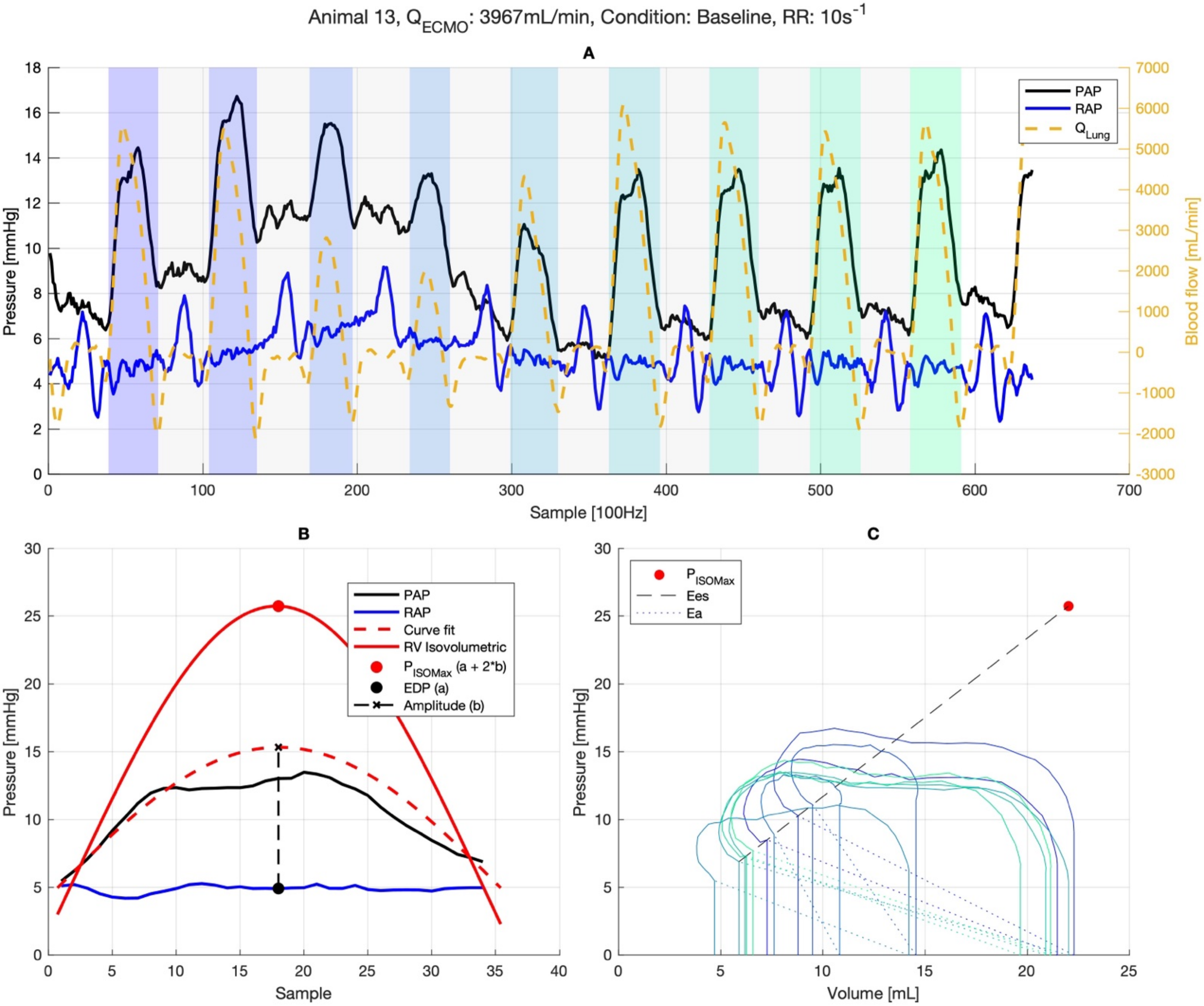

Animal 13, Q_{ECMO}: 3967mL/min, Condition: Baseline, RR: $10s^{-1}$
A
PAP
RAP
Q_{Lung}
Pressure [mmHg]
Blood flow [mL/min]
Sample [100Hz]
B
PAP
RAP
Curve fit
RV Isovolumetric
P_{ISOMax} (a + 2*b)
EDP (a)
Amplitude (b)
Pressure [mmHg]
Sample
C
P_{ISOMax}
Ees
Ea
Pressure [mmHg]
Volume [mL]

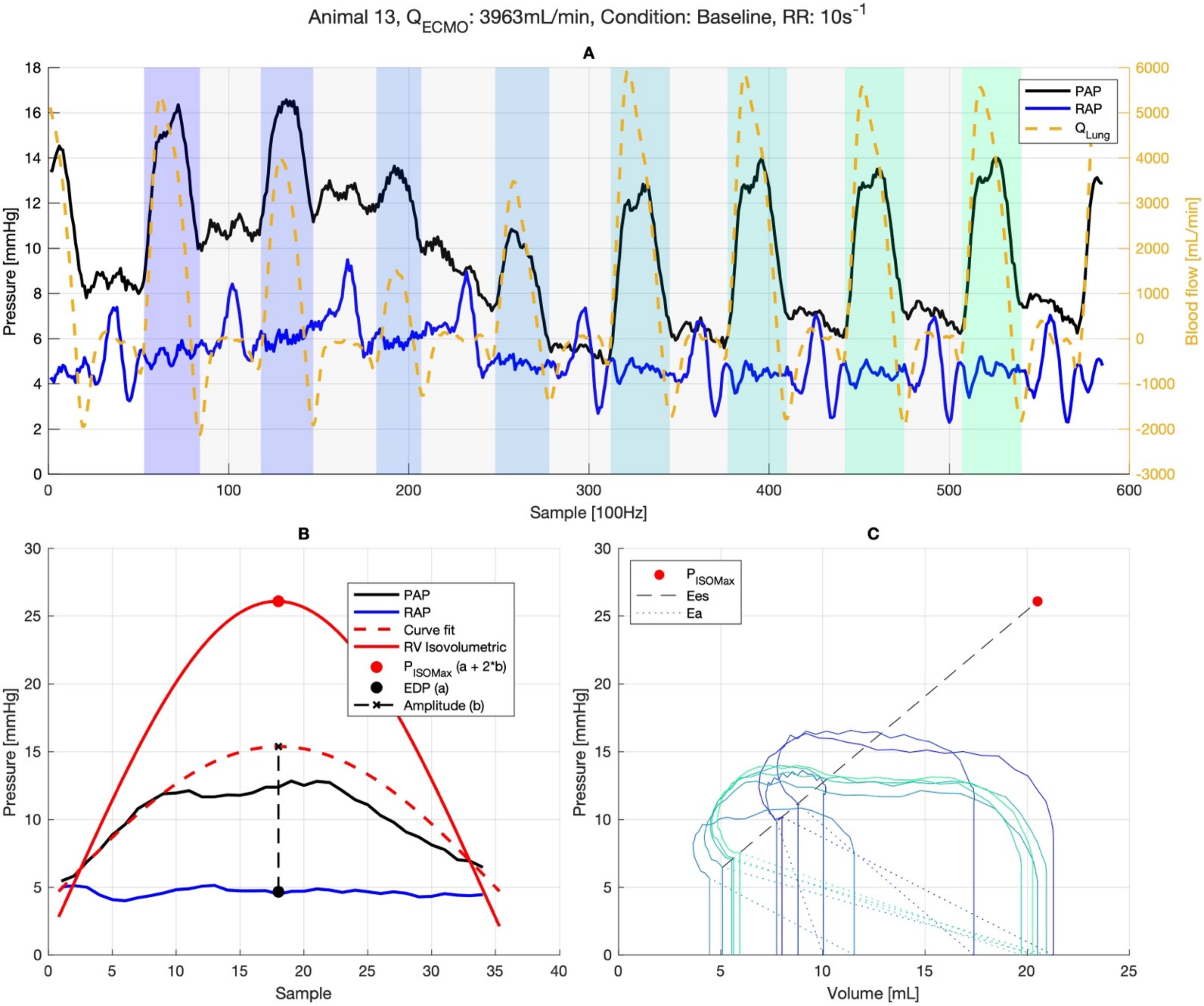

Animal 13, Q_{ECMO}: 3963mL/min, Condition: Baseline, RR: $10s^{-1}$
A
PAP
RAP
Q_{Lung}
Pressure [mmHg]
Blood flow [mL/min]
Sample [100Hz]
B
PAP
RAP
Curve fit
RV Isovolumetric
P_{ISOMax} (a + 2*b)
EDP (a)
Amplitude (b)
Pressure [mmHg]
Sample
C
P_{ISOMax}
Ees
Ea
Pressure [mmHg]
Volume [mL]

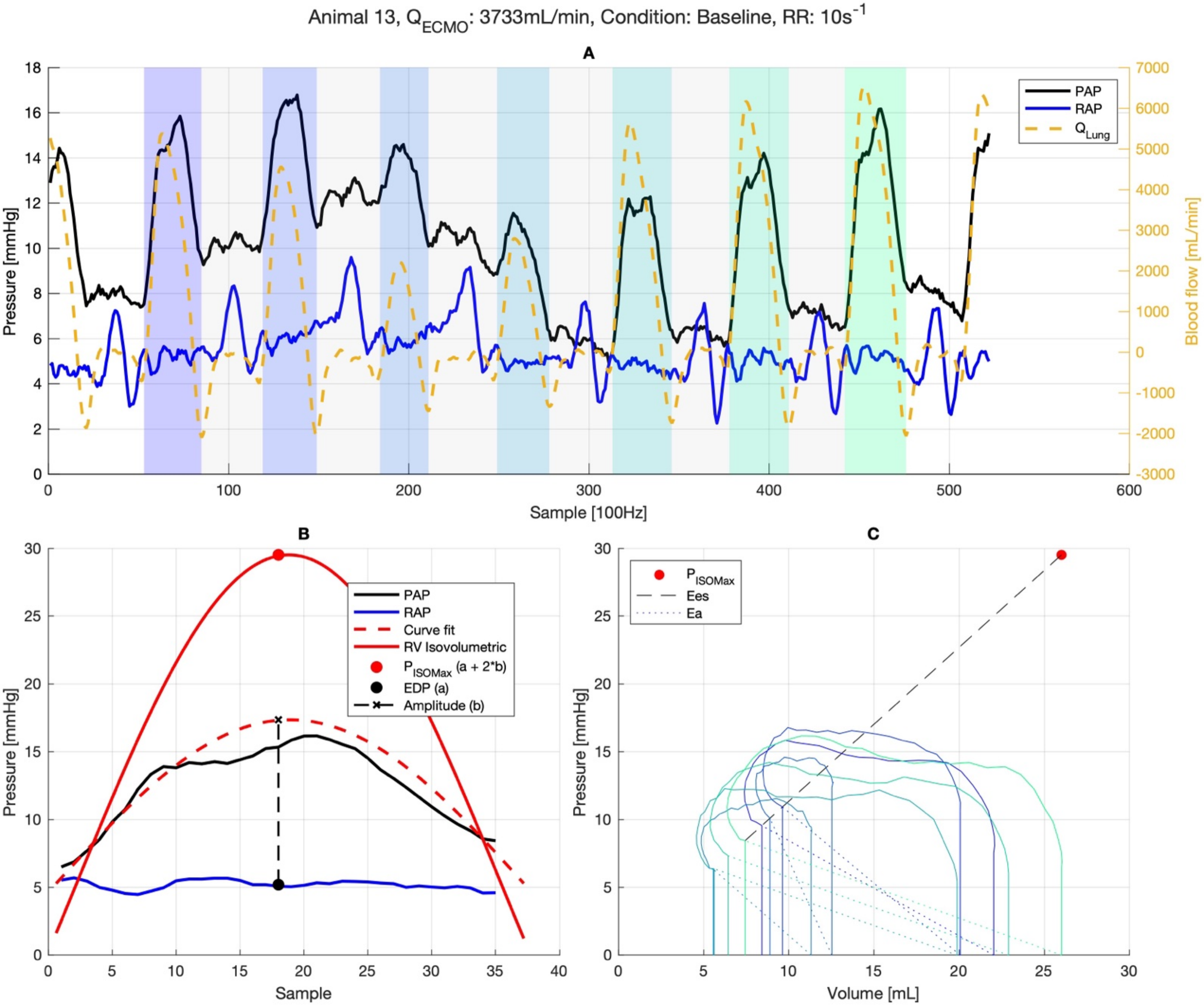

Animal 13, Q_ECMO: 3733mL/min, Condition: Baseline, RR: 10s^-1
A
Pressure [mmHg]
Blood flow [mL/min]
Sample [100Hz]
PAP
RAP
Q_Lung
B
Pressure [mmHg]
Sample
PAP
RAP
Curve fit
RV Isovolumetric
P_ISOMax (a + 2*b)
EDP (a)
Amplitude (b)
C
Pressure [mmHg]
Volume [mL]
P_ISOMax
Ees
Ea

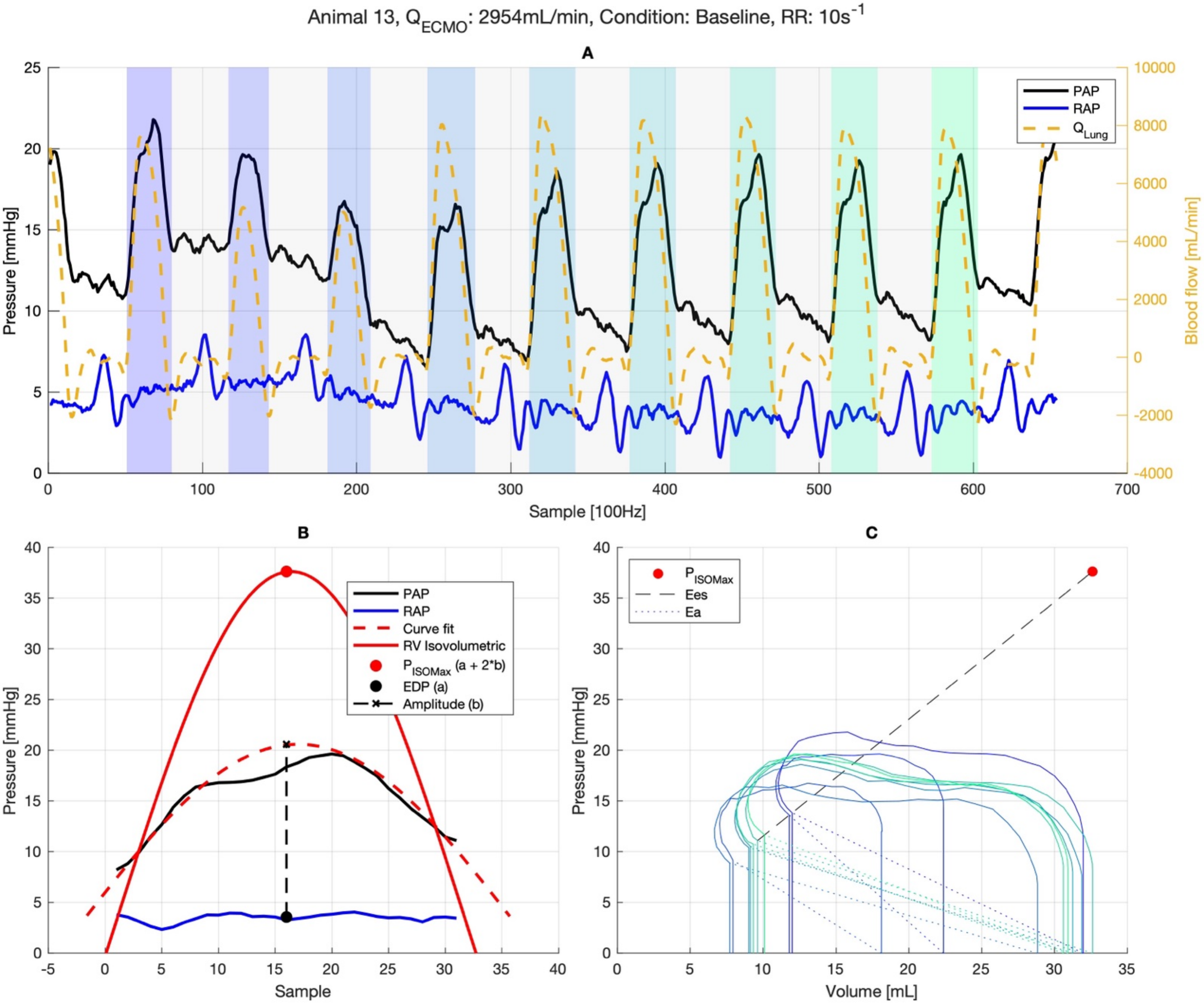

Animal 13, Q_ECMO: 2954mL/min, Condition: Baseline, RR: 10s^-1
A
PAP
RAP
Q_Lung
Pressure [mmHg]
Blood flow [mL/min]
Sample [100Hz]
B
PAP
RAP
Curve fit
RV Isovolumetric
P_ISOMax (a + 2*b)
EDP (a)
Amplitude (b)
Pressure [mmHg]
Sample
C
P_ISOMax
Ees
Ea
Pressure [mmHg]
Volume [mL]

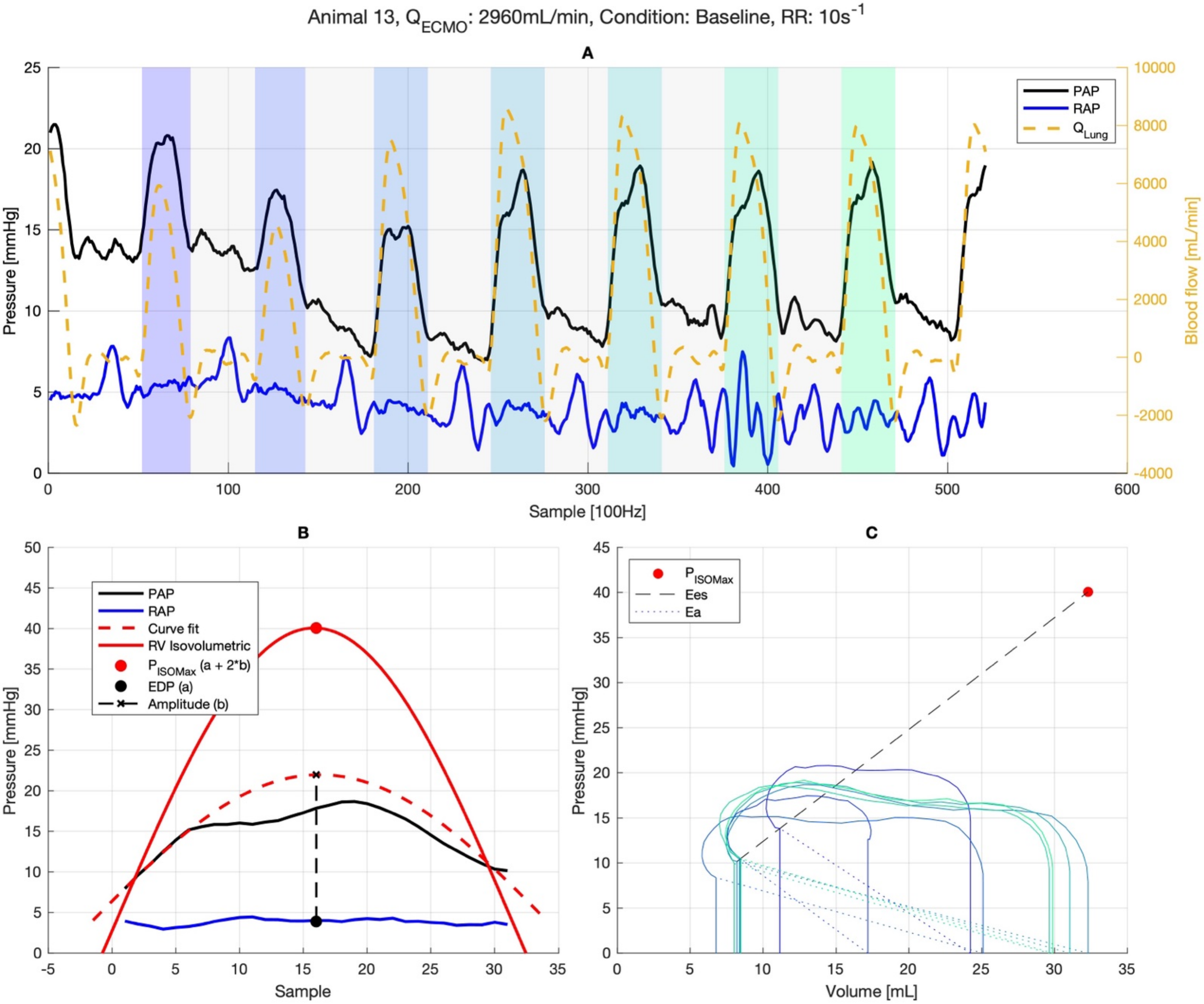
Animal 13, Q_ECMO: 2960mL/min, Condition: Baseline, RR: 10s^-1
A
PAP
RAP
Q_Lung
Pressure [mmHg]
Blood flow [mL/min]
Sample [100Hz]
B
PAP
RAP
Curve fit
RV Isovolumetric
P_ISOMax (a + 2*b)
EDP (a)
Amplitude (b)
Pressure [mmHg]
Sample
C
P_ISOMax
Ees
Ea
Pressure [mmHg]
Volume [mL]

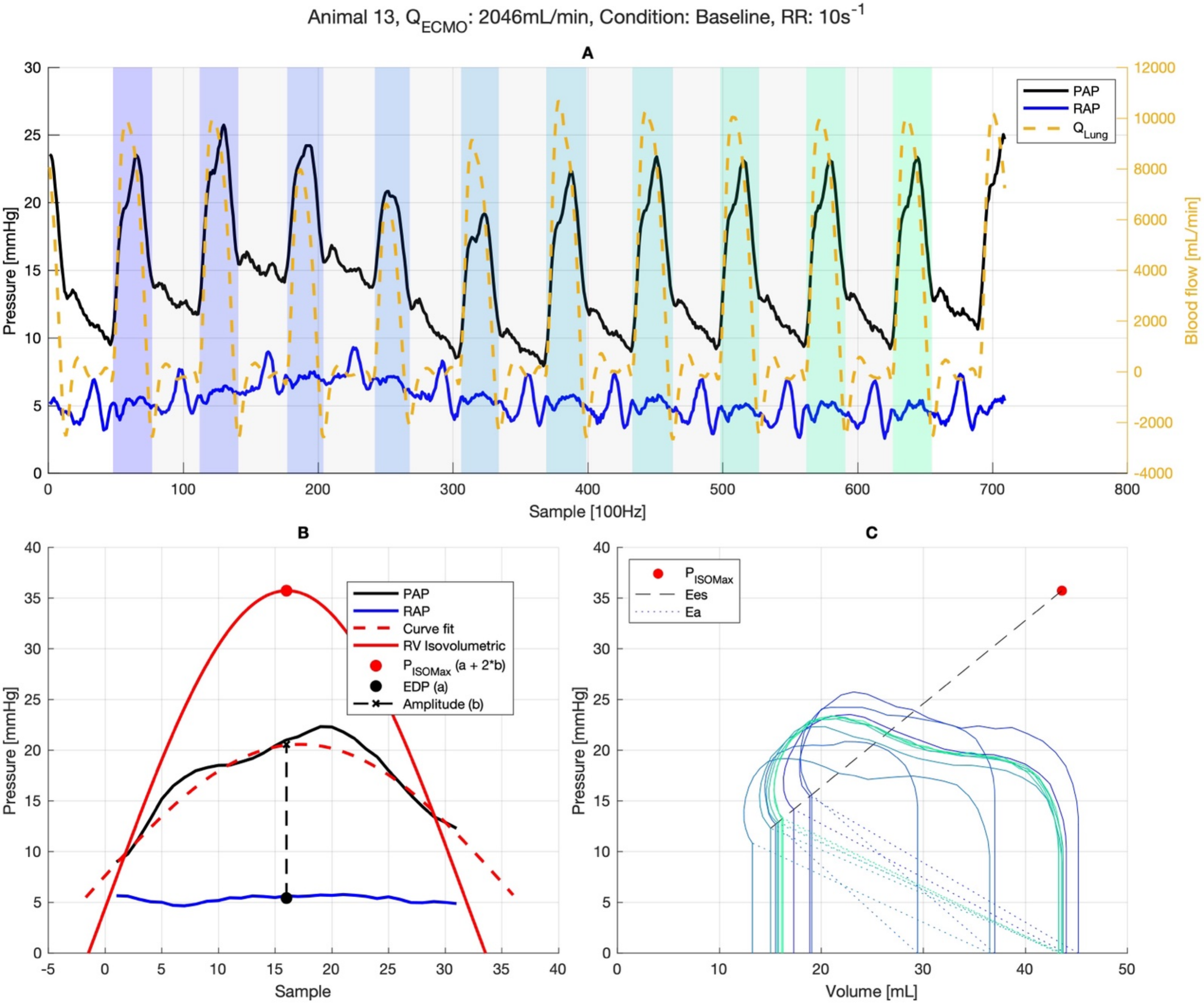
Animal 13, Q_{ECMO}: 2046mL/min, Condition: Baseline, RR: $10s^{-1}$
A
PAP
RAP
Q_{Lung}
Pressure [mmHg]
Blood flow [mL/min]
Sample [100Hz]
B
PAP
RAP
Curve fit
RV Isovolumetric
P_{ISOMax} (a + 2*b)
EDP (a)
Amplitude (b)
Pressure [mmHg]
Sample
C
P_{ISOMax}
Ees
Ea
Pressure [mmHg]
Volume [mL]

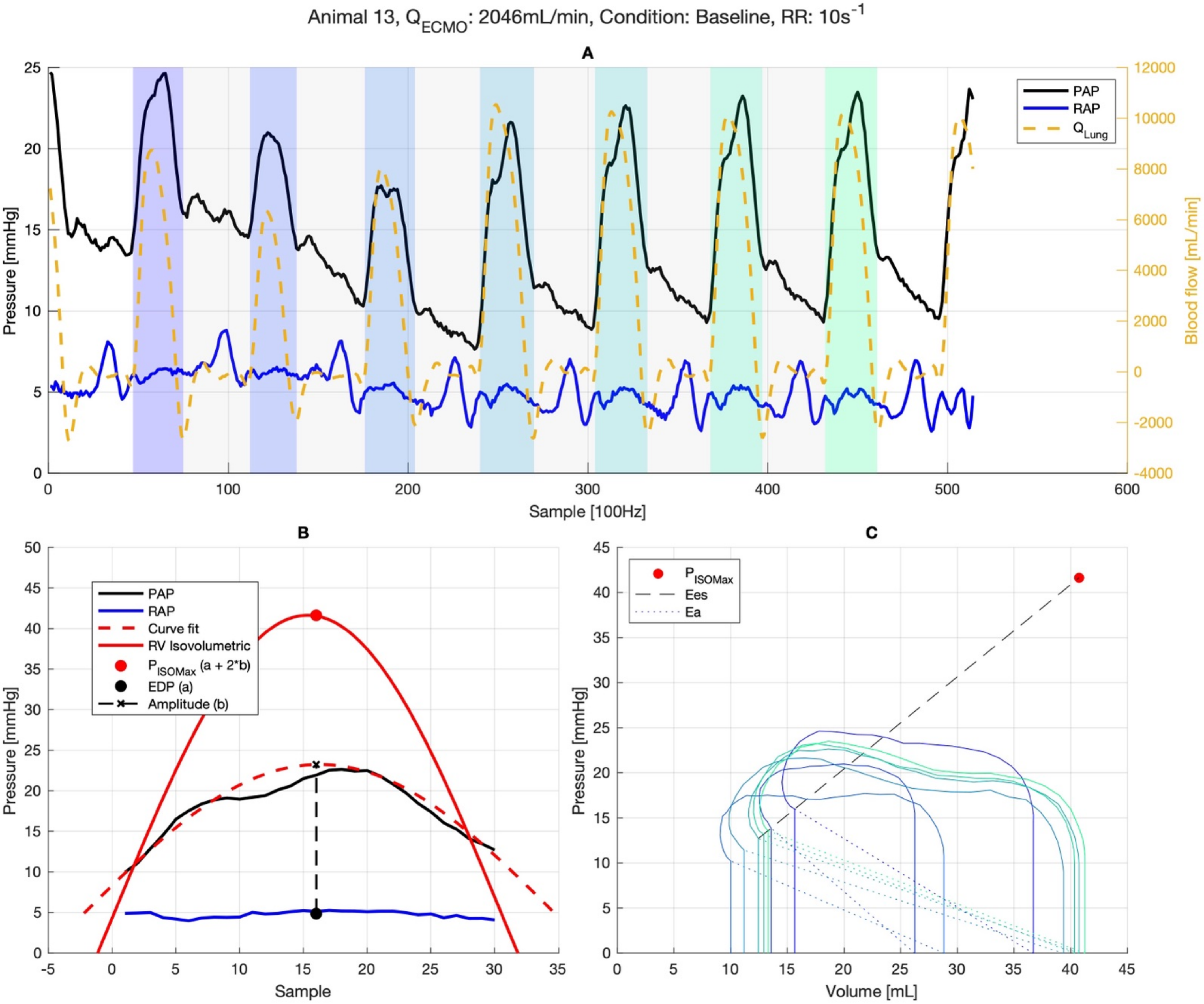

Animal 13, Q_ECMO: 2046mL/min, Condition: Baseline, RR: 10s^-1
A
Pressure [mmHg]
Blood flow [mL/min]
Sample [100Hz]
PAP
RAP
Q_Lung
B
PAP
RAP
Curve fit
RV Isovolumetric
P_ISOMax (a + 2*b)
EDP (a)
Amplitude (b)
Pressure [mmHg]
Sample
C
P_ISOMax
Ees
Ea
Pressure [mmHg]
Volume [mL]

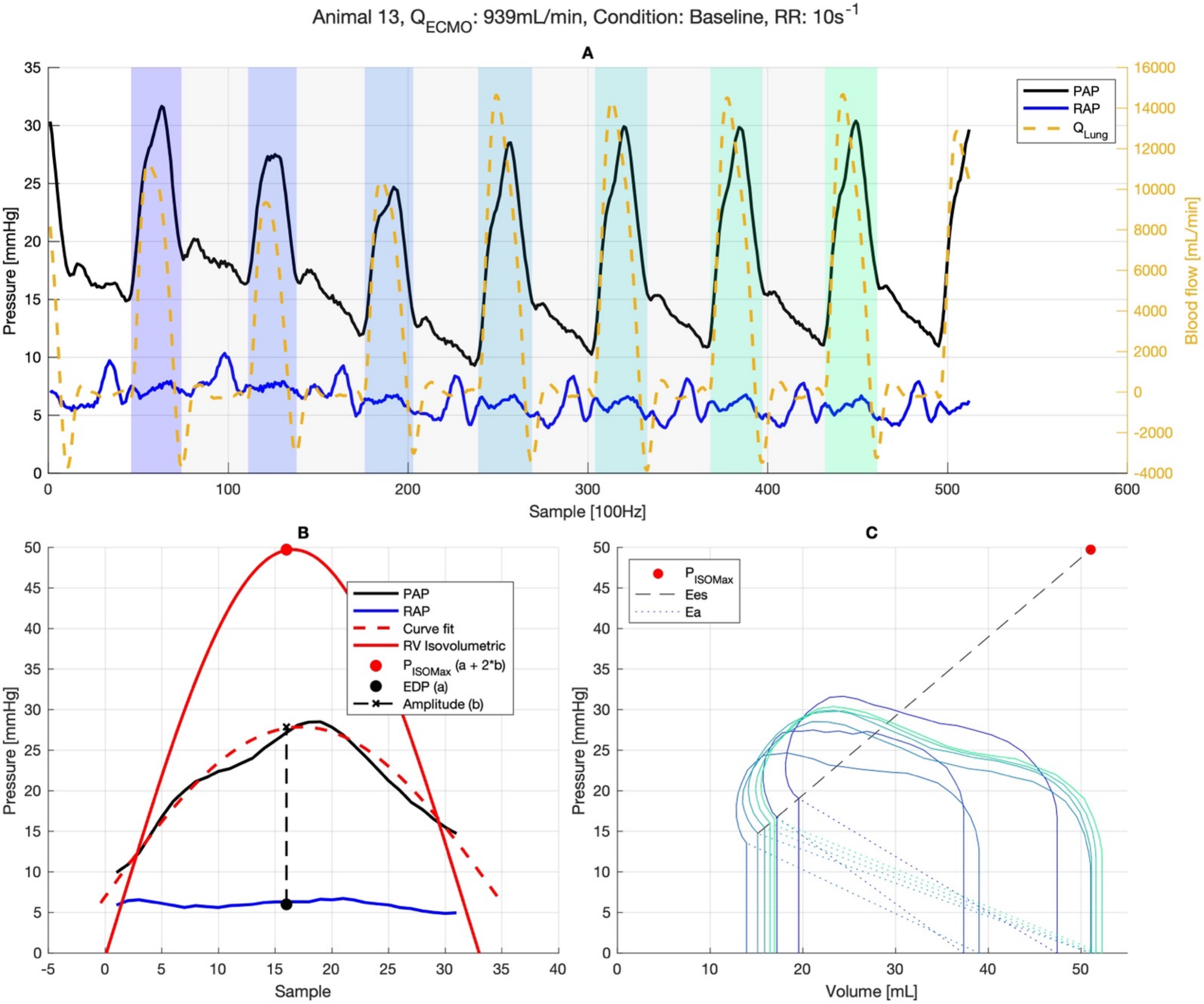

Animal 13, Q_ECMO: 939mL/min, Condition: Baseline, RR: 10s^-1
A
PAP
RAP
Q_Lung
Pressure [mmHg]
Blood flow [mL/min]
Sample [100Hz]
B
PAP
RAP
Curve fit
RV Isovolumetric
P_ISOMax (a + 2*b)
EDP (a)
Amplitude (b)
Pressure [mmHg]
Sample
C
P_ISOMax
Ees
Ea
Pressure [mmHg]
Volume [mL]

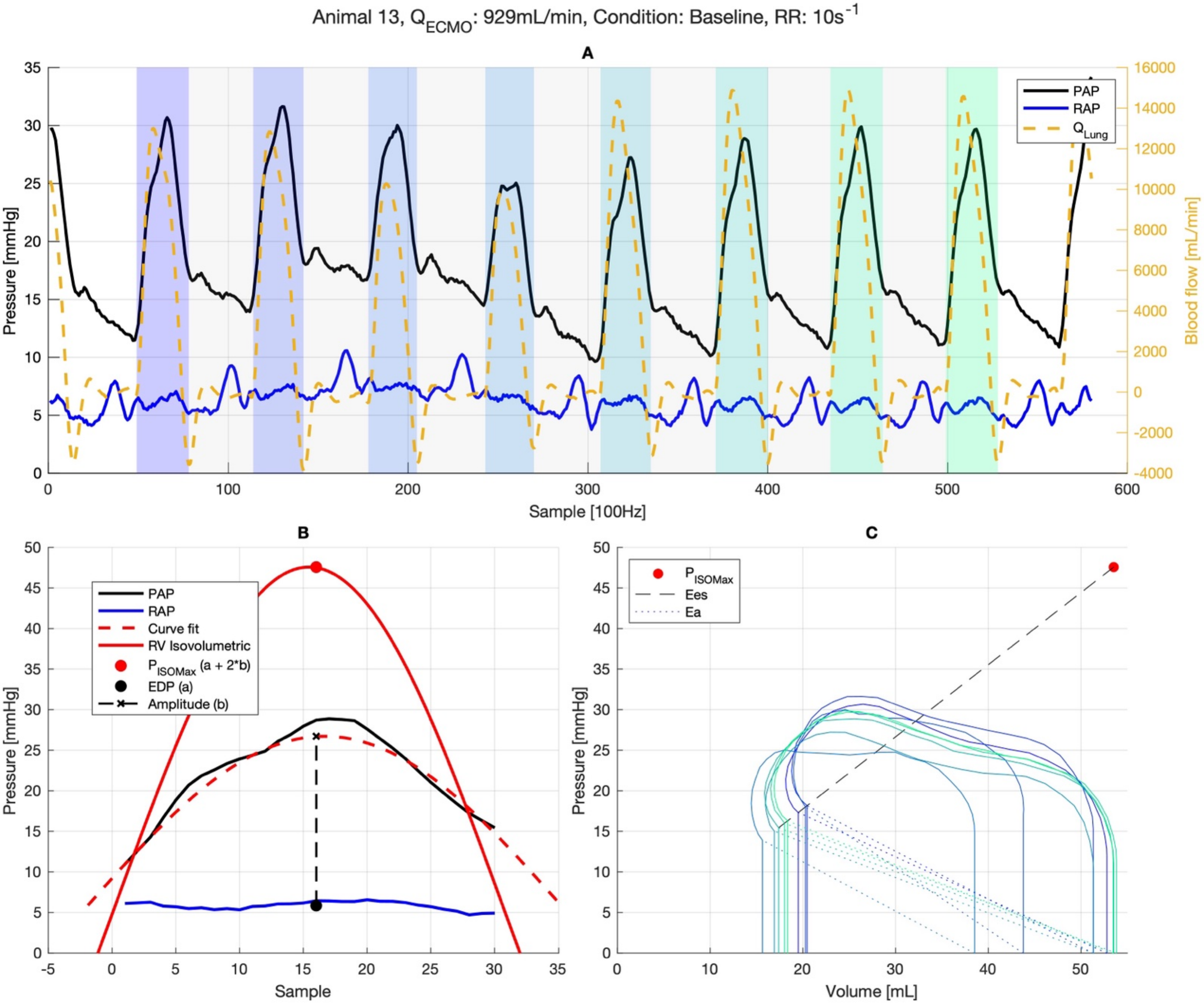

Animal 13, Q_{ECMO}: 929mL/min, Condition: Baseline, RR: $10s^{-1}$
A
PAP
RAP
Q_{Lung}
Pressure [mmHg]
Blood flow [mL/min]
Sample [100Hz]
B
PAP
RAP
Curve fit
RV Isovolumetric
P_{ISOMax} (a + 2*b)
EDP (a)
Amplitude (b)
Pressure [mmHg]
Sample
C
P_{ISOMax}
Ees
Ea
Pressure [mmHg]
Volume [mL]

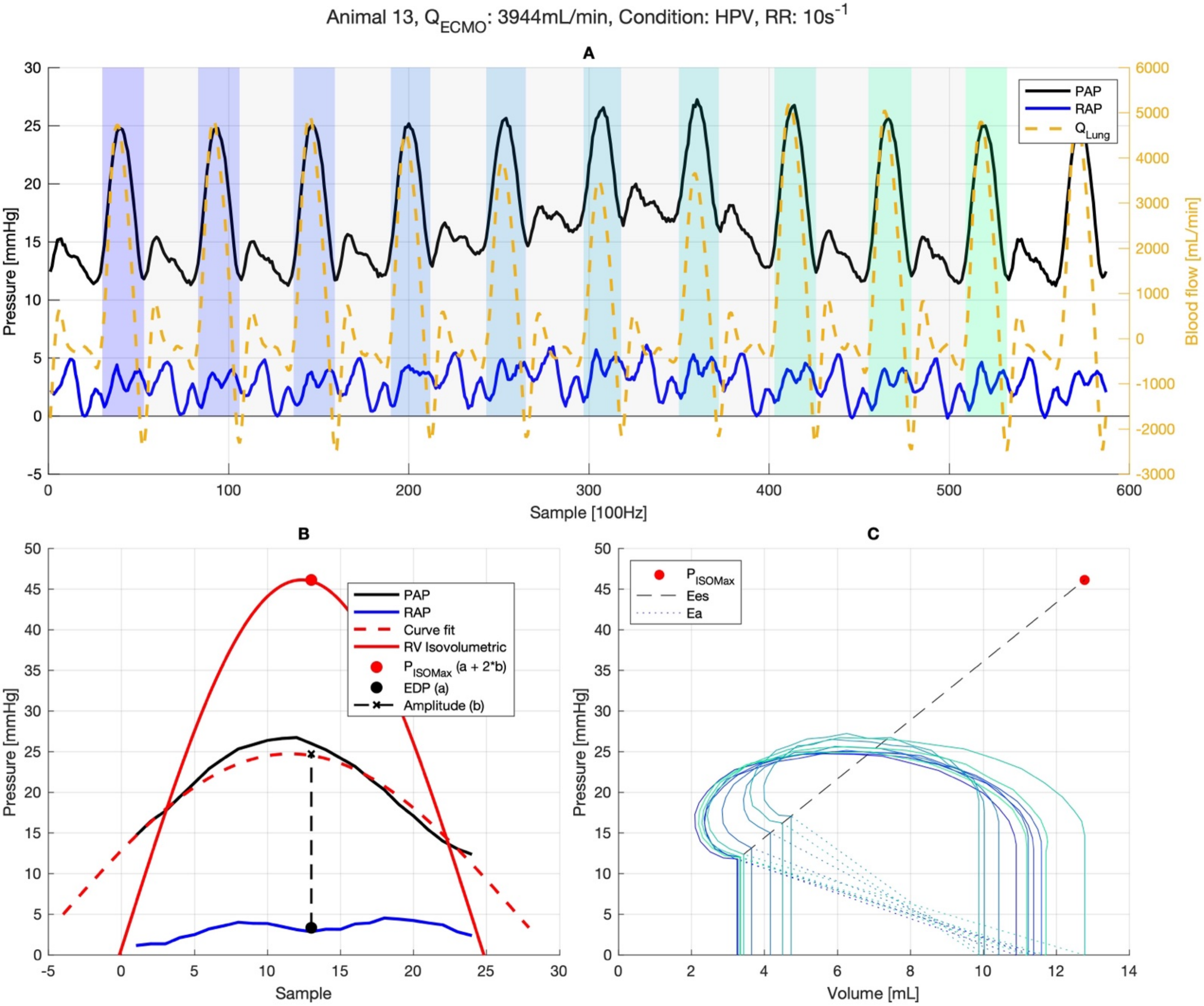
Animal 13, Q_ECMO: 3944mL/min, Condition: HPV, RR: 10s^-1
A
PAP
RAP
Q_Lung
Pressure [mmHg]
Blood flow [mL/min]
Sample [100Hz]
B
PAP
RAP
Curve fit
RV Isovolumetric
P_ISOMax (a + 2*b)
EDP (a)
Amplitude (b)
Pressure [mmHg]
Sample
C
P_ISOMax
Ees
Ea
Pressure [mmHg]
Volume [mL]

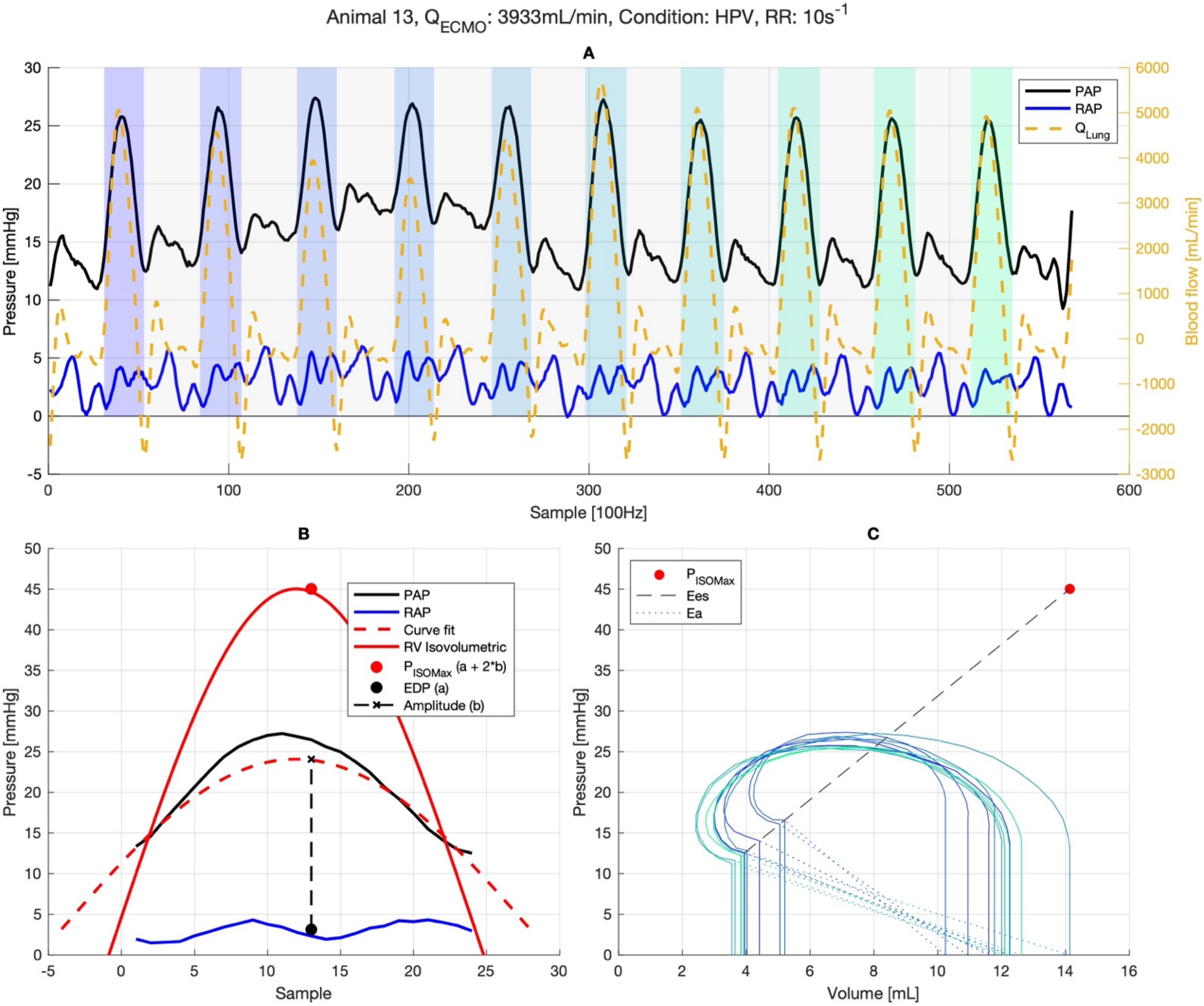

Animal 13, Q_ECMO: 3933mL/min, Condition: HPV, RR: 10s^-1
A
Pressure [mmHg]
Blood flow [mL/min]
Sample [100Hz]
PAP
RAP
Q_Lung
B
Pressure [mmHg]
Sample
PAP
RAP
Curve fit
RV Isovolumetric
P_ISOMax (a + 2*b)
EDP (a)
Amplitude (b)
C
Pressure [mmHg]
Volume [mL]
P_ISOMax
Ees
Ea

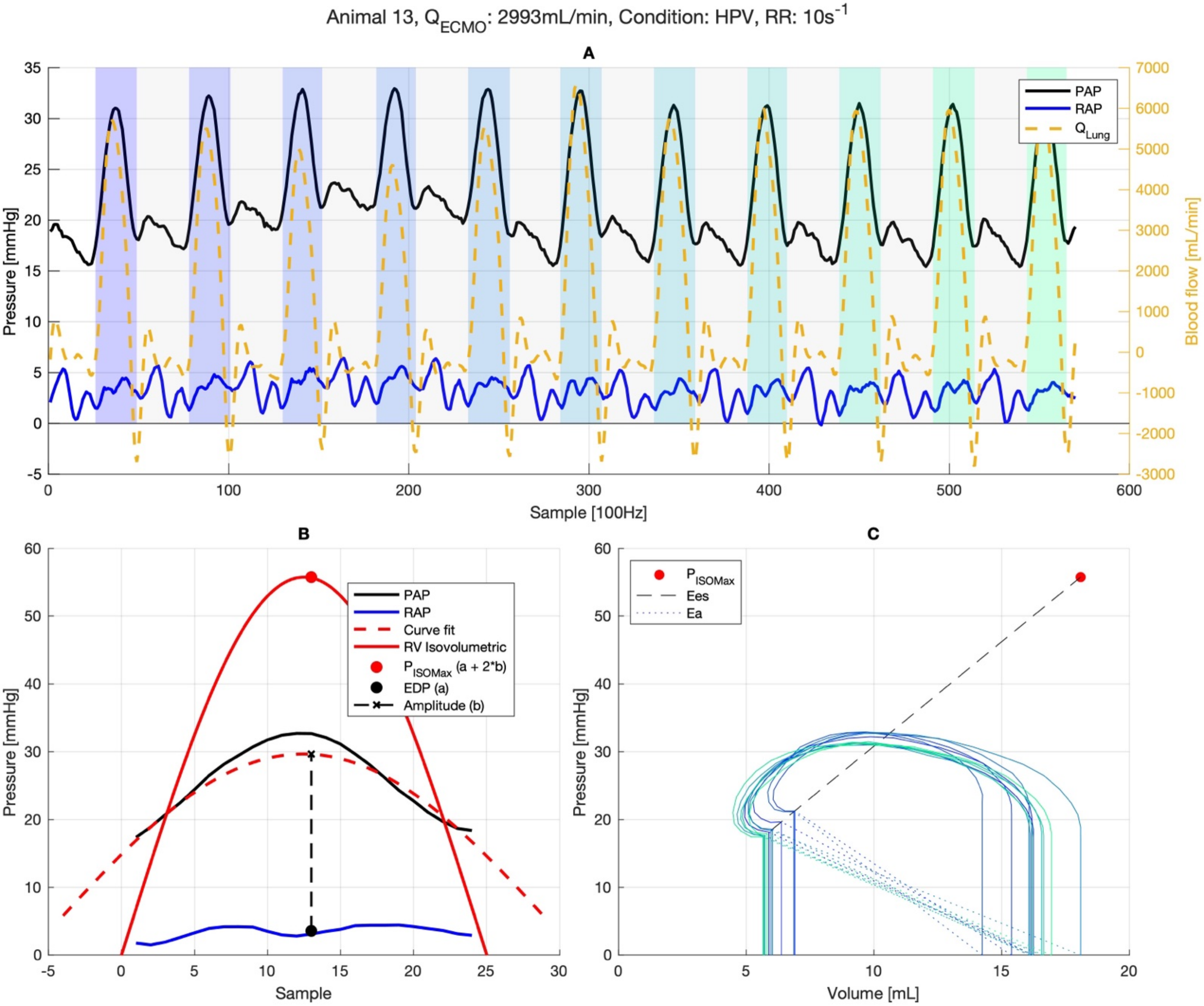
Animal 13, Q_{ECMO}: 2993mL/min, Condition: HPV, RR: $10s^{-1}$
A
PAP
RAP
Q_{Lung}
Pressure [mmHg]
Blood flow [mL/min]
Sample [100Hz]
B
PAP
RAP
Curve fit
RV Isovolumetric
P_{ISOMax} (a + 2*b)
EDP (a)
Amplitude (b)
Pressure [mmHg]
Sample
C
P_{ISOMax}
Ees
Ea
Pressure [mmHg]
Volume [mL]

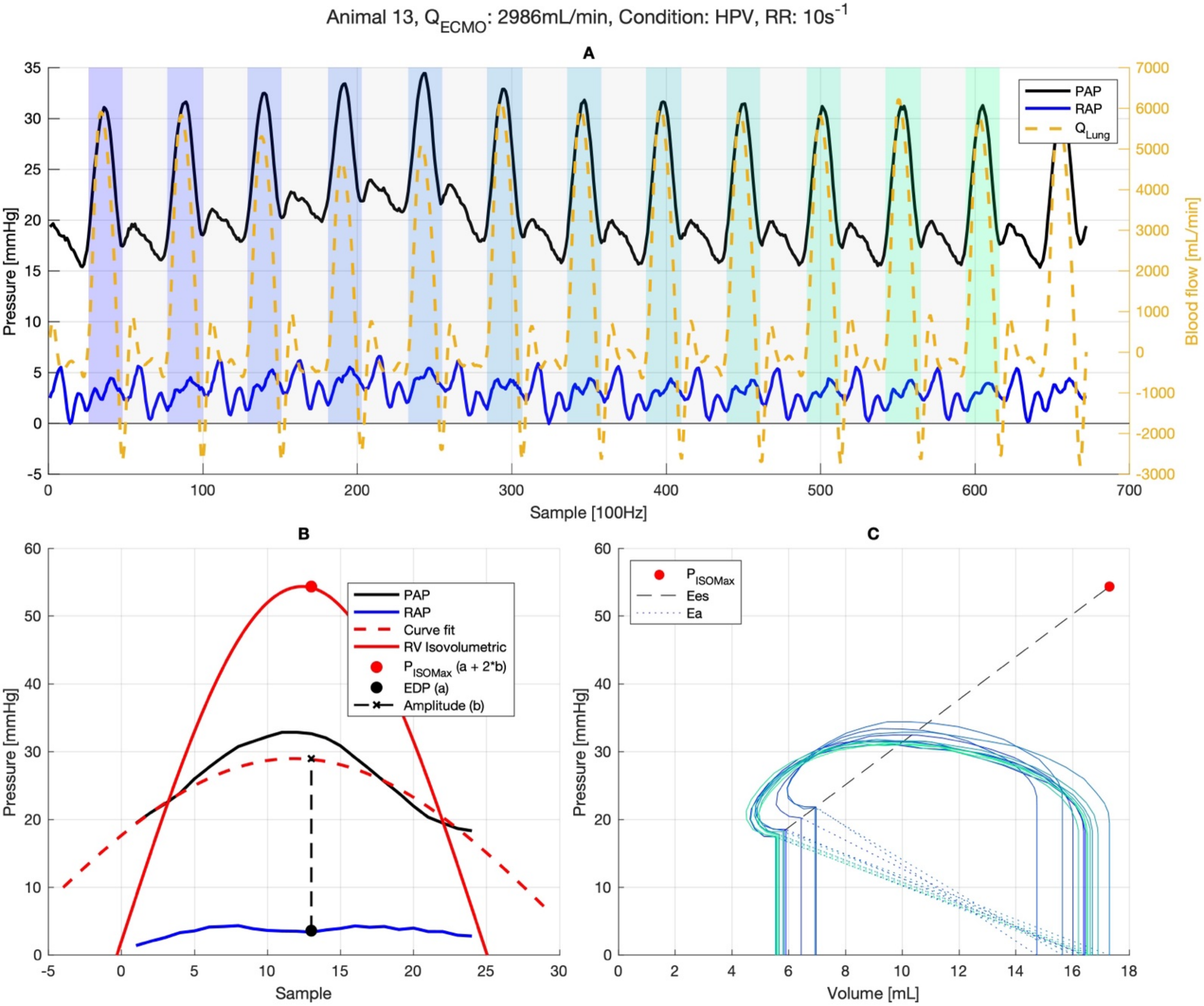
Animal 13, Q_{ECMO}: 2986mL/min, Condition: HPV, RR: 10s^{-1}
A
PAP
RAP
Q_{Lung}
Pressure [mmHg]
Blood flow [mL/min]
Sample [100Hz]
B
PAP
RAP
Curve fit
RV Isovolumetric
P_{ISOMax} (a + 2*b)
EDP (a)
Amplitude (b)
Pressure [mmHg]
Sample
C
P_{ISOMax}
Ees
Ea
Pressure [mmHg]
Volume [mL]

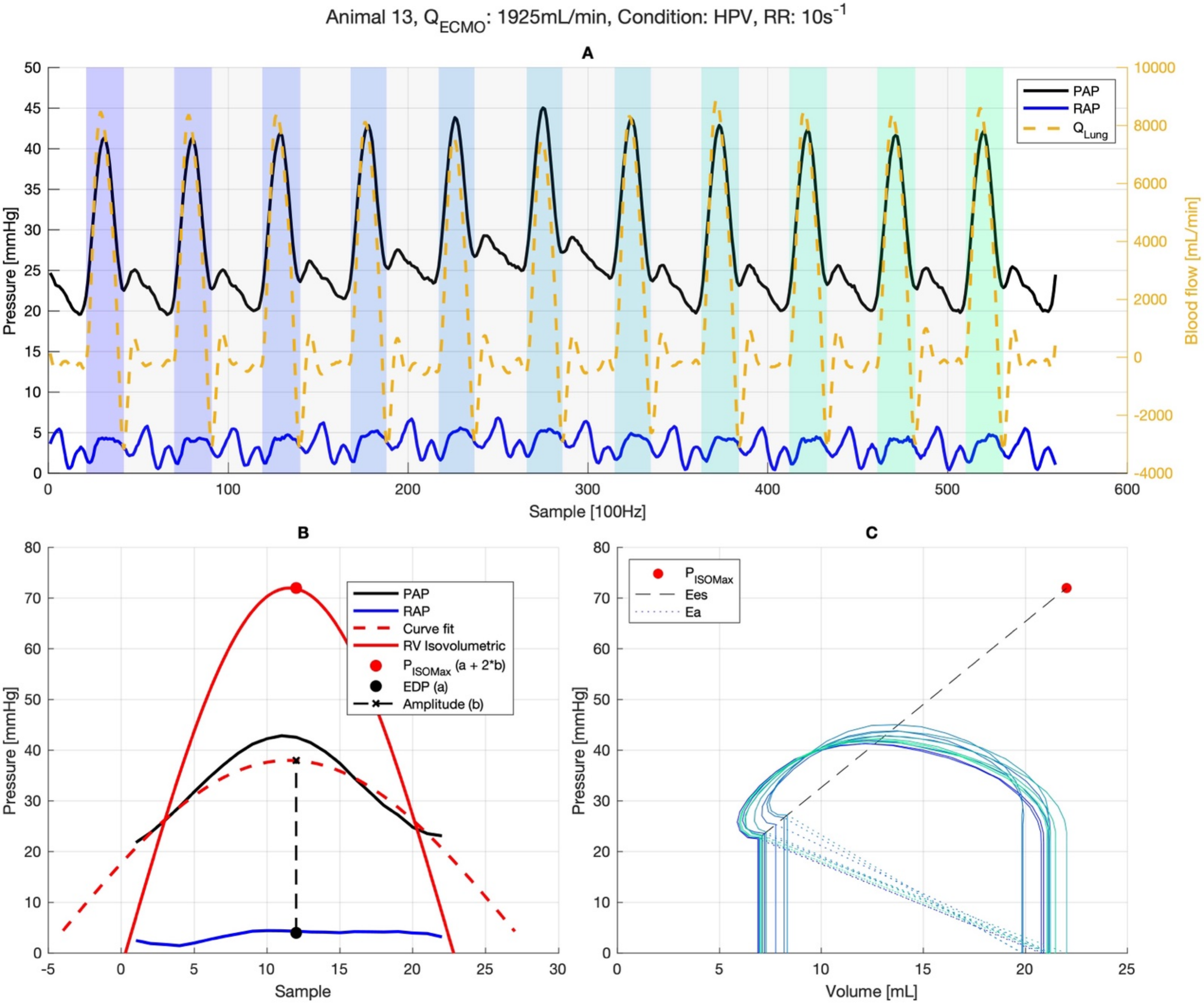

Animal 13, Q_{ECMO}: 1925mL/min, Condition: HPV, RR: $10s^{-1}$
A
PAP
RAP
Q_{Lung}
Pressure [mmHg]
Blood flow [mL/min]
Sample [100Hz]
B
PAP
RAP
Curve fit
RV Isovolumetric
P_{ISOMax} (a + 2*b)
EDP (a)
Amplitude (b)
Pressure [mmHg]
Sample
C
P_{ISOMax}
Ees
Ea
Pressure [mmHg]
Volume [mL]

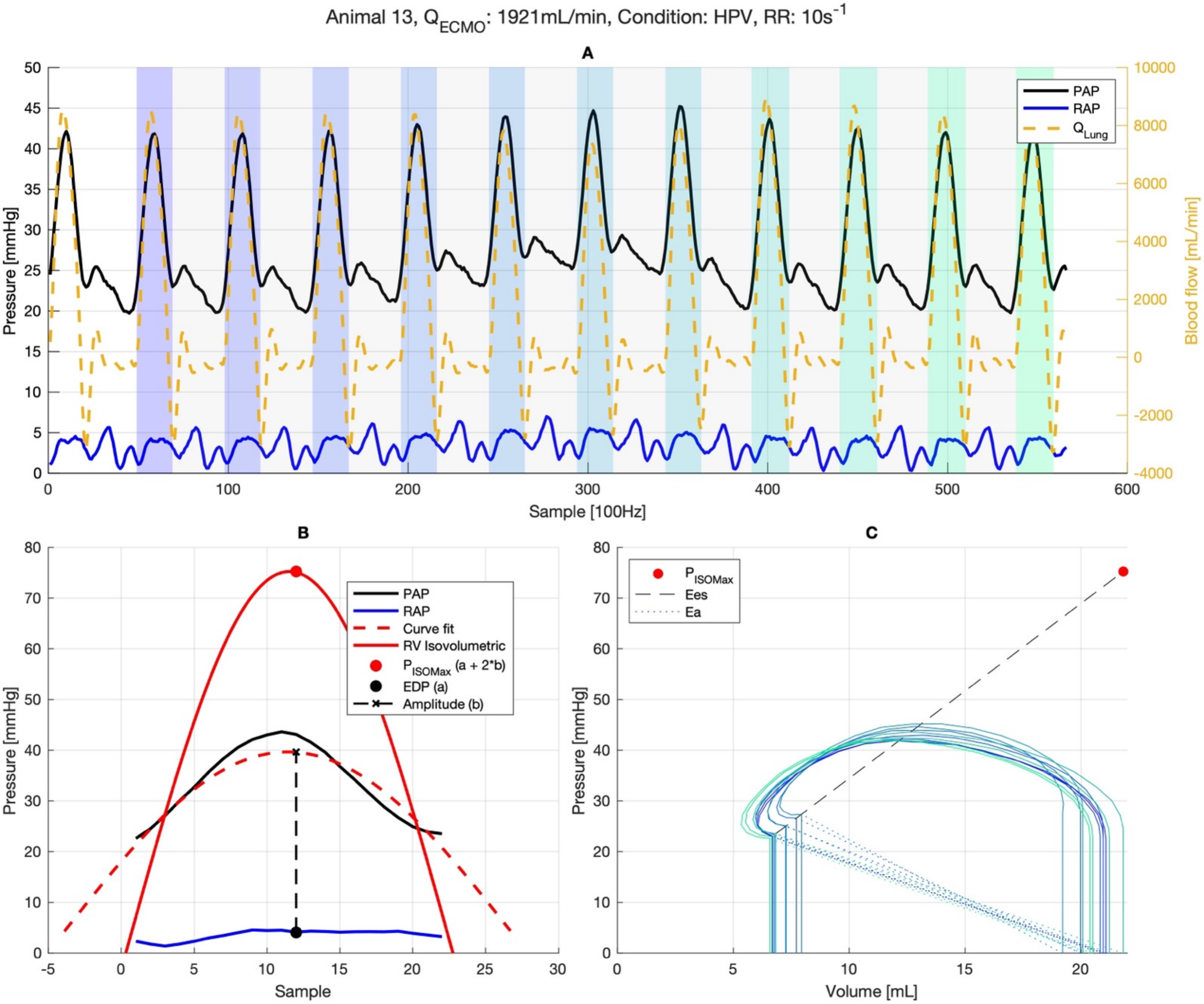
Animal 13, Q_ECMO: 1921mL/min, Condition: HPV, RR: 10s^-1
A
PAP
RAP
Q_Lung
Pressure [mmHg]
Blood flow [mL/min]
Sample [100Hz]
B
PAP
RAP
Curve fit
RV Isovolumetric
P_ISOMax (a + 2*b)
EDP (a)
Amplitude (b)
Pressure [mmHg]
Sample
C
P_ISOMax
Ees
Ea
Pressure [mmHg]
Volume [mL]

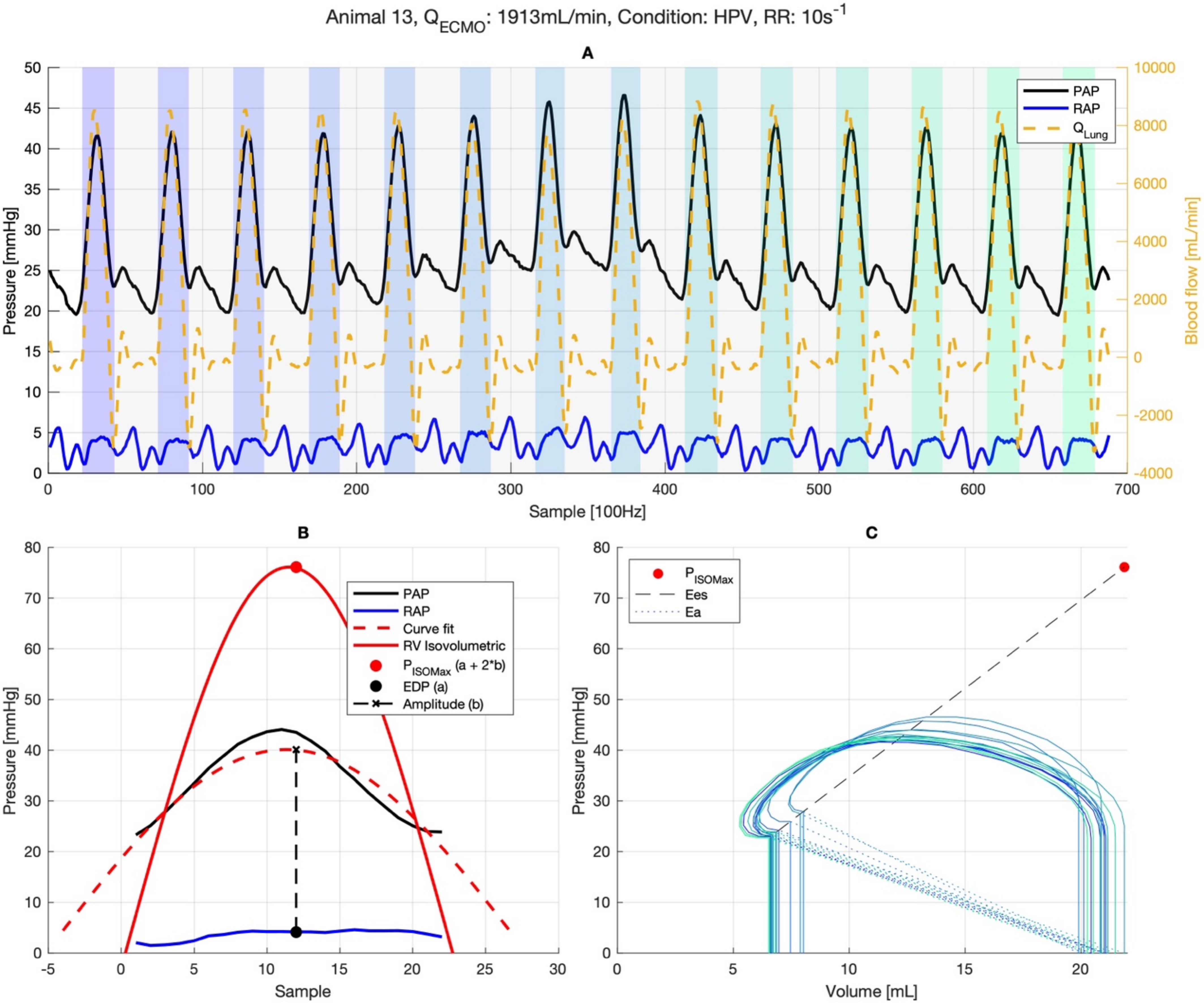

Animal 13, Q_{ECMO}: 1913mL/min, Condition: HPV, RR: $10s^{-1}$
A
PAP
RAP
Q_{Lung}
Pressure [mmHg]
Blood flow [mL/min]
Sample [100Hz]
B
PAP
RAP
Curve fit
RV Isovolumetric
P_{ISOMax} (a + 2*b)
EDP (a)
Amplitude (b)
Pressure [mmHg]
Sample
C
P_{ISOMax}
Ees
Ea
Pressure [mmHg]
Volume [mL]

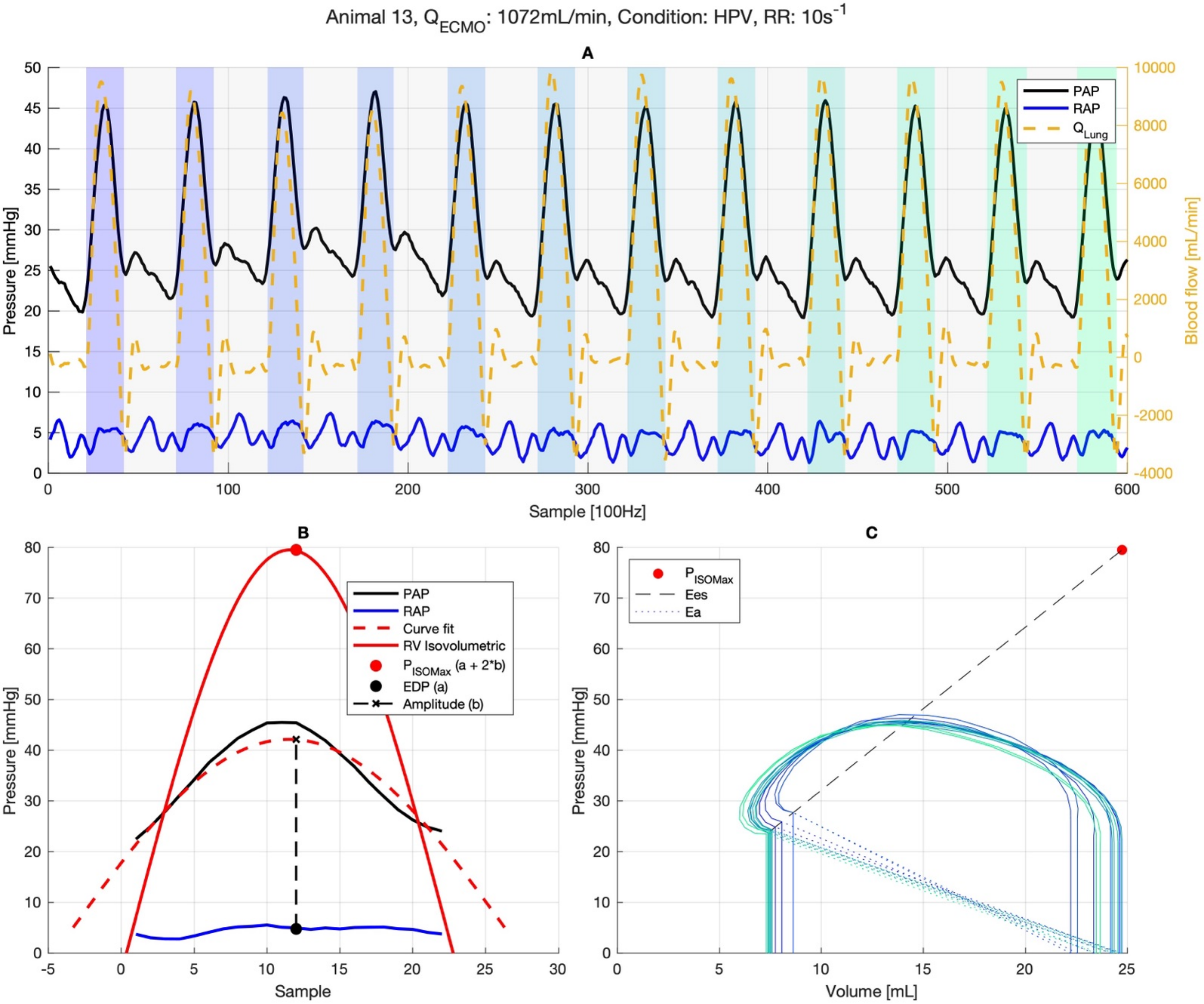

Animal 13, Q_{ECMO}: 1072mL/min, Condition: HPV, RR: $10s^{-1}$
A
PAP
RAP
Q_{Lung}
Pressure [mmHg]
Blood flow [mL/min]
Sample [100Hz]
B
PAP
RAP
Curve fit
RV Isovolumetric
P_{ISOMax} (a + 2*b)
EDP (a)
Amplitude (b)
Pressure [mmHg]
Sample
C
P_{ISOMax}
Ees
Ea
Pressure [mmHg]
Volume [mL]

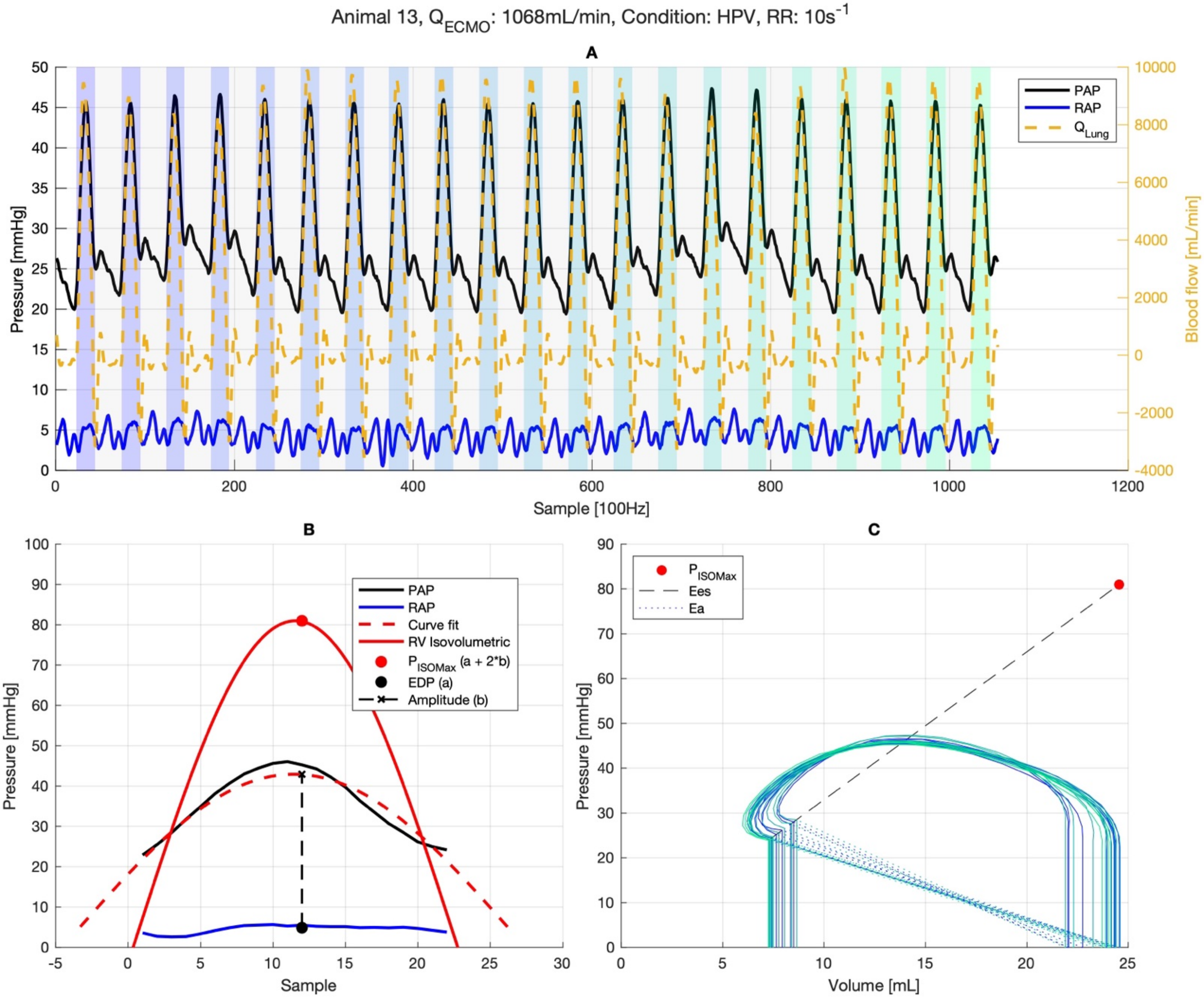

Animal 13, Q_{ECMO}: 1068mL/min, Condition: HPV, RR: $10s^{-1}$
A
PAP
RAP
Q_{Lung}
Pressure [mmHg]
Blood flow [mL/min]
Sample [100Hz]
B
PAP
RAP
Curve fit
RV Isovolumetric
P_{ISOMax} (a + 2*b)
EDP (a)
Amplitude (b)
Pressure [mmHg]
Sample
C
P_{ISOMax}
Ees
Ea
Pressure [mmHg]
Volume [mL]

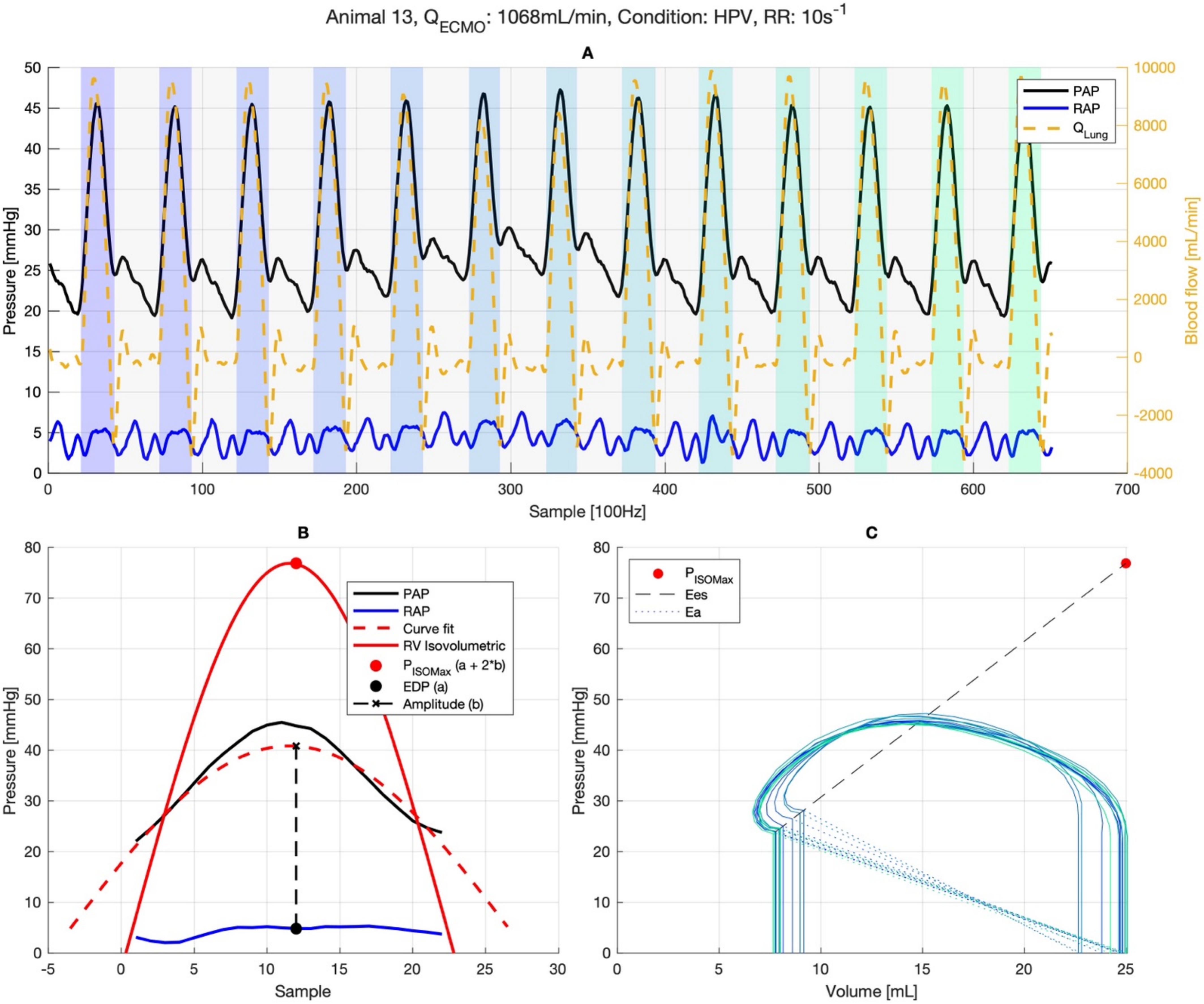

Animal 13, Q_ECMO: 1068mL/min, Condition: HPV, RR: 10s^-1
A
Pressure [mmHg]
Blood flow [mL/min]
Sample [100Hz]
PAP
RAP
Q_Lung
B
Pressure [mmHg]
Sample
PAP
RAP
Curve fit
RV Isovolumetric
P_ISOMax (a + 2*b)
EDP (a)
Amplitude (b)
C
Pressure [mmHg]
Volume [mL]
P_ISOMax
Ees
Ea

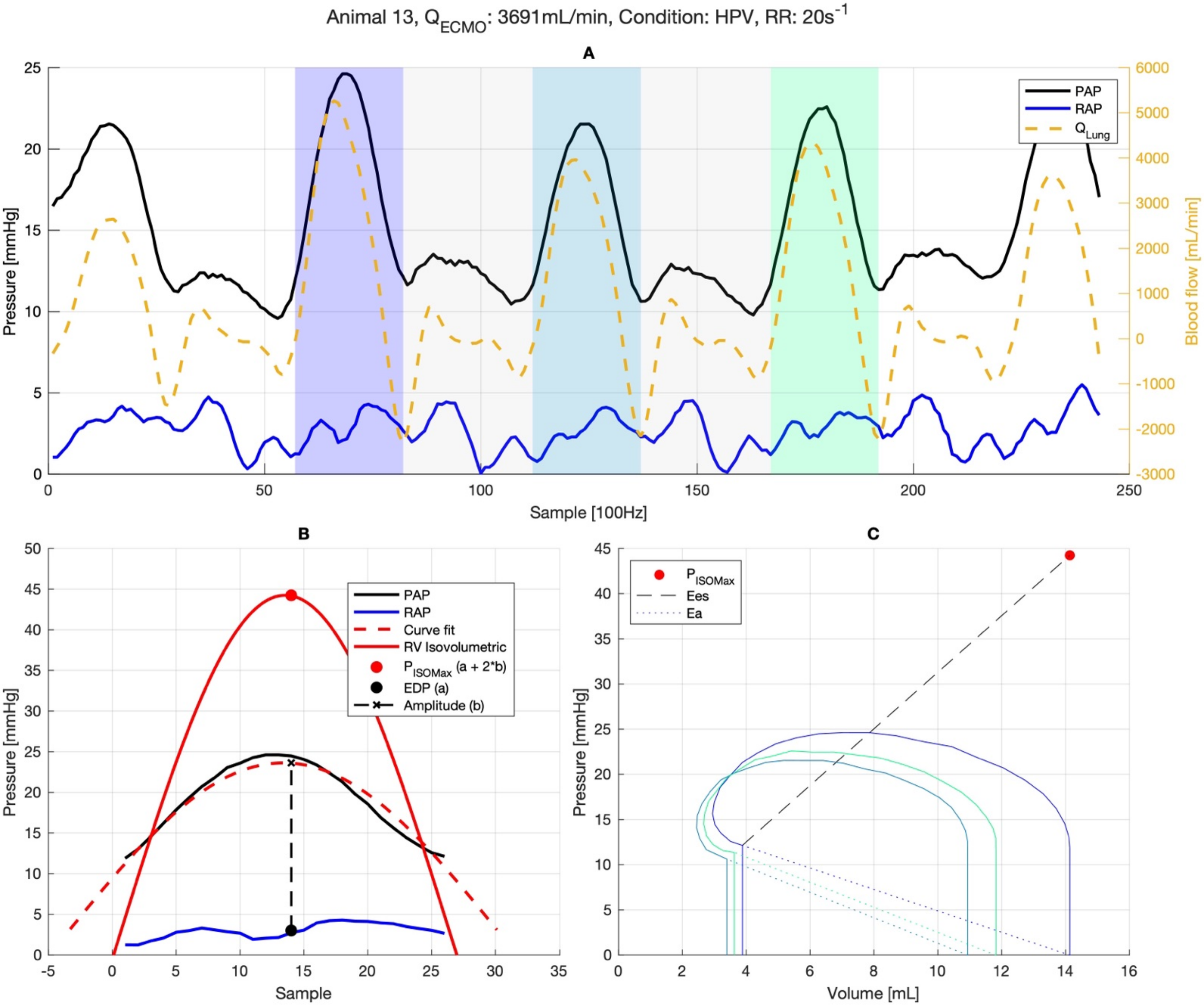

Animal 13, Q_{ECMO}: 3691mL/min, Condition: HPV, RR: $20s^{-1}$
A
PAP
RAP
Q_{Lung}
Pressure [mmHg]
Blood flow [mL/min]
Sample [100Hz]
B
PAP
RAP
Curve fit
RV Isovolumetric
P_{ISOMax} (a + 2*b)
EDP (a)
Amplitude (b)
Pressure [mmHg]
Sample
C
P_{ISOMax}
Ees
Ea
Pressure [mmHg]
Volume [mL]

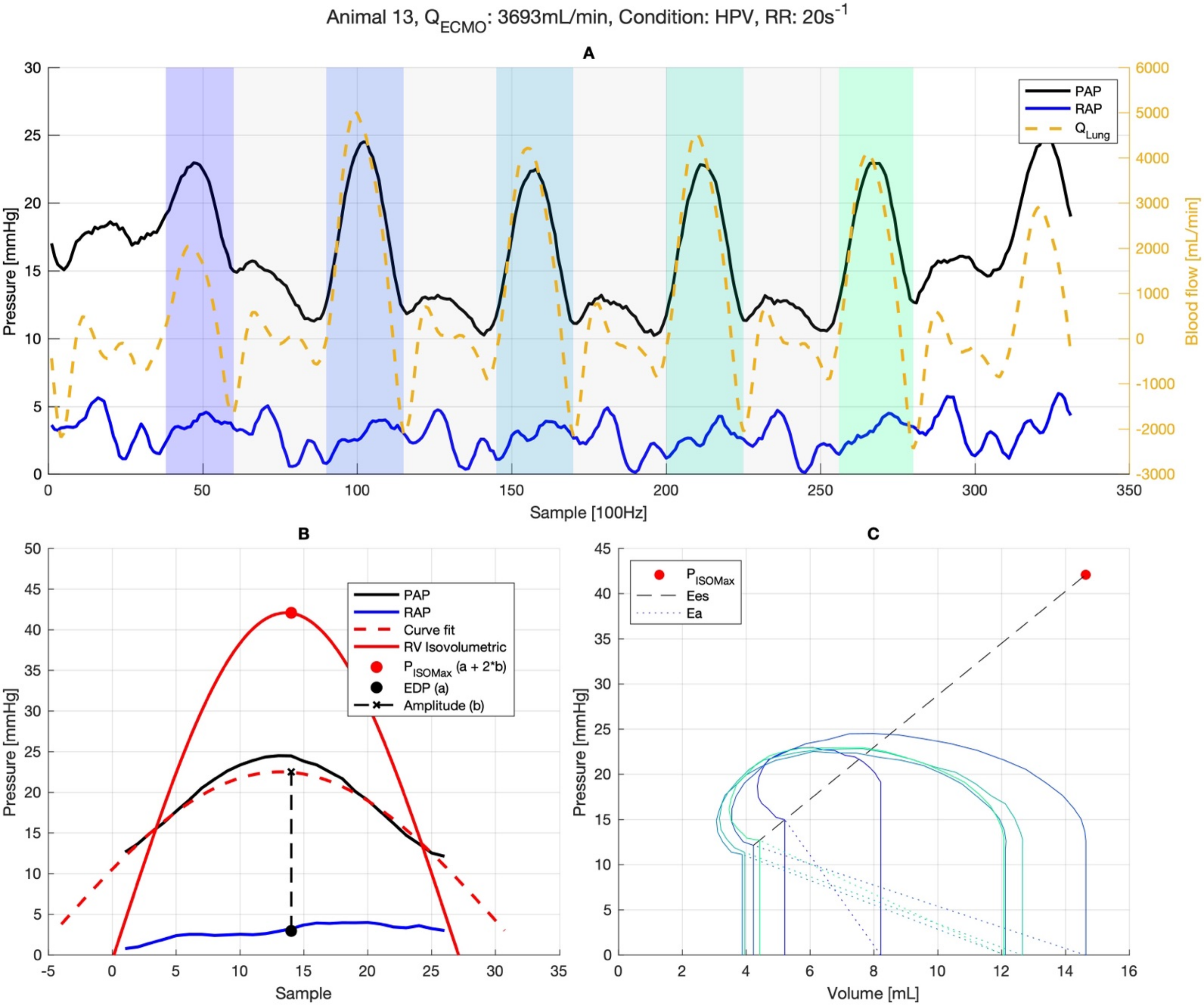

Animal 13, Q_ECMO: 3693mL/min, Condition: HPV, RR: 20s^-1
A
PAP
RAP
Q_Lung
Pressure [mmHg]
Blood flow [mL/min]
Sample [100Hz]
B
PAP
RAP
Curve fit
RV Isovolumetric
P_ISOMax (a + 2*b)
EDP (a)
Amplitude (b)
Pressure [mmHg]
Sample
C
P_ISOMax
Ees
Ea
Pressure [mmHg]
Volume [mL]

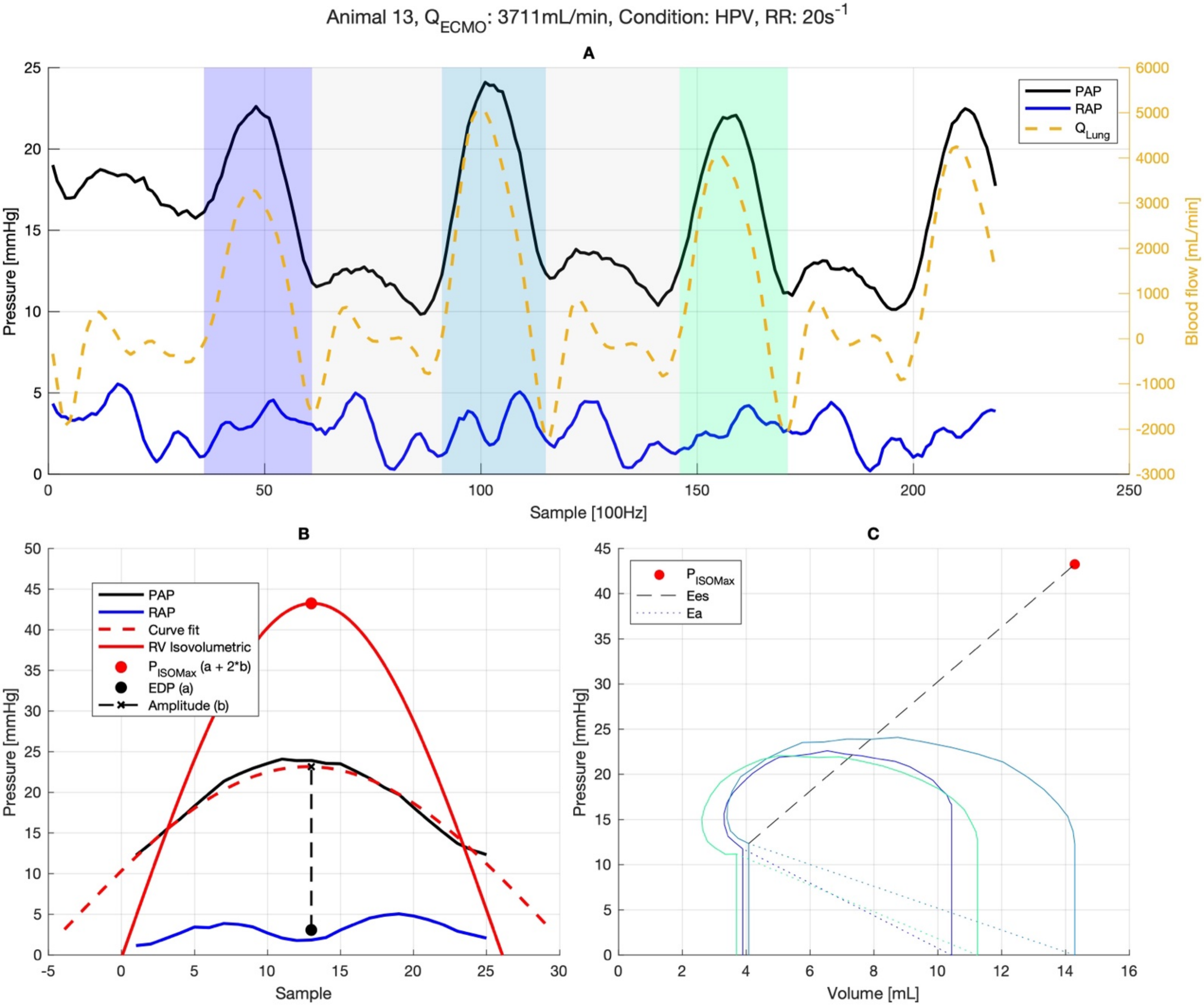

Animal 13, Q_{ECMO}: 3711mL/min, Condition: HPV, RR: $20s^{-1}$
A
PAP
RAP
Q_{Lung}
Pressure [mmHg]
Blood flow [mL/min]
Sample [100Hz]
B
PAP
RAP
Curve fit
RV Isovolumetric
P_{ISOMax} (a + 2*b)
EDP (a)
Amplitude (b)
Pressure [mmHg]
Sample
C
P_{ISOMax}
Ees
Ea
Pressure [mmHg]
Volume [mL]

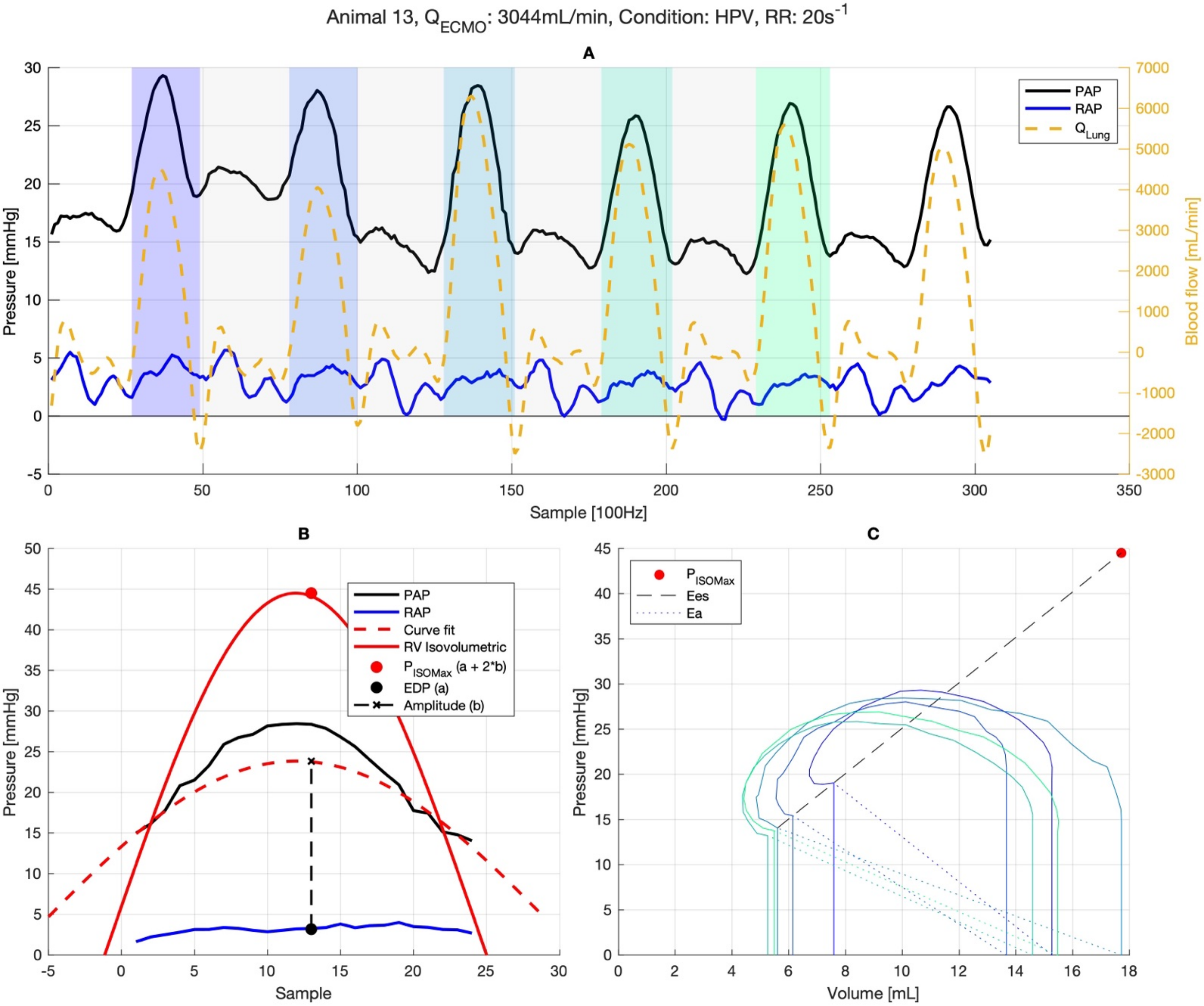

Animal 13, Q_{ECMO}: 3044mL/min, Condition: HPV, RR: 20s^{-1}
A
PAP
RAP
Q_{Lung}
Pressure [mmHg]
Blood flow [mL/min]
Sample [100Hz]
B
PAP
RAP
Curve fit
RV Isovolumetric
P_{ISOMax} (a + 2*b)
EDP (a)
Amplitude (b)
Pressure [mmHg]
Sample
C
P_{ISOMax}
Ees
Ea
Pressure [mmHg]
Volume [mL]

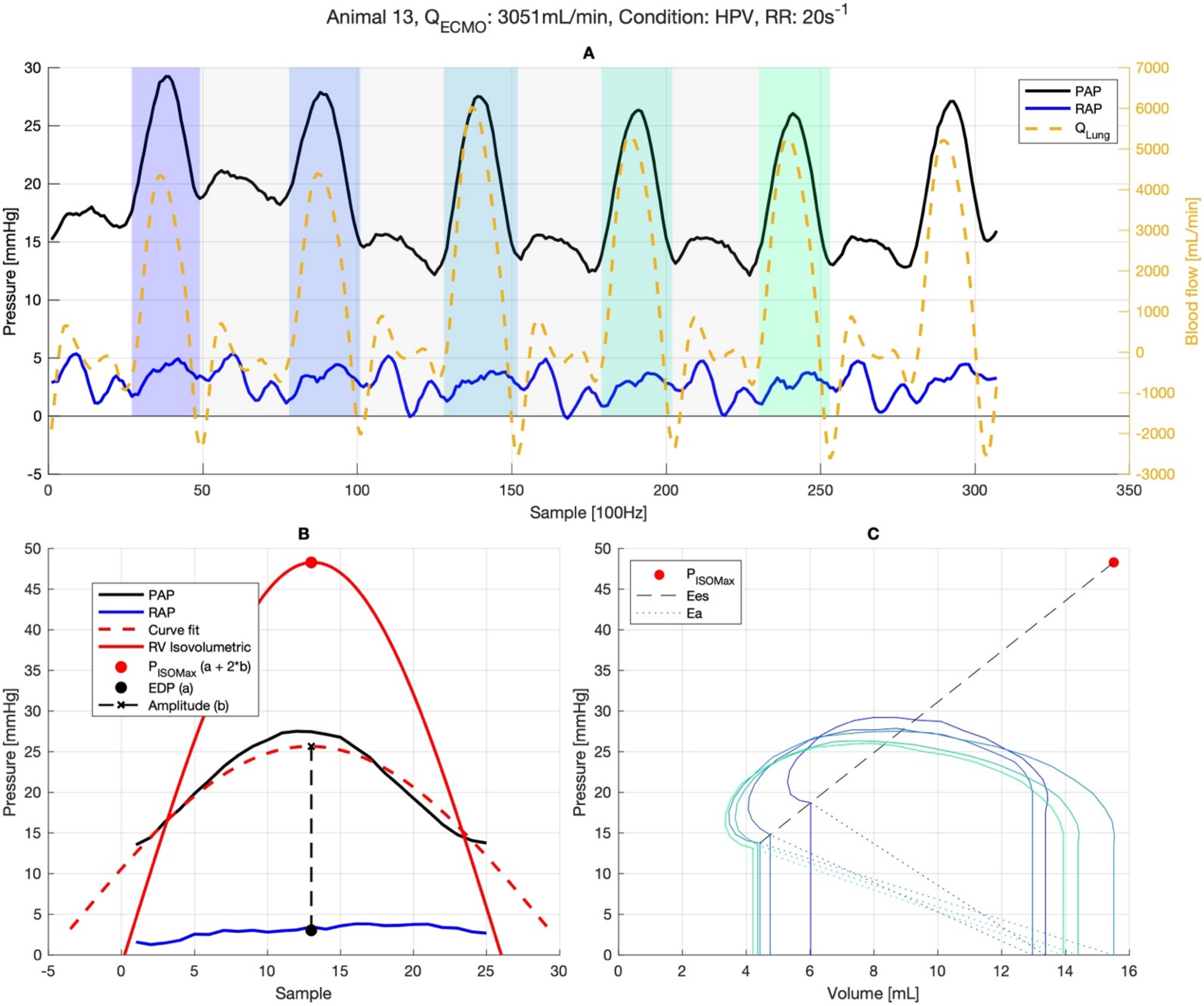

Animal 13, Q_{ECMO}: 3051mL/min, Condition: HPV, RR: 20s^{-1}
A
PAP
RAP
Q_{Lung}
Pressure [mmHg]
Blood flow [mL/min]
Sample [100Hz]
B
PAP
RAP
Curve fit
RV Isovolumetric
P_{ISOMax} (a + 2*b)
EDP (a)
Amplitude (b)
Pressure [mmHg]
Sample
C
P_{ISOMax}
Ees
Ea
Pressure [mmHg]
Volume [mL]

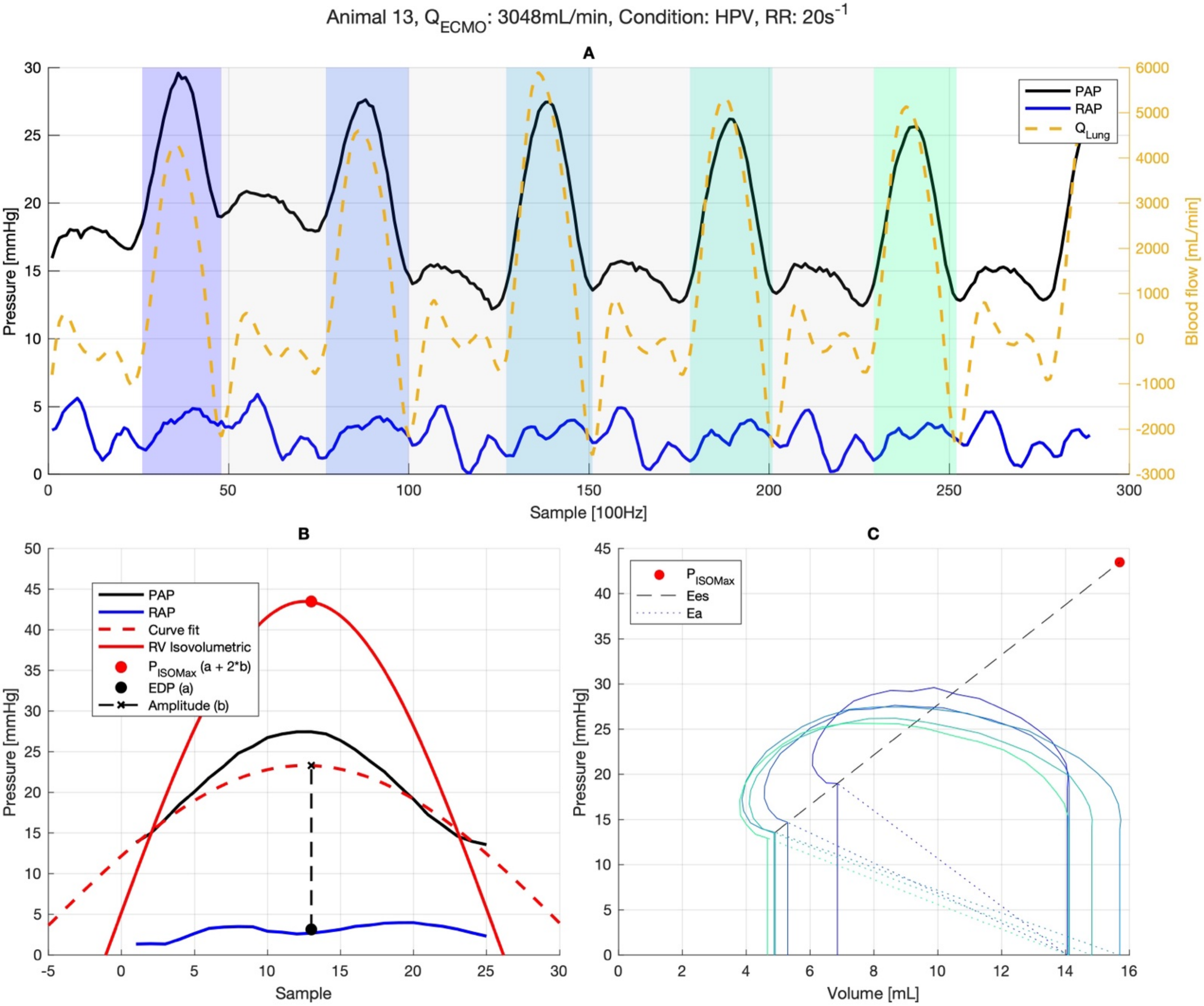

Animal 13, Q_ECMO: 3048mL/min, Condition: HPV, RR: 20s^-1
A
PAP
RAP
Q_Lung
Pressure [mmHg]
Blood flow [mL/min]
Sample [100Hz]
B
PAP
RAP
Curve fit
RV Isovolumetric
P_ISOMax (a + 2*b)
EDP (a)
Amplitude (b)
Pressure [mmHg]
Sample
C
P_ISOMax
Ees
Ea
Pressure [mmHg]
Volume [mL]

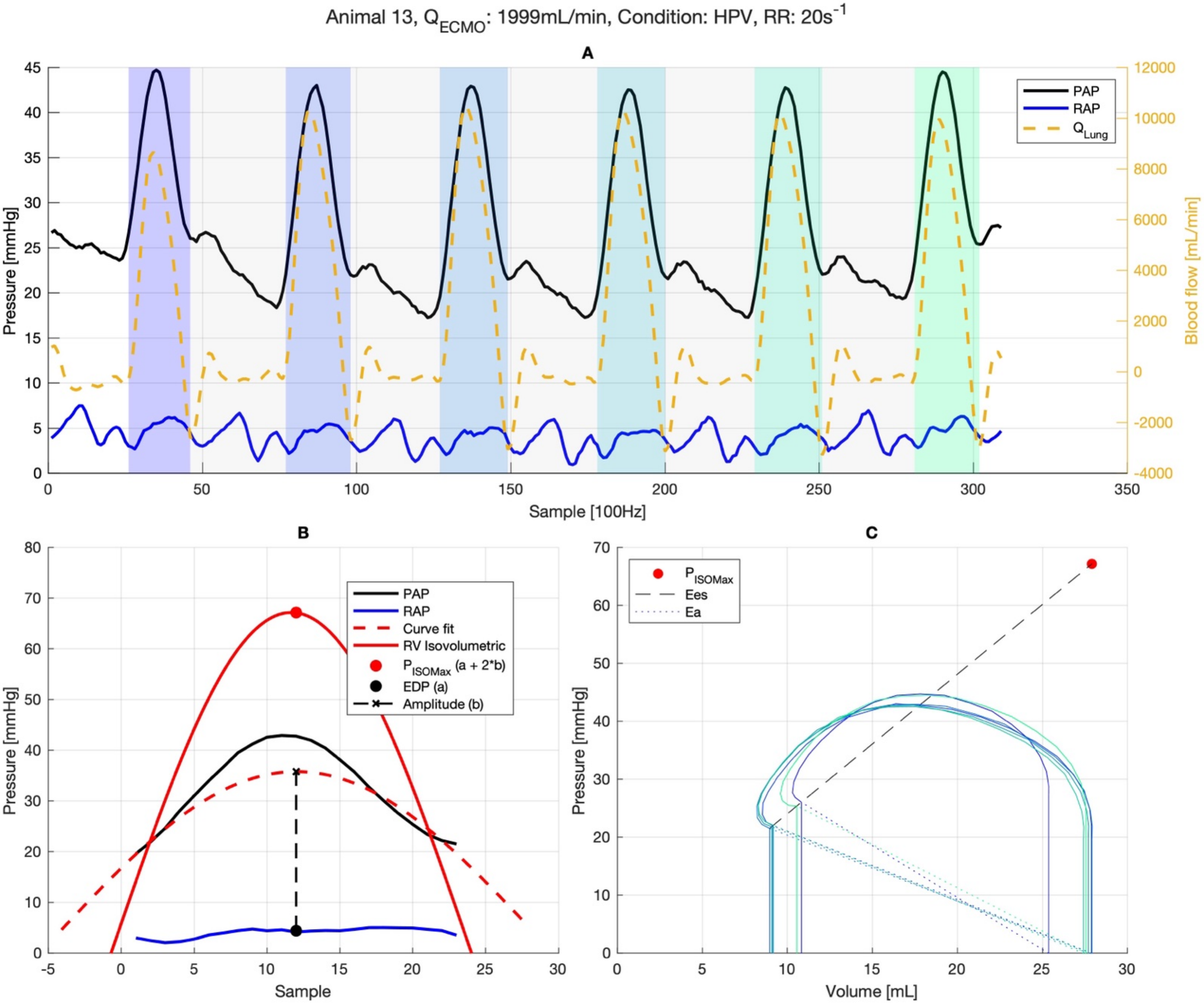

Animal 13, QECMO: 1999mL/min, Condition: HPV, RR: 20s-1
A
PAP
RAP
QLung
Pressure [mmHg]
Blood flow [mL/min]
Sample [100Hz]
B
PAP
RAP
Curve fit
RV Isovolumetric
PISOMax (a + 2*b)
EDP (a)
Amplitude (b)
Pressure [mmHg]
Sample
C
PISOMax
Ees
Ea
Pressure [mmHg]
Volume [mL]

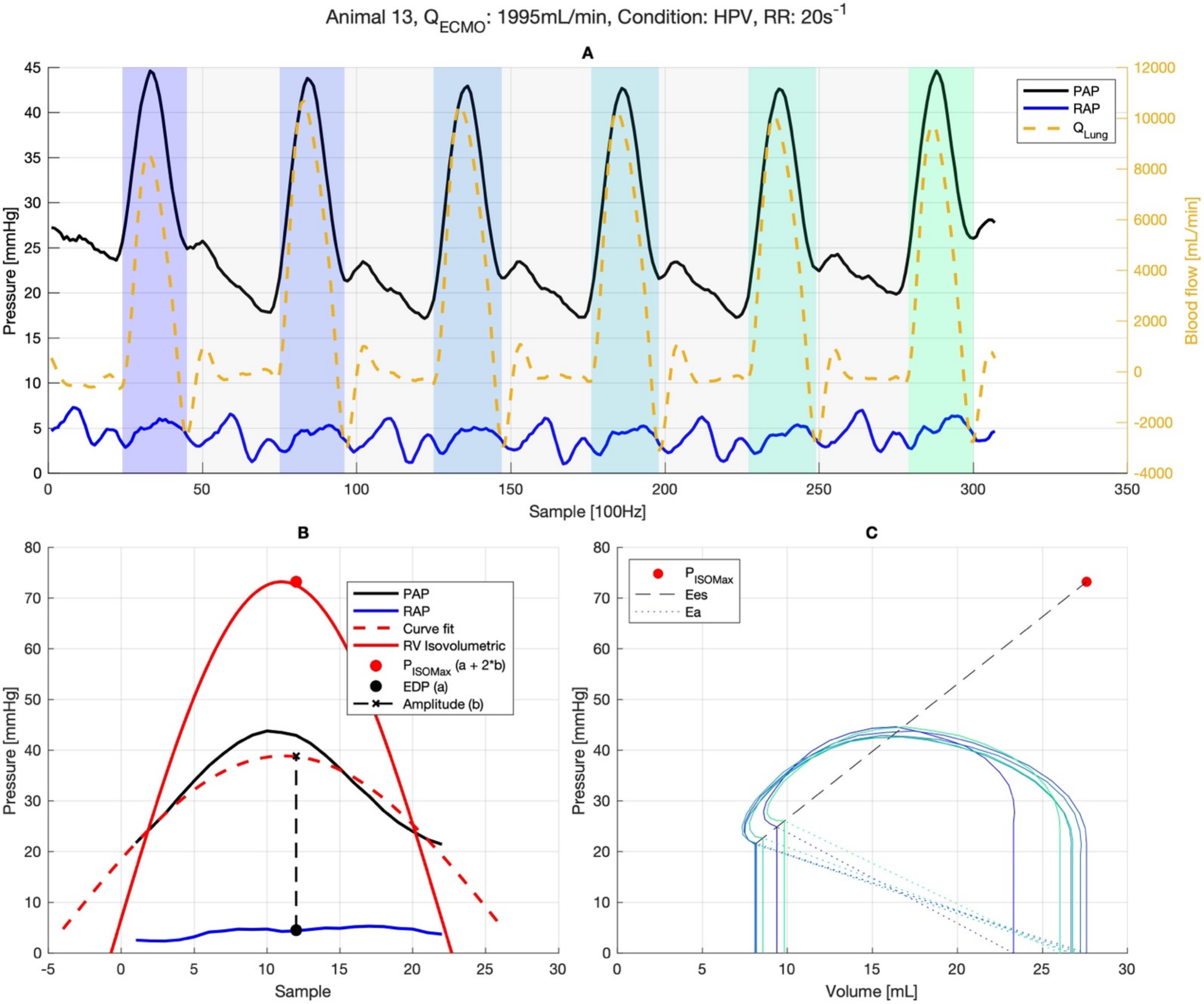

Animal 13, Q_{ECMO}: 1995mL/min, Condition: HPV, RR: 20s^{-1}
A
PAP
RAP
Q_{Lung}
Pressure [mmHg]
Blood flow [mL/min]
Sample [100Hz]
B
PAP
RAP
Curve fit
RV Isovolumetric
P_{ISOMax} (a + 2*b)
EDP (a)
Amplitude (b)
Pressure [mmHg]
Sample
C
P_{ISOMax}
Ees
Ea
Pressure [mmHg]
Volume [mL]

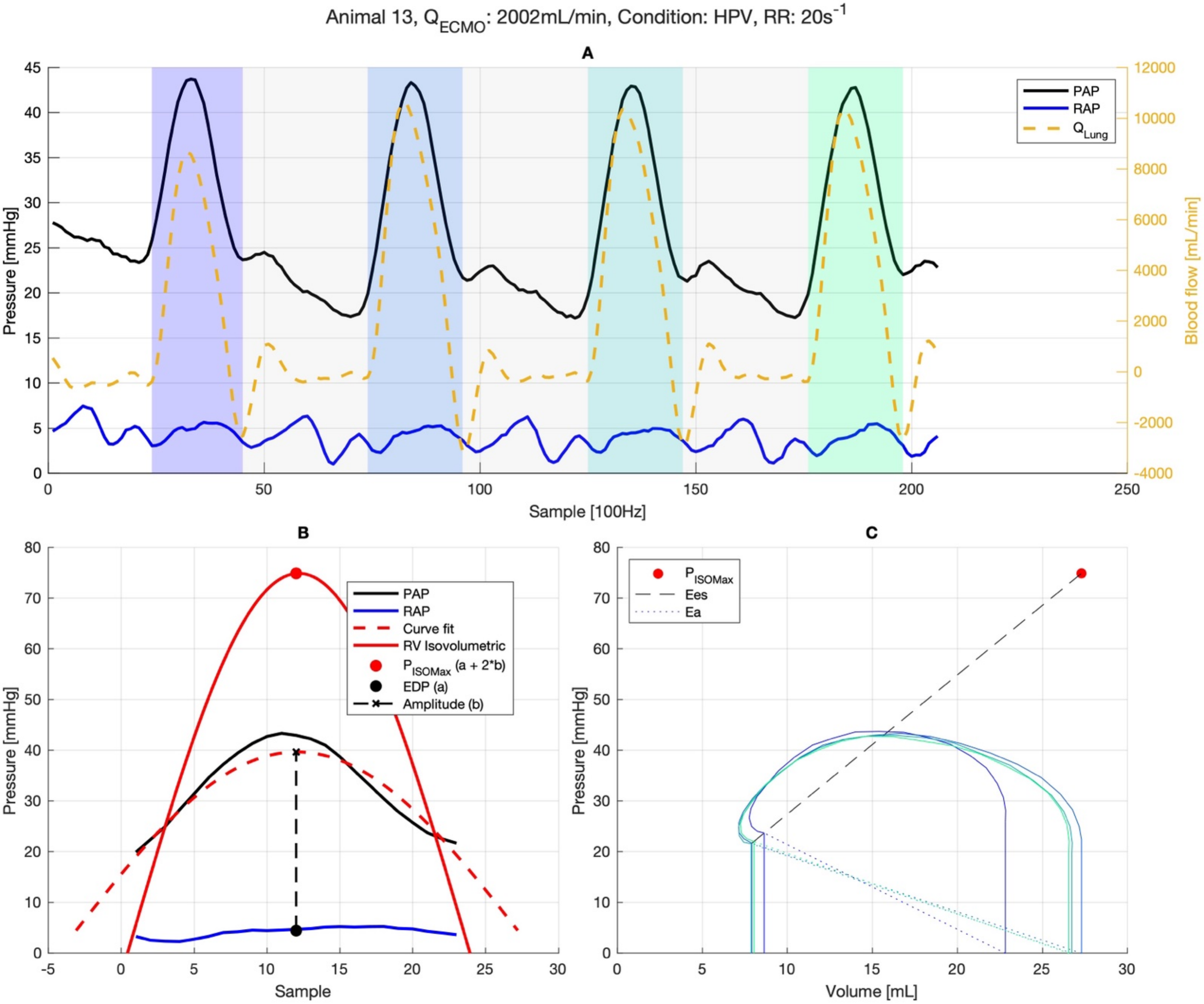

Animal 13, Q_ECMO: 2002mL/min, Condition: HPV, RR: 20s^-1
A
PAP
RAP
Q_Lung
Pressure [mmHg]
Blood flow [mL/min]
Sample [100Hz]
B
PAP
RAP
Curve fit
RV Isovolumetric
P_ISOMax (a + 2*b)
EDP (a)
Amplitude (b)
Pressure [mmHg]
Sample
C
P_ISOMax
Ees
Ea
Pressure [mmHg]
Volume [mL]

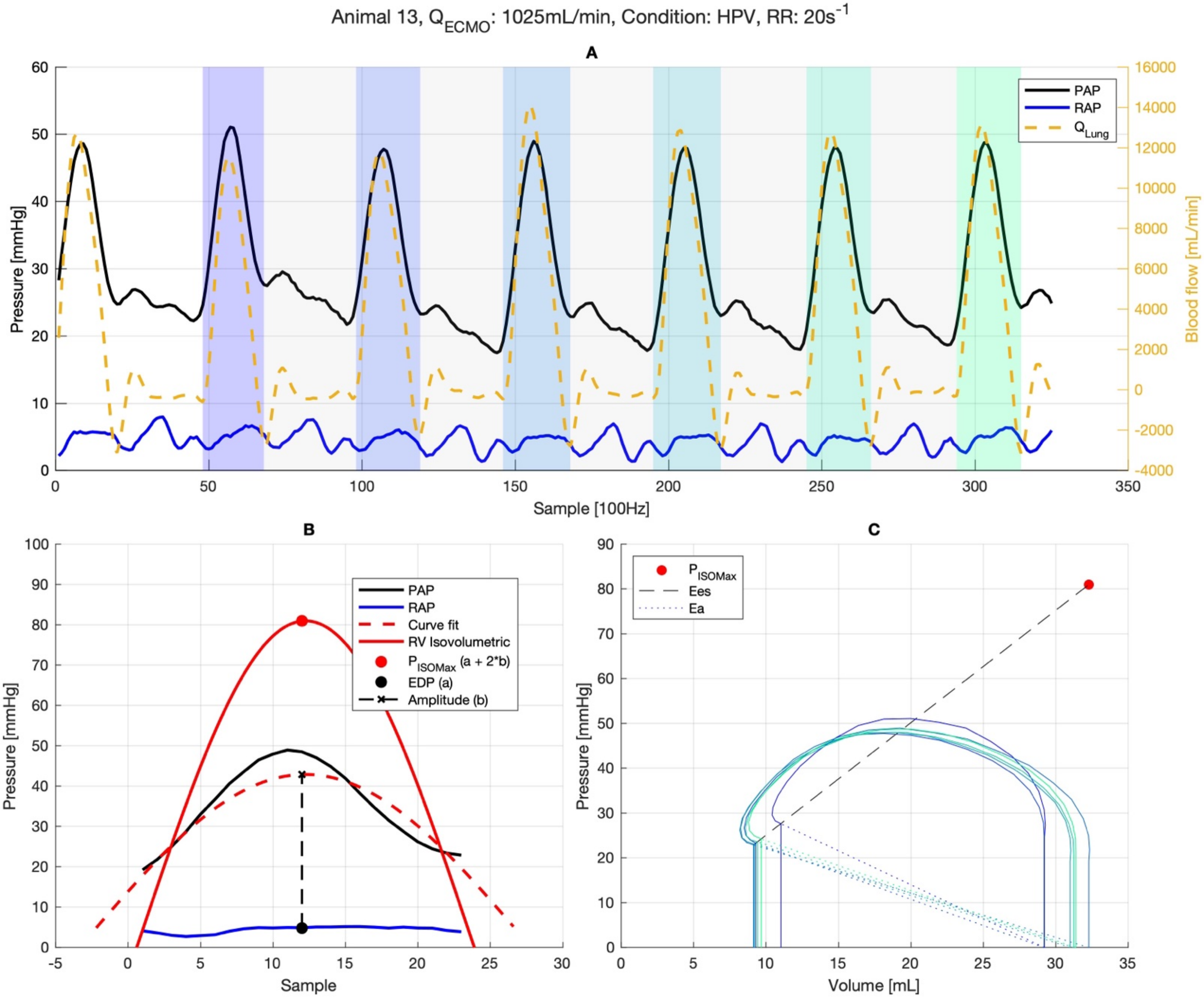
Animal 13, Q_{ECMO}: 1025mL/min, Condition: HPV, RR: $20s^{-1}$
A
Pressure [mmHg]
Blood flow [mL/min]
Sample [100Hz]
PAP
RAP
Q_{Lung}
B
Pressure [mmHg]
Sample
PAP
RAP
Curve fit
RV Isovolumetric
P_{ISOMax} (a + 2*b)
EDP (a)
Amplitude (b)
C
Pressure [mmHg]
Volume [mL]
P_{ISOMax}
Ees
Ea

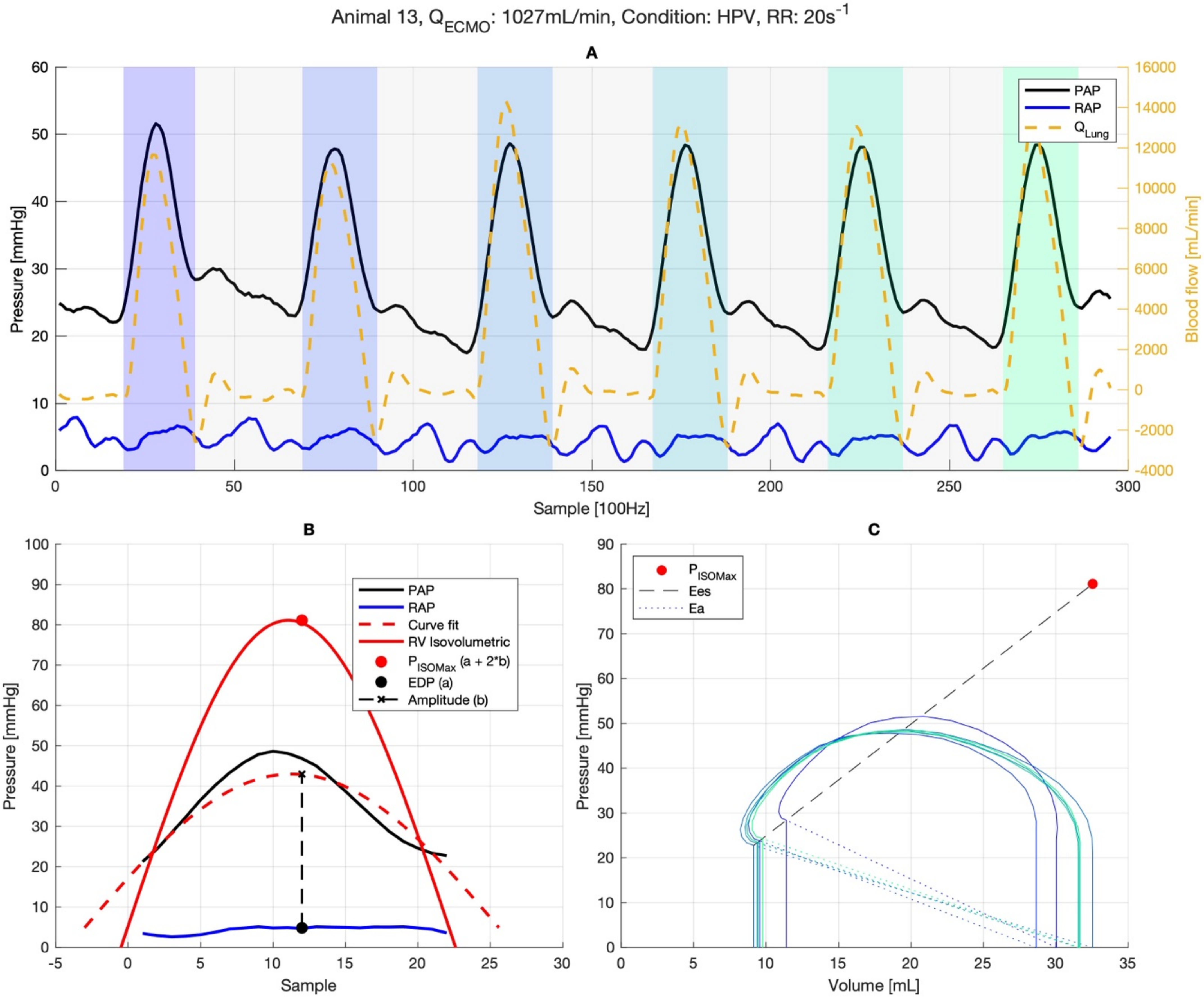

Animal 13, Q_ECMO: 1027mL/min, Condition: HPV, RR: 20s^-1
A
PAP
RAP
Q_Lung
Pressure [mmHg]
Blood flow [mL/min]
Sample [100Hz]
B
PAP
RAP
Curve fit
RV Isovolumetric
P_ISOMax (a + 2*b)
EDP (a)
Amplitude (b)
Pressure [mmHg]
Sample
C
P_ISOMax
Ees
Ea
Pressure [mmHg]
Volume [mL]

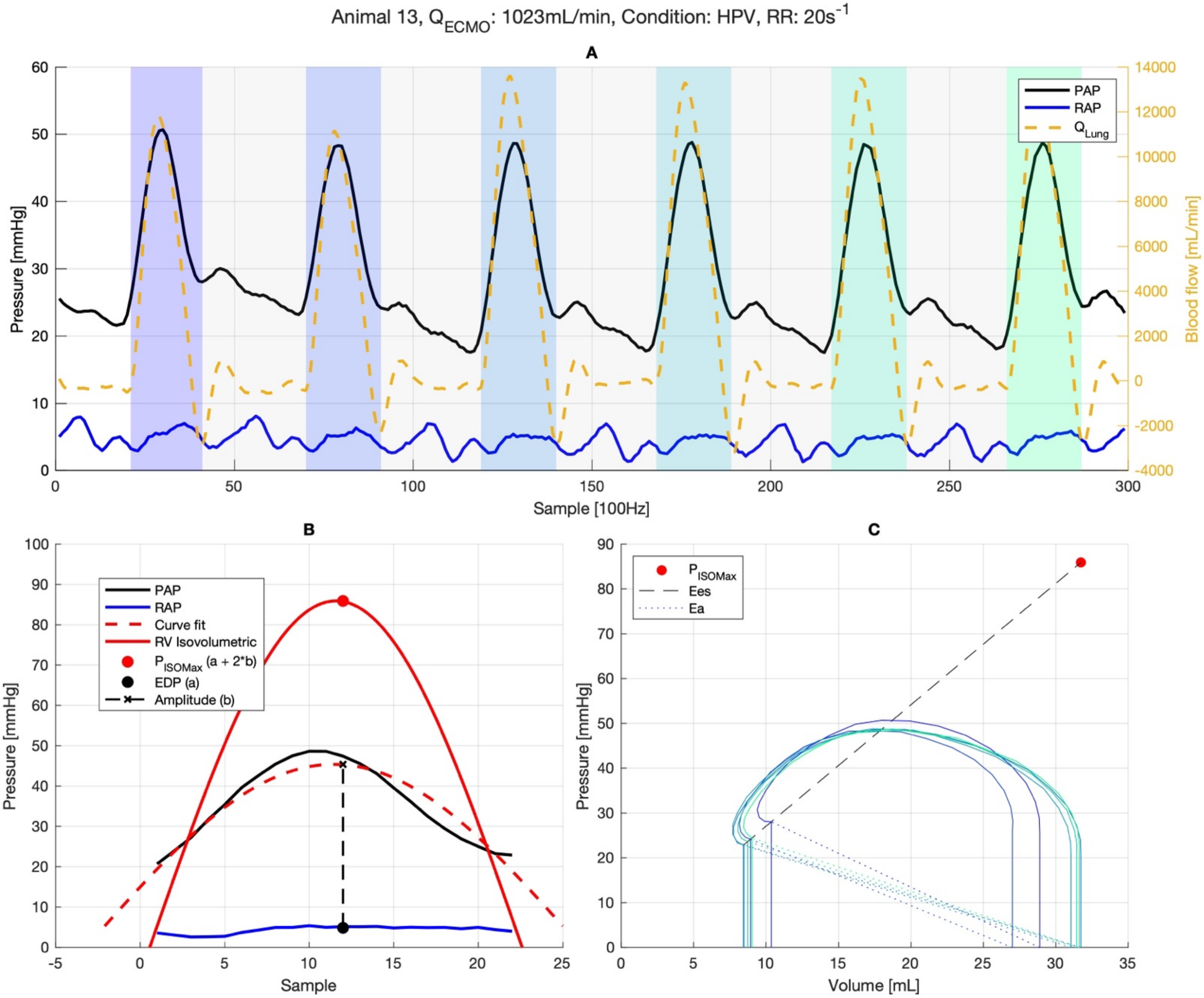
Animal 13, Q_ECMO: 1023mL/min, Condition: HPV, RR: 20s^-1
A
PAP
RAP
Q_Lung
Pressure [mmHg]
Blood flow [mL/min]
Sample [100Hz]
B
PAP
RAP
Curve fit
RV Isovolumetric
P_ISOMax (a + 2*b)
EDP (a)
Amplitude (b)
Pressure [mmHg]
Sample
C
P_ISOMax
Ees
Ea
Pressure [mmHg]
Volume [mL]

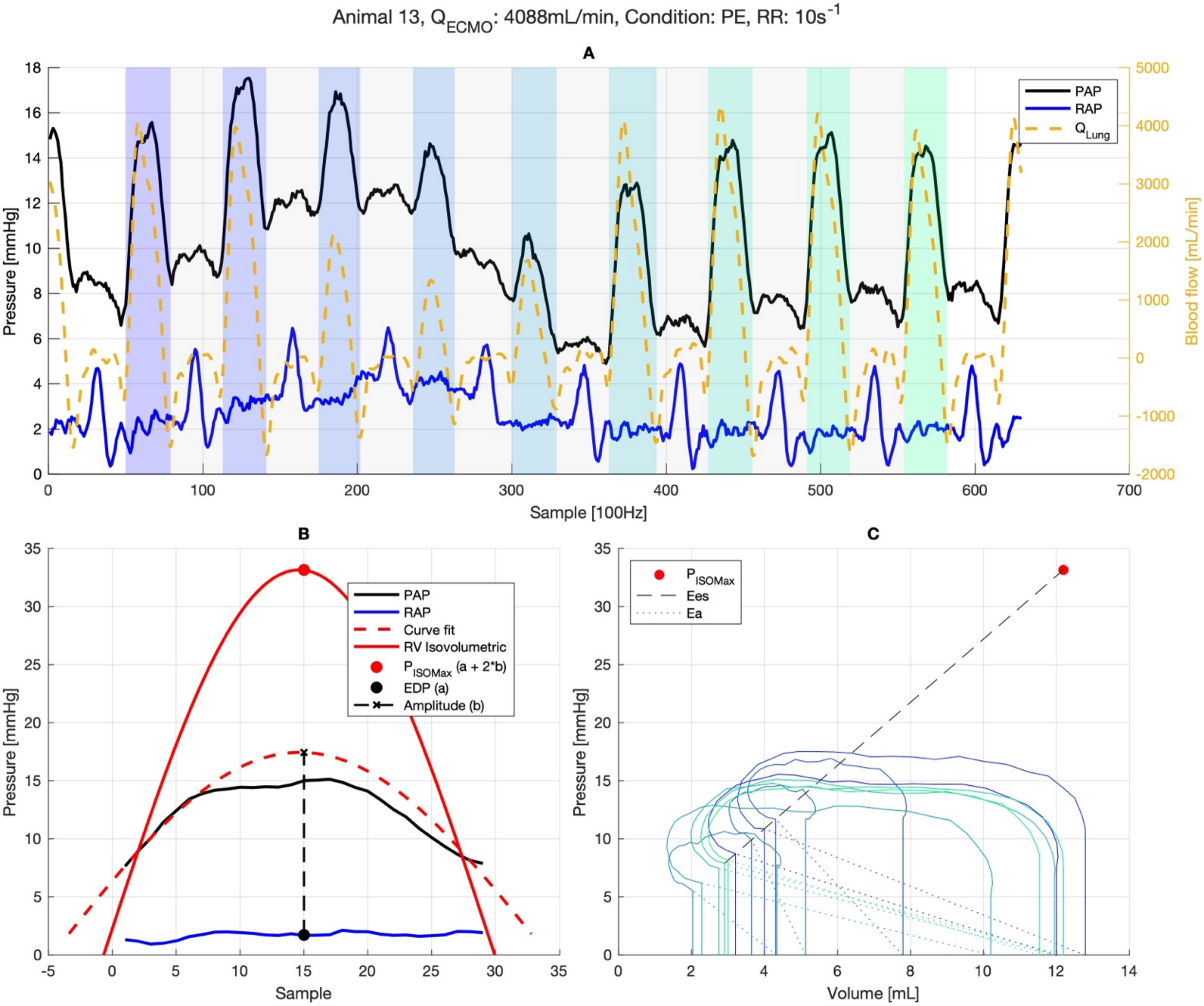

Animal 13, Q_{ECMO}: 4088mL/min, Condition: PE, RR: $10s^{-1}$
A
PAP
RAP
Q_{Lung}
Pressure [mmHg]
Blood flow [mL/min]
Sample [100Hz]
B
PAP
RAP
Curve fit
RV Isovolumetric
P_{ISOMax} (a + 2*b)
EDP (a)
Amplitude (b)
Pressure [mmHg]
Sample
C
P_{ISOMax}
Ees
Ea
Pressure [mmHg]
Volume [mL]

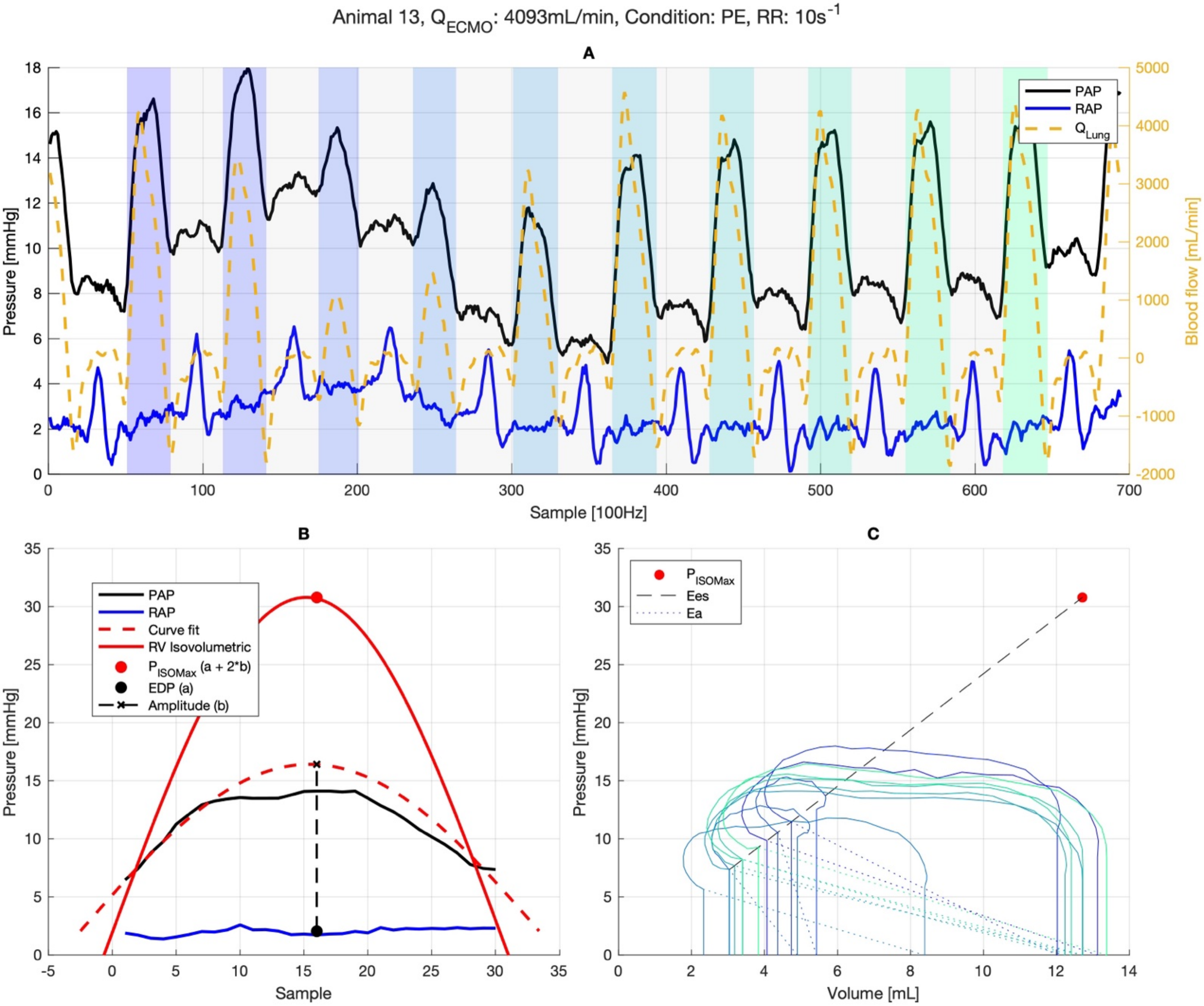

Animal 13, Q_{ECMO}: 4093mL/min, Condition: PE, RR: $10s^{-1}$
A
PAP
RAP
Q_{Lung}
Pressure [mmHg]
Blood flow [mL/min]
Sample [100Hz]
B
PAP
RAP
Curve fit
RV Isovolumetric
P_{ISOMax} (a + 2*b)
EDP (a)
Amplitude (b)
Pressure [mmHg]
Sample
C
P_{ISOMax}
Ees
Ea
Pressure [mmHg]
Volume [mL]

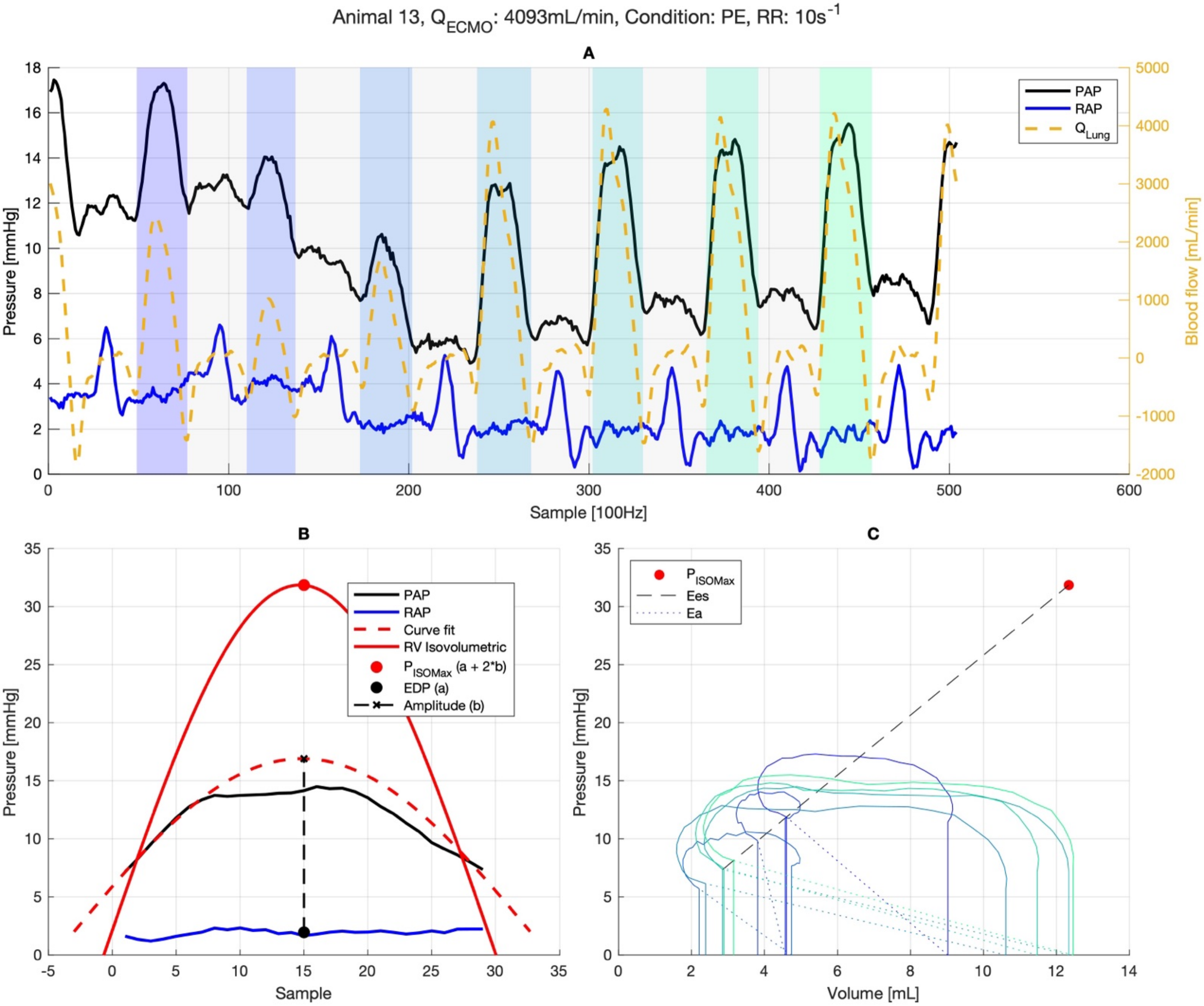

Animal 13, Q_{ECMO}: 4093mL/min, Condition: PE, RR: $10s^{-1}$
A
Pressure [mmHg]
Blood flow [mL/min]
Sample [100Hz]
PAP
RAP
Q_{Lung}
B
Pressure [mmHg]
Sample
PAP
RAP
Curve fit
RV Isovolumetric
P_{ISOMax} (a + 2*b)
EDP (a)
Amplitude (b)
C
Pressure [mmHg]
Volume [mL]
P_{ISOMax}
Ees
Ea

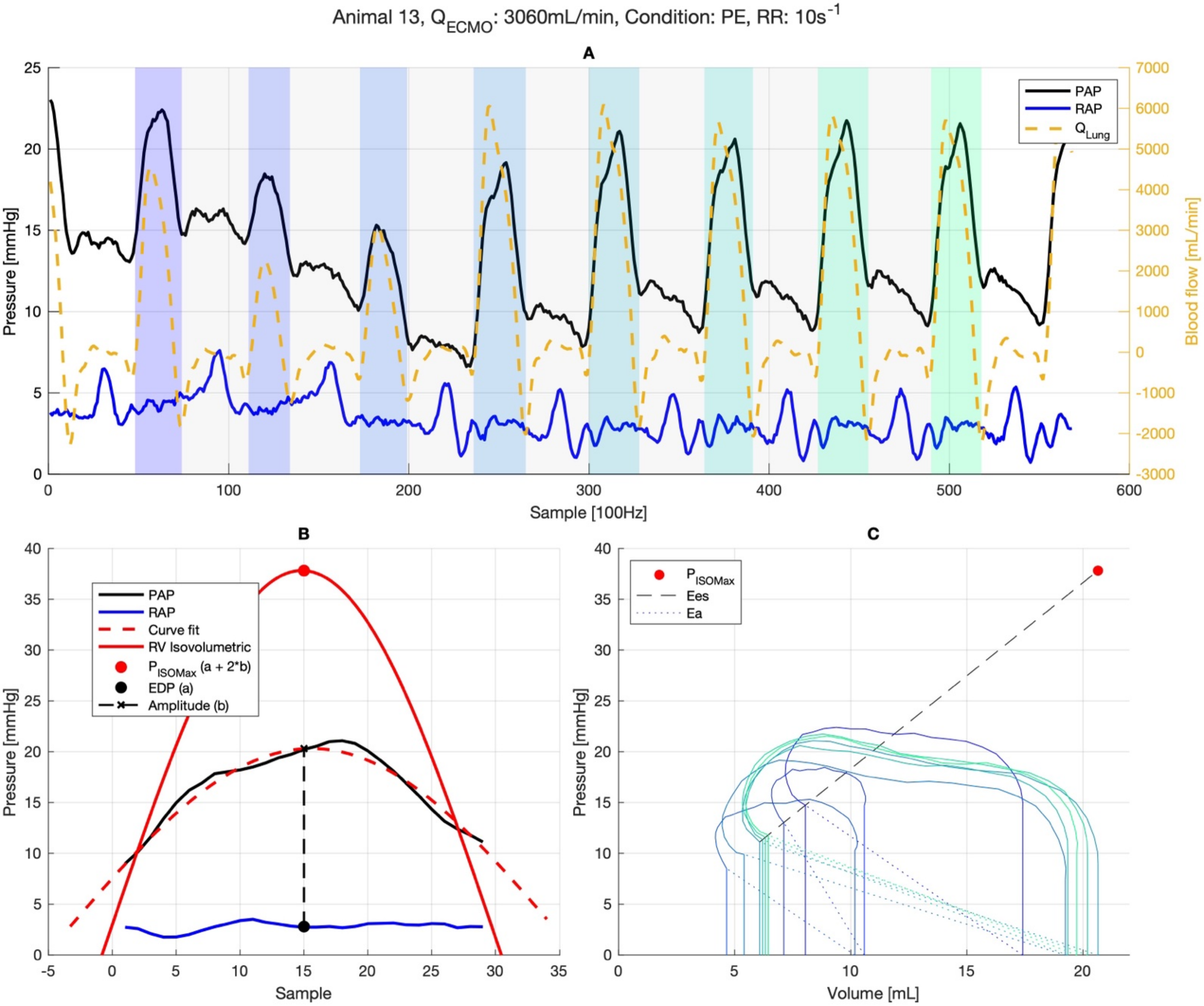

Animal 13, Q_{ECMO}: 3060mL/min, Condition: PE, RR: 10s^{-1}
A
Pressure [mmHg]
Blood flow [mL/min]
Sample [100Hz]
PAP
RAP
Q_{Lung}
B
Pressure [mmHg]
Sample
PAP
RAP
Curve fit
RV Isovolumetric
P_{ISOMax} (a + 2*b)
EDP (a)
Amplitude (b)
C
Pressure [mmHg]
Volume [mL]
P_{ISOMax}
Ees
Ea

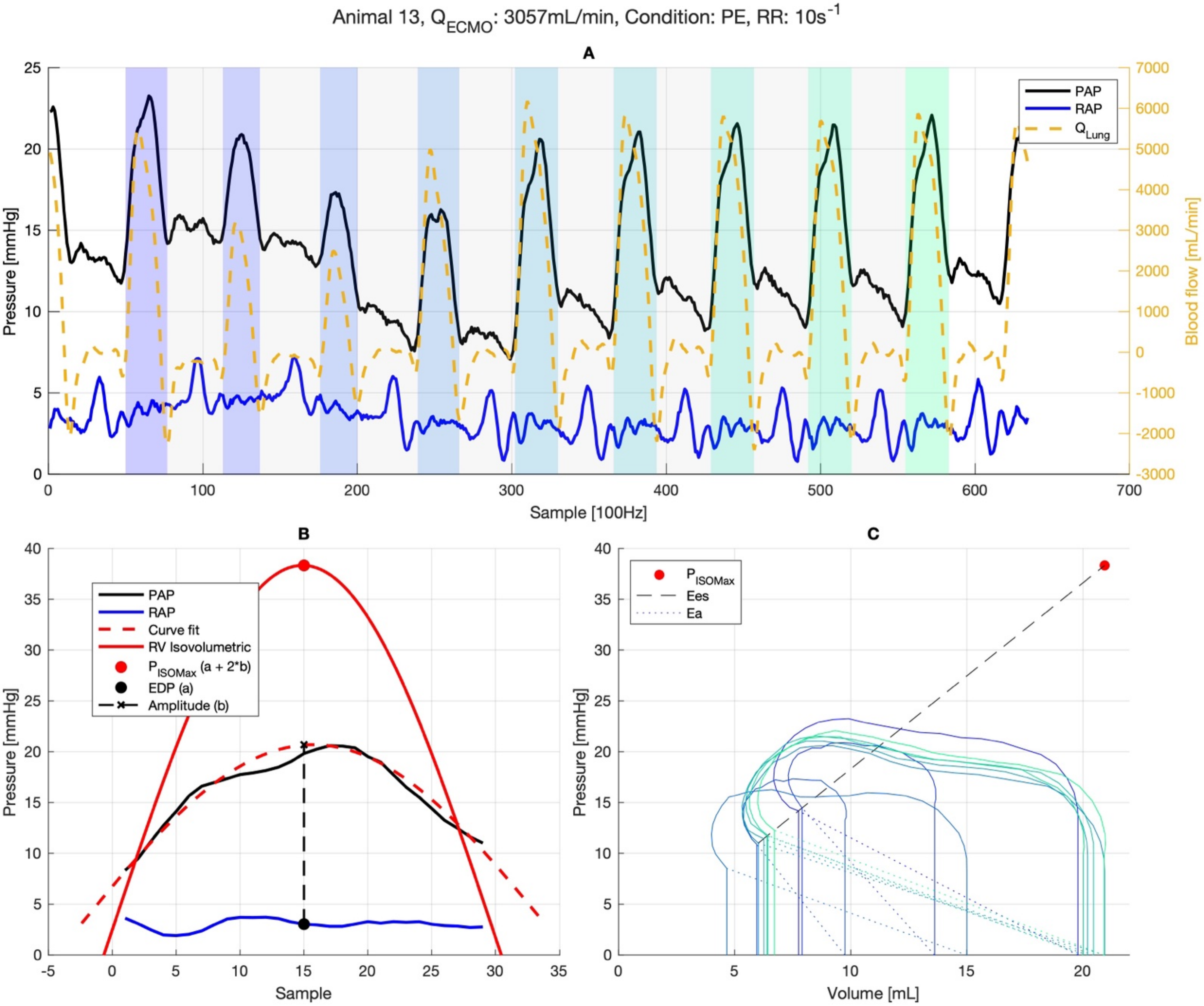

Animal 13, Q_ECMO: 3057mL/min, Condition: PE, RR: 10s^-1
A
Pressure [mmHg]
Blood flow [mL/min]
Sample [100Hz]
PAP
RAP
Q_Lung
B
PAP
RAP
Curve fit
RV Isovolumetric
P_ISOMax (a + 2*b)
EDP (a)
Amplitude (b)
Pressure [mmHg]
Sample
C
P_ISOMax
Ees
Ea
Pressure [mmHg]
Volume [mL]

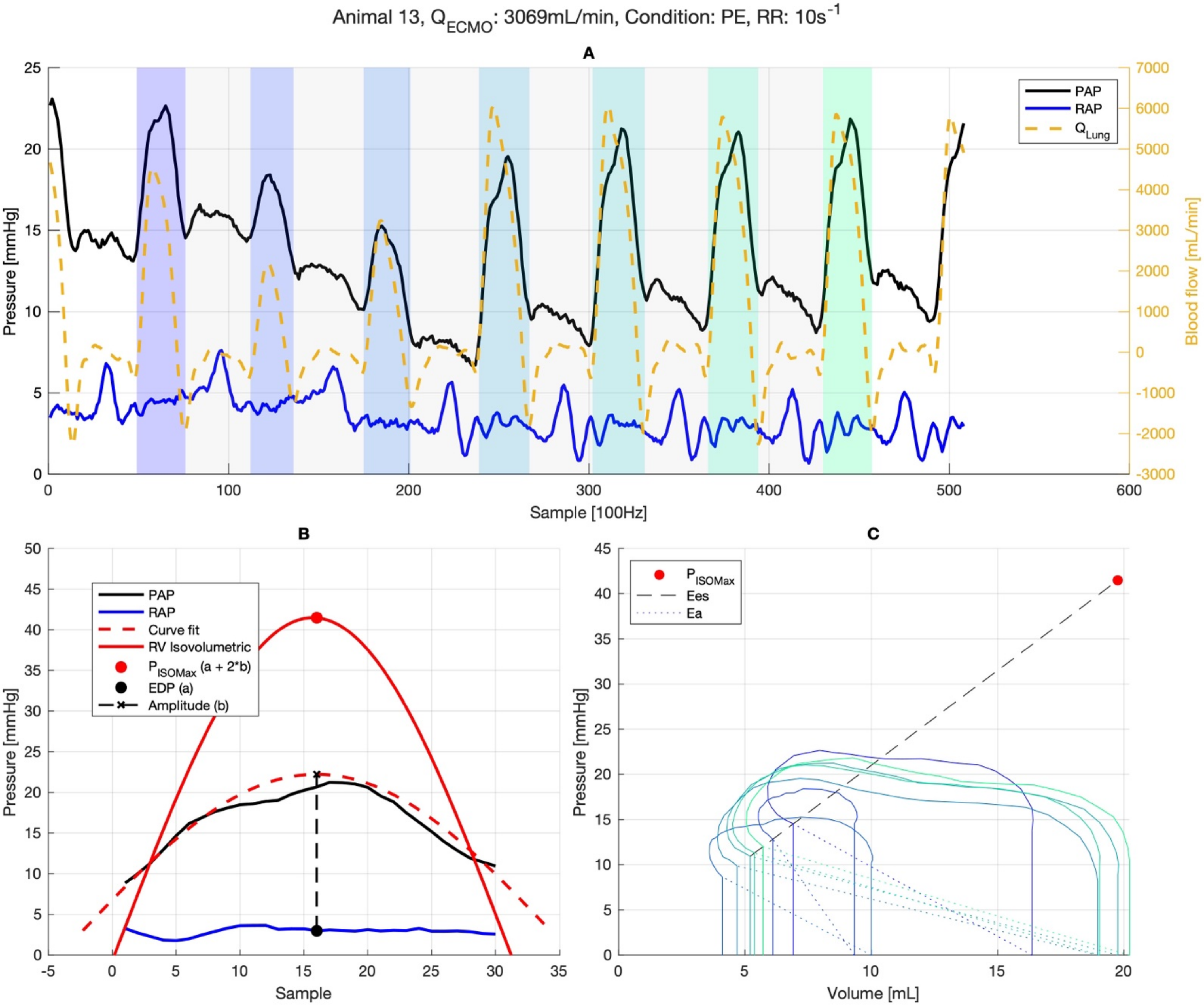

Animal 13, Q_ECMO: 3069mL/min, Condition: PE, RR: 10s^-1
A
PAP
RAP
Q_Lung
Pressure [mmHg]
Blood flow [mL/min]
Sample [100Hz]
B
PAP
RAP
Curve fit
RV Isovolumetric
P_ISOMax (a + 2*b)
EDP (a)
Amplitude (b)
Pressure [mmHg]
Sample
C
P_ISOMax
Ees
Ea
Pressure [mmHg]
Volume [mL]

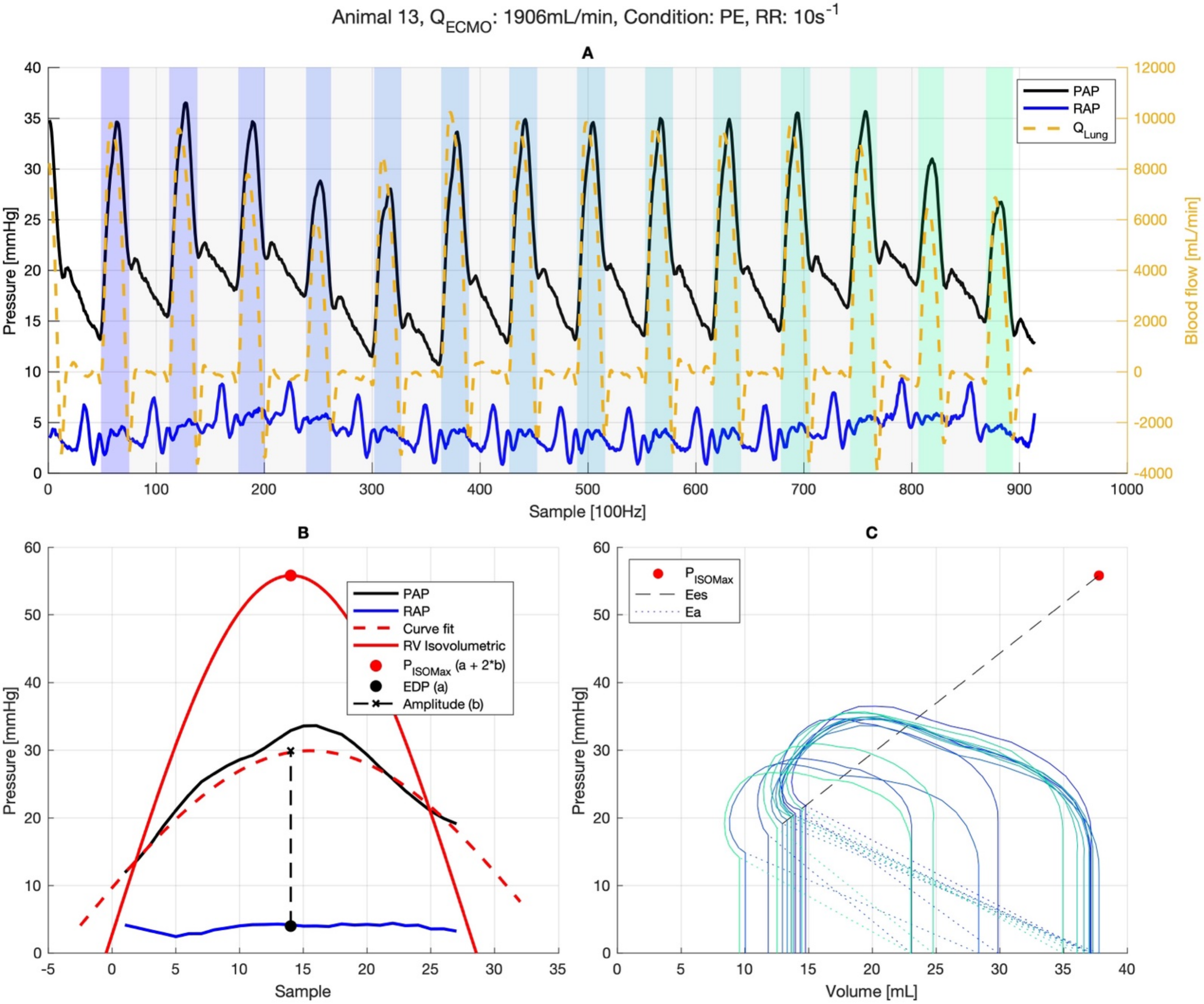

Animal 13, Q_{ECMO}: 1906mL/min, Condition: PE, RR: $10s^{-1}$
A
PAP
RAP
Q_{Lung}
Pressure [mmHg]
Blood flow [mL/min]
Sample [100Hz]
B
PAP
RAP
Curve fit
RV Isovolumetric
P_{ISOMax} (a + 2*b)
EDP (a)
Amplitude (b)
Pressure [mmHg]
Sample
C
P_{ISOMax}
Ees
Ea
Pressure [mmHg]
Volume [mL]

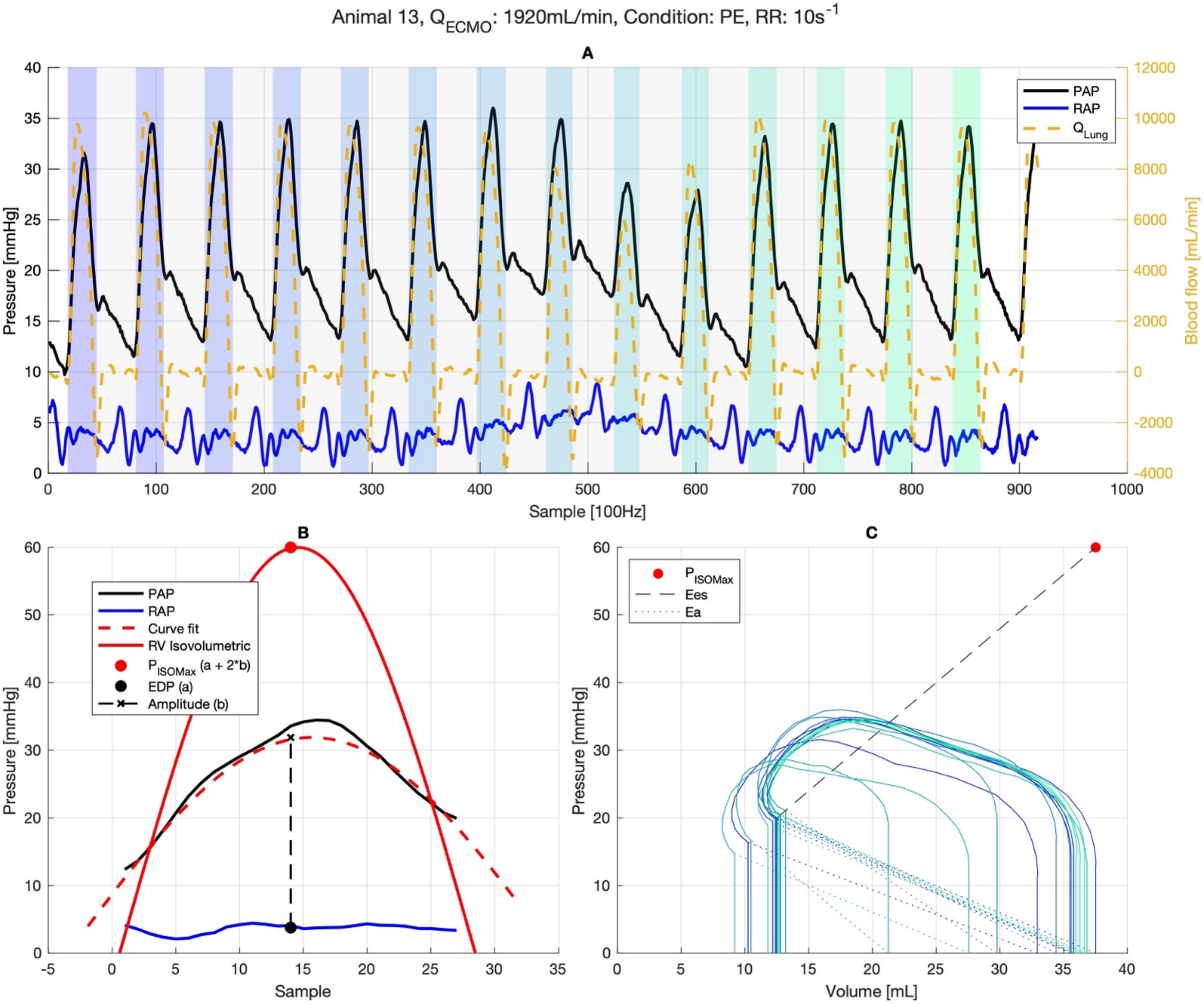

Animal 13, Q_{ECMO}: 1920mL/min, Condition: PE, RR: $10s^{-1}$
A
PAP
RAP
Q_{Lung}
Pressure [mmHg]
Blood flow [mL/min]
Sample [100Hz]
B
PAP
RAP
Curve fit
RV Isovolumetric
P_{ISOMax} (a + 2*b)
EDP (a)
Amplitude (b)
Pressure [mmHg]
Sample
C
P_{ISOMax}
Ees
Ea
Pressure [mmHg]
Volume [mL]

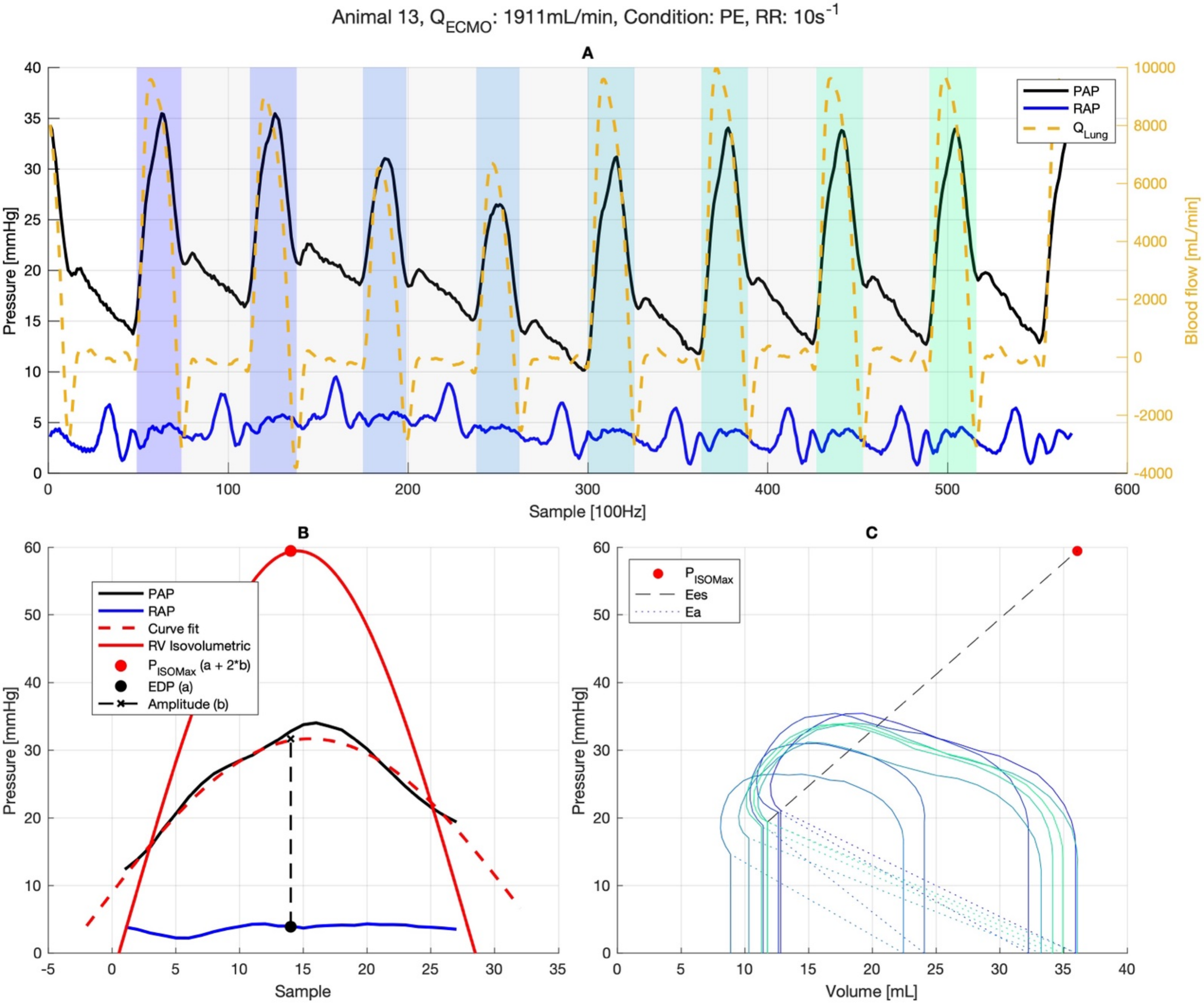

Animal 13, Q_{ECMO}: 1911mL/min, Condition: PE, RR: $10s^{-1}$
A
Pressure [mmHg]
Blood flow [mL/min]
Sample [100Hz]
PAP
RAP
Q_{Lung}
B
Pressure [mmHg]
Sample
PAP
RAP
Curve fit
RV Isovolumetric
P_{ISOMax} (a + 2*b)
EDP (a)
Amplitude (b)
C
Pressure [mmHg]
Volume [mL]
P_{ISOMax}
Ees
Ea

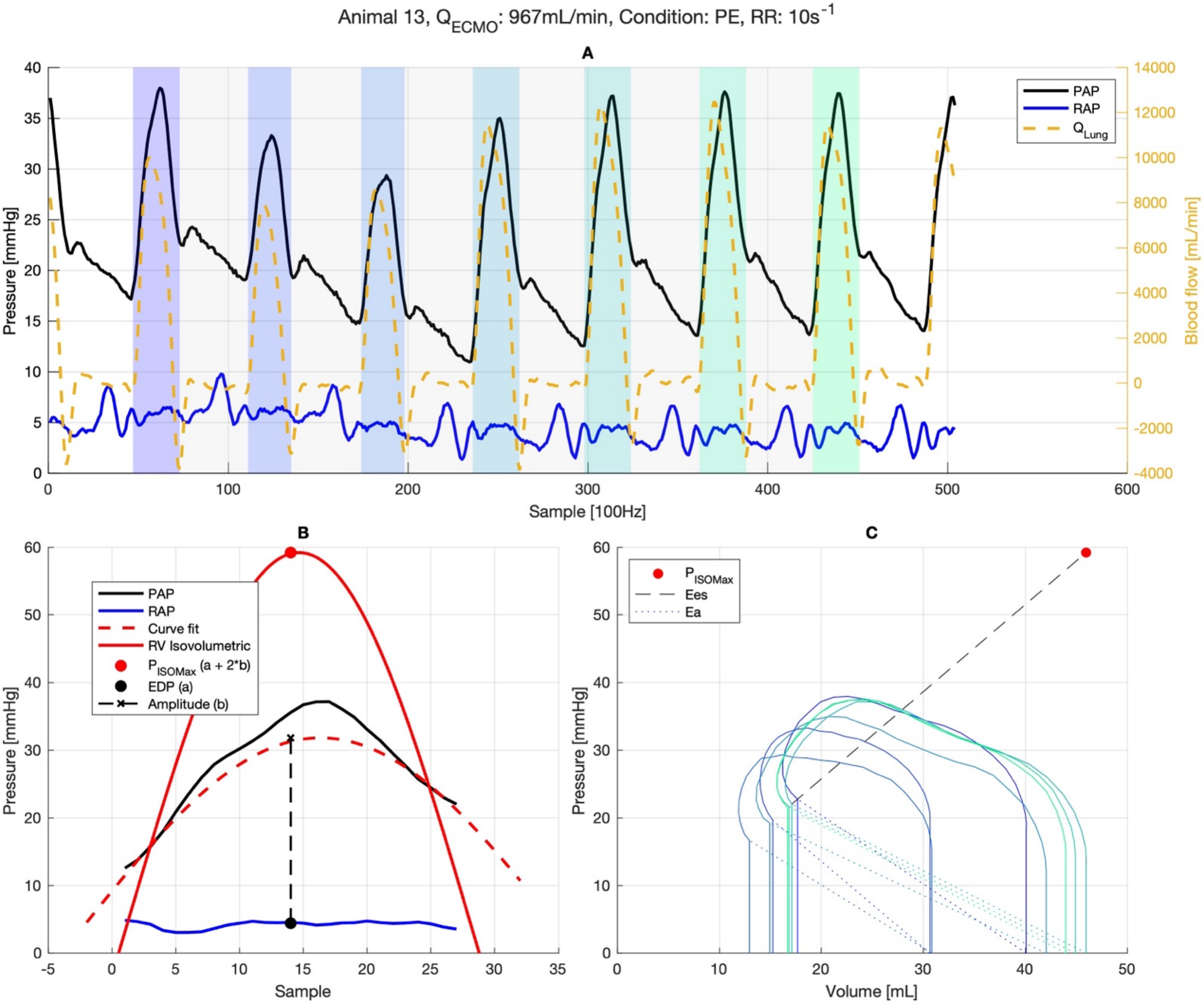

Animal 13, Q_{ECMO}: 967mL/min, Condition: PE, RR: $10s^{-1}$
A
PAP
RAP
Q_{Lung}
Pressure [mmHg]
Blood flow [mL/min]
Sample [100Hz]
B
PAP
RAP
Curve fit
RV Isovolumetric
P_{ISOMax} (a + 2*b)
EDP (a)
Amplitude (b)
Pressure [mmHg]
Sample
C
P_{ISOMax}
Ees
Ea
Pressure [mmHg]
Volume [mL]

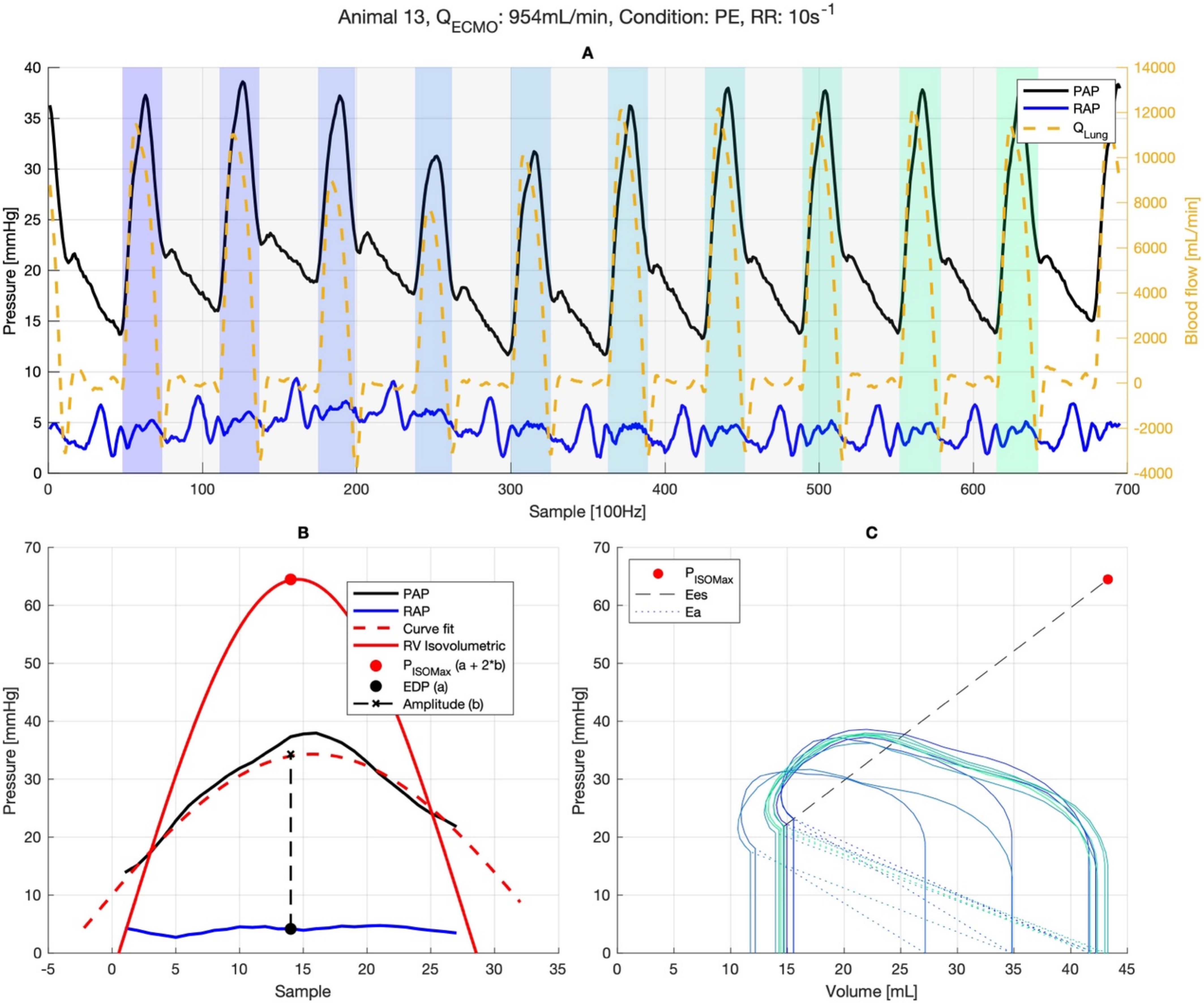
Animal 13, Q_{ECMO}: 954mL/min, Condition: PE, RR: 10s^{-1}
A
PAP
RAP
Q_{Lung}
Pressure [mmHg]
Blood flow [mL/min]
Sample [100Hz]
B
PAP
RAP
Curve fit
RV Isovolumetric
P_{ISOMax} (a + 2*b)
EDP (a)
Amplitude (b)
Pressure [mmHg]
Sample
C
P_{ISOMax}
Ees
Ea
Pressure [mmHg]
Volume [mL]

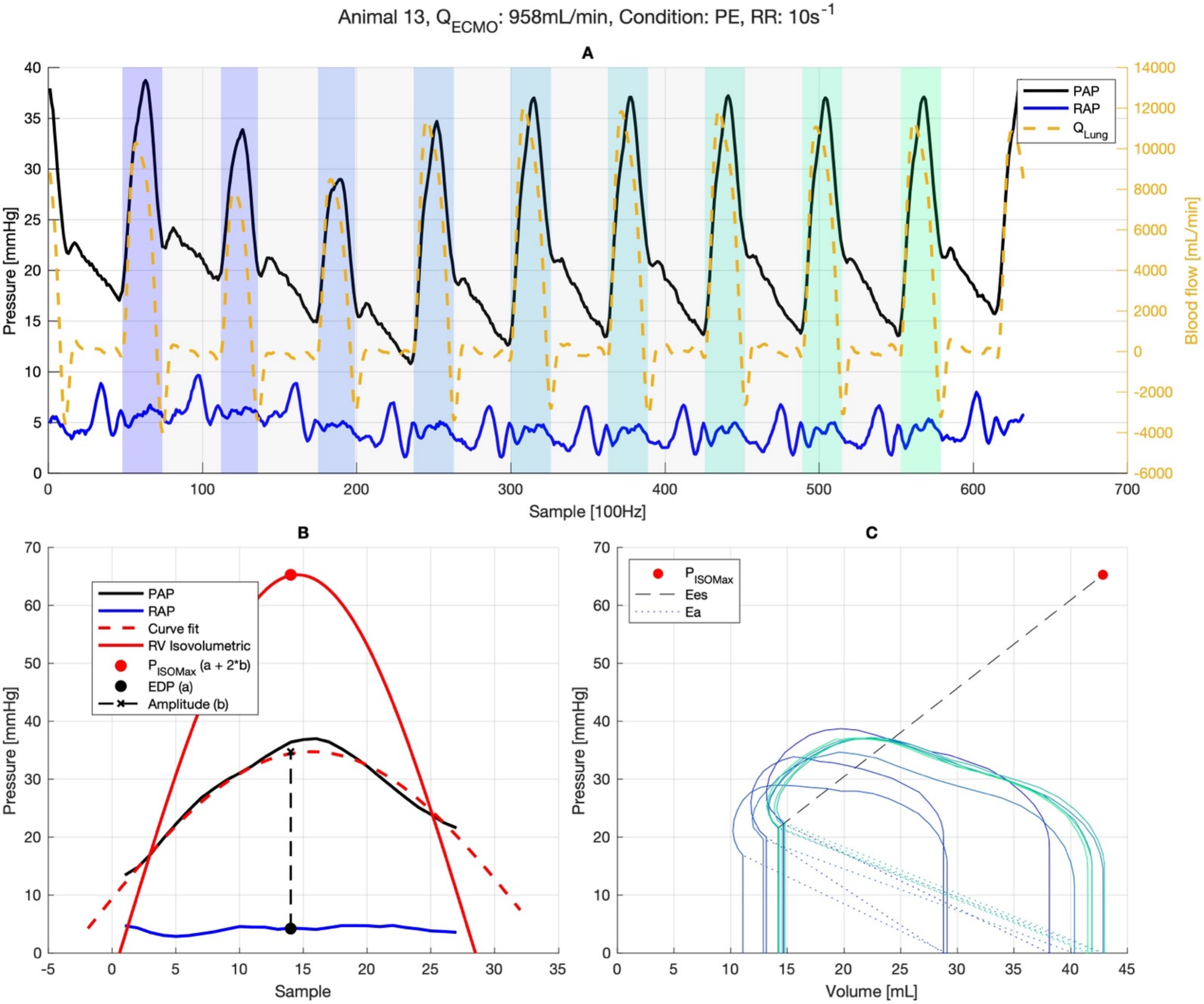

Animal 13, Q_ECMO: 958mL/min, Condition: PE, RR: 10s^-1
A
Pressure [mmHg]
Blood flow [mL/min]
Sample [100Hz]
PAP
RAP
Q_Lung
B
PAP
RAP
Curve fit
RV Isovolumetric
P_ISOMax (a + 2*b)
EDP (a)
Amplitude (b)
Pressure [mmHg]
Sample
C
P_ISOMax
Ees
Ea
Pressure [mmHg]
Volume [mL]

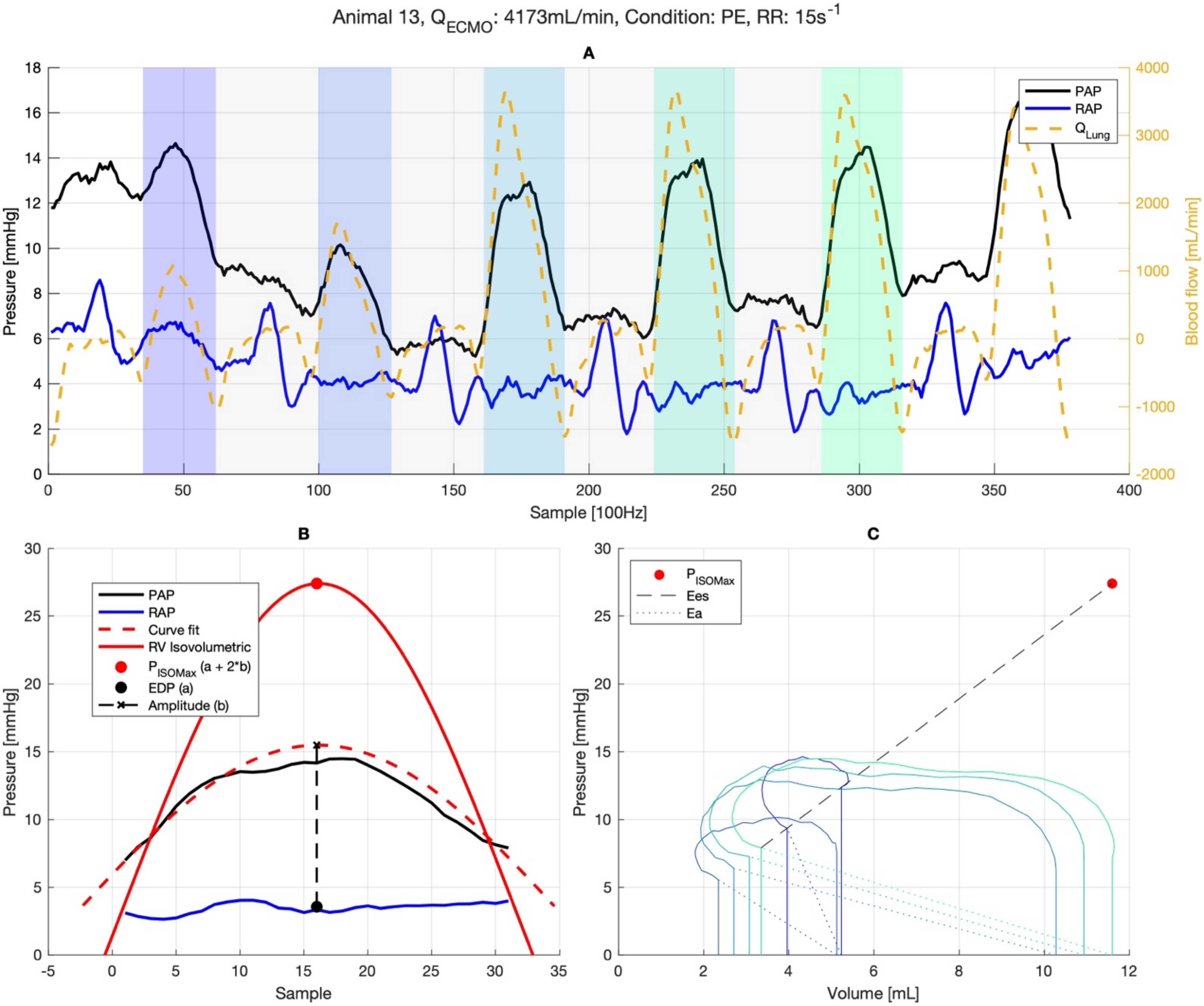

Animal 13, Q_{ECMO}: 4173mL/min, Condition: PE, RR: $15s^{-1}$
A
PAP
RAP
Q_{Lung}
Pressure [mmHg]
Blood flow [mL/min]
Sample [100Hz]
B
PAP
RAP
Curve fit
RV Isovolumetric
P_{ISOMax} (a + 2*b)
EDP (a)
Amplitude (b)
Pressure [mmHg]
Sample
C
P_{ISOMax}
Ees
Ea
Pressure [mmHg]
Volume [mL]

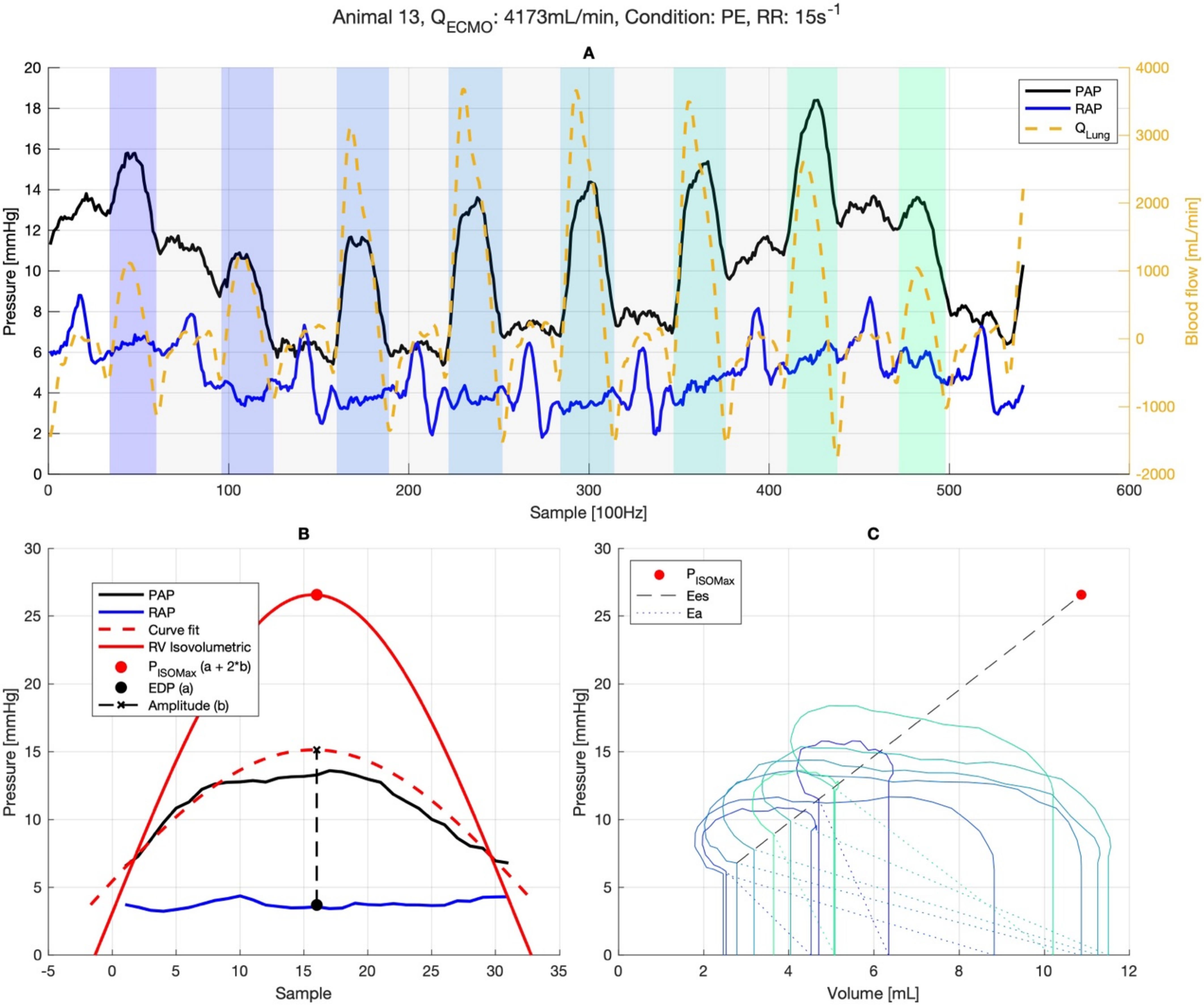

Animal 13, Q_{ECMO}: 4173mL/min, Condition: PE, RR: 15s^{-1}
A
PAP
RAP
Q_{Lung}
Pressure [mmHg]
Blood flow [mL/min]
Sample [100Hz]
B
PAP
RAP
Curve fit
RV Isovolumetric
P_{ISOMax} (a + 2*b)
EDP (a)
Amplitude (b)
Pressure [mmHg]
Sample
C
P_{ISOMax}
Ees
Ea
Pressure [mmHg]
Volume [mL]

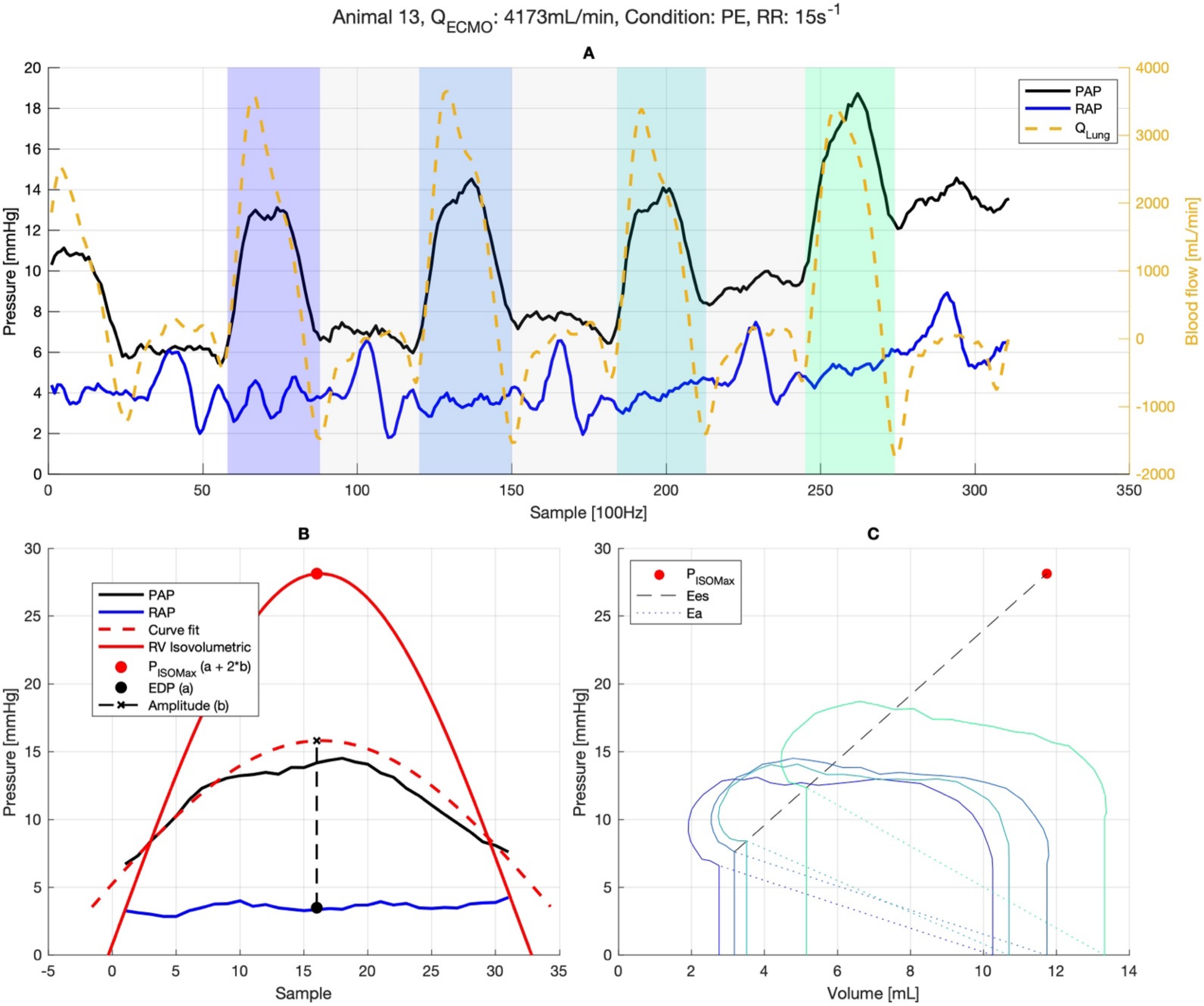

Animal 13, Q_{ECMO}: 4173mL/min, Condition: PE, RR: $15s^{-1}$
A
PAP
RAP
Q_{Lung}
Pressure [mmHg]
Blood flow [mL/min]
Sample [100Hz]
B
PAP
RAP
Curve fit
RV Isovolumetric
P_{ISOMax} (a + 2*b)
EDP (a)
Amplitude (b)
Pressure [mmHg]
Sample
C
P_{ISOMax}
Ees
Ea
Pressure [mmHg]
Volume [mL]

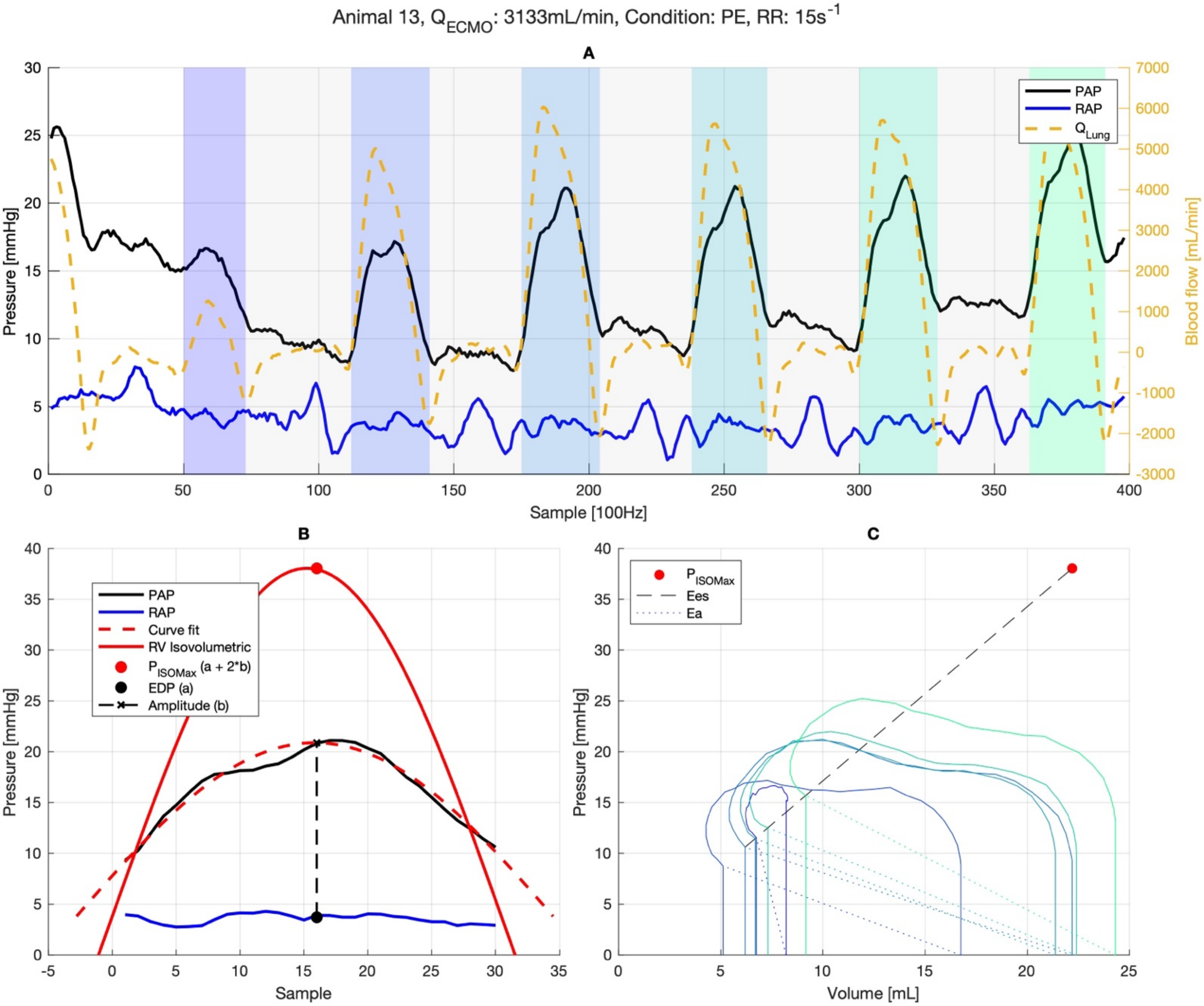

Animal 13, Q_{ECMO}: 3133mL/min, Condition: PE, RR: $15s^{-1}$
A
PAP
RAP
Q_{Lung}
Pressure [mmHg]
Blood flow [mL/min]
Sample [100Hz]
B
PAP
RAP
Curve fit
RV Isovolumetric
P_{ISOMax} (a + 2*b)
EDP (a)
Amplitude (b)
Pressure [mmHg]
Sample
C
P_{ISOMax}
Ees
Ea
Pressure [mmHg]
Volume [mL]

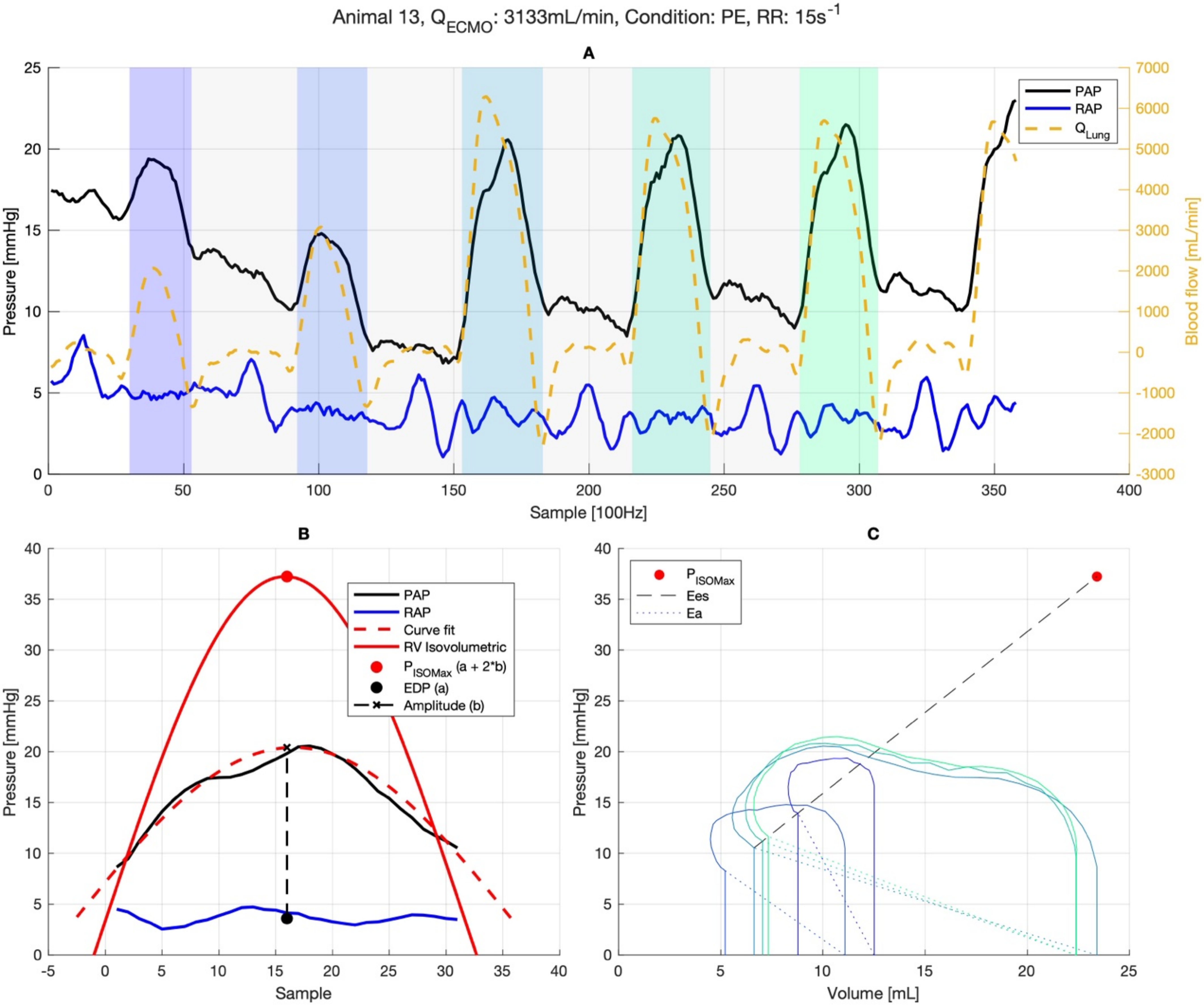

Animal 13, Q_{ECMO}: 3133mL/min, Condition: PE, RR: $15s^{-1}$
A
Pressure [mmHg]
Blood flow [mL/min]
Sample [100Hz]
PAP
RAP
Q_{Lung}
B
Pressure [mmHg]
Sample
PAP
RAP
Curve fit
RV Isovolumetric
P_{ISOMax} (a + 2*b)
EDP (a)
Amplitude (b)
C
Pressure [mmHg]
Volume [mL]
P_{ISOMax}
Ees
Ea

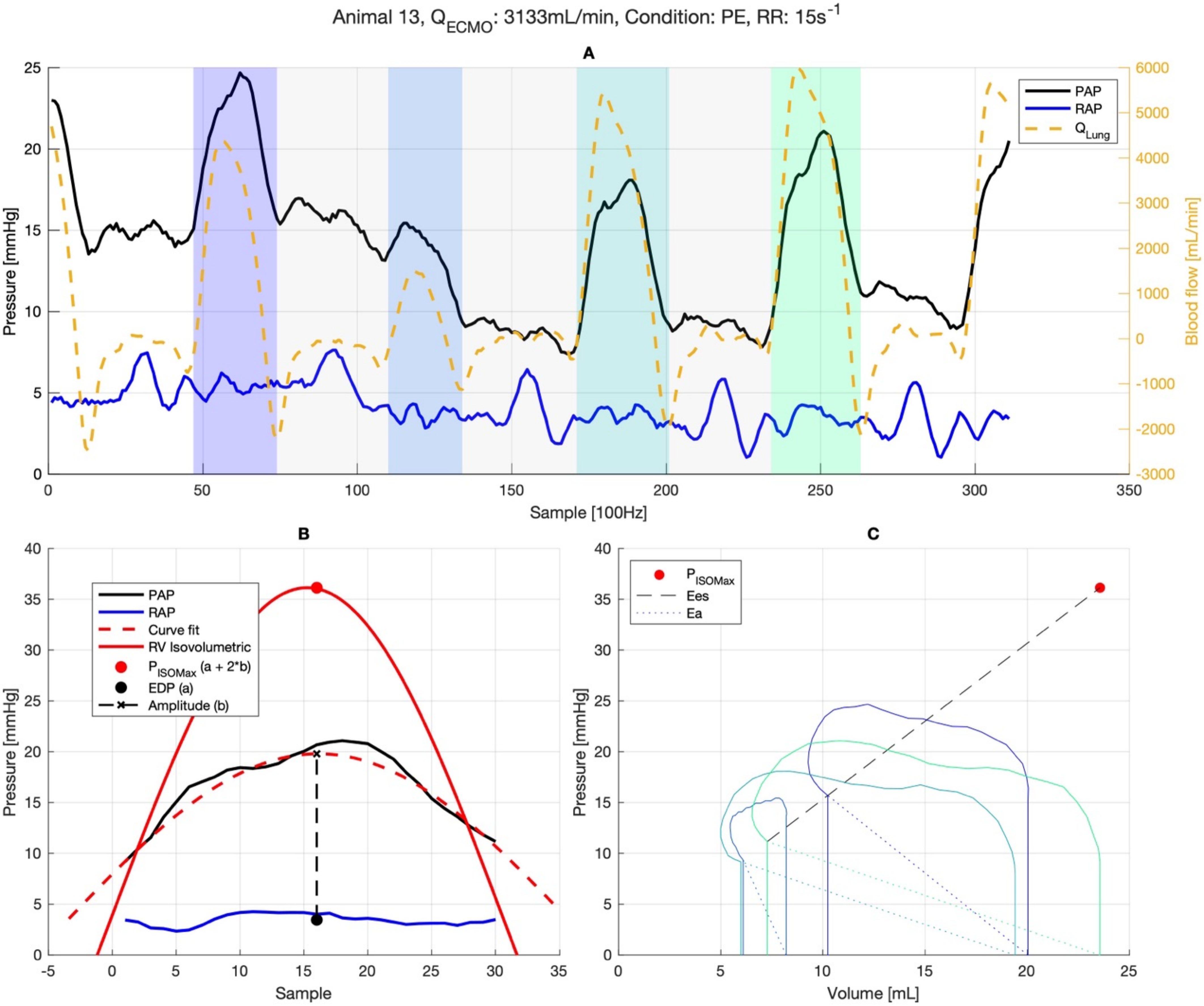

Animal 13, Q_{ECMO}: 3133mL/min, Condition: PE, RR: $15s^{-1}$
A
Pressure [mmHg]
Blood flow [mL/min]
Sample [100Hz]
PAP
RAP
Q_{Lung}
B
Pressure [mmHg]
Sample
PAP
RAP
Curve fit
RV Isovolumetric
P_{ISOMax} (a + 2*b)
EDP (a)
Amplitude (b)
C
Pressure [mmHg]
Volume [mL]
P_{ISOMax}
Ees
Ea

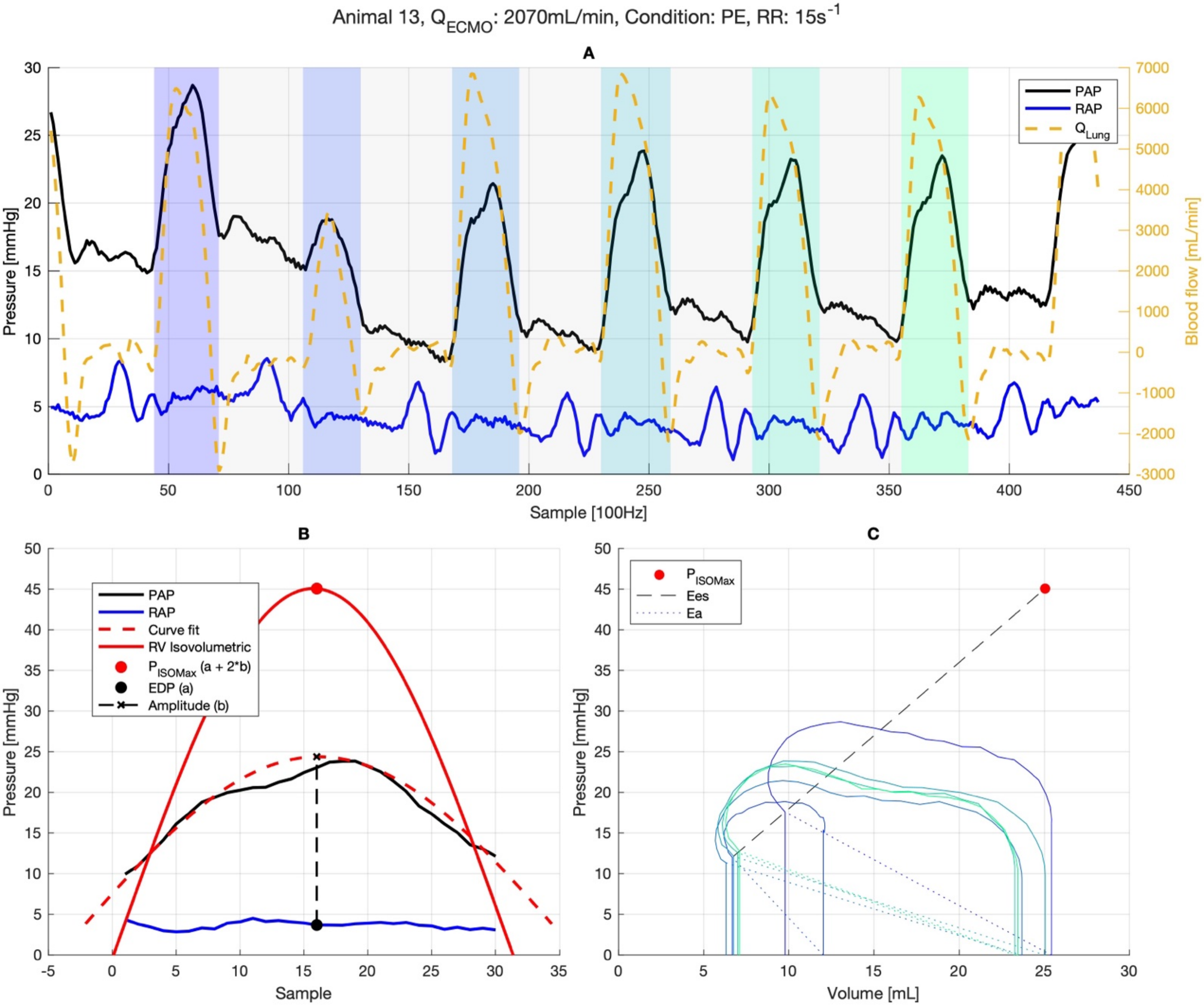

Animal 13, Q_ECMO: 2070mL/min, Condition: PE, RR: 15s^-1
A
PAP
RAP
Q_Lung
Pressure [mmHg]
Blood flow [mL/min]
Sample [100Hz]
B
PAP
RAP
Curve fit
RV Isovolumetric
P_ISOMax (a + 2*b)
EDP (a)
Amplitude (b)
Pressure [mmHg]
Sample
C
P_ISOMax
Ees
Ea
Pressure [mmHg]
Volume [mL]

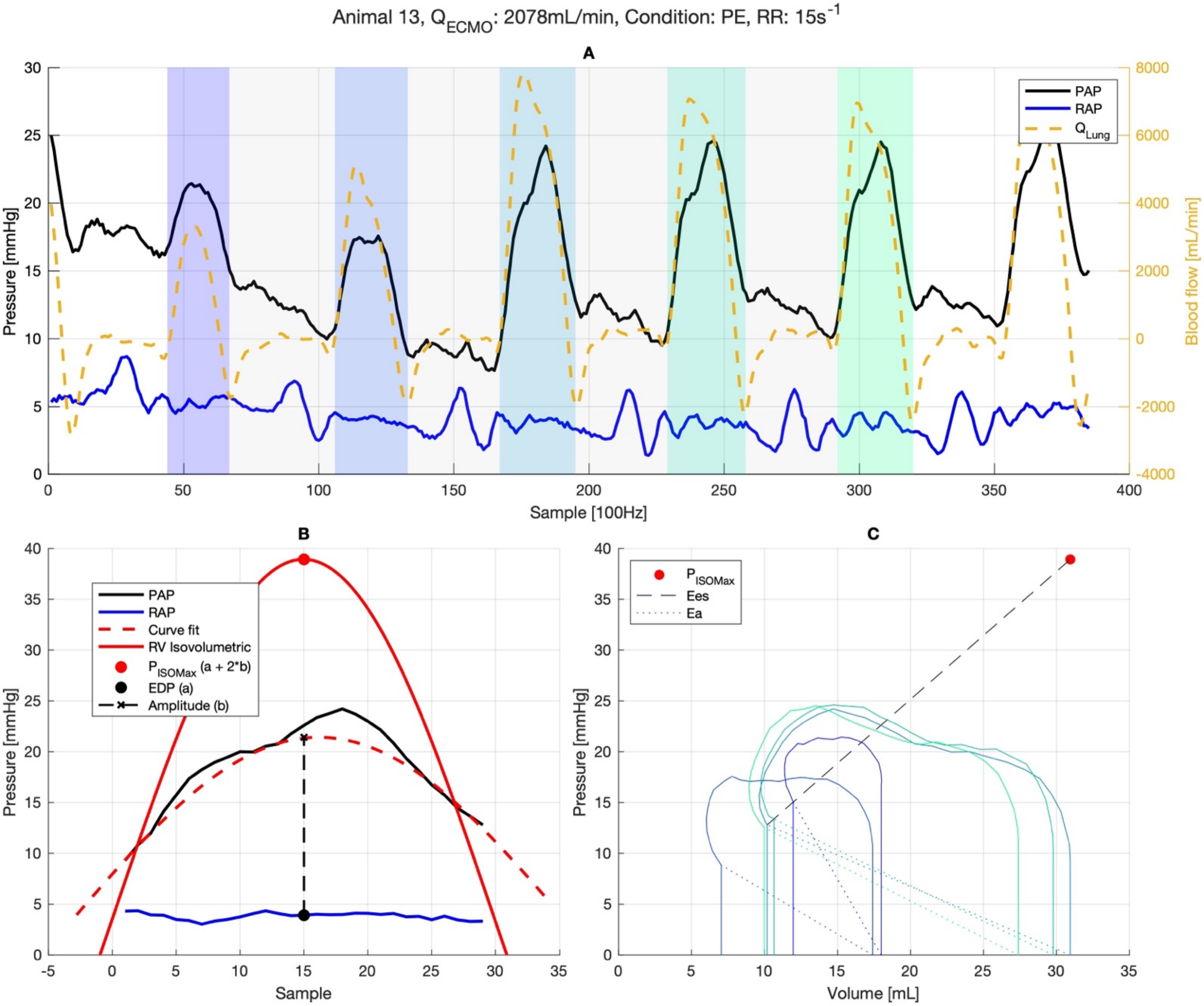

Animal 13, Q_{ECMO}: 2078mL/min, Condition: PE, RR: $15s^{-1}$
A
PAP
RAP
Q_{Lung}
Pressure [mmHg]
Blood flow [mL/min]
Sample [100Hz]
B
PAP
RAP
Curve fit
RV Isovolumetric
P_{ISOMax} (a + 2*b)
EDP (a)
Amplitude (b)
Pressure [mmHg]
Sample
C
P_{ISOMax}
Ees
Ea
Pressure [mmHg]
Volume [mL]

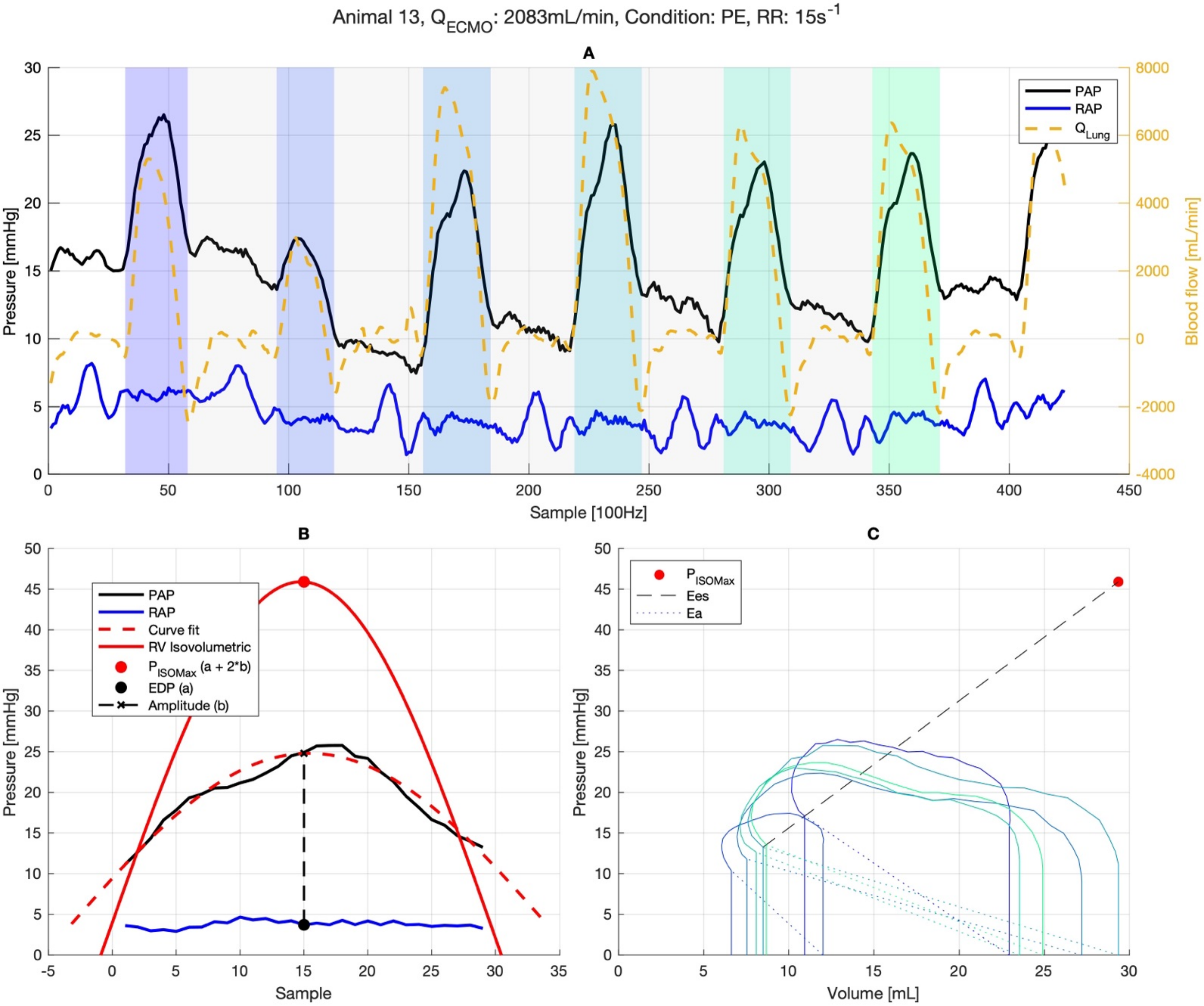

Animal 13, Q_ECMO: 2083mL/min, Condition: PE, RR: 15s^-1
A
PAP
RAP
Q_Lung
Pressure [mmHg]
Blood flow [mL/min]
Sample [100Hz]
B
PAP
RAP
Curve fit
RV Isovolumetric
P_ISOMax (a + 2*b)
EDP (a)
Amplitude (b)
Pressure [mmHg]
Sample
C
P_ISOMax
Ees
Ea
Pressure [mmHg]
Volume [mL]

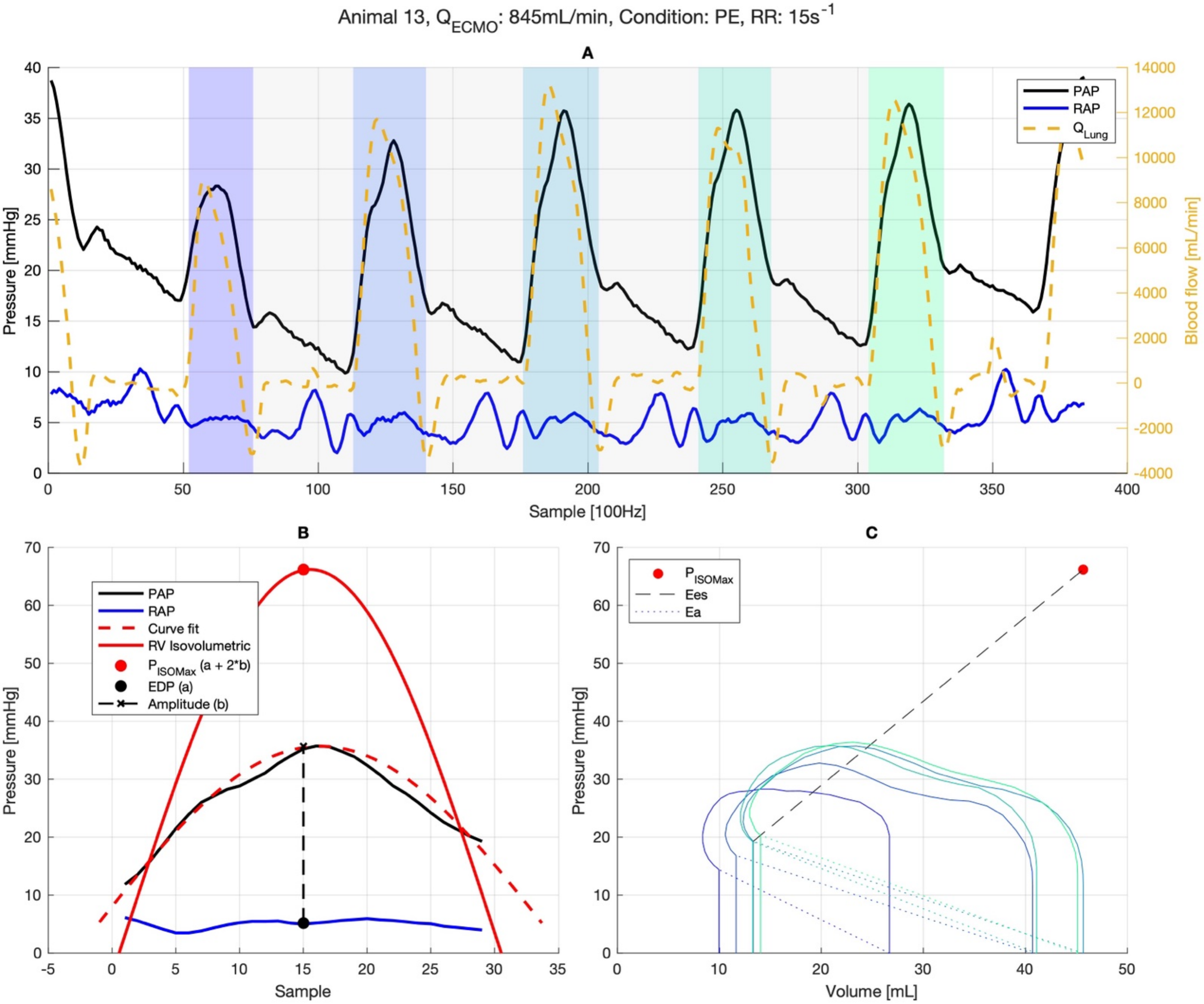
Animal 13, Q_{ECMO}: 845mL/min, Condition: PE, RR: 15s^{-1}
A
Pressure [mmHg]
Blood flow [mL/min]
Sample [100Hz]
PAP
RAP
Q_{Lung}
B
PAP
RAP
Curve fit
RV Isovolumetric
P_{ISOMax} (a + 2*b)
EDP (a)
Amplitude (b)
Pressure [mmHg]
Sample
C
P_{ISOMax}
Ees
Ea
Pressure [mmHg]
Volume [mL]

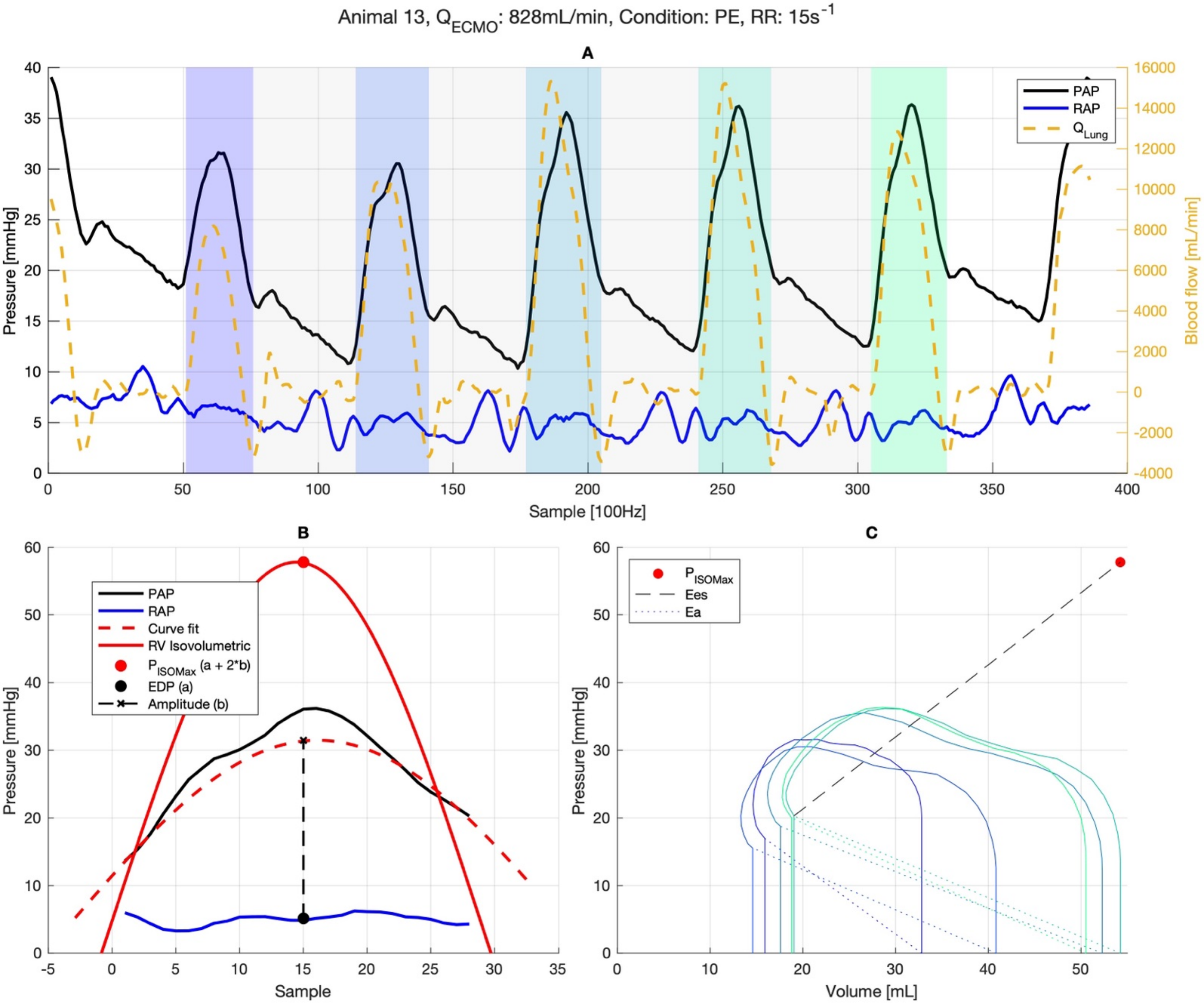

Animal 13, Q_{ECMO}: 828mL/min, Condition: PE, RR: $15s^{-1}$
A
PAP
RAP
Q_{Lung}
Pressure [mmHg]
Blood flow [mL/min]
Sample [100Hz]
B
PAP
RAP
Curve fit
RV Isovolumetric
P_{ISOMax} (a + 2*b)
EDP (a)
Amplitude (b)
Pressure [mmHg]
Sample
C
P_{ISOMax}
Ees
Ea
Pressure [mmHg]
Volume [mL]

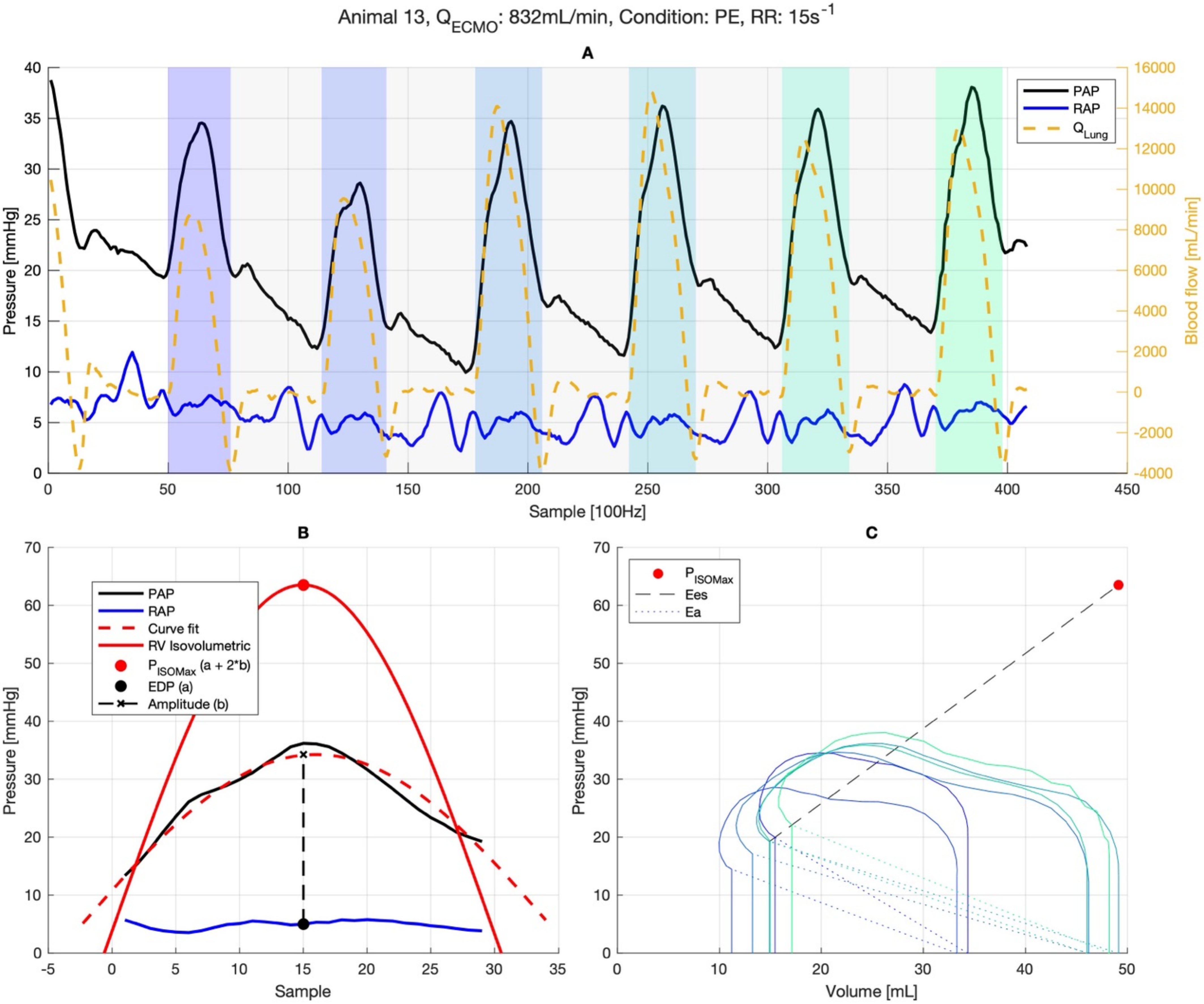

Animal 13, Q_{ECMO}: 832mL/min, Condition: PE, RR: 15s^{-1}
A
Pressure [mmHg]
Blood flow [mL/min]
Sample [100Hz]
PAP
RAP
Q_{Lung}
B
Pressure [mmHg]
Sample
PAP
RAP
Curve fit
RV Isovolumetric
P_{ISOMax} (a + 2*b)
EDP (a)
Amplitude (b)
C
Pressure [mmHg]
Volume [mL]
P_{ISOMax}
Ees
Ea
